# Characteristics of Minor Ions and Electrons in Flux Transfer Events Observed by the Magnetospheric Multiscale Mission

**DOI:** 10.1029/2020JA027778

**Published:** 2020-07-20

**Authors:** S. M. Petrinec, J. L. Burch, M. Chandler, C. J. Farrugia, S. A. Fuselier, B. L. Giles, R. G. Gomez, J. Mukherjee, W. R. Paterson, C. T. Russell, D. G. Sibeck, R. J. Strangeway, R. B. Torbert, K. J. Trattner, S. K. Vines, C. Zhao

**Affiliations:** ^1^ Lockheed Martin Advanced Technology Center Palo Alto CA USA; ^2^ Southwest Research Institute San Antonio TX USA; ^3^ NASA Marshall Space Flight Center Huntsville AL USA; ^4^ Space Science Center University of New Hampshire Durham NH USA; ^5^ Department of Physics and Astronomy University of Texas at San Antonio San Antonio TX USA; ^6^ NASA Goddard Space Flight Center Greenbelt MD USA; ^7^ Earth and Space Sciences University of California Los Angeles CA USA; ^8^ Laboratory for Atmospheric and Space Physics University of Colorado Boulder Boulder CO USA; ^9^ The Johns Hopkins University Applied Physics Laboratory Laurel MD USA

**Keywords:** magnetopause, flux transfer events, magnetic reconnection

## Abstract

In this study, the ion composition of flux transfer events (FTEs) observed within the magnetosheath proper is examined. These FTEs were observed just upstream of the Earth's postnoon magnetopause by the National Aeronautics and Space Administration (NASA) Magnetospheric Multiscale (MMS) spacecraft constellation. The minor ion characteristics are described using energy spectrograms, flux distributions, and ion moments as the constellation encountered each FTE. In conjunction with electron data and magnetic field observations, such observations provide important contextual information on the formation, topologies, and evolution of FTEs. In particular, minor ions, when combined with the field‐aligned streaming of electrons, are reliable indicators of FTE topology. The observations are also placed (i) in context of the solar wind magnetic field configuration, (ii) the connection of the sampled flux tube to the ionosphere, and (iii) the location relative to the modeled reconnection line at the magnetopause. While protons and alpha particles were often depleted within the FTEs relative to the surrounding magnetosheath plasma, the He^+^ and O^+^ populations showed clear enhancements either near the center or near the edges of the FTE, and the bulk plasma flow directions are consistent with magnetic reconnection northward of the spacecraft and convection from the dayside toward the flank magnetopause.

## Key Points

- Long‐duration magnetosheath FTEs observed under at postnoon local times and under similar solar wind conditions are compared and contrasted
- Minor ions from the magnetosphere are observed at higher energies at the edges than at the center of the FTEs
- The cores of the magnetosheath FTEs are on closed field lines

## 1 Introduction

Flux transfer events (FTEs) at the magnetopause are considered to be manifestations of transient magnetic reconnection between the interplanetary and magnetospheric magnetic fields. Various models have been proposed. Russell and Elphic (1978) suggested two elbow‐shaped magnetic flux tubes, which propagate south/north after reconnection. Scholer (1988) and Southwood et al. (1988) proposed bursty reconnection occurring at a finite single X‐line. A multiple X‐line model was put forward by Lee and Fu (1985, 1986). These models are reviewed in Scholer (1995).

From the time of initial identification of FTEs in International Sun‐Earth Explorer (ISEE) spacecraft magnetometer observations (Russell & Elphic, 1978, 1979), there have been many observational studies and numerical modeling efforts conducted to understand the properties and characteristics of this transient phenomenon, especially at the dayside terrestrial magnetopause. Many of these previous studies have focused on the occurrence frequency, dependence on the interplanetary magnetic field (IMF), and magnetic field configuration of FTEs (e.g., Berchem & Russell, 1984; Kawano & Russell, 1997; Pu et al., 2013; Rijnbeek et al., 1984; Sibeck, 2009). In the case of the Earth's magnetosphere and its interactions with the solar wind, FTEs have been preferentially observed during southward IMF conditions, with a mean occurrence interval of ~8 min (Rijnbeek et al., 1984; Russell et al., 1996). There is also an observed occurrence preference for the postnoon relative to the prenoon sector (Kawano & Russell, 1996). Several additional studies have examined the plasma characteristics (electrons and ions) of FTEs (e.g., Daly et al., 1981; Elphic, 1995; Farrugia et al., 2016; Hasegawa et al., 2016; Le et al., 1999; Paschmann et al., 1982). It has long been established that the in situ signatures of FTEs include a draping field region (Farrugia et al., 1987, 2016), and a central “core” region that comprises reconnected and twisted flux tube(s) containing a mixture of magnetosheath and magnetospheric plasmas (Paschmann et al., 1982; Thomsen et al., 1987). This mixture is often preferentially observed in the trailing portion of the FTE (Elphic, 1995; Le et al., 1999). Recent observational studies have used multipoint plasma (electron and undifferentiated ions of various energies) and field observations to describe in greater detail the physics and topology of flux ropes and FTEs (e.g., Fuselier, Vines, et al., 2017; Øieroset et al., 2011; Owen et al., 2001; Roux et al., 2015). It is found that FTEs are quite complex, often composed of a mixture of closed field regions (either plasmoids or field lines with both ends tied to the Earth), open field line regions (one foot tied to the Earth), and/or unconnected field line regions (e.g., of the magnetosheath proper). At times, colliding jets associated with magnetic reconnection at the magnetopause between the two active X‐lines flanking a flux rope are observed to result in symmetric, large guide field reconnection at the center of the flux rope (Øieroset et al., 2016).

The characteristics of observed ion and electron populations (energy and pitch angle) within FTEs are used to understand the topology and processes related to this phenomenon. However, even with such information, it can be difficult to unambiguously determine the sources. For example, while the appearance of bidirectional streaming accelerated electrons associated with a flux rope or an FTE is a strong indicator of closed magnetic field lines, it is challenging to determine if these field lines are magnetospheric field lines wrapped about the flux rope, or if reconnection occurring in multiple locations has closed the field line (cf., Fuselier, Vines, et al., 2017; Øieroset et al., 2011; Roux et al., 2015; Vines et al., 2017). The observation of minor ion species can be very useful in this context, as different species are associated with different sources and can thus lead to unambiguous identification of the source region (solar wind or magnetosphere). While protons are ubiquitous, alpha particles are observed primarily in the solar wind. They are also observed within the magnetosphere boundary layer (or low‐latitude boundary layer) as a consequence of solar wind entry via magnetic reconnection. The source of singly ionized helium and oxygen, however, is the ionosphere; they appear within the magnetosphere but are rarely observed in the solar wind except via magnetospheric escape. Therefore, examination of these minor ions in the context of FTEs and in conjunction with other plasma and field observations, such as direction of motion of field‐aligned electrons, provides a useful tool to help fully characterize and understand the nature of FTEs. This is the emphasis of the present paper.

One such examination of minor ion characteristics associated with FTEs within the outer terrestrial magnetosphere was conducted by Klumpar et al. (1990). For this investigation, AMPTE‐CCE/HPCE observations were utilized (Shelley et al., 1985). Although the time to sample a full 3‐D flux distribution using AMPTE‐CCE/HPCE was 2 min (longer than the typical FTE duration; cf., Kawano & Russell, 1996), composite plasma distributions were painstakingly created by carefully considering the orientation of the changing magnetic field at each sampling energy and angular bin (38‐ms sample resolution). It was generally concluded from this investigation that the ion composition within the FTE was unlike the magnetosheath, magnetosphere, or the boundary layers. Specifically, the alpha particle content was observed to be heated, but of reduced density relative to that of the low‐latitude boundary layer (LLBL) or within the magnetosheath. The He^+^ and O^+^ constituents relative to the proton population were smaller than expected when compared to the adjacent magnetospheric content.

Although several spacecraft during the past few decades have sampled in situ FTEs in the terrestrial magnetosheath, the magnetosphere, and at the magnetopause (as well as around other planetary magnetospheres such as Mercury; e.g., Imber et al., 2014; Russell & Walker, 1985; Slavin et al., 2009, 2010), Jupiter (e.g., Huddleston et al., 1997; Walker & Russell, 1985), and Saturn (Jasinski et al., 2016), the spacecraft in general have not been outfitted with instrumentation capable of discriminating various ion species. Although the minor ions do not contribute significantly to the large‐scale particle flux transfer, they provide important contextual information about the various population sources and transient physical processes associated with the FTE.

Using observations from the National Aeronautics and Space Administration (NASA)/Magnetospheric Multiscale (MMS) mission (Burch et al., 2016) and simultaneous solar wind observations as obtained from the Wind spacecraft near the Sun‐Earth L1 Lagrange point, it is now possible to examine in greater detail and at higher temporal resolution the characteristics and properties of the minor ion populations in relation to the more populous protons associated with FTEs. This is important for providing a more comprehensive understanding of particle flux transport of ion species between the solar wind, the magnetosphere, and the ionosphere resulting from transient magnetopause reconnection. For this initial effort, we limit ourselves to a case study approach. In this spirit, we compare and contrast the plasma characteristics of various ion species from four long‐duration (of a few minutes) FTEs observed in the magnetosheath by the MMS mission just upstream of the postnoon, low‐ to middle‐latitude magnetopause. These events were observed in nearly the same location (postnoon sector) and for similar solar wind conditions (i.e., southward IMF with a small to moderate Sun‐Earth (*B*_*x*_) component, and nominal solar wind density and solar wind speeds). The remainder of this paper compares and contrasts in detail the characteristics of these cases, with focus on the minor ion composition.

## 2 Instrumentation

Measurements of the magnetic field from the MMS Fluxgate Magnetometer (Russell et al., 2016) in conjunction with plasma observations from the Fast Plasma Investigation (FPI) (Pollock et al., 2016) and the Hot Plasma Composition Analyzer (HPCA) (Young et al., 2014) provide initial identification of potential magnetosheath FTEs (a more comprehensive description of this process is provided in the next section). The magnetometer observations are also used to determine the parallel and antiparallel directions for the electron measurements and to determine pitch angle for the 3‐D ion distributions. The FPI Dual Electron Spectrometer (DES) instruments measure full 3‐D electron distributions from 10 eV to 30 keV, and the electron fluxes parallel and antiparallel to the field are used to understand magnetic field topology as has been done in many previous studies (e.g., Fuselier et al., 1995, 1997, 2011, 2012, 2014; Lavraud et al., 2005, 2006; Pu et al., 2013; Roux et al., 2015; Russell et al., 2017). Ion densities from the FPI Dual Ion Spectrometer (DIS) instruments are also presented in this study. While the FPI ion observations do not provide mass resolution, the time resolution is better than that of HPCA.

The MMS HPCA instruments provide for each of the species H^+^, He^+^, He^++^, O^+^, and O^++^ full 3‐D plasma distribution functions at a 10‐s cadence (one half of the spacecraft spin period). A novel Radio Frequency (RF) system within the HPCA Electrostatic Analyzer is used to reduce the relatively large observed proton population by a known factor while allowing the full flux of minor ions to pass through the instrument and into the time‐of‐flight section of the instrument for mass discrimination (Burch et al., 2005). From these distribution functions, energy spectra time series and plasma moments are constructed. The plasma distribution functions are also rotated and translated into a magnetic field‐aligned coordinate system. Averaged magnetic field values and the proton velocity moments at 10‐s resolution are used for the coordinate transformations and translations of the HPCA distribution functions into field‐aligned coordinates where the velocity perpendicular to the magnetic field is 0.

Solar wind observations from the Wind spacecraft are first convected to the bow shock subsolar point (Farris & Russell, 1994) according to the observed solar wind speed. Additional time from the bow shock nose to the MMS location (using the difference along the *X* direction between the bow shock nose and the MMS location, divided by one quarter of the solar wind speed) is included. Convected solar wind magnetic field observations from the Wind Magnetic Field Investigation (MFI) in Geocentric Solar Magnetospheric (GSM) coordinates (Lepping et al., 1995) along with the observed Solar Wind Experiment (SWE) proton and alpha particle number densities and bulk flow speeds (Ogilvie et al., 1995) are then interpolated to 10‐s resolution to match that of the HPCA plasma moment resolution. The solar wind plasma moment data sets are essential for our investigation and are used to ascertain whether the proton and alpha particle density variations surrounding and within the FTEs as sampled by MMS are inherent to the structure or are affected by variations in the solar wind.

## 3 Event Selection

The prime science objective of the MMS mission is to sample ion, and especially electron, reconnection diffusion regions with a large complement of multipoint, high‐resolution measurements, and bring down the observations in burst mode for these events. The amount of data collected from all of the different instruments (25 on each of the four spacecraft) is such that only a small fraction of each orbit can be downlinked in full resolution burst mode. Burst mode intervals are selected by the “Scientist‐in‐the‐Loop” (SITL) for each orbit for downlink. Identified FTEs during the mission's dayside phase are usually ranked by the SITLs at a slightly lower priority than magnetopause crossings, and so (depending on buffer sizes and queue) not every FTE interval is brought down in burst mode; a few are instead brought down in fast survey mode at significantly reduced resolution. The identification of FTEs by the SITL was based primarily on magnetic field signatures (e.g., bipolar signature in at least one component coincident with intensity enhancement); associated accelerated flows (if present) were also often noted. While the SITLs have provided very useful comments describing their selection of interesting events as observed by MMS, it is important to independently verify candidate FTE encounters from the observations.

FTEs typically pass over sampling spacecraft in a few tens of seconds, and the MMS HPCA instruments observe a full 3‐D distribution in 10 s. Thus, for many FTEs only a very few complete HPCA distributions are available. To provide greater sampling within the magnetosheath FTE, the longest intervals have been preferentially selected. It is noted, however, that this selection criterion introduces the potential bias that minor ion composition of only the slowest moving and/or largest FTEs are examined. Nevertheless, the availability of complete HPCA distributions at such temporal resolution is a distinct enhancement over what was previously possible from other missions (e.g., Klumpar et al., 1990).

Properties and characteristics of four isolated FTEs observed within the magnetosheath close to the magnetopause are described in the next subsections. All four long‐duration FTEs were observed in the postnoon magnetosheath sector, south of the equatorial plane, and during intervals of steady southward IMF. Section 4 summarizes the similarities and differences of these long‐sampled FTEs.

### 3.1 Case 1: 23 October 2015, 12:48–12:52 UT

**Figure 1:**
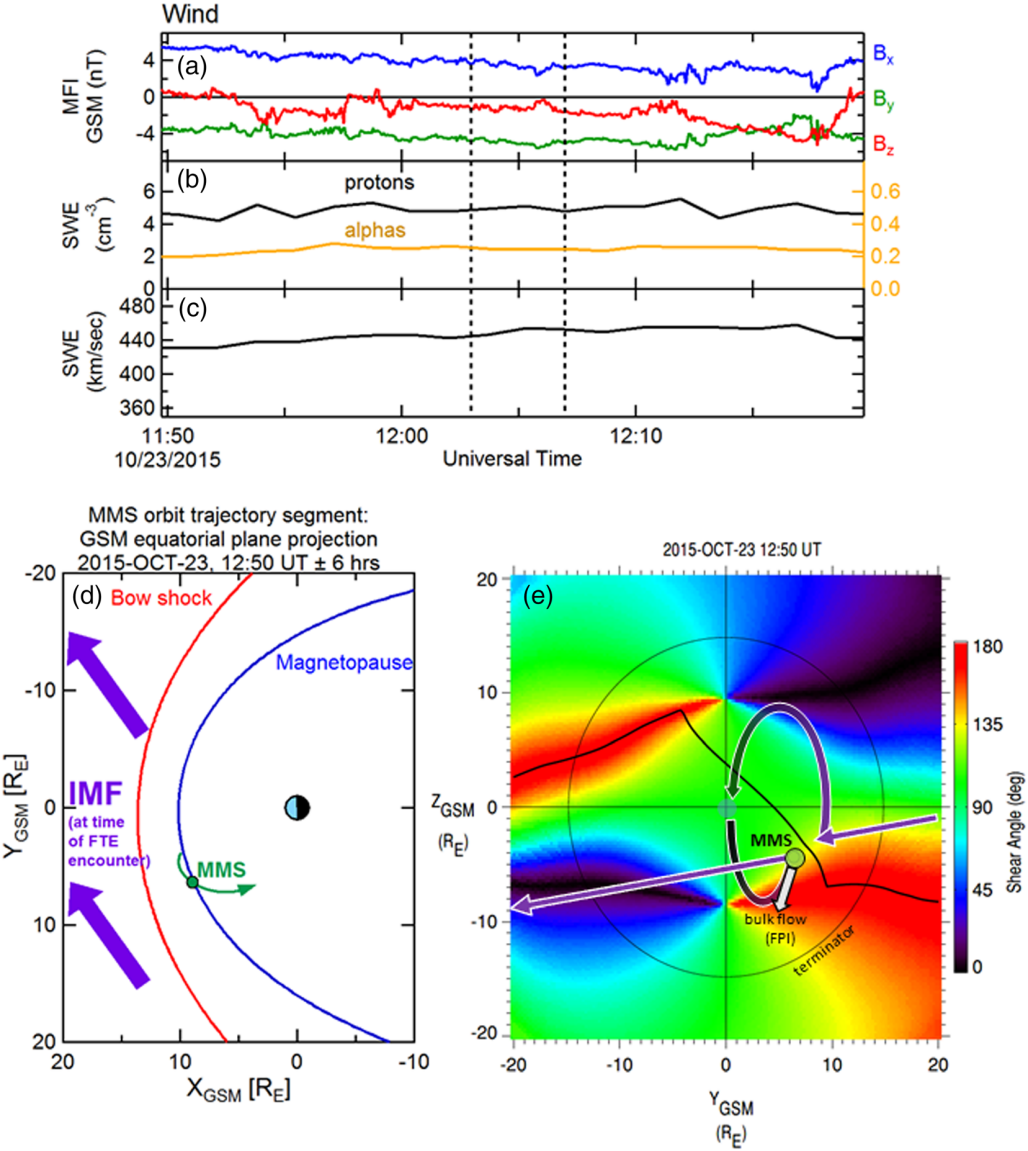
Contextual information for the FTE observed on 23 October 2015, 12:48–12:52 UT. (a) IMF observations from the Wind upstream monitor (shown at 3‐s resolution). (b) Proton (left‐hand scale) and alpha particle (right‐hand scale) densities as measured from Wind. (c) Solar wind bulk flow speed from Wind. Black vertical dashed lines demark the time at the Wind spacecraft that corresponds to the convected time of the FTE interval. (d) Projection of the MMS location and orbit segment (green) in the *XY*_GSM_ plane. The bow shock and magnetopause locations are shown for context. The IMF (purple) at this time was in a nominal Parker spiral configuration, pointing sunward and dawnward. (e) View of the magnetopause from the sun. the surface is colored by the magnetic shear angle between the magnetosheath and magnetospheric magnetic fields. The thin black circle denotes the terminator (*X* = 0) plane. The black line traces the loci of local maxima in magnetic shear, where reconnection is expected to be more likely to occur. The green circle marks the location of MMS in the magnetosheath. The white arrow illustrates the bulk flow velocity as measured by the FPI instrument during the FTE. The purple arrows and arcs represent the draped IMF and connection to the magnetospheric magnetic field, anchored at the ionosphere. In this case, it is suggested (based on the bulk plasma flow) that the sampled FTE was magnetically connected at least to the southern ionosphere.

Before providing a detailed examination of the magnetosheath FTE minor ions and electrons by MMS on 23 October 2015, 12:49–12:52 UT, contextual information is described. Figures 1a–1c shows the solar wind IMF and plasma parameters observed by the Wind spacecraft during this FTE encounter by MMS. The dashed vertical lines correspond to the FTE encounter interval, and the time labels refer to the observations at Wind, not to the convected time at MMS. The IMF in this case had strong *B*_*x*_ and *B*_*y*_ components of opposite sign; typical for a Parker spiral configuration. The *z* component was slightly negative (southward; with an IMF clock angle (tan^−1^(*B*_*y*_/*B*_*z*_) ≈ 257°)), which is conducive to the occurrence of reconnection and FTE production at the dayside magnetopause (e.g., Berchem & Russell, 1984; Rijnbeek et al., 1984). The solar wind proton number density (Figure 1b, black trace) was nominal at ~4–5 cm^−3^. The alpha particle number density is overlaid in orange within the same panel (Figure 1b) using the right‐hand scale (factors of 10 reduced from the left‐side scale) and was steady at ~0.2 cm^−3^. The number density ratio of solar wind alpha particles to protons of ~5% is also typical for the solar wind. Figure 1c shows the solar wind bulk flow speed; also nominal at ~440–450 km/s. The solar wind dynamic pressure during this time was a steady 1.9 nPa.

Figure 1d illustrates a 12‐hr orbit segment of the MMS constellation, centered at the time of the FTE observation and projected into the GSM equatorial plane. The parameterized location of the magnetopause (Shue et al., 1997) and bow shock (Farris et al., 1991), accounting for the average solar wind flow direction, are included. An outbound magnetopause crossing by the MMS spacecraft occurred about 4 hr prior to this event, and a brief encounter with the magnetosheath boundary layer (MSBL) occurred more than an hour after this sampled magnetosheath FTE. The convected IMF during this interval is also projected into the GSM equatorial plane (purple arrows).

Figure 1e shows the magnetic shear angle across the magnetopause surface, plotted as color contours as viewed from the Sun (the thin black circle denotes the terminator, where the magnetopause surface intersects the *X*_GSM_ = 0 plane). The shear angle values are determined from the magnetic fields on the magnetosheath side (Kobel & Flückiger, 1994) and the magnetospheric side (using the most recent International Geophysical Reference Field model within the Tsyganenko 1996 semiempirical magnetospheric magnetic field model; Tsyganenko, 1995) of the magnetopause. Based on the draped IMF, the dipole tilt angle (−5.8°), and dynamic pressure scaling of the magnetopause location, the loci of predicted magnetic reconnection sites are illustrated as a contiguous thick black line. This prediction is based upon the maximum magnetic shear model reconnection line as described in Trattner et al. (2007). The MMS spacecraft (depicted as a green circle) were located in the magnetosheath and were in close proximity to, but southward of, this predicted reconnection line. The thick purple arrows and arcs schematically depict the connection of the IMF to the magnetospheric magnetic field shortly after the reconnection process has altered the topology. This schematic provides only a crude representation; more details of the topologies are described in the next figures.

The white arrow originating from the MMS location in Figure 1e denotes the bulk plasma ion flow vector observed by FPI during the FTE. Its direction suggests that the motion of the observed FTE was southward but also toward the noon‐midnight meridian plane. Thus, the FTE motion included a component that was not along the ambient magnetosheath flow, which is (mostly) radial from the subsolar point. The FTE motion was likely due to forces (e.g., ***J*** × ***B*** tension and/or −**∇**P) related to the reconnection process.

**Figure 2:**
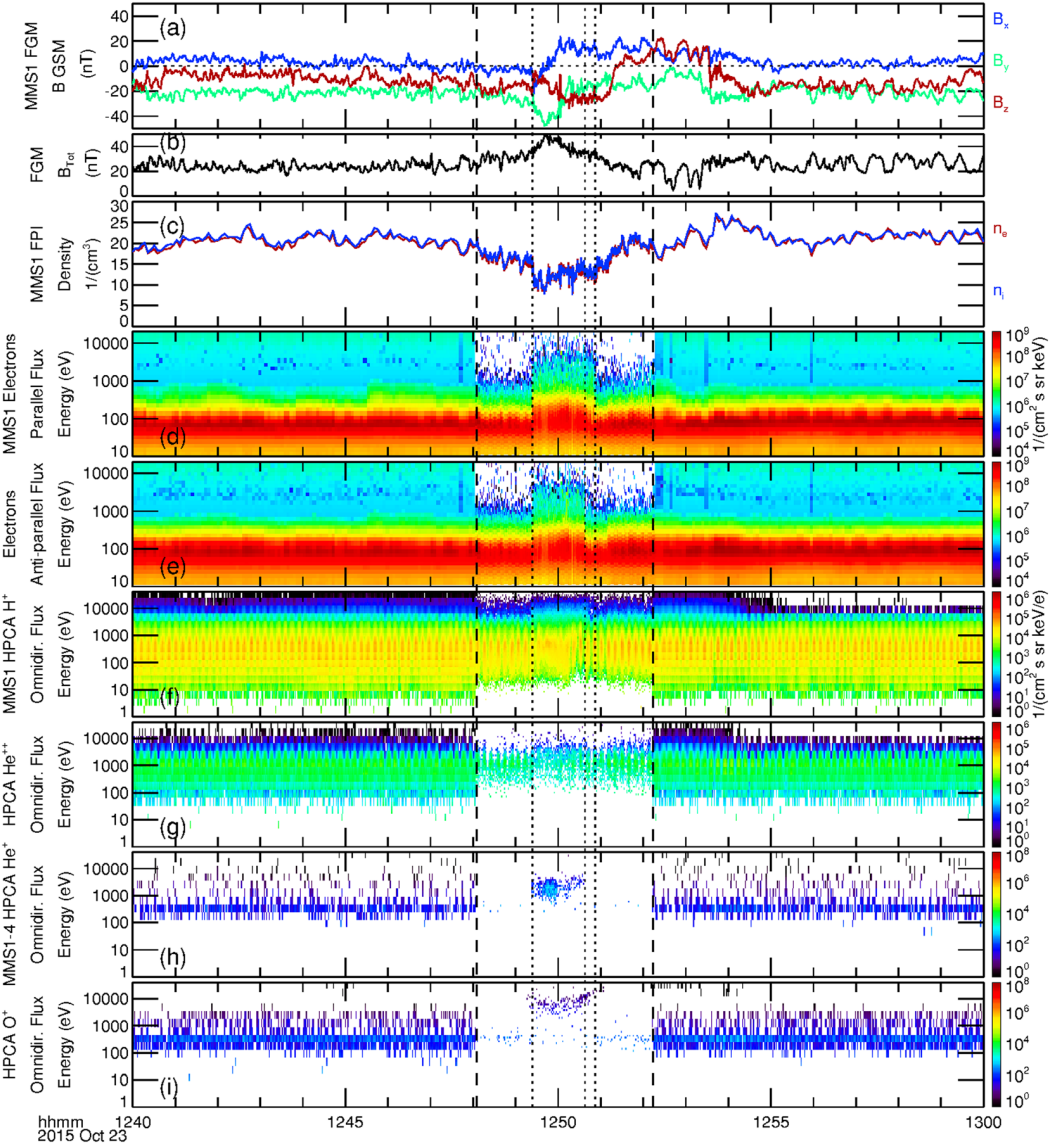
Display of a 20‐min time series during the observed magnetosheath FTE on 23 October 2015, 12:48–12:52 UT of (a) the magnetic field components (GSM), (b) magnetic field intensity, (c) the FPI ion and electron density, (d) energy spectrograms of electron flux parallel, and (e) antiparallel to the magnetic field, as observed by MMS1. Energy spectrograms of omnidirectional flux from HPCA observations of (f) protons, and (g) alpha particles from MMS1. Combined energy spectrograms of omnidirectional flux of (h) He^+^ ions and (i) O^+^ ions as sampled by the HPCA instruments on board all four MMS spacecraft. The selected burst mode segment spanning the FTE lies between the two vertical dashed lines. Features related to the “core” of the FTE are denoted by black dotted lines.

Figure 2a displays a 20‐min time series of the GSM magnetic field components as observed by the magnetometer on board MMS1. The MMS burst mode interval for all instruments on all four spacecraft is identical and lies between the two vertical dashed lines, fully enveloping the observed FTE. Figure 2b shows the magnetic field intensity during this time. Although displayed in GSM coordinates here, the magnetic field component variations were quite consistent with the passage of a spacecraft through a “typical” FTE (cf. Russell & Elphic, 1979); that is, a clear bipolar variation in one component, *B*_*x*_, with a coincident enhancement (of a factor of ~2) in the field intensity. The time of peak magnetic field intensity (approximately 12:49:45 UT) marks the time of closest encounter with the center of the FTE. The total ion (*n*_*i*_) and electron (*n*_*e*_) number densities as measured by the FPI instrument on board MMS1 are shown in Figure 2c. The FPI observations show a marked decrease of ~50% of both the ion and electron densities during the transit of this FTE. This reduction in plasma density coincides with the enhancement in the magnetic field intensity and could be the result of 3‐D dynamics such as the squeezing of plasma (as mentioned as a possible explanation in reference to other FTEs by Øieroset et al., 2011).

Figures 2d and 2e depict the energy spectrograms of electron number flux flowing parallel and antiparallel to the magnetic field, respectively. Vertical dotted lines demark the times of sudden changes in the electron fluxes and provide valuable topological information of the FTE. Higher‐energy (up to several keV) dispersionless bidirectional electrons first appeared at 23 October 2015/12:49:24 UT, at the leading edge of the FTE, indicating the first encounter of MMS with the outer boundary of the flux rope core (throughout this article, “leading edge” refers to the initial [earliest] sampling of the FTE by MMS, and “trailing edge” refers to the ending [latest] observation of the FTE). Just prior to this time, changes in the magnetic field are attributed to the draping of open (i.e., unattached to the magnetosphere) magnetic field lines of the magnetosheath around this flux rope (this will be schematically depicted in Figure 5). The enhancement of higher‐energy bidirectional electron flux beginning at 12:49:24 UT suggests that magnetic field lines comprising the core of the FTE were closed at the time of the MMS encounter, with both ends connected to the ionosphere. Such a scenario was described and schematically illustrated in another magnetosheath FTE case study by Roux et al. (2015) (based upon a detailed analysis of ion, electron, and magnetic field observations from Cluster) and was also schematically illustrated in Figure 2c′ of Pu et al. (2013) (based upon Cluster observations of differential energy fluxes of electrons as a function of pitch angle). The MMS spacecraft continued to sample the FTE core until 23 October 2015/12:50:38 UT, when the higher‐energy antiparallel electron flux disappeared while the higher‐energy parallel flux continued at the same intensity until 23 October 2015/12:50:52 UT. This behavior suggests that within this briefly sampled region the field lines had one foot in the southern ionosphere but were open to the magnetosheath (i.e., active magnetic reconnection was occurring here). Again, the overall sequence of observations and topology of the event is analogous to the description of the magnetosheath FTE described by Roux et al., 2015.

Figures 2f and 2g show energy spectrograms of the omnidirectional flux of protons and alpha particles from the HPCA instrument on MMS1. The burst mode HPCA data (between the dashed lines) were at full angular and energy resolution (16 azimuth × 8 polar angles × 64 energy bins), at a 10‐s cadence (fast survey mode data are at reduced angular and energy resolution, but at the same cadence). The energy spectrograms show a modest increase in proton and alpha particle fluxes at the higher energies within the flux rope core of the FTE (though the overall density decreased within—as was shown in Figure 2c), as compared to the surrounding magnetosheath. Figures 2h and 2i show energy spectrograms of the omnidirectional number fluxes of the relatively scarce He^+^ and O^+^ particles. In these two panels, the energy spectrograms displayed are the combined spectrograms as observed by the HPCA instruments from the four closely spaced MMS spacecraft. This is done to increase the low counting statistics of these species, to better understand the behavior of these ions. Prior to the first encounter with the core of the FTE at 12:49:24 UT, there were no energetic He^+^ ions. A few energetic O^+^ ions of energy >10 keV were observed immediately prior to this encounter (counts at lower energy are due to proton contamination, described in the next paragraph). Within the core of this event, energetic He^+^ and O^+^ ions were both observed. At the trailing edge, the He^+^ ions disappear coincident with the more energetic antiparallel electron fluxes, while O^+^ ions continued to be observed at higher energies until such time that the more energetic parallel electron fluxes disappeared. The appearance of higher‐energy He^+^ and O^+^ at the edges (especially at the trailing edge) of the core of the FTE relative to the center of the core region is directly related to the gyroradius of the ions. Higher‐energy particles have larger gyroradii, and O^+^ at the same energy has a larger gyroradius than He^+^; explaining the appearance of O^+^ outside of the core region and further from the FTE core region than the He^+^ ions.

It is noted here that the proton count rate has been reduced by a factor of two in the MMS1 and MMS2 HPCA instruments by the RF attenuation setting within the instrument, while MMS3 and MMS4 proton reduction by the RF attenuation is even more stringent (a factor of 10). This is done to suppress the large number of protons (especially within the magnetosheath) in order to better discriminate and observe the sparse minor ions. Nevertheless, some proton contamination still appears in the time‐of‐flight channels of heavier species at the lower energies.

To summarize the observations of Figure 2, the simultaneous presence of alpha particles along with He^+^ and O^+^ ions within the core of the FTE suggest that these field lines had undergone reconnection at an earlier time; though the presence of bidirectional energetic electrons indicate that the field lines of the core at the time of observation were closed. However, the continued presence of higher‐energy parallel electron flux after the higher‐energy antiparallel electron flux ceased to be observed indicate that reconnection to the southern ionosphere was still active at the trailing edge of the FTE. The minor ion densities and distribution functions during this FTE are next examined.

**Figure 3:**
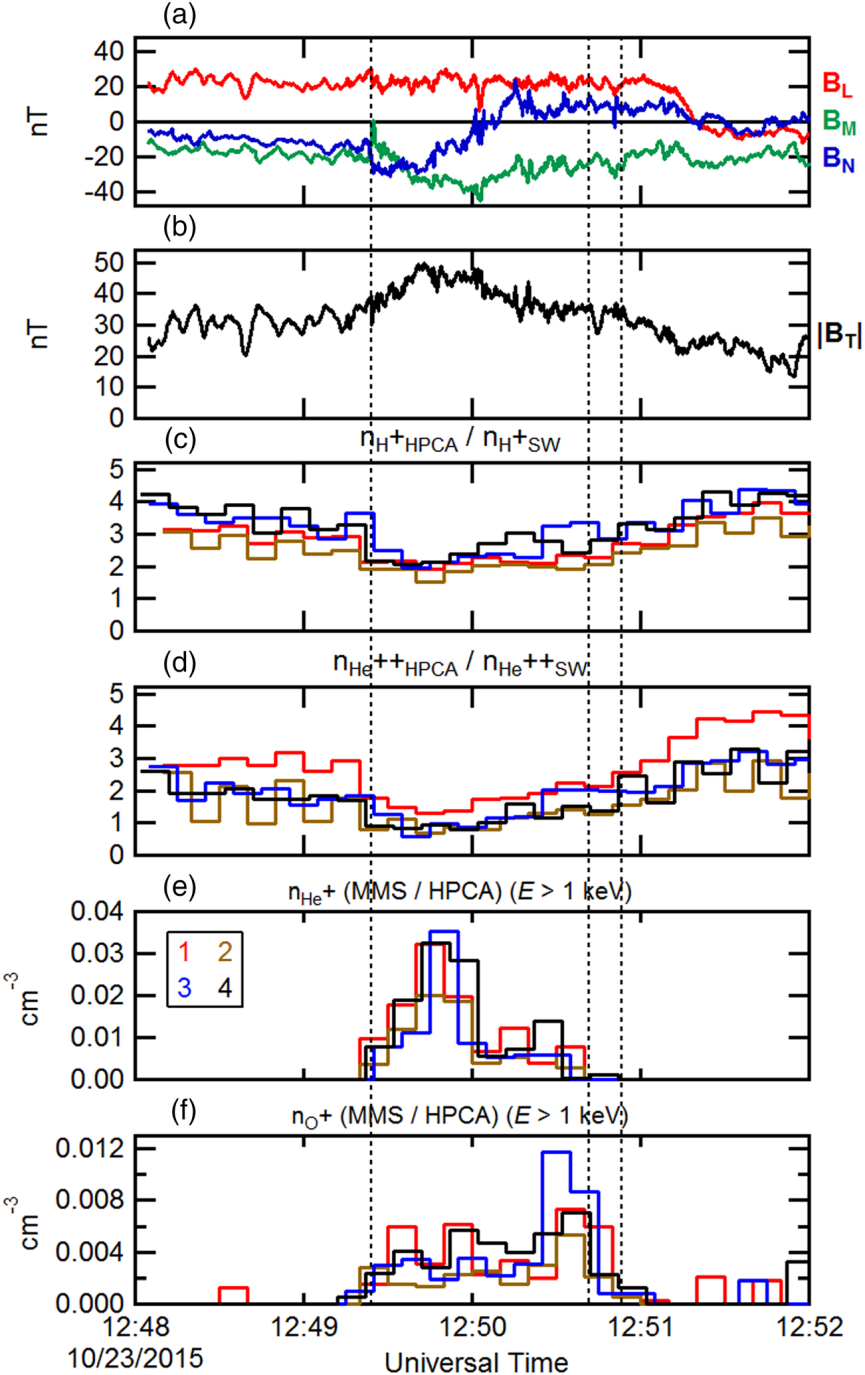
A 4‐min time series of the observed magnetosheath FTE of 23 October 2015 by MMS1. (a) The magnetic field components (rotated into an LMN coordinate system) and (b) magnetic field intensity. The HPCA number densities (normalized by the respective convected solar wind number densities) from all four MMS spacecraft for (c) proton and (d) alpha particles. The observed ion number densities of (e) He^+^ and (f) O^+^ from all four HPCA instruments. The black dotted lines correspond to the dotted lines in Figure 2.

Figure 3 shows ~4‐min interval of burst mode observations (between the dashed vertical lines of Figure 2). In Figure 3a, the magnetic field as observed by MMS1 has been rotated into an “LMN” boundary normal coordinate system by determining the Euler angles, which minimize the ratio between the minimum and maximum variances. In this coordinate system, the “L” direction denotes minimum variance, aligned along the axial field of the magnetosheath FTE. The orthogonal “M” and “N” directions correspond to the intermediate and maximum variance eigenvectors, respectively. The “N” direction corresponds to the component containing the most direct cut of the spacecraft through the FTE cross section, with maximal bipolar signature. Figure 3b again shows the magnetic field intensity for context. Dotted vertical lines are as in Figure 2. Figures 3c and 3d depict the H^+^ and He^++^ number densities determined from each of the HPCA instruments from all four MMS spacecraft (legend in Figure 3e) and normalized by the convected solar wind densities. Both species were depleted within the FTE, and this depletion was independent of any solar wind density variations. The differences in normalized densities between the different instruments are due primarily to the different RF attenuations employed, and because the half‐spin (10‐s) start times for each HPCA instrument are not coincident.

Figures 3e and 3f illustrate the time series of the He^+^ and O^+^ densities from all four HPCA instruments, respectively. As described above, despite the use of the RF to reduce proton contamination in the higher mass species, in the high‐density magnetosheath some lower energy protons still appeared in the He^+^ and O^+^ data products. This residual contamination is further reduced here by the use of partial densities. This is accomplished by constructing density moments using the number fluxes from energy channels above 1 keV. Figures 3e and 3f show that He^+^ and O^+^ were clearly present within this FTE core region and were nearly absent outside the FTE. In general, the He^+^ density was ~4 times larger than the O^+^ density. However, whereas the He^+^ density was greatest at the center of the FTE (inferred from the maximum magnetic field intensity), the O^+^ density was slightly larger near the trailing edge of the FTE. The differences in these smaller number densities between the different instruments are due to count statistics, and because the half‐spin start times for each HPCA instrument are not coincident.

**Figure 4:**
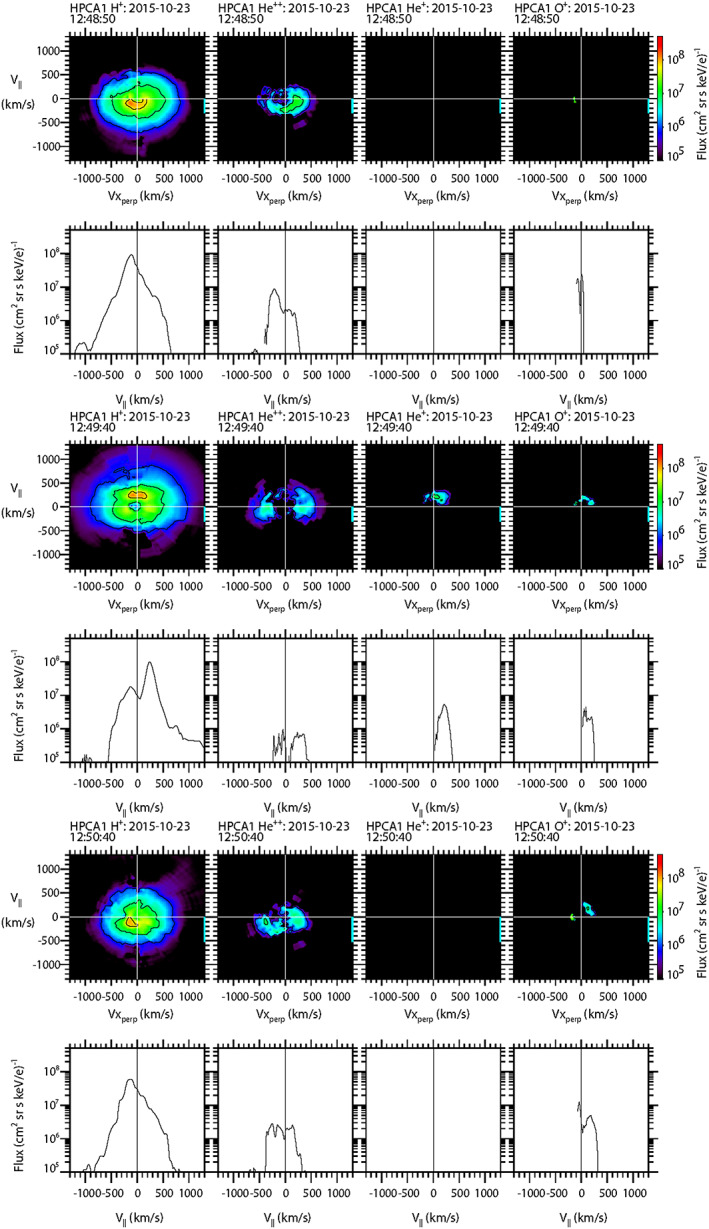
Two‐dimensional and 1‐D cuts of full 3‐D distribution functions as observed by the HPCA instrument on MMS1. Each column represents a different species {H^+^, He^++^, He^+^, and O^+^}, and each pair of rows represents a different 10‐s time interval. Top two rows: in the draped magnetosheath. Middle two rows: at the center of the FTE core. Bottom two rows: the intervals when unidirectional electrons were observed.

To further explore the characteristics of the populations of protons and the minor ion species to better understand the topological history of this event, sample 2‐D and 1‐D cuts of the full 3‐D particle flux distribution functions as measured by HPCA on board MMS1 are shown in Figure 4. For the 2‐D distribution cuts (e.g., top row of Figure 4), the magnetic field direction is aligned along the vertical axis, while for the corresponding (in time) 1‐D cuts (e.g., second row of Figure 4), the magnetic field direction lies along the horizontal axis. Each column represents an ion species (H^+^, He^++^, He^+^, and O^+^). The time labels denote the start time of the 10‐s interval. The complete set of distribution functions over this and the subsequent FTE burst mode intervals for all species and all four HPCA instruments are provided in the supporting information.

**Figure 5:**
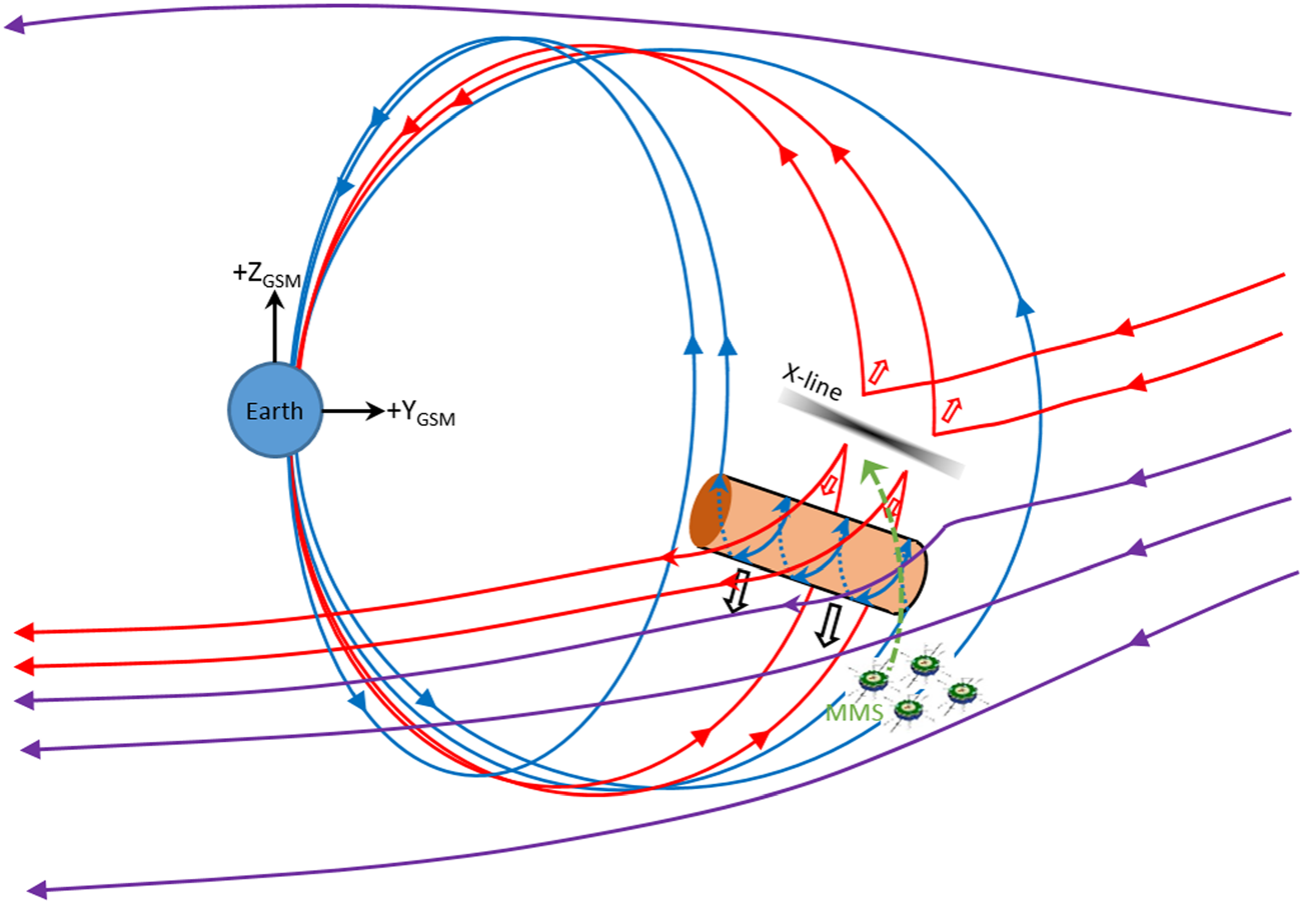
A schematic of a recently closed FTE event; with field lines (red) undergoing reconnection (one foot connected to the southern ionosphere; the other end connected to the draped IMF), and open field lines (purple draped IMF, unconnected to the magnetosphere). The green arrow depicts a possible path by which the MMS spacecraft passed through the southward moving flux rope and reconnection region. This schematic is similar to that of Roux et al. (2015) for an observed magnetosheath FTE.

The top two rows display the particle distributions along the open magnetosheath draped field region (12:48:50–12:49:00 UT), prior to the FTE core encounter. It is seen that both the protons and alpha particle fluxes peaked along the direction antiparallel to B, as would be expected for solar wind flow from the Sun since the event occurred within a “toward” sector (*B*_*x*_ > 0). The proton and alpha distributions are different because the shocked solar wind alpha population forms a shell distribution (Fuselier et al., 1988). No He^+^ or O^+^ flux was observed at this time. The next two rows display the particle flux distributions of all four species at the time of closest approach to the center of the FTE (12:49:40–12:49:50 UT), when higher‐energy bidirectional electrons were observed. At this time the proton flux is strongly peaked parallel to the magnetic field, with a smaller peak at lower energy traveling antiparallel to B. The alpha particles are mixed and much reduced; much of the flux appears to be perpendicular to the magnetic field. The He^+^ and O^+^ distributions both show significant number fluxes traveling parallel to the magnetic field. Thus, the proton, He^+^, and O^+^ populations all traveling parallel to the field at approximately the same velocity are all likely of ionospheric origin. This suggests that the FTE core was topologically connected to the magnetosphere and, based on the schematic of Figure 1e, the magnetospheric field lines were at least connected to the southern ionosphere. A more detailed topological schematic of this event is presented in Figure 5.

The last two rows show the particle distributions during the brief interval when higher‐energy unidirectional electrons were observed moving parallel to the magnetic field (12:50:40–12:50:50 UT). In contrast to the electrons, the proton distribution was very similar to that of the draped magnetosheath population described in the first two rows (i.e., peak flux was traveling ~100+ km/s antiparallel to the magnetic field). The alpha particle flux was more significant than in the core of the FTE but showed no clear peak relative to the magnetic field. He^+^ ion flux was not observed at this time; but there was still O^+^ ion flux traveling ~200 km/s parallel to the magnetic field (similar to the core of the FTE, but slightly more energetic).

Figure 5 shows a notional schematic of the topology of this FTE. Blue magnetic field lines are tied to the ionosphere at both ends, while the purple magnetic field lines represent the observed magnetosheath magnetic field orientation and are open. The red magnetic field lines have one end tied to the ionosphere and are actively undergoing magnetic reconnection. The observations suggest a scenario for which at least one twisted flux rope was formed at the magnetopause, with its northern edge collocated with the location of the maximum magnetic shear reconnection line. A second reconnection line at the southward edge of the flux rope shown in the figure would have been needed to initiate the formation of this FTE, with one foot connected to the southern ionosphere and the other end connected to the magnetosheath magnetic field. There may have been another flux rope formed north of the reconnection line (similar to the model of Lee & Fu, 1985); but this is unknown—and is not shown. The reconnection line southward of the flux rope (also not shown) likely became inactive after the FTE formation (due to the absence of unidirectional energetic electrons at the leading edge); and its cessation may be related to another change in topology such that both ends of the flux rope (FTE core) became tied to the ionosphere (as evidenced by the bidirectional energetic electrons) and described by Pu et al. (2013). The green dashed line symbolizes the MMS constellation motion through the magnetosheath FTE; in actuality it is the southward motion of the FTE moving past the spacecraft rather than any significant northward motion of the spacecraft. It is noted that it is possible that as MMS approached the FTE core, it passed through draped magnetosheath field lines that had never undergone magnetic reconnection (purple lines); if the spacecraft were close to the outer (i.e., duskward) edge of the FTE core.

The formed flux rope would convect southward due to the magnetosheath flow and influenced by the ***J*** × ***B*** and **∇**P forces due to reconnecting field lines originating at the still active reconnection line to the north; as evidenced by MMS observations of energetic electrons flowing only parallel to the magnetosheath magnetic field at the trailing edge of the flux rope (similar to the FTE event examined by Roux et al., 2015). At the time of the observation by MMS, the heavier ions within the FTE core have not yet traveled sufficiently far to be seen traveling in both directions relative to the now closed magnetic field.

### 3.2 Case 2: 8 November 2016, 11:15–11:25 UT

**Figure 6:**
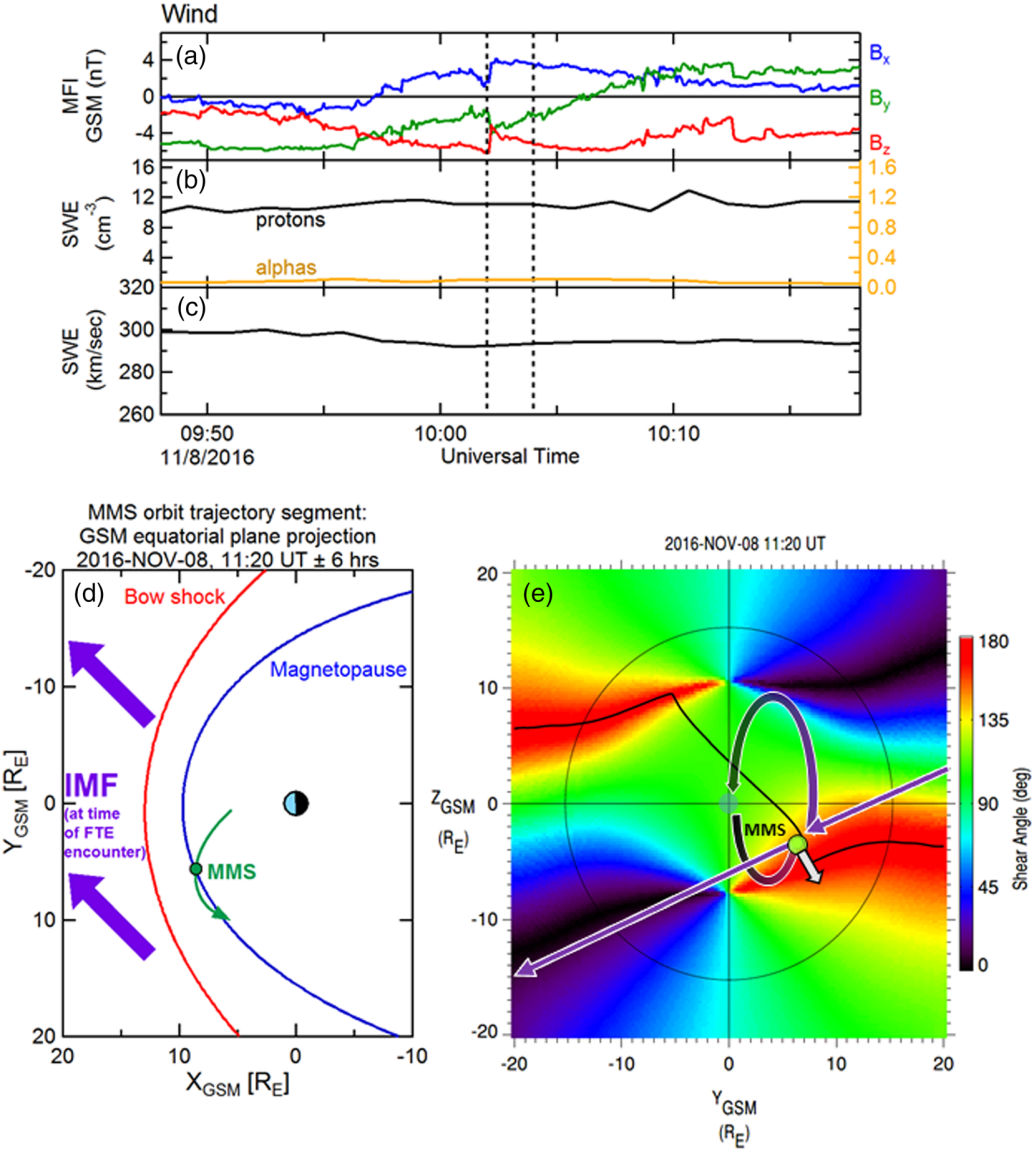
Contextual information for the FTE observed on 8 November 2016, 11:18–12:22 UT. The MMS location and the IMF configuration was very similar to the previous FTE case. The format of this figure is the same as in Figure 1.

The solar wind conditions pertaining to this observed FTE interval (8 November 2016, 11:19–11:22 UT) were very similar to those of the previous FTE discussed (section 3.1, Case 1: 23 October 2015), with significant positive *B*_*x*_ and negative *B*_*y*_ GSM components (as in Figure 1a, the solar wind conditions pertaining to the FTE interval are between the vertical dashed lines, and the time labels are at Wind—the convected time labels are not displayed here). As shown in Figure 6a, the IMF was more strongly southward than for the previous event, and the IMF was not as steady as in the previous case; the *B*_*y*_ component was very slowly changing from negative to positive values. Figures 6b and 6c show the solar wind plasma moments (number densities of protons and alpha particles, and bulk solar wind speed) as observed by Wind. The solar wind proton density was constant at 11 cm^−3^ while the alpha particle number density was <0.1 cm^−3^; only ~1% of the proton density. The solar wind bulk flow speed was very slow, at ~290 km/s.

This long‐duration magnetosheath FTE was observed by the MMS spacecraft in the postnoon sector, on the inbound trajectory. This sampling location close to the magnetopause is also very similar to the previous case. Figure 6d shows (in green) the projection into the GSM equatorial plane of the 12‐hr MMS orbit segment, along with the projected location of the MMS spacecraft at the time of the FTE observation relative to the model magnetopause and bow shock. The convected and projected IMF orientation is displayed as purple arrows (same format as in Figure 1d). Figure 6e shows the magnetic shear angle color contour map across the magnetopause surface, along with the predicted reconnection line relative to the MMS location (contiguous black line). Similar to the previous case, MMS (green circle) was in close proximity to, but just southward of, the predicted reconnection line. The FPI bulk plasma flow in this case (white arrow) is directed southward and away from the noon‐midnight meridian plane, in a direction that is more consistent with the expected ambient magnetosheath flow. Also similar to Figure 1e, the purple curves and arrows crudely depict the expected reconnection of IMF and magnetospheric magnetic field lines that resulted in the formation of the observed FTE.

**Figure 7:**
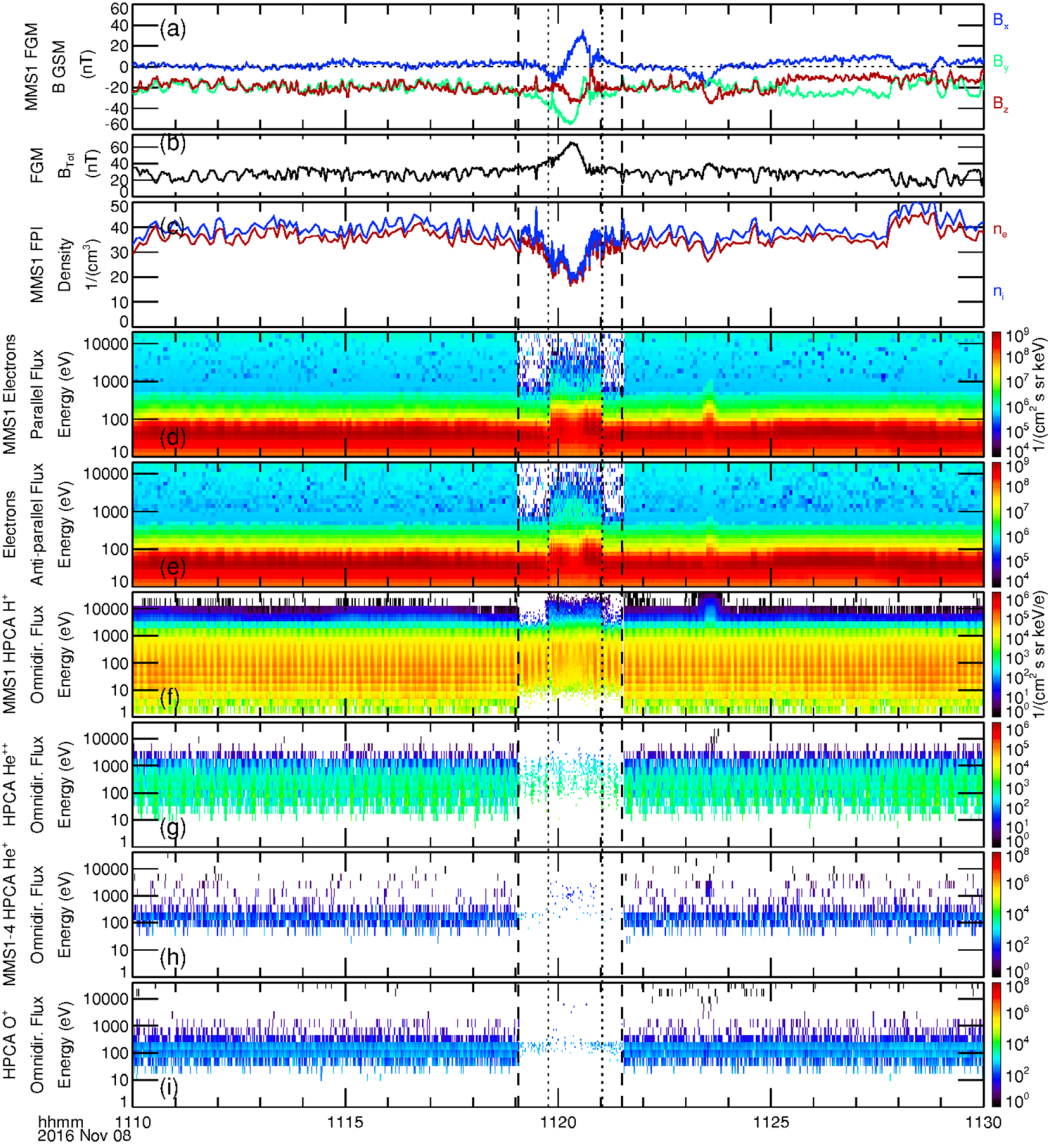
Display of a 20‐min time series during the observed magnetosheath FTE of 8 November 2016, 11:18–12:22 UT. The black dashed lines correspond to the burst mode interval of MMS. The dotted black lines represent the FTE core, as denoted by the enhancement in the more energetic electrons. The format of this figure is the same as in Figure 2.

The format of Figure 7 is similar to that of Figure 2. The magnetic field deviations in Figures 7a and 7b pertaining to the draped IMF and the FTE core are shorter in duration than in the first FTE case. The magnetic field intensity shown in Figure 7b increased by more than a factor of two at the center of the FTE, while the ion and electron densities (Figure 7c) decreased by a factor of 2. Enhancements in the higher‐energy electron fluxes were observed within the core of the flux rope (Figures 7d and 7e). These observed enhancements were dispersionless and bidirectional throughout, from the leading edge (8 November 2016/11:19:46 UT) to the trailing edge (8 November 2016/11:21:02 UT). These more energetic electrons again suggest that the magnetic field lines of the core of the FTE are closed in both the northern and southern ionosphere regions. In contrast to the previous case, however, there is no observed region of open field lines indicative of active magnetic reconnection (i.e., unidirectional higher‐energy electron enhancement).

The HPCA energy spectrogram of omnidirectional proton flux (Figure 7f) shows a more distinct enhancement of higher‐energy protons within the core of the FTE than was observed in the previous case. The leading edge shows a slight energy dispersion, which is likely due to the relatively larger gyroradius of the higher‐energy protons. The presence of proton dispersion at the trailing edge is not clear. Some higher‐energy protons were observed beyond the trailing edge of the FTE core (latter dotted line).

The alpha particle flux from MMS1 as shown in Figure 7g is sparse, which is expected since it was also sparse in the upstream solar wind. The higher‐energy fluxes of magnetospheric He^+^ within the FTE core from all four HPCA instruments is shown in Figure 7h. These ions were also sparse and only appeared within the FTE core, much like the He^+^ ions in the 23 October 2015 FTE. The higher‐energy O^+^ flux of Figure 7i was not significant.

**Figure 8:**
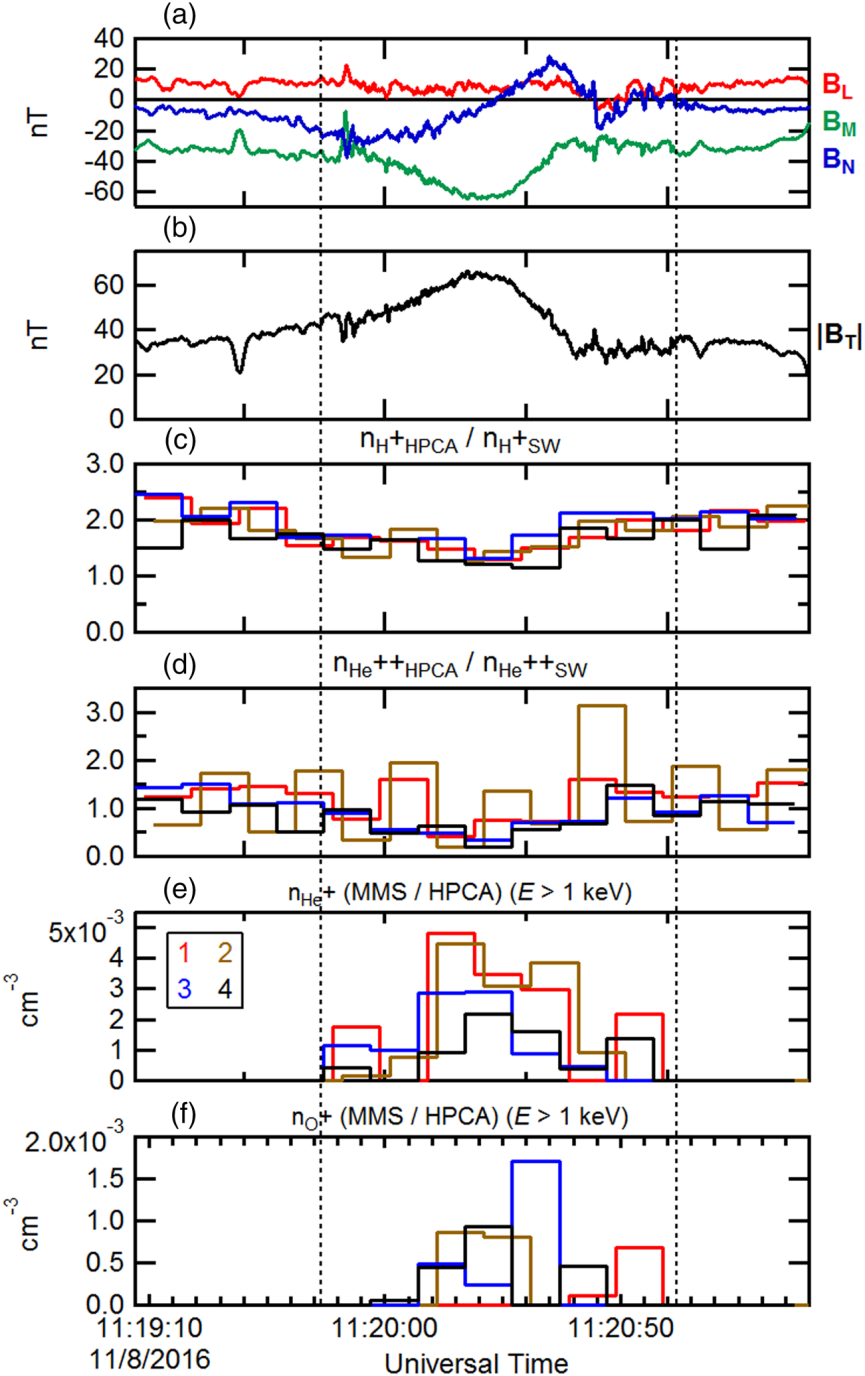
The ~2.5‐min burst mode time series of the observed magnetosheath FTE of 8 November 2016. The black dotted lines correspond to the dotted lines in Figure 7. The format is the same as in Figure 3.

Figure 8 is in the same format as Figure 3 and displays only the burst mode interval encompassing the sampled FTE. The magnetic field has been rotated into the “LMN” coordinate system in Figure 8a, and the magnetic field intensity is again displayed in Figure 8b. As with the previous FTE case, the proton and alpha number densities normalized to the convected solar wind densities were depressed within the FTE core, in comparison to the draped field region of the magnetosheath proper. He^+^ and O^+^ partial densities for energies >1 keV from the four HPCA instruments are shown in Figures 8e and 8f. The number densities of both species were enhanced within the FTE core but were about a factor ~6–10 smaller than in the previous FTE case.

**Figure 9:**
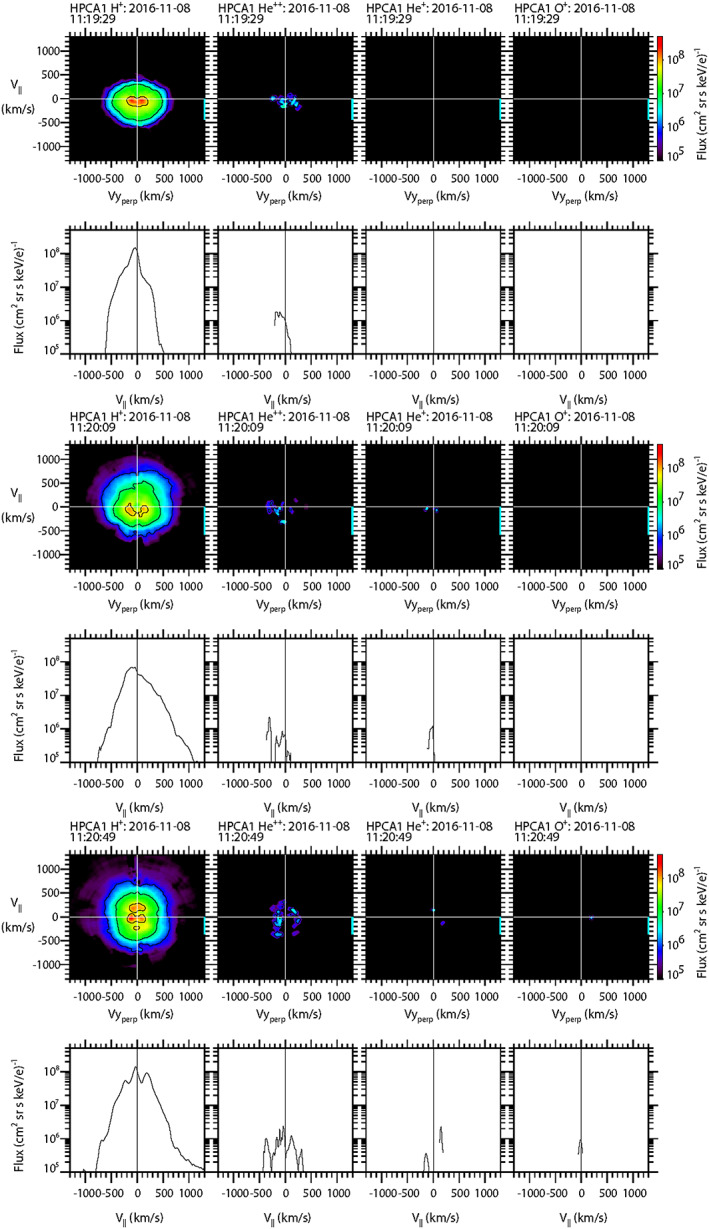
Two‐dimensional and 1‐D cuts of full 3‐D distribution functions as observed by the HPCA instrument on MMS1. Top two rows: in the draped magnetosheath. Middle two rows: at the time of maximum magnetic field intensity. Bottom two rows: the trailing edge of the FTE. The format is the same as in Figure 4.

Figure 9 is in the same format as Figure 4 and displays 2‐D and 1‐D cuts of particle distribution functions for each species (H^+^, He^++^, He^+^, and O^+^) constructed from HPCA observations during specific, individual half‐spins of the MMS1 spacecraft. The first two rows show the distribution functions observed prior to the encounter with the FTE core (11:19:29–11:19:39 UT). Protons were observed with the peak flux slowly traveling antiparallel to the magnetic field. The very slow speed associated with the peak of the proton flux is consistent with the slow solar wind speed observed at Wind. Alpha particles were also observed with a peak flux that was ~1% of the proton peak flux, also moving antiparallel to the magnetic field. He^+^ and O^+^ ions were not observed at this time.

The next two rows show particle distribution cuts for a time interval near the FTE core as estimated from the peak magnetic field intensity (11:20:19–11:20:29 UT). The proton peak flux remained antiparallel to the magnetic field (in contrast to the first FTE case), but there was apparent preferential heating of protons moving parallel to the magnetic field. Alpha particles from the solar wind continued to appear only in the antiparallel direction, while the distribution cuts of He^+^ and O^+^ ion fluxes were not significant.

The final two rows of Figure 9 show particle distribution cuts for a time interval at the trailing edge of the FTE core (11:20:49–11:20:59 UT). At this time, the protons and alpha particle fluxes were nearly isotropic, while the distribution cuts of He^+^ and O^+^ ion fluxes were again not significant.

The difference in magnetospheric He^+^ and O^+^ fluxes observed within this magnetosheath FTE from that observed in the first case may be due to whether a plasmaspheric plume (e.g., Fuselier, Burch, et al., 2017; Goldstein et al., 2004) and/or a significant warm plasma cloak existed (e.g., Chappell et al., 2008; Fuselier, Mukherjee, et al., 2019) close to the site of the FTE origination, or due to different conditions at the ionosphere (e.g., Fuselier, Trattner, et al., 2019; Gkioulidou et al., 2019; Yau et al., 1984). Because the MMS spacecraft did not encounter the magnetosphere proper for a significant time after these observations were made, the corresponding magnetospheric conditions and composition were not known.

From the electron and heavy ion observations from the MMS spacecraft during this FTE encounter, it is surmised that the connection and topology is similar to that described in Figure 5. The most significant difference is the absence of energetic unidirectional electrons at the trailing edge of the FTE core in the latter case. This suggests that active reconnection related to the formation of this FTE had recently ceased, and the structure had evolved to be a flux rope with both ends at the ionosphere.

### 3.3 Case 3: 20 October 2015, 15:45–15:51 UT

**Figure 10:**
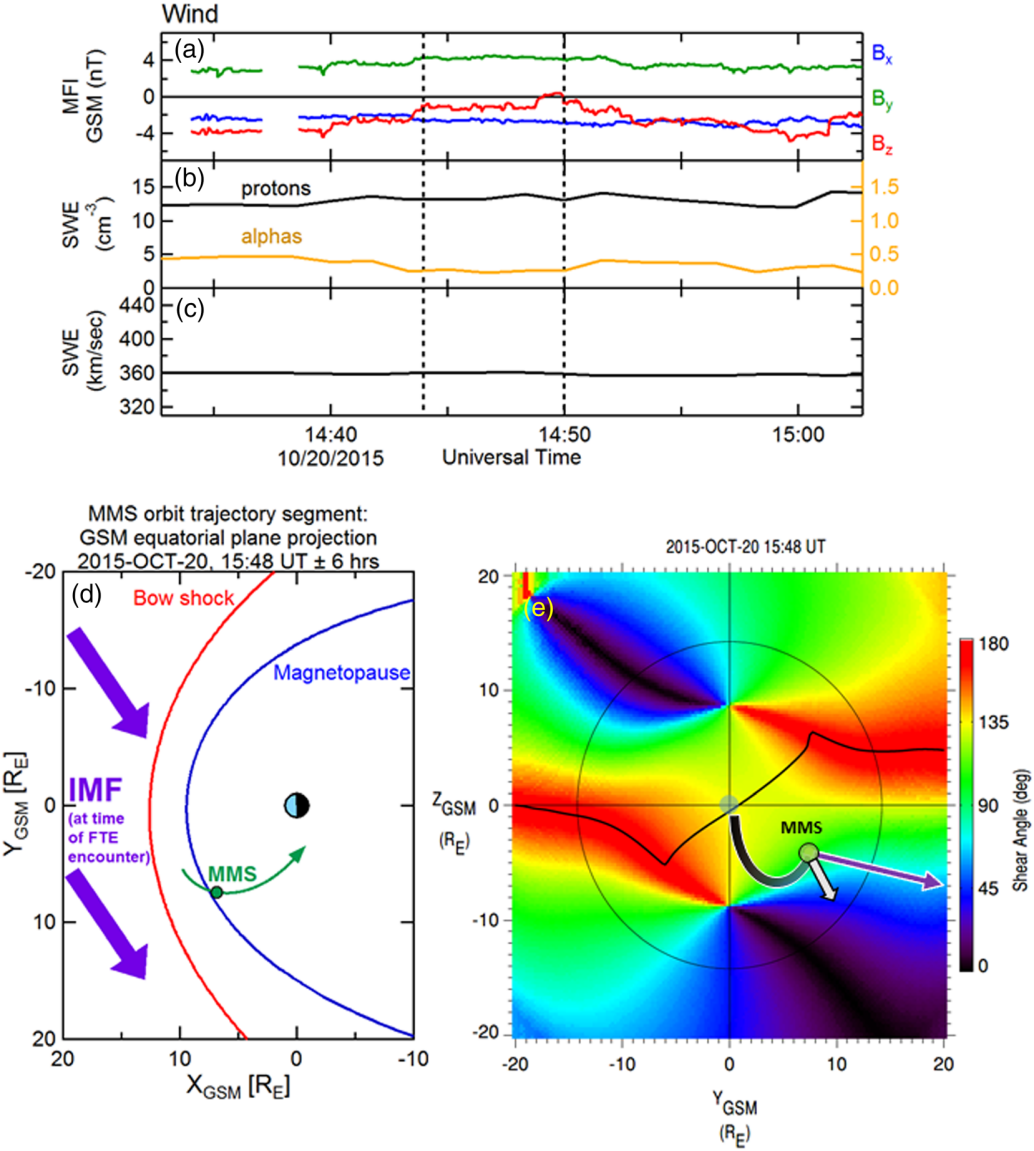
Contextual information for the FTE observed on 20 October 2015, 15:45–15:51 UT. The MMS location was similar to the previous FTE cases. Although the IMF was oriented along the typical Parker spiral angle as in the previous two cases, in this case the IMF was pointed toward the Earth and duskward. This orientation alters the magnetic shear angle map, the location of the predicted reconnection line, and the distance of MMS from this line. The format of this figure is the same as in Figure 1.

Similar to the previous two examined FTE cases, the IMF as observed by the upstream Wind spacecraft during this FTE interval (20 October 2015, 15:45–15:51 UT) was southward and oriented mainly along a typical Parker spiral configuration as depicted in Figure 10a (time labels are at Wind, and not the convected time at MMS. The time interval pertaining to the observed FTE lies between the two black vertical dashed lines). However, in contrast to the previous two examples, the IMF is directed antisunward (“away” sector, *B*_*x*_ < 0) and duskward (*B*_*y*_ < 0), and the IMF clock angle is ~101°. The solar wind proton and alpha particle number densities (Figure 10b) were relatively constant during this interval as measured by the Wind SWE (~12.0 and ~0.25 cm^−3^, respectively, for an alpha to proton density ratio of ~2%), as was the solar wind bulk speed of 360 km/s (Figure 10c). The solar wind speed in this case was intermediate—between the noted solar wind speeds of the previous two FTE cases.

This isolated, long‐duration magnetosheath FTE was observed by the MMS spacecraft after apogee (similar to the previous two examples), again in the postnoon sector south of the GSM equatorial plane. Figure 10d shows a 12‐hr projected MMS orbit segment centered in time about the location of the MMS constellation (green circle) during the magnetosheath FTE observation.

Figure 10e shows the magnetic shear angle color contour plot across the magnetopause surface, along with a black contiguous line depicting the predicted location of the reconnection line. The color contour map and predicted reconnection line are a near mirror image (dawn‐dusk) of the previous two examples due to the opposite sign of IMF *B*_y_. The location of MMS in this case (green circle) is therefore predicted to have been distant from the model reconnection line, and likely to have been distant from the FTE formation site as well. It is likely that this FTE had evolved and perhaps decayed significantly by the time it was encountered by MMS. The FPI ion bulk flow velocity (white arrow) observed near the center of the FTE core, drawn from the MMS location was nearly along the same direction as the ambient magnetosheath flow and consistent with an encounter with a reconnected flux tube that was preferentially connected to the southern ionosphere. The connection of the magnetosheath magnetic field with the magnetospheric field at MMS is crudely represented by the purple arrow and arc. In contrast to the first two cases, in this case the magnetosheath portion of the reconnected magnetic field vector points away from the noon meridian and points instead down the flank toward the magnetotail.

**Figure 11:**
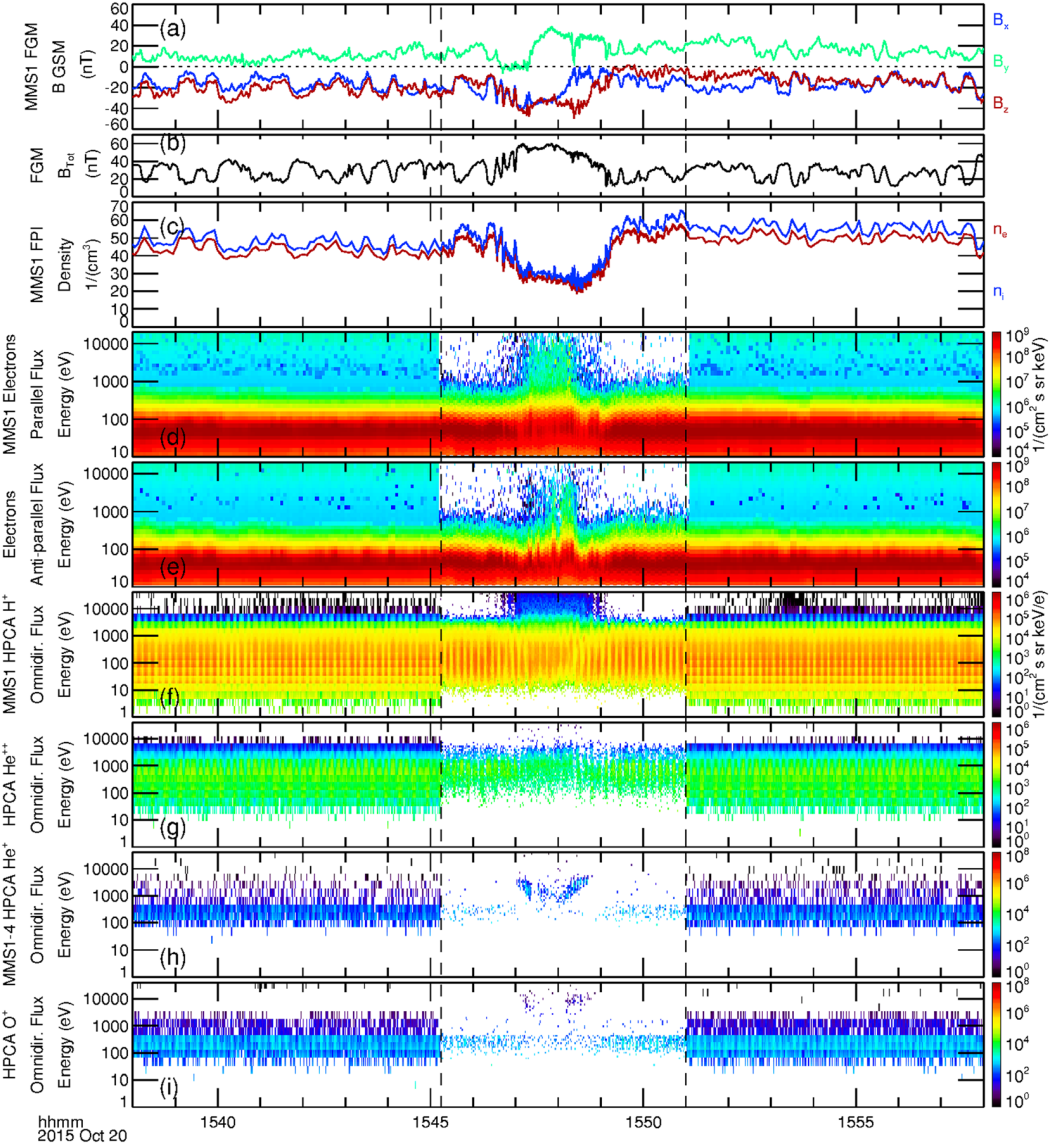
Display of a 20‐min time series during the observed magnetosheath FTE of 20 October 2015, 15:45–15:51 UT. The black dashed lines correspond to the burst mode interval of MMS. The format of this figure is the same as in Figure 2.

Figure 11 shows a 20‐min time series of observations that includes the burst mode magnetosheath FTE interval observed by MMS (between the vertical black dashed lines). The format is similar to that of Figure 2. The magnetic field GSM components and intensity as observed by MMS1 are displayed in Figures 11a and 11b. The time of initial increase in the magnetosheath magnetic field intensity is somewhat obscured by large oscillations. The magnetic field intensity then increased by a factor of 2–3 within the FTE, and then returned to ambient values ~15:49:25 UT. Similar oscillations of ion and electron number densities in antiphase with the magnetic field intensity were observed by the FPI on MMS1 (Figure 11c). Figures 11d and 11e depict the energy spectrograms of electron number flux flowing parallel and antiparallel to the magnetic field from the FPI instrument, respectively. Enhancement of the parallel energetic electron flux started just before ~15:47 UT and persisted until ~15:49 UT, with the largest higher‐energy parallel electron flux observed between ~15:47:20 and 15:48:30 UT. Significant parallel electron flux was seen above 10 keV. Enhancement of the antiparallel energetic electron flux was noticeably weaker, starting later and ending earlier than the parallel electron flux and did not extend to as high energies (up to several keV). The observation of both unidirectional and bidirectional accelerated electrons associated with this FTE indicate that the magnetic topology of this event is complicated and multiple field topologies may be involved; similar to the event described by Pu et al. (2013).

The energy flux spectrograms of omnidirectional proton and alpha particle flux as observed by the HPCA instrument on MMS1 are displayed in Figures 11f and 11g. A distinct energy dispersion of higher‐energy protons appeared near the leading edge of the FTE. Higher‐energy protons then continued to be enhanced during the interval ~15:47–15:49 UT. A much less pronounced energy dispersion of protons appeared at the trailing edge of this interval.

Figures 11h and 11i show the combined energy flux spectrograms of omnidirectional He^+^ and O^+^ from the HPCA instruments on board all four MMS spacecraft. Similar to the first FTE case (23 October 2015), the higher‐energy He^+^ ion flux shows a distinct energy dispersion wherein the highest energies appeared further from the center of the interval, and this is attributed to the ion gyroradius. The higher‐energy O^+^ ion flux showed a similar temporal variation but was less distinct. Observed counts at lower energies (less than ~1 keV) are proton contamination.

**Figure 12:**
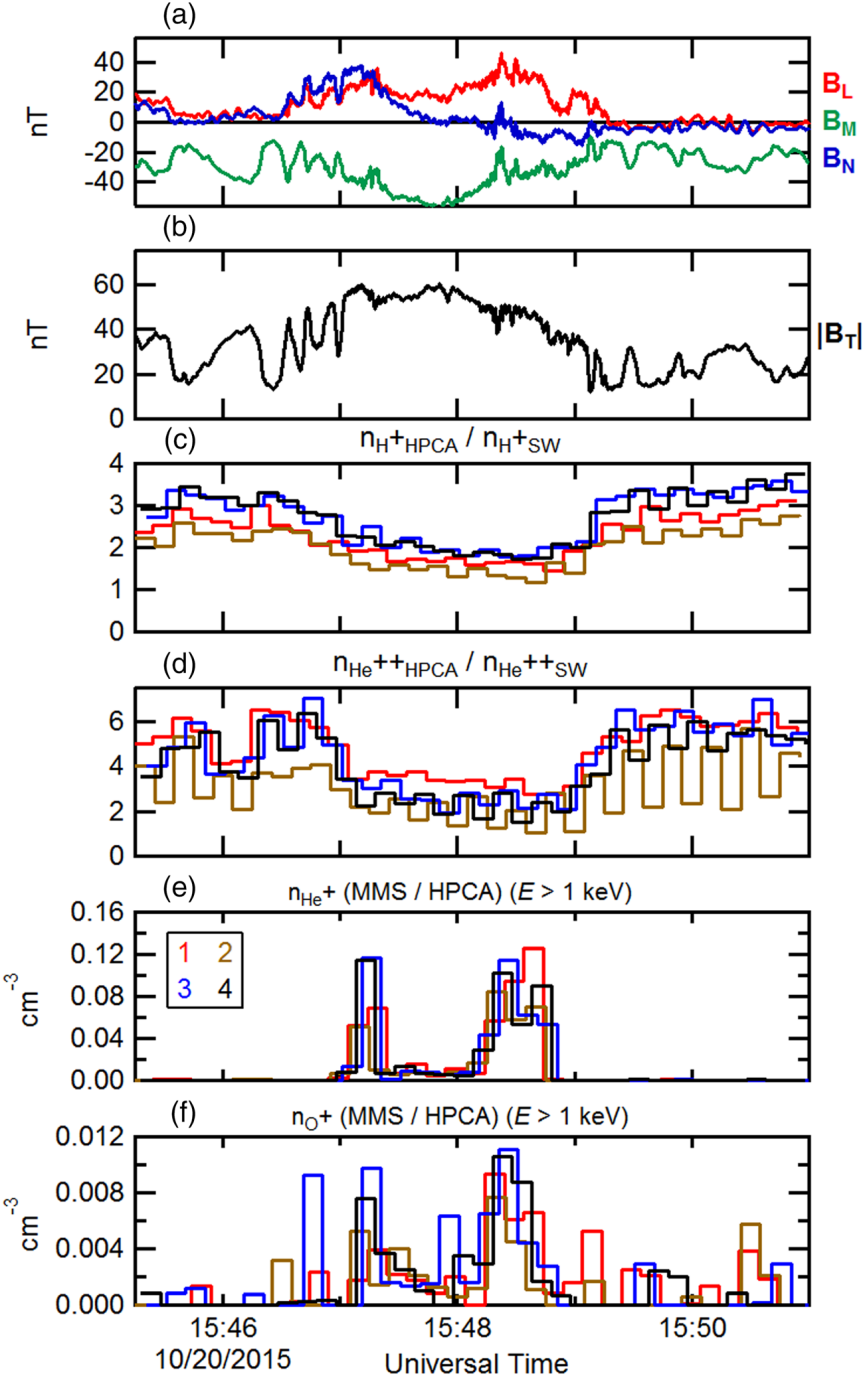
The ~6‐min burst mode time series of the observed magnetosheath FTE of 20 October 2016. The format is the same as in Figure 3.

Figure 12 shows the magnetic field and plasma moments for this FTE during the burst mode interval as measured by the instruments on board MMS. The format is similar to Figure 3. The magnetic field is displayed in Figures 12a and 12b in “LMN” coordinates. In the same manner as the previously examined FTE cases, the plasma proton and alpha number densities (normalized to the respective convected solar wind number densities) from all HPCA instruments from the four MMS spacecraft are presented in Figures 12c and 12d. The normalized proton and alpha densities were depleted relative to the magnetosheath proper outside of the FTE. He^+^ and O^+^ ion partial densities (for *E* > 1 keV) are shown across the sampled FTE in Figures 12e and 12f. In general the omnidirectional O^+^ density was a factor of ~10 smaller than the He^+^ density. In contrast to the earlier two cases, each of these magnetospheric ion species exhibit two distinct peaks at the edges of the FTE, with clear density depletions nearest the FTE center. It is noted that these peaks at the FTE edges were observed by all four HPCA instruments.

**Figure 13:**
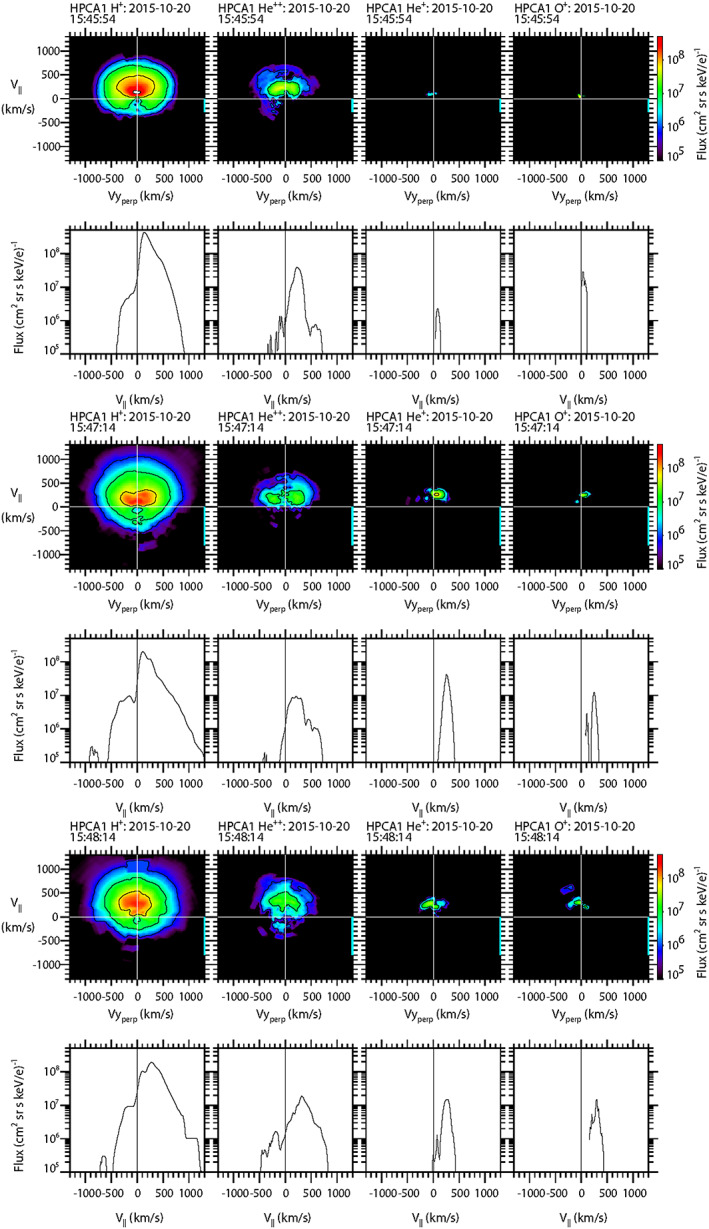
Two‐dimensional and 1‐D cuts of full 3‐D distribution functions as observed by the HPCA instrument on MMS1. Top two rows: in the magnetosheath. Middle two rows: at the time of the first peak in He^+^ and O^+^ densities. Bottom two rows: at the time of the second peak in He^+^ and O^+^ densities. The format is the same as in Figure 4.

Two‐dimensional and 1‐D cuts of the full 3‐D ion distributions from the HPCA instrument on board MMS1 are illustrated in Figure 13 for each of the four species {H^+^, He^++^, He^+^, and O^+^}. The first two rows show the ion distributions cuts in the magnetosheath (15:45:54–15:46:04 UT), prior to the encounter with the FTE core. For this case, the flux peaks from all four species were observed moving along the direction parallel to the magnetic field. Protons and alpha particles were clearly observed, with peak fluxes traveling parallel to the magnetic field. Although this is counter to the previous two cases, it is consistent with the bulk flow of plasma away from the Sun. Very narrow flux peaks of He^+^ and O^+^ ions are also noted, but at these low energies are likely just proton contamination.

The next two rows show particle distribution cuts from the time of the first density peak of minor ions within the FTE core (15:47:14–15:47:24 UT). The proton distribution shows some heating of the protons parallel to the magnetic field; and a minor component of the flux was moving antiparallel to the magnetic field. Alpha particles were observed moving parallel to the magnetic field. He^+^ and O^+^ ions were also both observed, also moving parallel to the magnetic field. These latter two species originated within the magnetosphere along field lines attached to the southern ionosphere.

The last two rows show particle distribution cuts from the time of the second density peak of minor ions within the FTE core (15:48:14–15:48:24 UT). The distribution cuts are very similar to the previous time interval; although there is an indication of an additional small flux peak parallel to the magnetic field in the alpha particle distribution cut.

The ion distributions of He^+^ and O^+^ moving parallel to the magnetic field are consistent with an FTE, which was magnetically connected to the southern ionosphere. In this respect, this reconnection scenario is similar to the first two FTE cases.

### 3.4 Case 4: 11 October 2015, 12:45–12:55 UT

**Figure 14:**
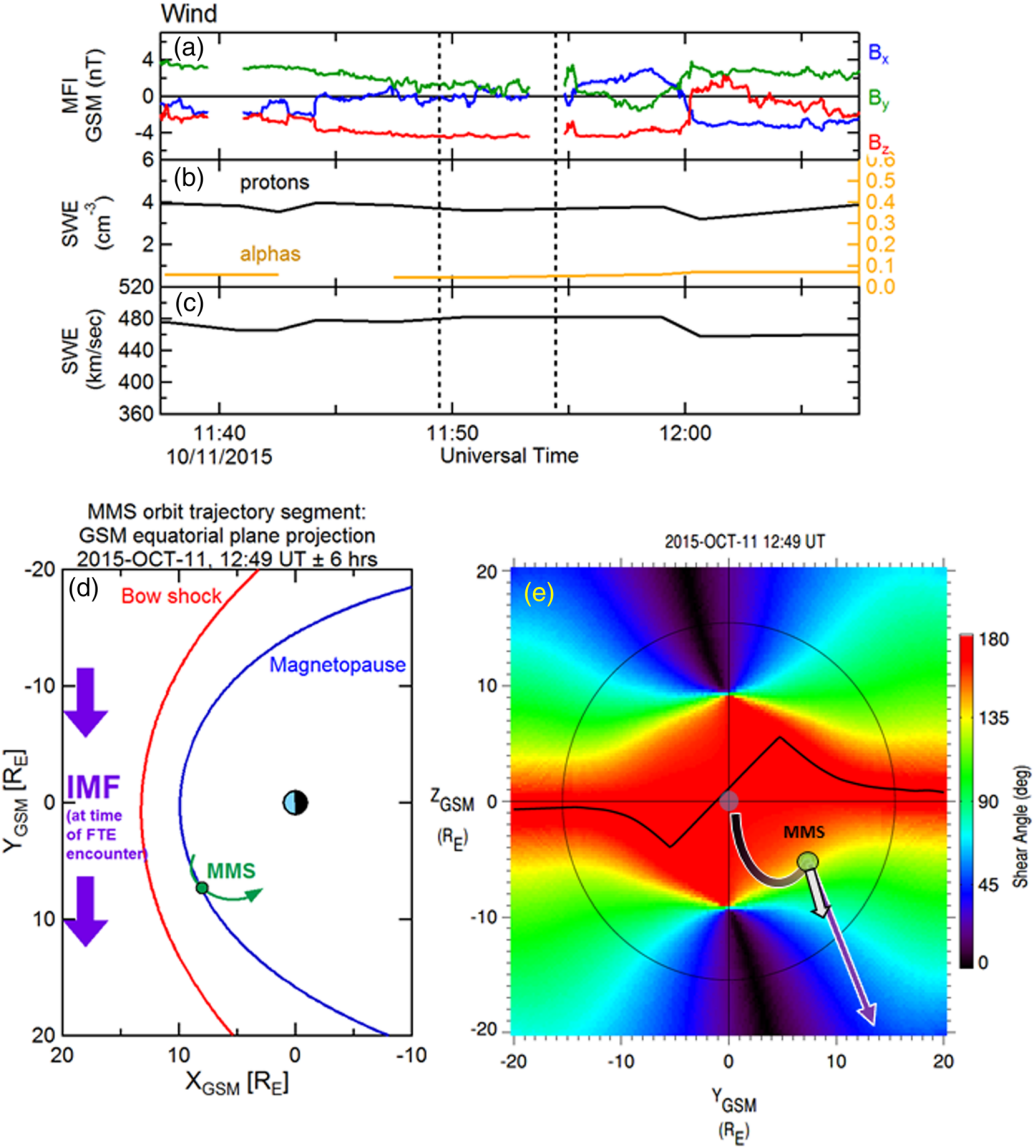
Contextual information for the FTE observed on 11 October 2015, 12:47–12:52 UT. The MMS location is similar to the previous FTE cases. Similar to the previous FTE case, the IMF during this time had a significant positive *B*_y‐GSM_ component. However, the IMF had an insignificant *B*_x‐GSM_ component and was strongly southward. The format of this figure is the same as in Figure 1.

During this FTE case, the IMF as observed by the Wind spacecraft was strongly southward, with a relatively small positive *B*_y_ component and a negligible *B*_x_ component (Figure 14a, between the dashed vertical lines. Time labels are at the Wind location, not the convected times of MMS). The solar wind proton number density was steady at ~4 cm^−3^ (Figure 14b), while the alpha particle number density in the solar wind was also steady and sparse at ~0.04 cm^−3^ (~1% of the proton number density). The solar wind speed was ~480 km/s (Figure 14c). Figure 14d shows a 12‐hr segment of the MMS trajectory projected into the GSM equatorial plane, along with the location of the MMS constellation location (green circle) within the magnetosheath but (as with the previous cases) close to the projected Shue et al. (1997) model magnetopause. Figure 14e shows the magnetic shear angle plot at the magnetopause as viewed from the Sun, along with the predicted reconnection line, the projected location of MMS (green circle), and the FPI bulk flow velocity (white arrow). Connection of the IMF to the magnetospheric magnetic field at the location of MMS is crudely depicted by the joined purple arrow and curve with a footpoint at the southern ionosphere. Similar to the previous case (and in contrast to the first two cases), the magnetosheath portion of the reconnected magnetic field vector points away from the noon meridian and points instead down the southern flank toward the magnetotail. As a consequence of the strongly southward IMF, large magnetic shear angles (>135°) occurred over much of the dayside magnetopause. Similar to the previous (20 October 2015) FTE case, this magnetosheath FTE observed by MMS was far from any predicted reconnection line (illustrated by a black contiguous line). If the FTE originated at or near the predicted reconnection line, it then likely evolved considerably by the time it reached MMS (Hasegawa et al., 2016) as the flux tube convected with the magnetosheath ambient flow and was influenced by the Lorentz force ***J*** × ***B*** and/or the pressure gradient force—Grad P associated with the reconnection process.

The large and steady southward IMF *B*_z_ condition during this interval is similar to the Geospace Environment Modeling (GEM) dayside kinetic challenge magnetopause crossing interval of 18 November 2015 as observed by MMS and previously analyzed by Kitamura et al. (2016). During that interval, MMS observed southward accelerated ion jets during encounters with the magnetopause boundary layers, indicating the presence of an active reconnection line north of the satellite. Also similar to the 11 October 2015 interval discussed here, the reconnection line was far from MMS during the GEM 18 November 2015 crossing (~5–7 R_E_), based on HPCA ion distributions (the reconnection line during the GEM interval then approached the MMS spacecraft as the IMF slightly rotated). A more comprehensive description of the relative distance between MMS and the magnetopause reconnection site during the GEM interval is provided in the companion paper of this special issue by Trattner et al. (2020).

**Figure 15:**
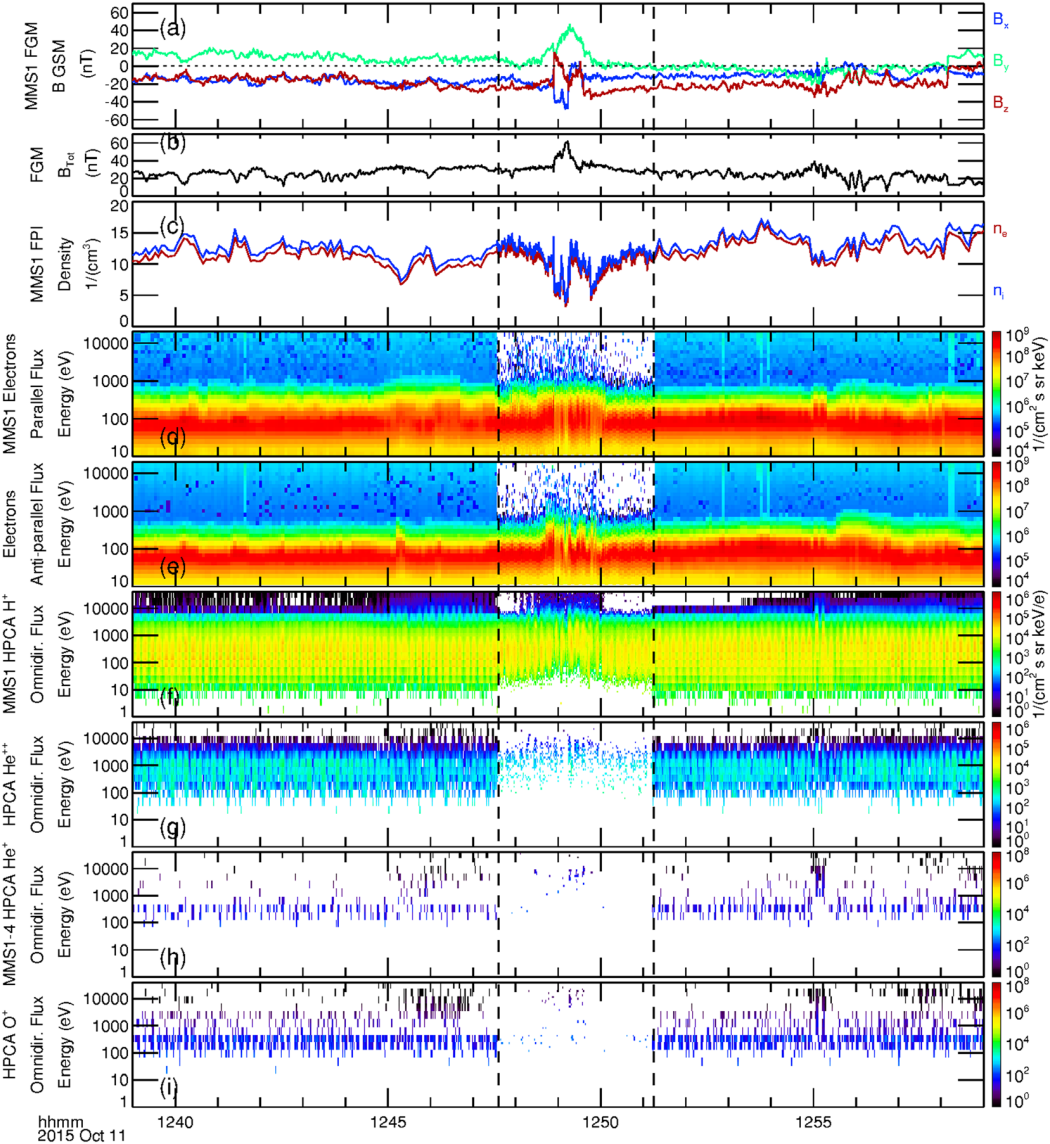
Display of a 20‐min time series during the observed magnetosheath FTE of 11 October 2015, 12:47–12:51 UT. The black dashed lines correspond to the burst mode interval of MMS. The format of this figure is the same as in Figure 2.

Figure 15 is in the same format as Figure 2 and shows a 20‐min interval centered upon the MMS burst mode segment within which this magnetosheath FTE was observed. Figures 15a and 15b show the magnetic field GSM components and intensity observed by MMS1, respectively. This interval of draped magnetosheath field and FTE core appears to be the shortest in duration of the four FTE cases, though the precise boundaries of the FTE core are unclear. The magnetosheath magnetic field rotated a bit during ~12:48:06–12:48:56 UT, but with no change in magnetic field intensity. At ~12:48:56 UT sudden and large deviations in the magnetic field components as well as a sudden enhancement in the field intensity were observed; the magnetic field intensity then became nearly twice the ambient value. The intensity then returned to ambient value ~12:49:30 UT; after which there continued be a rotation in the magnetic field components for another ~35 s; again with no significant change in the field intensity. While it is tempting to use the sudden changes in magnetic field intensity to demark the times of encounter with the FTE core (with the rotated magnetic field before and after this intensity increase due to the draping of open magnetosheath field lines), other plasma signatures (Figures 15d–15i) suggest that the FTE extended well beyond this interval. This discrepancy may be due to the evolution and decay of the FTE as it convected along the magnetopause.

The ion and electron densities from FPI in Figure 15c show general decreases (at least a factor of 2) from the ambient values, coincident with the rotations of the magnetic field surrounding the magnetic intensity increase. However, embedded within this larger decrease was an increase back to ambient densities (~12:49:10–12:49:48 UT), just after the peak in magnetic field intensity. Figures 15d and 15e show the energy spectrograms of electron fluxes parallel and antiparallel to the magnetic field. In contrast to the previous cases, the higher‐energy electron fluxes around this FTE were much less pronounced; the parallel flux was spread over ~2 min from about 12:48 to 12:50 UT, while the antiparallel flux was slightly enhanced between ~12:48:40 and 12:50:00 UT. The ion and electron behavior during this interval was significantly different from the previous three cases and suggests complex interactions within this FTE (including the possibility of interacting or coalescing FTEs, or a reconnecting current sheet).

**Figure 16:**
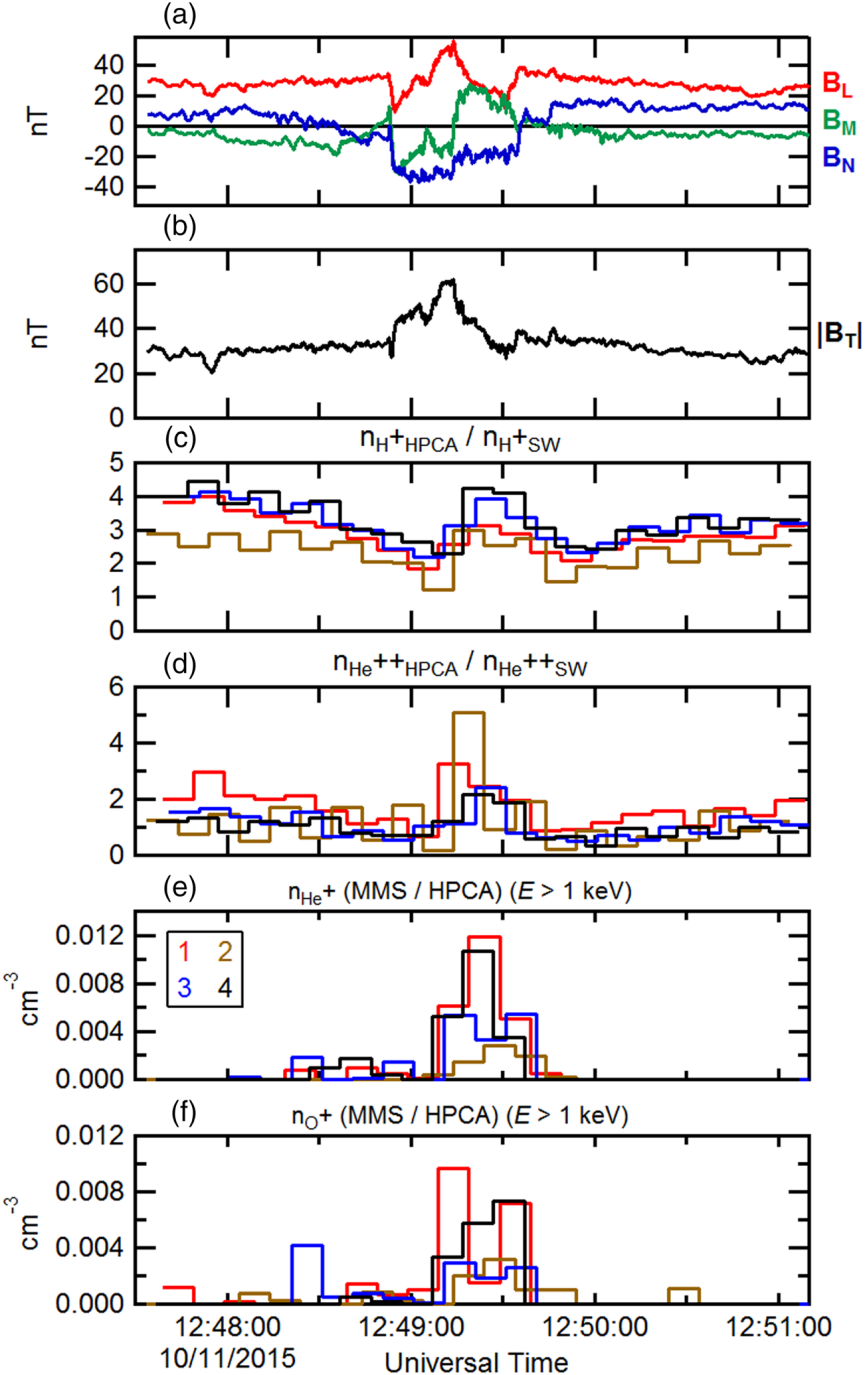
The ~3.5‐min burst mode time series of the observed magnetosheath FTE of 11 October 2016. The format is the same as in Figure 3.

Figure 15f shows the energy spectrogram of protons from the HPCA instrument on MMS1. Similar to the previous FTE case (20 October 2015), an energy dispersion of the proton flux at energies above several keV was observed prior to and during the initial magnetic field rotation (~12:47:50–12:48:30 UT) of the event. At the trailing edge (~12:50:00 UT) there was also a very brief energy dispersion of protons of ~10 s. The disappearance of higher‐energy protons just after 12:50 UT coincides well with the disappearance of higher‐energy electrons and denotes the trailing edge of the FTE. Figure 15g displays alpha particles observed by HPCA on MMS1, while Figures 15h and 15i show the combined observations from the HPCA instruments on all four MMS spacecraft of He^+^ and O^+^ ions. While sparse, these ions (*E* > 1 keV) were observed during the time interval of enhanced higher‐energy proton flux (Figure 15f). This is also noted in the partial number densities for these species in Figure 16.

Figures 16a and 16b show the magnetic field during the burst mode interval rotated into “LMN” coordinates, along with the field intensity for context. Figures 16c and 16d show the proton and alpha particle number densities from the HPCA instruments on all four MMS spacecraft, normalized to the convected solar wind proton and alpha particle density values respectively from Wind SWE. The observed enhancements in these two ion species within the FTE interval are not due to variations in the solar wind. While these enhancements do not align well with the magnetic field variations and intensity enhancement, they do align well with each other and with the enhancements in the He^+^ and O^+^ ion partial number densities (Figures 16e and 16f). The maximum observed density of the higher‐energy He^+^ was about a factor of 10 smaller than in the previous FTE case; both the He^+^ and O^+^ peak densities were of about the same magnitude (~0.008–.012 cm^−3^) and were both similar to the peak number density of higher‐energy O^+^ observed in the previous FTE case (20 October 2015).

**Figure 17:**
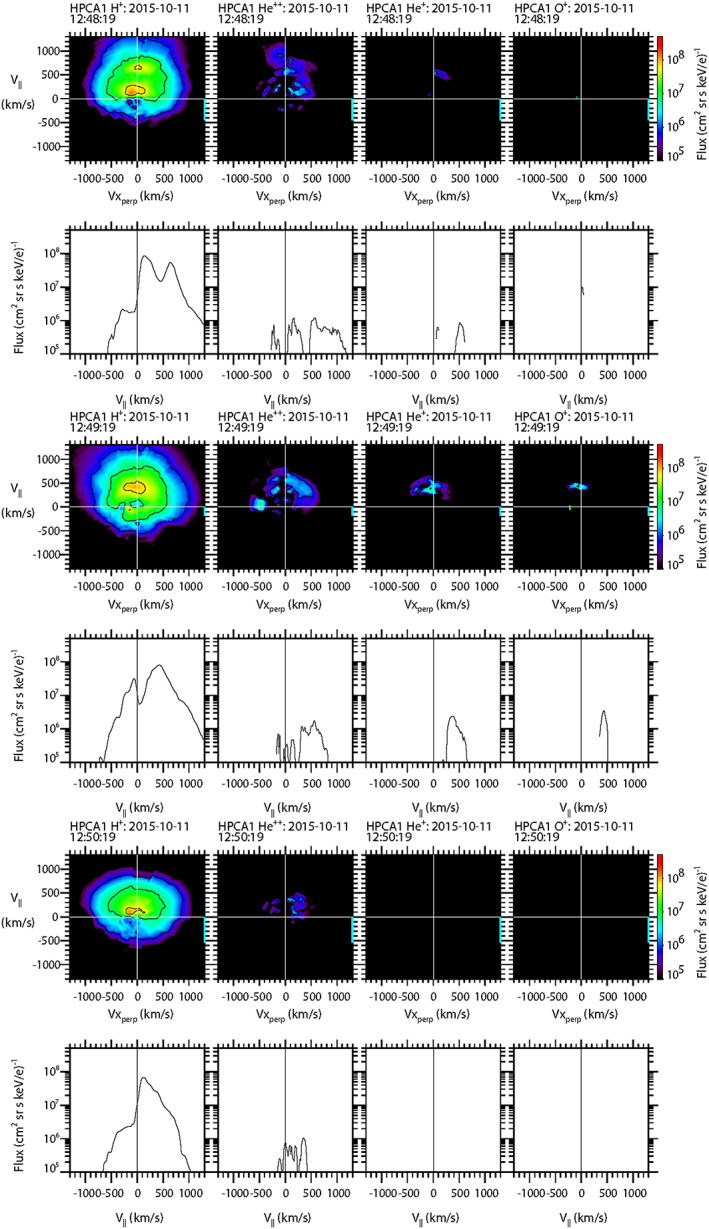
Two‐dimensional and 1‐D cuts of full 3‐D distribution functions as observed by the HPCA instrument on MMS1. Top two rows: in the draped magnetosheath. Middle two rows: at the time of peak number densities of all species. Bottom two rows: in the draped magnetosheath. The format is the same as in Figure 4.

Two‐dimensional and 1‐D cuts of the full 3‐D ion distributions from the HPCA instrument on board MMS1 are illustrated in Figure 17 for each of the four species {H^+^, He^++^, He^+^, and O^+^}. The first two rows show the ion distributions cuts near the observed start of the region of enhanced higher‐energy protons and electrons moving parallel to the magnetic field (12:48:19–12:48:29 UT). The distribution function of protons at this time was complex. A strong proton peak at a speed commensurate with the shocked solar wind flow (~100–200 km/s) was observed parallel to the magnetic field; similar to the previous FTE case. Another strong proton peak at ~700 km/s parallel to the magnetic field and represents protons reflected from the magnetopause at twice the local Alfven speed of ~210 km/s above the incident proton speed (cf. Fuselier et al., 1991; Vines et al., 2015). A third, much smaller peak was observed antiparallel to the magnetic field, much like what was observed during the first density peak of the previous FTE case. The alpha particles distribution indicated similar behavior as that of the protons; that is, two peaks parallel to the magnetic field, and a possible smaller peak antiparallel to the magnetic field. A He^+^ peak parallel to the magnetic field (~500 km/s) is also noted. This is consistent with magnetospheric ions exiting the magnetosphere and imply a connection of the local magnetic field to the southern ionosphere.

The next two rows of Figure 17 depict 3‐D distribution cuts of the ion constituents within the “core” of the FTE; just after the magnetic field intensity peak, as the ion and electron densities (FPI) increased to ambient levels (12:49:19–12:49:29 UT). The proton distribution at this time had a single defined peak parallel to the magnetic field, at a speed that was intermediate between the two peaks described at the earlier time (~400–500 km/s). There was also a peak moving antiparallel to the magnetic field at much slower speed (<100 km/s). The alpha particle distribution cut show that the flux again behaved similar to the protons, one peak instead of two moving along the magnetic field, observed at an intermediate speed The He^+^ and O^+^ fluxes were significant at this time, with a single peak parallel to the magnetic field at ~400–500 km/s.

The last two rows show the constituent distribution cuts shortly after the trailing edge of the FTE, in the magnetosheath (12:50:19–12:50:29 UT). The proton distribution at this time was very similar to the magnetosheath distribution in Figure 13 (top two rows). The alpha particles were of much lower flux and were observed moving parallel to the magnetic field. No He^+^ or O^+^ flux was observed at this time.

As with the previous intervals, the magnetospheric ion flows parallel to the magnetic field associated with this FTE are consistent with a magnetic connection to the southern ionosphere. However, the large distance of the observed interval from the model reconnection line and the relatively small He^+^ and O^+^ densities indicates that this FTE has evolved from the time of initiation to the point of observation. While the larger‐scale behavior of protons and alpha particles show a depletion within the FTE relative to the ambient magnetosheath, a localized enhancement of these populations exists within the FTE—but not at the location of maximum magnetic field intensity. The localized density enhancement suggests that this event is the result of a coalescence of two FTEs.

## 4 Summary

The ion composition in context with electron fluxes and magnetic field signatures of four long‐duration magnetosheath FTEs by the MMS mission occurring under similar steady or slowly varying solar wind conditions have been examined. All four events occurred during southward IMF. Three of the four cases were sampled during nominal Parker spiral conditions; while the fourth case was sampled during a time minimal IMF |*B*_*x*_|. The solar wind ion densities and solar wind bulk flow speeds were in the range 4–12 cm^−3^ and 290–480 km/s, respectively. The MMS spacecraft were situated in very similar locations during each encounter, that is, within the magnetosheath close to the magnetopause, in the midafternoon local time sector and southward of the GSM equator. Our main findings are as follows:

- The presence of ions of magnetospheric or solar wind origin within the FTEs has been discussed. From this their magnetic connectivity to the IMF and the ionosphere has been deduced.
- The FTE observations have been related to dayside reconnection and reconnection line locations along the magnetopause according to the maximum magnetic shear model. All four events were predicted to be southward of the reconnection line and hence to have some magnetic connection to the southern ionosphere. This prediction was supported by the observation of magnetospheric ions (He^+^ and O^+^) flowing parallel to the magnetic field within the “core” of each FTE.
- The simultaneous presence of alpha particles, and He^+^ and O^+^ ions, has been used to establish mixing. In one event this information was used to infer its structure and history. The observations indicated that it was connected to the ionosphere at both ends (i.e., on closed field lines), while the presence of a mixture of plasmas of both solar wind and ionospheric origin indicated that it had undergone reconnection in the past.
- Finite gyroradius effects in the time profiles of ions of ionospheric origin have been noted. This appeared in the form of energy dispersions. Thus, the most energetic ions, that is, those with the largest gyroradii, were typically observed in the outer regions of the FTEs.
- The variability of the minor ion content has been highlighted. Despite the similarities in solar wind conditions and the location of MMS, each of the four examined FTEs exhibited some unique features. The observed differences between these events demonstrate the challenges of understanding the occurrence, morphologies, topologies, and the evolution of FTEs. Table 1 summarizes the observations of the bulk plasma parameters as well as the minor ion species in relation to the magnetic field and region (i.e., draped magnetosheath field or the “core” of the FTE).

To conclude, although flux transfer events have often been found to exhibit complex behavior, ion composition measurements examined in conjunction with electron and magnetic field observations help to better understand interactions between the solar wind and magnetosphere in these transient phenomena. Specifically, the minor ions help determine connection to the solar wind, magnetosphere, or both. The minor ions also help establish connections to ionospheric footpoint(s) (northern and/or southern polar region), useful for understanding the magnetic topology associated with FTEs. A larger survey of observed minor ion content associated with FTEs is expected to provide a more comprehensive understanding of how such transient events topologically connect the plasmas of the shocked solar wind and the magnetosphere.

**Table 1:** Summary of Characteristics of Each Long‐Sampled FTE Case

	Case 1	Case 2	Case 3	Case 4
Distance of encounter from model reconnection line	Near	Near	Far	Far
Magnetic field magnitude	Core *B* field maximum ~1.5–2 times magnetosheath *B* field intensity	Core *B* field maximum ~2 times the magnetosheath *B* field intensity	Core *B* field maximum ~2–3 times the magnetosheath *B* field intensity	Core *B* field maximum ~2 times the magnetosheath *B* field intensity
Bulk ion flow velocity	Southward, toward noon	Southward, toward dusk	Southward, toward dusk	Southward, toward dusk
Electron density	Depletion in draped field region and in core region	Depletion in draped field region and in core region	Depletion in draped field region and in core region	Depletion in draped region, core region; large enhancement just after max of ***B***_T_
Higher‐energy electrons	Bidirectional within core; then only || to ***B***	Bidirectional within core	Mostly bidirectional, but || to ***B*** more enhanced, occurred earlier and later than antiparallel to ***B***	Moderate enhancement; mixed directions. || to ***B*** observed earlier—in draping region
Ion density	Depletion in draped field region and in core region	Depletion in draped field region and in core region	Depletion in draped field region and in core region	Depletion in draped region, core region; large enhancement just after max of ***B***_T_
Higher‐energy H+	Bidirectional in core region	Heated within core with peak in antiparallel direction; multiple peaks at trailing edge	Large peak || to ***B***; smaller peak antiparallel to ***B*** throughout	Multiple peaks || and antiparallel to ***B*** in draped region and within core
Higher‐energy He^++^	Bidirectional in core and trailing edge region	Low flux	Significant flux || to ***B*** in draped field region and throughout FTE	Multiple peaks || to ***B*** in draped region, single peak || to ***B*** within core
Higher‐energy He^+^	|| to ***B*** in core	Low flux	Significant flux || to ***B*** throughout FTE	Significant flux || to ***B*** within core
Higher‐energy O^+^	|| to ***B*** in core and trailing edge region	Low flux	Significant flux || to ***B*** throughout FTE	Significant flux || to ***B*** within core

## Supporting information

Supporting Information S1

Figure S1

Figure S2

Figure S3

Figure S4

## Data Availability

Solar wind data from the Wind spacecraft and MMS data sets are publicly available at the CDAWeb site (http://cdaweb.gsfc.nasa.gov/istp_public/). IDL routines for display of MMS data are also publicly available in the current SPEDAS software package, which can be found through the MMS Science Data Center and through the THEMIS TDAS website (at http://themis.ssl.berkeley.edu/software.shtml). The MMS HPCA data presented in the supporting information are publicly available at the CDAWeb site.
