# Supplementary material for: Characteristics of Minor Ions and Electrons in Flux Transfer Events Observed by the Magnetospheric Multiscale Mission
Source: J Geophys Res Space Phys. 2020 Jul 20;125(7):e2020JA027778. doi: 10.1029/2020JA027778 (PMC7507212; doi:10.1029/2020JA027778)
Supplement: Supplementary file 1 — Supporting Information S1 [file JGRA-125-e2020JA027778-s001.pdf]

Supporting Information:

Journal of Geophysical Research – Space Physics

"Supporting Information for Characteristics of Minor Ions and Electrons in Flux Transfer Events Observed by the Magnetospheric Multiscale Mission"

S. M. Petrinec[1], J. L. Burch[2], M. Chandler[3], C. J. Farrugia[4], S. A. Fuselier[2,5], B. L. Giles[6], R. G. Gomez[2,5], J. Mukherjee[2], W. R. Paterson[6], C. T. Russell[7], D. G. Sibeck[6], R. J. Strangeway[7], R. B. Torbert[4], K. J. Trattner[8], S. K. Vines[9], and C. Zhao[7]

[1]*Lockheed Martin Advanced Technology Center, Palo Alto, CA, USA*

[2]*Southwest Research Institute, San Antonio, TX, USA*

[3]*NASA Marshall Space Flight Center, Huntsville, AL, USA*

[4]*Space Science Center, University of New Hampshire, Durham, NH, USA*

[5]*University of Texas at San Antonio, San Antonio, TX, USA*

[6]*NASA Goddard Space Flight Center, Greenbelt, MD, USA*

[7]*Earth and Space Sciences, University of California, Los Angeles, CA, USA*

[8]*Laboratory for Atmospheric and Space Physics, University of Colorado Boulder, Boulder, CO, USA*

[9]*The Johns Hopkins University Applied Physics Laboratory, Laurel, MD, USA*

Figure Captions:

Fig. SI-1: 2D and 1D flux distribution cuts (in a magnetic field coordinate system) from all four MMS HPCA instruments, for four species {H^+, He^{++}, He^+, and O^+}, during the FTE burst mode of 2015-10-23.

Fig. SI-2: 2D and 1D flux distribution cuts (in a magnetic field coordinate system) from all four MMS HPCA instruments, for four species {H^+, He^{++}, He^+, and O^+}, during the FTE burst mode of 2016-11-08.

Fig. SI-3: 2D and 1D flux distribution cuts (in a magnetic field coordinate system) from all four MMS HPCA instruments, for four species {H^+, He^{++}, He^+, and O^+}, during the FTE burst mode of 2015-10-20.

Fig. SI-4: 2D and 1D flux distribution cuts (in a magnetic field coordinate system) from all four MMS HPCA instruments, for four species {H^+, He^{++}, He^+, and O^+}, during the FTE burst mode of 2015-10-11.
