# Supplementary material for: Characteristics of Minor Ions and Electrons in Flux Transfer Events Observed by the Magnetospheric Multiscale Mission
Source: J Geophys Res Space Phys. 2020 Jul 20;125(7):e2020JA027778. doi: 10.1029/2020JA027778 (PMC7507212; doi:10.1029/2020JA027778)
Supplement: Supplementary file 2 — Figure S1 [file JGRA-125-e2020JA027778-s002.pdf]

Supporting Information:

Journal of Geophysical Research – Space Physics

"Supporting Information for Characteristics of Minor Ions and Electrons in Flux Transfer Events Observed by the Magnetospheric Multiscale Mission"

S. M. Petrinec[1], J. L. Burch[2], M. Chandler[3], C. J. Farrugia[4], S. A. Fuselier[2,5], B. L. Giles[6], R. G. Gomez[2,5], J. Mukherjee[2], W. R. Paterson[6], C. T. Russell[7], D. G. Sibeck[6], R. J. Strangeway[7], R. B. Torbert[4], K. J. Trattner[8], S. K. Vines[9], and C. Zhao[7]

[1]*Lockheed Martin Advanced Technology Center, Palo Alto, CA, USA*

[2]*Southwest Research Institute, San Antonio, TX, USA*

[3]*NASA Marshall Space Flight Center, Huntsville, AL, USA*

[4]*Space Science Center, University of New Hampshire, Durham, NH, USA*

[5]*University of Texas at San Antonio, San Antonio, TX, USA*

[6]*NASA Goddard Space Flight Center, Greenbelt, MD, USA*

[7]*Earth and Space Sciences, University of California, Los Angeles, CA, USA*

[8]*Laboratory for Atmospheric and Space Physics, University of Colorado Boulder, Boulder, CO, USA*

[9]*The Johns Hopkins University Applied Physics Laboratory, Laurel, MD, USA*

2D and 1D flux distribution cuts (in a magnetic field coordinate system) from all four MMS HPCA instruments, for four species {H^+, He^{++}, He^+, and O^+}, during the FTE burst mode of 2015-10-23.

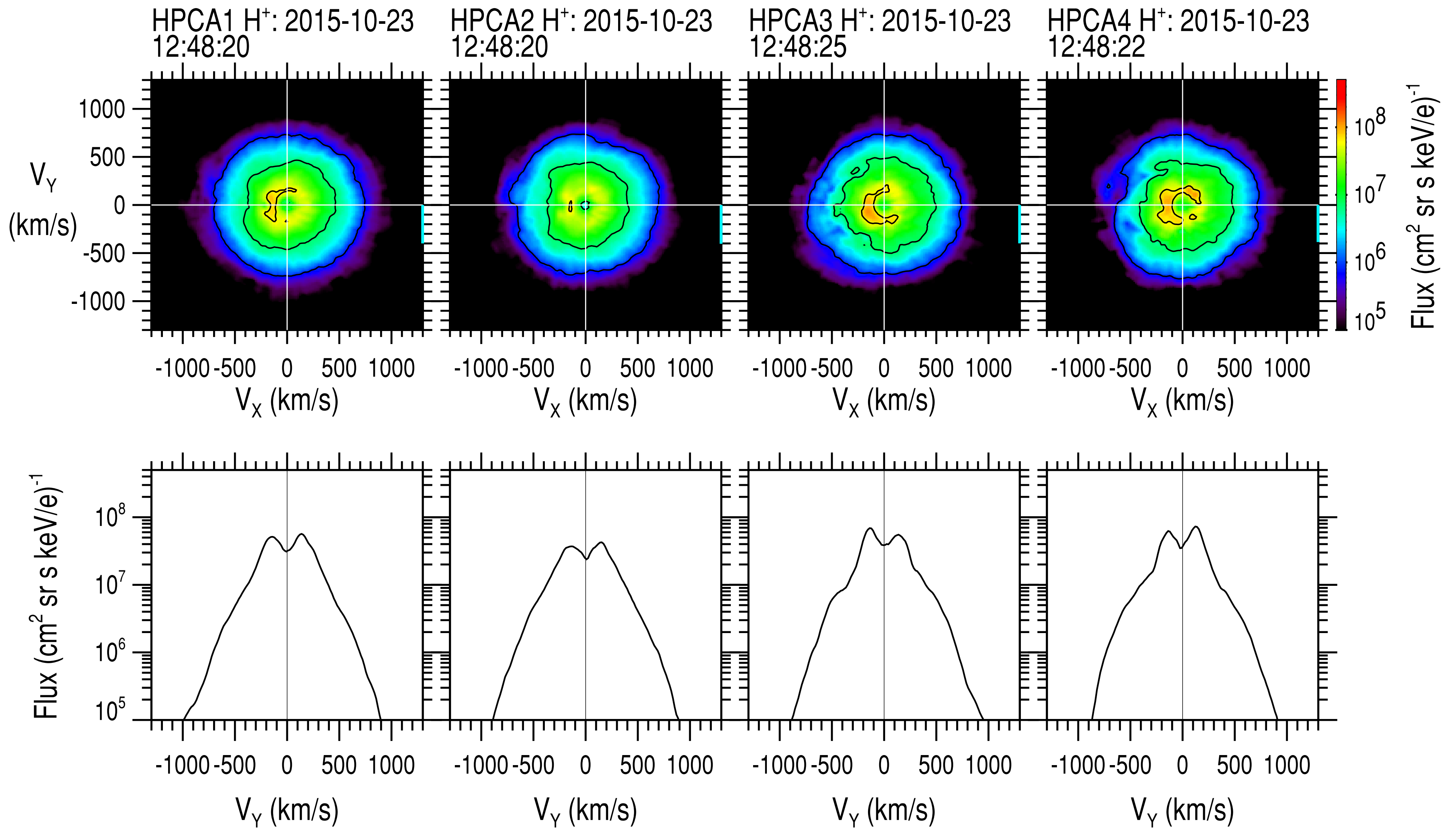

HPCA1 H+: 2015-10-23
12:48:20
HPCA2 H+: 2015-10-23
12:48:20
HPCA3 H+: 2015-10-23
12:48:25
HPCA4 H+: 2015-10-23
12:48:22
V_Y (km/s)
V_X (km/s)
-1000 -500 0 500 1000
Flux (cm^2 sr s keV/e)^-1
10^5 10^6 10^7 10^8

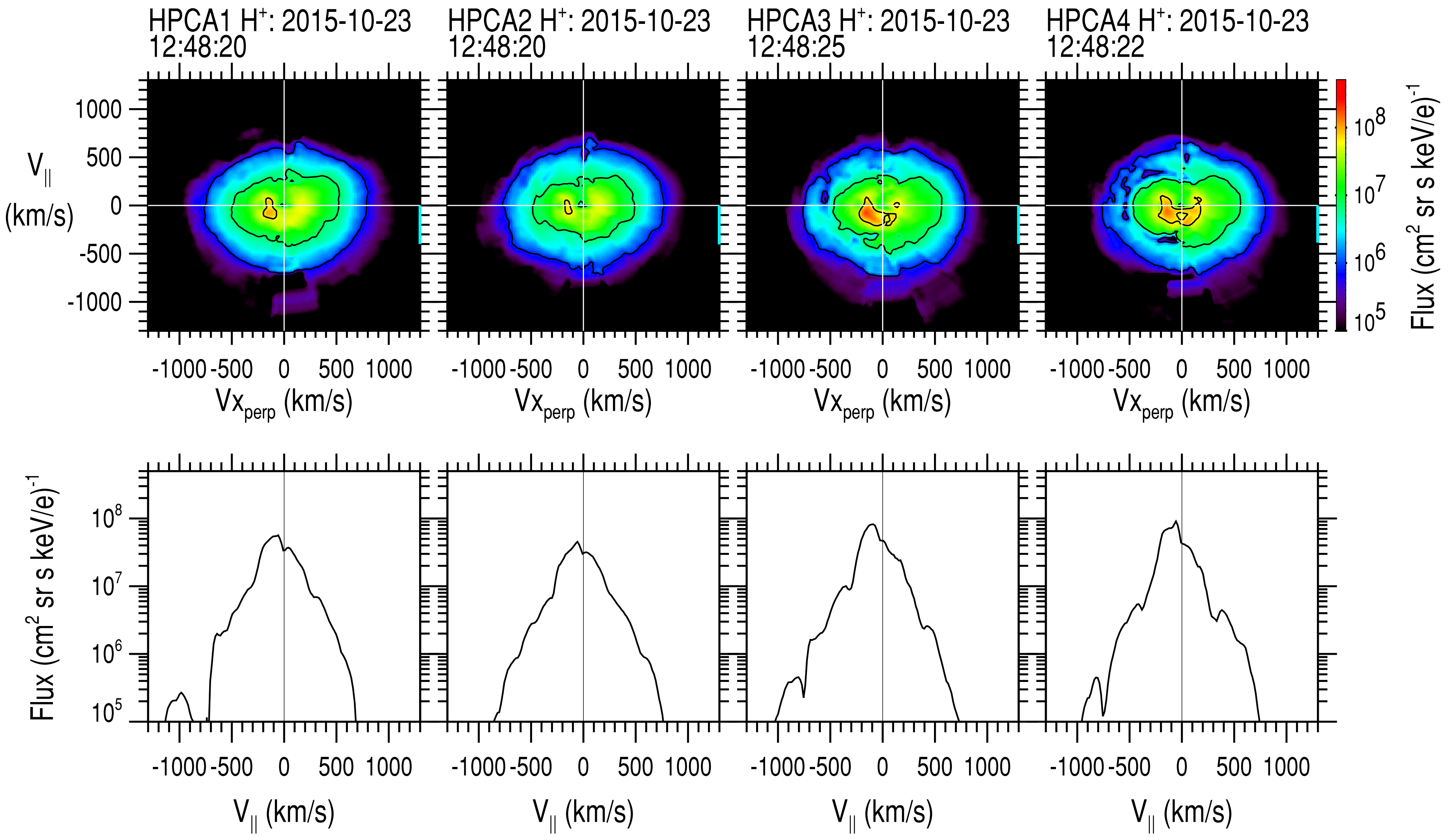
HPCA1 H+: 2015-10-23
12:48:20
HPCA2 H+: 2015-10-23
12:48:20
HPCA3 H+: 2015-10-23
12:48:25
HPCA4 H+: 2015-10-23
12:48:22
V|| (km/s)
Vxperp (km/s)
Flux (cm2 sr s keV/e)-1
V|| (km/s)

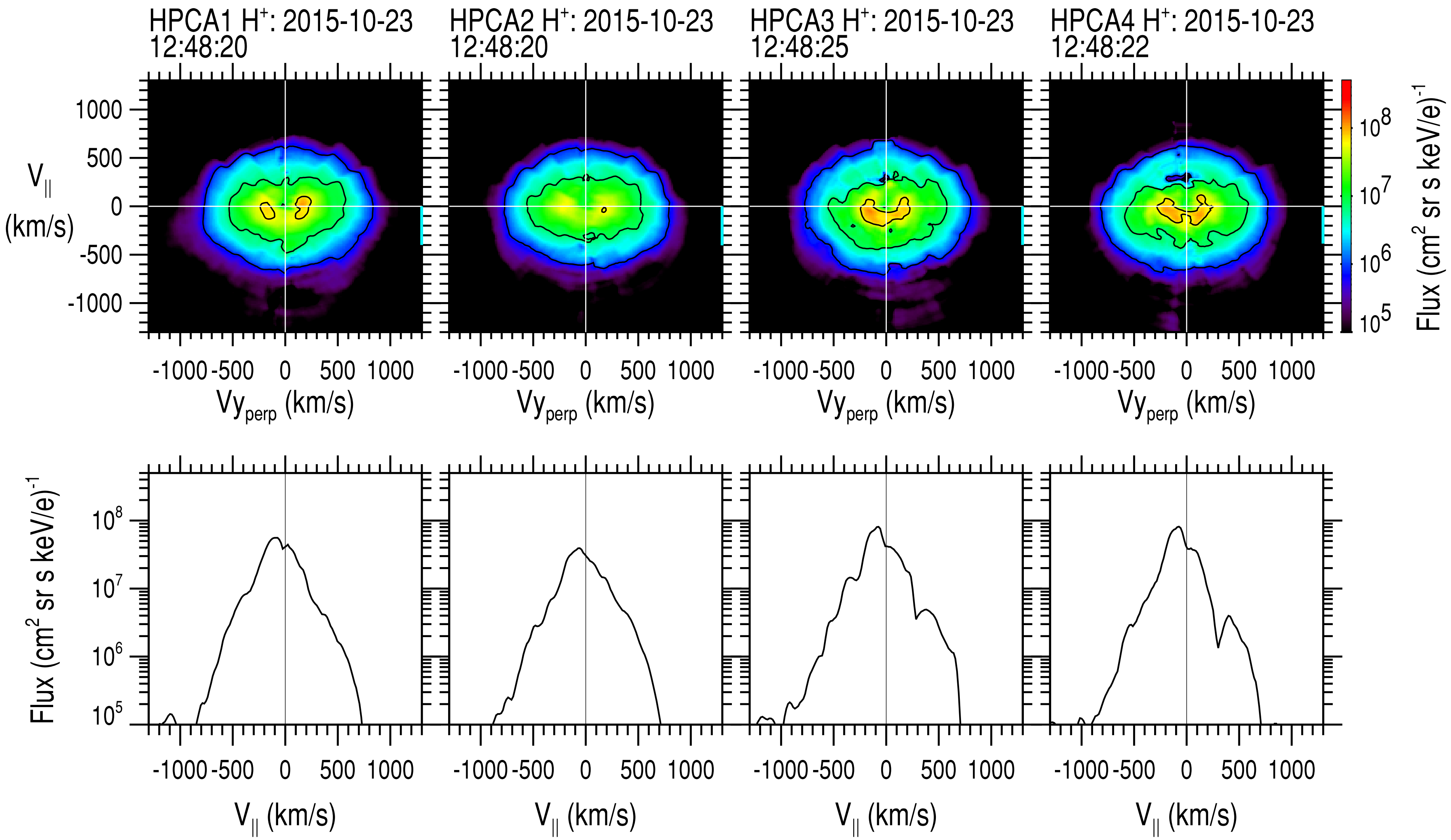

HPCA1 H+: 2015-10-23 12:48:20
HPCA2 H+: 2015-10-23 12:48:20
HPCA3 H+: 2015-10-23 12:48:25
HPCA4 H+: 2015-10-23 12:48:22
V|| (km/s)
Vyperp (km/s)
Flux (cm2 sr s keV/e)-1
-1000 -500 0 500 1000
10^5 10^6 10^7 10^8

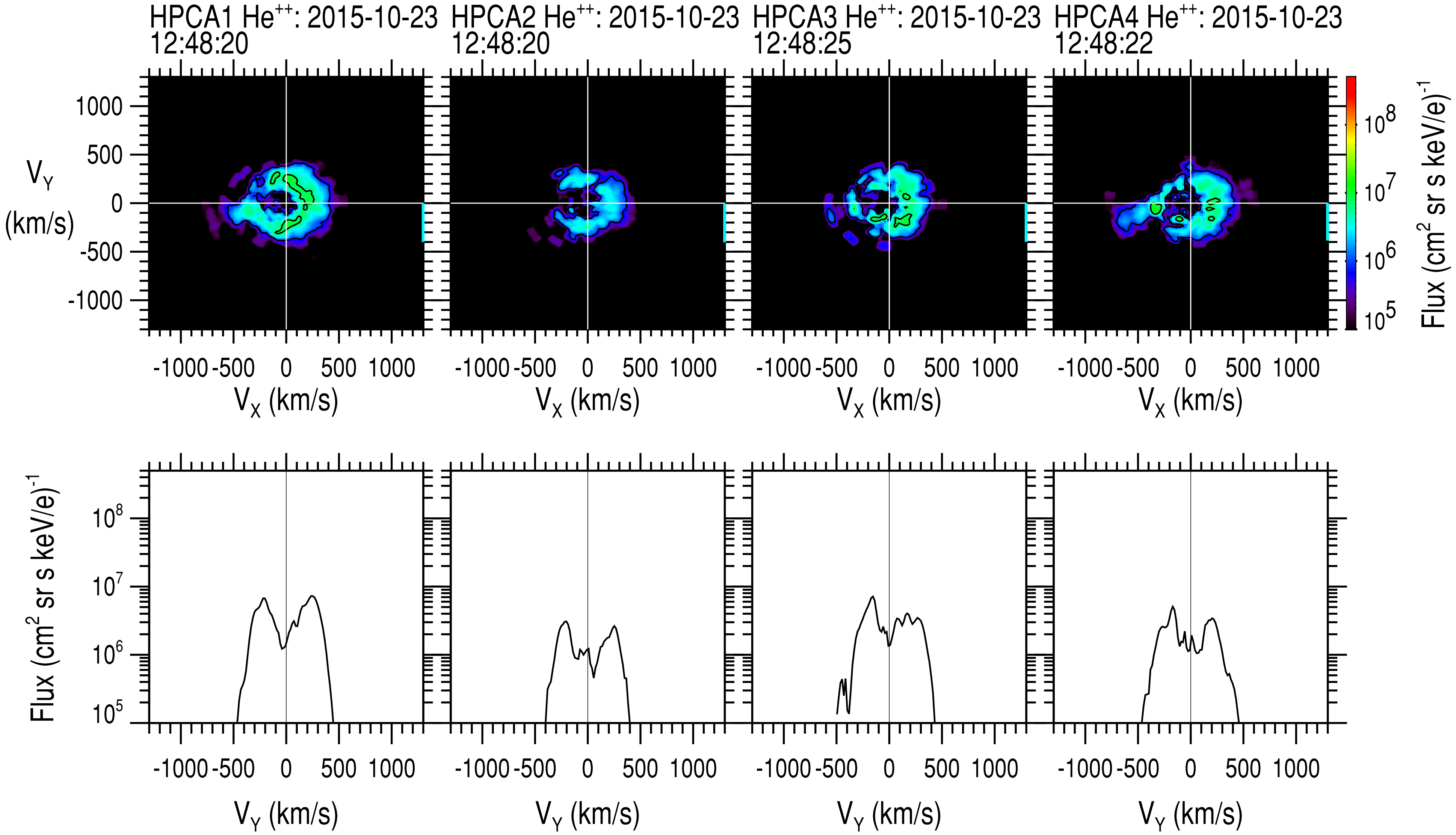

HPCA1 He⁺⁺: 2015-10-23 12:48:20
HPCA2 He⁺⁺: 2015-10-23 12:48:20
HPCA3 He⁺⁺: 2015-10-23 12:48:25
HPCA4 He⁺⁺: 2015-10-23 12:48:22
V_Y (km/s)
V_X (km/s)
Flux (cm^2 sr s keV/e)^-1

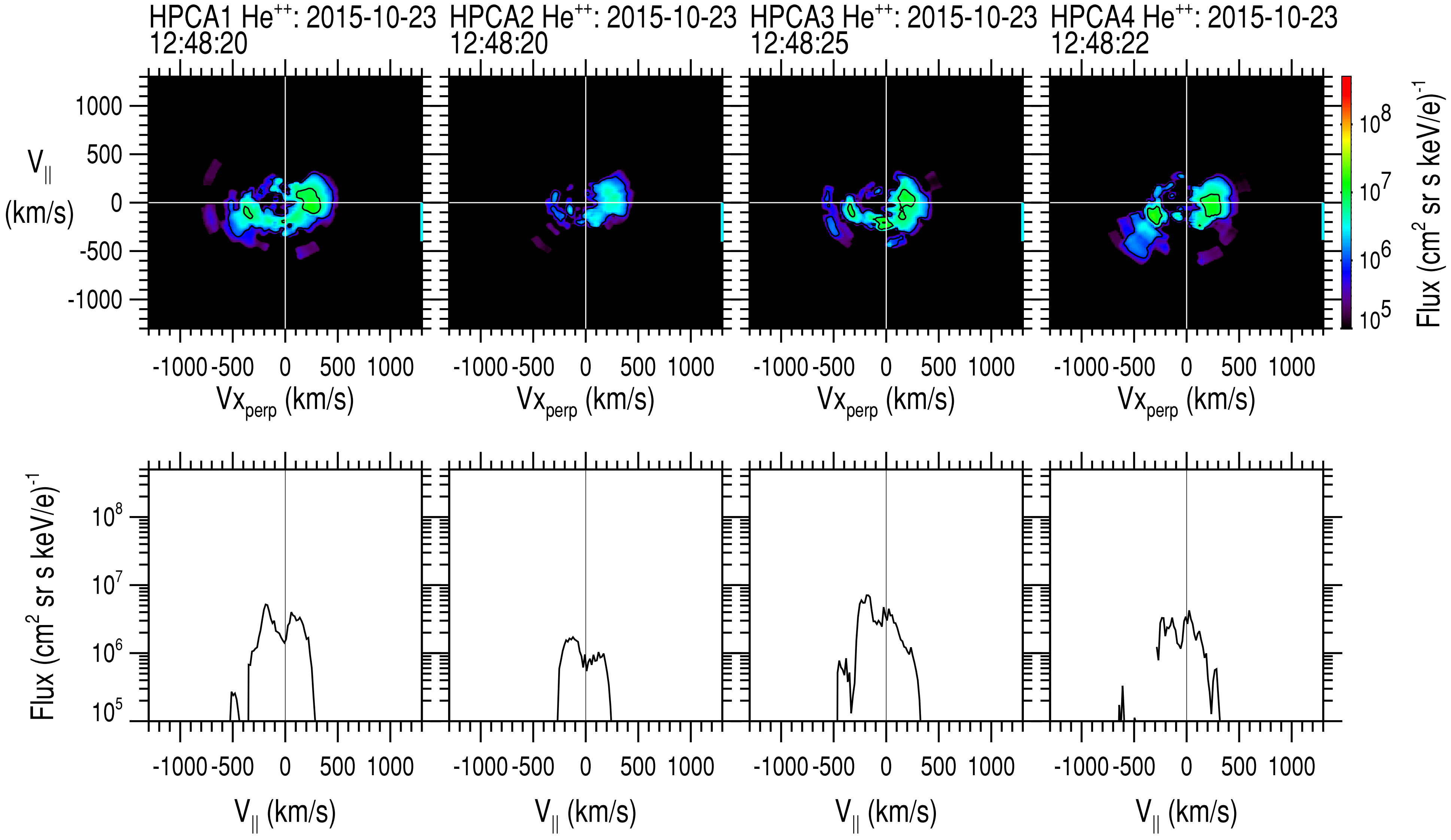

HPCA1 He++: 2015-10-23
12:48:20
HPCA2 He++: 2015-10-23
12:48:20
HPCA3 He++: 2015-10-23
12:48:25
HPCA4 He++: 2015-10-23
12:48:22
$V_{||}$ (km/s)
1000
500
0
-500
-1000
Vx_{perp} (km/s)
-1000 -500 0 500 1000
Flux $(cm^2\ sr\ s\ keV/e)^{-1}$
10^5
10^6
10^7
10^8
$V_{||}$ (km/s)

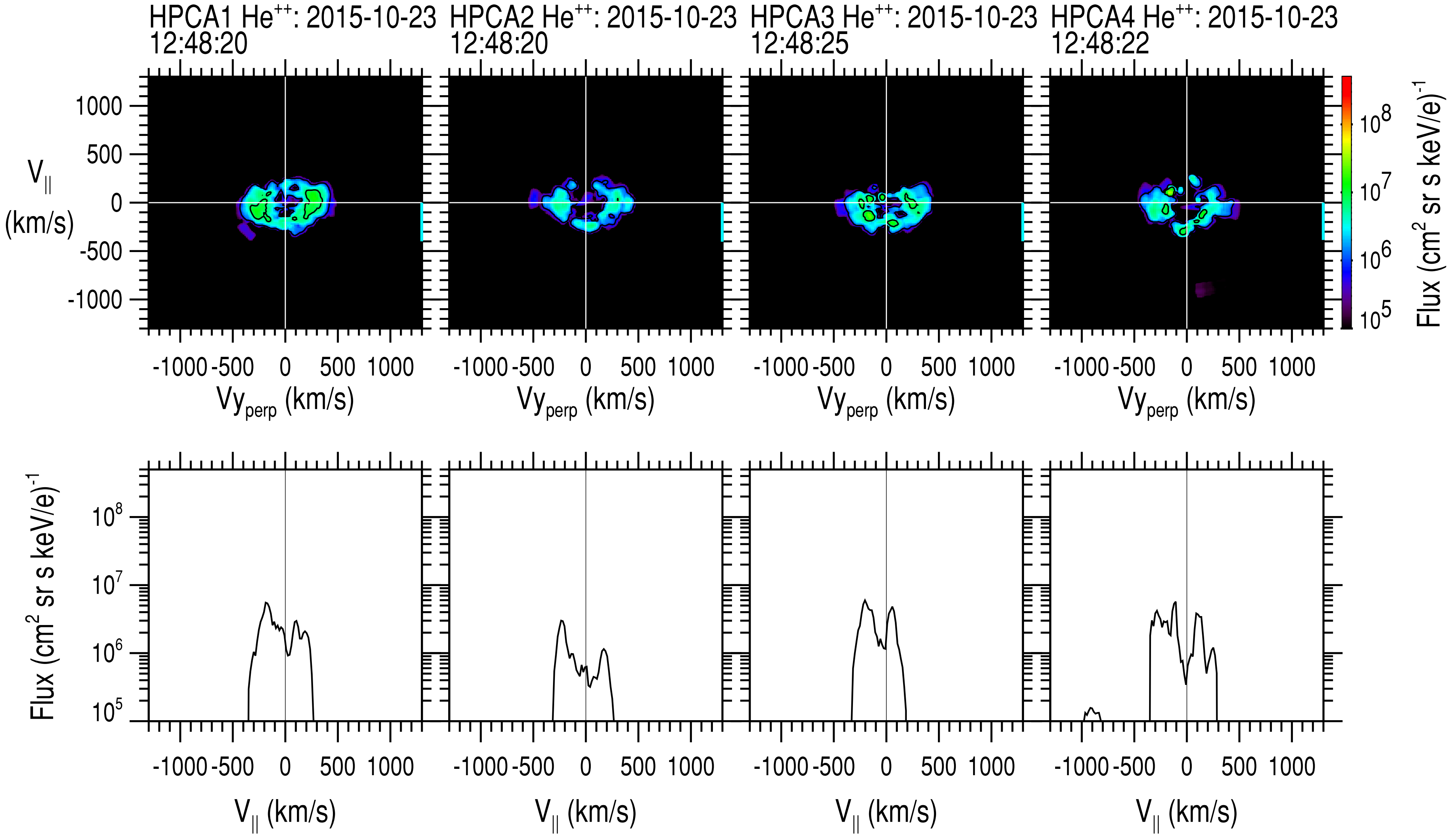
HPCA1 He++: 2015-10-23 12:48:20
HPCA2 He++: 2015-10-23 12:48:20
HPCA3 He++: 2015-10-23 12:48:25
HPCA4 He++: 2015-10-23 12:48:22
V|| (km/s)
Vyperp (km/s)
Flux (cm2 sr s keV/e)-1
V|| (km/s)

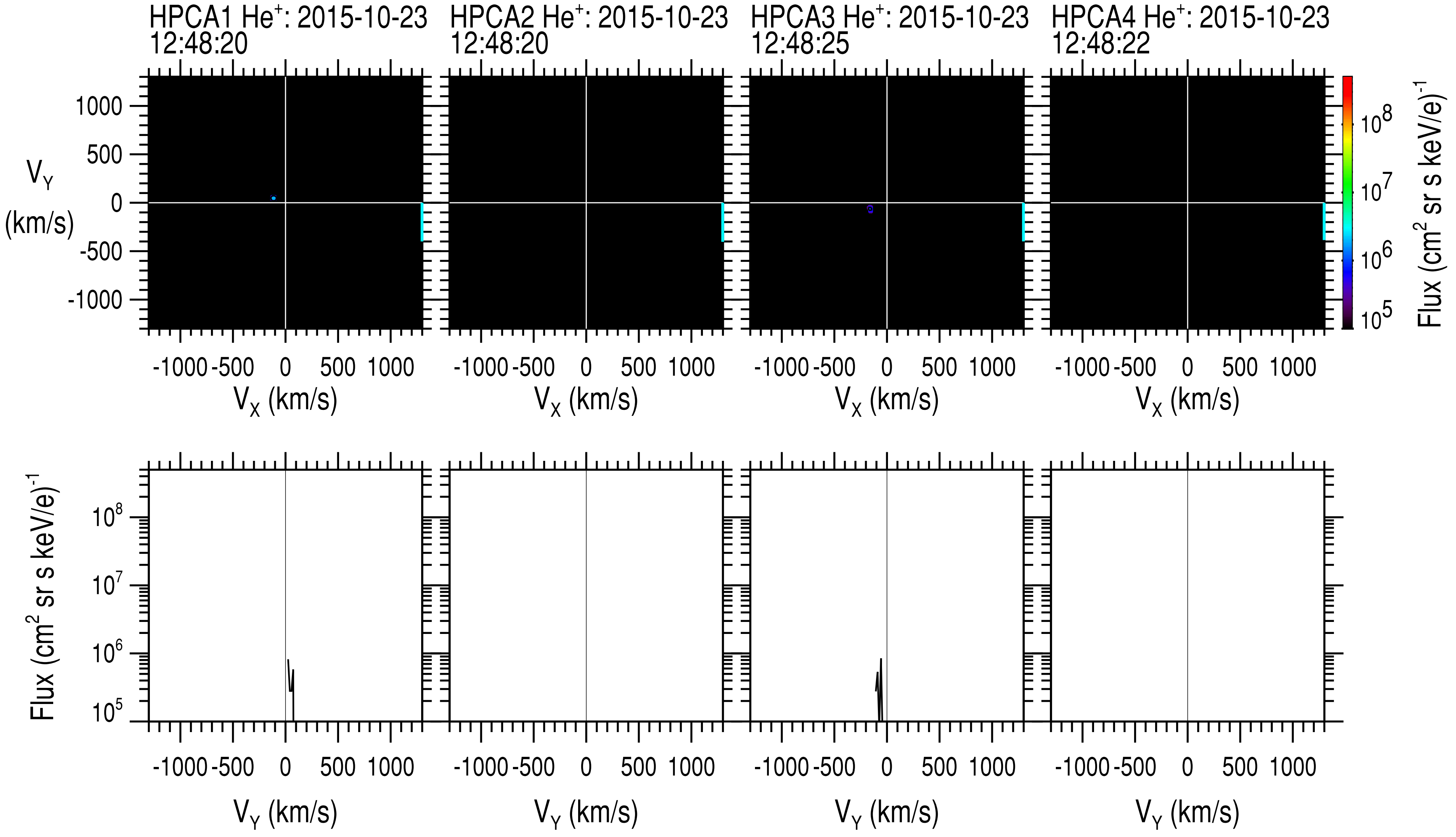

HPCA1 He+: 2015-10-23 12:48:20
HPCA2 He+: 2015-10-23 12:48:20
HPCA3 He+: 2015-10-23 12:48:25
HPCA4 He+: 2015-10-23 12:48:22
VY (km/s)
VX (km/s)
Flux (cm2 sr s keV/e)-1
-1000 -500 0 500 1000
10^5 10^6 10^7 10^8

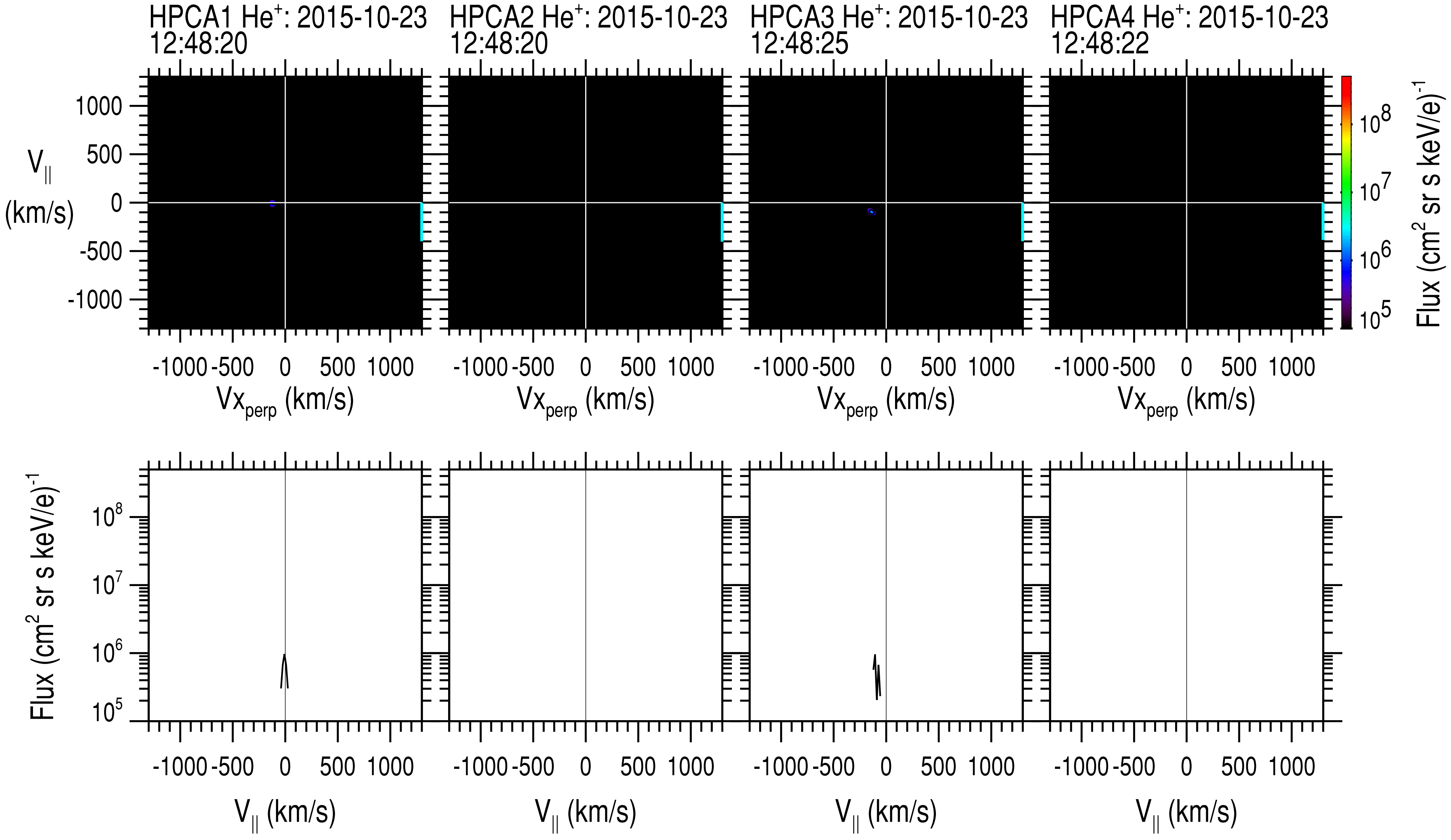
HPCA1 He+: 2015-10-23 12:48:20
HPCA2 He+: 2015-10-23 12:48:20
HPCA3 He+: 2015-10-23 12:48:25
HPCA4 He+: 2015-10-23 12:48:22
V|| (km/s)
Vxperp (km/s)
Flux (cm2 sr s keV/e)-1
-1000 -500 0 500 1000
10^5 10^6 10^7 10^8

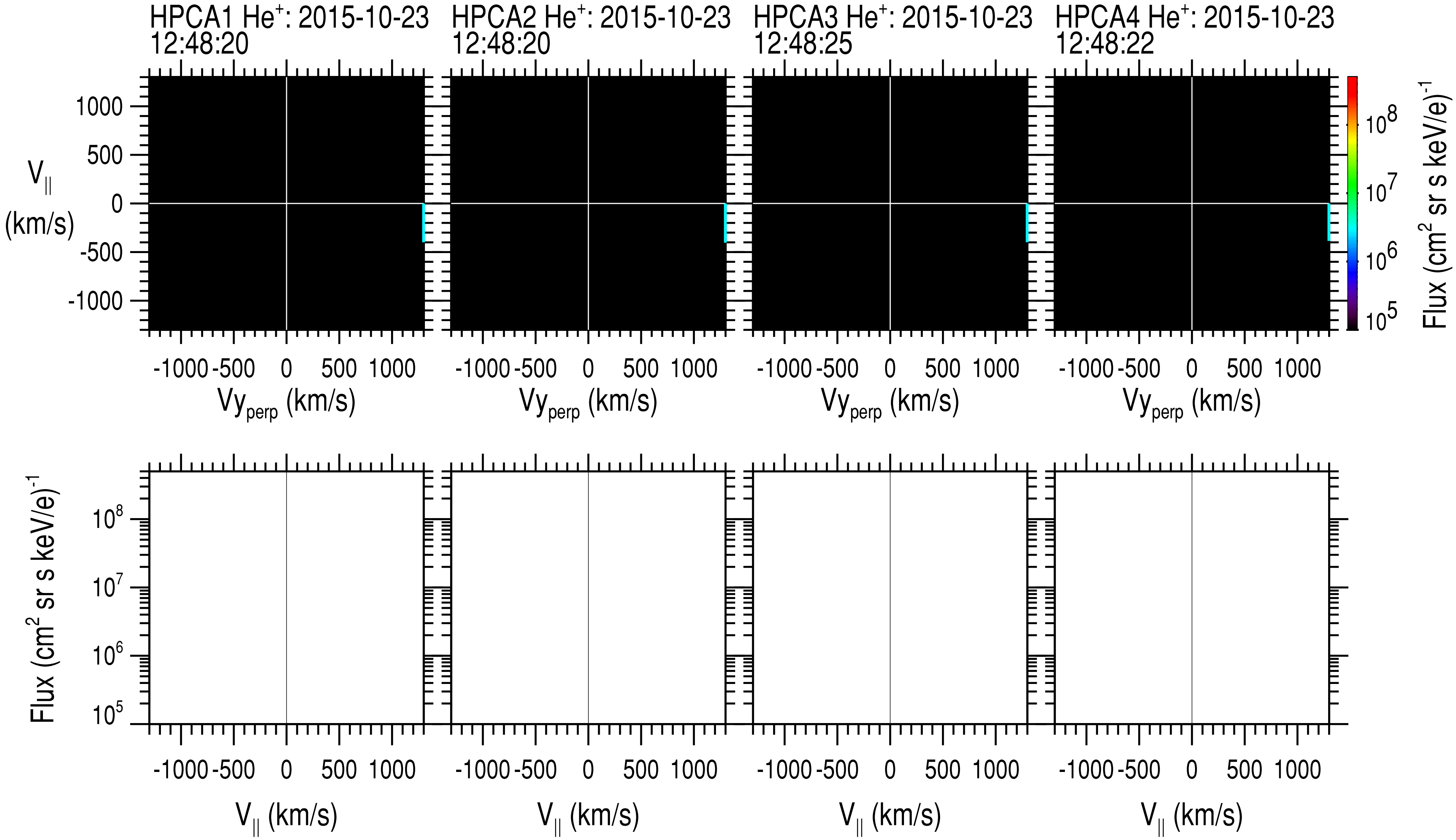

HPCA1 He+: 2015-10-23 12:48:20
HPCA2 He+: 2015-10-23 12:48:20
HPCA3 He+: 2015-10-23 12:48:25
HPCA4 He+: 2015-10-23 12:48:22
V|| (km/s)
1000
500
0
-500
-1000
-1000 -500 0 500 1000
Vyperp (km/s)
Flux (cm2 sr s keV/e)-1
105
106
107
108
V|| (km/s)

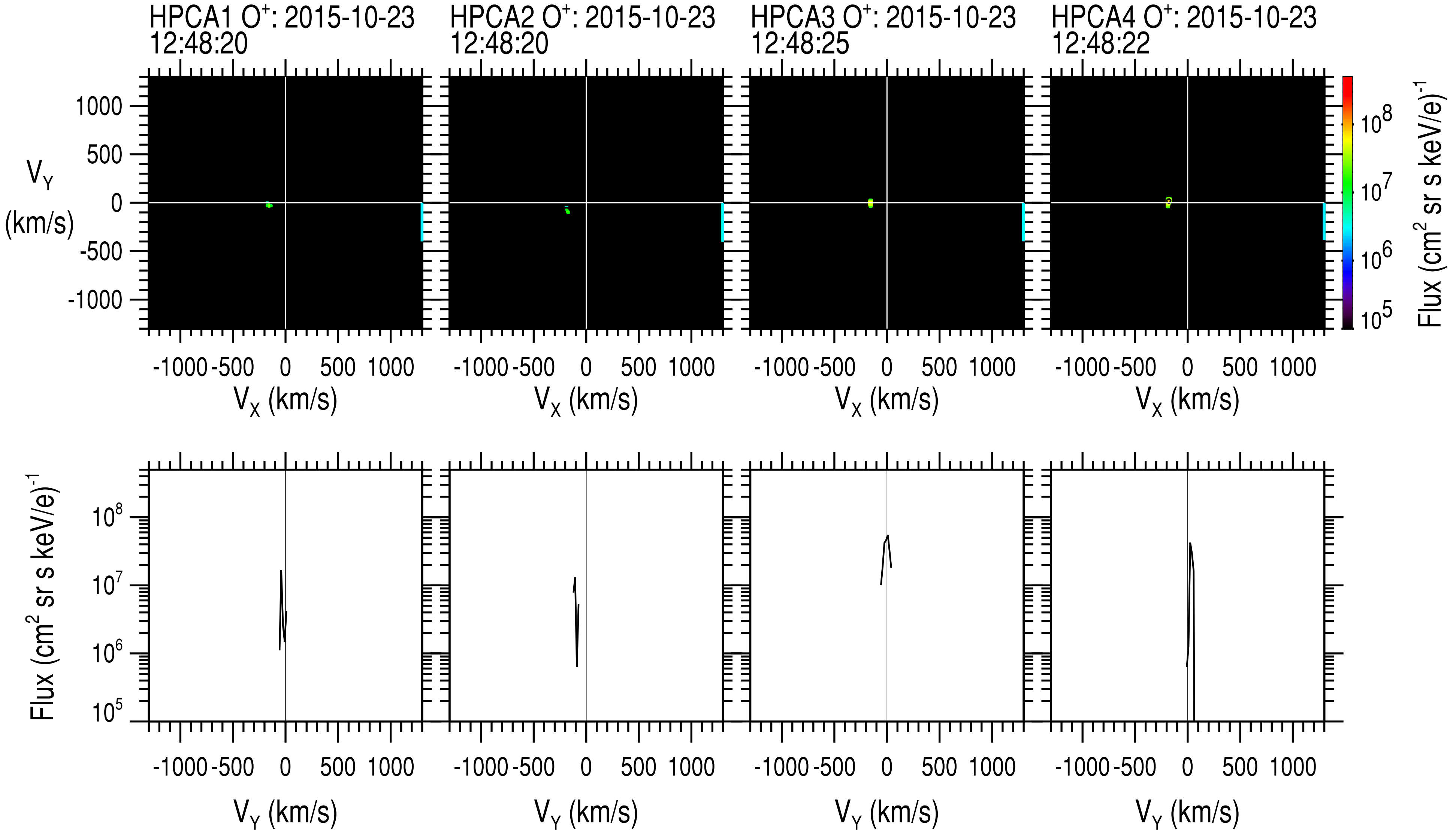

HPCA1 O+: 2015-10-23 12:48:20
HPCA2 O+: 2015-10-23 12:48:20
HPCA3 O+: 2015-10-23 12:48:25
HPCA4 O+: 2015-10-23 12:48:22
VY (km/s)
VX (km/s)
Flux (cm2 sr s keV/e)-1

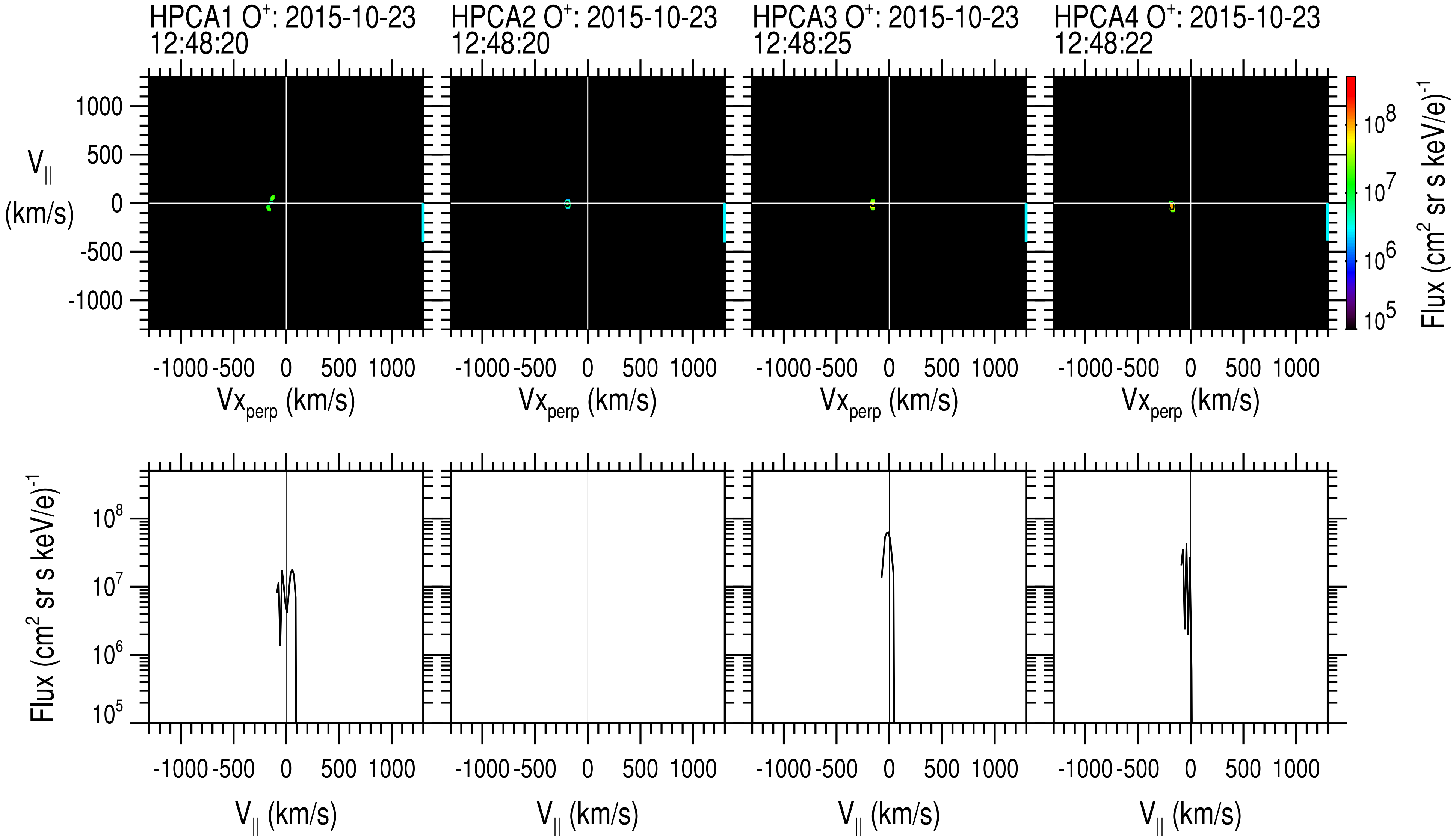

HPCA1 O+: 2015-10-23
12:48:20
HPCA2 O+: 2015-10-23
12:48:20
HPCA3 O+: 2015-10-23
12:48:25
HPCA4 O+: 2015-10-23
12:48:22
V|| (km/s)
Vxperp (km/s)
Flux (cm2 sr s keV/e)-1
V|| (km/s)

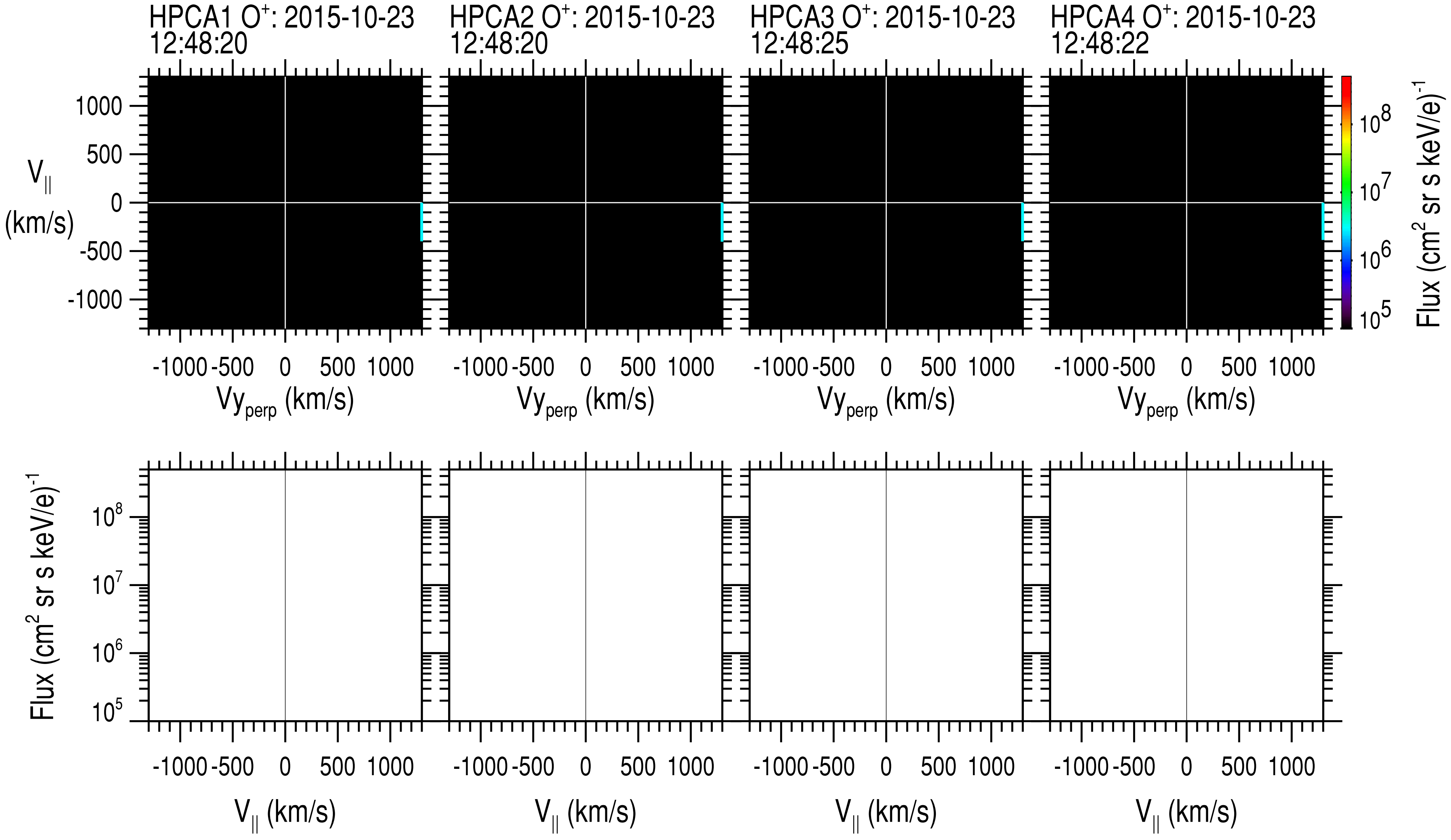
HPCA1 O+: 2015-10-23 12:48:20
HPCA2 O+: 2015-10-23 12:48:20
HPCA3 O+: 2015-10-23 12:48:25
HPCA4 O+: 2015-10-23 12:48:22
V|| (km/s)
Vyperp (km/s)
Flux (cm2 sr s keV/e)-1
10^5 10^6 10^7 10^8
-1000 -500 0 500 1000
V|| (km/s)

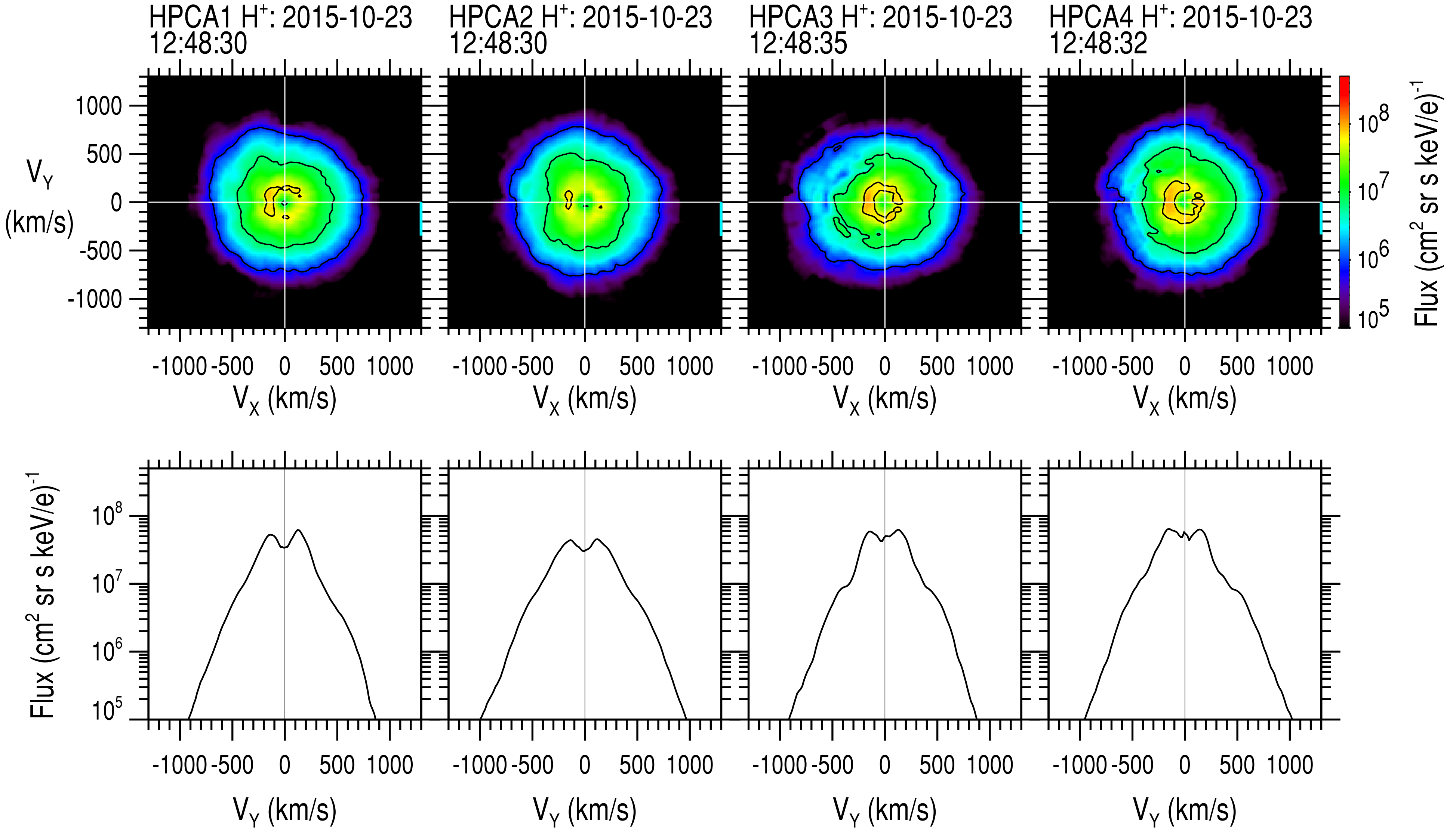
HPCA1 H+: 2015-10-23 12:48:30
HPCA2 H+: 2015-10-23 12:48:30
HPCA3 H+: 2015-10-23 12:48:35
HPCA4 H+: 2015-10-23 12:48:32
V_Y (km/s)
V_X (km/s)
Flux (cm^2 sr s keV/e)^-1
V_Y (km/s)

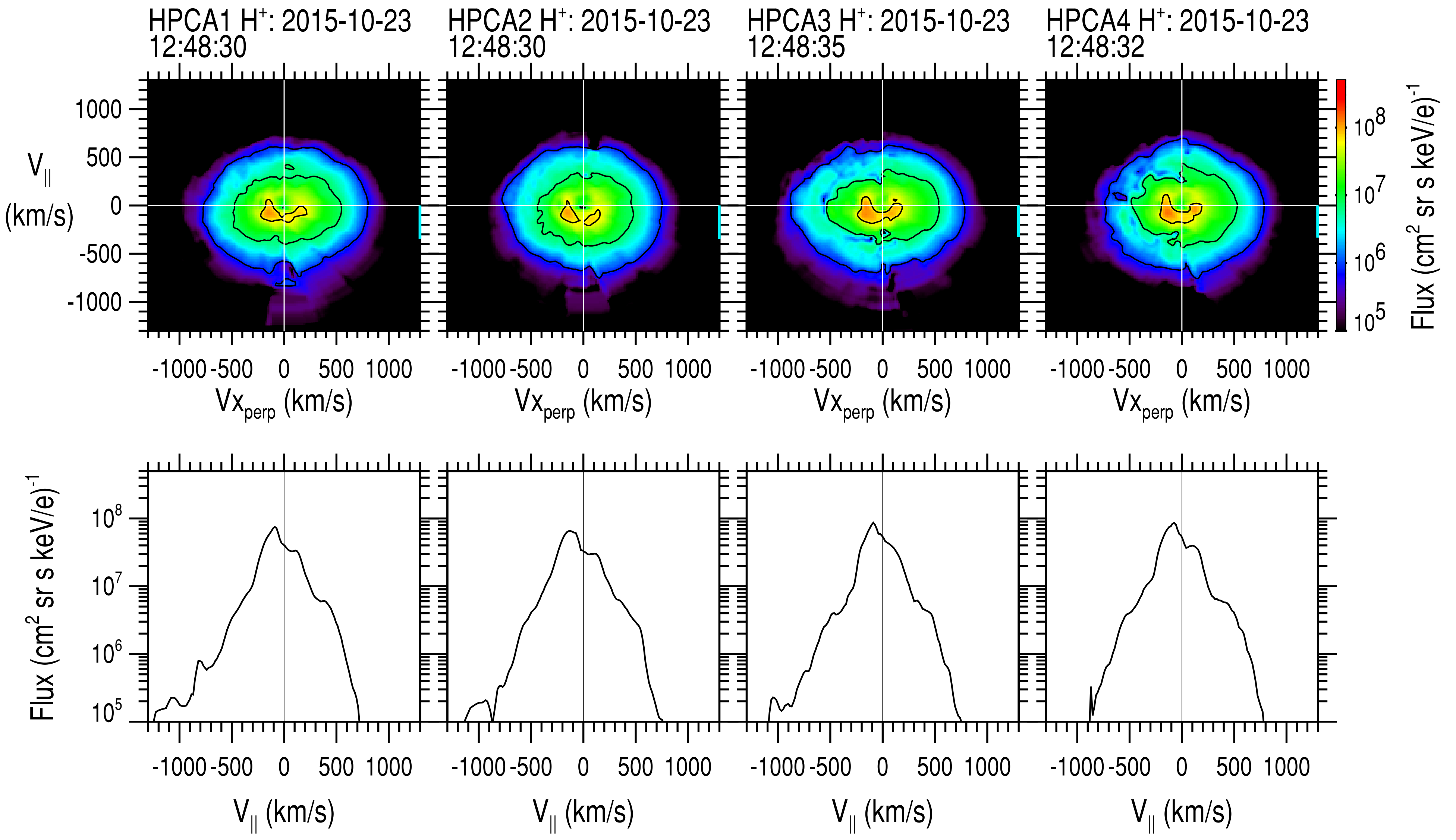
HPCA1 H+: 2015-10-23 12:48:30
HPCA2 H+: 2015-10-23 12:48:30
HPCA3 H+: 2015-10-23 12:48:35
HPCA4 H+: 2015-10-23 12:48:32
V|| (km/s)
Vxperp (km/s)
Flux (cm2 sr s keV/e)-1
V|| (km/s)

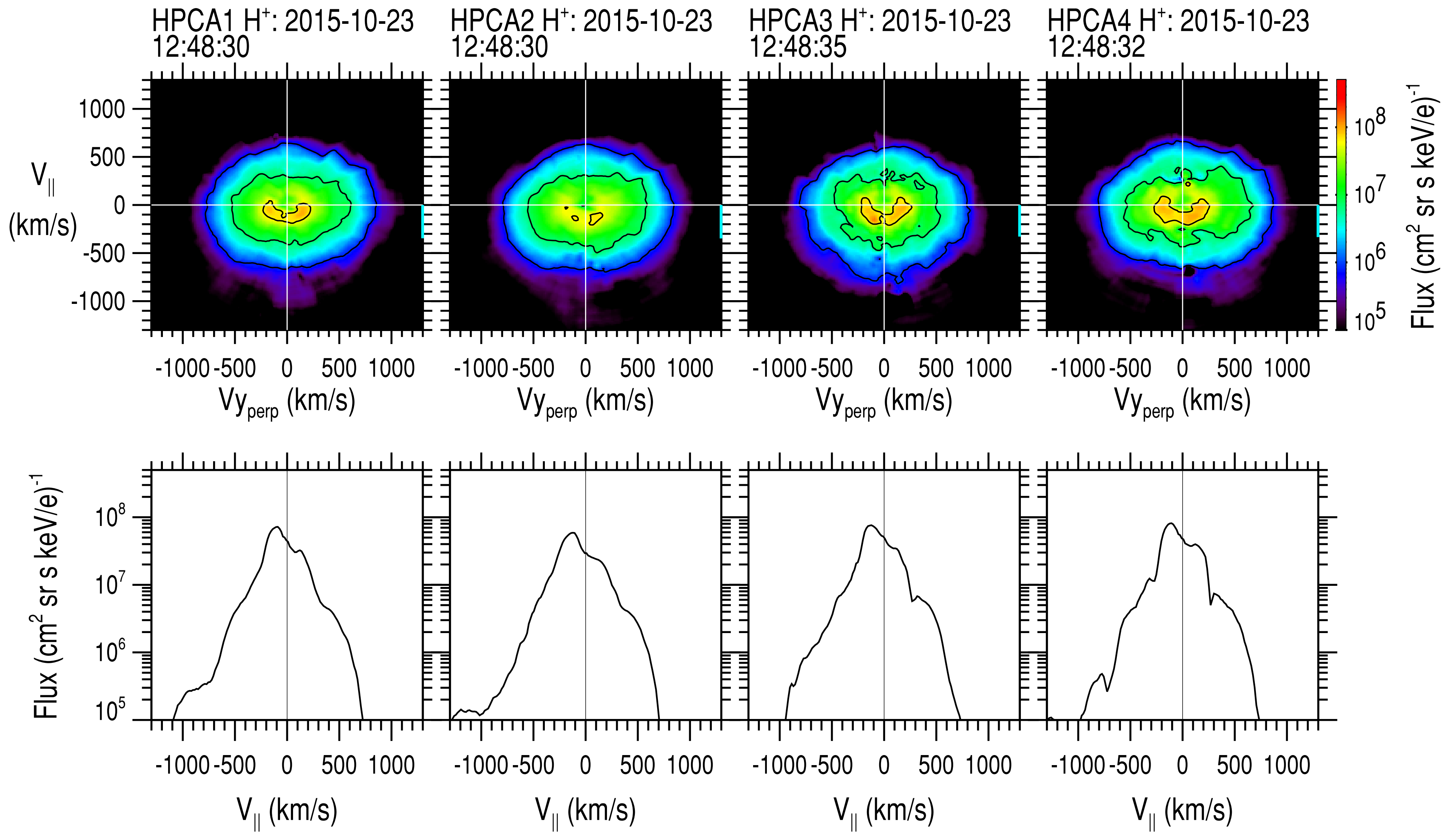
HPCA1 H+: 2015-10-23
12:48:30
HPCA2 H+: 2015-10-23
12:48:30
HPCA3 H+: 2015-10-23
12:48:35
HPCA4 H+: 2015-10-23
12:48:32
V|| (km/s)
Vyperp (km/s)
Flux (cm2 sr s keV/e)-1
V|| (km/s)

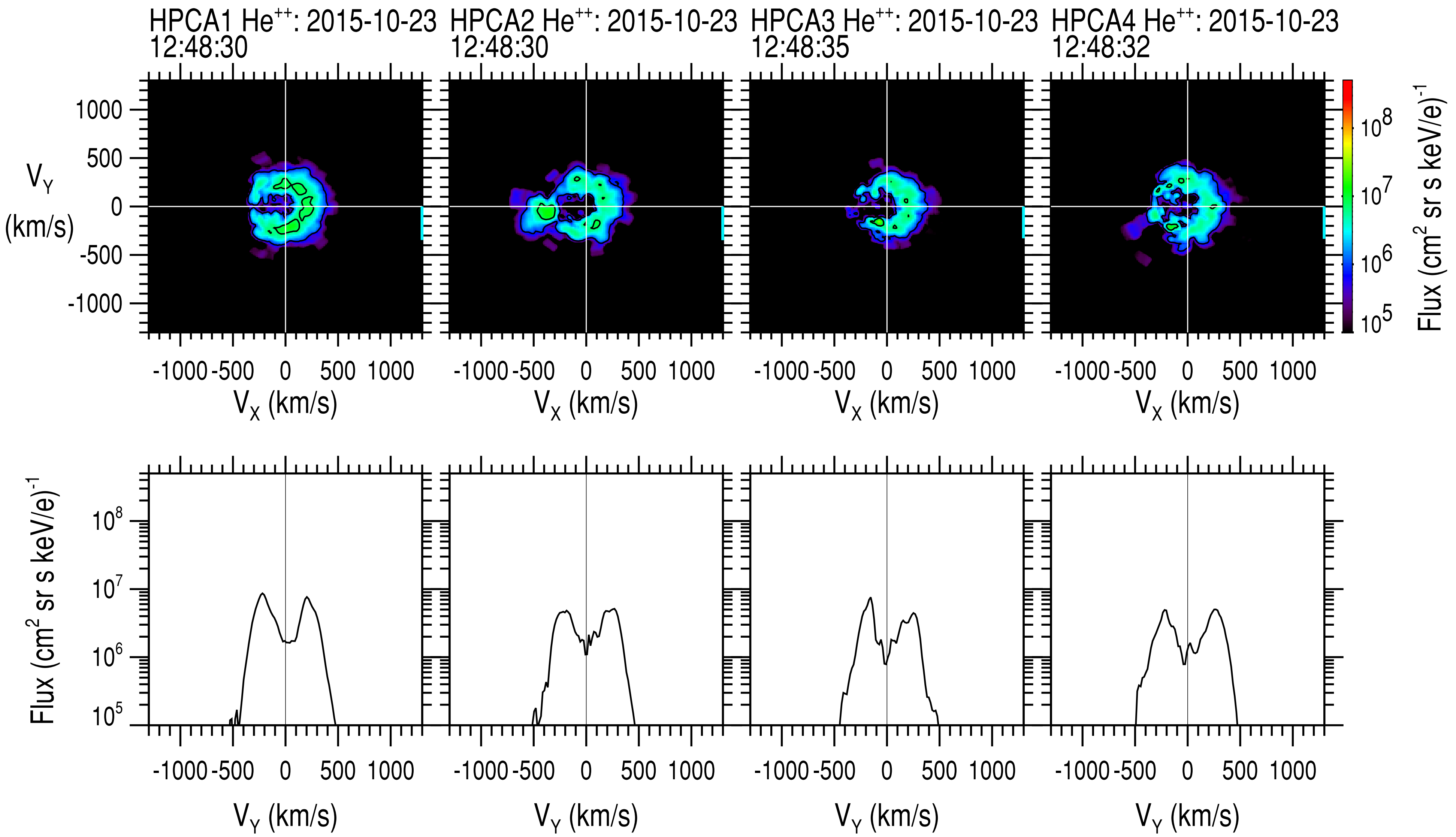

HPCA1 He++: 2015-10-23 12:48:30
HPCA2 He++: 2015-10-23 12:48:30
HPCA3 He++: 2015-10-23 12:48:35
HPCA4 He++: 2015-10-23 12:48:32
V_Y (km/s)
V_X (km/s)
Flux (cm^2 sr s keV/e)^-1
V_Y (km/s)

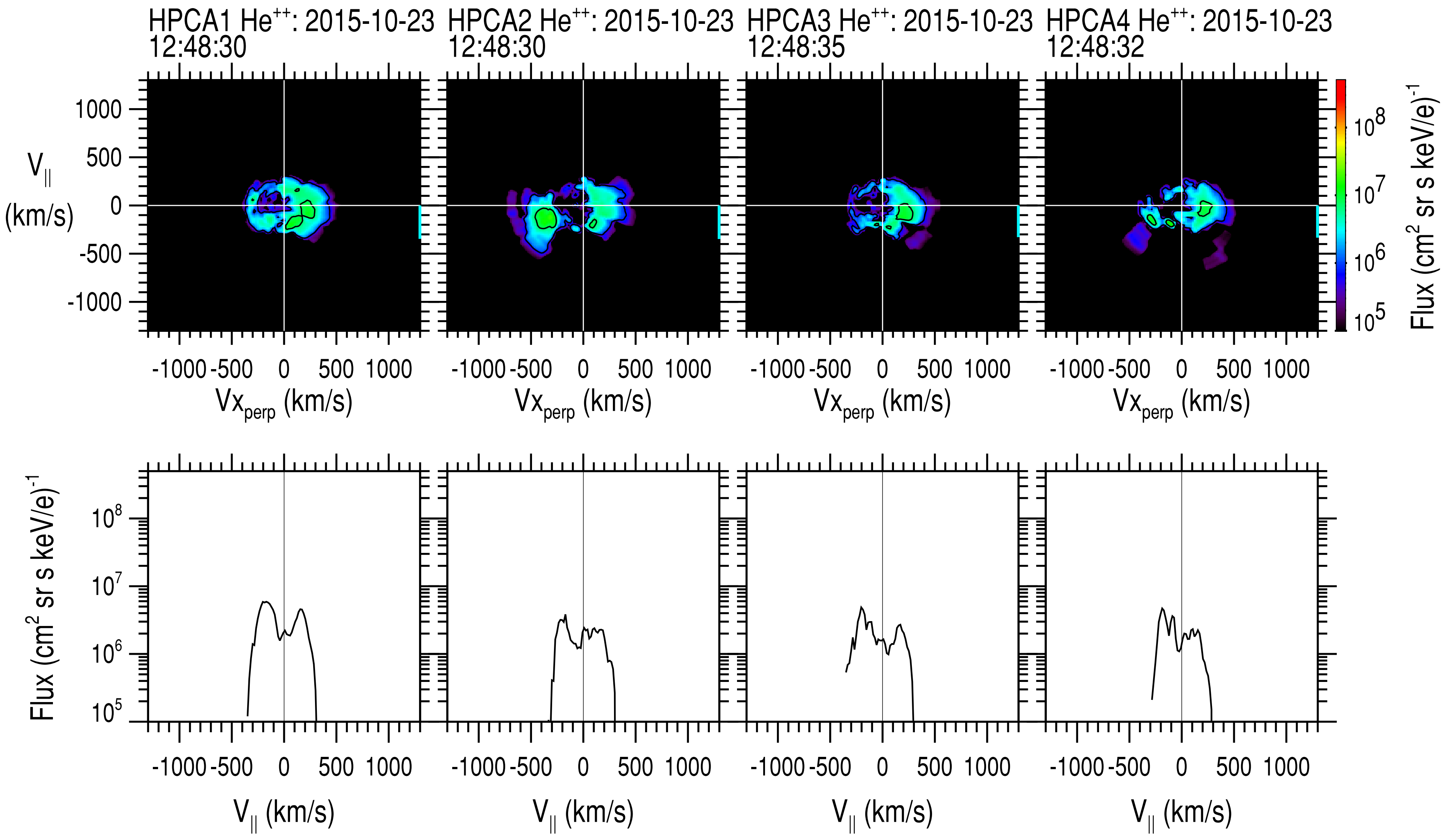

HPCA1 He++: 2015-10-23 12:48:30
HPCA2 He++: 2015-10-23 12:48:30
HPCA3 He++: 2015-10-23 12:48:35
HPCA4 He++: 2015-10-23 12:48:32
V|| (km/s)
Vxperp (km/s)
Flux (cm2 sr s keV/e)-1
V|| (km/s)

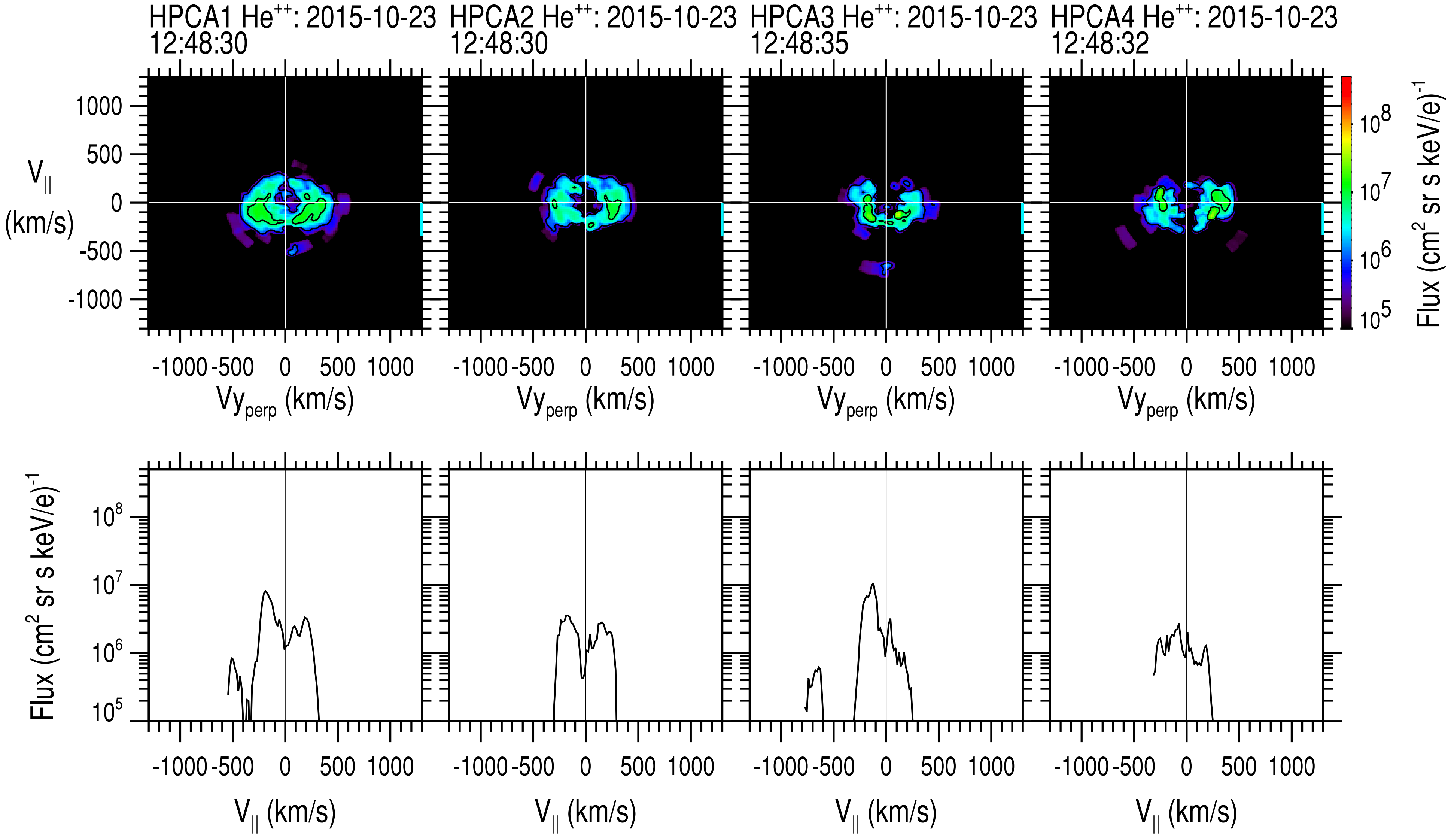
HPCA1 He++: 2015-10-23 12:48:30
HPCA2 He++: 2015-10-23 12:48:30
HPCA3 He++: 2015-10-23 12:48:35
HPCA4 He++: 2015-10-23 12:48:32
V|| (km/s)
Vyperp (km/s)
Flux (cm2 sr s keV/e)-1

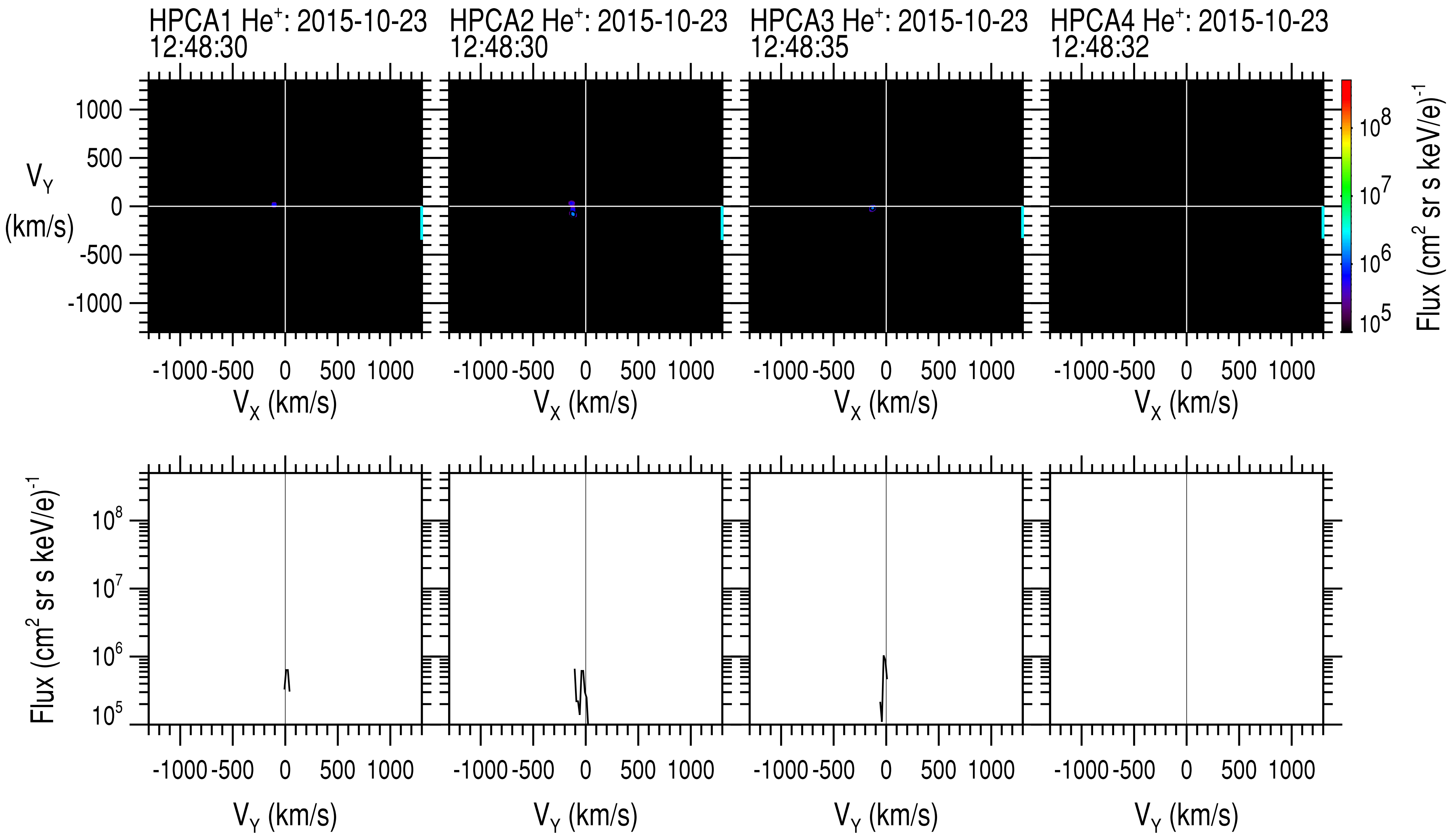
HPCA1 He+: 2015-10-23 12:48:30
HPCA2 He+: 2015-10-23 12:48:30
HPCA3 He+: 2015-10-23 12:48:35
HPCA4 He+: 2015-10-23 12:48:32
V_Y (km/s)
V_X (km/s)
Flux (cm^2 sr s keV/e)^-1
V_Y (km/s)

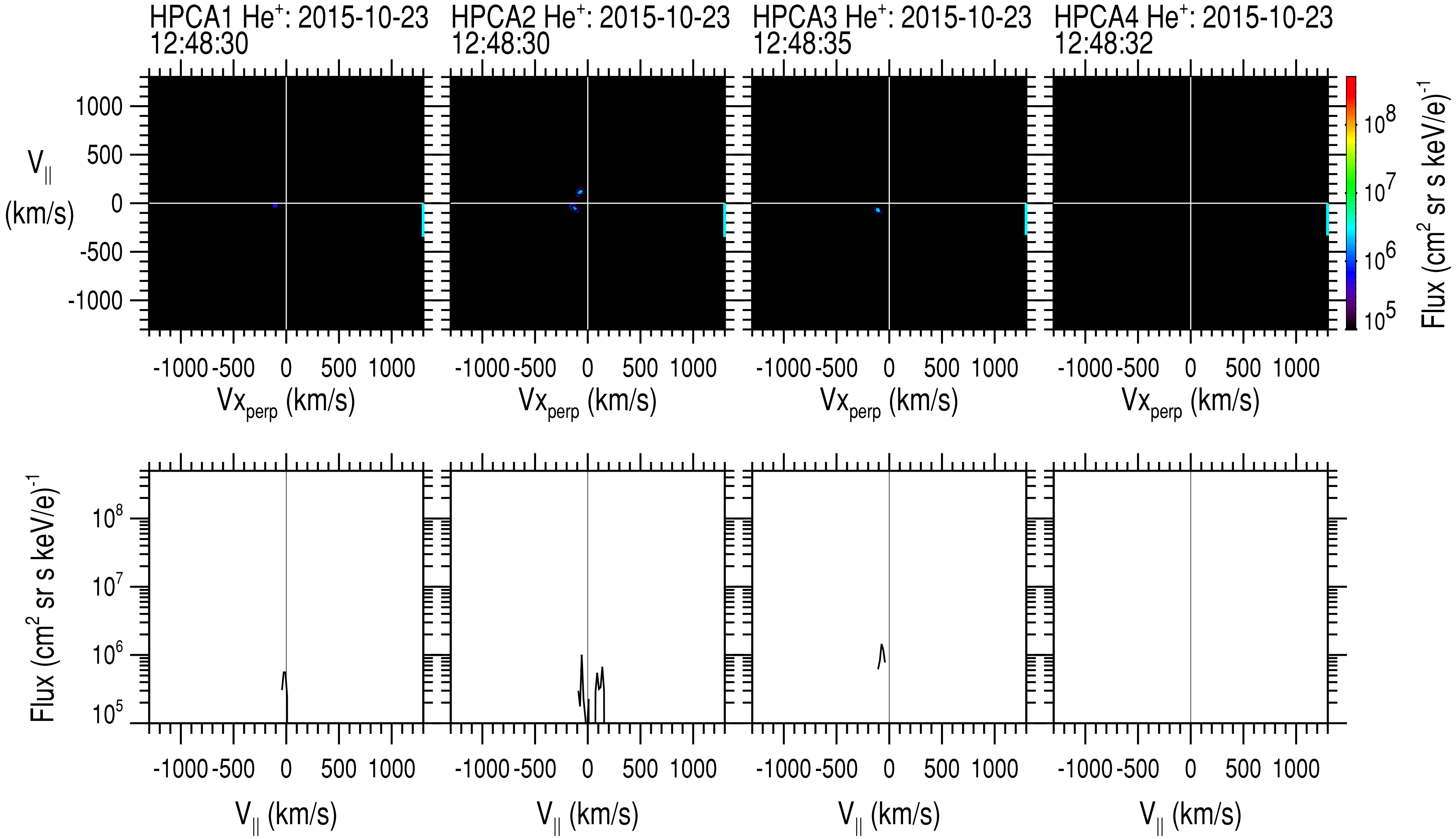
HPCA1 He+: 2015-10-23 12:48:30
HPCA2 He+: 2015-10-23 12:48:30
HPCA3 He+: 2015-10-23 12:48:35
HPCA4 He+: 2015-10-23 12:48:32
V|| (km/s)
Vxperp (km/s)
Flux (cm2 sr s keV/e)-1
1000
500
0
-500
-1000
105
106
107
108

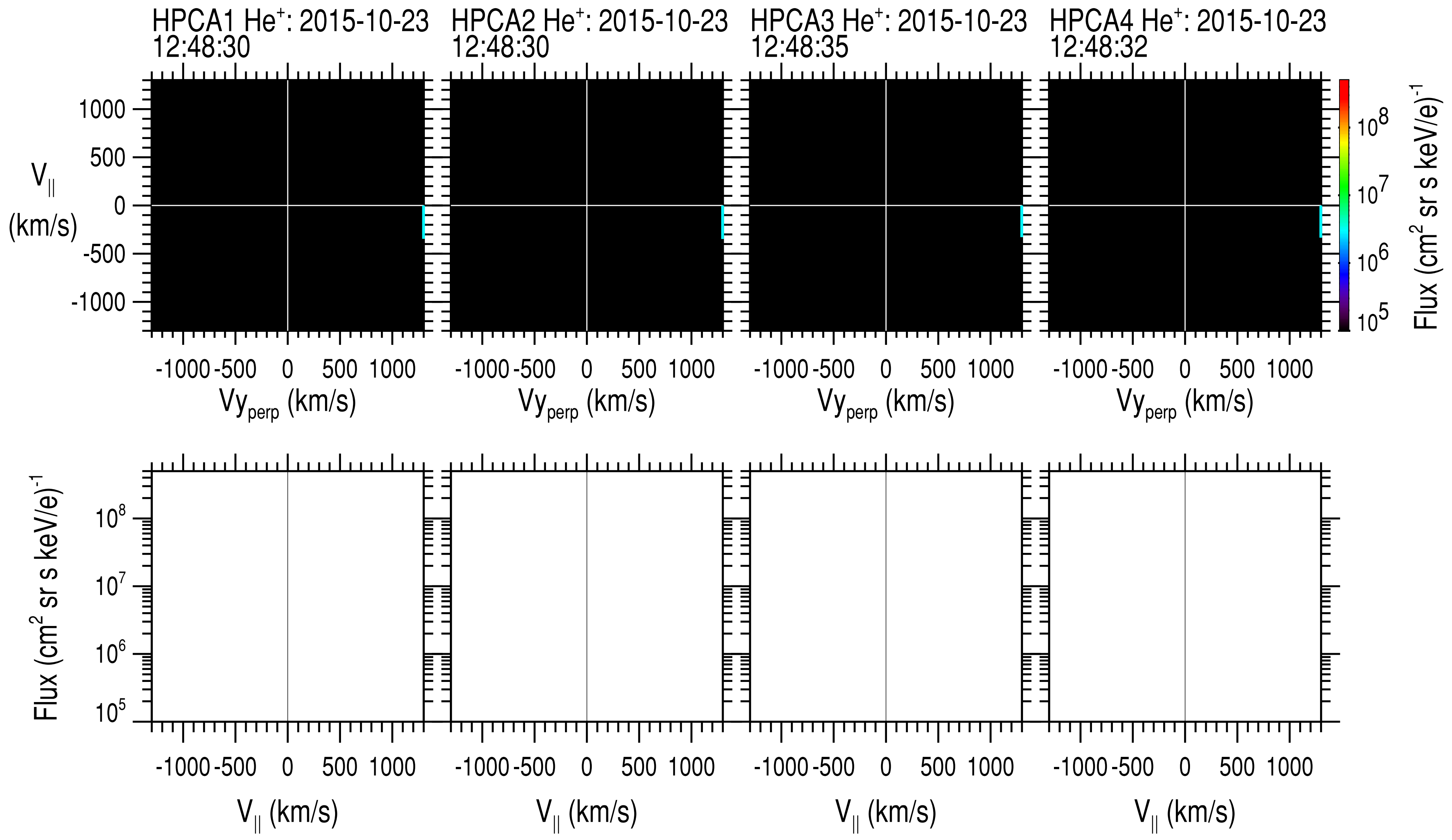

HPCA1 He+: 2015-10-23 12:48:30
HPCA2 He+: 2015-10-23 12:48:30
HPCA3 He+: 2015-10-23 12:48:35
HPCA4 He+: 2015-10-23 12:48:32
V|| (km/s)
Vyperp (km/s)
Flux (cm2 sr s keV/e)-1
-1000 -500 0 500 1000
10^5 10^6 10^7 10^8

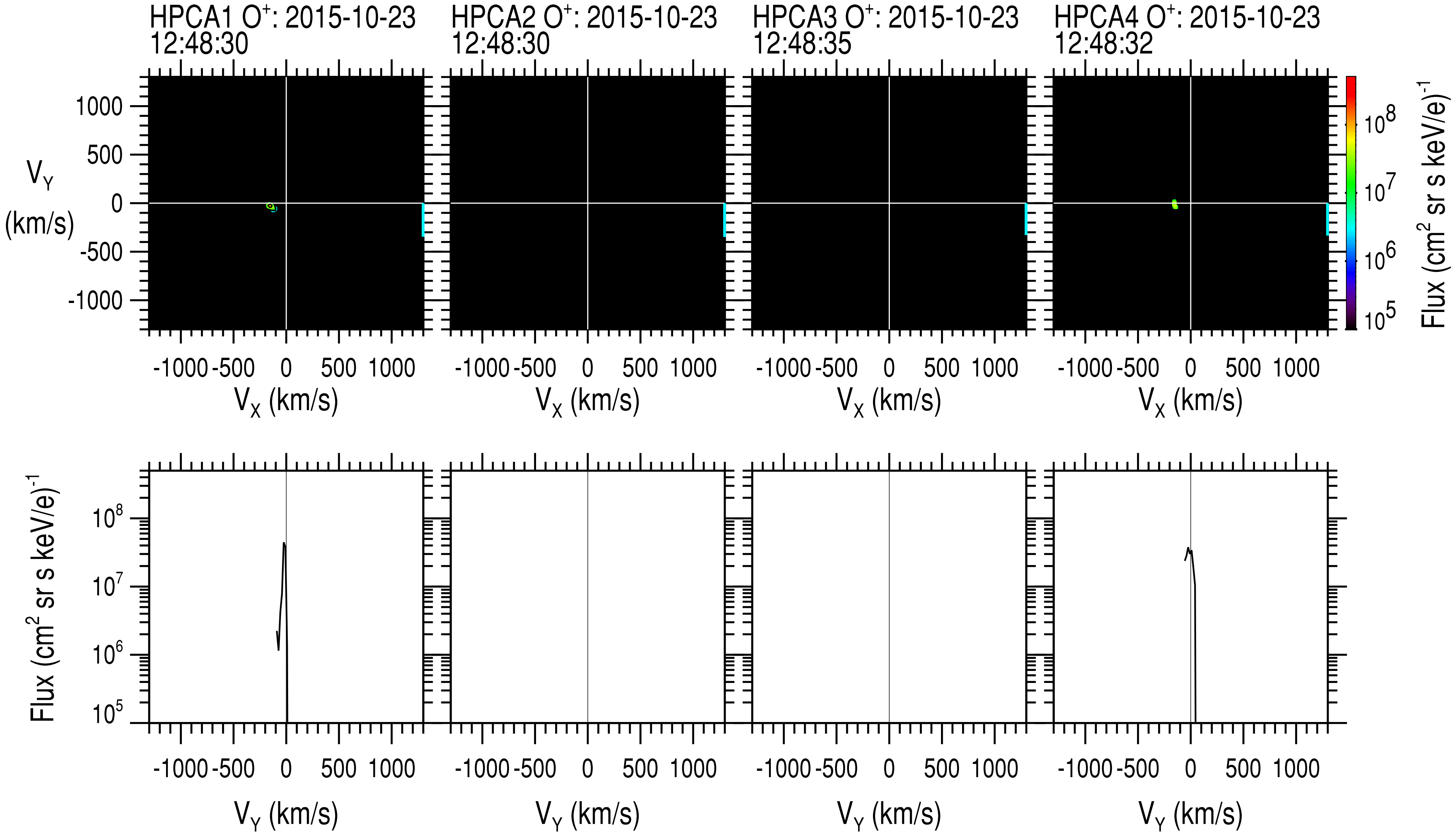

HPCA1 O+: 2015-10-23 12:48:30
HPCA2 O+: 2015-10-23 12:48:30
HPCA3 O+: 2015-10-23 12:48:35
HPCA4 O+: 2015-10-23 12:48:32
VY (km/s)
VX (km/s)
Flux (cm2 sr s keV/e)-1
VY (km/s)

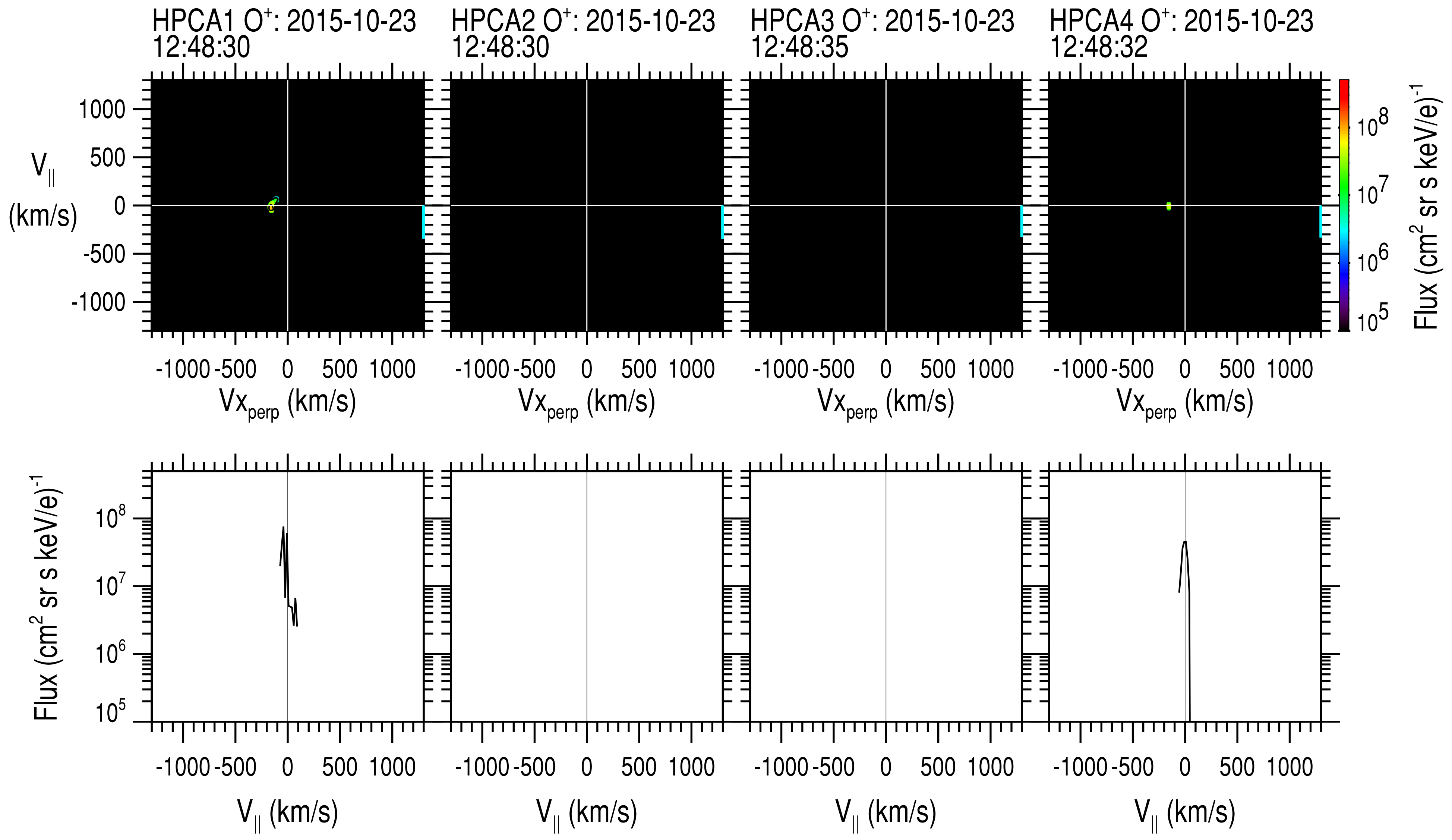
HPCA1 O+: 2015-10-23 12:48:30
HPCA2 O+: 2015-10-23 12:48:30
HPCA3 O+: 2015-10-23 12:48:35
HPCA4 O+: 2015-10-23 12:48:32
V|| (km/s)
Vxperp (km/s)
Flux (cm2 sr s keV/e)-1
1000
500
0
-500
-1000
105
106
107
108

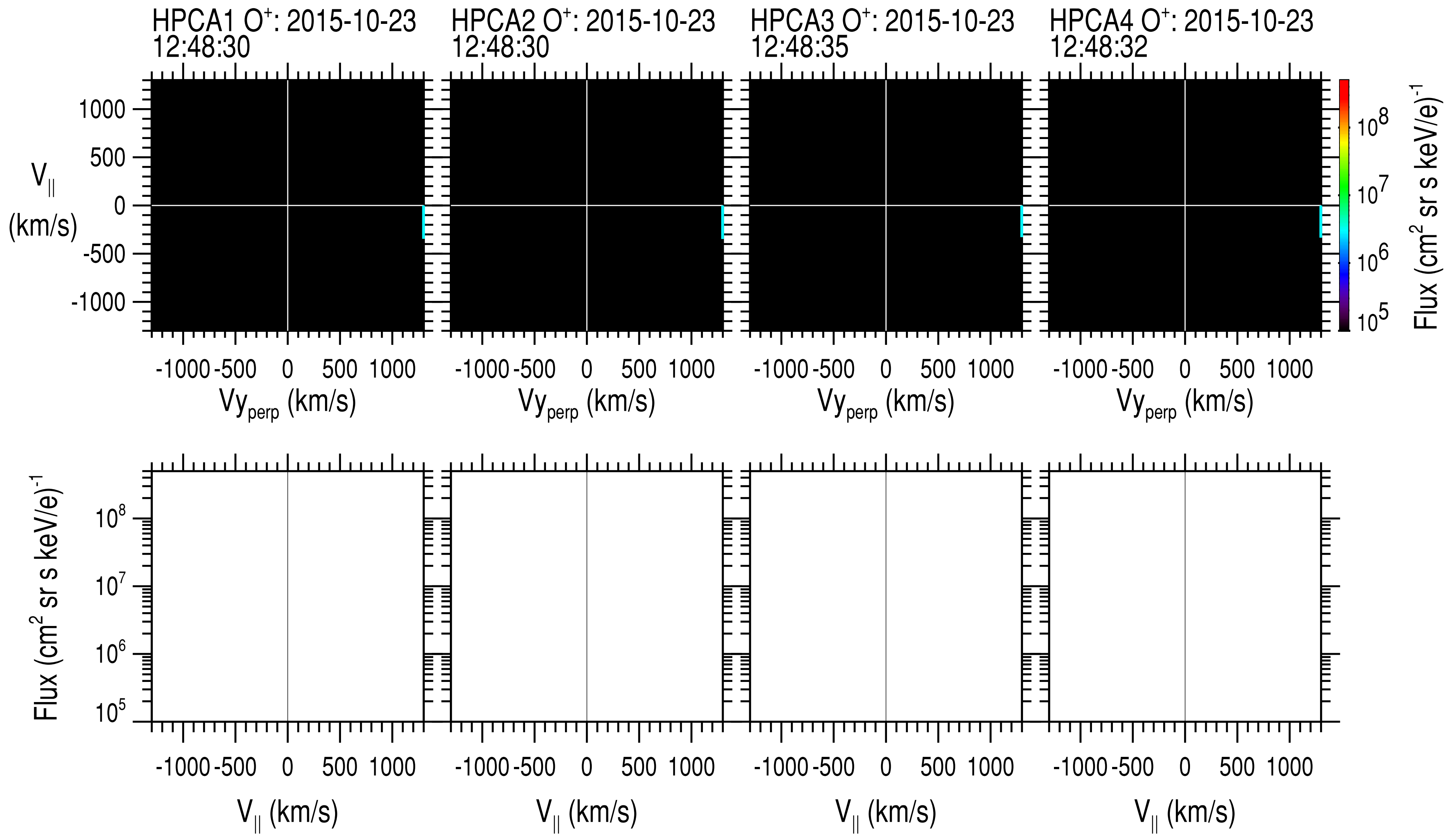

HPCA1 O⁺: 2015-10-23 12:48:30
HPCA2 O⁺: 2015-10-23 12:48:30
HPCA3 O⁺: 2015-10-23 12:48:35
HPCA4 O⁺: 2015-10-23 12:48:32
V|| (km/s)
Vy perp (km/s)
Flux (cm² sr s keV/e)⁻¹
1000 500 0 -500 -1000
-1000 -500 0 500 1000
10⁵ 10⁶ 10⁷ 10⁸

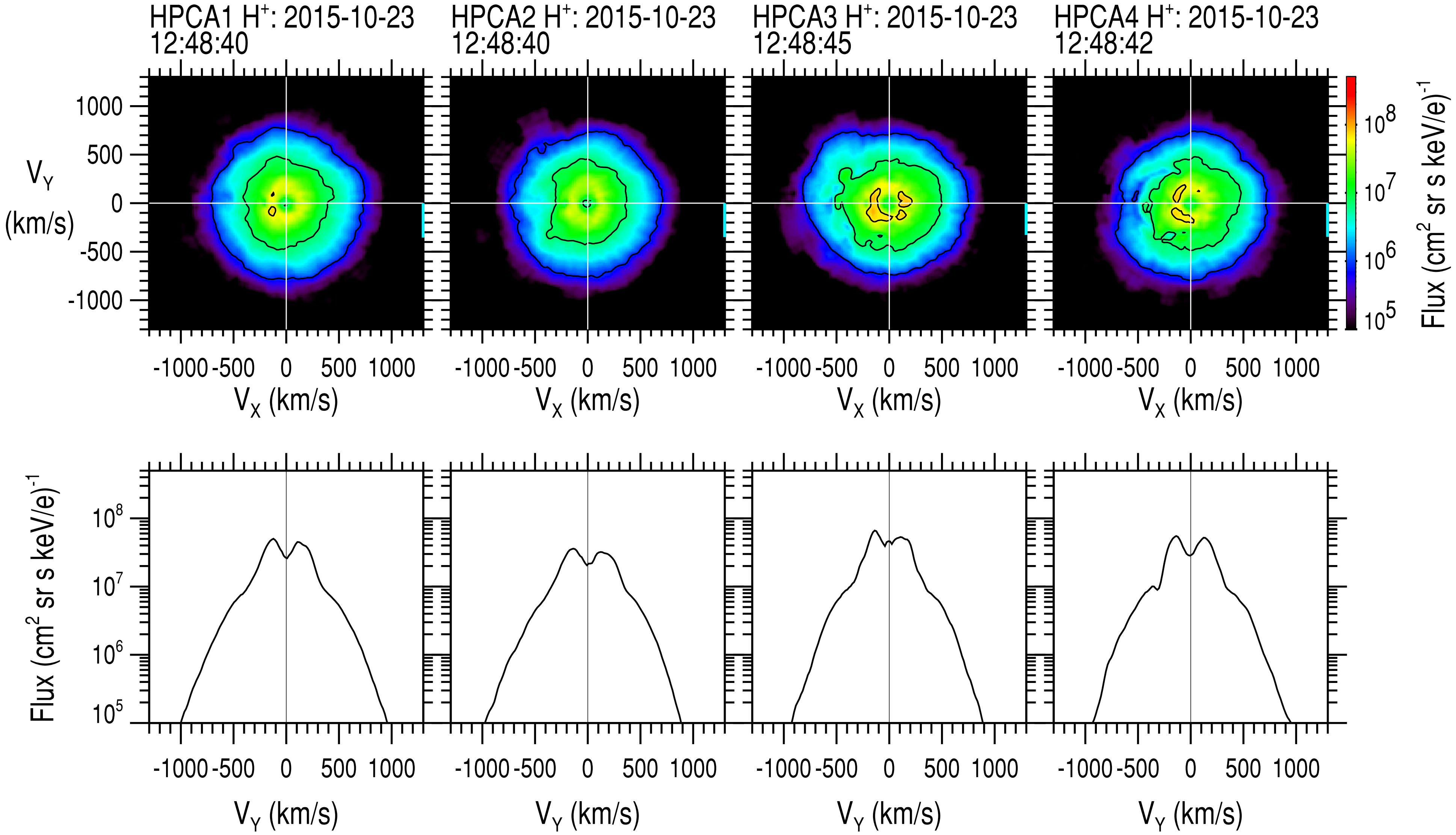
HPCA1 H+: 2015-10-23 12:48:40
HPCA2 H+: 2015-10-23 12:48:40
HPCA3 H+: 2015-10-23 12:48:45
HPCA4 H+: 2015-10-23 12:48:42
V_Y (km/s)
V_X (km/s)
Flux (cm^2 sr s keV/e)^-1
V_Y (km/s)

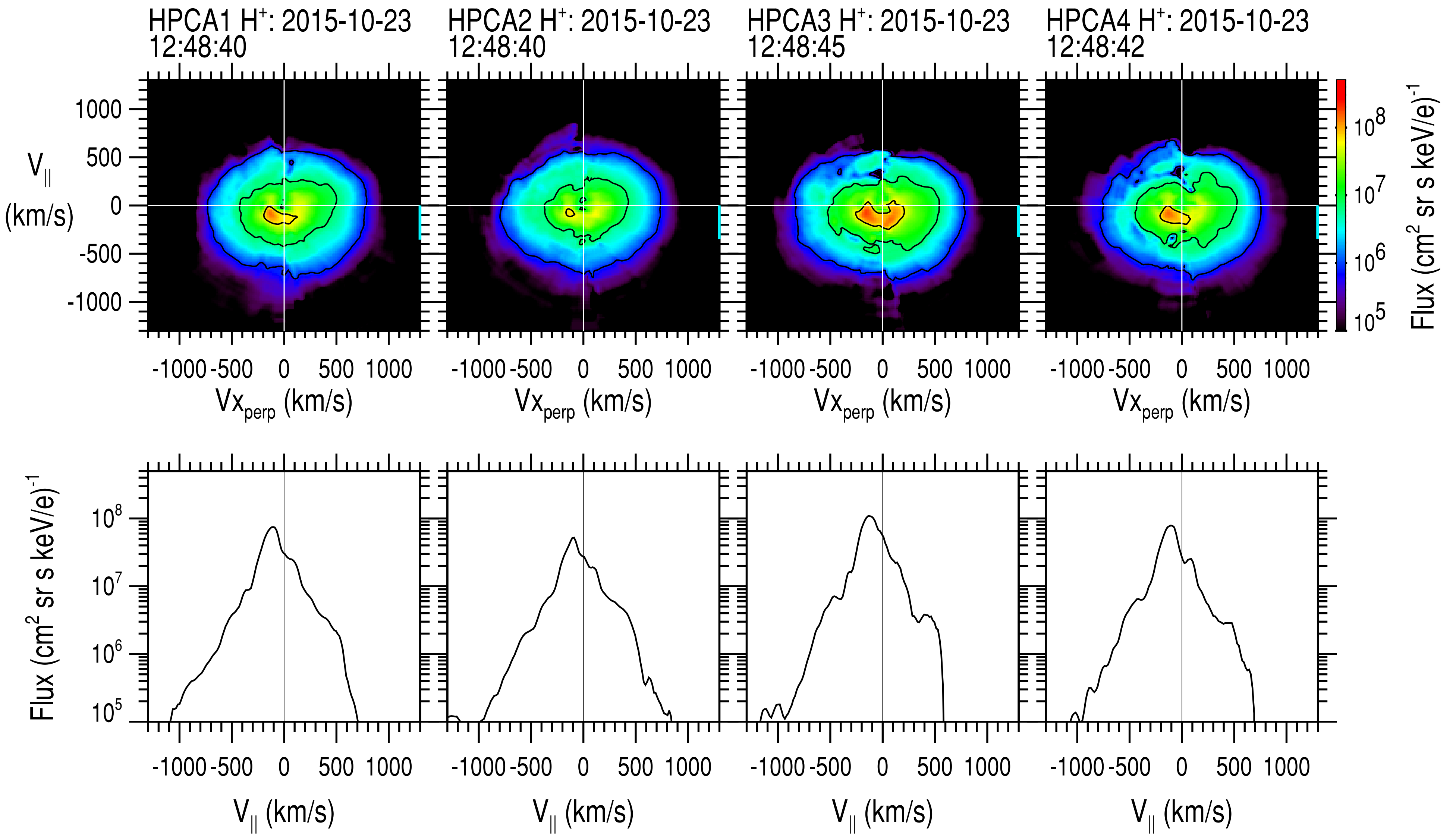

HPCA1 H⁺: 2015-10-23 12:48:40
HPCA2 H⁺: 2015-10-23 12:48:40
HPCA3 H⁺: 2015-10-23 12:48:45
HPCA4 H⁺: 2015-10-23 12:48:42
V|| (km/s)
Vx_perp (km/s)
Flux (cm² sr s keV/e)⁻¹
-1000 -500 0 500 1000
10⁵ 10⁶ 10⁷ 10⁸

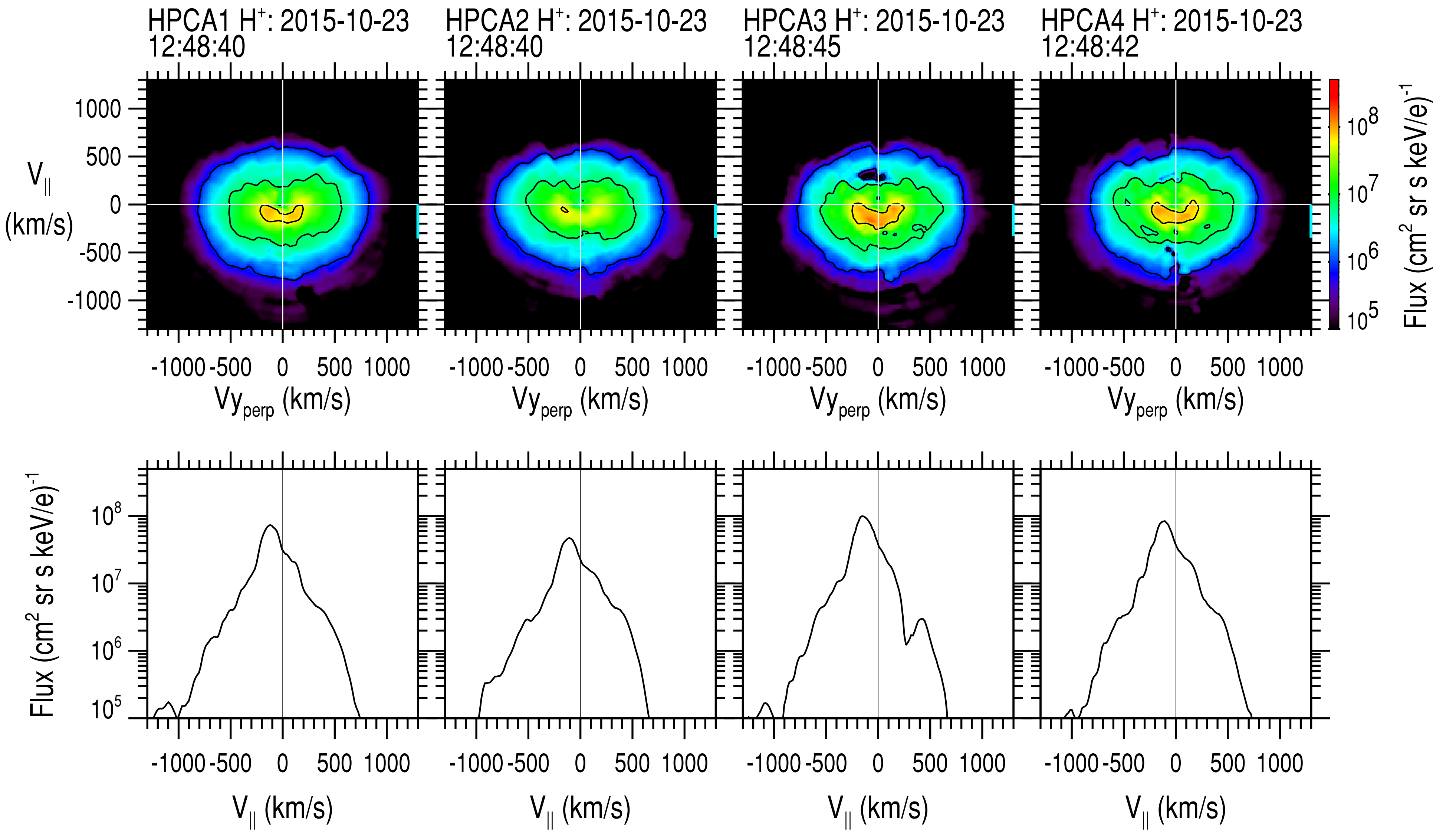

HPCA1 H+: 2015-10-23 12:48:40
HPCA2 H+: 2015-10-23 12:48:40
HPCA3 H+: 2015-10-23 12:48:45
HPCA4 H+: 2015-10-23 12:48:42
V|| (km/s)
Vyperp (km/s)
Flux (cm2 sr s keV/e)-1

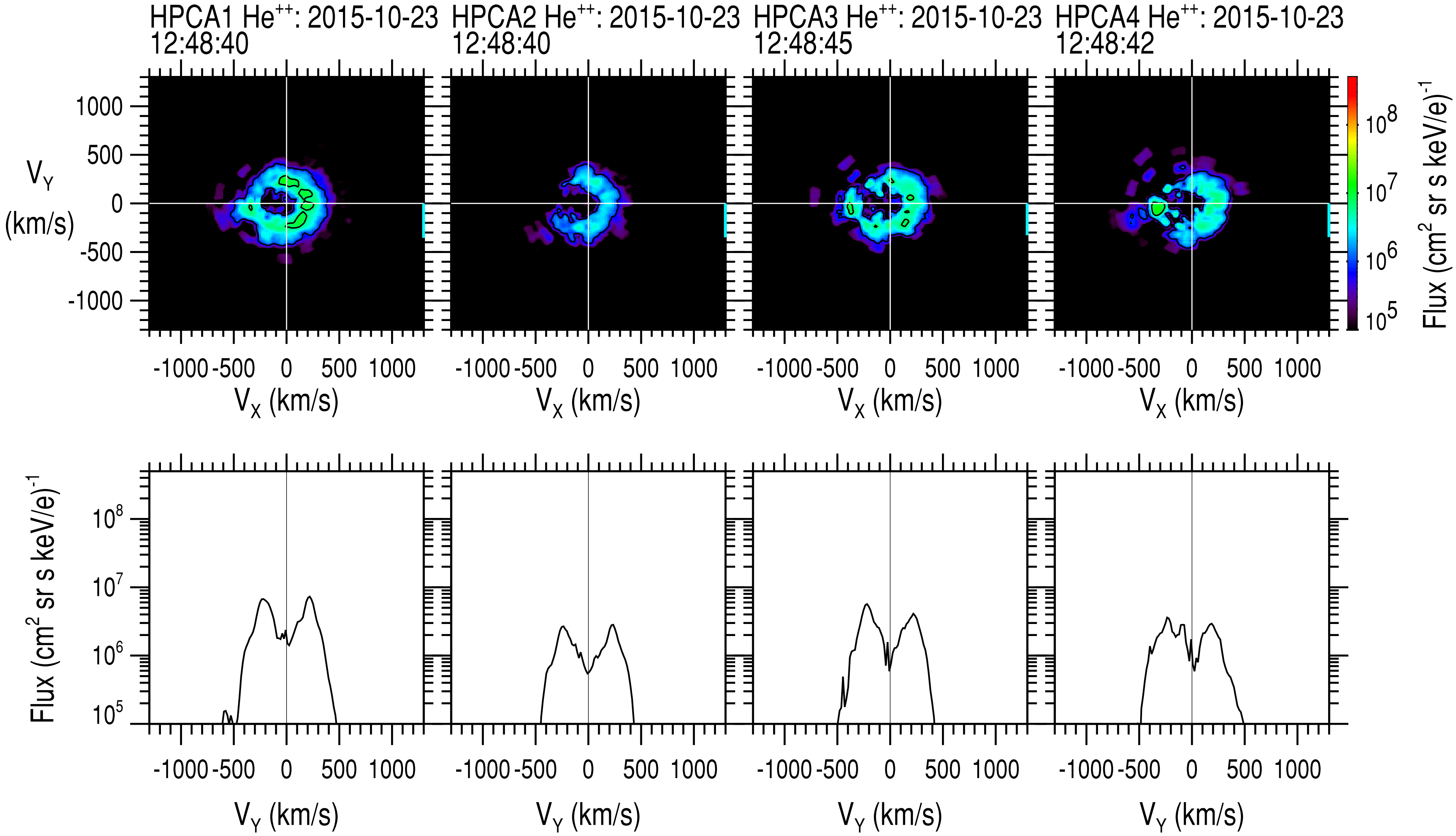
HPCA1 He++: 2015-10-23
12:48:40
HPCA2 He++: 2015-10-23
12:48:40
HPCA3 He++: 2015-10-23
12:48:45
HPCA4 He++: 2015-10-23
12:48:42
V_Y (km/s)
V_X (km/s)
Flux (cm^2 sr s keV/e)^-1
V_Y (km/s)

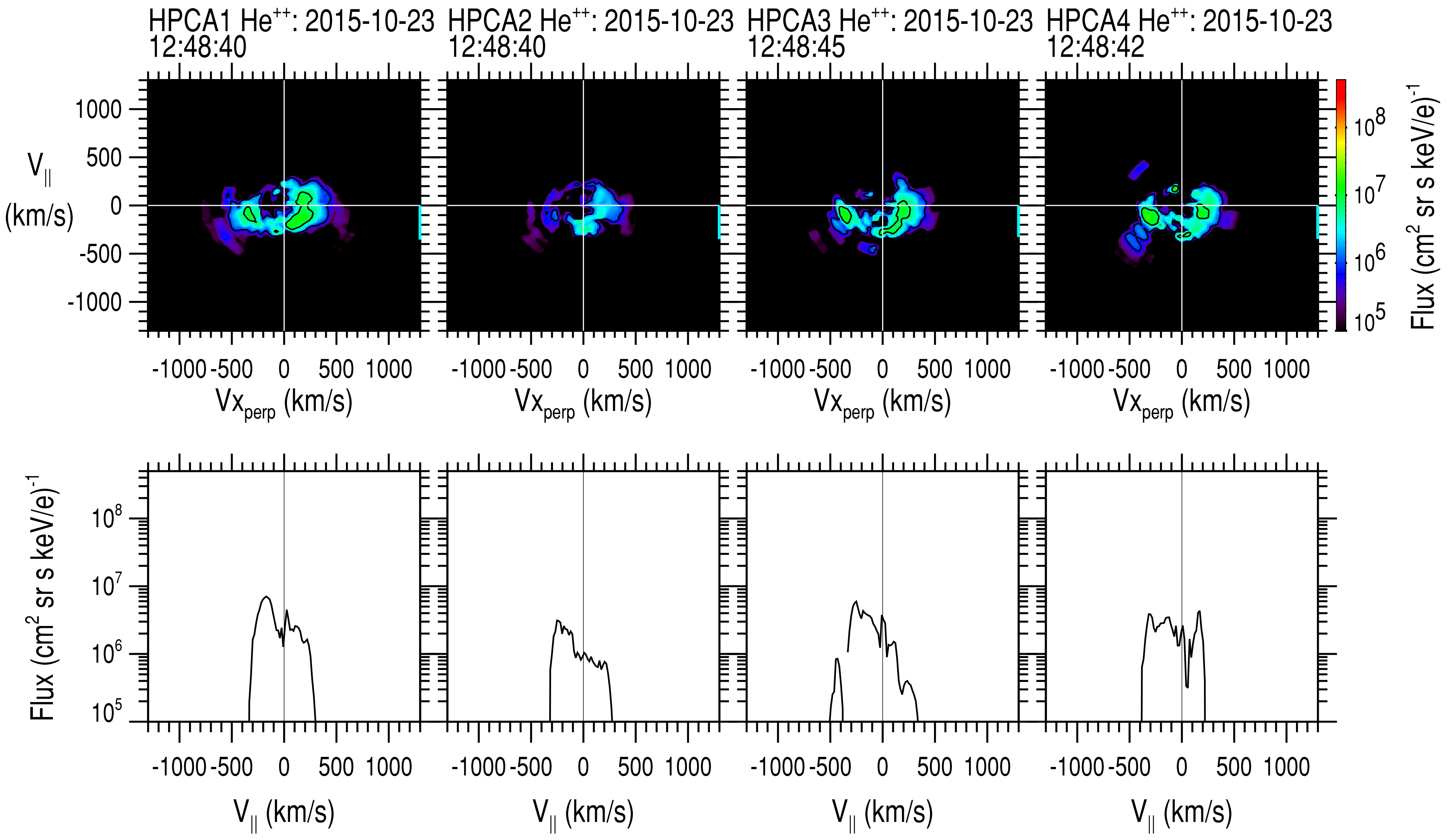
HPCA1 He++: 2015-10-23 12:48:40
HPCA2 He++: 2015-10-23 12:48:40
HPCA3 He++: 2015-10-23 12:48:45
HPCA4 He++: 2015-10-23 12:48:42
V|| (km/s)
Vxperp (km/s)
Flux (cm2 sr s keV/e)-1
-1000 -500 0 500 1000
10^5 10^6 10^7 10^8

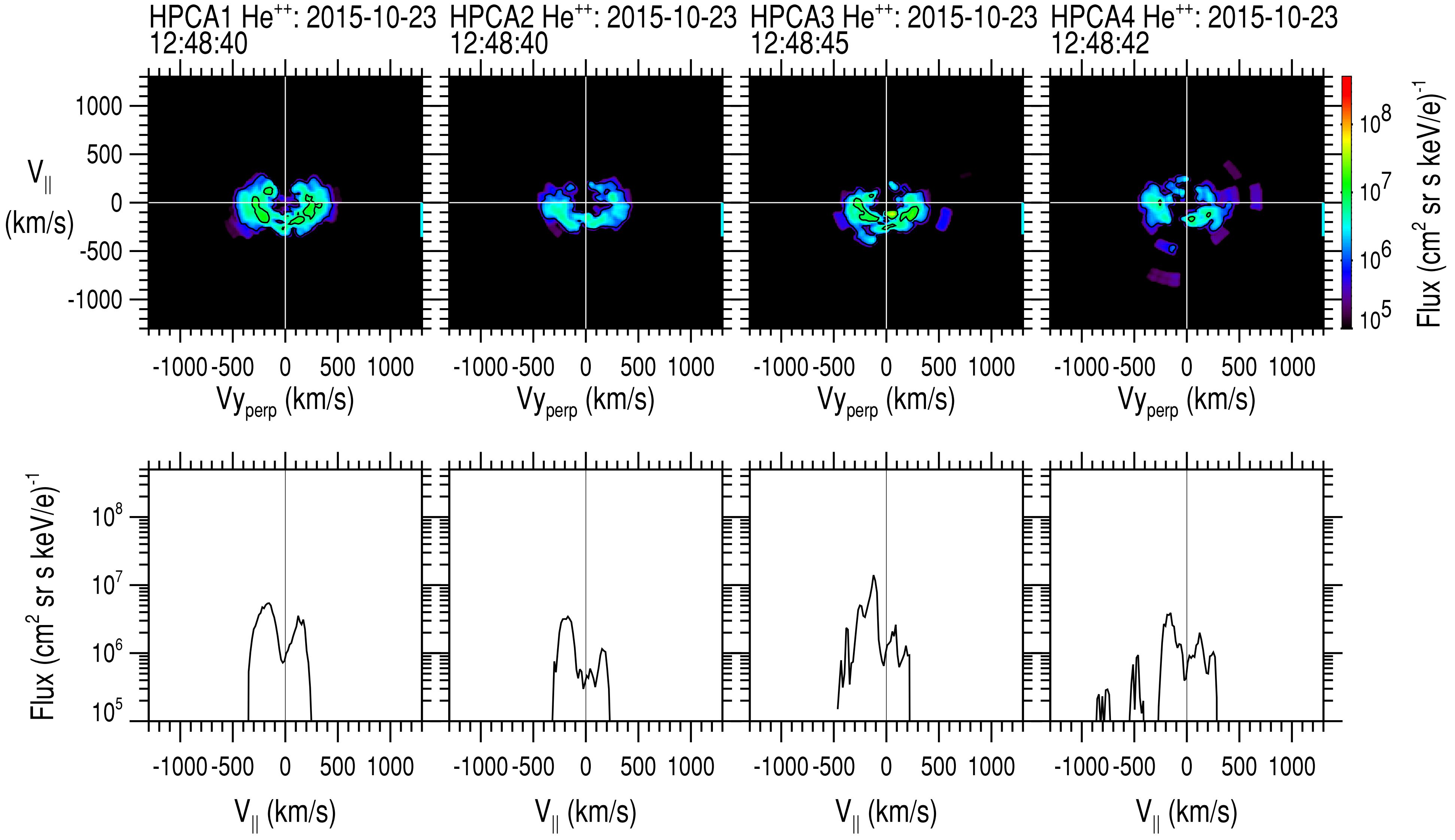

HPCA1 He++: 2015-10-23 12:48:40
HPCA2 He++: 2015-10-23 12:48:40
HPCA3 He++: 2015-10-23 12:48:45
HPCA4 He++: 2015-10-23 12:48:42
V|| (km/s)
Vyperp (km/s)
Flux (cm2 sr s keV/e)-1

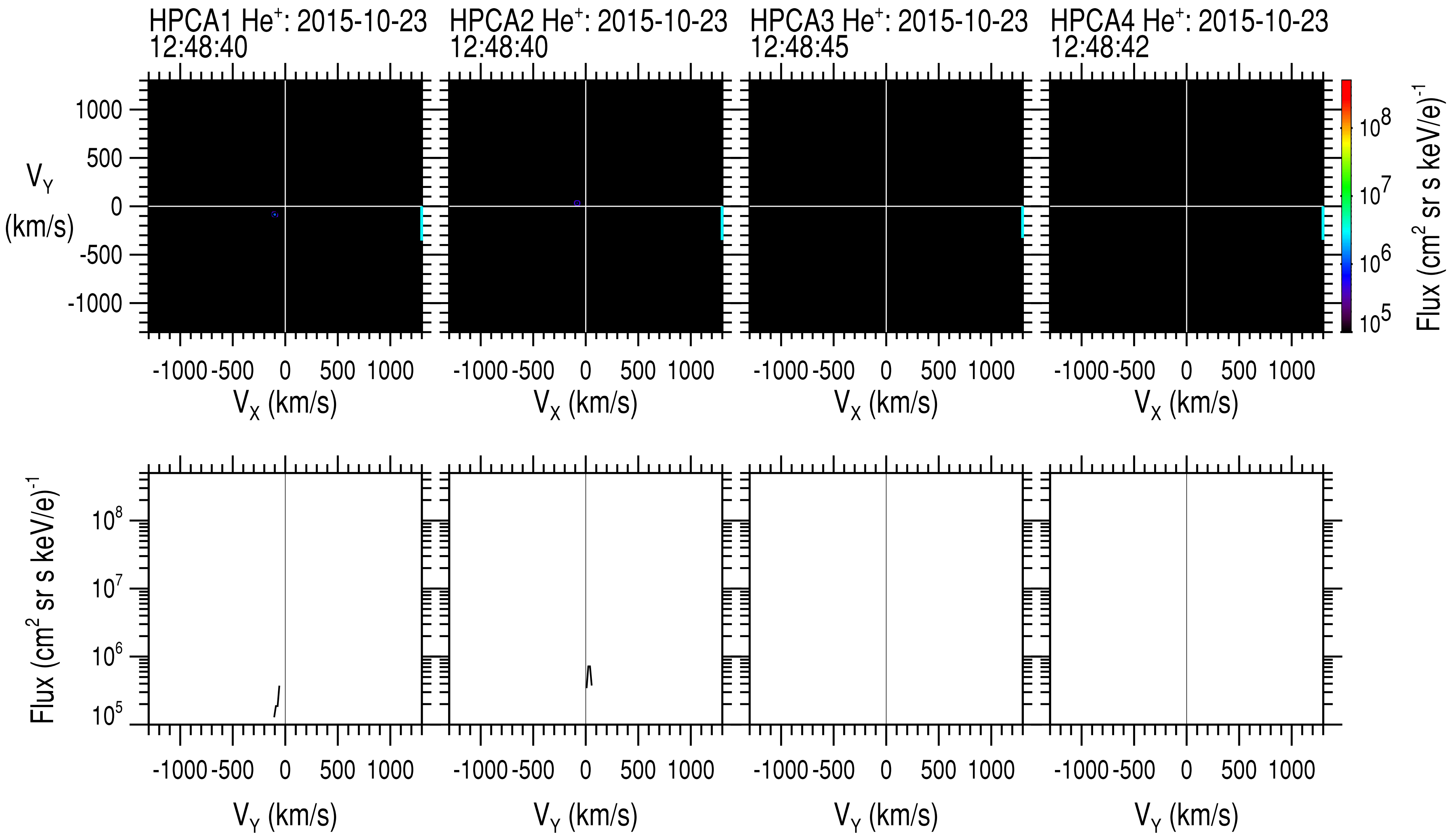

HPCA1 He+: 2015-10-23 12:48:40
HPCA2 He+: 2015-10-23 12:48:40
HPCA3 He+: 2015-10-23 12:48:45
HPCA4 He+: 2015-10-23 12:48:42
VY (km/s)
VX (km/s)
Flux (cm2 sr s keV/e)-1

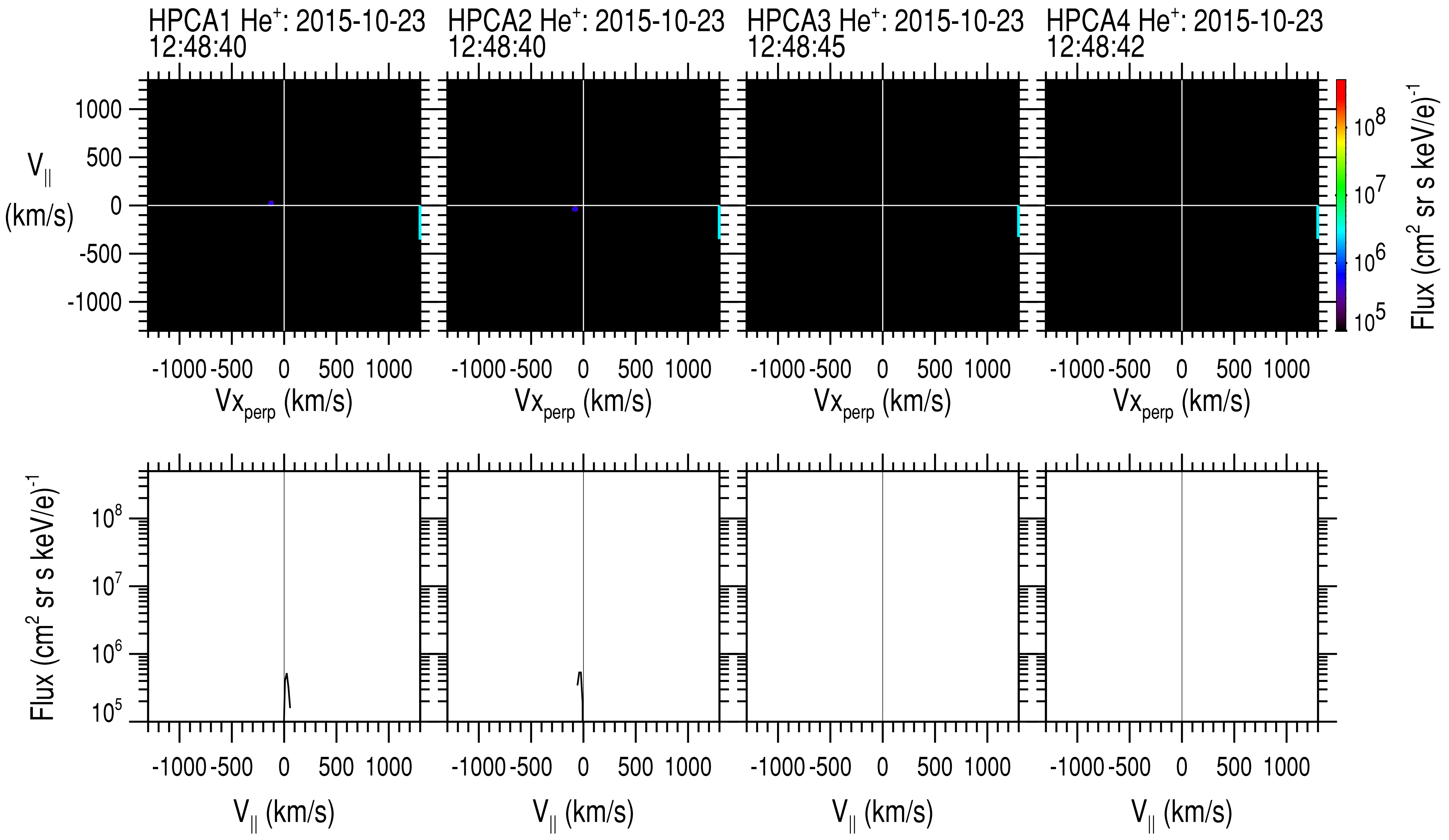
HPCA1 He+: 2015-10-23 12:48:40
HPCA2 He+: 2015-10-23 12:48:40
HPCA3 He+: 2015-10-23 12:48:45
HPCA4 He+: 2015-10-23 12:48:42
V|| (km/s)
Vxperp (km/s)
Flux (cm2 sr s keV/e)-1
V|| (km/s)

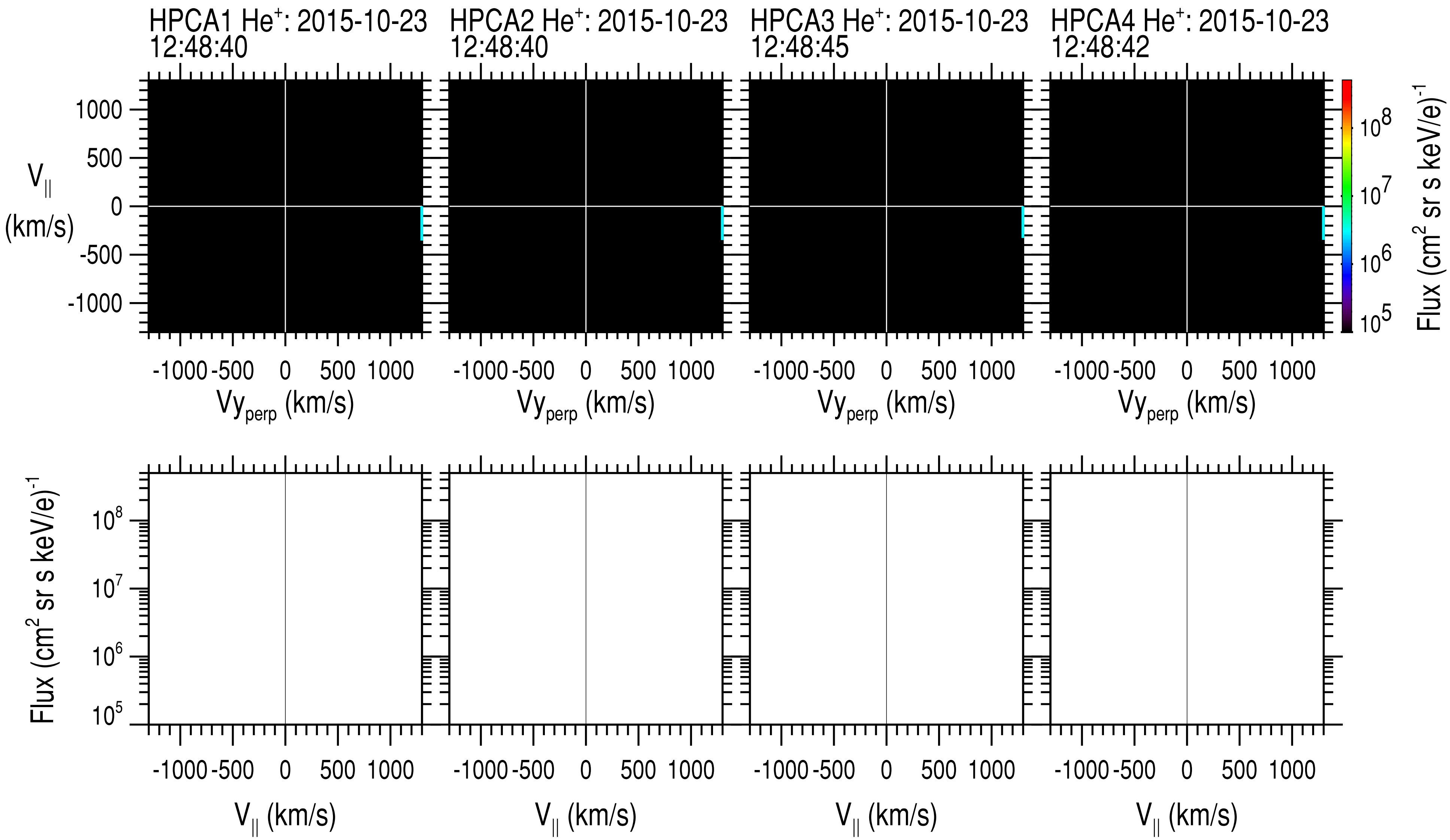
HPCA1 He+: 2015-10-23 12:48:40
HPCA2 He+: 2015-10-23 12:48:40
HPCA3 He+: 2015-10-23 12:48:45
HPCA4 He+: 2015-10-23 12:48:42
V|| (km/s)
Vyperp (km/s)
Flux (cm2 sr s keV/e)-1
-1000 -500 0 500 1000
10^5 10^6 10^7 10^8

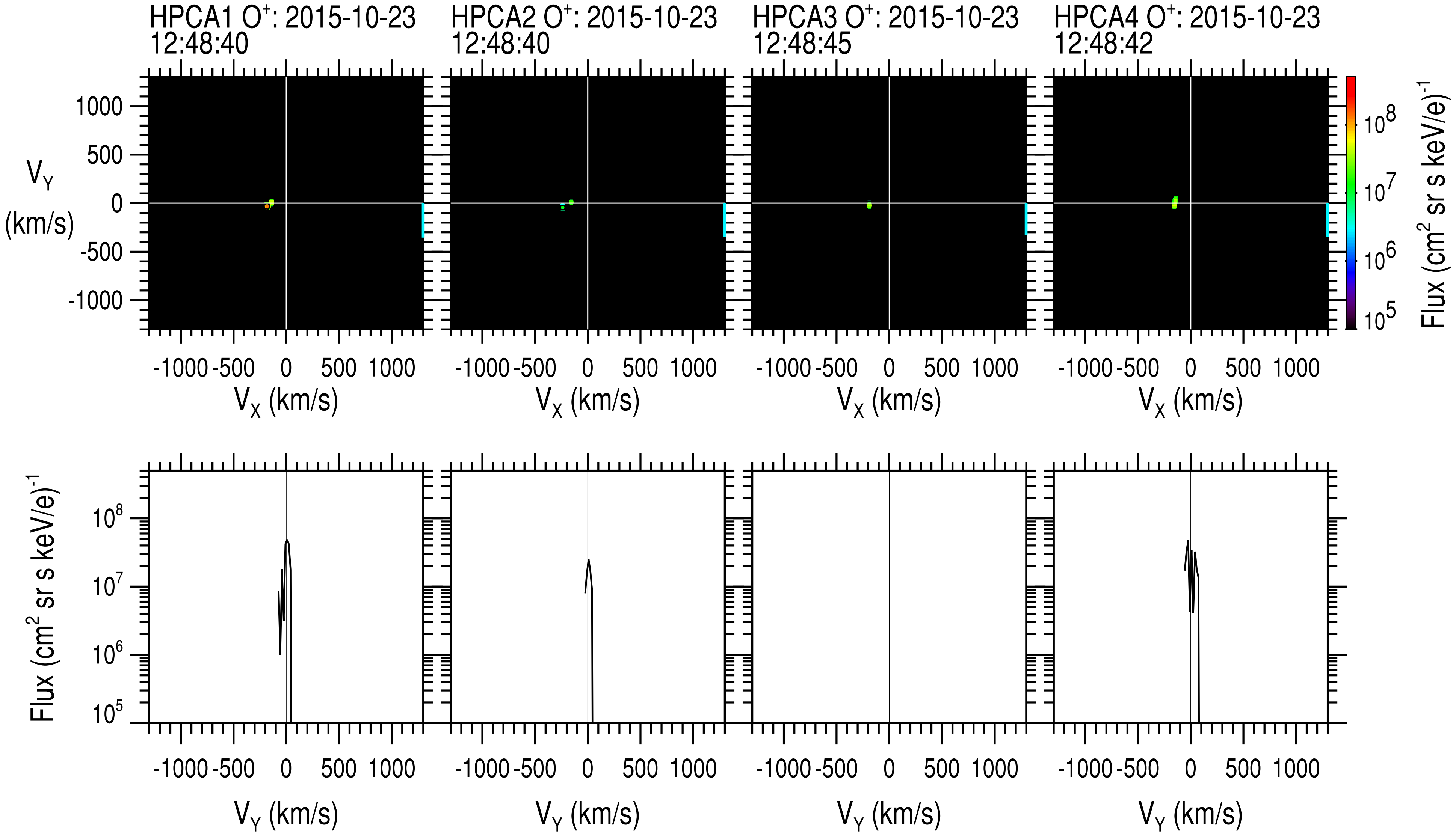
HPCA1 O+: 2015-10-23 12:48:40
HPCA2 O+: 2015-10-23 12:48:40
HPCA3 O+: 2015-10-23 12:48:45
HPCA4 O+: 2015-10-23 12:48:42
VY (km/s)
VX (km/s)
Flux (cm2 sr s keV/e)-1
VY (km/s)

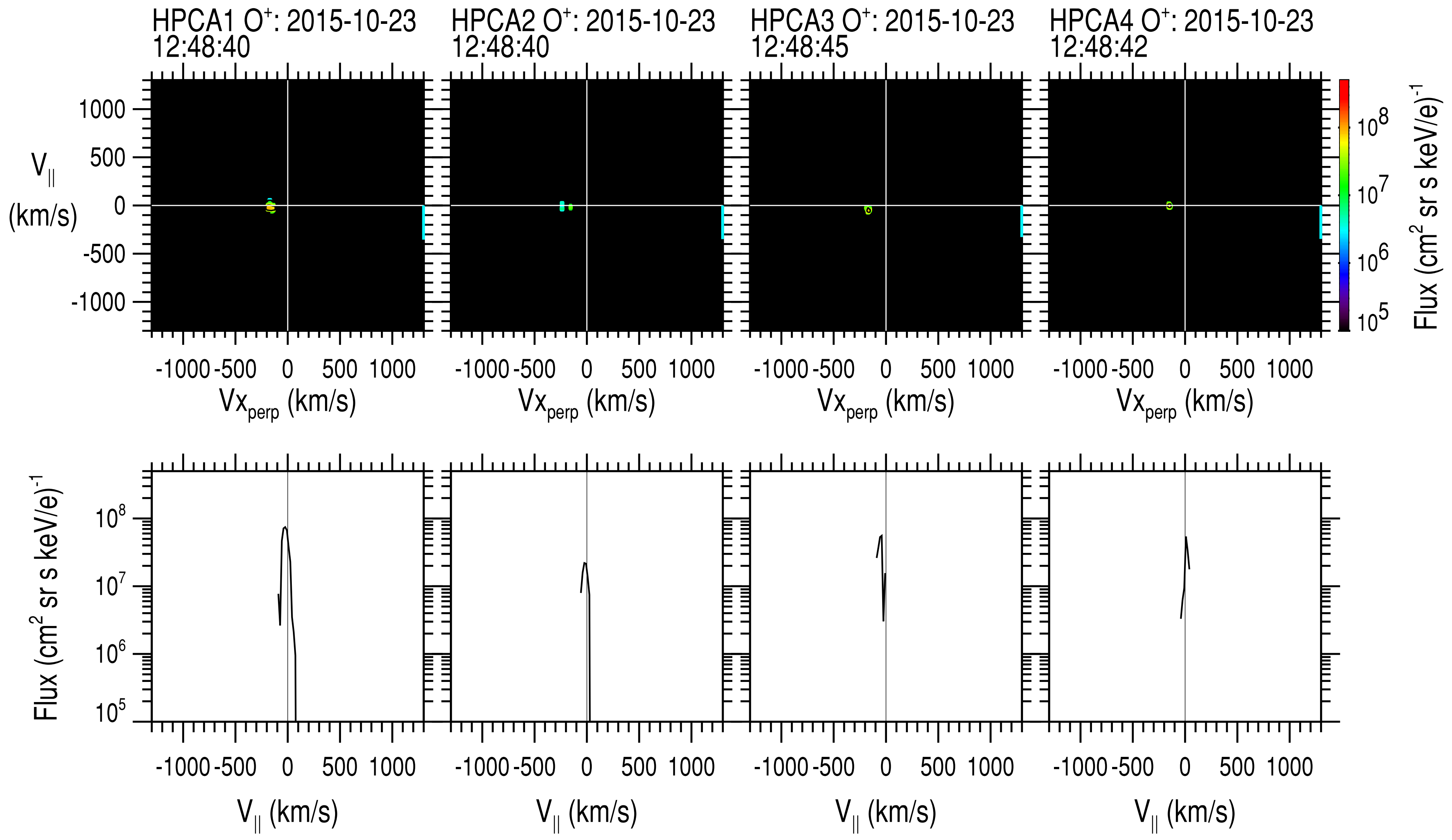
HPCA1 O+: 2015-10-23 12:48:40
HPCA2 O+: 2015-10-23 12:48:40
HPCA3 O+: 2015-10-23 12:48:45
HPCA4 O+: 2015-10-23 12:48:42
V|| (km/s)
Vxperp (km/s)
Flux (cm2 sr s keV/e)-1
-1000 -500 0 500 1000
10^5 10^6 10^7 10^8

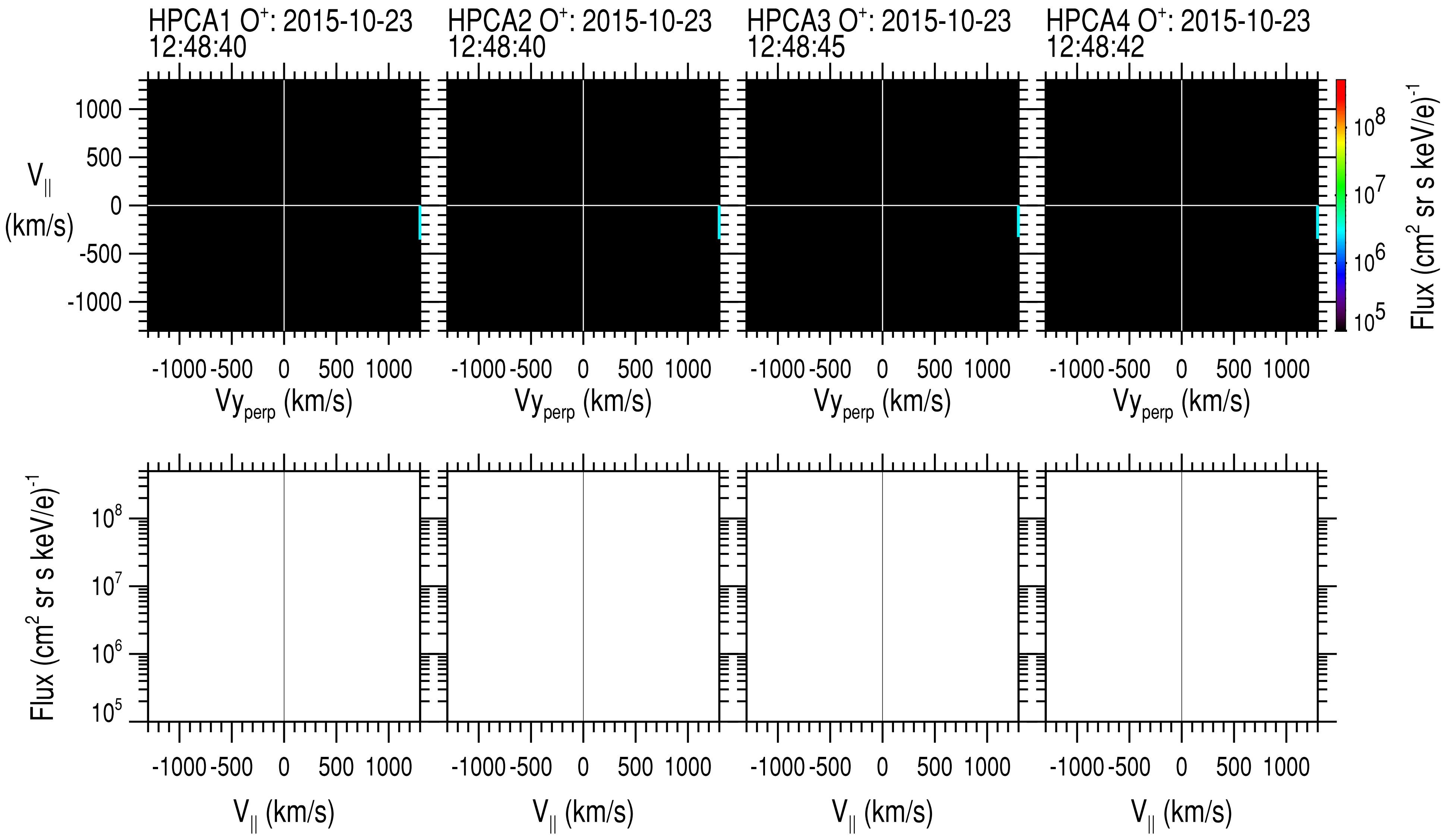
HPCA1 O+: 2015-10-23 12:48:40
HPCA2 O+: 2015-10-23 12:48:40
HPCA3 O+: 2015-10-23 12:48:45
HPCA4 O+: 2015-10-23 12:48:42
V|| (km/s)
Vyperp (km/s)
Flux (cm2 sr s keV/e)-1
V|| (km/s)

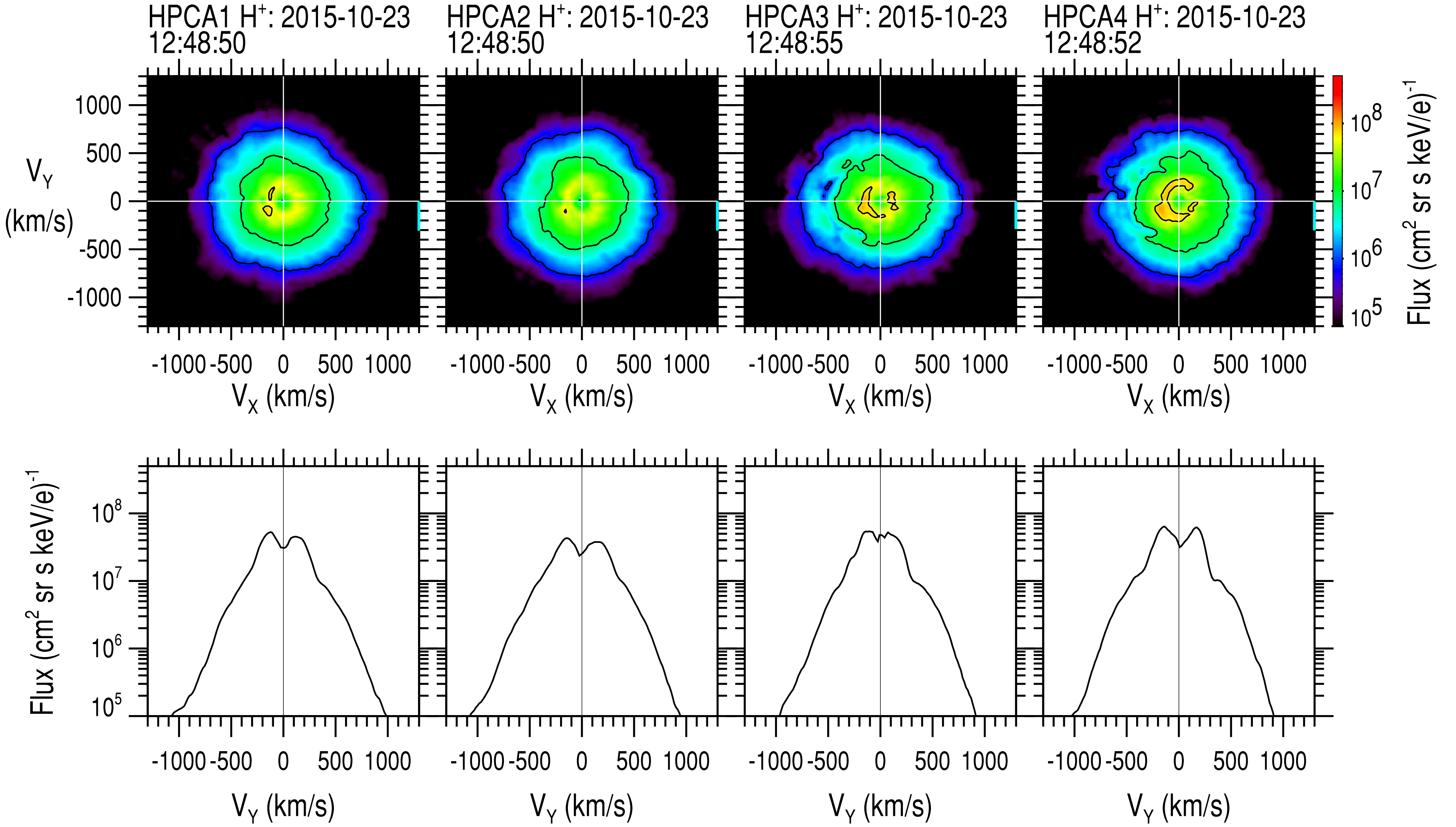

HPCA1 H+: 2015-10-23
12:48:50
HPCA2 H+: 2015-10-23
12:48:50
HPCA3 H+: 2015-10-23
12:48:55
HPCA4 H+: 2015-10-23
12:48:52
V_Y (km/s)
V_X (km/s)
Flux (cm^2 sr s keV/e)^-1
V_Y (km/s)

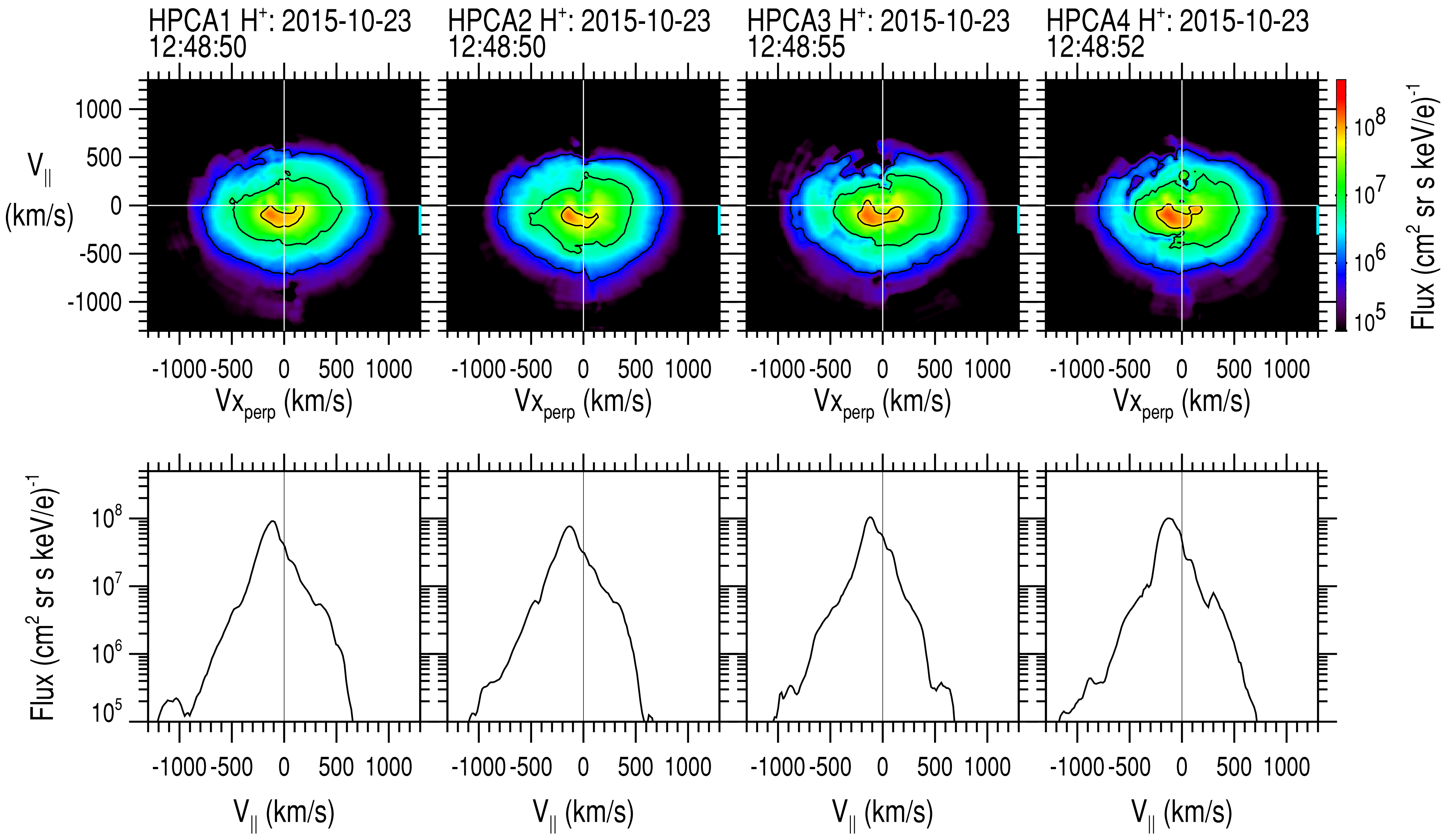
HPCA1 H+: 2015-10-23 12:48:50
HPCA2 H+: 2015-10-23 12:48:50
HPCA3 H+: 2015-10-23 12:48:55
HPCA4 H+: 2015-10-23 12:48:52
V|| (km/s)
Vxperp (km/s)
Flux (cm2 sr s keV/e)-1
V|| (km/s)
-1000 -500 0 500 1000
105 106 107 108

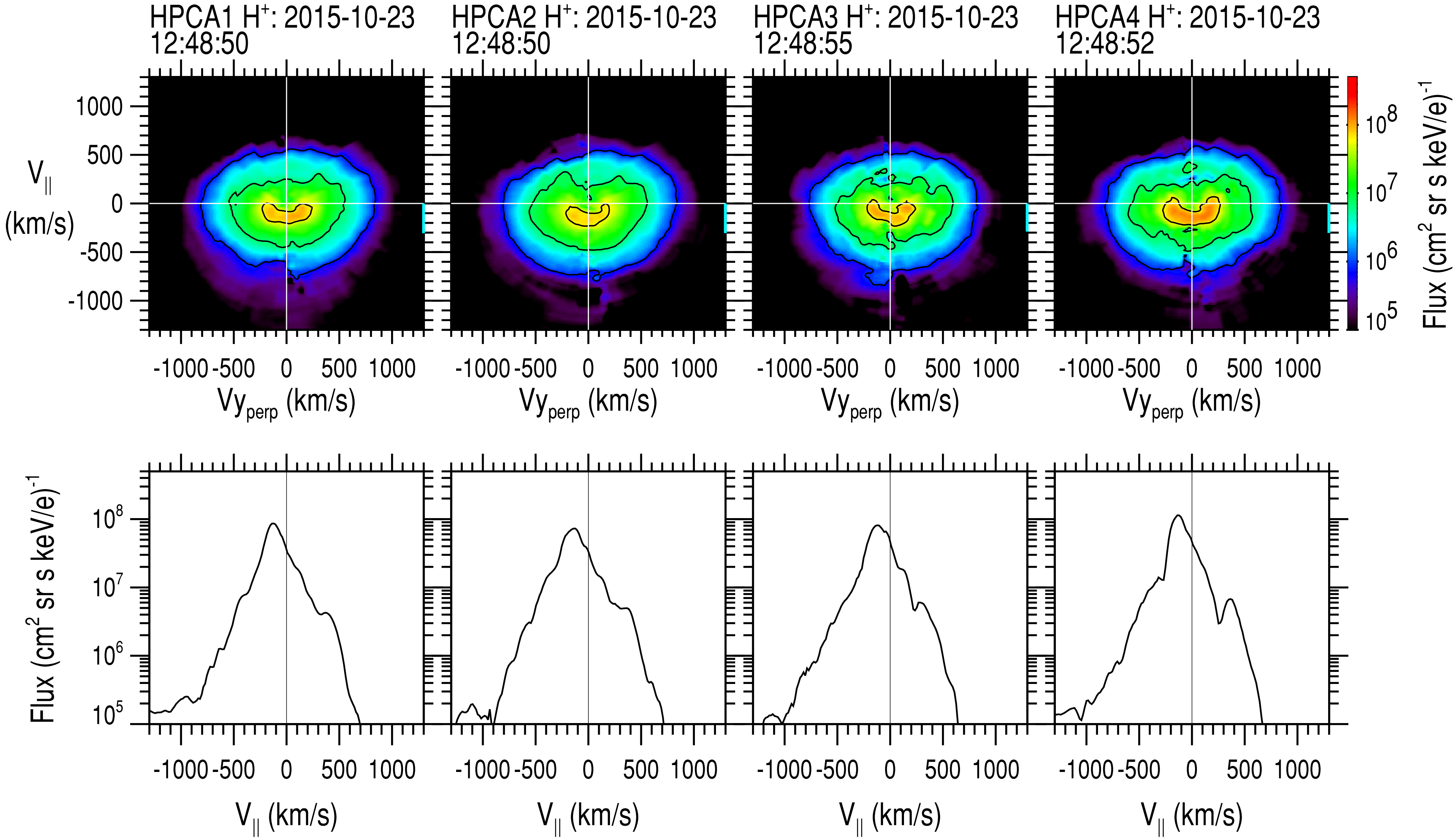
HPCA1 H+: 2015-10-23
12:48:50
HPCA2 H+: 2015-10-23
12:48:50
HPCA3 H+: 2015-10-23
12:48:55
HPCA4 H+: 2015-10-23
12:48:52
V||
(km/s)
Vyperp (km/s)
Flux (cm2 sr s keV/e)-1
V|| (km/s)

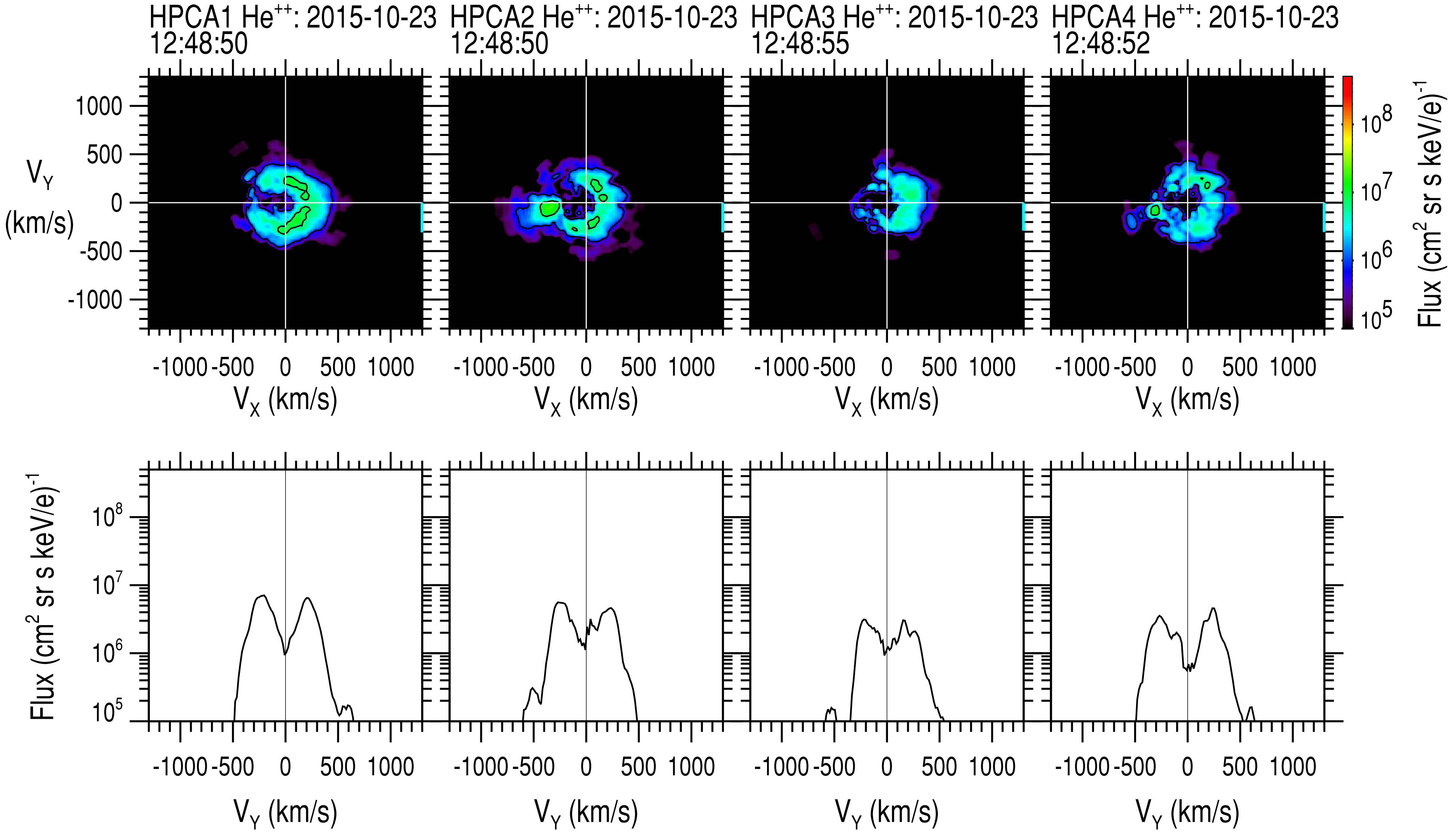

HPCA1 He++: 2015-10-23 12:48:50
HPCA2 He++: 2015-10-23 12:48:50
HPCA3 He++: 2015-10-23 12:48:55
HPCA4 He++: 2015-10-23 12:48:52
VY (km/s)
VX (km/s)
Flux (cm2 sr s keV/e)-1
VY (km/s)

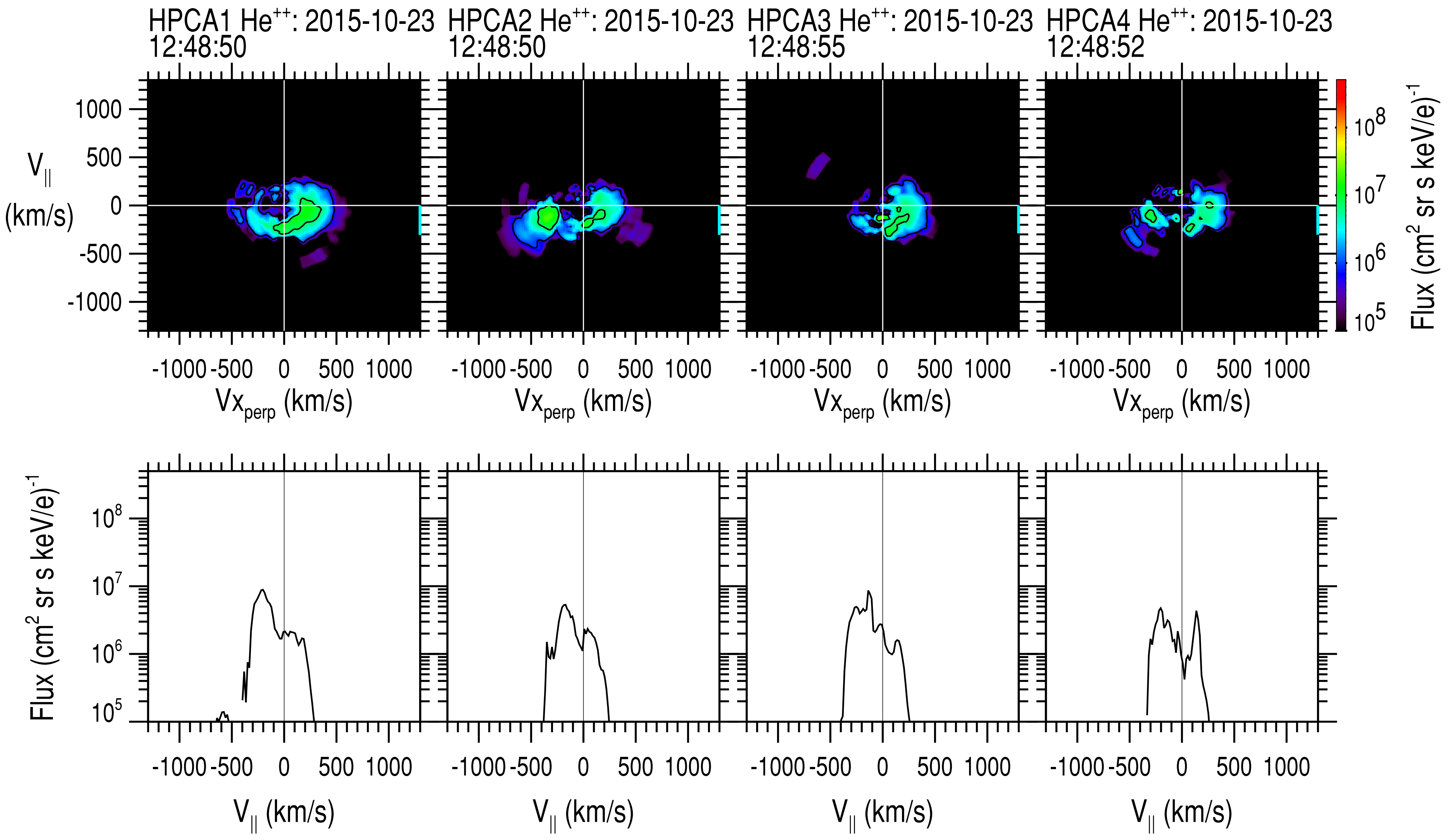
HPCA1 He++: 2015-10-23 12:48:50
HPCA2 He++: 2015-10-23 12:48:50
HPCA3 He++: 2015-10-23 12:48:55
HPCA4 He++: 2015-10-23 12:48:52
V|| (km/s)
Vxperp (km/s)
Flux (cm2 sr s keV/e)-1
V|| (km/s)

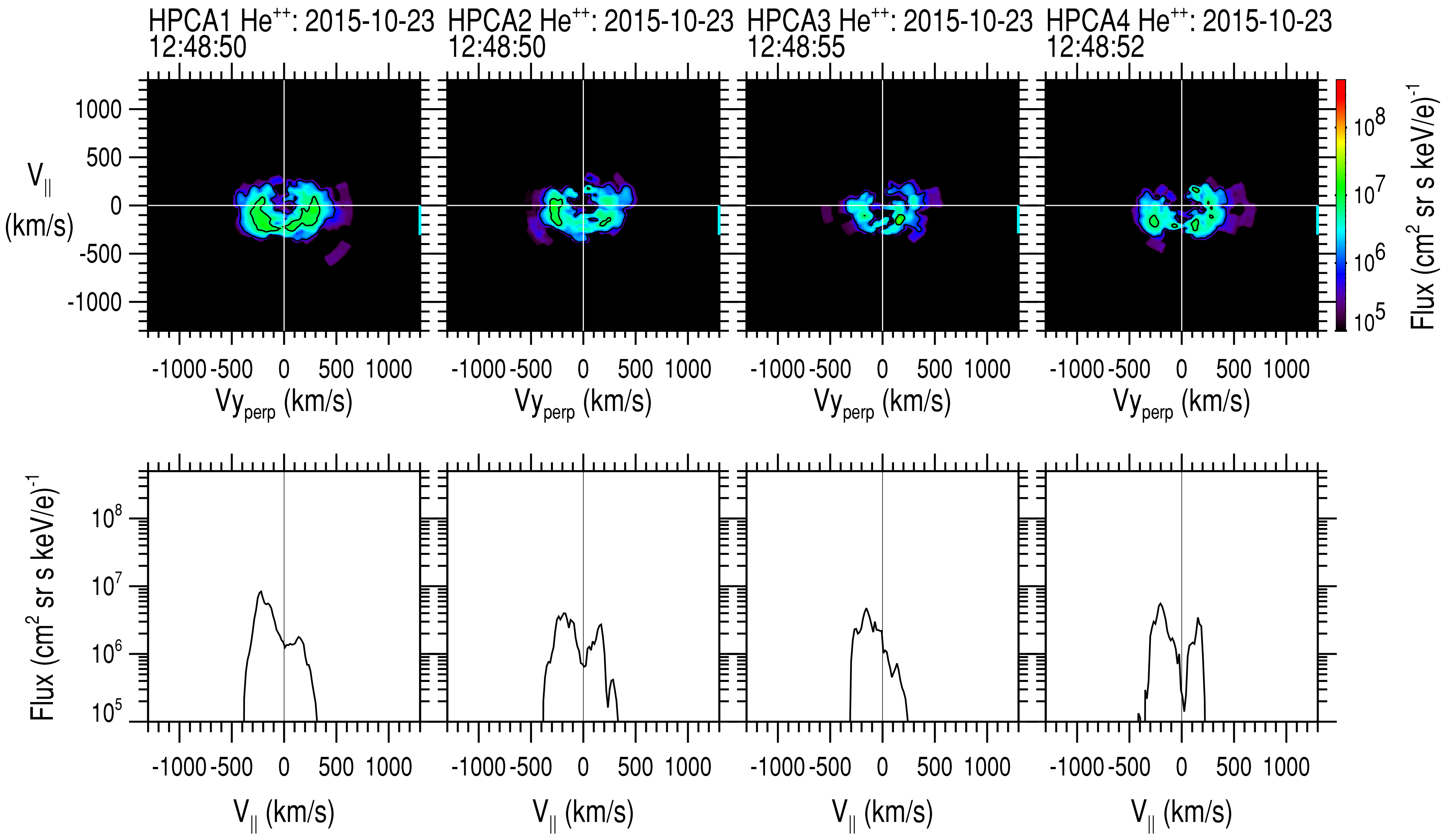
HPCA1 He++: 2015-10-23 12:48:50
HPCA2 He++: 2015-10-23 12:48:50
HPCA3 He++: 2015-10-23 12:48:55
HPCA4 He++: 2015-10-23 12:48:52
V|| (km/s)
Vyperp (km/s)
Flux (cm2 sr s keV/e)-1
V|| (km/s)

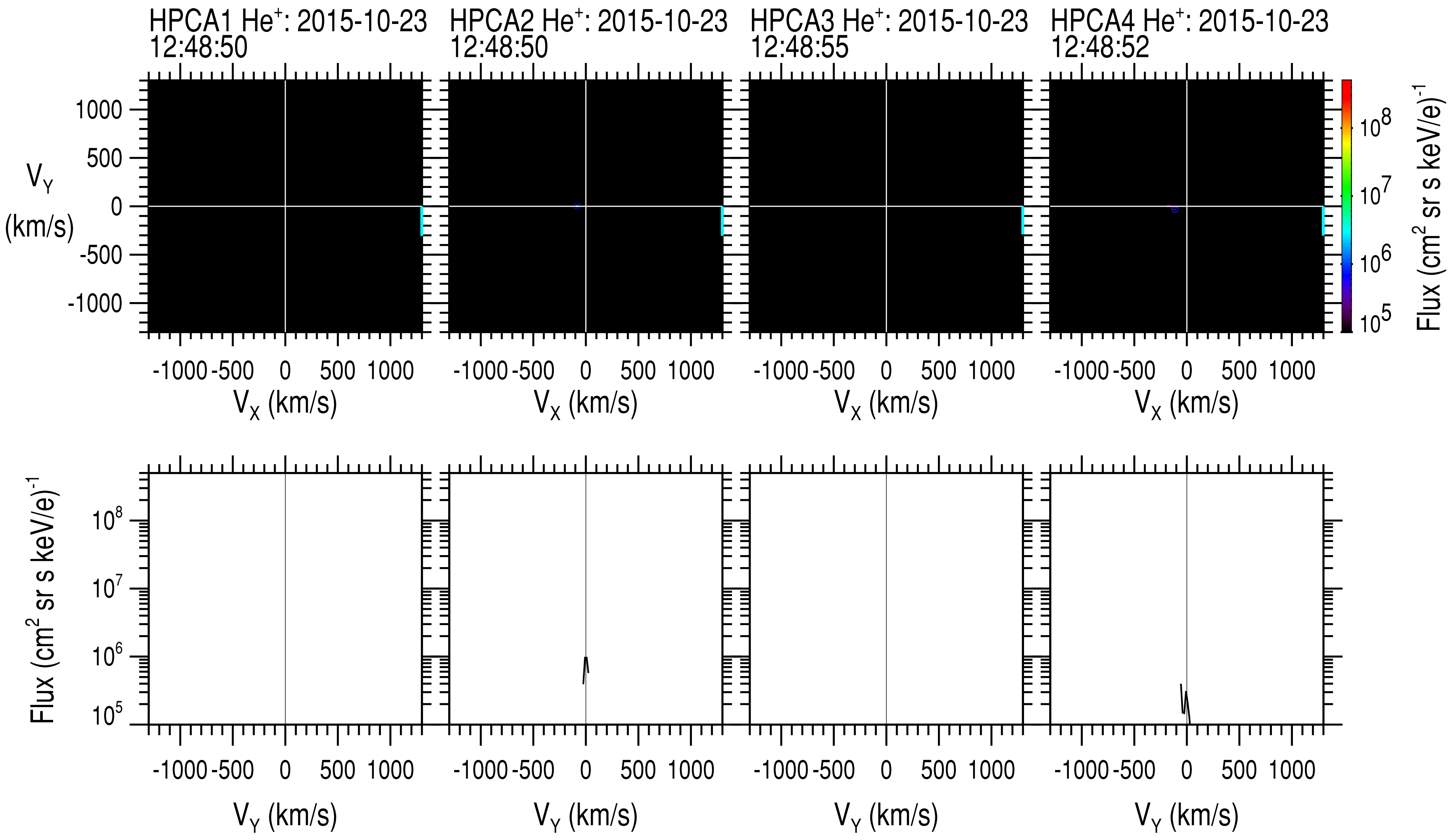
HPCA1 He+: 2015-10-23
12:48:50
HPCA2 He+: 2015-10-23
12:48:50
HPCA3 He+: 2015-10-23
12:48:55
HPCA4 He+: 2015-10-23
12:48:52
V_Y (km/s)
V_X (km/s)
-1000 -500 0 500 1000
Flux (cm^2 sr s keV/e)^-1
10^5 10^6 10^7 10^8

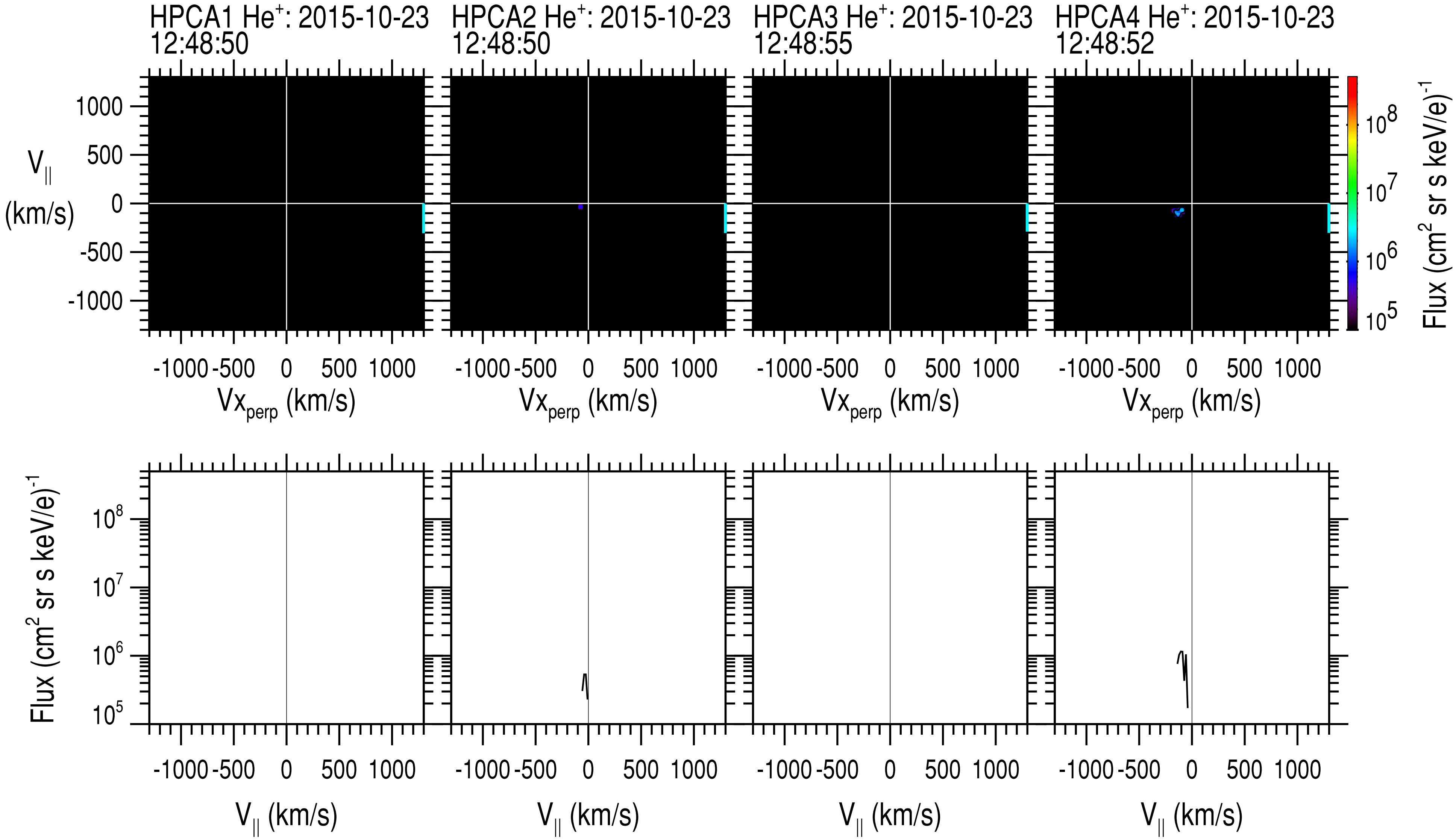
HPCA1 He+: 2015-10-23
12:48:50
HPCA2 He+: 2015-10-23
12:48:50
HPCA3 He+: 2015-10-23
12:48:55
HPCA4 He+: 2015-10-23
12:48:52
V|| (km/s)
Vxperp (km/s)
Flux (cm2 sr s keV/e)-1
-1000 -500 0 500 1000
10^5 10^6 10^7 10^8

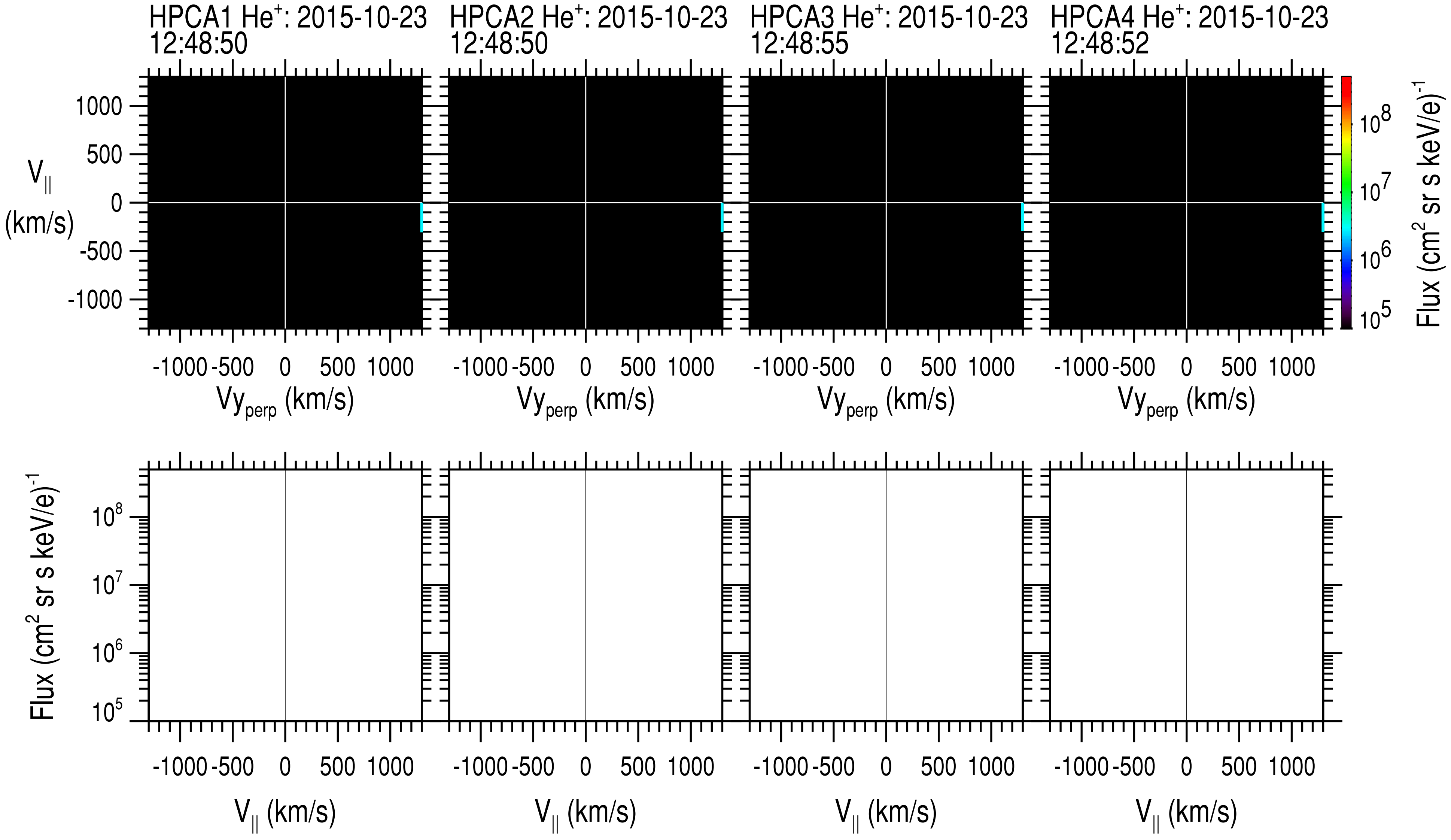
HPCA1 He+: 2015-10-23 12:48:50
HPCA2 He+: 2015-10-23 12:48:50
HPCA3 He+: 2015-10-23 12:48:55
HPCA4 He+: 2015-10-23 12:48:52
V|| (km/s)
Vyperp (km/s)
Flux (cm2 sr s keV/e)-1
-1000 -500 0 500 1000
10^5 10^6 10^7 10^8

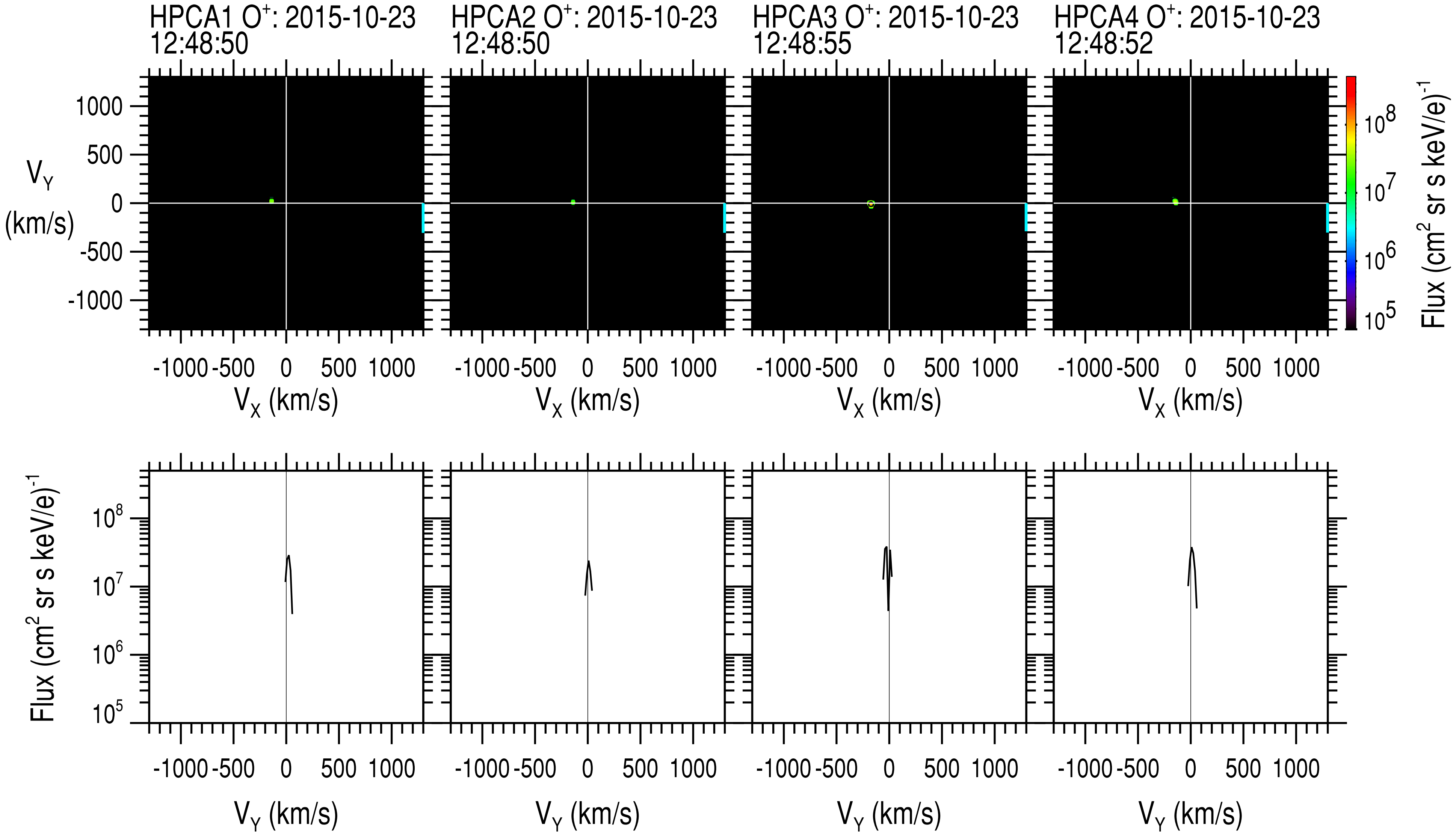
HPCA1 O+: 2015-10-23
12:48:50
HPCA2 O+: 2015-10-23
12:48:50
HPCA3 O+: 2015-10-23
12:48:55
HPCA4 O+: 2015-10-23
12:48:52
VY (km/s)
1000
500
0
-500
-1000
VX (km/s)
-1000 -500 0 500 1000
Flux (cm2 sr s keV/e)-1
105
106
107
108
VY (km/s)

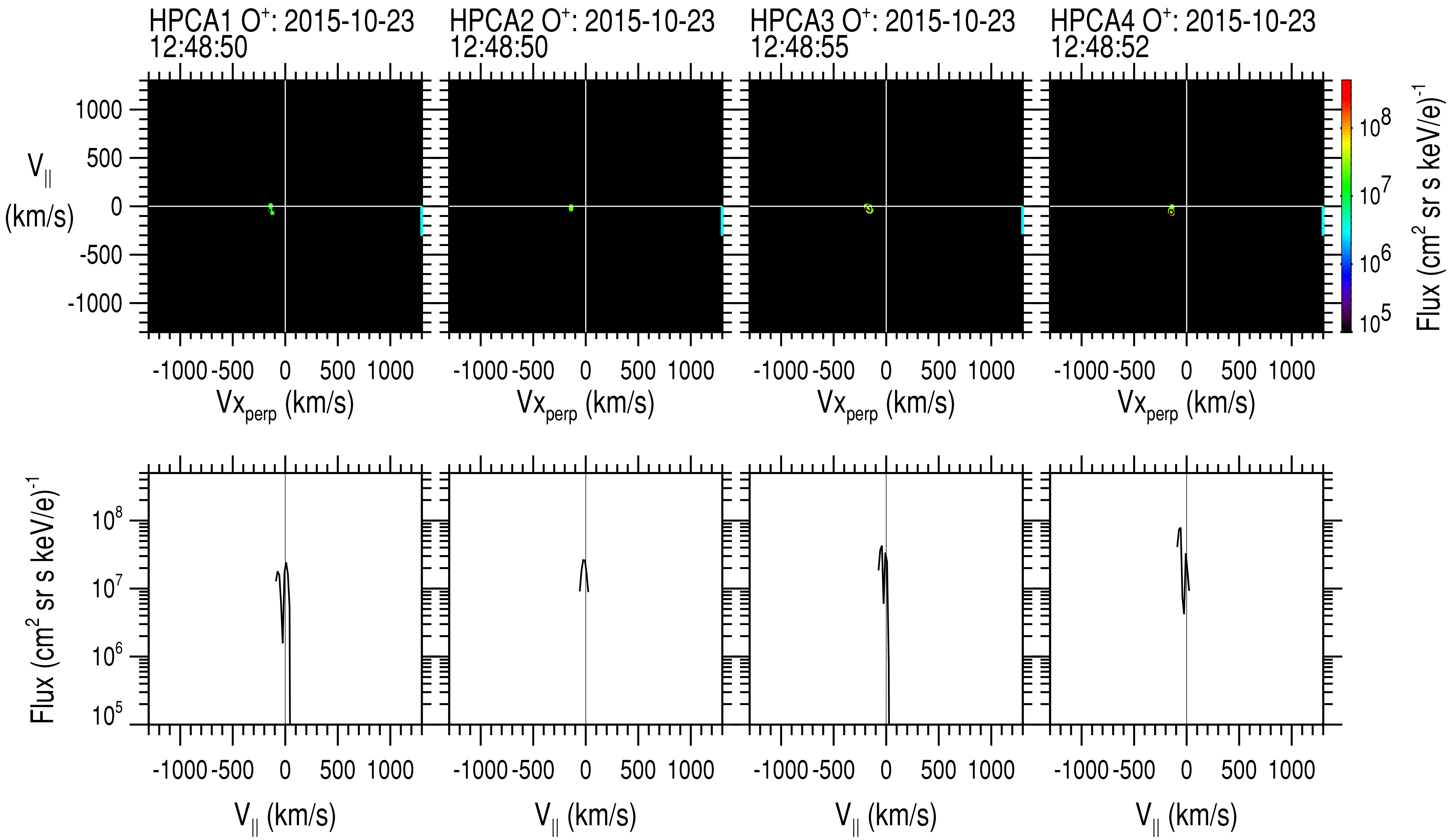
HPCA1 O+: 2015-10-23 12:48:50
HPCA2 O+: 2015-10-23 12:48:50
HPCA3 O+: 2015-10-23 12:48:55
HPCA4 O+: 2015-10-23 12:48:52
V|| (km/s)
Vxperp (km/s)
Flux (cm2 sr s keV/e)-1

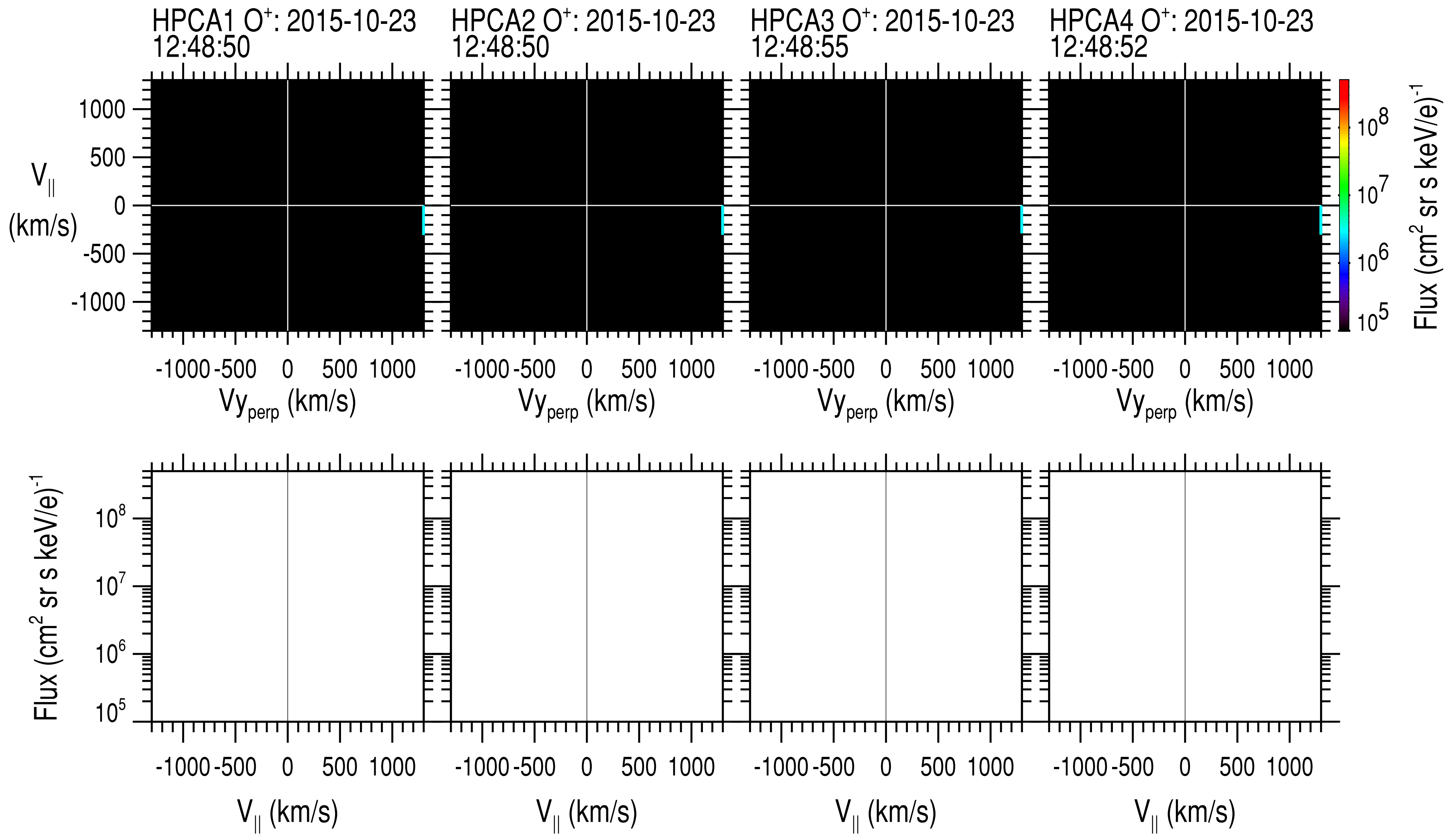
HPCA1 O+: 2015-10-23
12:48:50
HPCA2 O+: 2015-10-23
12:48:50
HPCA3 O+: 2015-10-23
12:48:55
HPCA4 O+: 2015-10-23
12:48:52
V|| (km/s)
1000
500
0
-500
-1000
Vy_perp (km/s)
Flux (cm2 sr s keV/e)-1
10^5
10^6
10^7
10^8

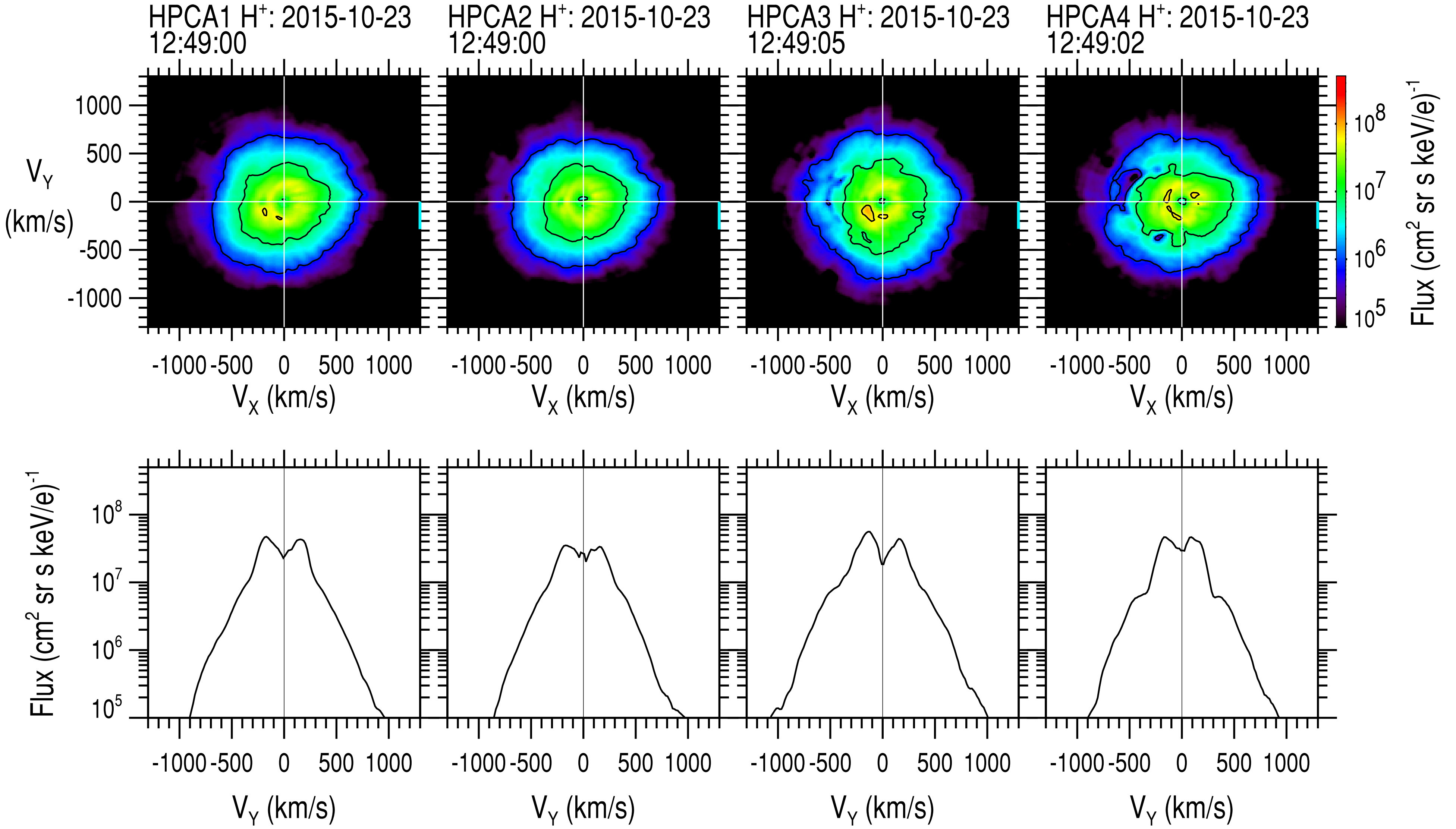

HPCA1 H+: 2015-10-23 12:49:00
HPCA2 H+: 2015-10-23 12:49:00
HPCA3 H+: 2015-10-23 12:49:05
HPCA4 H+: 2015-10-23 12:49:02
VY (km/s)
VX (km/s)
Flux (cm2 sr s keV/e)-1
VY (km/s)

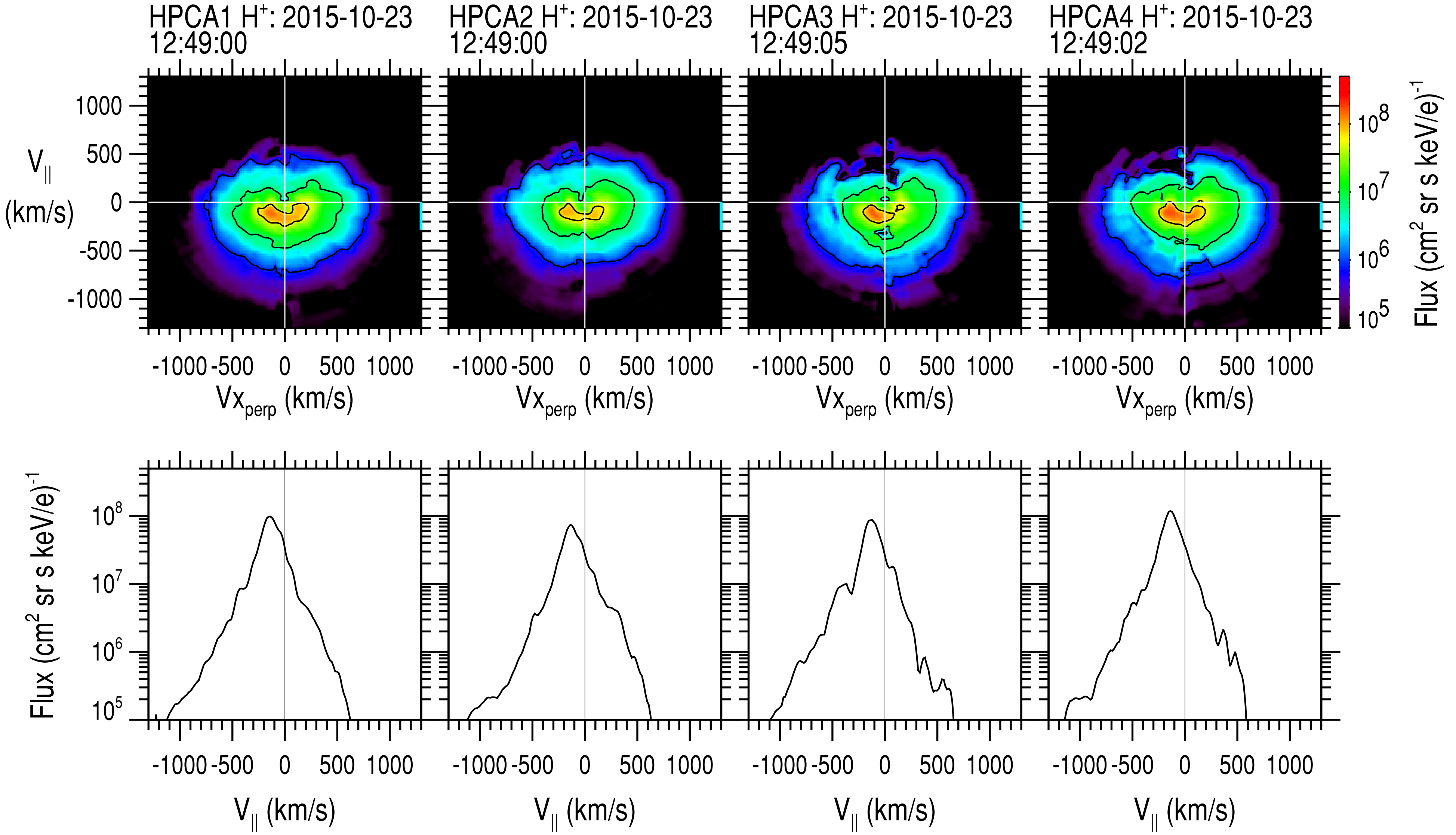

HPCA1 H+: 2015-10-23 12:49:00
HPCA2 H+: 2015-10-23 12:49:00
HPCA3 H+: 2015-10-23 12:49:05
HPCA4 H+: 2015-10-23 12:49:02
V|| (km/s)
Vxperp (km/s)
Flux (cm2 sr s keV/e)-1
-1000 -500 0 500 1000
10^5 10^6 10^7 10^8

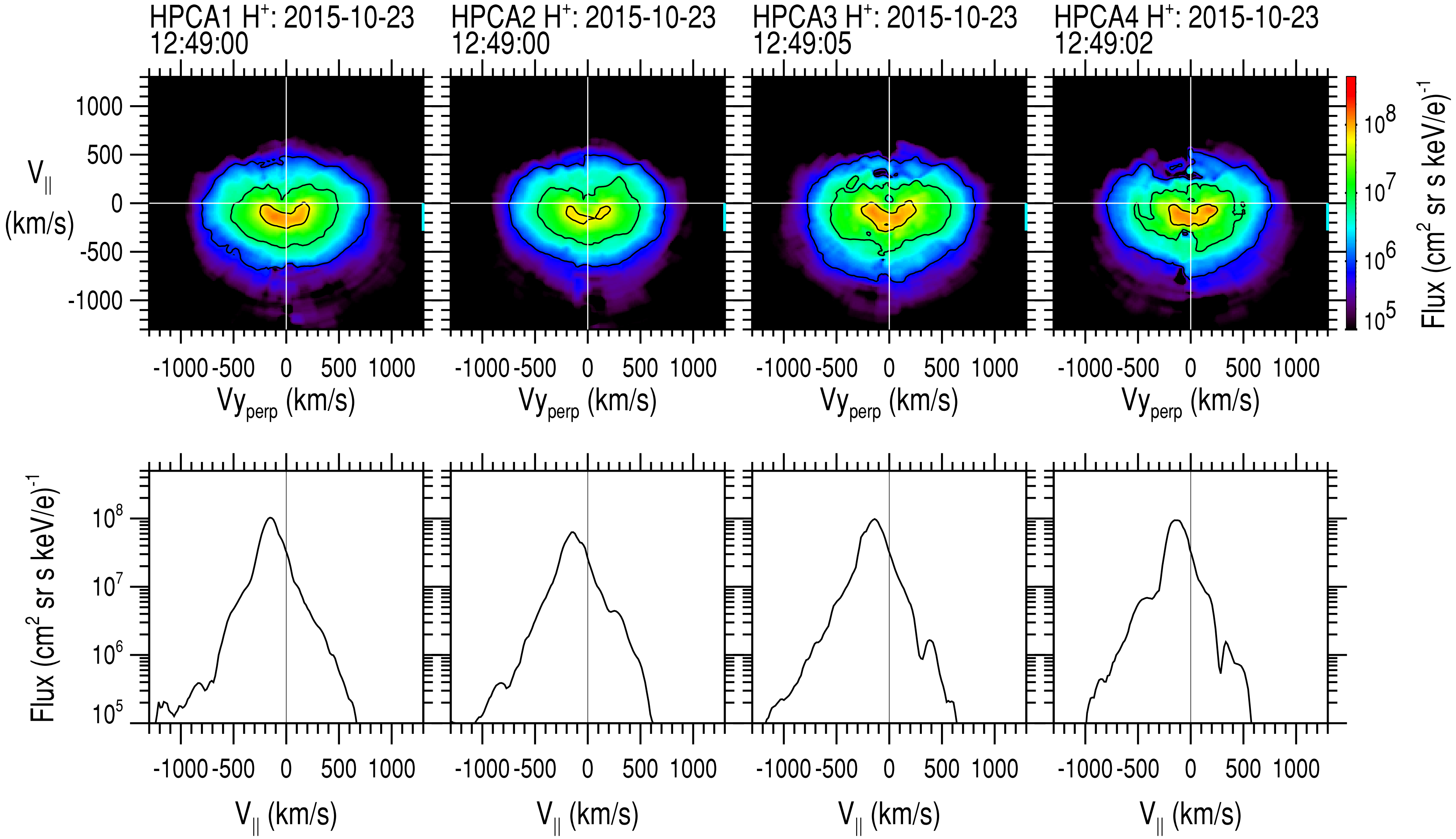

HPCA1 H+: 2015-10-23
12:49:00
HPCA2 H+: 2015-10-23
12:49:00
HPCA3 H+: 2015-10-23
12:49:05
HPCA4 H+: 2015-10-23
12:49:02
V|| (km/s)
Vyperp (km/s)
Flux (cm2 sr s keV/e)-1
V|| (km/s)

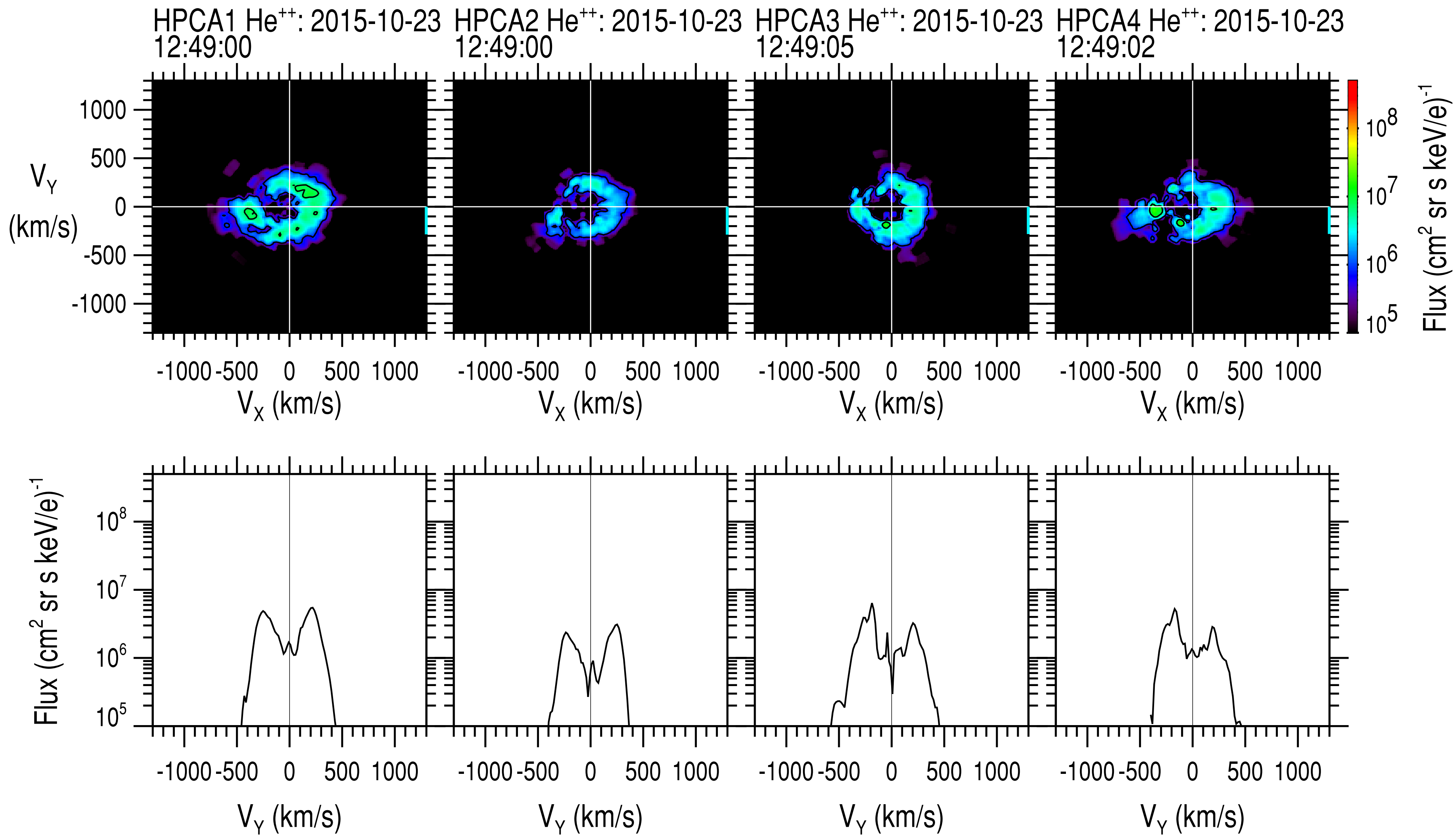

HPCA1 He++: 2015-10-23 12:49:00
HPCA2 He++: 2015-10-23 12:49:00
HPCA3 He++: 2015-10-23 12:49:05
HPCA4 He++: 2015-10-23 12:49:02
VY (km/s)
VX (km/s)
Flux (cm2 sr s keV/e)-1
VY (km/s)

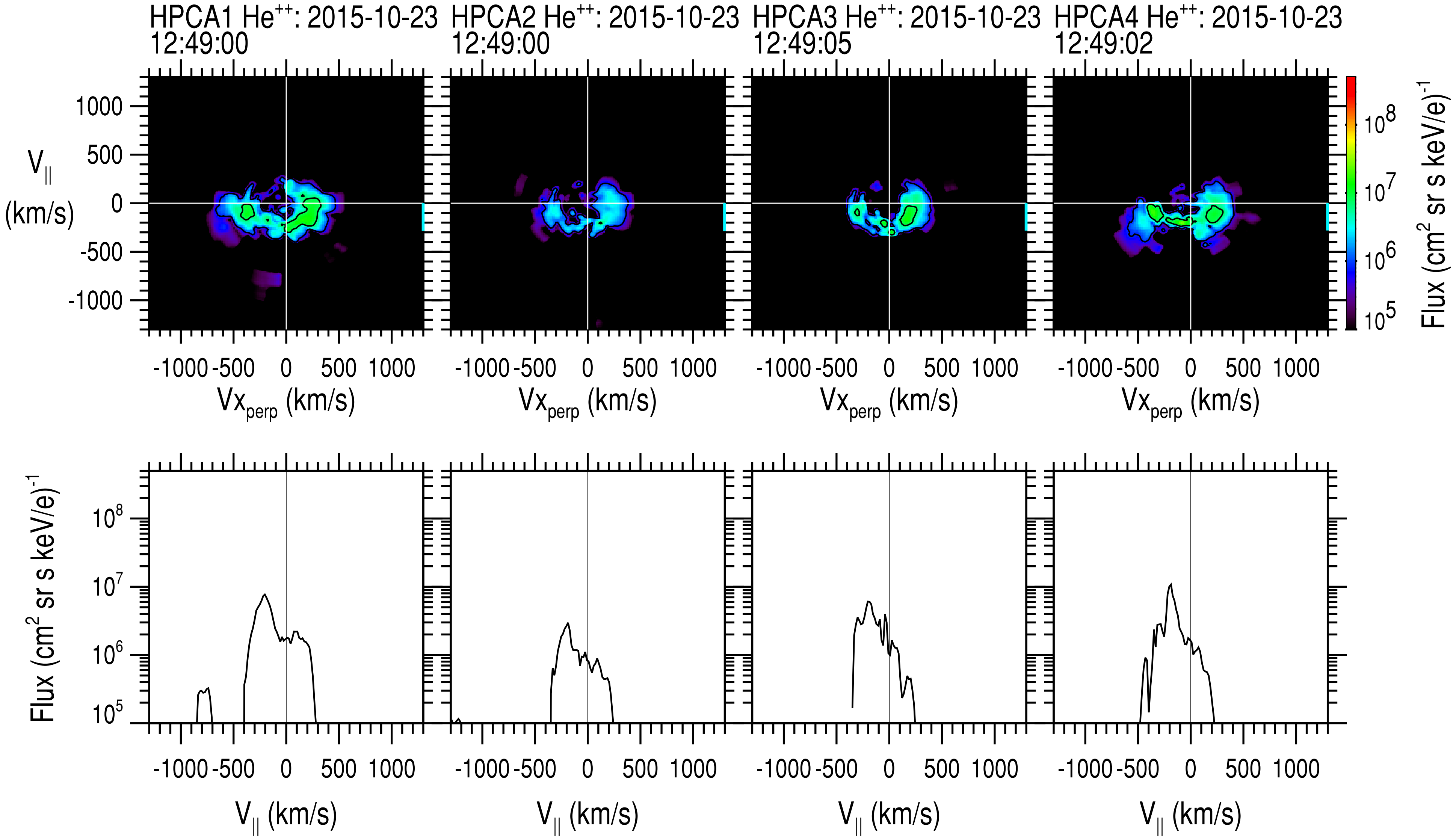

HPCA1 He++: 2015-10-23 12:49:00
HPCA2 He++: 2015-10-23 12:49:00
HPCA3 He++: 2015-10-23 12:49:05
HPCA4 He++: 2015-10-23 12:49:02
V|| (km/s)
Vxperp (km/s)
Flux (cm2 sr s keV/e)-1

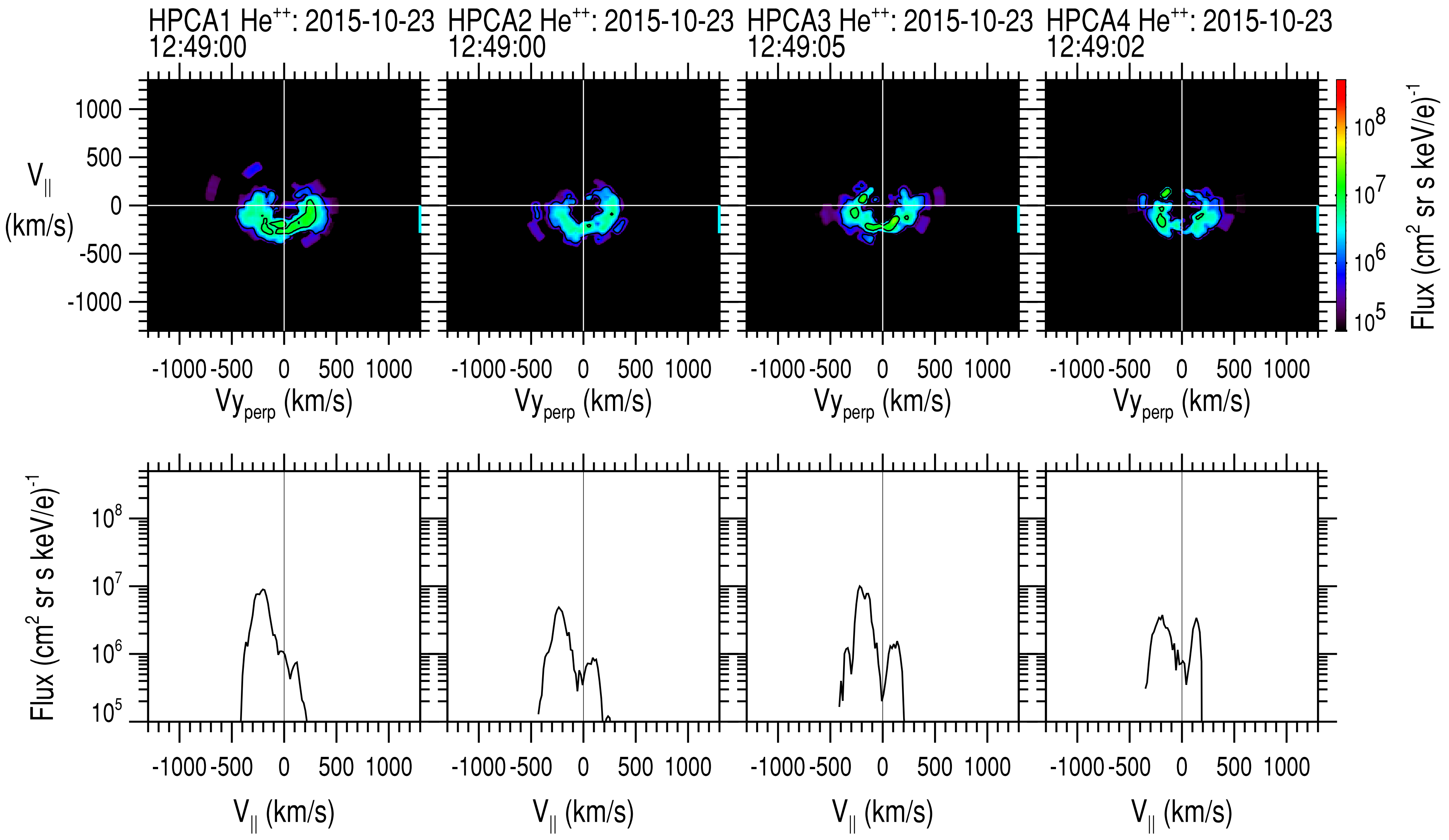
HPCA1 He++: 2015-10-23
12:49:00
HPCA2 He++: 2015-10-23
12:49:00
HPCA3 He++: 2015-10-23
12:49:05
HPCA4 He++: 2015-10-23
12:49:02
V|| (km/s)
Vyperp (km/s)
Flux (cm2 sr s keV/e)-1
V|| (km/s)

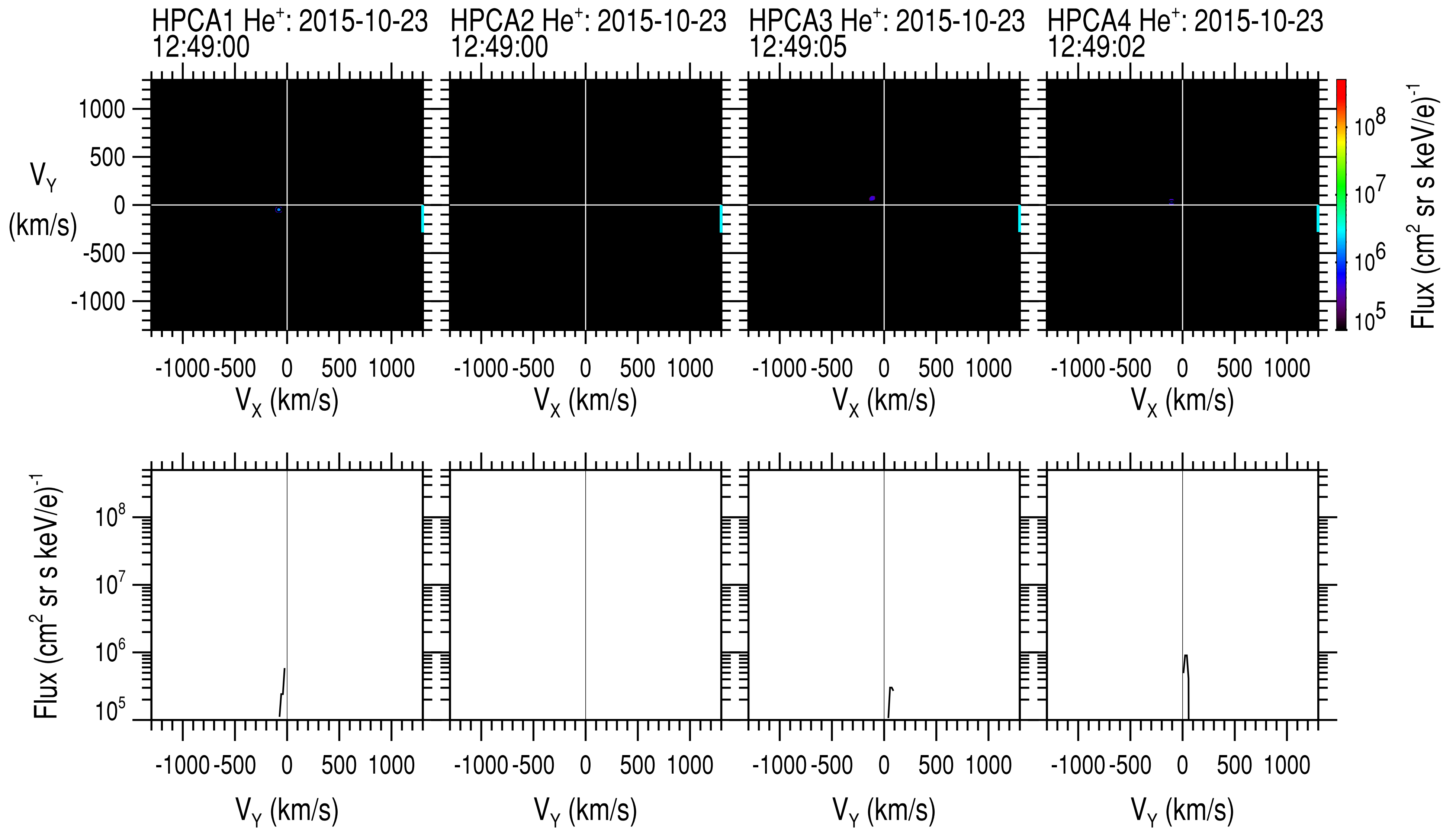
HPCA1 He+: 2015-10-23 12:49:00
HPCA2 He+: 2015-10-23 12:49:00
HPCA3 He+: 2015-10-23 12:49:05
HPCA4 He+: 2015-10-23 12:49:02
VY (km/s)
VX (km/s)
Flux (cm2 sr s keV/e)-1
1000
500
0
-500
-1000
108
107
106
105

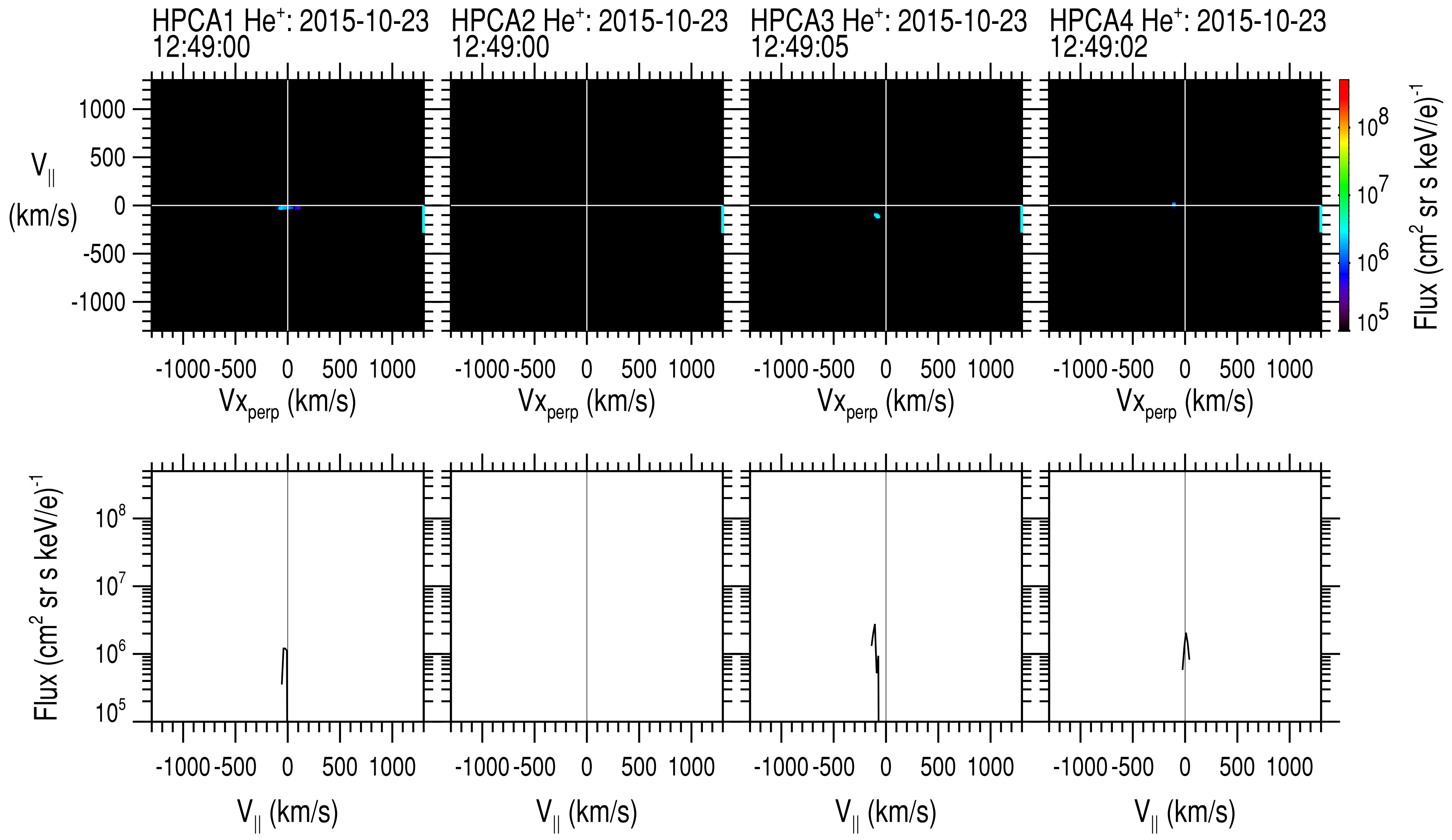
HPCA1 He+: 2015-10-23 12:49:00
HPCA2 He+: 2015-10-23 12:49:00
HPCA3 He+: 2015-10-23 12:49:05
HPCA4 He+: 2015-10-23 12:49:02
V|| (km/s)
Vxperp (km/s)
Flux (cm2 sr s keV/e)-1
-1000 -500 0 500 1000
10^5 10^6 10^7 10^8

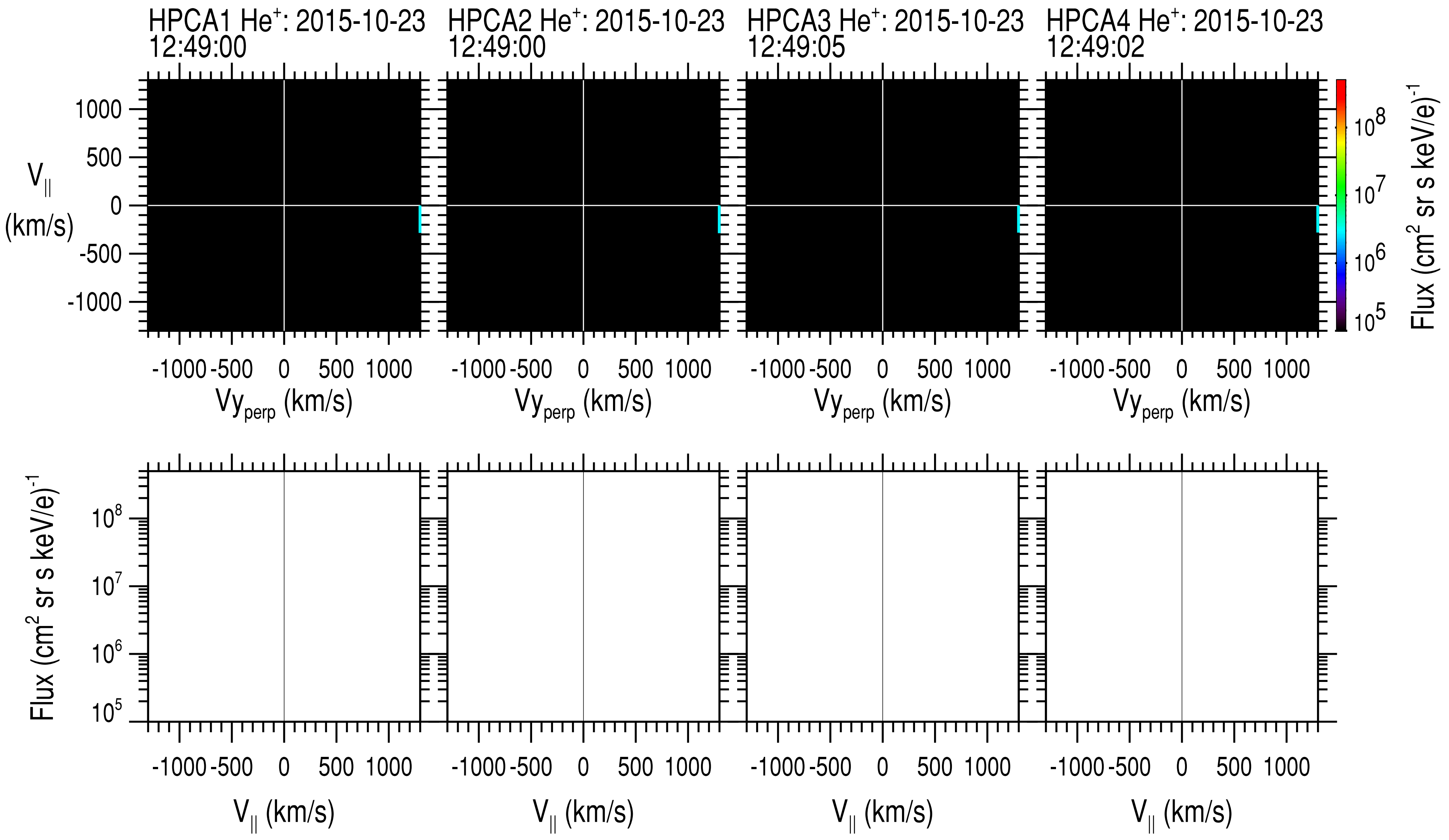

HPCA1 He+: 2015-10-23 12:49:00
HPCA2 He+: 2015-10-23 12:49:00
HPCA3 He+: 2015-10-23 12:49:05
HPCA4 He+: 2015-10-23 12:49:02
V|| (km/s)
Vyperp (km/s)
Flux (cm2 sr s keV/e)-1
1000
500
0
-500
-1000
105
106
107
108

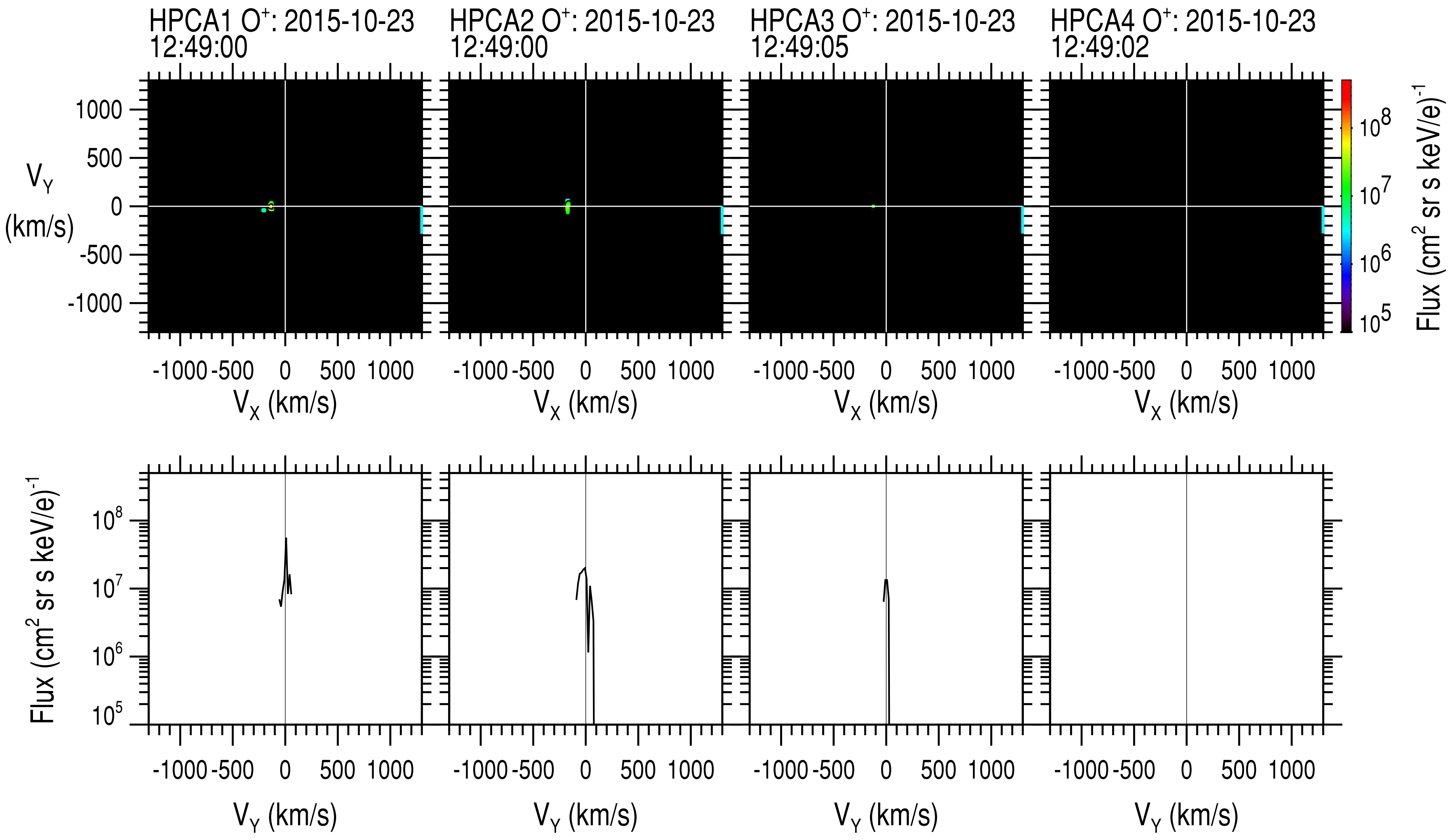

HPCA1 O+: 2015-10-23
12:49:00
HPCA2 O+: 2015-10-23
12:49:00
HPCA3 O+: 2015-10-23
12:49:05
HPCA4 O+: 2015-10-23
12:49:02
VY (km/s)
VX (km/s)
Flux (cm2 sr s keV/e)-1
VY (km/s)

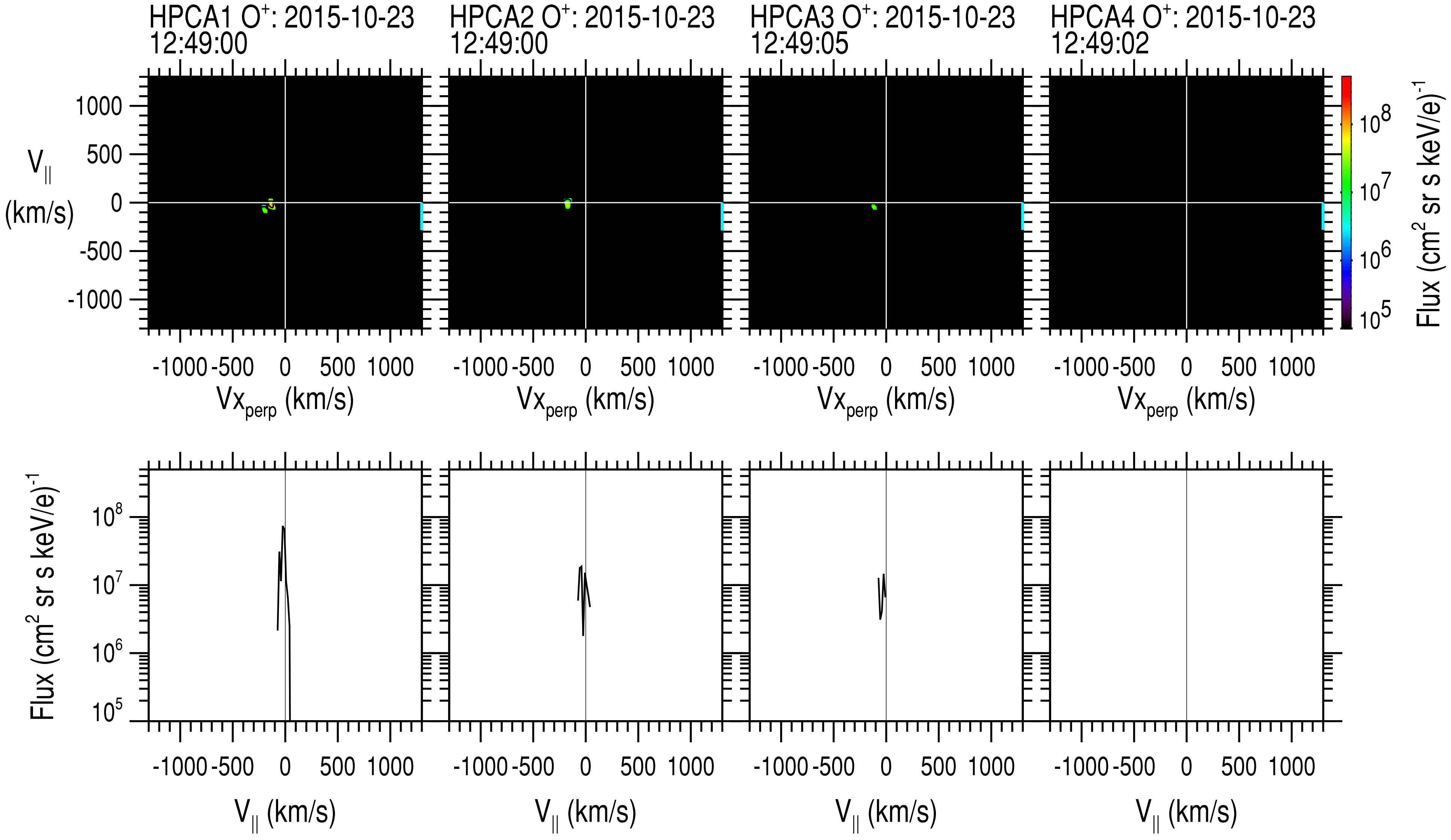
HPCA1 O+: 2015-10-23 12:49:00
HPCA2 O+: 2015-10-23 12:49:00
HPCA3 O+: 2015-10-23 12:49:05
HPCA4 O+: 2015-10-23 12:49:02
V|| (km/s)
Vxperp (km/s)
Flux (cm2 sr s keV/e)-1
-1000
-500
0
500
1000
10^5
10^6
10^7
10^8

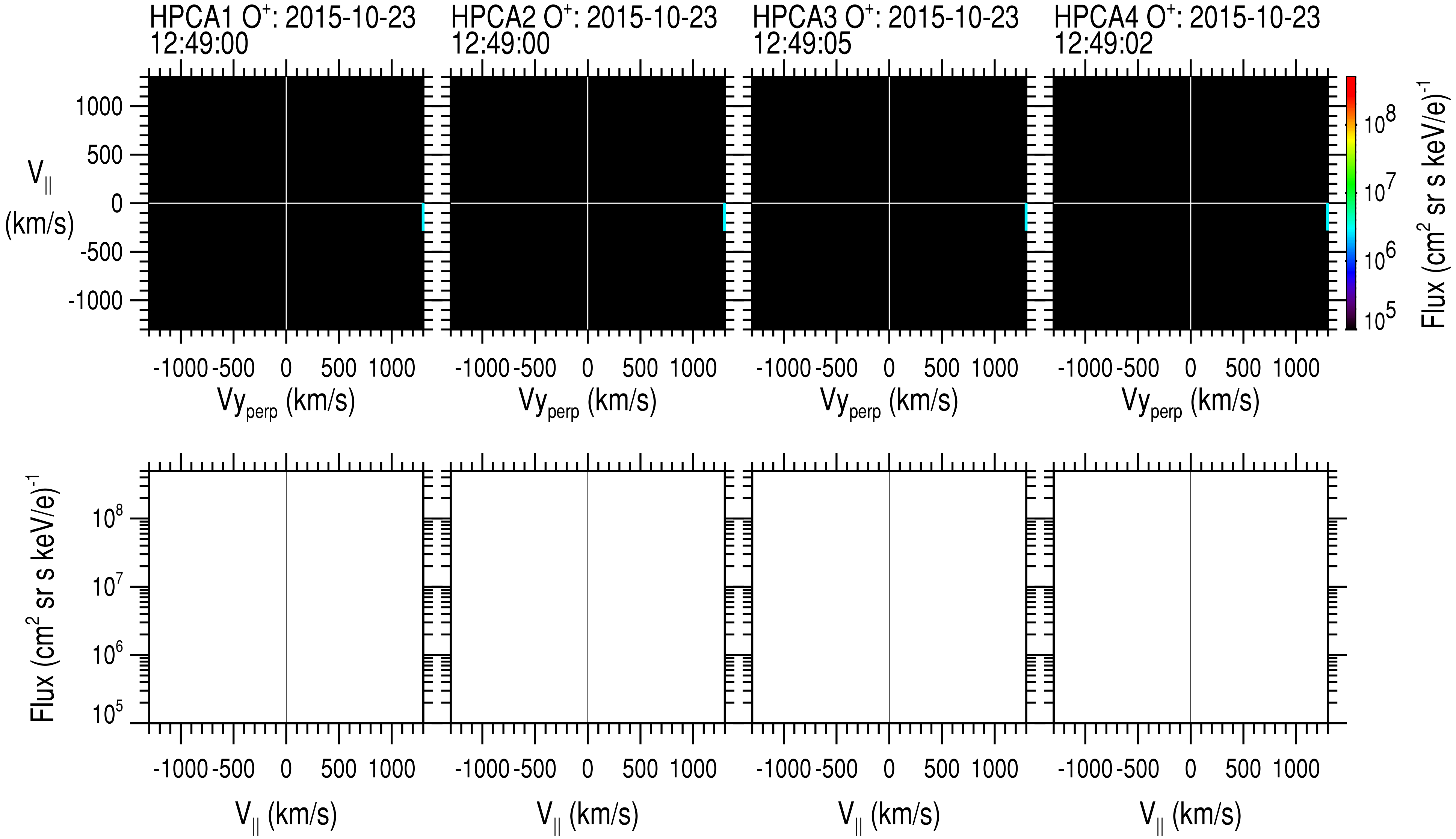
HPCA1 O+: 2015-10-23
12:49:00
HPCA2 O+: 2015-10-23
12:49:00
HPCA3 O+: 2015-10-23
12:49:05
HPCA4 O+: 2015-10-23
12:49:02
V|| (km/s)
Vyperp (km/s)
Flux (cm2 sr s keV/e)-1
-1000 -500 0 500 1000
10^5 10^6 10^7 10^8

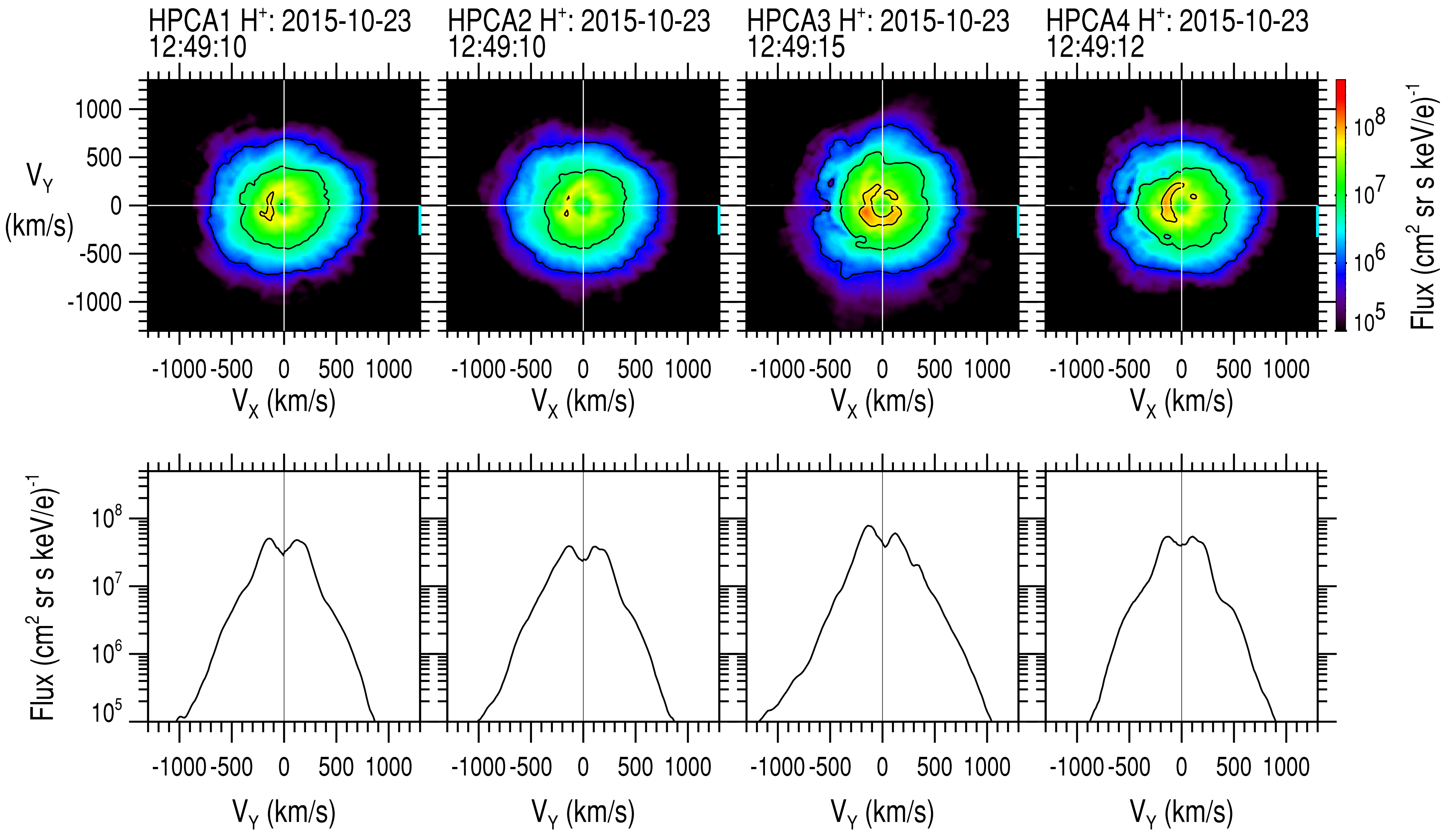
HPCA1 H+: 2015-10-23 12:49:10
HPCA2 H+: 2015-10-23 12:49:10
HPCA3 H+: 2015-10-23 12:49:15
HPCA4 H+: 2015-10-23 12:49:12
V_Y (km/s)
V_X (km/s)
Flux (cm^2 sr s keV/e)^-1
V_Y (km/s)

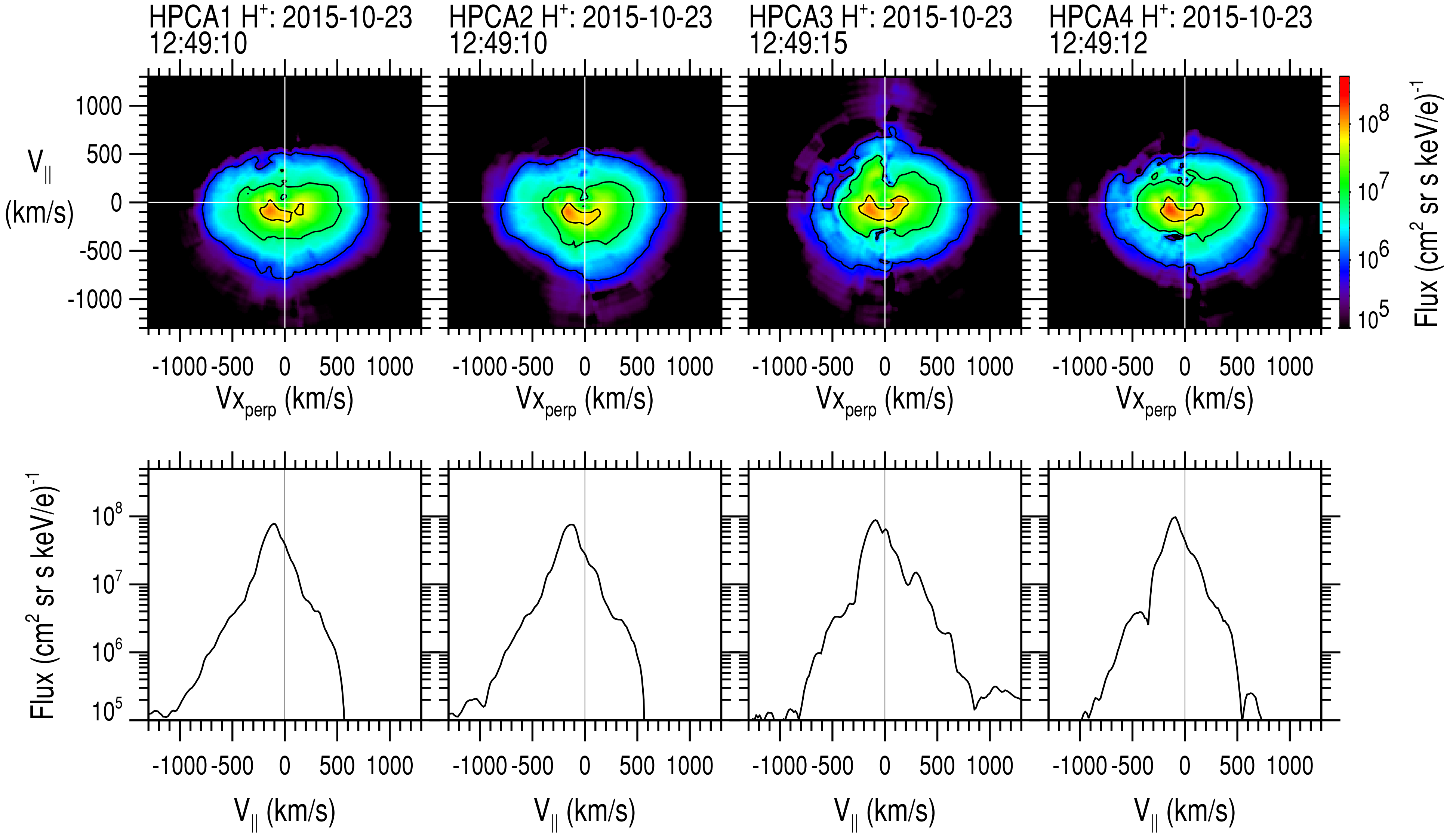

HPCA1 H+: 2015-10-23
12:49:10
HPCA2 H+: 2015-10-23
12:49:10
HPCA3 H+: 2015-10-23
12:49:15
HPCA4 H+: 2015-10-23
12:49:12
V|| (km/s)
Vxperp (km/s)
Flux (cm2 sr s keV/e)-1

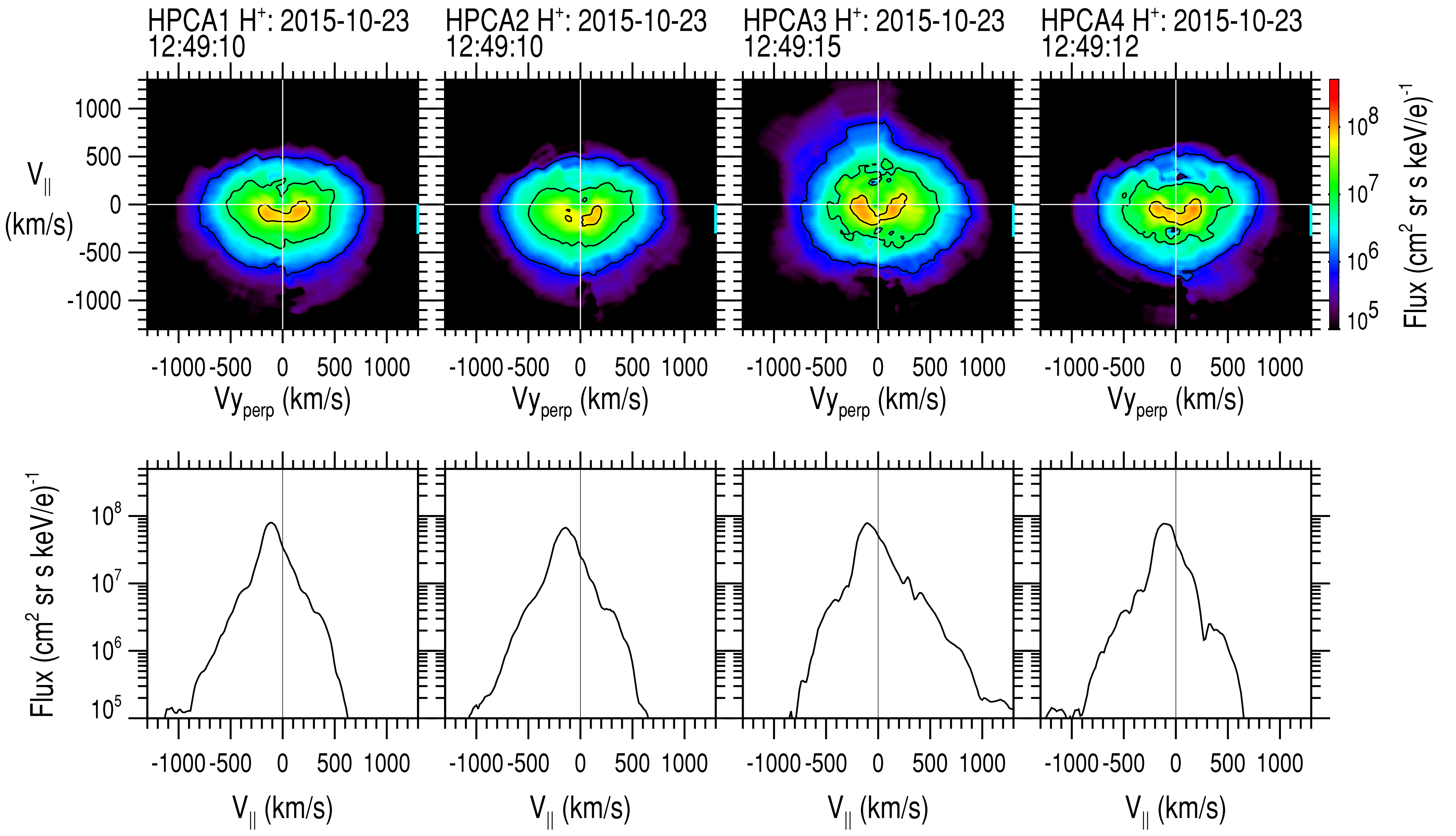
HPCA1 H+: 2015-10-23 12:49:10
HPCA2 H+: 2015-10-23 12:49:10
HPCA3 H+: 2015-10-23 12:49:15
HPCA4 H+: 2015-10-23 12:49:12
V|| (km/s)
Vyperp (km/s)
Flux (cm2 sr s keV/e)-1
V|| (km/s)
Flux (cm2 sr s keV/e)-1

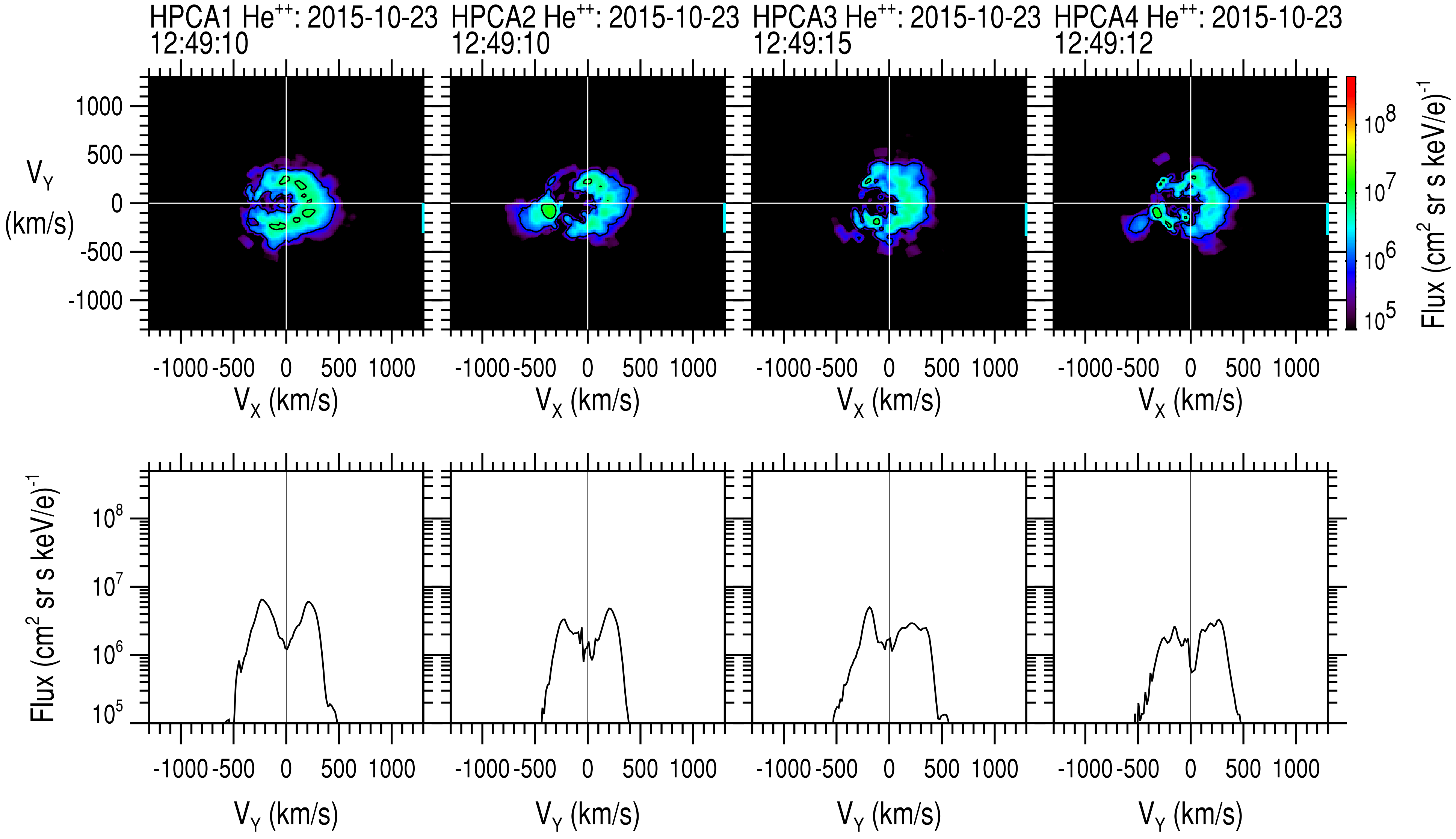

HPCA1 He++: 2015-10-23 12:49:10
HPCA2 He++: 2015-10-23 12:49:10
HPCA3 He++: 2015-10-23 12:49:15
HPCA4 He++: 2015-10-23 12:49:12
V_Y (km/s)
V_X (km/s)
Flux (cm^2 sr s keV/e)^-1
V_Y (km/s)

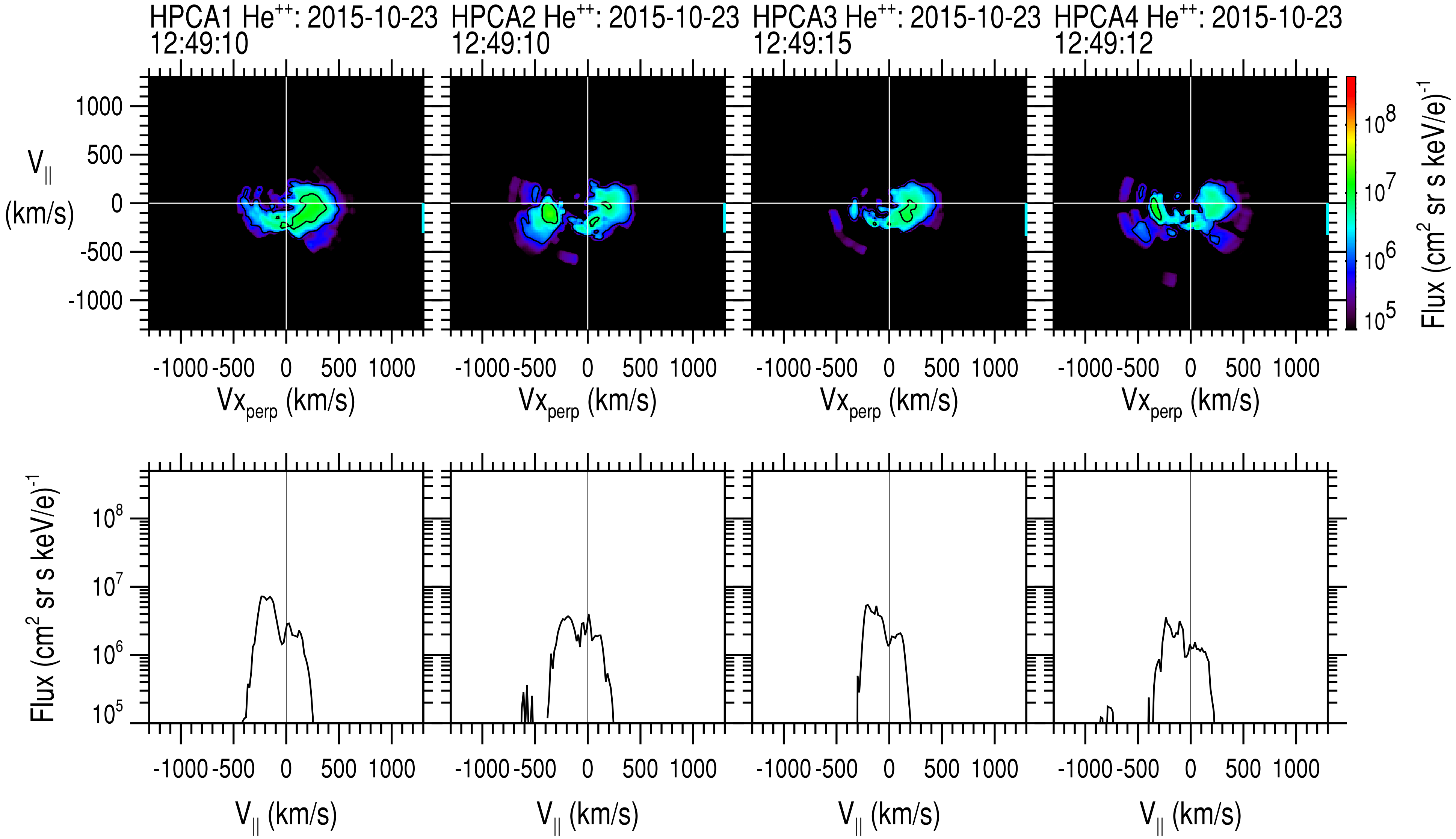
HPCA1 He++: 2015-10-23 12:49:10
HPCA2 He++: 2015-10-23 12:49:10
HPCA3 He++: 2015-10-23 12:49:15
HPCA4 He++: 2015-10-23 12:49:12
V|| (km/s)
Vxperp (km/s)
Flux (cm2 sr s keV/e)-1
V|| (km/s)

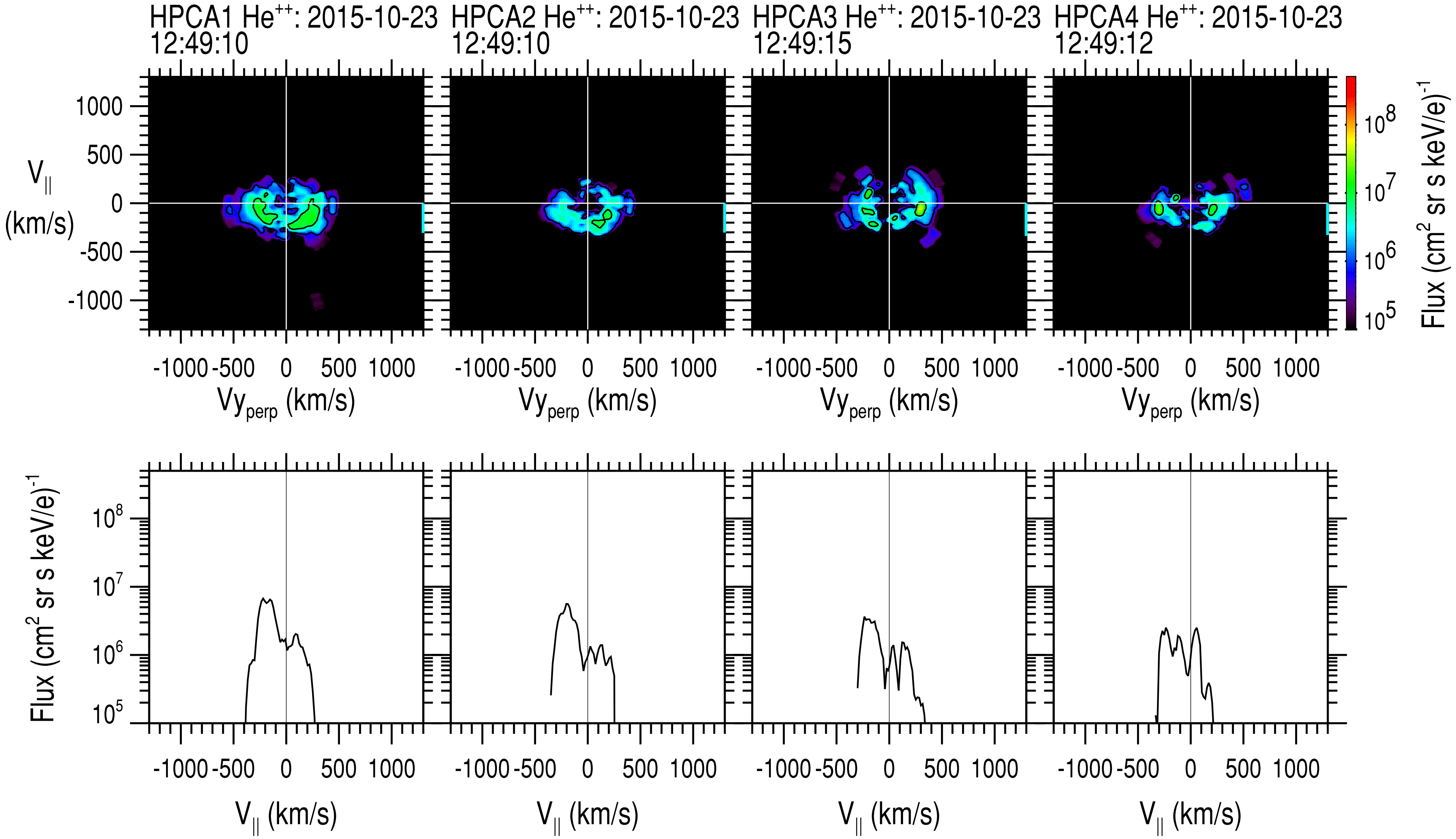
HPCA1 He++: 2015-10-23 12:49:10
HPCA2 He++: 2015-10-23 12:49:10
HPCA3 He++: 2015-10-23 12:49:15
HPCA4 He++: 2015-10-23 12:49:12
V|| (km/s)
Vyperp (km/s)
Flux (cm2 sr s keV/e)-1
V|| (km/s)

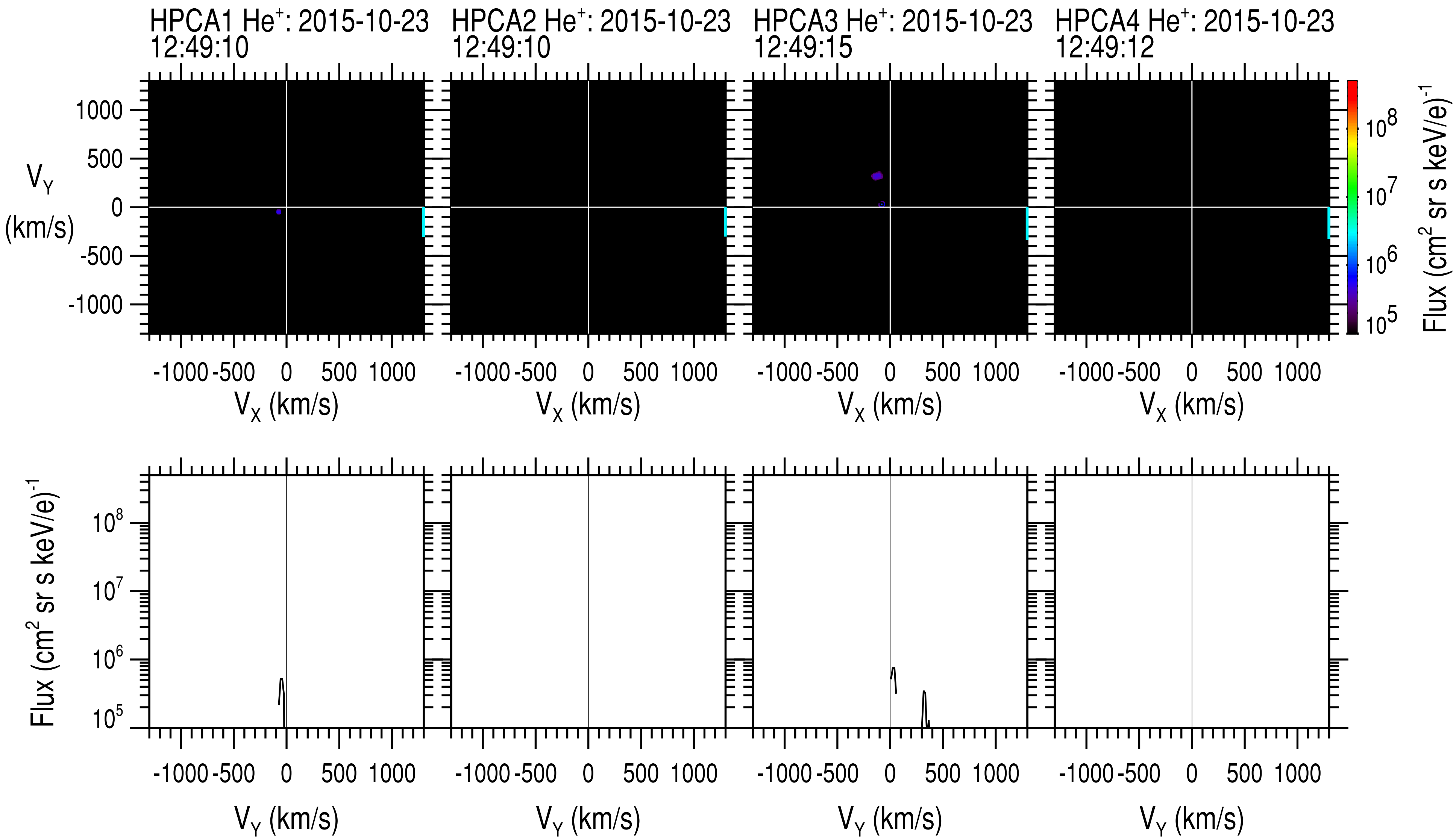
HPCA1 He+: 2015-10-23 12:49:10
HPCA2 He+: 2015-10-23 12:49:10
HPCA3 He+: 2015-10-23 12:49:15
HPCA4 He+: 2015-10-23 12:49:12
VY (km/s)
VX (km/s)
Flux (cm2 sr s keV/e)-1
VY (km/s)
Flux (cm2 sr s keV/e)-1

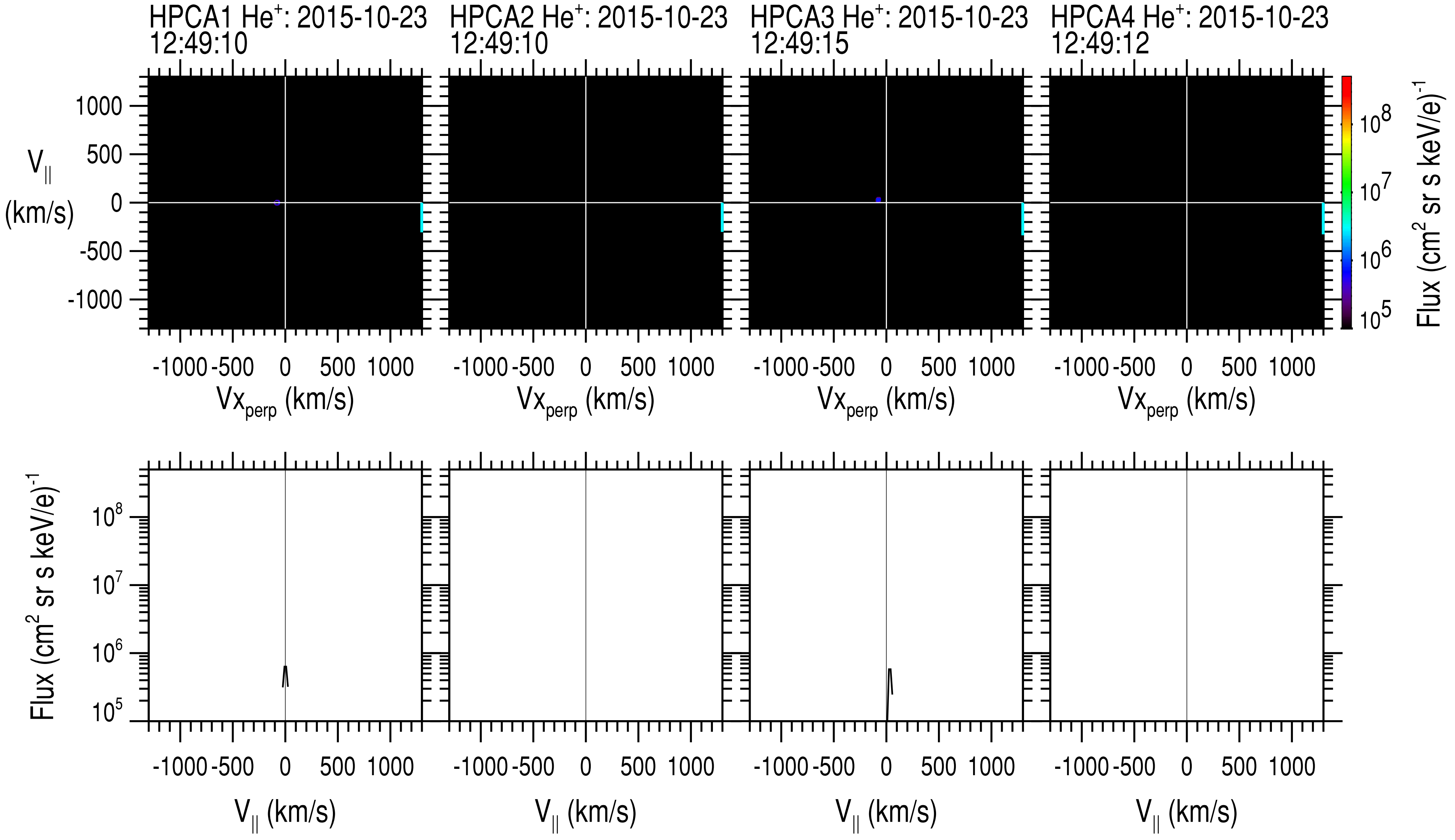

HPCA1 He+: 2015-10-23 12:49:10
HPCA2 He+: 2015-10-23 12:49:10
HPCA3 He+: 2015-10-23 12:49:15
HPCA4 He+: 2015-10-23 12:49:12
V|| (km/s)
Vxperp (km/s)
Flux (cm2 sr s keV/e)-1

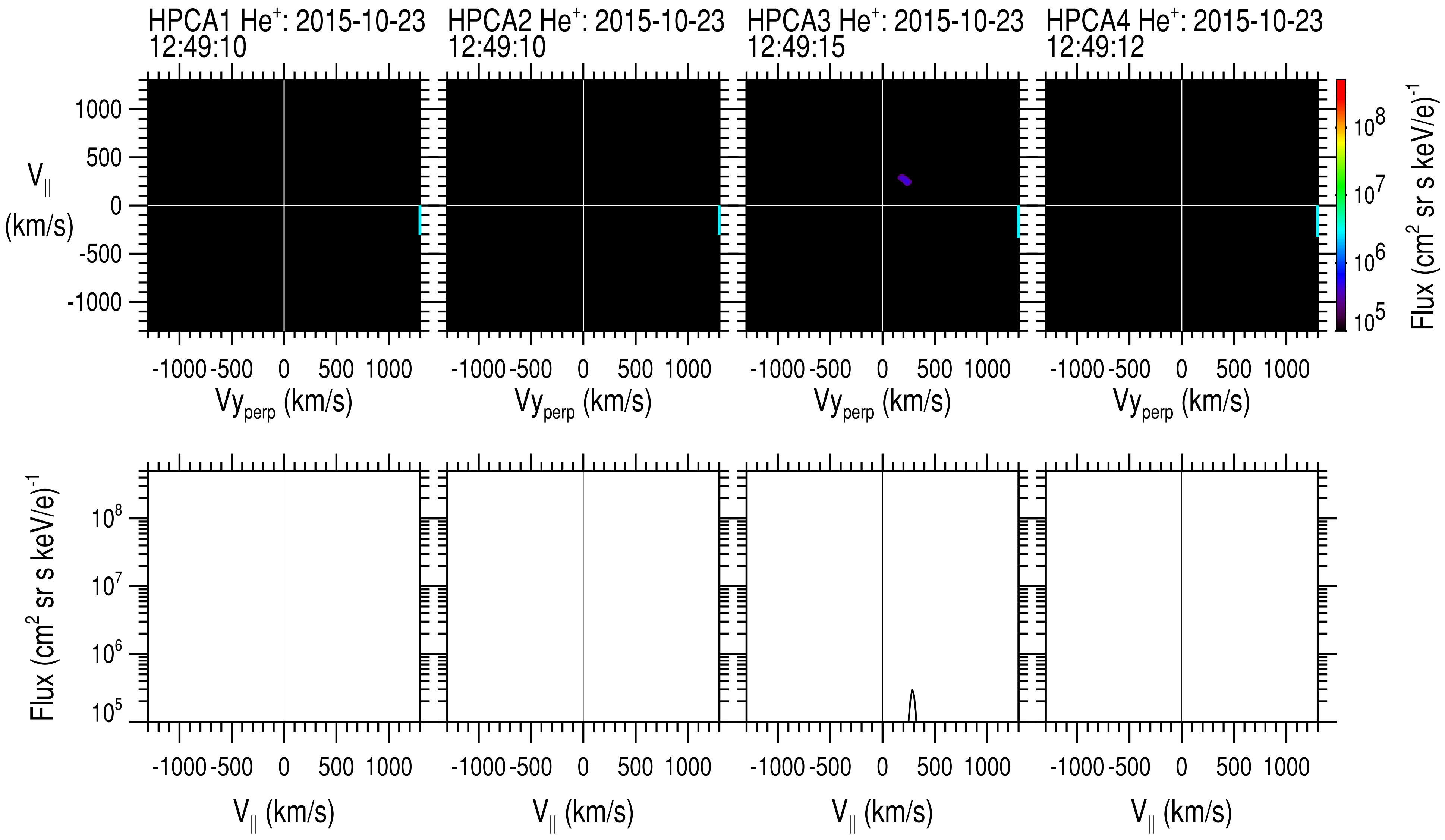
HPCA1 He+: 2015-10-23 12:49:10
HPCA2 He+: 2015-10-23 12:49:10
HPCA3 He+: 2015-10-23 12:49:15
HPCA4 He+: 2015-10-23 12:49:12
V|| (km/s)
Vyperp (km/s)
Flux (cm2 sr s keV/e)-1
1000
500
0
-500
-1000
10^5
10^6
10^7
10^8

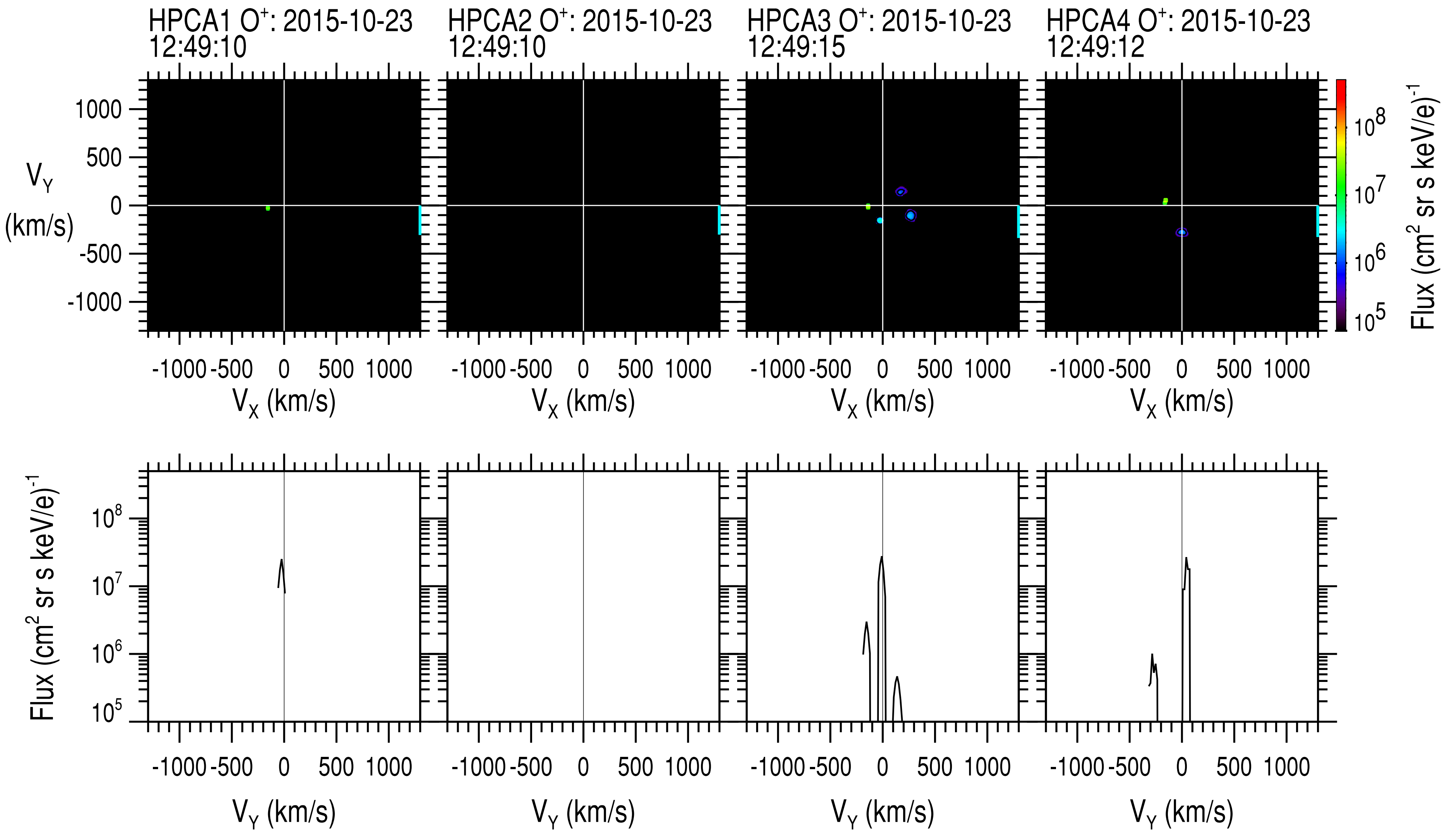
HPCA1 O+: 2015-10-23
12:49:10
HPCA2 O+: 2015-10-23
12:49:10
HPCA3 O+: 2015-10-23
12:49:15
HPCA4 O+: 2015-10-23
12:49:12
V_Y (km/s)
V_X (km/s)
Flux (cm^2 sr s keV/e)^-1
-1000 -500 0 500 1000
10^5 10^6 10^7 10^8

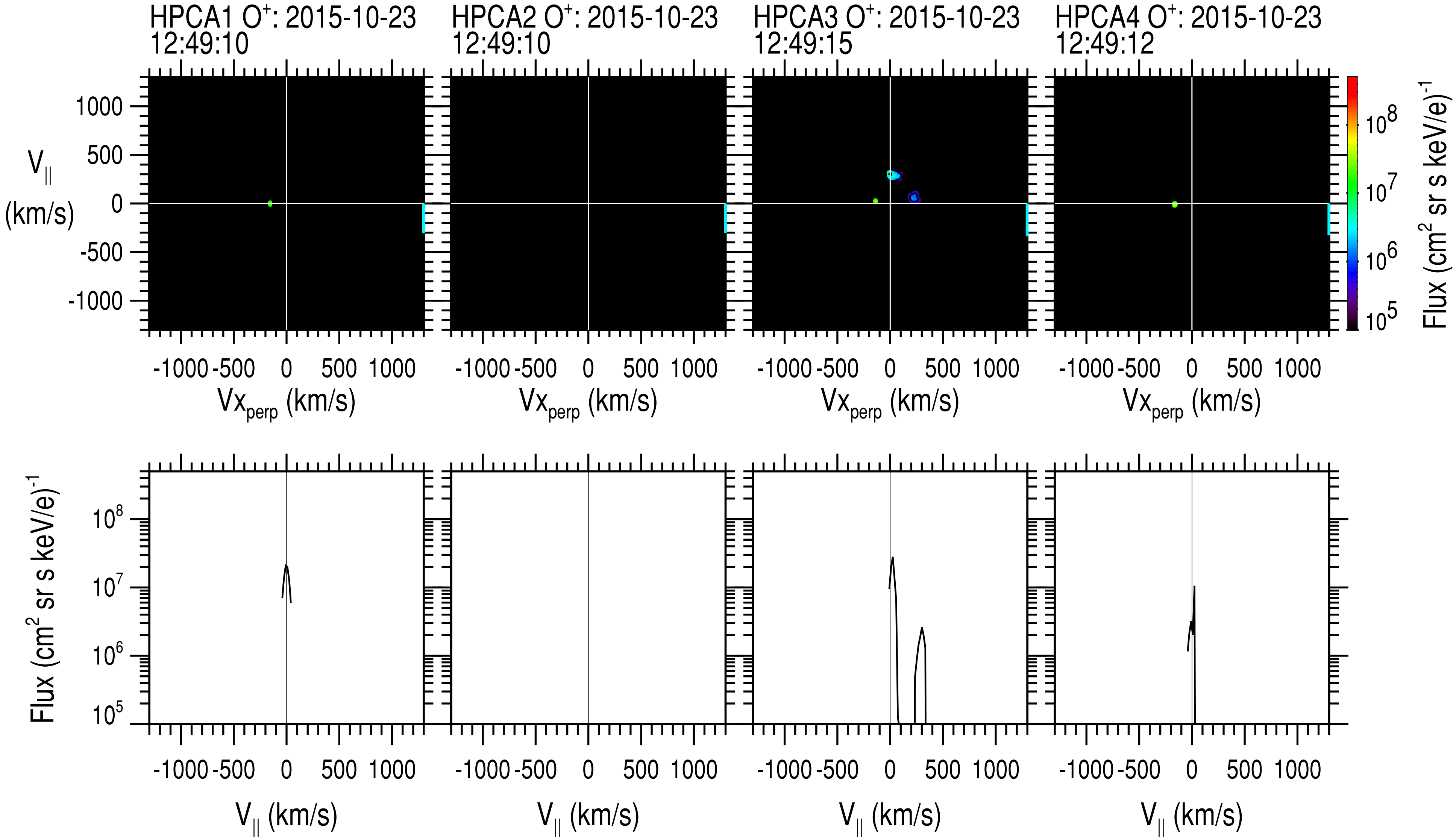

HPCA1 O+: 2015-10-23 12:49:10
HPCA2 O+: 2015-10-23 12:49:10
HPCA3 O+: 2015-10-23 12:49:15
HPCA4 O+: 2015-10-23 12:49:12
V|| (km/s)
Vxperp (km/s)
Flux (cm2 sr s keV/e)-1

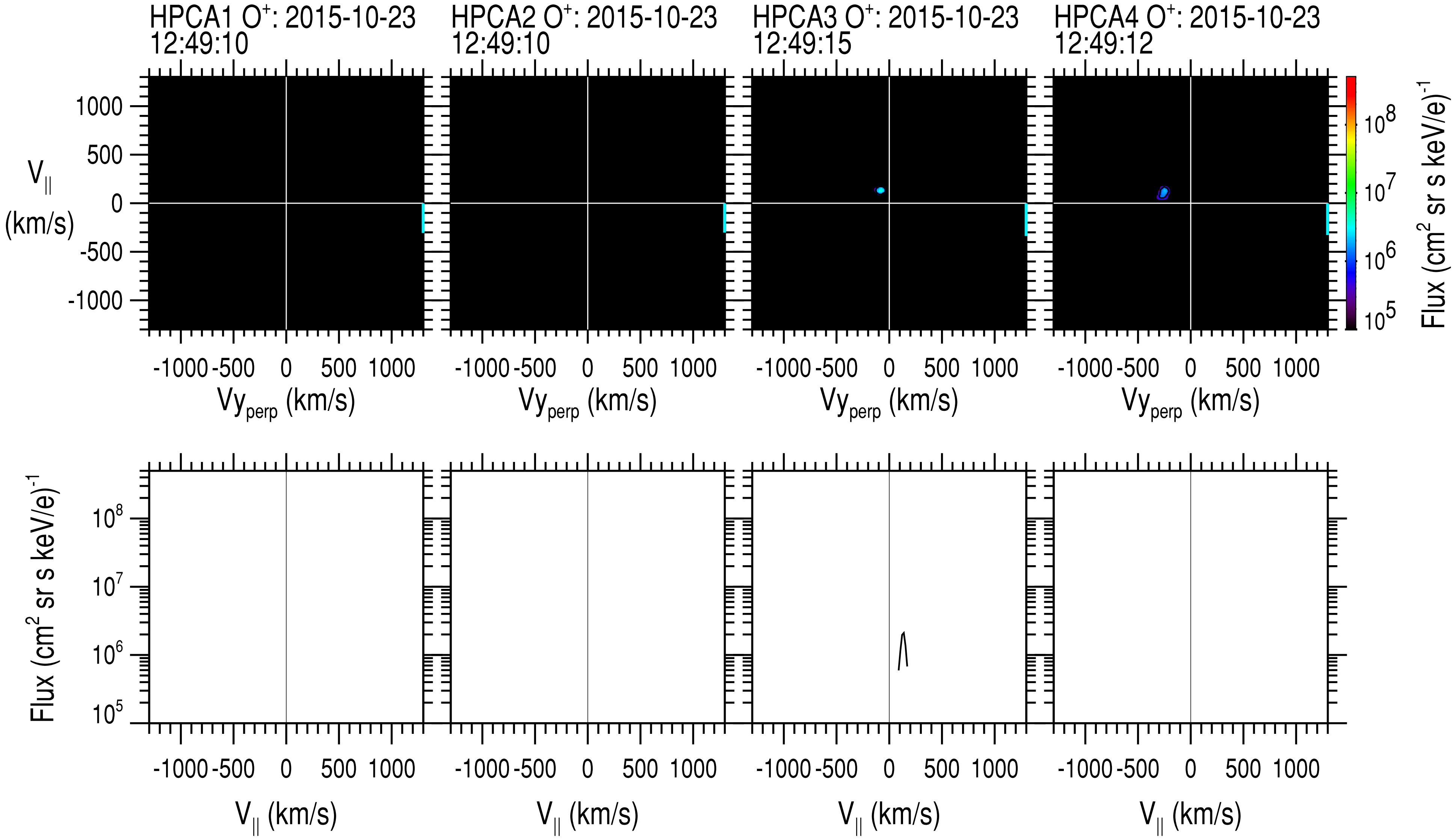
HPCA1 O+: 2015-10-23 12:49:10
HPCA2 O+: 2015-10-23 12:49:10
HPCA3 O+: 2015-10-23 12:49:15
HPCA4 O+: 2015-10-23 12:49:12
V|| (km/s)
Vyperp (km/s)
Flux (cm2 sr s keV/e)-1
-1000 -500 0 500 1000
10^5 10^6 10^7 10^8

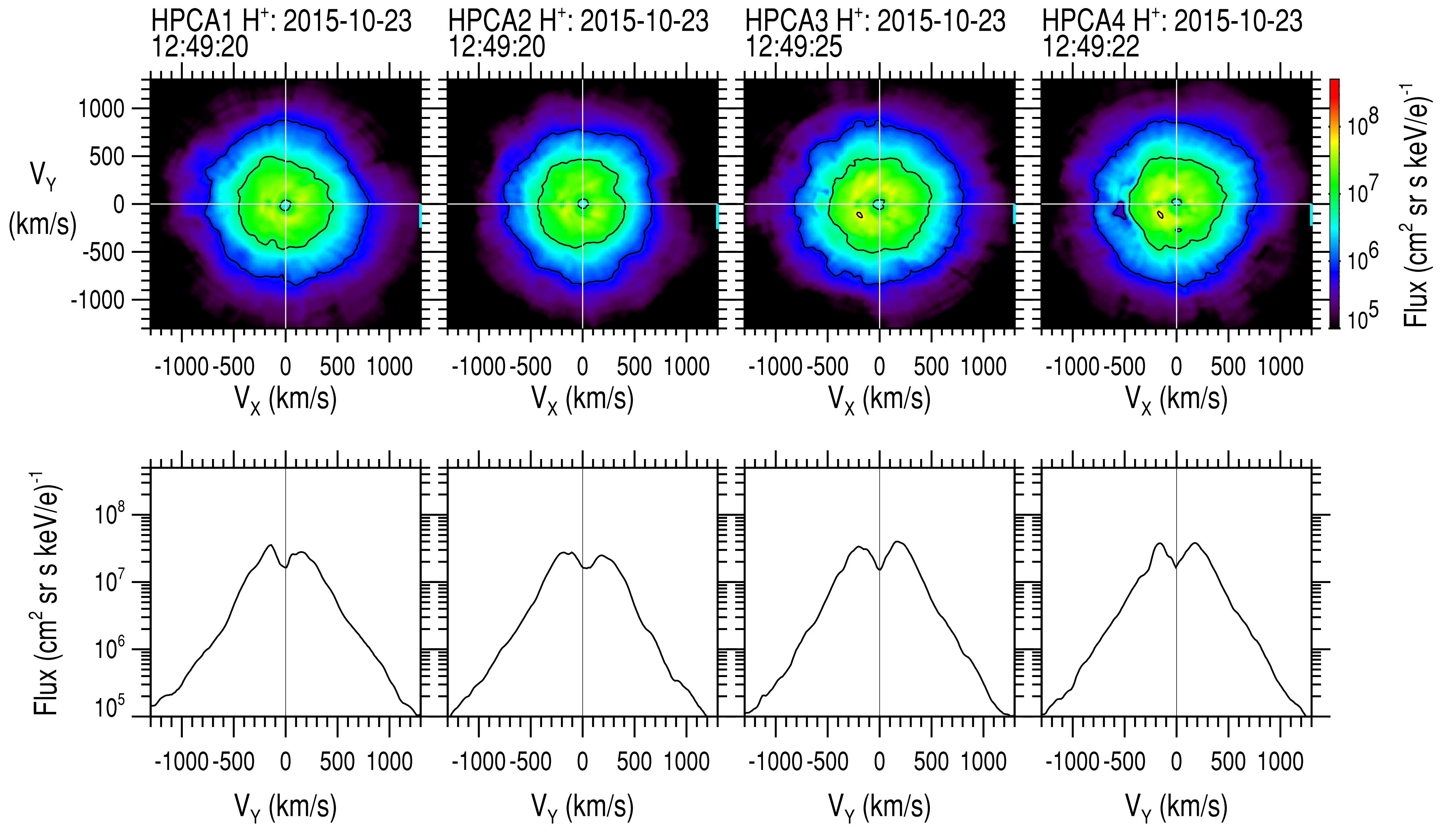

HPCA1 H+: 2015-10-23 12:49:20
HPCA2 H+: 2015-10-23 12:49:20
HPCA3 H+: 2015-10-23 12:49:25
HPCA4 H+: 2015-10-23 12:49:22
V_Y (km/s)
V_X (km/s)
Flux (cm^2 sr s keV/e)^-1
V_Y (km/s)

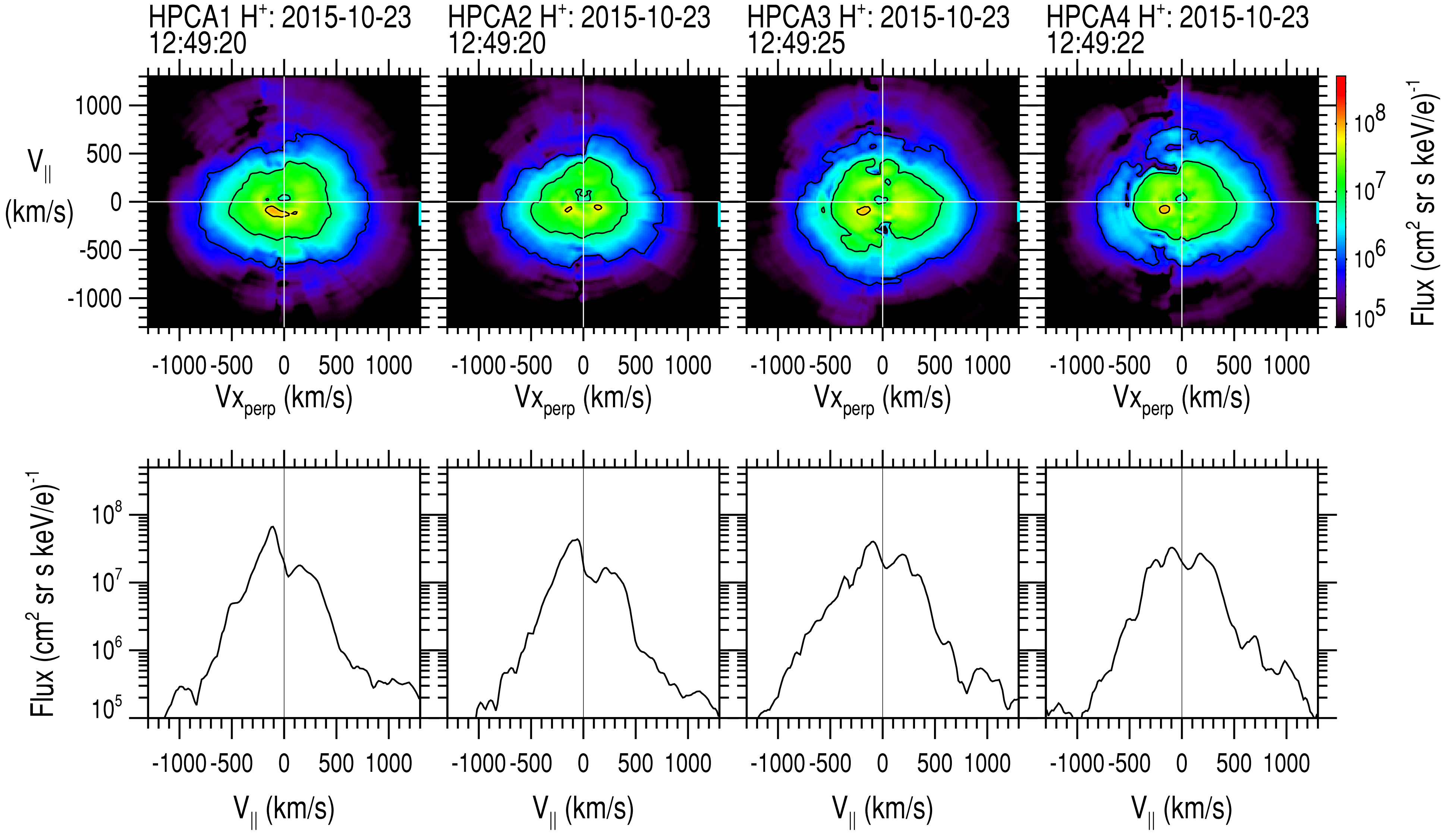

HPCA1 H+: 2015-10-23 12:49:20
HPCA2 H+: 2015-10-23 12:49:20
HPCA3 H+: 2015-10-23 12:49:25
HPCA4 H+: 2015-10-23 12:49:22
V|| (km/s)
Vxperp (km/s)
Flux (cm2 sr s keV/e)-1

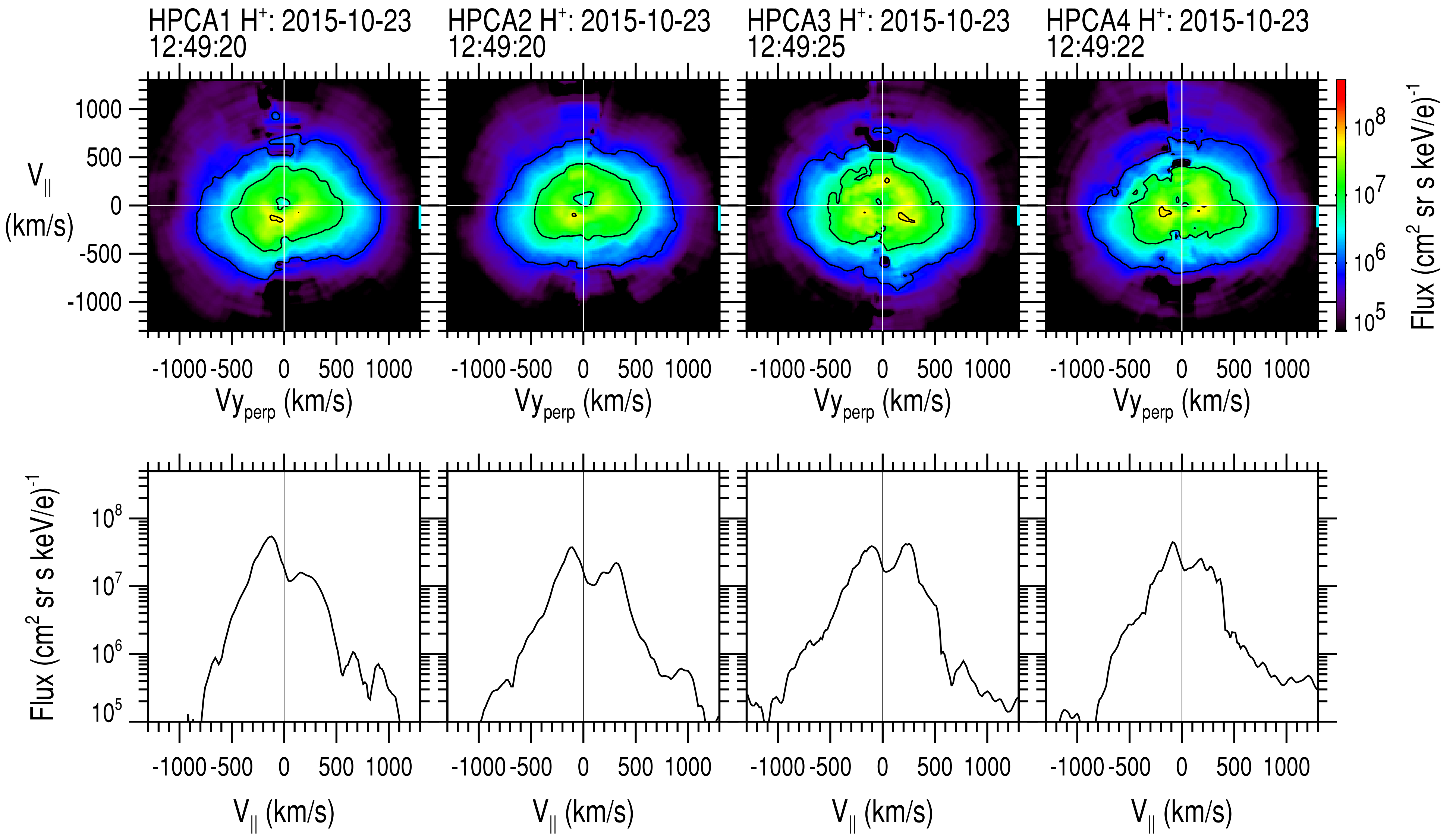

HPCA1 H+: 2015-10-23
12:49:20
HPCA2 H+: 2015-10-23
12:49:20
HPCA3 H+: 2015-10-23
12:49:25
HPCA4 H+: 2015-10-23
12:49:22
V|| (km/s)
Vyperp (km/s)
Flux (cm2 sr s keV/e)-1
V|| (km/s)

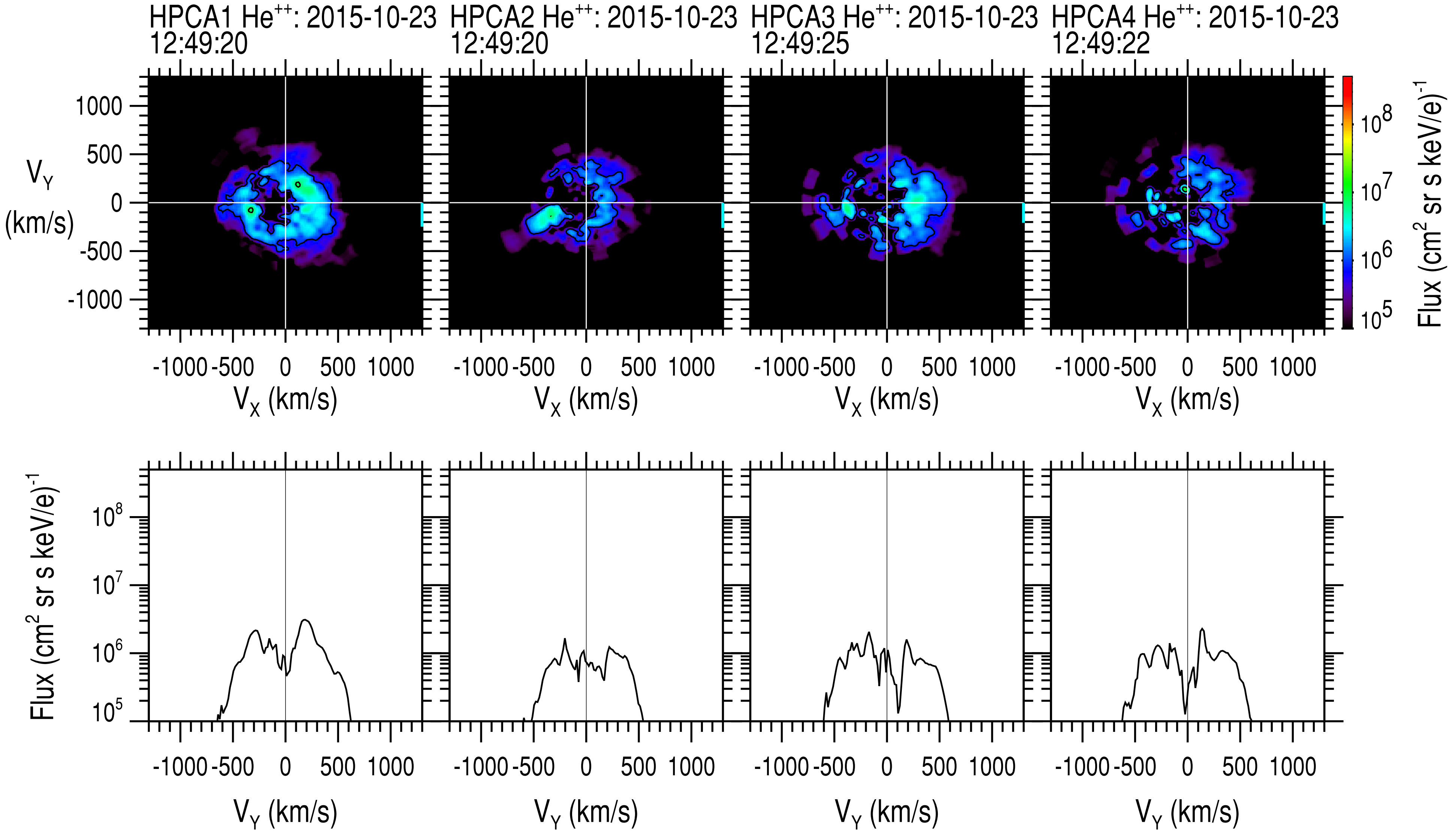
HPCA1 He++: 2015-10-23
12:49:20
HPCA2 He++: 2015-10-23
12:49:20
HPCA3 He++: 2015-10-23
12:49:25
HPCA4 He++: 2015-10-23
12:49:22
VY (km/s)
VX (km/s)
Flux (cm2 sr s keV/e)-1
-1000 -500 0 500 1000
10^5 10^6 10^7 10^8

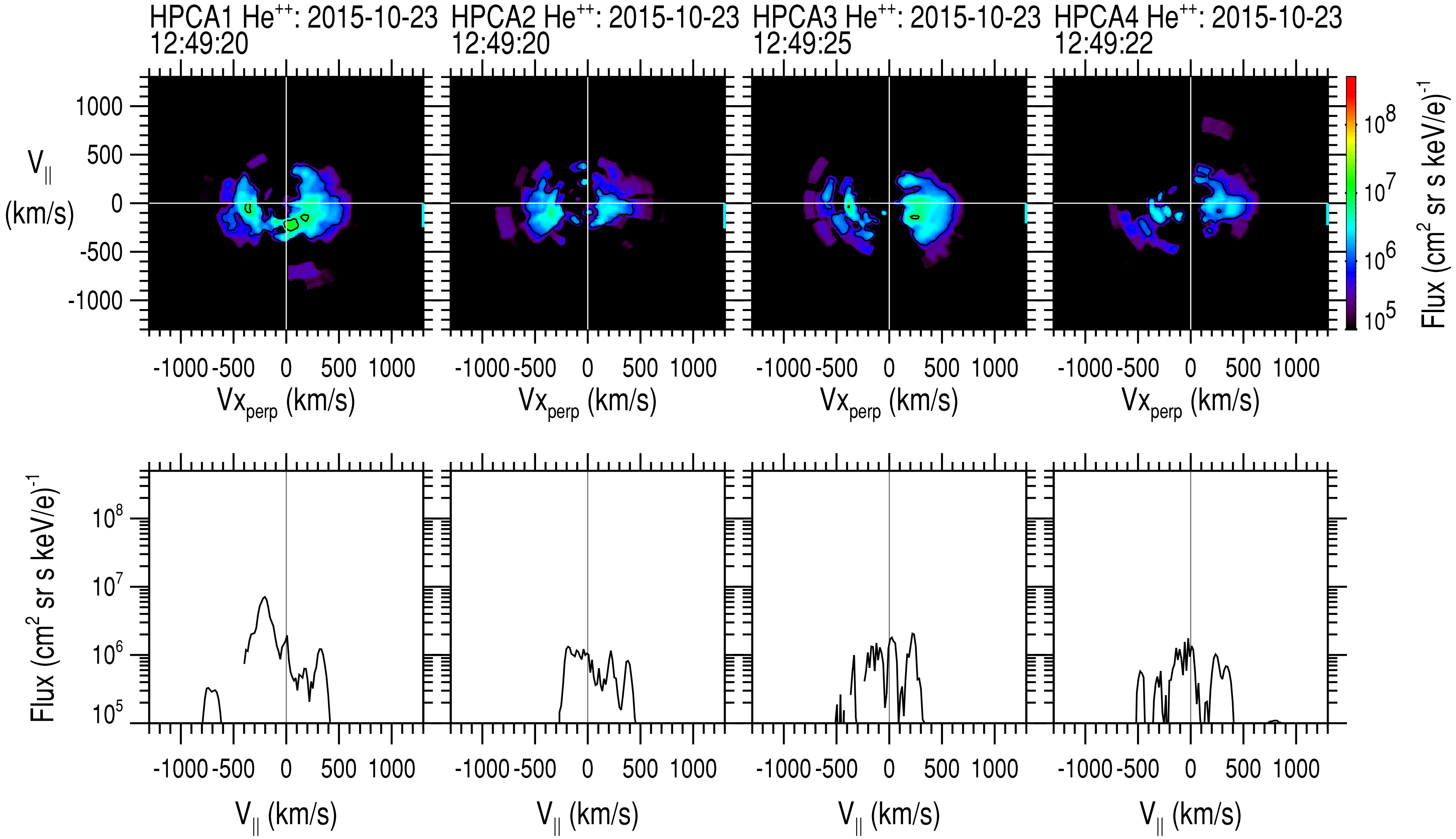

HPCA1 He++: 2015-10-23 12:49:20
HPCA2 He++: 2015-10-23 12:49:20
HPCA3 He++: 2015-10-23 12:49:25
HPCA4 He++: 2015-10-23 12:49:22
V|| (km/s)
Vxperp (km/s)
Flux (cm2 sr s keV/e)-1

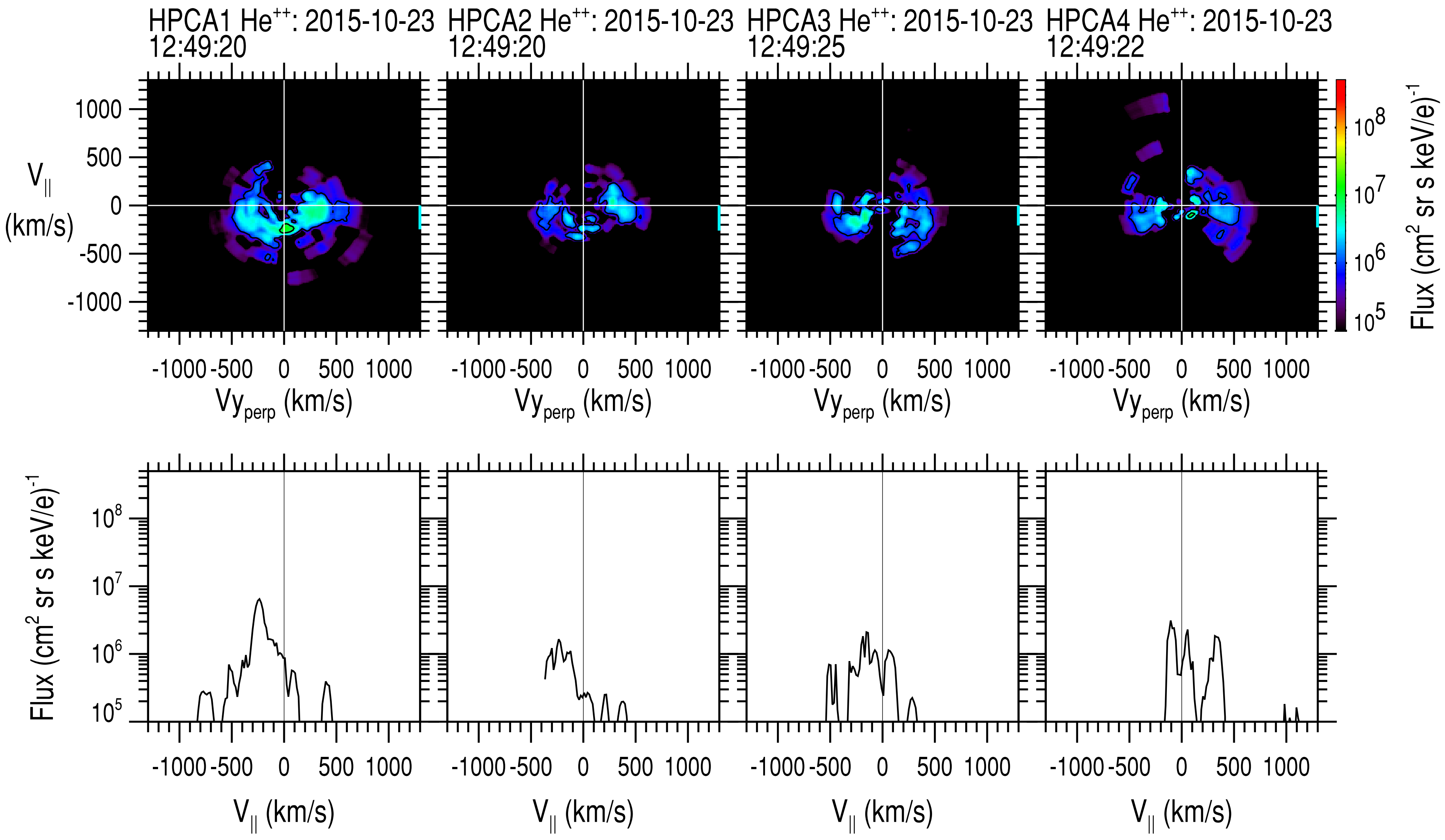
HPCA1 He++: 2015-10-23 12:49:20
HPCA2 He++: 2015-10-23 12:49:20
HPCA3 He++: 2015-10-23 12:49:25
HPCA4 He++: 2015-10-23 12:49:22
V|| (km/s)
Vyperp (km/s)
Flux (cm2 sr s keV/e)-1
V|| (km/s)

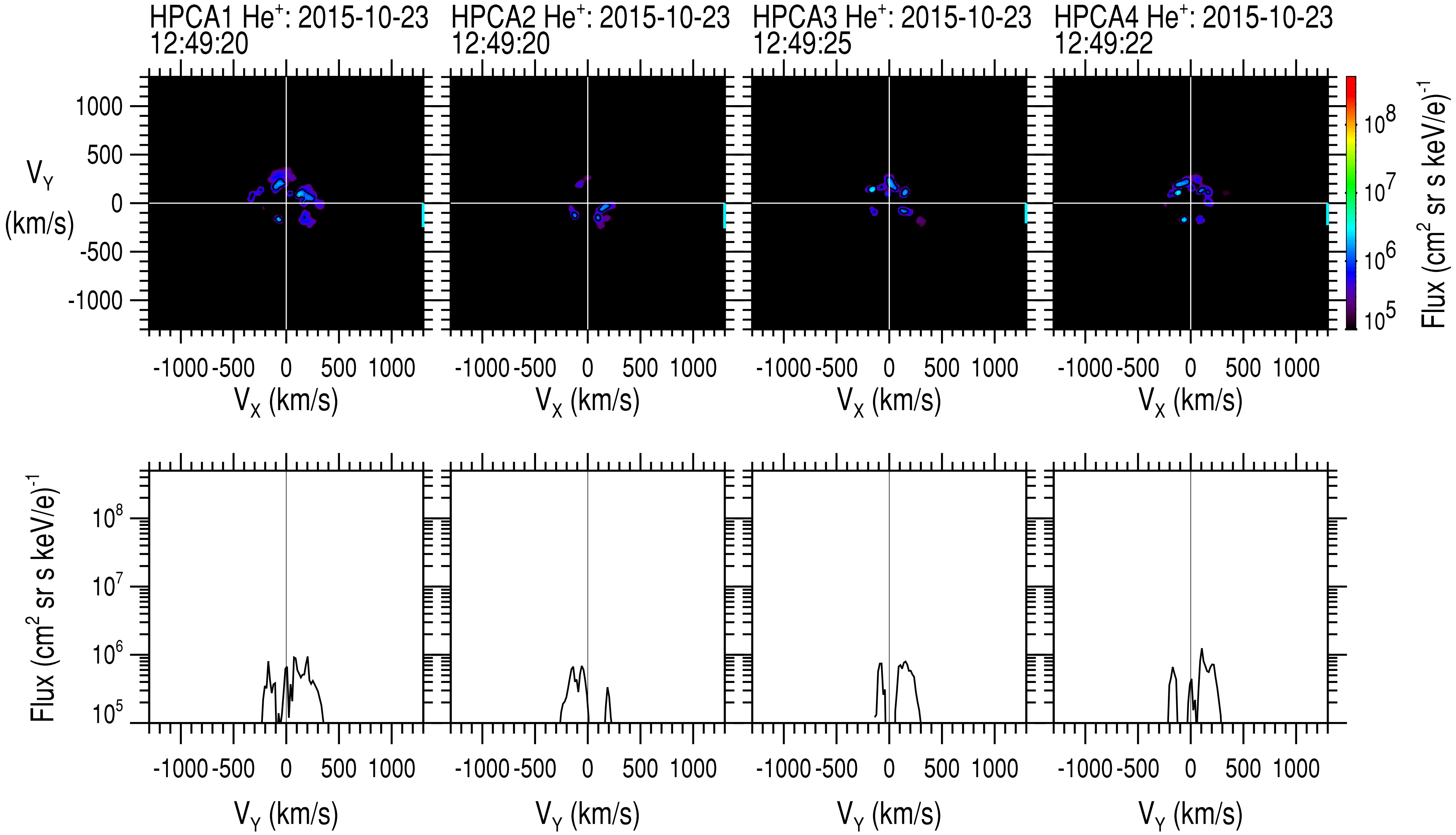

HPCA1 He+: 2015-10-23
12:49:20
HPCA2 He+: 2015-10-23
12:49:20
HPCA3 He+: 2015-10-23
12:49:25
HPCA4 He+: 2015-10-23
12:49:22
VY (km/s)
VX (km/s)
Flux (cm2 sr s keV/e)-1
VY (km/s)

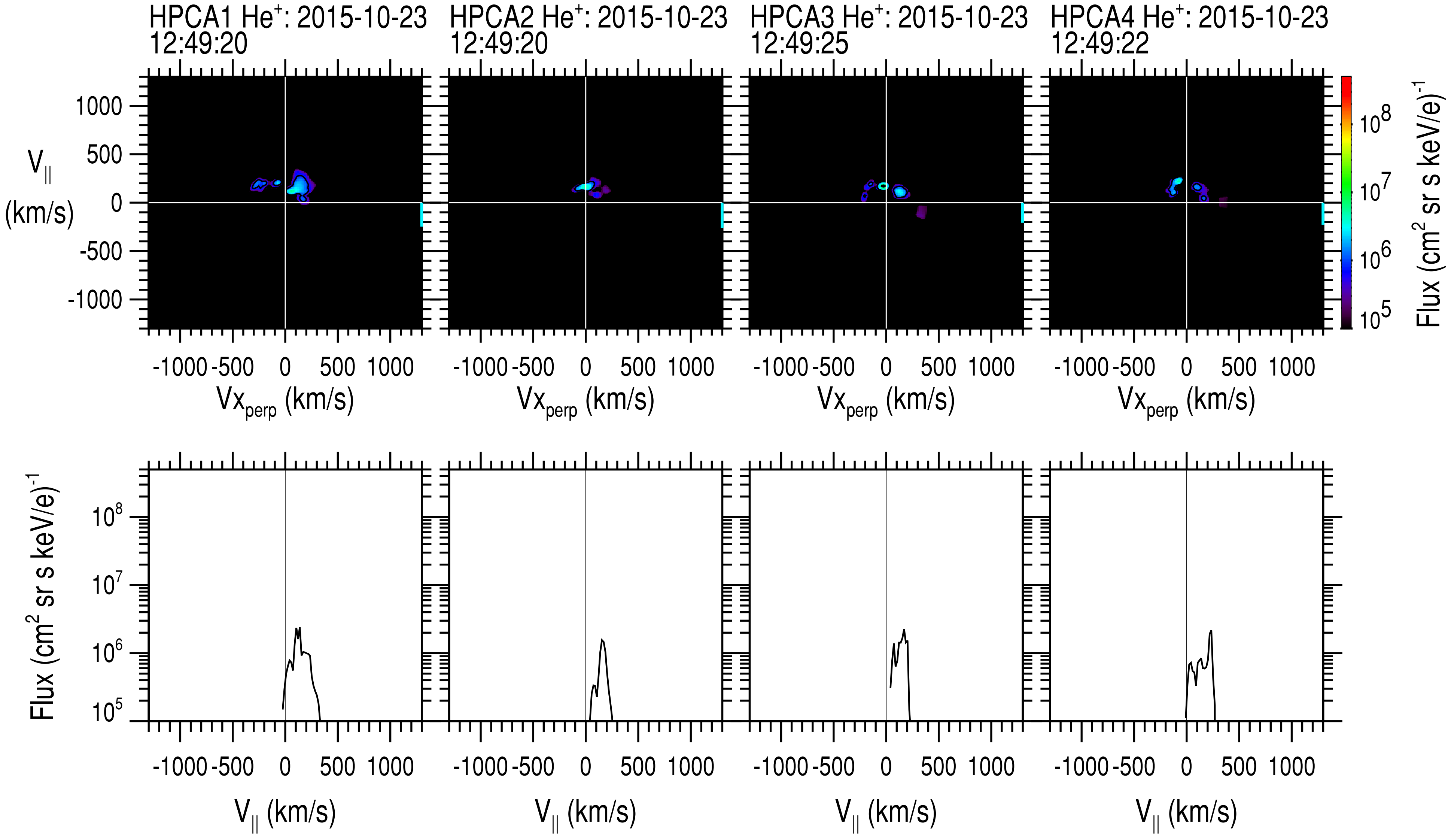
HPCA1 He+: 2015-10-23 12:49:20
HPCA2 He+: 2015-10-23 12:49:20
HPCA3 He+: 2015-10-23 12:49:25
HPCA4 He+: 2015-10-23 12:49:22
V|| (km/s)
Vxperp (km/s)
Flux (cm2 sr s keV/e)-1
1000
500
0
-500
-1000
10^5
10^6
10^7
10^8

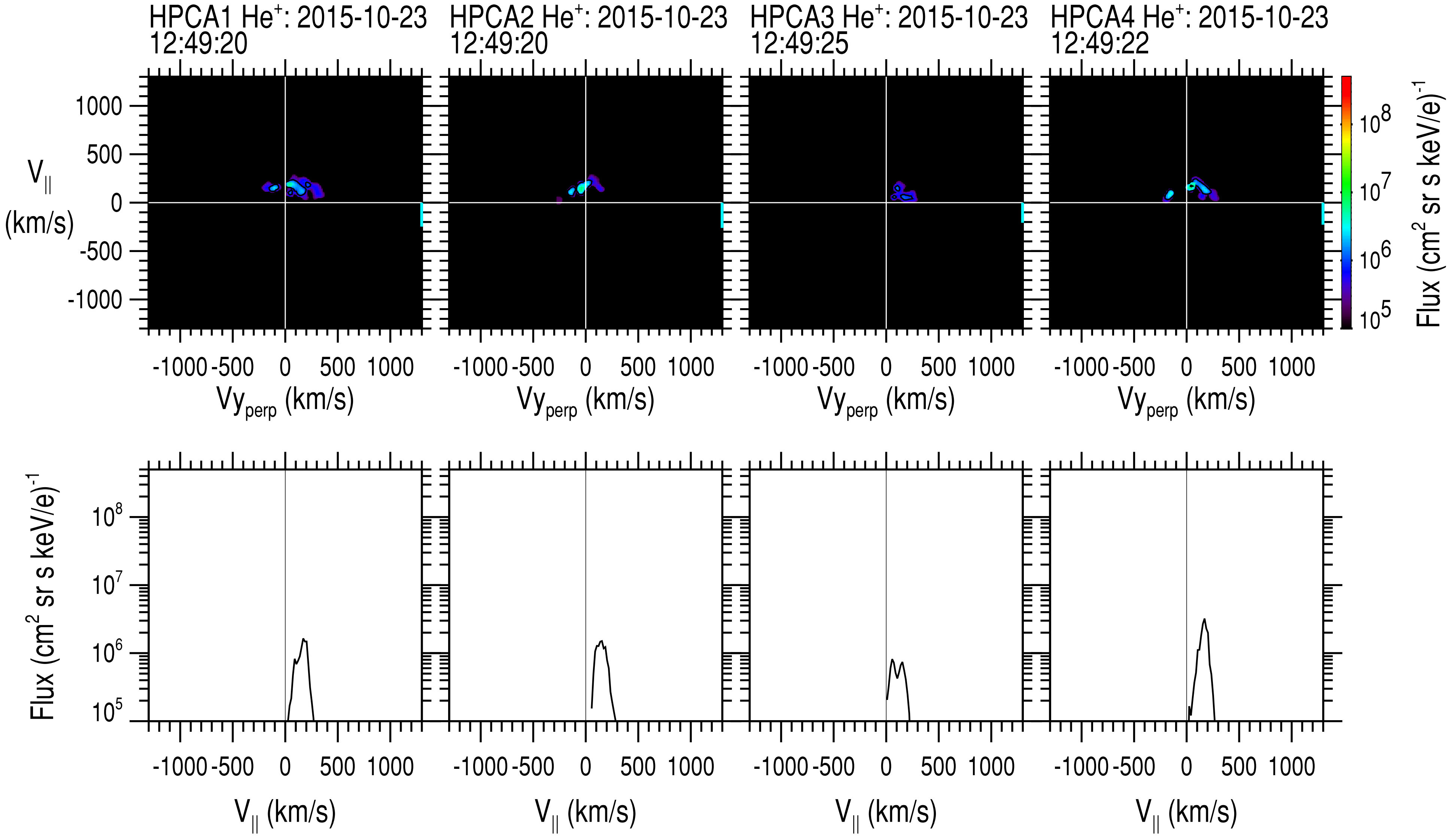
HPCA1 He+: 2015-10-23 12:49:20
HPCA2 He+: 2015-10-23 12:49:20
HPCA3 He+: 2015-10-23 12:49:25
HPCA4 He+: 2015-10-23 12:49:22
V|| (km/s)
Vyperp (km/s)
Flux (cm2 sr s keV/e)-1
V|| (km/s)

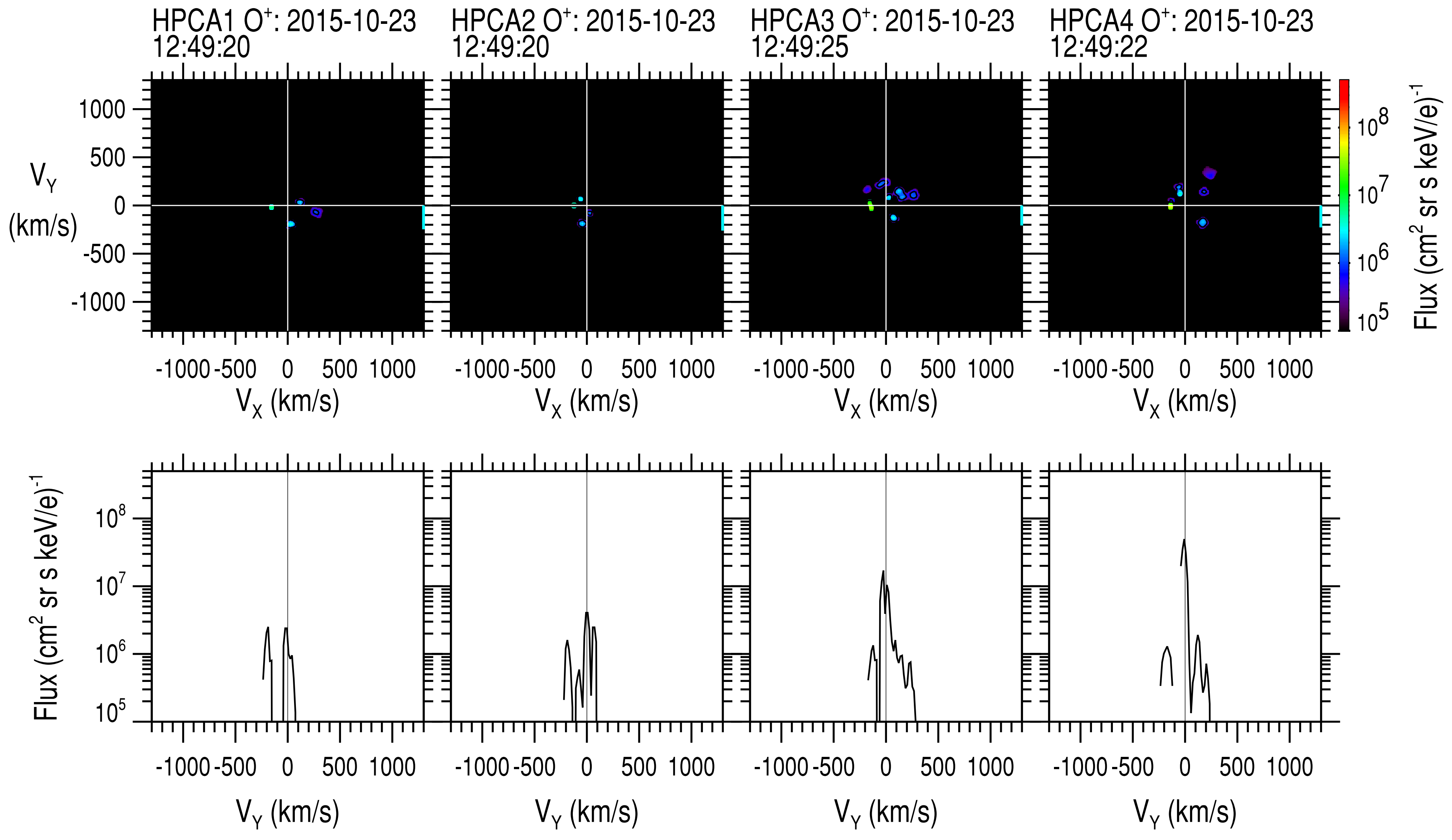
HPCA1 O+: 2015-10-23
12:49:20
HPCA2 O+: 2015-10-23
12:49:20
HPCA3 O+: 2015-10-23
12:49:25
HPCA4 O+: 2015-10-23
12:49:22
VY (km/s)
VX (km/s)
Flux (cm2 sr s keV/e)-1
-1000 -500 0 500 1000
10^5 10^6 10^7 10^8

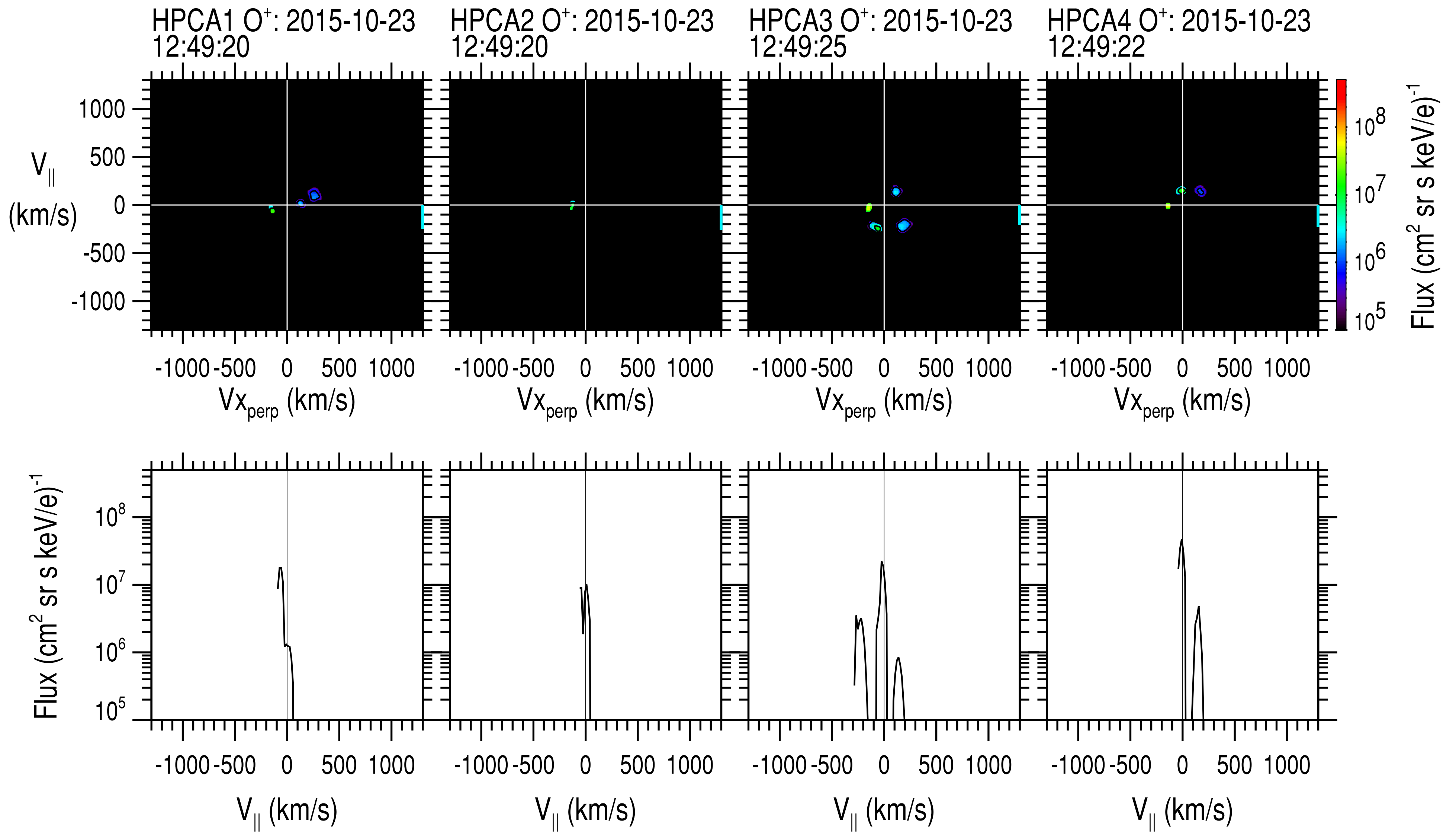
HPCA1 O+: 2015-10-23 12:49:20
HPCA2 O+: 2015-10-23 12:49:20
HPCA3 O+: 2015-10-23 12:49:25
HPCA4 O+: 2015-10-23 12:49:22
V|| (km/s)
Vxperp (km/s)
Flux (cm2 sr s keV/e)-1
1000
500
0
-500
-1000
10^5
10^6
10^7
10^8

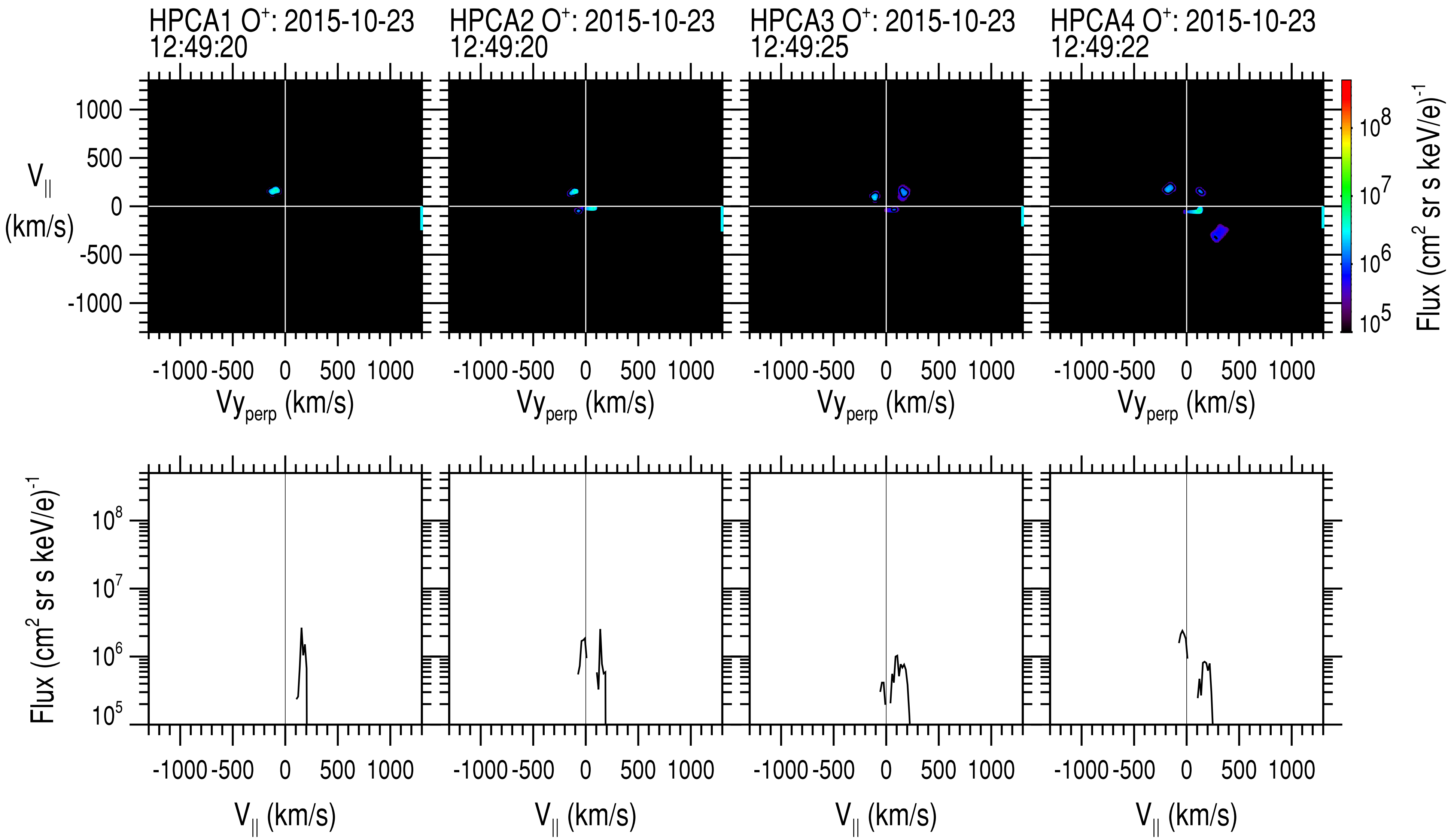
HPCA1 O+: 2015-10-23 12:49:20
HPCA2 O+: 2015-10-23 12:49:20
HPCA3 O+: 2015-10-23 12:49:25
HPCA4 O+: 2015-10-23 12:49:22
V|| (km/s)
Vyperp (km/s)
Flux (cm2 sr s keV/e)-1
V|| (km/s)
Flux (cm2 sr s keV/e)-1

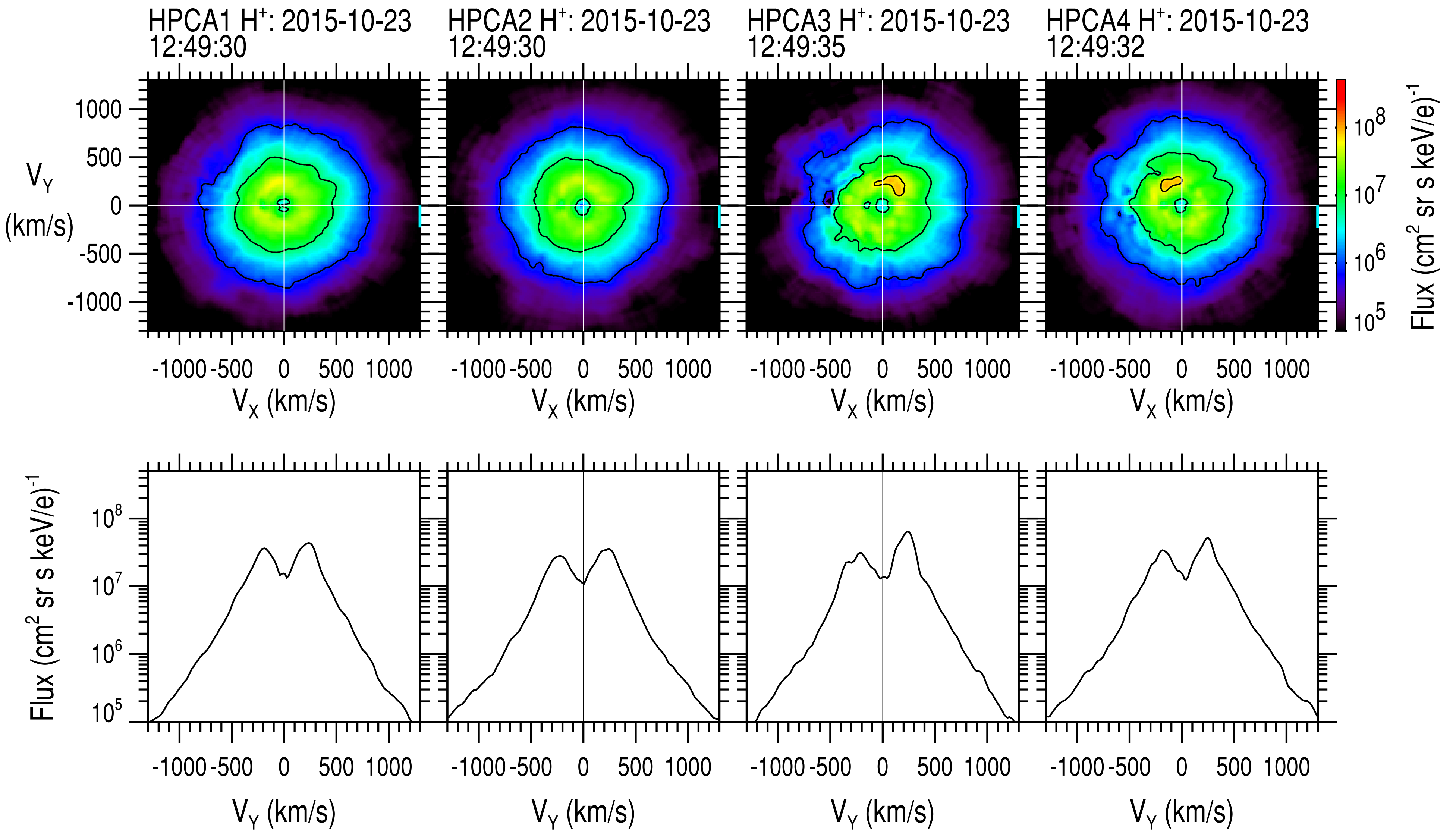
HPCA1 H+: 2015-10-23
12:49:30
HPCA2 H+: 2015-10-23
12:49:30
HPCA3 H+: 2015-10-23
12:49:35
HPCA4 H+: 2015-10-23
12:49:32
VY (km/s)
VX (km/s)
Flux (cm2 sr s keV/e)-1
VY (km/s)

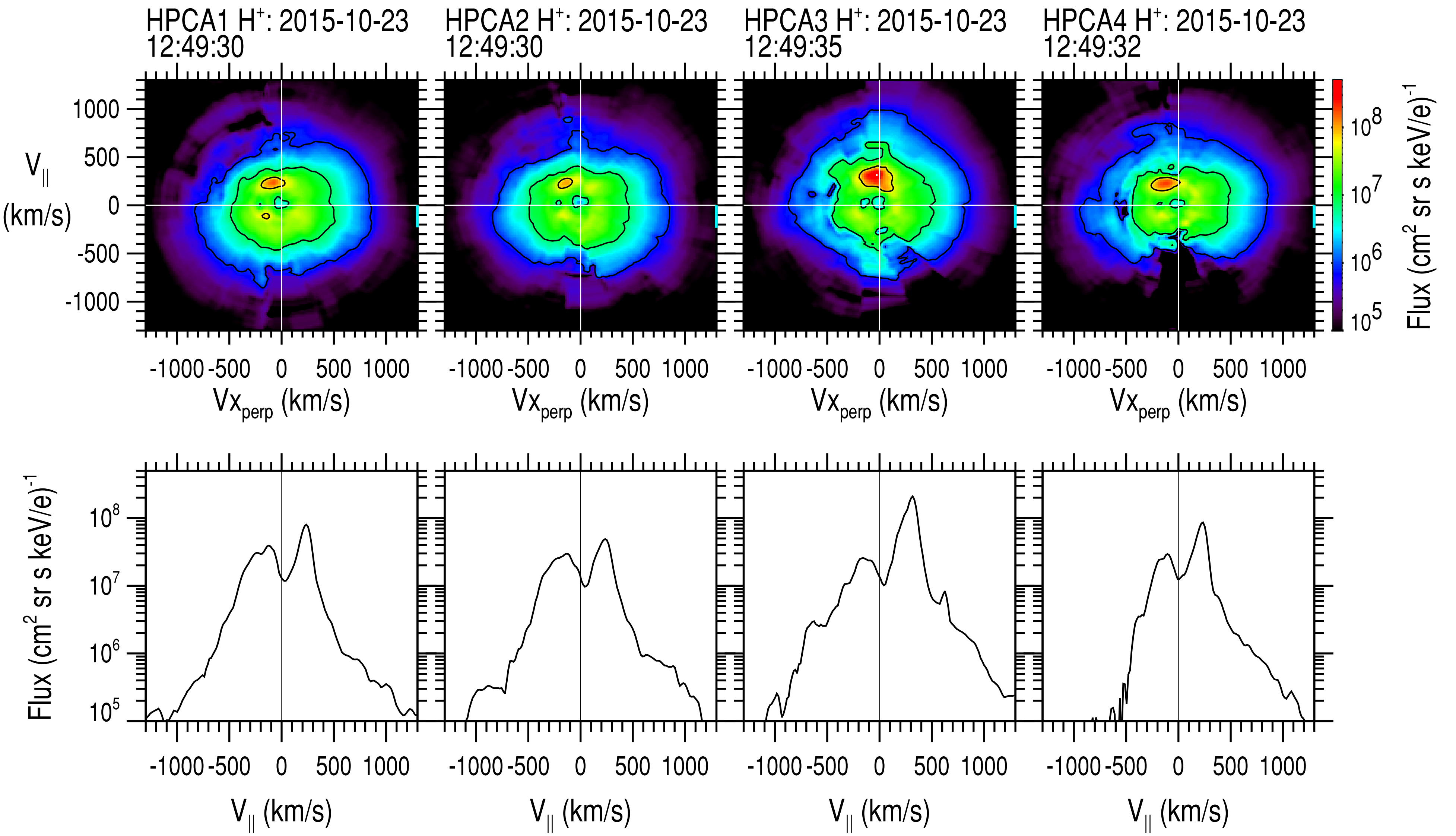
HPCA1 H+: 2015-10-23 12:49:30
HPCA2 H+: 2015-10-23 12:49:30
HPCA3 H+: 2015-10-23 12:49:35
HPCA4 H+: 2015-10-23 12:49:32
V|| (km/s)
Vxperp (km/s)
Flux (cm2 sr s keV/e)-1
V|| (km/s)

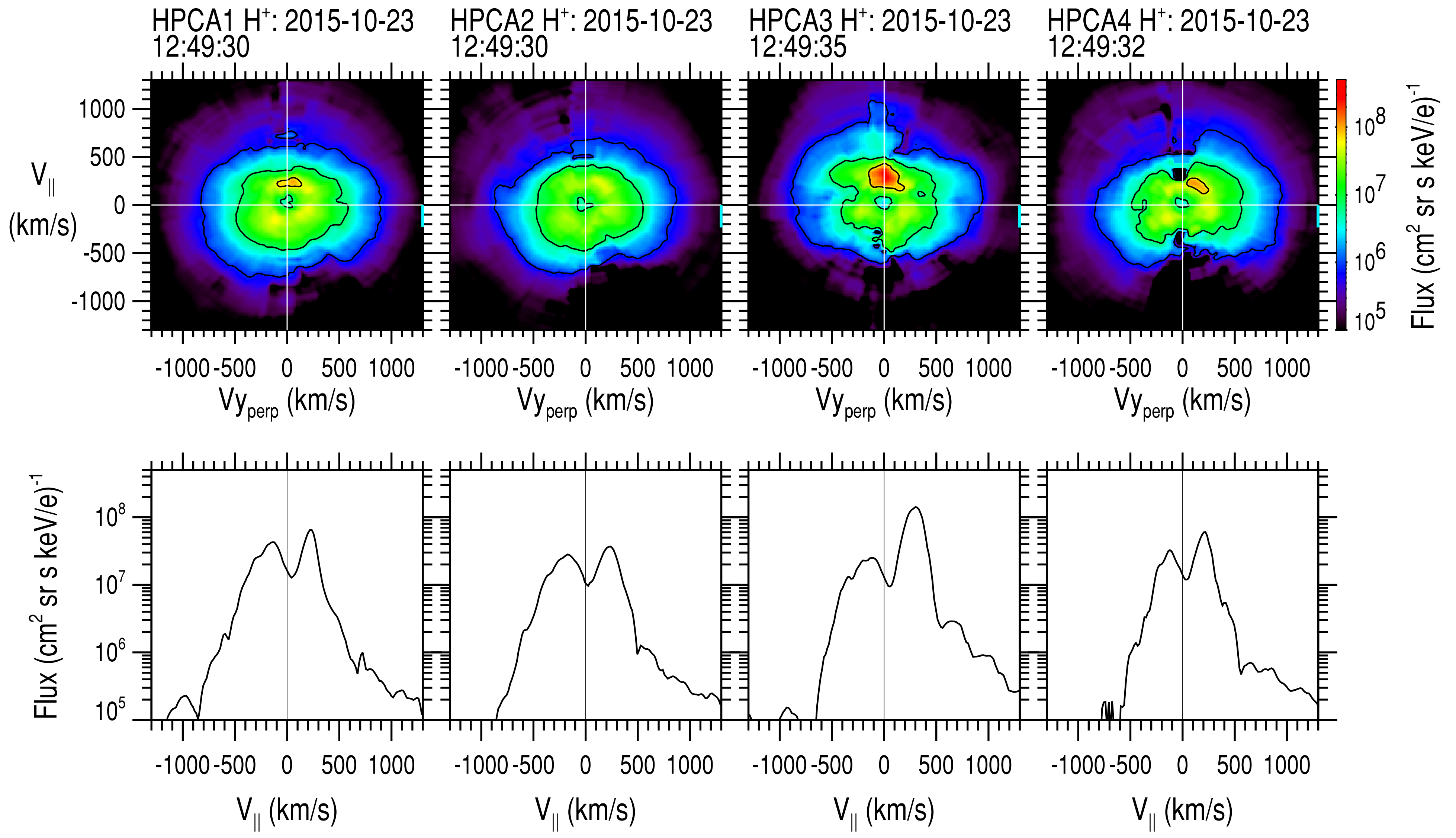

HPCA1 H+: 2015-10-23
12:49:30
HPCA2 H+: 2015-10-23
12:49:30
HPCA3 H+: 2015-10-23
12:49:35
HPCA4 H+: 2015-10-23
12:49:32
V|| (km/s)
Vyperp (km/s)
Flux (cm2 sr s keV/e)-1
10^5
10^6
10^7
10^8
1000
500
0
-500
-1000

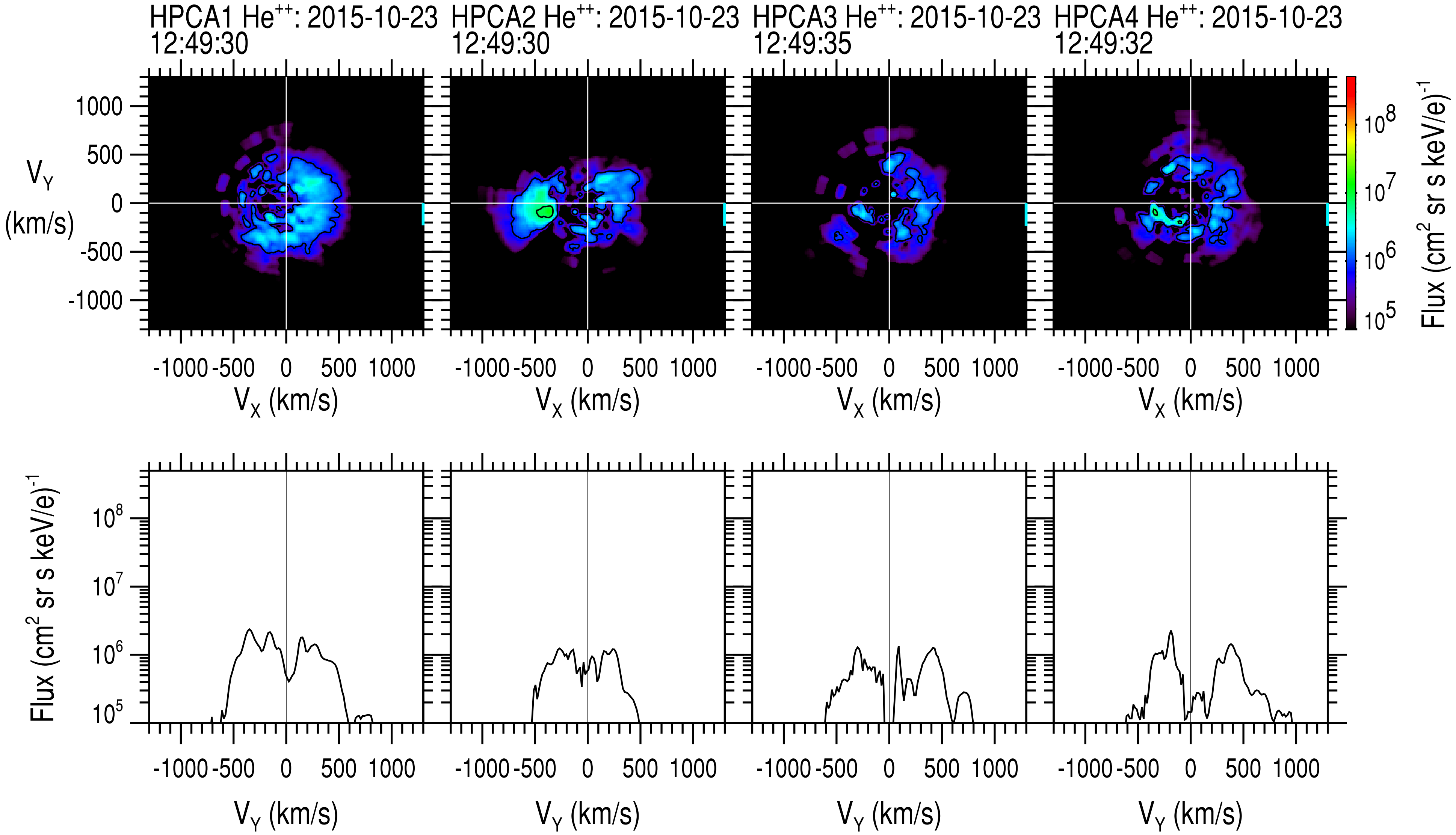

HPCA1 He++: 2015-10-23 12:49:30
HPCA2 He++: 2015-10-23 12:49:30
HPCA3 He++: 2015-10-23 12:49:35
HPCA4 He++: 2015-10-23 12:49:32
V_Y (km/s)
V_X (km/s)
Flux (cm^2 sr s keV/e)^-1
V_Y (km/s)

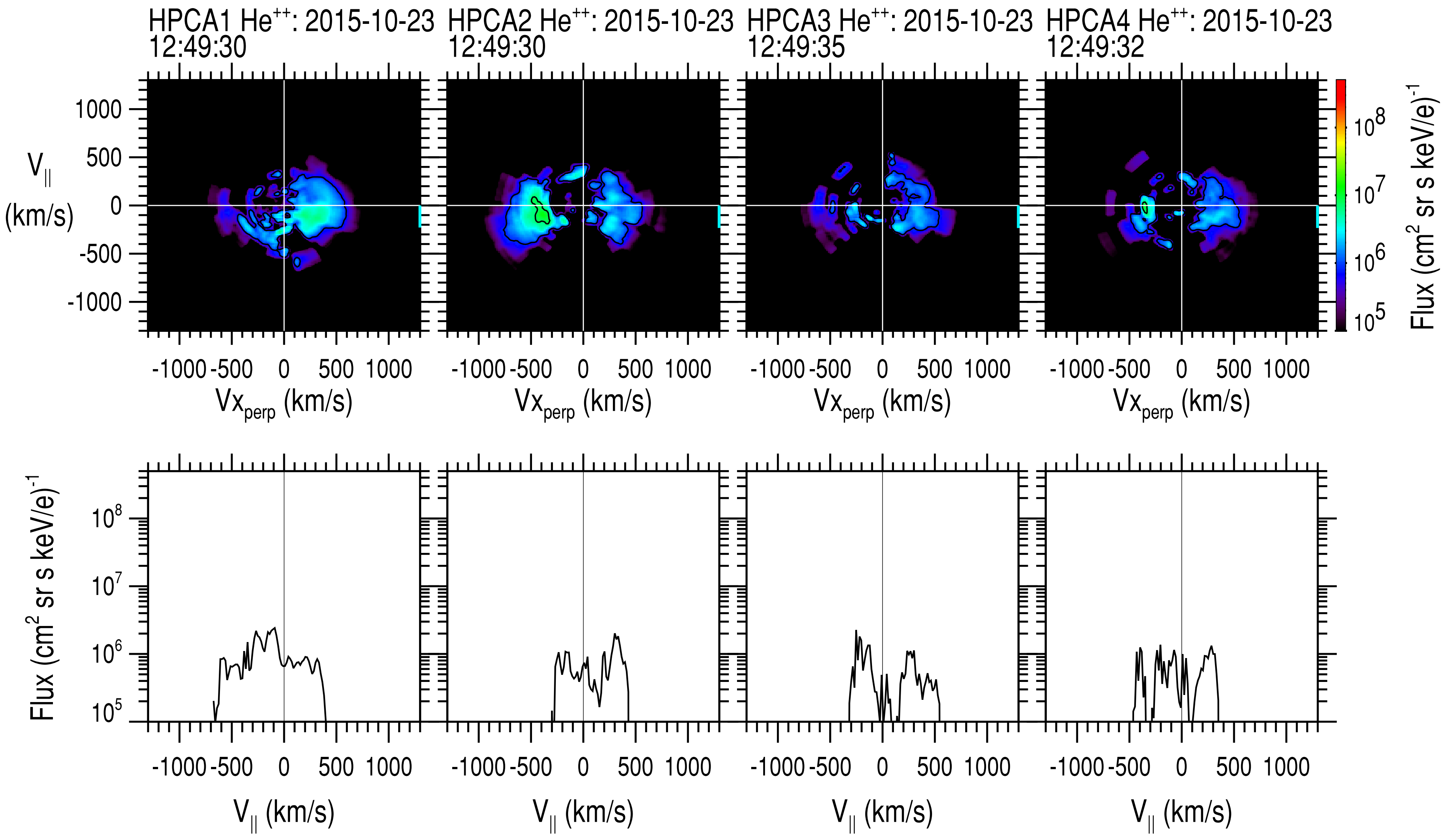
HPCA1 He++: 2015-10-23 12:49:30
HPCA2 He++: 2015-10-23 12:49:30
HPCA3 He++: 2015-10-23 12:49:35
HPCA4 He++: 2015-10-23 12:49:32
V|| (km/s)
Vxperp (km/s)
Flux (cm2 sr s keV/e)-1
-1000 -500 0 500 1000
10^5 10^6 10^7 10^8

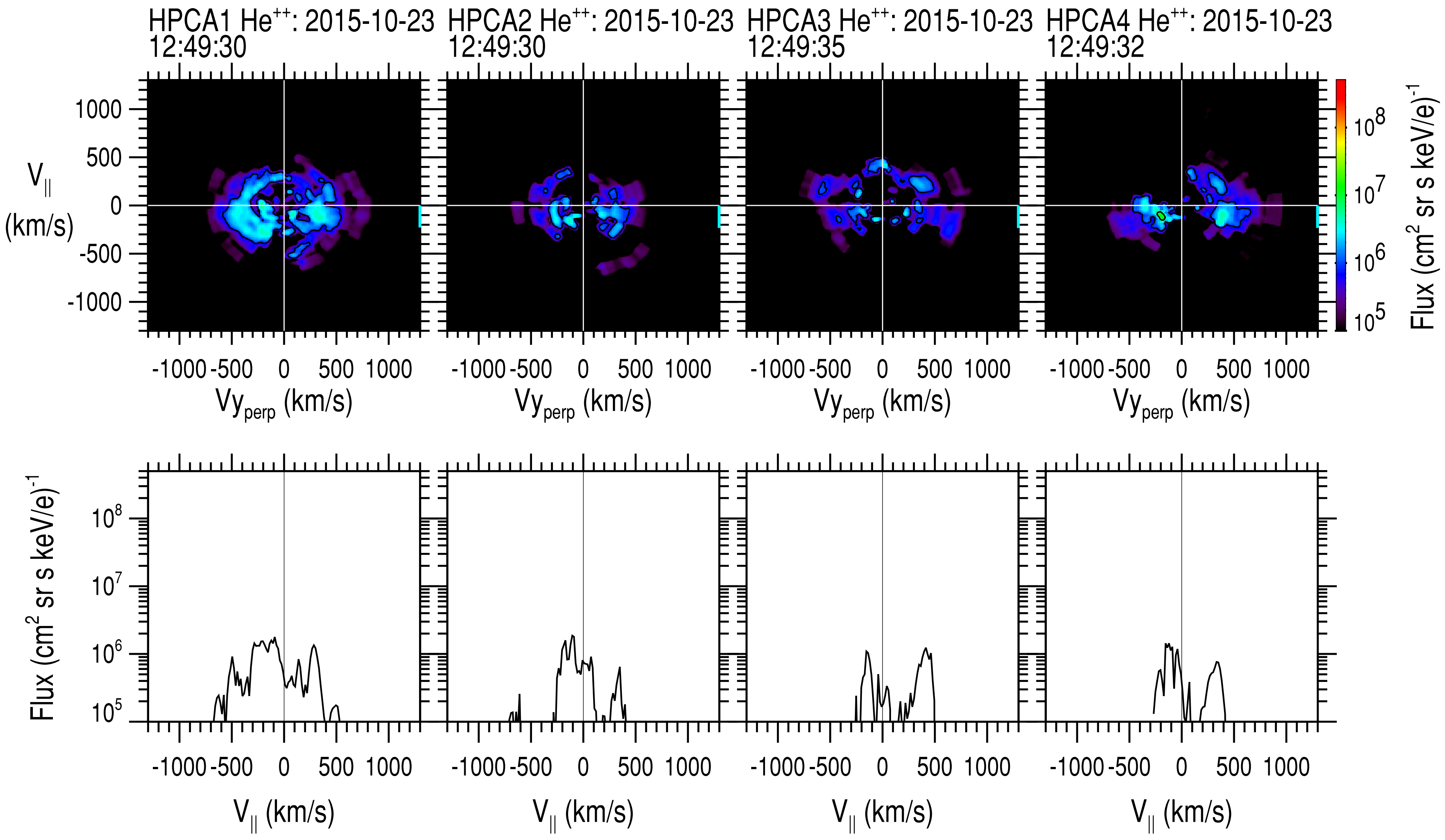
HPCA1 He++: 2015-10-23
12:49:30
HPCA2 He++: 2015-10-23
12:49:30
HPCA3 He++: 2015-10-23
12:49:35
HPCA4 He++: 2015-10-23
12:49:32
V||
(km/s)
Vyperp (km/s)
Flux (cm2 sr s keV/e)-1
V|| (km/s)

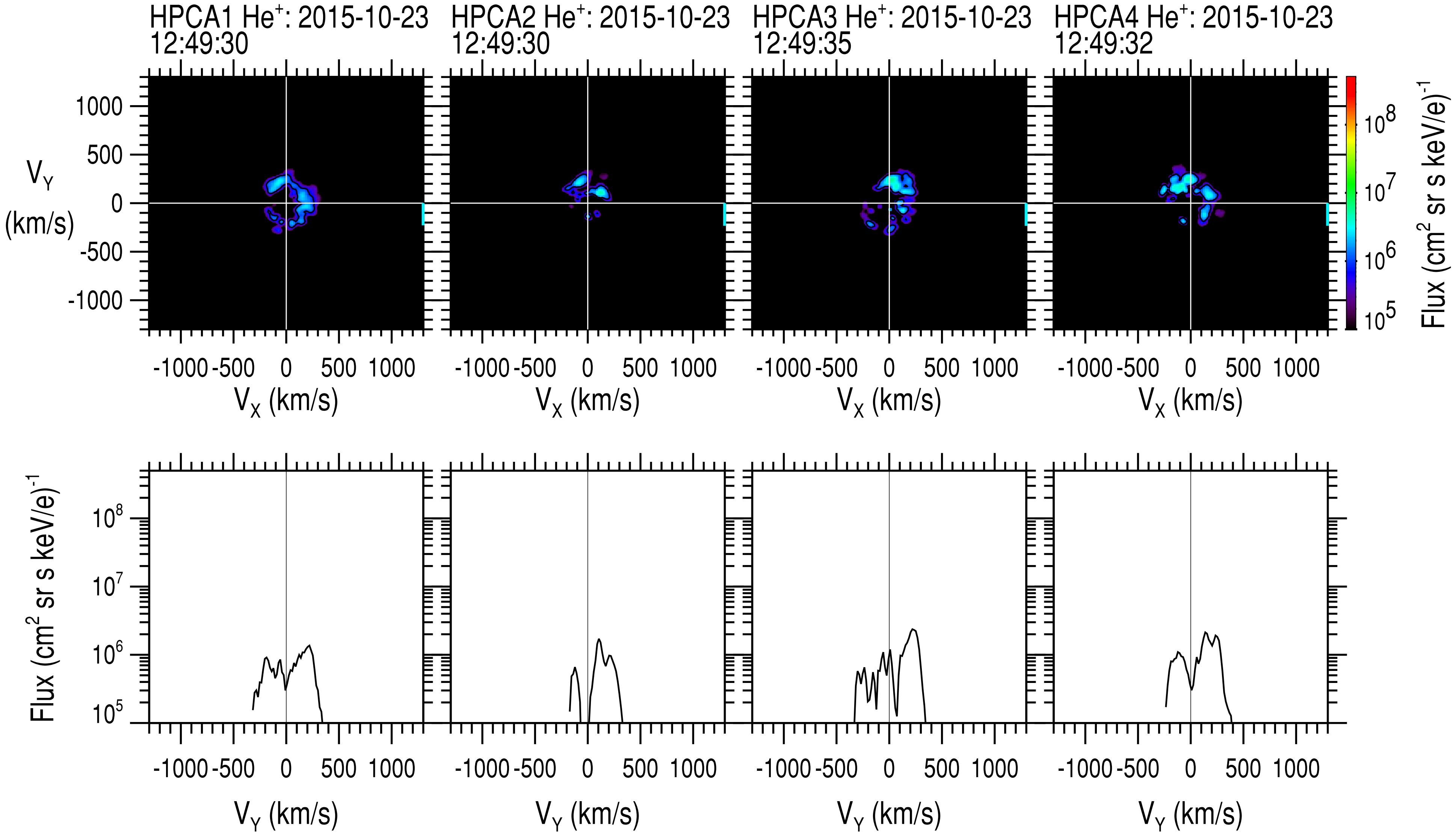
HPCA1 He+: 2015-10-23 12:49:30
HPCA2 He+: 2015-10-23 12:49:30
HPCA3 He+: 2015-10-23 12:49:35
HPCA4 He+: 2015-10-23 12:49:32
VY (km/s)
VX (km/s)
Flux (cm2 sr s keV/e)-1
1000
500
0
-500
-1000
105
106
107
108

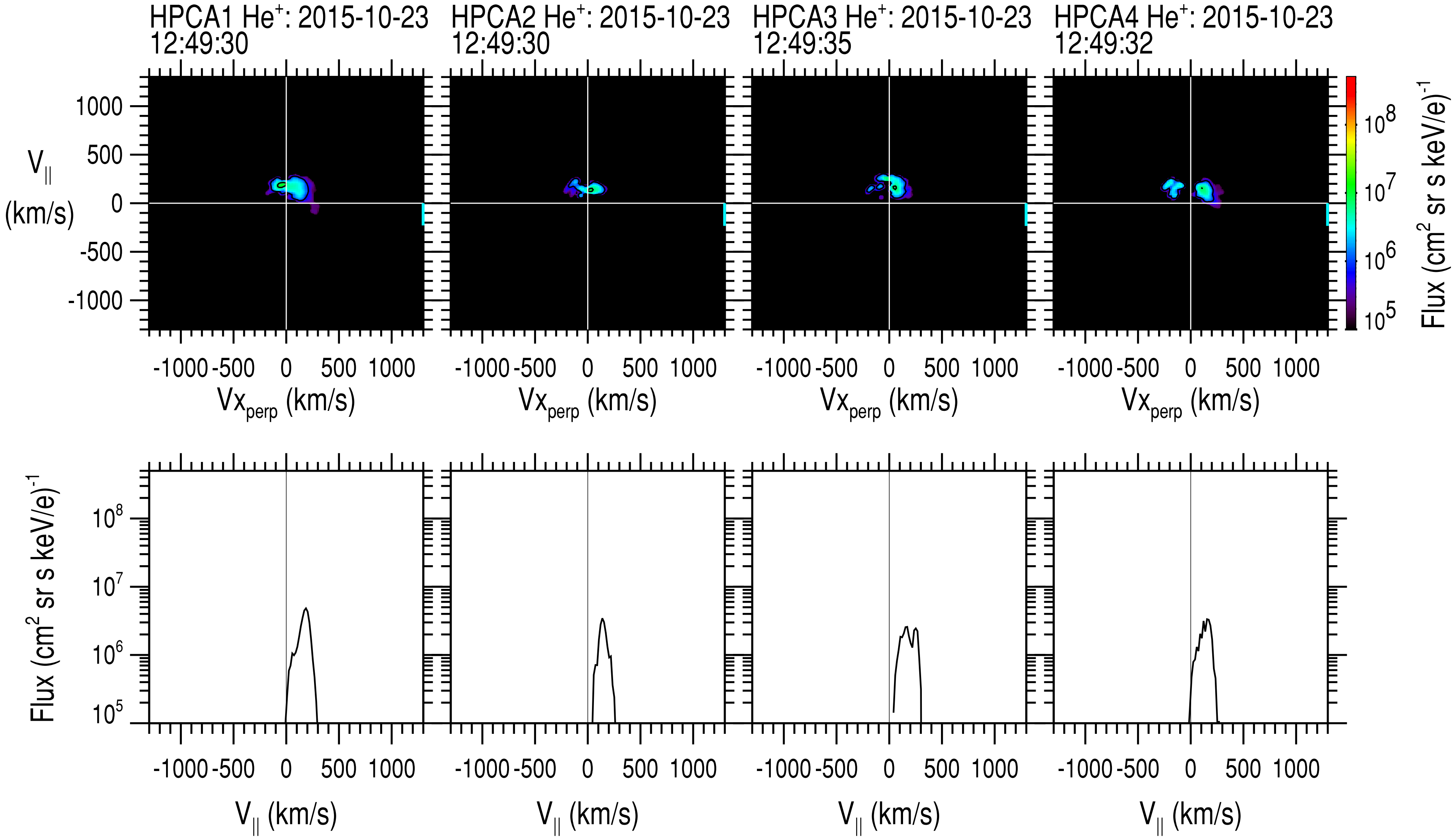
HPCA1 He+: 2015-10-23
12:49:30
HPCA2 He+: 2015-10-23
12:49:30
HPCA3 He+: 2015-10-23
12:49:35
HPCA4 He+: 2015-10-23
12:49:32
V|| (km/s)
Vxperp (km/s)
Flux (cm2 sr s keV/e)-1
V|| (km/s)

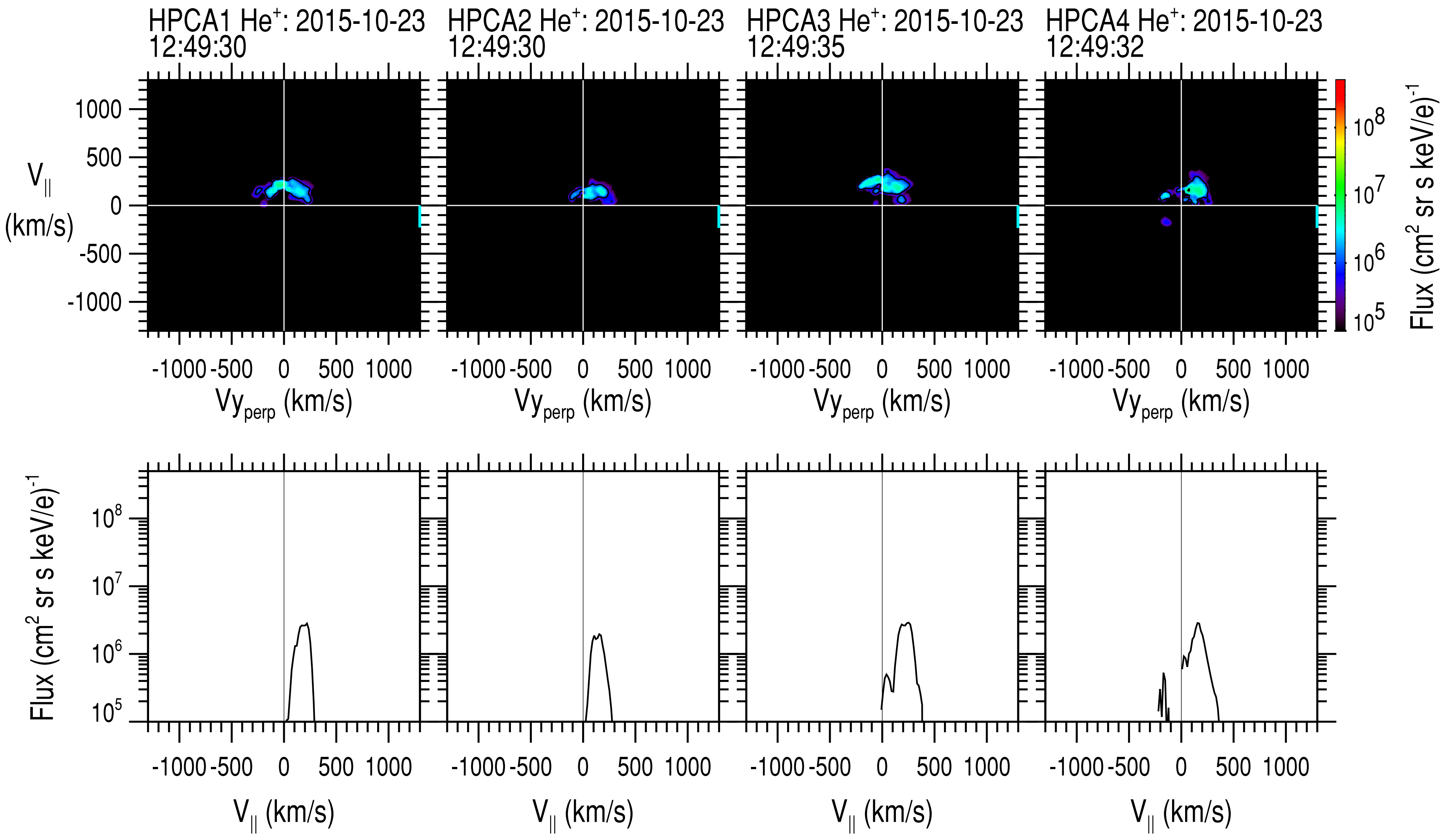

HPCA1 He+: 2015-10-23
12:49:30
HPCA2 He+: 2015-10-23
12:49:30
HPCA3 He+: 2015-10-23
12:49:35
HPCA4 He+: 2015-10-23
12:49:32
V|| (km/s)
Vyperp (km/s)
Flux (cm2 sr s keV/e)-1
V|| (km/s)

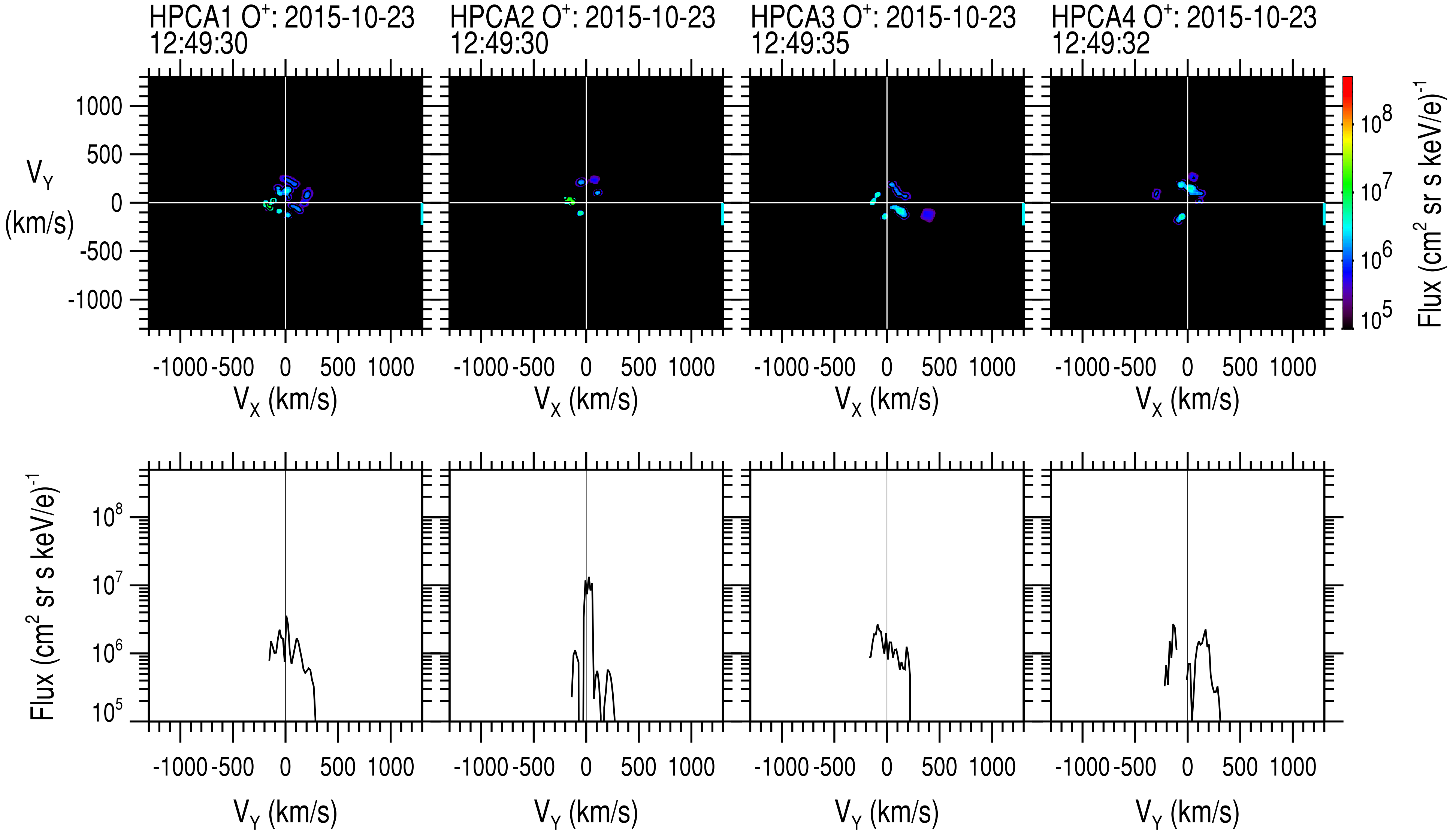

HPCA1 O+: 2015-10-23 12:49:30
HPCA2 O+: 2015-10-23 12:49:30
HPCA3 O+: 2015-10-23 12:49:35
HPCA4 O+: 2015-10-23 12:49:32
V_Y (km/s)
V_X (km/s)
Flux (cm^2 sr s keV/e)^-1
V_Y (km/s)

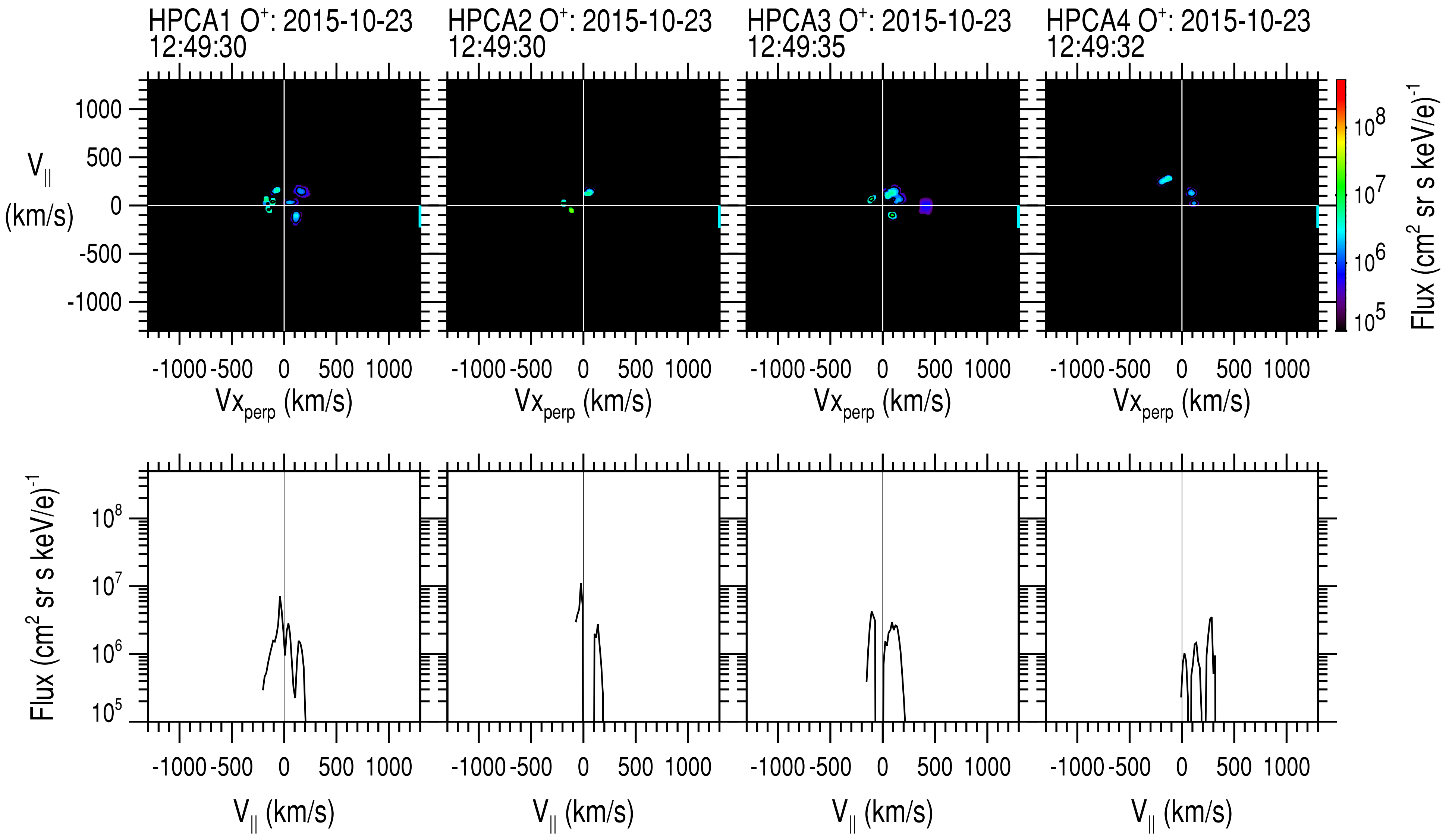

HPCA1 O+: 2015-10-23 12:49:30
HPCA2 O+: 2015-10-23 12:49:30
HPCA3 O+: 2015-10-23 12:49:35
HPCA4 O+: 2015-10-23 12:49:32
V|| (km/s)
Vxperp (km/s)
Flux (cm2 sr s keV/e)-1

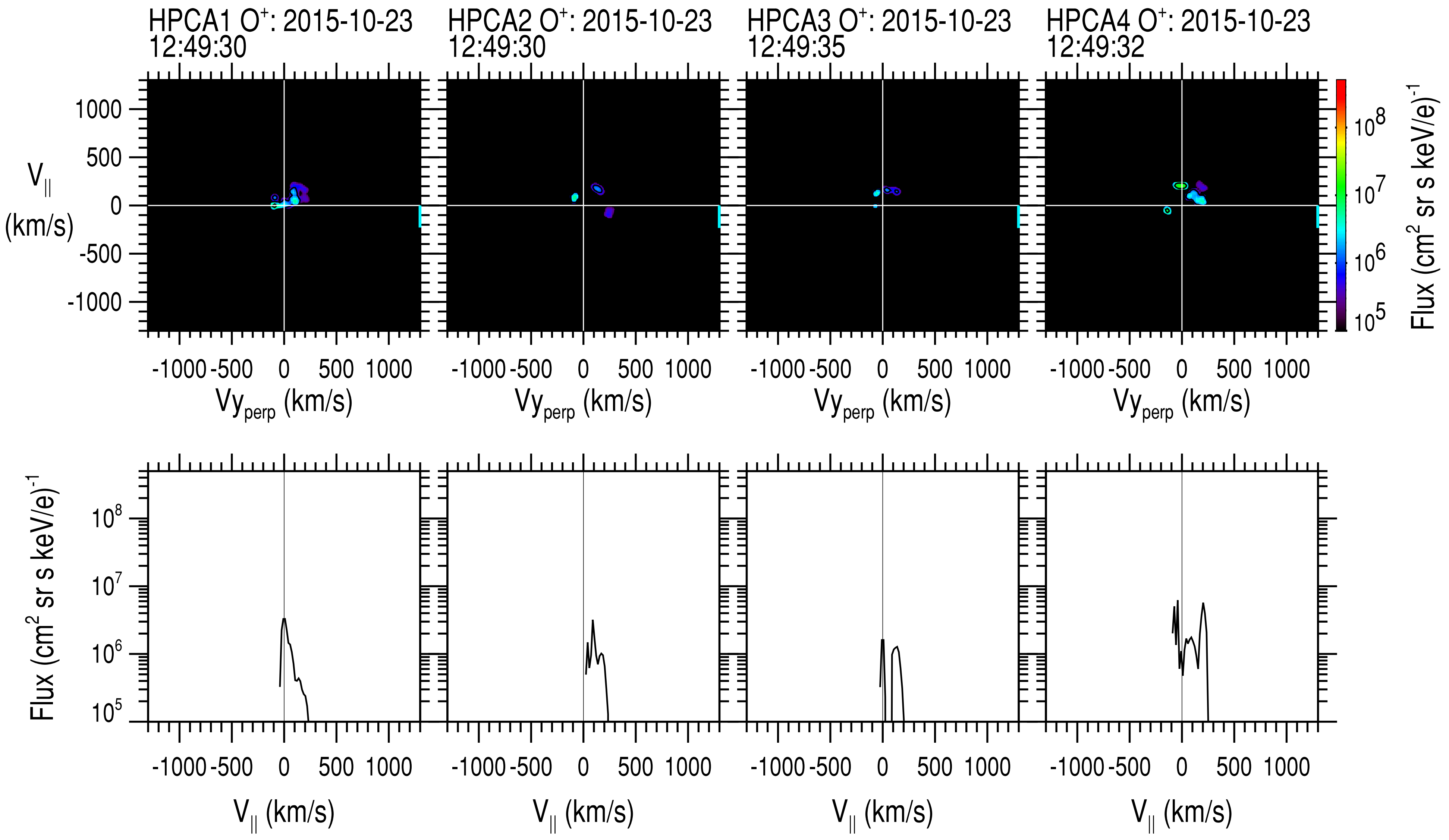

HPCA1 O+: 2015-10-23 12:49:30
HPCA2 O+: 2015-10-23 12:49:30
HPCA3 O+: 2015-10-23 12:49:35
HPCA4 O+: 2015-10-23 12:49:32
V|| (km/s)
Vyperp (km/s)
Flux (cm2 sr s keV/e)-1
V|| (km/s)

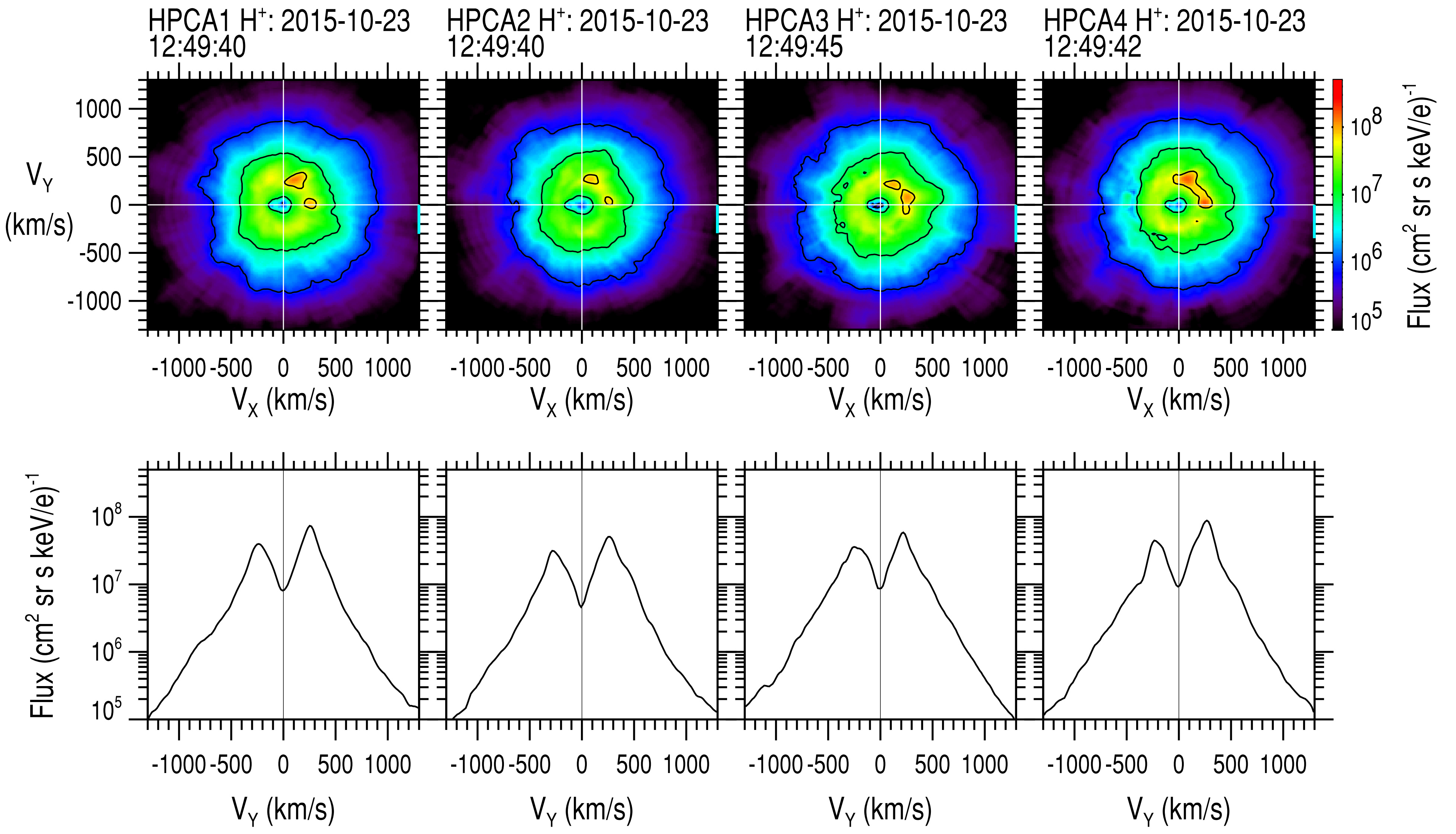
HPCA1 H+: 2015-10-23
12:49:40
HPCA2 H+: 2015-10-23
12:49:40
HPCA3 H+: 2015-10-23
12:49:45
HPCA4 H+: 2015-10-23
12:49:42
V_Y (km/s)
V_X (km/s)
Flux (cm^2 sr s keV/e)^-1
V_Y (km/s)

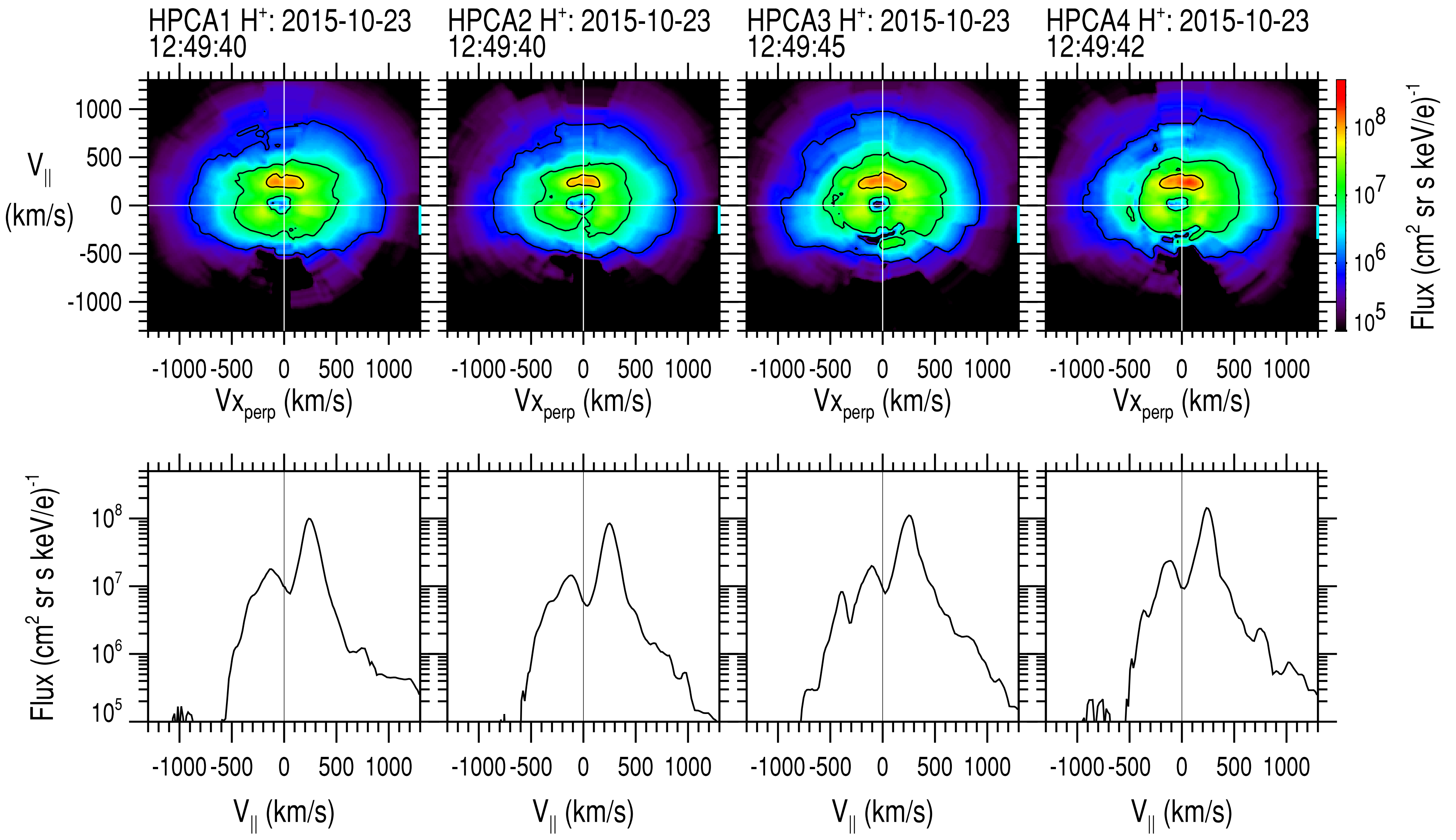
HPCA1 H+: 2015-10-23 12:49:40
HPCA2 H+: 2015-10-23 12:49:40
HPCA3 H+: 2015-10-23 12:49:45
HPCA4 H+: 2015-10-23 12:49:42
V|| (km/s)
Vxperp (km/s)
Flux (cm2 sr s keV/e)-1
V|| (km/s)

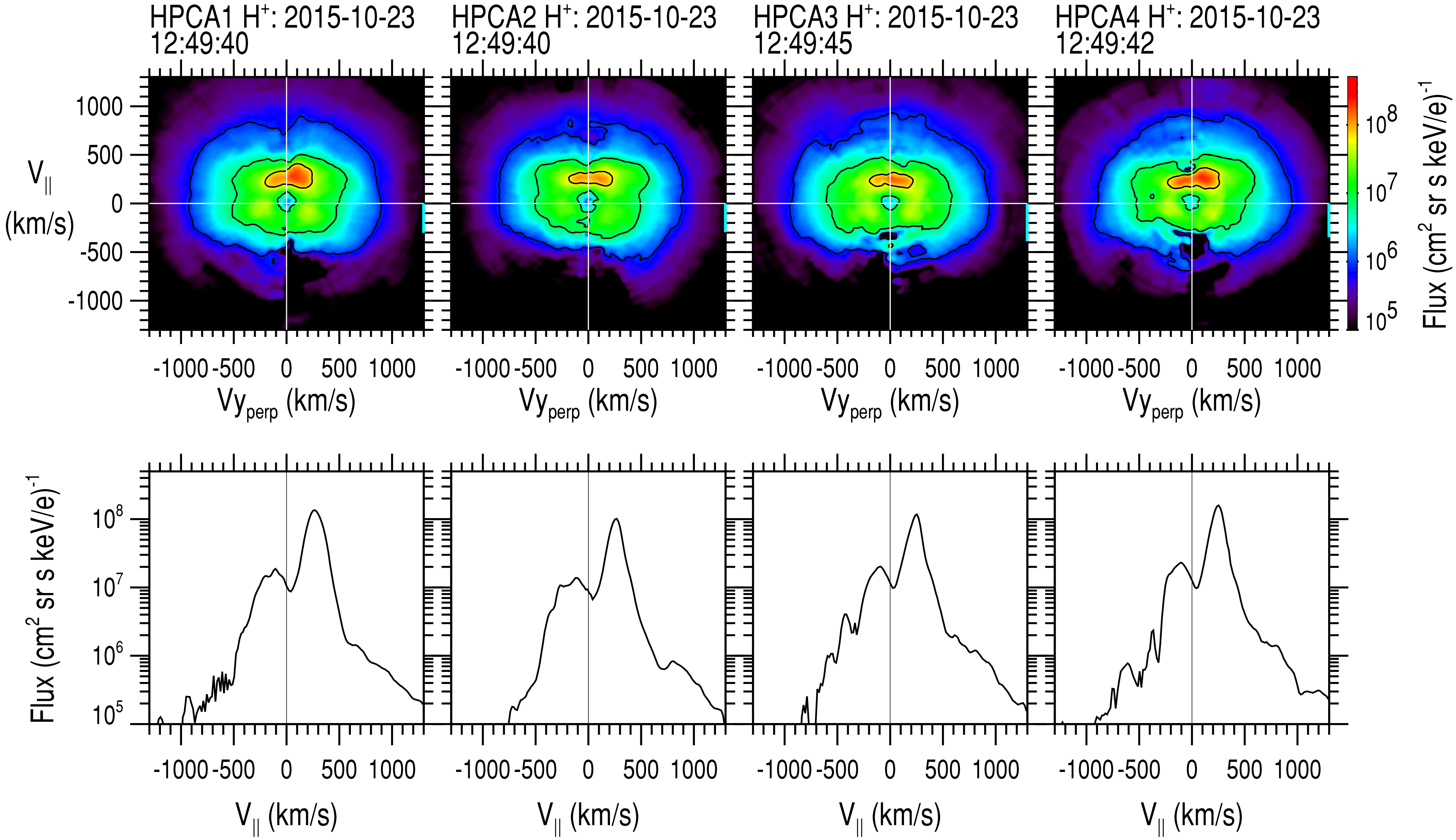
HPCA1 H+: 2015-10-23 12:49:40
HPCA2 H+: 2015-10-23 12:49:40
HPCA3 H+: 2015-10-23 12:49:45
HPCA4 H+: 2015-10-23 12:49:42
V|| (km/s)
Vyperp (km/s)
Flux (cm2 sr s keV/e)-1
V|| (km/s)

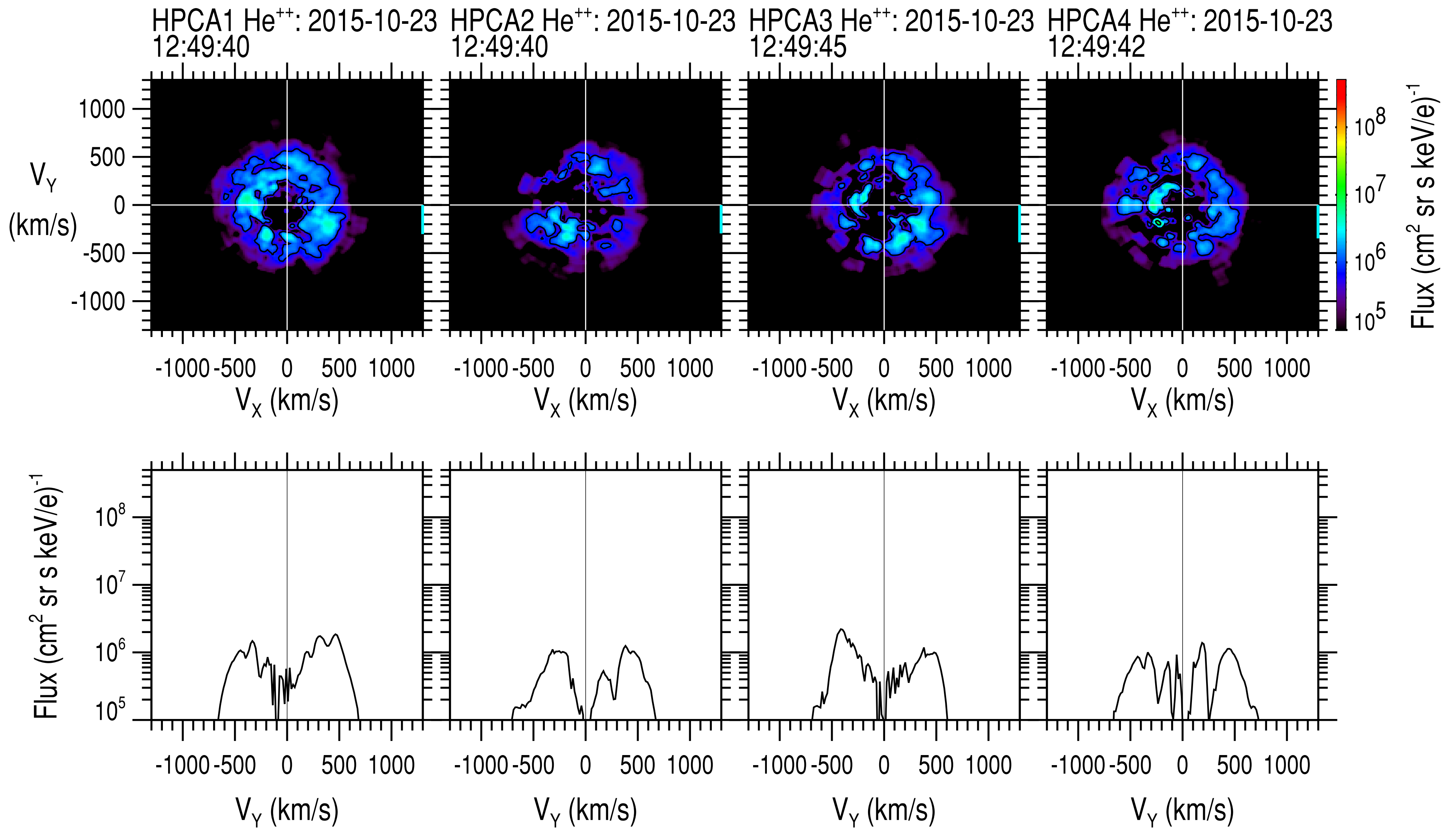

HPCA1 He++: 2015-10-23 12:49:40
HPCA2 He++: 2015-10-23 12:49:40
HPCA3 He++: 2015-10-23 12:49:45
HPCA4 He++: 2015-10-23 12:49:42
VY (km/s)
VX (km/s)
Flux (cm2 sr s keV/e)-1
VY (km/s)

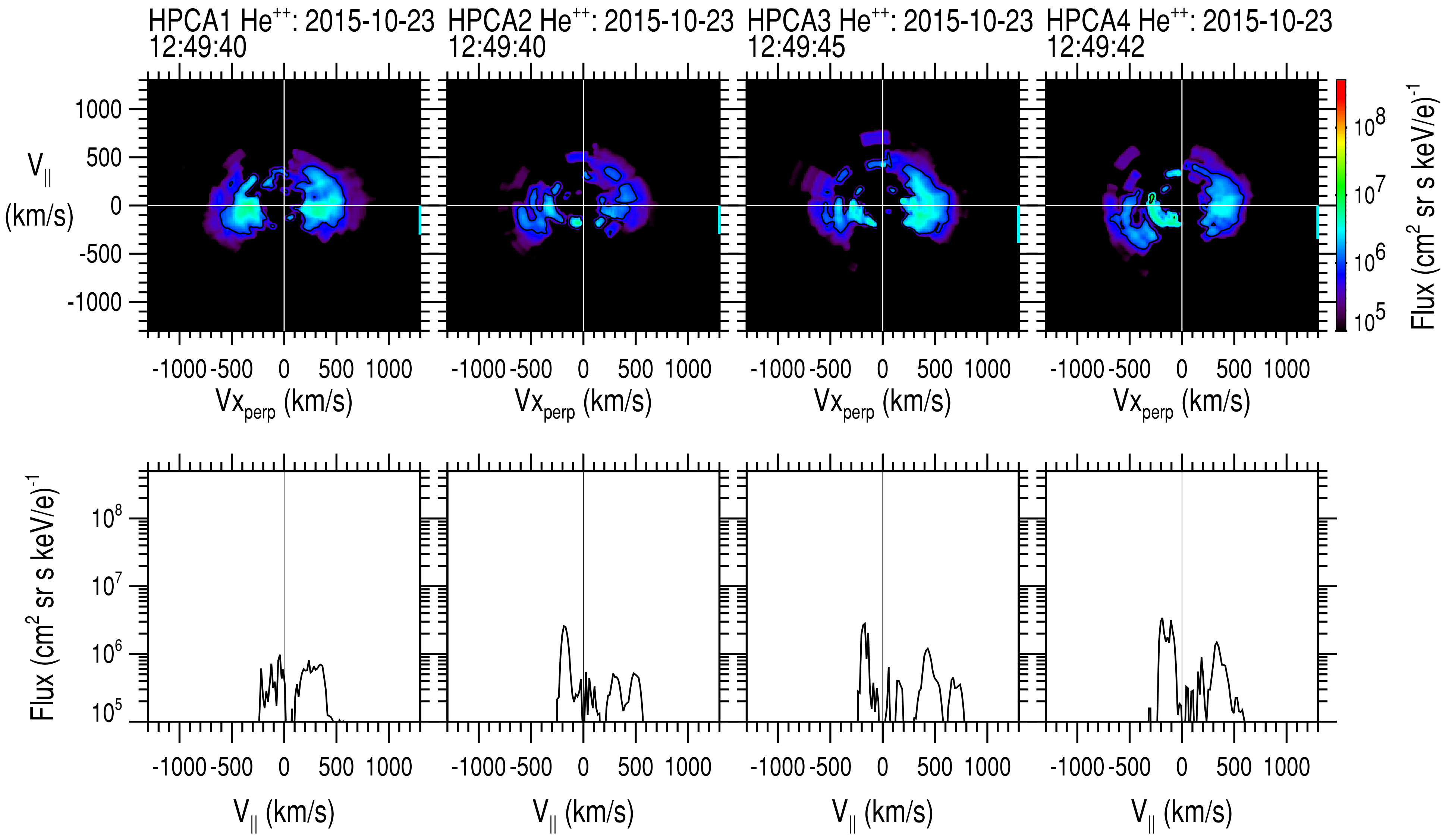
HPCA1 He++: 2015-10-23
12:49:40
HPCA2 He++: 2015-10-23
12:49:40
HPCA3 He++: 2015-10-23
12:49:45
HPCA4 He++: 2015-10-23
12:49:42
V|| (km/s)
Vxperp (km/s)
Flux (cm2 sr s keV/e)-1
V|| (km/s)

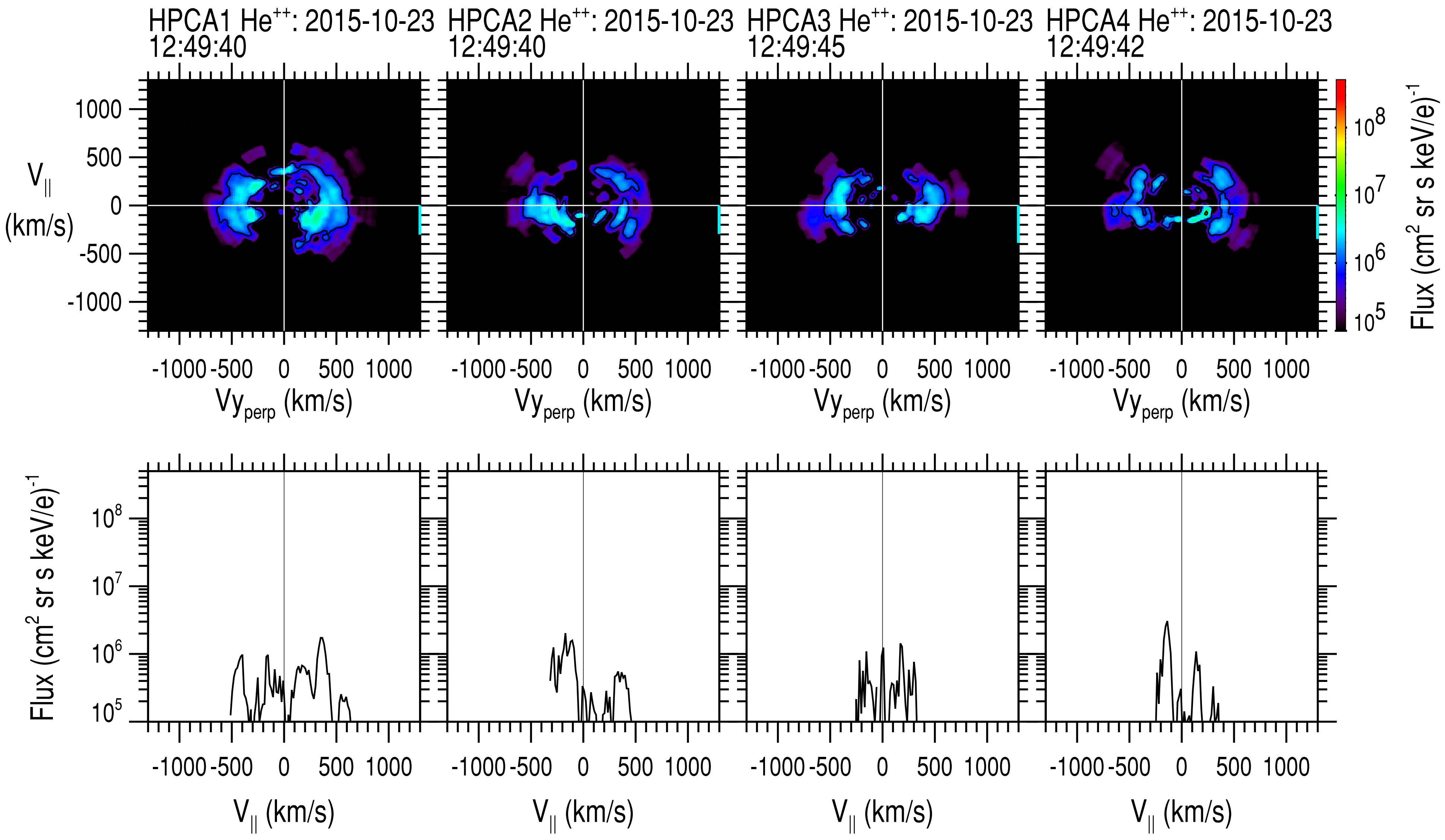
HPCA1 He++: 2015-10-23 12:49:40
HPCA2 He++: 2015-10-23 12:49:40
HPCA3 He++: 2015-10-23 12:49:45
HPCA4 He++: 2015-10-23 12:49:42
V|| (km/s)
Vyperp (km/s)
Flux (cm2 sr s keV/e)-1
-1000 -500 0 500 1000
105 106 107 108

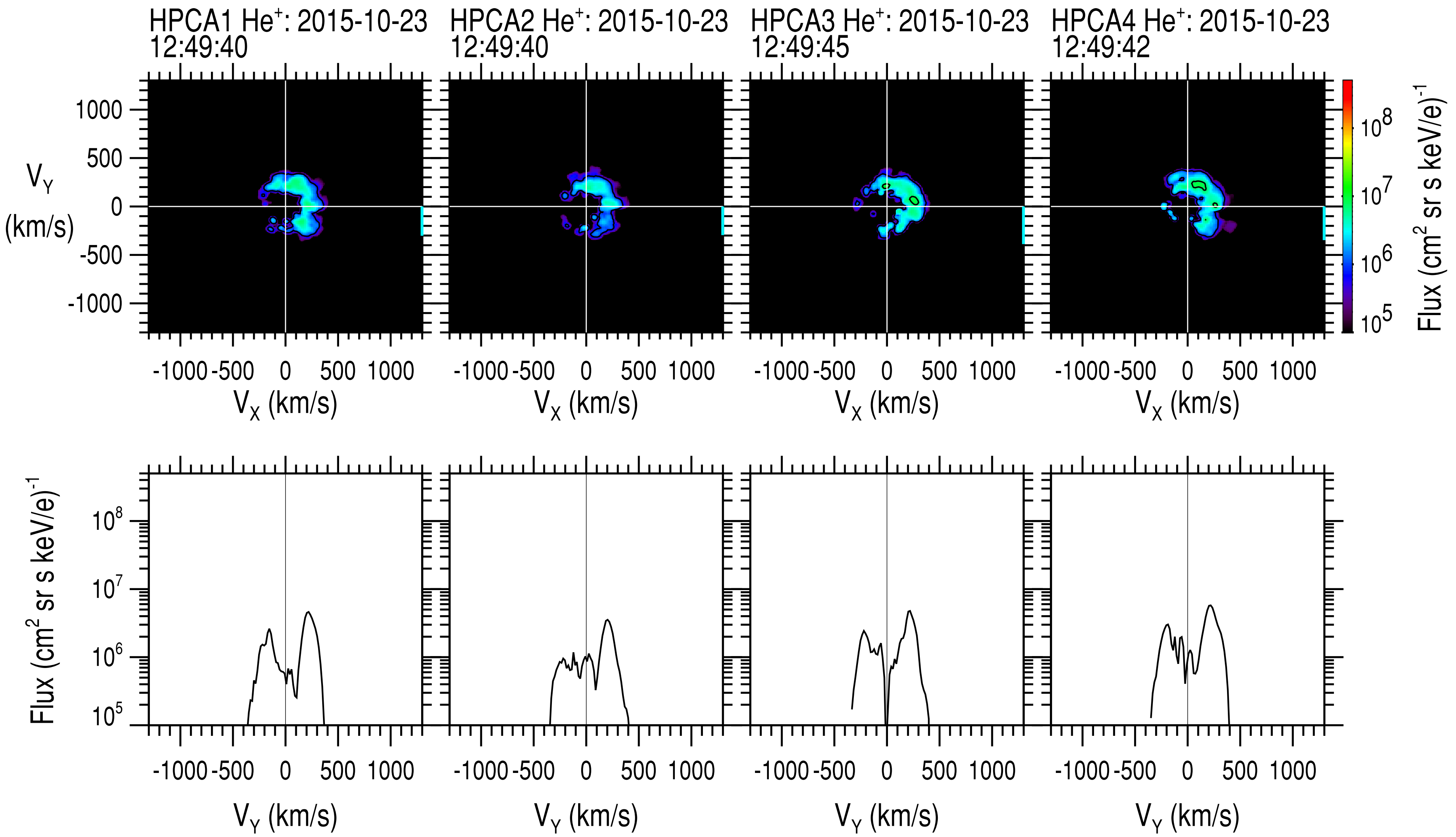

HPCA1 He+: 2015-10-23 12:49:40
HPCA2 He+: 2015-10-23 12:49:40
HPCA3 He+: 2015-10-23 12:49:45
HPCA4 He+: 2015-10-23 12:49:42
VY (km/s)
VX (km/s)
Flux (cm2 sr s keV/e)-1

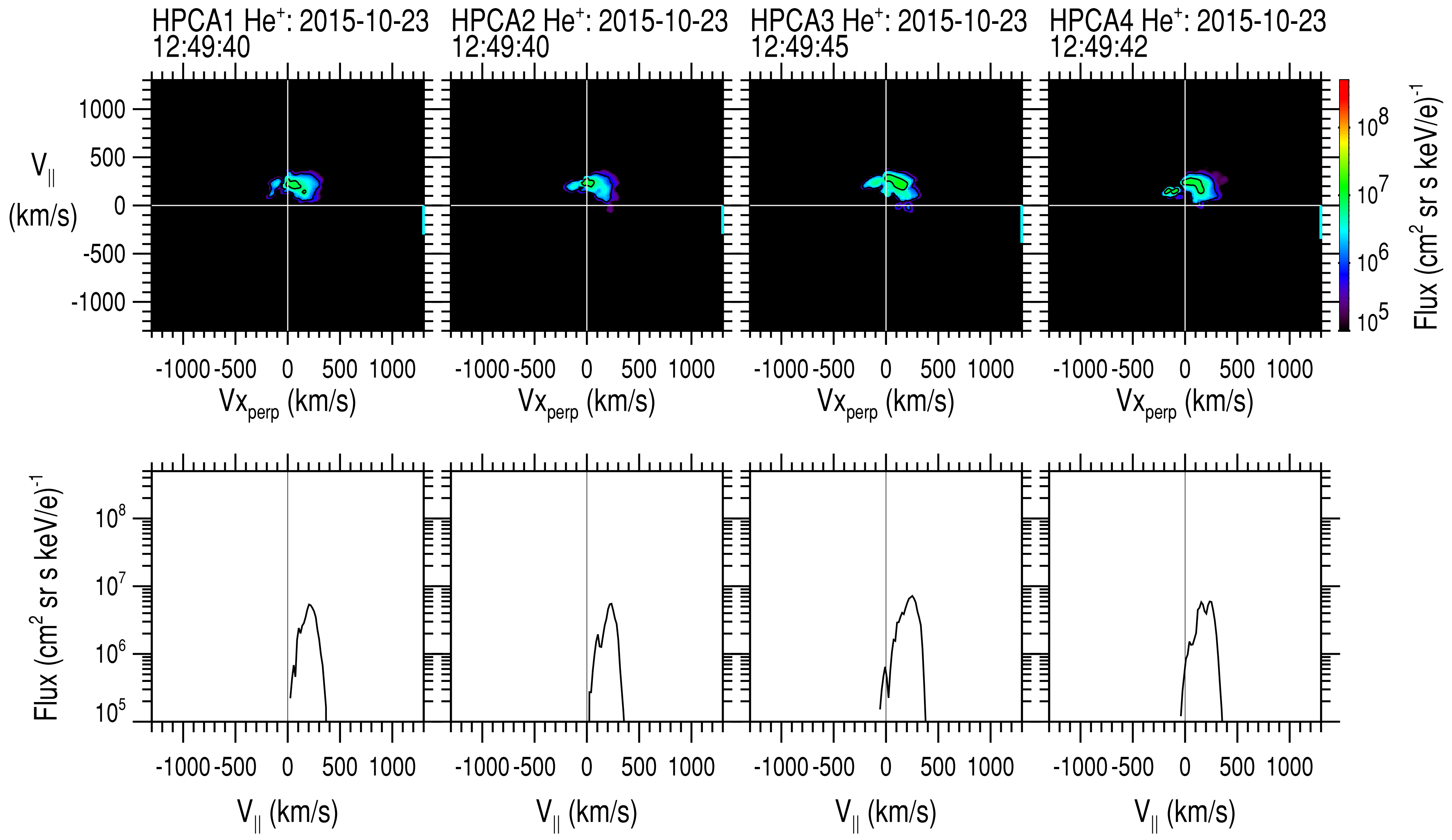

HPCA1 He+: 2015-10-23 12:49:40
HPCA2 He+: 2015-10-23 12:49:40
HPCA3 He+: 2015-10-23 12:49:45
HPCA4 He+: 2015-10-23 12:49:42
V|| (km/s)
Vxperp (km/s)
Flux (cm2 sr s keV/e)-1
V|| (km/s)
-1000 -500 0 500 1000
10^5 10^6 10^7 10^8

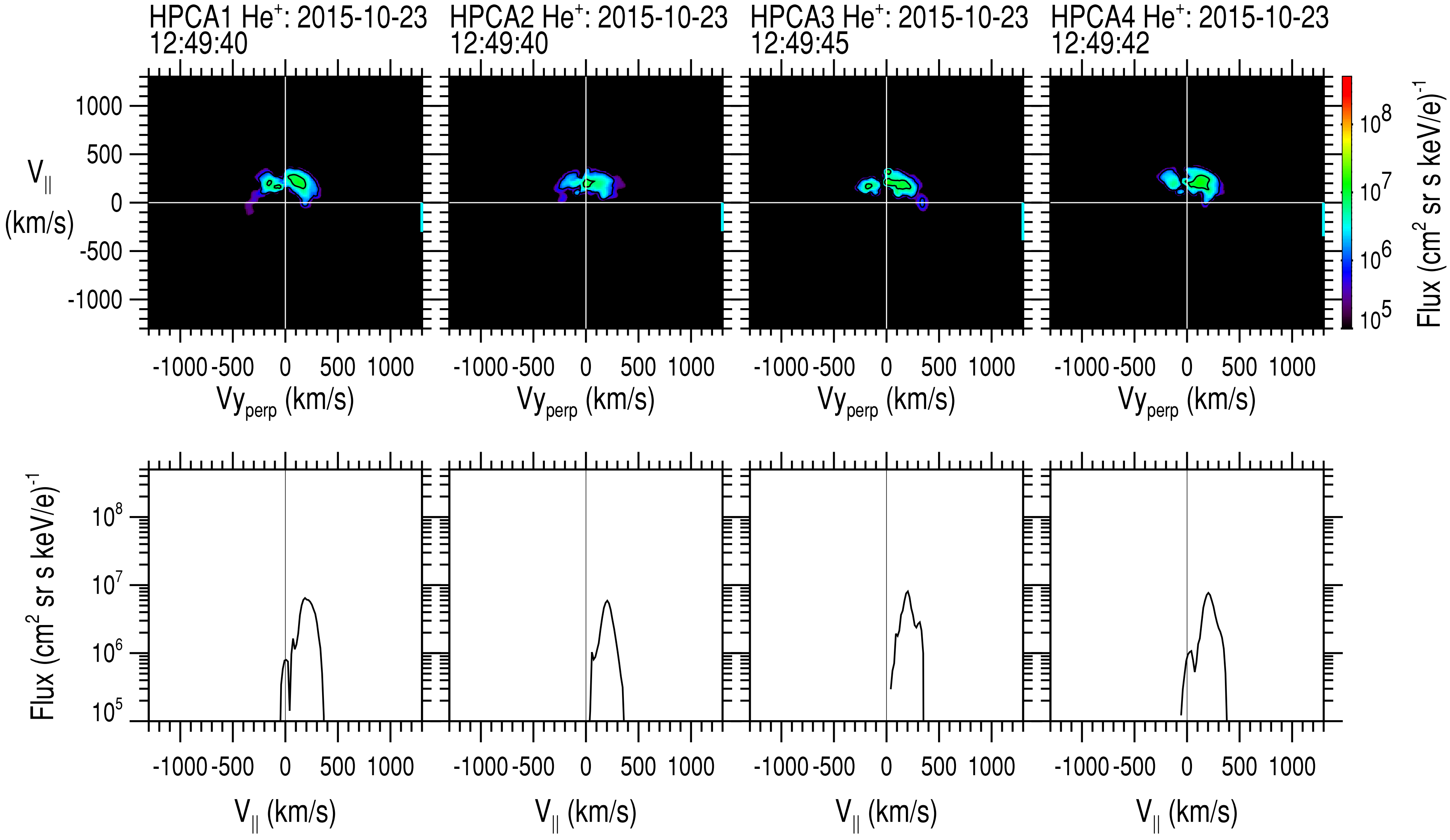
HPCA1 He+: 2015-10-23 12:49:40
HPCA2 He+: 2015-10-23 12:49:40
HPCA3 He+: 2015-10-23 12:49:45
HPCA4 He+: 2015-10-23 12:49:42
V|| (km/s)
Vyperp (km/s)
Flux (cm2 sr s keV/e)-1
V|| (km/s)

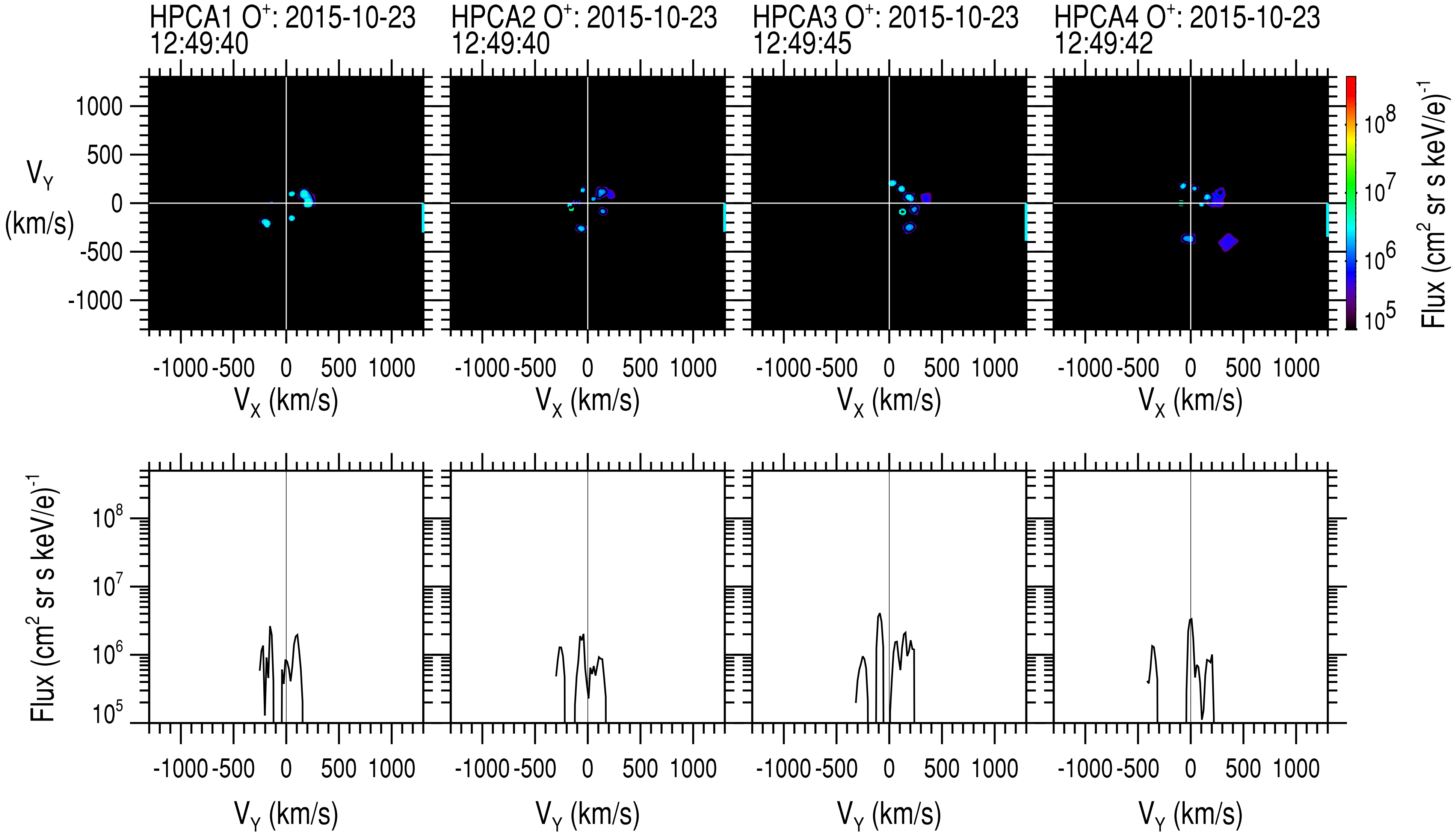
HPCA1 O+: 2015-10-23
12:49:40
HPCA2 O+: 2015-10-23
12:49:40
HPCA3 O+: 2015-10-23
12:49:45
HPCA4 O+: 2015-10-23
12:49:42
VY (km/s)
VX (km/s)
Flux (cm2 sr s keV/e)-1
VY (km/s)

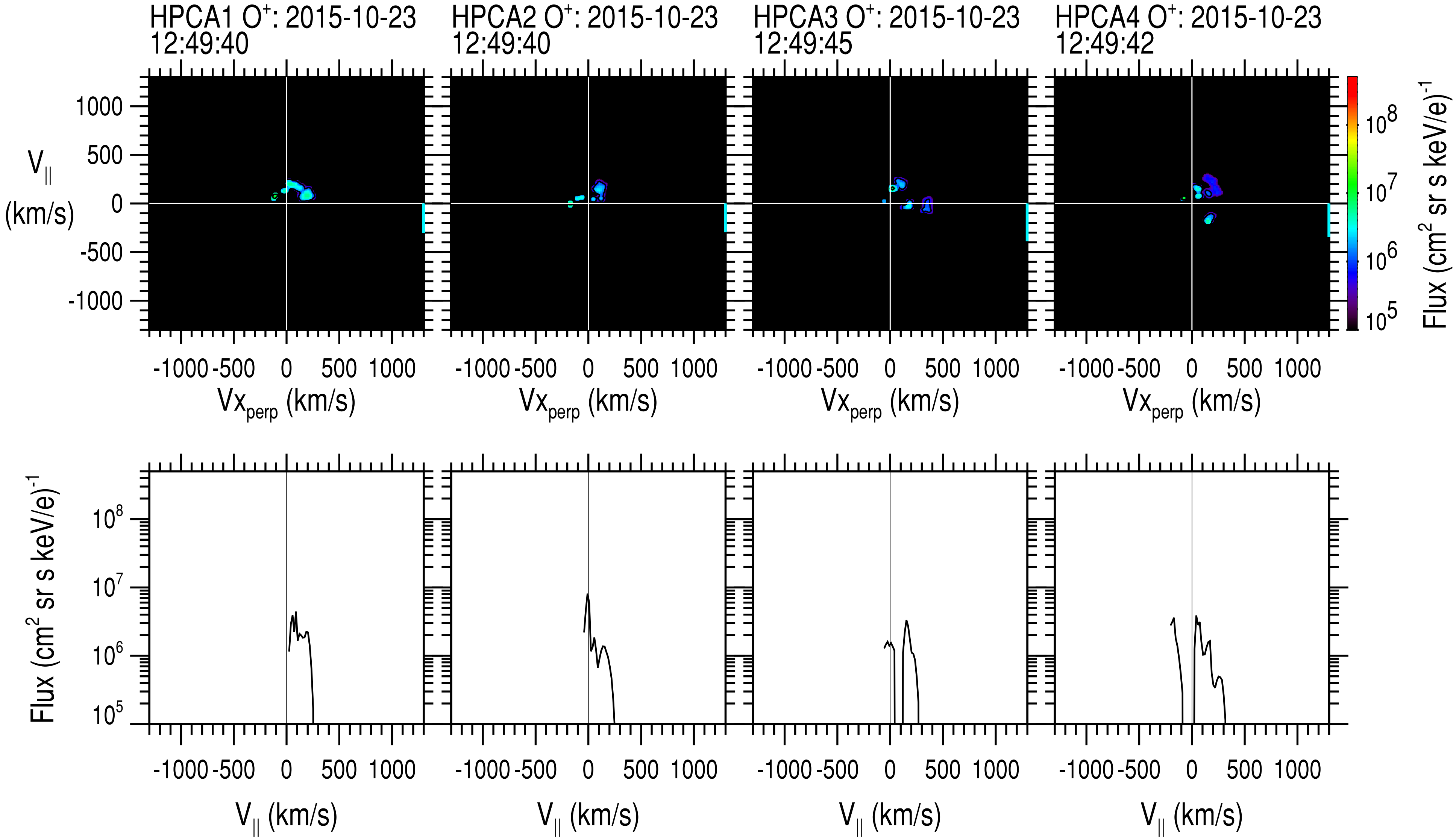

HPCA1 O+: 2015-10-23 12:49:40
HPCA2 O+: 2015-10-23 12:49:40
HPCA3 O+: 2015-10-23 12:49:45
HPCA4 O+: 2015-10-23 12:49:42
V|| (km/s)
Vxperp (km/s)
Flux (cm2 sr s keV/e)-1

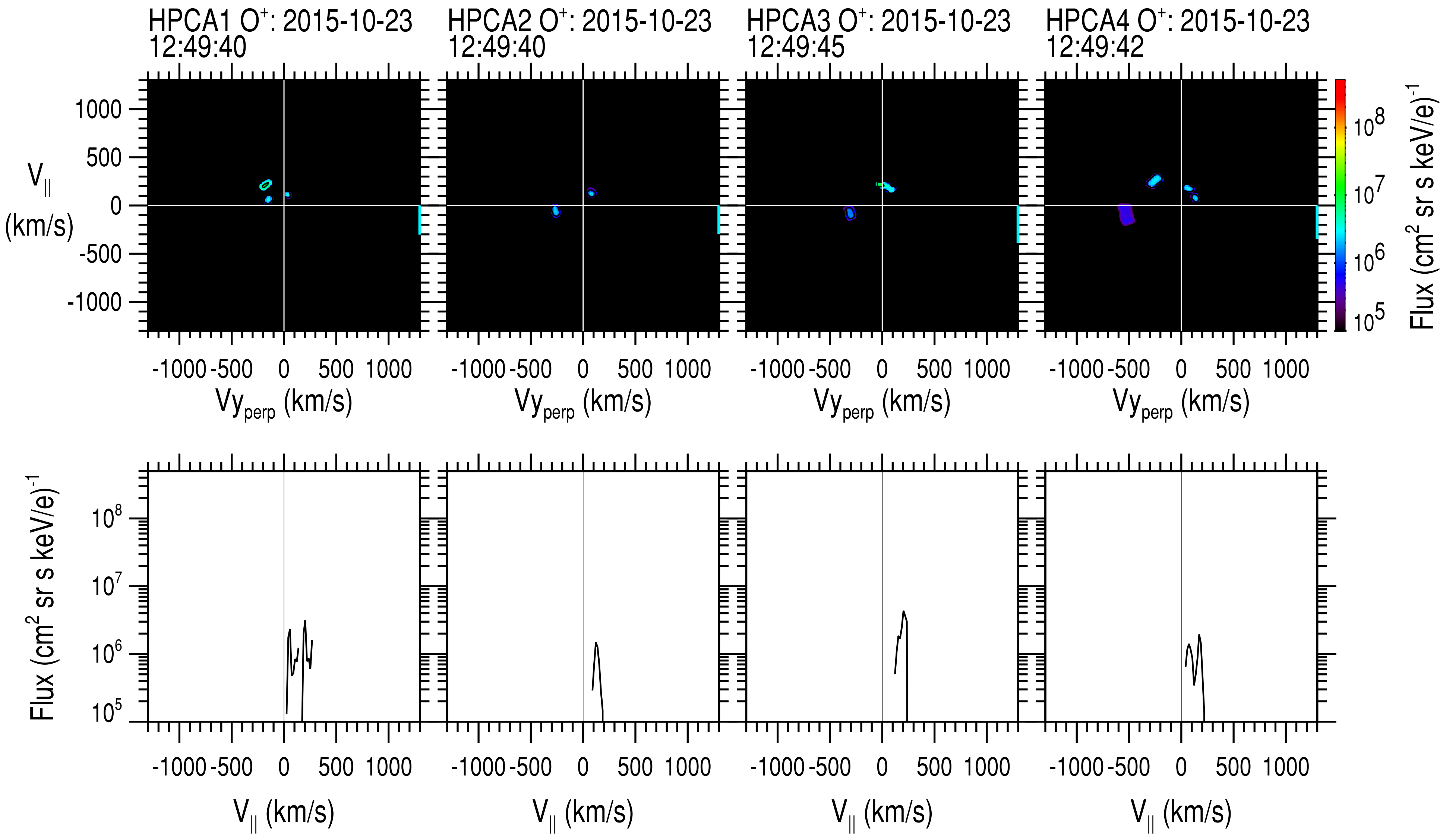

HPCA1 O+: 2015-10-23 12:49:40
HPCA2 O+: 2015-10-23 12:49:40
HPCA3 O+: 2015-10-23 12:49:45
HPCA4 O+: 2015-10-23 12:49:42
V|| (km/s)
Vyperp (km/s)
Flux (cm2 sr s keV/e)-1

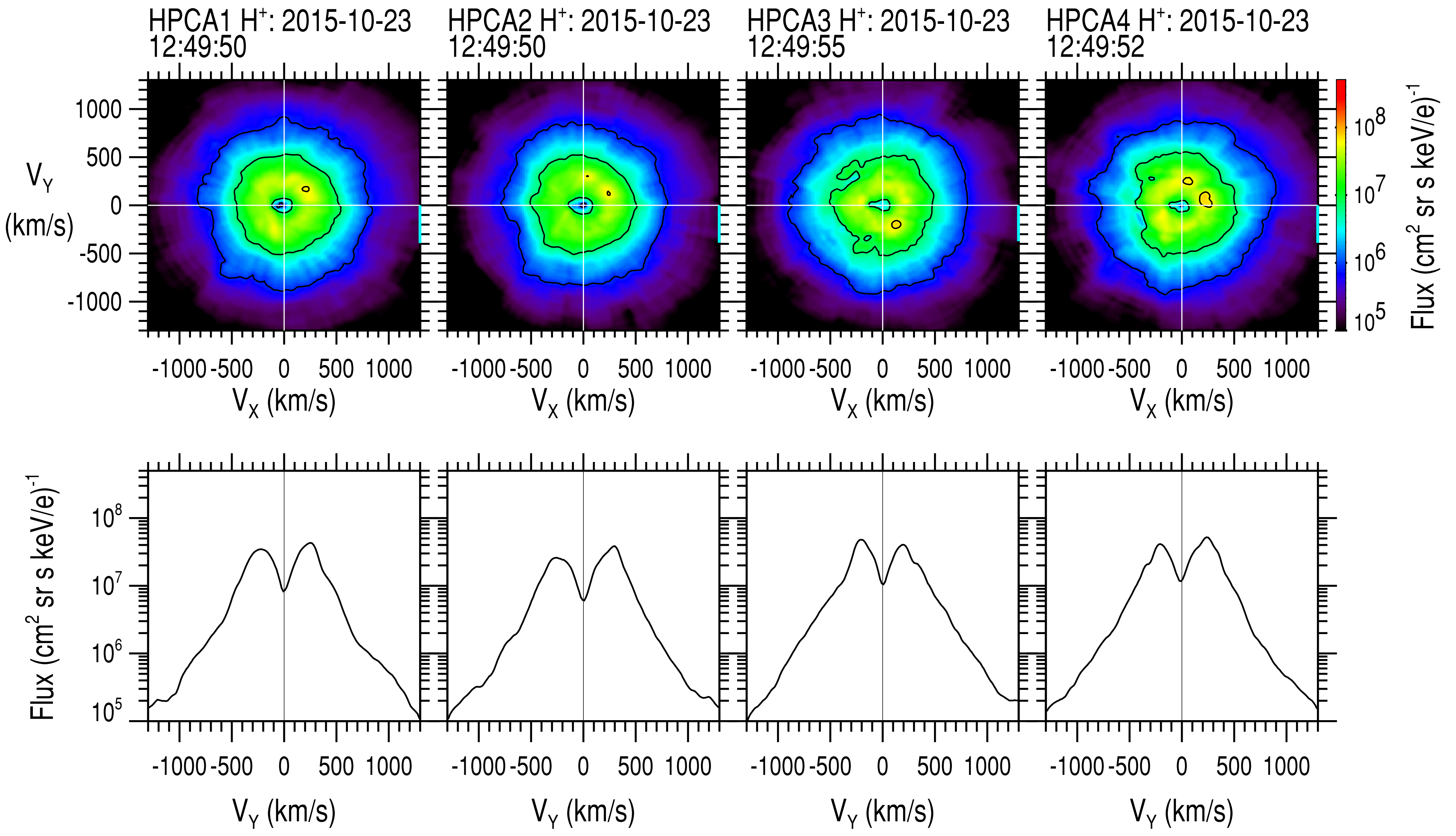

HPCA1 H+: 2015-10-23 12:49:50
HPCA2 H+: 2015-10-23 12:49:50
HPCA3 H+: 2015-10-23 12:49:55
HPCA4 H+: 2015-10-23 12:49:52
V_Y (km/s)
V_X (km/s)
Flux (cm^2 sr s keV/e)^-1
-1000 -500 0 500 1000
10^5 10^6 10^7 10^8

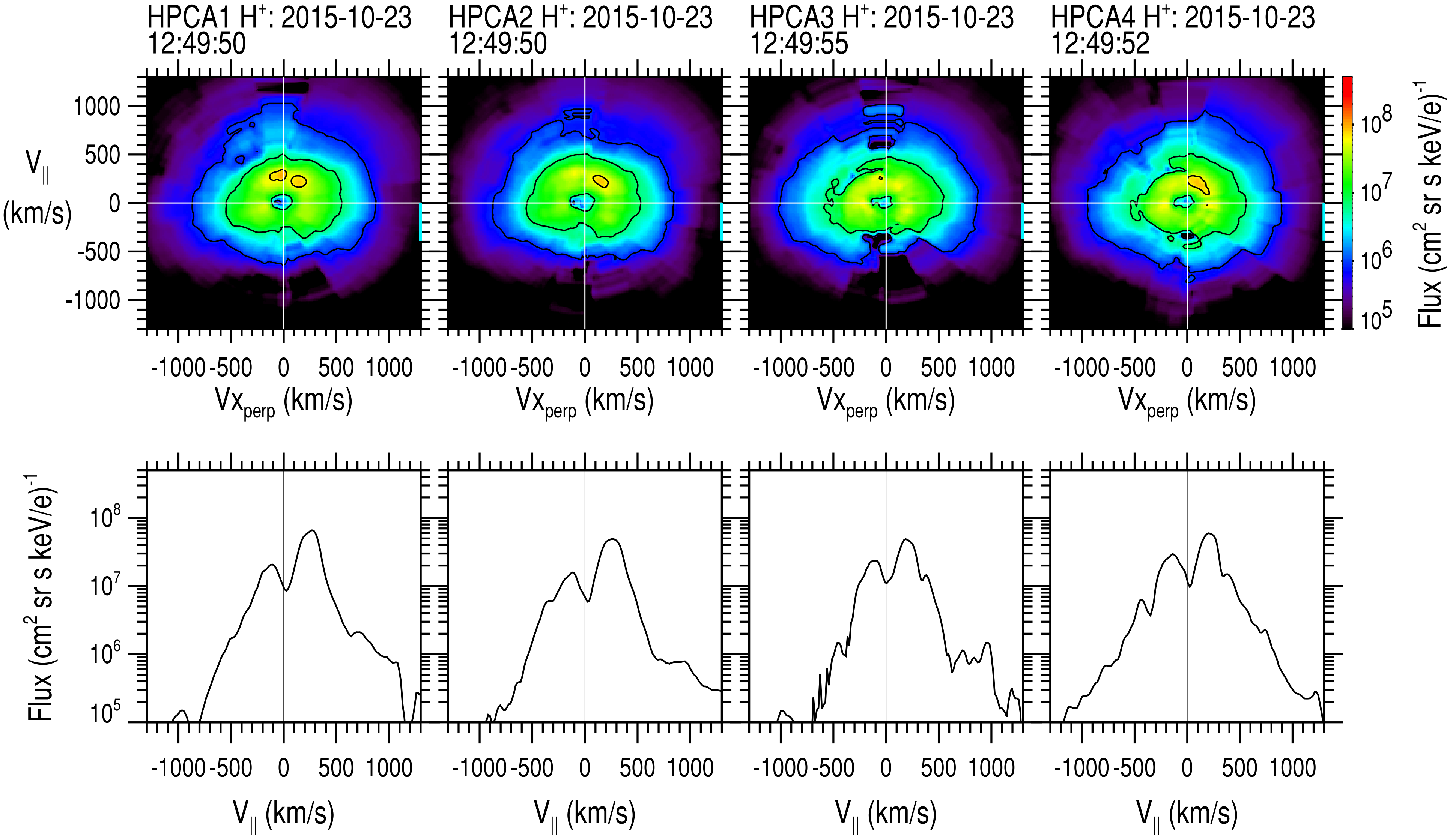

HPCA1 H+: 2015-10-23
12:49:50
HPCA2 H+: 2015-10-23
12:49:50
HPCA3 H+: 2015-10-23
12:49:55
HPCA4 H+: 2015-10-23
12:49:52
V|| (km/s)
Vxperp (km/s)
Flux (cm2 sr s keV/e)-1

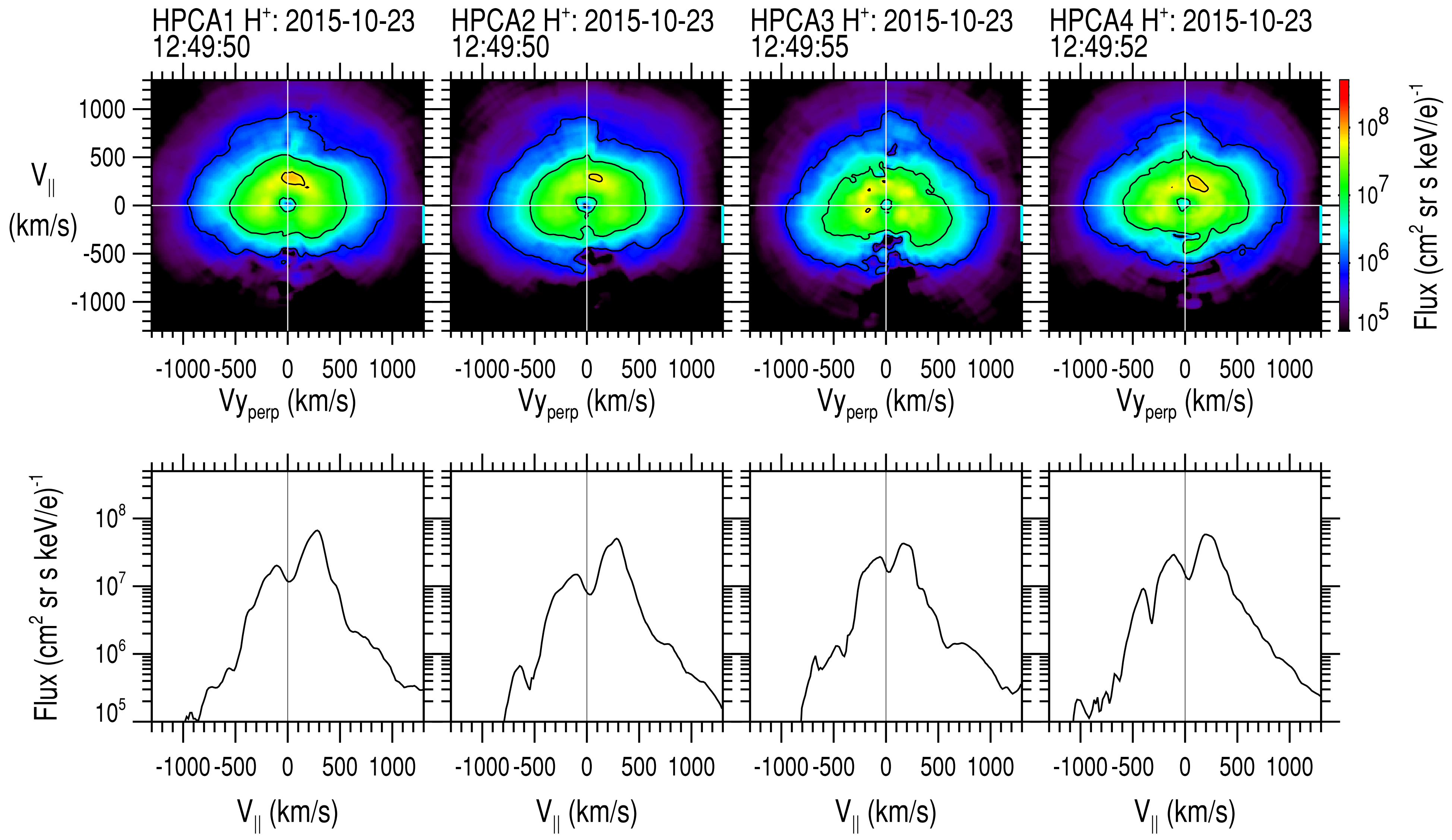

HPCA1 H+: 2015-10-23 12:49:50
HPCA2 H+: 2015-10-23 12:49:50
HPCA3 H+: 2015-10-23 12:49:55
HPCA4 H+: 2015-10-23 12:49:52
V|| (km/s)
Vyperp (km/s)
Flux (cm2 sr s keV/e)-1
-1000 -500 0 500 1000
10^5 10^6 10^7 10^8

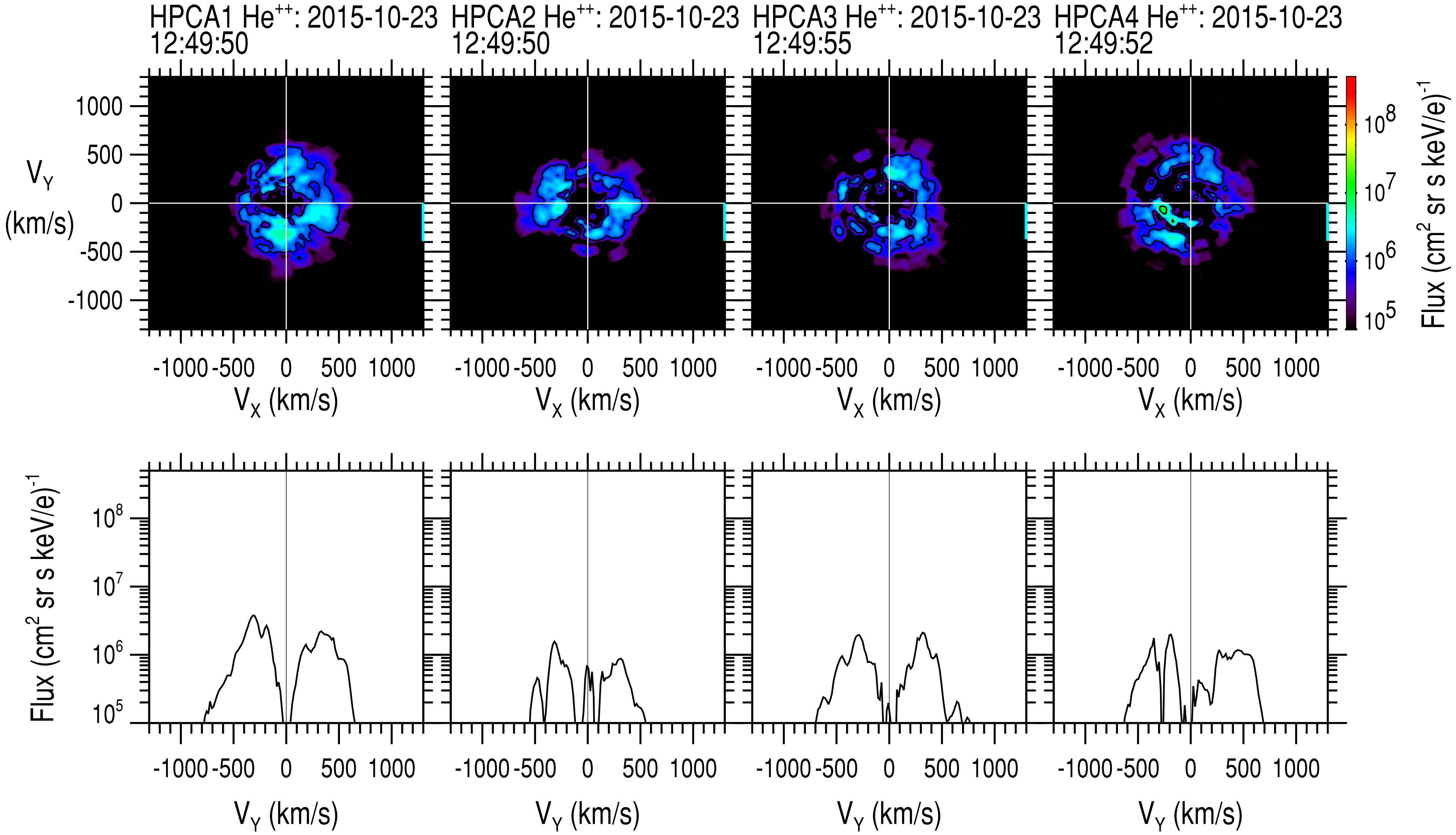

HPCA1 He++: 2015-10-23 12:49:50
HPCA2 He++: 2015-10-23 12:49:50
HPCA3 He++: 2015-10-23 12:49:55
HPCA4 He++: 2015-10-23 12:49:52
V_Y (km/s)
V_X (km/s)
Flux (cm^2 sr s keV/e)^-1

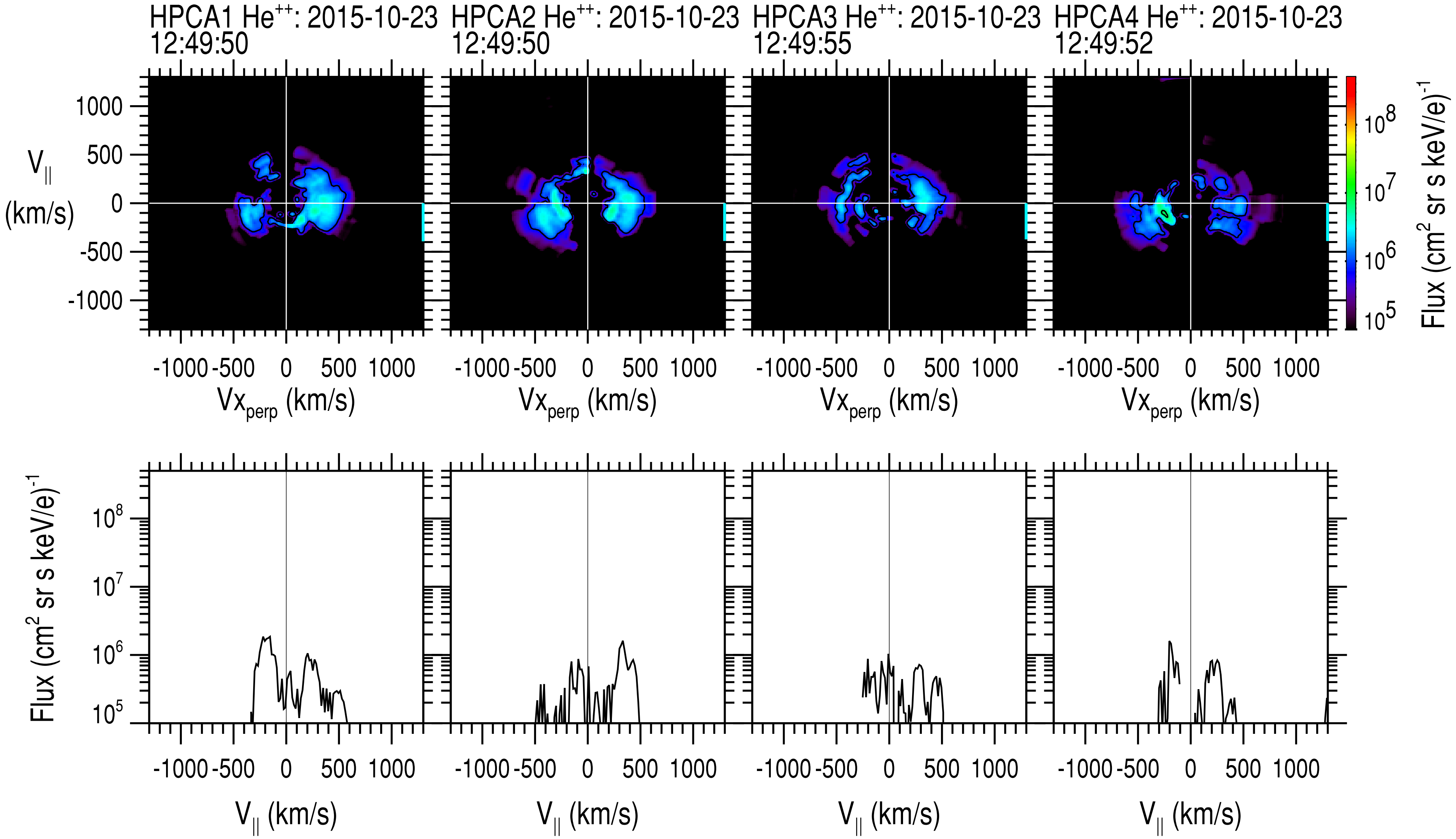
HPCA1 He++: 2015-10-23 12:49:50
HPCA2 He++: 2015-10-23 12:49:50
HPCA3 He++: 2015-10-23 12:49:55
HPCA4 He++: 2015-10-23 12:49:52
V|| (km/s)
Vxperp (km/s)
Flux (cm2 sr s keV/e)-1
-1000 -500 0 500 1000
10^5 10^6 10^7 10^8

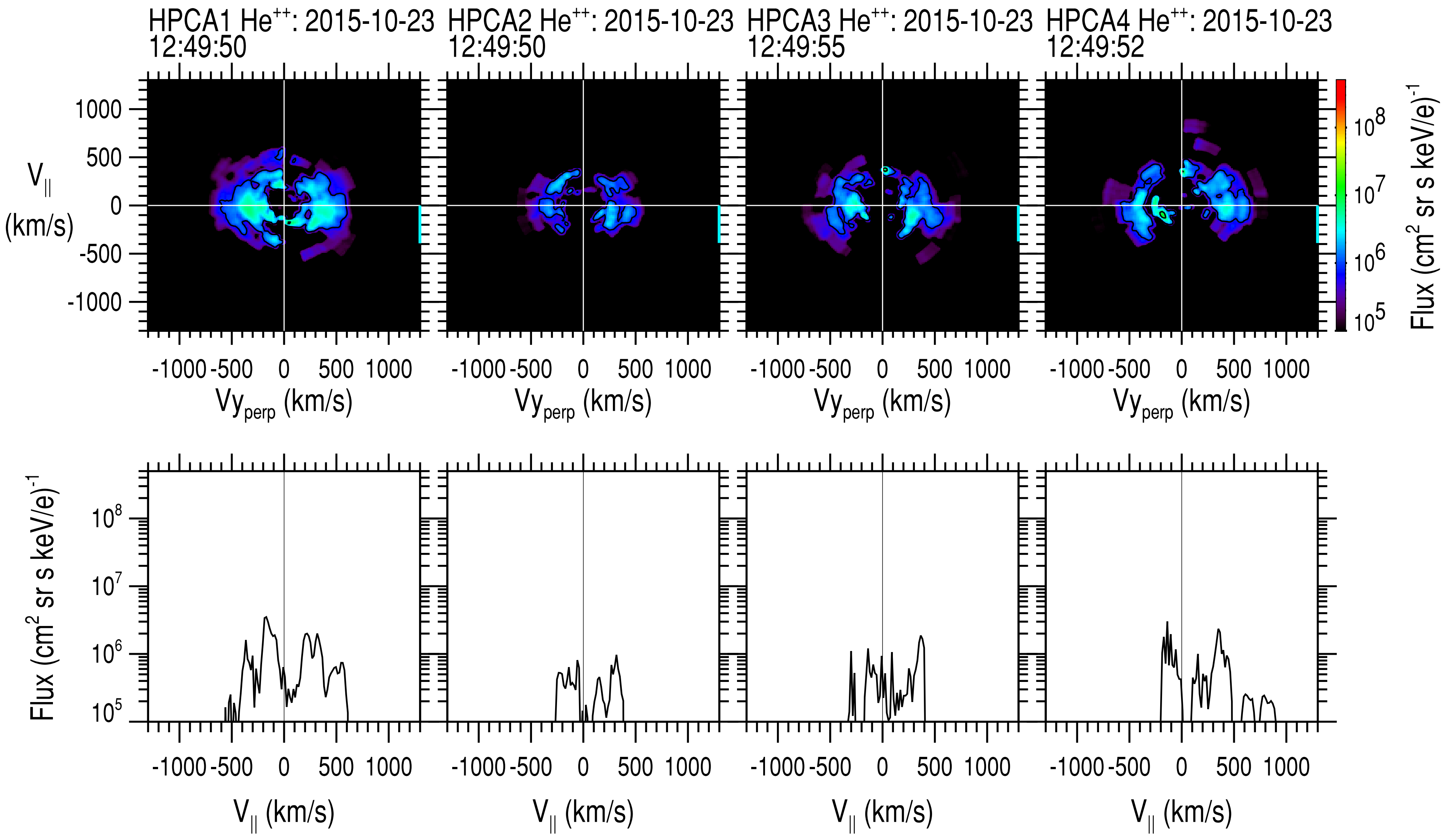

HPCA1 He++: 2015-10-23 12:49:50
HPCA2 He++: 2015-10-23 12:49:50
HPCA3 He++: 2015-10-23 12:49:55
HPCA4 He++: 2015-10-23 12:49:52
V|| (km/s)
Vyperp (km/s)
Flux (cm2 sr s keV/e)-1

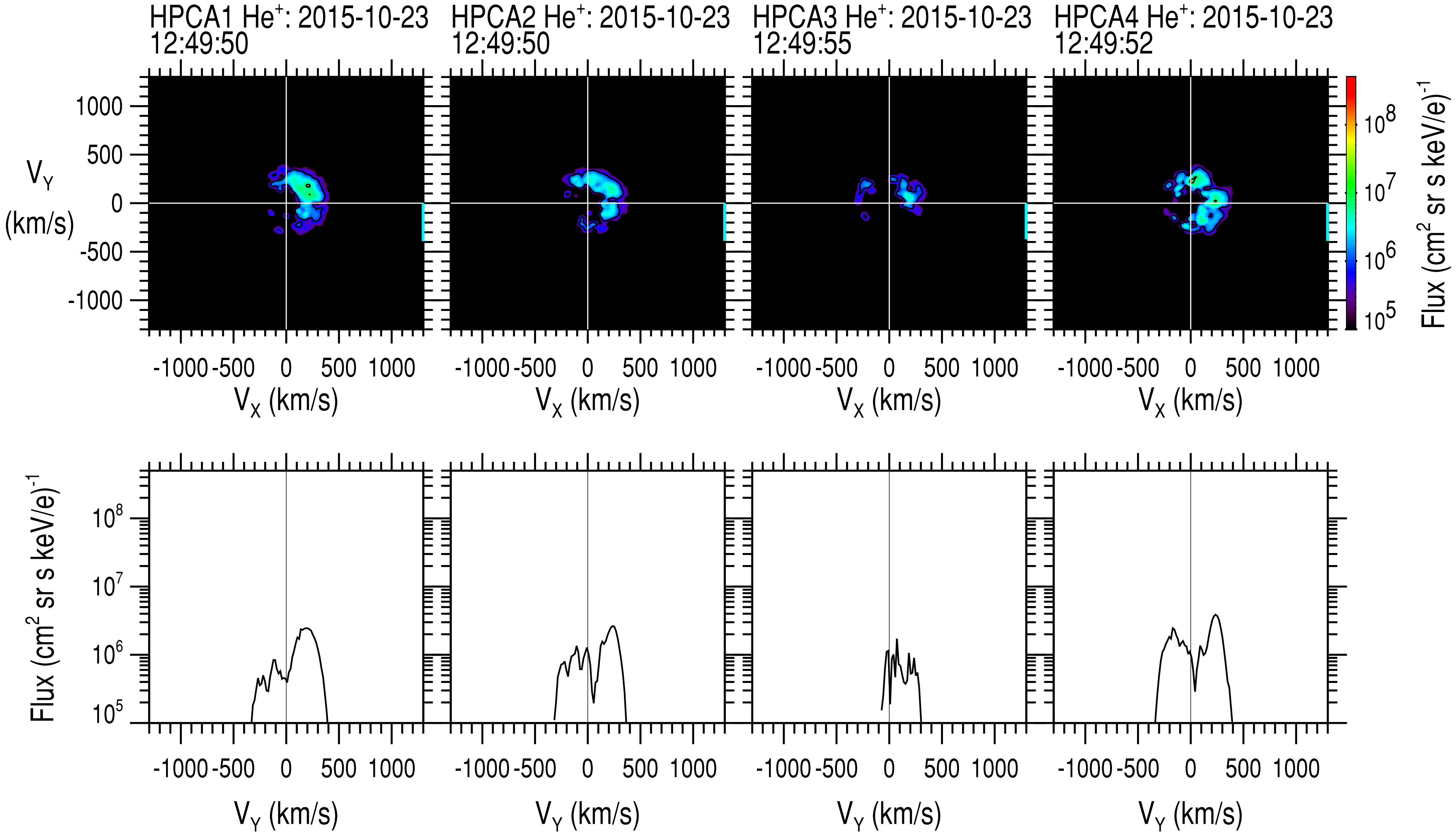
HPCA1 He+: 2015-10-23
12:49:50
HPCA2 He+: 2015-10-23
12:49:50
HPCA3 He+: 2015-10-23
12:49:55
HPCA4 He+: 2015-10-23
12:49:52
V_Y (km/s)
V_X (km/s)
Flux (cm2 sr s keV/e)-1
V_Y (km/s)

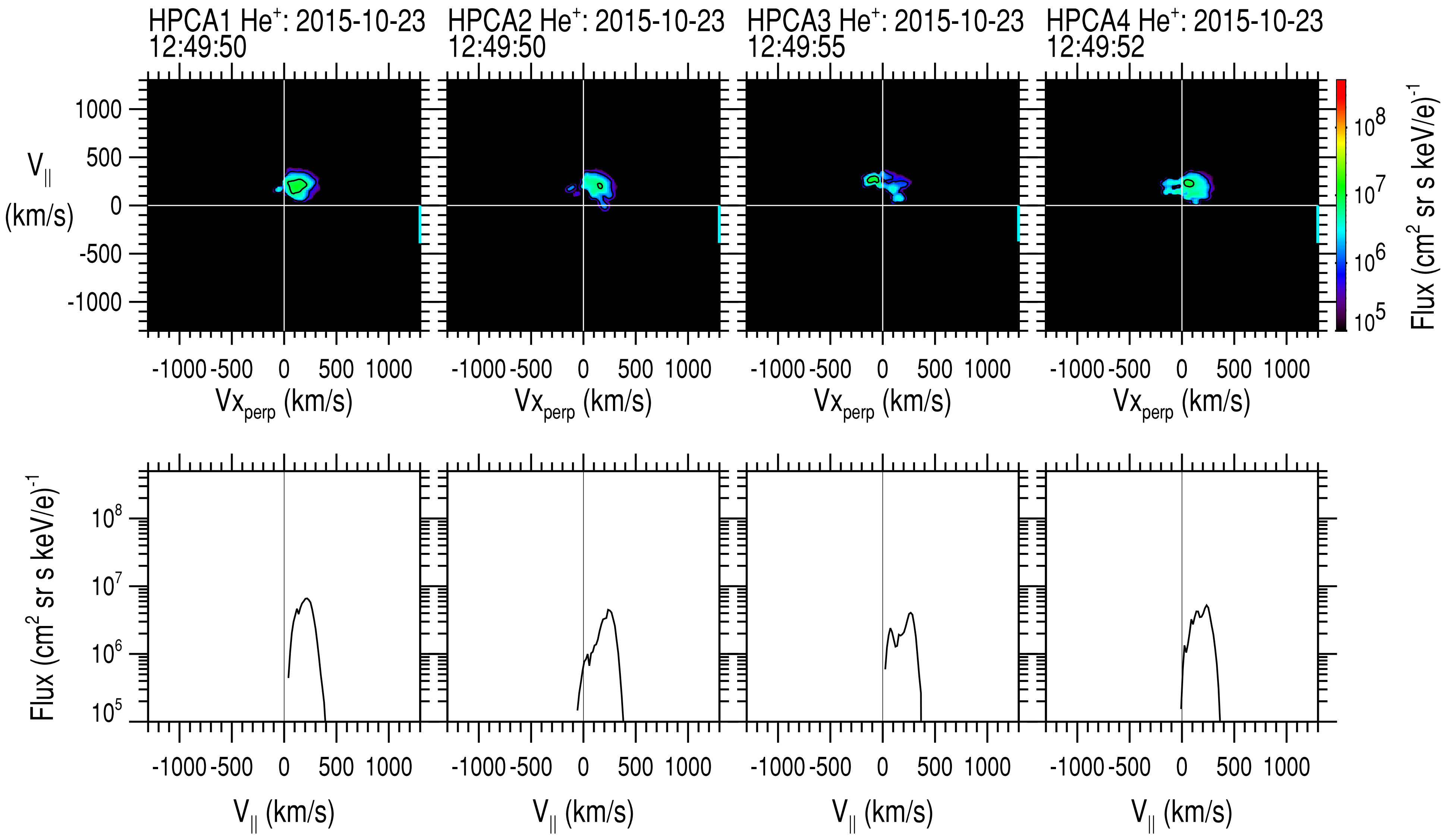
HPCA1 He+: 2015-10-23 12:49:50
HPCA2 He+: 2015-10-23 12:49:50
HPCA3 He+: 2015-10-23 12:49:55
HPCA4 He+: 2015-10-23 12:49:52
V|| (km/s)
Vxperp (km/s)
Flux (cm2 sr s keV/e)-1
1000
500
0
-500
-1000
10^5
10^6
10^7
10^8

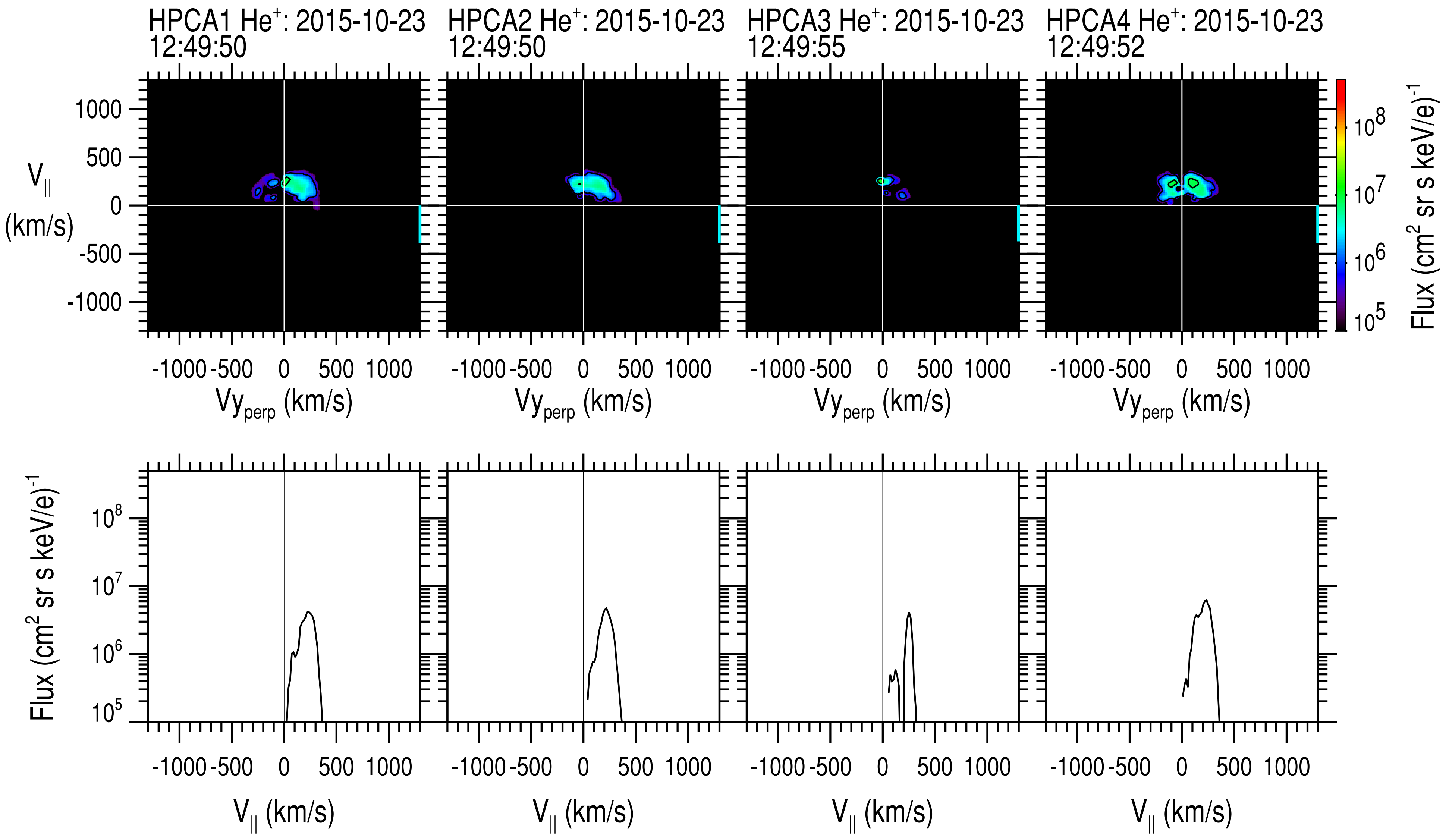

HPCA1 He+: 2015-10-23 12:49:50
HPCA2 He+: 2015-10-23 12:49:50
HPCA3 He+: 2015-10-23 12:49:55
HPCA4 He+: 2015-10-23 12:49:52
V|| (km/s)
Vyperp (km/s)
Flux (cm2 sr s keV/e)-1

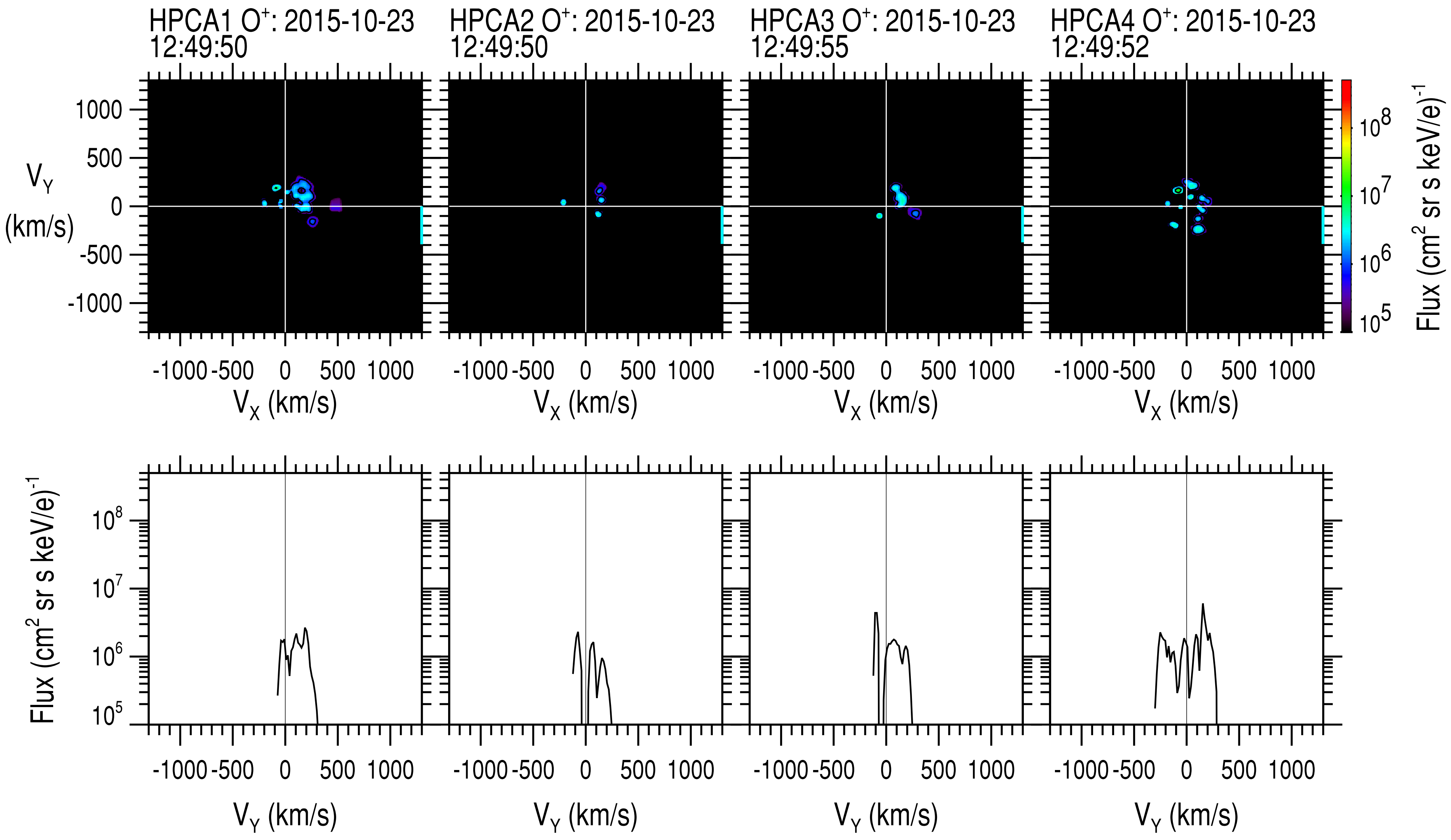
HPCA1 O+: 2015-10-23
12:49:50
HPCA2 O+: 2015-10-23
12:49:50
HPCA3 O+: 2015-10-23
12:49:55
HPCA4 O+: 2015-10-23
12:49:52
V_Y (km/s)
V_X (km/s)
Flux (cm^2 sr s keV/e)^-1
V_Y (km/s)
1000
500
0
-500
-1000
-1000 -500 0 500 1000
10^5
10^6
10^7
10^8

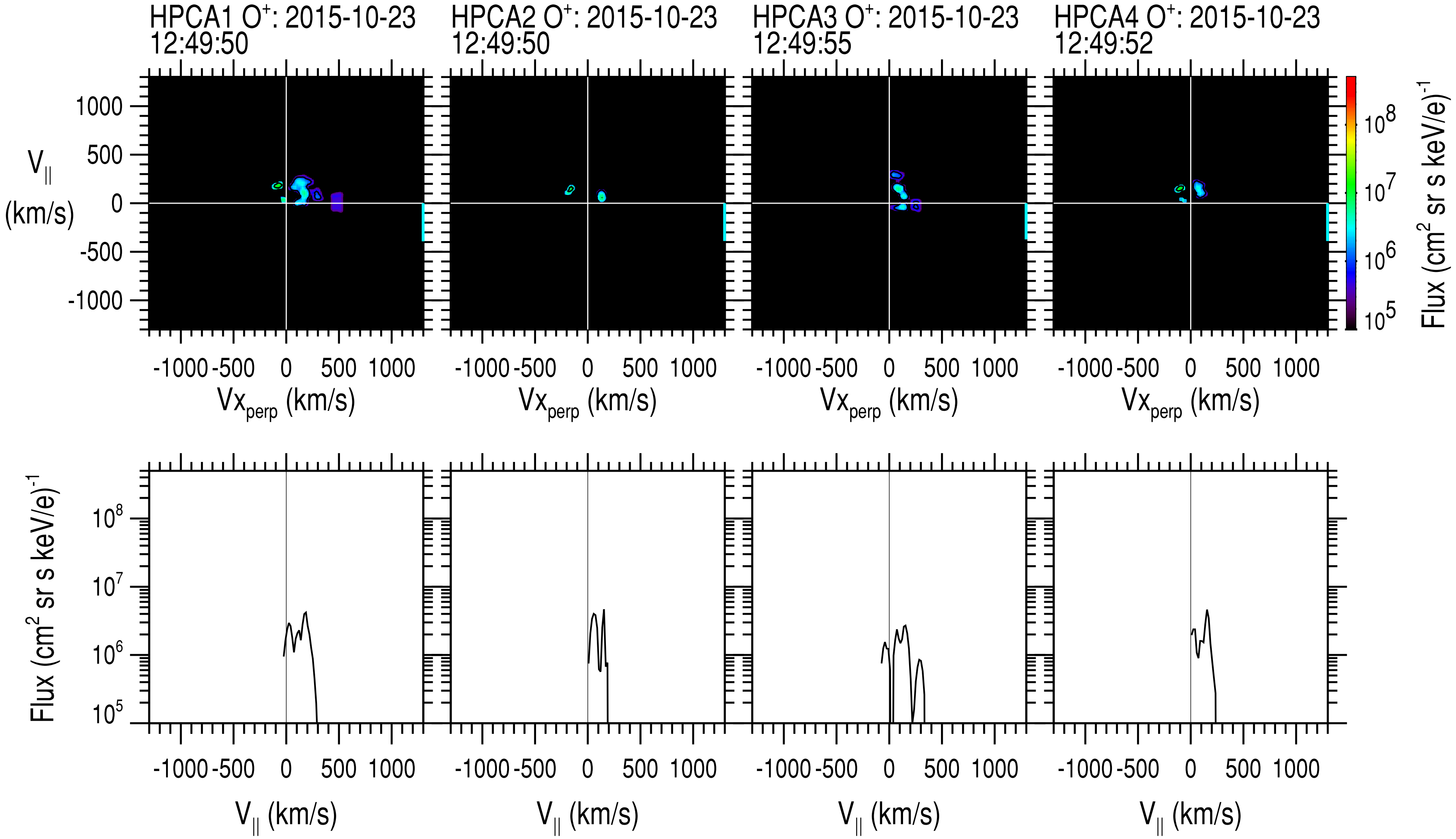
HPCA1 O+: 2015-10-23
12:49:50
HPCA2 O+: 2015-10-23
12:49:50
HPCA3 O+: 2015-10-23
12:49:55
HPCA4 O+: 2015-10-23
12:49:52
V|| (km/s)
1000
500
0
-500
-1000
Vxperp (km/s)
-1000 -500 0 500 1000
Flux (cm2 sr s keV/e)-1
10^5
10^6
10^7
10^8
V|| (km/s)

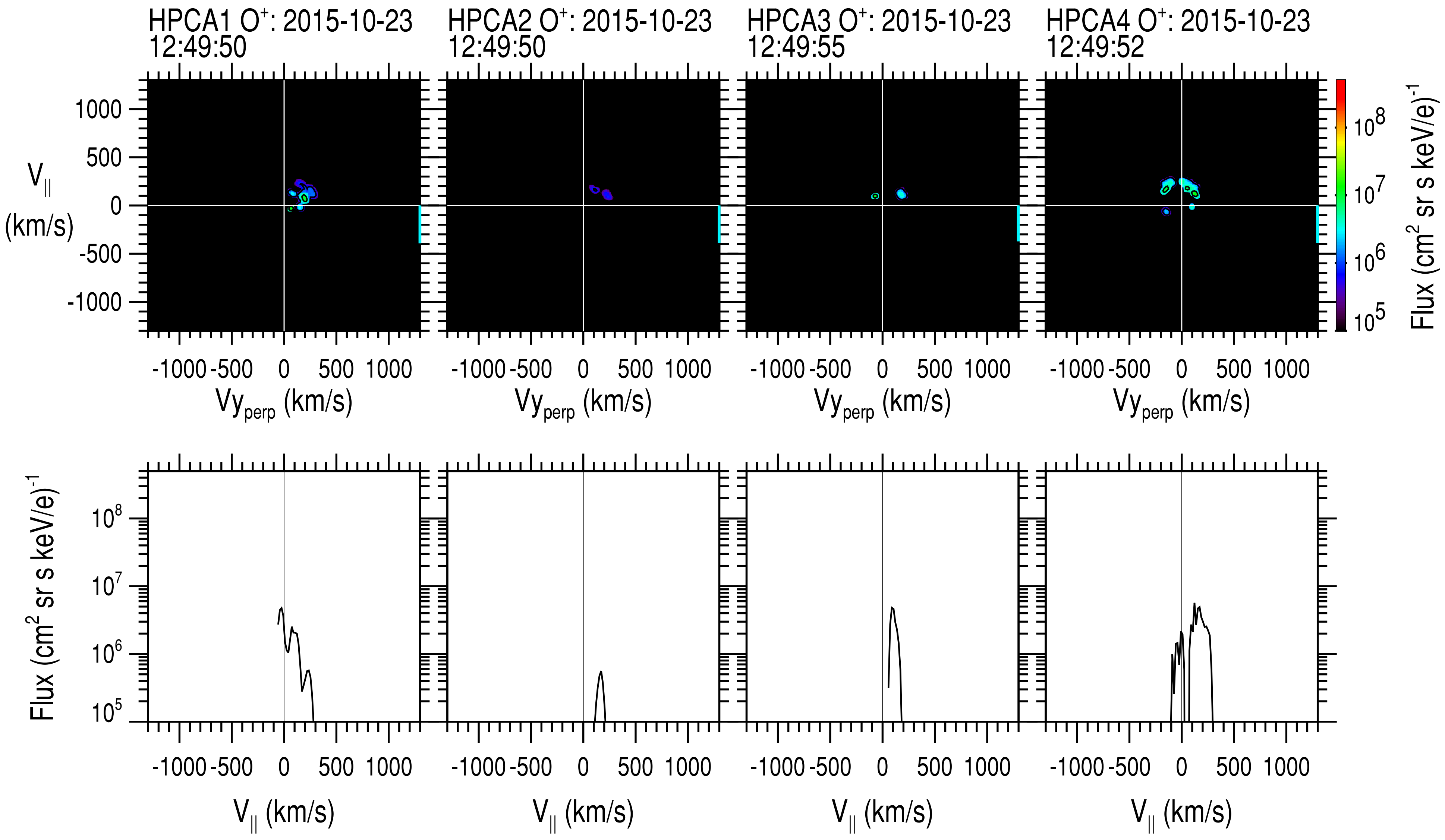
HPCA1 O+: 2015-10-23 12:49:50
HPCA2 O+: 2015-10-23 12:49:50
HPCA3 O+: 2015-10-23 12:49:55
HPCA4 O+: 2015-10-23 12:49:52
V|| (km/s)
Vy_perp (km/s)
Flux (cm2 sr s keV/e)-1
V|| (km/s)

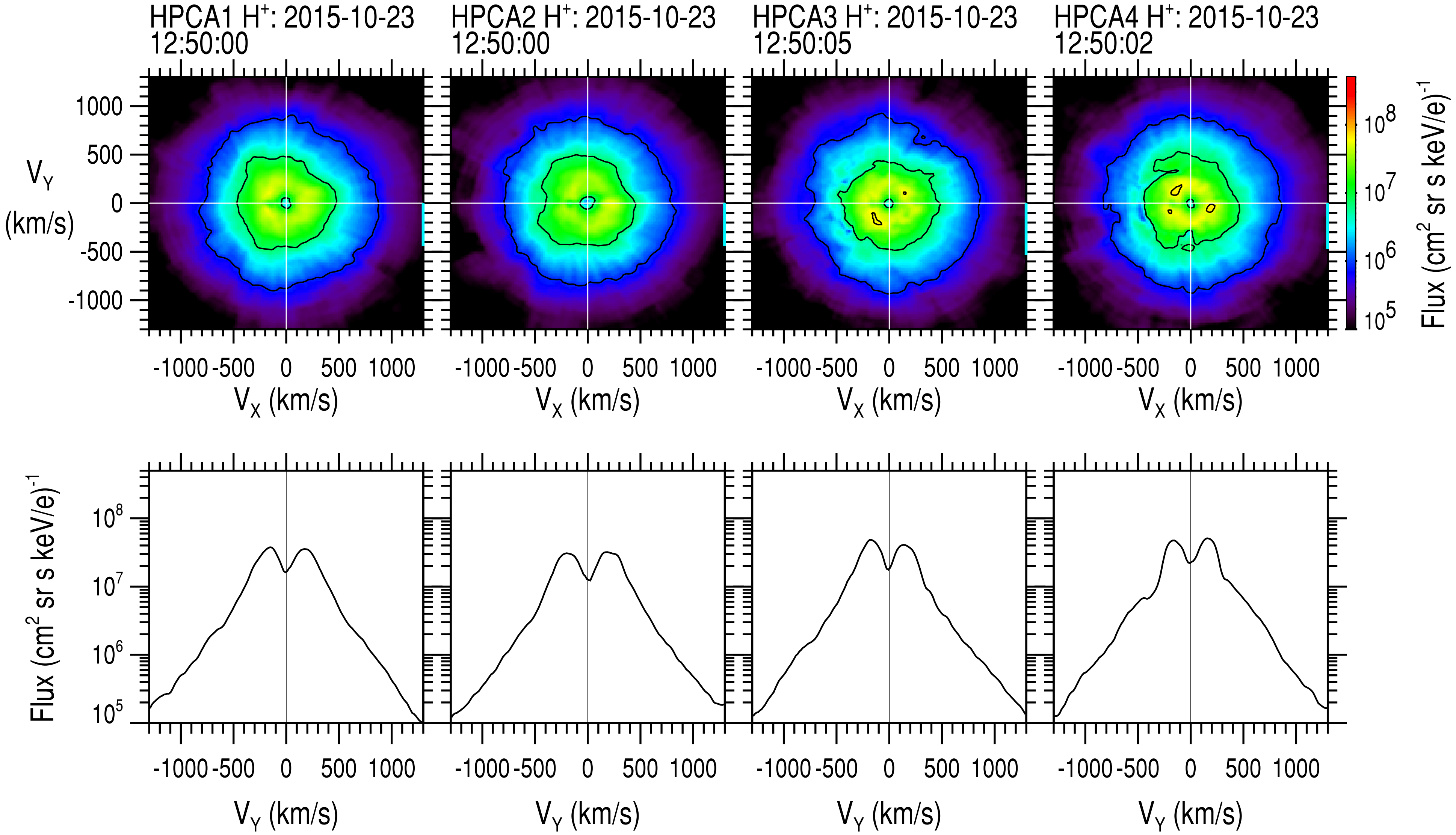
HPCA1 H+: 2015-10-23 12:50:00
HPCA2 H+: 2015-10-23 12:50:00
HPCA3 H+: 2015-10-23 12:50:05
HPCA4 H+: 2015-10-23 12:50:02
V_Y (km/s)
V_X (km/s)
-1000 -500 0 500 1000
Flux (cm^2 sr s keV/e)^-1
10^5 10^6 10^7 10^8

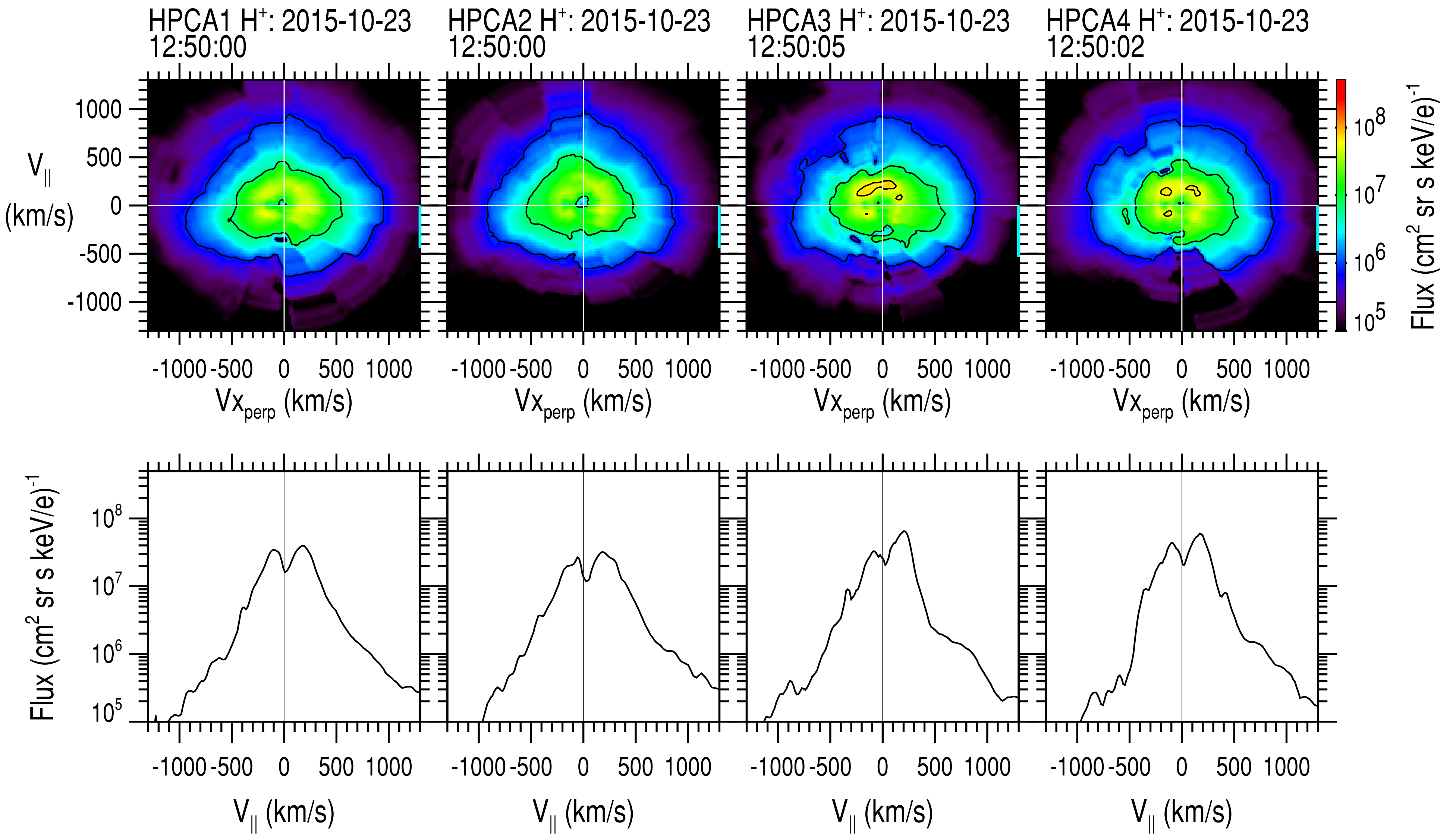

HPCA1 H+: 2015-10-23
12:50:00
HPCA2 H+: 2015-10-23
12:50:00
HPCA3 H+: 2015-10-23
12:50:05
HPCA4 H+: 2015-10-23
12:50:02
V|| (km/s)
Vxperp (km/s)
Flux (cm2 sr s keV/e)-1

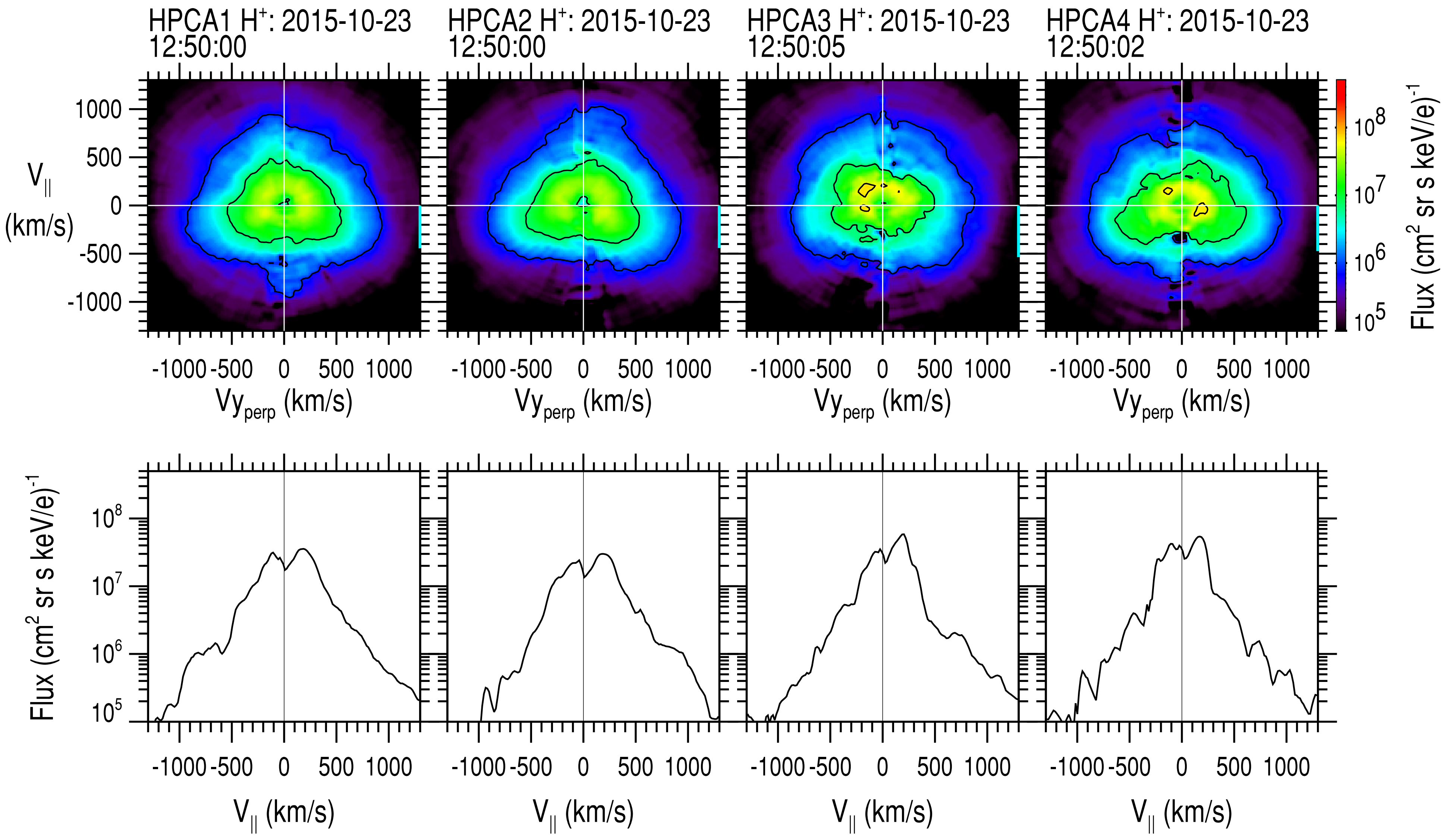
HPCA1 H+: 2015-10-23 12:50:00
HPCA2 H+: 2015-10-23 12:50:00
HPCA3 H+: 2015-10-23 12:50:05
HPCA4 H+: 2015-10-23 12:50:02
V|| (km/s)
Vyperp (km/s)
Flux (cm2 sr s keV/e)-1
-1000 -500 0 500 1000

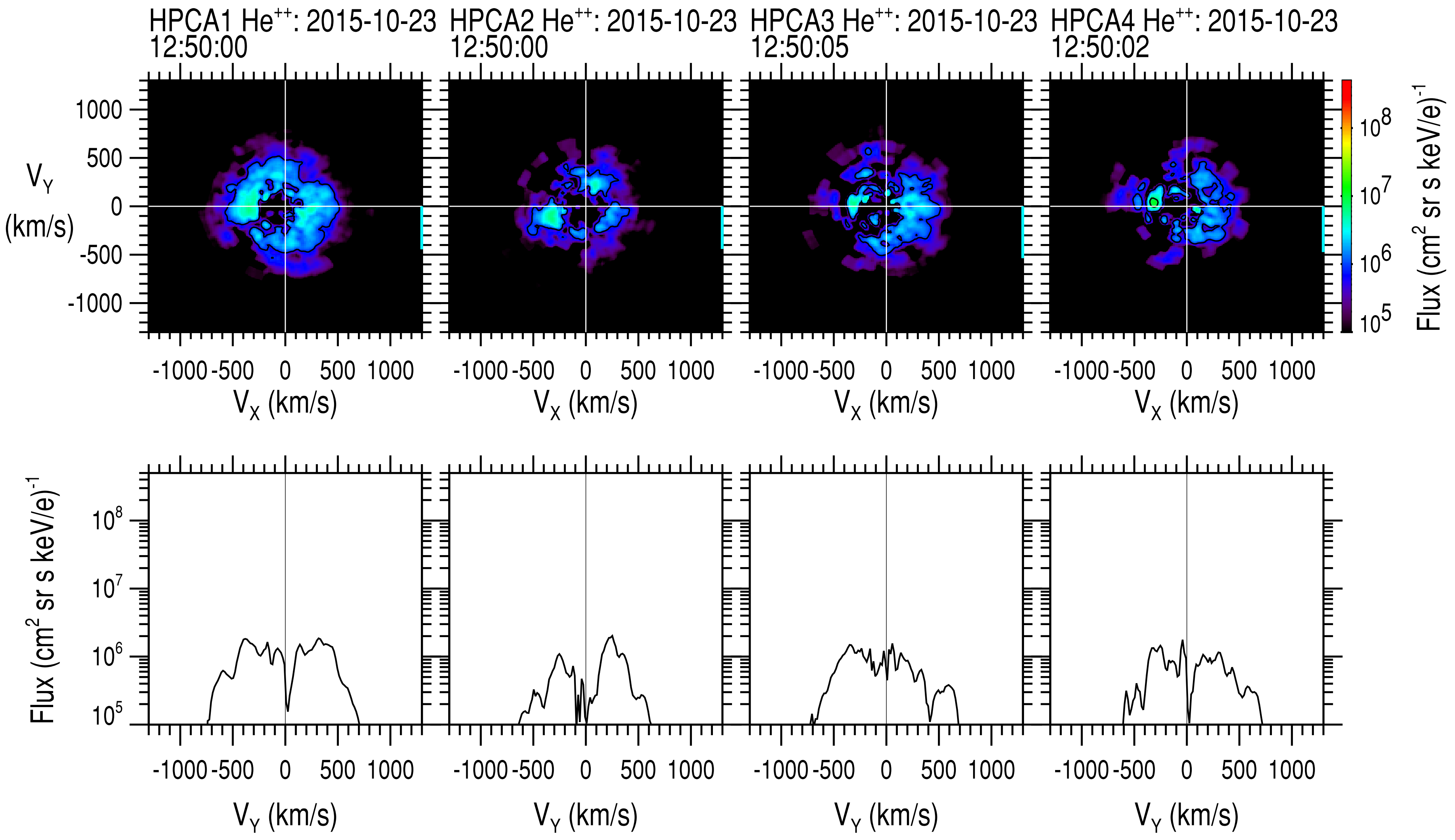
HPCA1 He^{++}: 2015-10-23 12:50:00
HPCA2 He^{++}: 2015-10-23 12:50:00
HPCA3 He^{++}: 2015-10-23 12:50:05
HPCA4 He^{++}: 2015-10-23 12:50:02
V_Y (km/s)
V_X (km/s)
Flux (cm^2 sr s keV/e)$^{-1}$
-1000 -500 0 500 1000
10^5 10^6 10^7 10^8

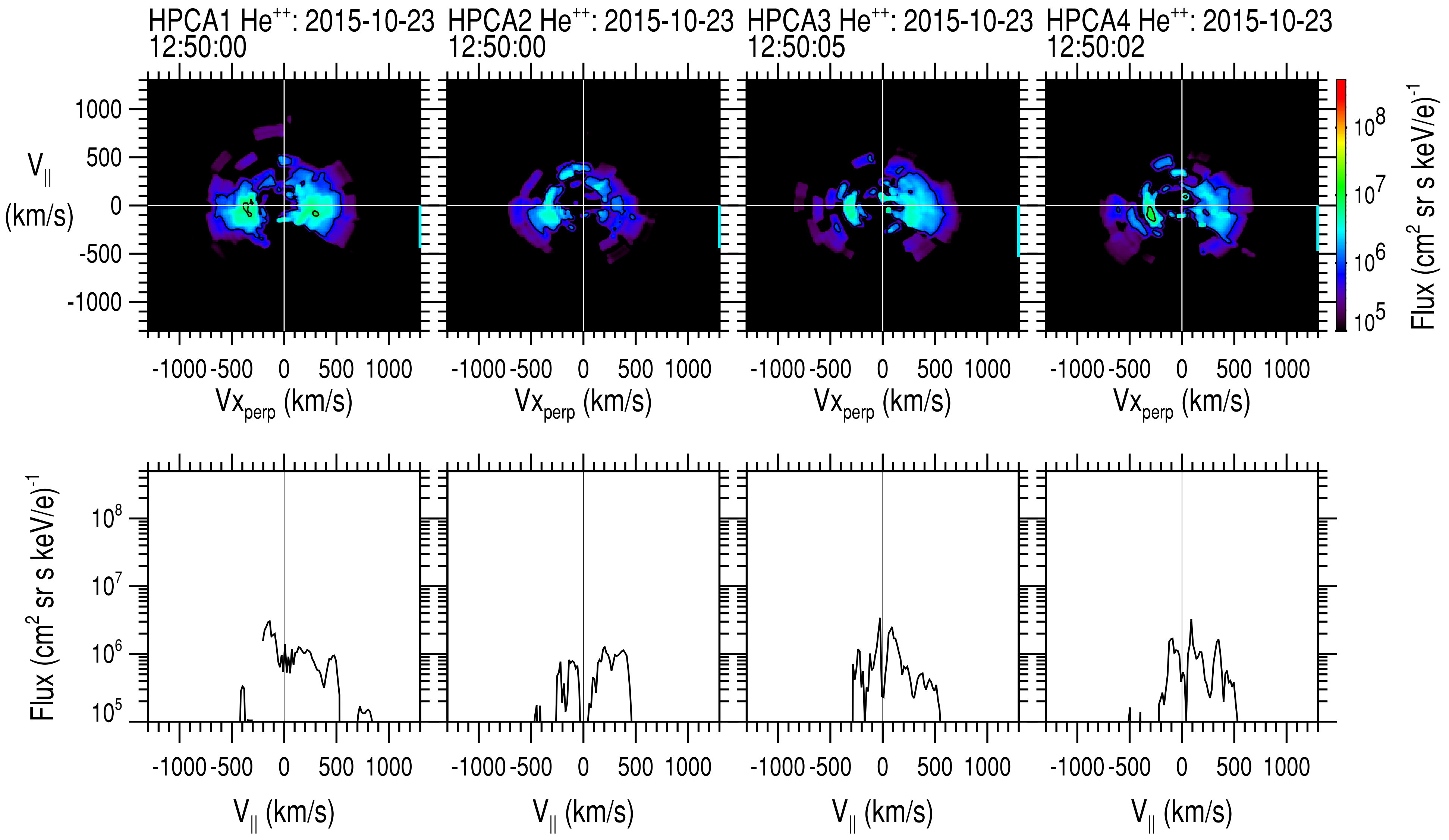
HPCA1 He++: 2015-10-23 12:50:00
HPCA2 He++: 2015-10-23 12:50:00
HPCA3 He++: 2015-10-23 12:50:05
HPCA4 He++: 2015-10-23 12:50:02
V|| (km/s)
Vxperp (km/s)
Flux (cm2 sr s keV/e)-1
1000
500
0
-500
-1000
10^5
10^6
10^7
10^8

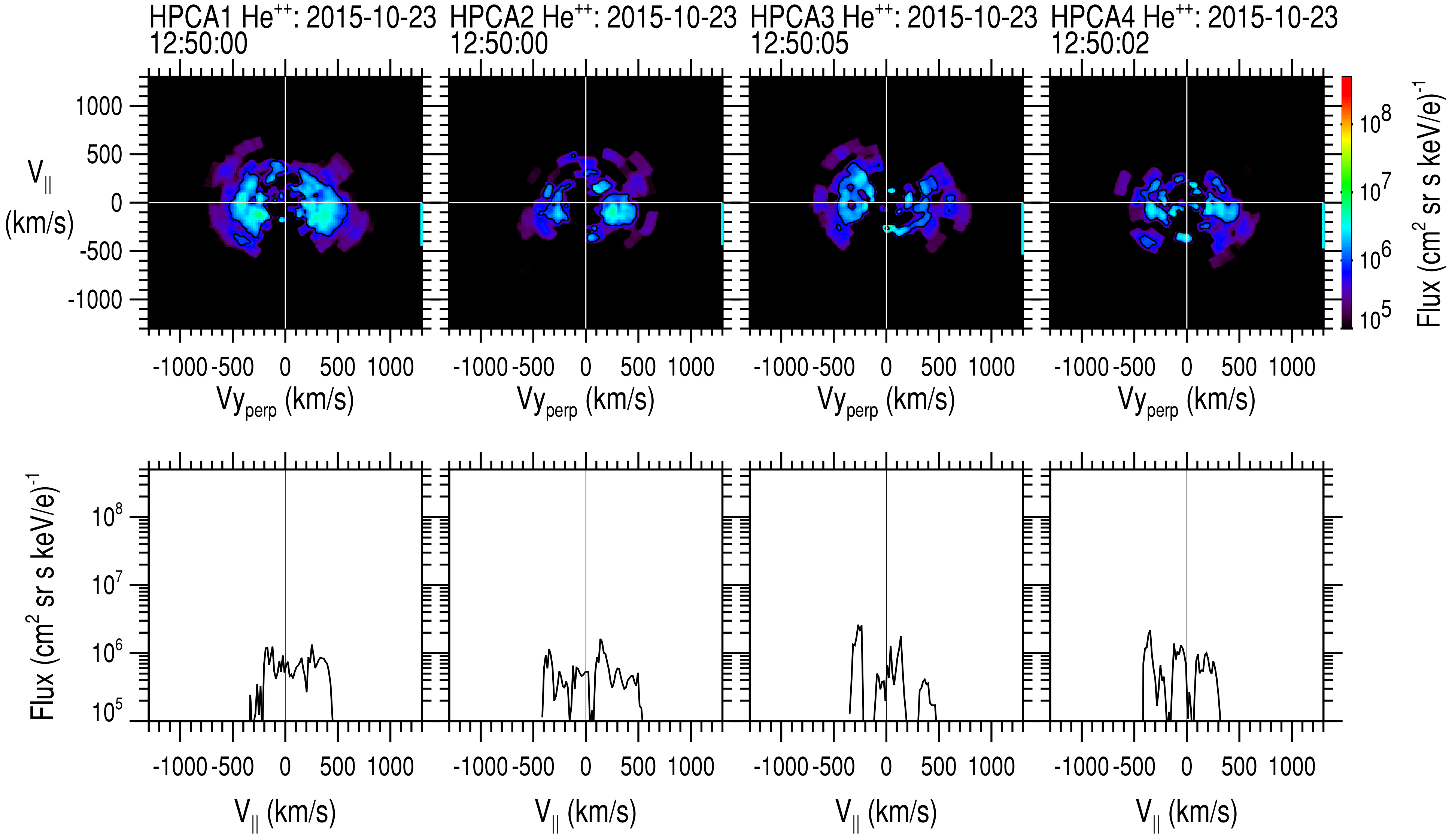

HPCA1 He++: 2015-10-23
12:50:00
HPCA2 He++: 2015-10-23
12:50:00
HPCA3 He++: 2015-10-23
12:50:05
HPCA4 He++: 2015-10-23
12:50:02
V||
(km/s)
1000
500
0
-500
-1000
-1000 -500 0 500 1000
Vyperp (km/s)
Flux (cm2 sr s keV/e)-1
10^5
10^6
10^7
10^8
V|| (km/s)

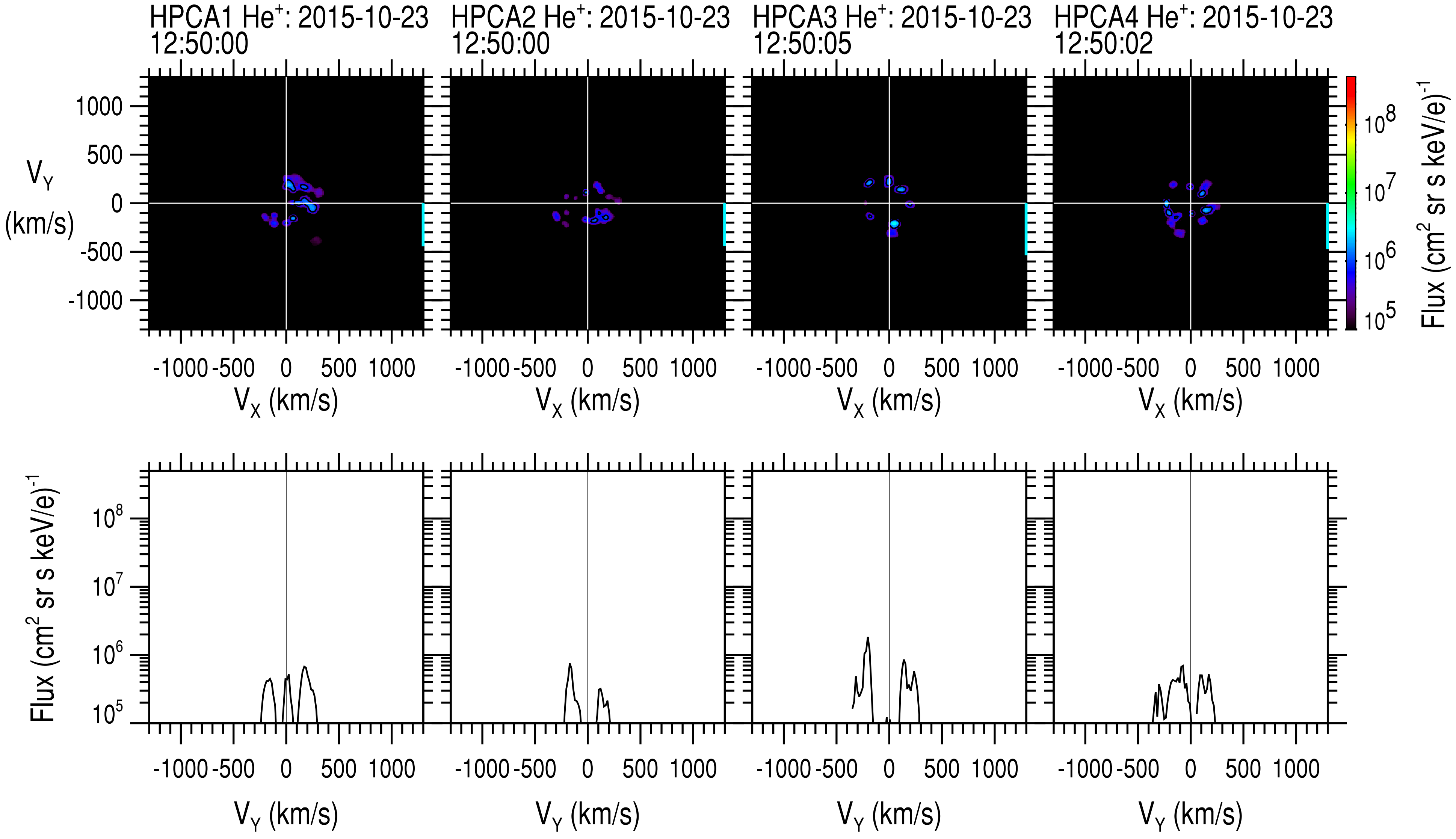
HPCA1 He+: 2015-10-23 12:50:00
HPCA2 He+: 2015-10-23 12:50:00
HPCA3 He+: 2015-10-23 12:50:05
HPCA4 He+: 2015-10-23 12:50:02
VY (km/s)
VX (km/s)
Flux (cm2 sr s keV/e)-1
VY (km/s)

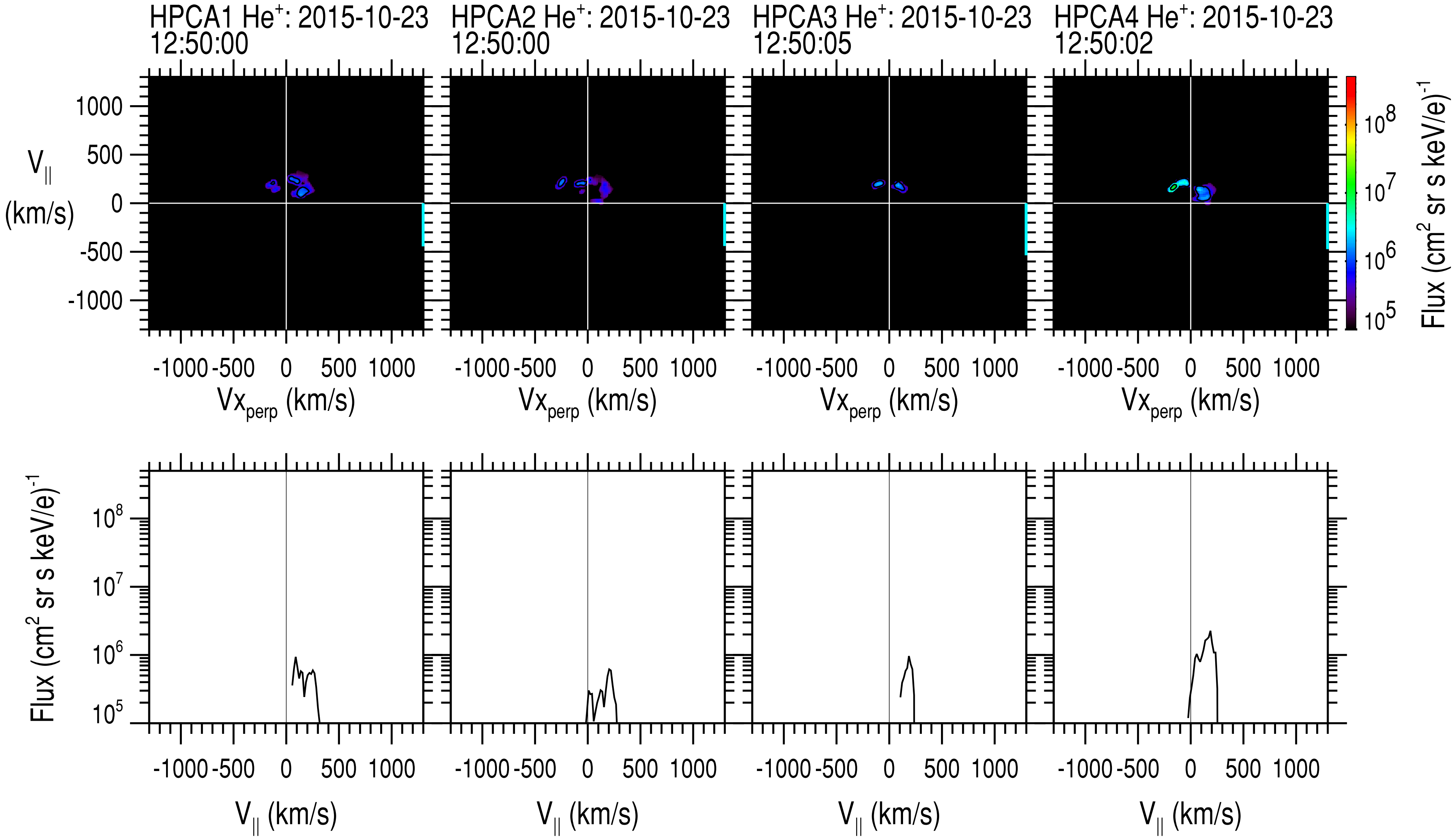
HPCA1 He+: 2015-10-23 12:50:00
HPCA2 He+: 2015-10-23 12:50:00
HPCA3 He+: 2015-10-23 12:50:05
HPCA4 He+: 2015-10-23 12:50:02
V|| (km/s)
Vxperp (km/s)
Flux (cm2 sr s keV/e)-1
V|| (km/s)

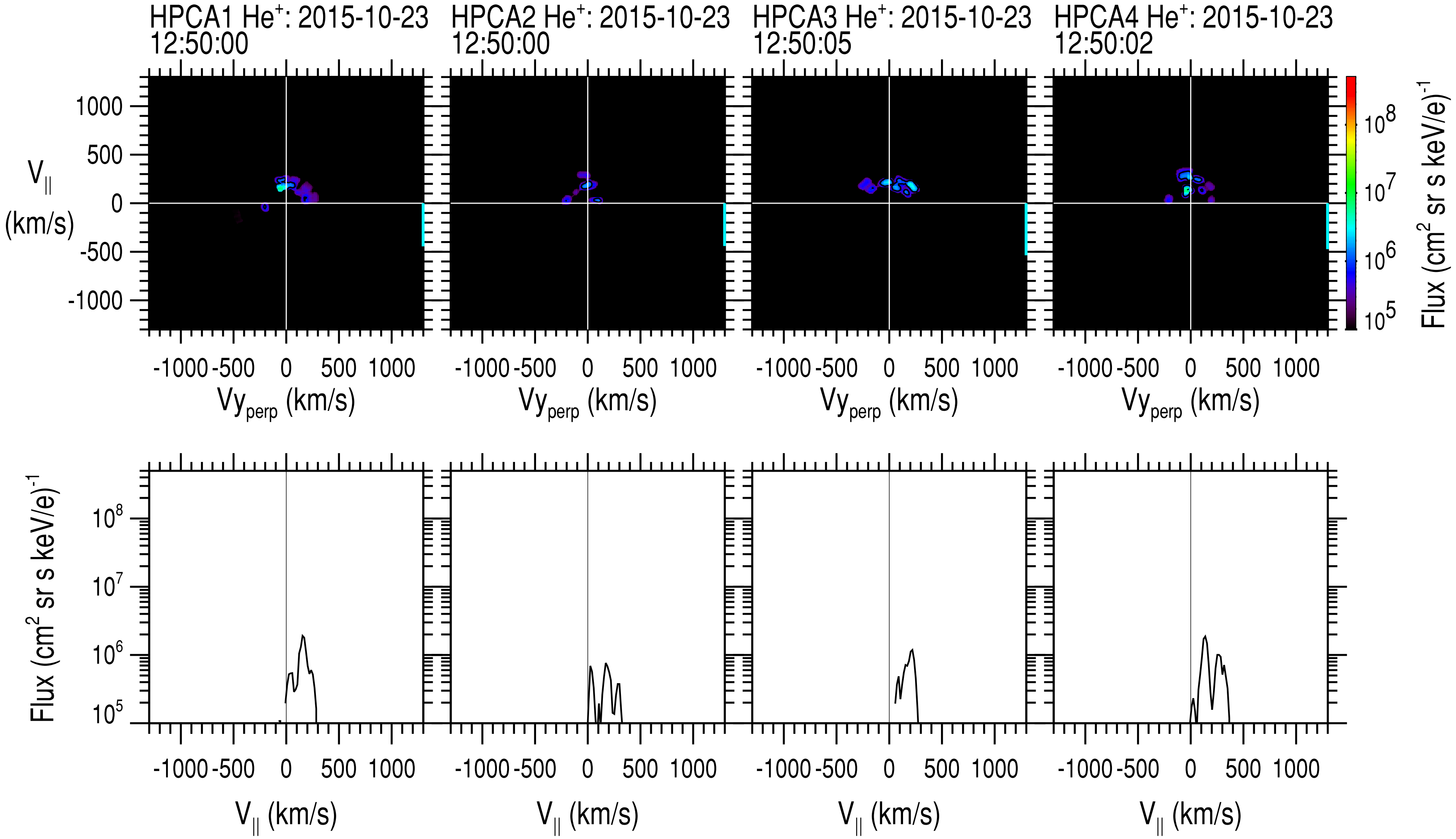
HPCA1 He+: 2015-10-23 12:50:00
HPCA2 He+: 2015-10-23 12:50:00
HPCA3 He+: 2015-10-23 12:50:05
HPCA4 He+: 2015-10-23 12:50:02
V|| (km/s)
Vyperp (km/s)
Flux (cm2 sr s keV/e)-1
V|| (km/s)

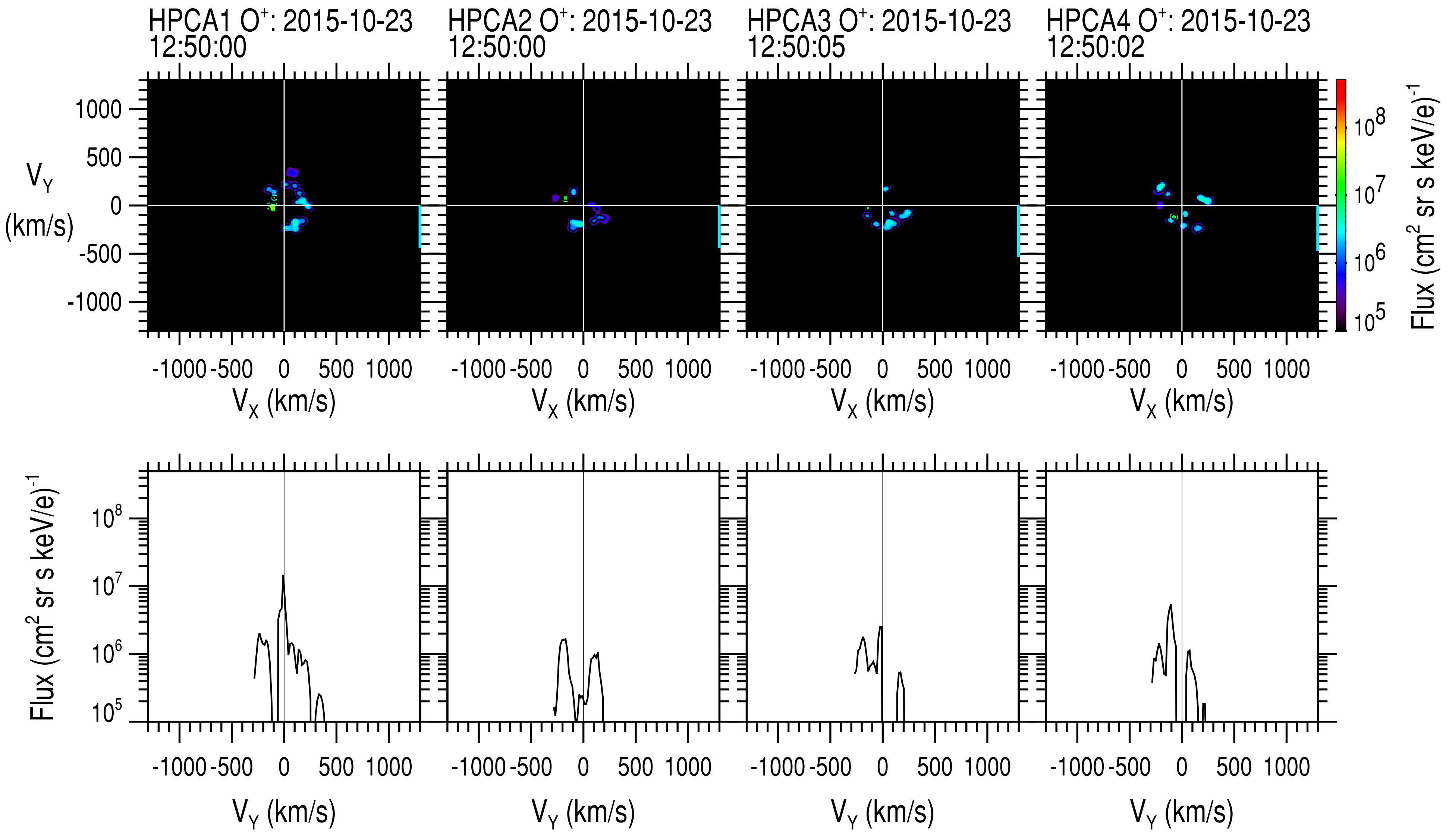

HPCA1 O+: 2015-10-23 12:50:00
HPCA2 O+: 2015-10-23 12:50:00
HPCA3 O+: 2015-10-23 12:50:05
HPCA4 O+: 2015-10-23 12:50:02
VY (km/s)
VX (km/s)
Flux (cm2 sr s keV/e)-1

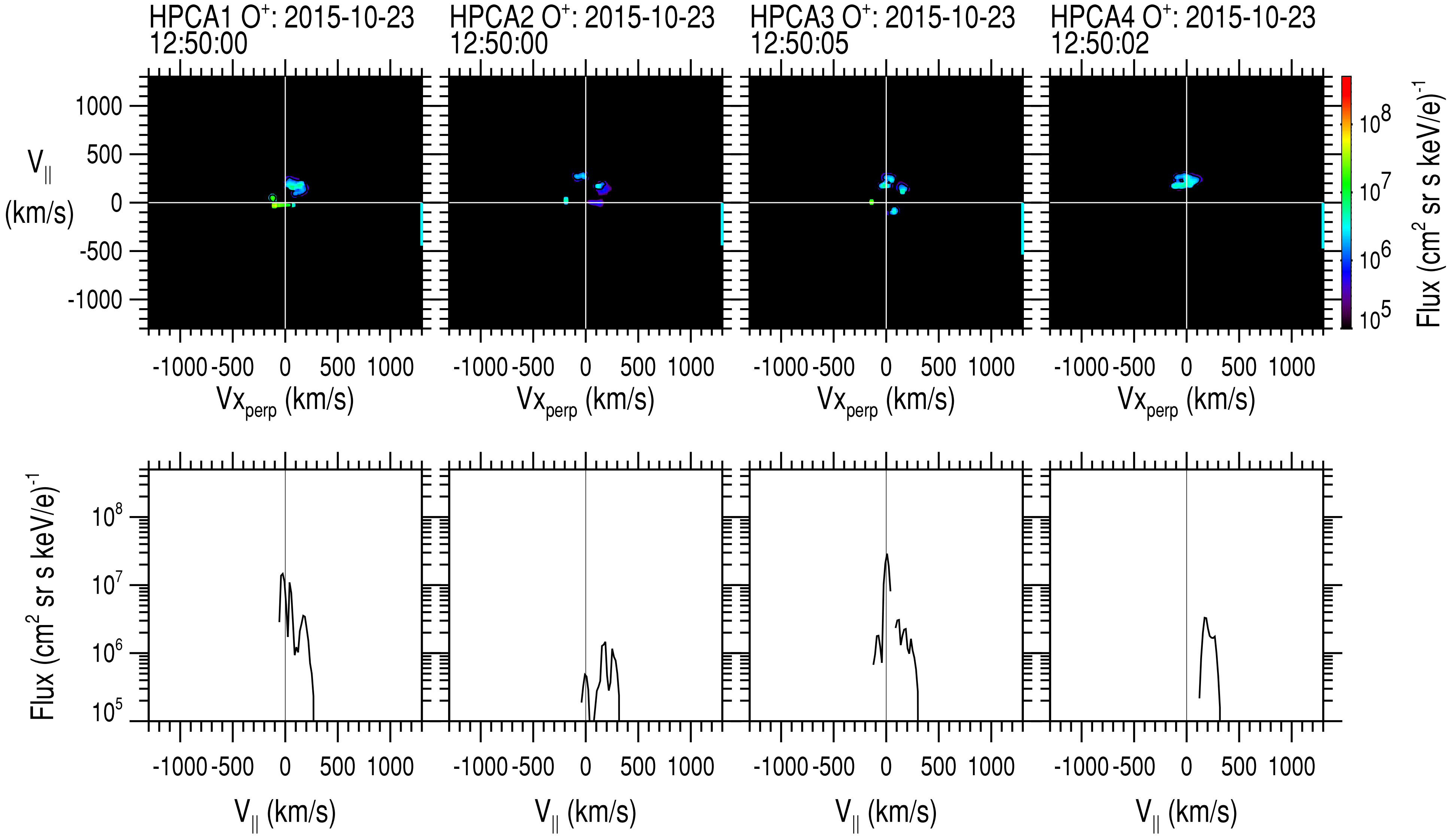

HPCA1 O+: 2015-10-23 12:50:00
HPCA2 O+: 2015-10-23 12:50:00
HPCA3 O+: 2015-10-23 12:50:05
HPCA4 O+: 2015-10-23 12:50:02
V|| (km/s)
Vxperp (km/s)
Flux (cm2 sr s keV/e)-1

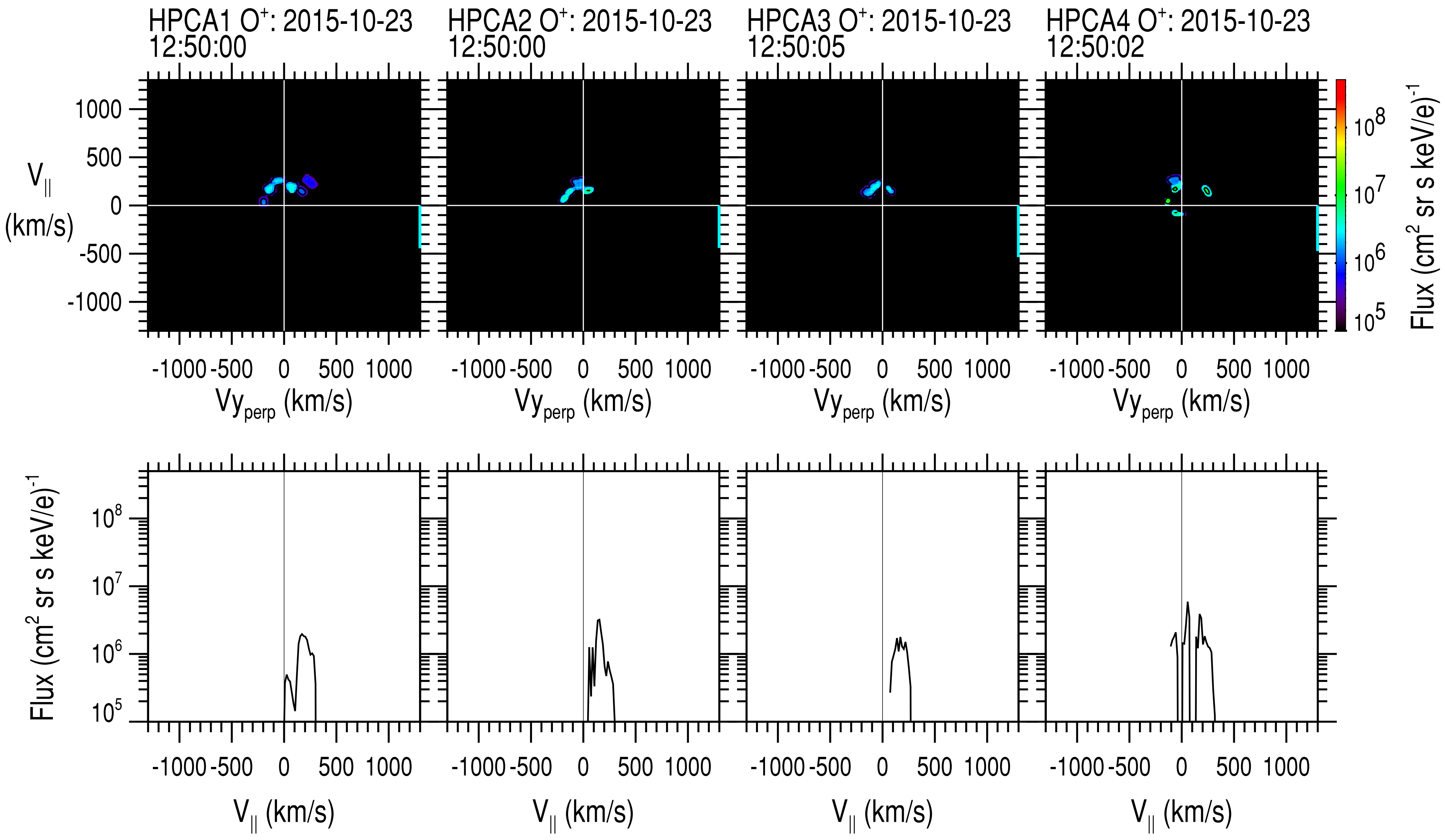
HPCA1 O+: 2015-10-23 12:50:00
HPCA2 O+: 2015-10-23 12:50:00
HPCA3 O+: 2015-10-23 12:50:05
HPCA4 O+: 2015-10-23 12:50:02
V|| (km/s)
Vyperp (km/s)
Flux (cm2 sr s keV/e)-1
V|| (km/s)

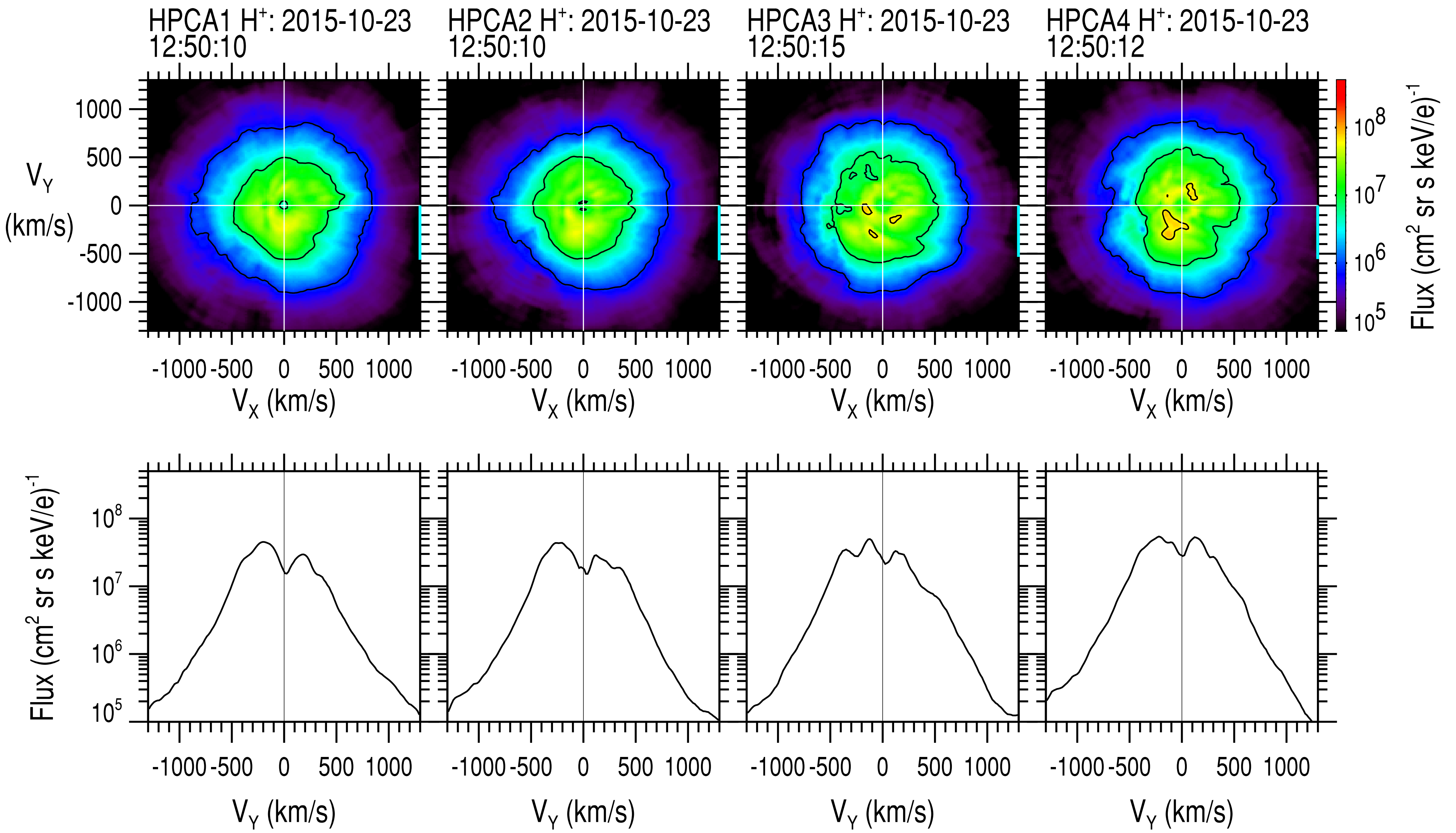

HPCA1 H+: 2015-10-23 12:50:10
HPCA2 H+: 2015-10-23 12:50:10
HPCA3 H+: 2015-10-23 12:50:15
HPCA4 H+: 2015-10-23 12:50:12
VY (km/s)
VX (km/s)
Flux (cm2 sr s keV/e)-1

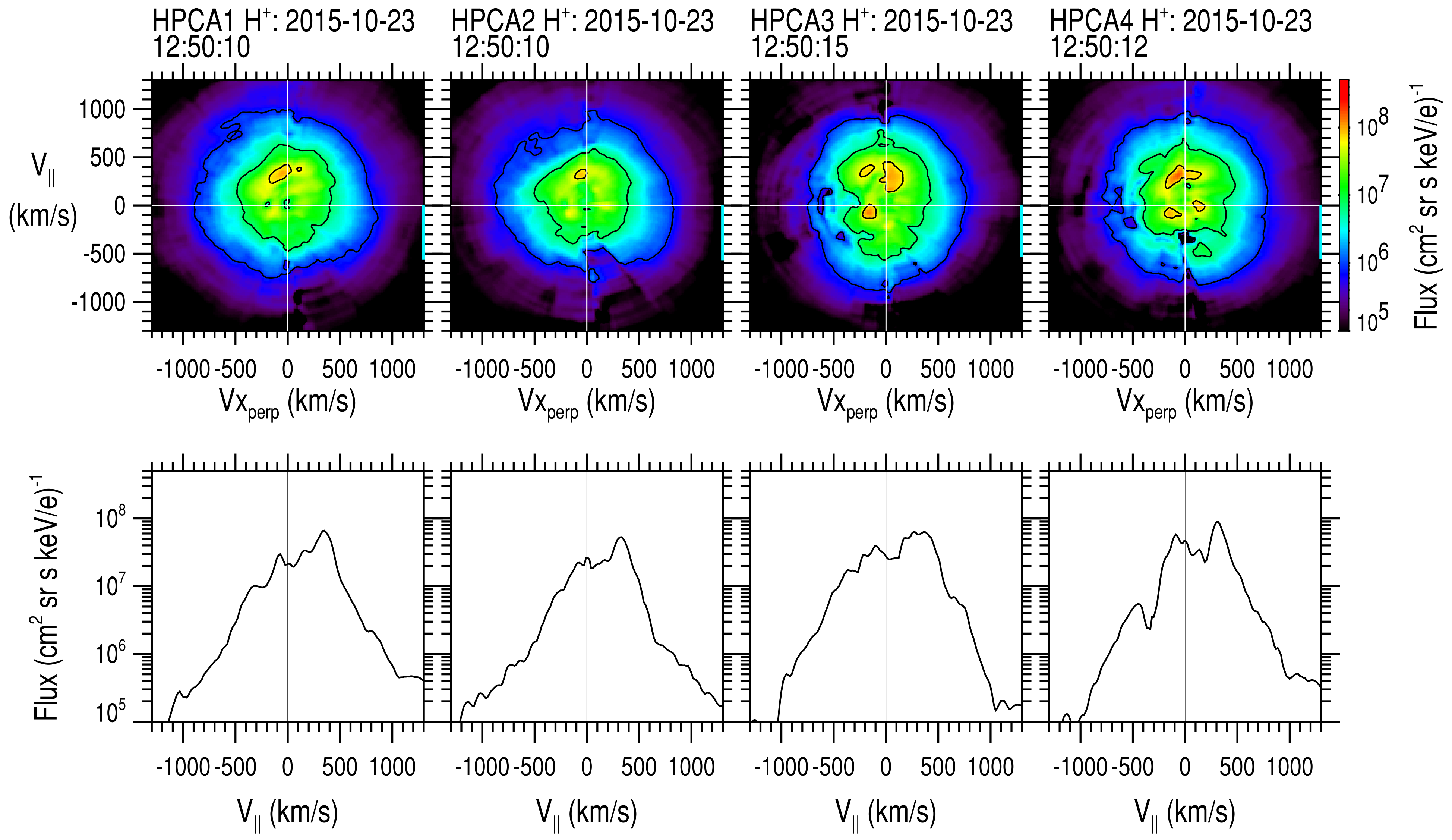
HPCA1 H⁺: 2015-10-23 12:50:10
HPCA2 H⁺: 2015-10-23 12:50:10
HPCA3 H⁺: 2015-10-23 12:50:15
HPCA4 H⁺: 2015-10-23 12:50:12
V|| (km/s)
Vxperp (km/s)
Flux (cm2 sr s keV/e)-1
105
106
107
108
-1000
-500
0
500
1000

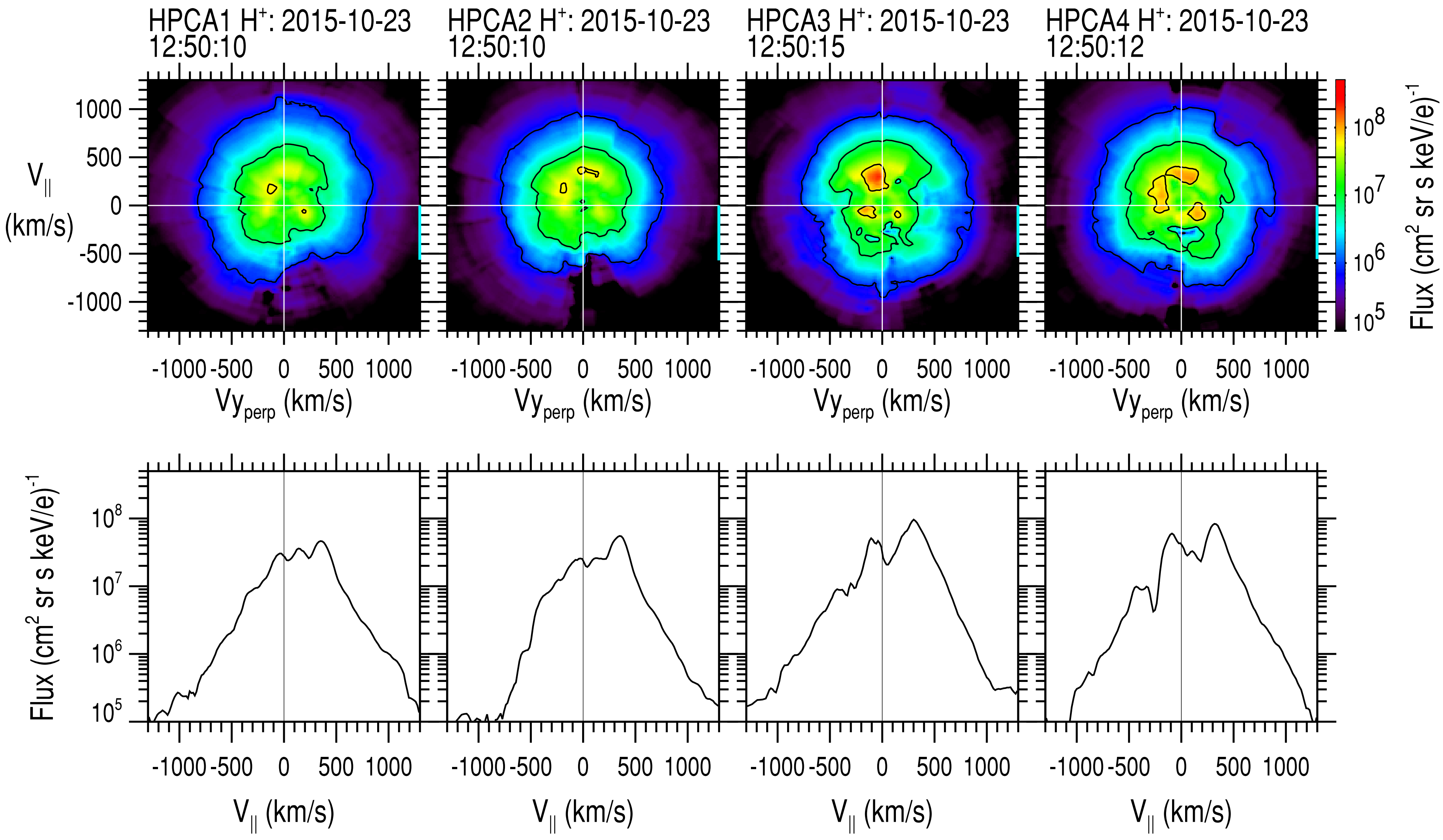
HPCA1 H+: 2015-10-23
12:50:10
HPCA2 H+: 2015-10-23
12:50:10
HPCA3 H+: 2015-10-23
12:50:15
HPCA4 H+: 2015-10-23
12:50:12
V||
(km/s)
1000
500
0
-500
-1000
-1000 -500 0 500 1000
Vyperp (km/s)
Flux (cm2 sr s keV/e)-1
105
106
107
108
V|| (km/s)

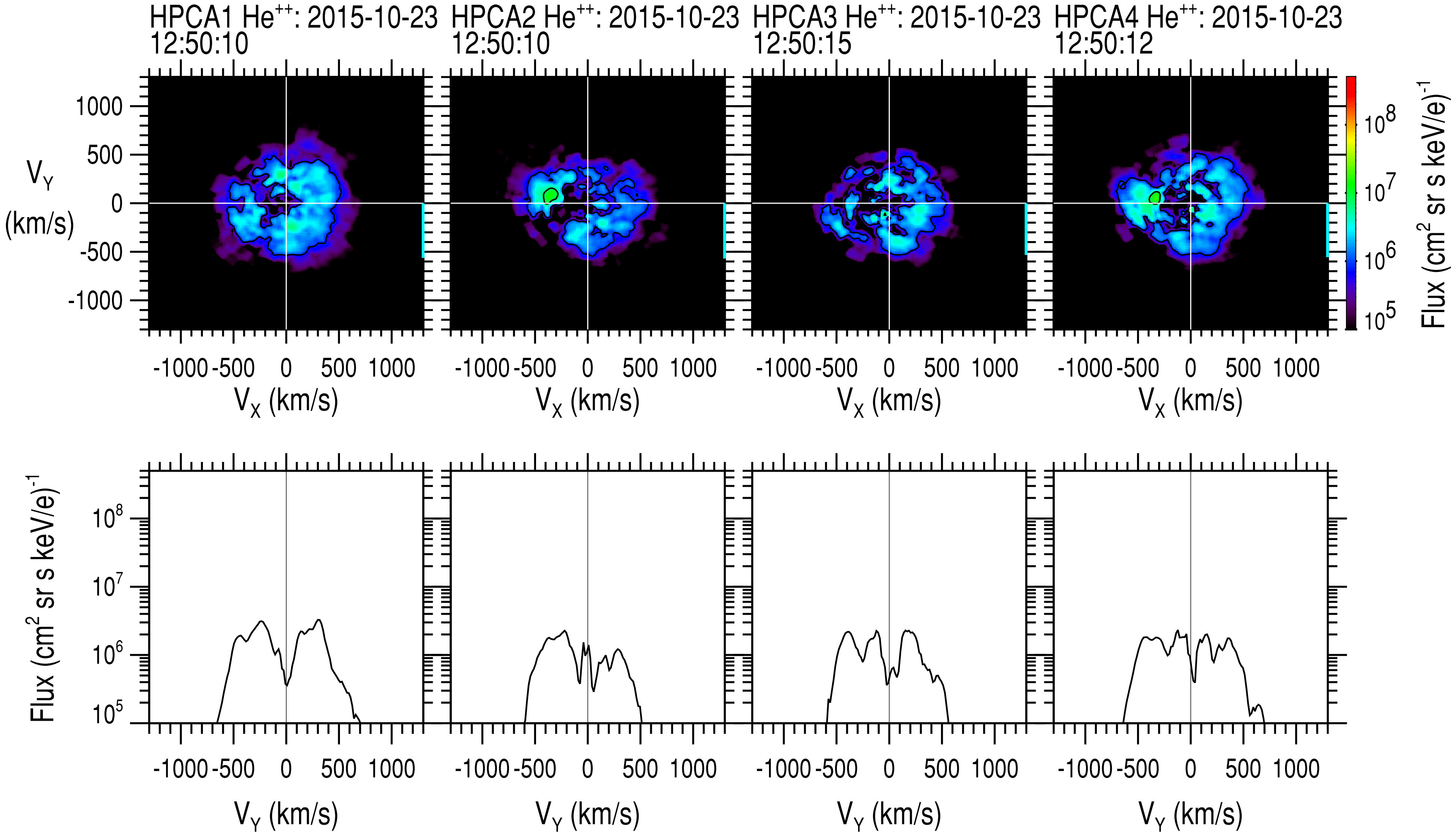

HPCA1 He++: 2015-10-23 12:50:10
HPCA2 He++: 2015-10-23 12:50:10
HPCA3 He++: 2015-10-23 12:50:15
HPCA4 He++: 2015-10-23 12:50:12
V_Y (km/s)
1000
500
0
-500
-1000
-1000 -500 0 500 1000
V_X (km/s)
Flux (cm^2 sr s keV/e)^-1
10^5
10^6
10^7
10^8
V_Y (km/s)

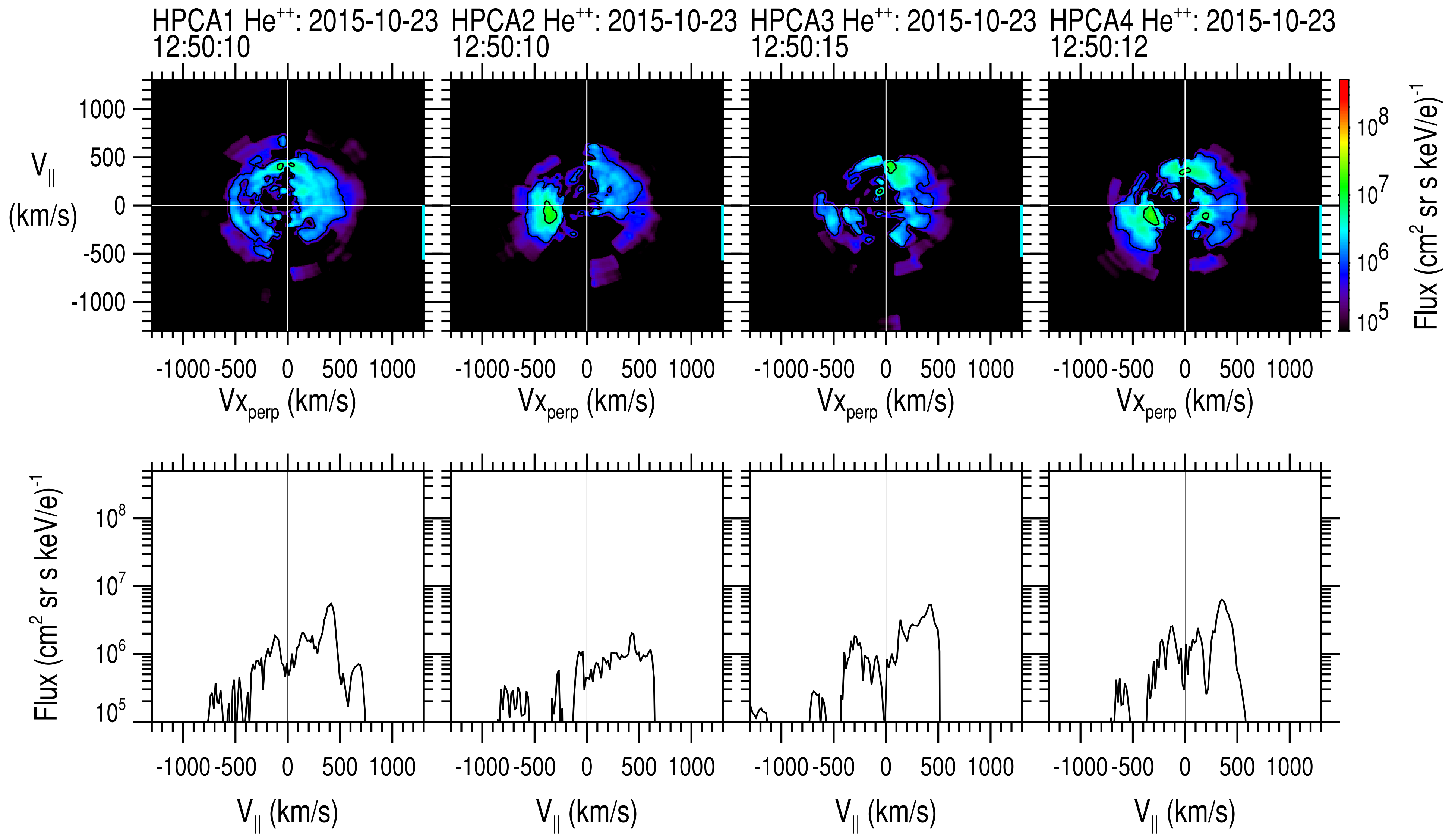
HPCA1 He++: 2015-10-23 12:50:10
HPCA2 He++: 2015-10-23 12:50:10
HPCA3 He++: 2015-10-23 12:50:15
HPCA4 He++: 2015-10-23 12:50:12
V|| (km/s)
Vxperp (km/s)
Flux (cm2 sr s keV/e)-1
-1000 -500 0 500 1000
10^5 10^6 10^7 10^8

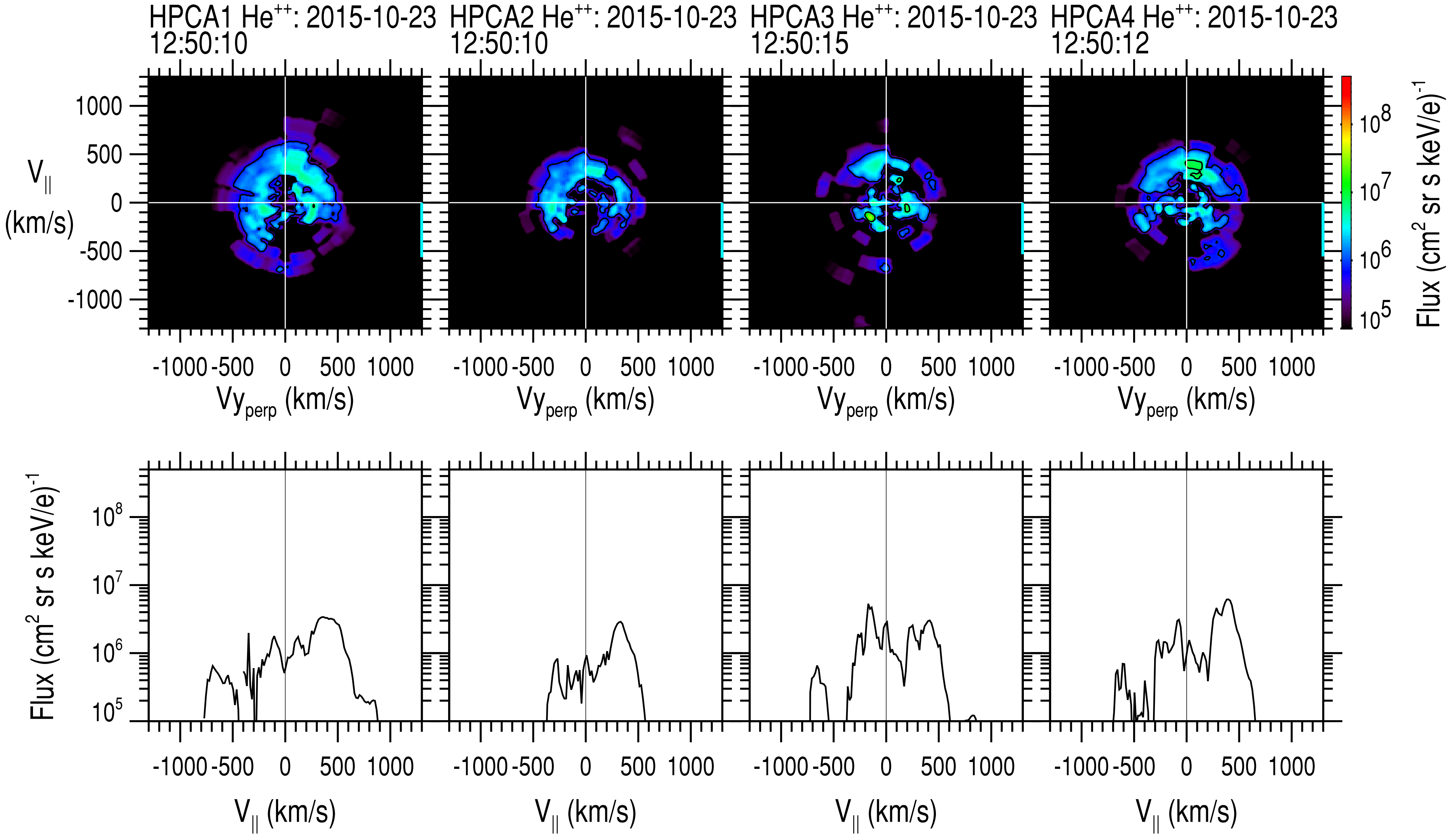
HPCA1 He++: 2015-10-23 12:50:10
HPCA2 He++: 2015-10-23 12:50:10
HPCA3 He++: 2015-10-23 12:50:15
HPCA4 He++: 2015-10-23 12:50:12
V|| (km/s)
Vyperp (km/s)
Flux (cm2 sr s keV/e)-1
V|| (km/s)
Flux (cm2 sr s keV/e)-1

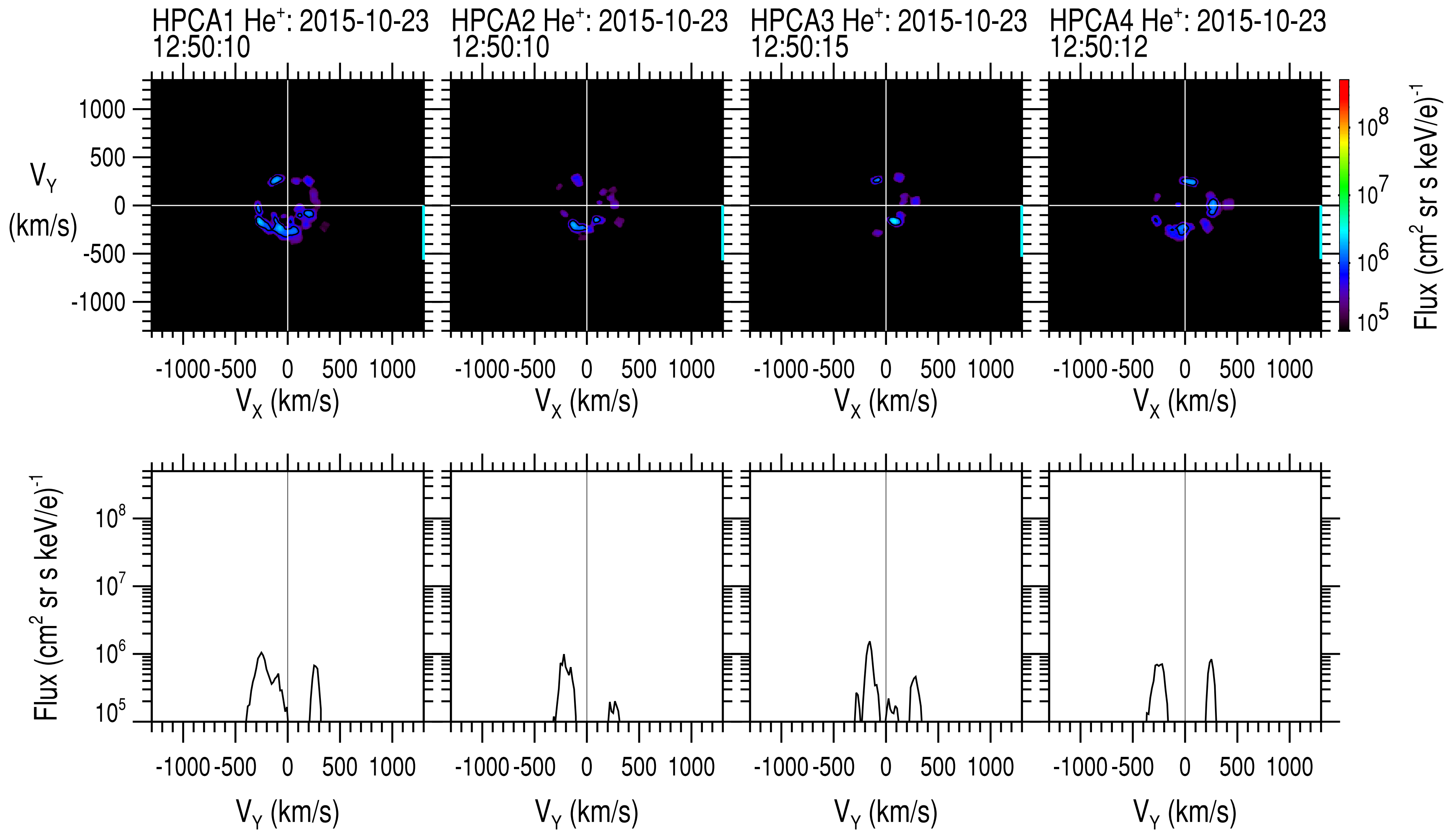

HPCA1 He+: 2015-10-23
12:50:10
HPCA2 He+: 2015-10-23
12:50:10
HPCA3 He+: 2015-10-23
12:50:15
HPCA4 He+: 2015-10-23
12:50:12
VY (km/s)
VX (km/s)
Flux (cm2 sr s keV/e)-1
VY (km/s)

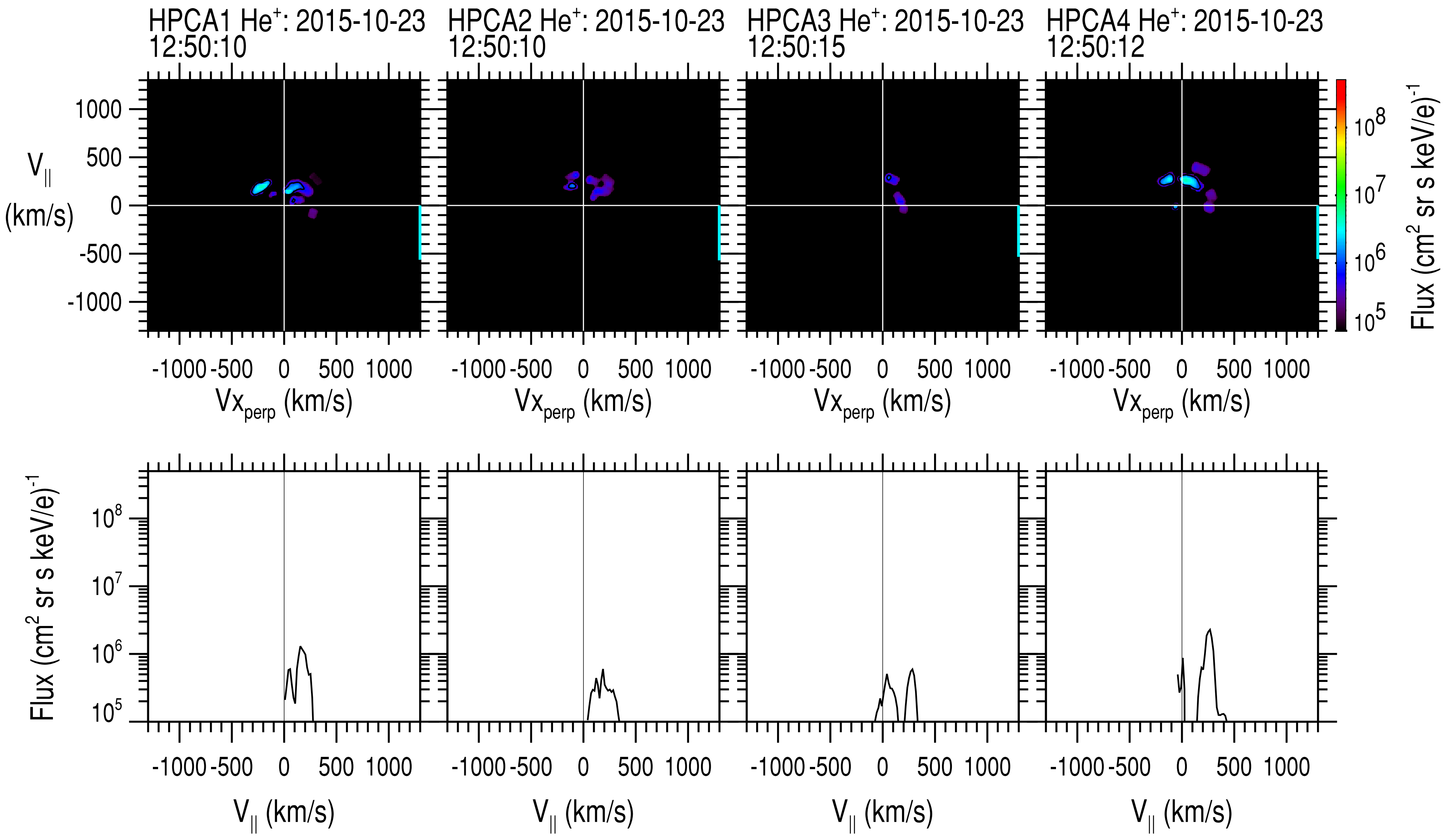

HPCA1 He+: 2015-10-23 12:50:10
HPCA2 He+: 2015-10-23 12:50:10
HPCA3 He+: 2015-10-23 12:50:15
HPCA4 He+: 2015-10-23 12:50:12
V|| (km/s)
Vxperp (km/s)
Flux (cm2 sr s keV/e)-1

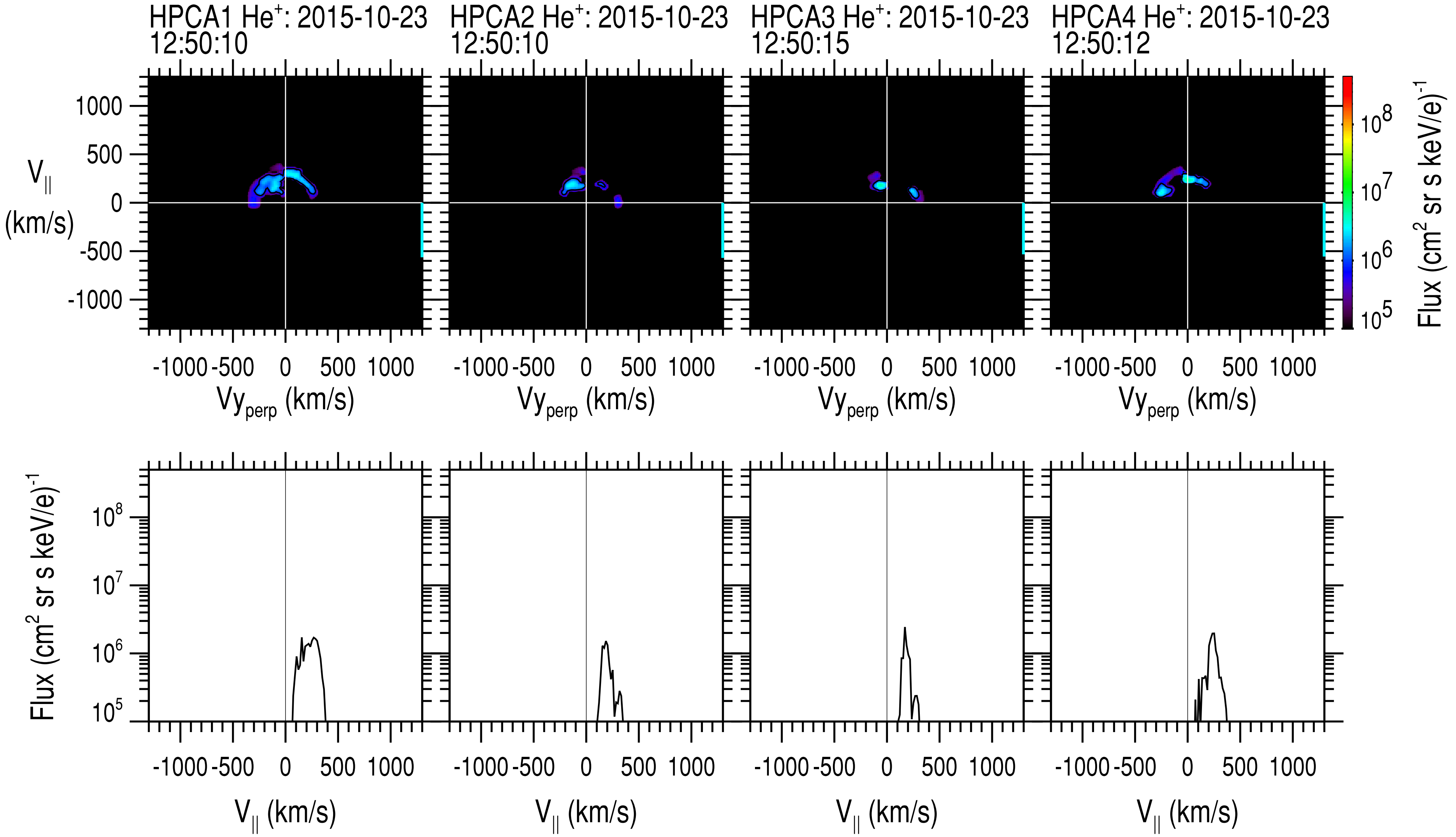

HPCA1 He+: 2015-10-23 12:50:10
HPCA2 He+: 2015-10-23 12:50:10
HPCA3 He+: 2015-10-23 12:50:15
HPCA4 He+: 2015-10-23 12:50:12
V|| (km/s)
Vyperp (km/s)
Flux (cm2 sr s keV/e)-1

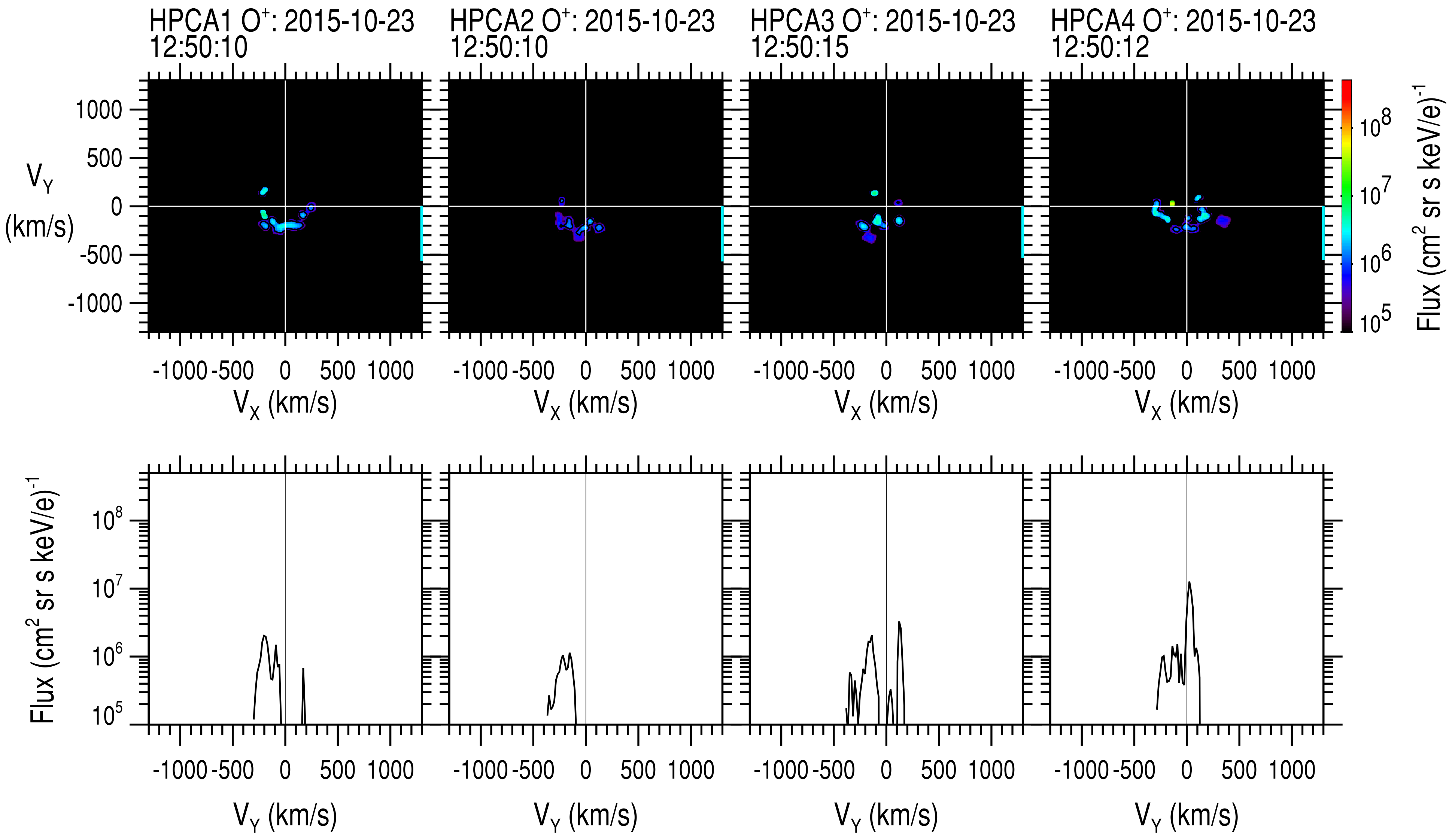
HPCA1 O+: 2015-10-23 12:50:10
HPCA2 O+: 2015-10-23 12:50:10
HPCA3 O+: 2015-10-23 12:50:15
HPCA4 O+: 2015-10-23 12:50:12
V_Y (km/s)
V_X (km/s)
Flux (cm^2 sr s keV/e)^-1
-1000 -500 0 500 1000
10^5 10^6 10^7 10^8

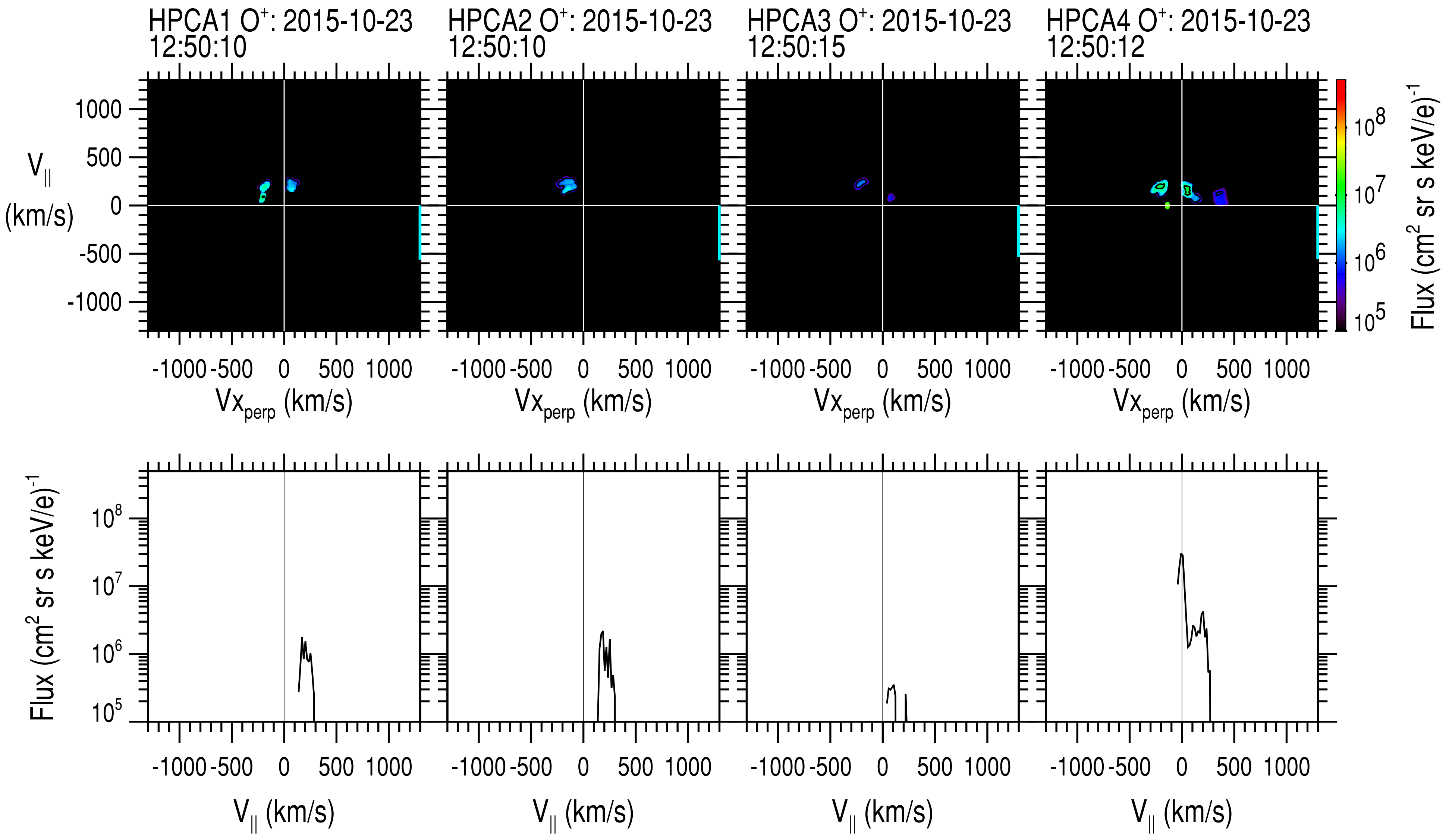
HPCA1 O+: 2015-10-23 12:50:10
HPCA2 O+: 2015-10-23 12:50:10
HPCA3 O+: 2015-10-23 12:50:15
HPCA4 O+: 2015-10-23 12:50:12
V|| (km/s)
Vxperp (km/s)
Flux (cm2 sr s keV/e)-1
V|| (km/s)

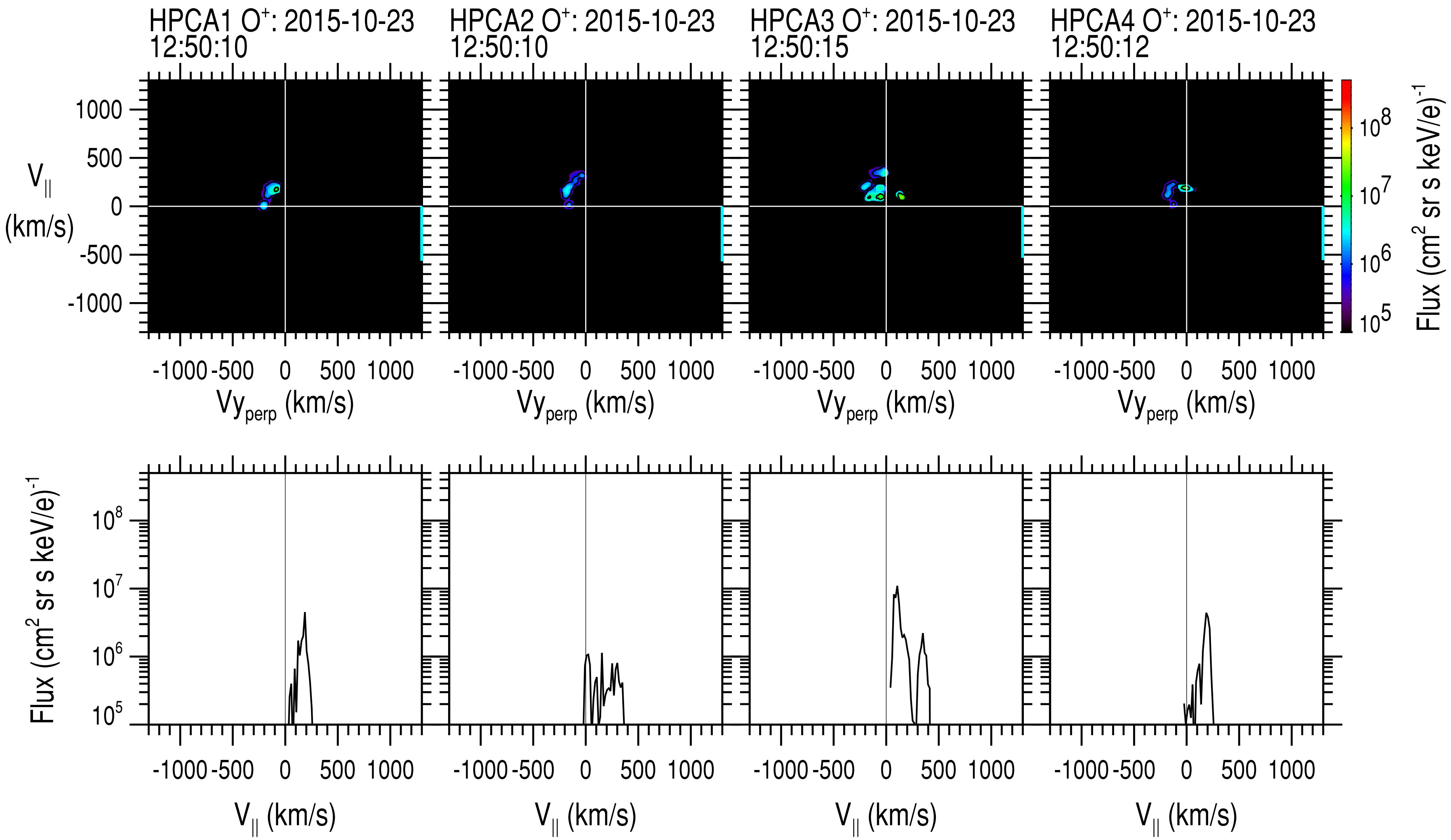
HPCA1 O+: 2015-10-23 12:50:10
HPCA2 O+: 2015-10-23 12:50:10
HPCA3 O+: 2015-10-23 12:50:15
HPCA4 O+: 2015-10-23 12:50:12
V|| (km/s)
Vyperp (km/s)
Flux (cm2 sr s keV/e)-1
1000
500
0
-500
-1000
10^5
10^6
10^7
10^8

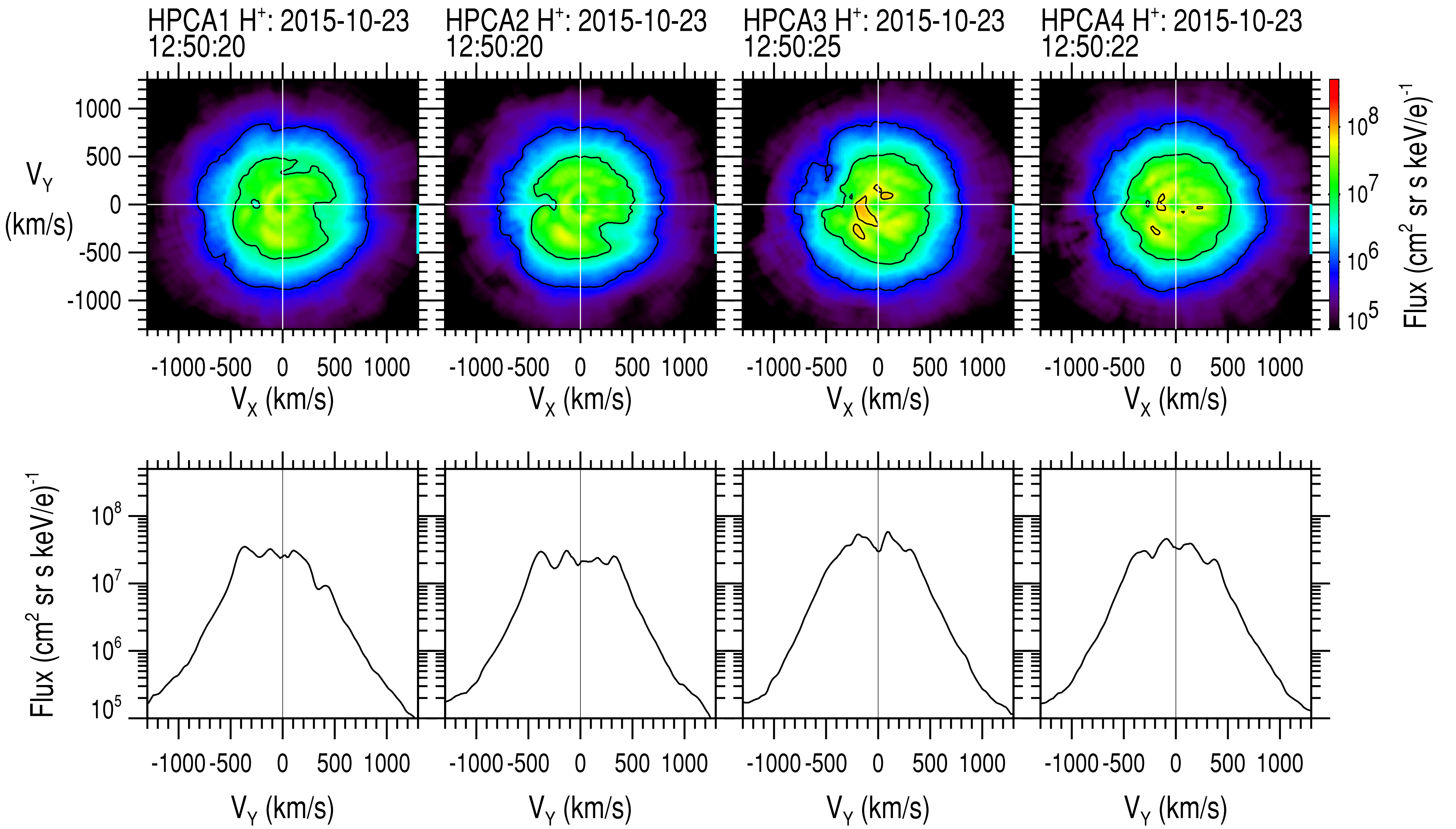

HPCA1 H+: 2015-10-23 12:50:20
HPCA2 H+: 2015-10-23 12:50:20
HPCA3 H+: 2015-10-23 12:50:25
HPCA4 H+: 2015-10-23 12:50:22
VY (km/s)
VX (km/s)
Flux (cm2 sr s keV/e)-1
VY (km/s)

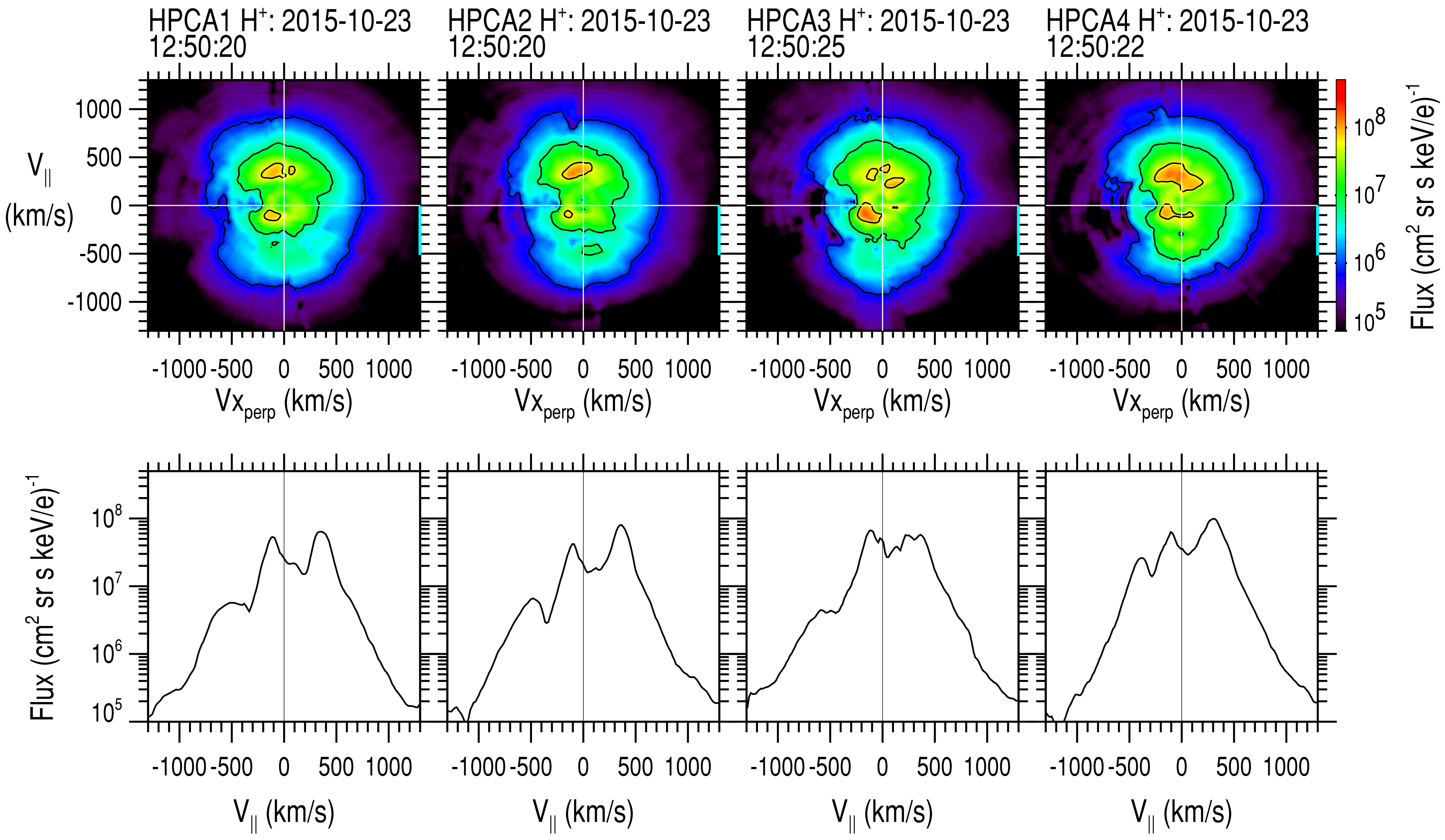
HPCA1 H+: 2015-10-23
12:50:20
HPCA2 H+: 2015-10-23
12:50:20
HPCA3 H+: 2015-10-23
12:50:25
HPCA4 H+: 2015-10-23
12:50:22
V||
(km/s)
Vxperp (km/s)
Flux (cm2 sr s keV/e)-1
V|| (km/s)

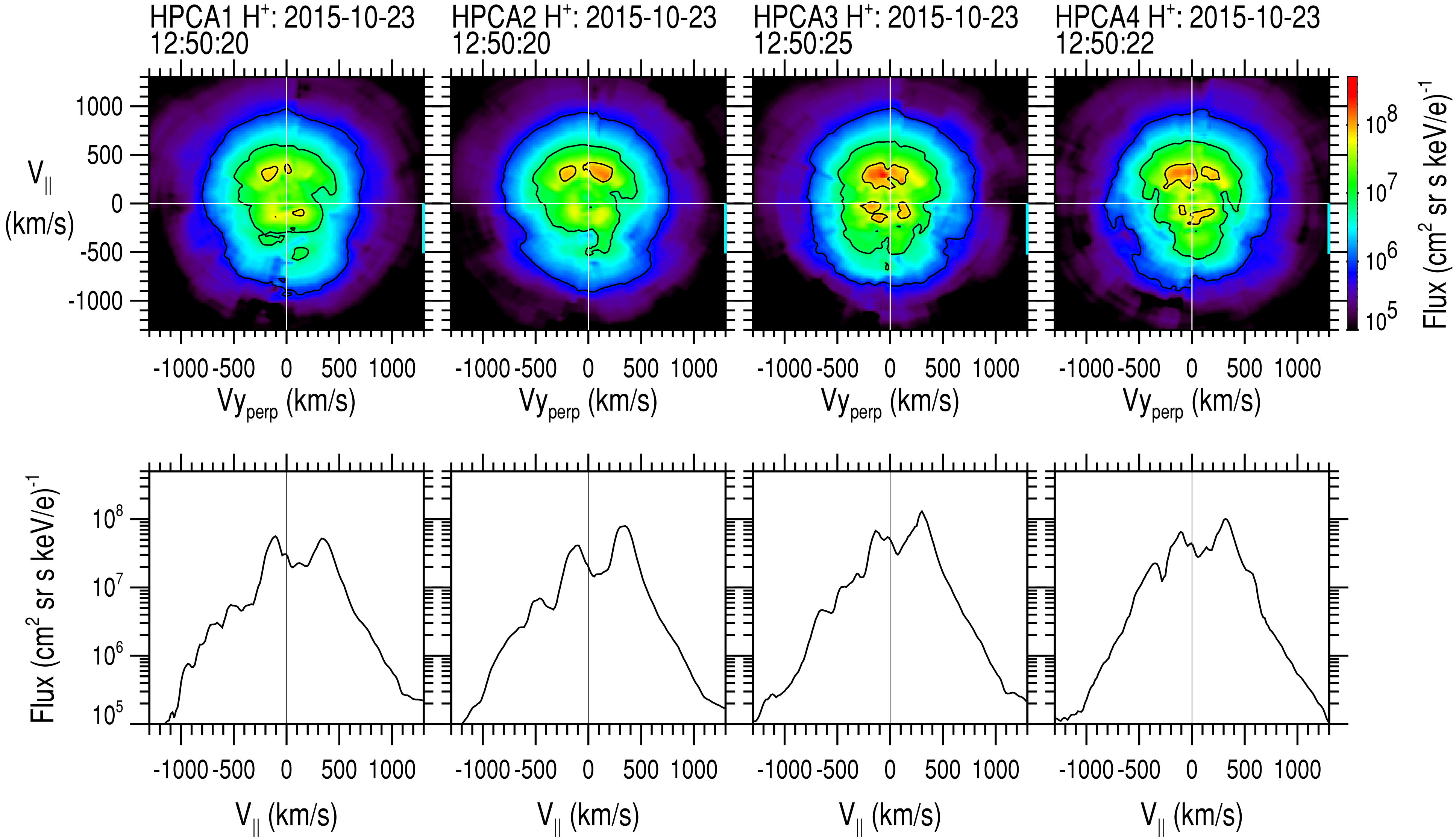

HPCA1 H+: 2015-10-23
12:50:20
HPCA2 H+: 2015-10-23
12:50:20
HPCA3 H+: 2015-10-23
12:50:25
HPCA4 H+: 2015-10-23
12:50:22
V|| (km/s)
Vyperp (km/s)
Flux (cm2 sr s keV/e)-1

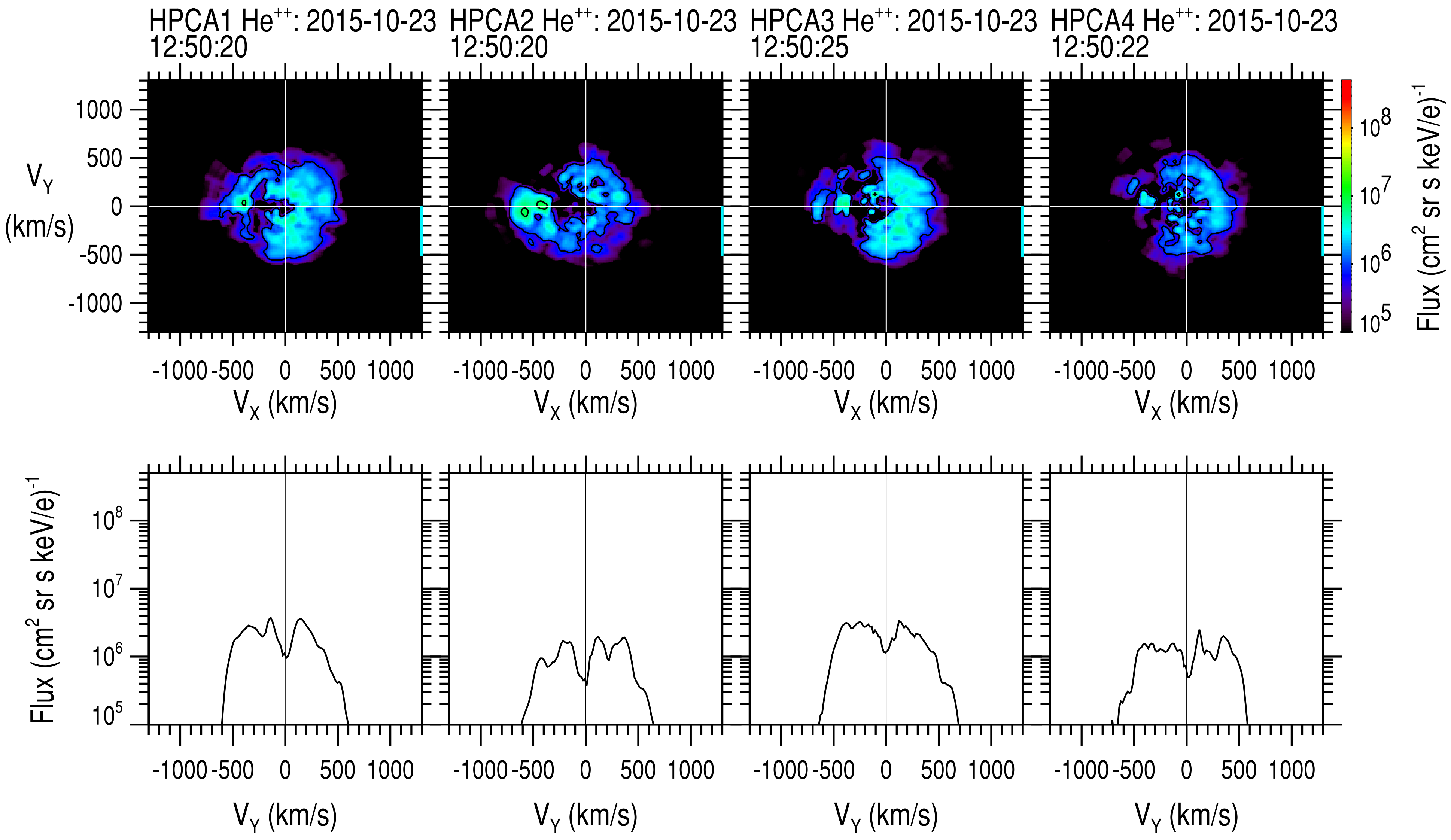

HPCA1 He++: 2015-10-23 12:50:20
HPCA2 He++: 2015-10-23 12:50:20
HPCA3 He++: 2015-10-23 12:50:25
HPCA4 He++: 2015-10-23 12:50:22
VY (km/s)
VX (km/s)
Flux (cm2 sr s keV/e)-1
VY (km/s)

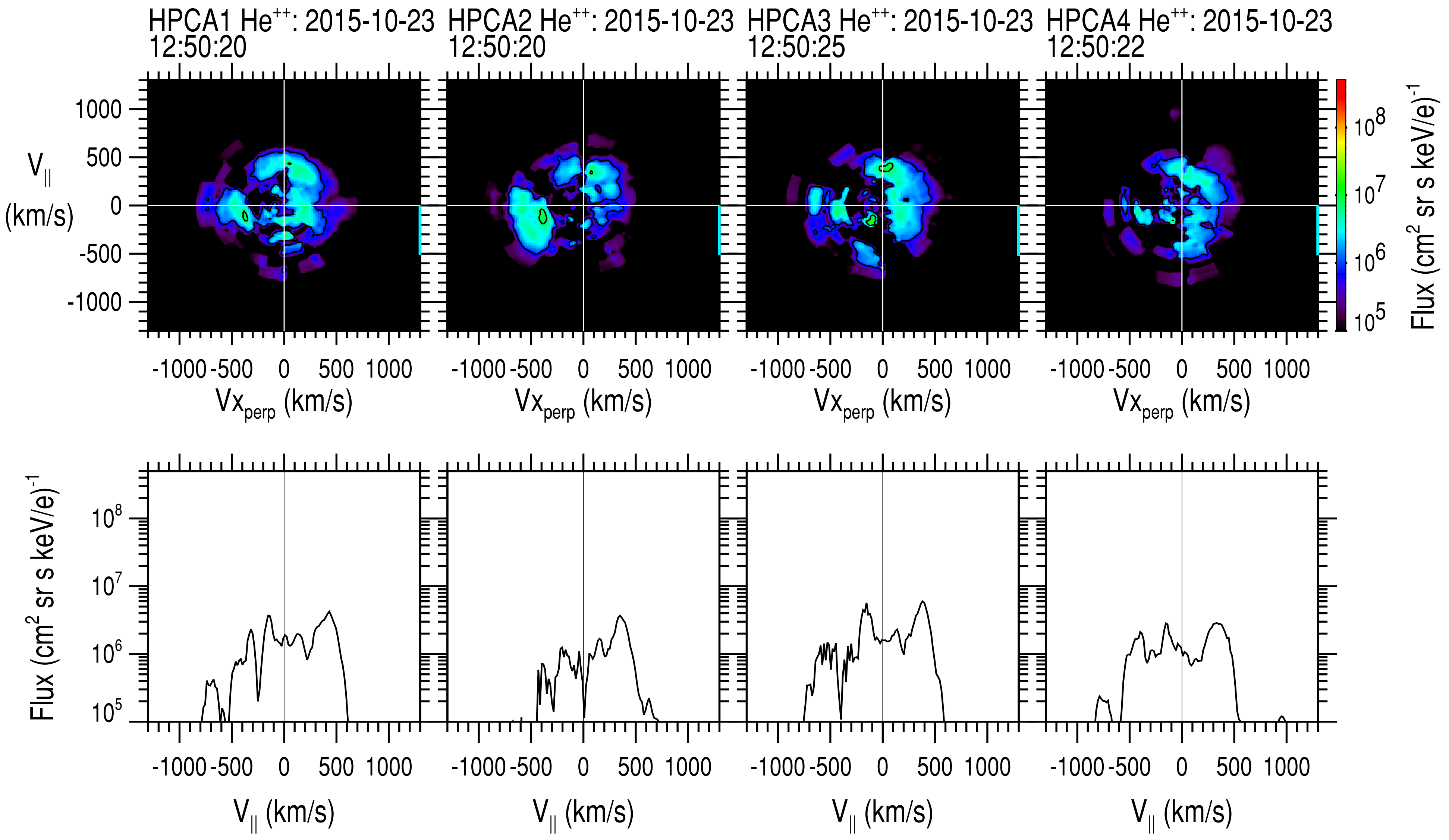
HPCA1 He++: 2015-10-23 12:50:20
HPCA2 He++: 2015-10-23 12:50:20
HPCA3 He++: 2015-10-23 12:50:25
HPCA4 He++: 2015-10-23 12:50:22
V|| (km/s)
Vxperp (km/s)
Flux (cm2 sr s keV/e)-1

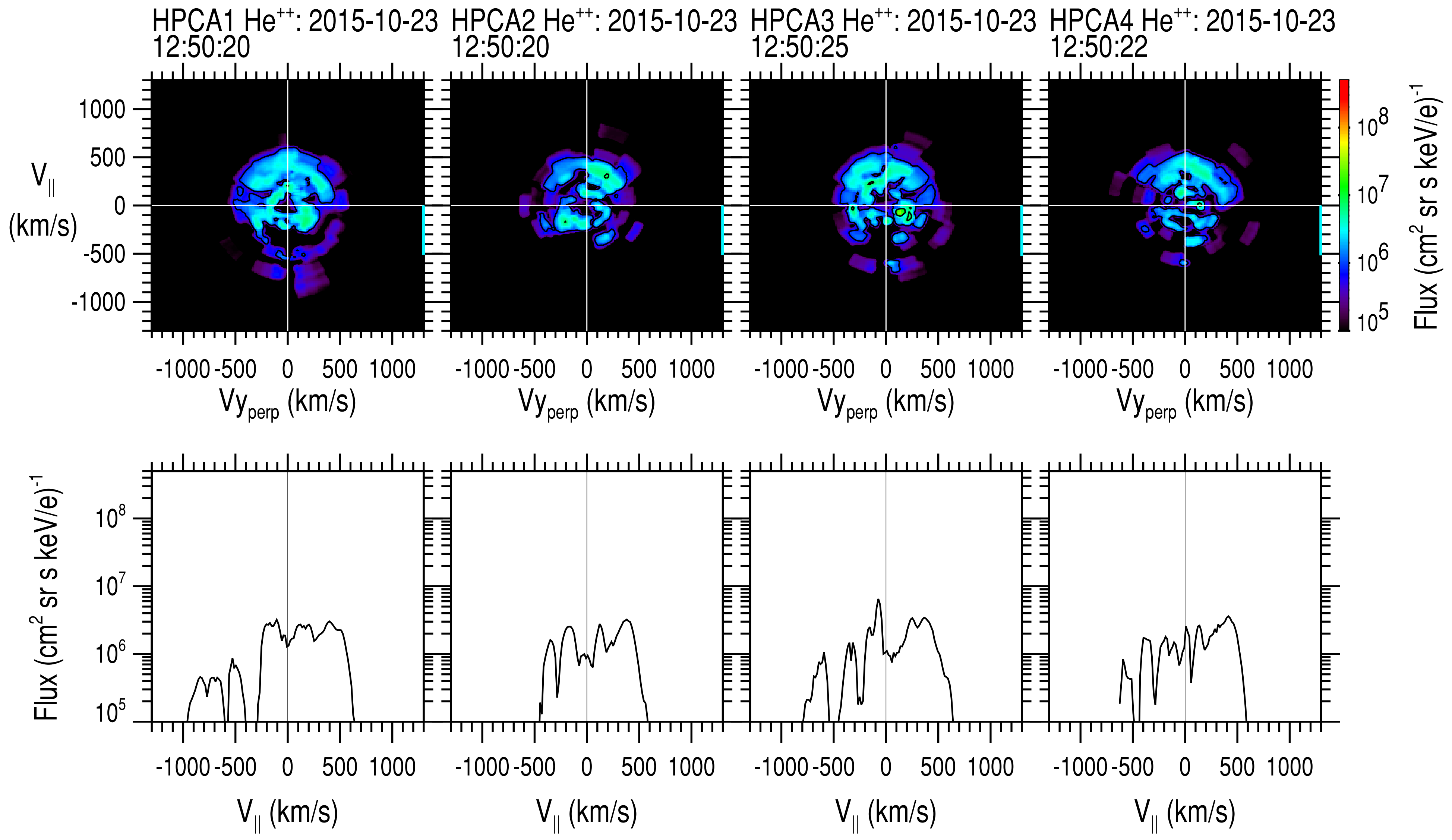
HPCA1 He++: 2015-10-23 12:50:20
HPCA2 He++: 2015-10-23 12:50:20
HPCA3 He++: 2015-10-23 12:50:25
HPCA4 He++: 2015-10-23 12:50:22
V|| (km/s)
Vyperp (km/s)
Flux (cm2 sr s keV/e)-1
V|| (km/s)

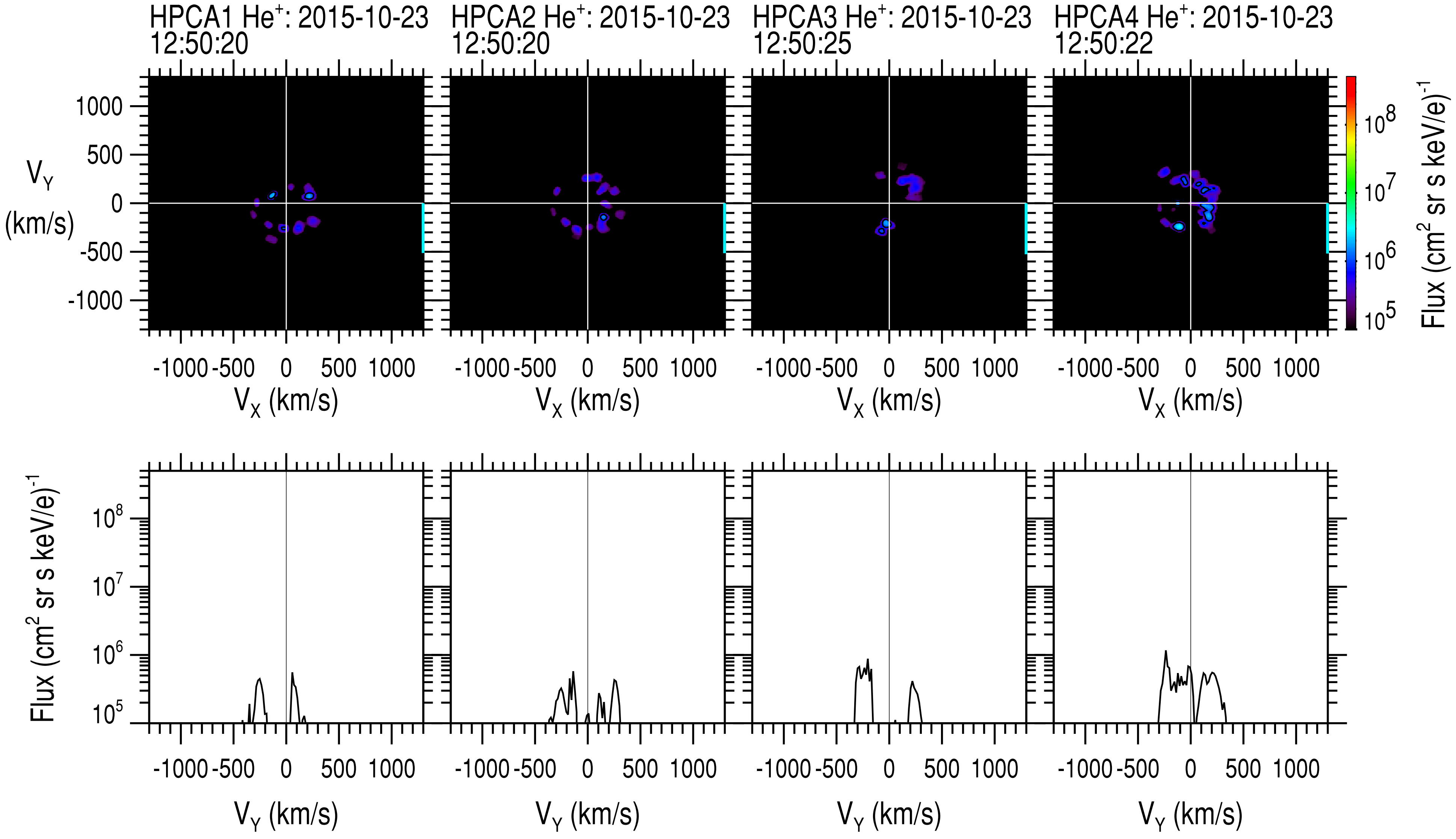

HPCA1 He+: 2015-10-23
12:50:20
HPCA2 He+: 2015-10-23
12:50:20
HPCA3 He+: 2015-10-23
12:50:25
HPCA4 He+: 2015-10-23
12:50:22
VY (km/s)
VX (km/s)
Flux (cm2 sr s keV/e)-1

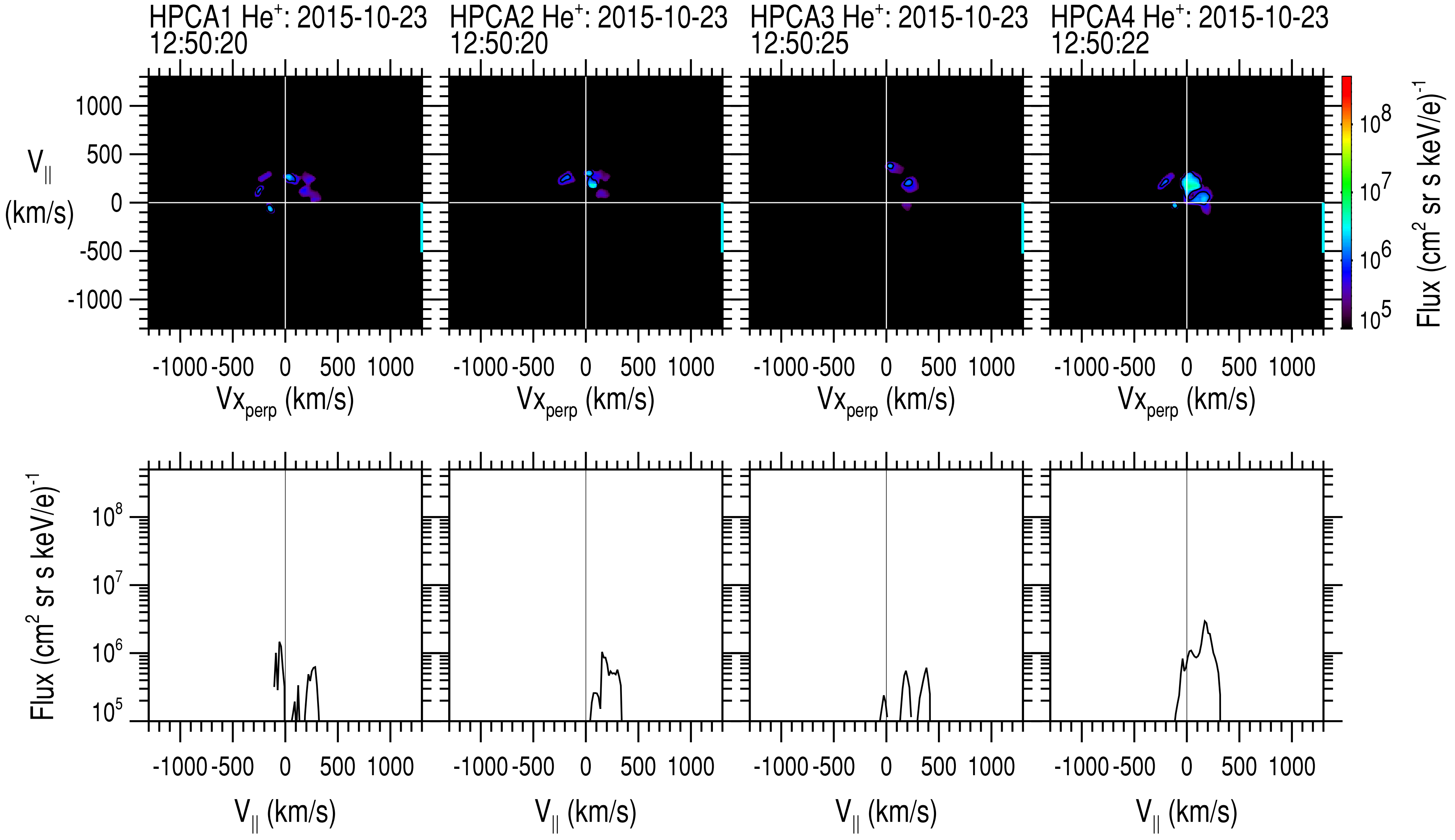
HPCA1 He+: 2015-10-23
12:50:20
HPCA2 He+: 2015-10-23
12:50:20
HPCA3 He+: 2015-10-23
12:50:25
HPCA4 He+: 2015-10-23
12:50:22
V|| (km/s)
Vxperp (km/s)
Flux (cm2 sr s keV/e)-1
1000
500
0
-500
-1000
-1000 -500 0 500 1000
10^5
10^6
10^7
10^8
V|| (km/s)

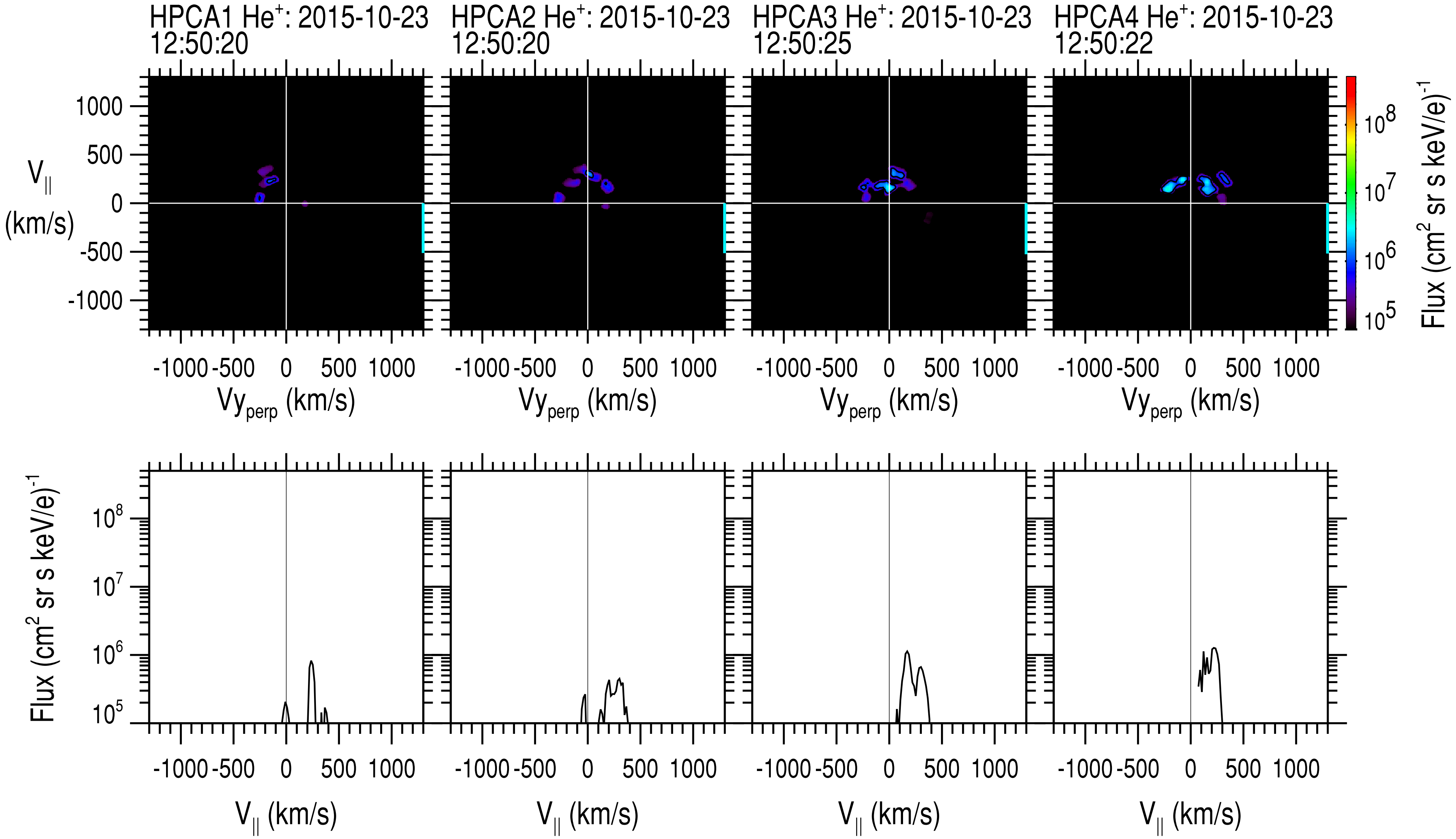

HPCA1 He+: 2015-10-23 12:50:20
HPCA2 He+: 2015-10-23 12:50:20
HPCA3 He+: 2015-10-23 12:50:25
HPCA4 He+: 2015-10-23 12:50:22
V|| (km/s)
Vyperp (km/s)
Flux (cm2 sr s keV/e)-1

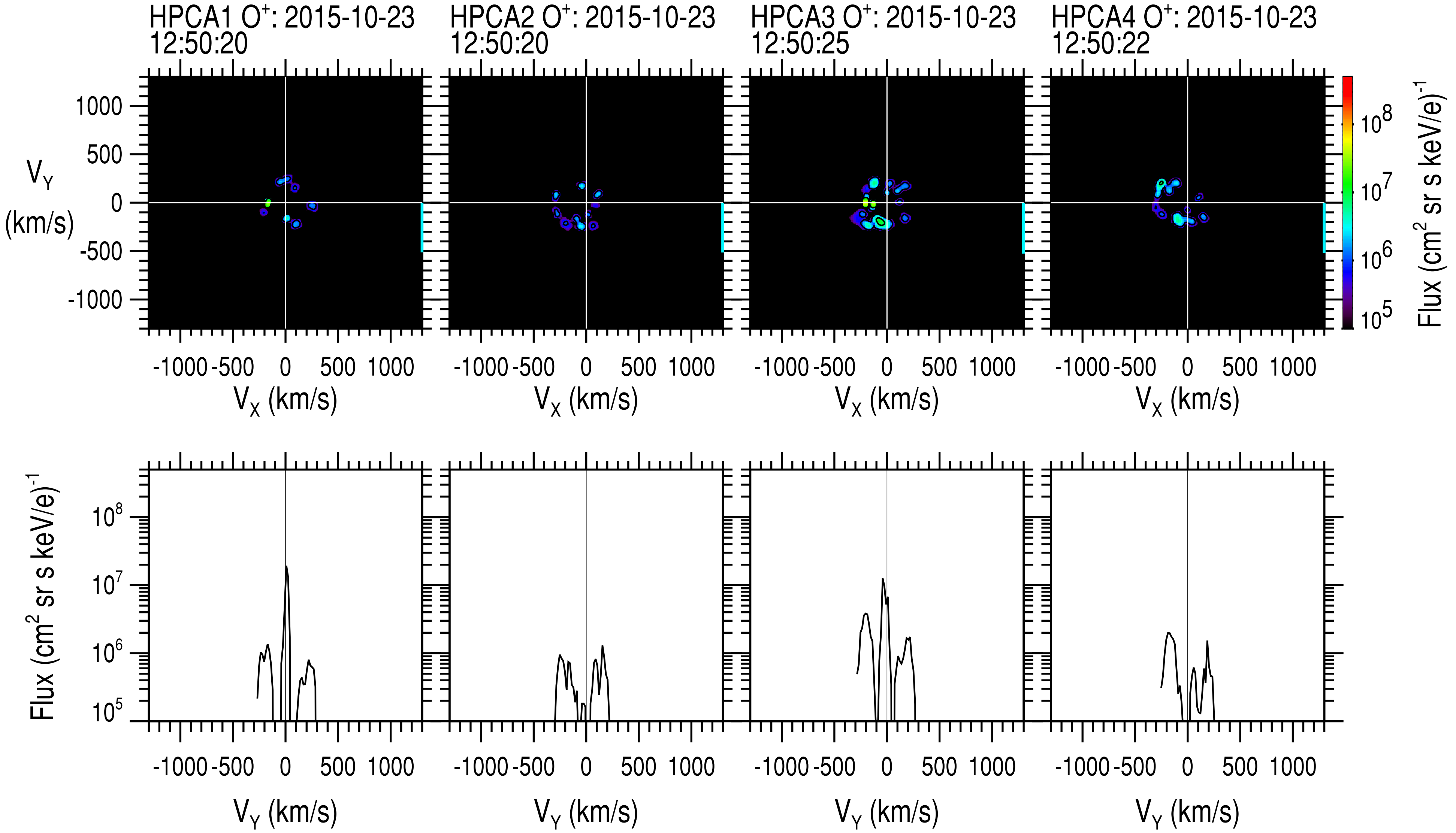
HPCA1 O+: 2015-10-23
12:50:20
HPCA2 O+: 2015-10-23
12:50:20
HPCA3 O+: 2015-10-23
12:50:25
HPCA4 O+: 2015-10-23
12:50:22
VY (km/s)
VX (km/s)
Flux (cm2 sr s keV/e)-1
-1000 -500 0 500 1000
10^5 10^6 10^7 10^8

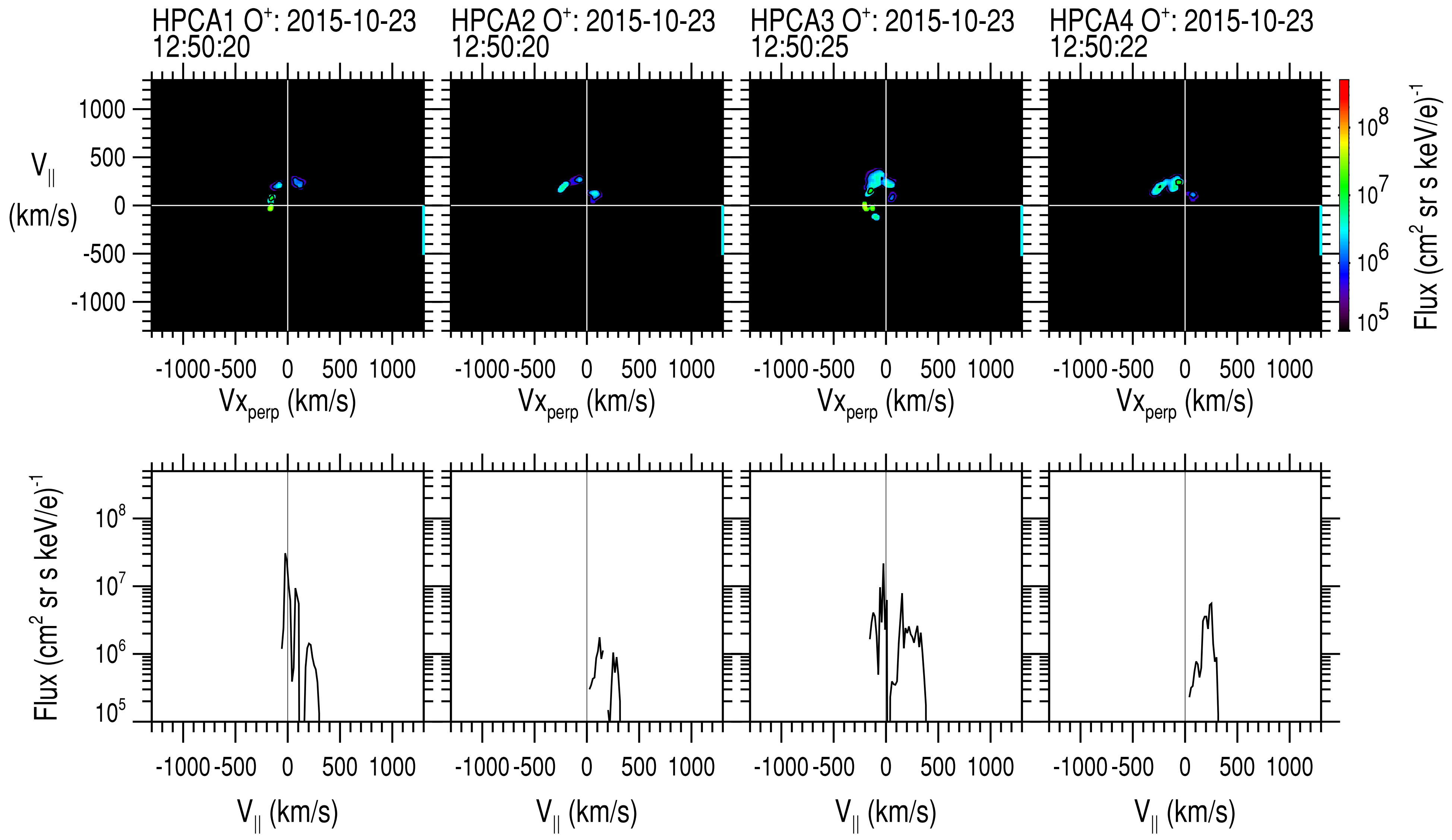

HPCA1 O+: 2015-10-23 12:50:20
HPCA2 O+: 2015-10-23 12:50:20
HPCA3 O+: 2015-10-23 12:50:25
HPCA4 O+: 2015-10-23 12:50:22
V|| (km/s)
Vxperp (km/s)
Flux (cm2 sr s keV/e)-1
V|| (km/s)

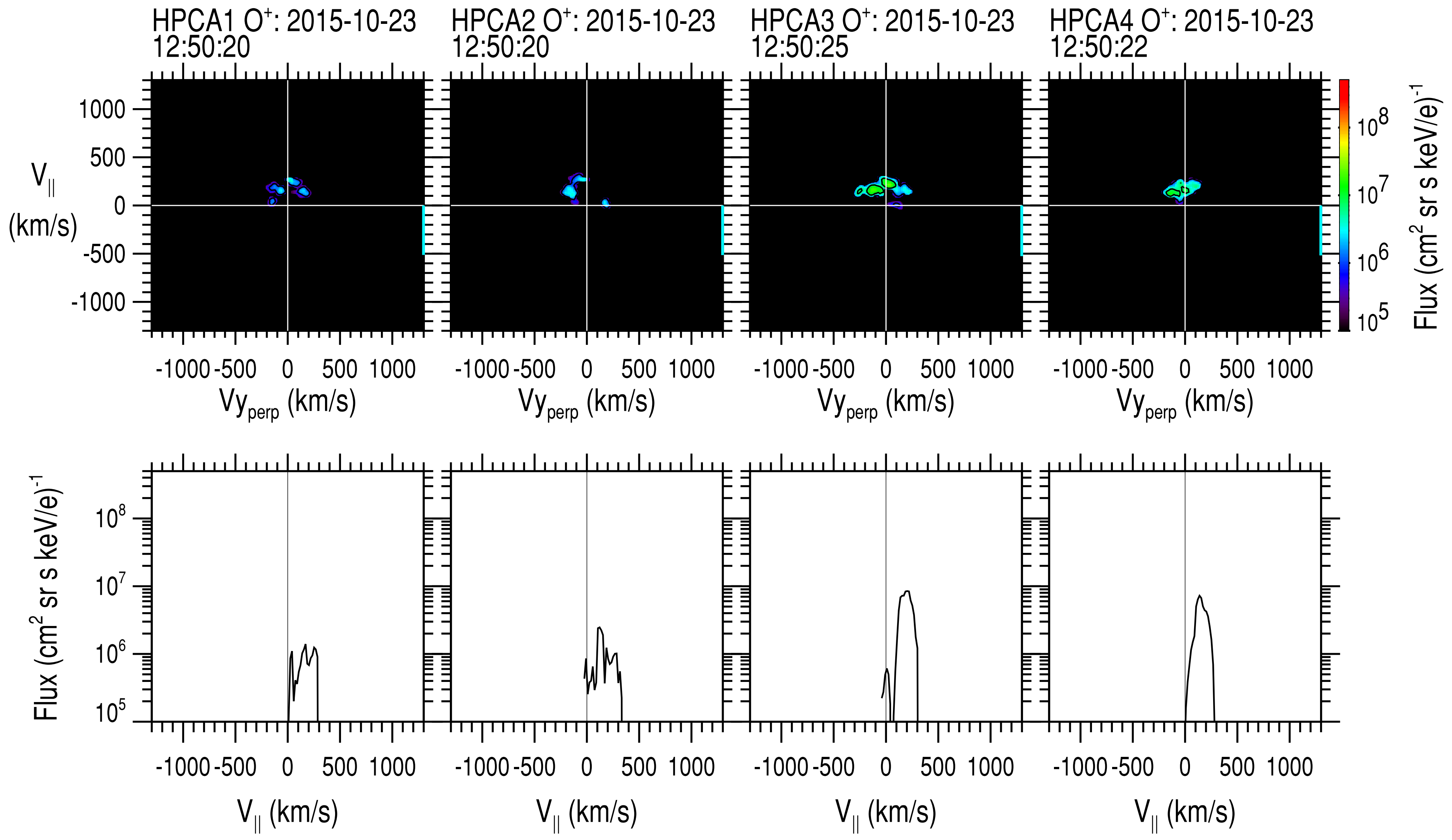
HPCA1 O+: 2015-10-23 12:50:20
HPCA2 O+: 2015-10-23 12:50:20
HPCA3 O+: 2015-10-23 12:50:25
HPCA4 O+: 2015-10-23 12:50:22
V|| (km/s)
Vyperp (km/s)
Flux (cm2 sr s keV/e)-1
V|| (km/s)

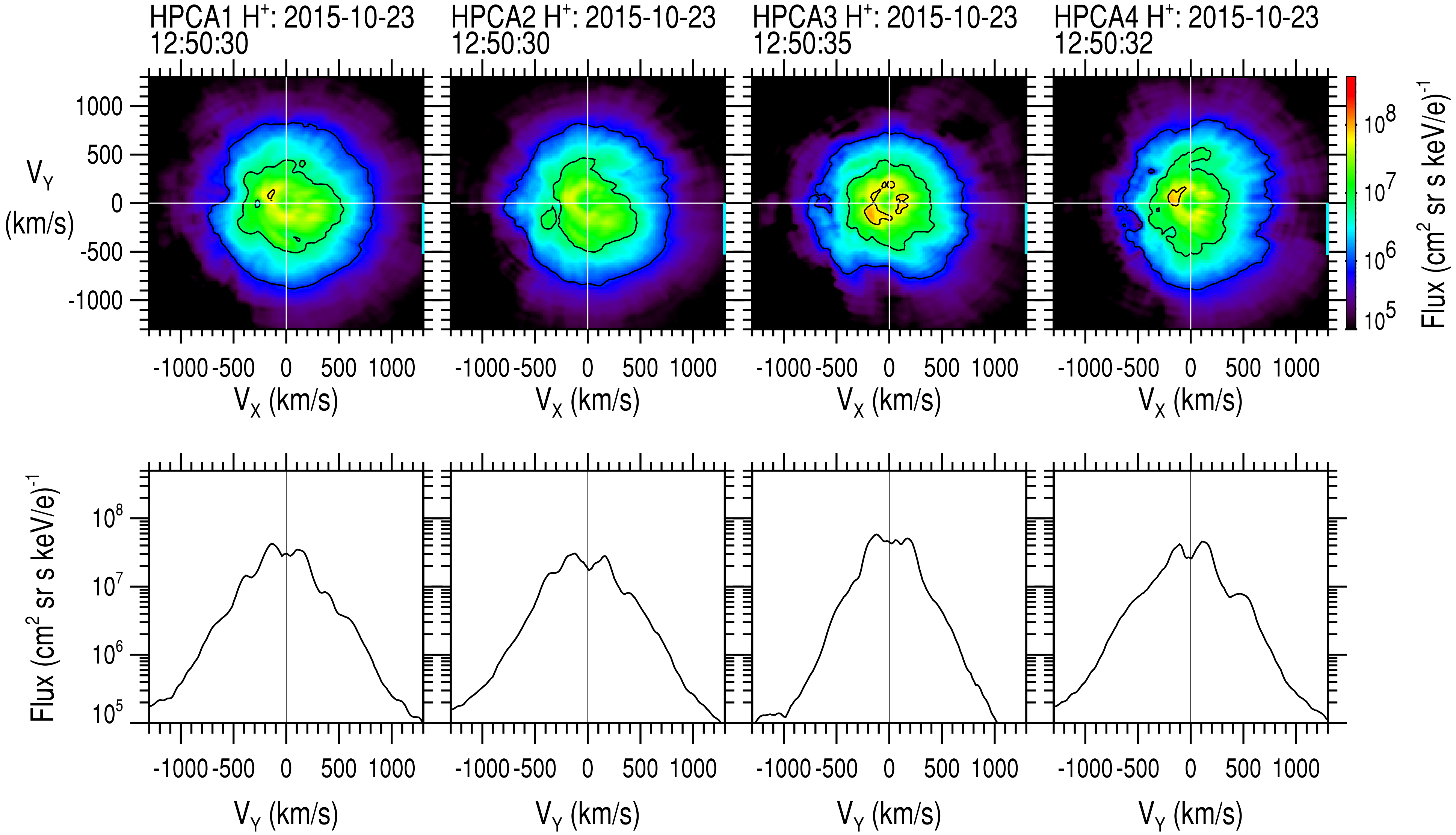
HPCA1 H+: 2015-10-23 12:50:30
HPCA2 H+: 2015-10-23 12:50:30
HPCA3 H+: 2015-10-23 12:50:35
HPCA4 H+: 2015-10-23 12:50:32
V_Y (km/s)
V_X (km/s)
Flux (cm^2 sr s keV/e)^-1
V_Y (km/s)
Flux (cm^2 sr s keV/e)^-1

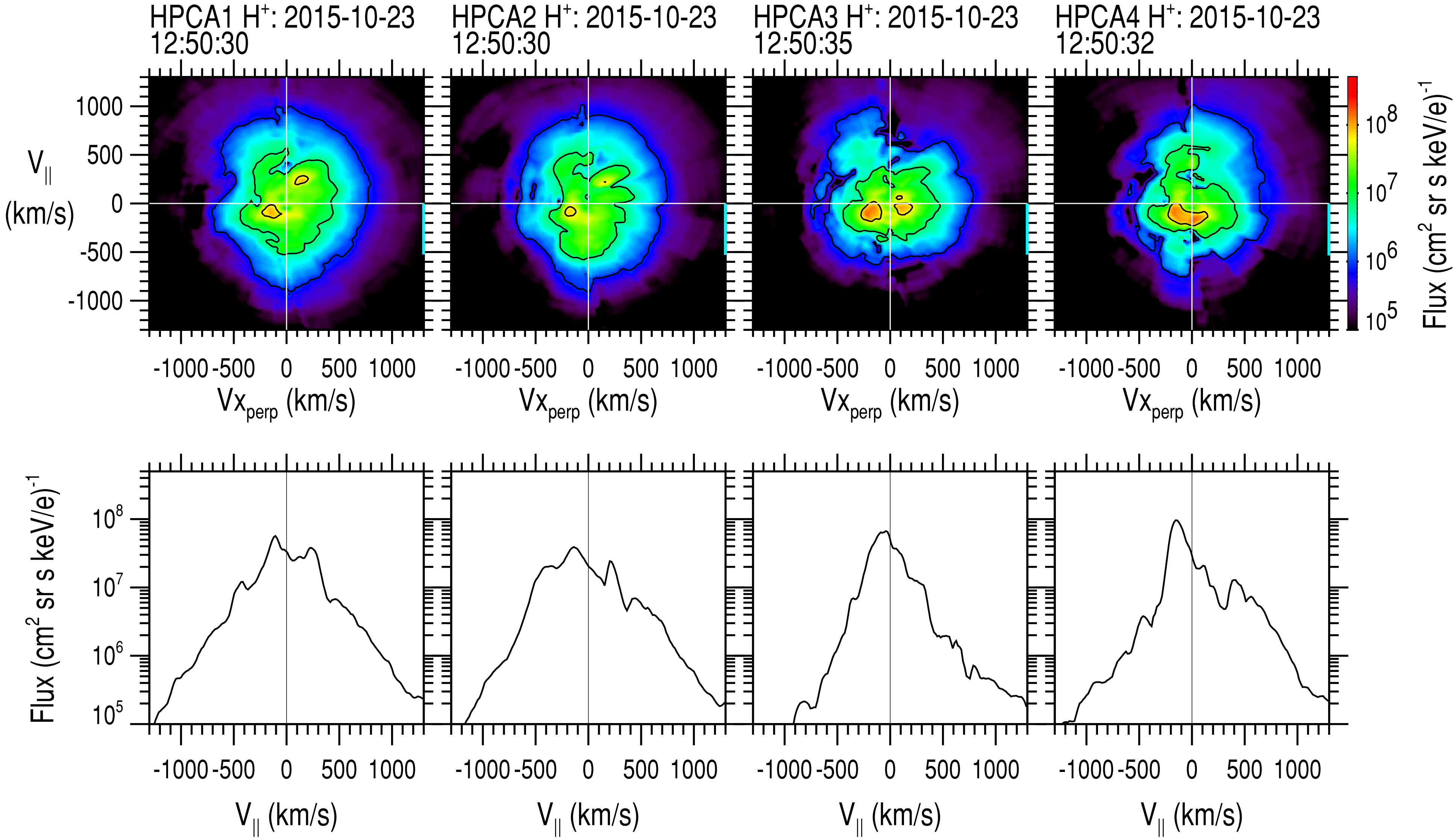

HPCA1 H+: 2015-10-23 12:50:30
HPCA2 H+: 2015-10-23 12:50:30
HPCA3 H+: 2015-10-23 12:50:35
HPCA4 H+: 2015-10-23 12:50:32
V|| (km/s)
Vxperp (km/s)
Flux (cm2 sr s keV/e)-1

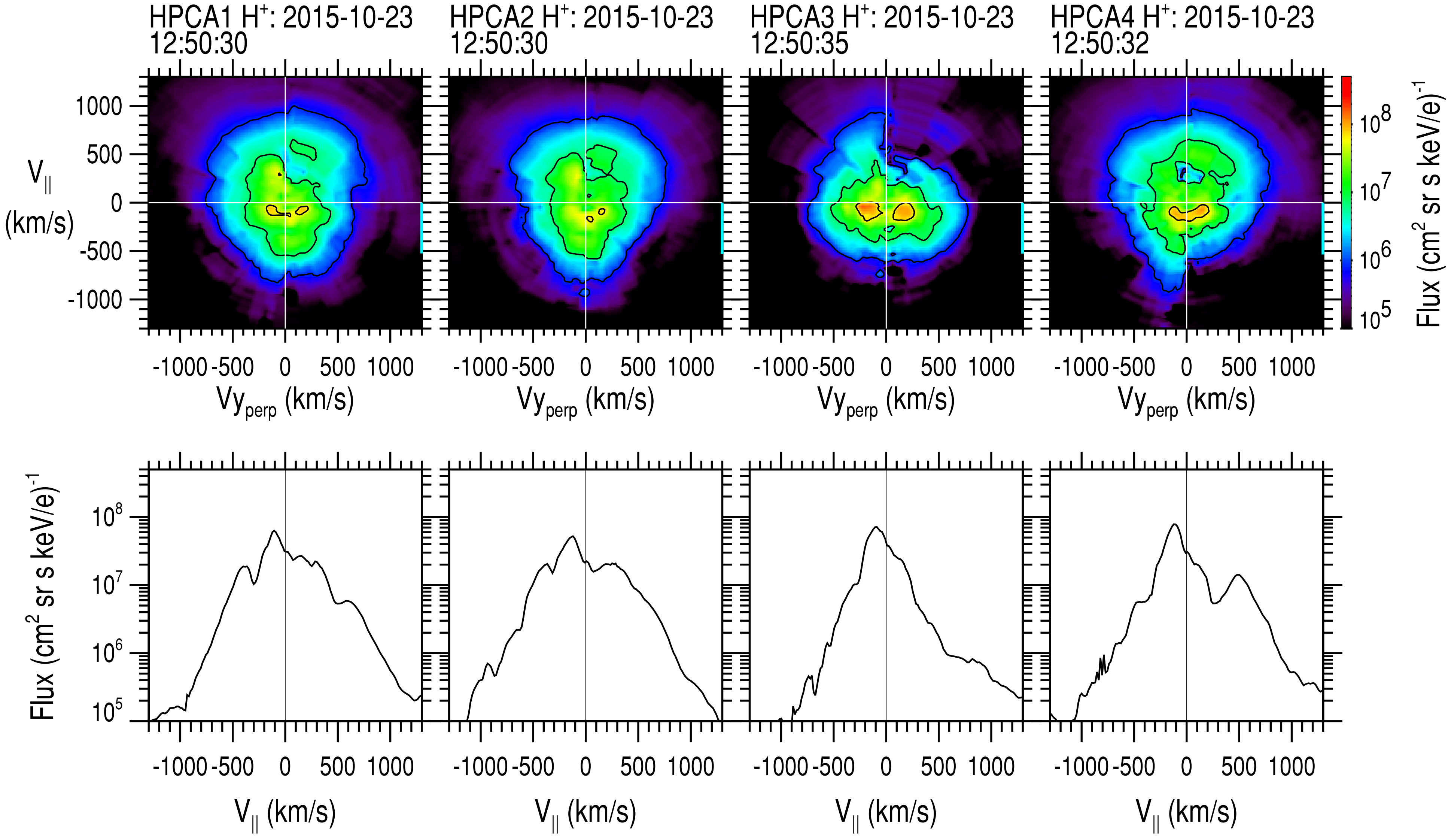
HPCA1 H+: 2015-10-23
12:50:30
HPCA2 H+: 2015-10-23
12:50:30
HPCA3 H+: 2015-10-23
12:50:35
HPCA4 H+: 2015-10-23
12:50:32
V|| (km/s)
1000
500
0
-500
-1000
-1000 -500 0 500 1000
Vyperp (km/s)
Flux (cm2 sr s keV/e)-1
10^5
10^6
10^7
10^8
V|| (km/s)

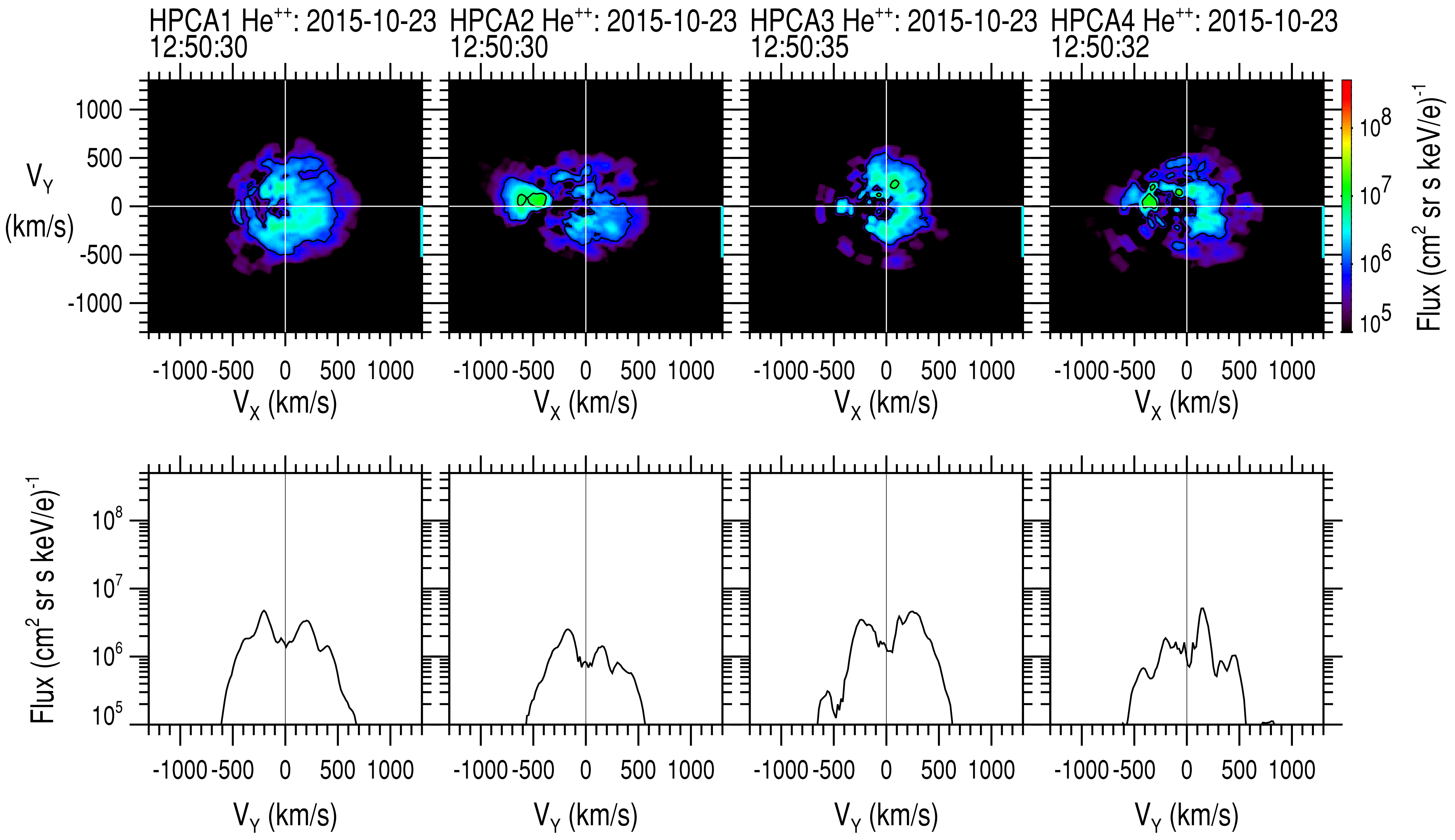
HPCA1 He++: 2015-10-23 12:50:30
HPCA2 He++: 2015-10-23 12:50:30
HPCA3 He++: 2015-10-23 12:50:35
HPCA4 He++: 2015-10-23 12:50:32
V_Y (km/s)
V_X (km/s)
Flux (cm^2 sr s keV/e)^-1
-1000 -500 0 500 1000
10^5 10^6 10^7 10^8

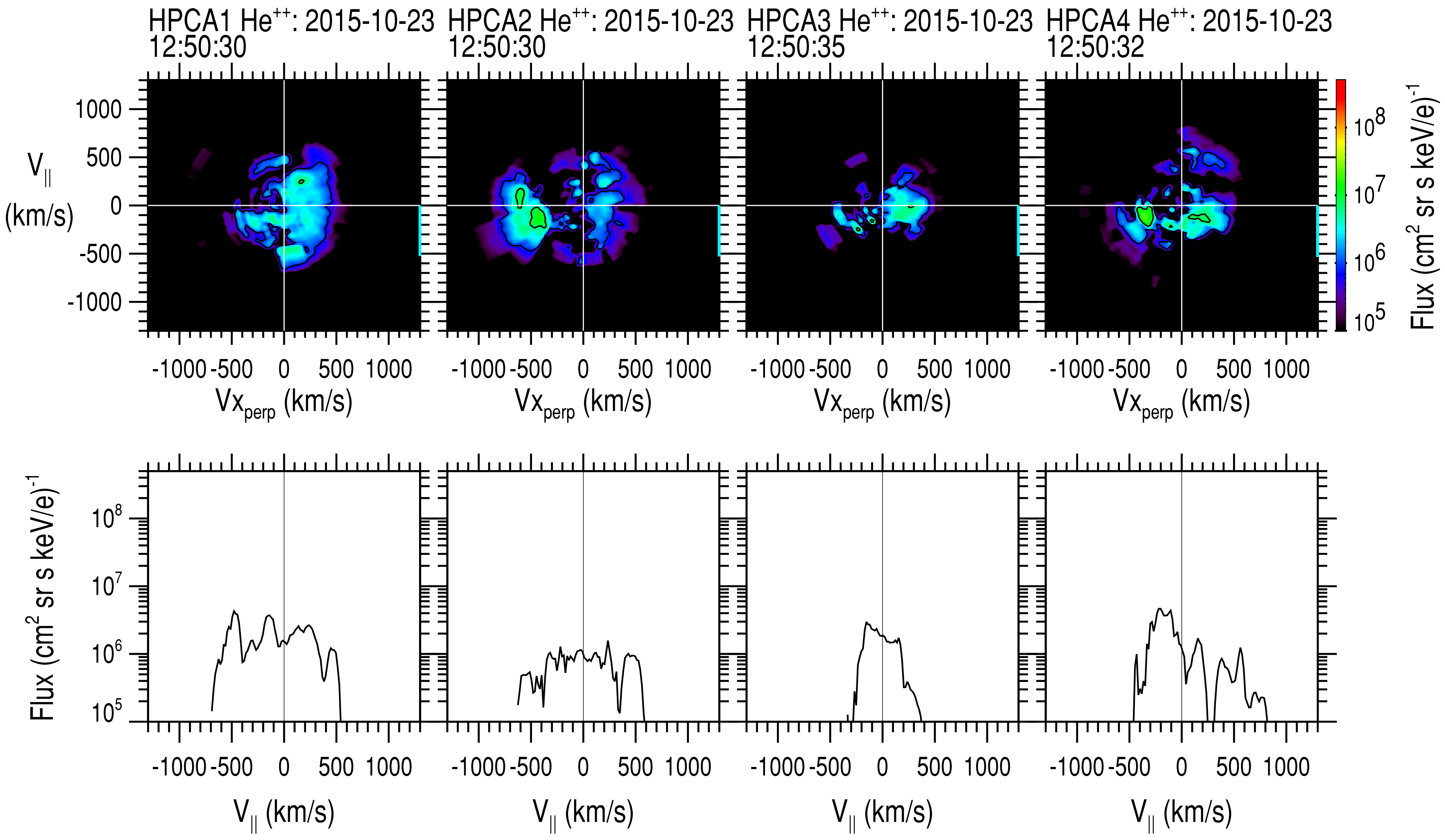
HPCA1 He++: 2015-10-23 12:50:30
HPCA2 He++: 2015-10-23 12:50:30
HPCA3 He++: 2015-10-23 12:50:35
HPCA4 He++: 2015-10-23 12:50:32
V|| (km/s)
Vxperp (km/s)
Flux (cm2 sr s keV/e)-1
V|| (km/s)

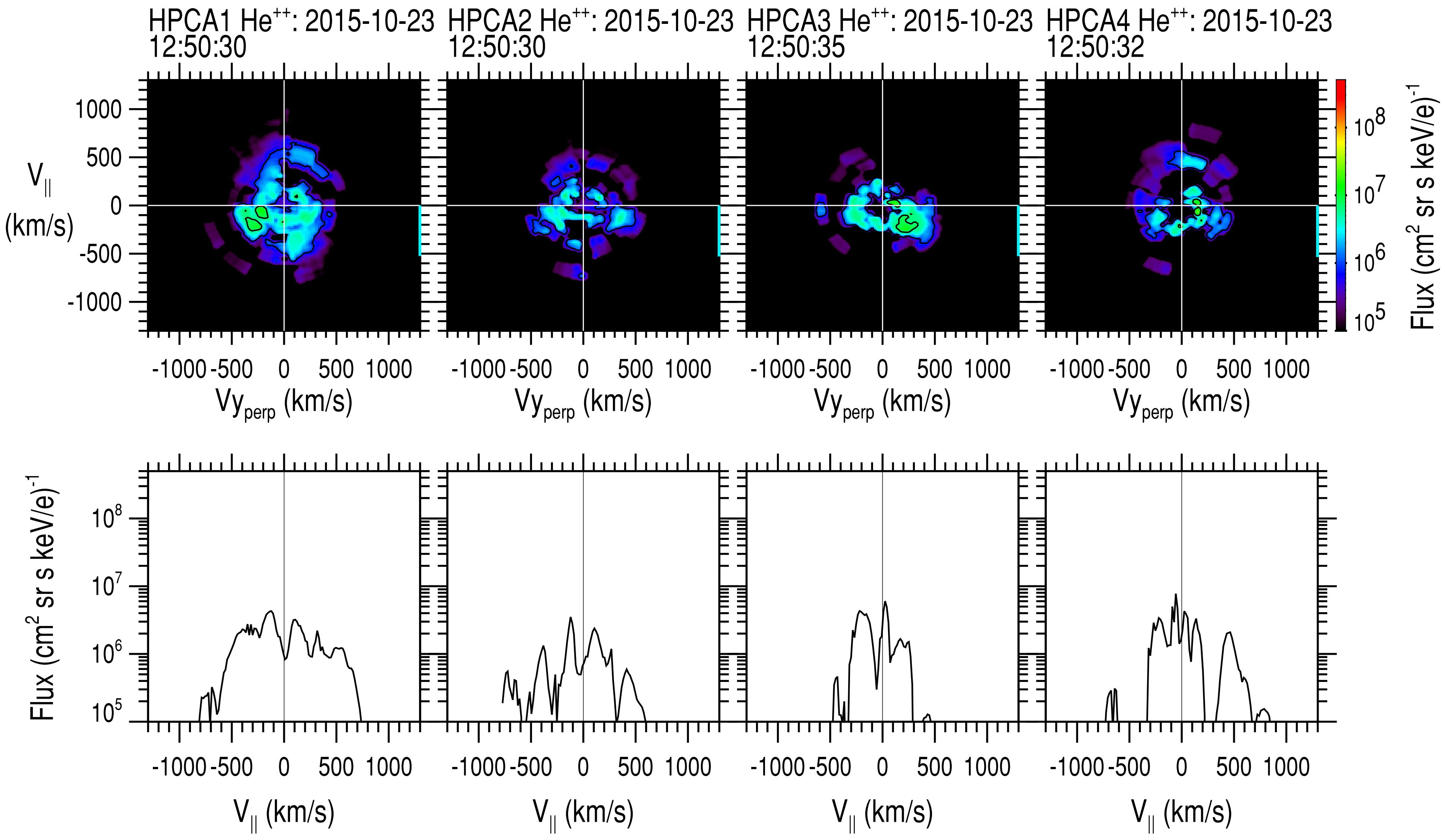
HPCA1 He++: 2015-10-23 12:50:30
HPCA2 He++: 2015-10-23 12:50:30
HPCA3 He++: 2015-10-23 12:50:35
HPCA4 He++: 2015-10-23 12:50:32
V|| (km/s)
Vyperp (km/s)
Flux (cm2 sr s keV/e)-1
V|| (km/s)
-1000 -500 0 500 1000
10^5 10^6 10^7 10^8

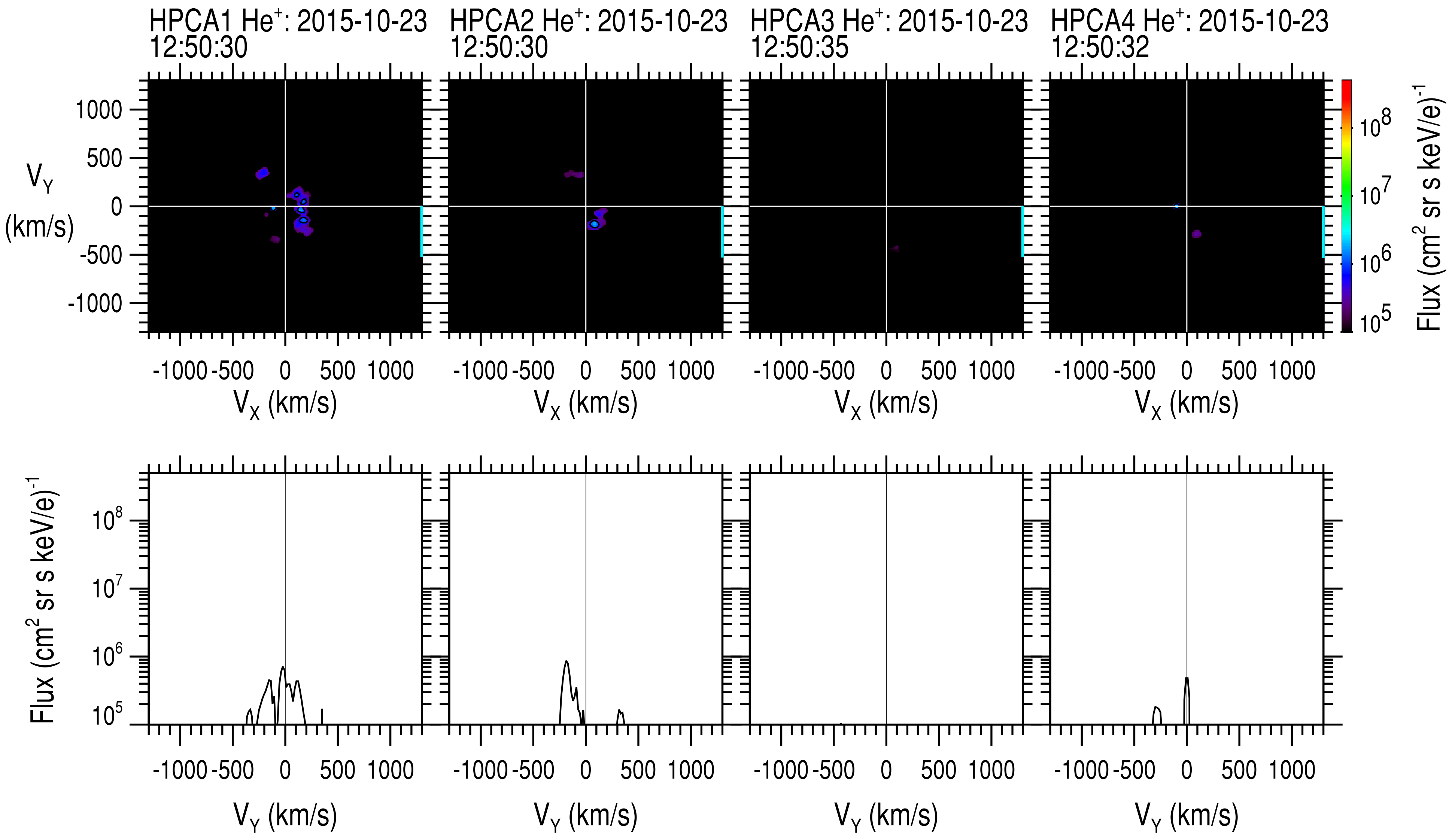

HPCA1 He+: 2015-10-23 12:50:30
HPCA2 He+: 2015-10-23 12:50:30
HPCA3 He+: 2015-10-23 12:50:35
HPCA4 He+: 2015-10-23 12:50:32
VY (km/s)
VX (km/s)
Flux (cm2 sr s keV/e)-1
1000
500
0
-500
-1000
10^5
10^6
10^7
10^8

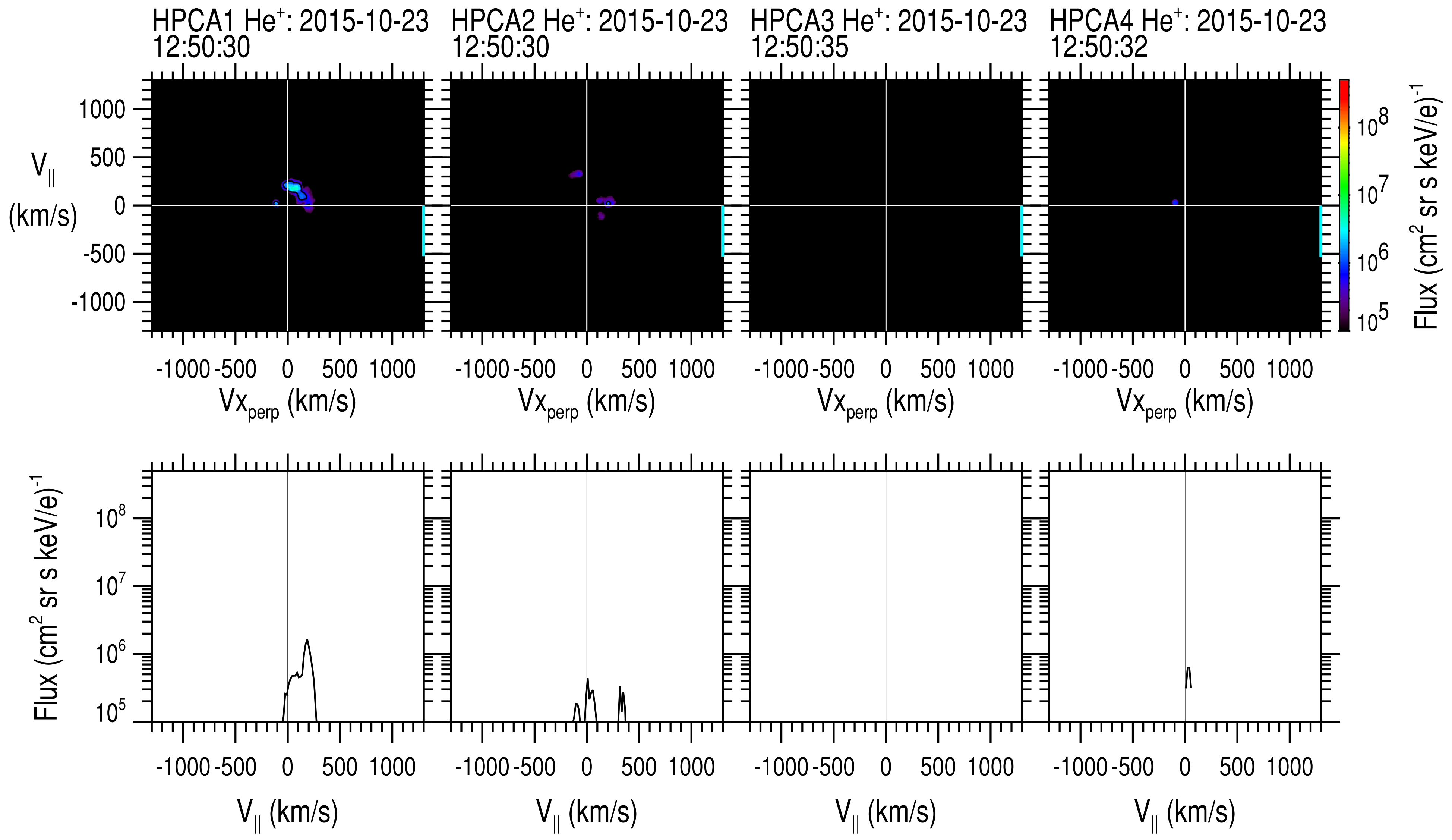

HPCA1 He+: 2015-10-23 12:50:30
HPCA2 He+: 2015-10-23 12:50:30
HPCA3 He+: 2015-10-23 12:50:35
HPCA4 He+: 2015-10-23 12:50:32
V|| (km/s)
Vxperp (km/s)
Flux (cm2 sr s keV/e)-1
1000
500
0
-500
-1000
105
106
107
108

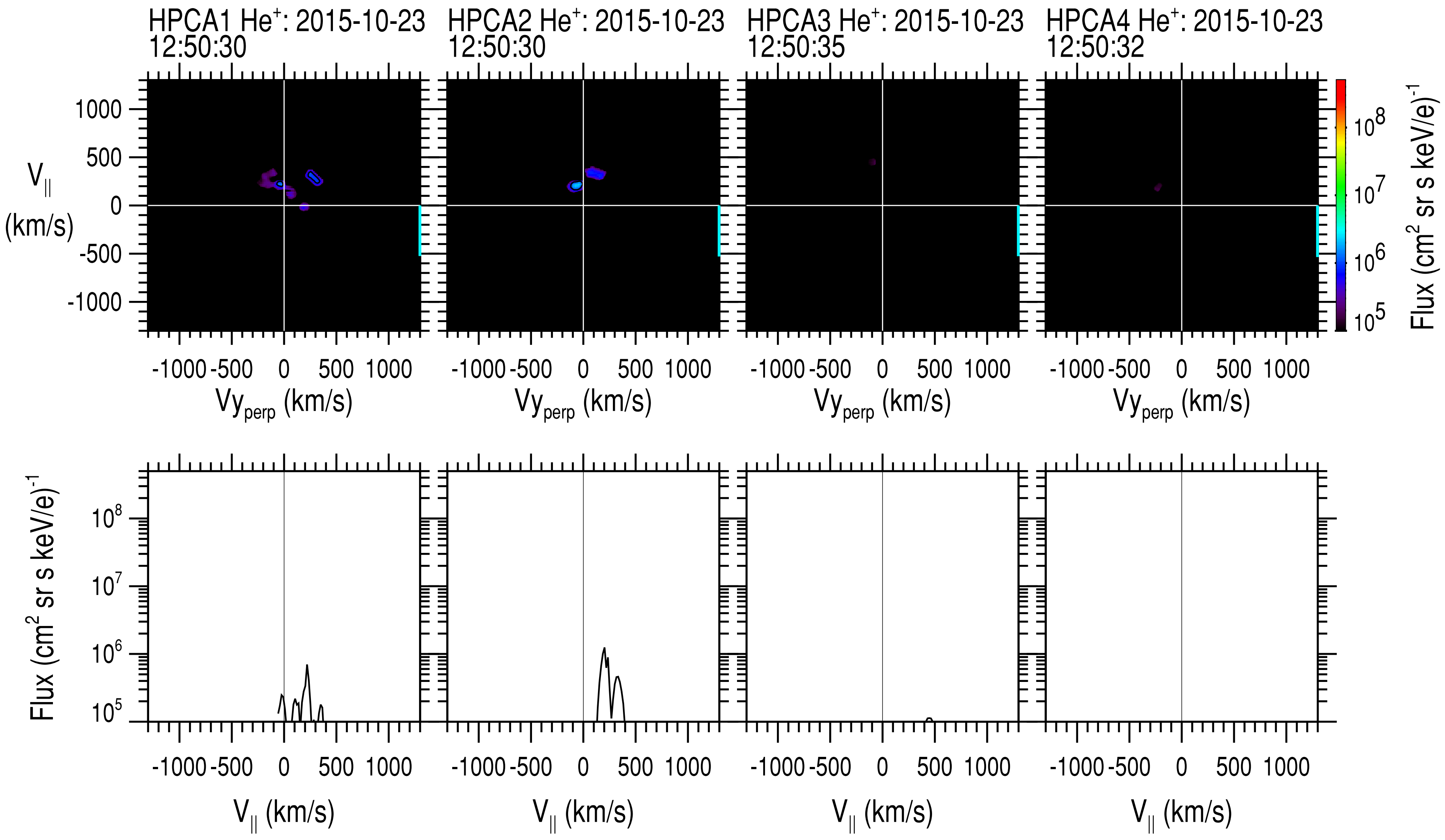

HPCA1 He+: 2015-10-23 12:50:30
HPCA2 He+: 2015-10-23 12:50:30
HPCA3 He+: 2015-10-23 12:50:35
HPCA4 He+: 2015-10-23 12:50:32
V|| (km/s)
Vy perp (km/s)
Flux (cm2 sr s keV/e)-1

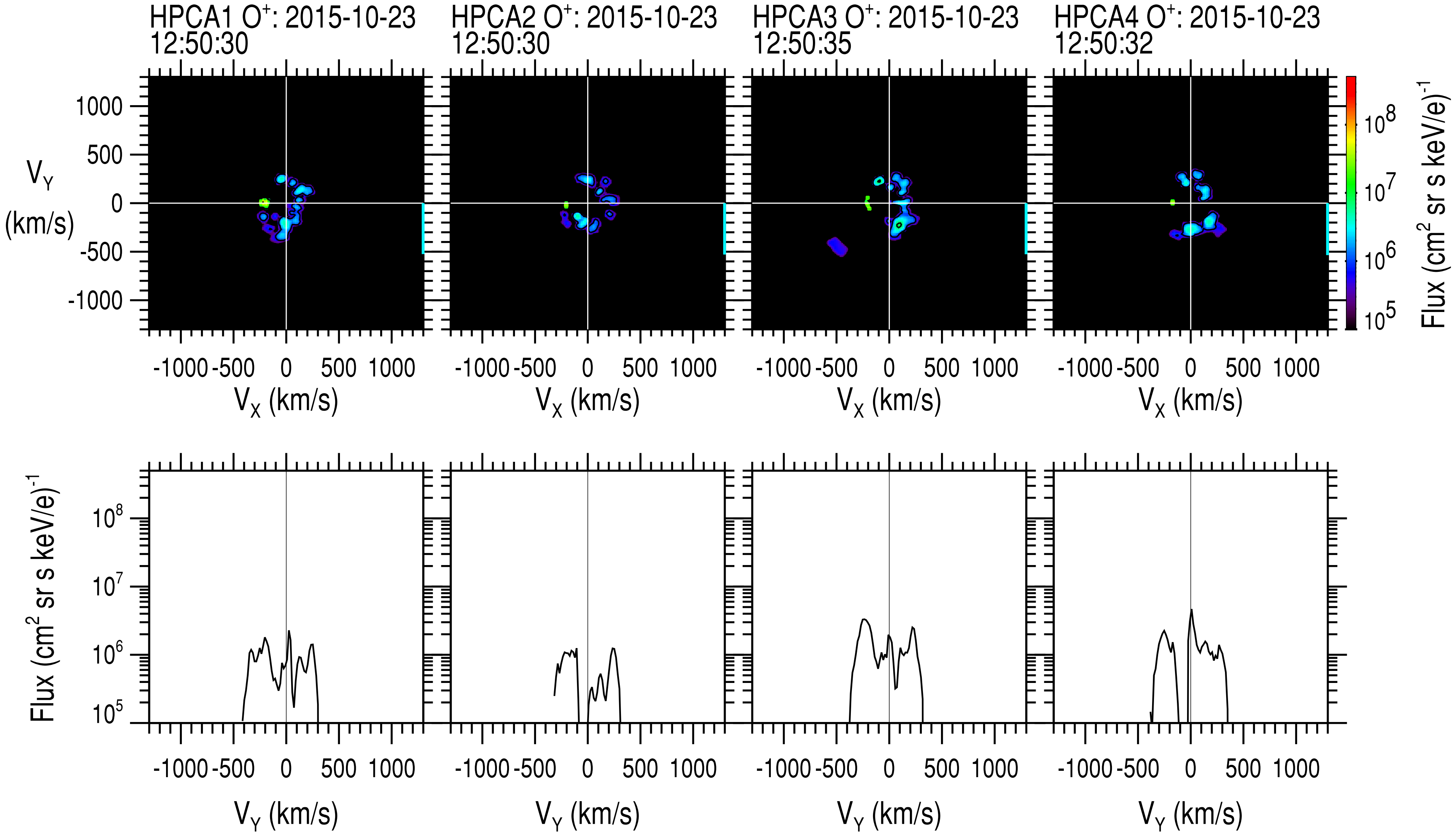

HPCA1 O+: 2015-10-23
12:50:30
HPCA2 O+: 2015-10-23
12:50:30
HPCA3 O+: 2015-10-23
12:50:35
HPCA4 O+: 2015-10-23
12:50:32
VY (km/s)
VX (km/s)
Flux (cm2 sr s keV/e)-1

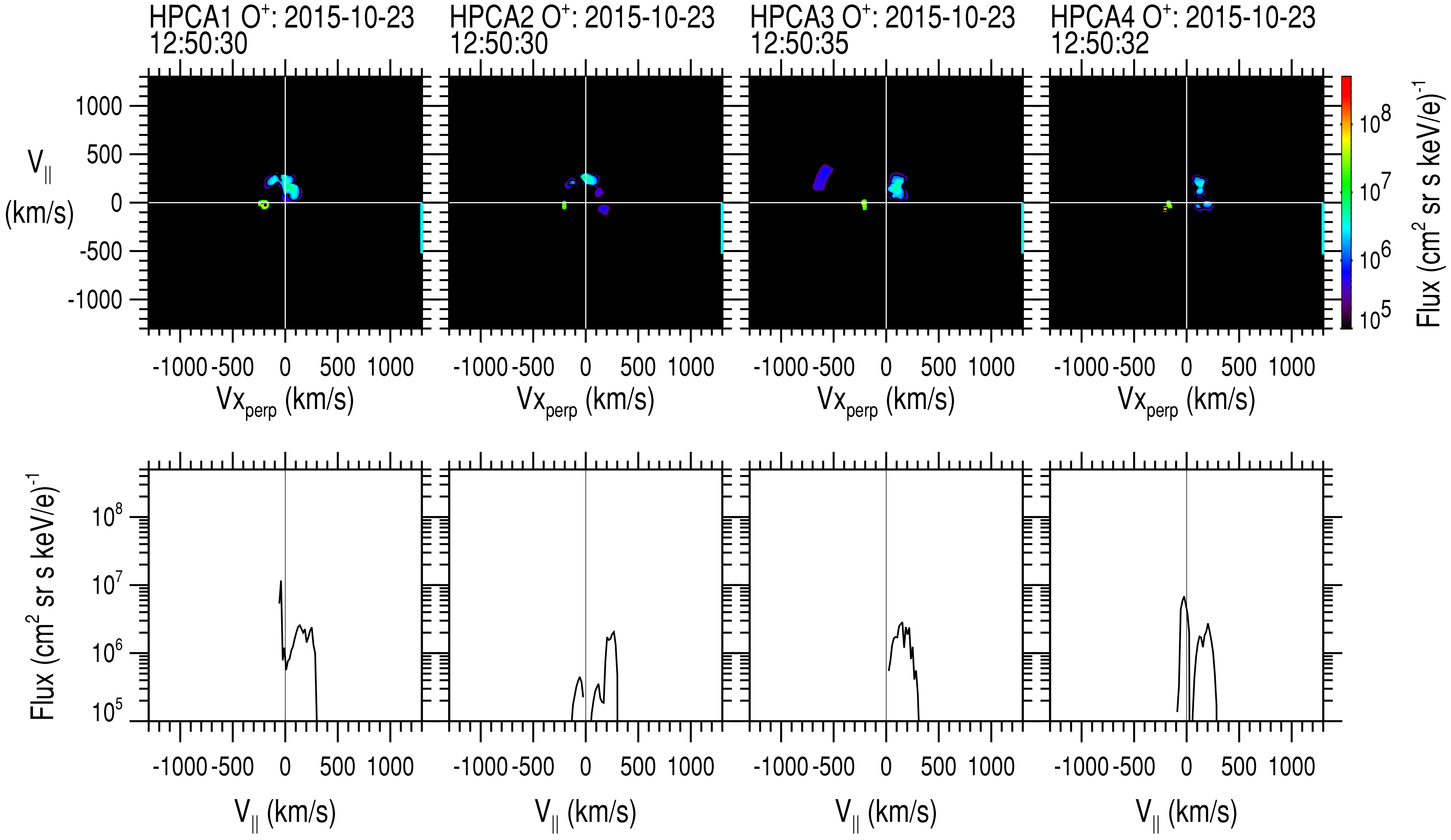

HPCA1 O+: 2015-10-23 12:50:30
HPCA2 O+: 2015-10-23 12:50:30
HPCA3 O+: 2015-10-23 12:50:35
HPCA4 O+: 2015-10-23 12:50:32
V|| (km/s)
Vxperp (km/s)
Flux (cm2 sr s keV/e)-1

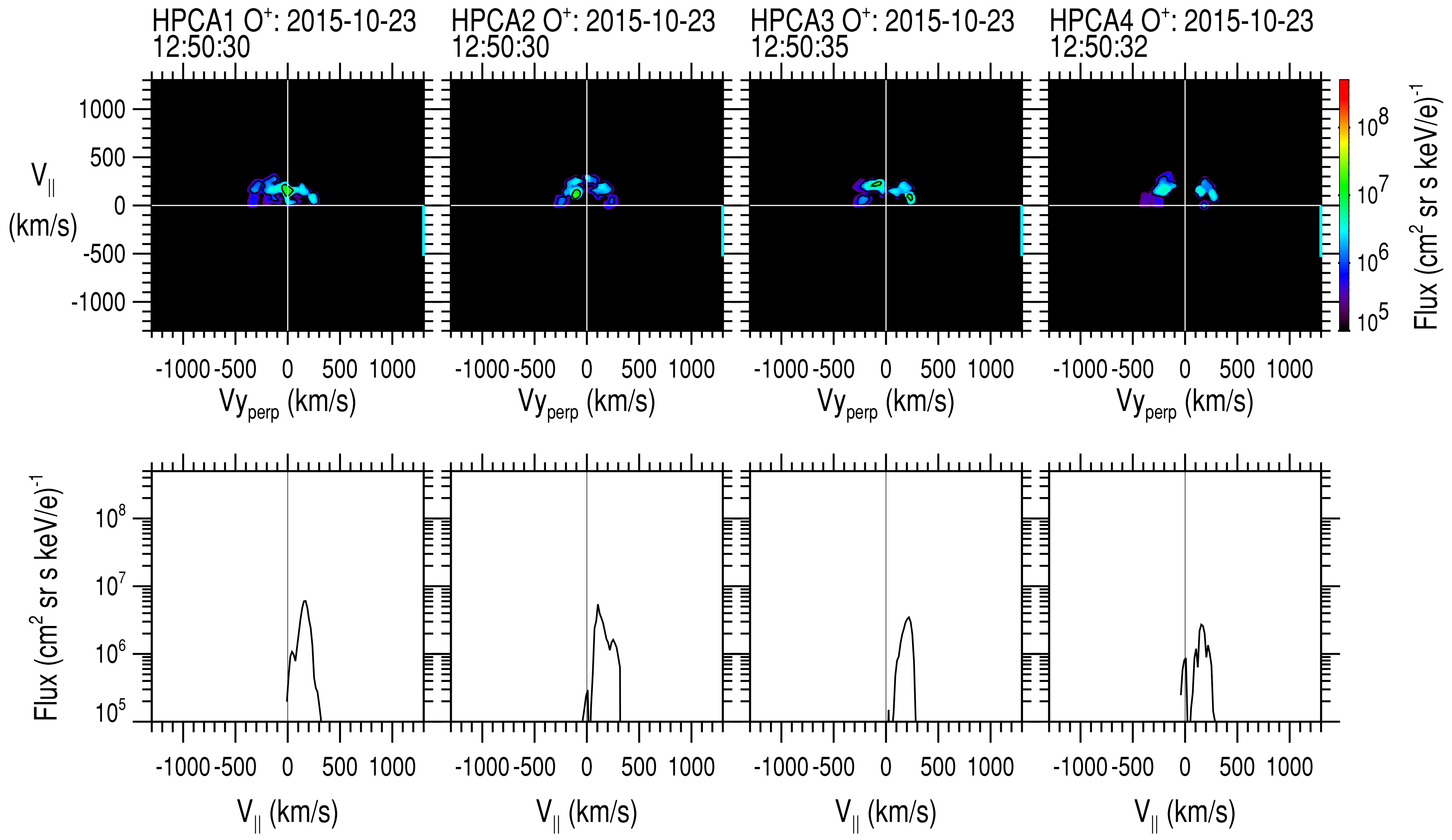

HPCA1 O+: 2015-10-23
12:50:30
HPCA2 O+: 2015-10-23
12:50:30
HPCA3 O+: 2015-10-23
12:50:35
HPCA4 O+: 2015-10-23
12:50:32
V|| (km/s)
Vyperp (km/s)
Flux (cm2 sr s keV/e)-1

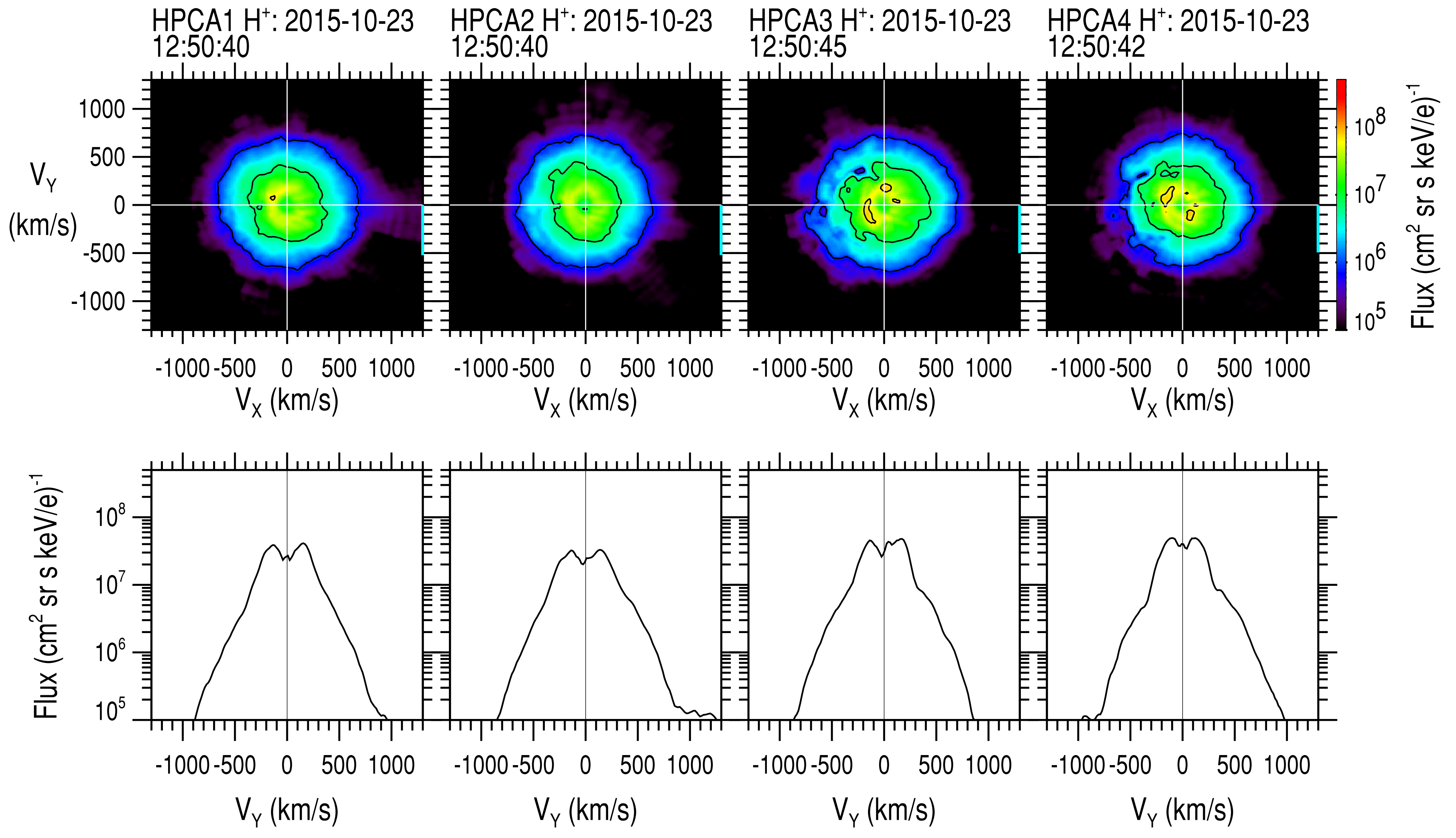

HPCA1 H+: 2015-10-23
12:50:40
HPCA2 H+: 2015-10-23
12:50:40
HPCA3 H+: 2015-10-23
12:50:45
HPCA4 H+: 2015-10-23
12:50:42
V_Y (km/s)
V_X (km/s)
Flux (cm^2 sr s keV/e)^-1

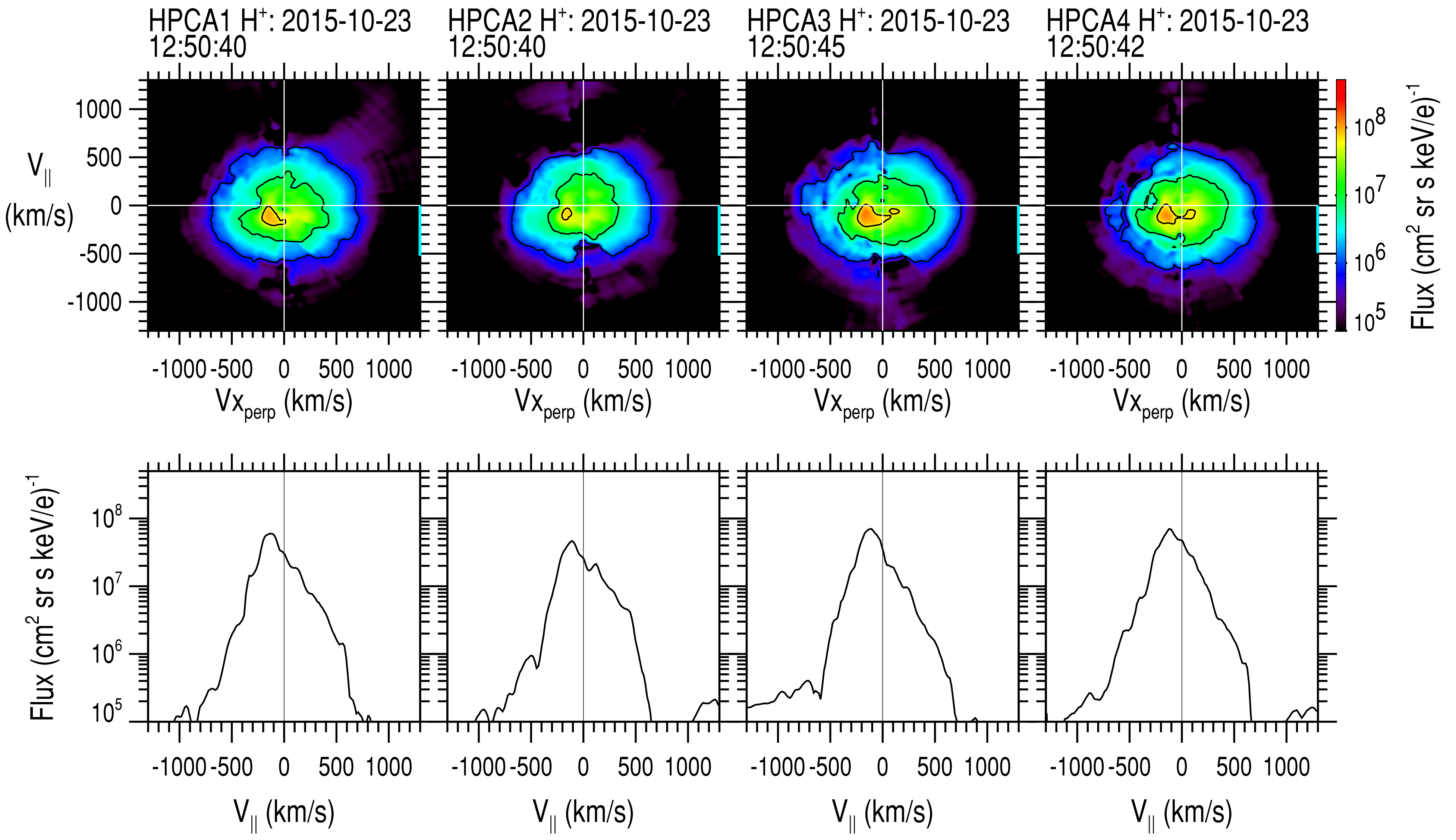

HPCA1 H+: 2015-10-23 12:50:40
HPCA2 H+: 2015-10-23 12:50:40
HPCA3 H+: 2015-10-23 12:50:45
HPCA4 H+: 2015-10-23 12:50:42
V|| (km/s)
Vxperp (km/s)
Flux (cm2 sr s keV/e)-1
V|| (km/s)

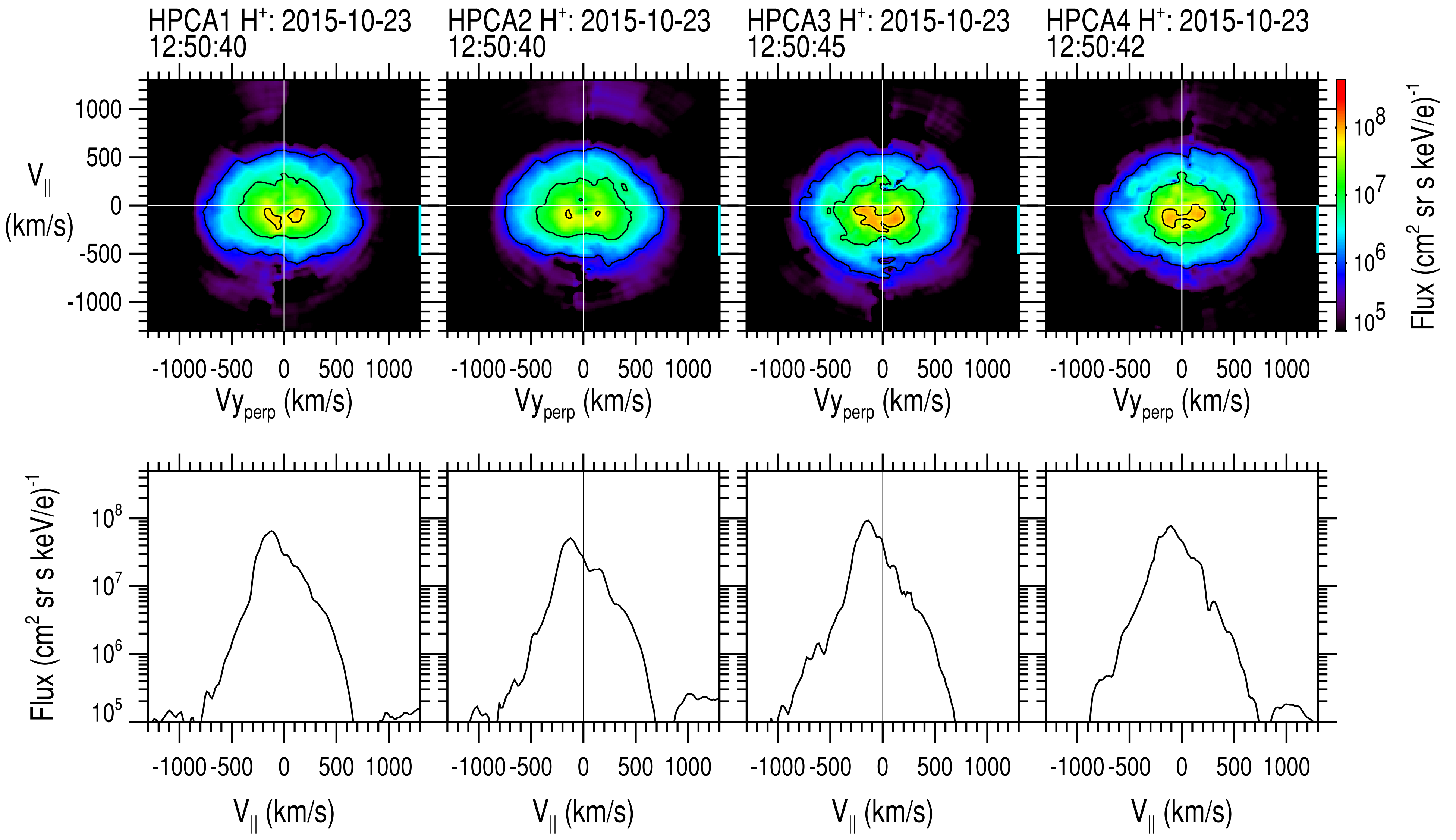

HPCA1 H+: 2015-10-23 12:50:40
HPCA2 H+: 2015-10-23 12:50:40
HPCA3 H+: 2015-10-23 12:50:45
HPCA4 H+: 2015-10-23 12:50:42
V|| (km/s)
Vyperp (km/s)
Flux (cm2 sr s keV/e)-1
-1000 -500 0 500 1000
10^5 10^6 10^7 10^8

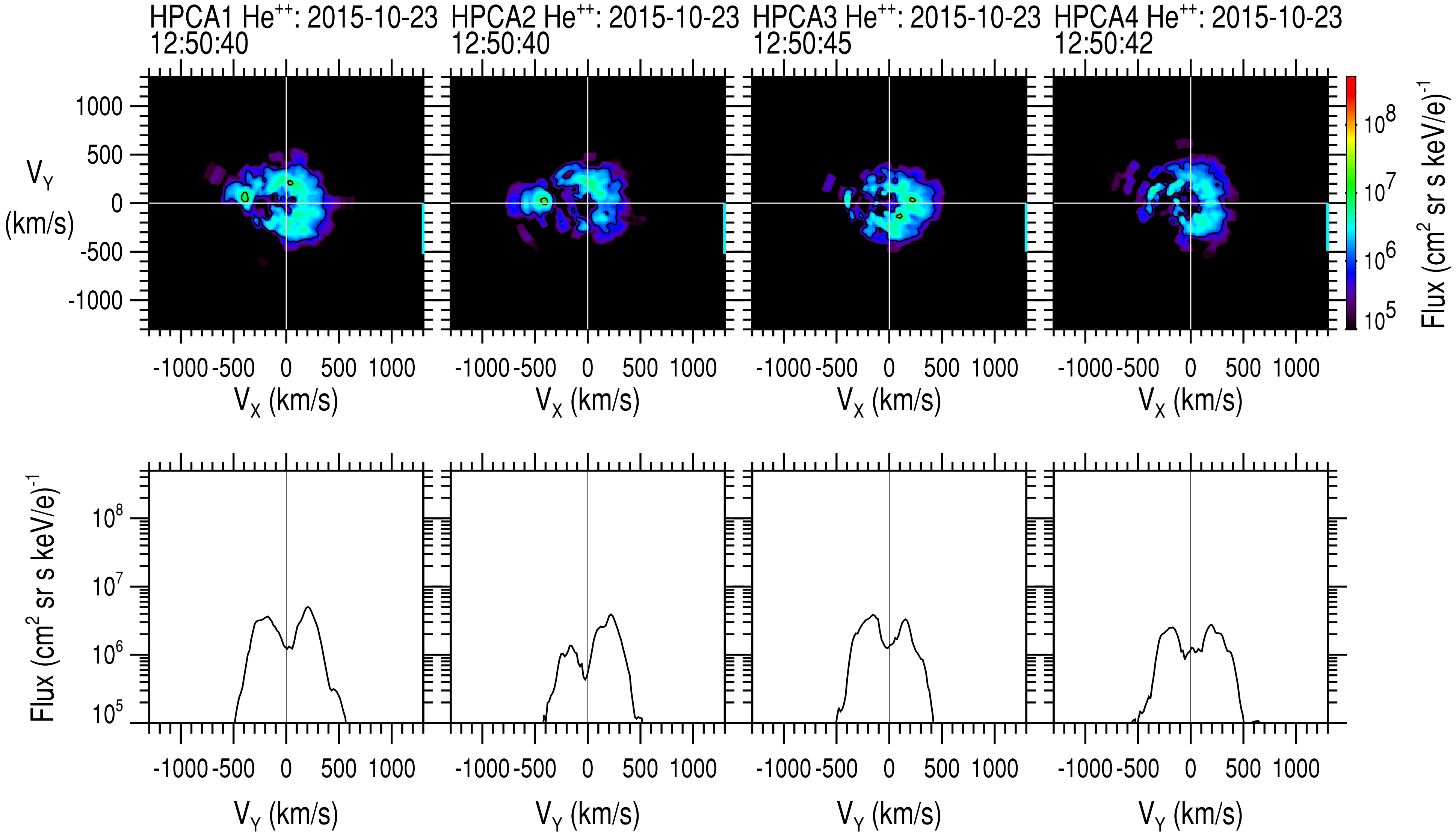
HPCA1 He++: 2015-10-23 12:50:40
HPCA2 He++: 2015-10-23 12:50:40
HPCA3 He++: 2015-10-23 12:50:45
HPCA4 He++: 2015-10-23 12:50:42
V_Y (km/s)
V_X (km/s)
Flux (cm^2 sr s keV/e)^-1

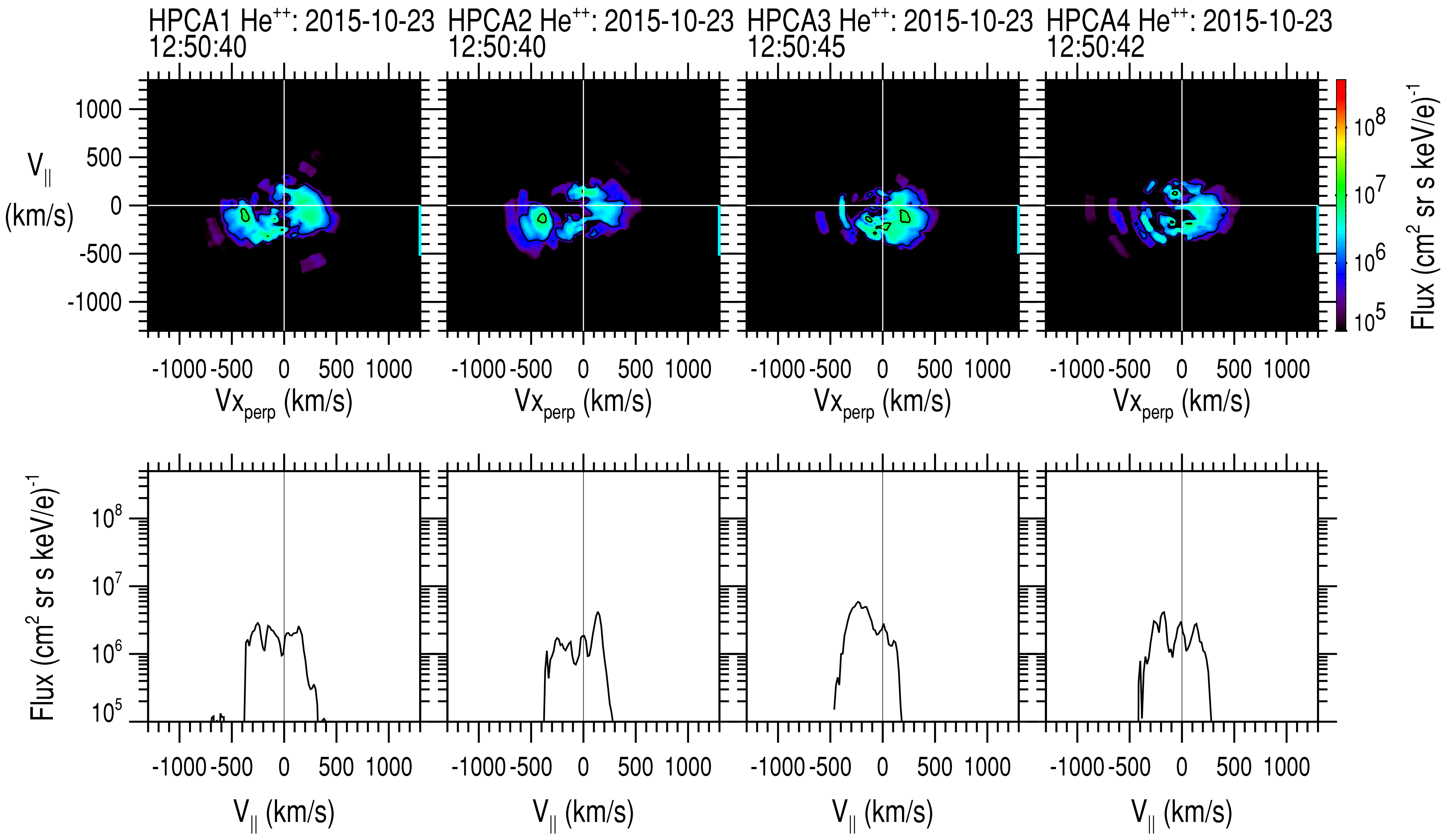
HPCA1 He++: 2015-10-23 12:50:40
HPCA2 He++: 2015-10-23 12:50:40
HPCA3 He++: 2015-10-23 12:50:45
HPCA4 He++: 2015-10-23 12:50:42
V|| (km/s)
Vxperp (km/s)
Flux (cm2 sr s keV/e)-1
V|| (km/s)

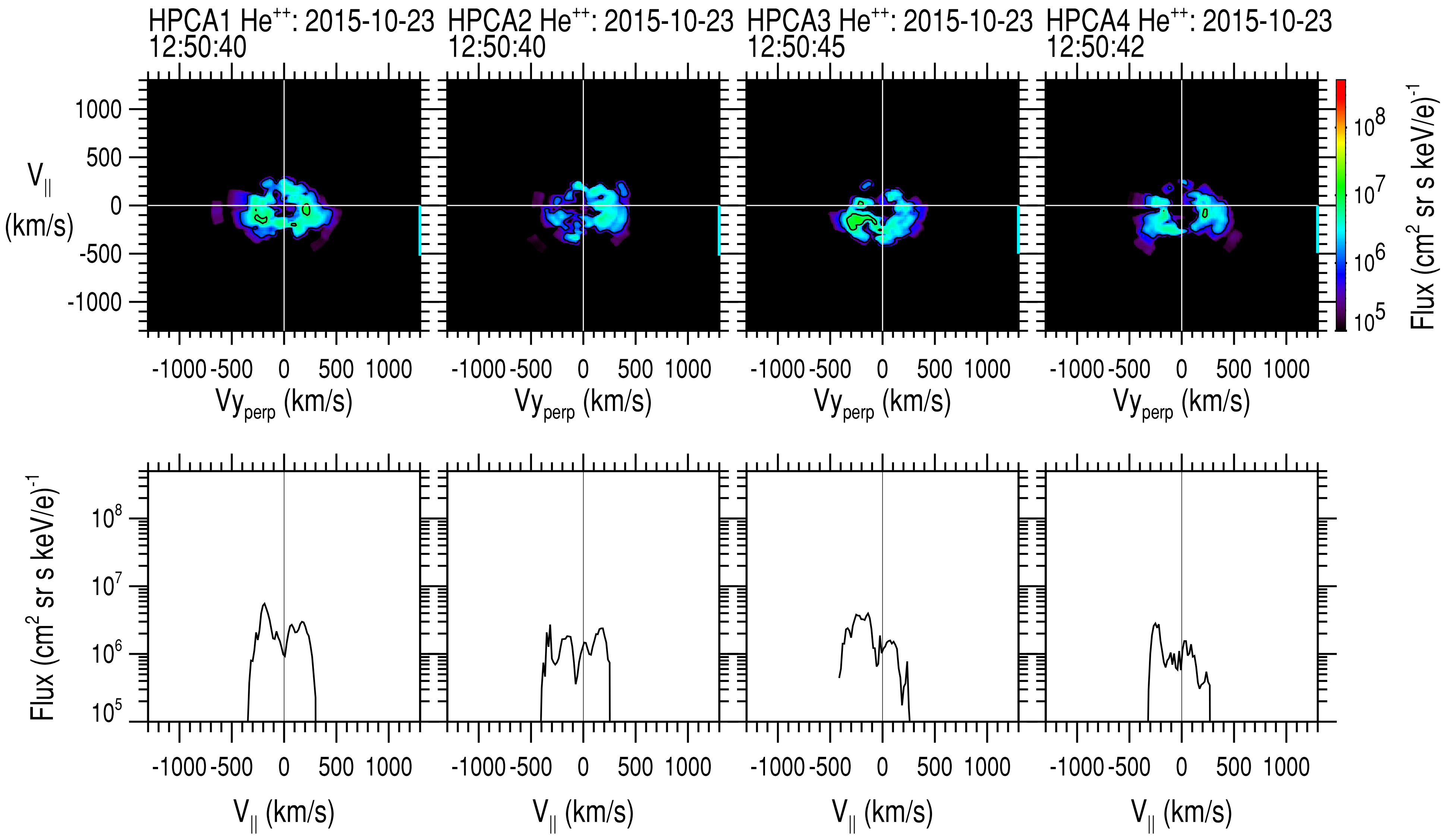
HPCA1 He++: 2015-10-23 12:50:40
HPCA2 He++: 2015-10-23 12:50:40
HPCA3 He++: 2015-10-23 12:50:45
HPCA4 He++: 2015-10-23 12:50:42
V|| (km/s)
Vyperp (km/s)
Flux (cm2 sr s keV/e)-1

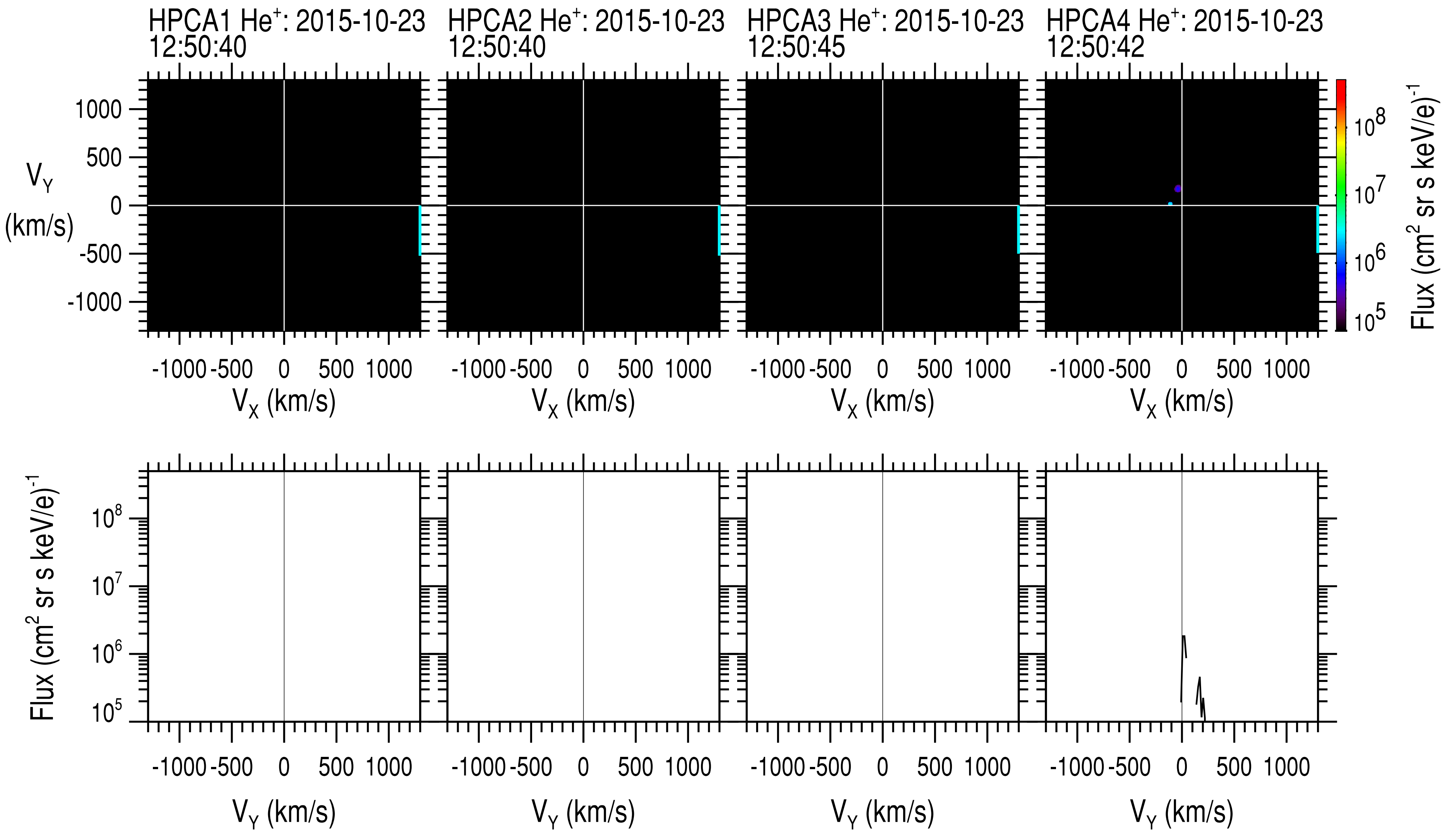
HPCA1 He+: 2015-10-23 12:50:40
HPCA2 He+: 2015-10-23 12:50:40
HPCA3 He+: 2015-10-23 12:50:45
HPCA4 He+: 2015-10-23 12:50:42
VY (km/s)
VX (km/s)
Flux (cm2 sr s keV/e)-1
VY (km/s)

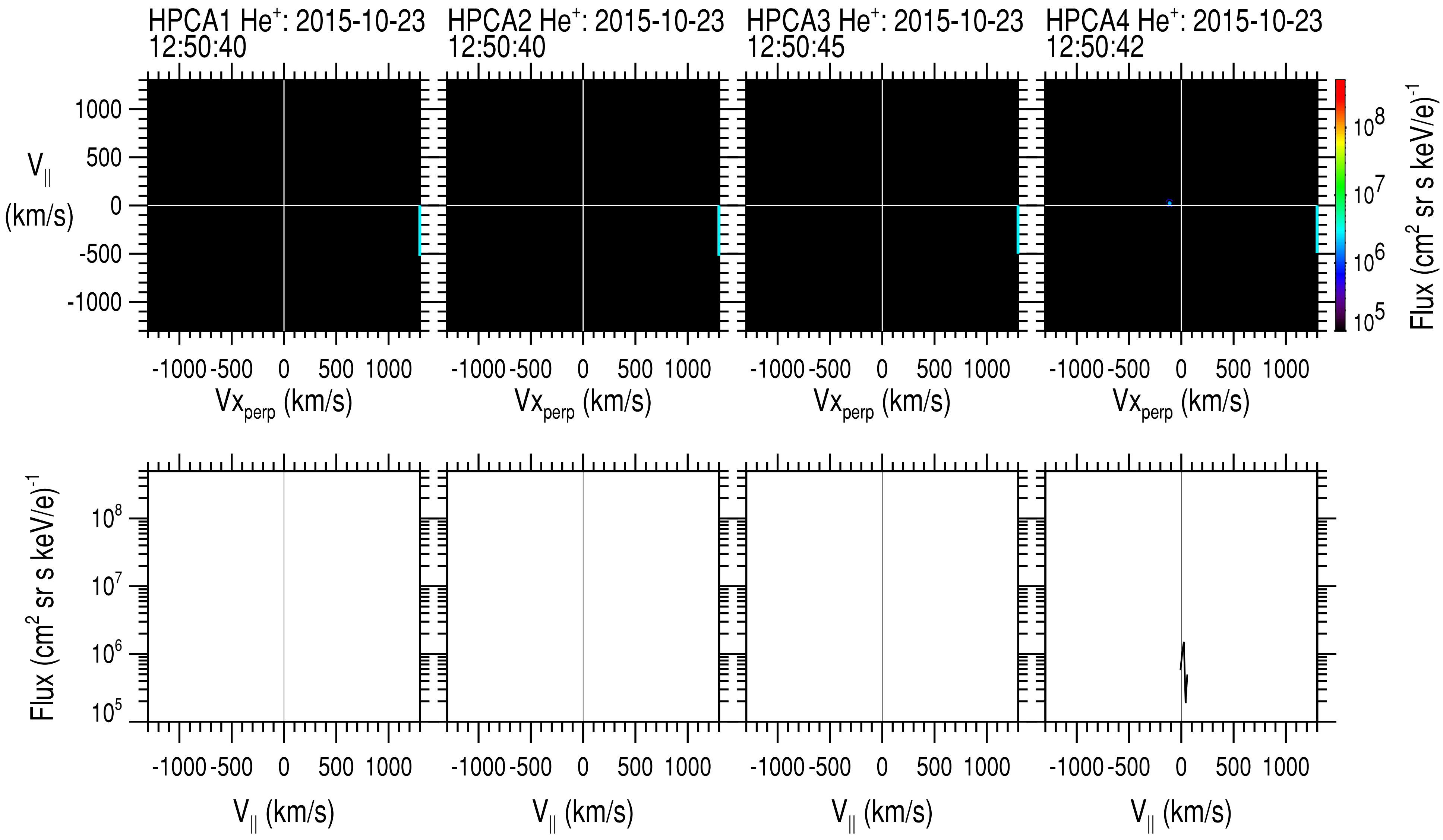

HPCA1 He+: 2015-10-23 12:50:40
HPCA2 He+: 2015-10-23 12:50:40
HPCA3 He+: 2015-10-23 12:50:45
HPCA4 He+: 2015-10-23 12:50:42
V|| (km/s)
Vxperp (km/s)
Flux (cm2 sr s keV/e)-1

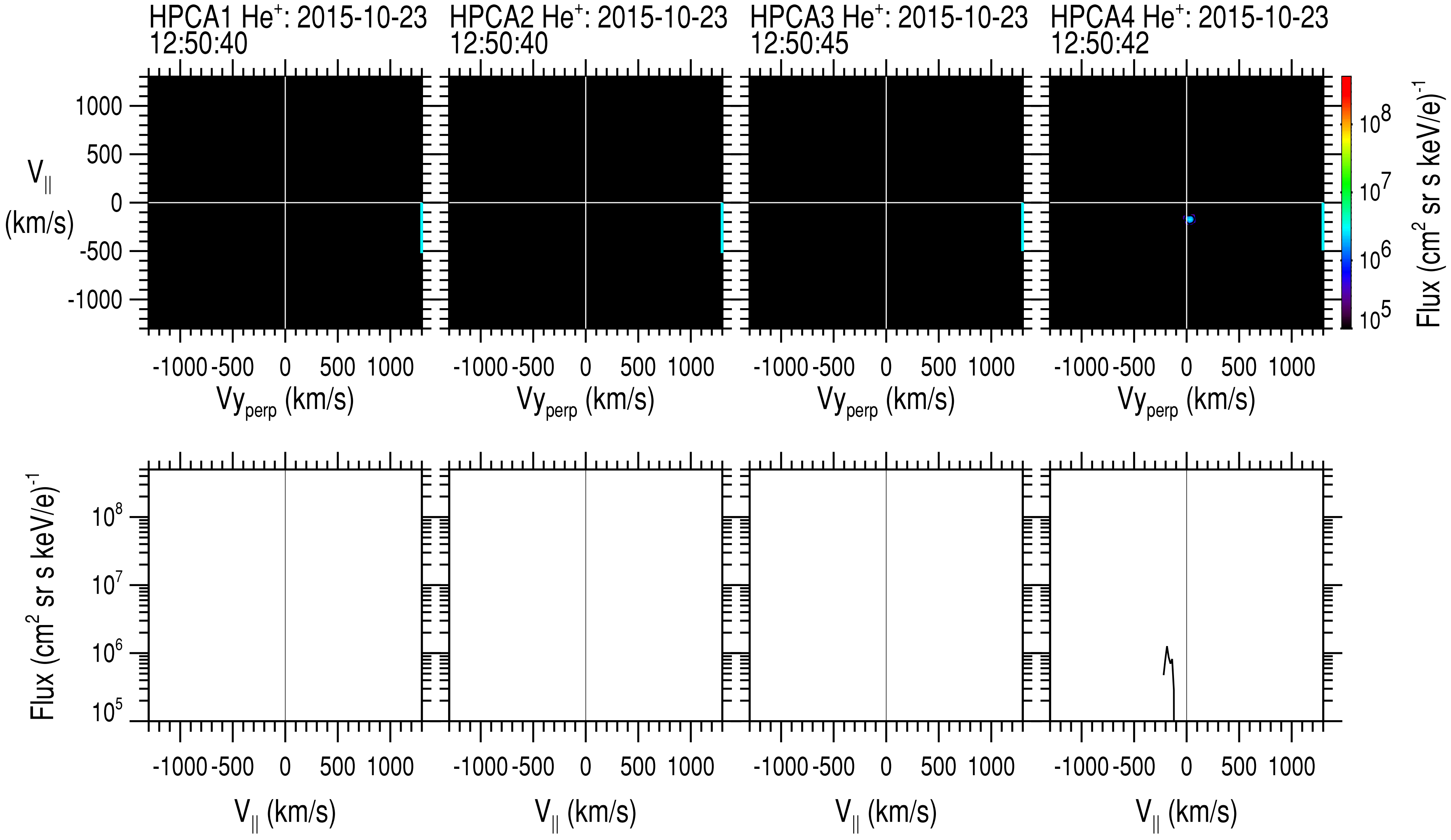

HPCA1 He+: 2015-10-23 12:50:40
HPCA2 He+: 2015-10-23 12:50:40
HPCA3 He+: 2015-10-23 12:50:45
HPCA4 He+: 2015-10-23 12:50:42
V|| (km/s)
Vyperp (km/s)
Flux (cm2 sr s keV/e)-1

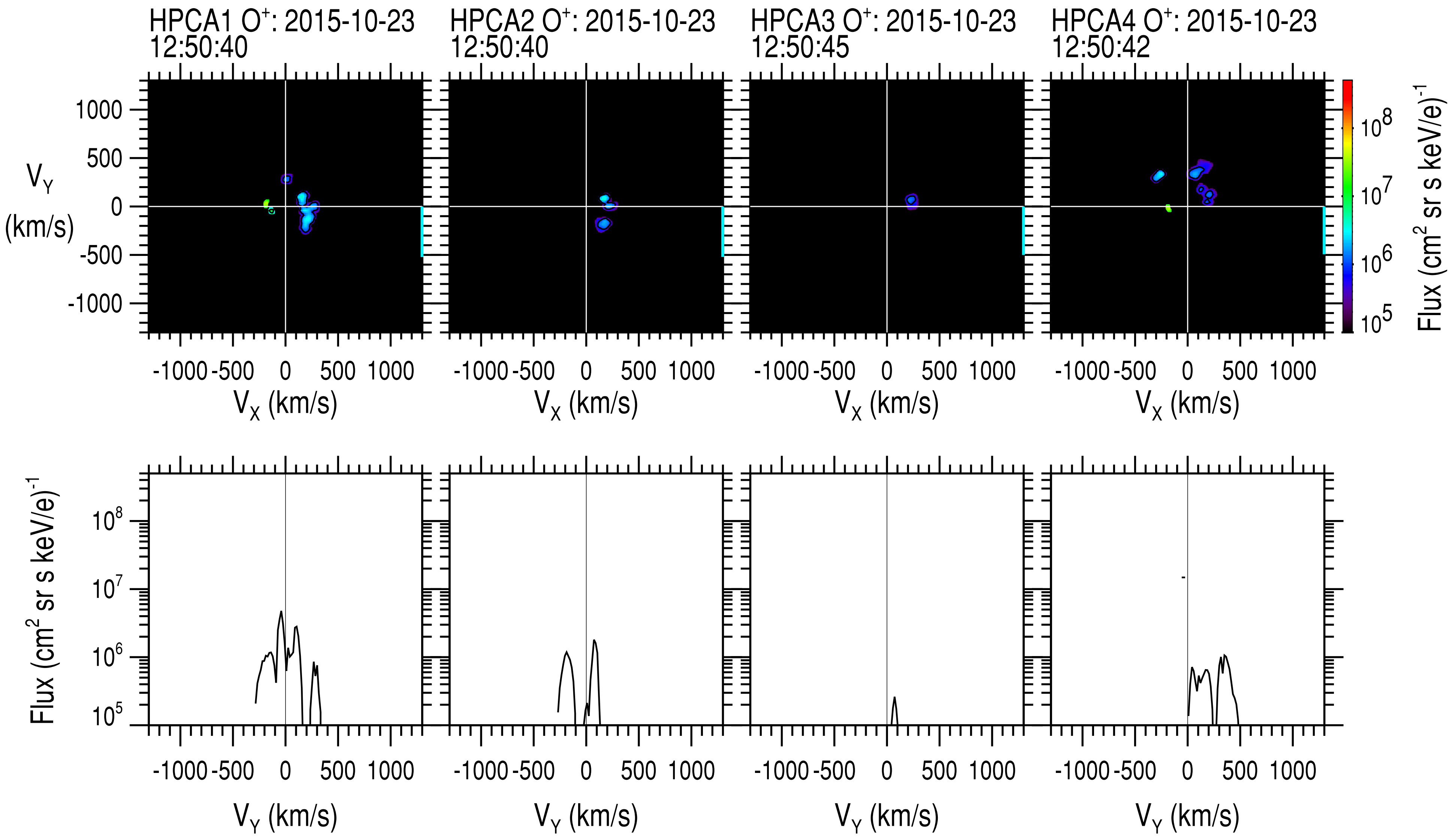
HPCA1 O+: 2015-10-23 12:50:40
HPCA2 O+: 2015-10-23 12:50:40
HPCA3 O+: 2015-10-23 12:50:45
HPCA4 O+: 2015-10-23 12:50:42
V_Y (km/s)
V_X (km/s)
Flux (cm^2 sr s keV/e)^-1

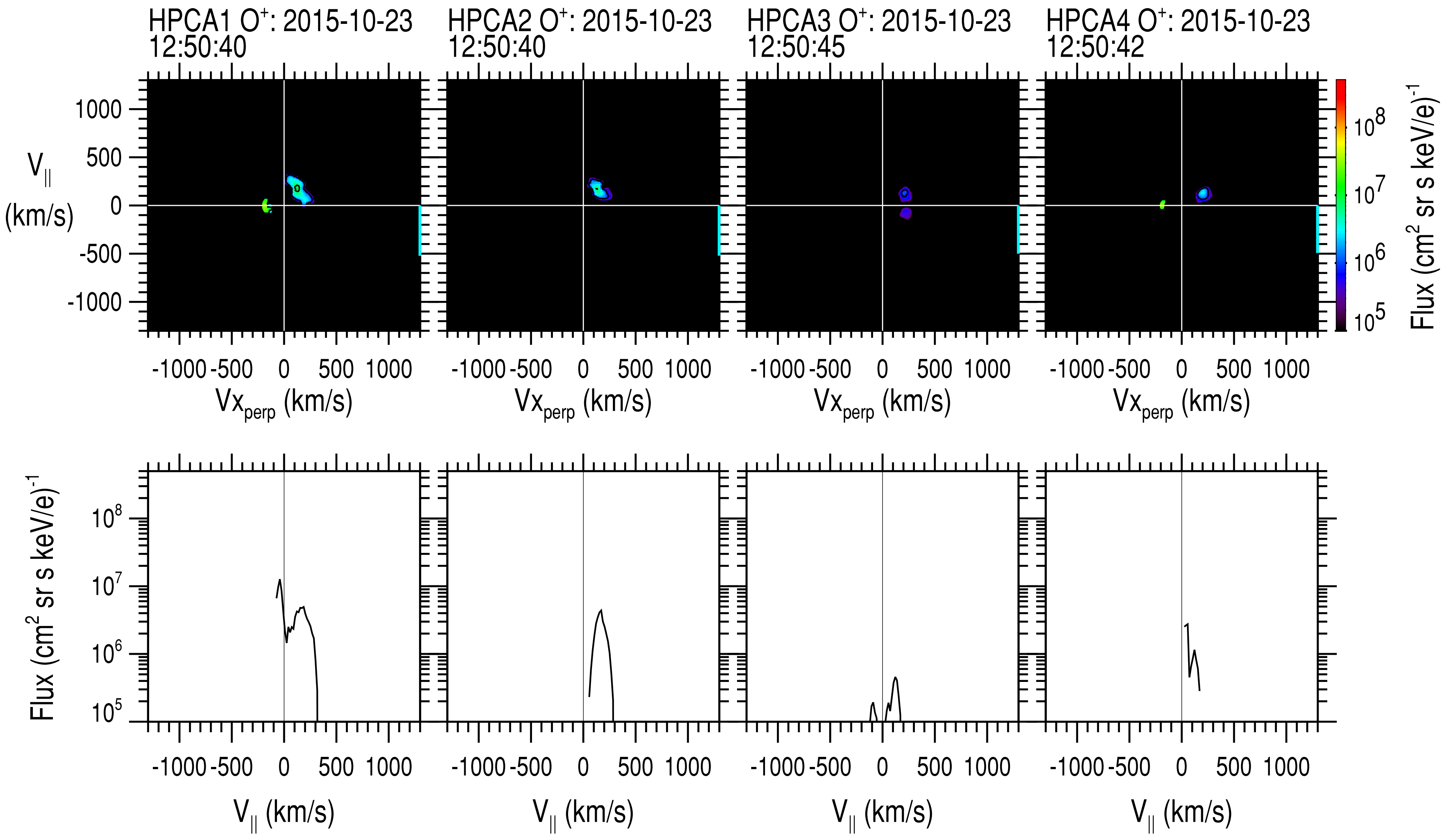
HPCA1 O+: 2015-10-23 12:50:40
HPCA2 O+: 2015-10-23 12:50:40
HPCA3 O+: 2015-10-23 12:50:45
HPCA4 O+: 2015-10-23 12:50:42
V|| (km/s)
Vxperp (km/s)
Flux (cm2 sr s keV/e)-1
-1000 -500 0 500 1000

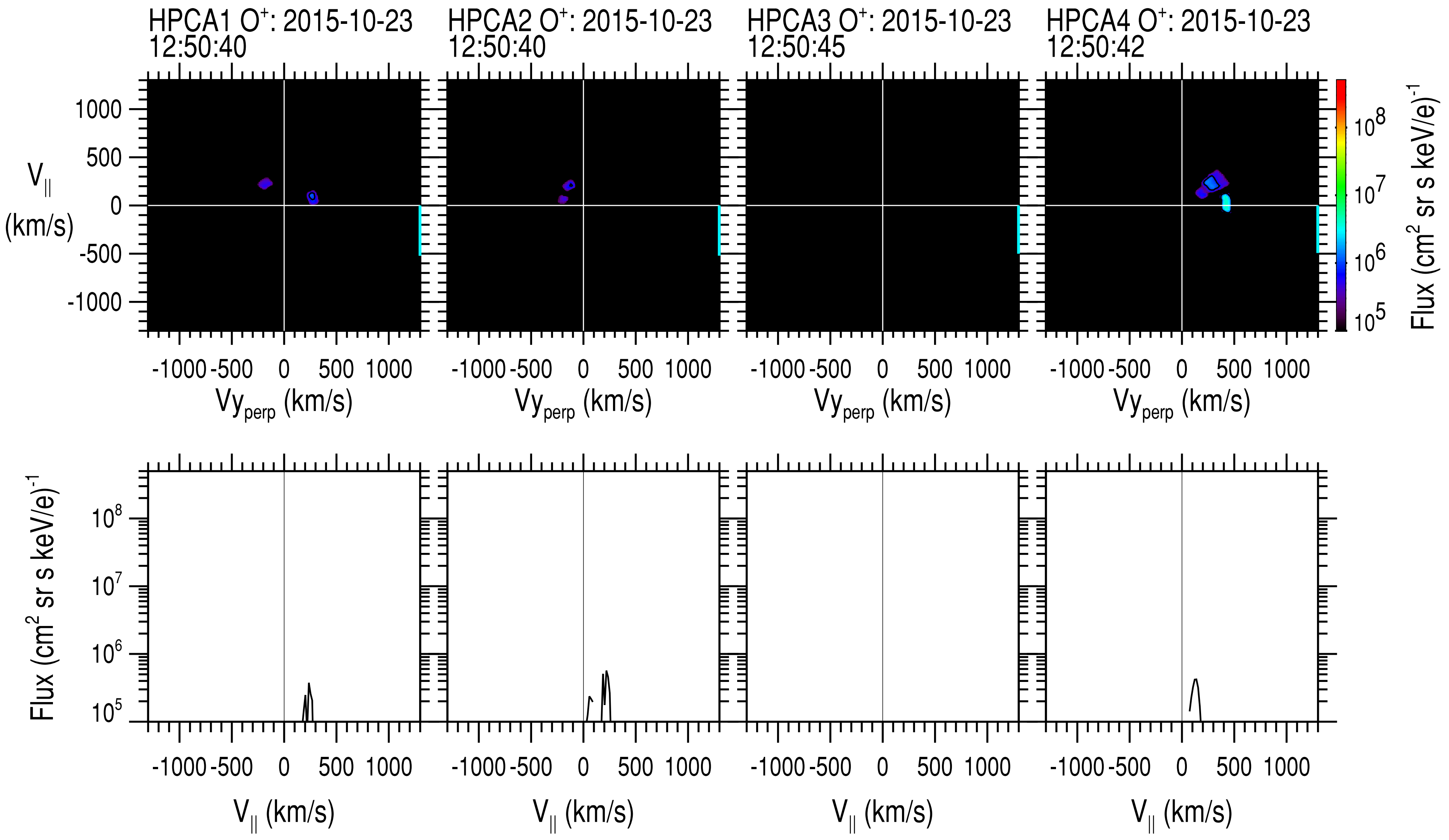
HPCA1 O+: 2015-10-23 12:50:40
HPCA2 O+: 2015-10-23 12:50:40
HPCA3 O+: 2015-10-23 12:50:45
HPCA4 O+: 2015-10-23 12:50:42
V|| (km/s)
1000
500
0
-500
-1000
Vyperp (km/s)
Flux (cm2 sr s keV/e)-1
10^5
10^6
10^7
10^8

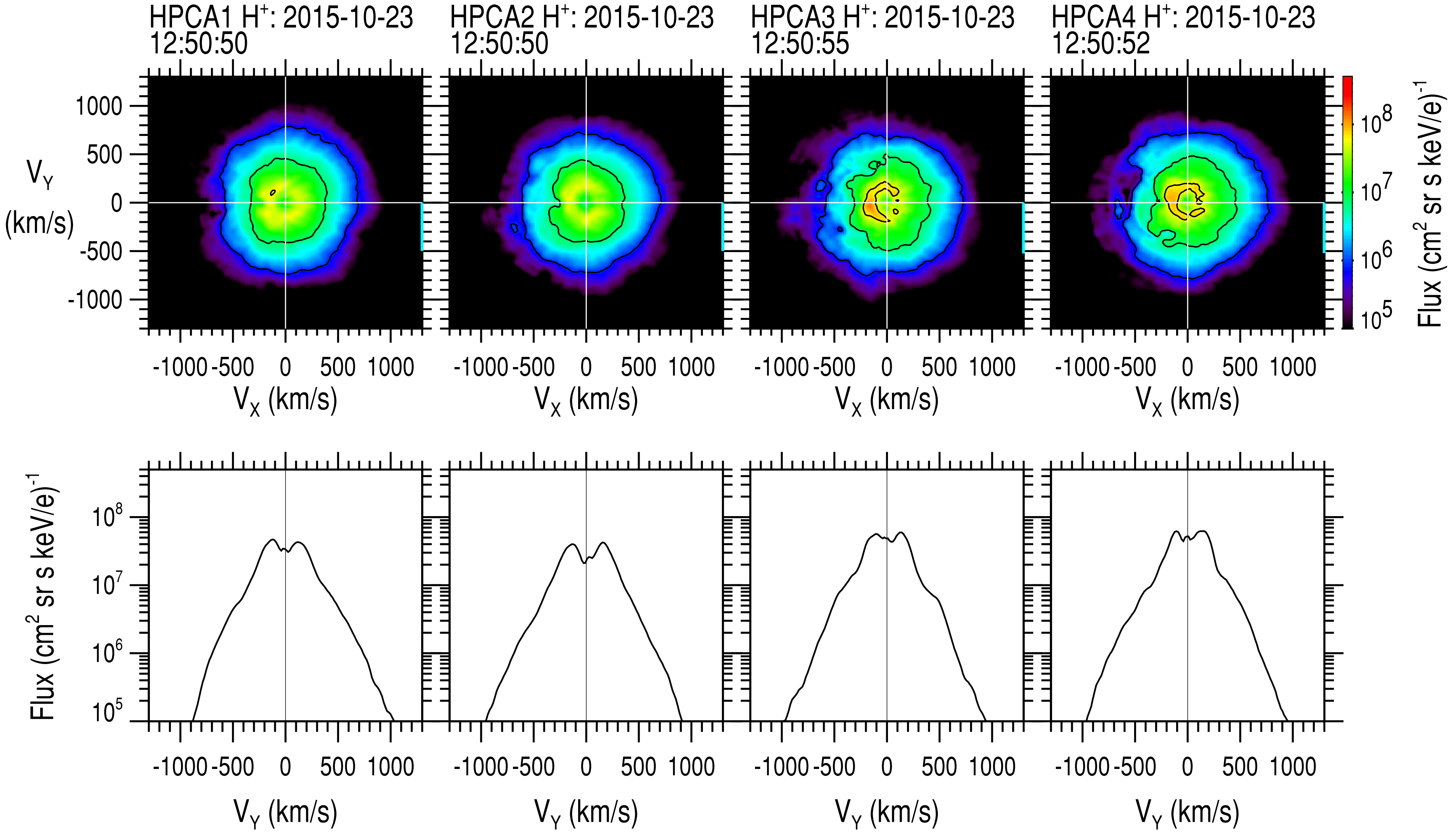

HPCA1 H+: 2015-10-23
12:50:50
HPCA2 H+: 2015-10-23
12:50:50
HPCA3 H+: 2015-10-23
12:50:55
HPCA4 H+: 2015-10-23
12:50:52
V_Y (km/s)
1000
500
0
-500
-1000
-1000 -500 0 500 1000
V_X (km/s)
Flux (cm^2 sr s keV/e)^-1
10^5
10^6
10^7
10^8
V_Y (km/s)

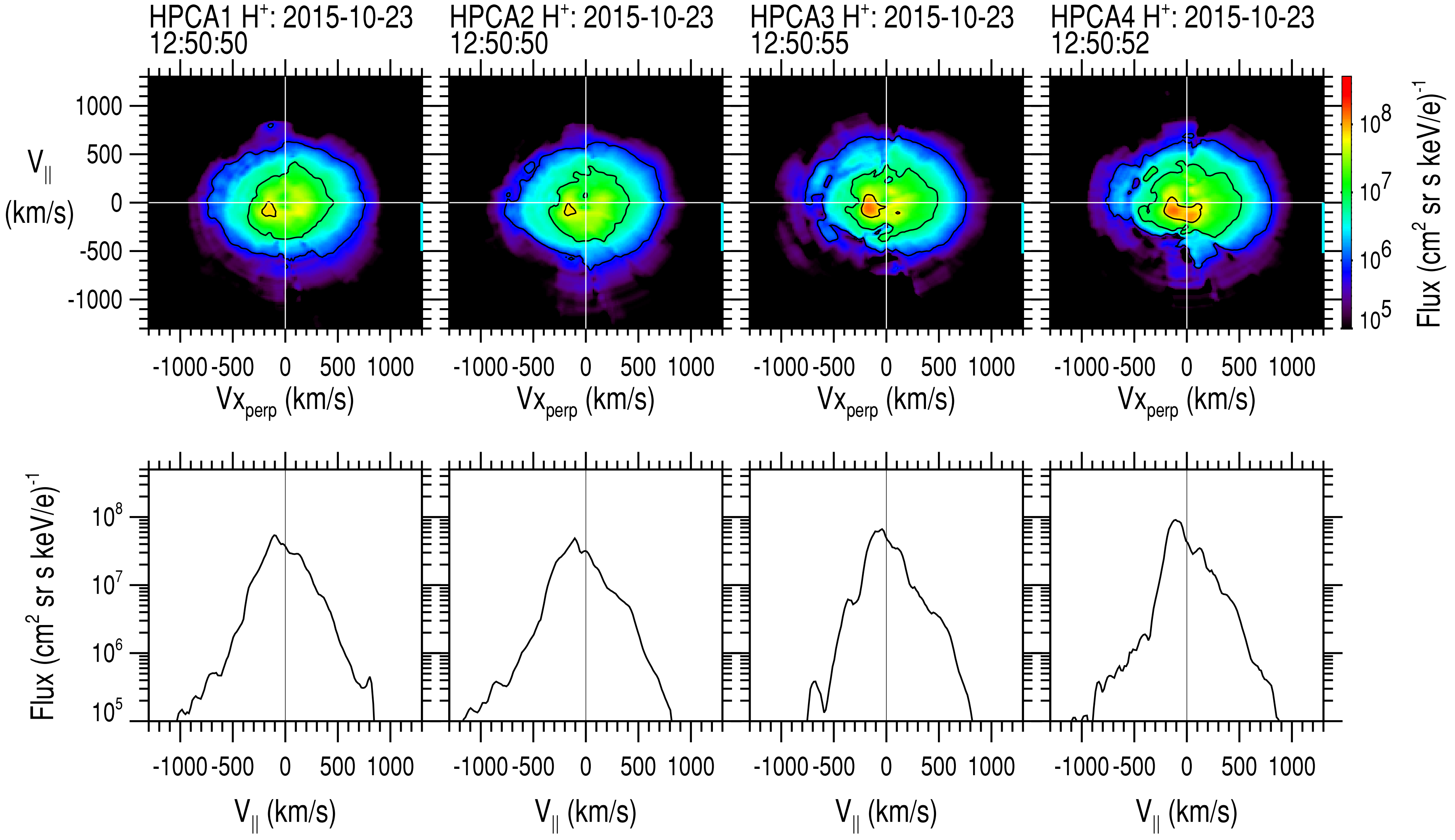

HPCA1 H+: 2015-10-23 12:50:50
HPCA2 H+: 2015-10-23 12:50:50
HPCA3 H+: 2015-10-23 12:50:55
HPCA4 H+: 2015-10-23 12:50:52
V|| (km/s)
Vxperp (km/s)
Flux (cm2 sr s keV/e)-1

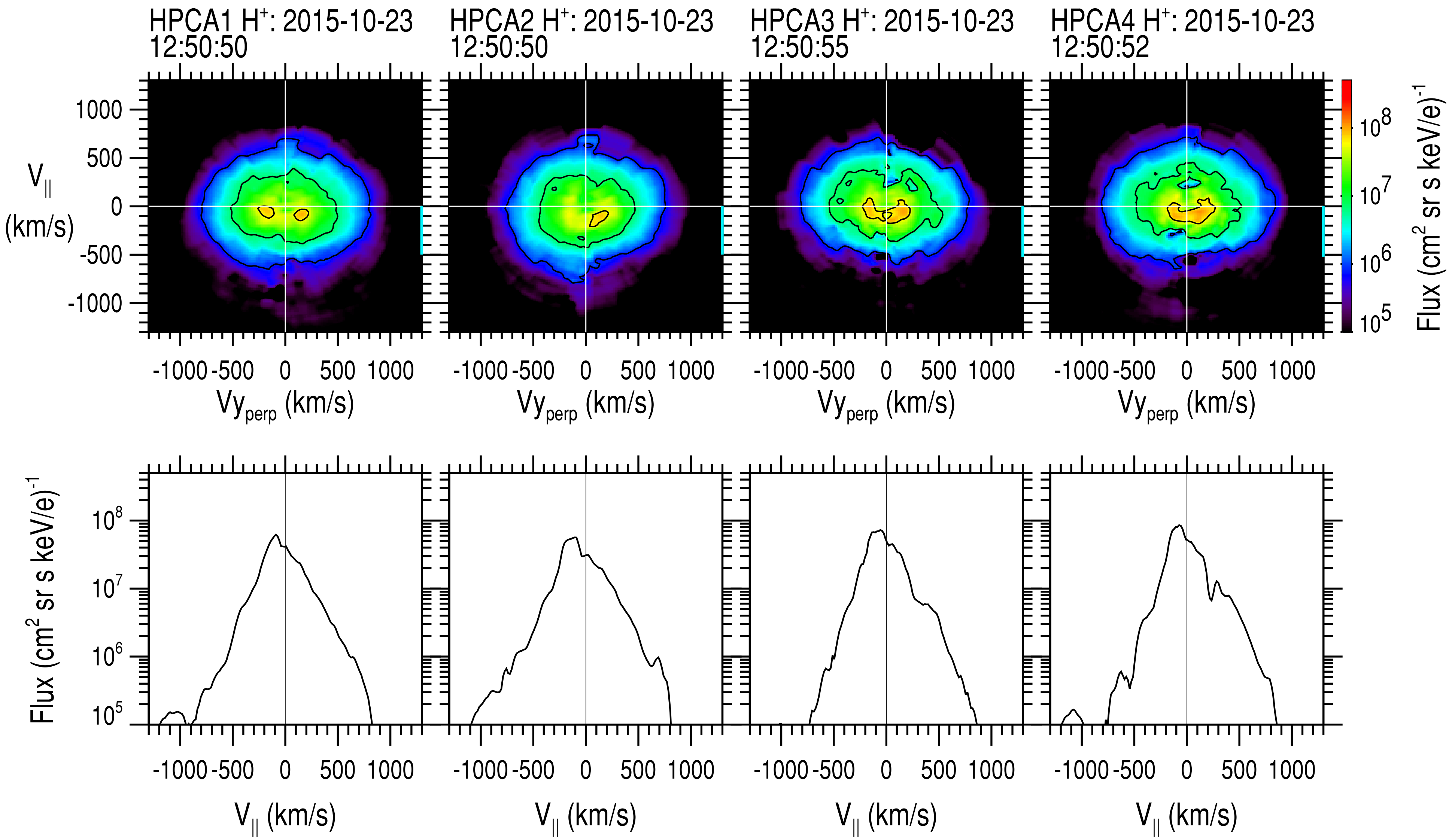

HPCA1 H+: 2015-10-23 12:50:50
HPCA2 H+: 2015-10-23 12:50:50
HPCA3 H+: 2015-10-23 12:50:55
HPCA4 H+: 2015-10-23 12:50:52
V|| (km/s)
Vyperp (km/s)
Flux (cm2 sr s keV/e)-1
V|| (km/s)

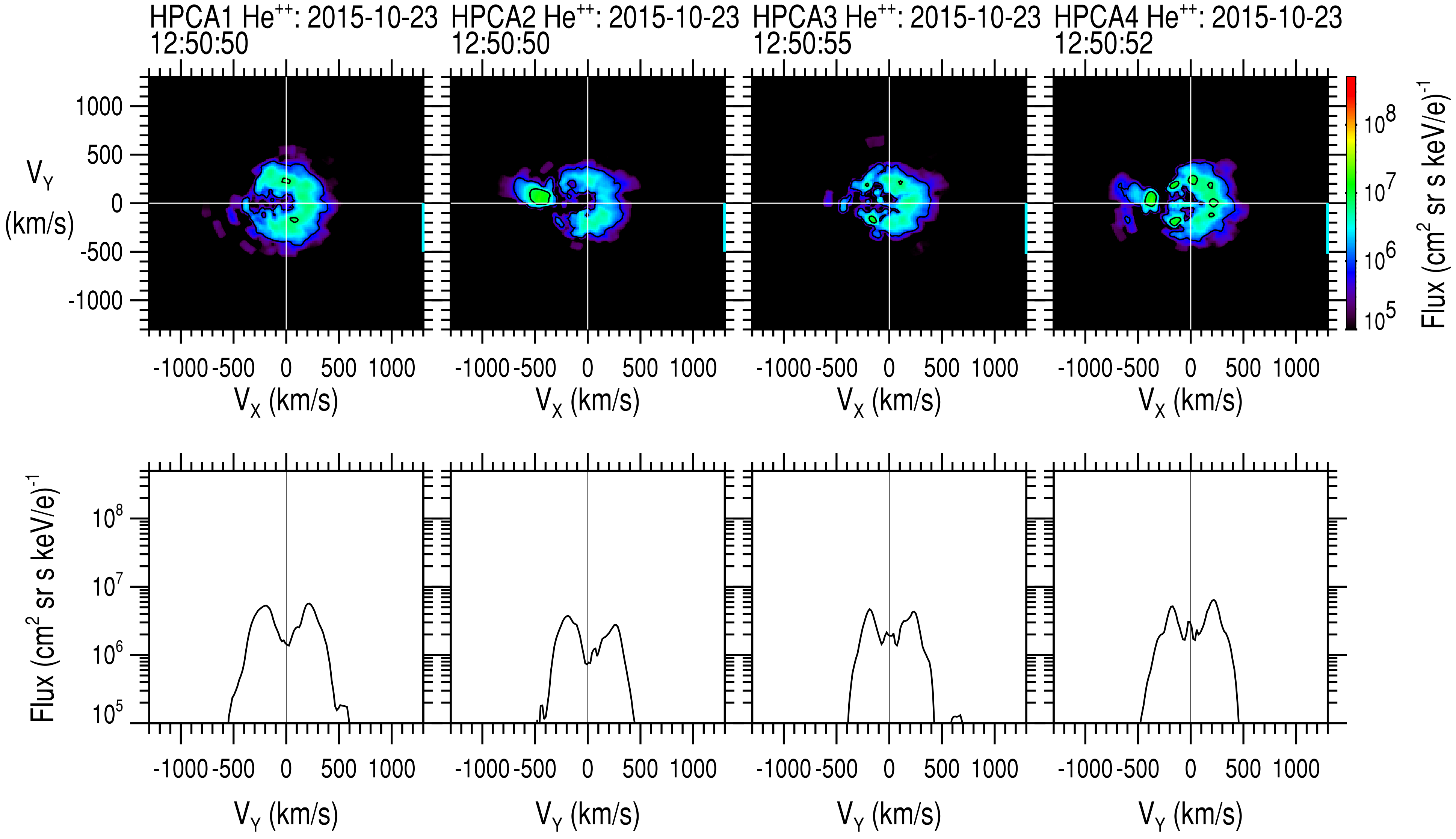

HPCA1 He++: 2015-10-23 12:50:50
HPCA2 He++: 2015-10-23 12:50:50
HPCA3 He++: 2015-10-23 12:50:55
HPCA4 He++: 2015-10-23 12:50:52
V_Y (km/s)
V_X (km/s)
Flux (cm^2 sr s keV/e)^-1

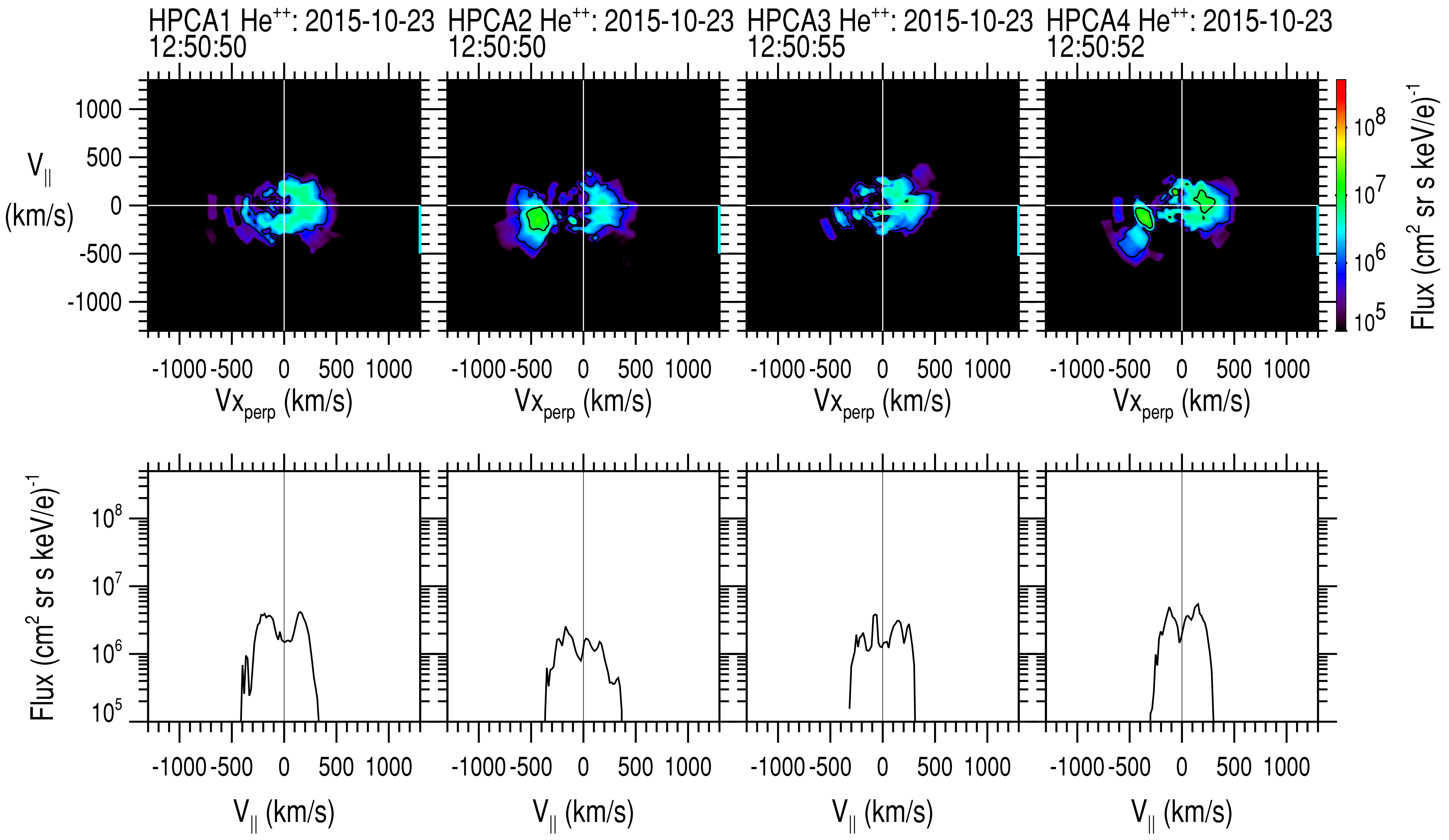

HPCA1 He++: 2015-10-23 12:50:50
HPCA2 He++: 2015-10-23 12:50:50
HPCA3 He++: 2015-10-23 12:50:55
HPCA4 He++: 2015-10-23 12:50:52
V|| (km/s)
Vxperp (km/s)
Flux (cm2 sr s keV/e)-1

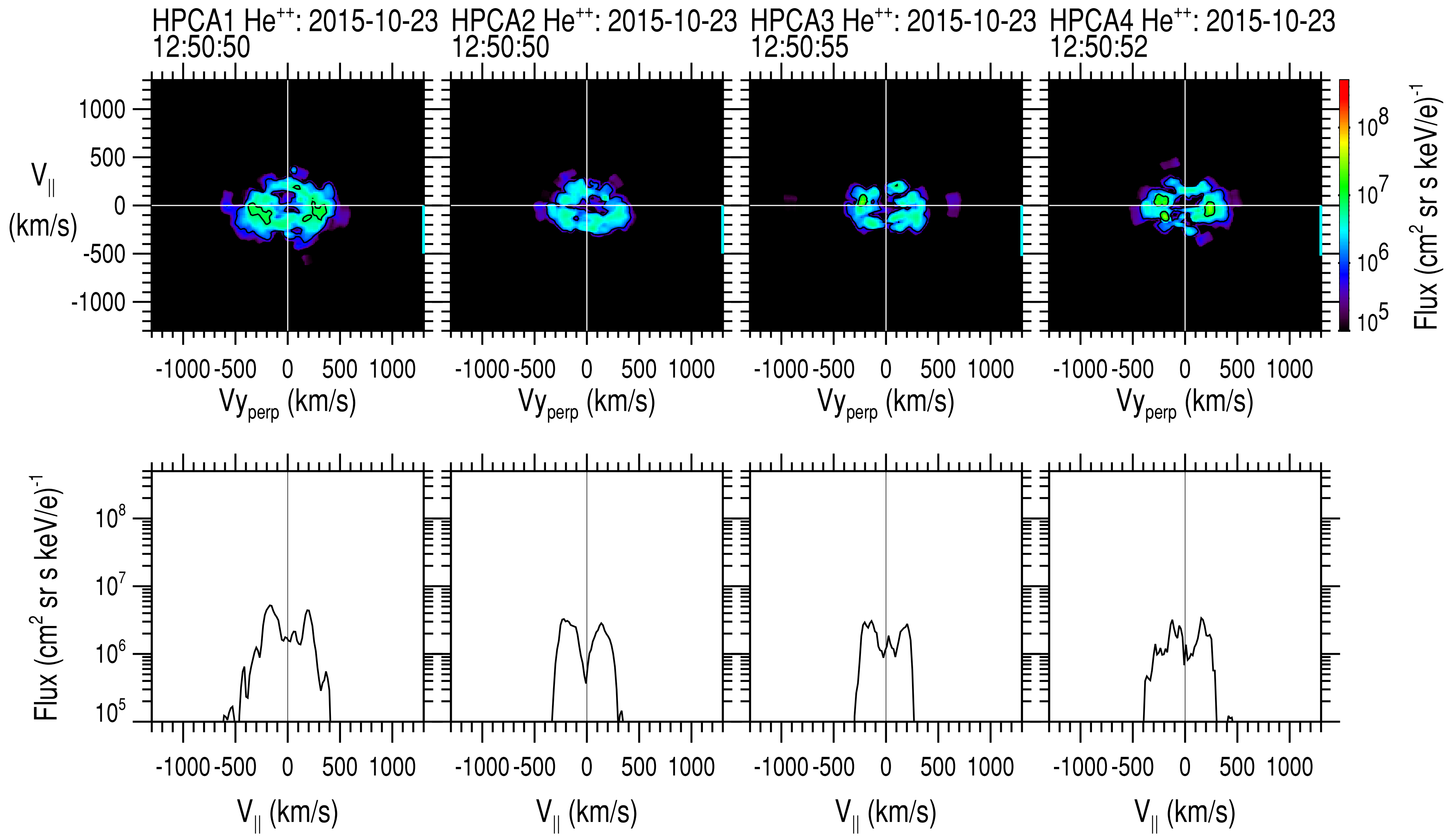
HPCA1 He++: 2015-10-23 12:50:50
HPCA2 He++: 2015-10-23 12:50:50
HPCA3 He++: 2015-10-23 12:50:55
HPCA4 He++: 2015-10-23 12:50:52
V|| (km/s)
Vyperp (km/s)
Flux (cm2 sr s keV/e)-1
V|| (km/s)

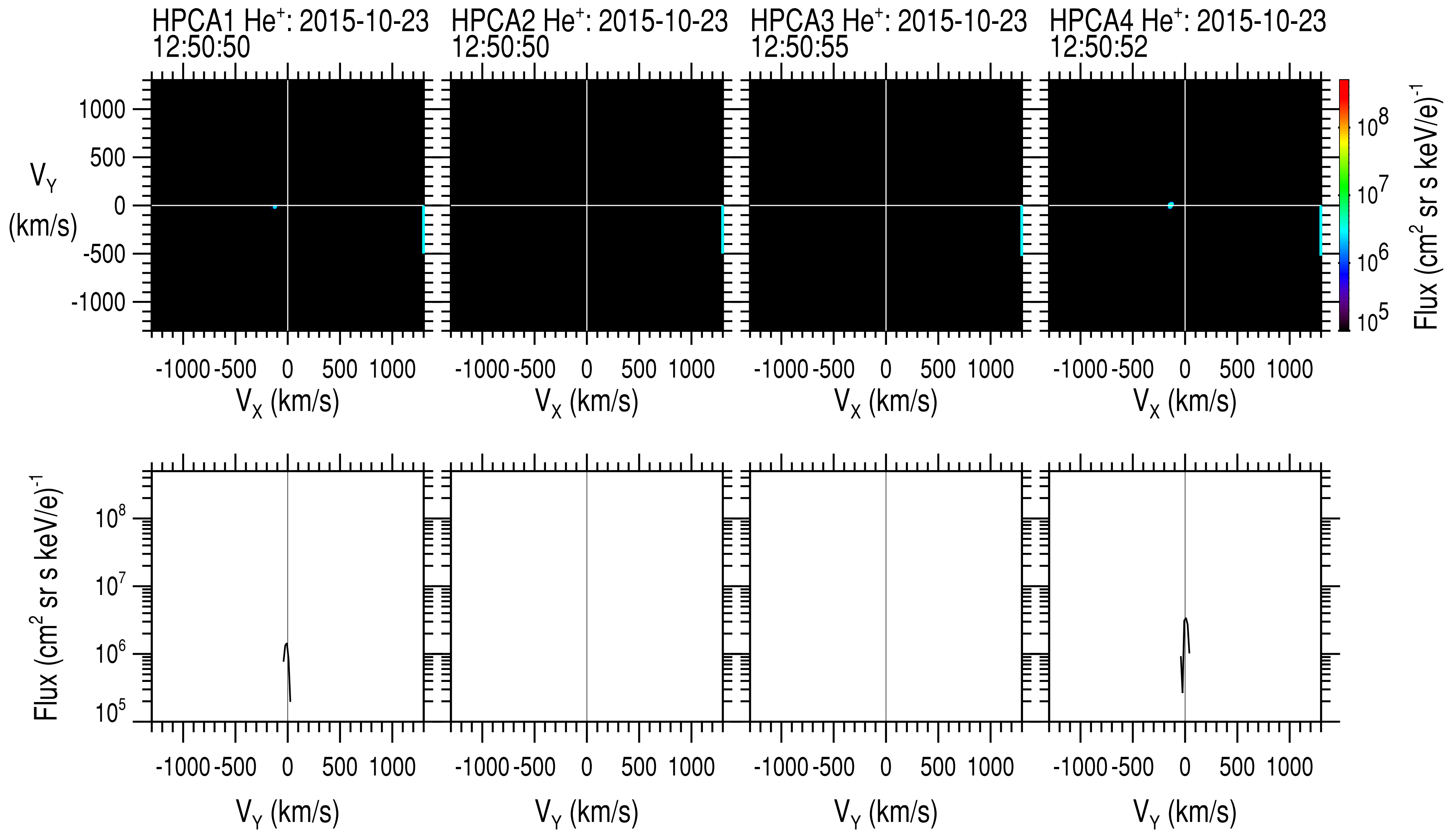
HPCA1 He+: 2015-10-23 12:50:50
HPCA2 He+: 2015-10-23 12:50:50
HPCA3 He+: 2015-10-23 12:50:55
HPCA4 He+: 2015-10-23 12:50:52
VY (km/s)
VX (km/s)
Flux (cm2 sr s keV/e)-1
1000
500
0
-500
-1000
10^5
10^6
10^7
10^8

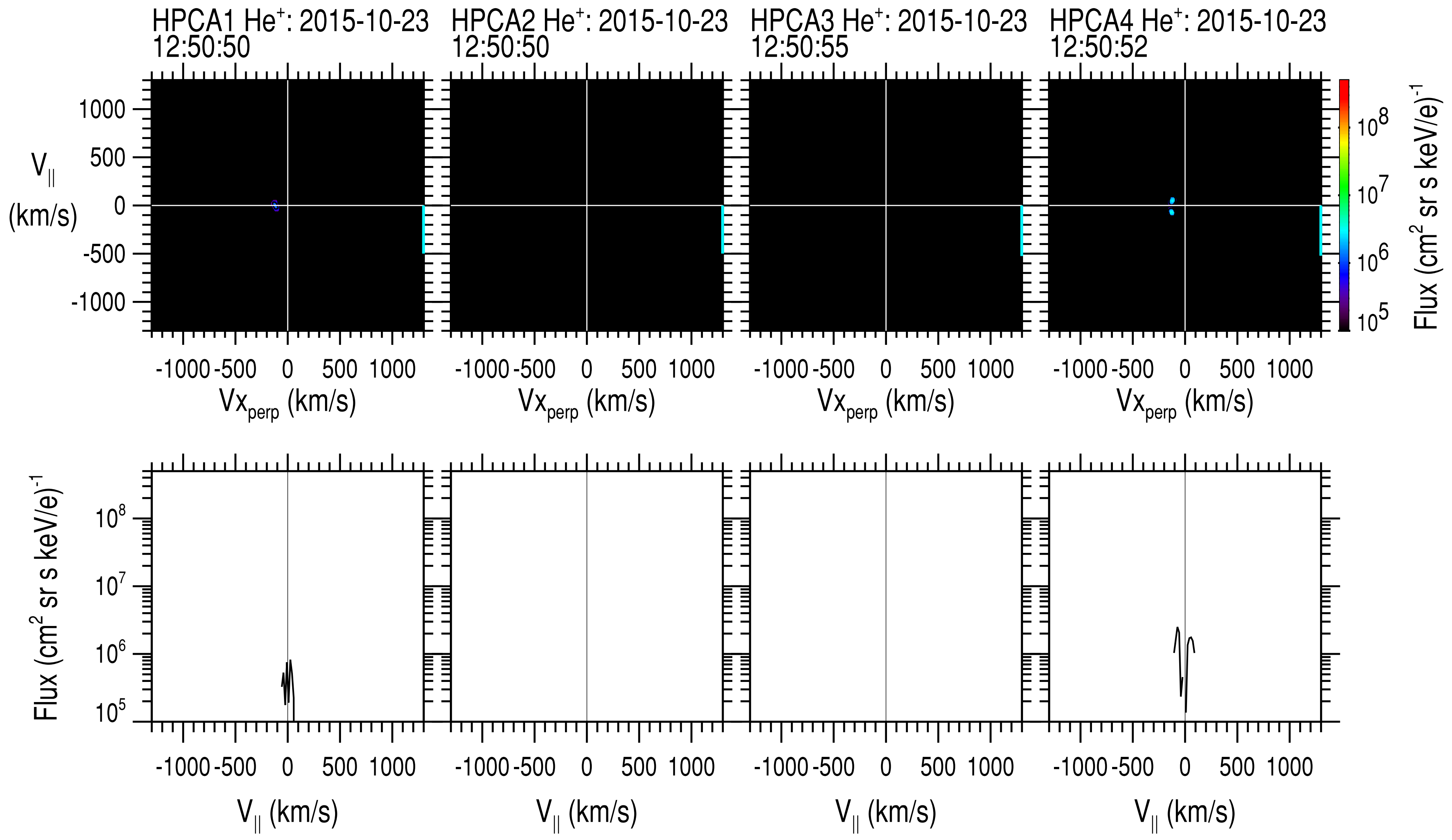
HPCA1 He+: 2015-10-23 12:50:50
HPCA2 He+: 2015-10-23 12:50:50
HPCA3 He+: 2015-10-23 12:50:55
HPCA4 He+: 2015-10-23 12:50:52
V|| (km/s)
1000
500
0
-500
-1000
-1000 -500 0 500 1000
Vxperp (km/s)
Flux (cm2 sr s keV/e)-1
10^5
10^6
10^7
10^8
V|| (km/s)

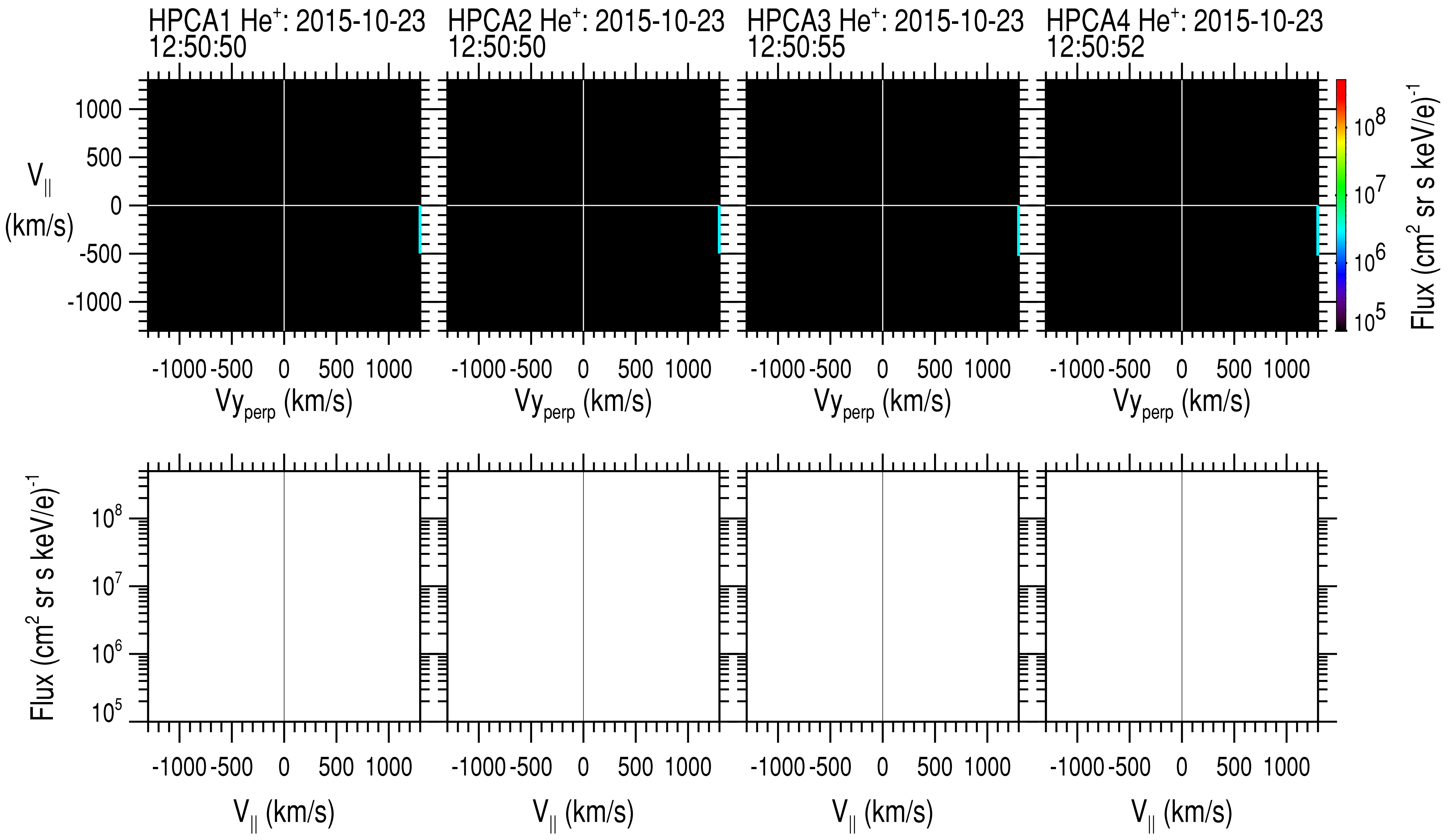
HPCA1 He+: 2015-10-23 12:50:50
HPCA2 He+: 2015-10-23 12:50:50
HPCA3 He+: 2015-10-23 12:50:55
HPCA4 He+: 2015-10-23 12:50:52
V|| (km/s)
Vyperp (km/s)
Flux (cm2 sr s keV/e)-1
1000
500
0
-500
-1000
105
106
107
108

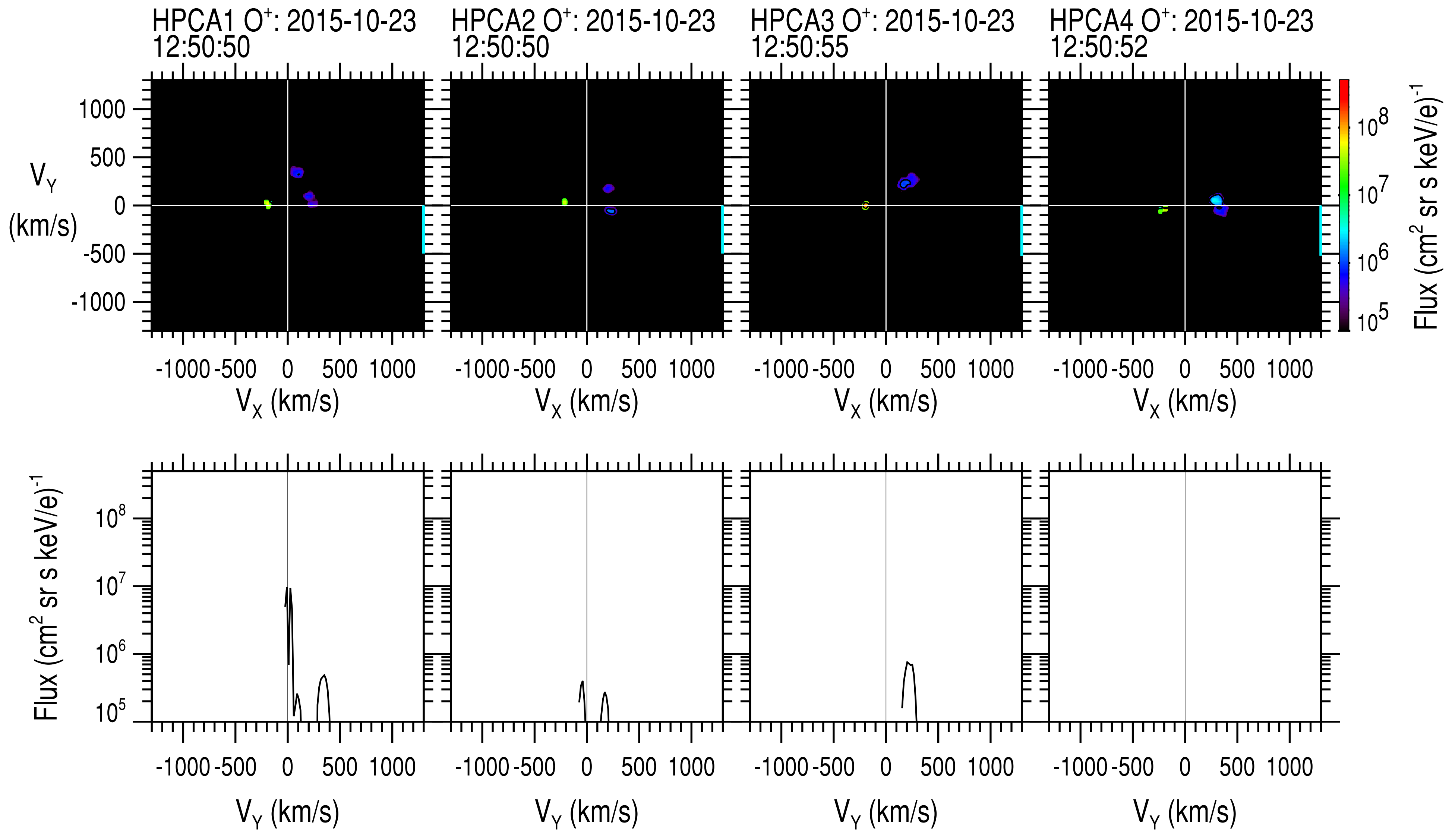
HPCA1 O+: 2015-10-23 12:50:50
HPCA2 O+: 2015-10-23 12:50:50
HPCA3 O+: 2015-10-23 12:50:55
HPCA4 O+: 2015-10-23 12:50:52
VY (km/s)
VX (km/s)
Flux (cm2 sr s keV/e)-1
VY (km/s)
Flux (cm2 sr s keV/e)-1

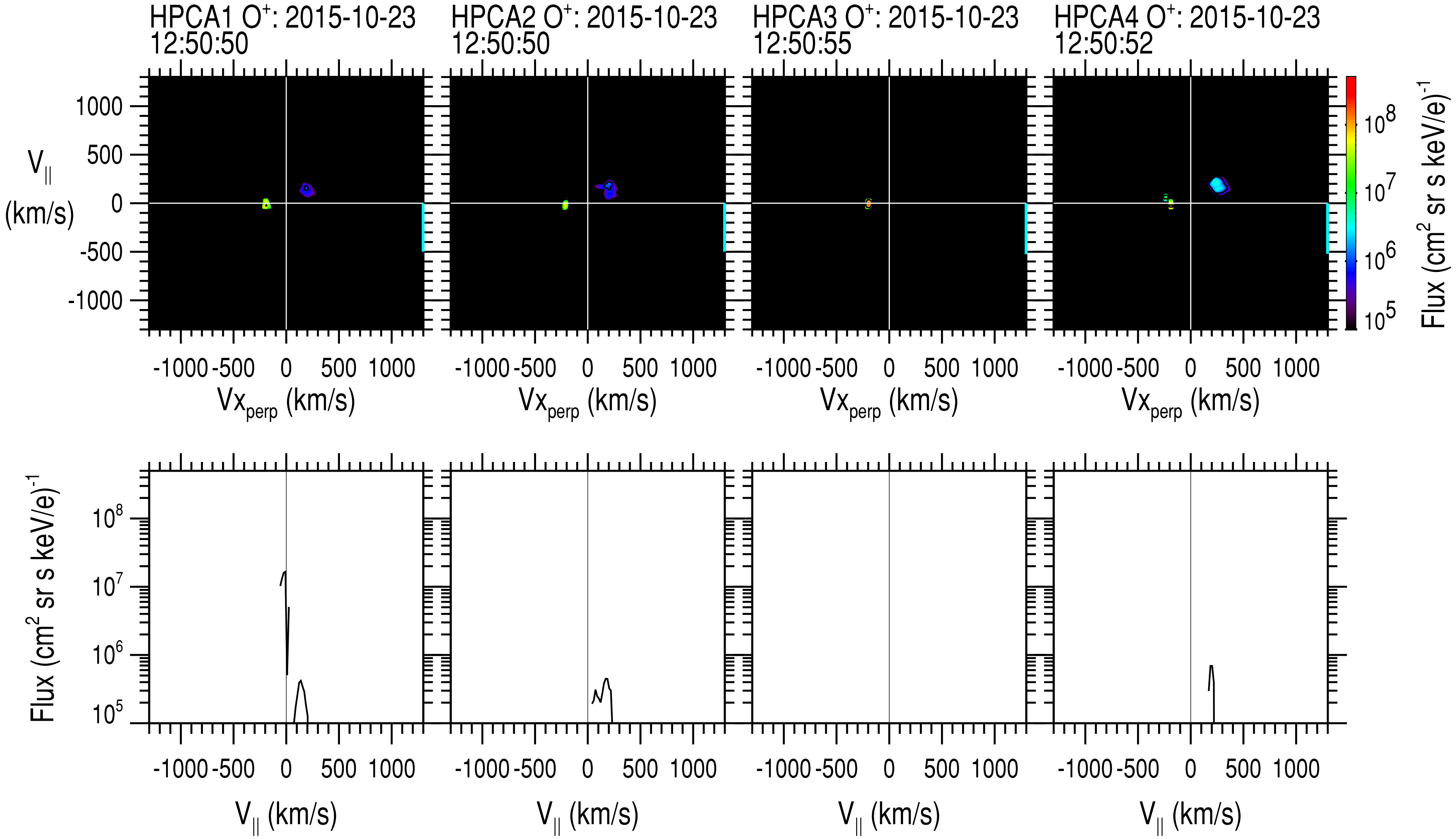
HPCA1 O+: 2015-10-23 12:50:50
HPCA2 O+: 2015-10-23 12:50:50
HPCA3 O+: 2015-10-23 12:50:55
HPCA4 O+: 2015-10-23 12:50:52
V|| (km/s)
Vxperp (km/s)
Flux (cm2 sr s keV/e)-1

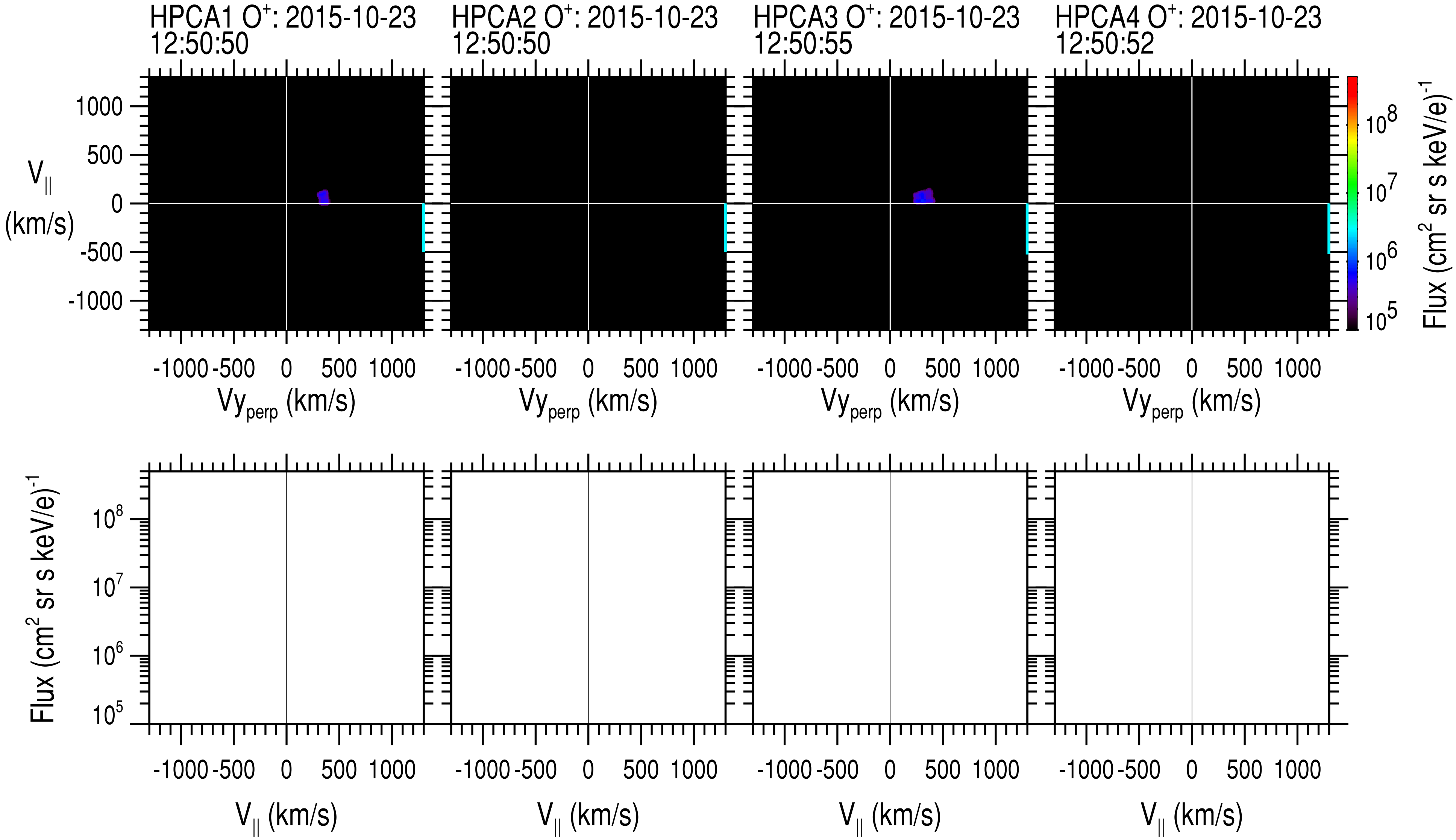

HPCA1 O+: 2015-10-23
12:50:50
HPCA2 O+: 2015-10-23
12:50:50
HPCA3 O+: 2015-10-23
12:50:55
HPCA4 O+: 2015-10-23
12:50:52
V|| (km/s)
1000
500
0
-500
-1000
Vyperp (km/s)
Flux (cm2 sr s keV/e)-1
10^5
10^6
10^7
10^8
V|| (km/s)

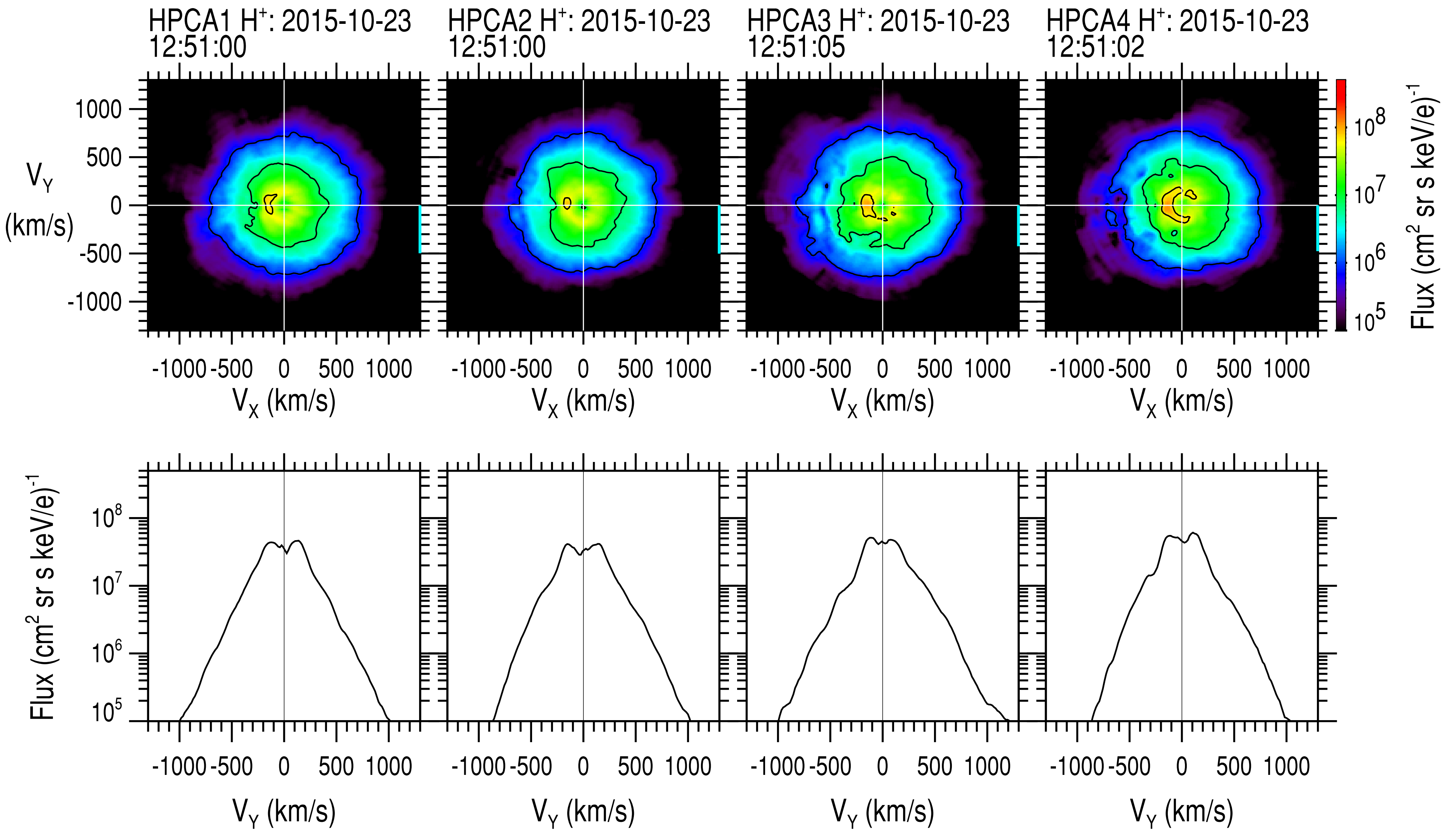

HPCA1 H+: 2015-10-23 12:51:00
HPCA2 H+: 2015-10-23 12:51:00
HPCA3 H+: 2015-10-23 12:51:05
HPCA4 H+: 2015-10-23 12:51:02
V_Y (km/s)
V_X (km/s)
Flux (cm2 sr s keV/e)-1

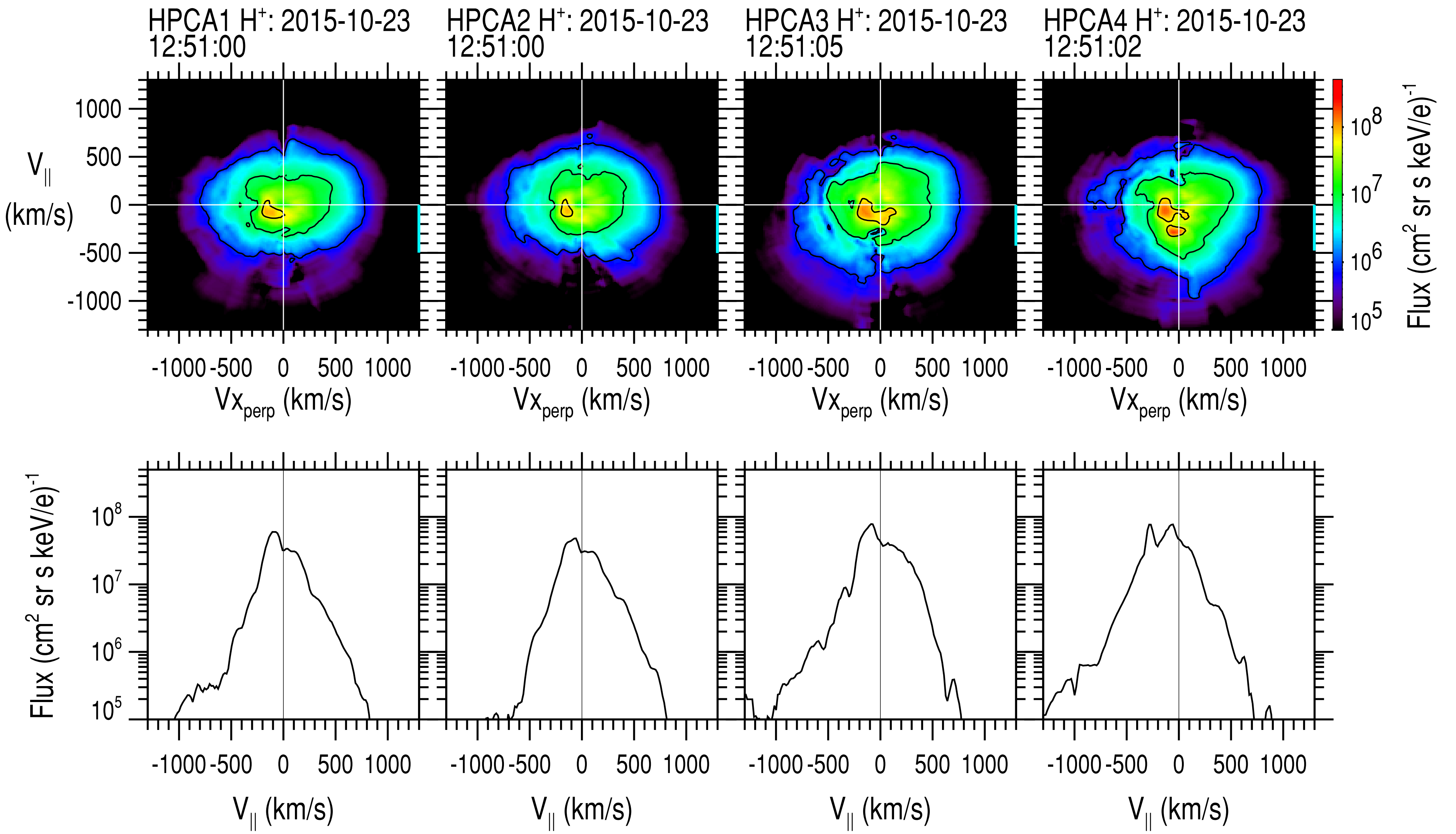

HPCA1 H+: 2015-10-23 12:51:00
HPCA2 H+: 2015-10-23 12:51:00
HPCA3 H+: 2015-10-23 12:51:05
HPCA4 H+: 2015-10-23 12:51:02
V|| (km/s)
Vx_perp (km/s)
Flux (cm2 sr s keV/e)-1

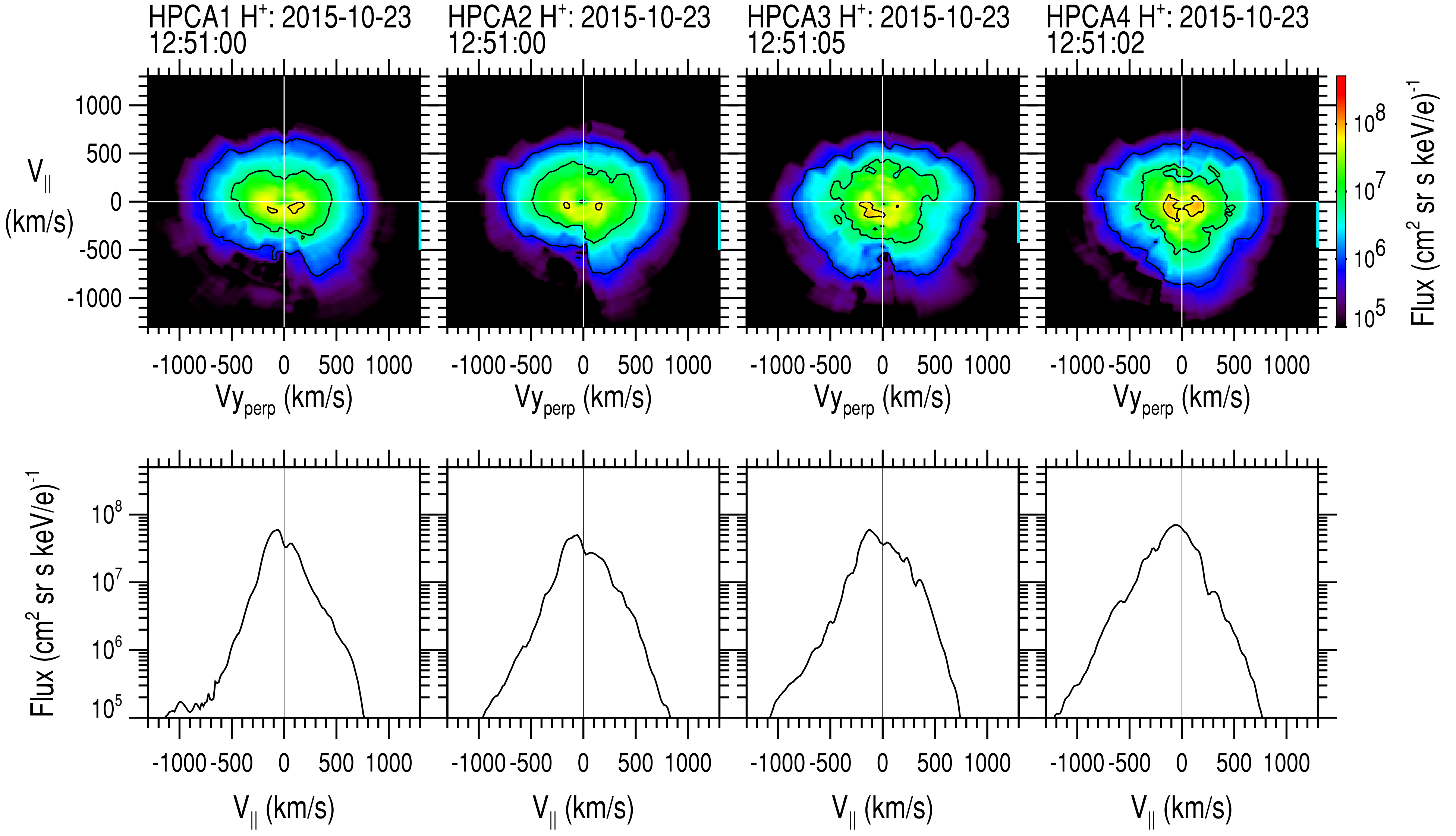
HPCA1 H+: 2015-10-23 12:51:00
HPCA2 H+: 2015-10-23 12:51:00
HPCA3 H+: 2015-10-23 12:51:05
HPCA4 H+: 2015-10-23 12:51:02
V|| (km/s)
Vyperp (km/s)
Flux (cm2 sr s keV/e)-1
V|| (km/s)

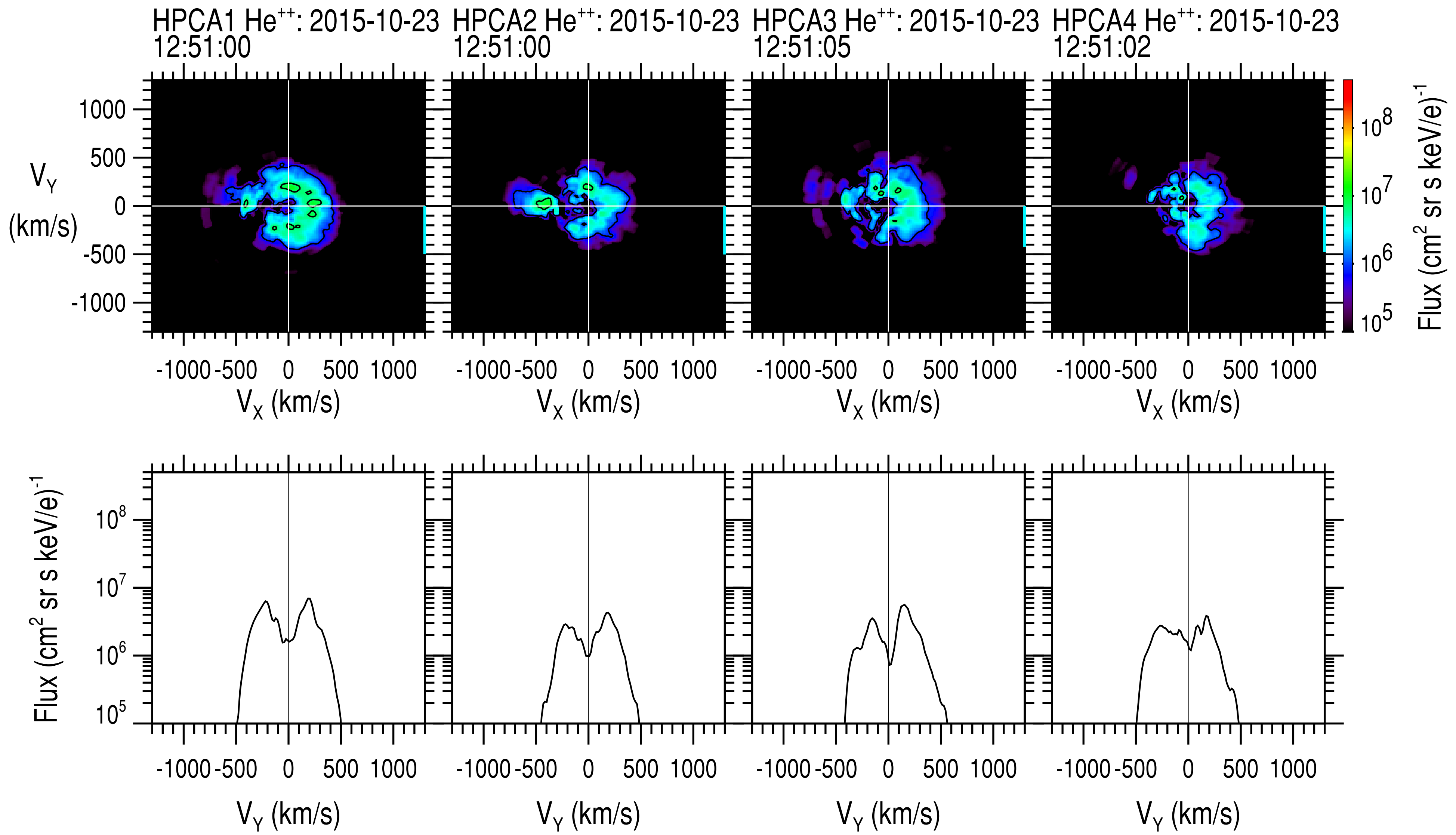

HPCA1 He++: 2015-10-23 12:51:00
HPCA2 He++: 2015-10-23 12:51:00
HPCA3 He++: 2015-10-23 12:51:05
HPCA4 He++: 2015-10-23 12:51:02
V_Y (km/s)
V_X (km/s)
Flux (cm2 sr s keV/e)-1
V_Y (km/s)

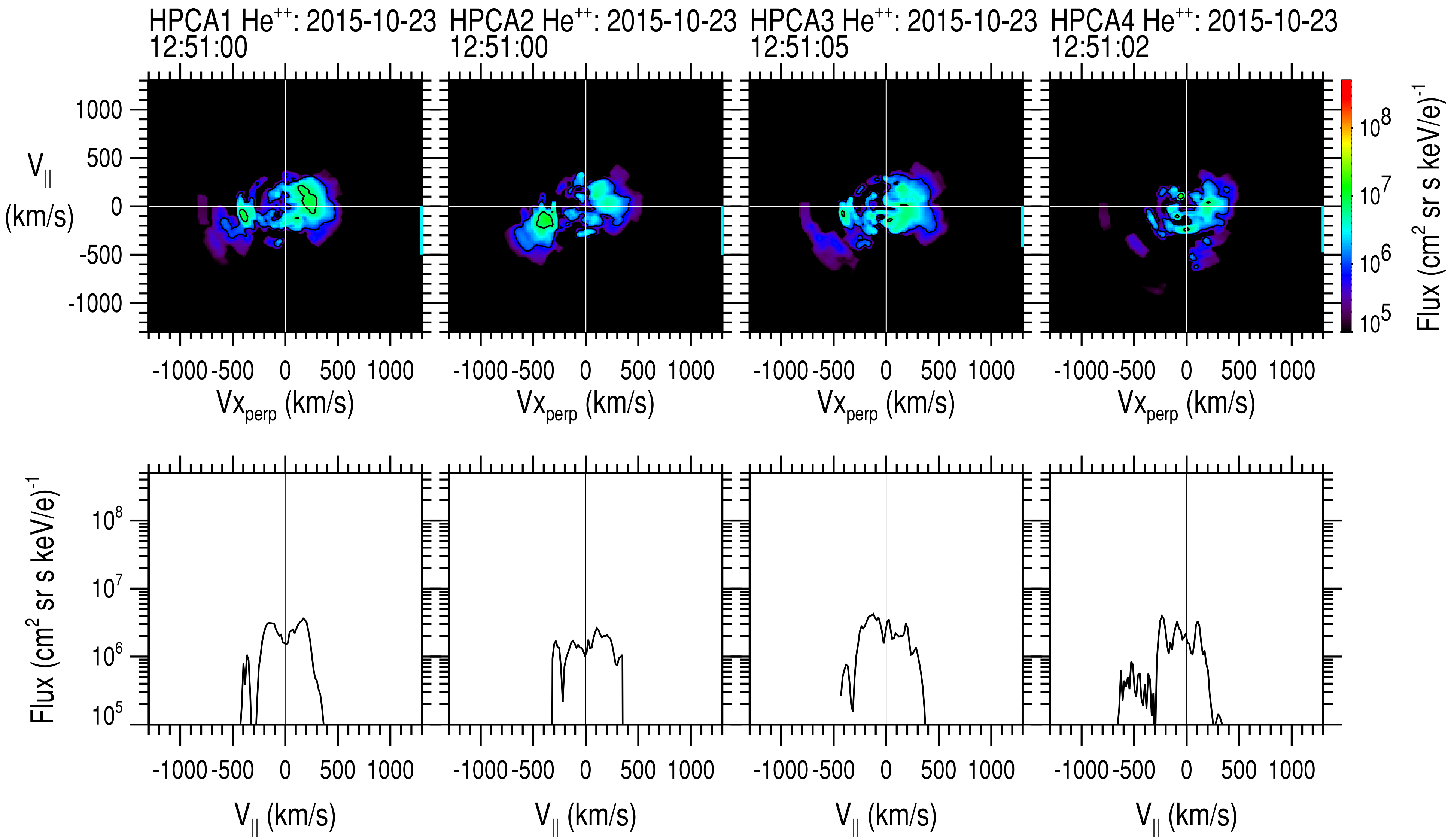
HPCA1 He++: 2015-10-23 12:51:00
HPCA2 He++: 2015-10-23 12:51:00
HPCA3 He++: 2015-10-23 12:51:05
HPCA4 He++: 2015-10-23 12:51:02
V|| (km/s)
Vxperp (km/s)
Flux (cm2 sr s keV/e)-1
V|| (km/s)

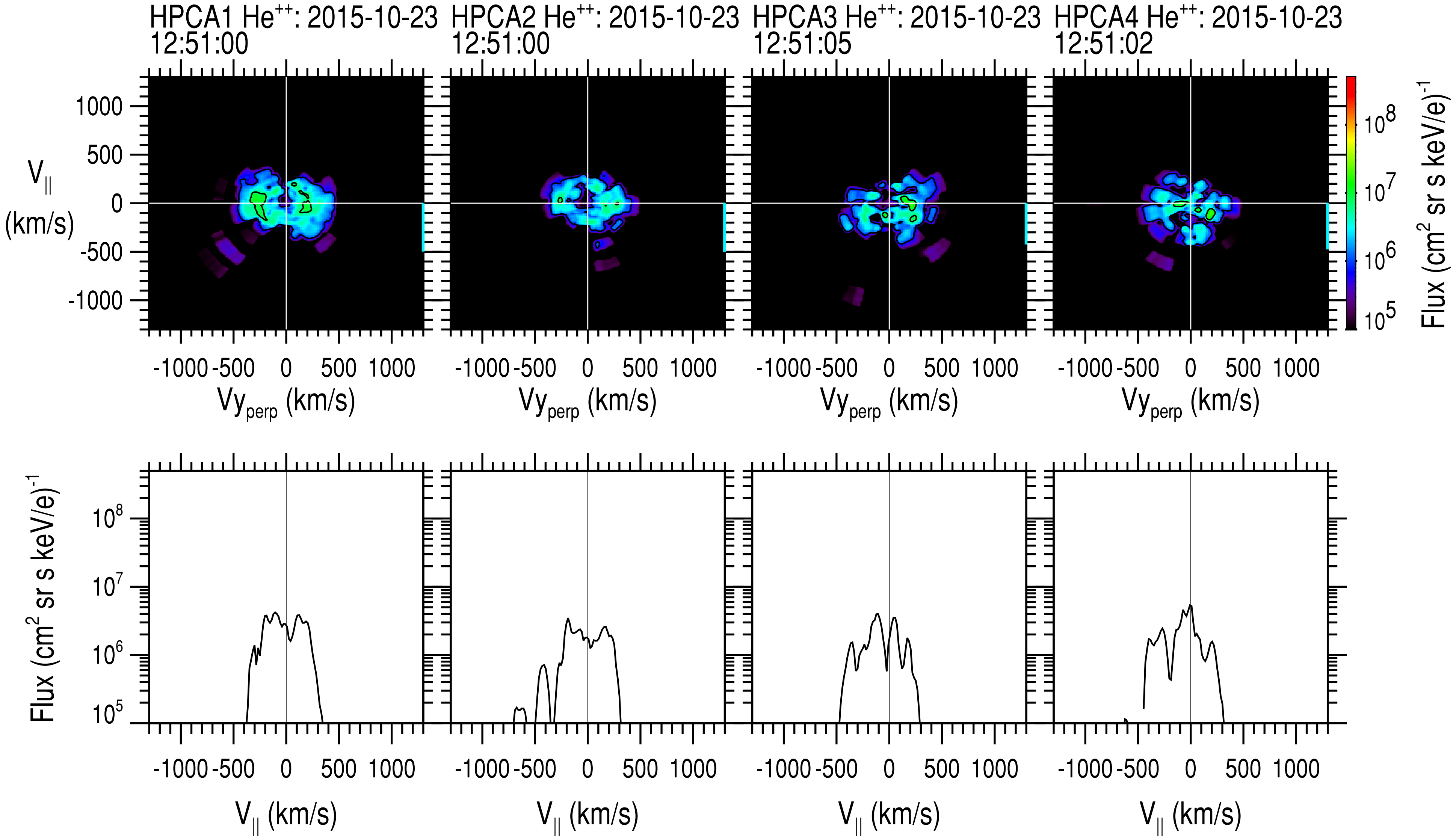
HPCA1 He++: 2015-10-23 12:51:00
HPCA2 He++: 2015-10-23 12:51:00
HPCA3 He++: 2015-10-23 12:51:05
HPCA4 He++: 2015-10-23 12:51:02
V|| (km/s)
Vyperp (km/s)
Flux (cm2 sr s keV/e)-1
1000
500
0
-500
-1000
10^5
10^6
10^7
10^8

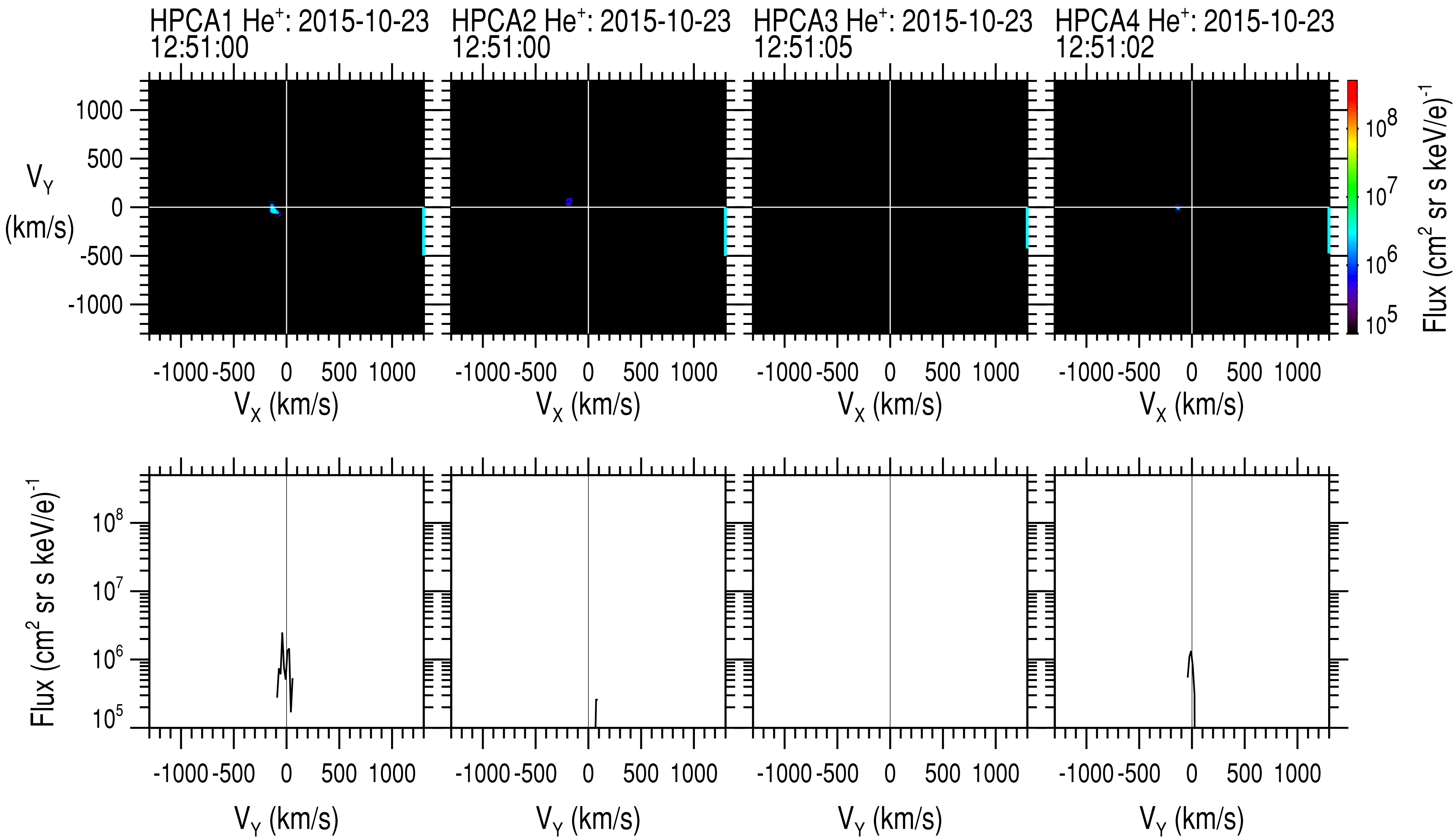
HPCA1 He+: 2015-10-23 12:51:00
HPCA2 He+: 2015-10-23 12:51:00
HPCA3 He+: 2015-10-23 12:51:05
HPCA4 He+: 2015-10-23 12:51:02
VY (km/s)
VX (km/s)
Flux (cm2 sr s keV/e)-1

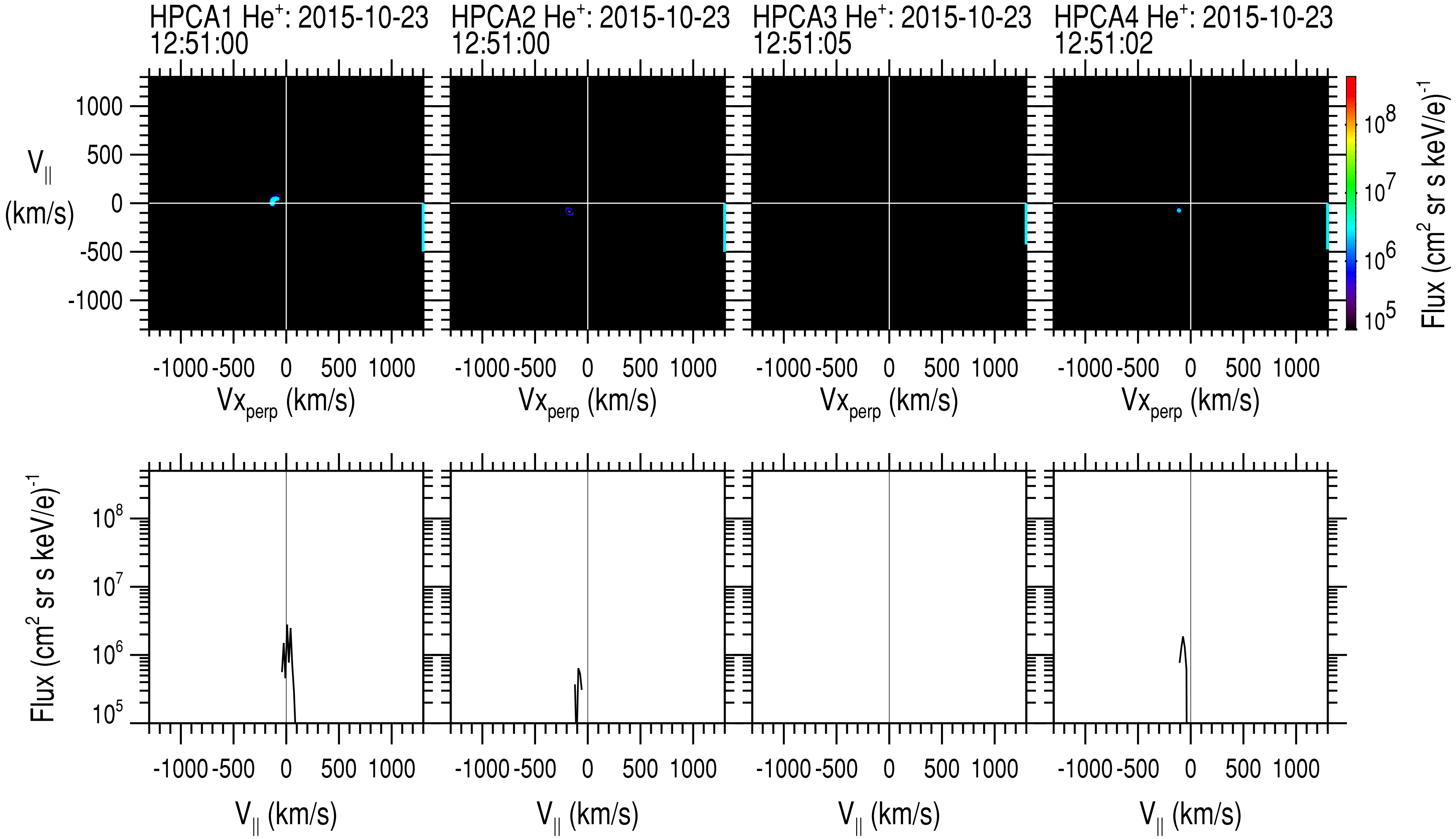
HPCA1 He⁺: 2015-10-23 12:51:00
HPCA2 He⁺: 2015-10-23 12:51:00
HPCA3 He⁺: 2015-10-23 12:51:05
HPCA4 He⁺: 2015-10-23 12:51:02
V|| (km/s)
Vx perp (km/s)
Flux (cm2 sr s keV/e)-1
V|| (km/s)

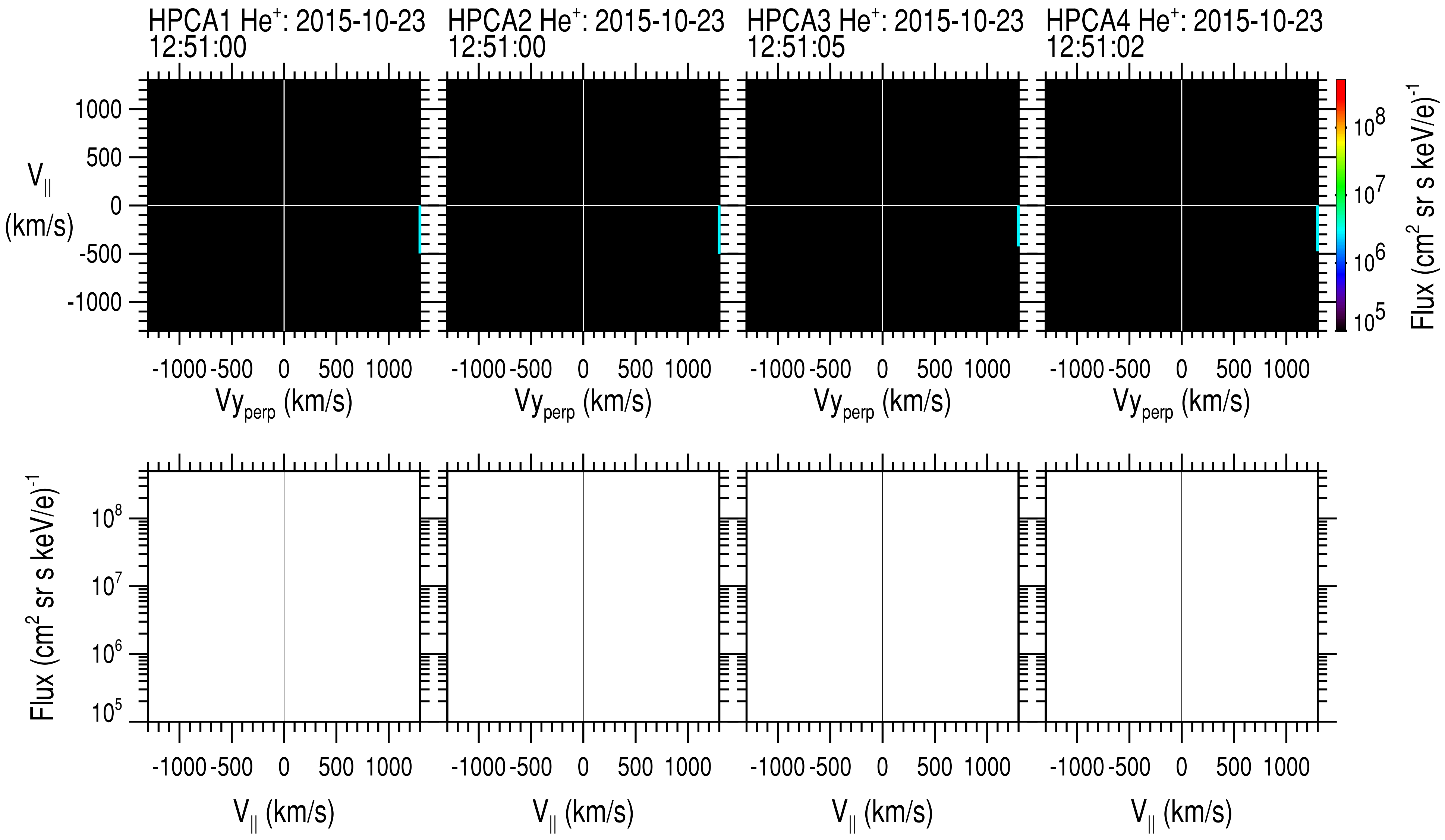
HPCA1 He+: 2015-10-23 12:51:00
HPCA2 He+: 2015-10-23 12:51:00
HPCA3 He+: 2015-10-23 12:51:05
HPCA4 He+: 2015-10-23 12:51:02
V|| (km/s)
Vyperp (km/s)
Flux (cm2 sr s keV/e)-1
V|| (km/s)
Flux (cm2 sr s keV/e)-1

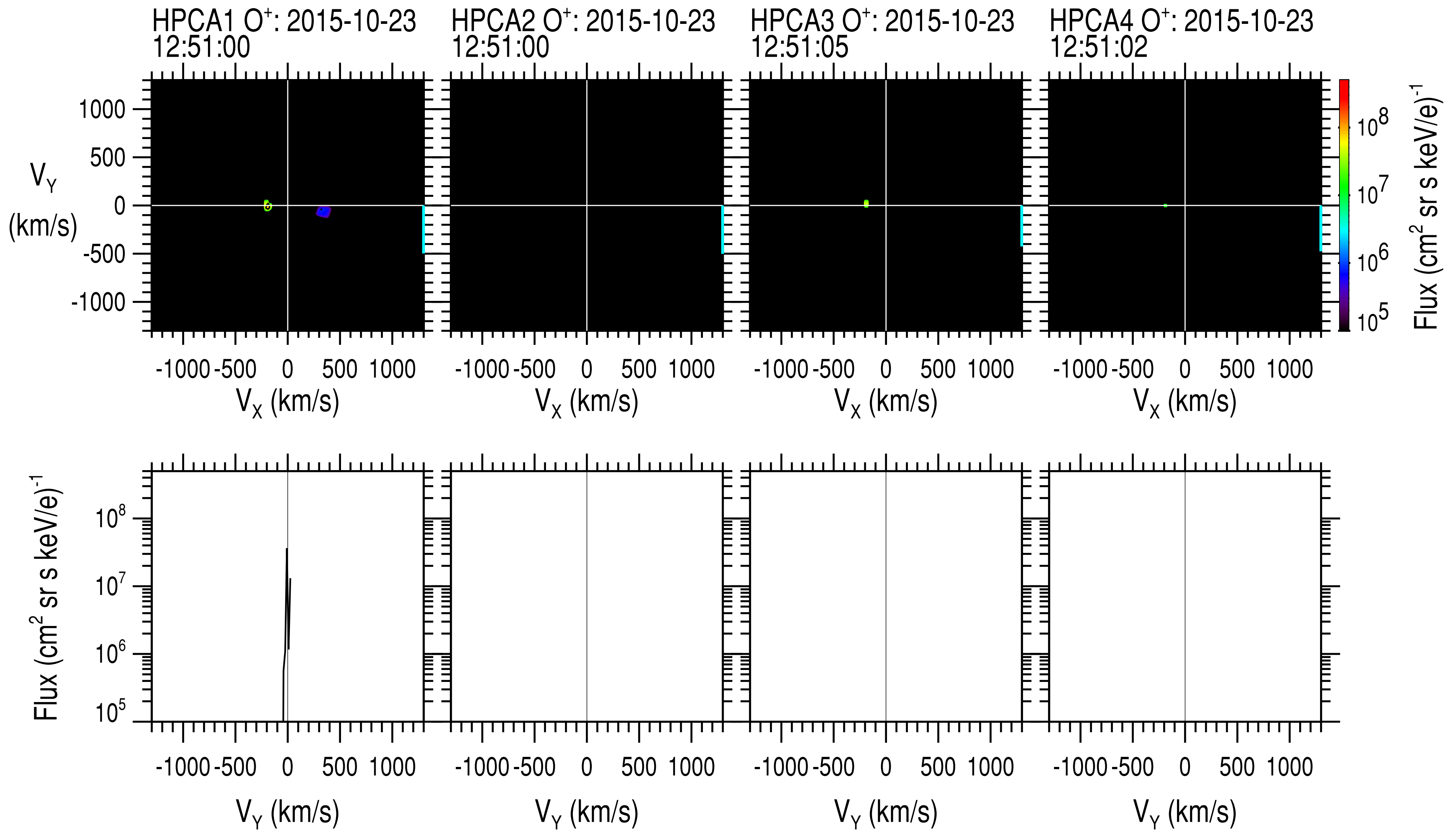
HPCA1 O+: 2015-10-23
12:51:00
HPCA2 O+: 2015-10-23
12:51:00
HPCA3 O+: 2015-10-23
12:51:05
HPCA4 O+: 2015-10-23
12:51:02
VY (km/s)
VX (km/s)
Flux (cm2 sr s keV/e)-1
1000
500
0
-500
-1000
10^5
10^6
10^7
10^8

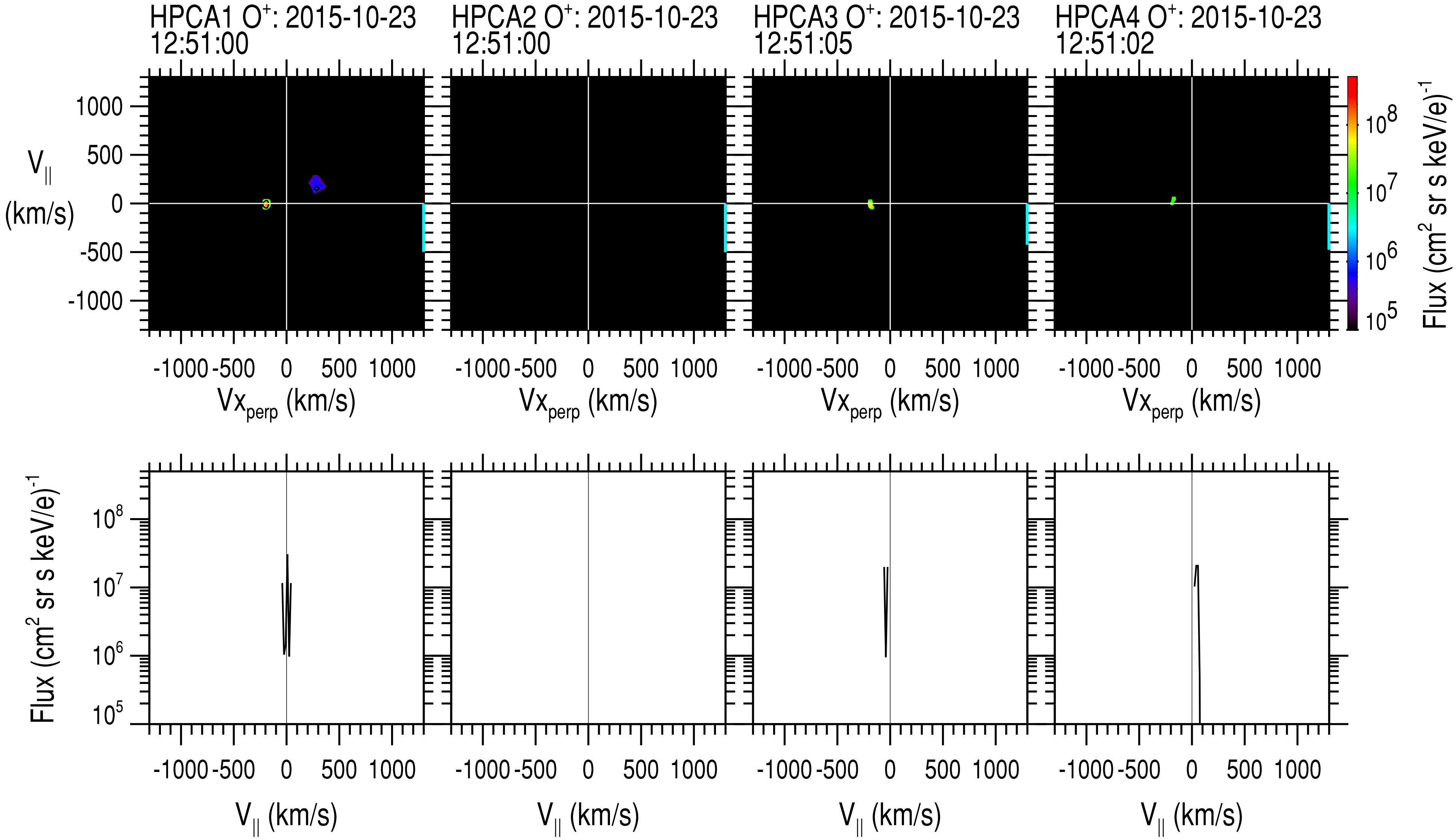
HPCA1 O+: 2015-10-23 12:51:00
HPCA2 O+: 2015-10-23 12:51:00
HPCA3 O+: 2015-10-23 12:51:05
HPCA4 O+: 2015-10-23 12:51:02
V|| (km/s)
Vxperp (km/s)
Flux (cm2 sr s keV/e)-1
V|| (km/s)

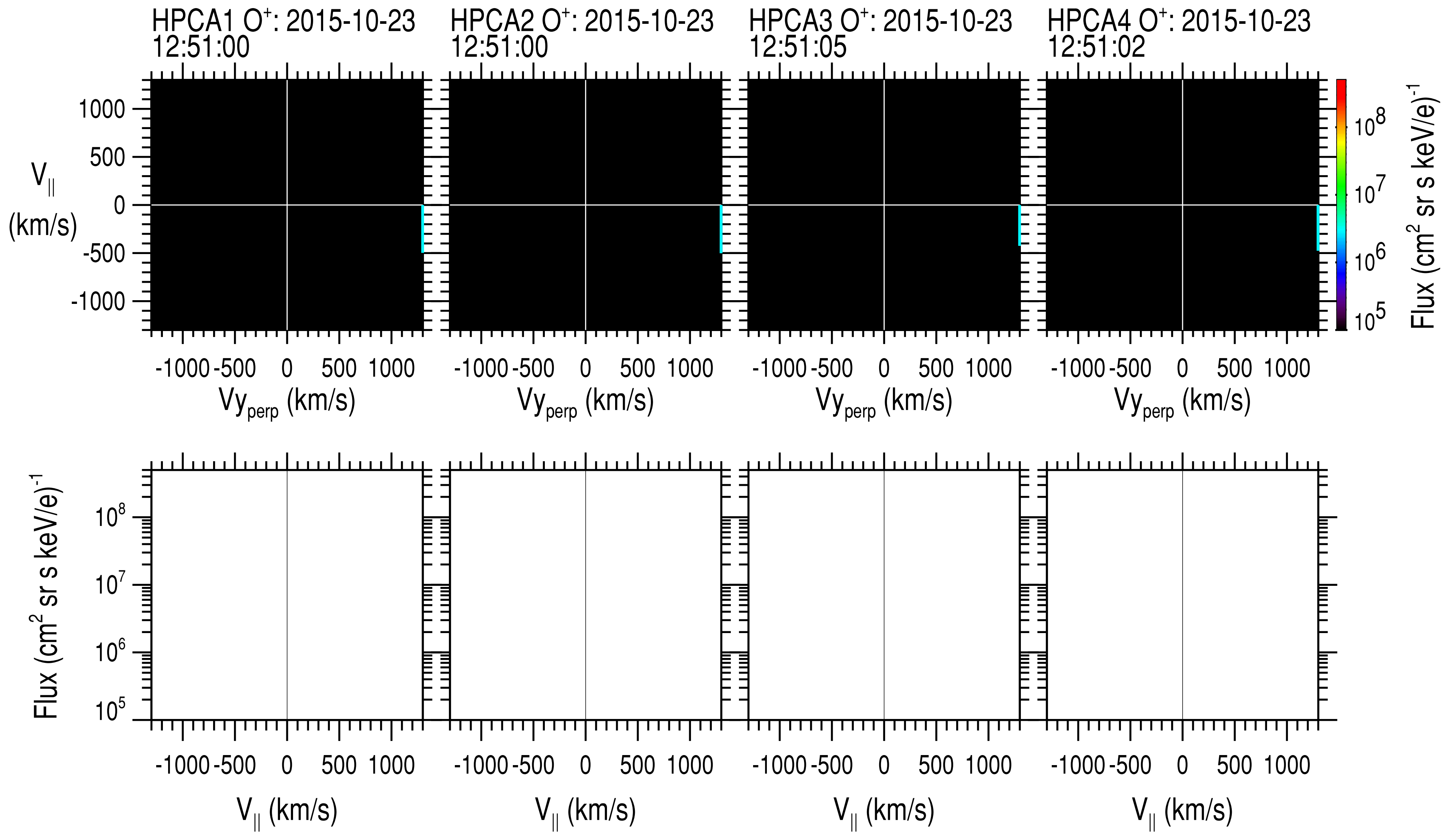
HPCA1 O+: 2015-10-23
12:51:00
HPCA2 O+: 2015-10-23
12:51:00
HPCA3 O+: 2015-10-23
12:51:05
HPCA4 O+: 2015-10-23
12:51:02
V|| (km/s)
1000
500
0
-500
-1000
-1000 -500 0 500 1000
Vyperp (km/s)
Flux (cm2 sr s keV/e)-1
10^5
10^6
10^7
10^8
V|| (km/s)

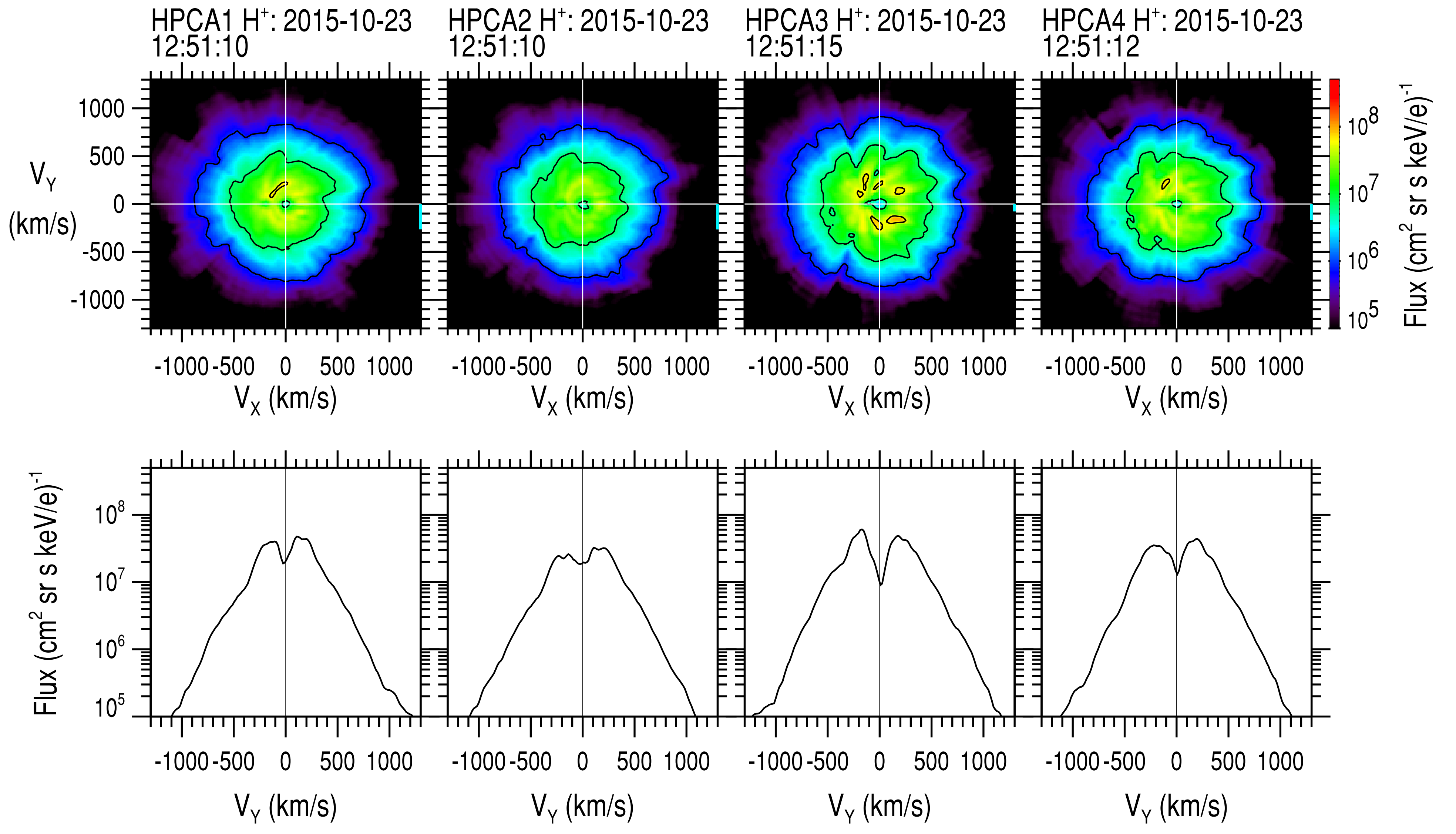
HPCA1 H+: 2015-10-23 12:51:10
HPCA2 H+: 2015-10-23 12:51:10
HPCA3 H+: 2015-10-23 12:51:15
HPCA4 H+: 2015-10-23 12:51:12
VY (km/s)
VX (km/s)
Flux (cm2 sr s keV/e)-1
-1000 -500 0 500 1000
10^5 10^6 10^7 10^8

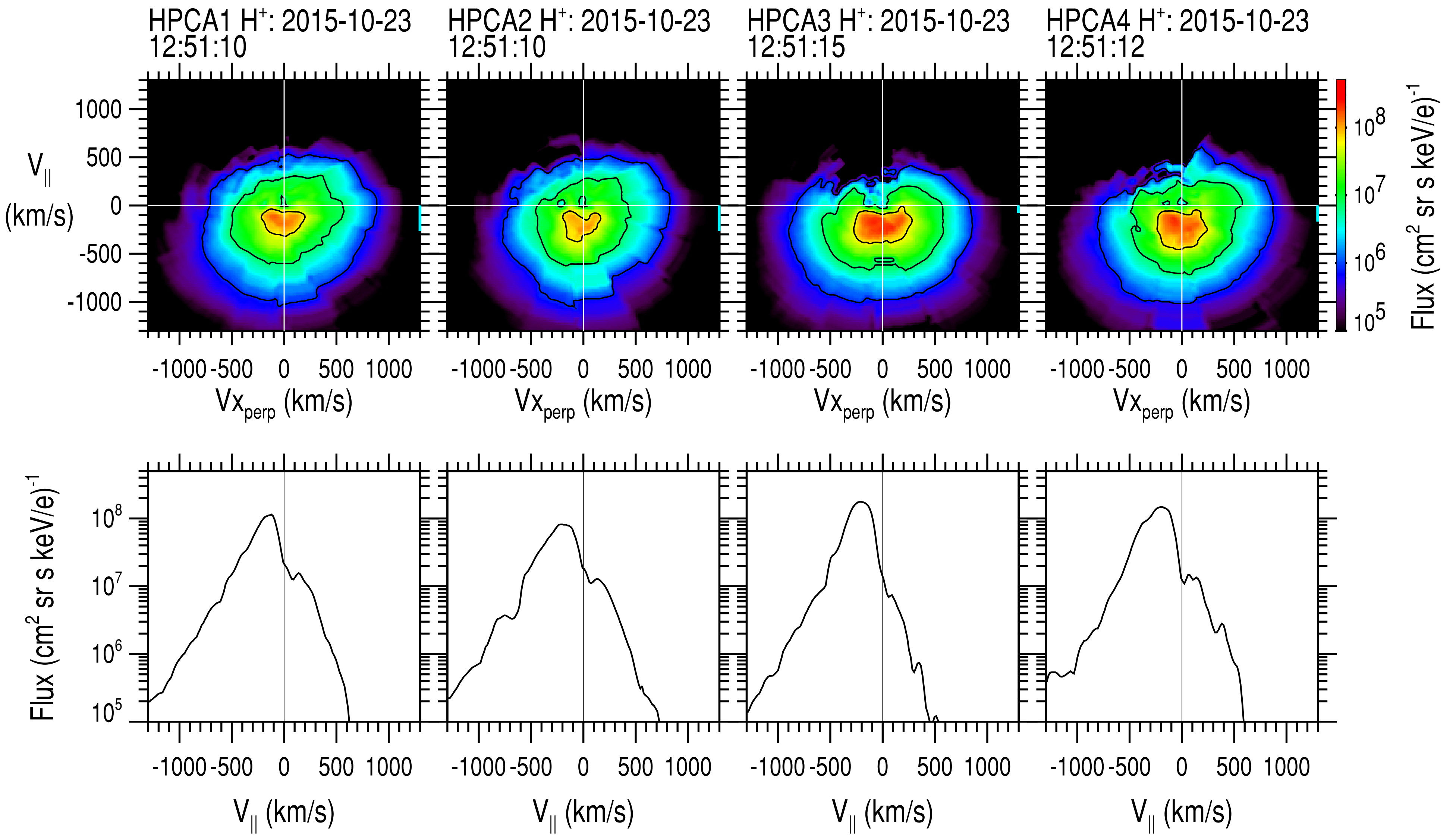

HPCA1 H+: 2015-10-23 12:51:10
HPCA2 H+: 2015-10-23 12:51:10
HPCA3 H+: 2015-10-23 12:51:15
HPCA4 H+: 2015-10-23 12:51:12
V|| (km/s)
Vxperp (km/s)
Flux (cm2 sr s keV/e)-1

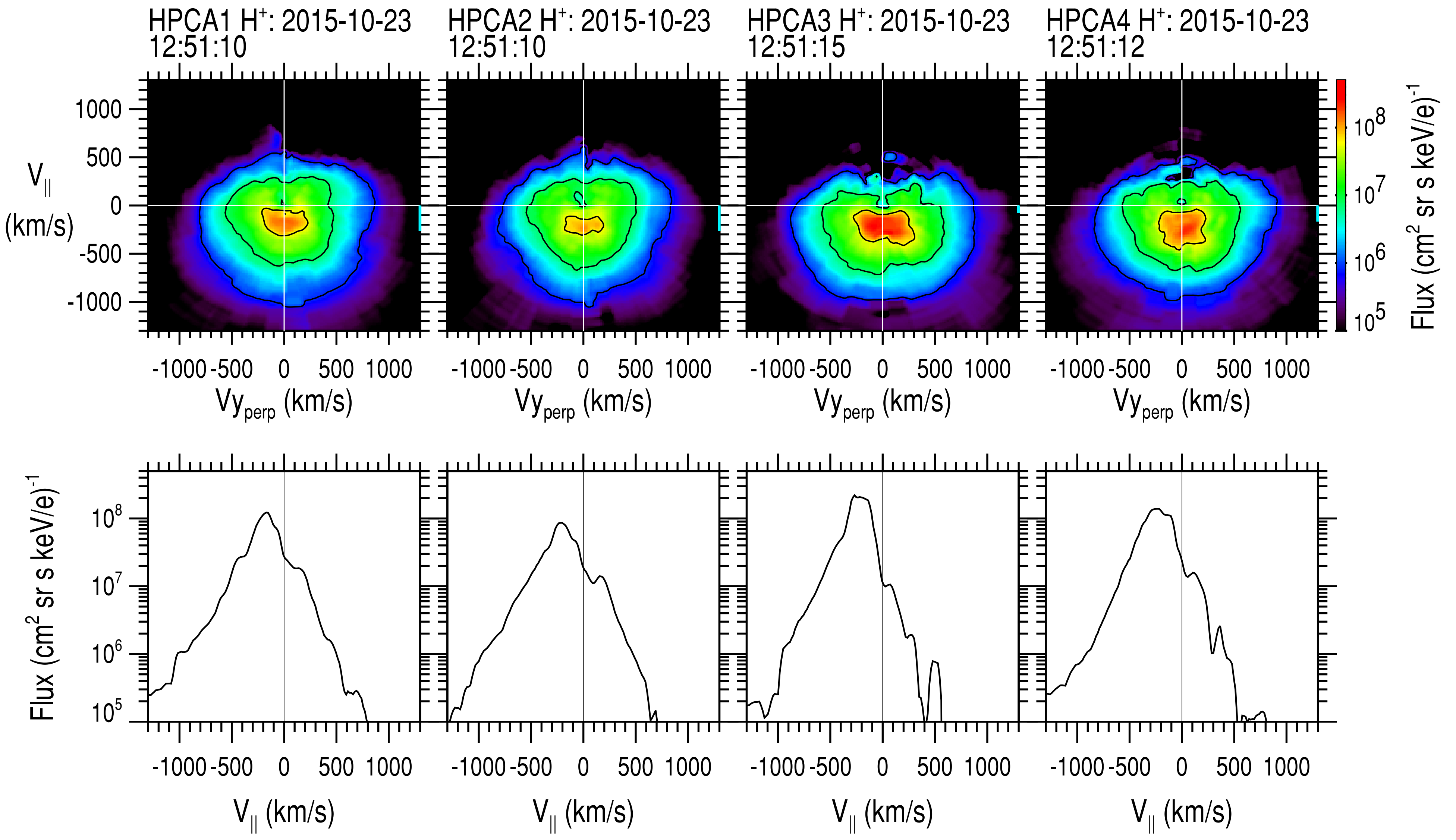
HPCA1 H+: 2015-10-23 12:51:10
HPCA2 H+: 2015-10-23 12:51:10
HPCA3 H+: 2015-10-23 12:51:15
HPCA4 H+: 2015-10-23 12:51:12
V|| (km/s)
Vyperp (km/s)
Flux (cm2 sr s keV/e)-1
V|| (km/s)

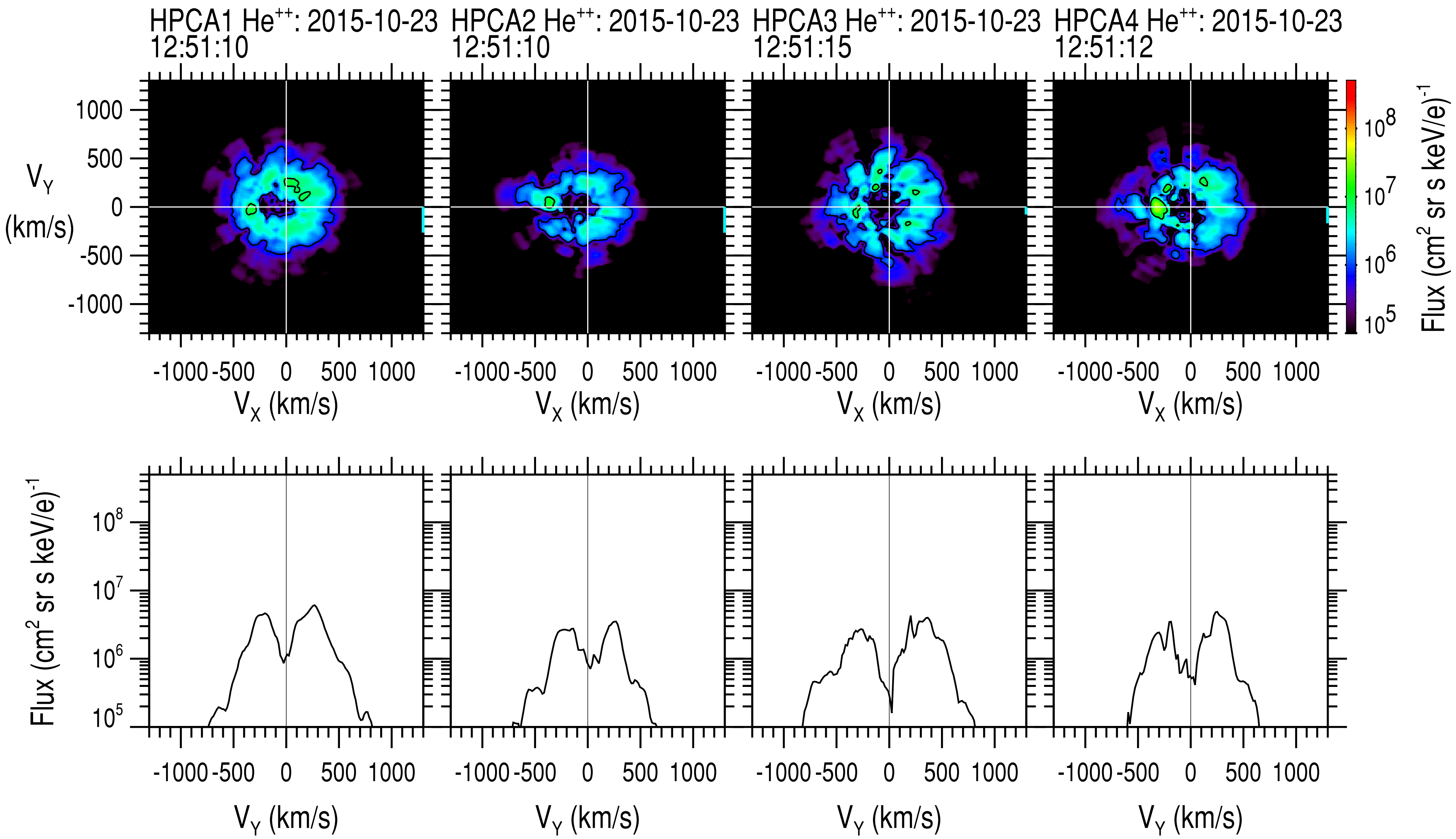
HPCA1 He++: 2015-10-23 12:51:10
HPCA2 He++: 2015-10-23 12:51:10
HPCA3 He++: 2015-10-23 12:51:15
HPCA4 He++: 2015-10-23 12:51:12
VY (km/s)
VX (km/s)
Flux (cm2 sr s keV/e)-1
VY (km/s)

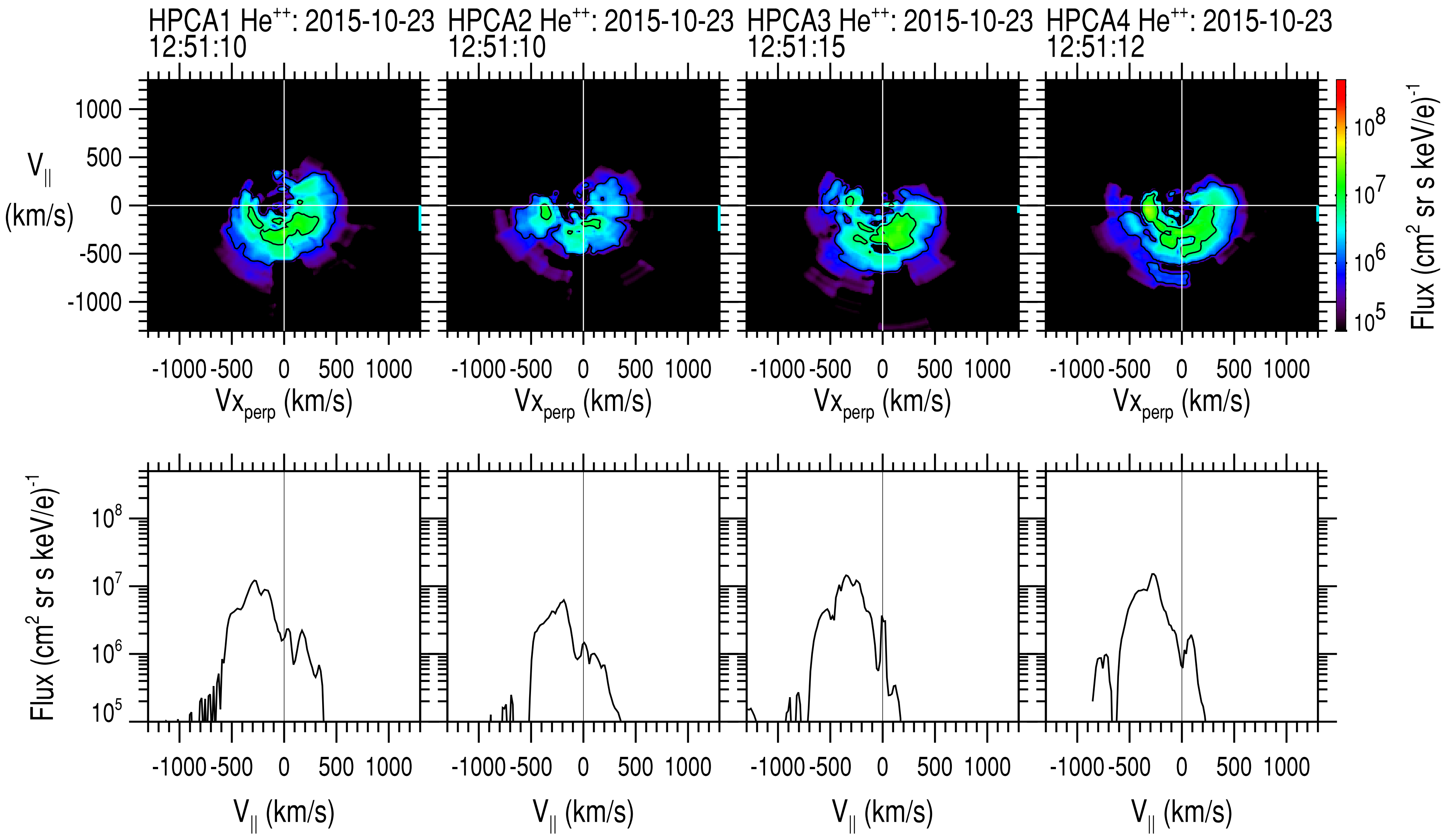
HPCA1 He++: 2015-10-23 12:51:10
HPCA2 He++: 2015-10-23 12:51:10
HPCA3 He++: 2015-10-23 12:51:15
HPCA4 He++: 2015-10-23 12:51:12
V|| (km/s)
Vxperp (km/s)
Flux (cm2 sr s keV/e)-1
V|| (km/s)

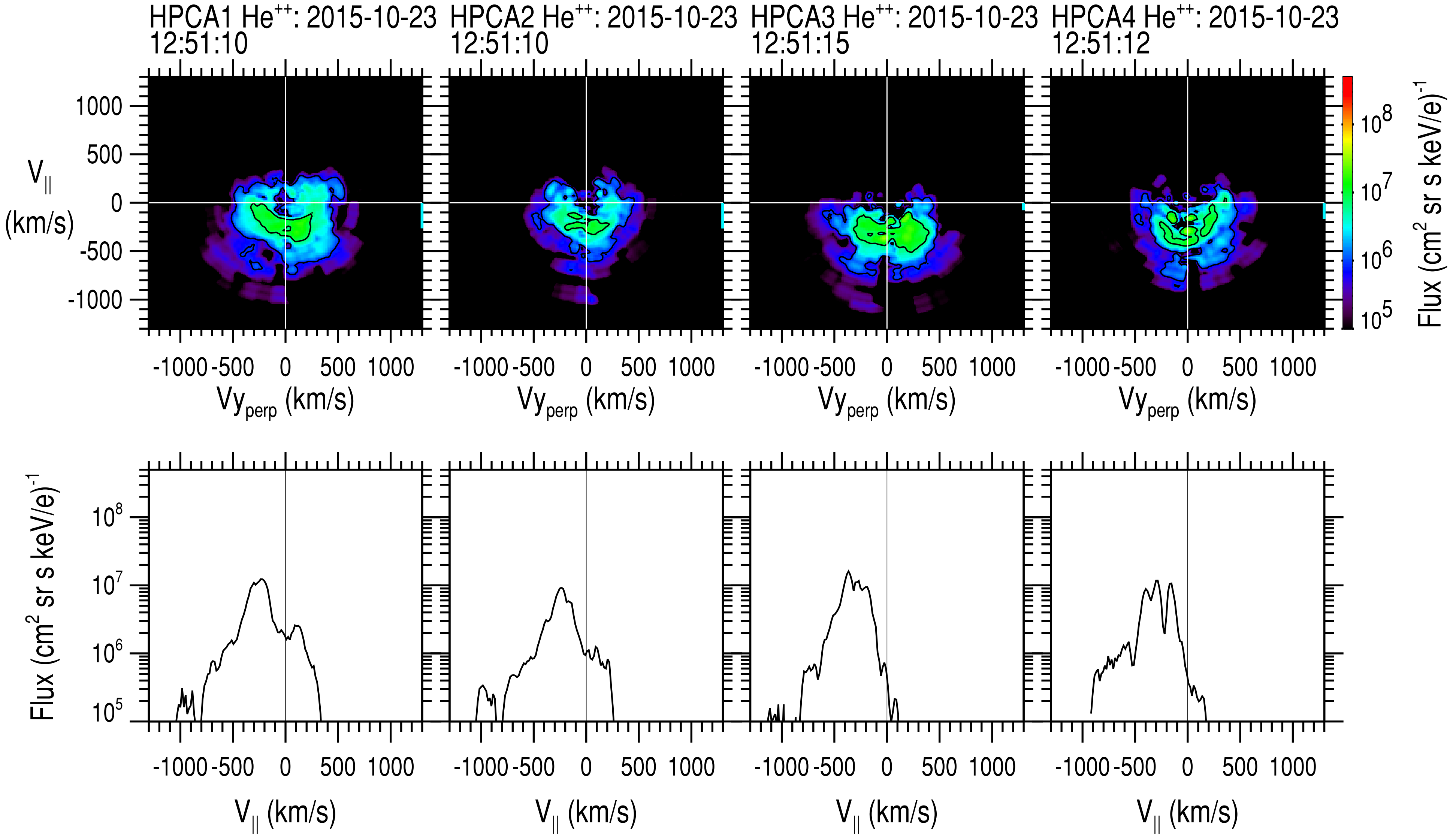

HPCA1 He++: 2015-10-23 12:51:10
HPCA2 He++: 2015-10-23 12:51:10
HPCA3 He++: 2015-10-23 12:51:15
HPCA4 He++: 2015-10-23 12:51:12
V|| (km/s)
Vyperp (km/s)
Flux (cm2 sr s keV/e)-1
1000
500
0
-500
-1000
10^5
10^6
10^7
10^8

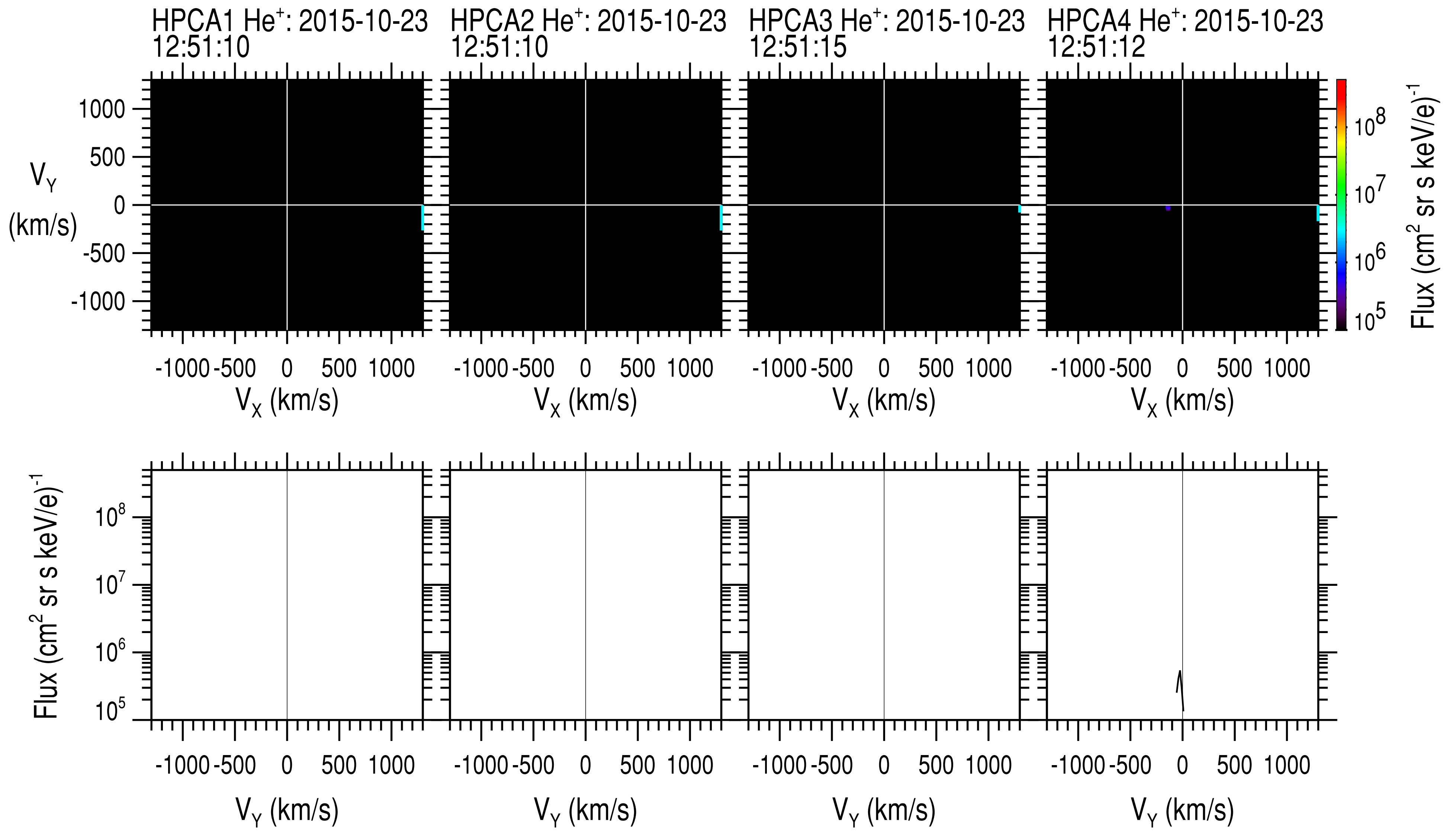

HPCA1 He+: 2015-10-23 12:51:10
HPCA2 He+: 2015-10-23 12:51:10
HPCA3 He+: 2015-10-23 12:51:15
HPCA4 He+: 2015-10-23 12:51:12
VY (km/s)
VX (km/s)
Flux (cm2 sr s keV/e)-1
-1000 -500 0 500 1000
10^5 10^6 10^7 10^8

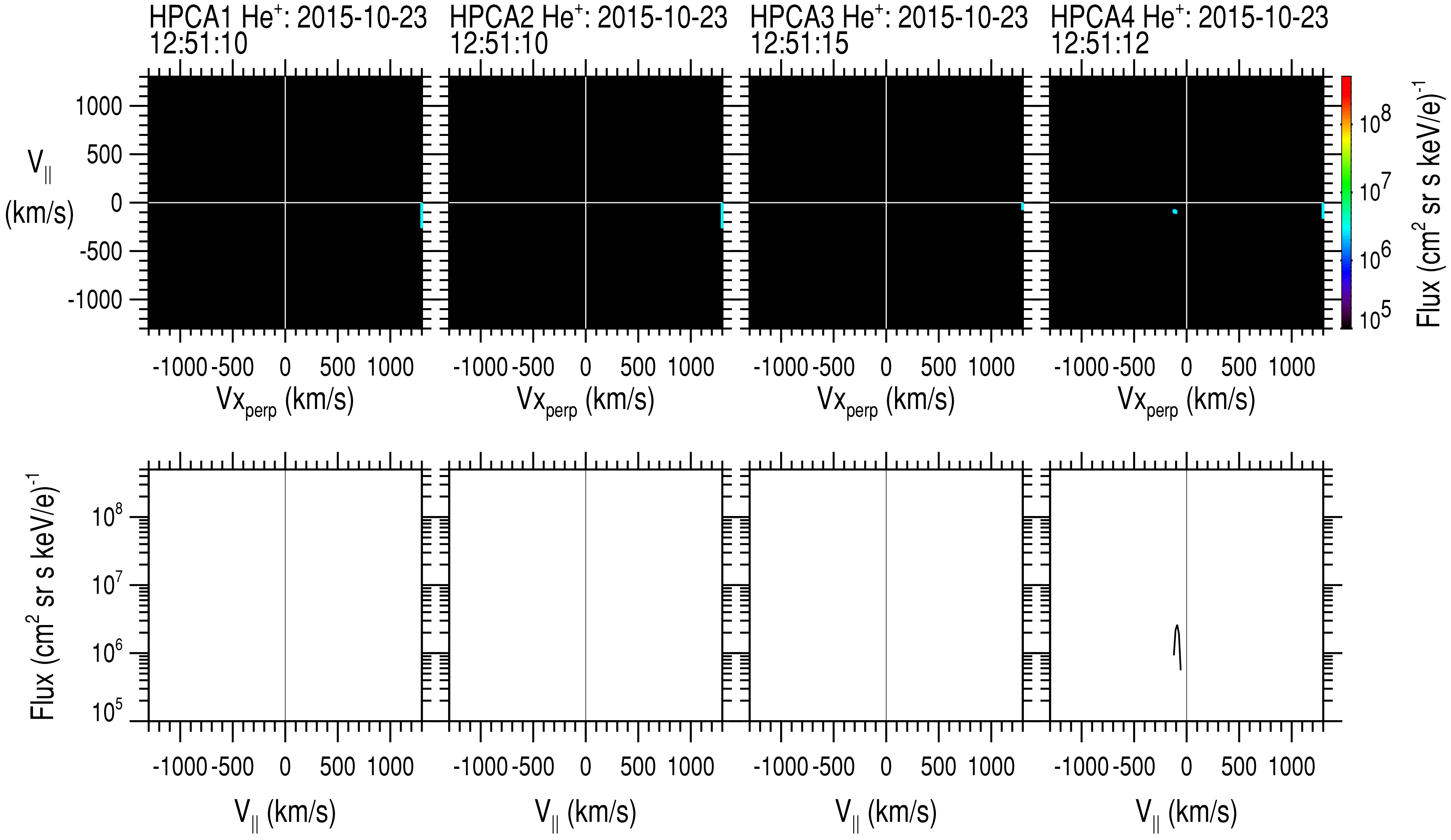
HPCA1 He+: 2015-10-23 12:51:10
HPCA2 He+: 2015-10-23 12:51:10
HPCA3 He+: 2015-10-23 12:51:15
HPCA4 He+: 2015-10-23 12:51:12
V|| (km/s)
Vxperp (km/s)
Flux (cm2 sr s keV/e)-1
V|| (km/s)

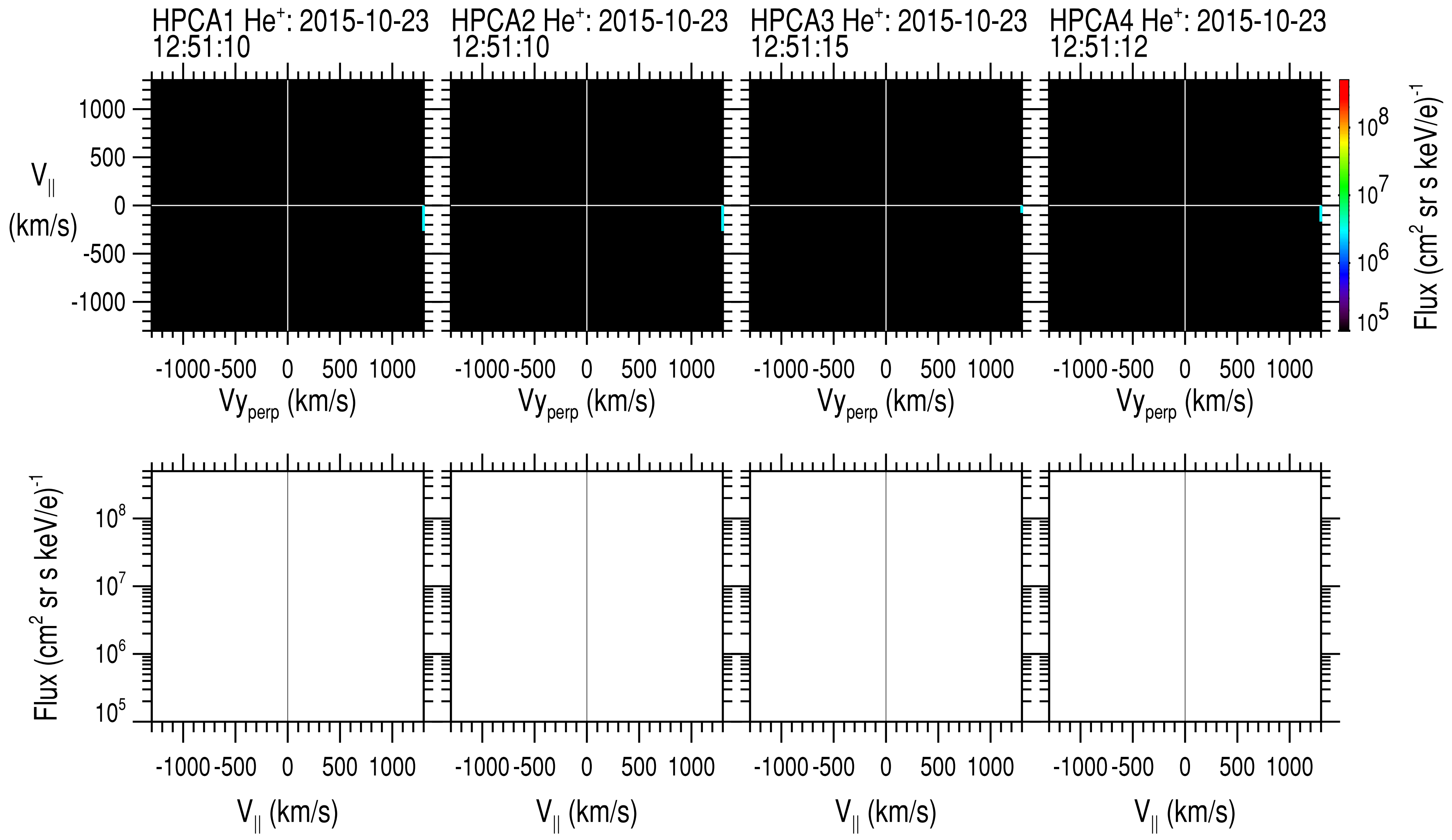
HPCA1 He+: 2015-10-23 12:51:10
HPCA2 He+: 2015-10-23 12:51:10
HPCA3 He+: 2015-10-23 12:51:15
HPCA4 He+: 2015-10-23 12:51:12
V|| (km/s)
1000
500
0
-500
-1000
Vyperp (km/s)
Flux (cm2 sr s keV/e)-1
10^5
10^6
10^7
10^8

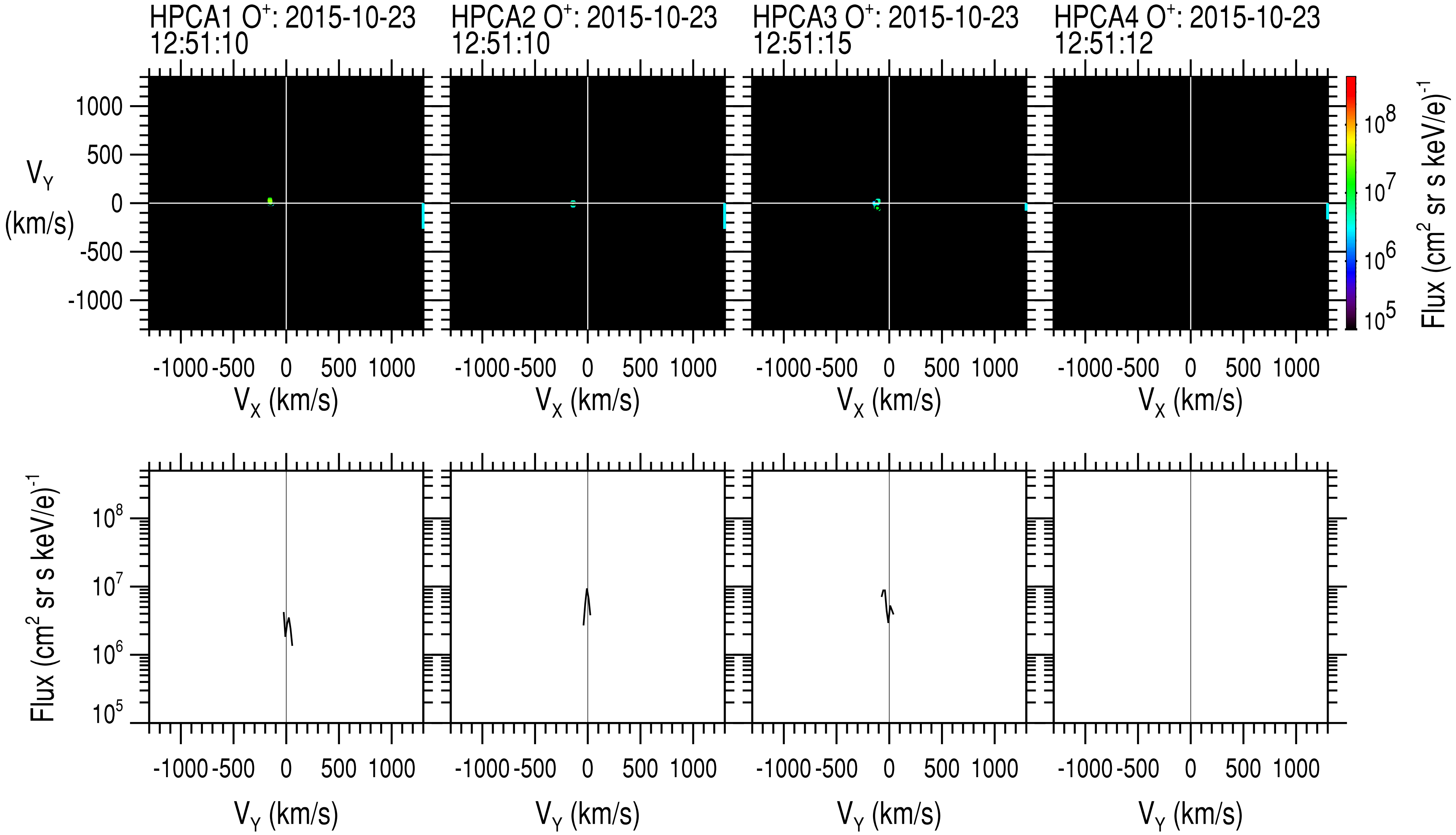

HPCA1 O+: 2015-10-23
12:51:10
HPCA2 O+: 2015-10-23
12:51:10
HPCA3 O+: 2015-10-23
12:51:15
HPCA4 O+: 2015-10-23
12:51:12
V_Y (km/s)
1000
500
0
-500
-1000
-1000 -500 0 500 1000
V_X (km/s)
Flux (cm^2 sr s keV/e)^-1
10^5
10^6
10^7
10^8
V_Y (km/s)

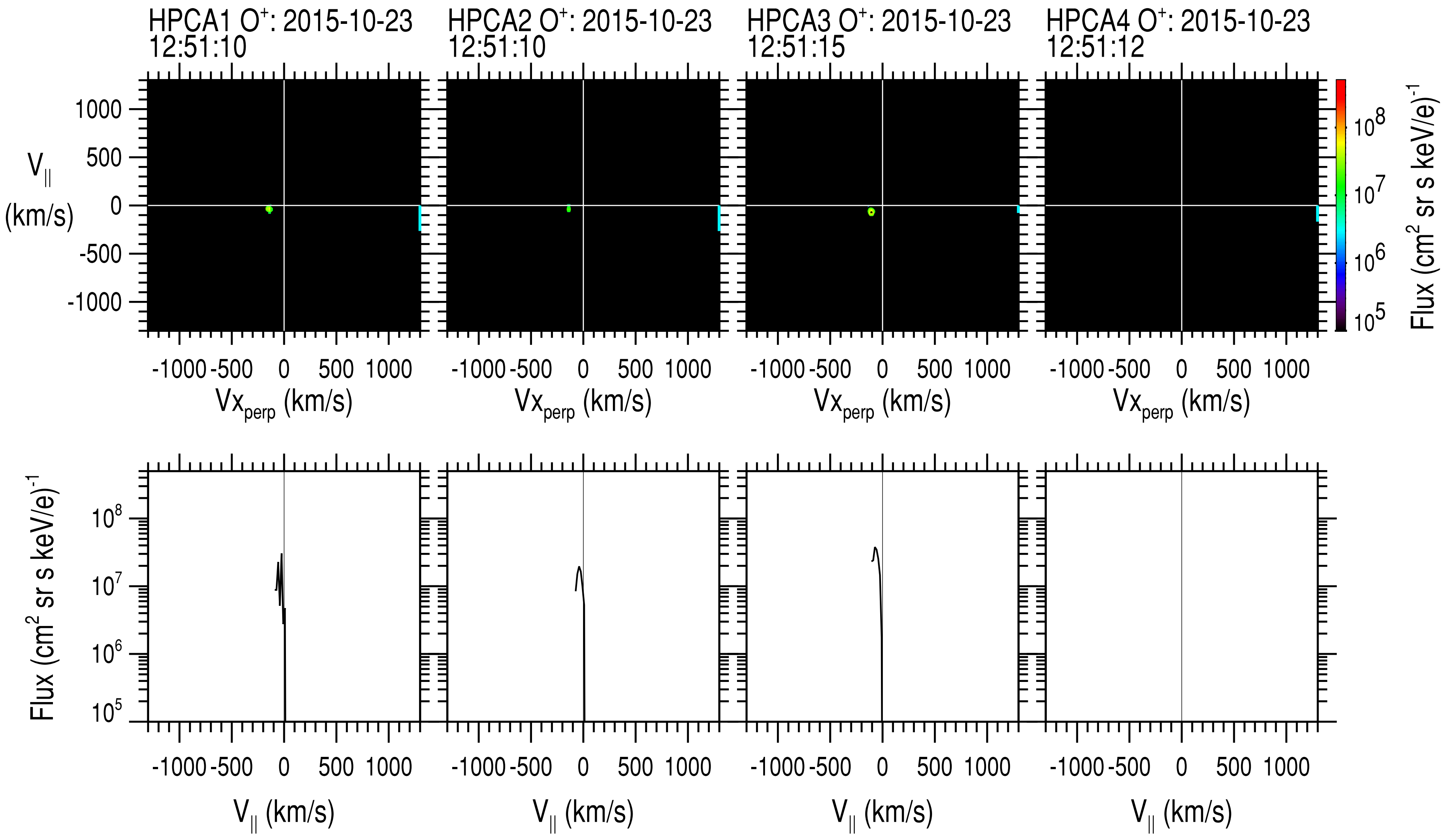
HPCA1 O+: 2015-10-23 12:51:10
HPCA2 O+: 2015-10-23 12:51:10
HPCA3 O+: 2015-10-23 12:51:15
HPCA4 O+: 2015-10-23 12:51:12
V|| (km/s)
1000
500
0
-500
-1000
Vxperp (km/s)
Flux (cm2 sr s keV/e)-1
10^5
10^6
10^7
10^8
V|| (km/s)

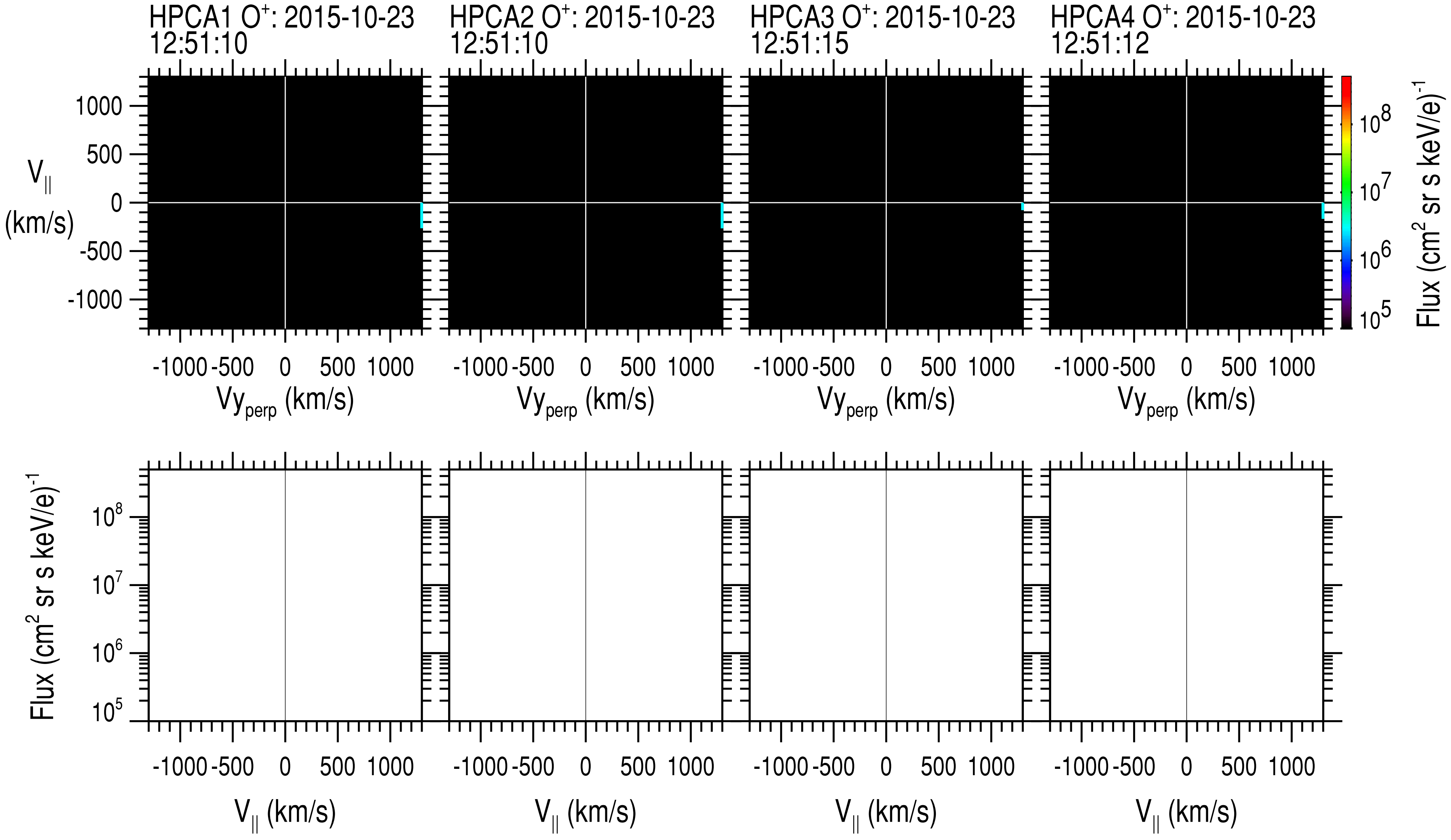
HPCA1 O+: 2015-10-23 12:51:10
HPCA2 O+: 2015-10-23 12:51:10
HPCA3 O+: 2015-10-23 12:51:15
HPCA4 O+: 2015-10-23 12:51:12
V|| (km/s)
Vyperp (km/s)
Flux (cm2 sr s keV/e)-1
-1000 -500 0 500 1000
10^5 10^6 10^7 10^8

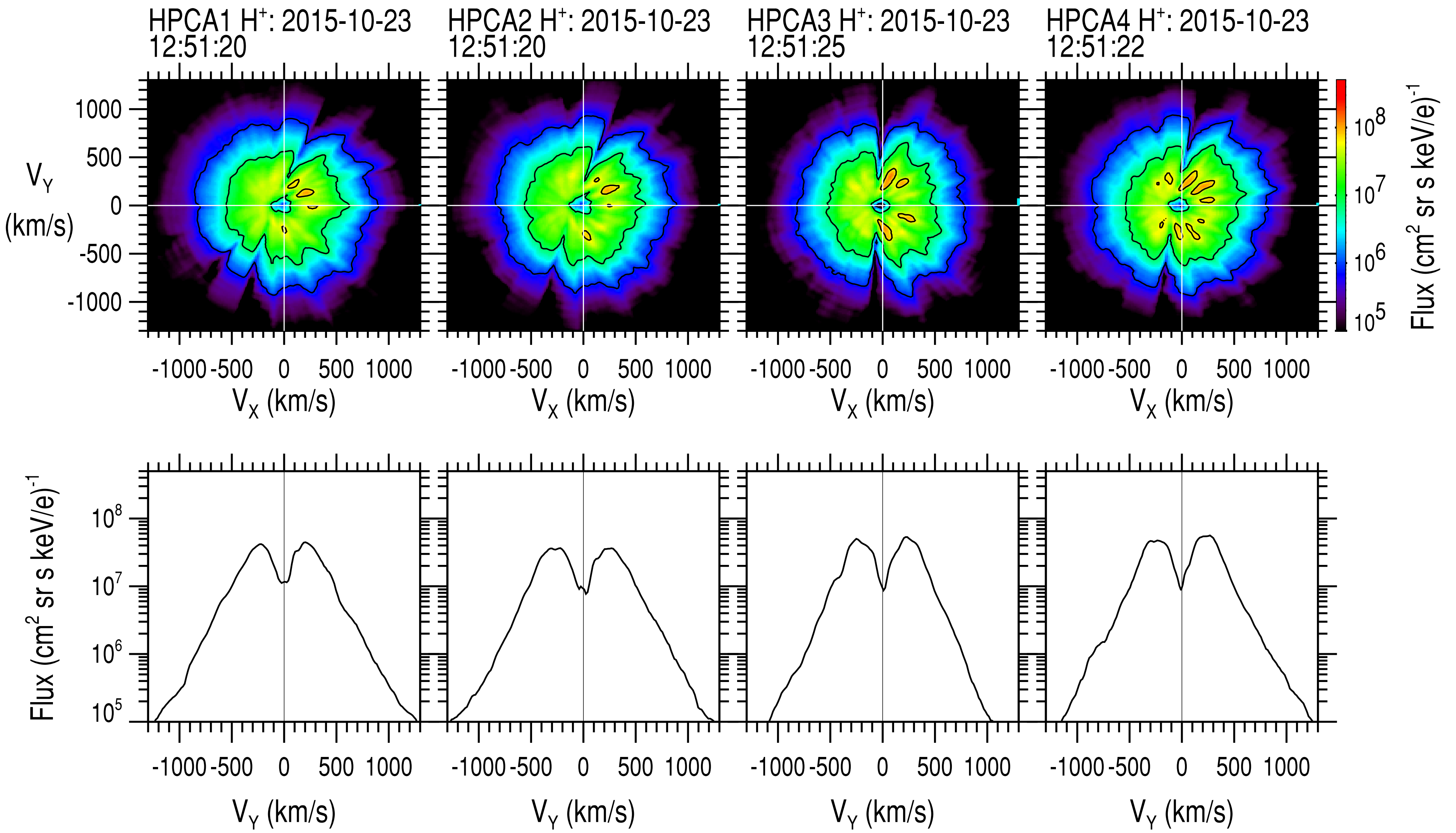

HPCA1 H+: 2015-10-23 12:51:20
HPCA2 H+: 2015-10-23 12:51:20
HPCA3 H+: 2015-10-23 12:51:25
HPCA4 H+: 2015-10-23 12:51:22
V_Y (km/s)
V_X (km/s)
Flux (cm^2 sr s keV/e)^-1
-1000 -500 0 500 1000
10^5 10^6 10^7 10^8

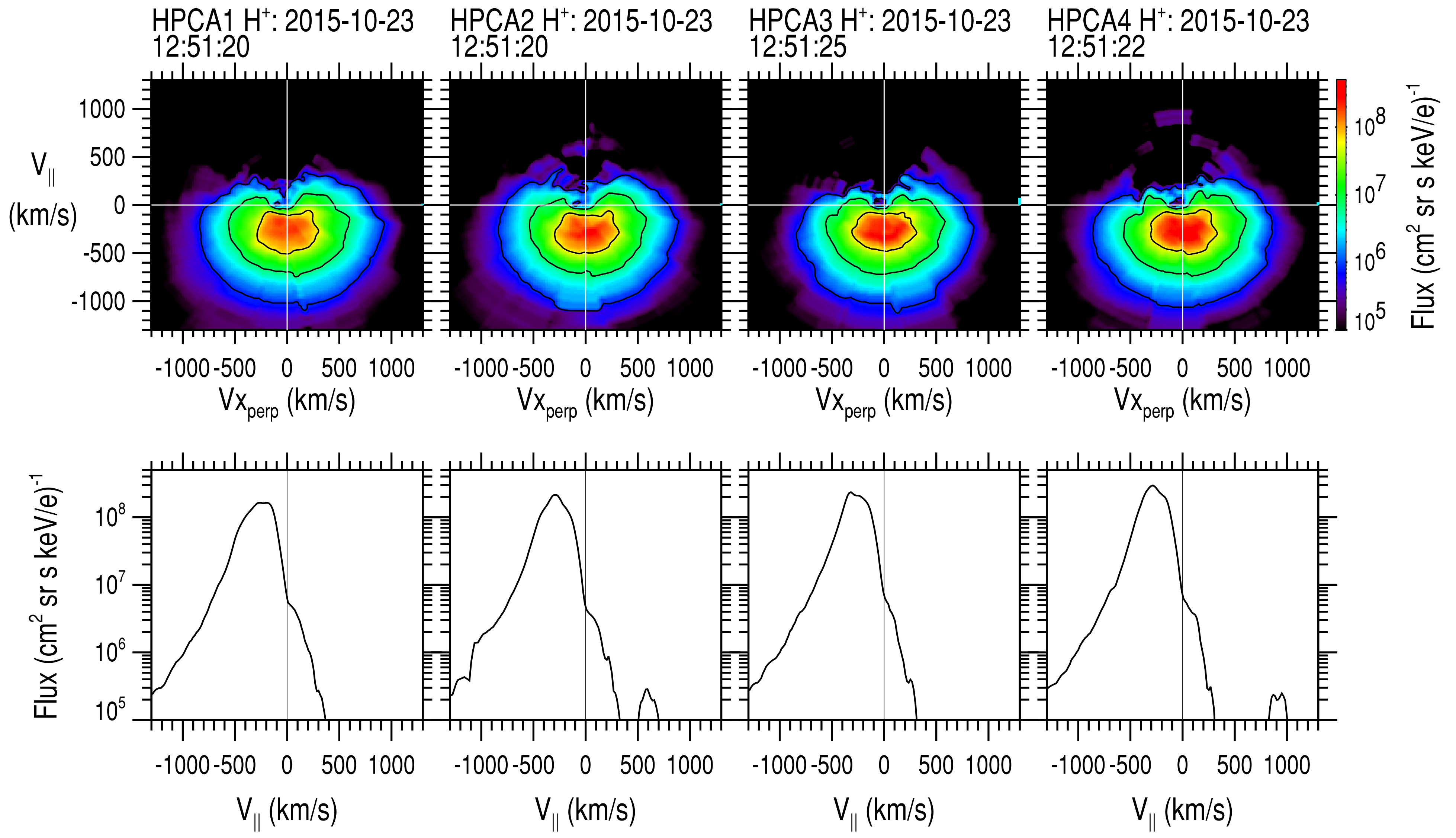
HPCA1 H+: 2015-10-23
12:51:20
HPCA2 H+: 2015-10-23
12:51:20
HPCA3 H+: 2015-10-23
12:51:25
HPCA4 H+: 2015-10-23
12:51:22
V|| (km/s)
1000
500
0
-500
-1000
-1000 -500 0 500 1000
Vxperp (km/s)
Flux (cm2 sr s keV/e)-1
10^5
10^6
10^7
10^8
V|| (km/s)

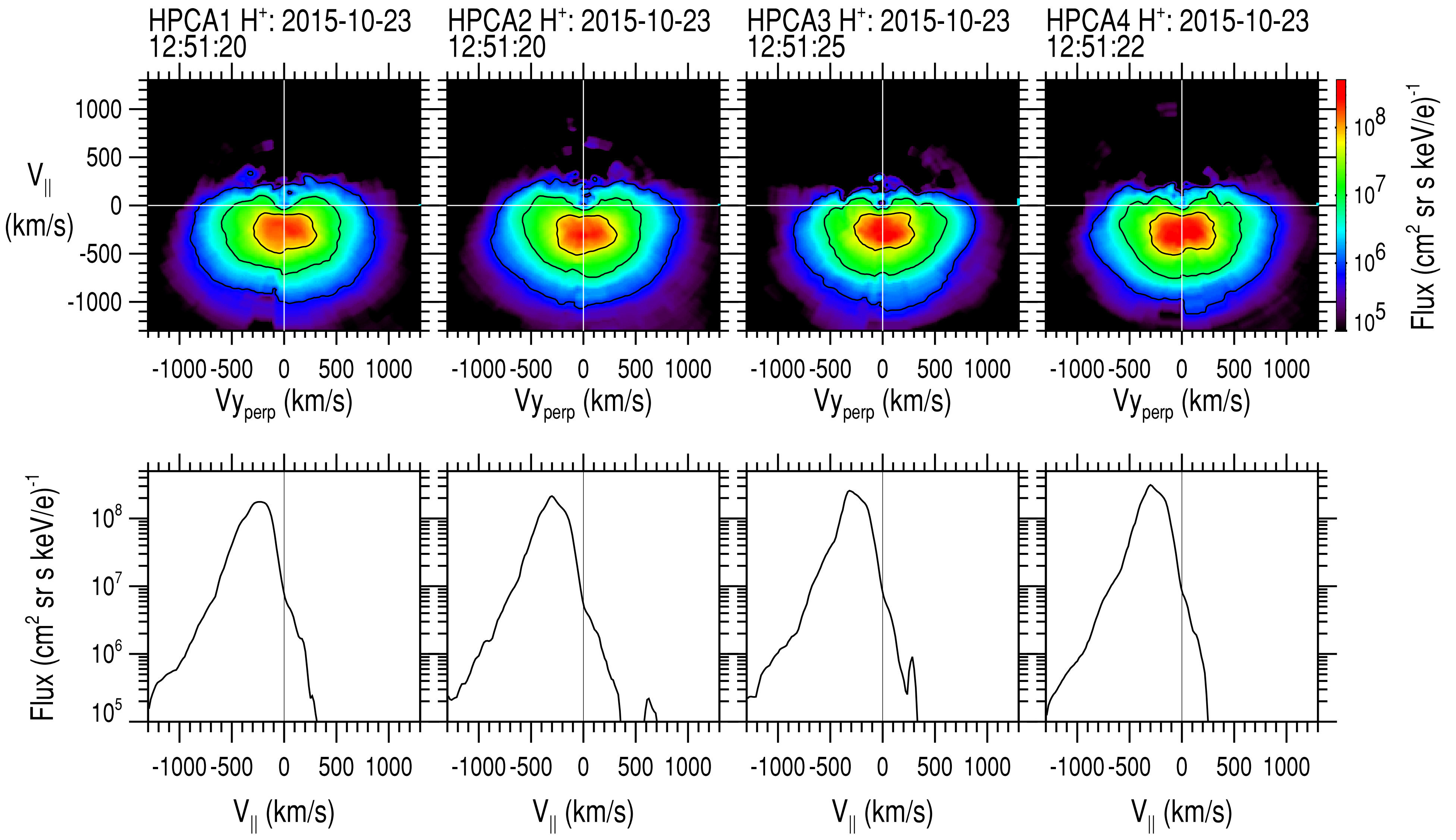
HPCA1 H+: 2015-10-23 12:51:20
HPCA2 H+: 2015-10-23 12:51:20
HPCA3 H+: 2015-10-23 12:51:25
HPCA4 H+: 2015-10-23 12:51:22
V|| (km/s)
Vyperp (km/s)
Flux (cm2 sr s keV/e)-1

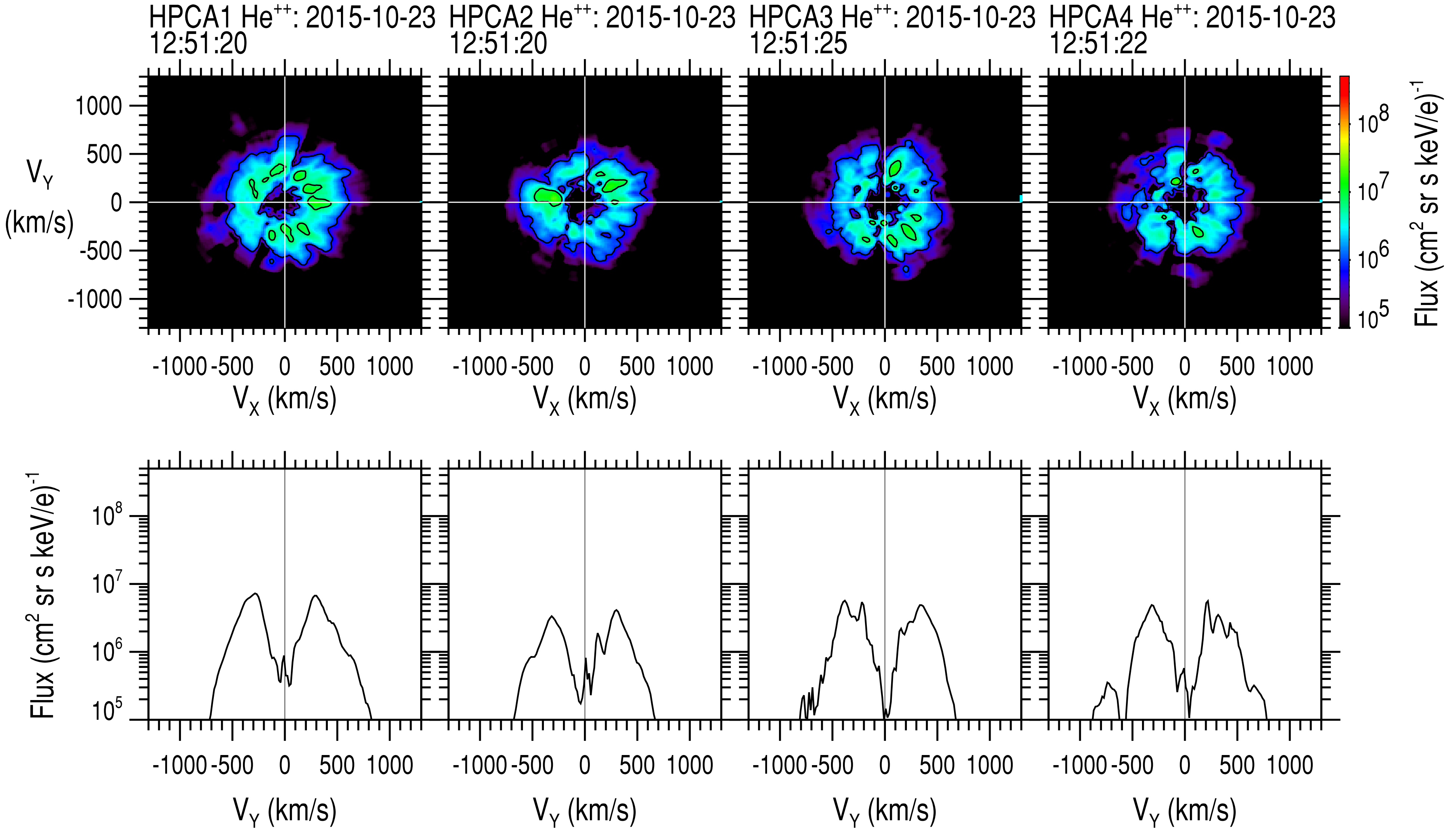
HPCA1 He++: 2015-10-23 12:51:20
HPCA2 He++: 2015-10-23 12:51:20
HPCA3 He++: 2015-10-23 12:51:25
HPCA4 He++: 2015-10-23 12:51:22
VY (km/s)
VX (km/s)
Flux (cm2 sr s keV/e)-1
VY (km/s)

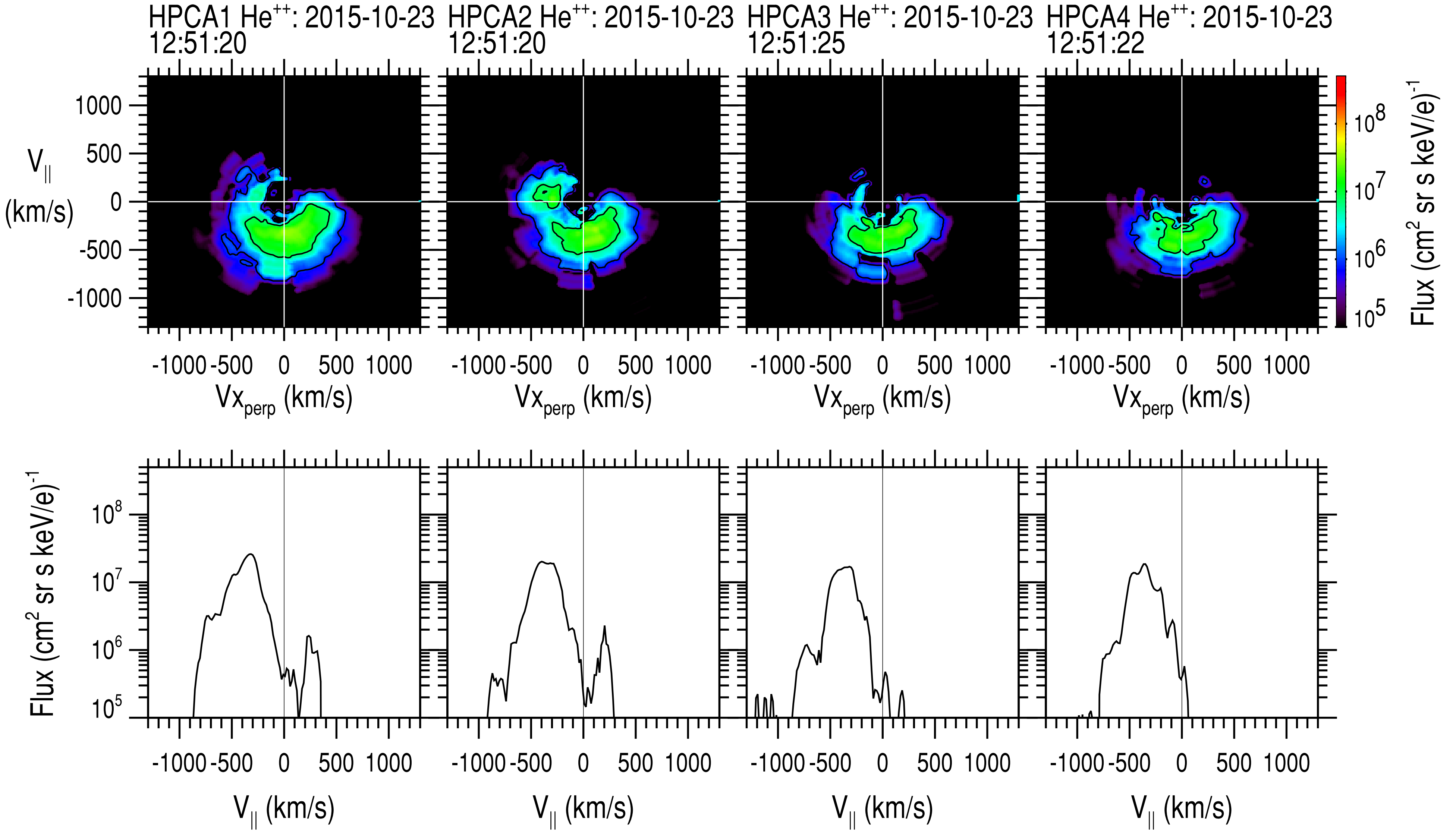
HPCA1 He++: 2015-10-23 12:51:20
HPCA2 He++: 2015-10-23 12:51:20
HPCA3 He++: 2015-10-23 12:51:25
HPCA4 He++: 2015-10-23 12:51:22
V|| (km/s)
Vxperp (km/s)
Flux (cm2 sr s keV/e)-1
105
106
107
108
-1000
-500
0
500
1000

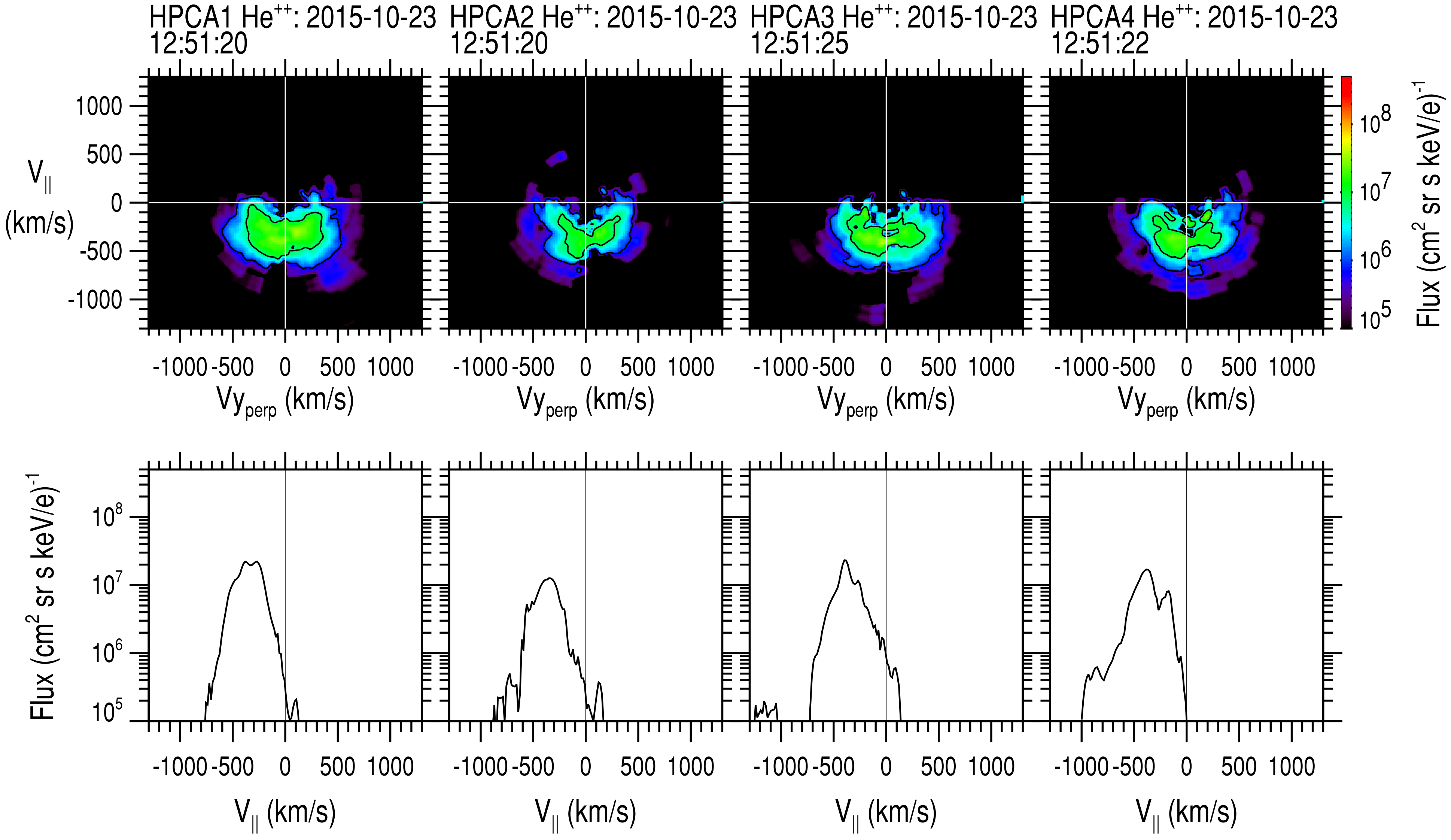
HPCA1 He++: 2015-10-23
12:51:20
HPCA2 He++: 2015-10-23
12:51:20
HPCA3 He++: 2015-10-23
12:51:25
HPCA4 He++: 2015-10-23
12:51:22
V|| (km/s)
Vyperp (km/s)
Flux (cm2 sr s keV/e)-1
V|| (km/s)

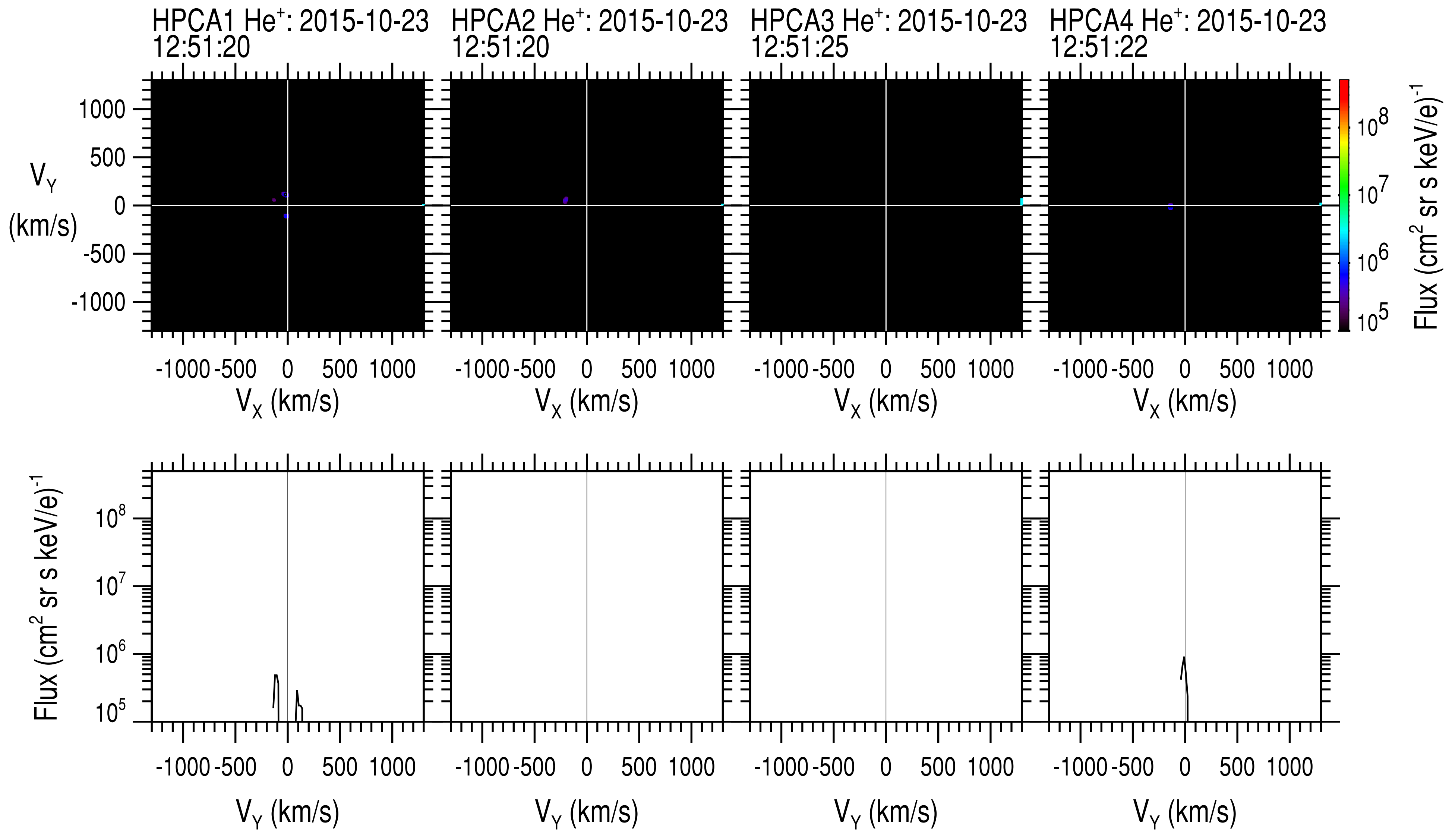
HPCA1 He+: 2015-10-23 12:51:20
HPCA2 He+: 2015-10-23 12:51:20
HPCA3 He+: 2015-10-23 12:51:25
HPCA4 He+: 2015-10-23 12:51:22
V_Y (km/s)
V_X (km/s)
Flux (cm^2 sr s keV/e)^-1
V_Y (km/s)
Flux (cm^2 sr s keV/e)^-1

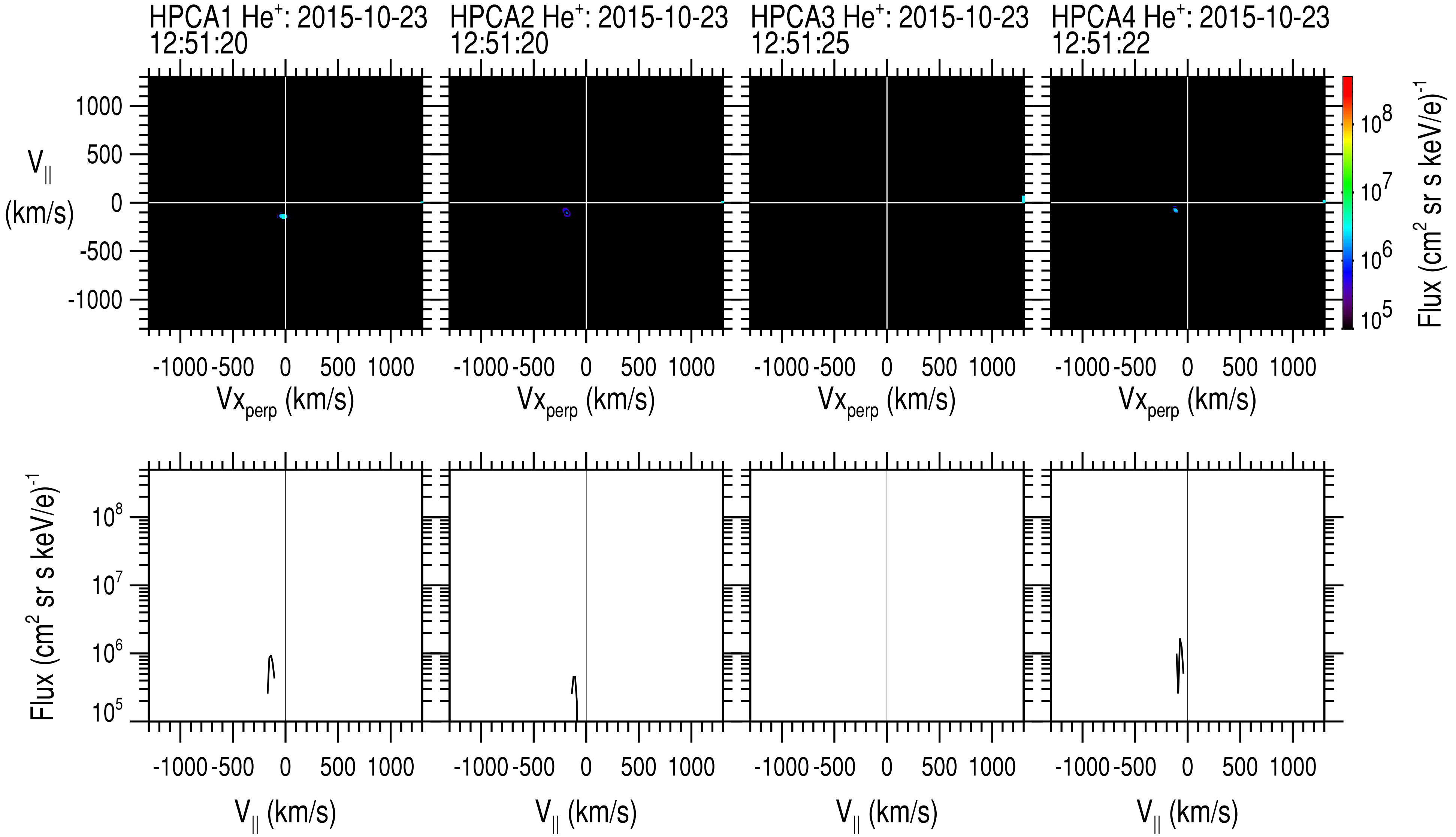
HPCA1 He+: 2015-10-23
12:51:20
HPCA2 He+: 2015-10-23
12:51:20
HPCA3 He+: 2015-10-23
12:51:25
HPCA4 He+: 2015-10-23
12:51:22
V|| (km/s)
Vxperp (km/s)
Flux (cm2 sr s keV/e)-1
V|| (km/s)

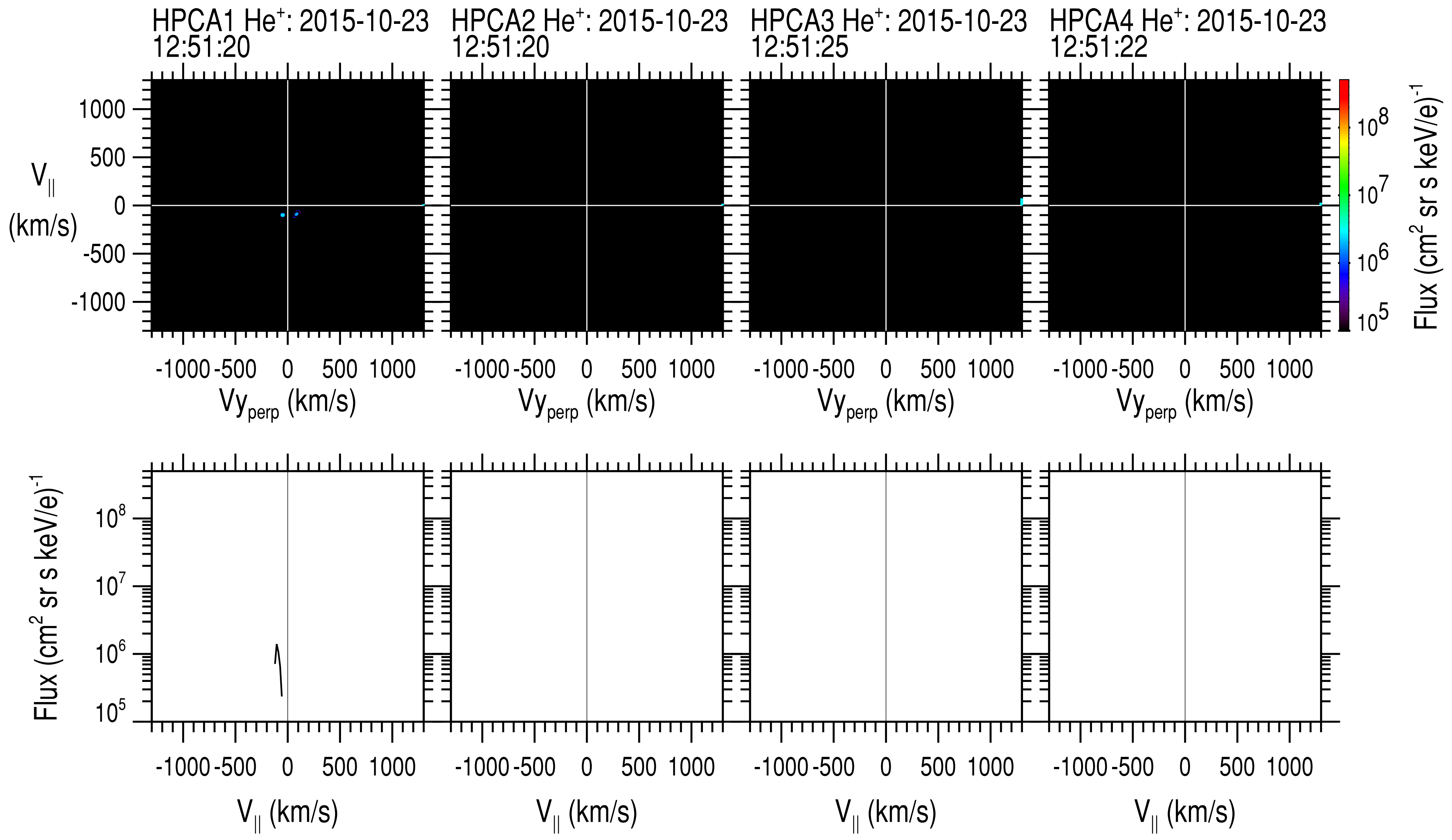

HPCA1 He+: 2015-10-23 12:51:20
HPCA2 He+: 2015-10-23 12:51:20
HPCA3 He+: 2015-10-23 12:51:25
HPCA4 He+: 2015-10-23 12:51:22
V|| (km/s)
Vyperp (km/s)
Flux (cm2 sr s keV/e)-1

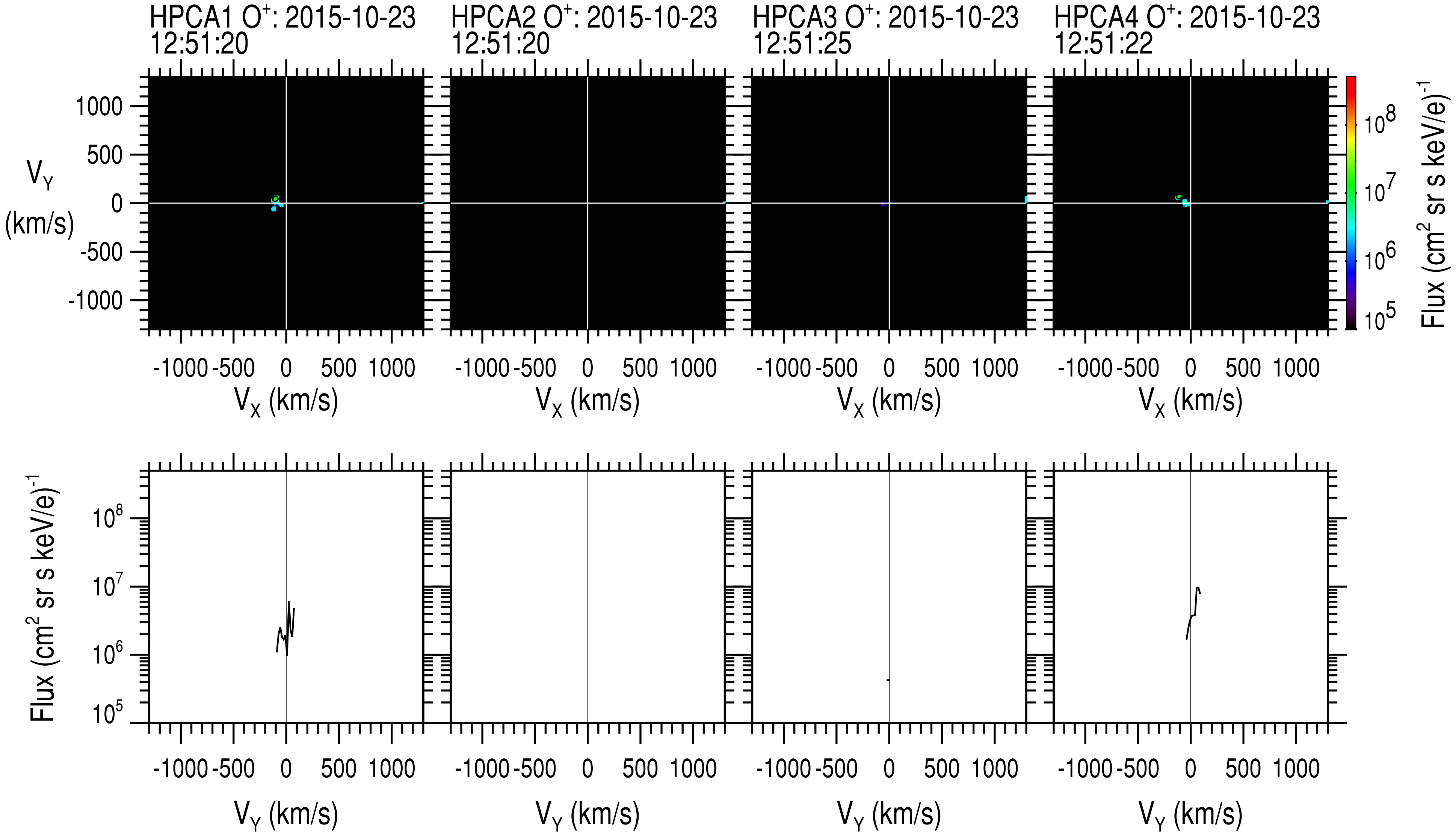
HPCA1 O+: 2015-10-23 12:51:20
HPCA2 O+: 2015-10-23 12:51:20
HPCA3 O+: 2015-10-23 12:51:25
HPCA4 O+: 2015-10-23 12:51:22
VY (km/s)
VX (km/s)
Flux (cm2 sr s keV/e)-1
VY (km/s)

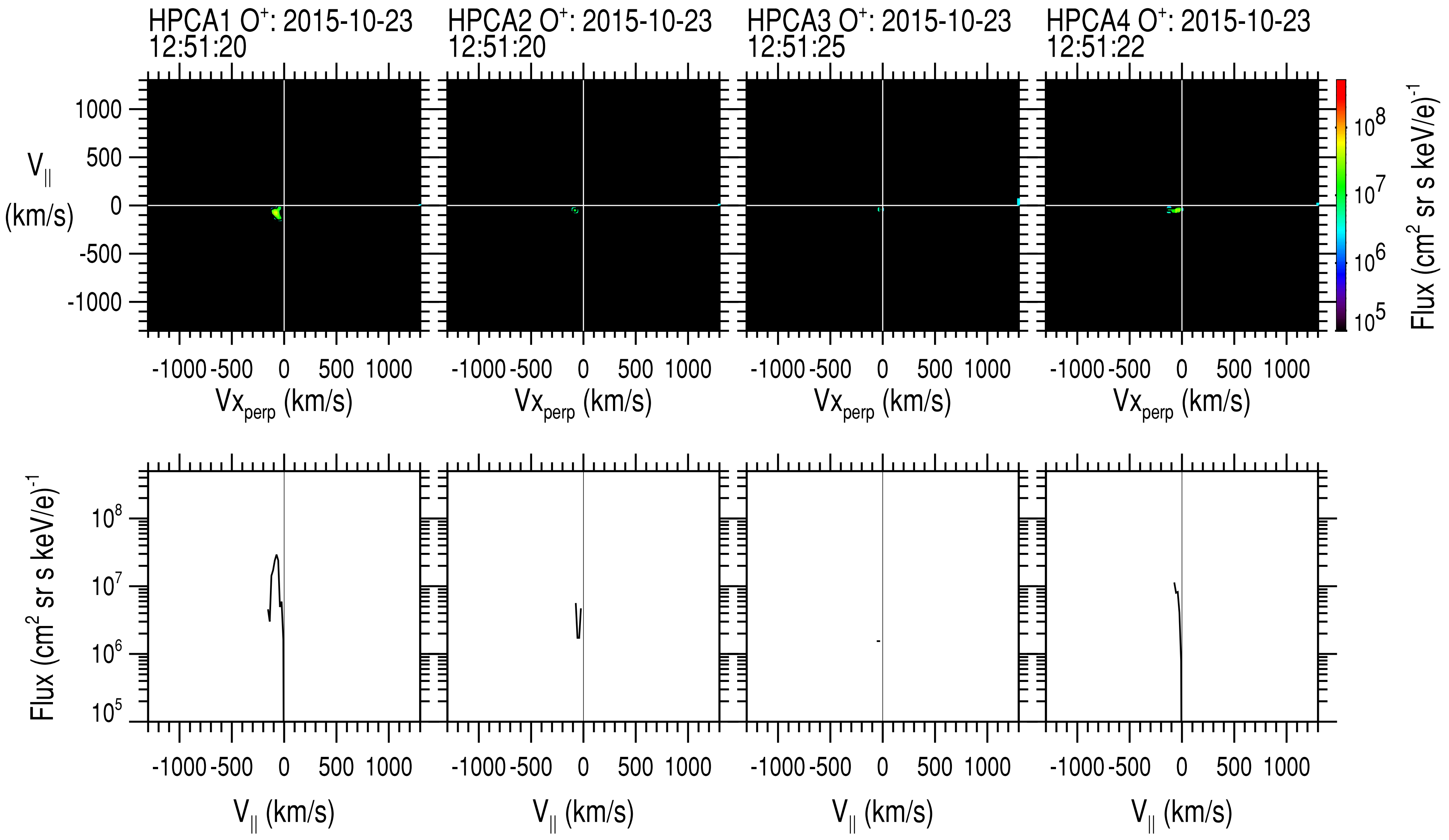
HPCA1 O+: 2015-10-23 12:51:20
HPCA2 O+: 2015-10-23 12:51:20
HPCA3 O+: 2015-10-23 12:51:25
HPCA4 O+: 2015-10-23 12:51:22
V|| (km/s)
Vxperp (km/s)
Flux (cm2 sr s keV/e)-1
V|| (km/s)

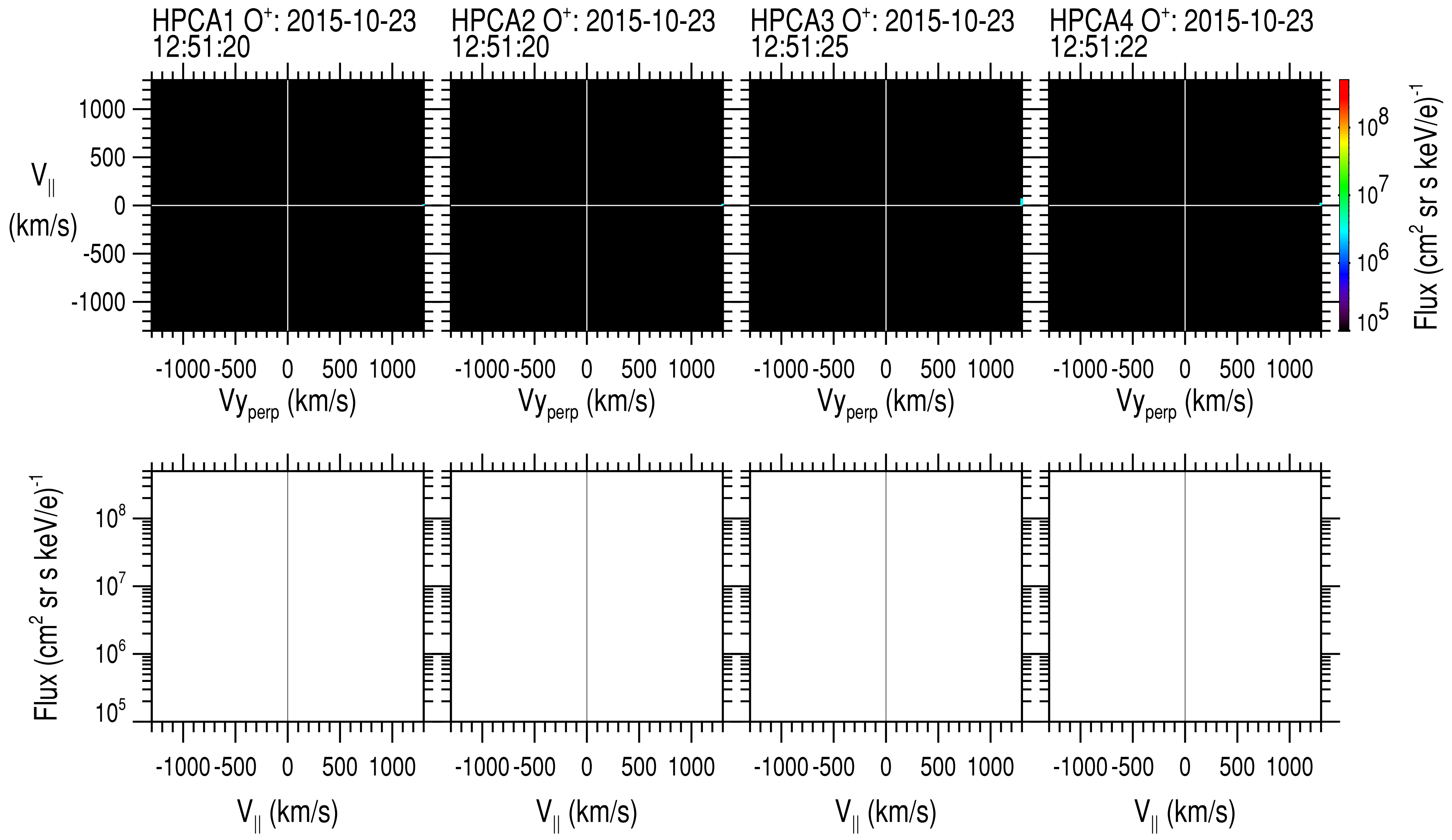
HPCA1 O+: 2015-10-23 12:51:20
HPCA2 O+: 2015-10-23 12:51:20
HPCA3 O+: 2015-10-23 12:51:25
HPCA4 O+: 2015-10-23 12:51:22
V|| (km/s)
Vyperp (km/s)
Flux (cm2 sr s keV/e)-1
-1000 -500 0 500 1000
10^5 10^6 10^7 10^8

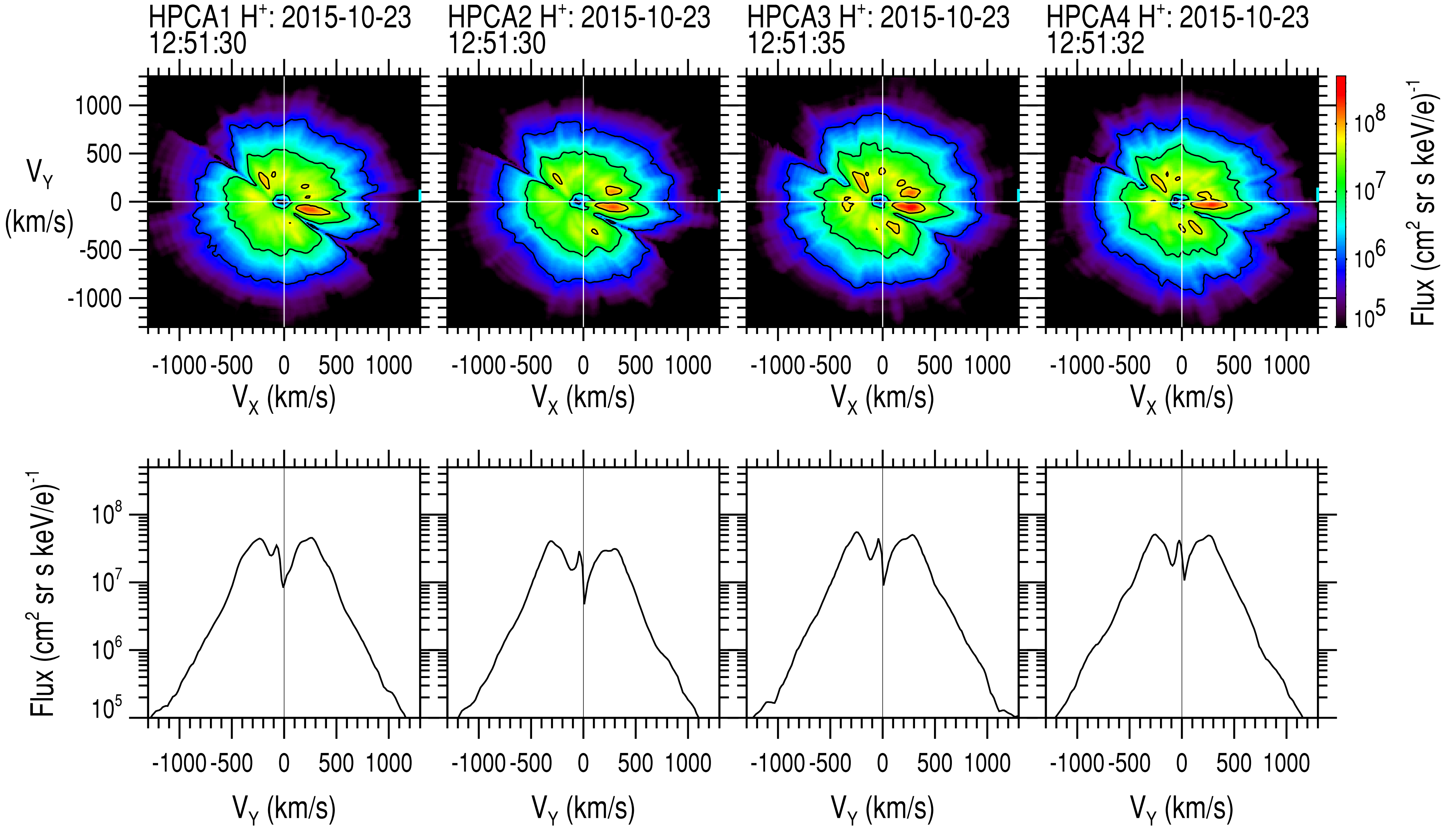
HPCA1 H$^+$: 2015-10-23 12:51:30
HPCA2 H$^+$: 2015-10-23 12:51:30
HPCA3 H$^+$: 2015-10-23 12:51:35
HPCA4 H$^+$: 2015-10-23 12:51:32
V_Y (km/s)
V_X (km/s)
Flux (cm^2 sr s keV/e)$^{-1}$
10^5 10^6 10^7 10^8
-1000 -500 0 500 1000

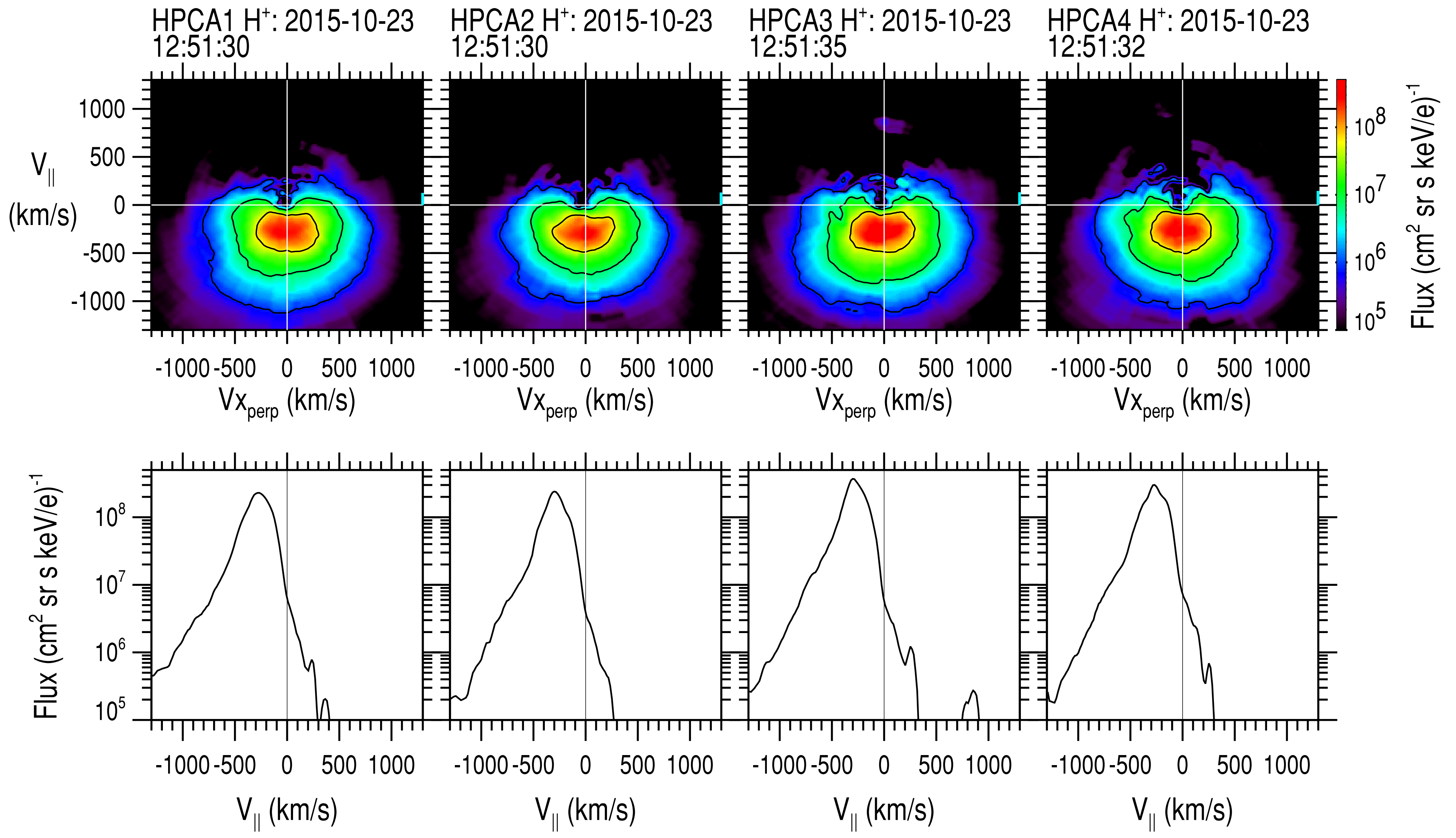
HPCA1 H+: 2015-10-23 12:51:30
HPCA2 H+: 2015-10-23 12:51:30
HPCA3 H+: 2015-10-23 12:51:35
HPCA4 H+: 2015-10-23 12:51:32
V|| (km/s)
Vxperp (km/s)
Flux (cm2 sr s keV/e)-1
-1000 -500 0 500 1000
105 106 107 108

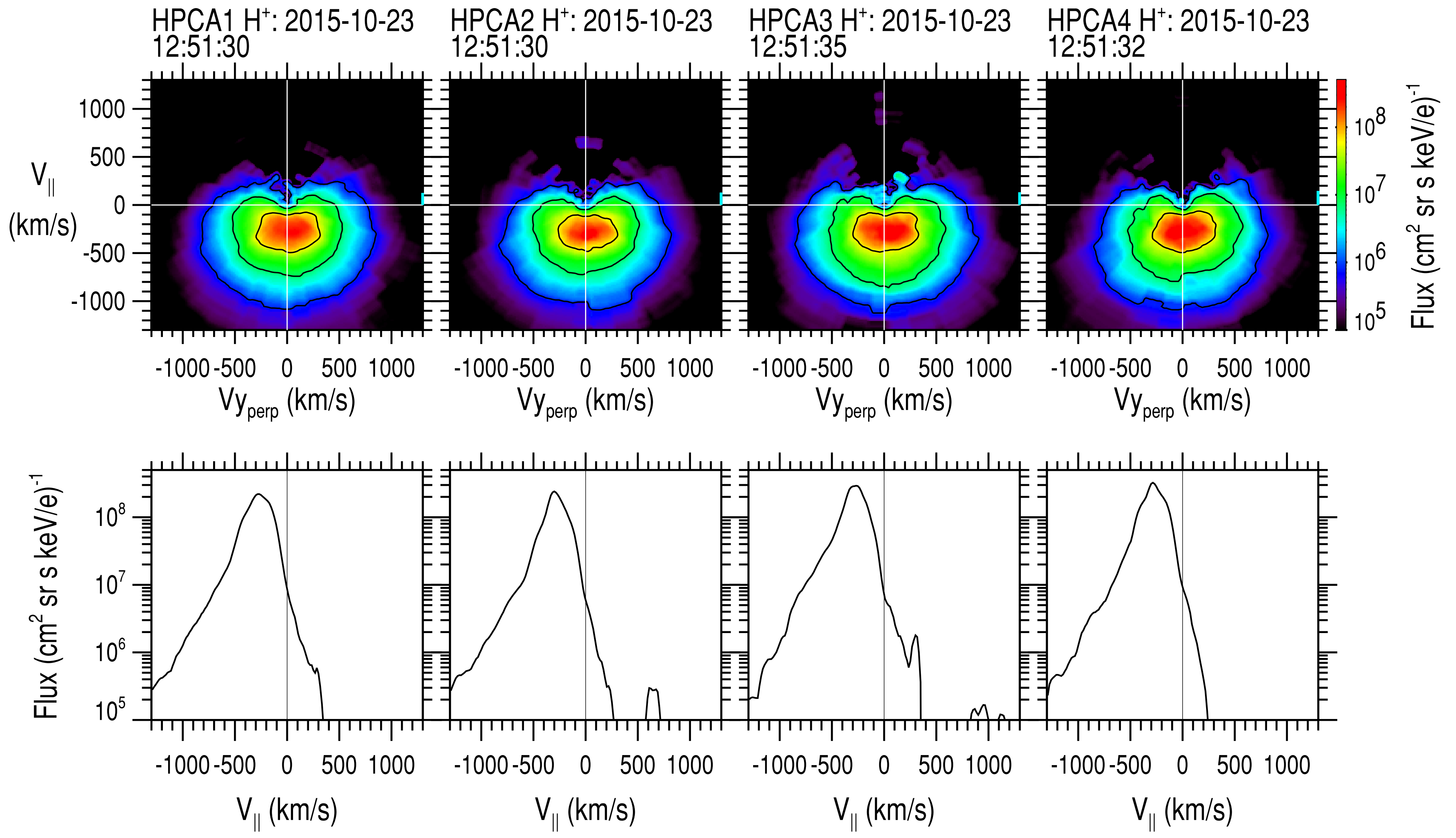

HPCA1 H+: 2015-10-23 12:51:30
HPCA2 H+: 2015-10-23 12:51:30
HPCA3 H+: 2015-10-23 12:51:35
HPCA4 H+: 2015-10-23 12:51:32
V|| (km/s)
Vyperp (km/s)
Flux (cm2 sr s keV/e)-1

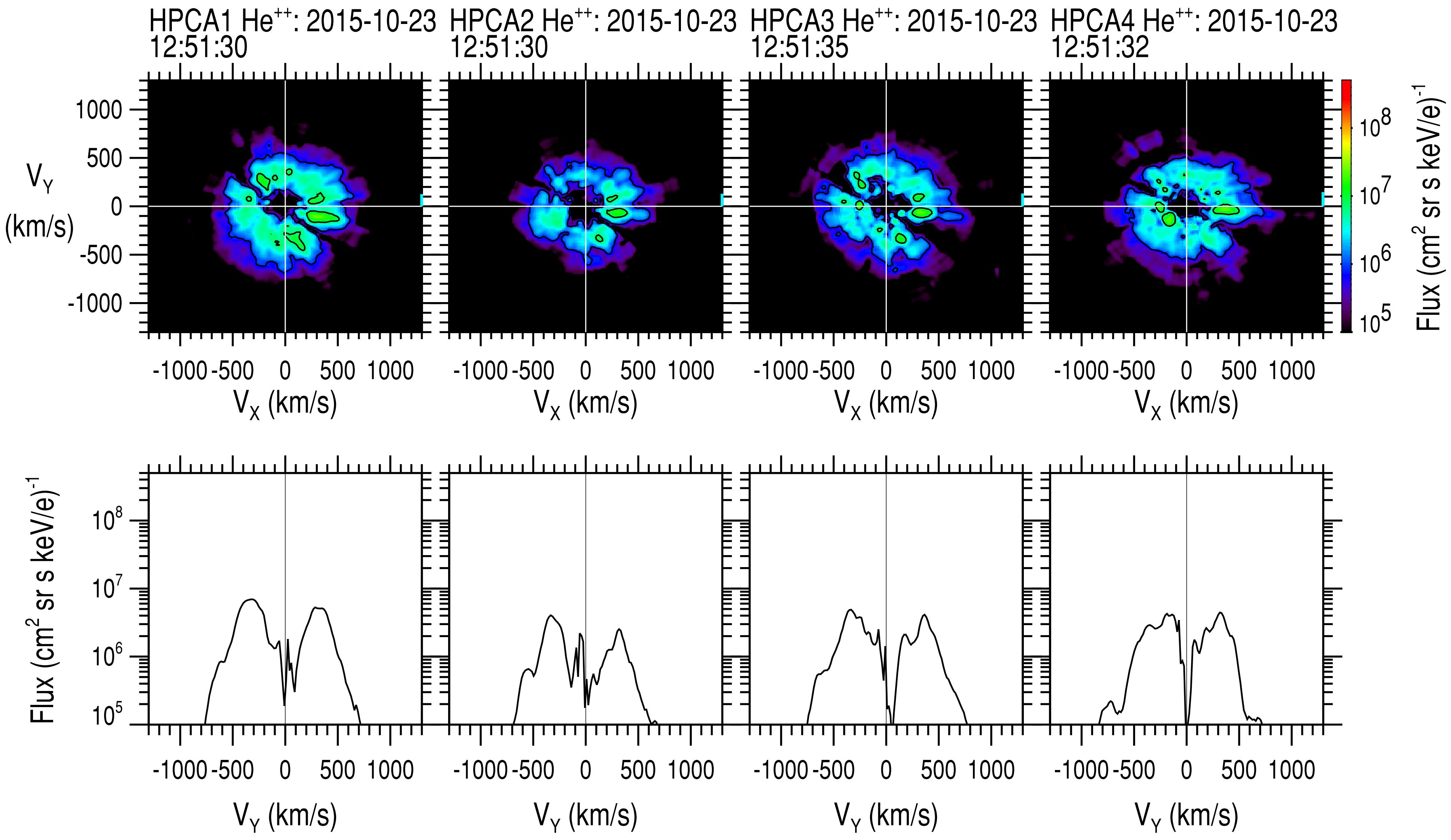

HPCA1 He++: 2015-10-23 12:51:30
HPCA2 He++: 2015-10-23 12:51:30
HPCA3 He++: 2015-10-23 12:51:35
HPCA4 He++: 2015-10-23 12:51:32
VY (km/s)
VX (km/s)
Flux (cm2 sr s keV/e)-1
VY (km/s)
-1000 -500 0 500 1000

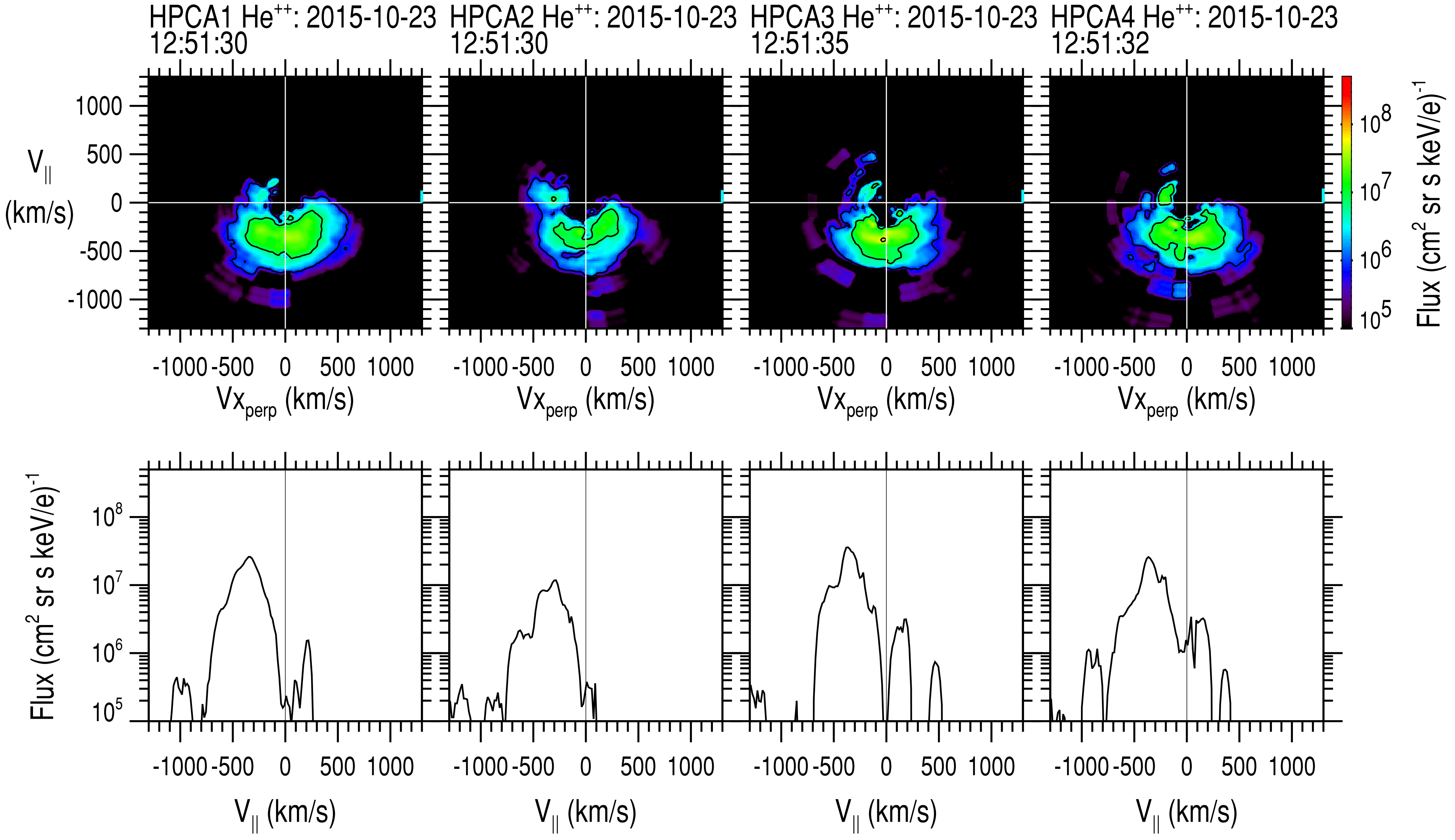
HPCA1 He++: 2015-10-23 12:51:30
HPCA2 He++: 2015-10-23 12:51:30
HPCA3 He++: 2015-10-23 12:51:35
HPCA4 He++: 2015-10-23 12:51:32
V|| (km/s)
Vxperp (km/s)
Flux (cm2 sr s keV/e)-1
V|| (km/s)

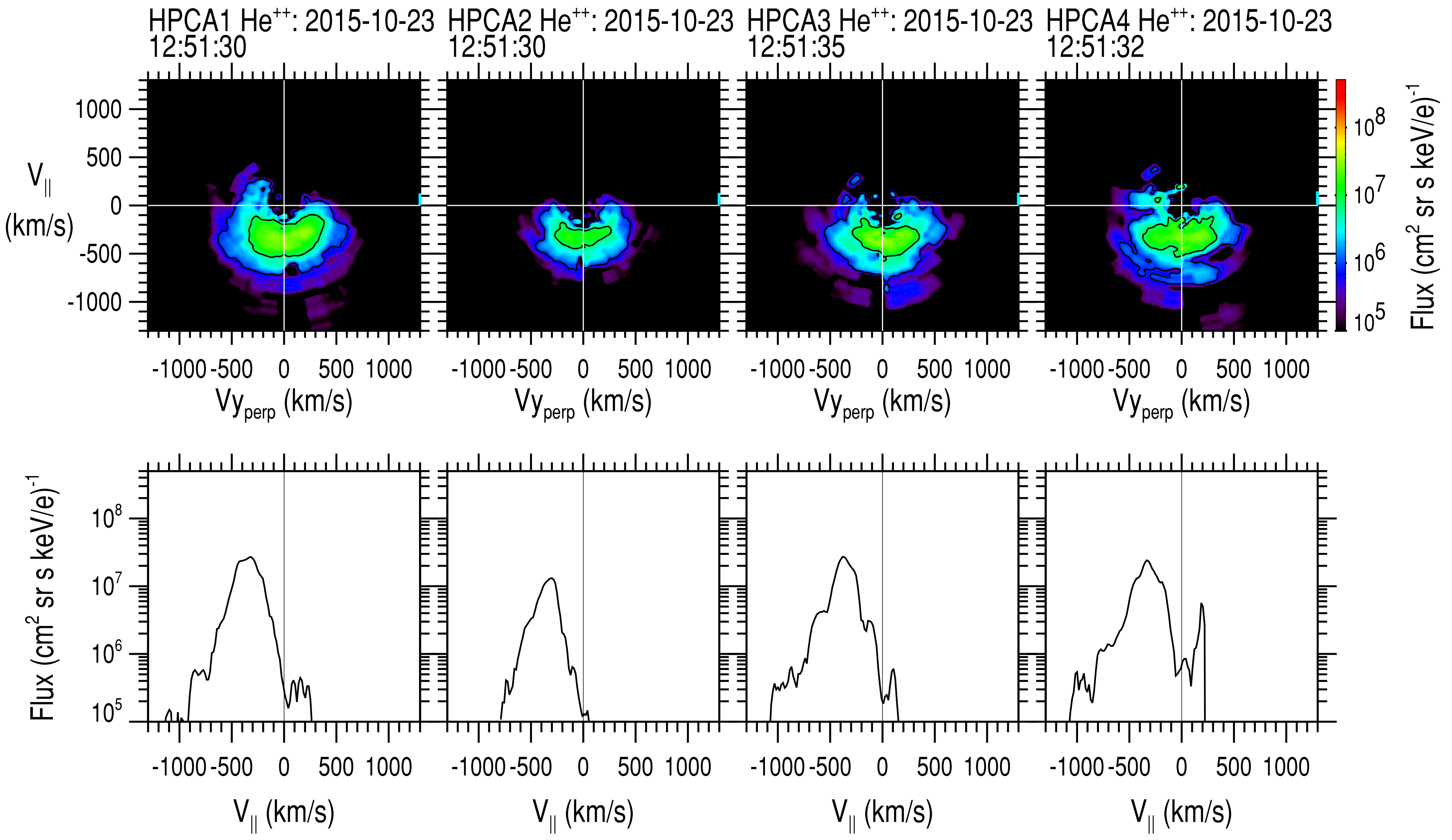
HPCA1 He++: 2015-10-23 12:51:30
HPCA2 He++: 2015-10-23 12:51:30
HPCA3 He++: 2015-10-23 12:51:35
HPCA4 He++: 2015-10-23 12:51:32
V|| (km/s)
Vyperp (km/s)
Flux (cm2 sr s keV/e)-1
V|| (km/s)

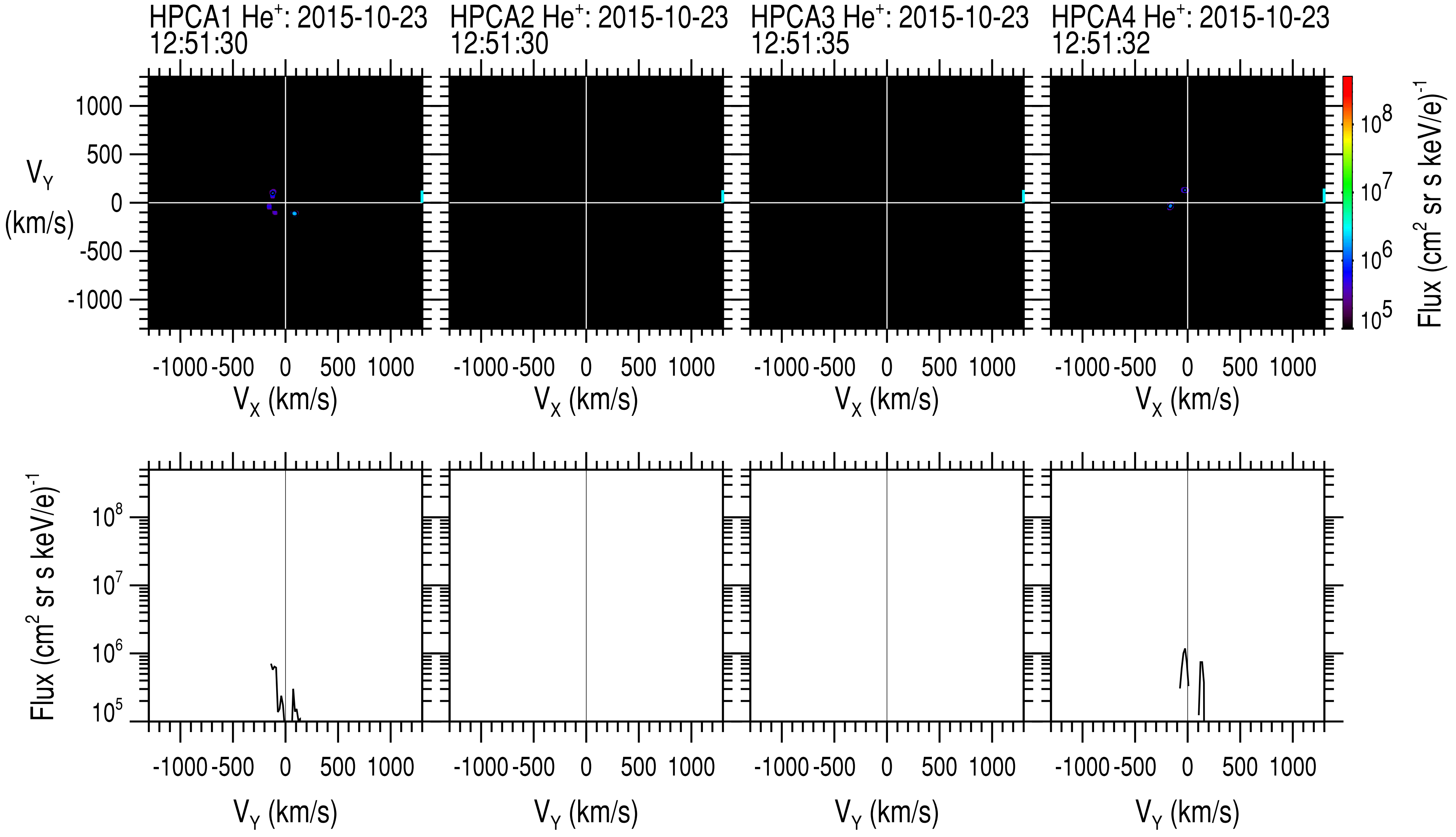
HPCA1 He+: 2015-10-23 12:51:30
HPCA2 He+: 2015-10-23 12:51:30
HPCA3 He+: 2015-10-23 12:51:35
HPCA4 He+: 2015-10-23 12:51:32
VY (km/s)
VX (km/s)
Flux (cm2 sr s keV/e)-1
-1000 -500 0 500 1000
10^5 10^6 10^7 10^8

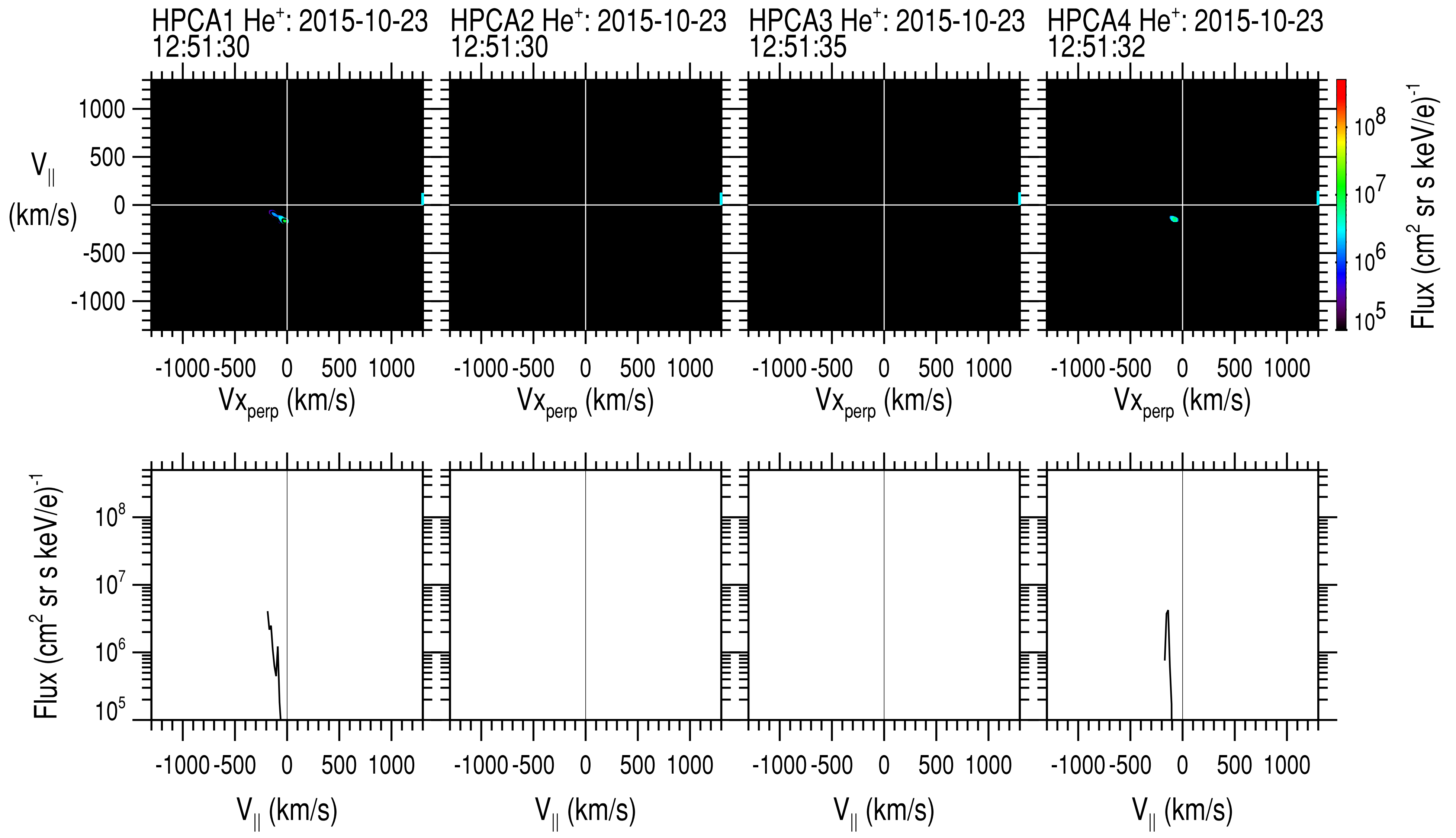
HPCA1 He⁺: 2015-10-23 12:51:30
HPCA2 He⁺: 2015-10-23 12:51:30
HPCA3 He⁺: 2015-10-23 12:51:35
HPCA4 He⁺: 2015-10-23 12:51:32
V|| (km/s)
Vx_perp (km/s)
Flux (cm² sr s keV/e)⁻¹
V|| (km/s)

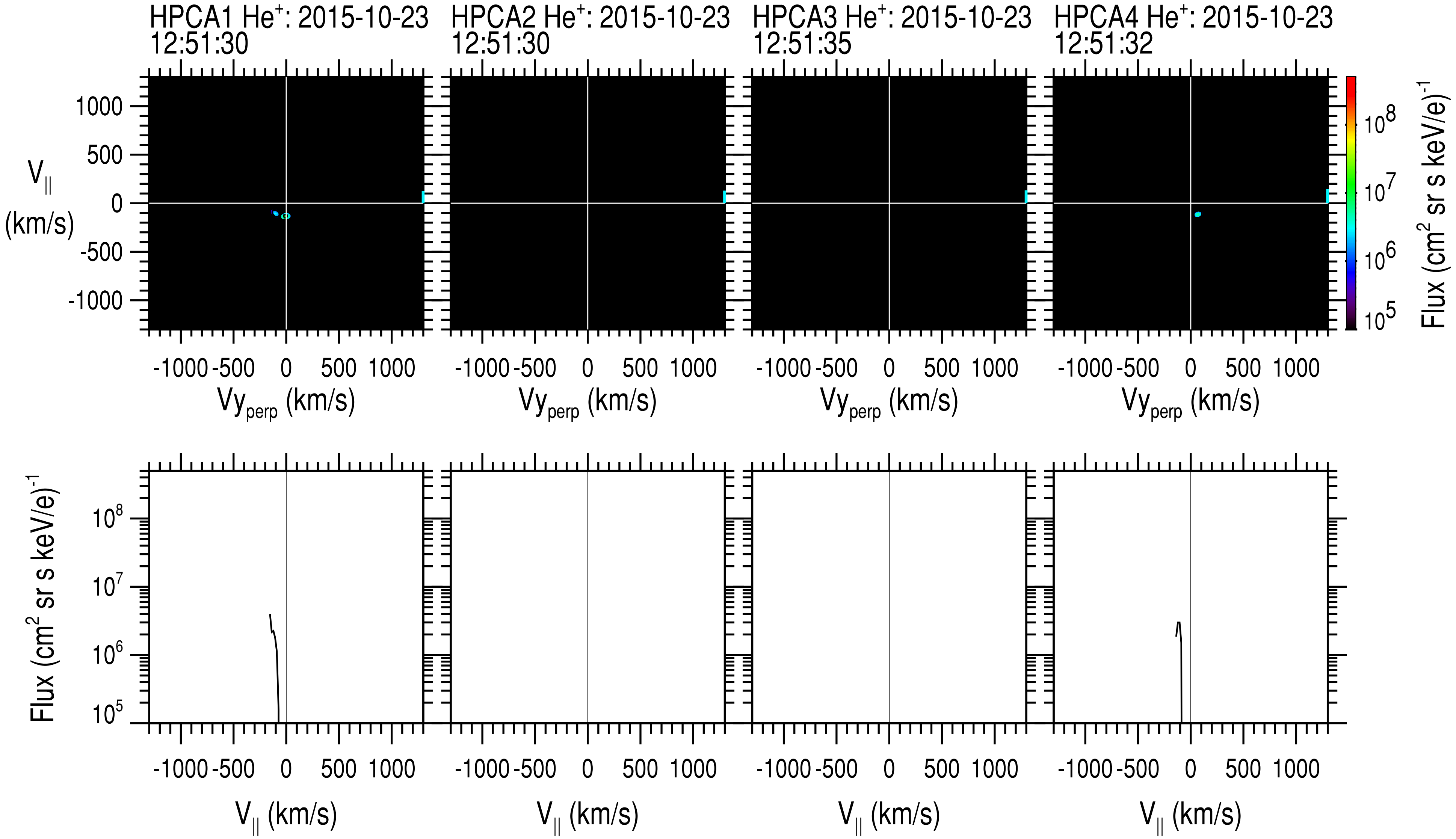
HPCA1 He+: 2015-10-23
12:51:30
HPCA2 He+: 2015-10-23
12:51:30
HPCA3 He+: 2015-10-23
12:51:35
HPCA4 He+: 2015-10-23
12:51:32
V|| (km/s)
Vyperp (km/s)
Flux (cm2 sr s keV/e)-1
1000
500
0
-500
-1000
10^5
10^6
10^7
10^8

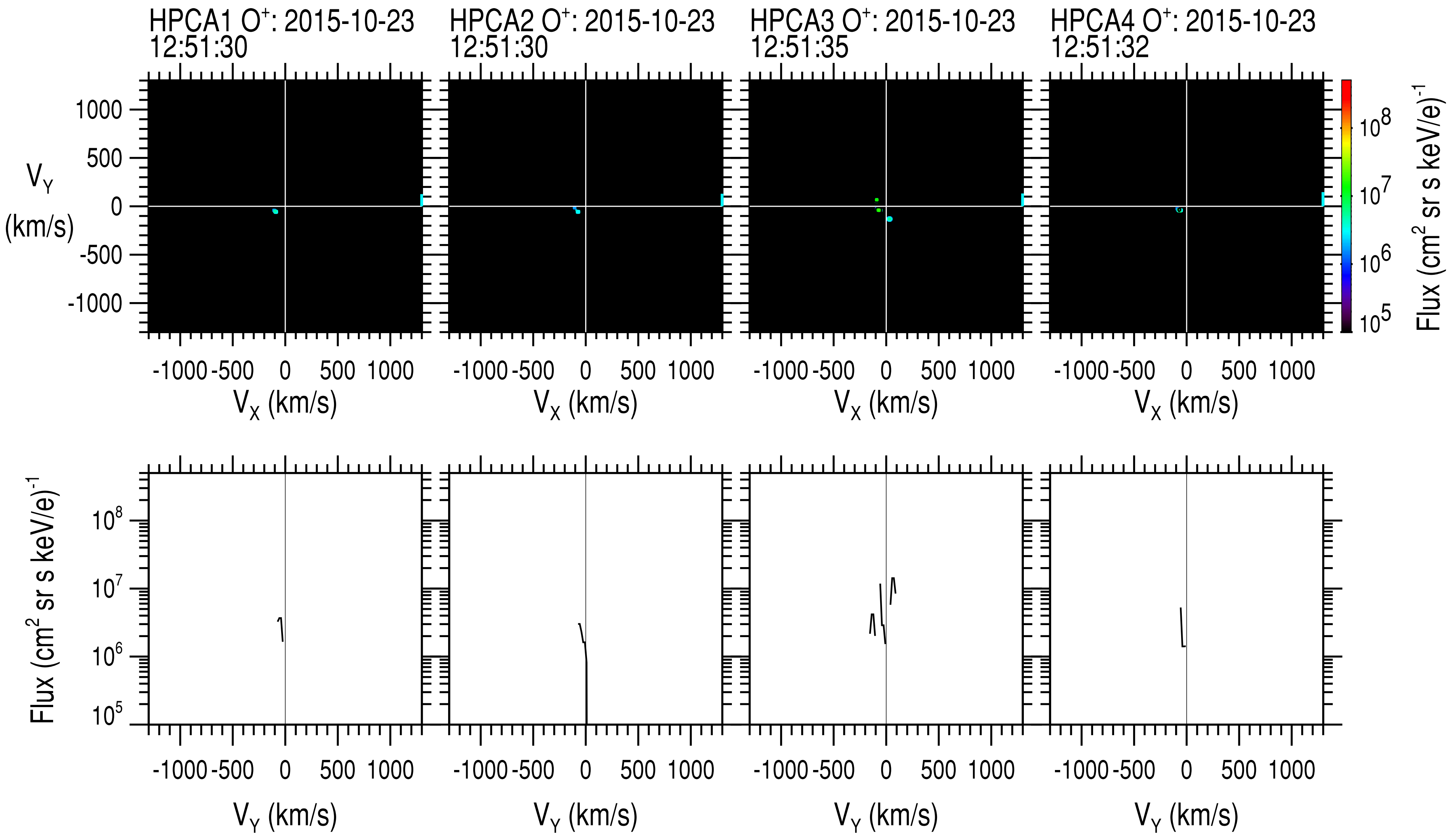
HPCA1 O+: 2015-10-23
12:51:30
HPCA2 O+: 2015-10-23
12:51:30
HPCA3 O+: 2015-10-23
12:51:35
HPCA4 O+: 2015-10-23
12:51:32
V_Y (km/s)
V_X (km/s)
Flux (cm^2 sr s keV/e)^-1
V_Y (km/s)

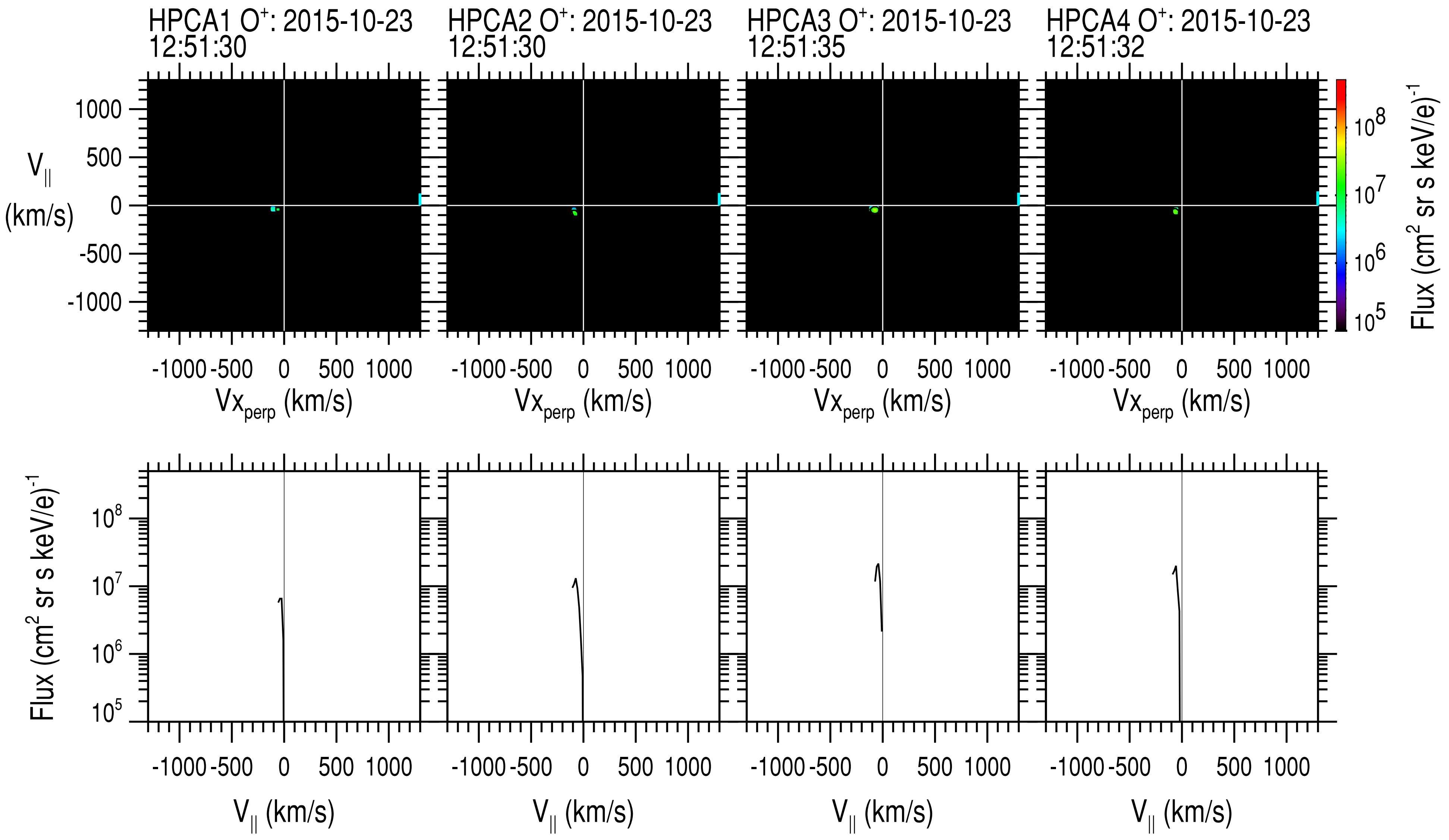
HPCA1 O+: 2015-10-23
12:51:30
HPCA2 O+: 2015-10-23
12:51:30
HPCA3 O+: 2015-10-23
12:51:35
HPCA4 O+: 2015-10-23
12:51:32
V||
(km/s)
Vxperp (km/s)
Flux (cm2 sr s keV/e)-1
V|| (km/s)

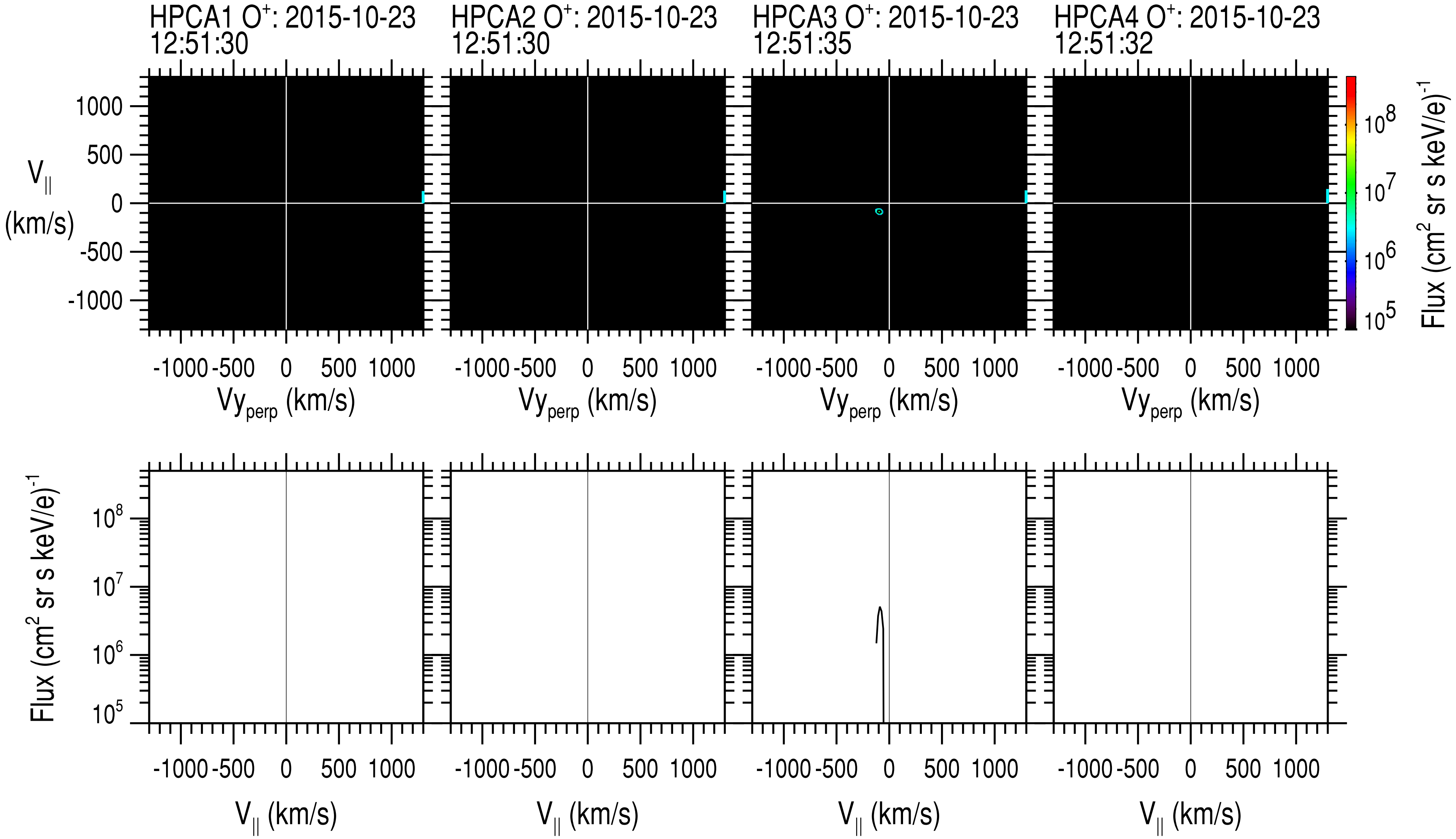
HPCA1 O+: 2015-10-23
12:51:30
HPCA2 O+: 2015-10-23
12:51:30
HPCA3 O+: 2015-10-23
12:51:35
HPCA4 O+: 2015-10-23
12:51:32
V|| (km/s)
Vyperp (km/s)
Flux (cm2 sr s keV/e)-1
V|| (km/s)

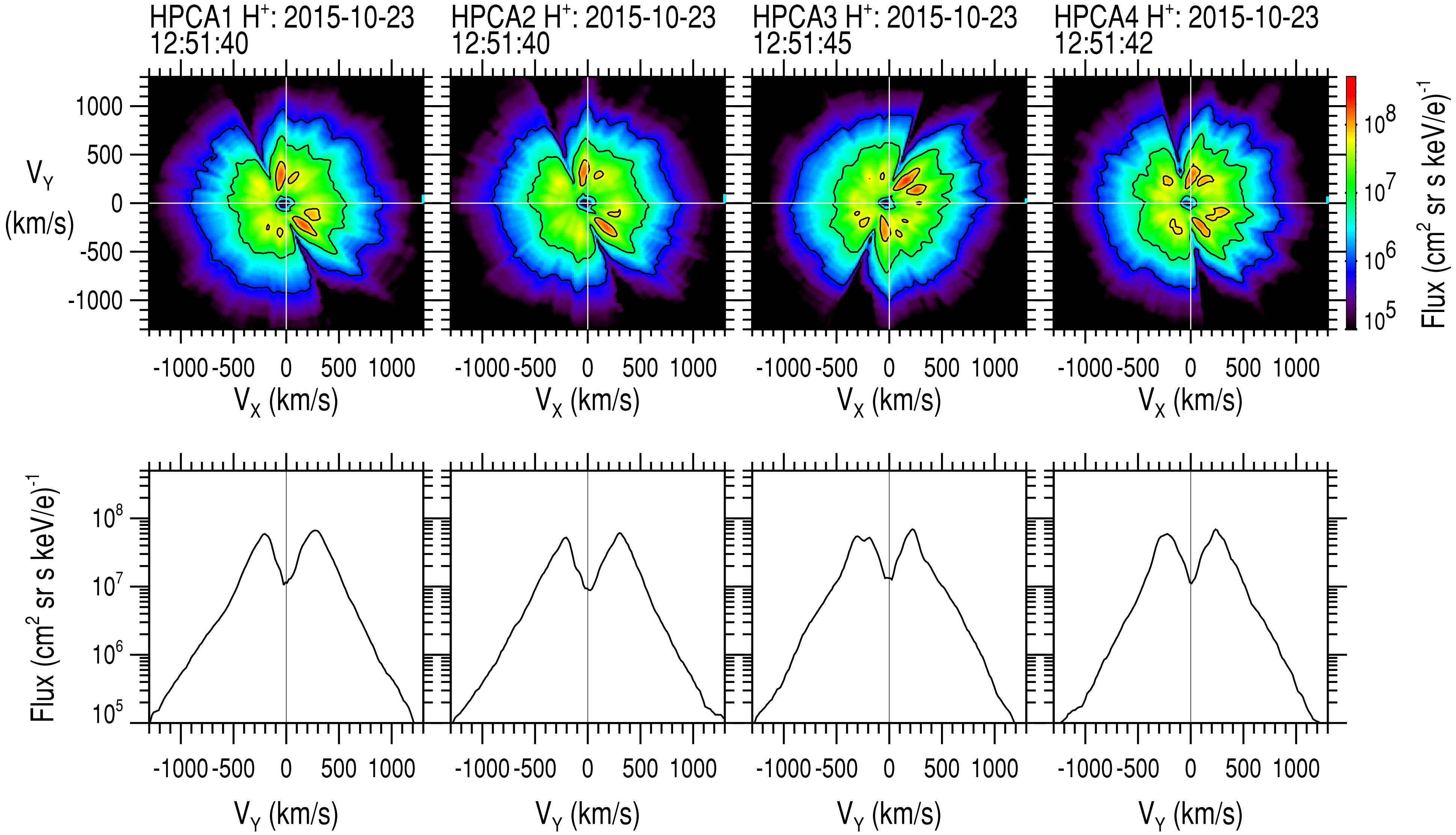
HPCA1 H+: 2015-10-23 12:51:40
HPCA2 H+: 2015-10-23 12:51:40
HPCA3 H+: 2015-10-23 12:51:45
HPCA4 H+: 2015-10-23 12:51:42
VY (km/s)
VX (km/s)
Flux (cm2 sr s keV/e)-1
-1000 -500 0 500 1000
105 106 107 108

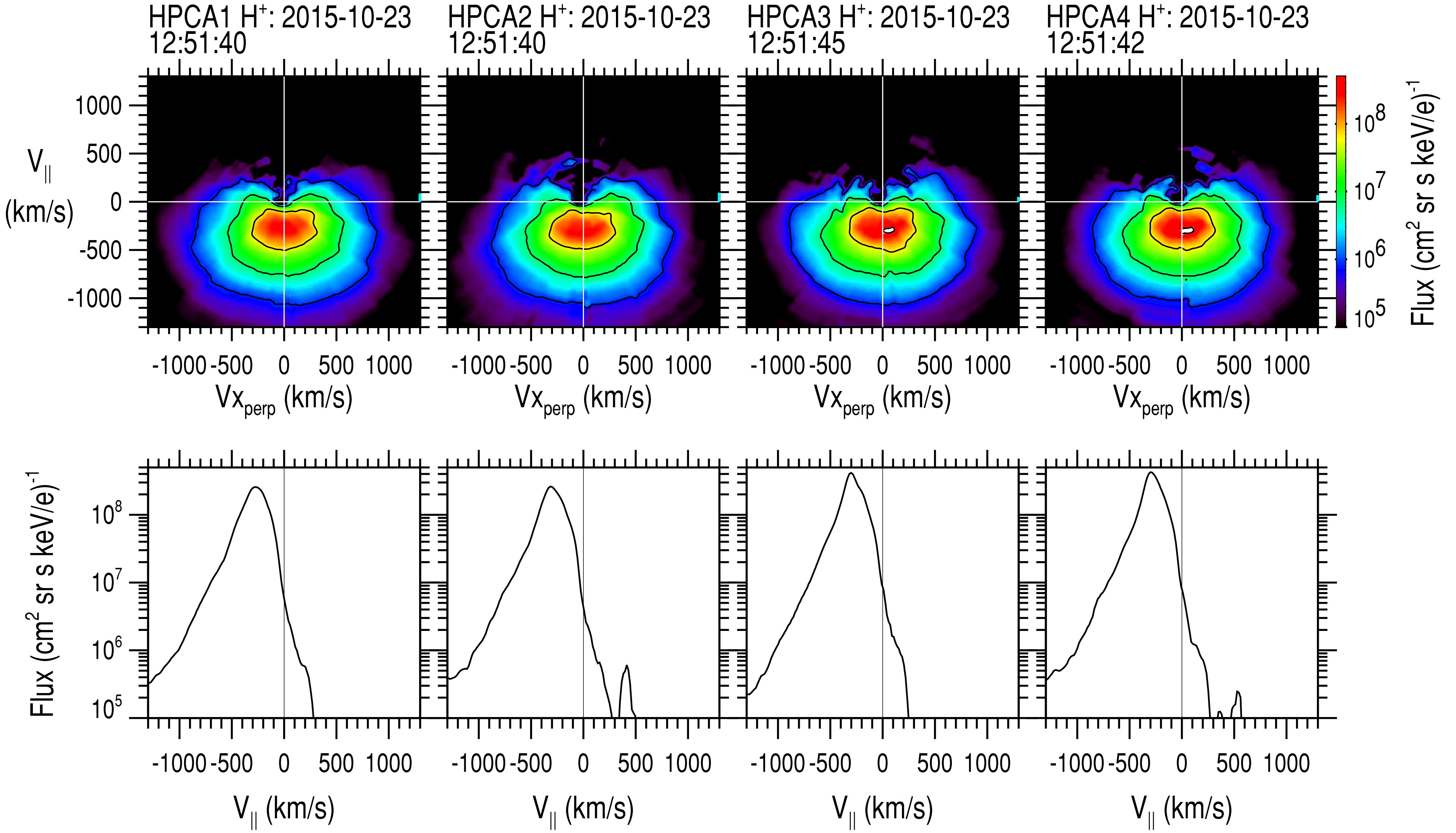

HPCA1 H+: 2015-10-23 12:51:40
HPCA2 H+: 2015-10-23 12:51:40
HPCA3 H+: 2015-10-23 12:51:45
HPCA4 H+: 2015-10-23 12:51:42
V|| (km/s)
Vxperp (km/s)
Flux (cm2 sr s keV/e)-1

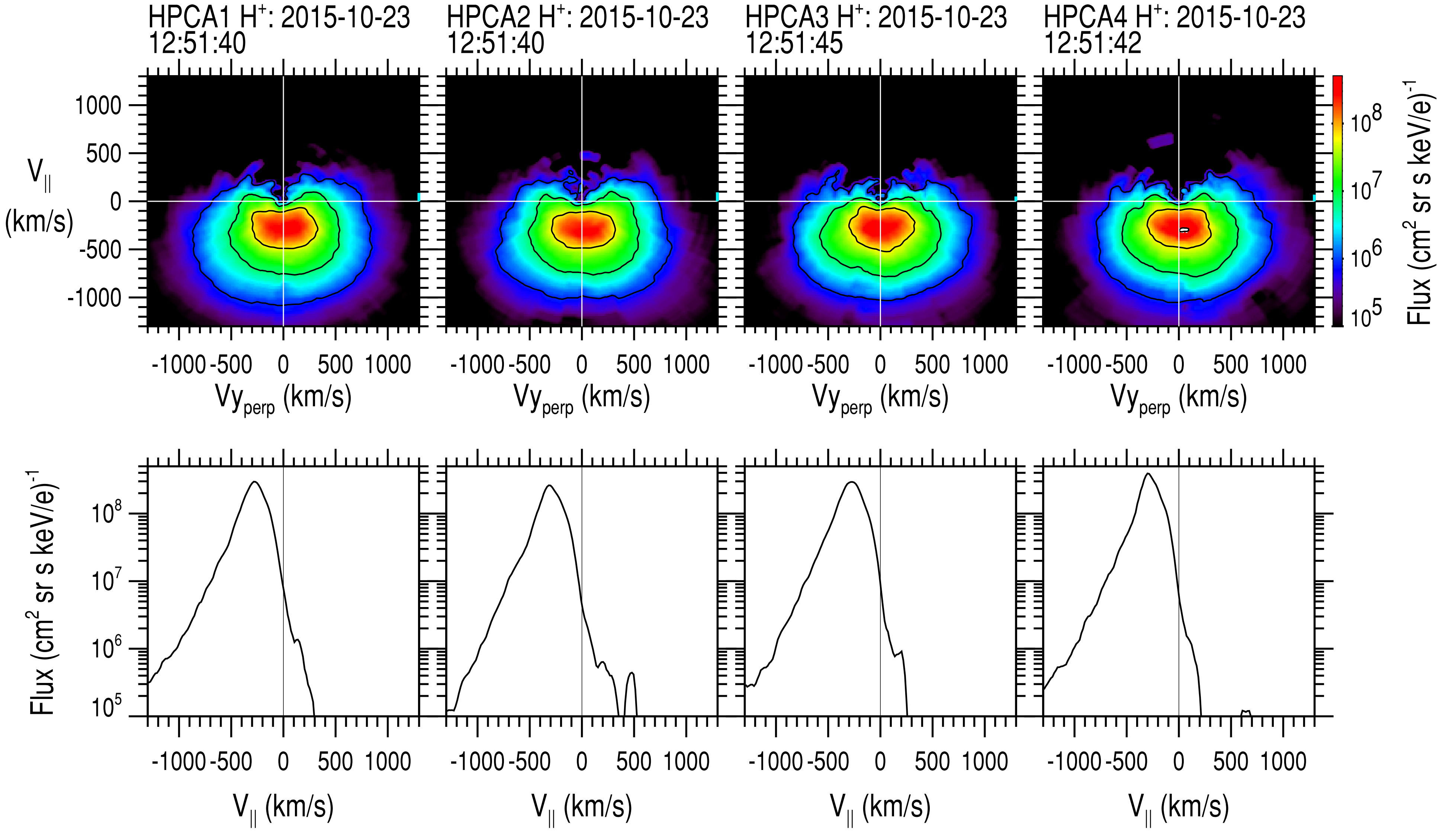

HPCA1 H+: 2015-10-23
12:51:40
HPCA2 H+: 2015-10-23
12:51:40
HPCA3 H+: 2015-10-23
12:51:45
HPCA4 H+: 2015-10-23
12:51:42
V|| (km/s)
Vyperp (km/s)
Flux (cm2 sr s keV/e)-1
V|| (km/s)

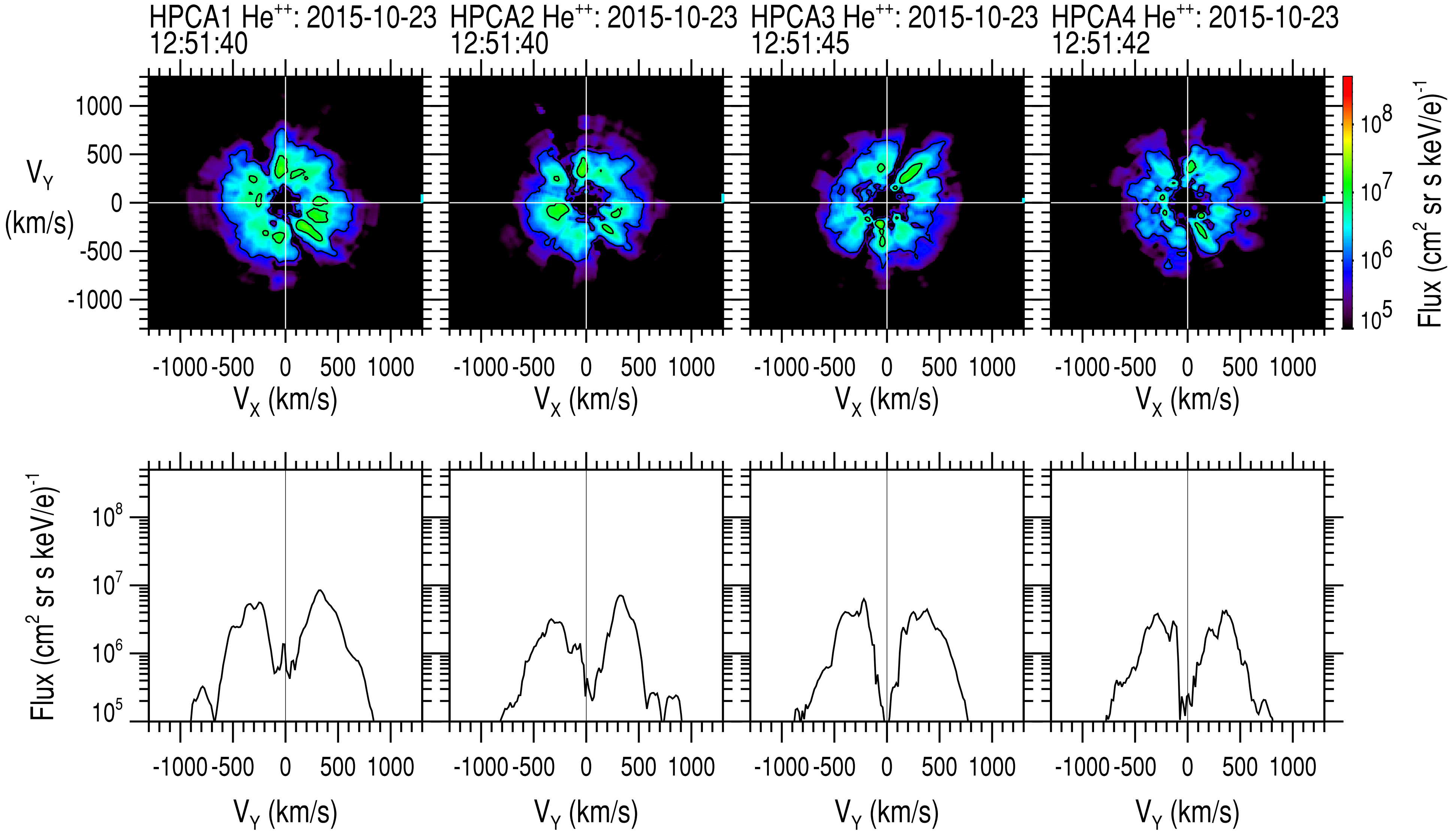

HPCA1 He++: 2015-10-23
12:51:40
HPCA2 He++: 2015-10-23
12:51:40
HPCA3 He++: 2015-10-23
12:51:45
HPCA4 He++: 2015-10-23
12:51:42
VY (km/s)
VX (km/s)
Flux (cm2 sr s keV/e)-1
105
106
107
108
-1000
-500
0
500
1000

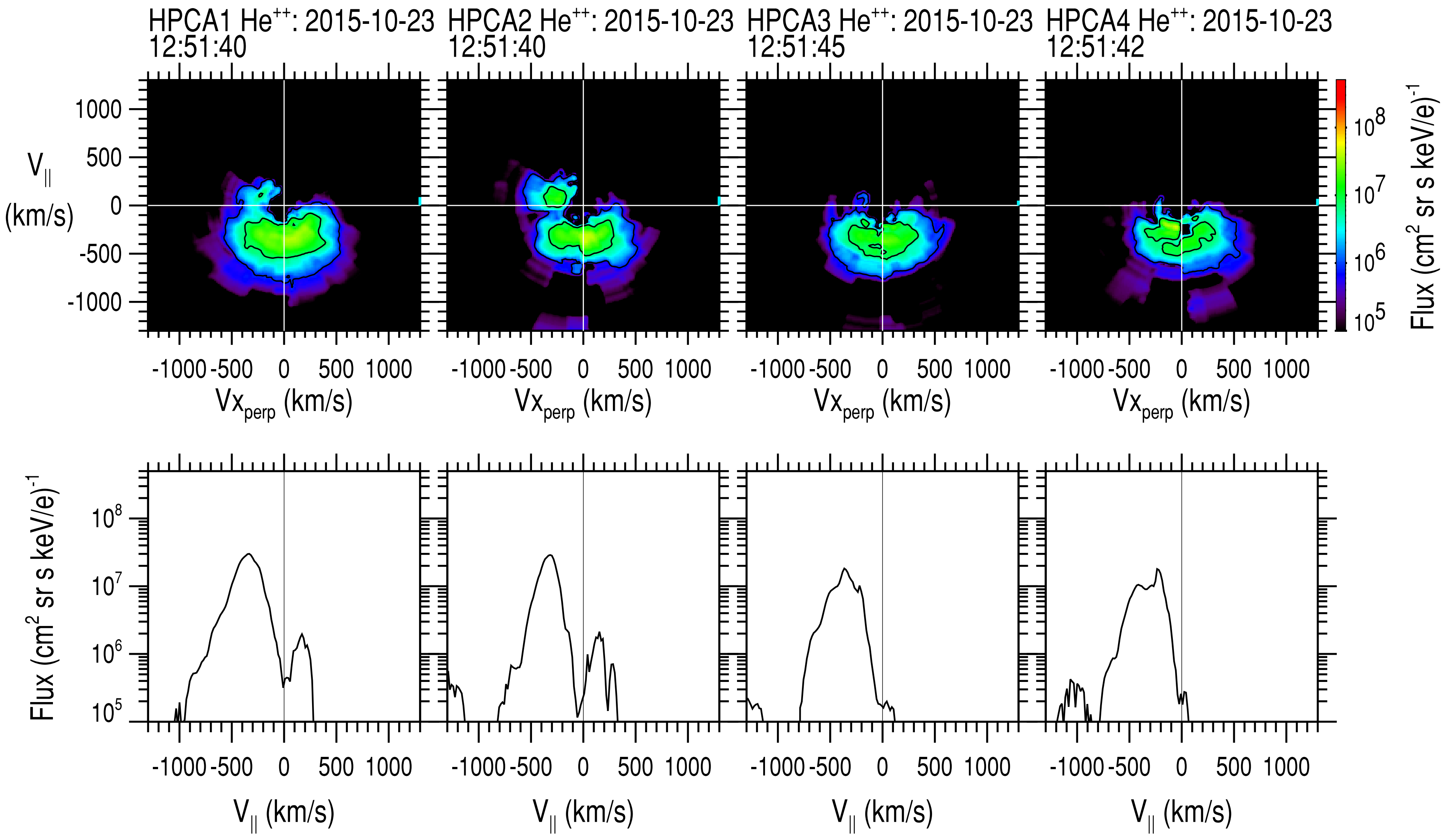
HPCA1 He++: 2015-10-23 12:51:40
HPCA2 He++: 2015-10-23 12:51:40
HPCA3 He++: 2015-10-23 12:51:45
HPCA4 He++: 2015-10-23 12:51:42
V|| (km/s)
Vxperp (km/s)
Flux (cm2 sr s keV/e)-1
V|| (km/s)

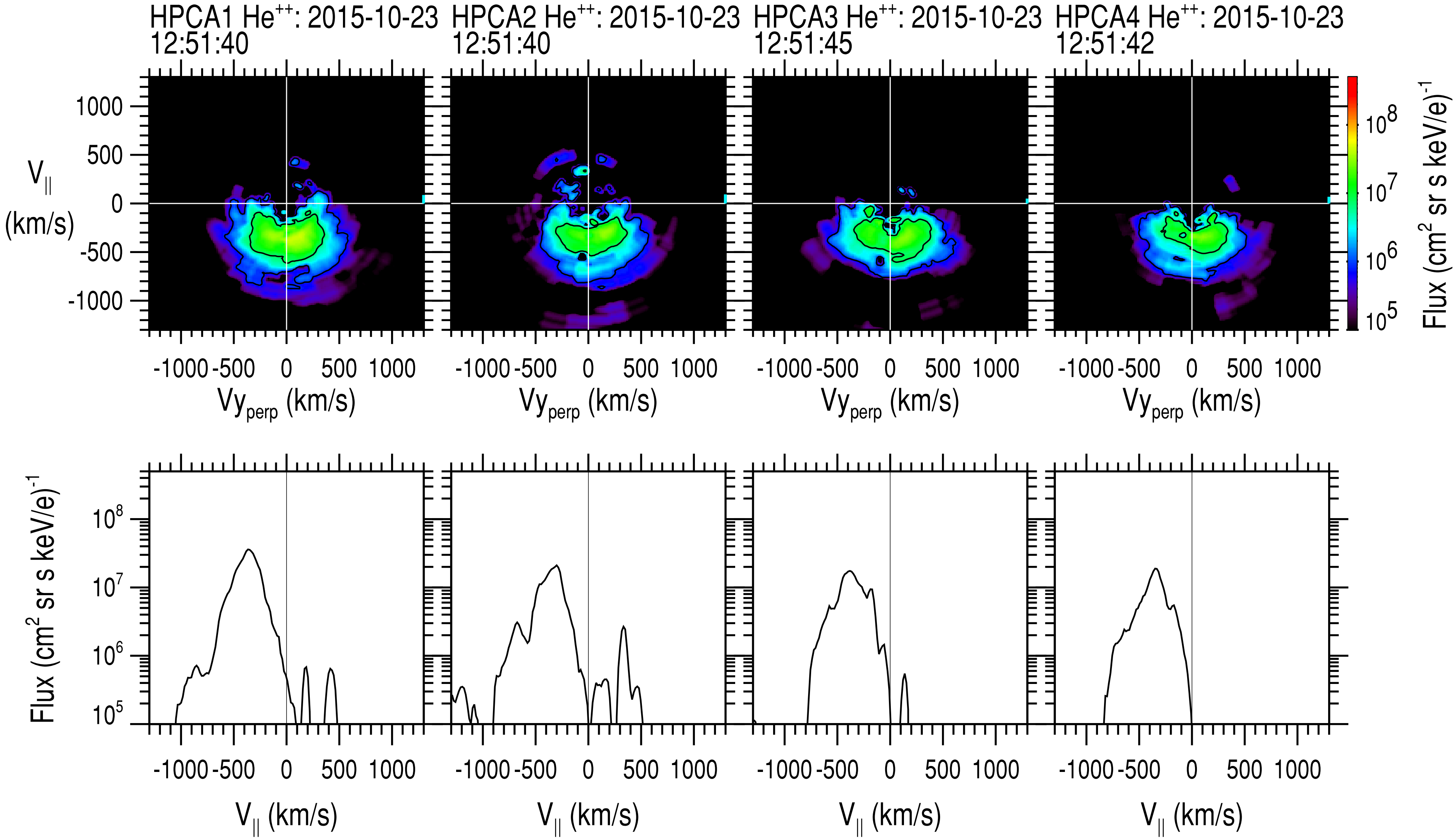
HPCA1 He++: 2015-10-23 12:51:40
HPCA2 He++: 2015-10-23 12:51:40
HPCA3 He++: 2015-10-23 12:51:45
HPCA4 He++: 2015-10-23 12:51:42
V|| (km/s)
Vyperp (km/s)
Flux (cm2 sr s keV/e)-1
V|| (km/s)

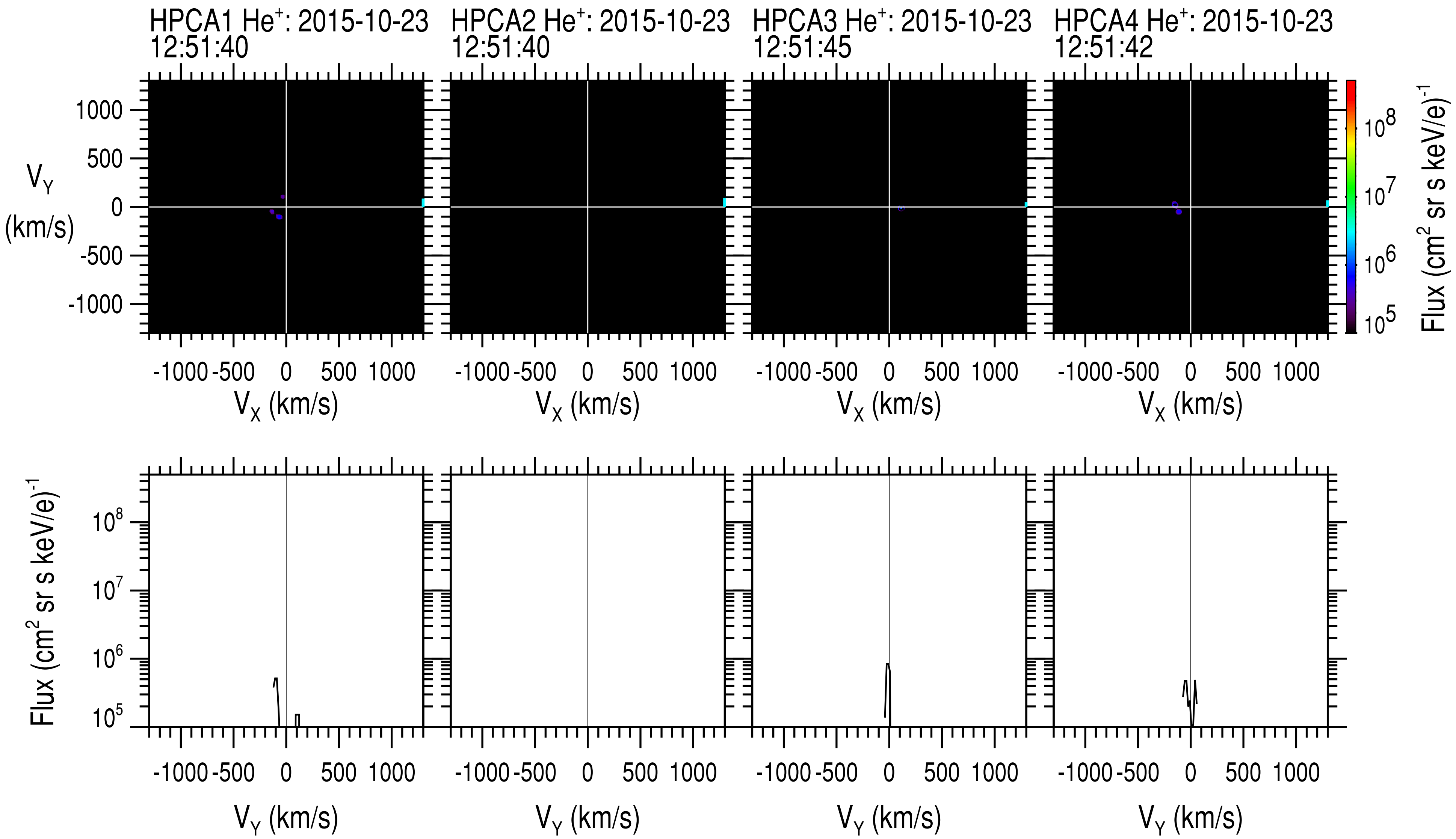

HPCA1 He+: 2015-10-23 12:51:40
HPCA2 He+: 2015-10-23 12:51:40
HPCA3 He+: 2015-10-23 12:51:45
HPCA4 He+: 2015-10-23 12:51:42
VY (km/s)
VX (km/s)
Flux (cm2 sr s keV/e)-1

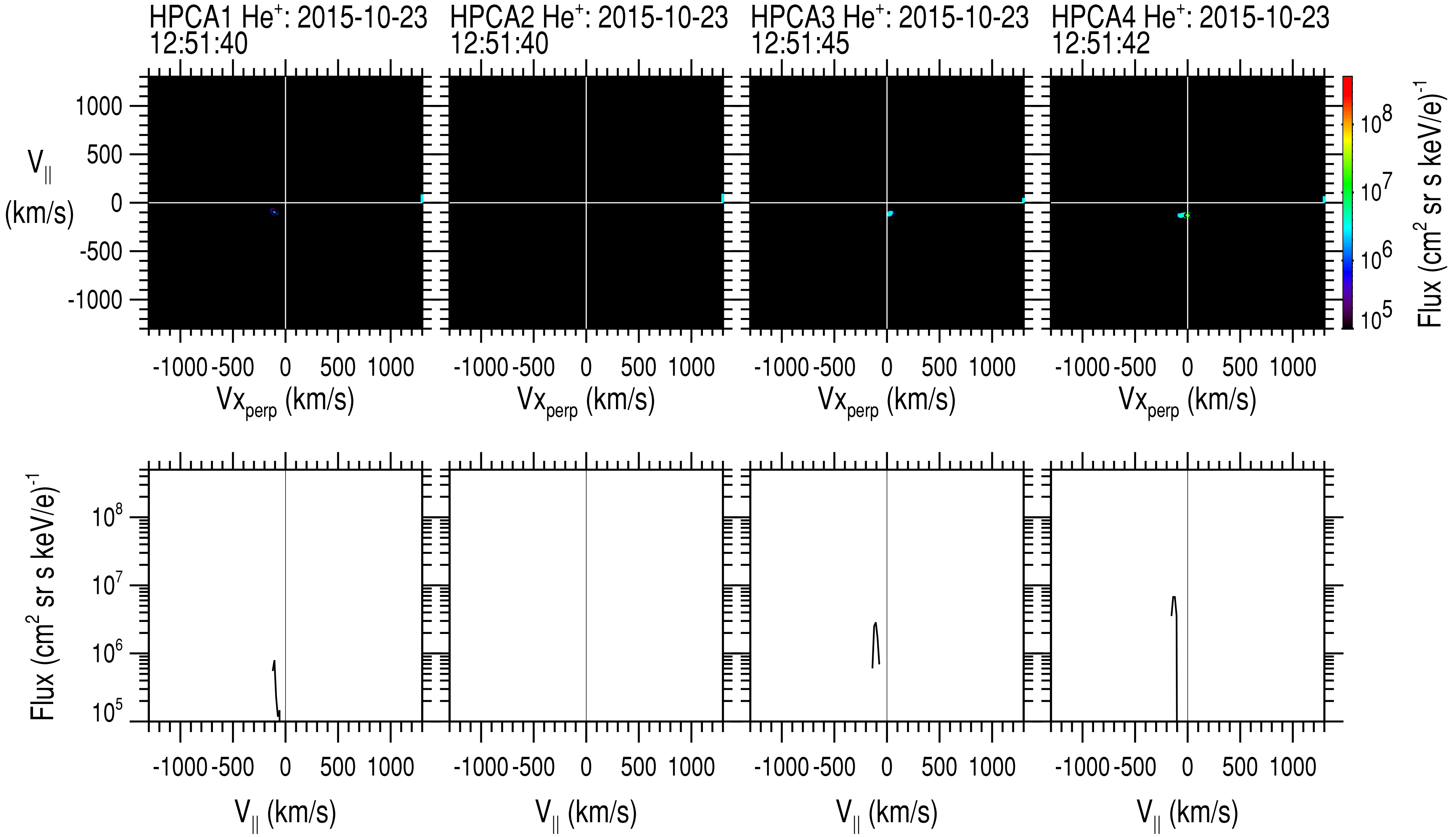
HPCA1 He+: 2015-10-23 12:51:40
HPCA2 He+: 2015-10-23 12:51:40
HPCA3 He+: 2015-10-23 12:51:45
HPCA4 He+: 2015-10-23 12:51:42
V|| (km/s)
1000
500
0
-500
-1000
-1000 -500 0 500 1000
Vxperp (km/s)
Flux (cm2 sr s keV/e)-1
10^5
10^6
10^7
10^8
V|| (km/s)

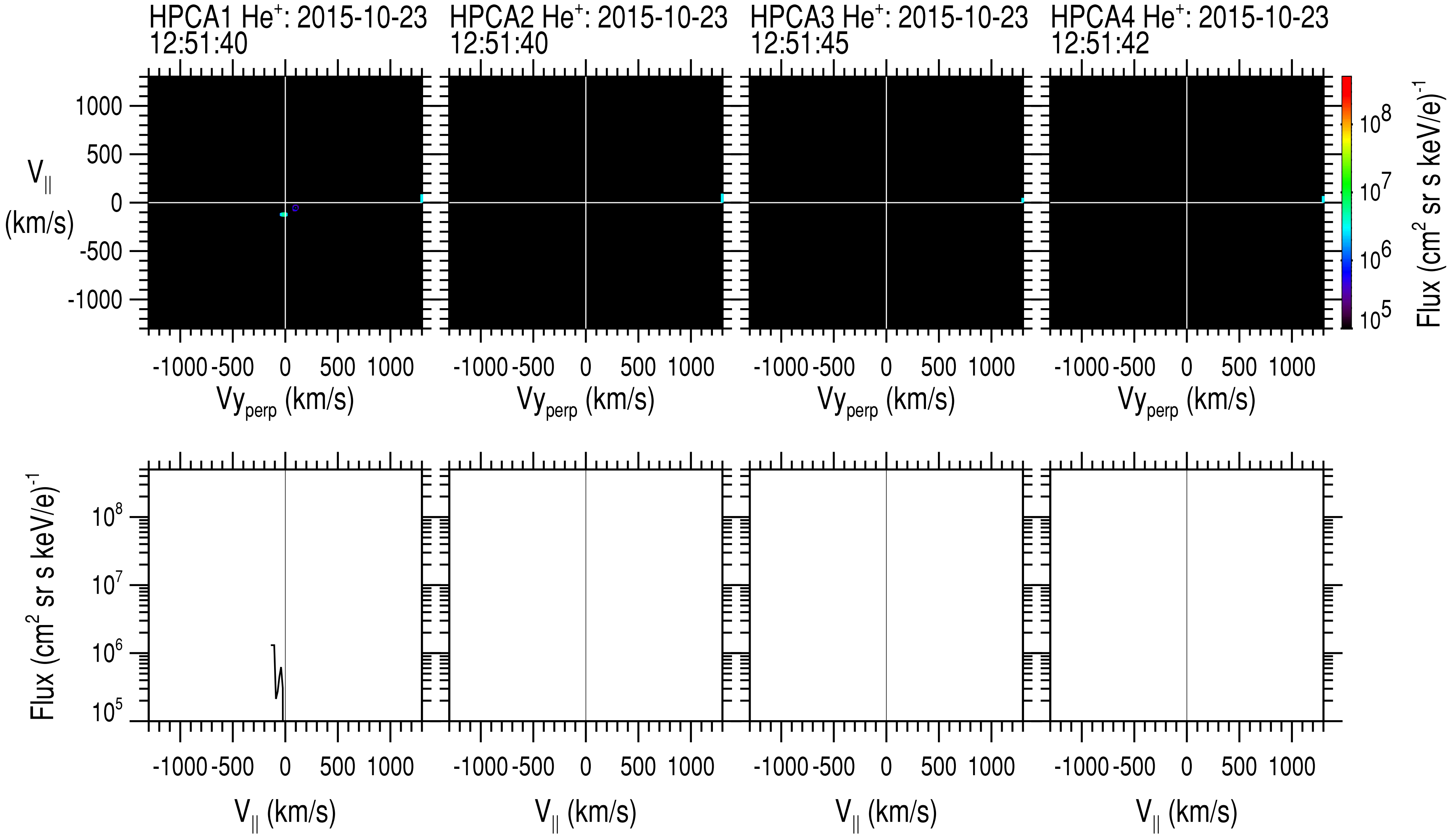

HPCA1 He+: 2015-10-23 12:51:40
HPCA2 He+: 2015-10-23 12:51:40
HPCA3 He+: 2015-10-23 12:51:45
HPCA4 He+: 2015-10-23 12:51:42
V|| (km/s)
Vyperp (km/s)
Flux (cm2 sr s keV/e)-1

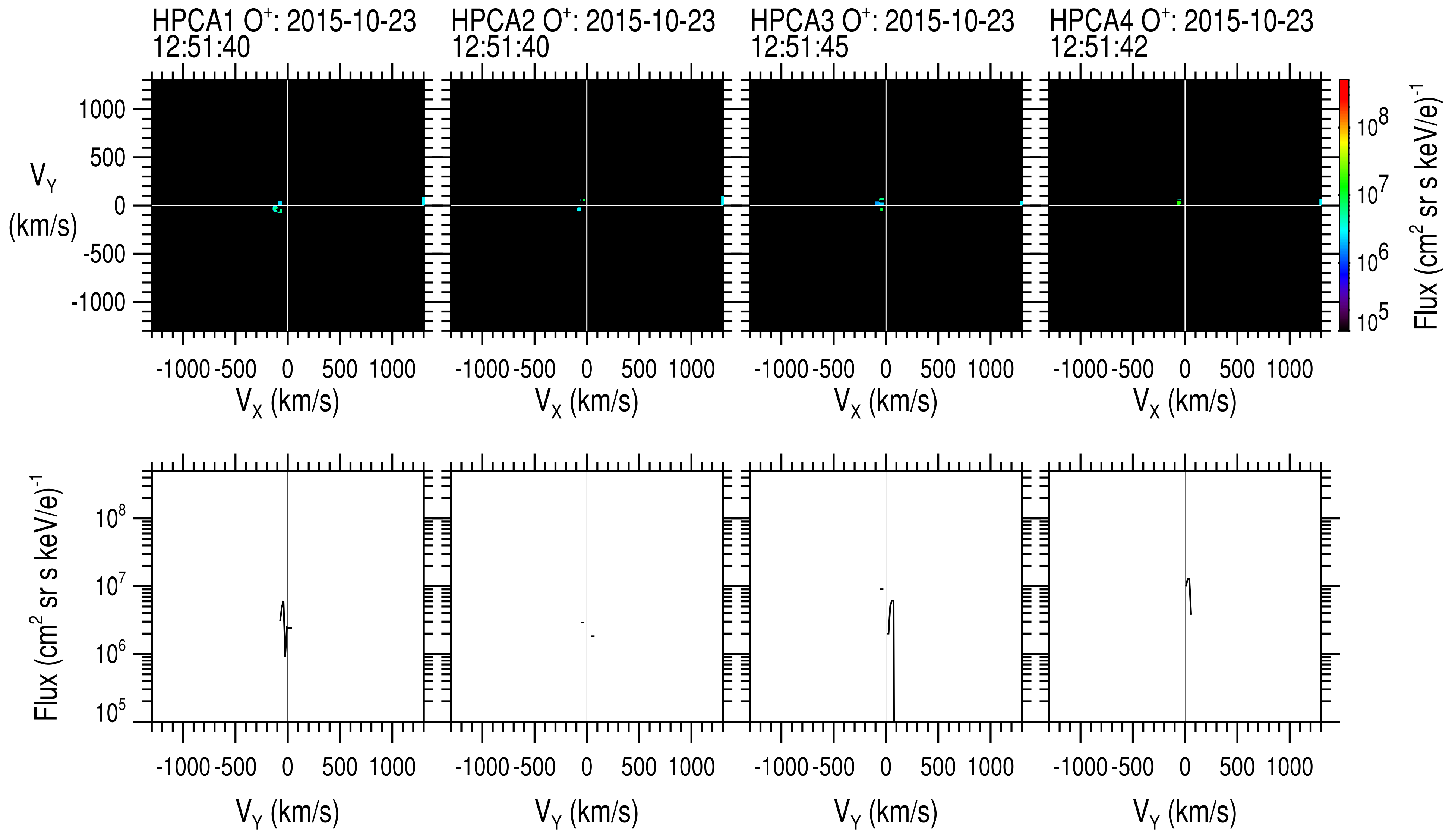
HPCA1 O+: 2015-10-23 12:51:40
HPCA2 O+: 2015-10-23 12:51:40
HPCA3 O+: 2015-10-23 12:51:45
HPCA4 O+: 2015-10-23 12:51:42
VY (km/s)
VX (km/s)
Flux (cm2 sr s keV/e)-1
-1000 -500 0 500 1000
10^5 10^6 10^7 10^8

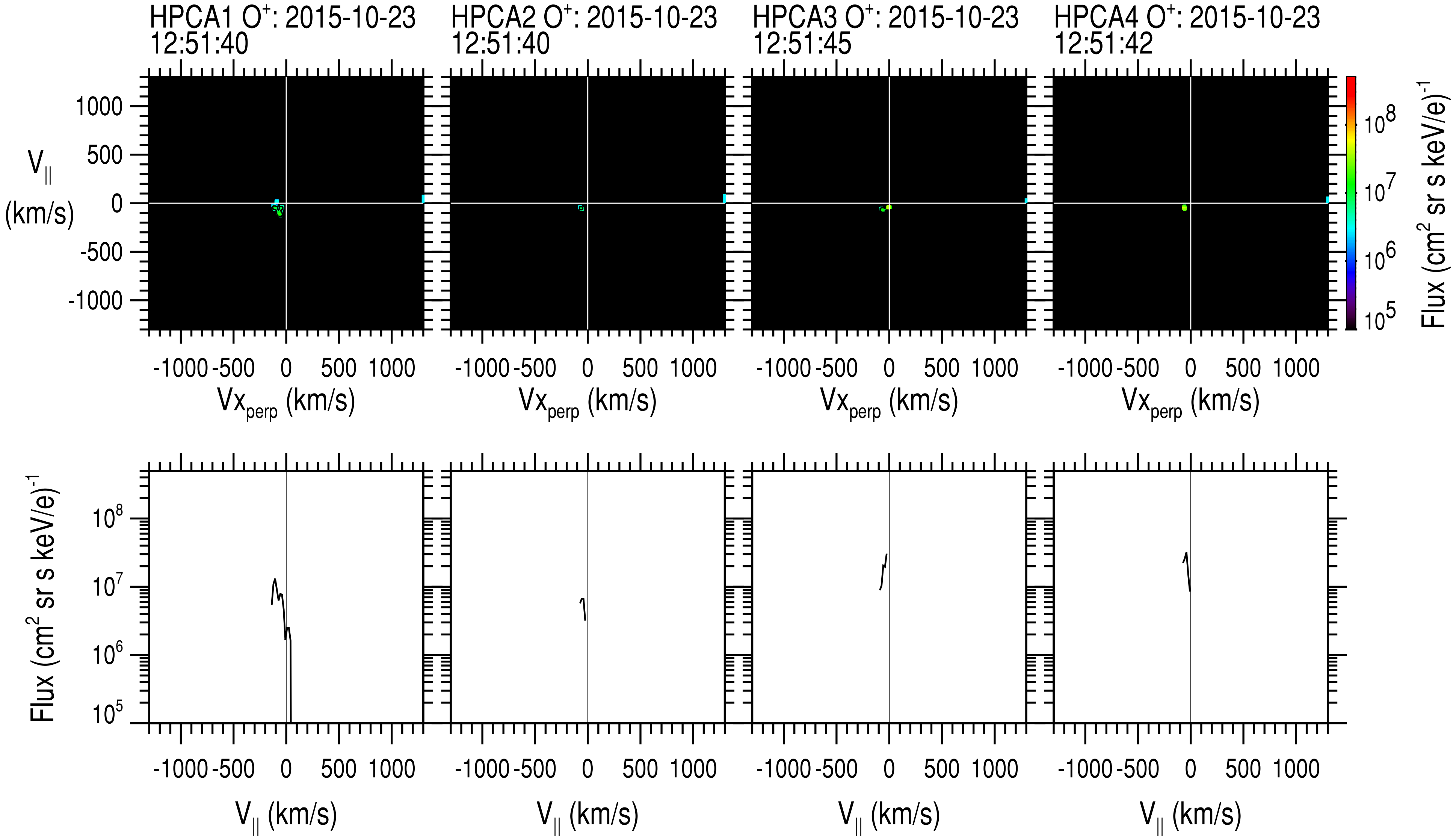
HPCA1 O+: 2015-10-23
12:51:40
HPCA2 O+: 2015-10-23
12:51:40
HPCA3 O+: 2015-10-23
12:51:45
HPCA4 O+: 2015-10-23
12:51:42
V|| (km/s)
Vxperp (km/s)
Flux (cm2 sr s keV/e)-1
V|| (km/s)

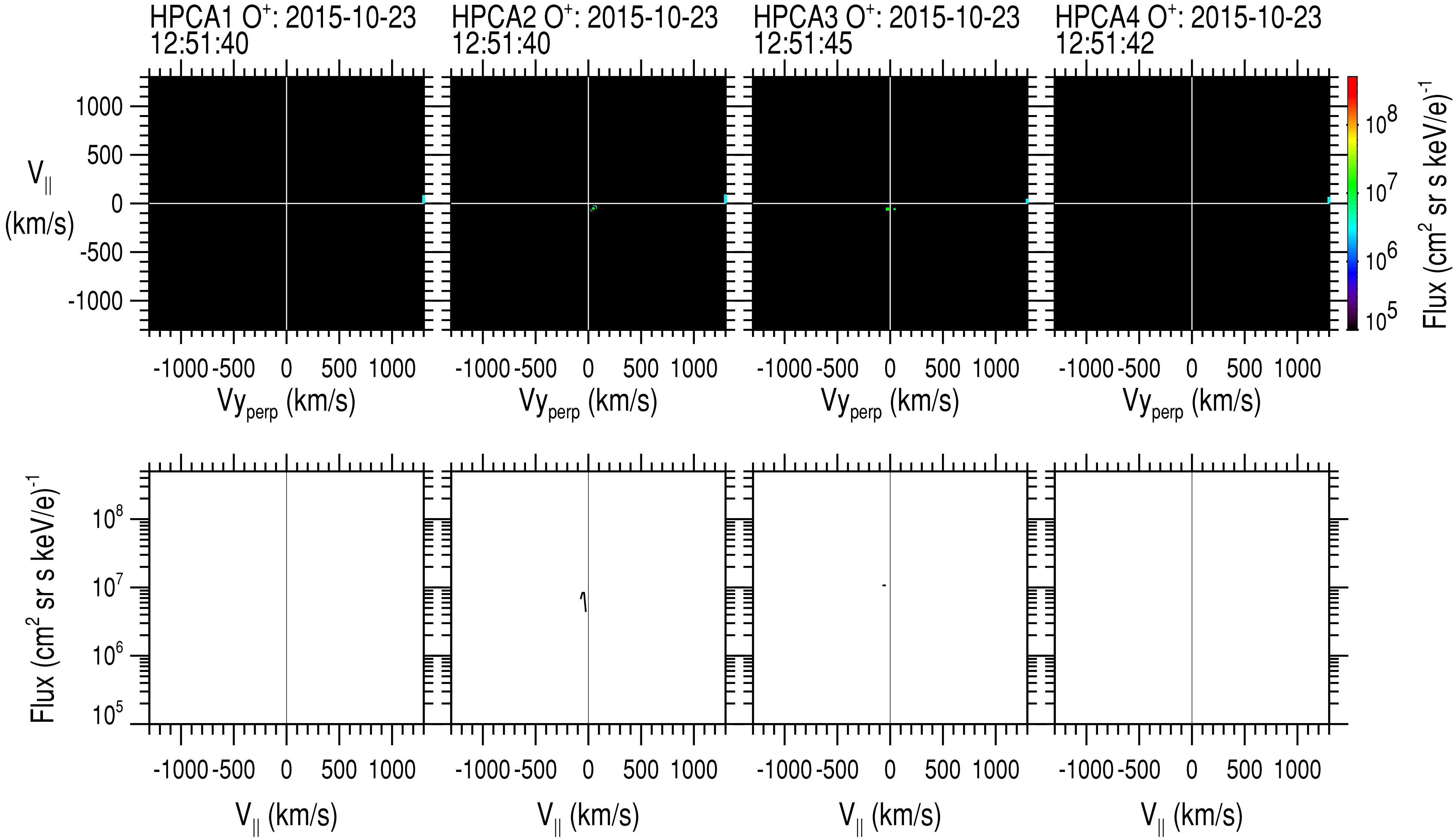
HPCA1 O+: 2015-10-23 12:51:40
HPCA2 O+: 2015-10-23 12:51:40
HPCA3 O+: 2015-10-23 12:51:45
HPCA4 O+: 2015-10-23 12:51:42
V|| (km/s)
Vyperp (km/s)
Flux (cm2 sr s keV/e)-1
-1000 -500 0 500 1000
10^5 10^6 10^7 10^8

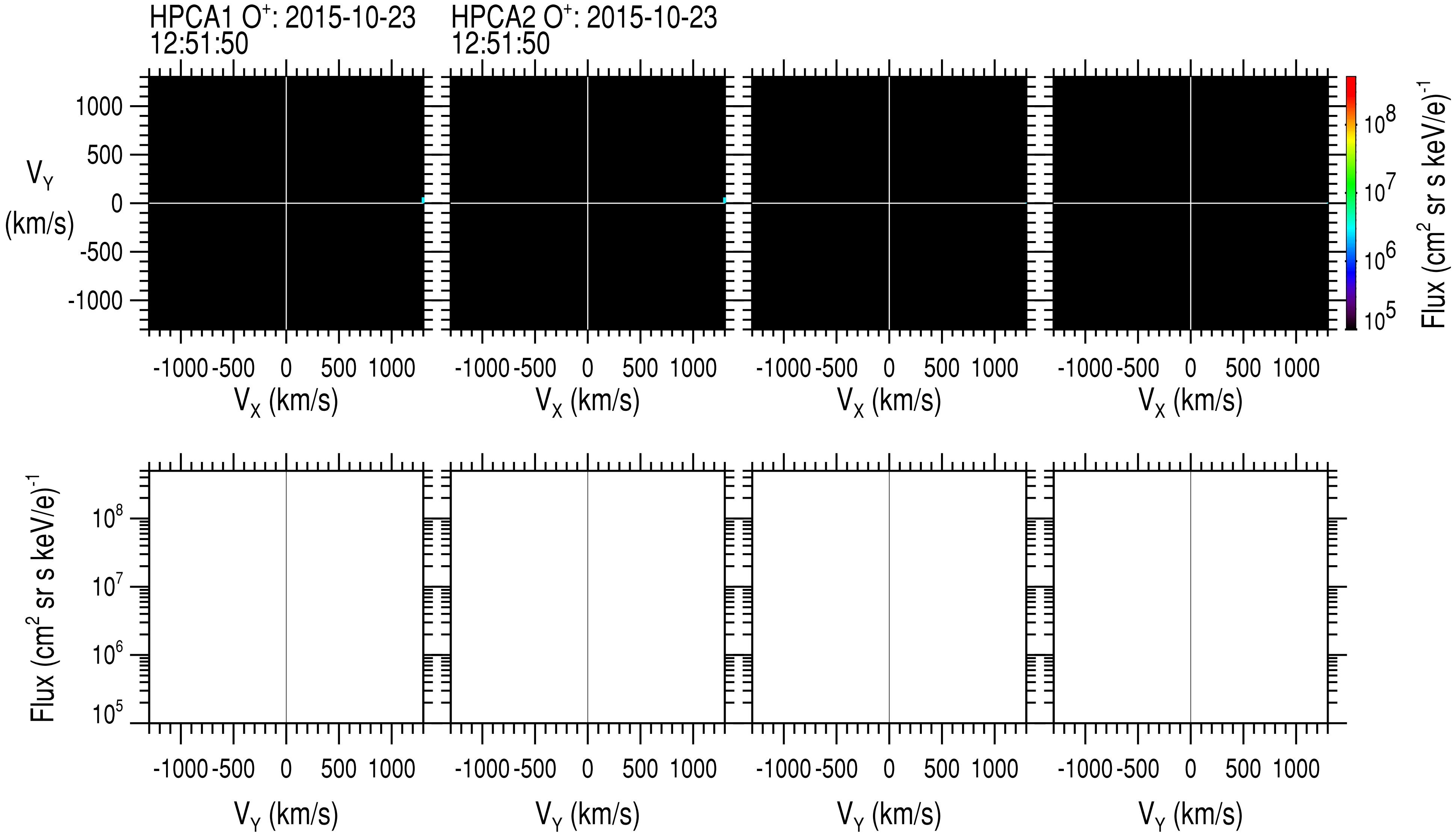
HPCA1 O+: 2015-10-23
12:51:50
HPCA2 O+: 2015-10-23
12:51:50
VY (km/s)
VX (km/s)
Flux (cm2 sr s keV/e)-1
VY (km/s)
Flux (cm2 sr s keV/e)-1

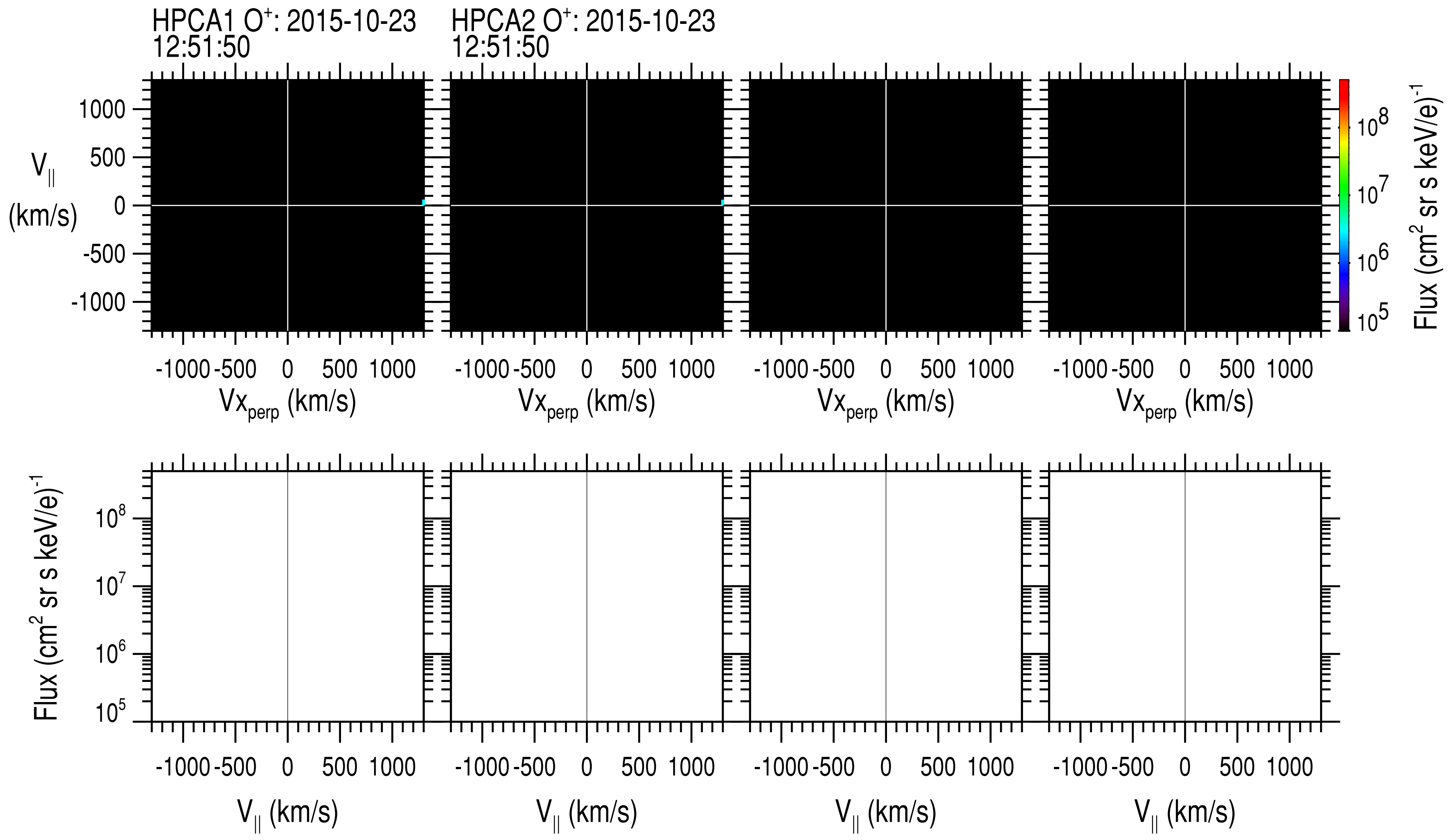
HPCA1 O+: 2015-10-23
12:51:50
HPCA2 O+: 2015-10-23
12:51:50
V|| (km/s)
1000
500
0
-500
-1000
Vx_perp (km/s)
Flux (cm2 sr s keV/e)-1
10^5
10^6
10^7
10^8

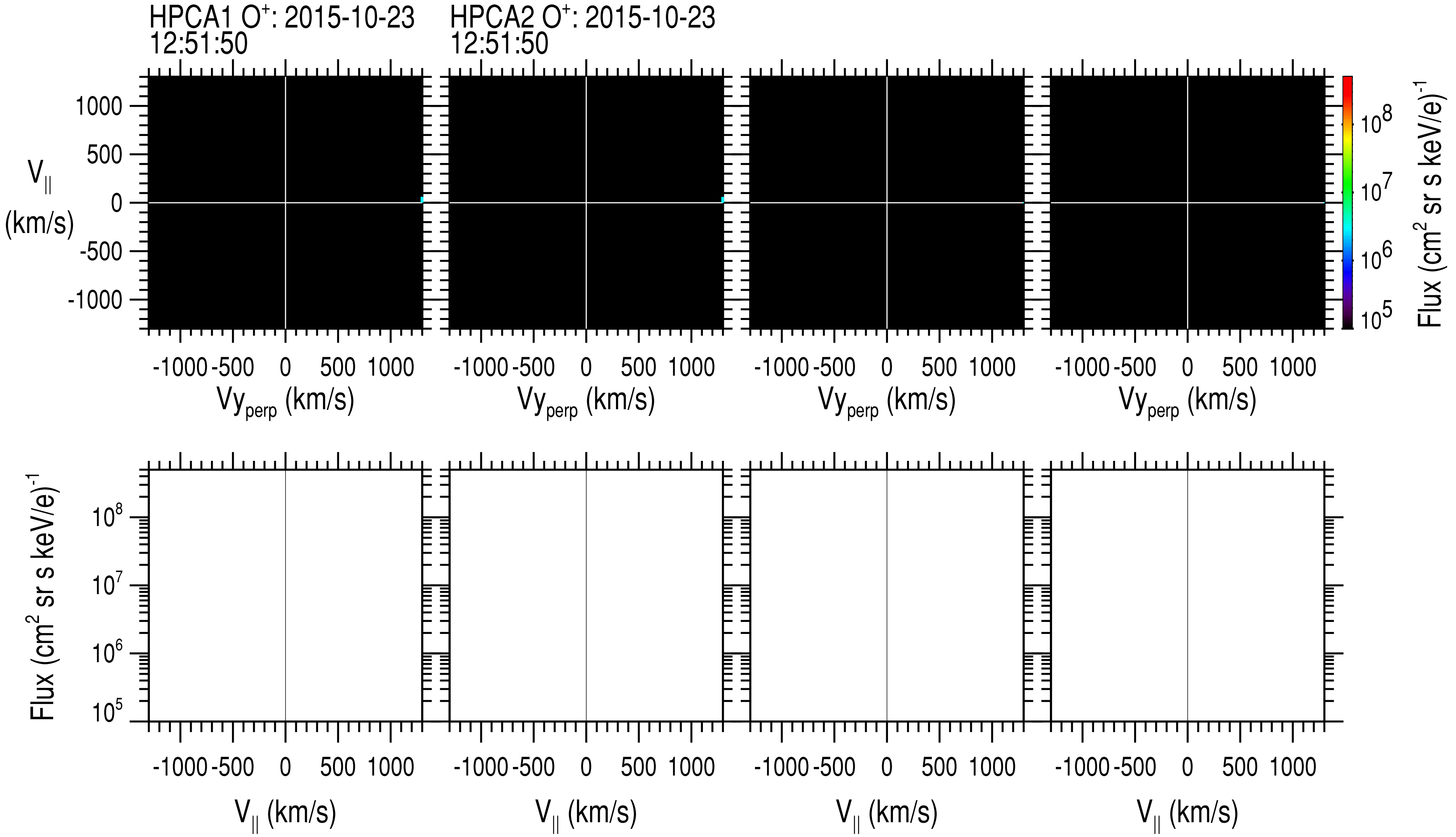

HPCA1 O+: 2015-10-23
12:51:50
HPCA2 O+: 2015-10-23
12:51:50
V|| (km/s)
1000
500
0
-500
-1000
-1000 -500 0 500 1000
Vyperp (km/s)
Flux (cm2 sr s keV/e)-1
10^5
10^6
10^7
10^8
V|| (km/s)
