# Supplementary material for: Characteristics of Minor Ions and Electrons in Flux Transfer Events Observed by the Magnetospheric Multiscale Mission
Source: J Geophys Res Space Phys. 2020 Jul 20;125(7):e2020JA027778. doi: 10.1029/2020JA027778 (PMC7507212; doi:10.1029/2020JA027778)
Supplement: Supplementary file 5 — Figure S4 [file JGRA-125-e2020JA027778-s005.pdf]

Supporting Information:

Journal of Geophysical Research – Space Physics

"Supporting Information for Characteristics of Minor Ions and Electrons in Flux Transfer Events Observed by the Magnetospheric Multiscale Mission"

S. M. Petrinec[1], J. L. Burch[2], M. Chandler[3], C. J. Farrugia[4], S. A. Fuselier[2,5], B. L. Giles[6], R. G. Gomez[2,5], J. Mukherjee[2], W. R. Paterson[6], C. T. Russell[7], D. G. Sibeck[6], R. J. Strangeway[7], R. B. Torbert[4], K. J. Trattner[8], S. K. Vines[9], and C. Zhao[7]

[1]*Lockheed Martin Advanced Technology Center, Palo Alto, CA, USA*

[2]*Southwest Research Institute, San Antonio, TX, USA*

[3]*NASA Marshall Space Flight Center, Huntsville, AL, USA*

[4]*Space Science Center, University of New Hampshire, Durham, NH, USA*

[5]*University of Texas at San Antonio, San Antonio, TX, USA*

[6]*NASA Goddard Space Flight Center, Greenbelt, MD, USA*

[7]*Earth and Space Sciences, University of California, Los Angeles, CA, USA*

[8]*Laboratory for Atmospheric and Space Physics, University of Colorado Boulder, Boulder, CO, USA*

[9]*The Johns Hopkins University Applied Physics Laboratory, Laurel, MD, USA*

2D and 1D flux distribution cuts (in a magnetic field coordinate system) from all four MMS HPCA instruments, for four species {H^+, He^{++}, He^+, and O^+}, during the FTE burst mode of 2015-10-11.

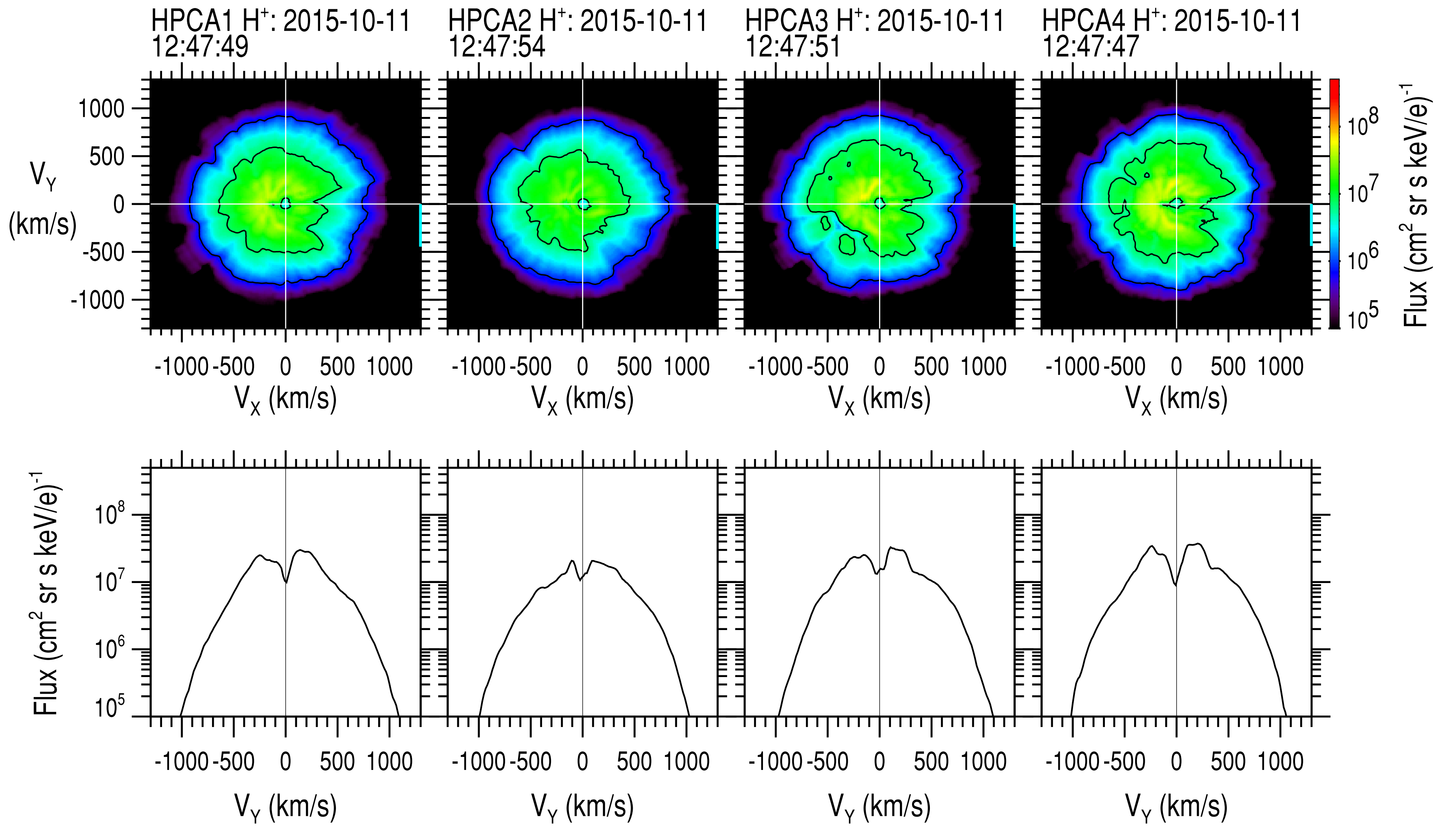

HPCA1 H+: 2015-10-11 12:47:49
HPCA2 H+: 2015-10-11 12:47:54
HPCA3 H+: 2015-10-11 12:47:51
HPCA4 H+: 2015-10-11 12:47:47
V_Y (km/s)
V_X (km/s)
Flux (cm^2 sr s keV/e)^-1
V_Y (km/s)

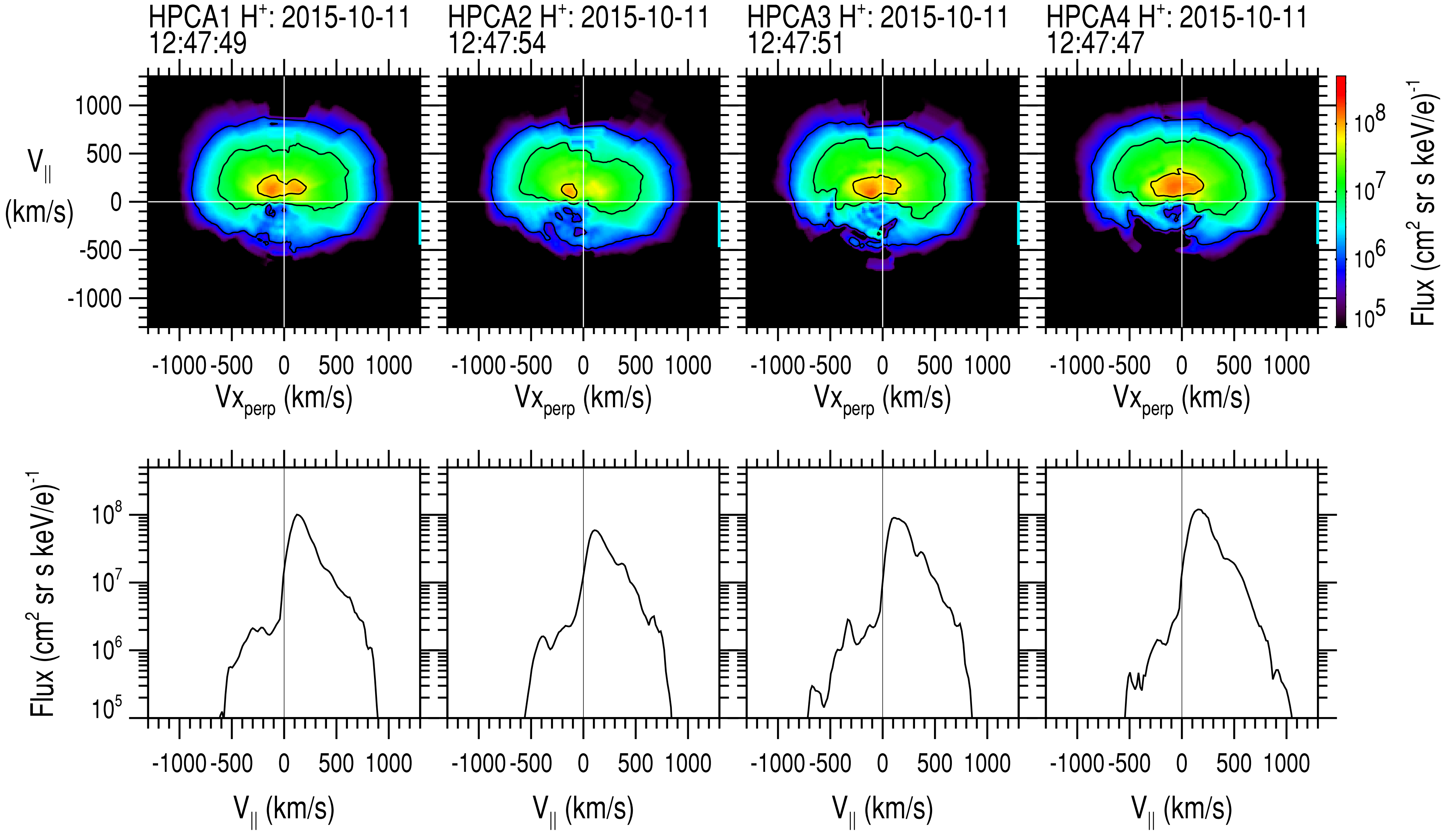

HPCA1 H+: 2015-10-11
12:47:49
HPCA2 H+: 2015-10-11
12:47:54
HPCA3 H+: 2015-10-11
12:47:51
HPCA4 H+: 2015-10-11
12:47:47
V|| (km/s)
Vxperp (km/s)
Flux (cm2 sr s keV/e)-1

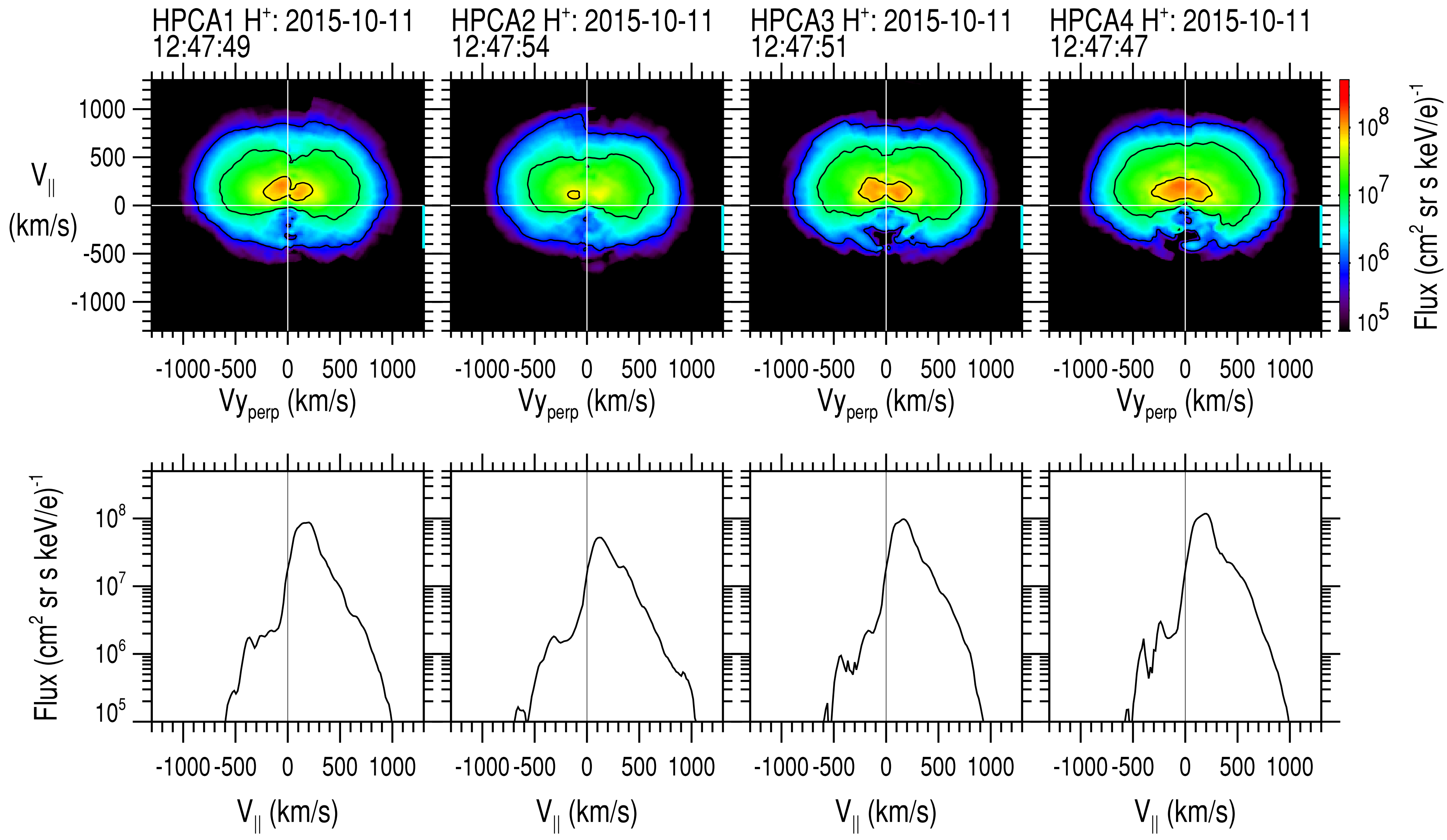

HPCA1 H+: 2015-10-11 12:47:49
HPCA2 H+: 2015-10-11 12:47:54
HPCA3 H+: 2015-10-11 12:47:51
HPCA4 H+: 2015-10-11 12:47:47
V|| (km/s)
Vyperp (km/s)
Flux (cm2 sr s keV/e)-1
-1000 -500 0 500 1000
105 106 107 108

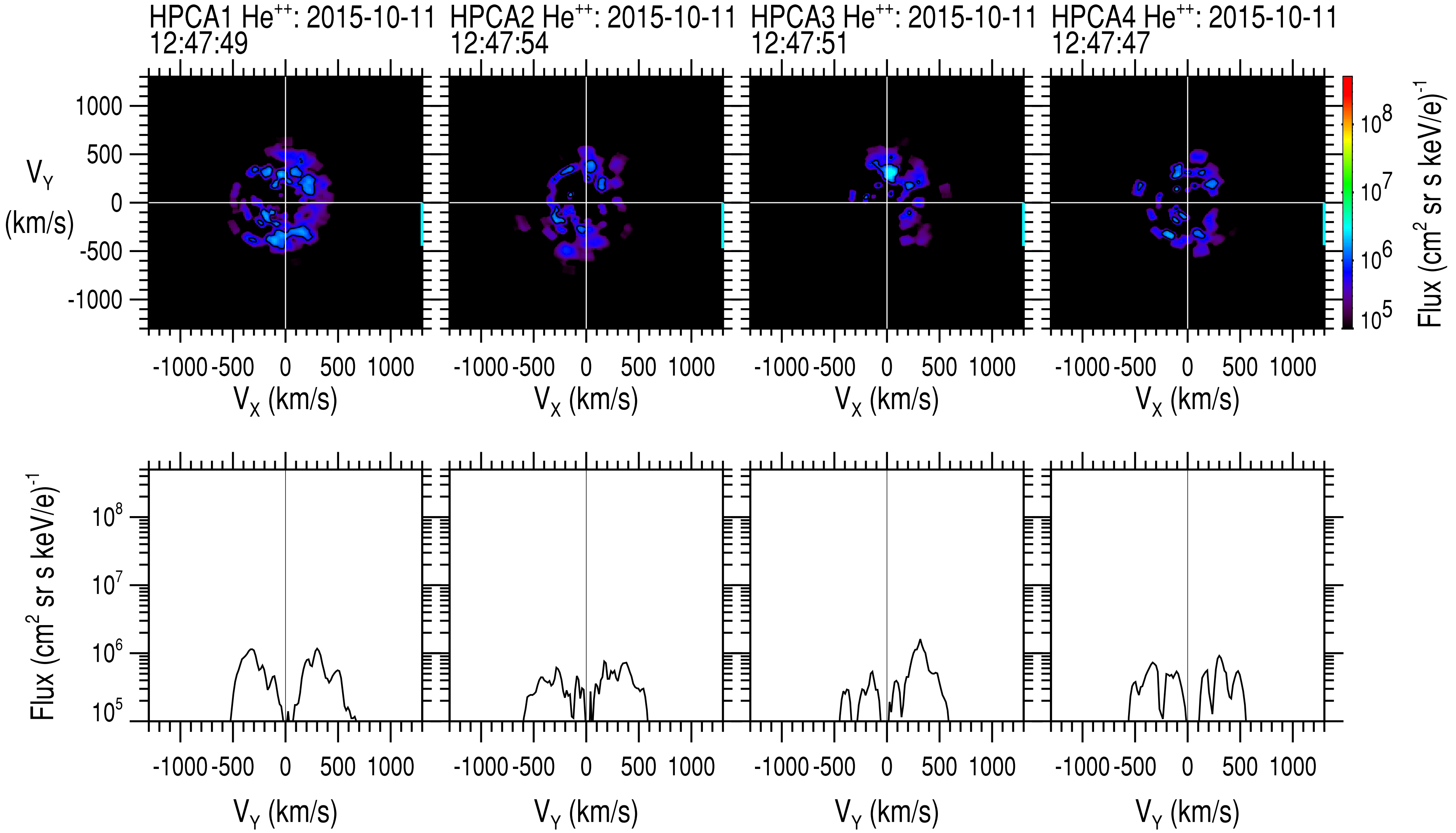

HPCA1 He++: 2015-10-11 12:47:49
HPCA2 He++: 2015-10-11 12:47:54
HPCA3 He++: 2015-10-11 12:47:51
HPCA4 He++: 2015-10-11 12:47:47
V_Y (km/s)
V_X (km/s)
Flux (cm^2 sr s keV/e)^-1

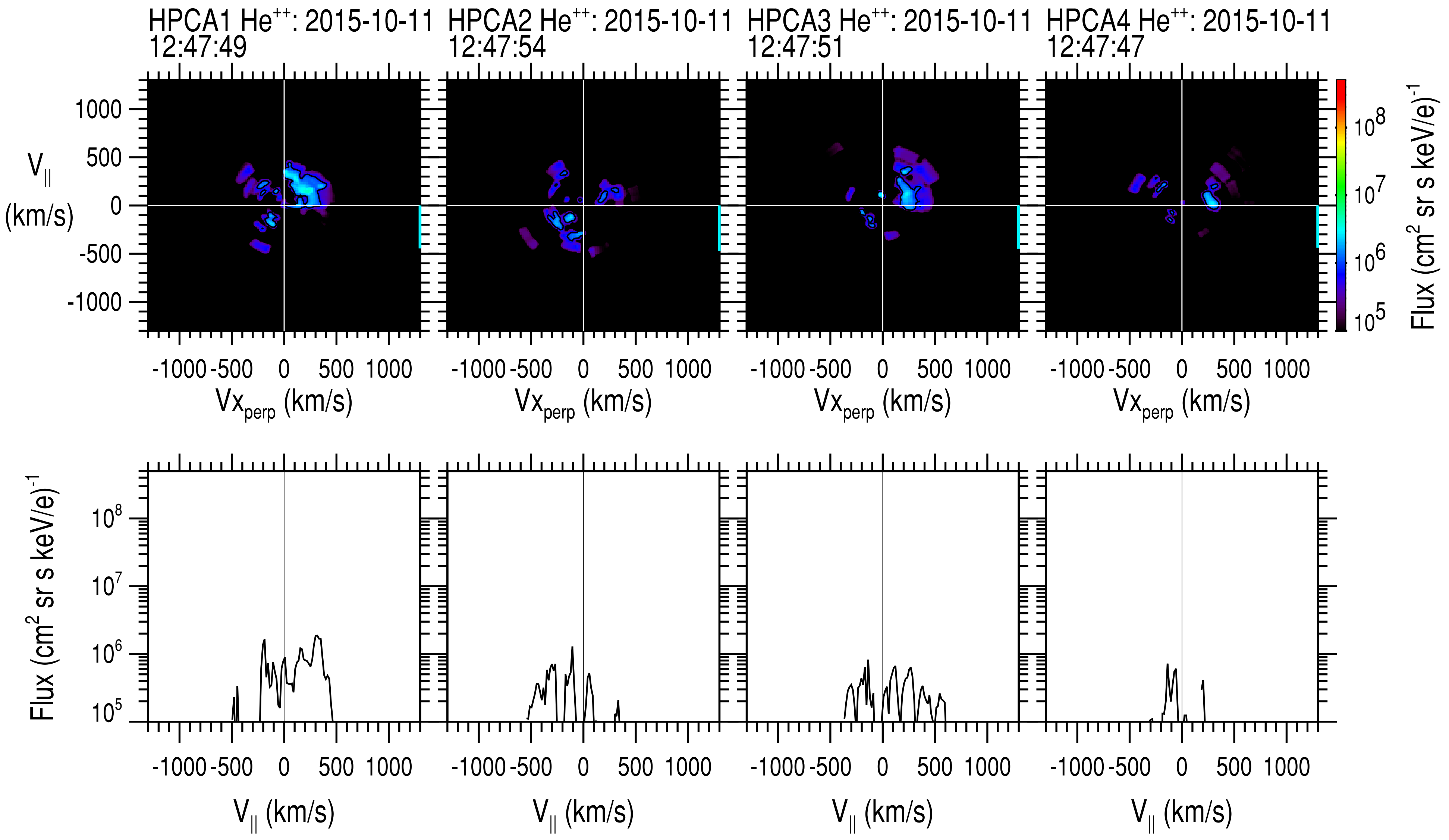
HPCA1 He++: 2015-10-11 12:47:49
HPCA2 He++: 2015-10-11 12:47:54
HPCA3 He++: 2015-10-11 12:47:51
HPCA4 He++: 2015-10-11 12:47:47
V|| (km/s)
Vxperp (km/s)
Flux (cm2 sr s keV/e)-1

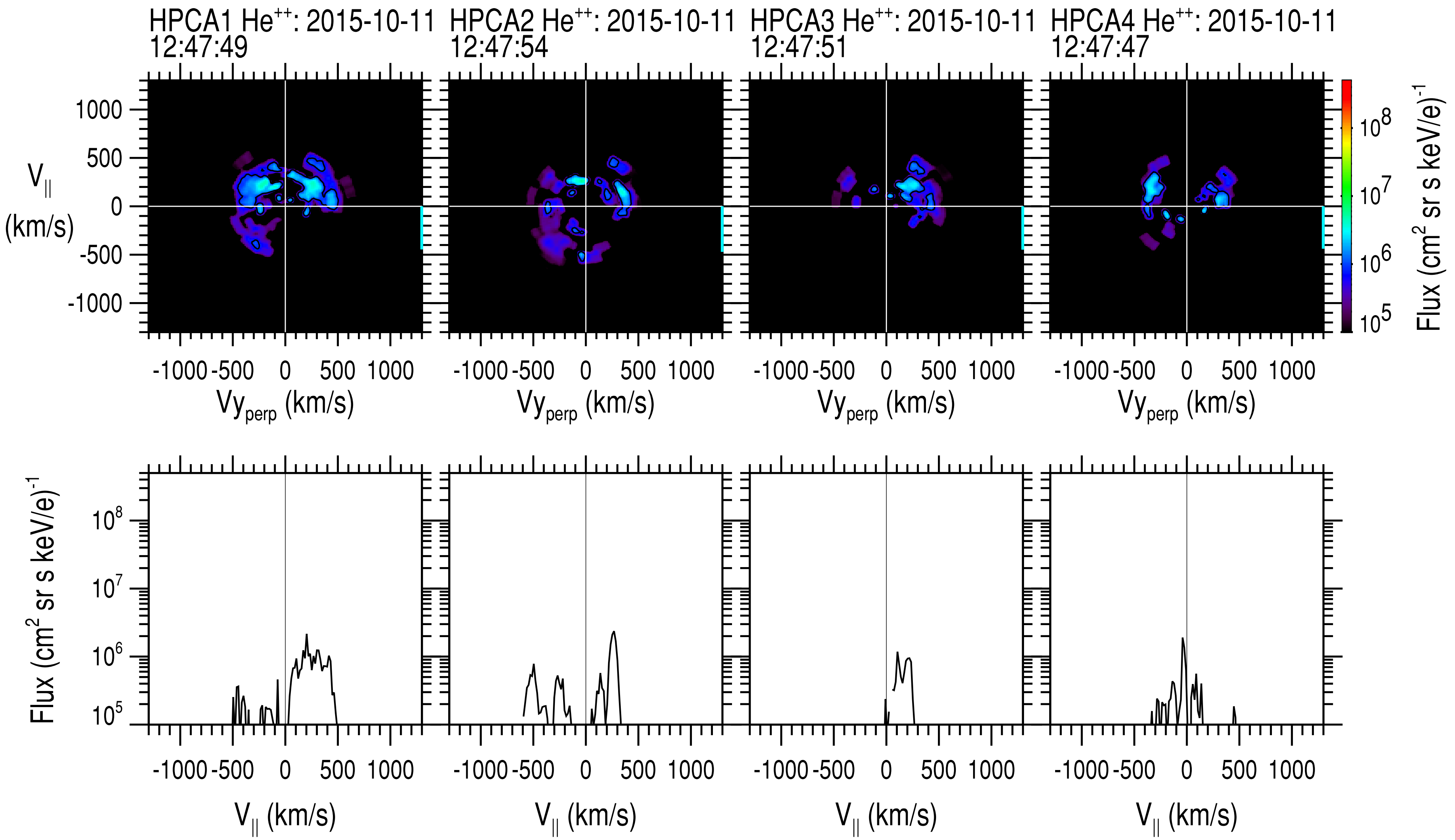
HPCA1 He++: 2015-10-11 12:47:49
HPCA2 He++: 2015-10-11 12:47:54
HPCA3 He++: 2015-10-11 12:47:51
HPCA4 He++: 2015-10-11 12:47:47
V|| (km/s)
Vyperp (km/s)
Flux (cm2 sr s keV/e)-1
V|| (km/s)

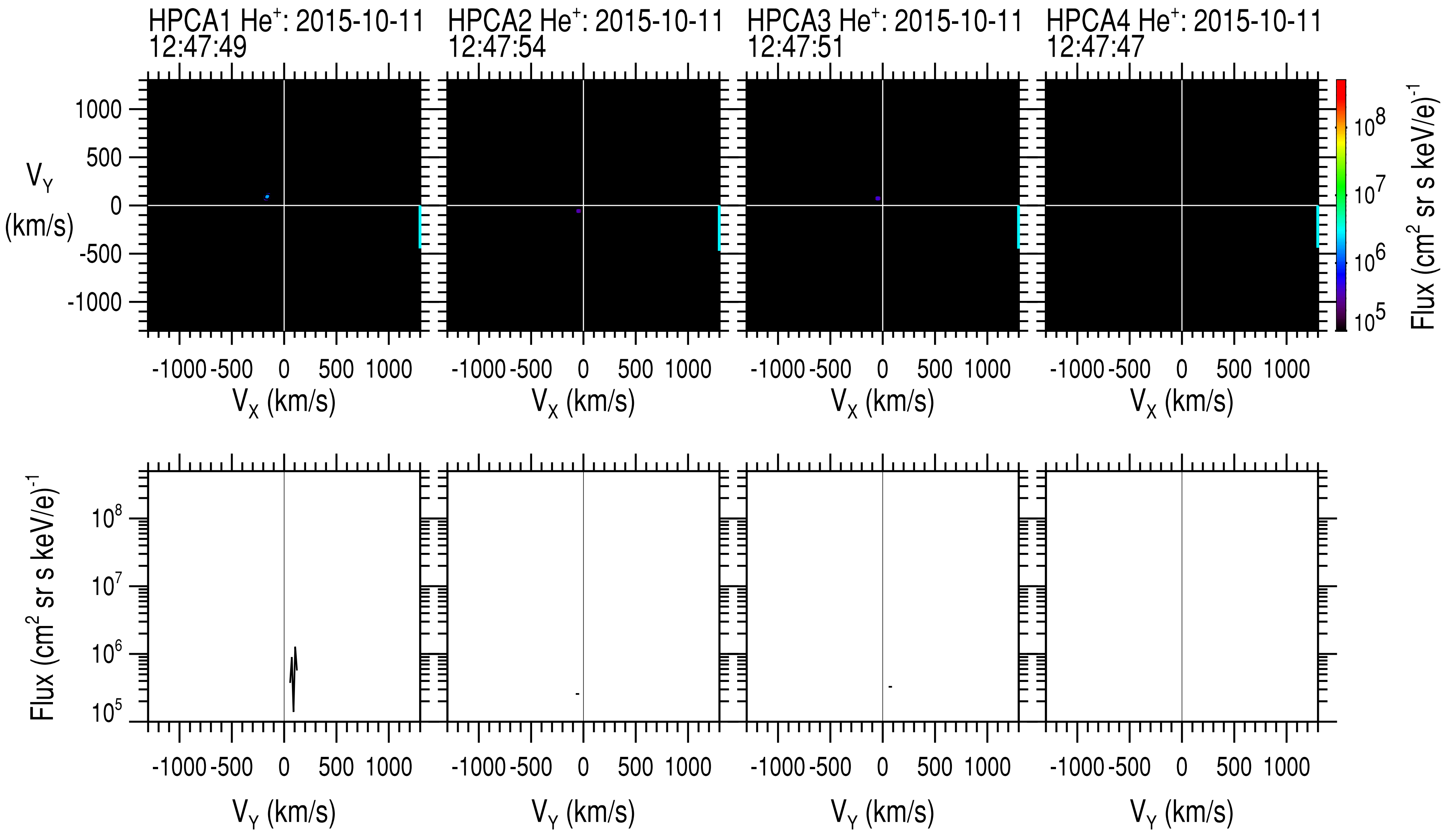

HPCA1 He+: 2015-10-11 12:47:49
HPCA2 He+: 2015-10-11 12:47:54
HPCA3 He+: 2015-10-11 12:47:51
HPCA4 He+: 2015-10-11 12:47:47
VY (km/s)
VX (km/s)
Flux (cm2 sr s keV/e)-1
VY (km/s)

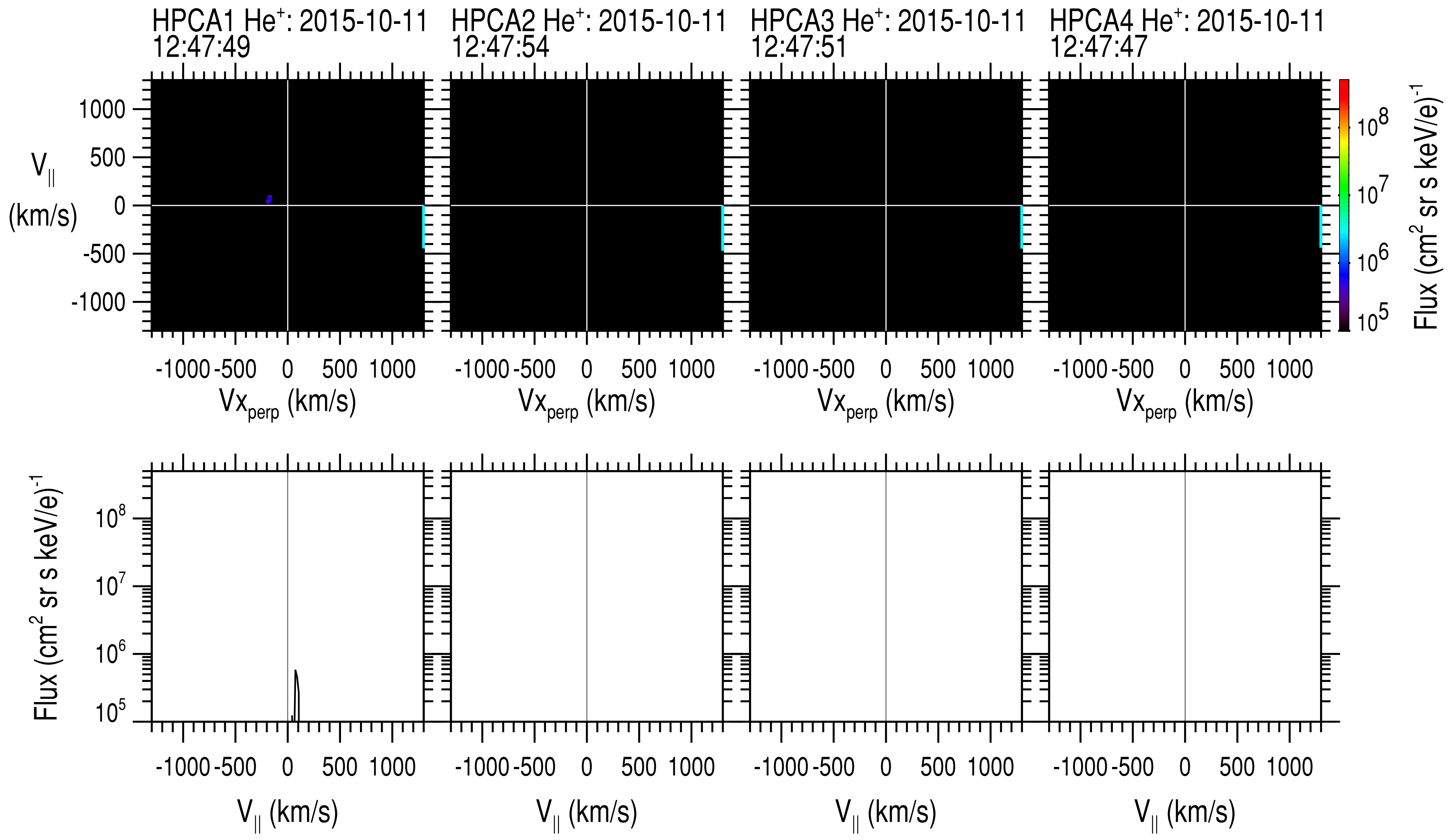

HPCA1 He+: 2015-10-11 12:47:49
HPCA2 He+: 2015-10-11 12:47:54
HPCA3 He+: 2015-10-11 12:47:51
HPCA4 He+: 2015-10-11 12:47:47
V|| (km/s)
Vxperp (km/s)
Flux (cm2 sr s keV/e)-1

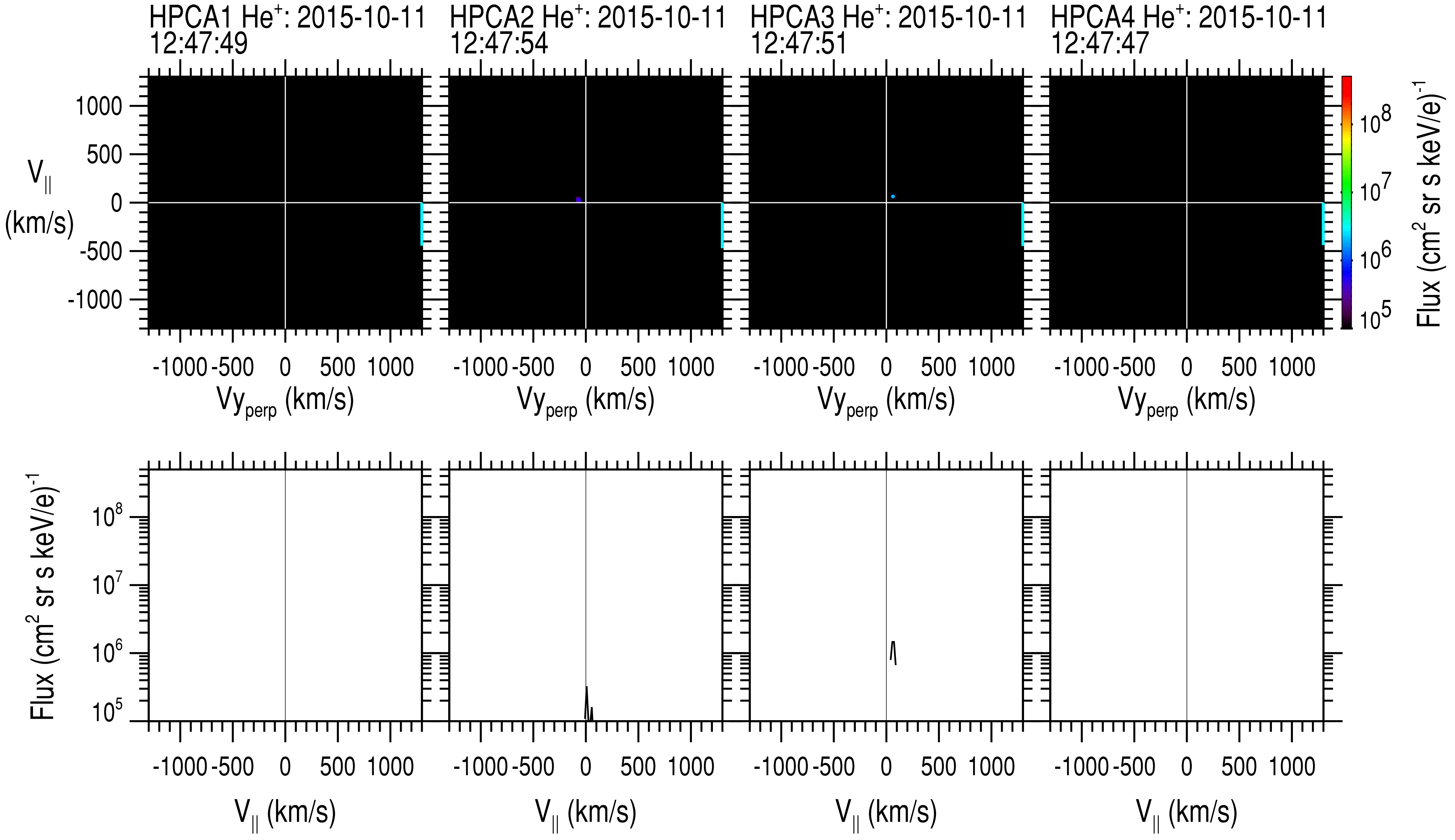

HPCA1 He+: 2015-10-11 12:47:49
HPCA2 He+: 2015-10-11 12:47:54
HPCA3 He+: 2015-10-11 12:47:51
HPCA4 He+: 2015-10-11 12:47:47
V|| (km/s)
Vyperp (km/s)
Flux (cm2 sr s keV/e)-1

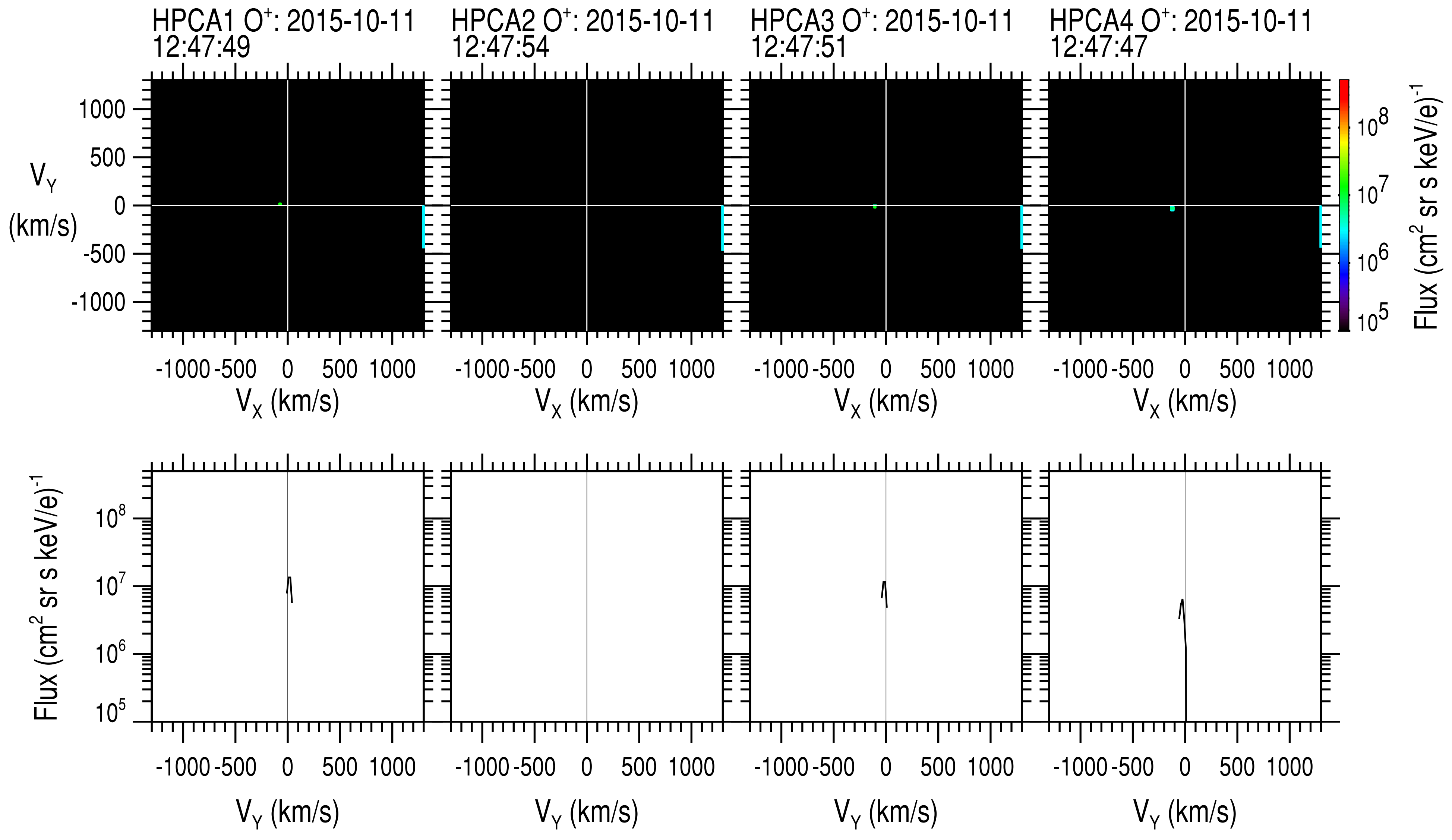

HPCA1 O+: 2015-10-11 12:47:49
HPCA2 O+: 2015-10-11 12:47:54
HPCA3 O+: 2015-10-11 12:47:51
HPCA4 O+: 2015-10-11 12:47:47
VY (km/s)
VX (km/s)
Flux (cm2 sr s keV/e)-1

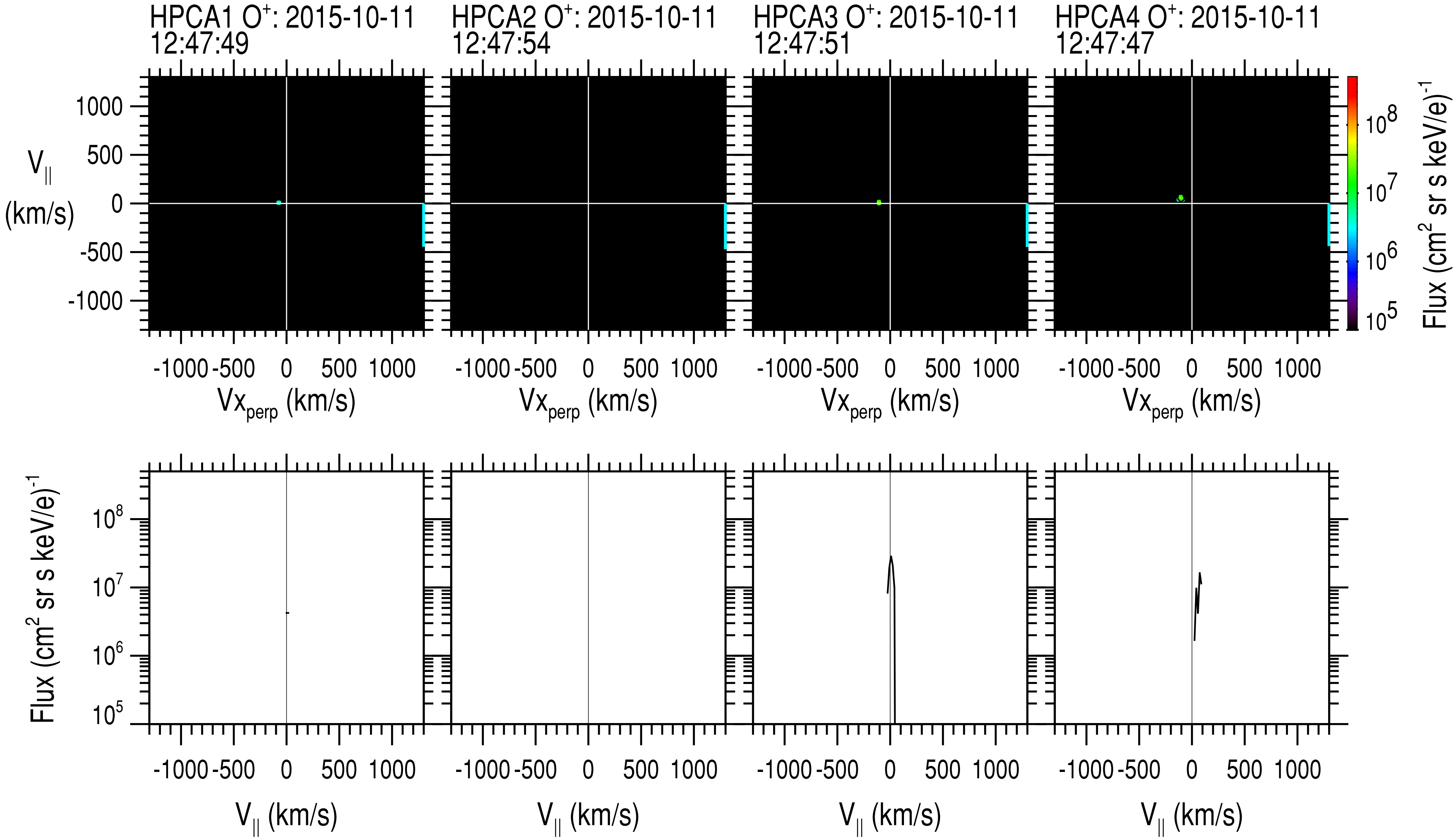

HPCA1 O+: 2015-10-11 12:47:49
HPCA2 O+: 2015-10-11 12:47:54
HPCA3 O+: 2015-10-11 12:47:51
HPCA4 O+: 2015-10-11 12:47:47
V|| (km/s)
1000
500
0
-500
-1000
Vxperp (km/s)
Flux (cm2 sr s keV/e)-1
10^5
10^6
10^7
10^8
V|| (km/s)

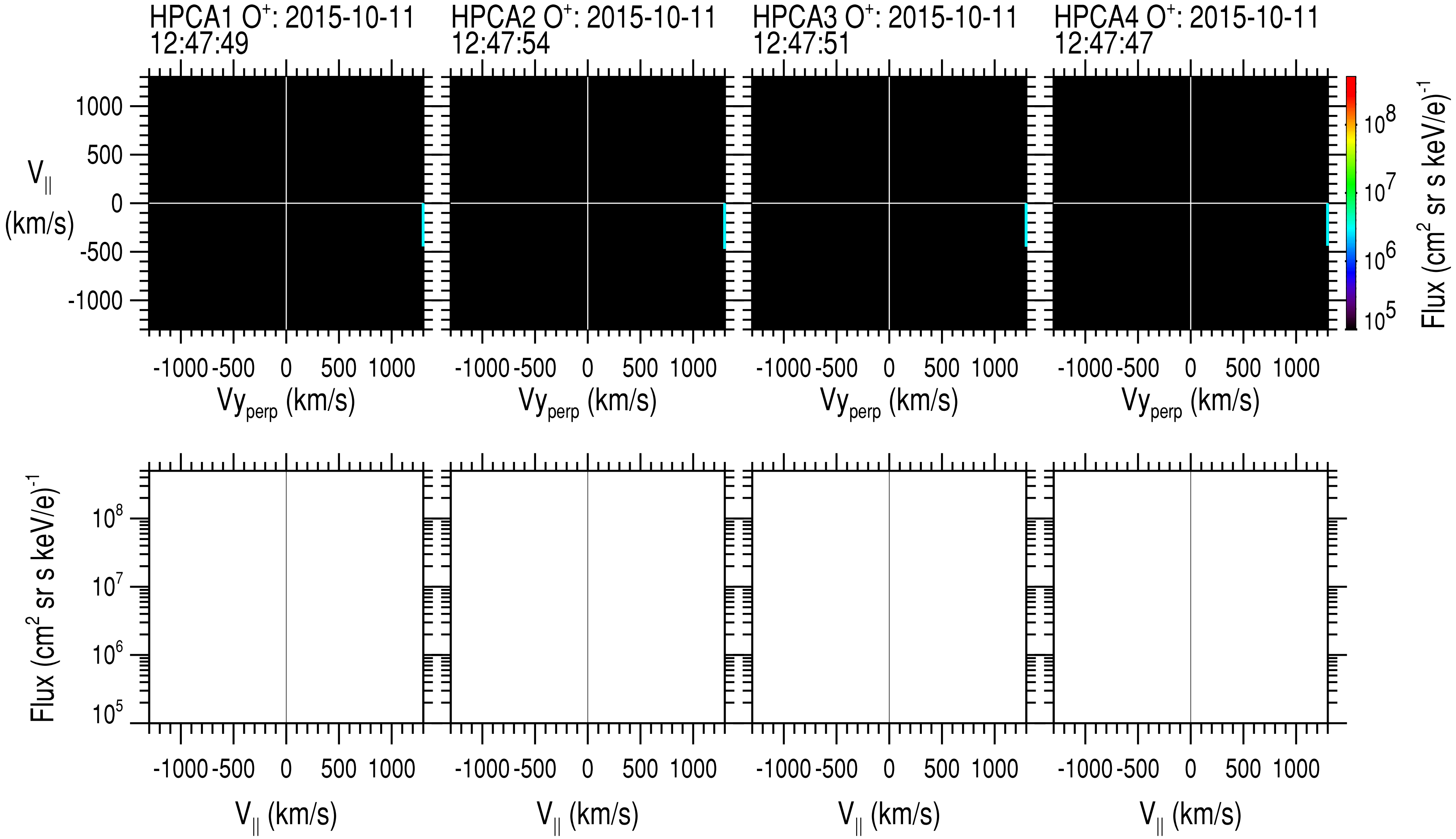
HPCA1 O+: 2015-10-11
12:47:49
HPCA2 O+: 2015-10-11
12:47:54
HPCA3 O+: 2015-10-11
12:47:51
HPCA4 O+: 2015-10-11
12:47:47
V|| (km/s)
Vyperp (km/s)
Flux (cm2 sr s keV/e)-1
V|| (km/s)

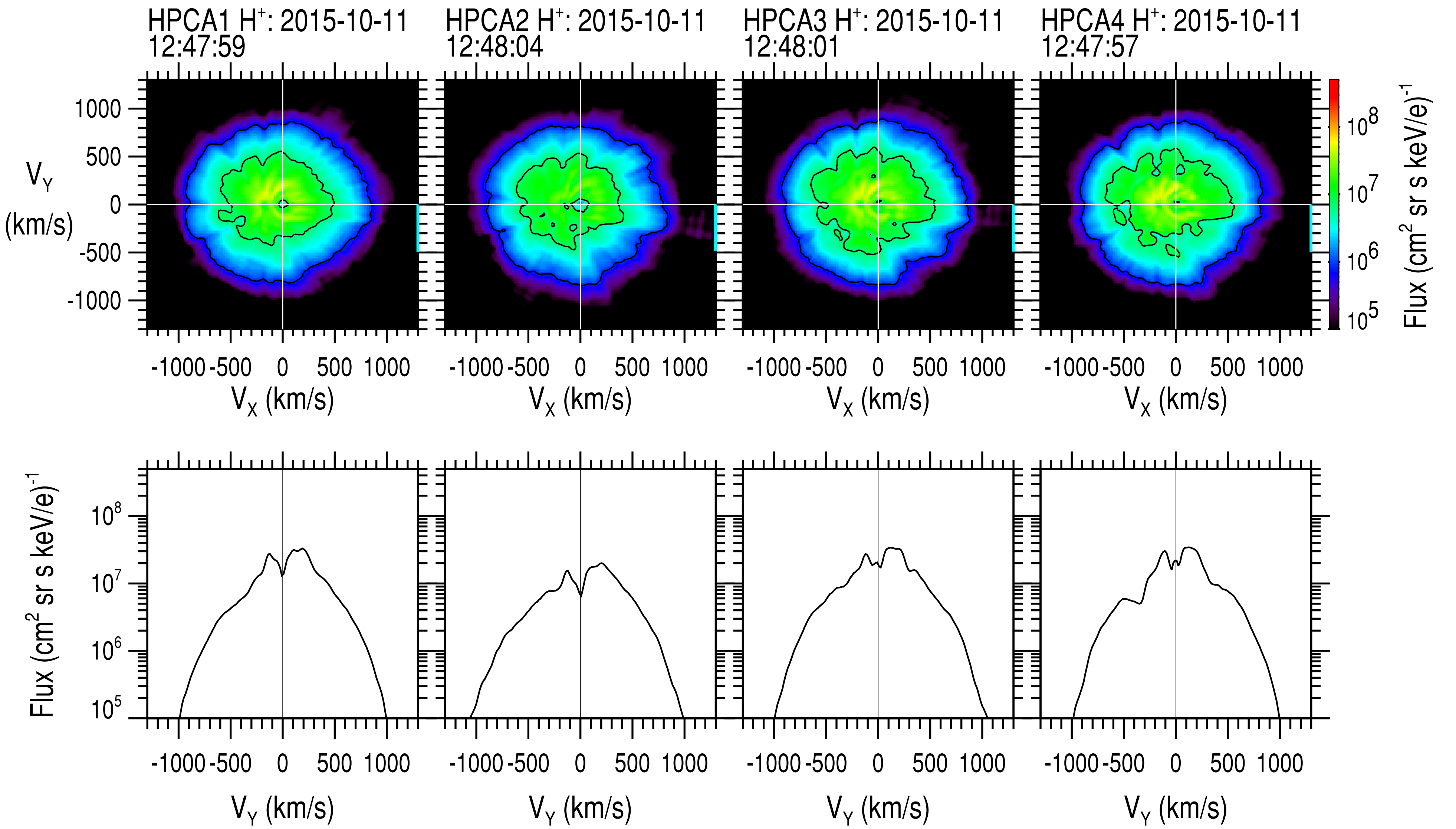
HPCA1 H+: 2015-10-11
12:47:59
HPCA2 H+: 2015-10-11
12:48:04
HPCA3 H+: 2015-10-11
12:48:01
HPCA4 H+: 2015-10-11
12:47:57
V_Y (km/s)
V_X (km/s)
Flux (cm^2 sr s keV/e)^-1
V_Y (km/s)

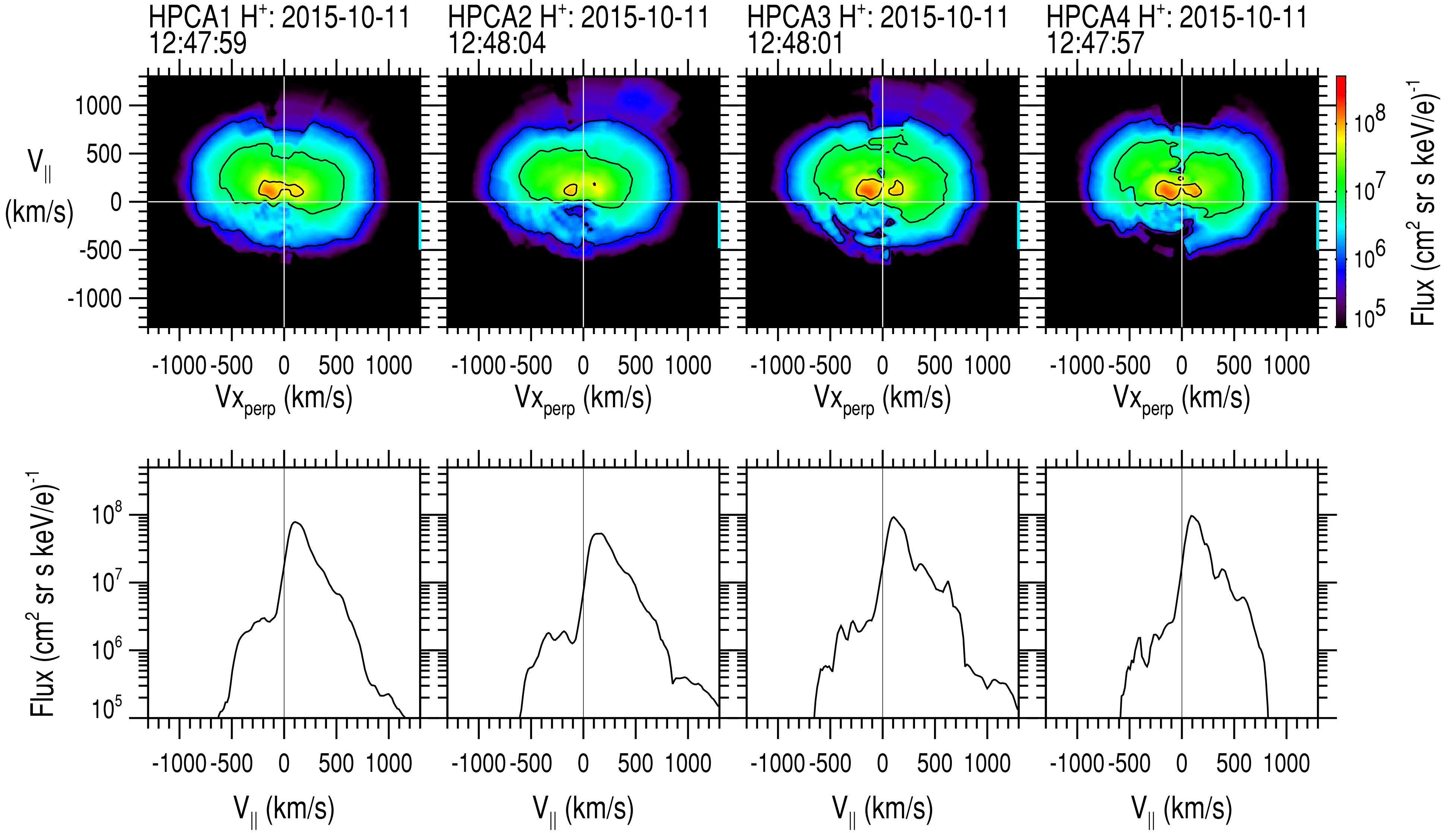

HPCA1 H⁺: 2015-10-11 12:47:59
HPCA2 H⁺: 2015-10-11 12:48:04
HPCA3 H⁺: 2015-10-11 12:48:01
HPCA4 H⁺: 2015-10-11 12:47:57
V|| (km/s)
Vx_perp (km/s)
Flux (cm² sr s keV/e)⁻¹
V|| (km/s)

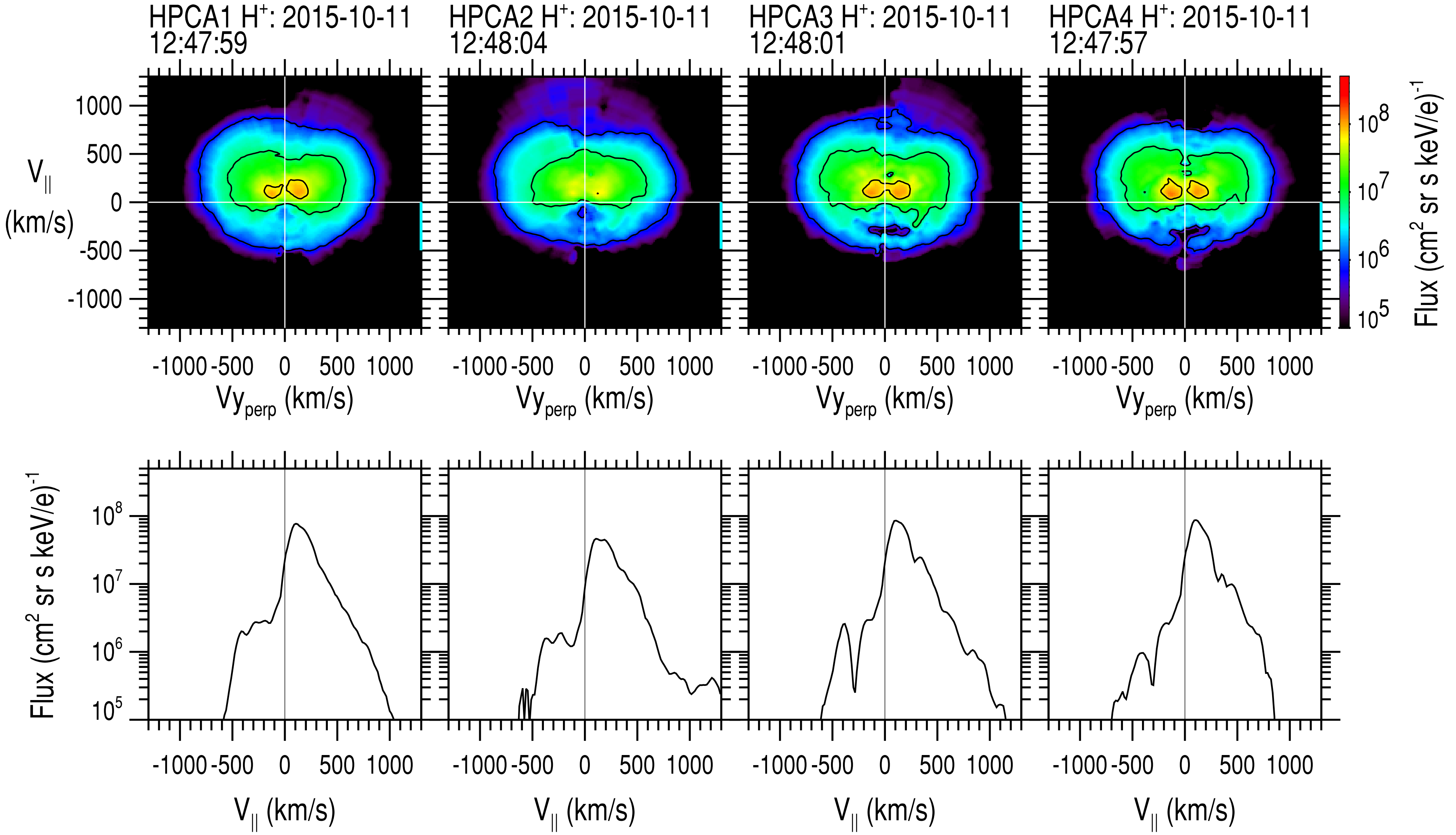

HPCA1 H+: 2015-10-11 12:47:59
HPCA2 H+: 2015-10-11 12:48:04
HPCA3 H+: 2015-10-11 12:48:01
HPCA4 H+: 2015-10-11 12:47:57
V|| (km/s)
Vyperp (km/s)
Flux (cm2 sr s keV/e)-1

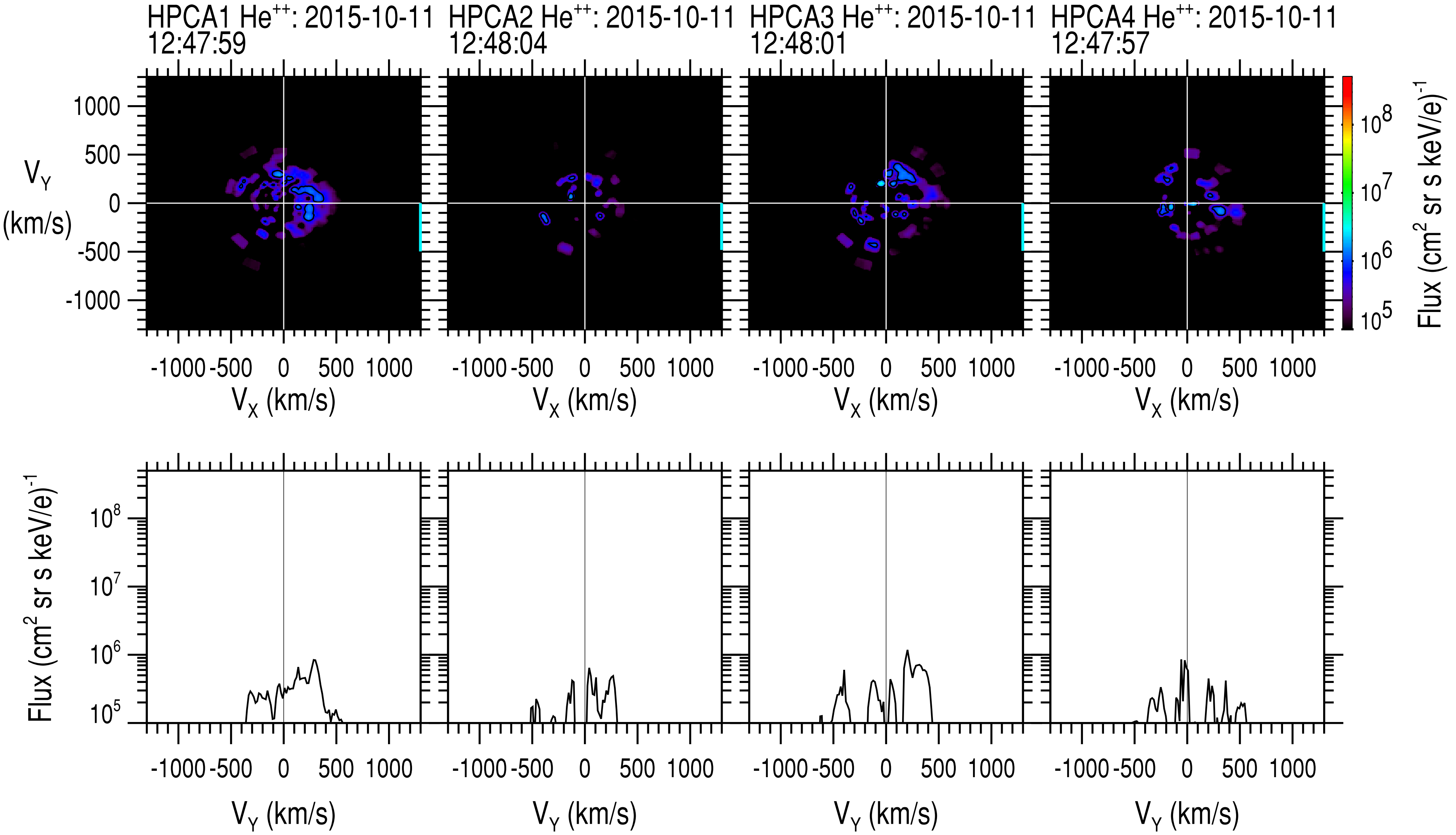

HPCA1 He^{++}: 2015-10-11 12:47:59
HPCA2 He^{++}: 2015-10-11 12:48:04
HPCA3 He^{++}: 2015-10-11 12:48:01
HPCA4 He^{++}: 2015-10-11 12:47:57
V_Y (km/s)
V_X (km/s)
Flux $(cm^2\ sr\ s\ keV/e)^{-1}$

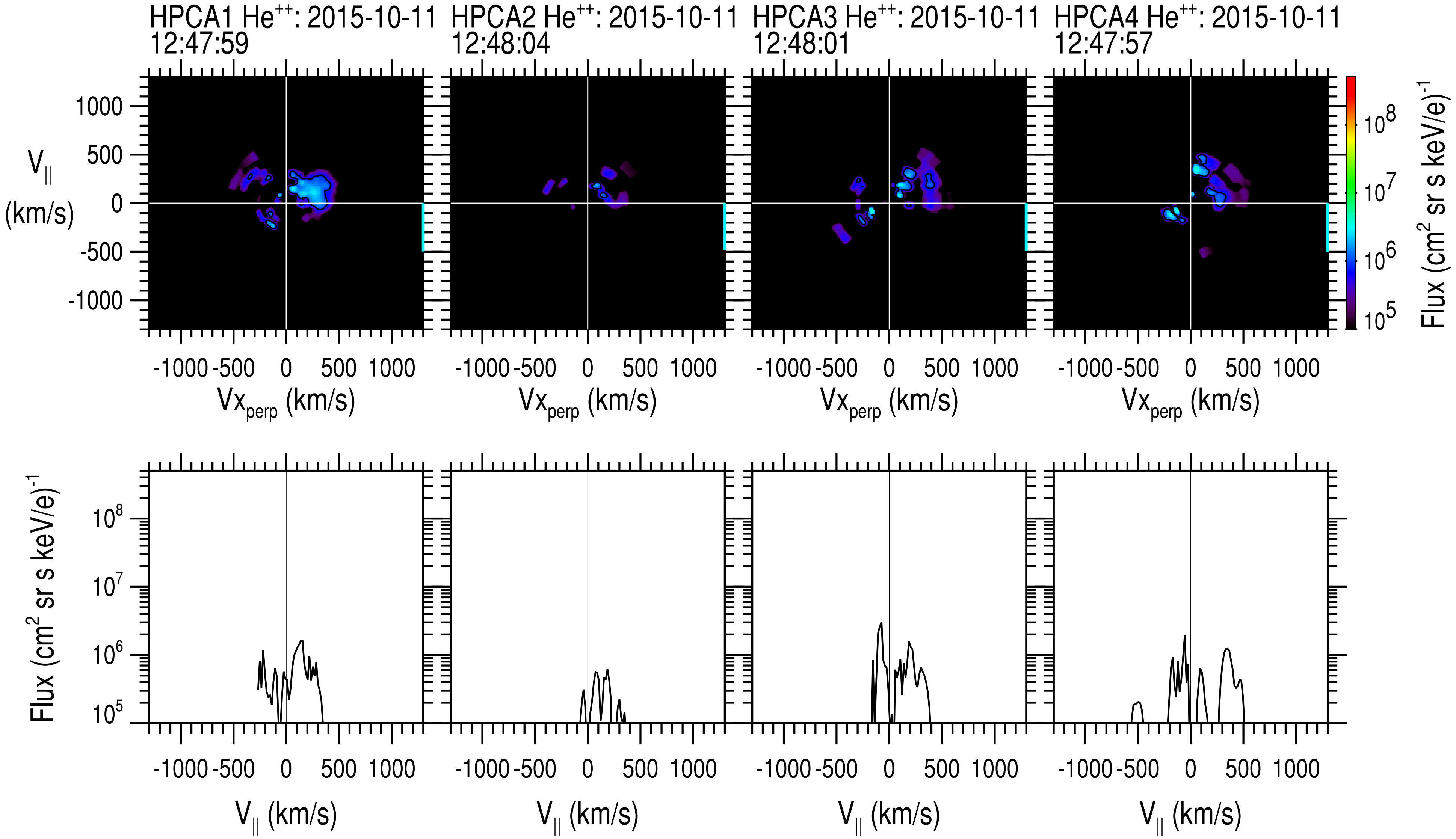

HPCA1 He++: 2015-10-11 12:47:59
HPCA2 He++: 2015-10-11 12:48:04
HPCA3 He++: 2015-10-11 12:48:01
HPCA4 He++: 2015-10-11 12:47:57
V|| (km/s)
Vxperp (km/s)
Flux (cm2 sr s keV/e)-1

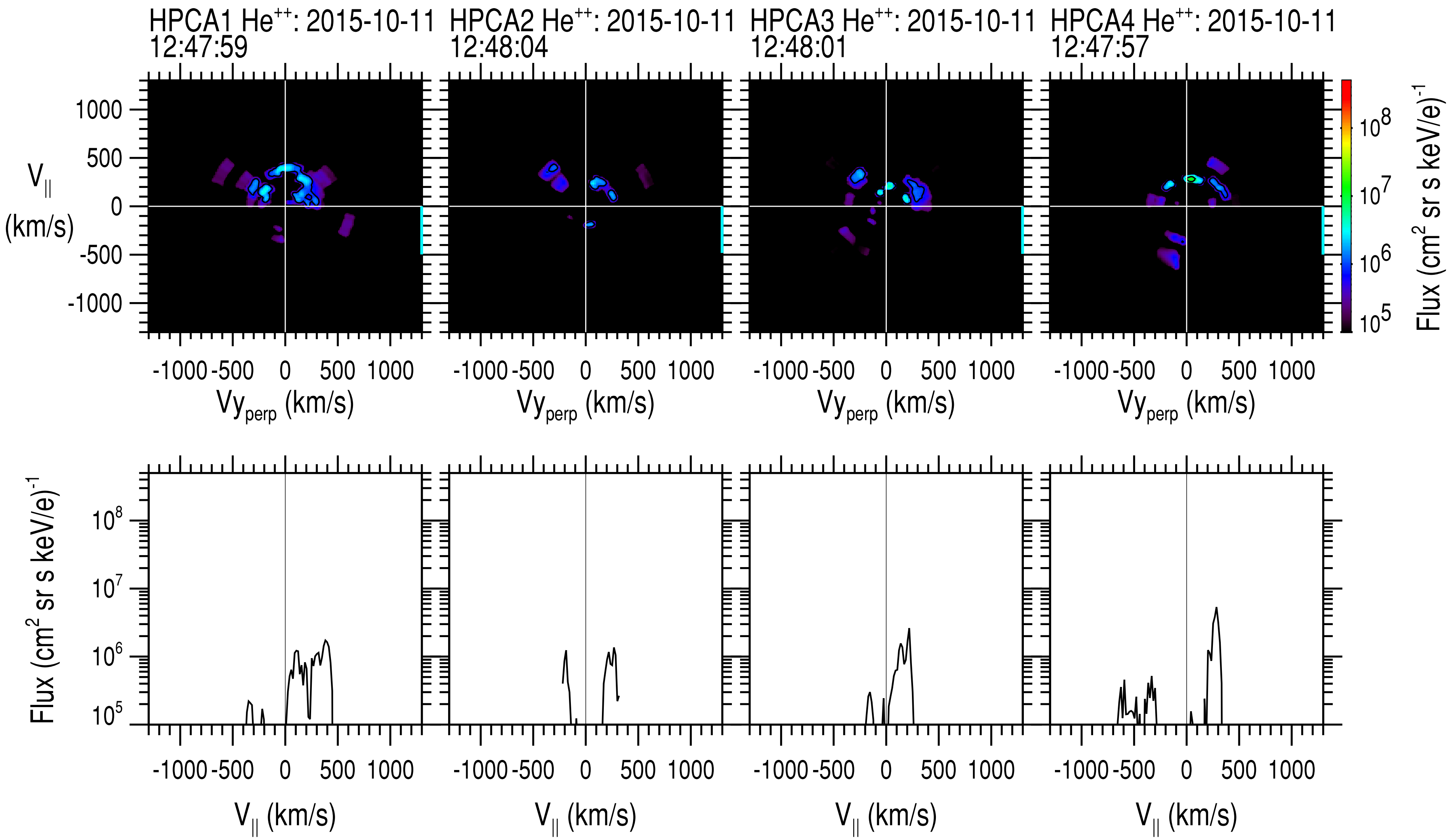
HPCA1 He++: 2015-10-11 12:47:59
HPCA2 He++: 2015-10-11 12:48:04
HPCA3 He++: 2015-10-11 12:48:01
HPCA4 He++: 2015-10-11 12:47:57
V|| (km/s)
Vyperp (km/s)
Flux (cm2 sr s keV/e)-1
V|| (km/s)

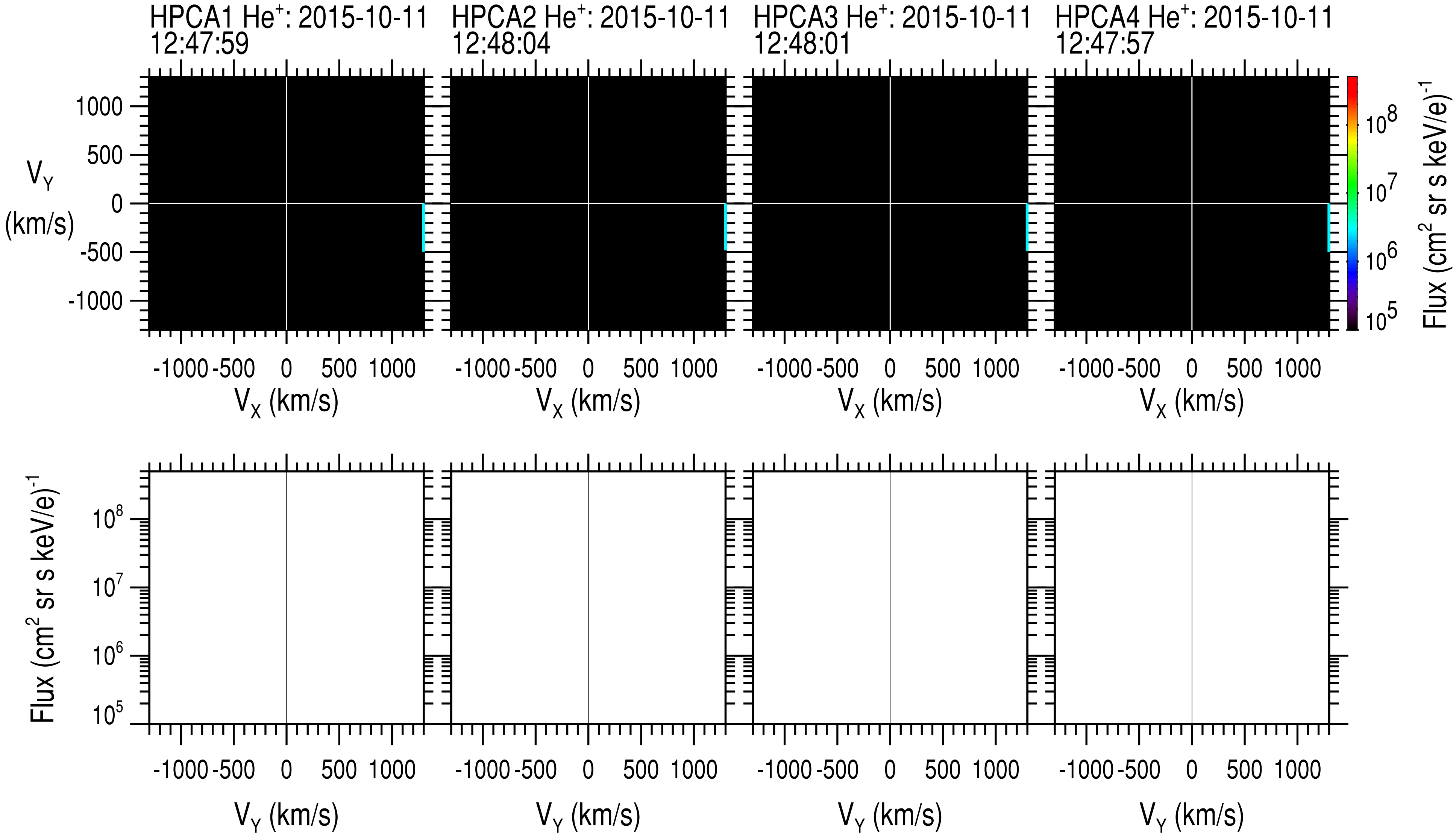

HPCA1 He+: 2015-10-11 12:47:59
HPCA2 He+: 2015-10-11 12:48:04
HPCA3 He+: 2015-10-11 12:48:01
HPCA4 He+: 2015-10-11 12:47:57
V_Y (km/s)
V_X (km/s)
Flux (cm^2 sr s keV/e)^-1
-1000 -500 0 500 1000
10^5 10^6 10^7 10^8

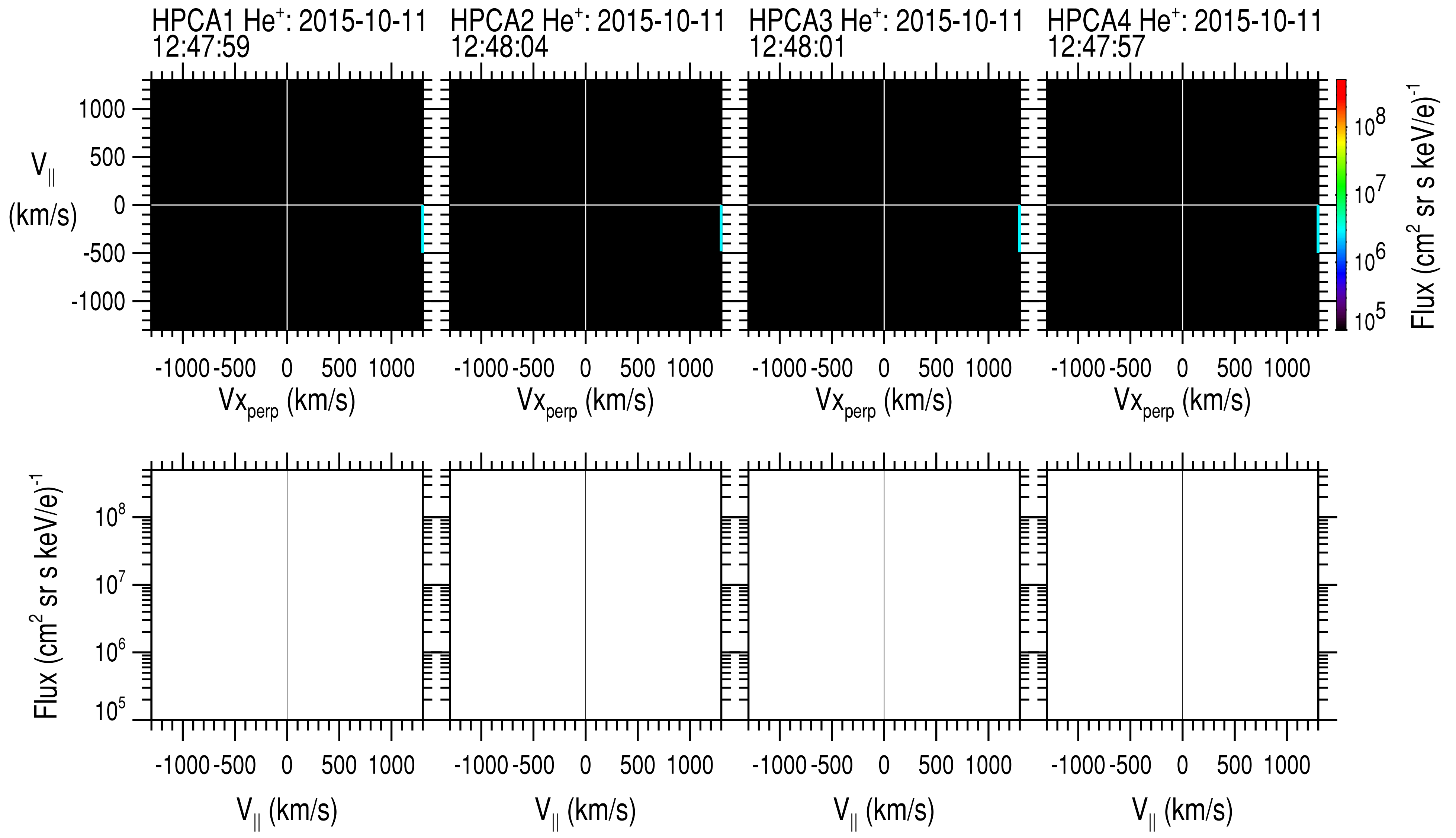
HPCA1 He+: 2015-10-11
12:47:59
HPCA2 He+: 2015-10-11
12:48:04
HPCA3 He+: 2015-10-11
12:48:01
HPCA4 He+: 2015-10-11
12:47:57
V|| (km/s)
1000
500
0
-500
-1000
Vxperp (km/s)
Flux (cm2 sr s keV/e)-1
10^5
10^6
10^7
10^8
V|| (km/s)

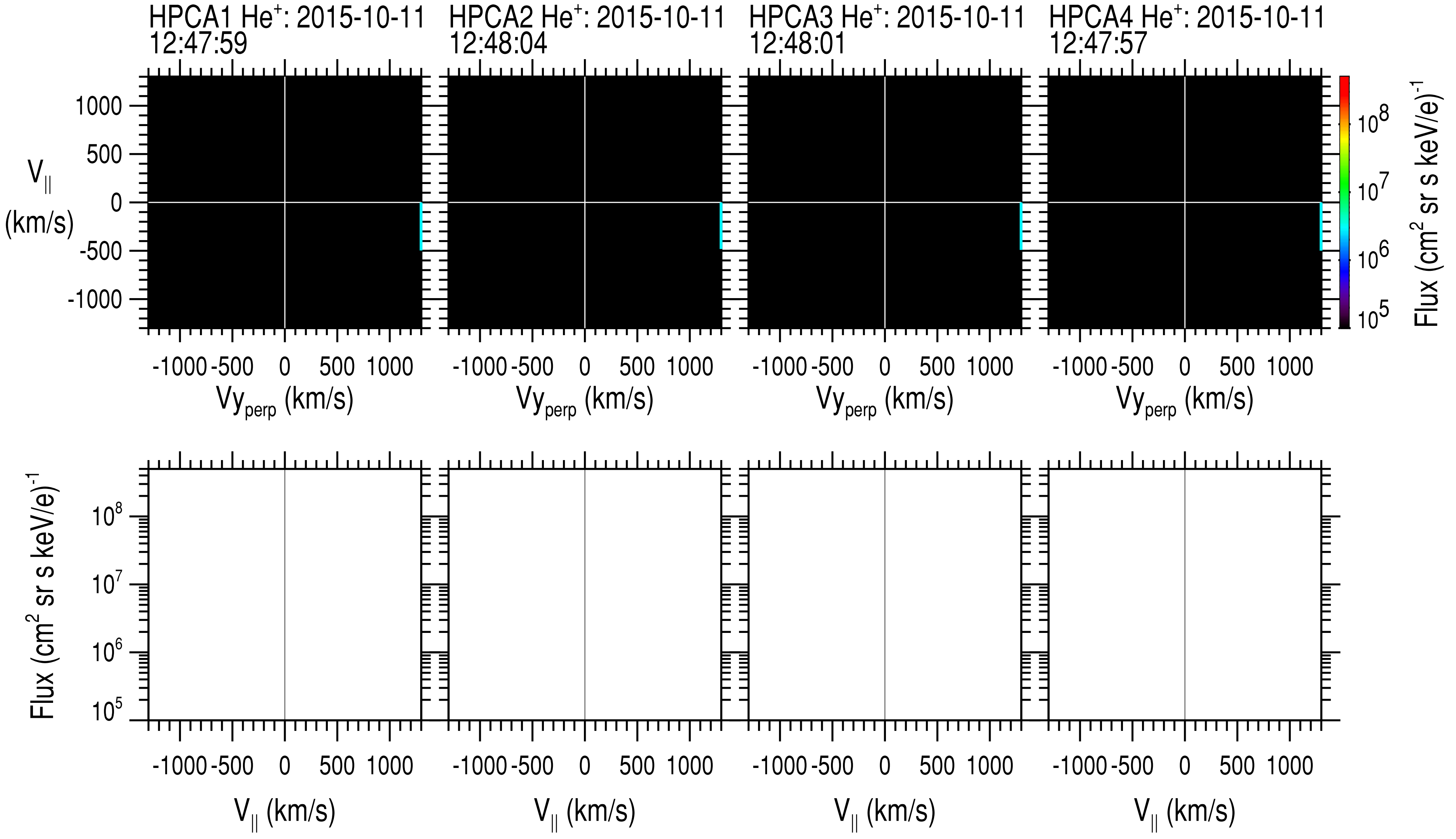

HPCA1 He+: 2015-10-11 12:47:59
HPCA2 He+: 2015-10-11 12:48:04
HPCA3 He+: 2015-10-11 12:48:01
HPCA4 He+: 2015-10-11 12:47:57
V|| (km/s)
Vyperp (km/s)
Flux (cm2 sr s keV/e)-1

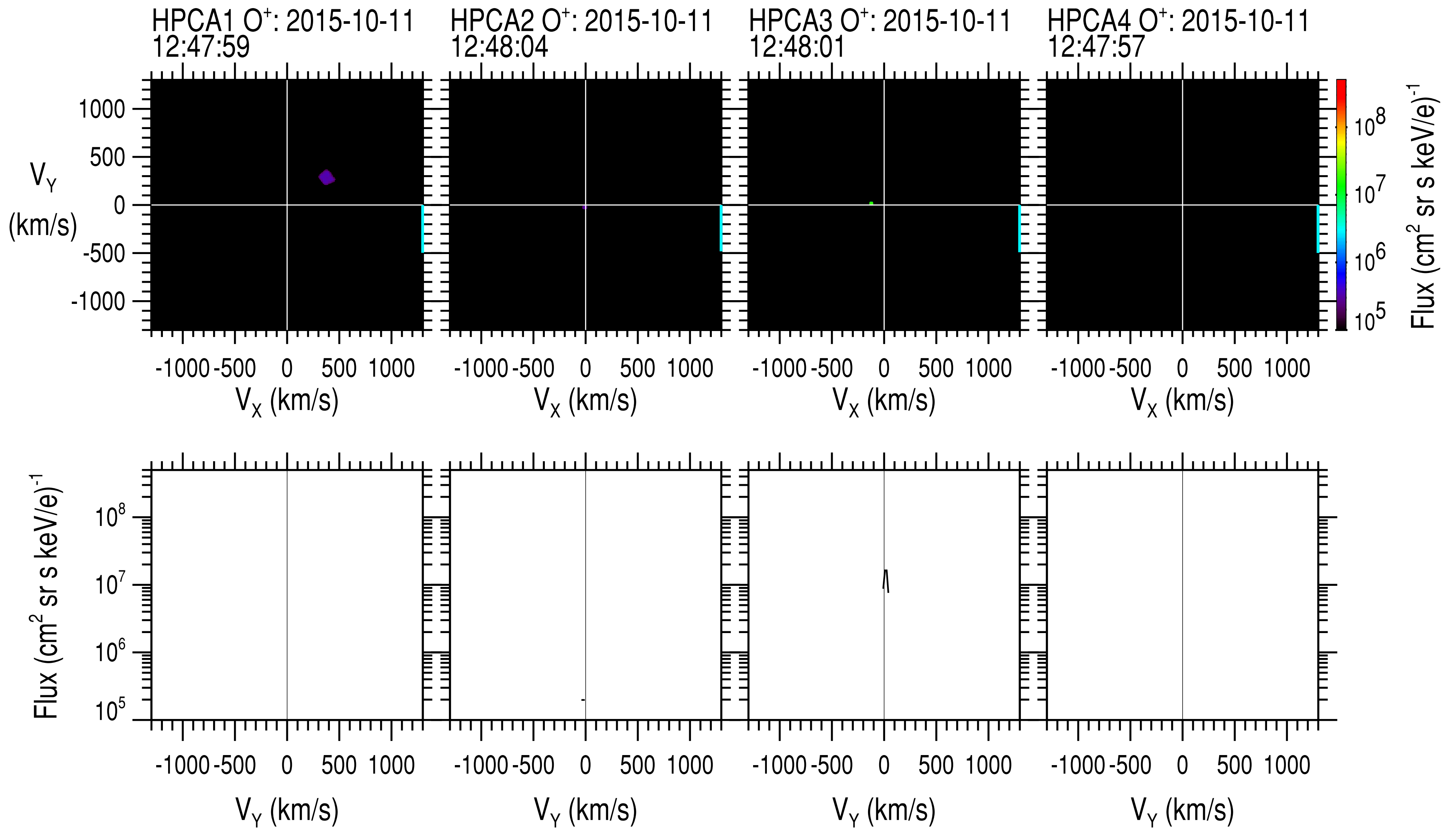

HPCA1 O+: 2015-10-11
12:47:59
HPCA2 O+: 2015-10-11
12:48:04
HPCA3 O+: 2015-10-11
12:48:01
HPCA4 O+: 2015-10-11
12:47:57
VY (km/s)
VX (km/s)
Flux (cm2 sr s keV/e)-1
VY (km/s)

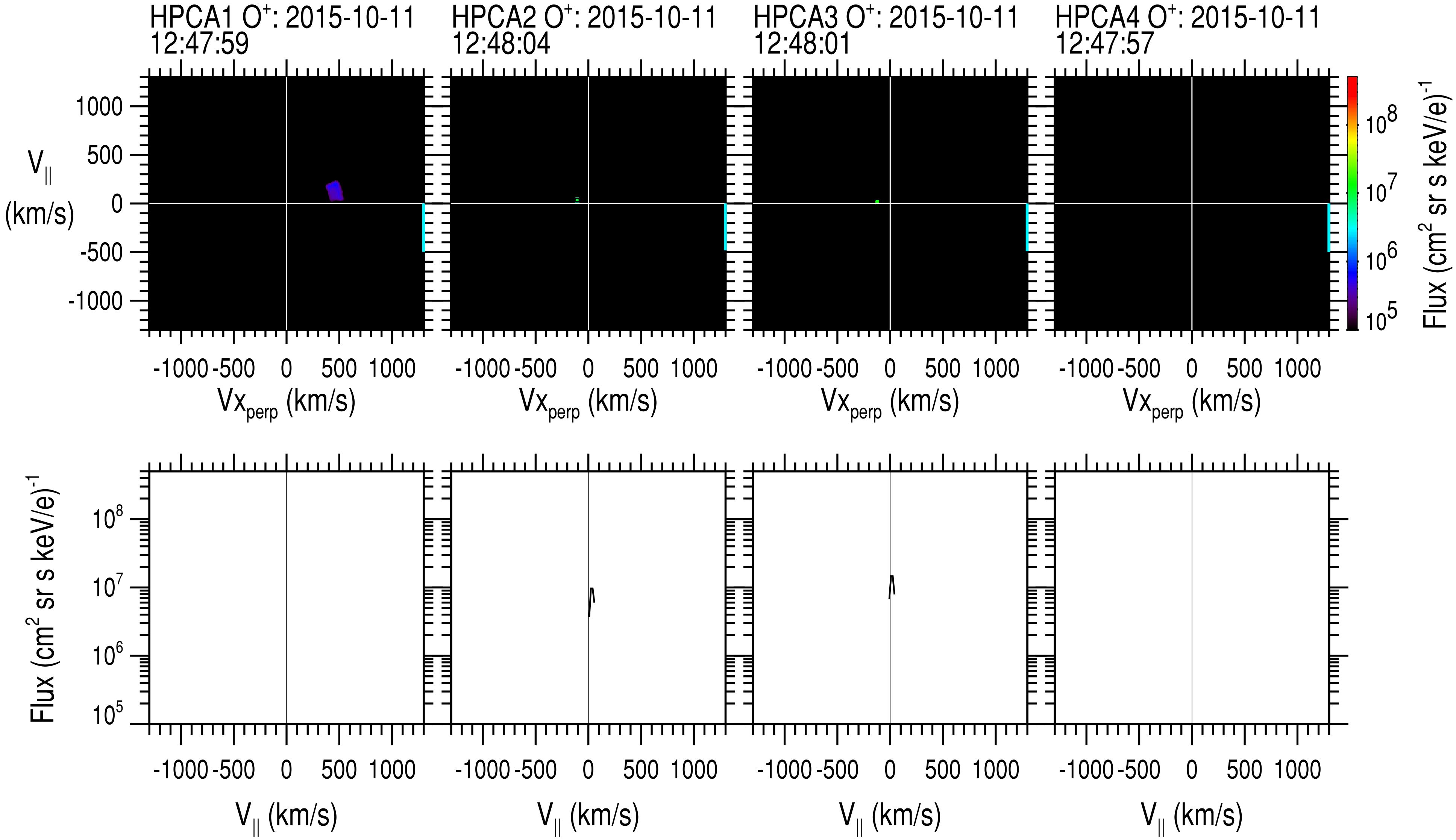
HPCA1 O+: 2015-10-11 12:47:59
HPCA2 O+: 2015-10-11 12:48:04
HPCA3 O+: 2015-10-11 12:48:01
HPCA4 O+: 2015-10-11 12:47:57
V|| (km/s)
Vxperp (km/s)
Flux (cm2 sr s keV/e)-1
Flux (cm2 sr s keV/e)-1
V|| (km/s)

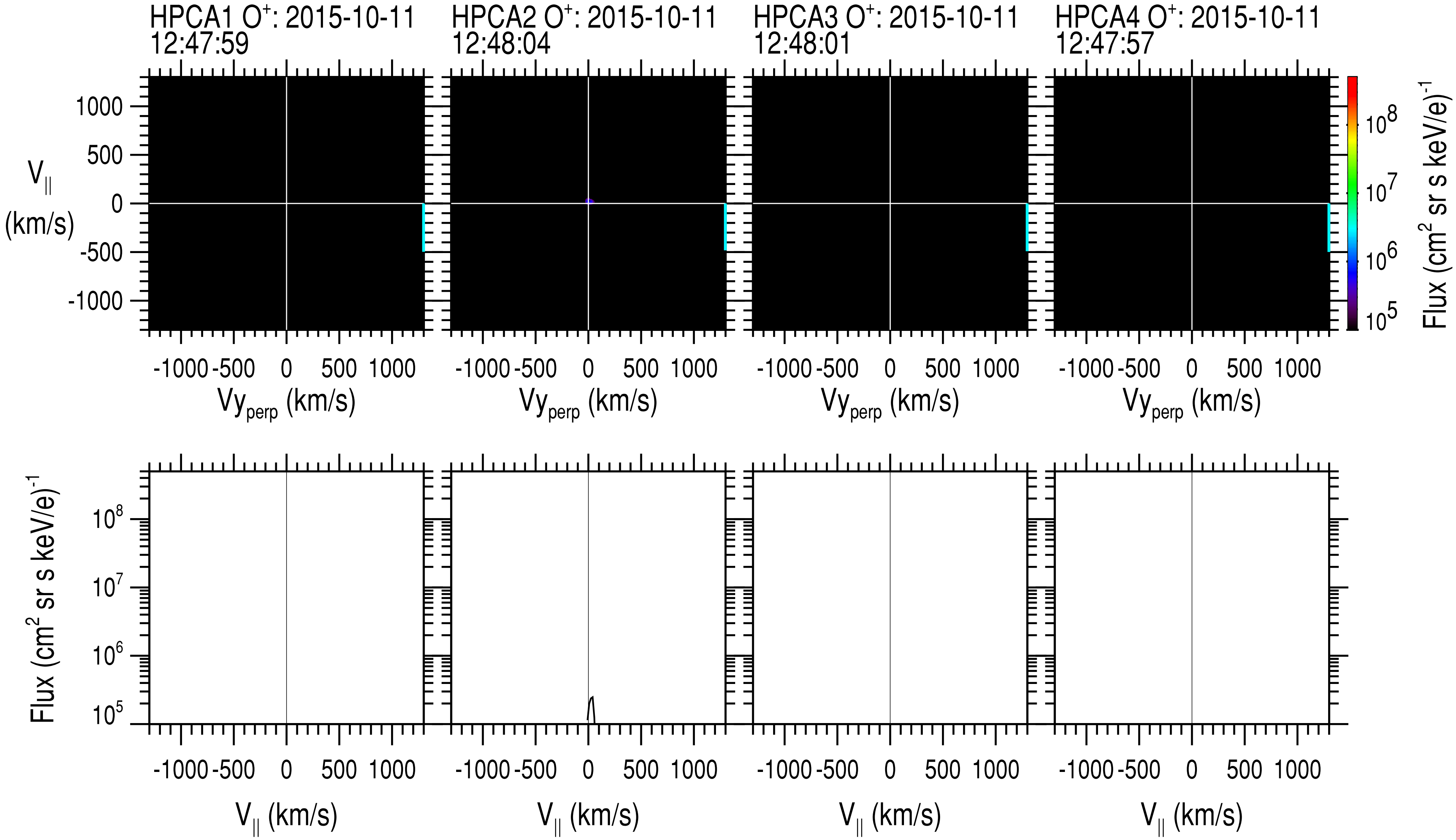
HPCA1 O+: 2015-10-11 12:47:59
HPCA2 O+: 2015-10-11 12:48:04
HPCA3 O+: 2015-10-11 12:48:01
HPCA4 O+: 2015-10-11 12:47:57
V|| (km/s)
Vyperp (km/s)
Flux (cm2 sr s keV/e)-1

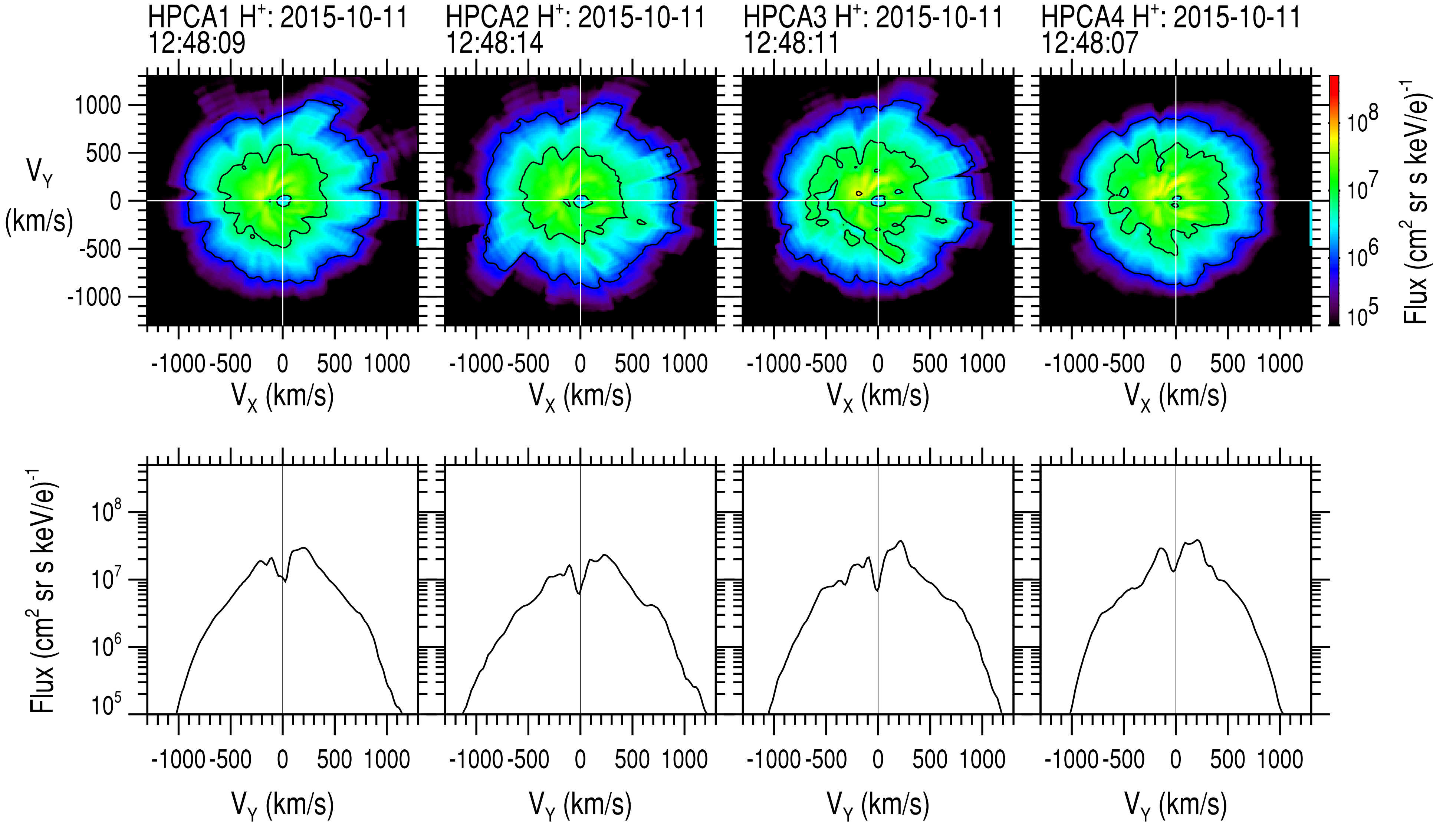
HPCA1 H+: 2015-10-11 12:48:09
HPCA2 H+: 2015-10-11 12:48:14
HPCA3 H+: 2015-10-11 12:48:11
HPCA4 H+: 2015-10-11 12:48:07
VY (km/s)
VX (km/s)
Flux (cm2 sr s keV/e)-1
VY (km/s)

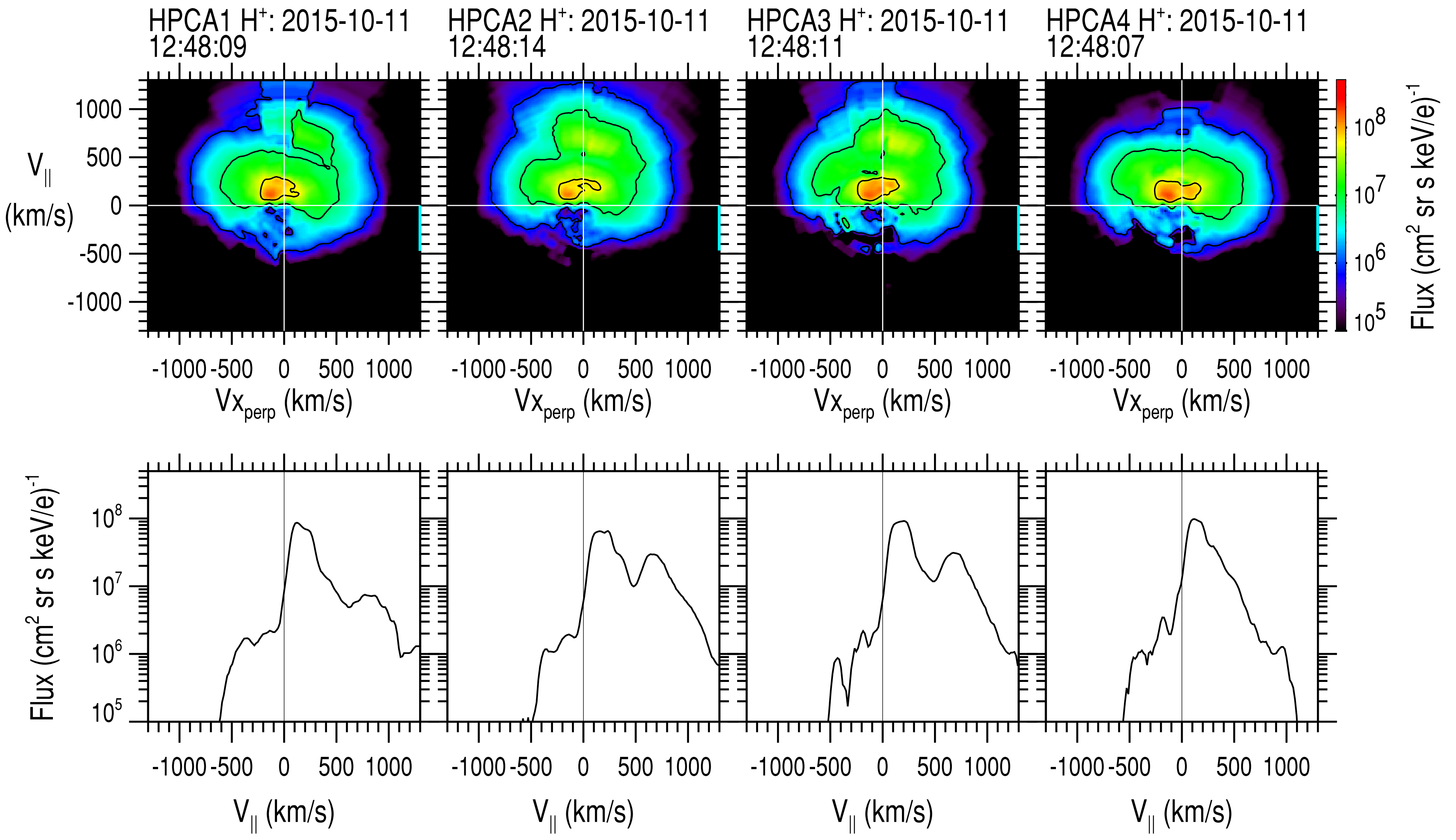

HPCA1 H+: 2015-10-11 12:48:09
HPCA2 H+: 2015-10-11 12:48:14
HPCA3 H+: 2015-10-11 12:48:11
HPCA4 H+: 2015-10-11 12:48:07
V|| (km/s)
Vxperp (km/s)
Flux (cm2 sr s keV/e)-1

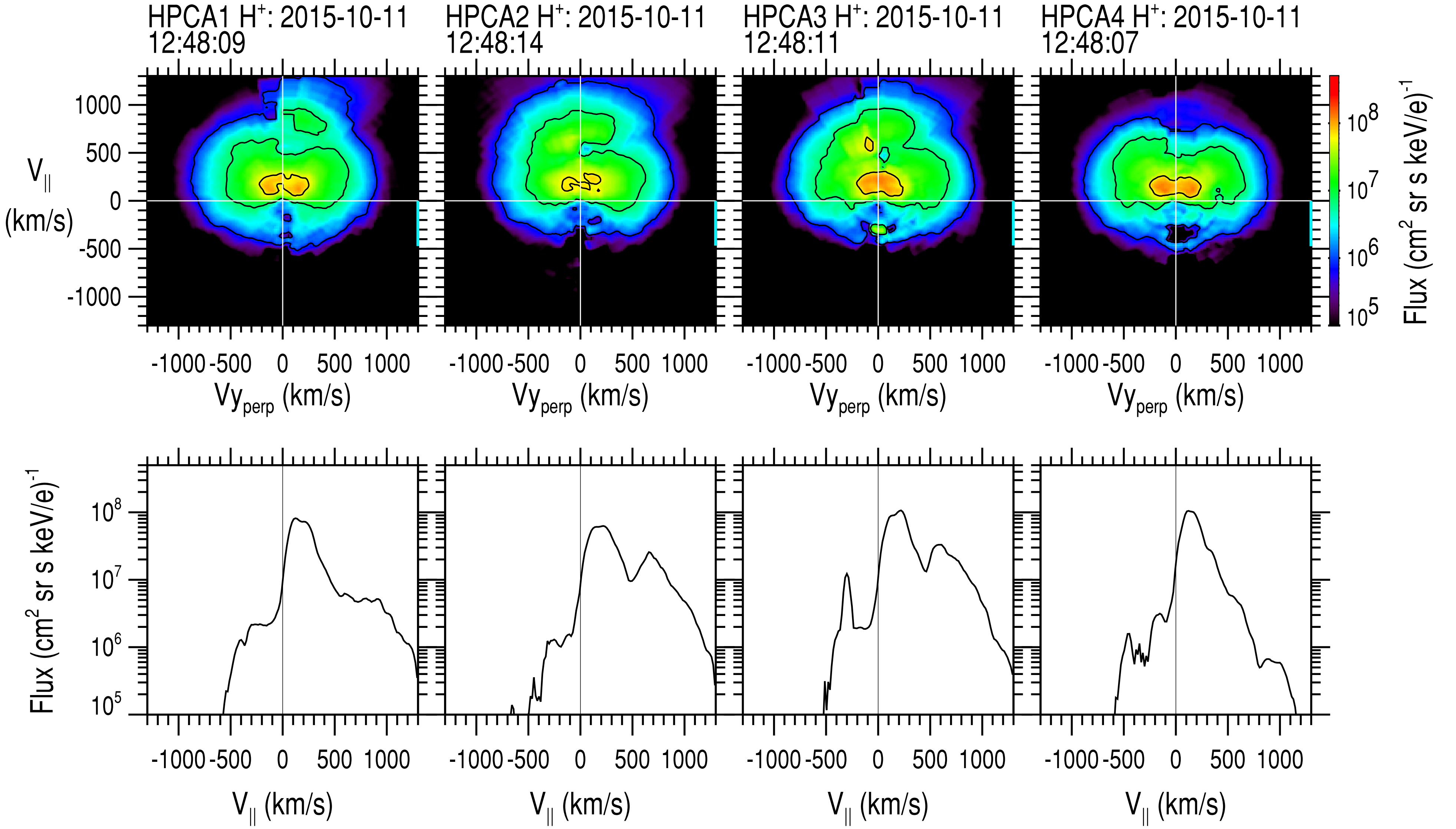

HPCA1 H+: 2015-10-11 12:48:09
HPCA2 H+: 2015-10-11 12:48:14
HPCA3 H+: 2015-10-11 12:48:11
HPCA4 H+: 2015-10-11 12:48:07
V|| (km/s)
Vyperp (km/s)
Flux (cm2 sr s keV/e)-1

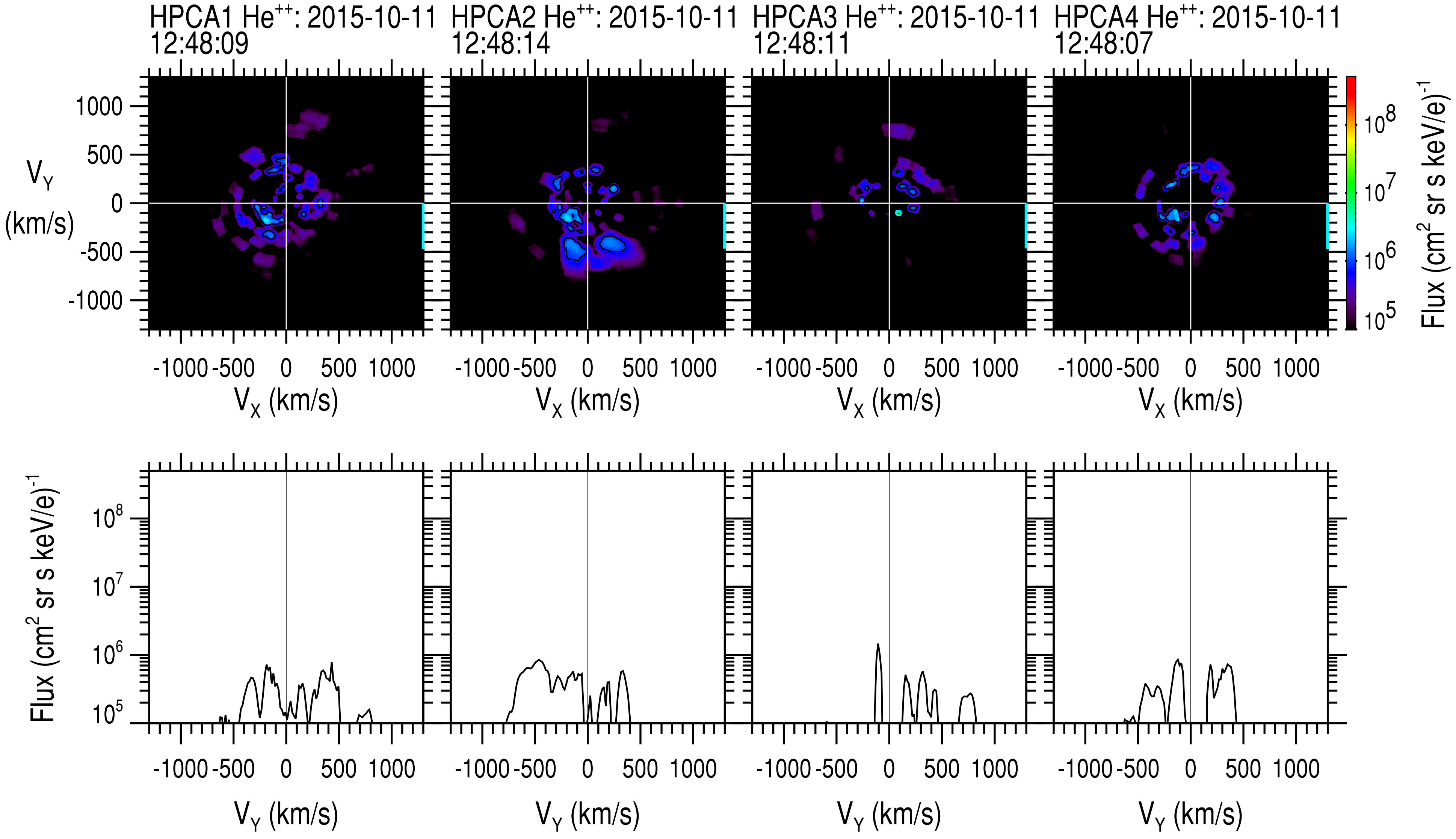

HPCA1 He++: 2015-10-11 12:48:09
HPCA2 He++: 2015-10-11 12:48:14
HPCA3 He++: 2015-10-11 12:48:11
HPCA4 He++: 2015-10-11 12:48:07
VY (km/s)
VX (km/s)
Flux (cm2 sr s keV/e)-1
VY (km/s)

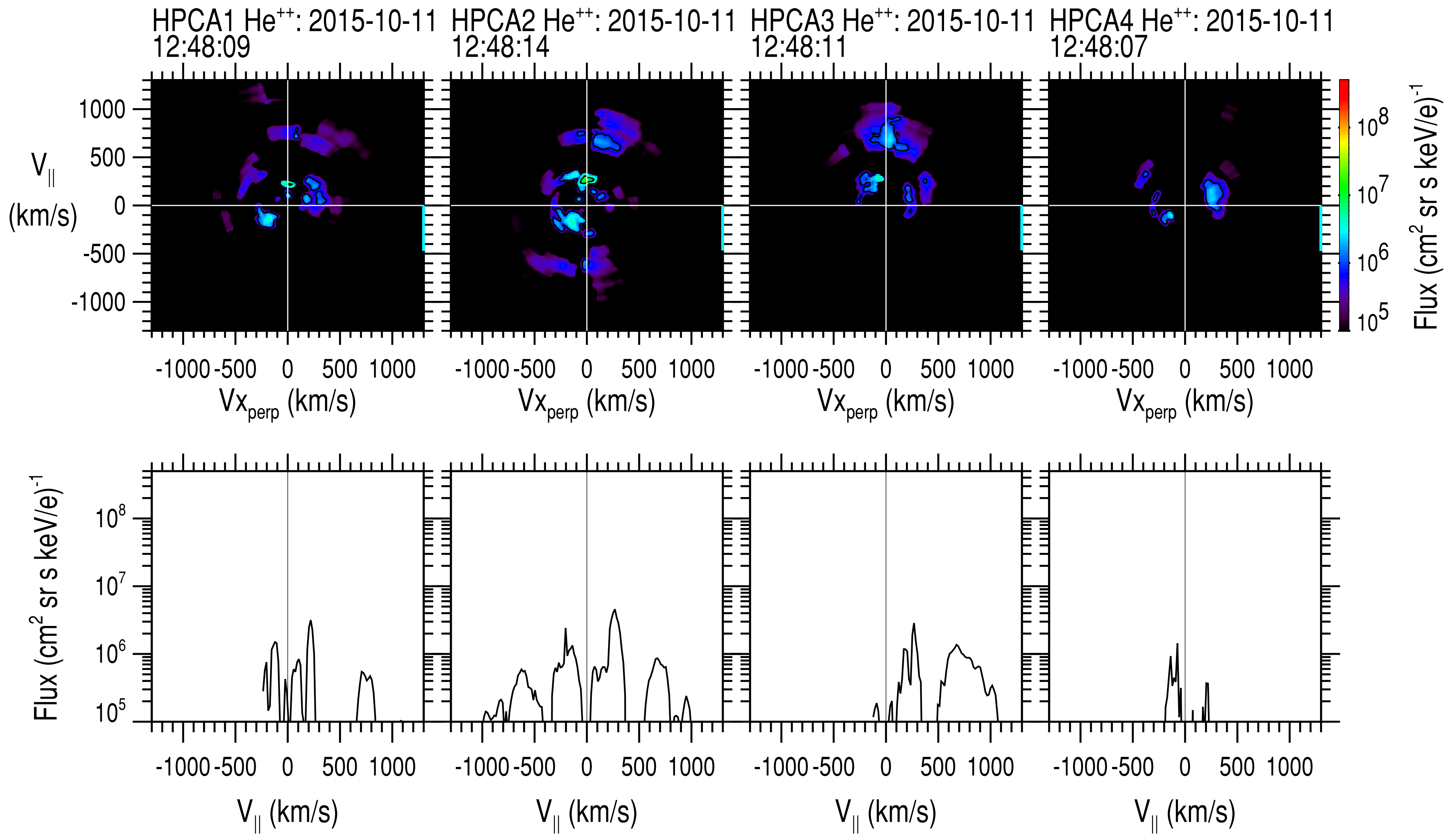

HPCA1 He++: 2015-10-11 12:48:09
HPCA2 He++: 2015-10-11 12:48:14
HPCA3 He++: 2015-10-11 12:48:11
HPCA4 He++: 2015-10-11 12:48:07
V|| (km/s)
Vxperp (km/s)
Flux (cm2 sr s keV/e)-1

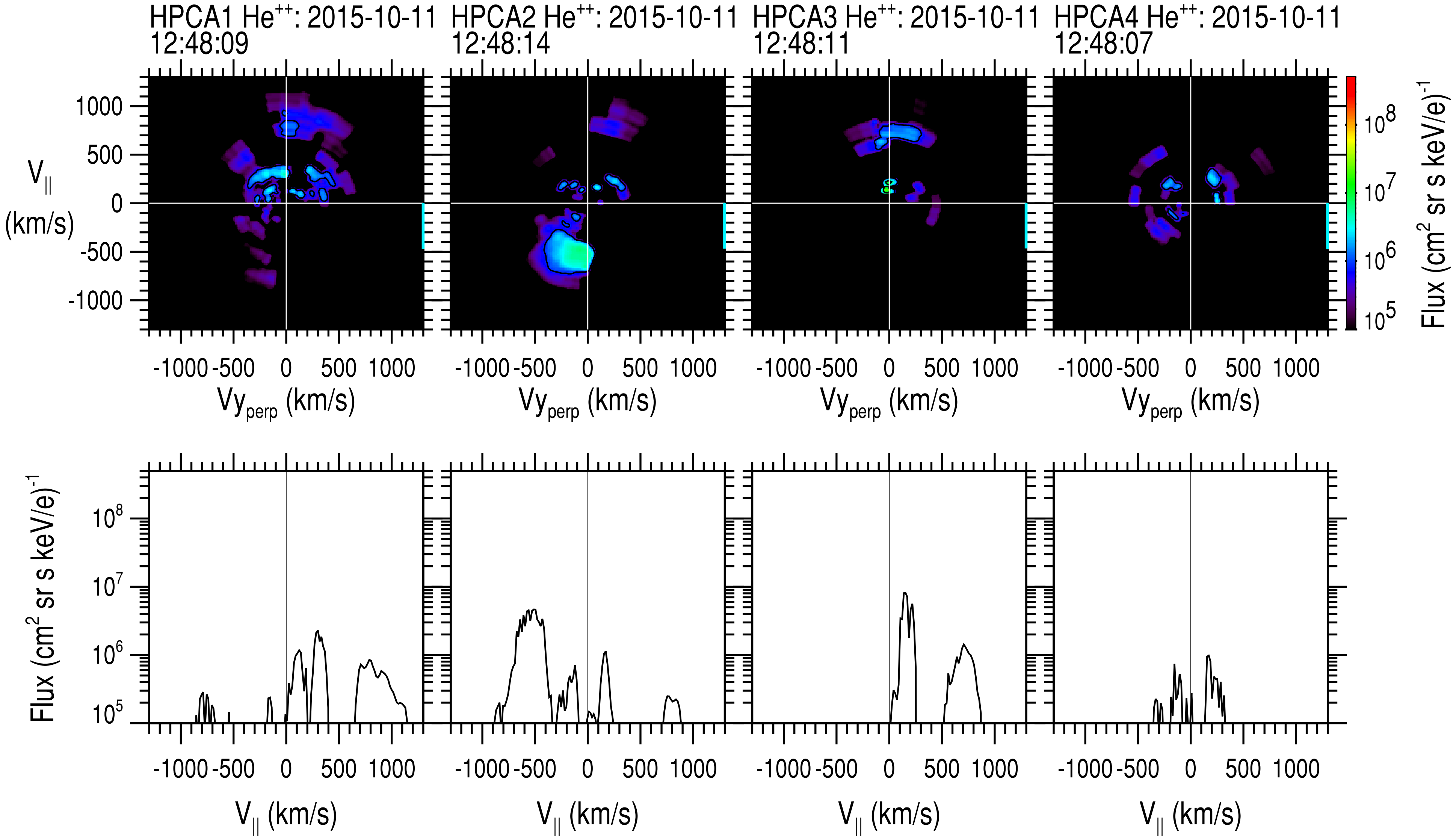

HPCA1 He++: 2015-10-11 12:48:09
HPCA2 He++: 2015-10-11 12:48:14
HPCA3 He++: 2015-10-11 12:48:11
HPCA4 He++: 2015-10-11 12:48:07
V|| (km/s)
Vyperp (km/s)
Flux (cm2 sr s keV/e)-1

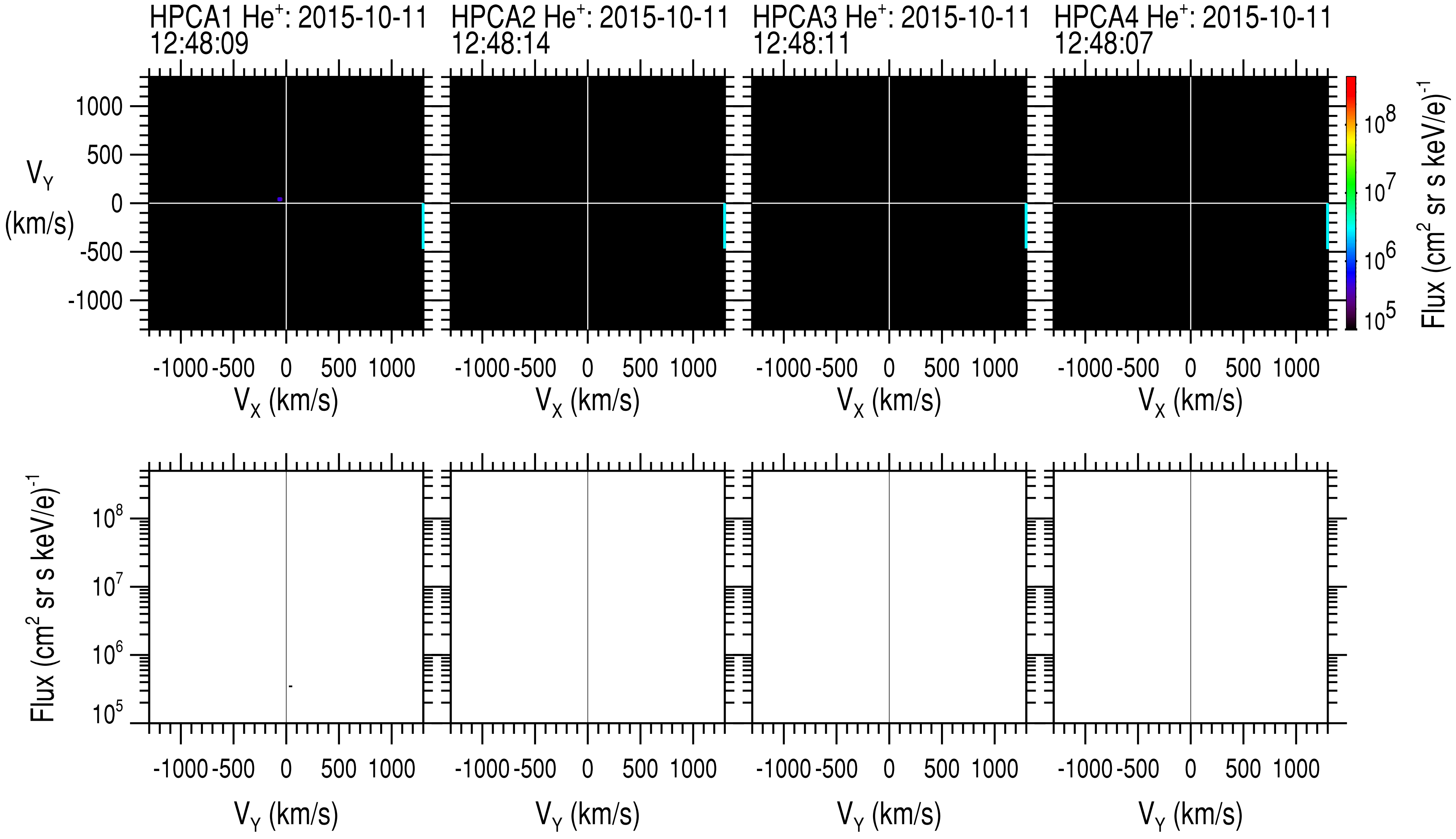
HPCA1 He+: 2015-10-11 12:48:09
HPCA2 He+: 2015-10-11 12:48:14
HPCA3 He+: 2015-10-11 12:48:11
HPCA4 He+: 2015-10-11 12:48:07
VY (km/s)
VX (km/s)
Flux (cm2 sr s keV/e)-1
VY (km/s)

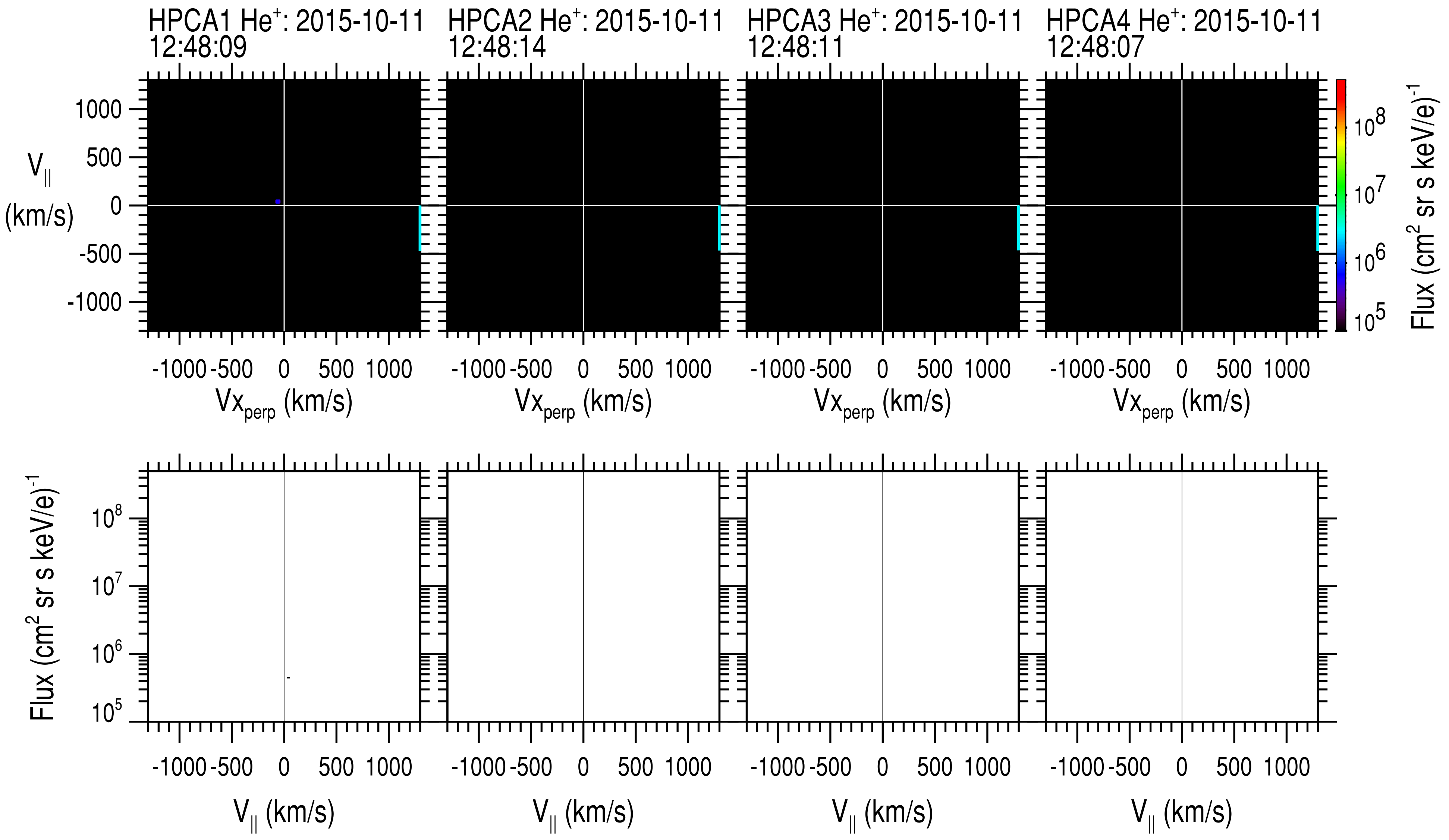
HPCA1 He+: 2015-10-11 12:48:09
HPCA2 He+: 2015-10-11 12:48:14
HPCA3 He+: 2015-10-11 12:48:11
HPCA4 He+: 2015-10-11 12:48:07
V|| (km/s)
Vxperp (km/s)
Flux (cm2 sr s keV/e)-1
V|| (km/s)
-1000 -500 0 500 1000
10^5 10^6 10^7 10^8

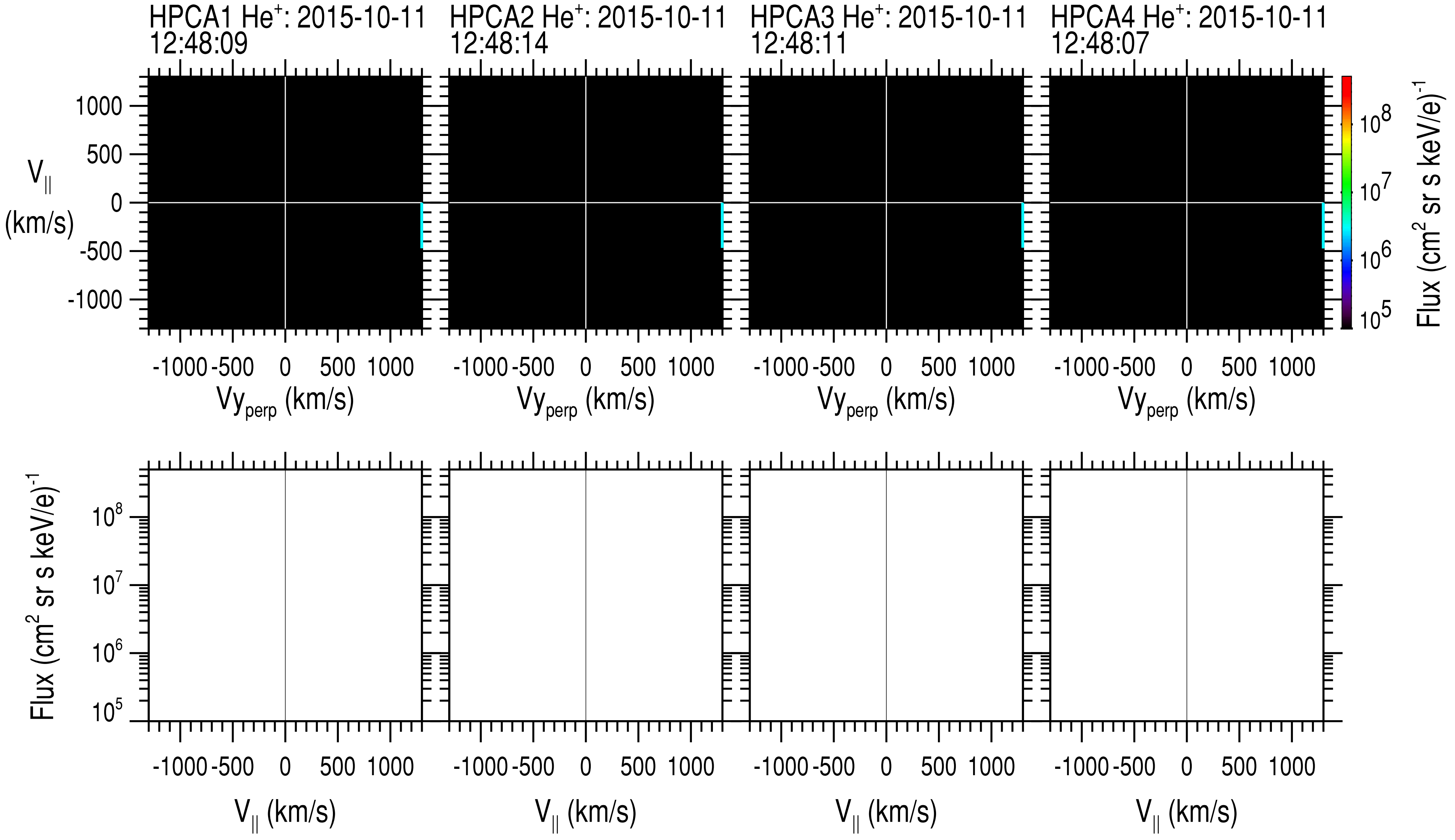
HPCA1 He+: 2015-10-11 12:48:09
HPCA2 He+: 2015-10-11 12:48:14
HPCA3 He+: 2015-10-11 12:48:11
HPCA4 He+: 2015-10-11 12:48:07
V|| (km/s)
Vyperp (km/s)
Flux (cm2 sr s keV/e)-1
-1000 -500 0 500 1000
10^5 10^6 10^7 10^8

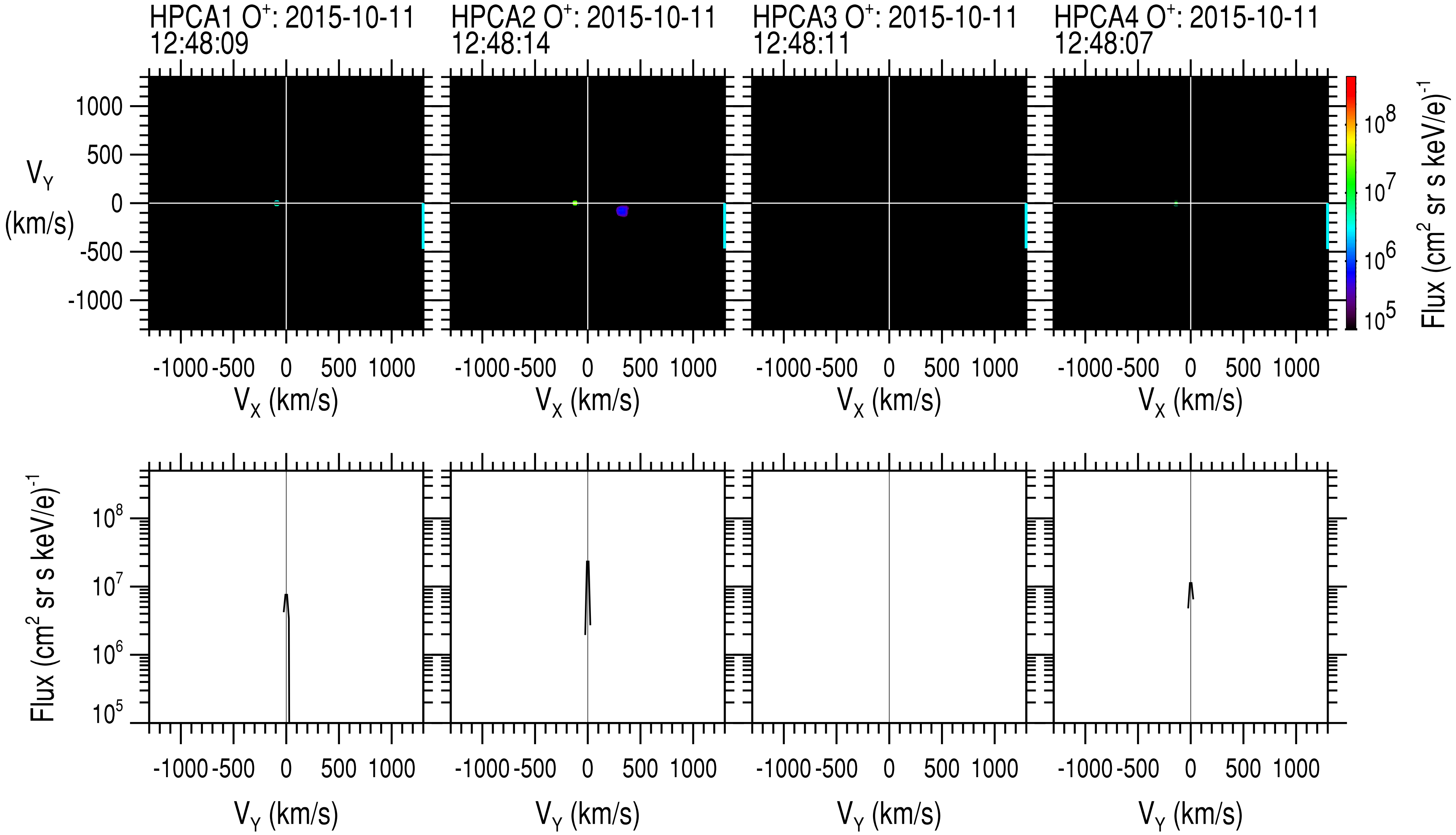

HPCA1 O+: 2015-10-11 12:48:09
HPCA2 O+: 2015-10-11 12:48:14
HPCA3 O+: 2015-10-11 12:48:11
HPCA4 O+: 2015-10-11 12:48:07
VY (km/s)
VX (km/s)
Flux (cm2 sr s keV/e)-1

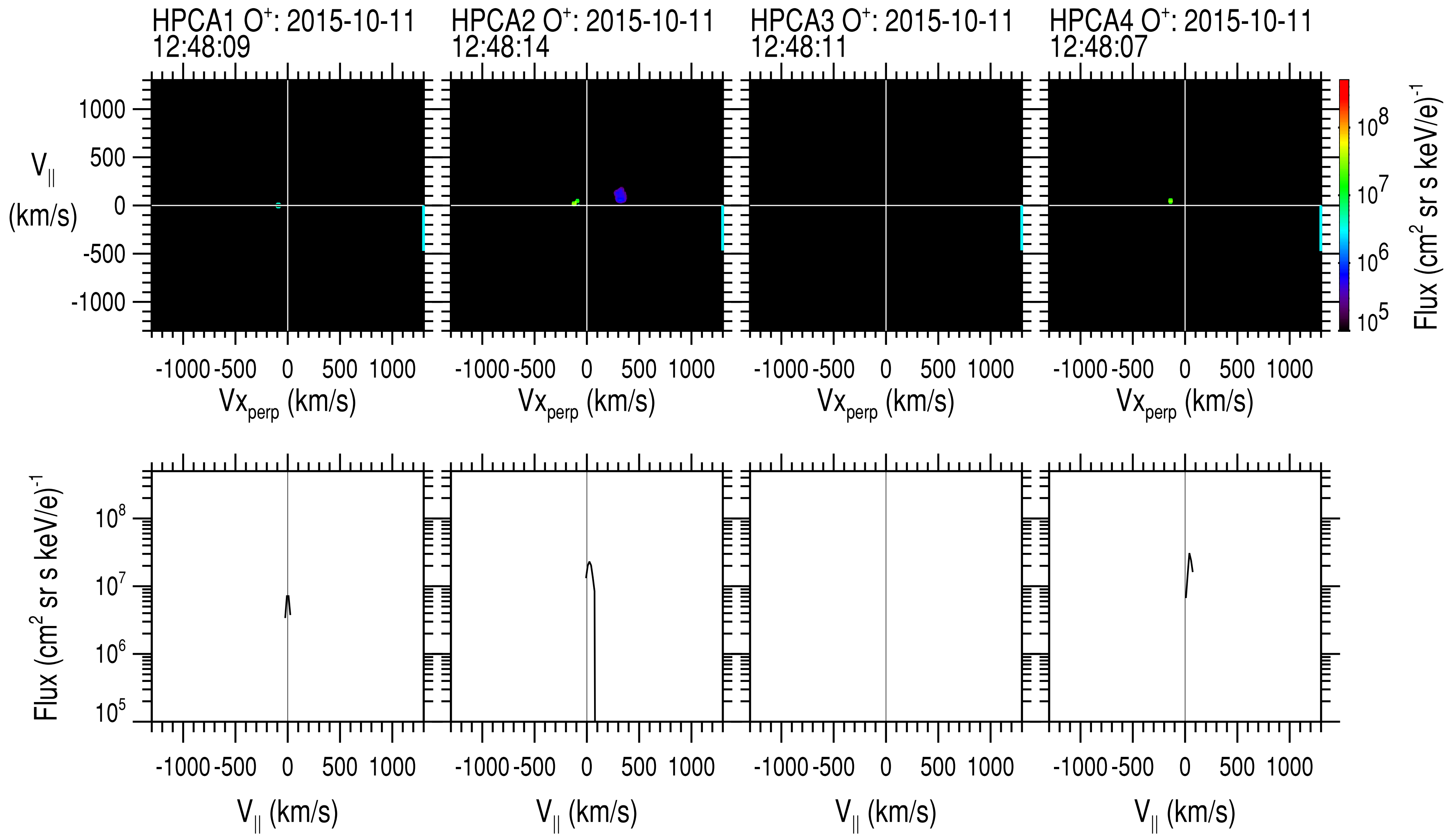
HPCA1 O+: 2015-10-11
12:48:09
HPCA2 O+: 2015-10-11
12:48:14
HPCA3 O+: 2015-10-11
12:48:11
HPCA4 O+: 2015-10-11
12:48:07
V|| (km/s)
Vxperp (km/s)
Flux (cm2 sr s keV/e)-1
V|| (km/s)

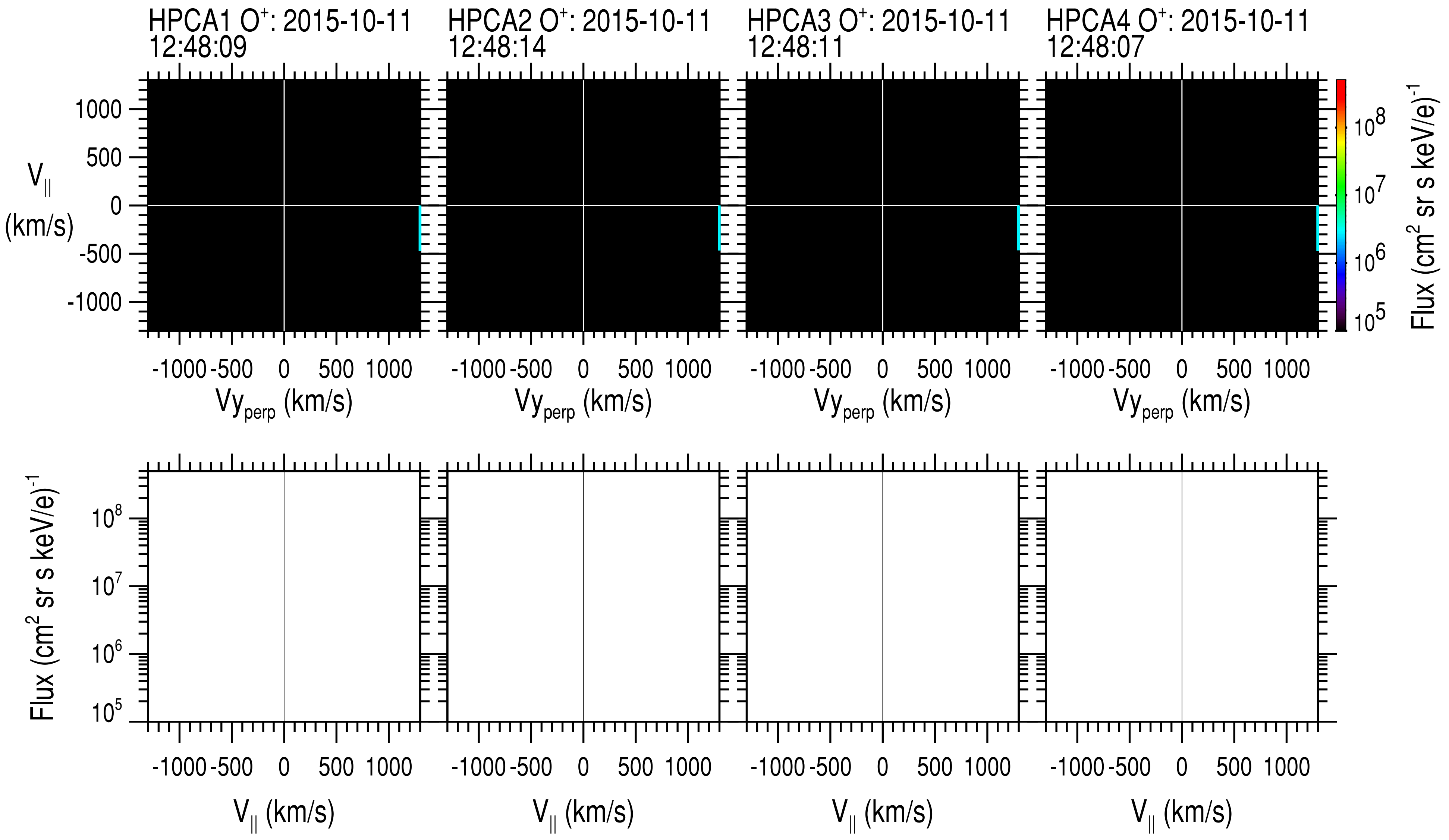

HPCA1 O+: 2015-10-11 12:48:09
HPCA2 O+: 2015-10-11 12:48:14
HPCA3 O+: 2015-10-11 12:48:11
HPCA4 O+: 2015-10-11 12:48:07
V|| (km/s)
Vy_perp (km/s)
Flux (cm2 sr s keV/e)-1
-1000 -500 0 500 1000
10^5 10^6 10^7 10^8

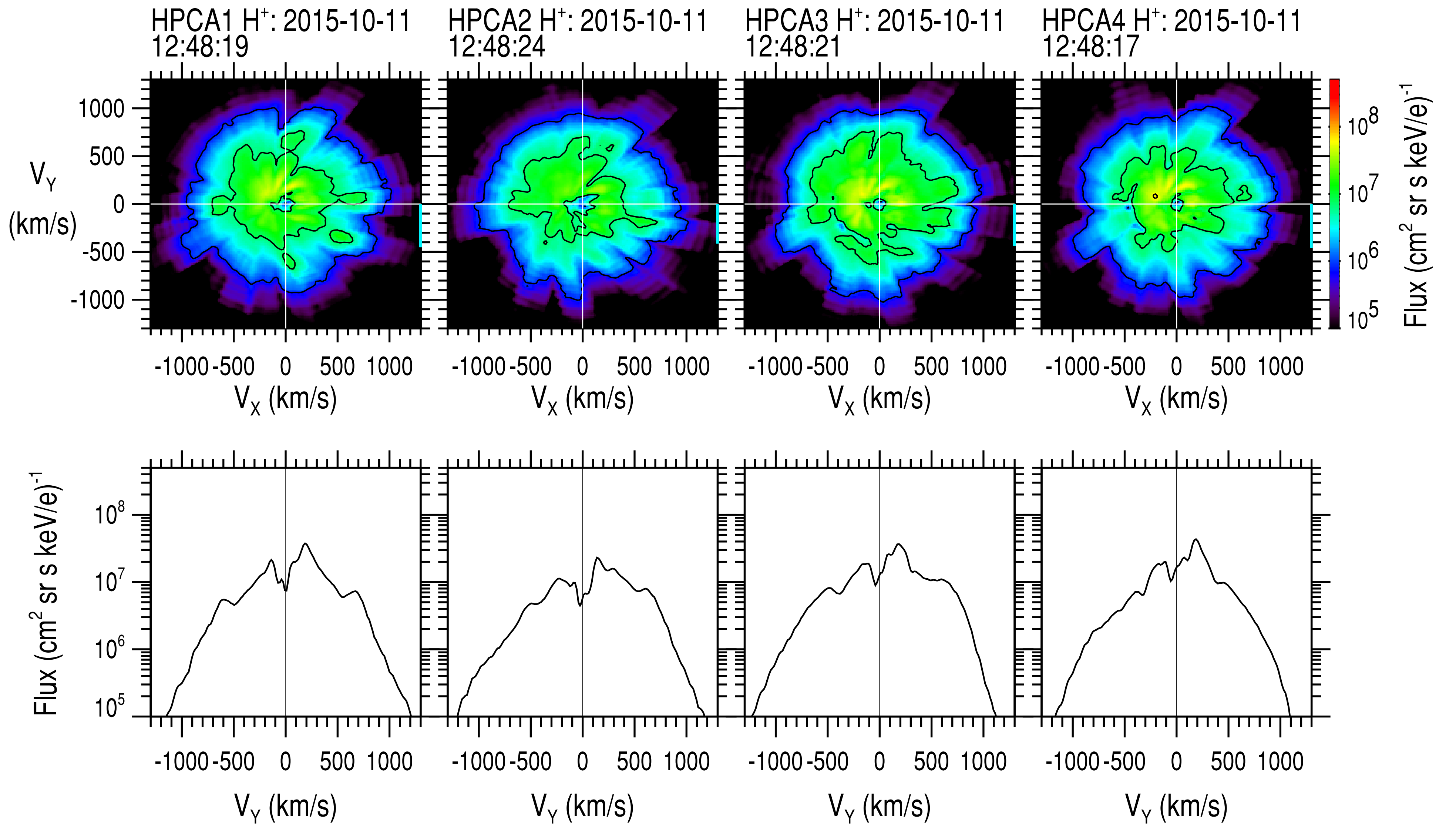
HPCA1 H+: 2015-10-11 12:48:19
HPCA2 H+: 2015-10-11 12:48:24
HPCA3 H+: 2015-10-11 12:48:21
HPCA4 H+: 2015-10-11 12:48:17
V_Y (km/s)
V_X (km/s)
Flux (cm^2 sr s keV/e)^-1
V_Y (km/s)

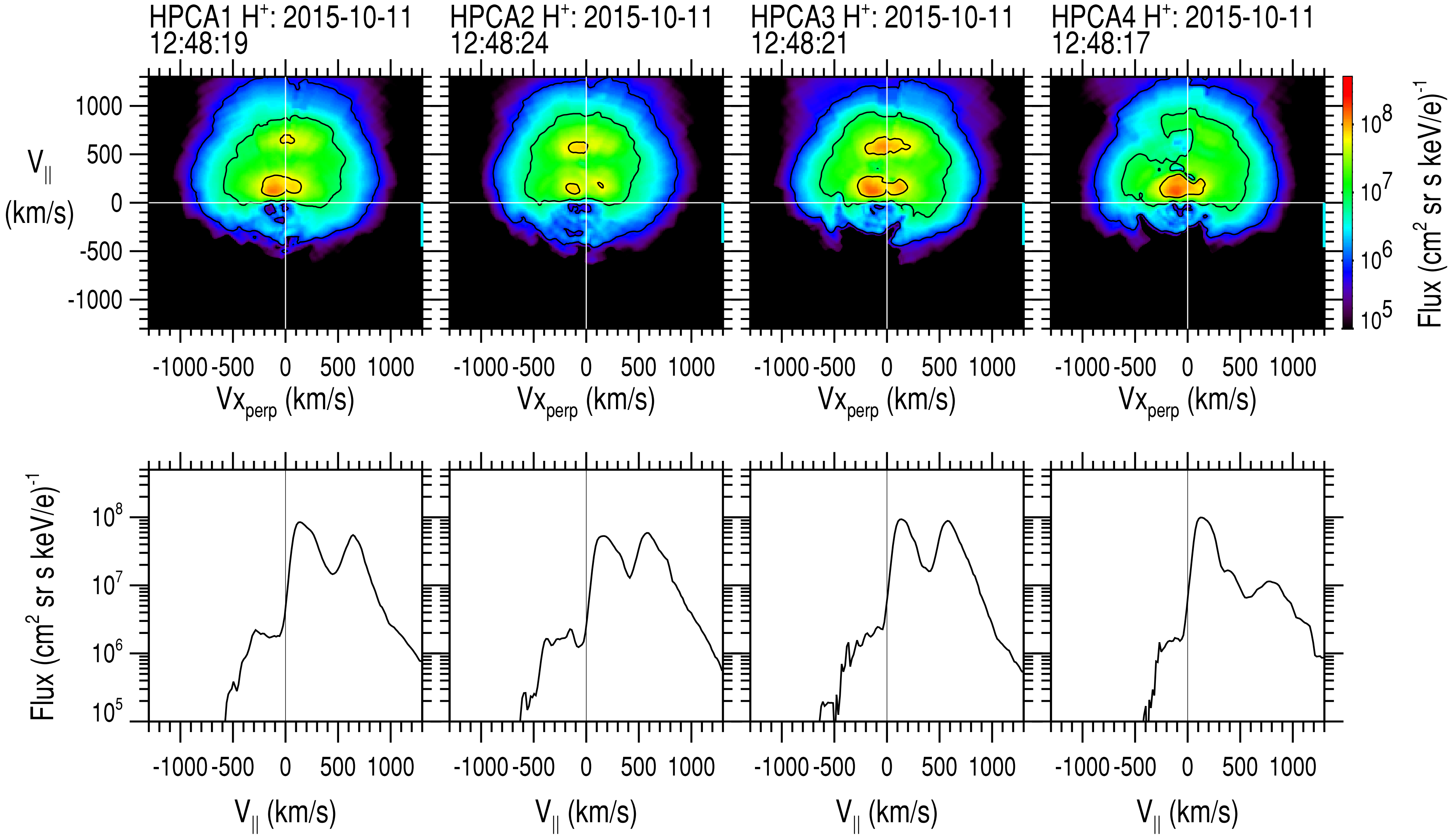
HPCA1 H+: 2015-10-11 12:48:19
HPCA2 H+: 2015-10-11 12:48:24
HPCA3 H+: 2015-10-11 12:48:21
HPCA4 H+: 2015-10-11 12:48:17
V|| (km/s)
Vxperp (km/s)
Flux (cm2 sr s keV/e)-1
V|| (km/s)

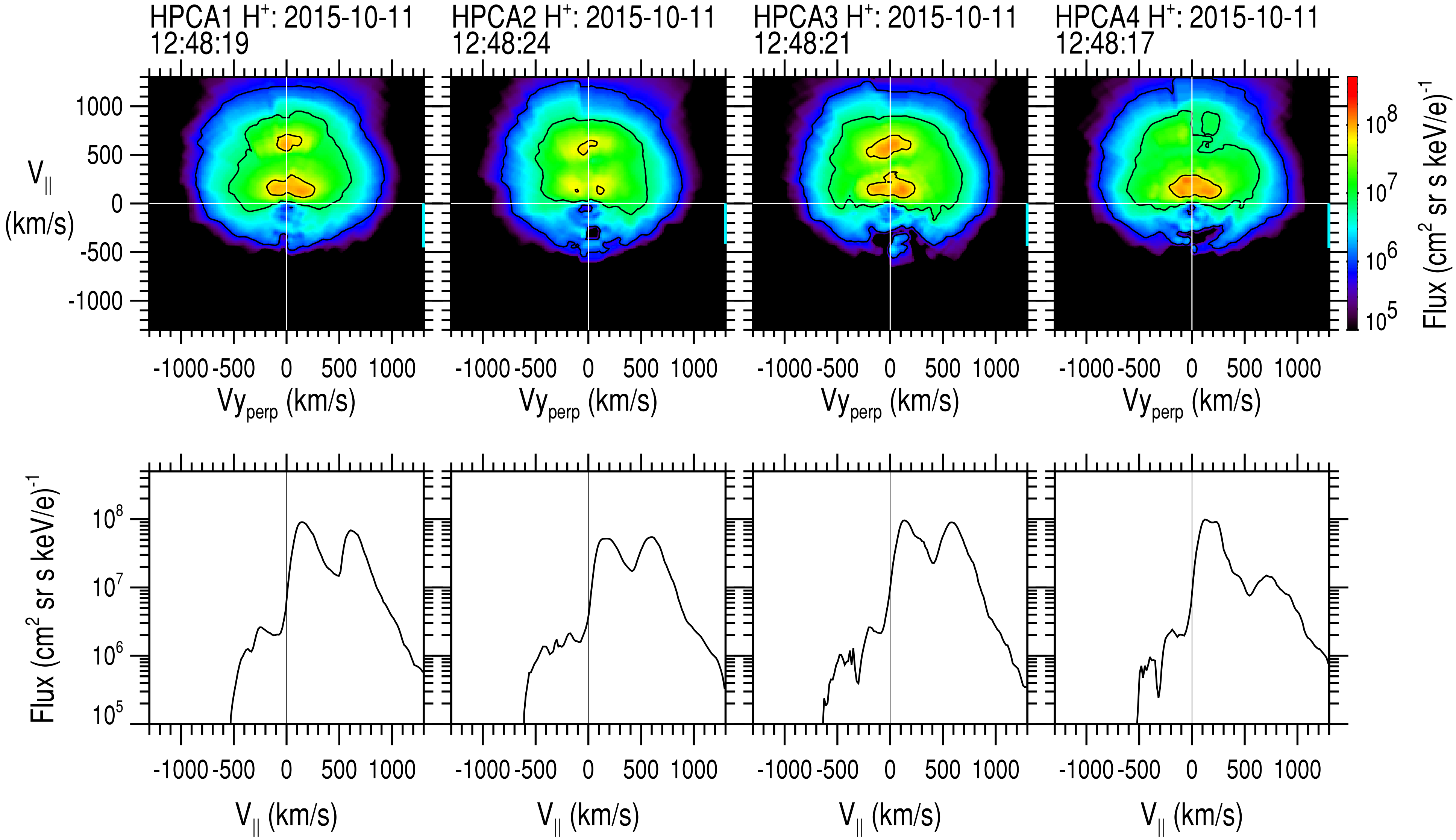
HPCA1 H+: 2015-10-11
12:48:19
HPCA2 H+: 2015-10-11
12:48:24
HPCA3 H+: 2015-10-11
12:48:21
HPCA4 H+: 2015-10-11
12:48:17
V|| (km/s)
Vyperp (km/s)
Flux (cm2 sr s keV/e)-1
V|| (km/s)

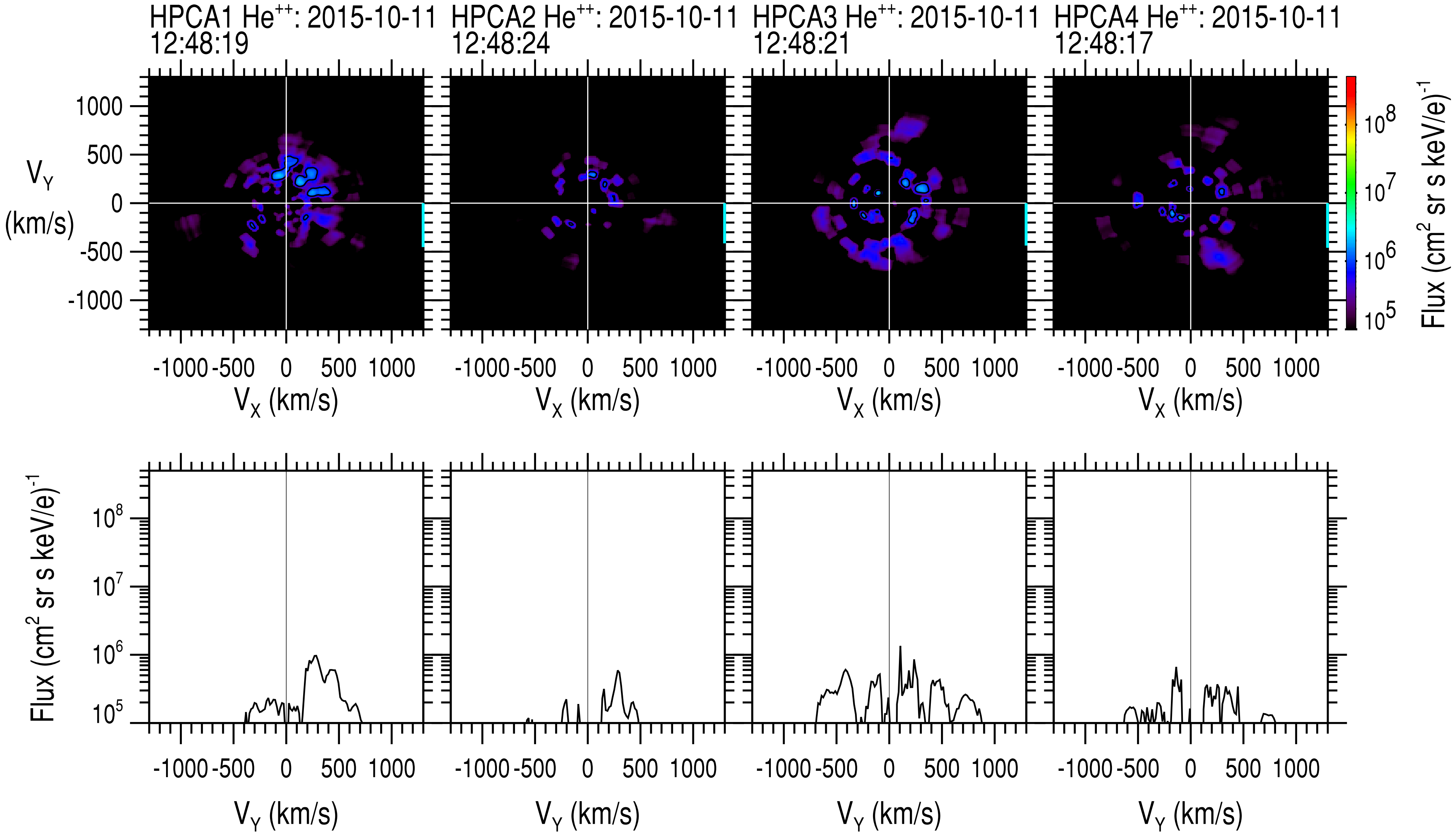
HPCA1 He++: 2015-10-11 12:48:19
HPCA2 He++: 2015-10-11 12:48:24
HPCA3 He++: 2015-10-11 12:48:21
HPCA4 He++: 2015-10-11 12:48:17
VY (km/s)
VX (km/s)
Flux (cm2 sr s keV/e)-1
VY (km/s)

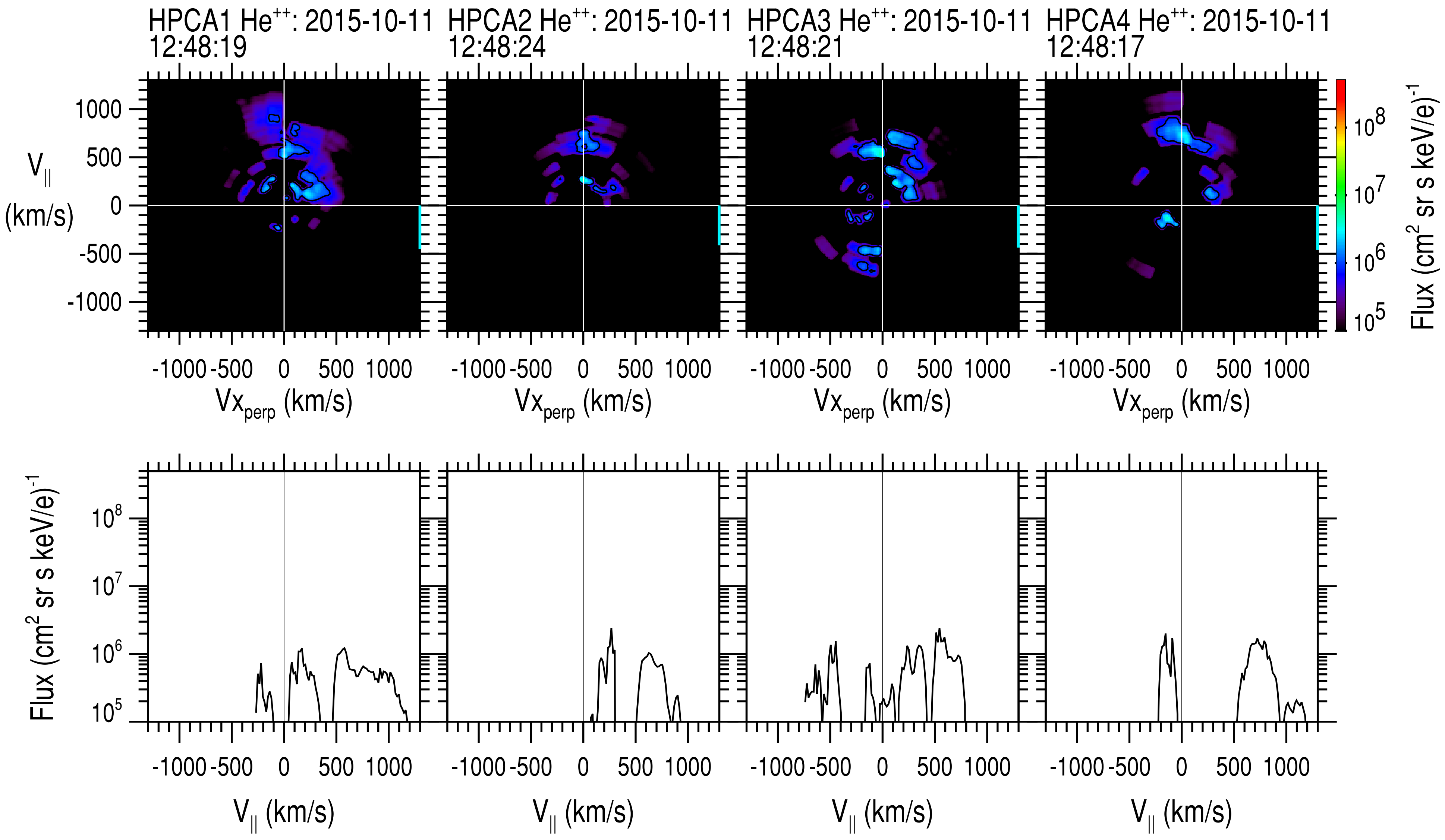
HPCA1 He++: 2015-10-11 12:48:19
HPCA2 He++: 2015-10-11 12:48:24
HPCA3 He++: 2015-10-11 12:48:21
HPCA4 He++: 2015-10-11 12:48:17
V|| (km/s)
Vxperp (km/s)
Flux (cm2 sr s keV/e)-1

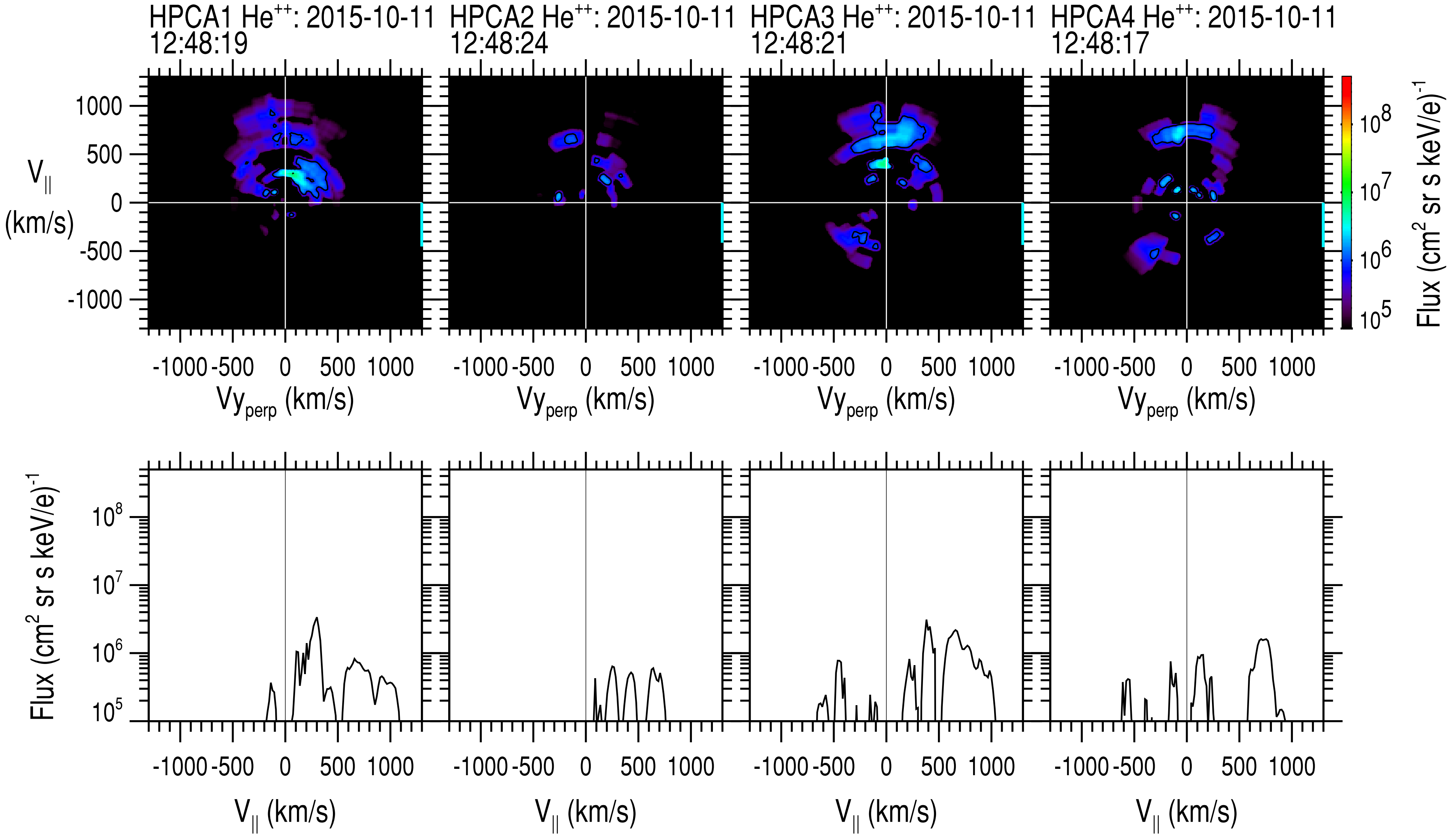

HPCA1 He++: 2015-10-11 12:48:19
HPCA2 He++: 2015-10-11 12:48:24
HPCA3 He++: 2015-10-11 12:48:21
HPCA4 He++: 2015-10-11 12:48:17
V|| (km/s)
1000
500
0
-500
-1000
-1000 -500 0 500 1000
Vyperp (km/s)
Flux (cm2 sr s keV/e)-1
10^5
10^6
10^7
10^8
V|| (km/s)

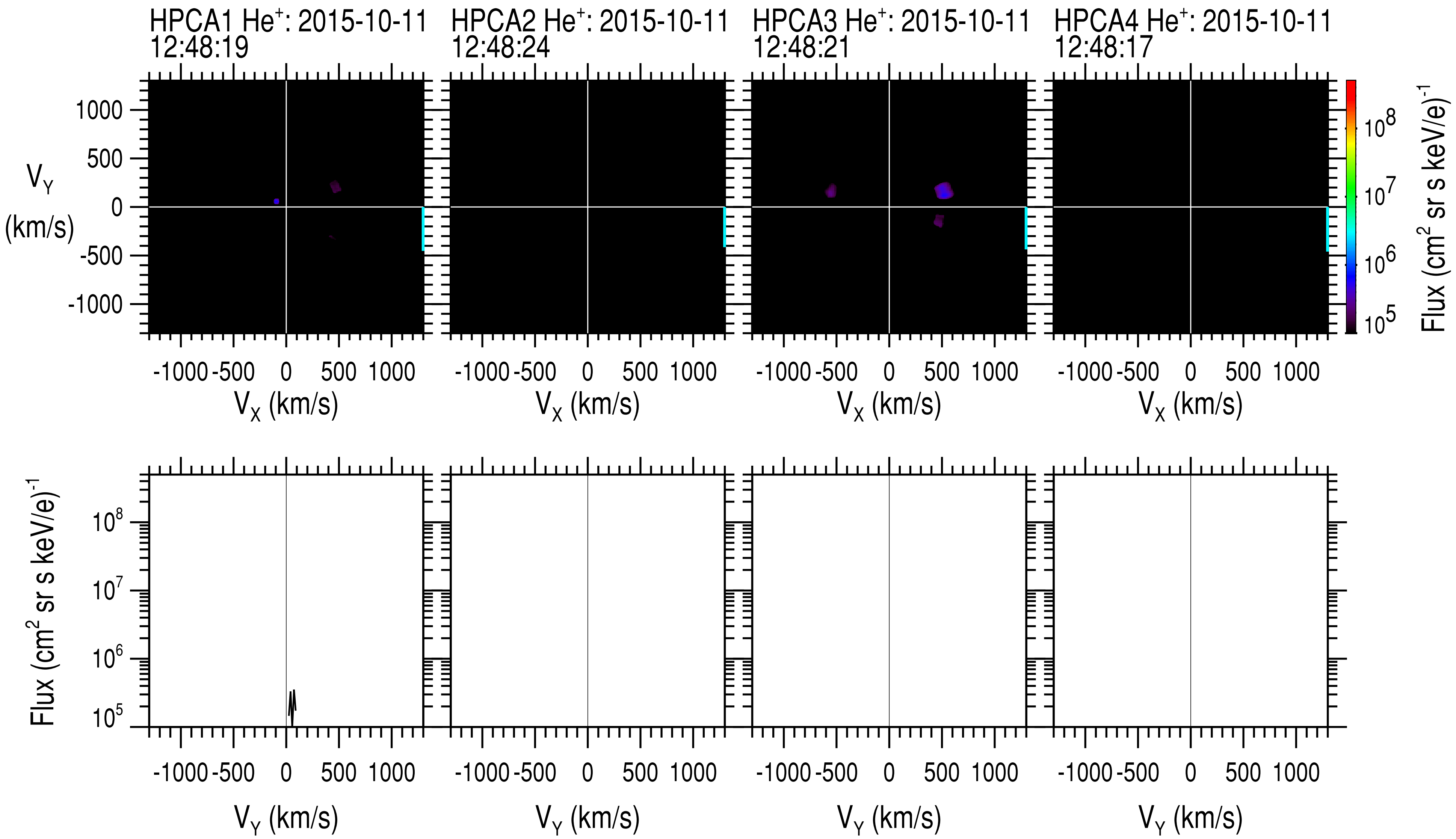
HPCA1 He+: 2015-10-11 12:48:19
HPCA2 He+: 2015-10-11 12:48:24
HPCA3 He+: 2015-10-11 12:48:21
HPCA4 He+: 2015-10-11 12:48:17
V_Y (km/s)
V_X (km/s)
Flux (cm^2 sr s keV/e)^-1
V_Y (km/s)

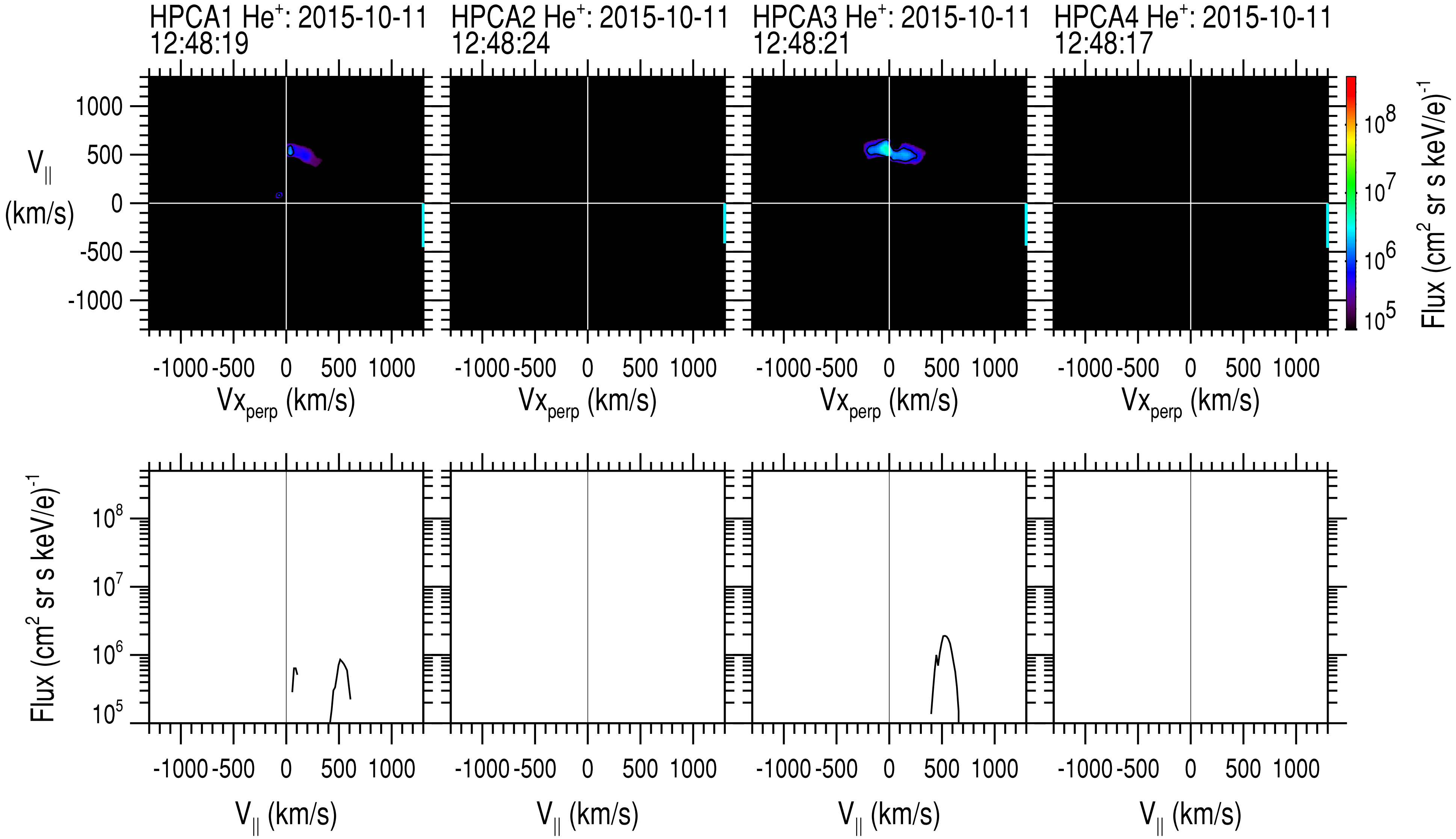

HPCA1 He+: 2015-10-11 12:48:19
HPCA2 He+: 2015-10-11 12:48:24
HPCA3 He+: 2015-10-11 12:48:21
HPCA4 He+: 2015-10-11 12:48:17
V|| (km/s)
Vxperp (km/s)
Flux (cm2 sr s keV/e)-1

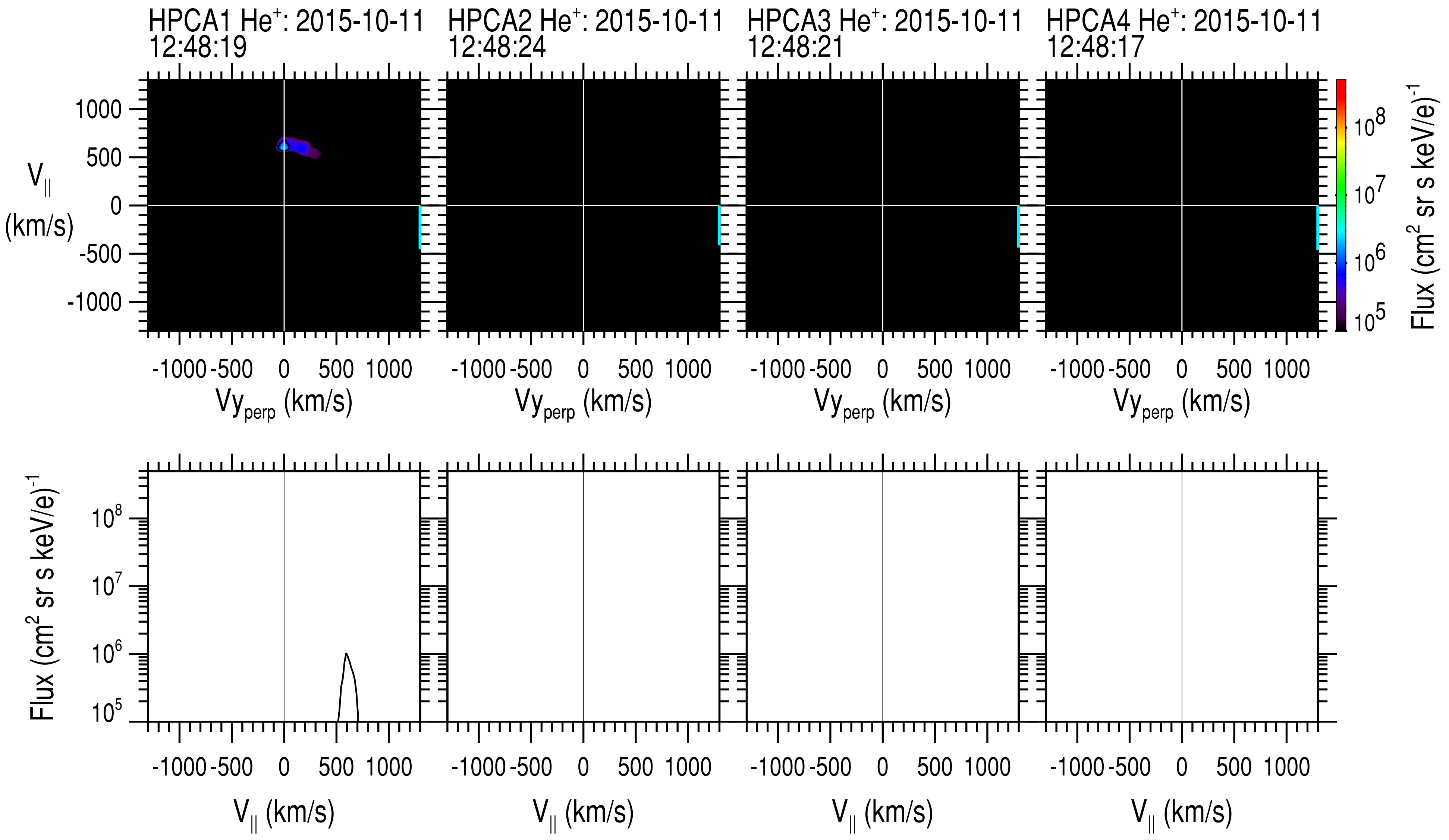

HPCA1 He+: 2015-10-11 12:48:19
HPCA2 He+: 2015-10-11 12:48:24
HPCA3 He+: 2015-10-11 12:48:21
HPCA4 He+: 2015-10-11 12:48:17
V|| (km/s)
Vyperp (km/s)
Flux (cm2 sr s keV/e)-1

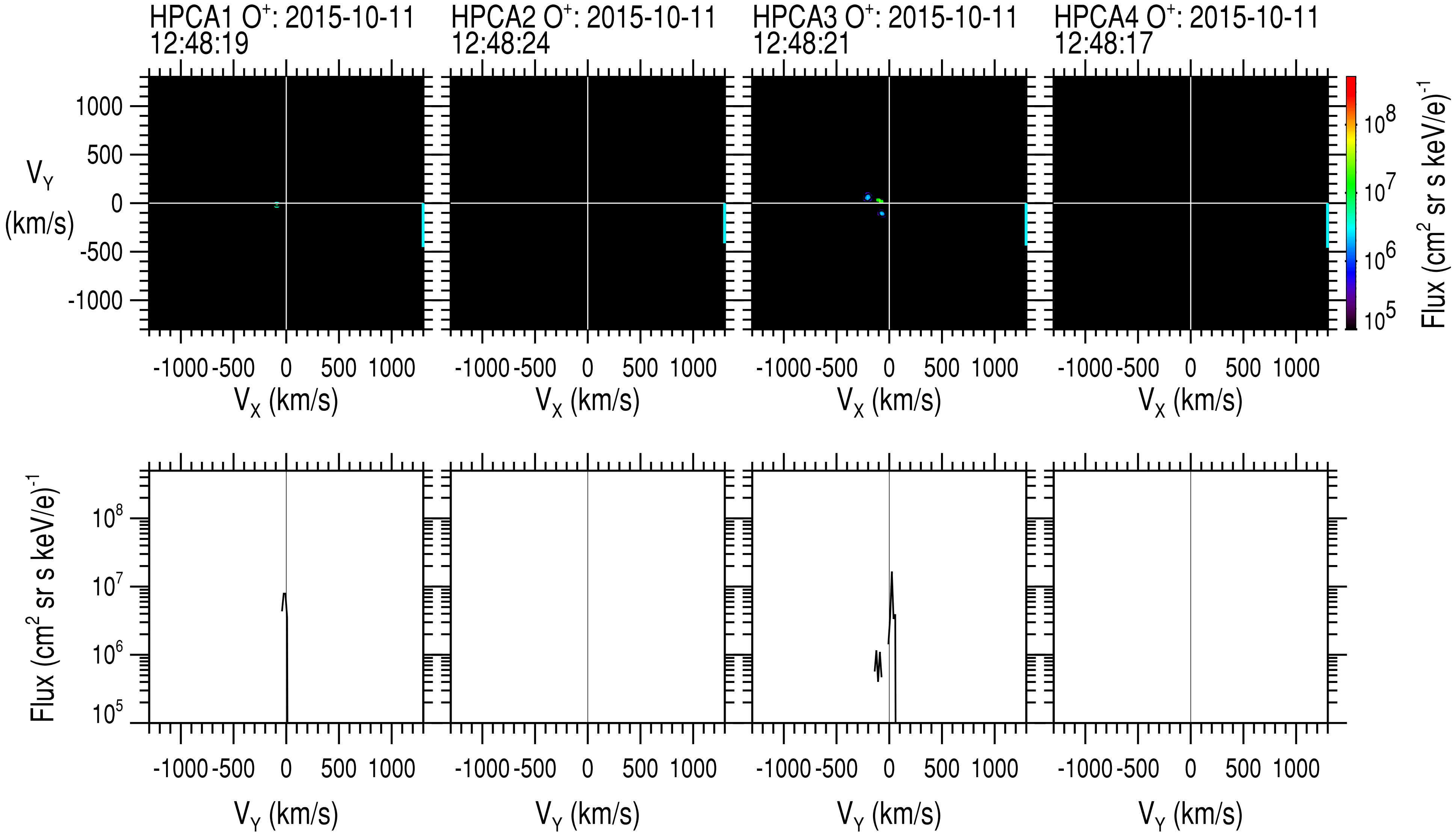

HPCA1 O+: 2015-10-11 12:48:19
HPCA2 O+: 2015-10-11 12:48:24
HPCA3 O+: 2015-10-11 12:48:21
HPCA4 O+: 2015-10-11 12:48:17
VY (km/s)
1000
500
0
-500
-1000
-1000 -500 0 500 1000
VX (km/s)
Flux (cm2 sr s keV/e)-1
10^5
10^6
10^7
10^8
VY (km/s)

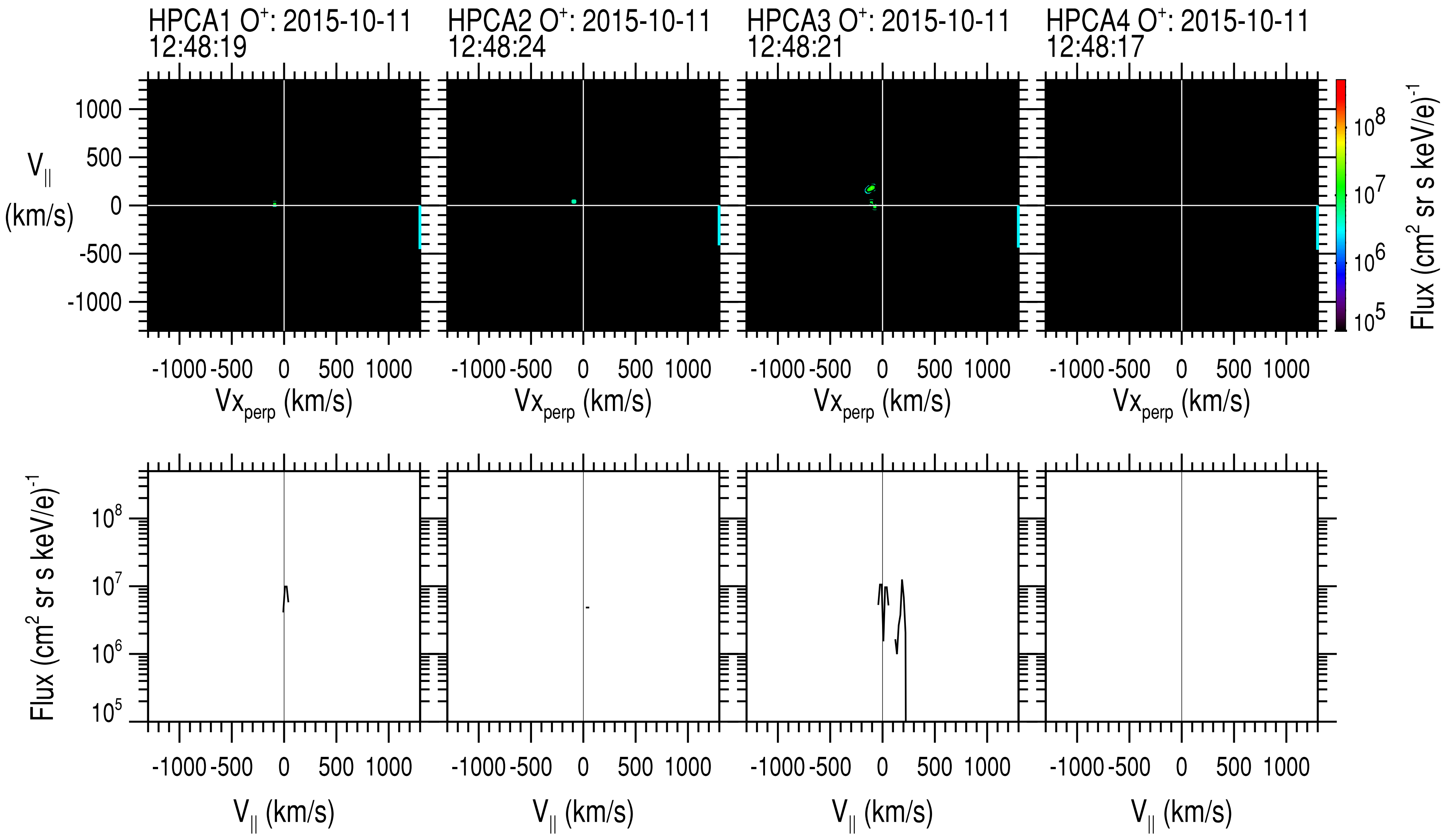

HPCA1 O+: 2015-10-11
12:48:19
HPCA2 O+: 2015-10-11
12:48:24
HPCA3 O+: 2015-10-11
12:48:21
HPCA4 O+: 2015-10-11
12:48:17
V|| (km/s)
1000
500
0
-500
-1000
-1000 -500 0 500 1000
Vxperp (km/s)
Flux (cm2 sr s keV/e)-1
10^8
10^7
10^6
10^5
V|| (km/s)

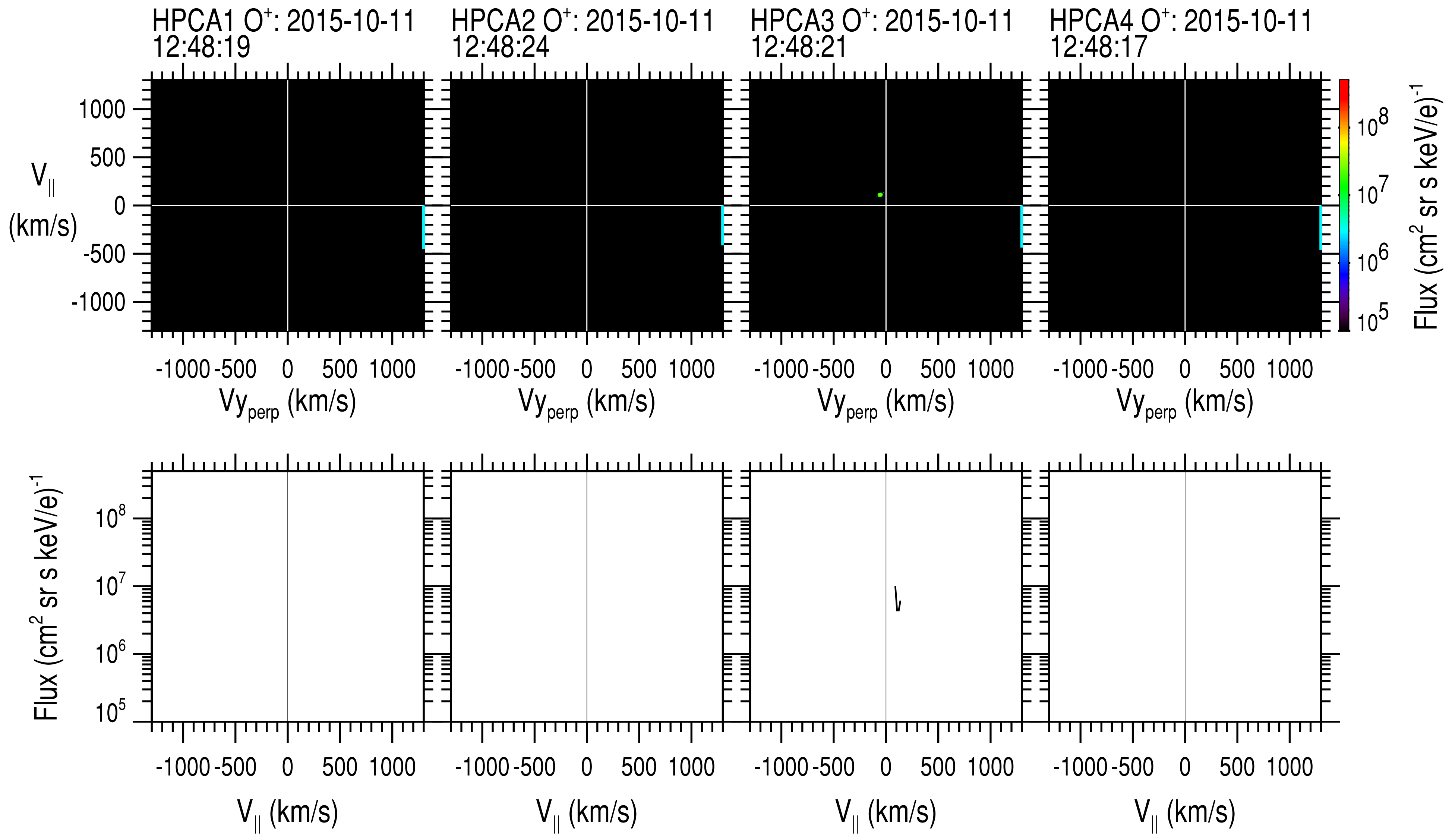

HPCA1 O+: 2015-10-11 12:48:19
HPCA2 O+: 2015-10-11 12:48:24
HPCA3 O+: 2015-10-11 12:48:21
HPCA4 O+: 2015-10-11 12:48:17
V|| (km/s)
Vyperp (km/s)
Flux (cm2 sr s keV/e)-1
-1000 -500 0 500 1000
105 106 107 108

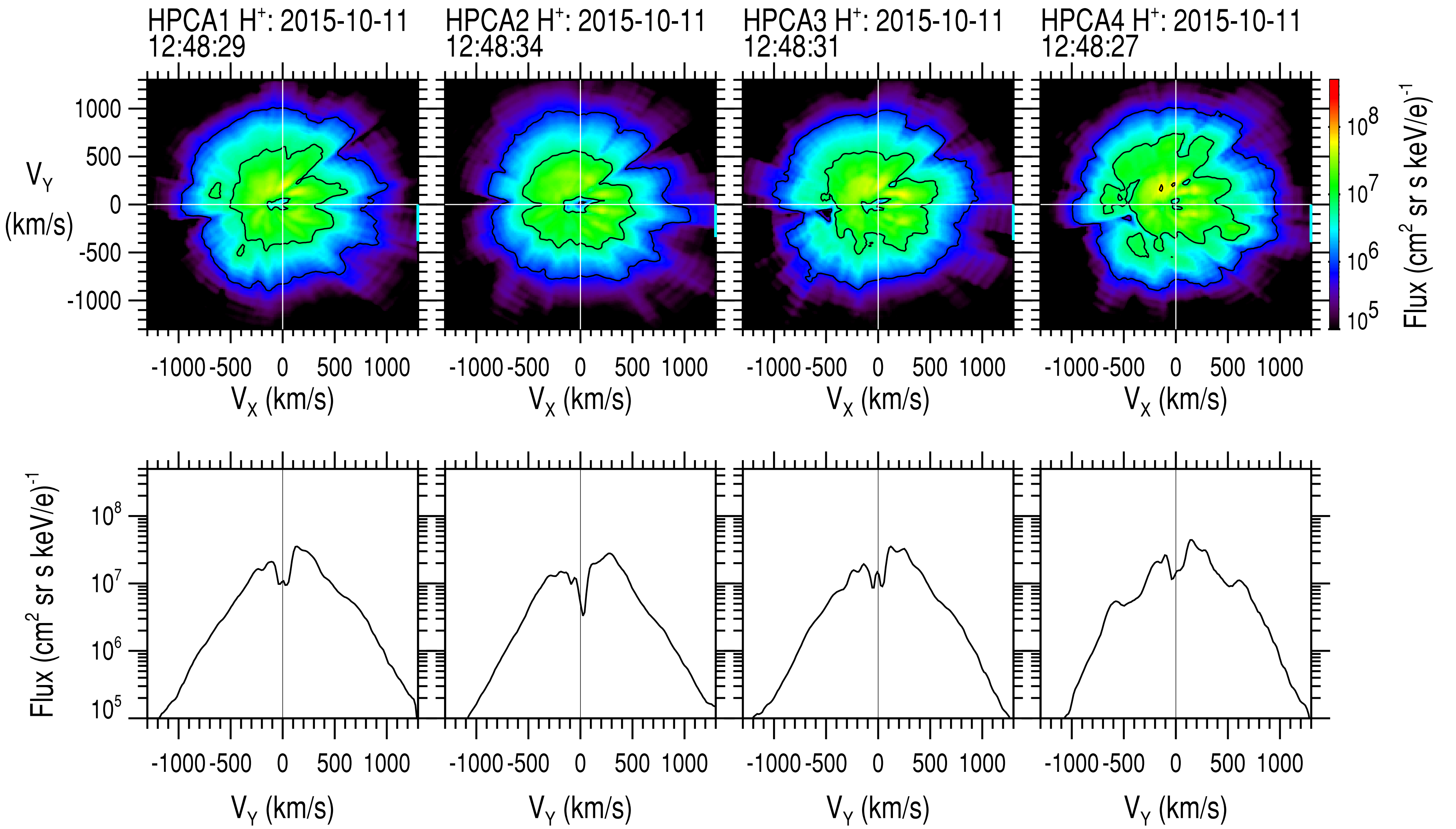

HPCA1 H+: 2015-10-11 12:48:29
HPCA2 H+: 2015-10-11 12:48:34
HPCA3 H+: 2015-10-11 12:48:31
HPCA4 H+: 2015-10-11 12:48:27
V_Y (km/s)
V_X (km/s)
Flux (cm^2 sr s keV/e)^-1

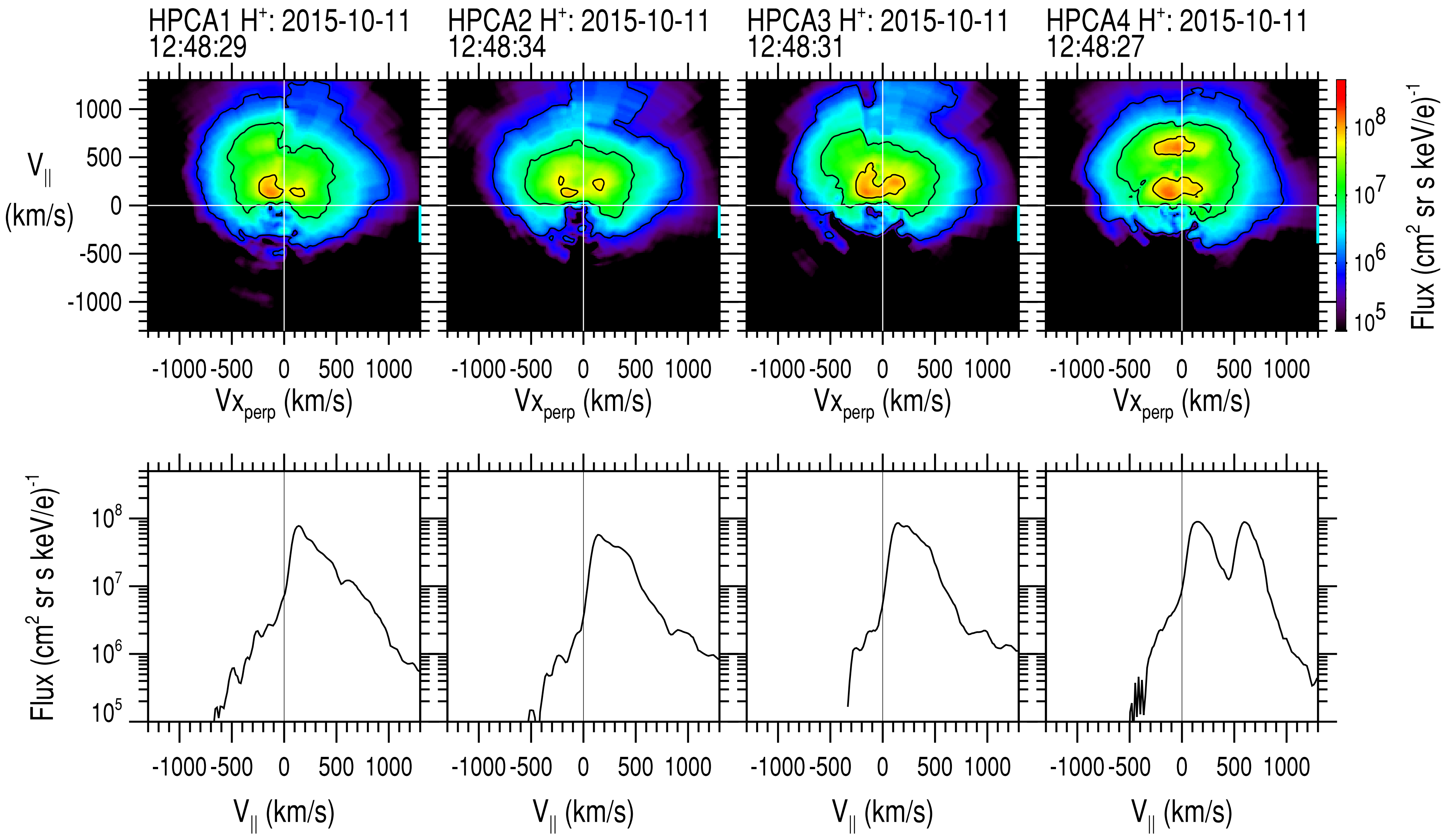
HPCA1 H+: 2015-10-11 12:48:29
HPCA2 H+: 2015-10-11 12:48:34
HPCA3 H+: 2015-10-11 12:48:31
HPCA4 H+: 2015-10-11 12:48:27
V|| (km/s)
Vxperp (km/s)
Flux (cm2 sr s keV/e)-1
-1000 -500 0 500 1000
10^5 10^6 10^7 10^8

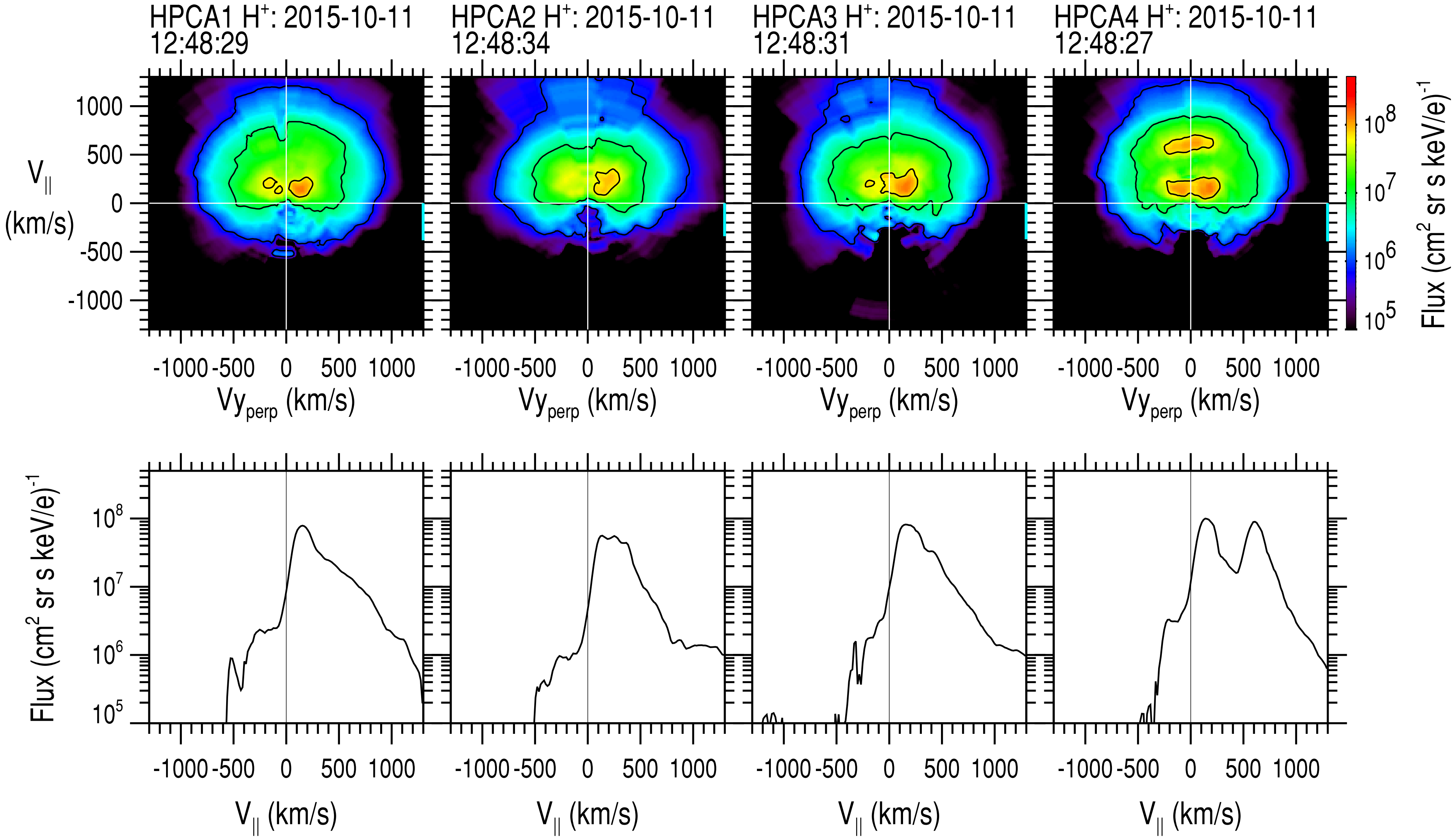

HPCA1 H+: 2015-10-11
12:48:29
HPCA2 H+: 2015-10-11
12:48:34
HPCA3 H+: 2015-10-11
12:48:31
HPCA4 H+: 2015-10-11
12:48:27
V|| (km/s)
Vyperp (km/s)
Flux (cm2 sr s keV/e)-1

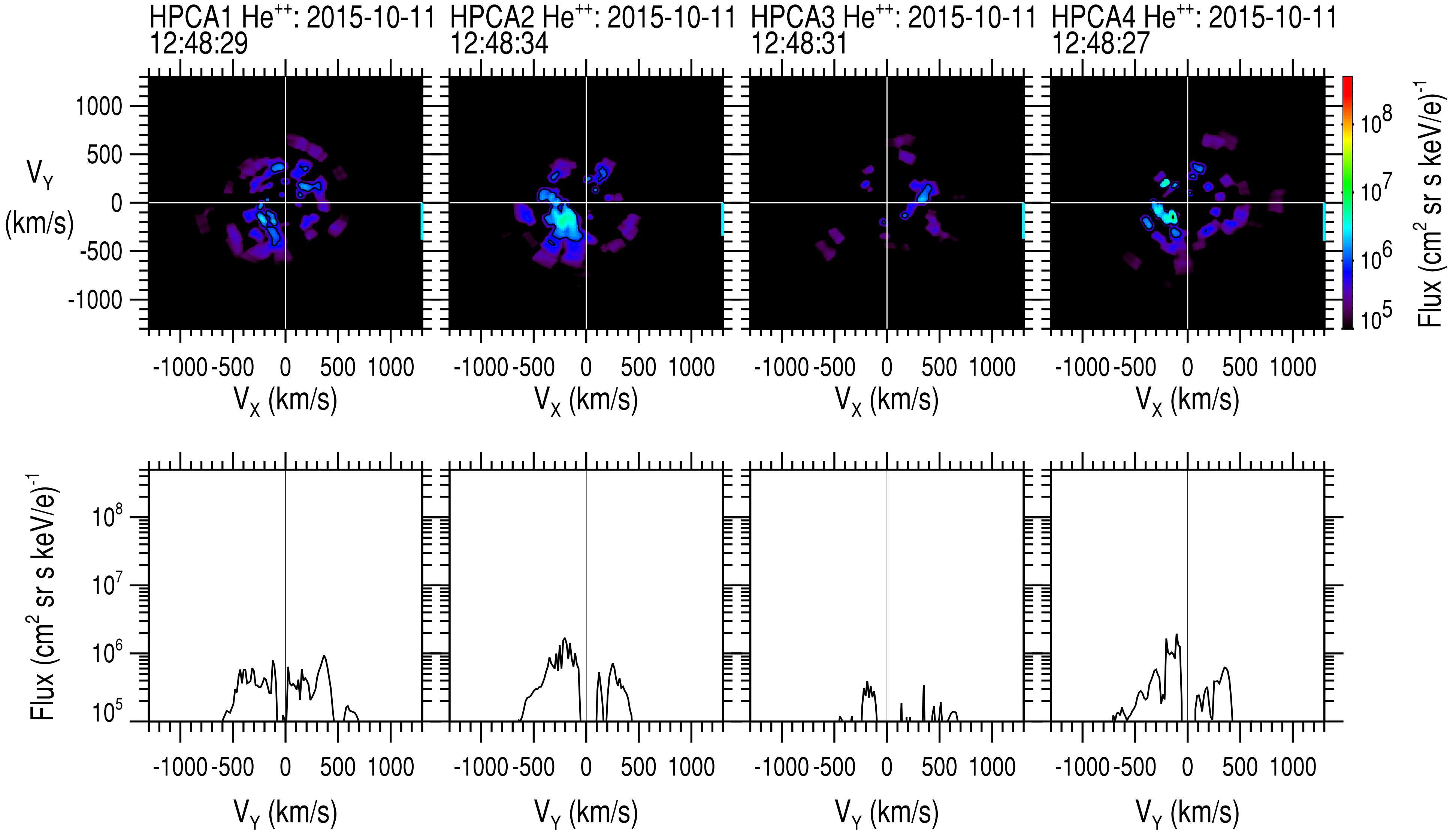

HPCA1 He++: 2015-10-11
12:48:29
HPCA2 He++: 2015-10-11
12:48:34
HPCA3 He++: 2015-10-11
12:48:31
HPCA4 He++: 2015-10-11
12:48:27
V_Y (km/s)
V_X (km/s)
Flux (cm^2 sr s keV/e)^-1
-1000 -500 0 500 1000
10^5 10^6 10^7 10^8

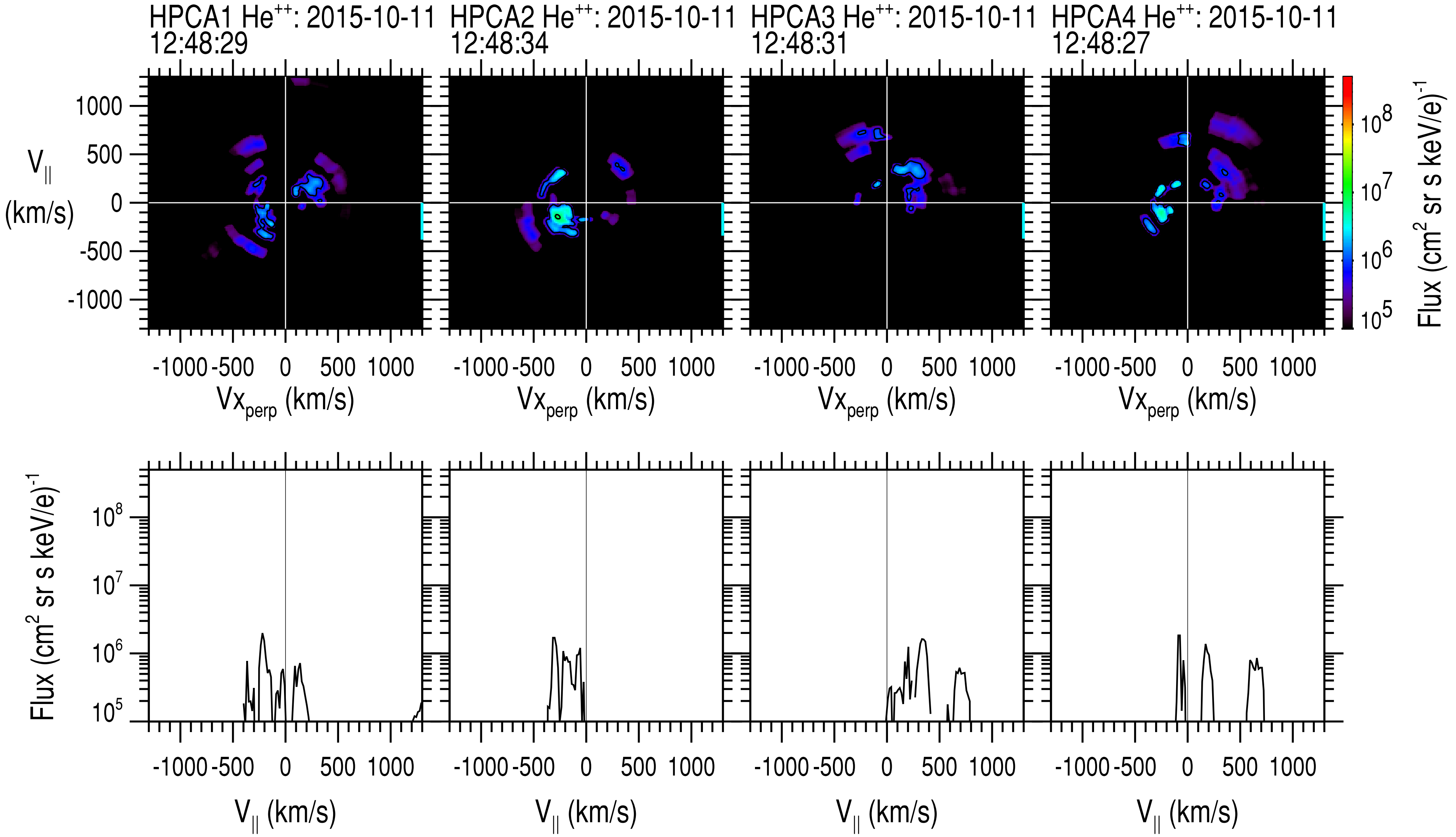
HPCA1 He++: 2015-10-11 12:48:29
HPCA2 He++: 2015-10-11 12:48:34
HPCA3 He++: 2015-10-11 12:48:31
HPCA4 He++: 2015-10-11 12:48:27
V|| (km/s)
Vxperp (km/s)
Flux (cm2 sr s keV/e)-1
-1000 -500 0 500 1000
10^5 10^6 10^7 10^8
V|| (km/s)

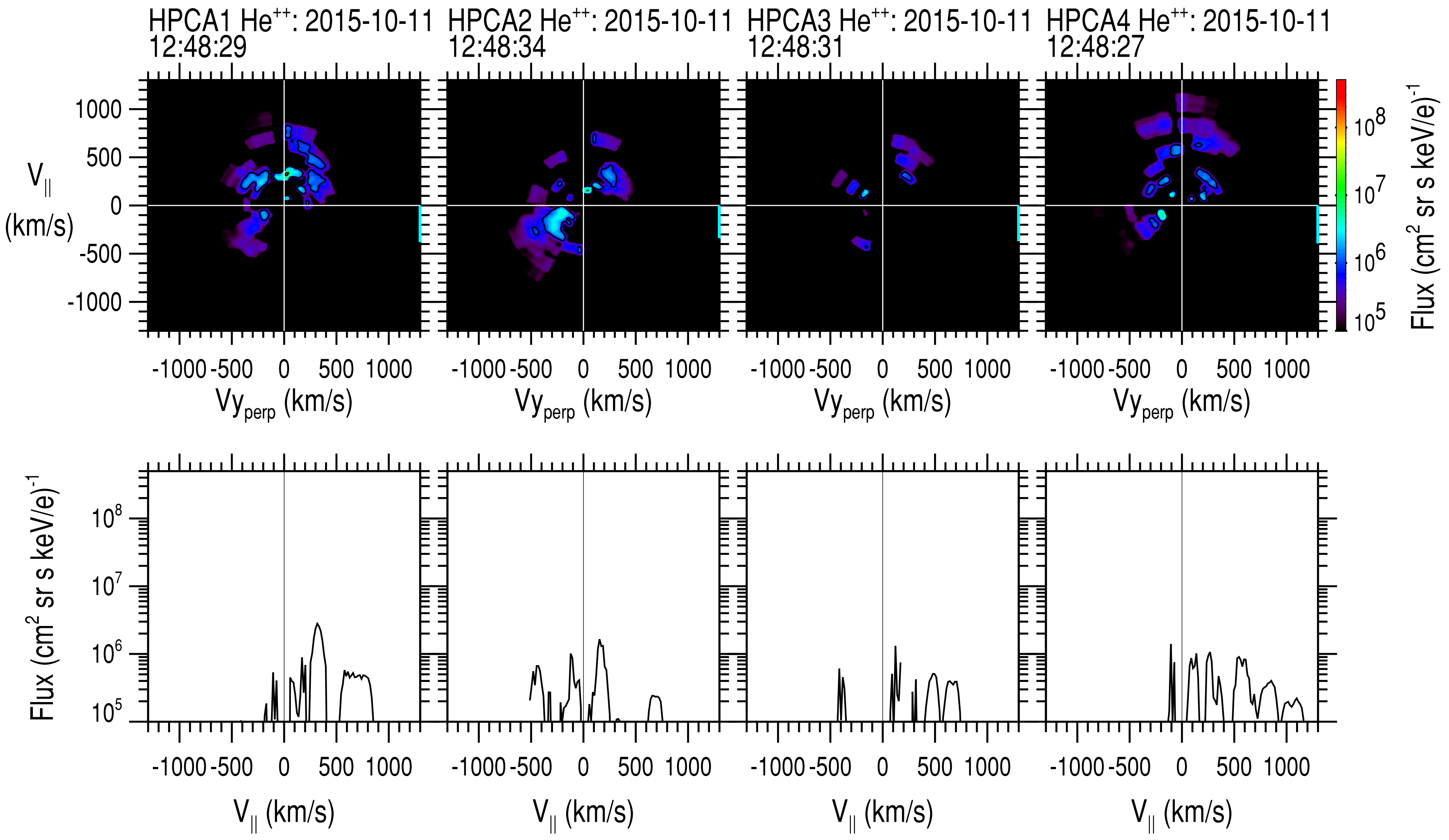

HPCA1 He++: 2015-10-11 12:48:29
HPCA2 He++: 2015-10-11 12:48:34
HPCA3 He++: 2015-10-11 12:48:31
HPCA4 He++: 2015-10-11 12:48:27
V|| (km/s)
Vyperp (km/s)
Flux (cm2 sr s keV/e)-1
V|| (km/s)

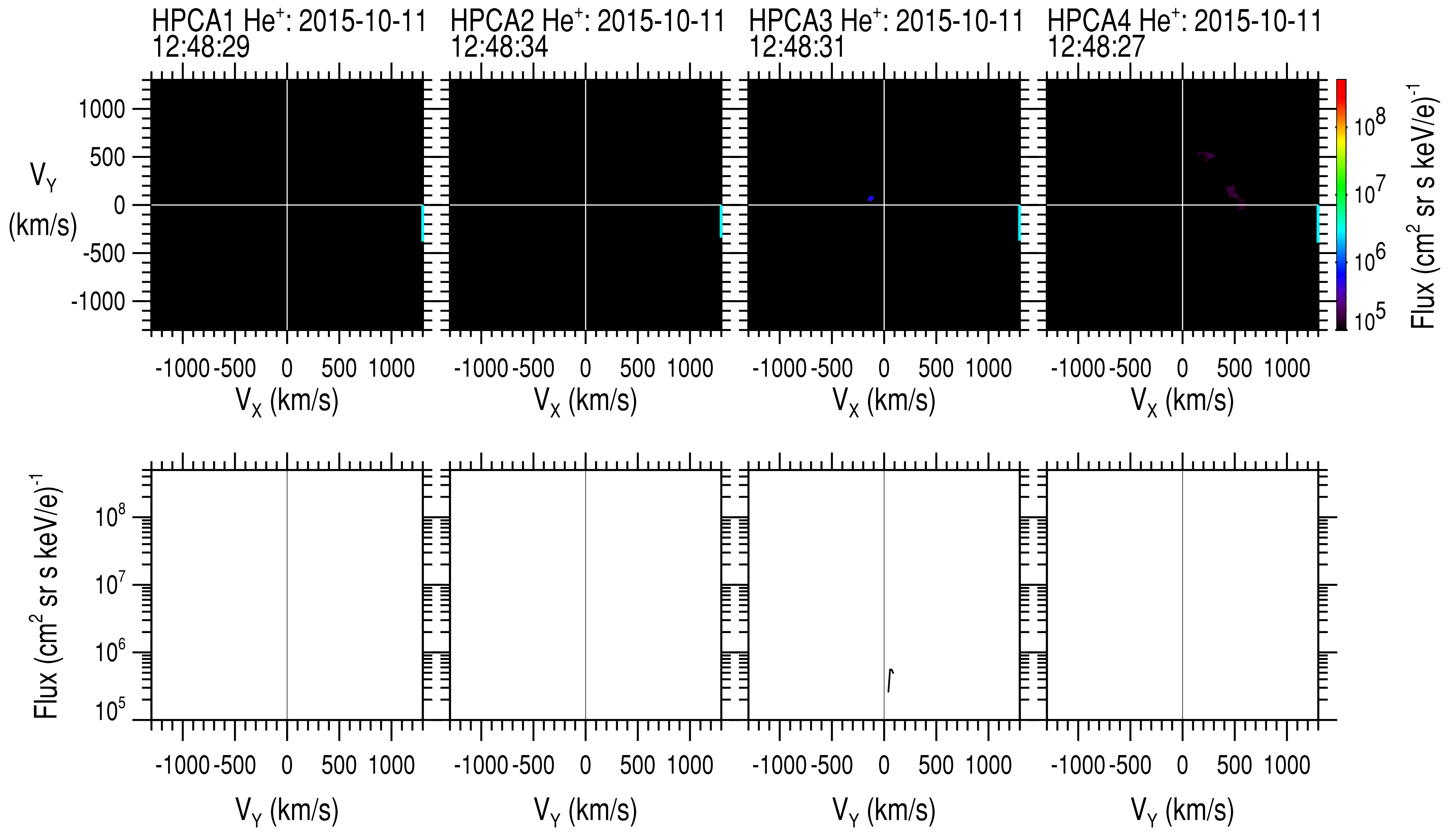

HPCA1 He+: 2015-10-11 12:48:29
HPCA2 He+: 2015-10-11 12:48:34
HPCA3 He+: 2015-10-11 12:48:31
HPCA4 He+: 2015-10-11 12:48:27
VY (km/s)
VX (km/s)
Flux (cm2 sr s keV/e)-1

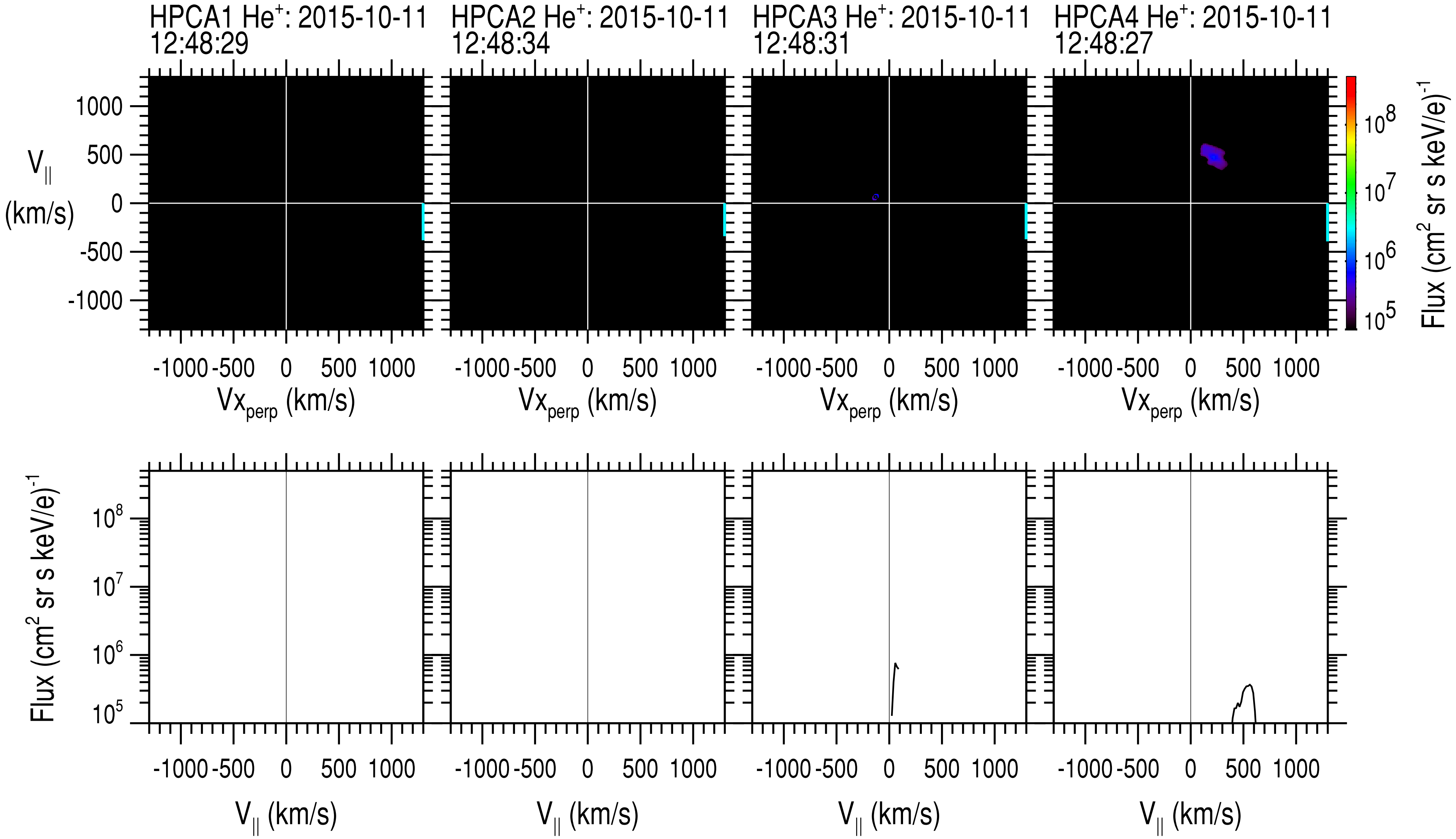

HPCA1 He+: 2015-10-11
12:48:29
HPCA2 He+: 2015-10-11
12:48:34
HPCA3 He+: 2015-10-11
12:48:31
HPCA4 He+: 2015-10-11
12:48:27
V|| (km/s)
Vxperp (km/s)
Flux (cm2 sr s keV/e)-1

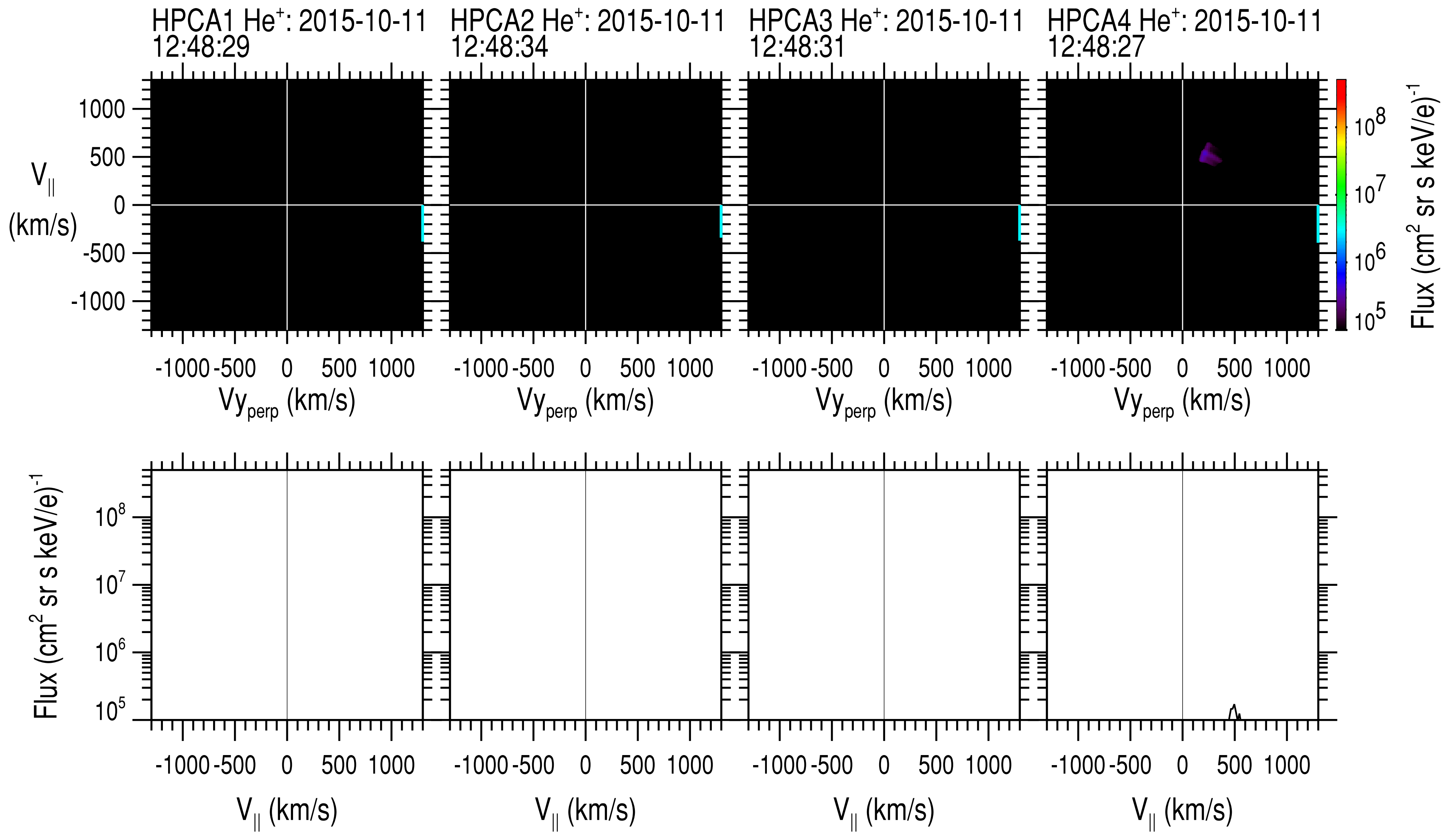
HPCA1 He+: 2015-10-11 12:48:29
HPCA2 He+: 2015-10-11 12:48:34
HPCA3 He+: 2015-10-11 12:48:31
HPCA4 He+: 2015-10-11 12:48:27
V|| (km/s)
Vyperp (km/s)
Flux (cm2 sr s keV/e)-1
-1000 -500 0 500 1000
10^5 10^6 10^7 10^8
V|| (km/s)

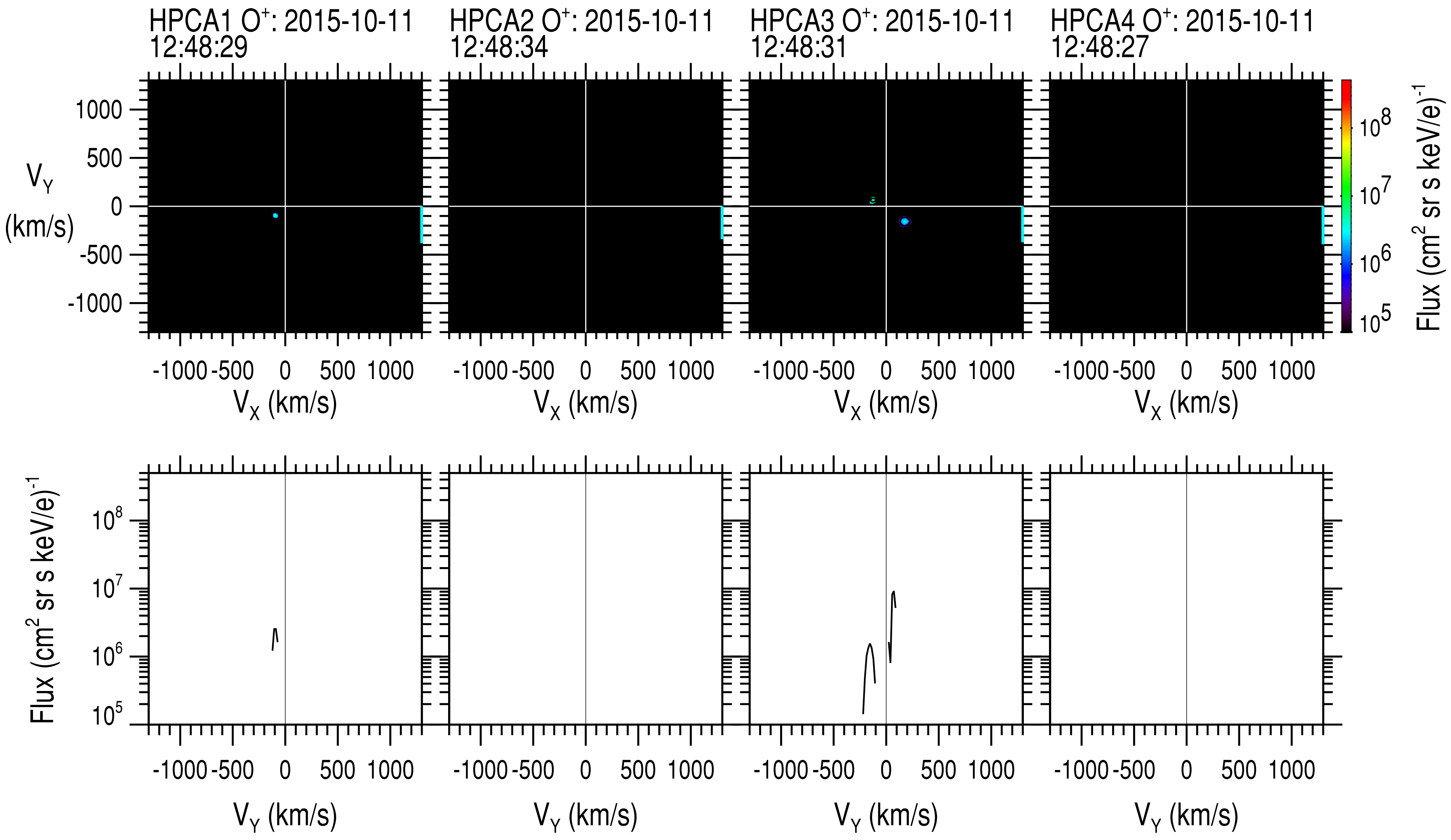
HPCA1 O+: 2015-10-11
12:48:29
HPCA2 O+: 2015-10-11
12:48:34
HPCA3 O+: 2015-10-11
12:48:31
HPCA4 O+: 2015-10-11
12:48:27
VY (km/s)
VX (km/s)
Flux (cm2 sr s keV/e)-1

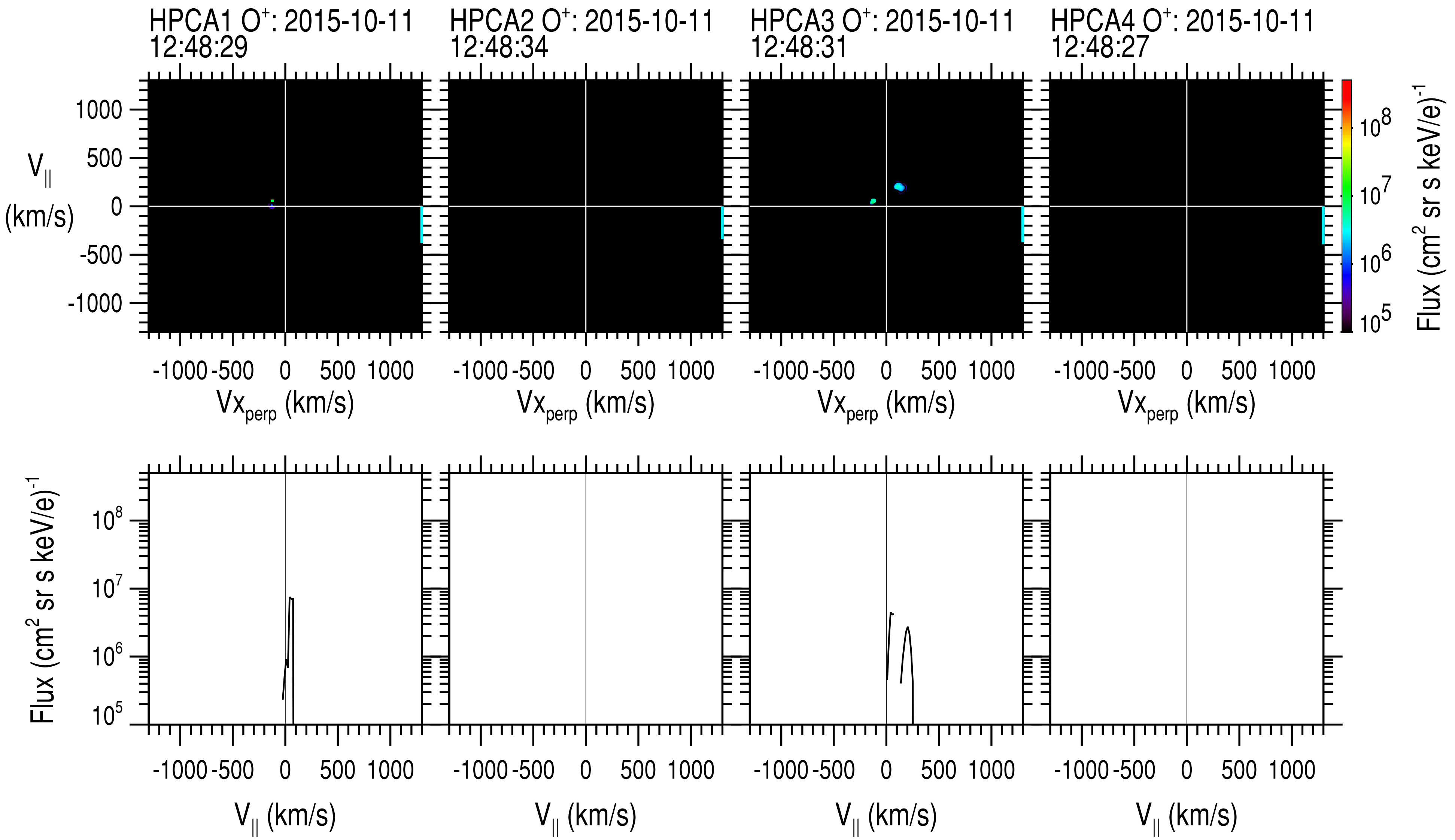

HPCA1 O+: 2015-10-11 12:48:29
HPCA2 O+: 2015-10-11 12:48:34
HPCA3 O+: 2015-10-11 12:48:31
HPCA4 O+: 2015-10-11 12:48:27
V|| (km/s)
Vxperp (km/s)
Flux (cm2 sr s keV/e)-1

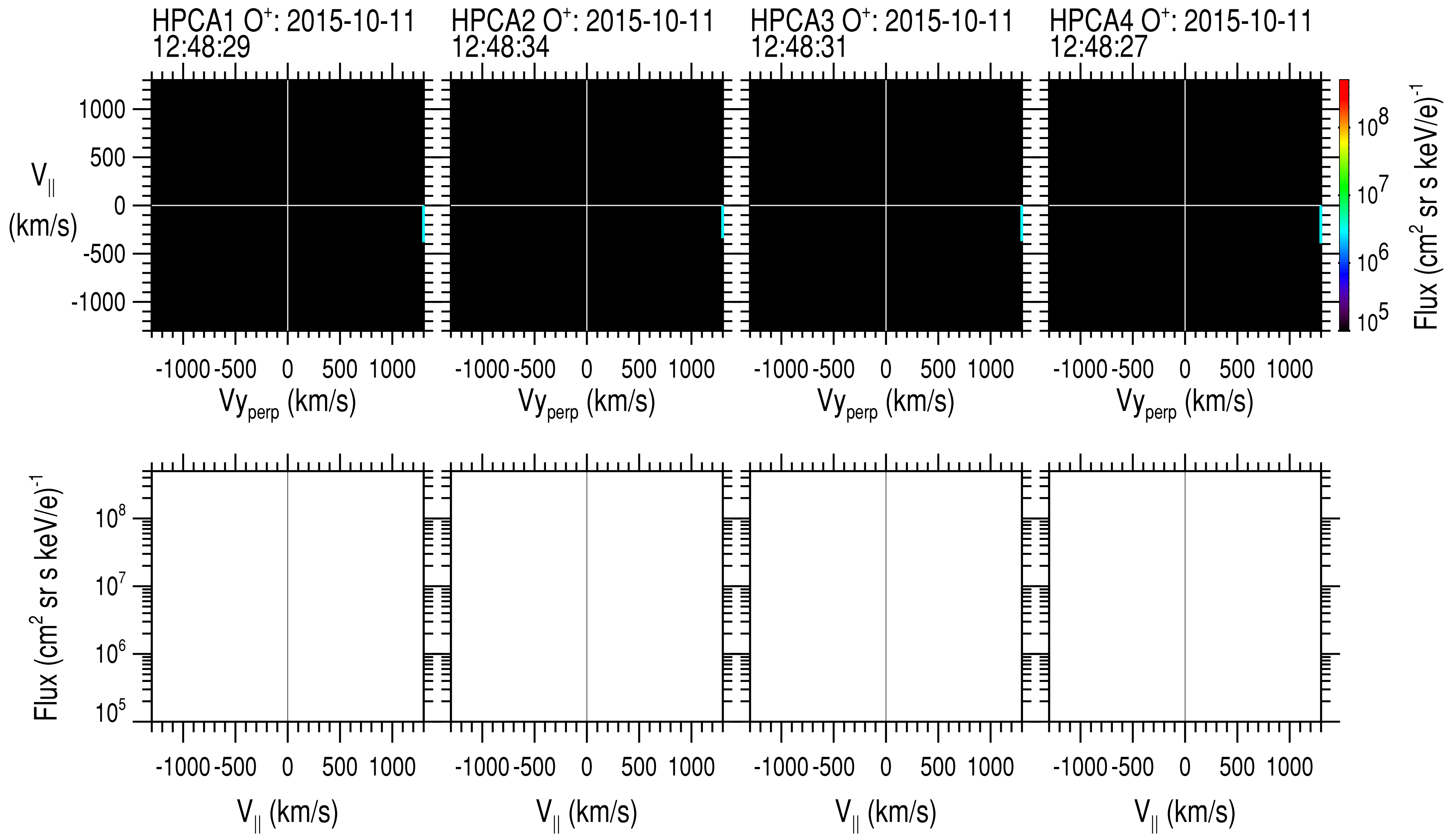

HPCA1 O+: 2015-10-11 12:48:29
HPCA2 O+: 2015-10-11 12:48:34
HPCA3 O+: 2015-10-11 12:48:31
HPCA4 O+: 2015-10-11 12:48:27
V|| (km/s)
Vyperp (km/s)
Flux (cm2 sr s keV/e)-1

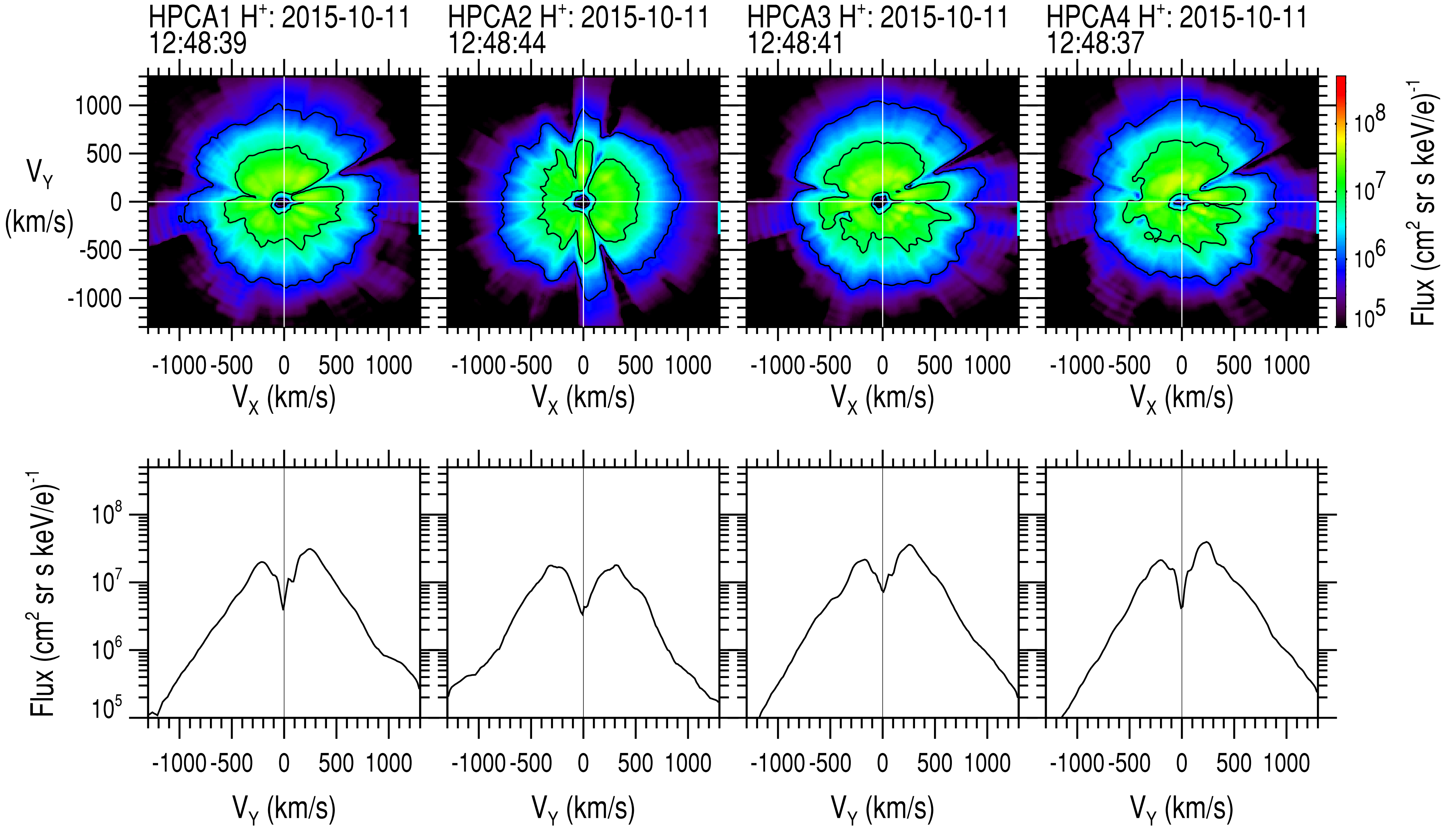
HPCA1 H+: 2015-10-11 12:48:39
HPCA2 H+: 2015-10-11 12:48:44
HPCA3 H+: 2015-10-11 12:48:41
HPCA4 H+: 2015-10-11 12:48:37
VY (km/s)
VX (km/s)
VY (km/s)
Flux (cm2 sr s keV/e)-1

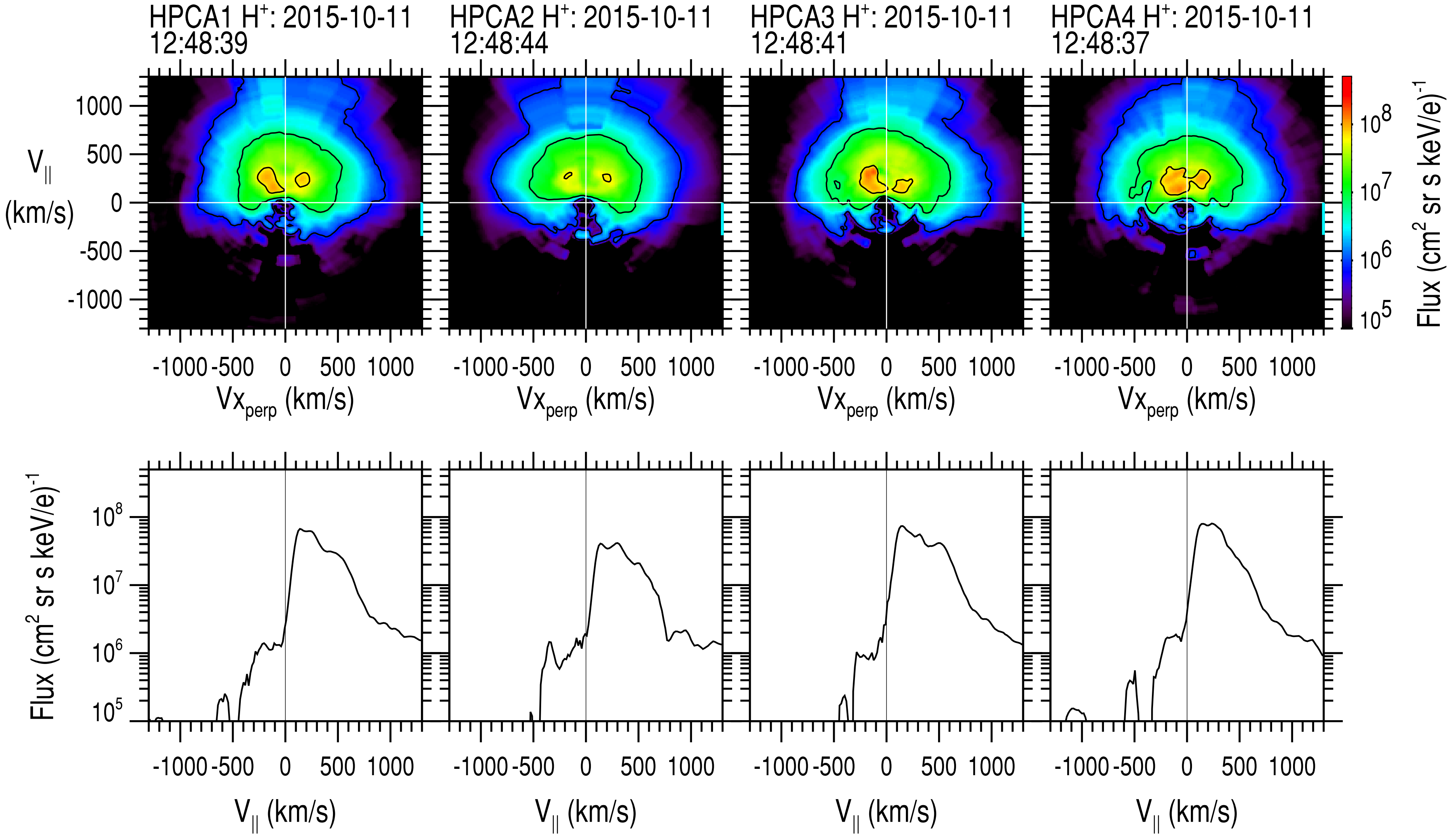
HPCA1 H+: 2015-10-11 12:48:39
HPCA2 H+: 2015-10-11 12:48:44
HPCA3 H+: 2015-10-11 12:48:41
HPCA4 H+: 2015-10-11 12:48:37
V|| (km/s)
1000
500
0
-500
-1000
-1000 -500 0 500 1000
Vxperp (km/s)
Flux (cm2 sr s keV/e)-1
105
106
107
108
V|| (km/s)

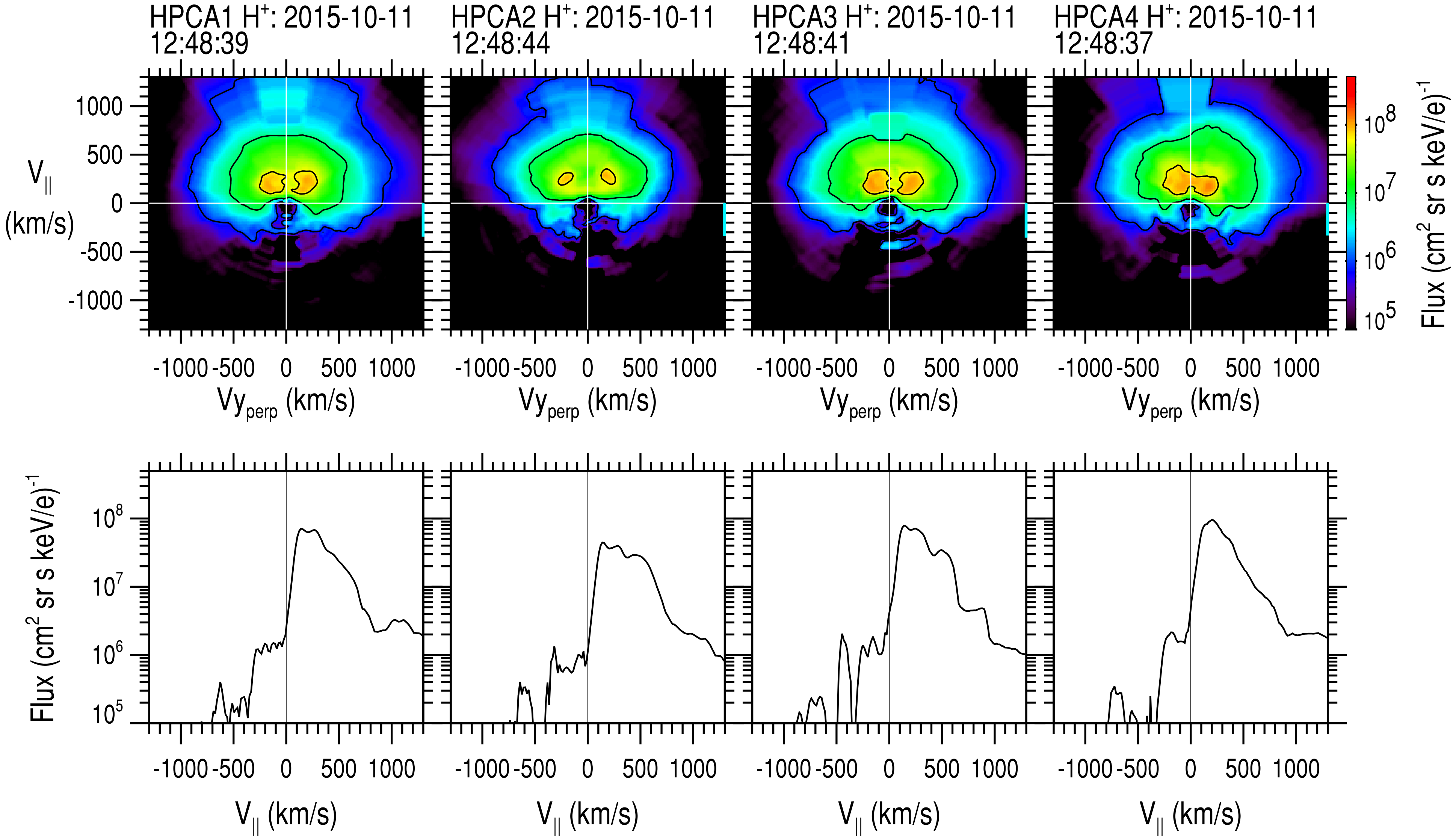
HPCA1 H+: 2015-10-11 12:48:39
HPCA2 H+: 2015-10-11 12:48:44
HPCA3 H+: 2015-10-11 12:48:41
HPCA4 H+: 2015-10-11 12:48:37
V|| (km/s)
Vyperp (km/s)
Flux (cm2 sr s keV/e)-1
V|| (km/s)

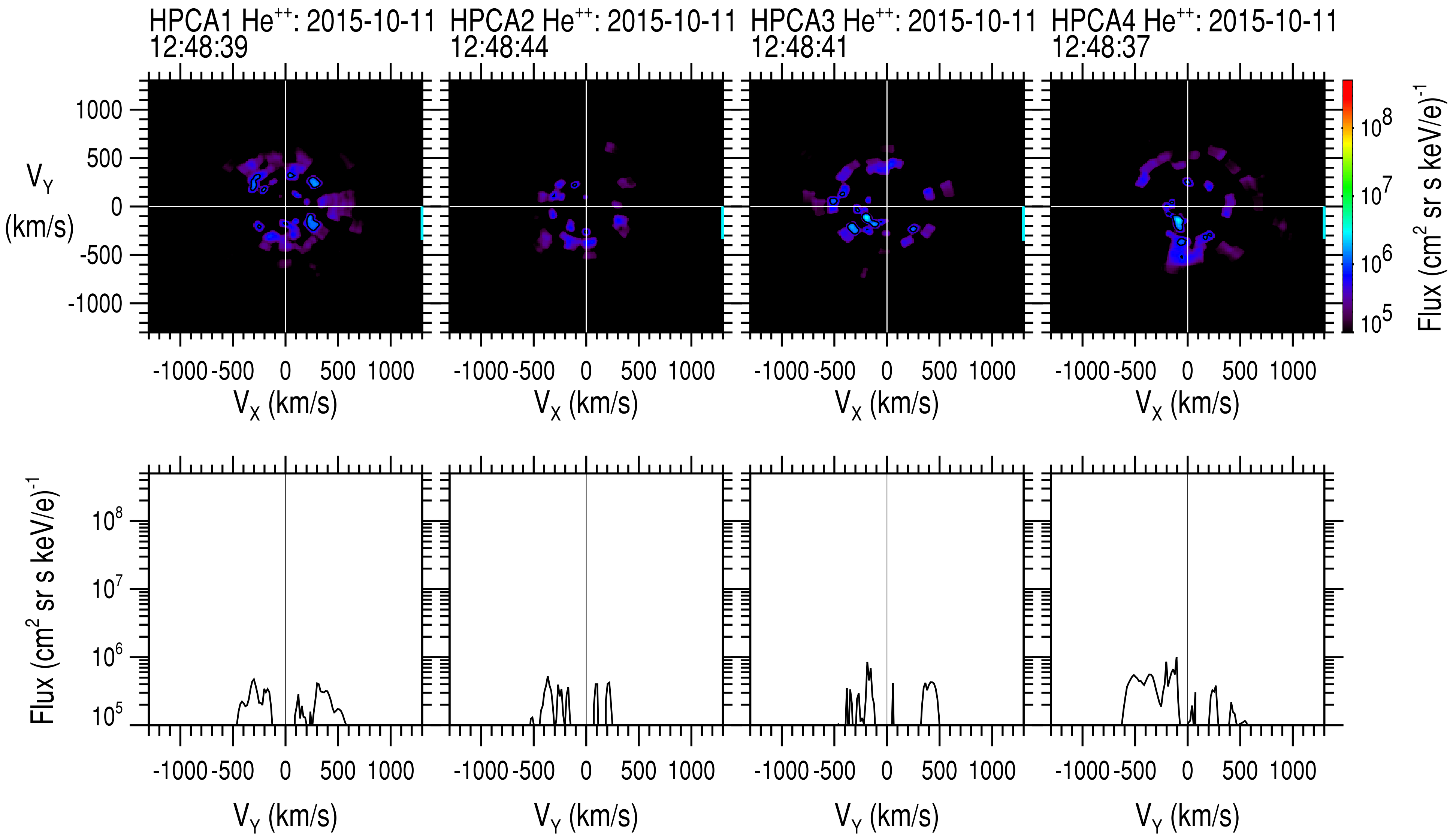
HPCA1 He++: 2015-10-11
12:48:39
HPCA2 He++: 2015-10-11
12:48:44
HPCA3 He++: 2015-10-11
12:48:41
HPCA4 He++: 2015-10-11
12:48:37
V_Y (km/s)
V_X (km/s)
Flux (cm^2 sr s keV/e)^-1
V_Y (km/s)

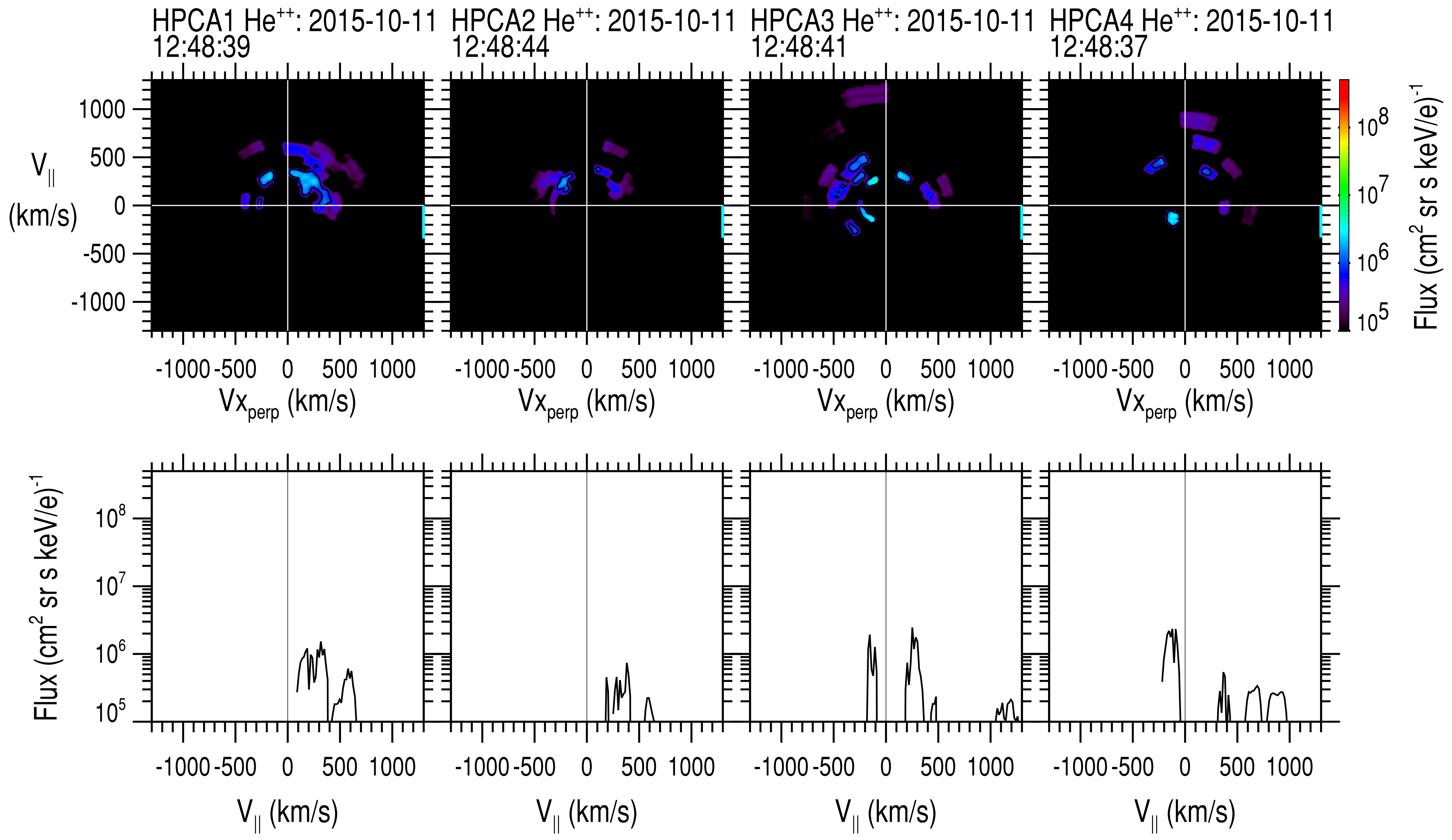
HPCA1 He++: 2015-10-11 12:48:39
HPCA2 He++: 2015-10-11 12:48:44
HPCA3 He++: 2015-10-11 12:48:41
HPCA4 He++: 2015-10-11 12:48:37
V|| (km/s)
Vxperp (km/s)
Flux (cm2 sr s keV/e)-1
1000
500
0
-500
-1000
10^5
10^6
10^7
10^8

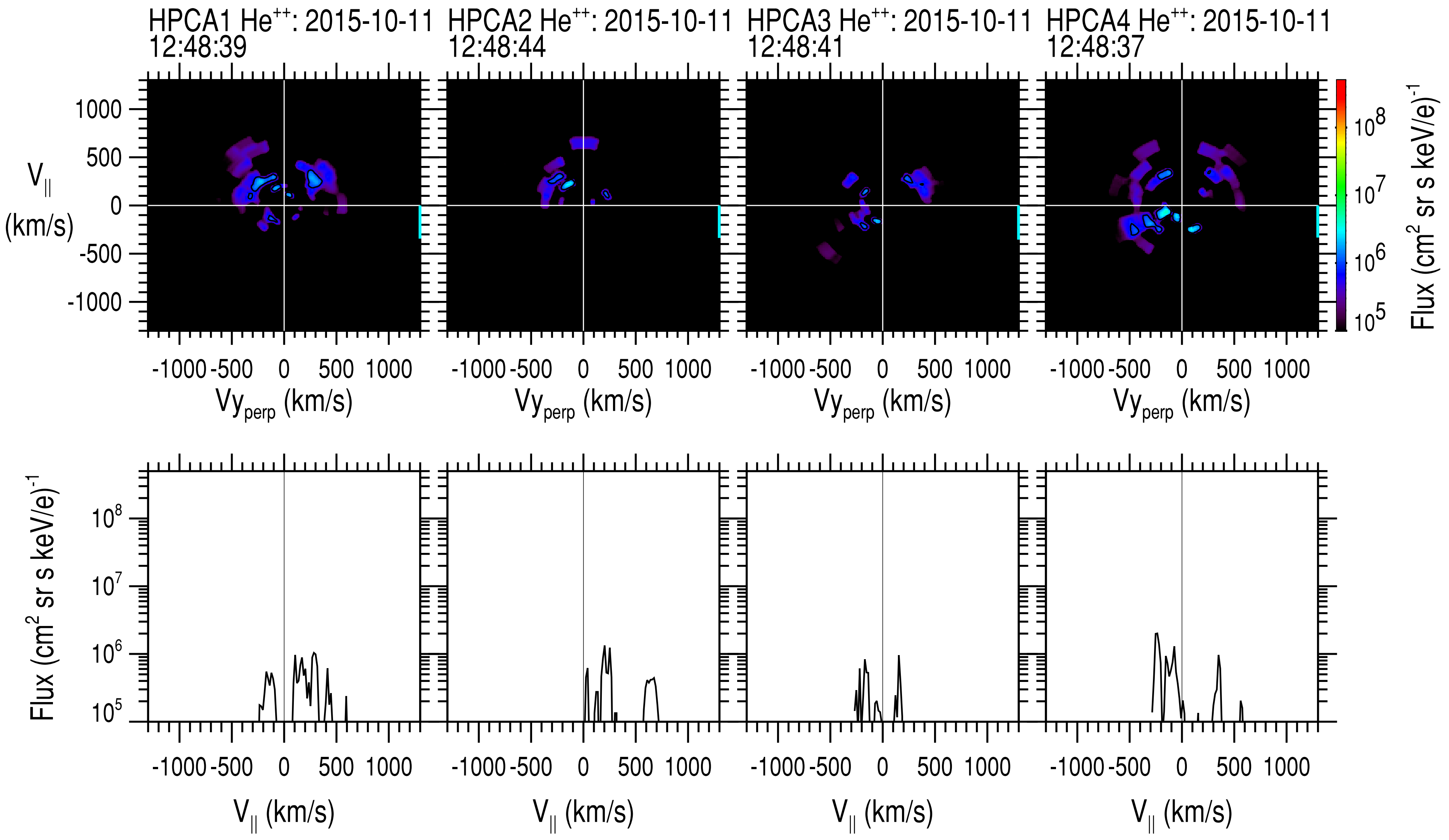

HPCA1 He++: 2015-10-11 12:48:39
HPCA2 He++: 2015-10-11 12:48:44
HPCA3 He++: 2015-10-11 12:48:41
HPCA4 He++: 2015-10-11 12:48:37
V|| (km/s)
Vyperp (km/s)
Flux (cm2 sr s keV/e)-1

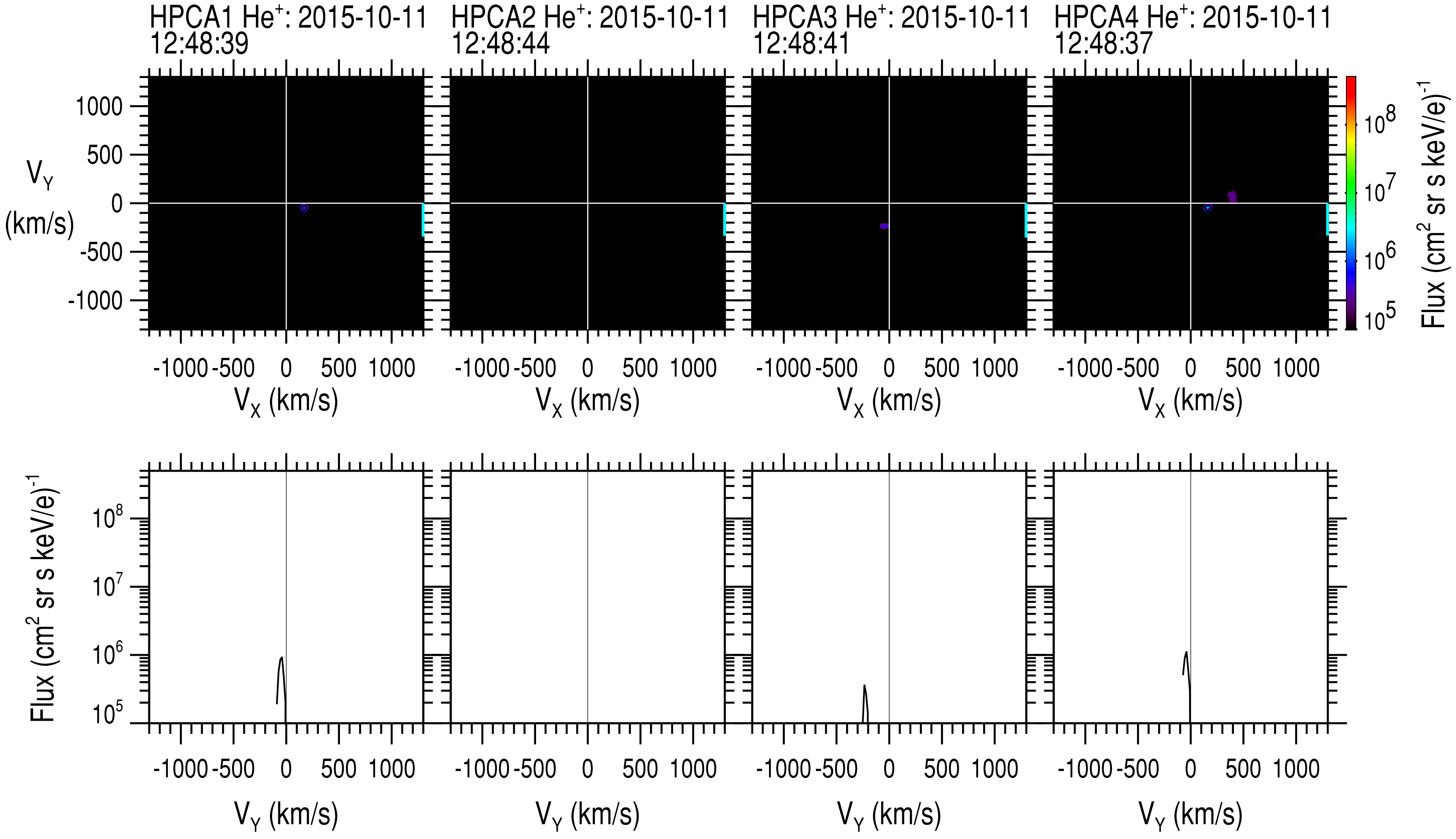

HPCA1 He+: 2015-10-11 12:48:39
HPCA2 He+: 2015-10-11 12:48:44
HPCA3 He+: 2015-10-11 12:48:41
HPCA4 He+: 2015-10-11 12:48:37
VY (km/s)
VX (km/s)
Flux (cm2 sr s keV/e)-1
VY (km/s)

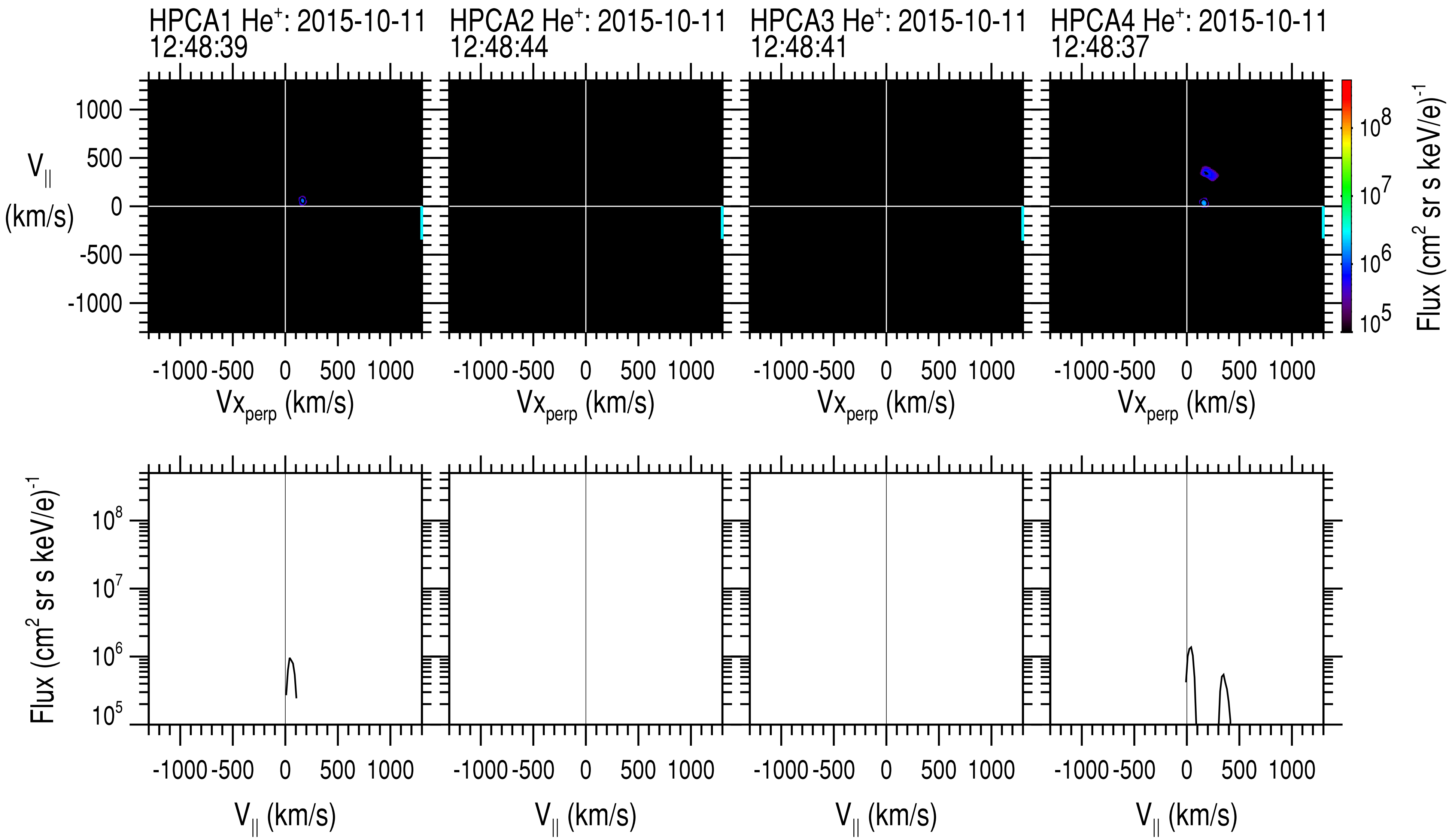

HPCA1 He+: 2015-10-11 12:48:39
HPCA2 He+: 2015-10-11 12:48:44
HPCA3 He+: 2015-10-11 12:48:41
HPCA4 He+: 2015-10-11 12:48:37
V|| (km/s)
Vxperp (km/s)
Flux (cm2 sr s keV/e)-1

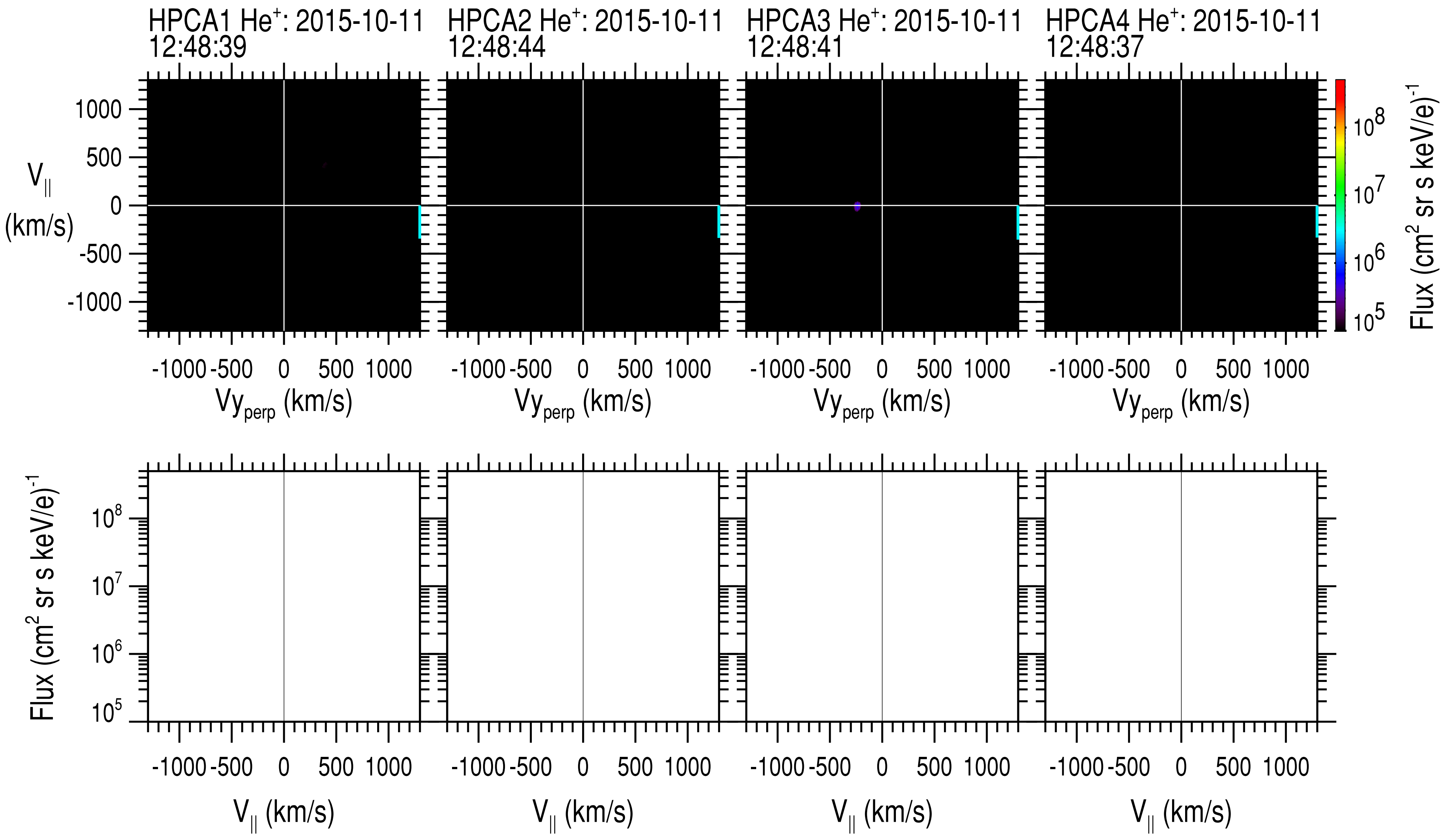
HPCA1 He+: 2015-10-11 12:48:39
HPCA2 He+: 2015-10-11 12:48:44
HPCA3 He+: 2015-10-11 12:48:41
HPCA4 He+: 2015-10-11 12:48:37
V|| (km/s)
Vyperp (km/s)
Flux (cm2 sr s keV/e)-1

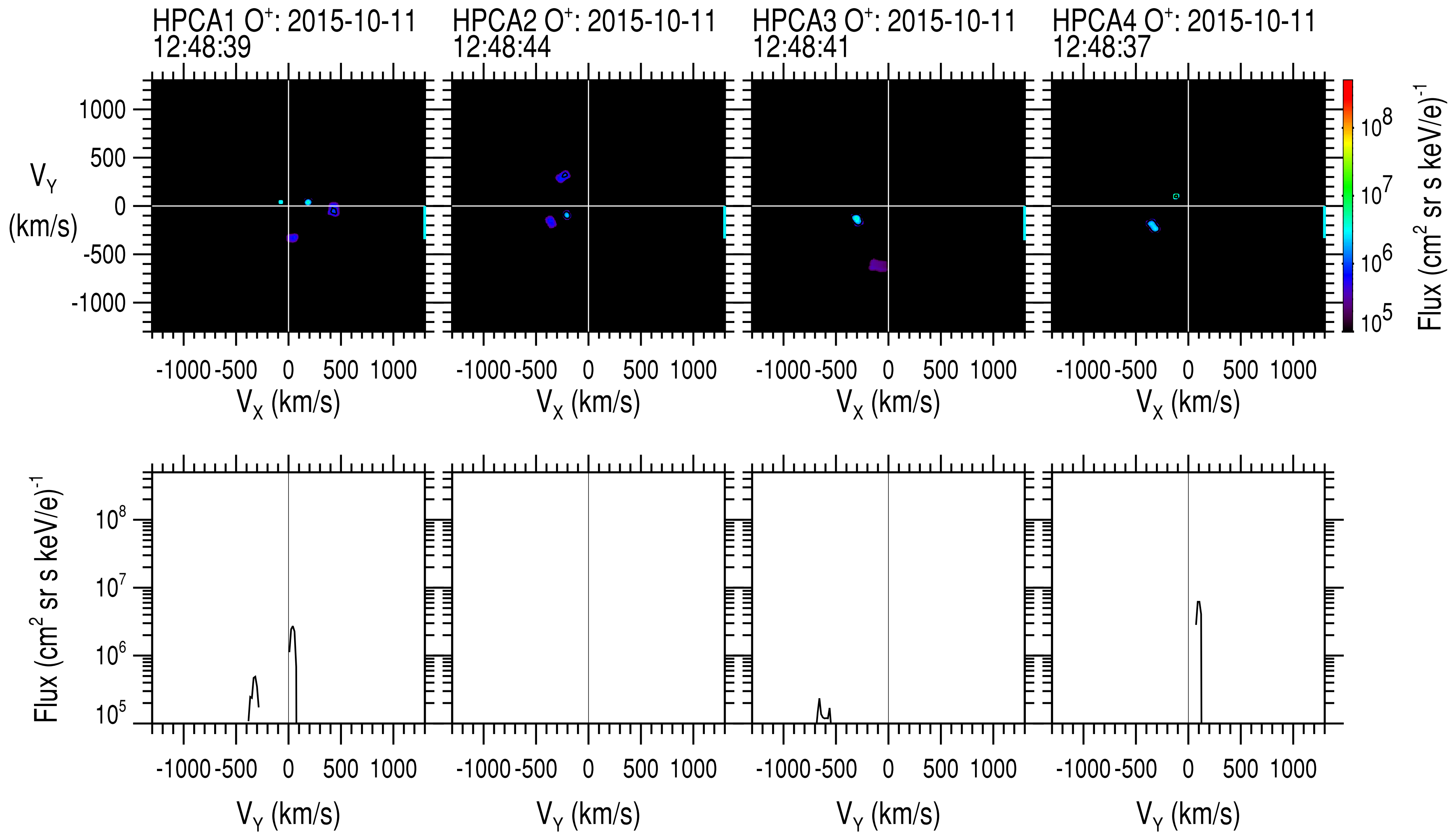

HPCA1 O+: 2015-10-11 12:48:39
HPCA2 O+: 2015-10-11 12:48:44
HPCA3 O+: 2015-10-11 12:48:41
HPCA4 O+: 2015-10-11 12:48:37
V_Y (km/s)
V_X (km/s)
Flux (cm^2 sr s keV/e)^-1
-1000 -500 0 500 1000
10^5 10^6 10^7 10^8

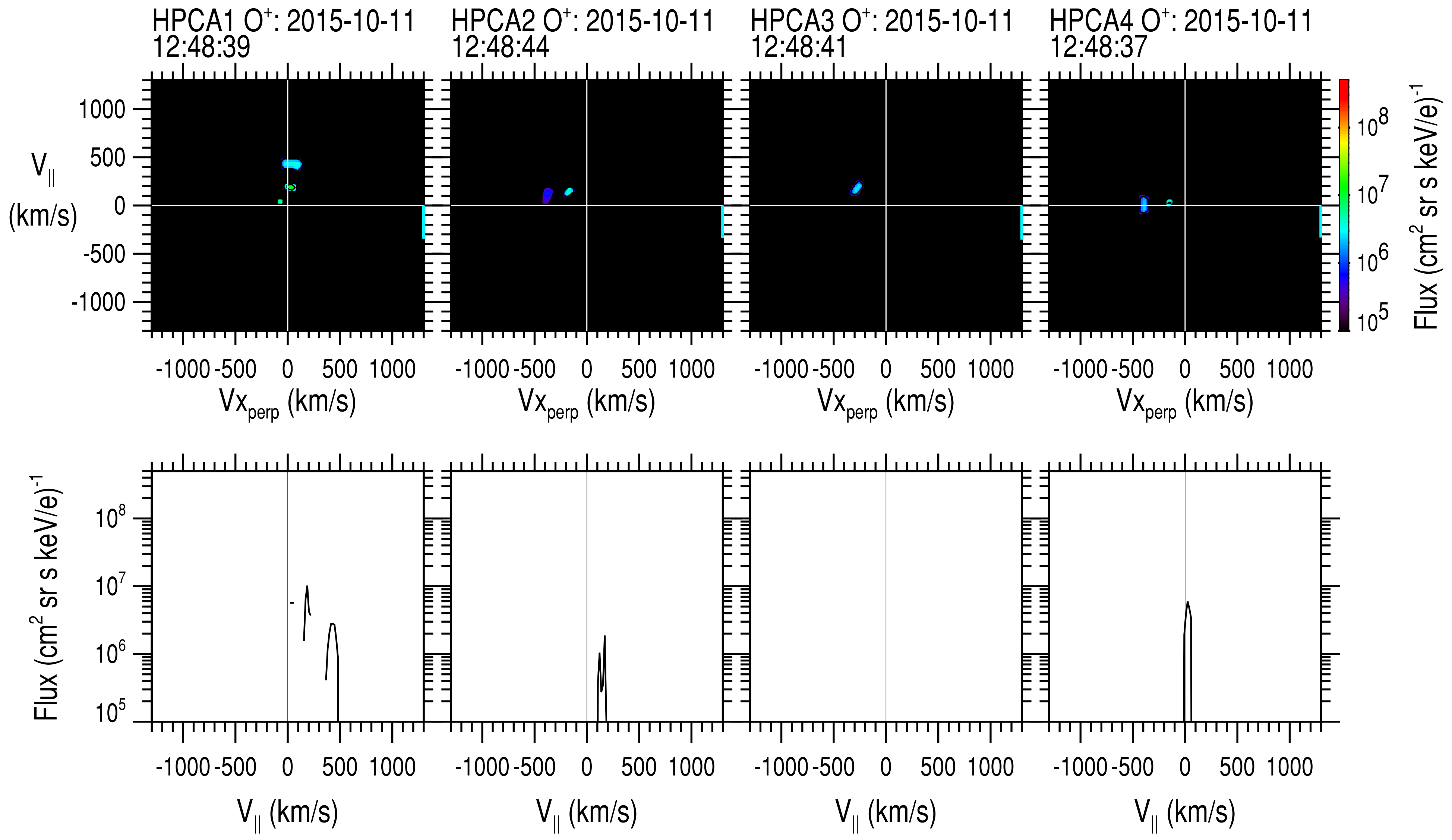
HPCA1 O+: 2015-10-11 12:48:39
HPCA2 O+: 2015-10-11 12:48:44
HPCA3 O+: 2015-10-11 12:48:41
HPCA4 O+: 2015-10-11 12:48:37
V|| (km/s)
Vxperp (km/s)
Flux (cm2 sr s keV/e)-1

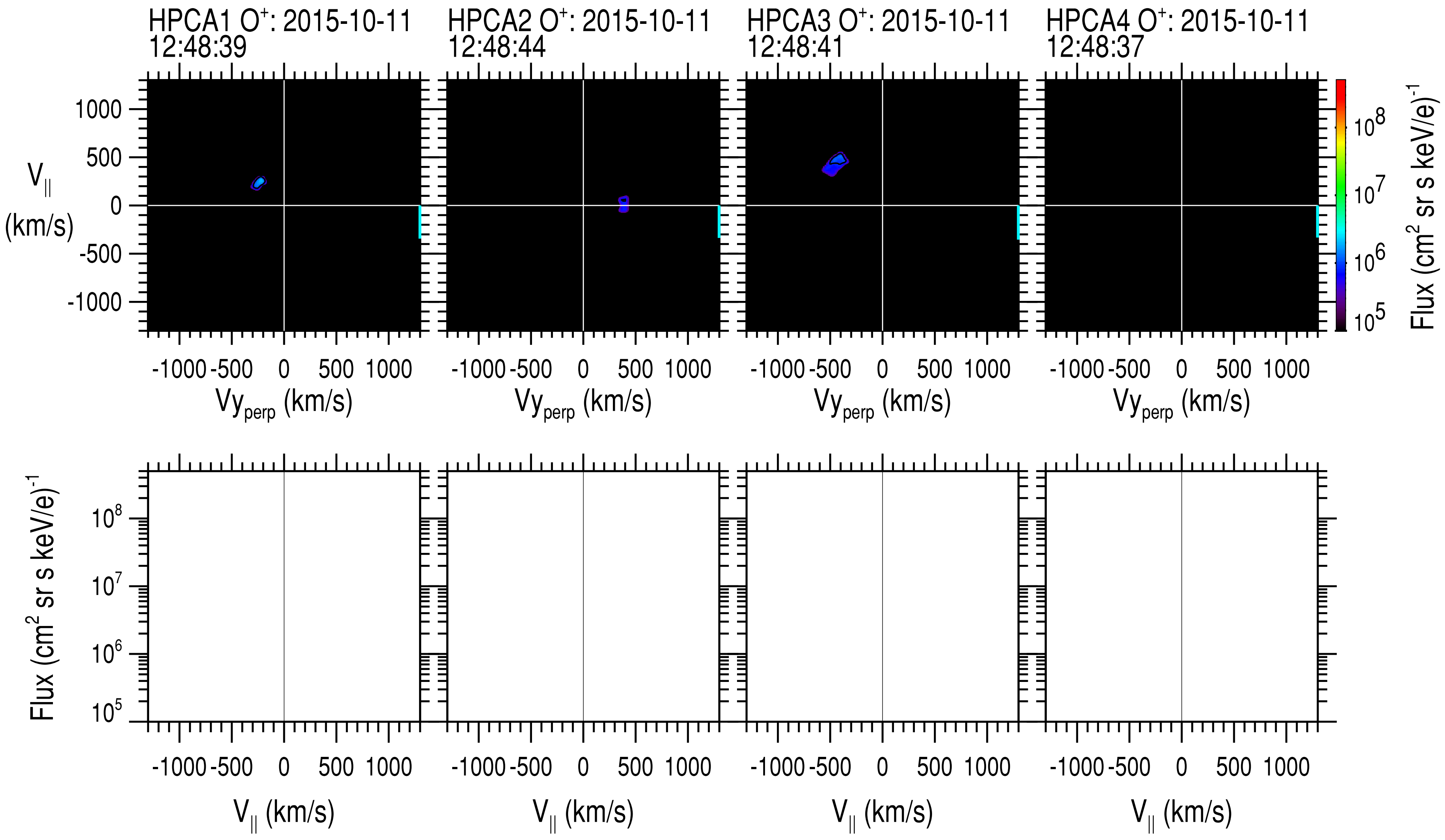

HPCA1 O+: 2015-10-11
12:48:39
HPCA2 O+: 2015-10-11
12:48:44
HPCA3 O+: 2015-10-11
12:48:41
HPCA4 O+: 2015-10-11
12:48:37
V|| (km/s)
1000
500
0
-500
-1000
Vyperp (km/s)
Flux (cm2 sr s keV/e)-1
10^5
10^6
10^7
10^8

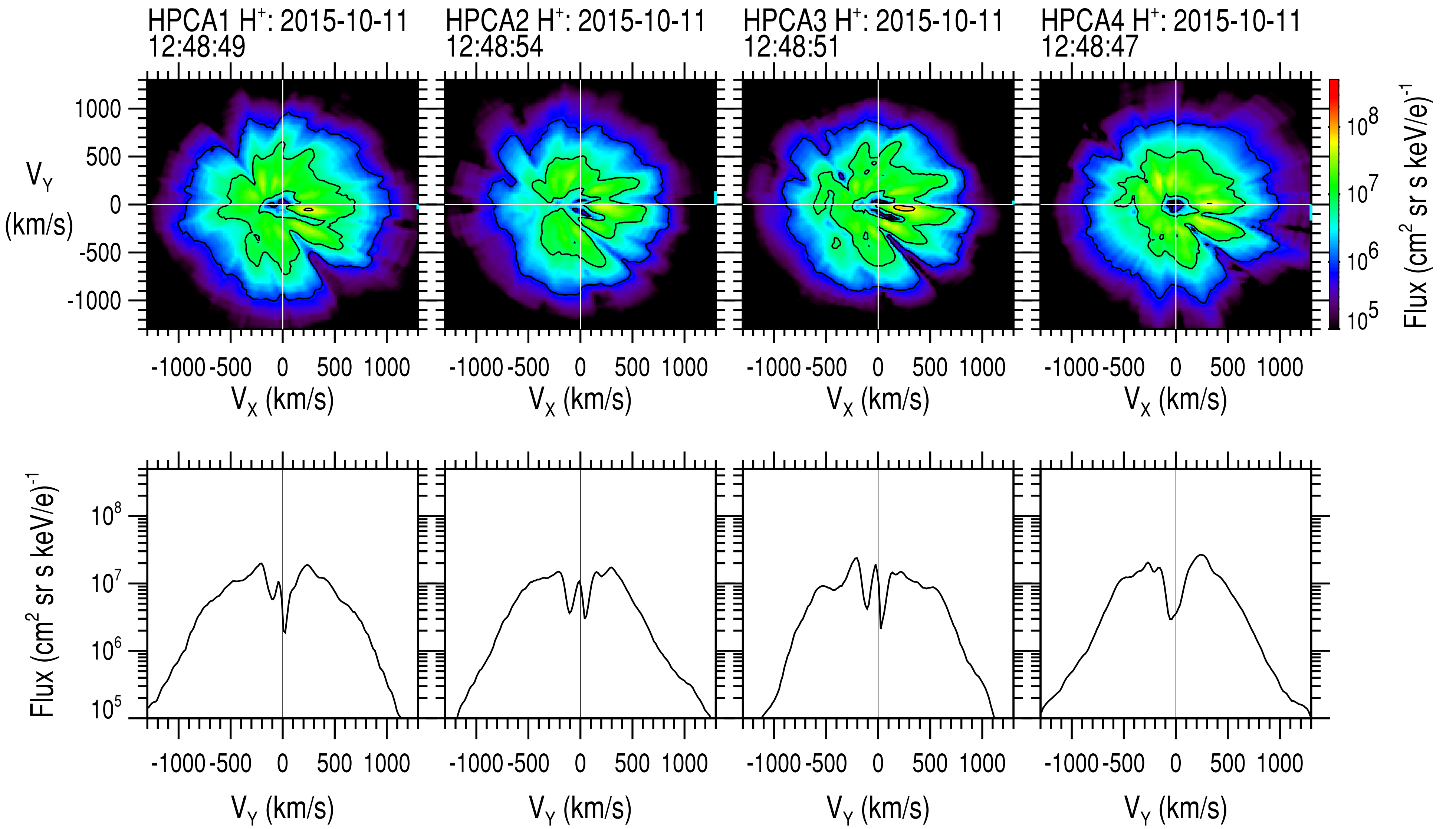

HPCA1 H+: 2015-10-11 12:48:49
HPCA2 H+: 2015-10-11 12:48:54
HPCA3 H+: 2015-10-11 12:48:51
HPCA4 H+: 2015-10-11 12:48:47
V_Y (km/s)
V_X (km/s)
Flux (cm^2 sr s keV/e)^-1

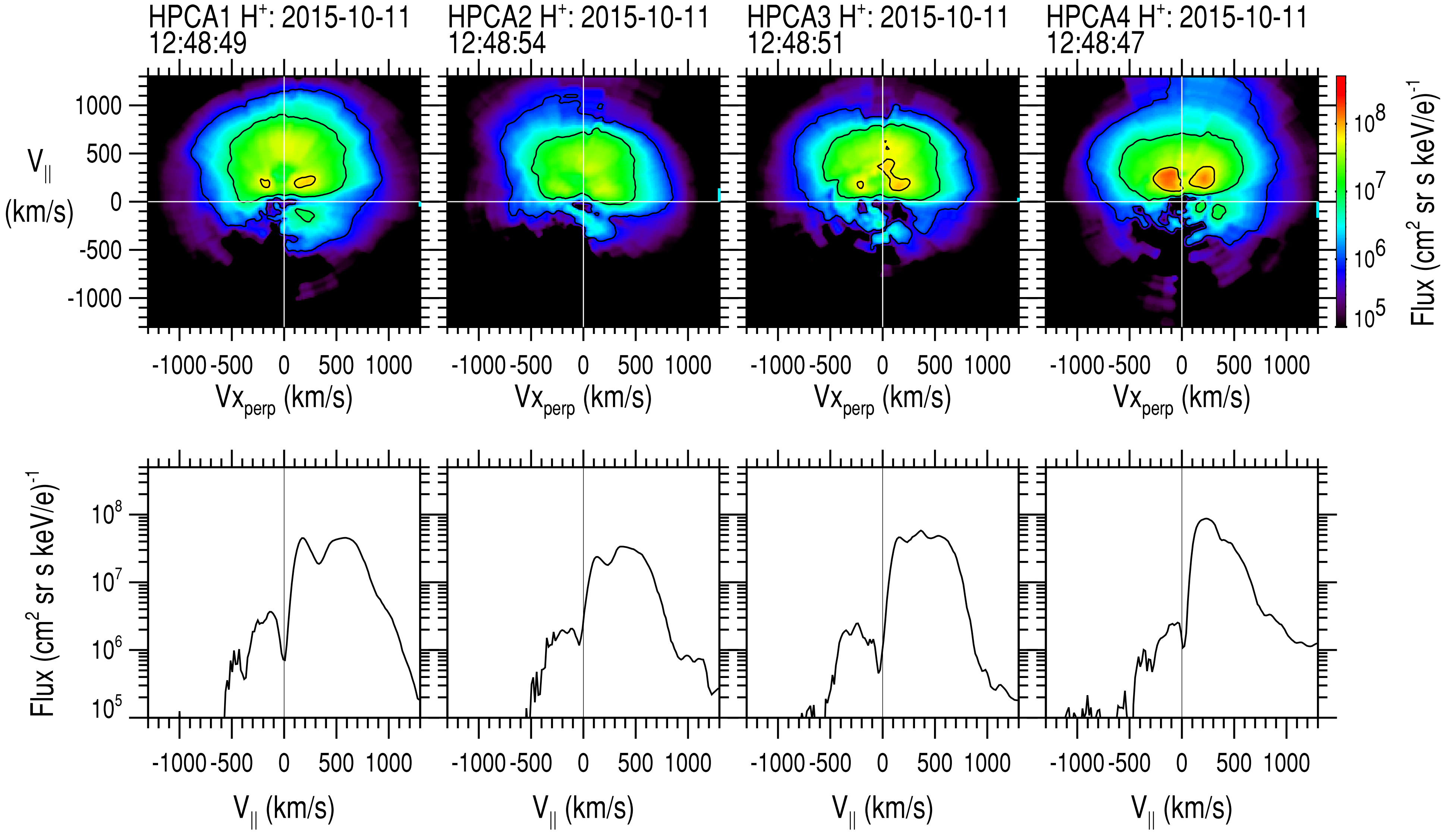

HPCA1 H+: 2015-10-11 12:48:49
HPCA2 H+: 2015-10-11 12:48:54
HPCA3 H+: 2015-10-11 12:48:51
HPCA4 H+: 2015-10-11 12:48:47
V|| (km/s)
Vxperp (km/s)
Flux (cm2 sr s keV/e)-1
V|| (km/s)

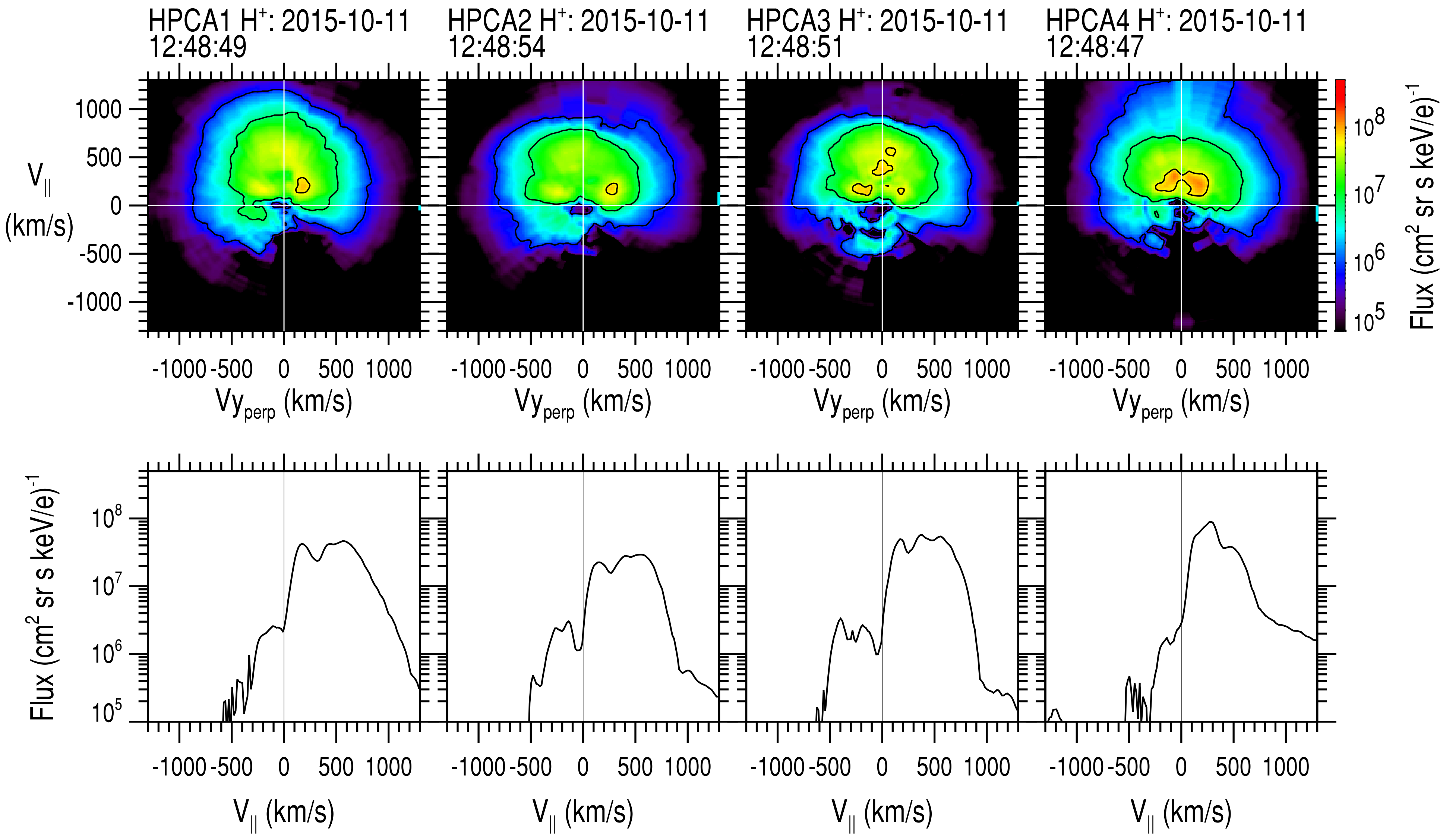

HPCA1 H+: 2015-10-11 12:48:49
HPCA2 H+: 2015-10-11 12:48:54
HPCA3 H+: 2015-10-11 12:48:51
HPCA4 H+: 2015-10-11 12:48:47
V|| (km/s)
Vyperp (km/s)
Flux (cm2 sr s keV/e)-1

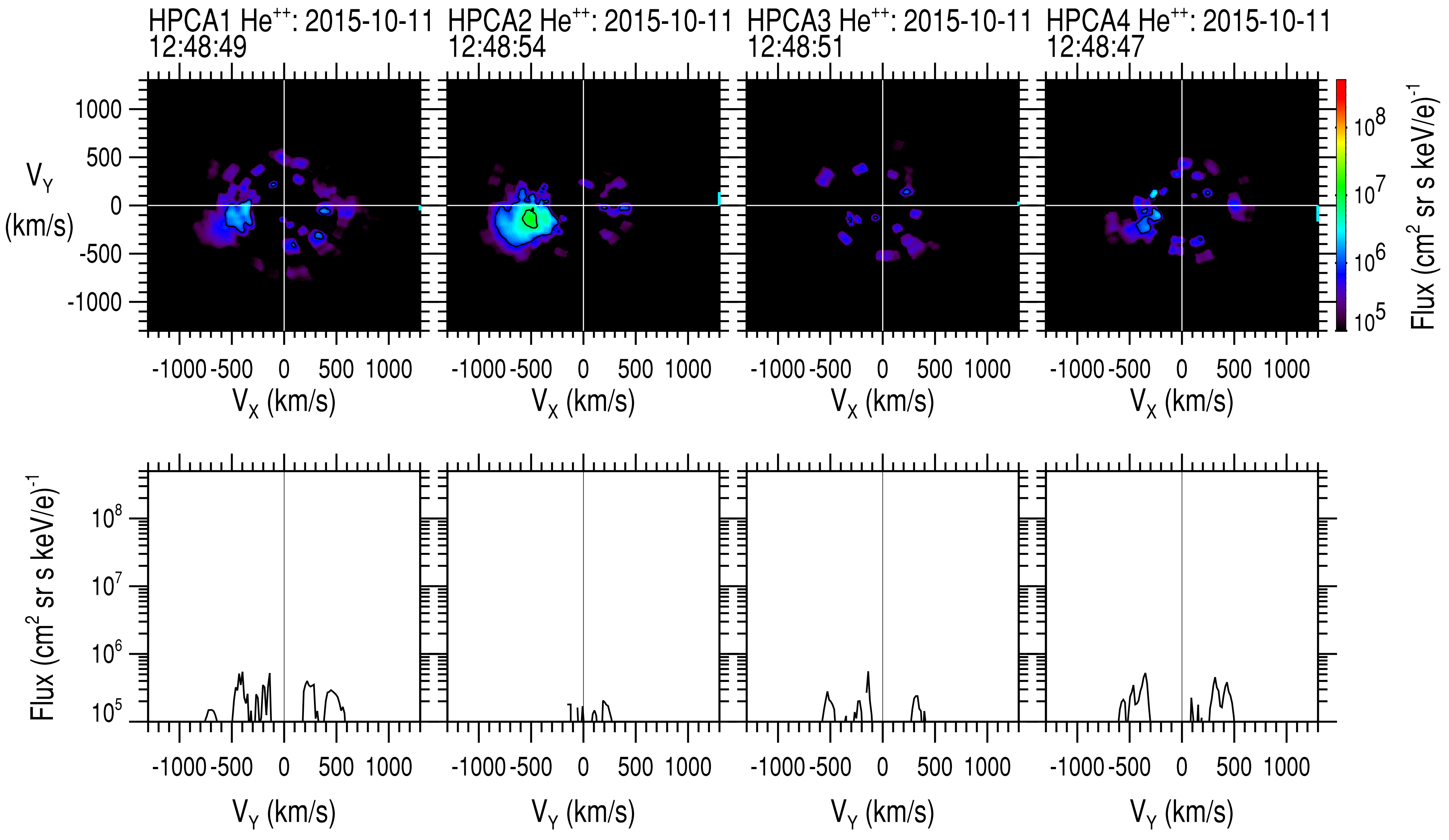

HPCA1 He++: 2015-10-11 12:48:49
HPCA2 He++: 2015-10-11 12:48:54
HPCA3 He++: 2015-10-11 12:48:51
HPCA4 He++: 2015-10-11 12:48:47
V_Y (km/s)
V_X (km/s)
Flux (cm^2 sr s keV/e)^-1

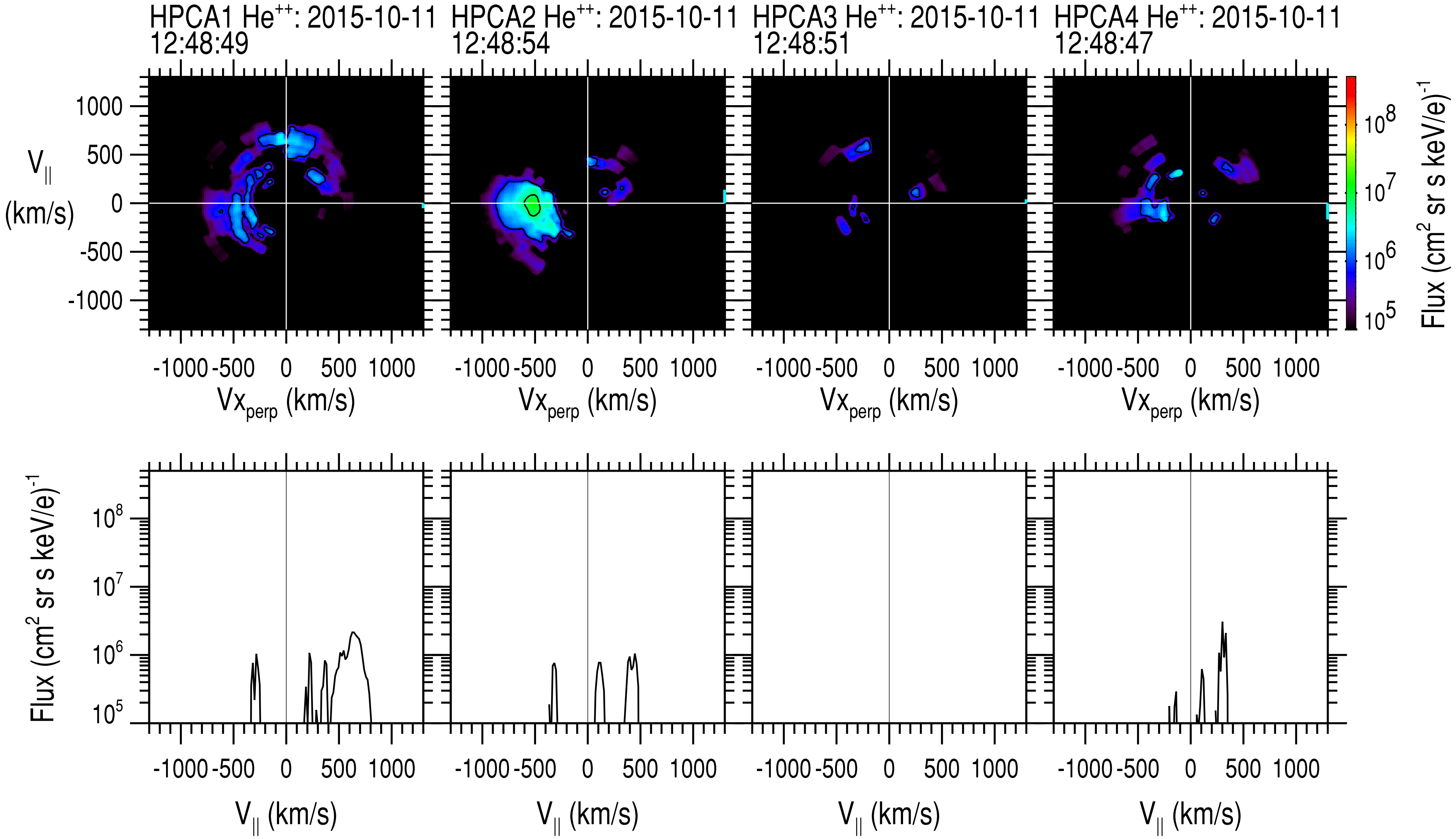

HPCA1 He++: 2015-10-11 12:48:49
HPCA2 He++: 2015-10-11 12:48:54
HPCA3 He++: 2015-10-11 12:48:51
HPCA4 He++: 2015-10-11 12:48:47
V|| (km/s)
1000
500
0
-500
-1000
Vxperp (km/s)
Flux (cm2 sr s keV/e)-1
10^5
10^6
10^7
10^8

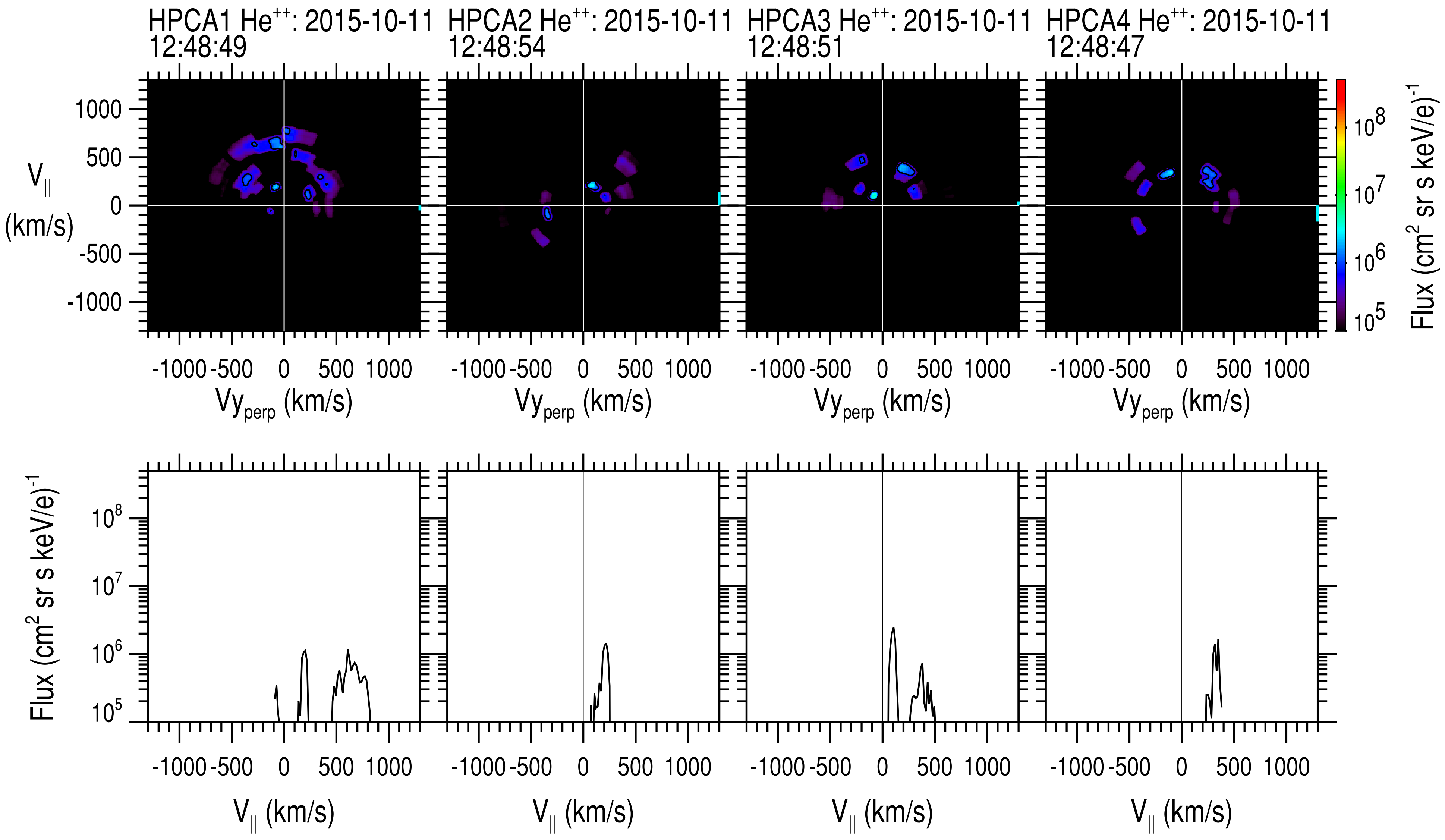

HPCA1 He++: 2015-10-11 12:48:49
HPCA2 He++: 2015-10-11 12:48:54
HPCA3 He++: 2015-10-11 12:48:51
HPCA4 He++: 2015-10-11 12:48:47
V|| (km/s)
Vyperp (km/s)
Flux (cm2 sr s keV/e)-1

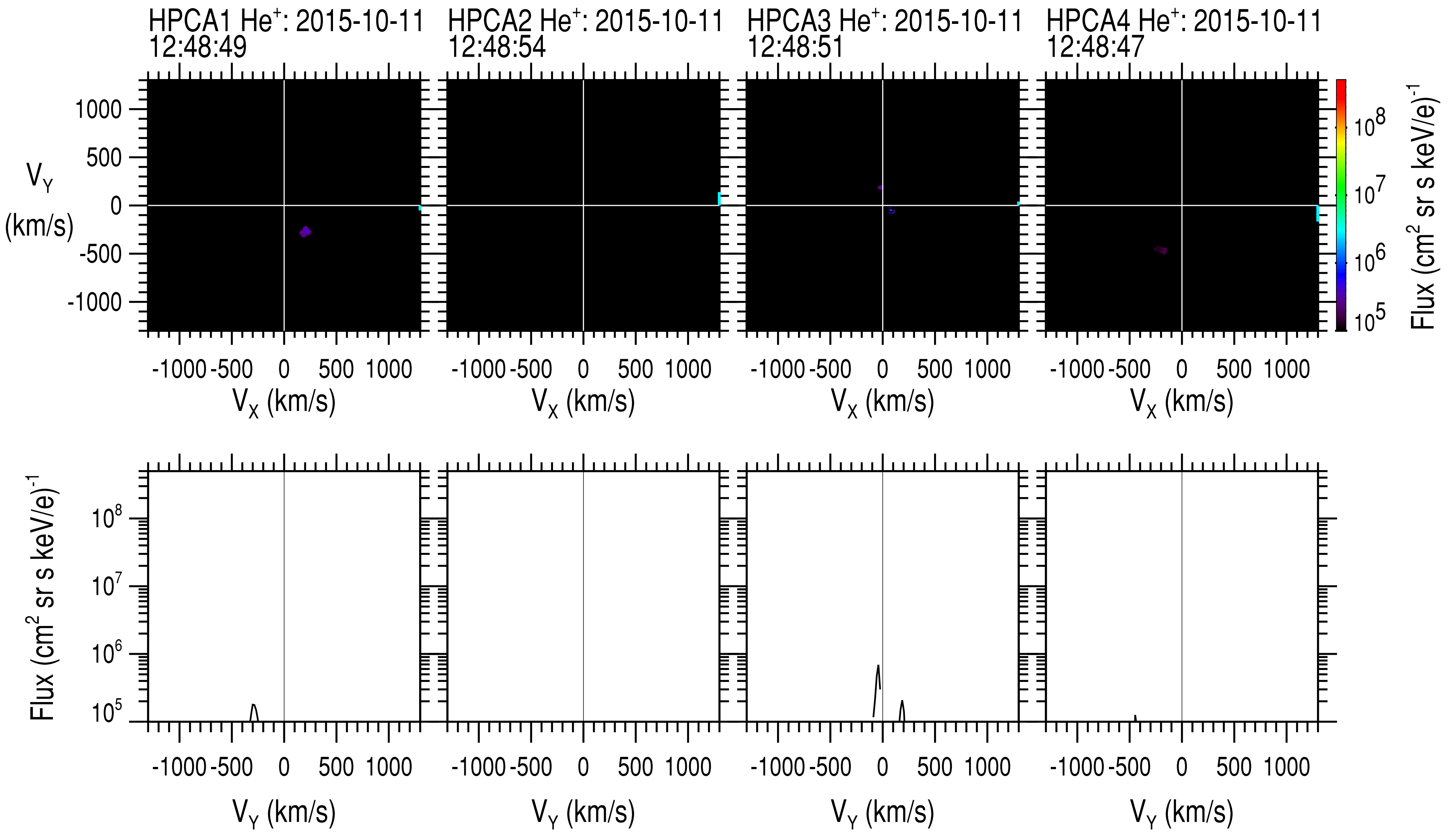

HPCA1 He+: 2015-10-11 12:48:49
HPCA2 He+: 2015-10-11 12:48:54
HPCA3 He+: 2015-10-11 12:48:51
HPCA4 He+: 2015-10-11 12:48:47
VY (km/s)
VX (km/s)
Flux (cm2 sr s keV/e)-1
-1000 -500 0 500 1000
10^5 10^6 10^7 10^8

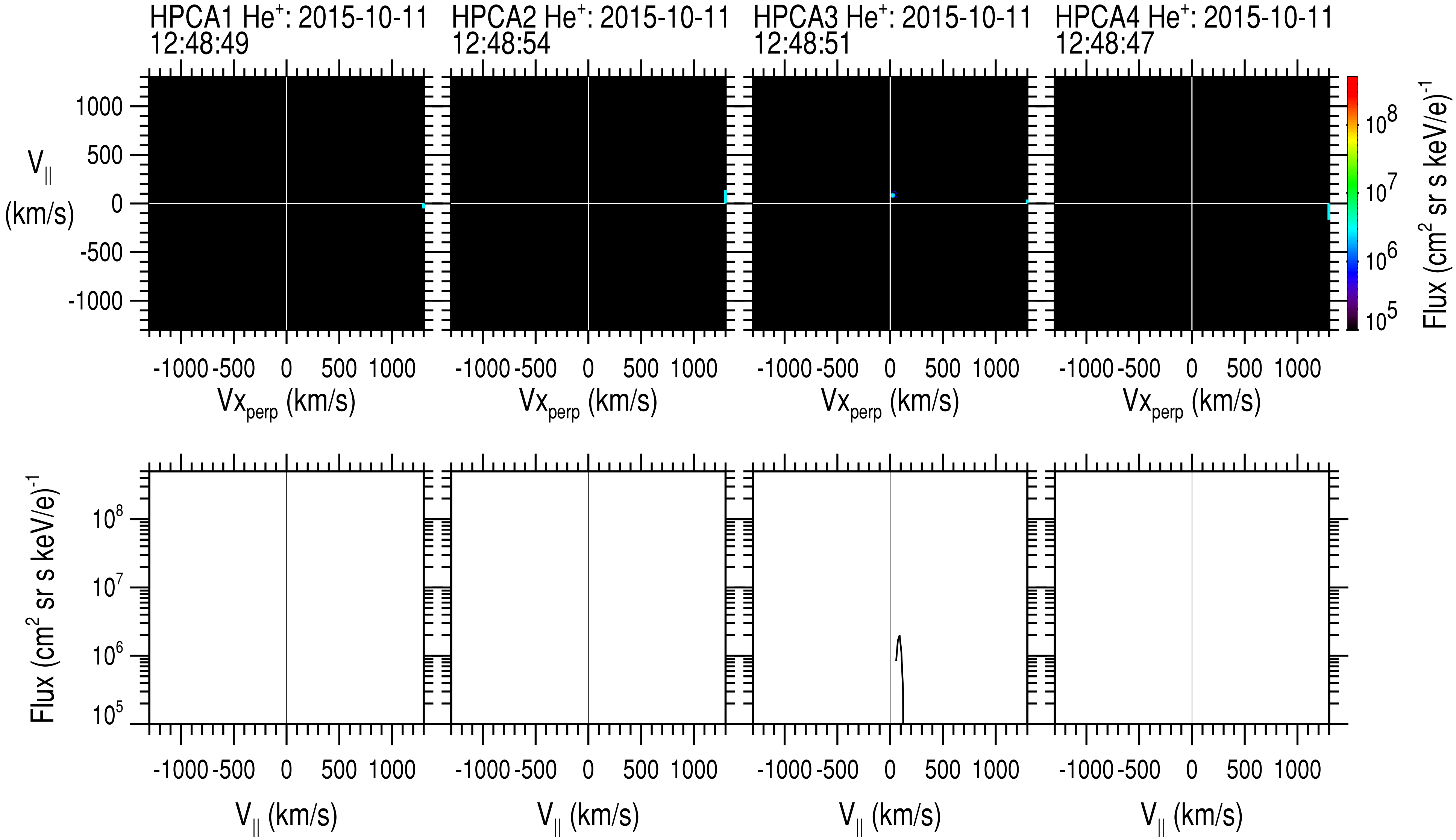

HPCA1 He+: 2015-10-11 12:48:49
HPCA2 He+: 2015-10-11 12:48:54
HPCA3 He+: 2015-10-11 12:48:51
HPCA4 He+: 2015-10-11 12:48:47
V|| (km/s)
Vxperp (km/s)
Flux (cm2 sr s keV/e)-1

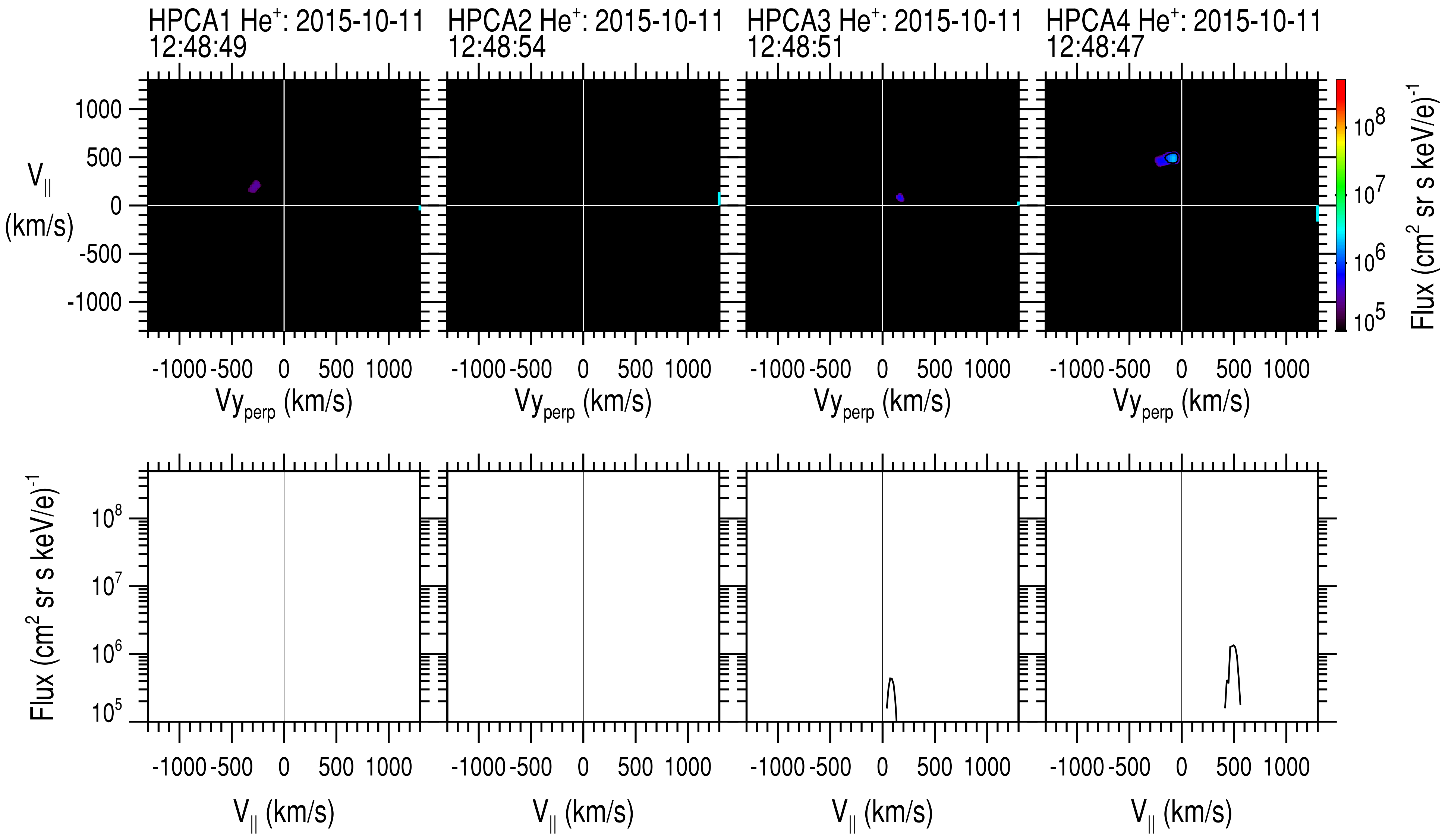

HPCA1 He+: 2015-10-11 12:48:49
HPCA2 He+: 2015-10-11 12:48:54
HPCA3 He+: 2015-10-11 12:48:51
HPCA4 He+: 2015-10-11 12:48:47
V|| (km/s)
Vyperp (km/s)
Flux (cm2 sr s keV/e)-1

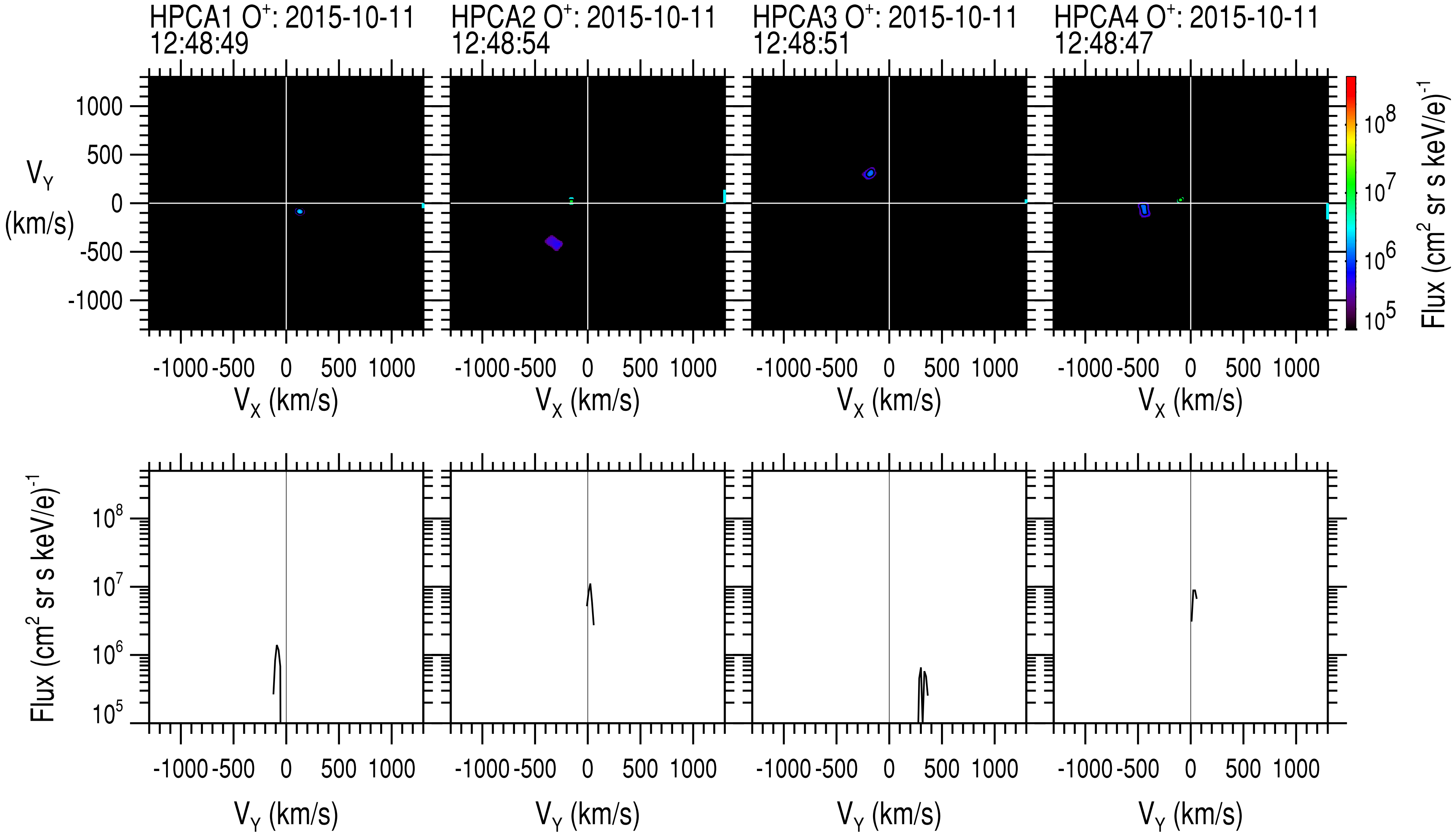
HPCA1 O+: 2015-10-11 12:48:49
HPCA2 O+: 2015-10-11 12:48:54
HPCA3 O+: 2015-10-11 12:48:51
HPCA4 O+: 2015-10-11 12:48:47
VY (km/s)
VX (km/s)
Flux (cm2 sr s keV/e)-1
VY (km/s)

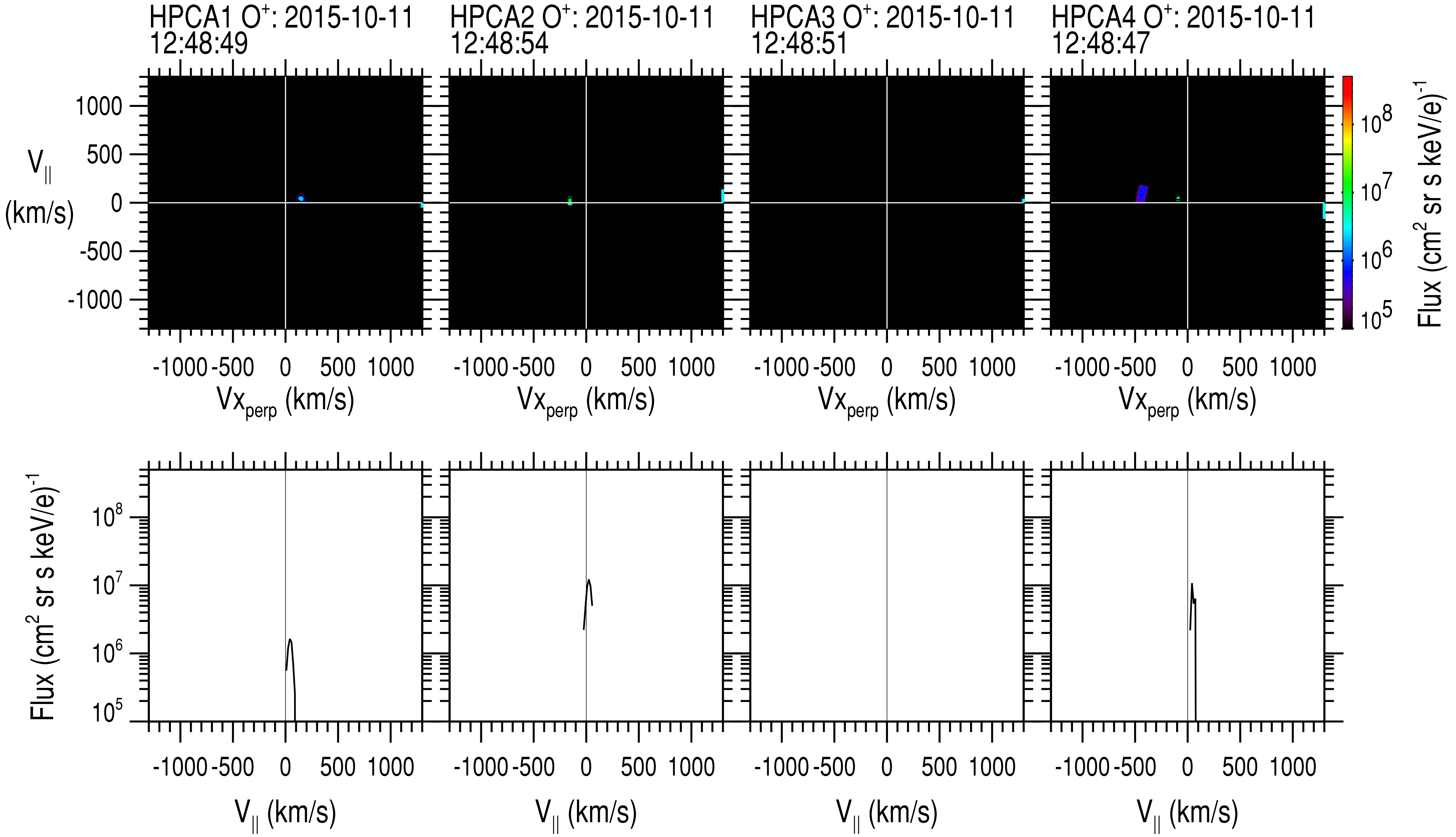

HPCA1 O+: 2015-10-11 12:48:49
HPCA2 O+: 2015-10-11 12:48:54
HPCA3 O+: 2015-10-11 12:48:51
HPCA4 O+: 2015-10-11 12:48:47
V|| (km/s)
Vxperp (km/s)
Flux (cm2 sr s keV/e)-1

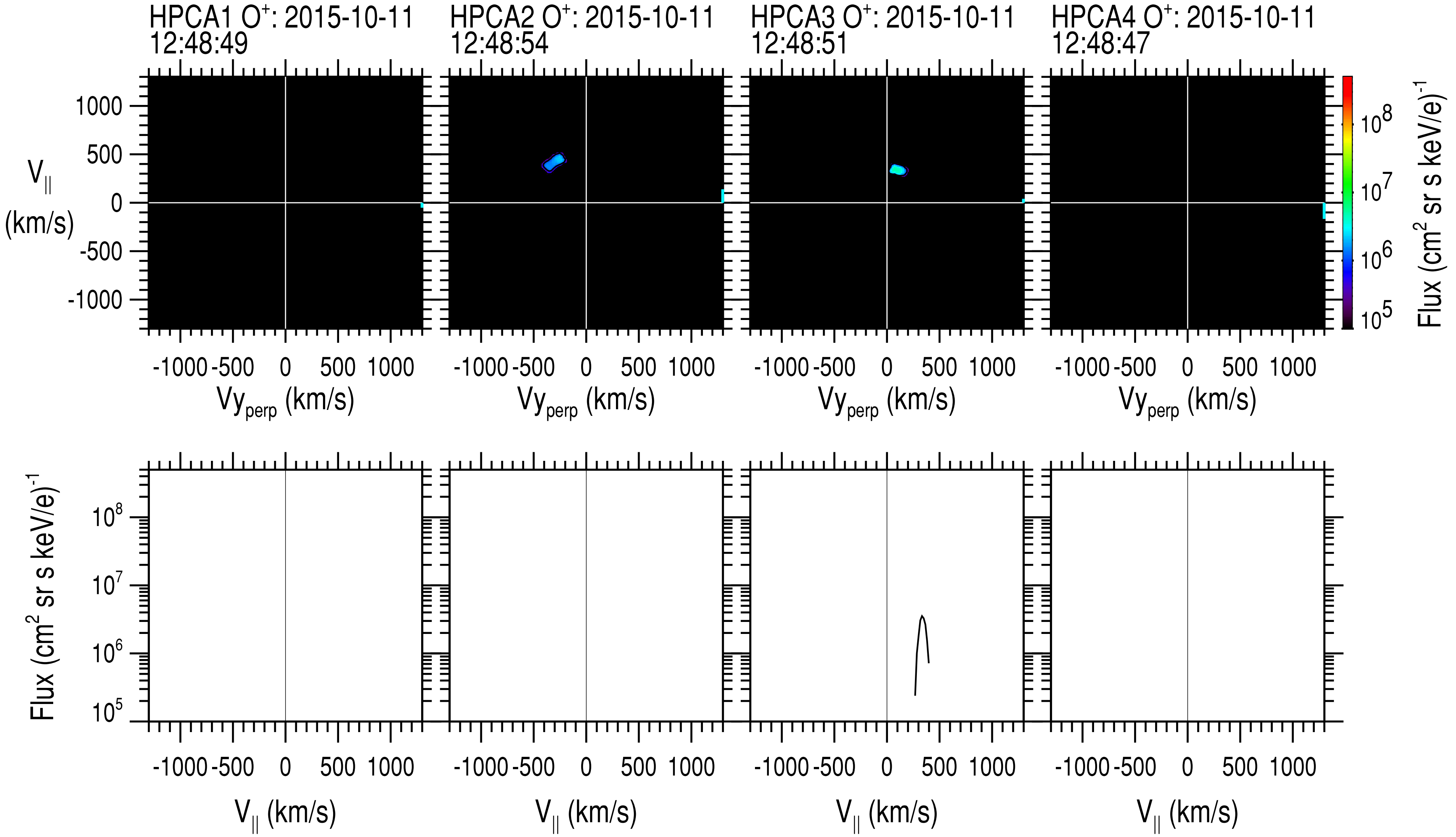
HPCA1 O+: 2015-10-11
12:48:49
HPCA2 O+: 2015-10-11
12:48:54
HPCA3 O+: 2015-10-11
12:48:51
HPCA4 O+: 2015-10-11
12:48:47
V|| (km/s)
Vy_perp (km/s)
Flux (cm2 sr s keV/e)-1
V|| (km/s)
-1000 -500 0 500 1000

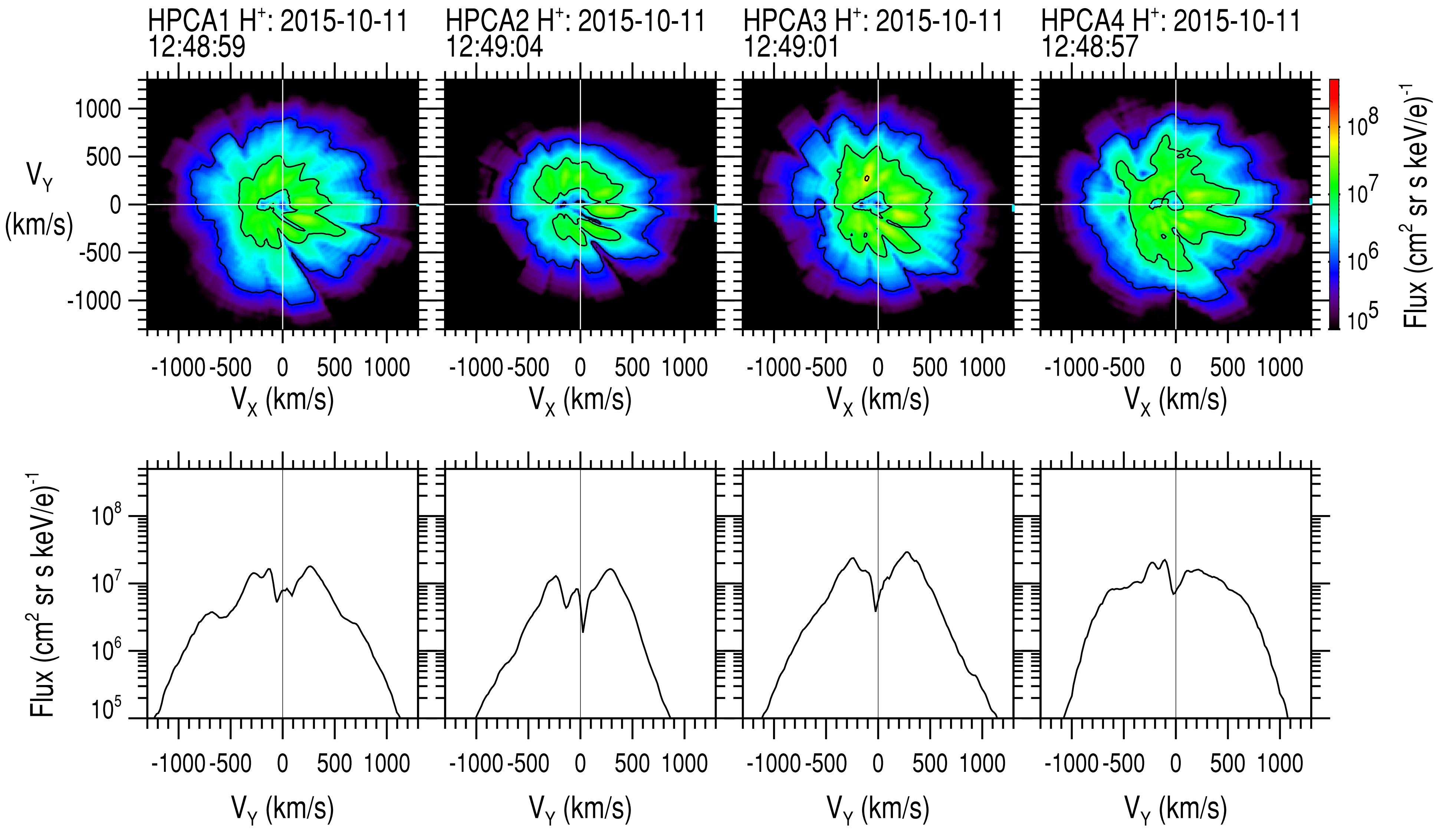

HPCA1 H+: 2015-10-11
12:48:59
HPCA2 H+: 2015-10-11
12:49:04
HPCA3 H+: 2015-10-11
12:49:01
HPCA4 H+: 2015-10-11
12:48:57
VY (km/s)
VX (km/s)
Flux (cm2 sr s keV/e)-1
VY (km/s)

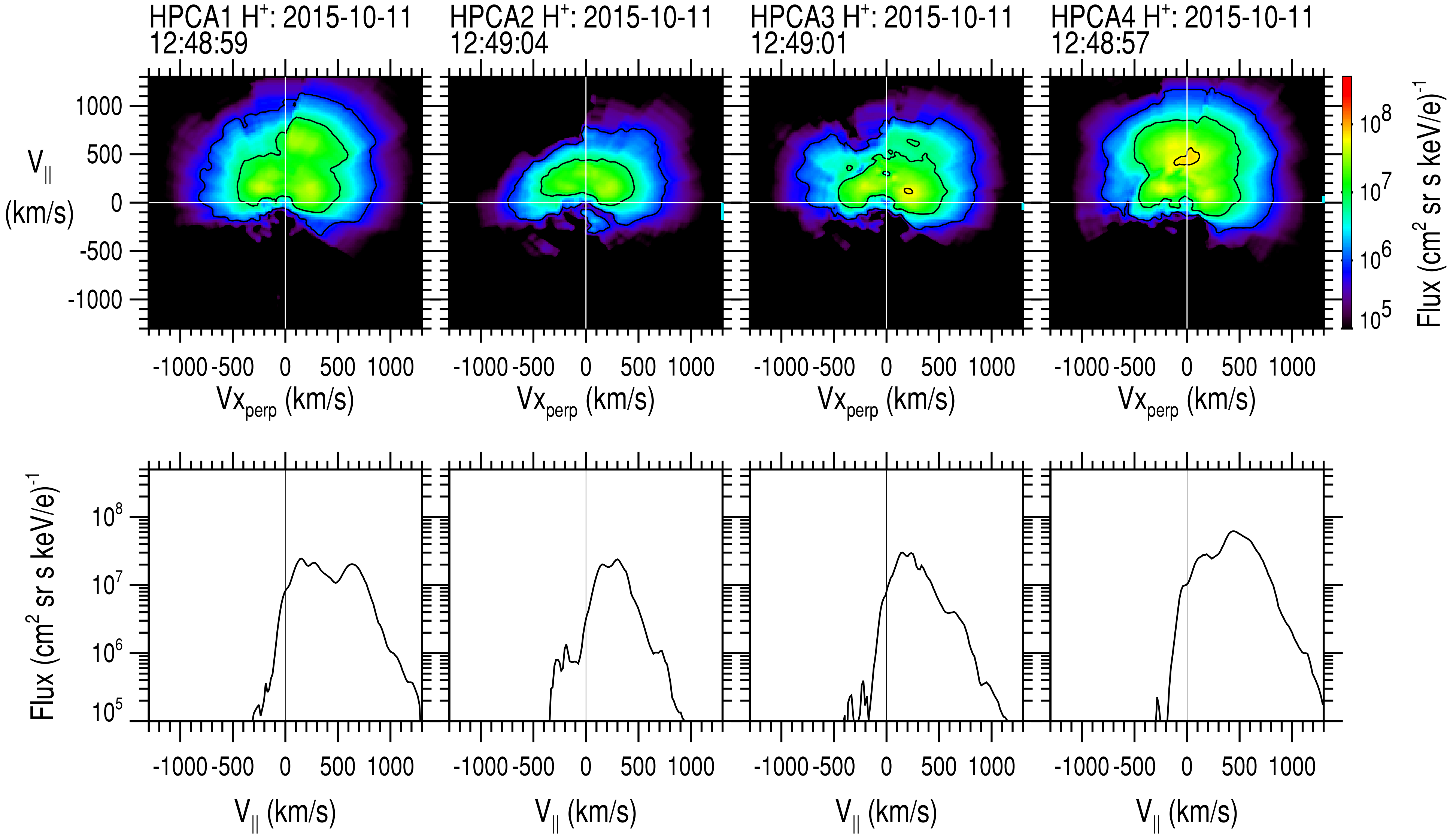
HPCA1 H+: 2015-10-11
12:48:59
HPCA2 H+: 2015-10-11
12:49:04
HPCA3 H+: 2015-10-11
12:49:01
HPCA4 H+: 2015-10-11
12:48:57
V|| (km/s)
Vxperp (km/s)
Flux (cm2 sr s keV/e)-1
-1000 -500 0 500 1000
105 106 107 108

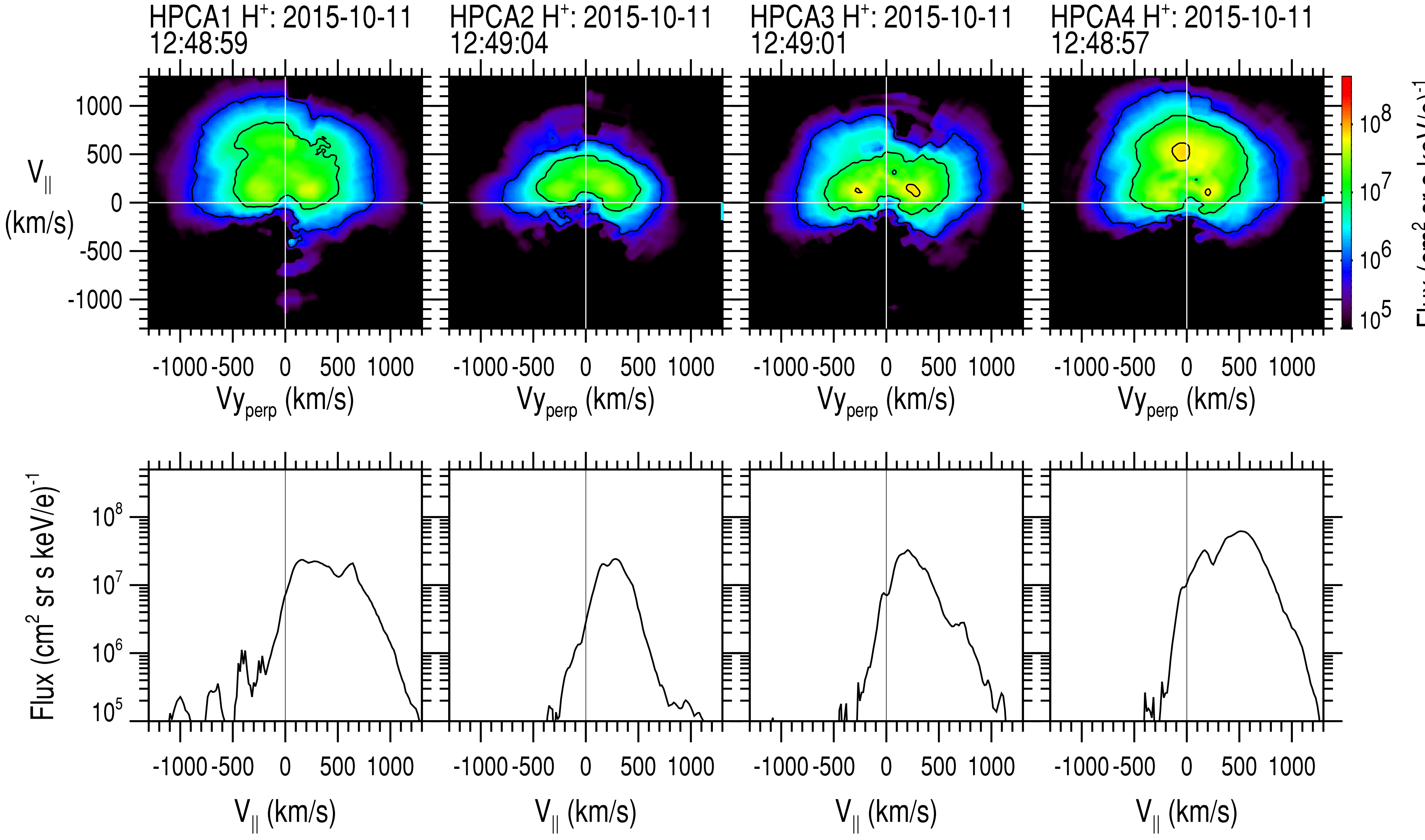
HPCA1 H+: 2015-10-11
12:48:59
HPCA2 H+: 2015-10-11
12:49:04
HPCA3 H+: 2015-10-11
12:49:01
HPCA4 H+: 2015-10-11
12:48:57
V||
(km/s)
Vyperp (km/s)
Flux (cm2 sr s keV/e)-1
V|| (km/s)

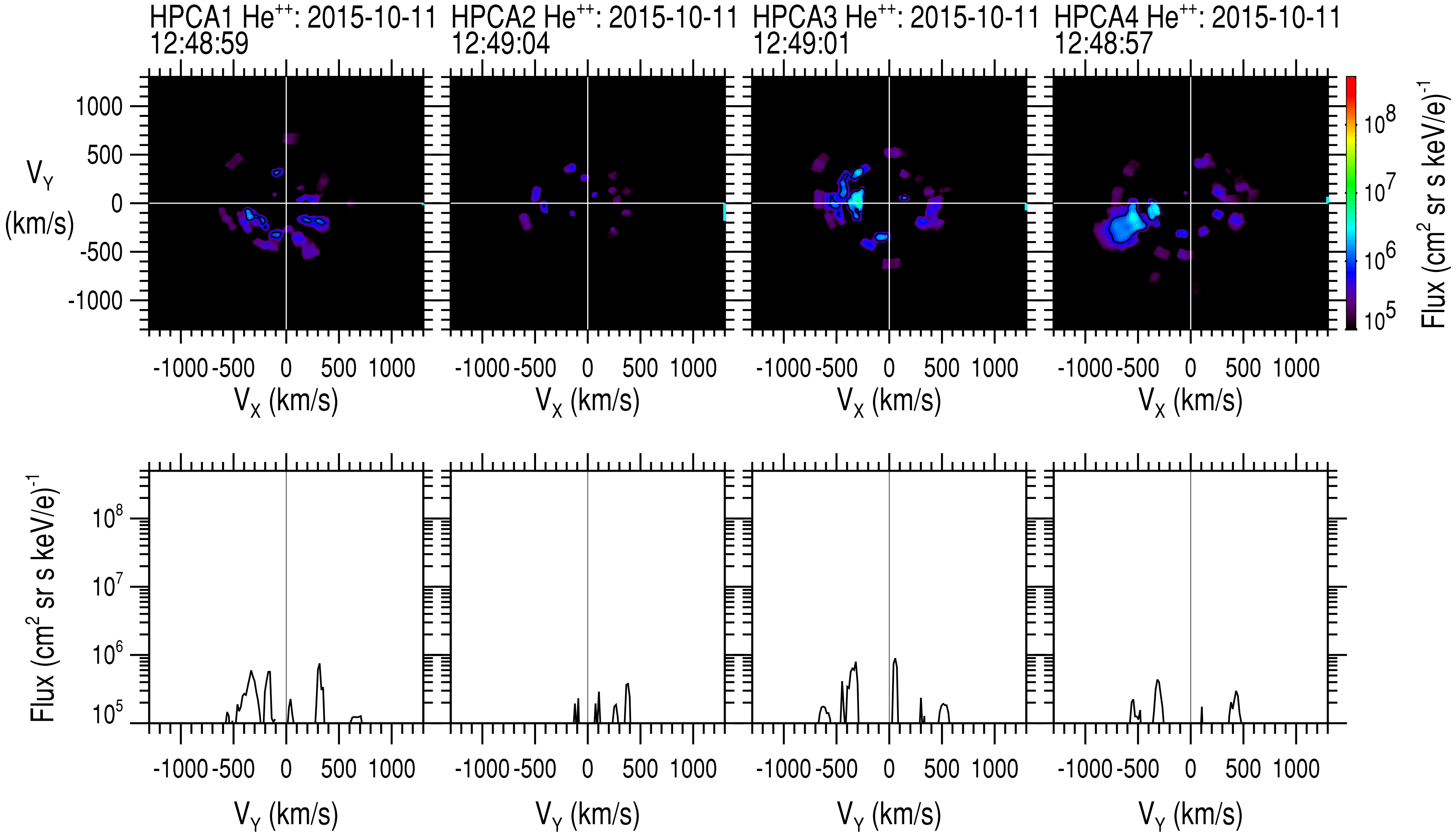

HPCA1 He++: 2015-10-11 12:48:59
HPCA2 He++: 2015-10-11 12:49:04
HPCA3 He++: 2015-10-11 12:49:01
HPCA4 He++: 2015-10-11 12:48:57
V_Y (km/s)
V_X (km/s)
Flux (cm^2 sr s keV/e)^-1
V_Y (km/s)
1000
500
0
-500
-1000
10^5
10^6
10^7
10^8

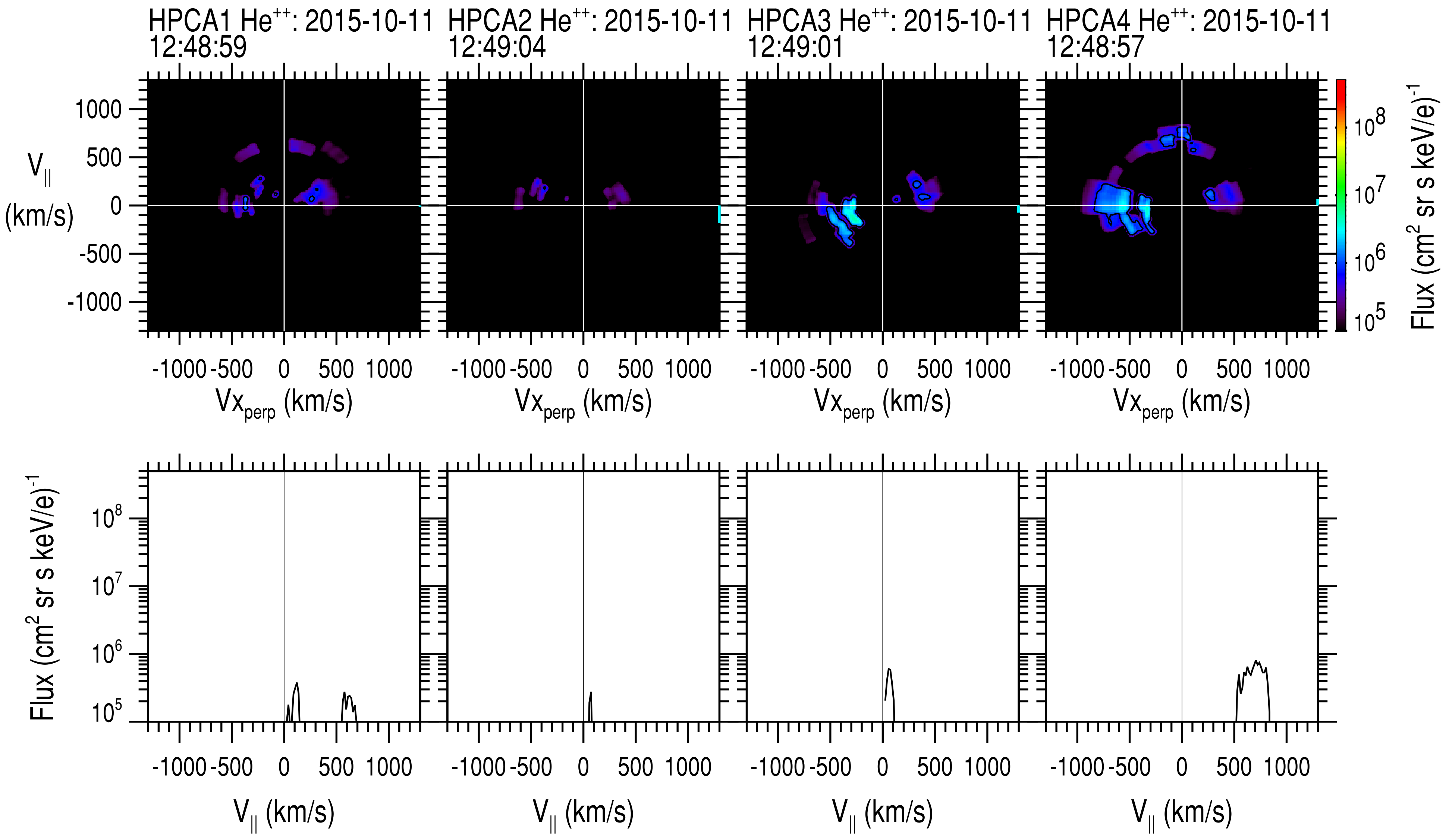
HPCA1 He++: 2015-10-11 12:48:59
HPCA2 He++: 2015-10-11 12:49:04
HPCA3 He++: 2015-10-11 12:49:01
HPCA4 He++: 2015-10-11 12:48:57
V|| (km/s)
Vxperp (km/s)
Flux (cm2 sr s keV/e)-1
V|| (km/s)

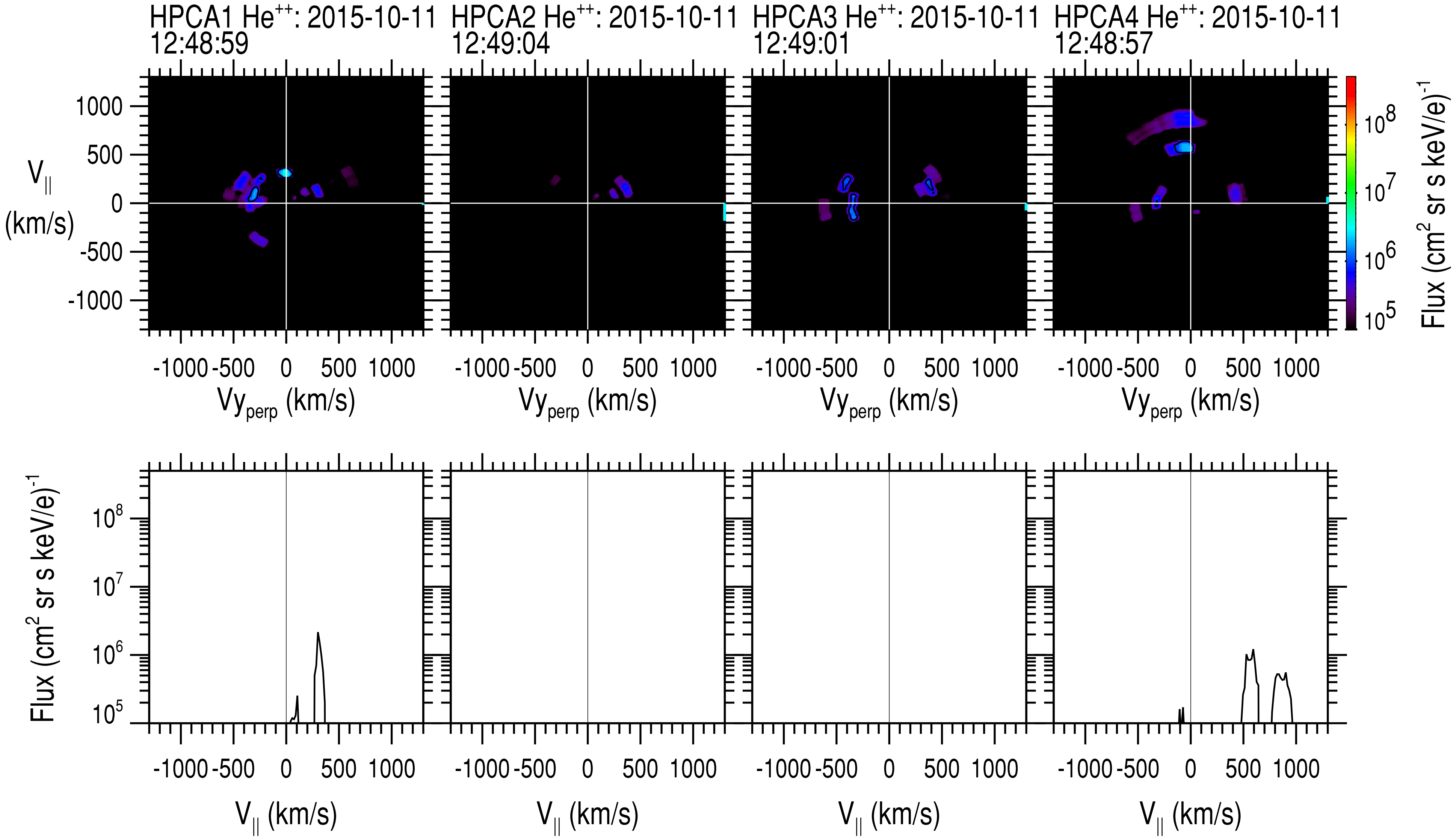

HPCA1 He++: 2015-10-11 12:48:59
HPCA2 He++: 2015-10-11 12:49:04
HPCA3 He++: 2015-10-11 12:49:01
HPCA4 He++: 2015-10-11 12:48:57
V|| (km/s)
Vyperp (km/s)
Flux (cm2 sr s keV/e)-1

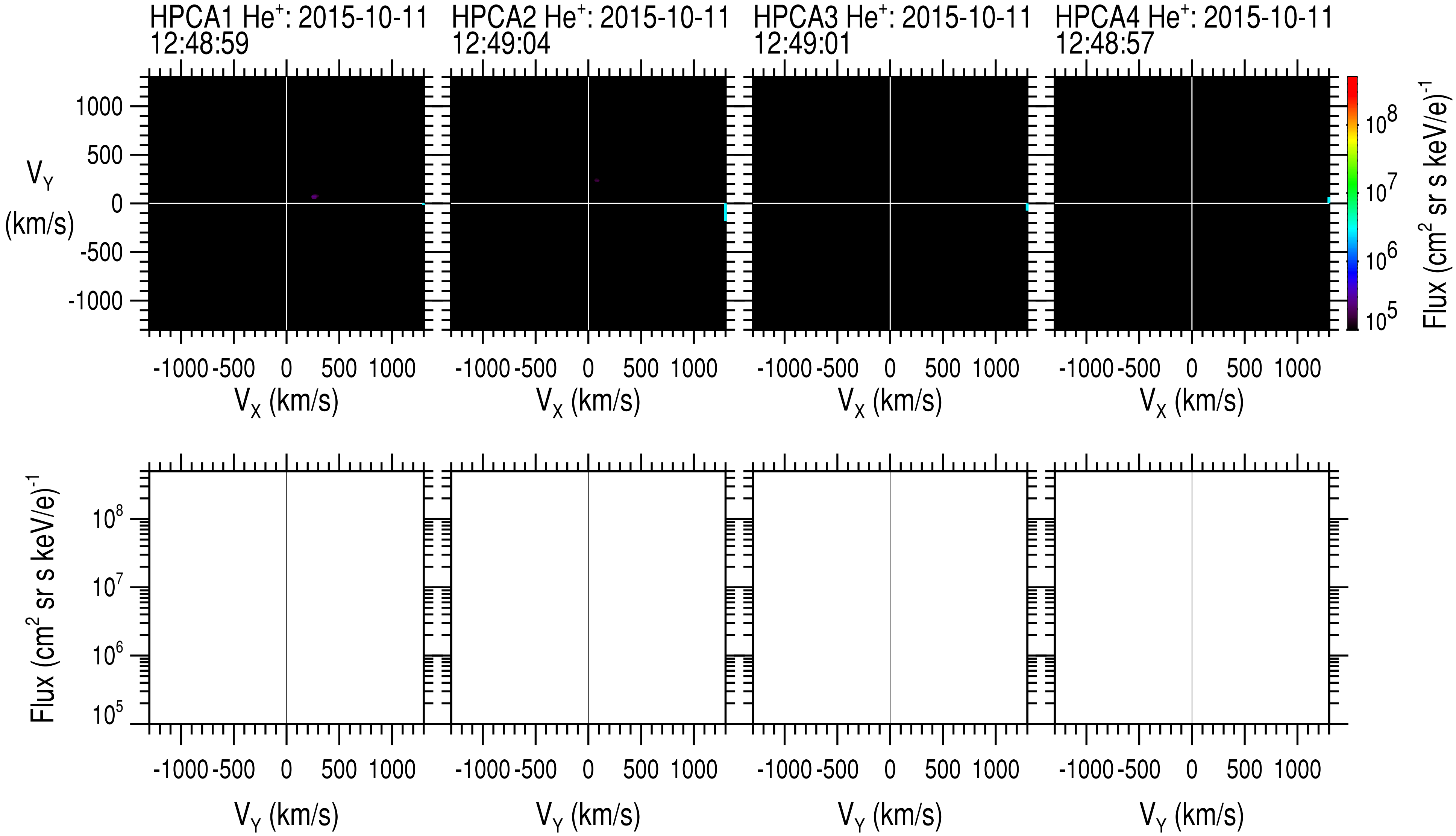
HPCA1 He+: 2015-10-11 12:48:59
HPCA2 He+: 2015-10-11 12:49:04
HPCA3 He+: 2015-10-11 12:49:01
HPCA4 He+: 2015-10-11 12:48:57
V_Y (km/s)
1000
500
0
-500
-1000
-1000 -500 0 500 1000
V_X (km/s)
Flux (cm^2 sr s keV/e)^-1
10^5
10^6
10^7
10^8
V_Y (km/s)

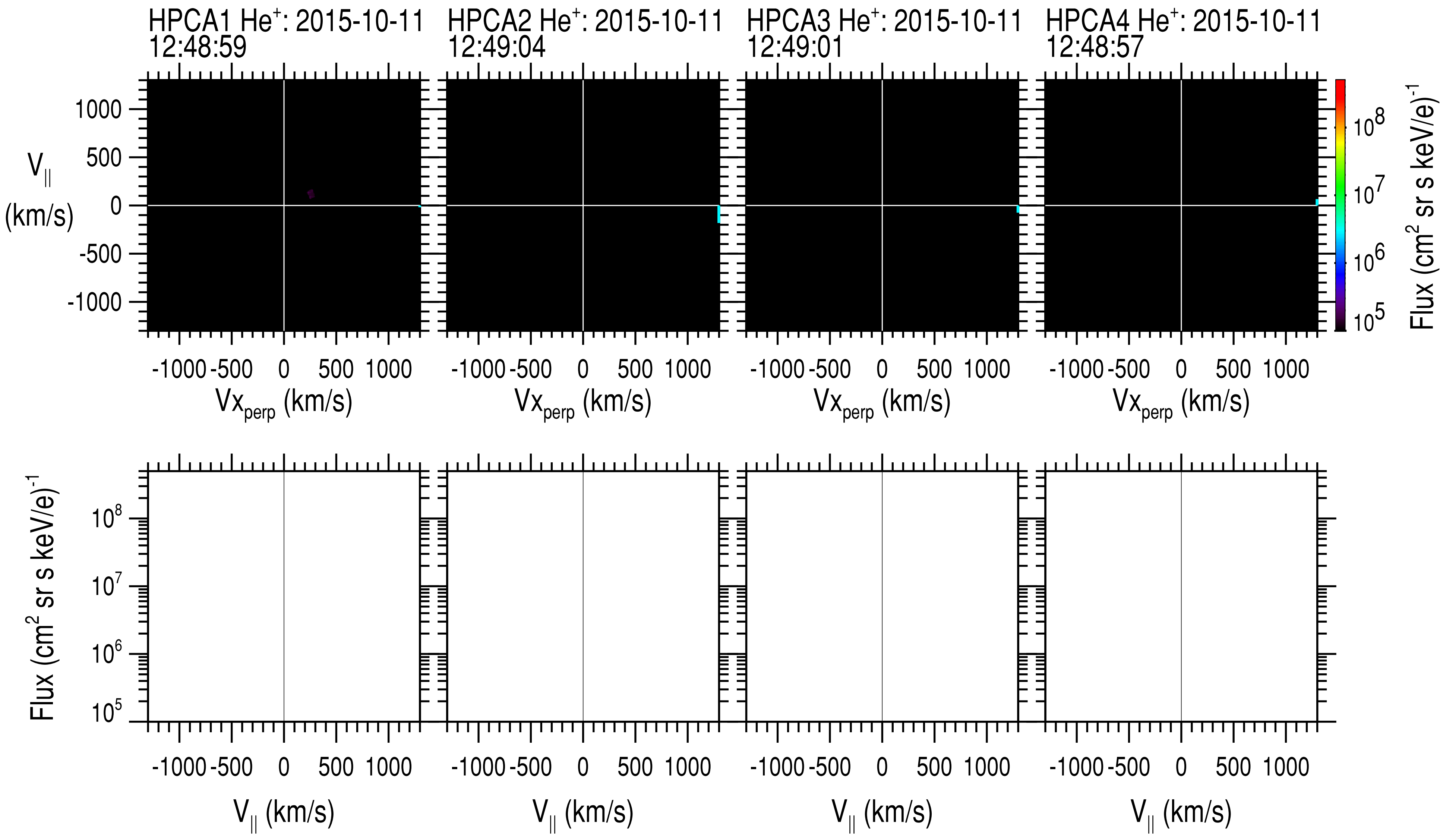
HPCA1 He+: 2015-10-11 12:48:59
HPCA2 He+: 2015-10-11 12:49:04
HPCA3 He+: 2015-10-11 12:49:01
HPCA4 He+: 2015-10-11 12:48:57
V|| (km/s)
Vxperp (km/s)
Flux (cm2 sr s keV/e)-1
-1000 -500 0 500 1000
10^5 10^6 10^7 10^8

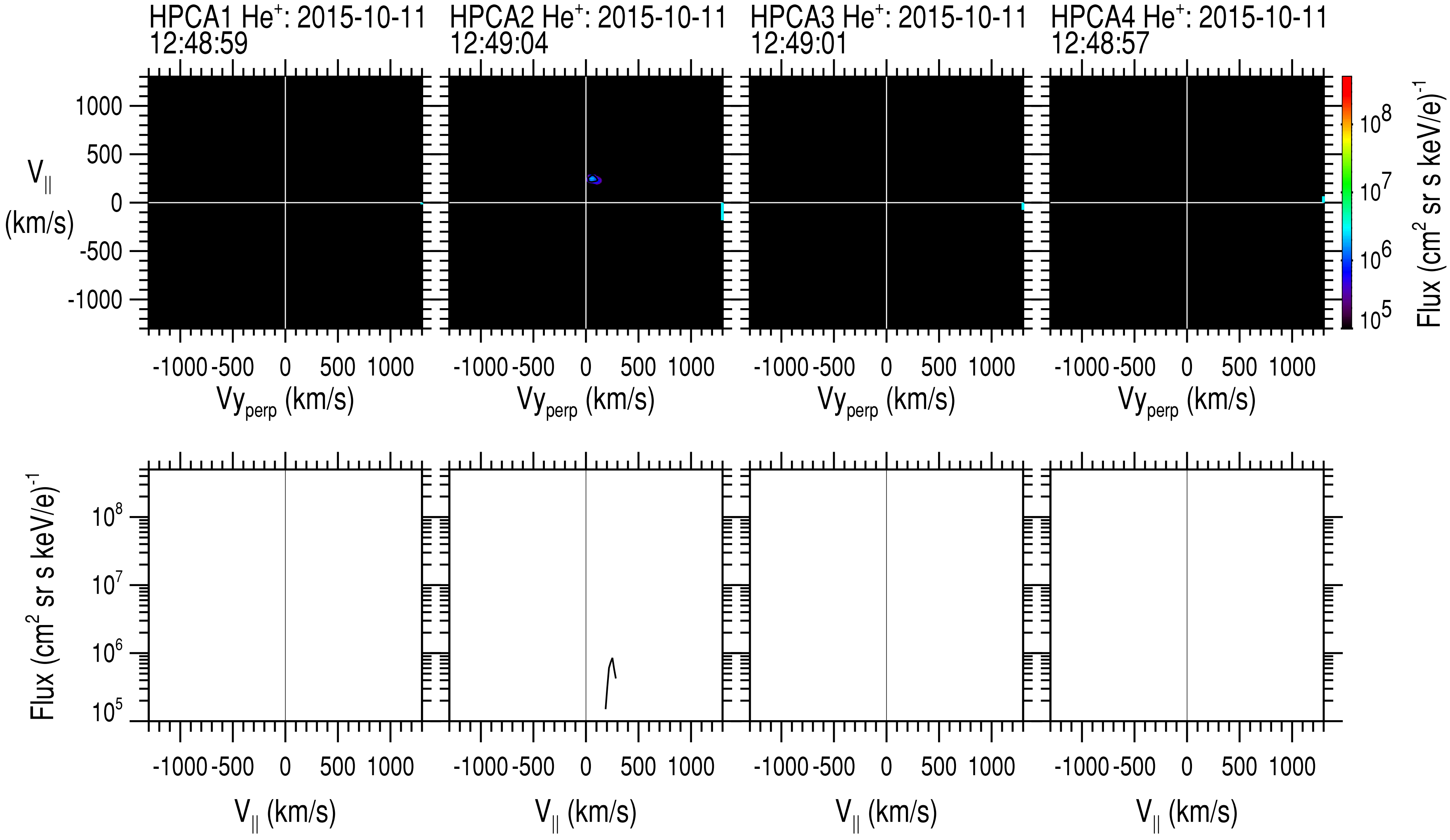

HPCA1 He+: 2015-10-11 12:48:59
HPCA2 He+: 2015-10-11 12:49:04
HPCA3 He+: 2015-10-11 12:49:01
HPCA4 He+: 2015-10-11 12:48:57
V|| (km/s)
Vyperp (km/s)
Flux (cm2 sr s keV/e)-1

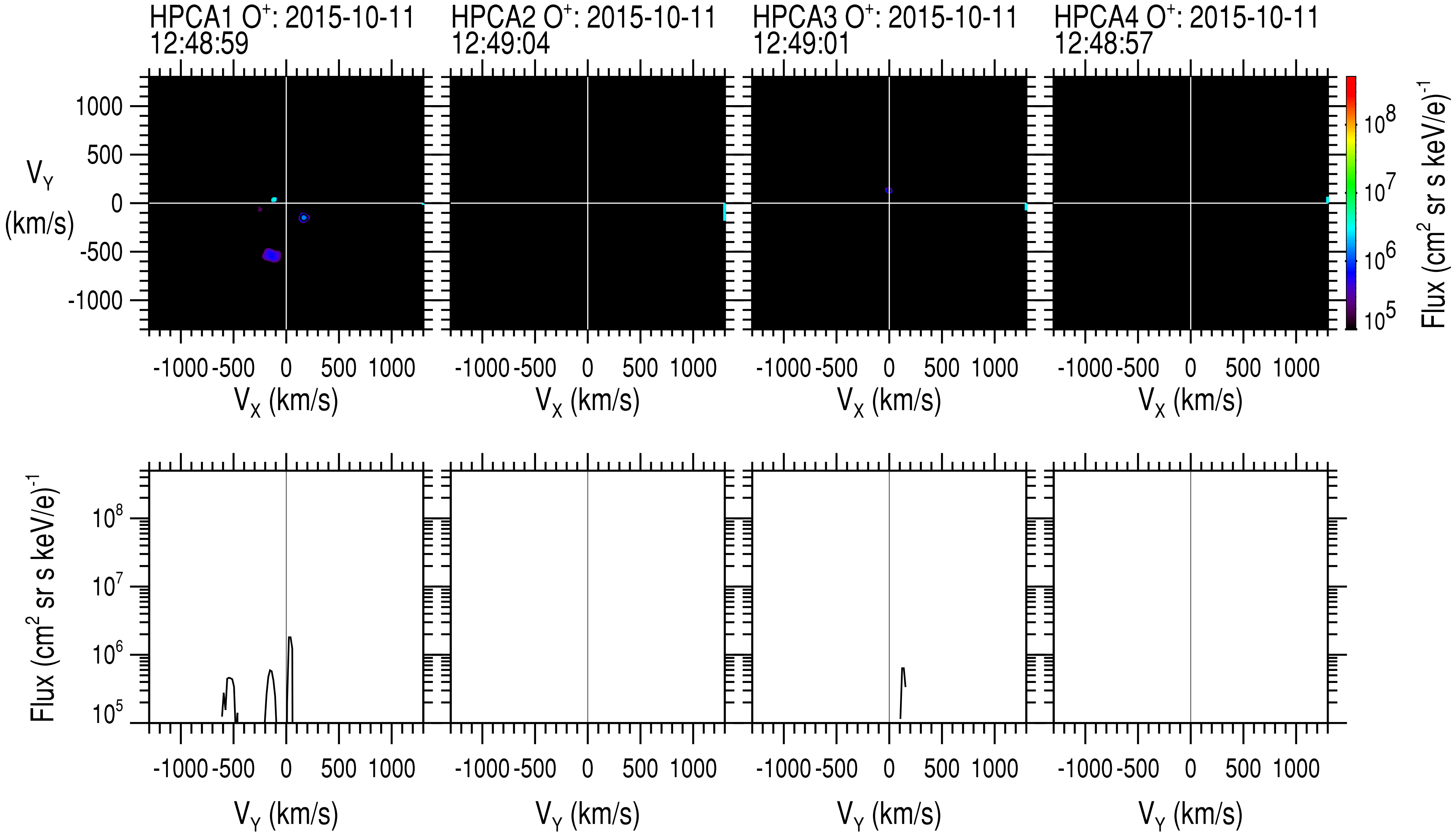

HPCA1 O+: 2015-10-11 12:48:59
HPCA2 O+: 2015-10-11 12:49:04
HPCA3 O+: 2015-10-11 12:49:01
HPCA4 O+: 2015-10-11 12:48:57
VY (km/s)
VX (km/s)
Flux (cm2 sr s keV/e)-1

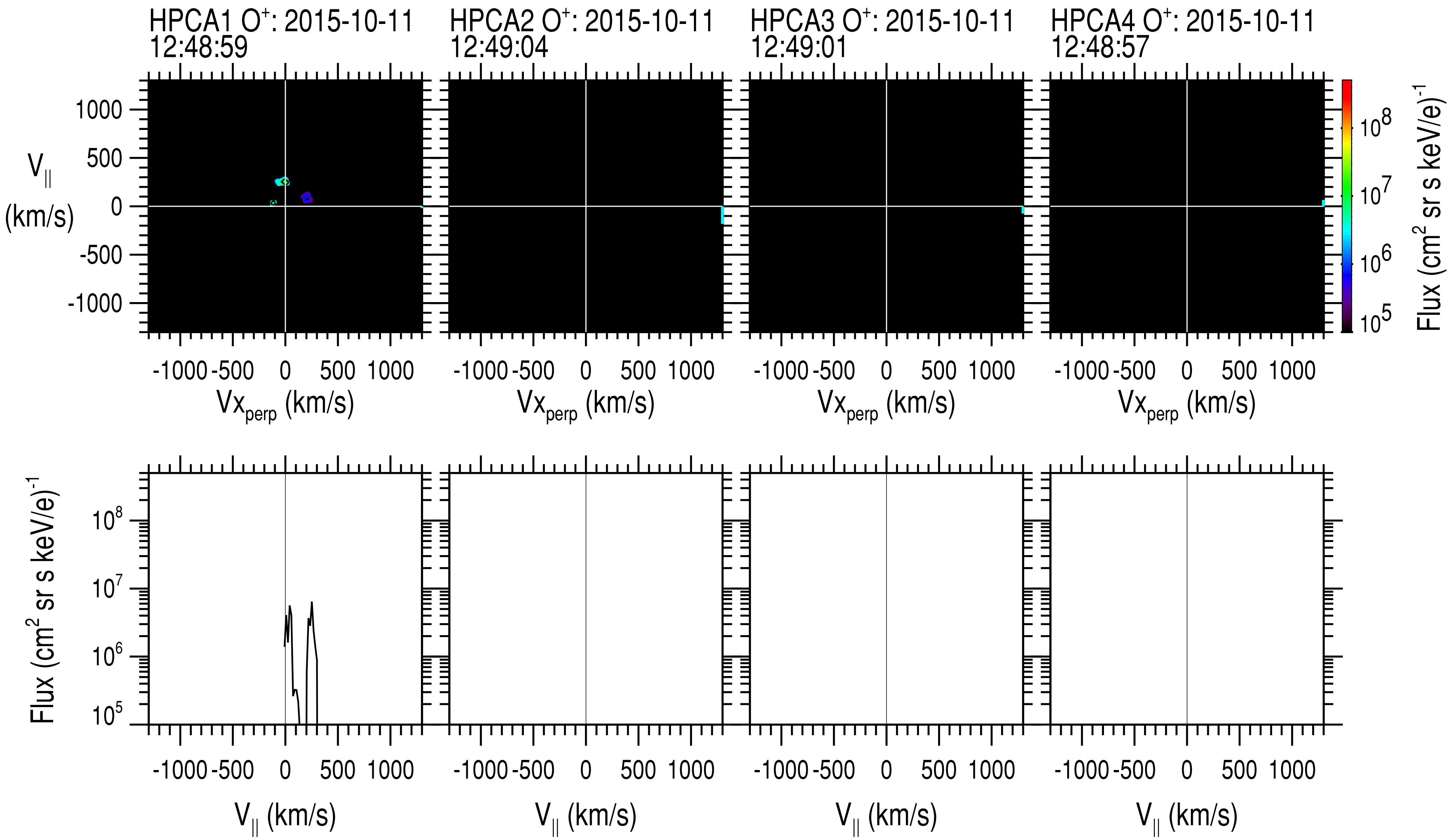
HPCA1 O+: 2015-10-11 12:48:59
HPCA2 O+: 2015-10-11 12:49:04
HPCA3 O+: 2015-10-11 12:49:01
HPCA4 O+: 2015-10-11 12:48:57
V|| (km/s)
Vxperp (km/s)
Flux (cm2 sr s keV/e)-1
-1000 -500 0 500 1000
10^5 10^6 10^7 10^8

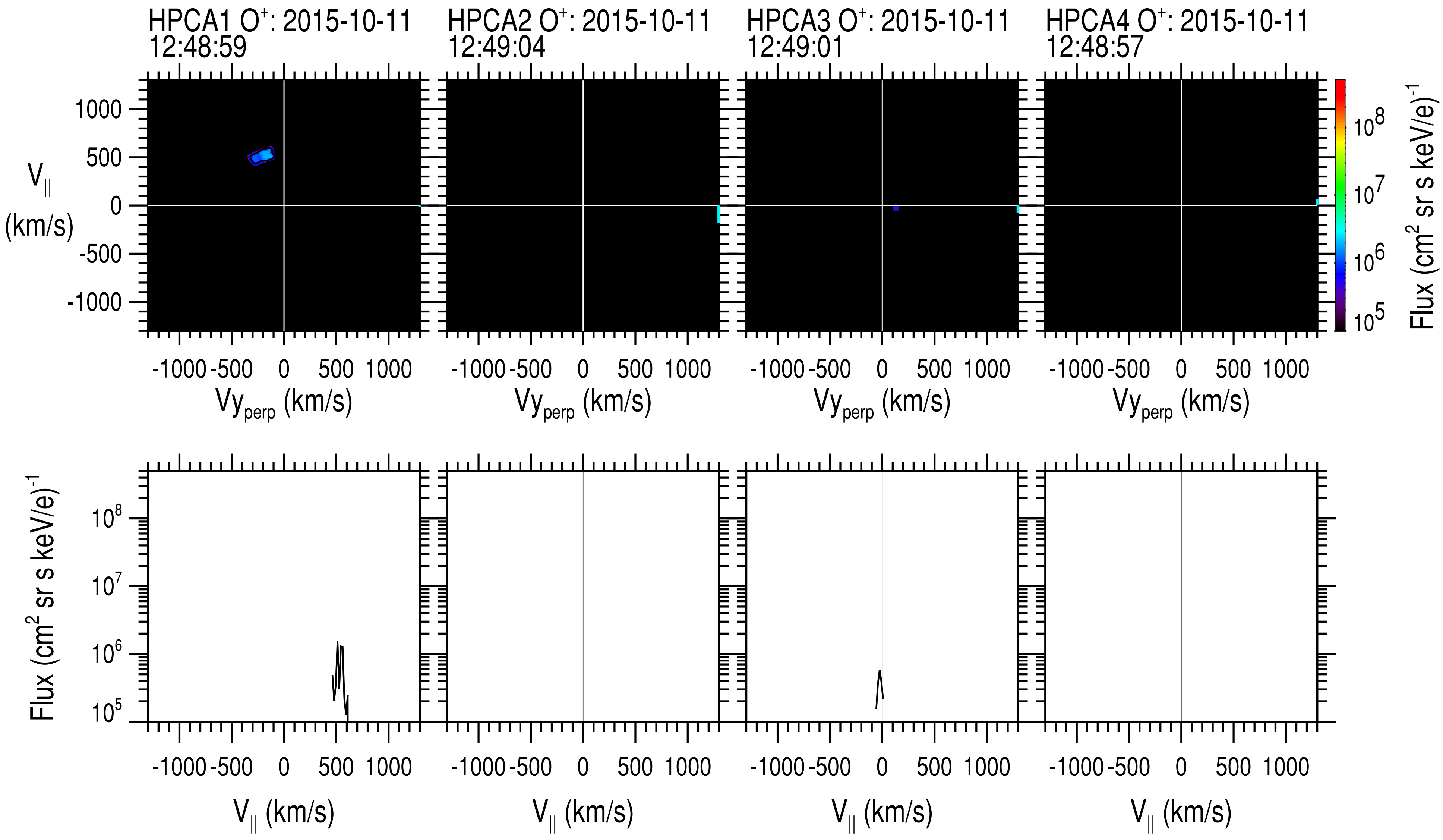

HPCA1 O+: 2015-10-11 12:48:59
HPCA2 O+: 2015-10-11 12:49:04
HPCA3 O+: 2015-10-11 12:49:01
HPCA4 O+: 2015-10-11 12:48:57
V|| (km/s)
Vyperp (km/s)
Flux (cm2 sr s keV/e)-1

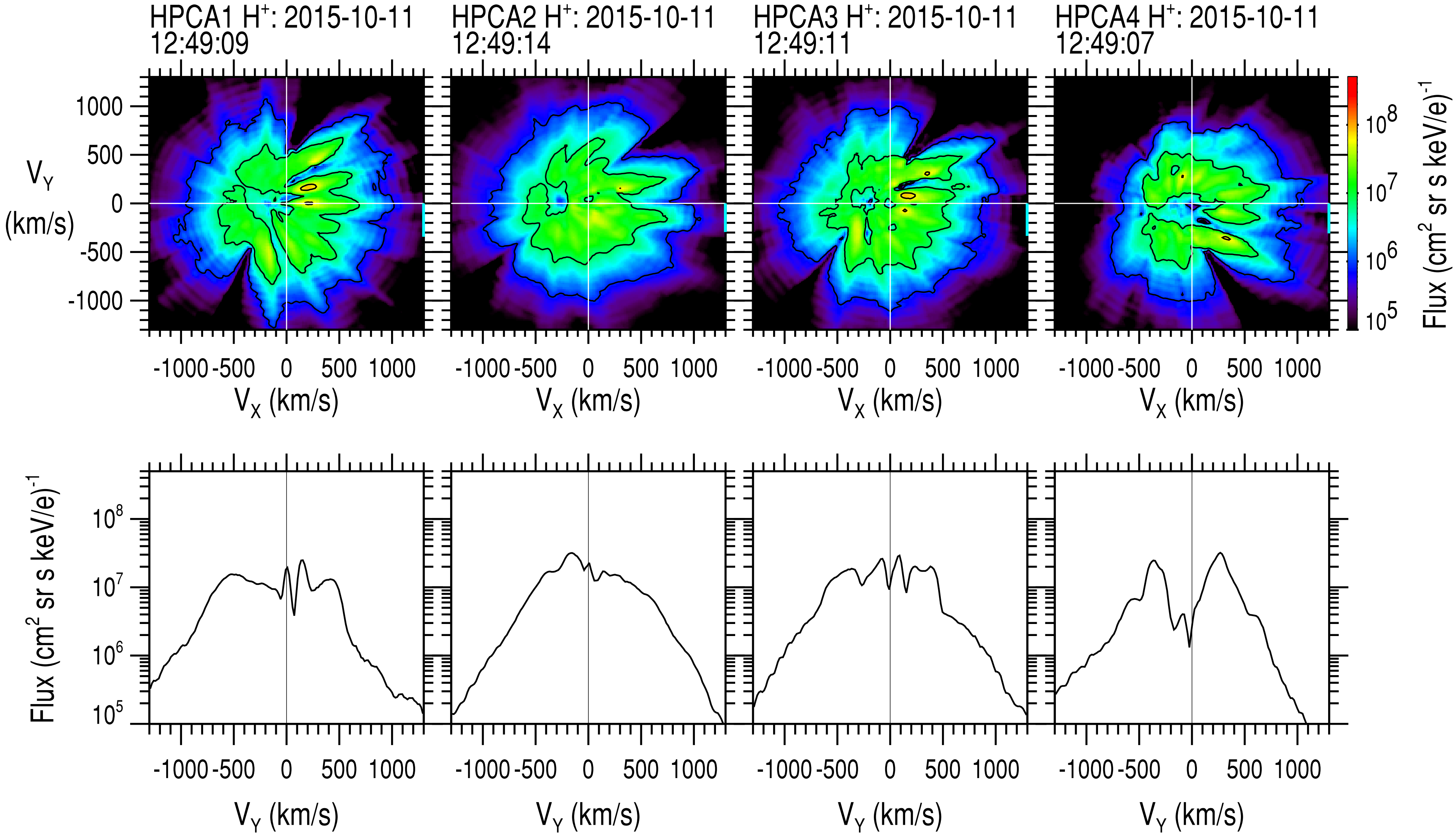
HPCA1 H+: 2015-10-11 12:49:09
HPCA2 H+: 2015-10-11 12:49:14
HPCA3 H+: 2015-10-11 12:49:11
HPCA4 H+: 2015-10-11 12:49:07
V_Y (km/s)
V_X (km/s)
Flux (cm^2 sr s keV/e)^-1

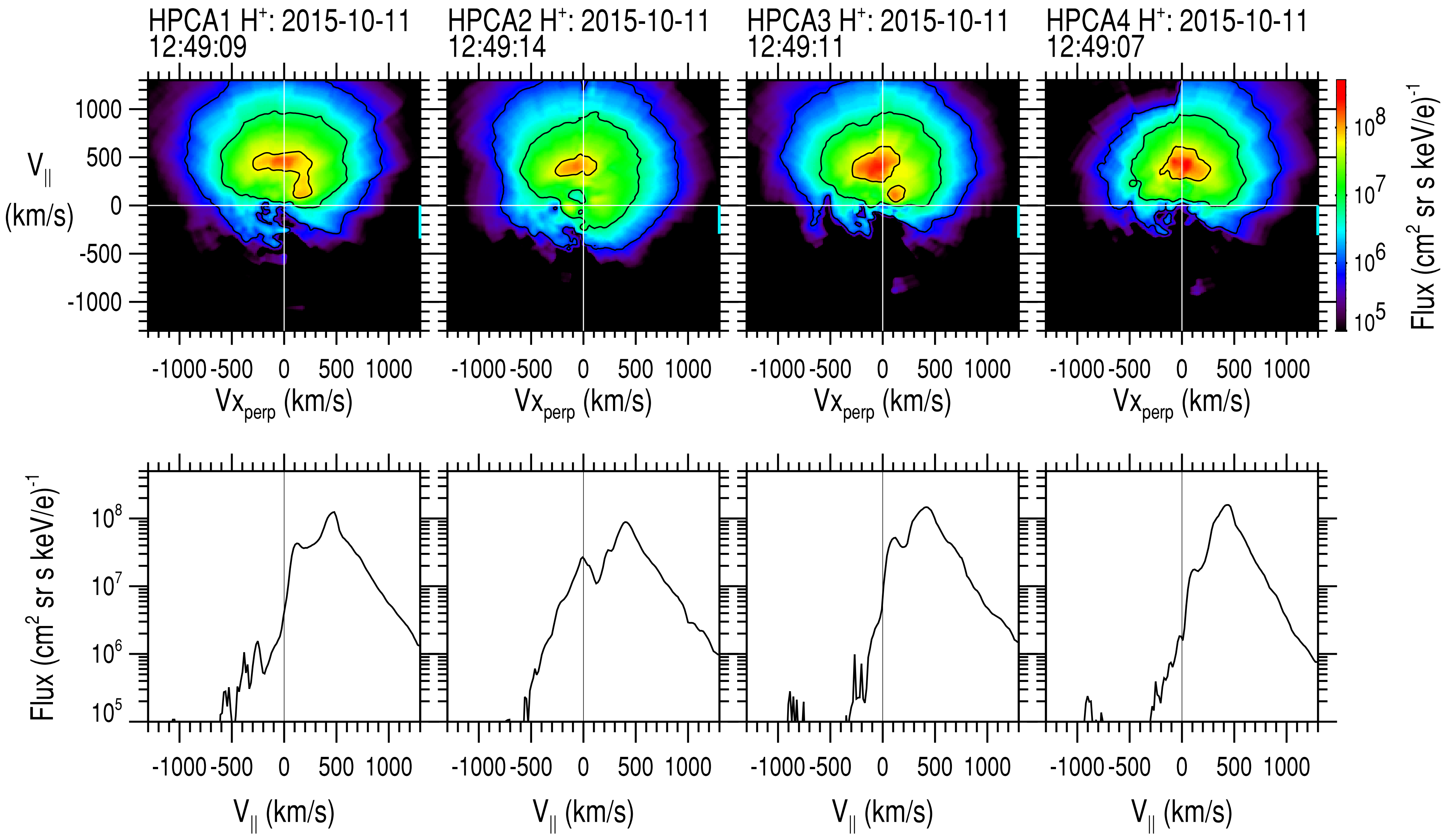

HPCA1 H+: 2015-10-11 12:49:09
HPCA2 H+: 2015-10-11 12:49:14
HPCA3 H+: 2015-10-11 12:49:11
HPCA4 H+: 2015-10-11 12:49:07
V|| (km/s)
1000
500
0
-500
-1000
Vxperp (km/s)
Flux (cm2 sr s keV/e)-1
105
106
107
108
V|| (km/s)

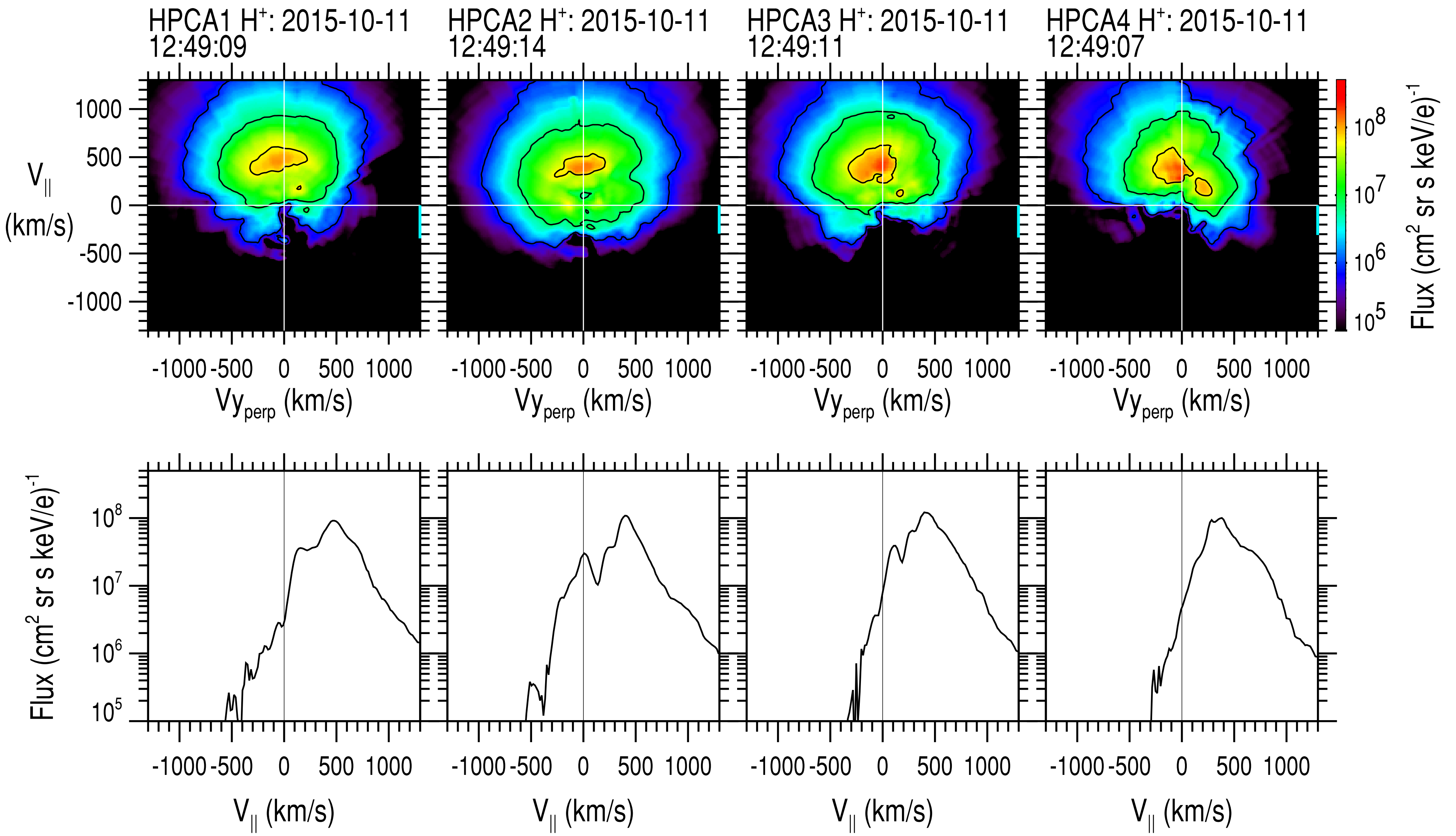

HPCA1 H+: 2015-10-11
12:49:09
HPCA2 H+: 2015-10-11
12:49:14
HPCA3 H+: 2015-10-11
12:49:11
HPCA4 H+: 2015-10-11
12:49:07
V|| (km/s)
Vyperp (km/s)
Flux (cm2 sr s keV/e)-1
V|| (km/s)

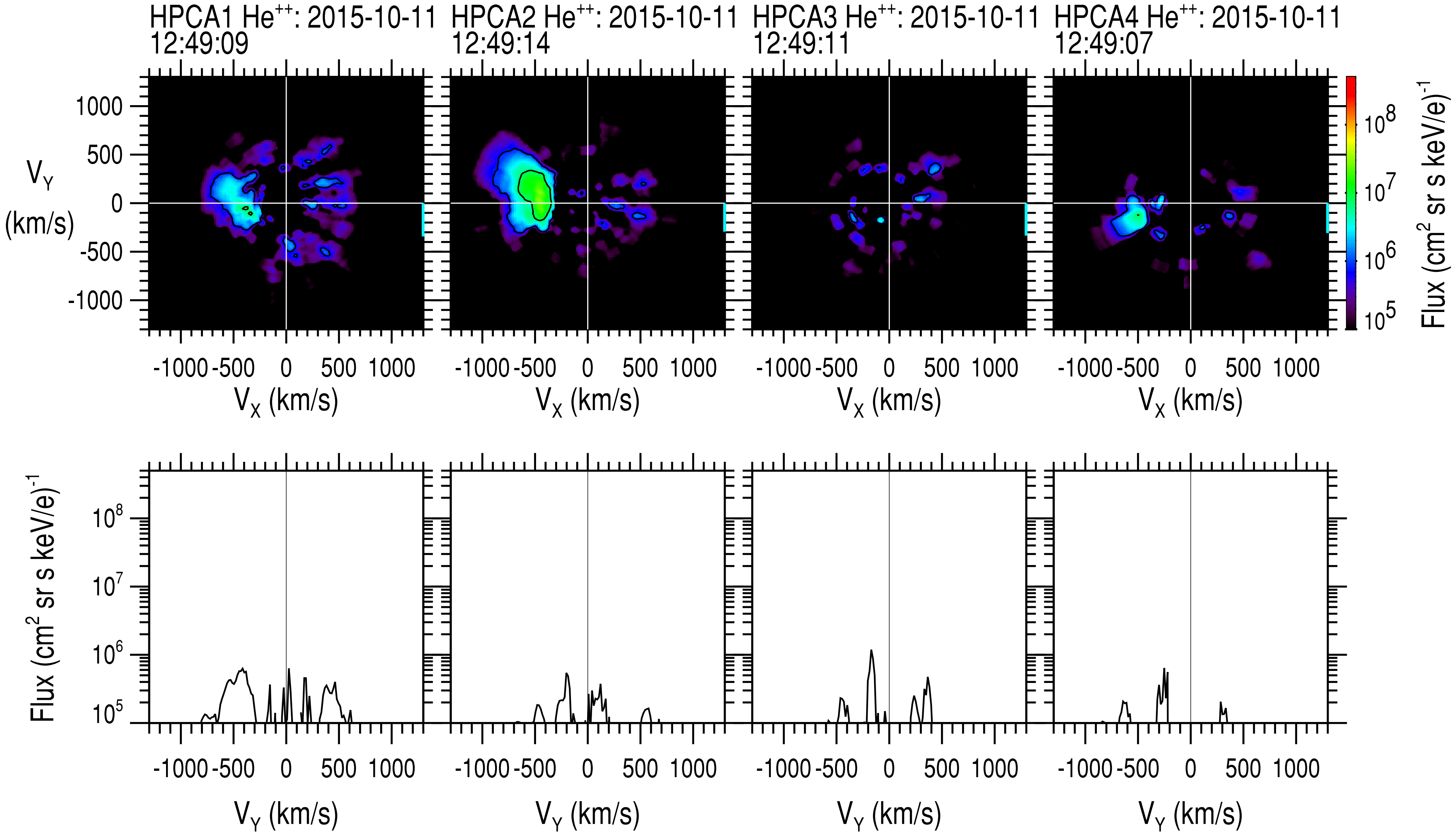

HPCA1 He++: 2015-10-11 12:49:09
HPCA2 He++: 2015-10-11 12:49:14
HPCA3 He++: 2015-10-11 12:49:11
HPCA4 He++: 2015-10-11 12:49:07
VY (km/s)
VX (km/s)
Flux (cm2 sr s keV/e)-1
VY (km/s)

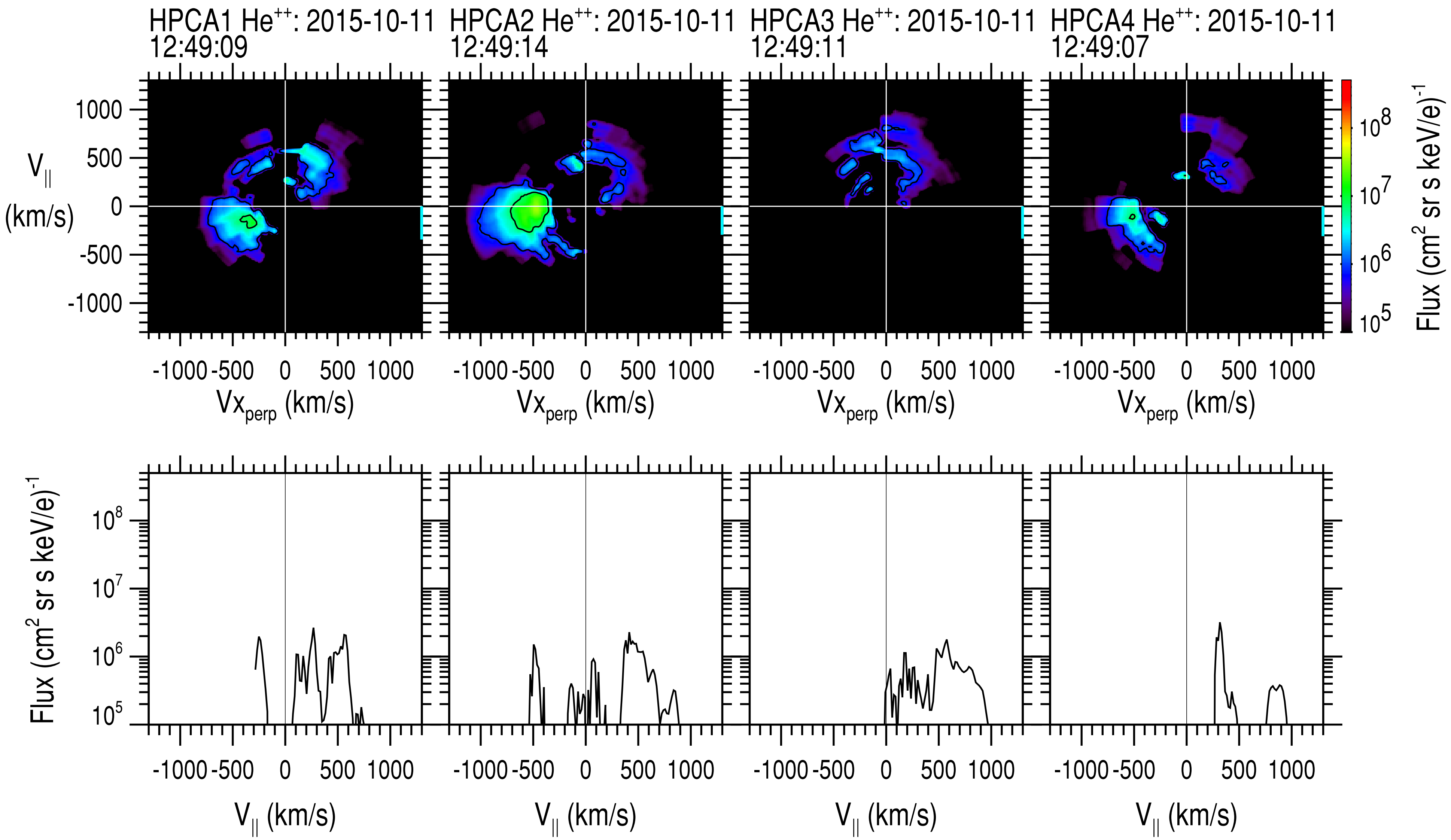
HPCA1 He++: 2015-10-11 12:49:09
HPCA2 He++: 2015-10-11 12:49:14
HPCA3 He++: 2015-10-11 12:49:11
HPCA4 He++: 2015-10-11 12:49:07
V|| (km/s)
1000
500
0
-500
-1000
Vxperp (km/s)
Flux (cm2 sr s keV/e)-1
108
107
106
105
V|| (km/s)

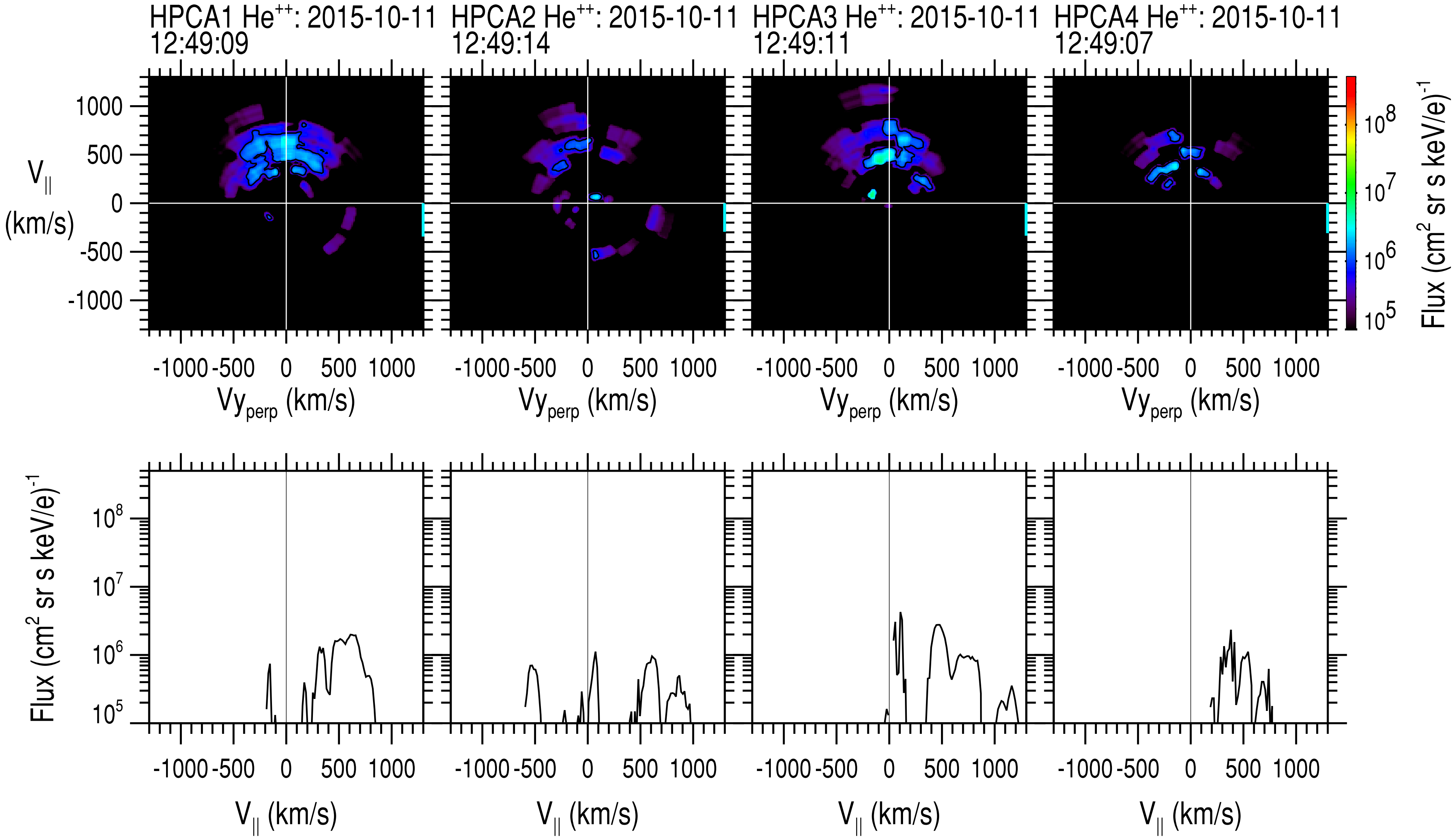

HPCA1 He++: 2015-10-11 12:49:09
HPCA2 He++: 2015-10-11 12:49:14
HPCA3 He++: 2015-10-11 12:49:11
HPCA4 He++: 2015-10-11 12:49:07
V|| (km/s)
1000
500
0
-500
-1000
-1000 -500 0 500 1000
Vyperp (km/s)
Flux (cm2 sr s keV/e)-1
105
106
107
108
V|| (km/s)

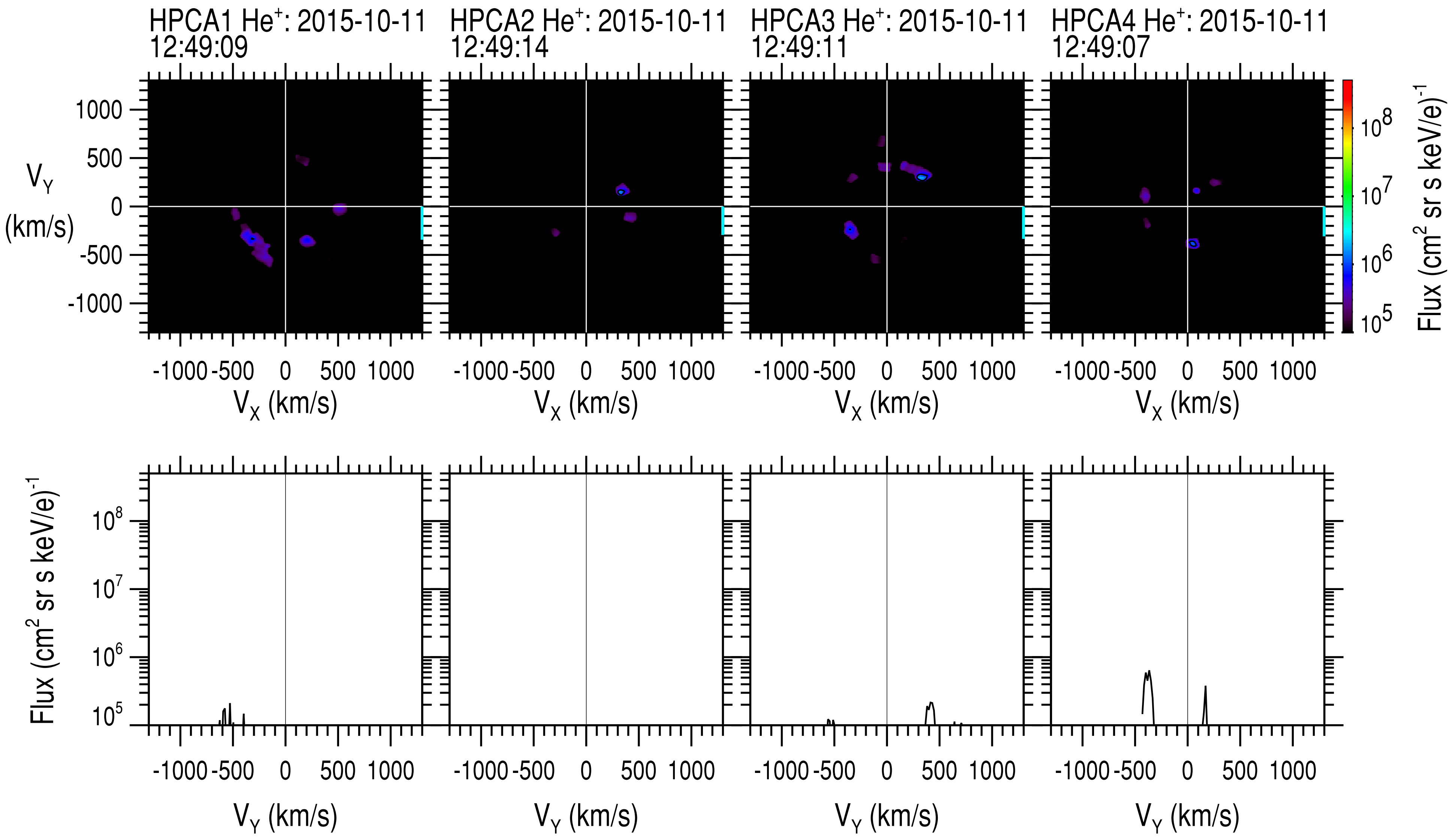

HPCA1 He+: 2015-10-11 12:49:09
HPCA2 He+: 2015-10-11 12:49:14
HPCA3 He+: 2015-10-11 12:49:11
HPCA4 He+: 2015-10-11 12:49:07
VY (km/s)
VX (km/s)
Flux (cm2 sr s keV/e)-1

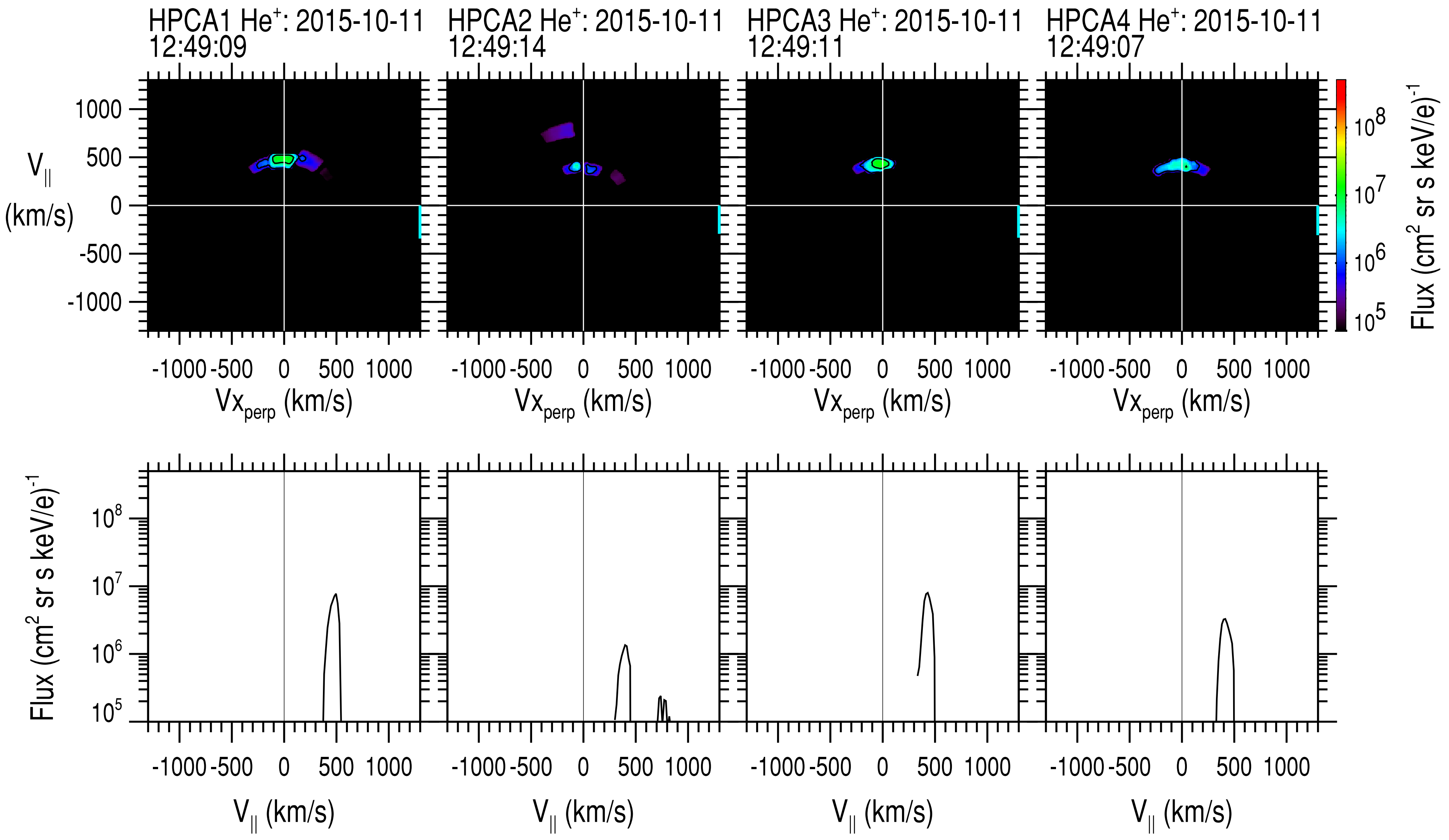
HPCA1 He+: 2015-10-11
12:49:09
HPCA2 He+: 2015-10-11
12:49:14
HPCA3 He+: 2015-10-11
12:49:11
HPCA4 He+: 2015-10-11
12:49:07
V|| (km/s)
Vxperp (km/s)
Flux (cm2 sr s keV/e)-1
-1000 -500 0 500 1000
10^5 10^6 10^7 10^8

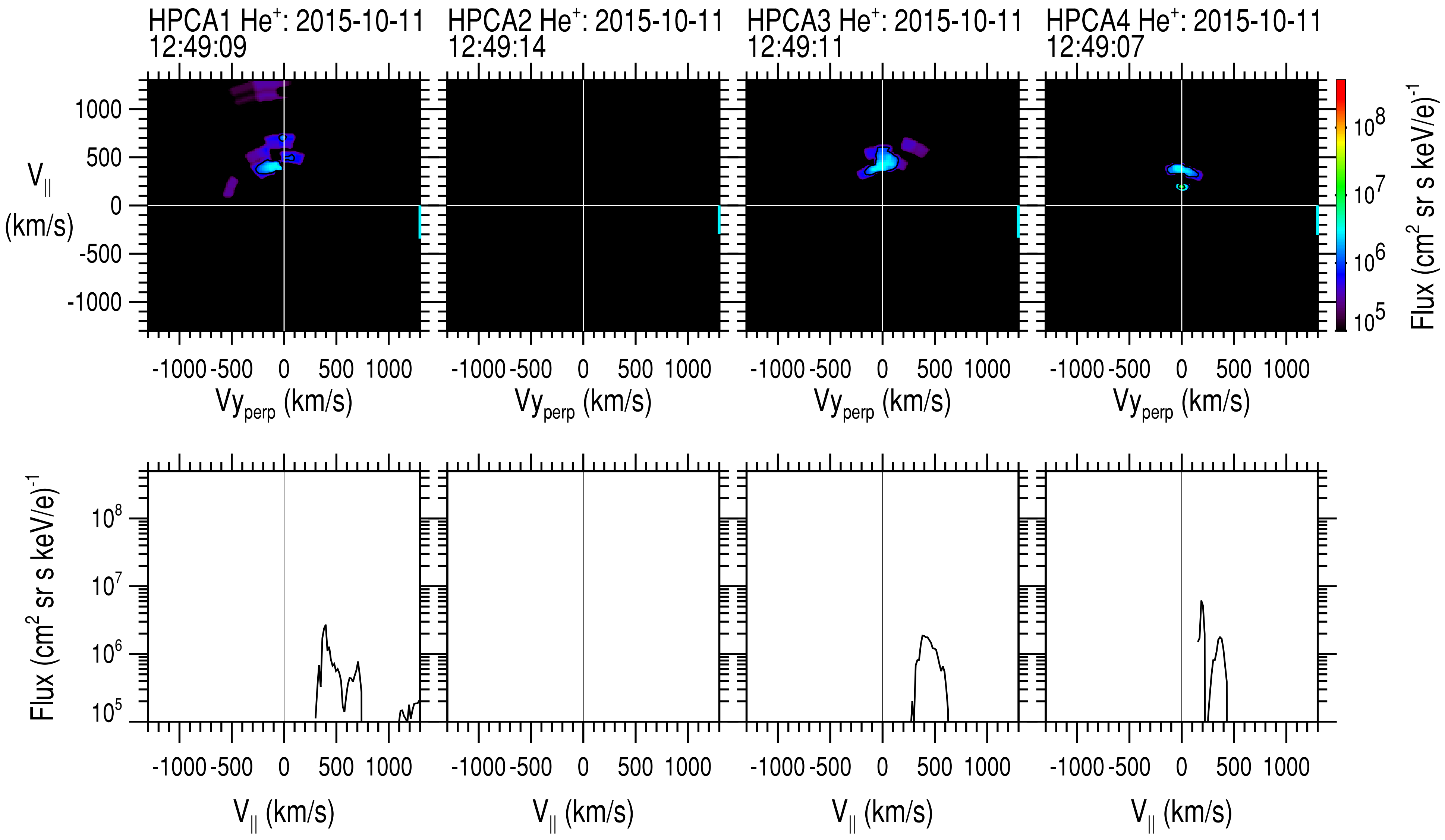

HPCA1 He+: 2015-10-11 12:49:09
HPCA2 He+: 2015-10-11 12:49:14
HPCA3 He+: 2015-10-11 12:49:11
HPCA4 He+: 2015-10-11 12:49:07
V|| (km/s)
Vyperp (km/s)
Flux (cm2 sr s keV/e)-1

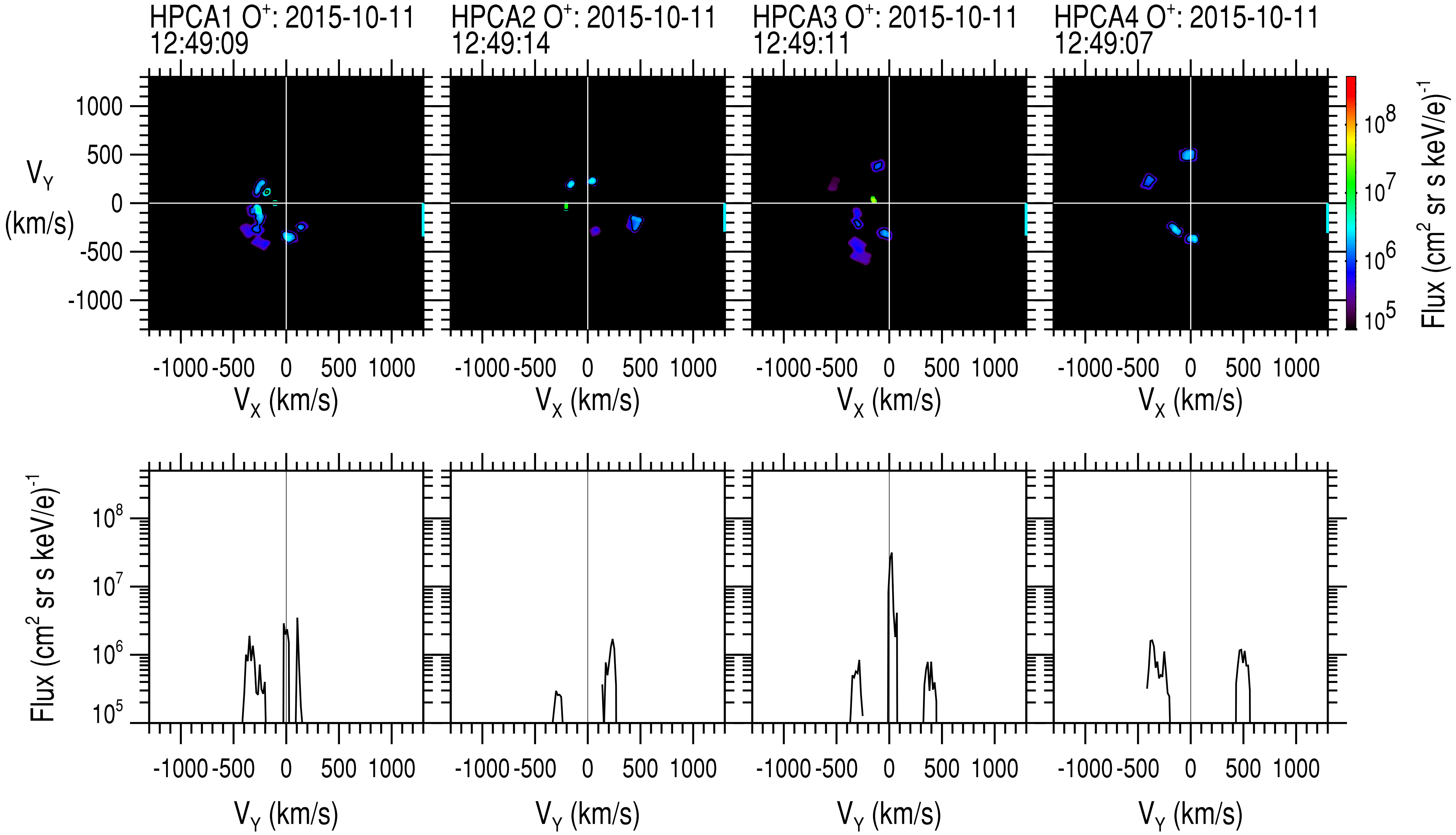
HPCA1 O+: 2015-10-11
12:49:09
HPCA2 O+: 2015-10-11
12:49:14
HPCA3 O+: 2015-10-11
12:49:11
HPCA4 O+: 2015-10-11
12:49:07
VY (km/s)
VX (km/s)
Flux (cm2 sr s keV/e)-1
VY (km/s)

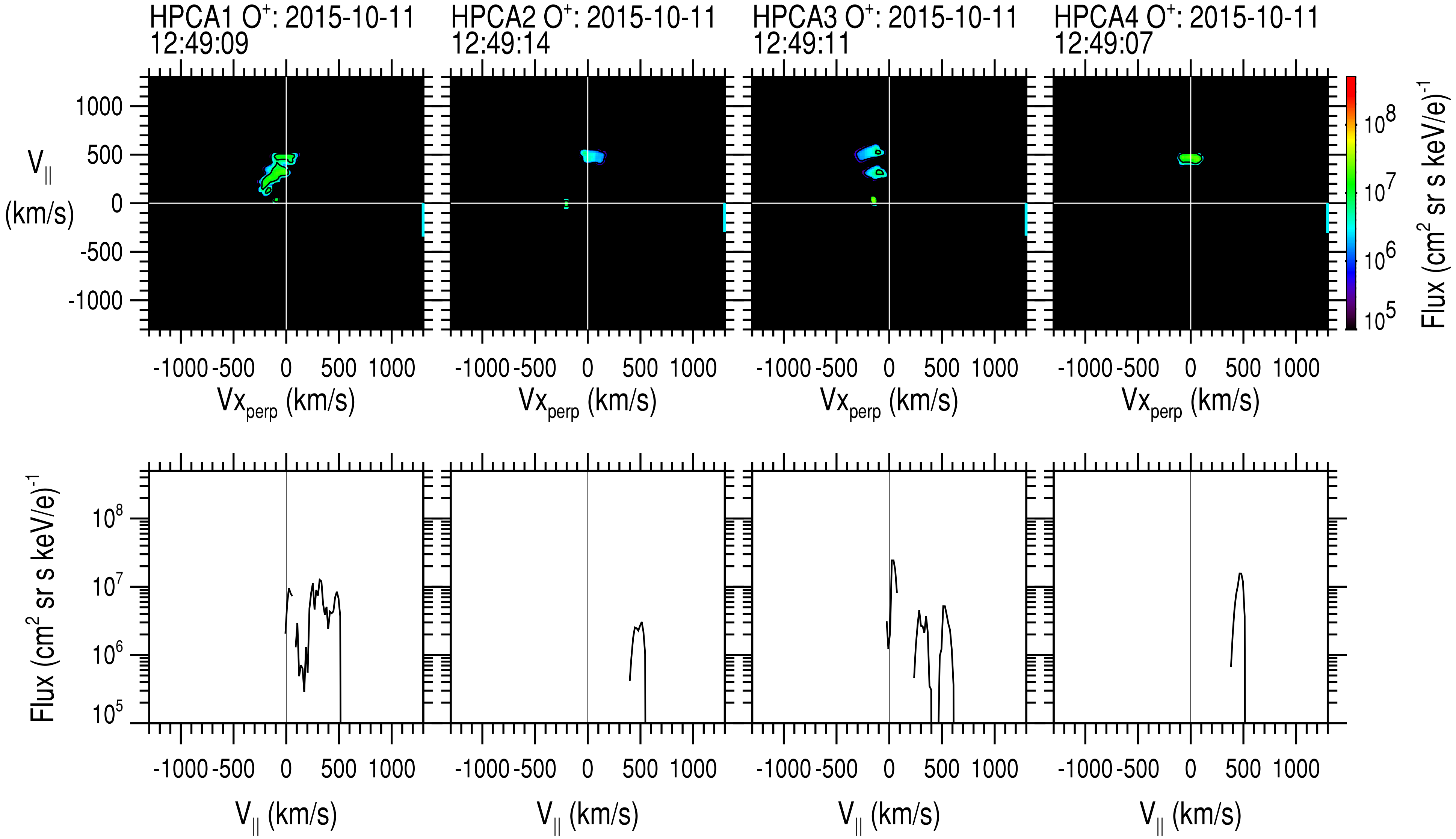

HPCA1 O+: 2015-10-11 12:49:09
HPCA2 O+: 2015-10-11 12:49:14
HPCA3 O+: 2015-10-11 12:49:11
HPCA4 O+: 2015-10-11 12:49:07
V|| (km/s)
Vxperp (km/s)
Flux (cm2 sr s keV/e)-1

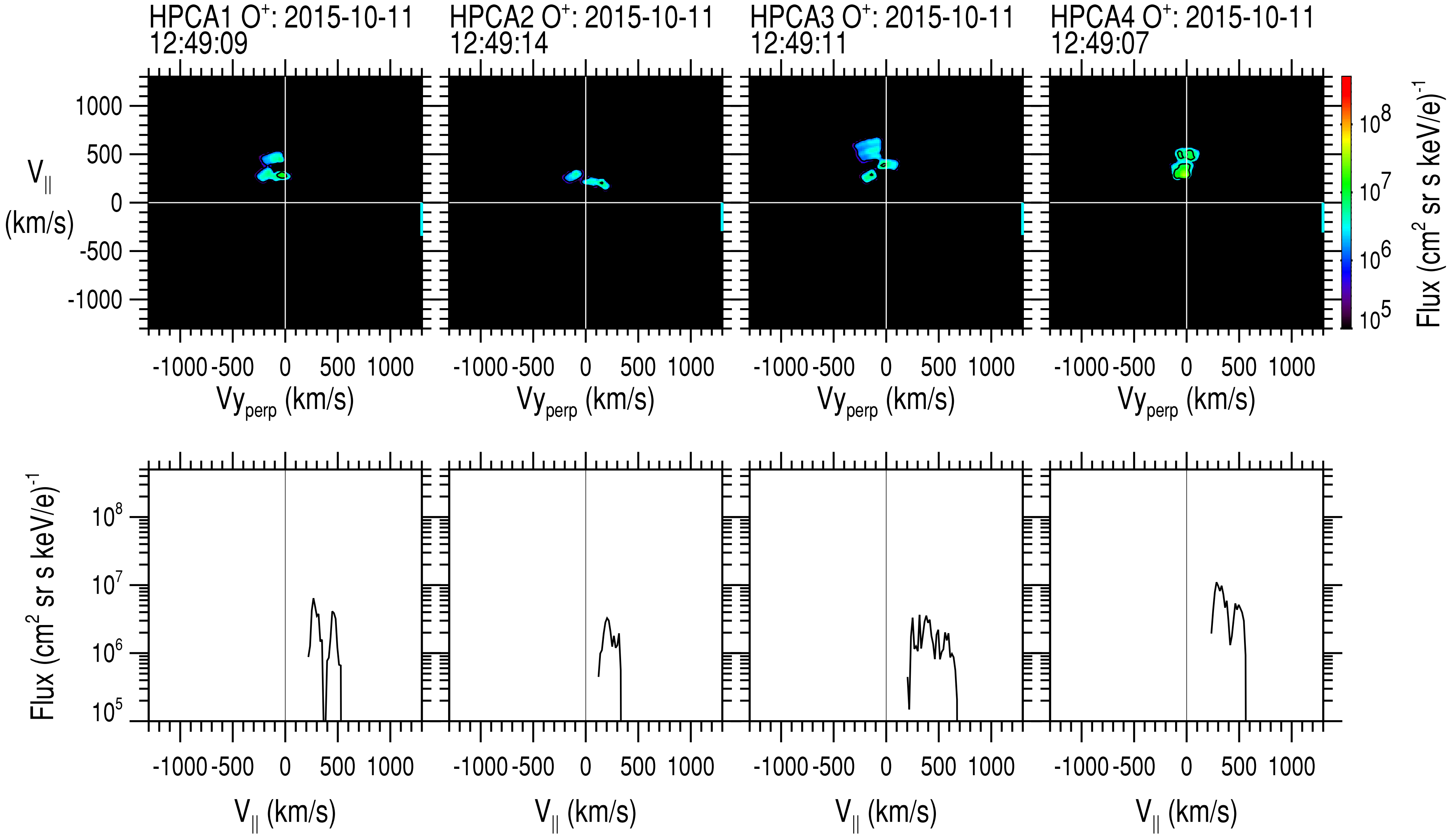
HPCA1 O+: 2015-10-11
12:49:09
HPCA2 O+: 2015-10-11
12:49:14
HPCA3 O+: 2015-10-11
12:49:11
HPCA4 O+: 2015-10-11
12:49:07
V|| (km/s)
Vyperp (km/s)
Flux (cm2 sr s keV/e)-1
-1000 -500 0 500 1000
10^5 10^6 10^7 10^8

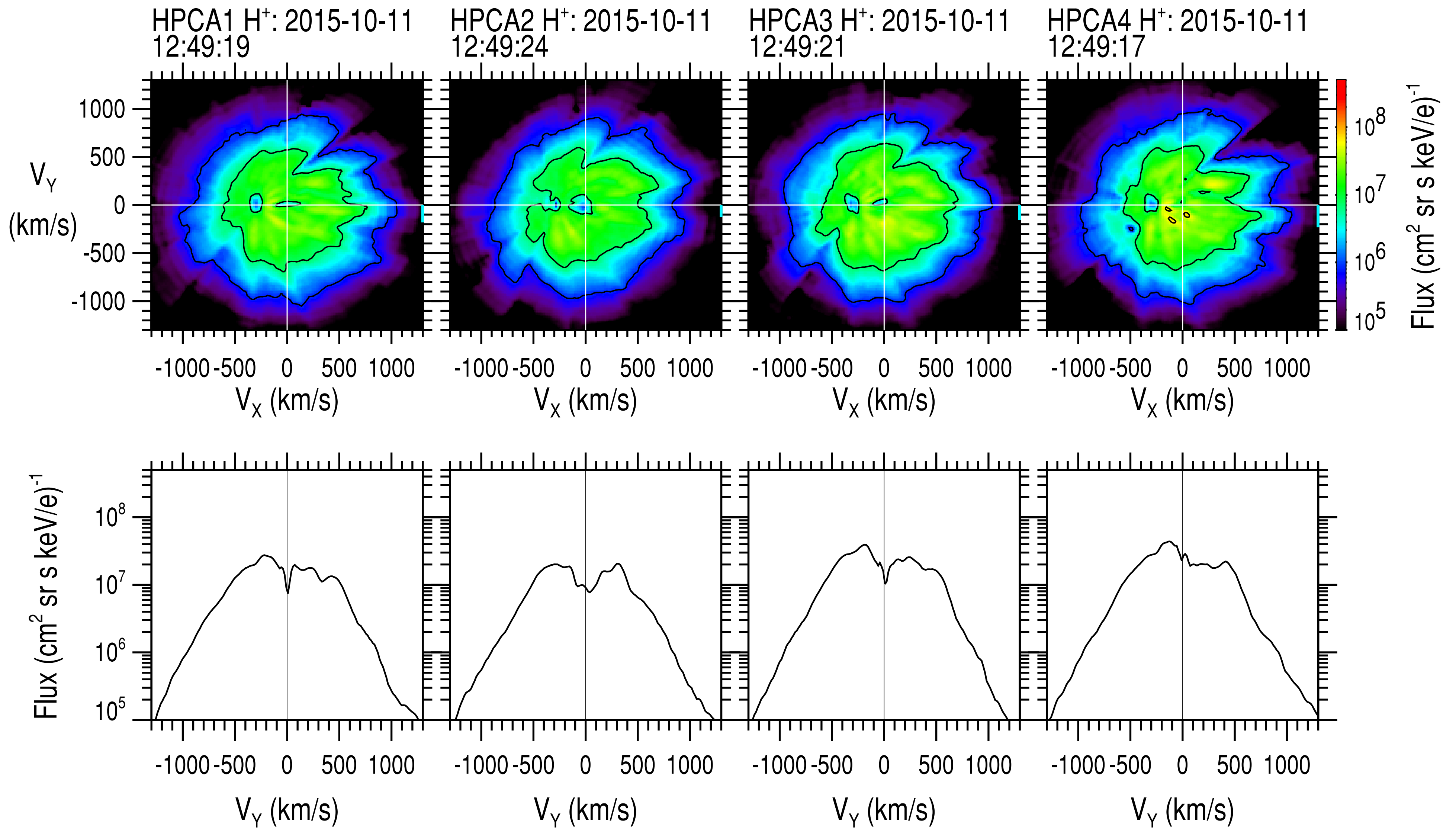

HPCA1 H+: 2015-10-11
12:49:19
HPCA2 H+: 2015-10-11
12:49:24
HPCA3 H+: 2015-10-11
12:49:21
HPCA4 H+: 2015-10-11
12:49:17
V_Y (km/s)
V_X (km/s)
Flux (cm^2 sr s keV/e)^-1
V_Y (km/s)

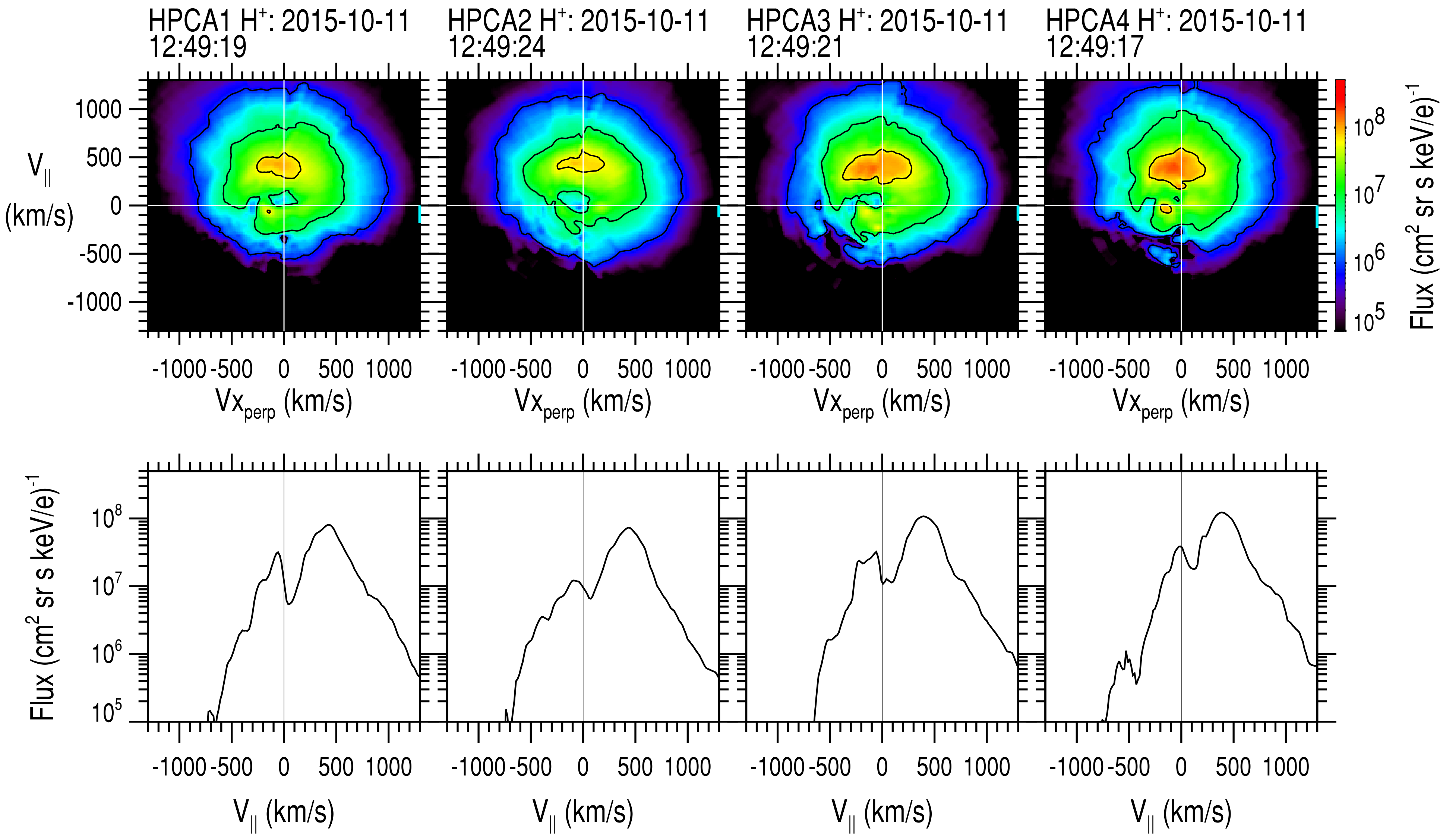

HPCA1 H+: 2015-10-11
12:49:19
HPCA2 H+: 2015-10-11
12:49:24
HPCA3 H+: 2015-10-11
12:49:21
HPCA4 H+: 2015-10-11
12:49:17
V|| (km/s)
Vxperp (km/s)
Flux (cm2 sr s keV/e)-1
V|| (km/s)

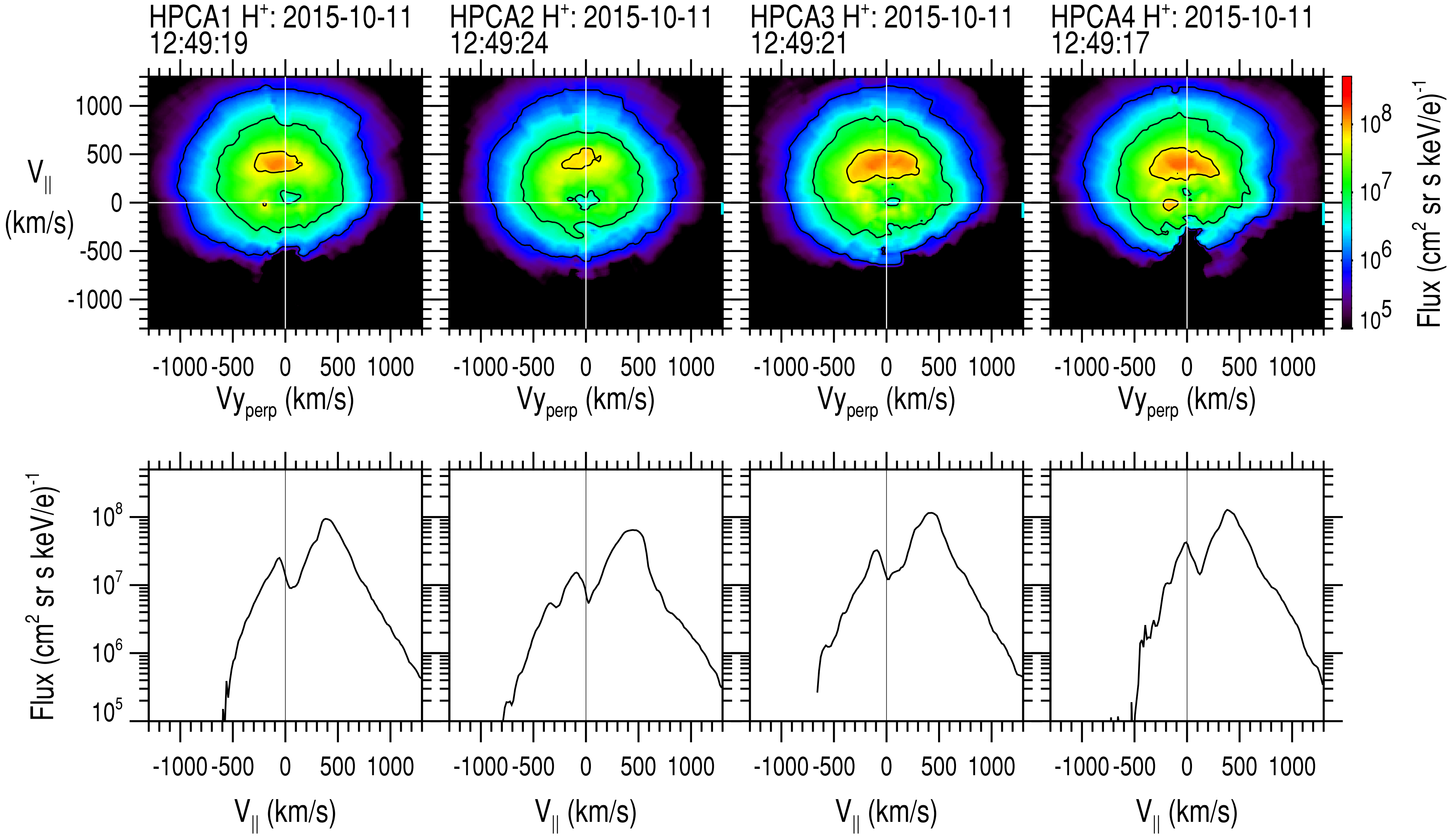

HPCA1 H+: 2015-10-11
12:49:19
HPCA2 H+: 2015-10-11
12:49:24
HPCA3 H+: 2015-10-11
12:49:21
HPCA4 H+: 2015-10-11
12:49:17
V|| (km/s)
Vyperp (km/s)
Flux (cm2 sr s keV/e)-1

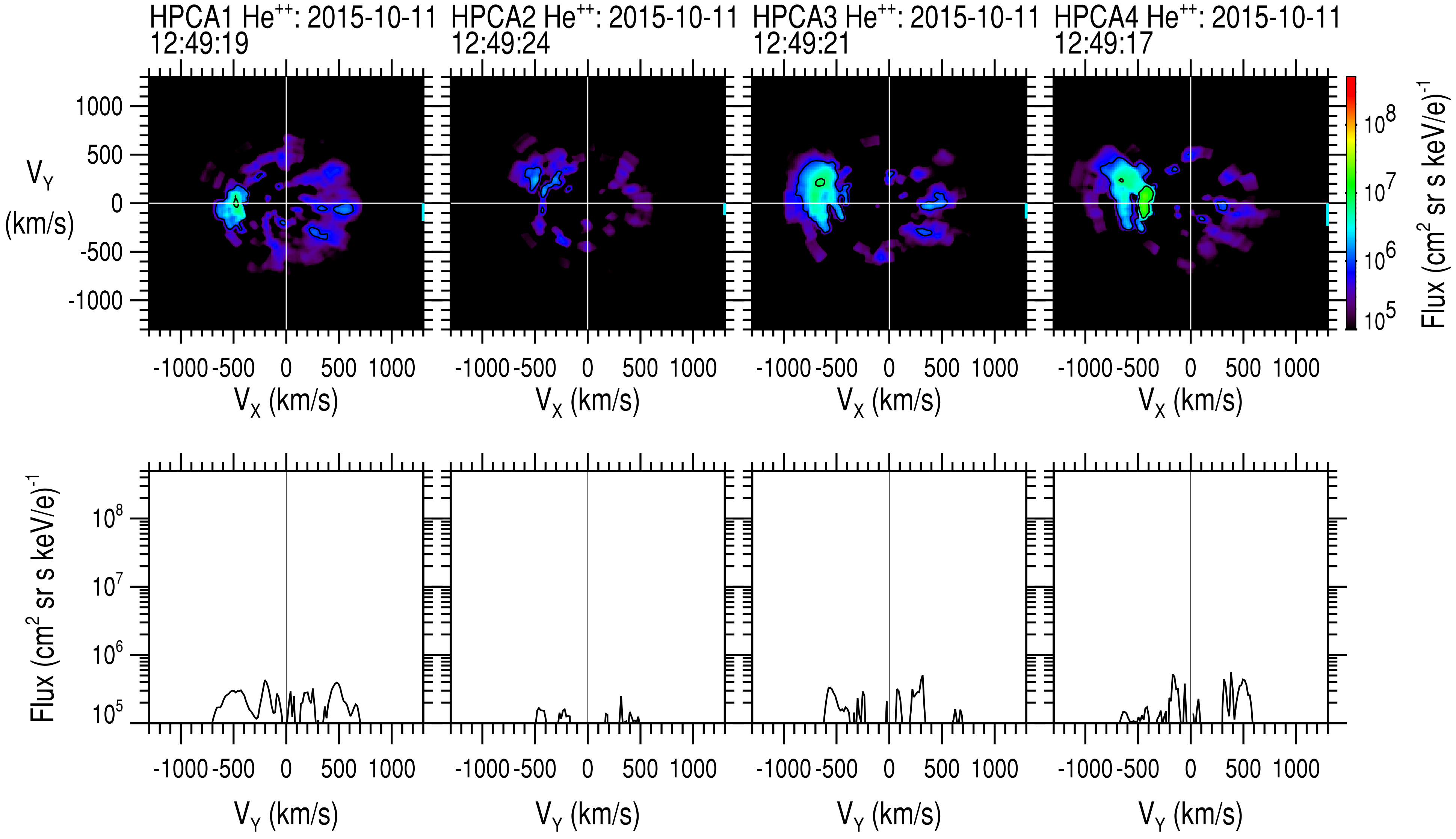

HPCA1 He++: 2015-10-11 12:49:19
HPCA2 He++: 2015-10-11 12:49:24
HPCA3 He++: 2015-10-11 12:49:21
HPCA4 He++: 2015-10-11 12:49:17
VY (km/s)
VX (km/s)
Flux (cm2 sr s keV/e)-1

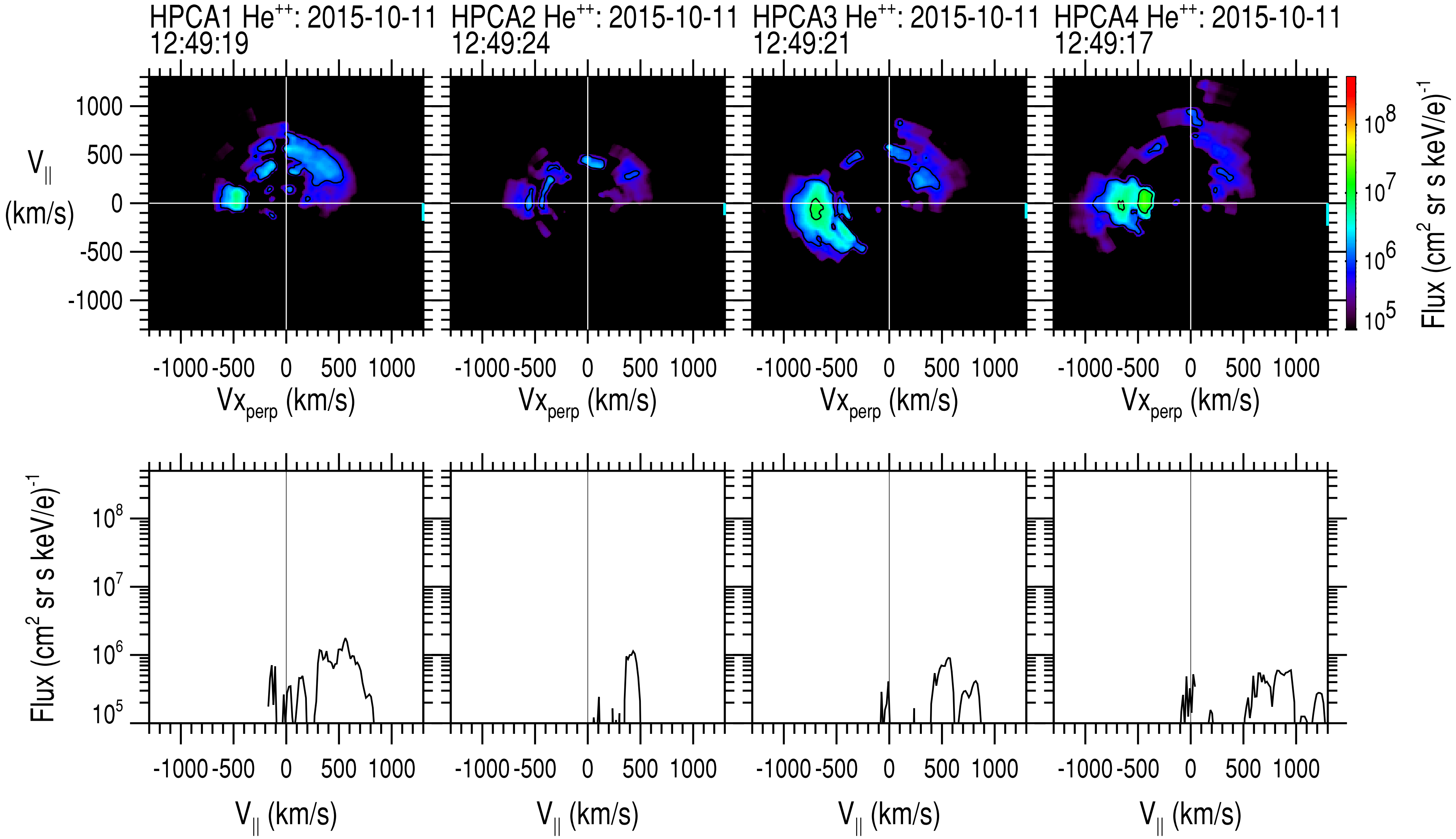

HPCA1 He++: 2015-10-11 12:49:19
HPCA2 He++: 2015-10-11 12:49:24
HPCA3 He++: 2015-10-11 12:49:21
HPCA4 He++: 2015-10-11 12:49:17
V|| (km/s)
Vxperp (km/s)
Flux (cm2 sr s keV/e)-1

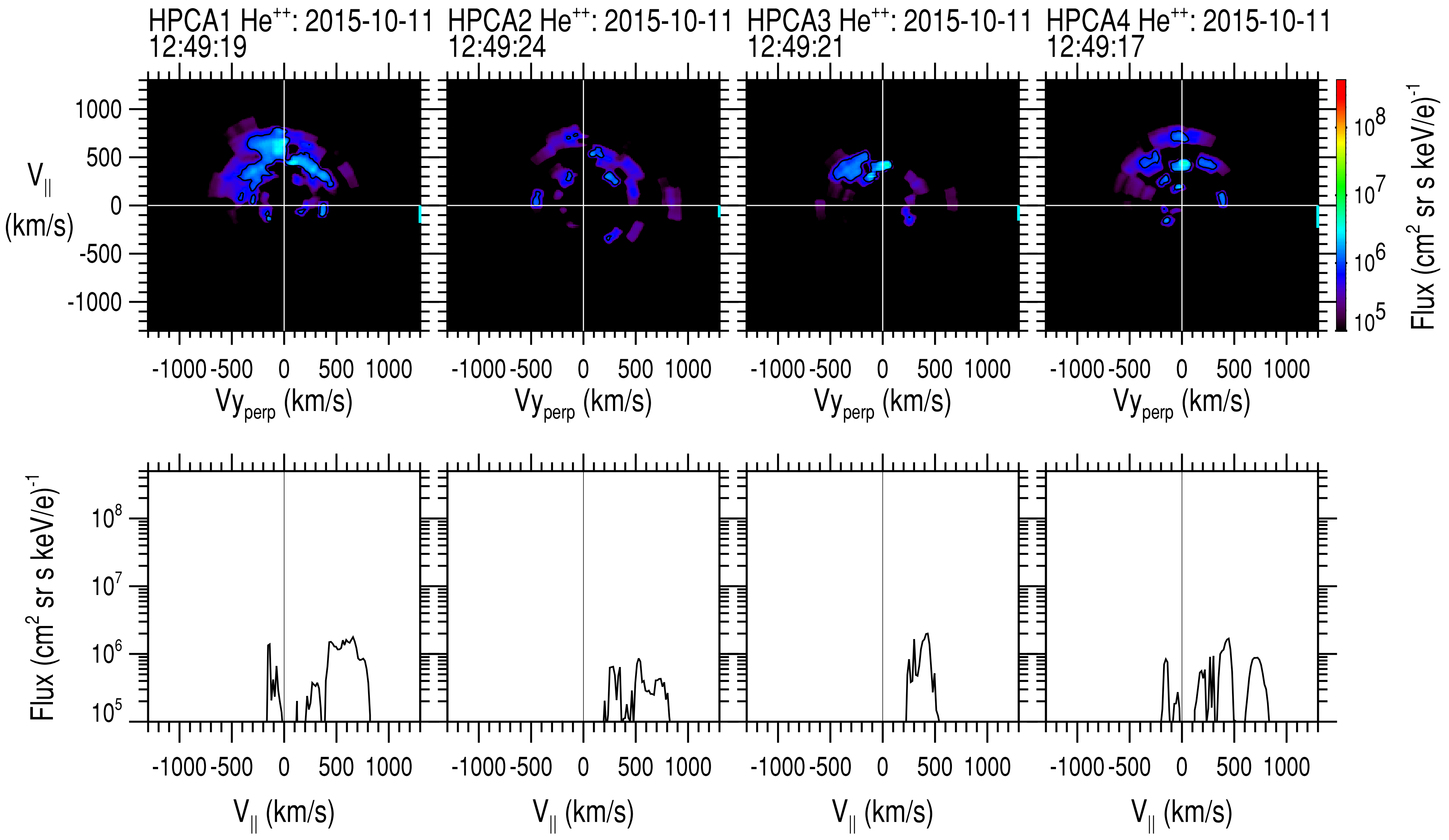

HPCA1 He++: 2015-10-11 12:49:19
HPCA2 He++: 2015-10-11 12:49:24
HPCA3 He++: 2015-10-11 12:49:21
HPCA4 He++: 2015-10-11 12:49:17
V|| (km/s)
Vyperp (km/s)
Flux (cm2 sr s keV/e)-1

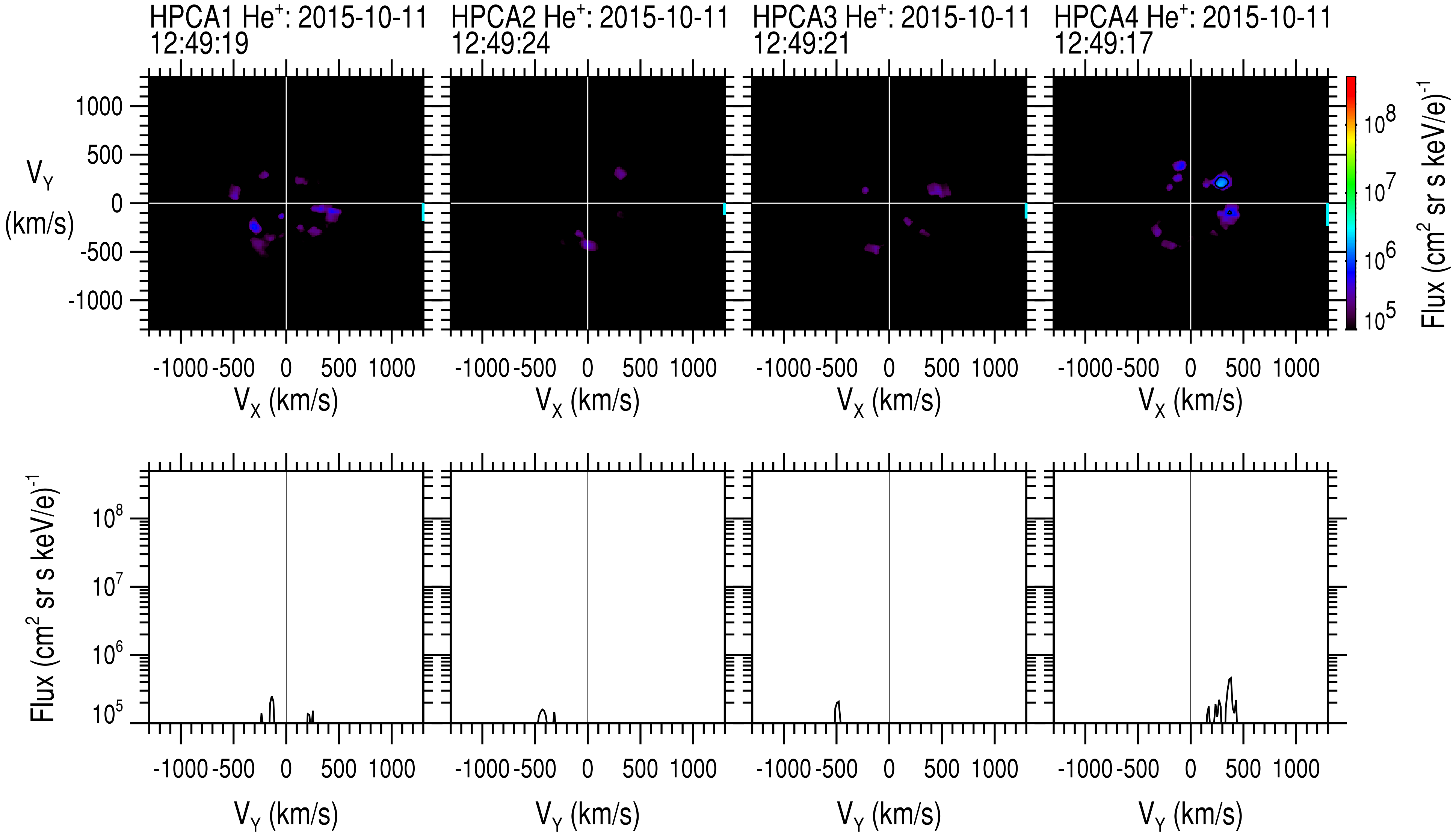
HPCA1 He+: 2015-10-11
12:49:19
HPCA2 He+: 2015-10-11
12:49:24
HPCA3 He+: 2015-10-11
12:49:21
HPCA4 He+: 2015-10-11
12:49:17
V_Y (km/s)
V_X (km/s)
Flux (cm2 sr s keV/e)-1
V_Y (km/s)

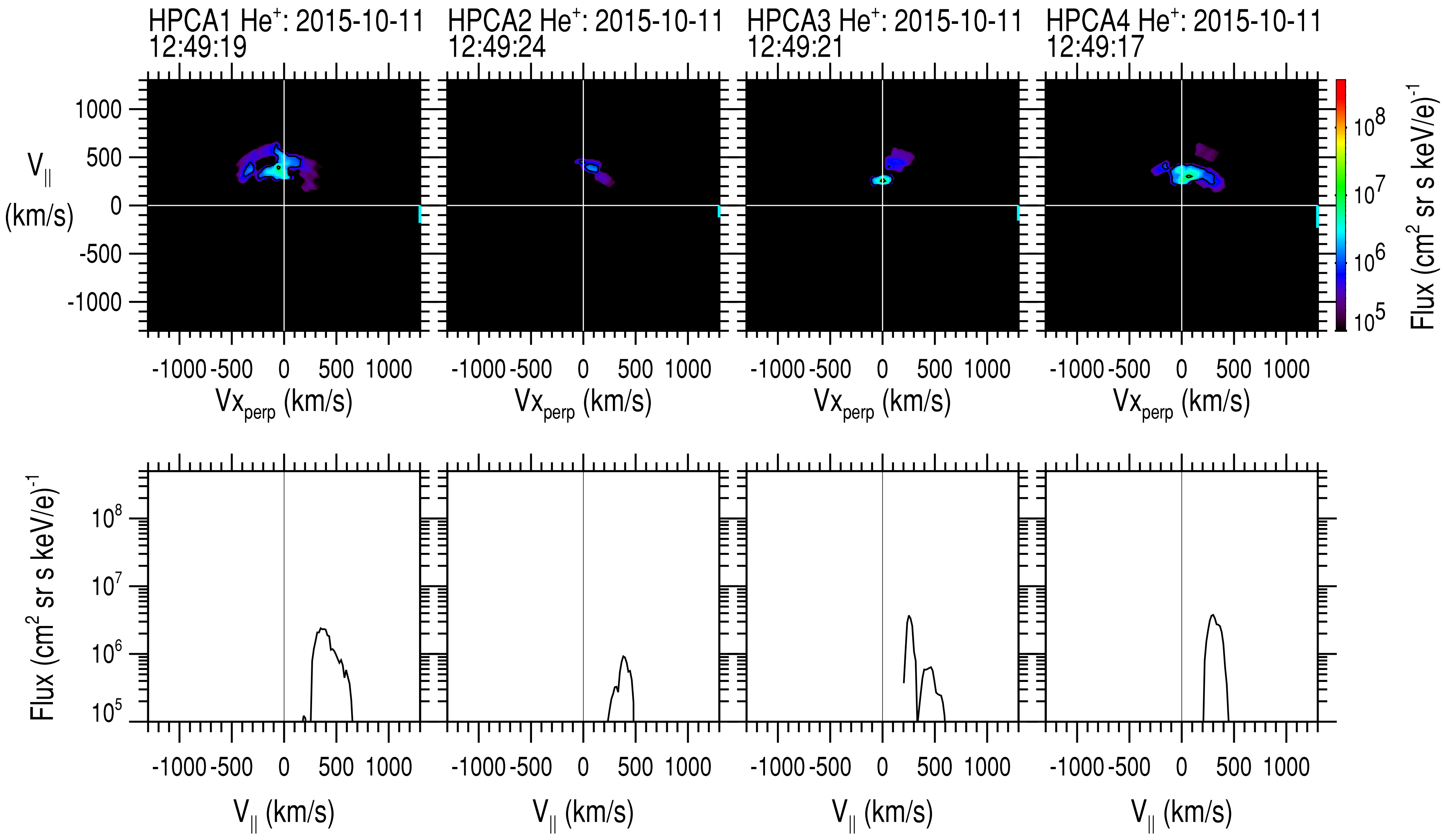

HPCA1 He+: 2015-10-11
12:49:19
HPCA2 He+: 2015-10-11
12:49:24
HPCA3 He+: 2015-10-11
12:49:21
HPCA4 He+: 2015-10-11
12:49:17
V|| (km/s)
Vxperp (km/s)
Flux (cm2 sr s keV/e)-1

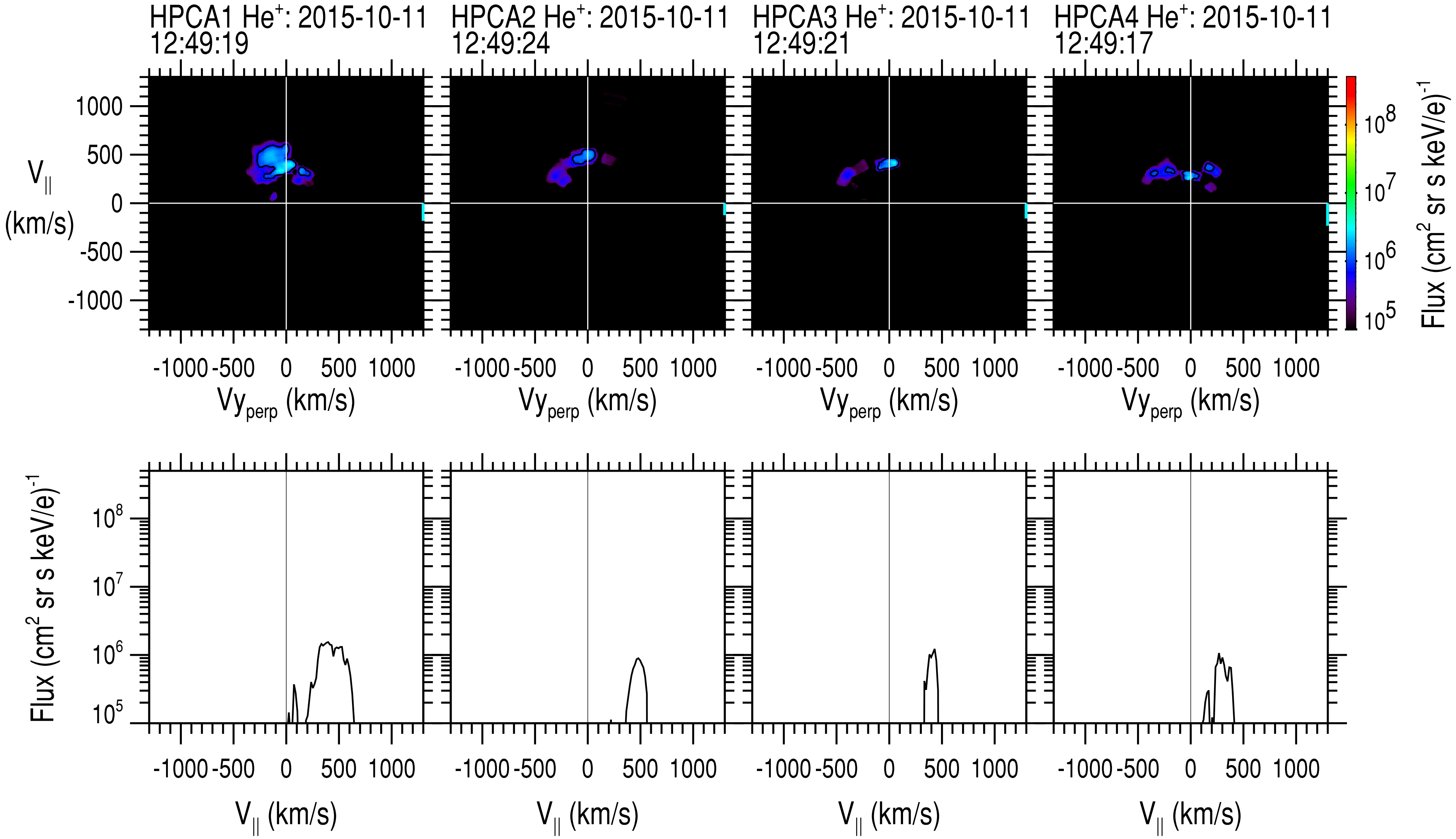

HPCA1 He+: 2015-10-11 12:49:19
HPCA2 He+: 2015-10-11 12:49:24
HPCA3 He+: 2015-10-11 12:49:21
HPCA4 He+: 2015-10-11 12:49:17
V|| (km/s)
Vyperp (km/s)
Flux (cm2 sr s keV/e)-1
-1000 -500 0 500 1000
10^5 10^6 10^7 10^8

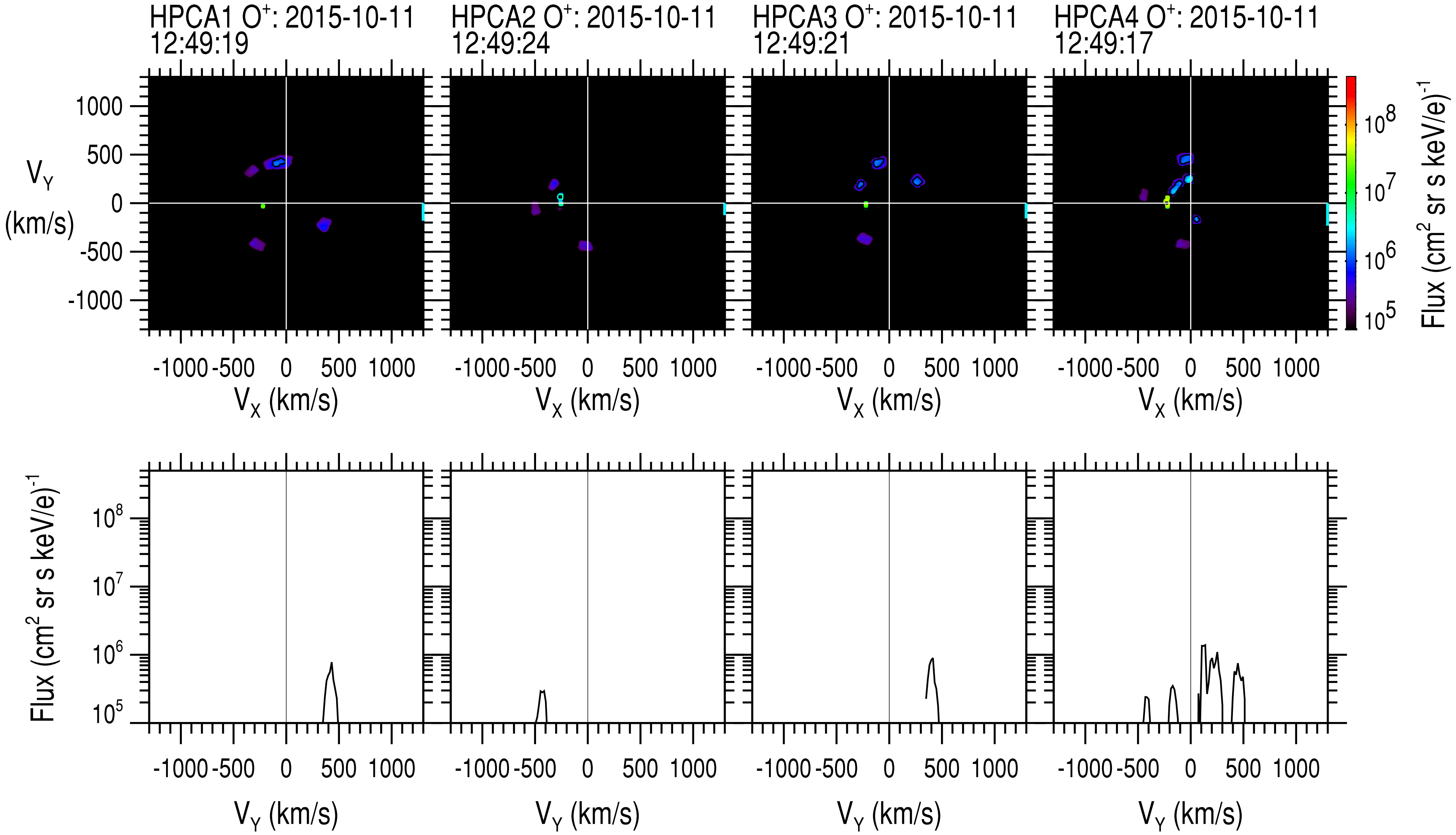

HPCA1 O+: 2015-10-11
12:49:19
HPCA2 O+: 2015-10-11
12:49:24
HPCA3 O+: 2015-10-11
12:49:21
HPCA4 O+: 2015-10-11
12:49:17
V_Y (km/s)
V_X (km/s)
Flux (cm^2 sr s keV/e)^-1
V_Y (km/s)

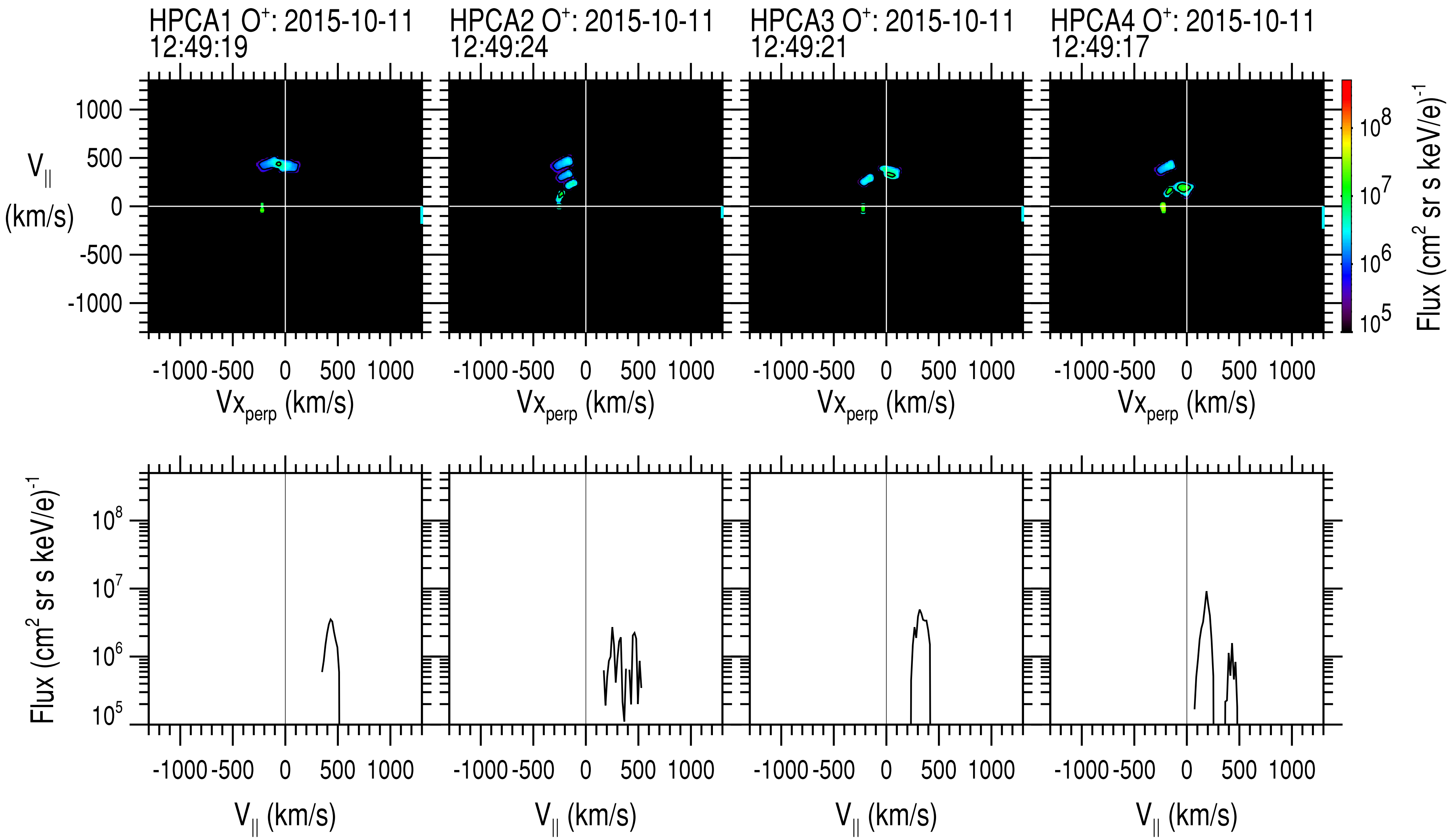

HPCA1 O+: 2015-10-11
12:49:19
HPCA2 O+: 2015-10-11
12:49:24
HPCA3 O+: 2015-10-11
12:49:21
HPCA4 O+: 2015-10-11
12:49:17
V|| (km/s)
1000
500
0
-500
-1000
-1000 -500 0 500 1000
Vxperp (km/s)
Flux (cm2 sr s keV/e)-1
10^5
10^6
10^7
10^8
V|| (km/s)

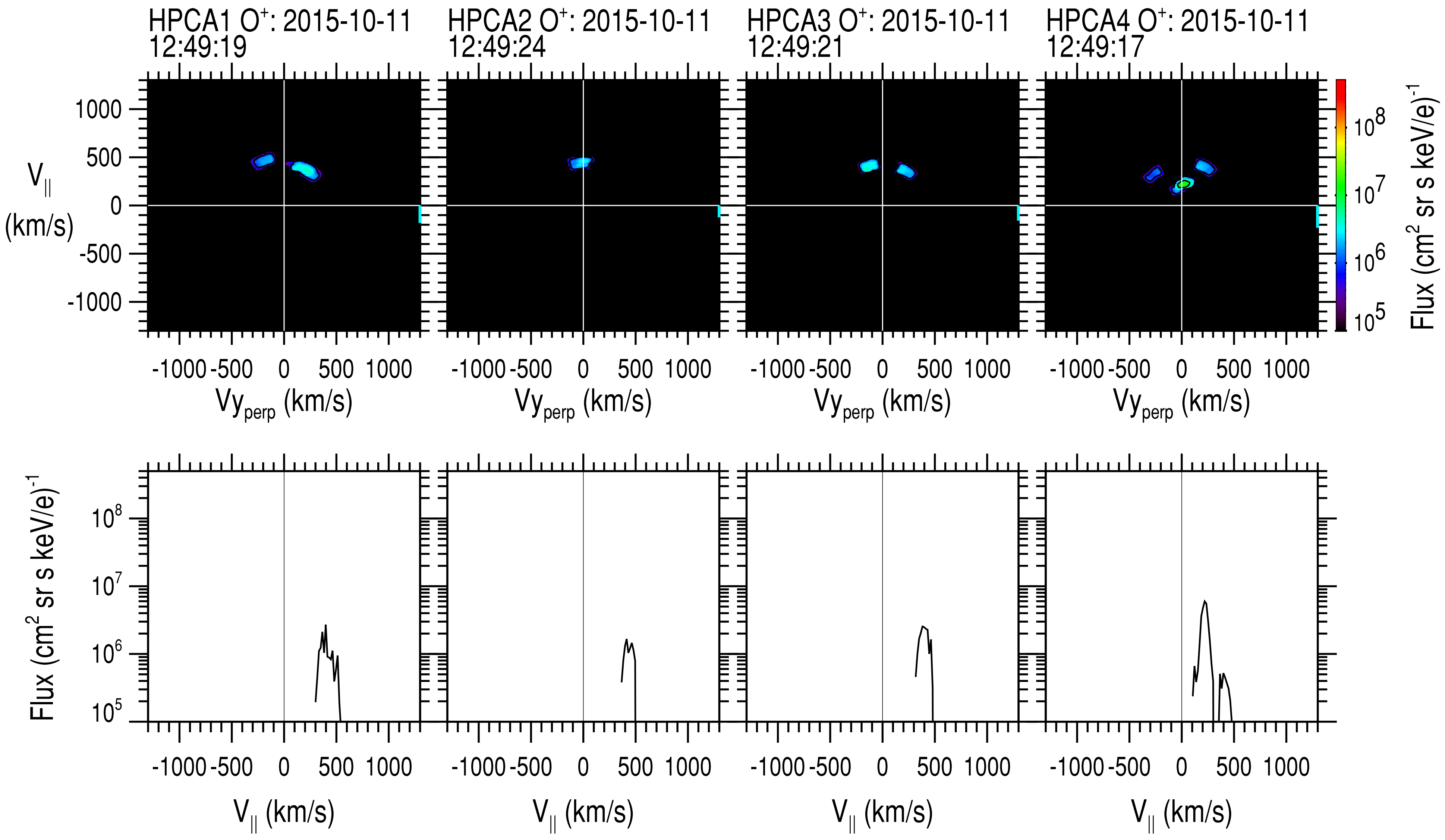
HPCA1 O+: 2015-10-11 12:49:19
HPCA2 O+: 2015-10-11 12:49:24
HPCA3 O+: 2015-10-11 12:49:21
HPCA4 O+: 2015-10-11 12:49:17
V|| (km/s)
Vyperp (km/s)
Flux (cm2 sr s keV/e)-1
-1000 -500 0 500 1000
10^5 10^6 10^7 10^8

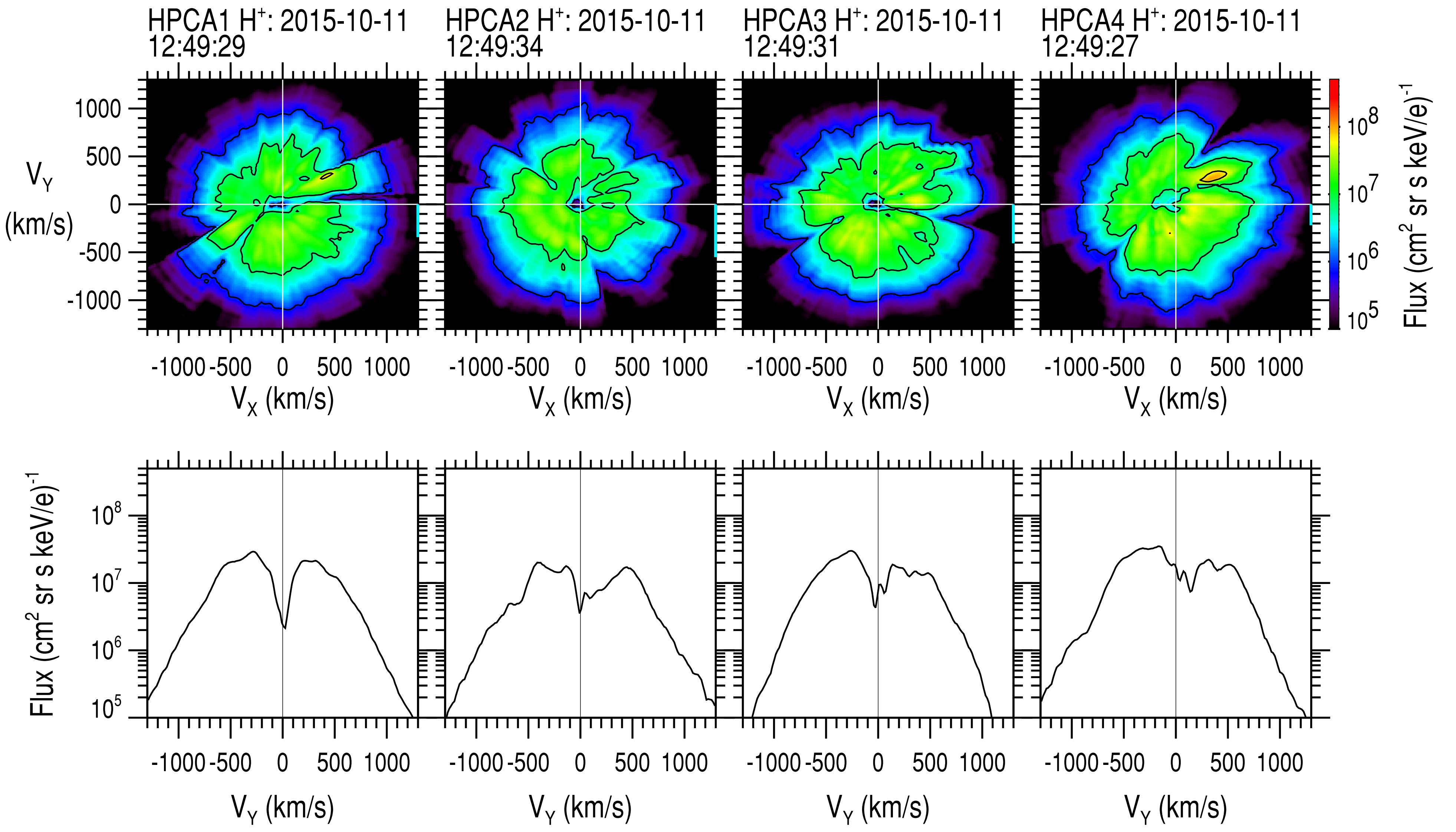

HPCA1 H+: 2015-10-11
12:49:29
HPCA2 H+: 2015-10-11
12:49:34
HPCA3 H+: 2015-10-11
12:49:31
HPCA4 H+: 2015-10-11
12:49:27
V_Y (km/s)
V_X (km/s)
Flux (cm2 sr s keV/e)-1
V_Y (km/s)

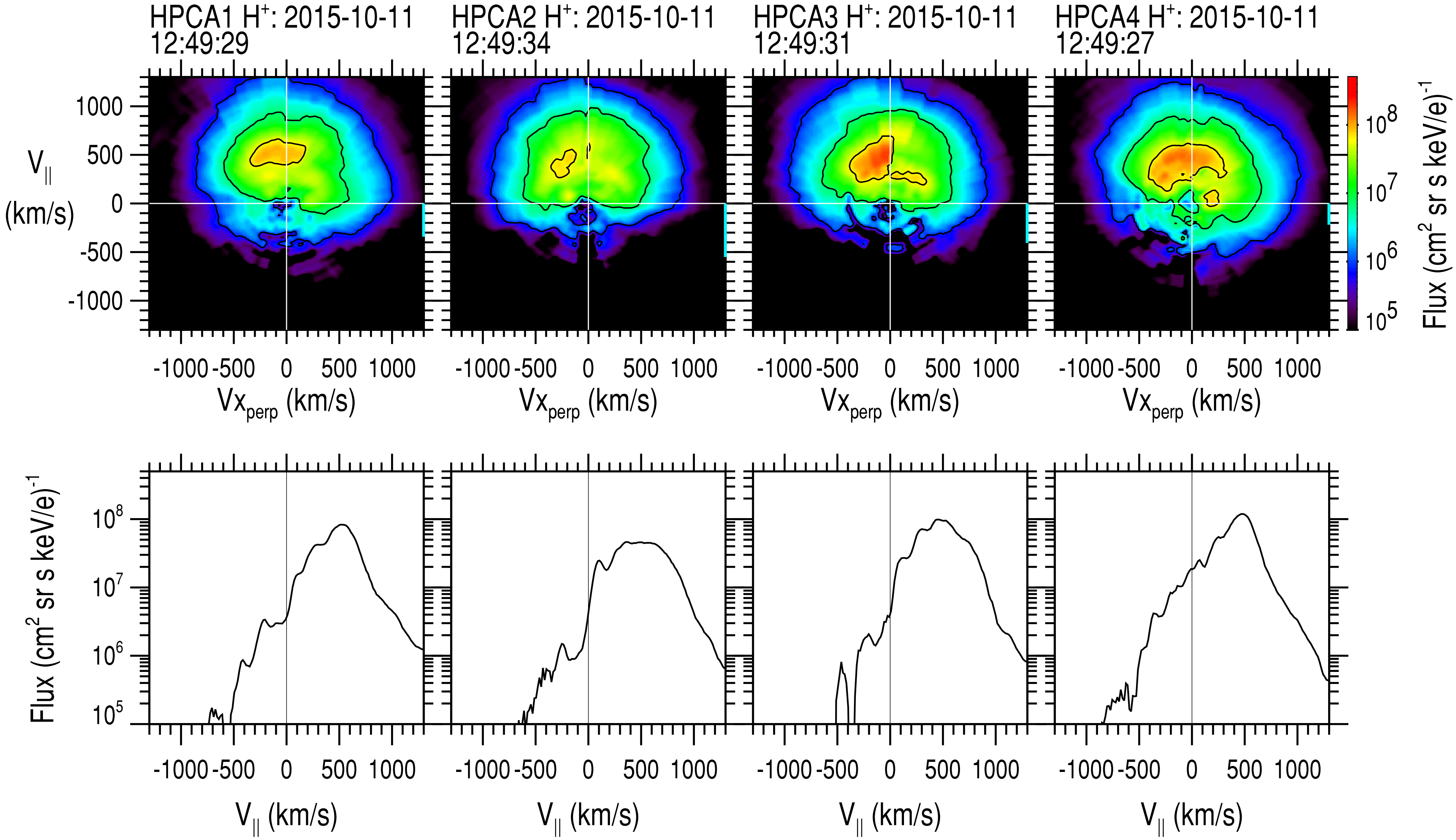

HPCA1 H+: 2015-10-11
12:49:29
HPCA2 H+: 2015-10-11
12:49:34
HPCA3 H+: 2015-10-11
12:49:31
HPCA4 H+: 2015-10-11
12:49:27
V|| (km/s)
Vxperp (km/s)
Flux (cm2 sr s keV/e)-1
V|| (km/s)

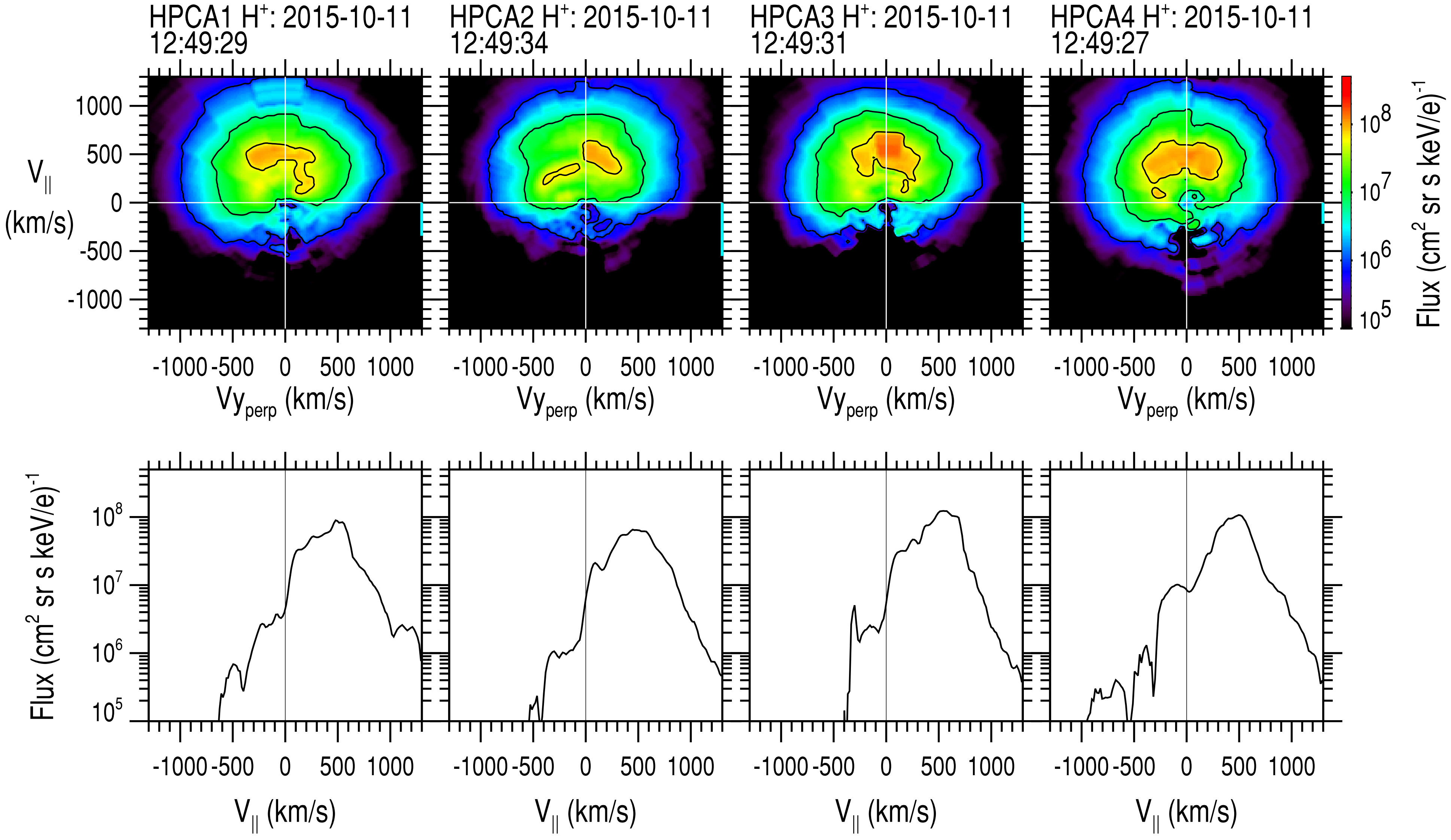

HPCA1 H+: 2015-10-11 12:49:29
HPCA2 H+: 2015-10-11 12:49:34
HPCA3 H+: 2015-10-11 12:49:31
HPCA4 H+: 2015-10-11 12:49:27
V|| (km/s)
Vyperp (km/s)
Flux (cm2 sr s keV/e)-1

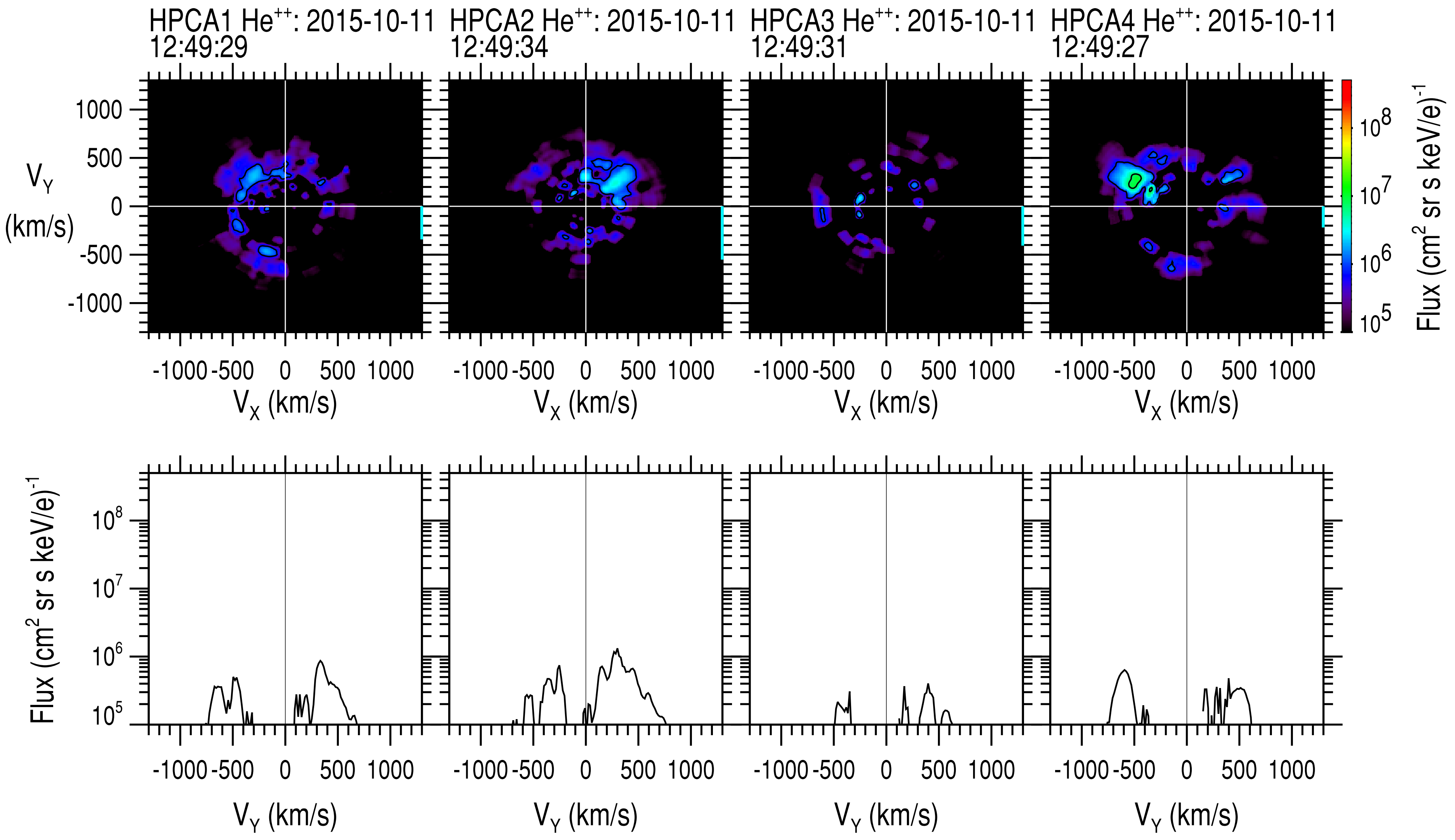

HPCA1 He++: 2015-10-11 12:49:29
HPCA2 He++: 2015-10-11 12:49:34
HPCA3 He++: 2015-10-11 12:49:31
HPCA4 He++: 2015-10-11 12:49:27
V_Y (km/s)
V_X (km/s)
-1000 -500 0 500 1000
Flux (cm^2 sr s keV/e)^-1
10^5 10^6 10^7 10^8
V_Y (km/s)

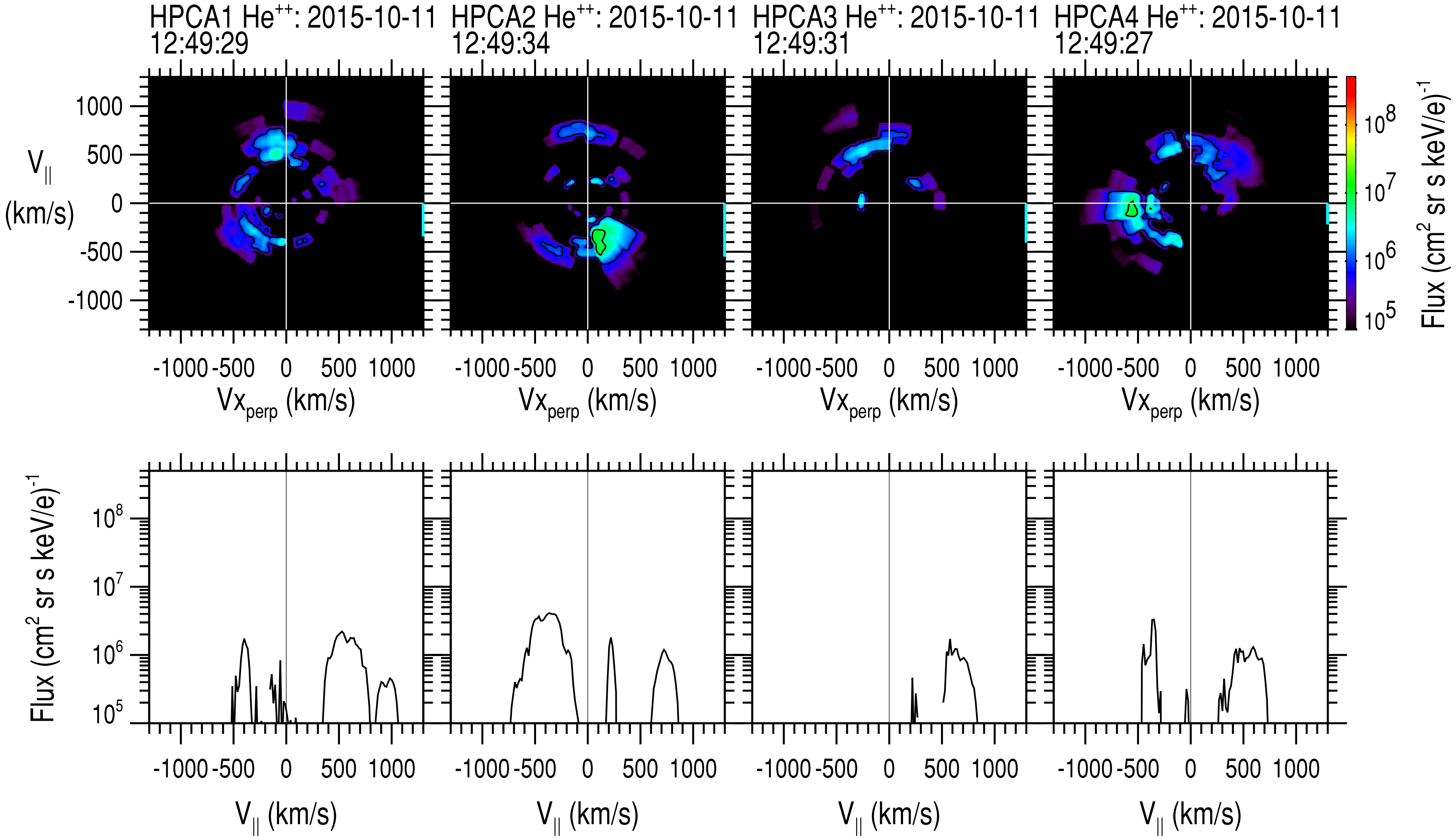

HPCA1 He++: 2015-10-11 12:49:29
HPCA2 He++: 2015-10-11 12:49:34
HPCA3 He++: 2015-10-11 12:49:31
HPCA4 He++: 2015-10-11 12:49:27
V|| (km/s)
1000
500
0
-500
-1000
-1000 -500 0 500 1000
Vxperp (km/s)
Flux (cm2 sr s keV/e)-1
10^5
10^6
10^7
10^8
V|| (km/s)

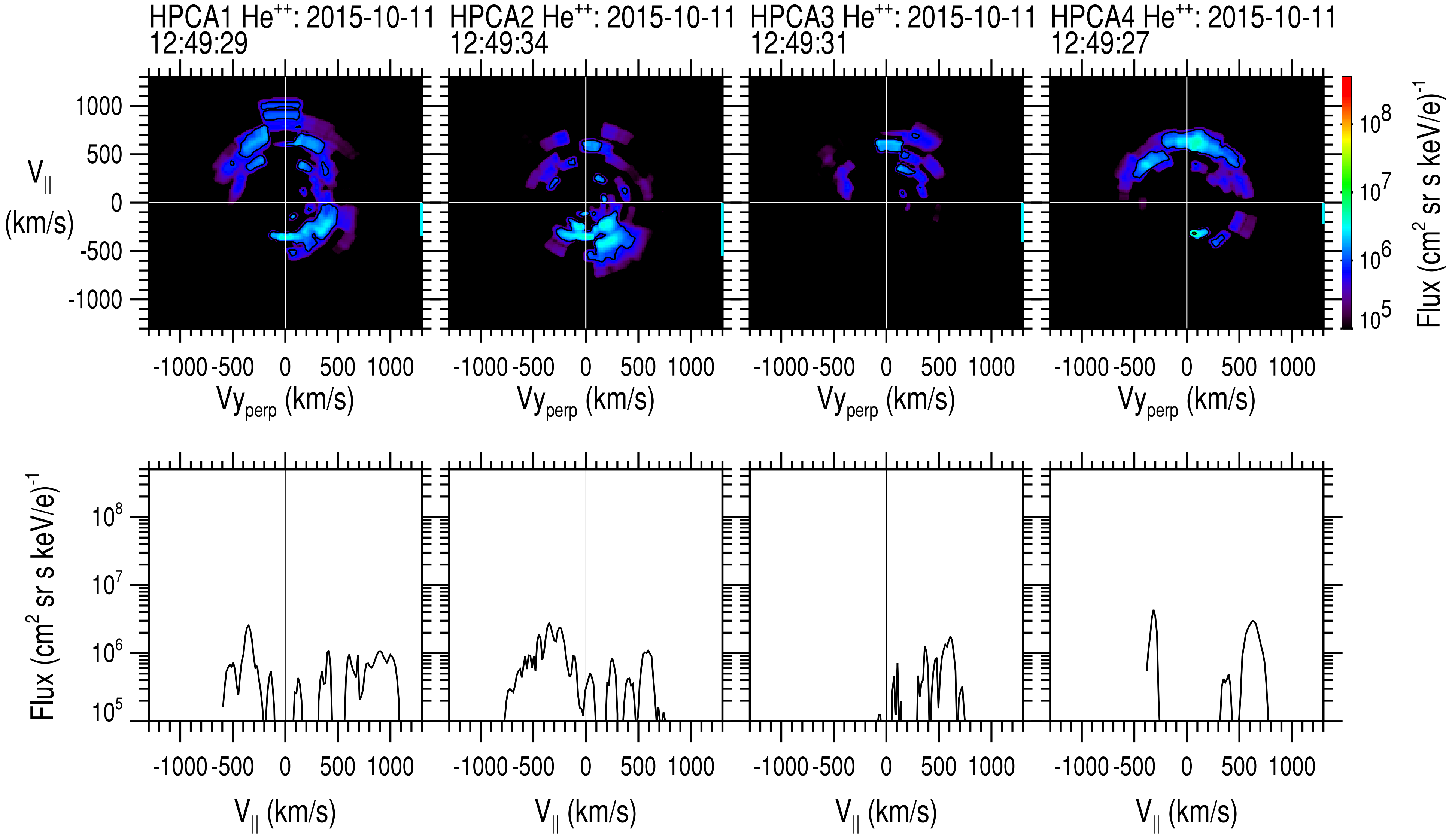

HPCA1 He++: 2015-10-11 12:49:29
HPCA2 He++: 2015-10-11 12:49:34
HPCA3 He++: 2015-10-11 12:49:31
HPCA4 He++: 2015-10-11 12:49:27
V|| (km/s)
Vyperp (km/s)
Flux (cm2 sr s keV/e)-1
-1000 -500 0 500 1000
105 106 107 108

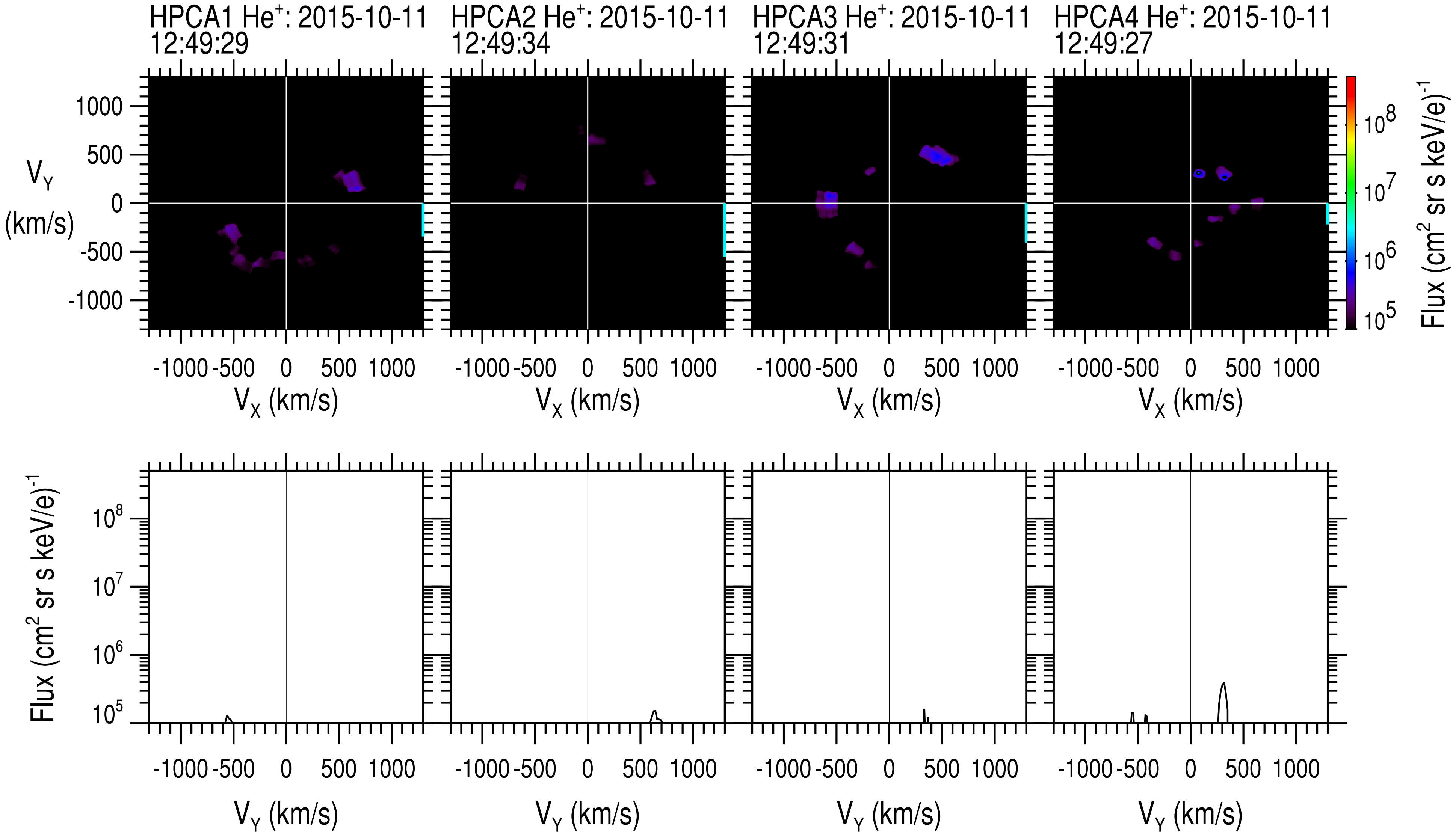

HPCA1 He+: 2015-10-11
12:49:29
HPCA2 He+: 2015-10-11
12:49:34
HPCA3 He+: 2015-10-11
12:49:31
HPCA4 He+: 2015-10-11
12:49:27
VY (km/s)
VX (km/s)
Flux (cm2 sr s keV/e)-1

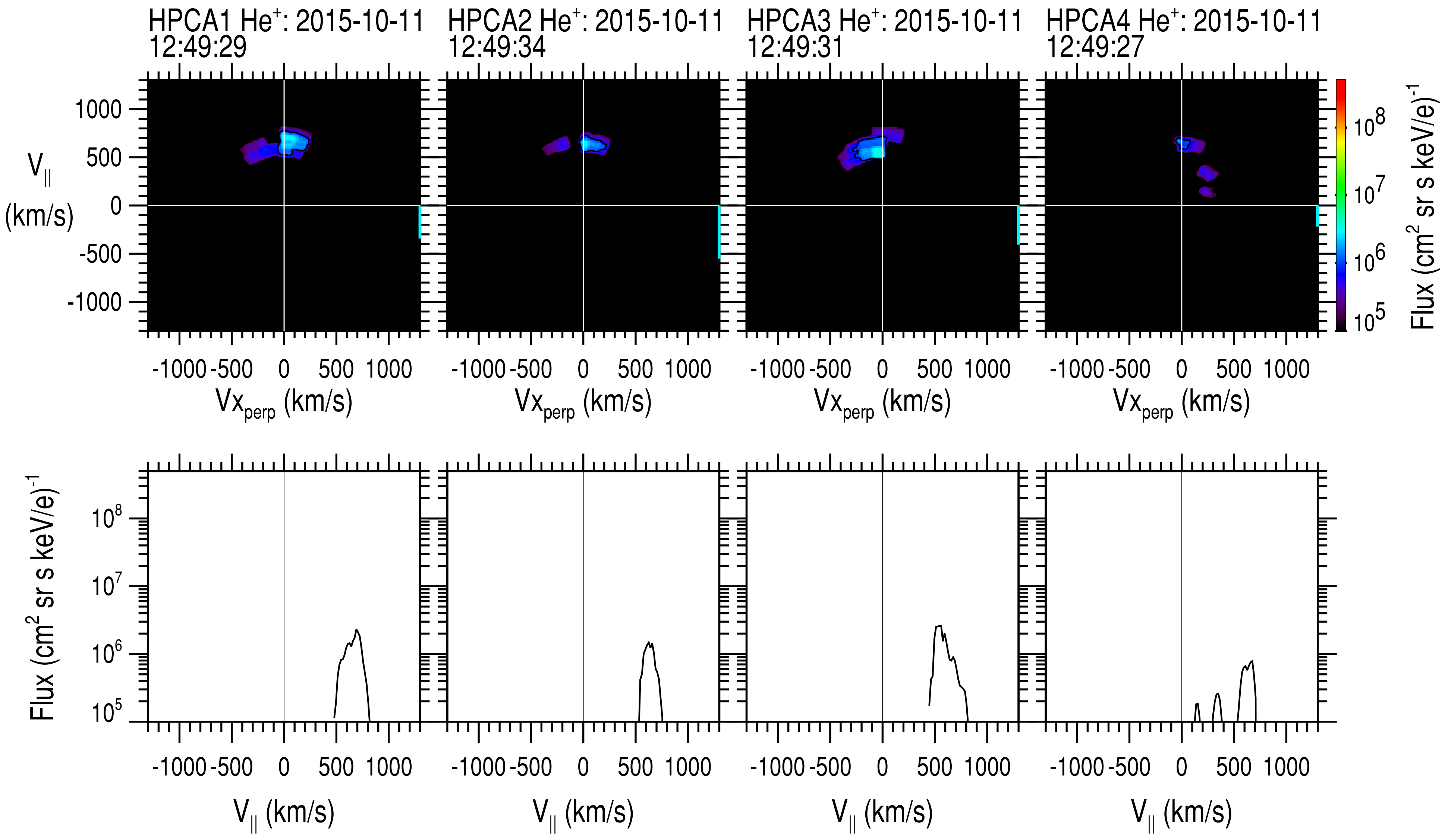
HPCA1 He+: 2015-10-11 12:49:29
HPCA2 He+: 2015-10-11 12:49:34
HPCA3 He+: 2015-10-11 12:49:31
HPCA4 He+: 2015-10-11 12:49:27
V|| (km/s)
Vxperp (km/s)
Flux (cm2 sr s keV/e)-1
V|| (km/s)

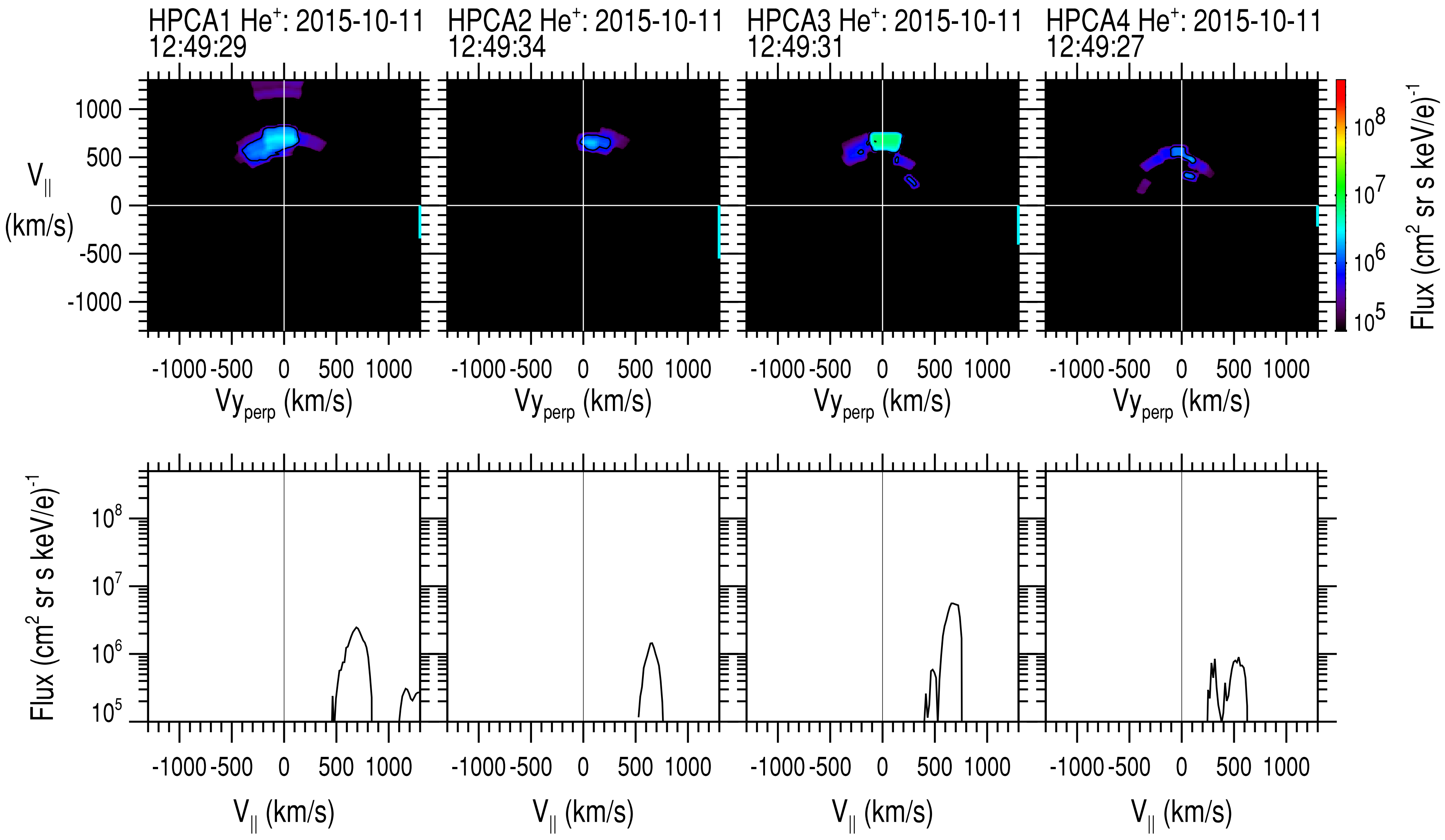
HPCA1 He+: 2015-10-11 12:49:29
HPCA2 He+: 2015-10-11 12:49:34
HPCA3 He+: 2015-10-11 12:49:31
HPCA4 He+: 2015-10-11 12:49:27
V|| (km/s)
Vyperp (km/s)
Flux (cm2 sr s keV/e)-1

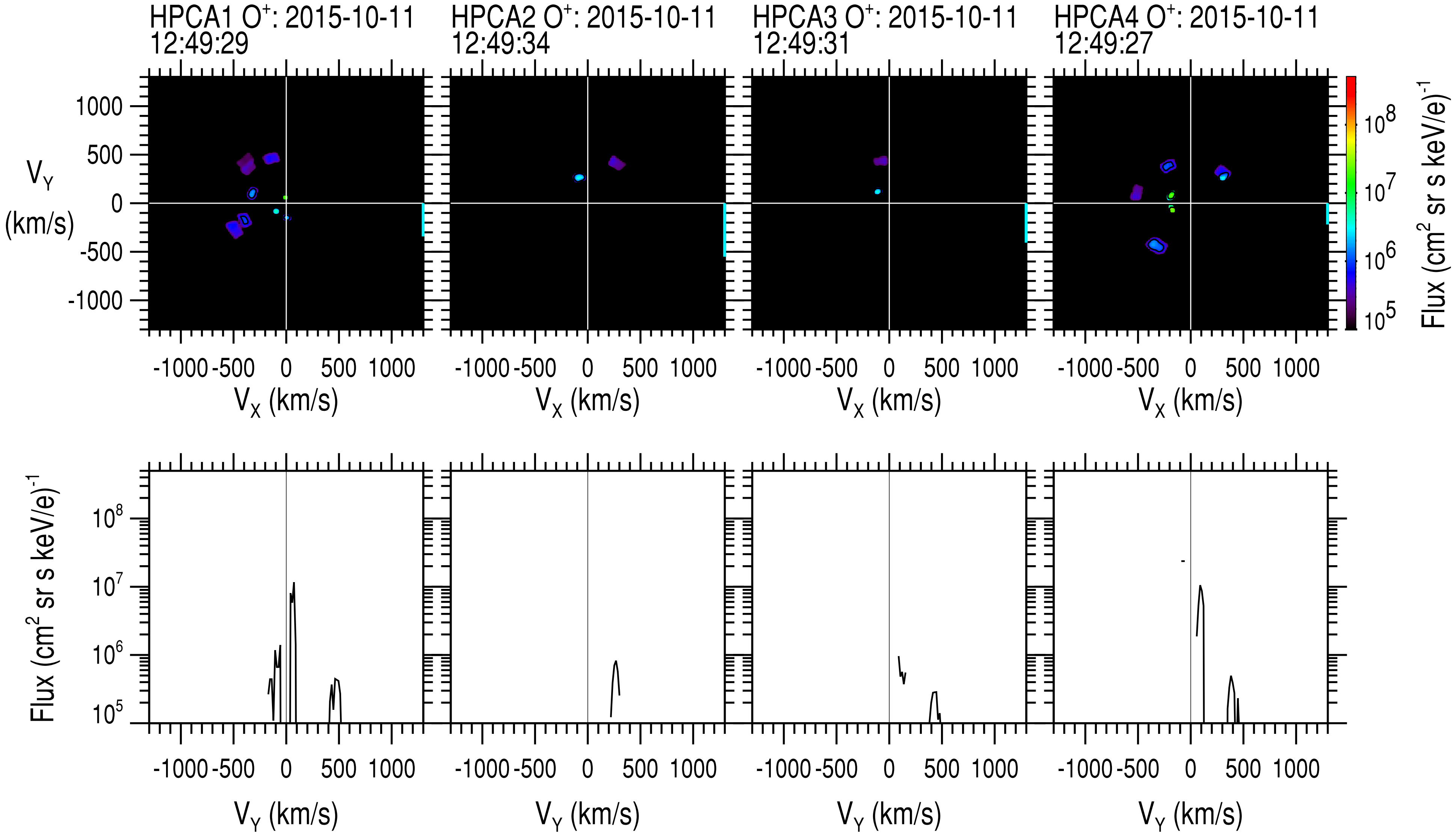
HPCA1 O+: 2015-10-11 12:49:29
HPCA2 O+: 2015-10-11 12:49:34
HPCA3 O+: 2015-10-11 12:49:31
HPCA4 O+: 2015-10-11 12:49:27
VY (km/s)
VX (km/s)
Flux (cm2 sr s keV/e)-1
-1000 -500 0 500 1000
10^5 10^6 10^7 10^8

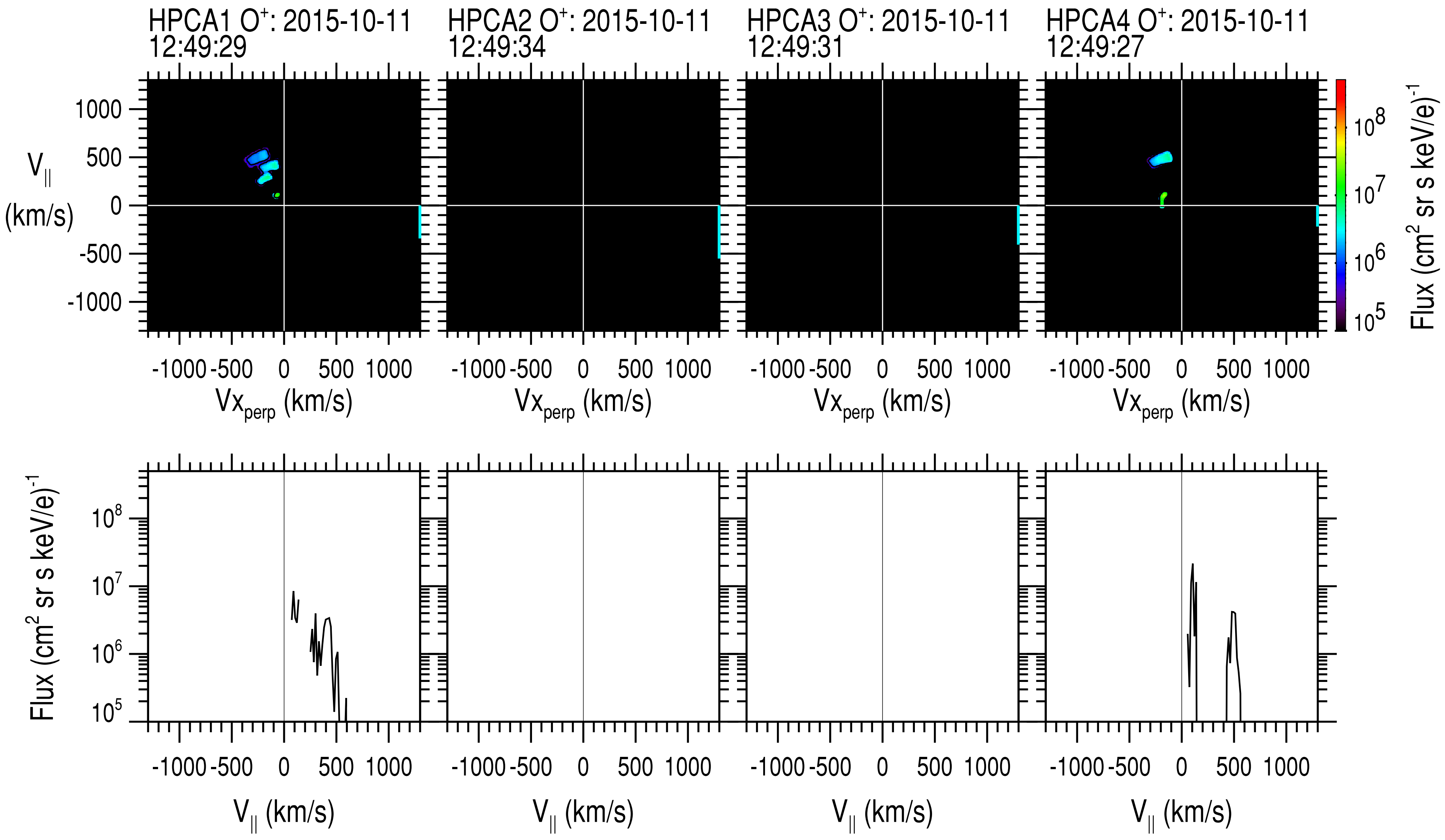

HPCA1 O+: 2015-10-11 12:49:29
HPCA2 O+: 2015-10-11 12:49:34
HPCA3 O+: 2015-10-11 12:49:31
HPCA4 O+: 2015-10-11 12:49:27
V|| (km/s)
Vxperp (km/s)
Flux (cm2 sr s keV/e)-1

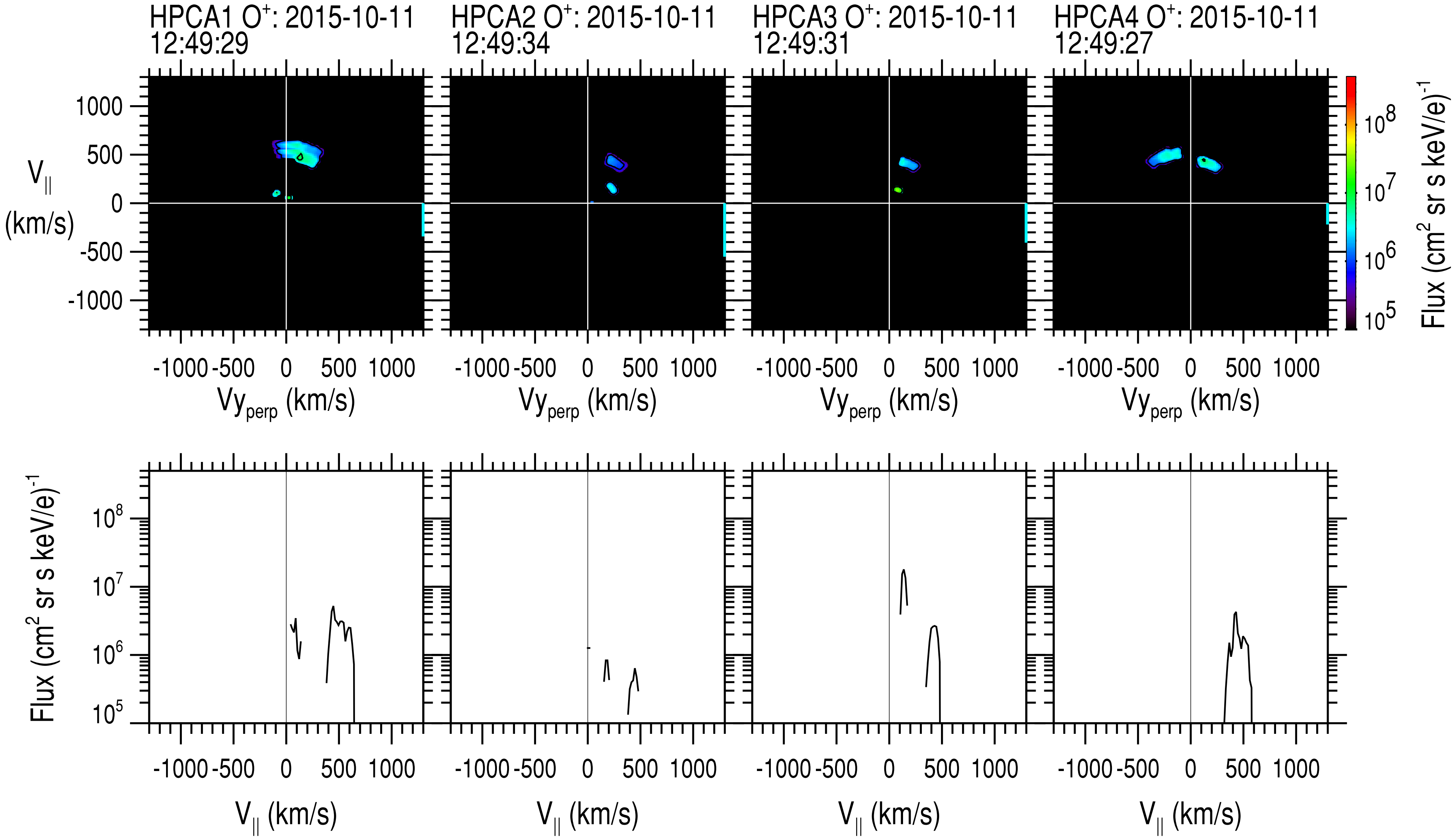

HPCA1 O+: 2015-10-11
12:49:29
HPCA2 O+: 2015-10-11
12:49:34
HPCA3 O+: 2015-10-11
12:49:31
HPCA4 O+: 2015-10-11
12:49:27
V|| (km/s)
Vyperp (km/s)
Flux (cm2 sr s keV/e)-1

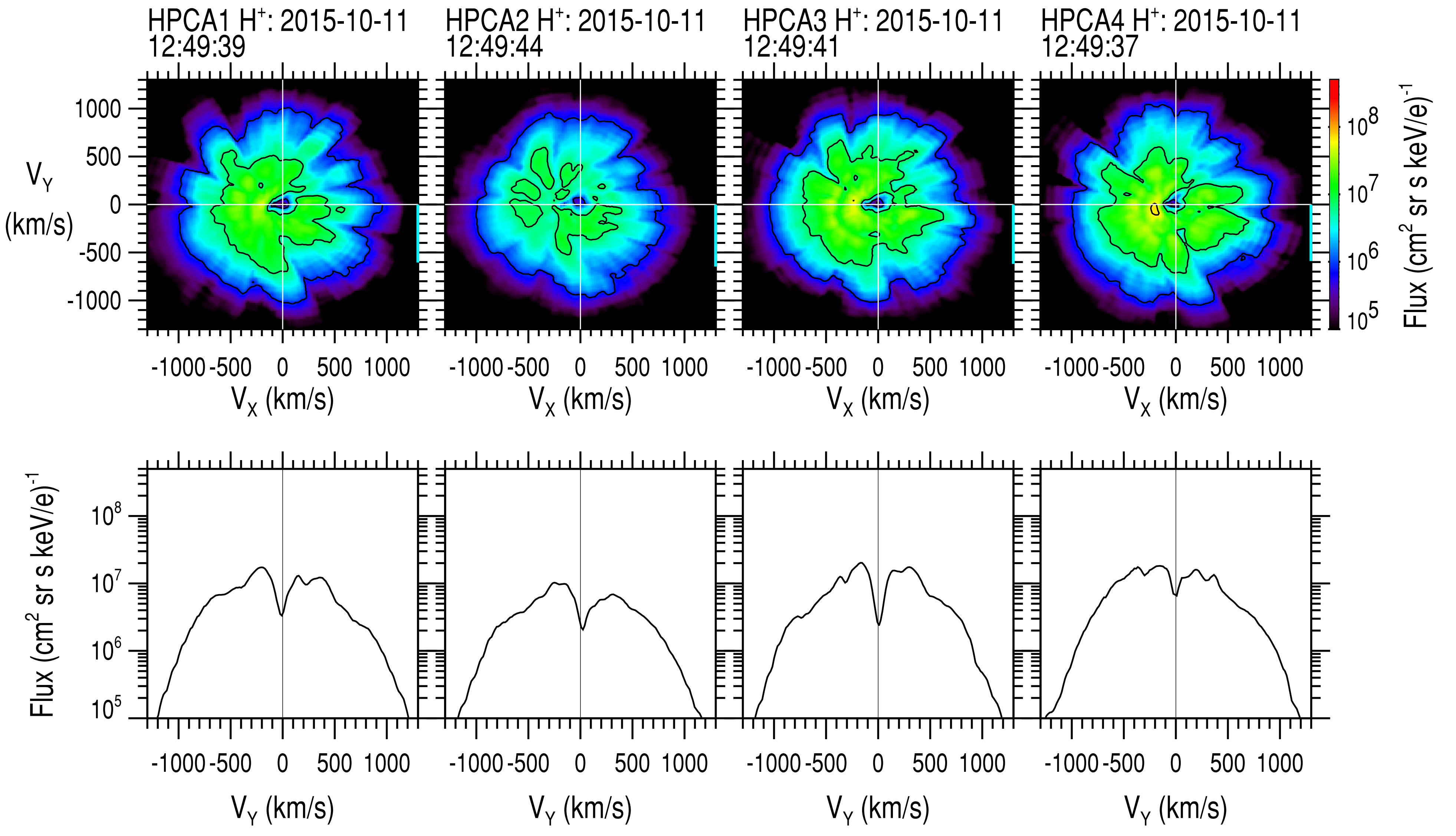
HPCA1 H+: 2015-10-11 12:49:39
HPCA2 H+: 2015-10-11 12:49:44
HPCA3 H+: 2015-10-11 12:49:41
HPCA4 H+: 2015-10-11 12:49:37
V_Y (km/s)
V_X (km/s)
Flux (cm^2 sr s keV/e)^-1
V_Y (km/s)

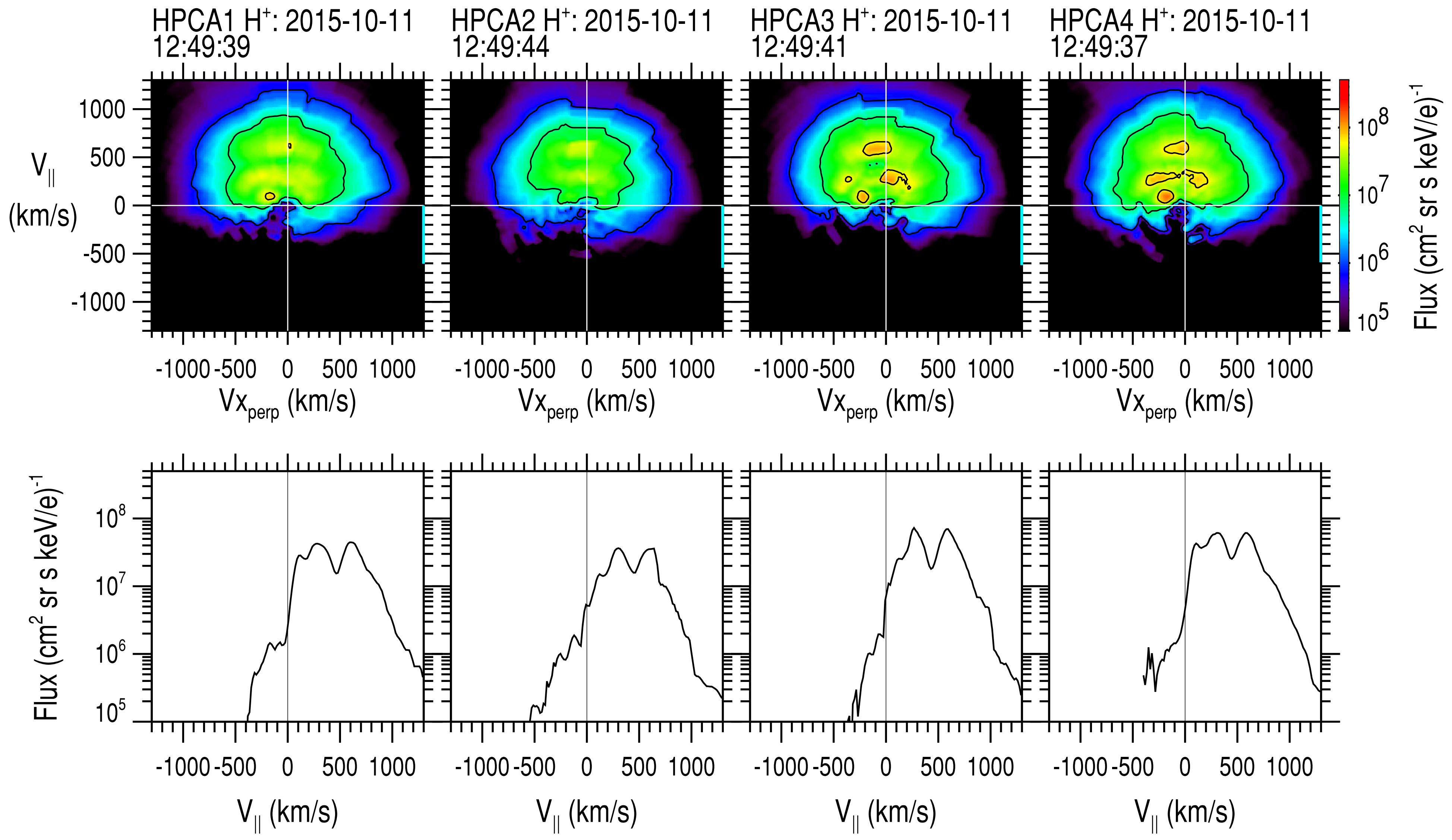

HPCA1 H+: 2015-10-11
12:49:39
HPCA2 H+: 2015-10-11
12:49:44
HPCA3 H+: 2015-10-11
12:49:41
HPCA4 H+: 2015-10-11
12:49:37
V|| (km/s)
Vxperp (km/s)
Flux (cm2 sr s keV/e)-1

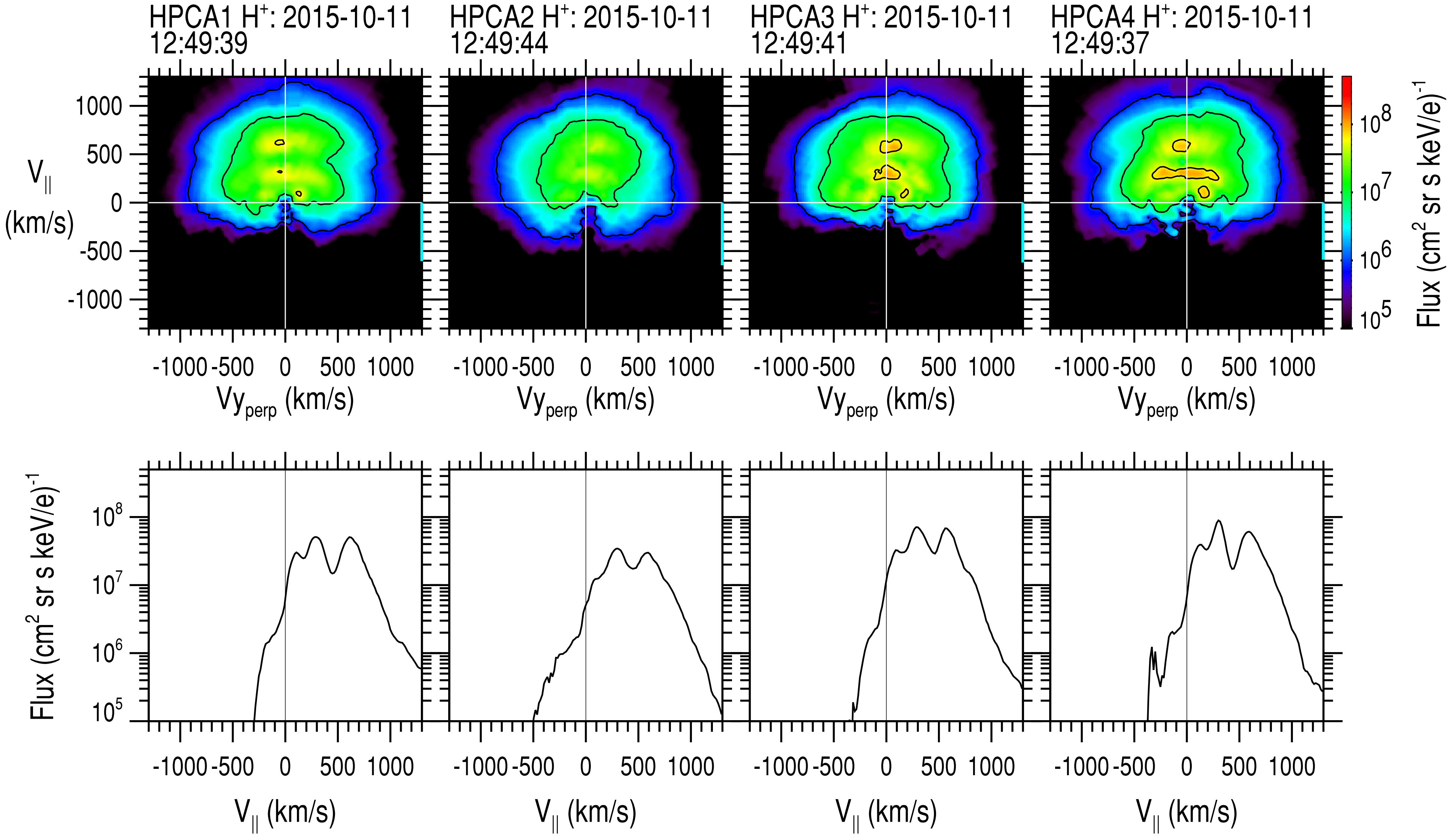

HPCA1 H+: 2015-10-11
12:49:39
HPCA2 H+: 2015-10-11
12:49:44
HPCA3 H+: 2015-10-11
12:49:41
HPCA4 H+: 2015-10-11
12:49:37
V||
(km/s)
1000
500
0
-500
-1000
-1000 -500 0 500 1000
Vy_perp (km/s)
10^5
10^6
10^7
10^8
Flux (cm^2 sr s keV/e)^-1
V|| (km/s)

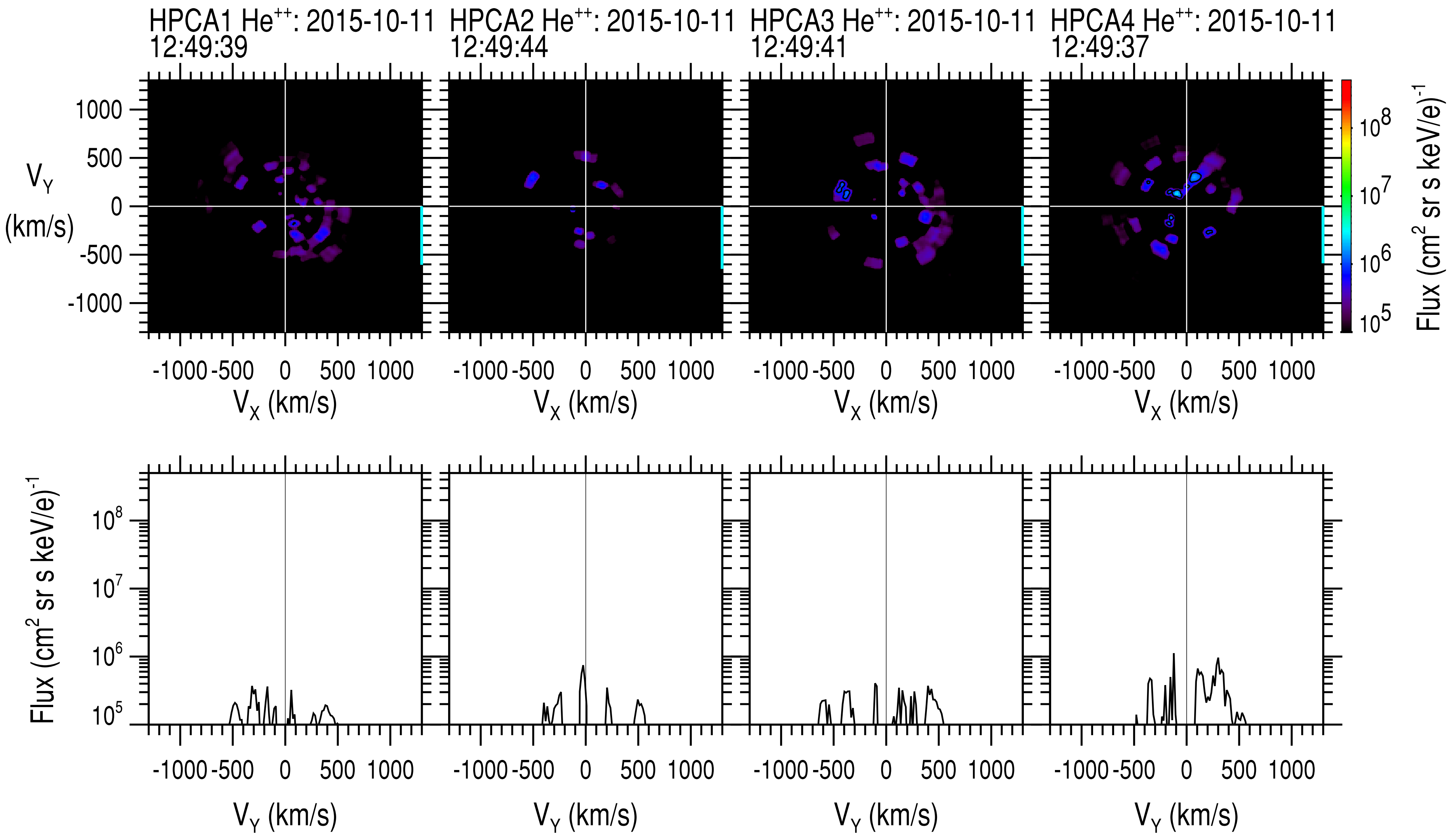

HPCA1 He++: 2015-10-11 12:49:39
HPCA2 He++: 2015-10-11 12:49:44
HPCA3 He++: 2015-10-11 12:49:41
HPCA4 He++: 2015-10-11 12:49:37
V_Y (km/s)
V_X (km/s)
Flux (cm2 sr s keV/e)-1

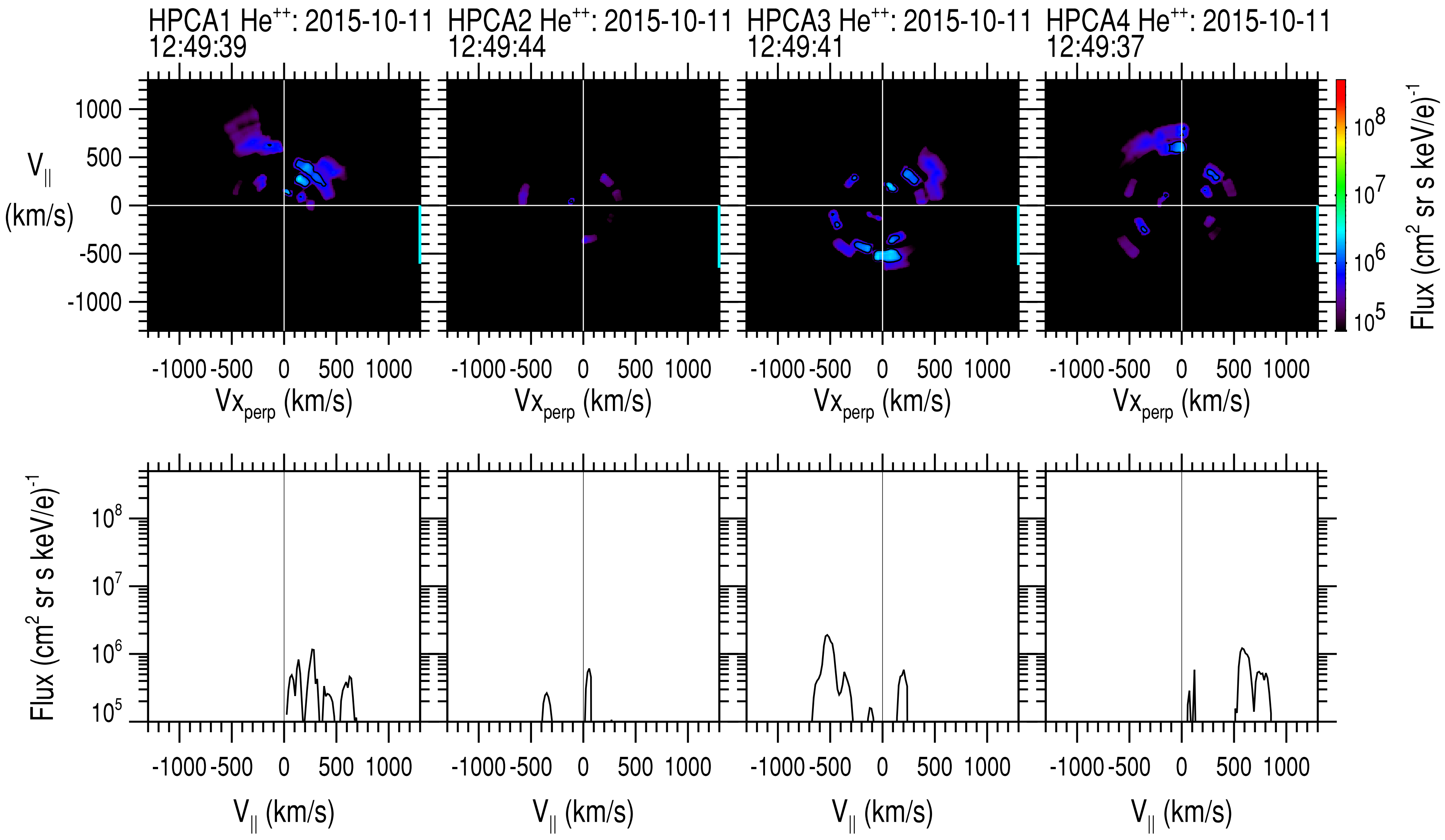

HPCA1 He++: 2015-10-11 12:49:39
HPCA2 He++: 2015-10-11 12:49:44
HPCA3 He++: 2015-10-11 12:49:41
HPCA4 He++: 2015-10-11 12:49:37
V|| (km/s)
Vxperp (km/s)
Flux (cm2 sr s keV/e)-1

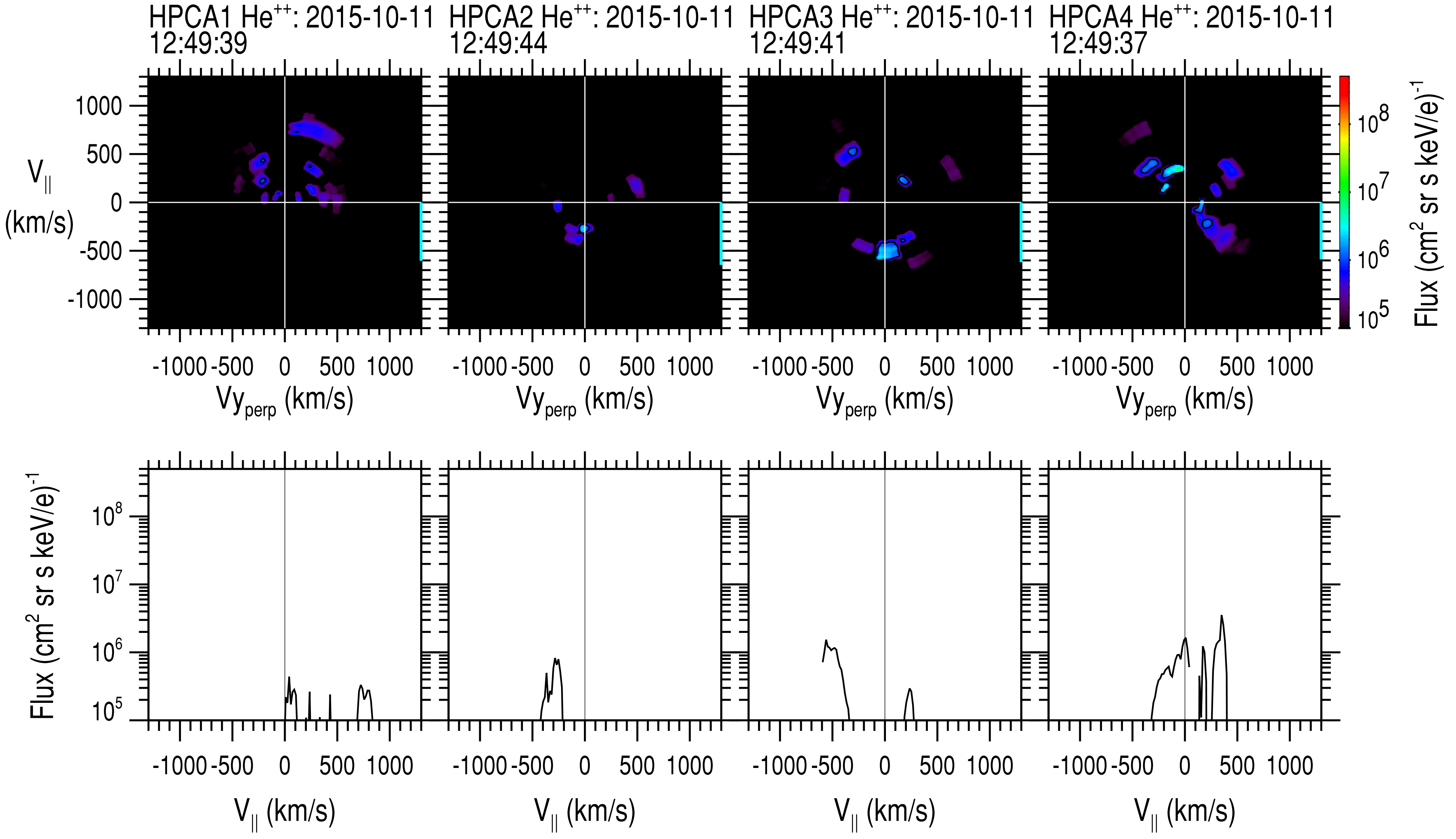

HPCA1 He++: 2015-10-11 12:49:39
HPCA2 He++: 2015-10-11 12:49:44
HPCA3 He++: 2015-10-11 12:49:41
HPCA4 He++: 2015-10-11 12:49:37
V|| (km/s)
Vyperp (km/s)
Flux (cm2 sr s keV/e)-1

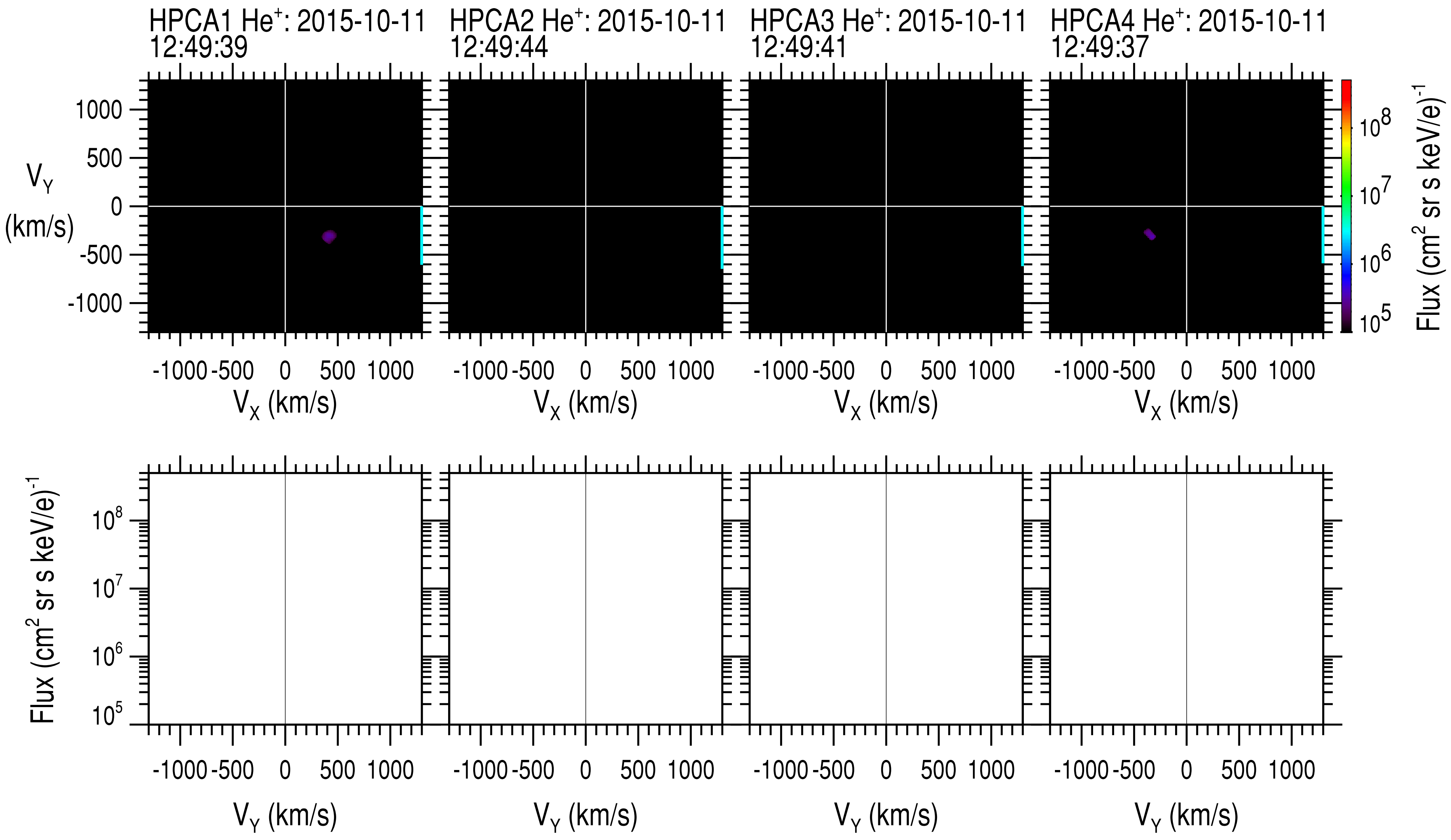

HPCA1 He+: 2015-10-11 12:49:39
HPCA2 He+: 2015-10-11 12:49:44
HPCA3 He+: 2015-10-11 12:49:41
HPCA4 He+: 2015-10-11 12:49:37
V_Y (km/s)
V_X (km/s)
Flux (cm^2 sr s keV/e)^-1

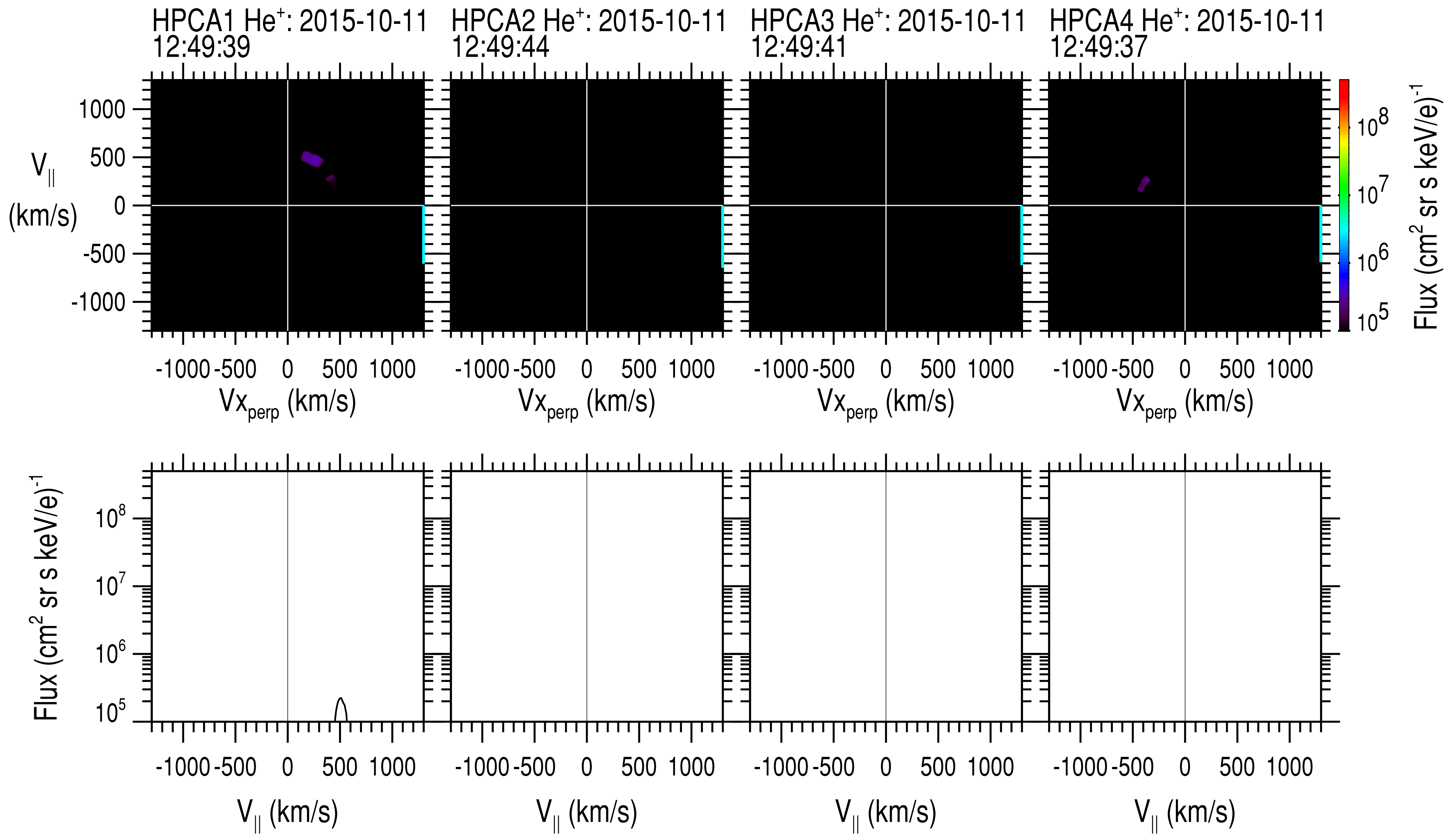

HPCA1 He+: 2015-10-11 12:49:39
HPCA2 He+: 2015-10-11 12:49:44
HPCA3 He+: 2015-10-11 12:49:41
HPCA4 He+: 2015-10-11 12:49:37
V|| (km/s)
Vxperp (km/s)
Flux (cm2 sr s keV/e)-1

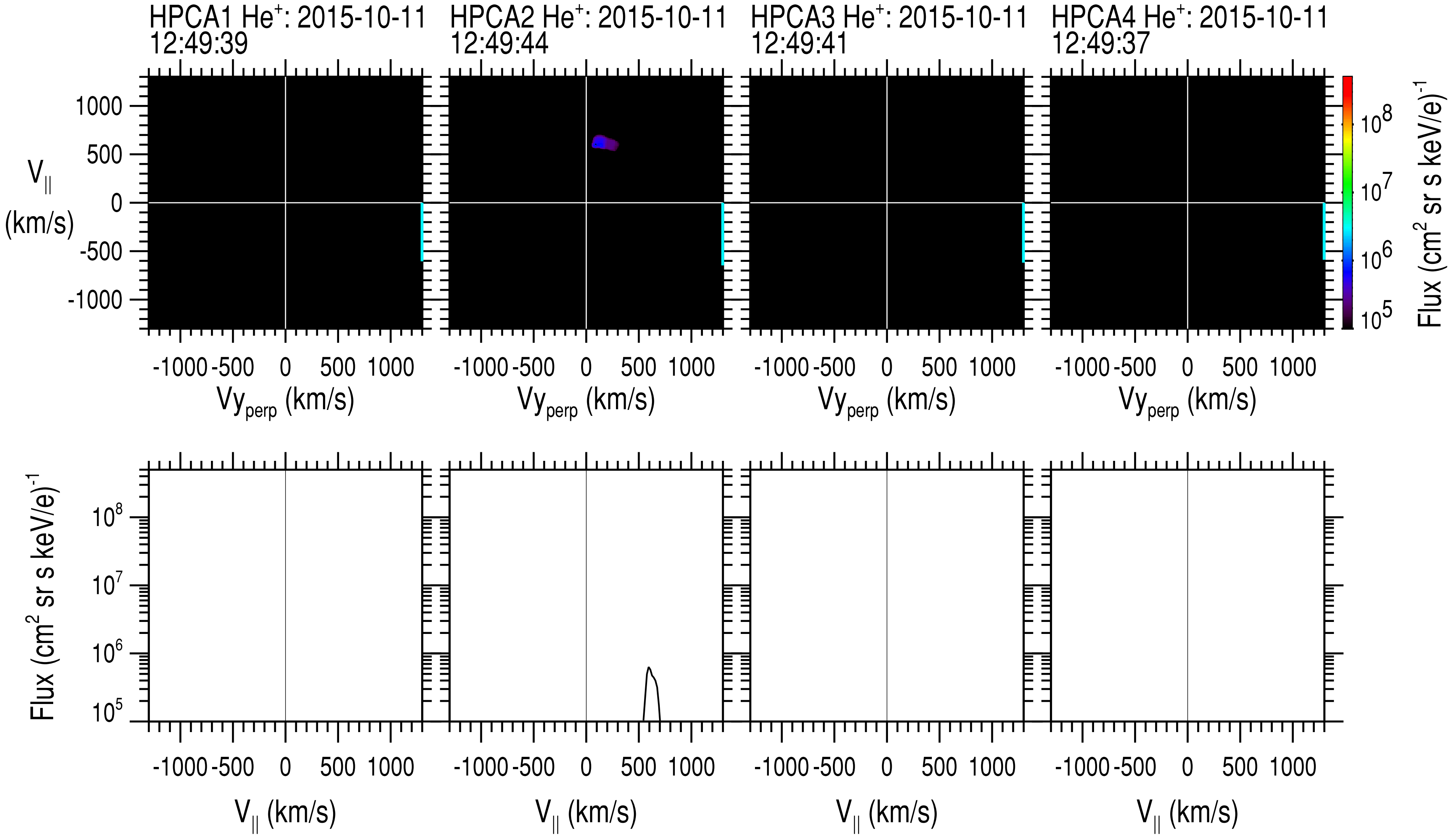

HPCA1 He+: 2015-10-11 12:49:39
HPCA2 He+: 2015-10-11 12:49:44
HPCA3 He+: 2015-10-11 12:49:41
HPCA4 He+: 2015-10-11 12:49:37
V|| (km/s)
Vyperp (km/s)
Flux (cm2 sr s keV/e)-1

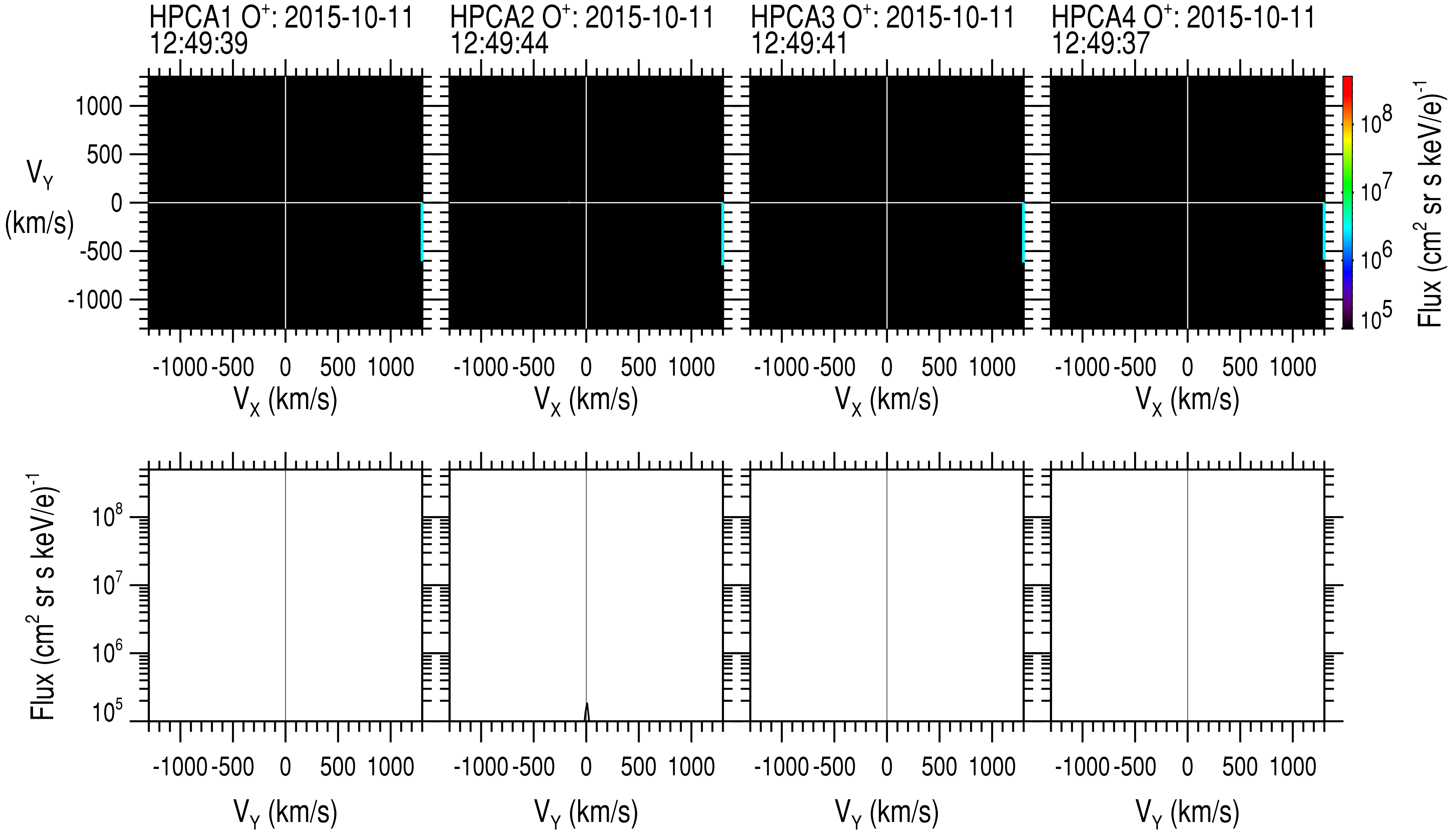
HPCA1 O+: 2015-10-11 12:49:39
HPCA2 O+: 2015-10-11 12:49:44
HPCA3 O+: 2015-10-11 12:49:41
HPCA4 O+: 2015-10-11 12:49:37
VY (km/s)
VX (km/s)
Flux (cm2 sr s keV/e)-1
VY (km/s)

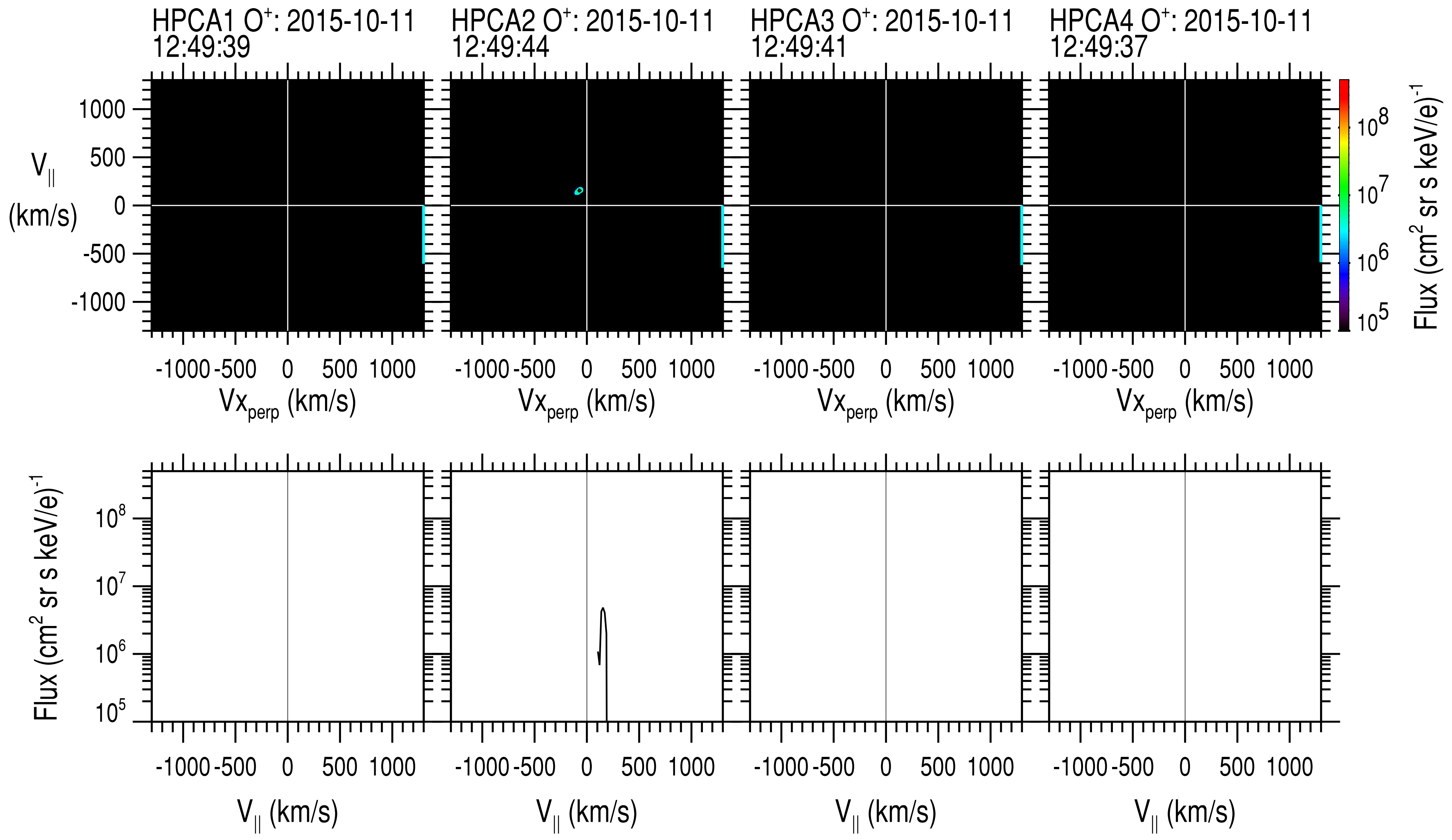

HPCA1 O+: 2015-10-11 12:49:39
HPCA2 O+: 2015-10-11 12:49:44
HPCA3 O+: 2015-10-11 12:49:41
HPCA4 O+: 2015-10-11 12:49:37
V|| (km/s)
Vxperp (km/s)
Flux (cm2 sr s keV/e)-1

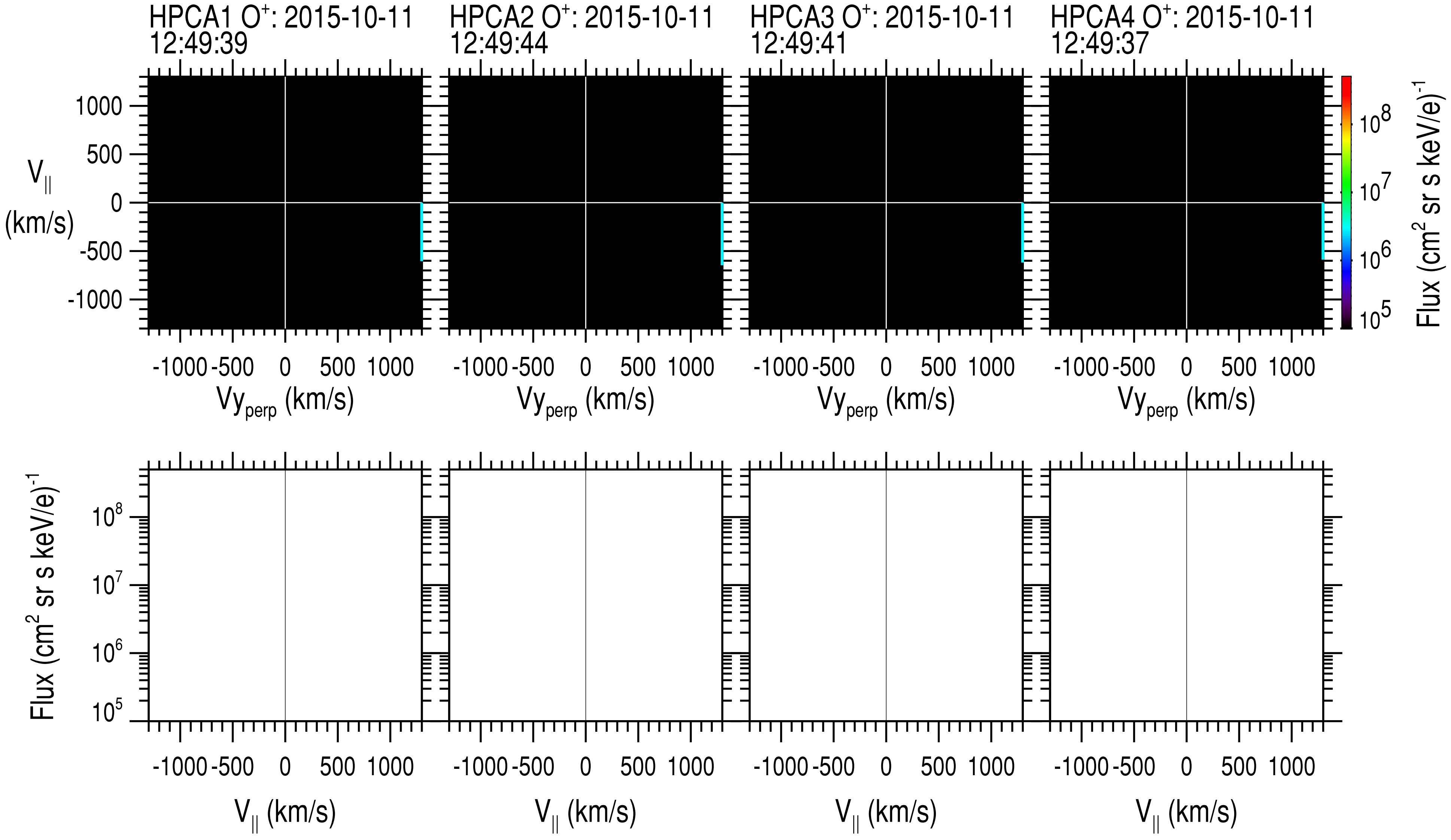

HPCA1 O+: 2015-10-11 12:49:39
HPCA2 O+: 2015-10-11 12:49:44
HPCA3 O+: 2015-10-11 12:49:41
HPCA4 O+: 2015-10-11 12:49:37
V|| (km/s)
1000
500
0
-500
-1000
Vyperp (km/s)
Flux (cm2 sr s keV/e)-1
10^5
10^6
10^7
10^8

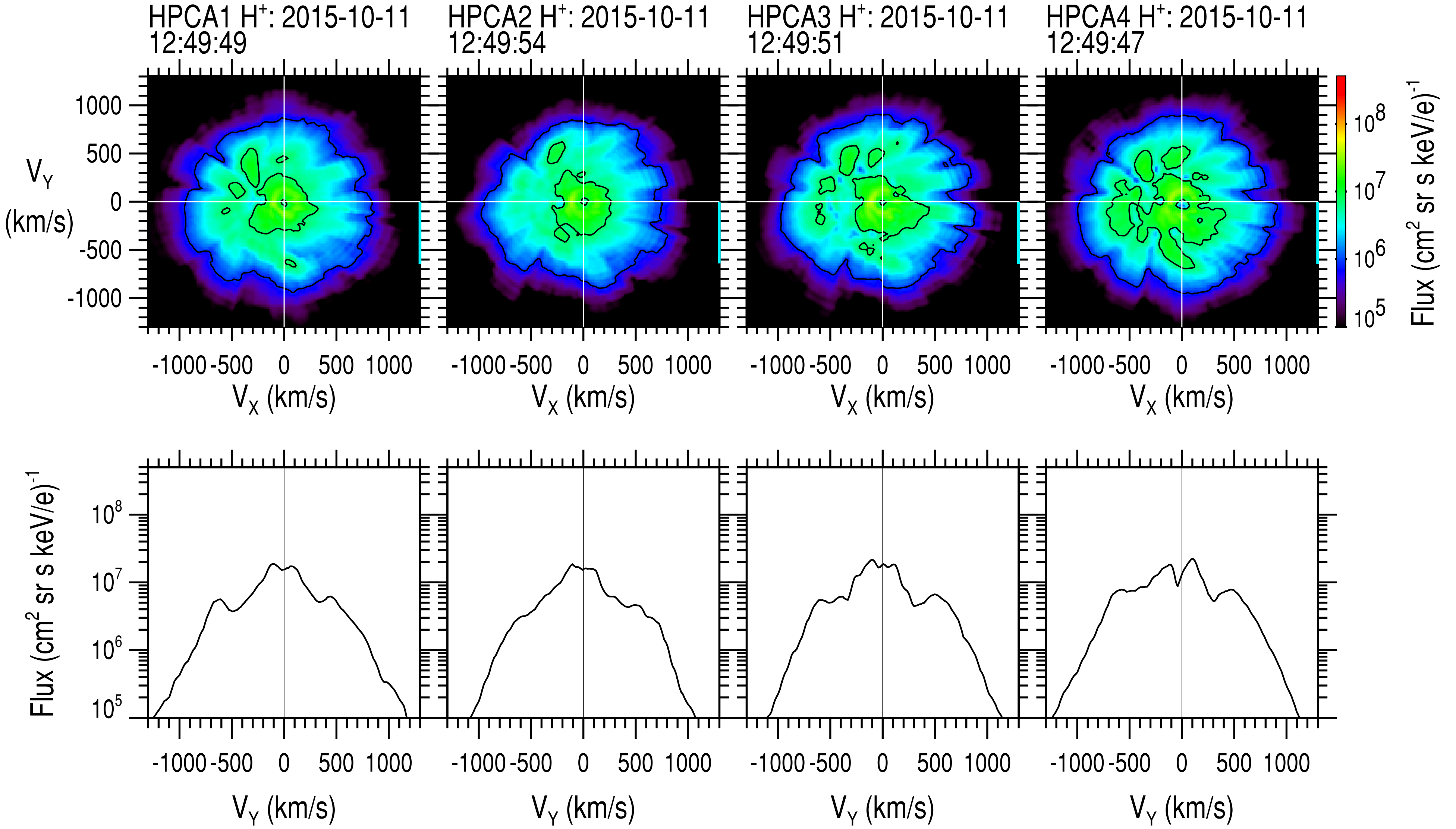

HPCA1 H+: 2015-10-11
12:49:49
HPCA2 H+: 2015-10-11
12:49:54
HPCA3 H+: 2015-10-11
12:49:51
HPCA4 H+: 2015-10-11
12:49:47
V_Y (km/s)
V_X (km/s)
Flux (cm^2 sr s keV/e)^-1
V_Y (km/s)

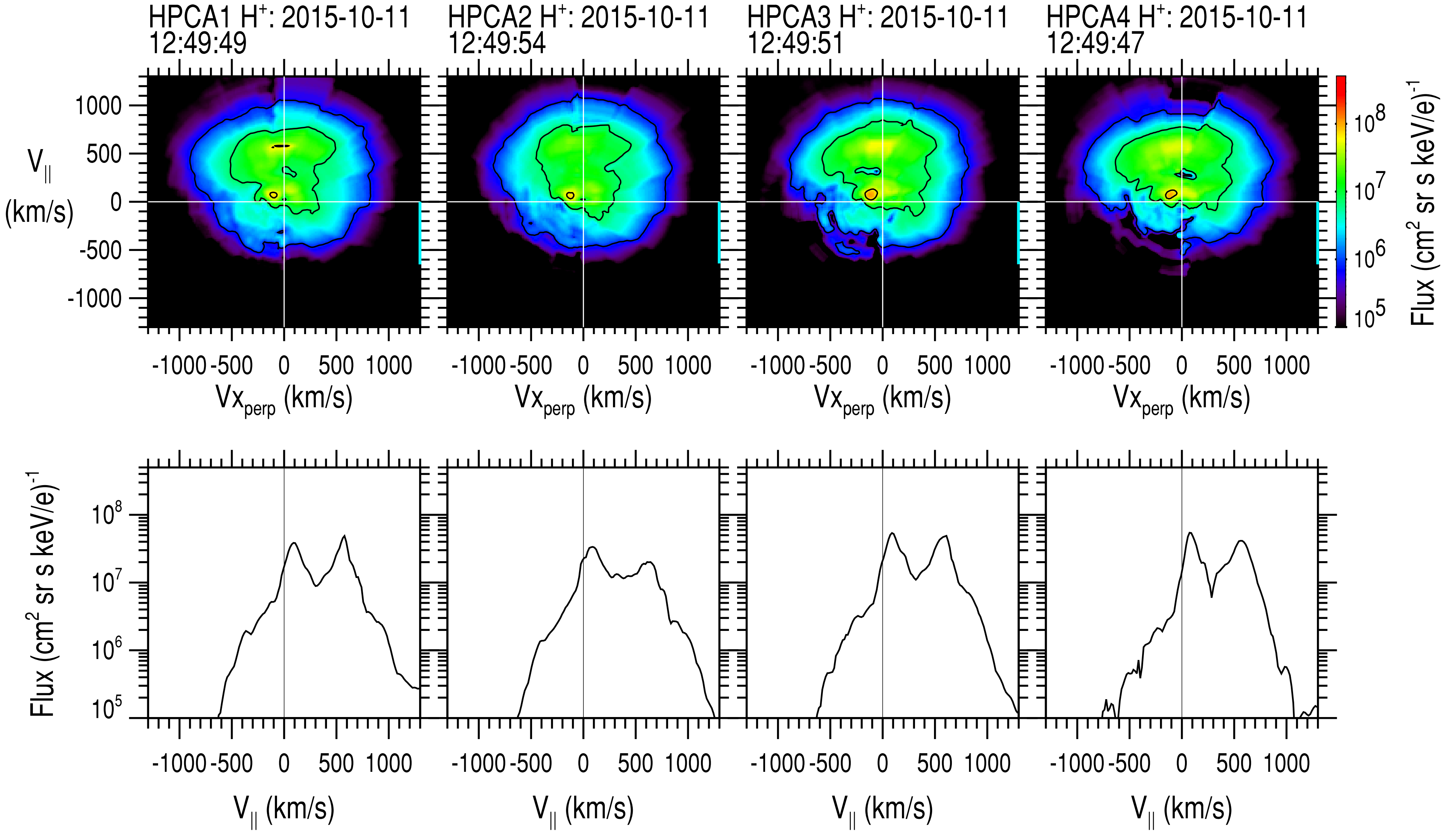

HPCA1 H+: 2015-10-11 12:49:49
HPCA2 H+: 2015-10-11 12:49:54
HPCA3 H+: 2015-10-11 12:49:51
HPCA4 H+: 2015-10-11 12:49:47
V|| (km/s)
Vxperp (km/s)
Flux (cm2 sr s keV/e)-1

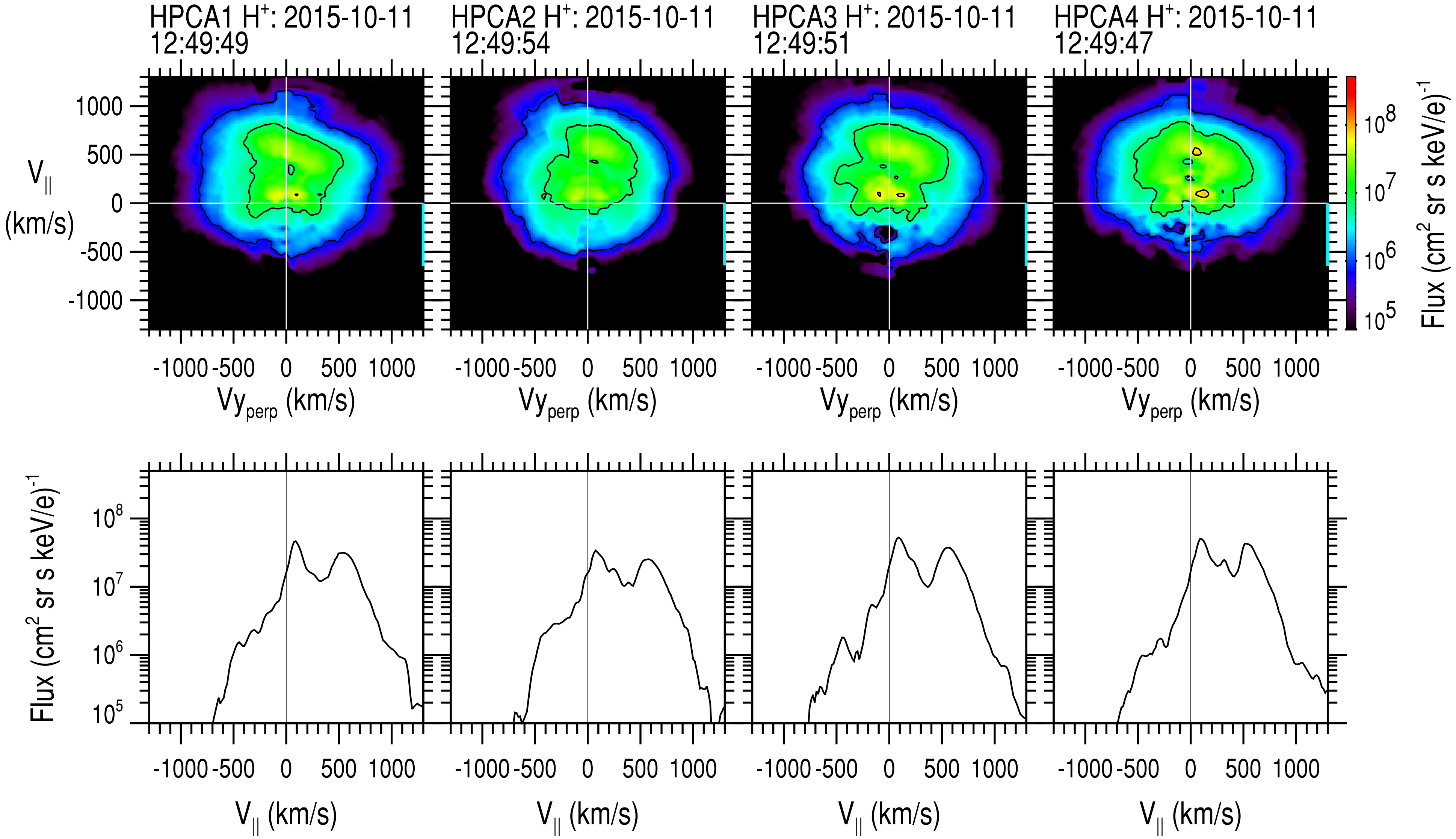

HPCA1 H+: 2015-10-11 12:49:49
HPCA2 H+: 2015-10-11 12:49:54
HPCA3 H+: 2015-10-11 12:49:51
HPCA4 H+: 2015-10-11 12:49:47
V|| (km/s)
Vyperp (km/s)
Flux (cm2 sr s keV/e)-1

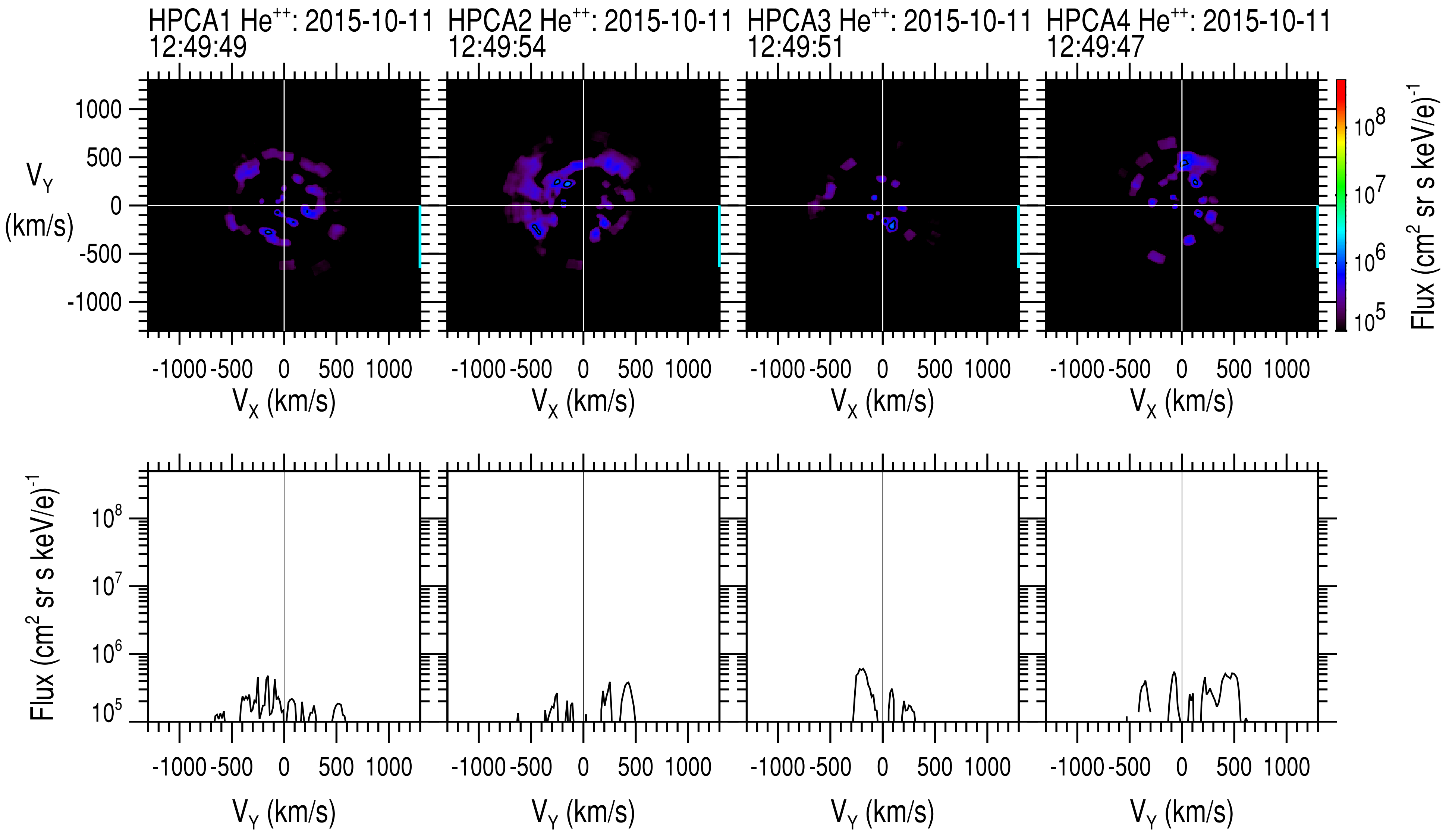
HPCA1 He++: 2015-10-11 12:49:49
HPCA2 He++: 2015-10-11 12:49:54
HPCA3 He++: 2015-10-11 12:49:51
HPCA4 He++: 2015-10-11 12:49:47
VY (km/s)
VX (km/s)
Flux (cm2 sr s keV/e)-1
VY (km/s)

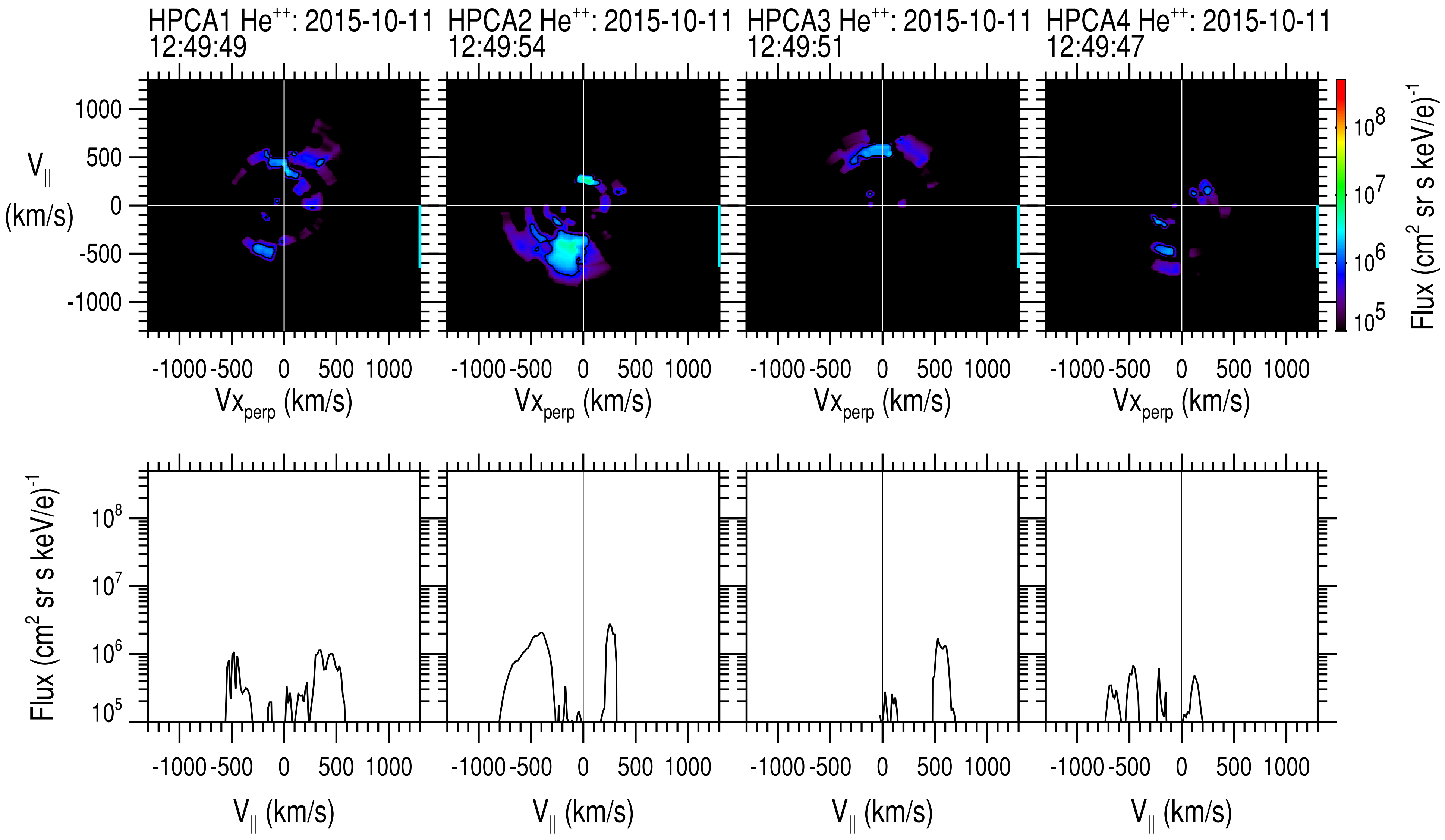
HPCA1 He++: 2015-10-11
12:49:49
HPCA2 He++: 2015-10-11
12:49:54
HPCA3 He++: 2015-10-11
12:49:51
HPCA4 He++: 2015-10-11
12:49:47
V|| (km/s)
Vxperp (km/s)
Flux (cm2 sr s keV/e)-1
V|| (km/s)

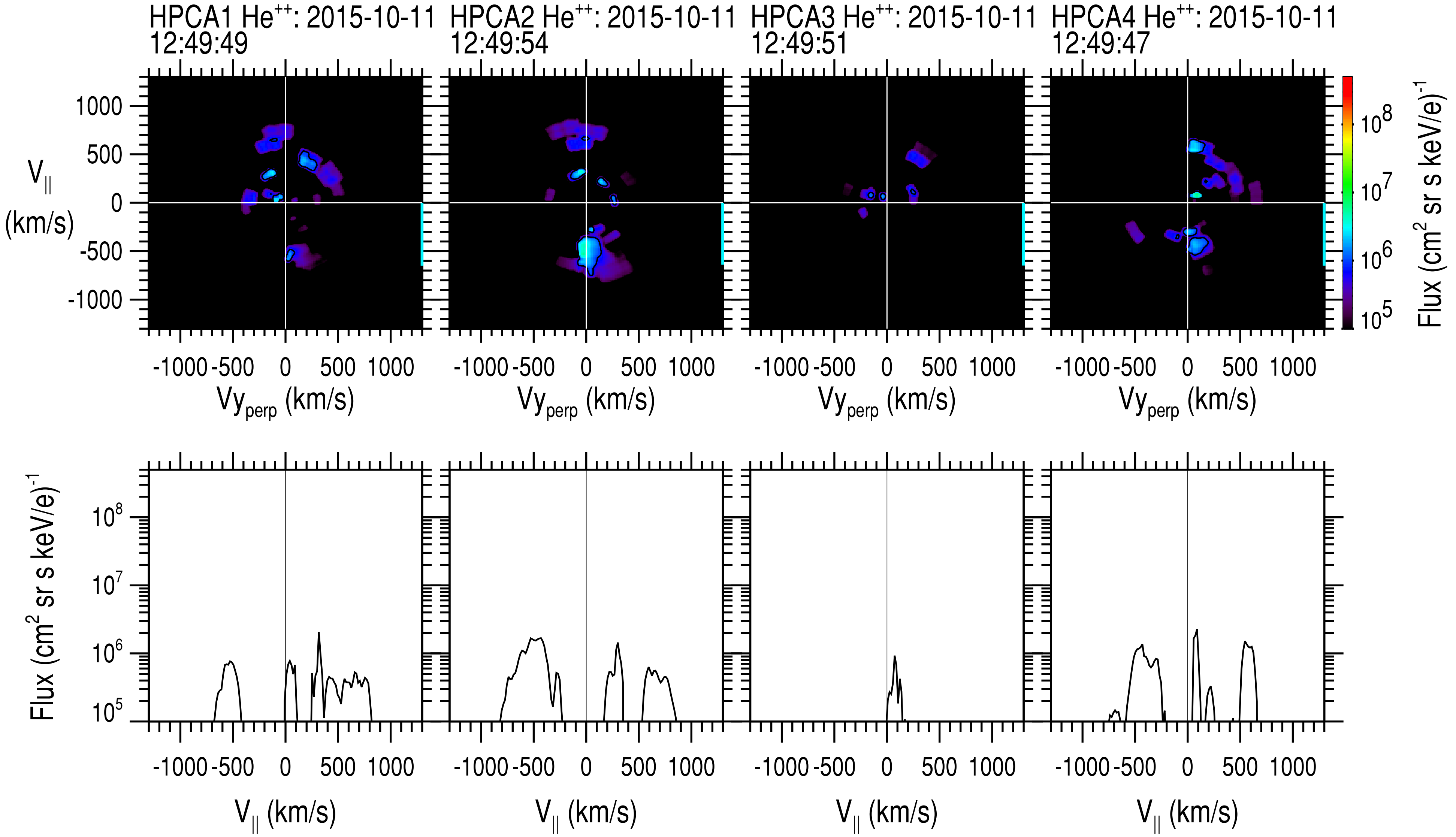

HPCA1 He++: 2015-10-11 12:49:49
HPCA2 He++: 2015-10-11 12:49:54
HPCA3 He++: 2015-10-11 12:49:51
HPCA4 He++: 2015-10-11 12:49:47
V|| (km/s)
Vyperp (km/s)
Flux (cm2 sr s keV/e)-1

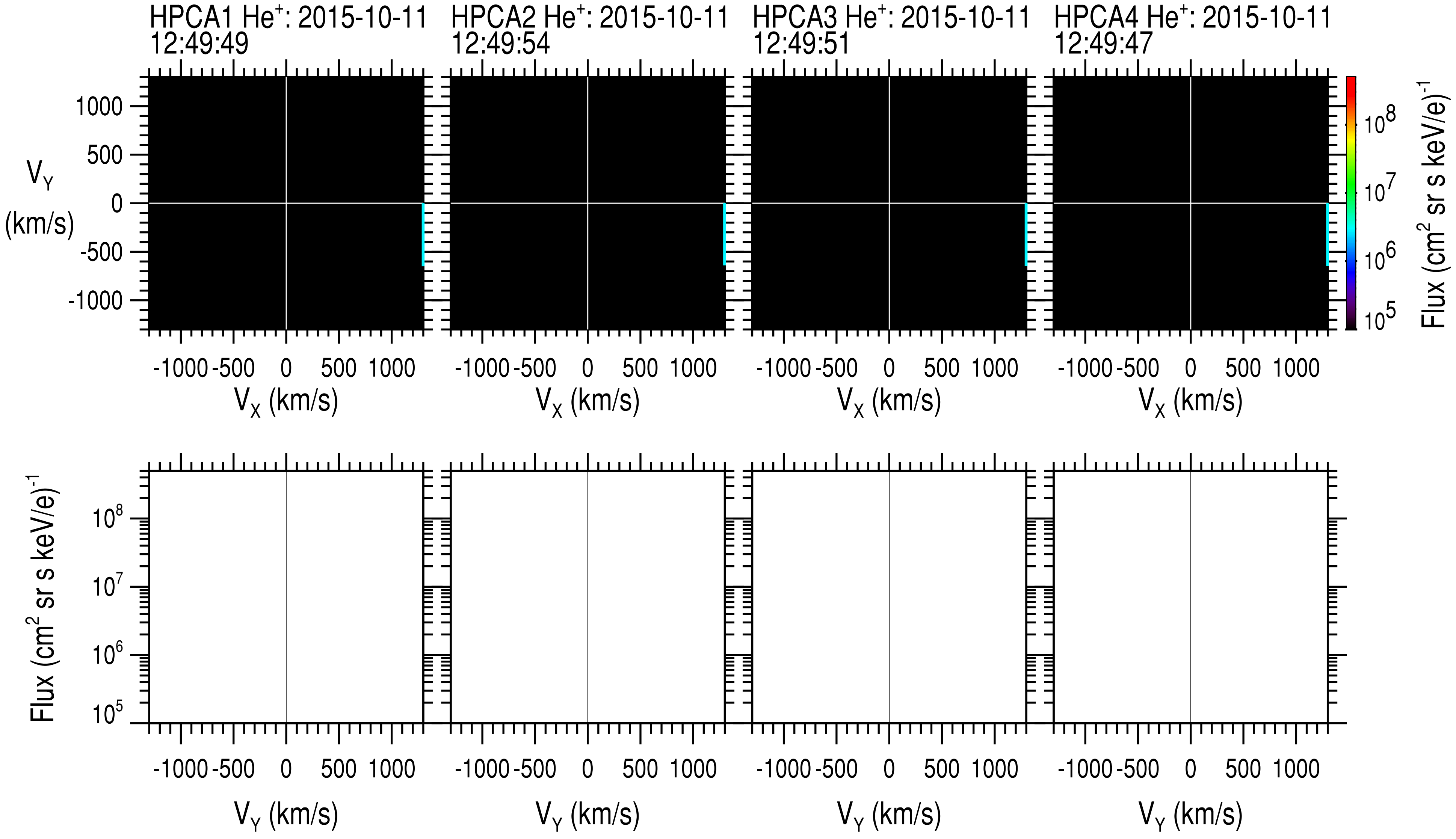

HPCA1 He+: 2015-10-11 12:49:49
HPCA2 He+: 2015-10-11 12:49:54
HPCA3 He+: 2015-10-11 12:49:51
HPCA4 He+: 2015-10-11 12:49:47
VY (km/s)
VX (km/s)
Flux (cm2 sr s keV/e)-1

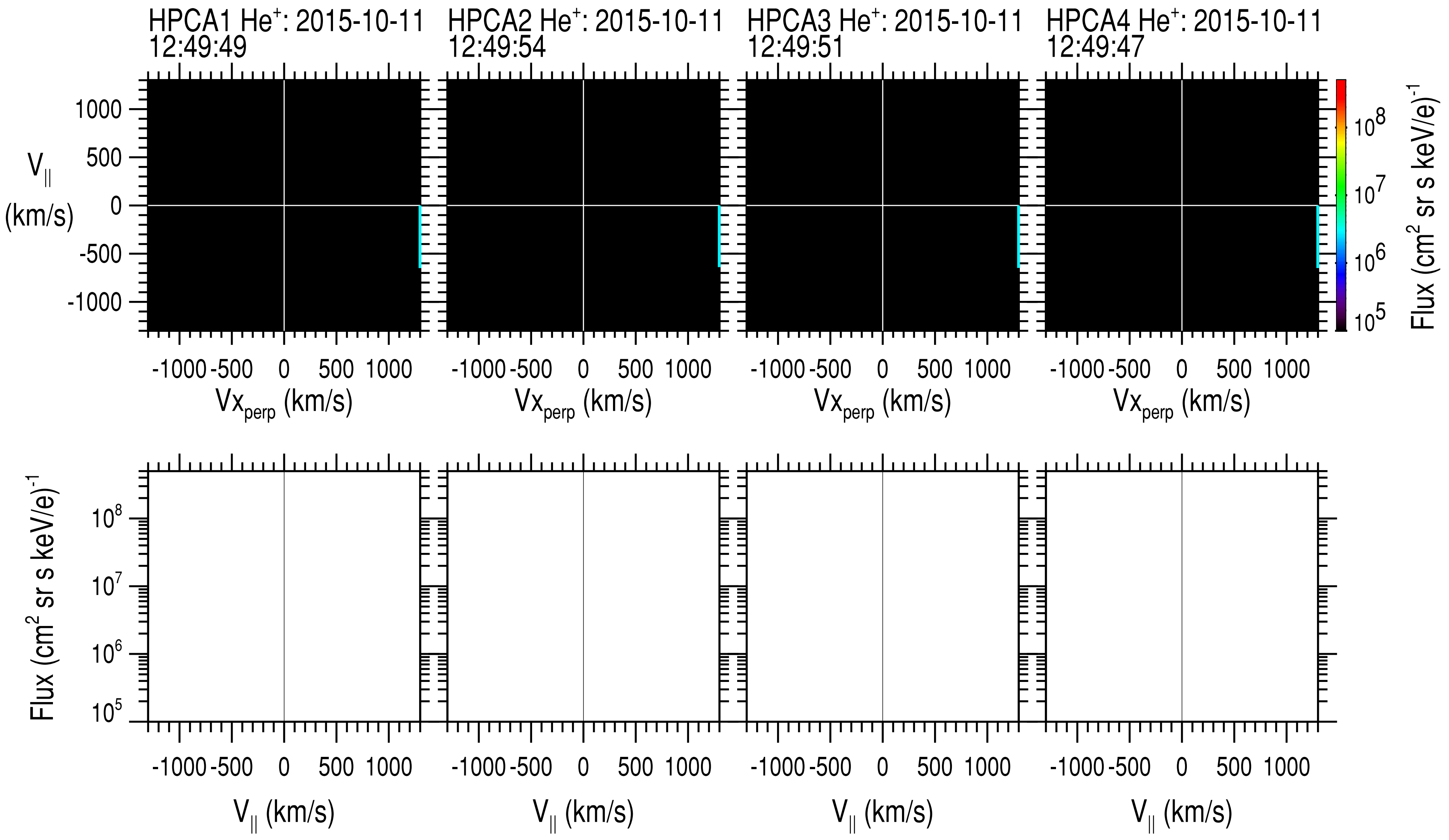
HPCA1 He+: 2015-10-11
12:49:49
HPCA2 He+: 2015-10-11
12:49:54
HPCA3 He+: 2015-10-11
12:49:51
HPCA4 He+: 2015-10-11
12:49:47
V|| (km/s)
1000
500
0
-500
-1000
-1000 -500 0 500 1000
Vxperp (km/s)
Flux (cm2 sr s keV/e)-1
10^5
10^6
10^7
10^8
V|| (km/s)

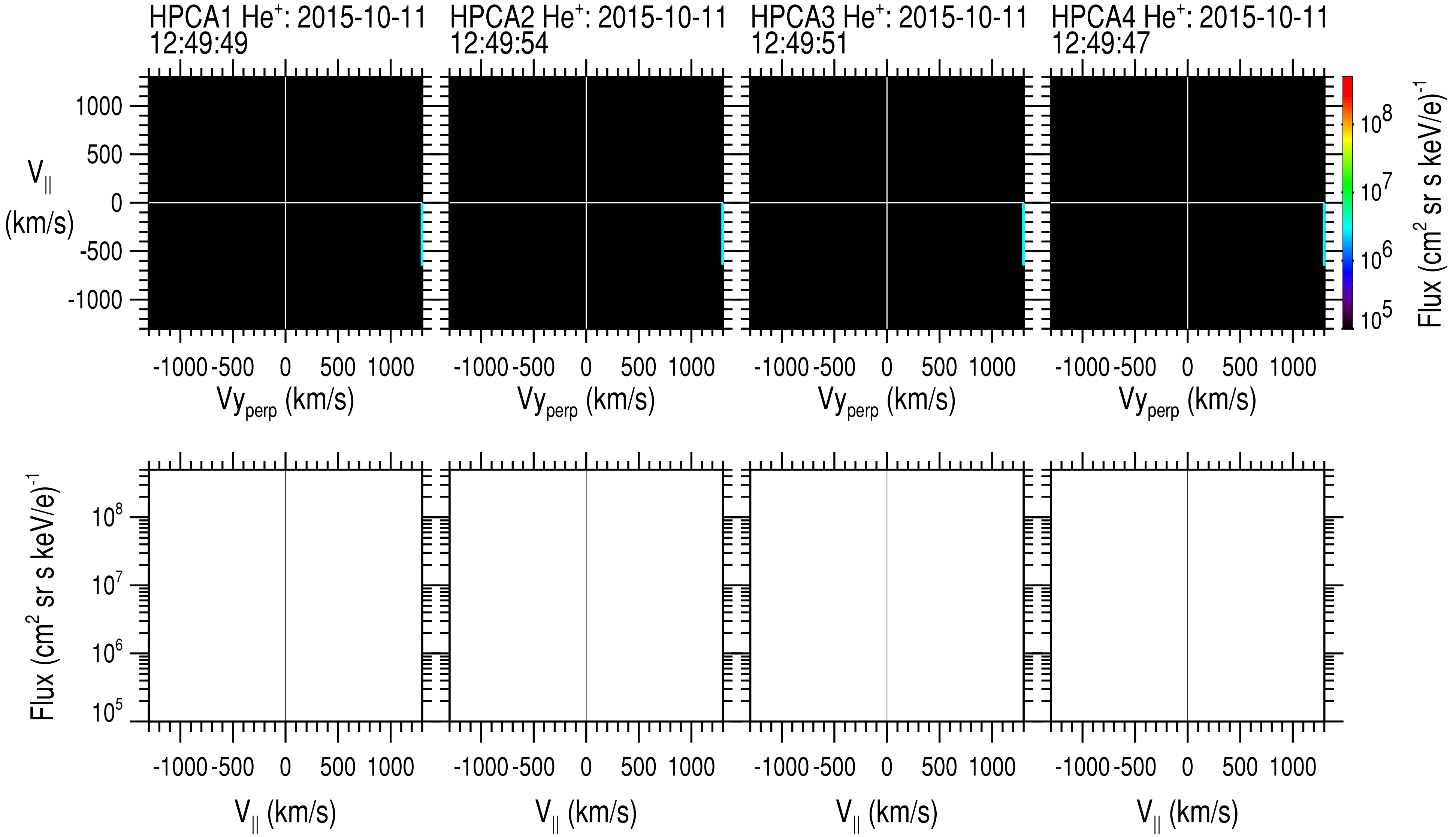

HPCA1 He+: 2015-10-11 12:49:49
HPCA2 He+: 2015-10-11 12:49:54
HPCA3 He+: 2015-10-11 12:49:51
HPCA4 He+: 2015-10-11 12:49:47
V|| (km/s)
Vyperp (km/s)
Flux (cm2 sr s keV/e)-1

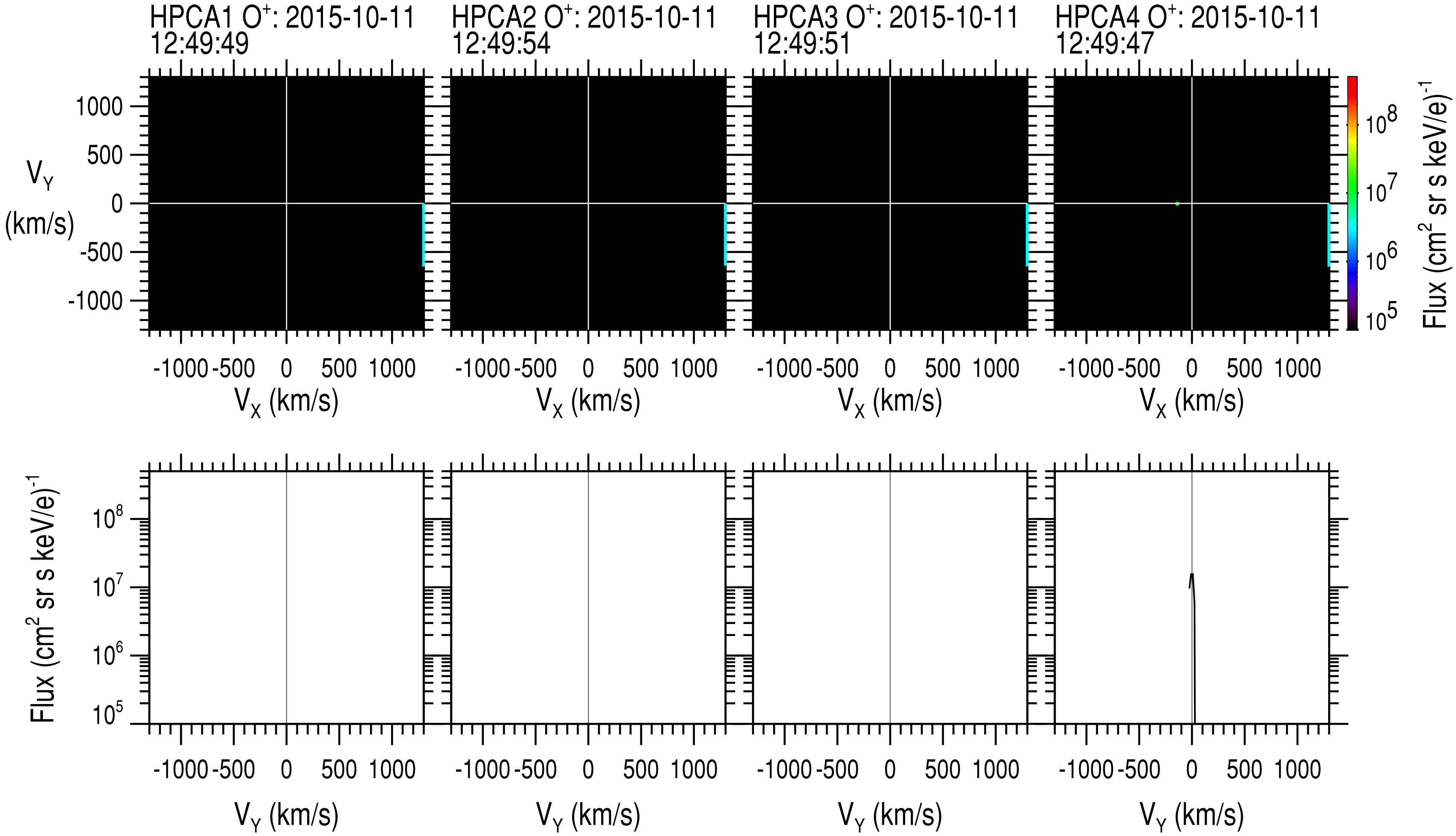

HPCA1 O+: 2015-10-11 12:49:49
HPCA2 O+: 2015-10-11 12:49:54
HPCA3 O+: 2015-10-11 12:49:51
HPCA4 O+: 2015-10-11 12:49:47
VY (km/s)
VX (km/s)
Flux (cm2 sr s keV/e)-1

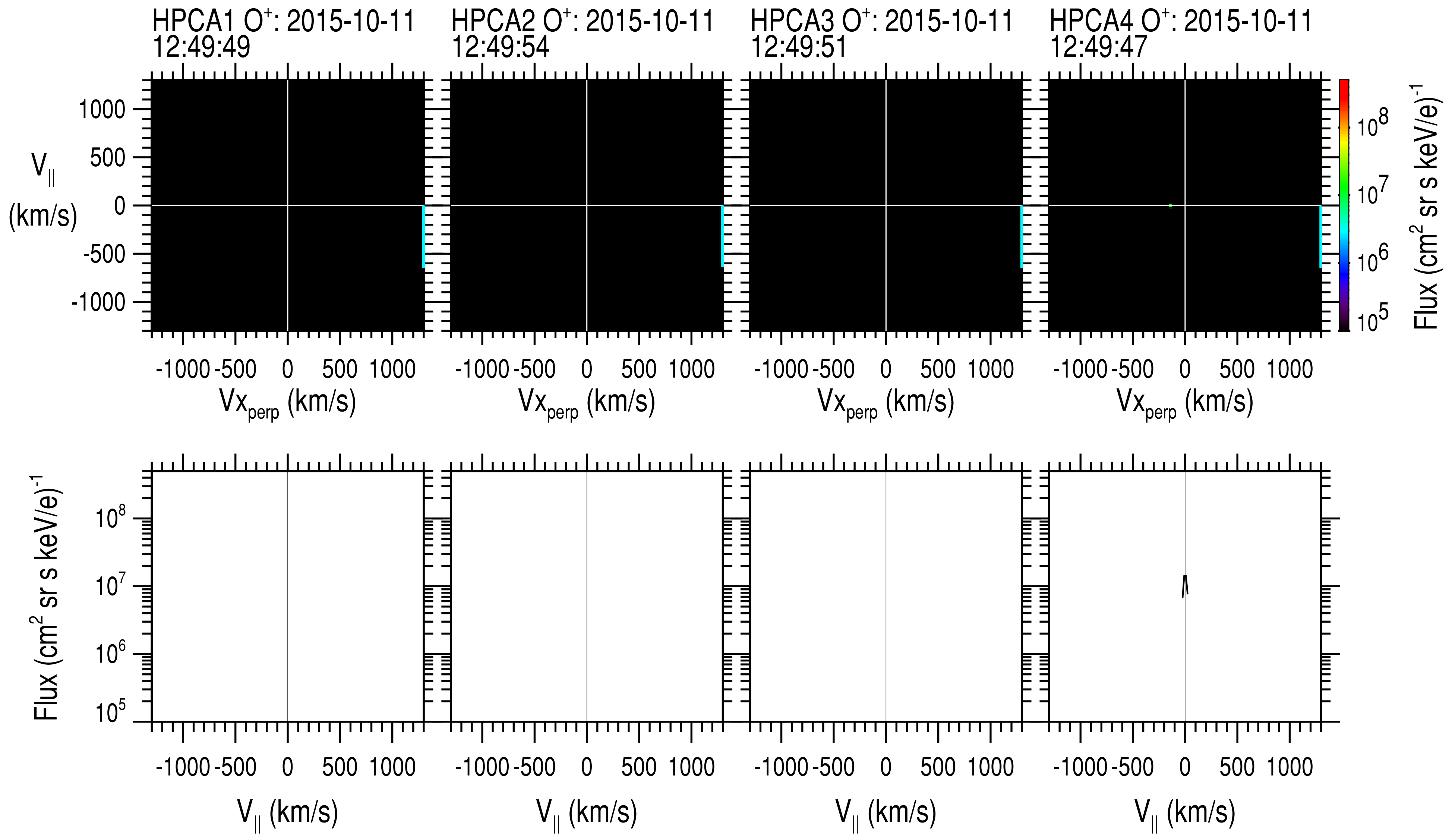

HPCA1 O+: 2015-10-11 12:49:49
HPCA2 O+: 2015-10-11 12:49:54
HPCA3 O+: 2015-10-11 12:49:51
HPCA4 O+: 2015-10-11 12:49:47
V|| (km/s)
Vxperp (km/s)
Flux (cm2 sr s keV/e)-1
-1000 -500 0 500 1000
10^5 10^6 10^7 10^8

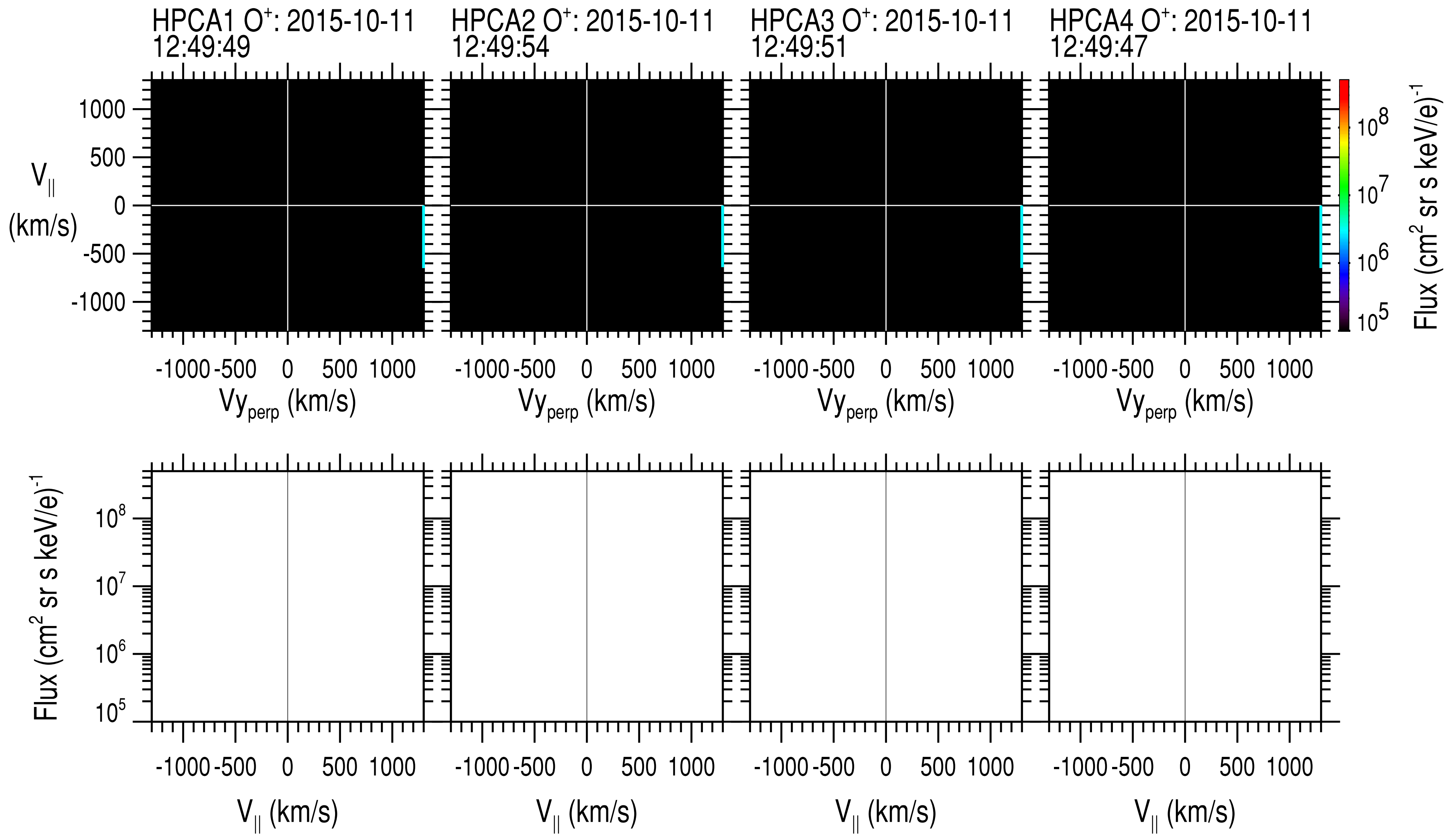

HPCA1 O+: 2015-10-11 12:49:49
HPCA2 O+: 2015-10-11 12:49:54
HPCA3 O+: 2015-10-11 12:49:51
HPCA4 O+: 2015-10-11 12:49:47
V|| (km/s)
1000
500
0
-500
-1000
Vyperp (km/s)
Flux (cm2 sr s keV/e)-1
10^5
10^6
10^7
10^8
V|| (km/s)

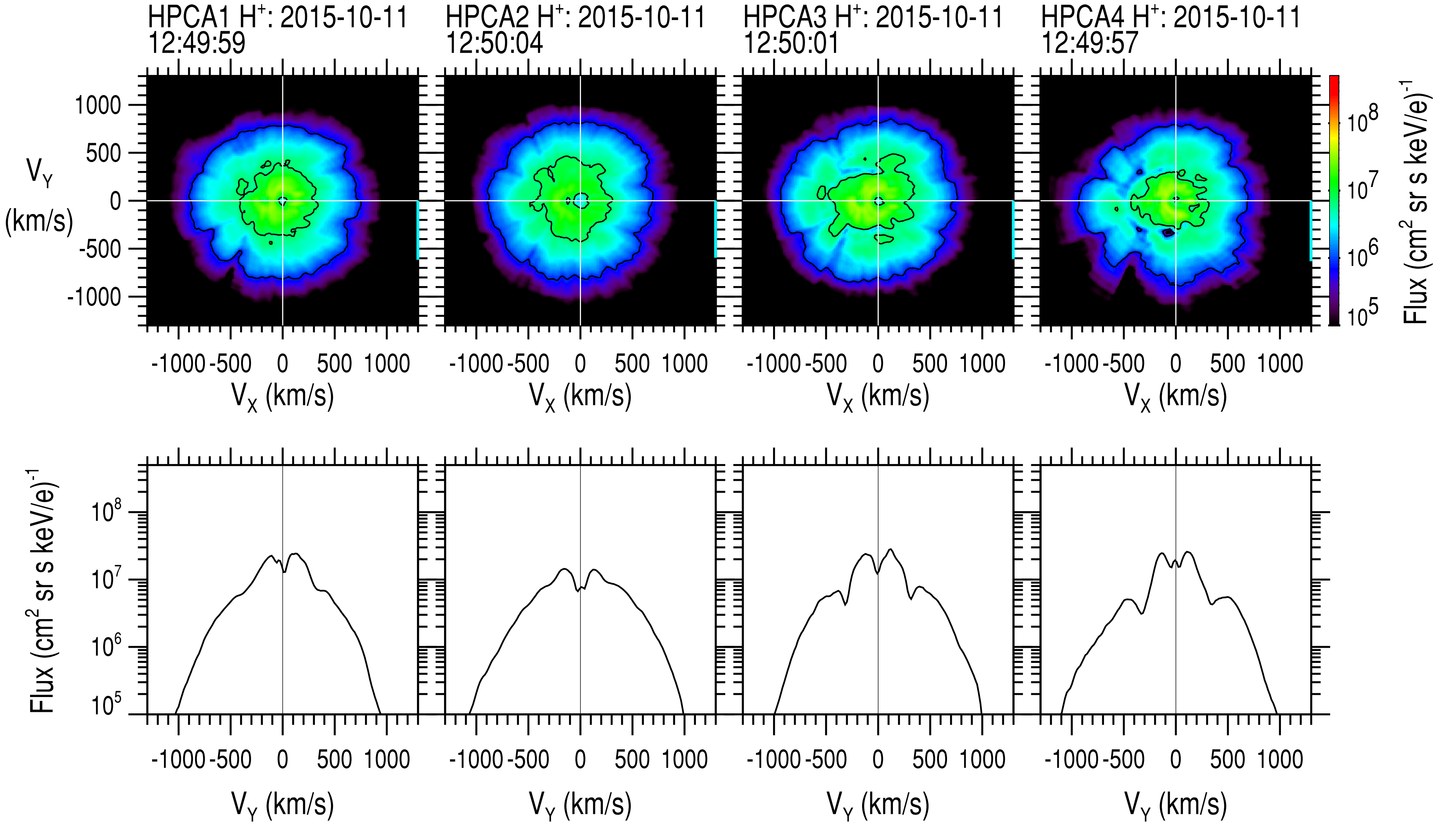
HPCA1 H+: 2015-10-11
12:49:59
HPCA2 H+: 2015-10-11
12:50:04
HPCA3 H+: 2015-10-11
12:50:01
HPCA4 H+: 2015-10-11
12:49:57
V_Y (km/s)
V_X (km/s)
Flux (cm2 sr s keV/e)-1
V_Y (km/s)

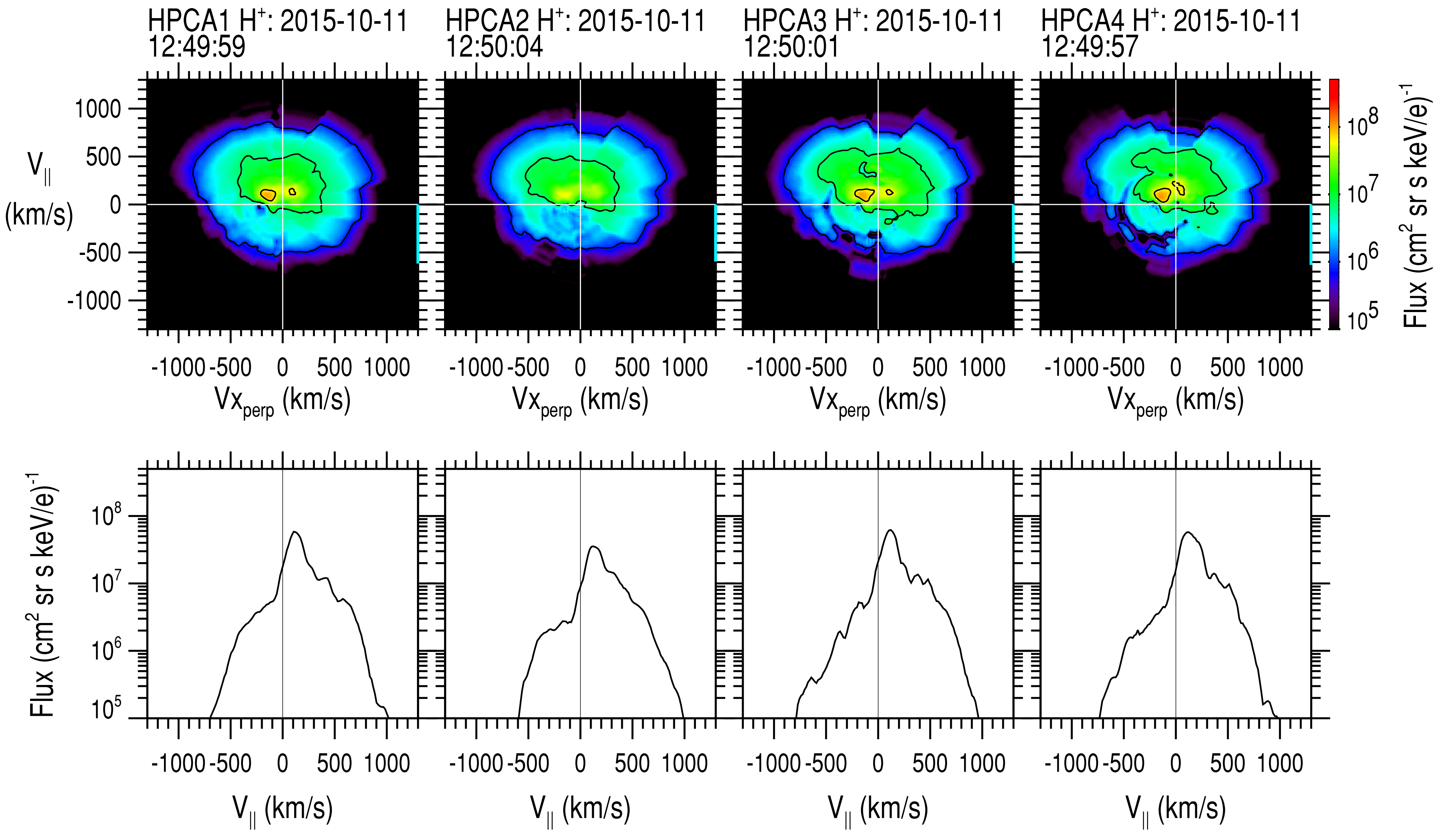
HPCA1 H+: 2015-10-11 12:49:59
HPCA2 H+: 2015-10-11 12:50:04
HPCA3 H+: 2015-10-11 12:50:01
HPCA4 H+: 2015-10-11 12:49:57
V|| (km/s)
Vxperp (km/s)
Flux (cm2 sr s keV/e)-1
V|| (km/s)

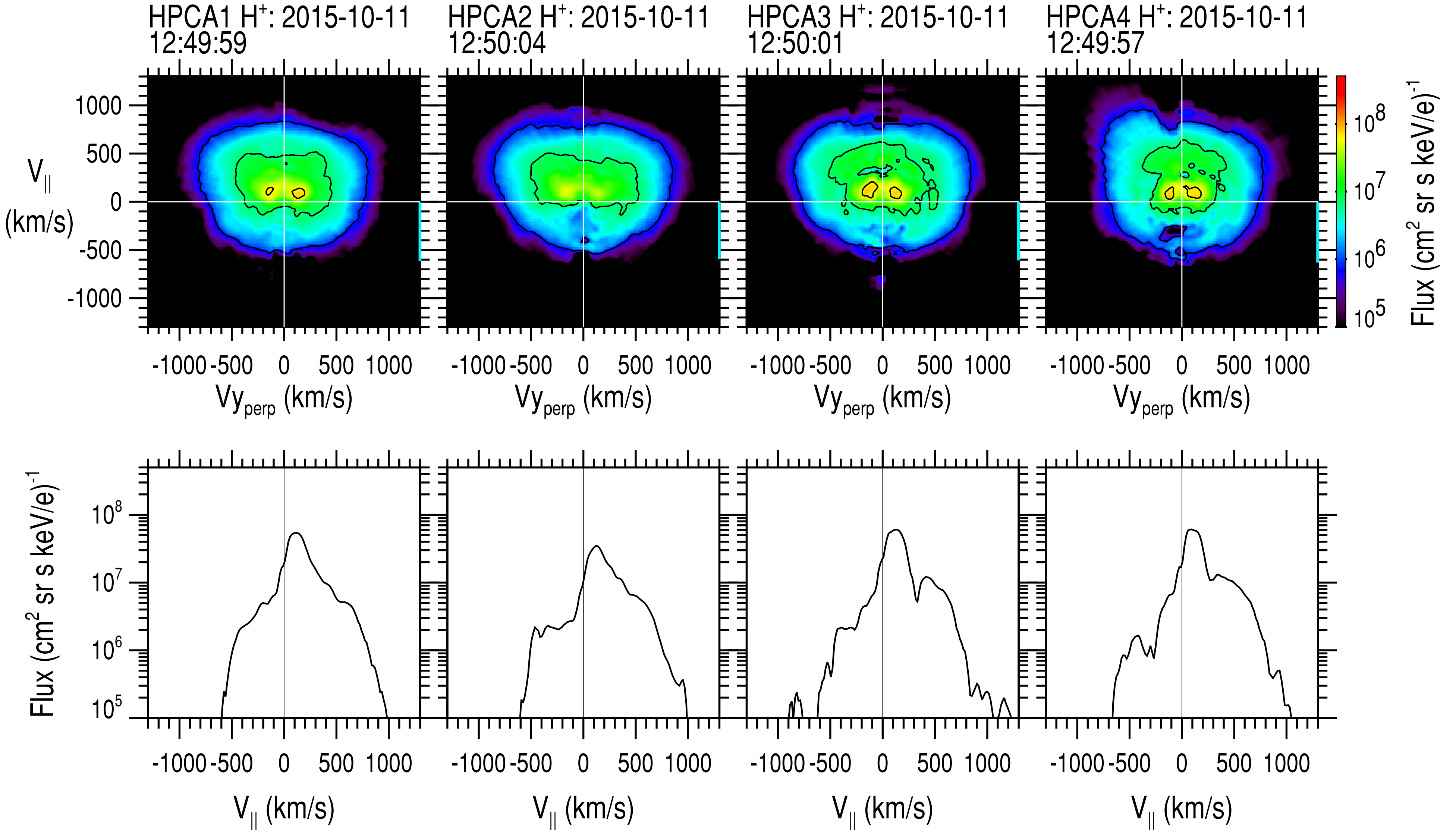

HPCA1 H+: 2015-10-11 12:49:59
HPCA2 H+: 2015-10-11 12:50:04
HPCA3 H+: 2015-10-11 12:50:01
HPCA4 H+: 2015-10-11 12:49:57
V|| (km/s)
Vyperp (km/s)
Flux (cm2 sr s keV/e)-1

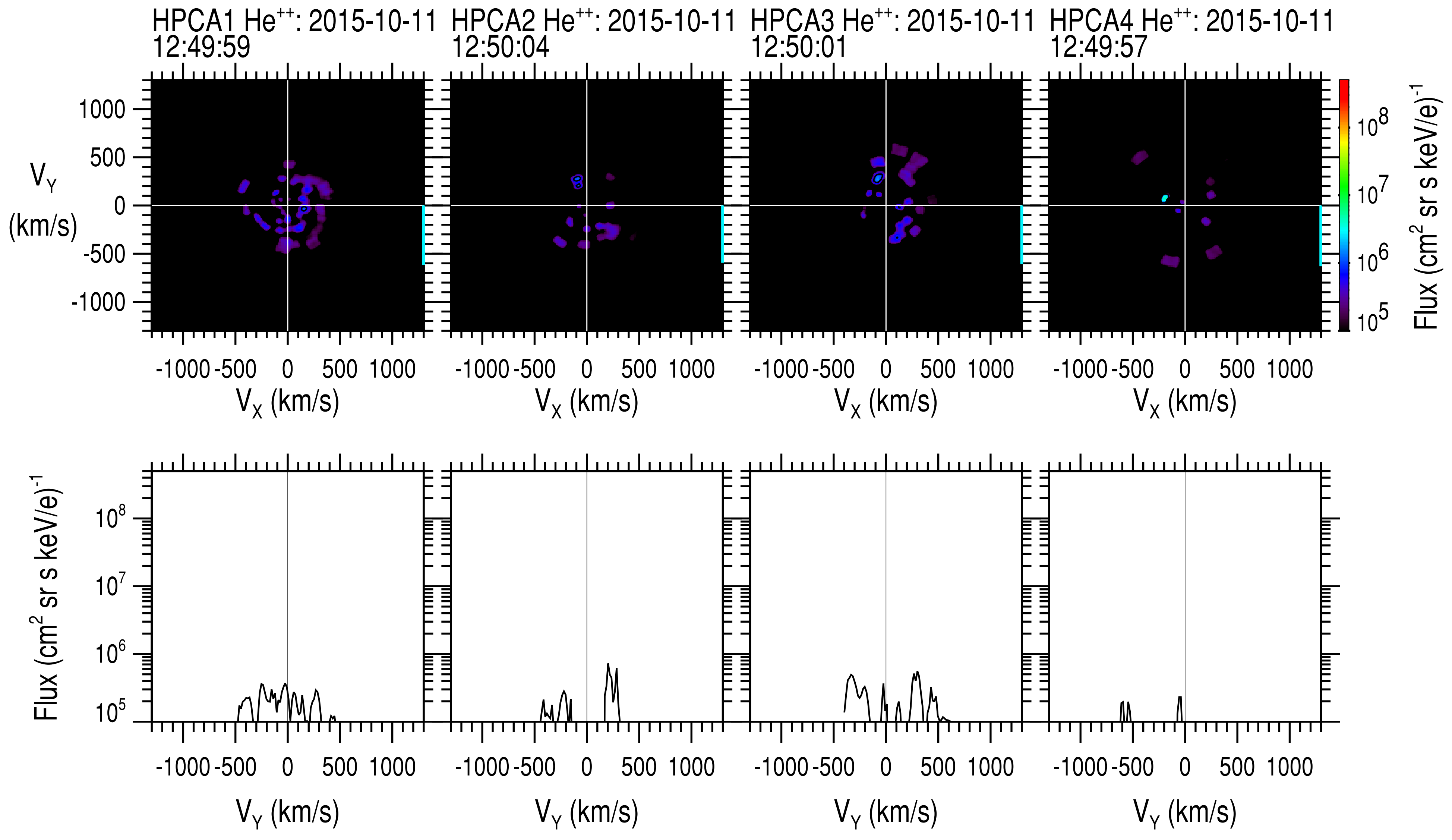

HPCA1 He++: 2015-10-11 12:49:59
HPCA2 He++: 2015-10-11 12:50:04
HPCA3 He++: 2015-10-11 12:50:01
HPCA4 He++: 2015-10-11 12:49:57
VY (km/s)
VX (km/s)
Flux (cm2 sr s keV/e)-1
-1000
-500
0
500
1000
10^5
10^6
10^7
10^8

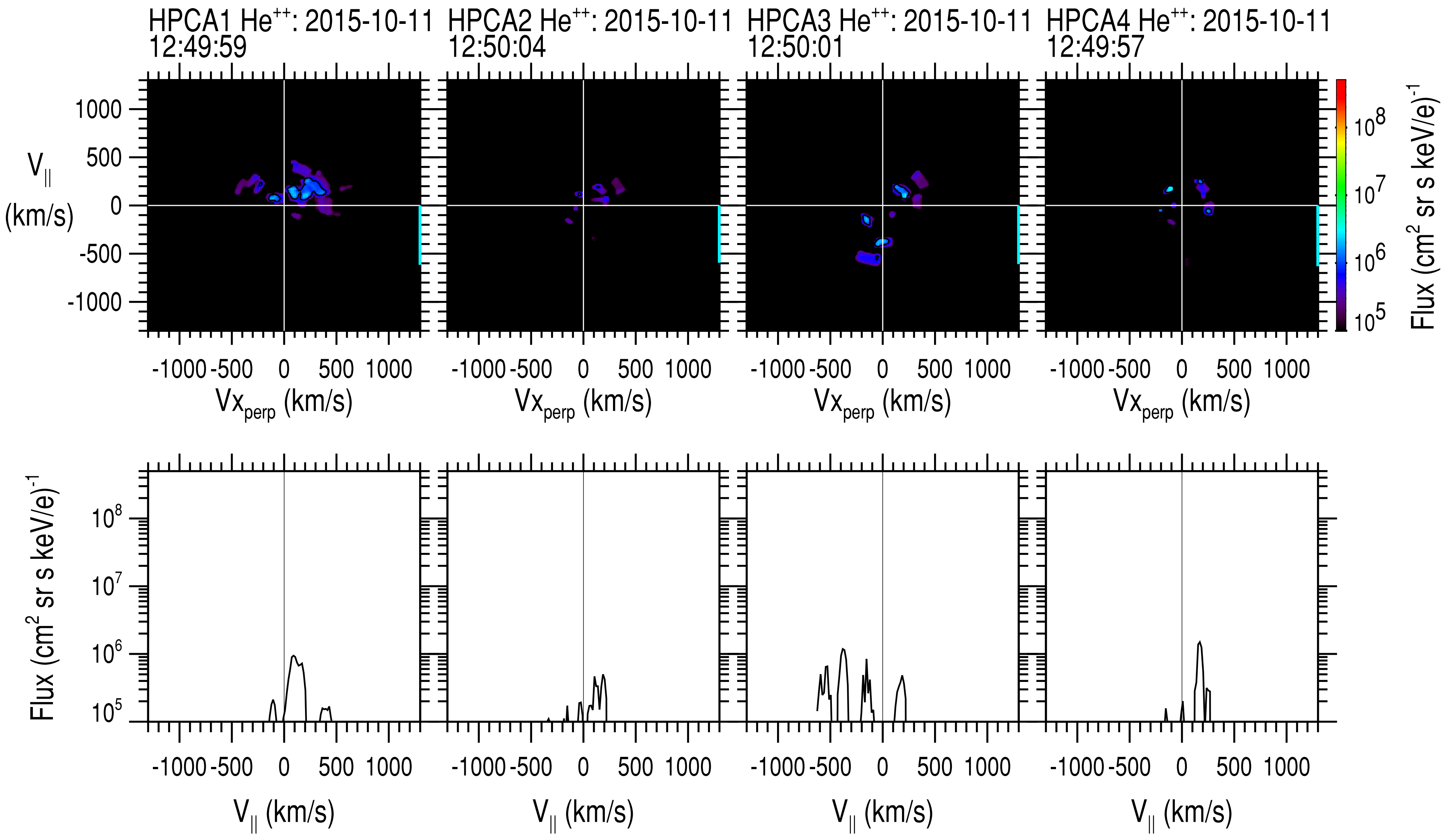

HPCA1 He++: 2015-10-11 12:49:59
HPCA2 He++: 2015-10-11 12:50:04
HPCA3 He++: 2015-10-11 12:50:01
HPCA4 He++: 2015-10-11 12:49:57
V|| (km/s)
Vxperp (km/s)
Flux (cm2 sr s keV/e)-1
-1000 -500 0 500 1000
10^5 10^6 10^7 10^8

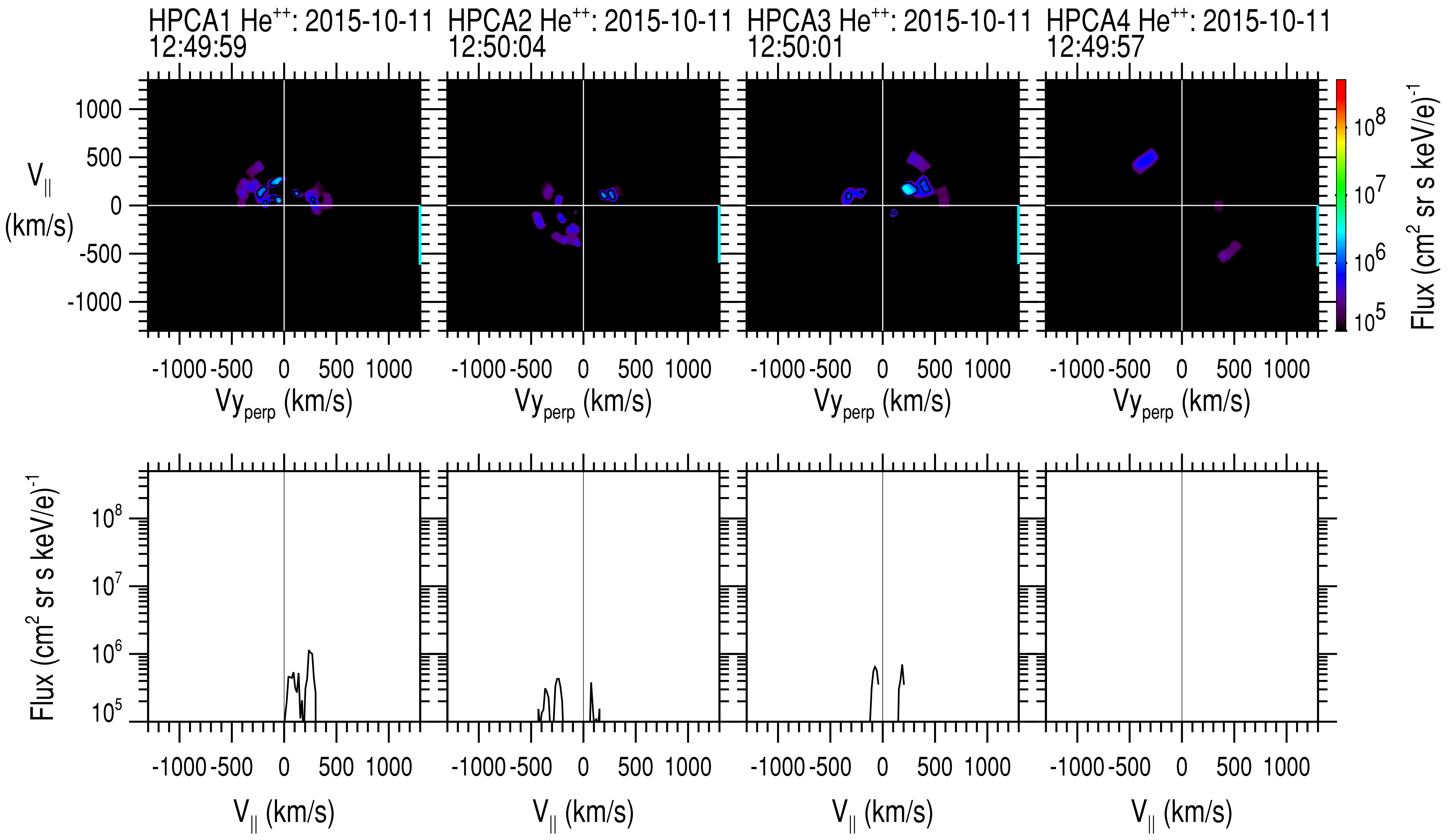

HPCA1 He++: 2015-10-11 12:49:59
HPCA2 He++: 2015-10-11 12:50:04
HPCA3 He++: 2015-10-11 12:50:01
HPCA4 He++: 2015-10-11 12:49:57
V|| (km/s)
Vyperp (km/s)
Flux (cm2 sr s keV/e)-1
V|| (km/s)

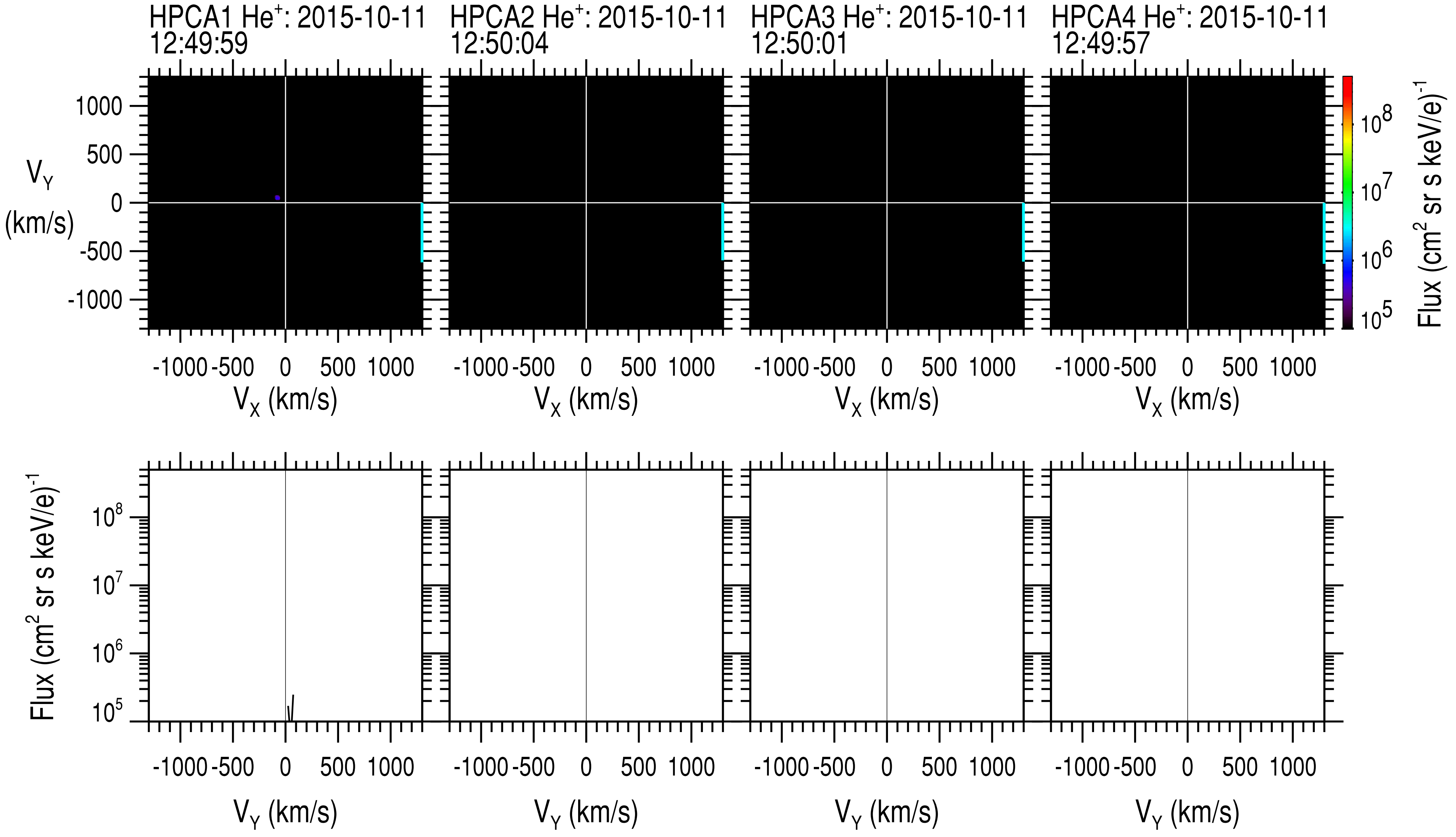

HPCA1 He+: 2015-10-11 12:49:59
HPCA2 He+: 2015-10-11 12:50:04
HPCA3 He+: 2015-10-11 12:50:01
HPCA4 He+: 2015-10-11 12:49:57
VY (km/s)
VX (km/s)
Flux (cm2 sr s keV/e)-1
VY (km/s)

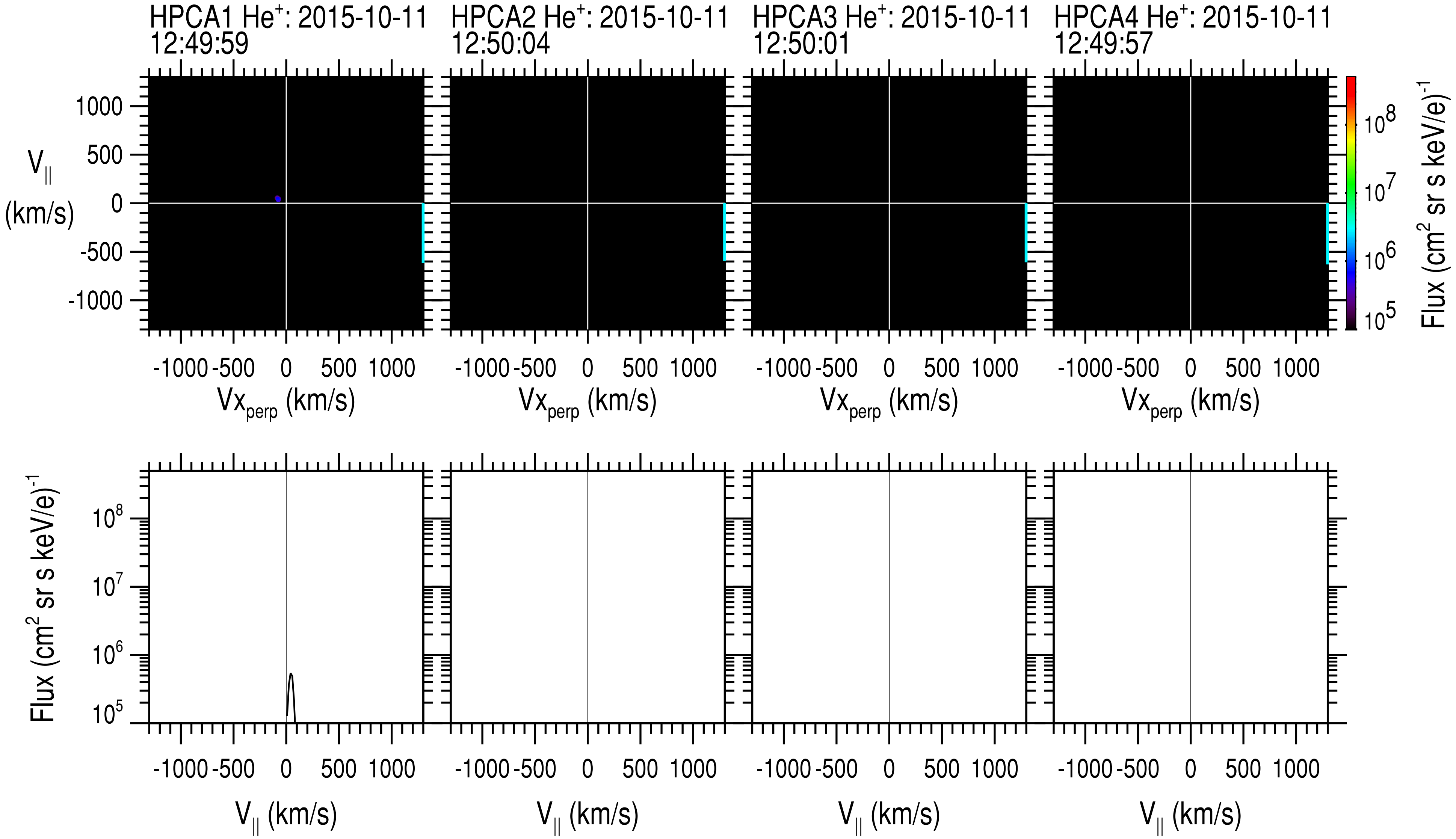

HPCA1 He+: 2015-10-11
12:49:59
HPCA2 He+: 2015-10-11
12:50:04
HPCA3 He+: 2015-10-11
12:50:01
HPCA4 He+: 2015-10-11
12:49:57
V|| (km/s)
Vxperp (km/s)
Flux (cm2 sr s keV/e)-1

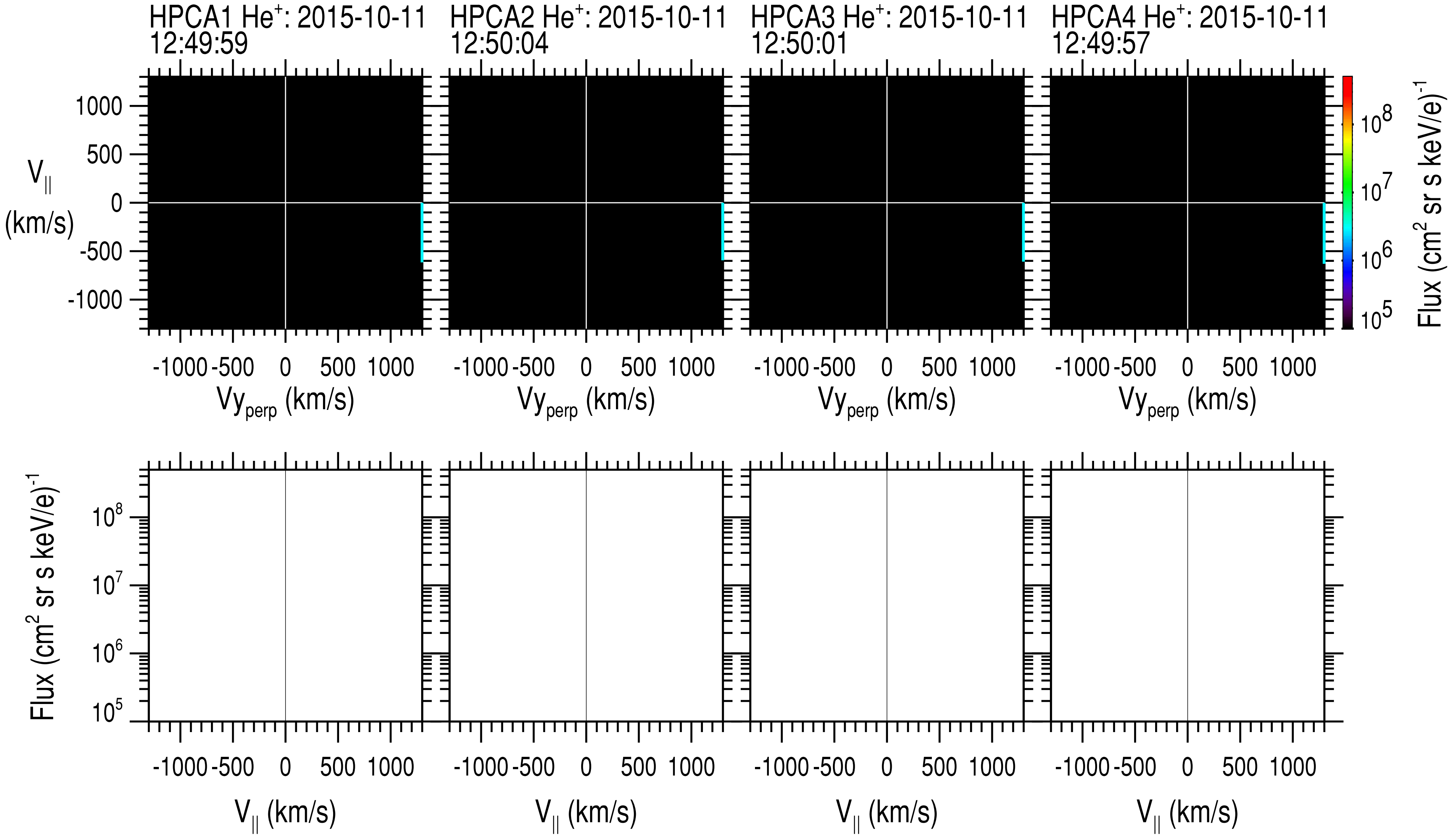
HPCA1 He+: 2015-10-11 12:49:59
HPCA2 He+: 2015-10-11 12:50:04
HPCA3 He+: 2015-10-11 12:50:01
HPCA4 He+: 2015-10-11 12:49:57
V|| (km/s)
1000
500
0
-500
-1000
Vyperp (km/s)
Flux (cm2 sr s keV/e)-1
10^5
10^6
10^7
10^8

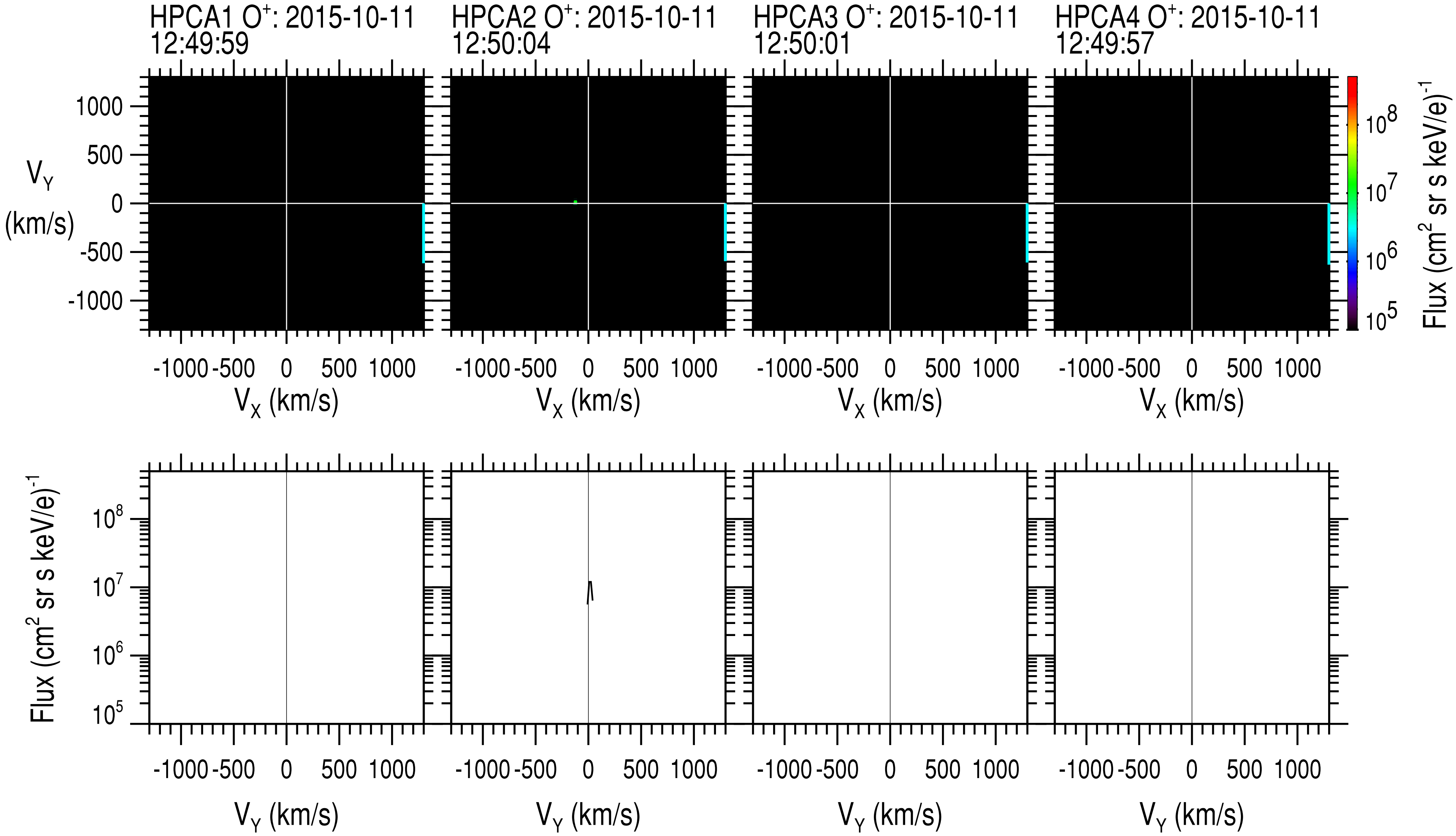

HPCA1 O+: 2015-10-11 12:49:59
HPCA2 O+: 2015-10-11 12:50:04
HPCA3 O+: 2015-10-11 12:50:01
HPCA4 O+: 2015-10-11 12:49:57
VY (km/s)
VX (km/s)
Flux (cm2 sr s keV/e)-1

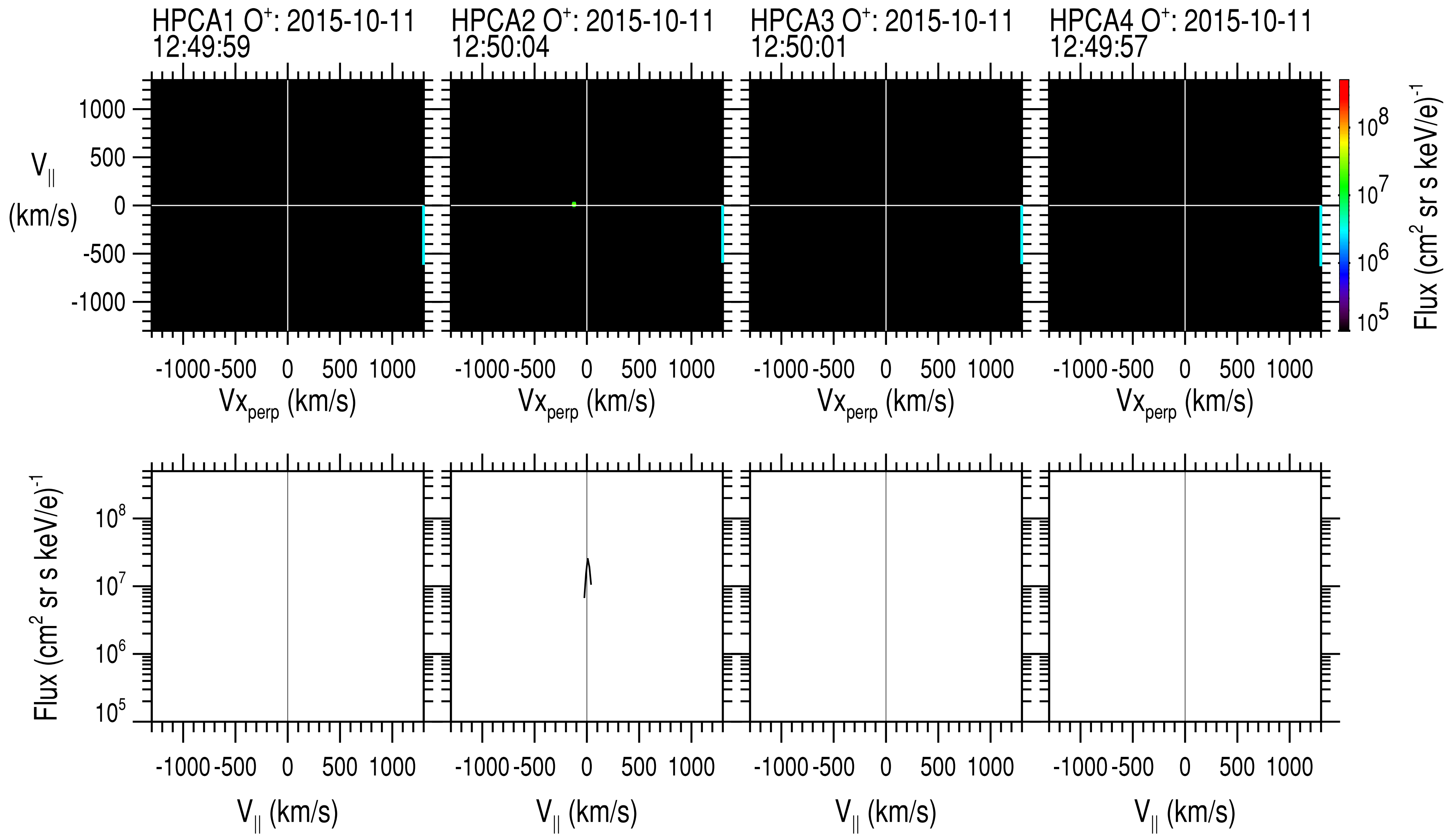
HPCA1 O+: 2015-10-11 12:49:59
HPCA2 O+: 2015-10-11 12:50:04
HPCA3 O+: 2015-10-11 12:50:01
HPCA4 O+: 2015-10-11 12:49:57
V|| (km/s)
Vxperp (km/s)
Flux (cm2 sr s keV/e)-1
V|| (km/s)

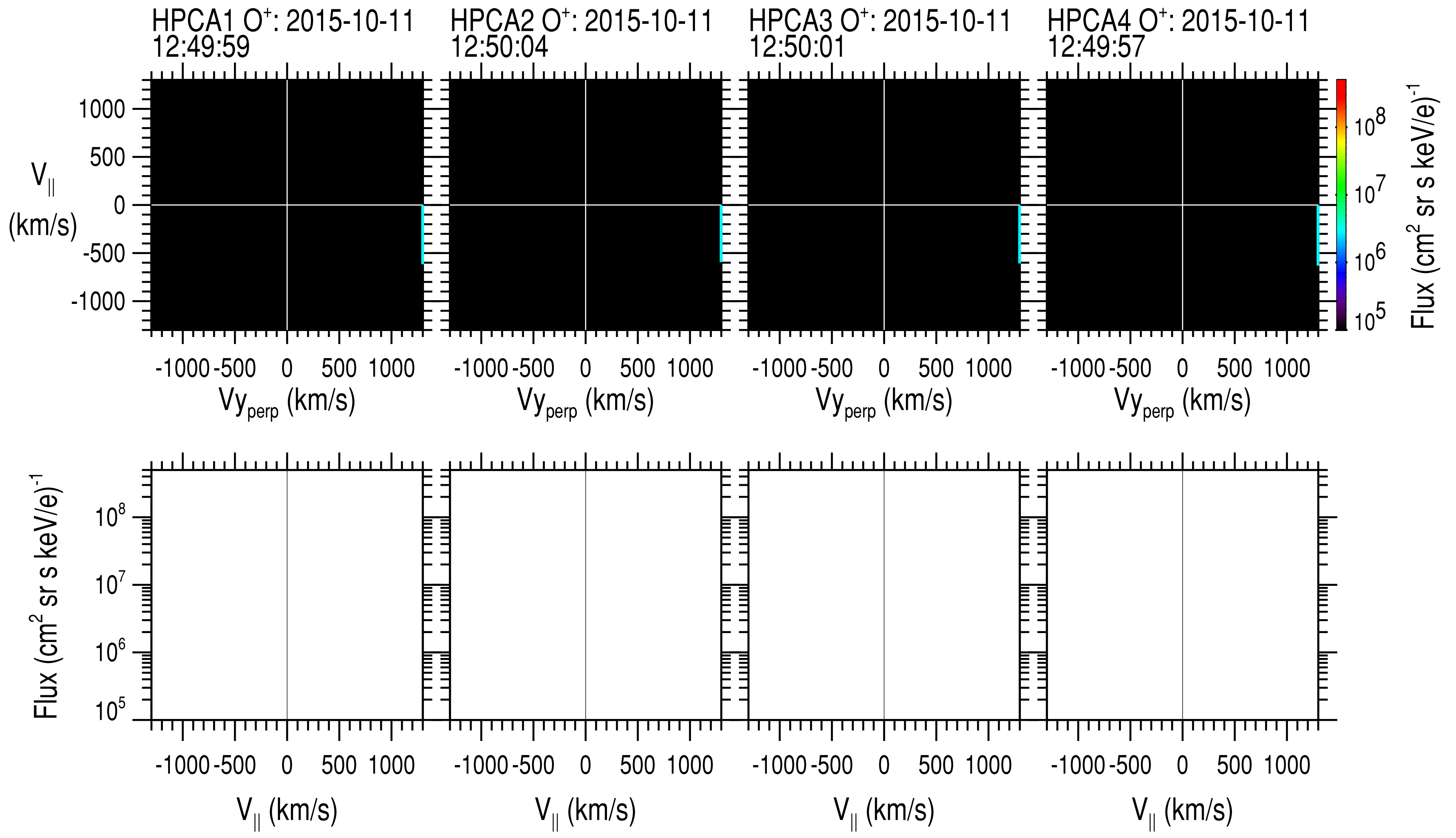

HPCA1 O+: 2015-10-11 12:49:59
HPCA2 O+: 2015-10-11 12:50:04
HPCA3 O+: 2015-10-11 12:50:01
HPCA4 O+: 2015-10-11 12:49:57
V|| (km/s)
Vyperp (km/s)
Flux (cm2 sr s keV/e)-1

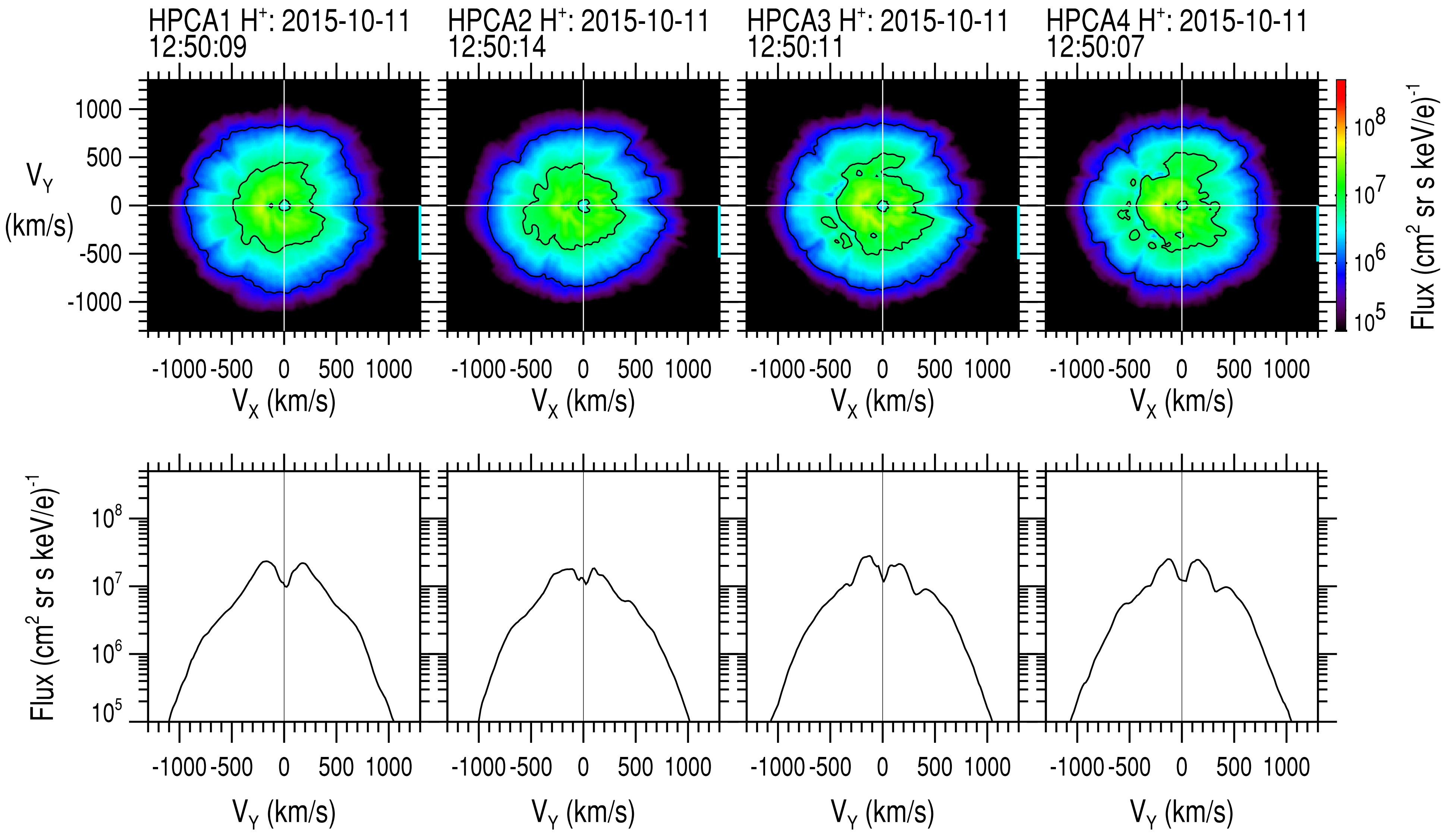
HPCA1 H+: 2015-10-11
12:50:09
HPCA2 H+: 2015-10-11
12:50:14
HPCA3 H+: 2015-10-11
12:50:11
HPCA4 H+: 2015-10-11
12:50:07
V_Y (km/s)
V_X (km/s)
Flux (cm^2 sr s keV/e)^-1
V_Y (km/s)
Flux (cm^2 sr s keV/e)^-1

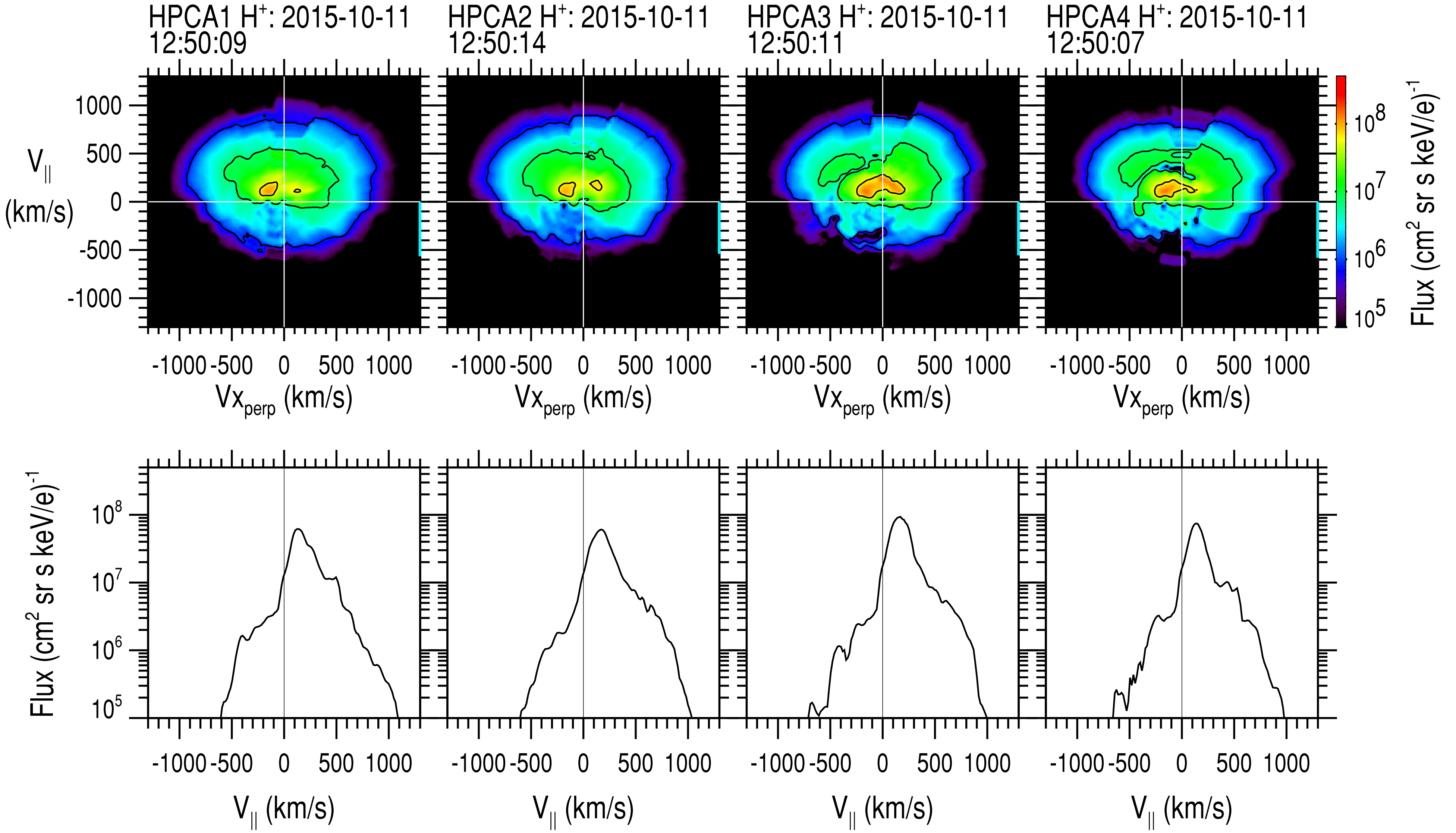

HPCA1 H+: 2015-10-11 12:50:09
HPCA2 H+: 2015-10-11 12:50:14
HPCA3 H+: 2015-10-11 12:50:11
HPCA4 H+: 2015-10-11 12:50:07
V|| (km/s)
Vxperp (km/s)
Flux (cm2 sr s keV/e)-1

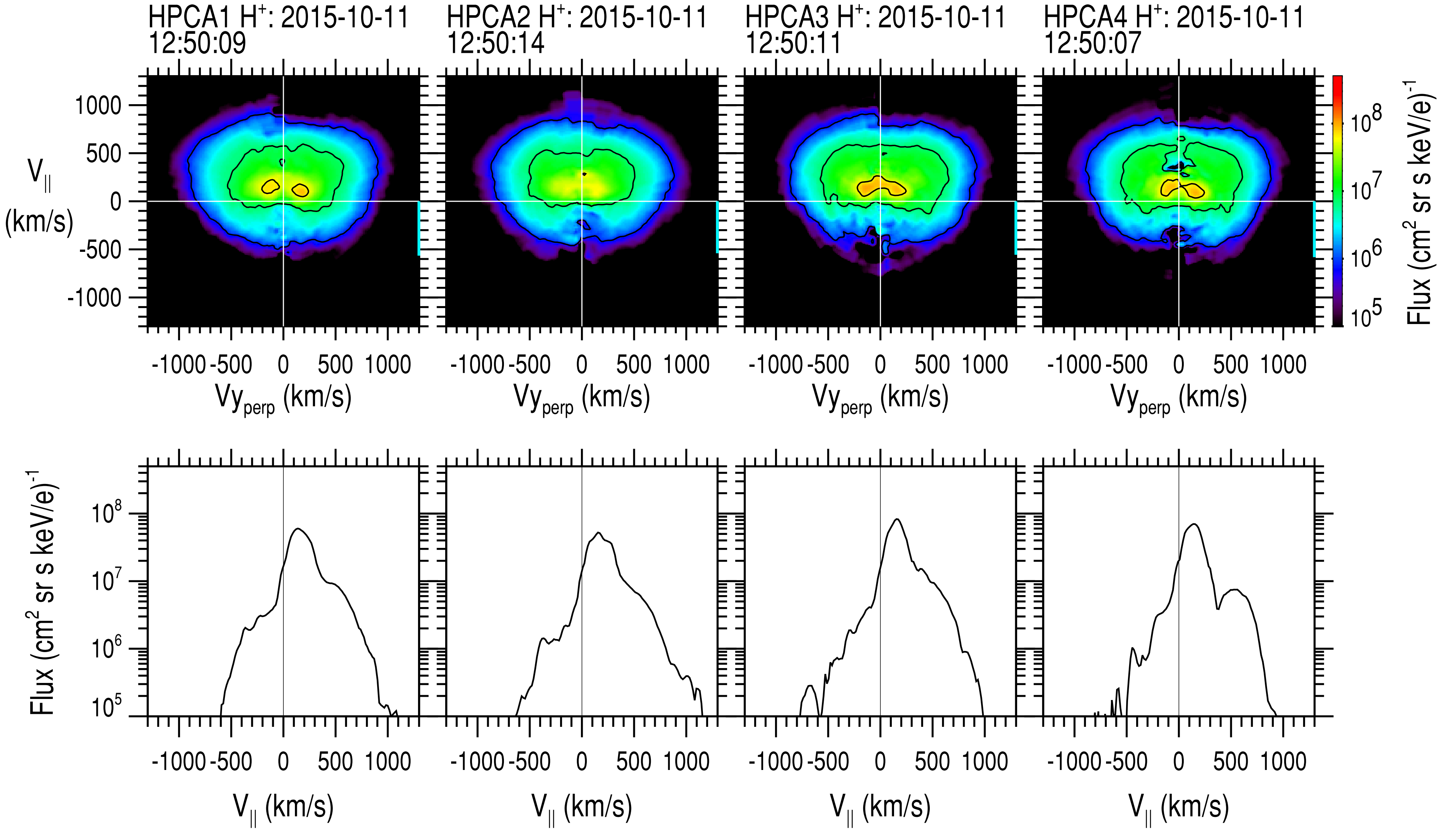

HPCA1 H+: 2015-10-11 12:50:09
HPCA2 H+: 2015-10-11 12:50:14
HPCA3 H+: 2015-10-11 12:50:11
HPCA4 H+: 2015-10-11 12:50:07
V|| (km/s)
Vyperp (km/s)
Flux (cm2 sr s keV/e)-1
-1000 -500 0 500 1000
10^5 10^6 10^7 10^8

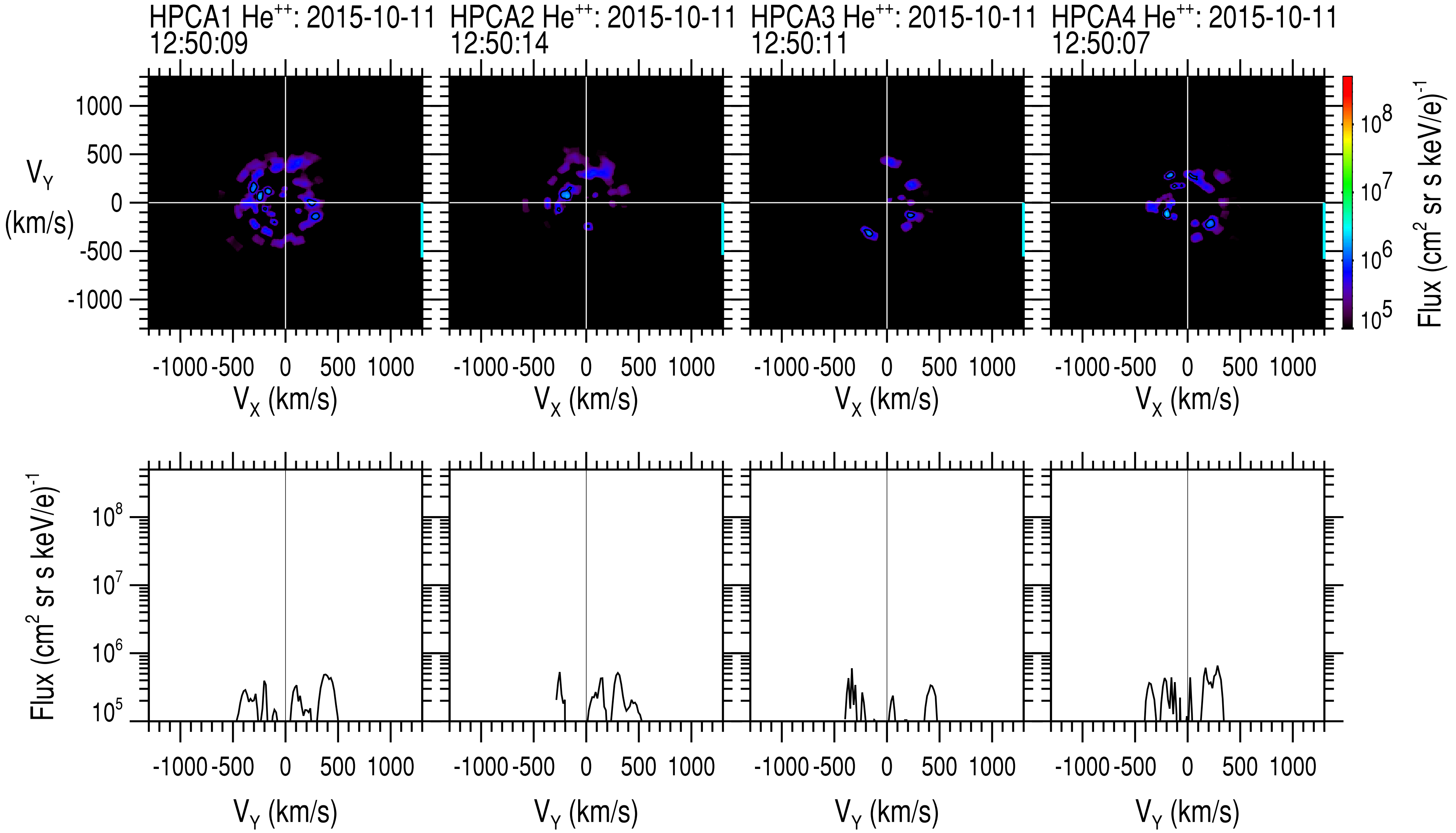

HPCA1 He^{++}: 2015-10-11 12:50:09
HPCA2 He^{++}: 2015-10-11 12:50:14
HPCA3 He^{++}: 2015-10-11 12:50:11
HPCA4 He^{++}: 2015-10-11 12:50:07
V_Y (km/s)
V_X (km/s)
Flux (cm^2 sr s keV/e)$^{-1}$

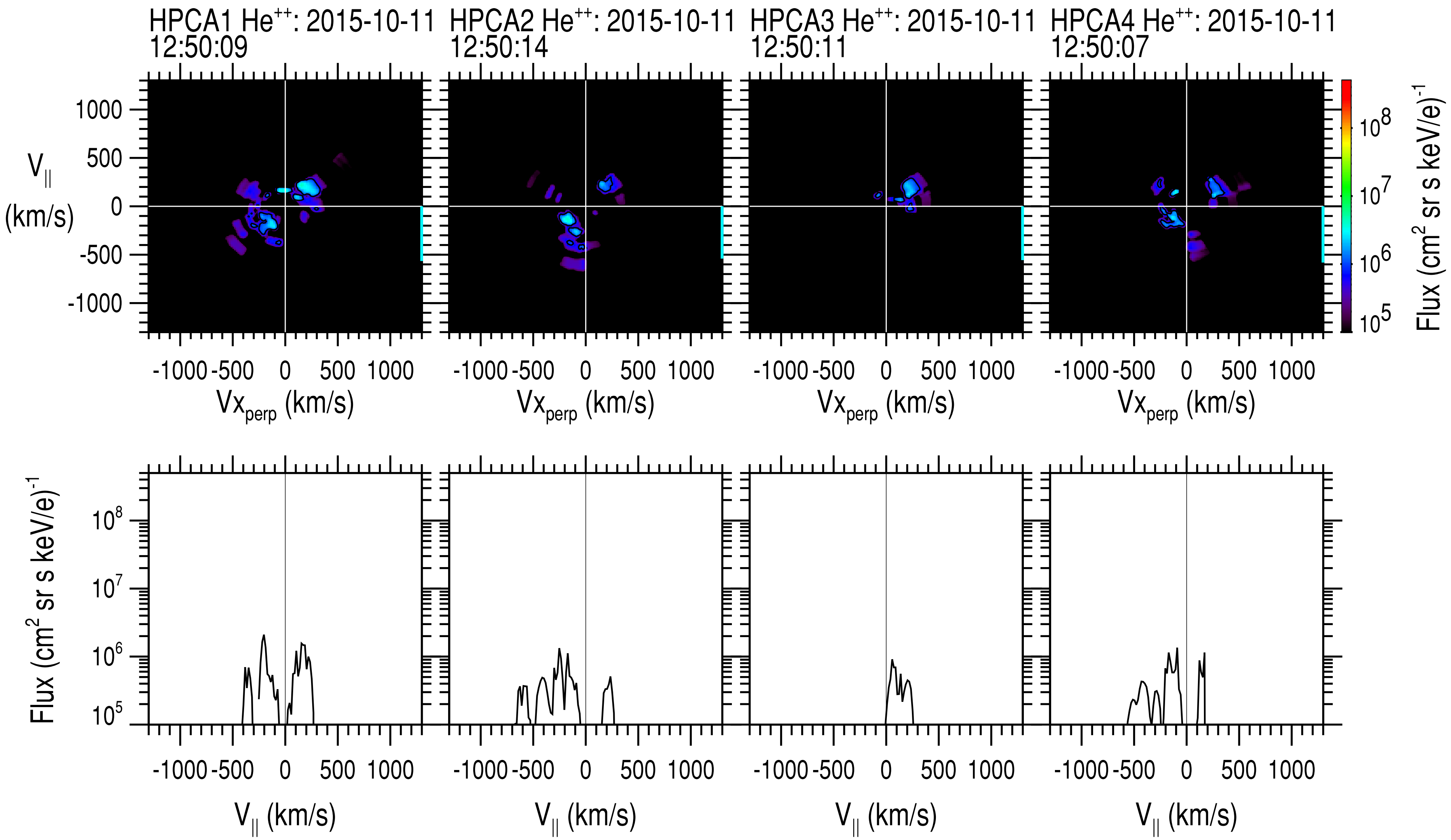

HPCA1 He++: 2015-10-11 12:50:09
HPCA2 He++: 2015-10-11 12:50:14
HPCA3 He++: 2015-10-11 12:50:11
HPCA4 He++: 2015-10-11 12:50:07
V|| (km/s)
Vxperp (km/s)
Flux (cm2 sr s keV/e)-1

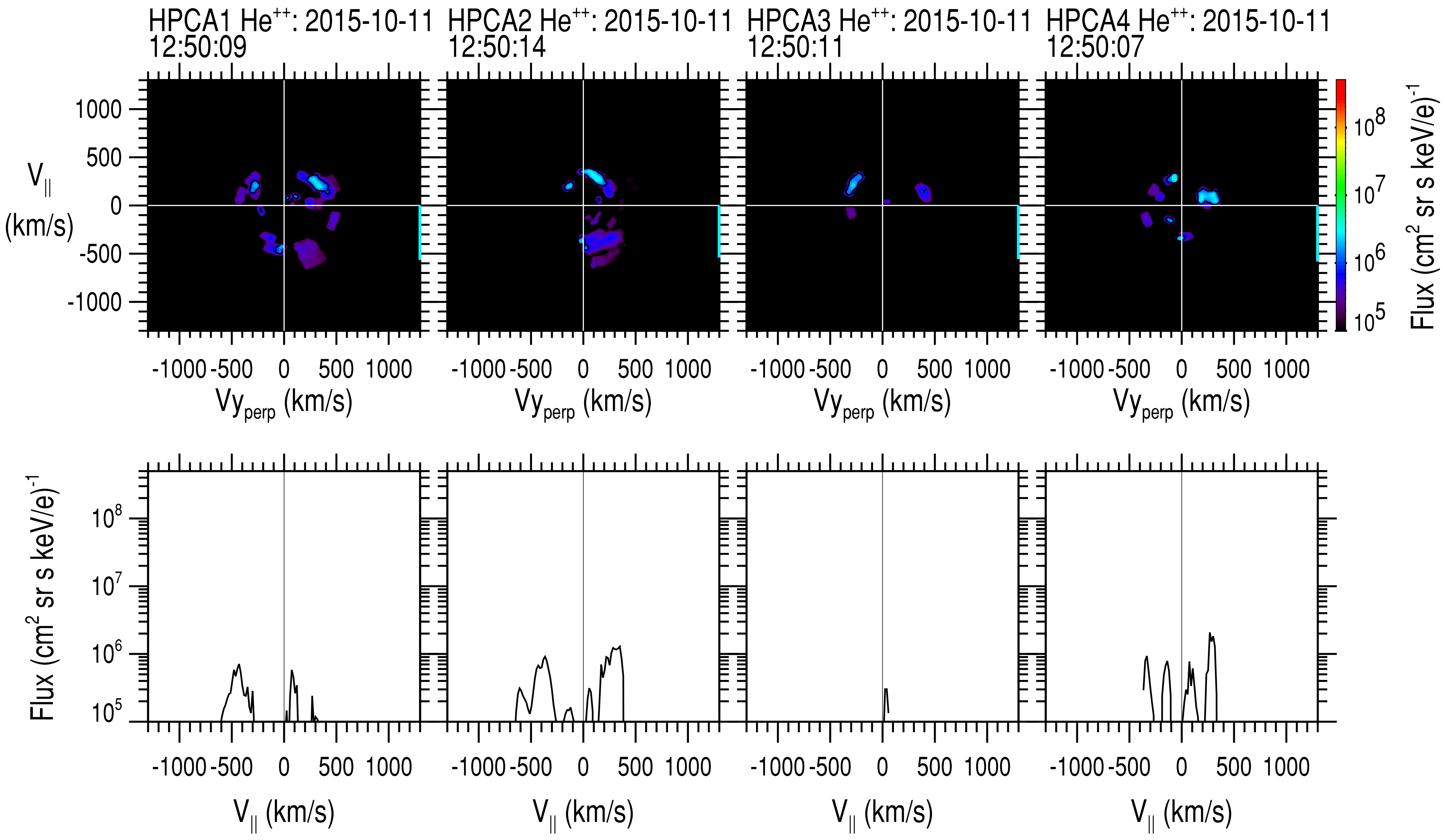

HPCA1 He++: 2015-10-11 12:50:09
HPCA2 He++: 2015-10-11 12:50:14
HPCA3 He++: 2015-10-11 12:50:11
HPCA4 He++: 2015-10-11 12:50:07
V|| (km/s)
Vyperp (km/s)
Flux (cm2 sr s keV/e)-1

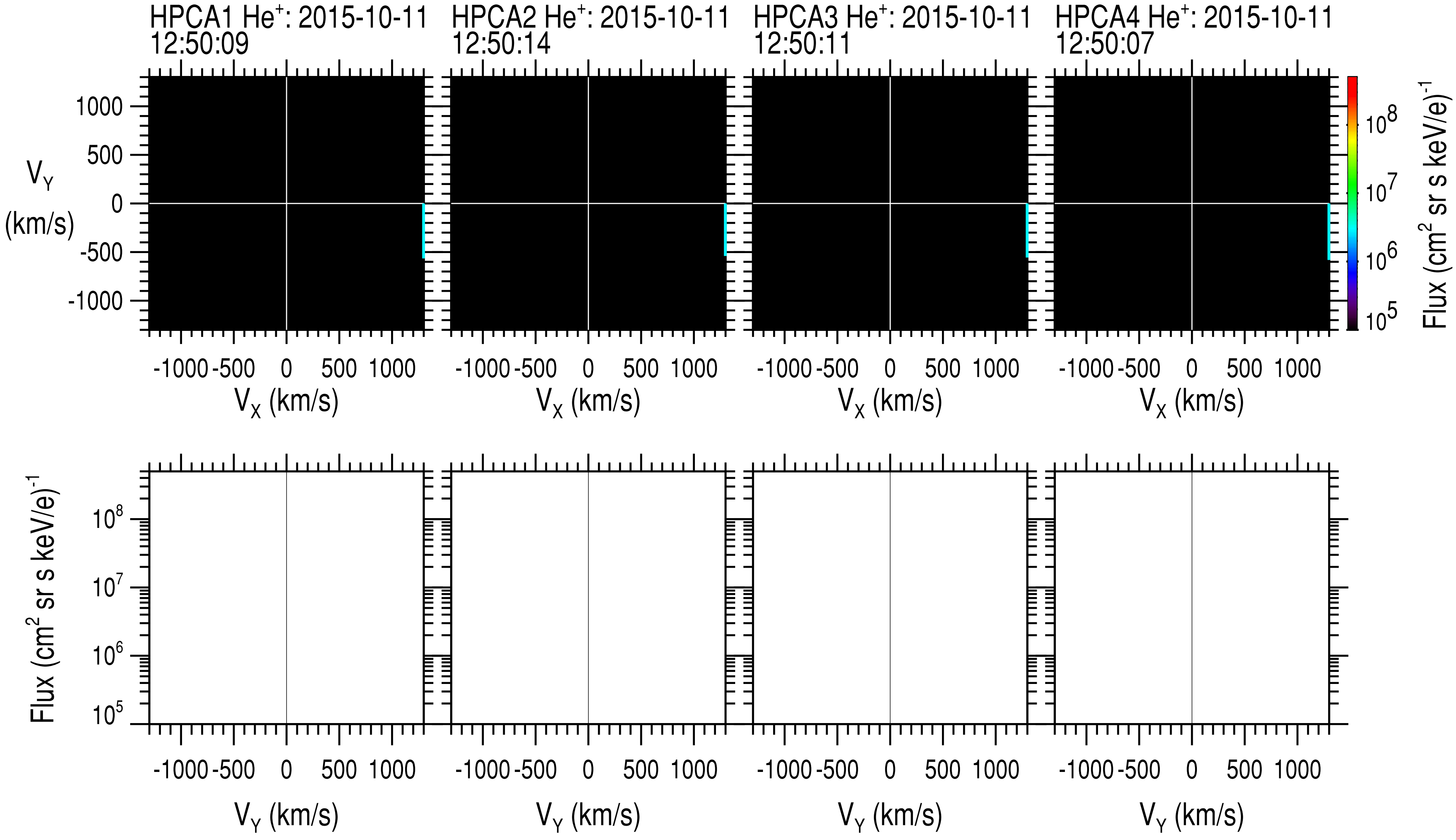

HPCA1 He+: 2015-10-11 12:50:09
HPCA2 He+: 2015-10-11 12:50:14
HPCA3 He+: 2015-10-11 12:50:11
HPCA4 He+: 2015-10-11 12:50:07
VY (km/s)
VX (km/s)
Flux (cm2 sr s keV/e)-1

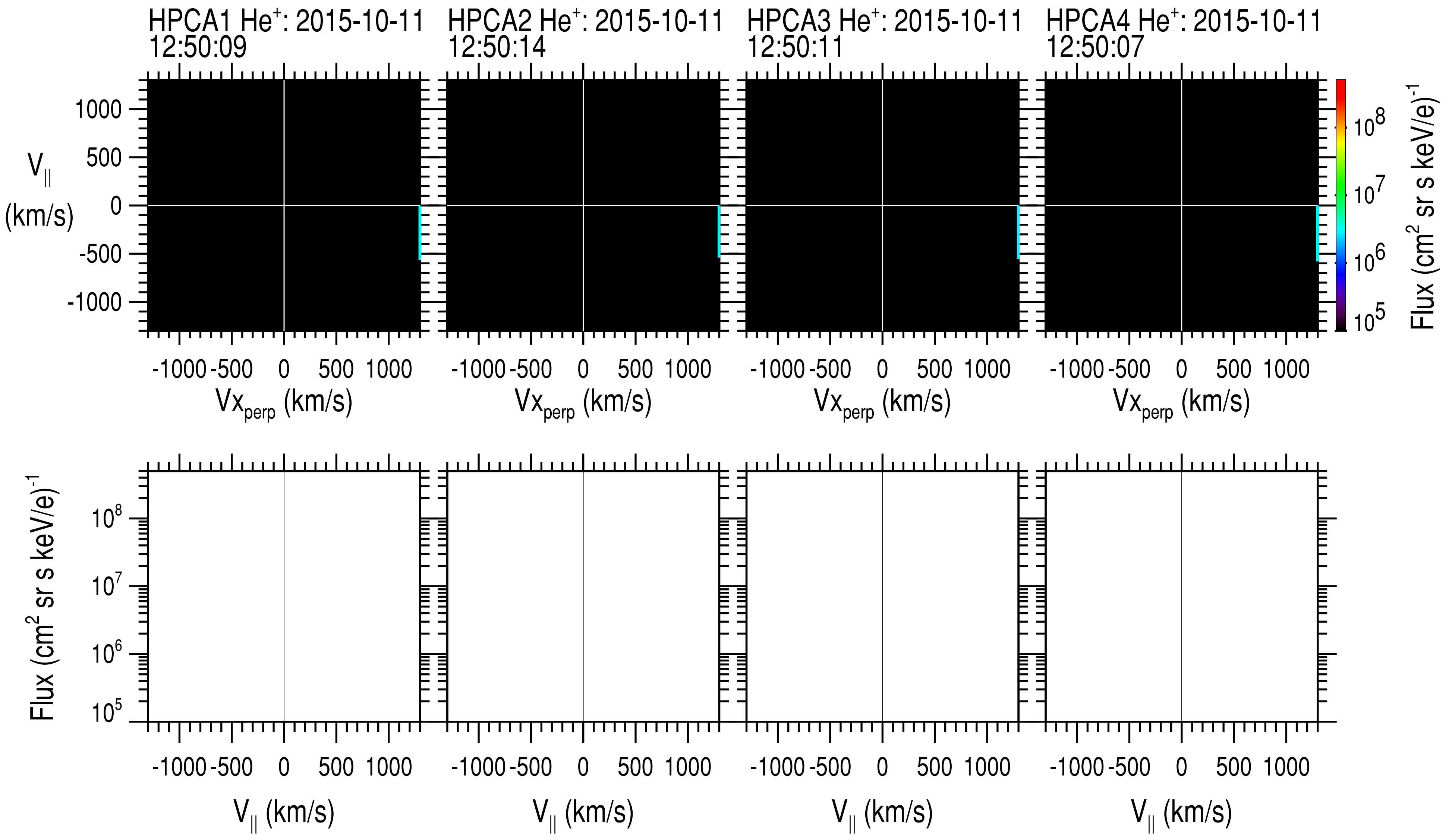
HPCA1 He+: 2015-10-11 12:50:09
HPCA2 He+: 2015-10-11 12:50:14
HPCA3 He+: 2015-10-11 12:50:11
HPCA4 He+: 2015-10-11 12:50:07
V|| (km/s)
Vxperp (km/s)
Flux (cm2 sr s keV/e)-1
-1000 -500 0 500 1000
10^5 10^6 10^7 10^8

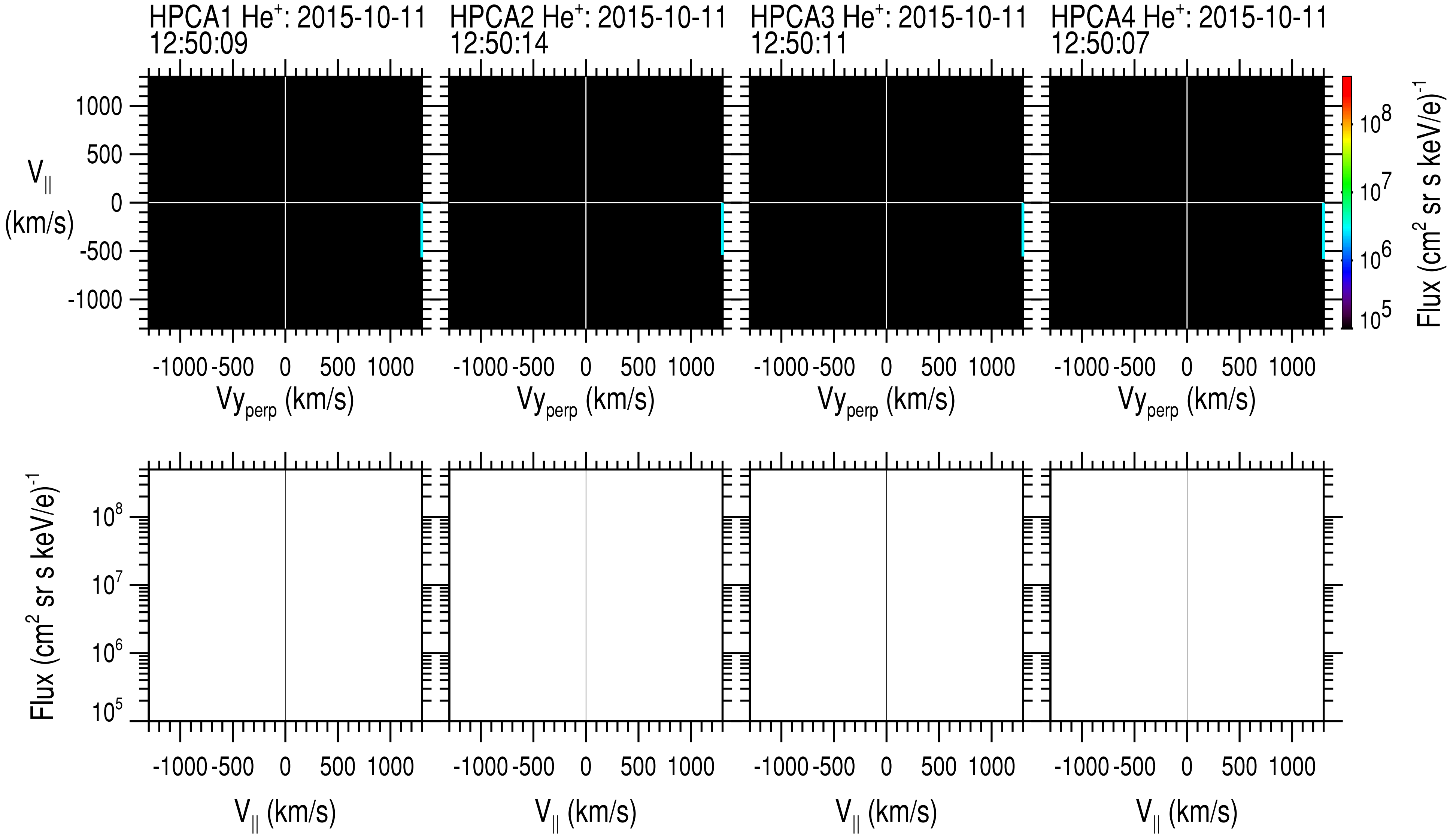

HPCA1 He+: 2015-10-11
12:50:09
HPCA2 He+: 2015-10-11
12:50:14
HPCA3 He+: 2015-10-11
12:50:11
HPCA4 He+: 2015-10-11
12:50:07
V|| (km/s)
1000
500
0
-500
-1000
-1000 -500 0 500 1000
Vyperp (km/s)
Flux (cm2 sr s keV/e)-1
10^5
10^6
10^7
10^8
V|| (km/s)

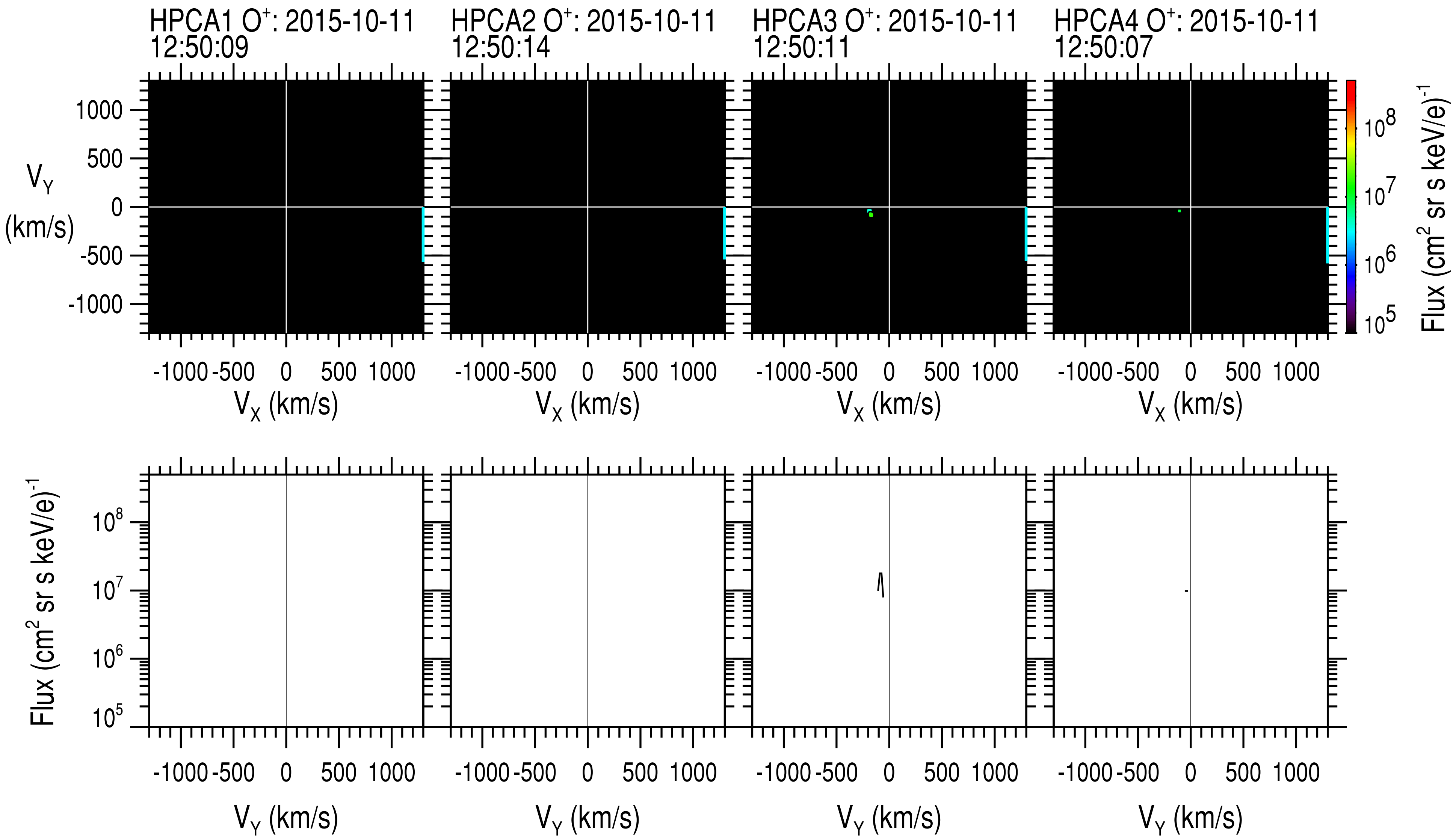
HPCA1 O+: 2015-10-11 12:50:09
HPCA2 O+: 2015-10-11 12:50:14
HPCA3 O+: 2015-10-11 12:50:11
HPCA4 O+: 2015-10-11 12:50:07
VY (km/s)
VX (km/s)
Flux (cm2 sr s keV/e)-1
VY (km/s)

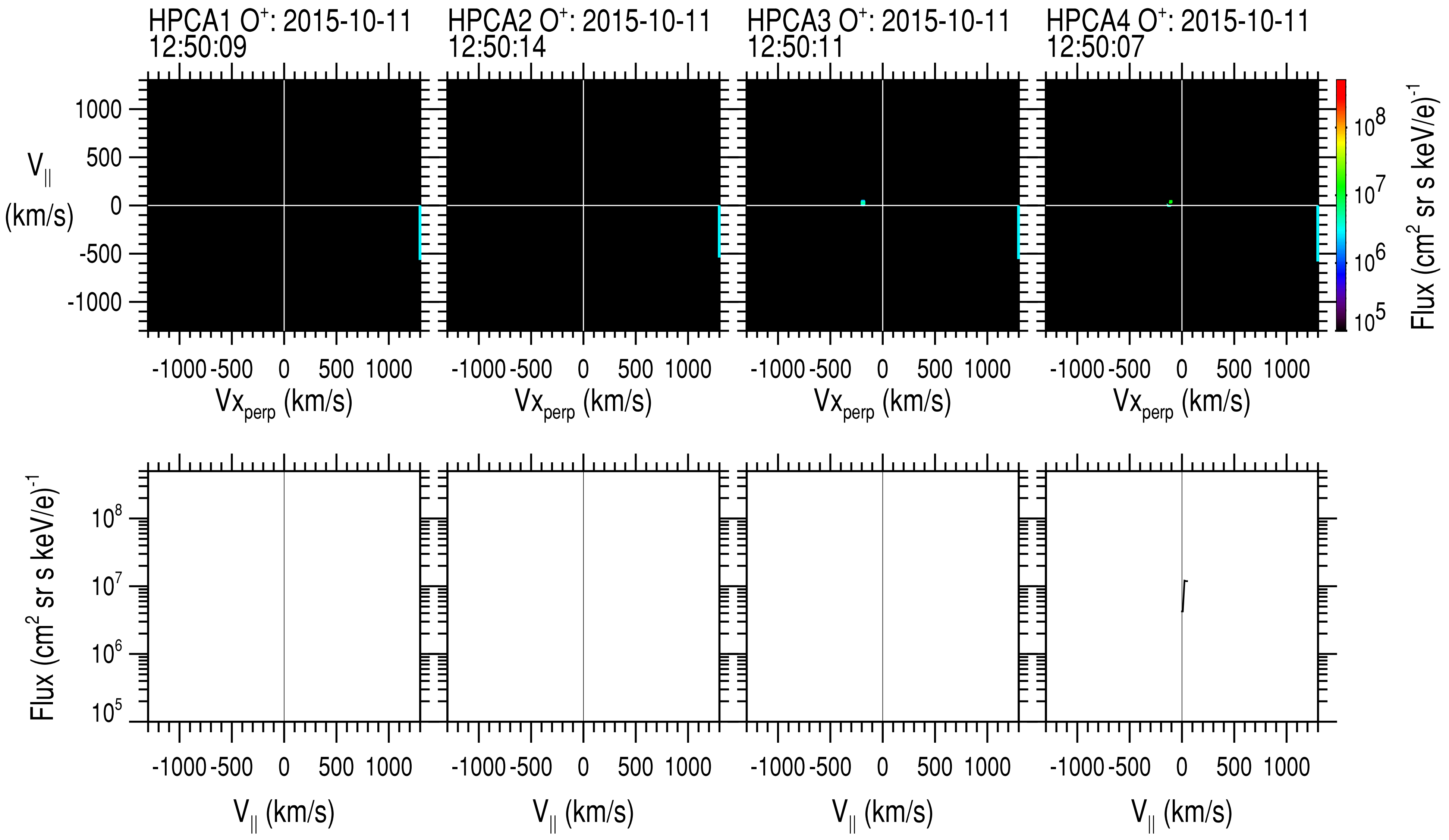
HPCA1 O+: 2015-10-11 12:50:09
HPCA2 O+: 2015-10-11 12:50:14
HPCA3 O+: 2015-10-11 12:50:11
HPCA4 O+: 2015-10-11 12:50:07
V|| (km/s)
Vxperp (km/s)
Flux (cm2 sr s keV/e)-1
V|| (km/s)
Flux (cm2 sr s keV/e)-1

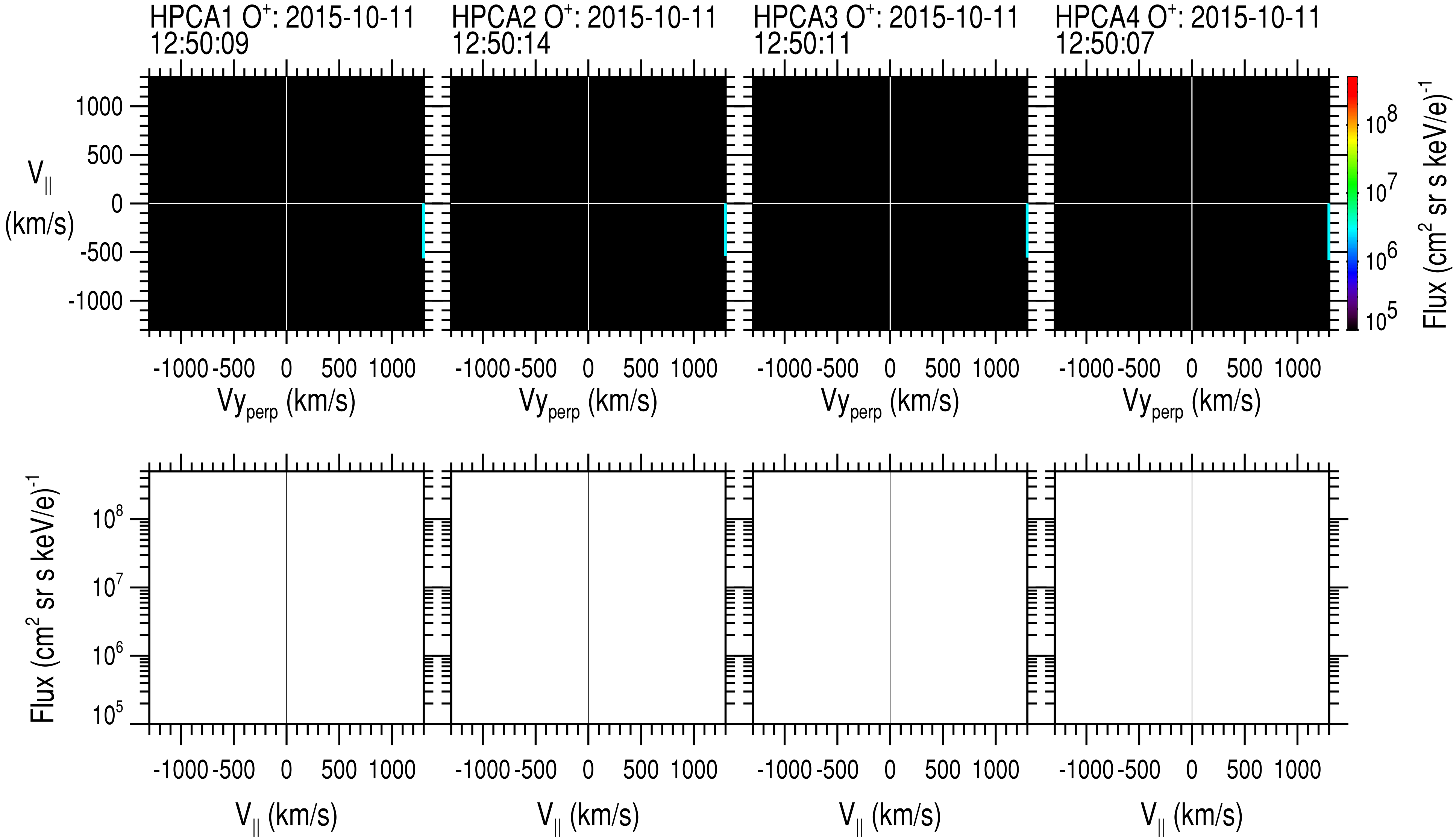

HPCA1 O+: 2015-10-11 12:50:09
HPCA2 O+: 2015-10-11 12:50:14
HPCA3 O+: 2015-10-11 12:50:11
HPCA4 O+: 2015-10-11 12:50:07
V|| (km/s)
1000
500
0
-500
-1000
Vyperp (km/s)
Flux (cm2 sr s keV/e)-1
10^5
10^6
10^7
10^8

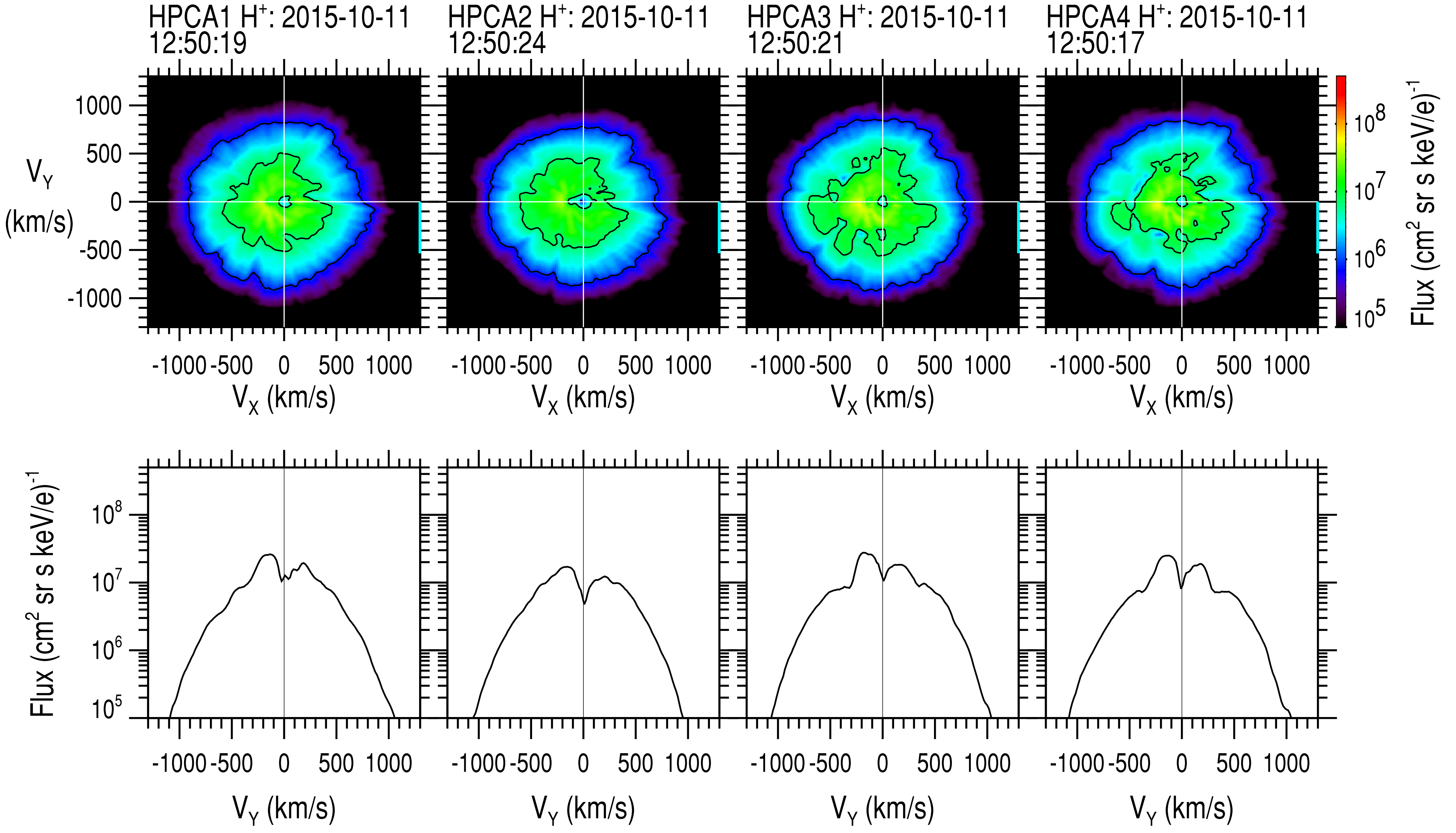

HPCA1 H+: 2015-10-11
12:50:19
HPCA2 H+: 2015-10-11
12:50:24
HPCA3 H+: 2015-10-11
12:50:21
HPCA4 H+: 2015-10-11
12:50:17
V_Y (km/s)
V_X (km/s)
Flux (cm^2 sr s keV/e)^-1
V_Y (km/s)

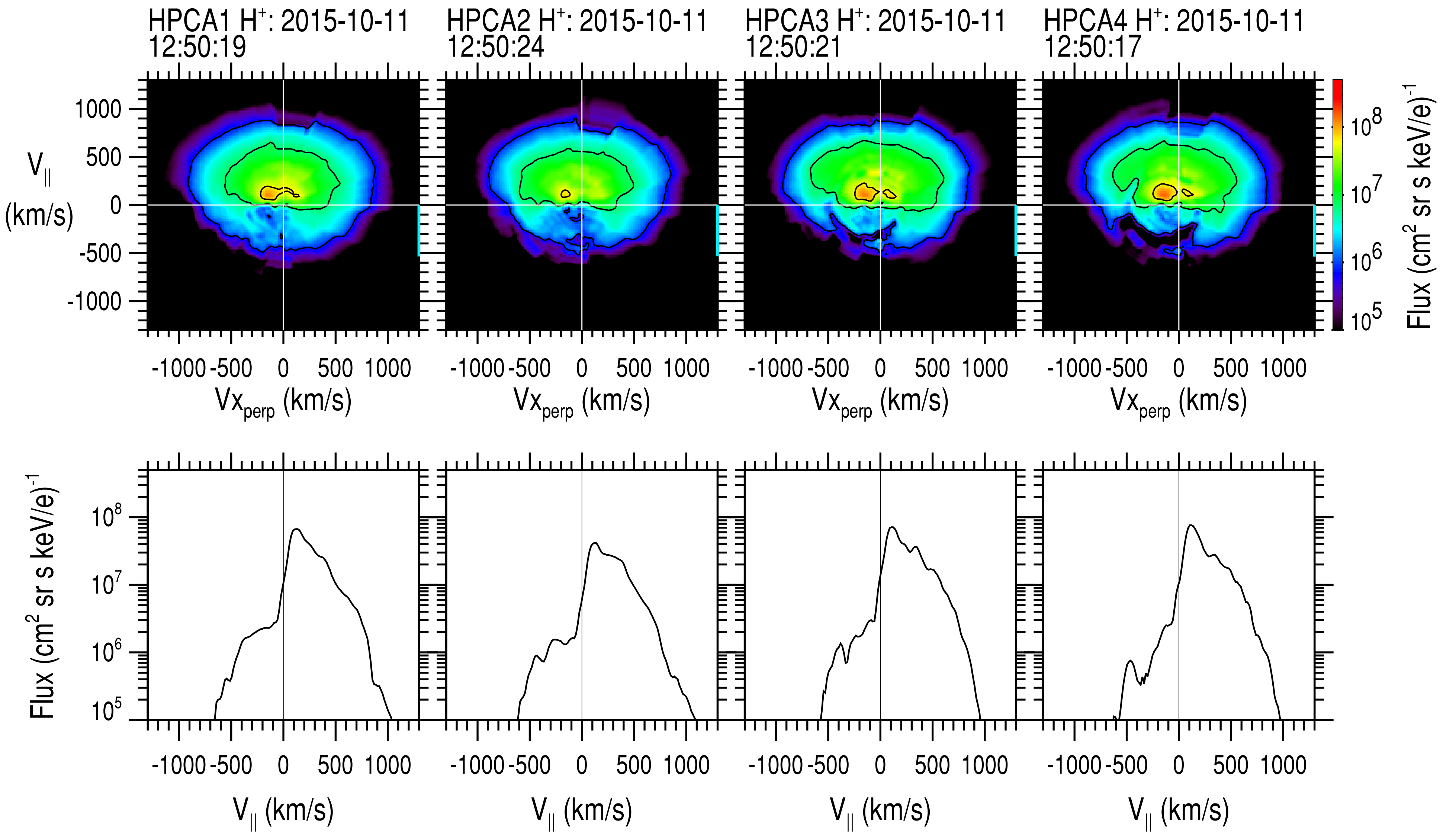

HPCA1 H+: 2015-10-11 12:50:19
HPCA2 H+: 2015-10-11 12:50:24
HPCA3 H+: 2015-10-11 12:50:21
HPCA4 H+: 2015-10-11 12:50:17
V|| (km/s)
Vxperp (km/s)
Flux (cm2 sr s keV/e)-1

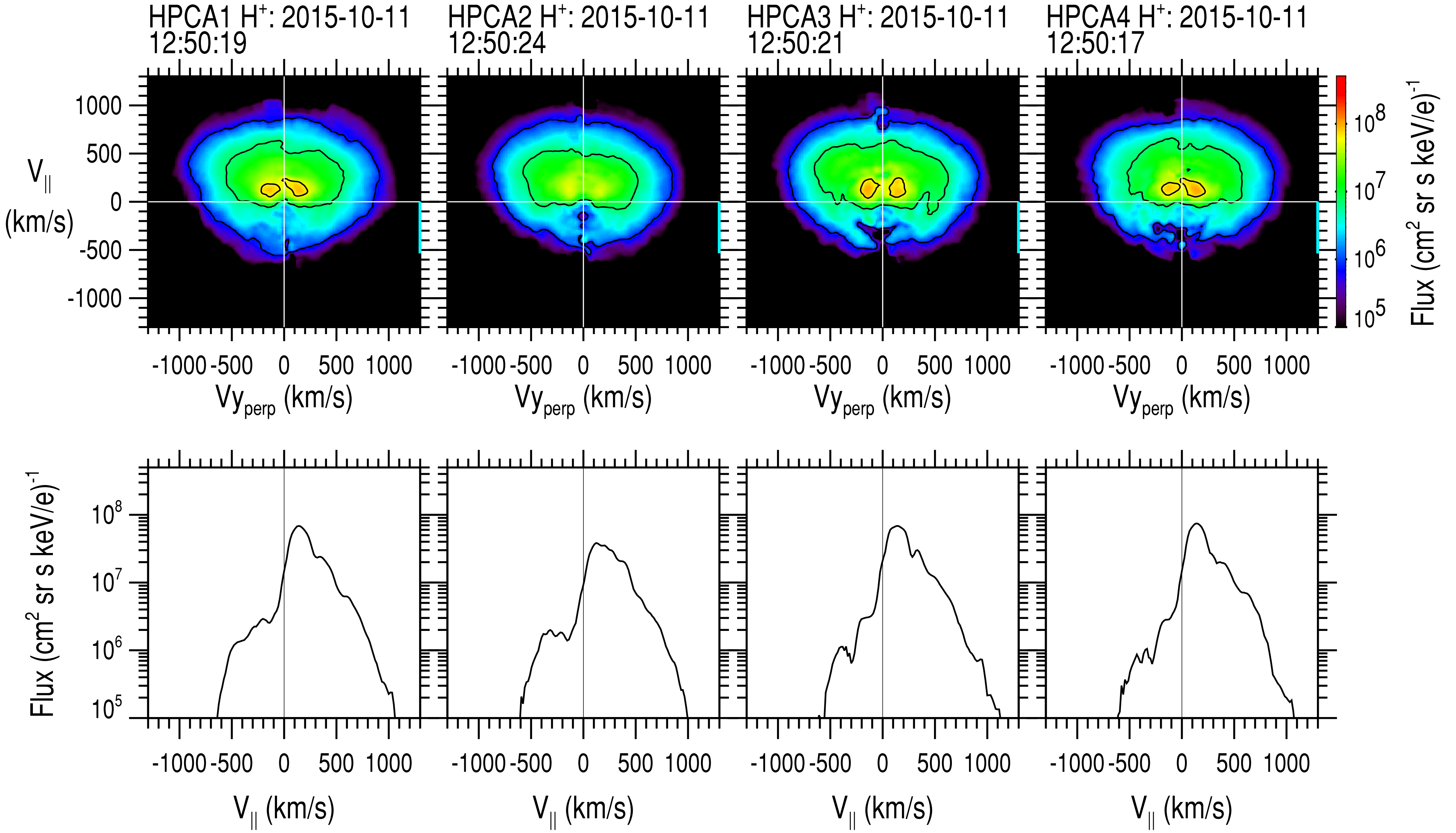
HPCA1 H+: 2015-10-11
12:50:19
HPCA2 H+: 2015-10-11
12:50:24
HPCA3 H+: 2015-10-11
12:50:21
HPCA4 H+: 2015-10-11
12:50:17
V|| (km/s)
Vyperp (km/s)
Flux (cm2 sr s keV/e)-1
V|| (km/s)

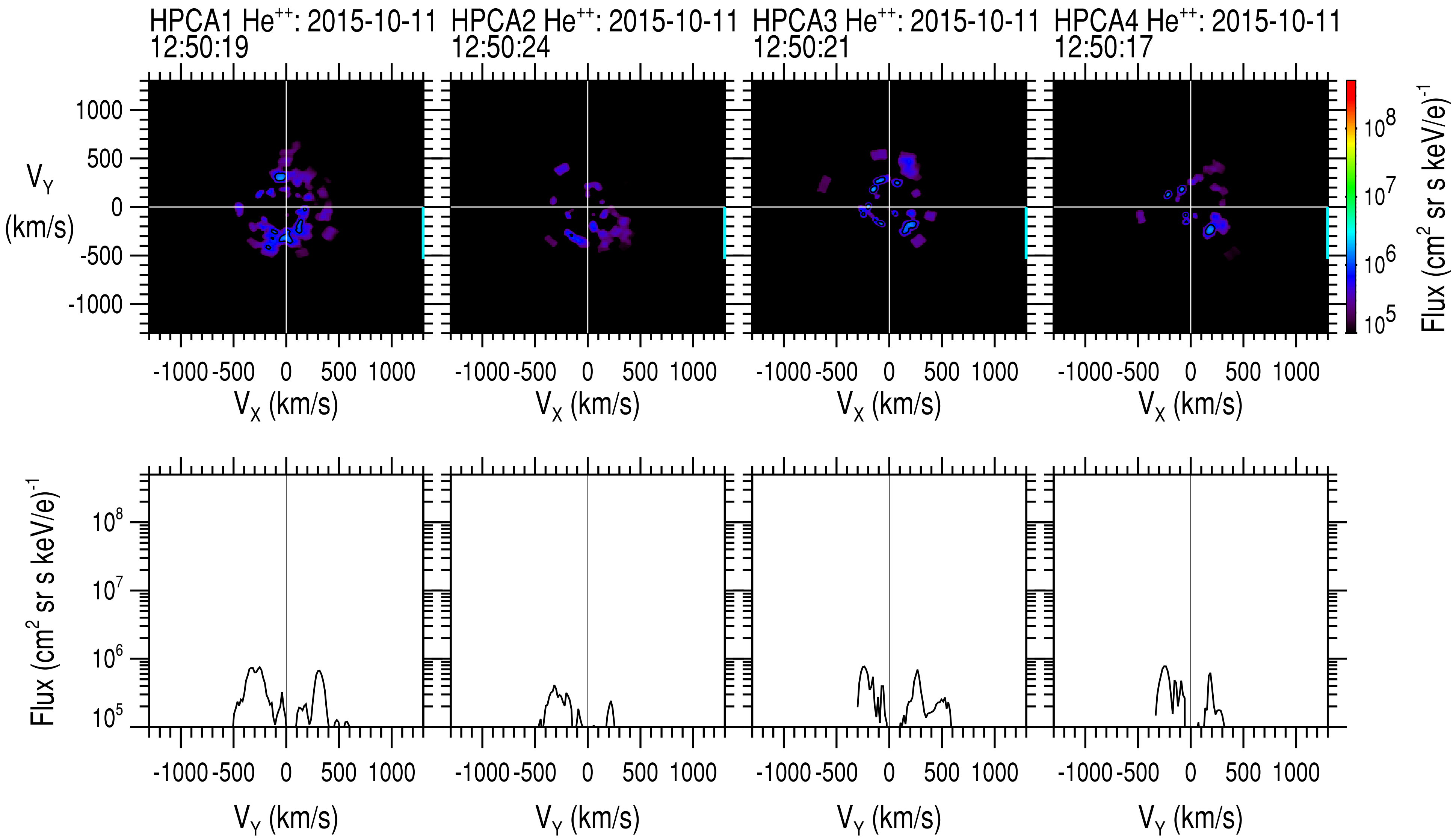

HPCA1 He++: 2015-10-11 12:50:19
HPCA2 He++: 2015-10-11 12:50:24
HPCA3 He++: 2015-10-11 12:50:21
HPCA4 He++: 2015-10-11 12:50:17
V_Y (km/s)
V_X (km/s)
Flux (cm2 sr s keV/e)-1
V_Y (km/s)

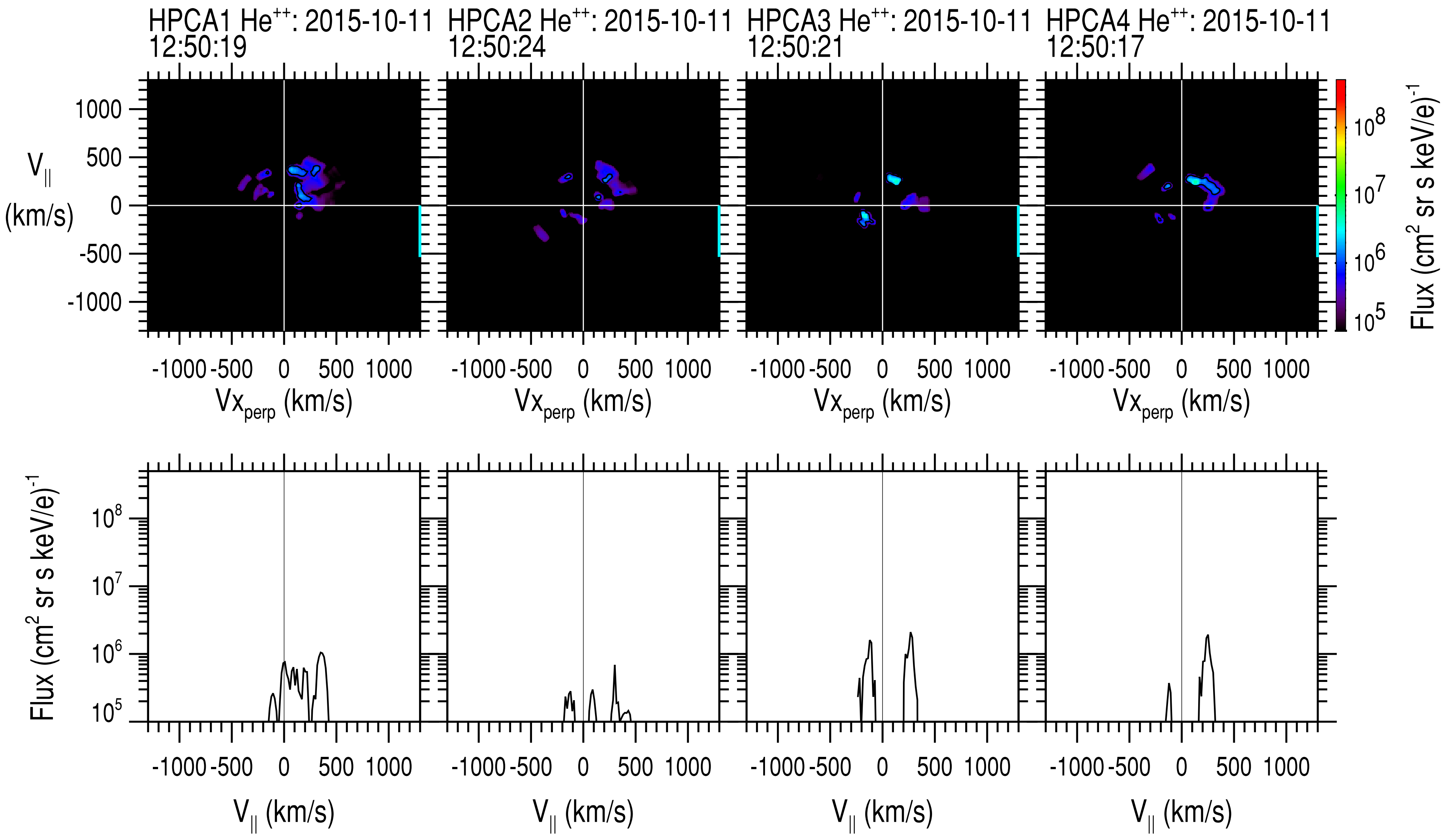

HPCA1 He++: 2015-10-11 12:50:19
HPCA2 He++: 2015-10-11 12:50:24
HPCA3 He++: 2015-10-11 12:50:21
HPCA4 He++: 2015-10-11 12:50:17
V|| (km/s)
Vxperp (km/s)
Flux (cm2 sr s keV/e)-1

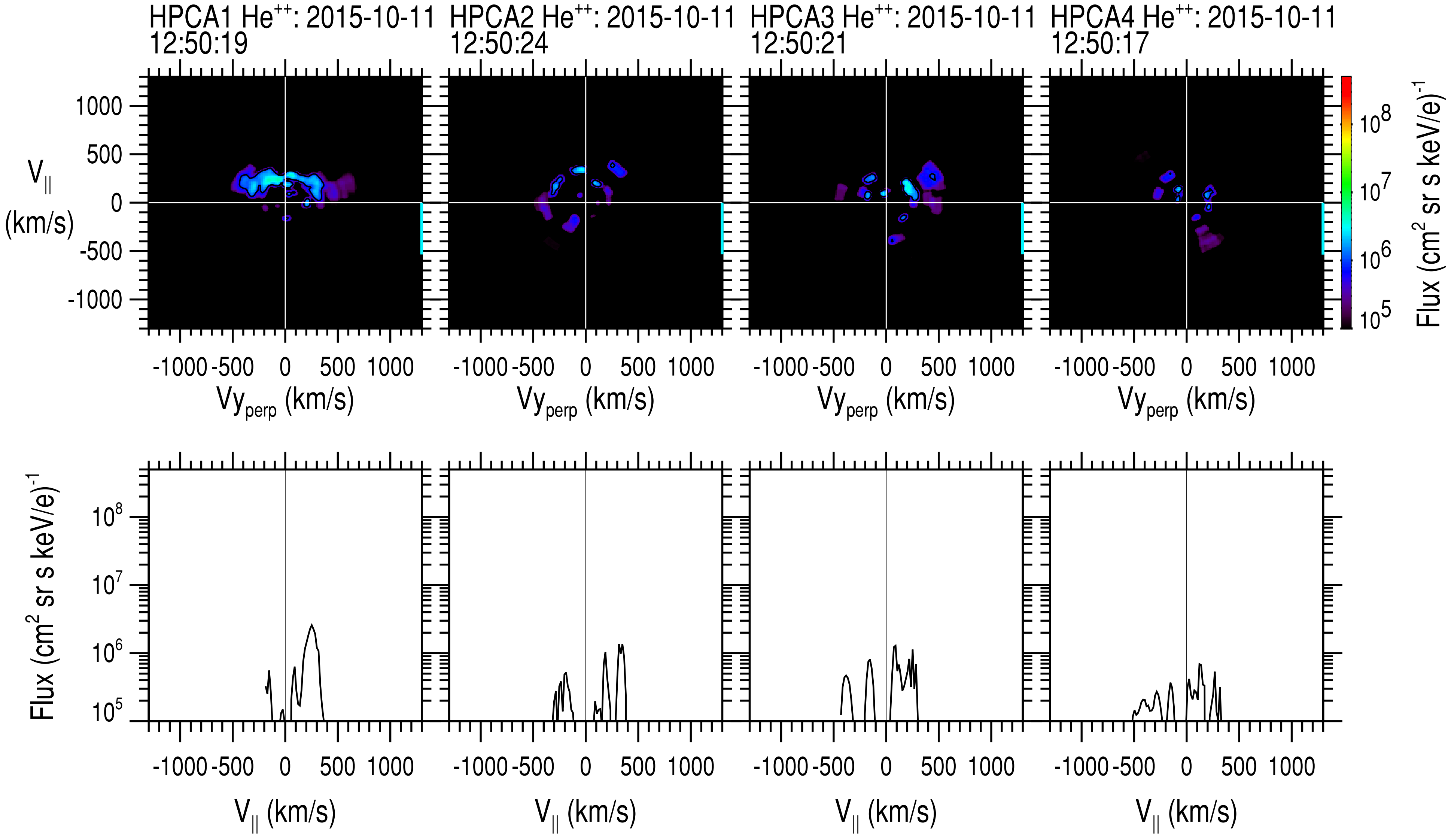

HPCA1 He++: 2015-10-11 12:50:19
HPCA2 He++: 2015-10-11 12:50:24
HPCA3 He++: 2015-10-11 12:50:21
HPCA4 He++: 2015-10-11 12:50:17
V|| (km/s)
Vyperp (km/s)
Flux (cm2 sr s keV/e)-1

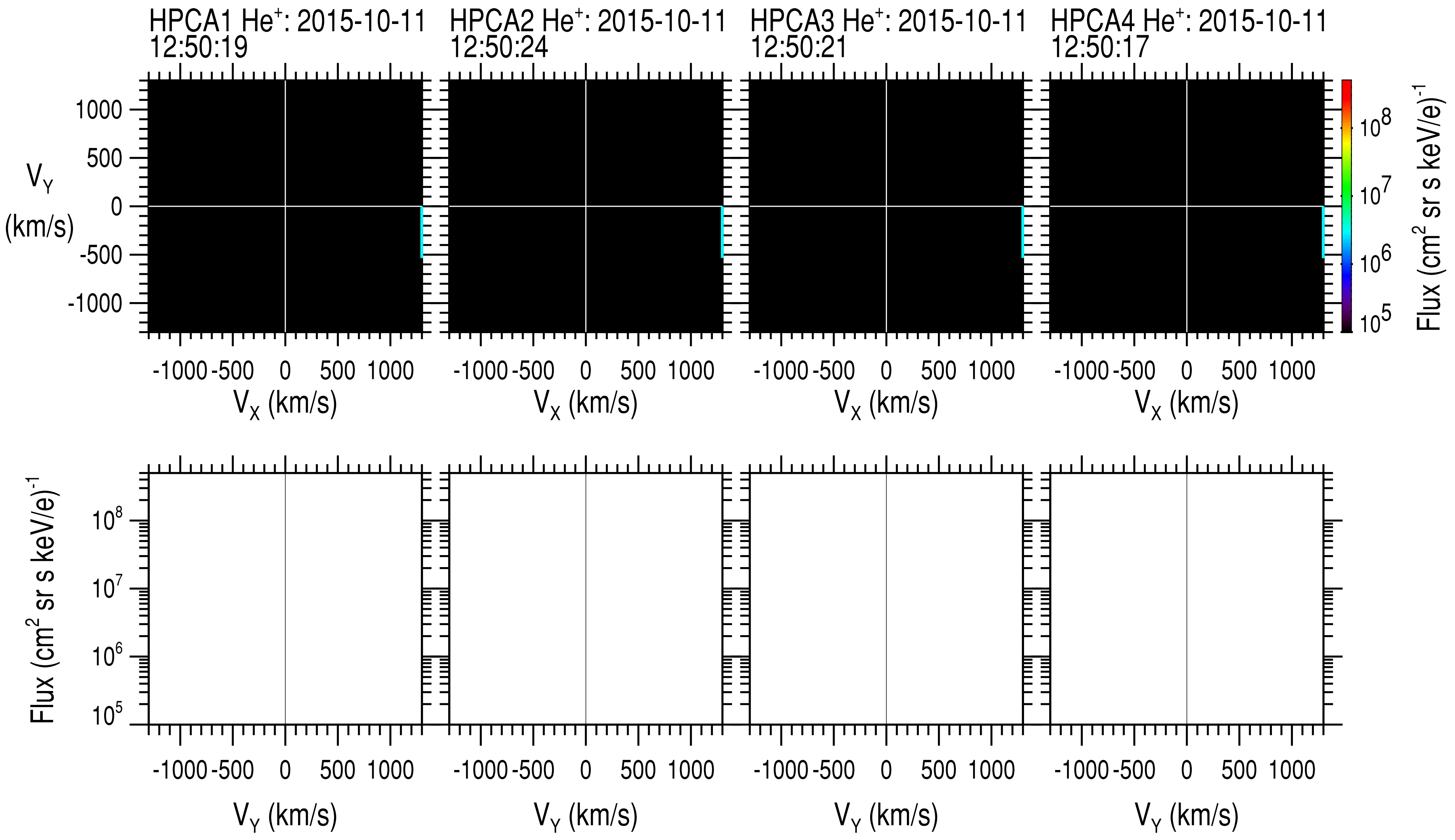
HPCA1 He⁺: 2015-10-11 12:50:19
HPCA2 He⁺: 2015-10-11 12:50:24
HPCA3 He⁺: 2015-10-11 12:50:21
HPCA4 He⁺: 2015-10-11 12:50:17
V_Y (km/s)
V_X (km/s)
Flux (cm^2 sr s keV/e)^-1
-1000 -500 0 500 1000
10^5 10^6 10^7 10^8

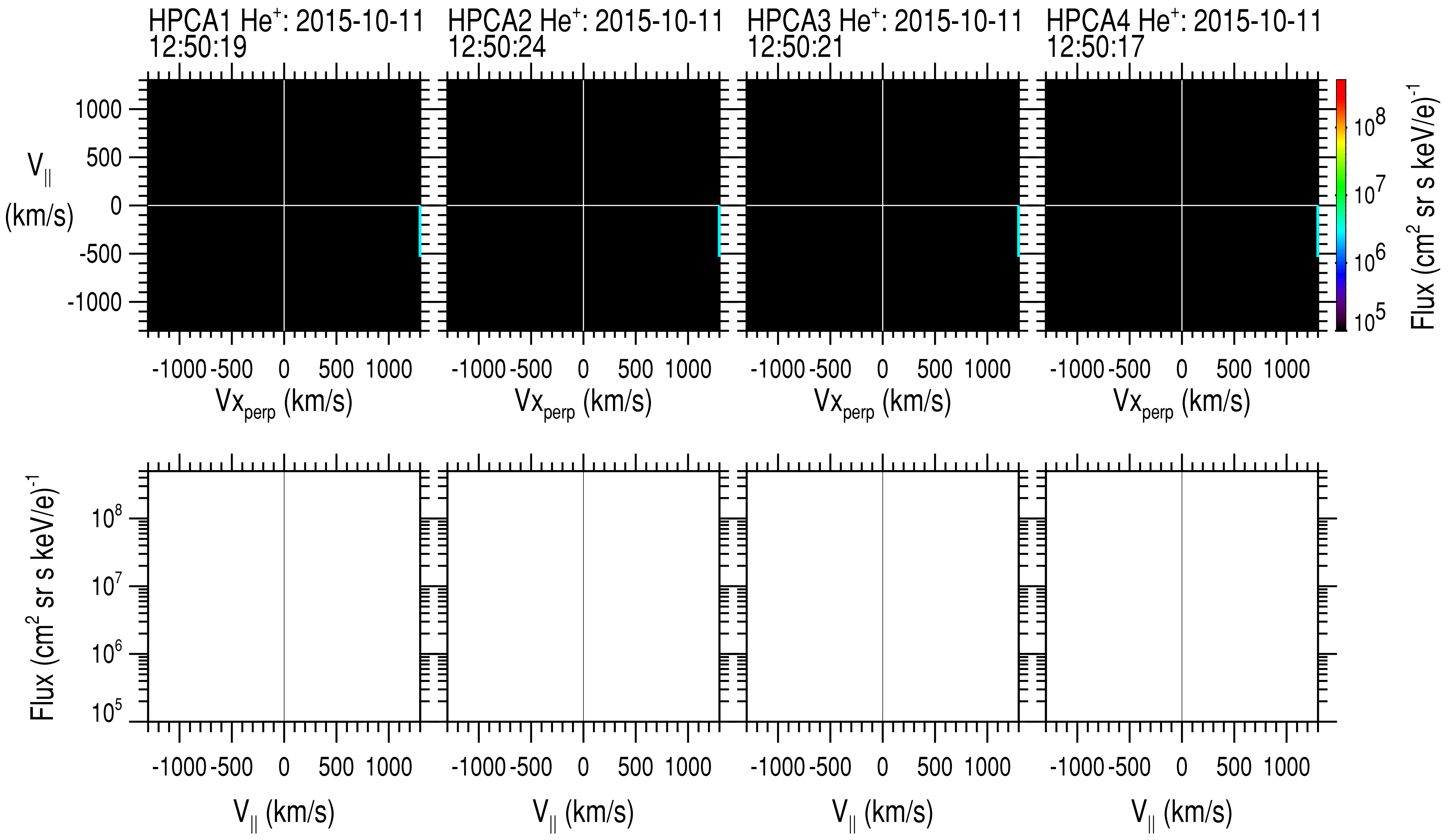
HPCA1 He+: 2015-10-11 12:50:19
HPCA2 He+: 2015-10-11 12:50:24
HPCA3 He+: 2015-10-11 12:50:21
HPCA4 He+: 2015-10-11 12:50:17
V|| (km/s)
Vxperp (km/s)
Flux (cm2 sr s keV/e)-1
-1000 -500 0 500 1000
10^5 10^6 10^7 10^8

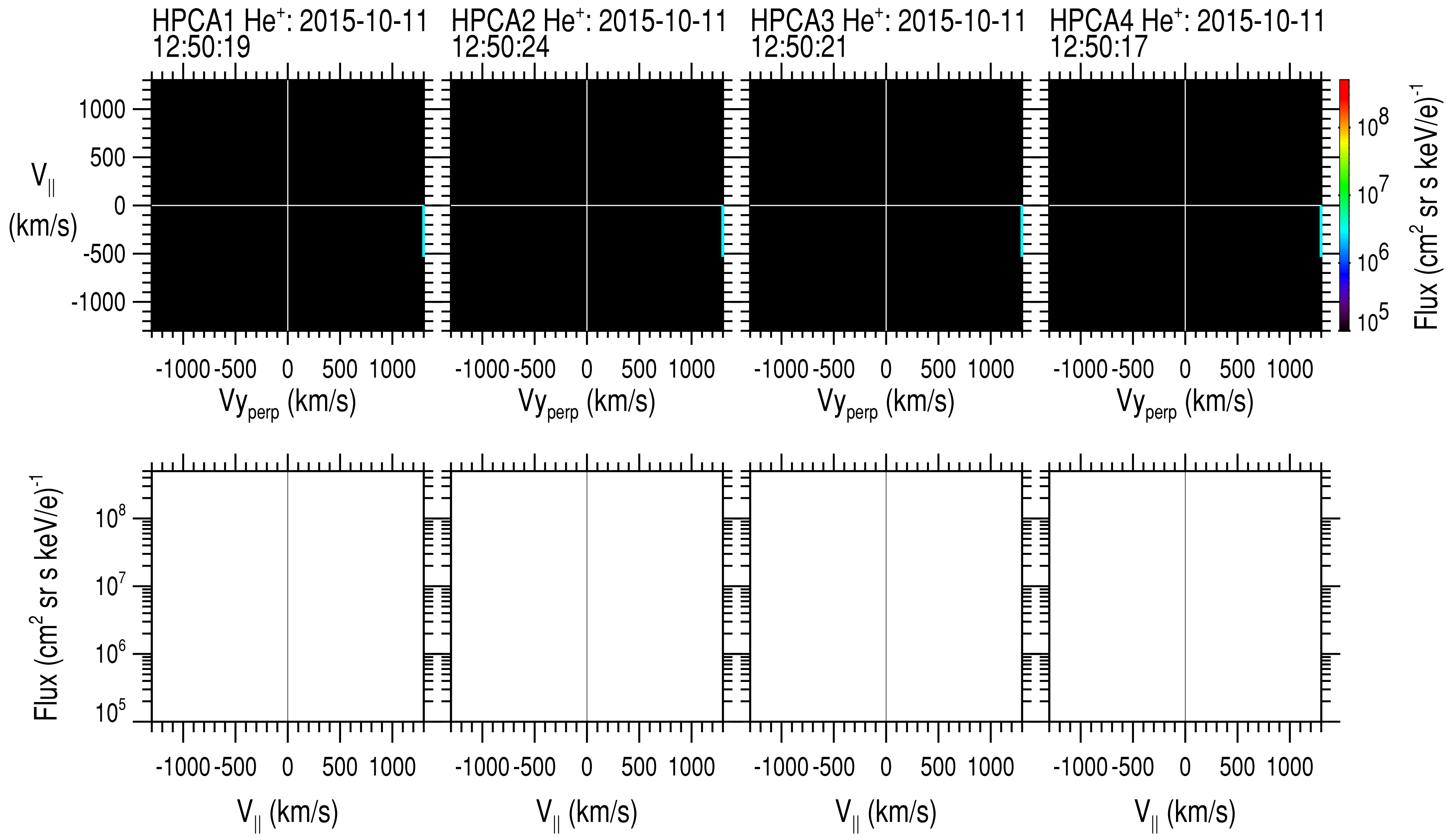

HPCA1 He+: 2015-10-11
12:50:19
HPCA2 He+: 2015-10-11
12:50:24
HPCA3 He+: 2015-10-11
12:50:21
HPCA4 He+: 2015-10-11
12:50:17
V|| (km/s)
1000
500
0
-500
-1000
Vyperp (km/s)
Flux (cm2 sr s keV/e)-1
10^5
10^6
10^7
10^8

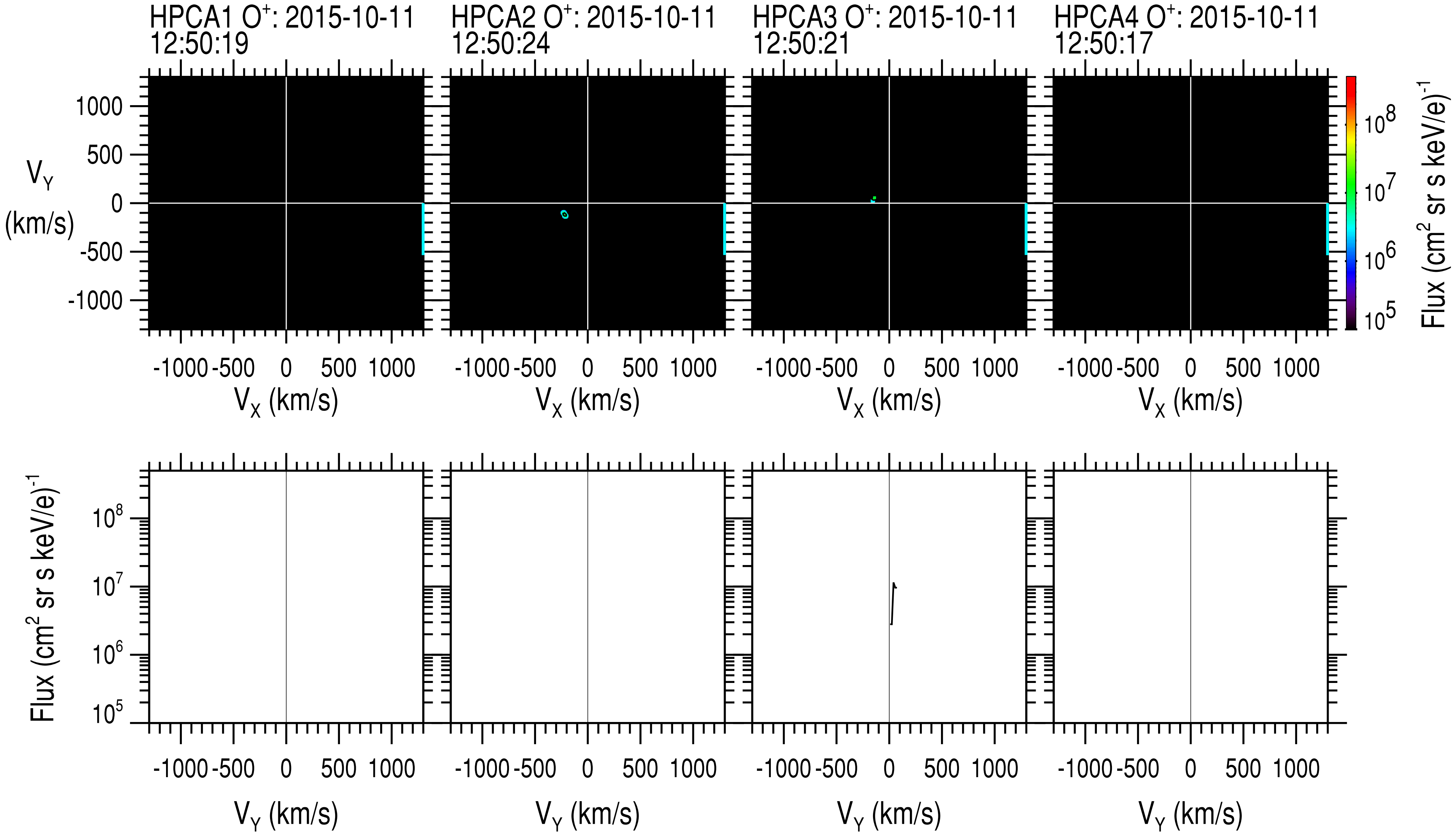

HPCA1 O+: 2015-10-11 12:50:19
HPCA2 O+: 2015-10-11 12:50:24
HPCA3 O+: 2015-10-11 12:50:21
HPCA4 O+: 2015-10-11 12:50:17
VY (km/s)
VX (km/s)
Flux (cm2 sr s keV/e)-1

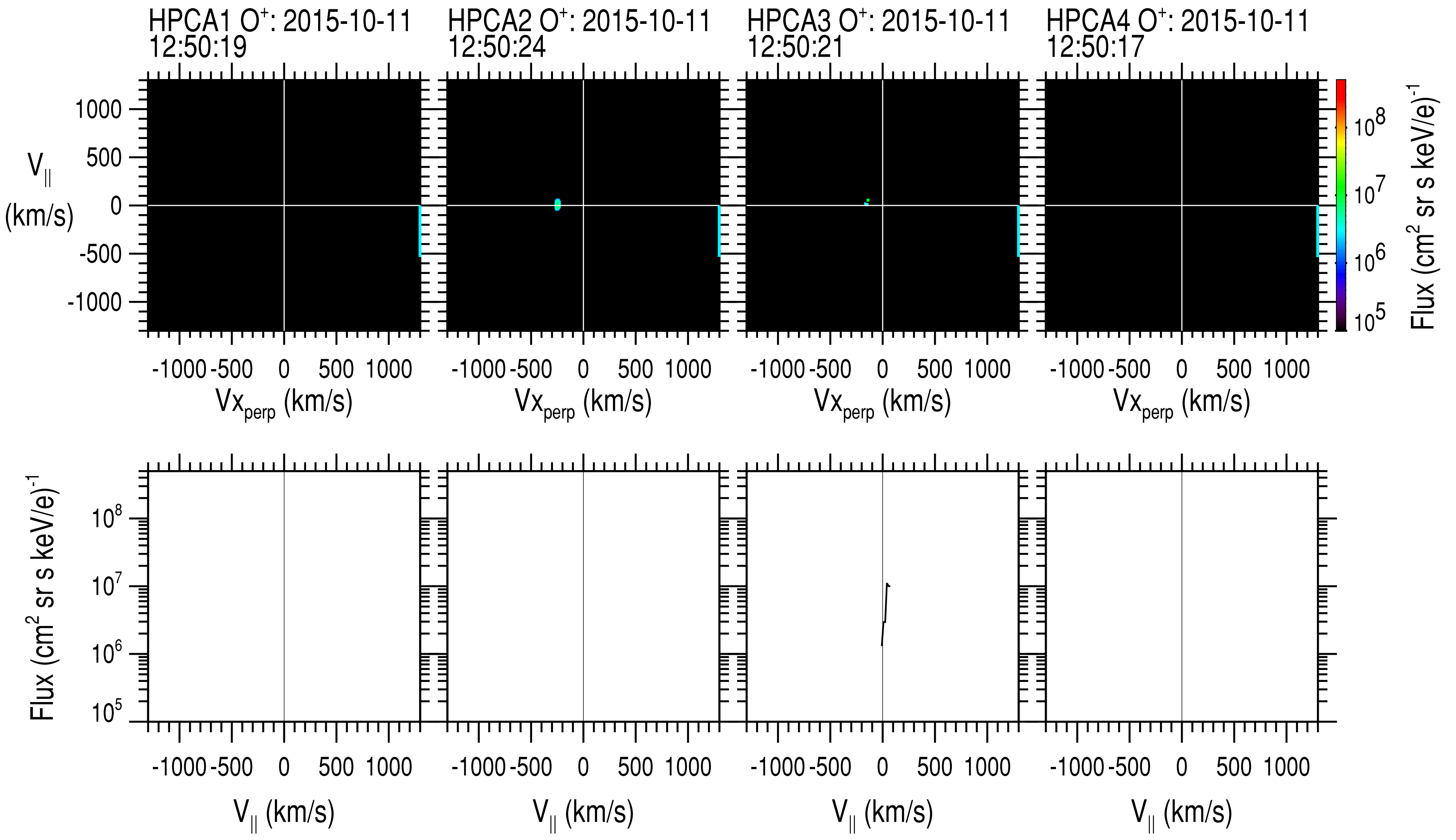
HPCA1 O+: 2015-10-11 12:50:19
HPCA2 O+: 2015-10-11 12:50:24
HPCA3 O+: 2015-10-11 12:50:21
HPCA4 O+: 2015-10-11 12:50:17
V|| (km/s)
Vxperp (km/s)
Flux (cm2 sr s keV/e)-1
-1000 -500 0 500 1000
10^5 10^6 10^7 10^8

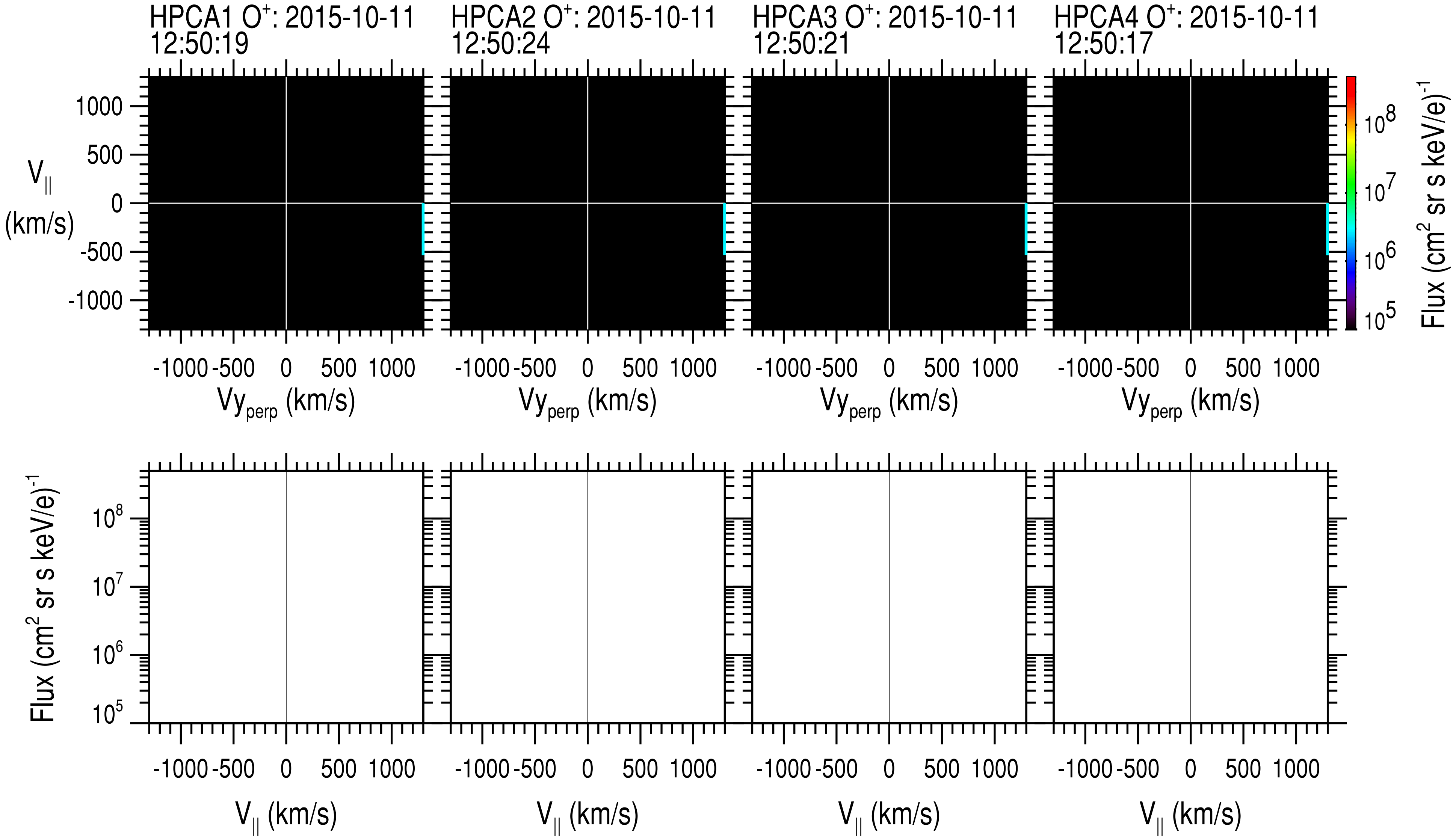

HPCA1 O+: 2015-10-11 12:50:19
HPCA2 O+: 2015-10-11 12:50:24
HPCA3 O+: 2015-10-11 12:50:21
HPCA4 O+: 2015-10-11 12:50:17
V|| (km/s)
Vyperp (km/s)
Flux (cm2 sr s keV/e)-1
1000
500
0
-500
-1000
10^5
10^6
10^7
10^8

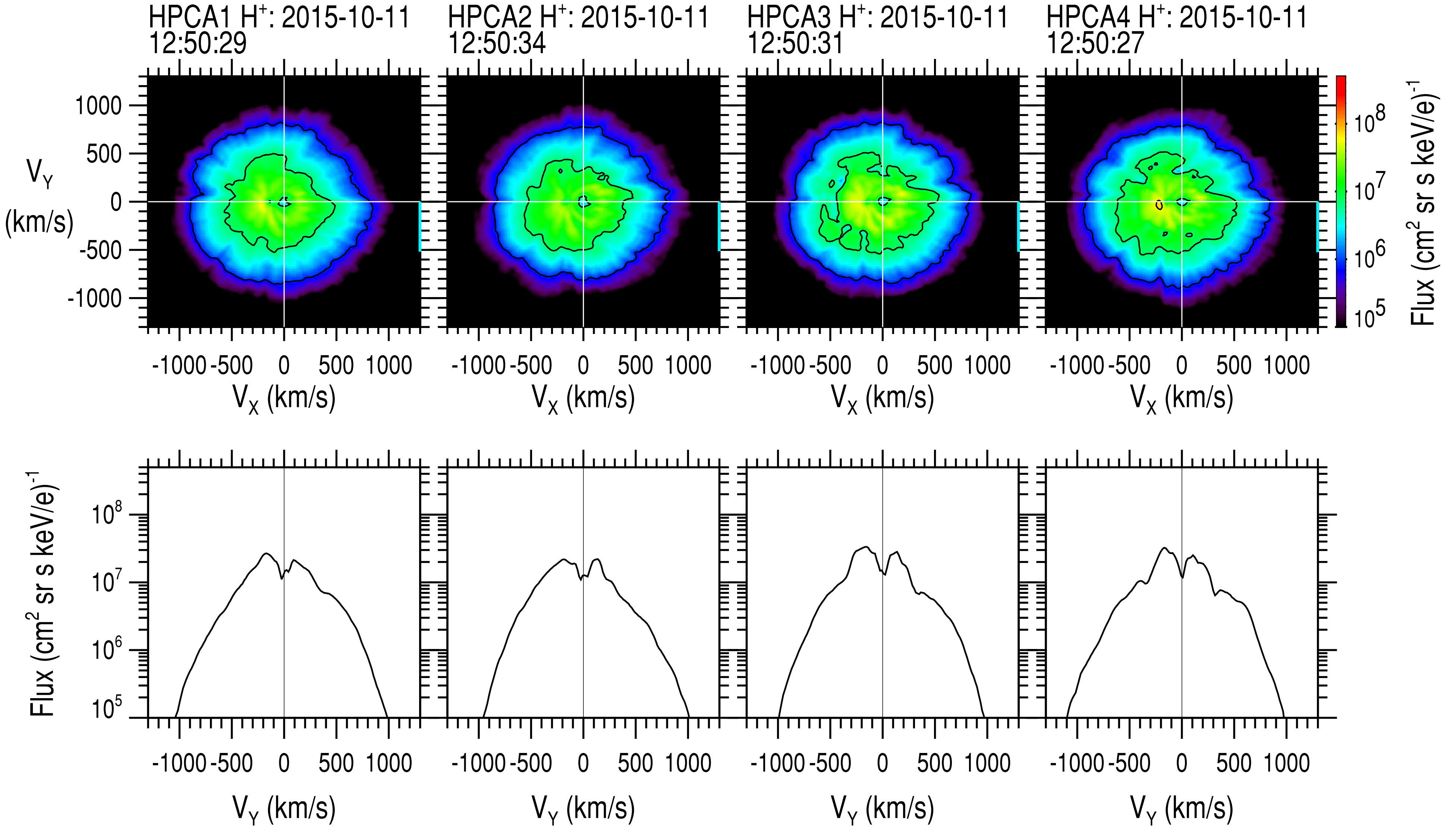
HPCA1 H+: 2015-10-11
12:50:29
HPCA2 H+: 2015-10-11
12:50:34
HPCA3 H+: 2015-10-11
12:50:31
HPCA4 H+: 2015-10-11
12:50:27
V_Y (km/s)
V_X (km/s)
Flux (cm^2 sr s keV/e)^-1
V_Y (km/s)

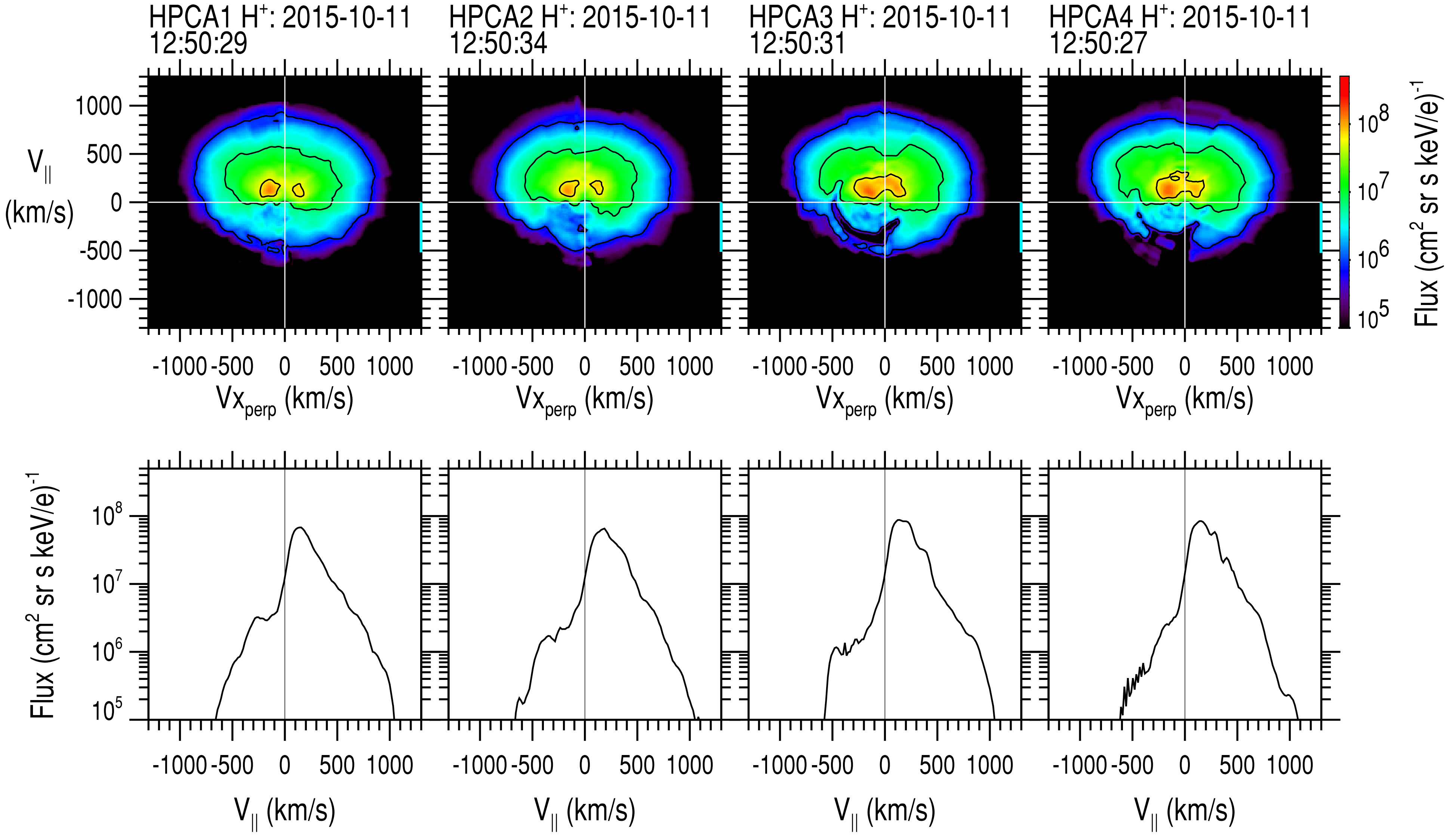

HPCA1 H+: 2015-10-11 12:50:29
HPCA2 H+: 2015-10-11 12:50:34
HPCA3 H+: 2015-10-11 12:50:31
HPCA4 H+: 2015-10-11 12:50:27
V|| (km/s)
Vxperp (km/s)
Flux (cm2 sr s keV/e)-1
V|| (km/s)

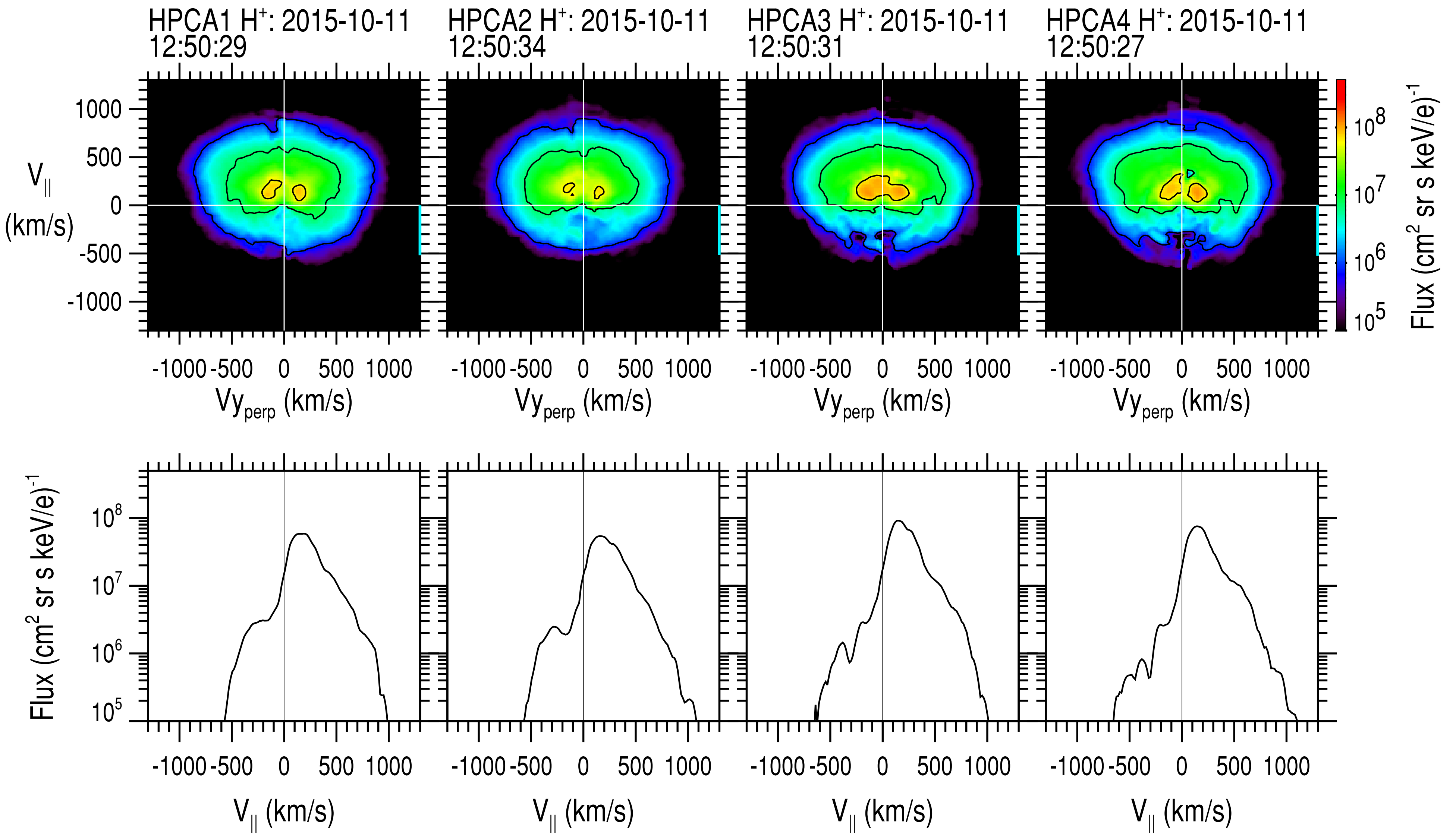

HPCA1 H+: 2015-10-11
12:50:29
HPCA2 H+: 2015-10-11
12:50:34
HPCA3 H+: 2015-10-11
12:50:31
HPCA4 H+: 2015-10-11
12:50:27
V|| (km/s)
Vyperp (km/s)
Flux (cm2 sr s keV/e)-1
V|| (km/s)

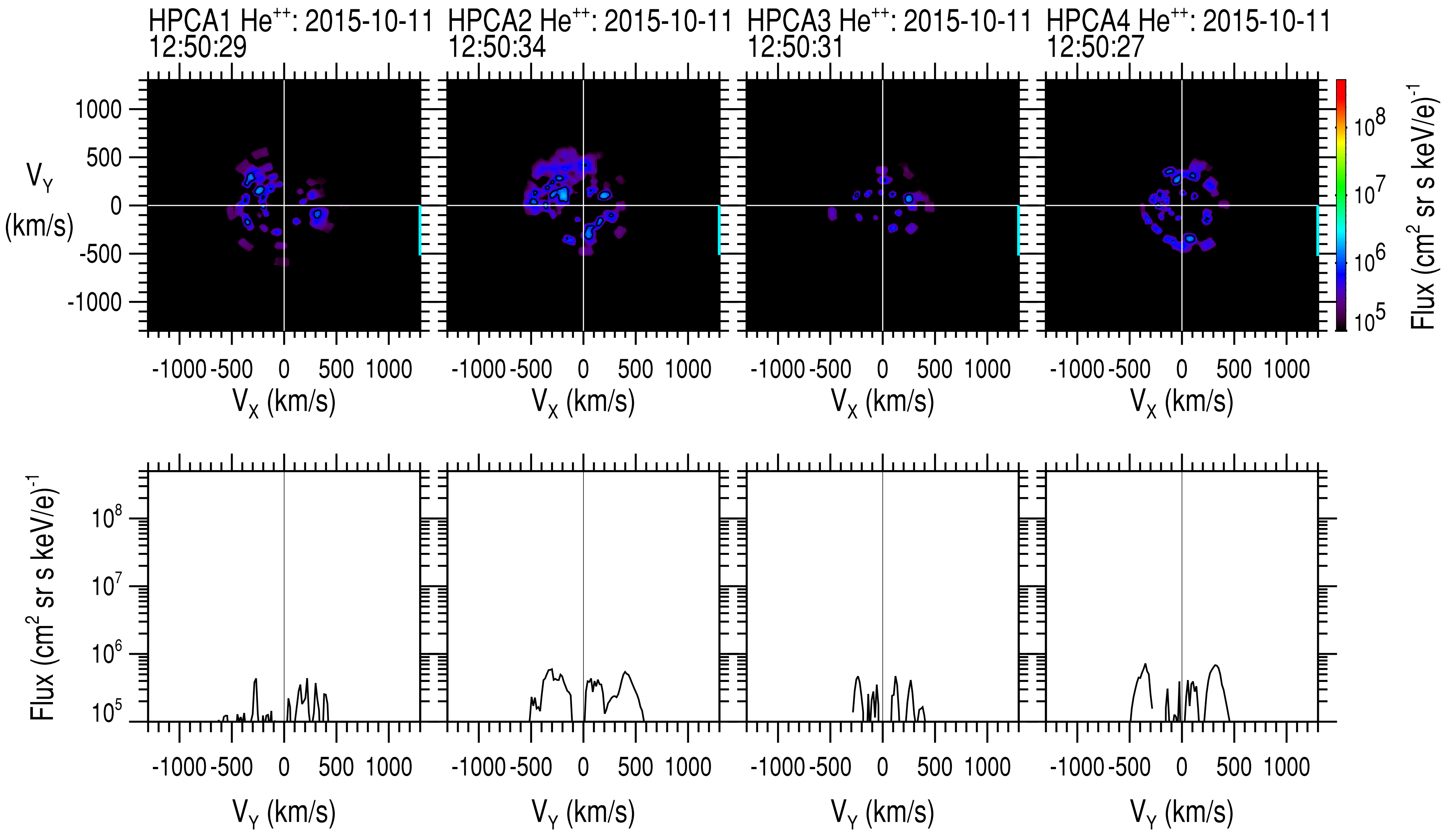
HPCA1 He++: 2015-10-11 12:50:29
HPCA2 He++: 2015-10-11 12:50:34
HPCA3 He++: 2015-10-11 12:50:31
HPCA4 He++: 2015-10-11 12:50:27
V_Y (km/s)
V_X (km/s)
Flux (cm^2 sr s keV/e)^-1
V_Y (km/s)
Flux (cm^2 sr s keV/e)^-1

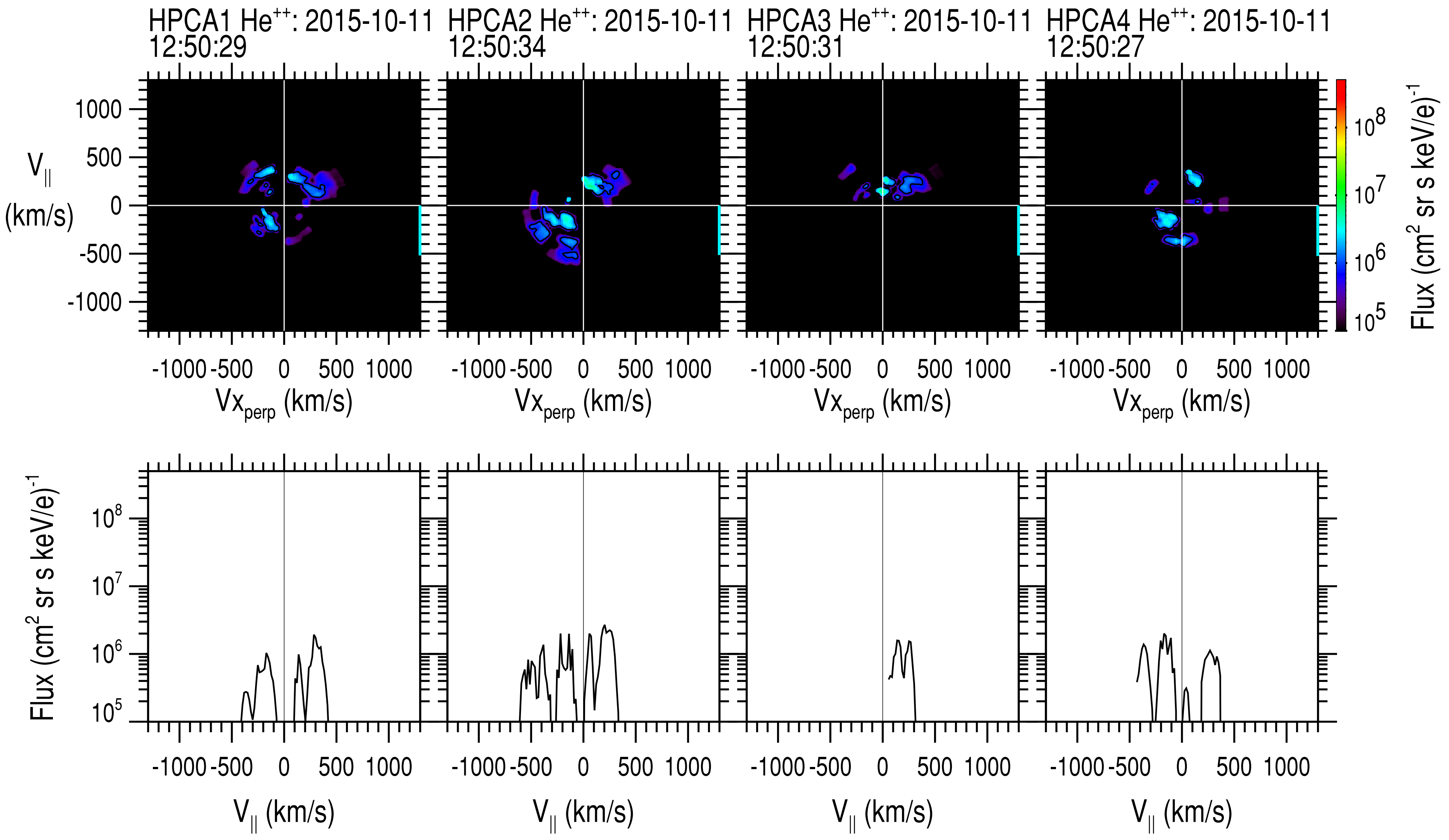

HPCA1 He++: 2015-10-11 12:50:29
HPCA2 He++: 2015-10-11 12:50:34
HPCA3 He++: 2015-10-11 12:50:31
HPCA4 He++: 2015-10-11 12:50:27
V|| (km/s)
Vxperp (km/s)
Flux (cm2 sr s keV/e)-1

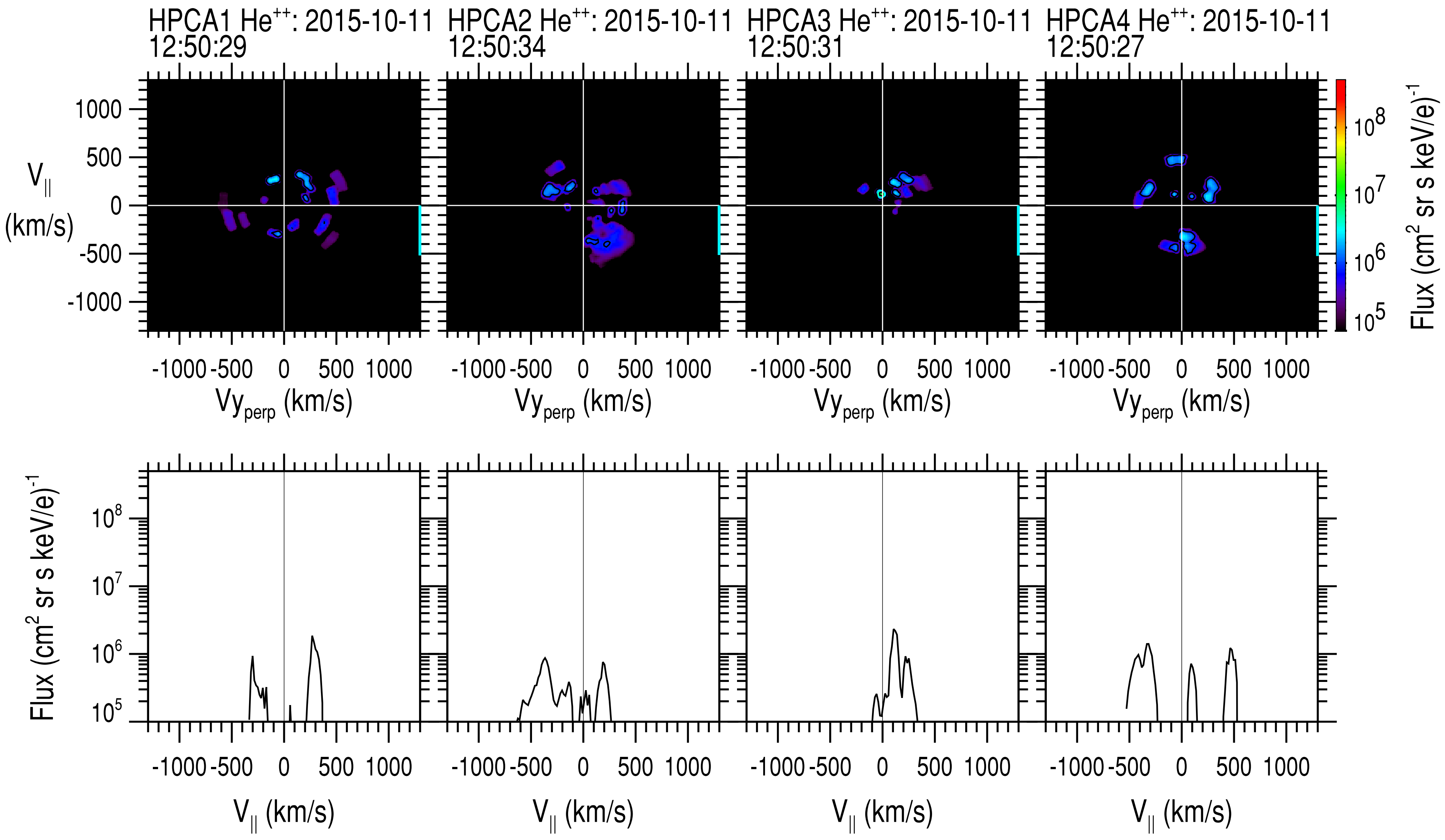

HPCA1 He++: 2015-10-11 12:50:29
HPCA2 He++: 2015-10-11 12:50:34
HPCA3 He++: 2015-10-11 12:50:31
HPCA4 He++: 2015-10-11 12:50:27
V|| (km/s)
Vyperp (km/s)
Flux (cm2 sr s keV/e)-1

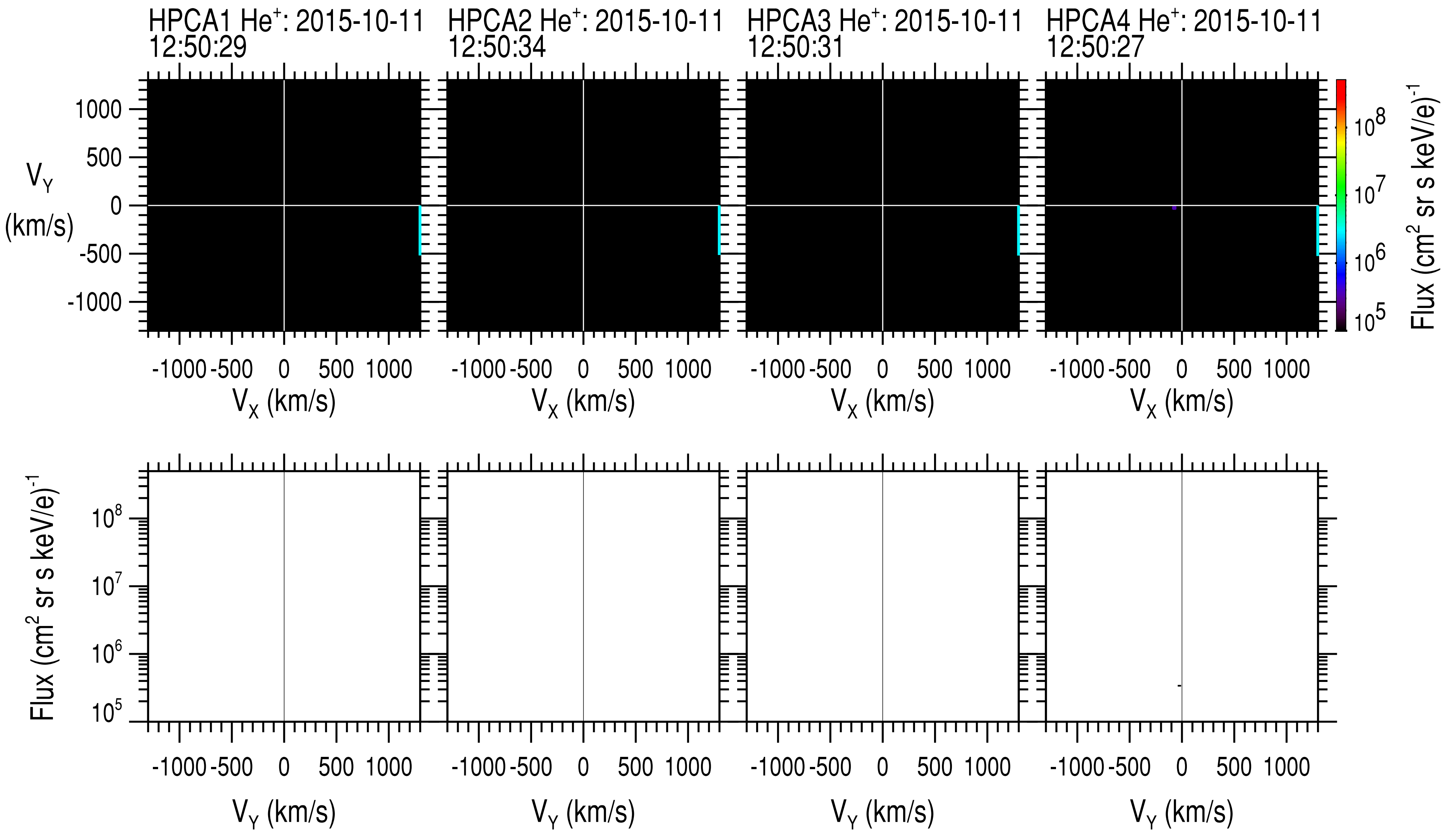

HPCA1 He+: 2015-10-11 12:50:29
HPCA2 He+: 2015-10-11 12:50:34
HPCA3 He+: 2015-10-11 12:50:31
HPCA4 He+: 2015-10-11 12:50:27
VY (km/s)
VX (km/s)
Flux (cm2 sr s keV/e)-1
-1000 -500 0 500 1000
10^5 10^6 10^7 10^8

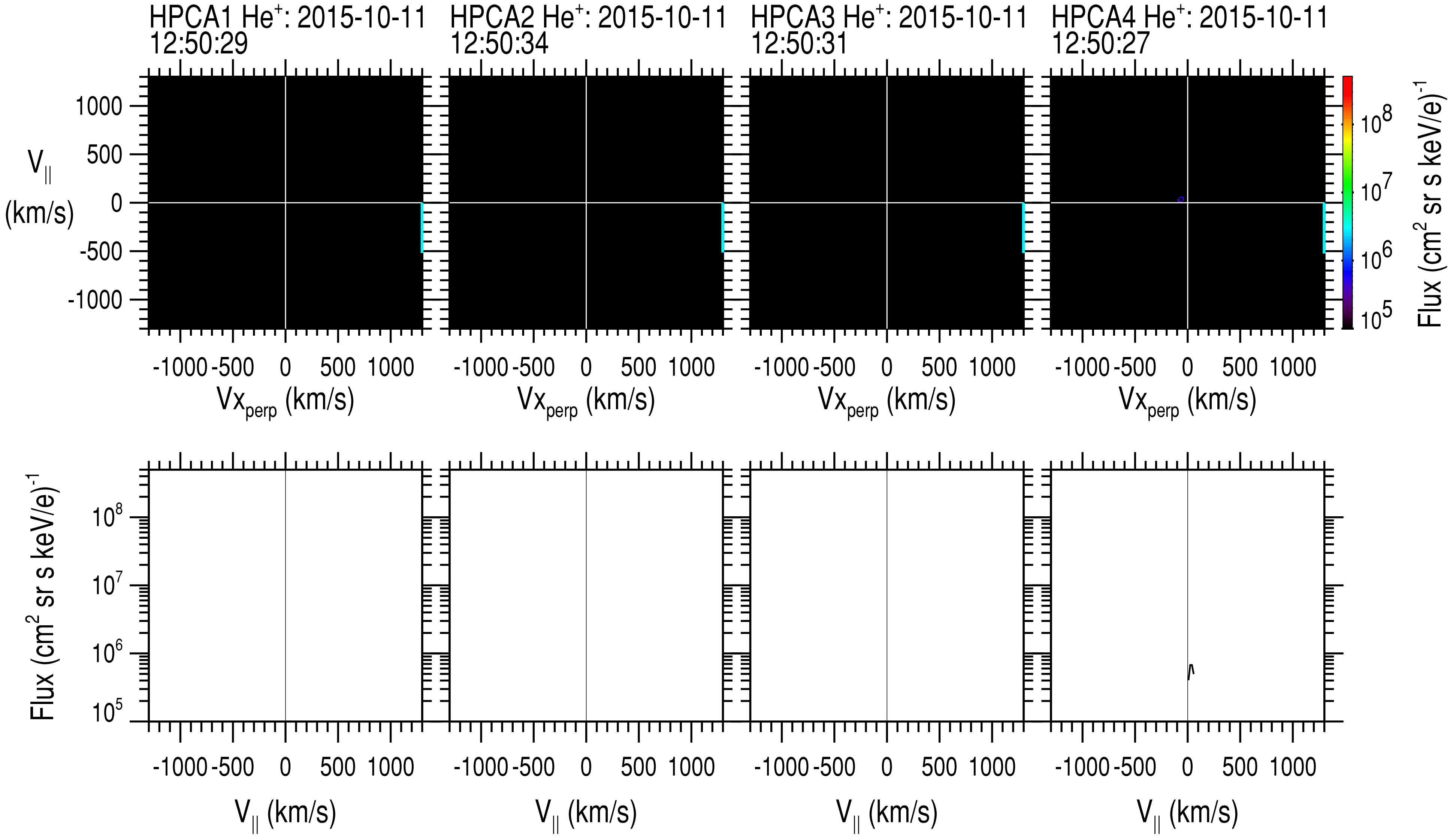

HPCA1 He+: 2015-10-11 12:50:29
HPCA2 He+: 2015-10-11 12:50:34
HPCA3 He+: 2015-10-11 12:50:31
HPCA4 He+: 2015-10-11 12:50:27
V|| (km/s)
Vx_perp (km/s)
Flux (cm2 sr s keV/e)-1

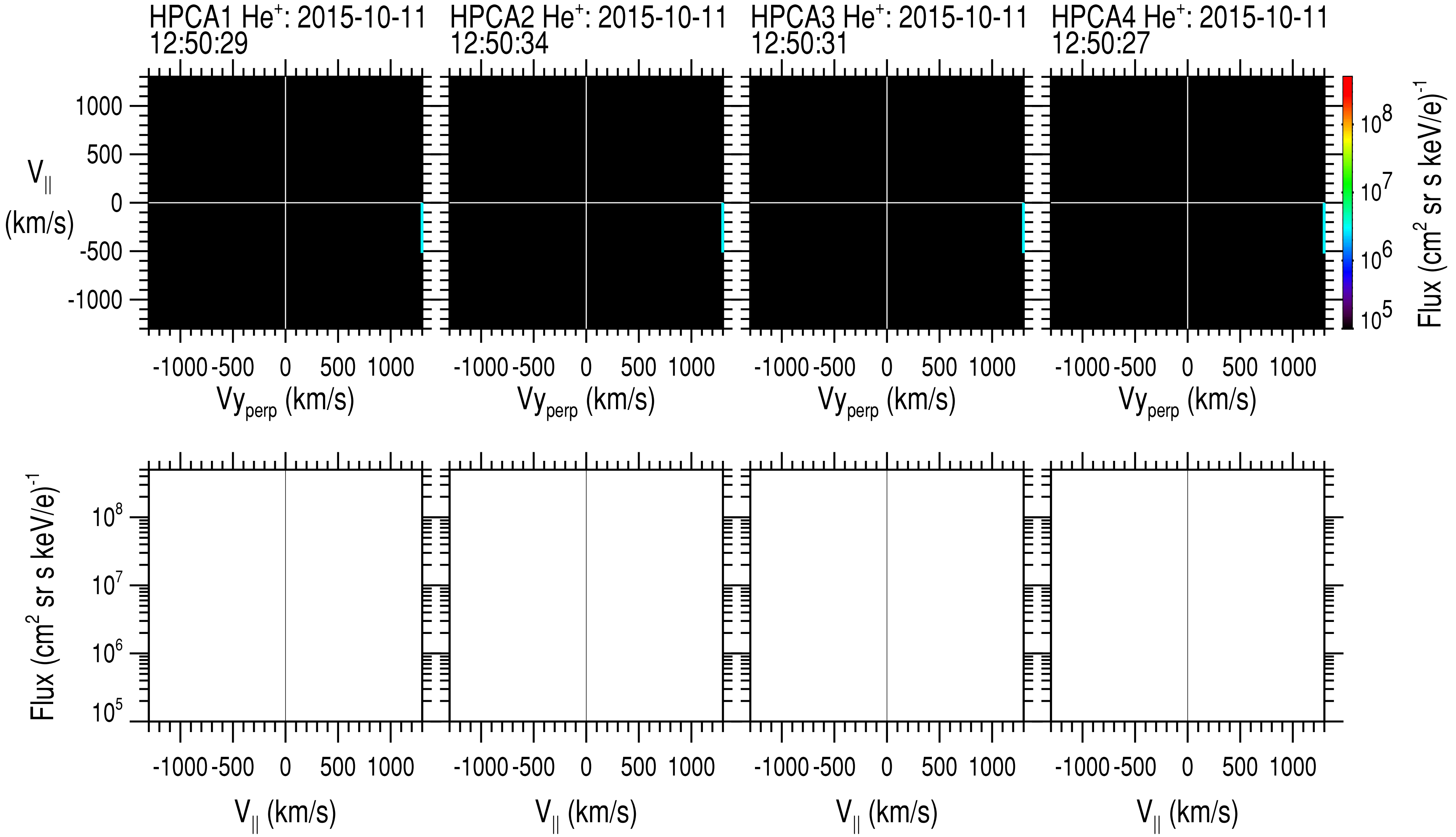
HPCA1 He+: 2015-10-11 12:50:29
HPCA2 He+: 2015-10-11 12:50:34
HPCA3 He+: 2015-10-11 12:50:31
HPCA4 He+: 2015-10-11 12:50:27
V|| (km/s)
1000
500
0
-500
-1000
Vyperp (km/s)
Flux (cm2 sr s keV/e)-1
10^5
10^6
10^7
10^8
V|| (km/s)

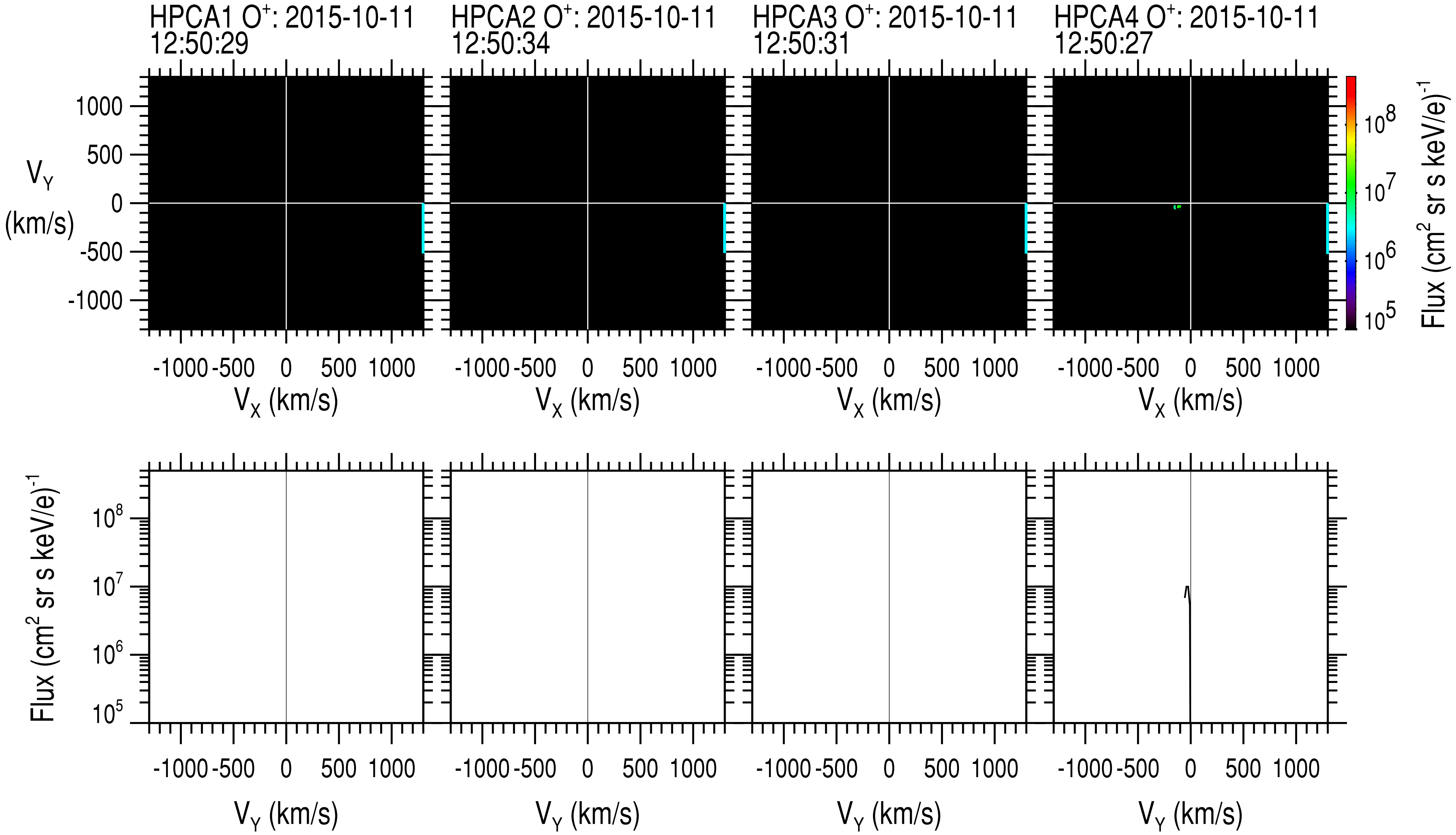

HPCA1 O+: 2015-10-11
12:50:29
HPCA2 O+: 2015-10-11
12:50:34
HPCA3 O+: 2015-10-11
12:50:31
HPCA4 O+: 2015-10-11
12:50:27
VY (km/s)
1000
500
0
-500
-1000
-1000 -500 0 500 1000
VX (km/s)
Flux (cm2 sr s keV/e)-1
10^5
10^6
10^7
10^8
VY (km/s)

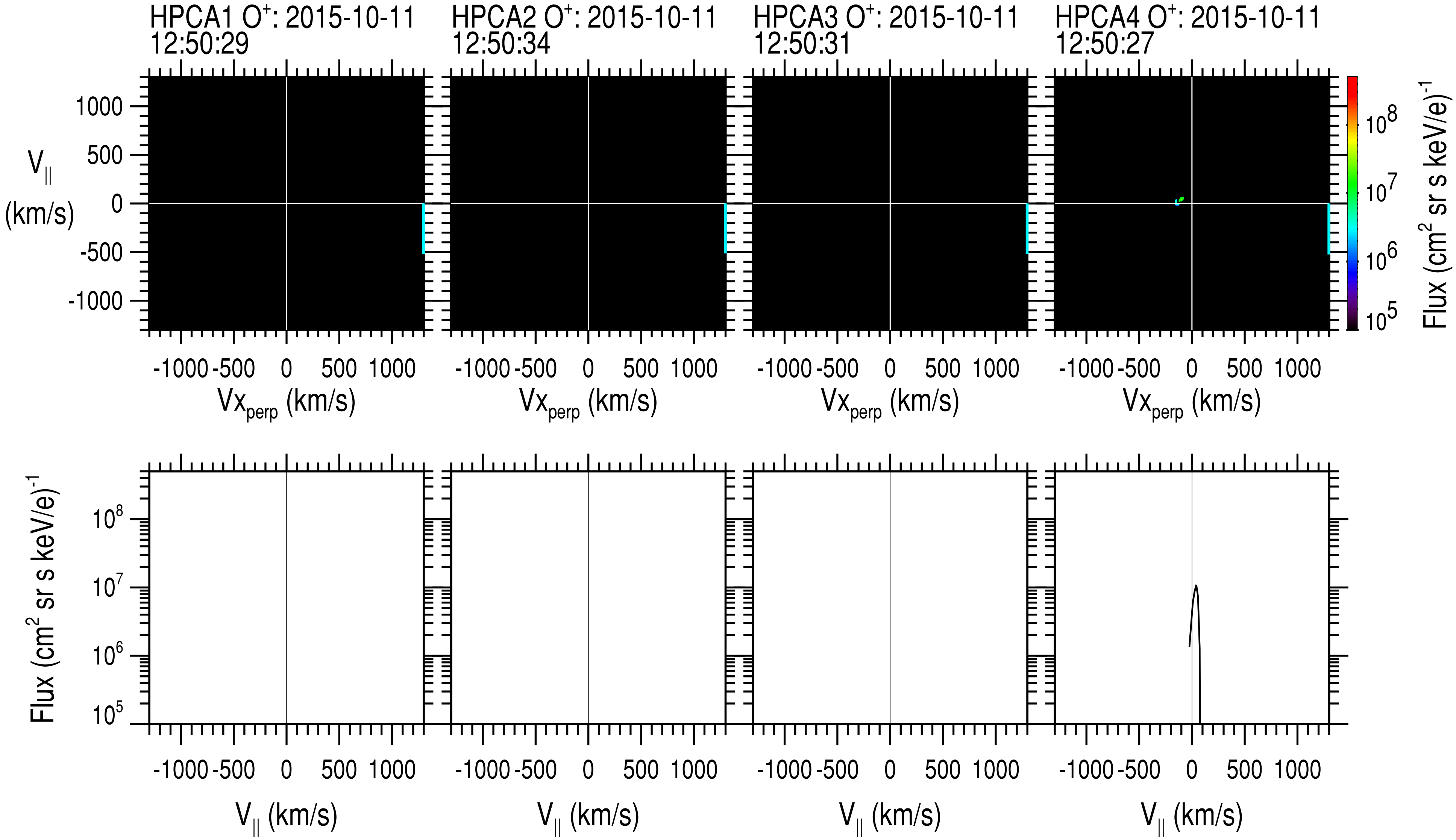

HPCA1 O+: 2015-10-11 12:50:29
HPCA2 O+: 2015-10-11 12:50:34
HPCA3 O+: 2015-10-11 12:50:31
HPCA4 O+: 2015-10-11 12:50:27
V|| (km/s)
Vxperp (km/s)
Flux (cm2 sr s keV/e)-1

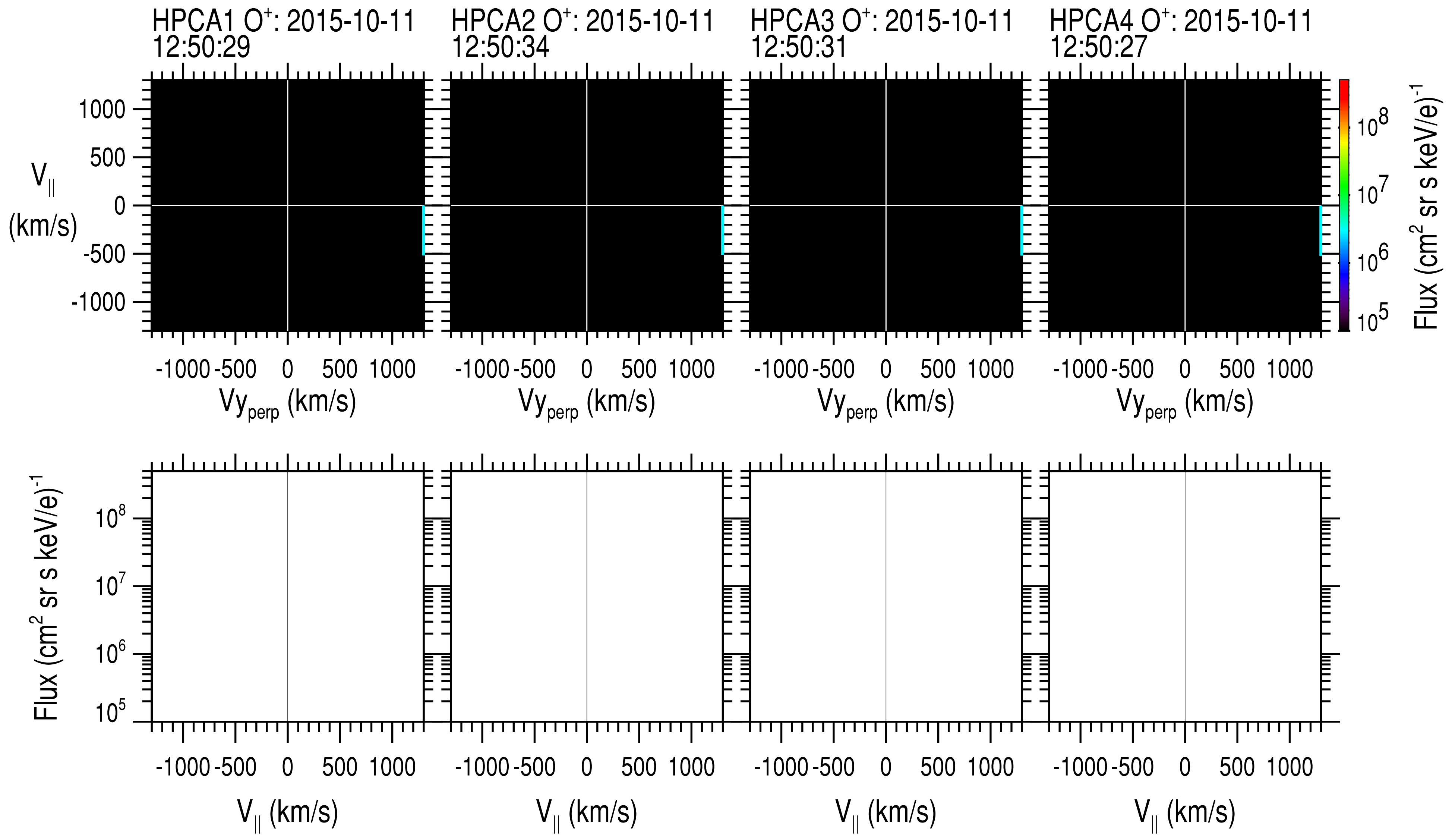
HPCA1 O+: 2015-10-11 12:50:29
HPCA2 O+: 2015-10-11 12:50:34
HPCA3 O+: 2015-10-11 12:50:31
HPCA4 O+: 2015-10-11 12:50:27
V|| (km/s)
1000
500
0
-500
-1000
Vyperp (km/s)
Flux (cm2 sr s keV/e)-1
10^5
10^6
10^7
10^8
V|| (km/s)

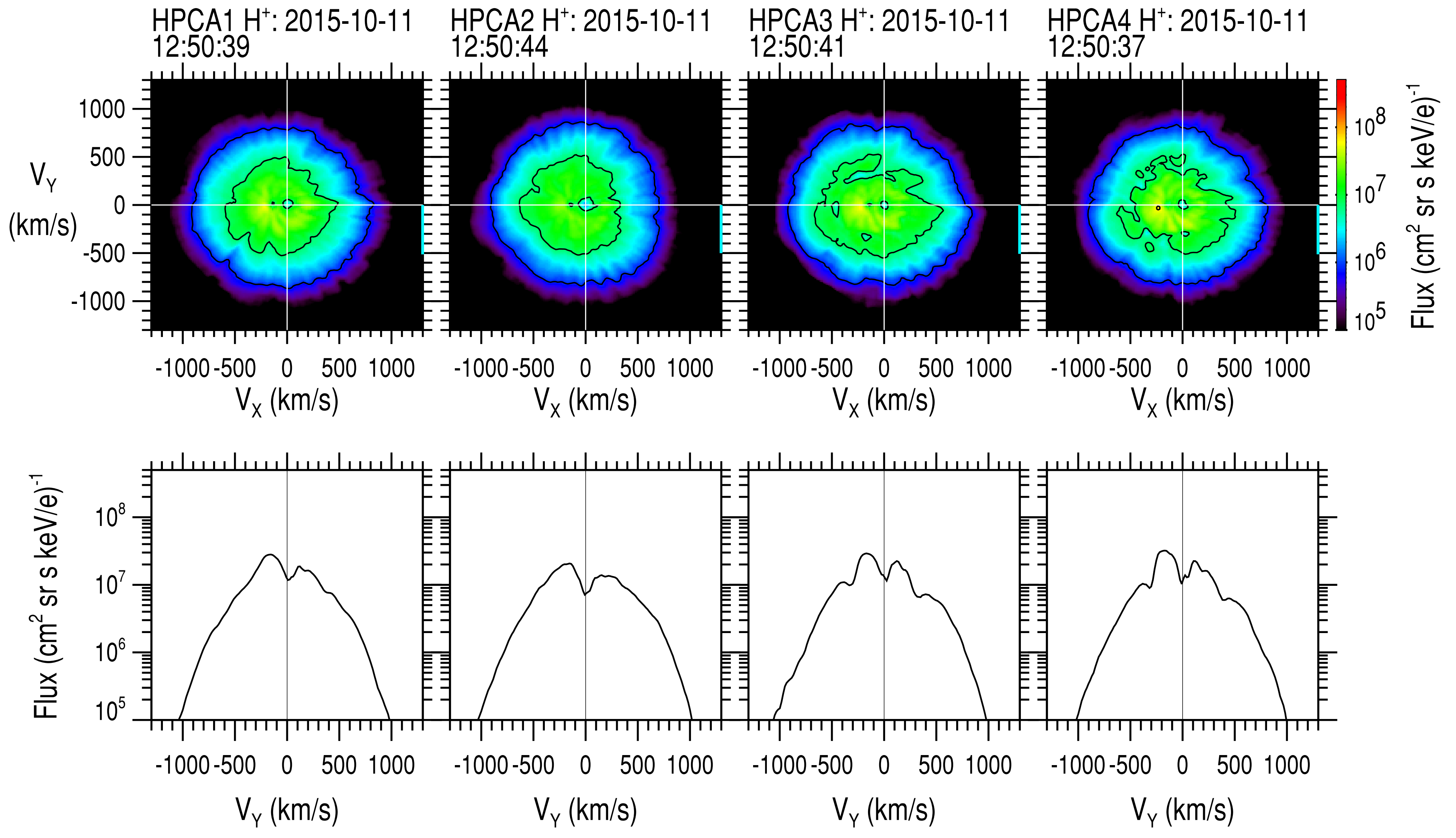
HPCA1 H+: 2015-10-11
12:50:39
HPCA2 H+: 2015-10-11
12:50:44
HPCA3 H+: 2015-10-11
12:50:41
HPCA4 H+: 2015-10-11
12:50:37
V_Y (km/s)
V_X (km/s)
Flux (cm^2 sr s keV/e)^-1
V_Y (km/s)

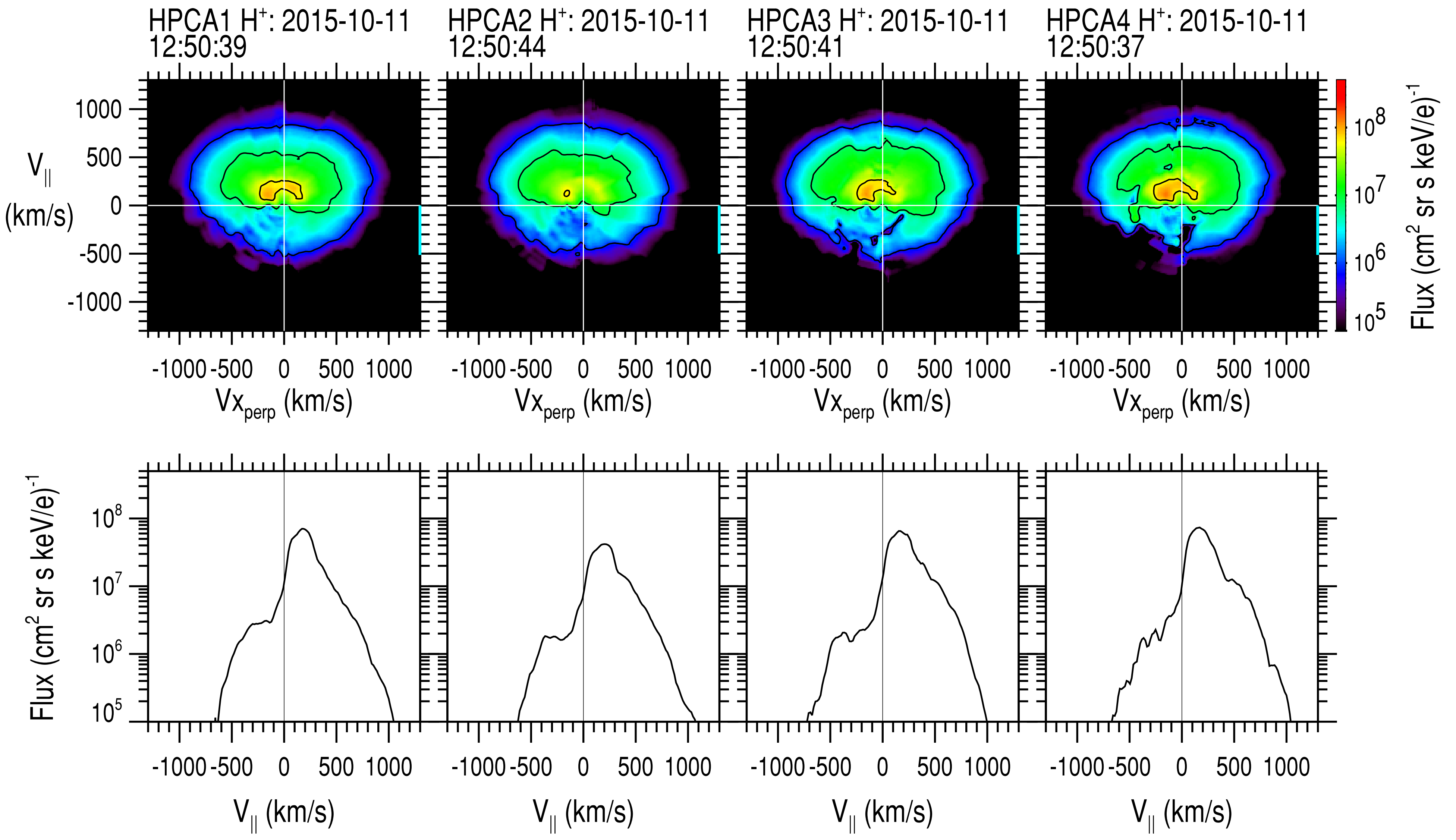

HPCA1 H+: 2015-10-11 12:50:39
HPCA2 H+: 2015-10-11 12:50:44
HPCA3 H+: 2015-10-11 12:50:41
HPCA4 H+: 2015-10-11 12:50:37
V|| (km/s)
Vxperp (km/s)
Flux (cm2 sr s keV/e)-1

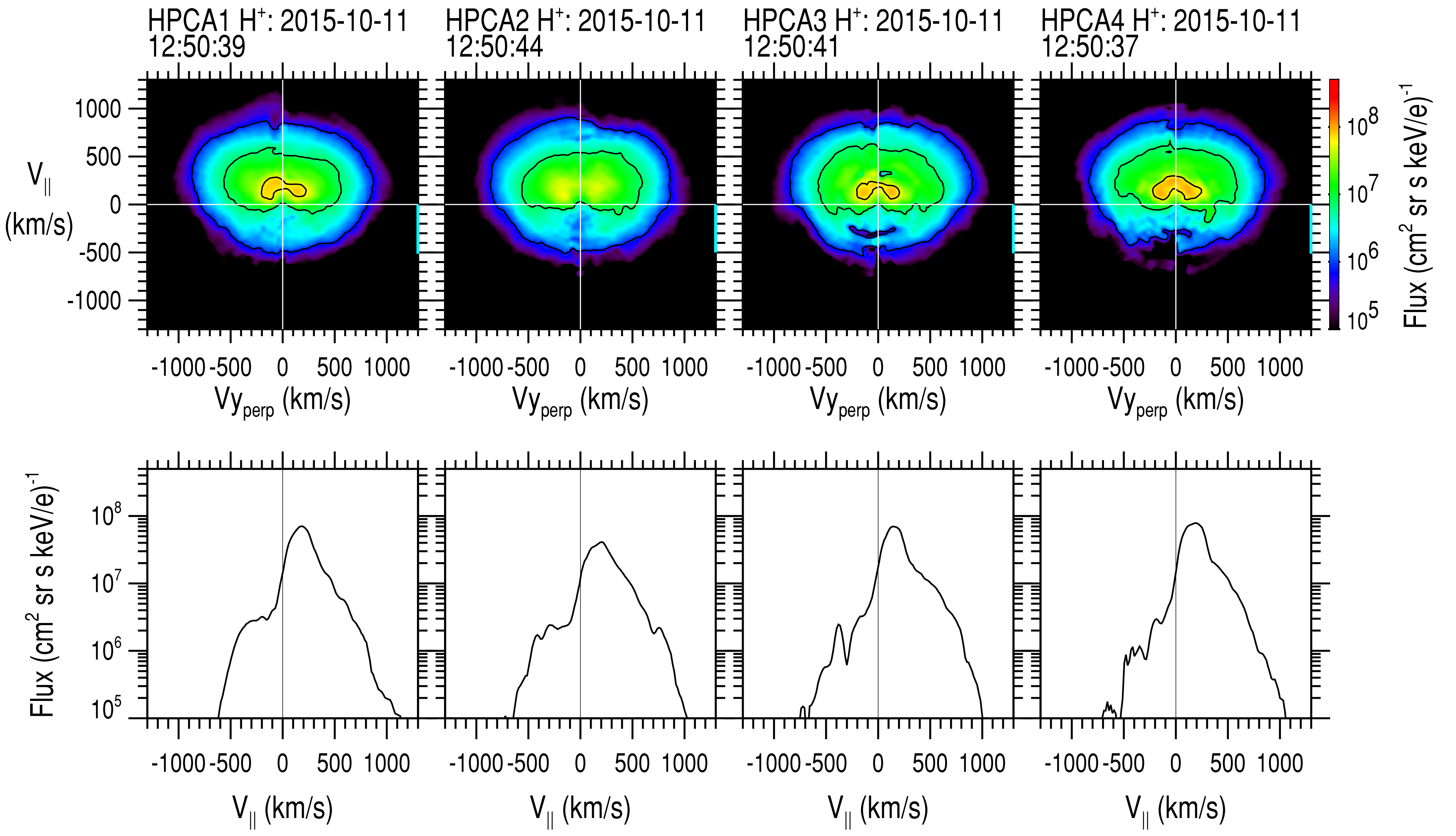

HPCA1 H+: 2015-10-11 12:50:39
HPCA2 H+: 2015-10-11 12:50:44
HPCA3 H+: 2015-10-11 12:50:41
HPCA4 H+: 2015-10-11 12:50:37
V|| (km/s)
Vyperp (km/s)
Flux (cm2 sr s keV/e)-1

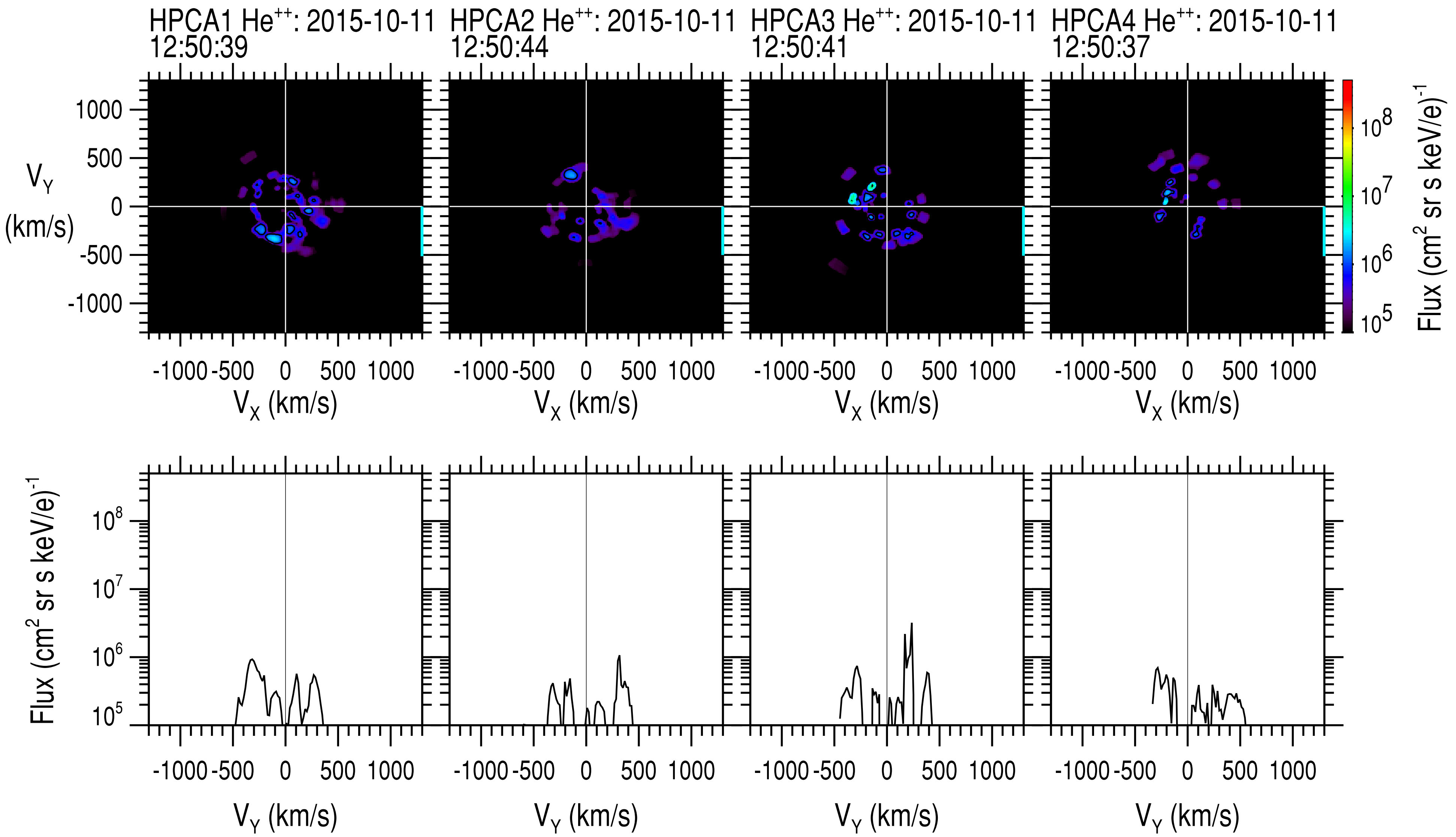

HPCA1 He⁺⁺: 2015-10-11 12:50:39
HPCA2 He⁺⁺: 2015-10-11 12:50:44
HPCA3 He⁺⁺: 2015-10-11 12:50:41
HPCA4 He⁺⁺: 2015-10-11 12:50:37
V_Y (km/s)
V_X (km/s)
Flux $(cm^2$ sr s keV/e$)^{-1}$
-1000 -500 0 500 1000
10^5 10^6 10^7 10^8

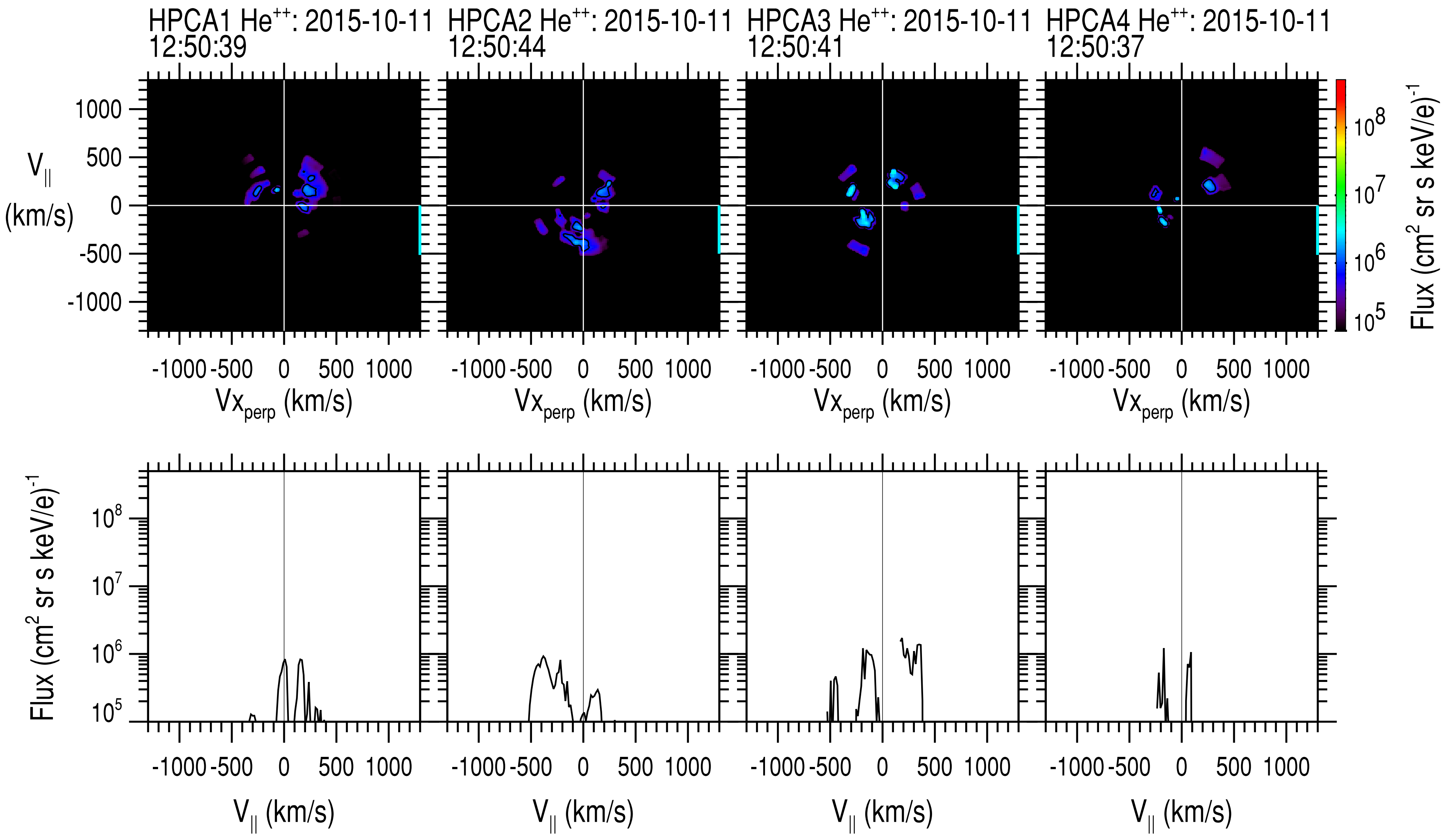

HPCA1 He++: 2015-10-11 12:50:39
HPCA2 He++: 2015-10-11 12:50:44
HPCA3 He++: 2015-10-11 12:50:41
HPCA4 He++: 2015-10-11 12:50:37
V|| (km/s)
Vxperp (km/s)
Flux (cm2 sr s keV/e)-1
V|| (km/s)

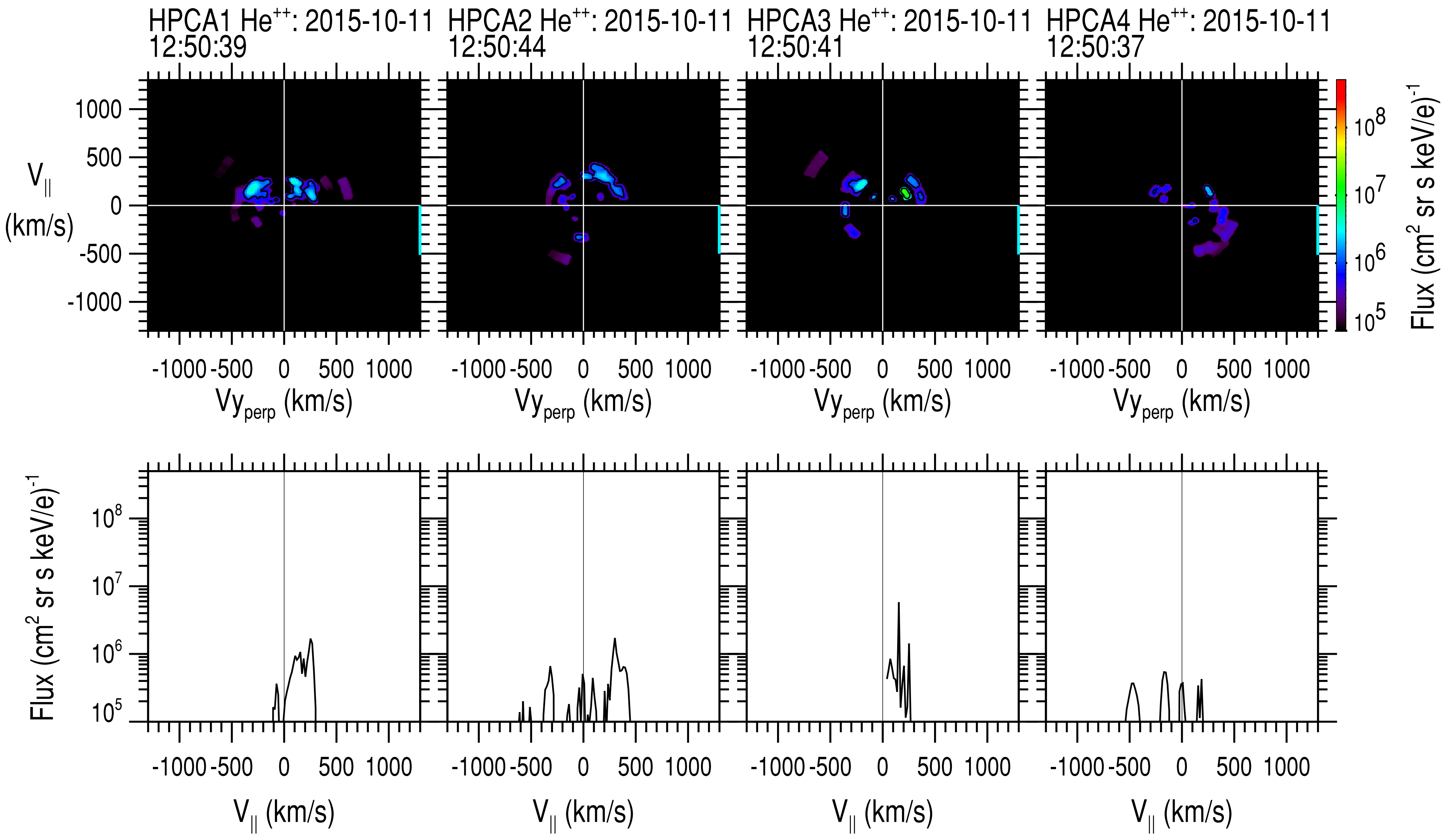
HPCA1 He++: 2015-10-11 12:50:39
HPCA2 He++: 2015-10-11 12:50:44
HPCA3 He++: 2015-10-11 12:50:41
HPCA4 He++: 2015-10-11 12:50:37
V|| (km/s)
Vyperp (km/s)
Flux (cm2 sr s keV/e)-1
1000
500
0
-500
-1000
10^5
10^6
10^7
10^8

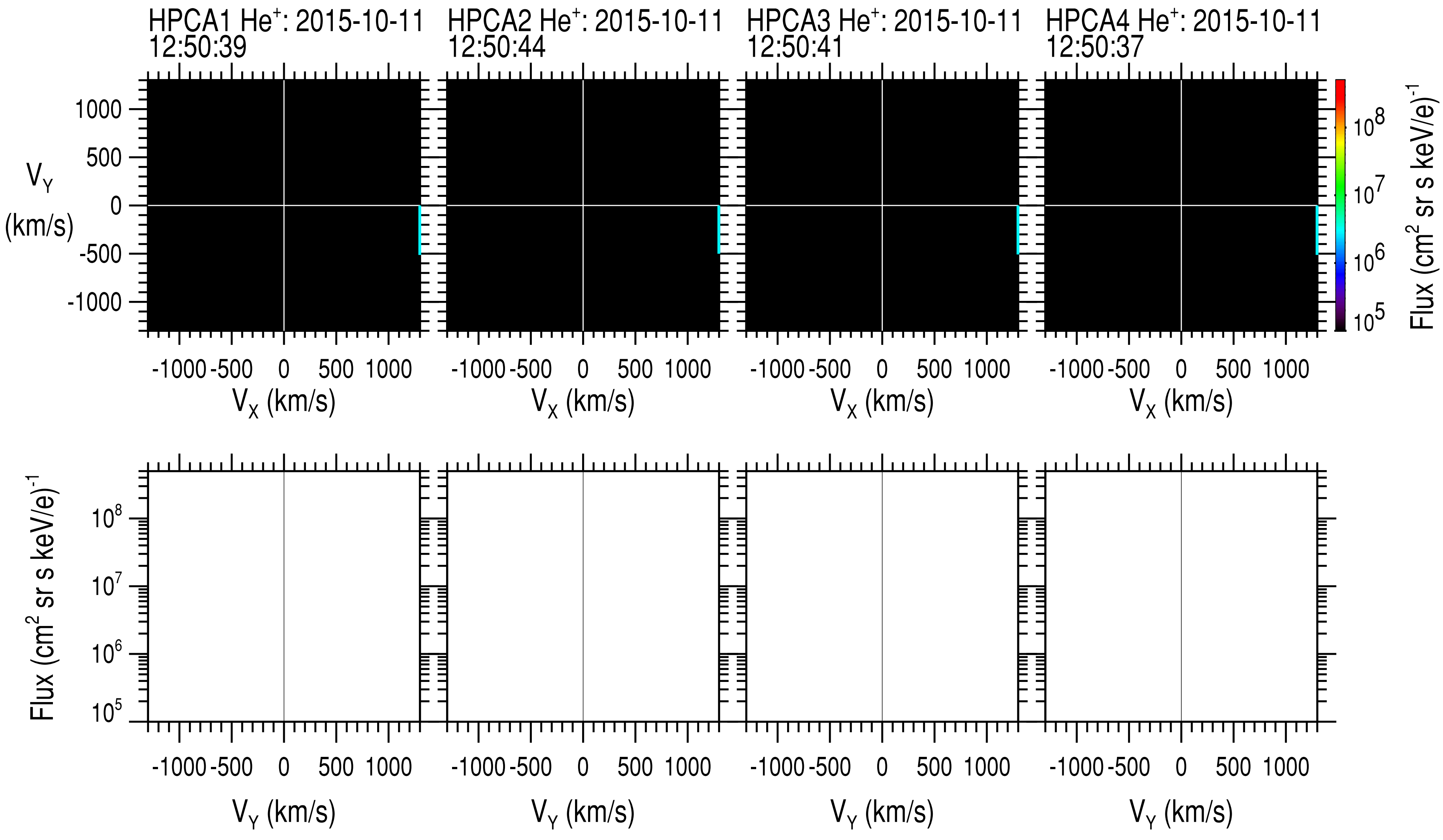
HPCA1 He+: 2015-10-11 12:50:39
HPCA2 He+: 2015-10-11 12:50:44
HPCA3 He+: 2015-10-11 12:50:41
HPCA4 He+: 2015-10-11 12:50:37
V_Y (km/s)
V_X (km/s)
Flux (cm^2 sr s keV/e)^-1
V_Y (km/s)

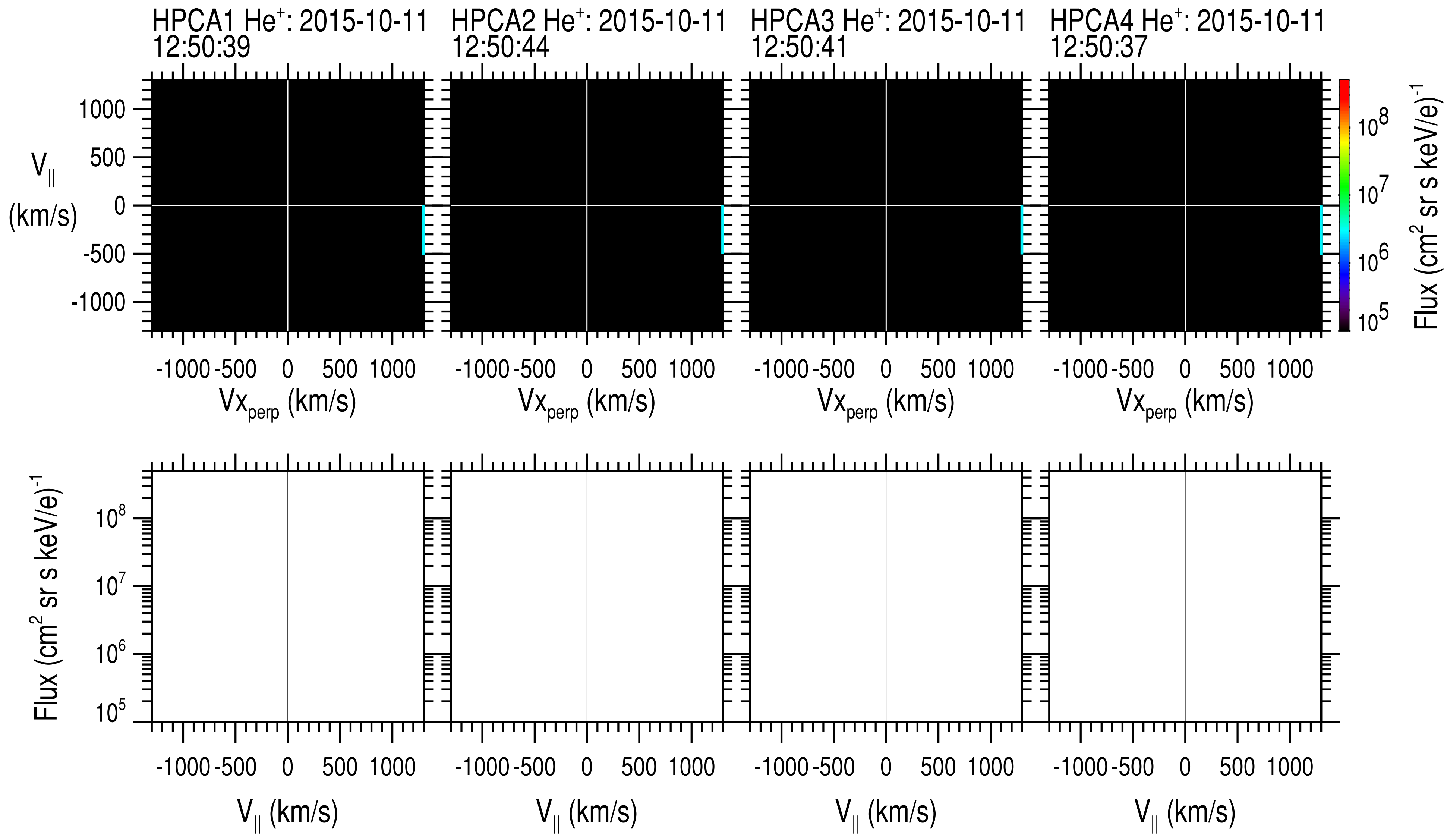
HPCA1 He+: 2015-10-11 12:50:39
HPCA2 He+: 2015-10-11 12:50:44
HPCA3 He+: 2015-10-11 12:50:41
HPCA4 He+: 2015-10-11 12:50:37
V|| (km/s)
1000
500
0
-500
-1000
Vxperp (km/s)
Flux (cm2 sr s keV/e)-1
10^5
10^6
10^7
10^8
V|| (km/s)

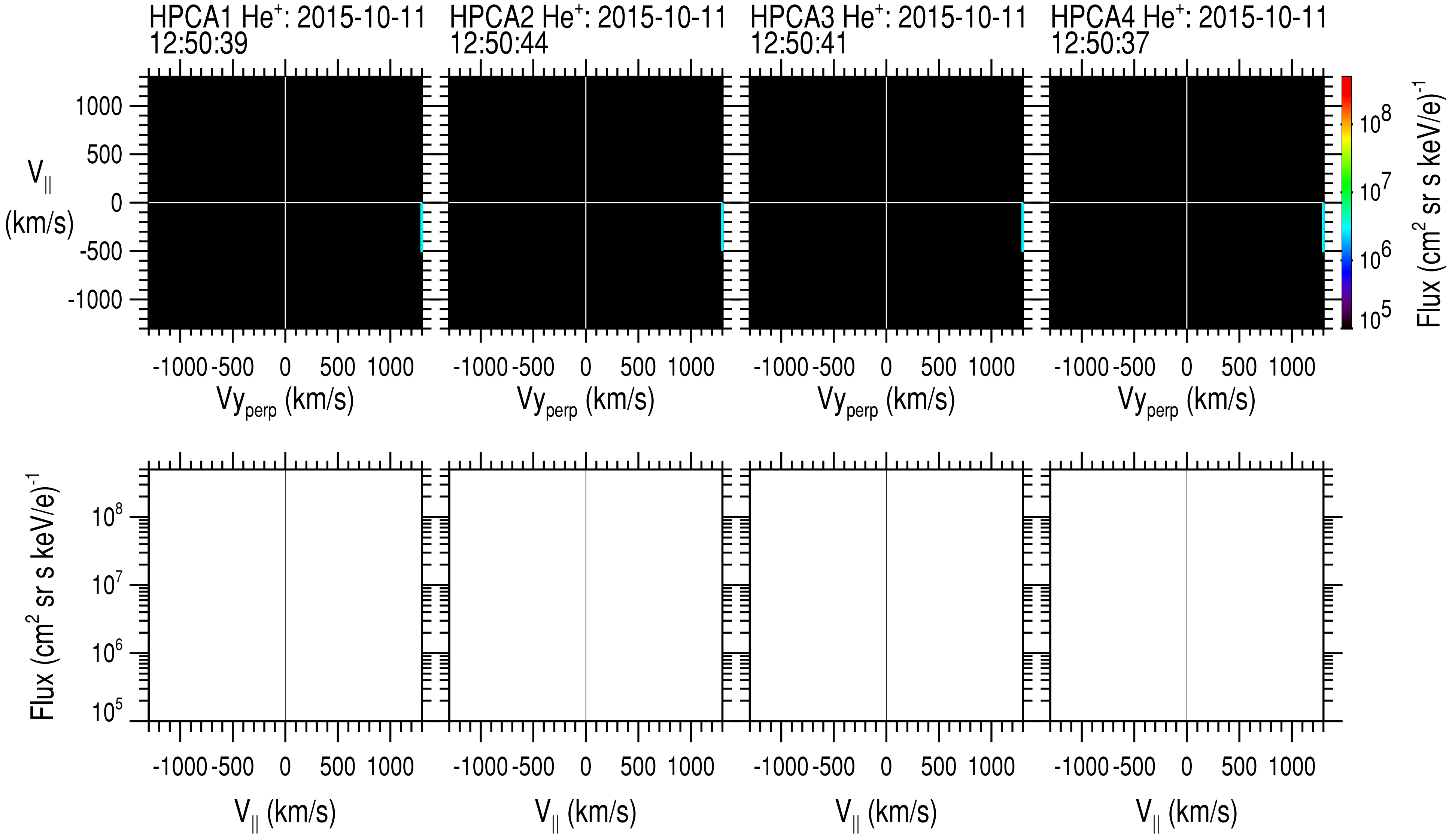
HPCA1 He$^+$: 2015-10-11
12:50:39
HPCA2 He$^+$: 2015-10-11
12:50:44
HPCA3 He$^+$: 2015-10-11
12:50:41
HPCA4 He$^+$: 2015-10-11
12:50:37
$V_{||}$ (km/s)
1000
500
0
-500
-1000
-1000 -500 0 500 1000
Vy_{perp} (km/s)
Flux (cm^2 sr s keV/e)$^{-1}$
10^5
10^6
10^7
10^8
$V_{||}$ (km/s)

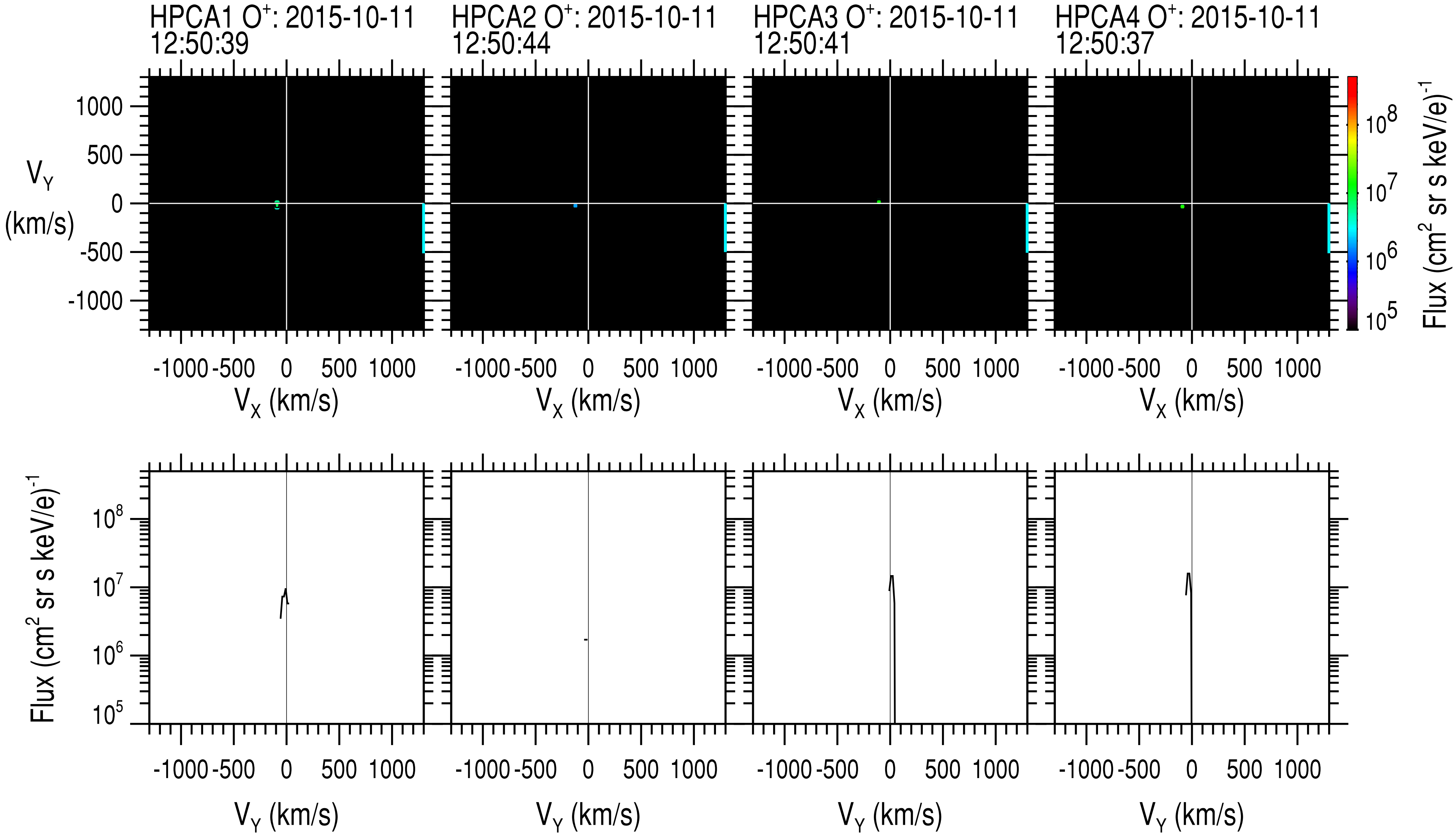
HPCA1 O+: 2015-10-11
12:50:39
HPCA2 O+: 2015-10-11
12:50:44
HPCA3 O+: 2015-10-11
12:50:41
HPCA4 O+: 2015-10-11
12:50:37
VY (km/s)
VX (km/s)
Flux (cm2 sr s keV/e)-1
VY (km/s)

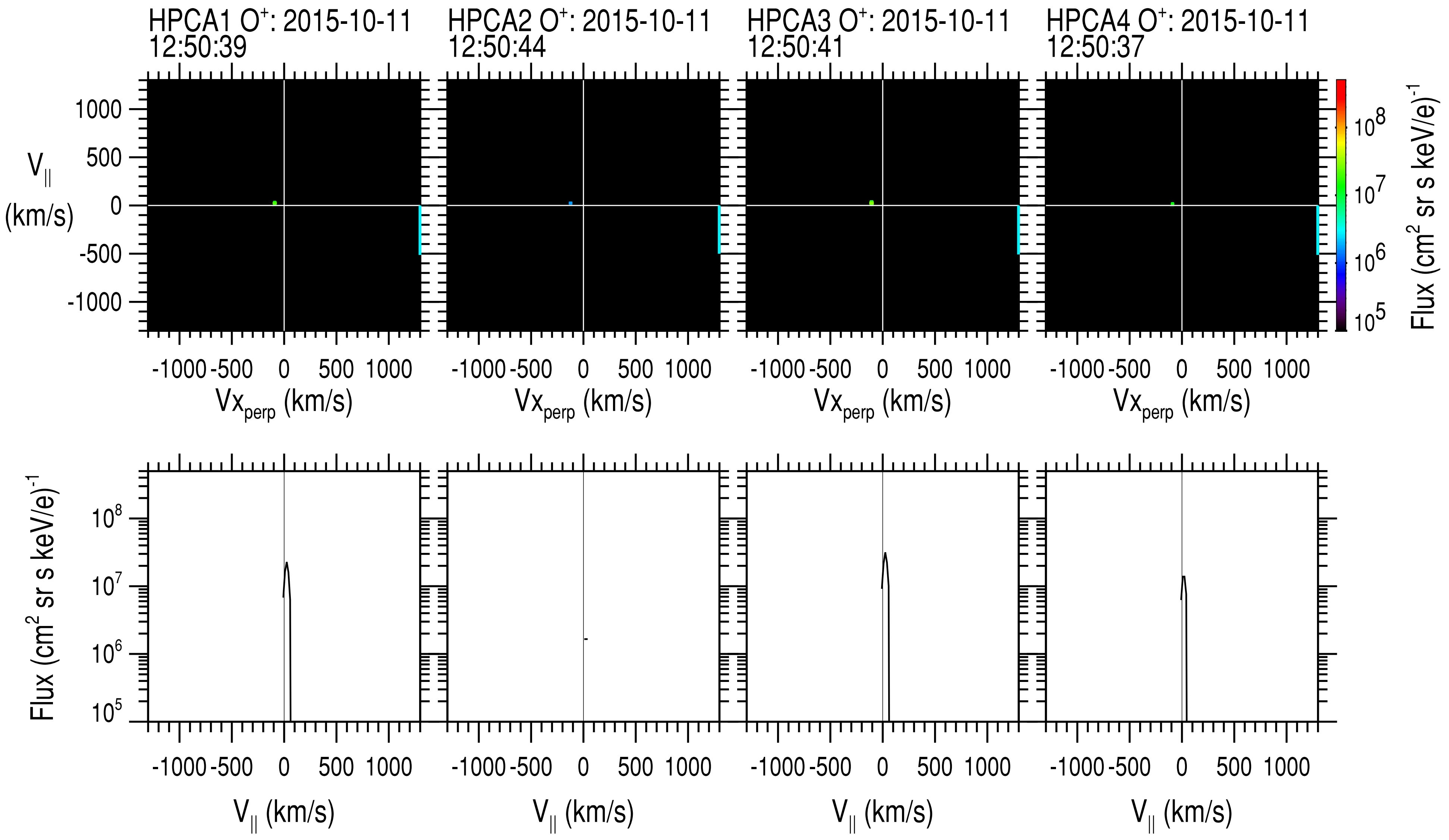
HPCA1 O+: 2015-10-11 12:50:39
HPCA2 O+: 2015-10-11 12:50:44
HPCA3 O+: 2015-10-11 12:50:41
HPCA4 O+: 2015-10-11 12:50:37
V|| (km/s)
Vxperp (km/s)
Flux (cm2 sr s keV/e)-1
1000
500
0
-500
-1000
10^5
10^6
10^7
10^8

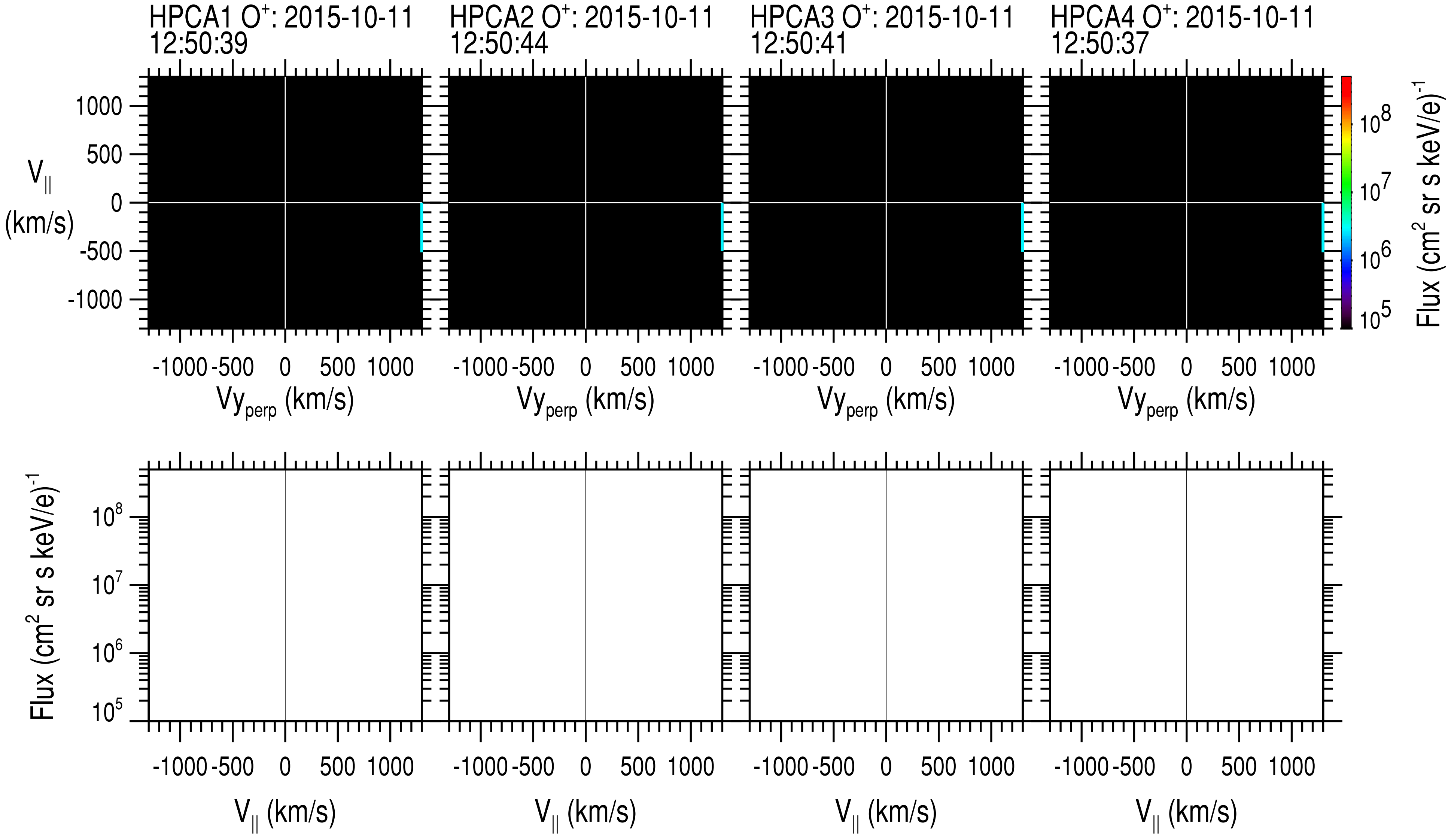
HPCA1 O+: 2015-10-11 12:50:39
HPCA2 O+: 2015-10-11 12:50:44
HPCA3 O+: 2015-10-11 12:50:41
HPCA4 O+: 2015-10-11 12:50:37
V|| (km/s)
Vyperp (km/s)
Flux (cm2 sr s keV/e)-1
V|| (km/s)
