# Supplementary material for: Characteristics of Minor Ions and Electrons in Flux Transfer Events Observed by the Magnetospheric Multiscale Mission
Source: J Geophys Res Space Phys. 2020 Jul 20;125(7):e2020JA027778. doi: 10.1029/2020JA027778 (PMC7507212; doi:10.1029/2020JA027778)
Supplement: Supplementary file 4 — Figure S3 [file JGRA-125-e2020JA027778-s004.pdf]

Supporting Information:

Journal of Geophysical Research – Space Physics

"Supporting Information for Characteristics of Minor Ions and Electrons in Flux Transfer Events Observed by the Magnetospheric Multiscale Mission"

S. M. Petrinec[1], J. L. Burch[2], M. Chandler[3], C. J. Farrugia[4], S. A. Fuselier[2,5], B. L. Giles[6], R. G. Gomez[2,5], J. Mukherjee[2], W. R. Paterson[6], C. T. Russell[7], D. G. Sibeck[6], R. J. Strangeway[7], R. B. Torbert[4], K. J. Trattner[8], S. K. Vines[9], and C. Zhao[7]

[1]*Lockheed Martin Advanced Technology Center, Palo Alto, CA, USA*

[2]*Southwest Research Institute, San Antonio, TX, USA*

[3]*NASA Marshall Space Flight Center, Huntsville, AL, USA*

[4]*Space Science Center, University of New Hampshire, Durham, NH, USA*

[5]*University of Texas at San Antonio, San Antonio, TX, USA*

[6]*NASA Goddard Space Flight Center, Greenbelt, MD, USA*

[7]*Earth and Space Sciences, University of California, Los Angeles, CA, USA*

[8]*Laboratory for Atmospheric and Space Physics, University of Colorado Boulder, Boulder, CO, USA*

[9]*The Johns Hopkins University Applied Physics Laboratory, Laurel, MD, USA*

2D and 1D flux distribution cuts (in a magnetic field coordinate system) from all four MMS HPCA instruments, for four species {H^+, He^{++}, He^+, and O^+}, during the FTE burst mode of 2015-10-20.

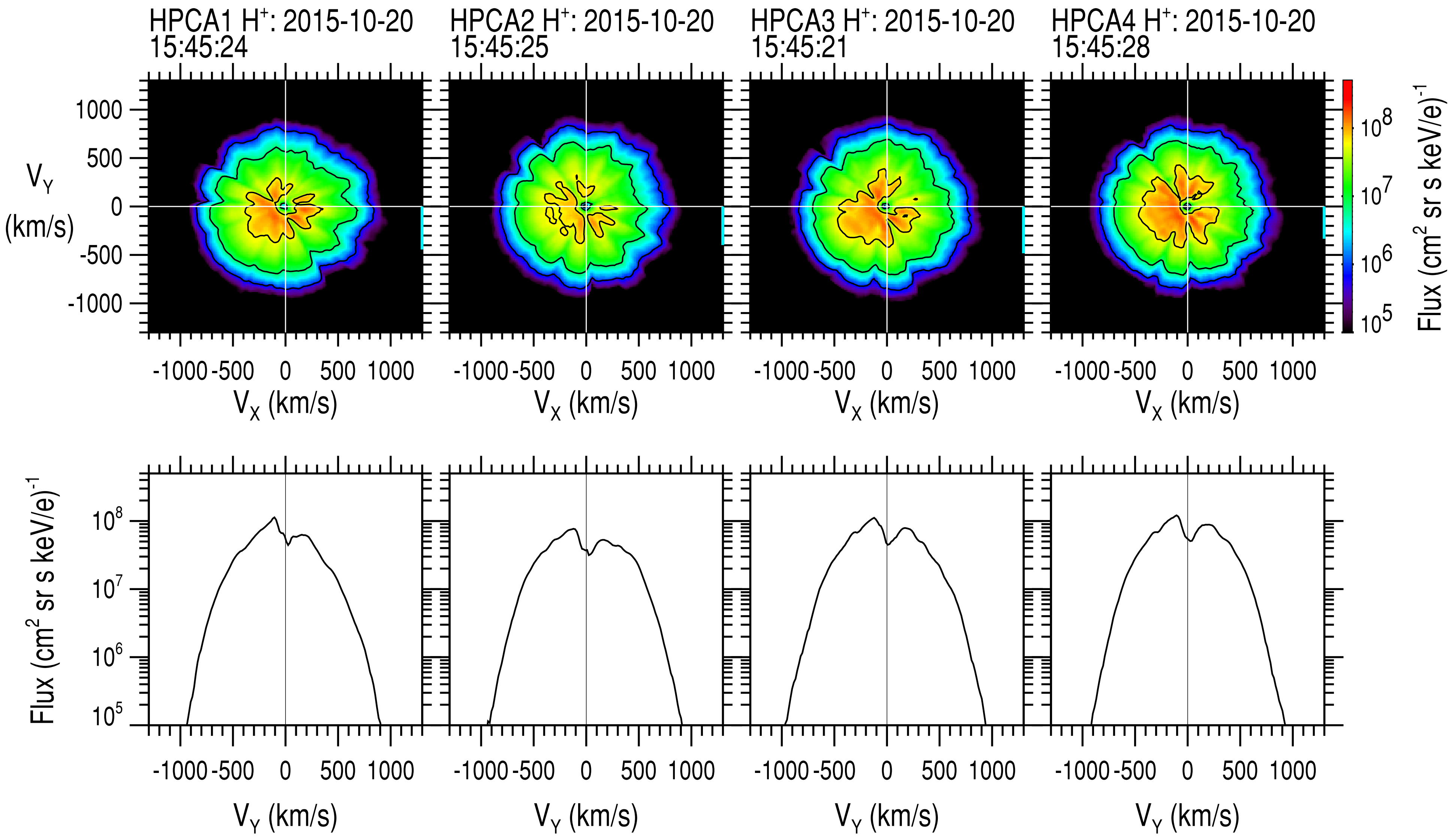

HPCA1 H+: 2015-10-20
15:45:24
HPCA2 H+: 2015-10-20
15:45:25
HPCA3 H+: 2015-10-20
15:45:21
HPCA4 H+: 2015-10-20
15:45:28
V_Y (km/s)
V_X (km/s)
Flux (cm^2 sr s keV/e)^-1
V_Y (km/s)

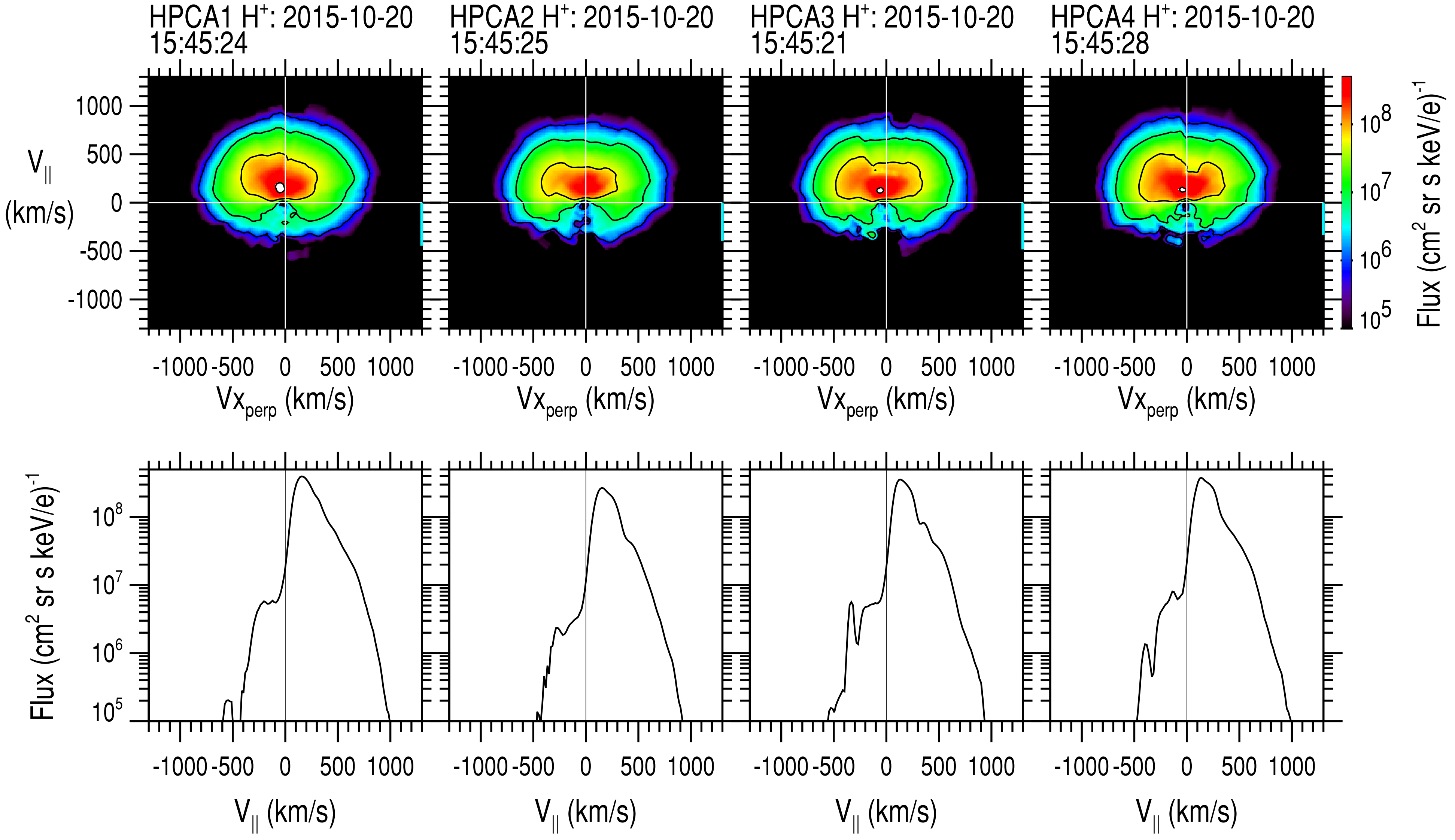

HPCA1 H+: 2015-10-20 15:45:24
HPCA2 H+: 2015-10-20 15:45:25
HPCA3 H+: 2015-10-20 15:45:21
HPCA4 H+: 2015-10-20 15:45:28
V|| (km/s)
Vxperp (km/s)
Flux (cm2 sr s keV/e)-1
-1000 -500 0 500 1000
10^5 10^6 10^7 10^8

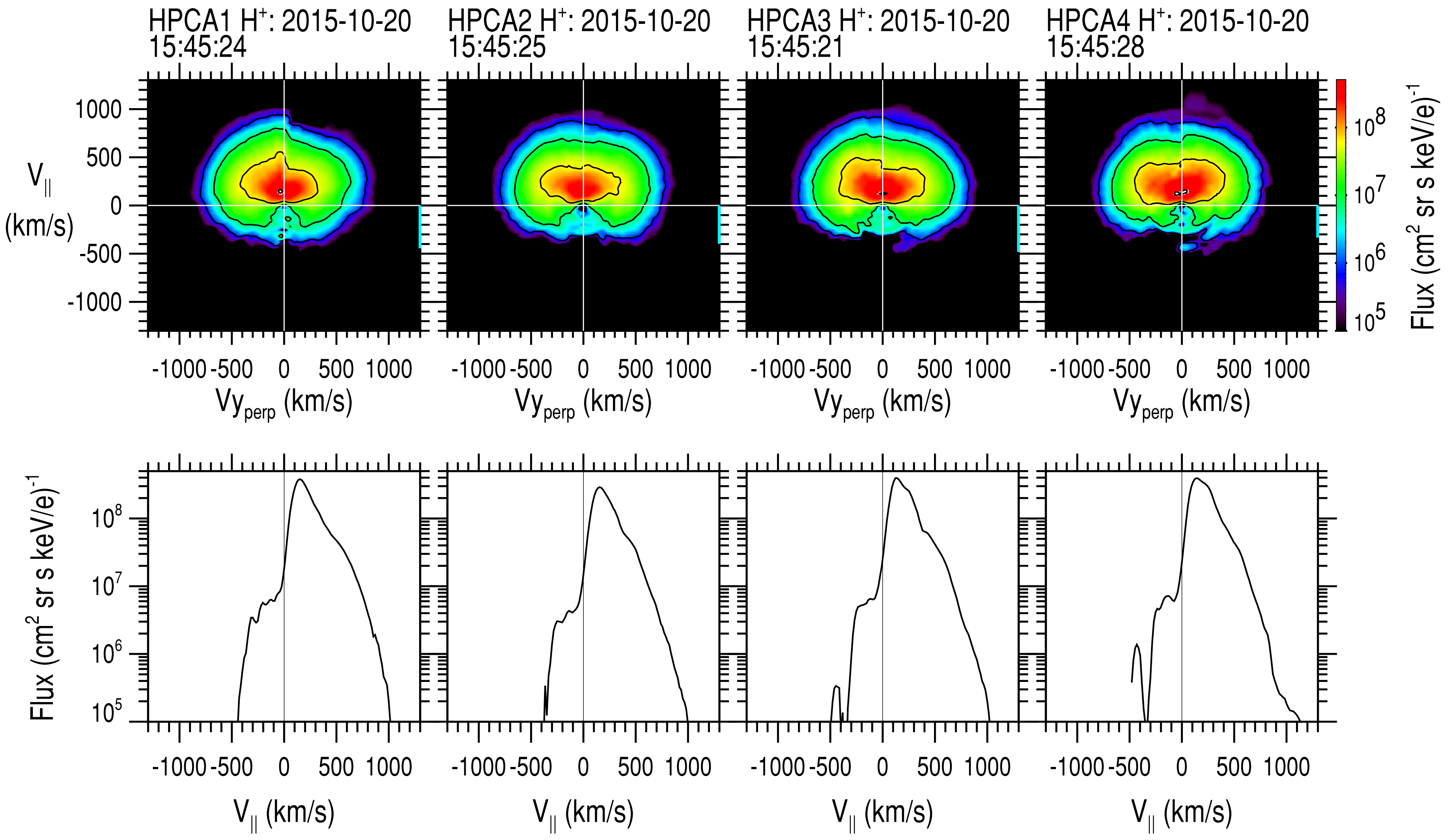

HPCA1 H⁺: 2015-10-20 15:45:24
HPCA2 H⁺: 2015-10-20 15:45:25
HPCA3 H⁺: 2015-10-20 15:45:21
HPCA4 H⁺: 2015-10-20 15:45:28
V|| (km/s)
Vyperp (km/s)
Flux (cm2 sr s keV/e)-1

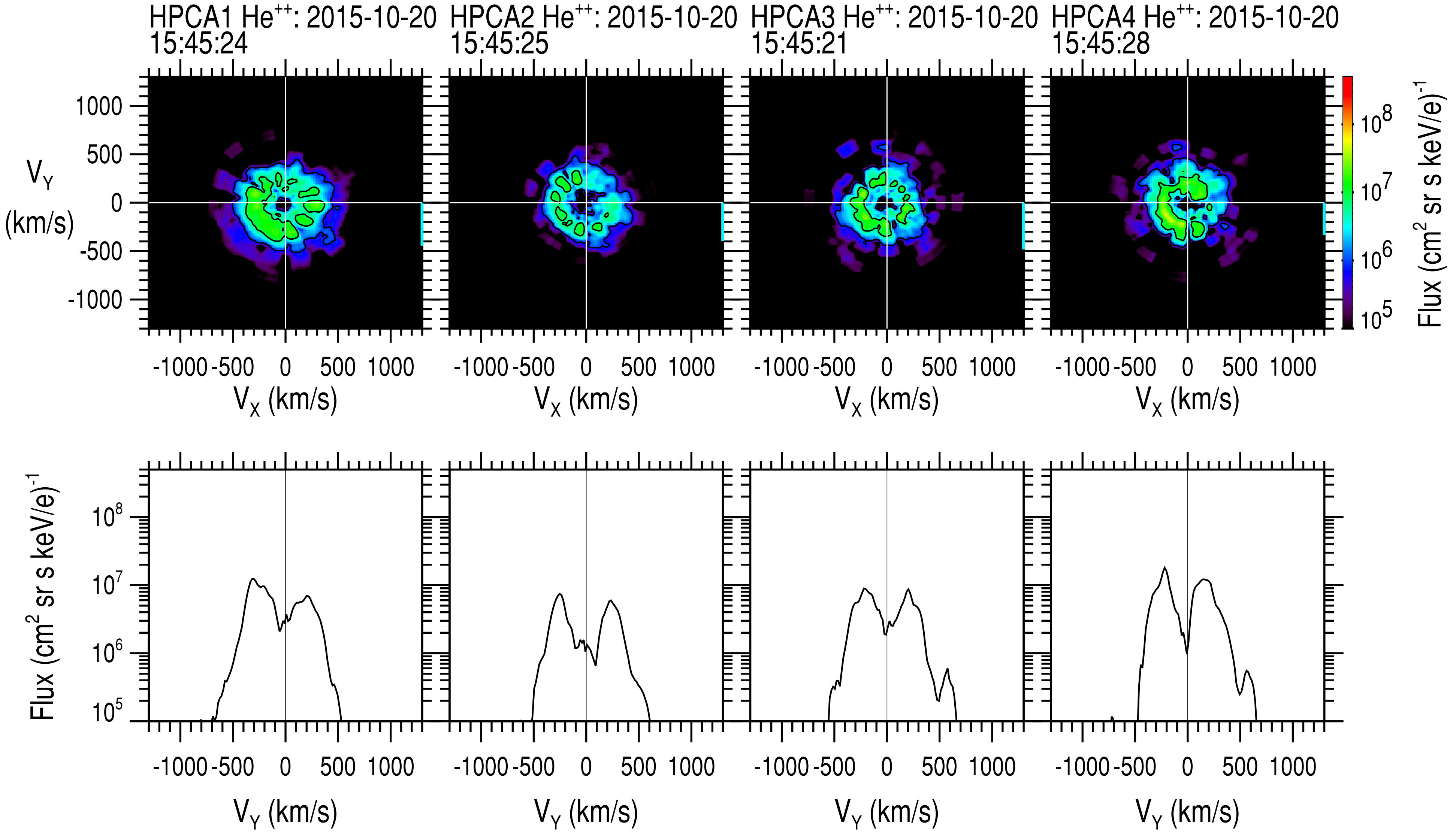
HPCA1 He++: 2015-10-20 15:45:24
HPCA2 He++: 2015-10-20 15:45:25
HPCA3 He++: 2015-10-20 15:45:21
HPCA4 He++: 2015-10-20 15:45:28
VY (km/s)
VX (km/s)
Flux (cm2 sr s keV/e)-1
VY (km/s)
Flux (cm2 sr s keV/e)-1

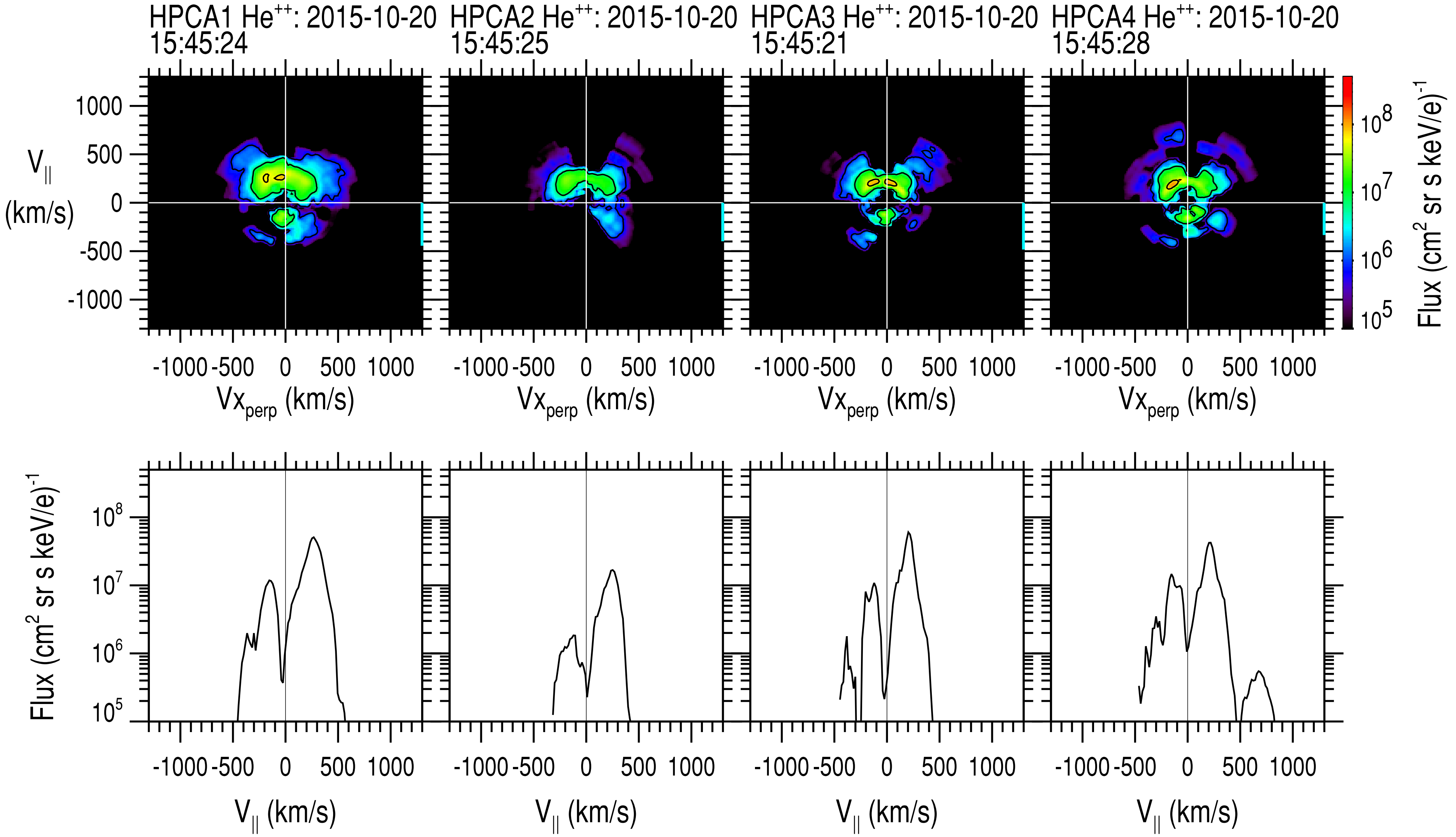
HPCA1 He++: 2015-10-20
15:45:24
HPCA2 He++: 2015-10-20
15:45:25
HPCA3 He++: 2015-10-20
15:45:21
HPCA4 He++: 2015-10-20
15:45:28
V|| (km/s)
Vxperp (km/s)
Flux (cm2 sr s keV/e)-1
V|| (km/s)

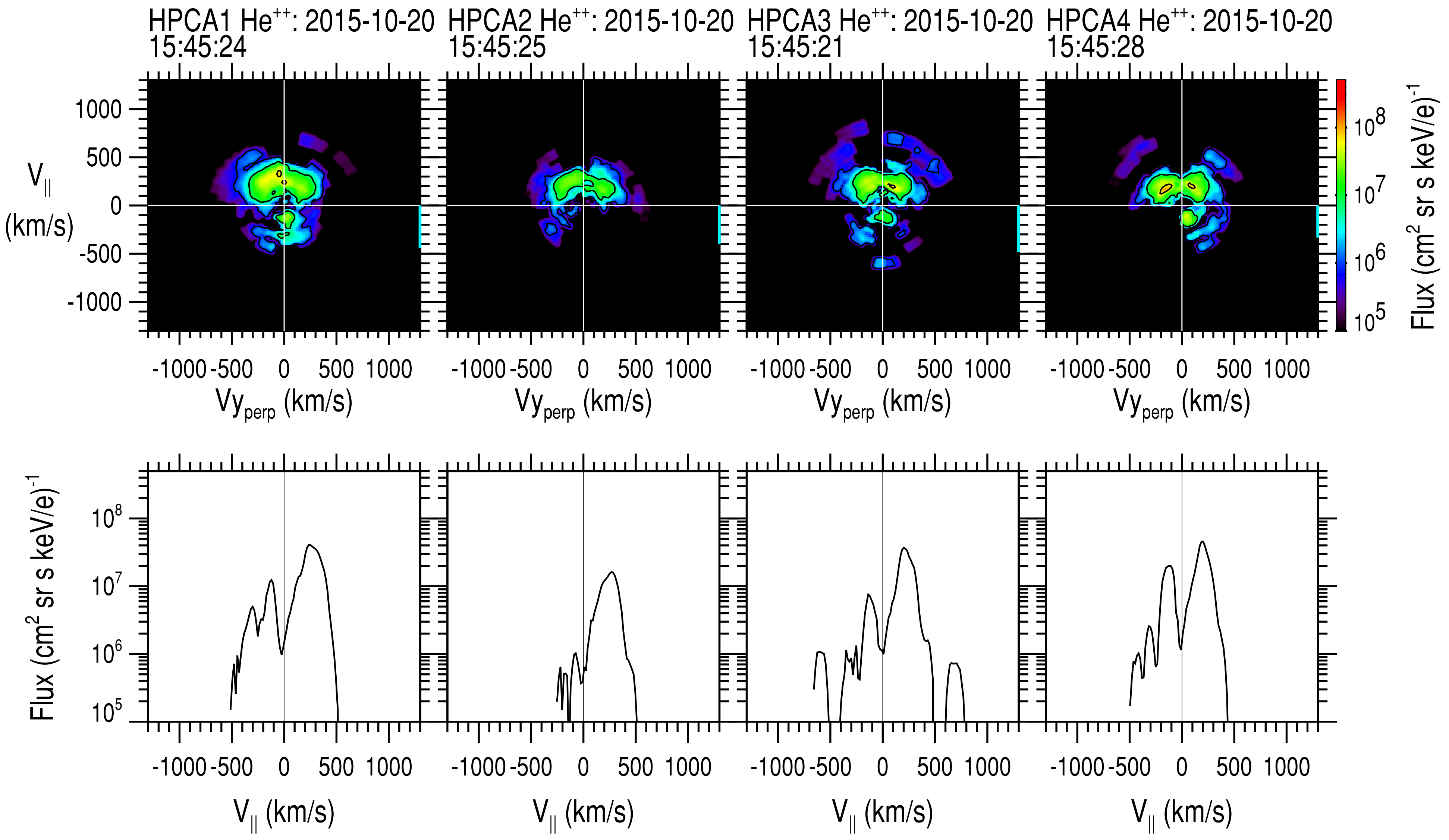
HPCA1 He++: 2015-10-20 15:45:24
HPCA2 He++: 2015-10-20 15:45:25
HPCA3 He++: 2015-10-20 15:45:21
HPCA4 He++: 2015-10-20 15:45:28
V|| (km/s)
Vyperp (km/s)
Flux (cm2 sr s keV/e)-1
V|| (km/s)
1000
500
0
-500
-1000
10^5
10^6
10^7
10^8

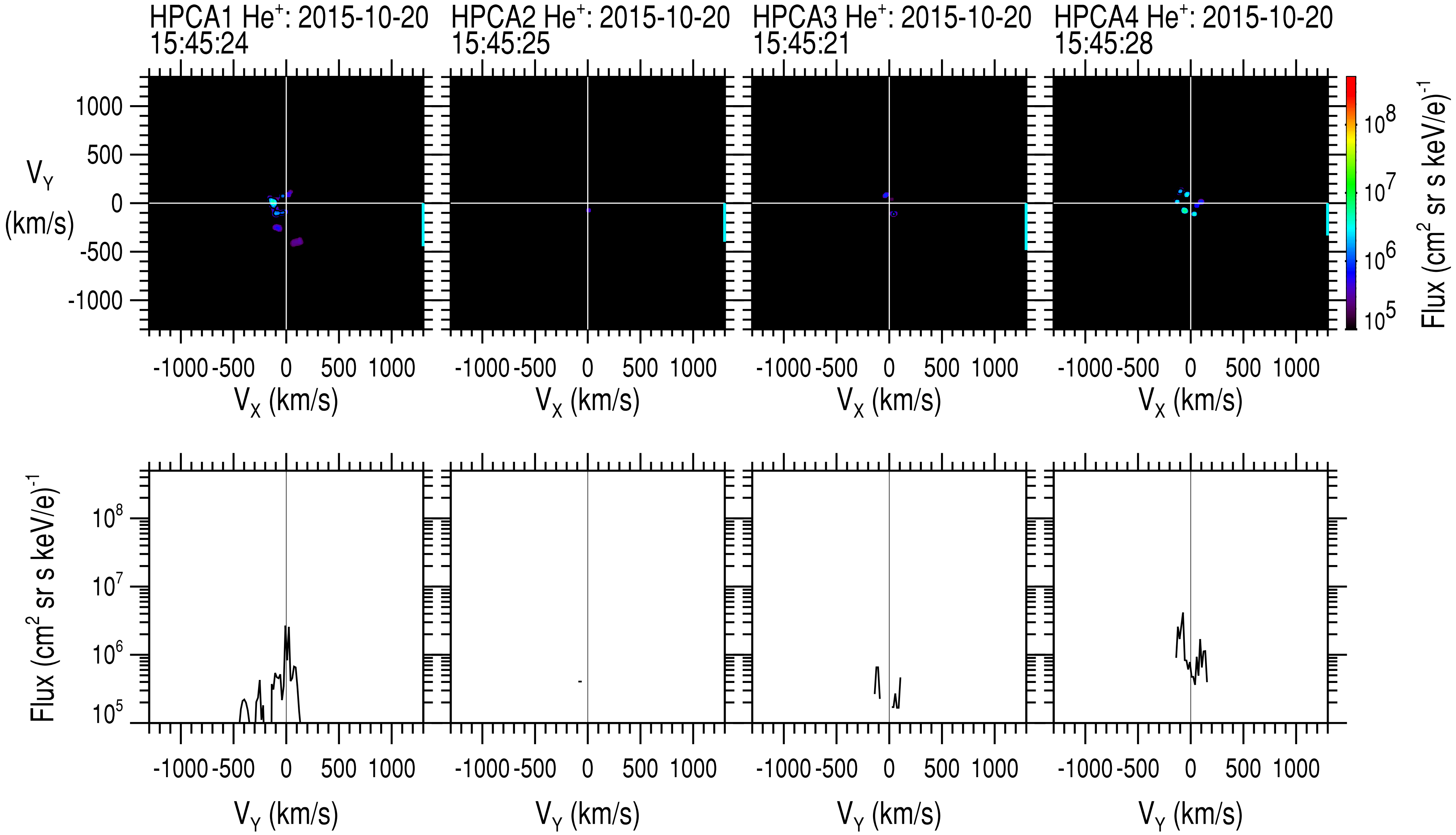
HPCA1 He+: 2015-10-20 15:45:24
HPCA2 He+: 2015-10-20 15:45:25
HPCA3 He+: 2015-10-20 15:45:21
HPCA4 He+: 2015-10-20 15:45:28
V_Y (km/s)
V_X (km/s)
Flux (cm^2 sr s keV/e)^-1
-1000 -500 0 500 1000
10^5 10^6 10^7 10^8

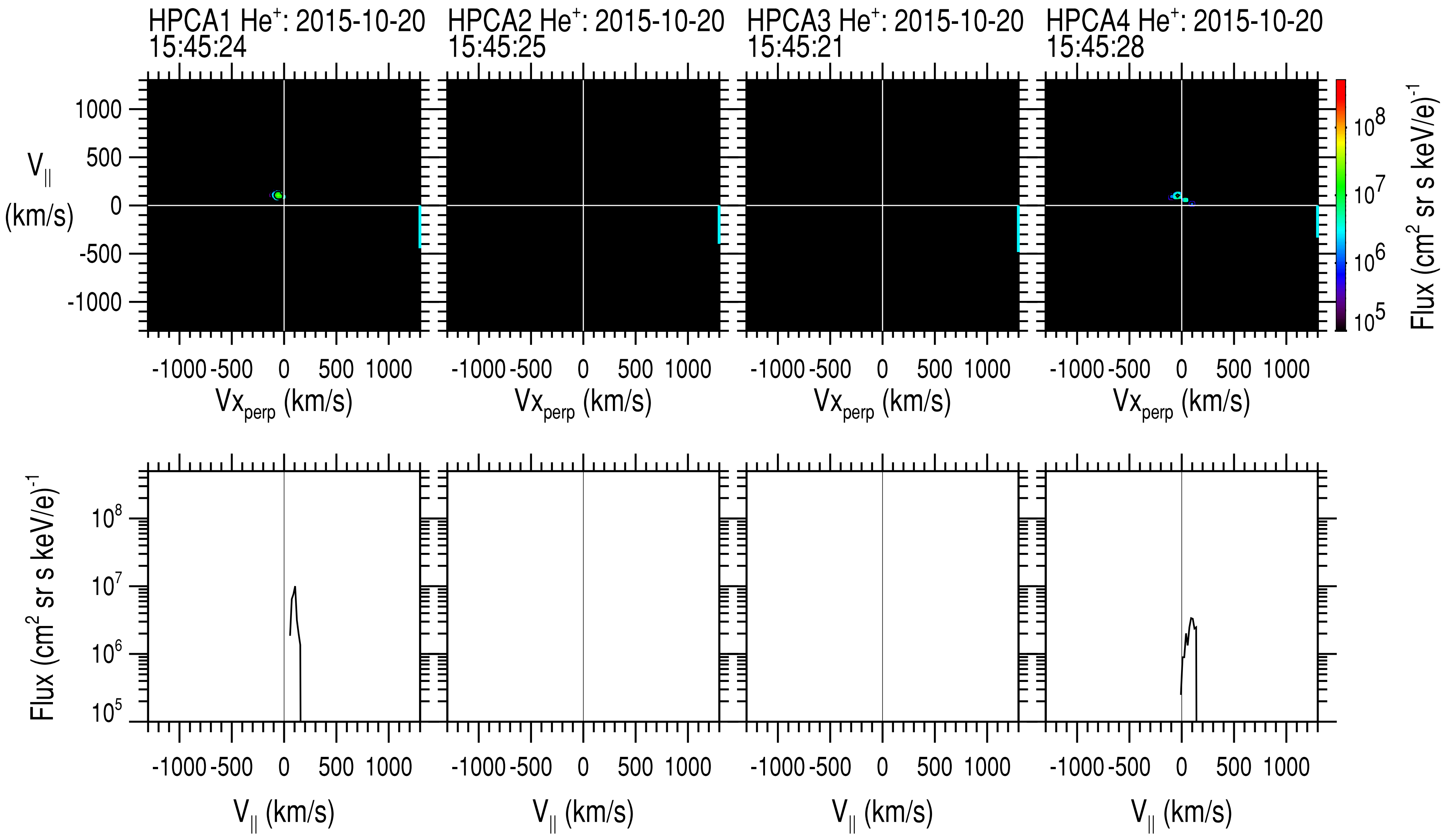

HPCA1 He+: 2015-10-20 15:45:24
HPCA2 He+: 2015-10-20 15:45:25
HPCA3 He+: 2015-10-20 15:45:21
HPCA4 He+: 2015-10-20 15:45:28
V|| (km/s)
Vxperp (km/s)
Flux (cm2 sr s keV/e)-1

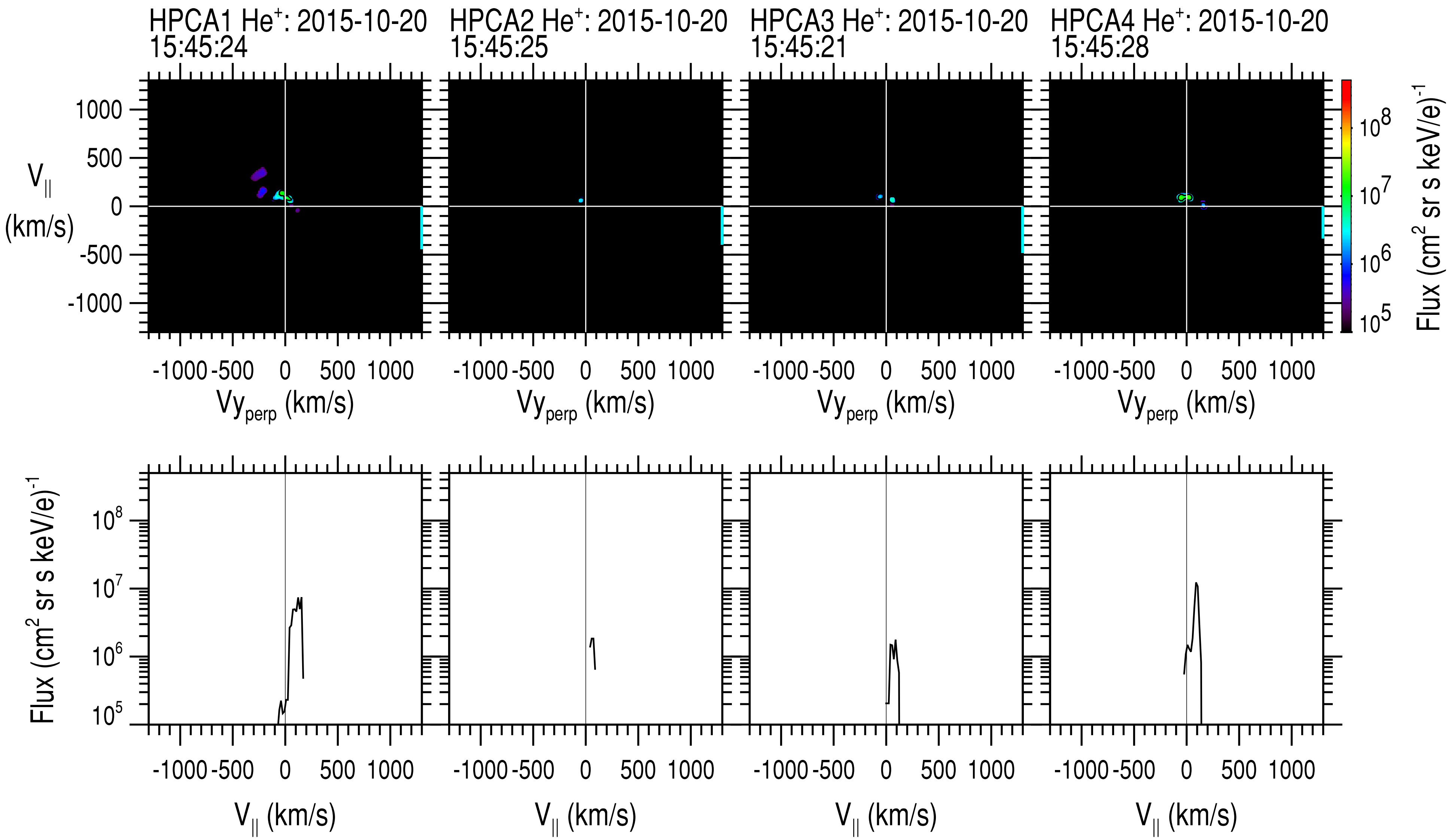
HPCA1 He⁺: 2015-10-20 15:45:24
HPCA2 He⁺: 2015-10-20 15:45:25
HPCA3 He⁺: 2015-10-20 15:45:21
HPCA4 He⁺: 2015-10-20 15:45:28
V|| (km/s)
Vyperp (km/s)
Flux (cm2 sr s keV/e)-1
1000
500
0
-500
-1000
105
106
107
108

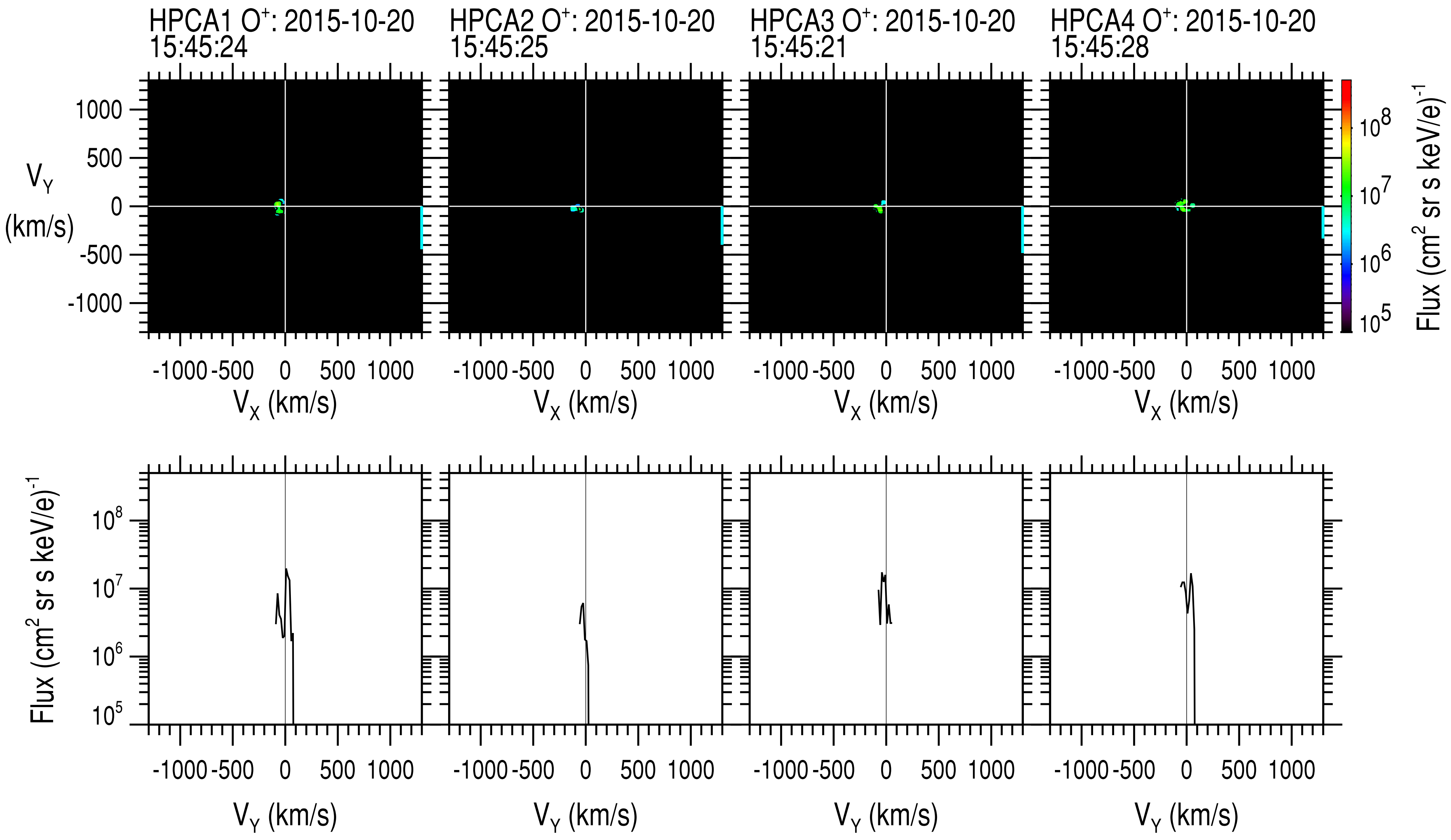

HPCA1 O+: 2015-10-20 15:45:24
HPCA2 O+: 2015-10-20 15:45:25
HPCA3 O+: 2015-10-20 15:45:21
HPCA4 O+: 2015-10-20 15:45:28
V_Y (km/s)
V_X (km/s)
Flux (cm^2 sr s keV/e)^-1
-1000 -500 0 500 1000
10^5 10^6 10^7 10^8

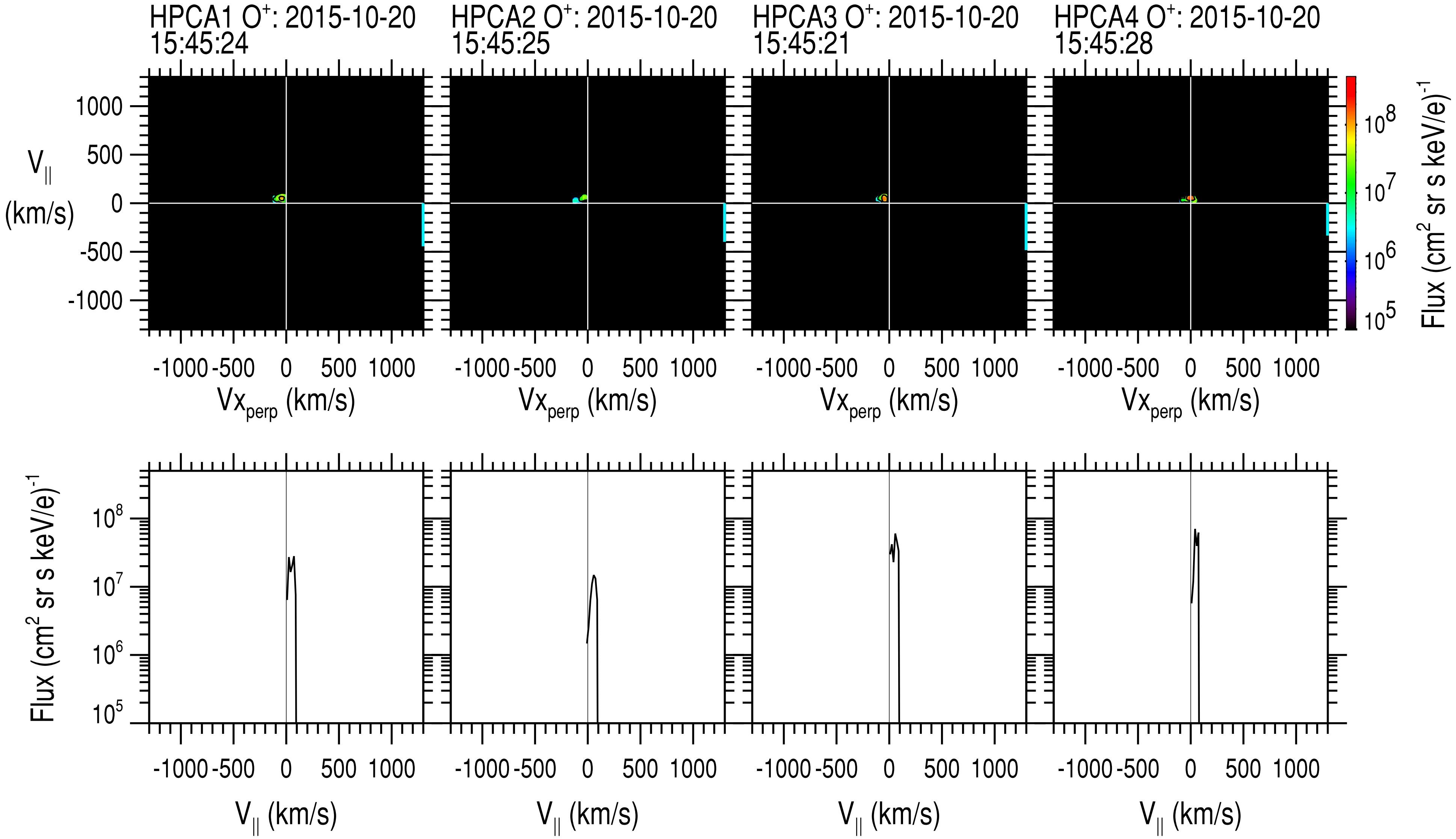
HPCA1 O+: 2015-10-20 15:45:24
HPCA2 O+: 2015-10-20 15:45:25
HPCA3 O+: 2015-10-20 15:45:21
HPCA4 O+: 2015-10-20 15:45:28
V|| (km/s)
Vxperp (km/s)
Flux (cm2 sr s keV/e)-1

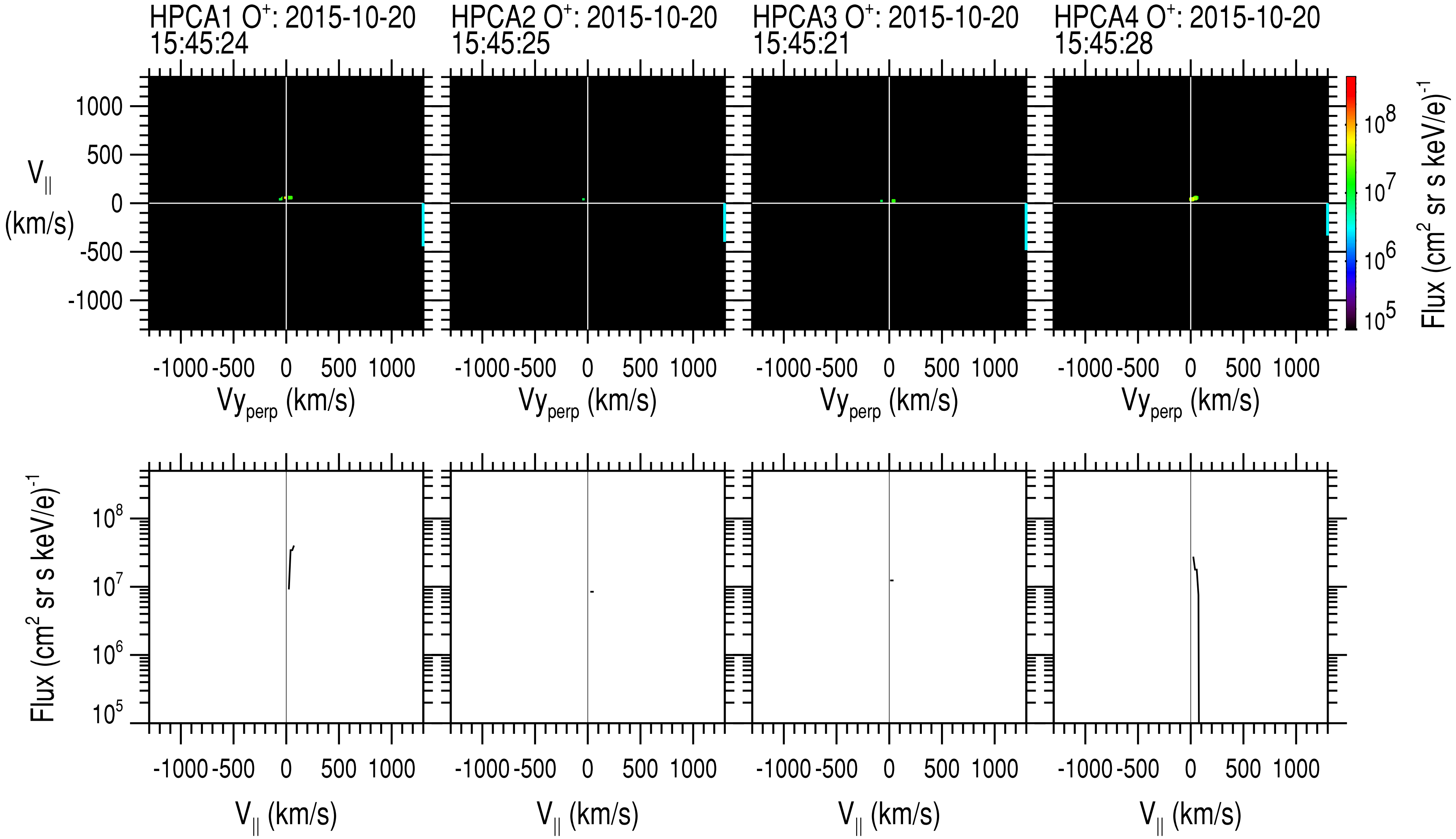
HPCA1 O+: 2015-10-20
15:45:24
HPCA2 O+: 2015-10-20
15:45:25
HPCA3 O+: 2015-10-20
15:45:21
HPCA4 O+: 2015-10-20
15:45:28
V||
(km/s)
1000
500
0
-500
-1000
-1000 -500 0 500 1000
Vy perp (km/s)
Flux (cm2 sr s keV/e)-1
10^8
10^7
10^6
10^5
V|| (km/s)

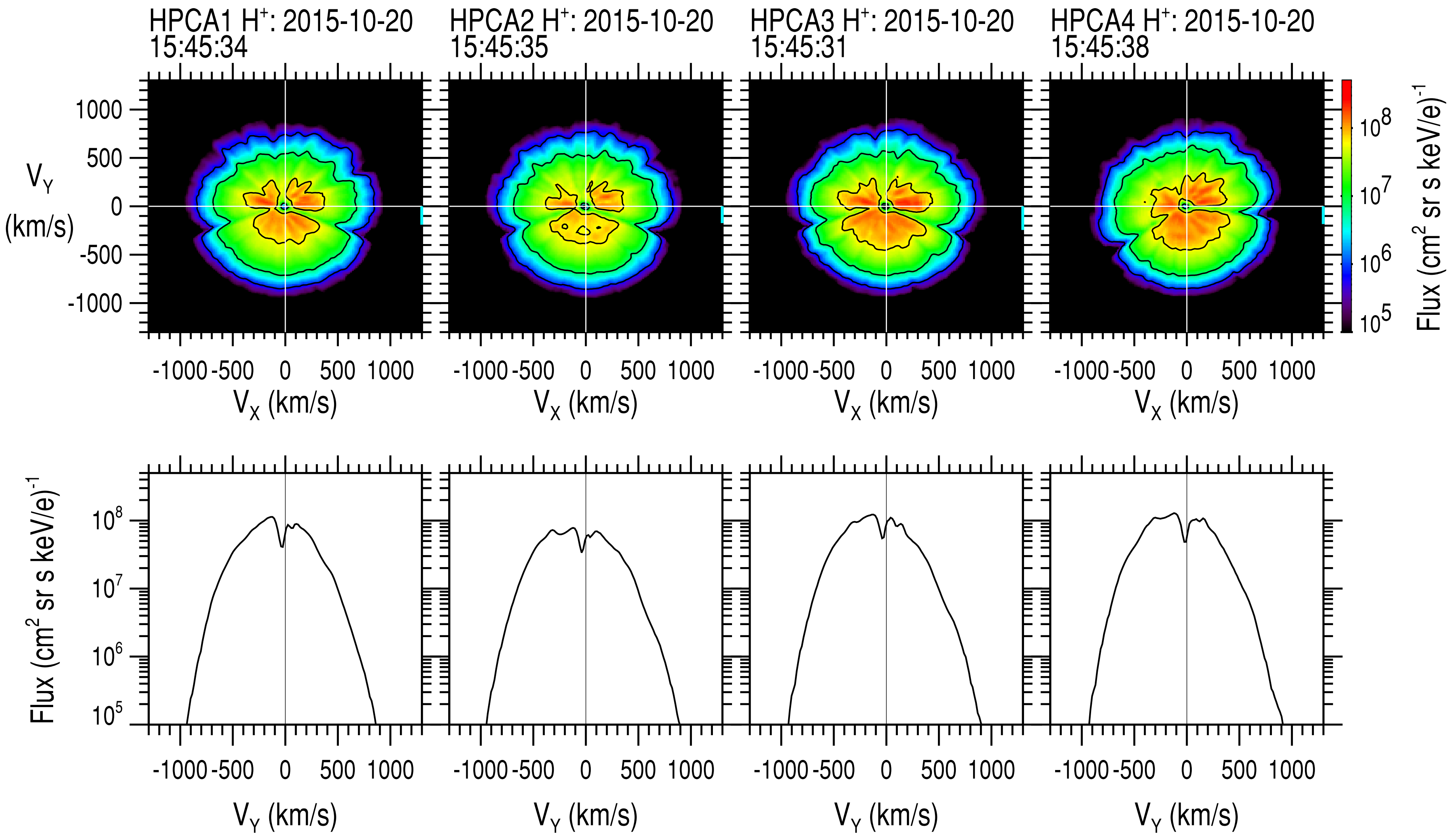

HPCA1 H+: 2015-10-20 15:45:34
HPCA2 H+: 2015-10-20 15:45:35
HPCA3 H+: 2015-10-20 15:45:31
HPCA4 H+: 2015-10-20 15:45:38
V_Y (km/s)
V_X (km/s)
Flux (cm^2 sr s keV/e)^-1
V_Y (km/s)

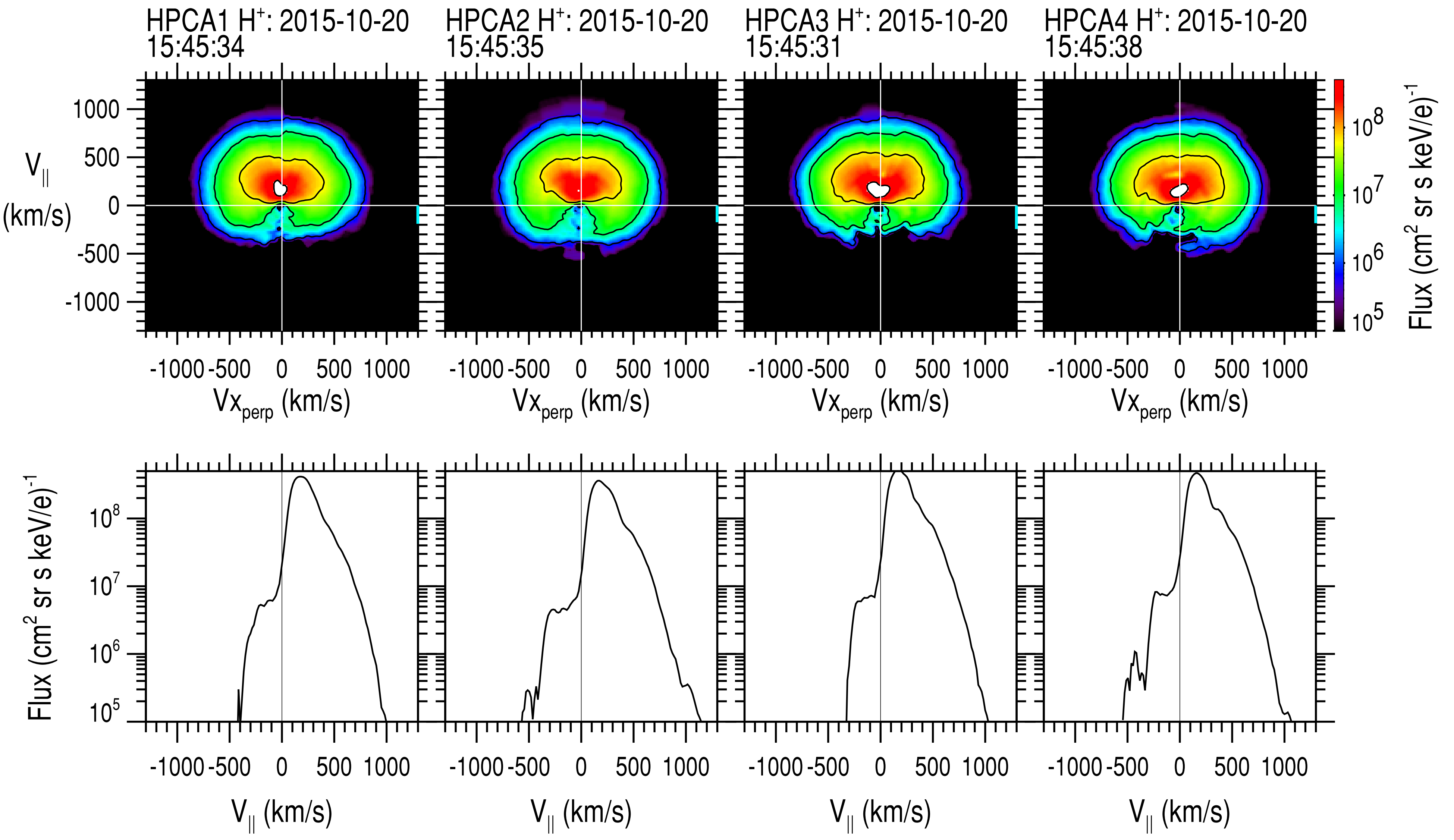
HPCA1 H+: 2015-10-20 15:45:34
HPCA2 H+: 2015-10-20 15:45:35
HPCA3 H+: 2015-10-20 15:45:31
HPCA4 H+: 2015-10-20 15:45:38
V|| (km/s)
Vxperp (km/s)
Flux (cm2 sr s keV/e)-1
-1000 -500 0 500 1000
10^5 10^6 10^7 10^8

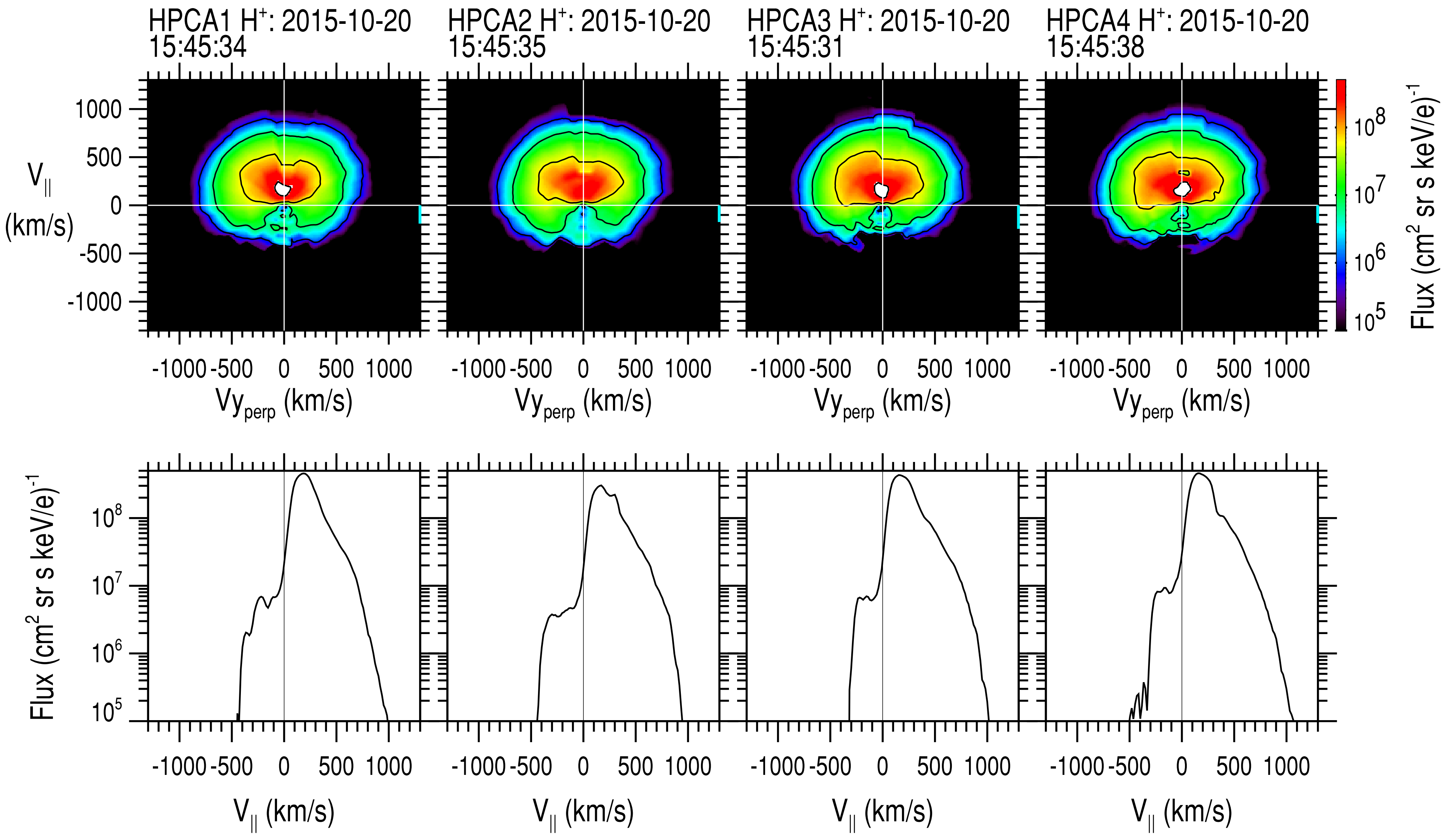
HPCA1 H+: 2015-10-20
15:45:34
HPCA2 H+: 2015-10-20
15:45:35
HPCA3 H+: 2015-10-20
15:45:31
HPCA4 H+: 2015-10-20
15:45:38
V||
(km/s)
Vyperp (km/s)
Flux (cm2 sr s keV/e)-1
V|| (km/s)

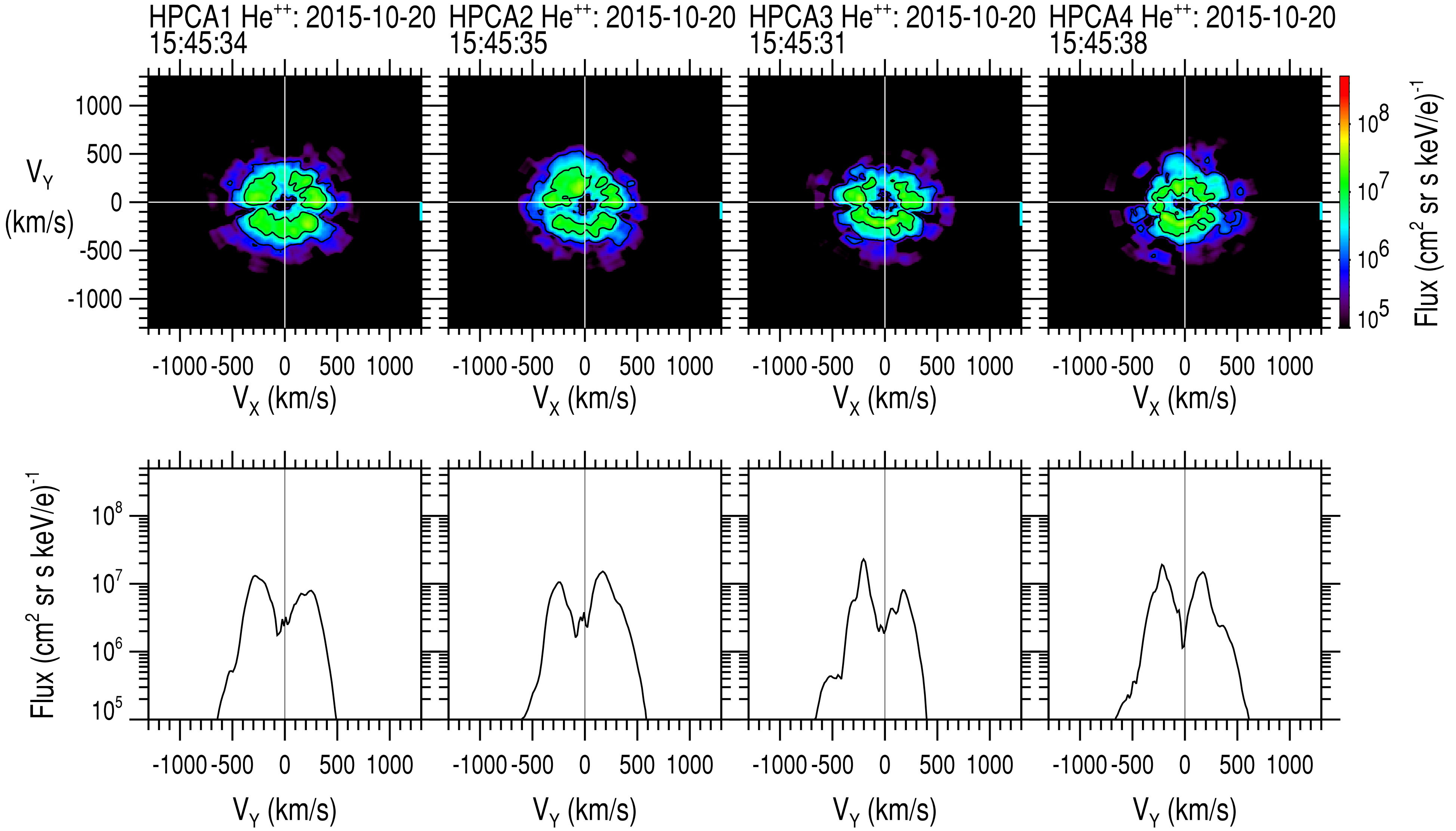

HPCA1 He++: 2015-10-20 15:45:34
HPCA2 He++: 2015-10-20 15:45:35
HPCA3 He++: 2015-10-20 15:45:31
HPCA4 He++: 2015-10-20 15:45:38
VY (km/s)
VX (km/s)
Flux (cm2 sr s keV/e)-1
VY (km/s)

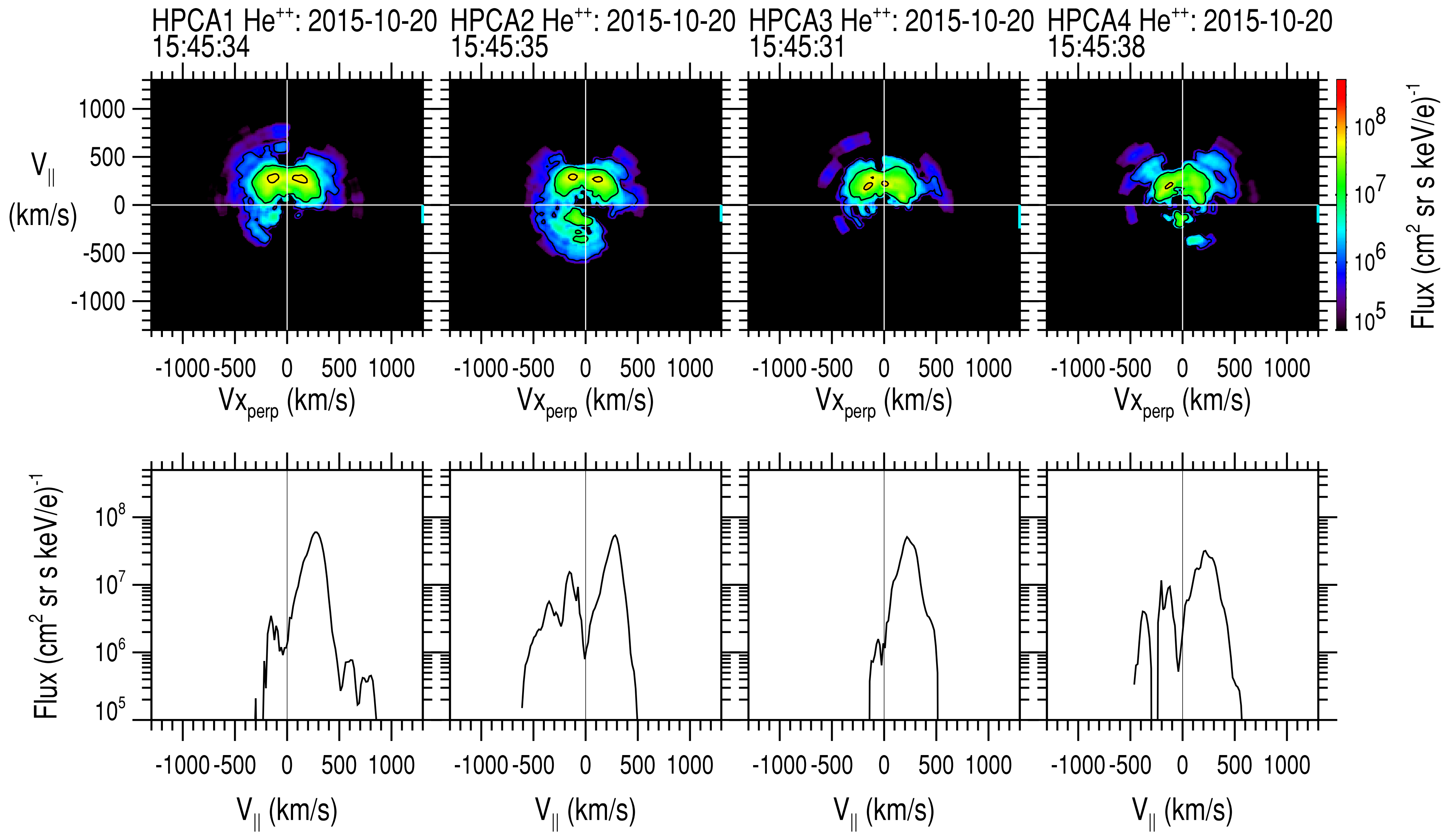

HPCA1 He++: 2015-10-20 15:45:34
HPCA2 He++: 2015-10-20 15:45:35
HPCA3 He++: 2015-10-20 15:45:31
HPCA4 He++: 2015-10-20 15:45:38
V|| (km/s)
Vxperp (km/s)
Flux (cm2 sr s keV/e)-1
-1000 -500 0 500 1000
10^5 10^6 10^7 10^8

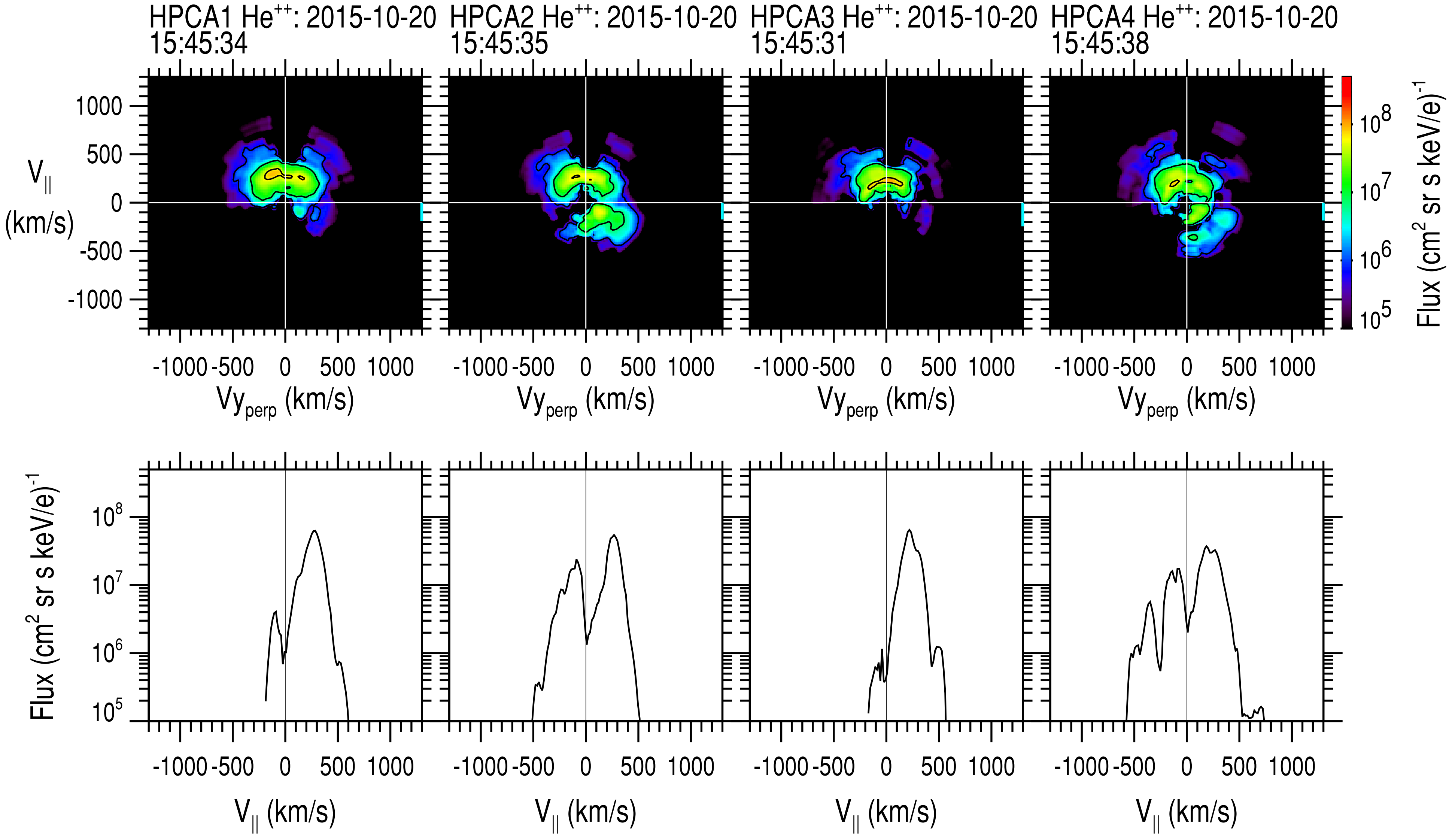

HPCA1 He++: 2015-10-20 15:45:34
HPCA2 He++: 2015-10-20 15:45:35
HPCA3 He++: 2015-10-20 15:45:31
HPCA4 He++: 2015-10-20 15:45:38
V|| (km/s)
Vy perp (km/s)
Flux (cm2 sr s keV/e)-1

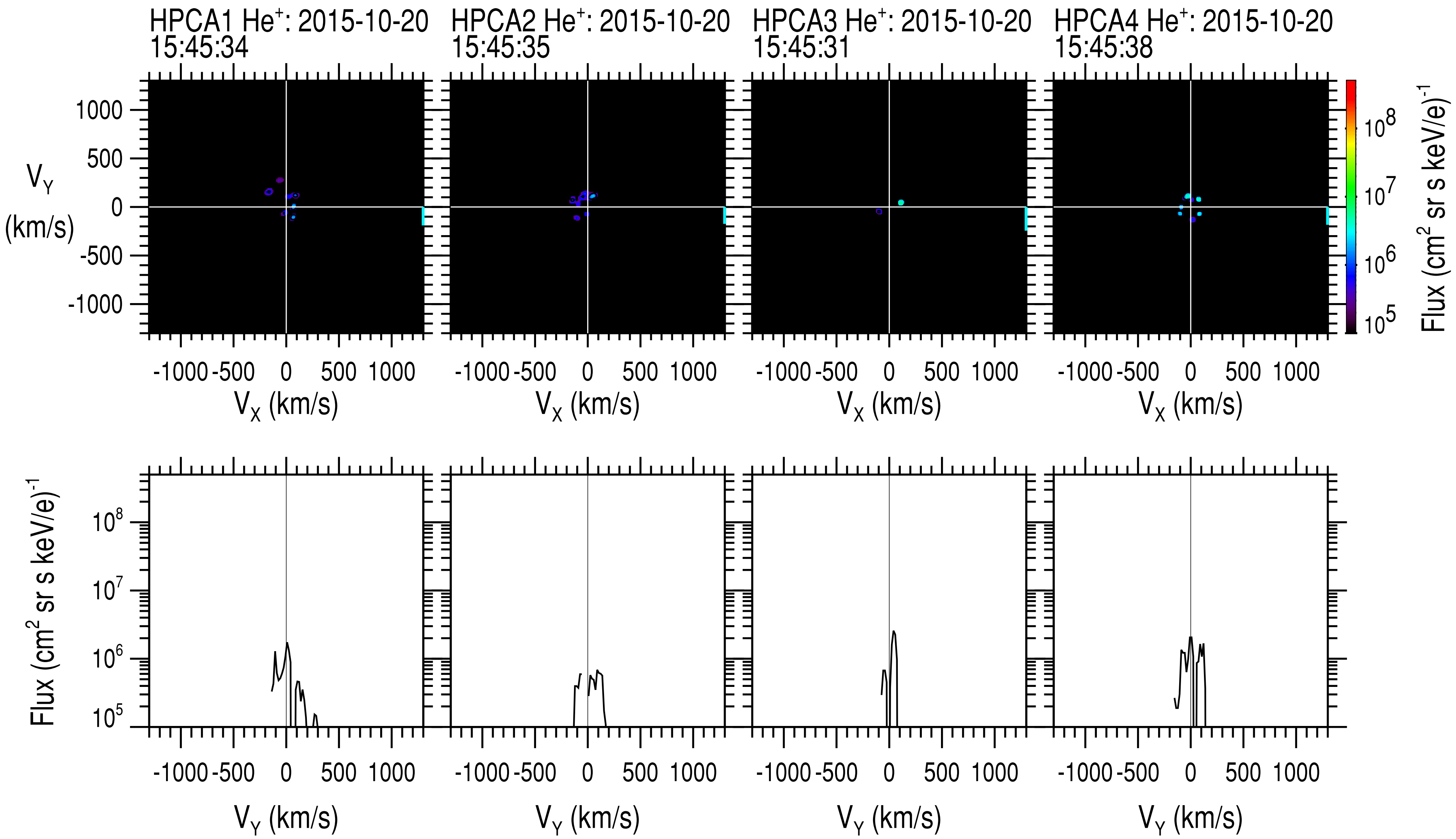
HPCA1 He+: 2015-10-20 15:45:34
HPCA2 He+: 2015-10-20 15:45:35
HPCA3 He+: 2015-10-20 15:45:31
HPCA4 He+: 2015-10-20 15:45:38
V_Y (km/s)
V_X (km/s)
Flux (cm^2 sr s keV/e)^-1

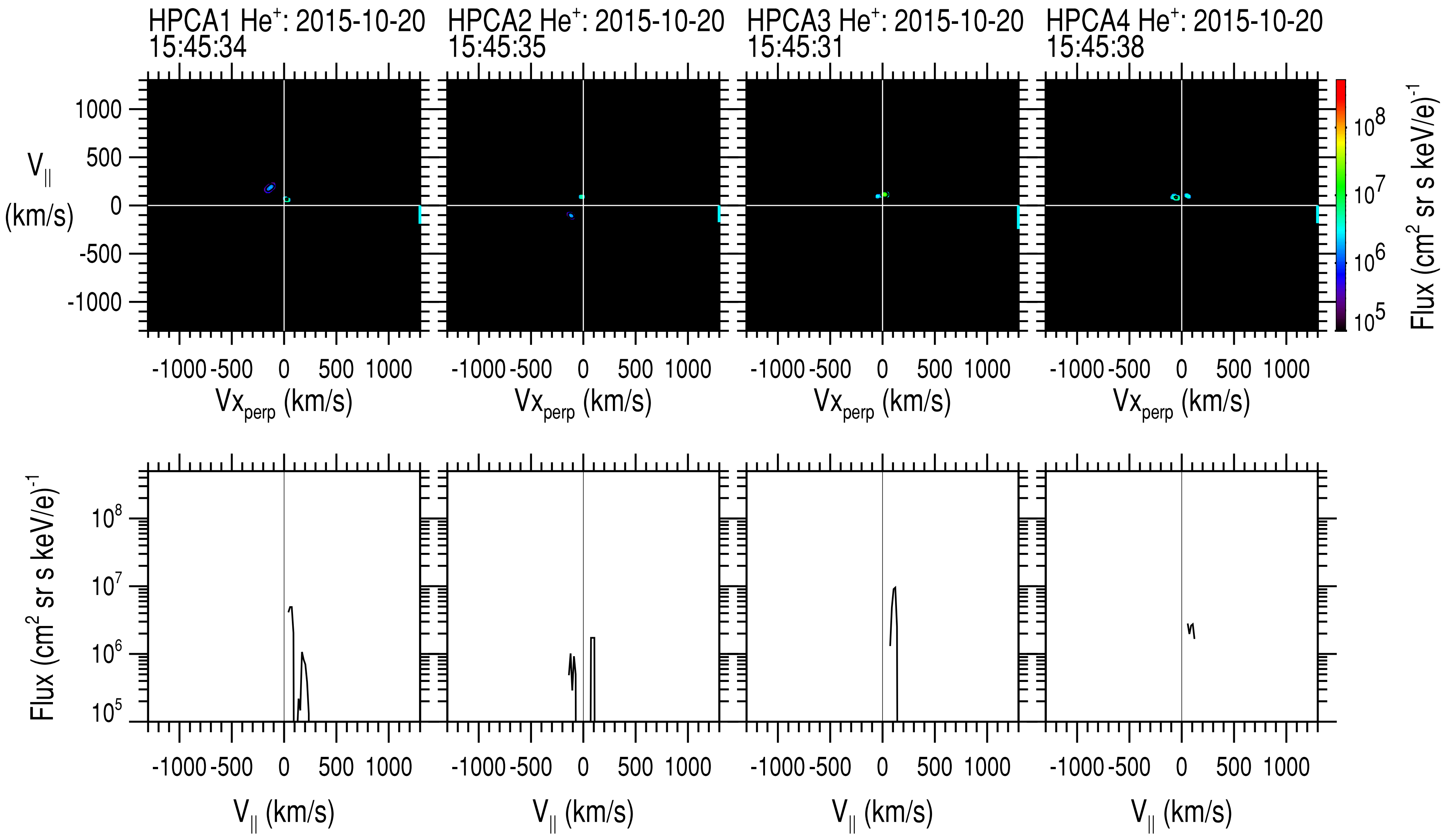

HPCA1 He+: 2015-10-20
15:45:34
HPCA2 He+: 2015-10-20
15:45:35
HPCA3 He+: 2015-10-20
15:45:31
HPCA4 He+: 2015-10-20
15:45:38
V|| (km/s)
Vxperp (km/s)
Flux (cm2 sr s keV/e)-1

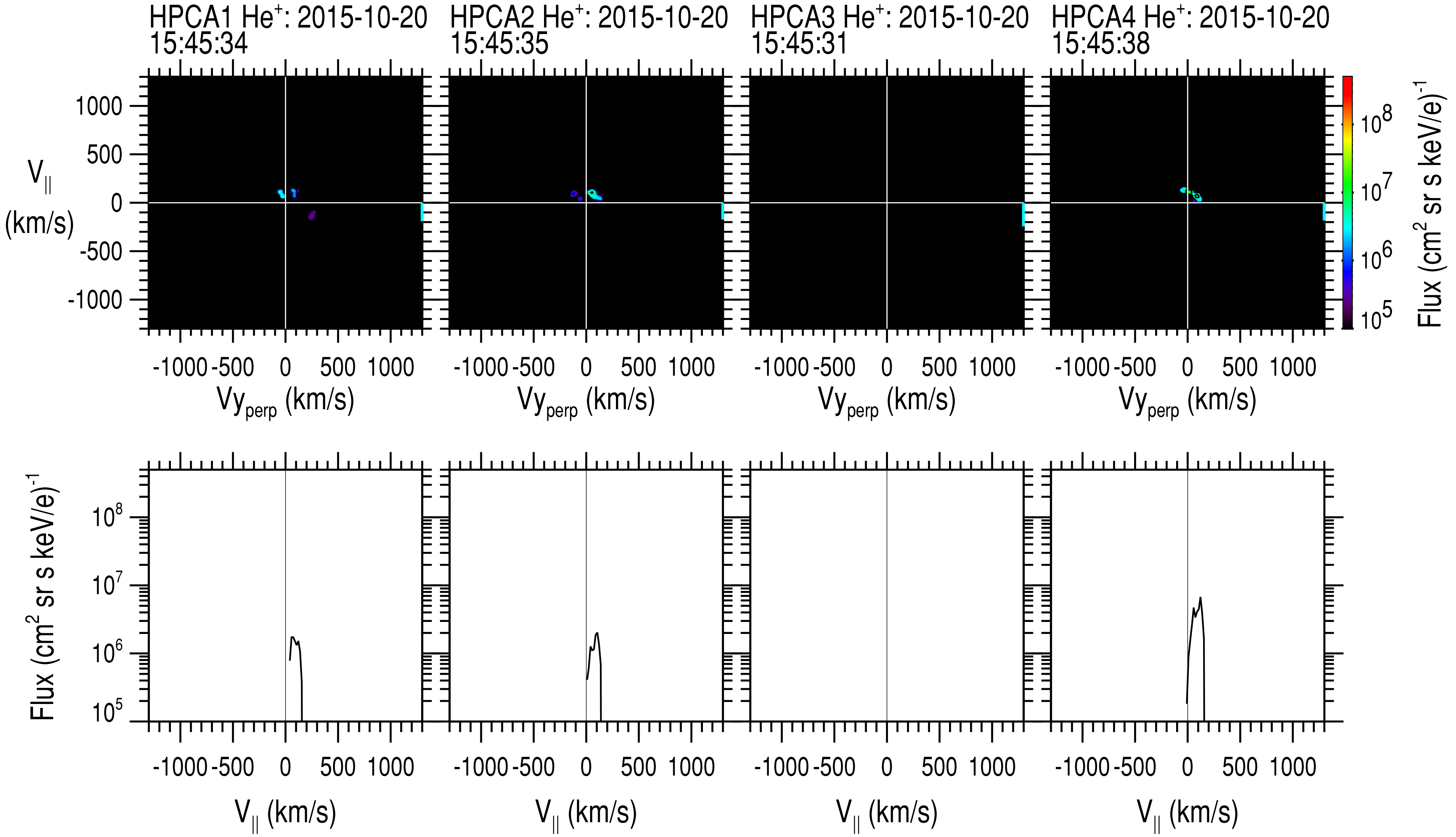
HPCA1 He+: 2015-10-20 15:45:34
HPCA2 He+: 2015-10-20 15:45:35
HPCA3 He+: 2015-10-20 15:45:31
HPCA4 He+: 2015-10-20 15:45:38
V|| (km/s)
Vyperp (km/s)
Flux (cm2 sr s keV/e)-1
-1000 -500 0 500 1000
10^5 10^6 10^7 10^8

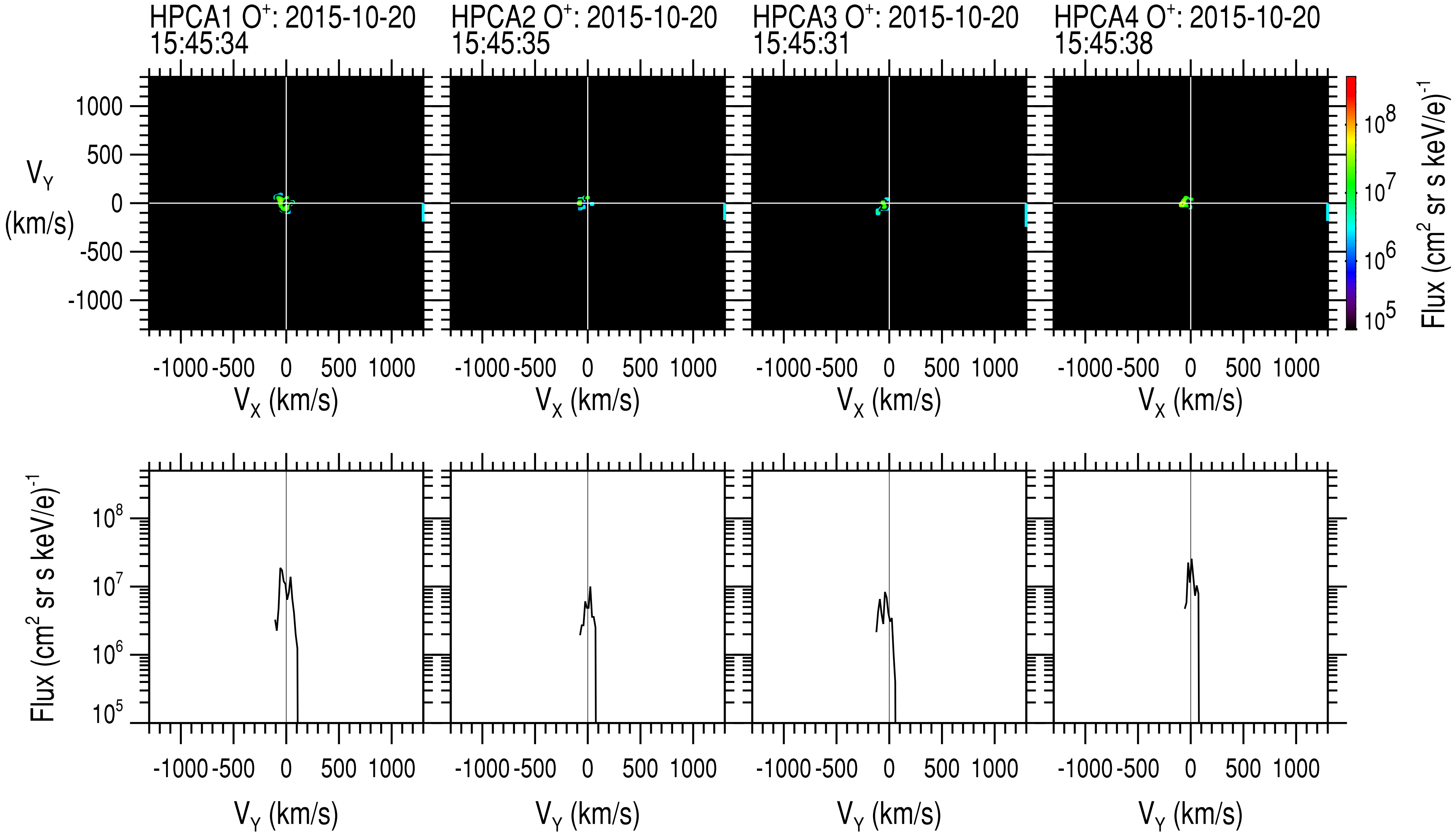
HPCA1 O+: 2015-10-20 15:45:34
HPCA2 O+: 2015-10-20 15:45:35
HPCA3 O+: 2015-10-20 15:45:31
HPCA4 O+: 2015-10-20 15:45:38
V_Y (km/s)
V_X (km/s)
Flux (cm^2 sr s keV/e)^-1

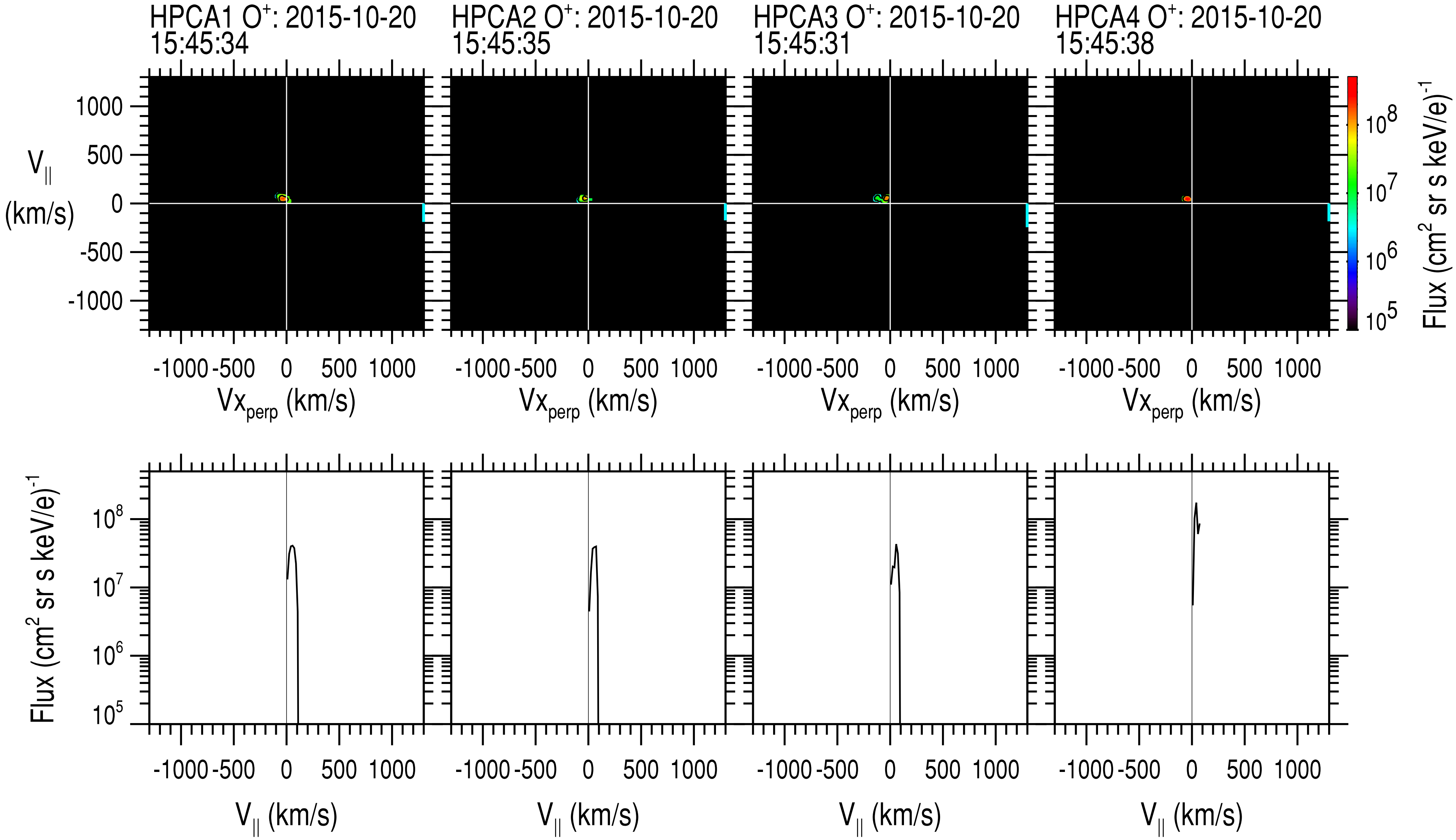
HPCA1 O+: 2015-10-20
15:45:34
HPCA2 O+: 2015-10-20
15:45:35
HPCA3 O+: 2015-10-20
15:45:31
HPCA4 O+: 2015-10-20
15:45:38
V|| (km/s)
Vxperp (km/s)
Flux (cm2 sr s keV/e)-1
1000
500
0
-500
-1000
10^5
10^6
10^7
10^8

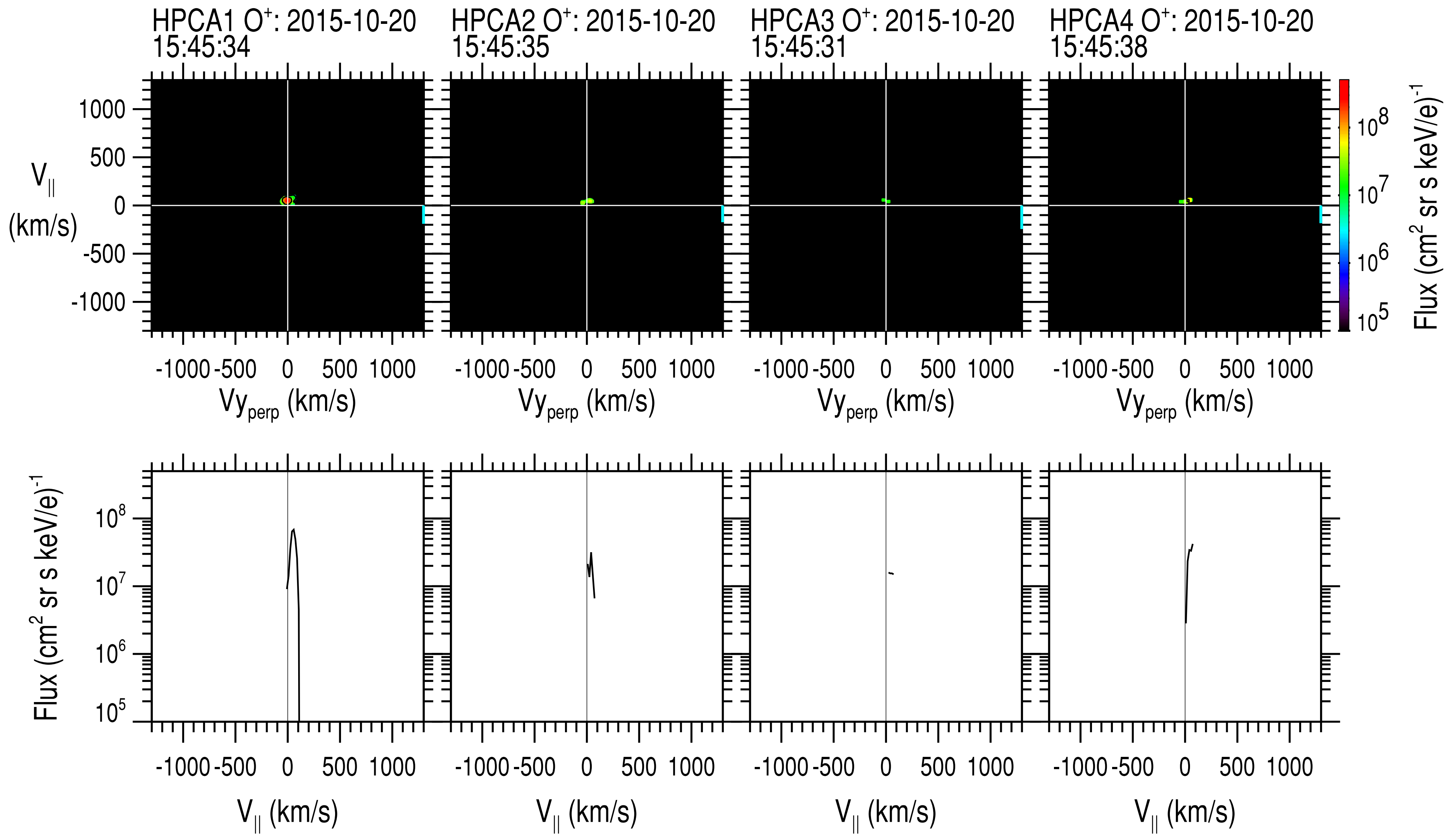
HPCA1 O+: 2015-10-20
15:45:34
HPCA2 O+: 2015-10-20
15:45:35
HPCA3 O+: 2015-10-20
15:45:31
HPCA4 O+: 2015-10-20
15:45:38
V|| (km/s)
Vyperp (km/s)
Flux (cm2 sr s keV/e)-1
V|| (km/s)

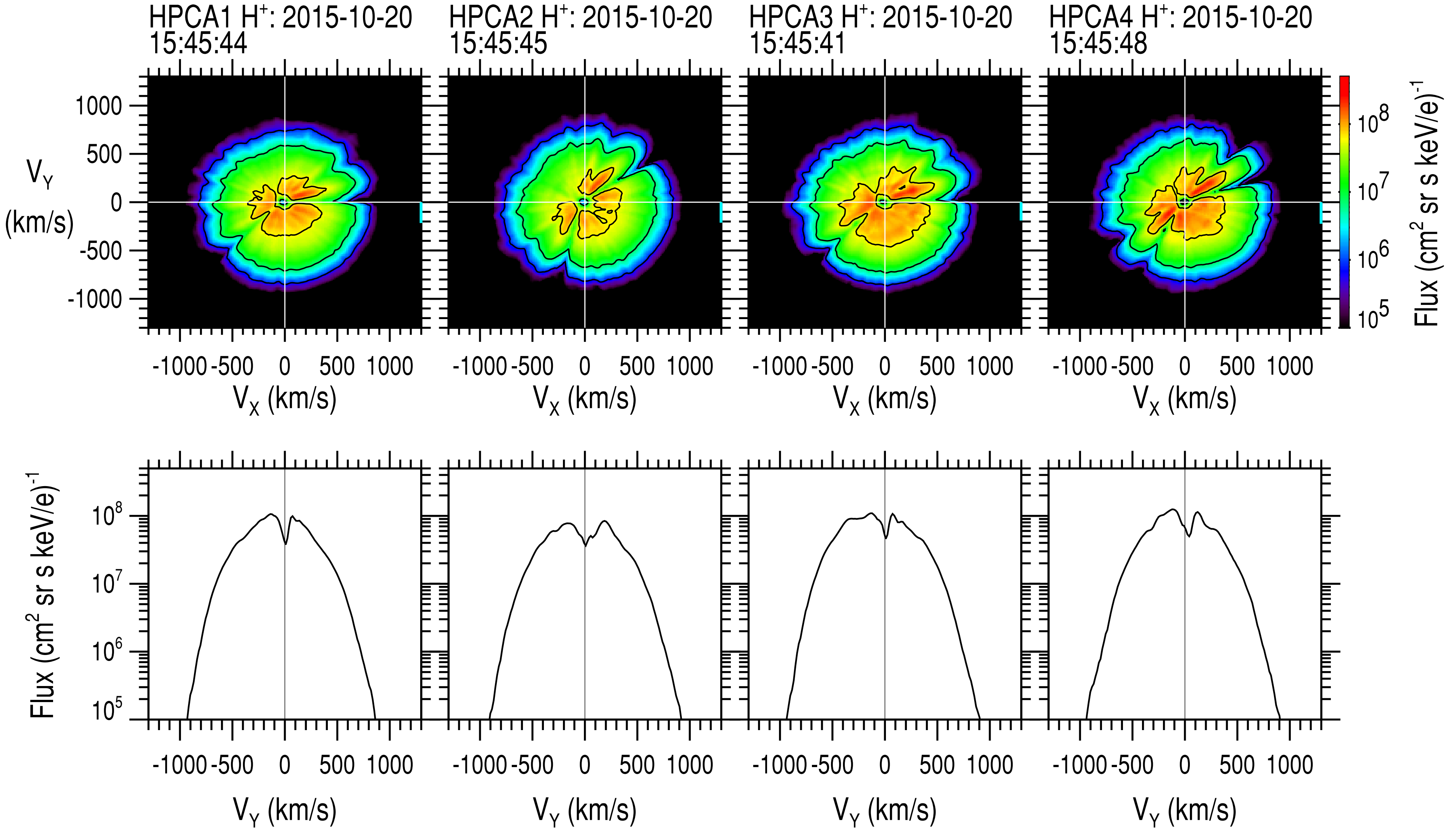
HPCA1 H+: 2015-10-20
15:45:44
HPCA2 H+: 2015-10-20
15:45:45
HPCA3 H+: 2015-10-20
15:45:41
HPCA4 H+: 2015-10-20
15:45:48
V_Y (km/s)
V_X (km/s)
Flux (cm^2 sr s keV/e)^-1
V_Y (km/s)
Flux (cm^2 sr s keV/e)^-1

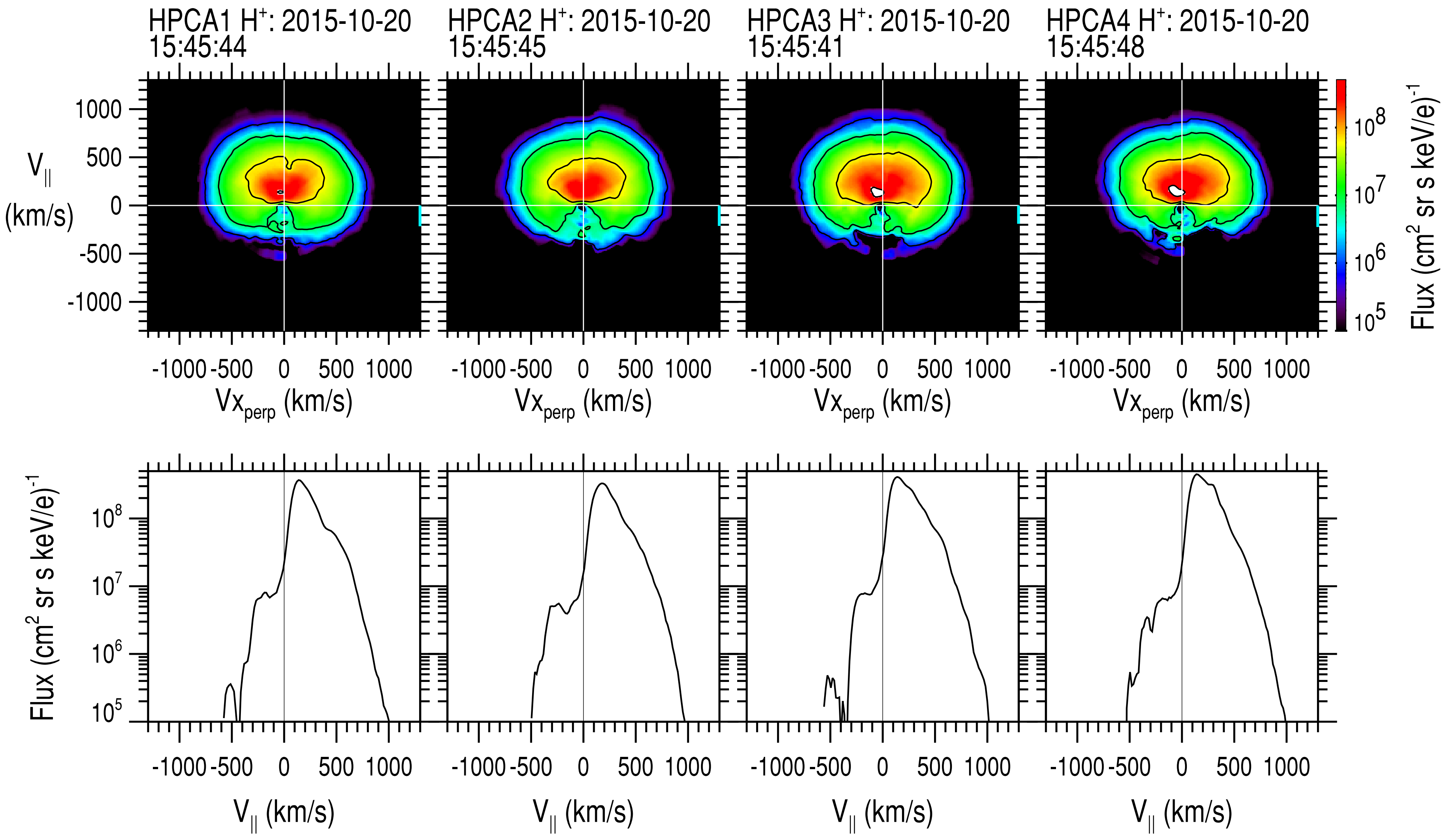
HPCA1 H+: 2015-10-20 15:45:44
HPCA2 H+: 2015-10-20 15:45:45
HPCA3 H+: 2015-10-20 15:45:41
HPCA4 H+: 2015-10-20 15:45:48
V|| (km/s)
Vxperp (km/s)
Flux (cm2 sr s keV/e)-1
V|| (km/s)

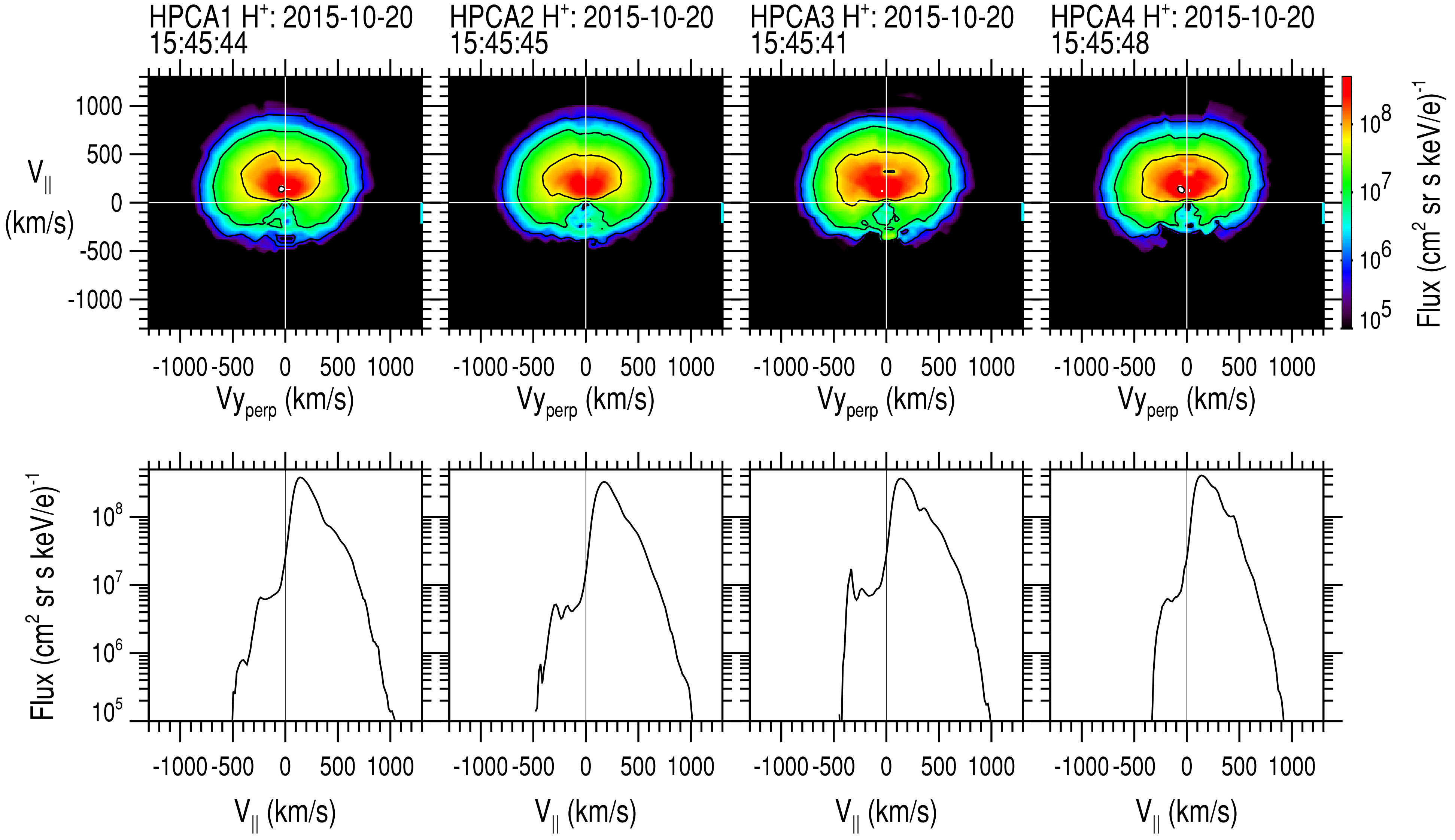
HPCA1 H+: 2015-10-20 15:45:44
HPCA2 H+: 2015-10-20 15:45:45
HPCA3 H+: 2015-10-20 15:45:41
HPCA4 H+: 2015-10-20 15:45:48
V|| (km/s)
Vyperp (km/s)
Flux (cm2 sr s keV/e)-1
-1000 -500 0 500 1000
10^5 10^6 10^7 10^8

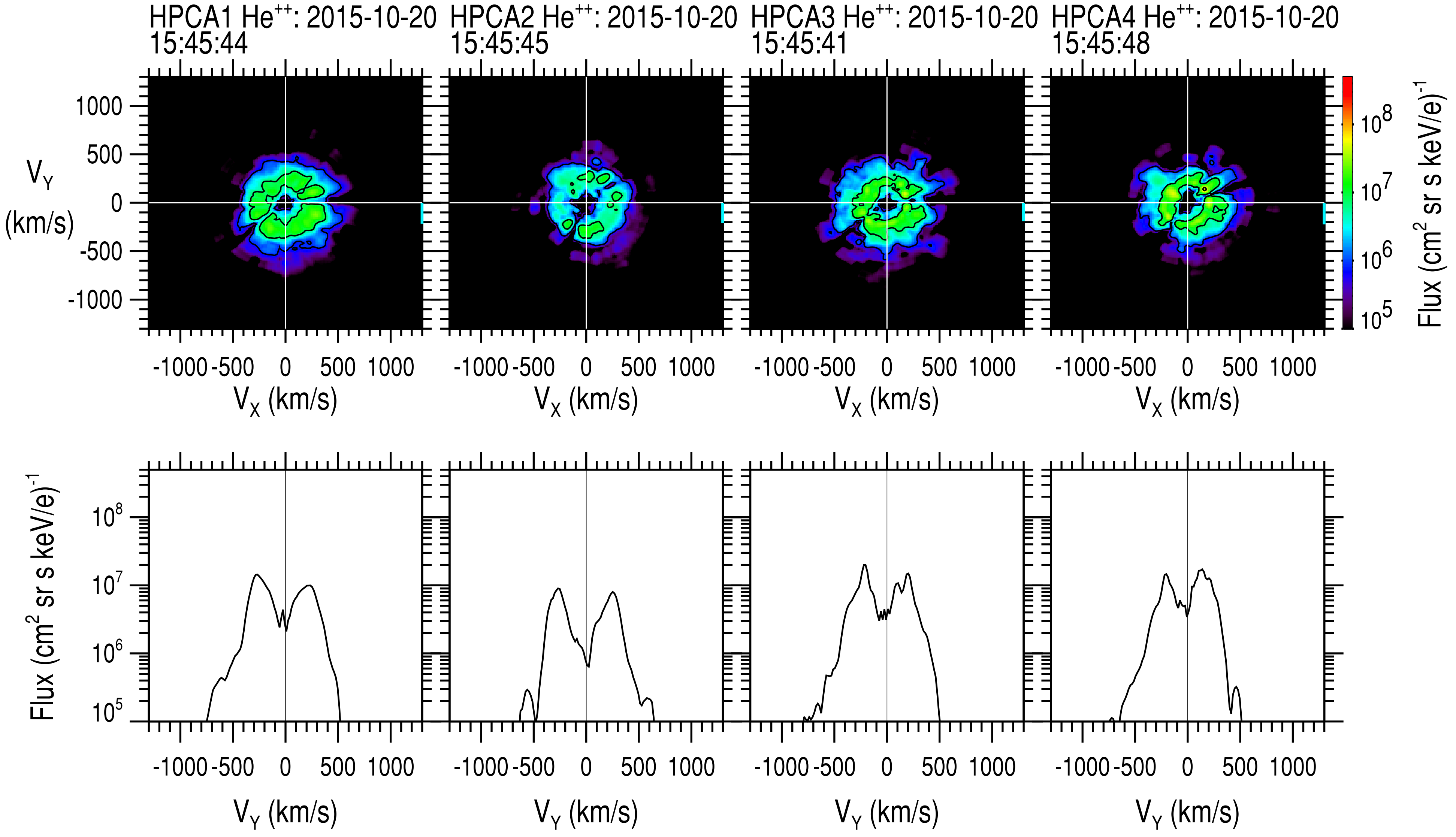
HPCA1 He++: 2015-10-20 15:45:44
HPCA2 He++: 2015-10-20 15:45:45
HPCA3 He++: 2015-10-20 15:45:41
HPCA4 He++: 2015-10-20 15:45:48
V_Y (km/s)
V_X (km/s)
Flux (cm^2 sr s keV/e)^-1
V_Y (km/s)

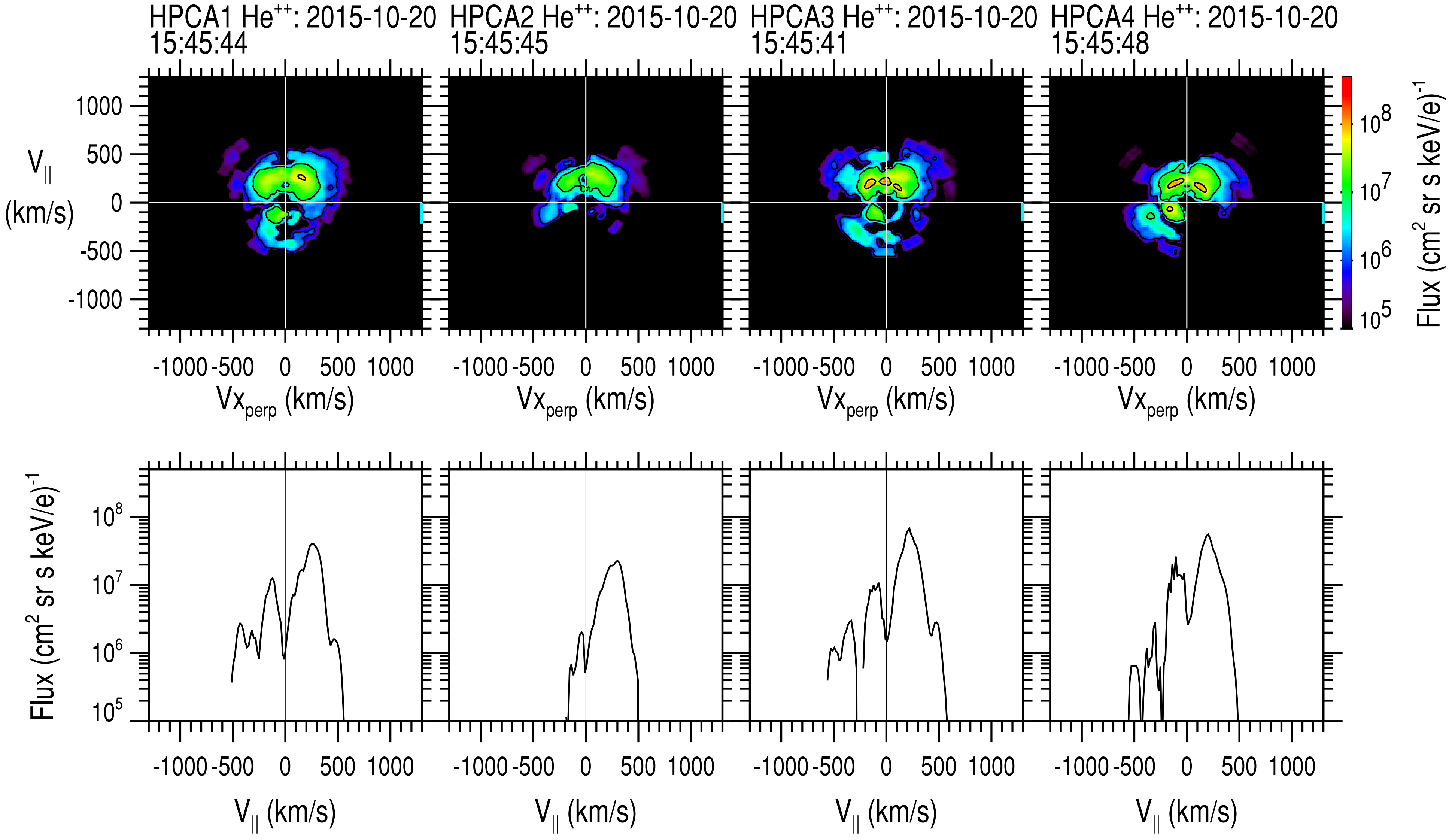

HPCA1 He++: 2015-10-20 15:45:44
HPCA2 He++: 2015-10-20 15:45:45
HPCA3 He++: 2015-10-20 15:45:41
HPCA4 He++: 2015-10-20 15:45:48
V|| (km/s)
Vxperp (km/s)
Flux (cm2 sr s keV/e)-1

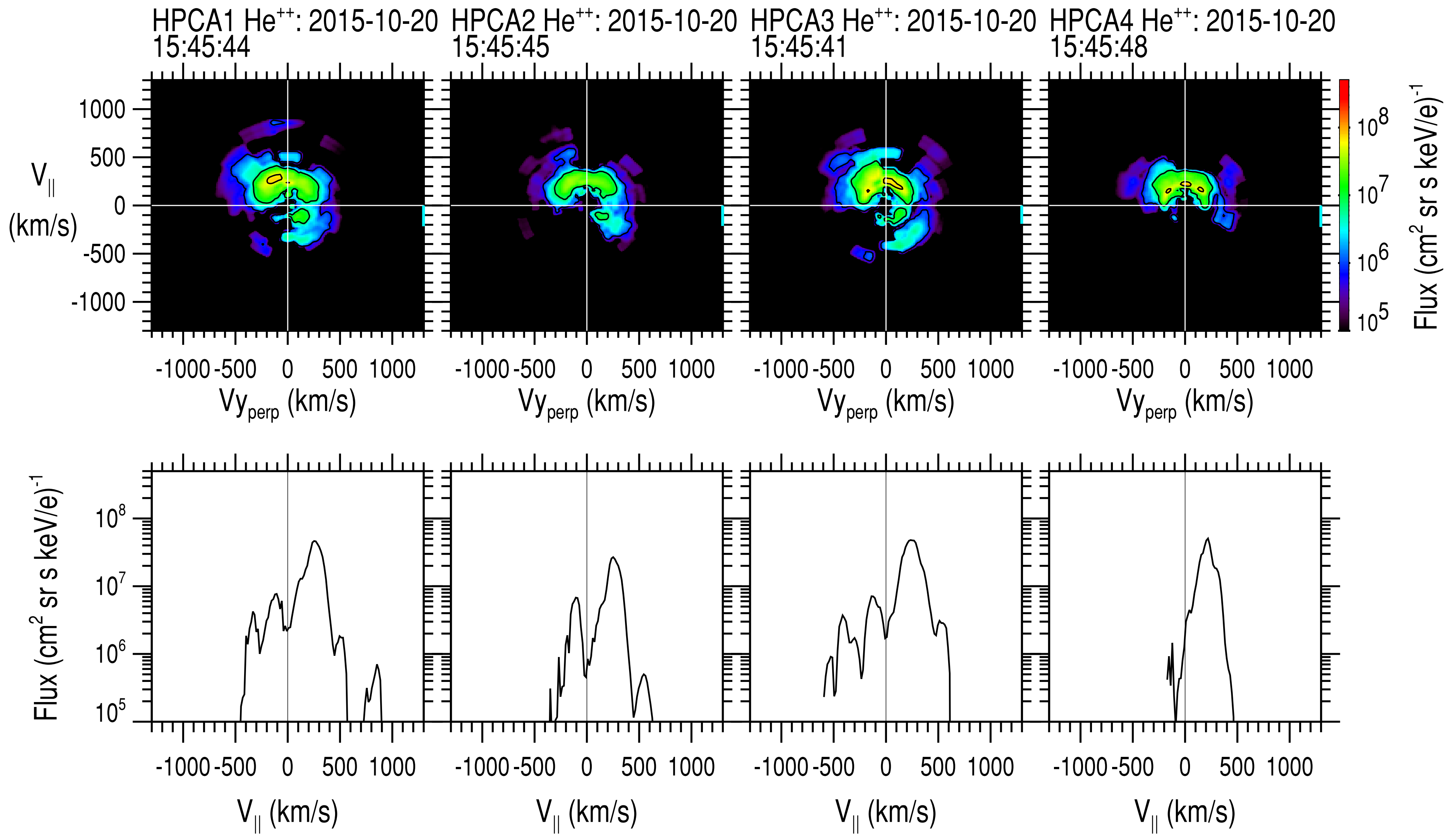

HPCA1 He++: 2015-10-20 15:45:44
HPCA2 He++: 2015-10-20 15:45:45
HPCA3 He++: 2015-10-20 15:45:41
HPCA4 He++: 2015-10-20 15:45:48
V|| (km/s)
1000
500
0
-500
-1000
Vyperp (km/s)
Flux (cm2 sr s keV/e)-1
10^5
10^6
10^7
10^8
V|| (km/s)

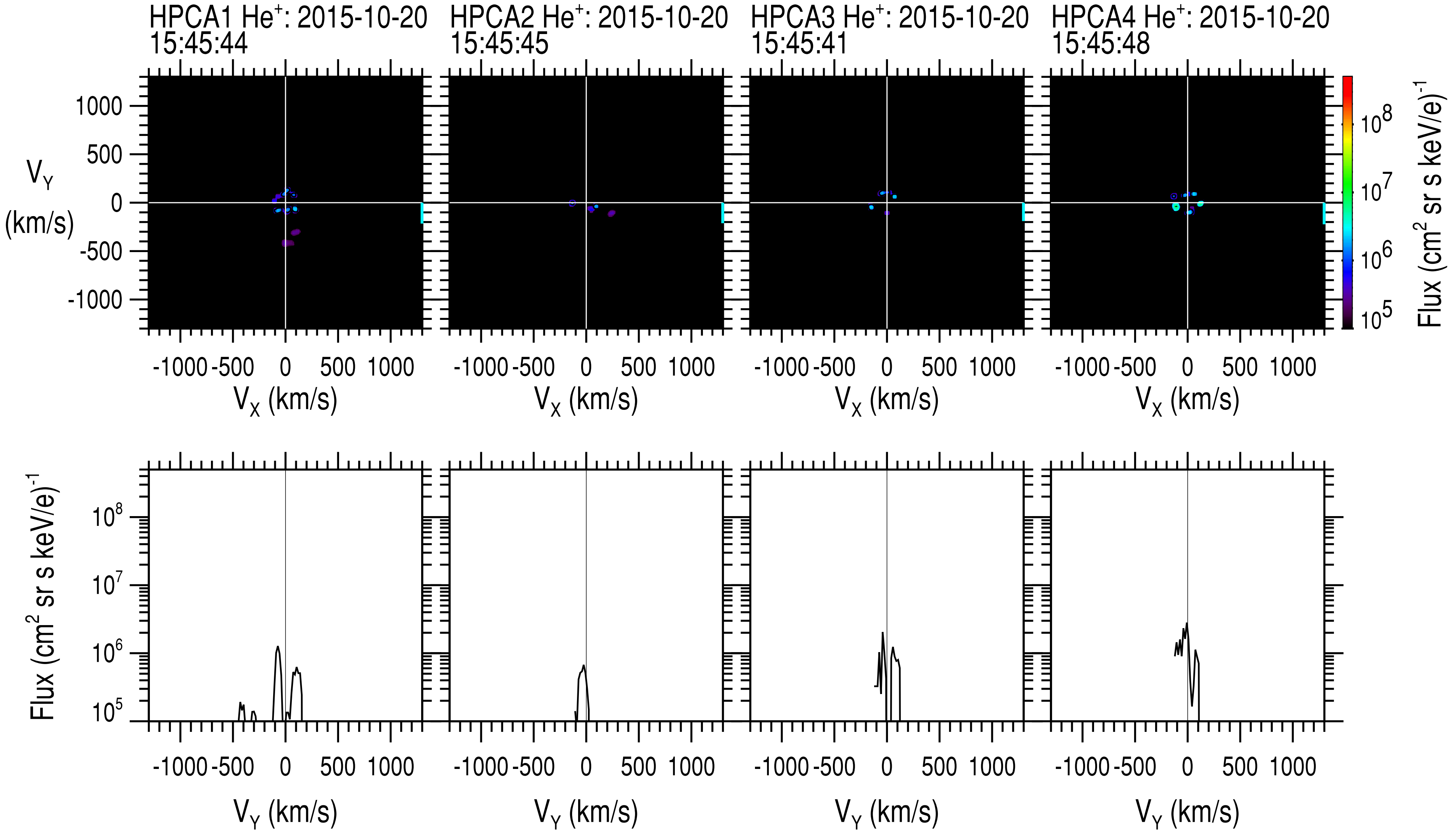
HPCA1 He+: 2015-10-20
15:45:44
HPCA2 He+: 2015-10-20
15:45:45
HPCA3 He+: 2015-10-20
15:45:41
HPCA4 He+: 2015-10-20
15:45:48
VY (km/s)
VX (km/s)
Flux (cm2 sr s keV/e)-1
-1000 -500 0 500 1000
10^5 10^6 10^7 10^8

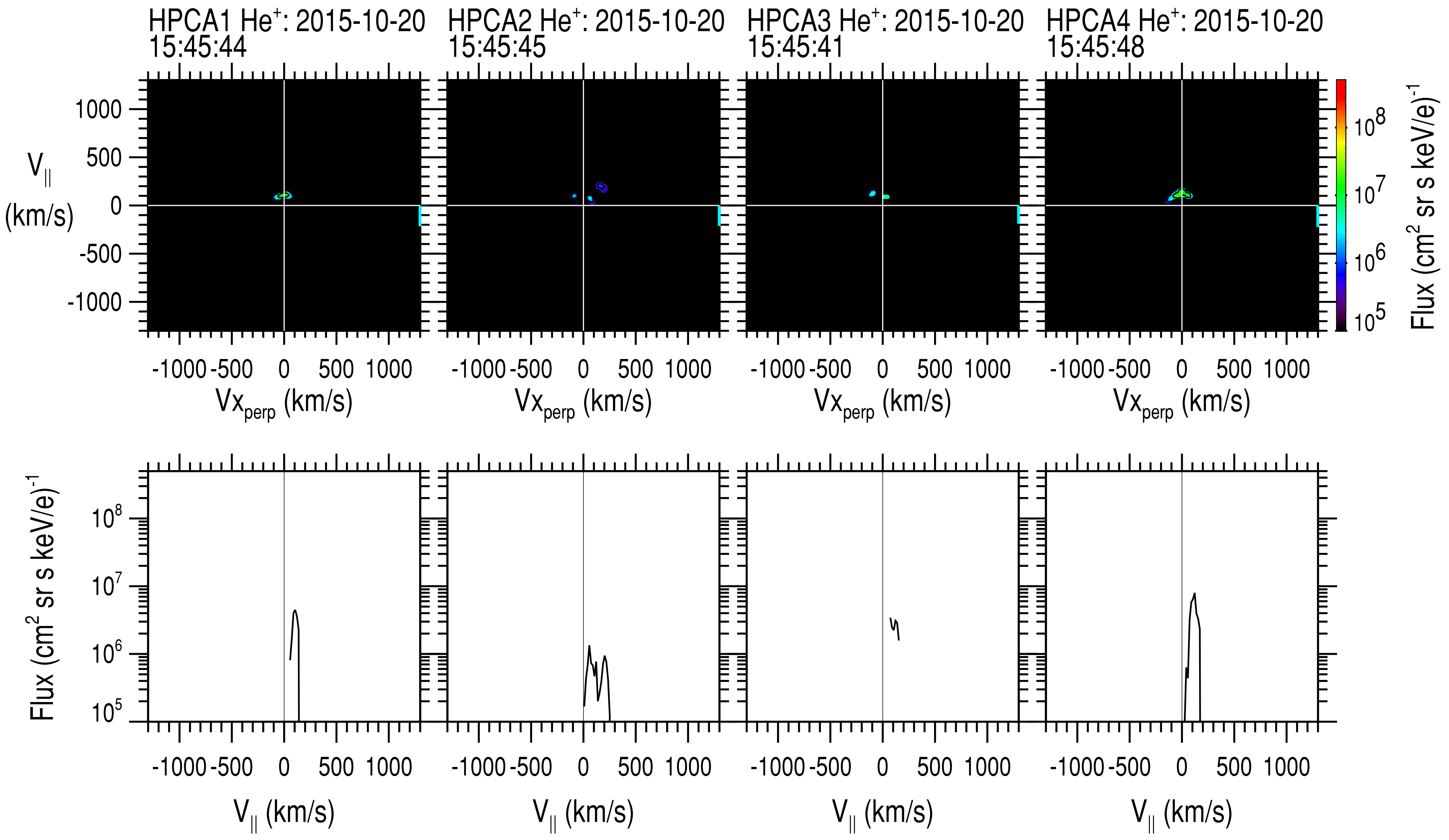

HPCA1 He+: 2015-10-20 15:45:44
HPCA2 He+: 2015-10-20 15:45:45
HPCA3 He+: 2015-10-20 15:45:41
HPCA4 He+: 2015-10-20 15:45:48
V|| (km/s)
Vxperp (km/s)
Flux (cm2 sr s keV/e)-1

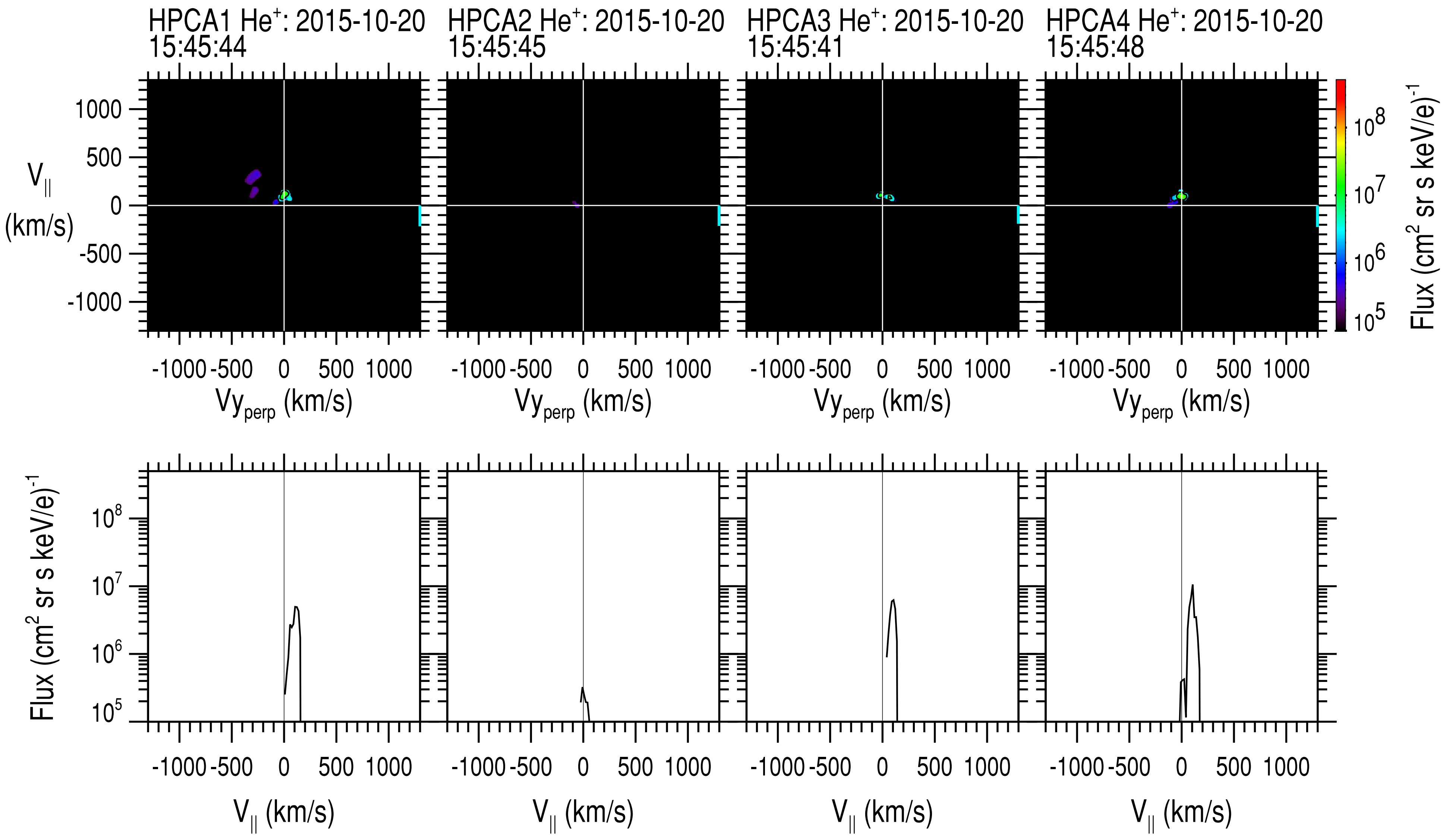
HPCA1 He+: 2015-10-20
15:45:44
HPCA2 He+: 2015-10-20
15:45:45
HPCA3 He+: 2015-10-20
15:45:41
HPCA4 He+: 2015-10-20
15:45:48
V|| (km/s)
Vyperp (km/s)
Flux (cm2 sr s keV/e)-1
-1000 -500 0 500 1000
10^5 10^6 10^7 10^8

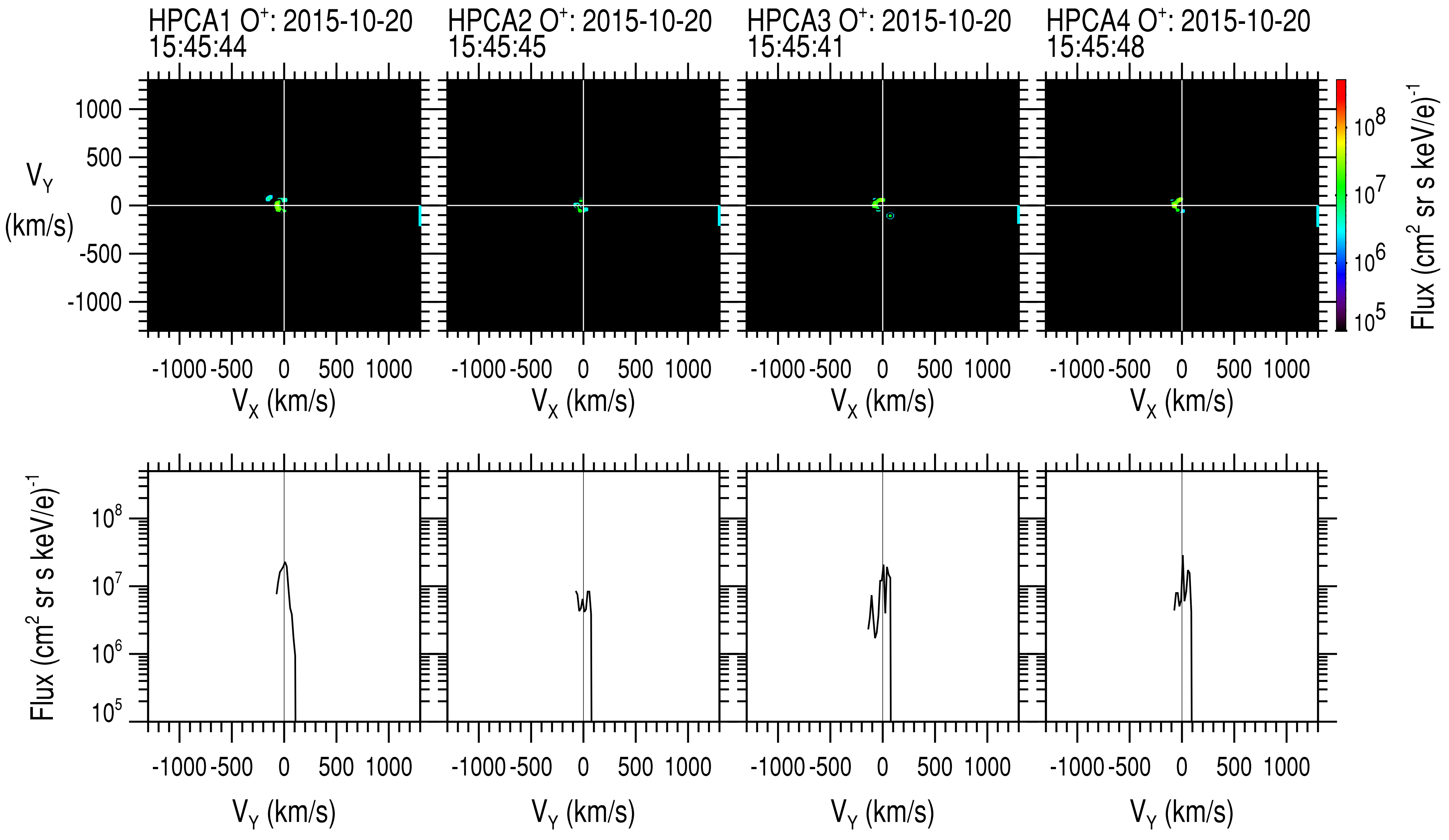
HPCA1 O+: 2015-10-20 15:45:44
HPCA2 O+: 2015-10-20 15:45:45
HPCA3 O+: 2015-10-20 15:45:41
HPCA4 O+: 2015-10-20 15:45:48
VY (km/s)
VX (km/s)
Flux (cm2 sr s keV/e)-1
-1000 -500 0 500 1000
105 106 107 108

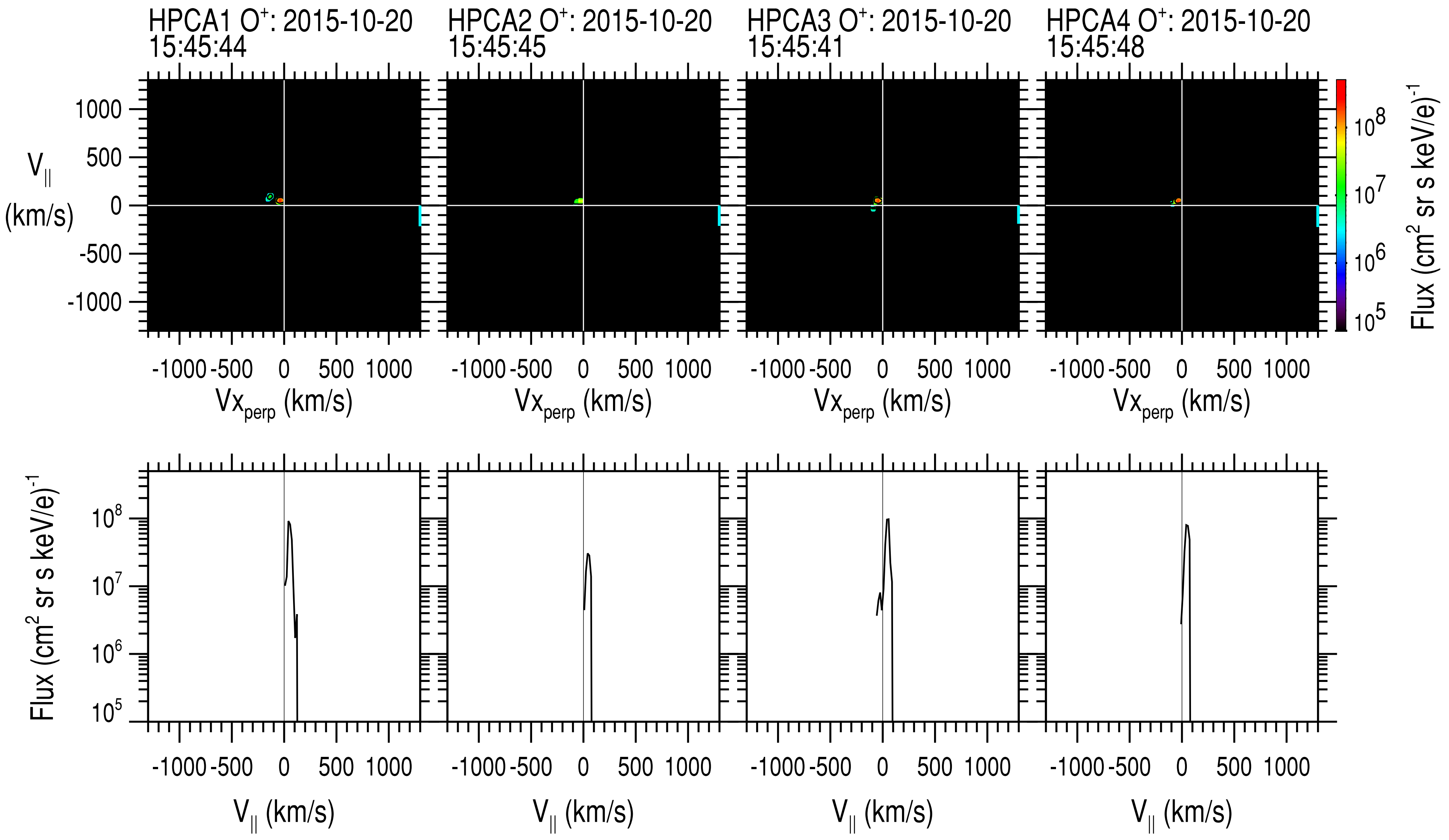
HPCA1 O+: 2015-10-20 15:45:44
HPCA2 O+: 2015-10-20 15:45:45
HPCA3 O+: 2015-10-20 15:45:41
HPCA4 O+: 2015-10-20 15:45:48
V|| (km/s)
Vx perp (km/s)
Flux (cm2 sr s keV/e)-1
V|| (km/s)

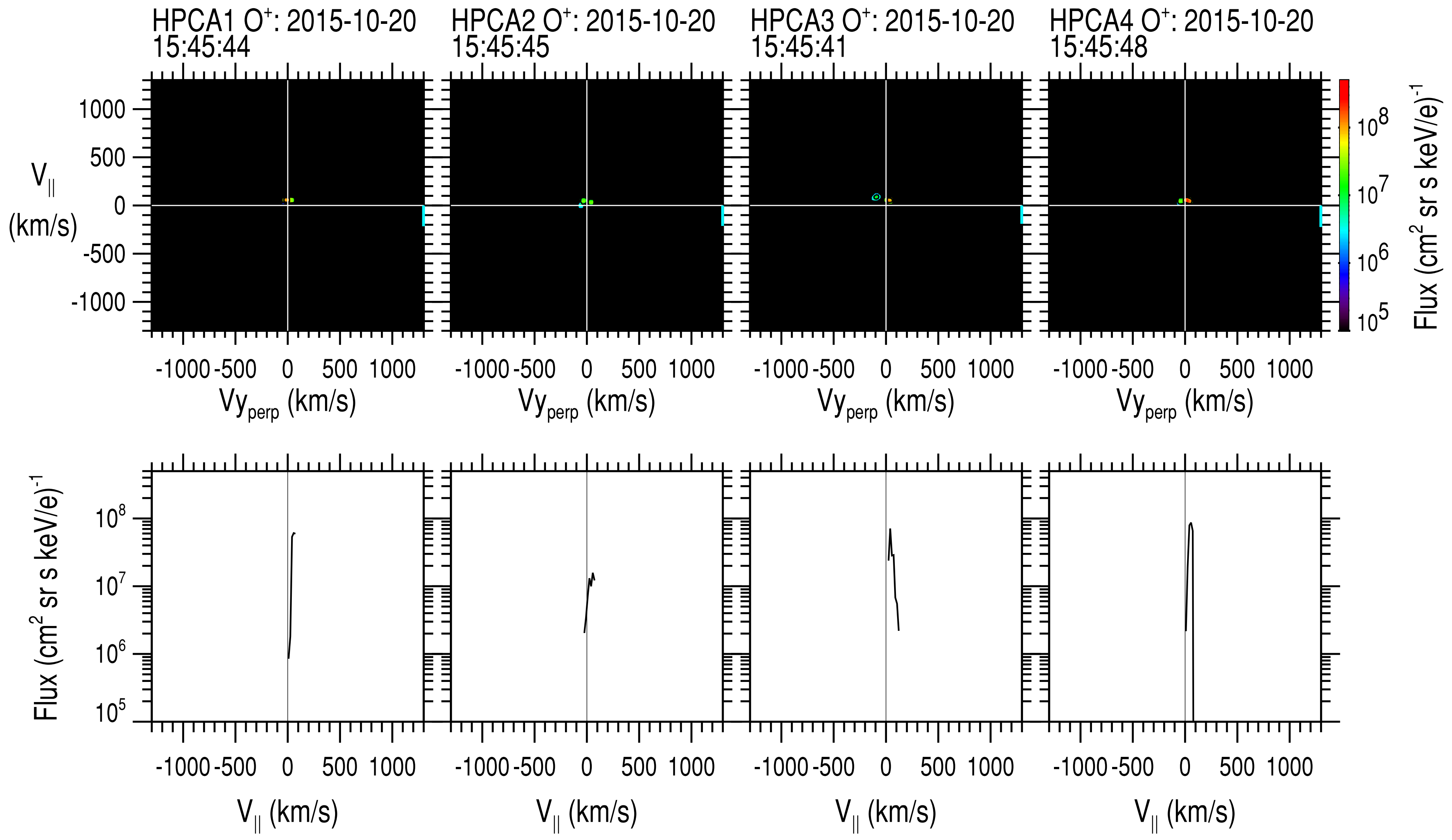

HPCA1 O+: 2015-10-20
15:45:44
HPCA2 O+: 2015-10-20
15:45:45
HPCA3 O+: 2015-10-20
15:45:41
HPCA4 O+: 2015-10-20
15:45:48
V|| (km/s)
Vyperp (km/s)
Flux (cm2 sr s keV/e)-1

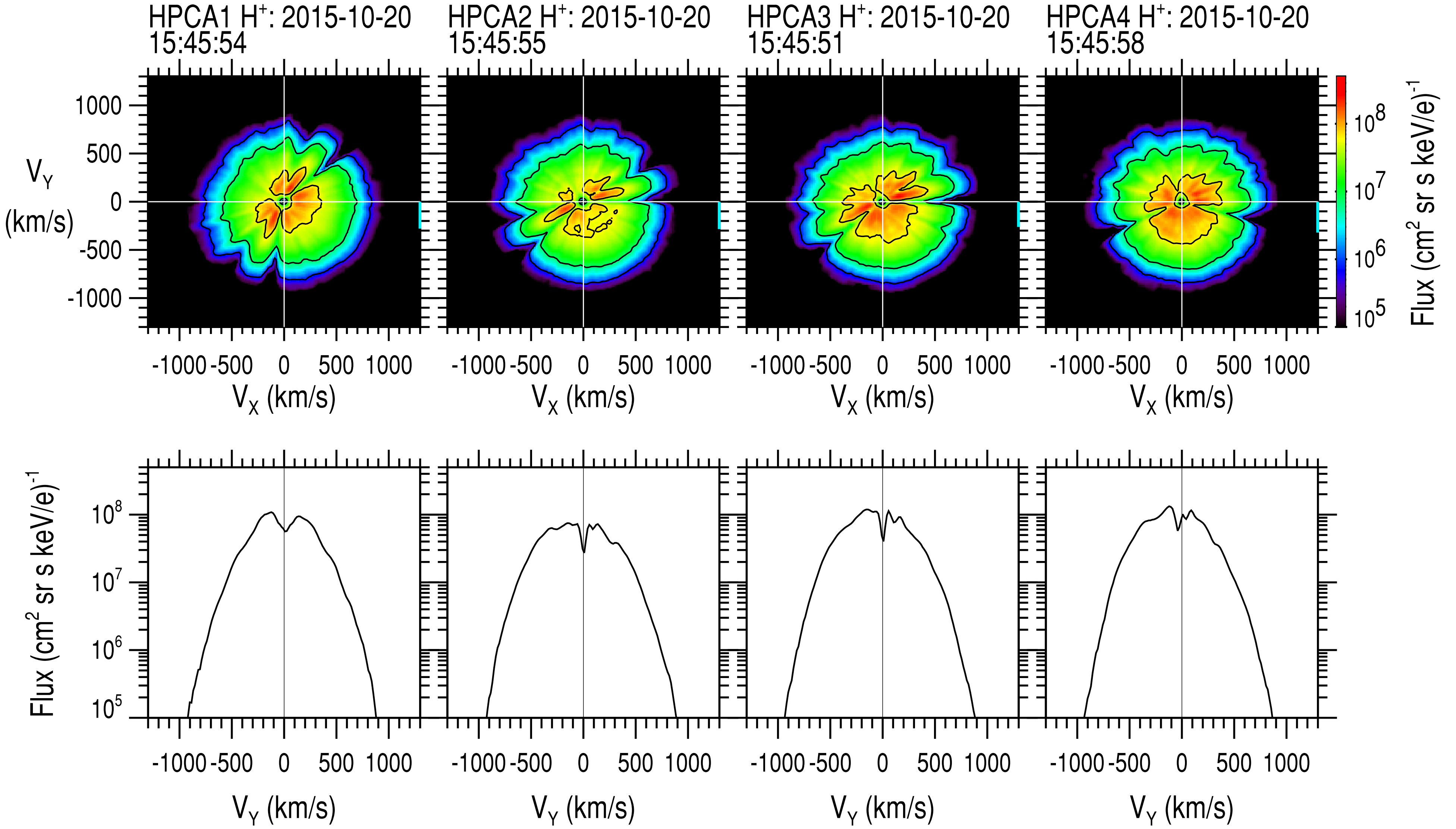

HPCA1 H+: 2015-10-20
15:45:54
HPCA2 H+: 2015-10-20
15:45:55
HPCA3 H+: 2015-10-20
15:45:51
HPCA4 H+: 2015-10-20
15:45:58
VY (km/s)
VX (km/s)
Flux (cm2 sr s keV/e)-1

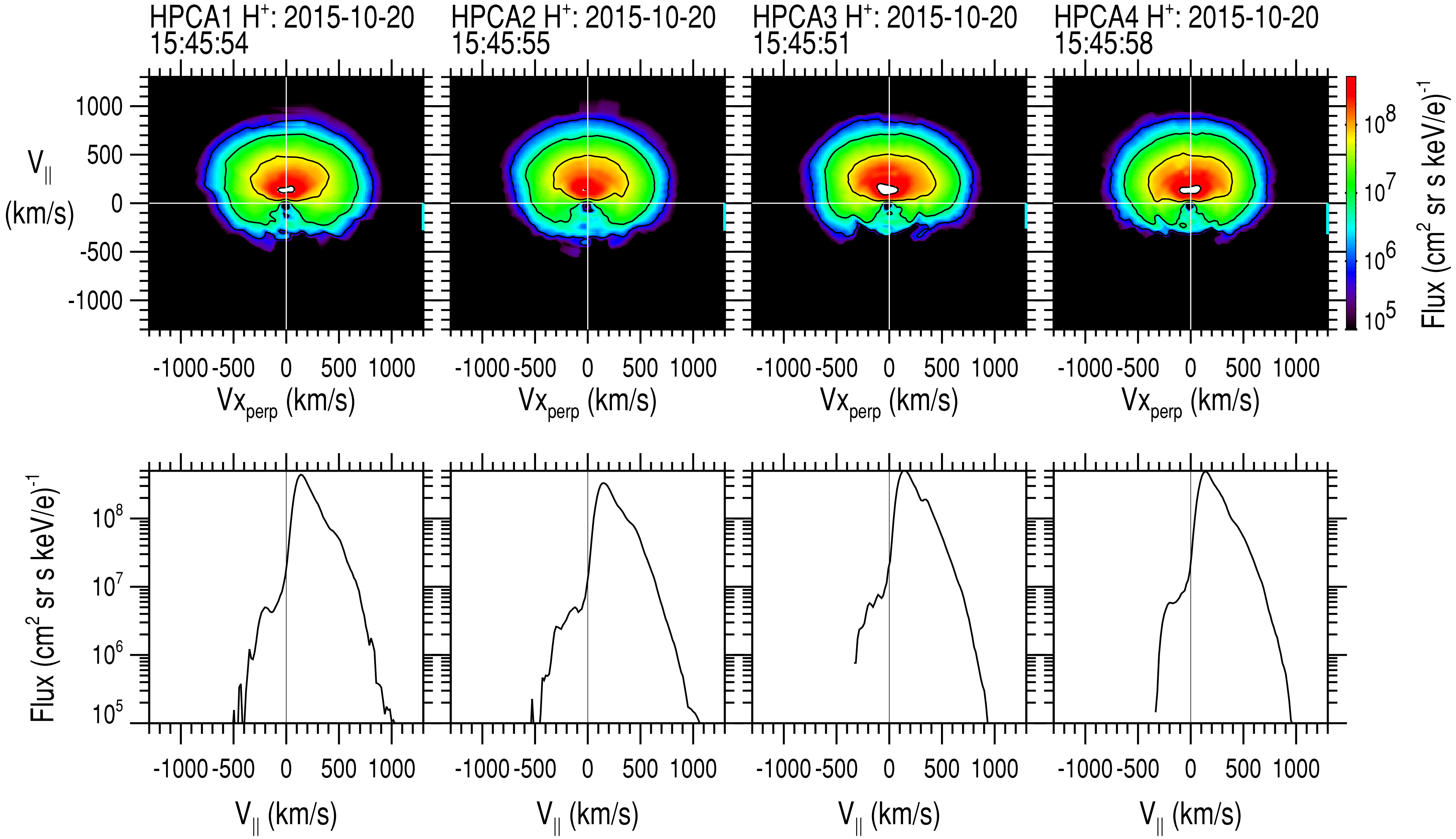

HPCA1 H+: 2015-10-20 15:45:54
HPCA2 H+: 2015-10-20 15:45:55
HPCA3 H+: 2015-10-20 15:45:51
HPCA4 H+: 2015-10-20 15:45:58
V|| (km/s)
Vxperp (km/s)
Flux (cm2 sr s keV/e)-1
-1000 -500 0 500 1000
10^5 10^6 10^7 10^8

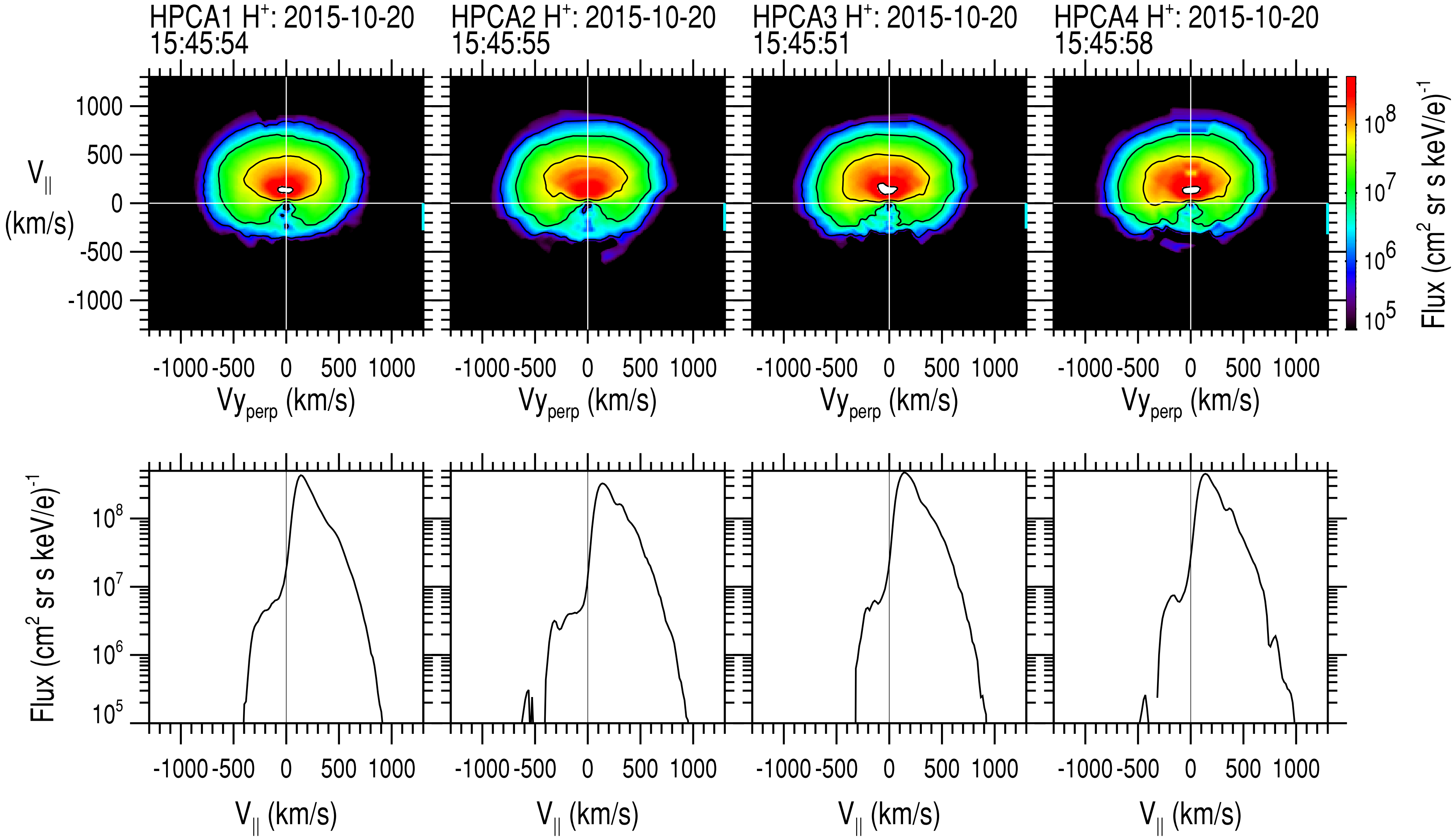
HPCA1 H+: 2015-10-20
15:45:54
HPCA2 H+: 2015-10-20
15:45:55
HPCA3 H+: 2015-10-20
15:45:51
HPCA4 H+: 2015-10-20
15:45:58
V|| (km/s)
Vyperp (km/s)
Flux (cm2 sr s keV/e)-1
-1000 -500 0 500 1000
10^5 10^6 10^7 10^8

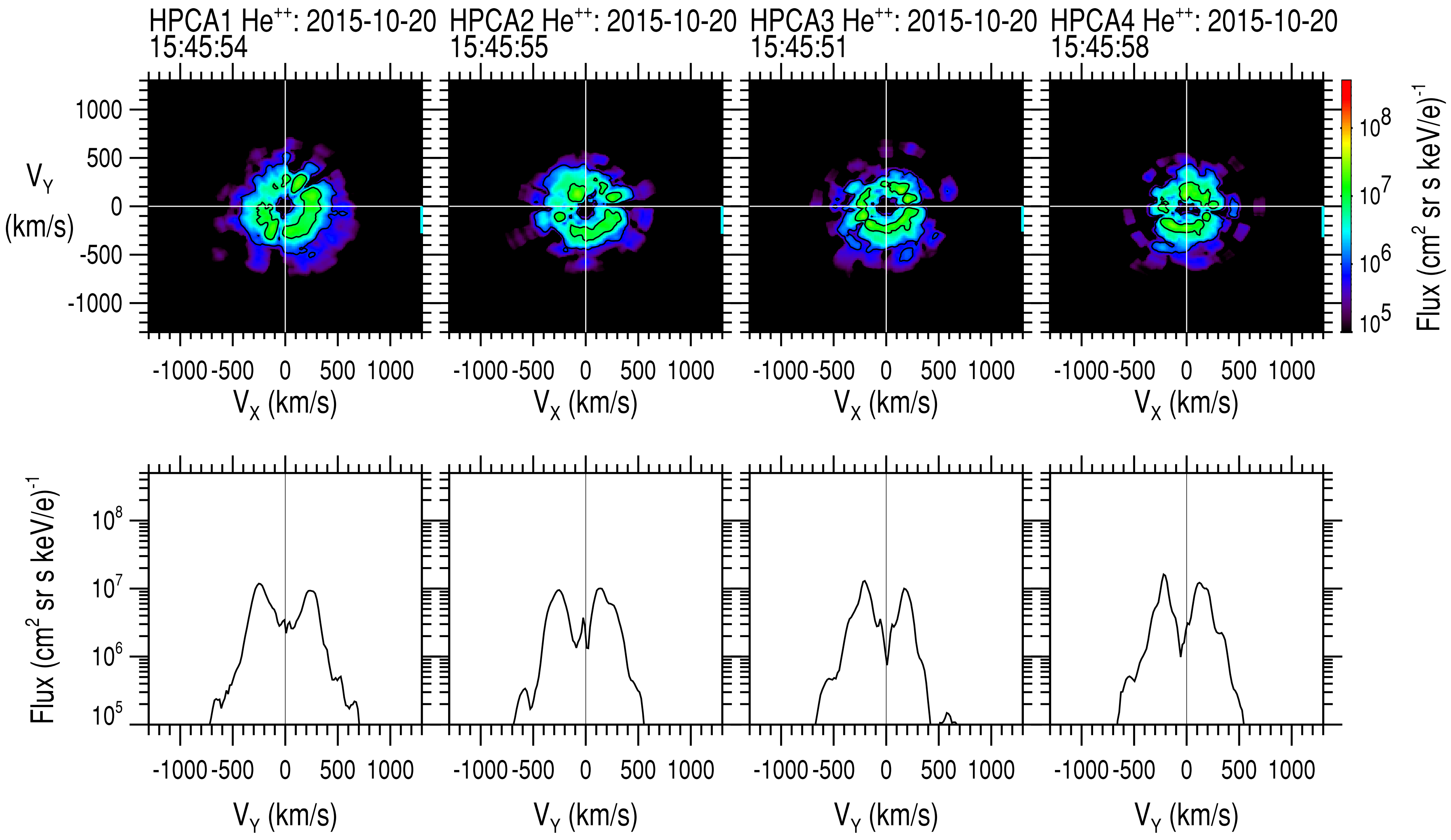
HPCA1 He++: 2015-10-20 15:45:54
HPCA2 He++: 2015-10-20 15:45:55
HPCA3 He++: 2015-10-20 15:45:51
HPCA4 He++: 2015-10-20 15:45:58
VY (km/s)
VX (km/s)
Flux (cm2 sr s keV/e)-1
-1000 -500 0 500 1000
105 106 107 108

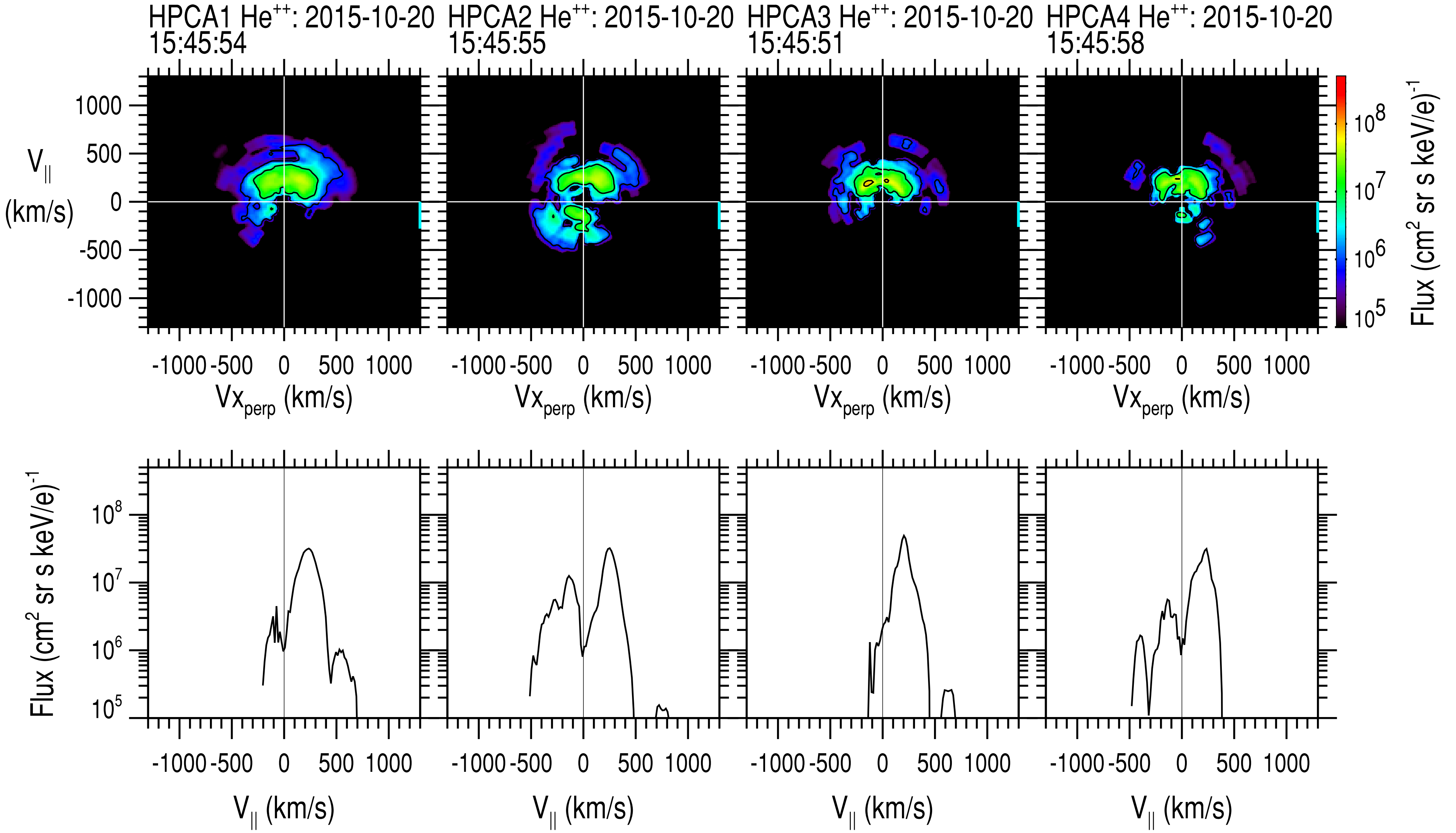
HPCA1 He++: 2015-10-20 15:45:54
HPCA2 He++: 2015-10-20 15:45:55
HPCA3 He++: 2015-10-20 15:45:51
HPCA4 He++: 2015-10-20 15:45:58
V|| (km/s)
Vxperp (km/s)
Flux (cm2 sr s keV/e)-1
V|| (km/s)

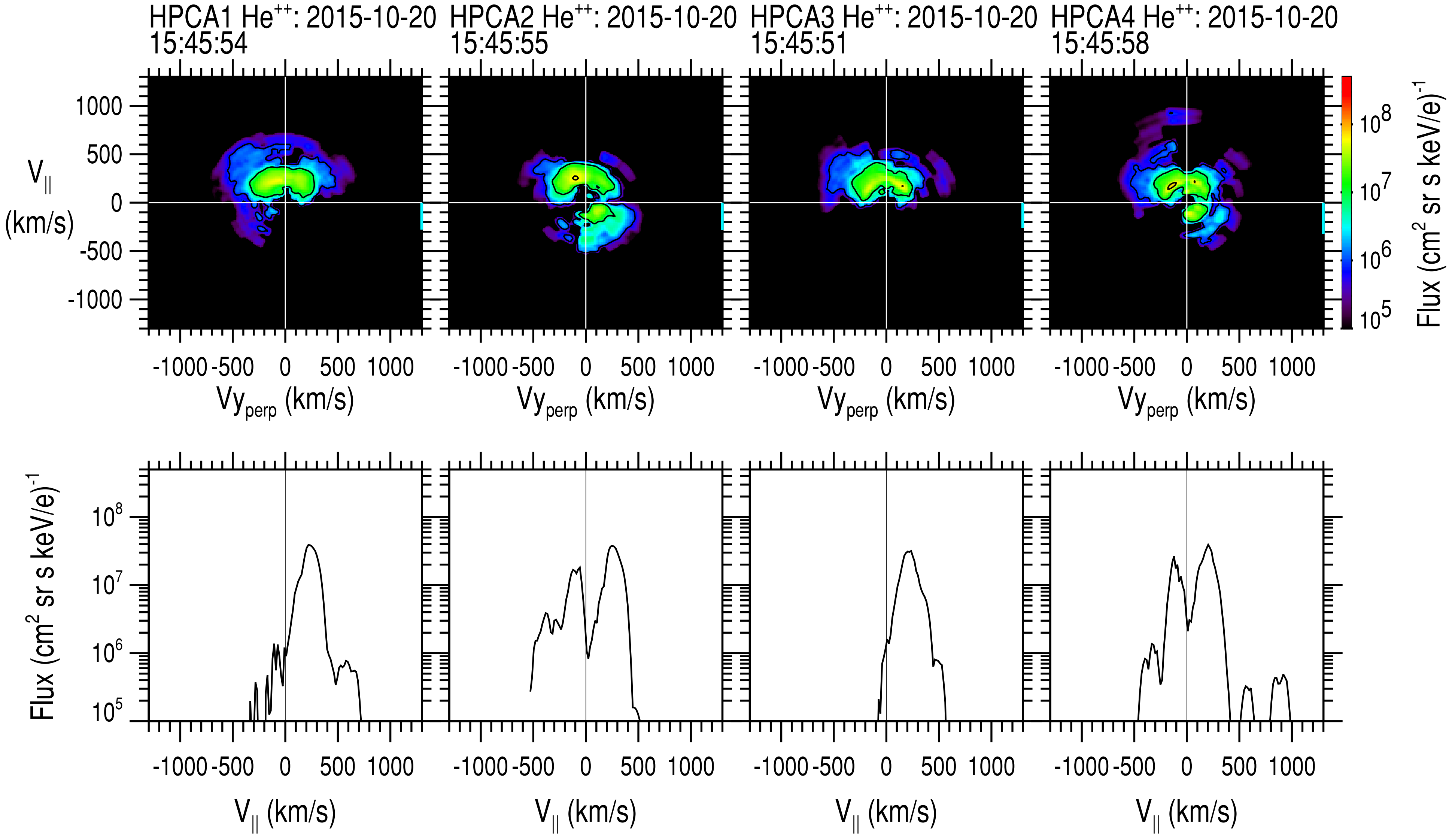
HPCA1 He++: 2015-10-20
15:45:54
HPCA2 He++: 2015-10-20
15:45:55
HPCA3 He++: 2015-10-20
15:45:51
HPCA4 He++: 2015-10-20
15:45:58
V|| (km/s)
Vy perp (km/s)
Flux (cm2 sr s keV/e)-1
-1000 -500 0 500 1000
10^5 10^6 10^7 10^8
V|| (km/s)

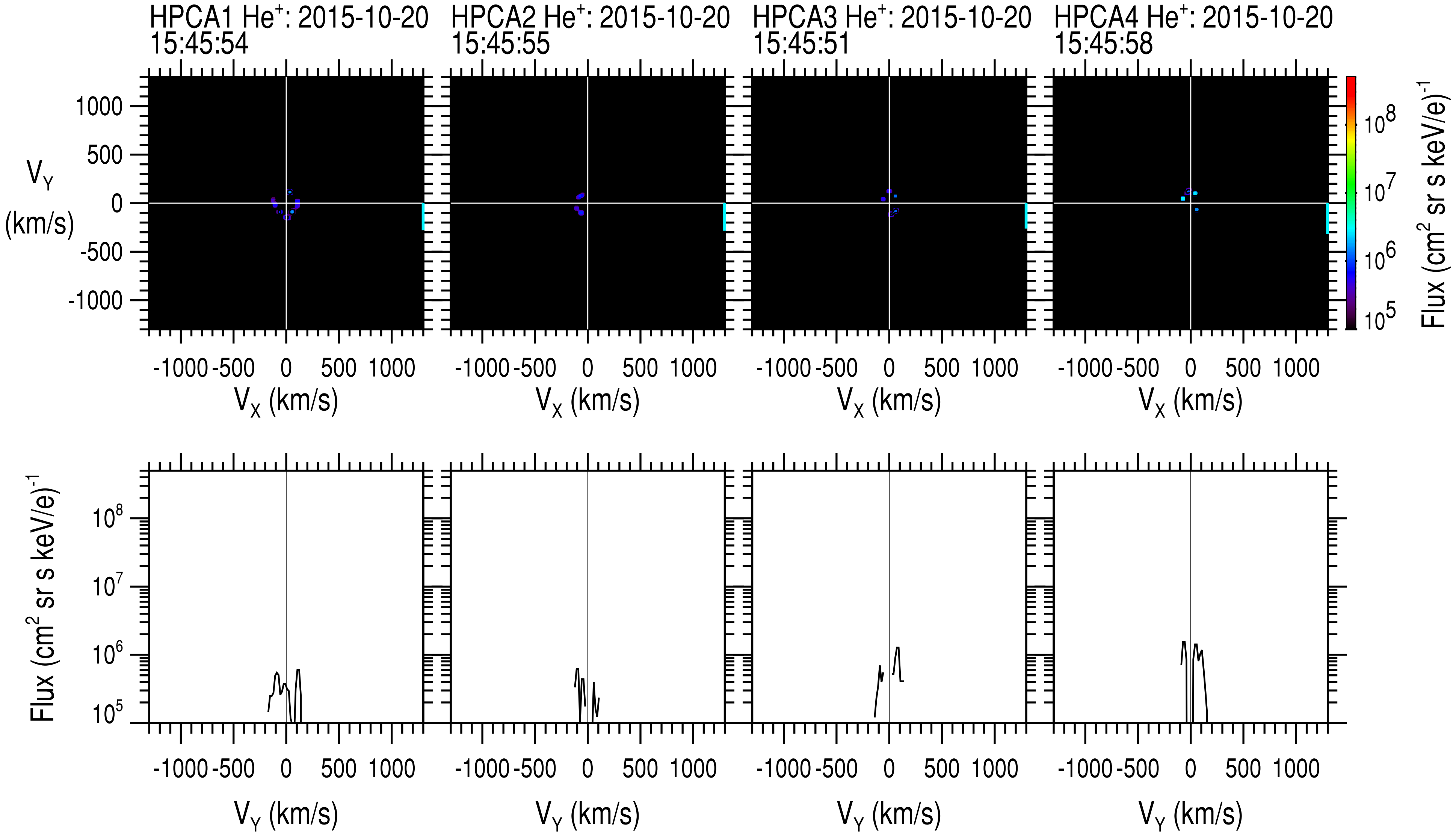
HPCA1 He+: 2015-10-20 15:45:54
HPCA2 He+: 2015-10-20 15:45:55
HPCA3 He+: 2015-10-20 15:45:51
HPCA4 He+: 2015-10-20 15:45:58
V_Y (km/s)
V_X (km/s)
Flux (cm^2 sr s keV/e)^-1

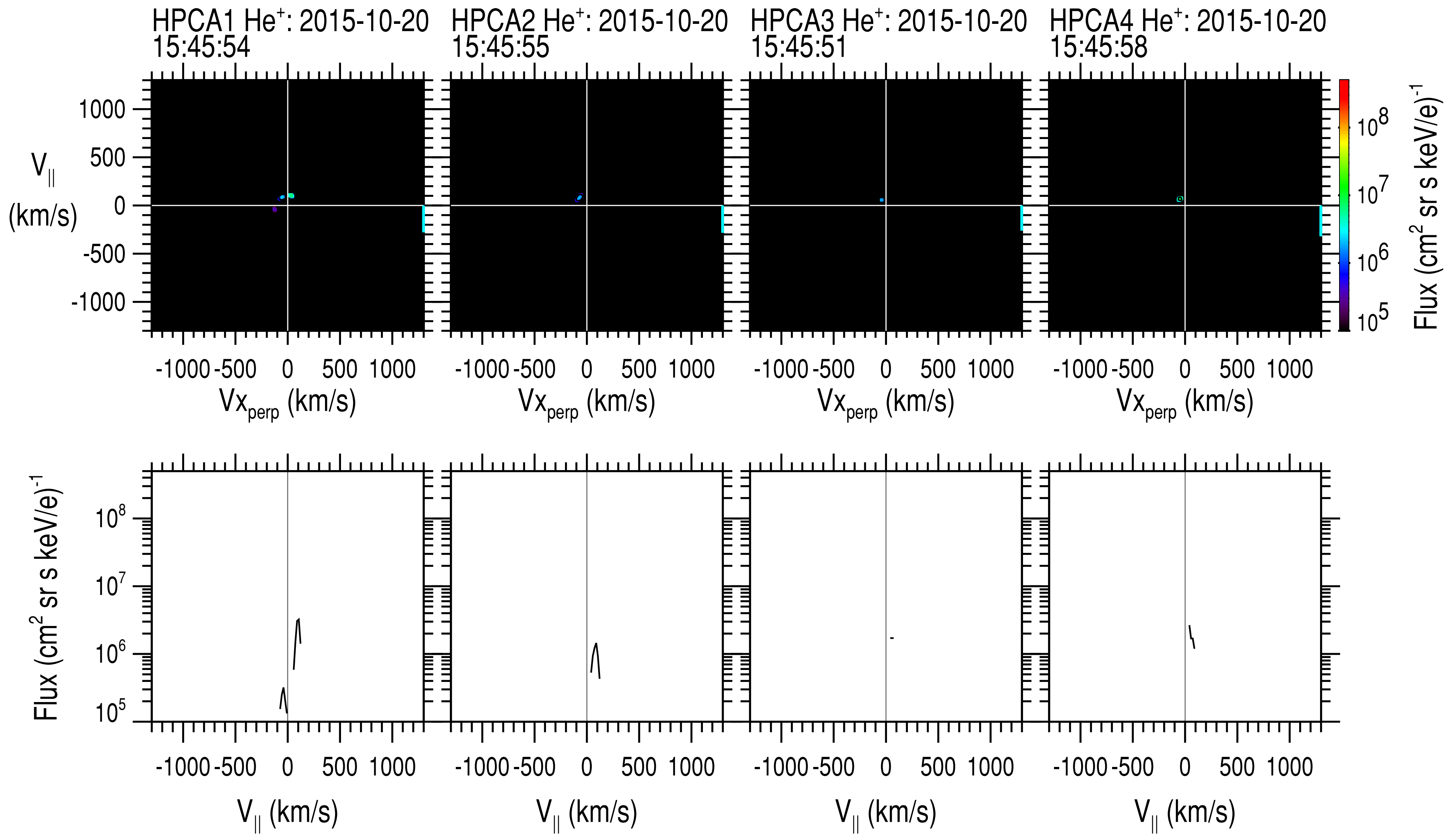

HPCA1 He+: 2015-10-20 15:45:54
HPCA2 He+: 2015-10-20 15:45:55
HPCA3 He+: 2015-10-20 15:45:51
HPCA4 He+: 2015-10-20 15:45:58
V|| (km/s)
1000
500
0
-500
-1000
-1000 -500 0 500 1000
Vxperp (km/s)
Flux (cm2 sr s keV/e)-1
10^5
10^6
10^7
10^8
V|| (km/s)

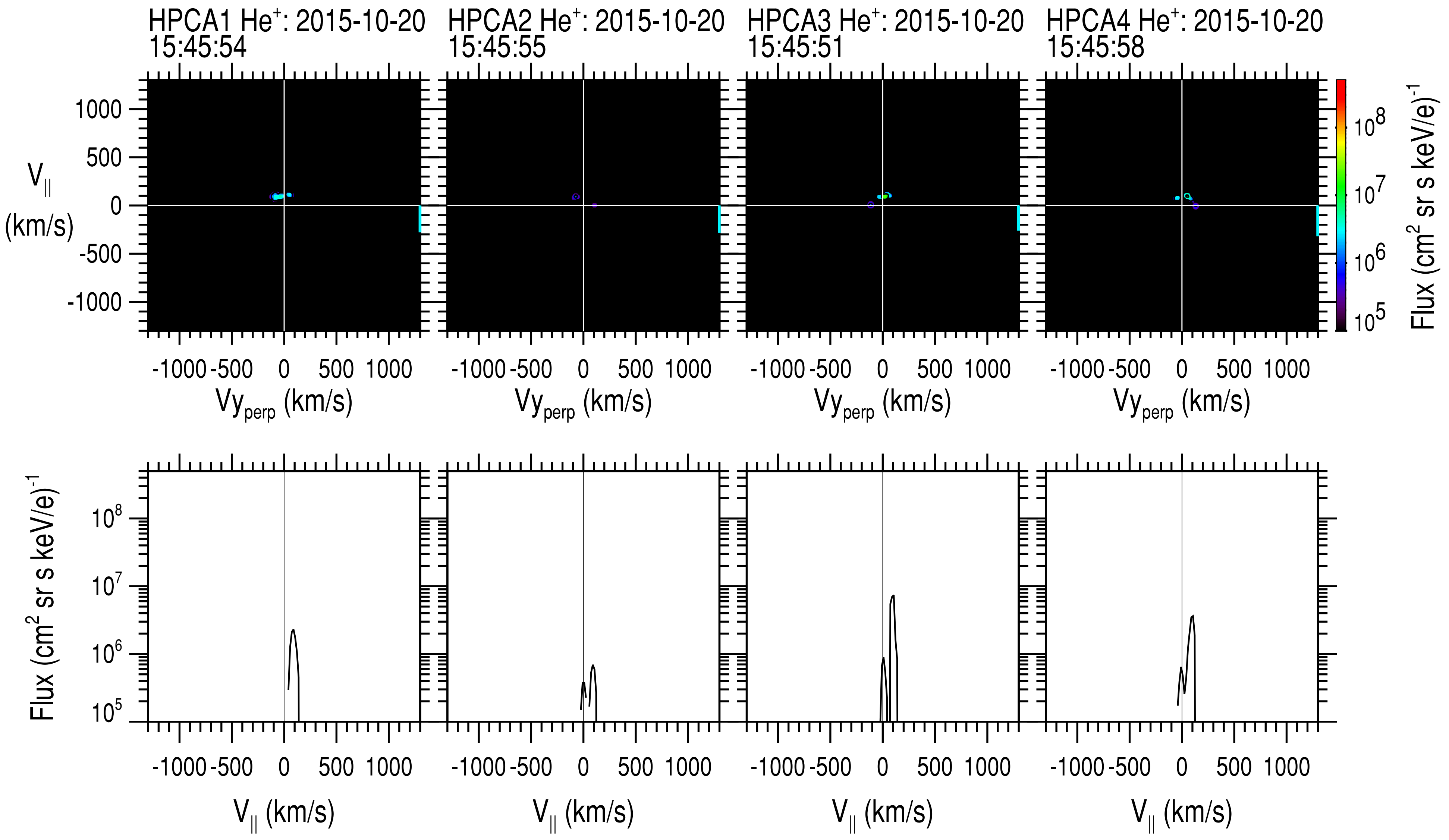
HPCA1 He+: 2015-10-20 15:45:54
HPCA2 He+: 2015-10-20 15:45:55
HPCA3 He+: 2015-10-20 15:45:51
HPCA4 He+: 2015-10-20 15:45:58
V|| (km/s)
1000
500
0
-500
-1000
-1000 -500 0 500 1000
Vyperp (km/s)
Flux (cm2 sr s keV/e)-1
10^5
10^6
10^7
10^8
V|| (km/s)

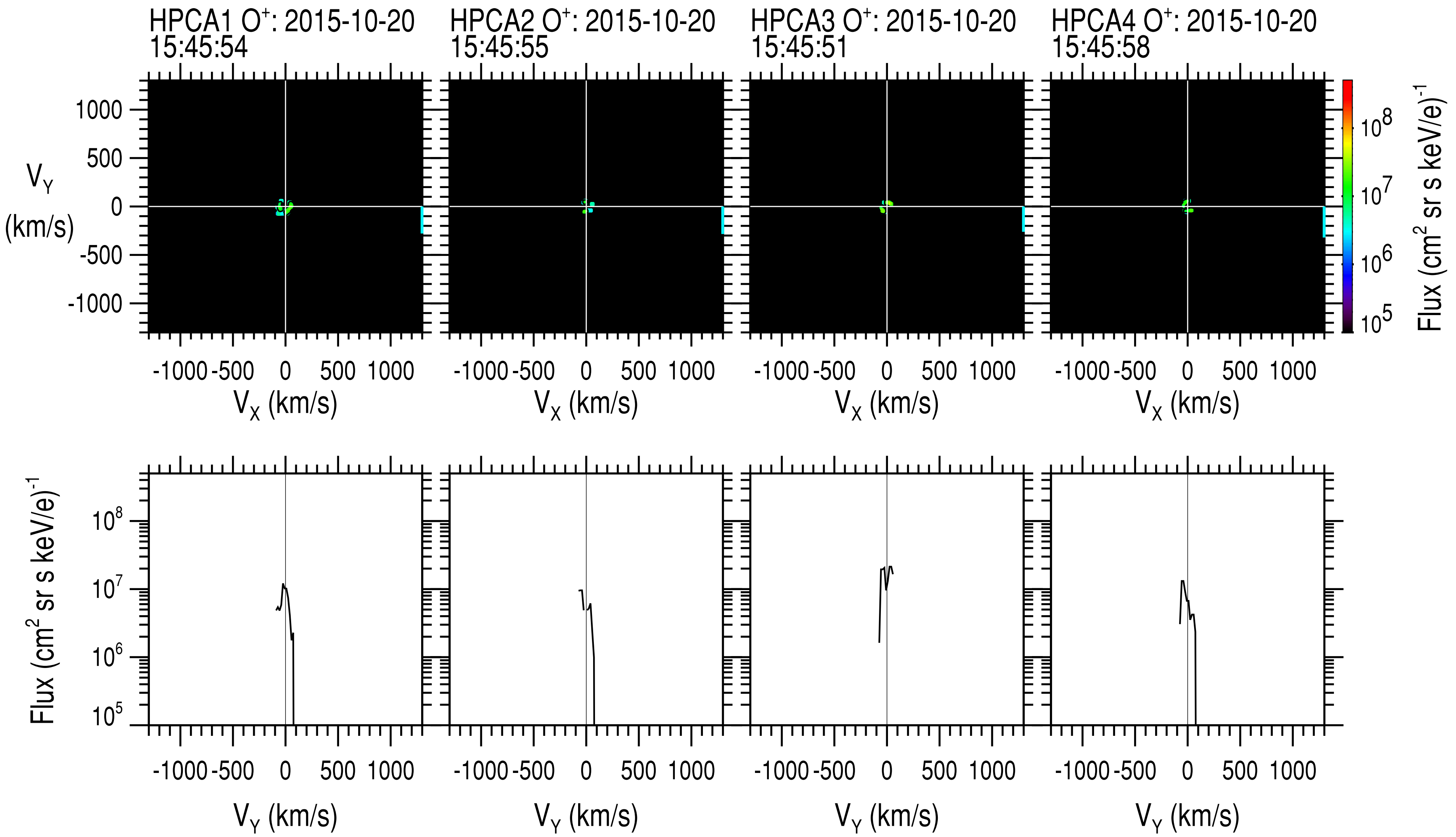
HPCA1 O+: 2015-10-20
15:45:54
HPCA2 O+: 2015-10-20
15:45:55
HPCA3 O+: 2015-10-20
15:45:51
HPCA4 O+: 2015-10-20
15:45:58
VY (km/s)
VX (km/s)
Flux (cm2 sr s keV/e)-1
VY (km/s)
1000
500
0
-500
-1000
105
106
107
108

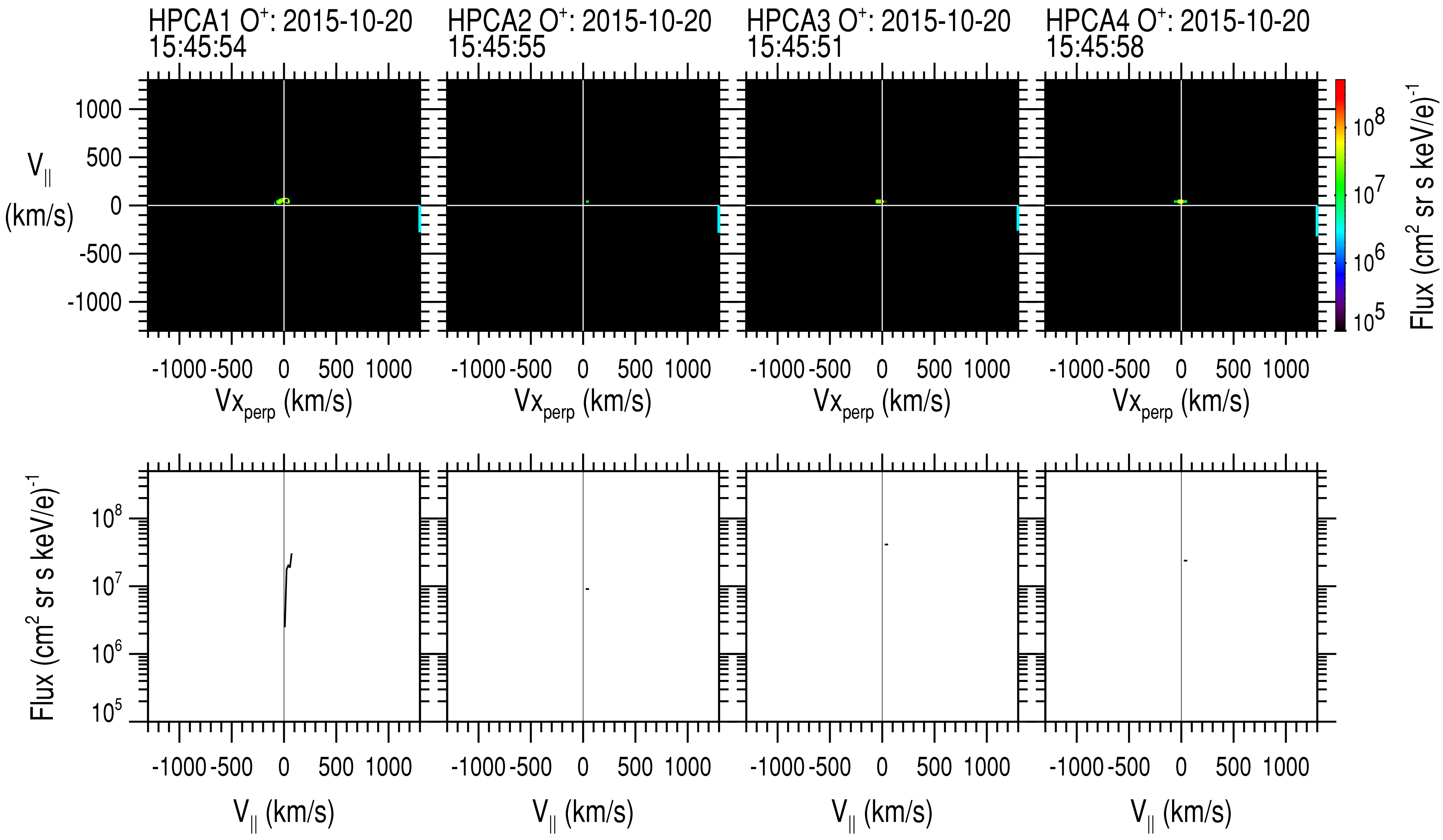

HPCA1 O+: 2015-10-20 15:45:54
HPCA2 O+: 2015-10-20 15:45:55
HPCA3 O+: 2015-10-20 15:45:51
HPCA4 O+: 2015-10-20 15:45:58
V|| (km/s)
Vxperp (km/s)
Flux (cm2 sr s keV/e)-1

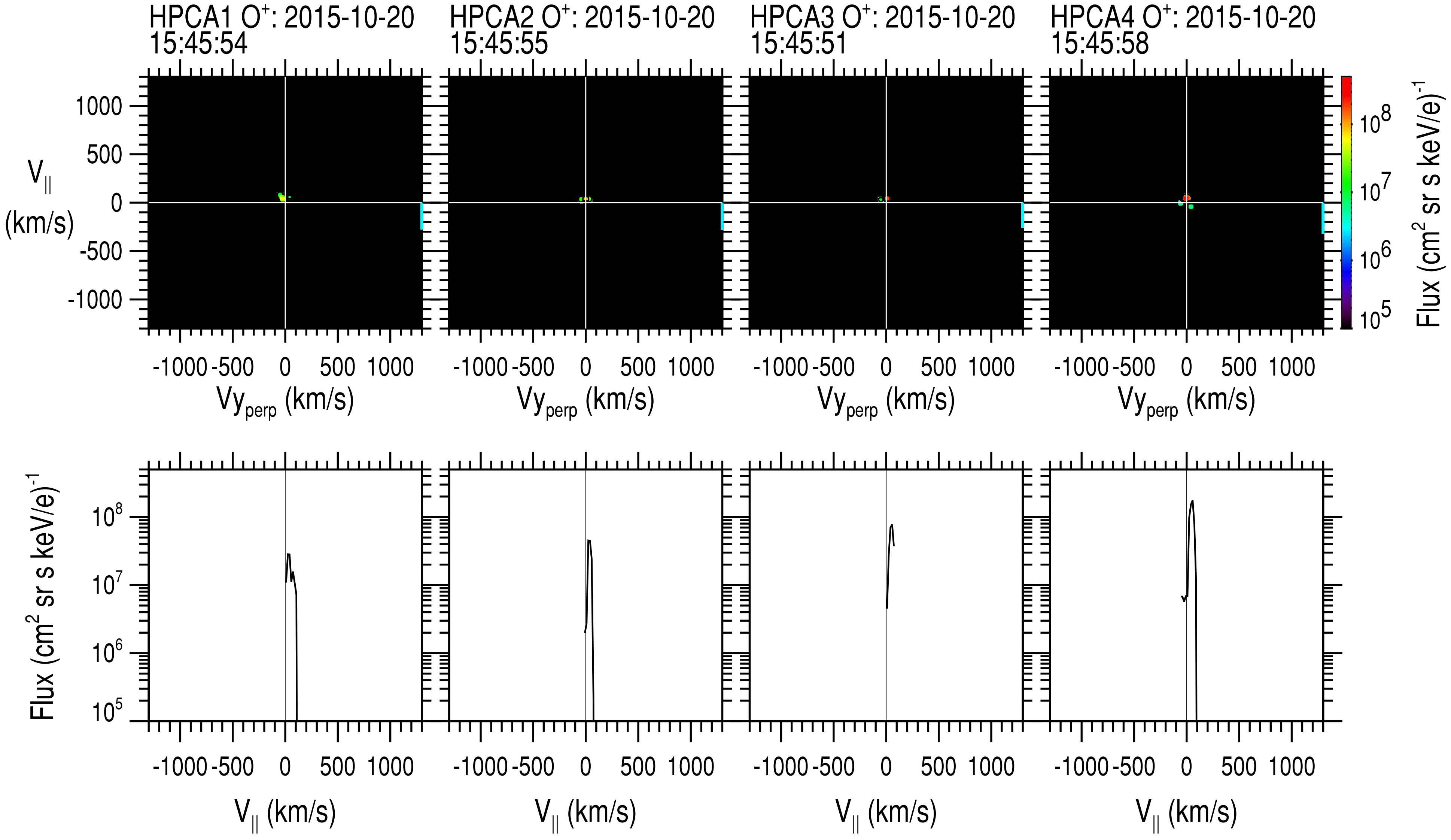
HPCA1 O+: 2015-10-20 15:45:54
HPCA2 O+: 2015-10-20 15:45:55
HPCA3 O+: 2015-10-20 15:45:51
HPCA4 O+: 2015-10-20 15:45:58
V|| (km/s)
Vyperp (km/s)
Flux (cm2 sr s keV/e)-1
V|| (km/s)

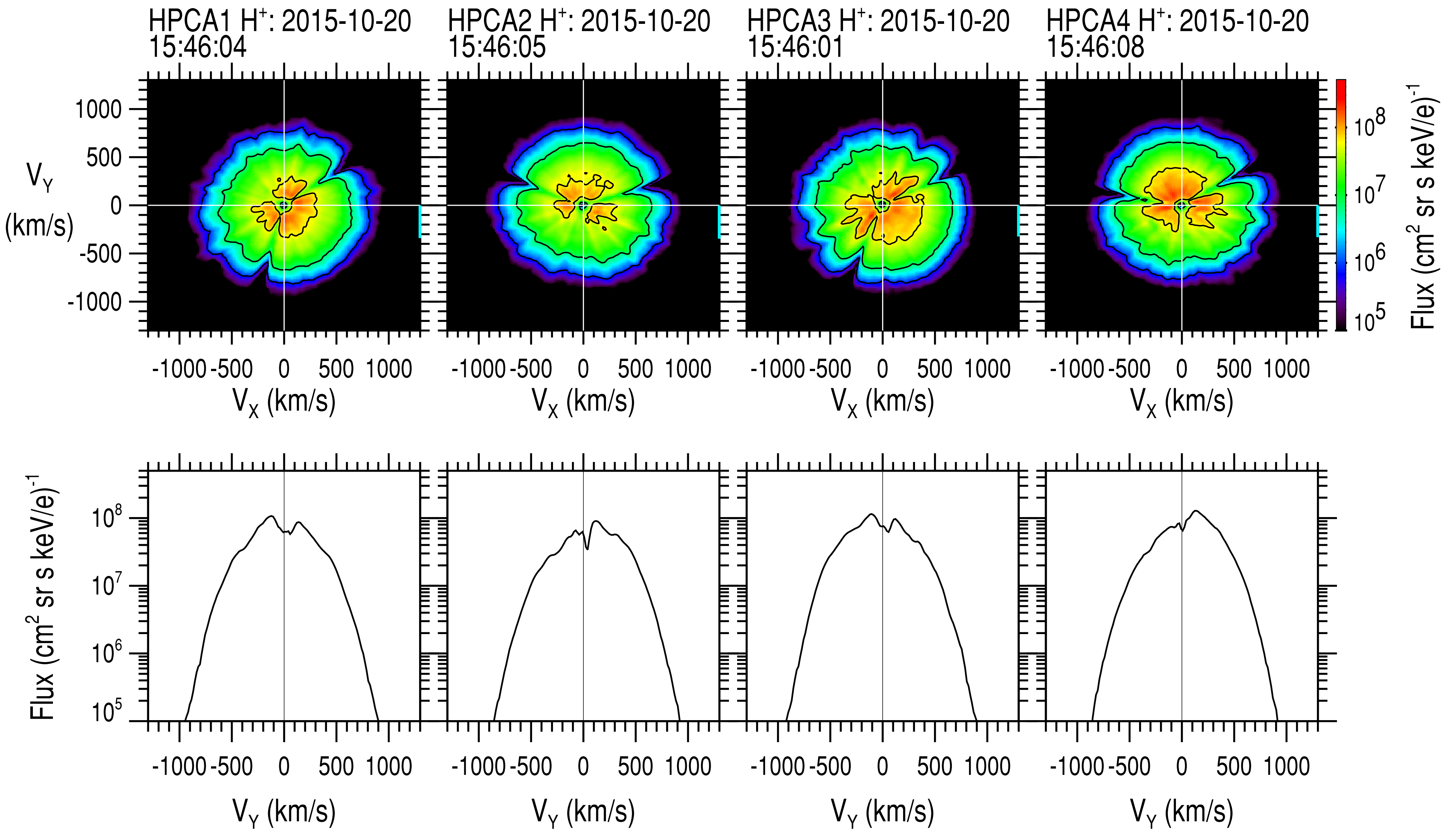

HPCA1 H+: 2015-10-20
15:46:04
HPCA2 H+: 2015-10-20
15:46:05
HPCA3 H+: 2015-10-20
15:46:01
HPCA4 H+: 2015-10-20
15:46:08
V_Y (km/s)
V_X (km/s)
1000
500
0
-500
-1000
Flux (cm2 sr s keV/e)-1
10^5
10^6
10^7
10^8
V_Y (km/s)

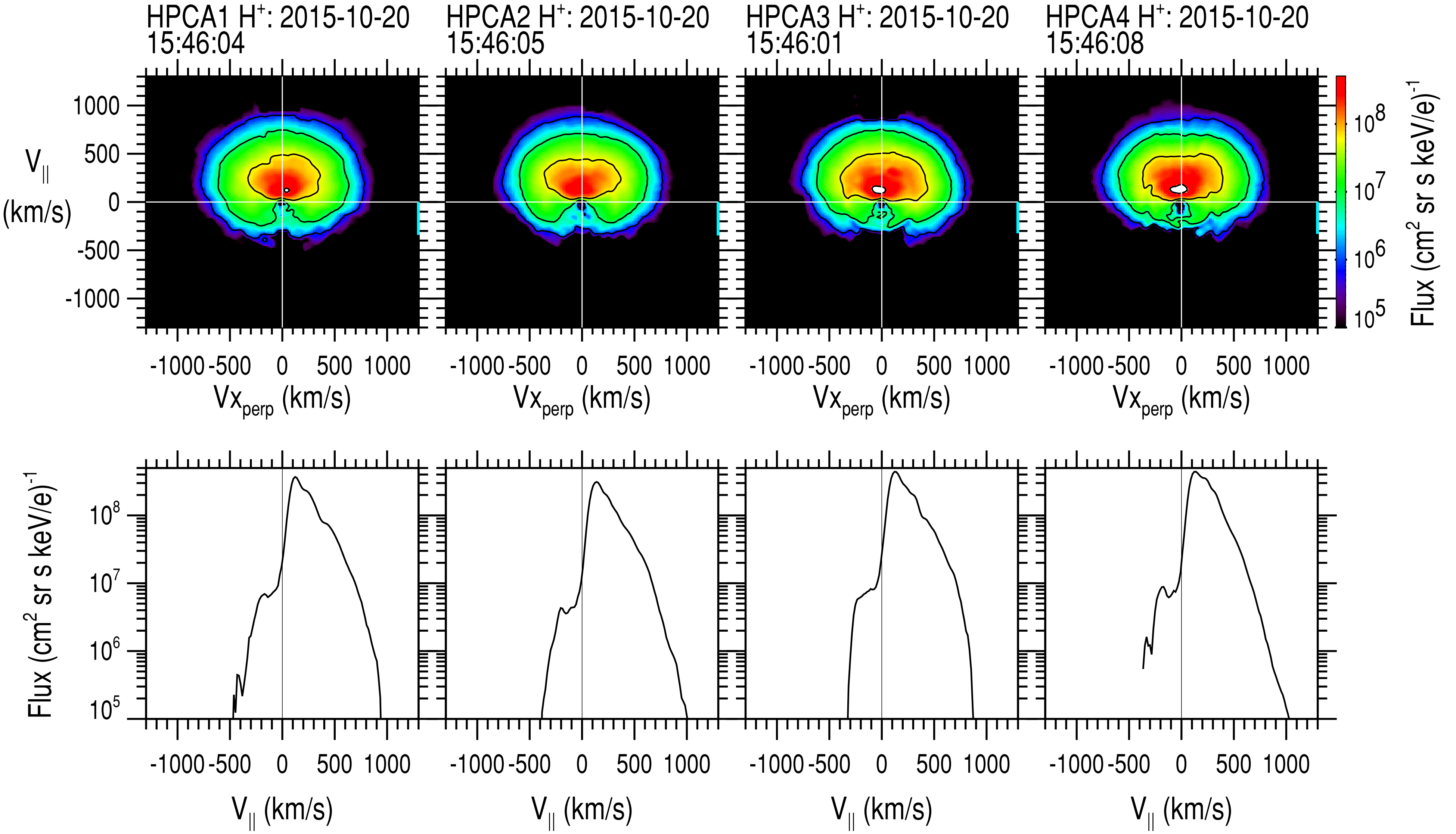
HPCA1 H+: 2015-10-20 15:46:04
HPCA2 H+: 2015-10-20 15:46:05
HPCA3 H+: 2015-10-20 15:46:01
HPCA4 H+: 2015-10-20 15:46:08
V|| (km/s)
Vxperp (km/s)
Flux (cm2 sr s keV/e)-1
V|| (km/s)

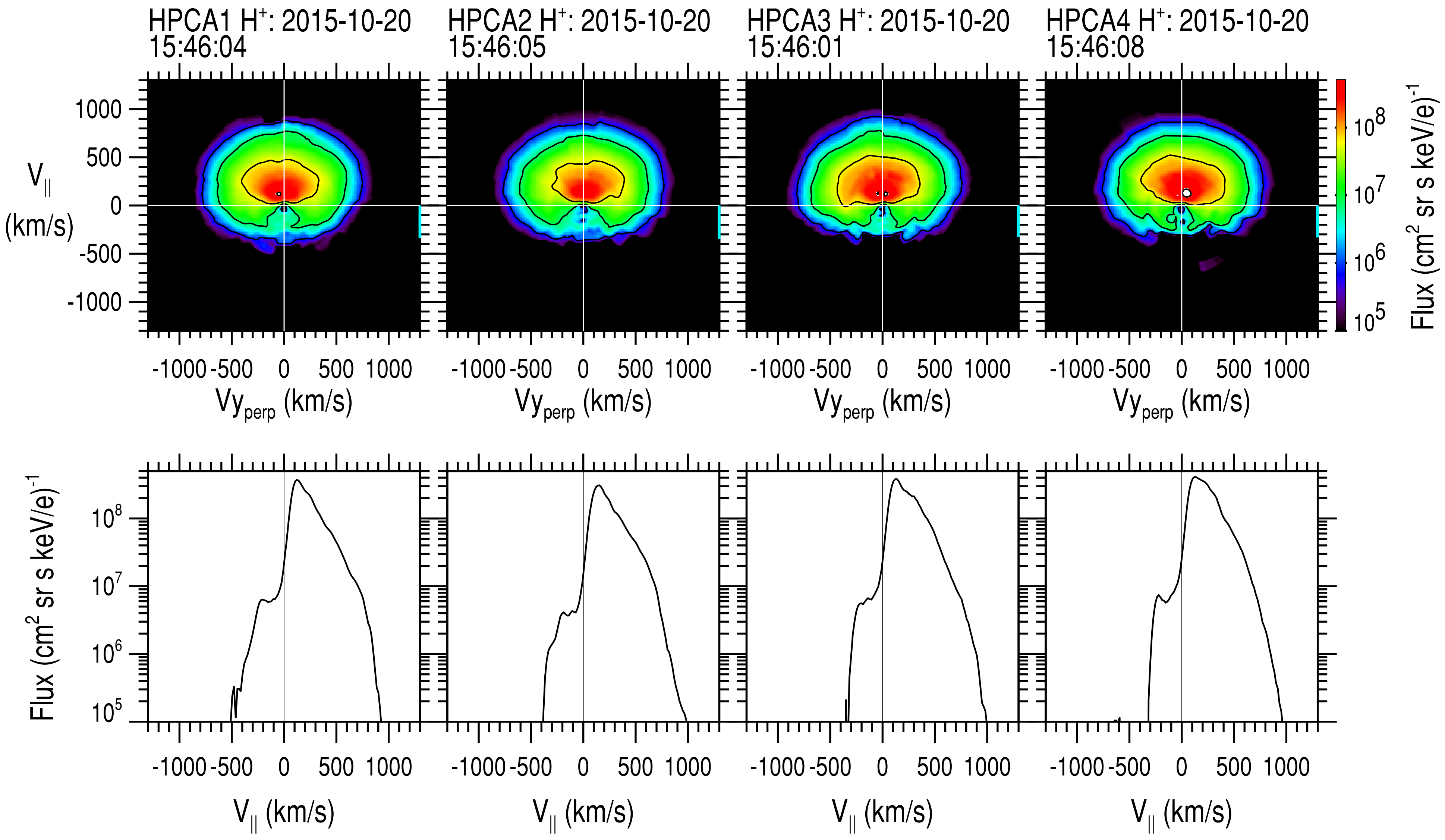
HPCA1 H+: 2015-10-20
15:46:04
HPCA2 H+: 2015-10-20
15:46:05
HPCA3 H+: 2015-10-20
15:46:01
HPCA4 H+: 2015-10-20
15:46:08
V|| (km/s)
Vyperp (km/s)
Flux (cm2 sr s keV/e)-1
V|| (km/s)

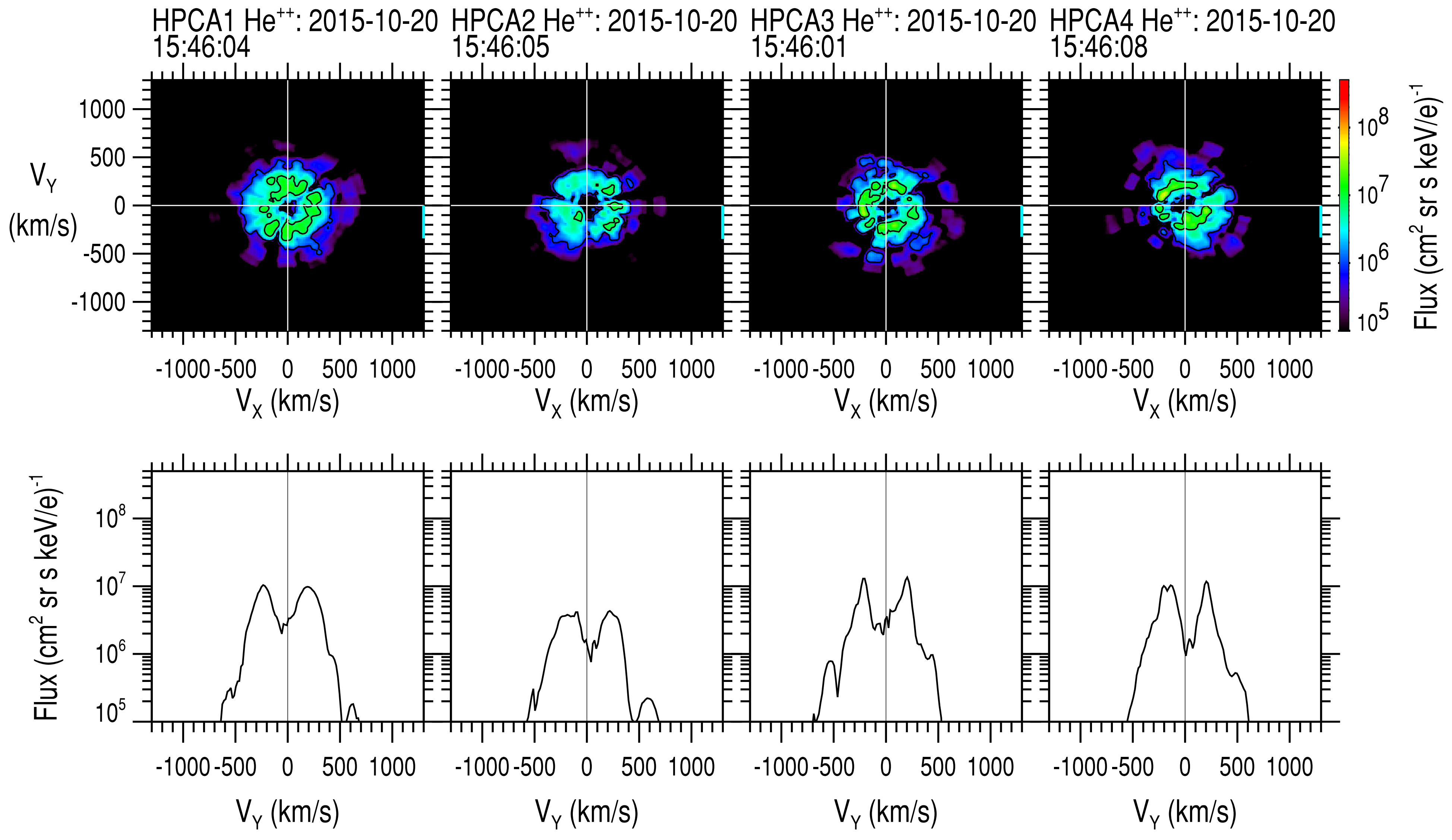

HPCA1 He++: 2015-10-20 15:46:04
HPCA2 He++: 2015-10-20 15:46:05
HPCA3 He++: 2015-10-20 15:46:01
HPCA4 He++: 2015-10-20 15:46:08
V_Y (km/s)
V_X (km/s)
Flux (cm^2 sr s keV/e)^-1
V_Y (km/s)
Flux (cm^2 sr s keV/e)^-1

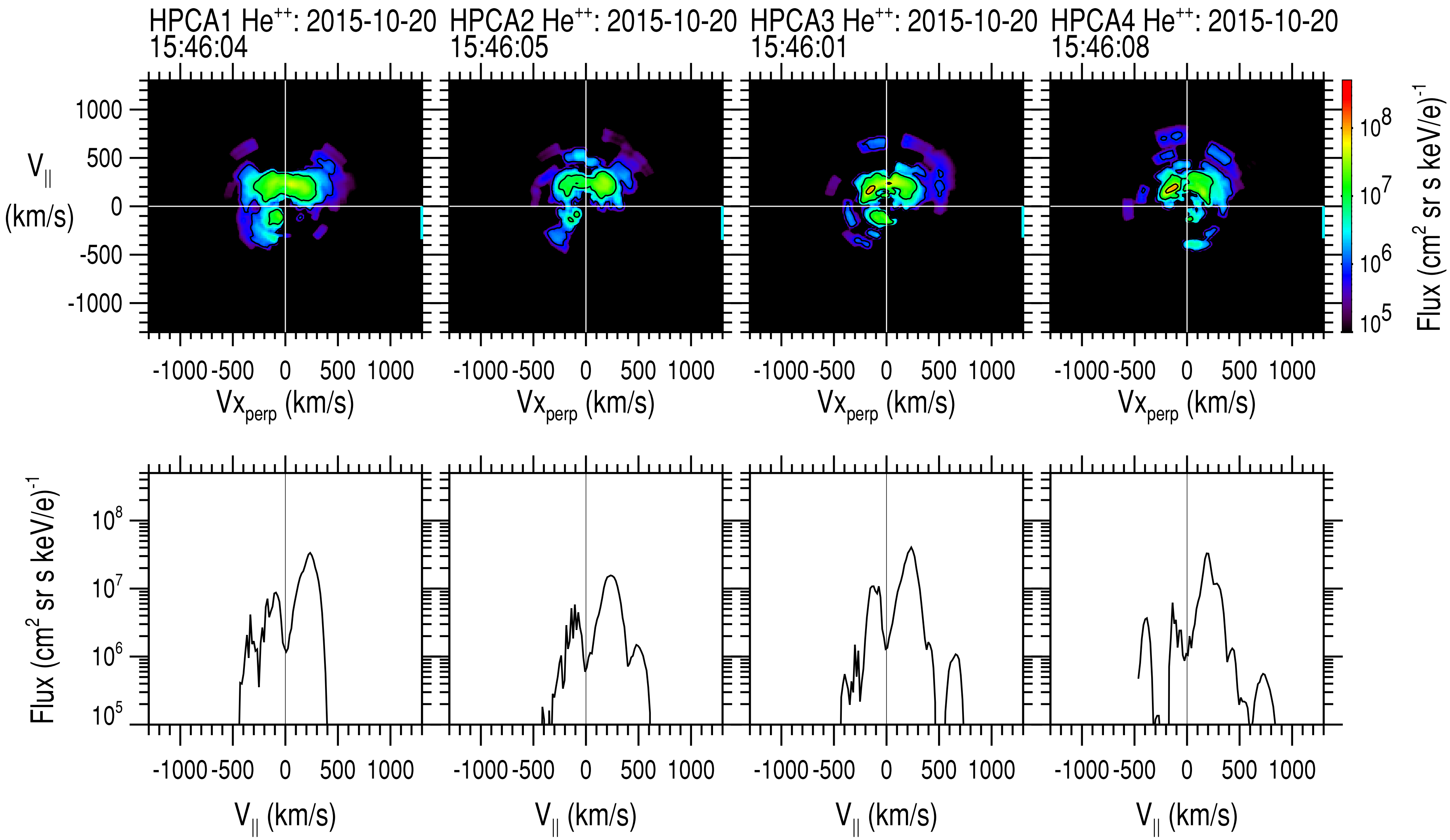
HPCA1 He^{++}: 2015-10-20 15:46:04
HPCA2 He^{++}: 2015-10-20 15:46:05
HPCA3 He^{++}: 2015-10-20 15:46:01
HPCA4 He^{++}: 2015-10-20 15:46:08
$V_{||}$ (km/s)
Vx_{perp} (km/s)
Flux (cm^2 sr s keV/e)$^{-1}$
$V_{||}$ (km/s)
Flux (cm^2 sr s keV/e)$^{-1}$

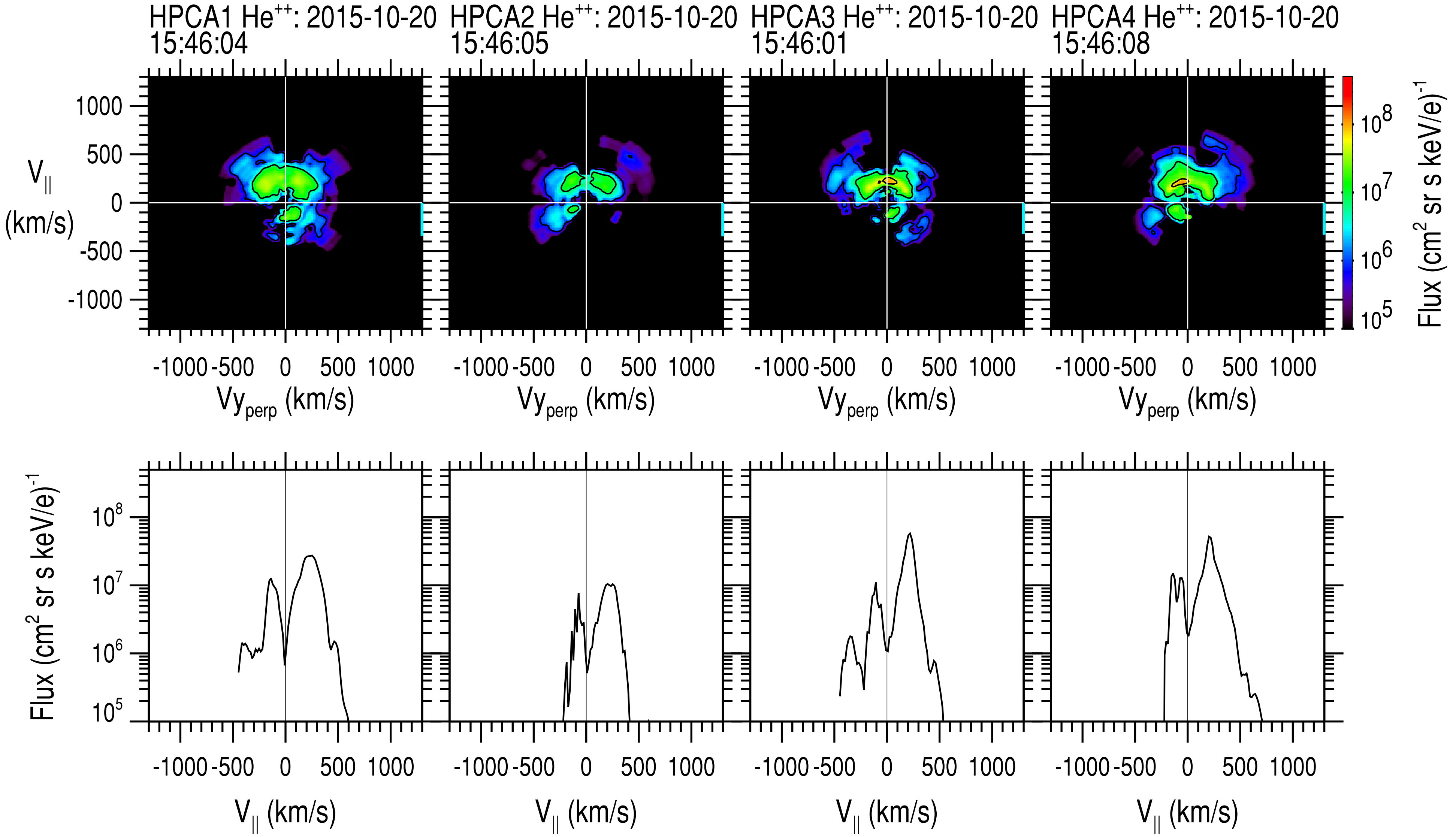

HPCA1 He++: 2015-10-20 15:46:04
HPCA2 He++: 2015-10-20 15:46:05
HPCA3 He++: 2015-10-20 15:46:01
HPCA4 He++: 2015-10-20 15:46:08
V|| (km/s)
Vyperp (km/s)
Flux (cm2 sr s keV/e)-1

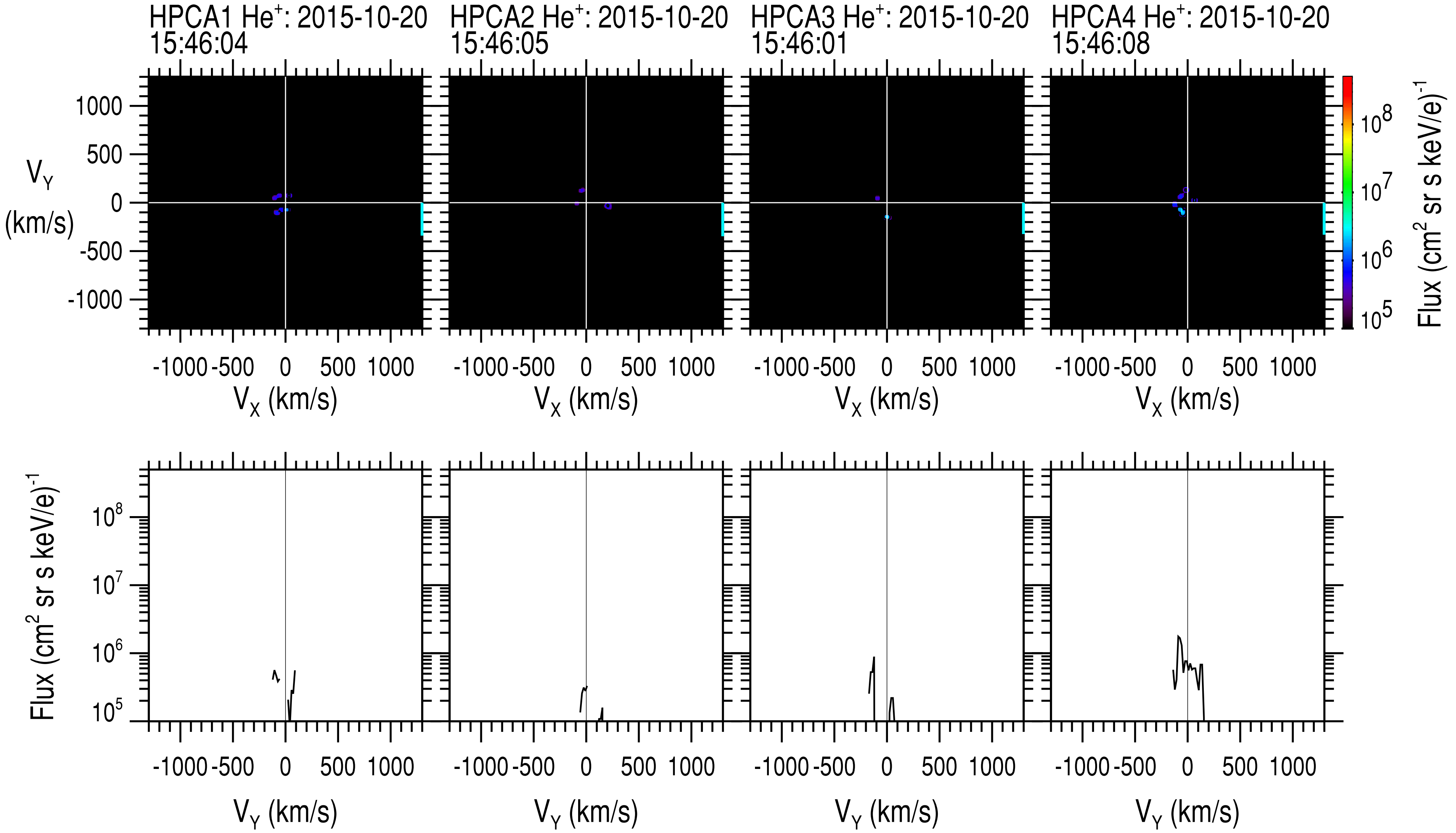
HPCA1 He+: 2015-10-20 15:46:04
HPCA2 He+: 2015-10-20 15:46:05
HPCA3 He+: 2015-10-20 15:46:01
HPCA4 He+: 2015-10-20 15:46:08
V_Y (km/s)
V_X (km/s)
Flux (cm^2 sr s keV/e)^-1

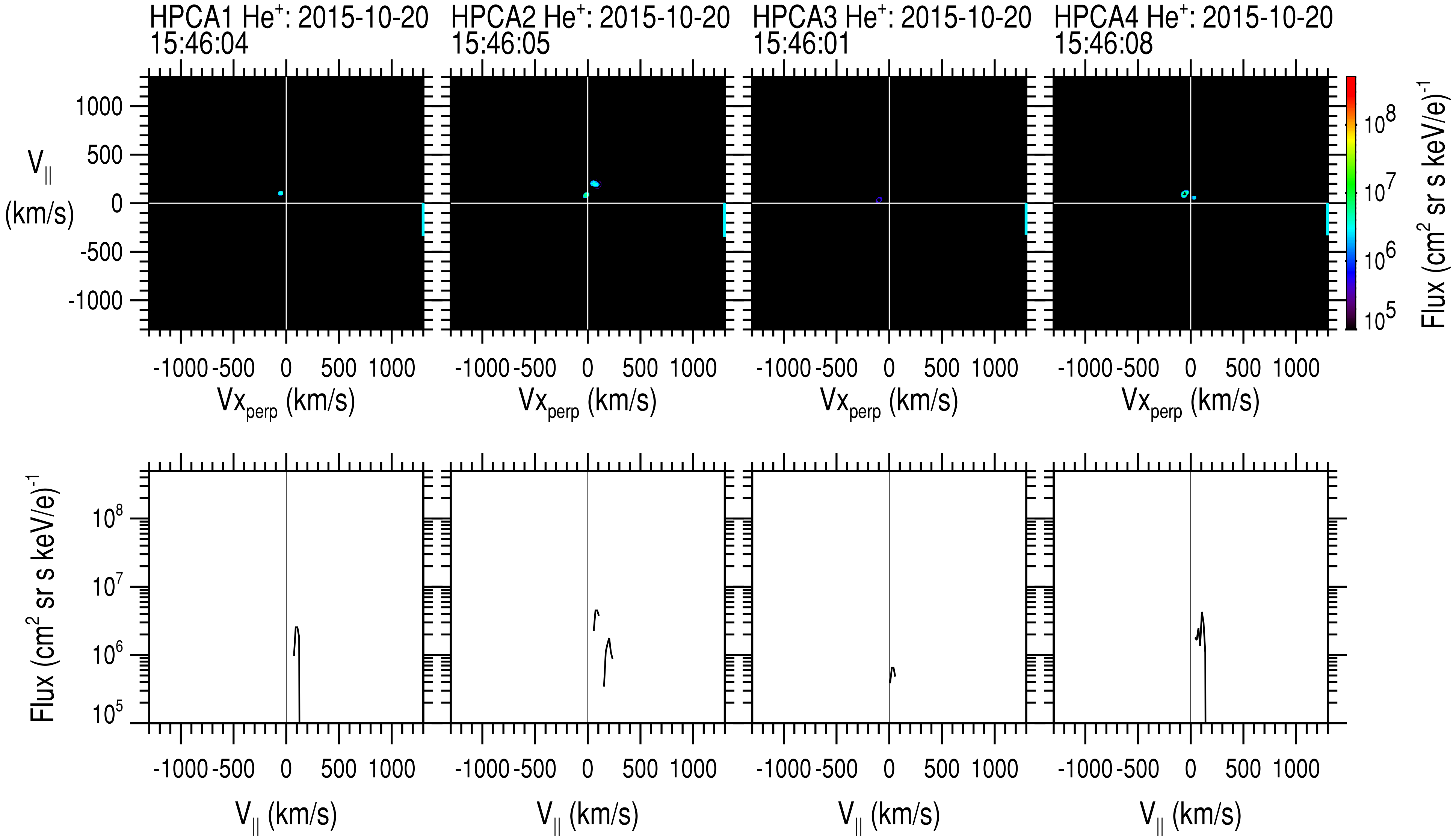
HPCA1 He+: 2015-10-20 15:46:04
HPCA2 He+: 2015-10-20 15:46:05
HPCA3 He+: 2015-10-20 15:46:01
HPCA4 He+: 2015-10-20 15:46:08
V|| (km/s)
Vxperp (km/s)
Flux (cm2 sr s keV/e)-1
1000
500
0
-500
-1000
10^5
10^6
10^7
10^8

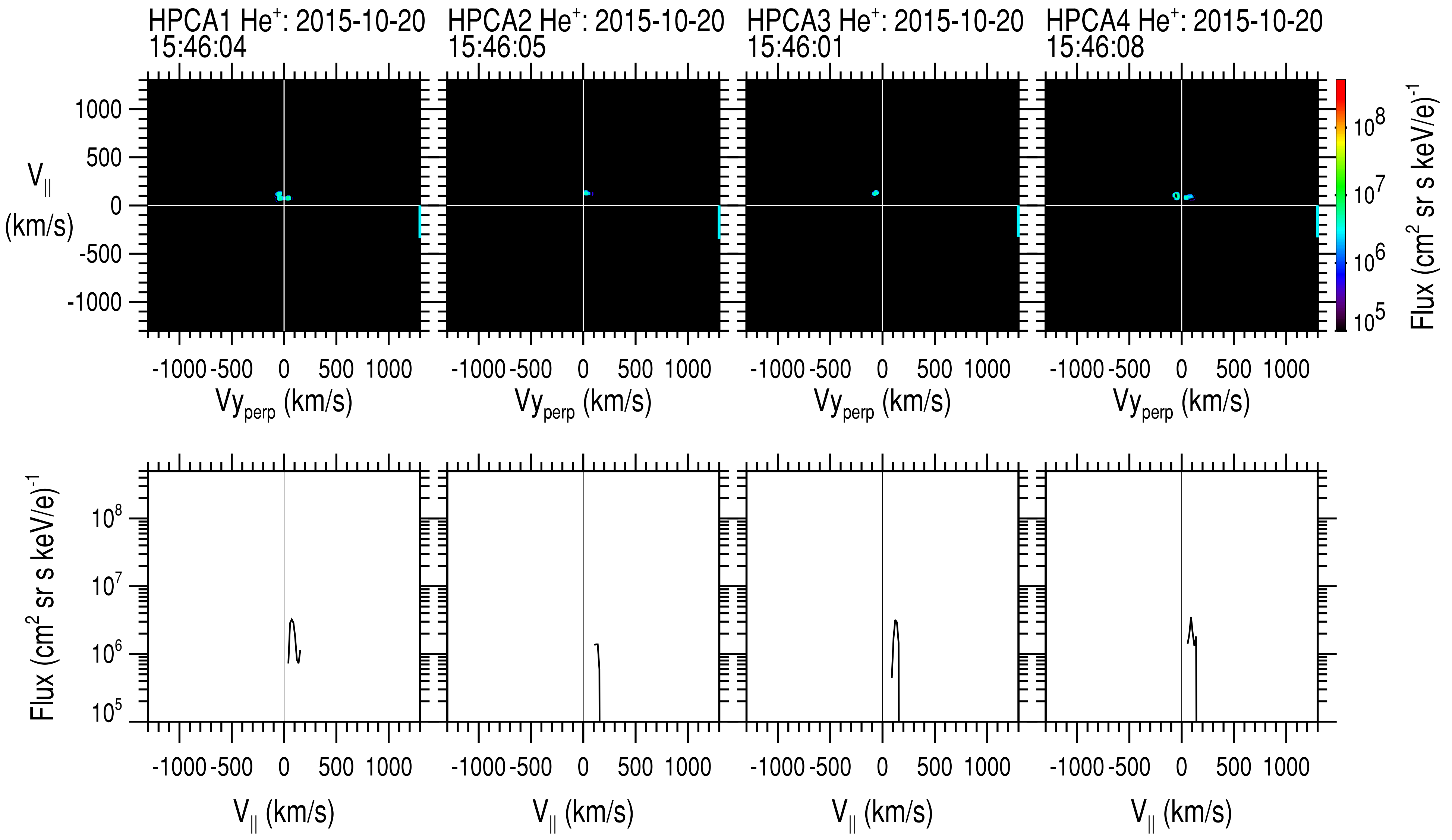
HPCA1 He+: 2015-10-20 15:46:04
HPCA2 He+: 2015-10-20 15:46:05
HPCA3 He+: 2015-10-20 15:46:01
HPCA4 He+: 2015-10-20 15:46:08
V|| (km/s)
1000
500
0
-500
-1000
Vyperp (km/s)
Flux (cm2 sr s keV/e)-1
10^5
10^6
10^7
10^8
V|| (km/s)

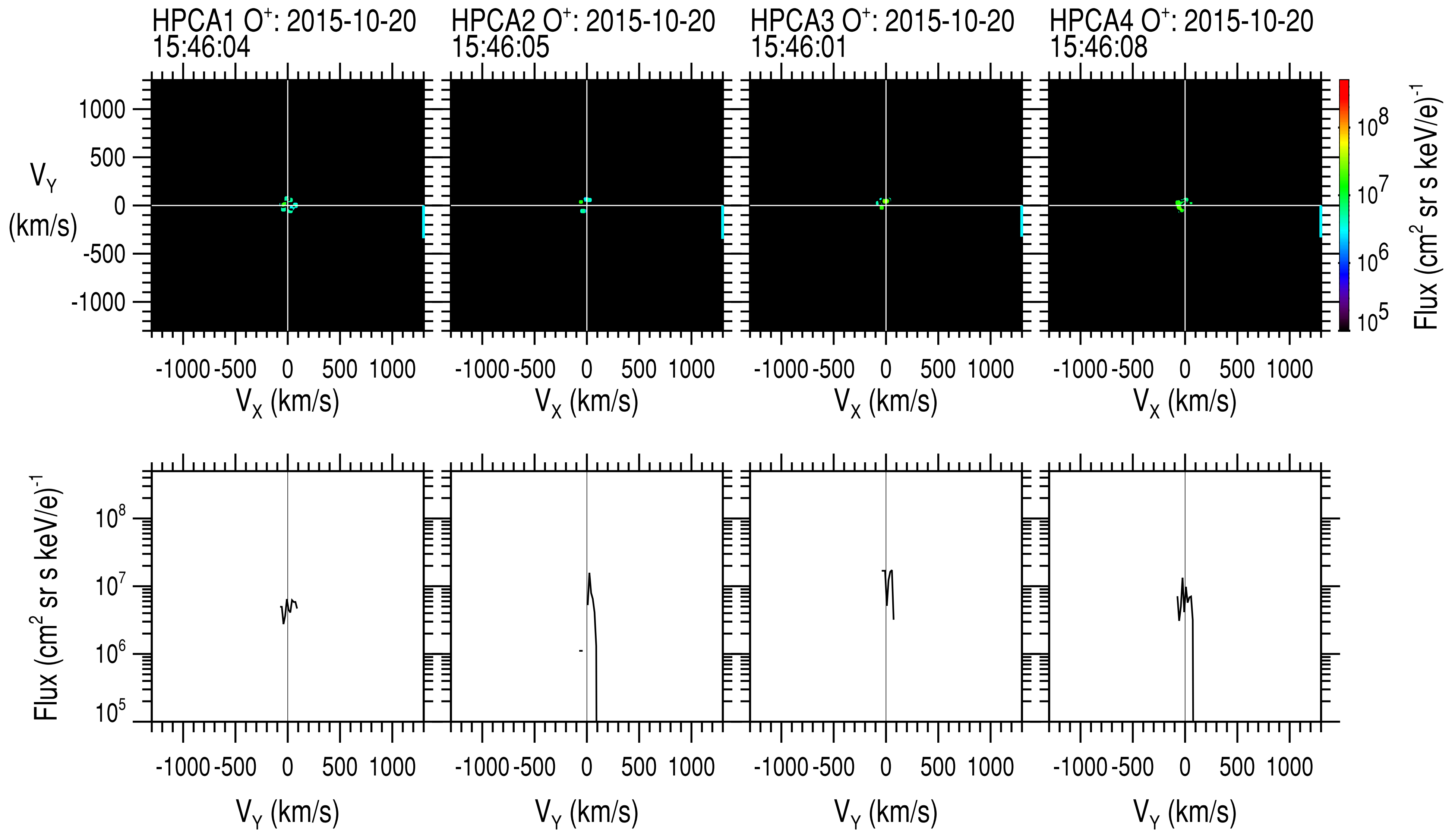
HPCA1 O$^+$: 2015-10-20 15:46:04
HPCA2 O$^+$: 2015-10-20 15:46:05
HPCA3 O$^+$: 2015-10-20 15:46:01
HPCA4 O$^+$: 2015-10-20 15:46:08
V_Y (km/s)
V_X (km/s)
Flux $(cm^2\ sr\ s\ keV/e)^{-1}$
10^5 10^6 10^7 10^8
-1000 -500 0 500 1000

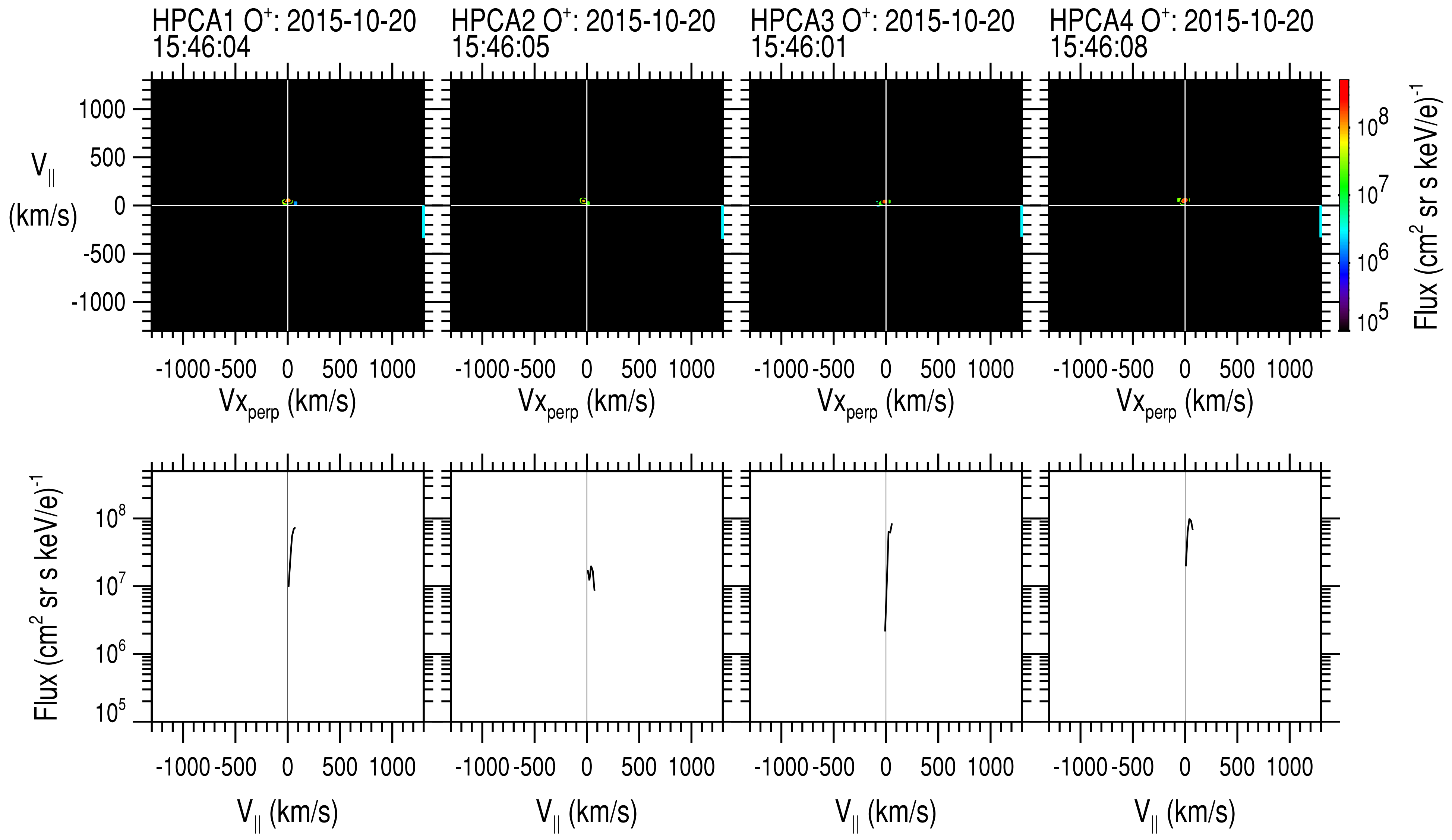
HPCA1 O+: 2015-10-20
15:46:04
HPCA2 O+: 2015-10-20
15:46:05
HPCA3 O+: 2015-10-20
15:46:01
HPCA4 O+: 2015-10-20
15:46:08
V|| (km/s)
1000
500
0
-500
-1000
-1000 -500 0 500 1000
Vx_perp (km/s)
Flux (cm2 sr s keV/e)-1
10^5
10^6
10^7
10^8
V|| (km/s)

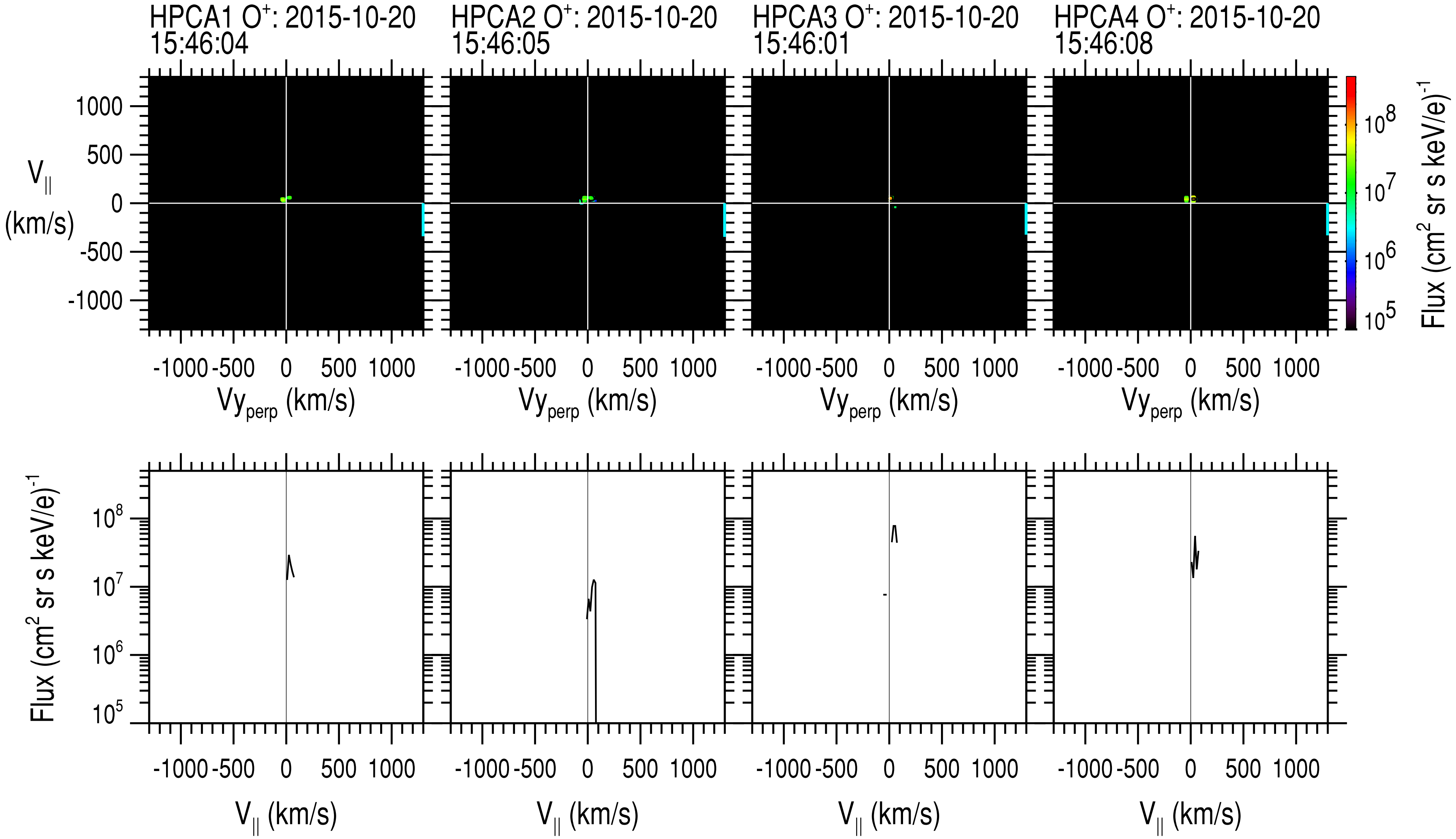
HPCA1 O+: 2015-10-20
15:46:04
HPCA2 O+: 2015-10-20
15:46:05
HPCA3 O+: 2015-10-20
15:46:01
HPCA4 O+: 2015-10-20
15:46:08
V|| (km/s)
1000
500
0
-500
-1000
-1000 -500 0 500 1000
Vyperp (km/s)
Flux (cm2 sr s keV/e)-1
10^5
10^6
10^7
10^8
V|| (km/s)

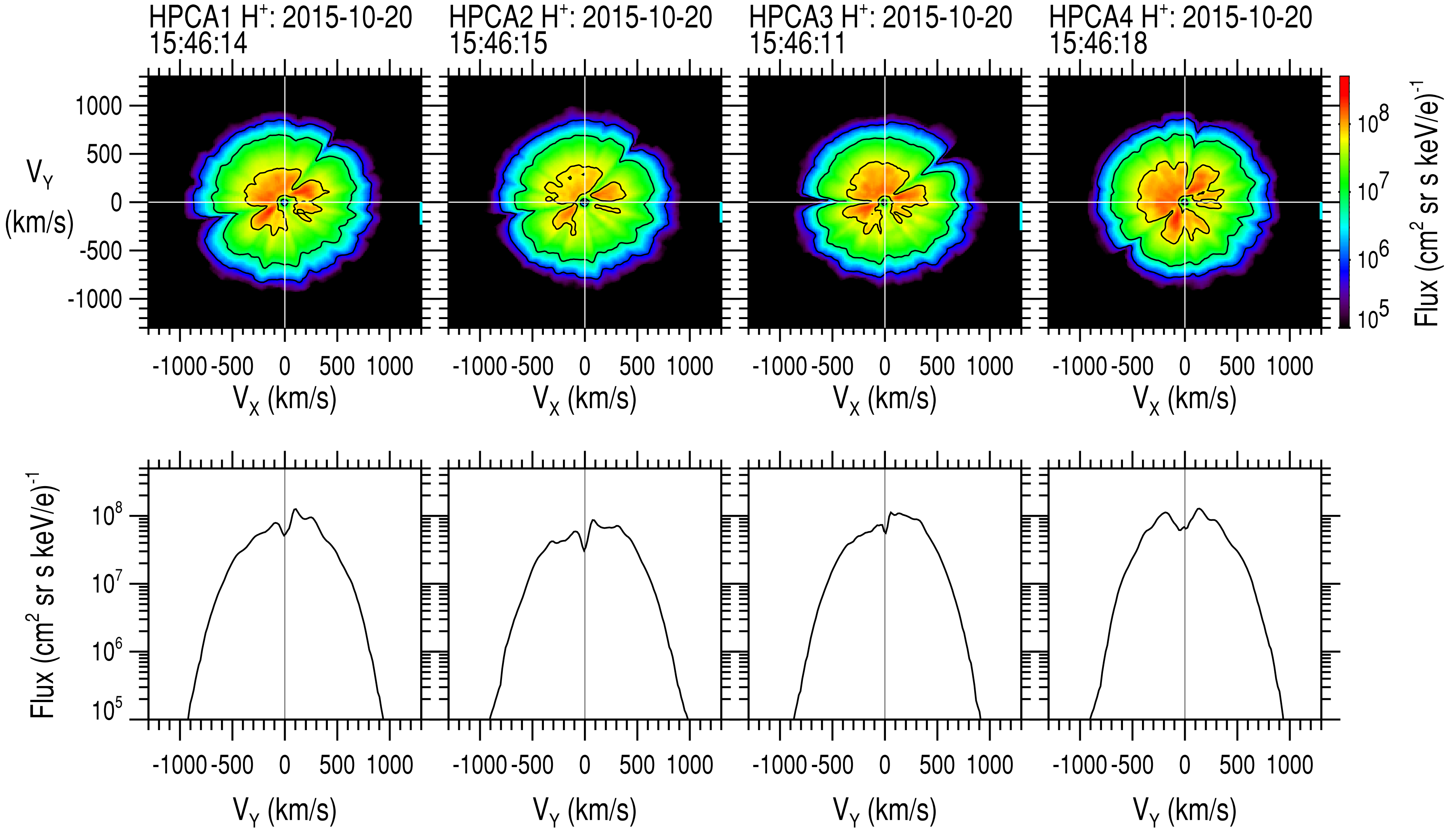

HPCA1 H+: 2015-10-20 15:46:14
HPCA2 H+: 2015-10-20 15:46:15
HPCA3 H+: 2015-10-20 15:46:11
HPCA4 H+: 2015-10-20 15:46:18
V_Y (km/s)
V_X (km/s)
Flux (cm^2 sr s keV/e)^-1

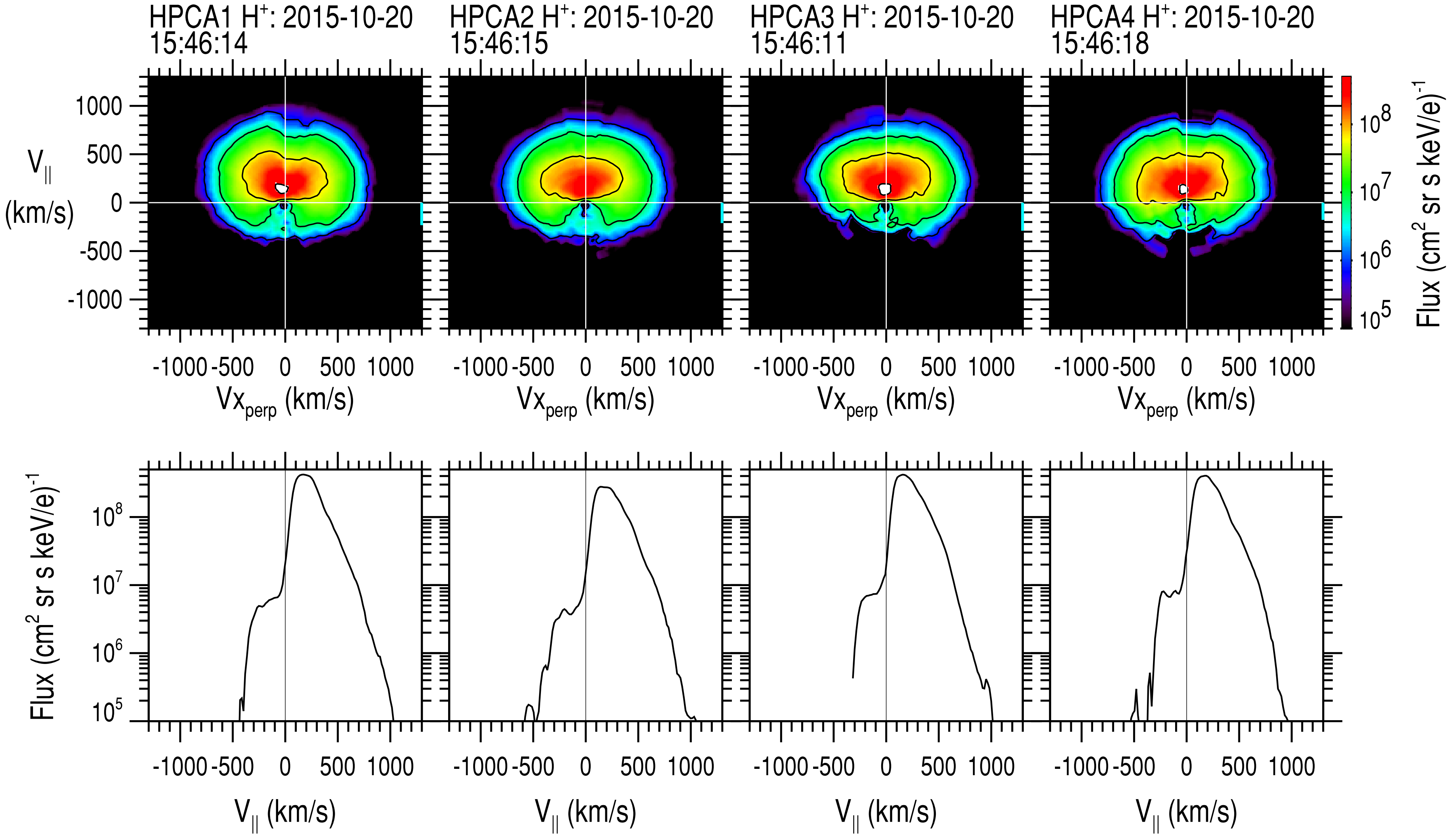

HPCA1 H+: 2015-10-20 15:46:14
HPCA2 H+: 2015-10-20 15:46:15
HPCA3 H+: 2015-10-20 15:46:11
HPCA4 H+: 2015-10-20 15:46:18
V|| (km/s)
Vxperp (km/s)
Flux (cm2 sr s keV/e)-1

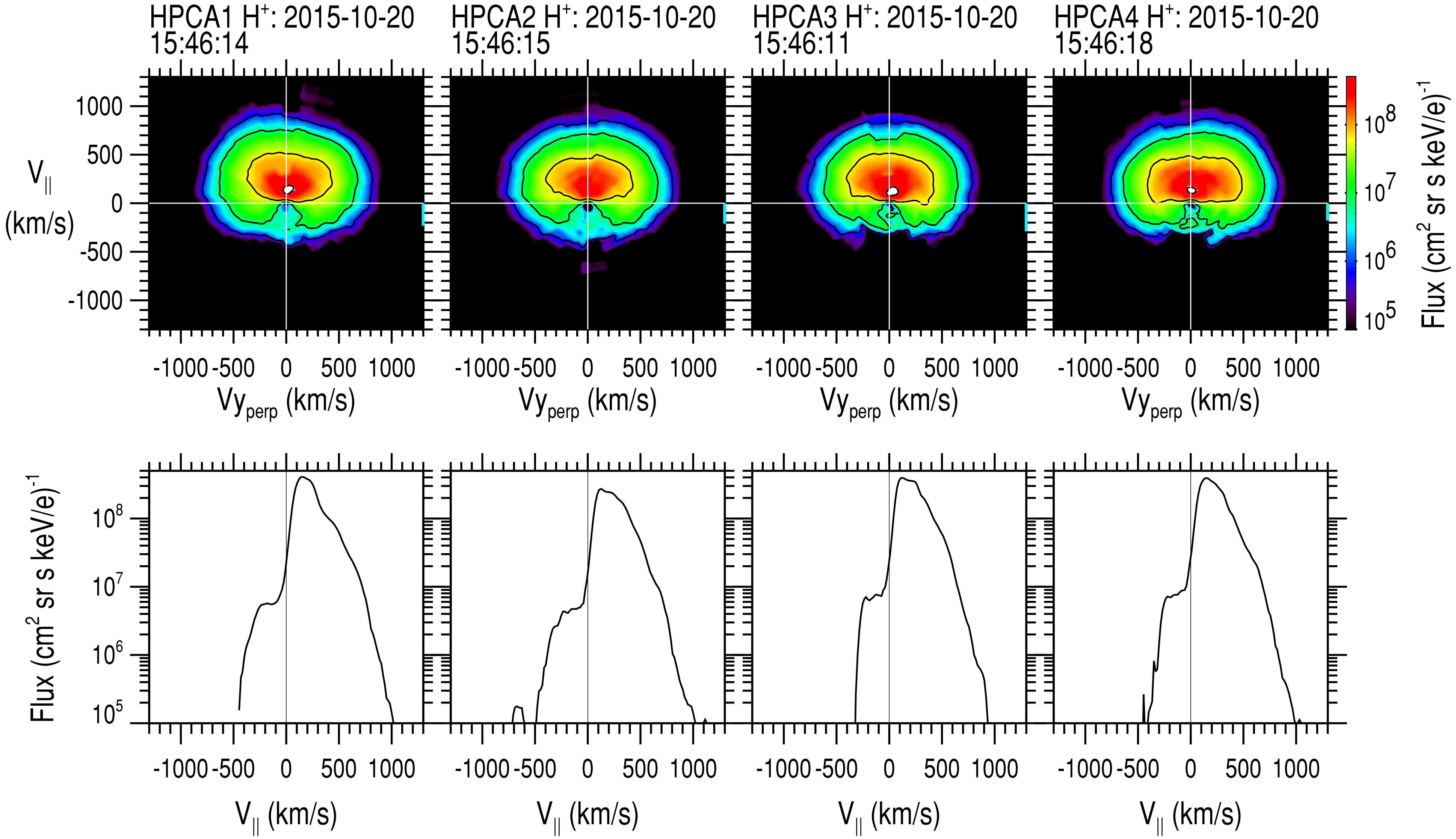
HPCA1 H+: 2015-10-20
15:46:14
HPCA2 H+: 2015-10-20
15:46:15
HPCA3 H+: 2015-10-20
15:46:11
HPCA4 H+: 2015-10-20
15:46:18
V||
(km/s)
Vyperp (km/s)
Flux (cm2 sr s keV/e)-1
V|| (km/s)

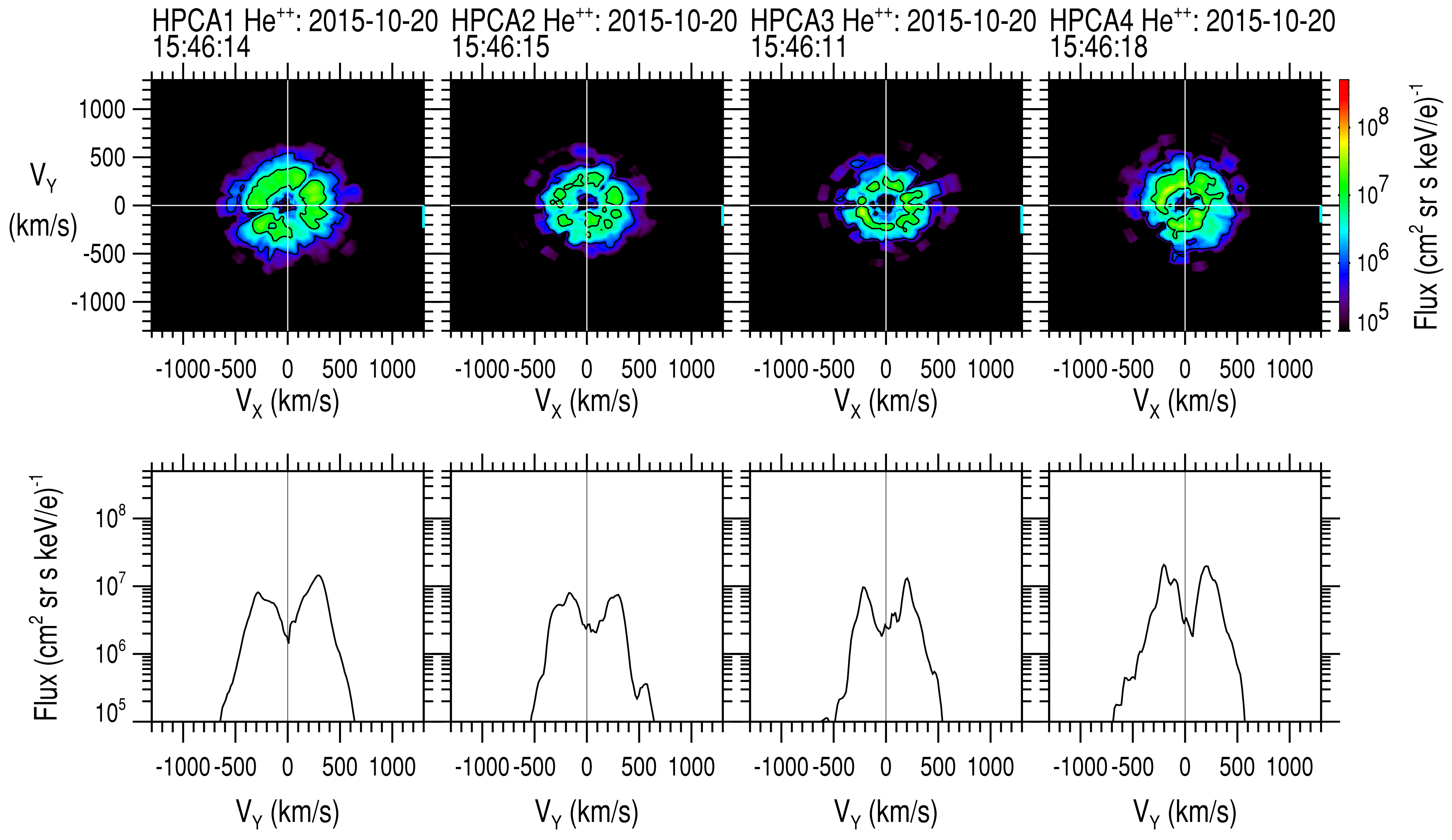

HPCA1 He++: 2015-10-20 15:46:14
HPCA2 He++: 2015-10-20 15:46:15
HPCA3 He++: 2015-10-20 15:46:11
HPCA4 He++: 2015-10-20 15:46:18
VY (km/s)
VX (km/s)
Flux (cm2 sr s keV/e)-1
VY (km/s)

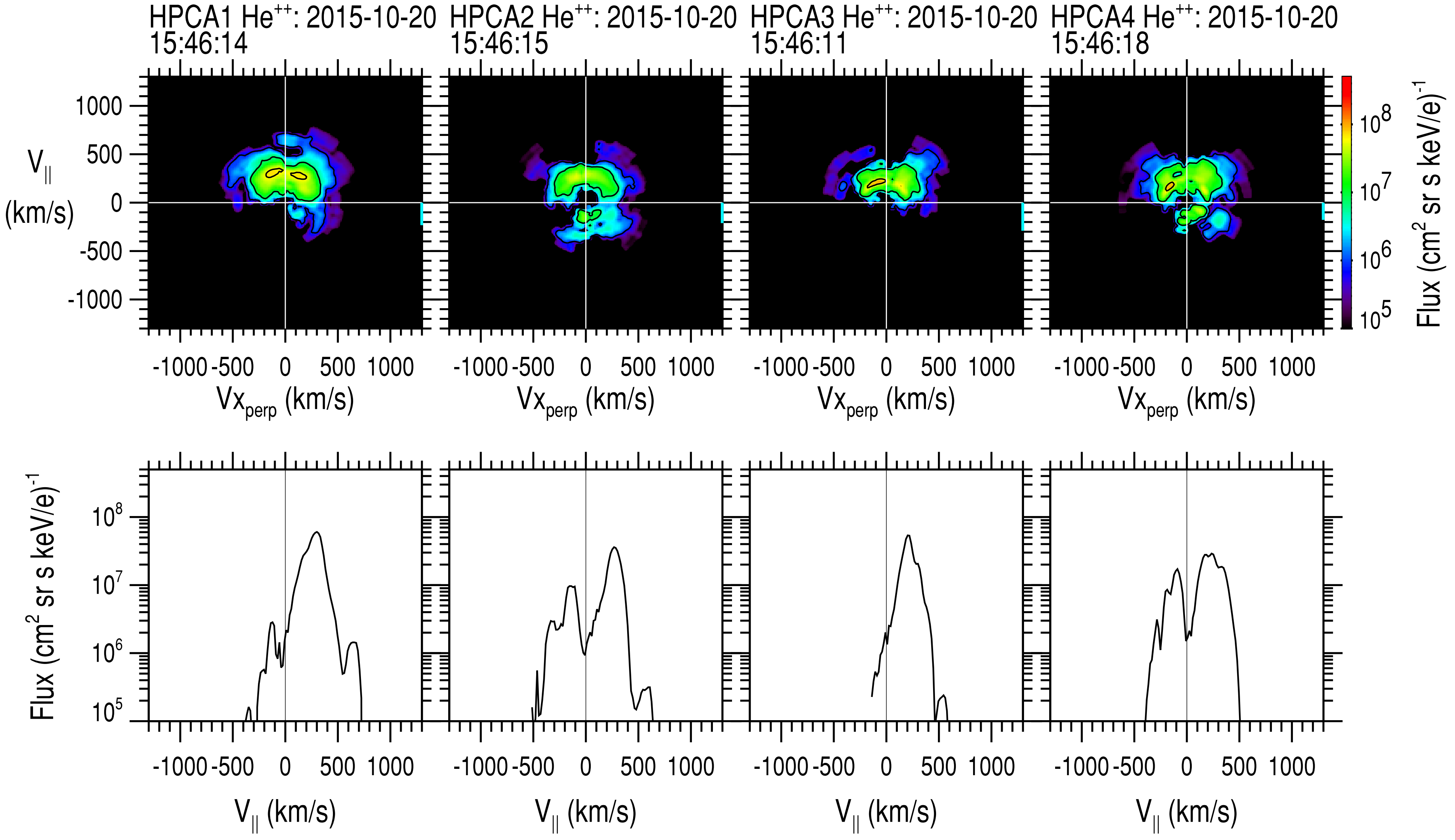

HPCA1 He++: 2015-10-20
15:46:14
HPCA2 He++: 2015-10-20
15:46:15
HPCA3 He++: 2015-10-20
15:46:11
HPCA4 He++: 2015-10-20
15:46:18
V|| (km/s)
Vxperp (km/s)
Flux (cm2 sr s keV/e)-1
1000
500
0
-500
-1000
-1000 -500 0 500 1000
10^5 10^6 10^7 10^8

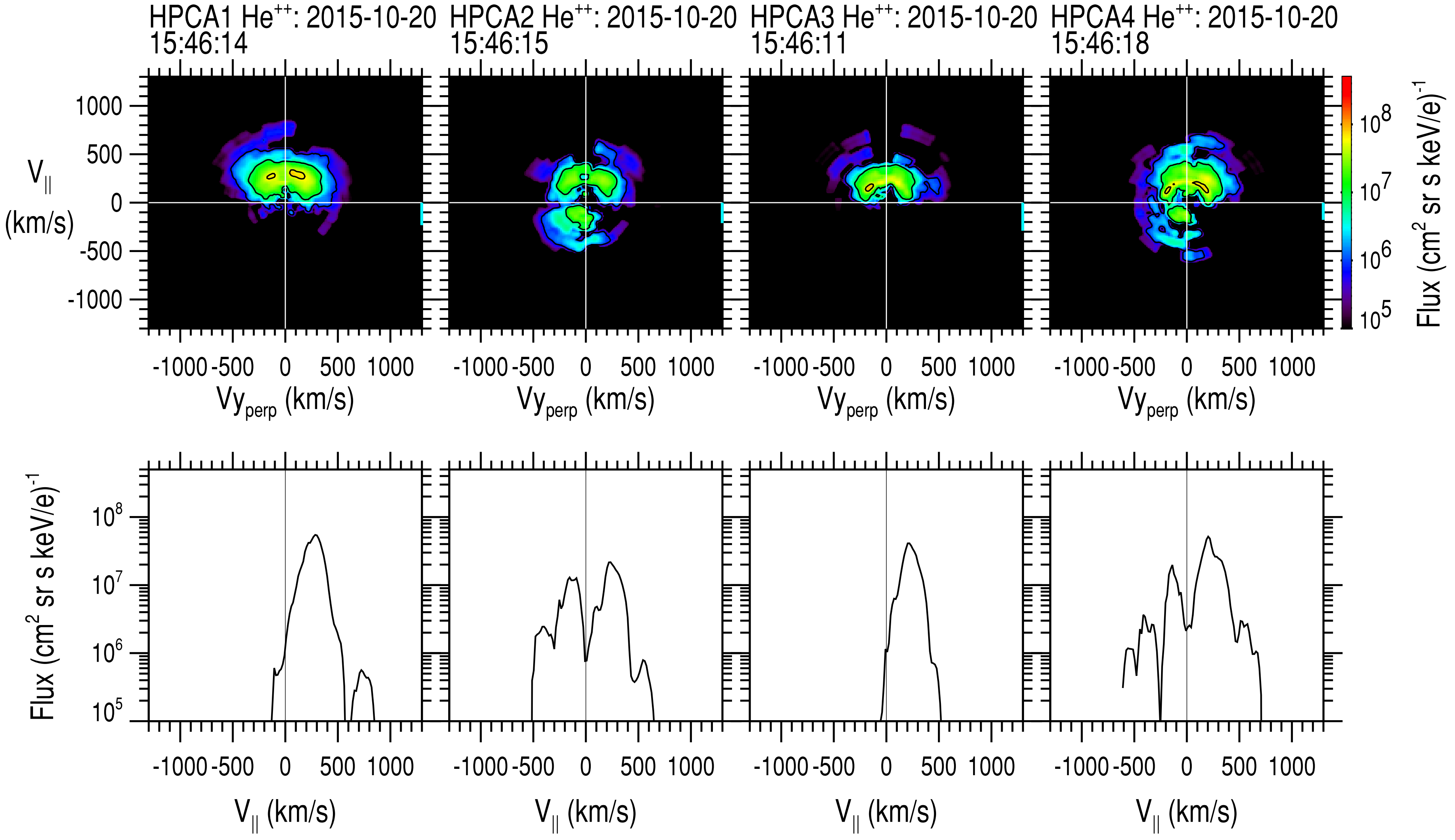
HPCA1 He++: 2015-10-20 15:46:14
HPCA2 He++: 2015-10-20 15:46:15
HPCA3 He++: 2015-10-20 15:46:11
HPCA4 He++: 2015-10-20 15:46:18
V|| (km/s)
Vyperp (km/s)
Flux (cm2 sr s keV/e)-1
-1000 -500 0 500 1000
10^5 10^6 10^7 10^8

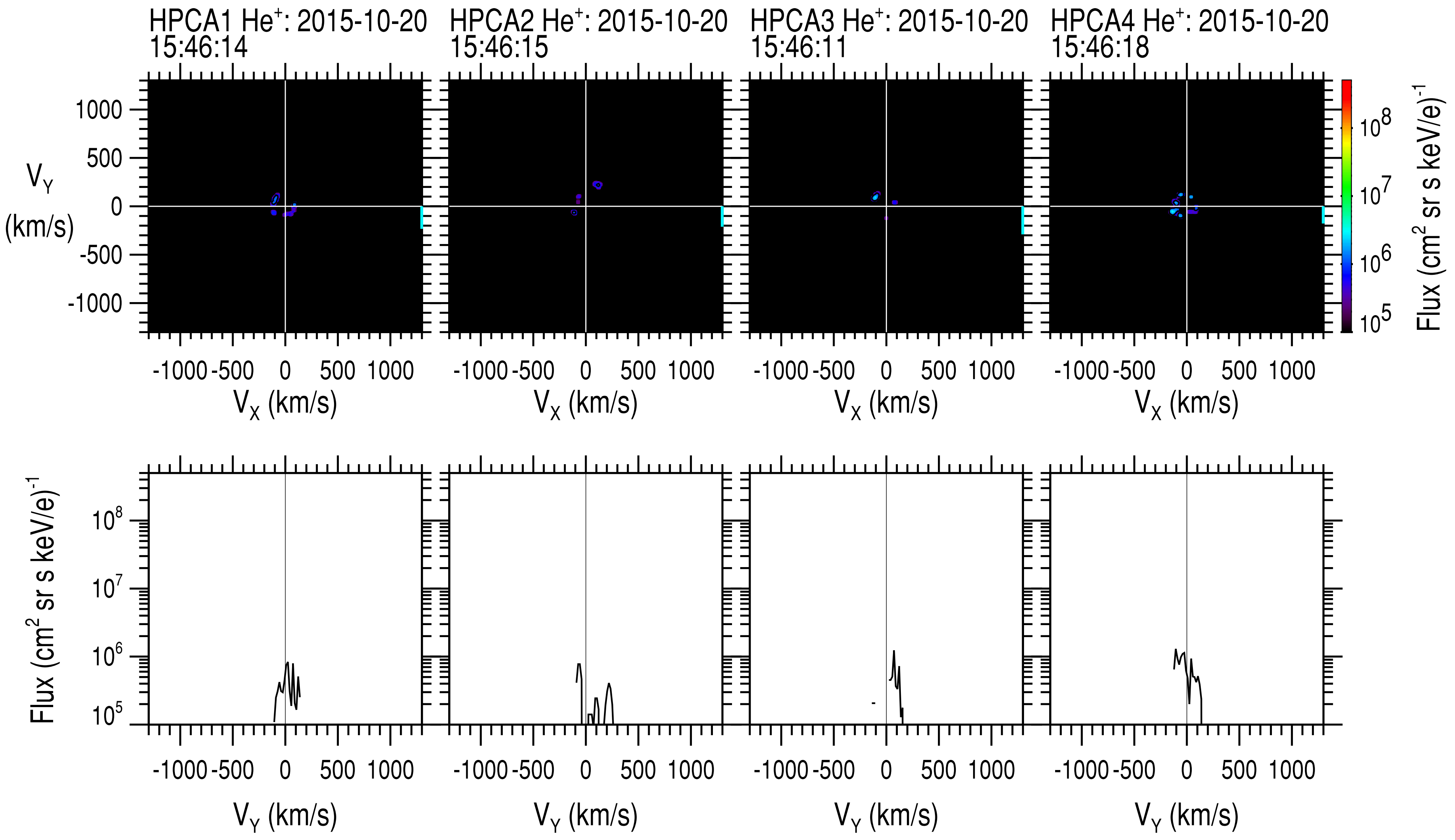

HPCA1 He+: 2015-10-20 15:46:14
HPCA2 He+: 2015-10-20 15:46:15
HPCA3 He+: 2015-10-20 15:46:11
HPCA4 He+: 2015-10-20 15:46:18
V_Y (km/s)
V_X (km/s)
Flux (cm^2 sr s keV/e)^-1

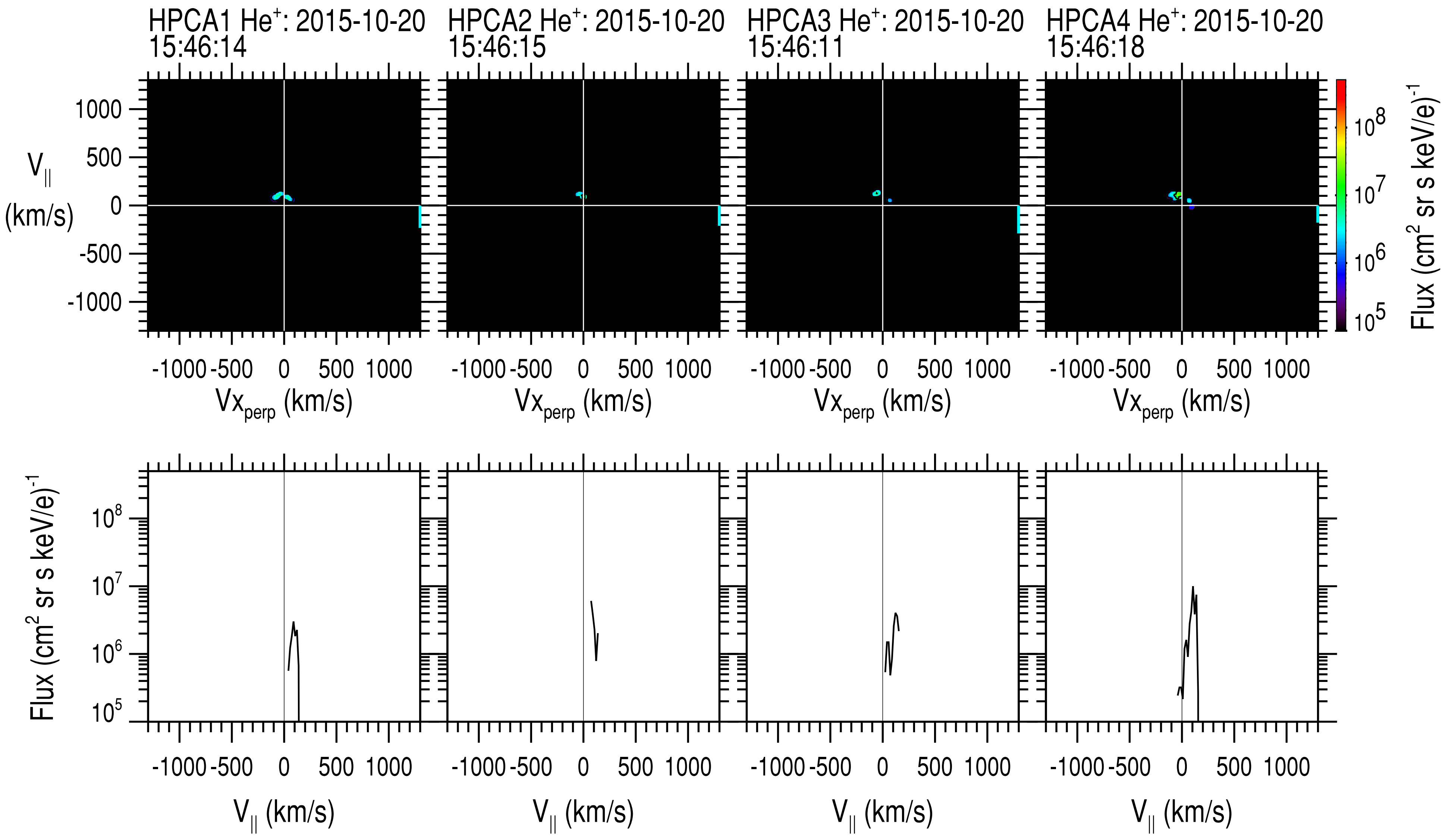

HPCA1 He+: 2015-10-20 15:46:14
HPCA2 He+: 2015-10-20 15:46:15
HPCA3 He+: 2015-10-20 15:46:11
HPCA4 He+: 2015-10-20 15:46:18
V|| (km/s)
Vxperp (km/s)
Flux (cm2 sr s keV/e)-1

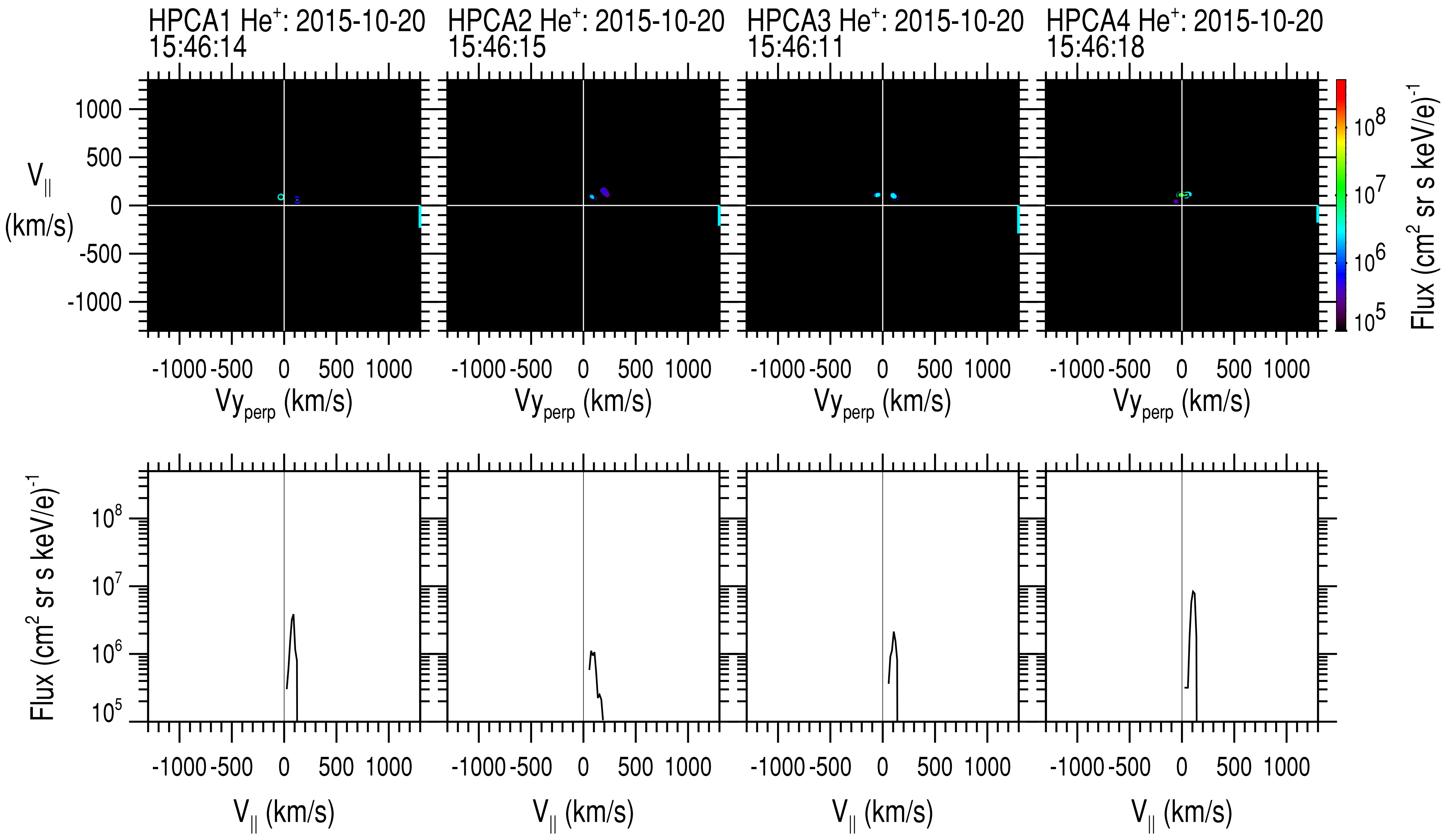
HPCA1 He+: 2015-10-20 15:46:14
HPCA2 He+: 2015-10-20 15:46:15
HPCA3 He+: 2015-10-20 15:46:11
HPCA4 He+: 2015-10-20 15:46:18
V|| (km/s)
Vyperp (km/s)
Flux (cm2 sr s keV/e)-1
1000
500
0
-500
-1000
10^5
10^6
10^7
10^8

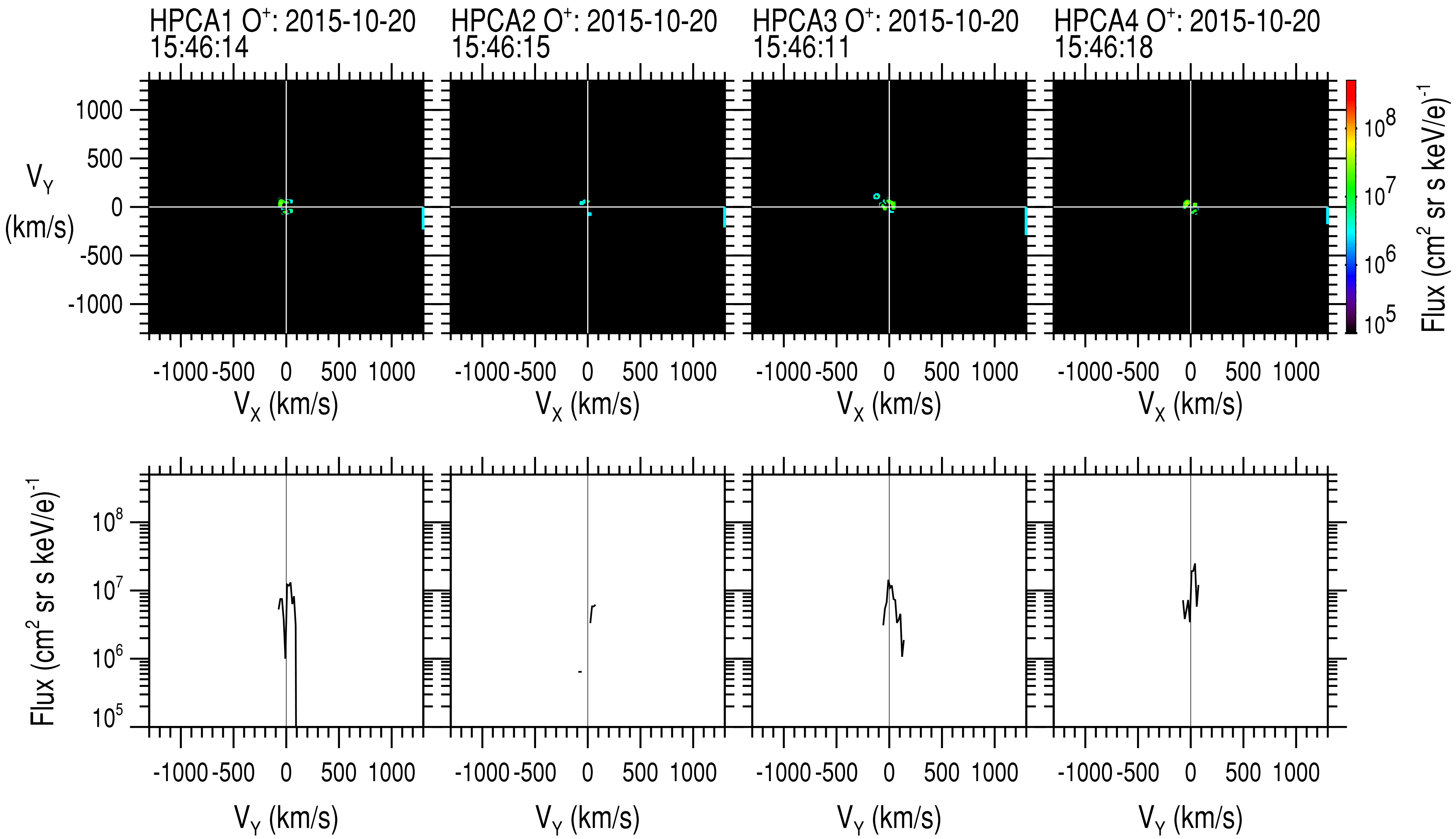

HPCA1 O+: 2015-10-20 15:46:14
HPCA2 O+: 2015-10-20 15:46:15
HPCA3 O+: 2015-10-20 15:46:11
HPCA4 O+: 2015-10-20 15:46:18
VY (km/s)
VX (km/s)
Flux (cm2 sr s keV/e)-1

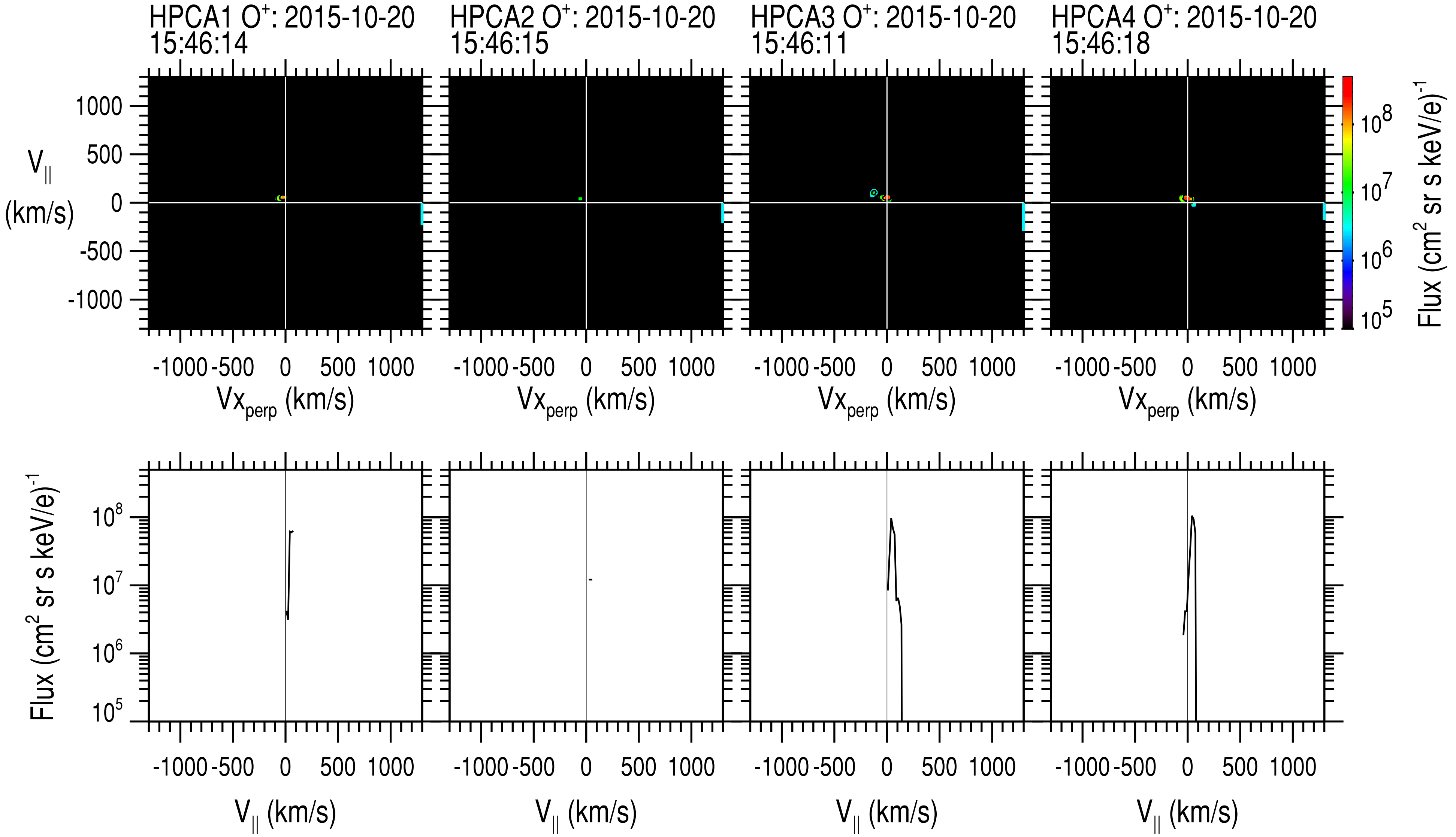
HPCA1 O+: 2015-10-20 15:46:14
HPCA2 O+: 2015-10-20 15:46:15
HPCA3 O+: 2015-10-20 15:46:11
HPCA4 O+: 2015-10-20 15:46:18
V|| (km/s)
Vxperp (km/s)
Flux (cm2 sr s keV/e)-1
V|| (km/s)
Flux (cm2 sr s keV/e)-1

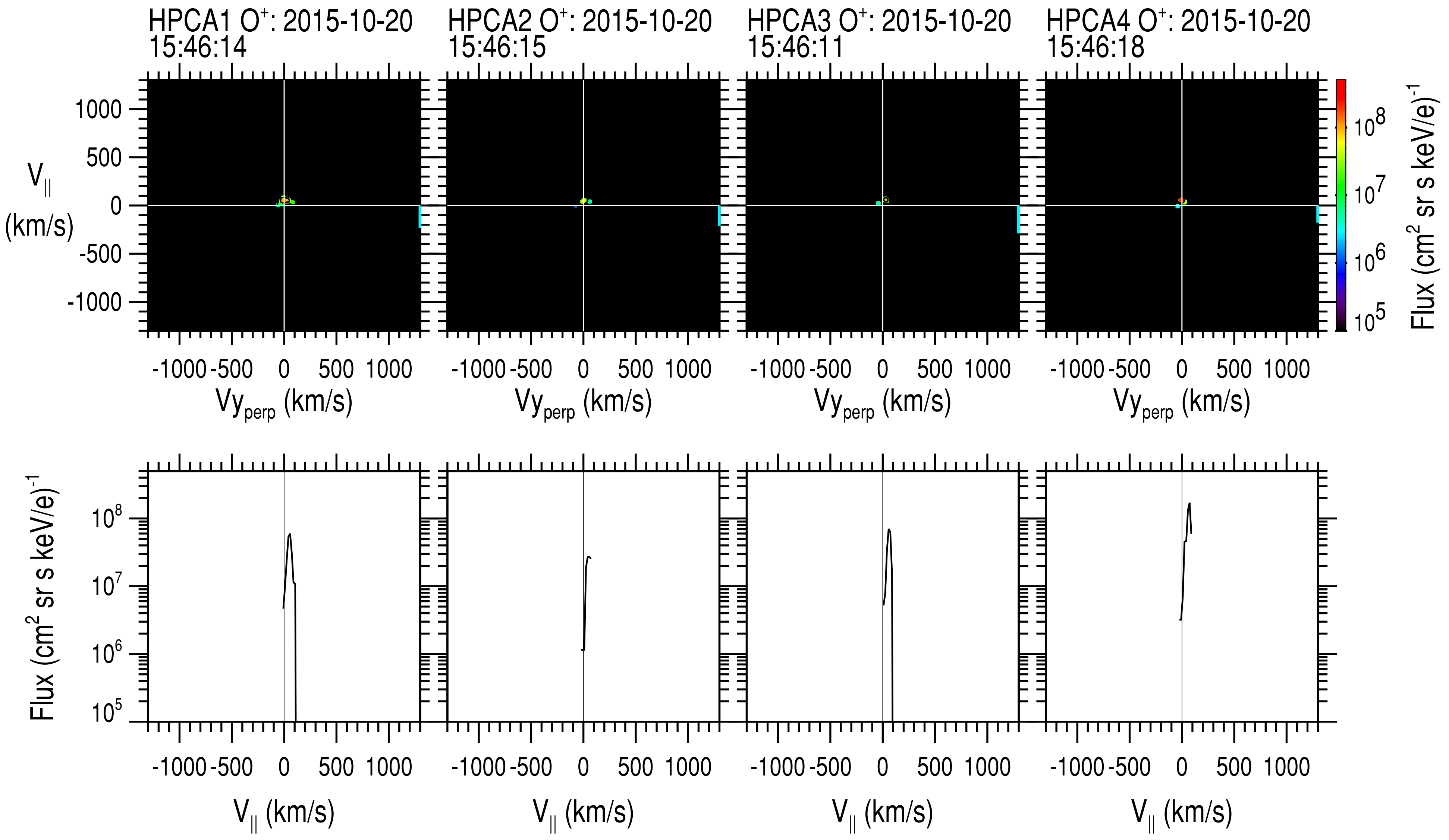
HPCA1 O+: 2015-10-20 15:46:14
HPCA2 O+: 2015-10-20 15:46:15
HPCA3 O+: 2015-10-20 15:46:11
HPCA4 O+: 2015-10-20 15:46:18
V|| (km/s)
Vyperp (km/s)
Flux (cm2 sr s keV/e)-1
-1000 -500 0 500 1000
10^5 10^6 10^7 10^8

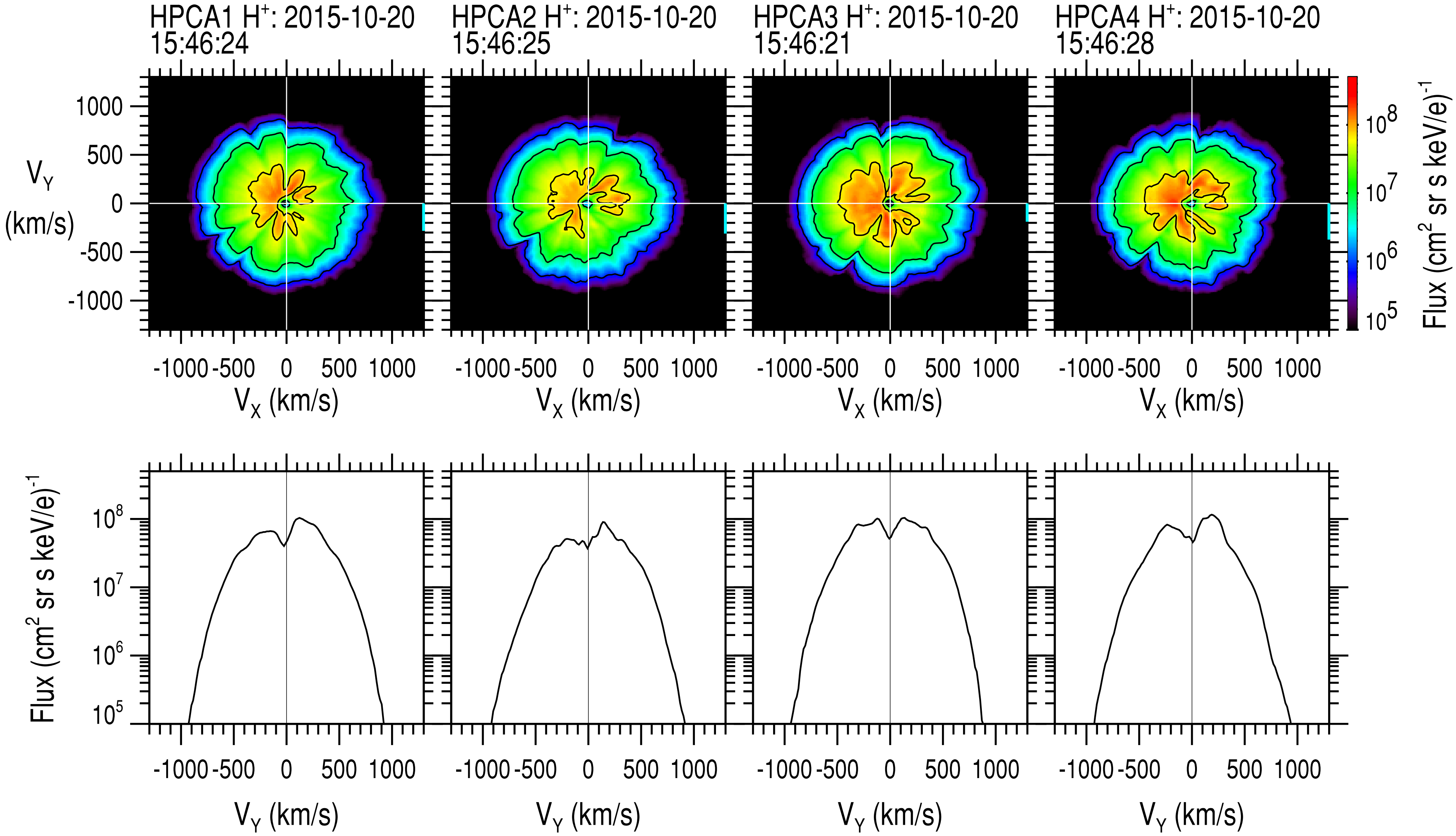
HPCA1 H+: 2015-10-20 15:46:24
HPCA2 H+: 2015-10-20 15:46:25
HPCA3 H+: 2015-10-20 15:46:21
HPCA4 H+: 2015-10-20 15:46:28
VY (km/s)
VX (km/s)
Flux (cm2 sr s keV/e)-1
VY (km/s)
Flux (cm2 sr s keV/e)-1

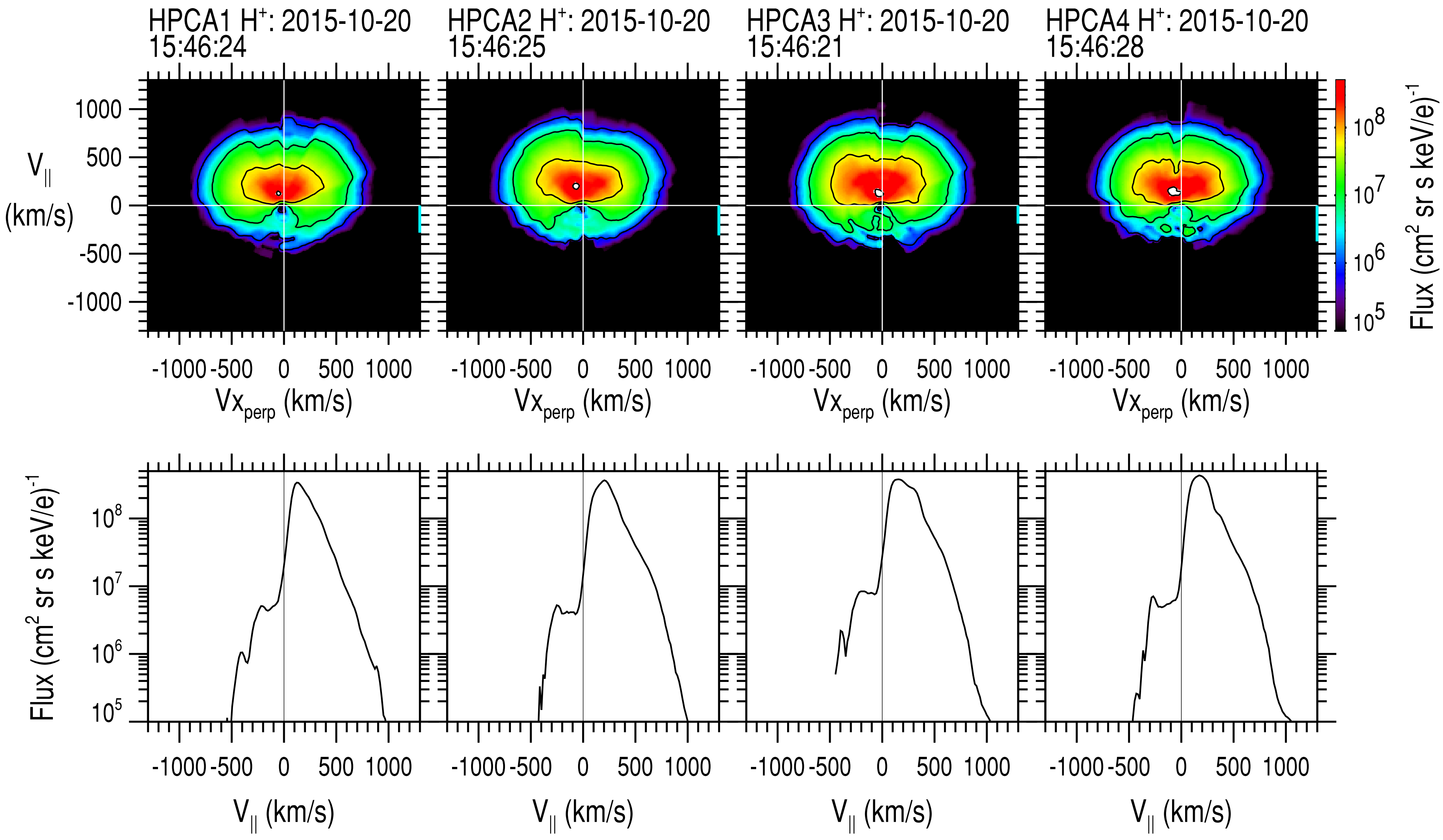
HPCA1 H+: 2015-10-20 15:46:24
HPCA2 H+: 2015-10-20 15:46:25
HPCA3 H+: 2015-10-20 15:46:21
HPCA4 H+: 2015-10-20 15:46:28
V|| (km/s)
Vx_perp (km/s)
Flux (cm2 sr s keV/e)-1
-1000 -500 0 500 1000
10^5 10^6 10^7 10^8

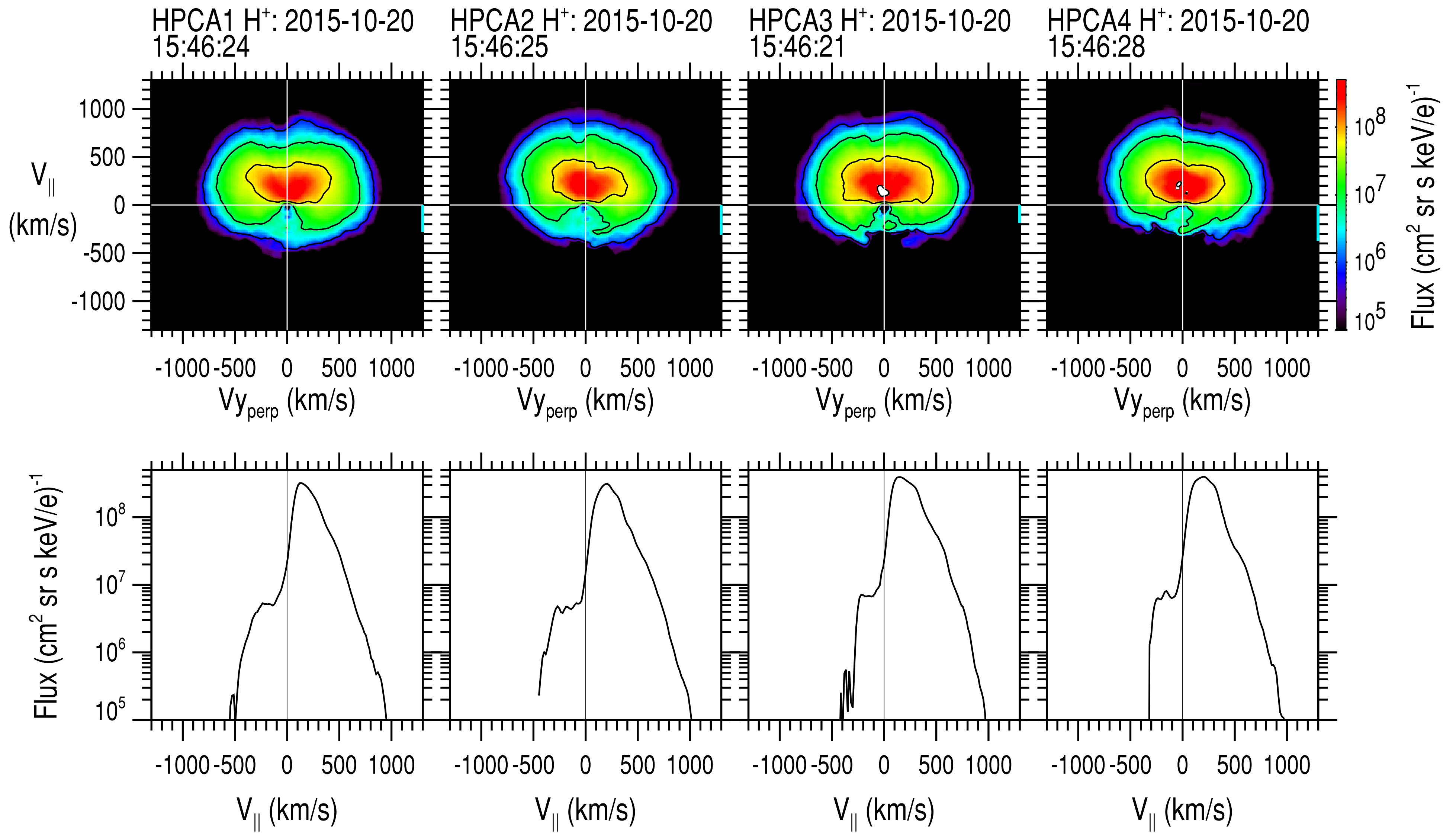

HPCA1 H+: 2015-10-20 15:46:24
HPCA2 H+: 2015-10-20 15:46:25
HPCA3 H+: 2015-10-20 15:46:21
HPCA4 H+: 2015-10-20 15:46:28
V|| (km/s)
Vyperp (km/s)
Flux (cm2 sr s keV/e)-1

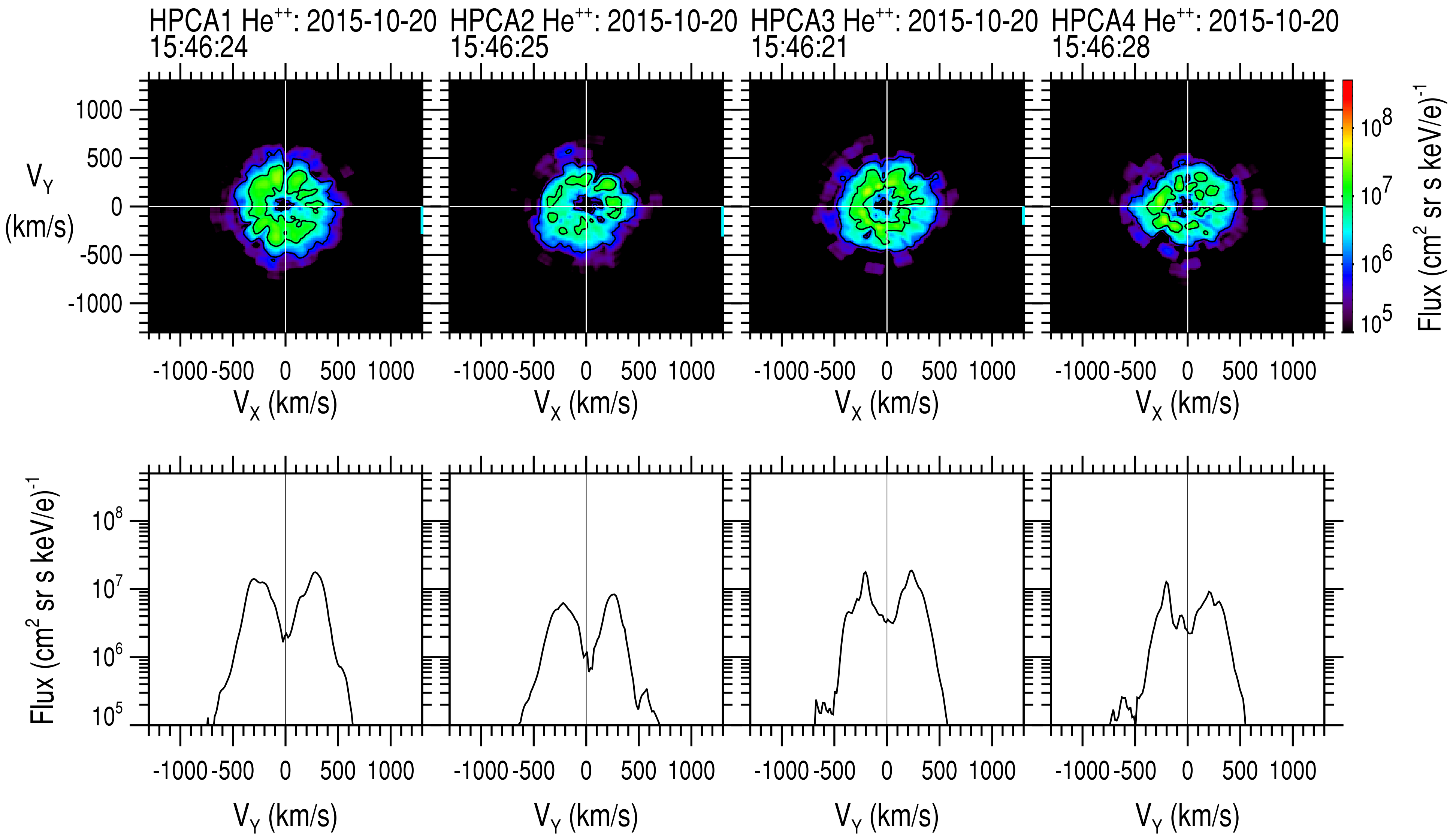
HPCA1 He++: 2015-10-20 15:46:24
HPCA2 He++: 2015-10-20 15:46:25
HPCA3 He++: 2015-10-20 15:46:21
HPCA4 He++: 2015-10-20 15:46:28
V_Y (km/s)
V_X (km/s)
Flux (cm^2 sr s keV/e)^-1
V_Y (km/s)

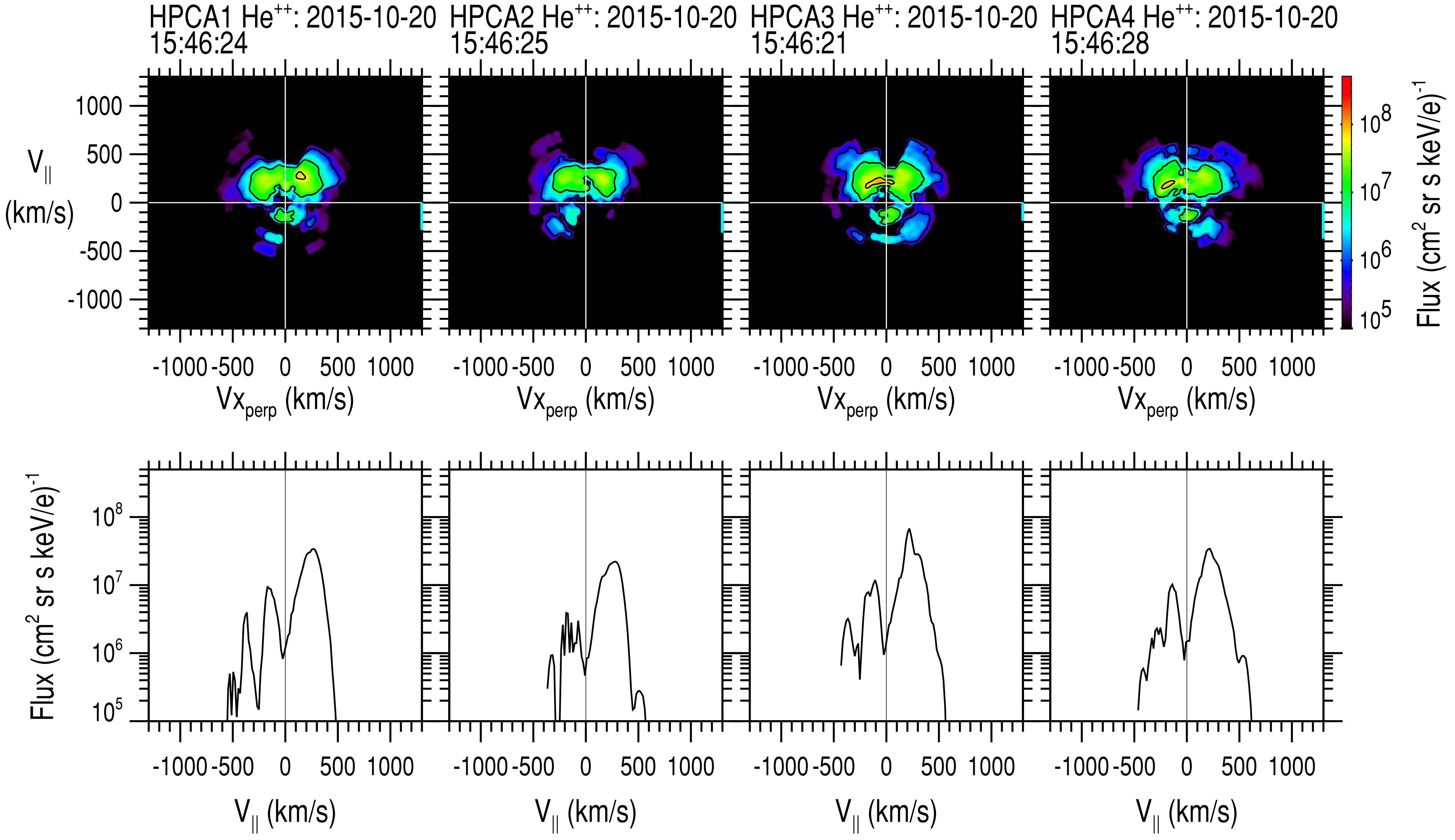
HPCA1 He++: 2015-10-20
15:46:24
HPCA2 He++: 2015-10-20
15:46:25
HPCA3 He++: 2015-10-20
15:46:21
HPCA4 He++: 2015-10-20
15:46:28
V|| (km/s)
Vxperp (km/s)
Flux (cm2 sr s keV/e)-1
V|| (km/s)

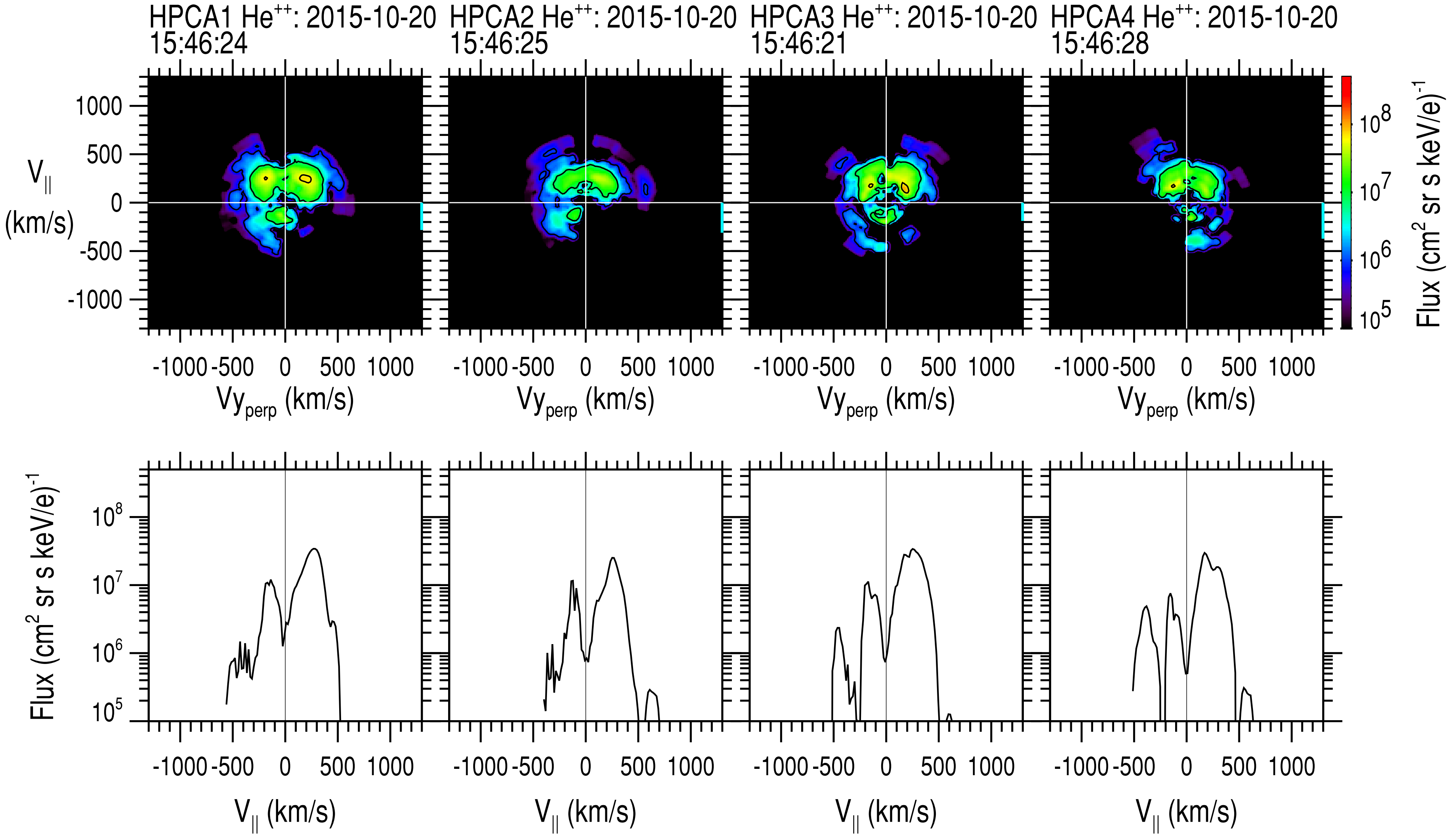

HPCA1 He++: 2015-10-20 15:46:24
HPCA2 He++: 2015-10-20 15:46:25
HPCA3 He++: 2015-10-20 15:46:21
HPCA4 He++: 2015-10-20 15:46:28
V|| (km/s)
Vyperp (km/s)
Flux (cm2 sr s keV/e)-1

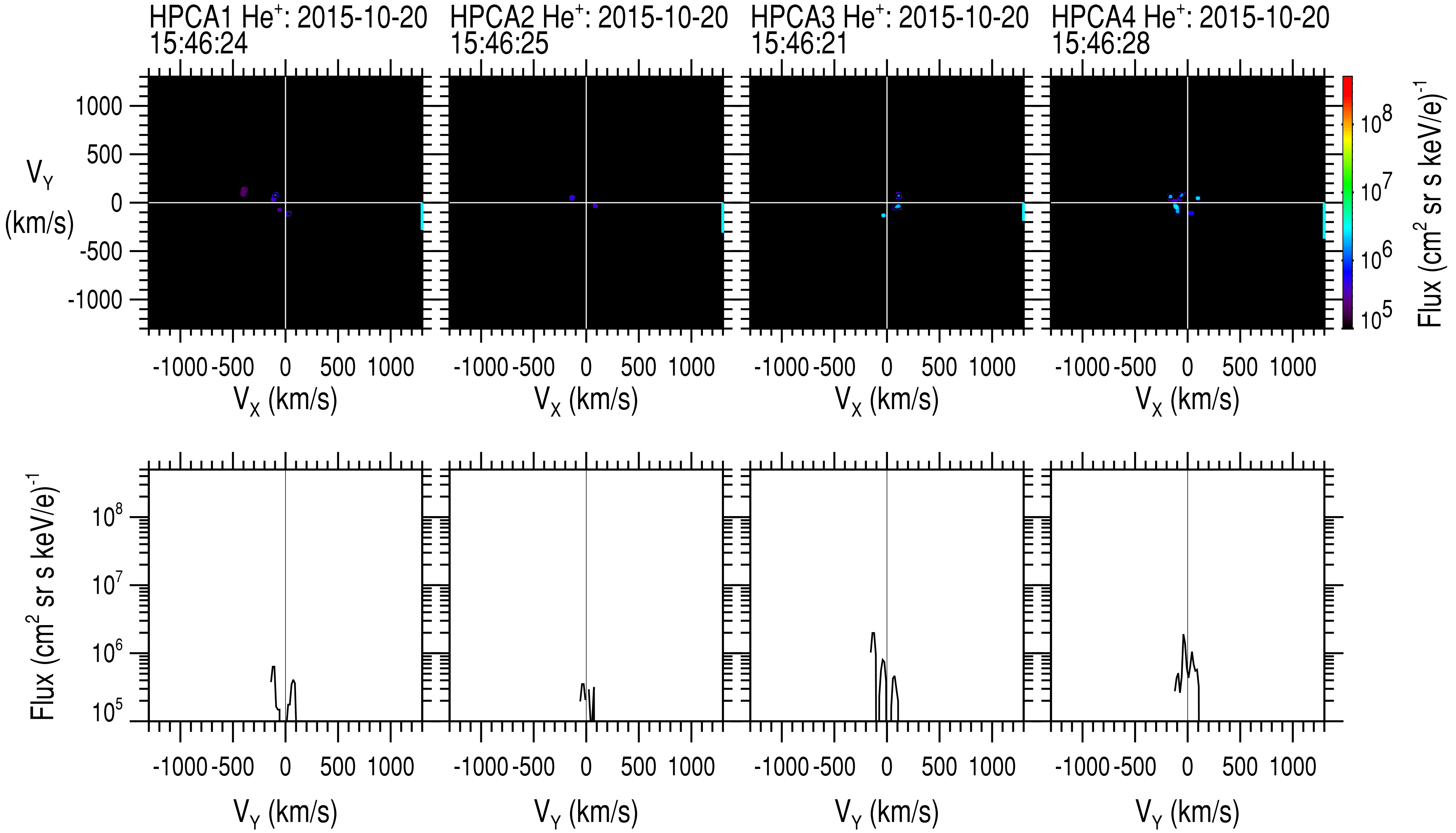
HPCA1 He+: 2015-10-20 15:46:24
HPCA2 He+: 2015-10-20 15:46:25
HPCA3 He+: 2015-10-20 15:46:21
HPCA4 He+: 2015-10-20 15:46:28
VY (km/s)
VX (km/s)
Flux (cm2 sr s keV/e)-1
VY (km/s)

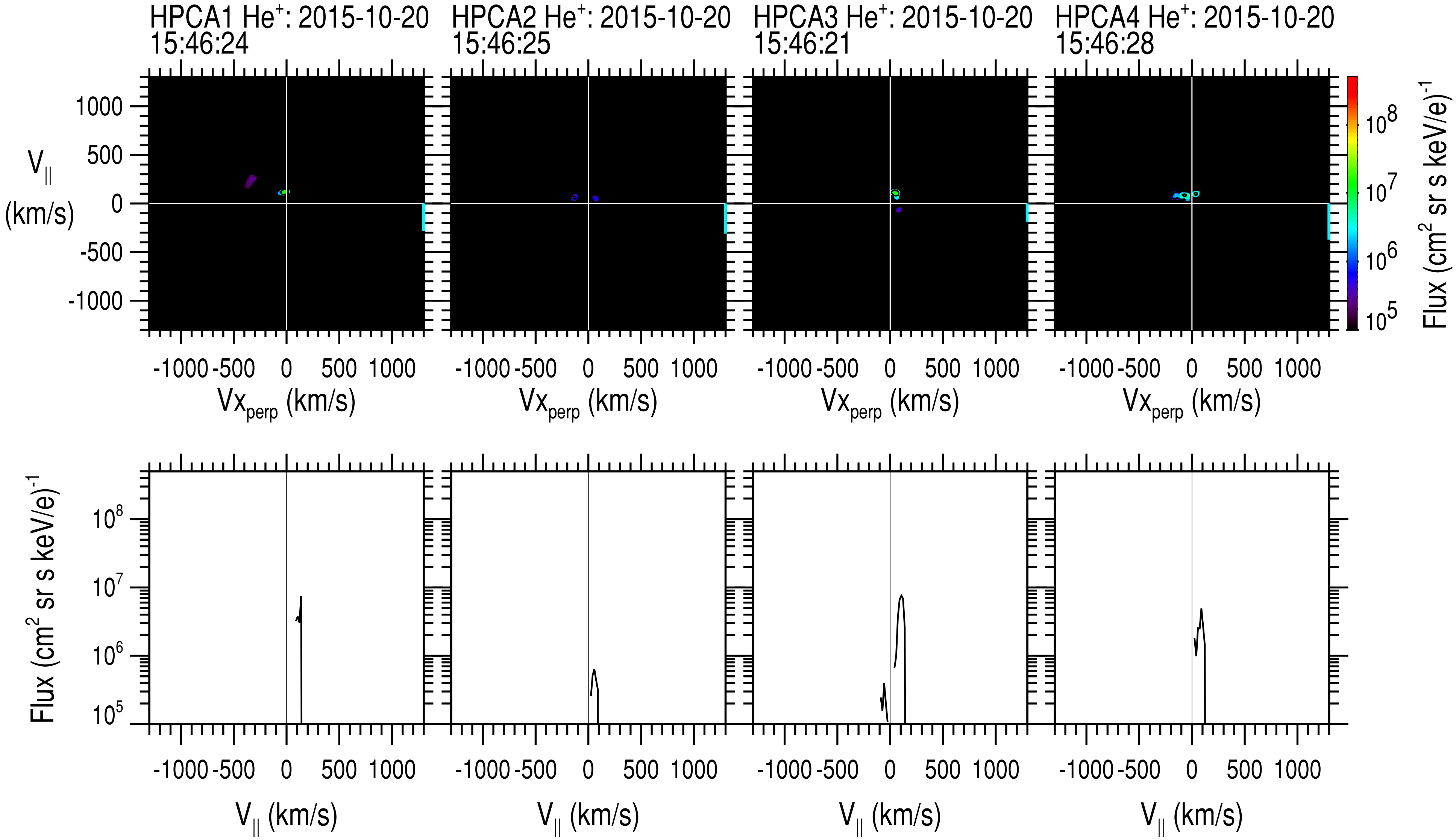

HPCA1 He+: 2015-10-20 15:46:24
HPCA2 He+: 2015-10-20 15:46:25
HPCA3 He+: 2015-10-20 15:46:21
HPCA4 He+: 2015-10-20 15:46:28
V|| (km/s)
Vxperp (km/s)
Flux (cm2 sr s keV/e)-1

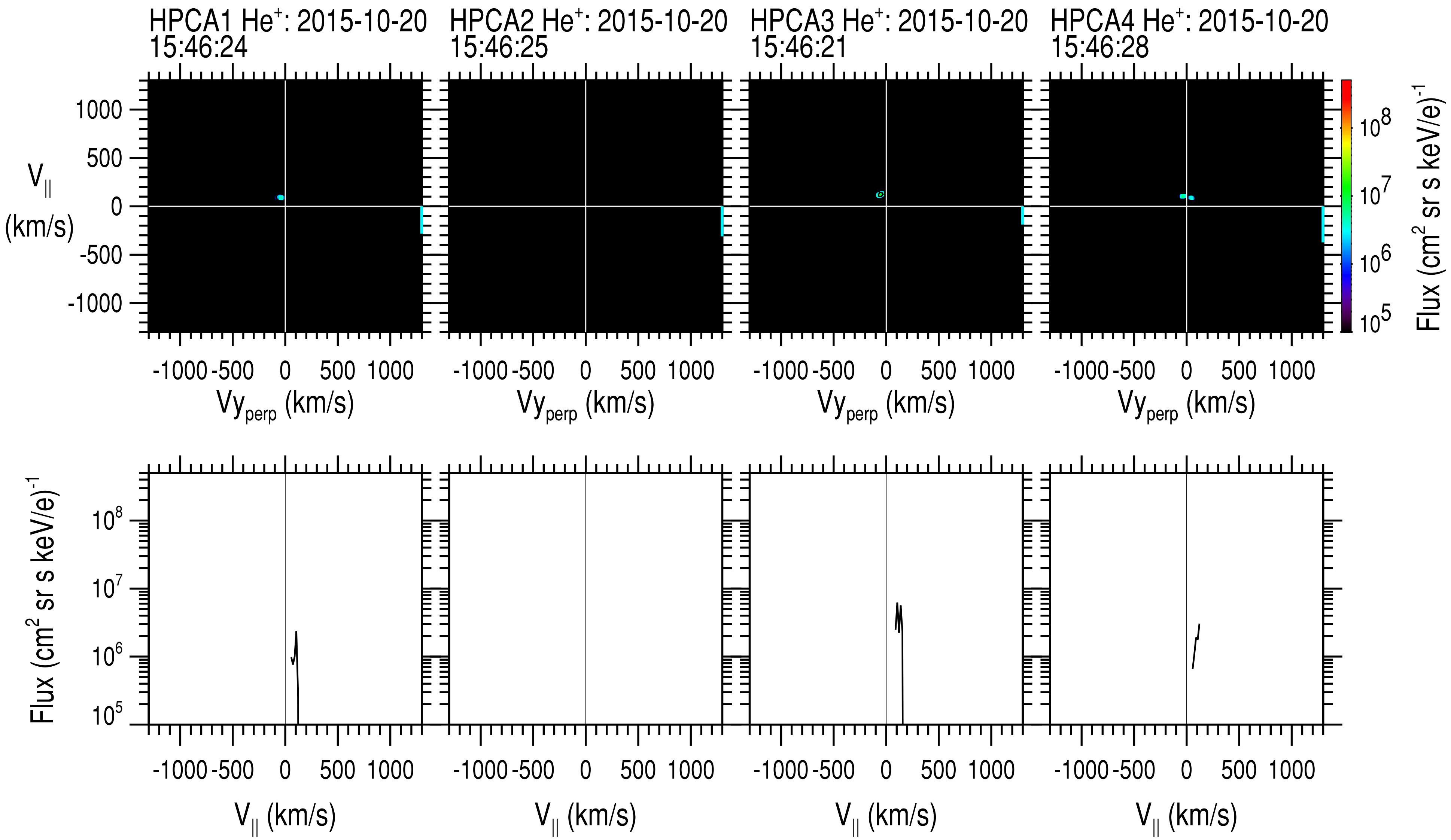
HPCA1 He+: 2015-10-20 15:46:24
HPCA2 He+: 2015-10-20 15:46:25
HPCA3 He+: 2015-10-20 15:46:21
HPCA4 He+: 2015-10-20 15:46:28
V|| (km/s)
Vyperp (km/s)
Flux (cm2 sr s keV/e)-1
V|| (km/s)

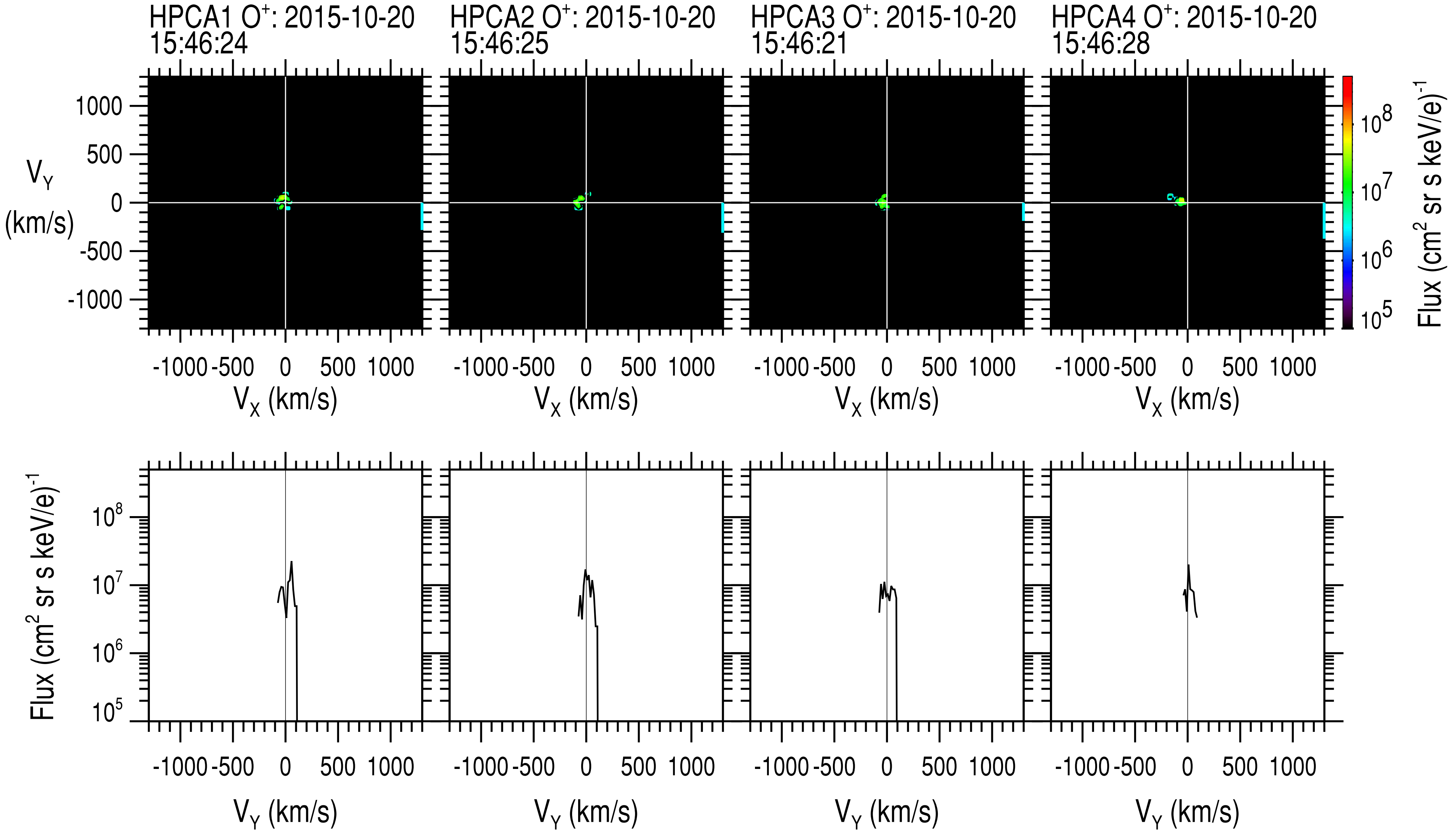
HPCA1 O+: 2015-10-20
15:46:24
HPCA2 O+: 2015-10-20
15:46:25
HPCA3 O+: 2015-10-20
15:46:21
HPCA4 O+: 2015-10-20
15:46:28
VY
(km/s)
VX (km/s)
VY (km/s)
Flux (cm2 sr s keV/e)-1

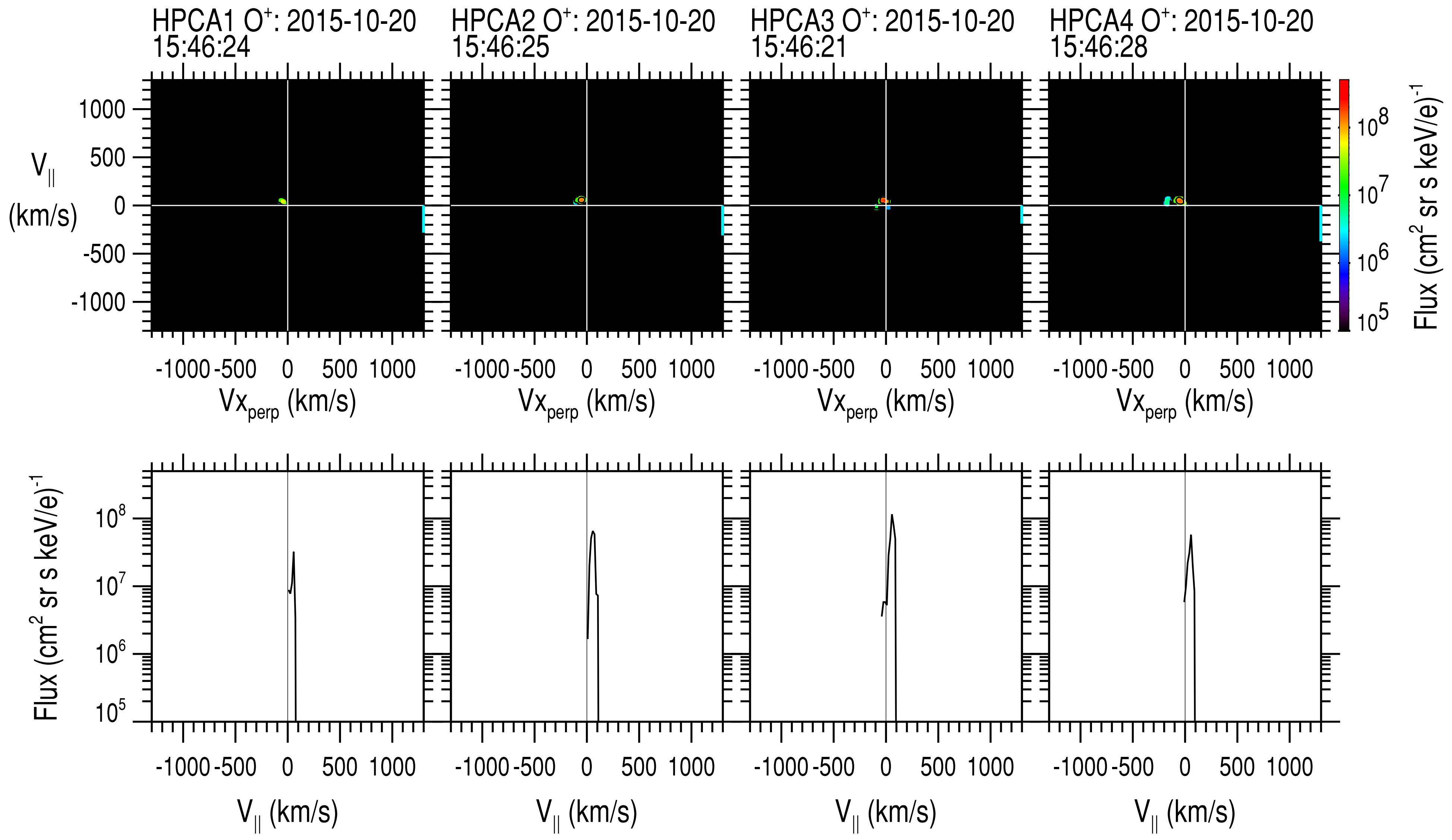

HPCA1 O+: 2015-10-20 15:46:24
HPCA2 O+: 2015-10-20 15:46:25
HPCA3 O+: 2015-10-20 15:46:21
HPCA4 O+: 2015-10-20 15:46:28
V|| (km/s)
Vxperp (km/s)
Flux (cm2 sr s keV/e)-1

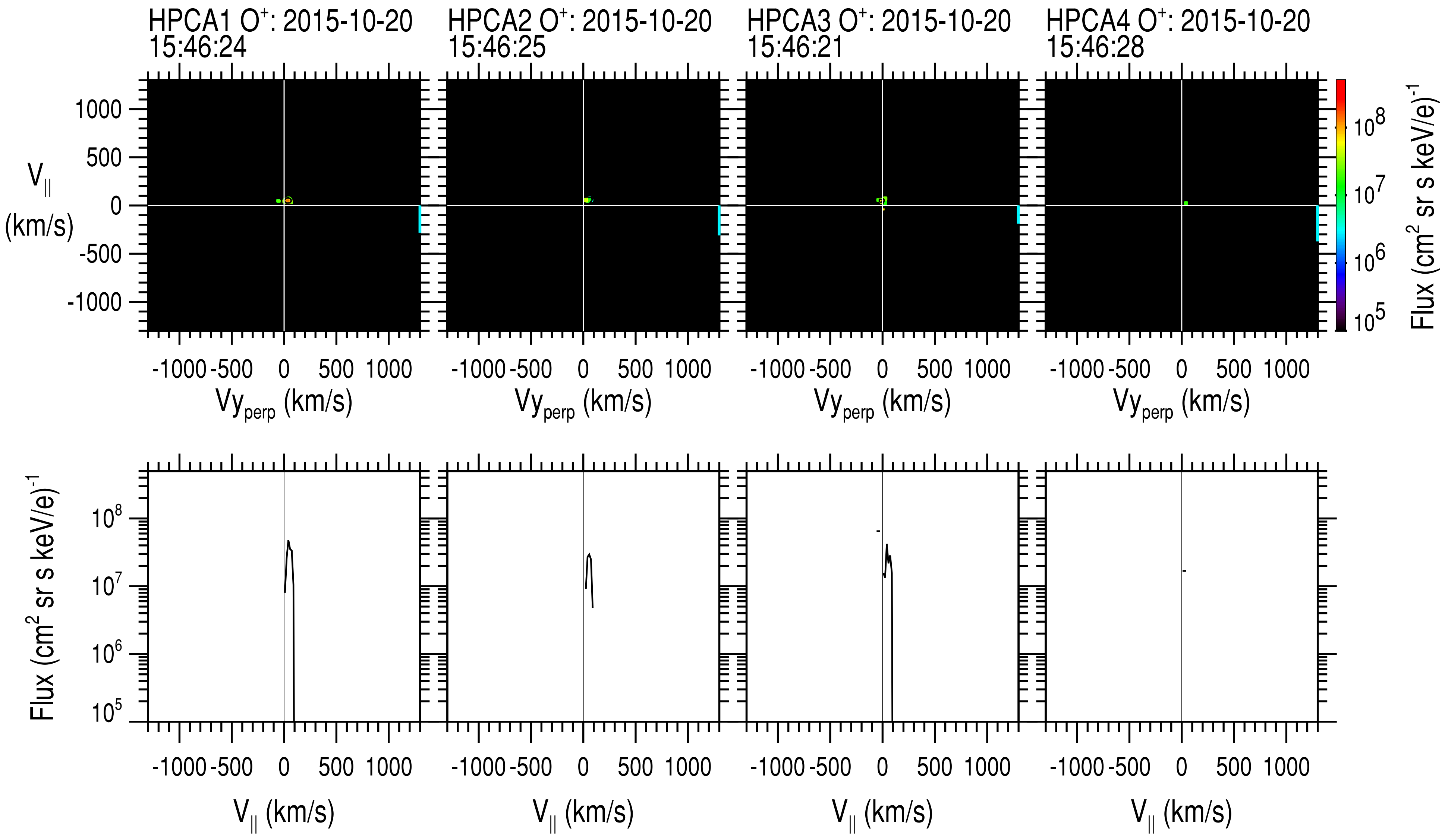
HPCA1 O+: 2015-10-20 15:46:24
HPCA2 O+: 2015-10-20 15:46:25
HPCA3 O+: 2015-10-20 15:46:21
HPCA4 O+: 2015-10-20 15:46:28
V|| (km/s)
Vyperp (km/s)
Flux (cm2 sr s keV/e)-1
1000
500
0
-500
-1000
10^5
10^6
10^7
10^8

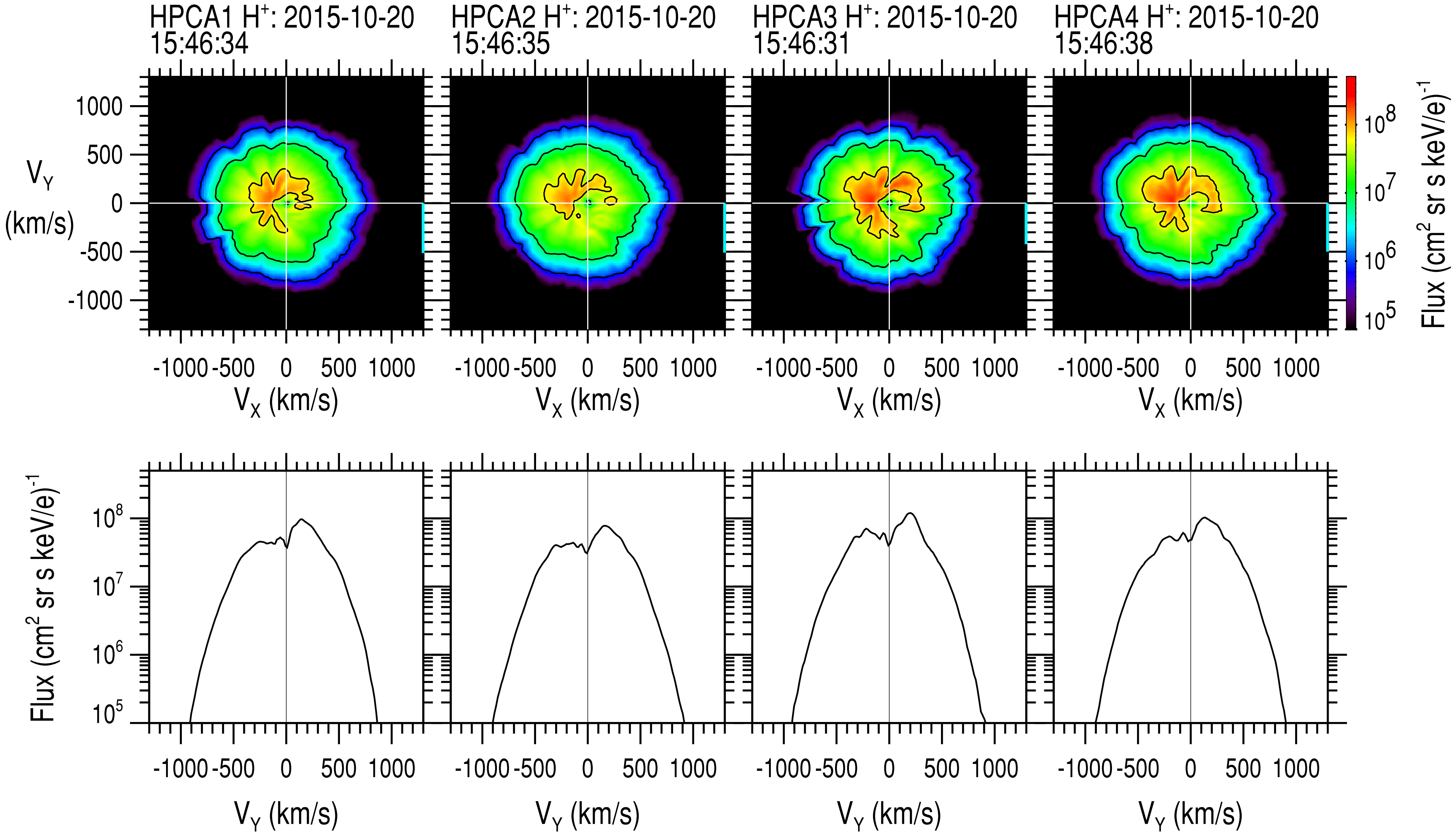

HPCA1 H+: 2015-10-20 15:46:34
HPCA2 H+: 2015-10-20 15:46:35
HPCA3 H+: 2015-10-20 15:46:31
HPCA4 H+: 2015-10-20 15:46:38
VY (km/s)
VX (km/s)
Flux (cm2 sr s keV/e)-1

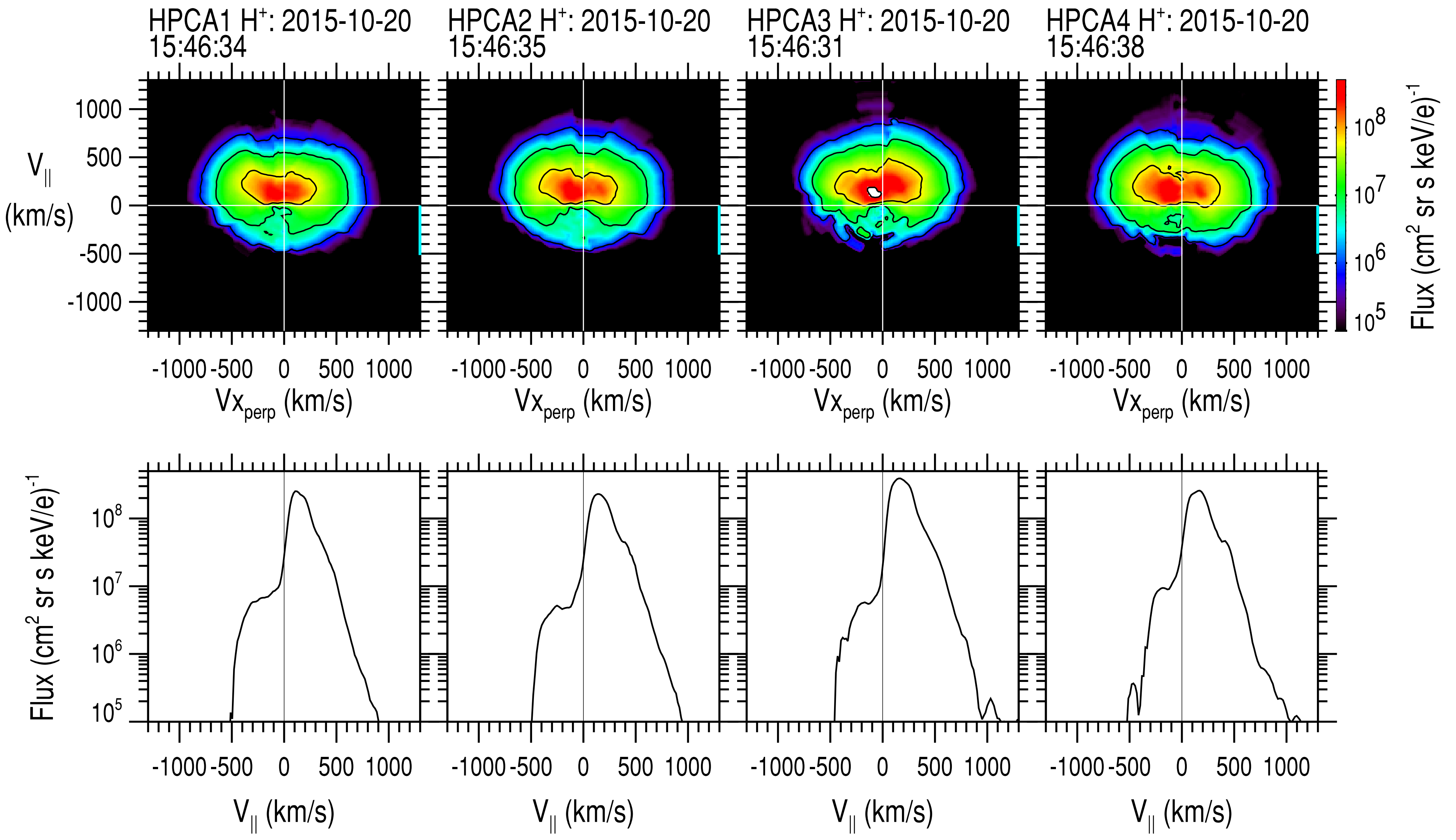
HPCA1 H+: 2015-10-20 15:46:34
HPCA2 H+: 2015-10-20 15:46:35
HPCA3 H+: 2015-10-20 15:46:31
HPCA4 H+: 2015-10-20 15:46:38
V|| (km/s)
Vxperp (km/s)
Flux (cm2 sr s keV/e)-1
1000
500
0
-500
-1000
10^5
10^6
10^7
10^8

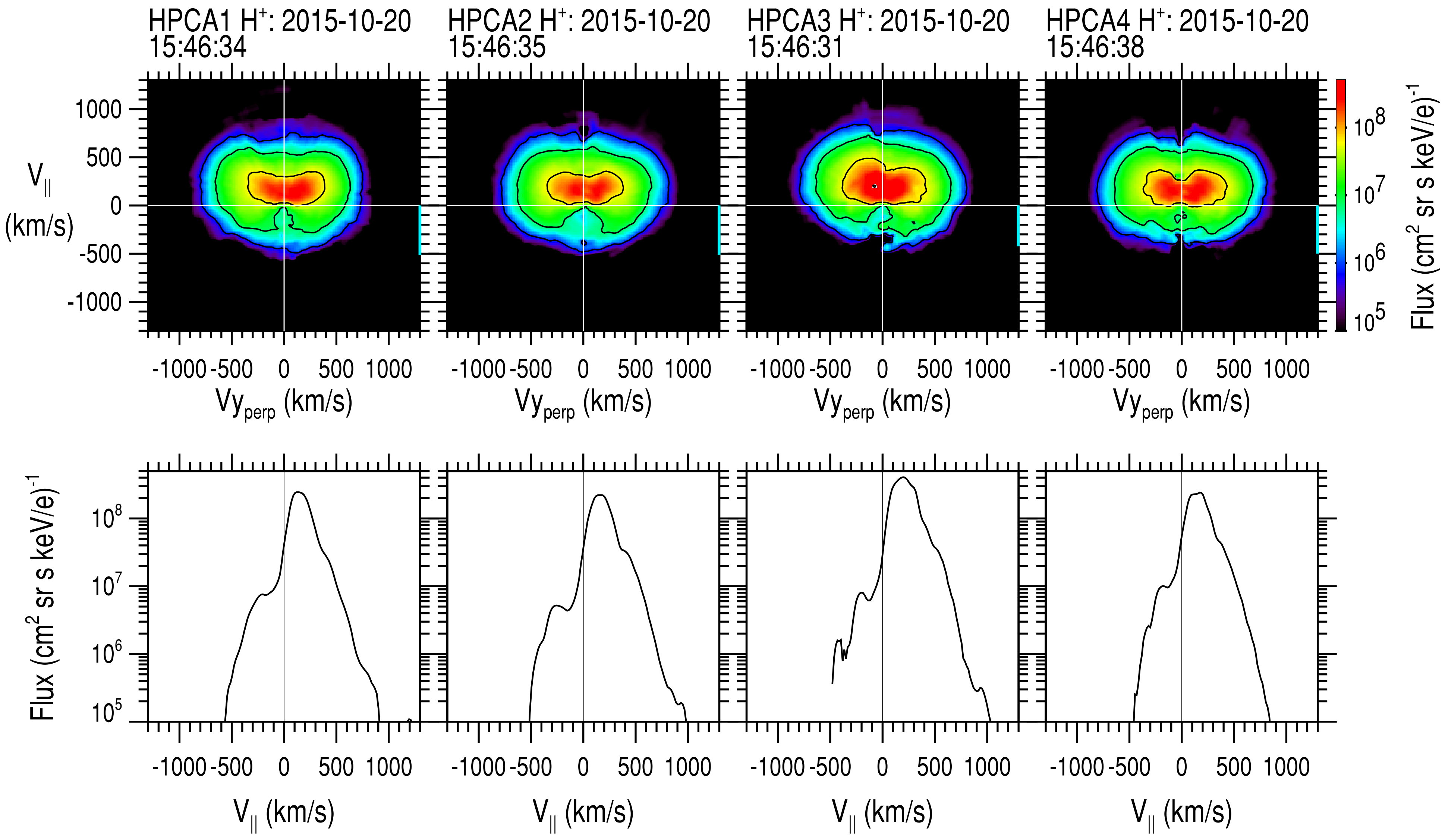
HPCA1 H+: 2015-10-20
15:46:34
HPCA2 H+: 2015-10-20
15:46:35
HPCA3 H+: 2015-10-20
15:46:31
HPCA4 H+: 2015-10-20
15:46:38
V|| (km/s)
Vyperp (km/s)
Flux (cm2 sr s keV/e)-1
V|| (km/s)

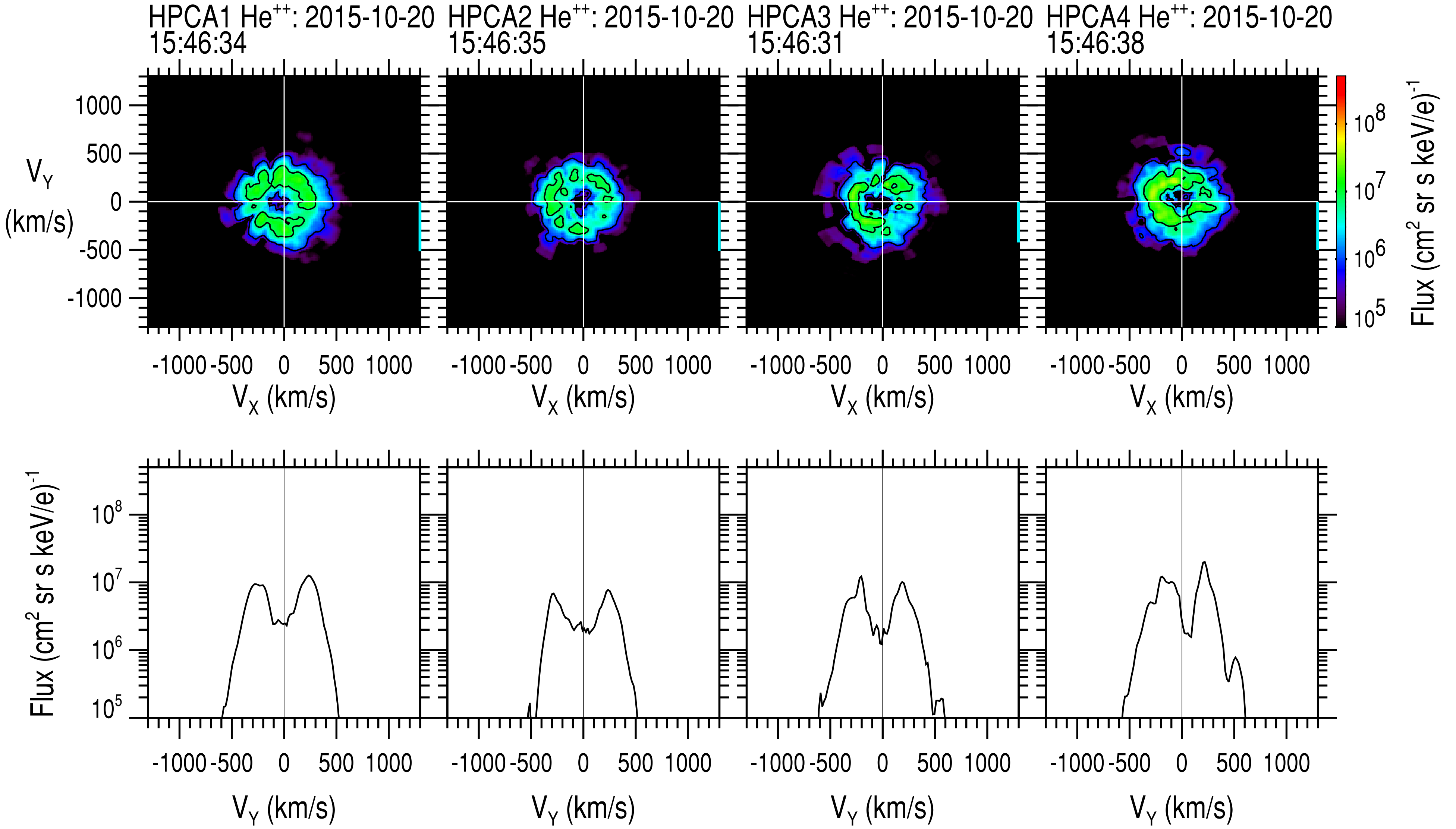
HPCA1 He++: 2015-10-20 15:46:34
HPCA2 He++: 2015-10-20 15:46:35
HPCA3 He++: 2015-10-20 15:46:31
HPCA4 He++: 2015-10-20 15:46:38
VY (km/s)
VX (km/s)
Flux (cm2 sr s keV/e)-1
VY (km/s)

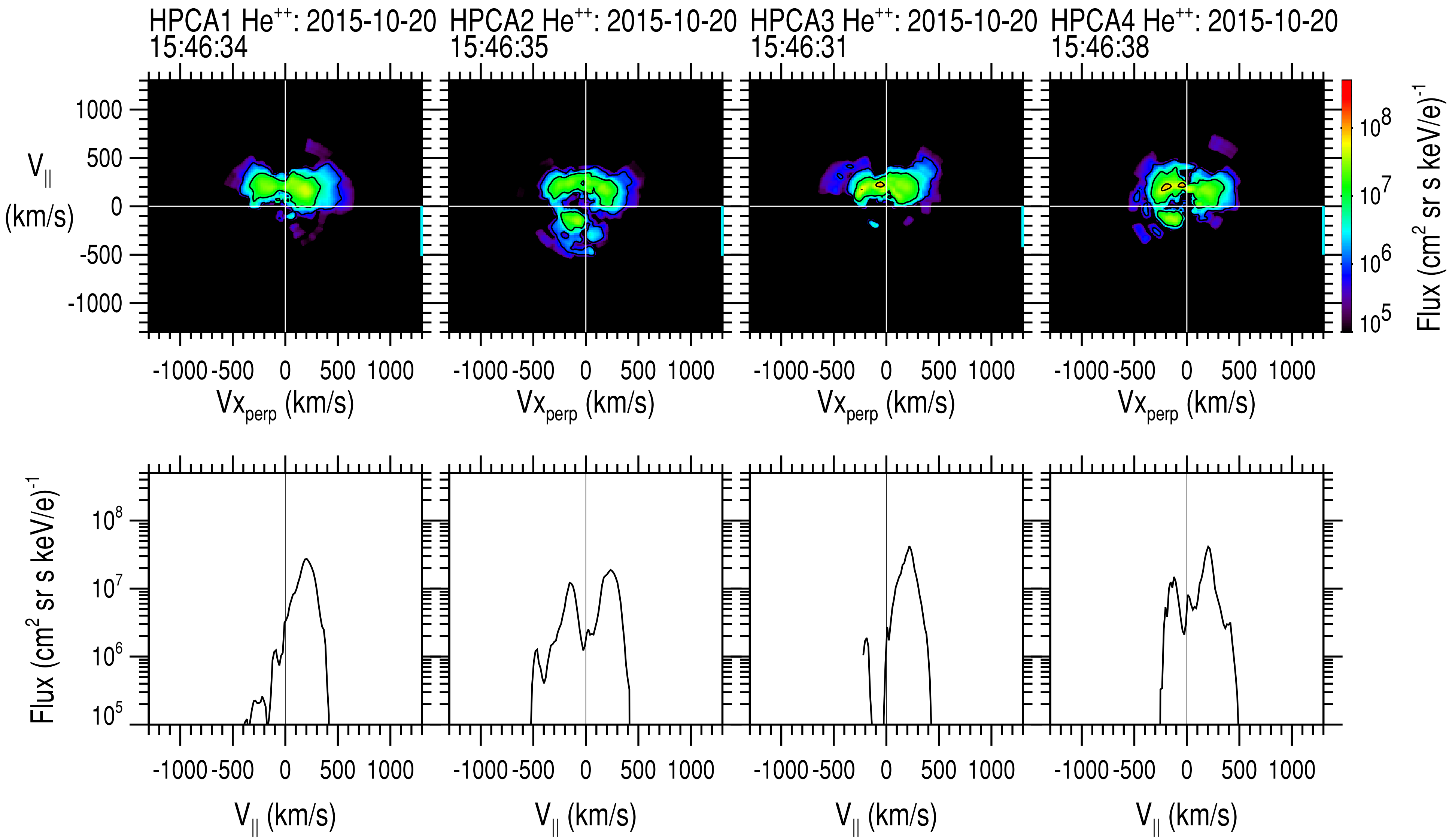

HPCA1 He++: 2015-10-20 15:46:34
HPCA2 He++: 2015-10-20 15:46:35
HPCA3 He++: 2015-10-20 15:46:31
HPCA4 He++: 2015-10-20 15:46:38
V|| (km/s)
Vxperp (km/s)
Flux (cm2 sr s keV/e)-1

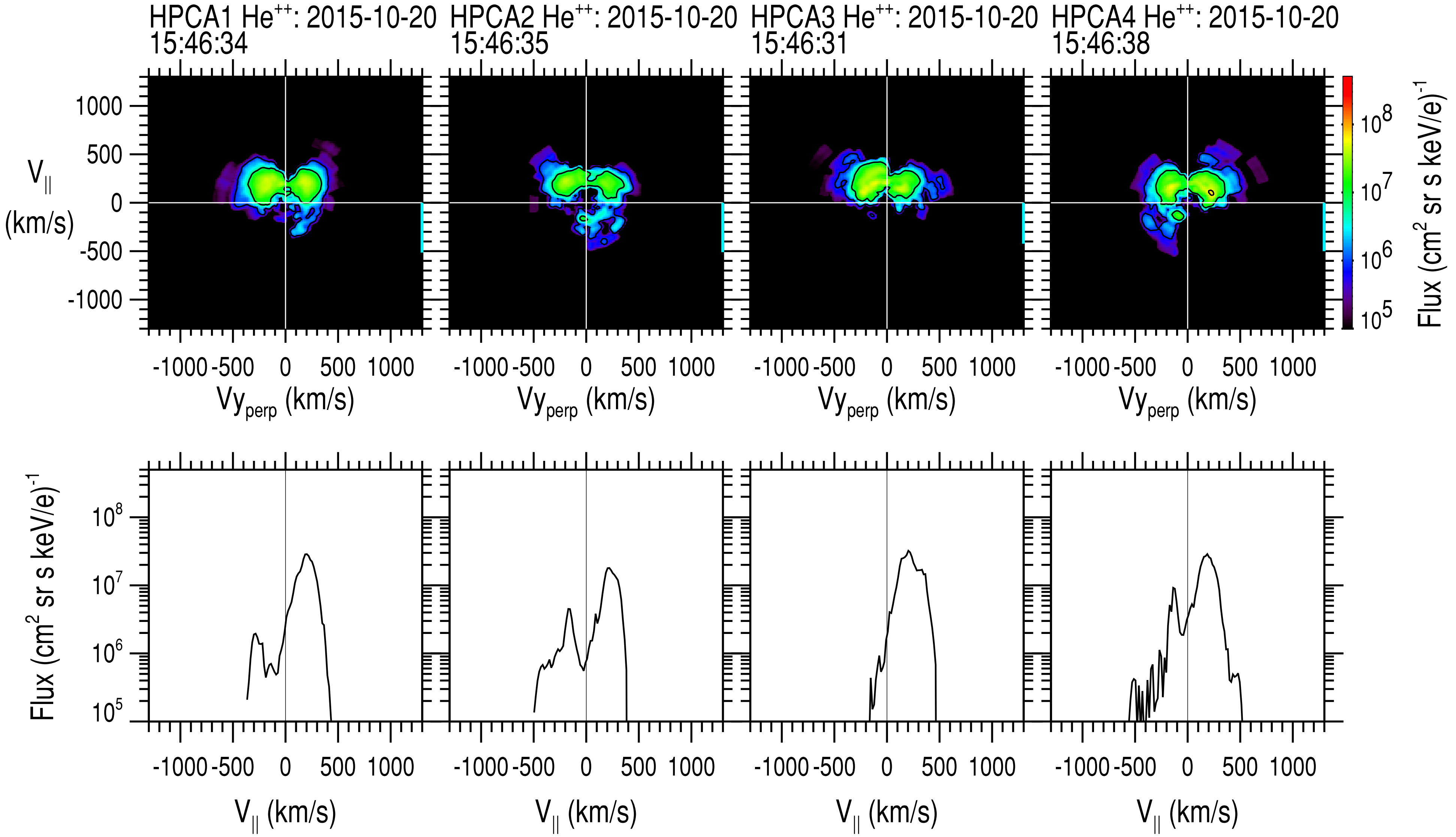
HPCA1 He++: 2015-10-20 15:46:34
HPCA2 He++: 2015-10-20 15:46:35
HPCA3 He++: 2015-10-20 15:46:31
HPCA4 He++: 2015-10-20 15:46:38
V|| (km/s)
Vyperp (km/s)
Flux (cm2 sr s keV/e)-1
V|| (km/s)

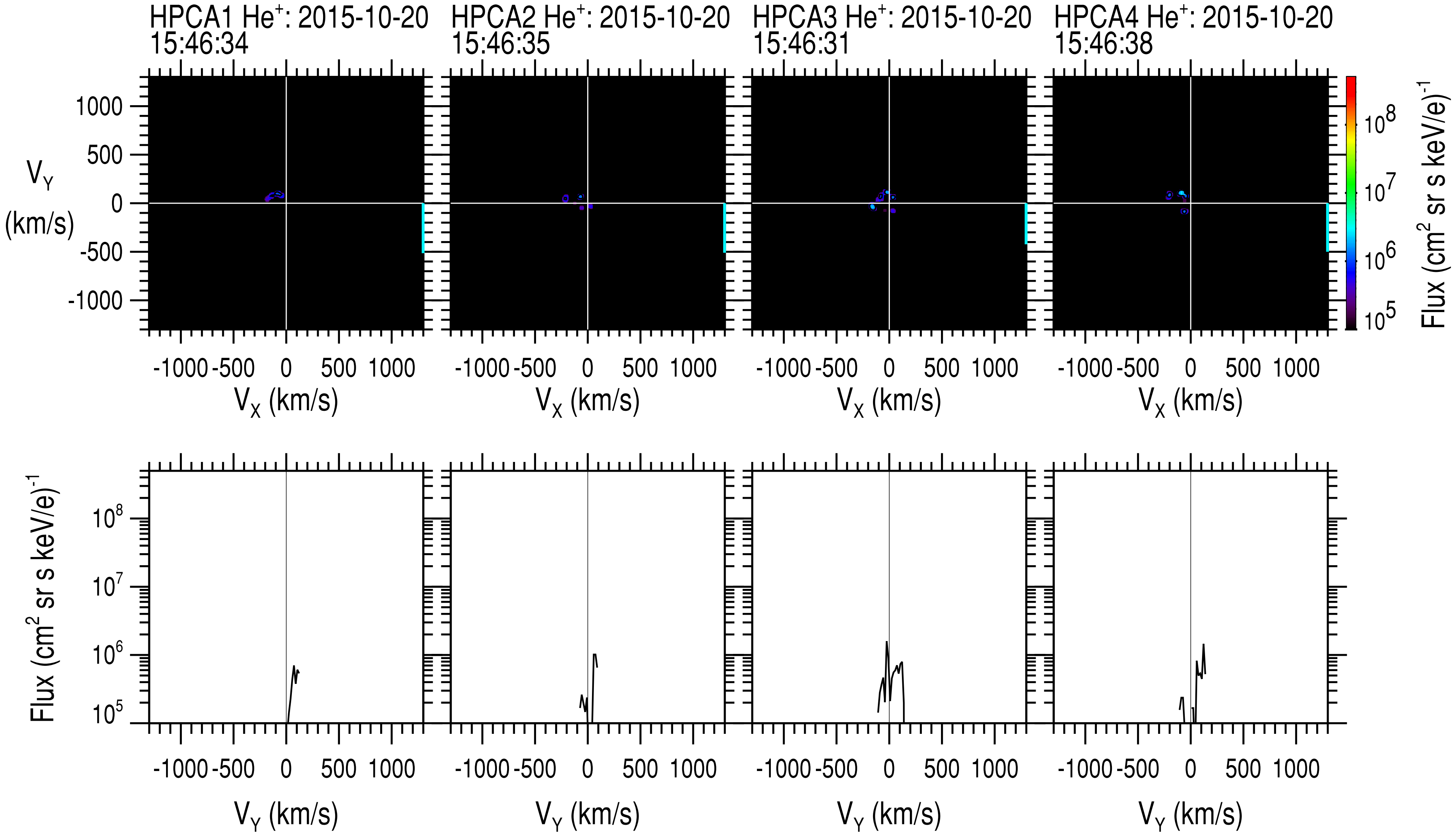
HPCA1 He+: 2015-10-20 15:46:34
HPCA2 He+: 2015-10-20 15:46:35
HPCA3 He+: 2015-10-20 15:46:31
HPCA4 He+: 2015-10-20 15:46:38
VY (km/s)
VX (km/s)
Flux (cm2 sr s keV/e)-1
VY (km/s)

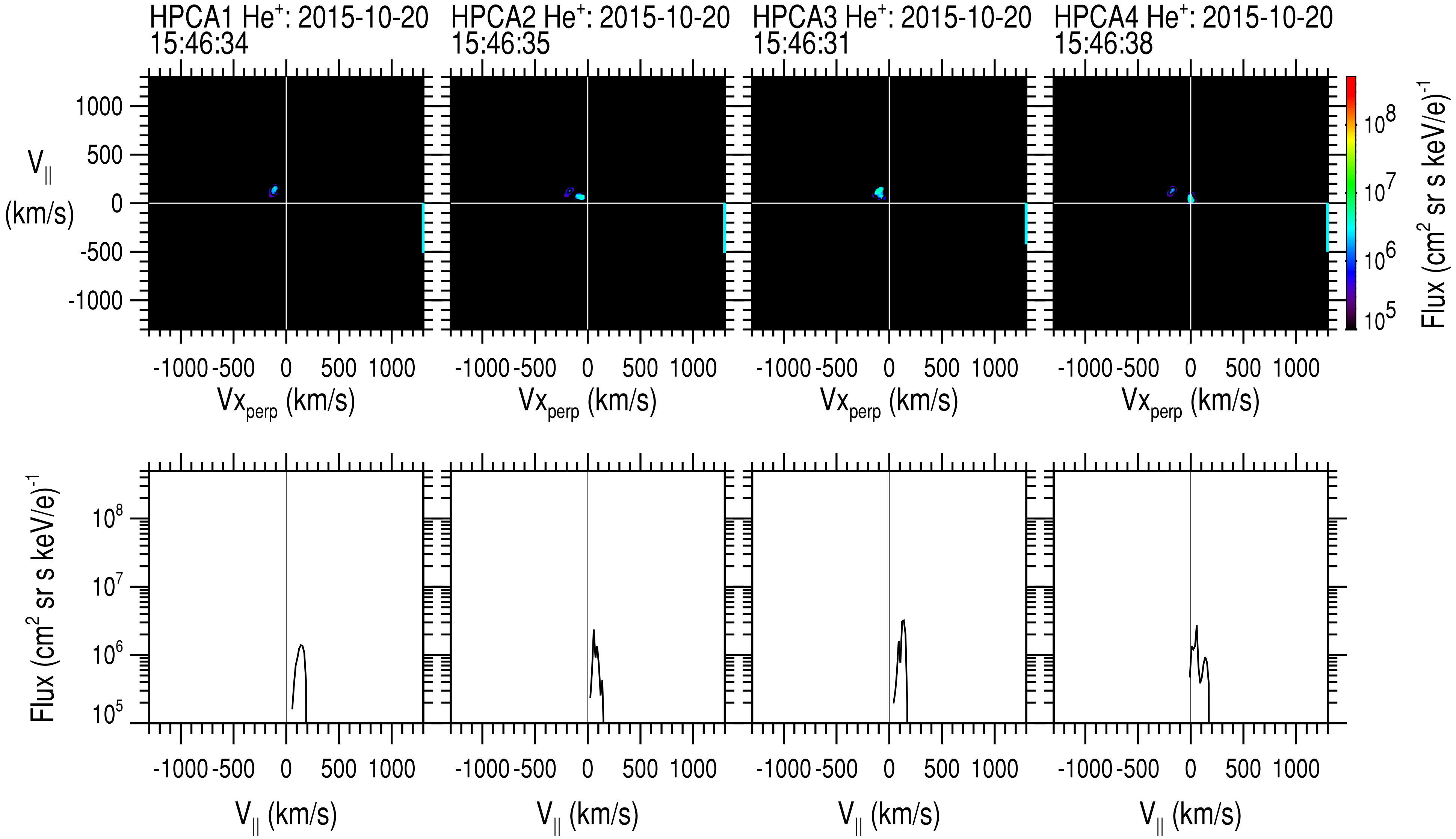
HPCA1 He+: 2015-10-20 15:46:34
HPCA2 He+: 2015-10-20 15:46:35
HPCA3 He+: 2015-10-20 15:46:31
HPCA4 He+: 2015-10-20 15:46:38
V|| (km/s)
Vxperp (km/s)
Flux (cm2 sr s keV/e)-1
V|| (km/s)

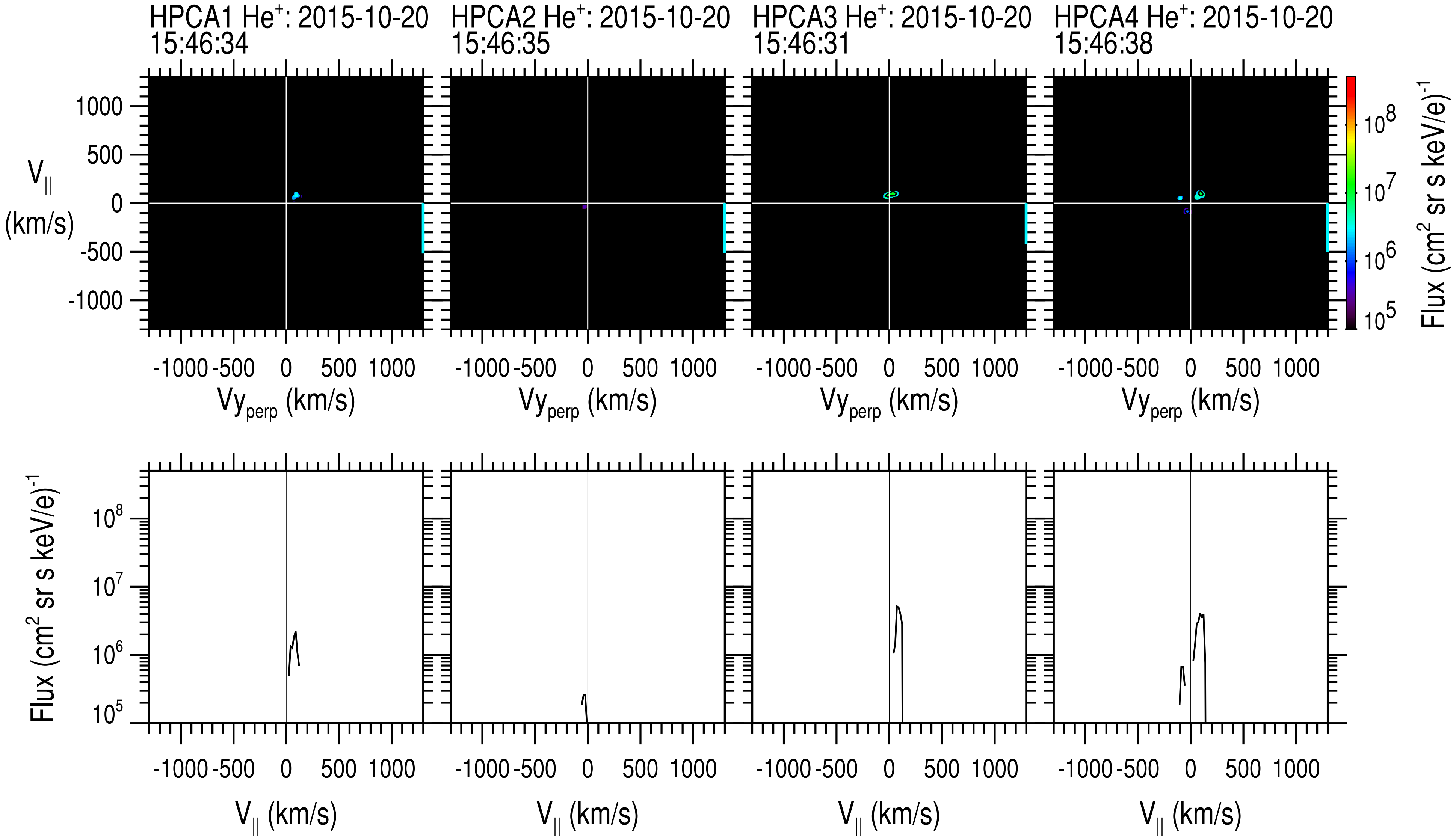

HPCA1 He+: 2015-10-20
15:46:34
HPCA2 He+: 2015-10-20
15:46:35
HPCA3 He+: 2015-10-20
15:46:31
HPCA4 He+: 2015-10-20
15:46:38
V|| (km/s)
Vyperp (km/s)
Flux (cm2 sr s keV/e)-1
-1000 -500 0 500 1000
10^5 10^6 10^7 10^8

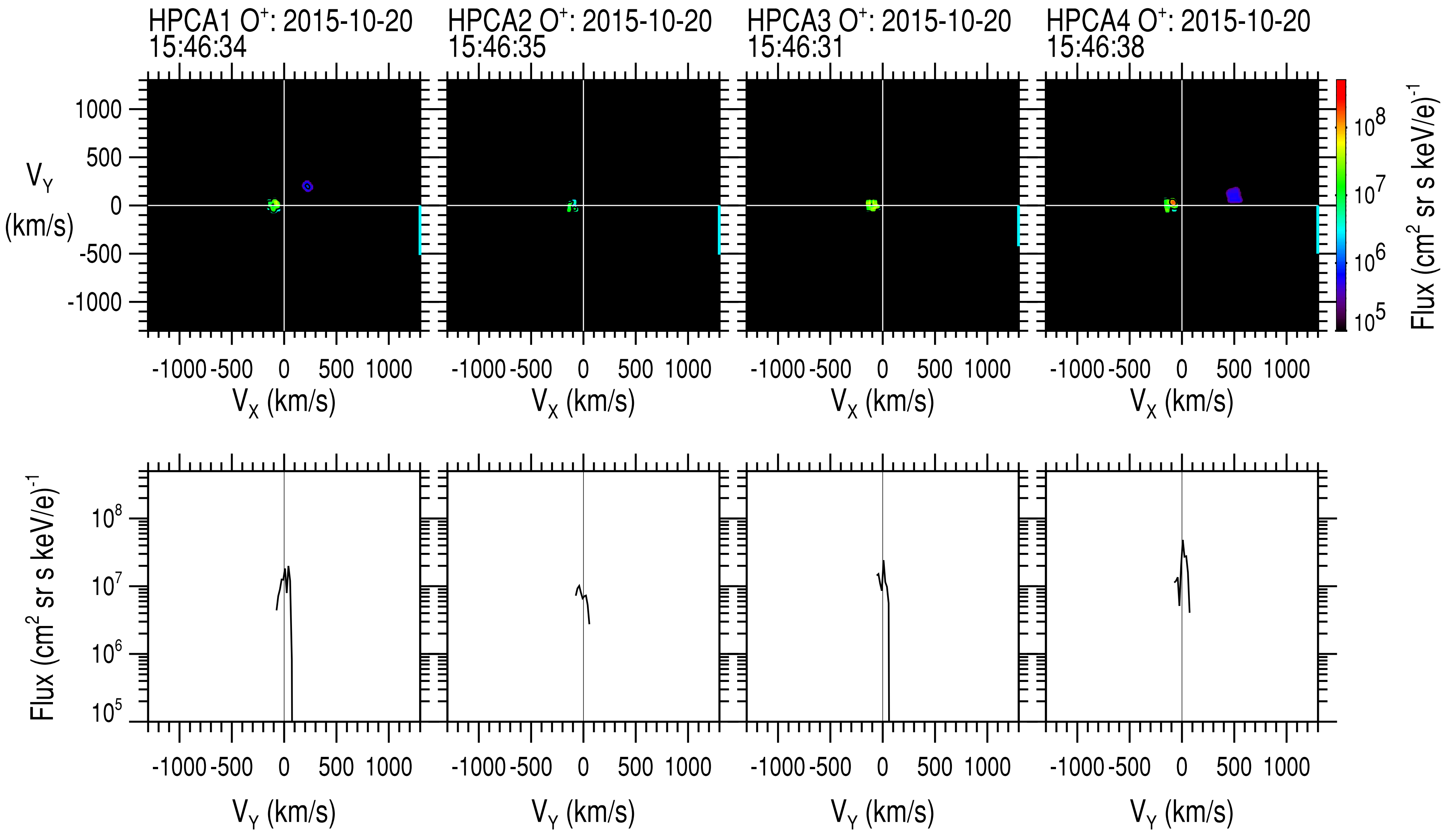
HPCA1 O+: 2015-10-20
15:46:34
HPCA2 O+: 2015-10-20
15:46:35
HPCA3 O+: 2015-10-20
15:46:31
HPCA4 O+: 2015-10-20
15:46:38
VY (km/s)
VX (km/s)
Flux (cm2 sr s keV/e)-1
-1000 -500 0 500 1000
10^5 10^6 10^7 10^8

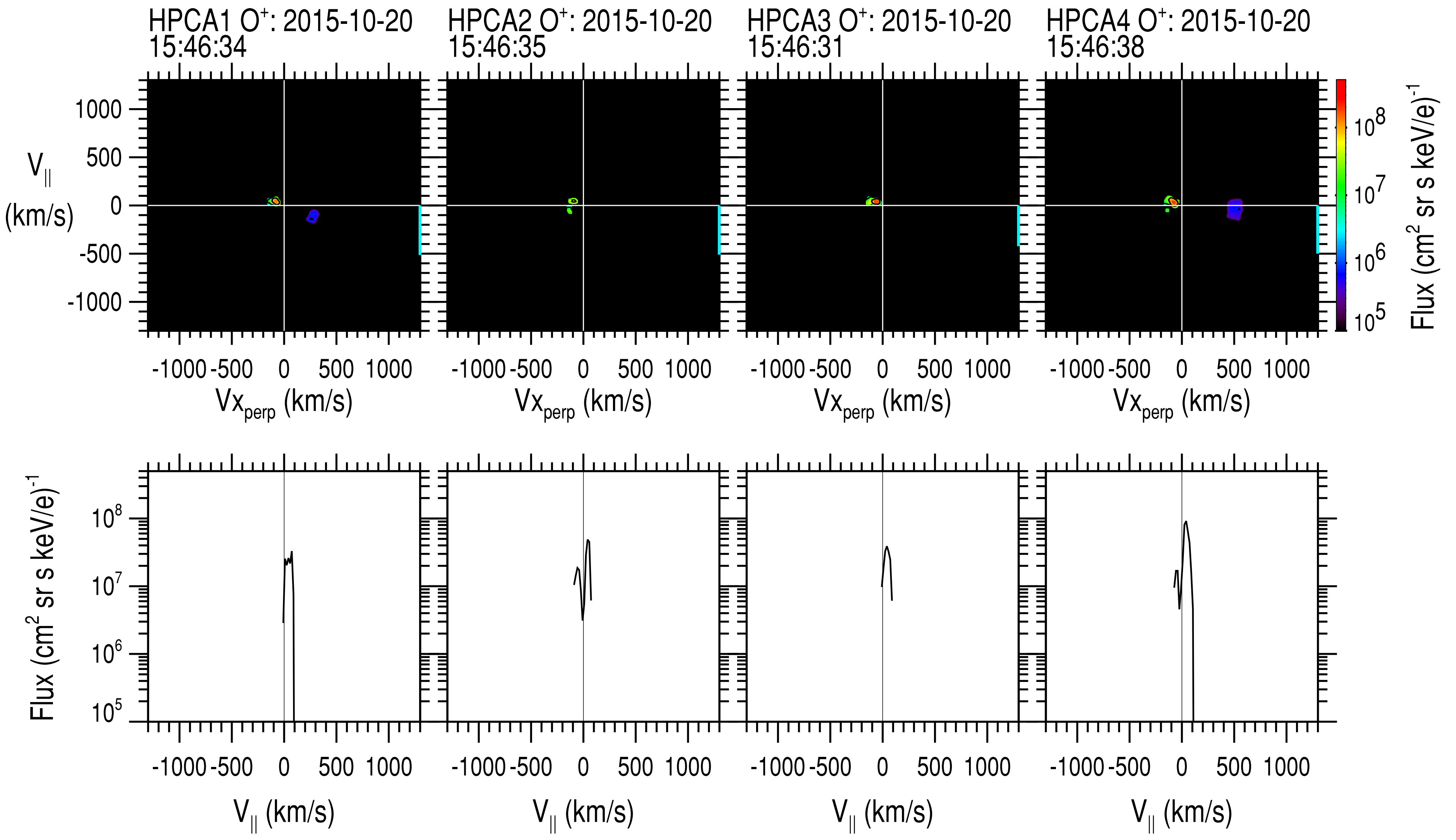

HPCA1 O+: 2015-10-20 15:46:34
HPCA2 O+: 2015-10-20 15:46:35
HPCA3 O+: 2015-10-20 15:46:31
HPCA4 O+: 2015-10-20 15:46:38
V|| (km/s)
Vx_perp (km/s)
Flux (cm2 sr s keV/e)-1
V|| (km/s)

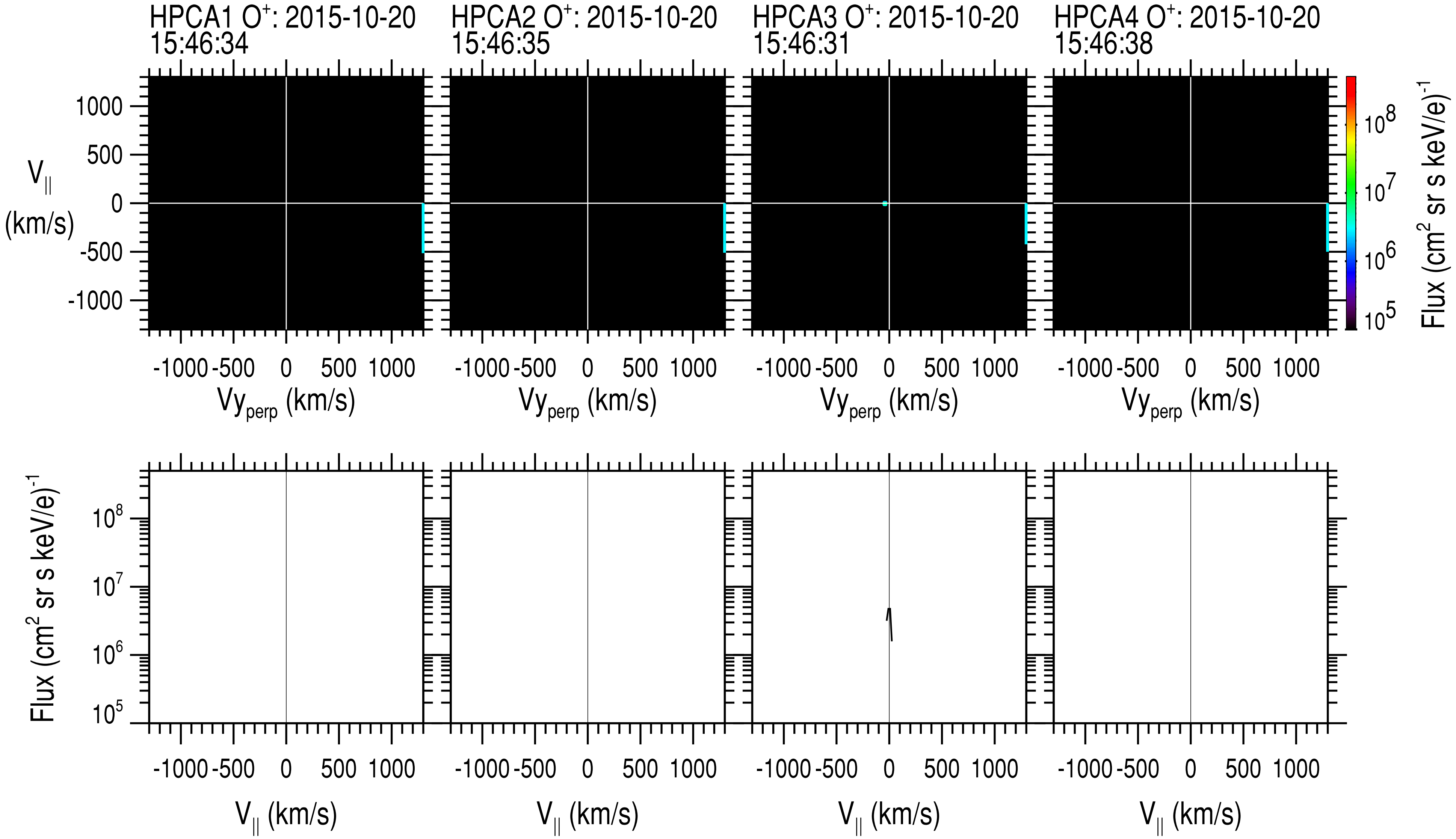
HPCA1 O+: 2015-10-20 15:46:34
HPCA2 O+: 2015-10-20 15:46:35
HPCA3 O+: 2015-10-20 15:46:31
HPCA4 O+: 2015-10-20 15:46:38
V|| (km/s)
Vyperp (km/s)
Flux (cm2 sr s keV/e)-1
1000
500
0
-500
-1000
105
106
107
108

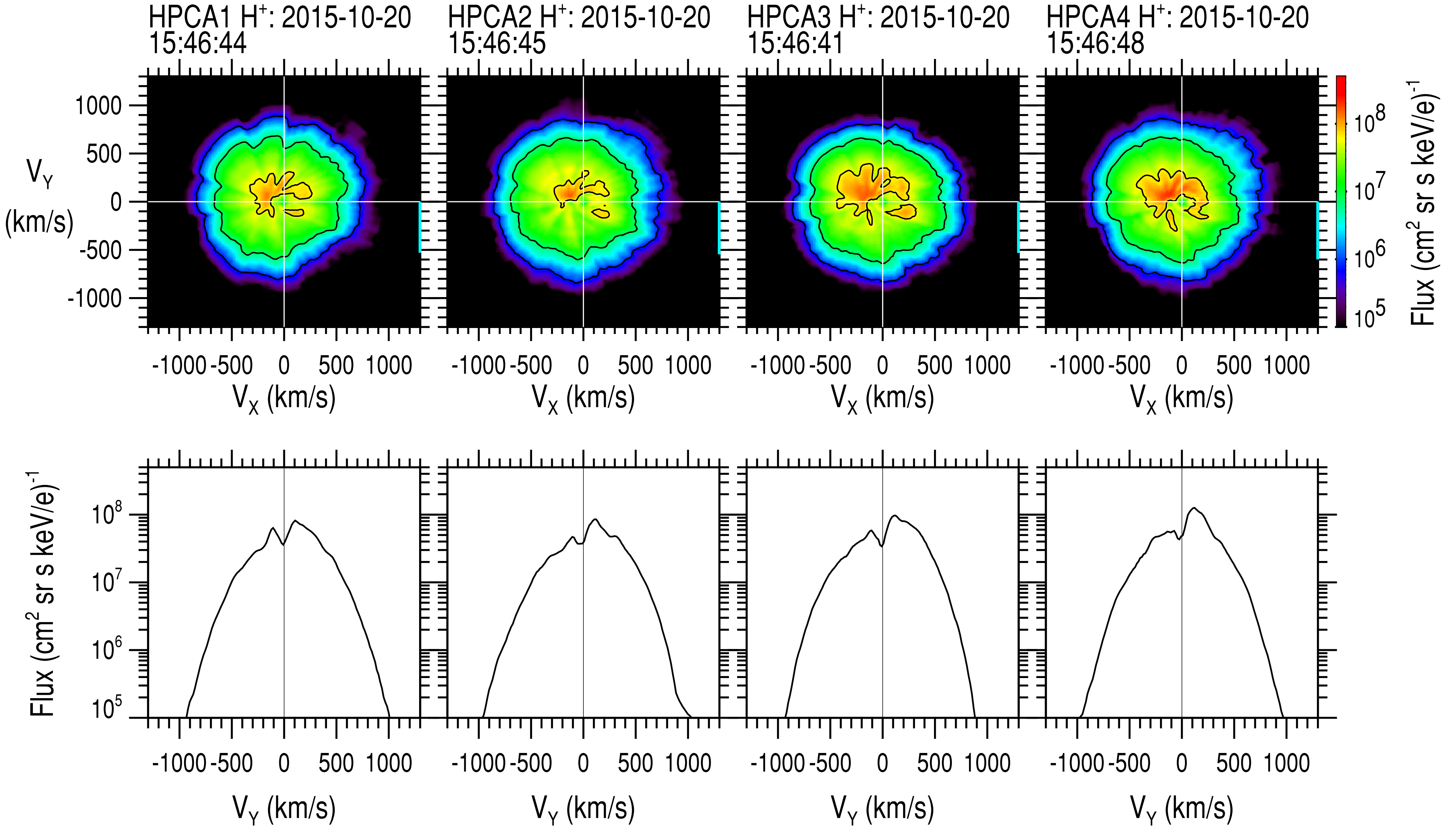
HPCA1 H+: 2015-10-20
15:46:44
HPCA2 H+: 2015-10-20
15:46:45
HPCA3 H+: 2015-10-20
15:46:41
HPCA4 H+: 2015-10-20
15:46:48
VY (km/s)
VX (km/s)
Flux (cm2 sr s keV/e)-1
VY (km/s)

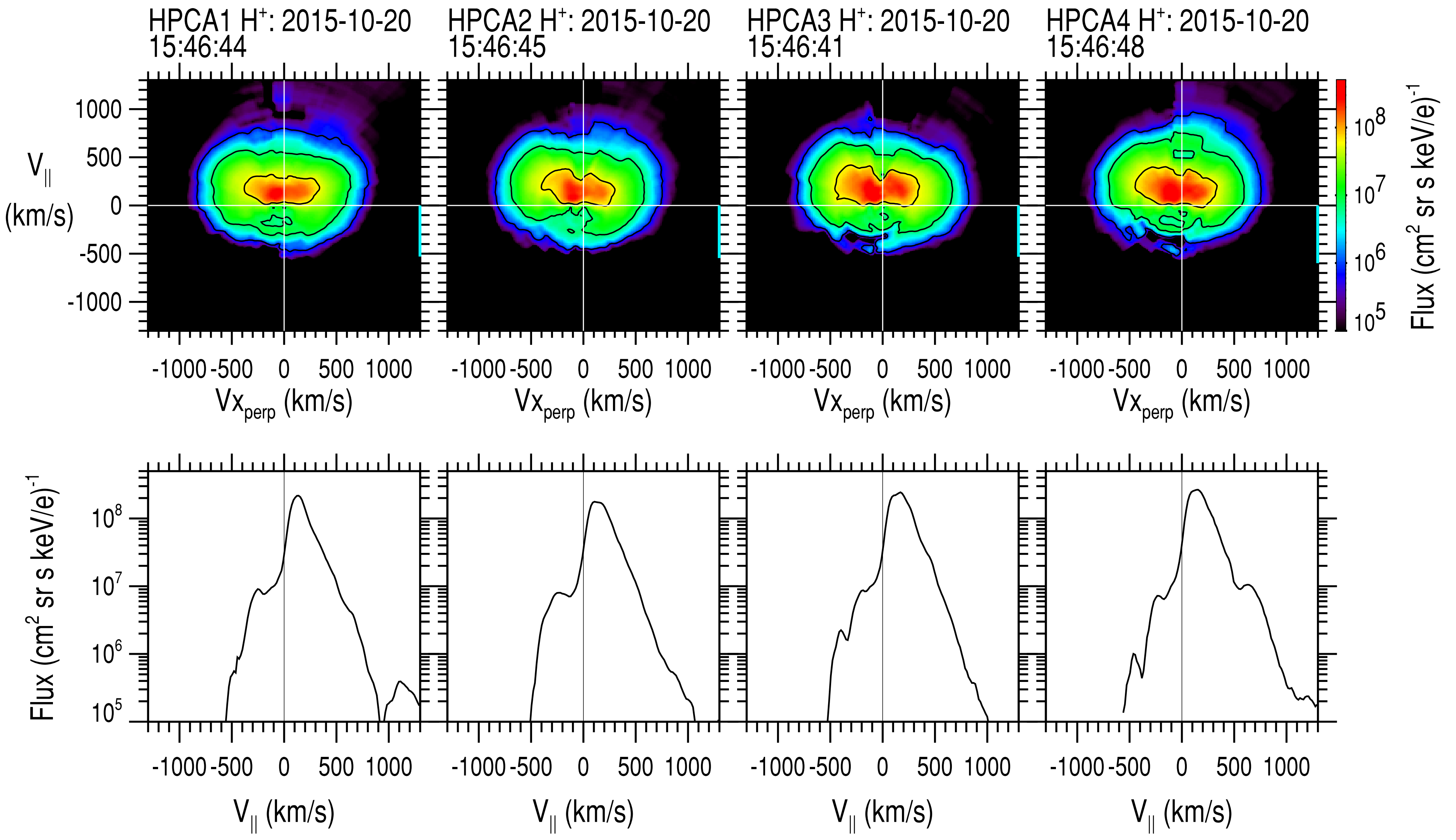
HPCA1 H+: 2015-10-20 15:46:44
HPCA2 H+: 2015-10-20 15:46:45
HPCA3 H+: 2015-10-20 15:46:41
HPCA4 H+: 2015-10-20 15:46:48
V|| (km/s)
Vxperp (km/s)
Flux (cm2 sr s keV/e)-1
V|| (km/s)

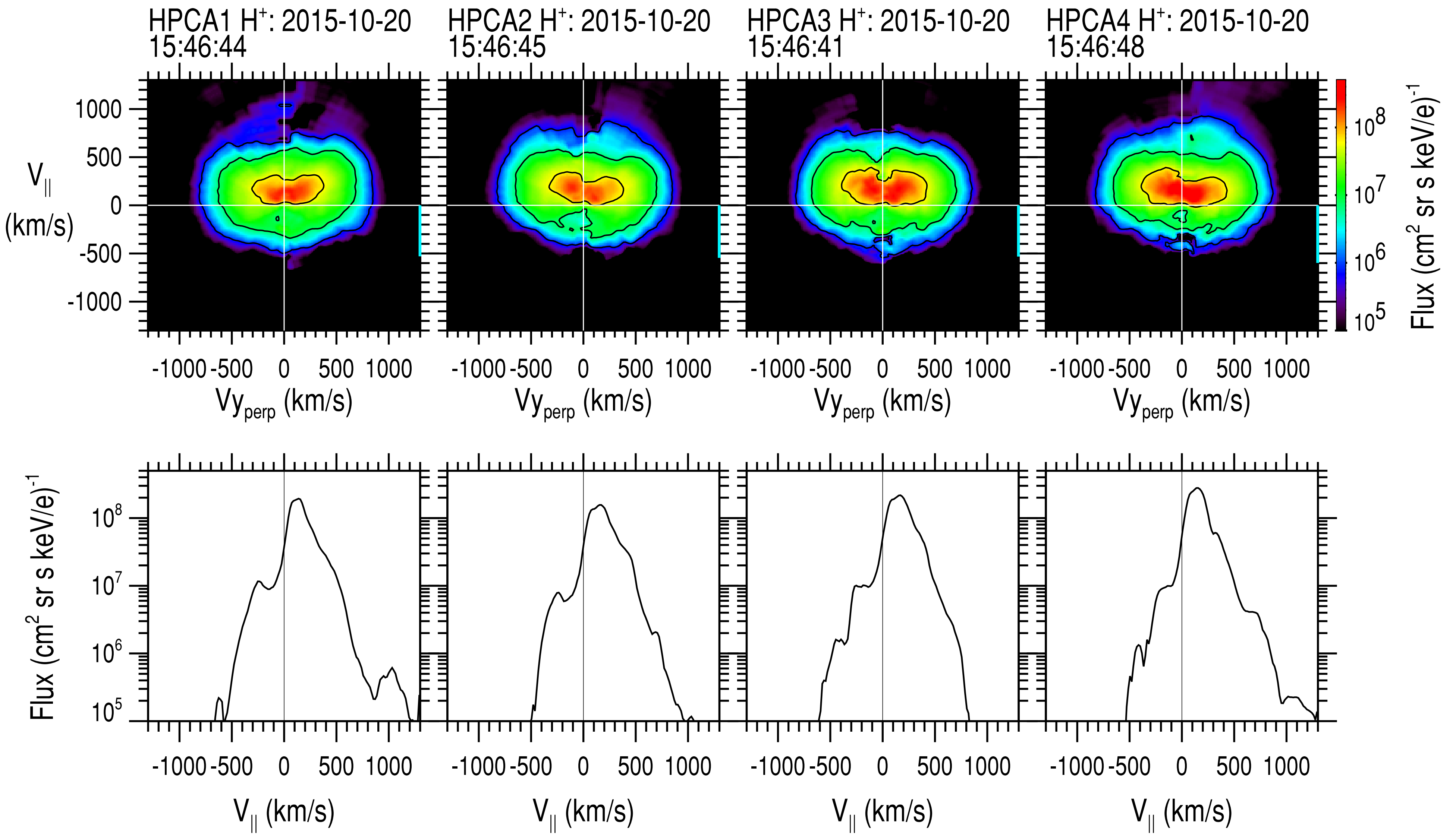
HPCA1 H+: 2015-10-20
15:46:44
HPCA2 H+: 2015-10-20
15:46:45
HPCA3 H+: 2015-10-20
15:46:41
HPCA4 H+: 2015-10-20
15:46:48
V|| (km/s)
Vyperp (km/s)
Flux (cm2 sr s keV/e)-1
V|| (km/s)

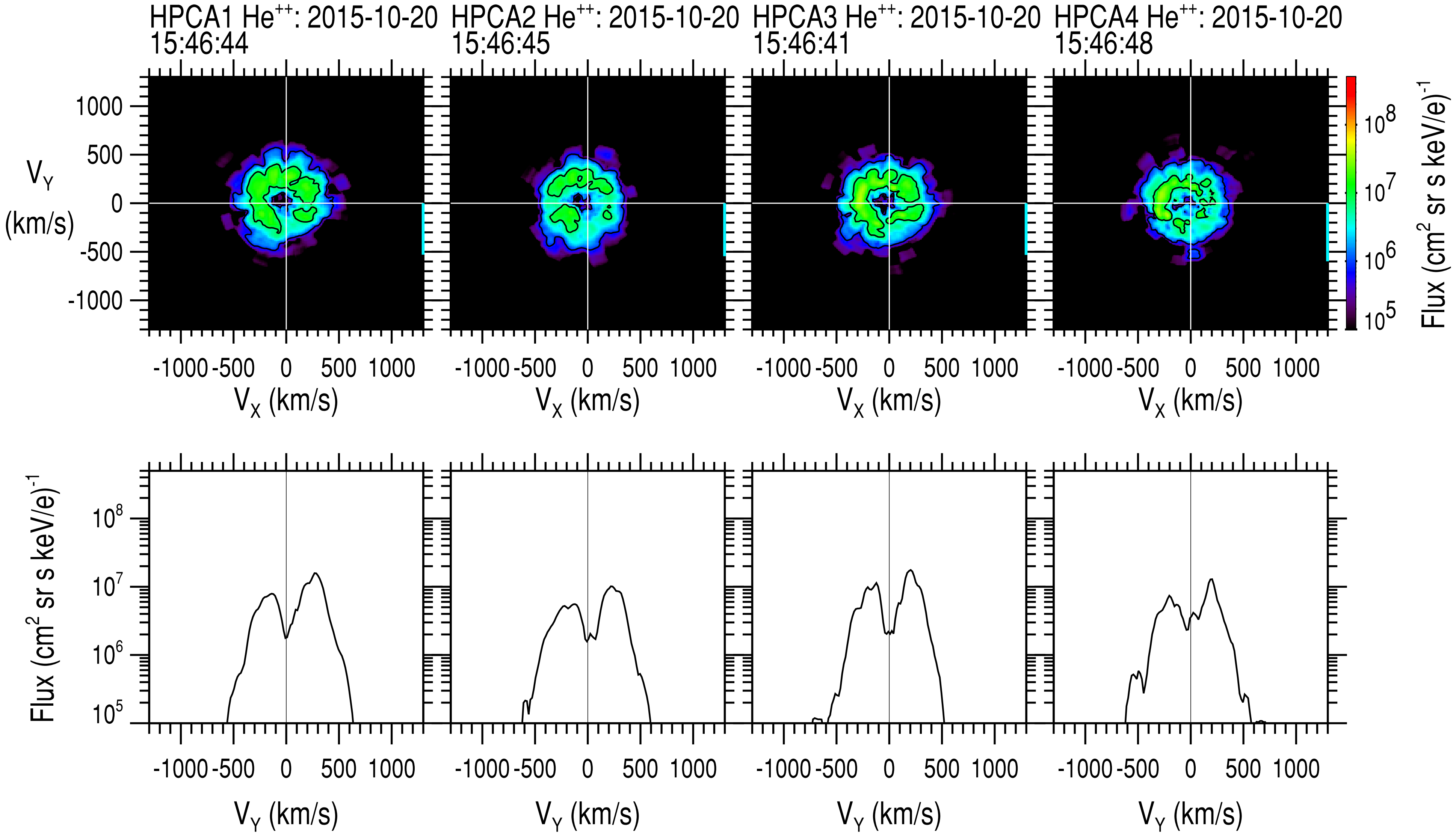
HPCA1 He++: 2015-10-20
15:46:44
HPCA2 He++: 2015-10-20
15:46:45
HPCA3 He++: 2015-10-20
15:46:41
HPCA4 He++: 2015-10-20
15:46:48
VY (km/s)
VX (km/s)
Flux (cm2 sr s keV/e)-1
-1000 -500 0 500 1000
105 106 107 108

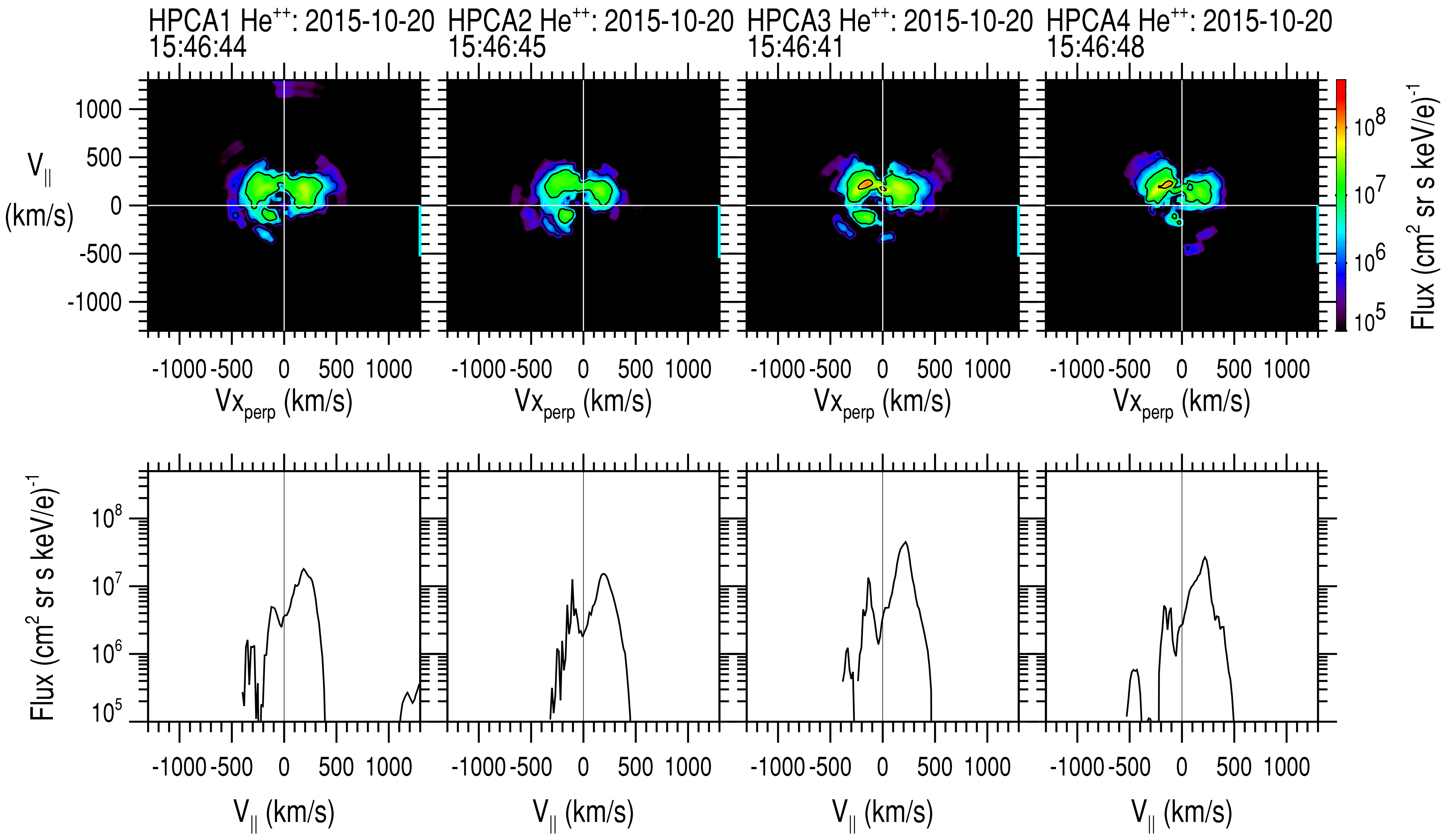
HPCA1 He++: 2015-10-20 15:46:44
HPCA2 He++: 2015-10-20 15:46:45
HPCA3 He++: 2015-10-20 15:46:41
HPCA4 He++: 2015-10-20 15:46:48
V|| (km/s)
Vxperp (km/s)
Flux (cm2 sr s keV/e)-1

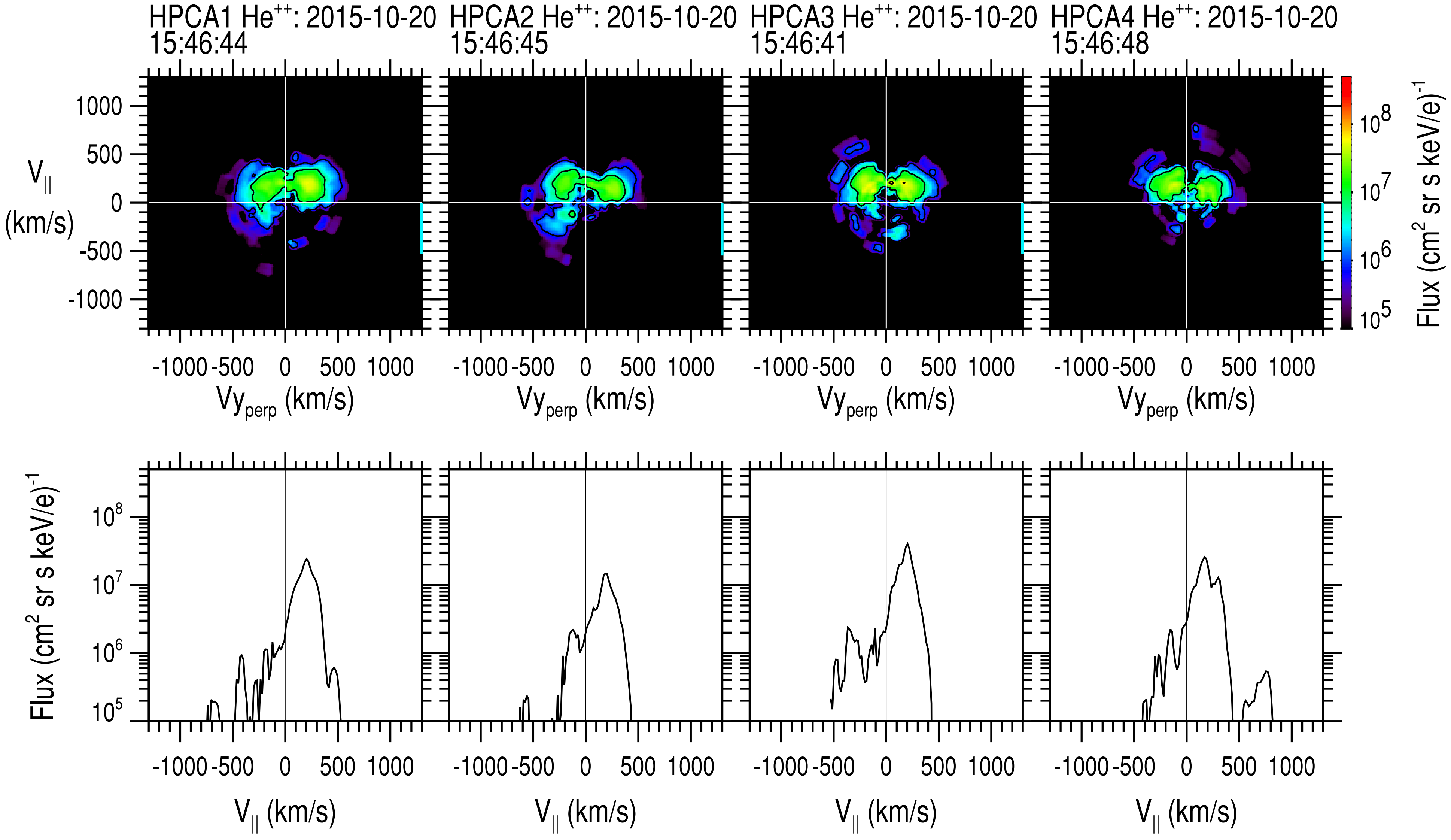

HPCA1 He++: 2015-10-20 15:46:44
HPCA2 He++: 2015-10-20 15:46:45
HPCA3 He++: 2015-10-20 15:46:41
HPCA4 He++: 2015-10-20 15:46:48
V|| (km/s)
Vyperp (km/s)
Flux (cm2 sr s keV/e)-1

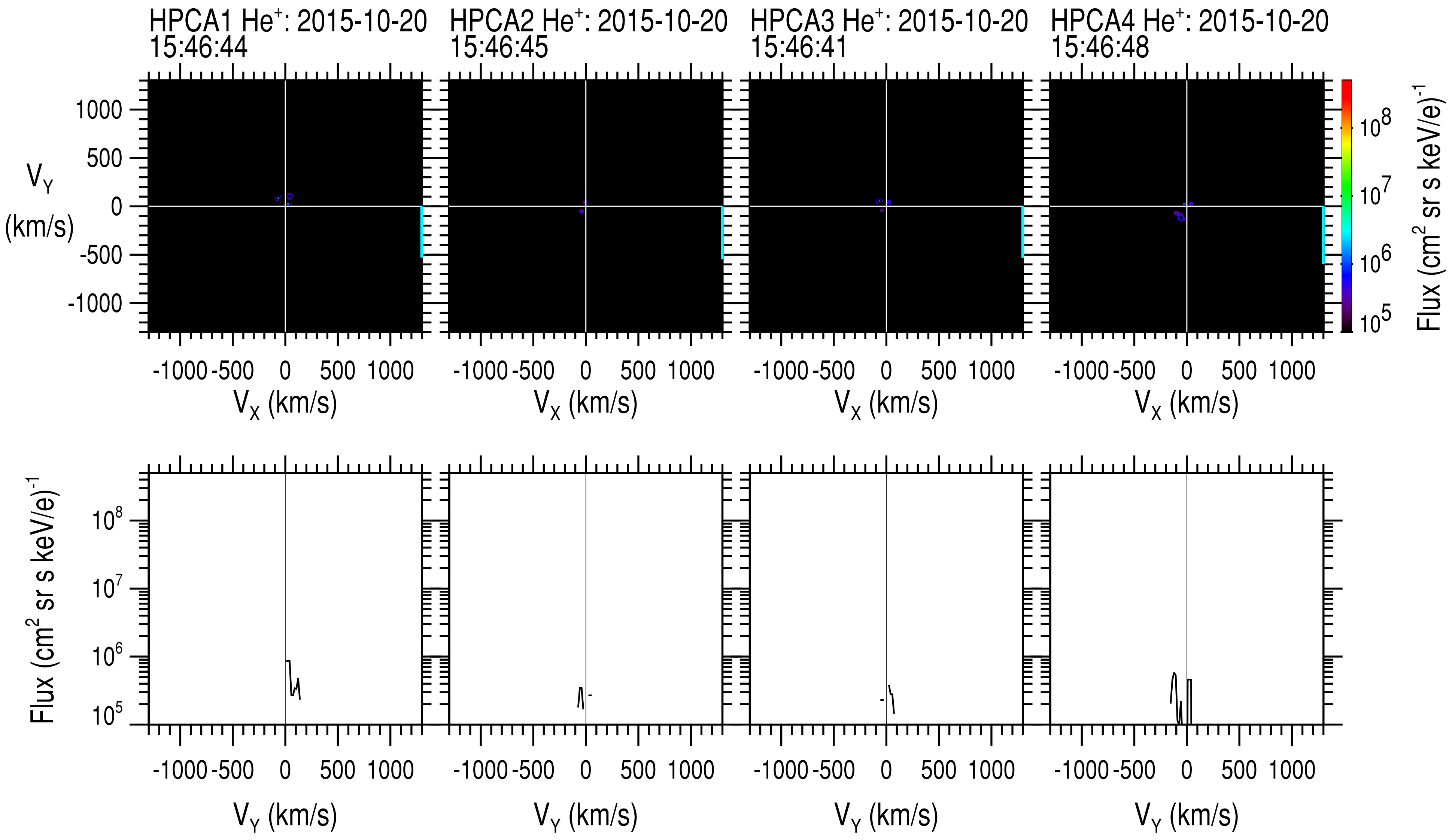
HPCA1 He+: 2015-10-20 15:46:44
HPCA2 He+: 2015-10-20 15:46:45
HPCA3 He+: 2015-10-20 15:46:41
HPCA4 He+: 2015-10-20 15:46:48
VY (km/s)
VX (km/s)
Flux (cm2 sr s keV/e)-1
VY (km/s)

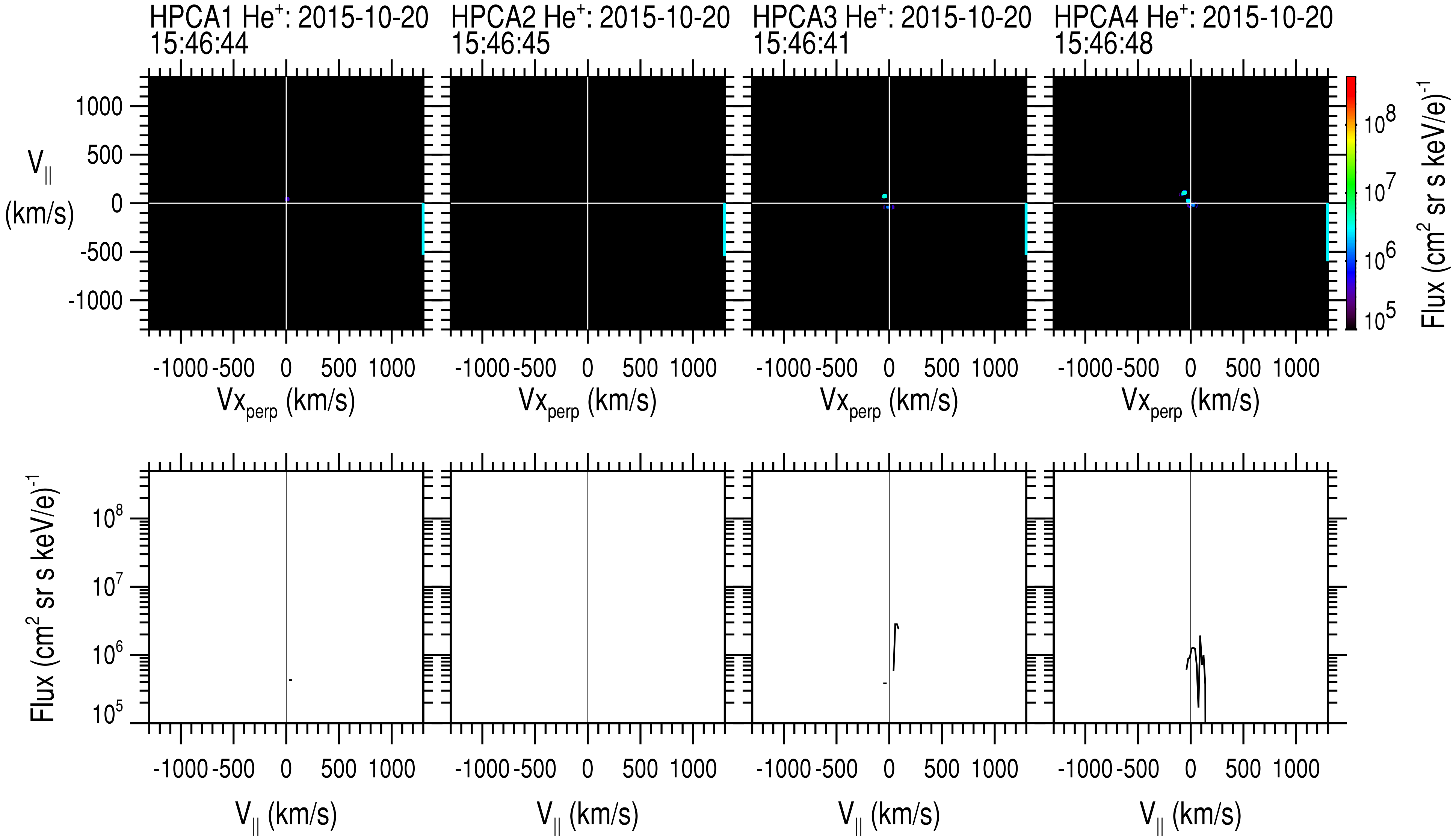
HPCA1 He+: 2015-10-20 15:46:44
HPCA2 He+: 2015-10-20 15:46:45
HPCA3 He+: 2015-10-20 15:46:41
HPCA4 He+: 2015-10-20 15:46:48
V|| (km/s)
Vxperp (km/s)
Flux (cm2 sr s keV/e)-1
-1000 -500 0 500 1000
10^5 10^6 10^7 10^8

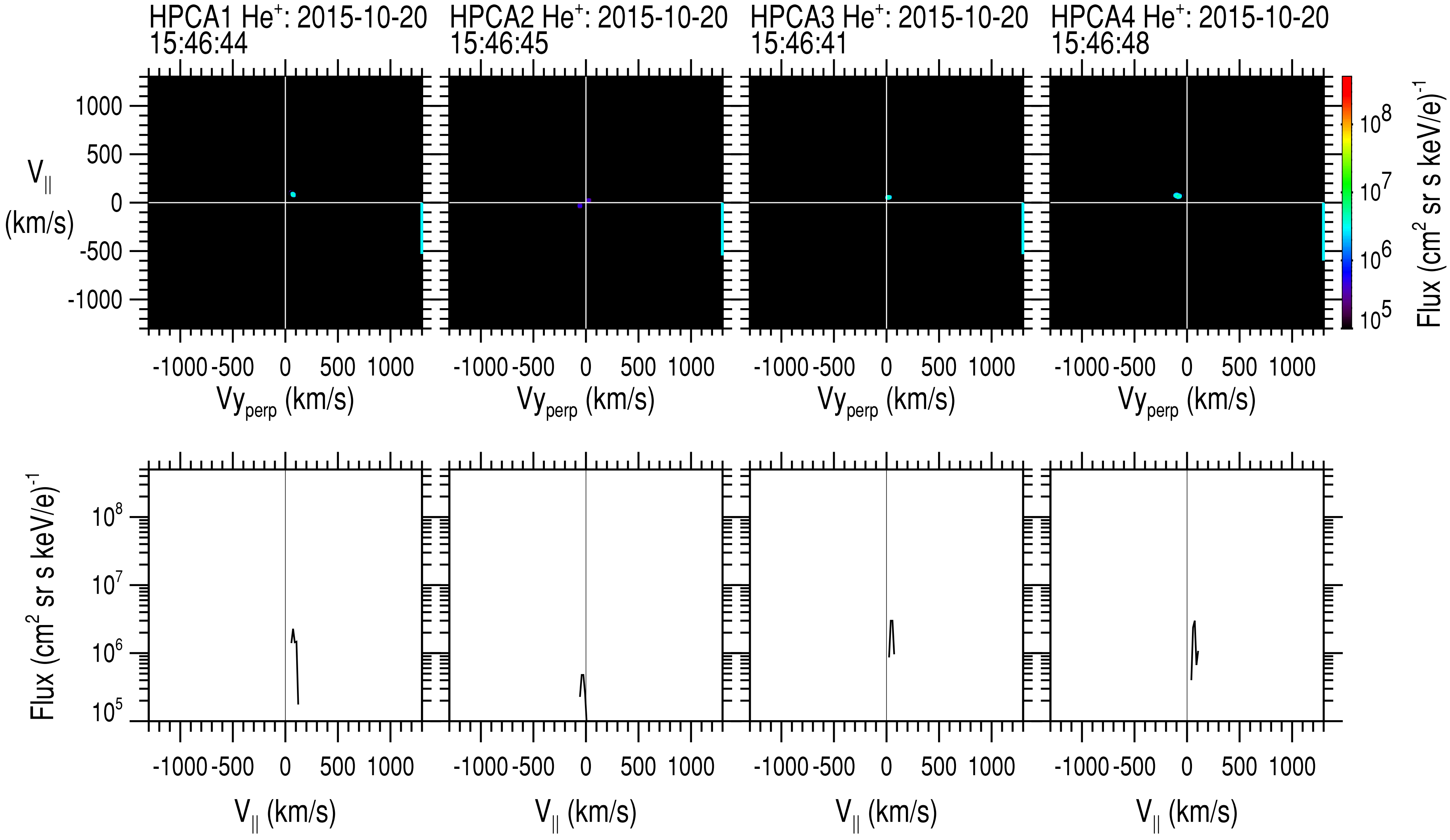
HPCA1 He+: 2015-10-20 15:46:44
HPCA2 He+: 2015-10-20 15:46:45
HPCA3 He+: 2015-10-20 15:46:41
HPCA4 He+: 2015-10-20 15:46:48
V|| (km/s)
Vyperp (km/s)
Flux (cm2 sr s keV/e)-1
-1000 -500 0 500 1000
10^5 10^6 10^7 10^8

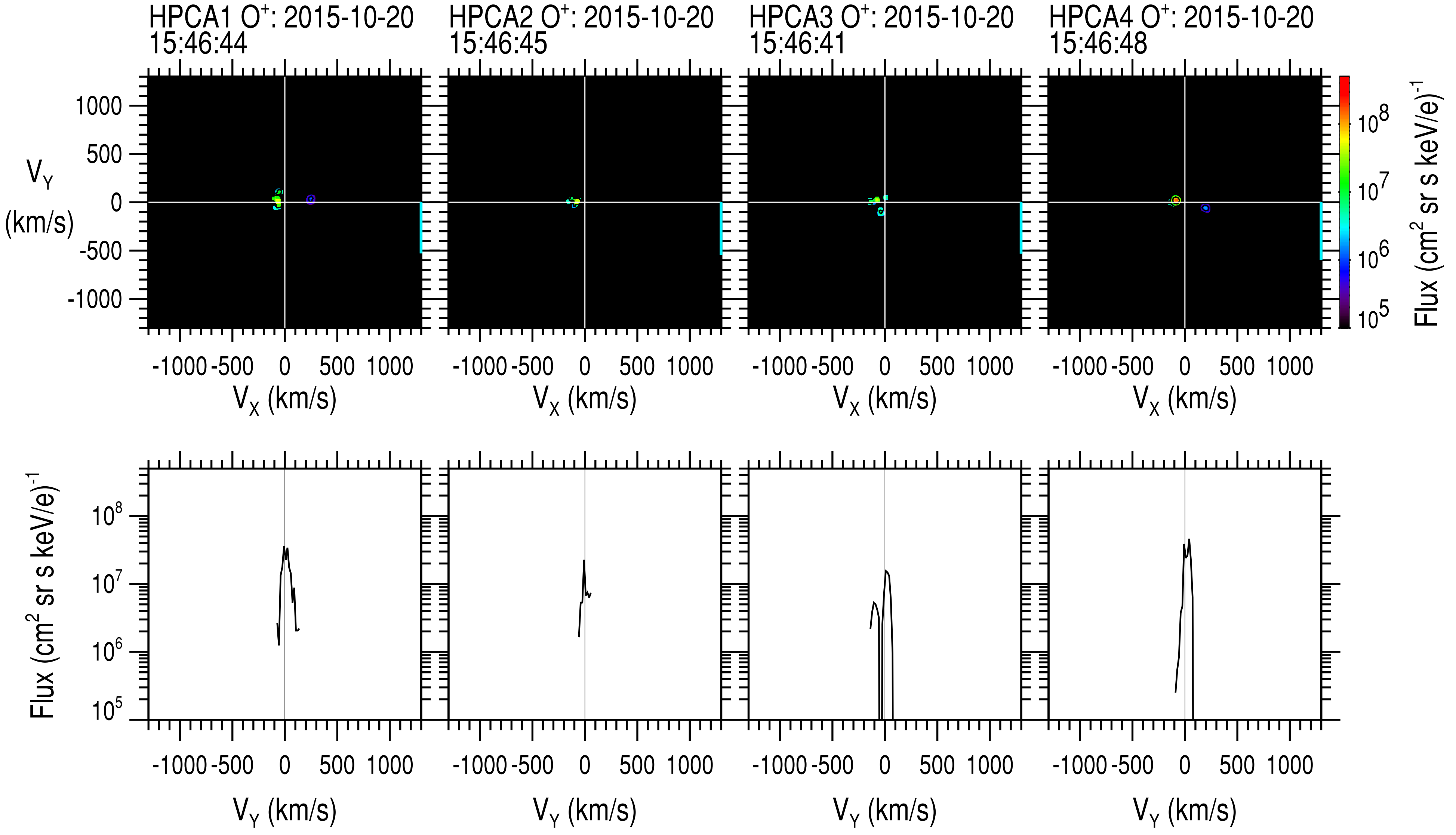

HPCA1 O+: 2015-10-20
15:46:44
HPCA2 O+: 2015-10-20
15:46:45
HPCA3 O+: 2015-10-20
15:46:41
HPCA4 O+: 2015-10-20
15:46:48
V_Y (km/s)
V_X (km/s)
Flux (cm² sr s keV/e)⁻¹
V_Y (km/s)
1000
500
0
-500
-1000
10⁵
10⁶
10⁷
10⁸

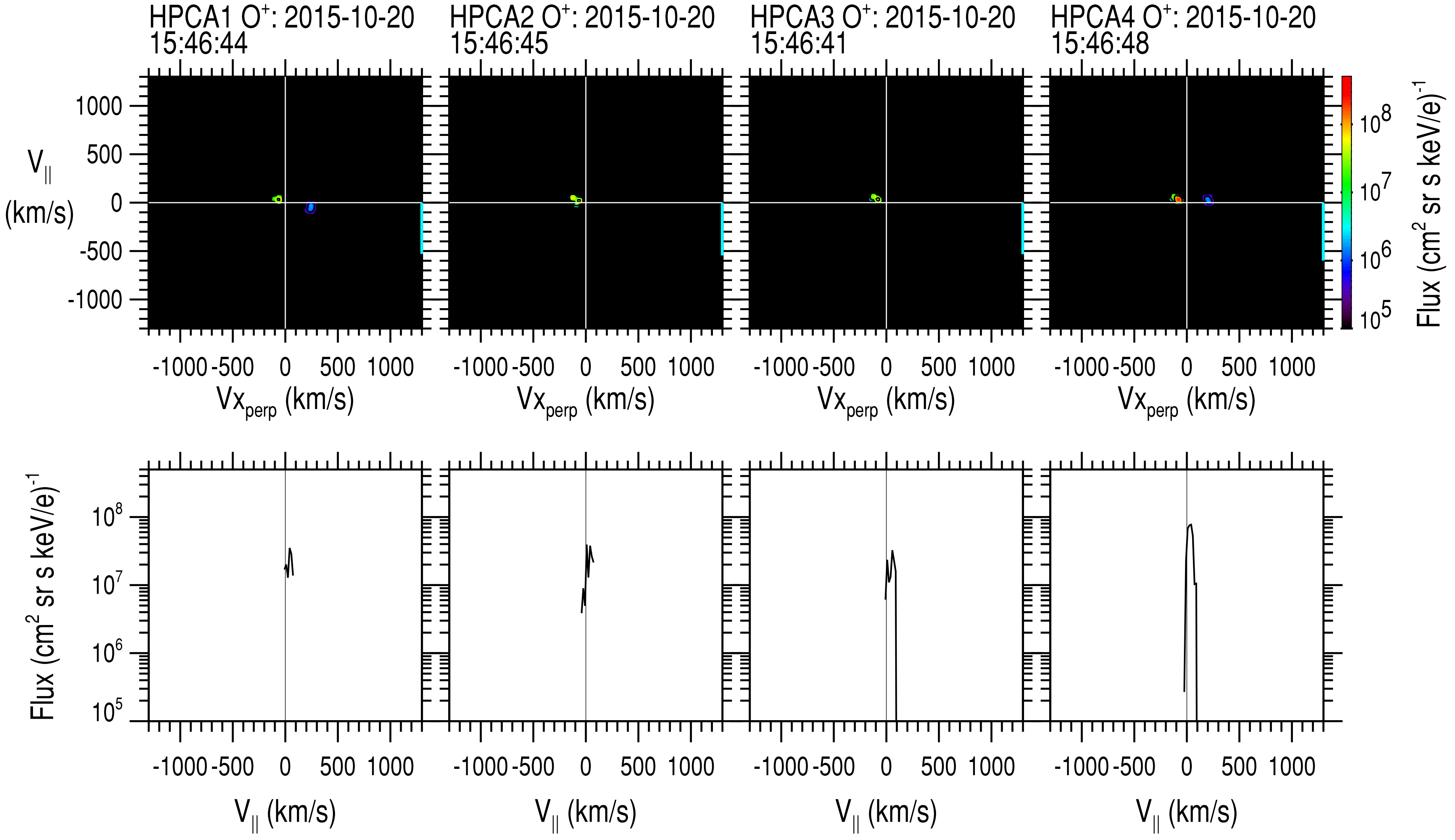

HPCA1 O+: 2015-10-20 15:46:44
HPCA2 O+: 2015-10-20 15:46:45
HPCA3 O+: 2015-10-20 15:46:41
HPCA4 O+: 2015-10-20 15:46:48
V|| (km/s)
Vxperp (km/s)
Flux (cm2 sr s keV/e)-1

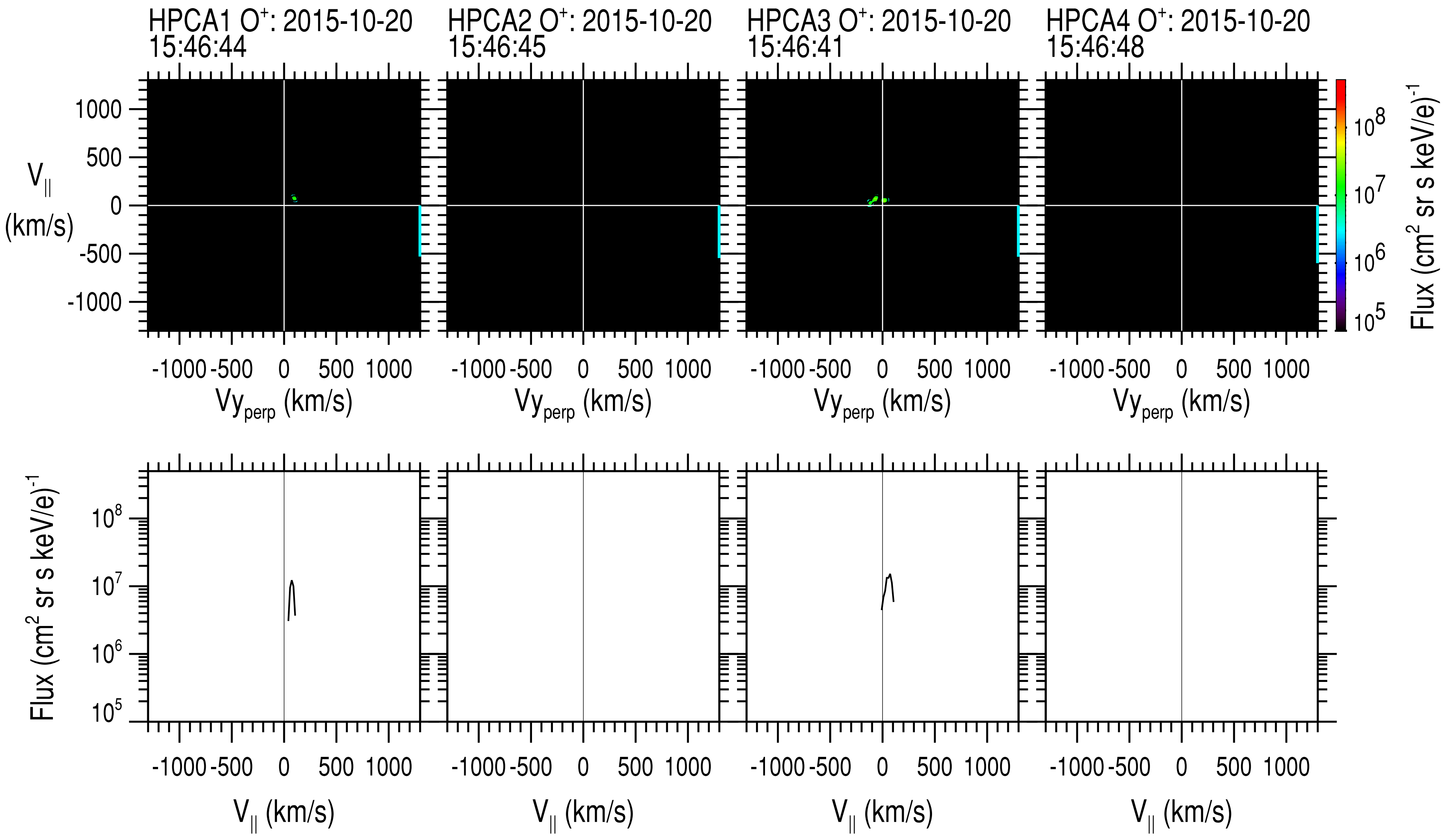
HPCA1 O+: 2015-10-20
15:46:44
HPCA2 O+: 2015-10-20
15:46:45
HPCA3 O+: 2015-10-20
15:46:41
HPCA4 O+: 2015-10-20
15:46:48
V|| (km/s)
Vyperp (km/s)
Flux (cm2 sr s keV/e)-1
-1000 -500 0 500 1000
10^5 10^6 10^7 10^8

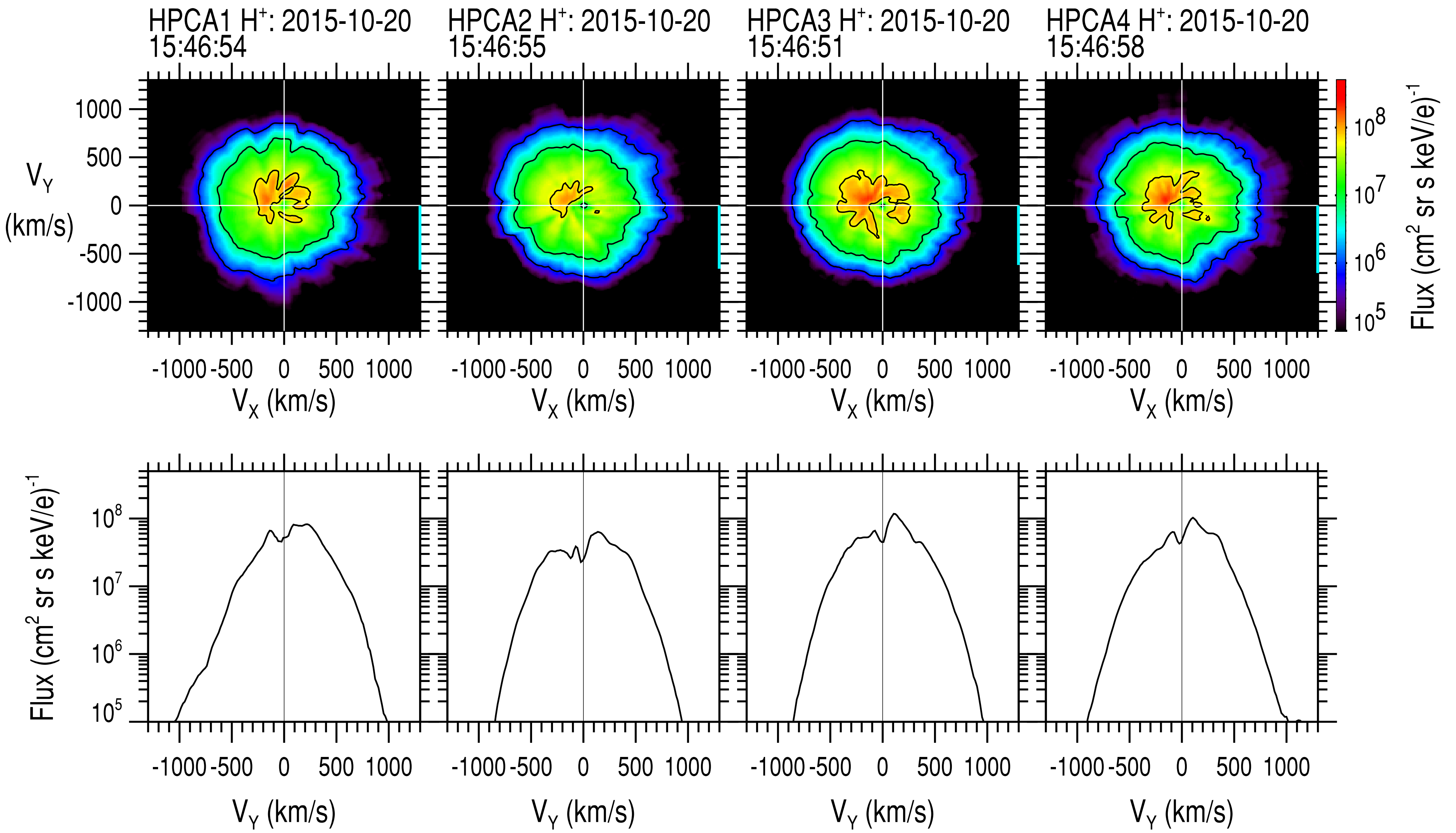
HPCA1 H+: 2015-10-20 15:46:54
HPCA2 H+: 2015-10-20 15:46:55
HPCA3 H+: 2015-10-20 15:46:51
HPCA4 H+: 2015-10-20 15:46:58
V_Y (km/s)
V_X (km/s)
Flux (cm^2 sr s keV/e)^-1

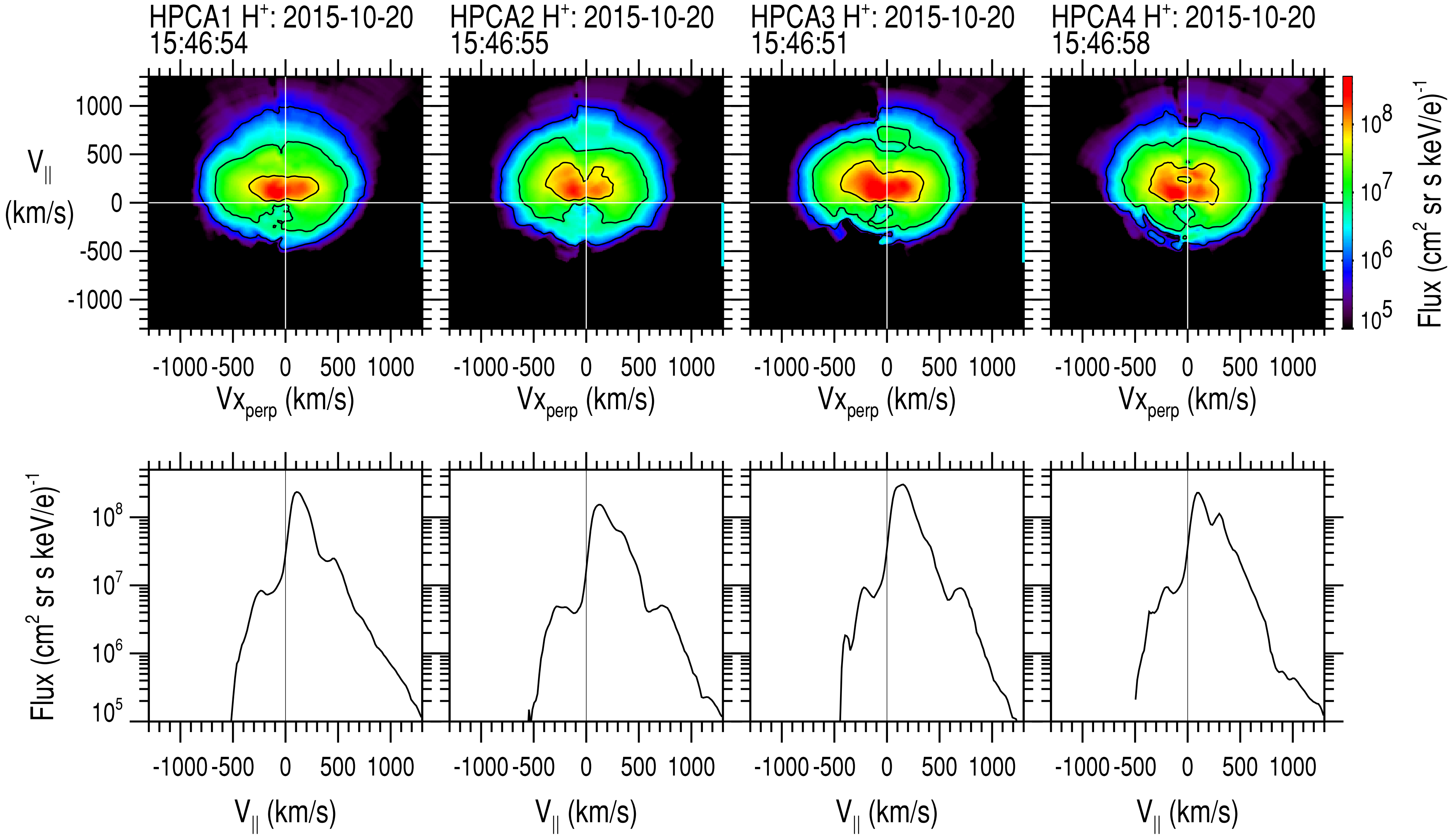

HPCA1 H+: 2015-10-20
15:46:54
HPCA2 H+: 2015-10-20
15:46:55
HPCA3 H+: 2015-10-20
15:46:51
HPCA4 H+: 2015-10-20
15:46:58
V|| (km/s)
Vx_perp (km/s)
Flux (cm^2 sr s keV/e)^-1

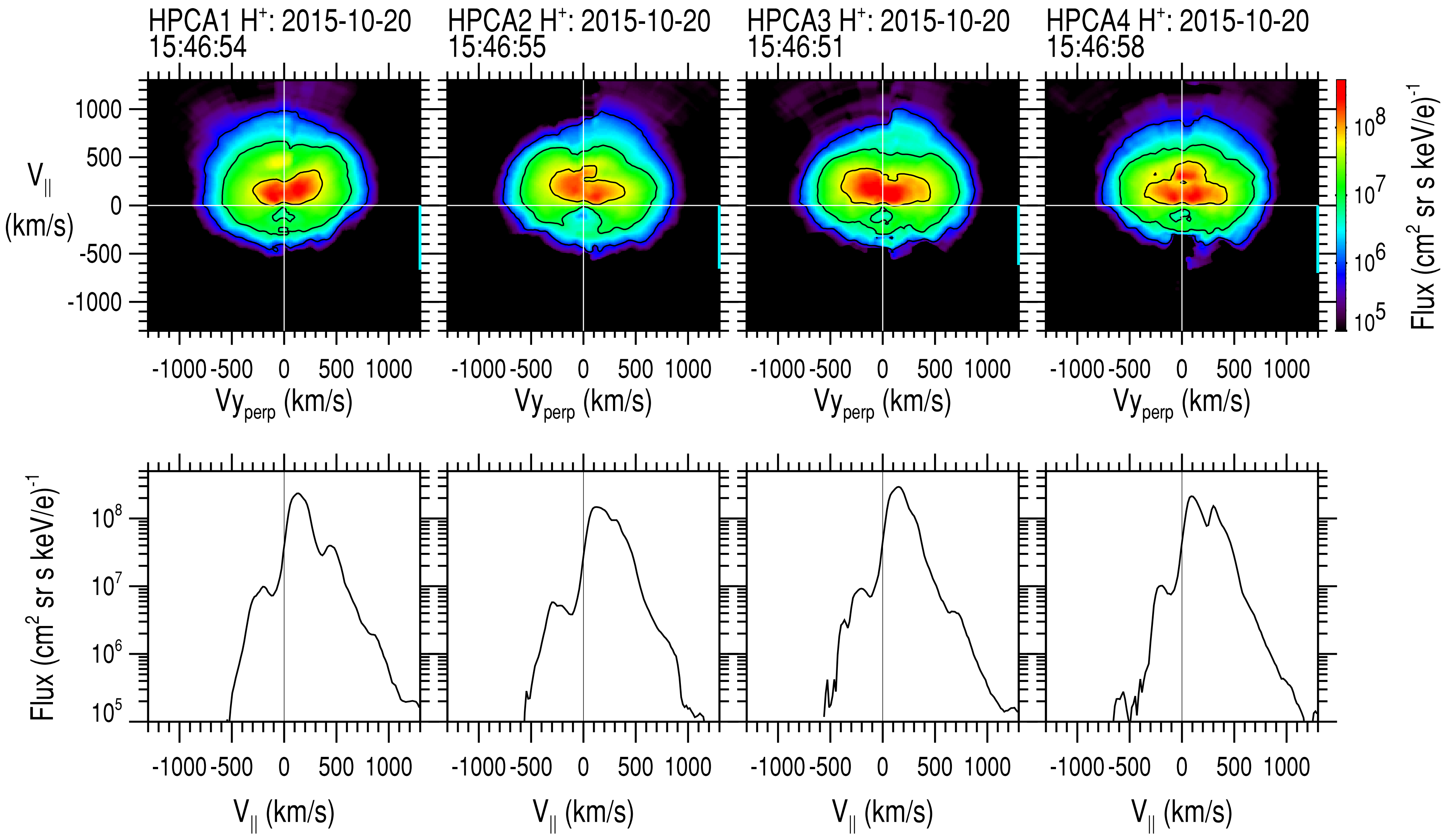

HPCA1 H+: 2015-10-20 15:46:54
HPCA2 H+: 2015-10-20 15:46:55
HPCA3 H+: 2015-10-20 15:46:51
HPCA4 H+: 2015-10-20 15:46:58
V|| (km/s)
Vyperp (km/s)
Flux (cm2 sr s keV/e)-1
V|| (km/s)

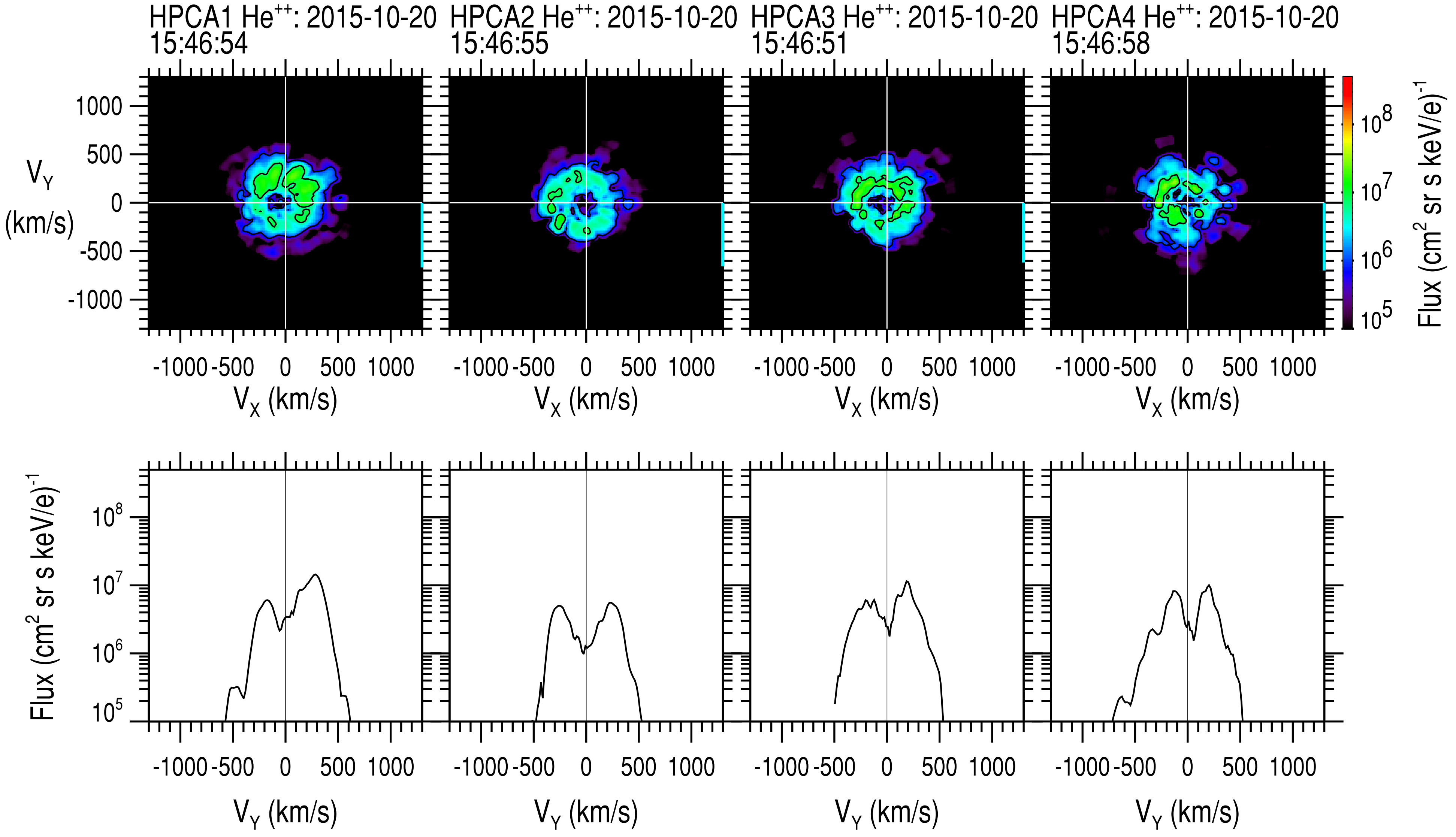

HPCA1 He++: 2015-10-20 15:46:54
HPCA2 He++: 2015-10-20 15:46:55
HPCA3 He++: 2015-10-20 15:46:51
HPCA4 He++: 2015-10-20 15:46:58
VY (km/s)
VX (km/s)
Flux (cm2 sr s keV/e)-1
VY (km/s)

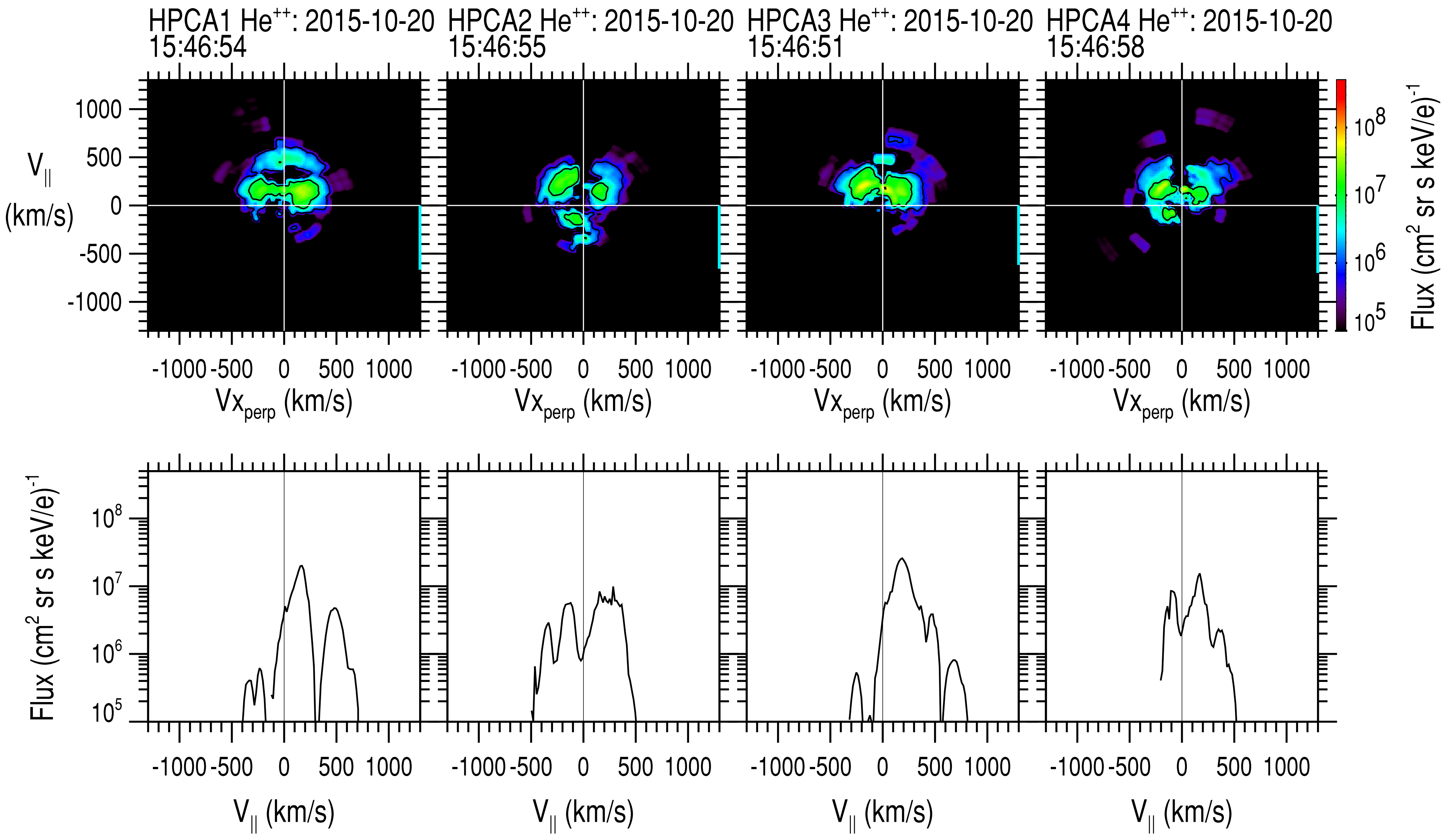
HPCA1 He++: 2015-10-20
15:46:54
HPCA2 He++: 2015-10-20
15:46:55
HPCA3 He++: 2015-10-20
15:46:51
HPCA4 He++: 2015-10-20
15:46:58
V|| (km/s)
Vxperp (km/s)
Flux (cm2 sr s keV/e)-1
V|| (km/s)

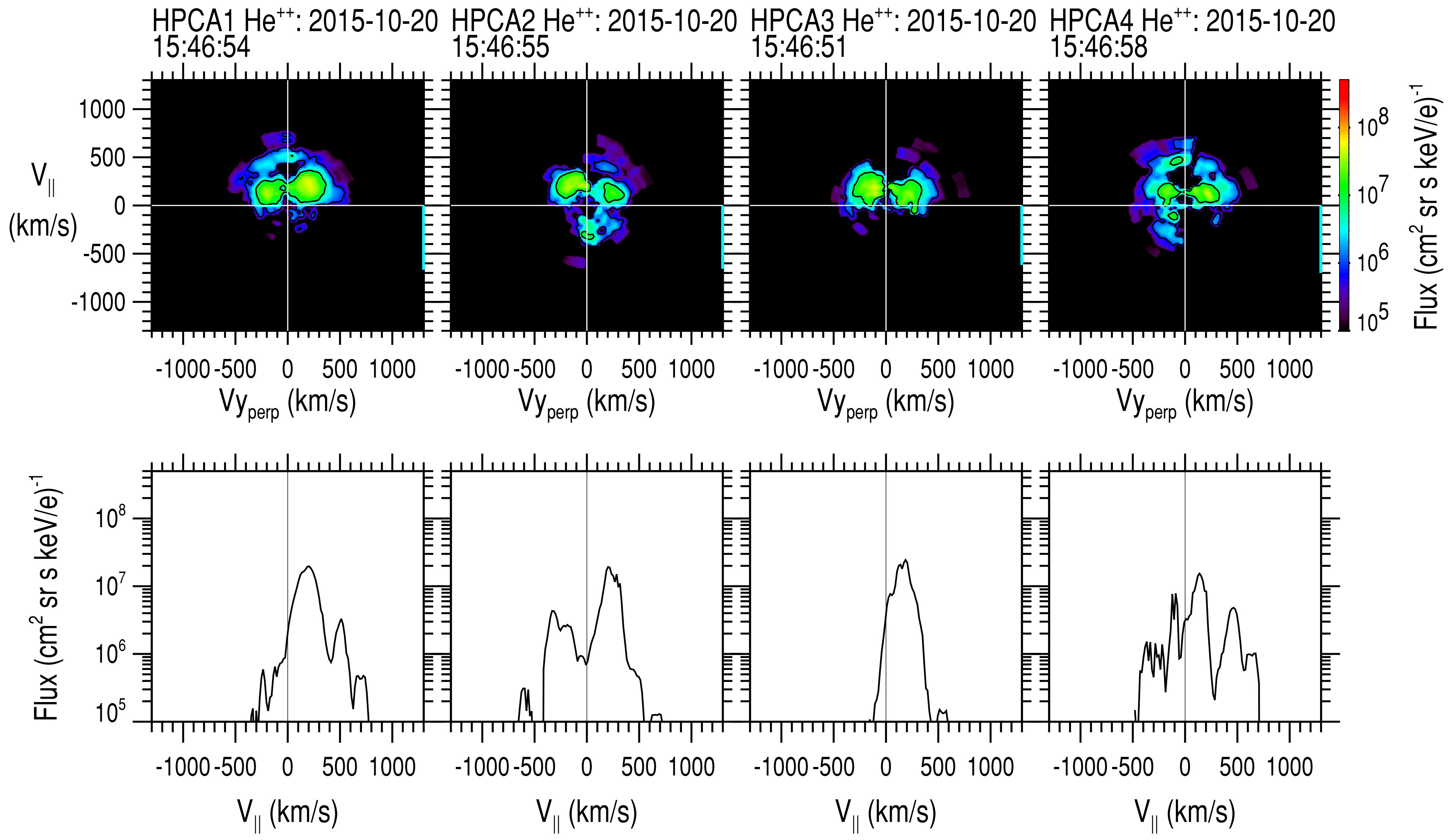
HPCA1 He++: 2015-10-20
15:46:54
HPCA2 He++: 2015-10-20
15:46:55
HPCA3 He++: 2015-10-20
15:46:51
HPCA4 He++: 2015-10-20
15:46:58
V|| (km/s)
Vyperp (km/s)
Flux (cm2 sr s keV/e)-1
V|| (km/s)

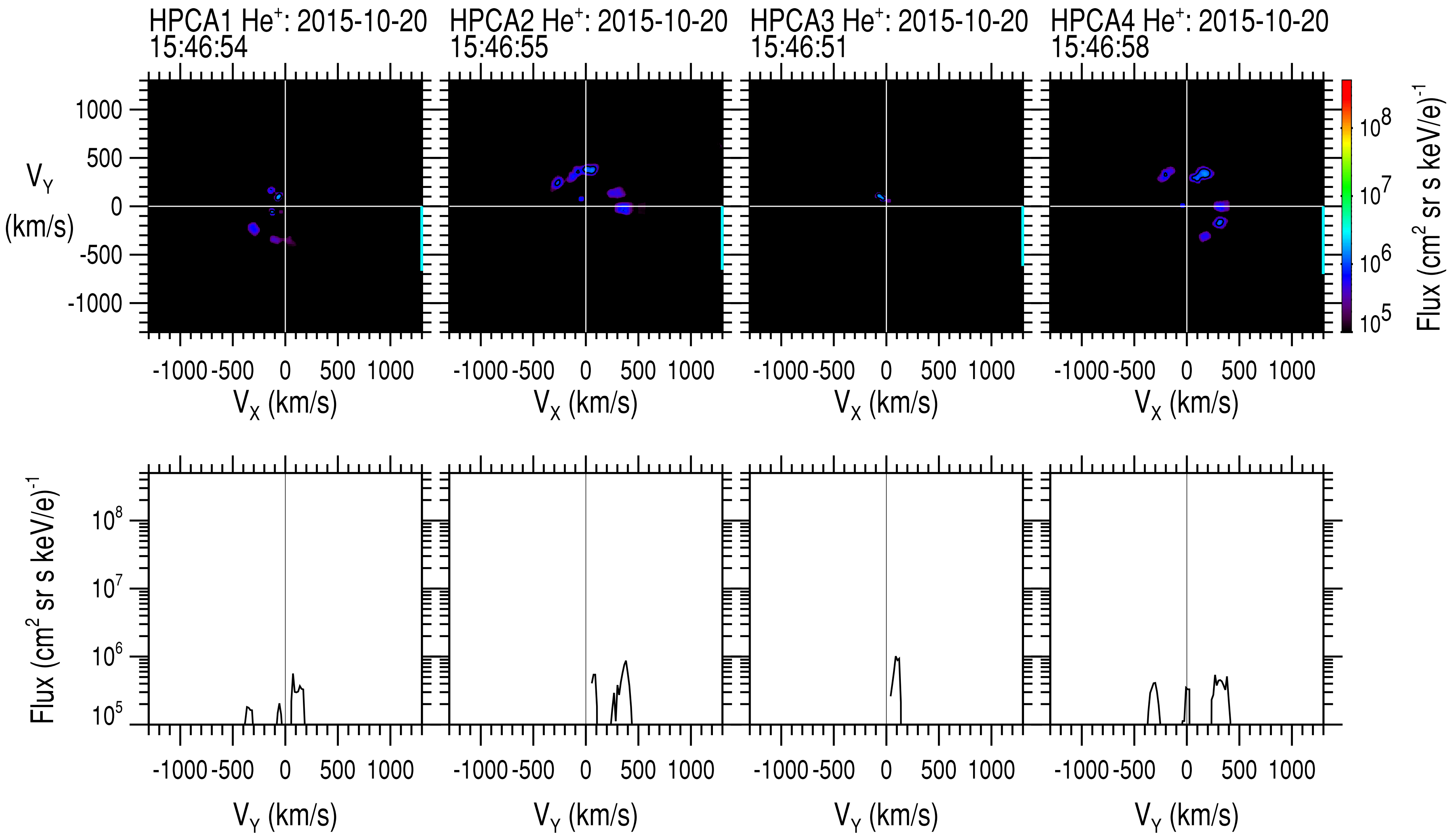

HPCA1 He+: 2015-10-20 15:46:54
HPCA2 He+: 2015-10-20 15:46:55
HPCA3 He+: 2015-10-20 15:46:51
HPCA4 He+: 2015-10-20 15:46:58
VY (km/s)
VX (km/s)
Flux (cm2 sr s keV/e)-1

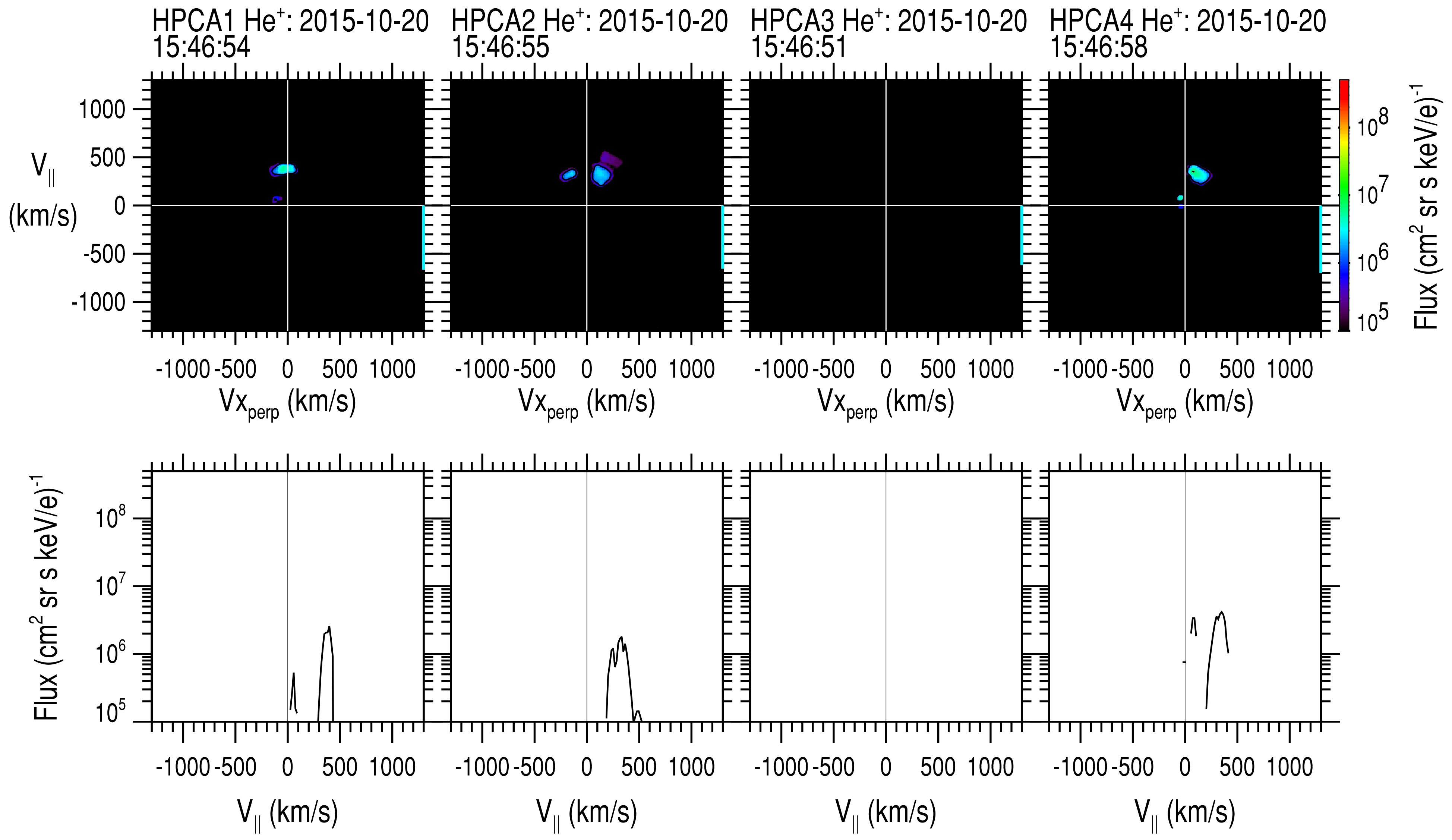
HPCA1 He+: 2015-10-20 15:46:54
HPCA2 He+: 2015-10-20 15:46:55
HPCA3 He+: 2015-10-20 15:46:51
HPCA4 He+: 2015-10-20 15:46:58
V|| (km/s)
Vxperp (km/s)
Flux (cm2 sr s keV/e)-1
-1000 -500 0 500 1000
10^5 10^6 10^7 10^8

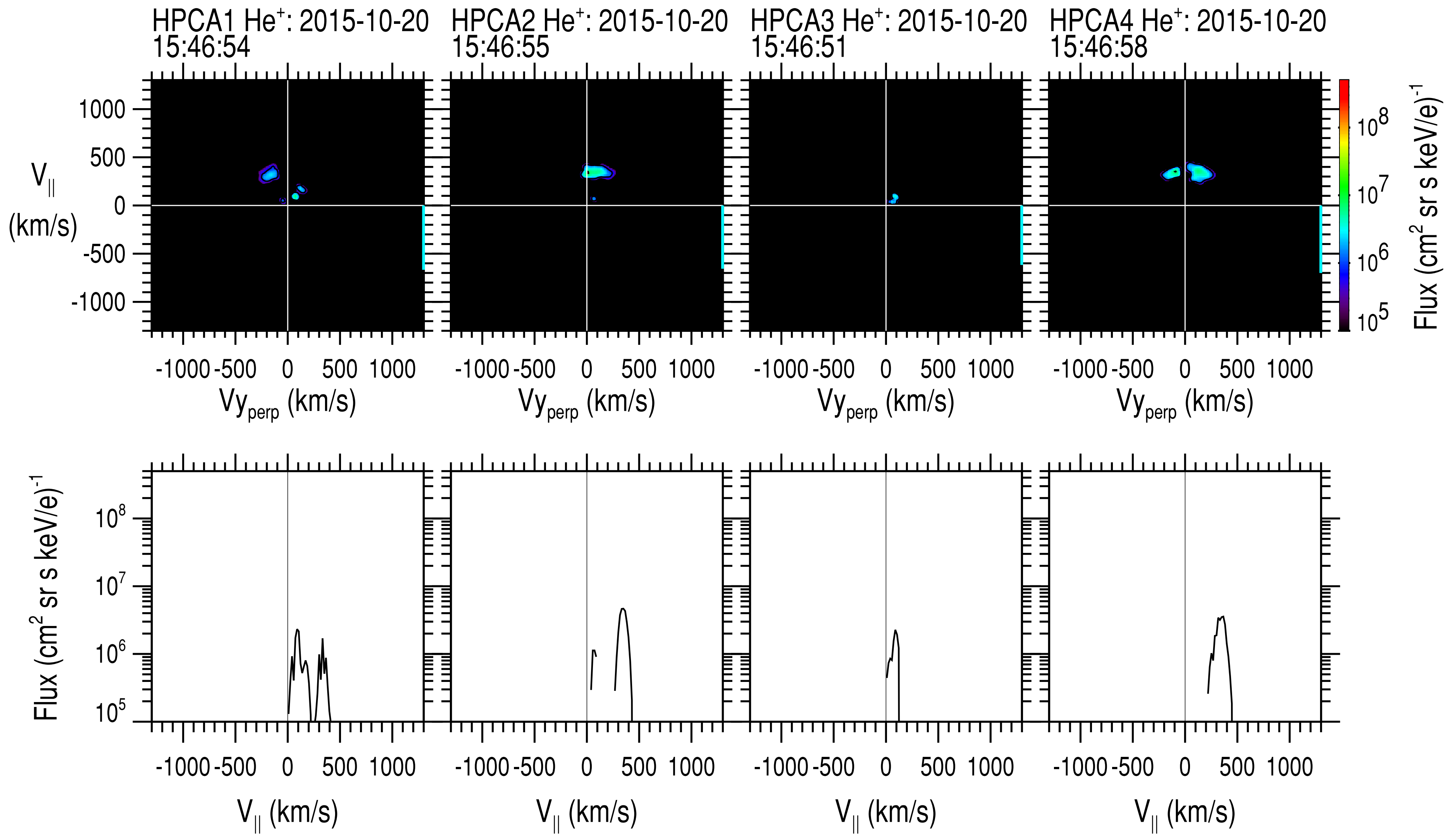

HPCA1 He+: 2015-10-20 15:46:54
HPCA2 He+: 2015-10-20 15:46:55
HPCA3 He+: 2015-10-20 15:46:51
HPCA4 He+: 2015-10-20 15:46:58
V|| (km/s)
Vyperp (km/s)
Flux (cm2 sr s keV/e)-1
-1000 -500 0 500 1000
10^5 10^6 10^7 10^8

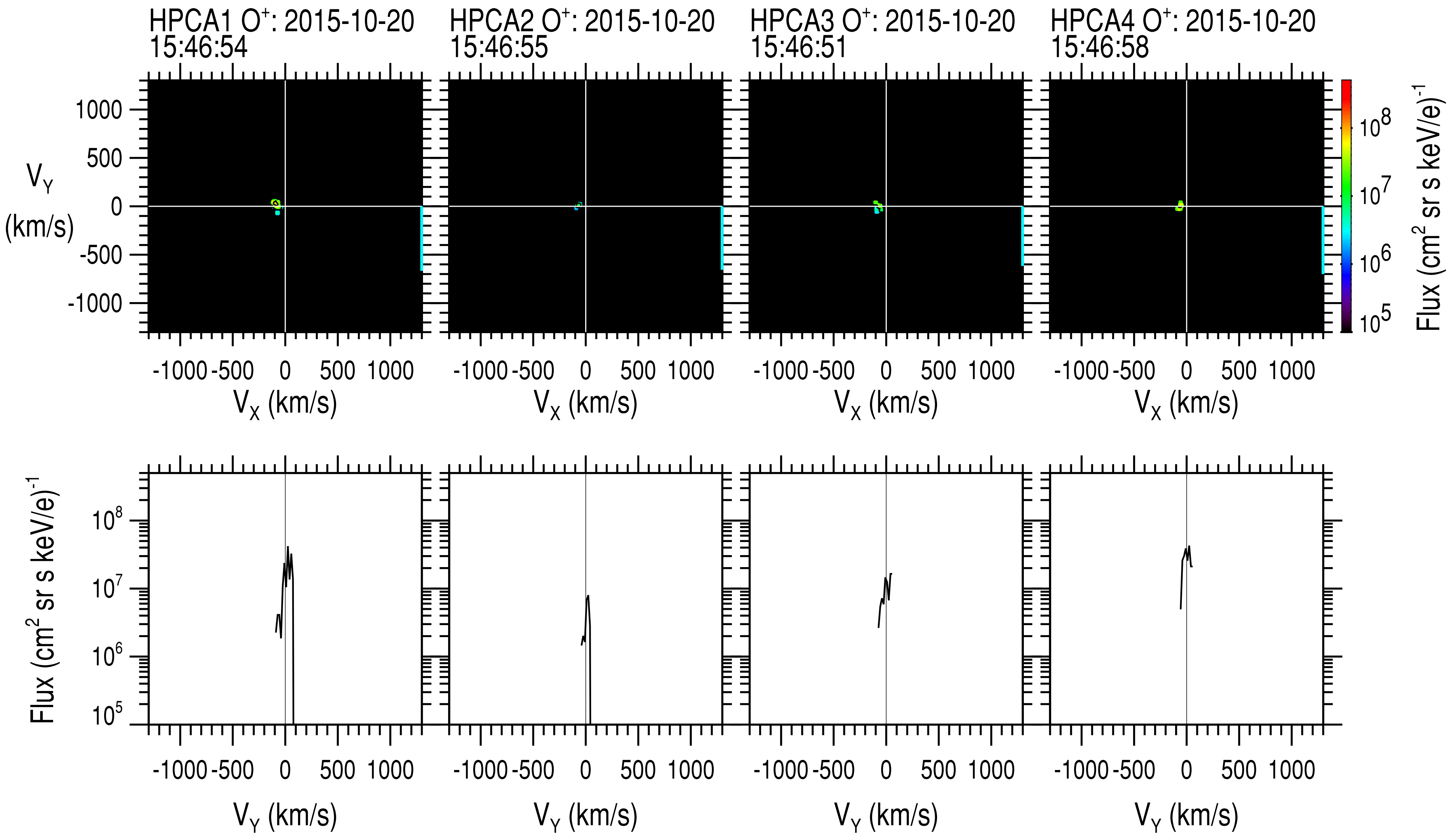
HPCA1 O+: 2015-10-20
15:46:54
HPCA2 O+: 2015-10-20
15:46:55
HPCA3 O+: 2015-10-20
15:46:51
HPCA4 O+: 2015-10-20
15:46:58
V_Y (km/s)
V_X (km/s)
Flux (cm2 sr s keV/e)-1
V_Y (km/s)

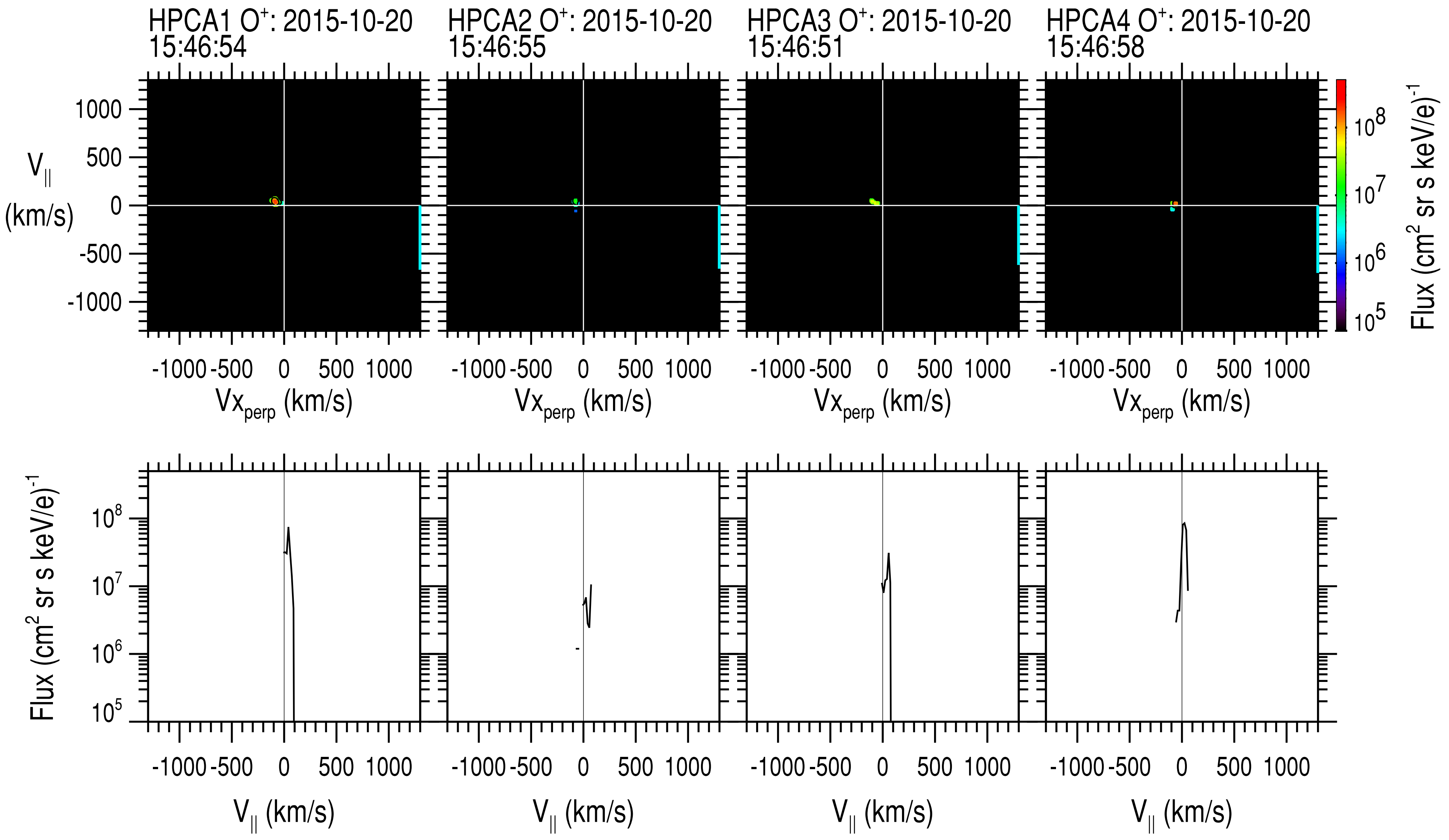
HPCA1 O+: 2015-10-20 15:46:54
HPCA2 O+: 2015-10-20 15:46:55
HPCA3 O+: 2015-10-20 15:46:51
HPCA4 O+: 2015-10-20 15:46:58
V|| (km/s)
Vxperp (km/s)
Flux (cm2 sr s keV/e)-1
-1000 -500 0 500 1000

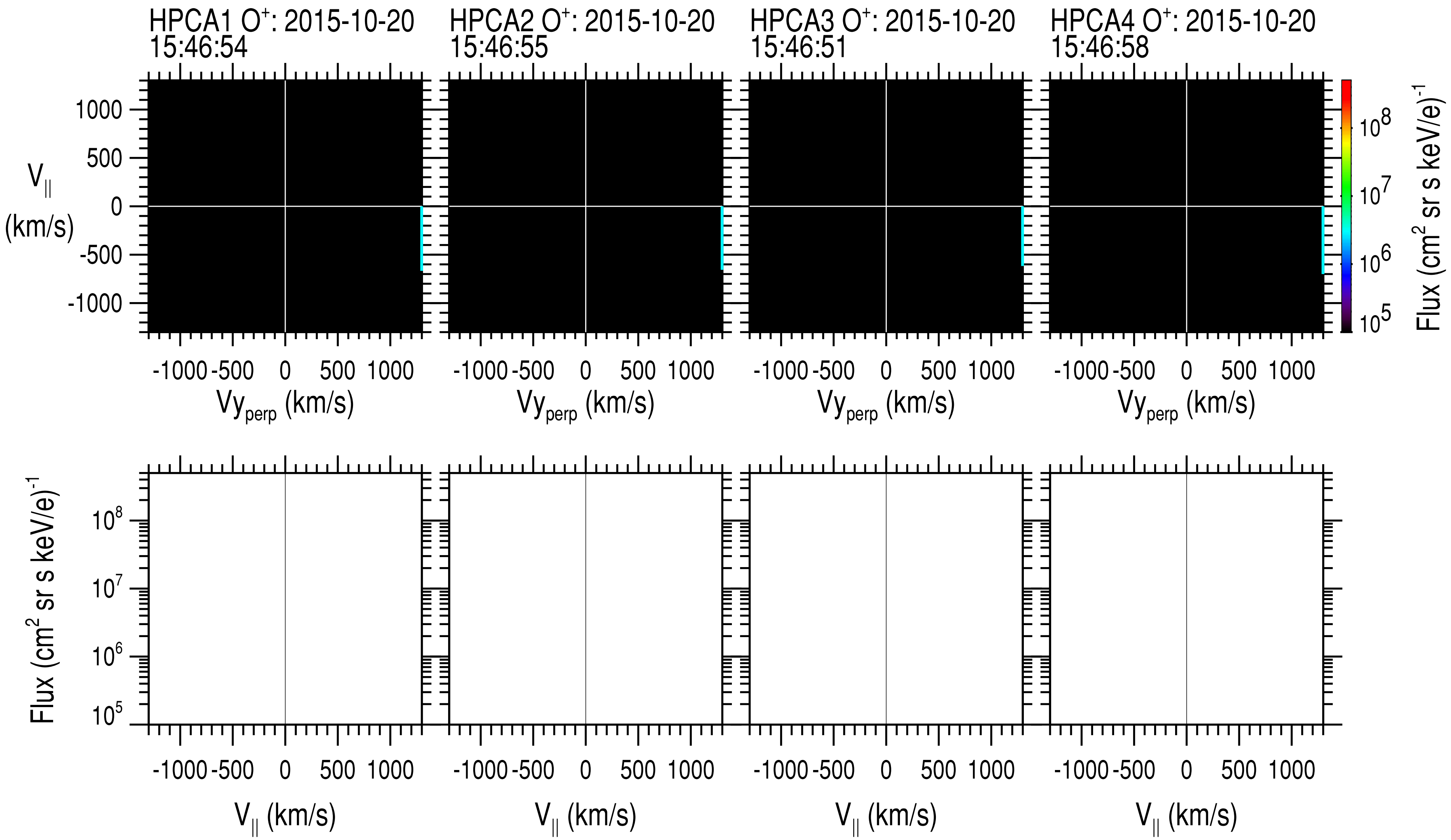

HPCA1 O+: 2015-10-20 15:46:54
HPCA2 O+: 2015-10-20 15:46:55
HPCA3 O+: 2015-10-20 15:46:51
HPCA4 O+: 2015-10-20 15:46:58
V|| (km/s)
1000
500
0
-500
-1000
Vyperp (km/s)
Flux (cm2 sr s keV/e)-1
10^5
10^6
10^7
10^8
V|| (km/s)

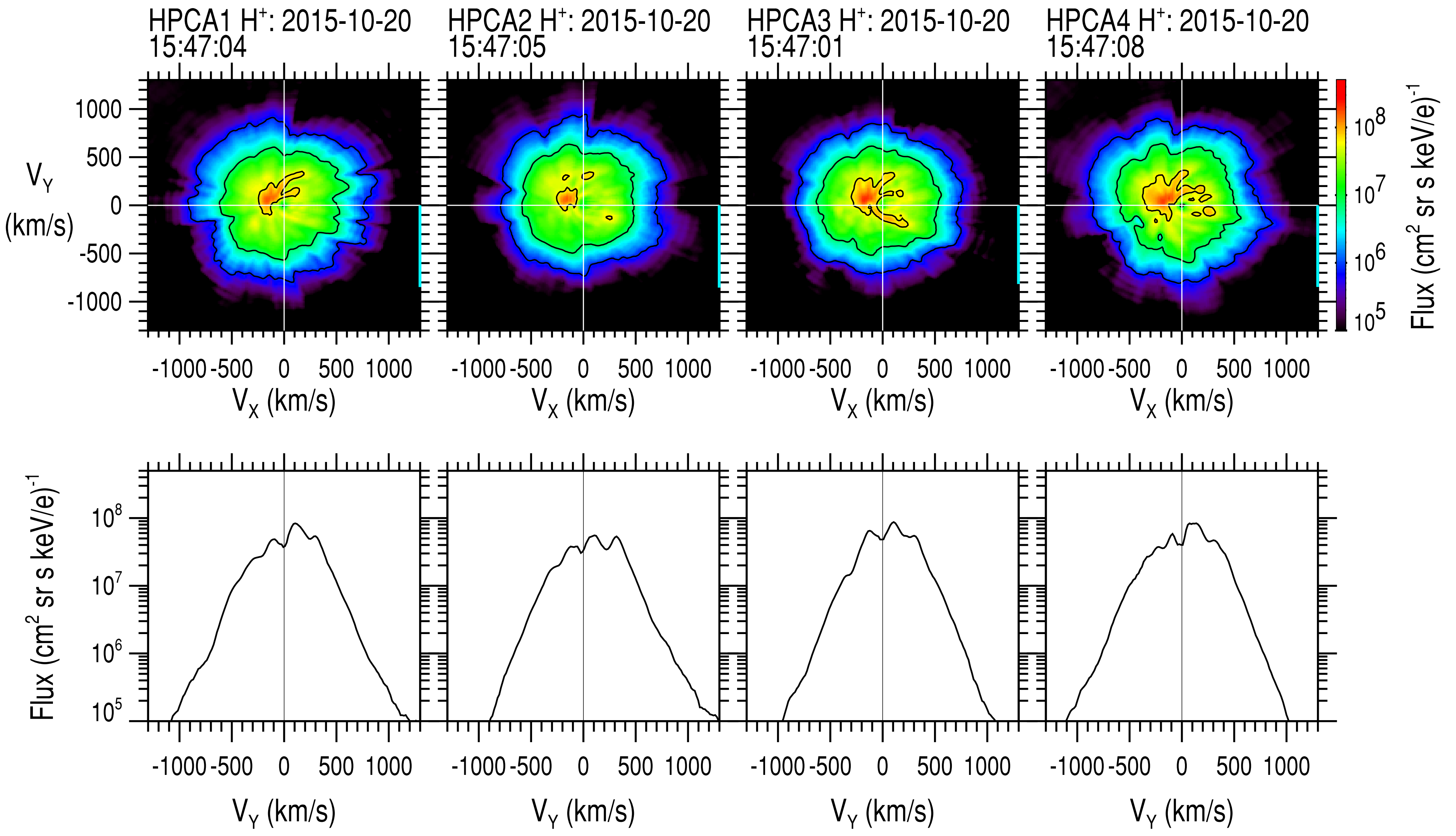
HPCA1 H+: 2015-10-20 15:47:04
HPCA2 H+: 2015-10-20 15:47:05
HPCA3 H+: 2015-10-20 15:47:01
HPCA4 H+: 2015-10-20 15:47:08
V_Y (km/s)
V_X (km/s)
Flux (cm^2 sr s keV/e)^-1
V_Y (km/s)
Flux (cm^2 sr s keV/e)^-1

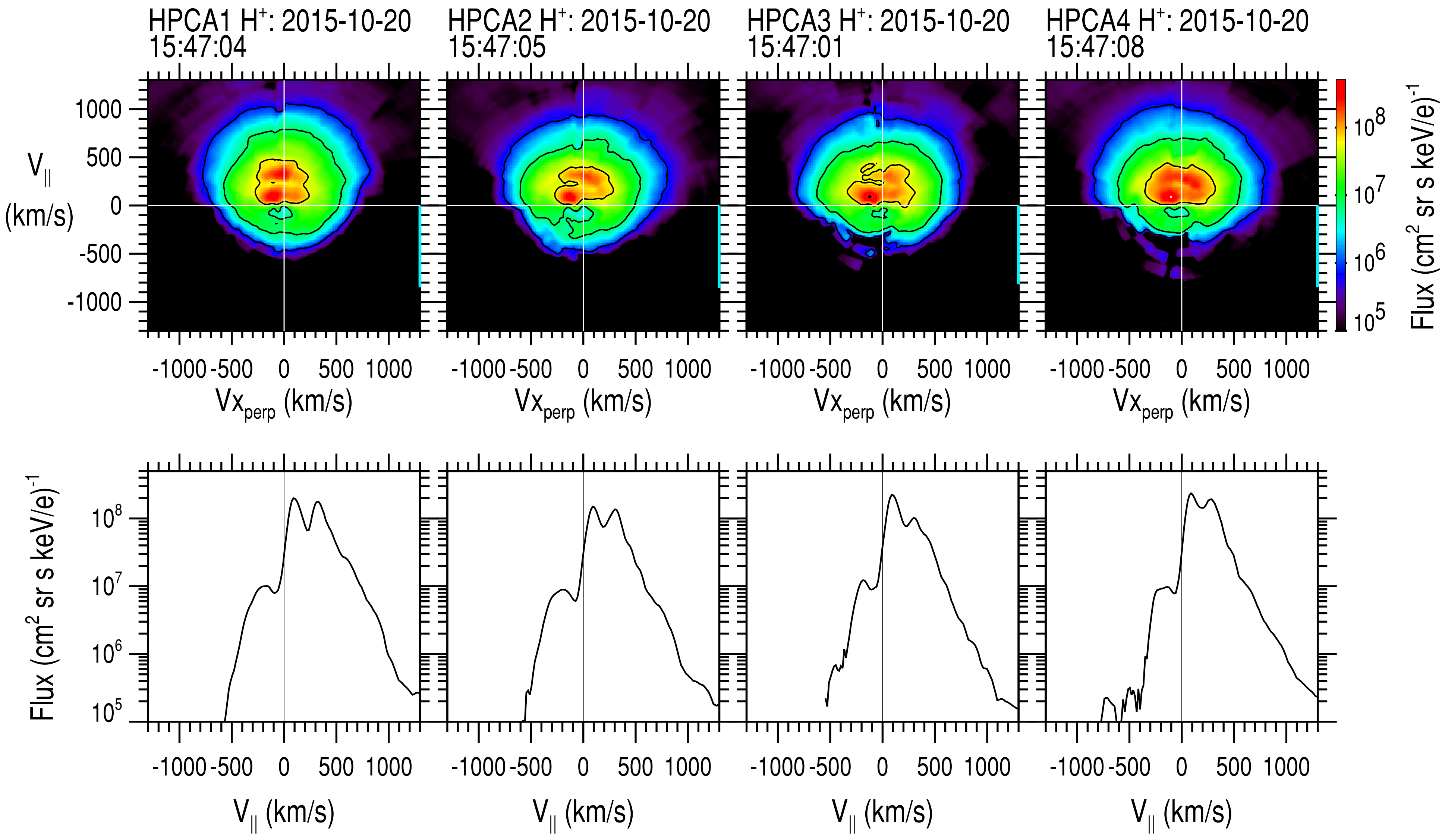
HPCA1 H+: 2015-10-20
15:47:04
HPCA2 H+: 2015-10-20
15:47:05
HPCA3 H+: 2015-10-20
15:47:01
HPCA4 H+: 2015-10-20
15:47:08
V|| (km/s)
Vxperp (km/s)
Flux (cm2 sr s keV/e)-1
V|| (km/s)
Flux (cm2 sr s keV/e)-1

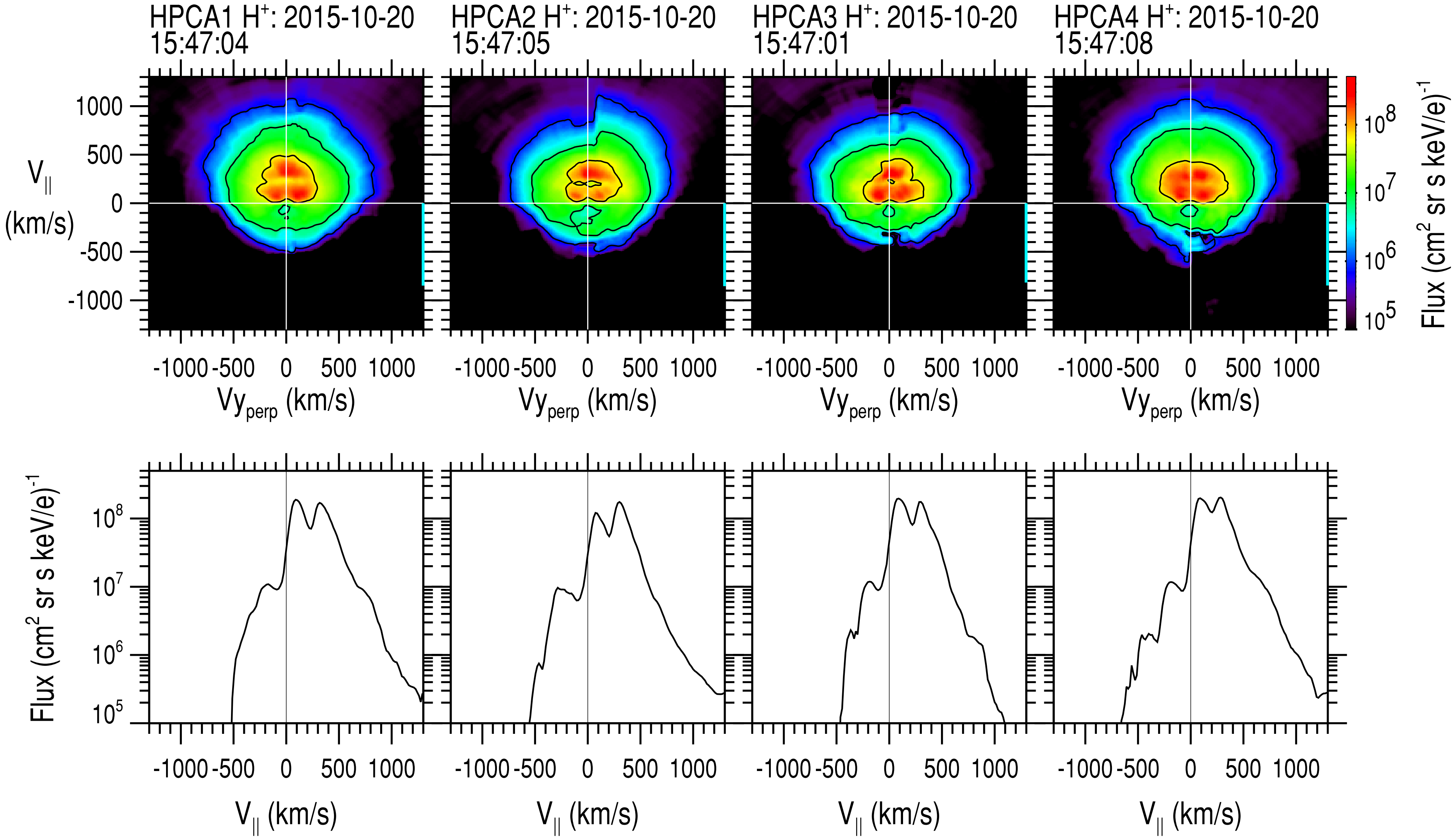
HPCA1 H+: 2015-10-20 15:47:04
HPCA2 H+: 2015-10-20 15:47:05
HPCA3 H+: 2015-10-20 15:47:01
HPCA4 H+: 2015-10-20 15:47:08
V|| (km/s)
Vyperp (km/s)
Flux (cm2 sr s keV/e)-1
-1000 -500 0 500 1000
105
106
107
108

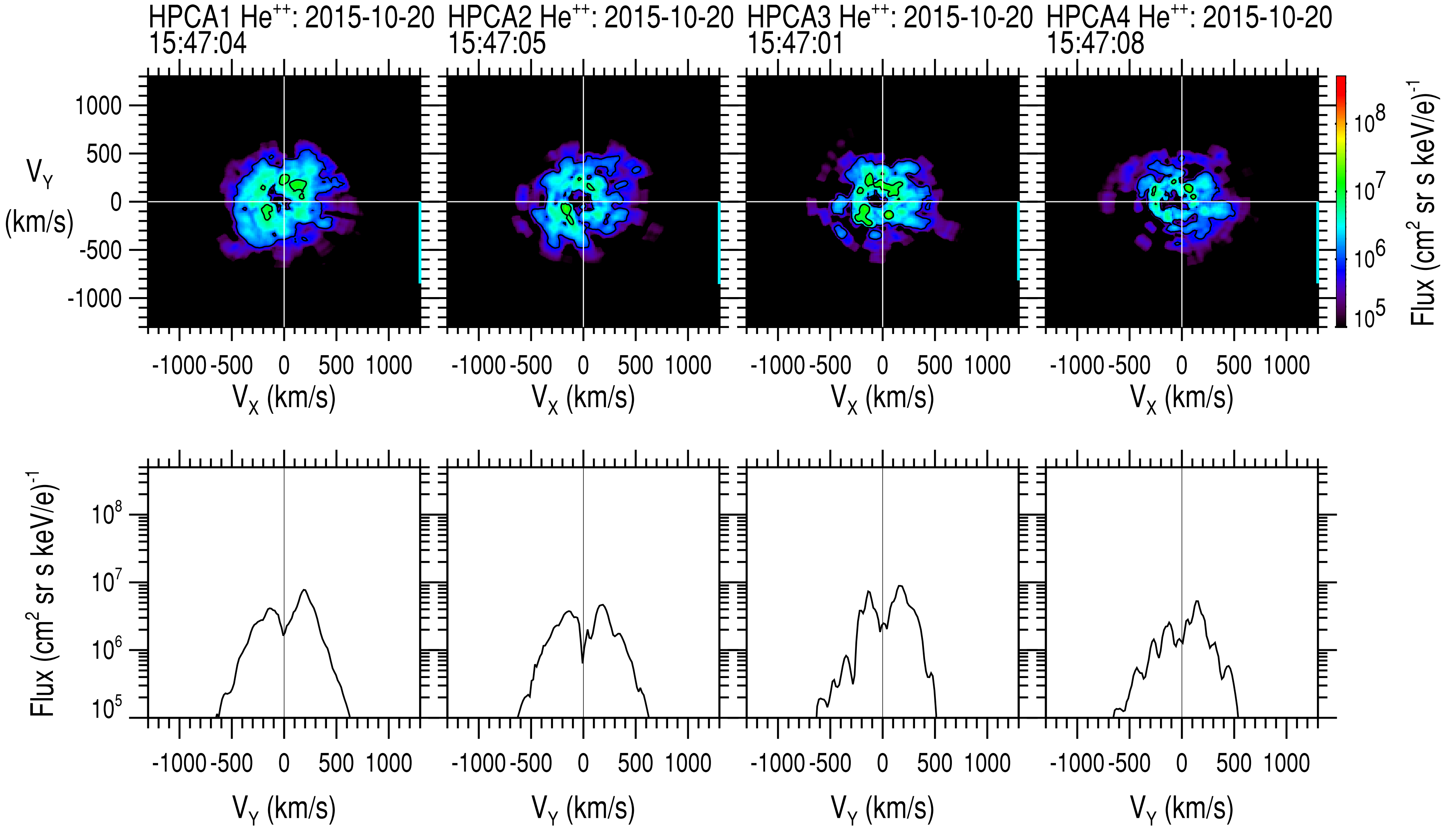
HPCA1 He++: 2015-10-20
15:47:04
HPCA2 He++: 2015-10-20
15:47:05
HPCA3 He++: 2015-10-20
15:47:01
HPCA4 He++: 2015-10-20
15:47:08
VY (km/s)
VX (km/s)
Flux (cm2 sr s keV/e)-1
-1000
-500
0
500
1000
10^5
10^6
10^7
10^8

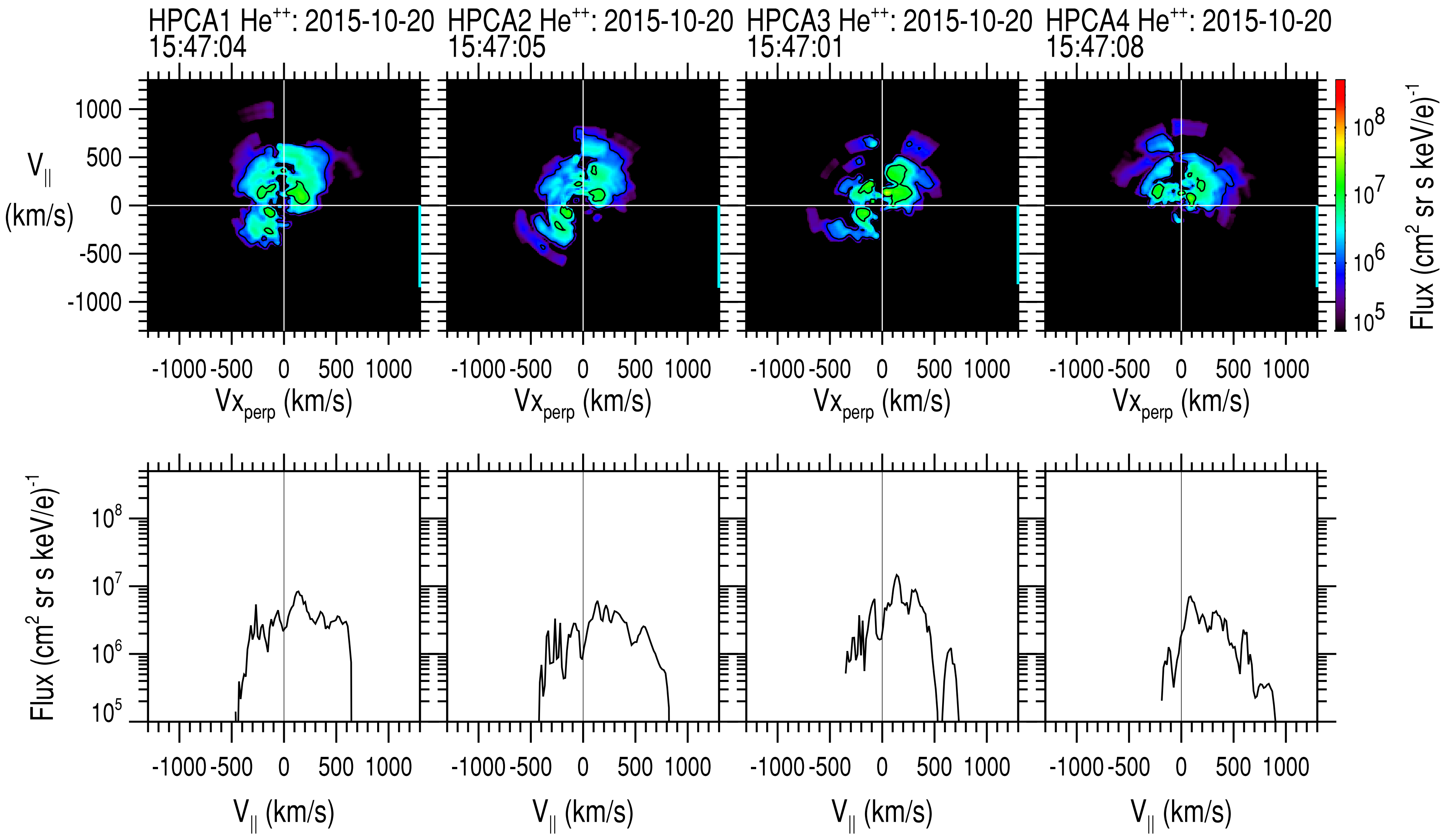
HPCA1 He++: 2015-10-20 15:47:04
HPCA2 He++: 2015-10-20 15:47:05
HPCA3 He++: 2015-10-20 15:47:01
HPCA4 He++: 2015-10-20 15:47:08
V|| (km/s)
Vxperp (km/s)
Flux (cm2 sr s keV/e)-1
V|| (km/s)

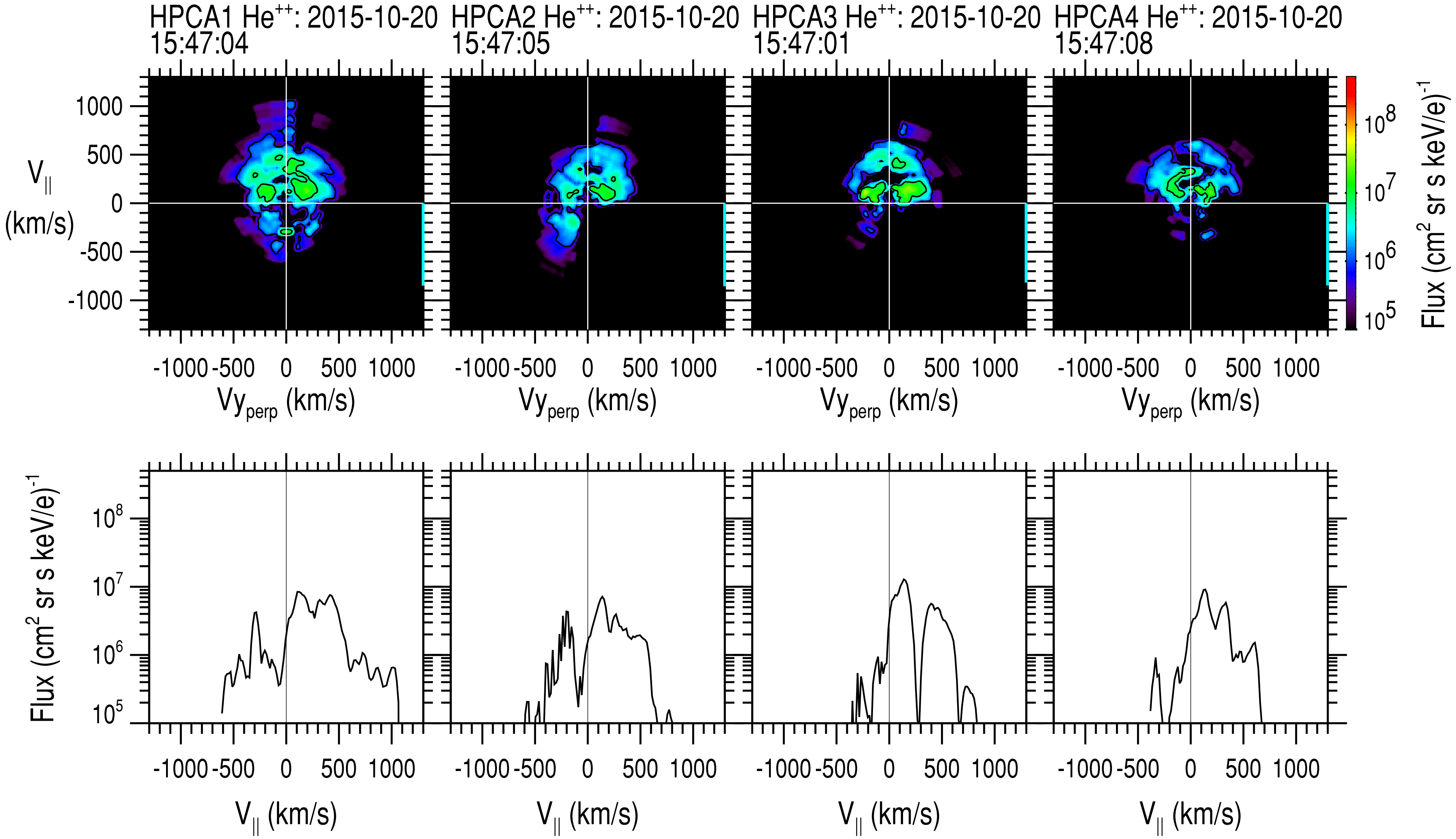
HPCA1 He++: 2015-10-20 15:47:04
HPCA2 He++: 2015-10-20 15:47:05
HPCA3 He++: 2015-10-20 15:47:01
HPCA4 He++: 2015-10-20 15:47:08
V|| (km/s)
Vyperp (km/s)
Flux (cm2 sr s keV/e)-1
V|| (km/s)

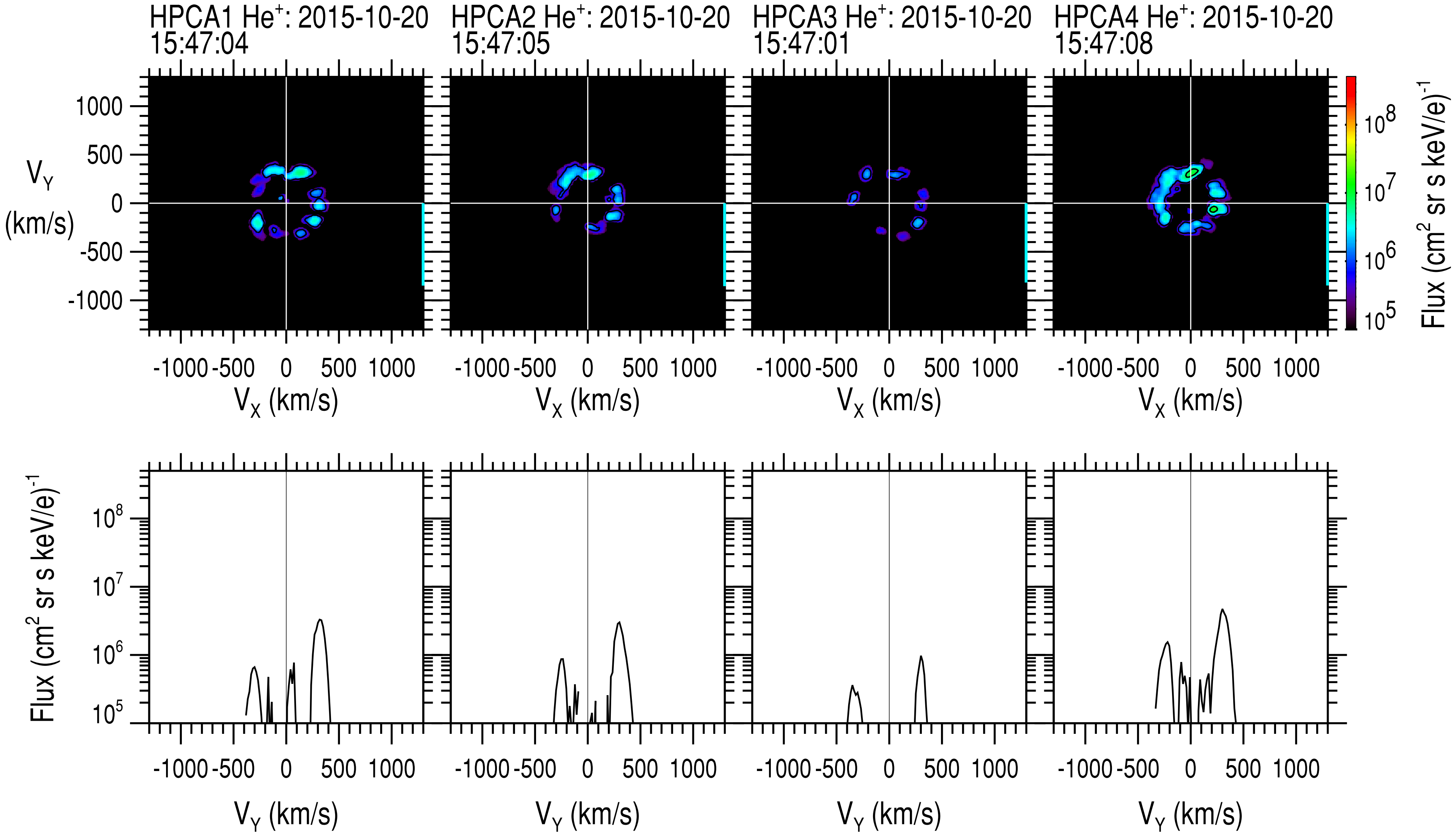
HPCA1 He+: 2015-10-20 15:47:04
HPCA2 He+: 2015-10-20 15:47:05
HPCA3 He+: 2015-10-20 15:47:01
HPCA4 He+: 2015-10-20 15:47:08
VY (km/s)
VX (km/s)
Flux (cm2 sr s keV/e)-1
VY (km/s)

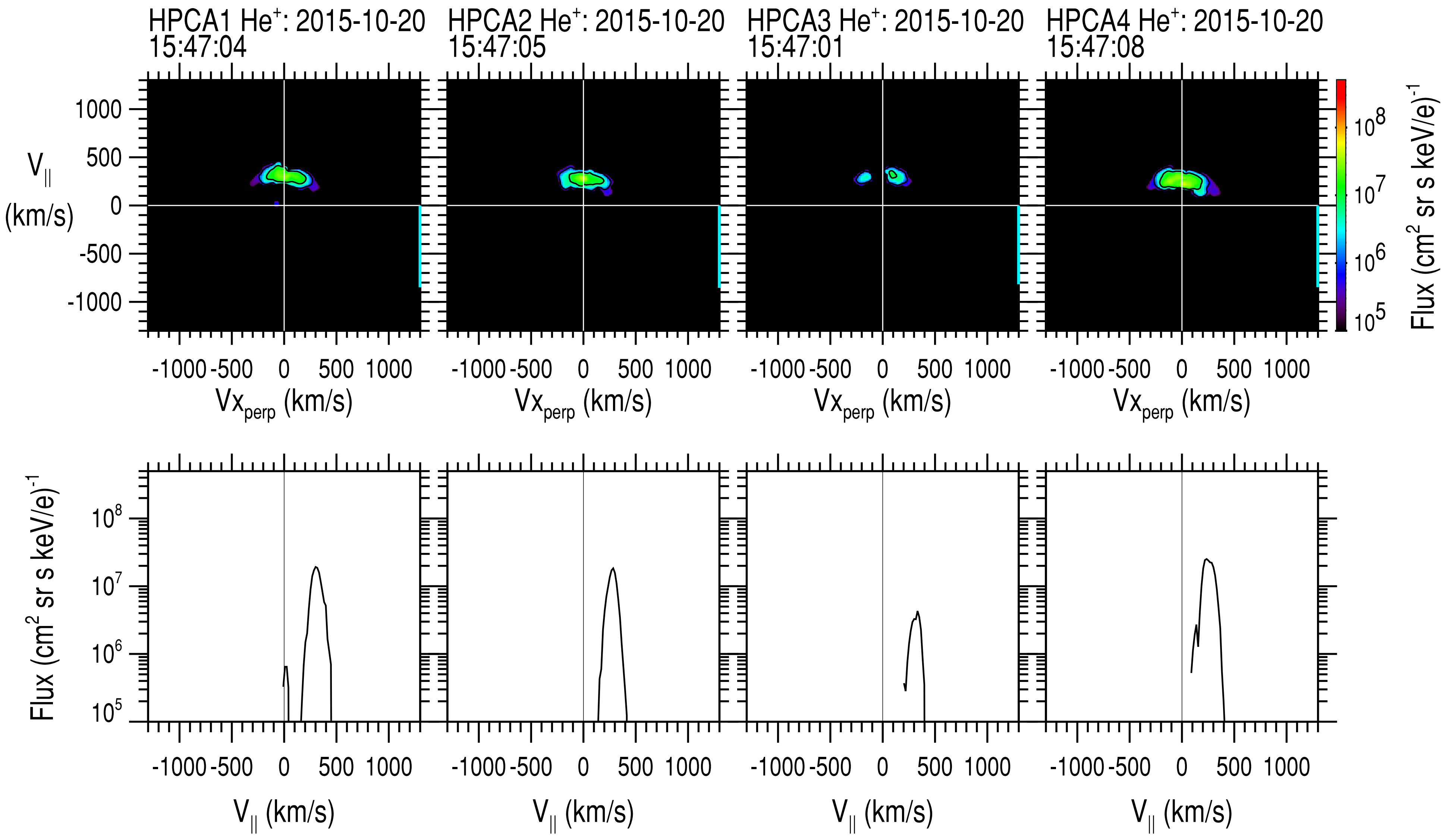

HPCA1 He+: 2015-10-20 15:47:04
HPCA2 He+: 2015-10-20 15:47:05
HPCA3 He+: 2015-10-20 15:47:01
HPCA4 He+: 2015-10-20 15:47:08
V|| (km/s)
Vxperp (km/s)
Flux (cm2 sr s keV/e)-1

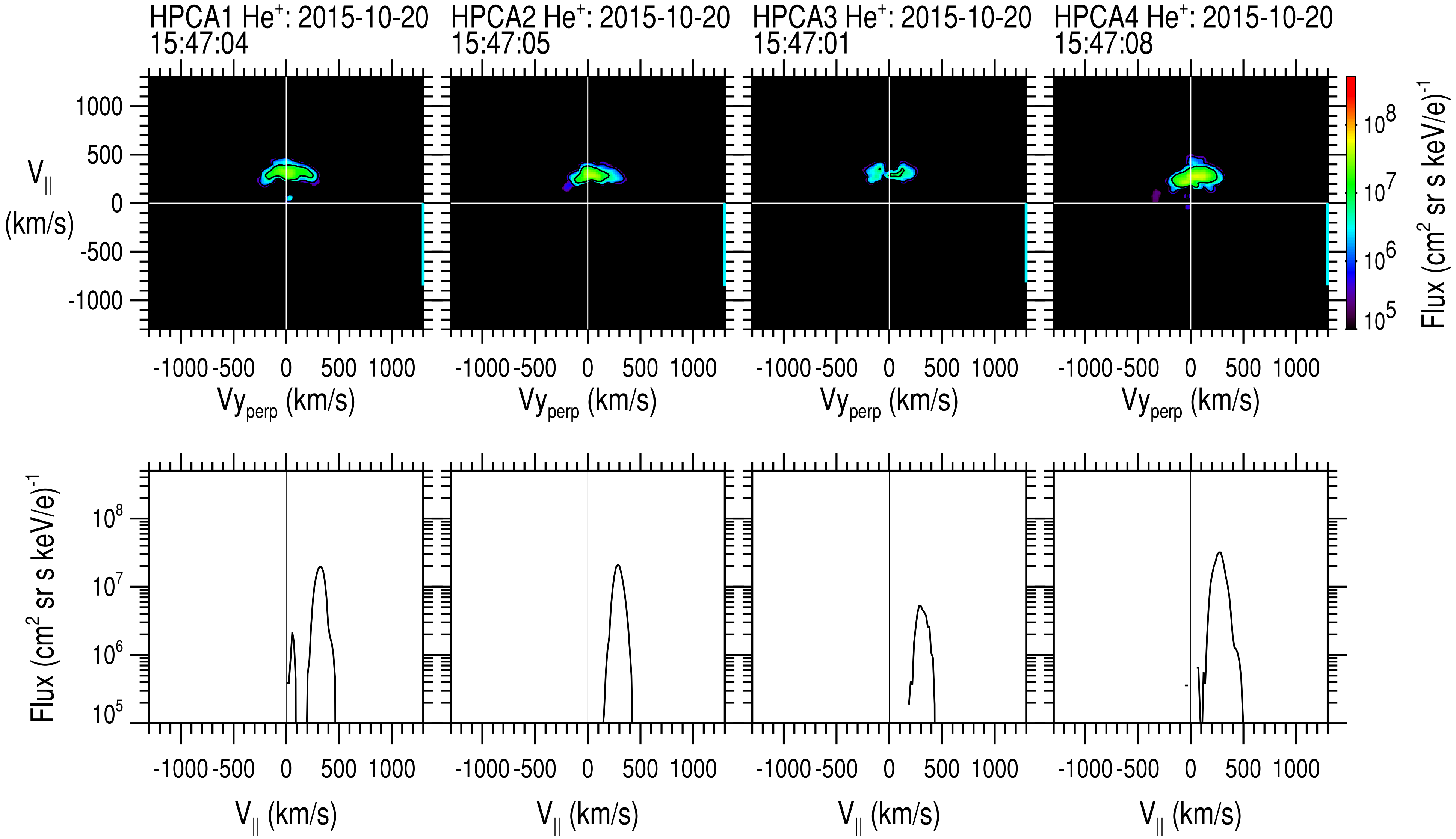
HPCA1 He+: 2015-10-20 15:47:04
HPCA2 He+: 2015-10-20 15:47:05
HPCA3 He+: 2015-10-20 15:47:01
HPCA4 He+: 2015-10-20 15:47:08
V|| (km/s)
Vyperp (km/s)
Flux (cm2 sr s keV/e)-1
V|| (km/s)

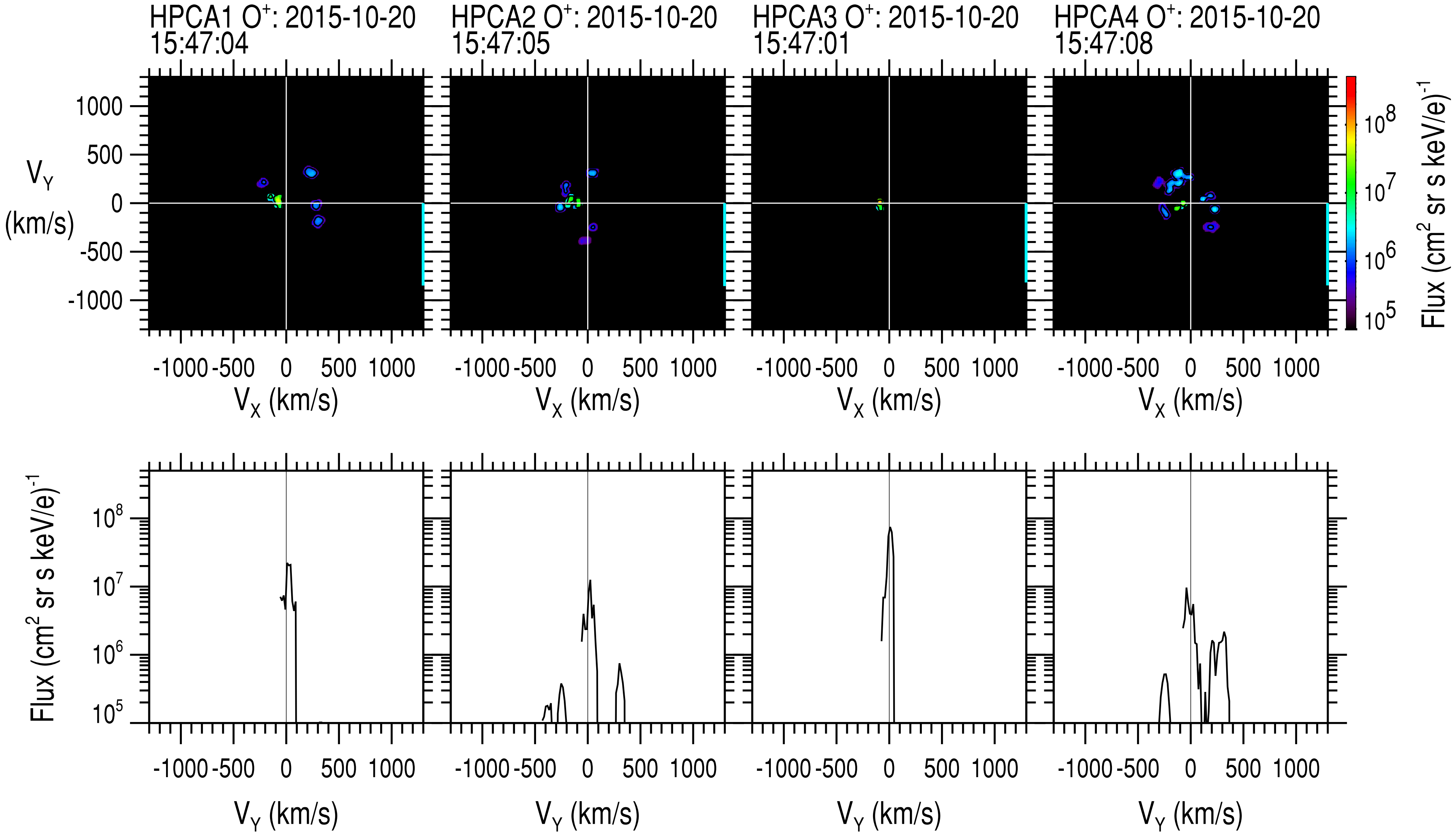

HPCA1 O+: 2015-10-20
15:47:04
HPCA2 O+: 2015-10-20
15:47:05
HPCA3 O+: 2015-10-20
15:47:01
HPCA4 O+: 2015-10-20
15:47:08
V_Y (km/s)
V_X (km/s)
Flux (cm^2 sr s keV/e)^-1

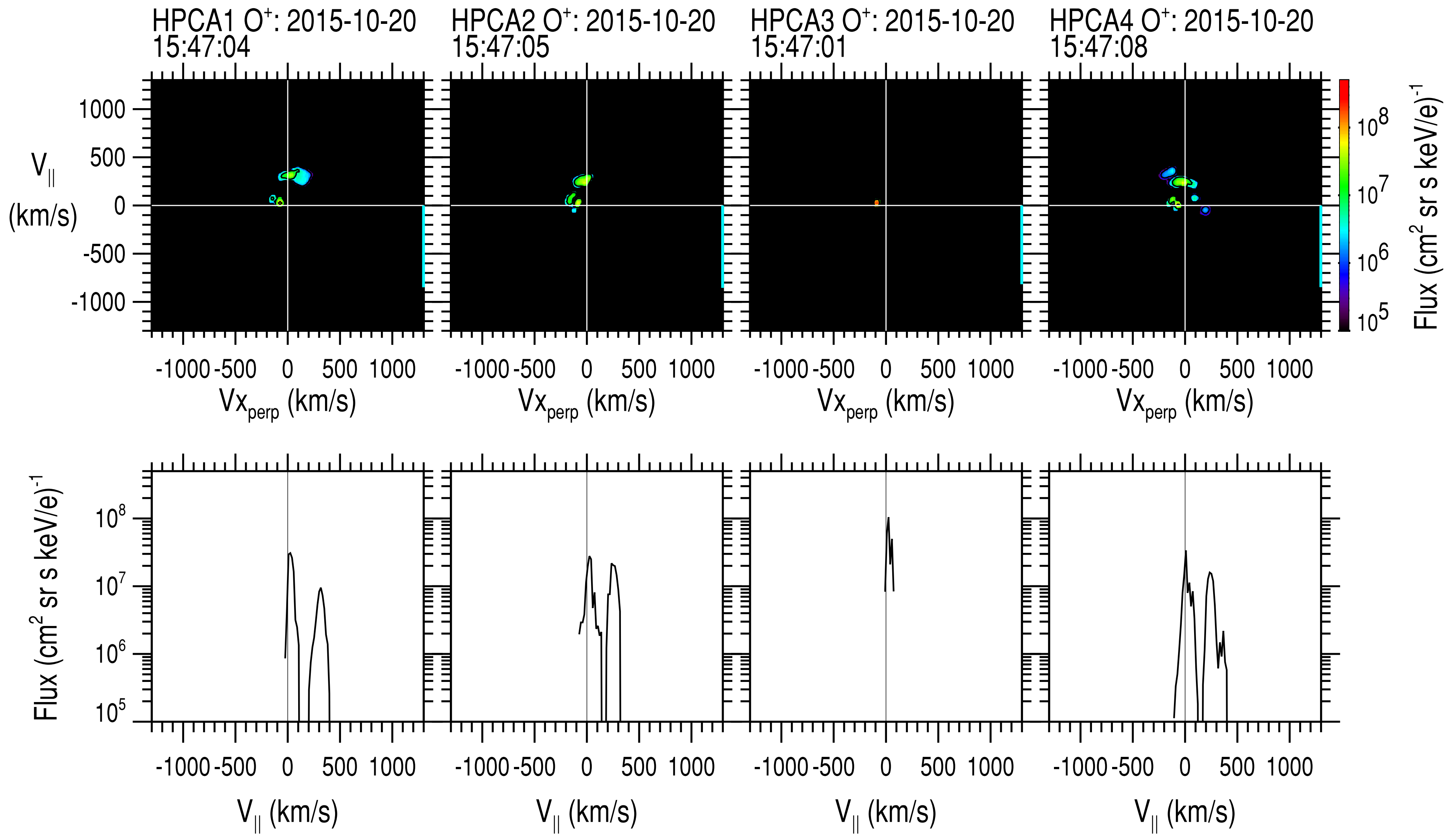

HPCA1 O+: 2015-10-20
15:47:04
HPCA2 O+: 2015-10-20
15:47:05
HPCA3 O+: 2015-10-20
15:47:01
HPCA4 O+: 2015-10-20
15:47:08
V|| (km/s)
Vxperp (km/s)
Flux (cm2 sr s keV/e)-1
1000
500
0
-500
-1000
10^5
10^6
10^7
10^8

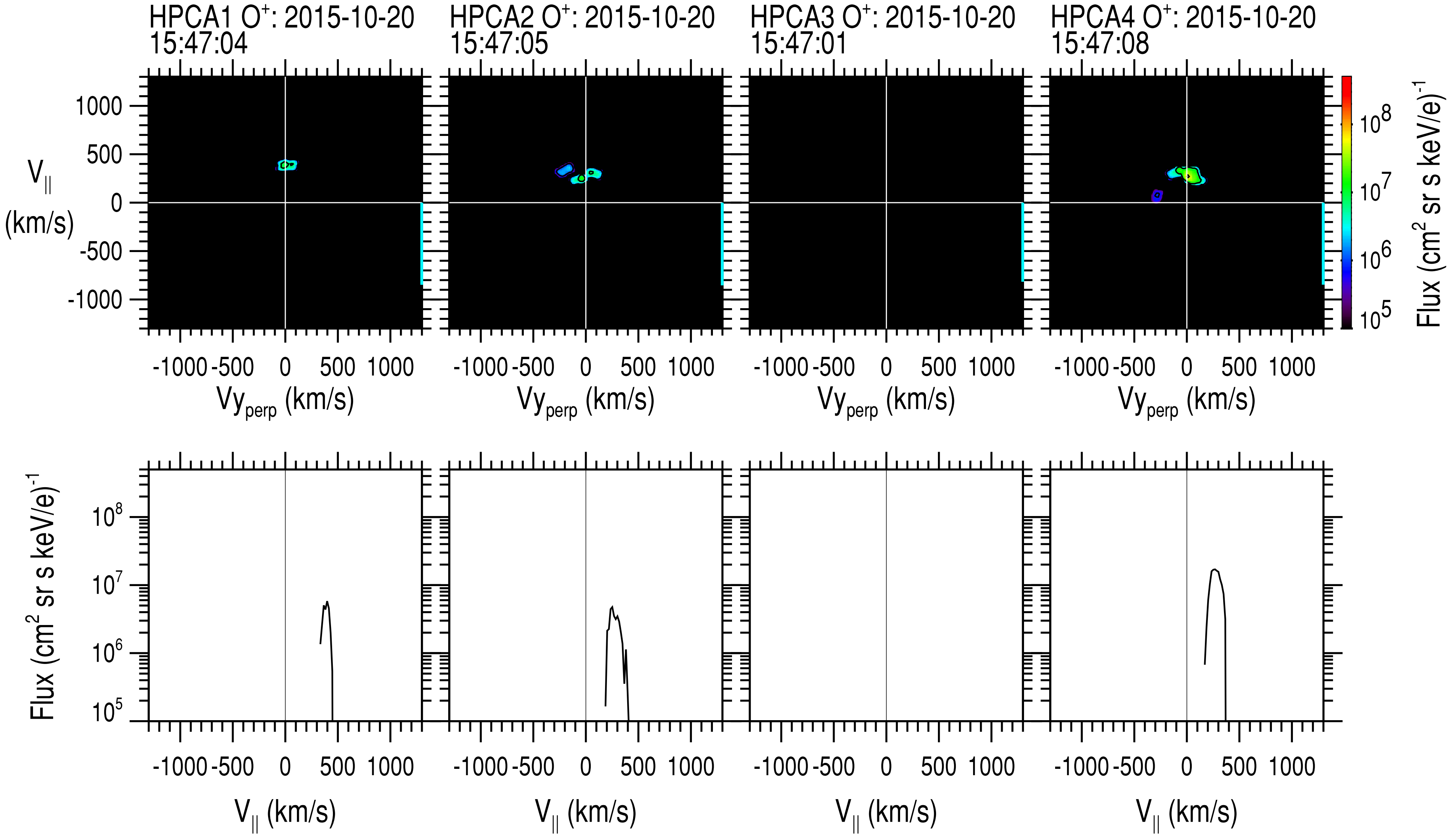
HPCA1 O+: 2015-10-20 15:47:04
HPCA2 O+: 2015-10-20 15:47:05
HPCA3 O+: 2015-10-20 15:47:01
HPCA4 O+: 2015-10-20 15:47:08
V|| (km/s)
Vyperp (km/s)
Flux (cm2 sr s keV/e)-1

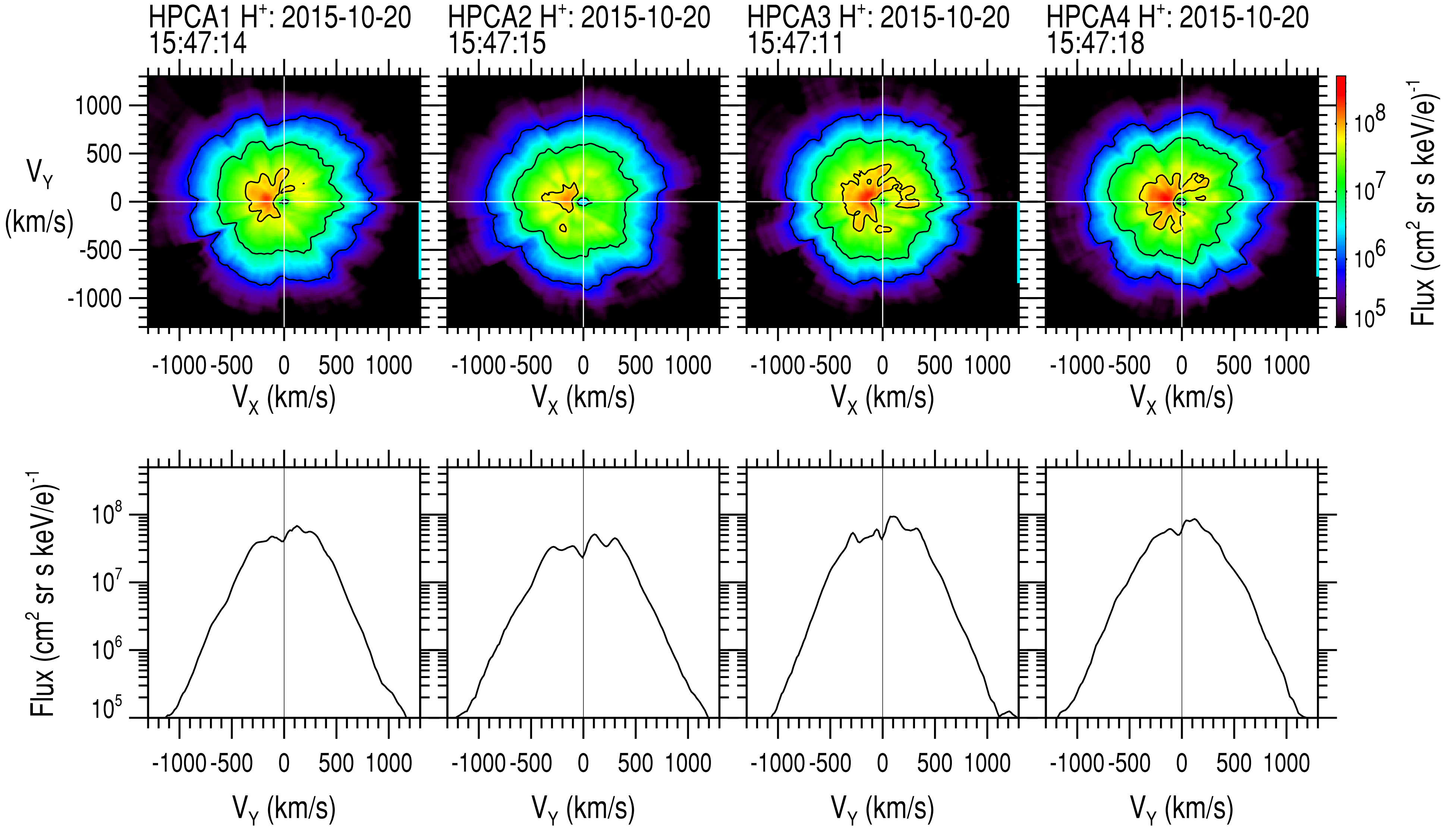
HPCA1 H+: 2015-10-20
15:47:14
HPCA2 H+: 2015-10-20
15:47:15
HPCA3 H+: 2015-10-20
15:47:11
HPCA4 H+: 2015-10-20
15:47:18
V_Y (km/s)
1000
500
0
-500
-1000
-1000 -500 0 500 1000
V_X (km/s)
Flux (cm^2 sr s keV/e)^-1
10^5
10^6
10^7
10^8
V_Y (km/s)

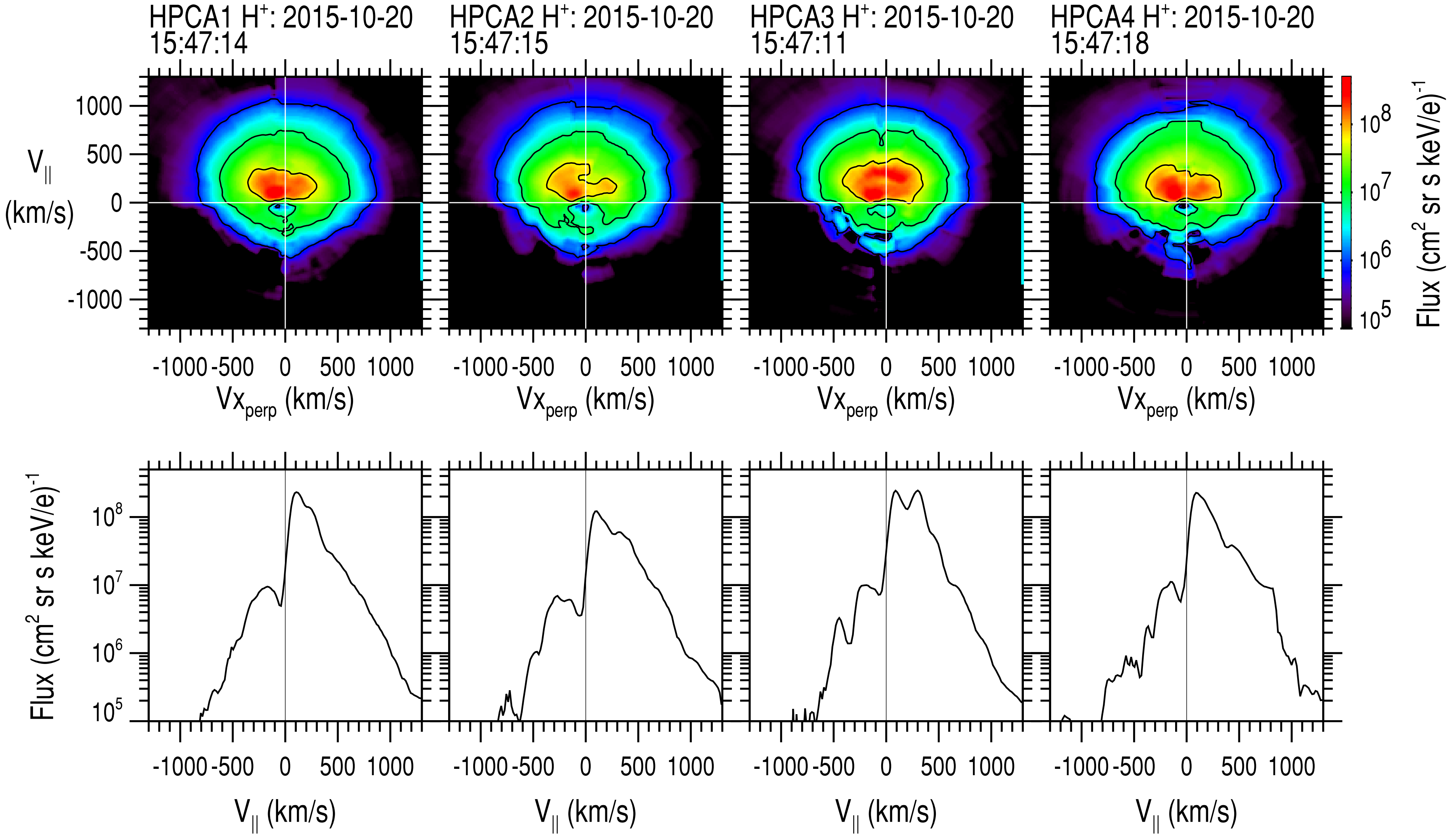

HPCA1 H+: 2015-10-20 15:47:14
HPCA2 H+: 2015-10-20 15:47:15
HPCA3 H+: 2015-10-20 15:47:11
HPCA4 H+: 2015-10-20 15:47:18
V|| (km/s)
Vxperp (km/s)
Flux (cm2 sr s keV/e)-1
V|| (km/s)

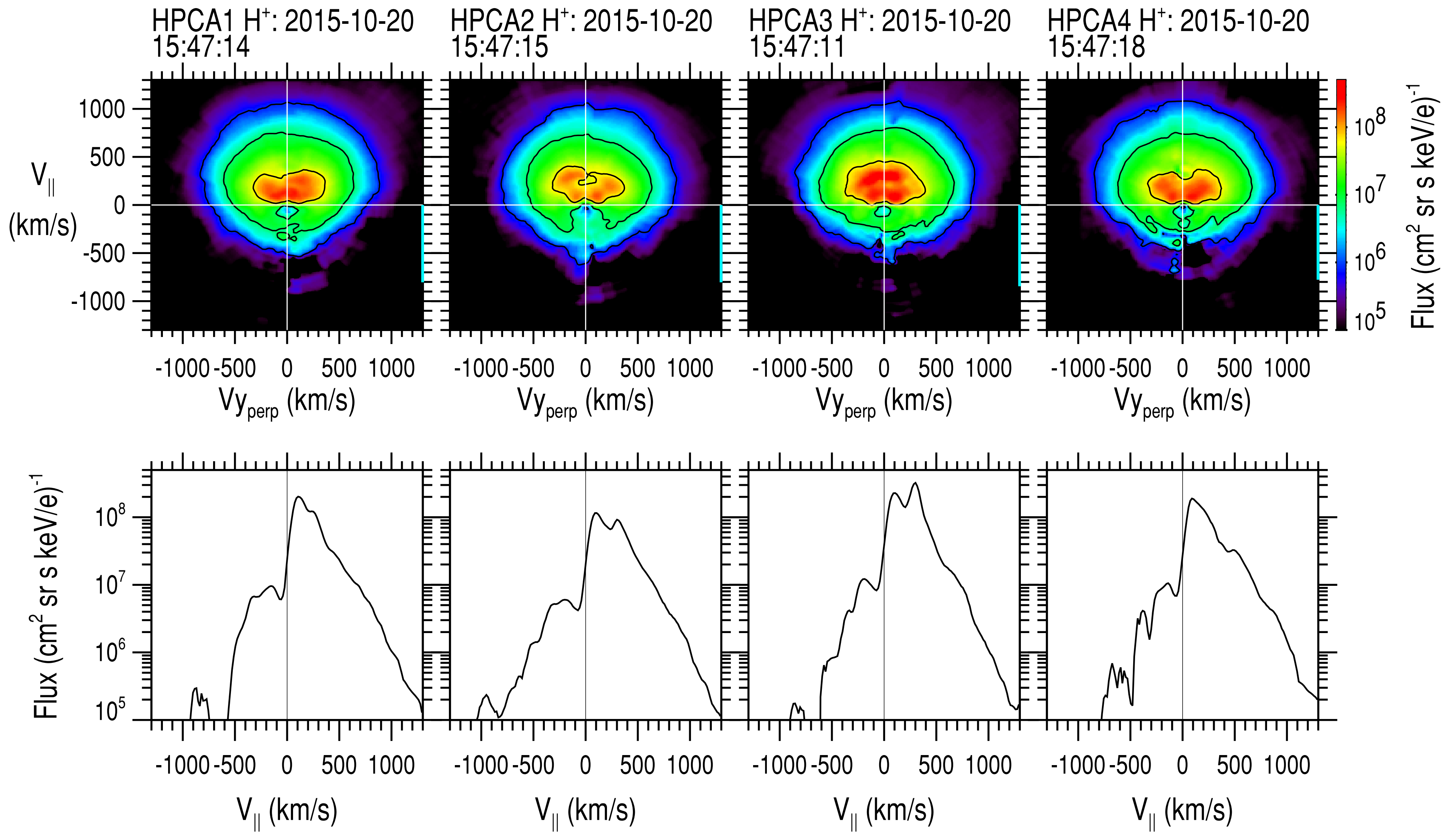
HPCA1 H+: 2015-10-20
15:47:14
HPCA2 H+: 2015-10-20
15:47:15
HPCA3 H+: 2015-10-20
15:47:11
HPCA4 H+: 2015-10-20
15:47:18
V|| (km/s)
Vyperp (km/s)
Flux (cm2 sr s keV/e)-1
V|| (km/s)
-1000 -500 0 500 1000
10^5 10^6 10^7 10^8

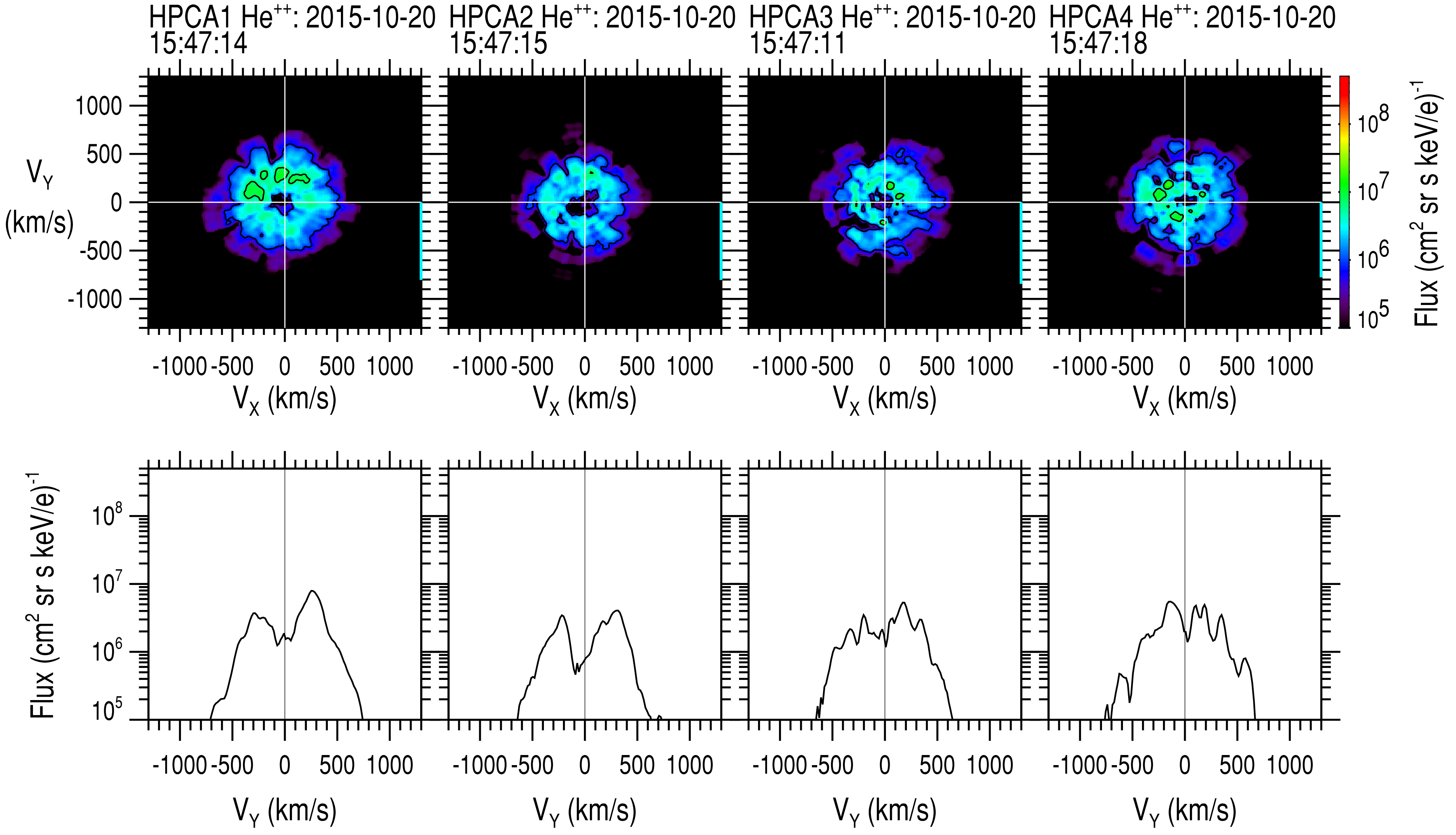
HPCA1 He++: 2015-10-20
15:47:14
HPCA2 He++: 2015-10-20
15:47:15
HPCA3 He++: 2015-10-20
15:47:11
HPCA4 He++: 2015-10-20
15:47:18
VY (km/s)
VX (km/s)
Flux (cm2 sr s keV/e)-1
105
106
107
108
-1000 -500 0 500 1000
VY (km/s)

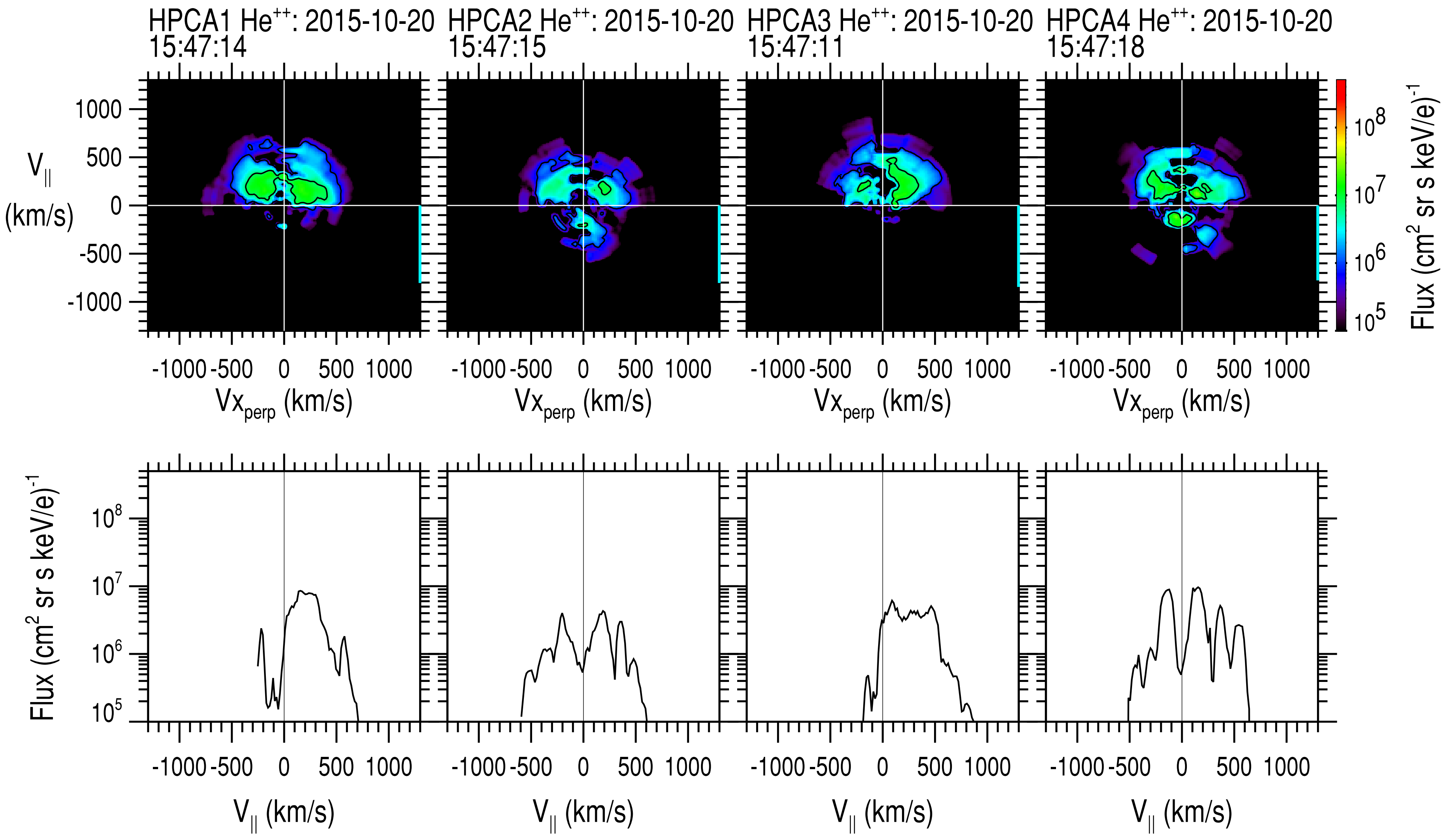
HPCA1 He++: 2015-10-20 15:47:14
HPCA2 He++: 2015-10-20 15:47:15
HPCA3 He++: 2015-10-20 15:47:11
HPCA4 He++: 2015-10-20 15:47:18
V|| (km/s)
1000
500
0
-500
-1000
-1000 -500 0 500 1000
Vxperp (km/s)
Flux (cm2 sr s keV/e)-1
10^5
10^6
10^7
10^8
V|| (km/s)

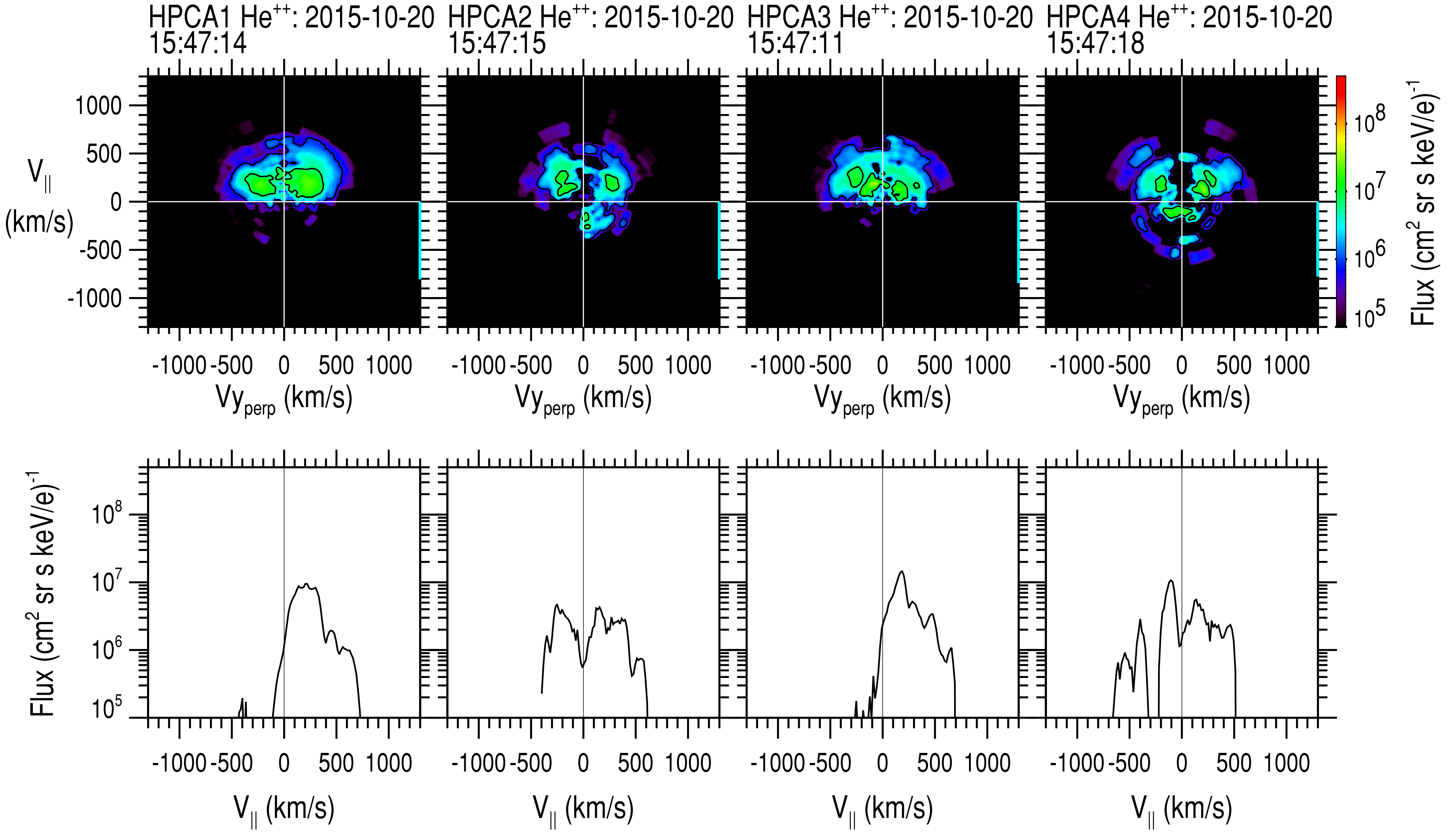
HPCA1 He++: 2015-10-20 15:47:14
HPCA2 He++: 2015-10-20 15:47:15
HPCA3 He++: 2015-10-20 15:47:11
HPCA4 He++: 2015-10-20 15:47:18
V|| (km/s)
Vyperp (km/s)
Flux (cm2 sr s keV/e)-1
V|| (km/s)

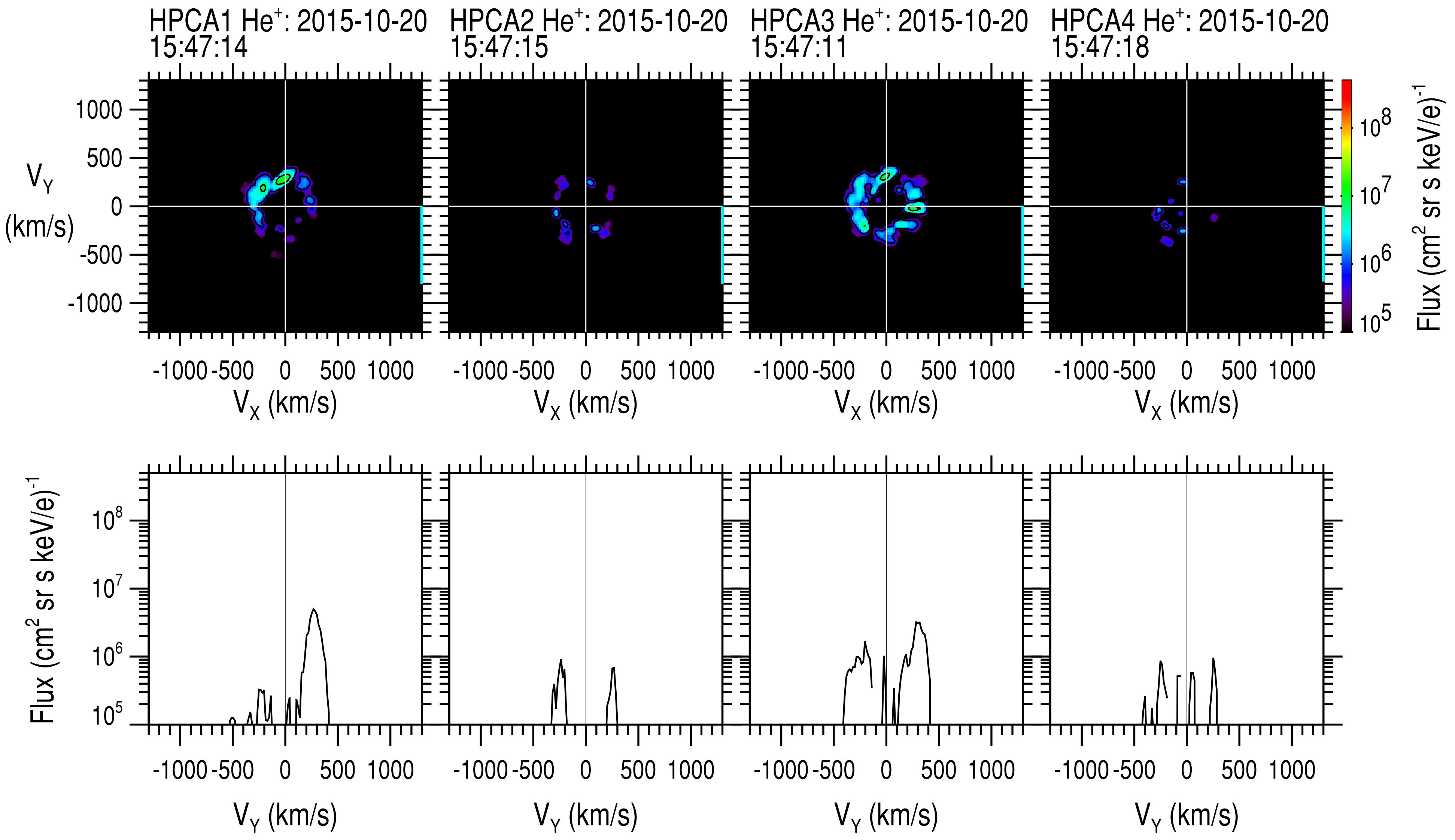
HPCA1 He+: 2015-10-20 15:47:14
HPCA2 He+: 2015-10-20 15:47:15
HPCA3 He+: 2015-10-20 15:47:11
HPCA4 He+: 2015-10-20 15:47:18
V_Y (km/s)
V_X (km/s)
Flux (cm^2 sr s keV/e)^-1
V_Y (km/s)
Flux (cm^2 sr s keV/e)^-1

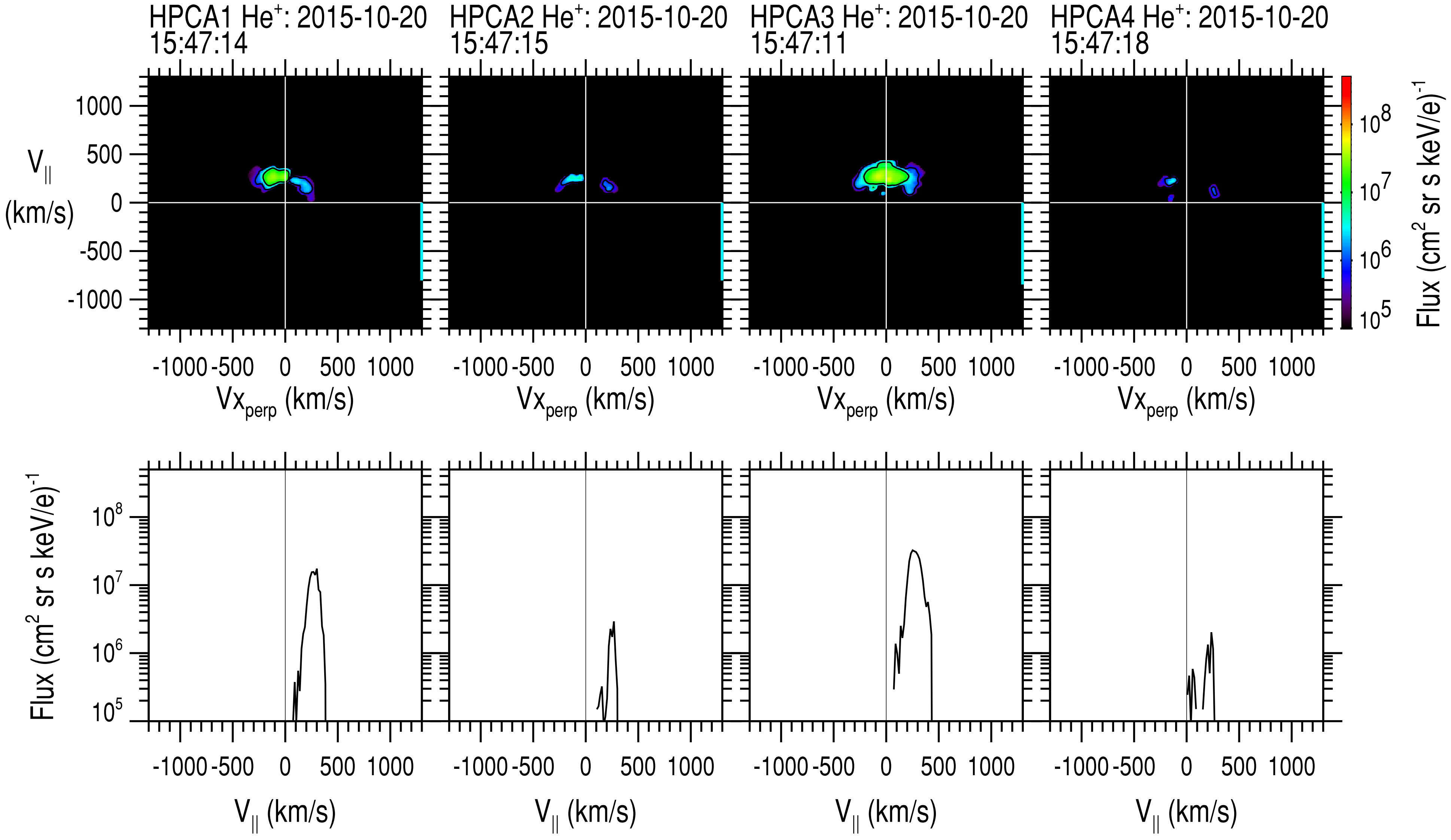
HPCA1 He+: 2015-10-20 15:47:14
HPCA2 He+: 2015-10-20 15:47:15
HPCA3 He+: 2015-10-20 15:47:11
HPCA4 He+: 2015-10-20 15:47:18
V|| (km/s)
Vxperp (km/s)
Flux (cm2 sr s keV/e)-1
1000
500
0
-500
-1000
10^5
10^6
10^7
10^8

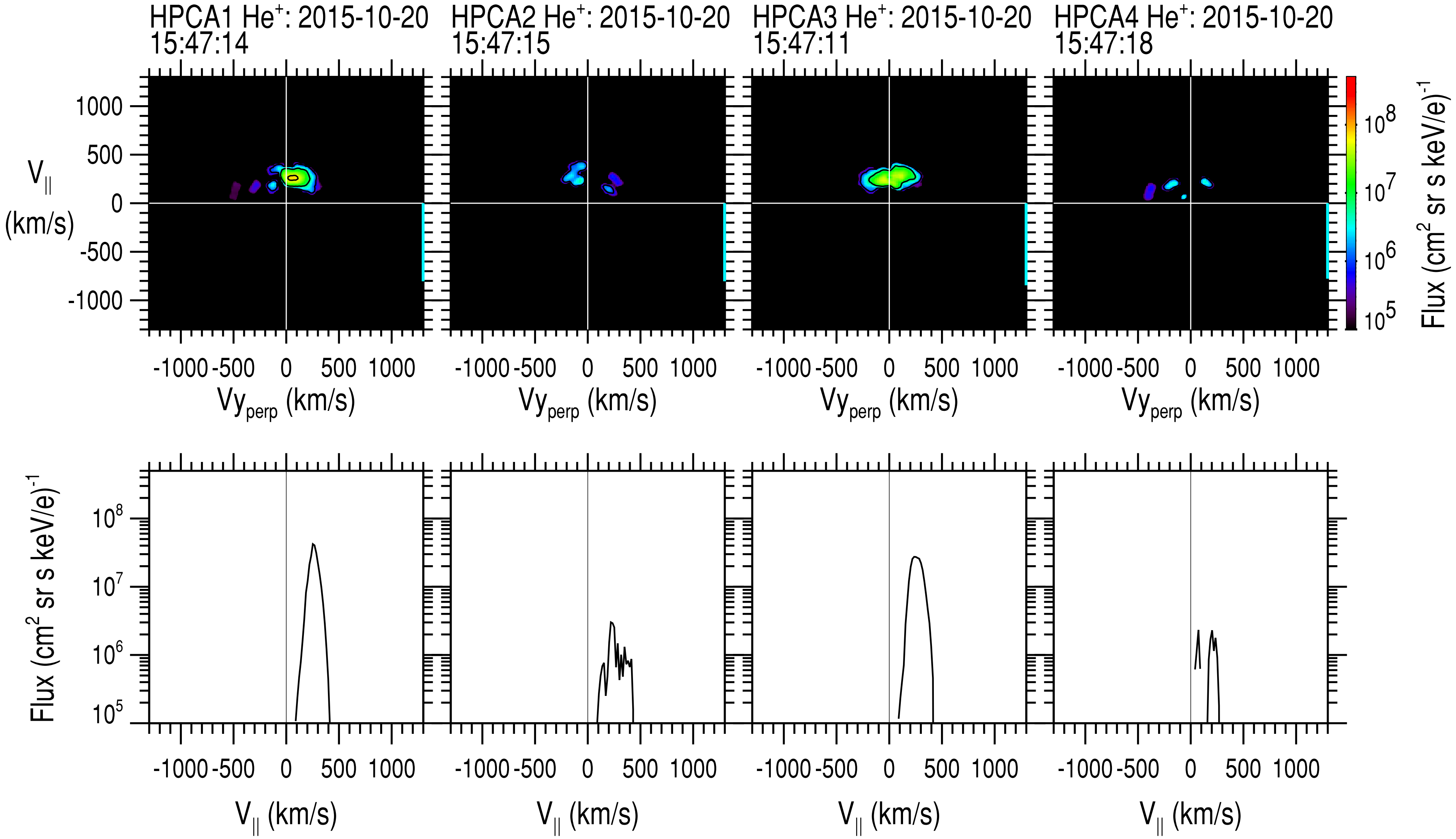
HPCA1 He+: 2015-10-20 15:47:14
HPCA2 He+: 2015-10-20 15:47:15
HPCA3 He+: 2015-10-20 15:47:11
HPCA4 He+: 2015-10-20 15:47:18
V|| (km/s)
Vyperp (km/s)
Flux (cm2 sr s keV/e)-1
1000
500
0
-500
-1000
10^5
10^6
10^7
10^8

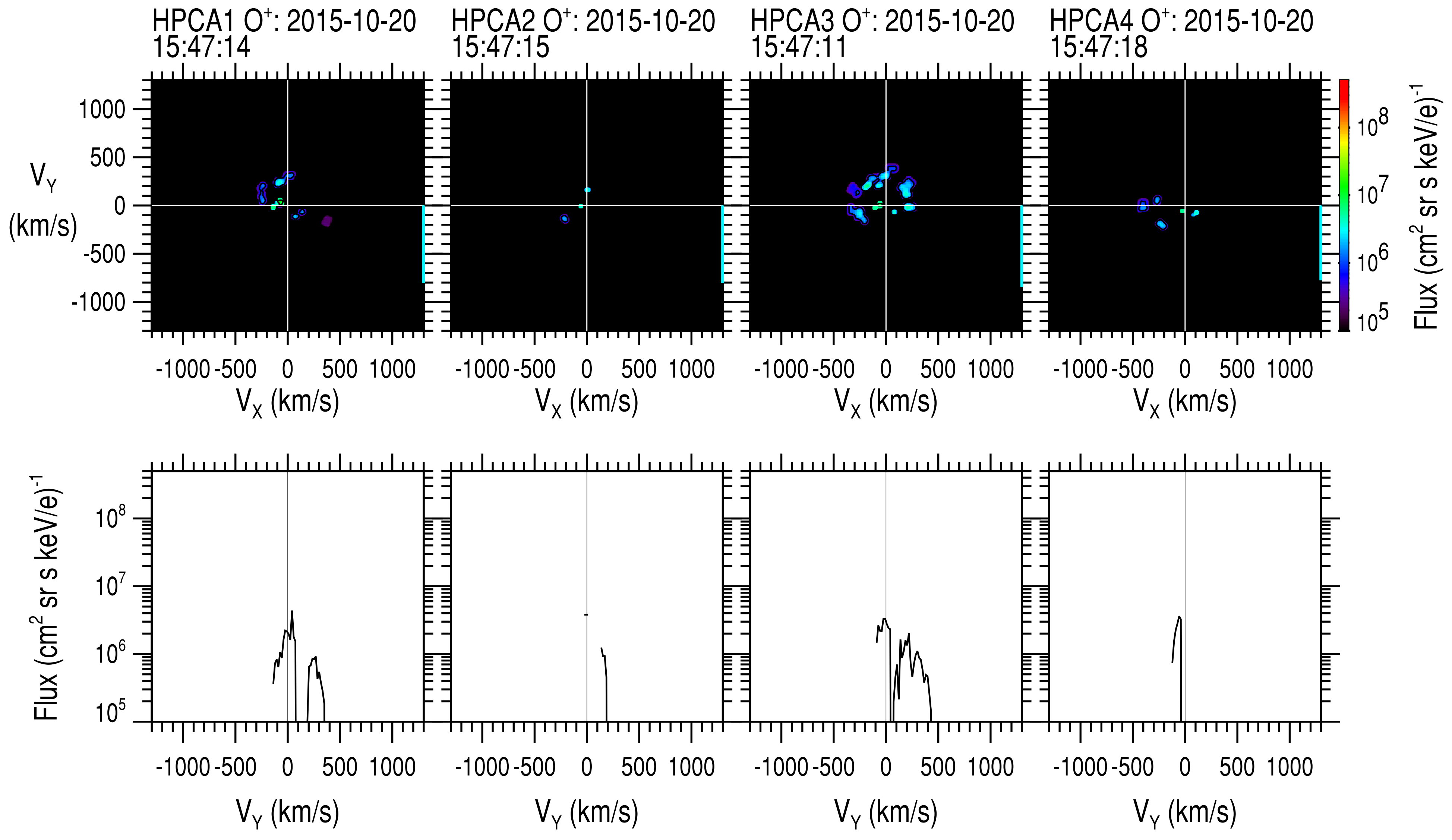

HPCA1 O+: 2015-10-20
15:47:14
HPCA2 O+: 2015-10-20
15:47:15
HPCA3 O+: 2015-10-20
15:47:11
HPCA4 O+: 2015-10-20
15:47:18
V_Y (km/s)
V_X (km/s)
Flux (cm^2 sr s keV/e)^-1
-1000 -500 0 500 1000
10^5 10^6 10^7 10^8

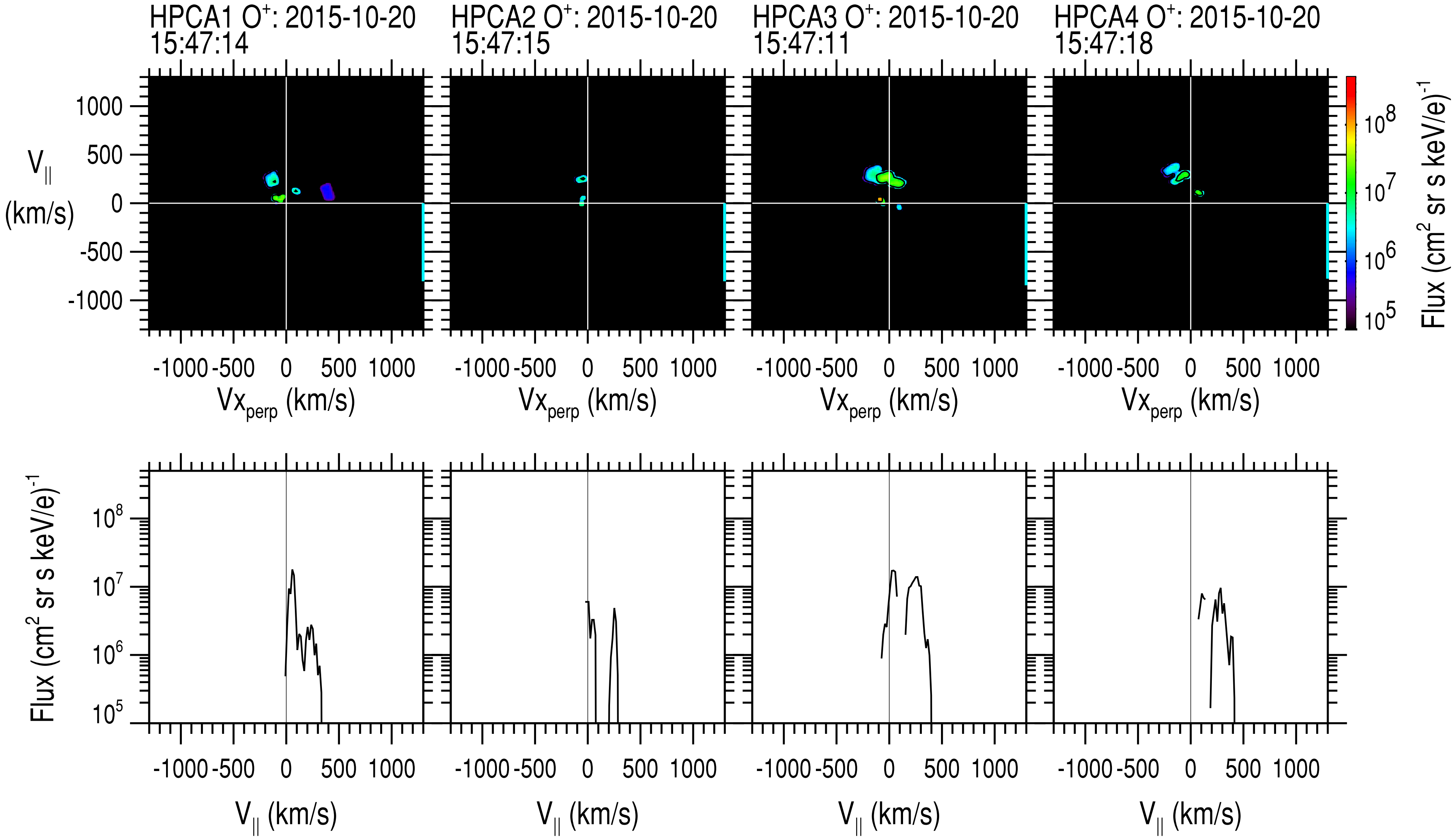
HPCA1 O+: 2015-10-20 15:47:14
HPCA2 O+: 2015-10-20 15:47:15
HPCA3 O+: 2015-10-20 15:47:11
HPCA4 O+: 2015-10-20 15:47:18
V|| (km/s)
Vxperp (km/s)
Flux (cm2 sr s keV/e)-1
V|| (km/s)

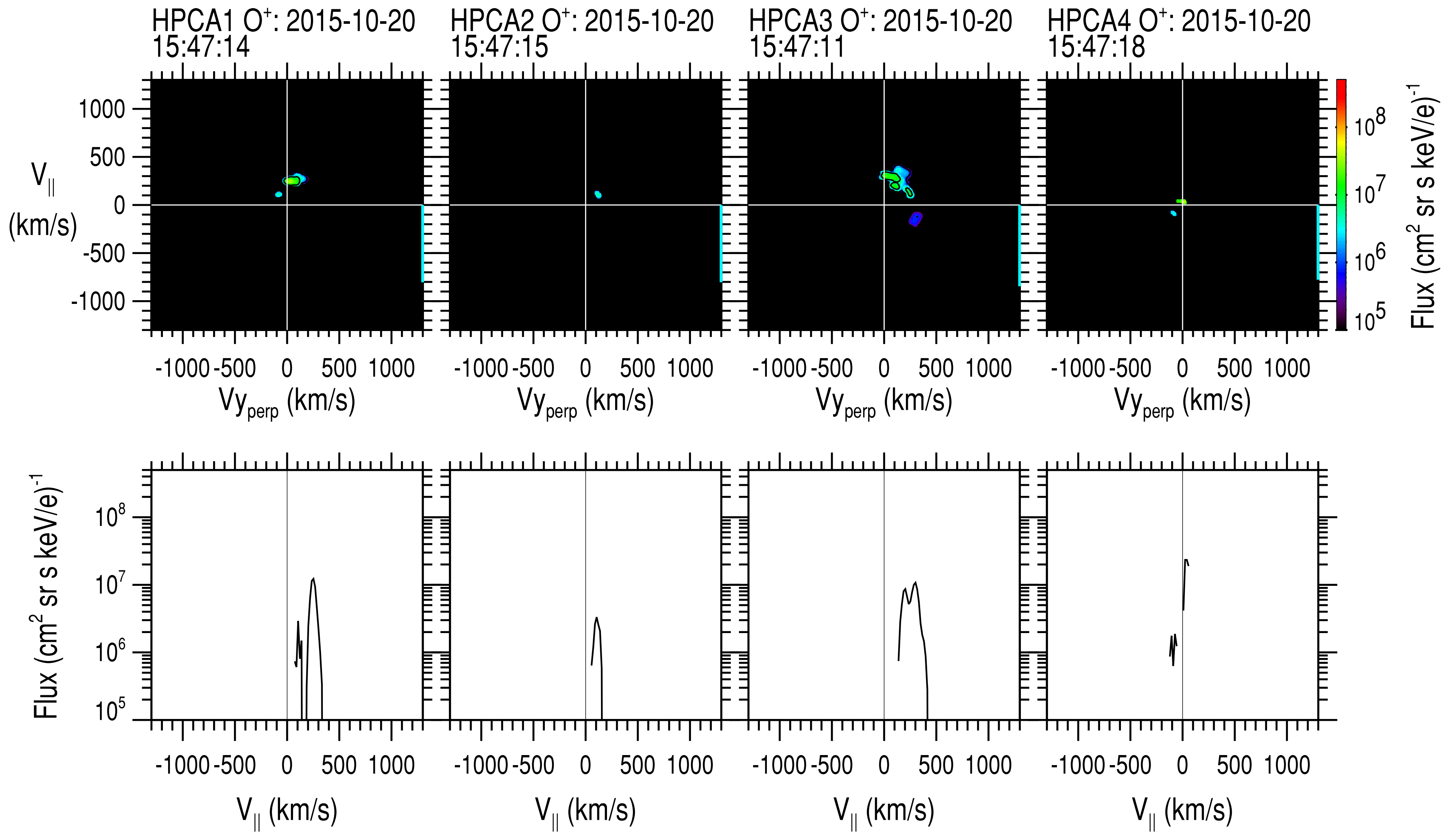

HPCA1 O+: 2015-10-20
15:47:14
HPCA2 O+: 2015-10-20
15:47:15
HPCA3 O+: 2015-10-20
15:47:11
HPCA4 O+: 2015-10-20
15:47:18
V|| (km/s)
Vyperp (km/s)
Flux (cm2 sr s keV/e)-1

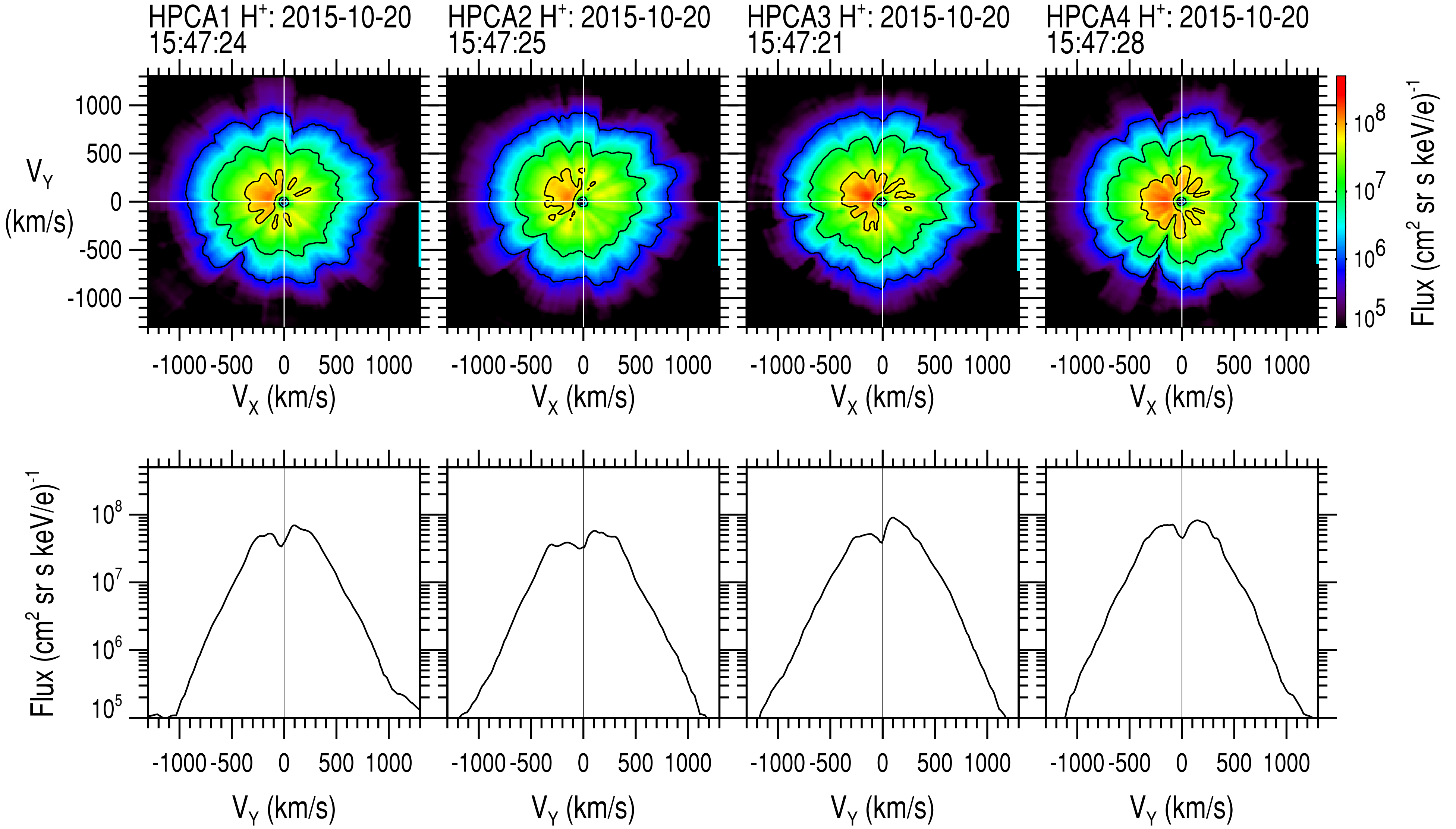

HPCA1 H+: 2015-10-20 15:47:24
HPCA2 H+: 2015-10-20 15:47:25
HPCA3 H+: 2015-10-20 15:47:21
HPCA4 H+: 2015-10-20 15:47:28
V_Y (km/s)
V_X (km/s)
Flux (cm^2 sr s keV/e)^-1

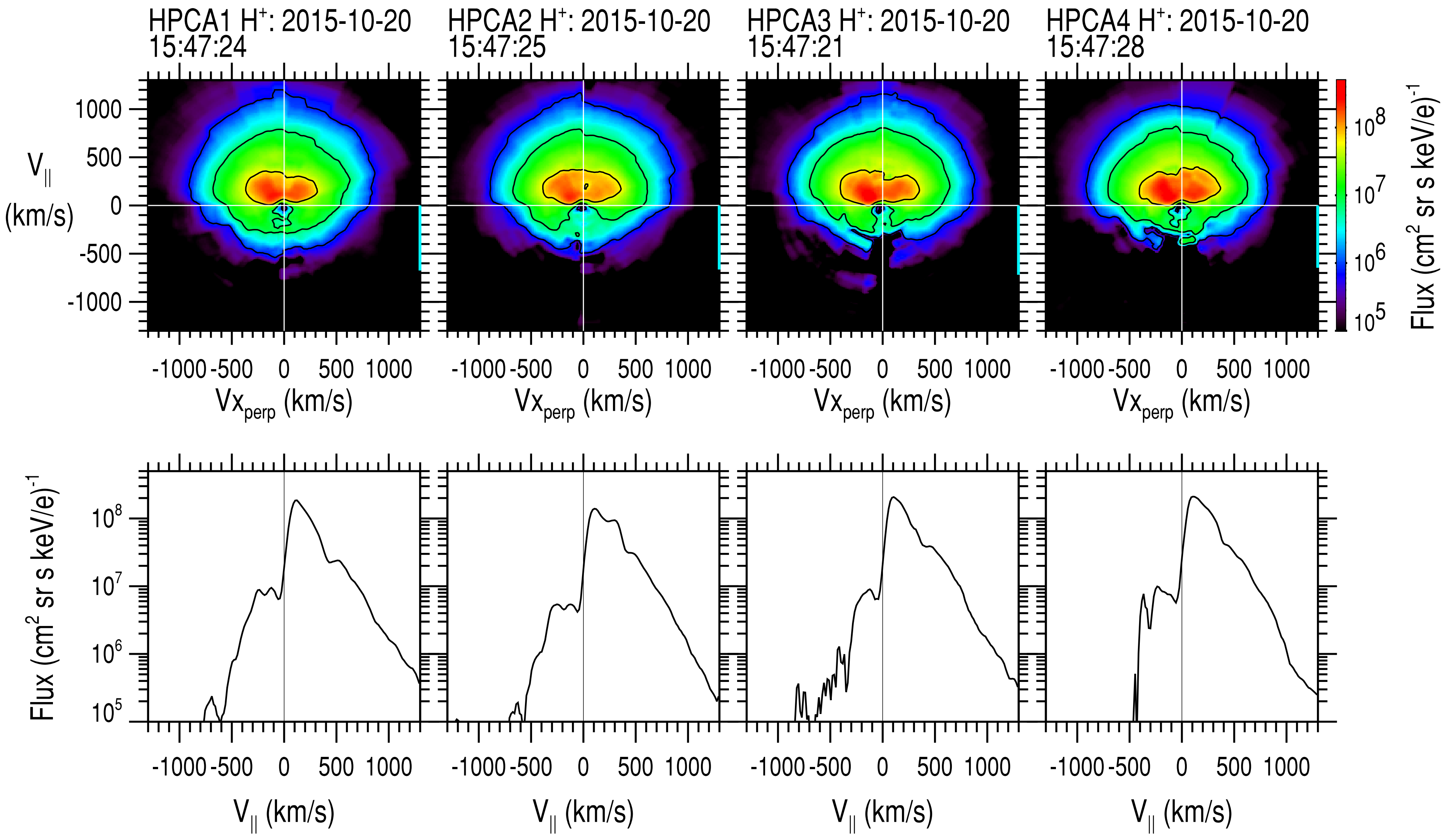
HPCA1 H+: 2015-10-20 15:47:24
HPCA2 H+: 2015-10-20 15:47:25
HPCA3 H+: 2015-10-20 15:47:21
HPCA4 H+: 2015-10-20 15:47:28
V|| (km/s)
Vxperp (km/s)
Flux (cm2 sr s keV/e)-1

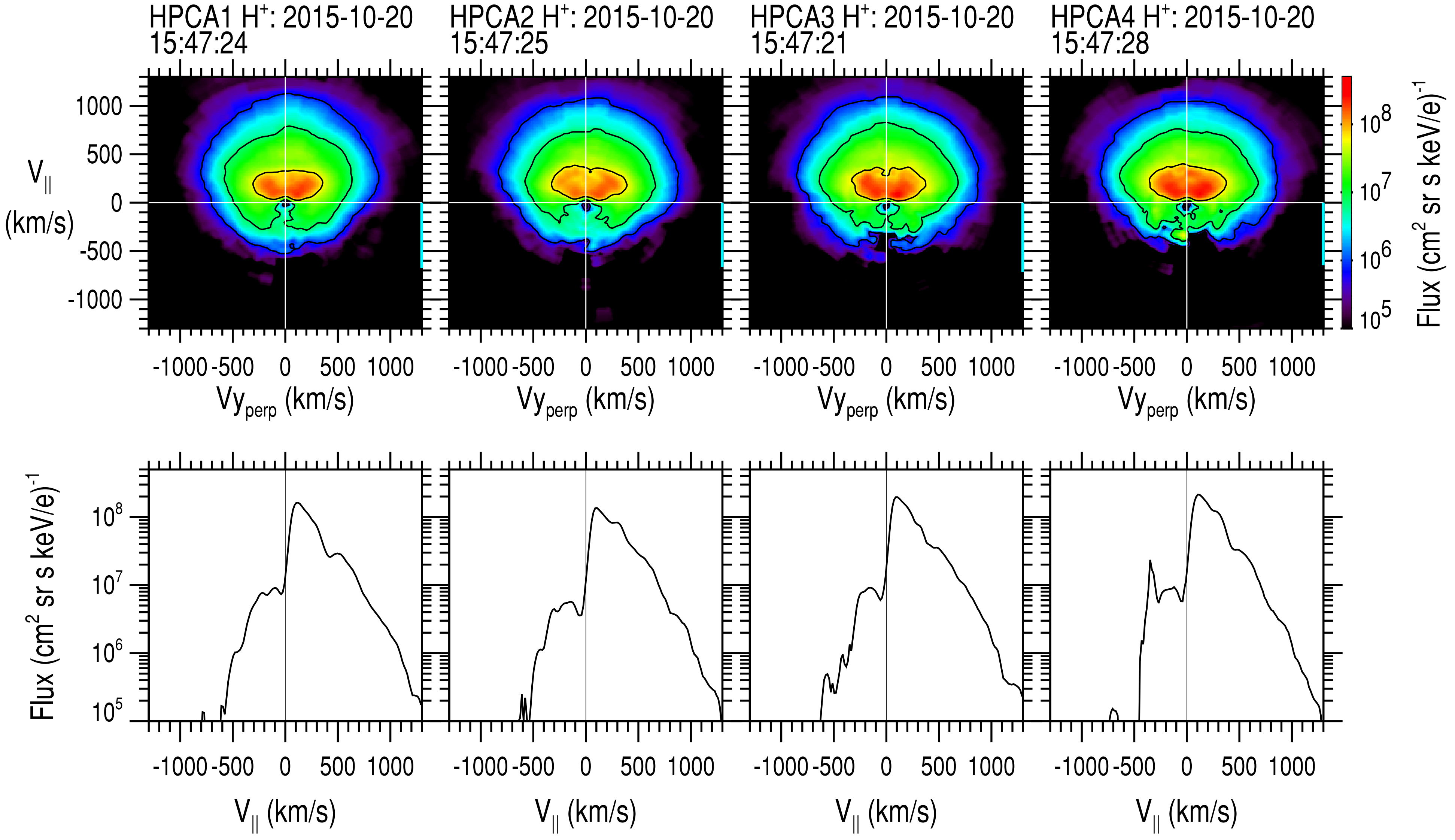

HPCA1 H+: 2015-10-20 15:47:24
HPCA2 H+: 2015-10-20 15:47:25
HPCA3 H+: 2015-10-20 15:47:21
HPCA4 H+: 2015-10-20 15:47:28
V|| (km/s)
Vyperp (km/s)
Flux (cm2 sr s keV/e)-1

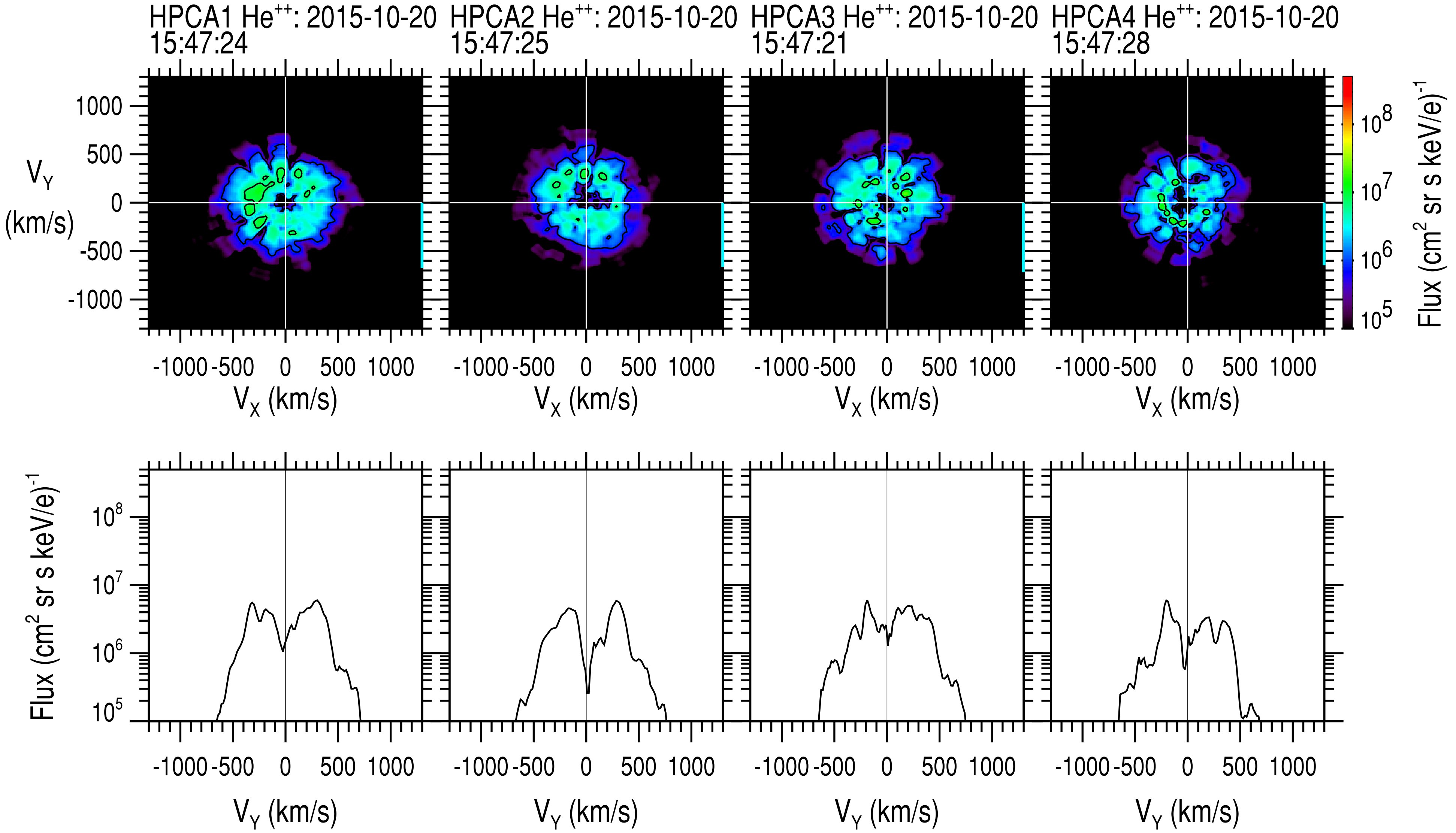

HPCA1 He++: 2015-10-20 15:47:24
HPCA2 He++: 2015-10-20 15:47:25
HPCA3 He++: 2015-10-20 15:47:21
HPCA4 He++: 2015-10-20 15:47:28
V_Y (km/s)
V_X (km/s)
Flux (cm^2 sr s keV/e)^-1

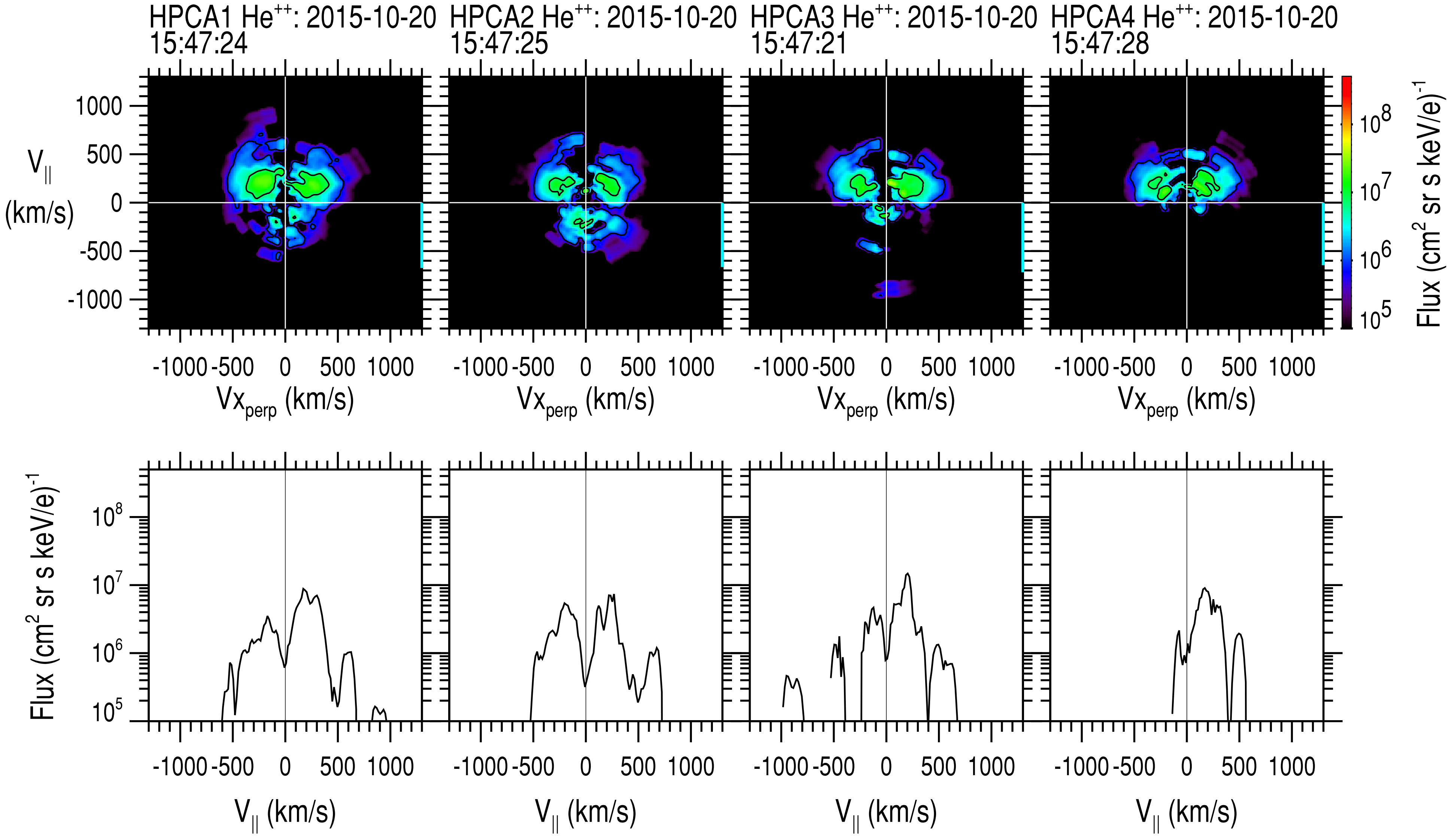

HPCA1 He++: 2015-10-20 15:47:24
HPCA2 He++: 2015-10-20 15:47:25
HPCA3 He++: 2015-10-20 15:47:21
HPCA4 He++: 2015-10-20 15:47:28
V|| (km/s)
Vxperp (km/s)
Flux (cm2 sr s keV/e)-1

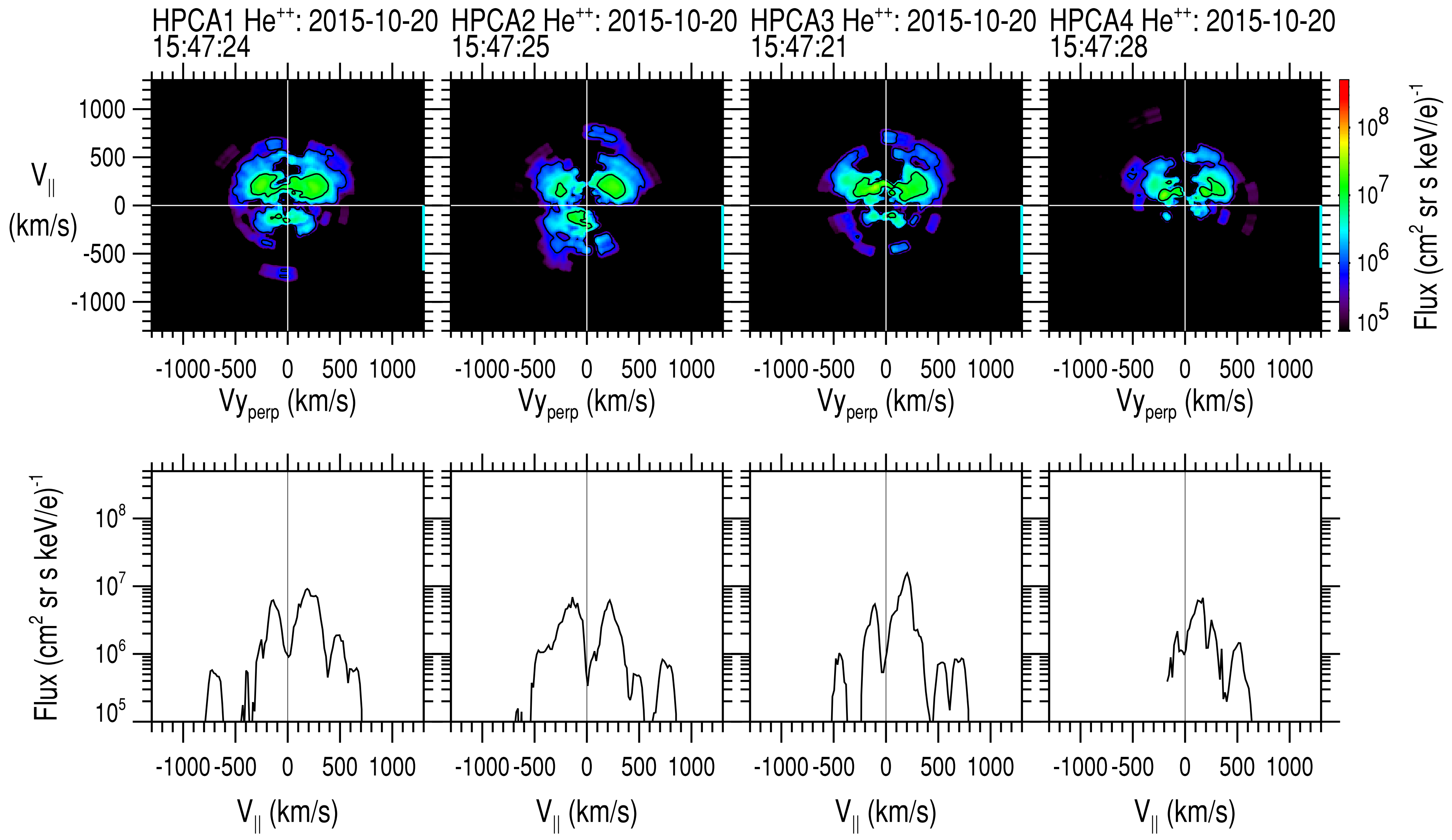

HPCA1 He++: 2015-10-20 15:47:24
HPCA2 He++: 2015-10-20 15:47:25
HPCA3 He++: 2015-10-20 15:47:21
HPCA4 He++: 2015-10-20 15:47:28
V|| (km/s)
Vyperp (km/s)
Flux (cm2 sr s keV/e)-1
1000
500
0
-500
-1000
105
106
107
108

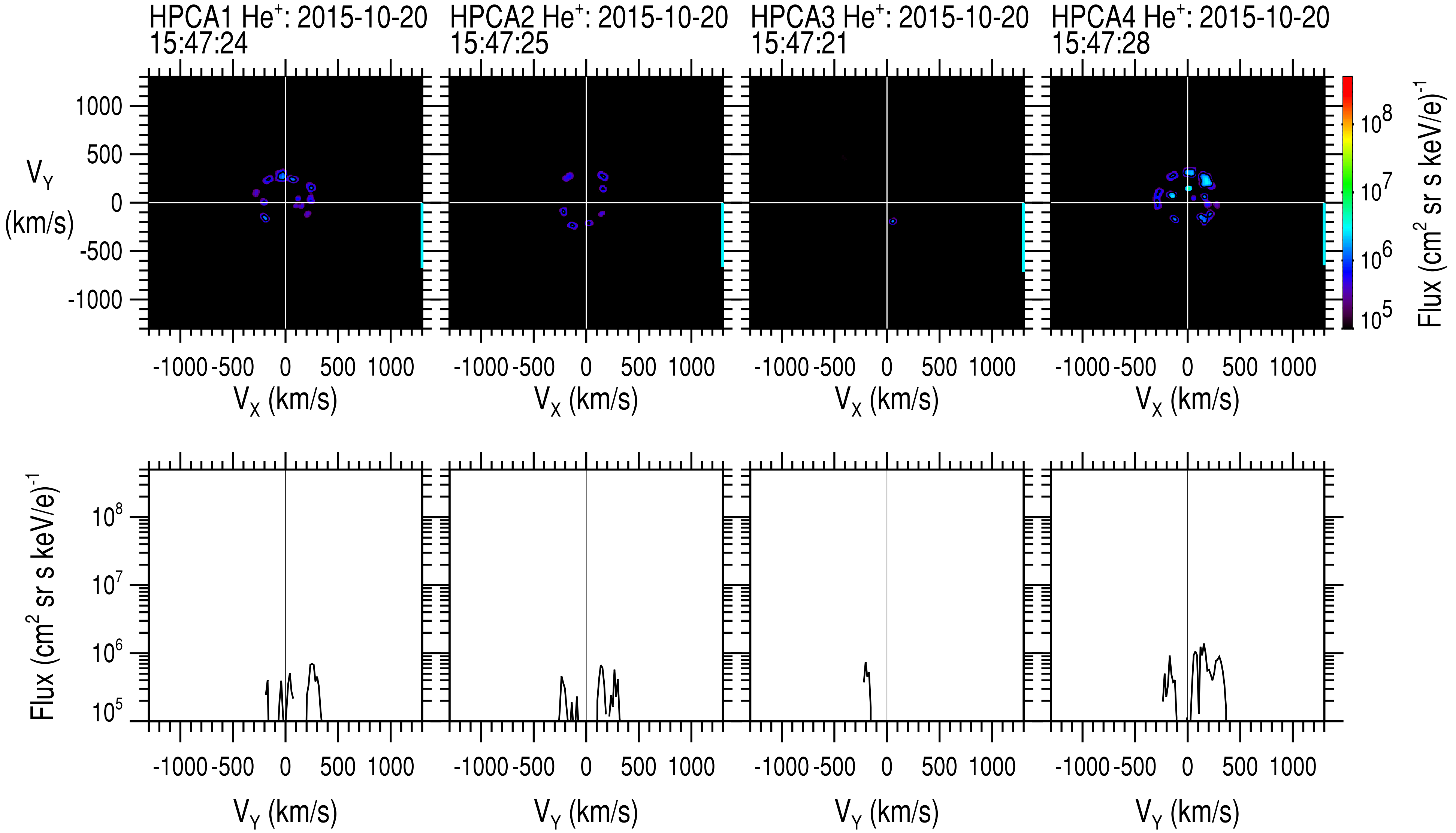
HPCA1 He+: 2015-10-20 15:47:24
HPCA2 He+: 2015-10-20 15:47:25
HPCA3 He+: 2015-10-20 15:47:21
HPCA4 He+: 2015-10-20 15:47:28
VY (km/s)
VX (km/s)
Flux (cm2 sr s keV/e)-1
VY (km/s)

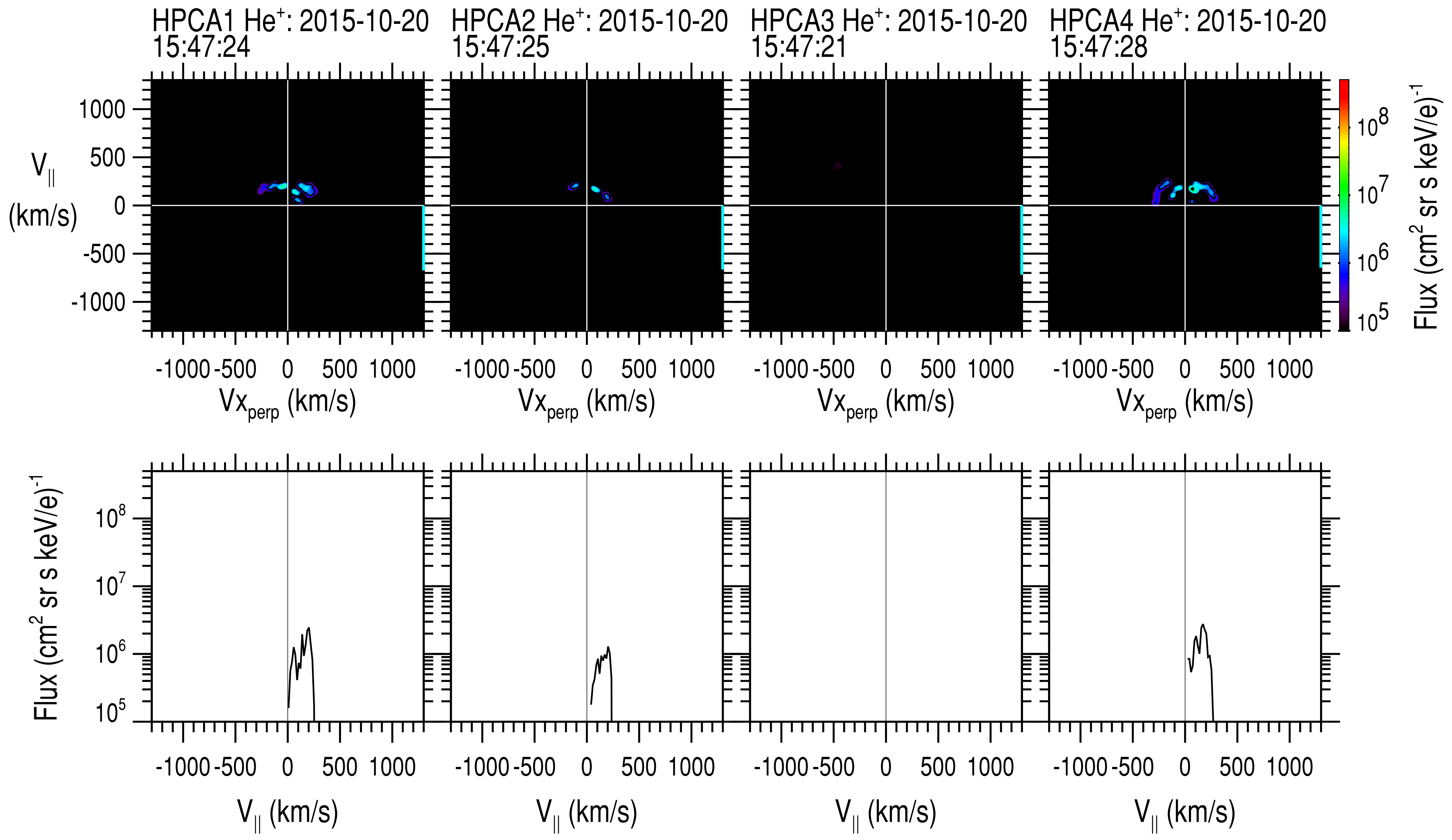
HPCA1 He+: 2015-10-20 15:47:24
HPCA2 He+: 2015-10-20 15:47:25
HPCA3 He+: 2015-10-20 15:47:21
HPCA4 He+: 2015-10-20 15:47:28
V|| (km/s)
Vxperp (km/s)
Flux (cm2 sr s keV/e)-1
-1000 -500 0 500 1000
10^5 10^6 10^7 10^8

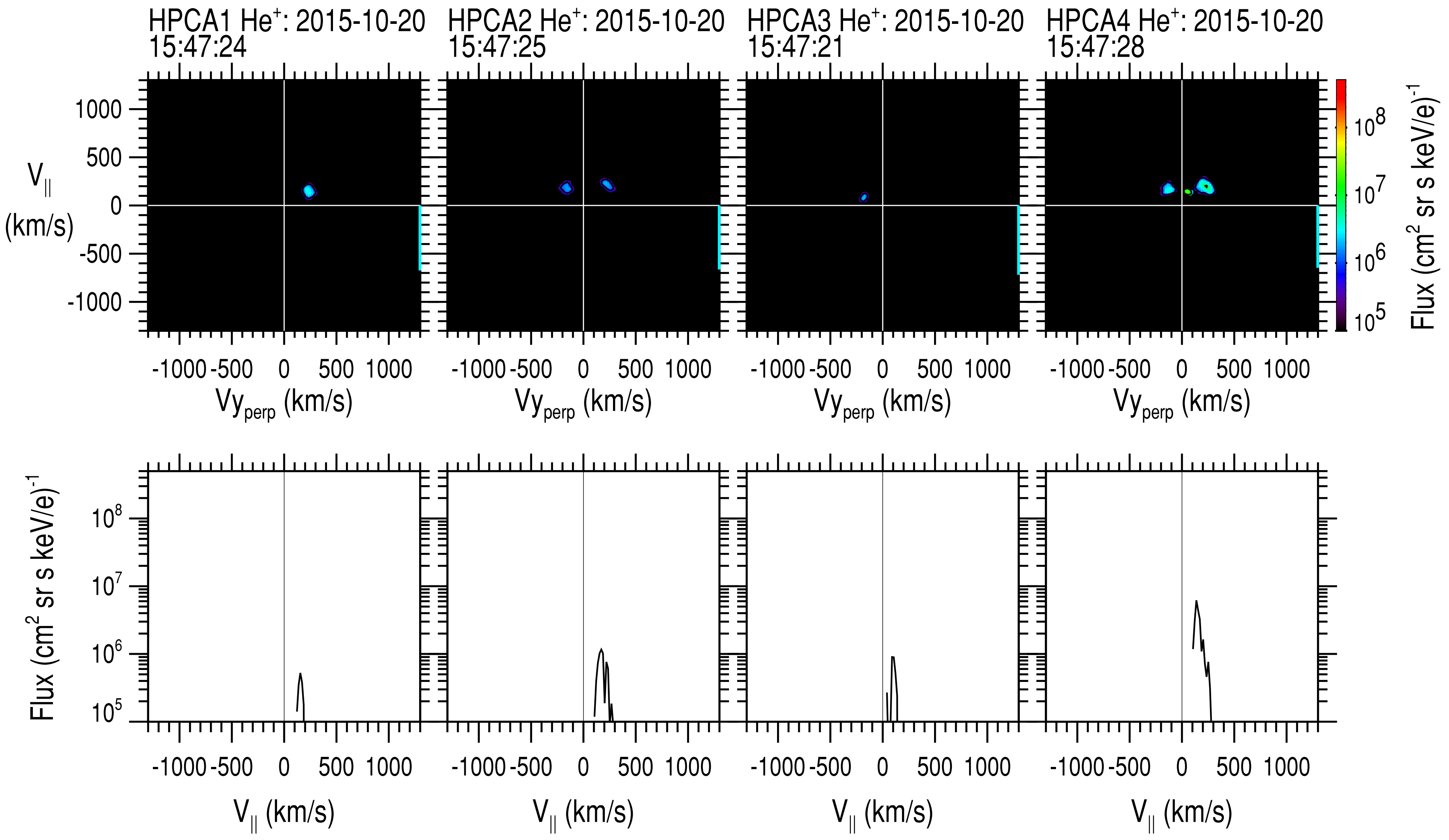
HPCA1 He+: 2015-10-20 15:47:24
HPCA2 He+: 2015-10-20 15:47:25
HPCA3 He+: 2015-10-20 15:47:21
HPCA4 He+: 2015-10-20 15:47:28
V|| (km/s)
Vyperp (km/s)
Flux (cm2 sr s keV/e)-1
1000
500
0
-500
-1000
10^5
10^6
10^7
10^8

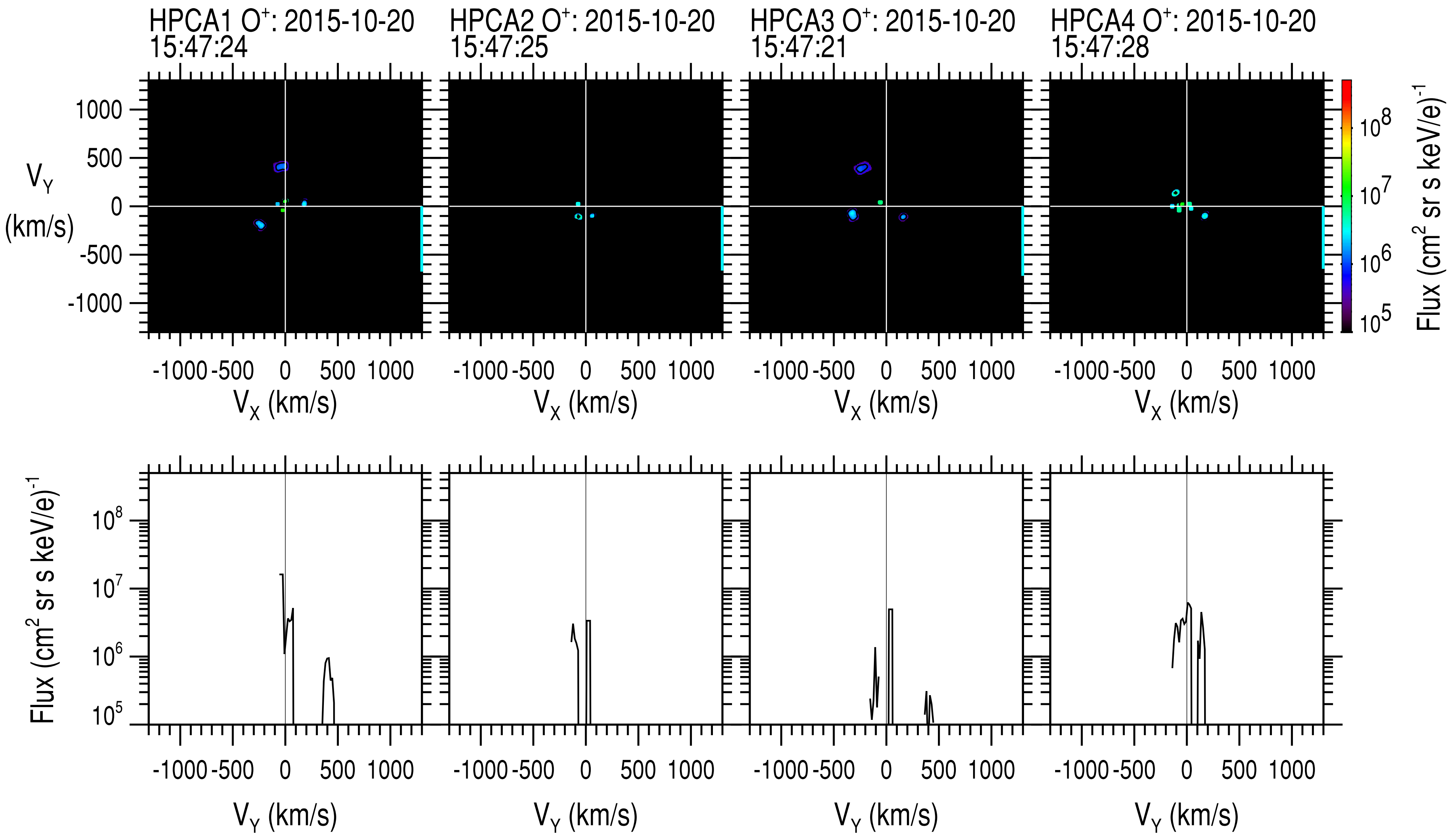

HPCA1 O+: 2015-10-20
15:47:24
HPCA2 O+: 2015-10-20
15:47:25
HPCA3 O+: 2015-10-20
15:47:21
HPCA4 O+: 2015-10-20
15:47:28
V_Y (km/s)
1000
500
0
-500
-1000
-1000 -500 0 500 1000
V_X (km/s)
Flux (cm^2 sr s keV/e)^-1
10^5
10^6
10^7
10^8
V_Y (km/s)

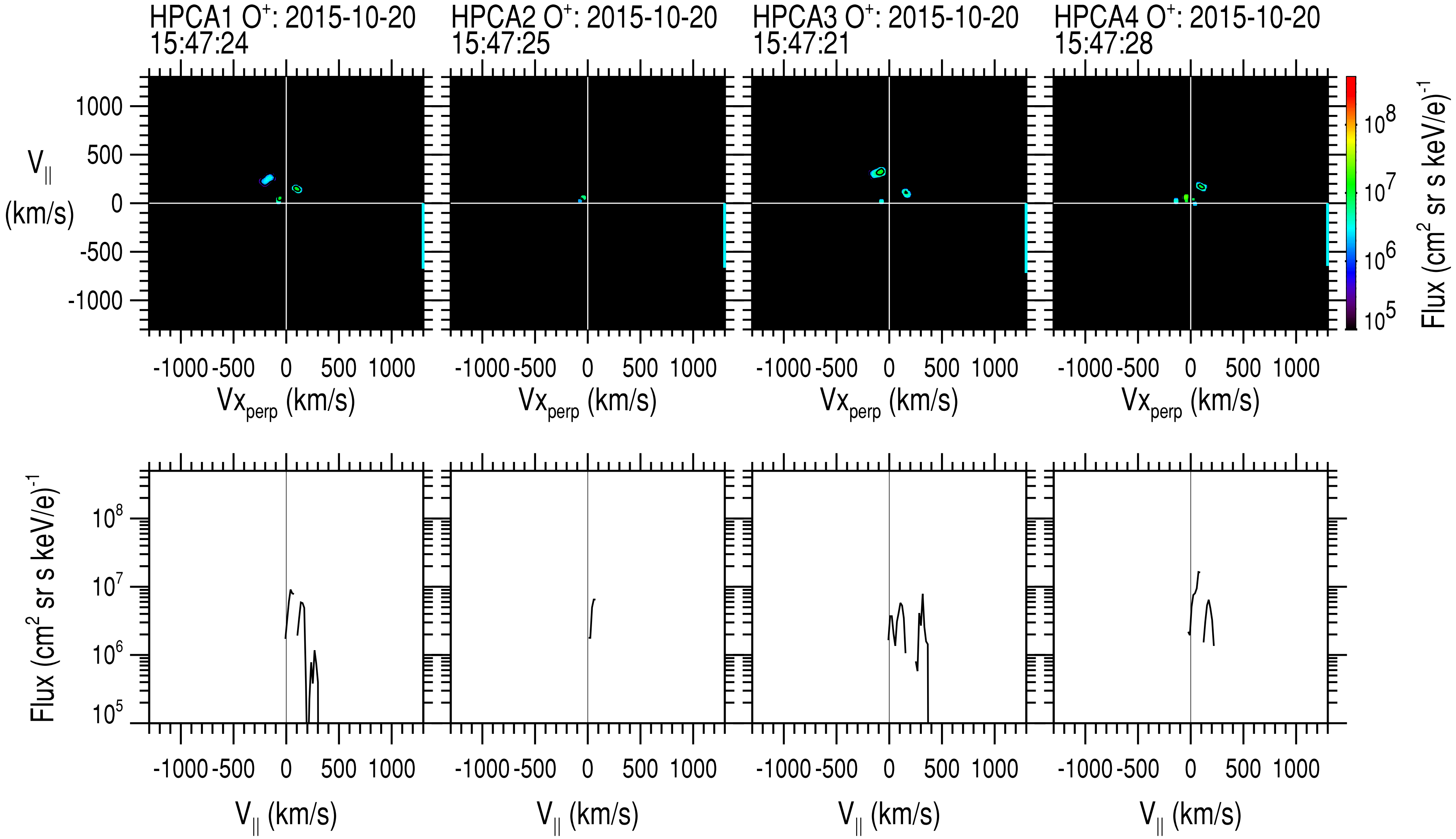
HPCA1 O+: 2015-10-20 15:47:24
HPCA2 O+: 2015-10-20 15:47:25
HPCA3 O+: 2015-10-20 15:47:21
HPCA4 O+: 2015-10-20 15:47:28
V|| (km/s)
Vxperp (km/s)
Flux (cm2 sr s keV/e)-1
1000
500
0
-500
-1000
105
106
107
108

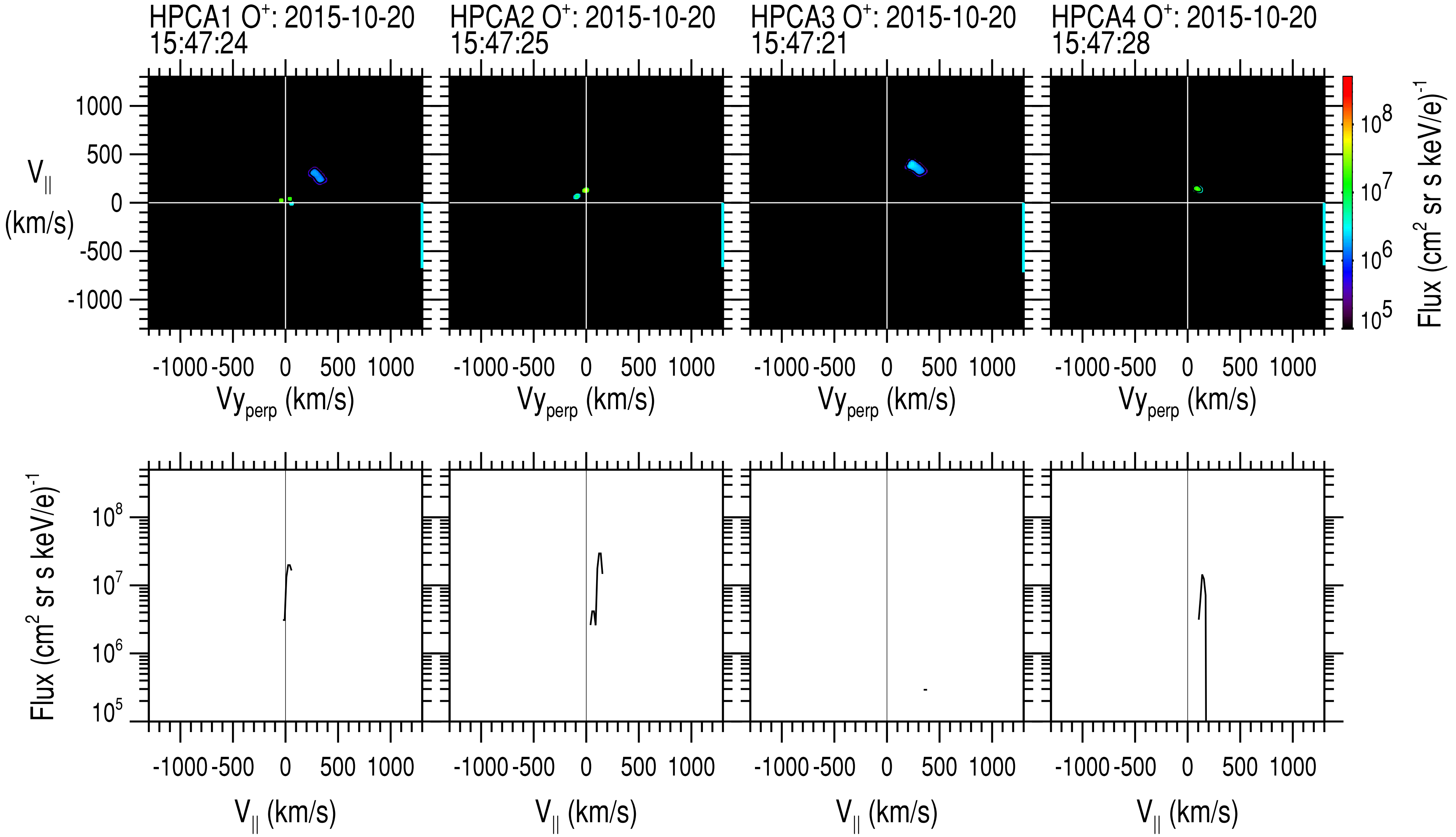

HPCA1 O+: 2015-10-20 15:47:24
HPCA2 O+: 2015-10-20 15:47:25
HPCA3 O+: 2015-10-20 15:47:21
HPCA4 O+: 2015-10-20 15:47:28
V|| (km/s)
Vyperp (km/s)
Flux (cm2 sr s keV/e)-1

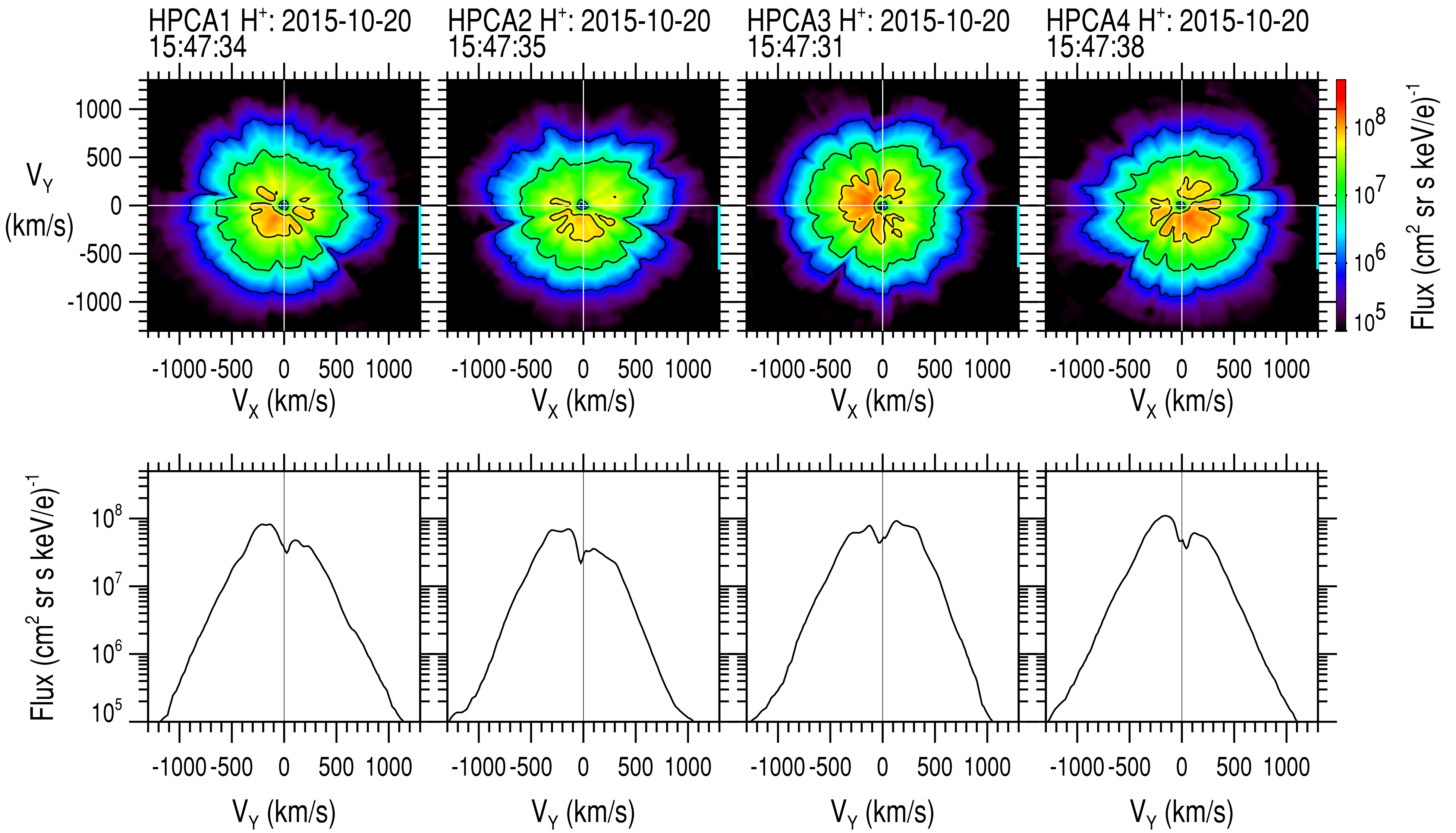

HPCA1 H+: 2015-10-20 15:47:34
HPCA2 H+: 2015-10-20 15:47:35
HPCA3 H+: 2015-10-20 15:47:31
HPCA4 H+: 2015-10-20 15:47:38
VY (km/s)
VX (km/s)
Flux (cm2 sr s keV/e)-1
VY (km/s)

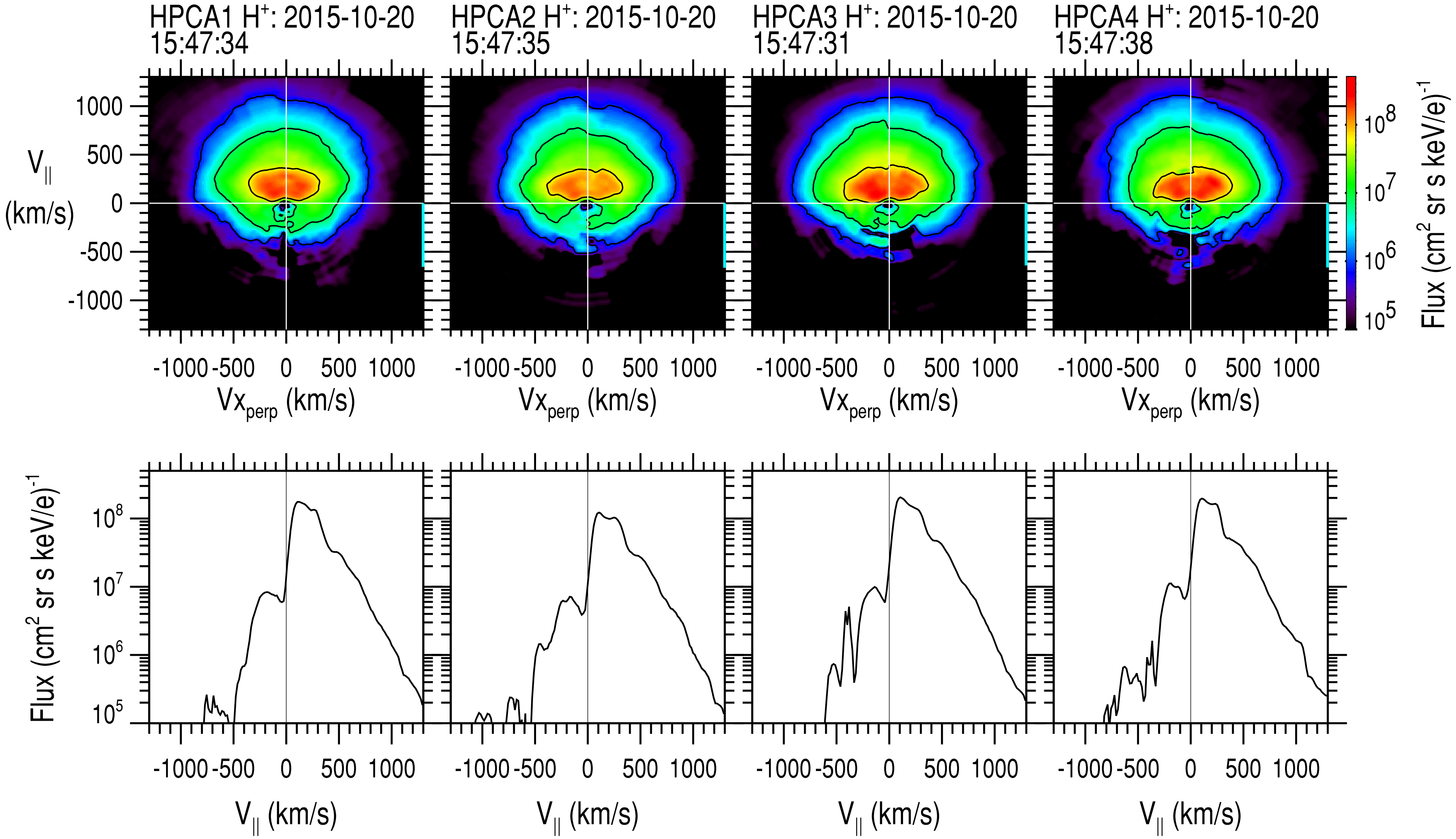
HPCA1 H+: 2015-10-20 15:47:34
HPCA2 H+: 2015-10-20 15:47:35
HPCA3 H+: 2015-10-20 15:47:31
HPCA4 H+: 2015-10-20 15:47:38
V|| (km/s)
Vxperp (km/s)
Flux (cm2 sr s keV/e)-1
V|| (km/s)

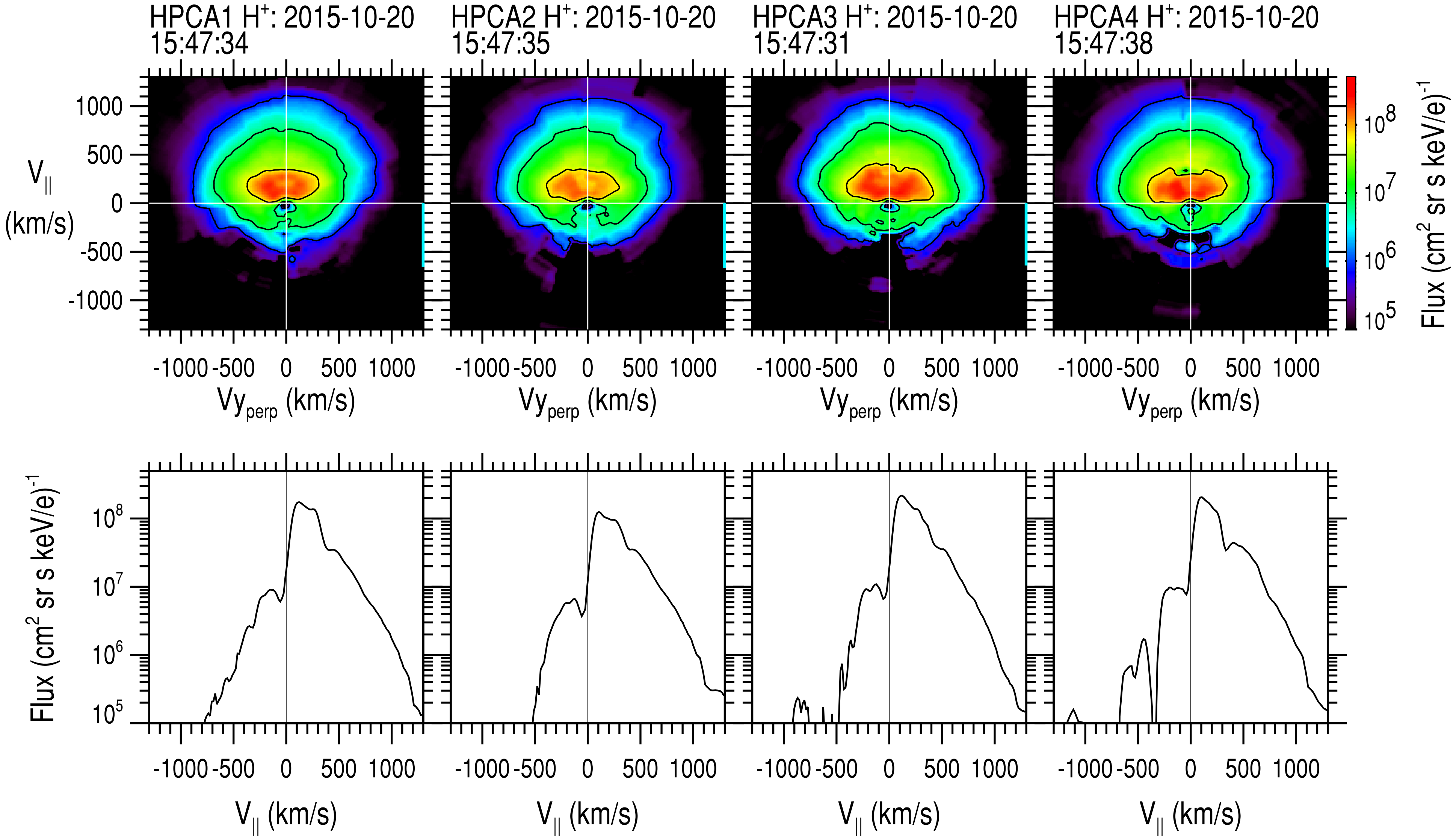
HPCA1 H+: 2015-10-20 15:47:34
HPCA2 H+: 2015-10-20 15:47:35
HPCA3 H+: 2015-10-20 15:47:31
HPCA4 H+: 2015-10-20 15:47:38
V|| (km/s)
Vyperp (km/s)
Flux (cm2 sr s keV/e)-1
V|| (km/s)

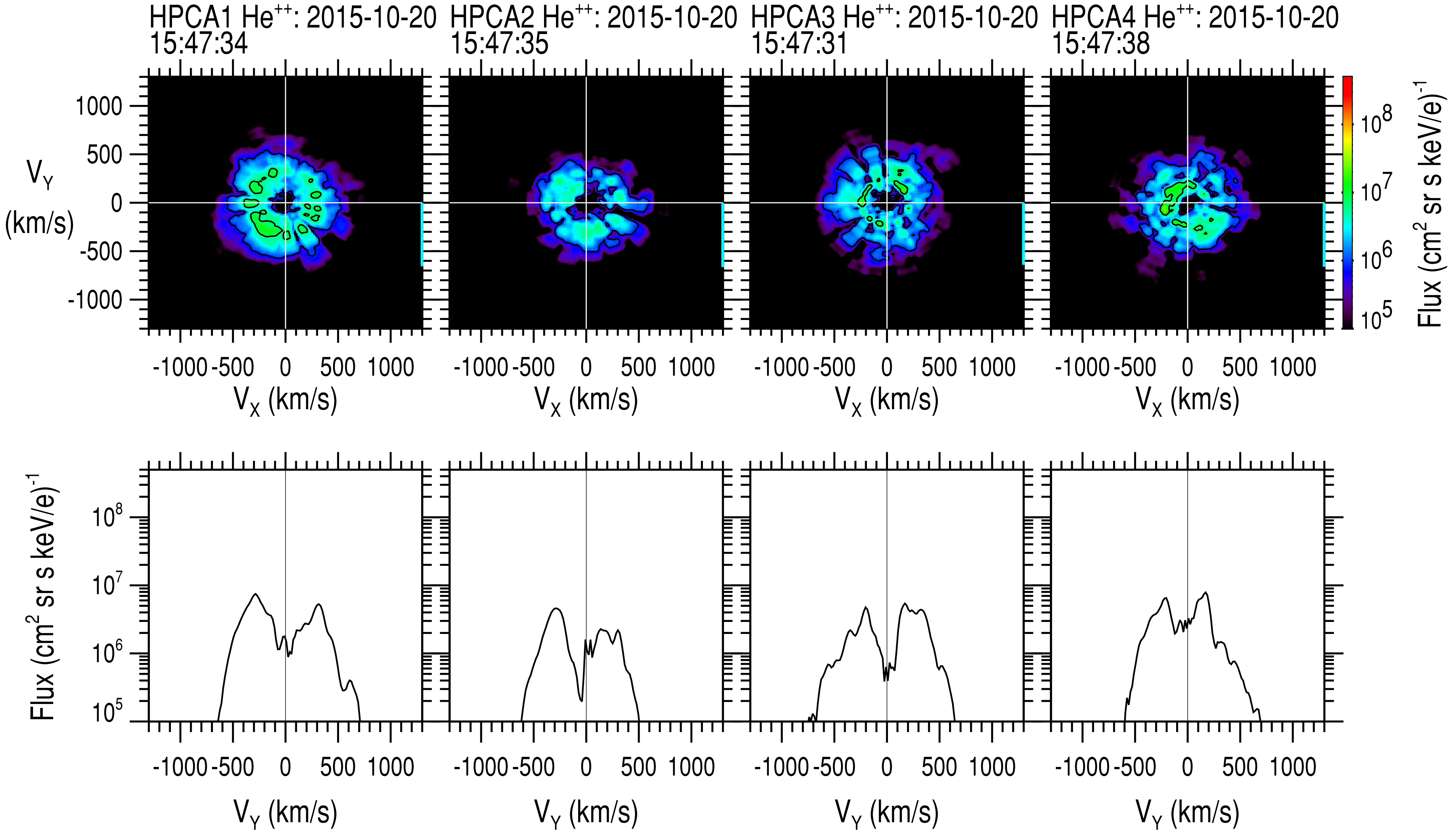
HPCA1 He++: 2015-10-20
15:47:34
HPCA2 He++: 2015-10-20
15:47:35
HPCA3 He++: 2015-10-20
15:47:31
HPCA4 He++: 2015-10-20
15:47:38
VY (km/s)
VX (km/s)
Flux (cm2 sr s keV/e)-1
VY (km/s)

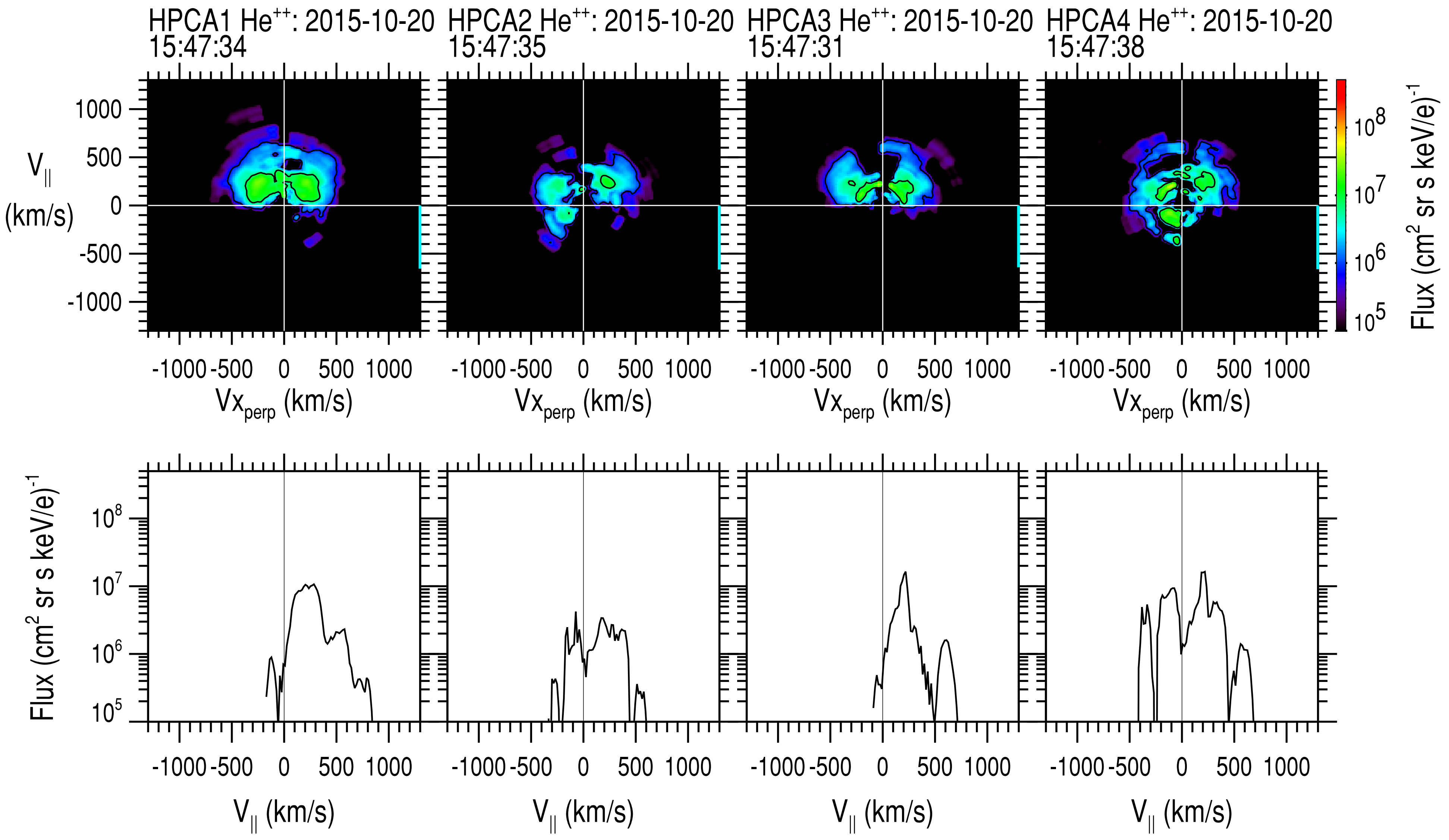
HPCA1 He++: 2015-10-20
15:47:34
HPCA2 He++: 2015-10-20
15:47:35
HPCA3 He++: 2015-10-20
15:47:31
HPCA4 He++: 2015-10-20
15:47:38
V|| (km/s)
Vxperp (km/s)
Flux (cm2 sr s keV/e)-1
V|| (km/s)

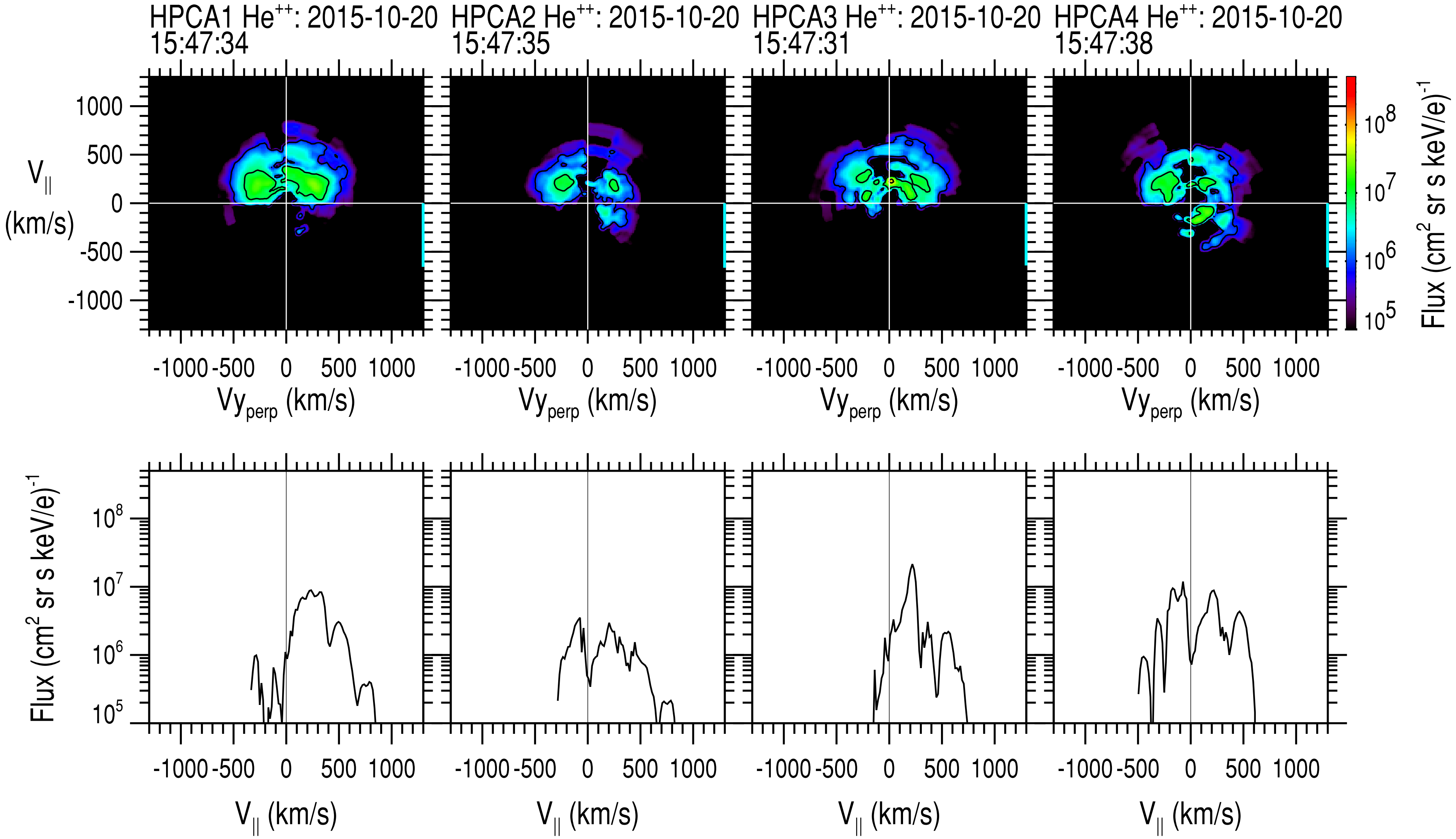

HPCA1 He++: 2015-10-20 15:47:34
HPCA2 He++: 2015-10-20 15:47:35
HPCA3 He++: 2015-10-20 15:47:31
HPCA4 He++: 2015-10-20 15:47:38
V|| (km/s)
Vyperp (km/s)
Flux (cm2 sr s keV/e)-1

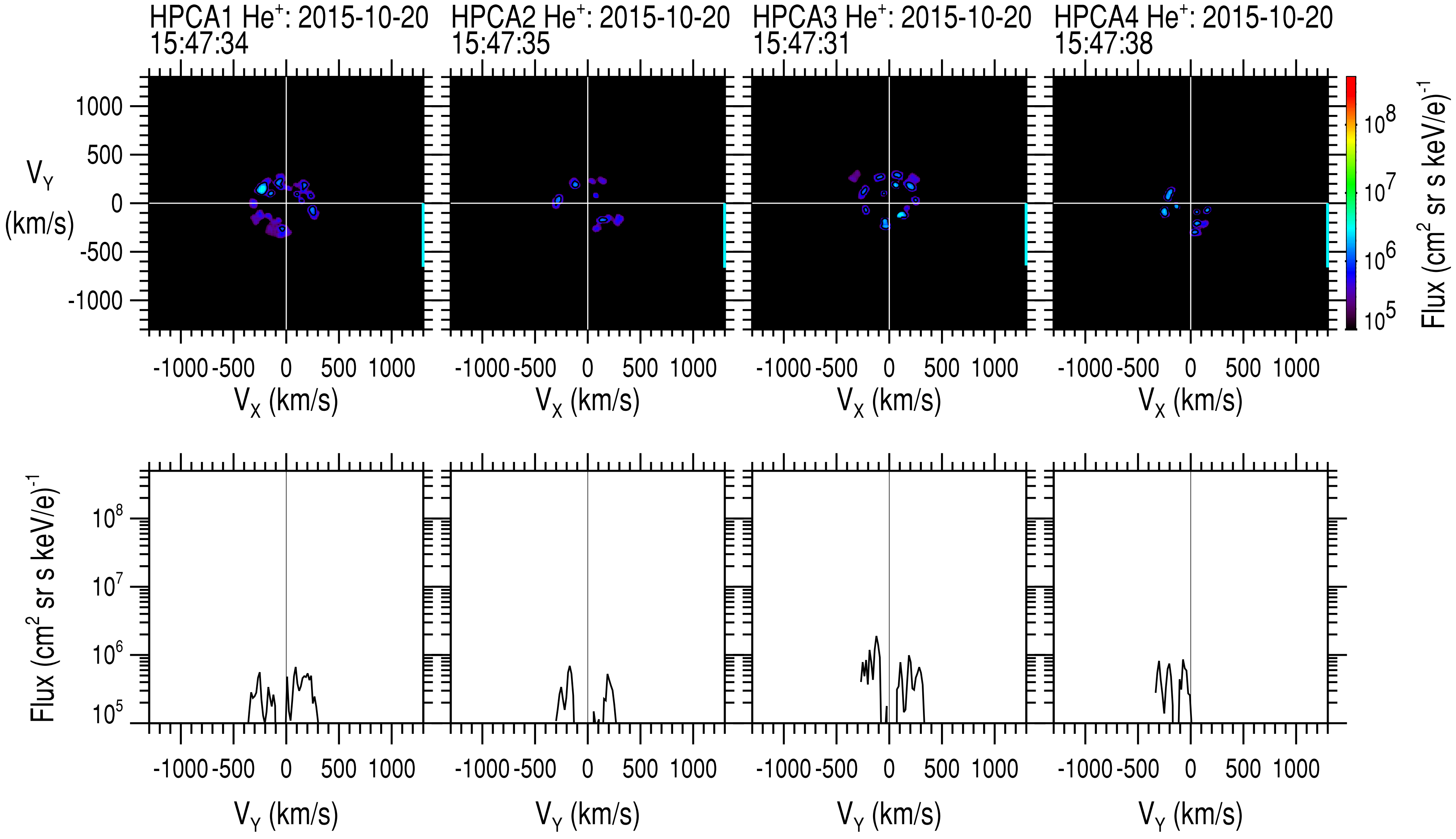

HPCA1 He+: 2015-10-20
15:47:34
HPCA2 He+: 2015-10-20
15:47:35
HPCA3 He+: 2015-10-20
15:47:31
HPCA4 He+: 2015-10-20
15:47:38
VY (km/s)
VX (km/s)
Flux (cm2 sr s keV/e)-1

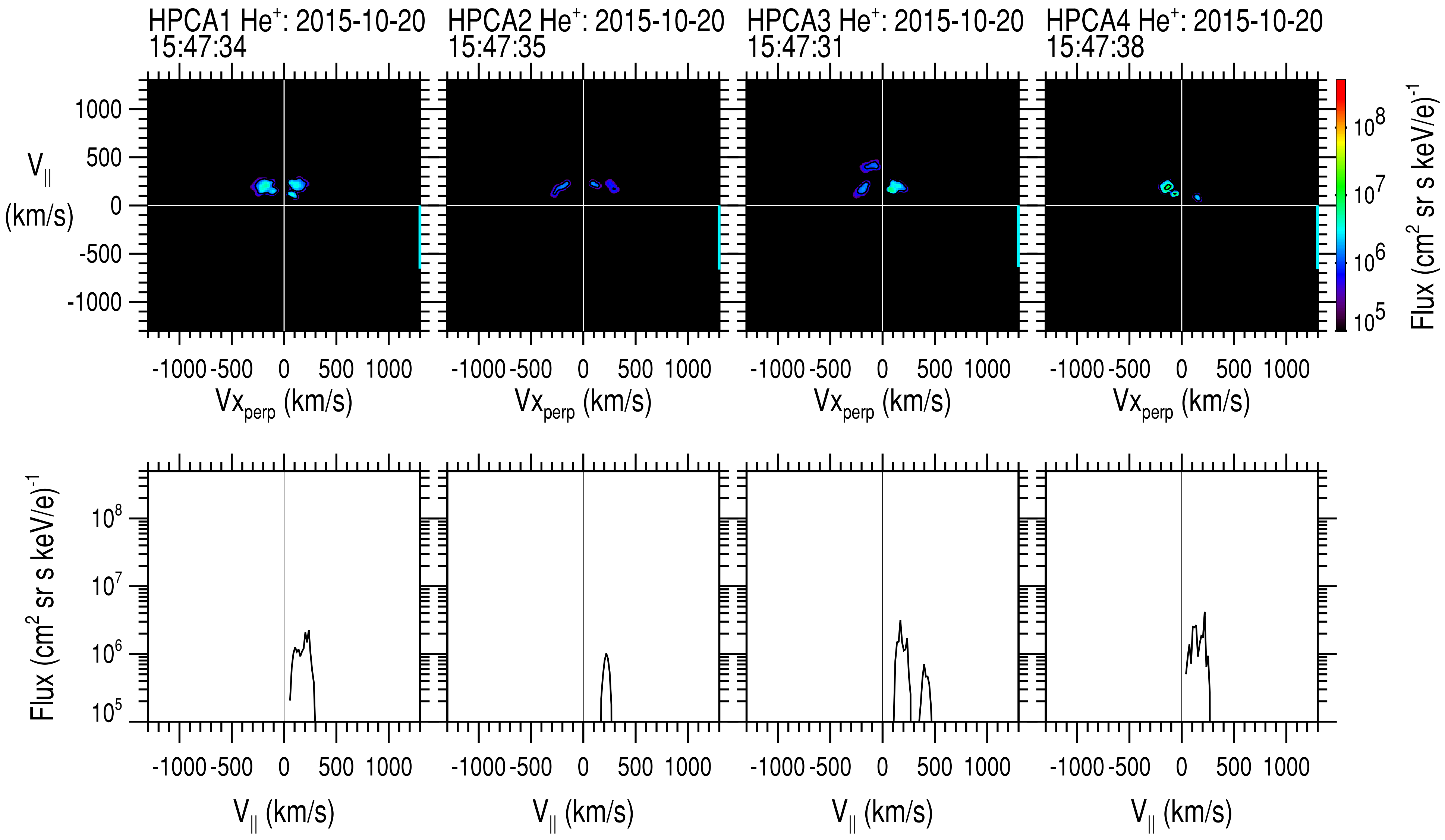
HPCA1 He+: 2015-10-20 15:47:34
HPCA2 He+: 2015-10-20 15:47:35
HPCA3 He+: 2015-10-20 15:47:31
HPCA4 He+: 2015-10-20 15:47:38
V|| (km/s)
Vxperp (km/s)
Flux (cm2 sr s keV/e)-1
V|| (km/s)

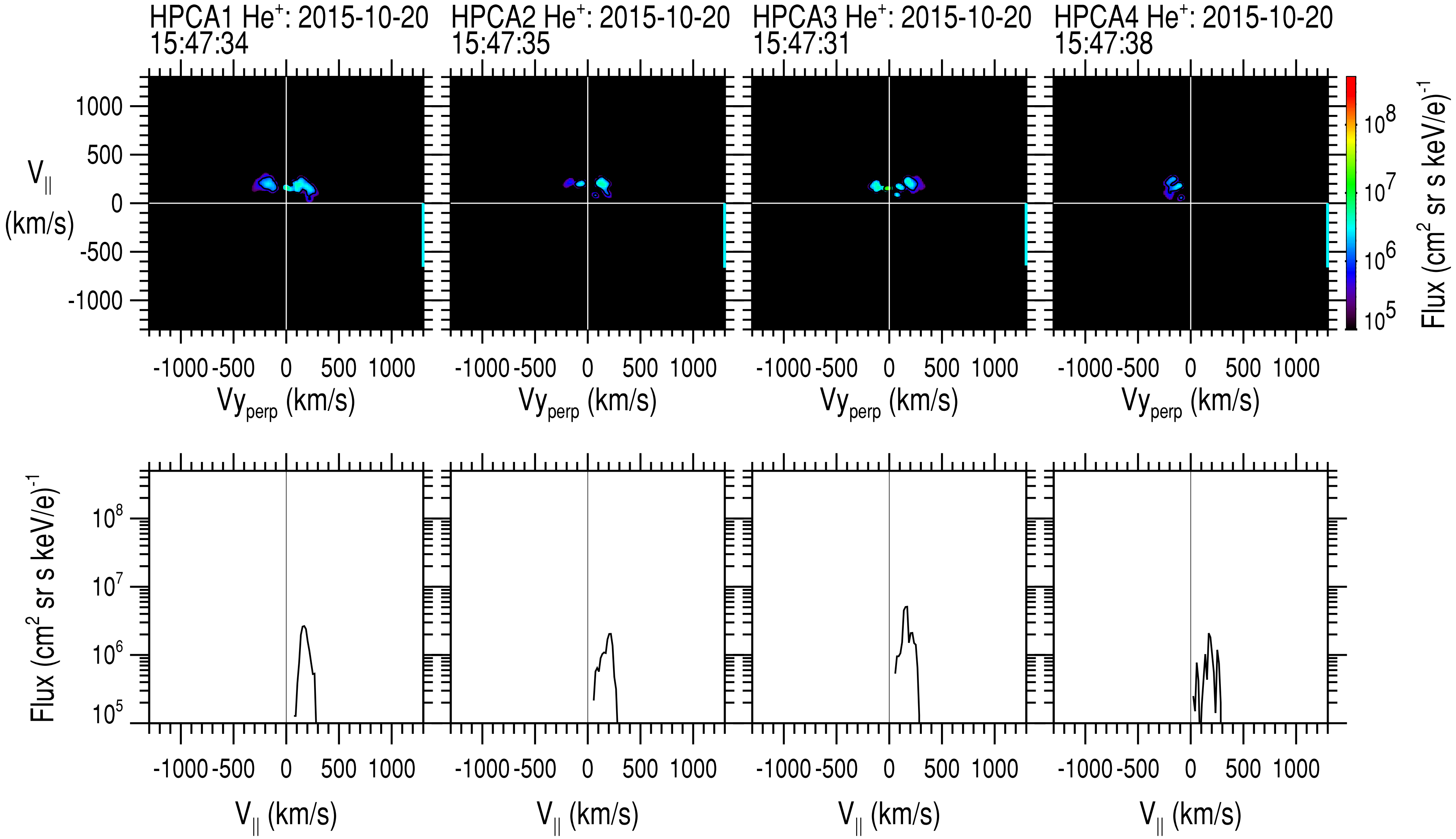

HPCA1 He+: 2015-10-20 15:47:34
HPCA2 He+: 2015-10-20 15:47:35
HPCA3 He+: 2015-10-20 15:47:31
HPCA4 He+: 2015-10-20 15:47:38
V|| (km/s)
Vyperp (km/s)
Flux (cm2 sr s keV/e)-1

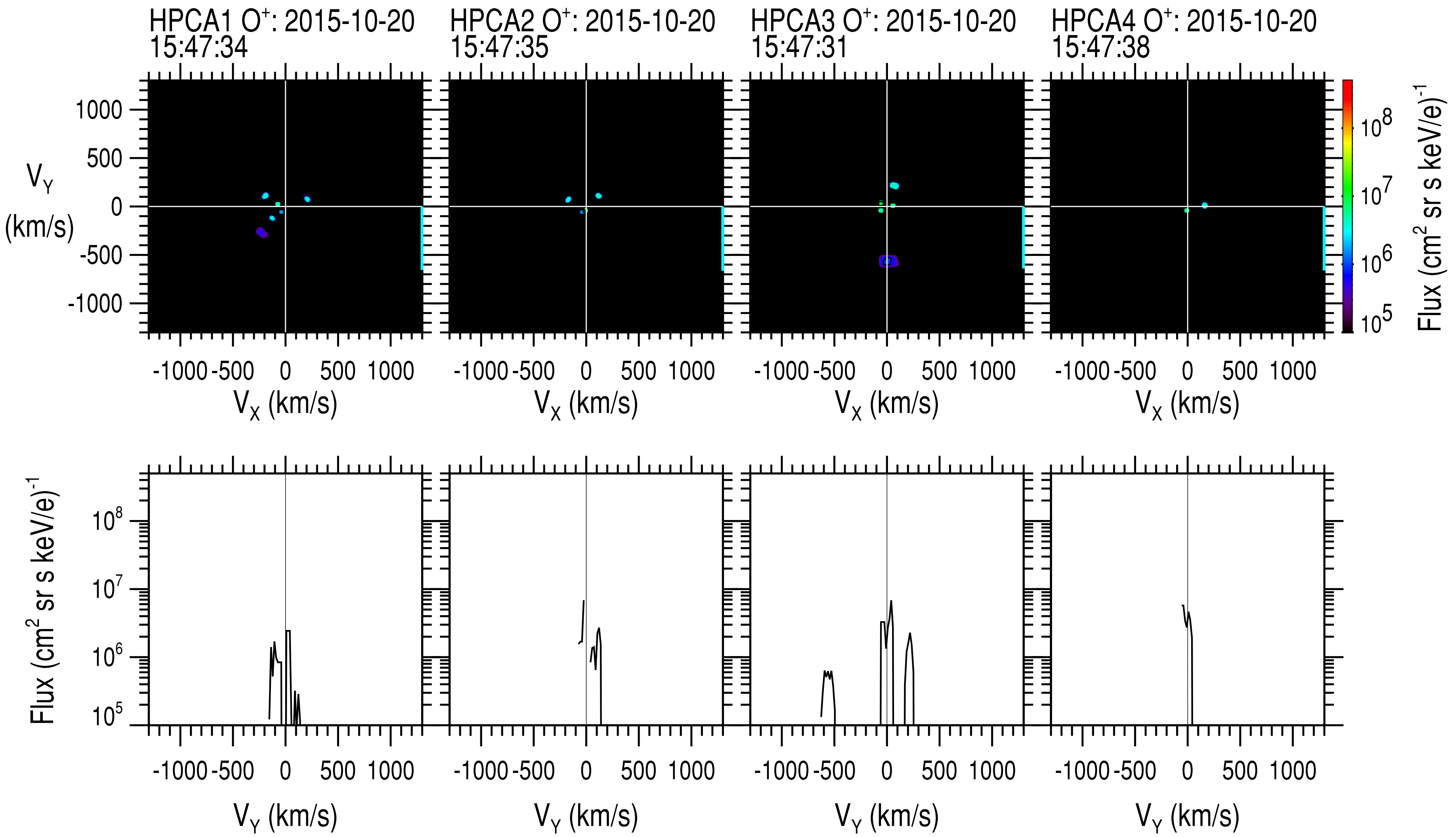
HPCA1 O+: 2015-10-20
15:47:34
HPCA2 O+: 2015-10-20
15:47:35
HPCA3 O+: 2015-10-20
15:47:31
HPCA4 O+: 2015-10-20
15:47:38
VY (km/s)
VX (km/s)
Flux (cm2 sr s keV/e)-1
VY (km/s)

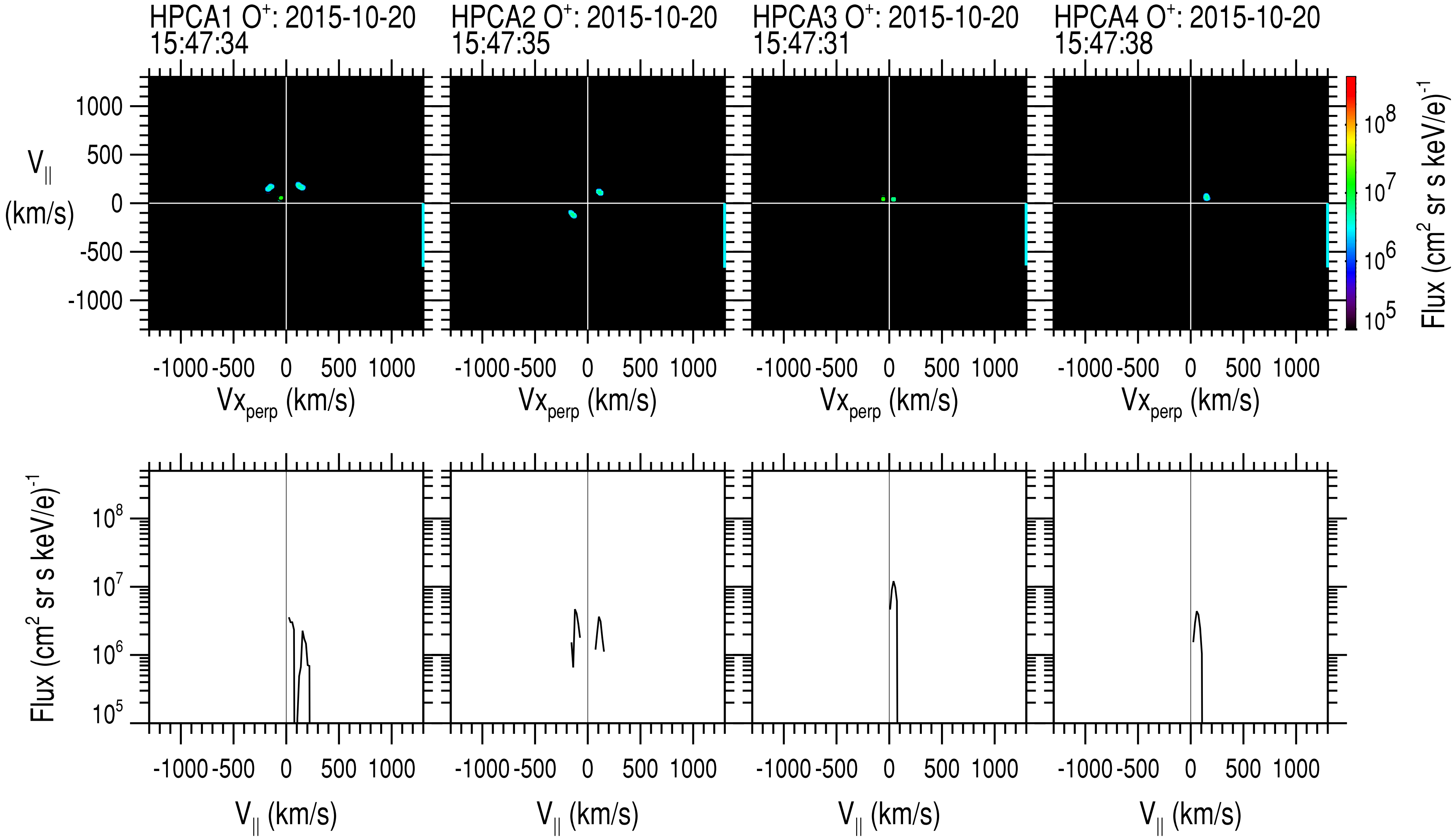
HPCA1 O+: 2015-10-20
15:47:34
HPCA2 O+: 2015-10-20
15:47:35
HPCA3 O+: 2015-10-20
15:47:31
HPCA4 O+: 2015-10-20
15:47:38
V|| (km/s)
Vxperp (km/s)
Flux (cm2 sr s keV/e)-1
1000
500
0
-500
-1000
105
106
107
108

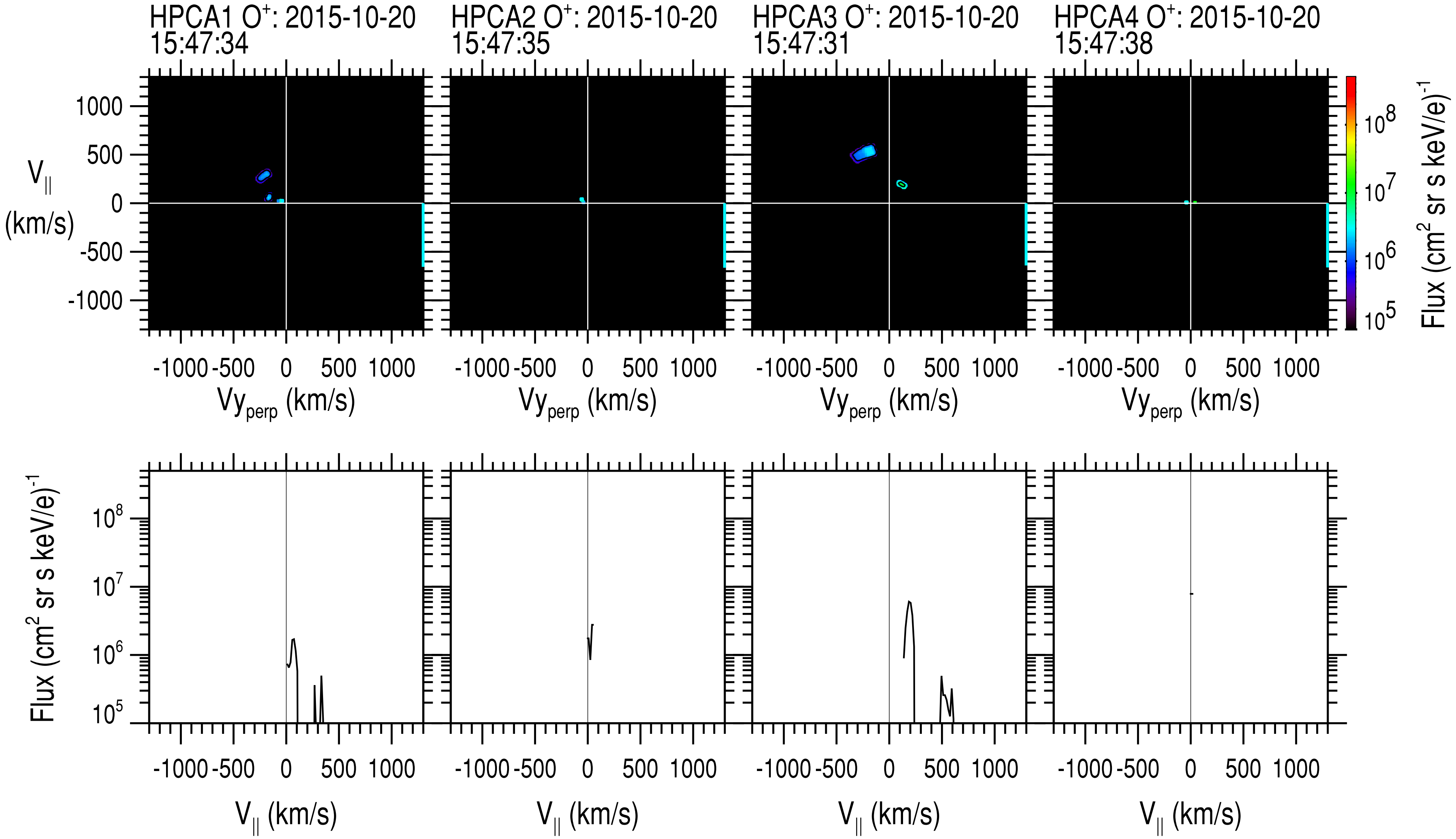

HPCA1 O+: 2015-10-20 15:47:34
HPCA2 O+: 2015-10-20 15:47:35
HPCA3 O+: 2015-10-20 15:47:31
HPCA4 O+: 2015-10-20 15:47:38
V|| (km/s)
Vyperp (km/s)
Flux (cm2 sr s keV/e)-1

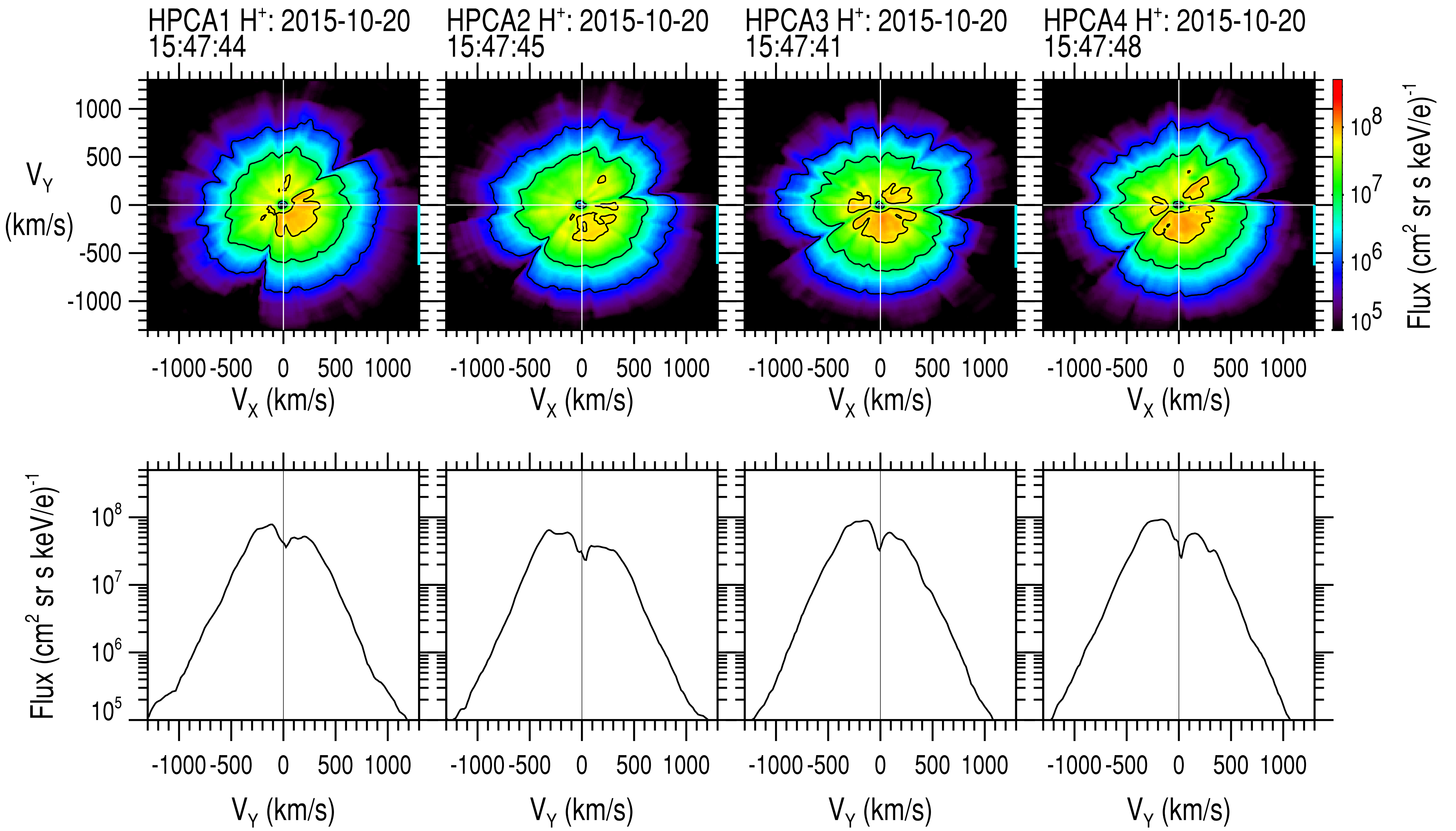

HPCA1 H⁺: 2015-10-20 15:47:44
HPCA2 H⁺: 2015-10-20 15:47:45
HPCA3 H⁺: 2015-10-20 15:47:41
HPCA4 H⁺: 2015-10-20 15:47:48
V_Y (km/s)
V_X (km/s)
Flux (cm² sr s keV/e)⁻¹
10⁵
10⁶
10⁷
10⁸
-1000
-500
0
500
1000

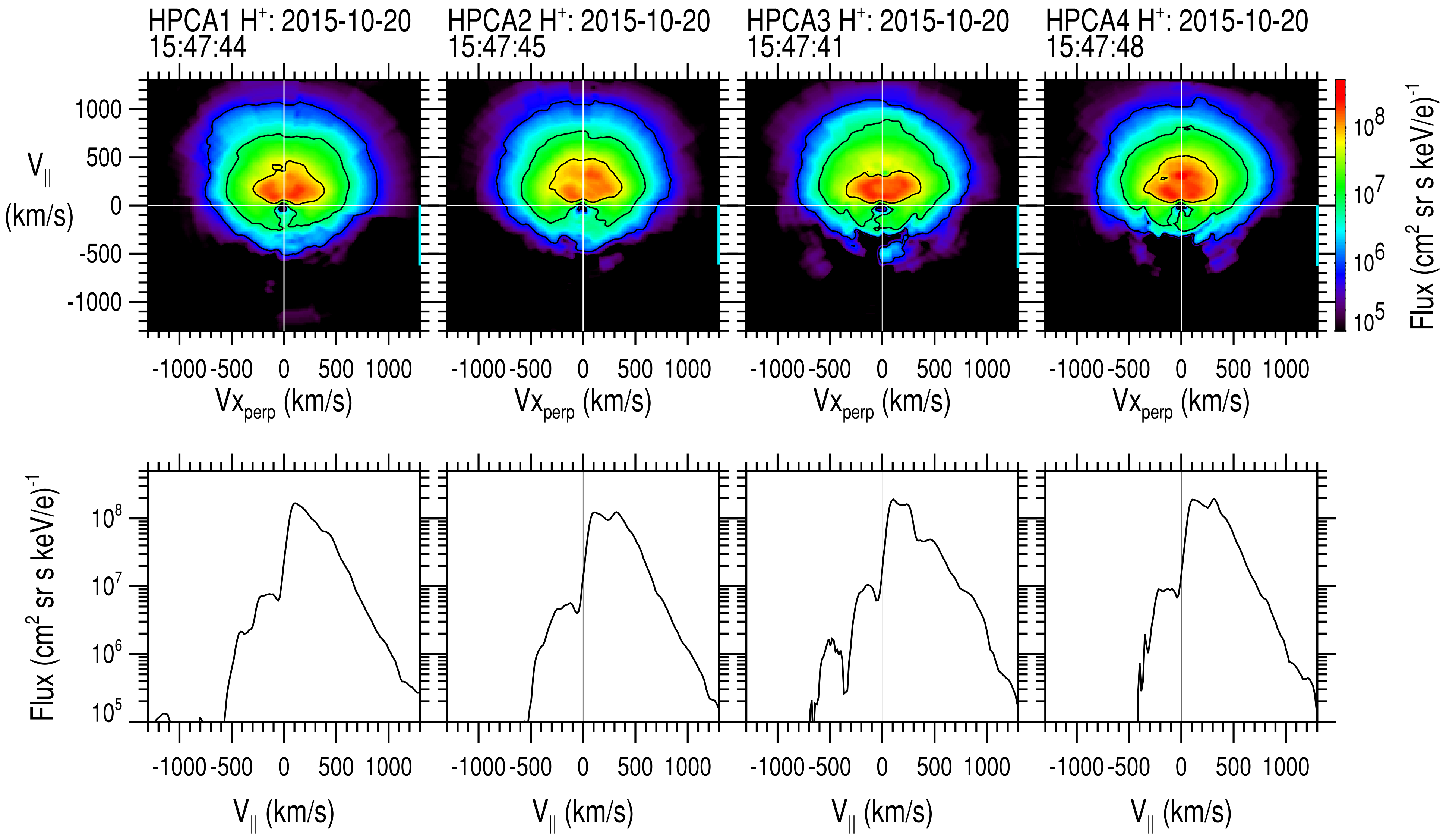

HPCA1 H+: 2015-10-20 15:47:44
HPCA2 H+: 2015-10-20 15:47:45
HPCA3 H+: 2015-10-20 15:47:41
HPCA4 H+: 2015-10-20 15:47:48
V|| (km/s)
Vxperp (km/s)
Flux (cm2 sr s keV/e)-1

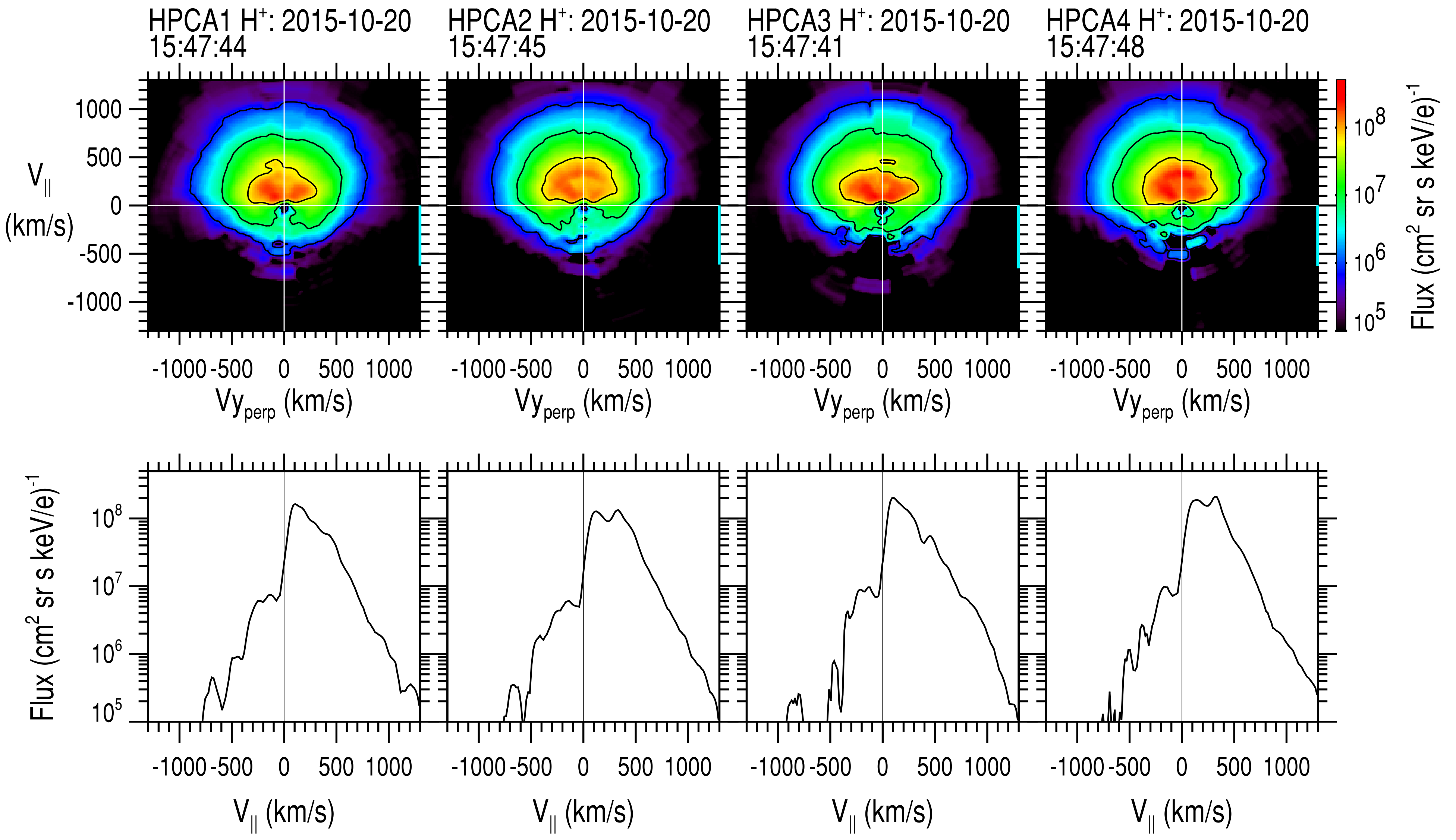
HPCA1 H+: 2015-10-20 15:47:44
HPCA2 H+: 2015-10-20 15:47:45
HPCA3 H+: 2015-10-20 15:47:41
HPCA4 H+: 2015-10-20 15:47:48
V|| (km/s)
Vyperp (km/s)
Flux (cm2 sr s keV/e)-1
V|| (km/s)

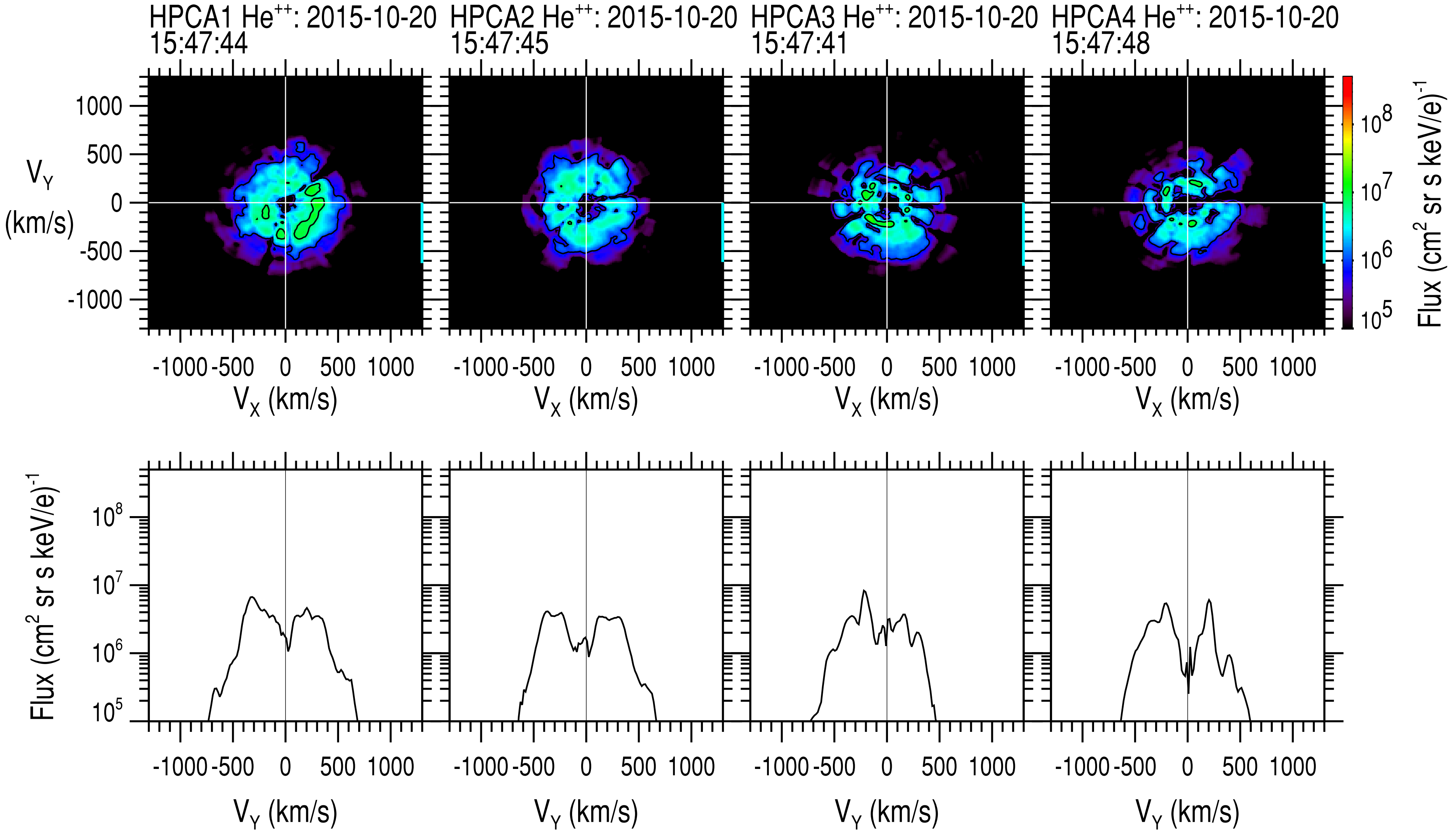

HPCA1 He++: 2015-10-20 15:47:44
HPCA2 He++: 2015-10-20 15:47:45
HPCA3 He++: 2015-10-20 15:47:41
HPCA4 He++: 2015-10-20 15:47:48
VY (km/s)
VX (km/s)
Flux (cm2 sr s keV/e)-1
VY (km/s)

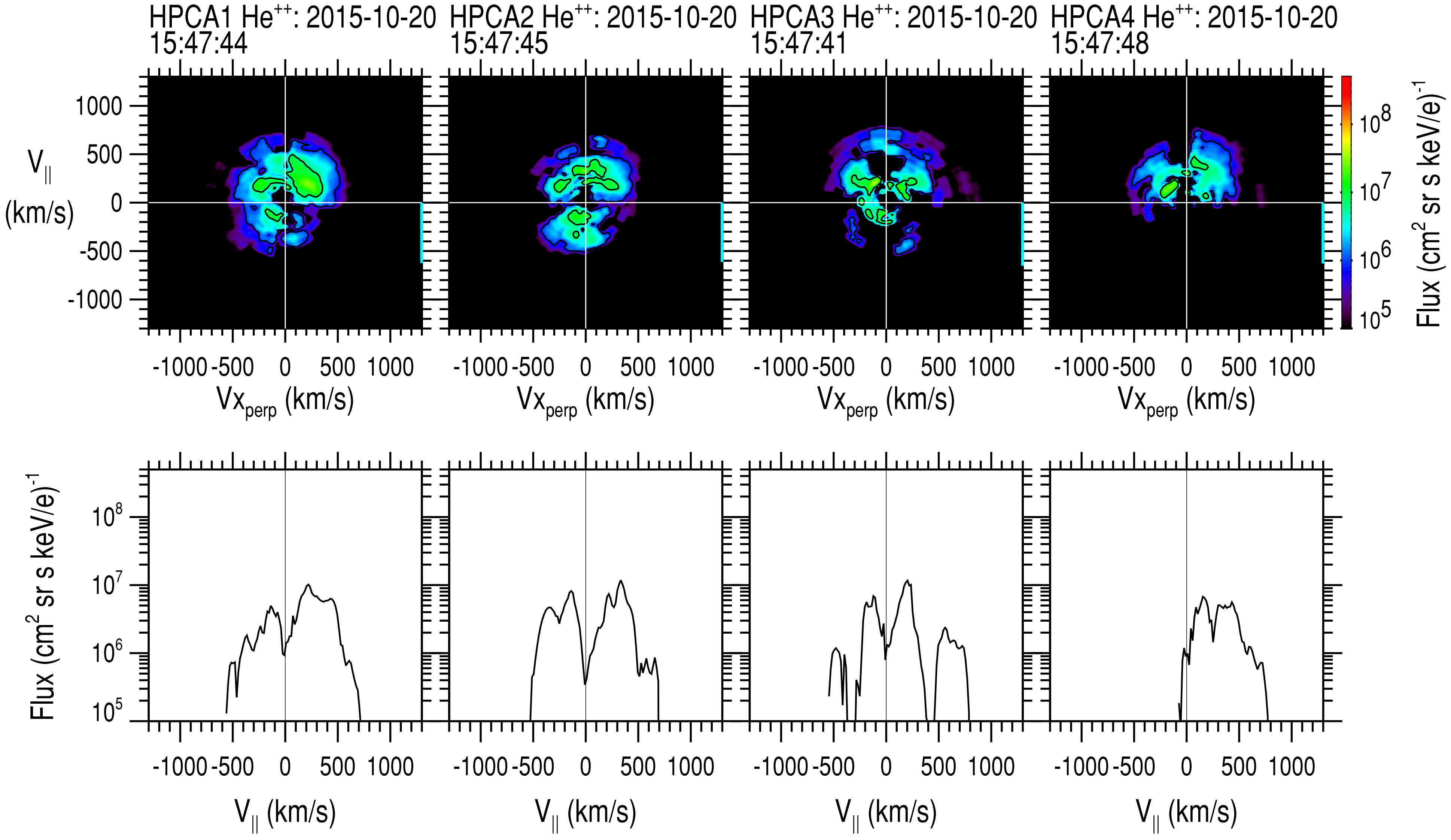
HPCA1 He++: 2015-10-20 15:47:44
HPCA2 He++: 2015-10-20 15:47:45
HPCA3 He++: 2015-10-20 15:47:41
HPCA4 He++: 2015-10-20 15:47:48
V|| (km/s)
Vxperp (km/s)
Flux (cm2 sr s keV/e)-1
V|| (km/s)

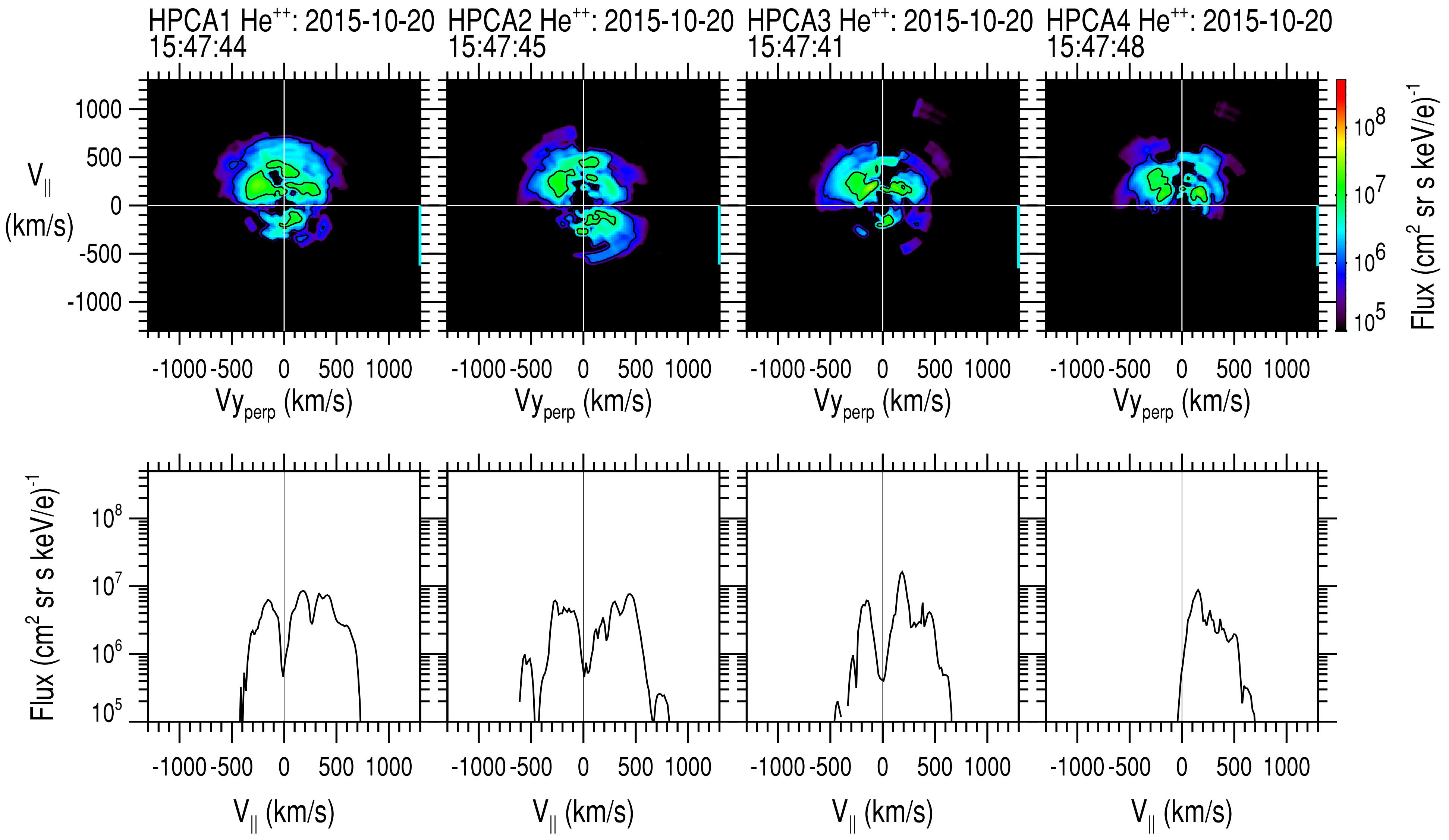
HPCA1 He++: 2015-10-20 15:47:44
HPCA2 He++: 2015-10-20 15:47:45
HPCA3 He++: 2015-10-20 15:47:41
HPCA4 He++: 2015-10-20 15:47:48
V|| (km/s)
Vyperp (km/s)
Flux (cm2 sr s keV/e)-1

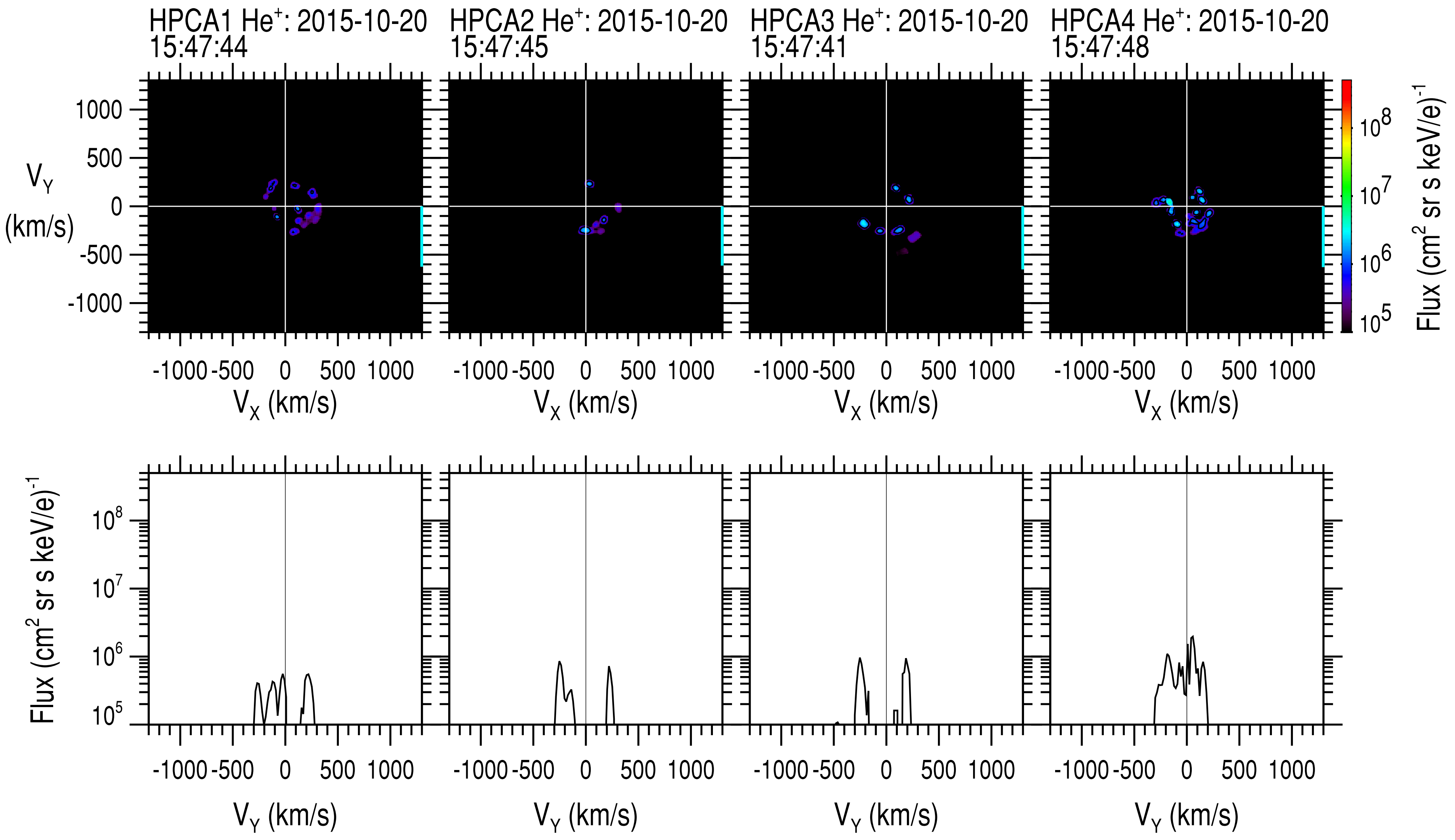

HPCA1 He+: 2015-10-20 15:47:44
HPCA2 He+: 2015-10-20 15:47:45
HPCA3 He+: 2015-10-20 15:47:41
HPCA4 He+: 2015-10-20 15:47:48
VY (km/s)
VX (km/s)
Flux (cm2 sr s keV/e)-1
VY (km/s)

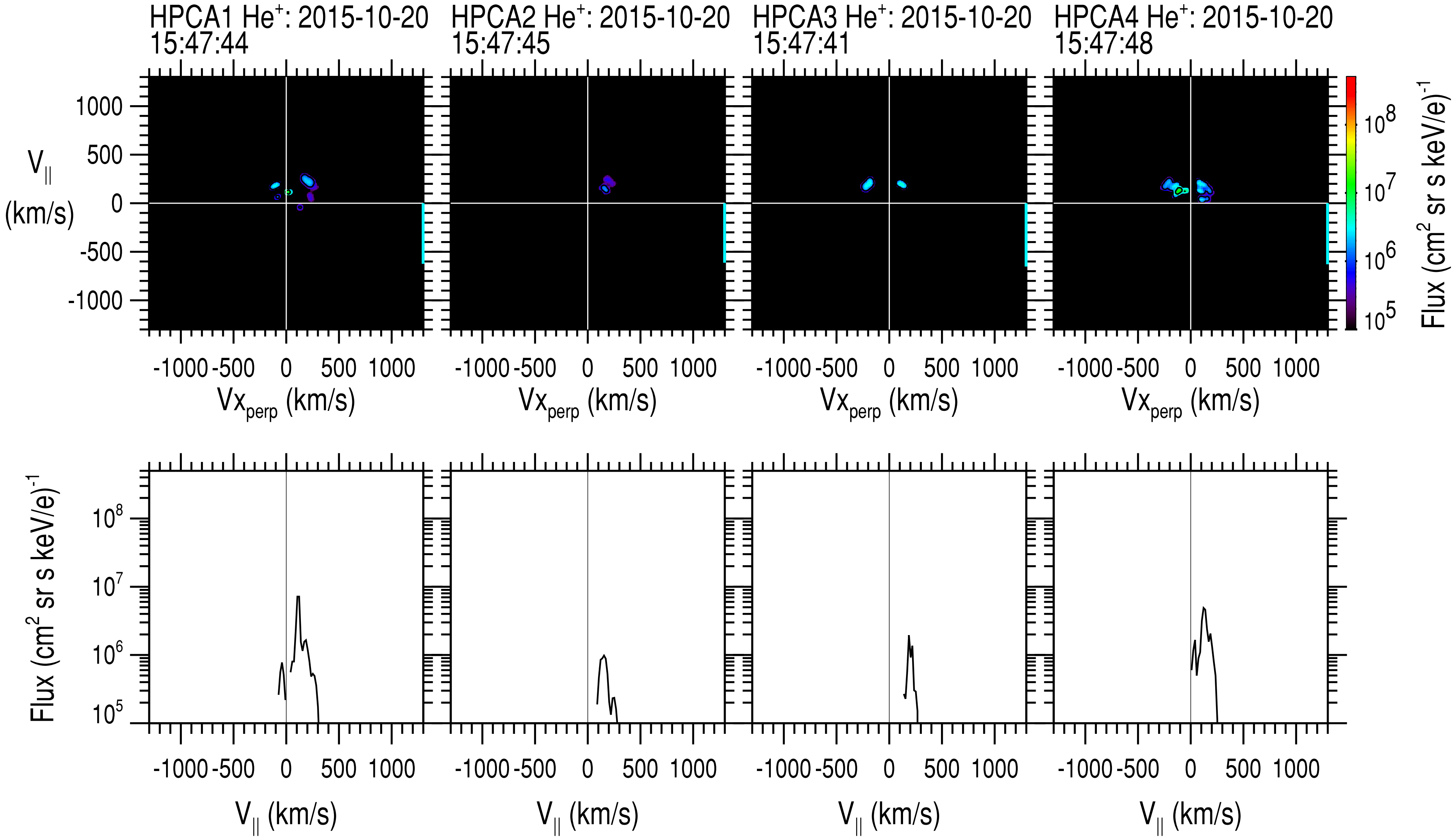
HPCA1 He+: 2015-10-20 15:47:44
HPCA2 He+: 2015-10-20 15:47:45
HPCA3 He+: 2015-10-20 15:47:41
HPCA4 He+: 2015-10-20 15:47:48
V|| (km/s)
Vxperp (km/s)
Flux (cm2 sr s keV/e)-1
1000
500
0
-500
-1000
10^5
10^6
10^7
10^8

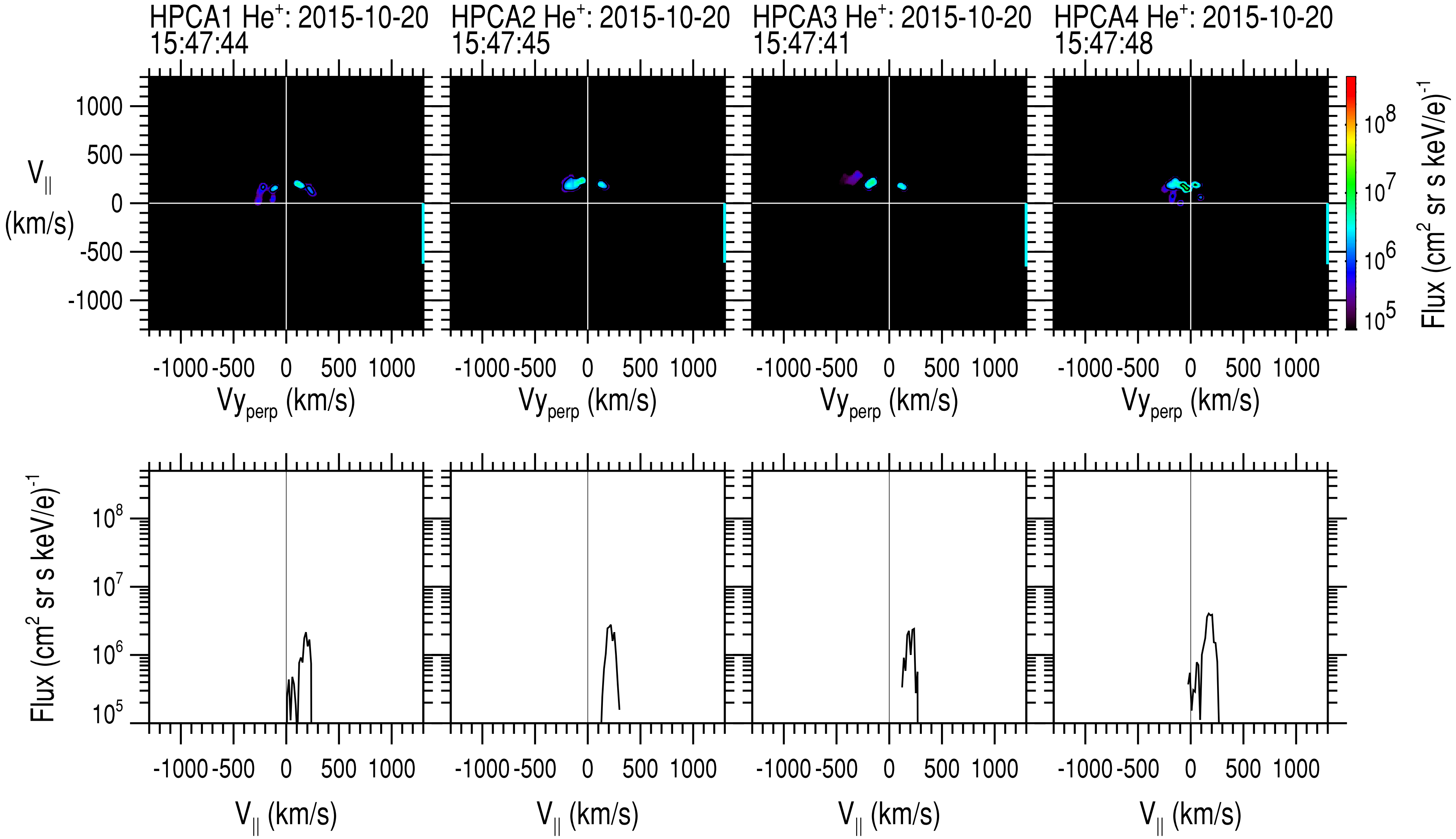

HPCA1 He+: 2015-10-20 15:47:44
HPCA2 He+: 2015-10-20 15:47:45
HPCA3 He+: 2015-10-20 15:47:41
HPCA4 He+: 2015-10-20 15:47:48
V|| (km/s)
Vyperp (km/s)
Flux (cm2 sr s keV/e)-1

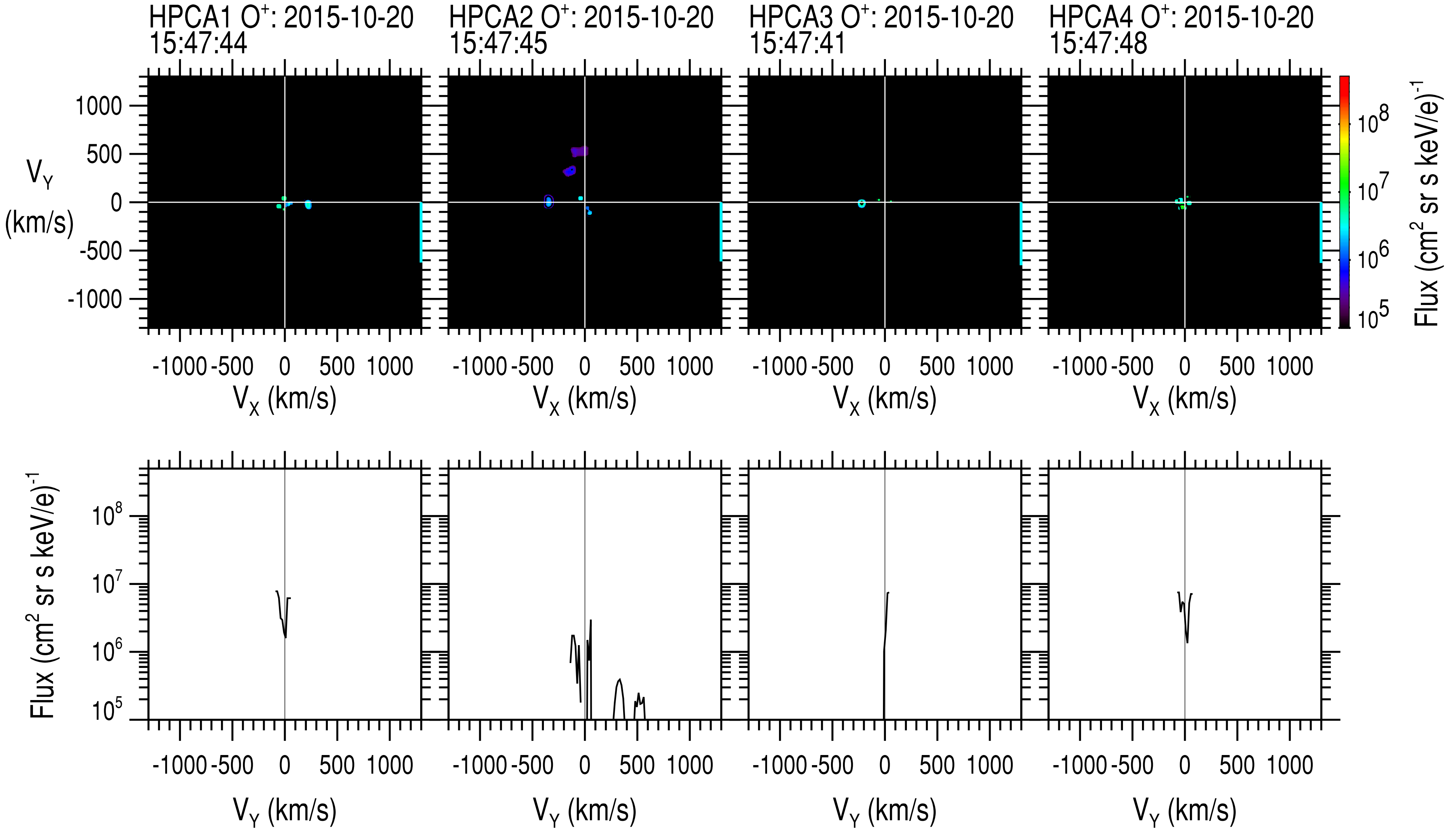
HPCA1 O+: 2015-10-20 15:47:44
HPCA2 O+: 2015-10-20 15:47:45
HPCA3 O+: 2015-10-20 15:47:41
HPCA4 O+: 2015-10-20 15:47:48
V_Y (km/s)
V_X (km/s)
Flux (cm2 sr s keV/e)-1
-1000 -500 0 500 1000
10^5 10^6 10^7 10^8

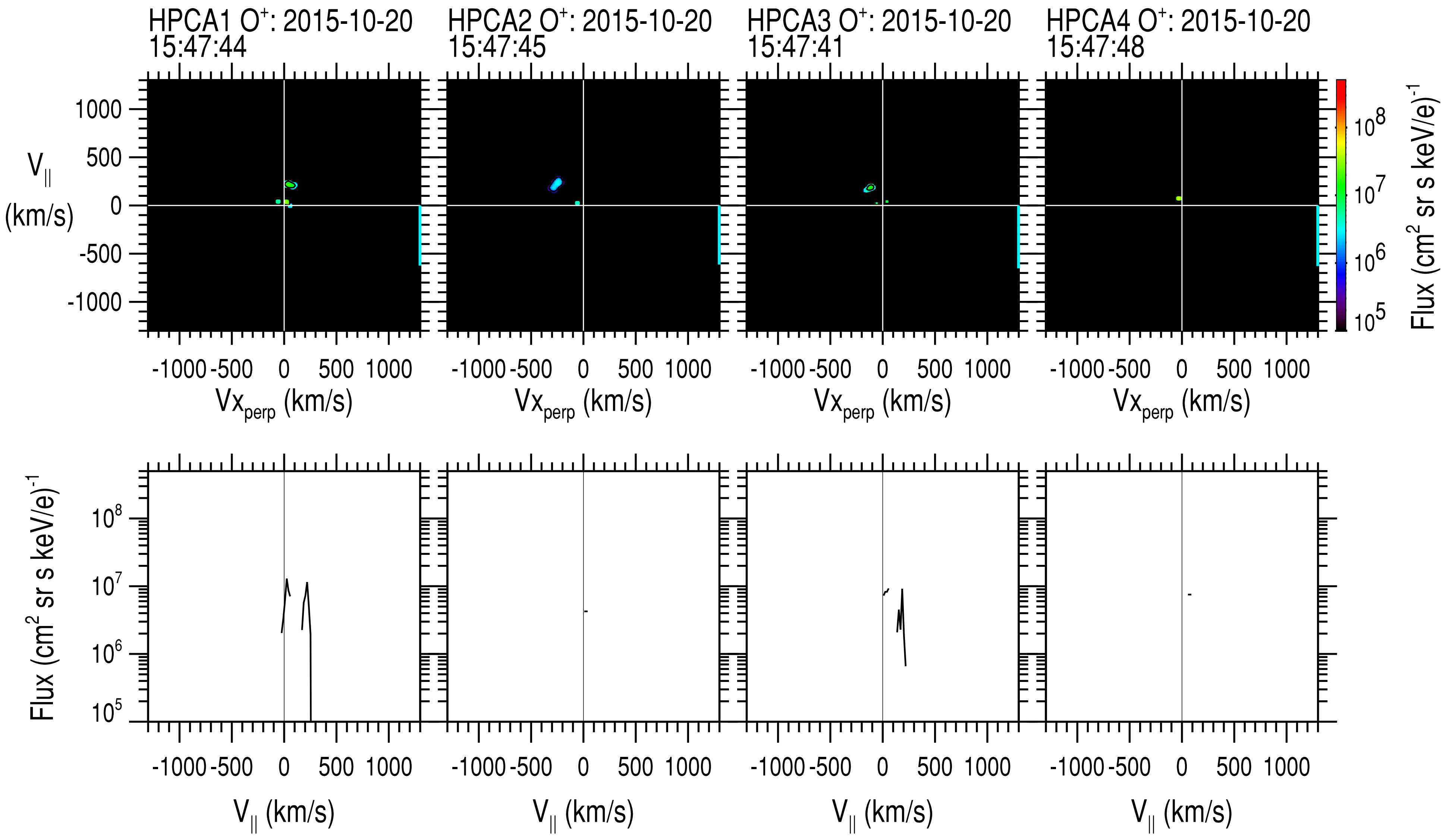
HPCA1 O+: 2015-10-20 15:47:44
HPCA2 O+: 2015-10-20 15:47:45
HPCA3 O+: 2015-10-20 15:47:41
HPCA4 O+: 2015-10-20 15:47:48
V|| (km/s)
Vxperp (km/s)
Flux (cm2 sr s keV/e)-1

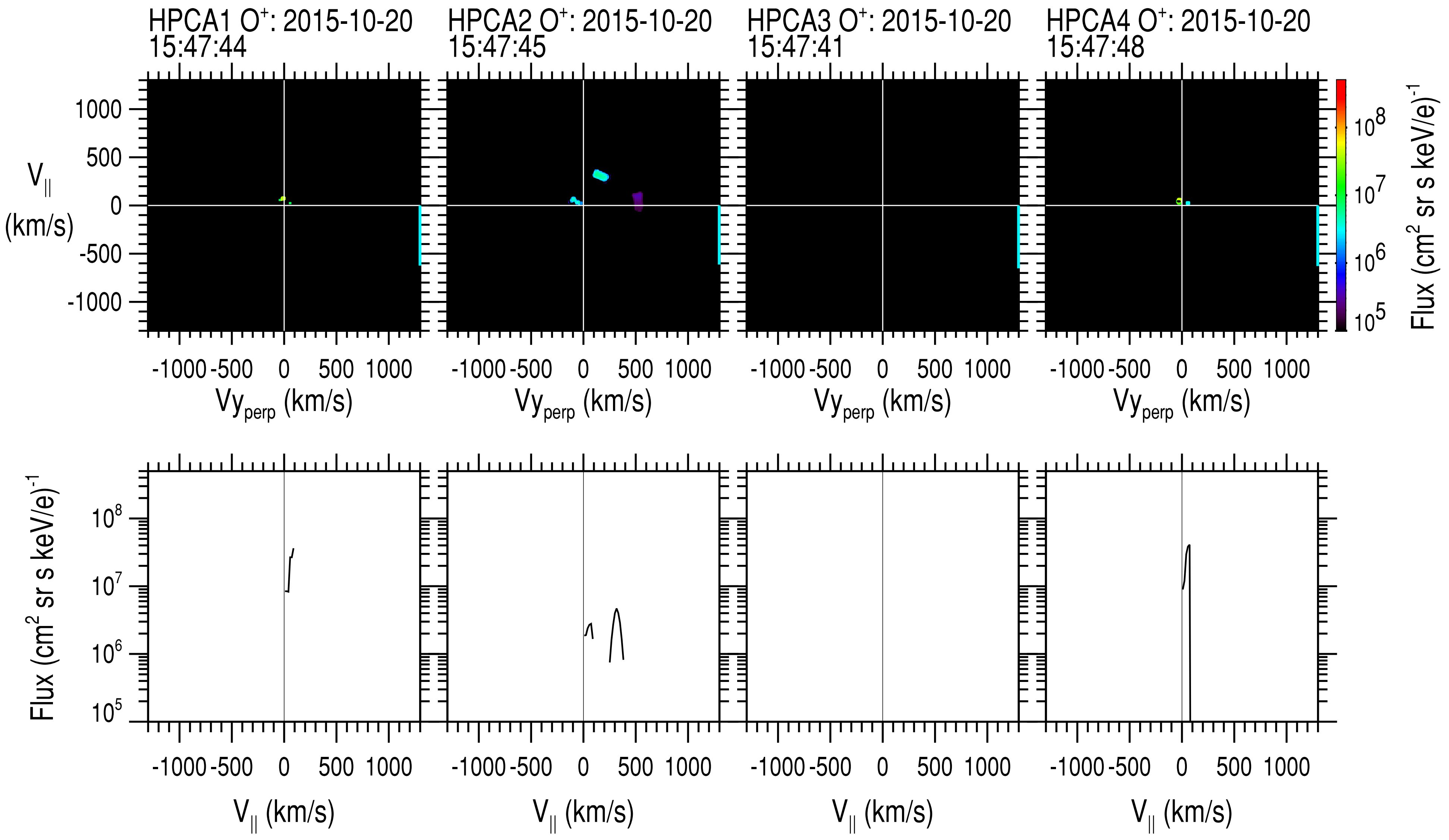
HPCA1 O+: 2015-10-20
15:47:44
HPCA2 O+: 2015-10-20
15:47:45
HPCA3 O+: 2015-10-20
15:47:41
HPCA4 O+: 2015-10-20
15:47:48
V|| (km/s)
Vyperp (km/s)
Flux (cm2 sr s keV/e)-1
-1000 -500 0 500 1000
10^5 10^6 10^7 10^8

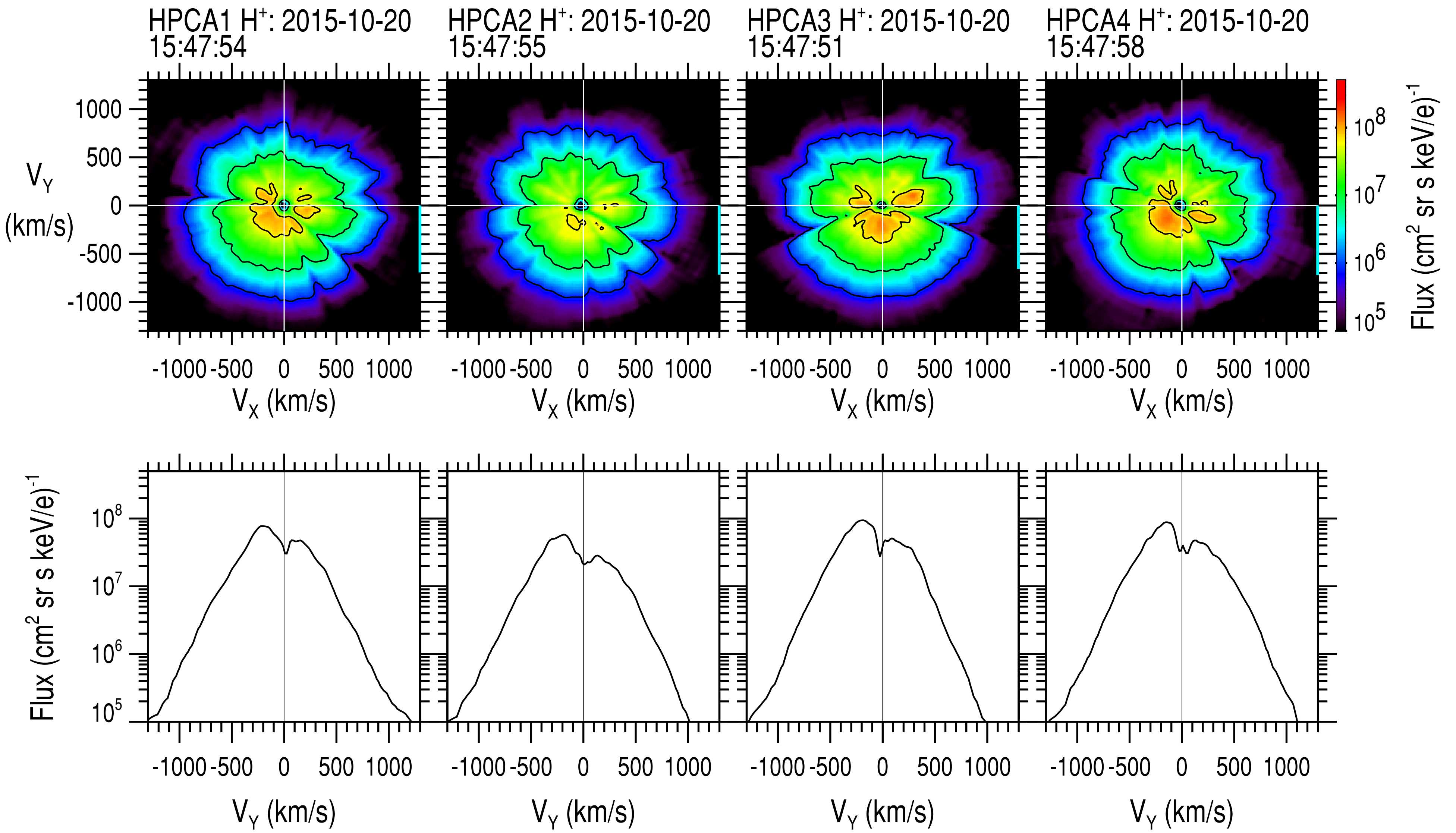

HPCA1 H+: 2015-10-20 15:47:54
HPCA2 H+: 2015-10-20 15:47:55
HPCA3 H+: 2015-10-20 15:47:51
HPCA4 H+: 2015-10-20 15:47:58
V_Y (km/s)
V_X (km/s)
Flux (cm^2 sr s keV/e)^-1

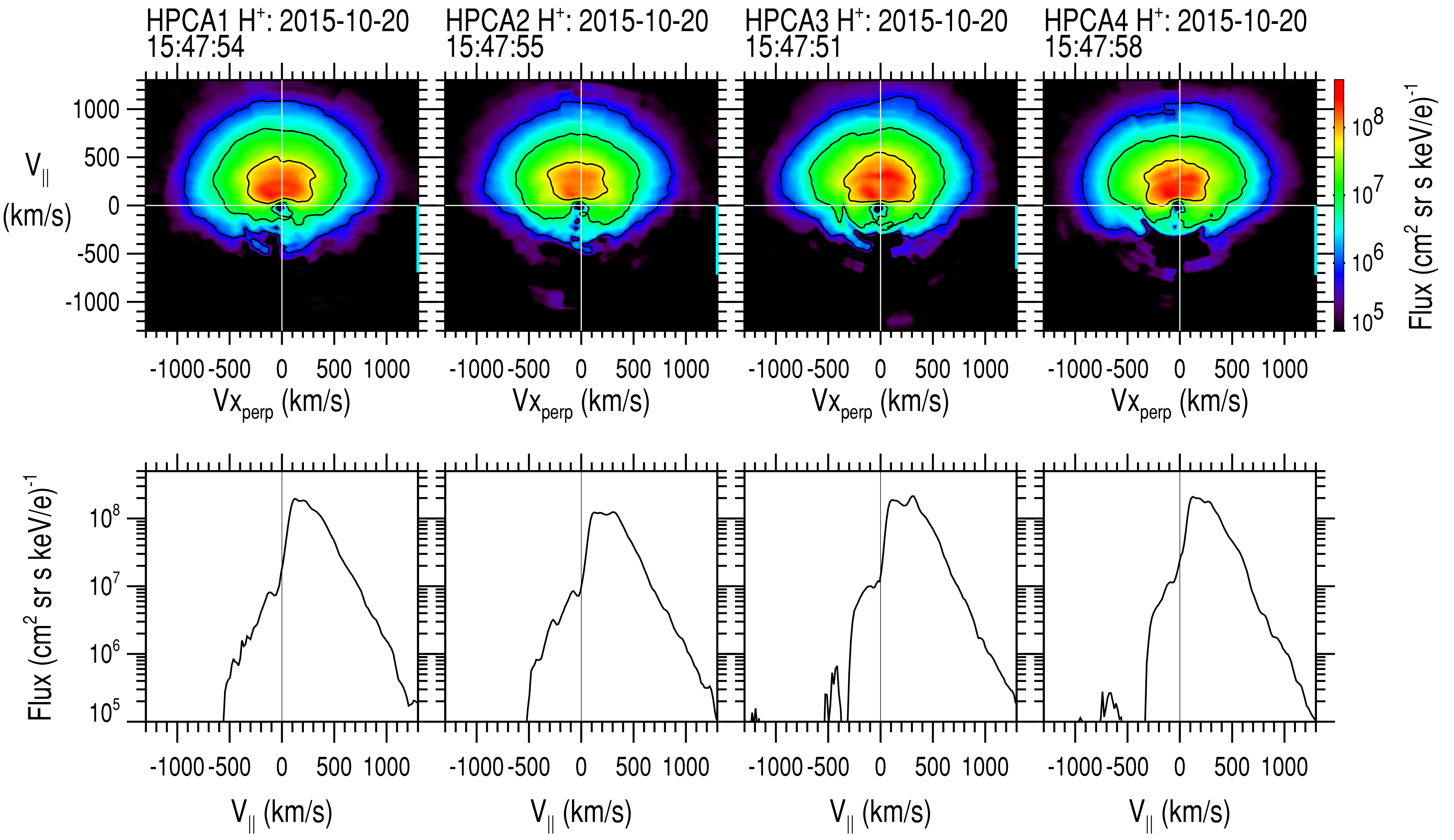
HPCA1 H+: 2015-10-20
15:47:54
HPCA2 H+: 2015-10-20
15:47:55
HPCA3 H+: 2015-10-20
15:47:51
HPCA4 H+: 2015-10-20
15:47:58
V|| (km/s)
Vxperp (km/s)
Flux (cm2 sr s keV/e)-1
V|| (km/s)
Flux (cm2 sr s keV/e)-1

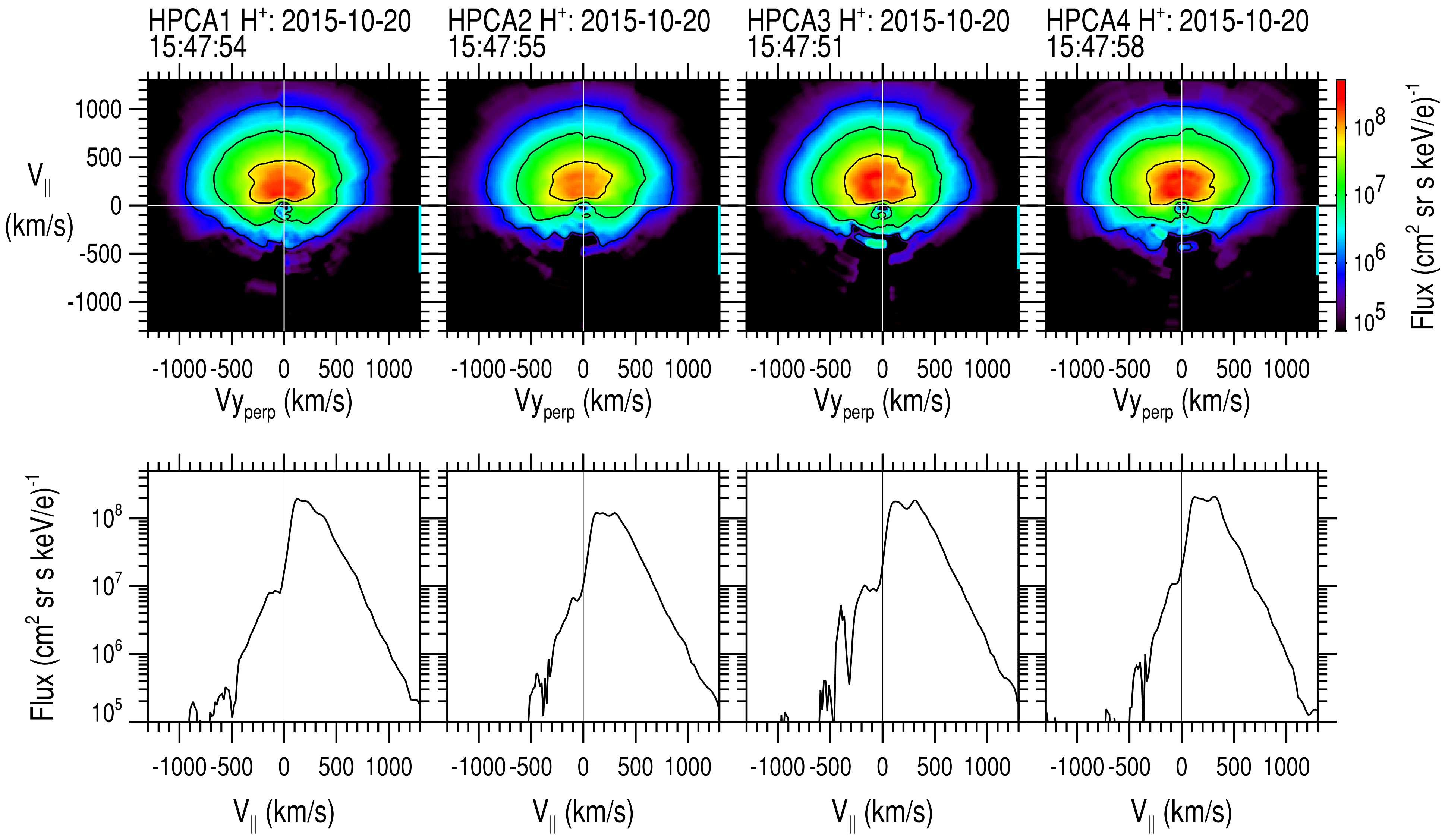

HPCA1 H+: 2015-10-20 15:47:54
HPCA2 H+: 2015-10-20 15:47:55
HPCA3 H+: 2015-10-20 15:47:51
HPCA4 H+: 2015-10-20 15:47:58
V|| (km/s)
Vyperp (km/s)
Flux (cm2 sr s keV/e)-1

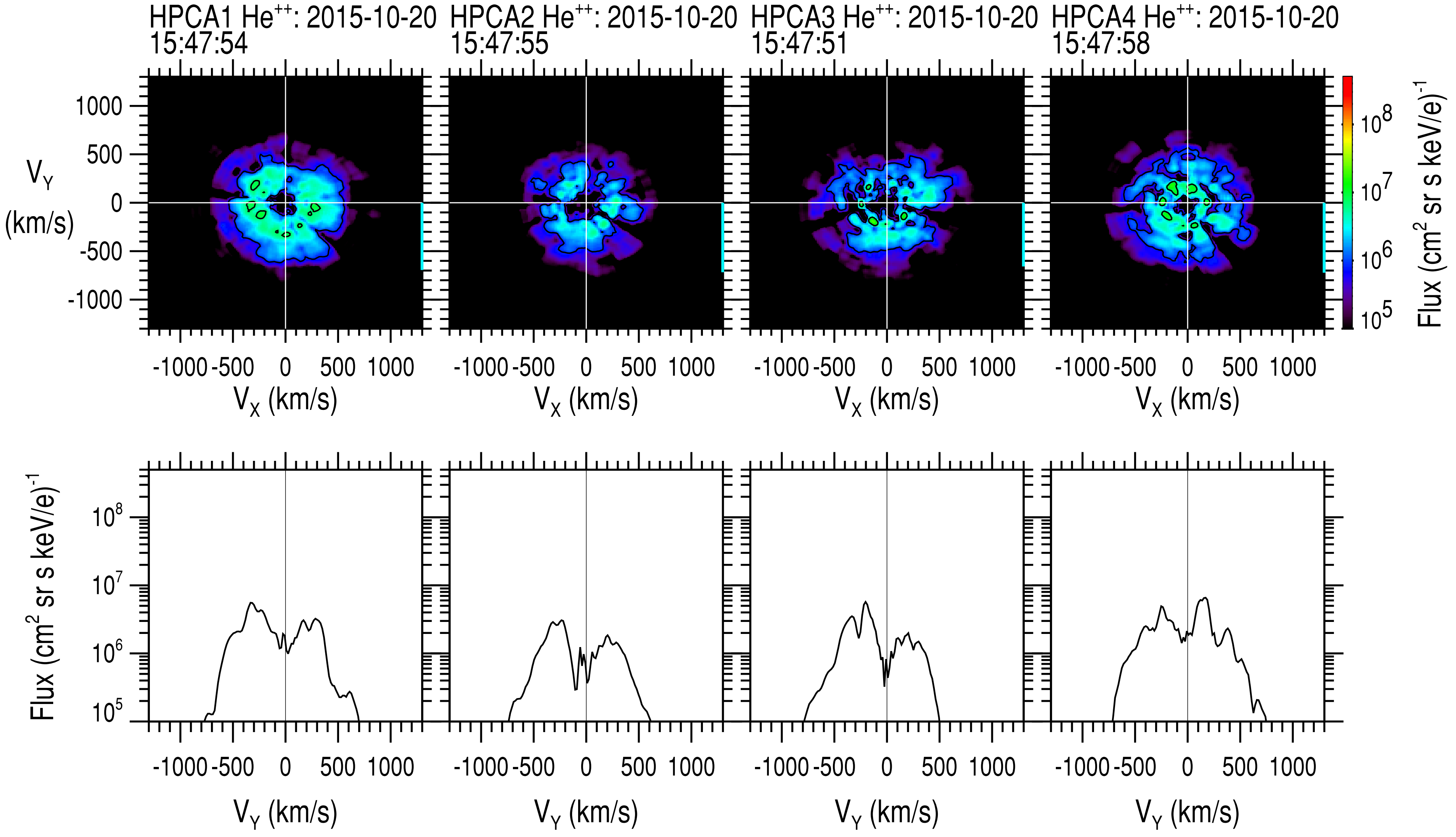

HPCA1 He++: 2015-10-20 15:47:54
HPCA2 He++: 2015-10-20 15:47:55
HPCA3 He++: 2015-10-20 15:47:51
HPCA4 He++: 2015-10-20 15:47:58
V_Y (km/s)
V_X (km/s)
V_Y (km/s)
Flux (cm^2 sr s keV/e)^-1
Flux (cm^2 sr s keV/e)^-1

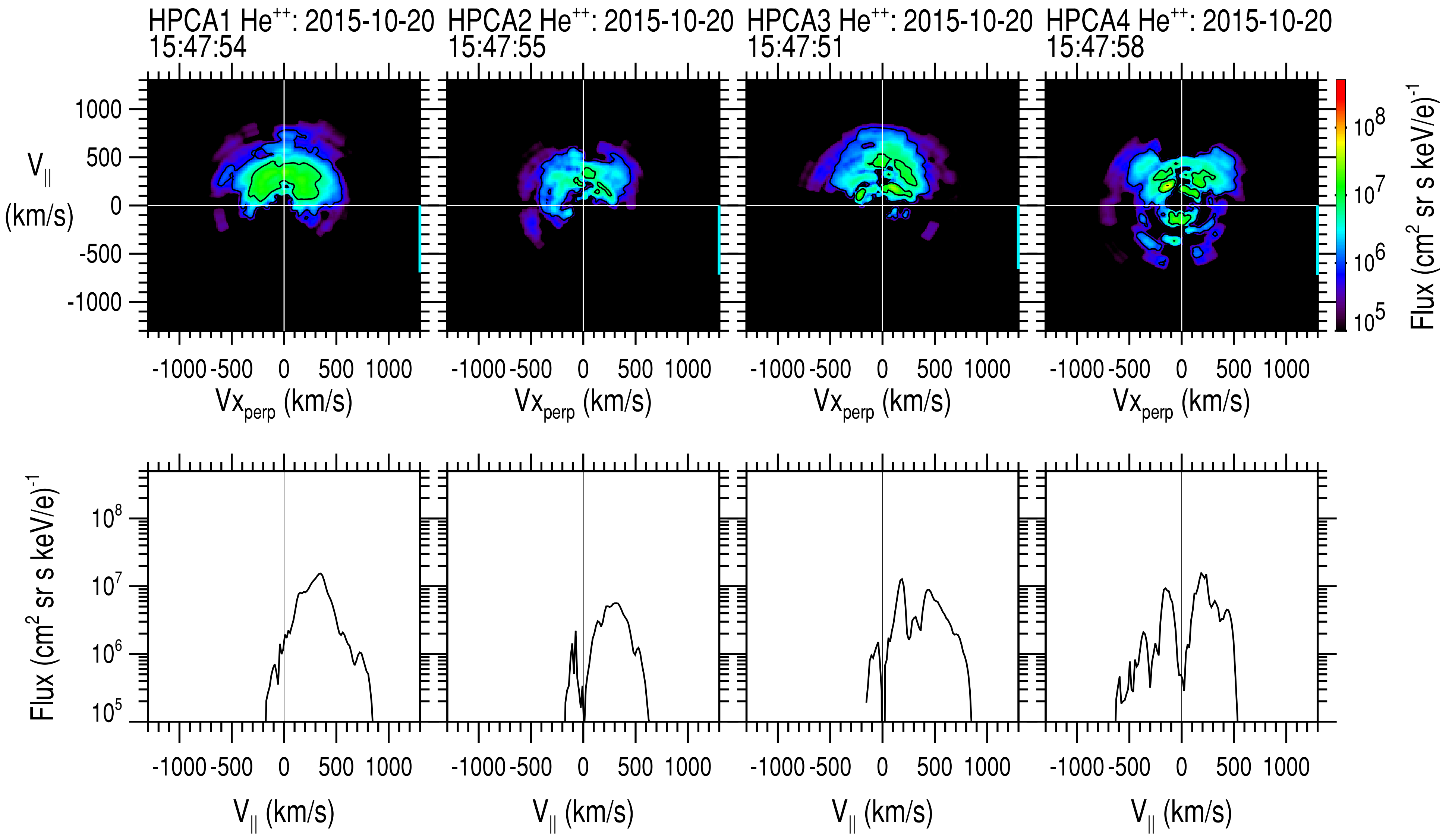
HPCA1 He++: 2015-10-20 15:47:54
HPCA2 He++: 2015-10-20 15:47:55
HPCA3 He++: 2015-10-20 15:47:51
HPCA4 He++: 2015-10-20 15:47:58
V|| (km/s)
Vxperp (km/s)
Flux (cm2 sr s keV/e)-1
V|| (km/s)
Flux (cm2 sr s keV/e)-1

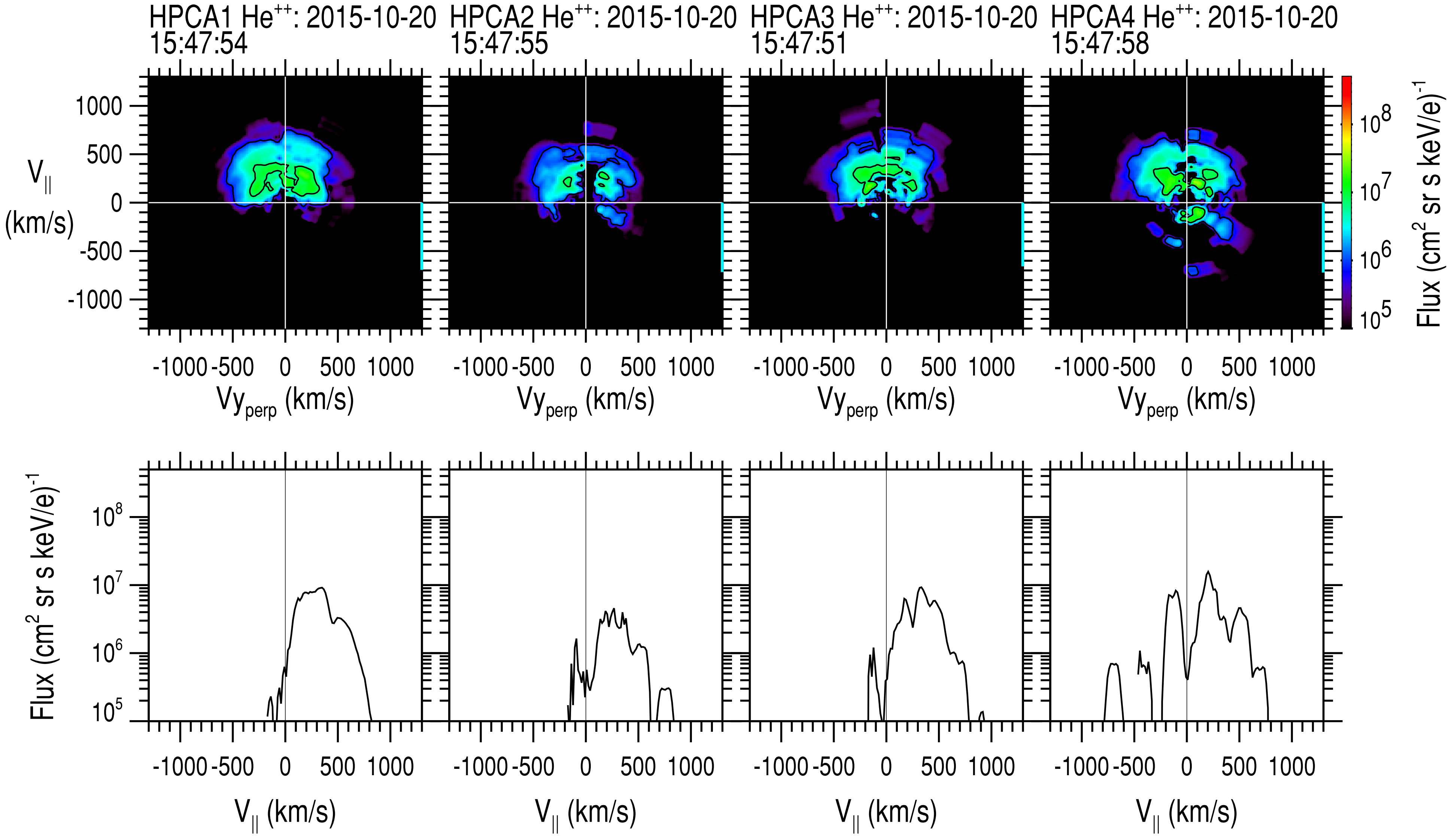
HPCA1 He++: 2015-10-20 15:47:54
HPCA2 He++: 2015-10-20 15:47:55
HPCA3 He++: 2015-10-20 15:47:51
HPCA4 He++: 2015-10-20 15:47:58
V|| (km/s)
Vyperp (km/s)
Flux (cm2 sr s keV/e)-1
V|| (km/s)

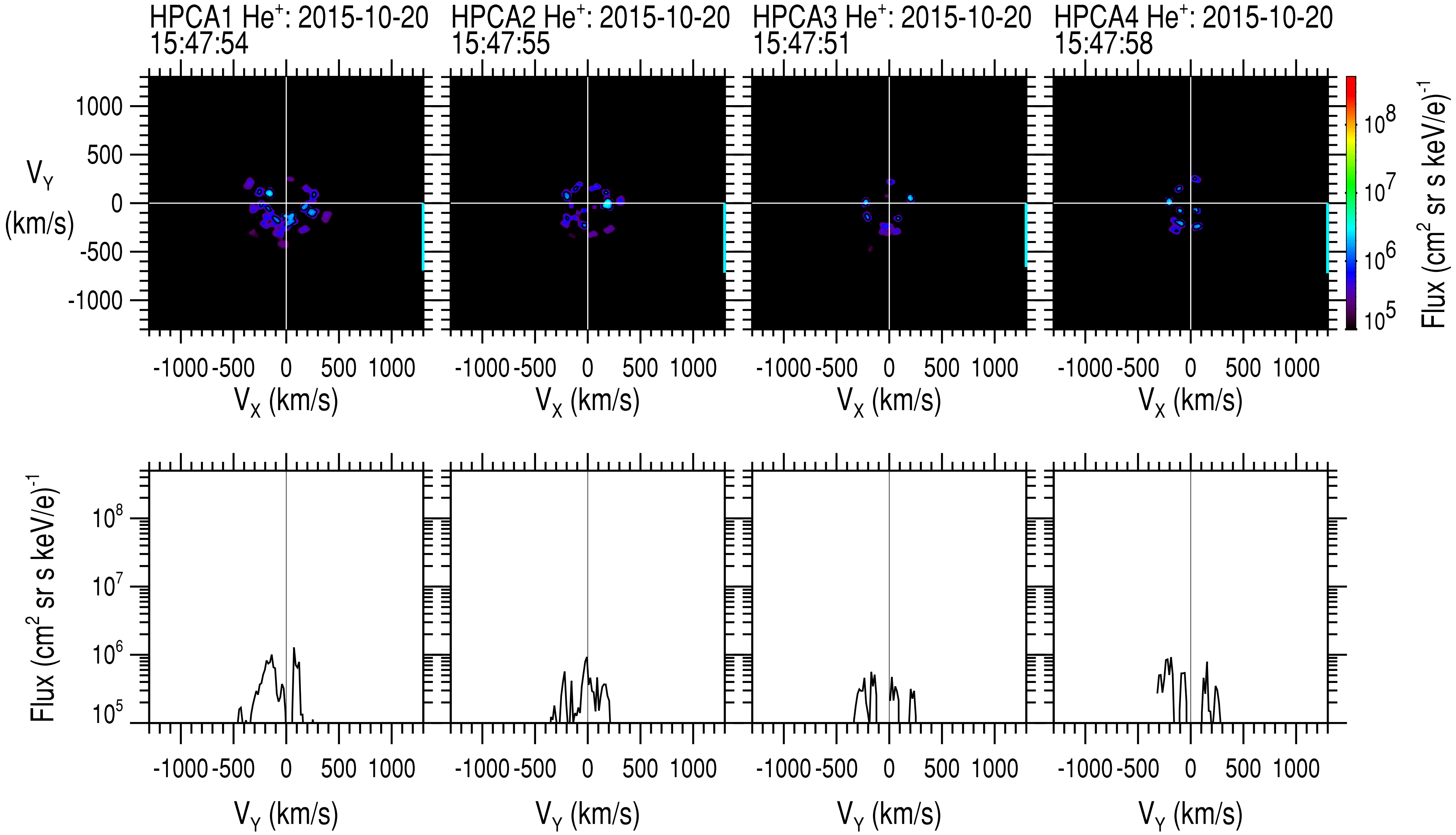
HPCA1 He+: 2015-10-20 15:47:54
HPCA2 He+: 2015-10-20 15:47:55
HPCA3 He+: 2015-10-20 15:47:51
HPCA4 He+: 2015-10-20 15:47:58
VY (km/s)
VX (km/s)
Flux (cm2 sr s keV/e)-1
VY (km/s)

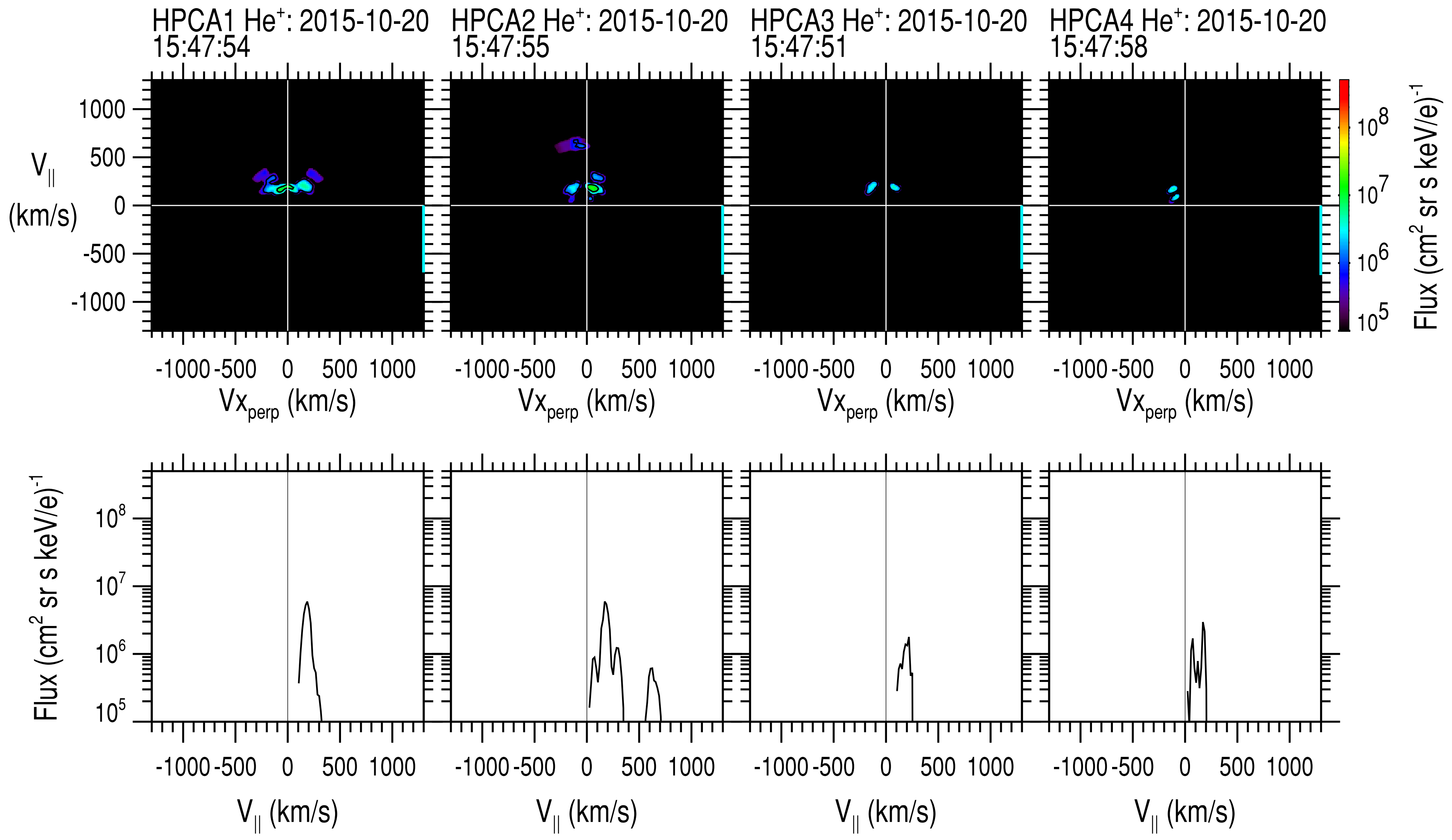

HPCA1 He+: 2015-10-20 15:47:54
HPCA2 He+: 2015-10-20 15:47:55
HPCA3 He+: 2015-10-20 15:47:51
HPCA4 He+: 2015-10-20 15:47:58
V|| (km/s)
Vxperp (km/s)
Flux (cm2 sr s keV/e)-1

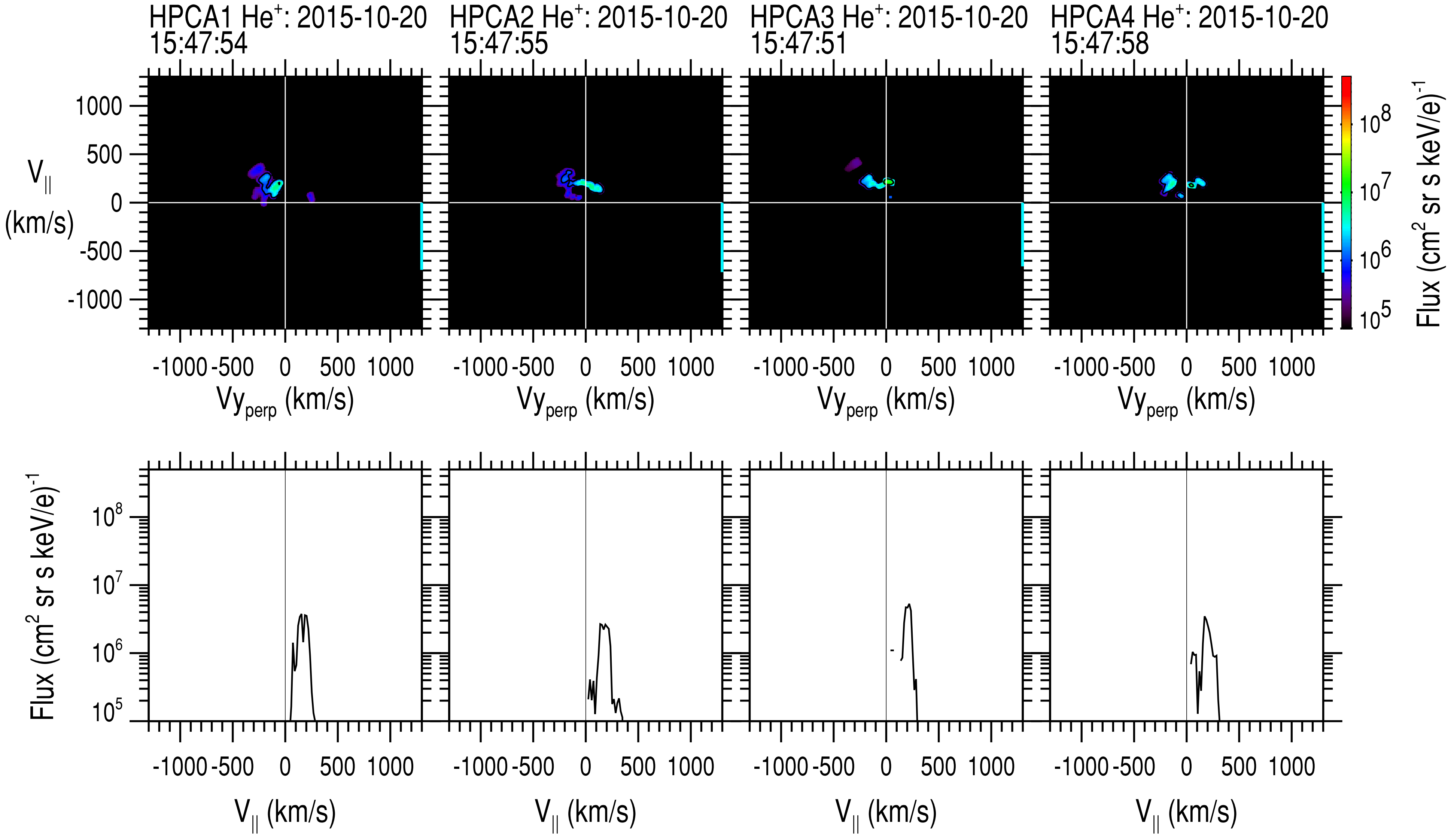
HPCA1 He+: 2015-10-20 15:47:54
HPCA2 He+: 2015-10-20 15:47:55
HPCA3 He+: 2015-10-20 15:47:51
HPCA4 He+: 2015-10-20 15:47:58
V|| (km/s)
Vyperp (km/s)
Flux (cm2 sr s keV/e)-1
V|| (km/s)

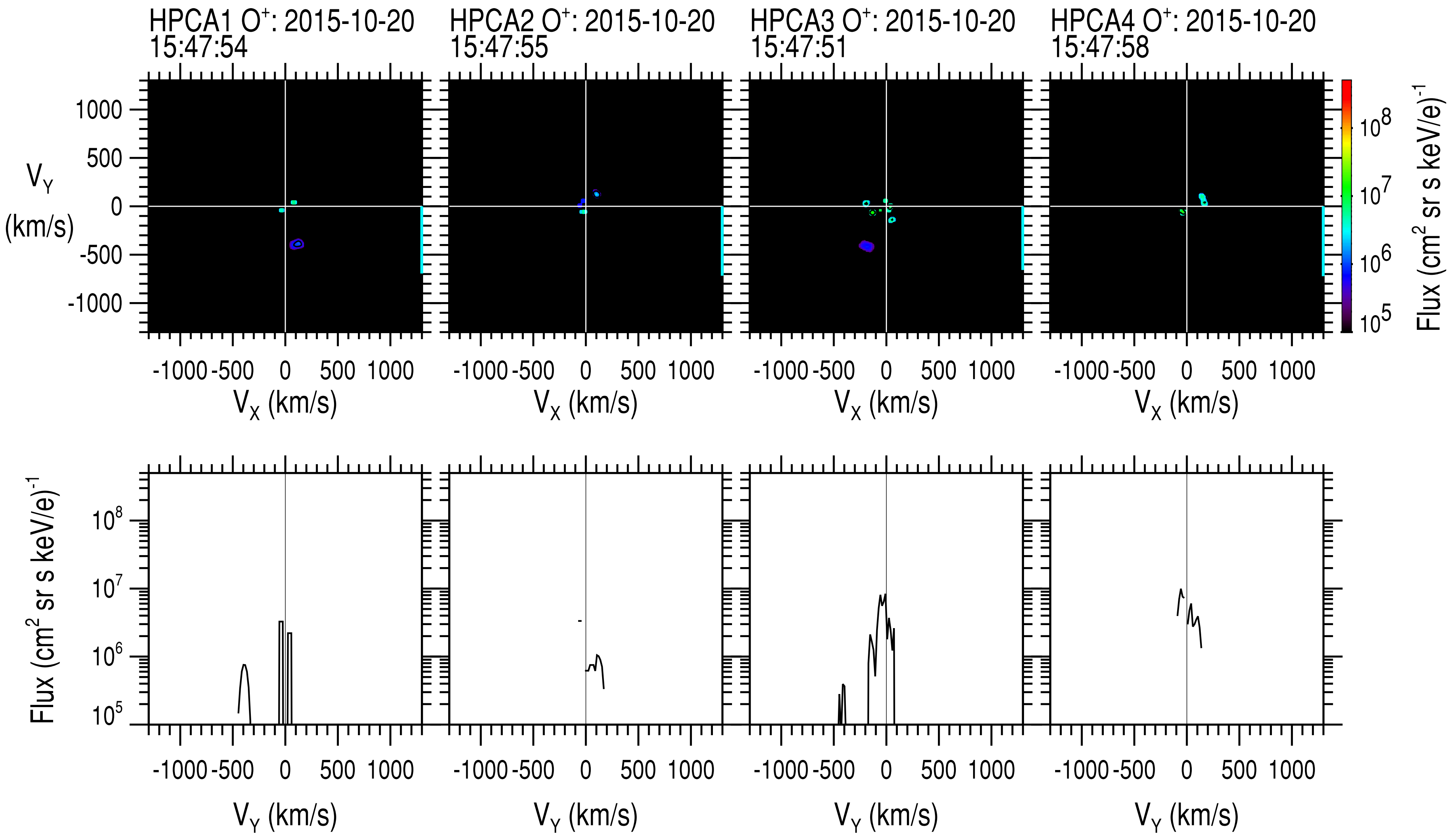
HPCA1 O+: 2015-10-20 15:47:54
HPCA2 O+: 2015-10-20 15:47:55
HPCA3 O+: 2015-10-20 15:47:51
HPCA4 O+: 2015-10-20 15:47:58
VY (km/s)
VX (km/s)
Flux (cm2 sr s keV/e)-1

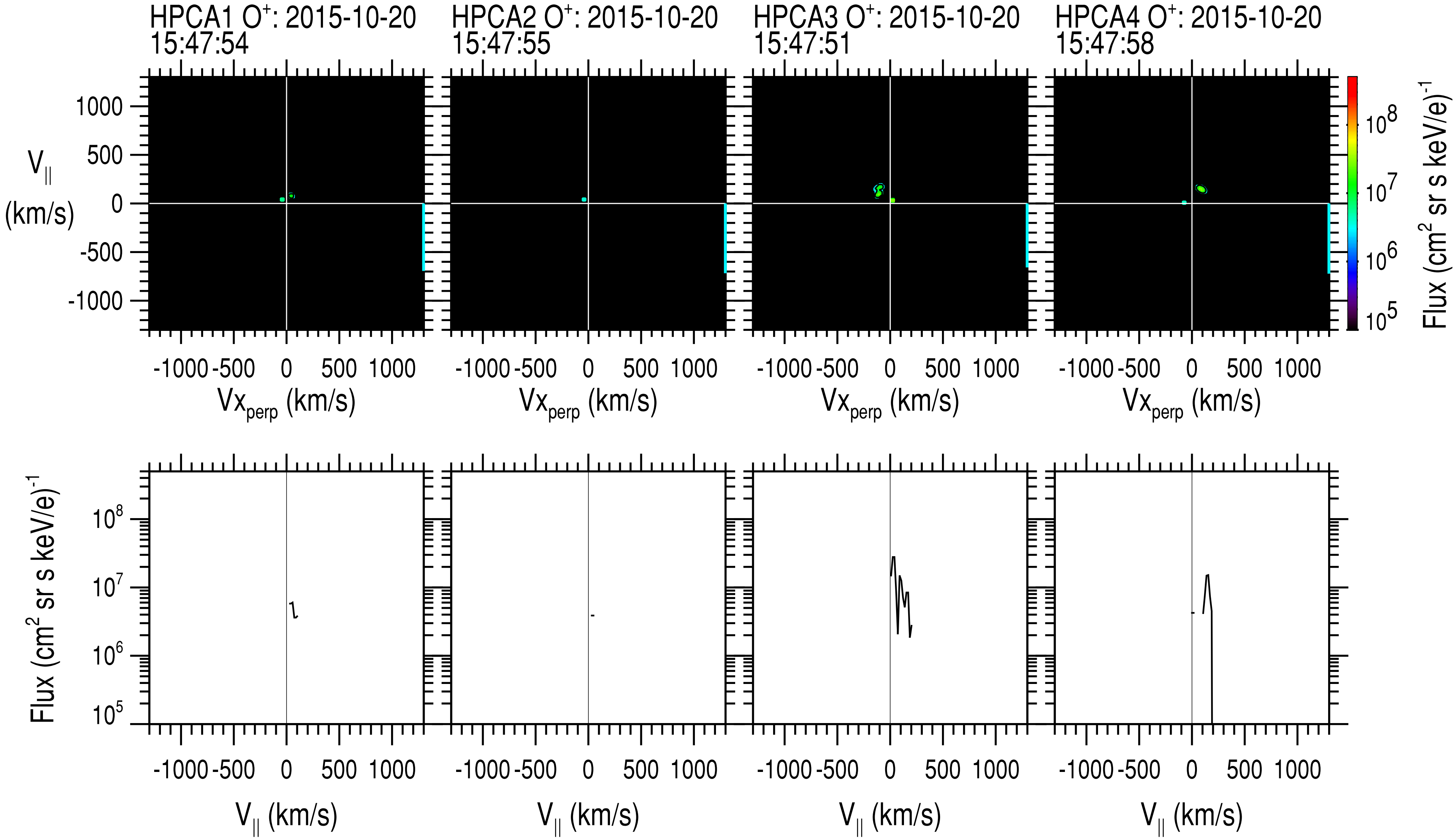

HPCA1 O+: 2015-10-20 15:47:54
HPCA2 O+: 2015-10-20 15:47:55
HPCA3 O+: 2015-10-20 15:47:51
HPCA4 O+: 2015-10-20 15:47:58
V|| (km/s)
Vxperp (km/s)
Flux (cm2 sr s keV/e)-1
-1000 -500 0 500 1000
10^5 10^6 10^7 10^8

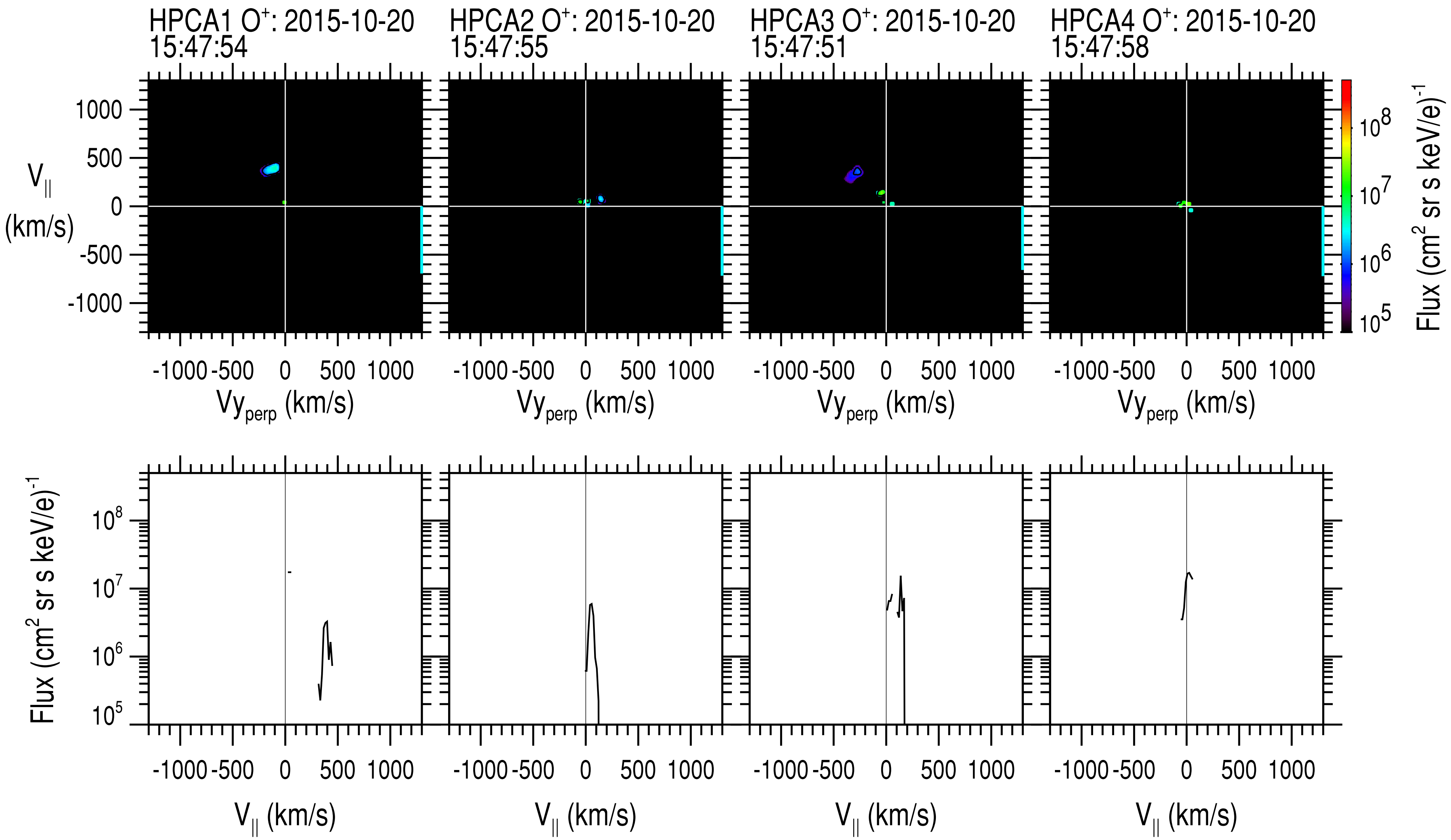
HPCA1 O+: 2015-10-20 15:47:54
HPCA2 O+: 2015-10-20 15:47:55
HPCA3 O+: 2015-10-20 15:47:51
HPCA4 O+: 2015-10-20 15:47:58
V|| (km/s)
Vyperp (km/s)
Flux (cm2 sr s keV/e)-1
V|| (km/s)

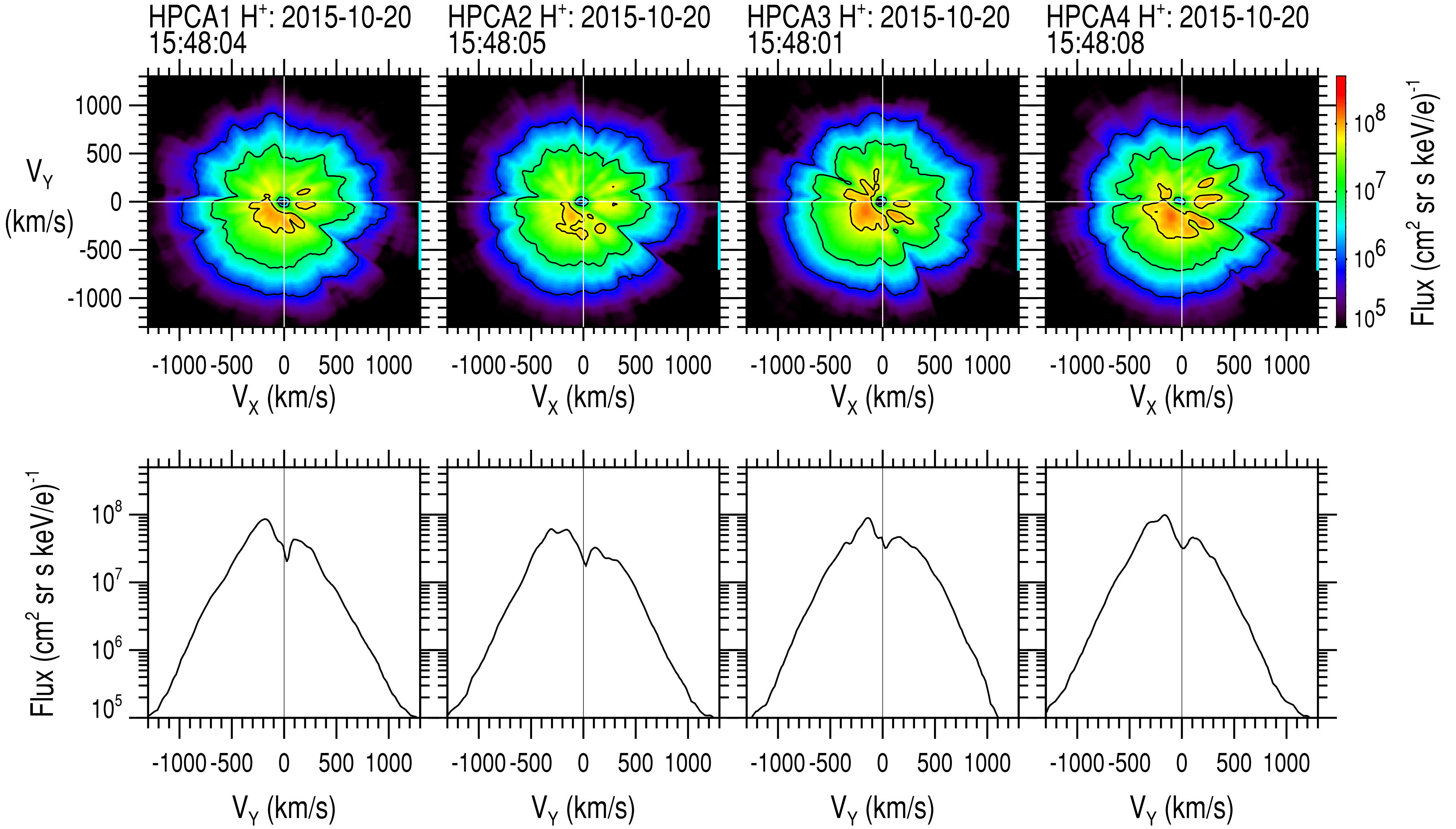

HPCA1 H+: 2015-10-20 15:48:04
HPCA2 H+: 2015-10-20 15:48:05
HPCA3 H+: 2015-10-20 15:48:01
HPCA4 H+: 2015-10-20 15:48:08
V_Y (km/s)
V_X (km/s)
Flux (cm^2 sr s keV/e)^-1

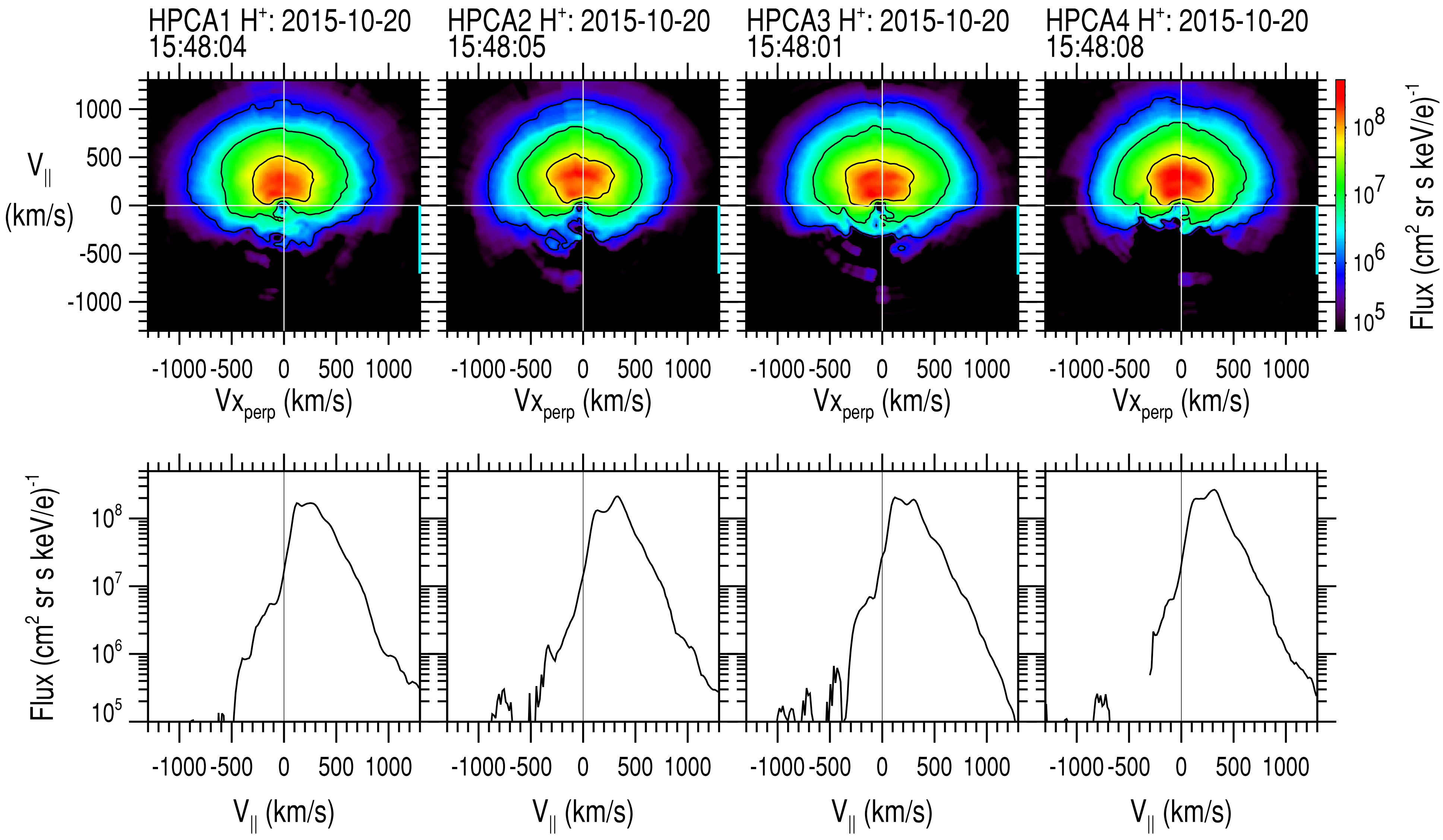

HPCA1 H+: 2015-10-20 15:48:04
HPCA2 H+: 2015-10-20 15:48:05
HPCA3 H+: 2015-10-20 15:48:01
HPCA4 H+: 2015-10-20 15:48:08
V|| (km/s)
Vxperp (km/s)
Flux (cm2 sr s keV/e)-1

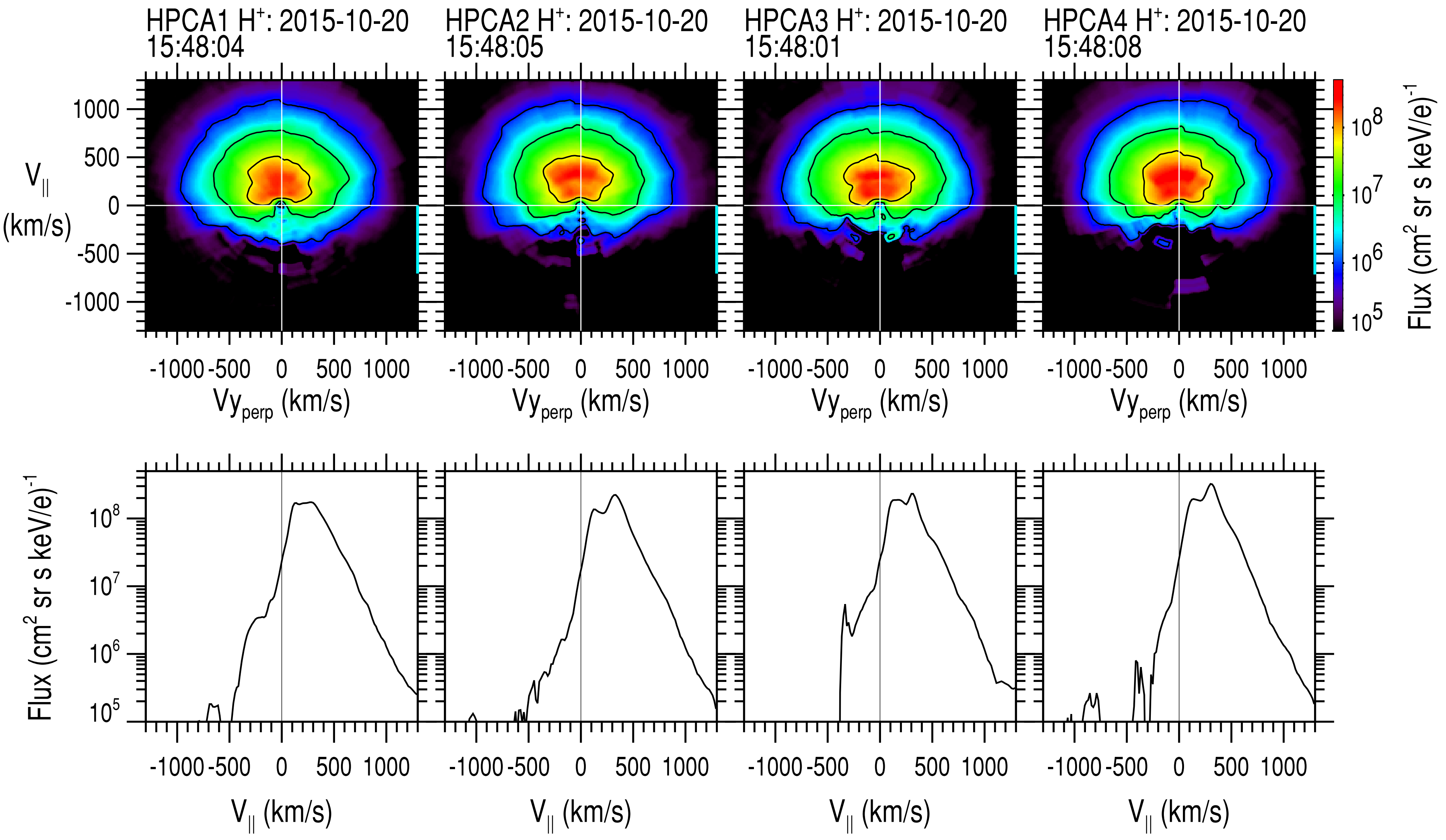
HPCA1 H+: 2015-10-20
15:48:04
HPCA2 H+: 2015-10-20
15:48:05
HPCA3 H+: 2015-10-20
15:48:01
HPCA4 H+: 2015-10-20
15:48:08
V|| (km/s)
Vyperp (km/s)
Flux (cm2 sr s keV/e)-1
10^5 10^6 10^7 10^8
-1000 -500 0 500 1000

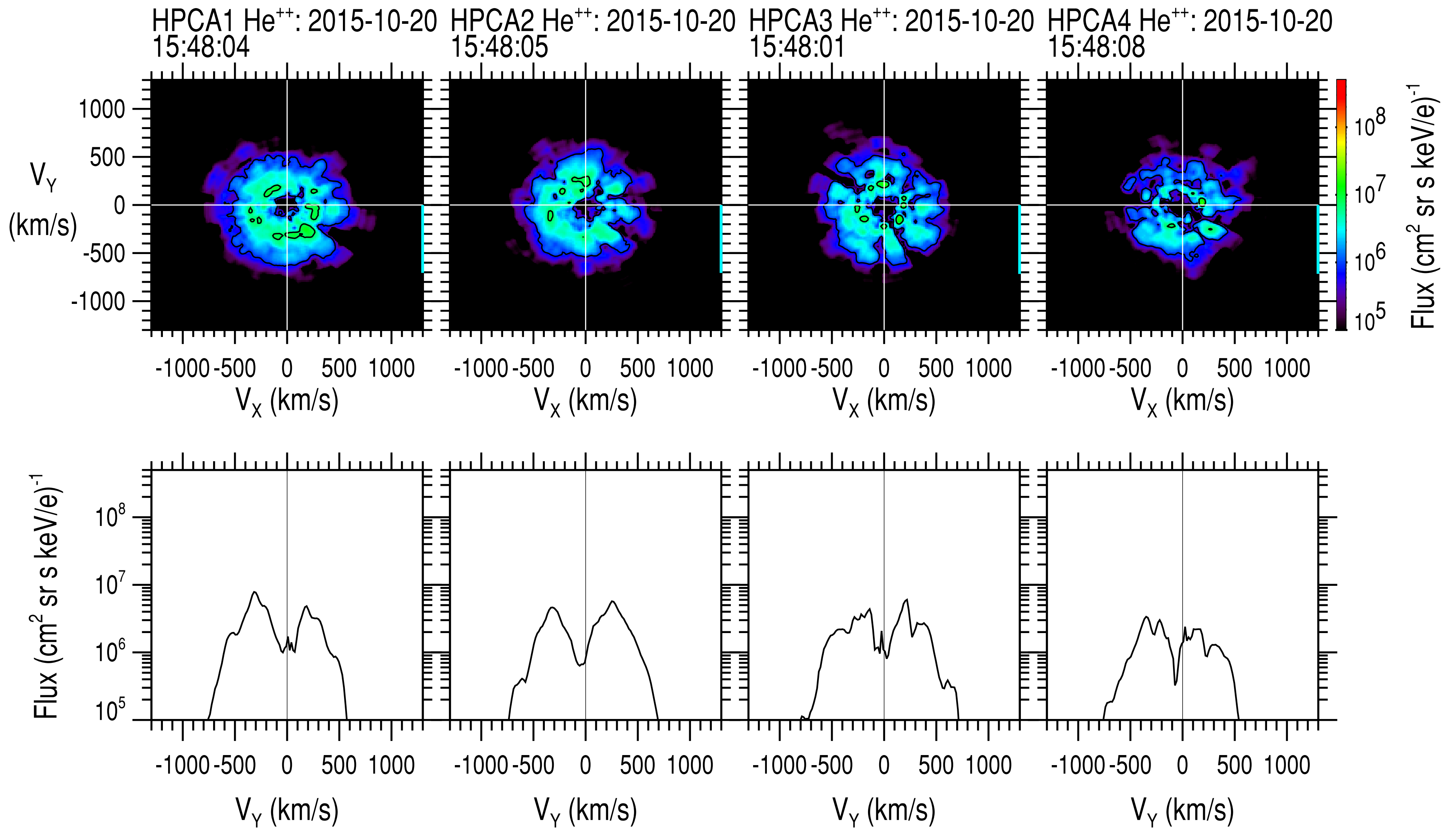
HPCA1 He++: 2015-10-20 15:48:04
HPCA2 He++: 2015-10-20 15:48:05
HPCA3 He++: 2015-10-20 15:48:01
HPCA4 He++: 2015-10-20 15:48:08
V_Y (km/s)
V_X (km/s)
Flux (cm^2 sr s keV/e)^-1
-1000 -500 0 500 1000
10^5 10^6 10^7 10^8

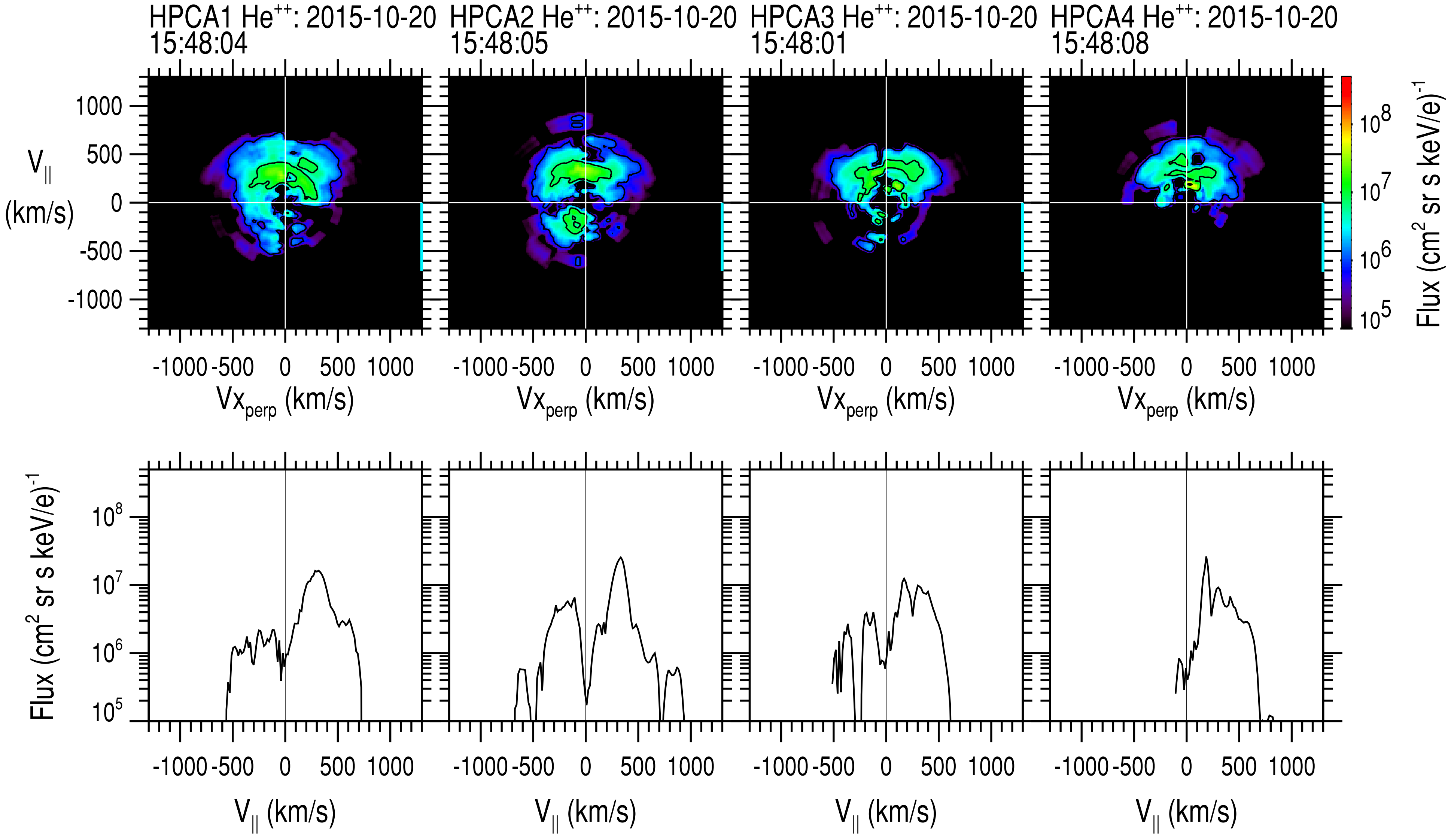

HPCA1 He++: 2015-10-20
15:48:04
HPCA2 He++: 2015-10-20
15:48:05
HPCA3 He++: 2015-10-20
15:48:01
HPCA4 He++: 2015-10-20
15:48:08
V|| (km/s)
1000
500
0
-500
-1000
-1000 -500 0 500 1000
Vxperp (km/s)
Flux (cm2 sr s keV/e)-1
105
106
107
108
V|| (km/s)

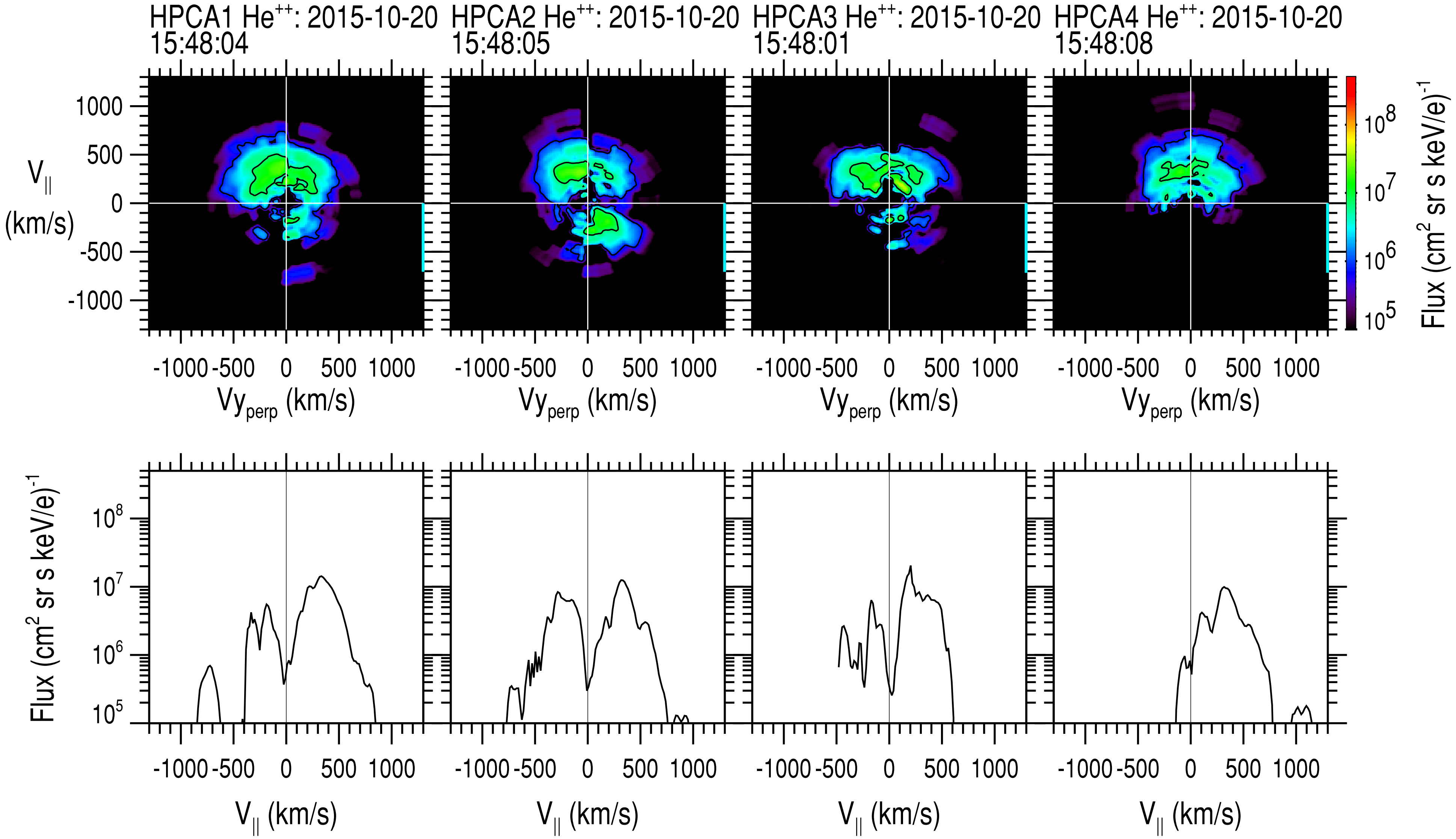

HPCA1 He++: 2015-10-20 15:48:04
HPCA2 He++: 2015-10-20 15:48:05
HPCA3 He++: 2015-10-20 15:48:01
HPCA4 He++: 2015-10-20 15:48:08
V|| (km/s)
Vyperp (km/s)
Flux (cm2 sr s keV/e)-1

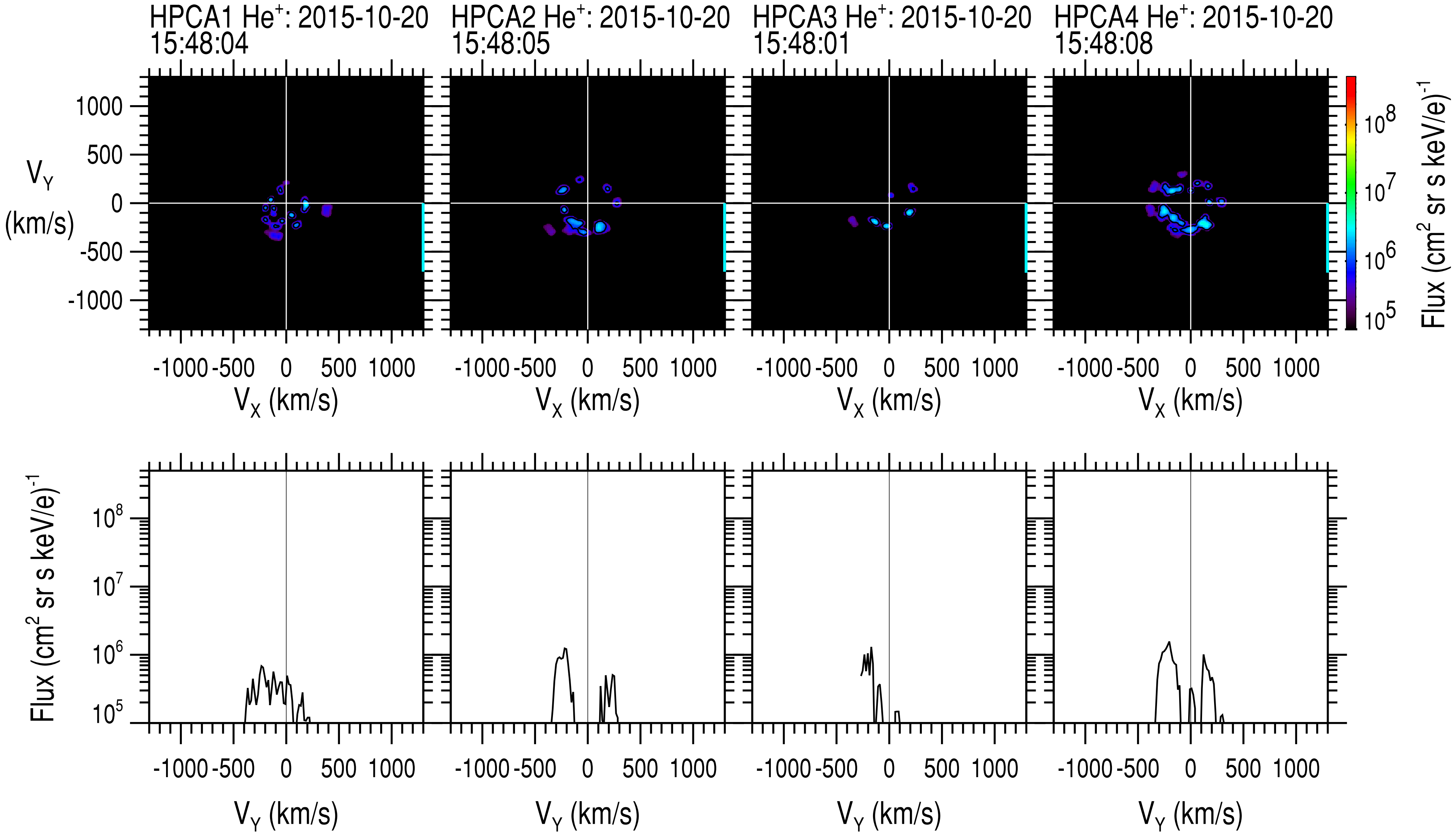
HPCA1 He+: 2015-10-20 15:48:04
HPCA2 He+: 2015-10-20 15:48:05
HPCA3 He+: 2015-10-20 15:48:01
HPCA4 He+: 2015-10-20 15:48:08
VY (km/s)
VX (km/s)
Flux (cm2 sr s keV/e)-1
VY (km/s)

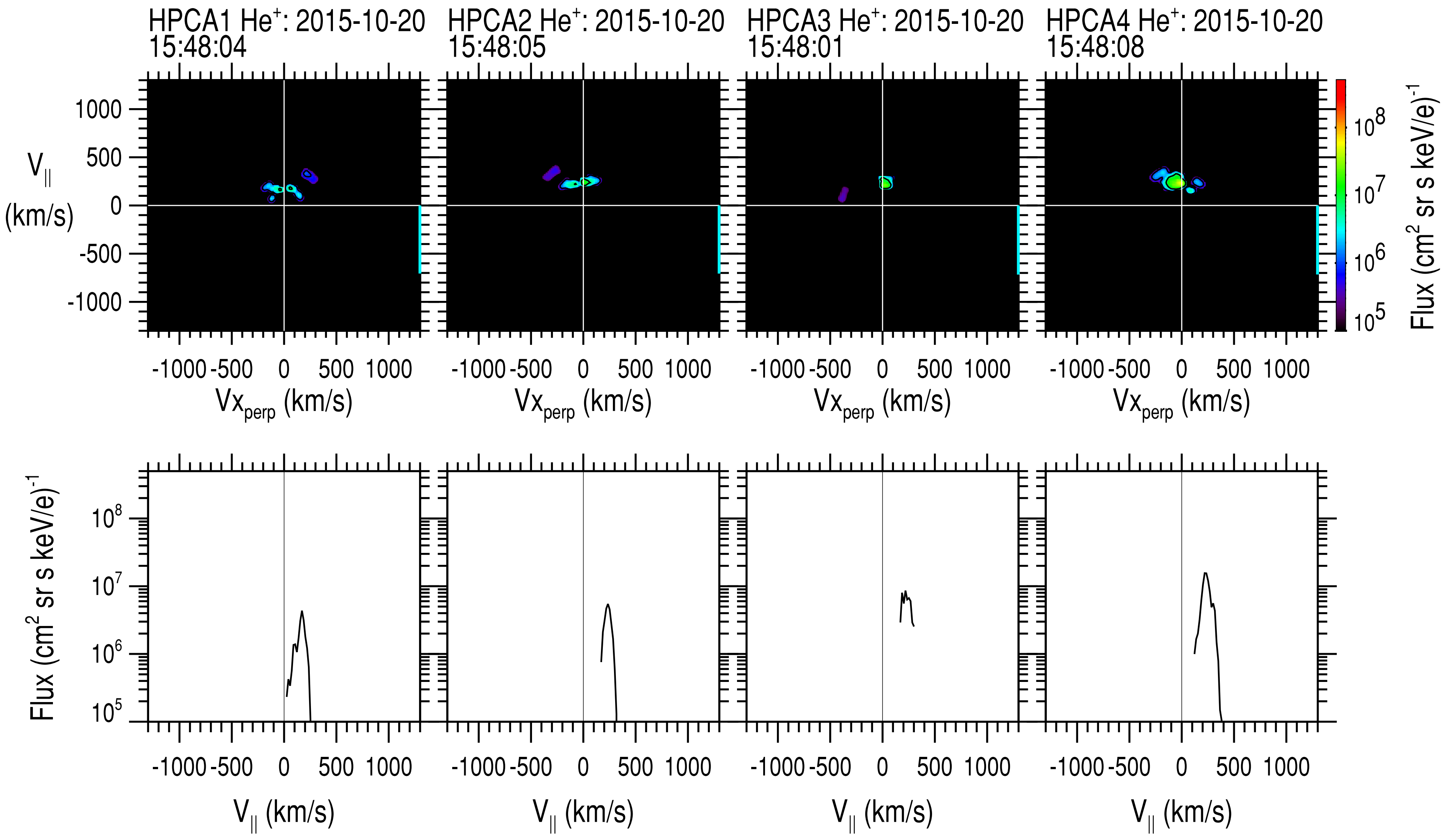
HPCA1 He+: 2015-10-20
15:48:04
HPCA2 He+: 2015-10-20
15:48:05
HPCA3 He+: 2015-10-20
15:48:01
HPCA4 He+: 2015-10-20
15:48:08
V|| (km/s)
Vxperp (km/s)
Flux (cm2 sr s keV/e)-1

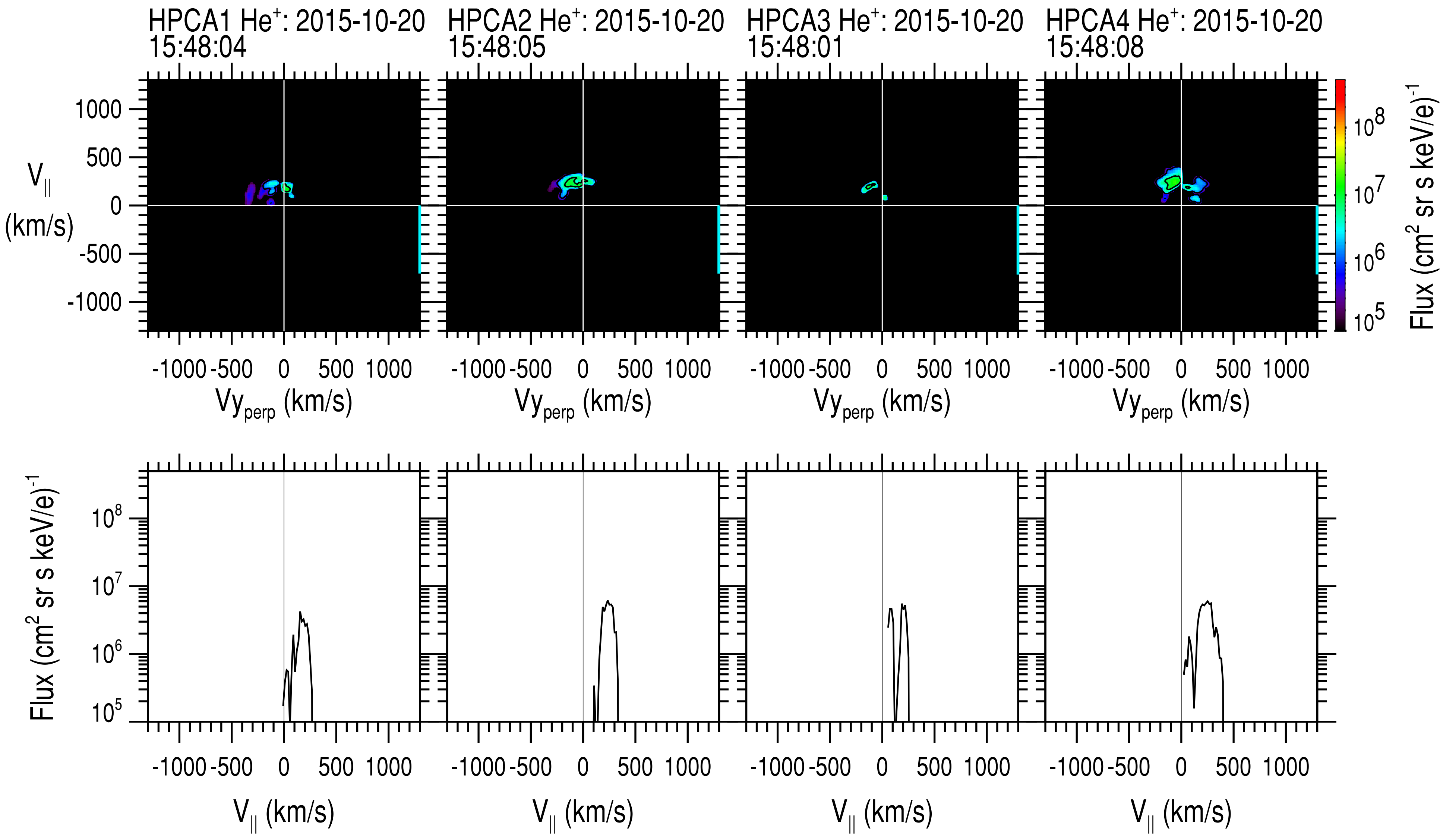
HPCA1 He+: 2015-10-20 15:48:04
HPCA2 He+: 2015-10-20 15:48:05
HPCA3 He+: 2015-10-20 15:48:01
HPCA4 He+: 2015-10-20 15:48:08
V|| (km/s)
Vyperp (km/s)
Flux (cm2 sr s keV/e)-1
V|| (km/s)

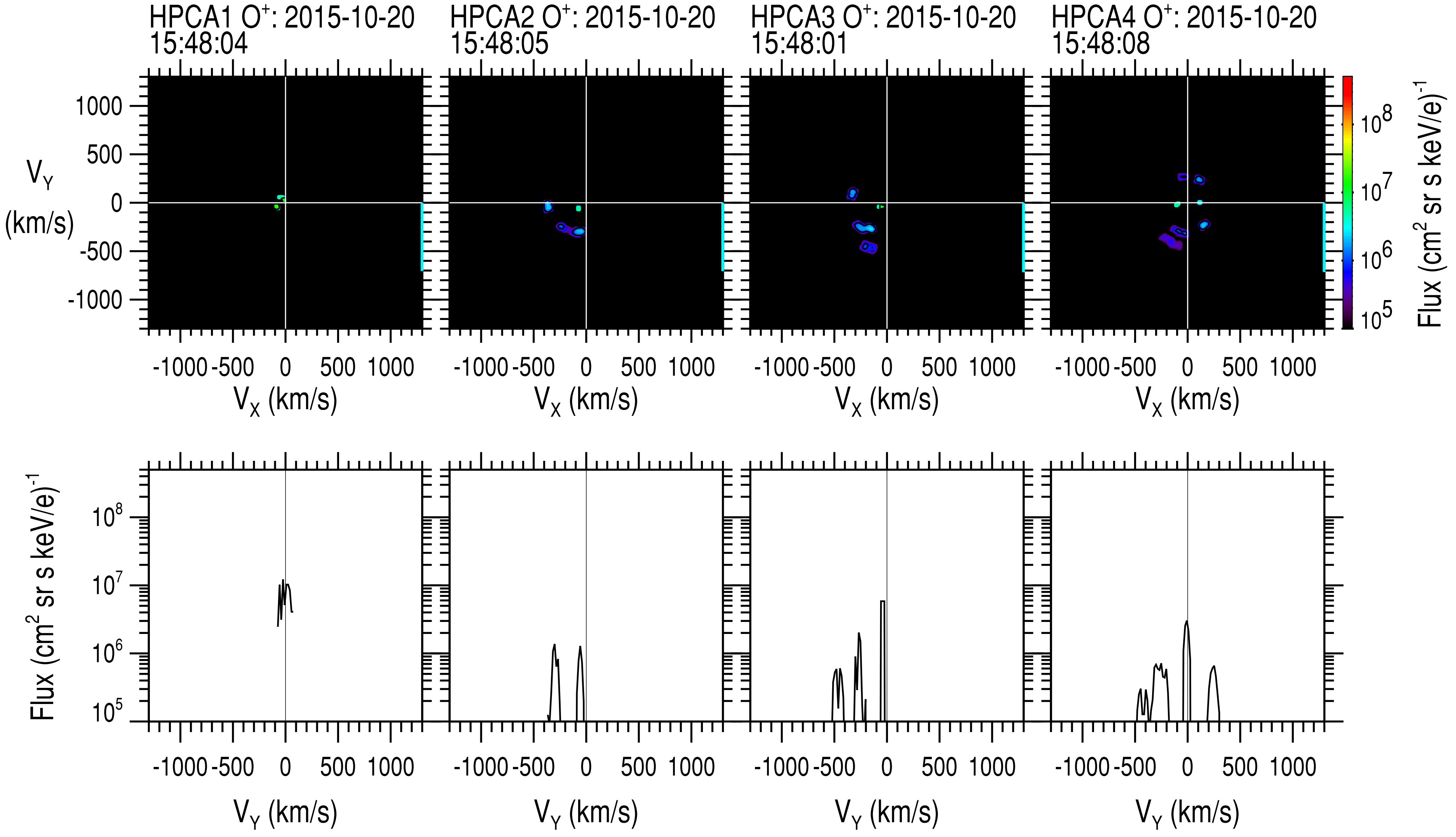
HPCA1 O$^+$: 2015-10-20 15:48:04
HPCA2 O$^+$: 2015-10-20 15:48:05
HPCA3 O$^+$: 2015-10-20 15:48:01
HPCA4 O$^+$: 2015-10-20 15:48:08
V_Y (km/s)
V_X (km/s)
Flux (cm^2 sr s keV/e)$^{-1}$
V_Y (km/s)

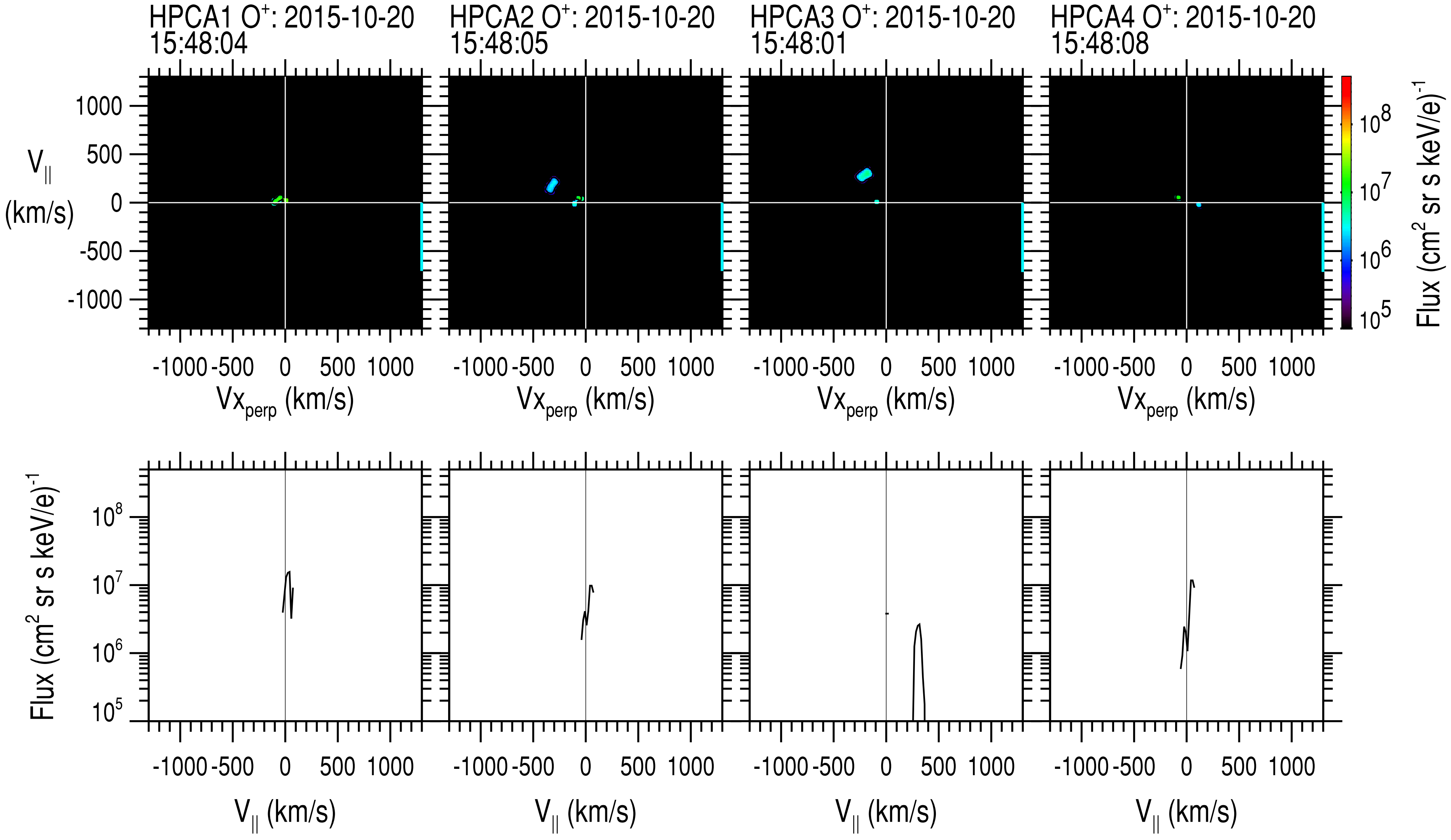
HPCA1 O+: 2015-10-20
15:48:04
HPCA2 O+: 2015-10-20
15:48:05
HPCA3 O+: 2015-10-20
15:48:01
HPCA4 O+: 2015-10-20
15:48:08
V|| (km/s)
1000
500
0
-500
-1000
-1000 -500 0 500 1000
Vxperp (km/s)
Flux (cm2 sr s keV/e)-1
105
106
107
108
V|| (km/s)

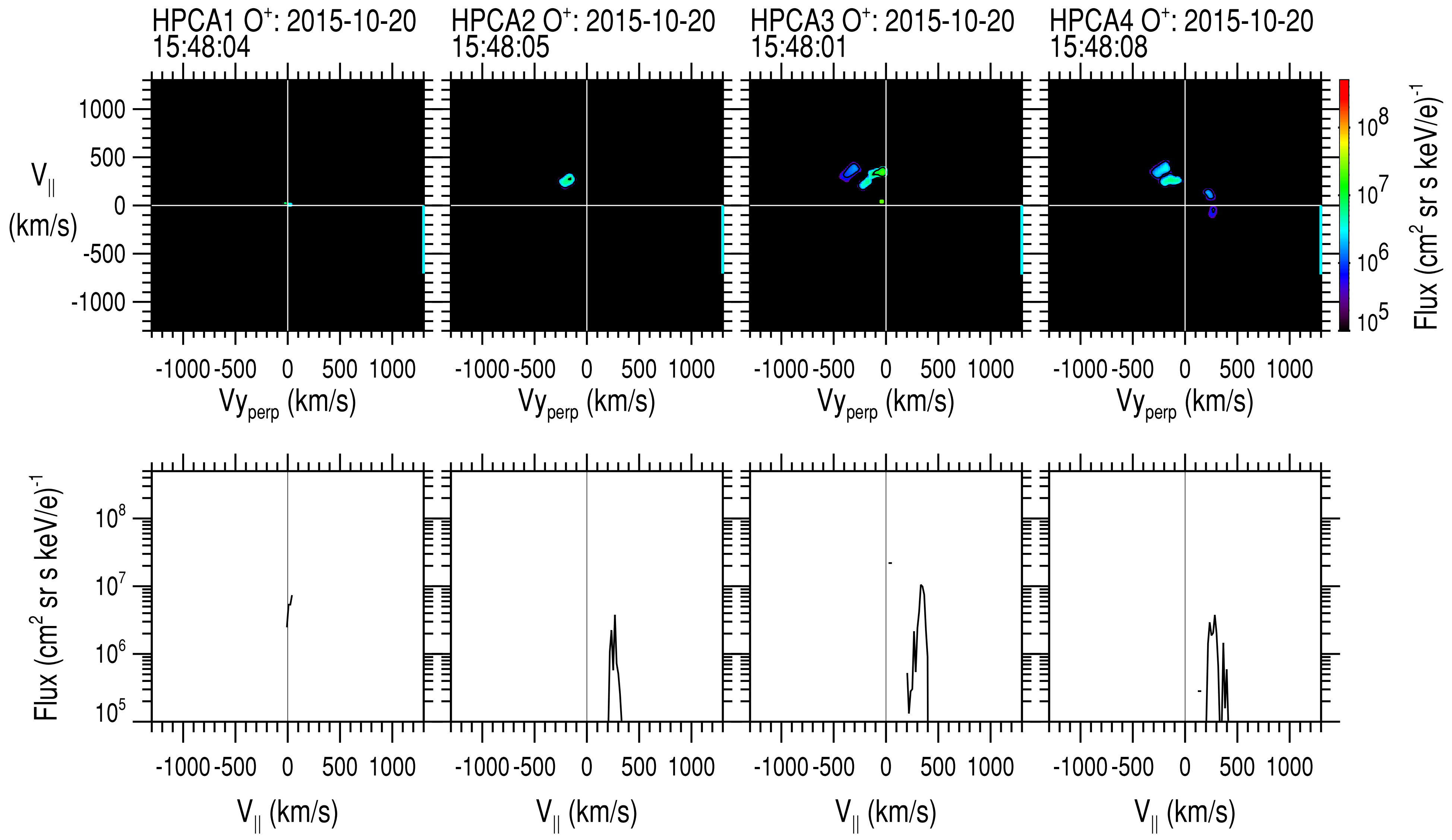

HPCA1 O+: 2015-10-20 15:48:04
HPCA2 O+: 2015-10-20 15:48:05
HPCA3 O+: 2015-10-20 15:48:01
HPCA4 O+: 2015-10-20 15:48:08
V|| (km/s)
Vyperp (km/s)
Flux (cm2 sr s keV/e)-1

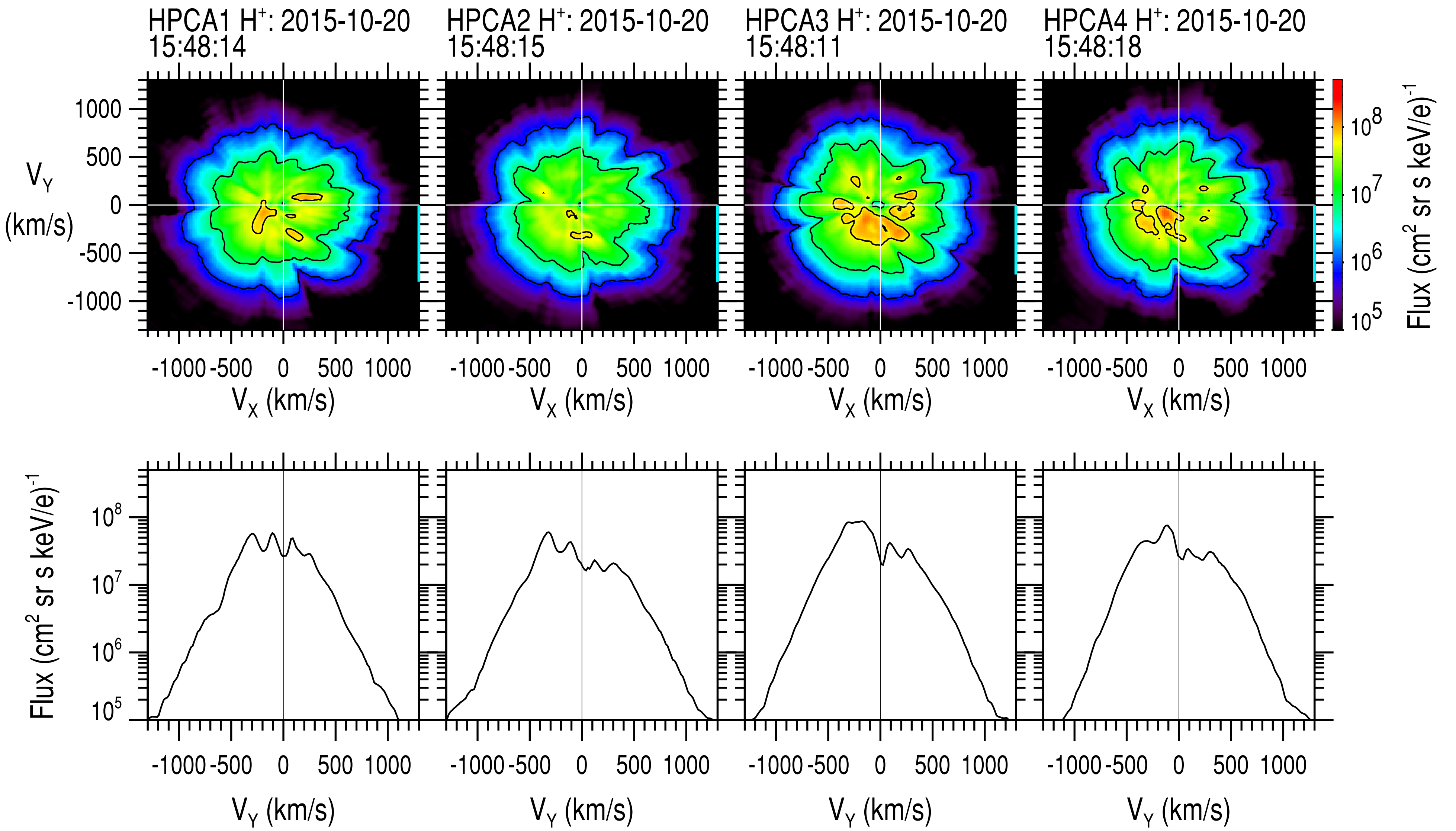
HPCA1 H+: 2015-10-20 15:48:14
HPCA2 H+: 2015-10-20 15:48:15
HPCA3 H+: 2015-10-20 15:48:11
HPCA4 H+: 2015-10-20 15:48:18
V_Y (km/s)
V_X (km/s)
Flux (cm^2 sr s keV/e)^-1
10^5
10^6
10^7
10^8
-1000 -500 0 500 1000
V_Y (km/s)

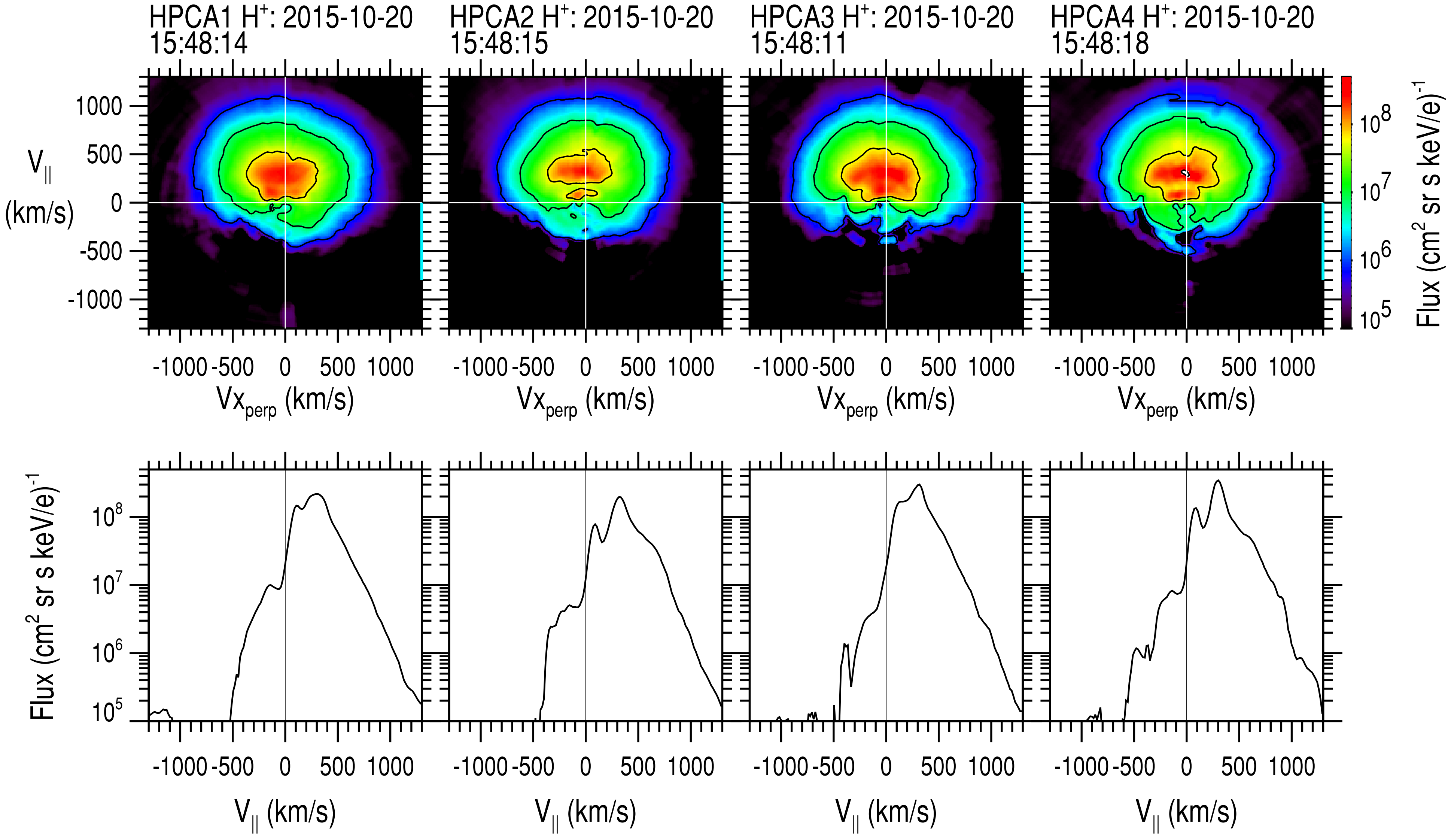
HPCA1 H+: 2015-10-20
15:48:14
HPCA2 H+: 2015-10-20
15:48:15
HPCA3 H+: 2015-10-20
15:48:11
HPCA4 H+: 2015-10-20
15:48:18
V||
(km/s)
Vxperp (km/s)
Flux (cm2 sr s keV/e)-1
V|| (km/s)

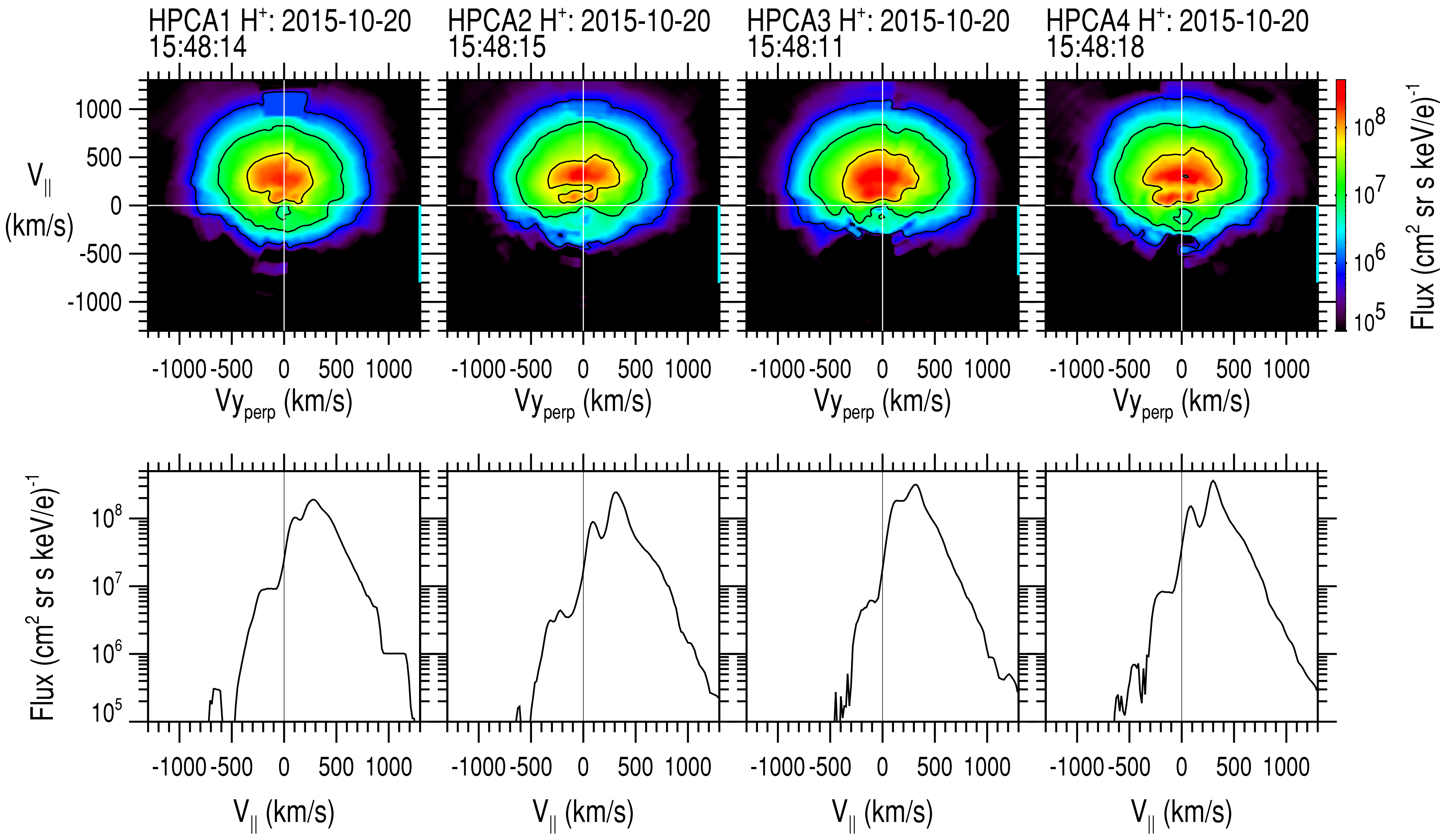

HPCA1 H+: 2015-10-20 15:48:14
HPCA2 H+: 2015-10-20 15:48:15
HPCA3 H+: 2015-10-20 15:48:11
HPCA4 H+: 2015-10-20 15:48:18
V|| (km/s)
Vyperp (km/s)
Flux (cm2 sr s keV/e)-1
10^5 10^6 10^7 10^8
-1000 -500 0 500 1000

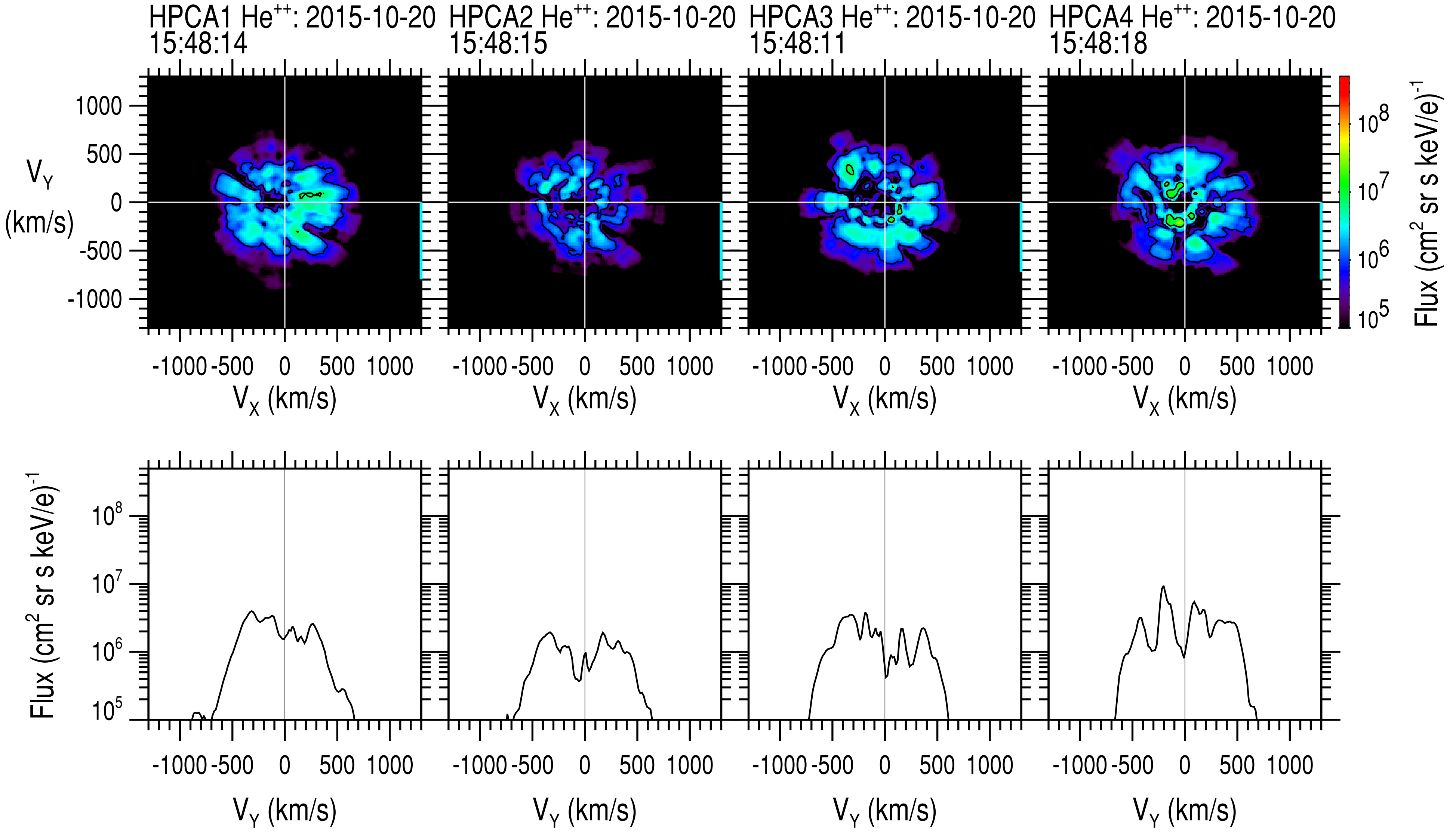
HPCA1 He++: 2015-10-20 15:48:14
HPCA2 He++: 2015-10-20 15:48:15
HPCA3 He++: 2015-10-20 15:48:11
HPCA4 He++: 2015-10-20 15:48:18
V_Y (km/s)
V_X (km/s)
Flux (cm^2 sr s keV/e)^-1
V_Y (km/s)

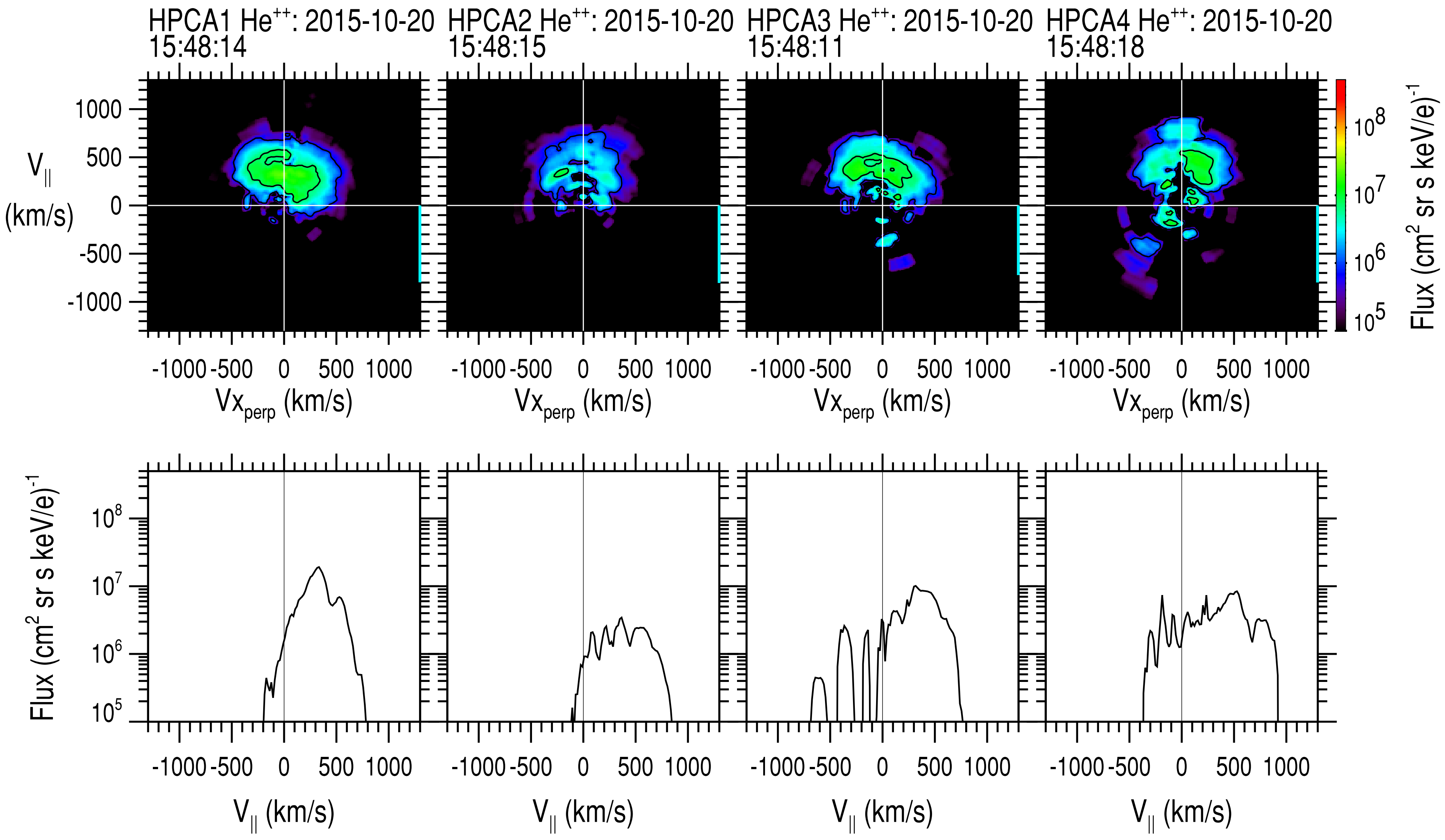

HPCA1 He++: 2015-10-20 15:48:14
HPCA2 He++: 2015-10-20 15:48:15
HPCA3 He++: 2015-10-20 15:48:11
HPCA4 He++: 2015-10-20 15:48:18
V|| (km/s)
Vxperp (km/s)
Flux (cm2 sr s keV/e)-1

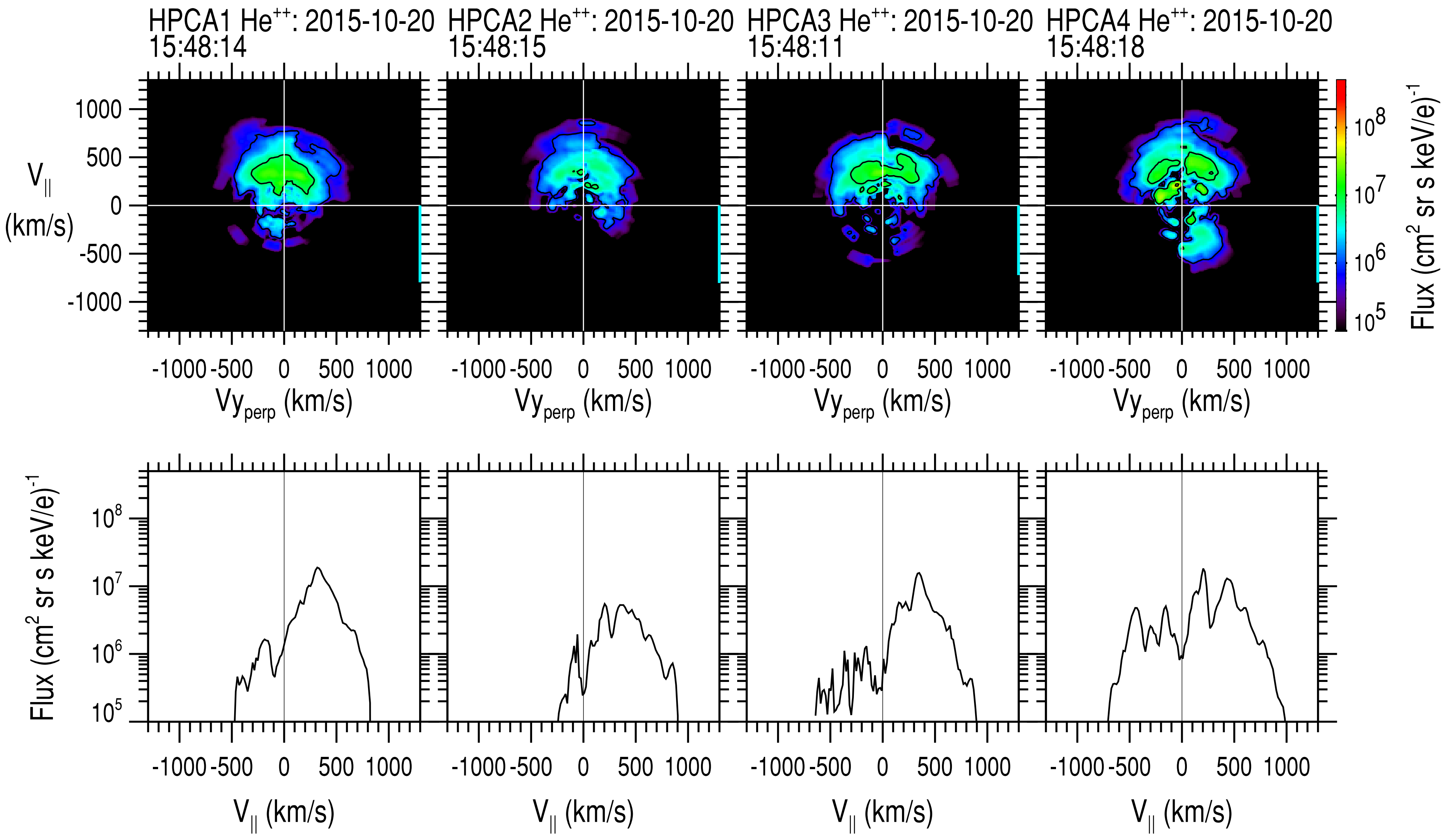

HPCA1 He++: 2015-10-20 15:48:14
HPCA2 He++: 2015-10-20 15:48:15
HPCA3 He++: 2015-10-20 15:48:11
HPCA4 He++: 2015-10-20 15:48:18
V|| (km/s)
Vyperp (km/s)
Flux (cm2 sr s keV/e)-1

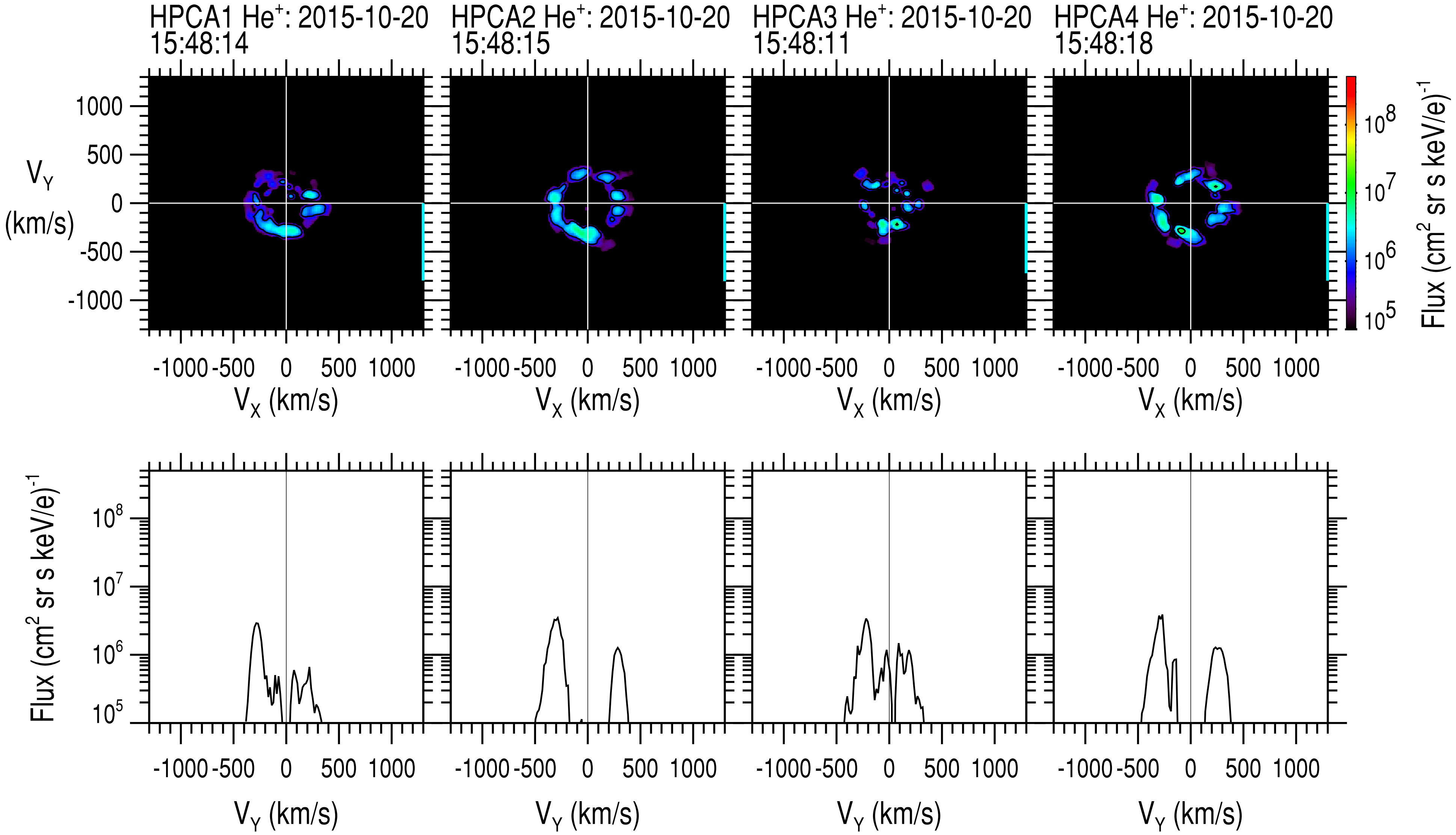
HPCA1 He+: 2015-10-20 15:48:14
HPCA2 He+: 2015-10-20 15:48:15
HPCA3 He+: 2015-10-20 15:48:11
HPCA4 He+: 2015-10-20 15:48:18
VY (km/s)
VX (km/s)
Flux (cm2 sr s keV/e)-1
VY (km/s)

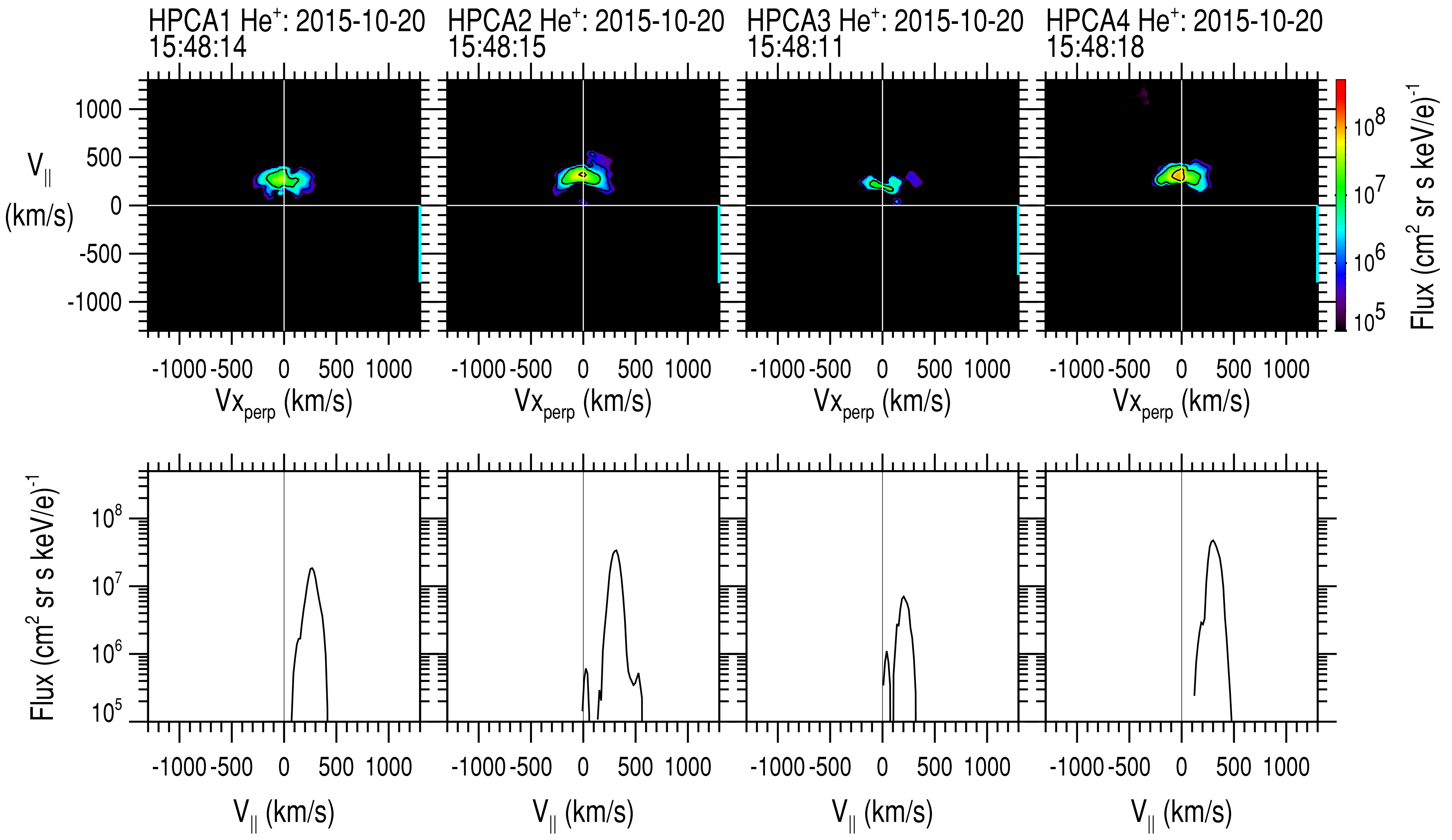

HPCA1 He+: 2015-10-20 15:48:14
HPCA2 He+: 2015-10-20 15:48:15
HPCA3 He+: 2015-10-20 15:48:11
HPCA4 He+: 2015-10-20 15:48:18
V|| (km/s)
Vxperp (km/s)
Flux (cm2 sr s keV/e)-1

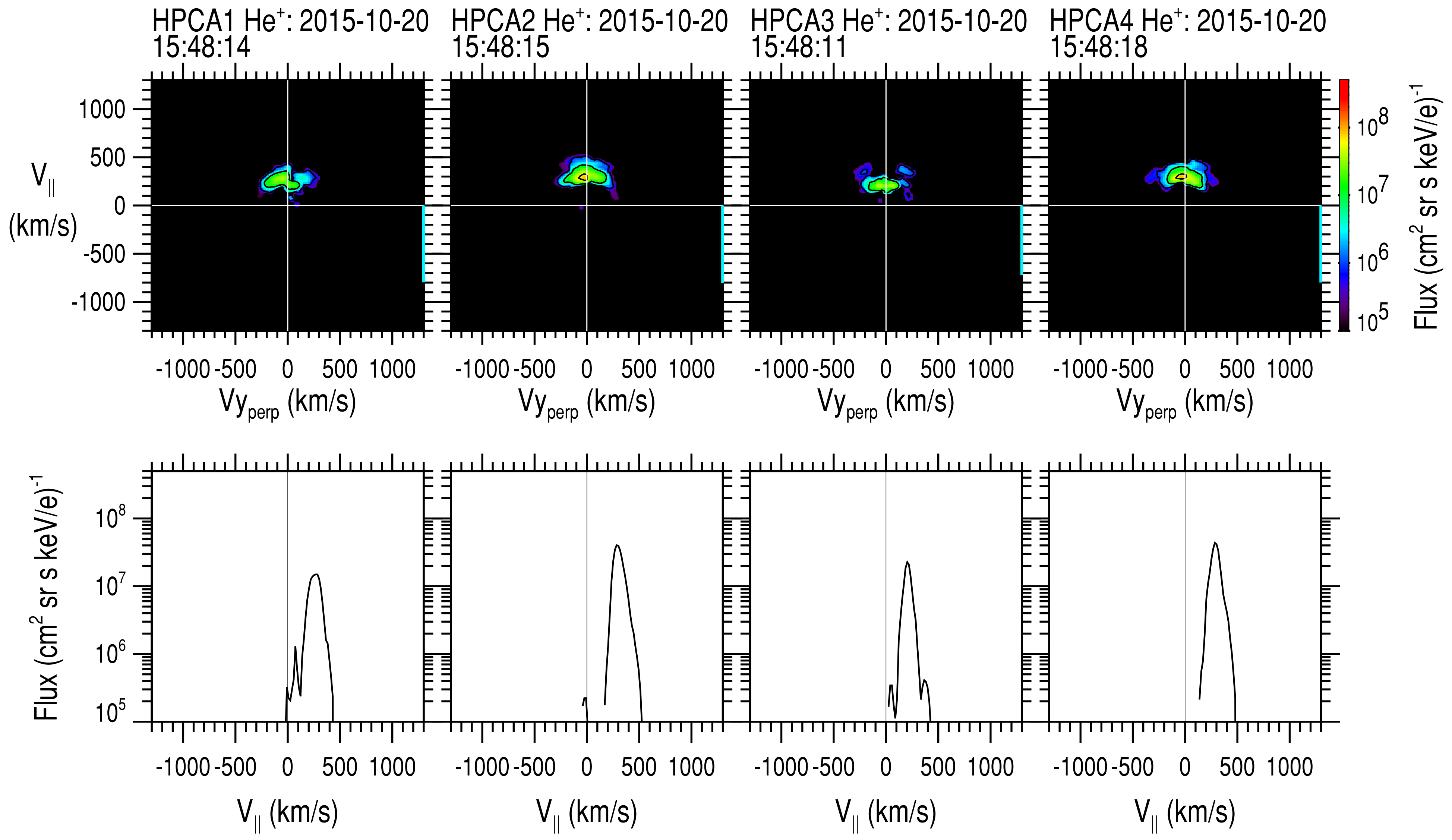
HPCA1 He^{+}: 2015-10-20 15:48:14
HPCA2 He^{+}: 2015-10-20 15:48:15
HPCA3 He^{+}: 2015-10-20 15:48:11
HPCA4 He^{+}: 2015-10-20 15:48:18
$V_{||}$ (km/s)
Vy_{perp} (km/s)
Flux (cm^2 sr s keV/e)$^{-1}$
$V_{||}$ (km/s)

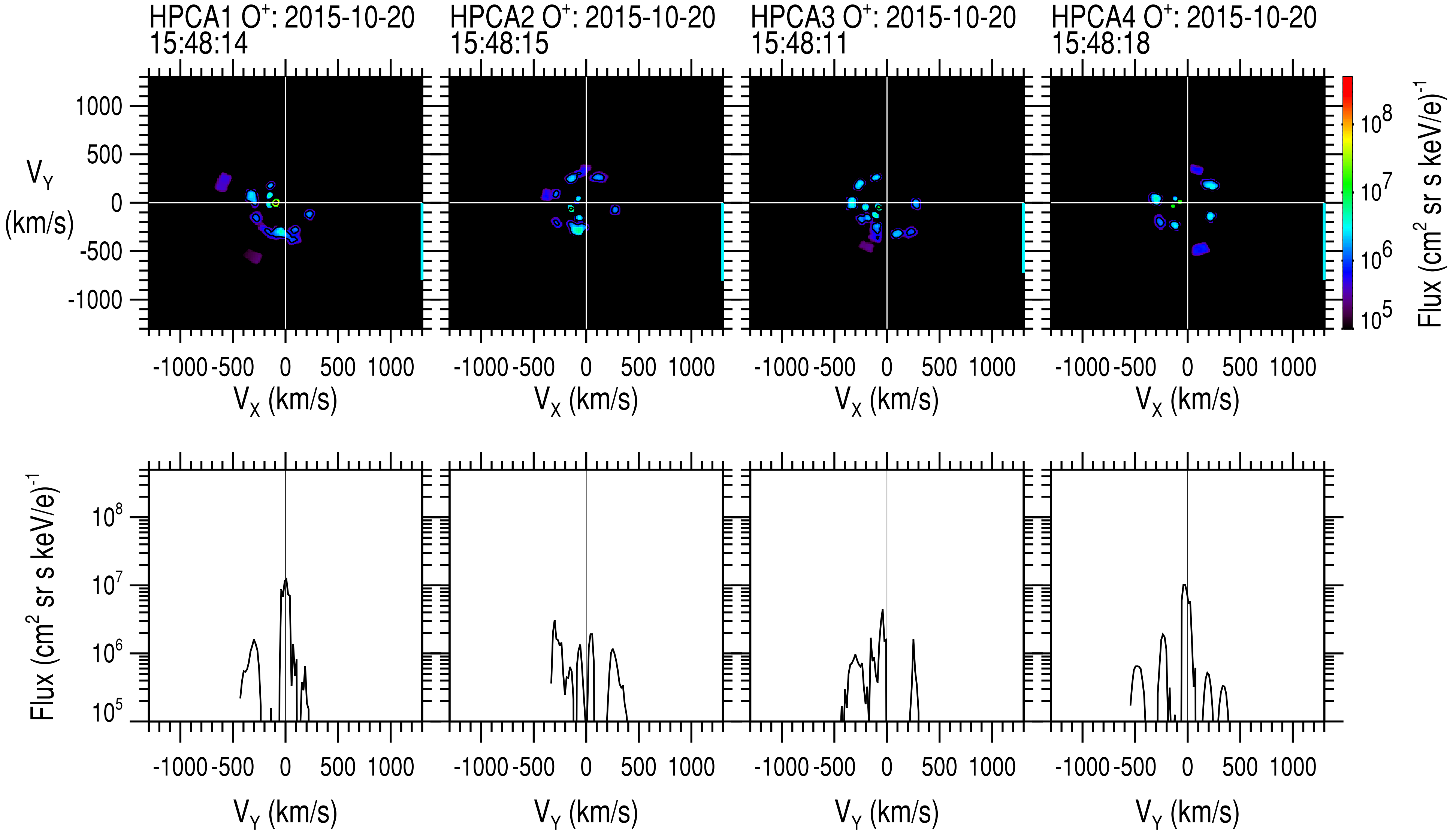

HPCA1 O+: 2015-10-20 15:48:14
HPCA2 O+: 2015-10-20 15:48:15
HPCA3 O+: 2015-10-20 15:48:11
HPCA4 O+: 2015-10-20 15:48:18
VY (km/s)
VX (km/s)
Flux (cm2 sr s keV/e)-1

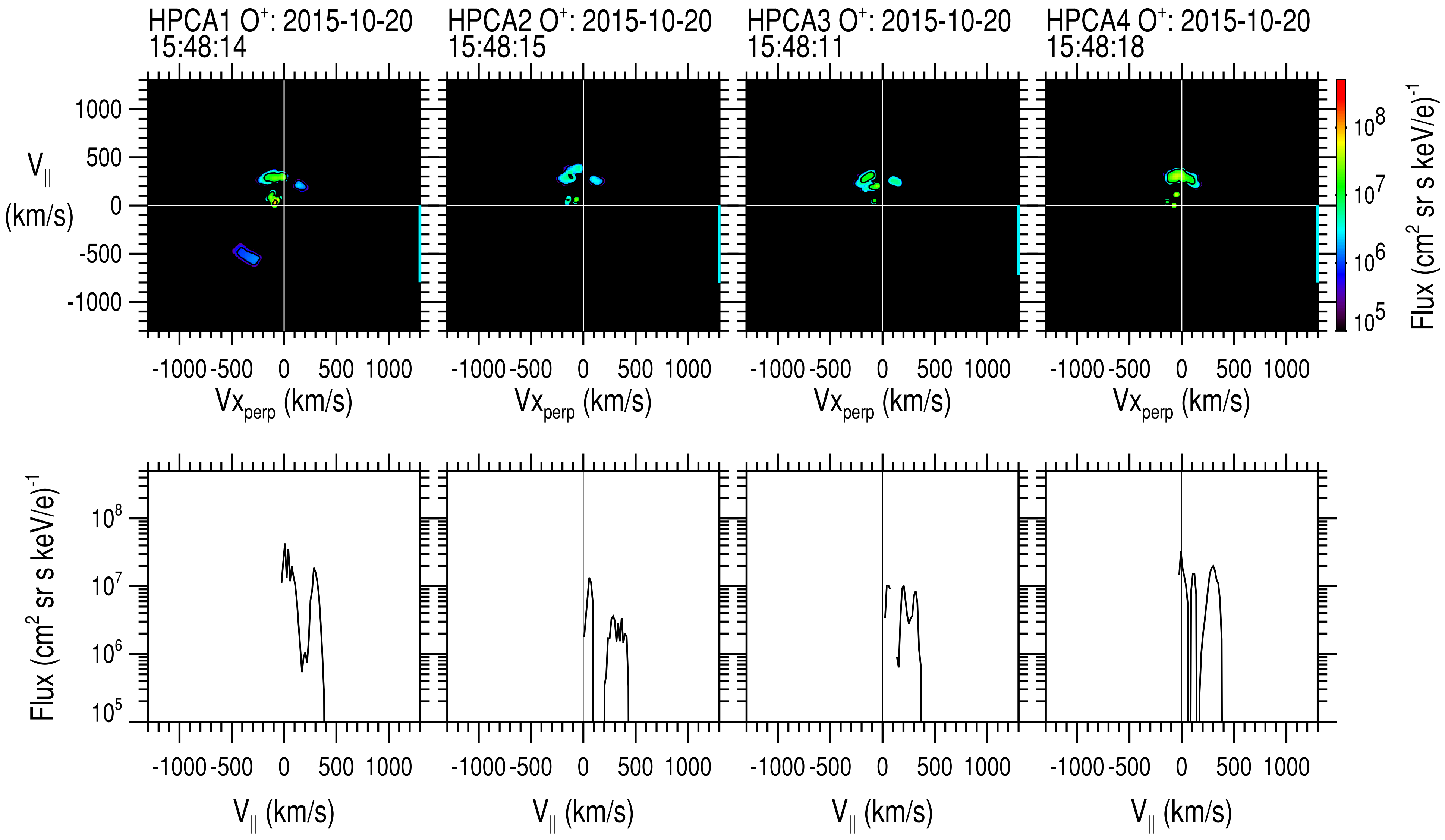
HPCA1 O+: 2015-10-20 15:48:14
HPCA2 O+: 2015-10-20 15:48:15
HPCA3 O+: 2015-10-20 15:48:11
HPCA4 O+: 2015-10-20 15:48:18
V|| (km/s)
Vxperp (km/s)
Flux (cm2 sr s keV/e)-1
V|| (km/s)

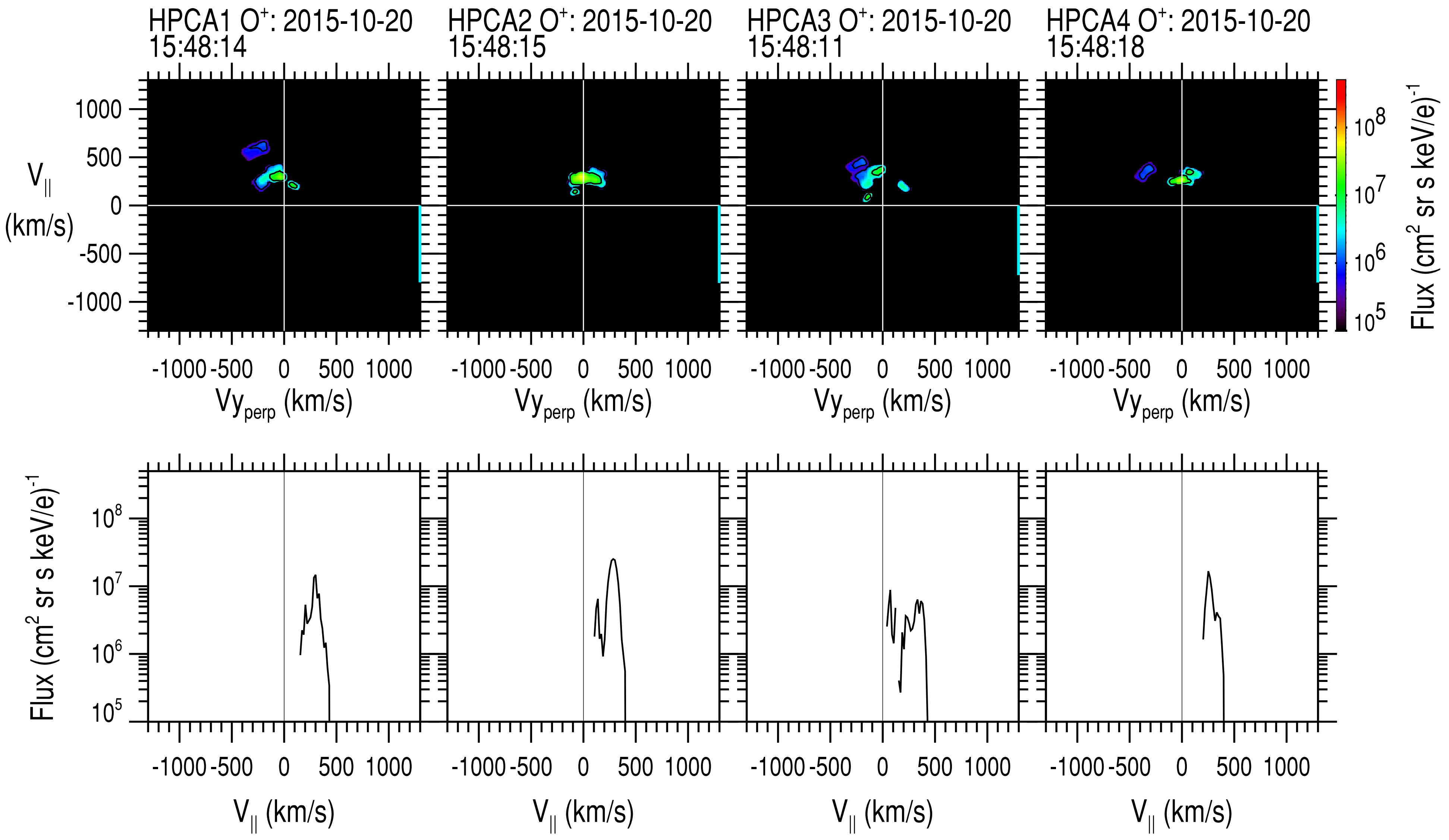

HPCA1 O+: 2015-10-20 15:48:14
HPCA2 O+: 2015-10-20 15:48:15
HPCA3 O+: 2015-10-20 15:48:11
HPCA4 O+: 2015-10-20 15:48:18
V|| (km/s)
Vyperp (km/s)
Flux (cm2 sr s keV/e)-1
V|| (km/s)

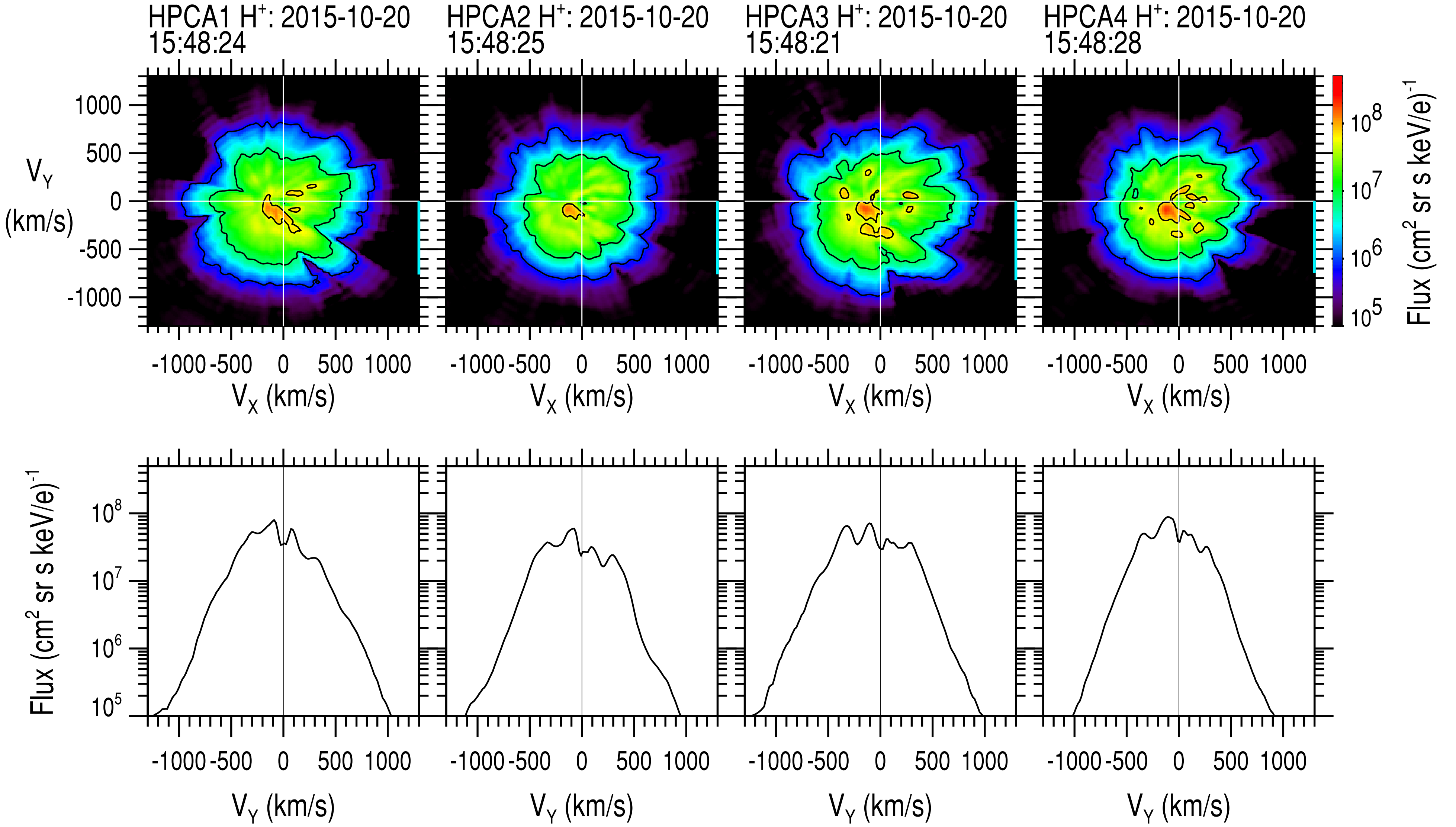
HPCA1 H⁺: 2015-10-20 15:48:24
HPCA2 H⁺: 2015-10-20 15:48:25
HPCA3 H⁺: 2015-10-20 15:48:21
HPCA4 H⁺: 2015-10-20 15:48:28
V_Y (km/s)
V_X (km/s)
Flux (cm^2 sr s keV/e)^-1
V_Y (km/s)

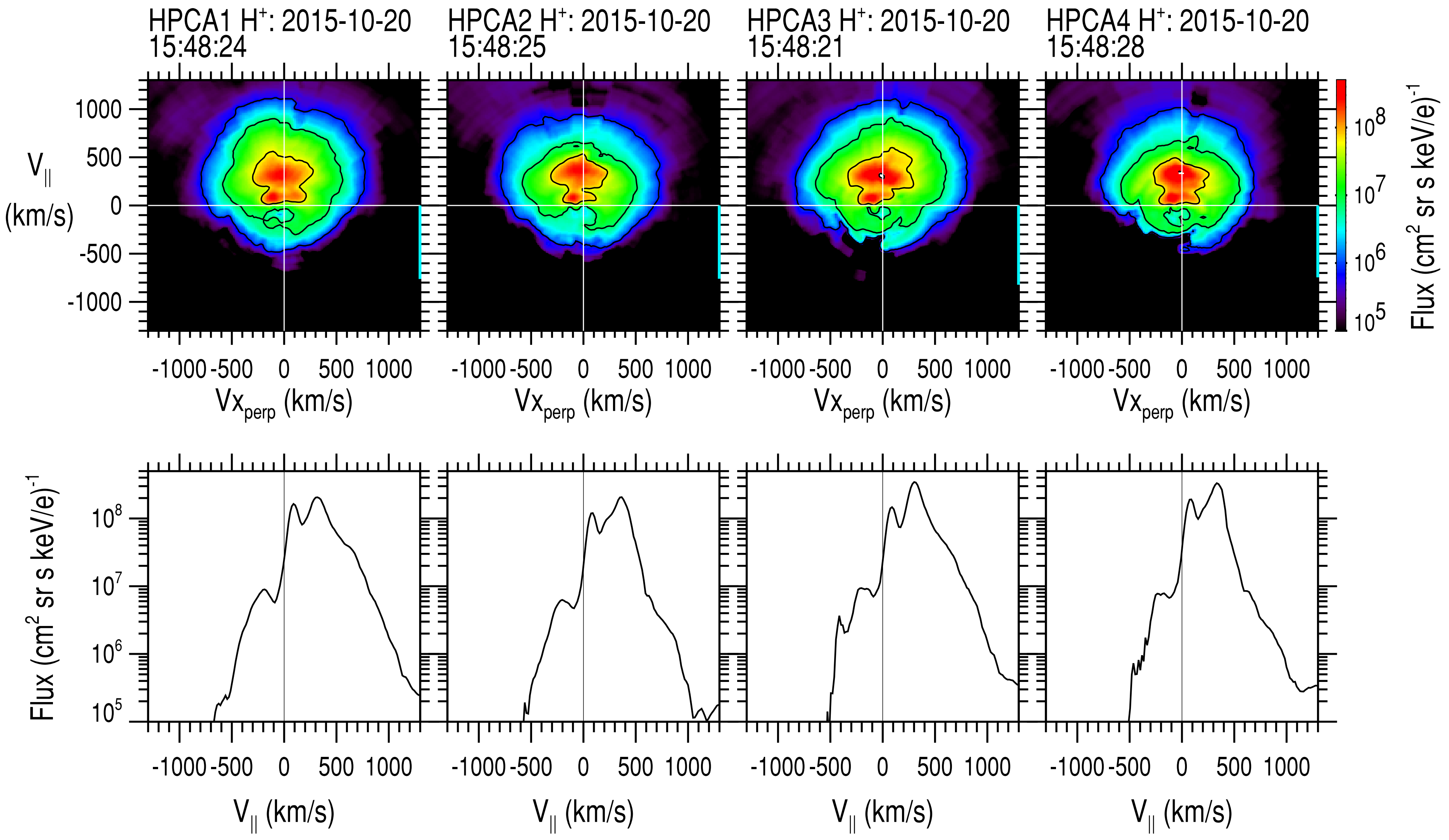

HPCA1 H^+: 2015-10-20 15:48:24
HPCA2 H^+: 2015-10-20 15:48:25
HPCA3 H^+: 2015-10-20 15:48:21
HPCA4 H^+: 2015-10-20 15:48:28
$V_{||}$ (km/s)
Vx_{perp} (km/s)
Flux $(cm^2\ sr\ s\ keV/e)^{-1}$
10^5
10^6
10^7
10^8
-1000
-500
0
500
1000
$V_{||}$ (km/s)

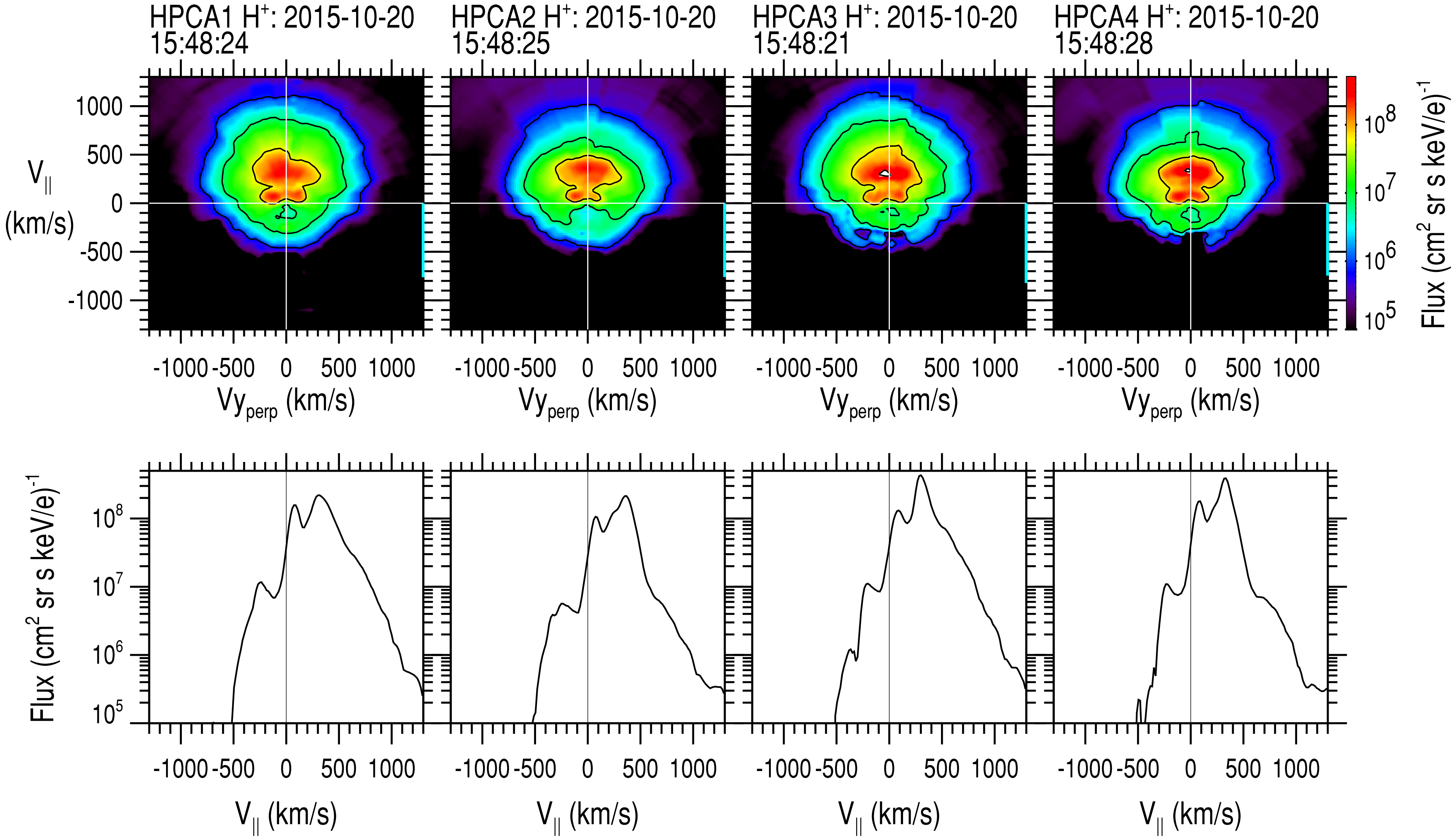
HPCA1 H+: 2015-10-20
15:48:24
HPCA2 H+: 2015-10-20
15:48:25
HPCA3 H+: 2015-10-20
15:48:21
HPCA4 H+: 2015-10-20
15:48:28
V|| (km/s)
Vyperp (km/s)
Flux (cm2 sr s keV/e)-1
V|| (km/s)

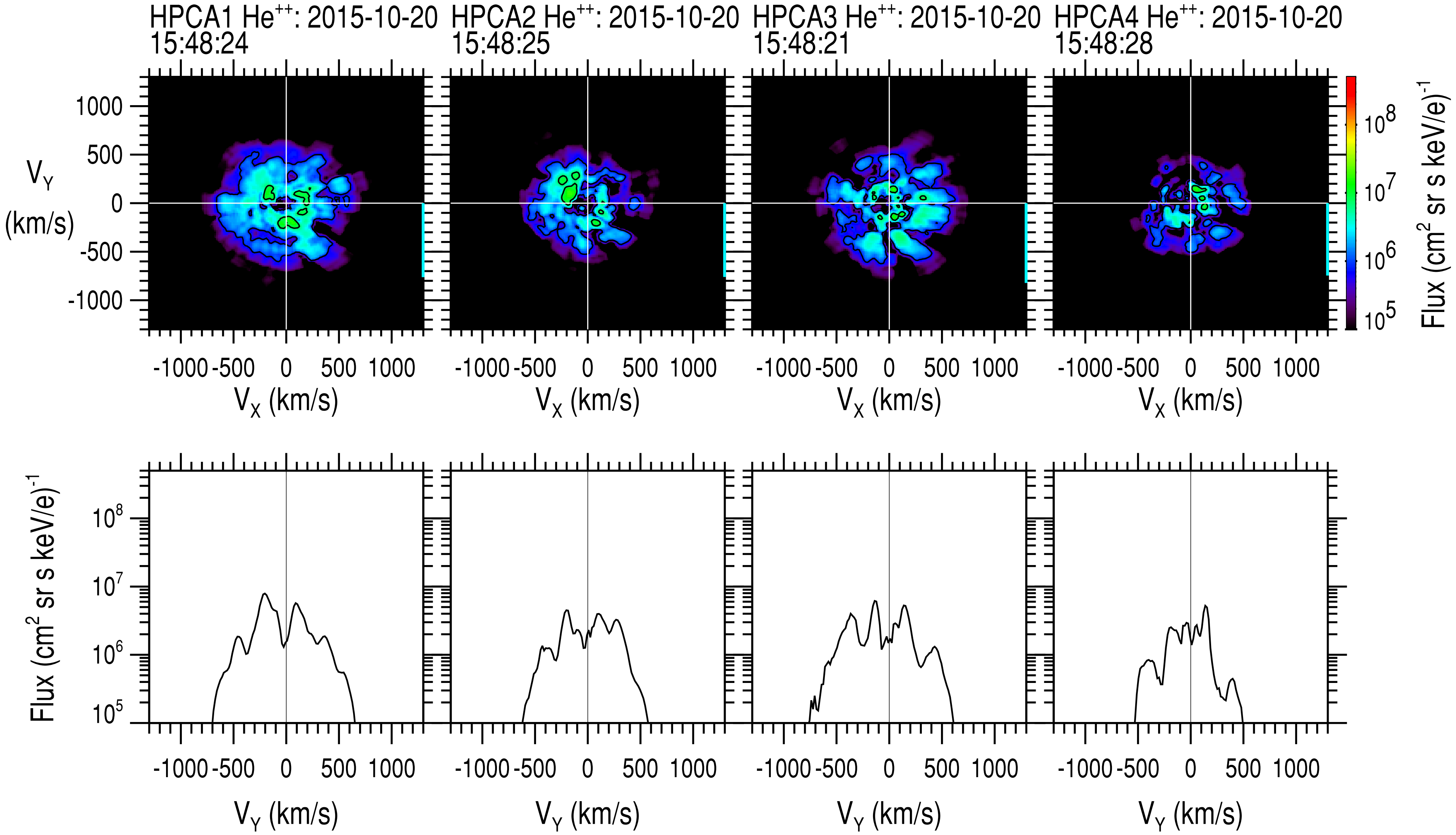

HPCA1 He++: 2015-10-20 15:48:24
HPCA2 He++: 2015-10-20 15:48:25
HPCA3 He++: 2015-10-20 15:48:21
HPCA4 He++: 2015-10-20 15:48:28
VY (km/s)
VX (km/s)
Flux (cm2 sr s keV/e)-1
VY (km/s)

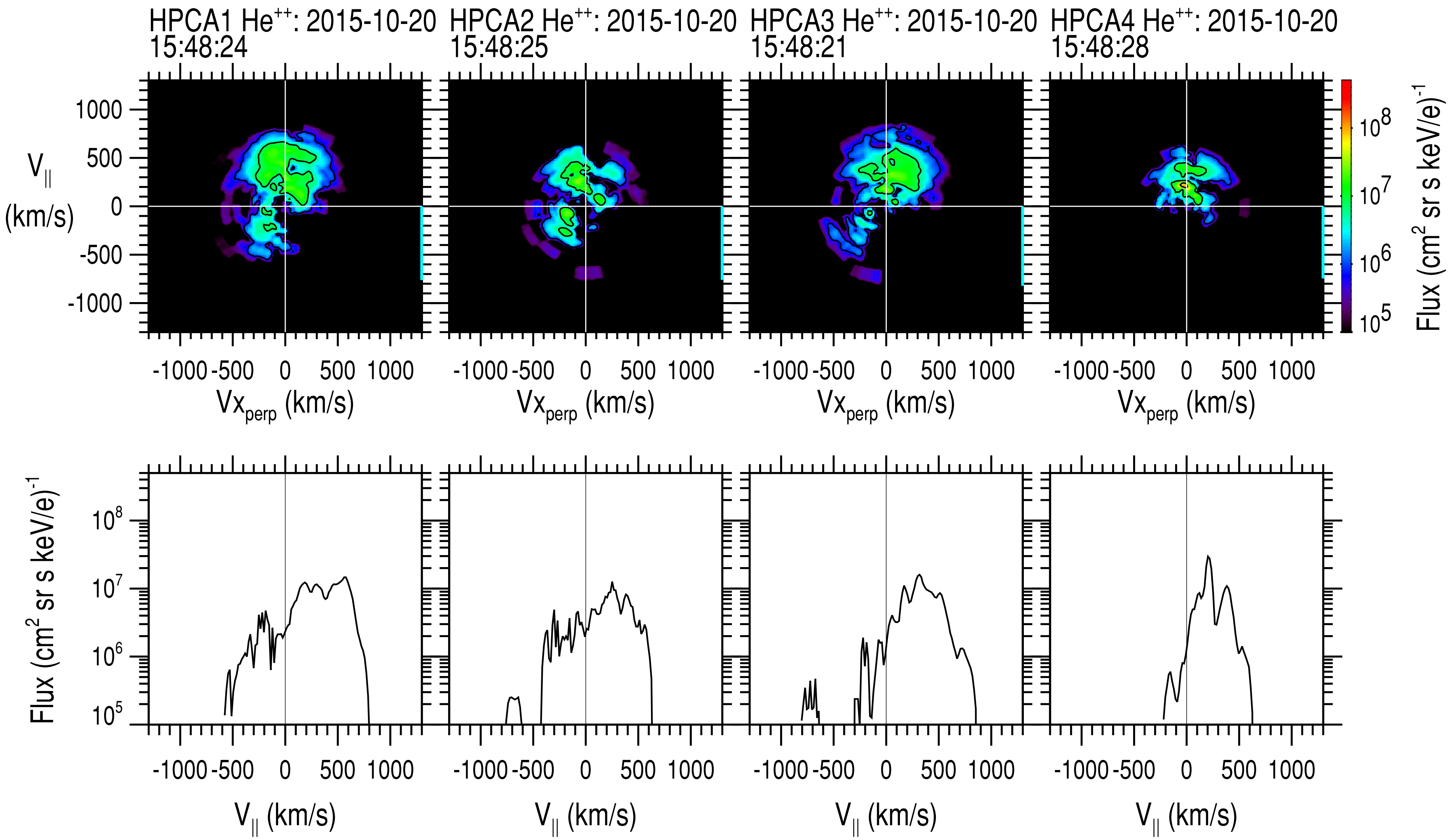
HPCA1 He++: 2015-10-20
15:48:24
HPCA2 He++: 2015-10-20
15:48:25
HPCA3 He++: 2015-10-20
15:48:21
HPCA4 He++: 2015-10-20
15:48:28
V|| (km/s)
Vxperp (km/s)
Flux (cm2 sr s keV/e)-1
V|| (km/s)

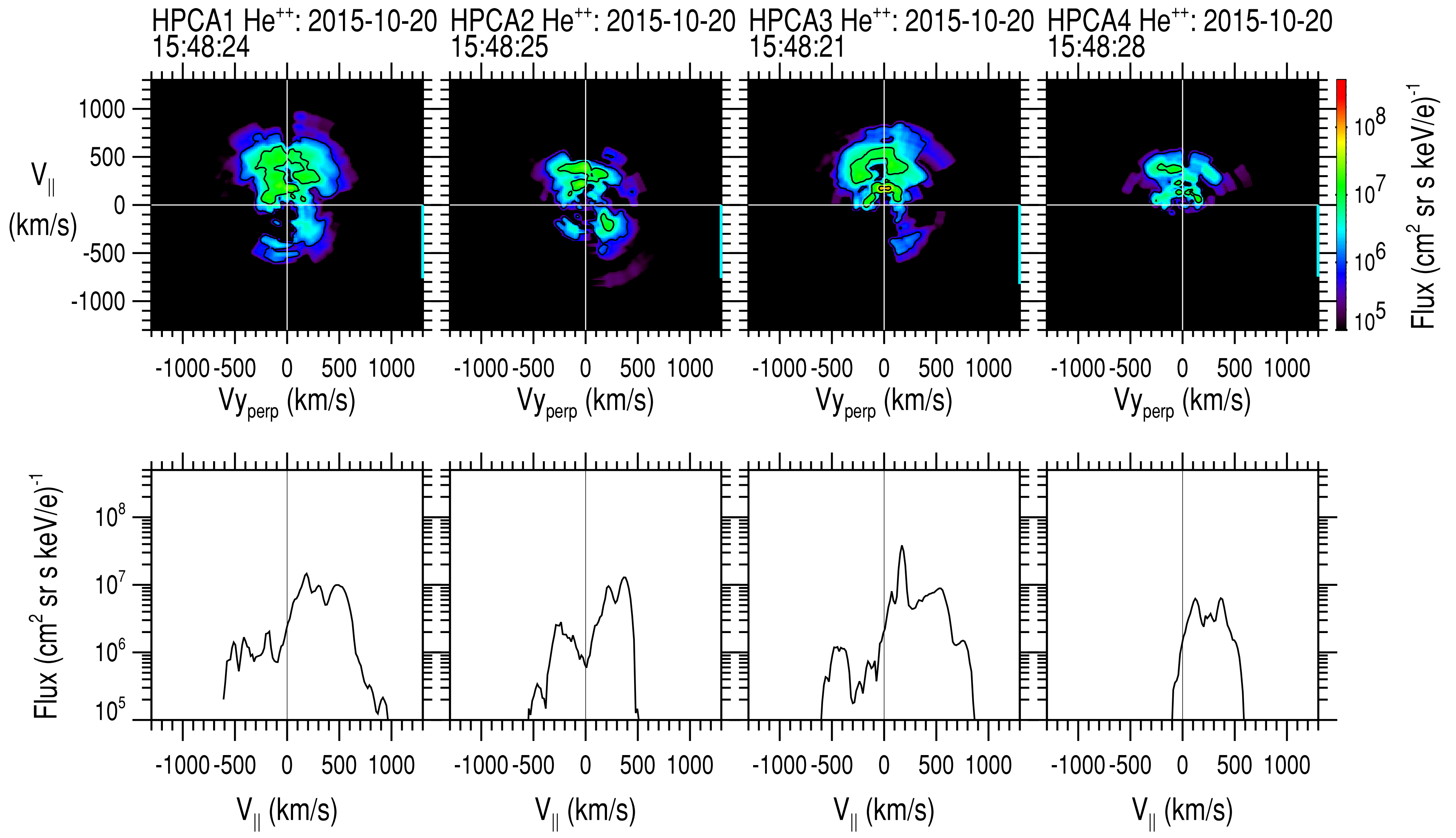

HPCA1 He++: 2015-10-20
15:48:24
HPCA2 He++: 2015-10-20
15:48:25
HPCA3 He++: 2015-10-20
15:48:21
HPCA4 He++: 2015-10-20
15:48:28
V|| (km/s)
Vyperp (km/s)
Flux (cm2 sr s keV/e)-1
1000
500
0
-500
-1000
10^5
10^6
10^7
10^8

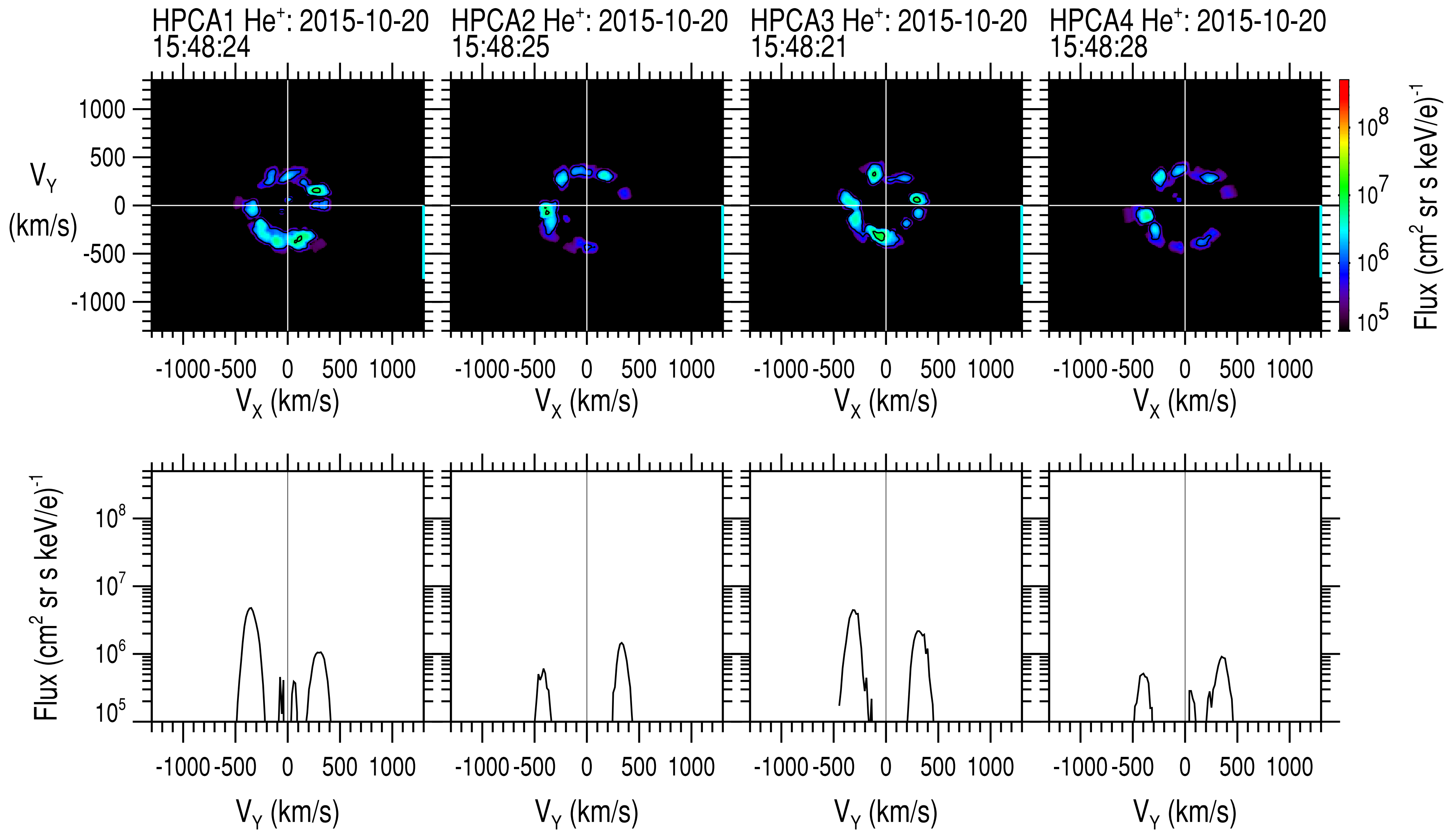
HPCA1 He+: 2015-10-20 15:48:24
HPCA2 He+: 2015-10-20 15:48:25
HPCA3 He+: 2015-10-20 15:48:21
HPCA4 He+: 2015-10-20 15:48:28
VY (km/s)
VX (km/s)
Flux (cm2 sr s keV/e)-1
VY (km/s)

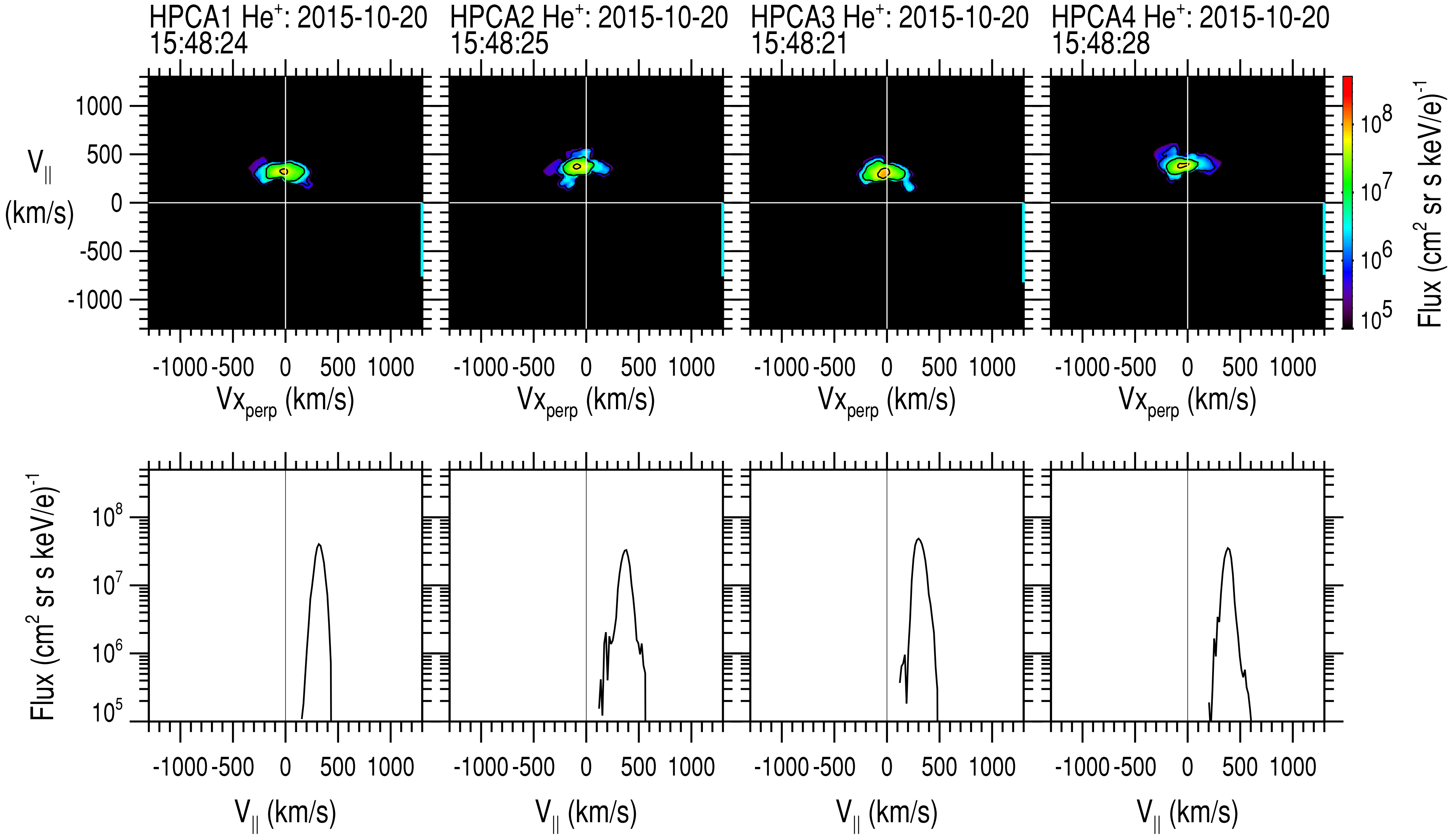
HPCA1 He+: 2015-10-20 15:48:24
HPCA2 He+: 2015-10-20 15:48:25
HPCA3 He+: 2015-10-20 15:48:21
HPCA4 He+: 2015-10-20 15:48:28
V|| (km/s)
Vxperp (km/s)
Flux (cm2 sr s keV/e)-1
V|| (km/s)
Flux (cm2 sr s keV/e)-1

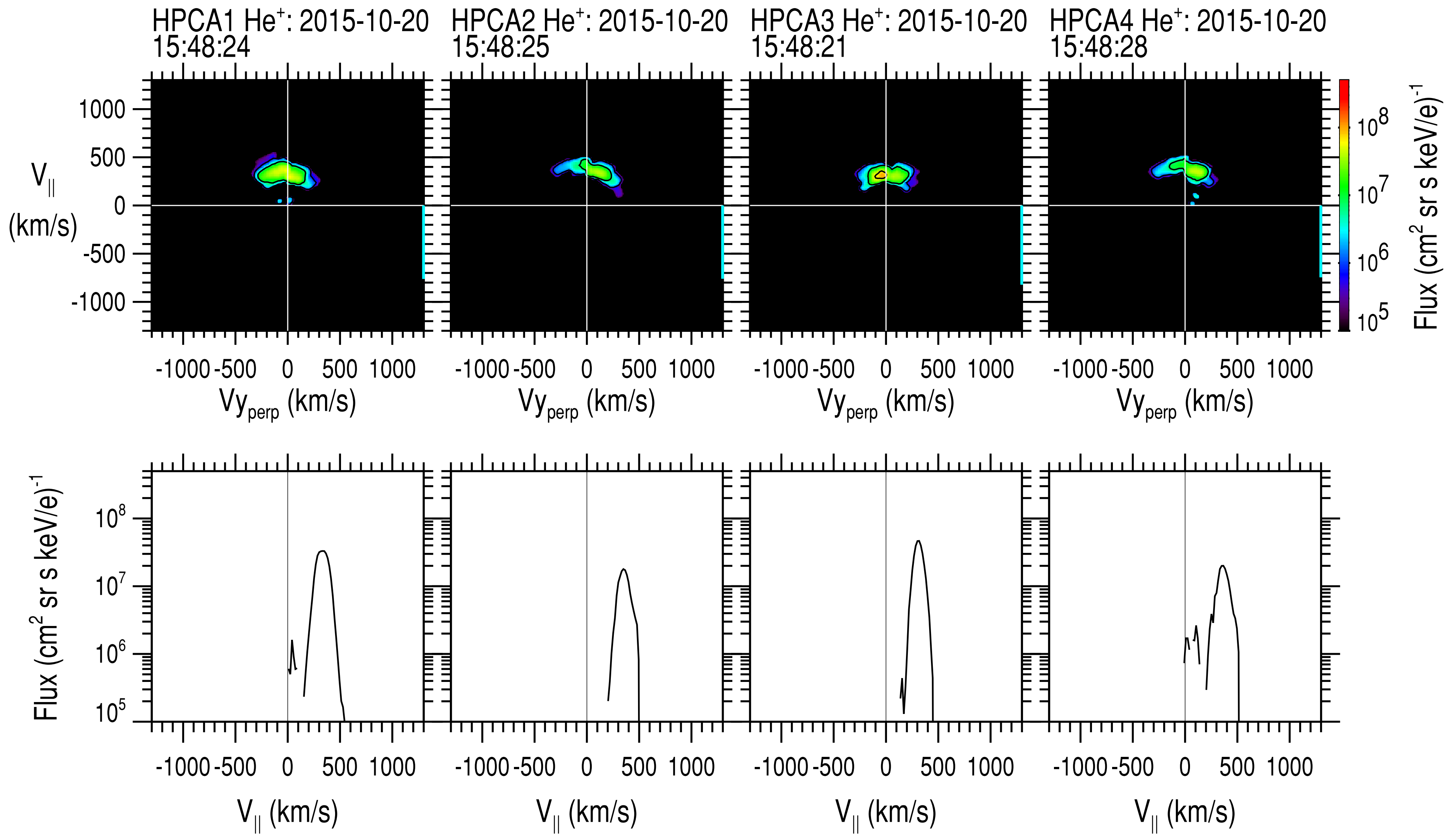
HPCA1 He+: 2015-10-20 15:48:24
HPCA2 He+: 2015-10-20 15:48:25
HPCA3 He+: 2015-10-20 15:48:21
HPCA4 He+: 2015-10-20 15:48:28
V|| (km/s)
Vyperp (km/s)
Flux (cm2 sr s keV/e)-1
-1000 -500 0 500 1000
10^5 10^6 10^7 10^8

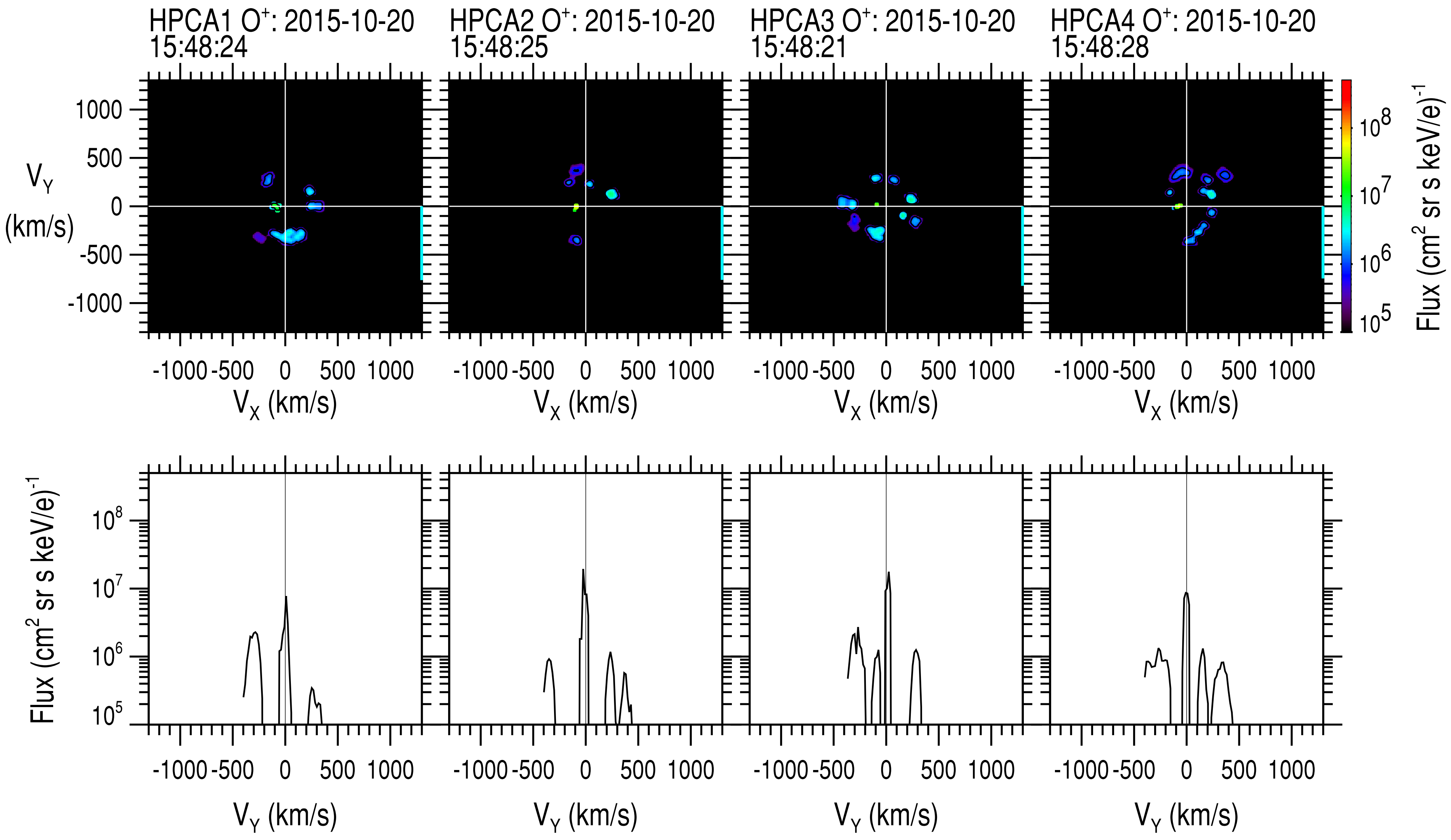
HPCA1 O+: 2015-10-20
15:48:24
HPCA2 O+: 2015-10-20
15:48:25
HPCA3 O+: 2015-10-20
15:48:21
HPCA4 O+: 2015-10-20
15:48:28
VY (km/s)
VX (km/s)
Flux (cm2 sr s keV/e)-1
VY (km/s)

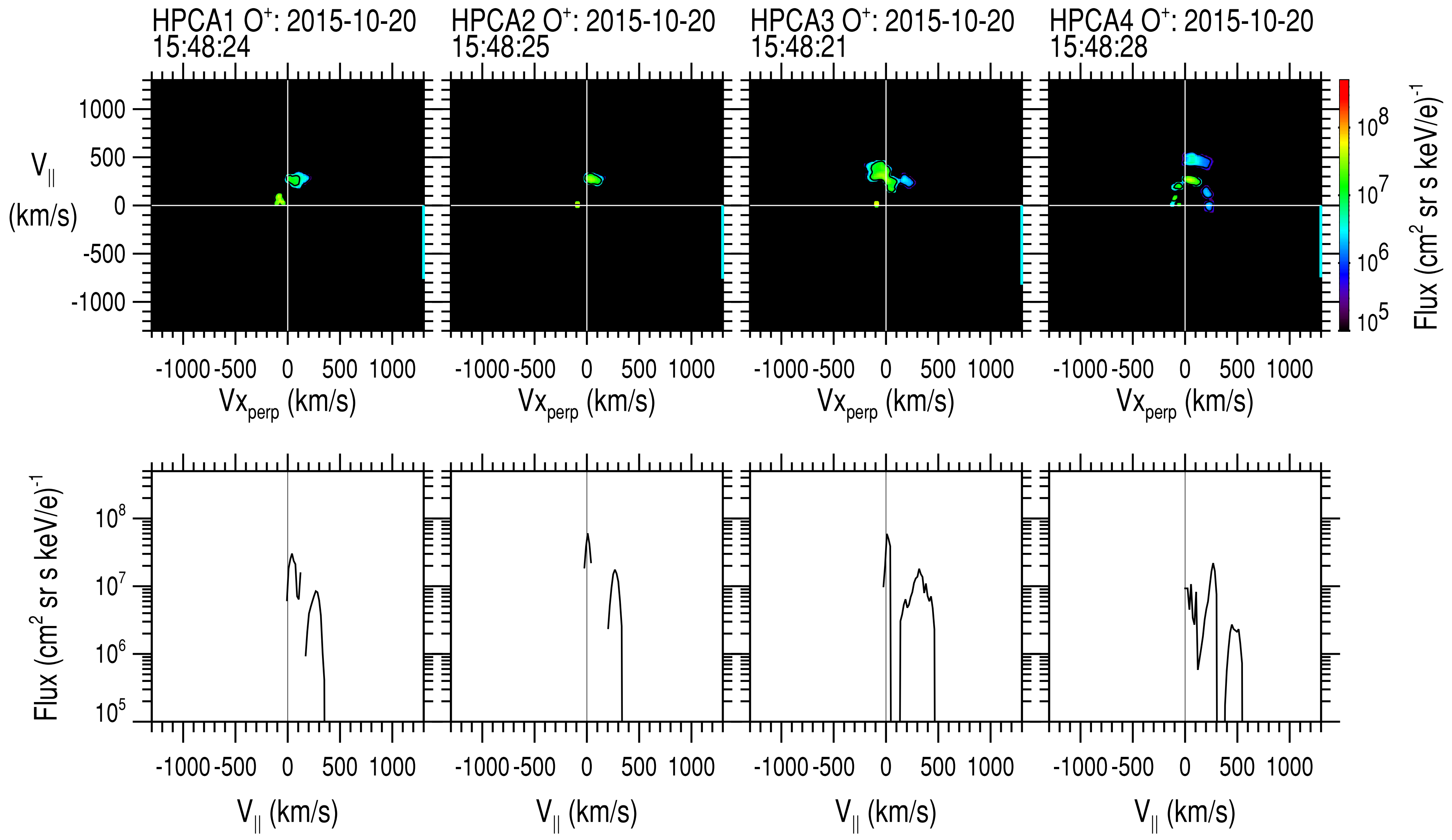
HPCA1 O+: 2015-10-20
15:48:24
HPCA2 O+: 2015-10-20
15:48:25
HPCA3 O+: 2015-10-20
15:48:21
HPCA4 O+: 2015-10-20
15:48:28
V|| (km/s)
Vxperp (km/s)
Flux (cm2 sr s keV/e)-1
1000
500
0
-500
-1000
-1000 -500 0 500 1000
10^5
10^6
10^7
10^8

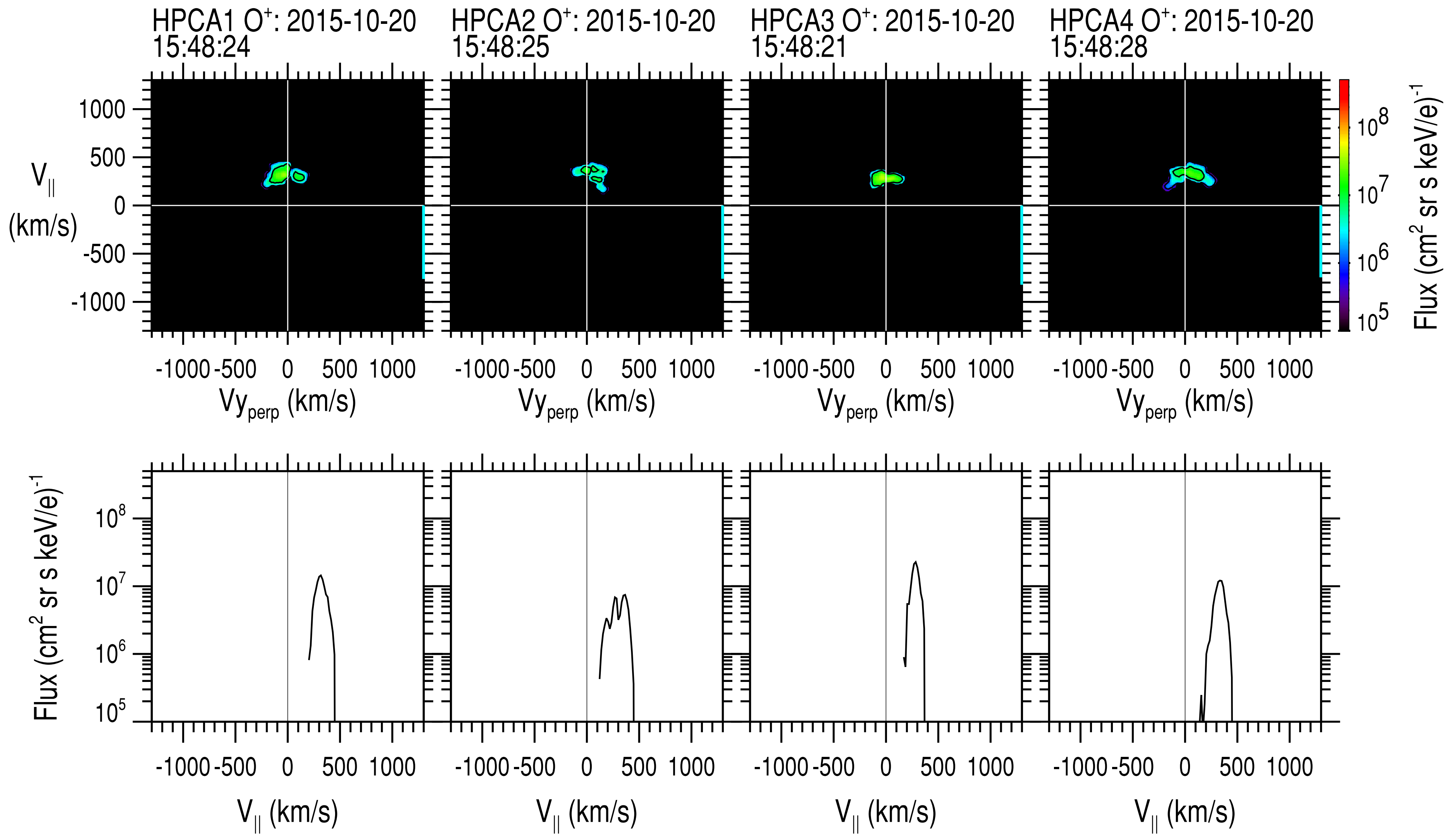
HPCA1 O+: 2015-10-20 15:48:24
HPCA2 O+: 2015-10-20 15:48:25
HPCA3 O+: 2015-10-20 15:48:21
HPCA4 O+: 2015-10-20 15:48:28
V|| (km/s)
Vyperp (km/s)
Flux (cm2 sr s keV/e)-1

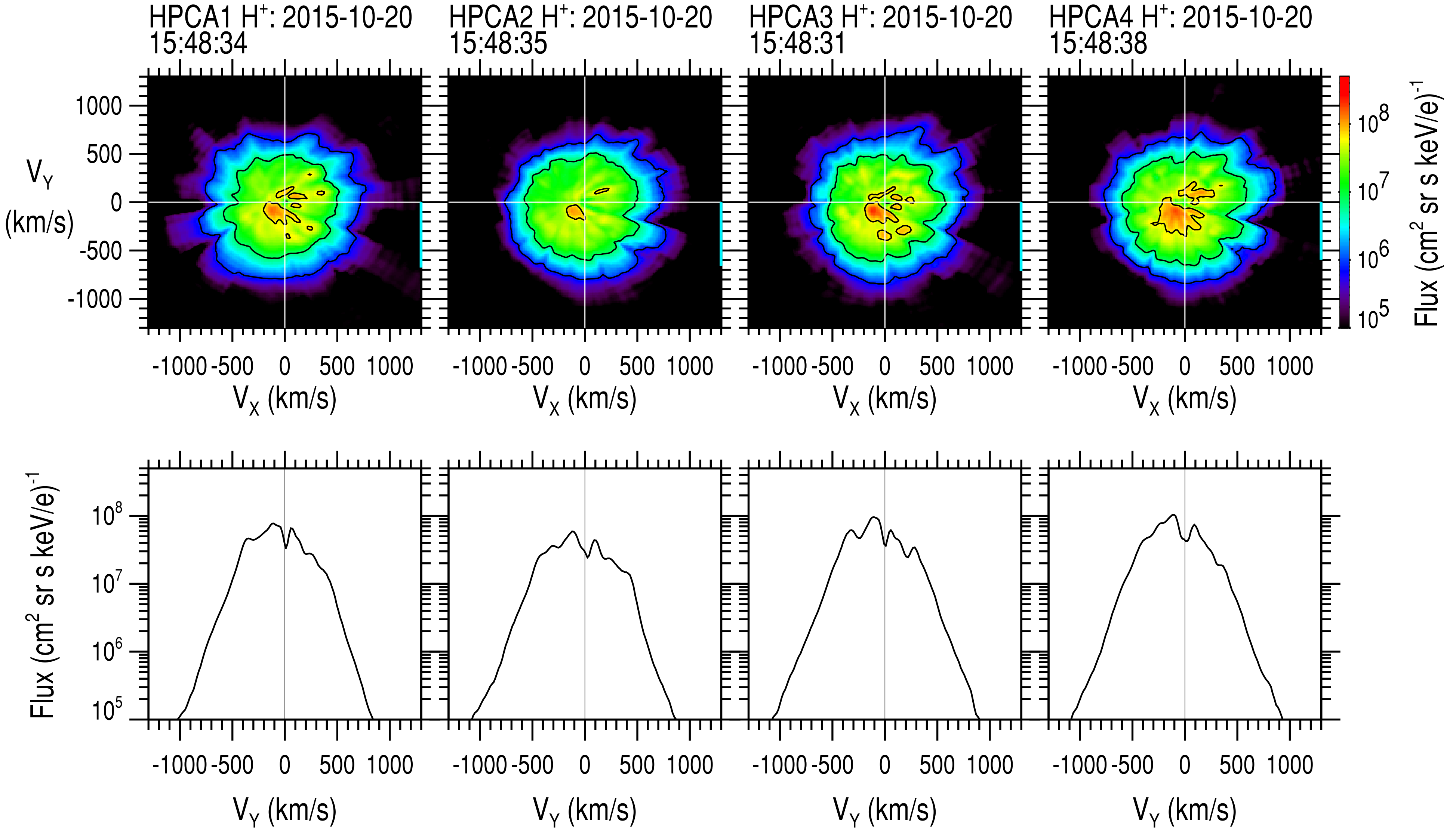

HPCA1 H+: 2015-10-20 15:48:34
HPCA2 H+: 2015-10-20 15:48:35
HPCA3 H+: 2015-10-20 15:48:31
HPCA4 H+: 2015-10-20 15:48:38
V_Y (km/s)
V_X (km/s)
Flux (cm2 sr s keV/e)-1

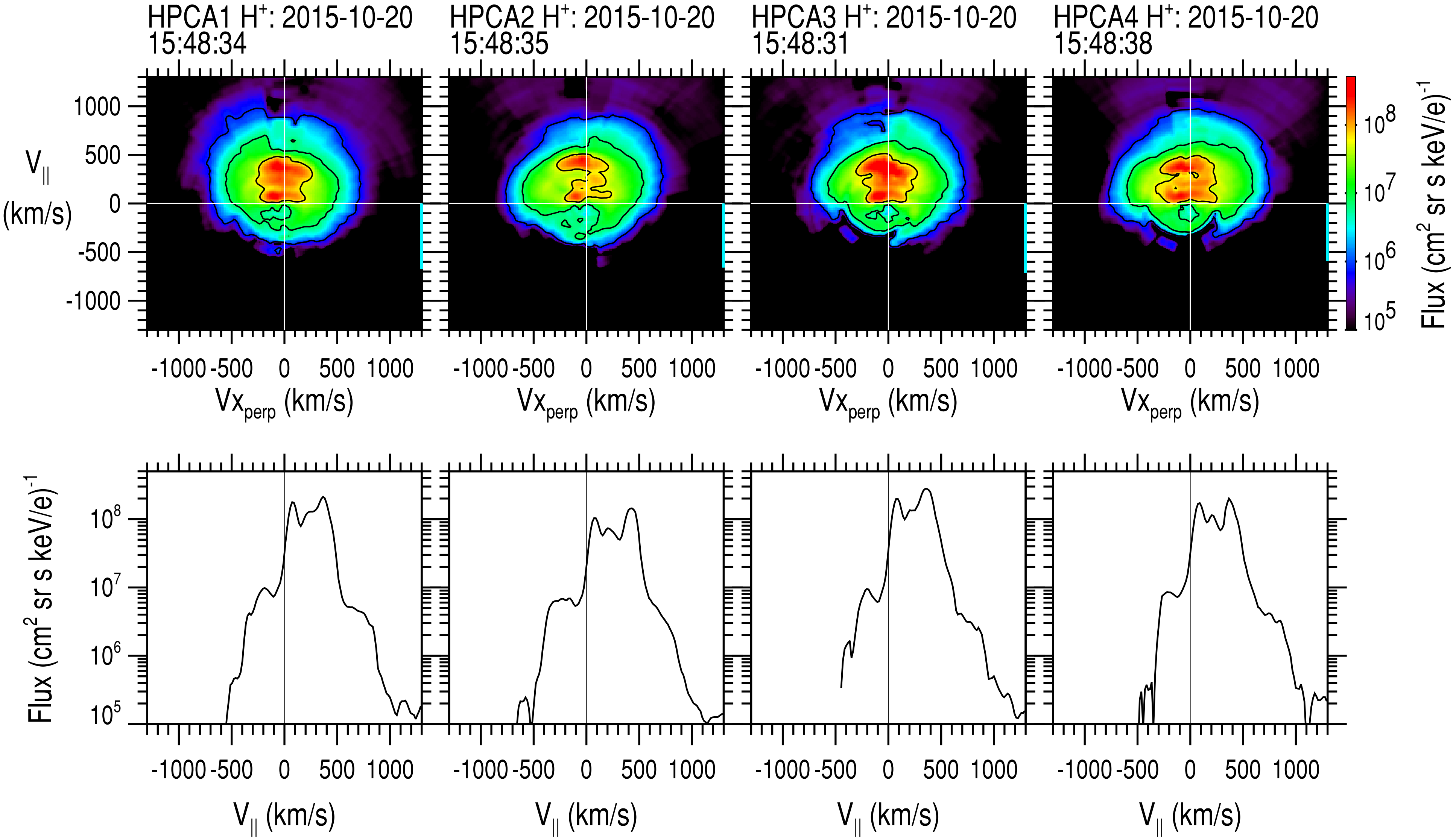
HPCA1 H+: 2015-10-20
15:48:34
HPCA2 H+: 2015-10-20
15:48:35
HPCA3 H+: 2015-10-20
15:48:31
HPCA4 H+: 2015-10-20
15:48:38
V|| (km/s)
Vxperp (km/s)
Flux (cm2 sr s keV/e)-1
V|| (km/s)

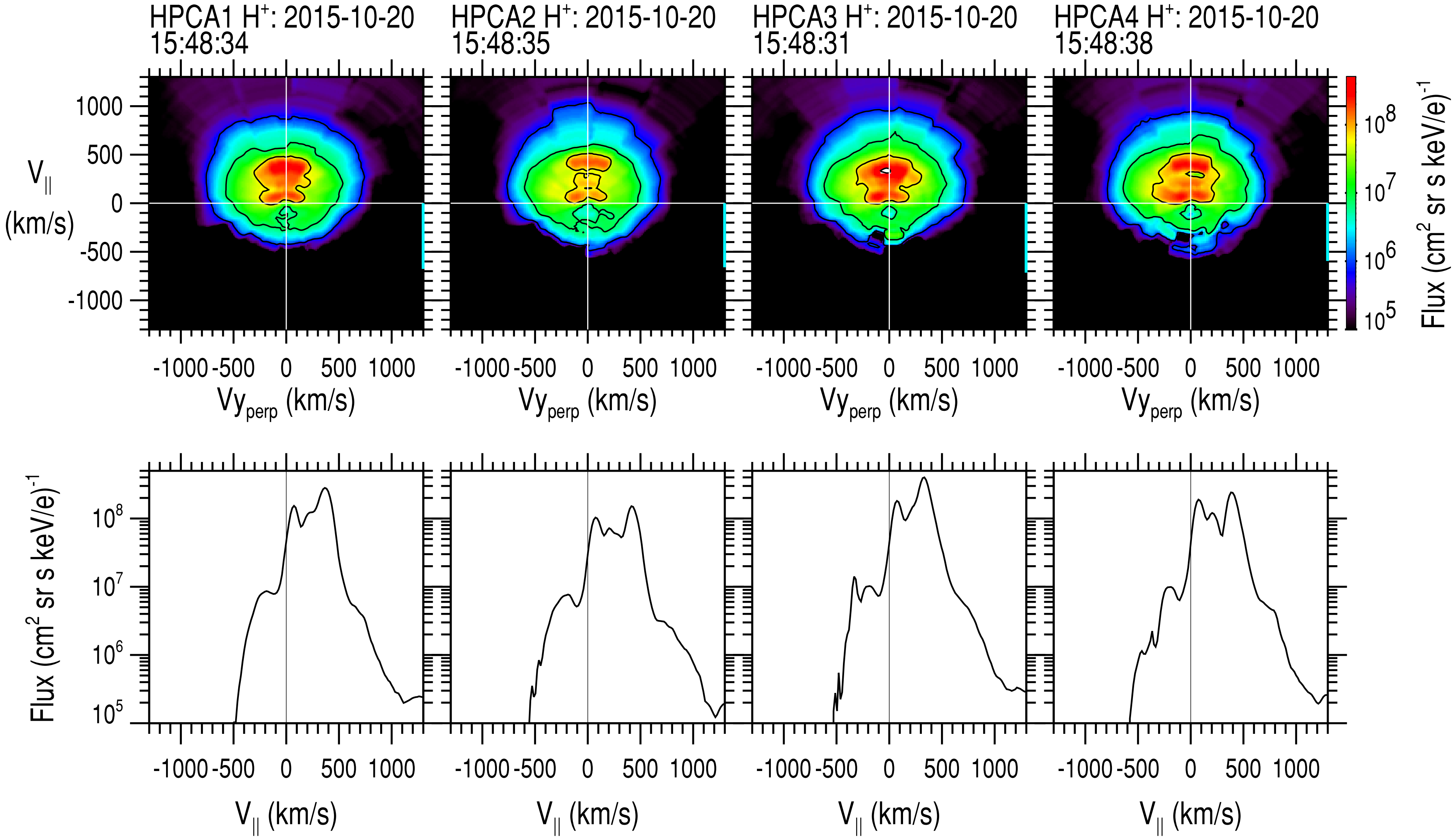
HPCA1 H+: 2015-10-20 15:48:34
HPCA2 H+: 2015-10-20 15:48:35
HPCA3 H+: 2015-10-20 15:48:31
HPCA4 H+: 2015-10-20 15:48:38
V|| (km/s)
Vyperp (km/s)
Flux (cm2 sr s keV/e)-1

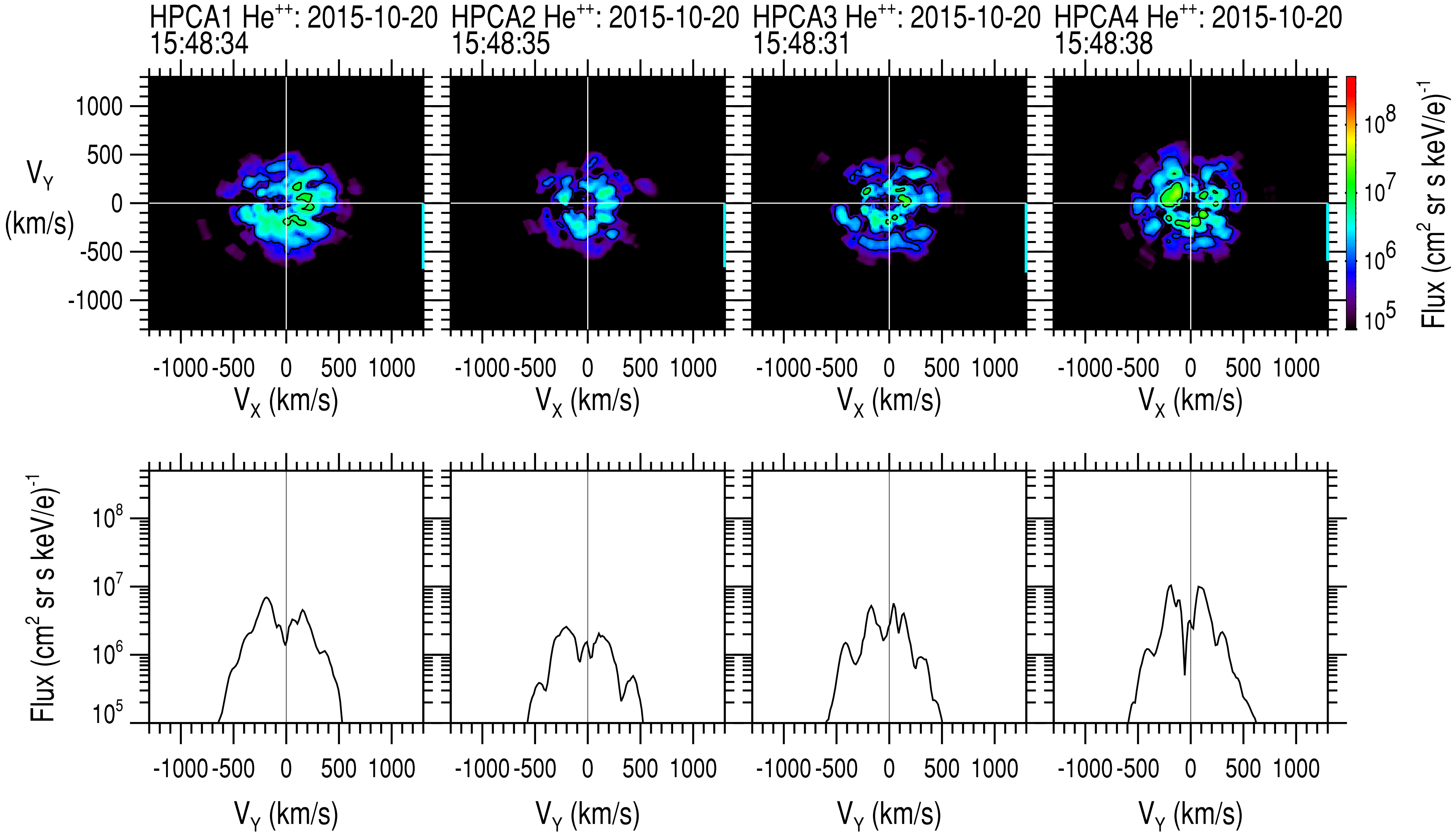

HPCA1 He++: 2015-10-20 15:48:34
HPCA2 He++: 2015-10-20 15:48:35
HPCA3 He++: 2015-10-20 15:48:31
HPCA4 He++: 2015-10-20 15:48:38
VY (km/s)
VX (km/s)
Flux (cm2 sr s keV/e)-1
VY (km/s)

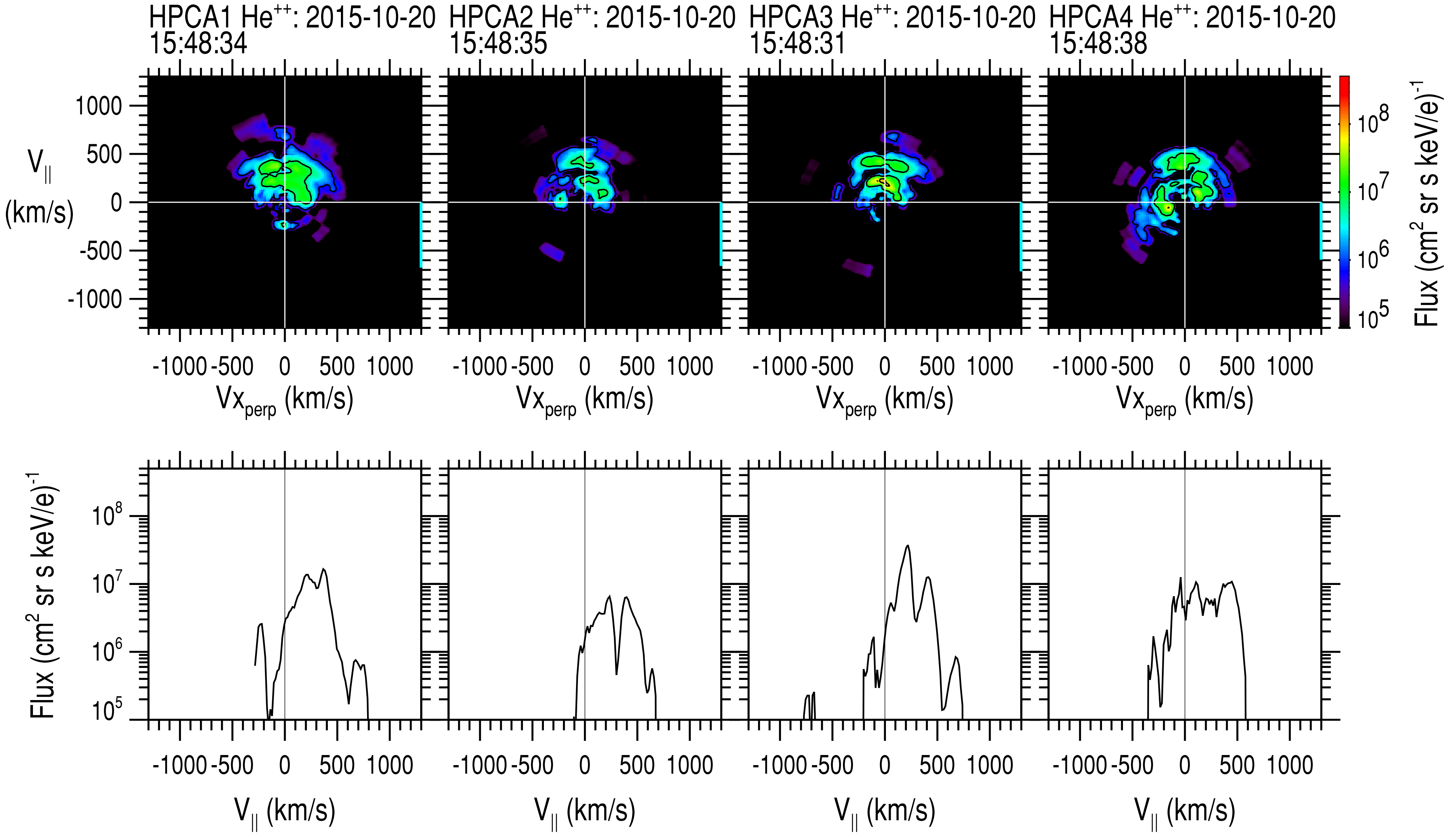
HPCA1 He++: 2015-10-20 15:48:34
HPCA2 He++: 2015-10-20 15:48:35
HPCA3 He++: 2015-10-20 15:48:31
HPCA4 He++: 2015-10-20 15:48:38
V|| (km/s)
Vxperp (km/s)
Flux (cm2 sr s keV/e)-1
V|| (km/s)

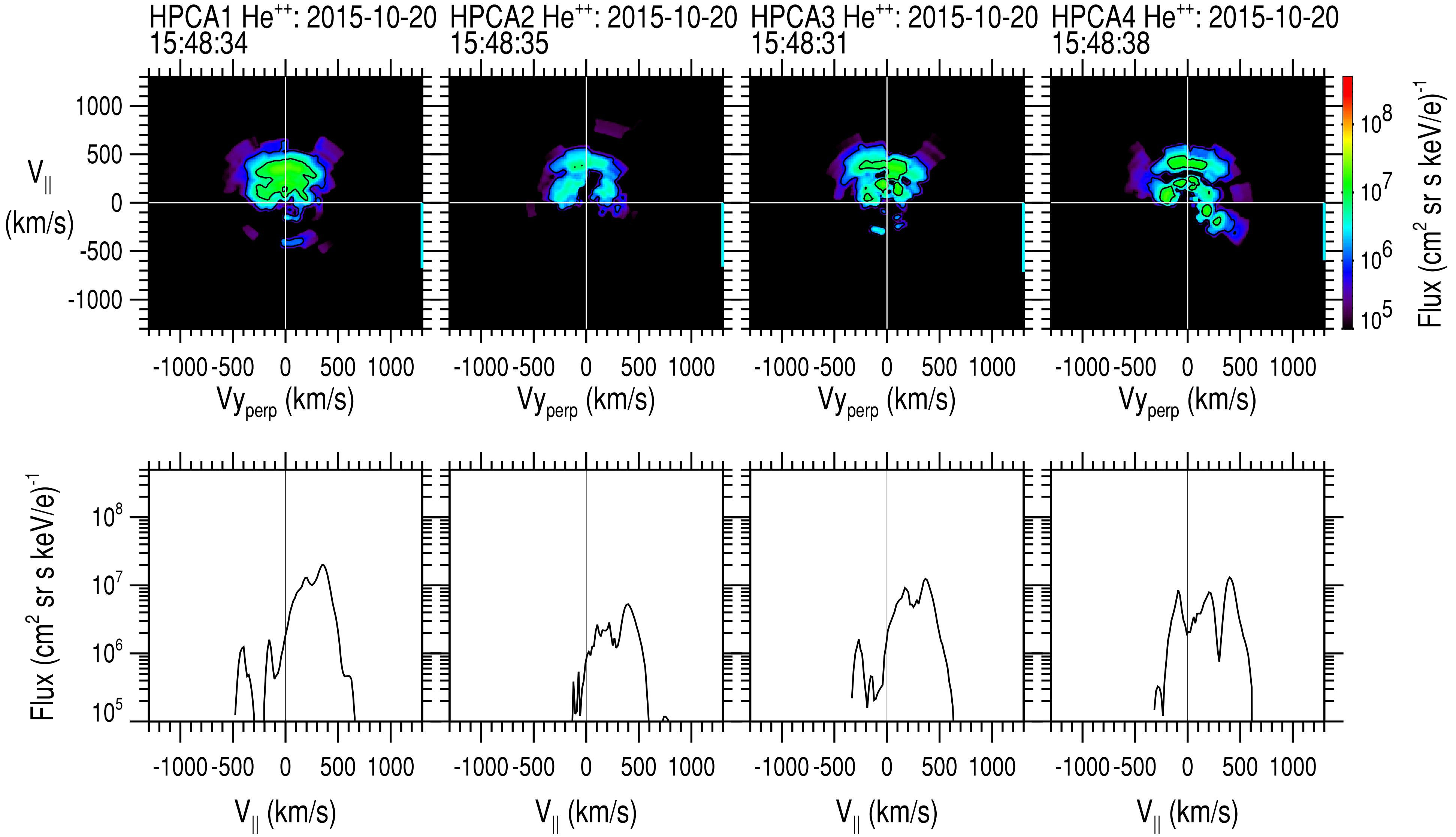
HPCA1 He++: 2015-10-20 15:48:34
HPCA2 He++: 2015-10-20 15:48:35
HPCA3 He++: 2015-10-20 15:48:31
HPCA4 He++: 2015-10-20 15:48:38
V|| (km/s)
Vyperp (km/s)
Flux (cm2 sr s keV/e)-1
-1000
-500
0
500
1000
10^5
10^6
10^7
10^8

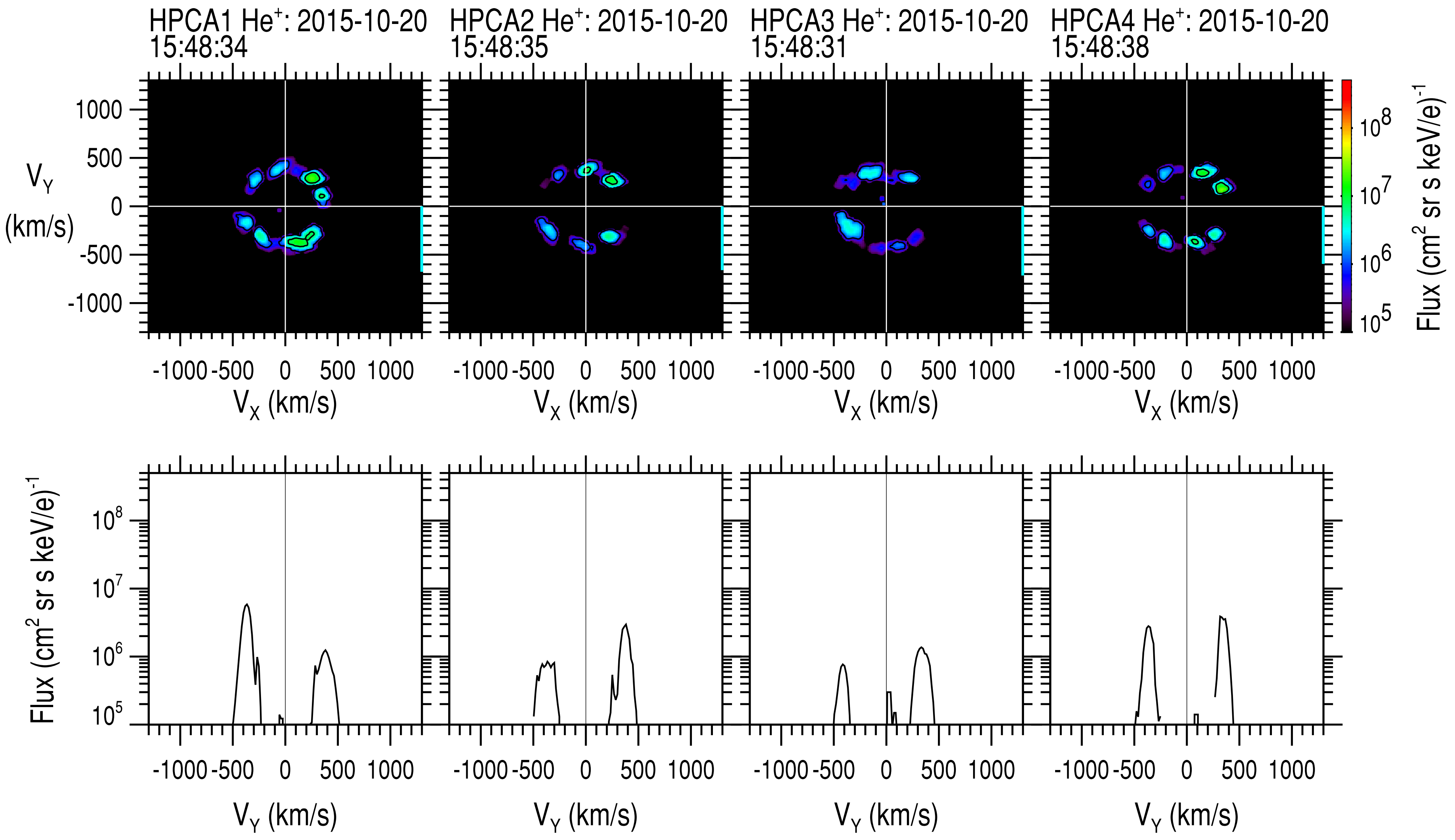
HPCA1 He+: 2015-10-20
15:48:34
HPCA2 He+: 2015-10-20
15:48:35
HPCA3 He+: 2015-10-20
15:48:31
HPCA4 He+: 2015-10-20
15:48:38
VY (km/s)
VX (km/s)
Flux (cm2 sr s keV/e)-1

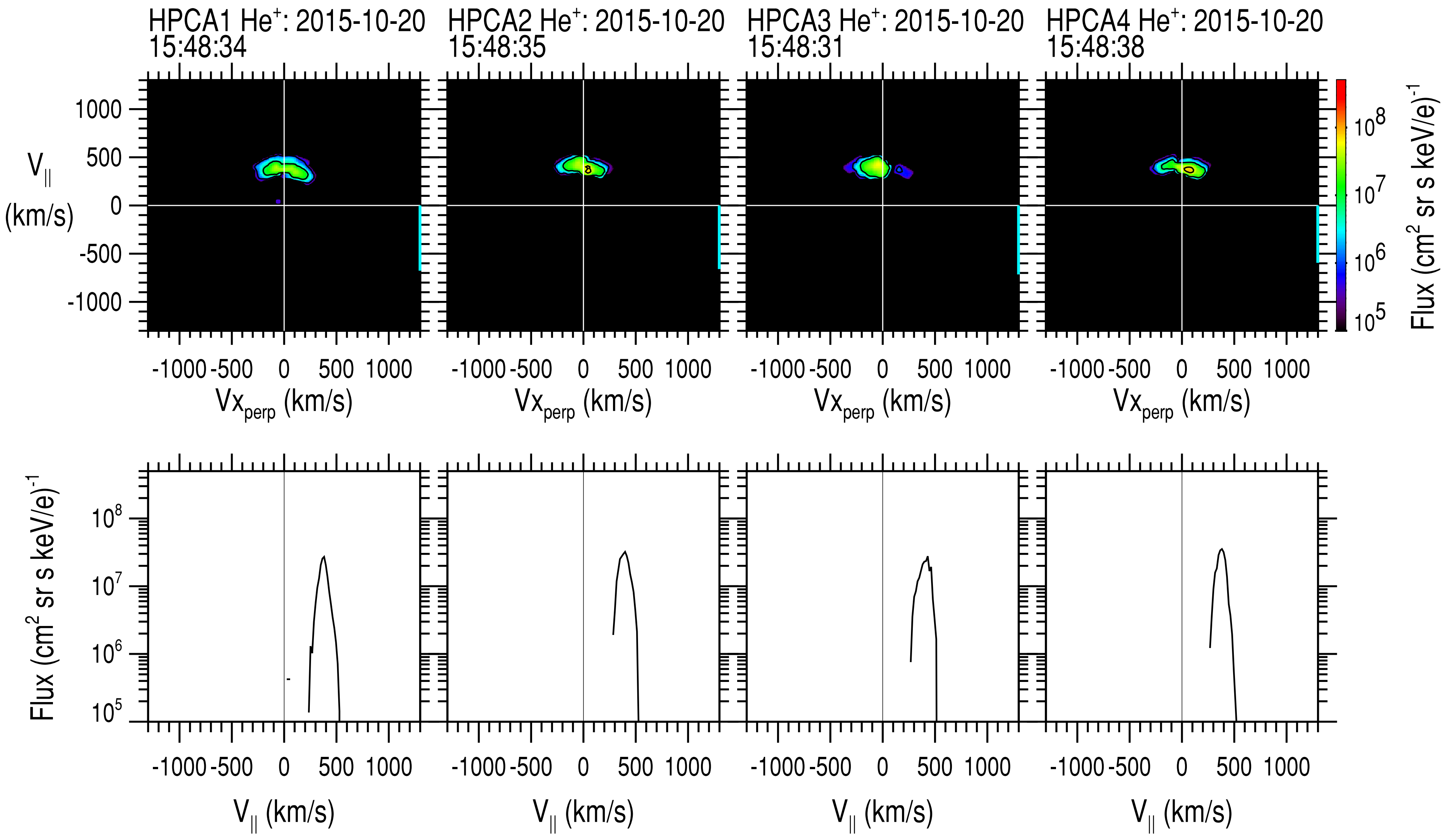

HPCA1 He+: 2015-10-20 15:48:34
HPCA2 He+: 2015-10-20 15:48:35
HPCA3 He+: 2015-10-20 15:48:31
HPCA4 He+: 2015-10-20 15:48:38
V|| (km/s)
Vxperp (km/s)
Flux (cm2 sr s keV/e)-1
-1000 -500 0 500 1000
10^5 10^6 10^7 10^8

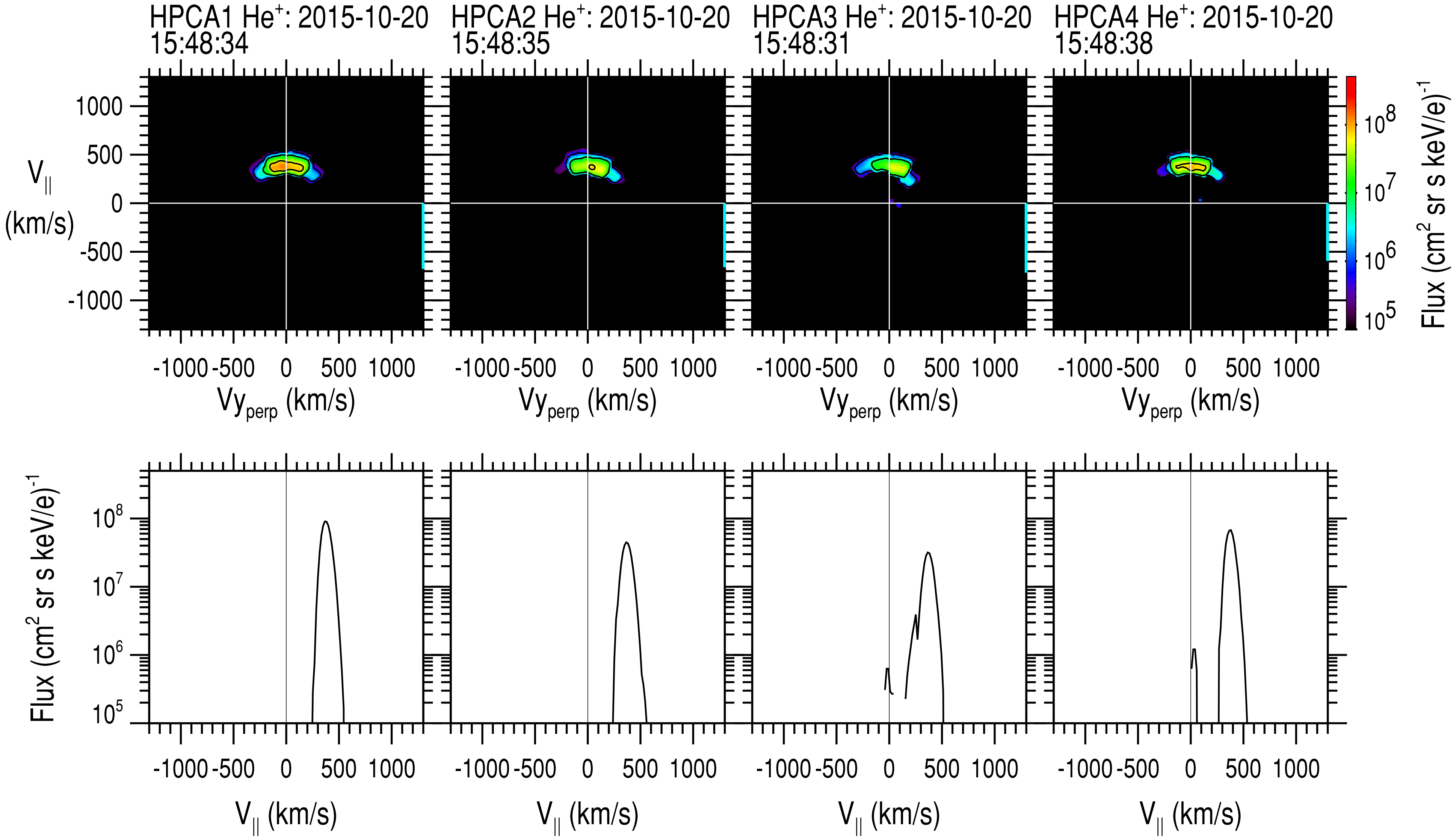
HPCA1 He+: 2015-10-20 15:48:34
HPCA2 He+: 2015-10-20 15:48:35
HPCA3 He+: 2015-10-20 15:48:31
HPCA4 He+: 2015-10-20 15:48:38
V|| (km/s)
Vyperp (km/s)
Flux (cm2 sr s keV/e)-1
1000
500
0
-500
-1000
105
106
107
108

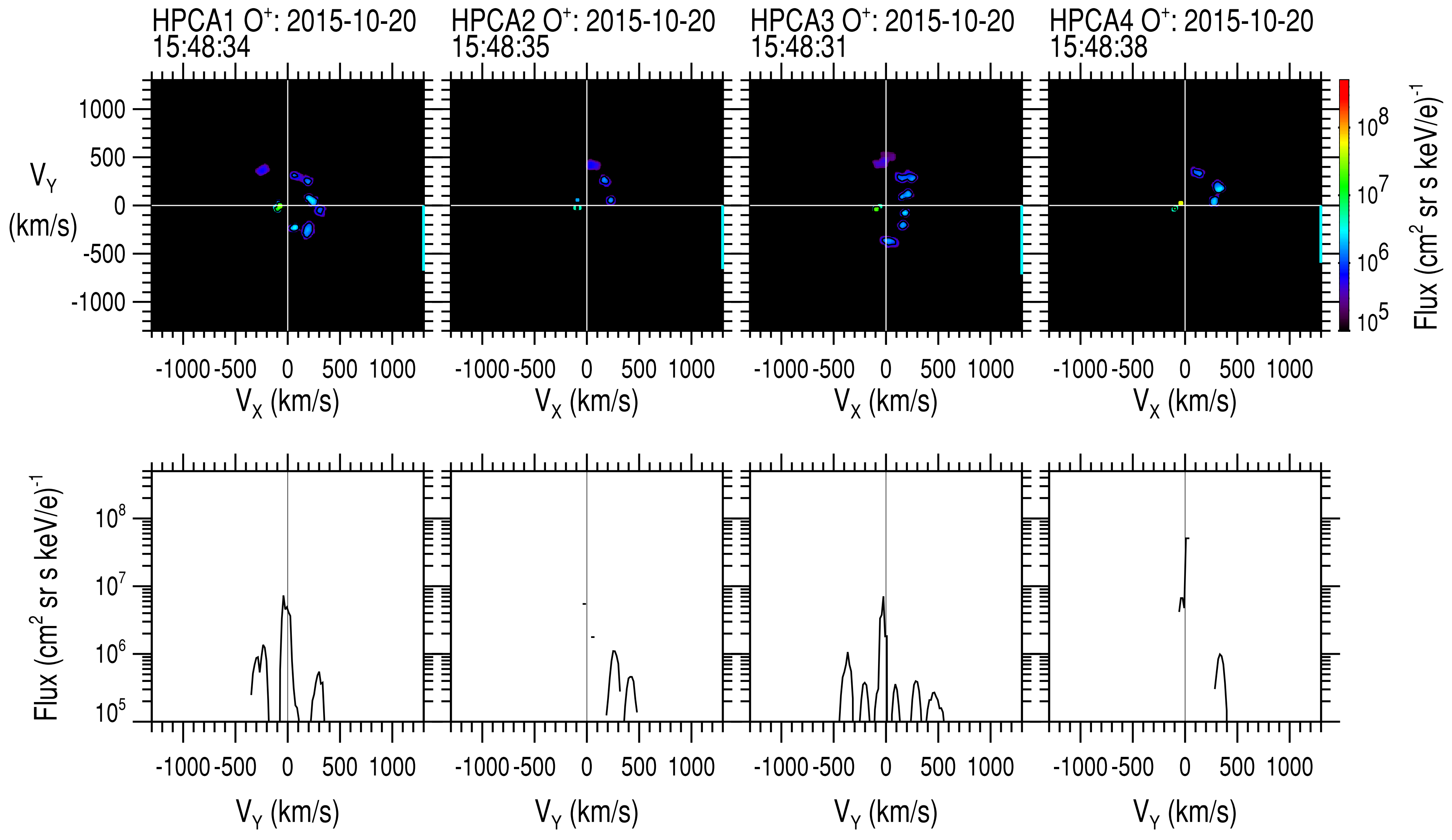

HPCA1 O⁺: 2015-10-20
15:48:34
HPCA2 O⁺: 2015-10-20
15:48:35
HPCA3 O⁺: 2015-10-20
15:48:31
HPCA4 O⁺: 2015-10-20
15:48:38
V_Y (km/s)
V_X (km/s)
V_Y (km/s)
Flux (cm² sr s keV/e)⁻¹
-1000 -500 0 500 1000
10⁵ 10⁶ 10⁷ 10⁸

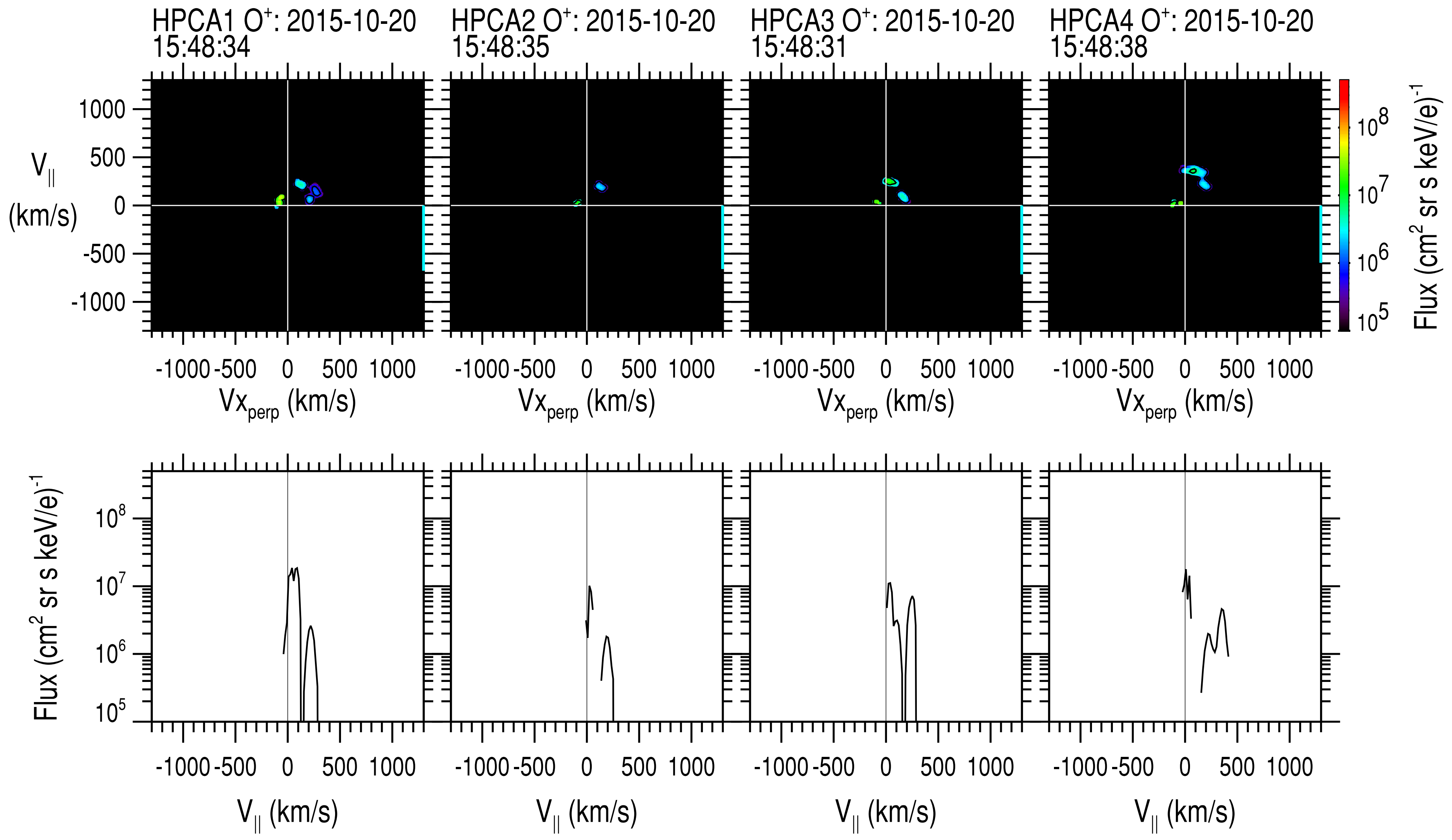
HPCA1 O+: 2015-10-20
15:48:34
HPCA2 O+: 2015-10-20
15:48:35
HPCA3 O+: 2015-10-20
15:48:31
HPCA4 O+: 2015-10-20
15:48:38
V|| (km/s)
Vxperp (km/s)
Flux (cm2 sr s keV/e)-1
1000
500
0
-500
-1000
-1000 -500 0 500 1000
10^5
10^6
10^7
10^8

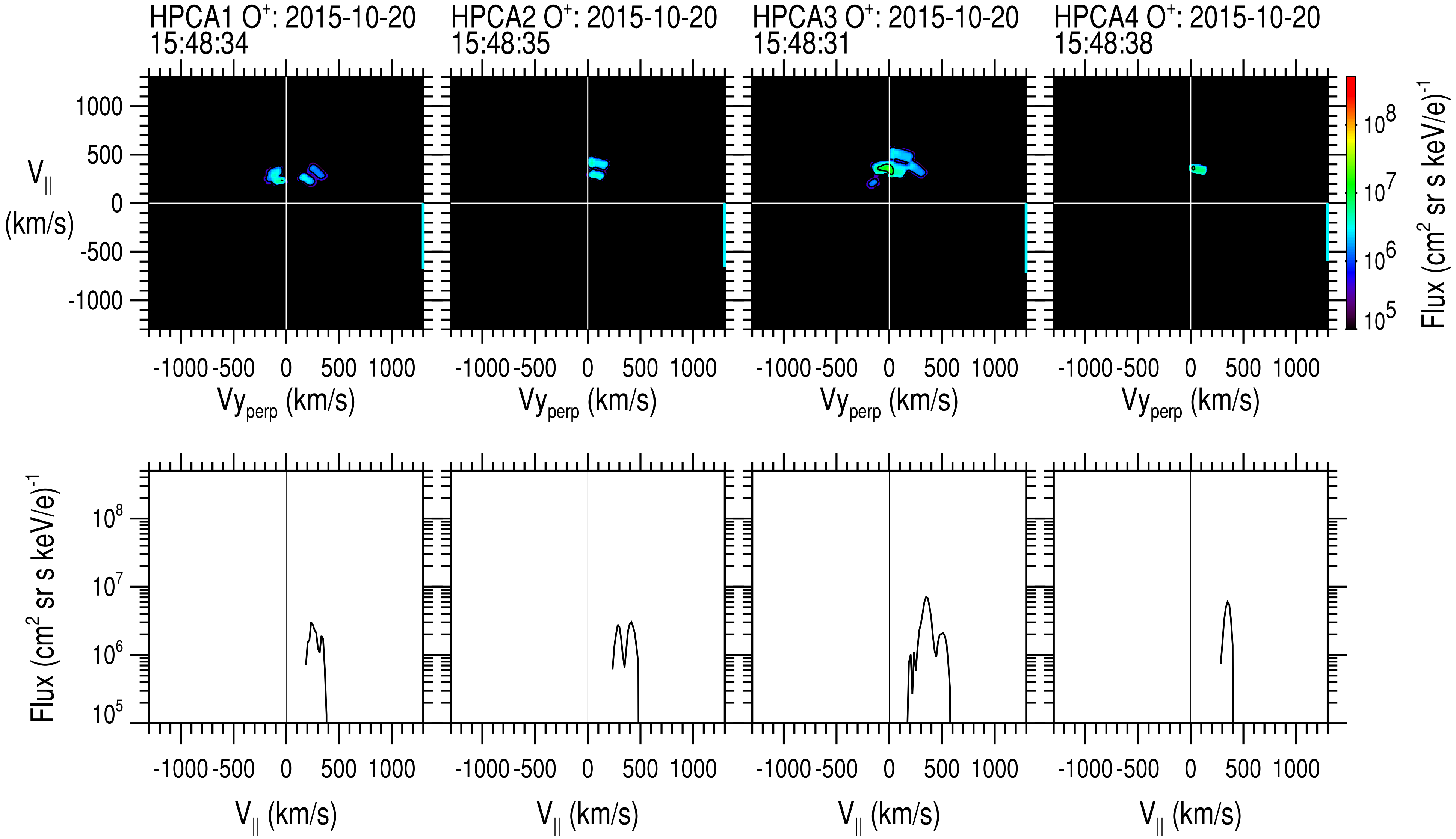

HPCA1 O+: 2015-10-20 15:48:34
HPCA2 O+: 2015-10-20 15:48:35
HPCA3 O+: 2015-10-20 15:48:31
HPCA4 O+: 2015-10-20 15:48:38
V|| (km/s)
Vyperp (km/s)
Flux (cm2 sr s keV/e)-1

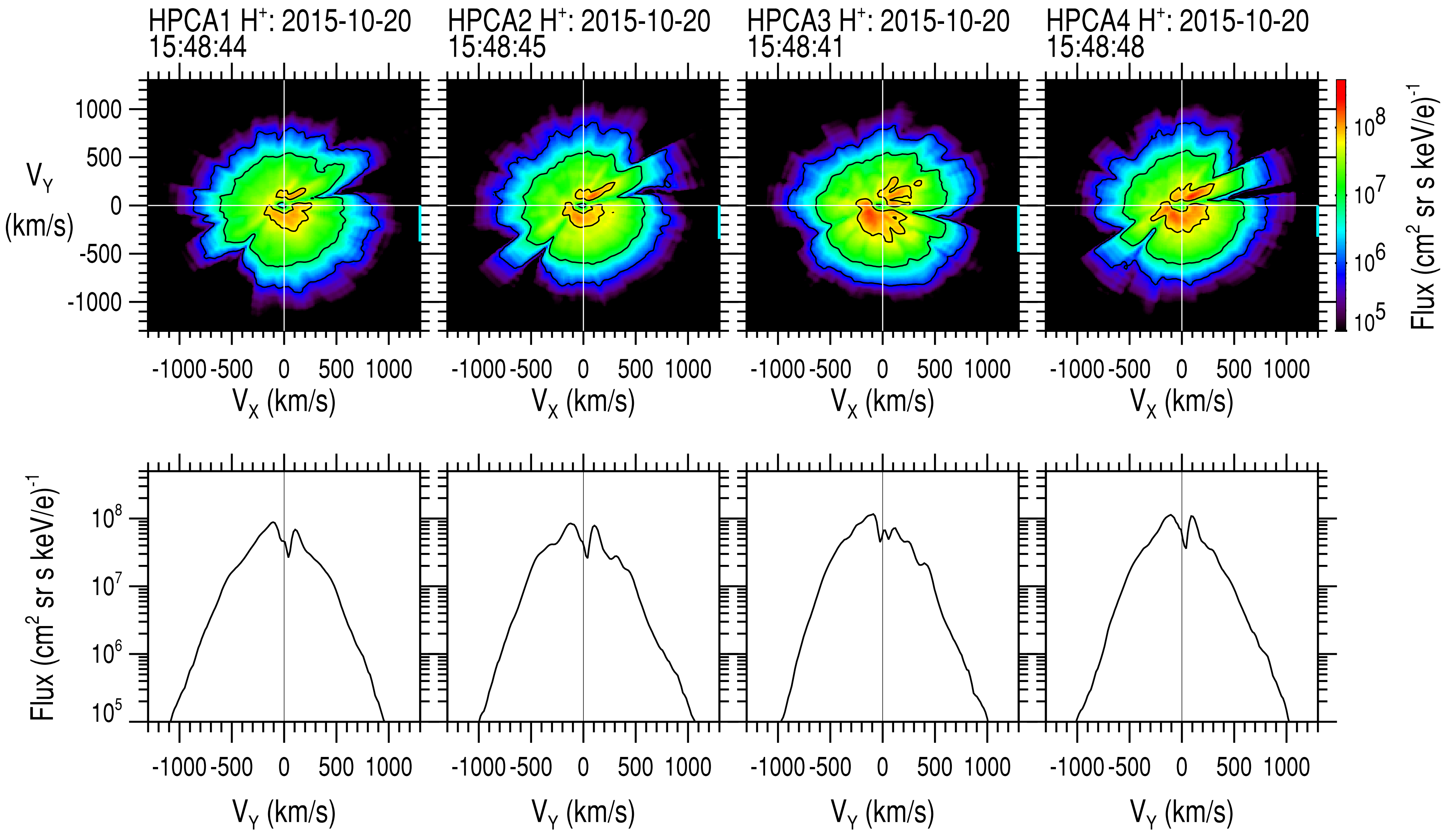

HPCA1 H+: 2015-10-20 15:48:44
HPCA2 H+: 2015-10-20 15:48:45
HPCA3 H+: 2015-10-20 15:48:41
HPCA4 H+: 2015-10-20 15:48:48
VY (km/s)
VX (km/s)
Flux (cm2 sr s keV/e)-1

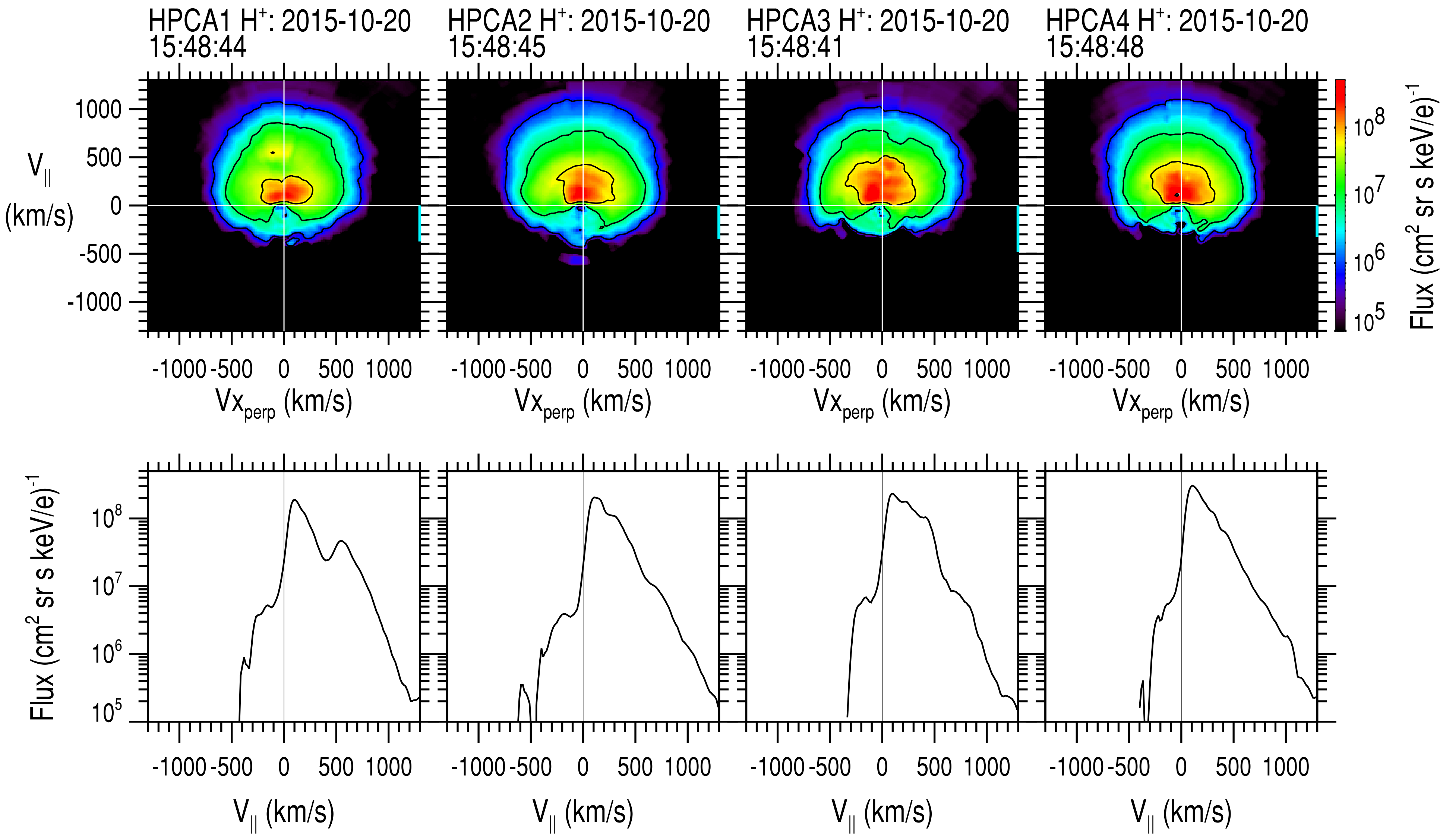

HPCA1 H+: 2015-10-20 15:48:44
HPCA2 H+: 2015-10-20 15:48:45
HPCA3 H+: 2015-10-20 15:48:41
HPCA4 H+: 2015-10-20 15:48:48
V|| (km/s)
Vx_perp (km/s)
Flux (cm2 sr s keV/e)-1

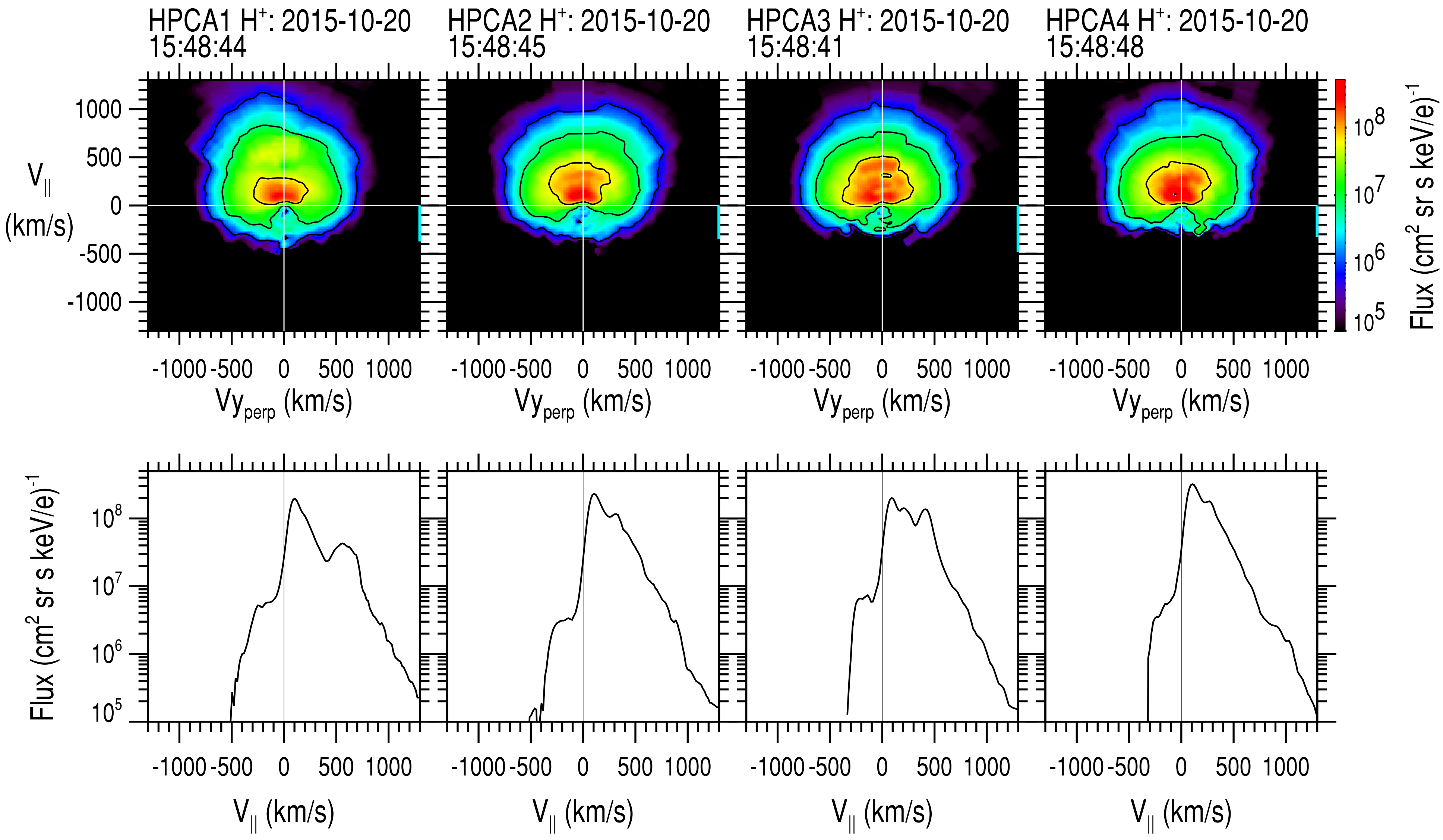

HPCA1 H+: 2015-10-20 15:48:44
HPCA2 H+: 2015-10-20 15:48:45
HPCA3 H+: 2015-10-20 15:48:41
HPCA4 H+: 2015-10-20 15:48:48
V|| (km/s)
Vyperp (km/s)
Flux (cm2 sr s keV/e)-1

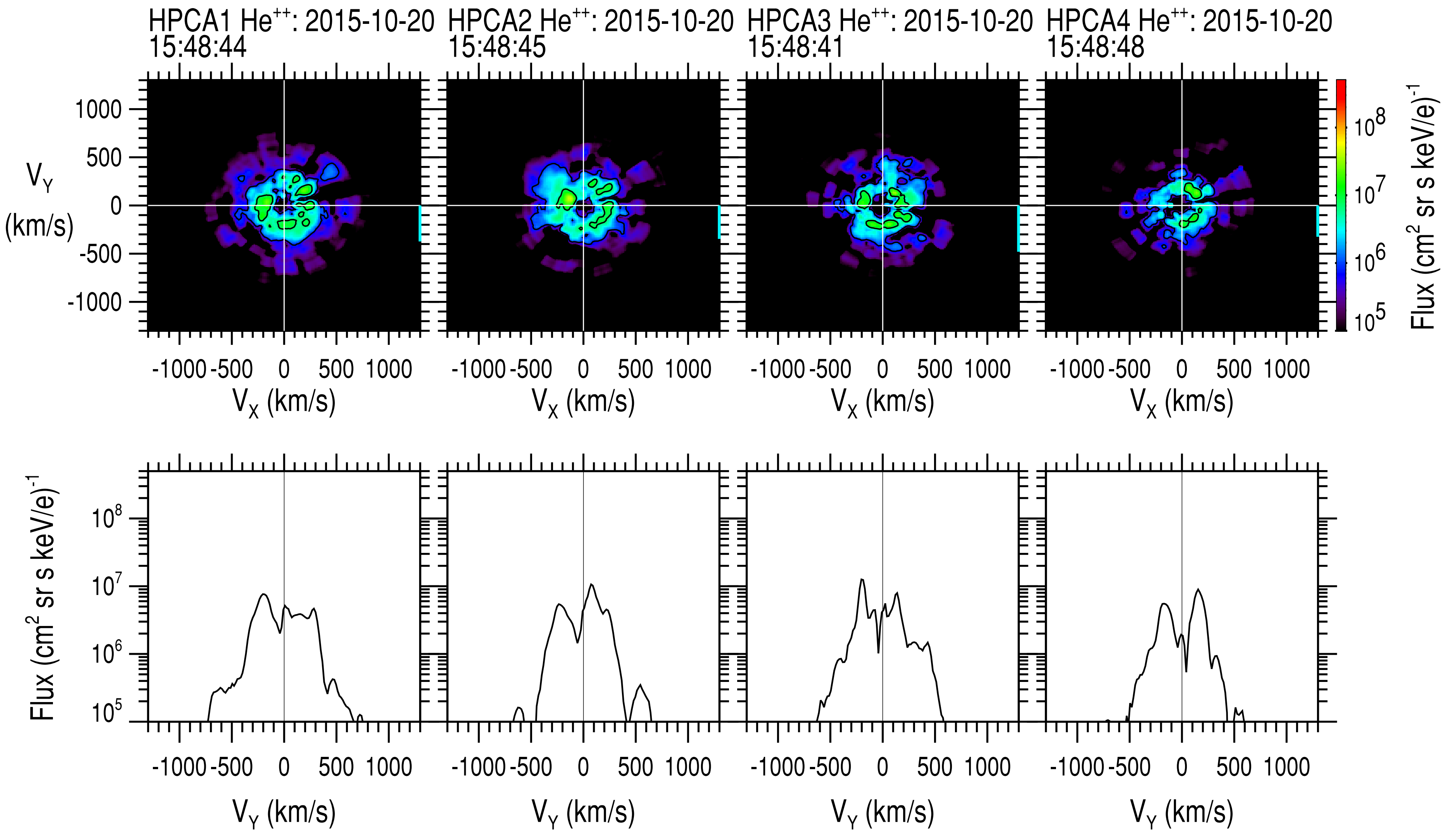

HPCA1 He++: 2015-10-20 15:48:44
HPCA2 He++: 2015-10-20 15:48:45
HPCA3 He++: 2015-10-20 15:48:41
HPCA4 He++: 2015-10-20 15:48:48
VY (km/s)
VX (km/s)
Flux (cm2 sr s keV/e)-1
VY (km/s)

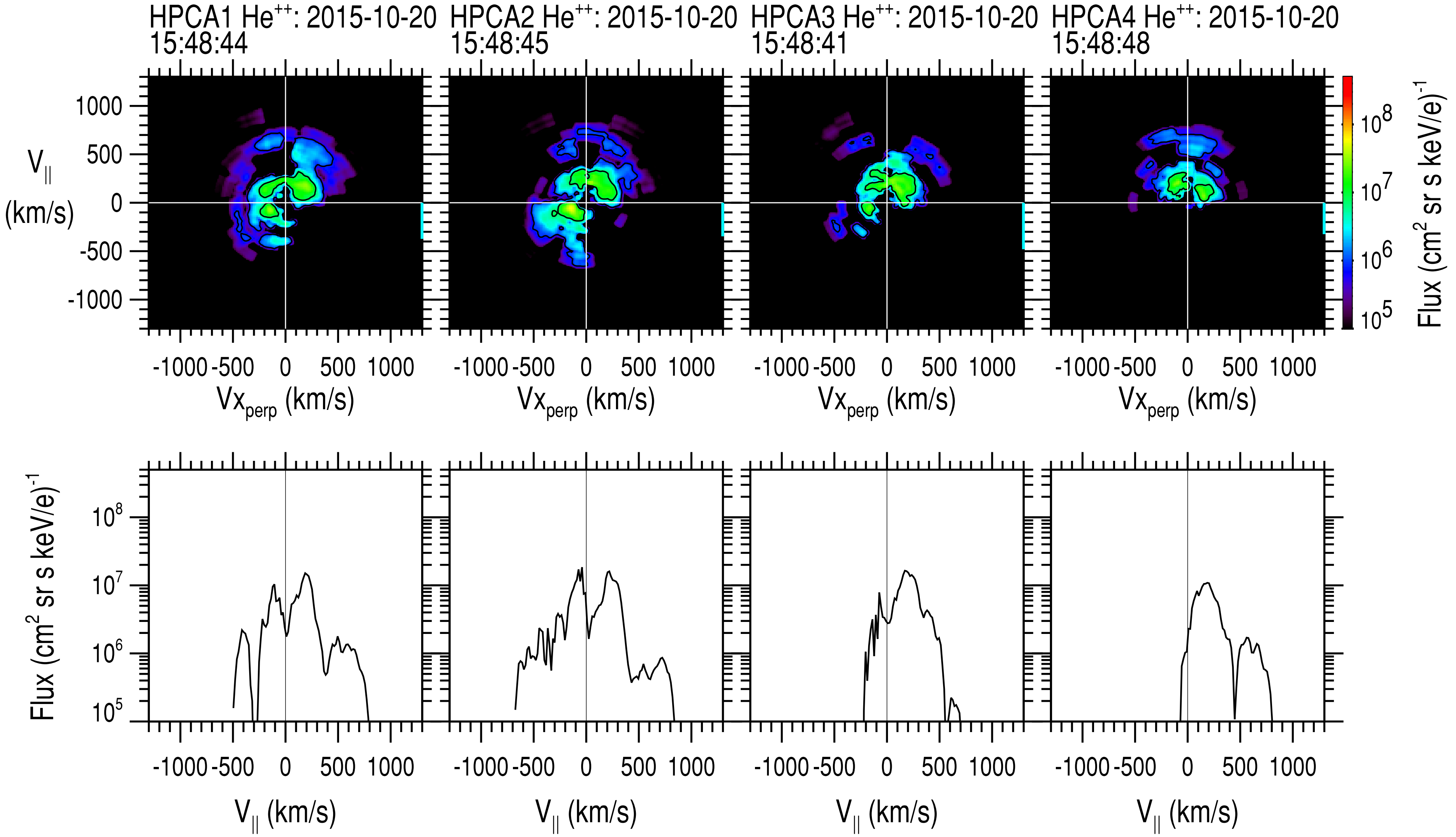
HPCA1 He++: 2015-10-20 15:48:44
HPCA2 He++: 2015-10-20 15:48:45
HPCA3 He++: 2015-10-20 15:48:41
HPCA4 He++: 2015-10-20 15:48:48
V|| (km/s)
Vxperp (km/s)
Flux (cm2 sr s keV/e)-1
V|| (km/s)
-1000 -500 0 500 1000
10^5 10^6 10^7 10^8

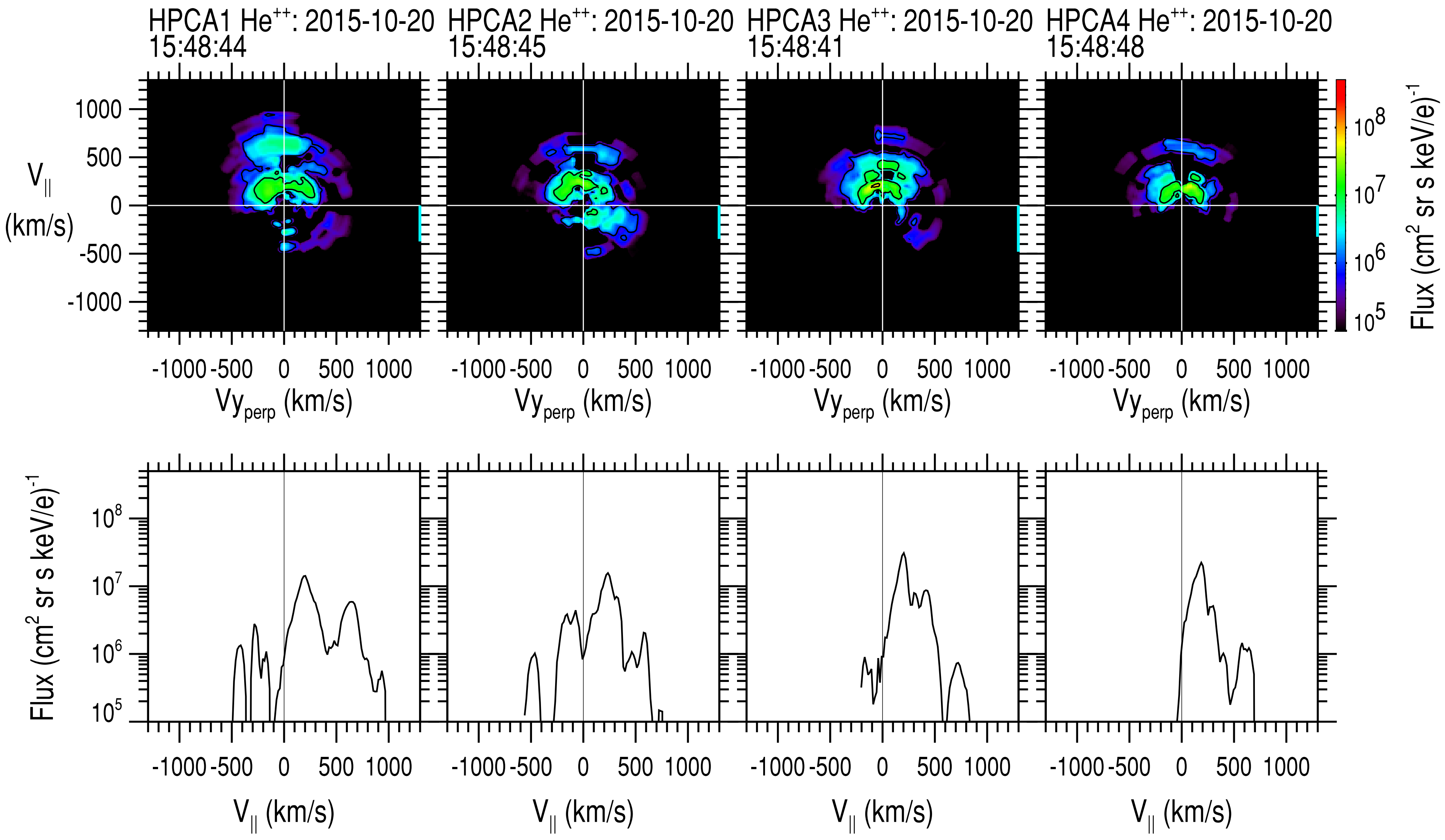
HPCA1 He++: 2015-10-20 15:48:44
HPCA2 He++: 2015-10-20 15:48:45
HPCA3 He++: 2015-10-20 15:48:41
HPCA4 He++: 2015-10-20 15:48:48
V|| (km/s)
Vyperp (km/s)
Flux (cm2 sr s keV/e)-1
V|| (km/s)

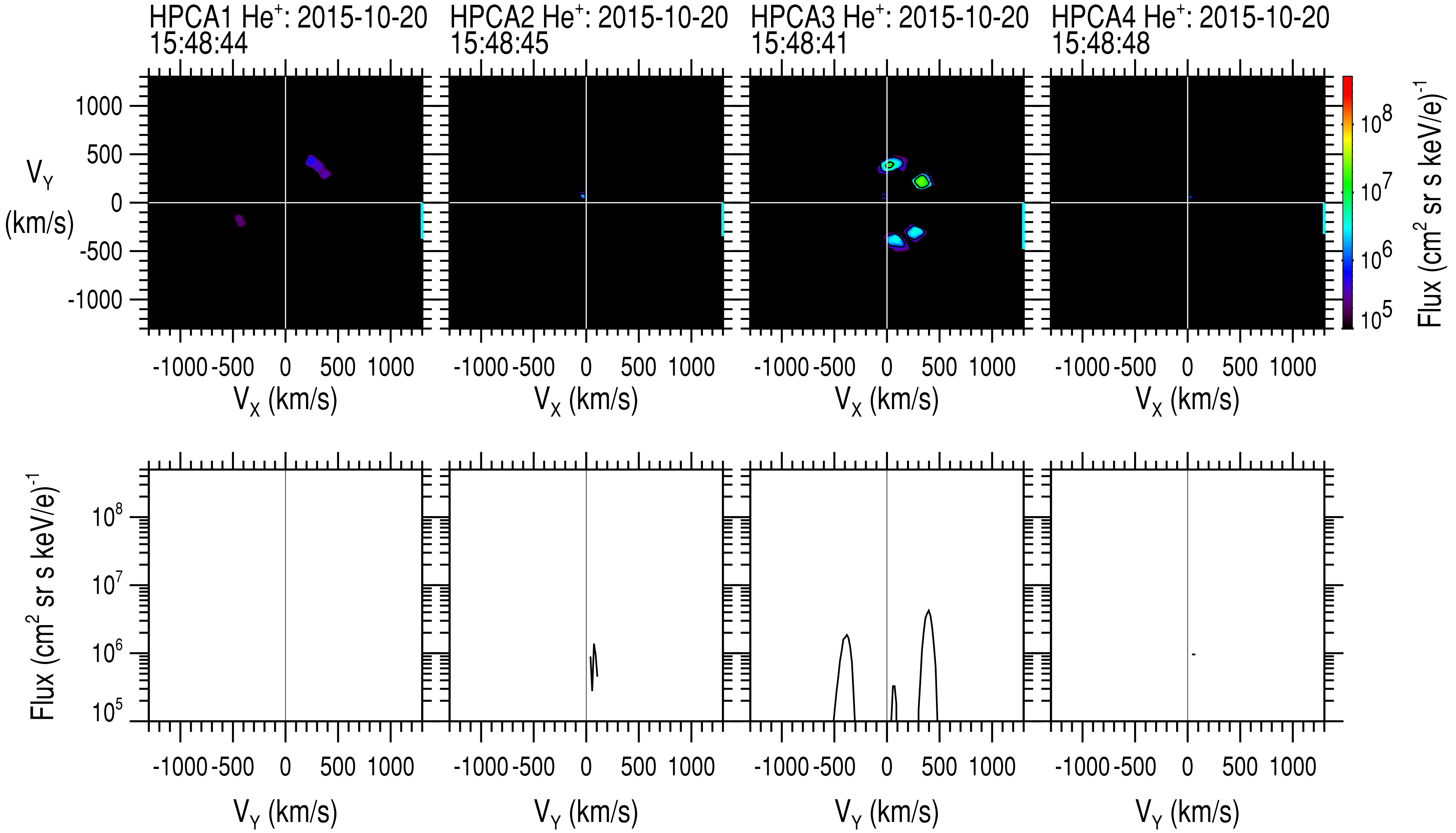
HPCA1 He+: 2015-10-20
15:48:44
HPCA2 He+: 2015-10-20
15:48:45
HPCA3 He+: 2015-10-20
15:48:41
HPCA4 He+: 2015-10-20
15:48:48
VY (km/s)
VX (km/s)
Flux (cm2 sr s keV/e)-1
VY (km/s)

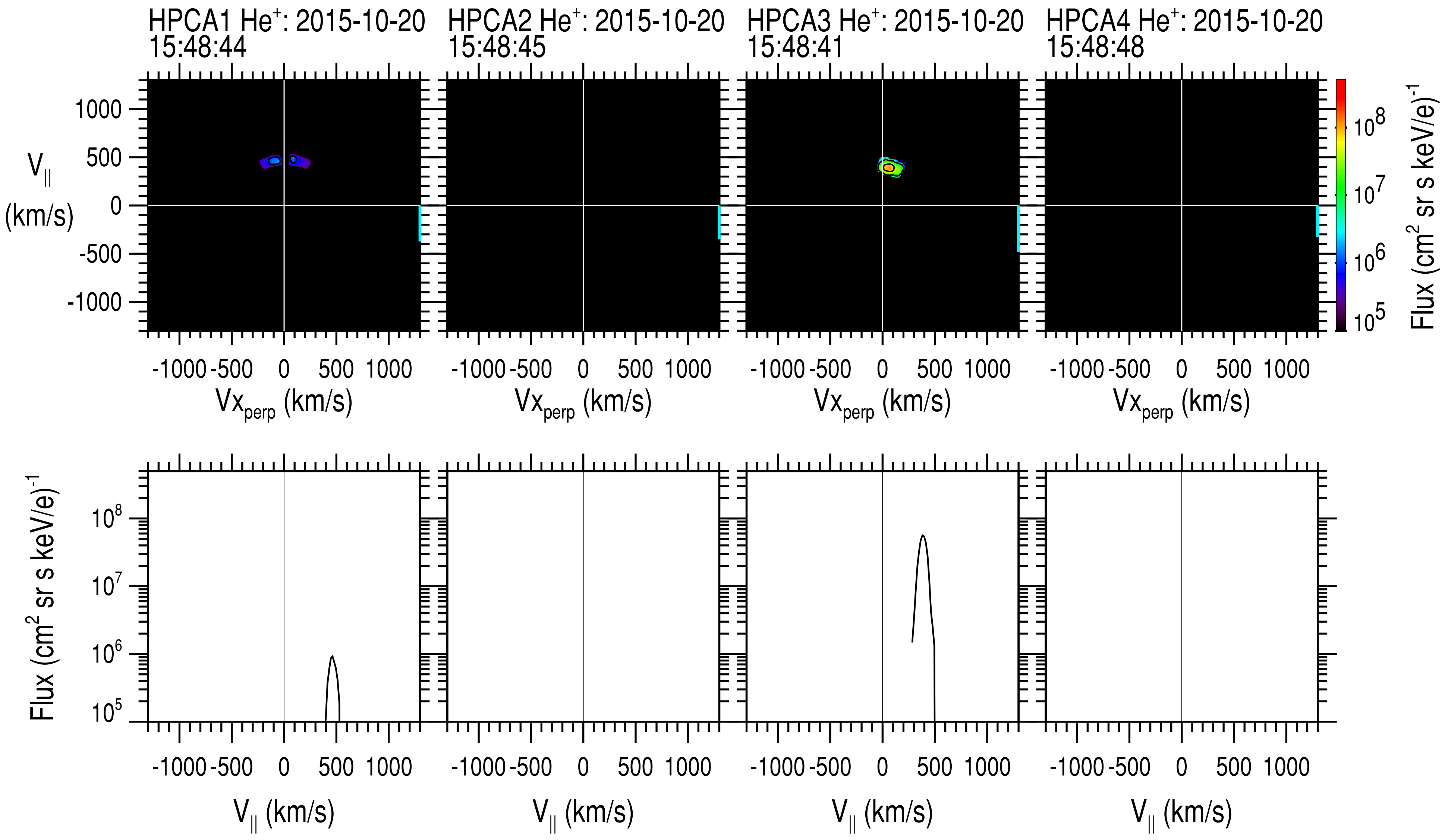
HPCA1 He+: 2015-10-20 15:48:44
HPCA2 He+: 2015-10-20 15:48:45
HPCA3 He+: 2015-10-20 15:48:41
HPCA4 He+: 2015-10-20 15:48:48
V|| (km/s)
Vxperp (km/s)
Flux (cm2 sr s keV/e)-1
-1000 -500 0 500 1000
10^5 10^6 10^7 10^8

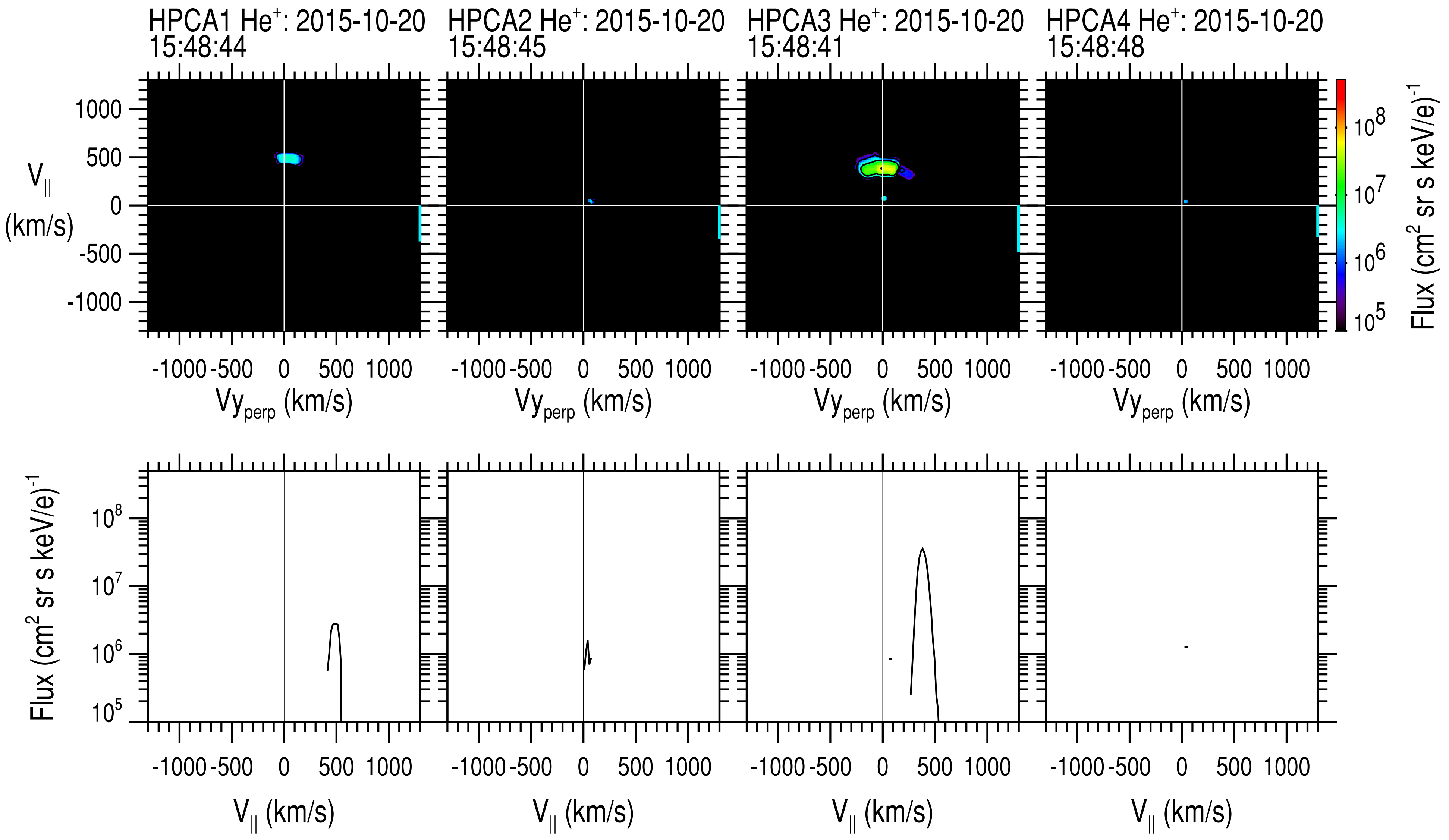
HPCA1 He+: 2015-10-20 15:48:44
HPCA2 He+: 2015-10-20 15:48:45
HPCA3 He+: 2015-10-20 15:48:41
HPCA4 He+: 2015-10-20 15:48:48
V|| (km/s)
Vyperp (km/s)
Flux (cm2 sr s keV/e)-1
1000
500
0
-500
-1000
10^5
10^6
10^7
10^8

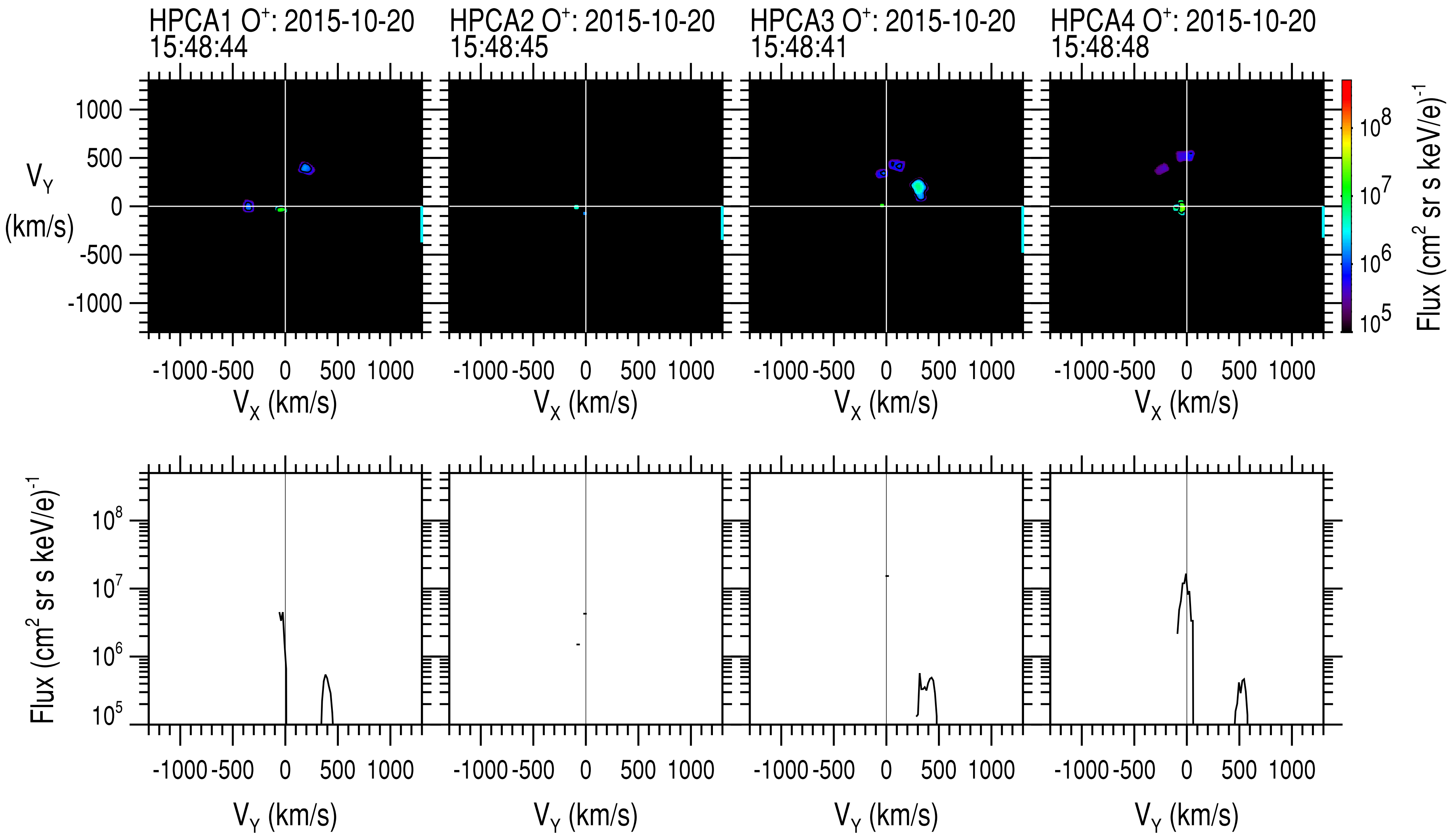
HPCA1 O+: 2015-10-20
15:48:44
HPCA2 O+: 2015-10-20
15:48:45
HPCA3 O+: 2015-10-20
15:48:41
HPCA4 O+: 2015-10-20
15:48:48
VY (km/s)
VX (km/s)
Flux (cm2 sr s keV/e)-1
VY (km/s)

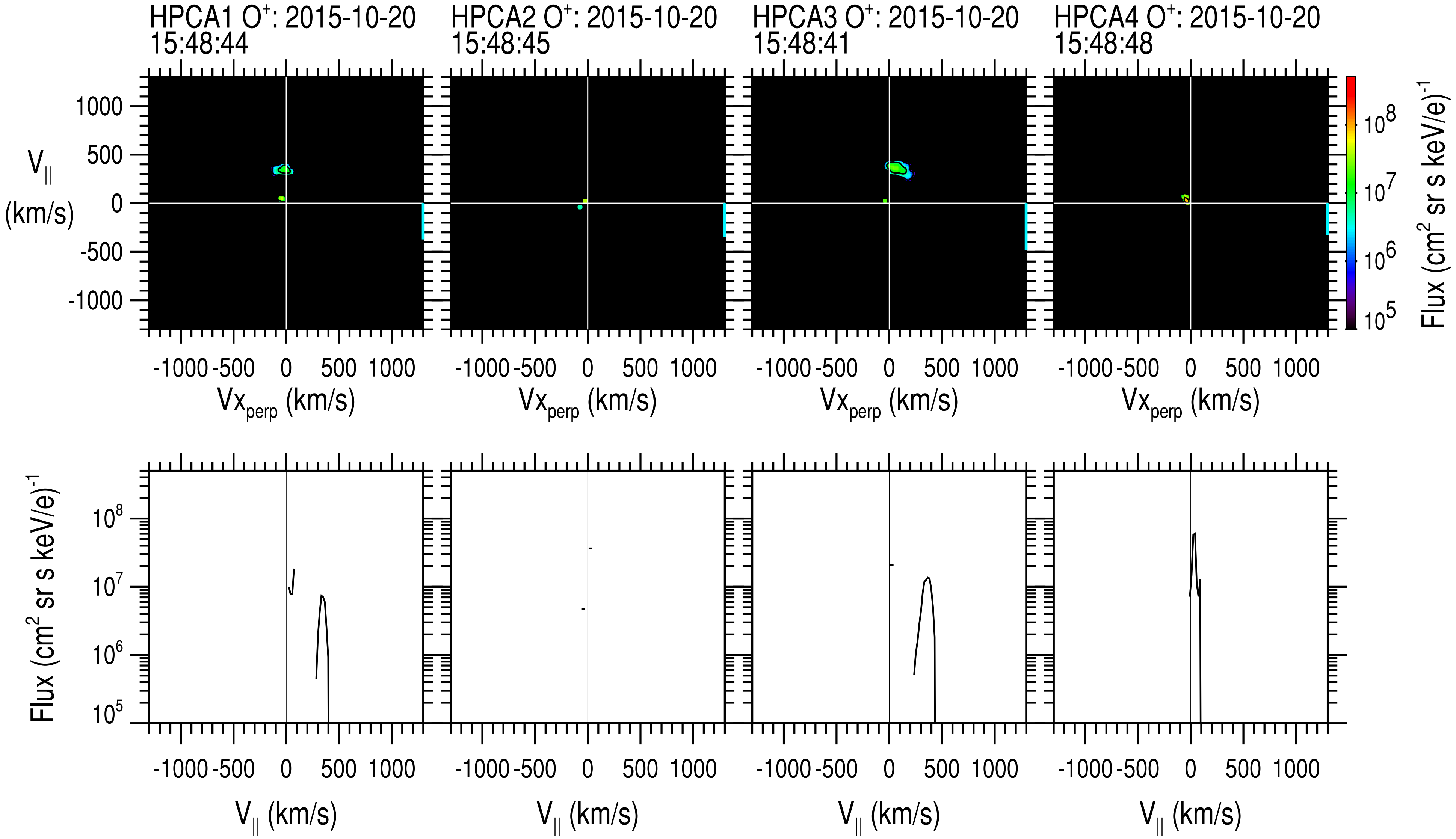

HPCA1 O+: 2015-10-20
15:48:44
HPCA2 O+: 2015-10-20
15:48:45
HPCA3 O+: 2015-10-20
15:48:41
HPCA4 O+: 2015-10-20
15:48:48
V|| (km/s)
1000
500
0
-500
-1000
-1000 -500 0 500 1000
Vxperp (km/s)
Flux (cm2 sr s keV/e)-1
105
106
107
108
V|| (km/s)

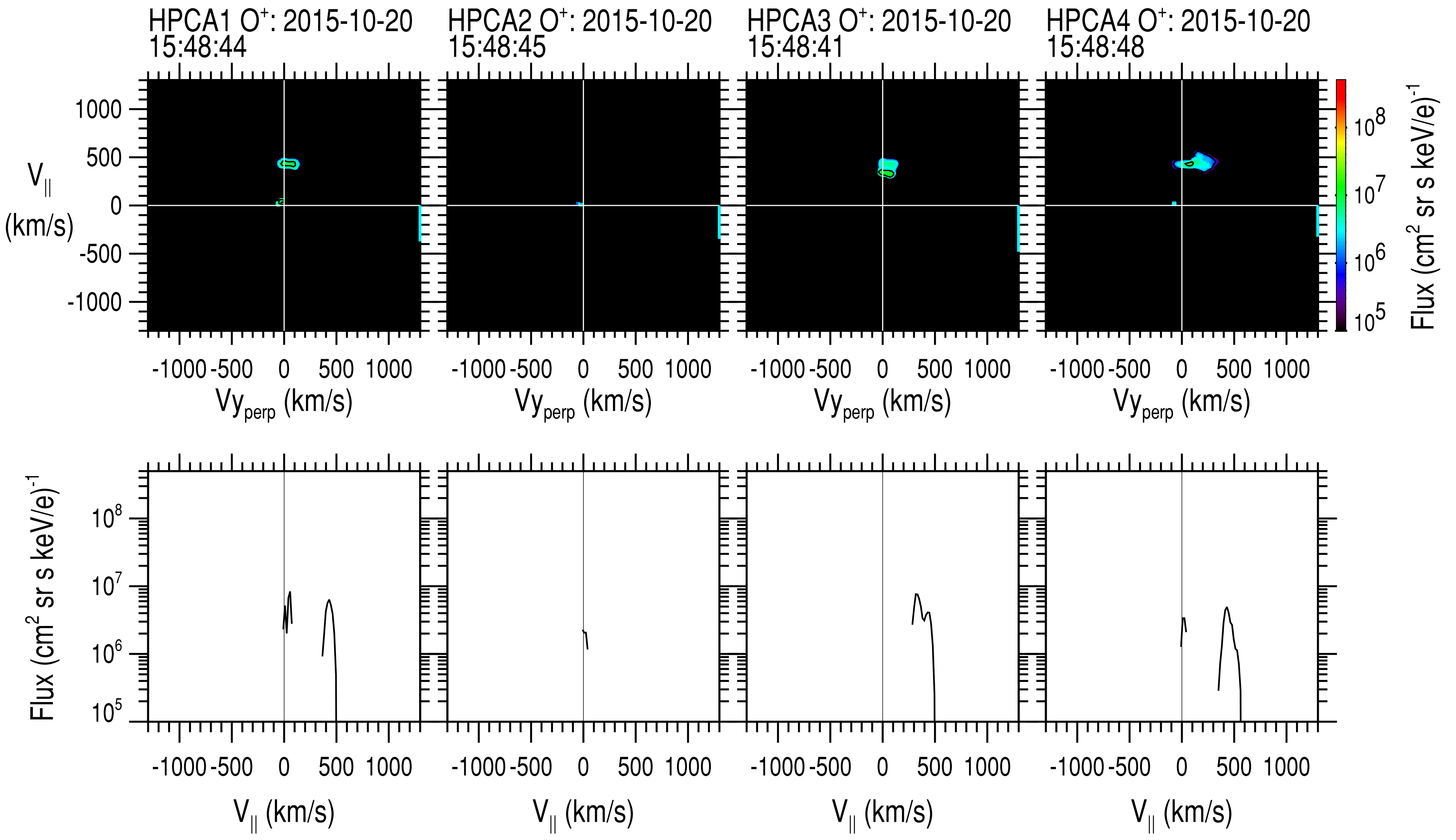
HPCA1 O+: 2015-10-20 15:48:44
HPCA2 O+: 2015-10-20 15:48:45
HPCA3 O+: 2015-10-20 15:48:41
HPCA4 O+: 2015-10-20 15:48:48
V|| (km/s)
Vyperp (km/s)
Flux (cm2 sr s keV/e)-1
V|| (km/s)
Flux (cm2 sr s keV/e)-1

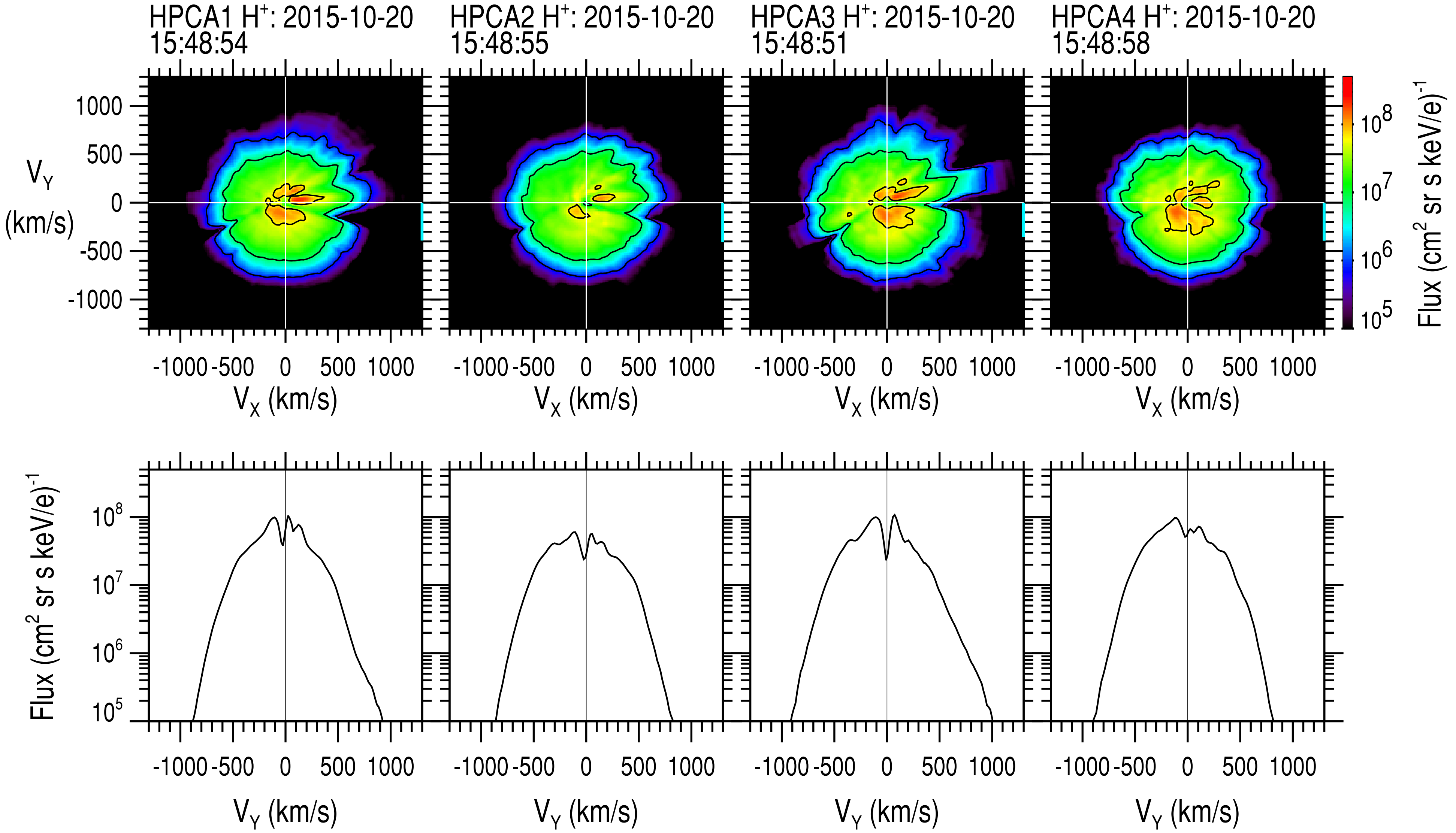

HPCA1 H+: 2015-10-20 15:48:54
HPCA2 H+: 2015-10-20 15:48:55
HPCA3 H+: 2015-10-20 15:48:51
HPCA4 H+: 2015-10-20 15:48:58
V_Y (km/s)
V_X (km/s)
Flux (cm2 sr s keV/e)-1

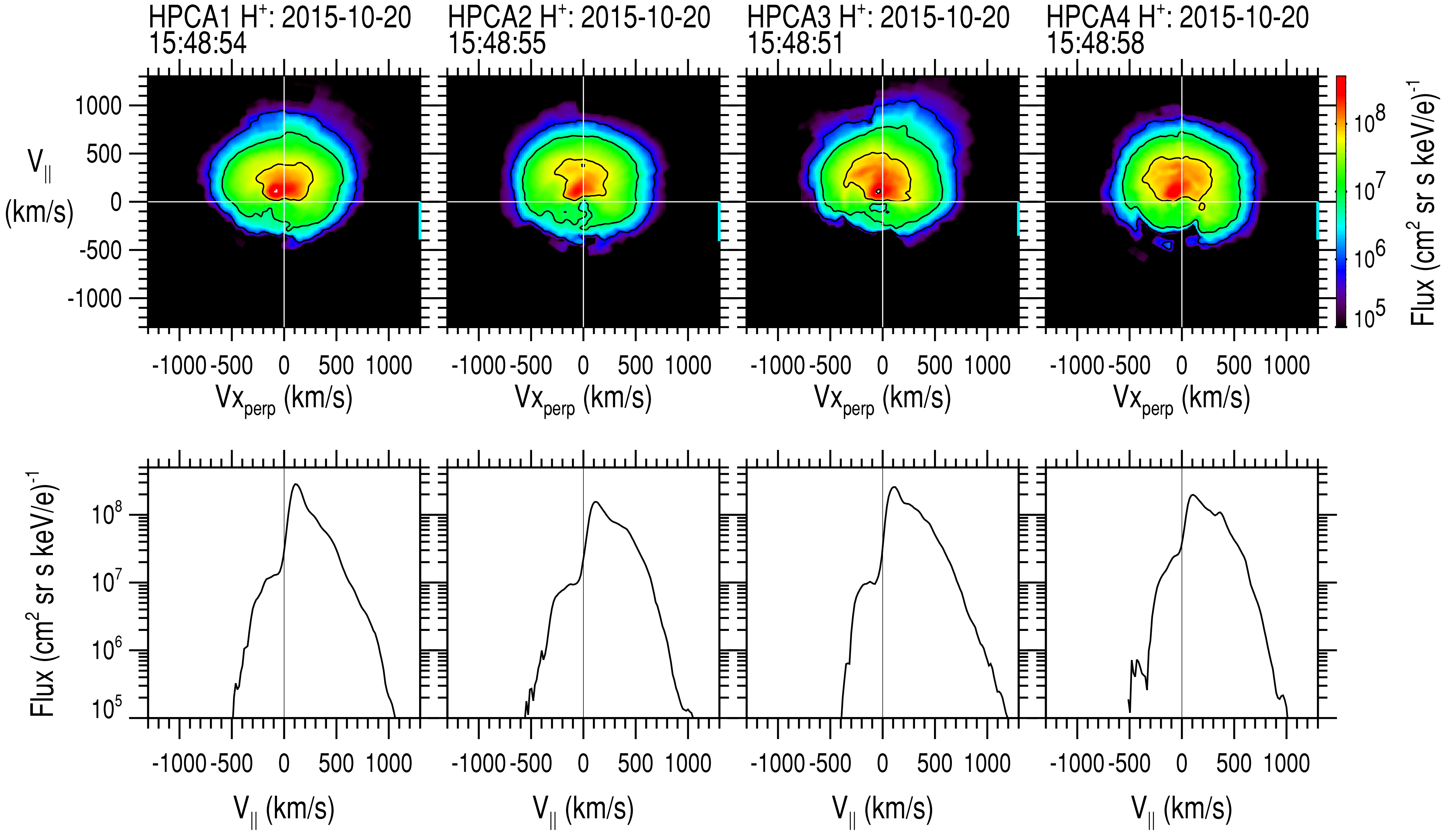
HPCA1 H+: 2015-10-20 15:48:54
HPCA2 H+: 2015-10-20 15:48:55
HPCA3 H+: 2015-10-20 15:48:51
HPCA4 H+: 2015-10-20 15:48:58
V|| (km/s)
Vxperp (km/s)
Flux (cm2 sr s keV/e)-1
V|| (km/s)

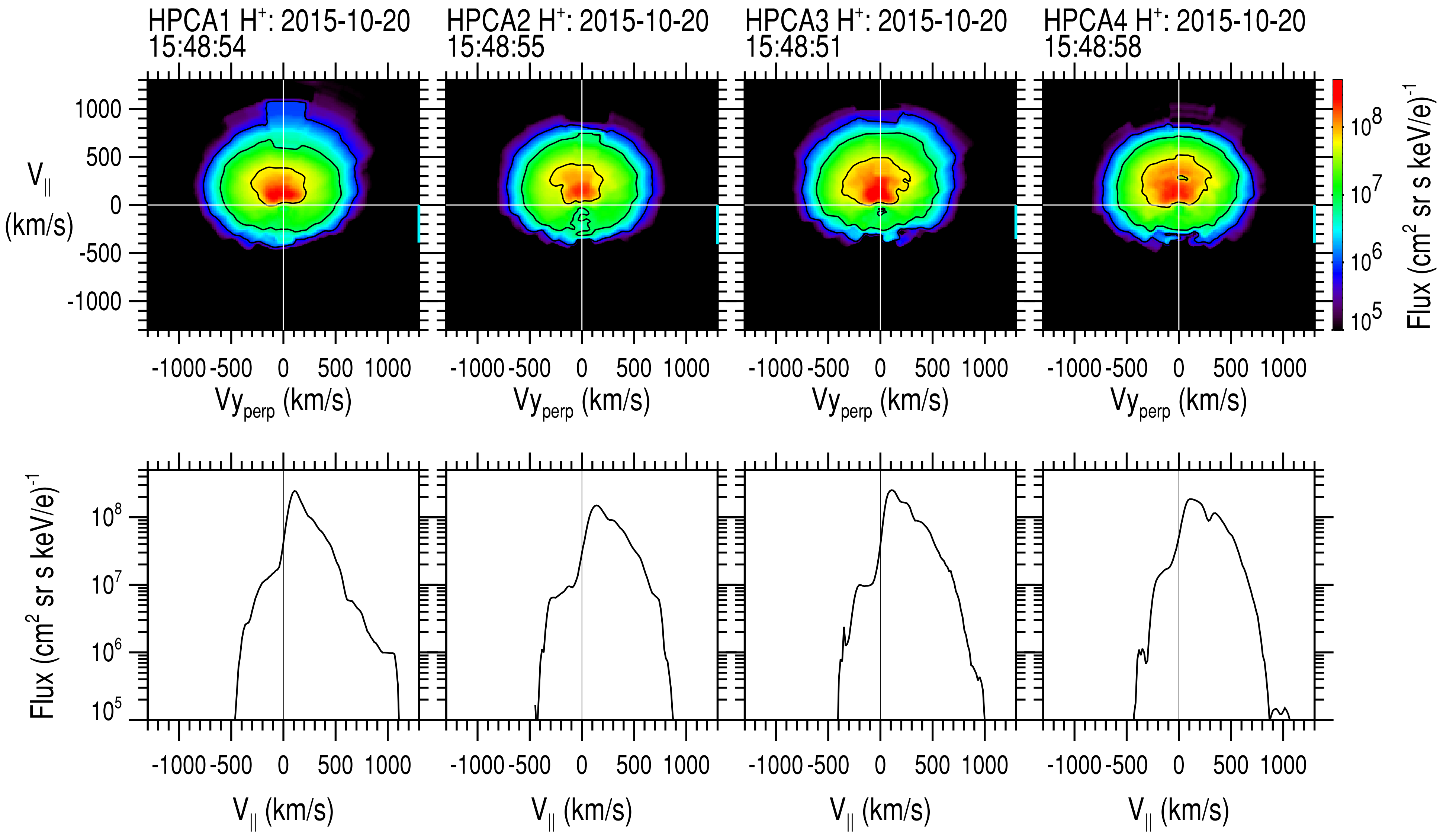
HPCA1 H+: 2015-10-20 15:48:54
HPCA2 H+: 2015-10-20 15:48:55
HPCA3 H+: 2015-10-20 15:48:51
HPCA4 H+: 2015-10-20 15:48:58
V|| (km/s)
Vyperp (km/s)
Flux (cm2 sr s keV/e)-1
V|| (km/s)

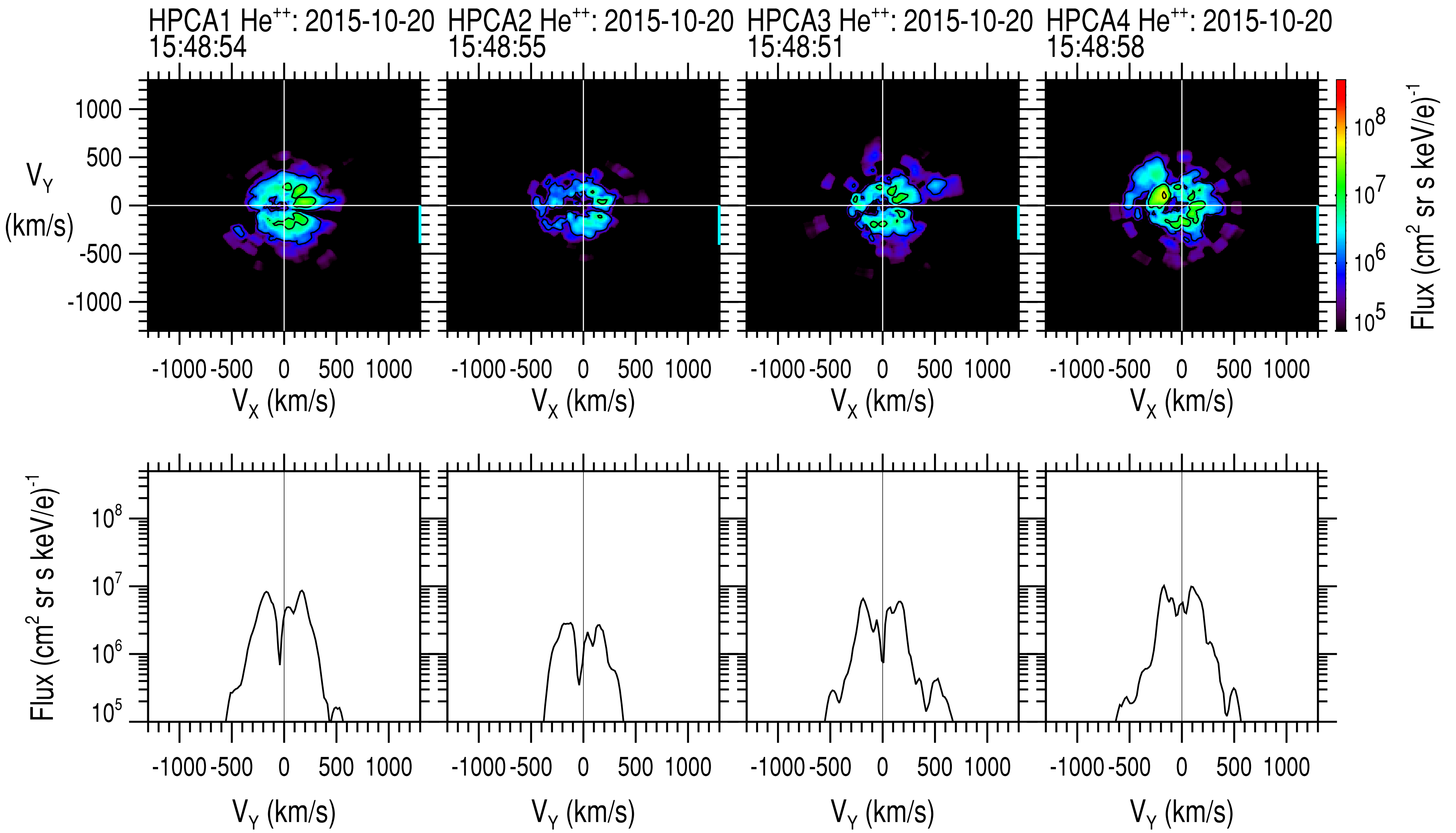
HPCA1 He++: 2015-10-20
15:48:54
HPCA2 He++: 2015-10-20
15:48:55
HPCA3 He++: 2015-10-20
15:48:51
HPCA4 He++: 2015-10-20
15:48:58
V_Y (km/s)
V_X (km/s)
Flux (cm^2 sr s keV/e)^-1
-1000 -500 0 500 1000
10^5 10^6 10^7 10^8

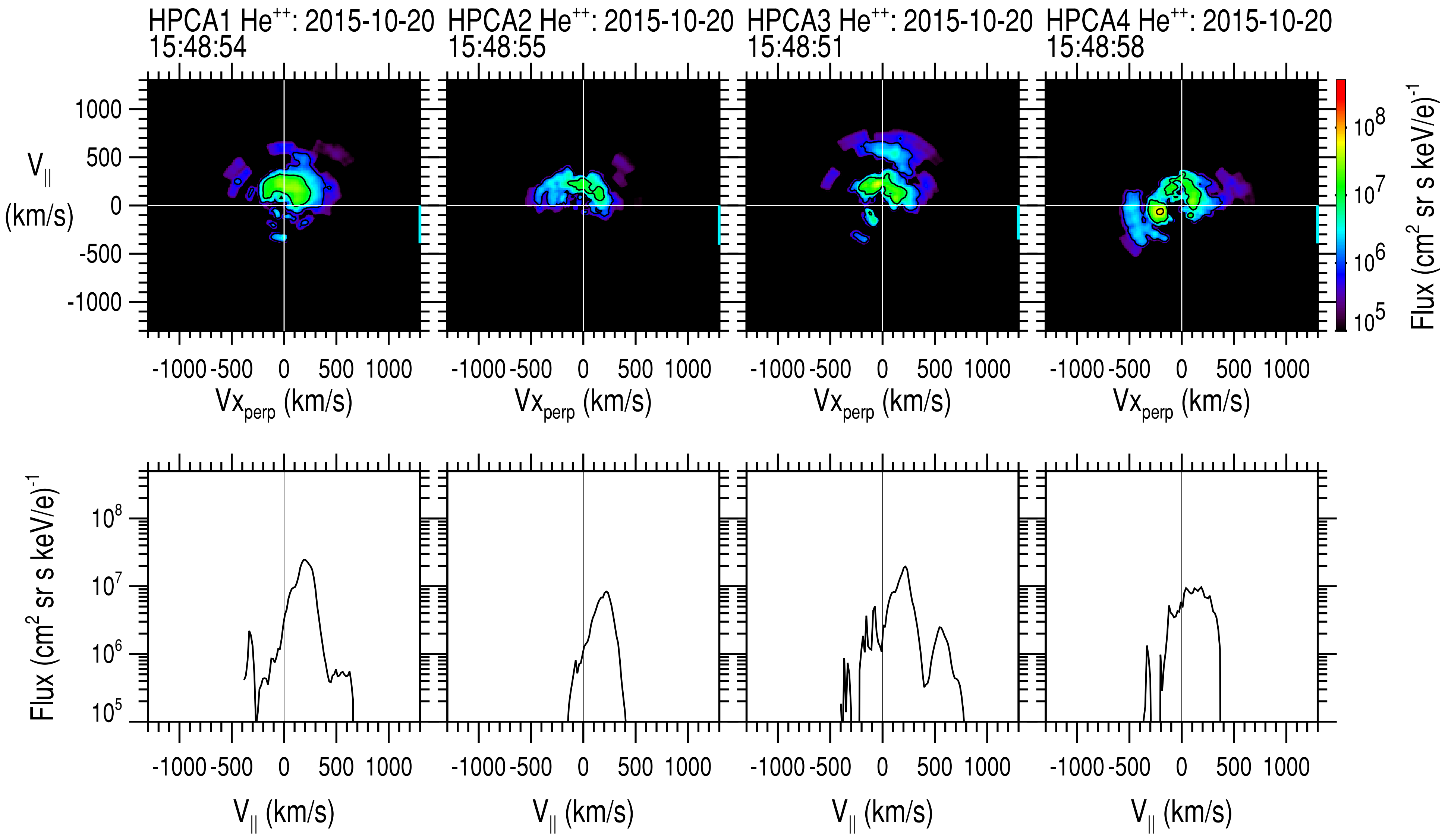
HPCA1 He++: 2015-10-20
15:48:54
HPCA2 He++: 2015-10-20
15:48:55
HPCA3 He++: 2015-10-20
15:48:51
HPCA4 He++: 2015-10-20
15:48:58
V||
(km/s)
1000
500
0
-500
-1000
-1000 -500 0 500 1000
Vxperp (km/s)
Flux (cm2 sr s keV/e)-1
10^5
10^6
10^7
10^8
V|| (km/s)

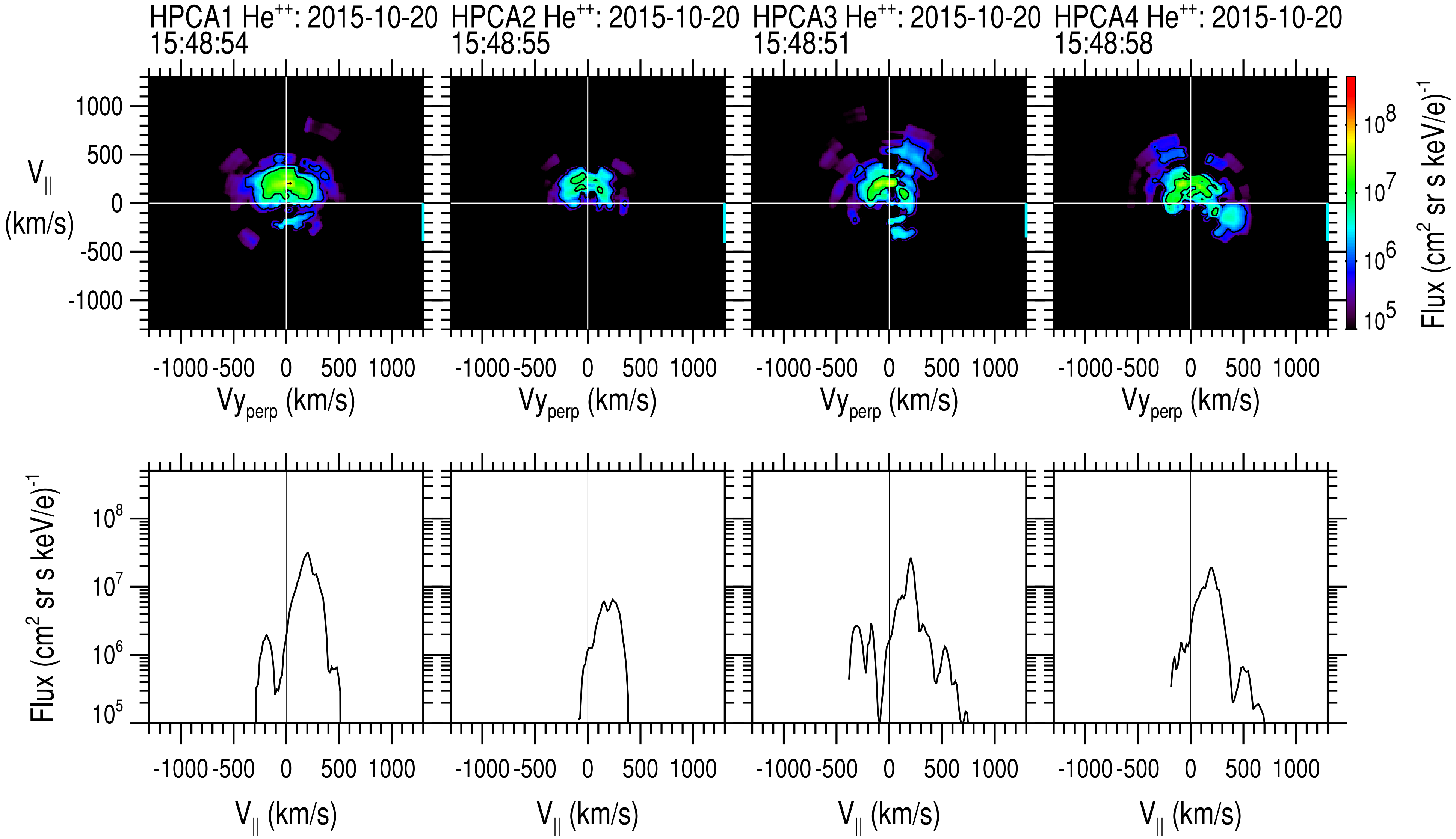
HPCA1 He++: 2015-10-20 15:48:54
HPCA2 He++: 2015-10-20 15:48:55
HPCA3 He++: 2015-10-20 15:48:51
HPCA4 He++: 2015-10-20 15:48:58
V|| (km/s)
Vyperp (km/s)
Flux (cm2 sr s keV/e)-1
V|| (km/s)

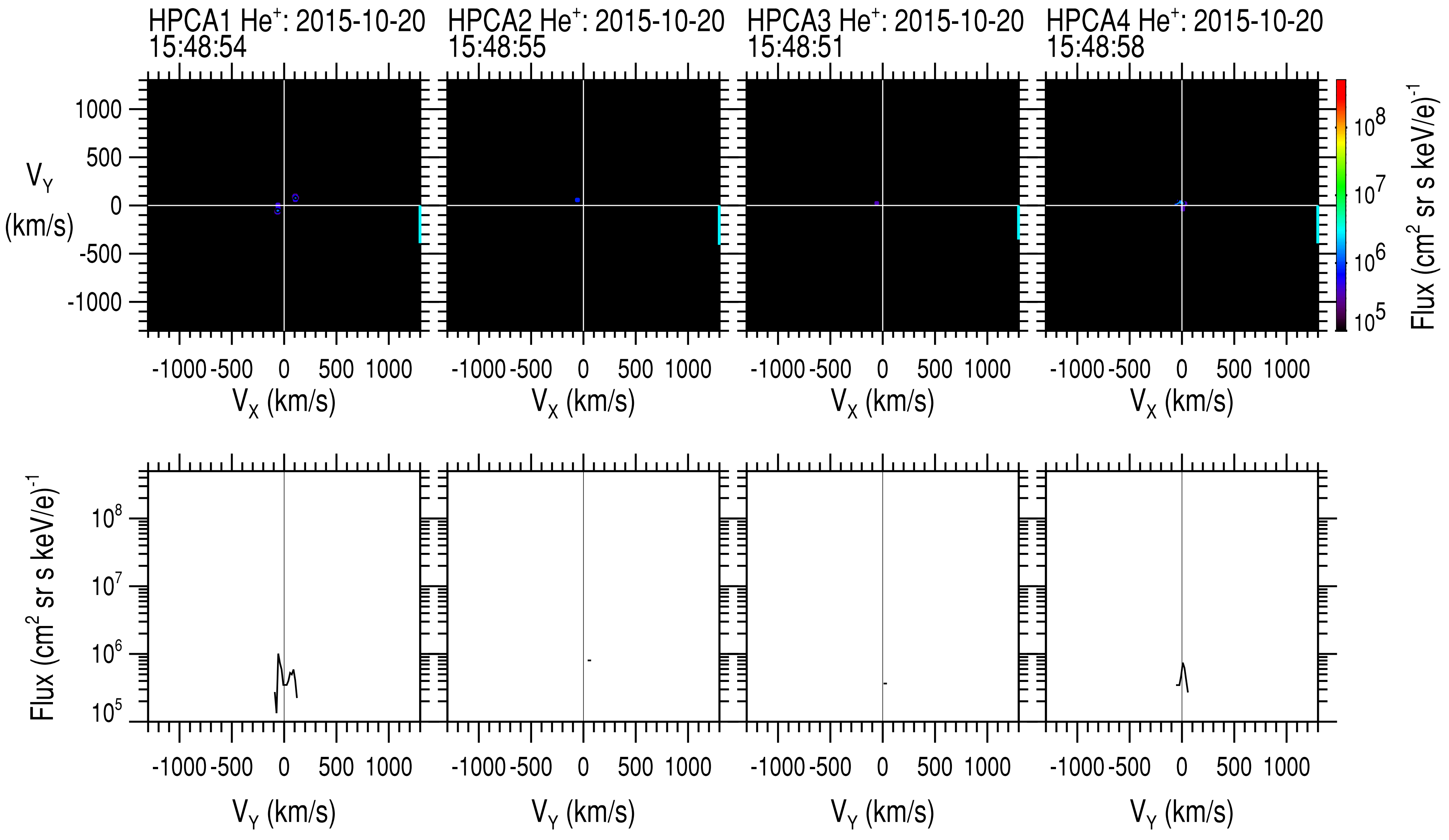

HPCA1 He+: 2015-10-20 15:48:54
HPCA2 He+: 2015-10-20 15:48:55
HPCA3 He+: 2015-10-20 15:48:51
HPCA4 He+: 2015-10-20 15:48:58
VY (km/s)
VX (km/s)
Flux (cm2 sr s keV/e)-1

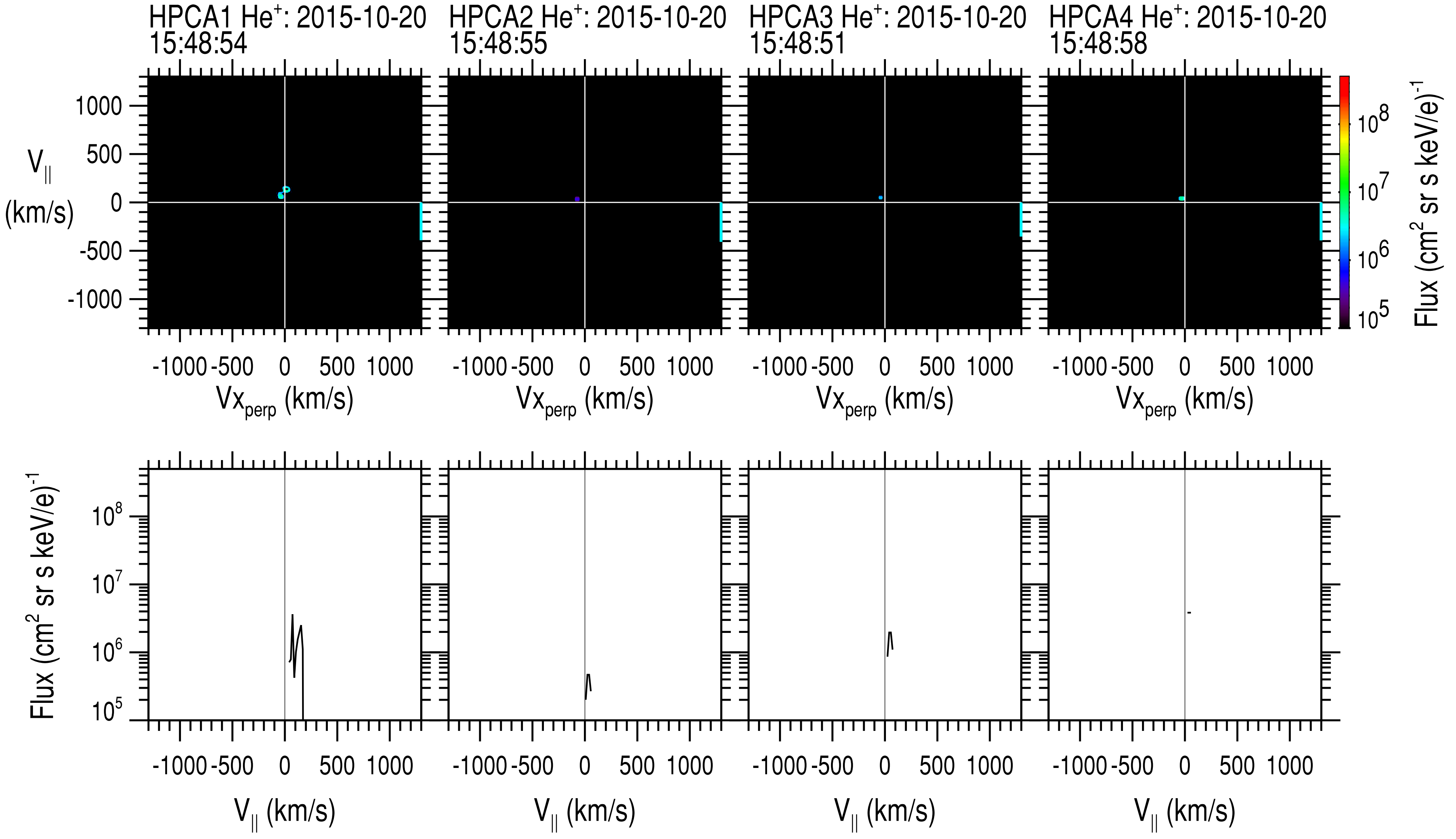
HPCA1 He+: 2015-10-20 15:48:54
HPCA2 He+: 2015-10-20 15:48:55
HPCA3 He+: 2015-10-20 15:48:51
HPCA4 He+: 2015-10-20 15:48:58
V|| (km/s)
Vxperp (km/s)
Flux (cm2 sr s keV/e)-1
-1000 -500 0 500 1000
10^5 10^6 10^7 10^8

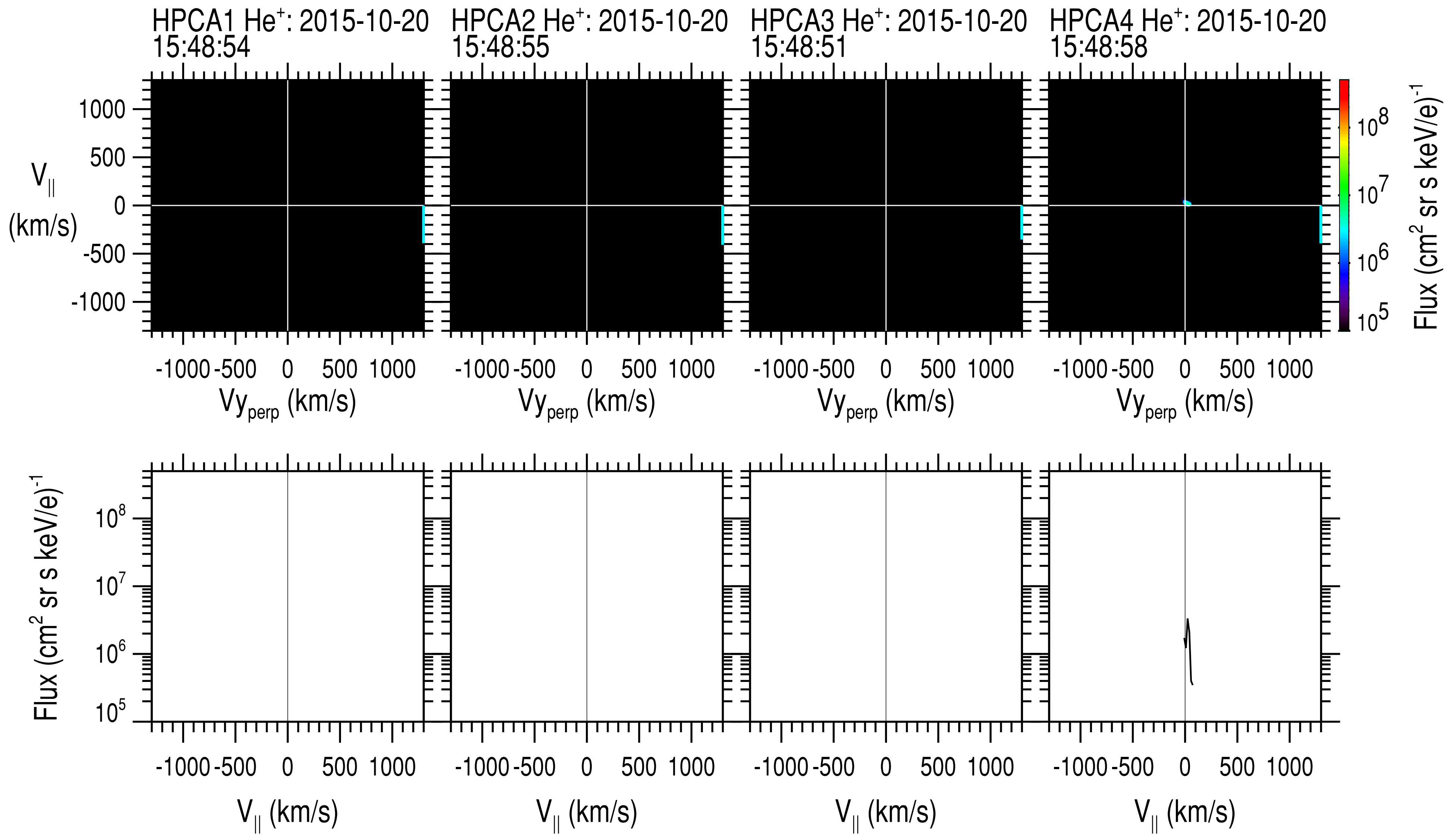

HPCA1 He+: 2015-10-20
15:48:54
HPCA2 He+: 2015-10-20
15:48:55
HPCA3 He+: 2015-10-20
15:48:51
HPCA4 He+: 2015-10-20
15:48:58
V|| (km/s)
Vyperp (km/s)
Flux (cm2 sr s keV/e)-1
-1000 -500 0 500 1000
10^5 10^6 10^7 10^8

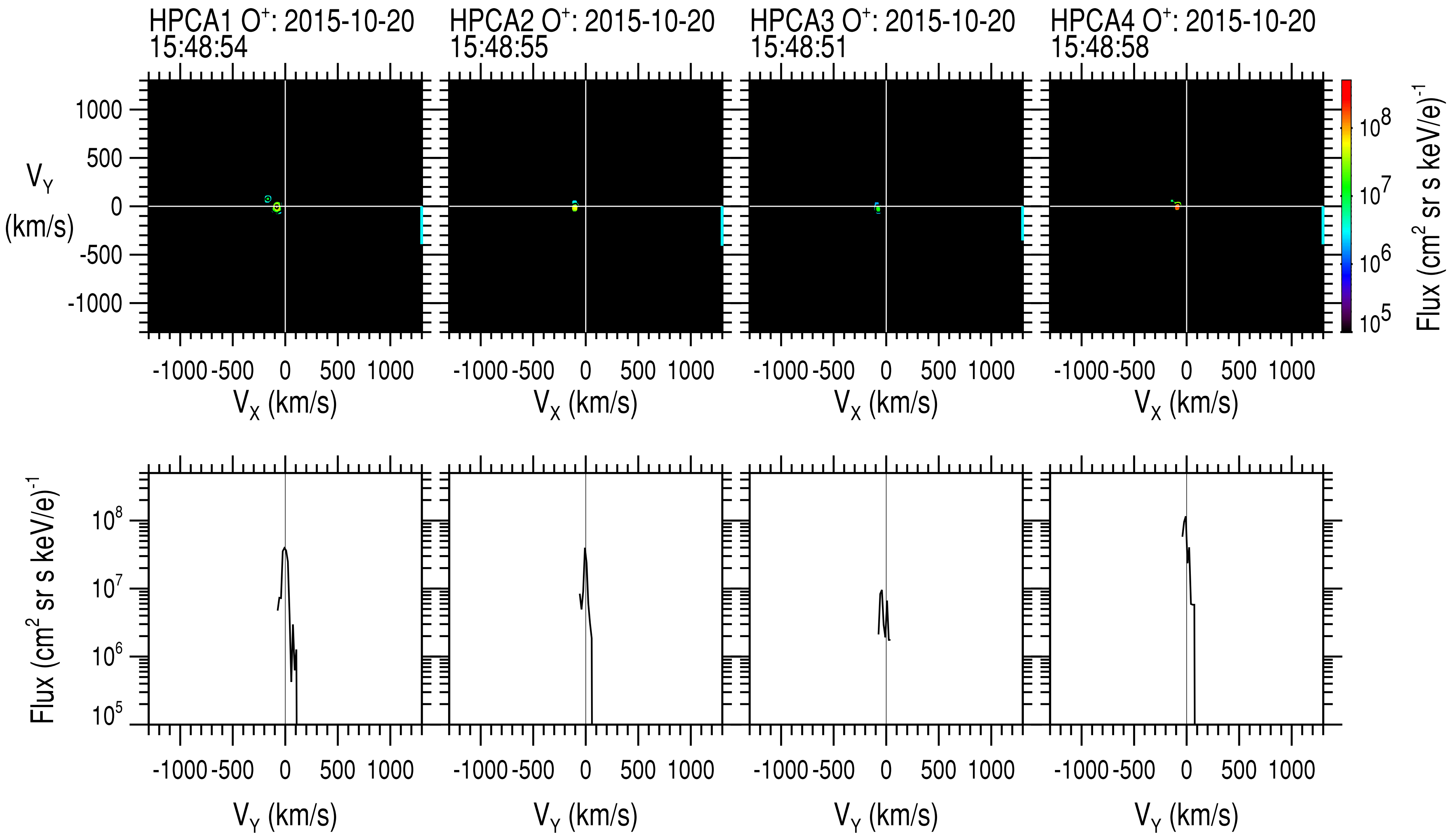

HPCA1 O+: 2015-10-20
15:48:54
HPCA2 O+: 2015-10-20
15:48:55
HPCA3 O+: 2015-10-20
15:48:51
HPCA4 O+: 2015-10-20
15:48:58
VY (km/s)
VX (km/s)
Flux (cm2 sr s keV/e)-1

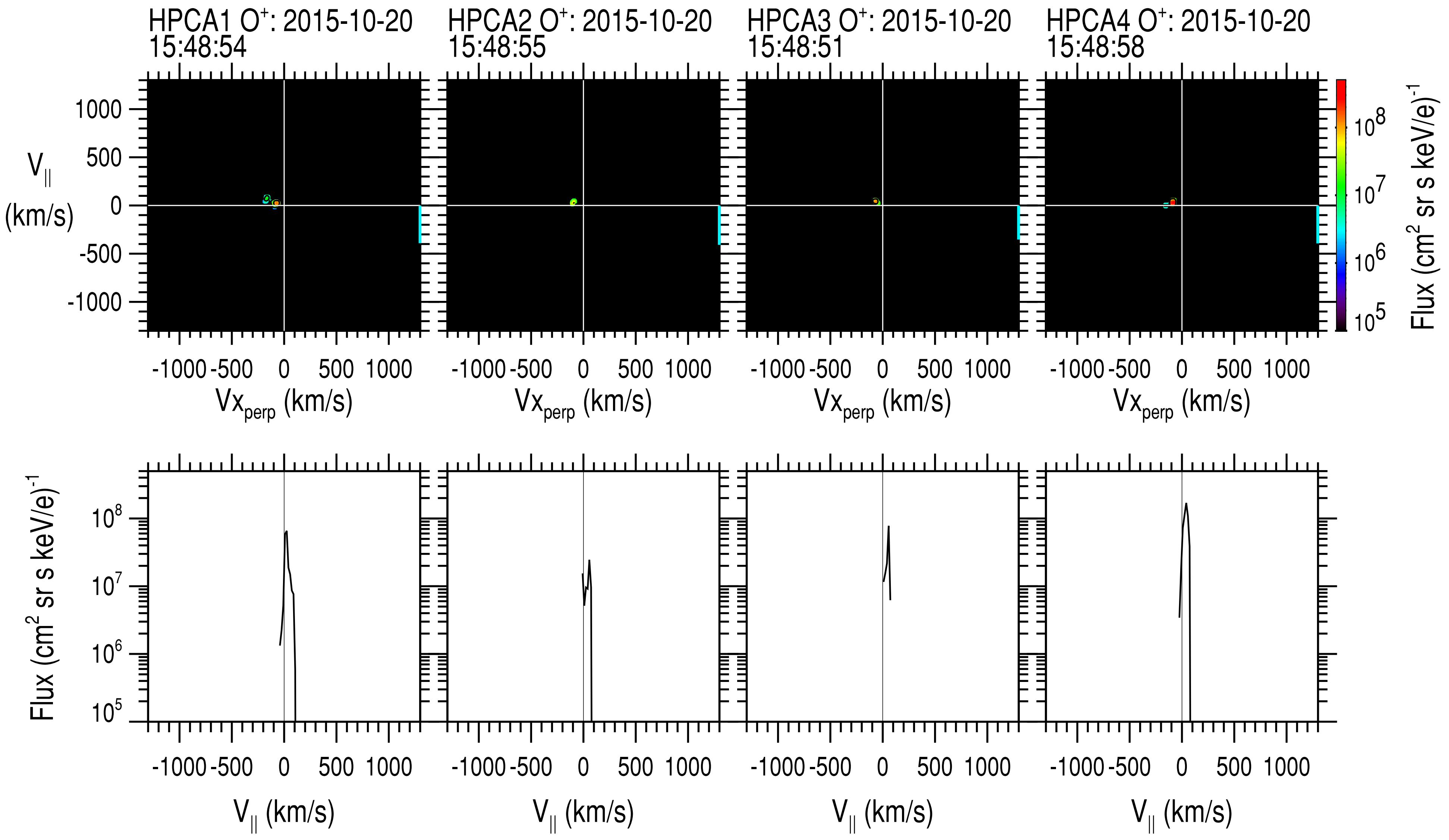
HPCA1 O+: 2015-10-20 15:48:54
HPCA2 O+: 2015-10-20 15:48:55
HPCA3 O+: 2015-10-20 15:48:51
HPCA4 O+: 2015-10-20 15:48:58
V|| (km/s)
Vxperp (km/s)
Flux (cm2 sr s keV/e)-1
-1000 -500 0 500 1000
10^5 10^6 10^7 10^8

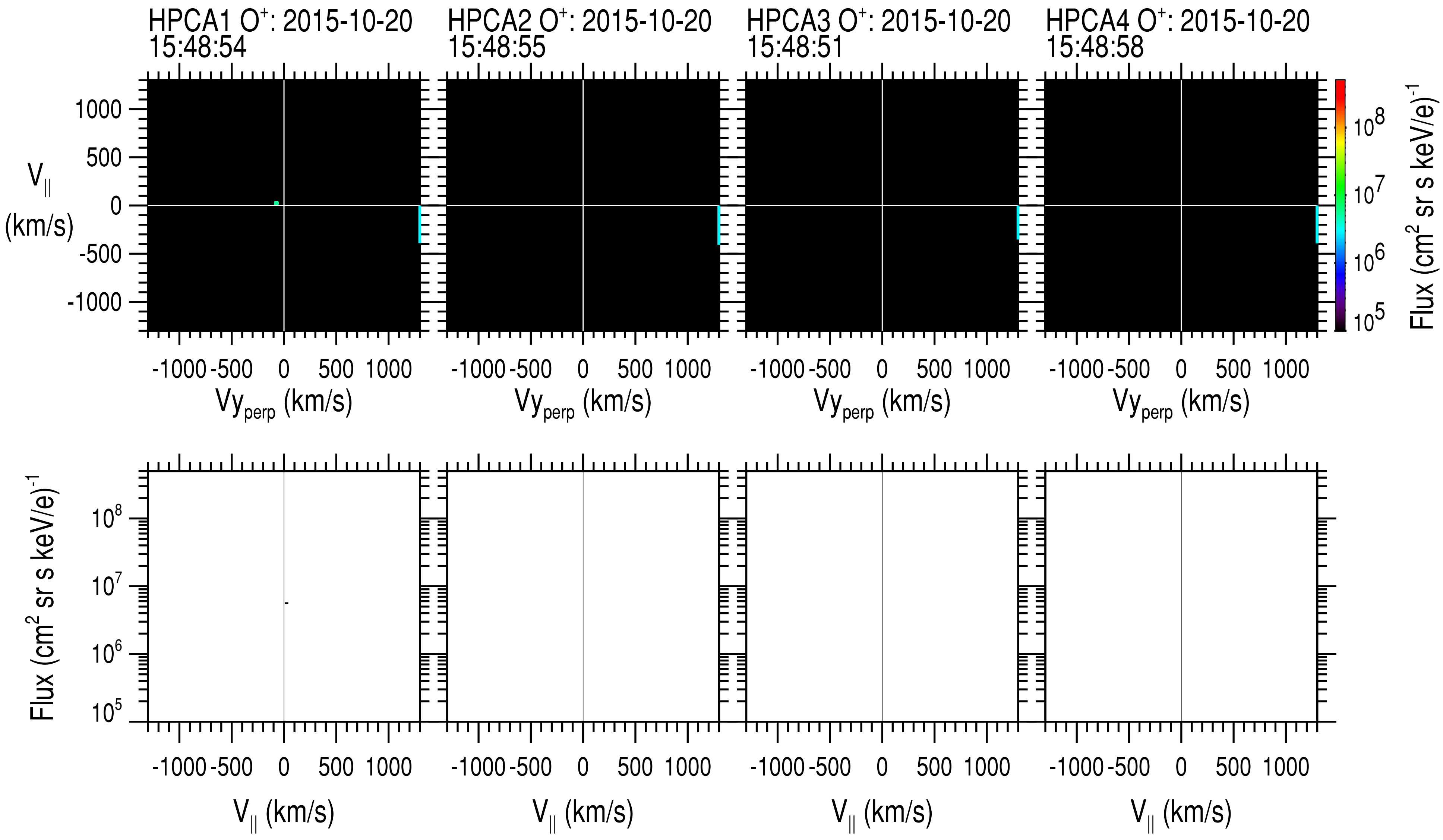
HPCA1 O+: 2015-10-20 15:48:54
HPCA2 O+: 2015-10-20 15:48:55
HPCA3 O+: 2015-10-20 15:48:51
HPCA4 O+: 2015-10-20 15:48:58
V|| (km/s)
Vyperp (km/s)
Flux (cm2 sr s keV/e)-1
-1000 -500 0 500 1000
10^5 10^6 10^7 10^8

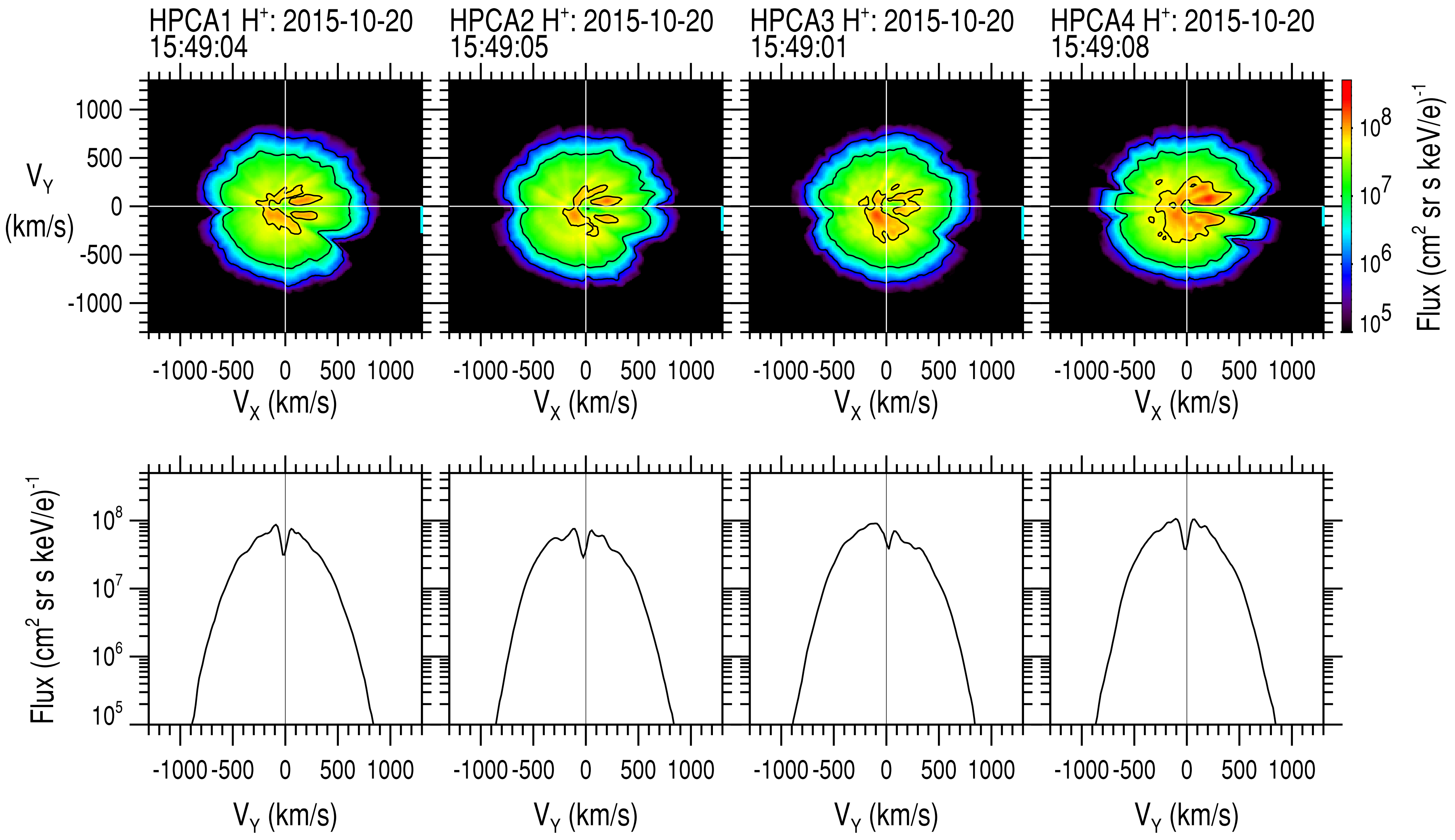
HPCA1 H+: 2015-10-20 15:49:04
HPCA2 H+: 2015-10-20 15:49:05
HPCA3 H+: 2015-10-20 15:49:01
HPCA4 H+: 2015-10-20 15:49:08
VY (km/s)
VX (km/s)
Flux (cm2 sr s keV/e)-1
VY (km/s)

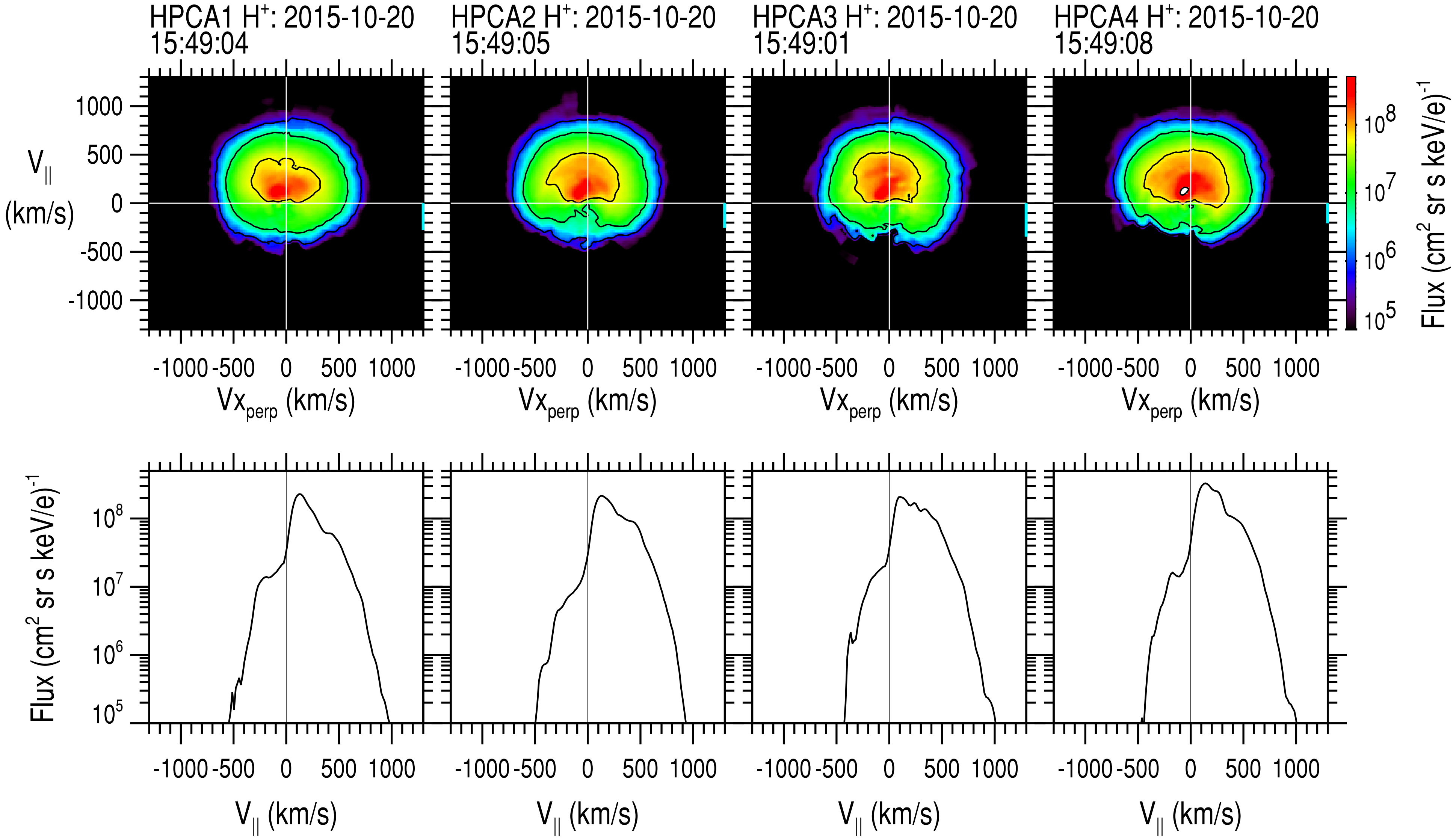
HPCA1 H+: 2015-10-20 15:49:04
HPCA2 H+: 2015-10-20 15:49:05
HPCA3 H+: 2015-10-20 15:49:01
HPCA4 H+: 2015-10-20 15:49:08
V|| (km/s)
Vxperp (km/s)
Flux (cm2 sr s keV/e)-1

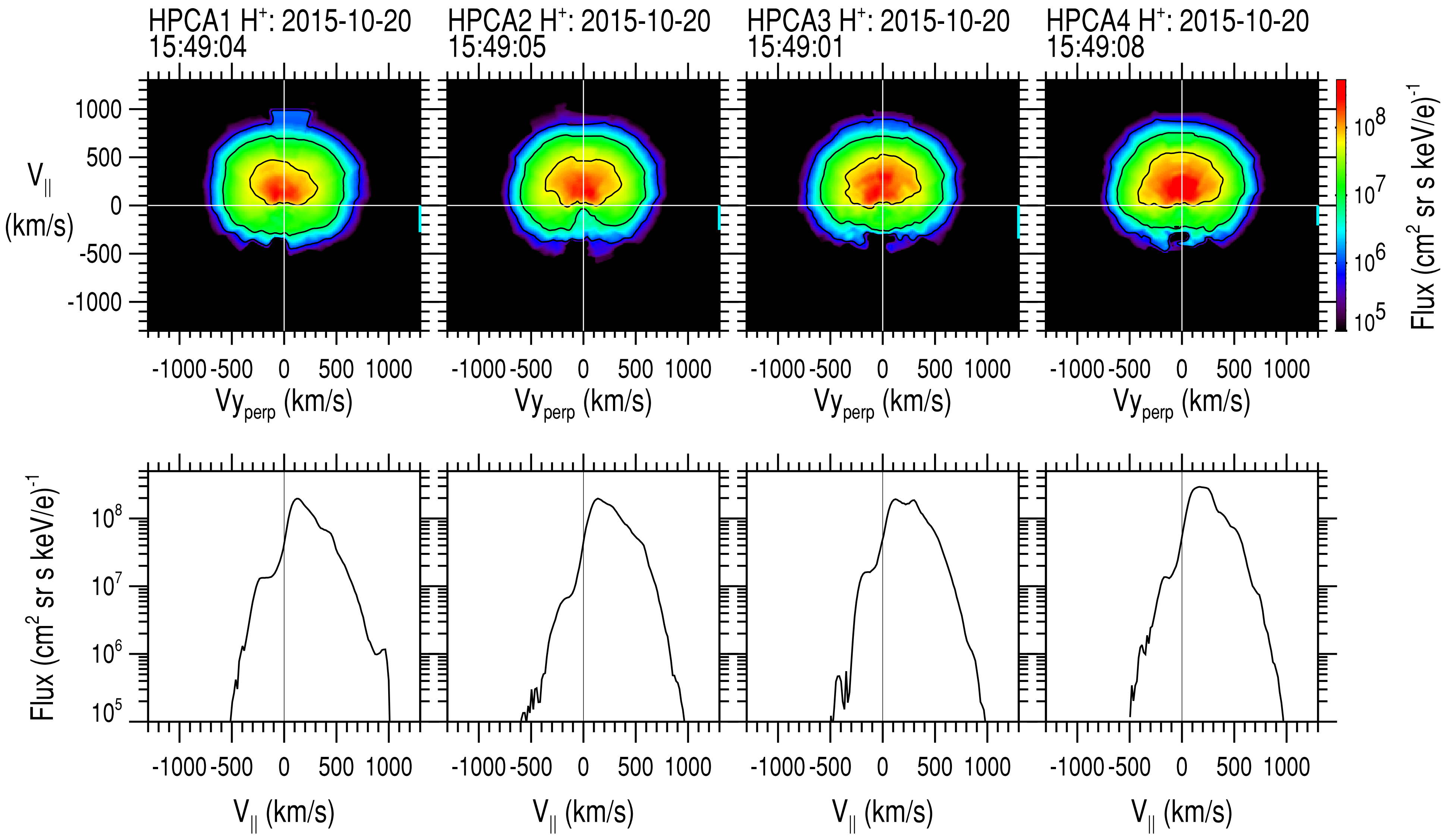
HPCA1 H+: 2015-10-20
15:49:04
HPCA2 H+: 2015-10-20
15:49:05
HPCA3 H+: 2015-10-20
15:49:01
HPCA4 H+: 2015-10-20
15:49:08
V|| (km/s)
Vyperp (km/s)
Flux (cm2 sr s keV/e)-1
V|| (km/s)

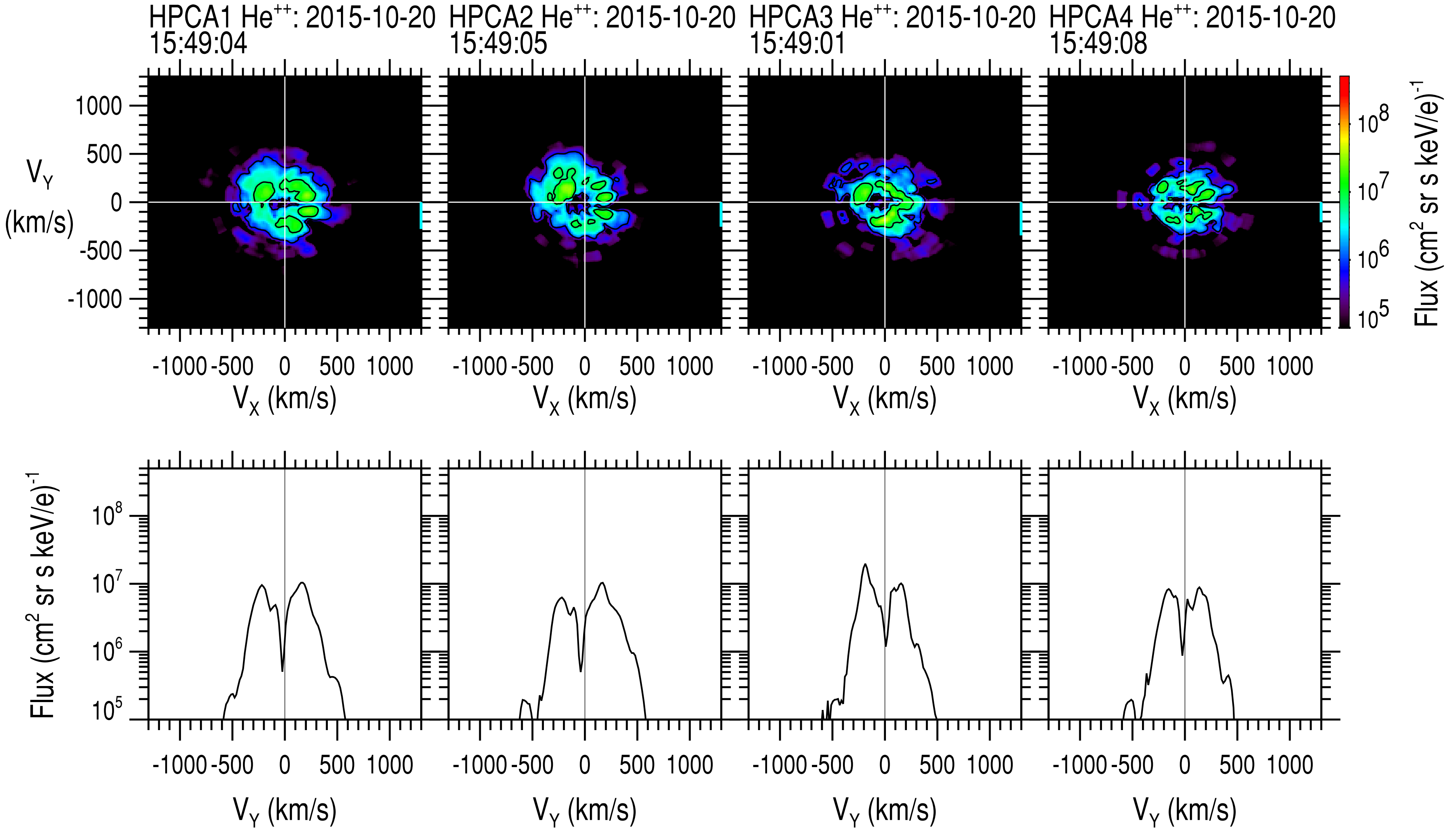
HPCA1 He++: 2015-10-20
15:49:04
HPCA2 He++: 2015-10-20
15:49:05
HPCA3 He++: 2015-10-20
15:49:01
HPCA4 He++: 2015-10-20
15:49:08
VY (km/s)
VX (km/s)
Flux (cm2 sr s keV/e)-1
VY (km/s)

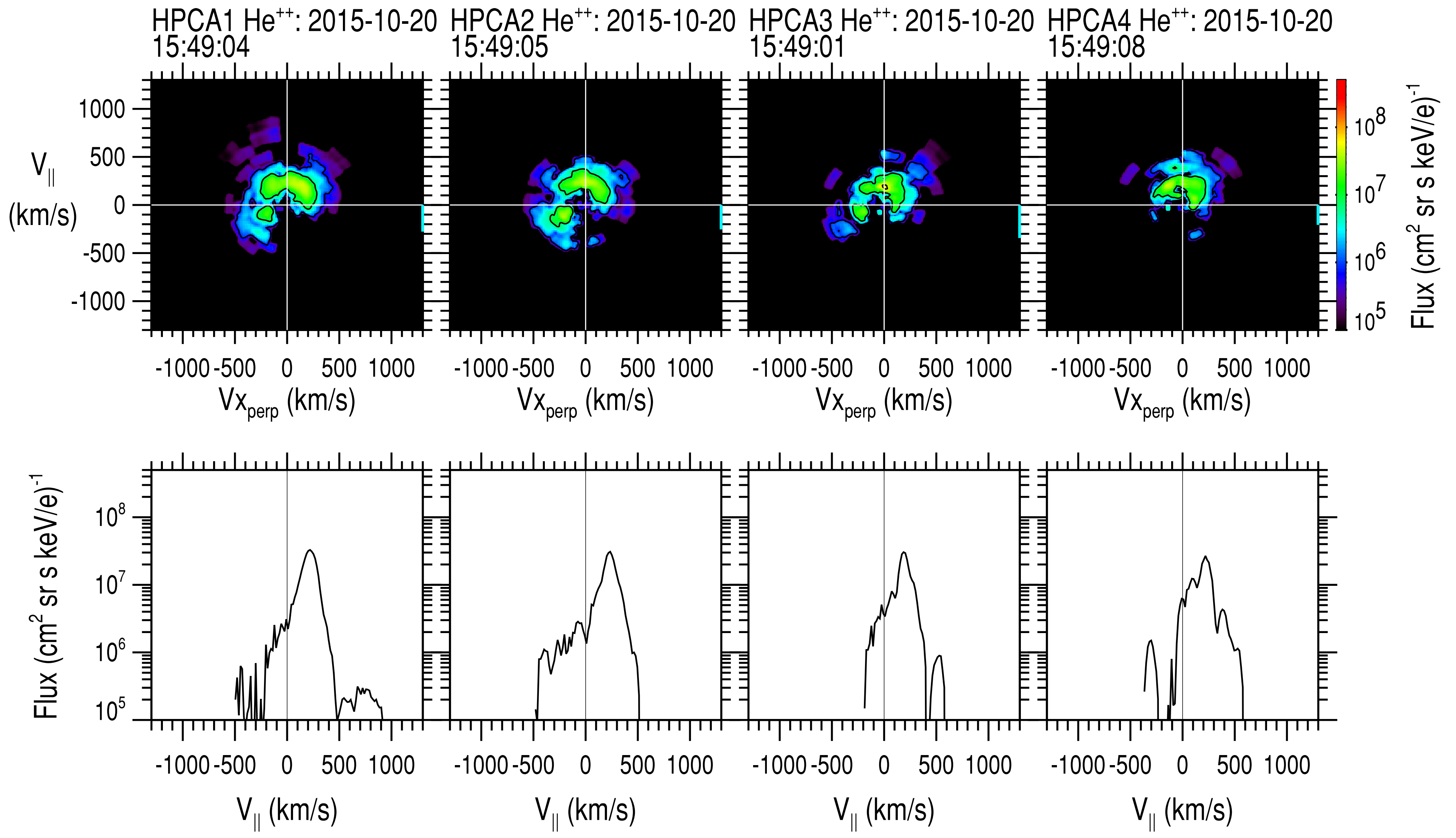
HPCA1 He++: 2015-10-20 15:49:04
HPCA2 He++: 2015-10-20 15:49:05
HPCA3 He++: 2015-10-20 15:49:01
HPCA4 He++: 2015-10-20 15:49:08
V|| (km/s)
Vxperp (km/s)
Flux (cm2 sr s keV/e)-1
-1000 -500 0 500 1000
10^5 10^6 10^7 10^8

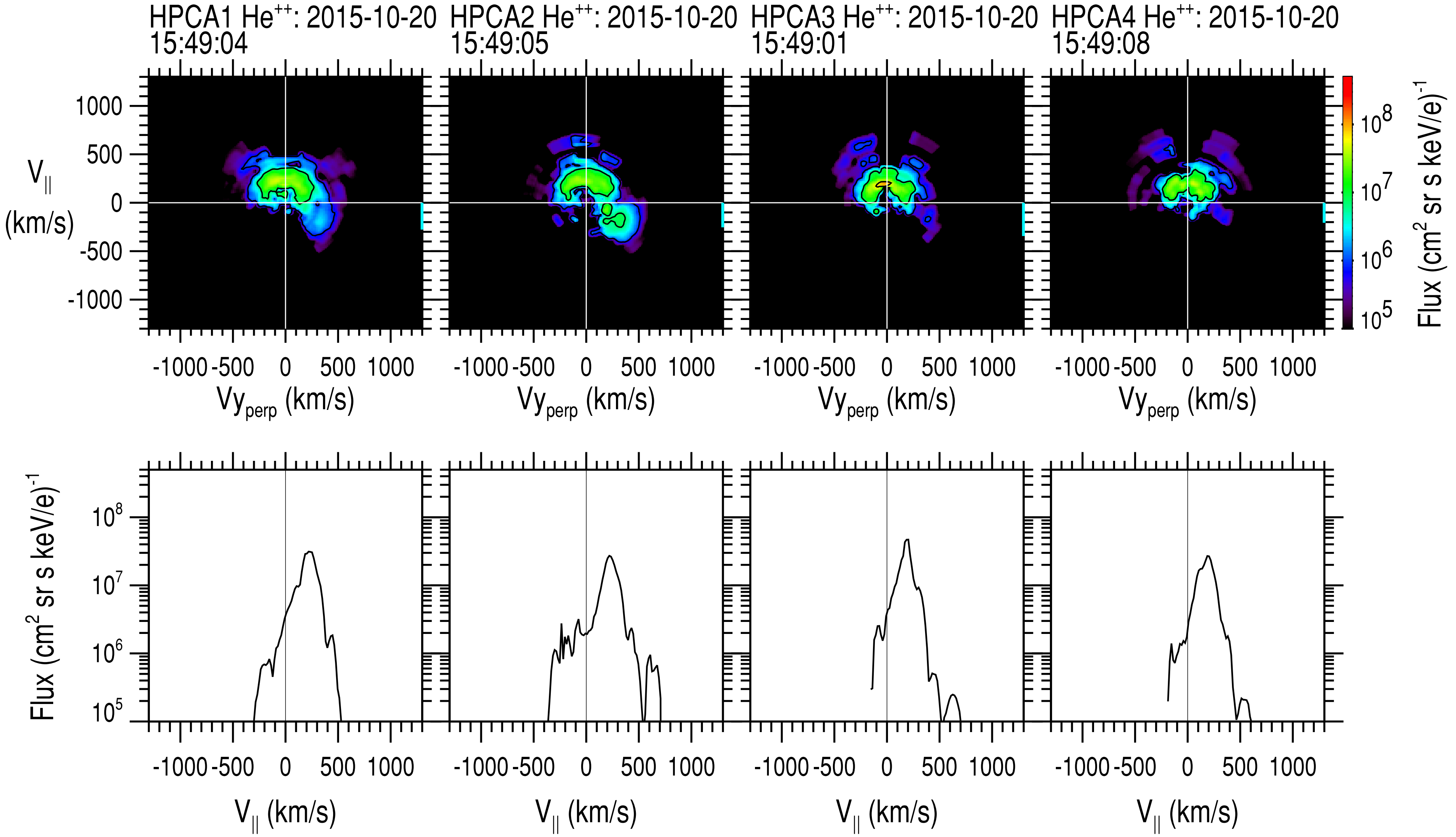

HPCA1 He++: 2015-10-20
15:49:04
HPCA2 He++: 2015-10-20
15:49:05
HPCA3 He++: 2015-10-20
15:49:01
HPCA4 He++: 2015-10-20
15:49:08
V|| (km/s)
Vyperp (km/s)
Flux (cm2 sr s keV/e)-1
V|| (km/s)

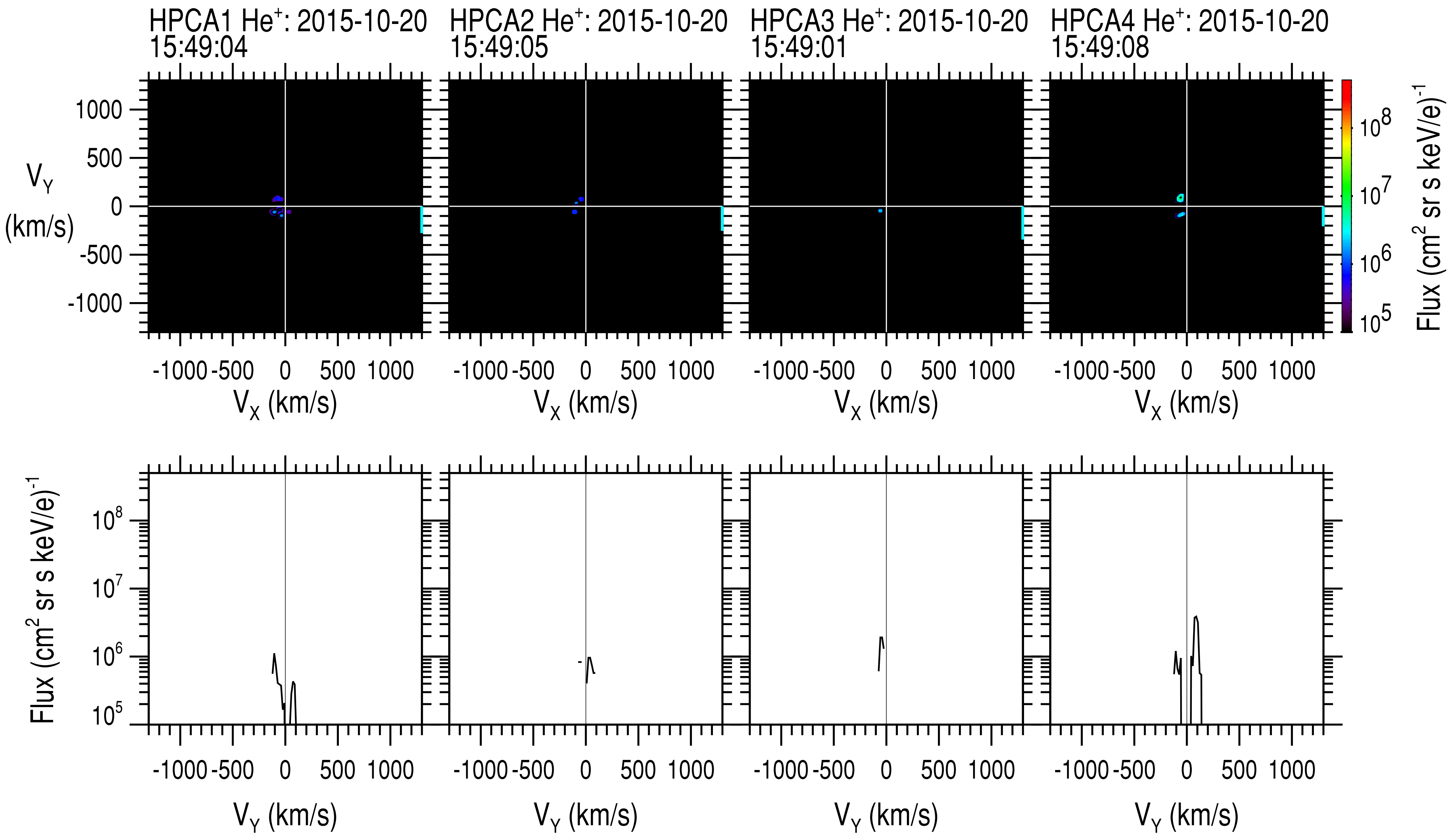

HPCA1 He+: 2015-10-20 15:49:04
HPCA2 He+: 2015-10-20 15:49:05
HPCA3 He+: 2015-10-20 15:49:01
HPCA4 He+: 2015-10-20 15:49:08
V_Y (km/s)
V_X (km/s)
Flux (cm^2 sr s keV/e)^-1
-1000 -500 0 500 1000
10^5 10^6 10^7 10^8

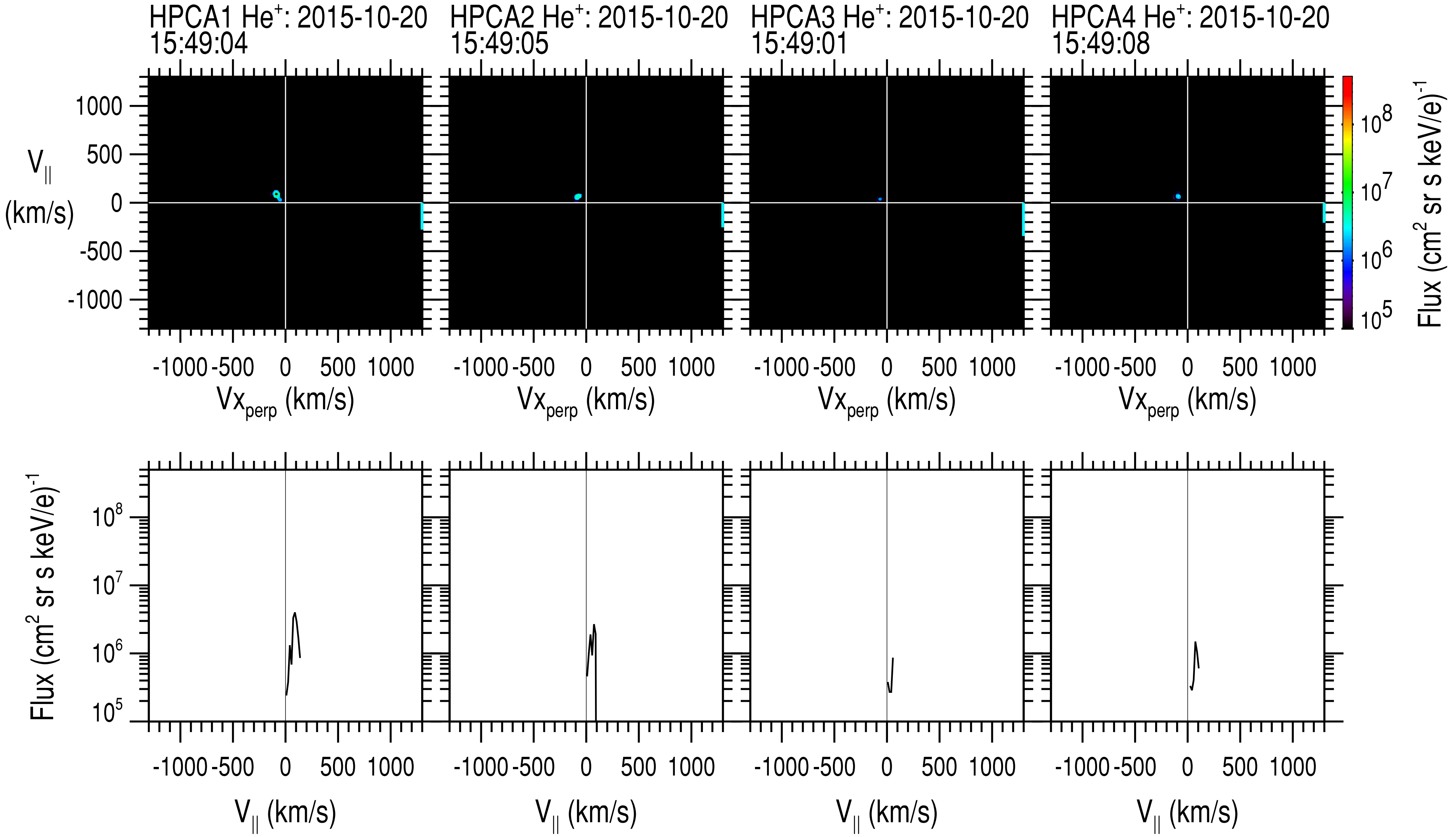
HPCA1 He+: 2015-10-20 15:49:04
HPCA2 He+: 2015-10-20 15:49:05
HPCA3 He+: 2015-10-20 15:49:01
HPCA4 He+: 2015-10-20 15:49:08
V|| (km/s)
Vxperp (km/s)
Flux (cm2 sr s keV/e)-1
V|| (km/s)

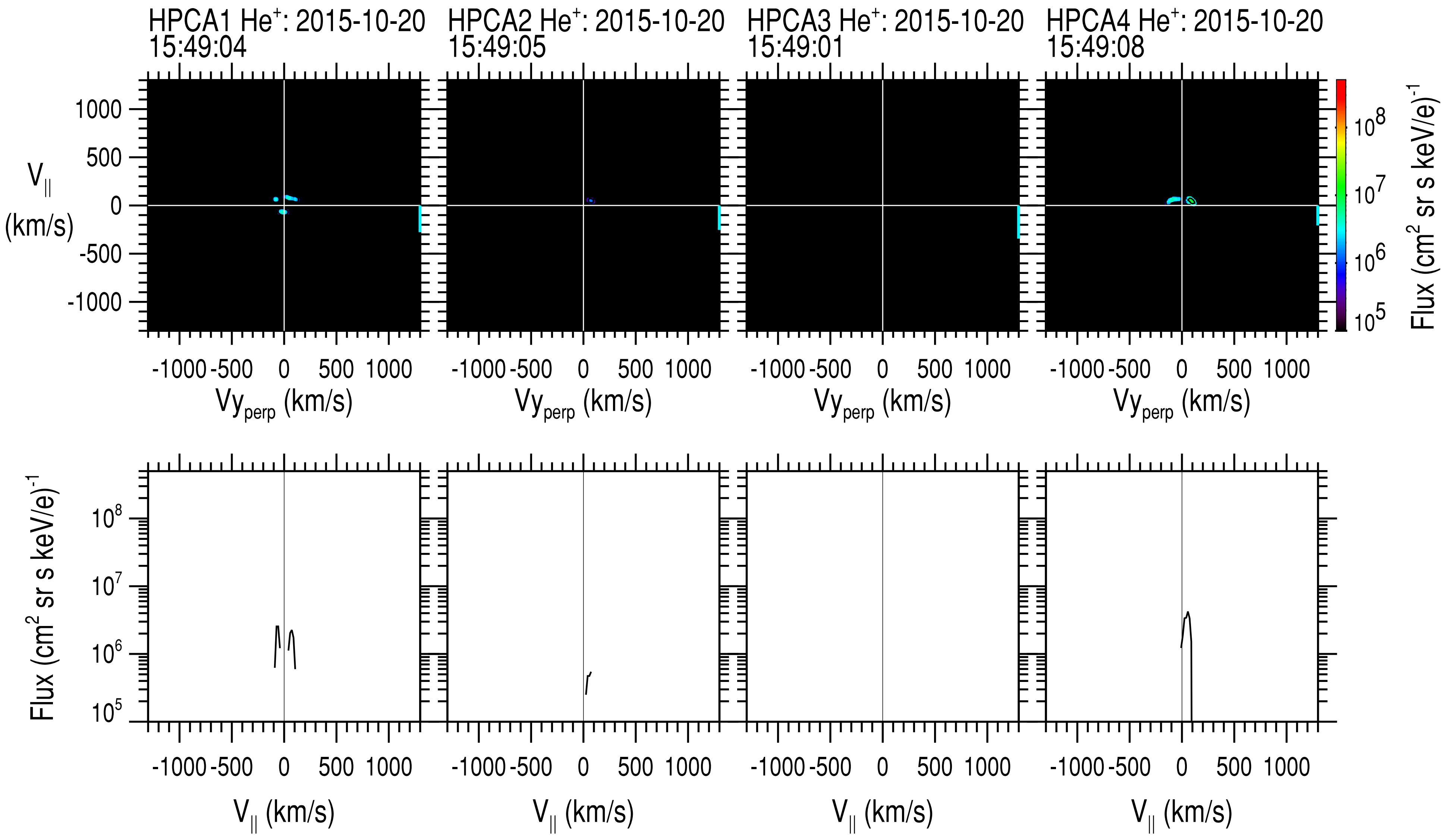

HPCA1 He+: 2015-10-20 15:49:04
HPCA2 He+: 2015-10-20 15:49:05
HPCA3 He+: 2015-10-20 15:49:01
HPCA4 He+: 2015-10-20 15:49:08
V|| (km/s)
Vyperp (km/s)
Flux (cm2 sr s keV/e)-1

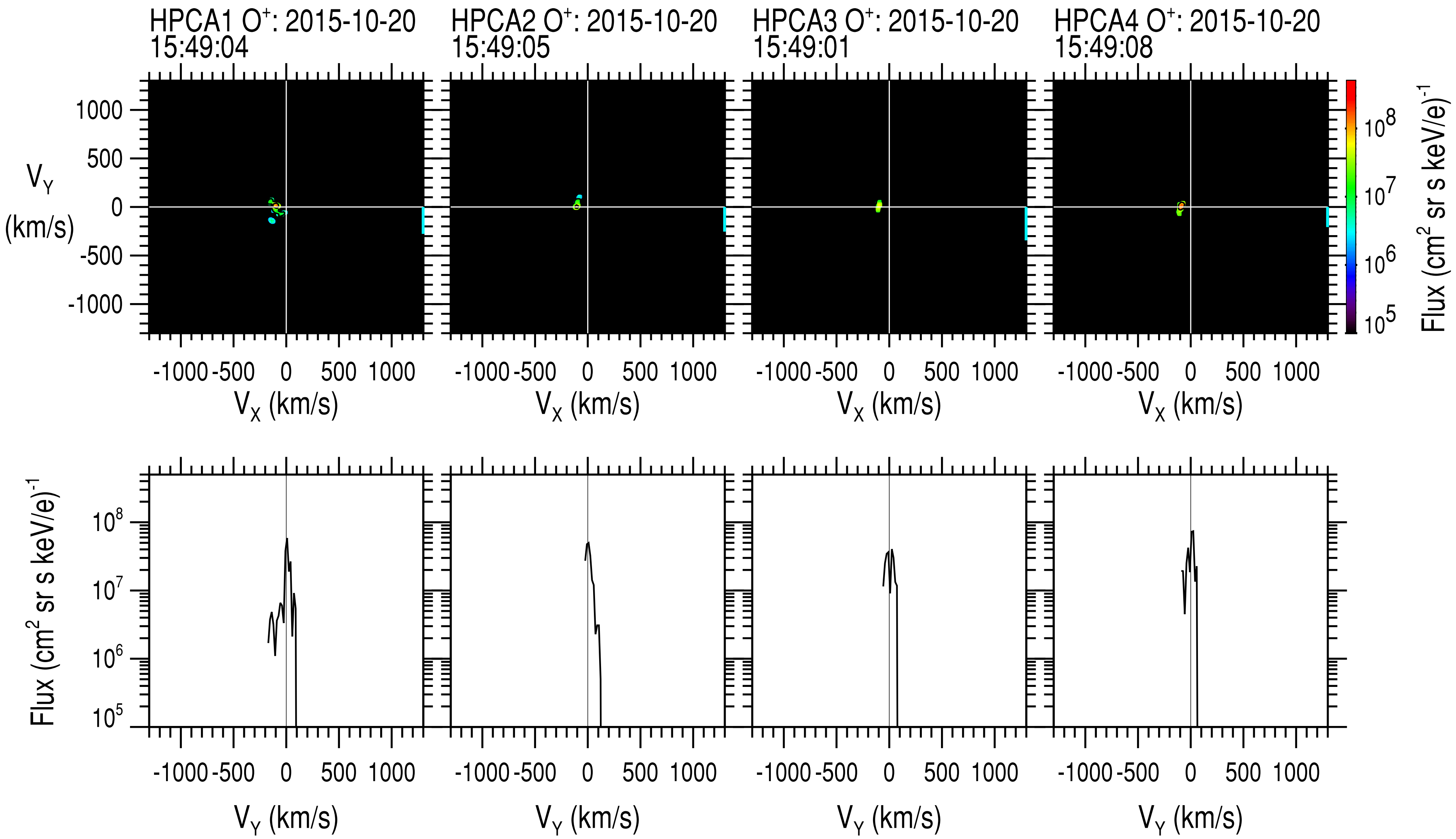

HPCA1 O+: 2015-10-20 15:49:04
HPCA2 O+: 2015-10-20 15:49:05
HPCA3 O+: 2015-10-20 15:49:01
HPCA4 O+: 2015-10-20 15:49:08
VY (km/s)
VX (km/s)
Flux (cm2 sr s keV/e)-1

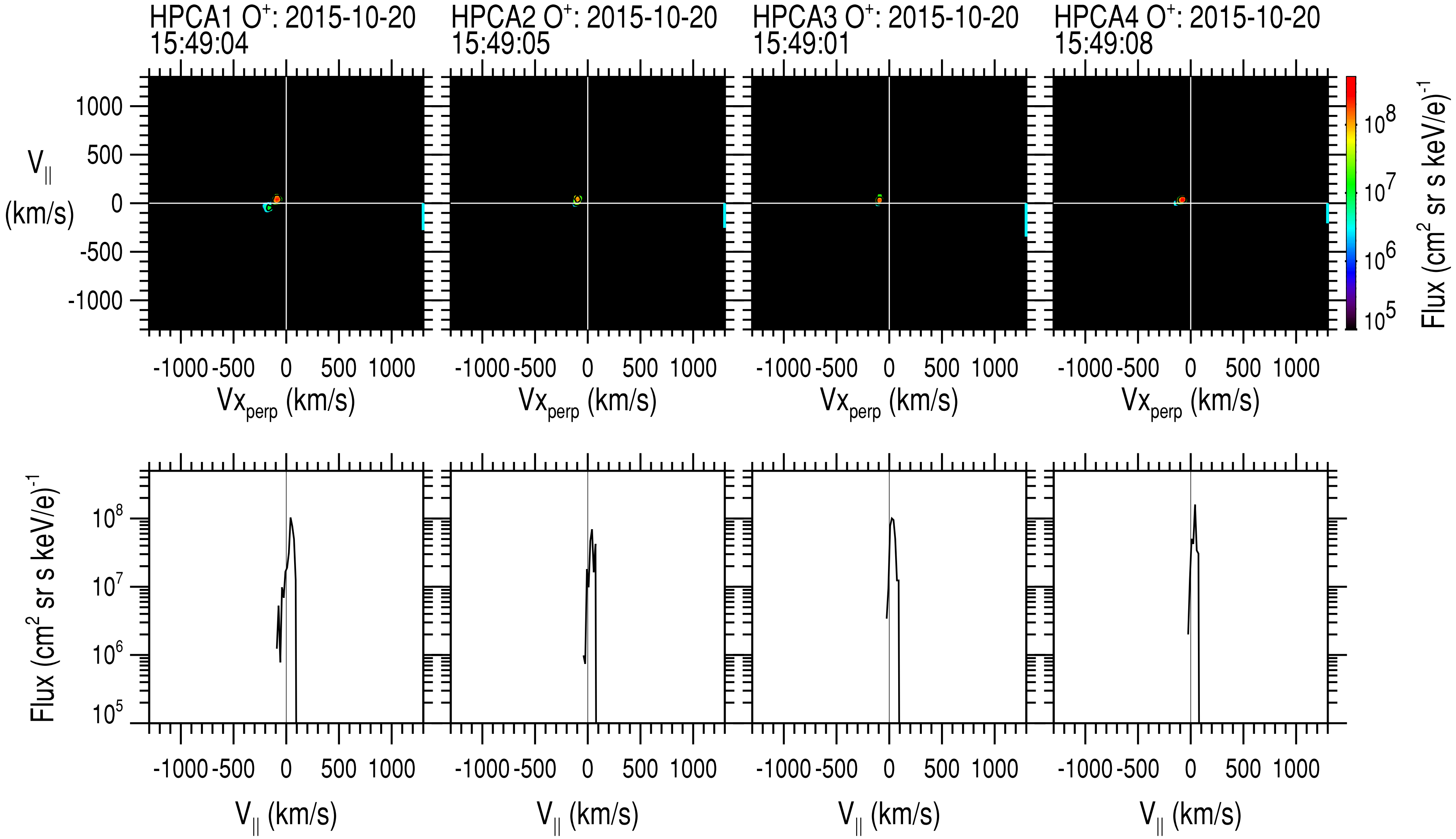
HPCA1 O+: 2015-10-20 15:49:04
HPCA2 O+: 2015-10-20 15:49:05
HPCA3 O+: 2015-10-20 15:49:01
HPCA4 O+: 2015-10-20 15:49:08
V|| (km/s)
Vxperp (km/s)
Flux (cm2 sr s keV/e)-1
V|| (km/s)

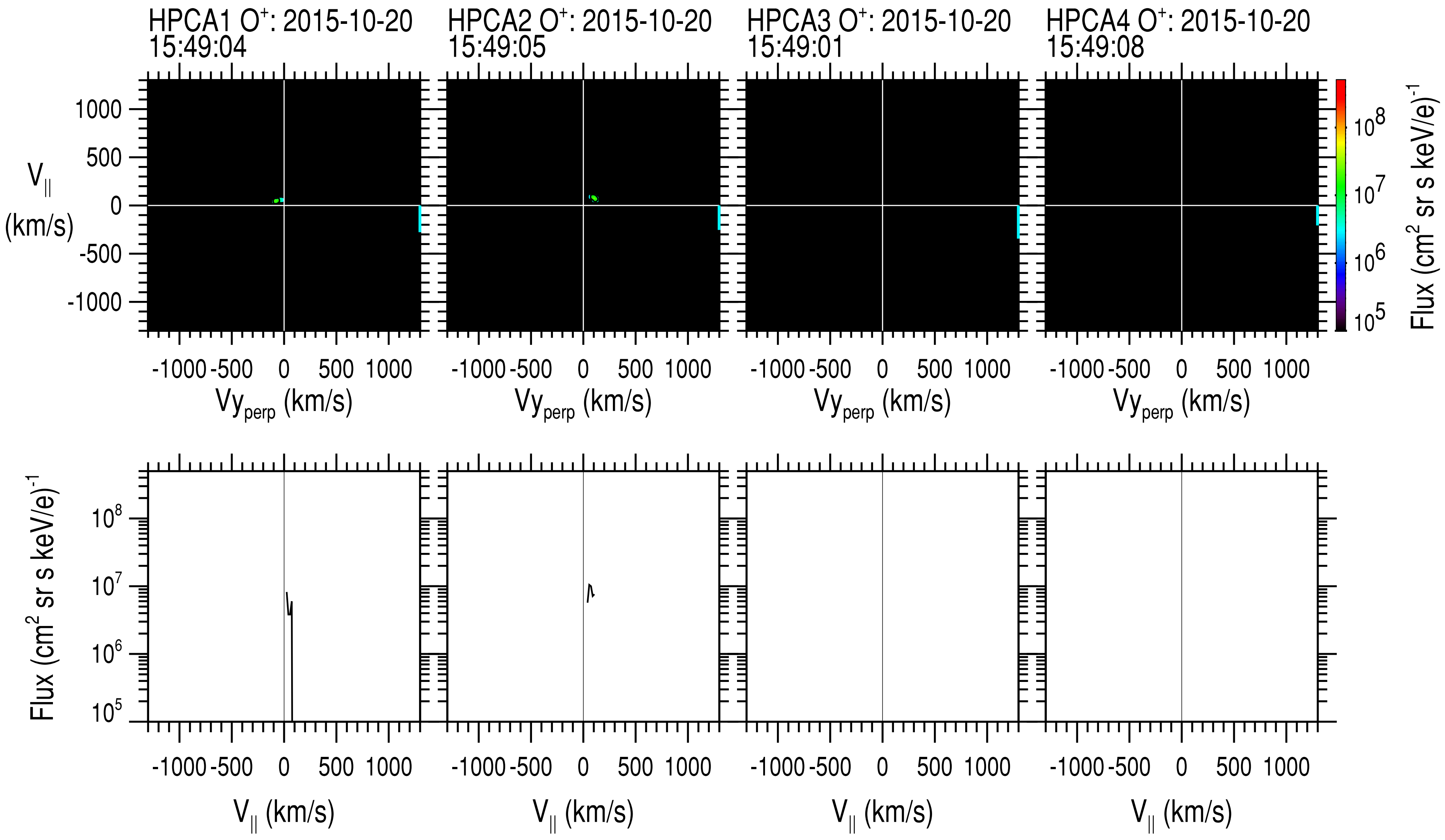
HPCA1 O+: 2015-10-20
15:49:04
HPCA2 O+: 2015-10-20
15:49:05
HPCA3 O+: 2015-10-20
15:49:01
HPCA4 O+: 2015-10-20
15:49:08
V|| (km/s)
Vyperp (km/s)
Flux (cm2 sr s keV/e)-1
-1000
-500
0
500
1000
10^5
10^6
10^7
10^8

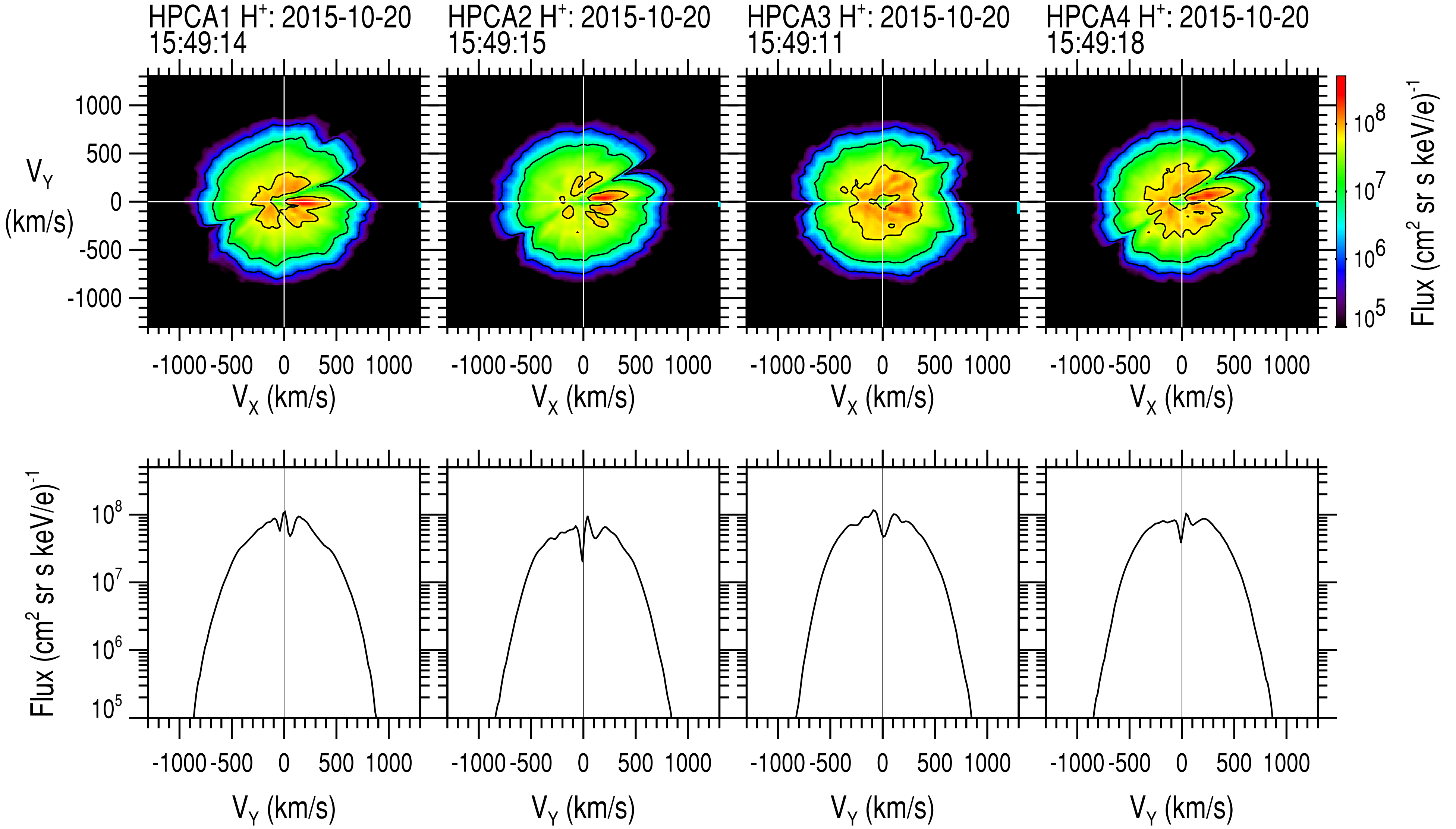
HPCA1 H+: 2015-10-20 15:49:14
HPCA2 H+: 2015-10-20 15:49:15
HPCA3 H+: 2015-10-20 15:49:11
HPCA4 H+: 2015-10-20 15:49:18
VY (km/s)
VX (km/s)
Flux (cm2 sr s keV/e)-1
-1000 -500 0 500 1000
10^5 10^6 10^7 10^8

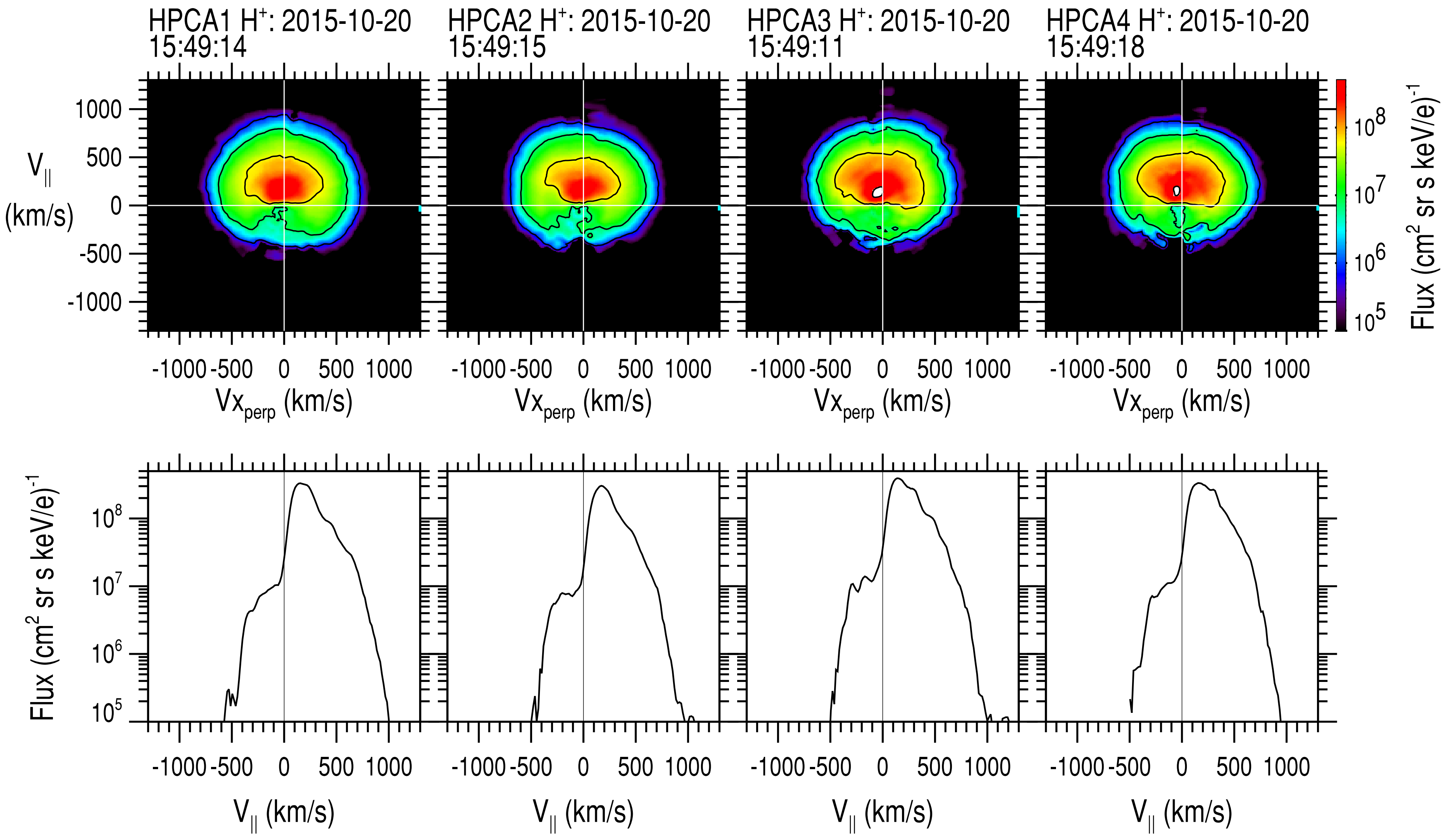
HPCA1 H+: 2015-10-20 15:49:14
HPCA2 H+: 2015-10-20 15:49:15
HPCA3 H+: 2015-10-20 15:49:11
HPCA4 H+: 2015-10-20 15:49:18
V|| (km/s)
Vxperp (km/s)
Flux (cm2 sr s keV/e)-1

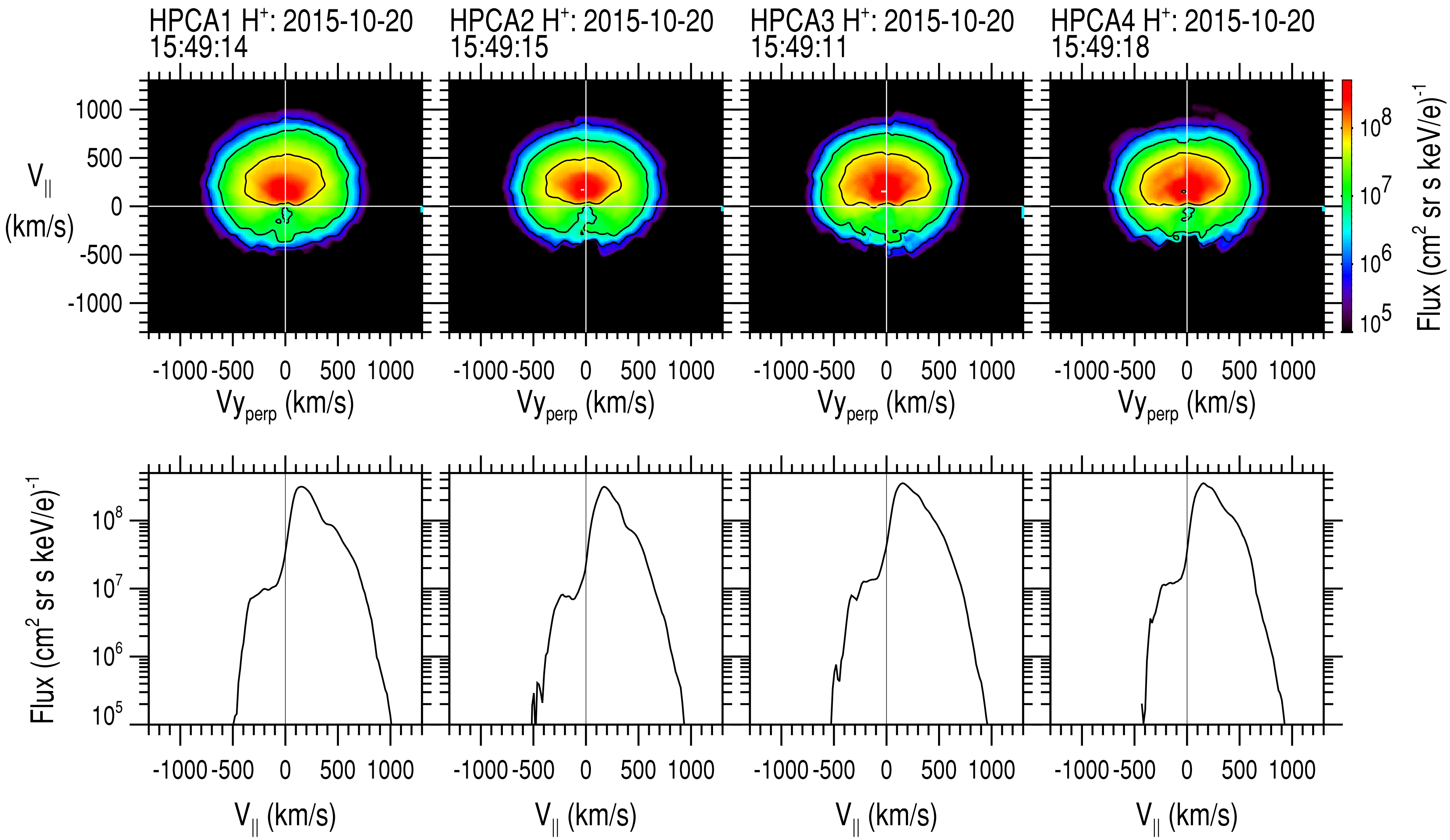
HPCA1 H+: 2015-10-20
15:49:14
HPCA2 H+: 2015-10-20
15:49:15
HPCA3 H+: 2015-10-20
15:49:11
HPCA4 H+: 2015-10-20
15:49:18
V||
(km/s)
Vyperp (km/s)
Flux (cm2 sr s keV/e)-1
V|| (km/s)

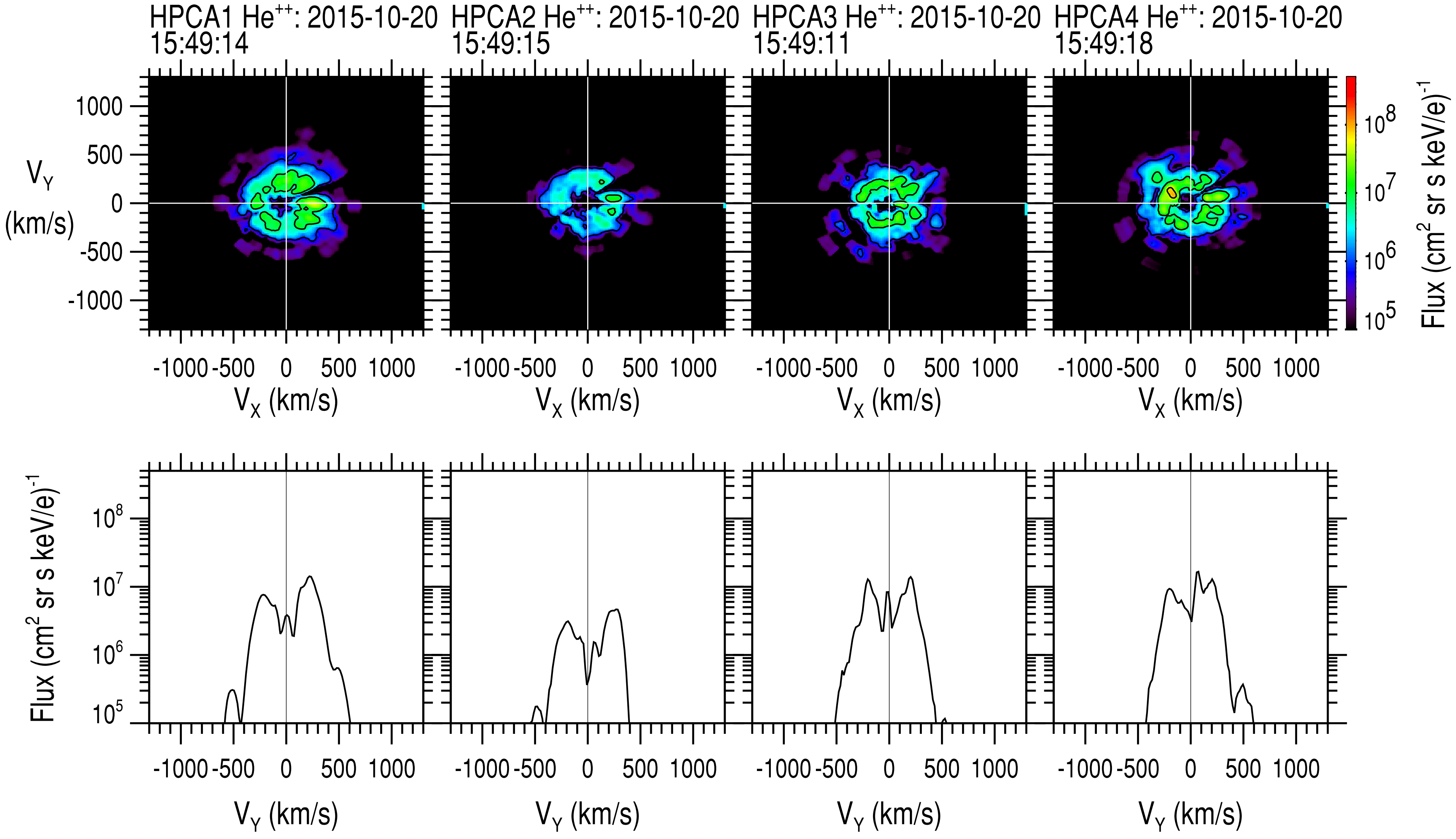
HPCA1 He++: 2015-10-20
15:49:14
HPCA2 He++: 2015-10-20
15:49:15
HPCA3 He++: 2015-10-20
15:49:11
HPCA4 He++: 2015-10-20
15:49:18
VY (km/s)
1000
500
0
-500
-1000
-1000 -500 0 500 1000
VX (km/s)
Flux (cm2 sr s keV/e)-1
105
106
107
108
VY (km/s)

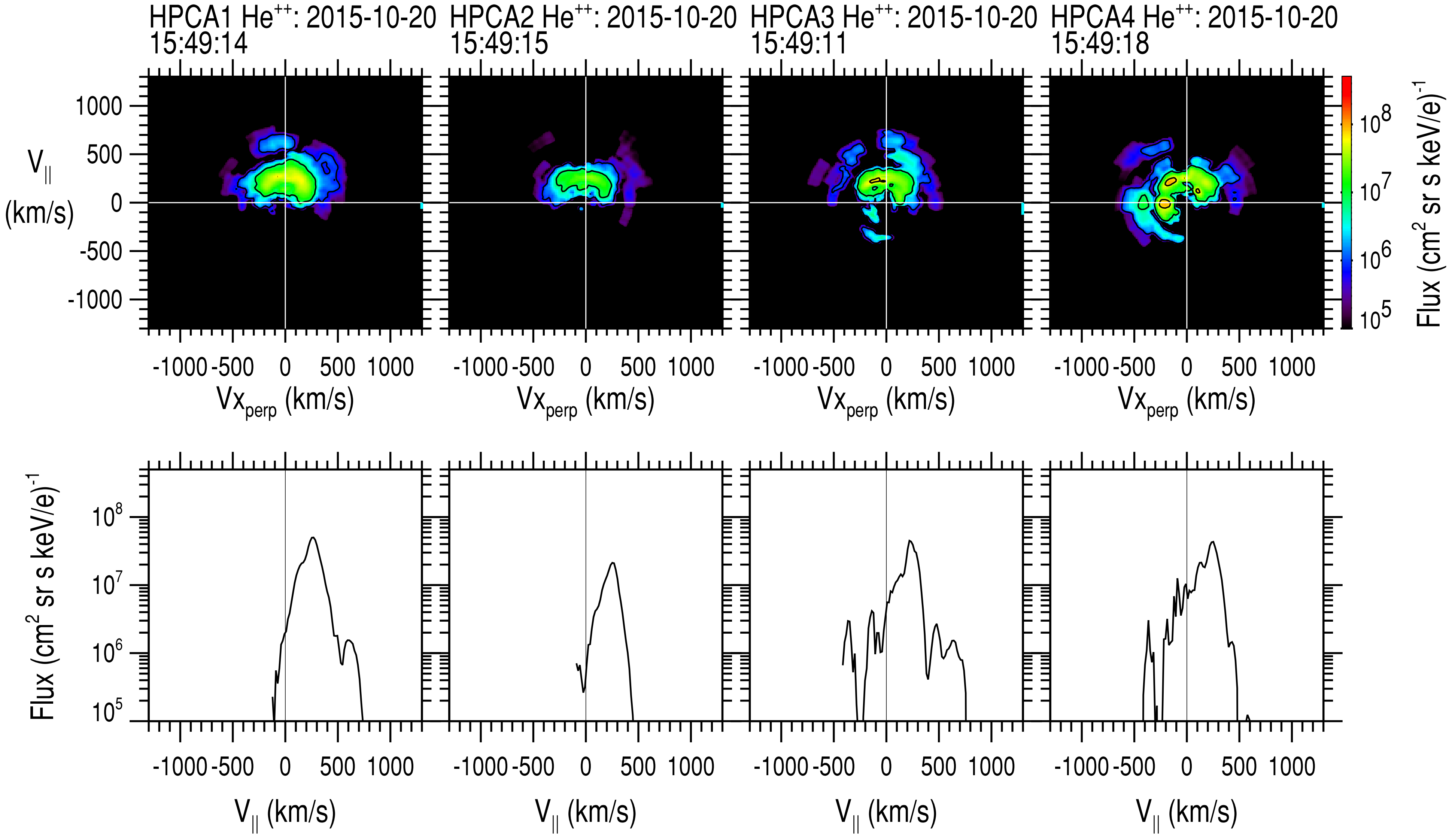

HPCA1 He++: 2015-10-20 15:49:14
HPCA2 He++: 2015-10-20 15:49:15
HPCA3 He++: 2015-10-20 15:49:11
HPCA4 He++: 2015-10-20 15:49:18
V|| (km/s)
Vxperp (km/s)
Flux (cm2 sr s keV/e)-1

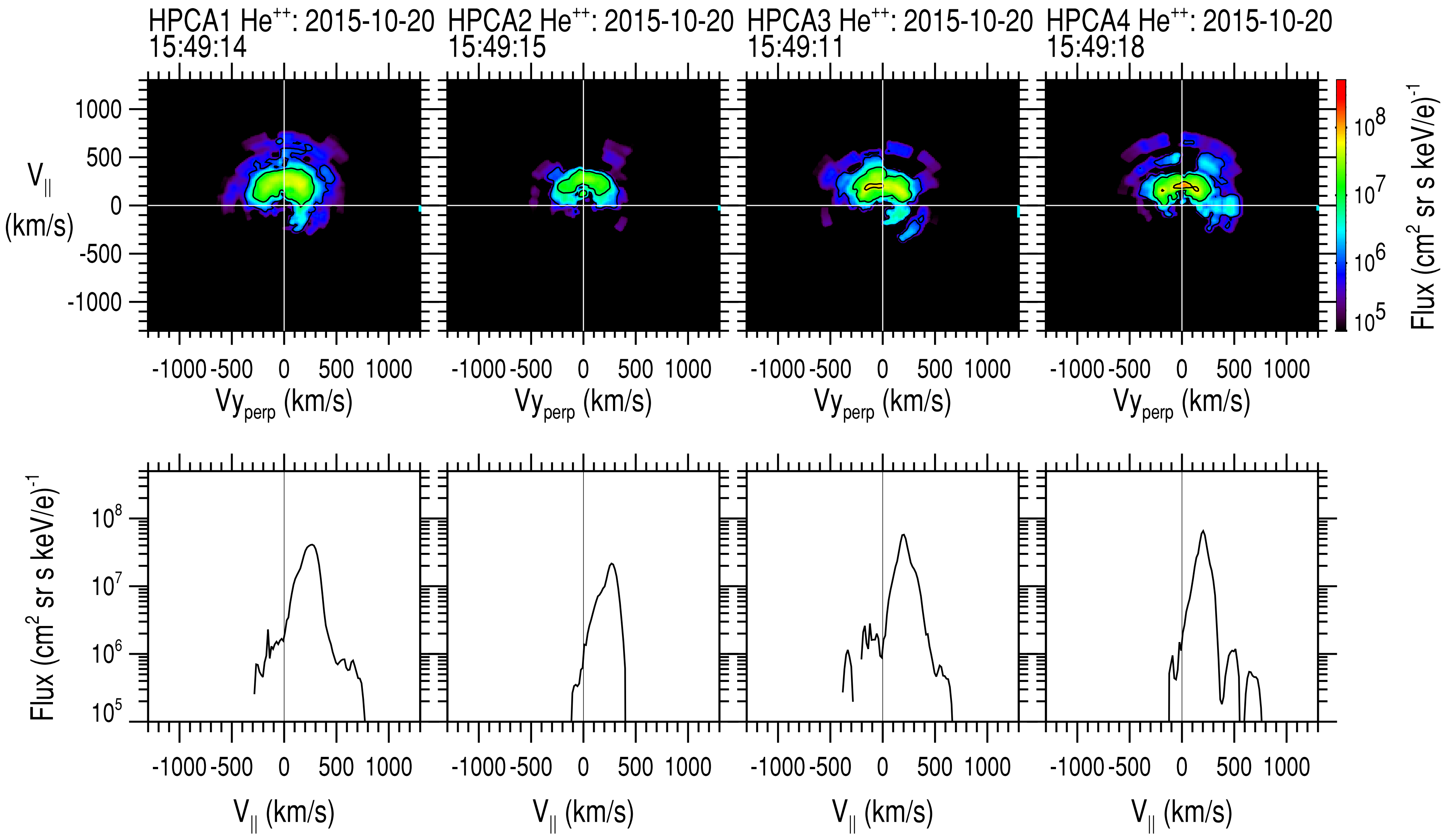
HPCA1 He++: 2015-10-20 15:49:14
HPCA2 He++: 2015-10-20 15:49:15
HPCA3 He++: 2015-10-20 15:49:11
HPCA4 He++: 2015-10-20 15:49:18
V|| (km/s)
Vyperp (km/s)
Flux (cm2 sr s keV/e)-1
V|| (km/s)

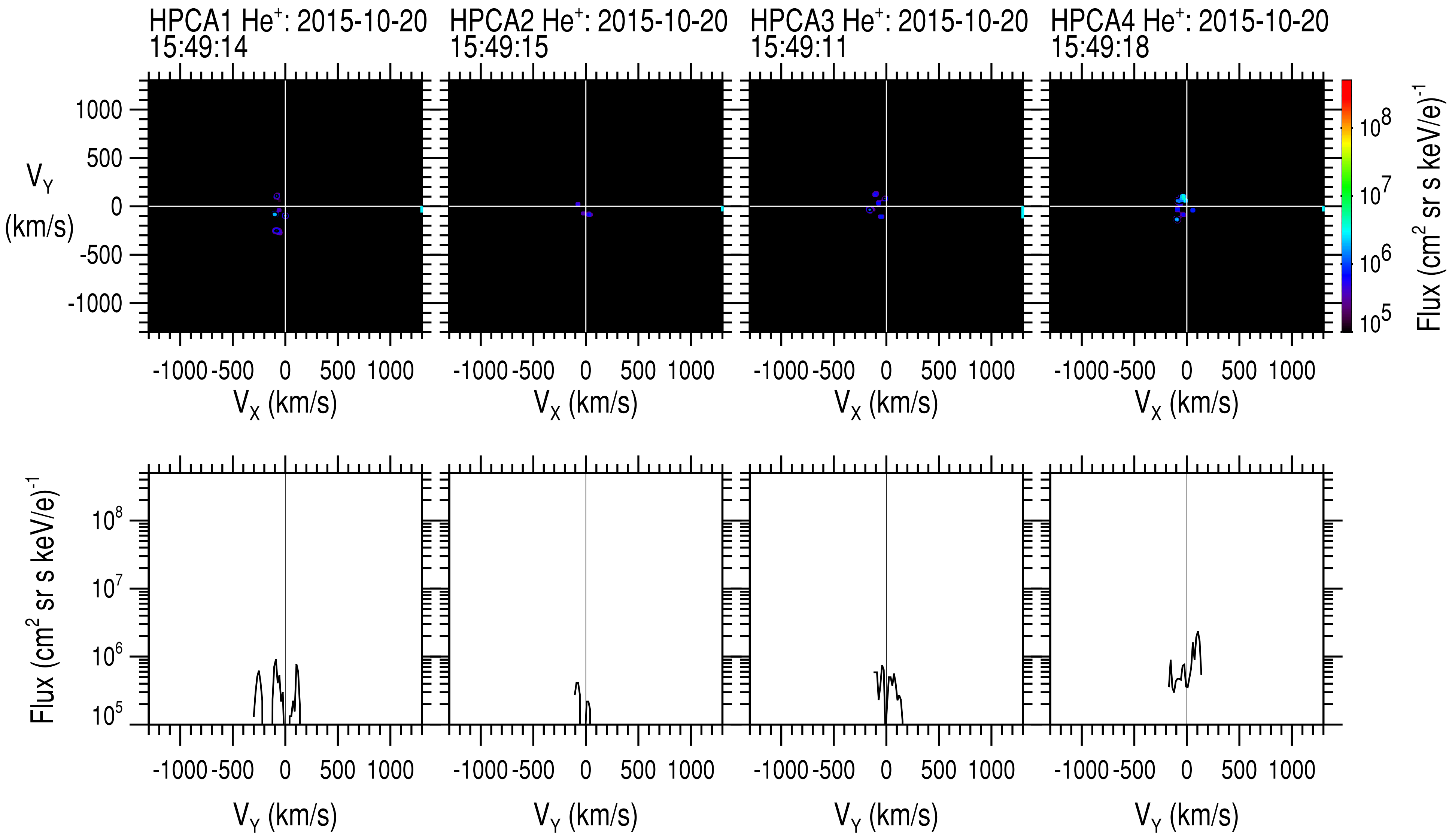
HPCA1 He+: 2015-10-20 15:49:14
HPCA2 He+: 2015-10-20 15:49:15
HPCA3 He+: 2015-10-20 15:49:11
HPCA4 He+: 2015-10-20 15:49:18
VY (km/s)
VX (km/s)
Flux (cm2 sr s keV/e)-1
-1000 -500 0 500 1000
10^5 10^6 10^7 10^8

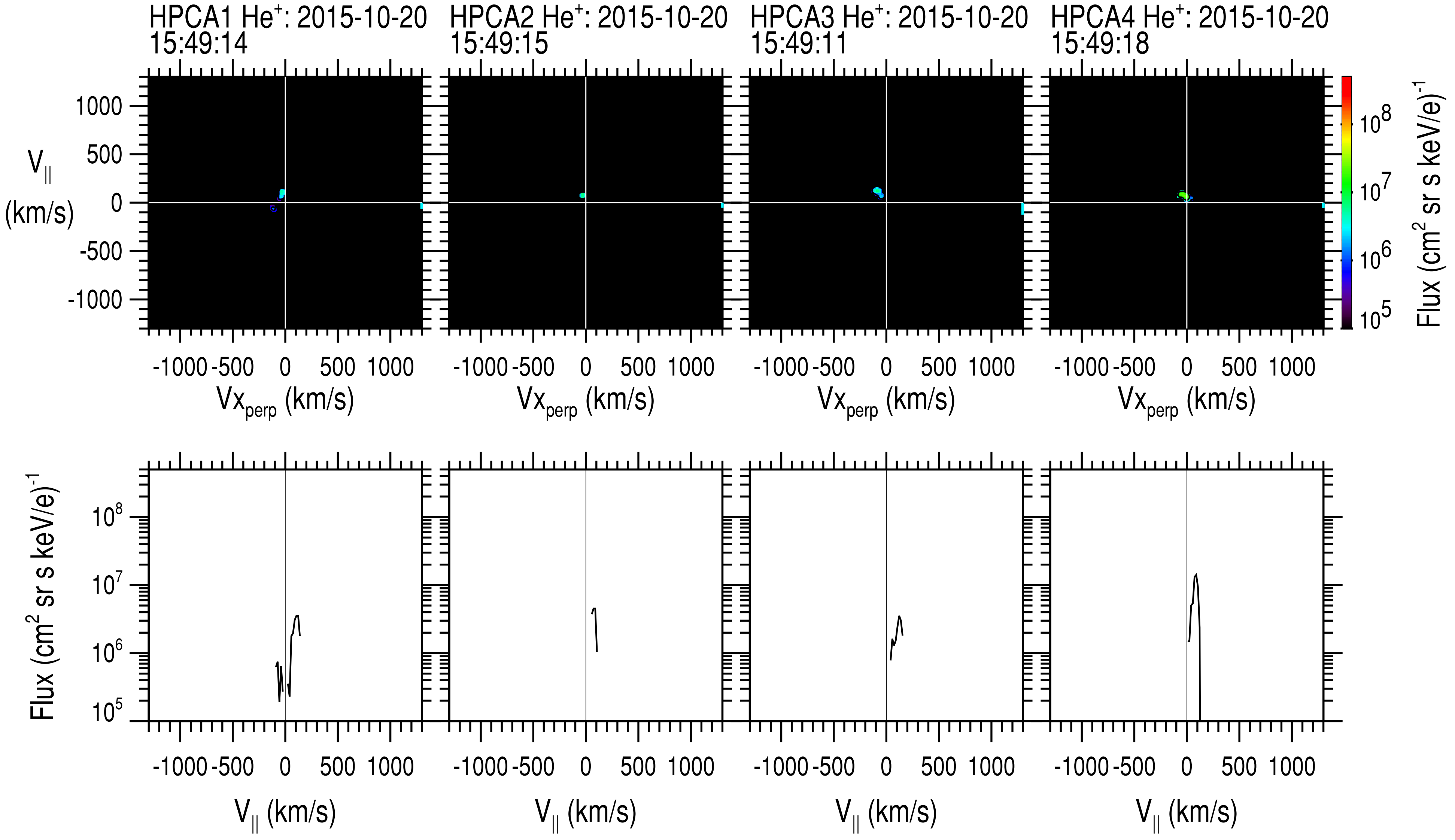
HPCA1 He+: 2015-10-20 15:49:14
HPCA2 He+: 2015-10-20 15:49:15
HPCA3 He+: 2015-10-20 15:49:11
HPCA4 He+: 2015-10-20 15:49:18
V|| (km/s)
Vxperp (km/s)
Flux (cm2 sr s keV/e)-1
-1000 -500 0 500 1000
10^5 10^6 10^7 10^8

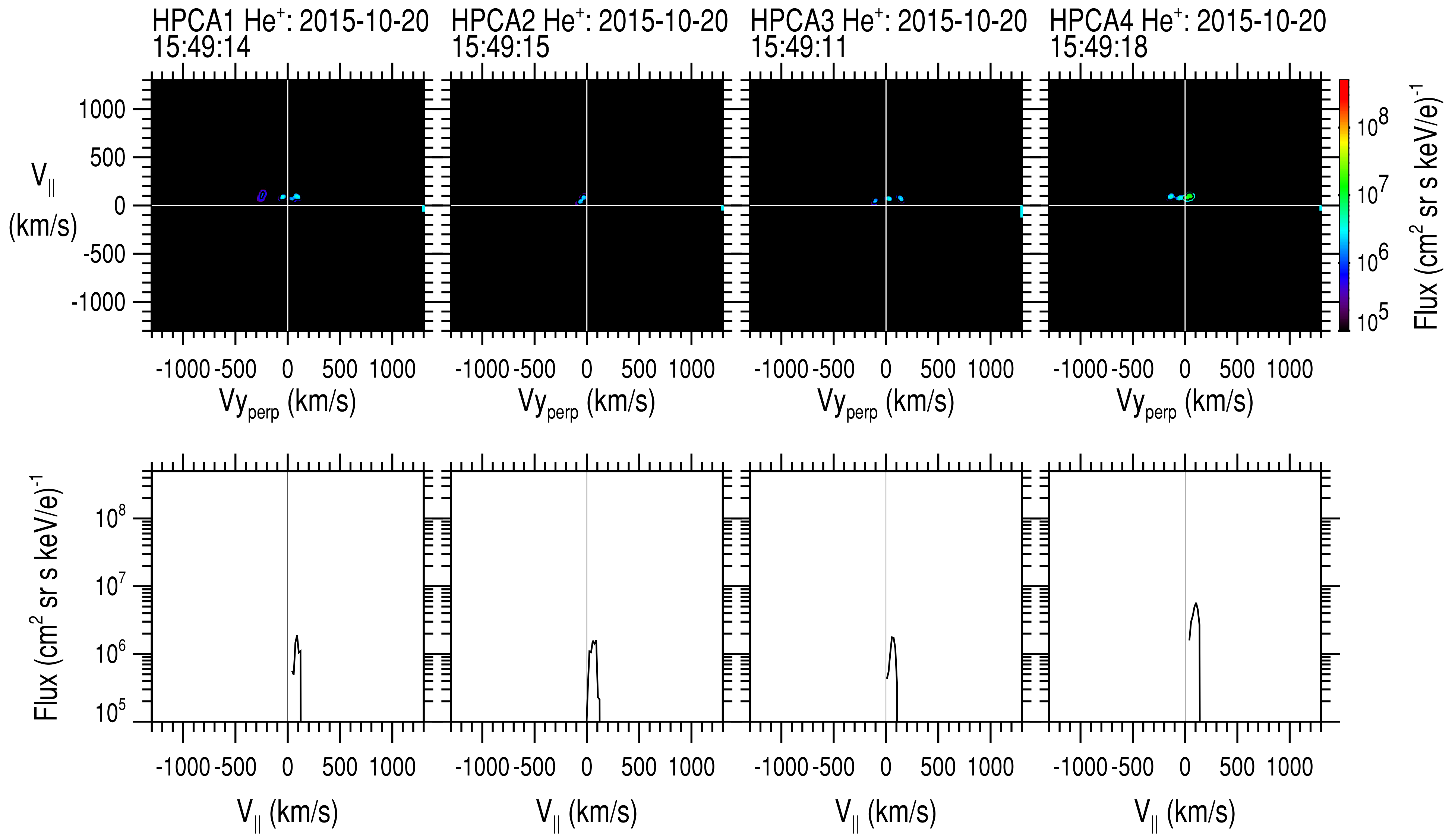

HPCA1 He+: 2015-10-20 15:49:14
HPCA2 He+: 2015-10-20 15:49:15
HPCA3 He+: 2015-10-20 15:49:11
HPCA4 He+: 2015-10-20 15:49:18
V|| (km/s)
Vyperp (km/s)
Flux (cm2 sr s keV/e)-1

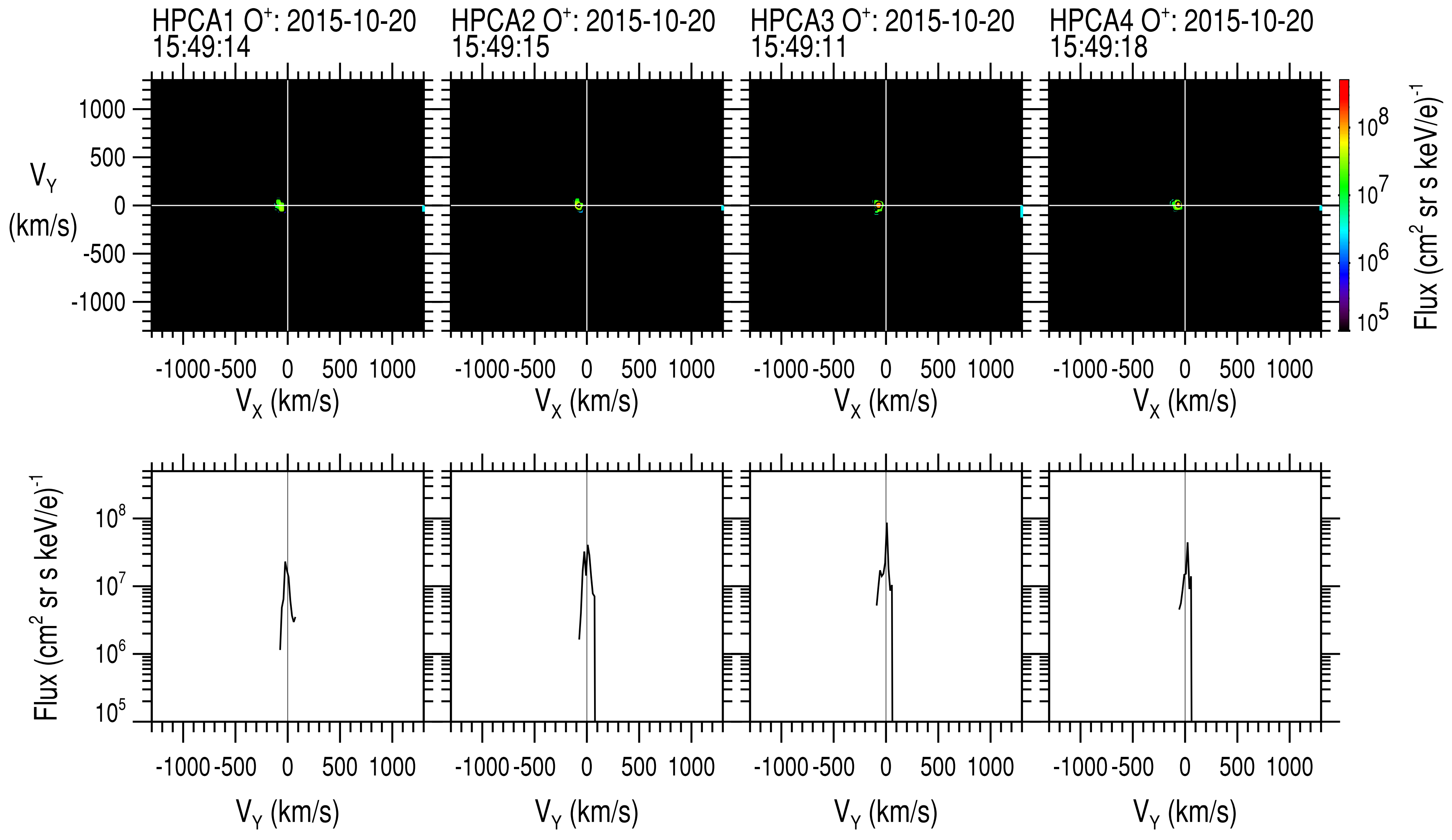

HPCA1 O+: 2015-10-20 15:49:14
HPCA2 O+: 2015-10-20 15:49:15
HPCA3 O+: 2015-10-20 15:49:11
HPCA4 O+: 2015-10-20 15:49:18
V_Y (km/s)
V_X (km/s)
Flux (cm^2 sr s keV/e)^-1

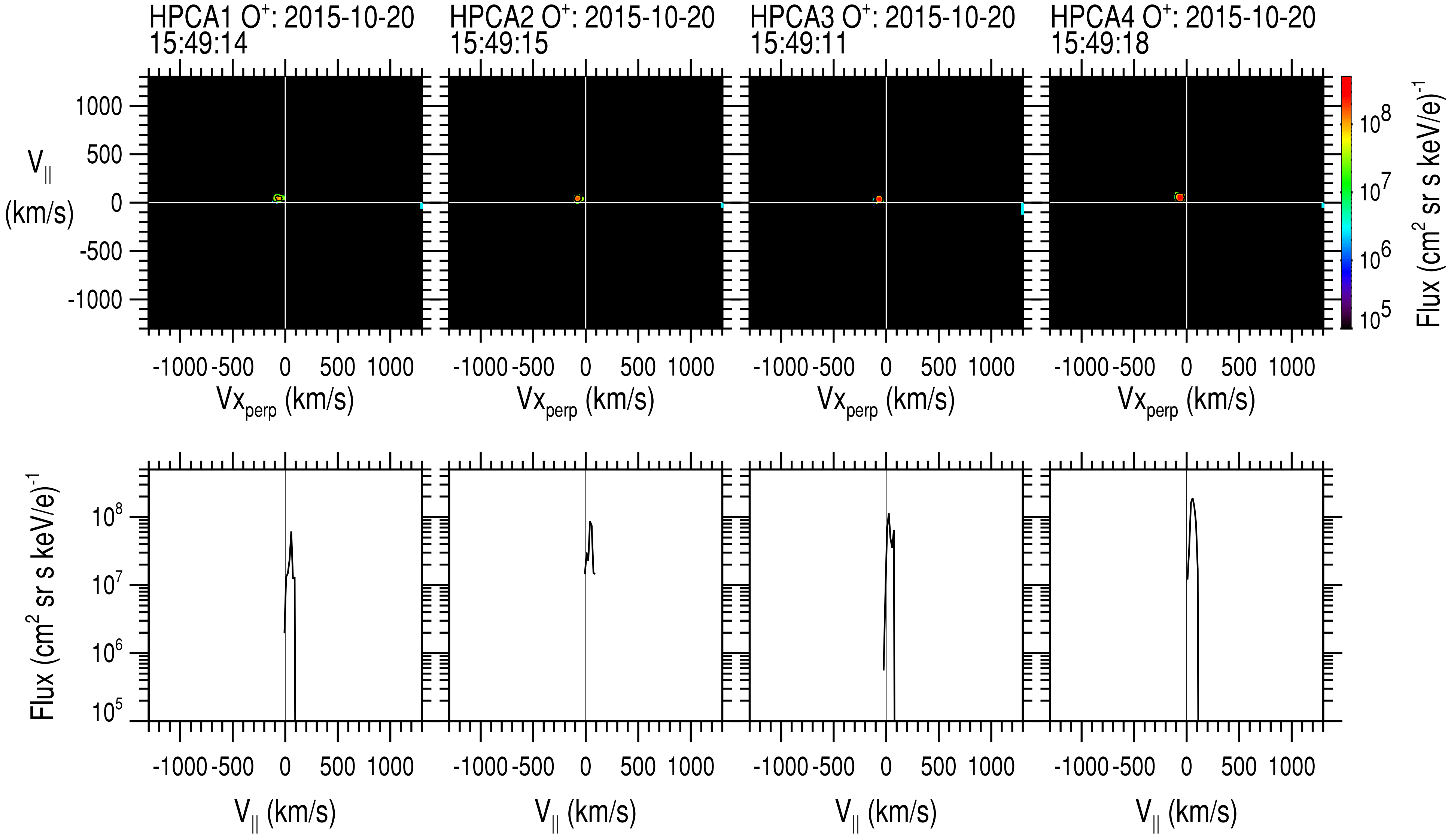
HPCA1 O+: 2015-10-20 15:49:14
HPCA2 O+: 2015-10-20 15:49:15
HPCA3 O+: 2015-10-20 15:49:11
HPCA4 O+: 2015-10-20 15:49:18
V|| (km/s)
Vxperp (km/s)
Flux (cm2 sr s keV/e)-1
1000
500
0
-500
-1000
10^5
10^6
10^7
10^8

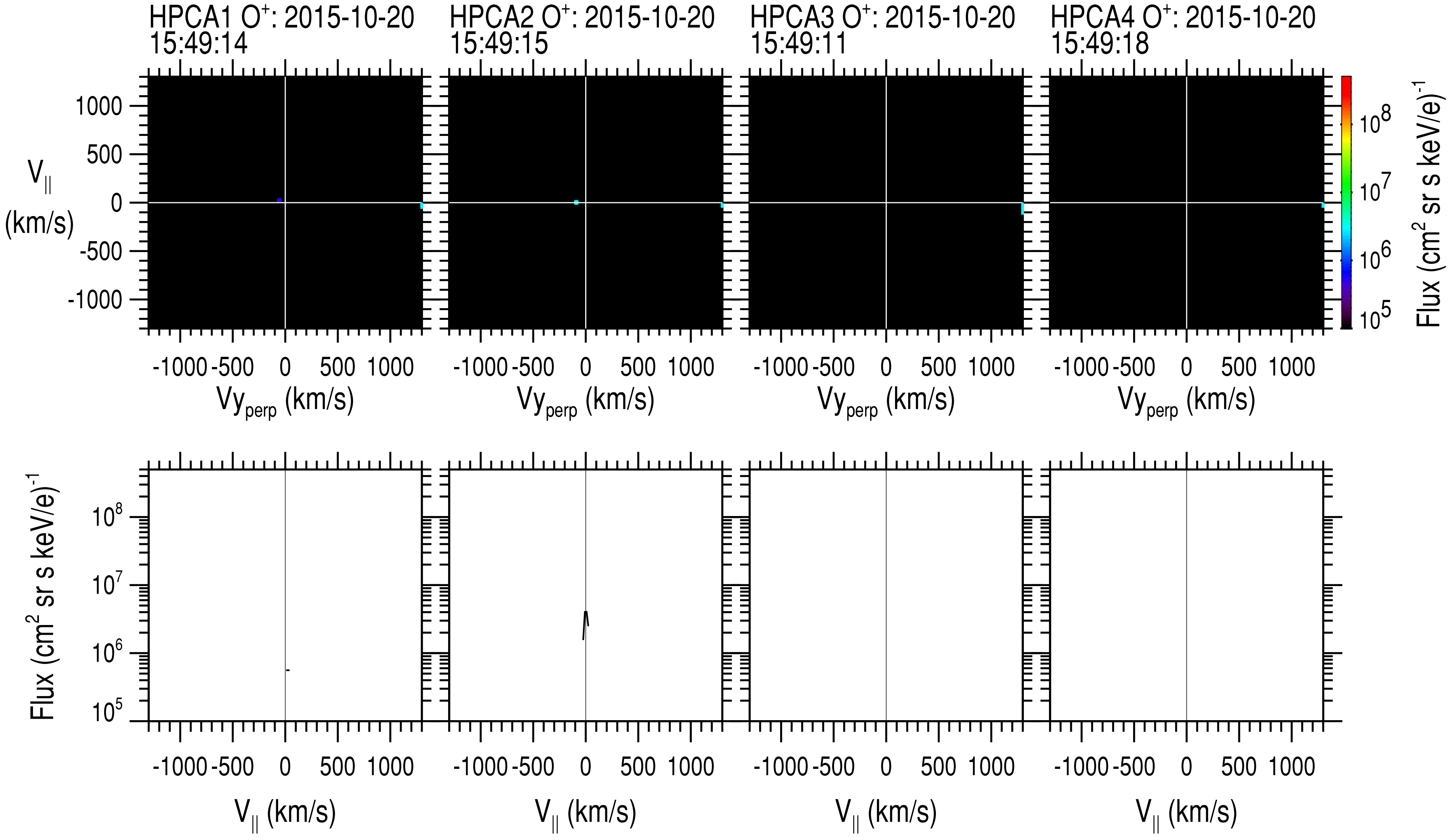
HPCA1 O+: 2015-10-20 15:49:14
HPCA2 O+: 2015-10-20 15:49:15
HPCA3 O+: 2015-10-20 15:49:11
HPCA4 O+: 2015-10-20 15:49:18
V|| (km/s)
1000
500
0
-500
-1000
Vyperp (km/s)
Flux (cm2 sr s keV/e)-1
10^5
10^6
10^7
10^8
-1000
-500
0
500
1000
V|| (km/s)

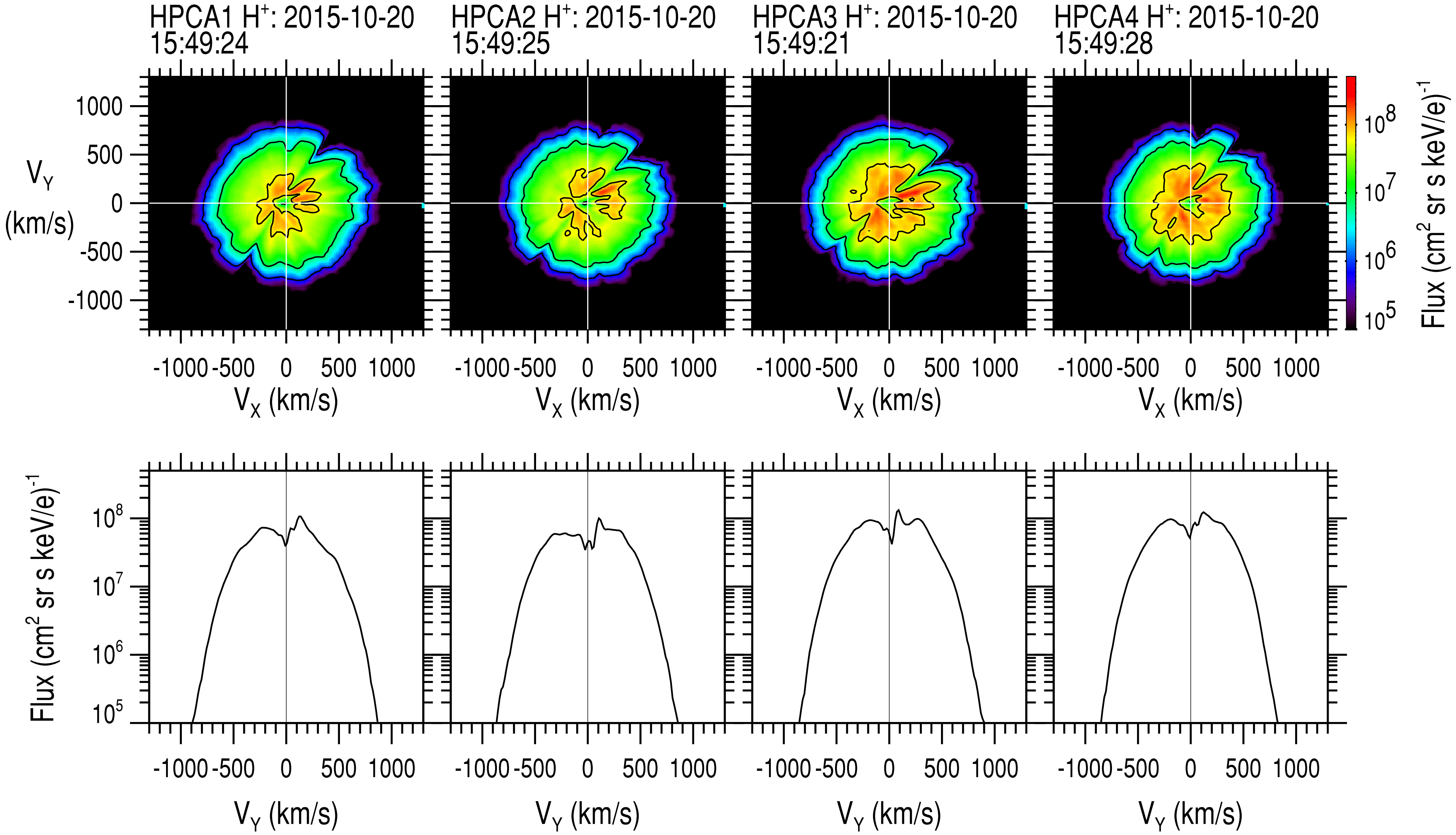
HPCA1 H+: 2015-10-20 15:49:24
HPCA2 H+: 2015-10-20 15:49:25
HPCA3 H+: 2015-10-20 15:49:21
HPCA4 H+: 2015-10-20 15:49:28
VY (km/s)
VX (km/s)
Flux (cm2 sr s keV/e)-1
VY (km/s)

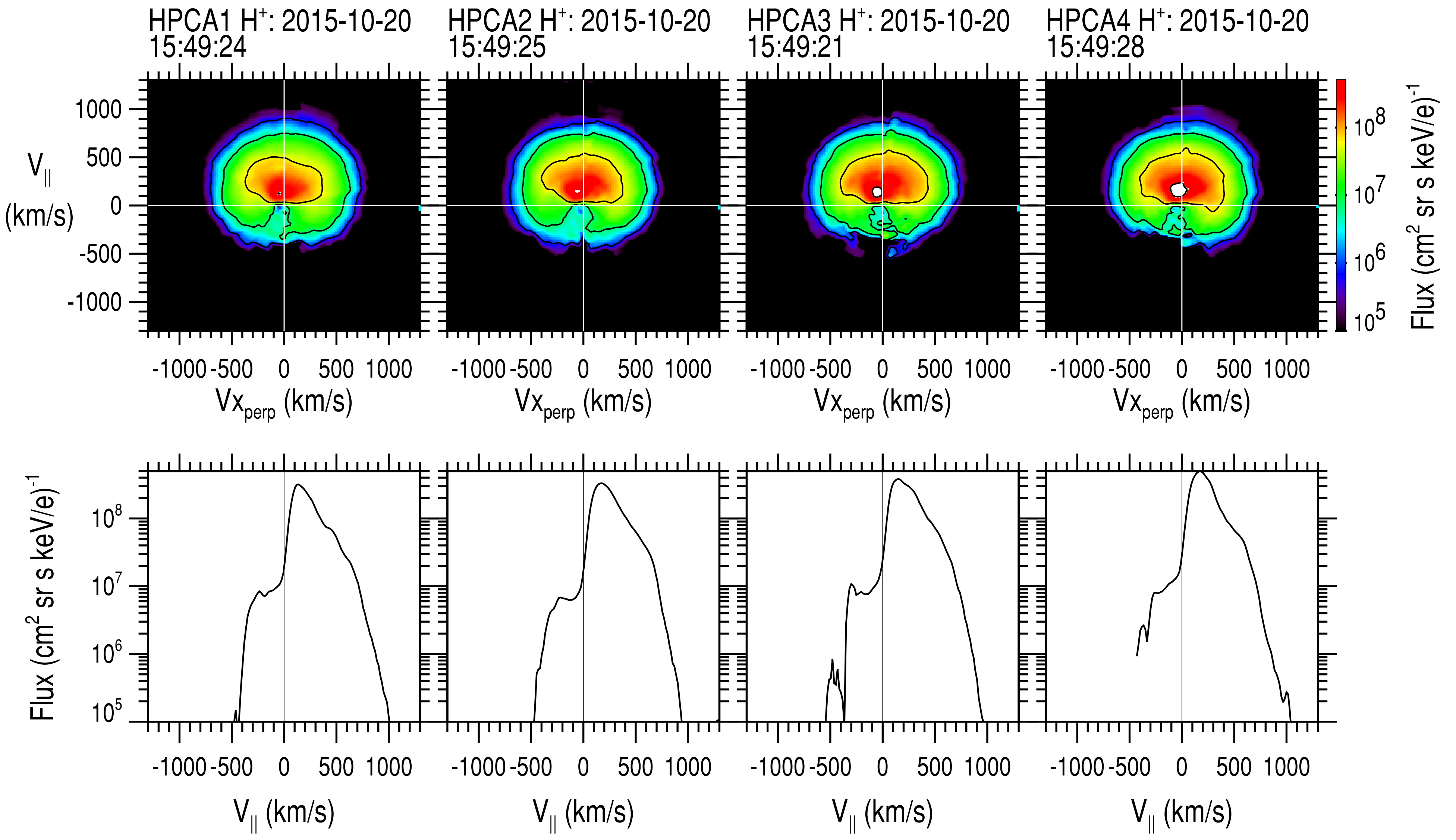
HPCA1 H+: 2015-10-20
15:49:24
HPCA2 H+: 2015-10-20
15:49:25
HPCA3 H+: 2015-10-20
15:49:21
HPCA4 H+: 2015-10-20
15:49:28
V|| (km/s)
Vxperp (km/s)
Flux (cm2 sr s keV/e)-1
V|| (km/s)

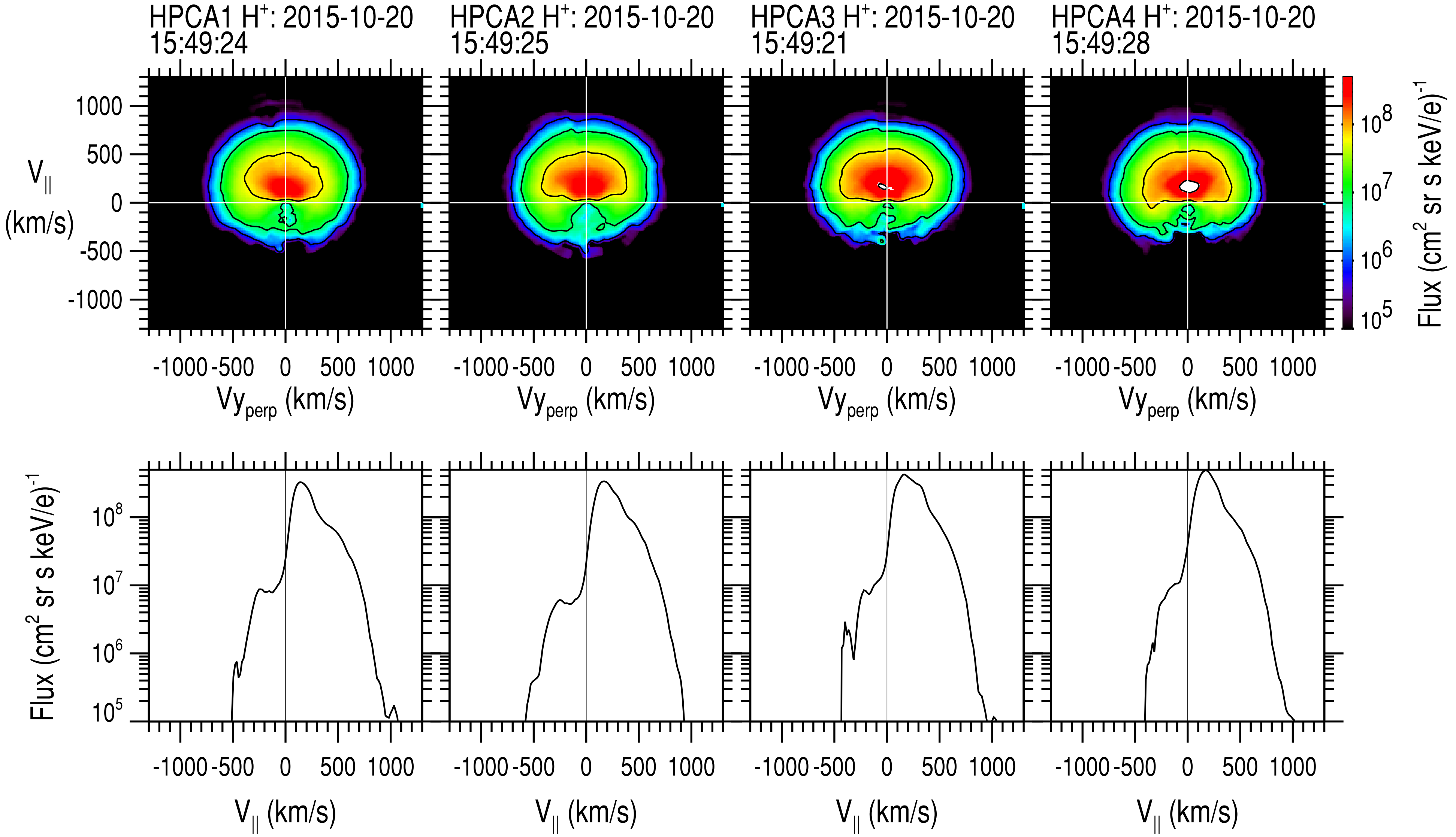
HPCA1 H+: 2015-10-20
15:49:24
HPCA2 H+: 2015-10-20
15:49:25
HPCA3 H+: 2015-10-20
15:49:21
HPCA4 H+: 2015-10-20
15:49:28
V|| (km/s)
Vyperp (km/s)
Flux (cm2 sr s keV/e)-1
V|| (km/s)

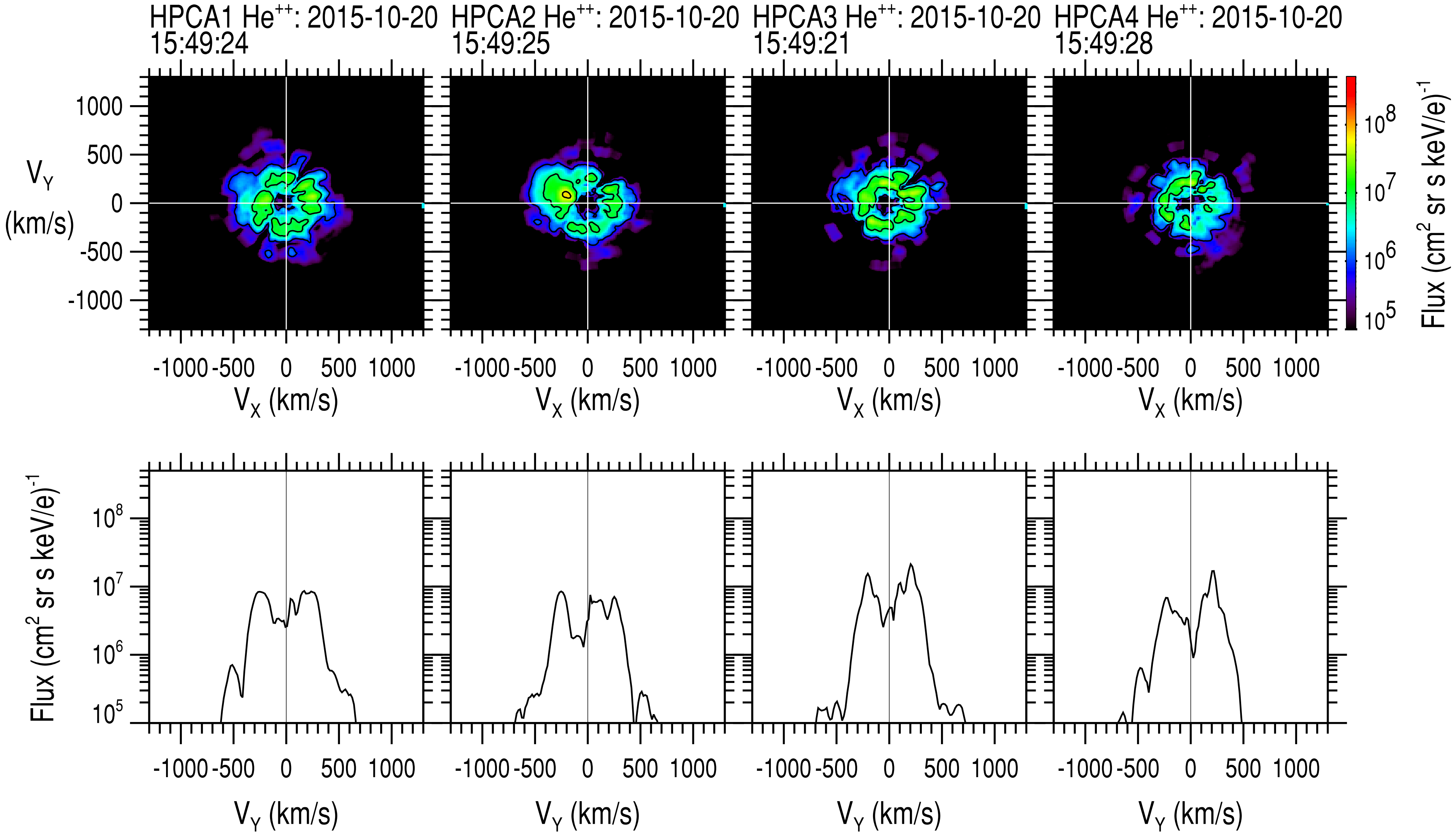

HPCA1 He++: 2015-10-20 15:49:24
HPCA2 He++: 2015-10-20 15:49:25
HPCA3 He++: 2015-10-20 15:49:21
HPCA4 He++: 2015-10-20 15:49:28
V_Y (km/s)
V_X (km/s)
Flux (cm^2 sr s keV/e)^-1
V_Y (km/s)

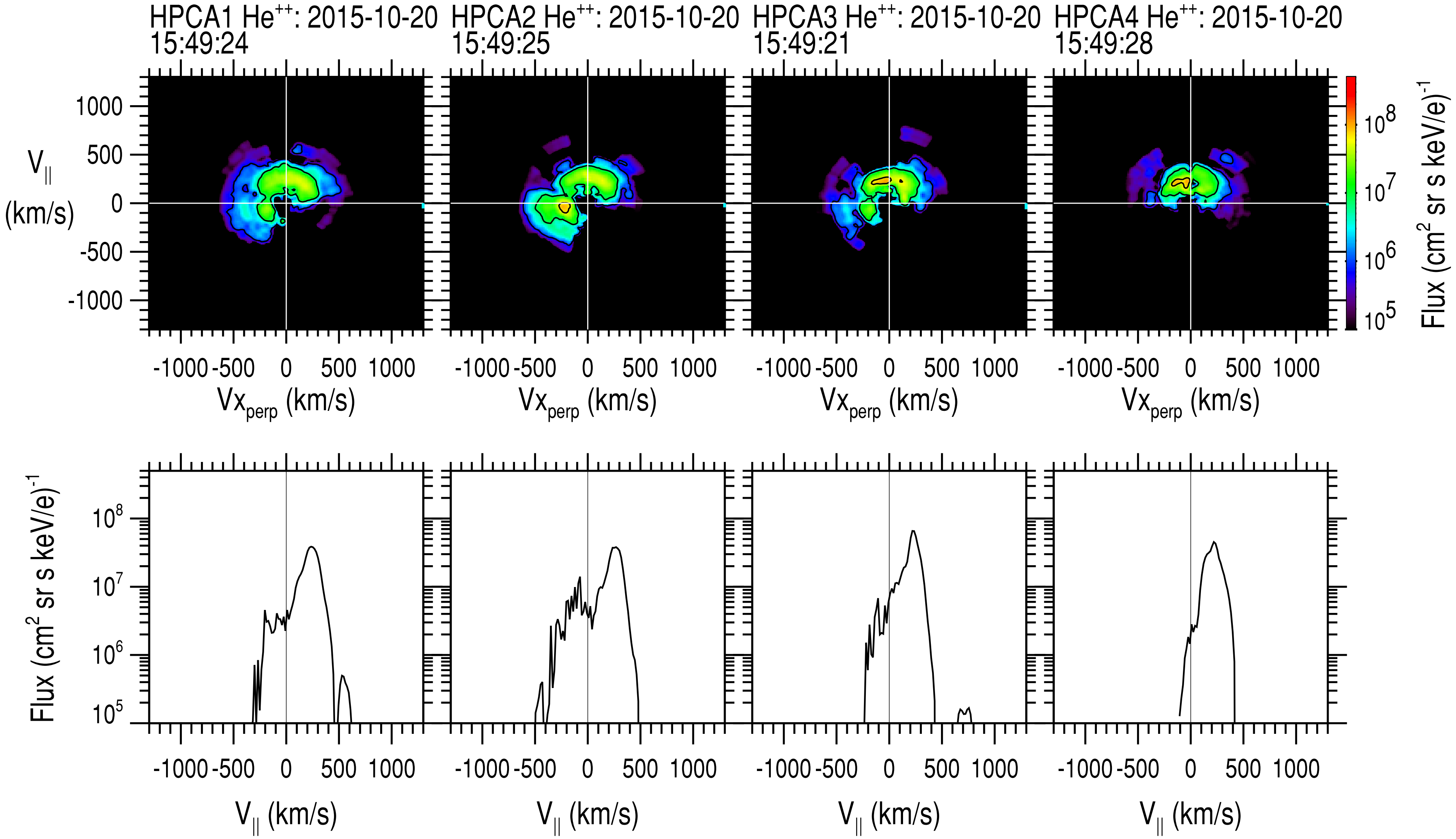
HPCA1 He++: 2015-10-20 15:49:24
HPCA2 He++: 2015-10-20 15:49:25
HPCA3 He++: 2015-10-20 15:49:21
HPCA4 He++: 2015-10-20 15:49:28
V|| (km/s)
Vxperp (km/s)
Flux (cm2 sr s keV/e)-1

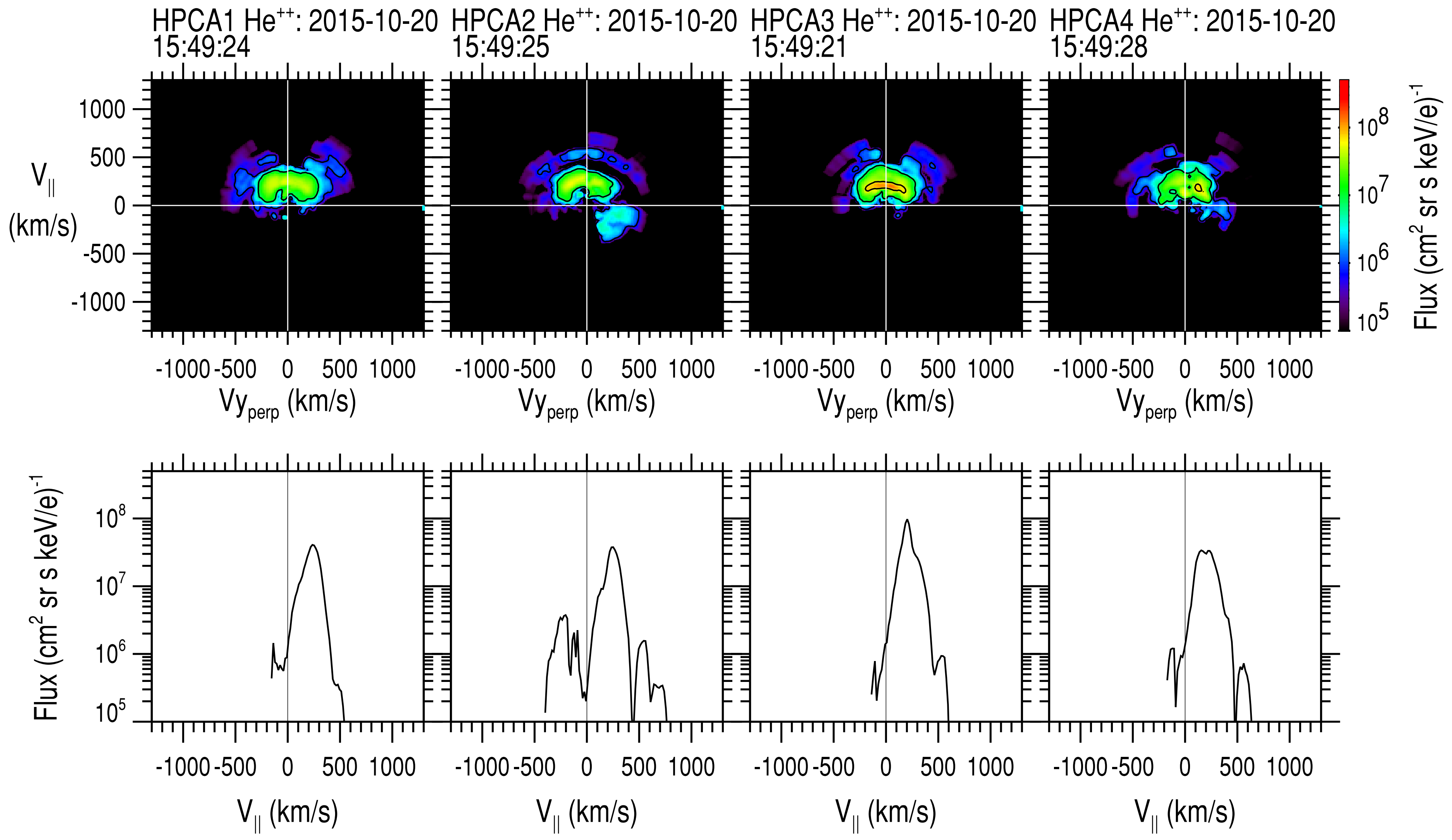
HPCA1 He++: 2015-10-20 15:49:24
HPCA2 He++: 2015-10-20 15:49:25
HPCA3 He++: 2015-10-20 15:49:21
HPCA4 He++: 2015-10-20 15:49:28
V|| (km/s)
Vyperp (km/s)
Flux (cm2 sr s keV/e)-1
1000
500
0
-500
-1000
105
106
107
108

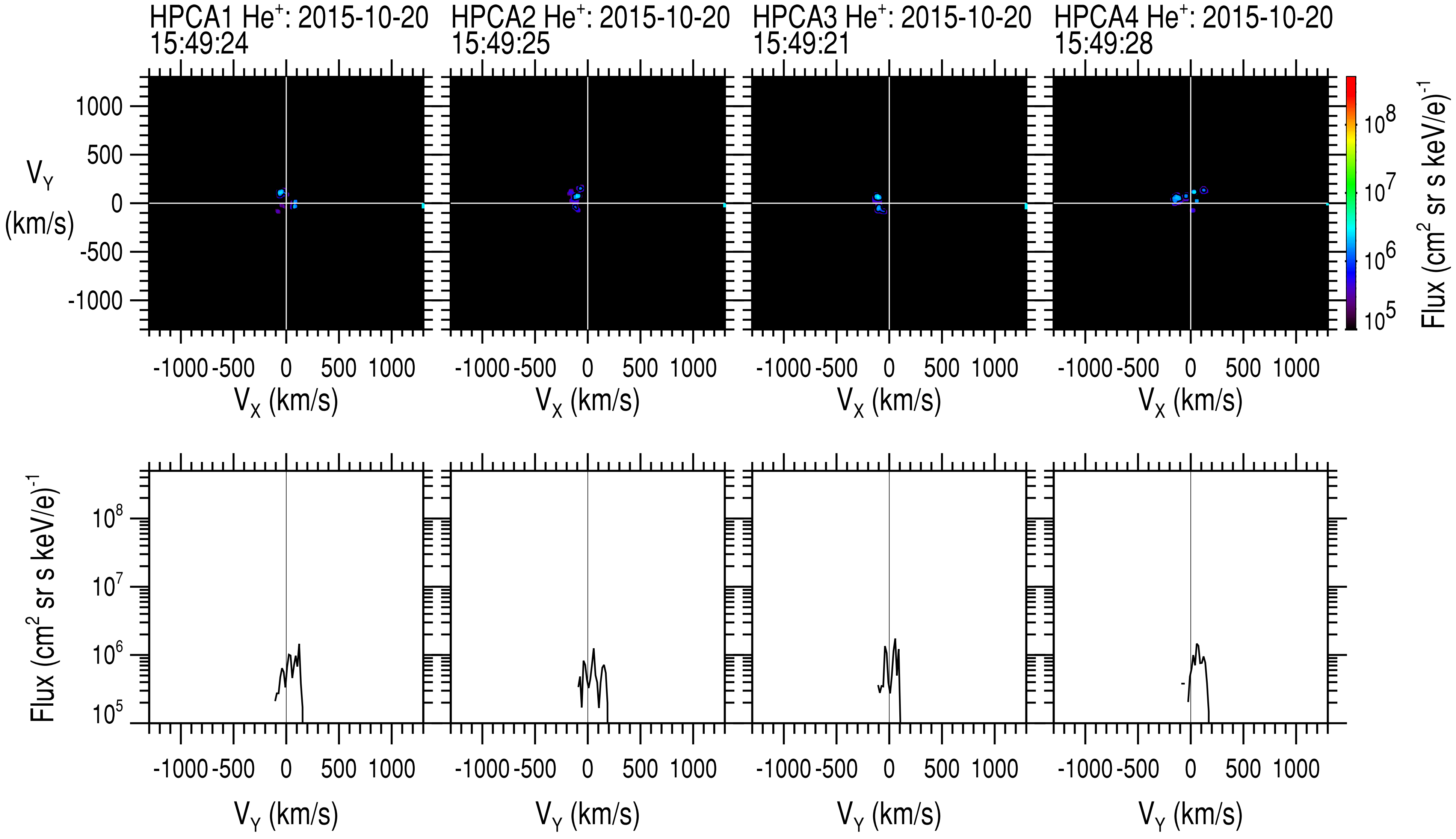
HPCA1 He+: 2015-10-20 15:49:24
HPCA2 He+: 2015-10-20 15:49:25
HPCA3 He+: 2015-10-20 15:49:21
HPCA4 He+: 2015-10-20 15:49:28
VY (km/s)
VX (km/s)
Flux (cm2 sr s keV/e)-1
VY (km/s)

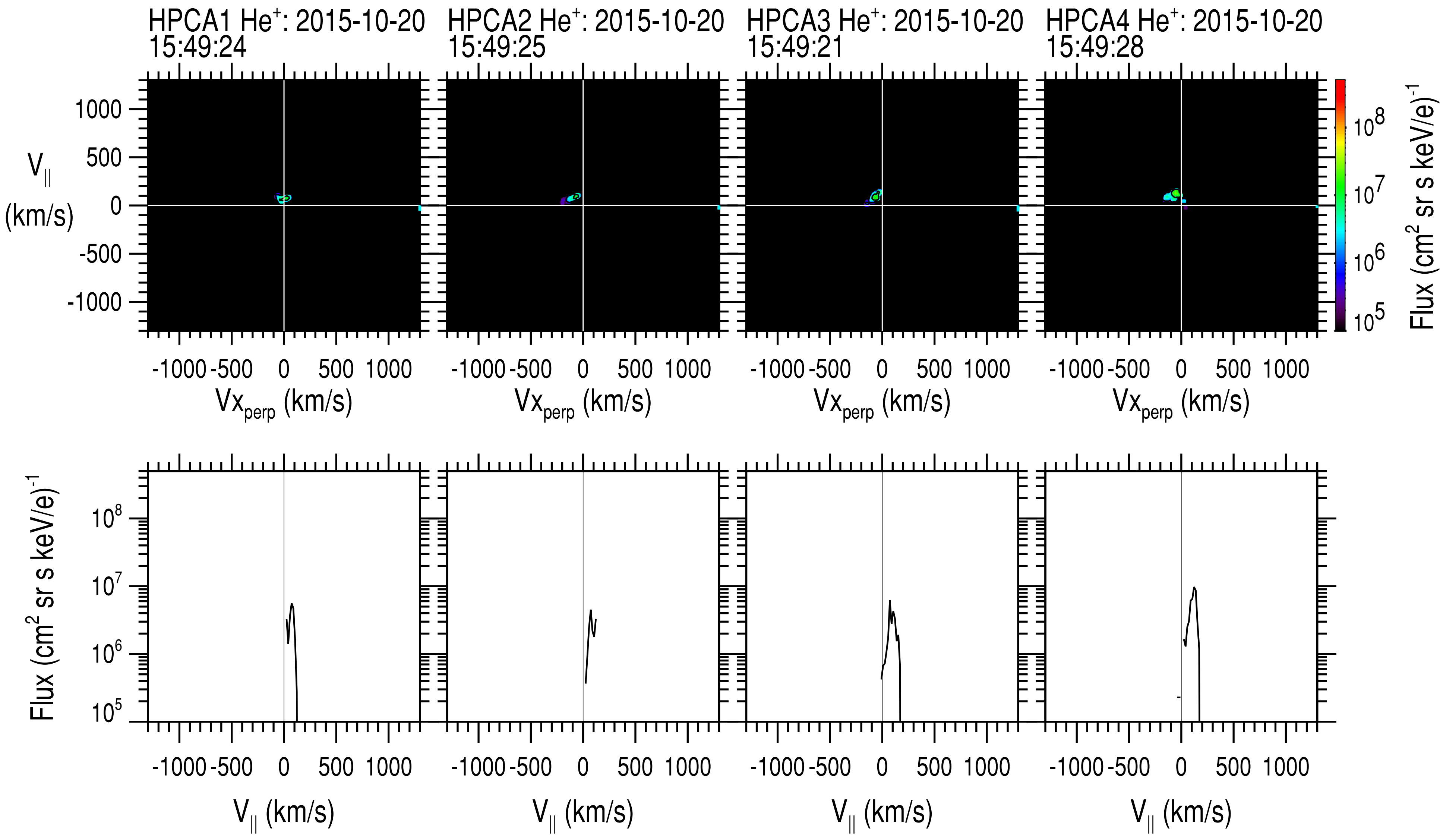
HPCA1 He+: 2015-10-20 15:49:24
HPCA2 He+: 2015-10-20 15:49:25
HPCA3 He+: 2015-10-20 15:49:21
HPCA4 He+: 2015-10-20 15:49:28
V|| (km/s)
Vxperp (km/s)
Flux (cm2 sr s keV/e)-1
V|| (km/s)

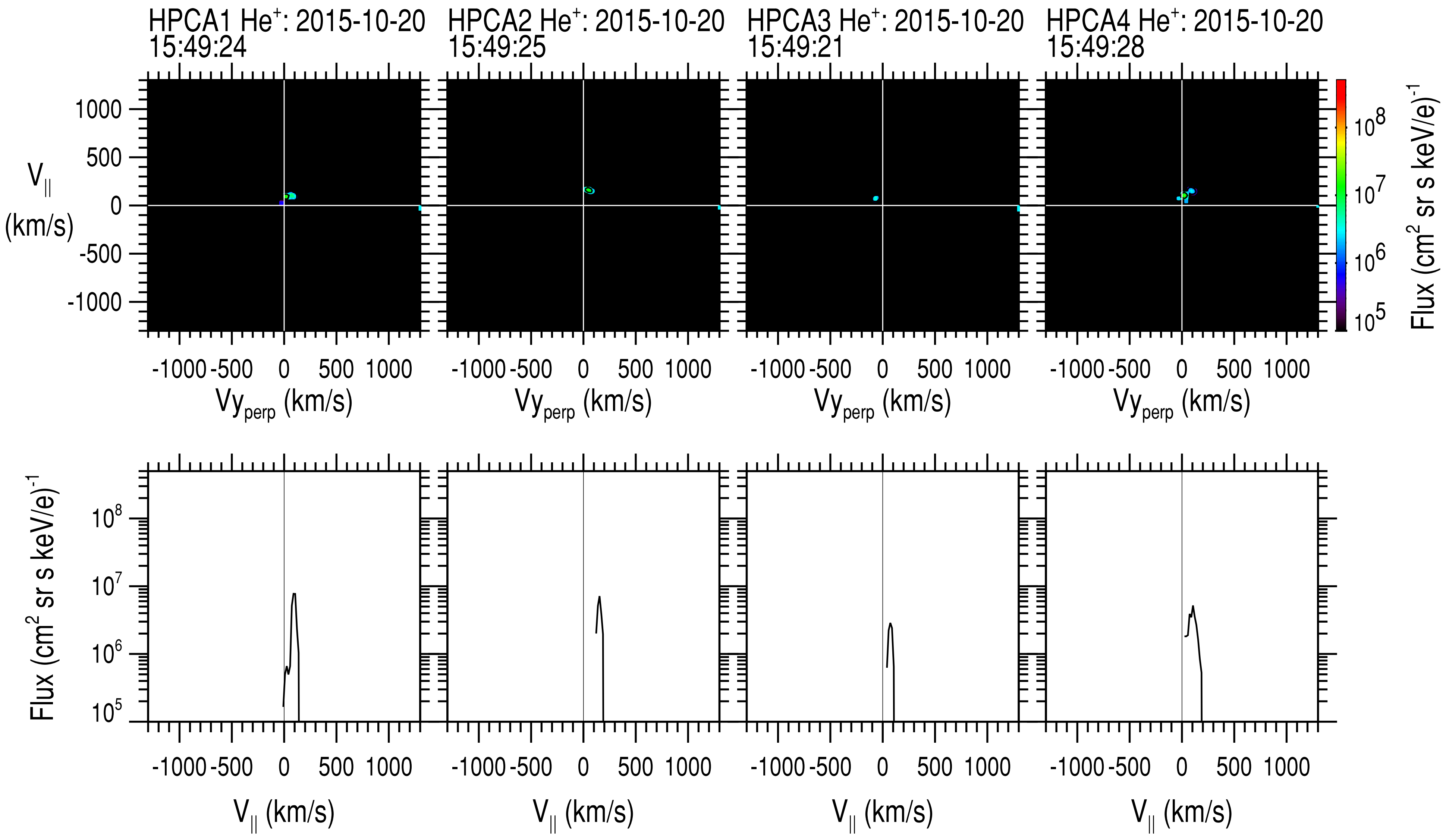

HPCA1 He+: 2015-10-20 15:49:24
HPCA2 He+: 2015-10-20 15:49:25
HPCA3 He+: 2015-10-20 15:49:21
HPCA4 He+: 2015-10-20 15:49:28
V|| (km/s)
Vyperp (km/s)
Flux (cm2 sr s keV/e)-1

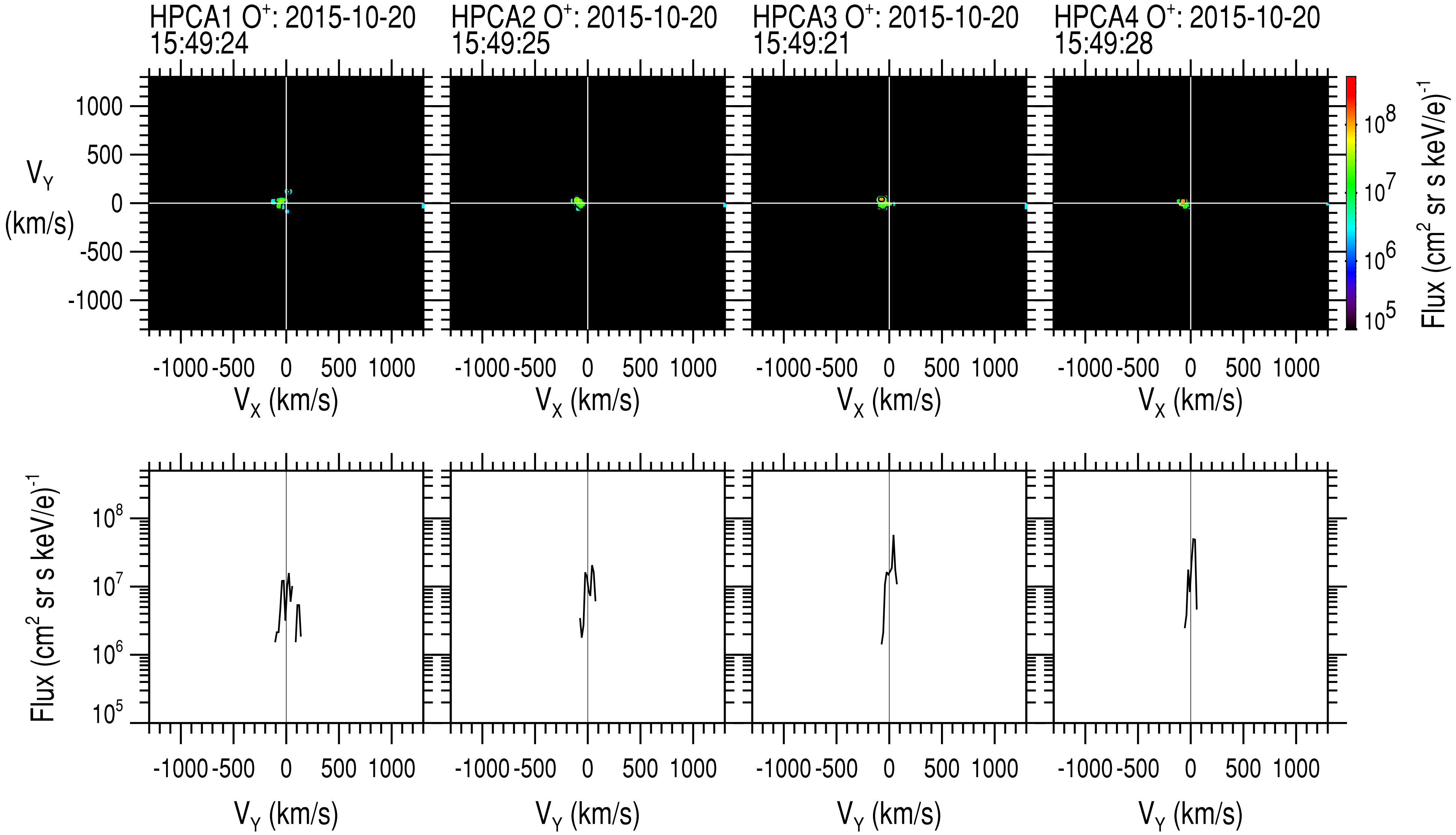

HPCA1 O+: 2015-10-20 15:49:24
HPCA2 O+: 2015-10-20 15:49:25
HPCA3 O+: 2015-10-20 15:49:21
HPCA4 O+: 2015-10-20 15:49:28
VY (km/s)
VX (km/s)
Flux (cm2 sr s keV/e)-1

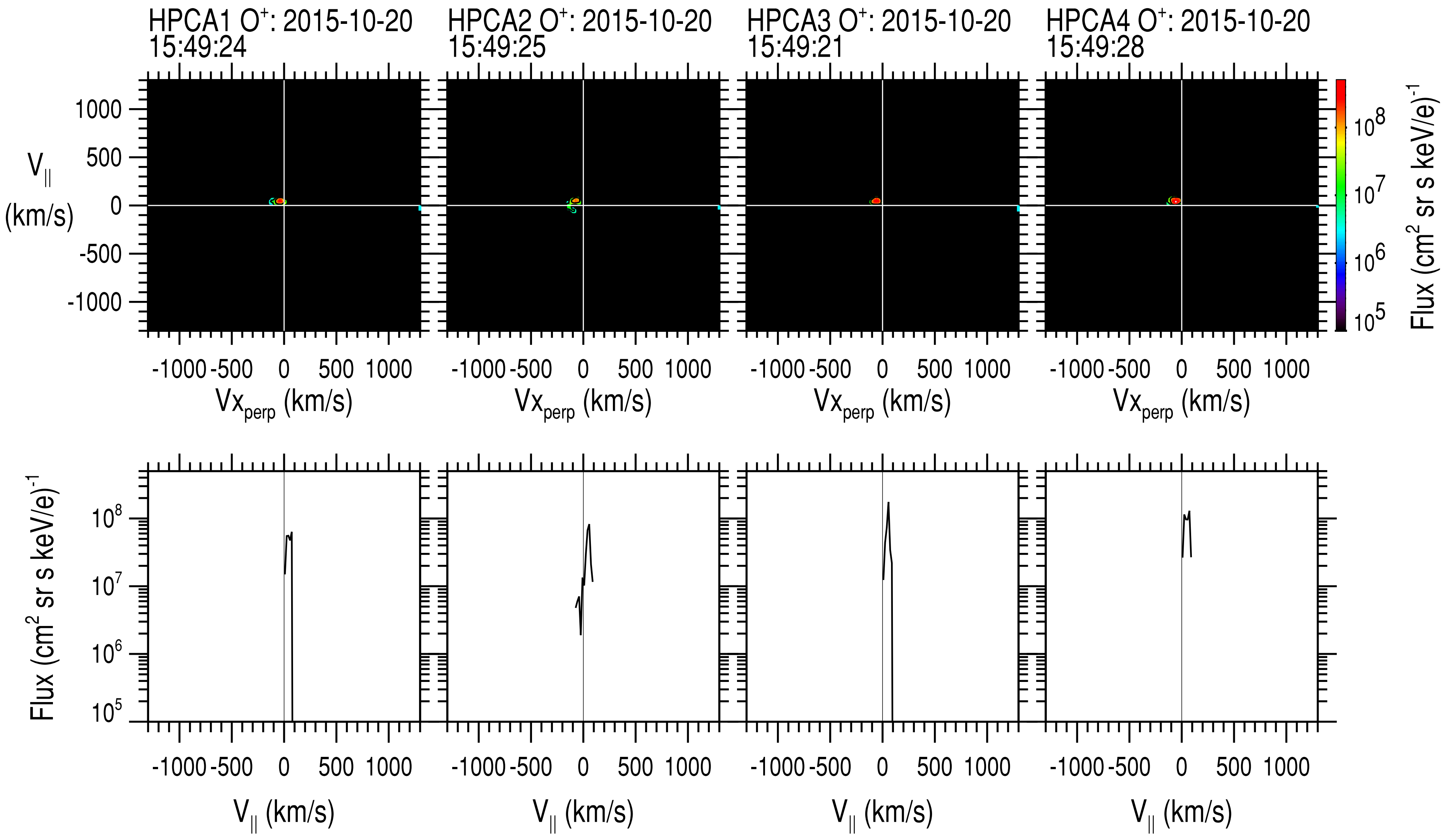
HPCA1 O+: 2015-10-20 15:49:24
HPCA2 O+: 2015-10-20 15:49:25
HPCA3 O+: 2015-10-20 15:49:21
HPCA4 O+: 2015-10-20 15:49:28
V|| (km/s)
1000
500
0
-500
-1000
-1000 -500 0 500 1000
Vxperp (km/s)
Flux (cm2 sr s keV/e)-1
10^5
10^6
10^7
10^8
V|| (km/s)

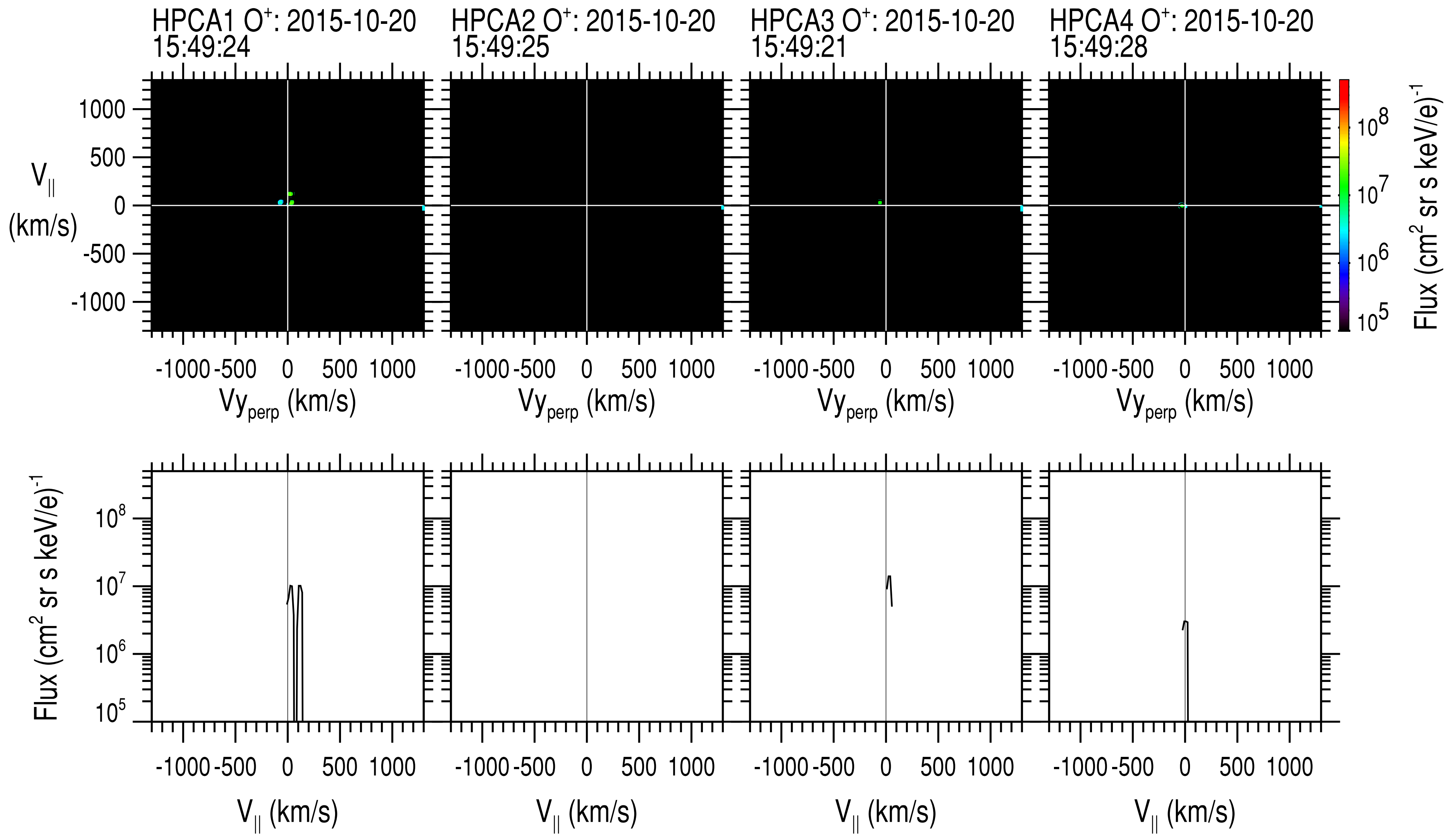
HPCA1 O+: 2015-10-20 15:49:24
HPCA2 O+: 2015-10-20 15:49:25
HPCA3 O+: 2015-10-20 15:49:21
HPCA4 O+: 2015-10-20 15:49:28
V|| (km/s)
Vyperp (km/s)
Flux (cm2 sr s keV/e)-1
V|| (km/s)

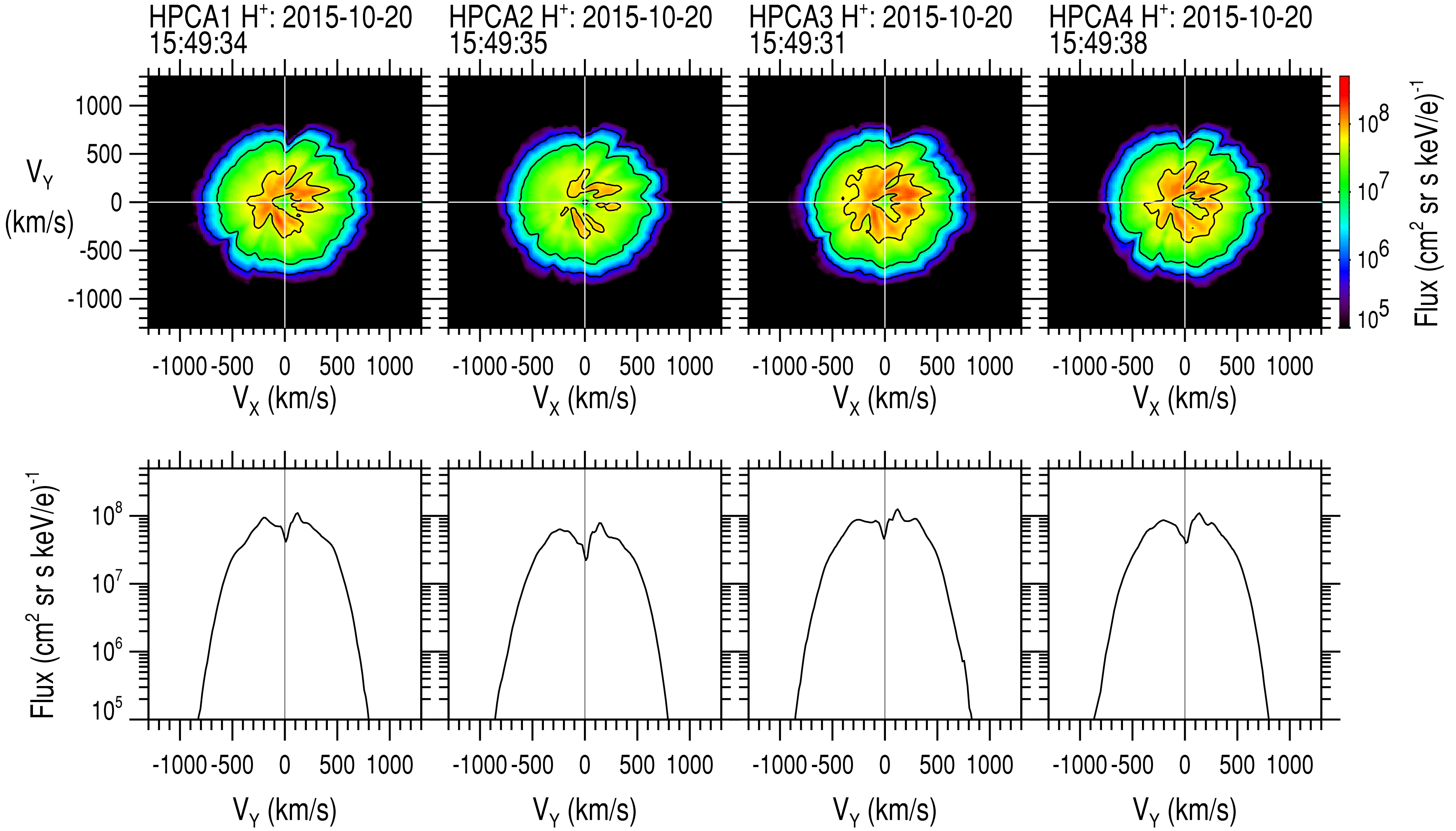
HPCA1 H+: 2015-10-20
15:49:34
HPCA2 H+: 2015-10-20
15:49:35
HPCA3 H+: 2015-10-20
15:49:31
HPCA4 H+: 2015-10-20
15:49:38
V_Y (km/s)
V_X (km/s)
Flux (cm^2 sr s keV/e)^-1
V_Y (km/s)
1000
500
0
-500
-1000
-1000 -500 0 500 1000
10^5
10^6
10^7
10^8

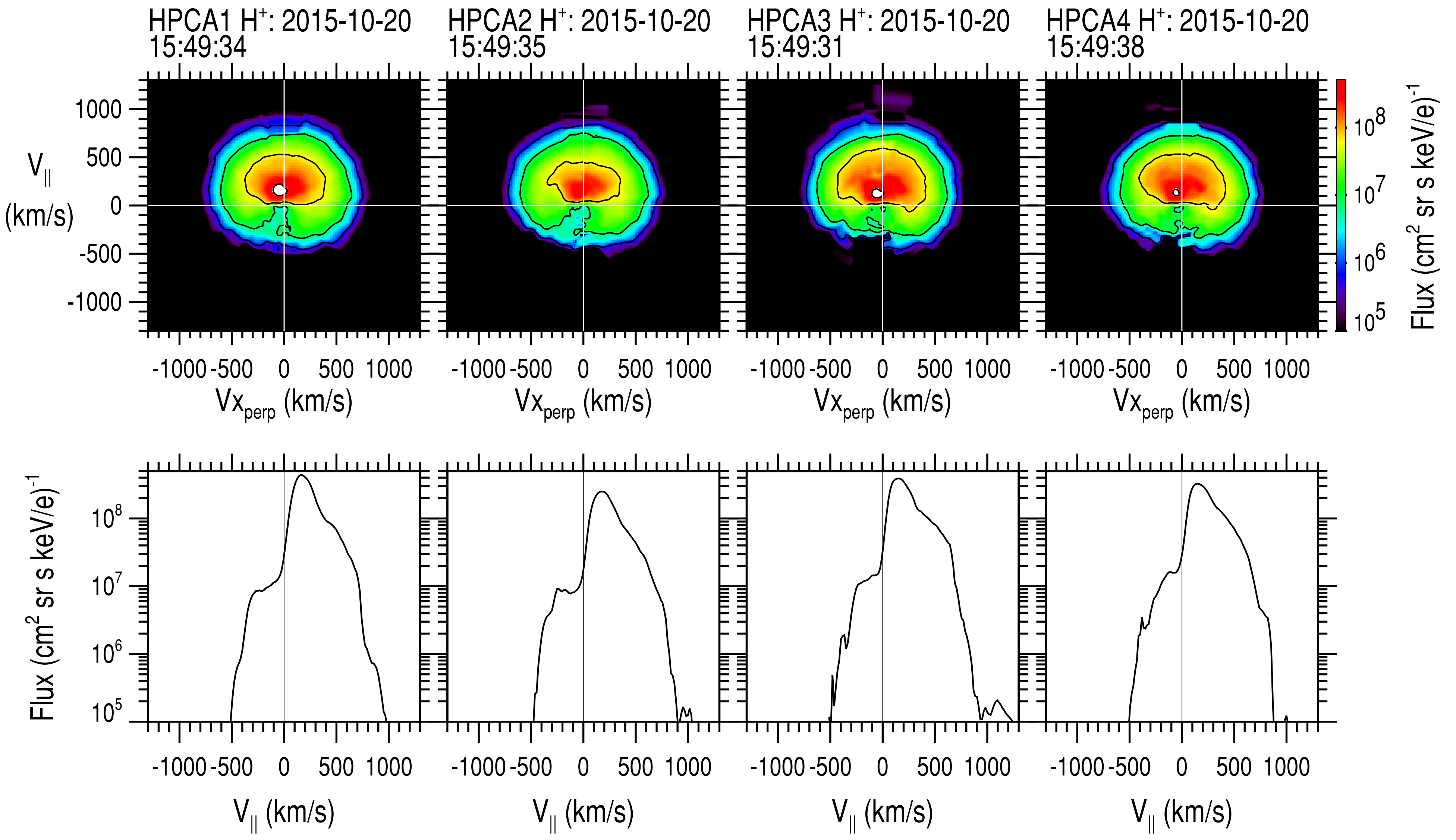
HPCA1 H+: 2015-10-20 15:49:34
HPCA2 H+: 2015-10-20 15:49:35
HPCA3 H+: 2015-10-20 15:49:31
HPCA4 H+: 2015-10-20 15:49:38
V|| (km/s)
Vxperp (km/s)
Flux (cm2 sr s keV/e)-1
-1000 -500 0 500 1000
10^5 10^6 10^7 10^8

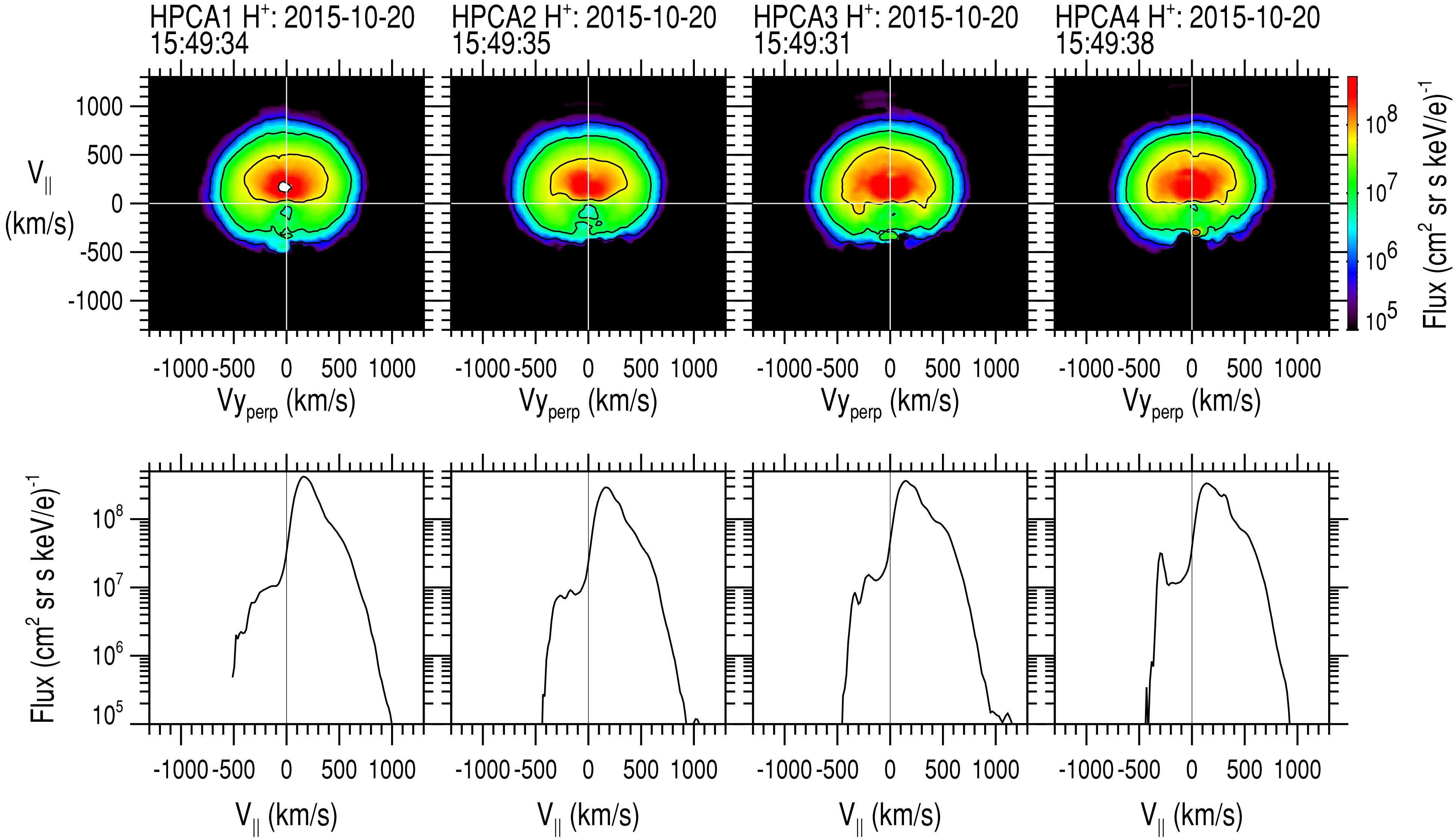

HPCA1 H+: 2015-10-20 15:49:34
HPCA2 H+: 2015-10-20 15:49:35
HPCA3 H+: 2015-10-20 15:49:31
HPCA4 H+: 2015-10-20 15:49:38
V|| (km/s)
Vyperp (km/s)
Flux (cm2 sr s keV/e)-1

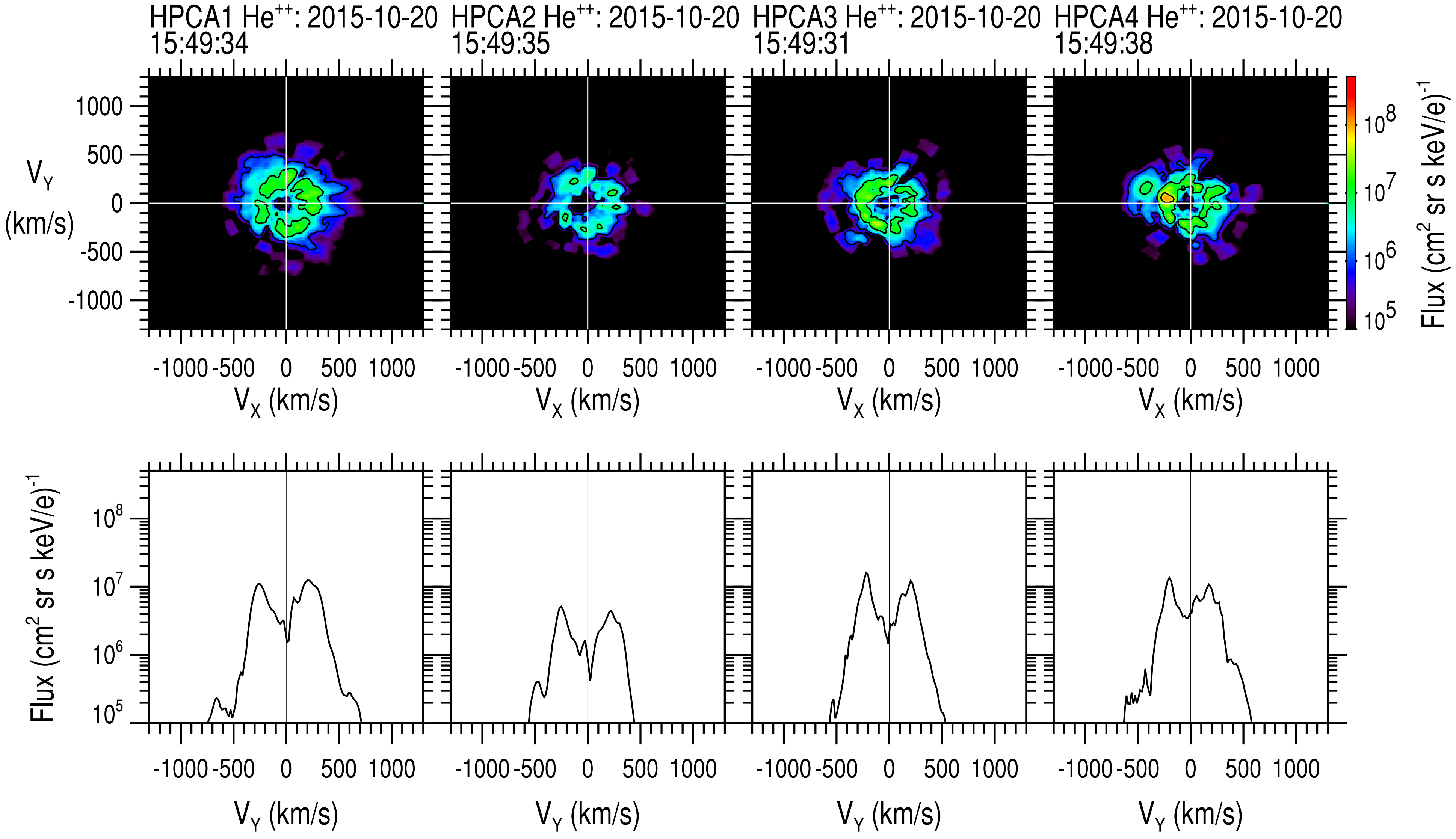
HPCA1 He++: 2015-10-20 15:49:34
HPCA2 He++: 2015-10-20 15:49:35
HPCA3 He++: 2015-10-20 15:49:31
HPCA4 He++: 2015-10-20 15:49:38
VY (km/s)
VX (km/s)
Flux (cm2 sr s keV/e)-1
VY (km/s)
Flux (cm2 sr s keV/e)-1

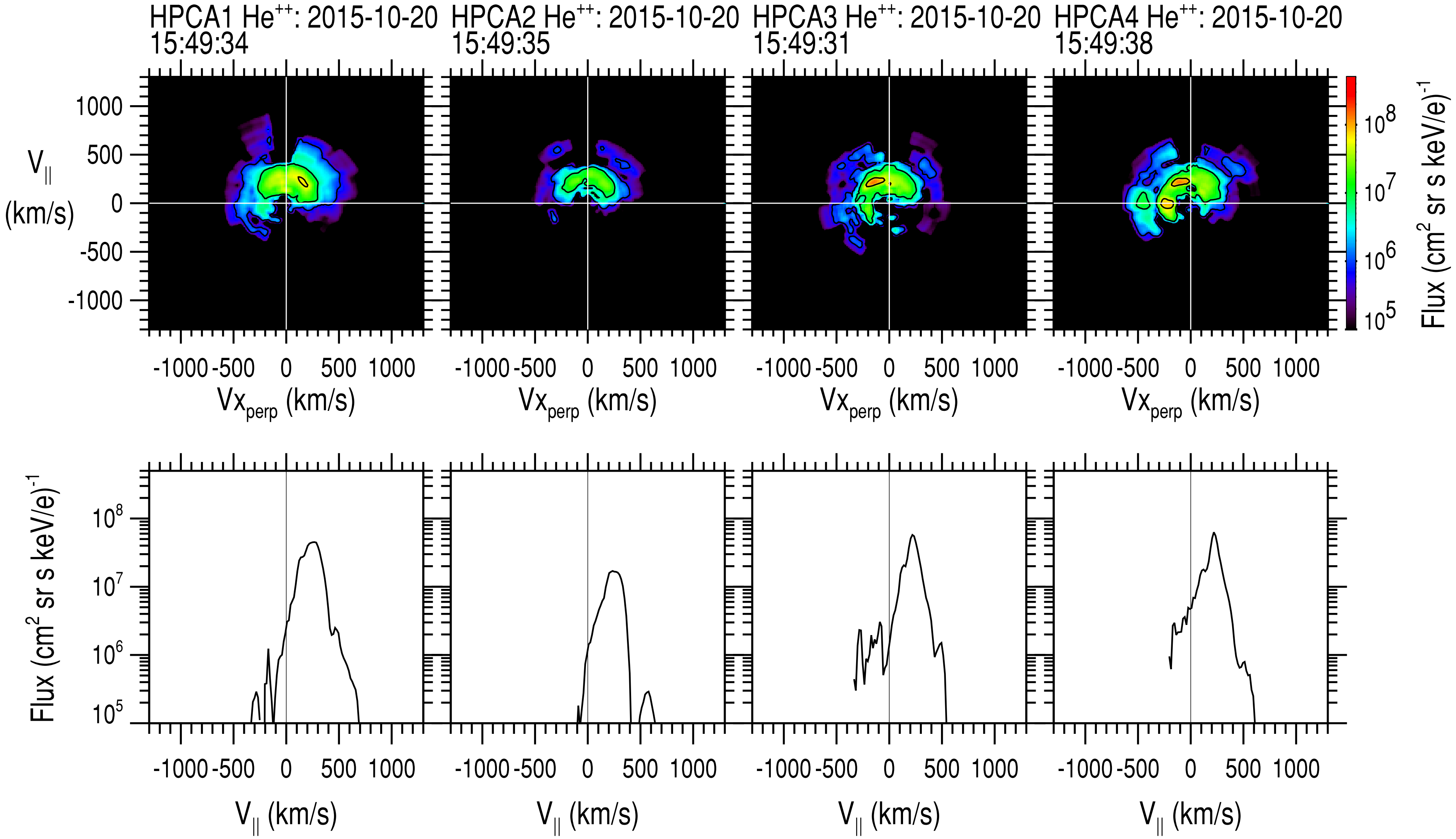

HPCA1 He++: 2015-10-20 15:49:34
HPCA2 He++: 2015-10-20 15:49:35
HPCA3 He++: 2015-10-20 15:49:31
HPCA4 He++: 2015-10-20 15:49:38
V|| (km/s)
Vxperp (km/s)
Flux (cm2 sr s keV/e)-1
V|| (km/s)

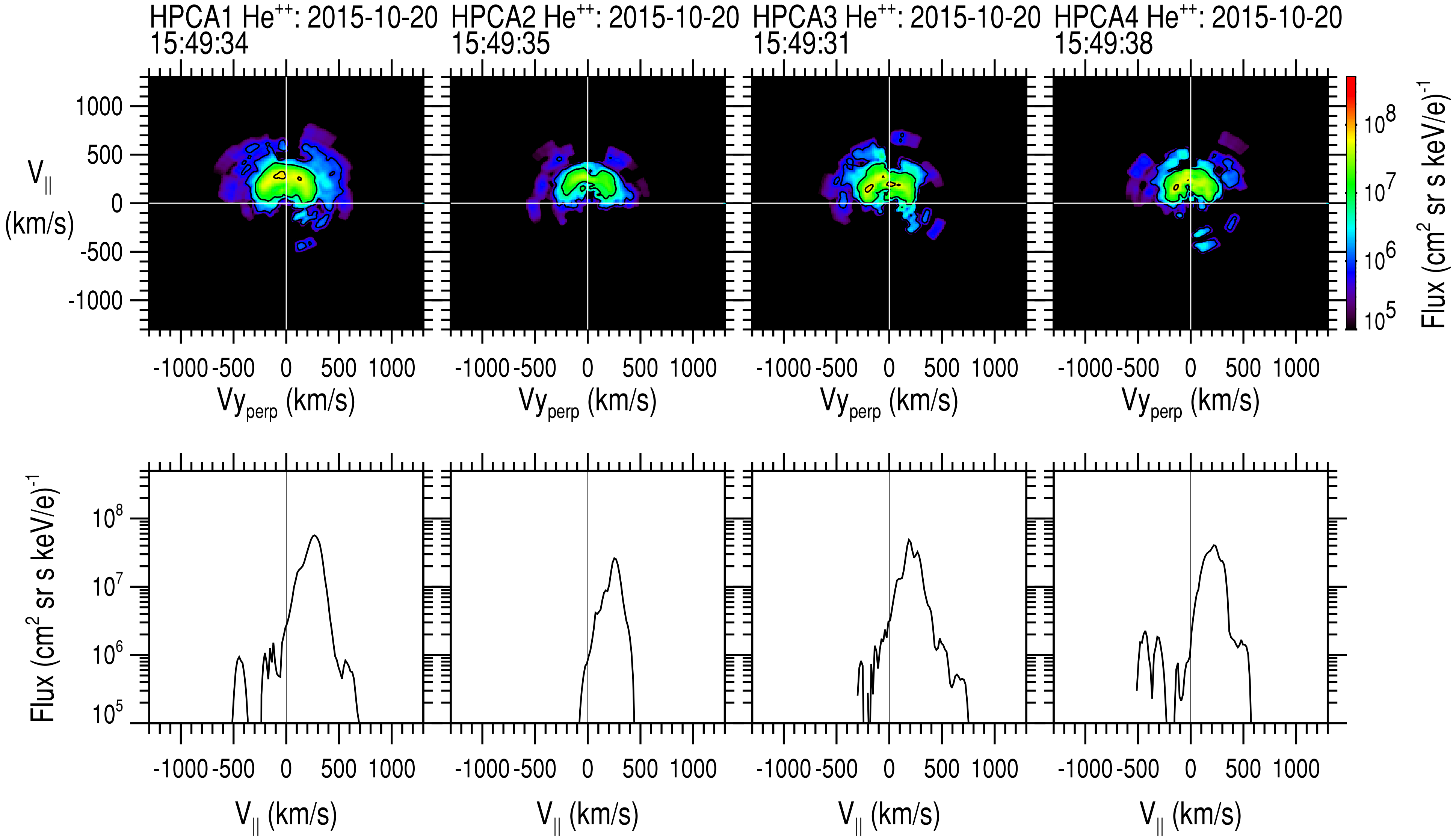

HPCA1 He++: 2015-10-20 15:49:34
HPCA2 He++: 2015-10-20 15:49:35
HPCA3 He++: 2015-10-20 15:49:31
HPCA4 He++: 2015-10-20 15:49:38
V|| (km/s)
Vyperp (km/s)
Flux (cm2 sr s keV/e)-1

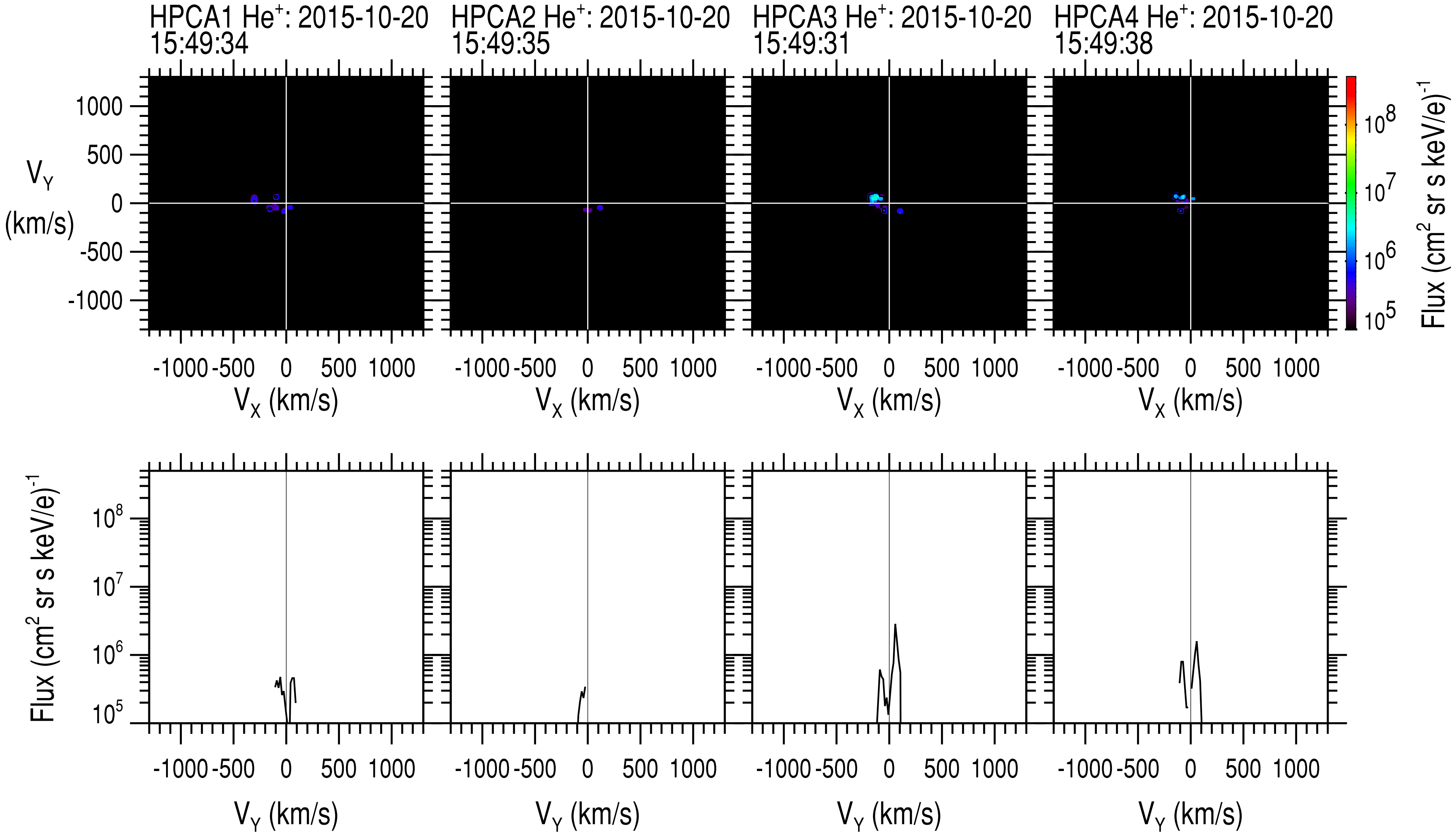
HPCA1 He+: 2015-10-20 15:49:34
HPCA2 He+: 2015-10-20 15:49:35
HPCA3 He+: 2015-10-20 15:49:31
HPCA4 He+: 2015-10-20 15:49:38
VY (km/s)
VX (km/s)
Flux (cm2 sr s keV/e)-1
-1000 -500 0 500 1000
10^5 10^6 10^7 10^8

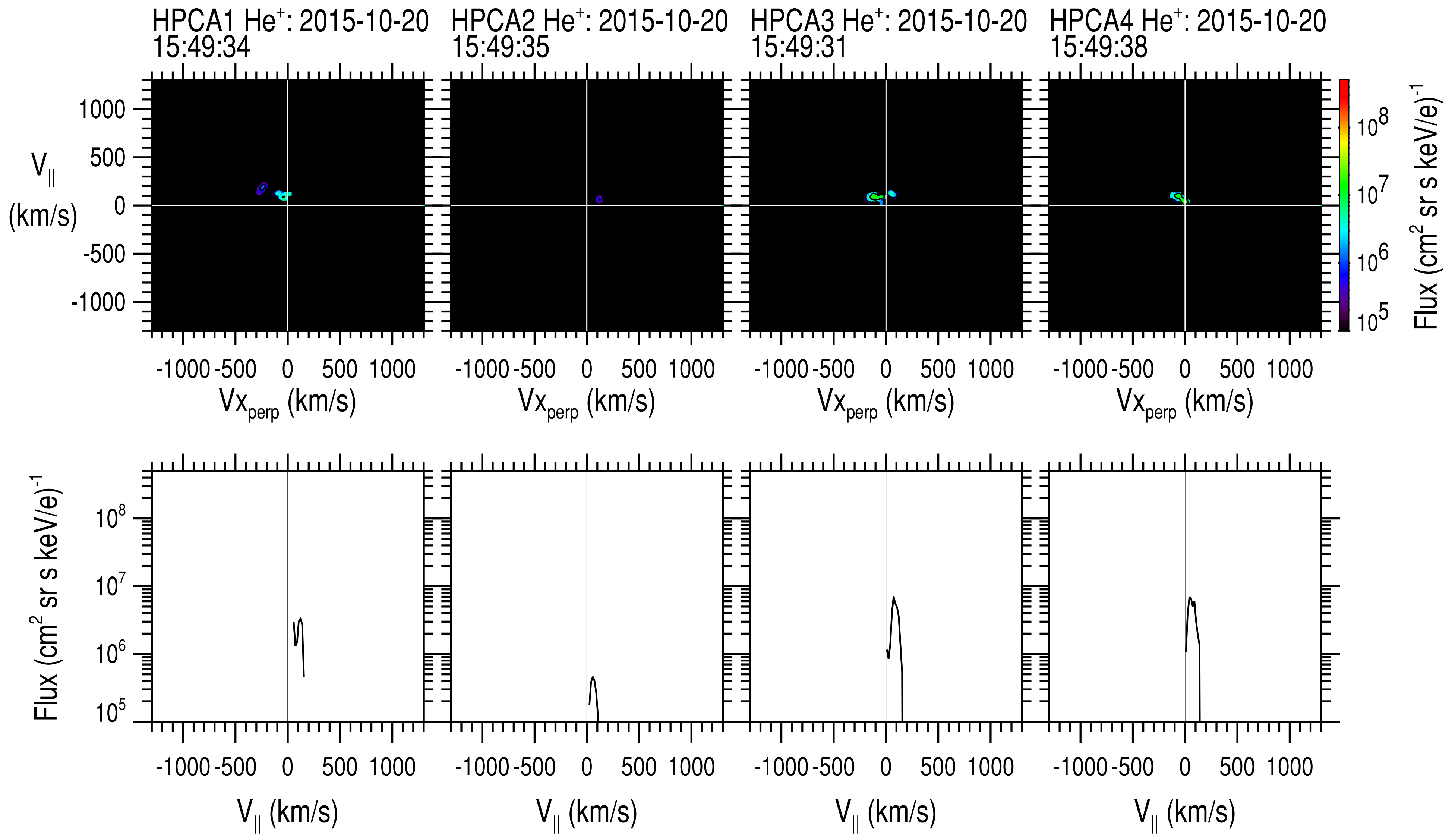
HPCA1 He+: 2015-10-20 15:49:34
HPCA2 He+: 2015-10-20 15:49:35
HPCA3 He+: 2015-10-20 15:49:31
HPCA4 He+: 2015-10-20 15:49:38
V|| (km/s)
Vxperp (km/s)
Flux (cm2 sr s keV/e)-1
1000
500
0
-500
-1000
10^5
10^6
10^7
10^8

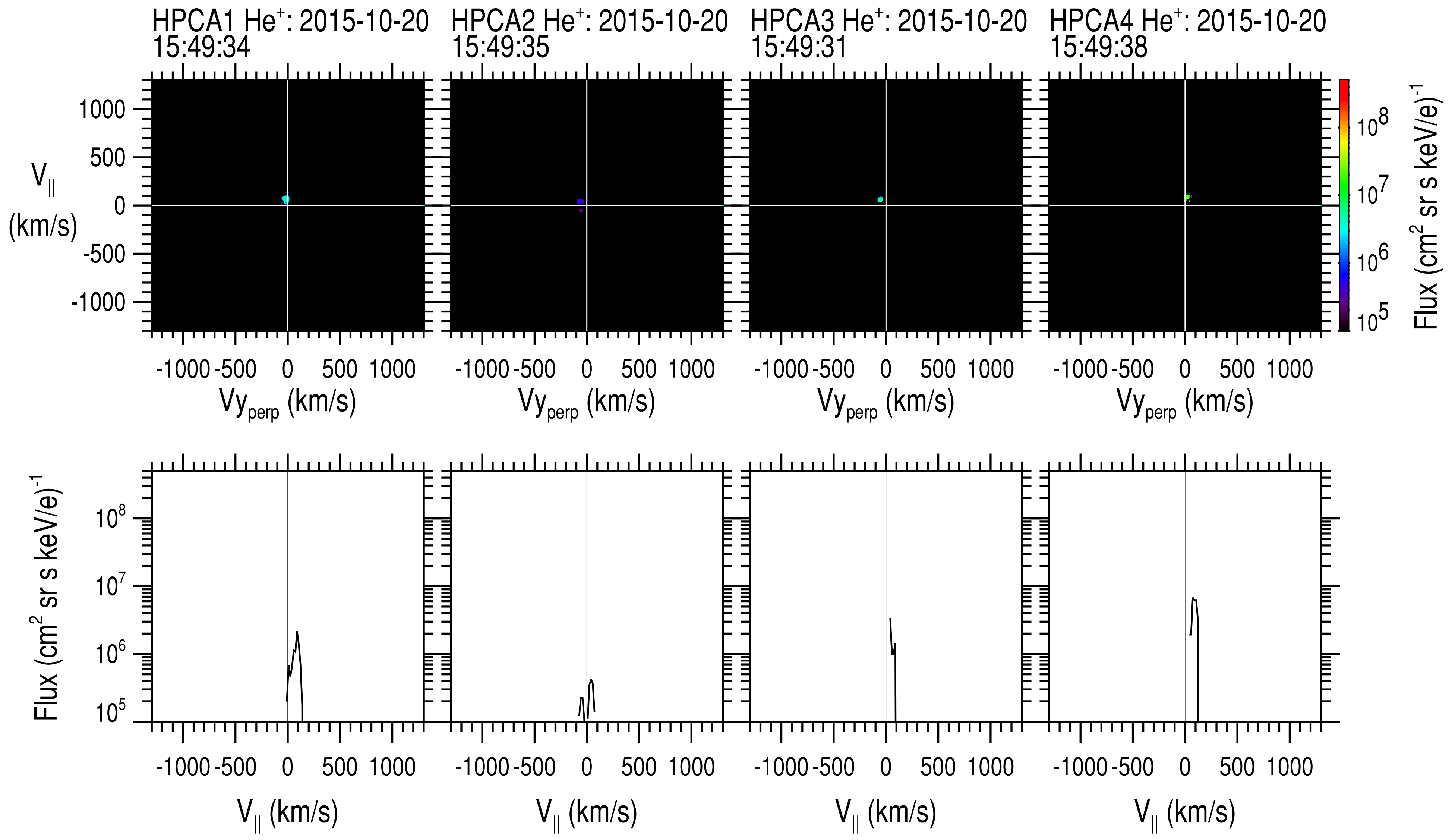
HPCA1 He+: 2015-10-20 15:49:34
HPCA2 He+: 2015-10-20 15:49:35
HPCA3 He+: 2015-10-20 15:49:31
HPCA4 He+: 2015-10-20 15:49:38
V|| (km/s)
Vyperp (km/s)
Flux (cm2 sr s keV/e)-1
1000
500
0
-500
-1000
10^5
10^6
10^7
10^8

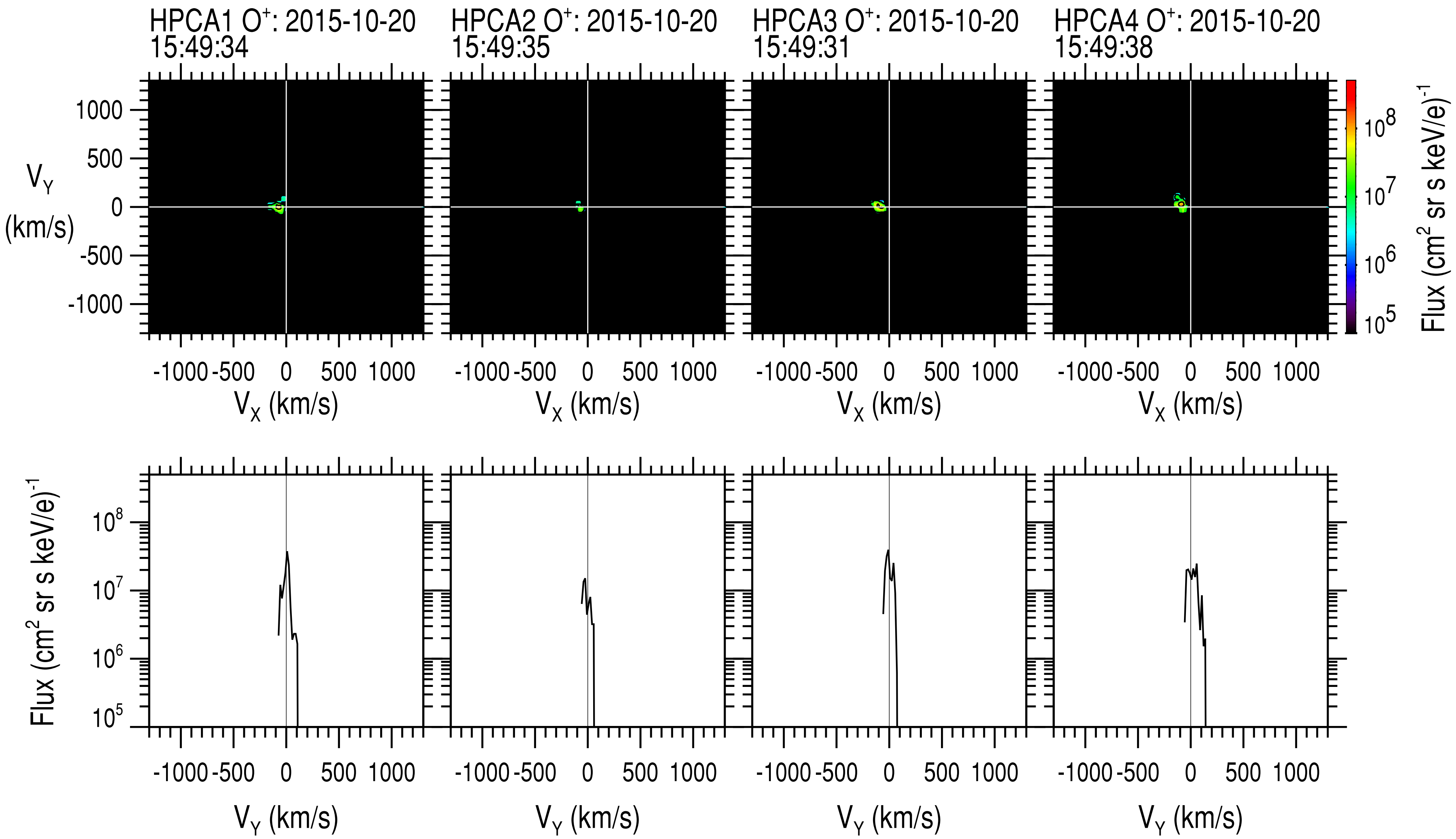
HPCA1 O+: 2015-10-20
15:49:34
HPCA2 O+: 2015-10-20
15:49:35
HPCA3 O+: 2015-10-20
15:49:31
HPCA4 O+: 2015-10-20
15:49:38
V_Y (km/s)
V_X (km/s)
Flux (cm^2 sr s keV/e)^-1
V_Y (km/s)

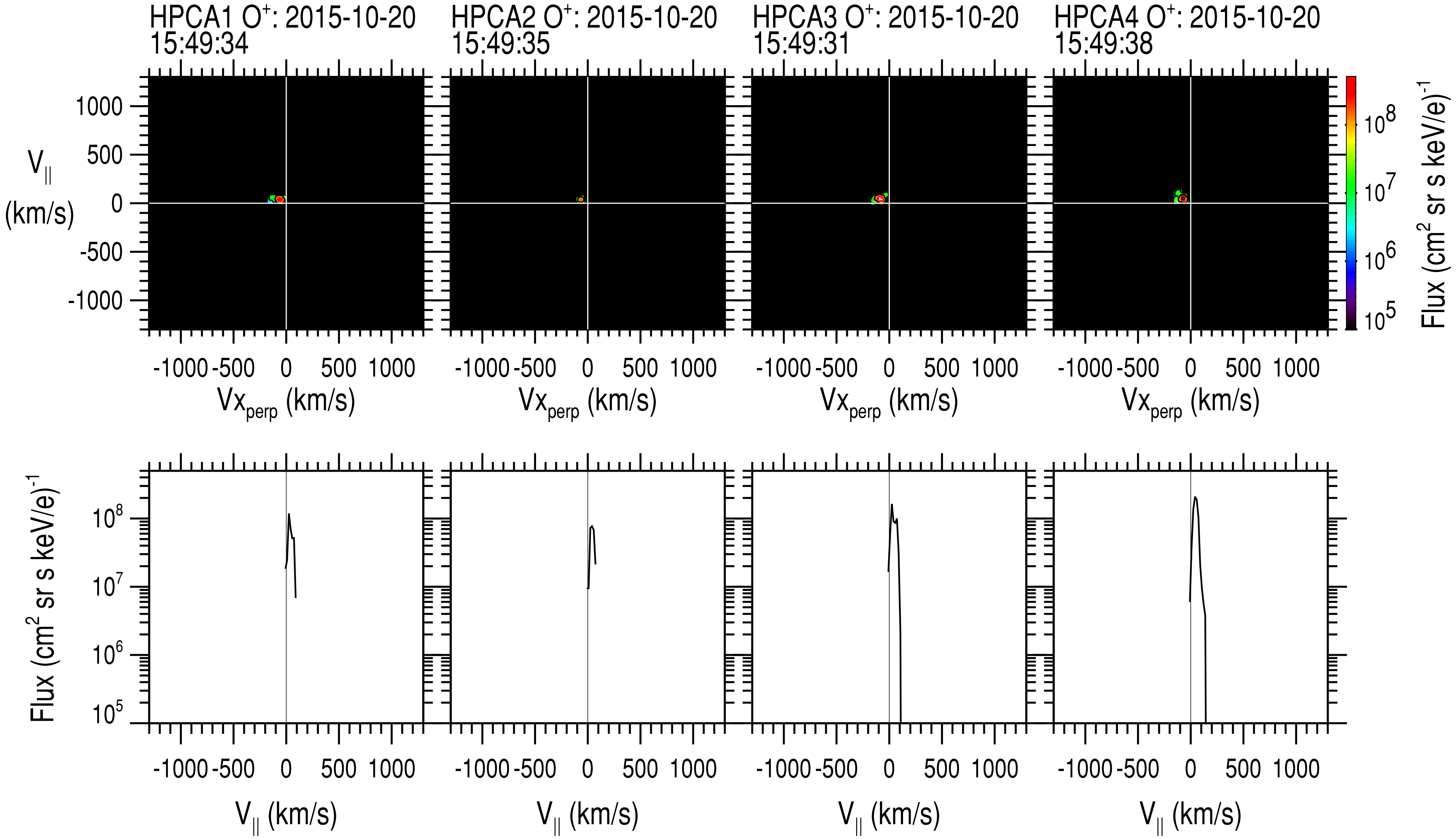
HPCA1 O+: 2015-10-20 15:49:34
HPCA2 O+: 2015-10-20 15:49:35
HPCA3 O+: 2015-10-20 15:49:31
HPCA4 O+: 2015-10-20 15:49:38
V|| (km/s)
Vxperp (km/s)
Flux (cm2 sr s keV/e)-1
V|| (km/s)
-1000 -500 0 500 1000
10^5 10^6 10^7 10^8

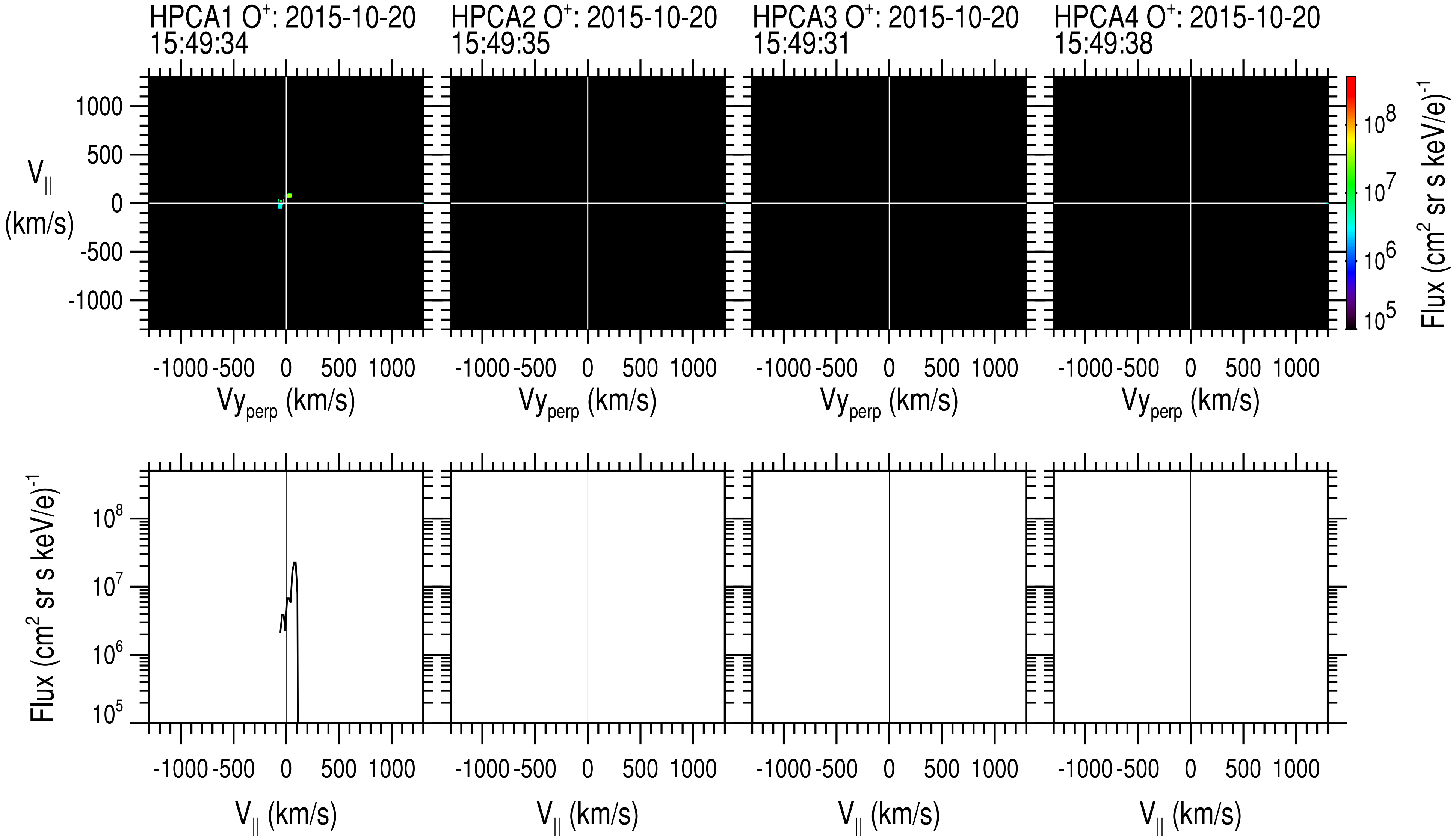
HPCA1 O+: 2015-10-20 15:49:34
HPCA2 O+: 2015-10-20 15:49:35
HPCA3 O+: 2015-10-20 15:49:31
HPCA4 O+: 2015-10-20 15:49:38
V|| (km/s)
Vyperp (km/s)
Flux (cm2 sr s keV/e)-1
V|| (km/s)
Flux (cm2 sr s keV/e)-1

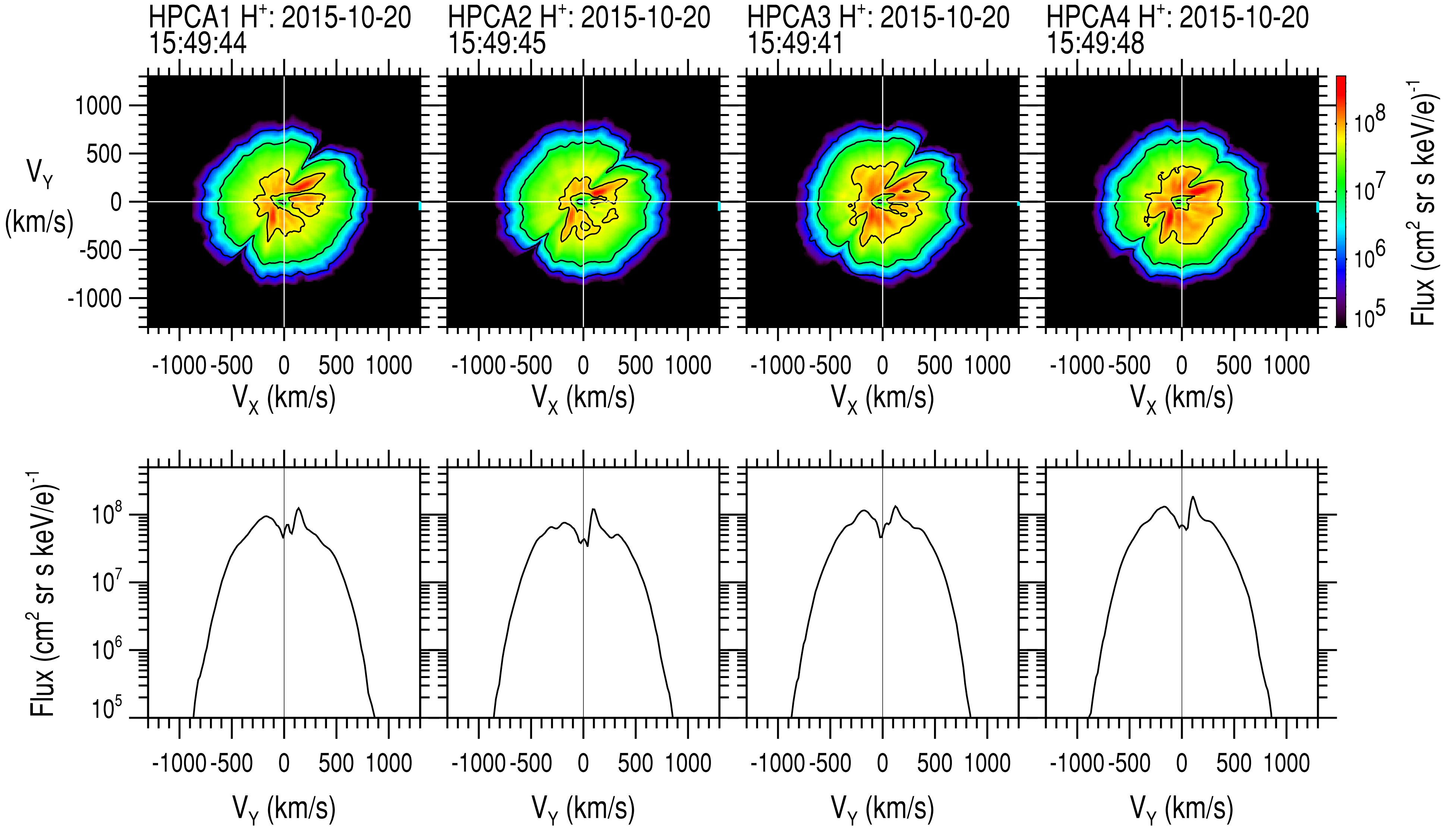
HPCA1 H+: 2015-10-20 15:49:44
HPCA2 H+: 2015-10-20 15:49:45
HPCA3 H+: 2015-10-20 15:49:41
HPCA4 H+: 2015-10-20 15:49:48
V_Y (km/s)
V_X (km/s)
Flux (cm^2 sr s keV/e)^-1
V_Y (km/s)

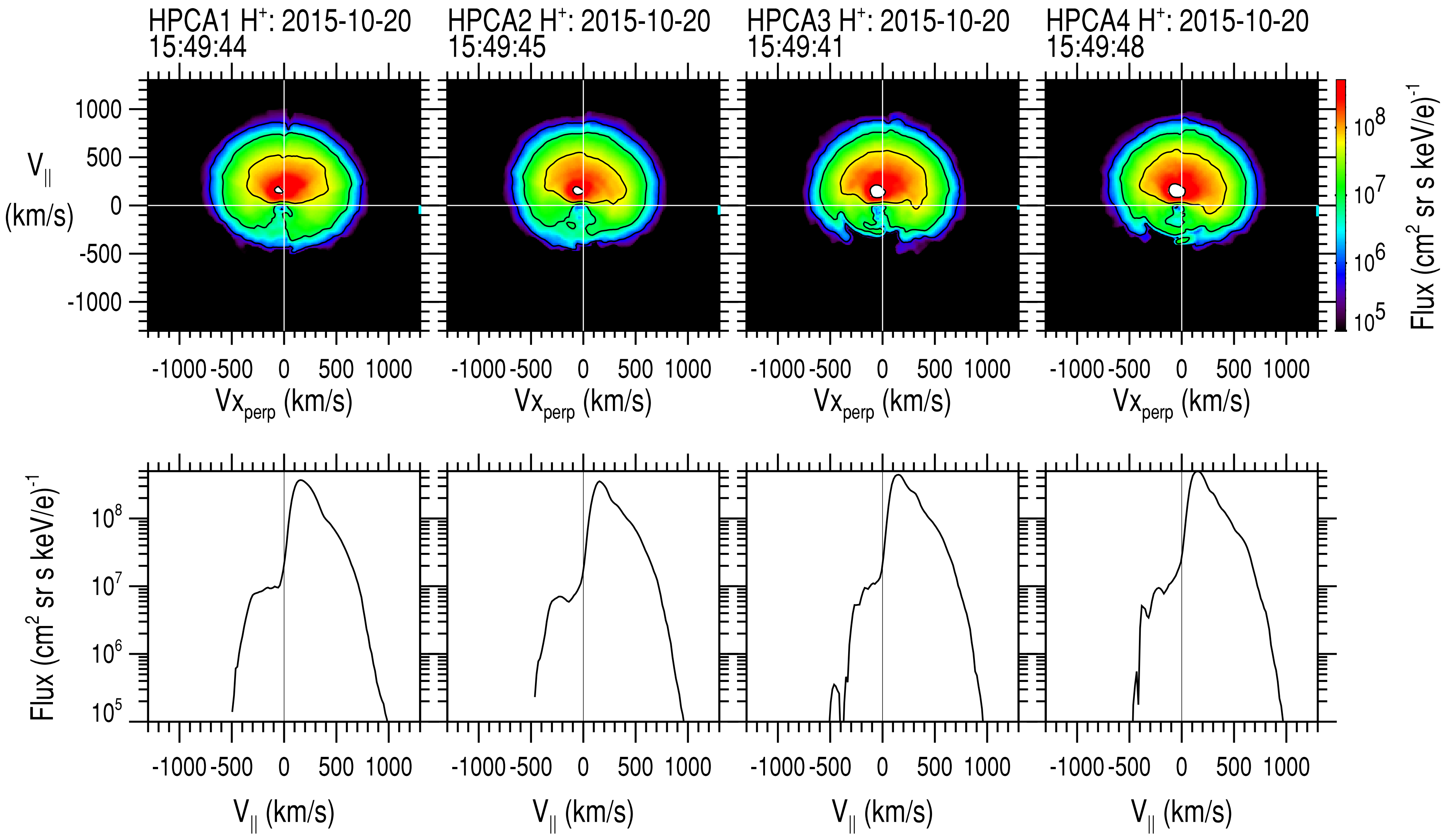

HPCA1 H+: 2015-10-20 15:49:44
HPCA2 H+: 2015-10-20 15:49:45
HPCA3 H+: 2015-10-20 15:49:41
HPCA4 H+: 2015-10-20 15:49:48
V|| (km/s)
Vxperp (km/s)
Flux (cm2 sr s keV/e)-1

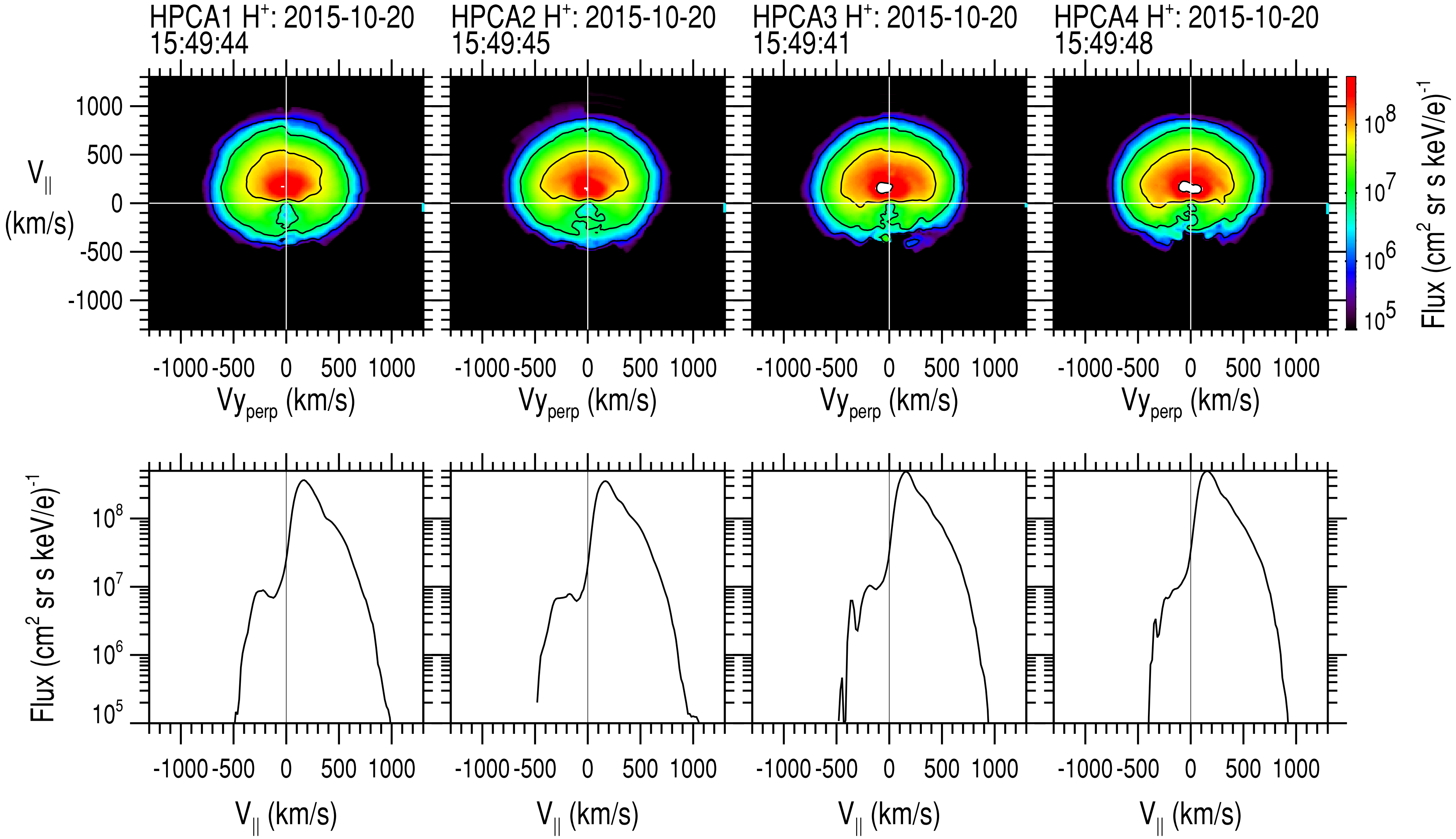

HPCA1 H+: 2015-10-20 15:49:44
HPCA2 H+: 2015-10-20 15:49:45
HPCA3 H+: 2015-10-20 15:49:41
HPCA4 H+: 2015-10-20 15:49:48
V|| (km/s)
Vyperp (km/s)
Flux (cm2 sr s keV/e)-1

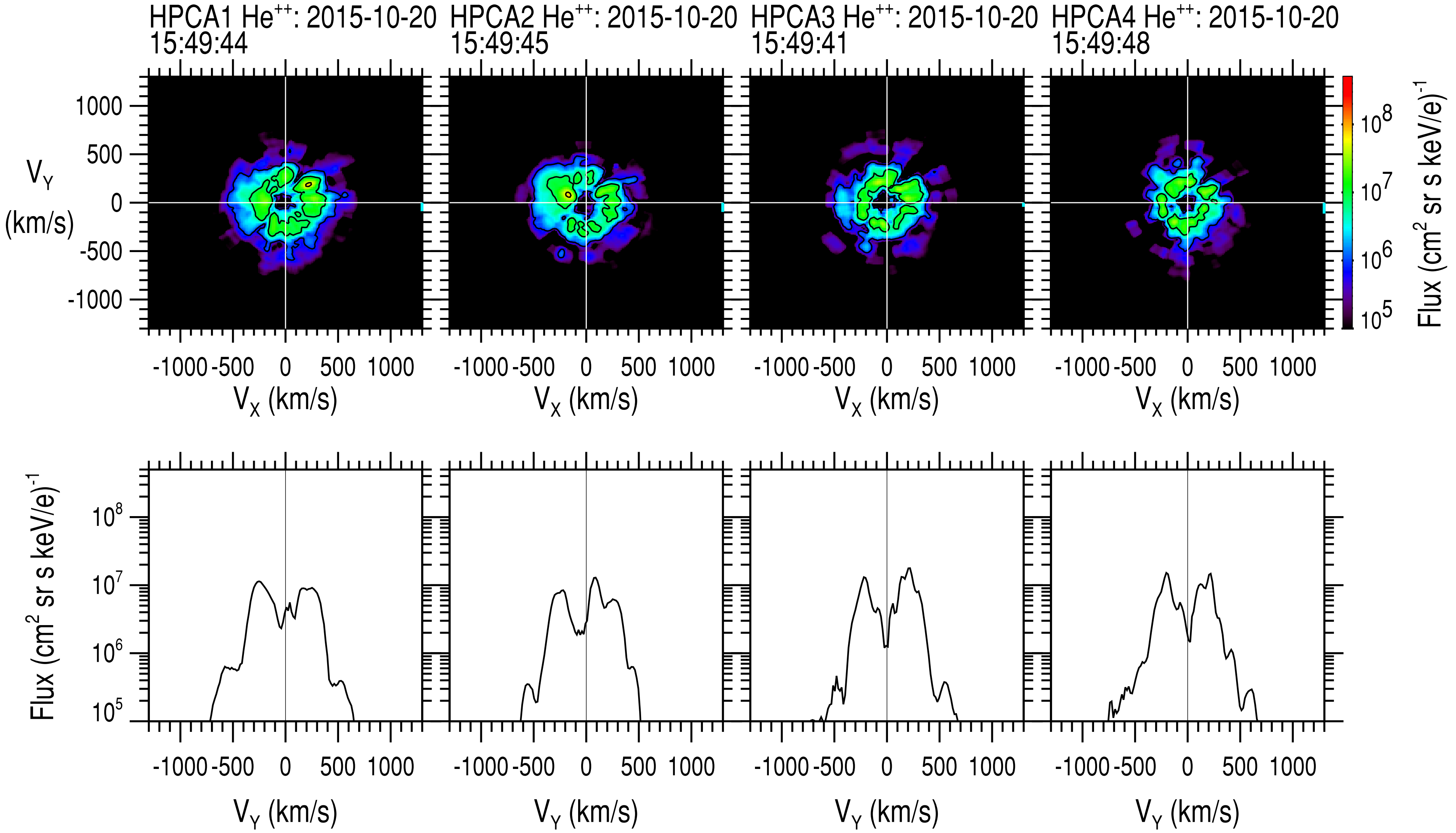
HPCA1 He++: 2015-10-20 15:49:44
HPCA2 He++: 2015-10-20 15:49:45
HPCA3 He++: 2015-10-20 15:49:41
HPCA4 He++: 2015-10-20 15:49:48
VY (km/s)
VX (km/s)
Flux (cm2 sr s keV/e)-1
VY (km/s)
Flux (cm2 sr s keV/e)-1

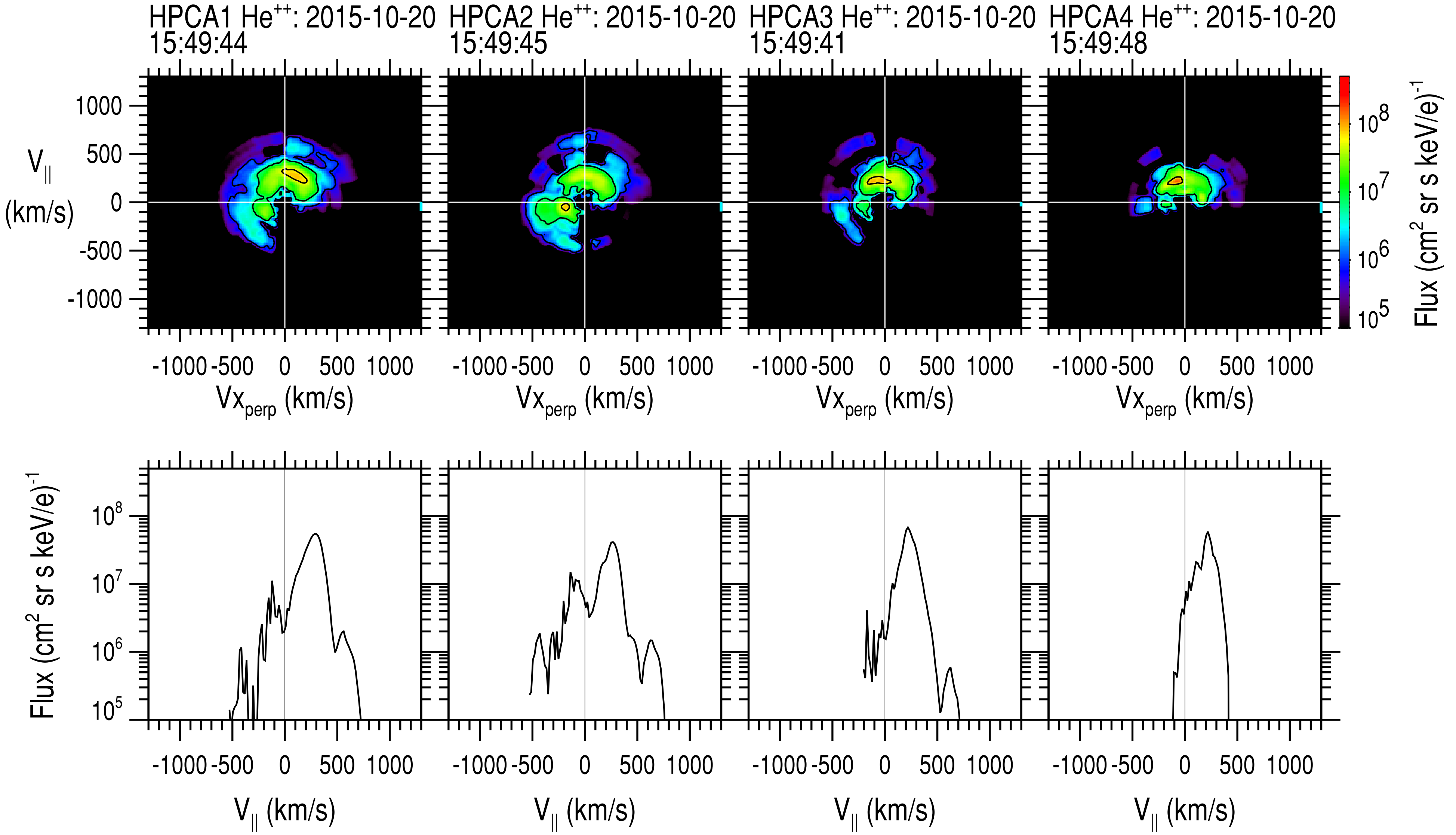

HPCA1 He++: 2015-10-20 15:49:44
HPCA2 He++: 2015-10-20 15:49:45
HPCA3 He++: 2015-10-20 15:49:41
HPCA4 He++: 2015-10-20 15:49:48
V|| (km/s)
Vxperp (km/s)
Flux (cm2 sr s keV/e)-1

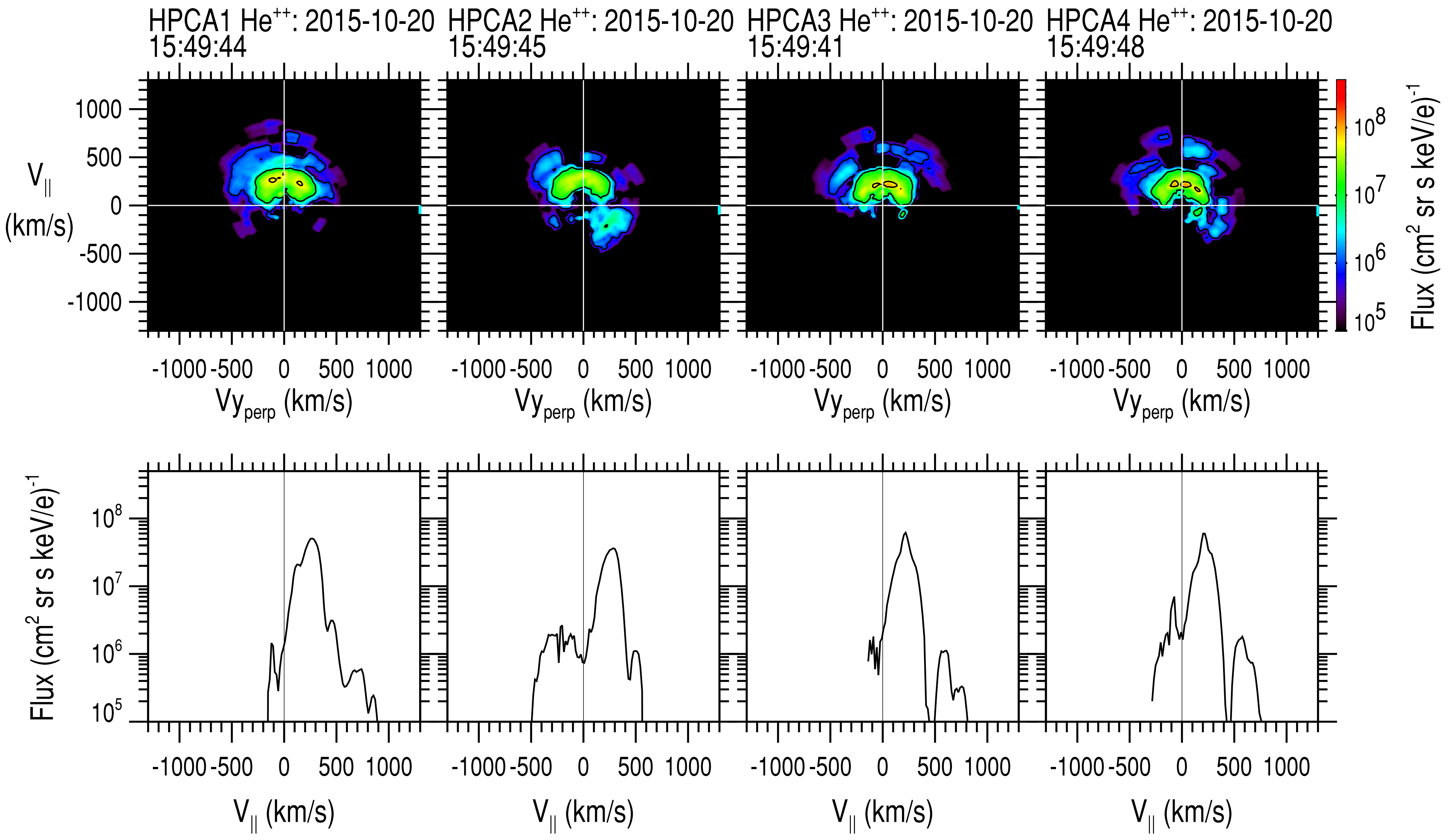

HPCA1 He++: 2015-10-20 15:49:44
HPCA2 He++: 2015-10-20 15:49:45
HPCA3 He++: 2015-10-20 15:49:41
HPCA4 He++: 2015-10-20 15:49:48
V|| (km/s)
Vyperp (km/s)
Flux (cm2 sr s keV/e)-1
-1000 -500 0 500 1000
10^5 10^6 10^7 10^8

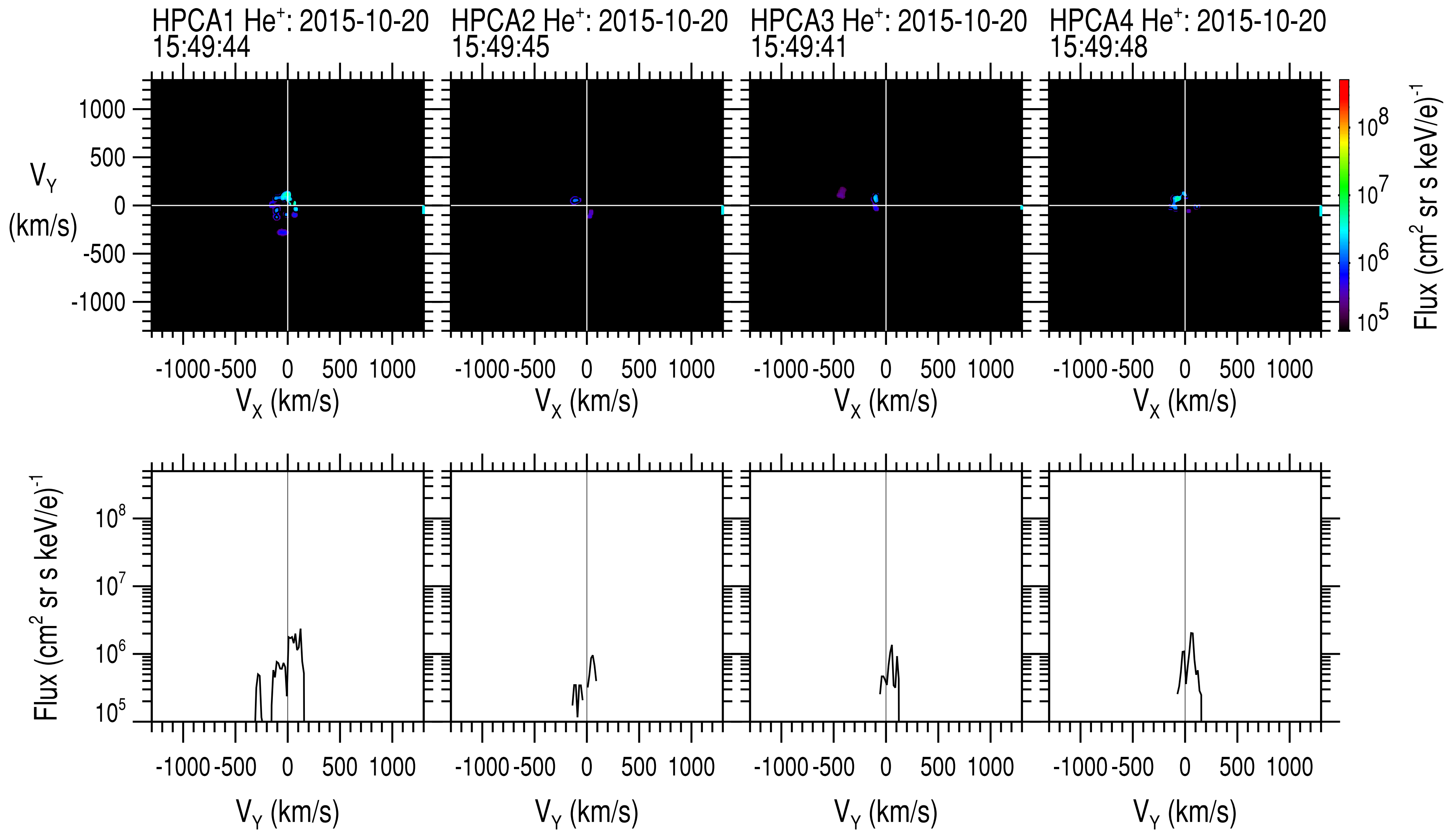
HPCA1 He+: 2015-10-20 15:49:44
HPCA2 He+: 2015-10-20 15:49:45
HPCA3 He+: 2015-10-20 15:49:41
HPCA4 He+: 2015-10-20 15:49:48
VY (km/s)
VX (km/s)
Flux (cm2 sr s keV/e)-1
VY (km/s)

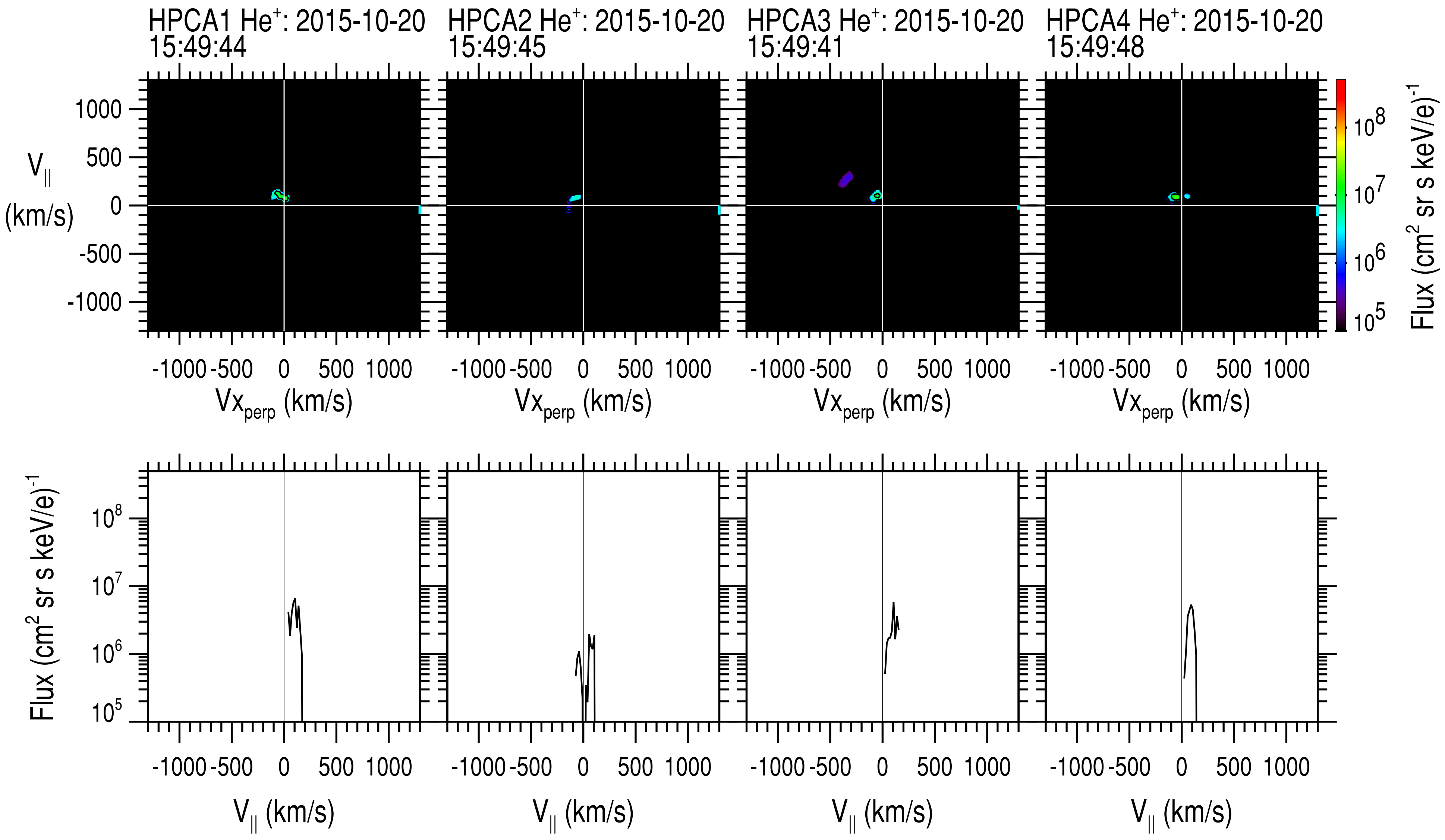

HPCA1 He+: 2015-10-20 15:49:44
HPCA2 He+: 2015-10-20 15:49:45
HPCA3 He+: 2015-10-20 15:49:41
HPCA4 He+: 2015-10-20 15:49:48
V|| (km/s)
Vxperp (km/s)
Flux (cm2 sr s keV/e)-1

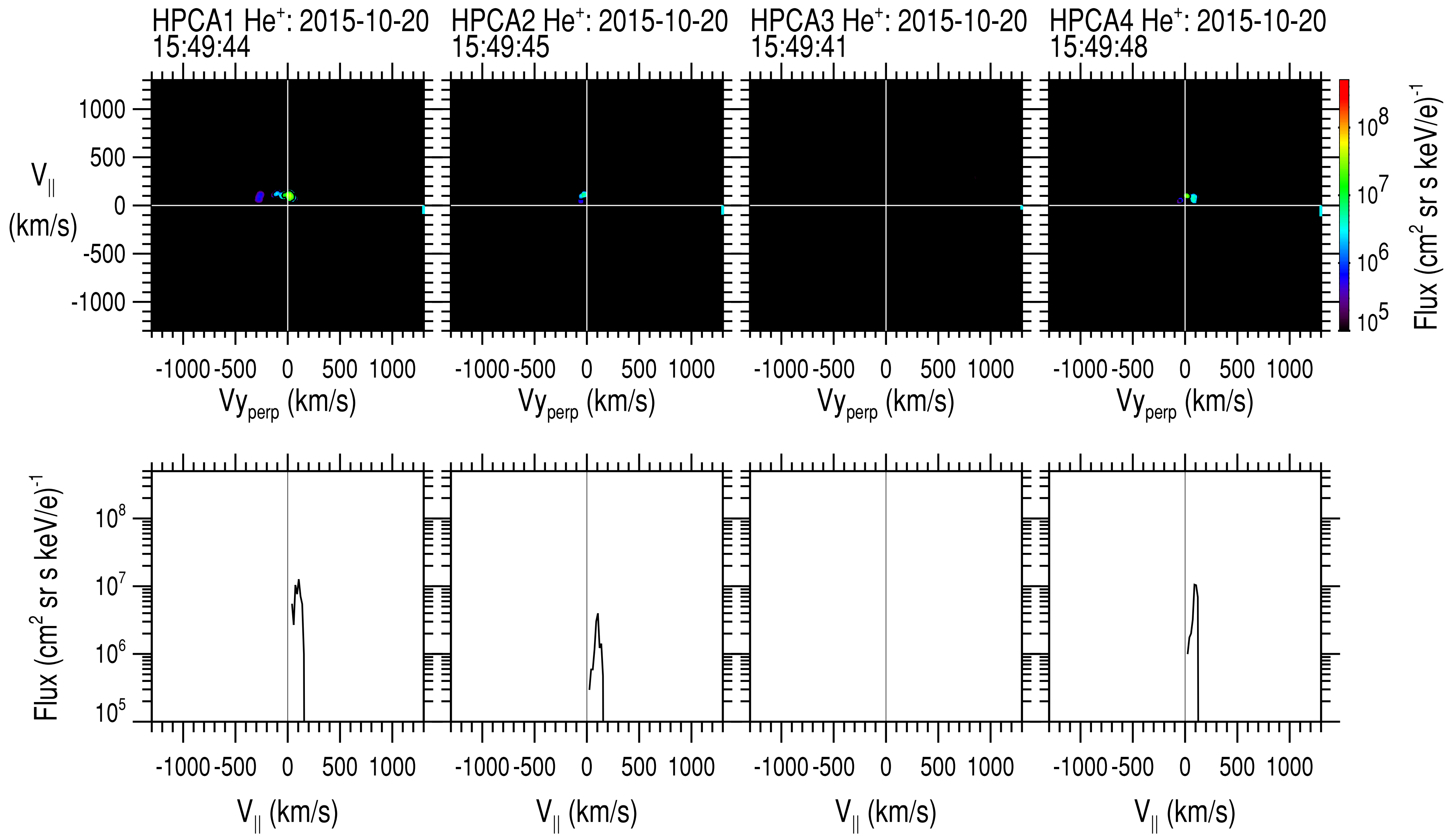
HPCA1 He+: 2015-10-20 15:49:44
HPCA2 He+: 2015-10-20 15:49:45
HPCA3 He+: 2015-10-20 15:49:41
HPCA4 He+: 2015-10-20 15:49:48
V|| (km/s)
Vyperp (km/s)
Flux (cm2 sr s keV/e)-1
1000
500
0
-500
-1000
10^5
10^6
10^7
10^8

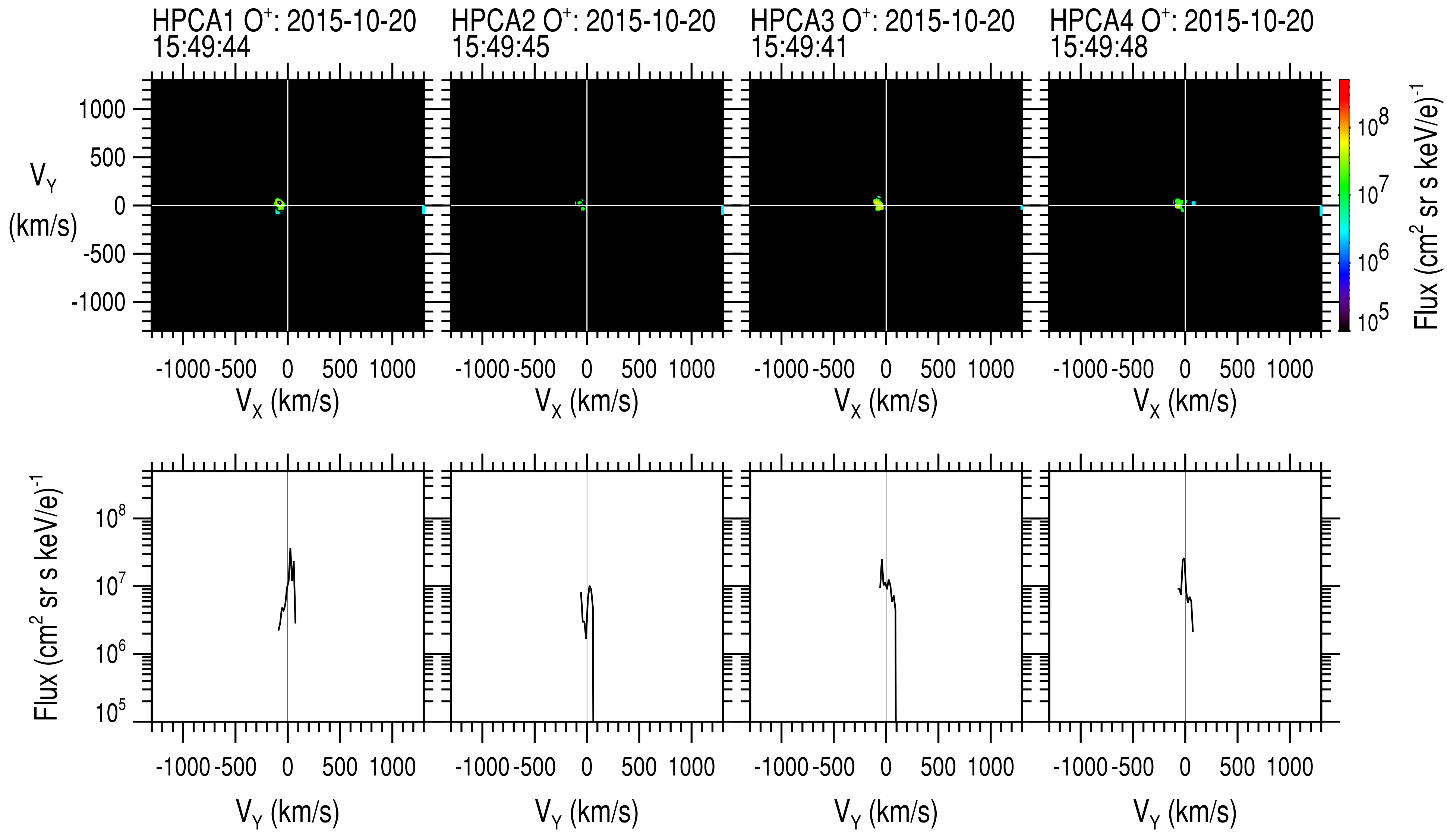
HPCA1 O+: 2015-10-20 15:49:44
HPCA2 O+: 2015-10-20 15:49:45
HPCA3 O+: 2015-10-20 15:49:41
HPCA4 O+: 2015-10-20 15:49:48
VY (km/s)
1000
500
0
-500
-1000
-1000 -500 0 500 1000
VX (km/s)
Flux (cm2 sr s keV/e)-1
10^5
10^6
10^7
10^8
VY (km/s)

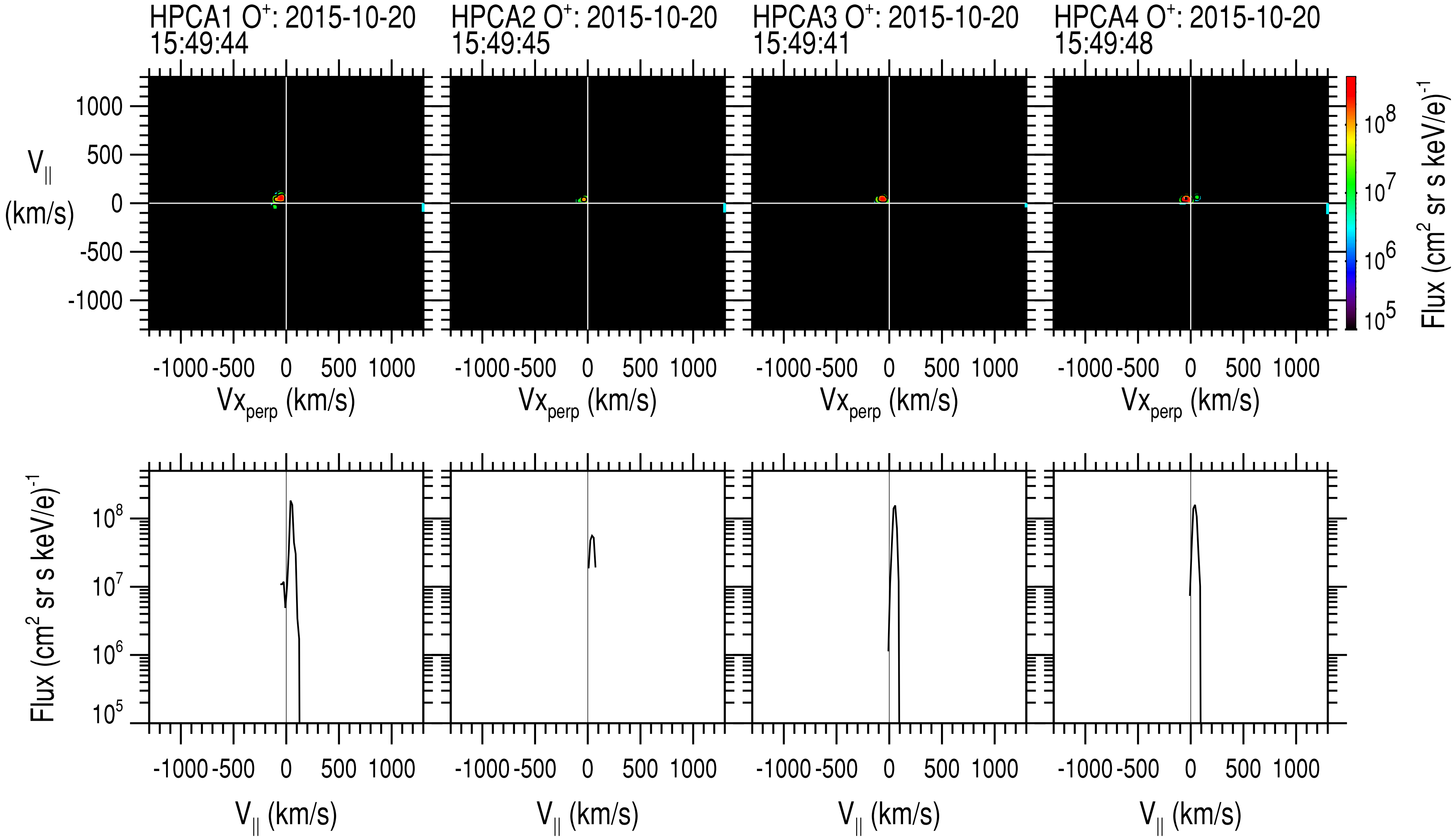
HPCA1 O+: 2015-10-20
15:49:44
HPCA2 O+: 2015-10-20
15:49:45
HPCA3 O+: 2015-10-20
15:49:41
HPCA4 O+: 2015-10-20
15:49:48
V|| (km/s)
Vxperp (km/s)
Flux (cm2 sr s keV/e)-1
-1000 -500 0 500 1000
10^5 10^6 10^7 10^8

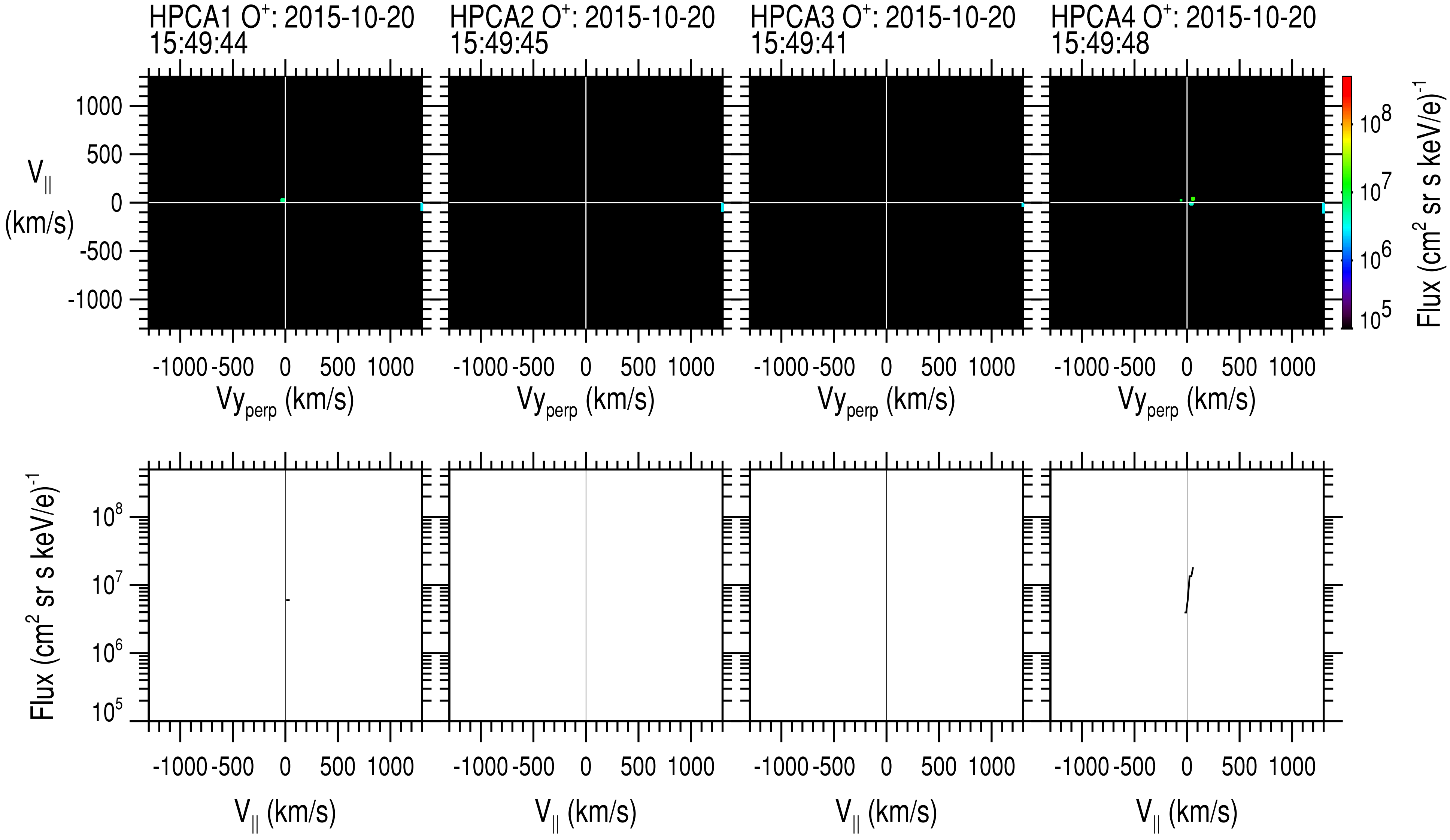

HPCA1 O+: 2015-10-20 15:49:44
HPCA2 O+: 2015-10-20 15:49:45
HPCA3 O+: 2015-10-20 15:49:41
HPCA4 O+: 2015-10-20 15:49:48
V|| (km/s)
1000
500
0
-500
-1000
Vyperp (km/s)
Flux (cm2 sr s keV/e)-1
10^5
10^6
10^7
10^8

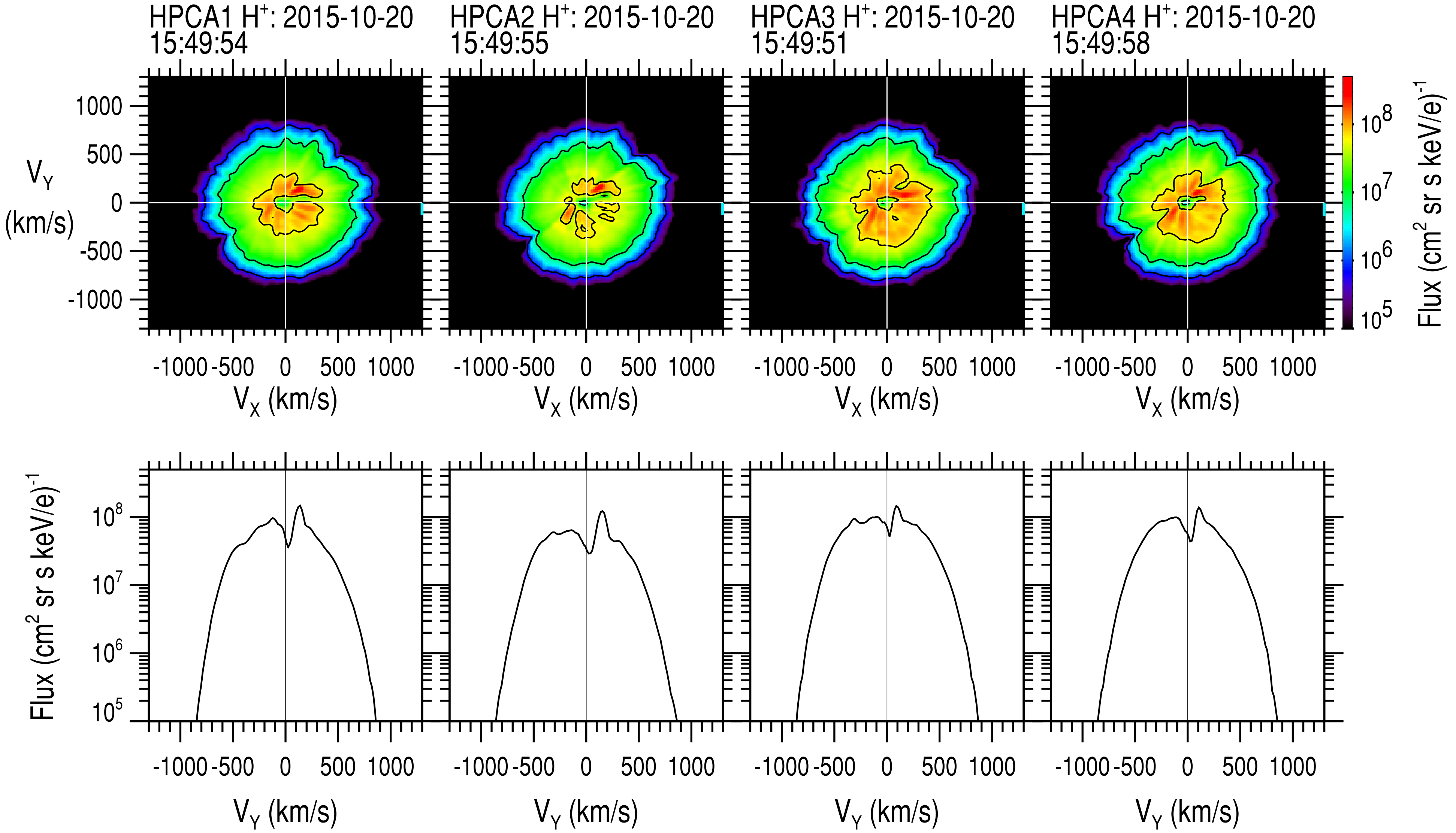
HPCA1 H+: 2015-10-20
15:49:54
HPCA2 H+: 2015-10-20
15:49:55
HPCA3 H+: 2015-10-20
15:49:51
HPCA4 H+: 2015-10-20
15:49:58
VY (km/s)
VX (km/s)
Flux (cm2 sr s keV/e)-1
VY (km/s)

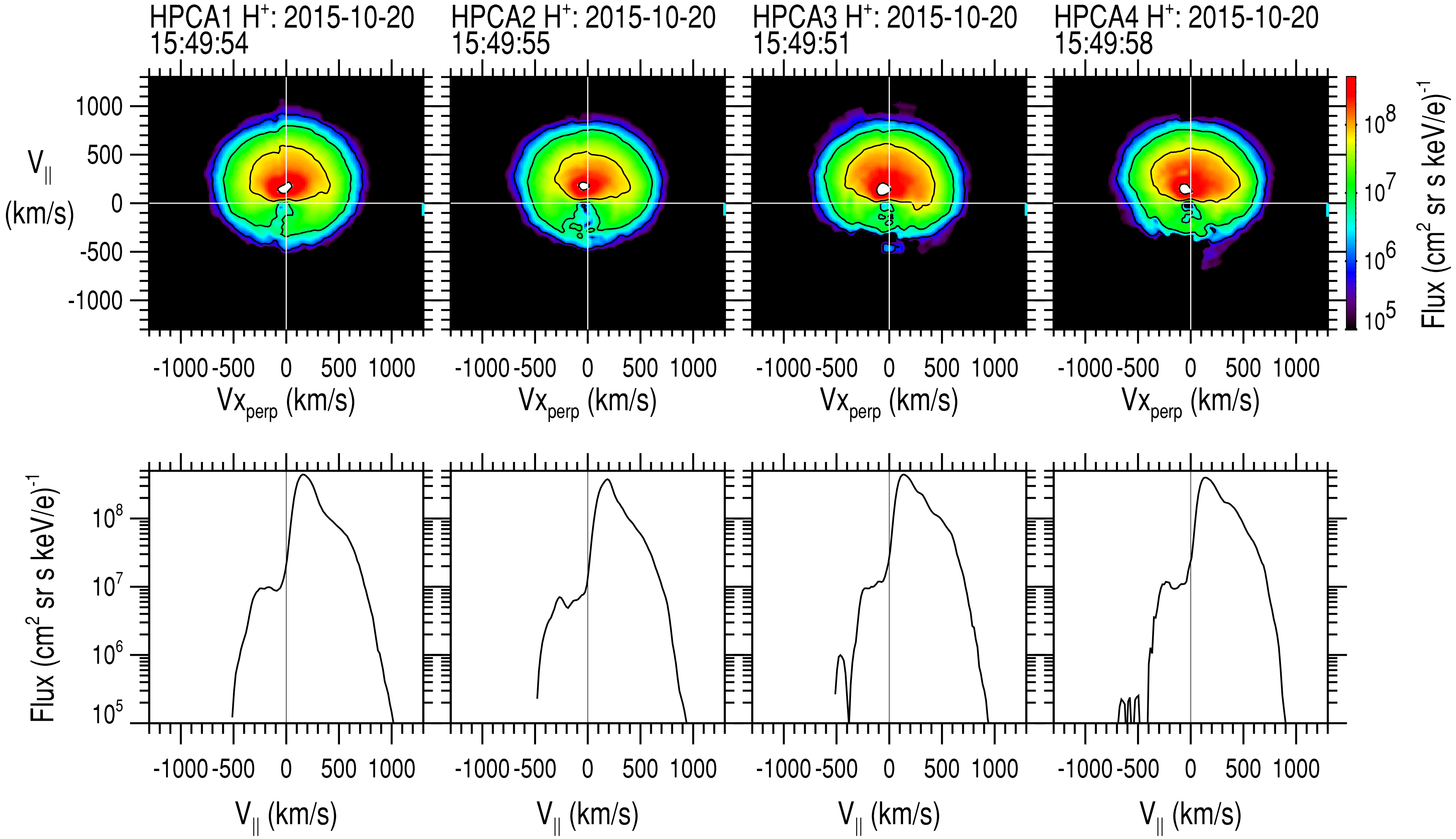
HPCA1 H+: 2015-10-20 15:49:54
HPCA2 H+: 2015-10-20 15:49:55
HPCA3 H+: 2015-10-20 15:49:51
HPCA4 H+: 2015-10-20 15:49:58
V|| (km/s)
Vxperp (km/s)
Flux (cm2 sr s keV/e)-1
V|| (km/s)

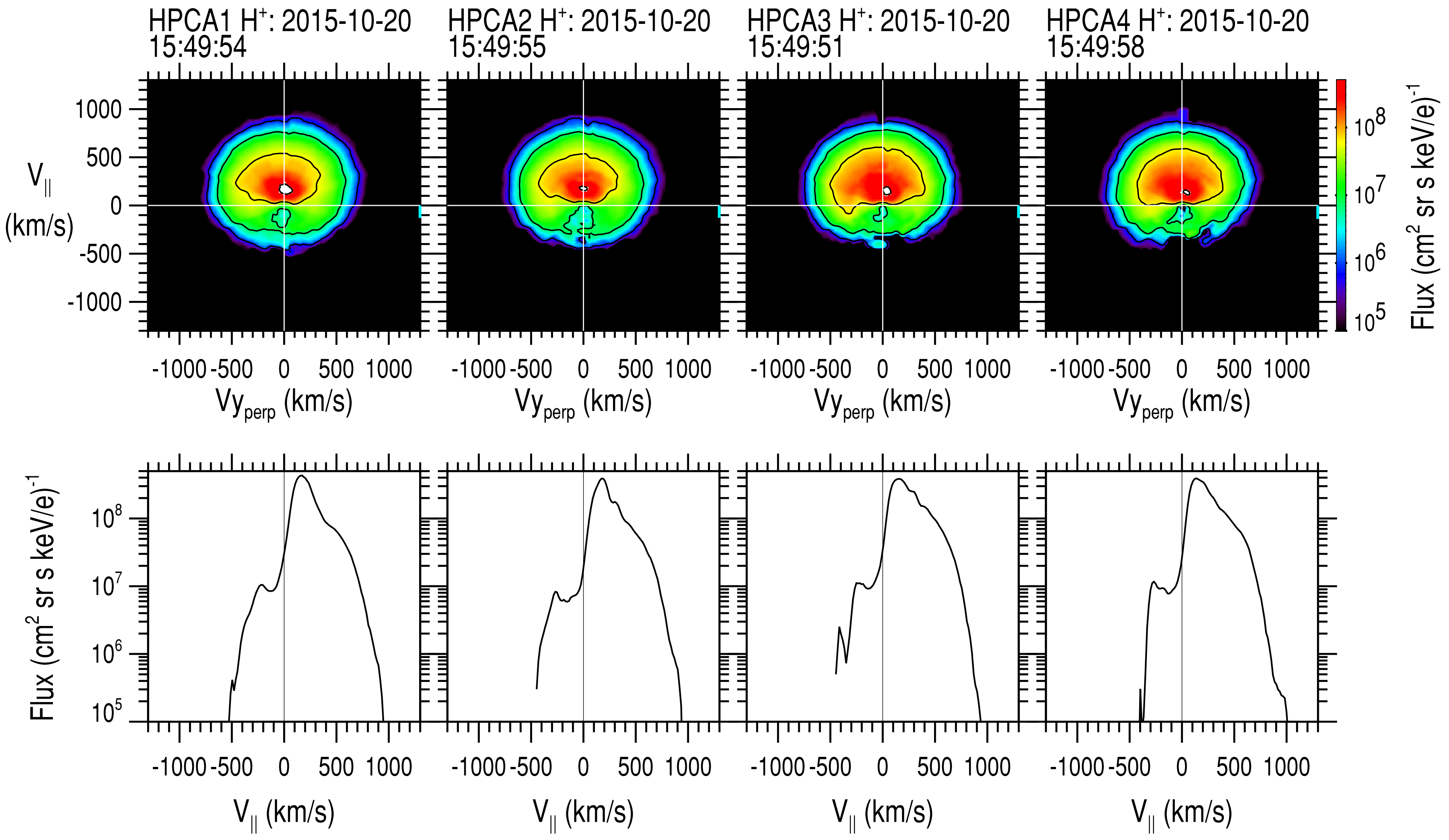
HPCA1 H+: 2015-10-20 15:49:54
HPCA2 H+: 2015-10-20 15:49:55
HPCA3 H+: 2015-10-20 15:49:51
HPCA4 H+: 2015-10-20 15:49:58
V|| (km/s)
Vyperp (km/s)
Flux (cm2 sr s keV/e)-1
V|| (km/s)

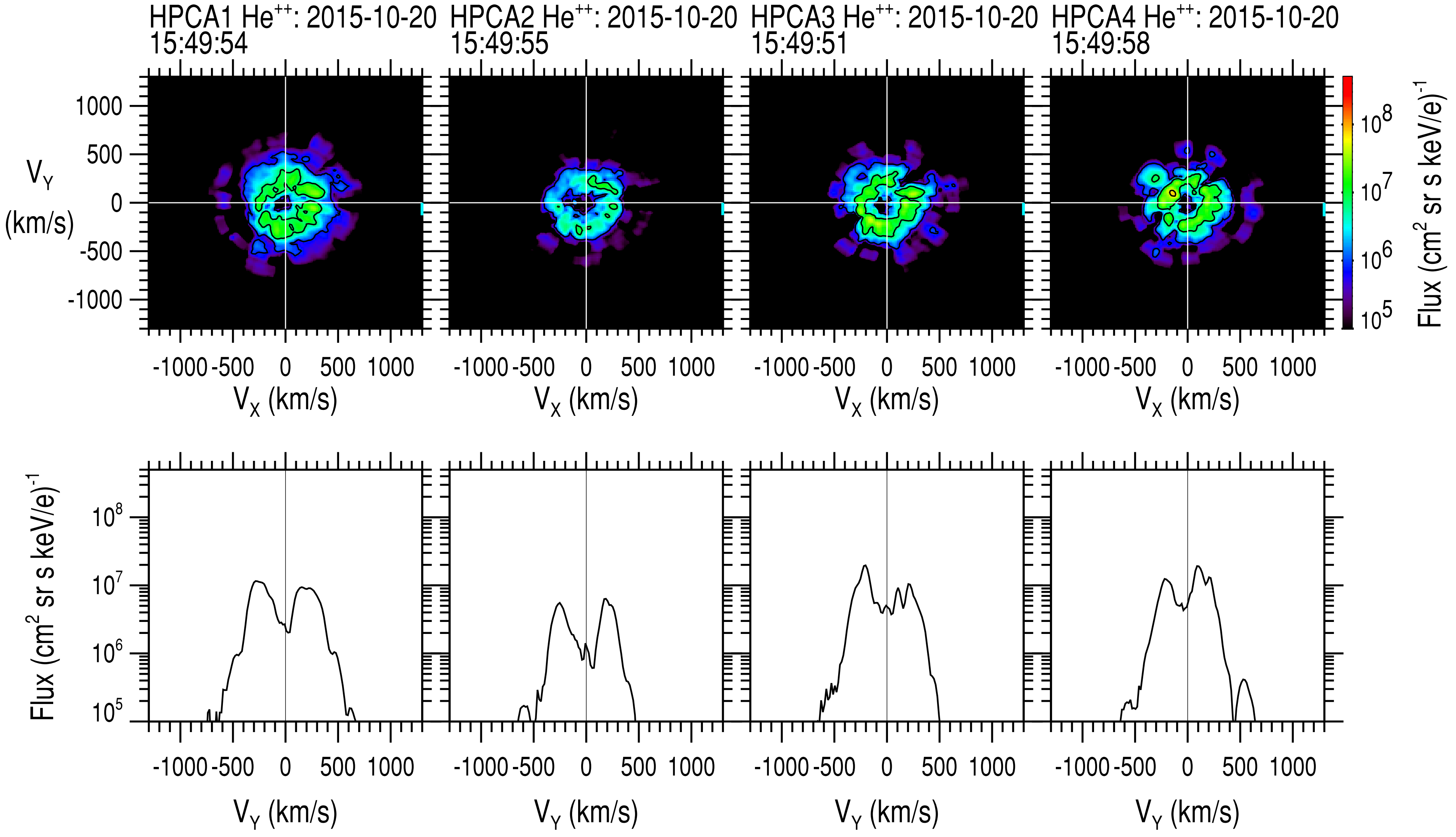

HPCA1 He++: 2015-10-20 15:49:54
HPCA2 He++: 2015-10-20 15:49:55
HPCA3 He++: 2015-10-20 15:49:51
HPCA4 He++: 2015-10-20 15:49:58
V_Y (km/s)
V_X (km/s)
Flux (cm^2 sr s keV/e)^-1
V_Y (km/s)

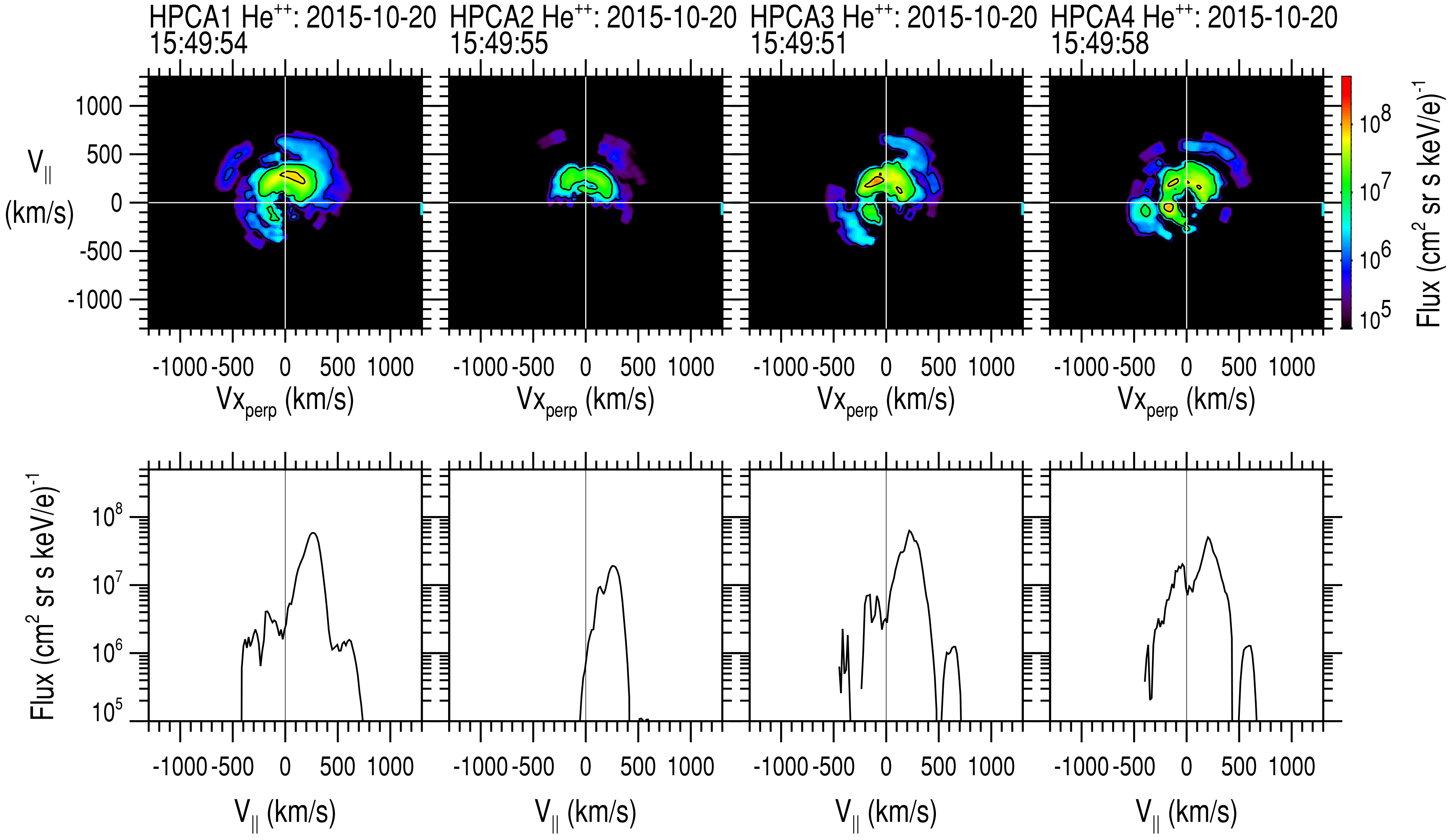
HPCA1 He++: 2015-10-20 15:49:54
HPCA2 He++: 2015-10-20 15:49:55
HPCA3 He++: 2015-10-20 15:49:51
HPCA4 He++: 2015-10-20 15:49:58
V|| (km/s)
Vxperp (km/s)
Flux (cm2 sr s keV/e)-1
V|| (km/s)

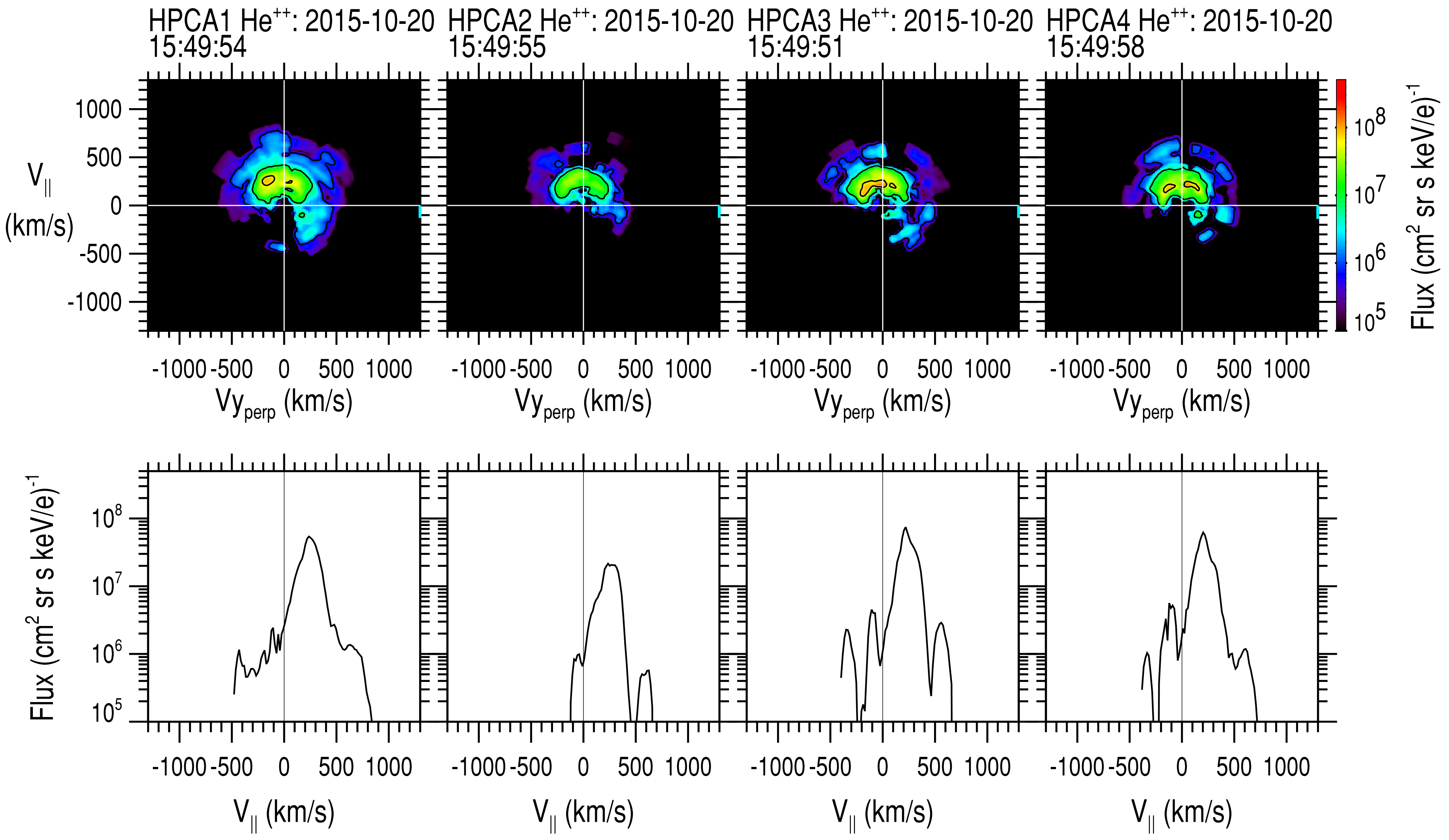

HPCA1 He++: 2015-10-20 15:49:54
HPCA2 He++: 2015-10-20 15:49:55
HPCA3 He++: 2015-10-20 15:49:51
HPCA4 He++: 2015-10-20 15:49:58
V|| (km/s)
Vyperp (km/s)
Flux (cm2 sr s keV/e)-1

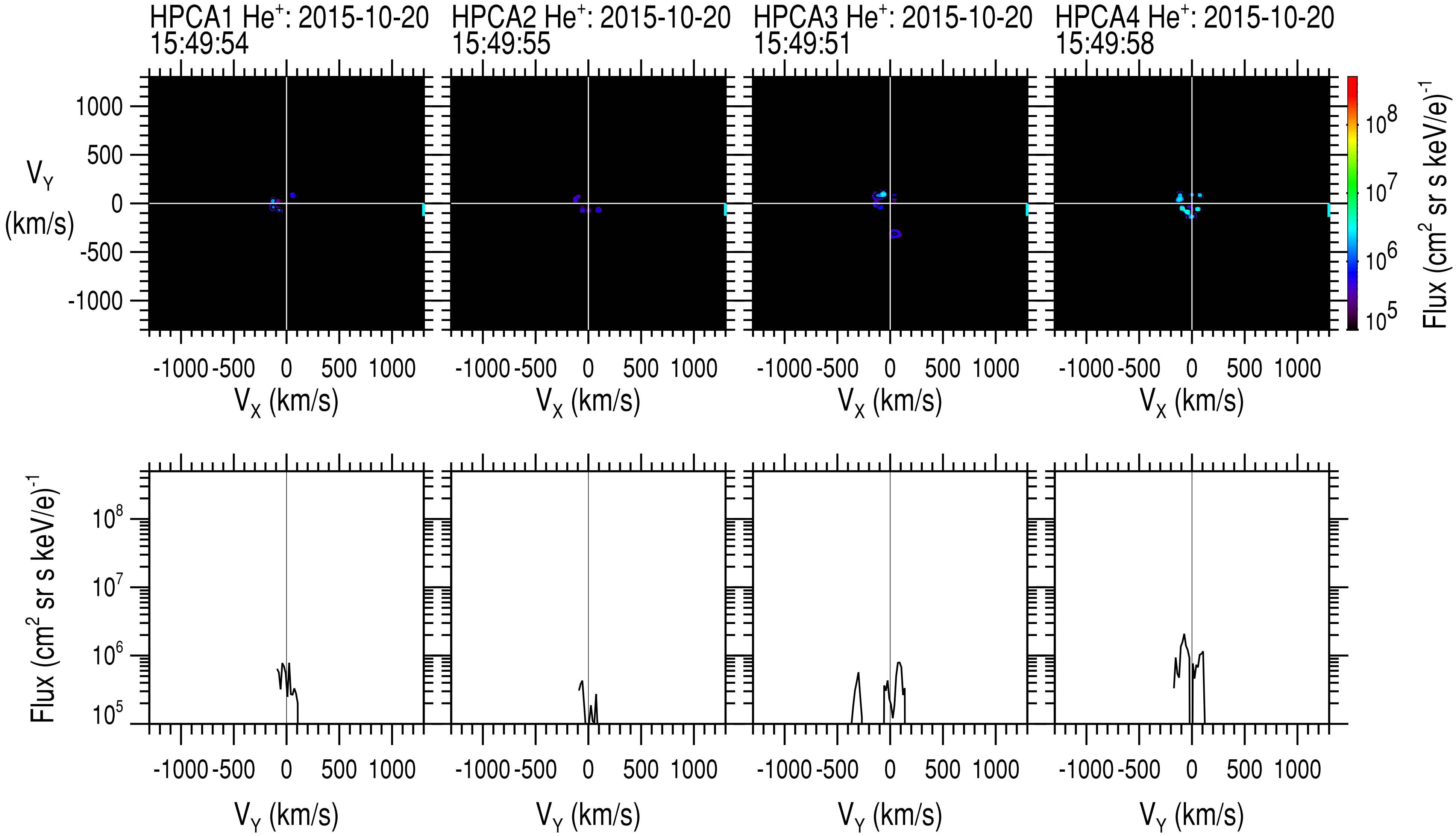

HPCA1 He+: 2015-10-20
15:49:54
HPCA2 He+: 2015-10-20
15:49:55
HPCA3 He+: 2015-10-20
15:49:51
HPCA4 He+: 2015-10-20
15:49:58
VY (km/s)
VX (km/s)
Flux (cm2 sr s keV/e)-1
VY (km/s)
Flux (cm2 sr s keV/e)-1

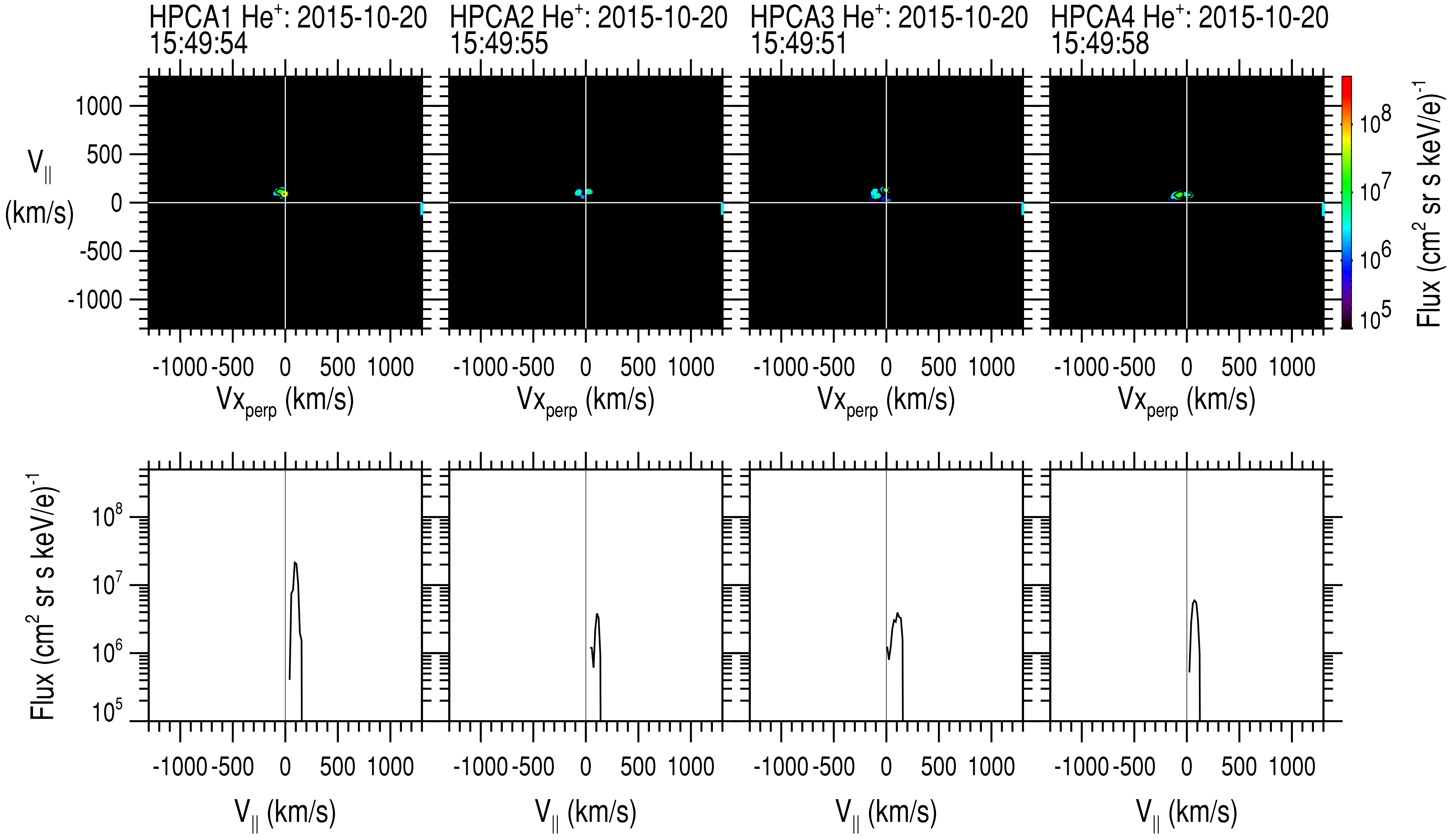
HPCA1 He+: 2015-10-20 15:49:54
HPCA2 He+: 2015-10-20 15:49:55
HPCA3 He+: 2015-10-20 15:49:51
HPCA4 He+: 2015-10-20 15:49:58
V|| (km/s)
Vxperp (km/s)
Flux (cm2 sr s keV/e)-1
-1000 -500 0 500 1000
105 106 107 108

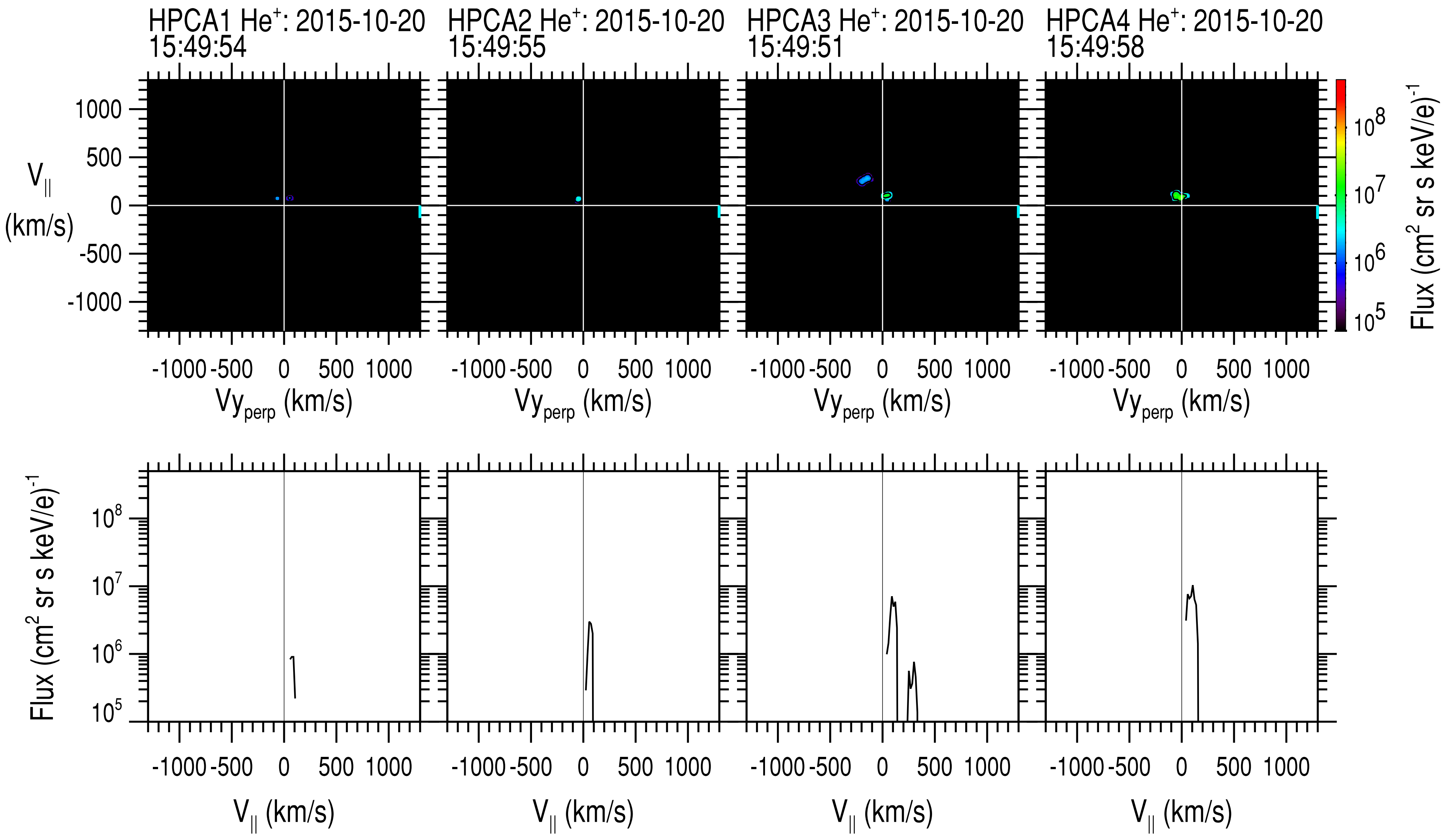
HPCA1 He+: 2015-10-20
15:49:54
HPCA2 He+: 2015-10-20
15:49:55
HPCA3 He+: 2015-10-20
15:49:51
HPCA4 He+: 2015-10-20
15:49:58
V|| (km/s)
Vyperp (km/s)
Flux (cm2 sr s keV/e)-1
-1000 -500 0 500 1000
10^5 10^6 10^7 10^8

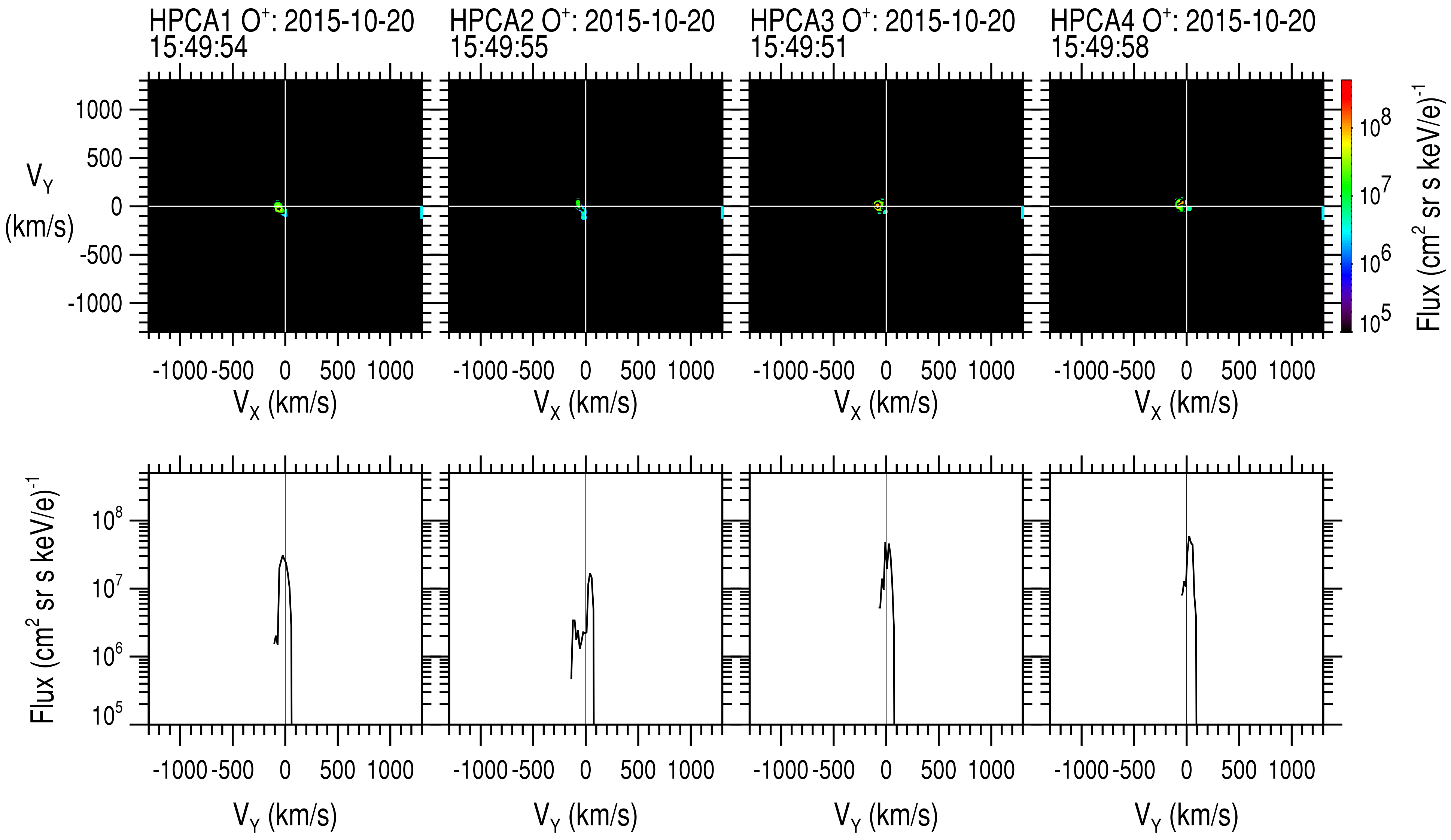

HPCA1 O+: 2015-10-20
15:49:54
HPCA2 O+: 2015-10-20
15:49:55
HPCA3 O+: 2015-10-20
15:49:51
HPCA4 O+: 2015-10-20
15:49:58
VY (km/s)
VX (km/s)
VY (km/s)
Flux (cm2 sr s keV/e)-1
-1000 -500 0 500 1000
10^5 10^6 10^7 10^8

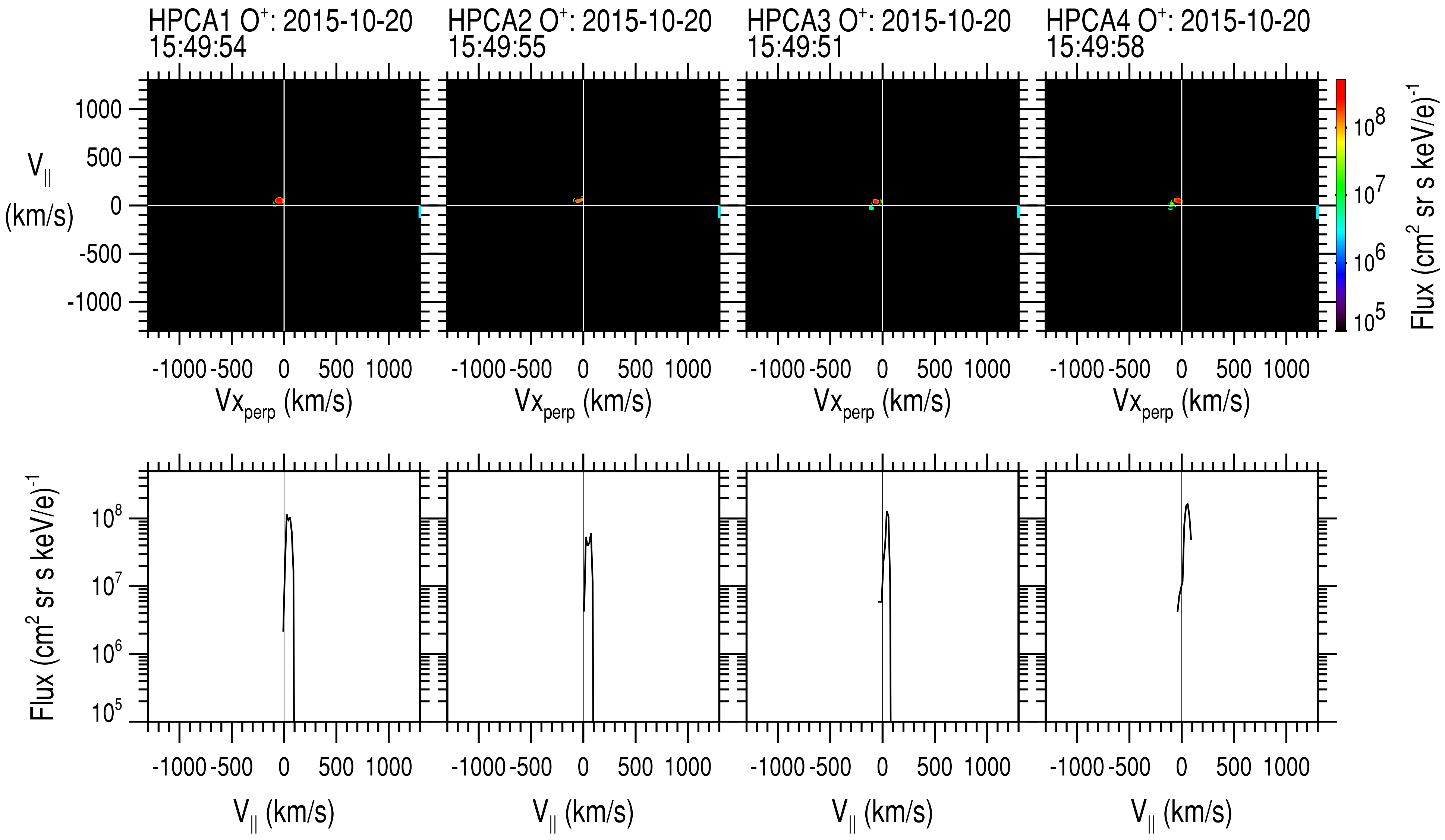
HPCA1 O+: 2015-10-20 15:49:54
HPCA2 O+: 2015-10-20 15:49:55
HPCA3 O+: 2015-10-20 15:49:51
HPCA4 O+: 2015-10-20 15:49:58
V|| (km/s)
Vxperp (km/s)
Flux (cm2 sr s keV/e)-1
V|| (km/s)

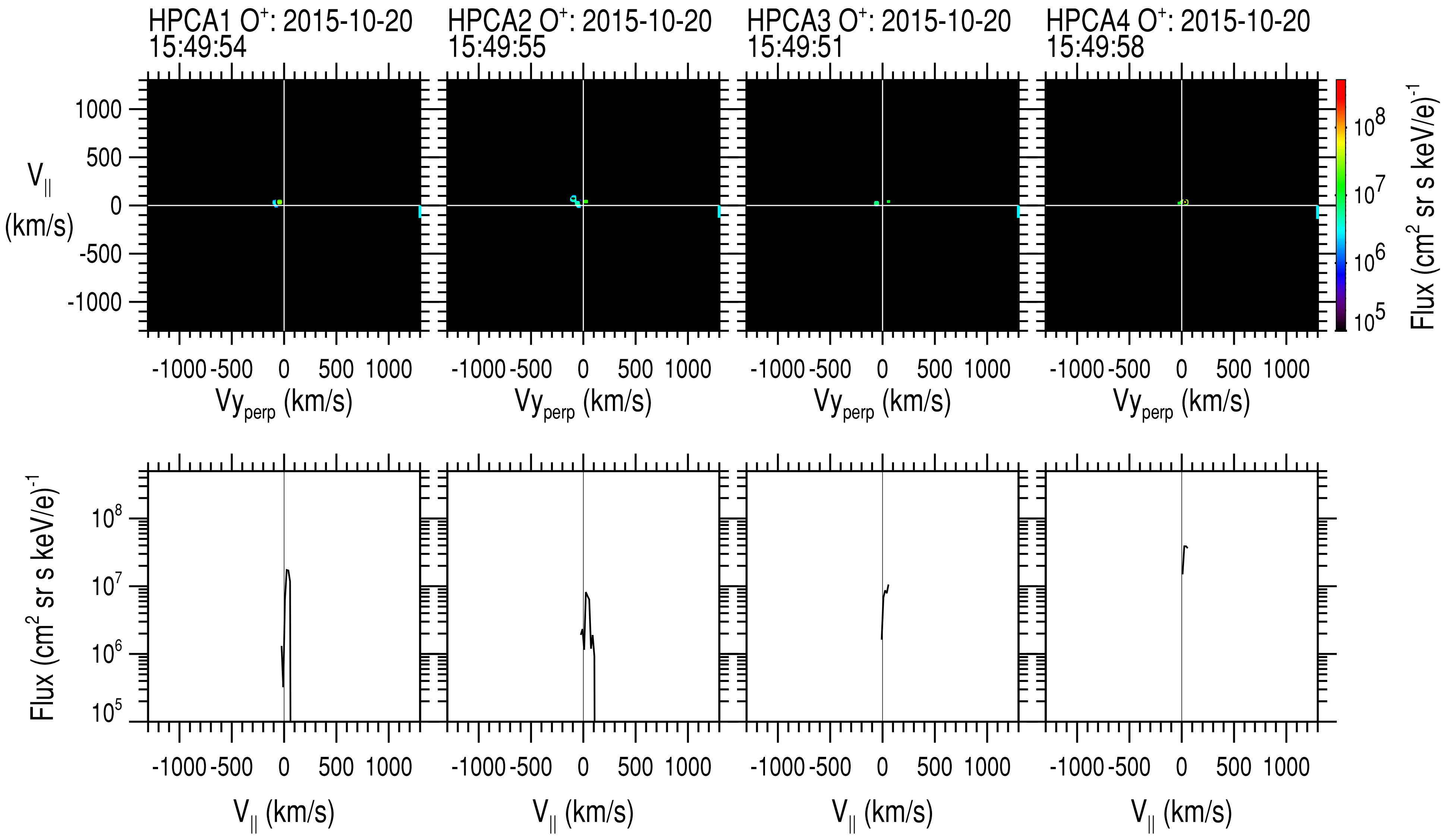
HPCA1 O+: 2015-10-20 15:49:54
HPCA2 O+: 2015-10-20 15:49:55
HPCA3 O+: 2015-10-20 15:49:51
HPCA4 O+: 2015-10-20 15:49:58
V|| (km/s)
Vyperp (km/s)
Flux (cm2 sr s keV/e)-1
1000
500
0
-500
-1000
10^5
10^6
10^7
10^8

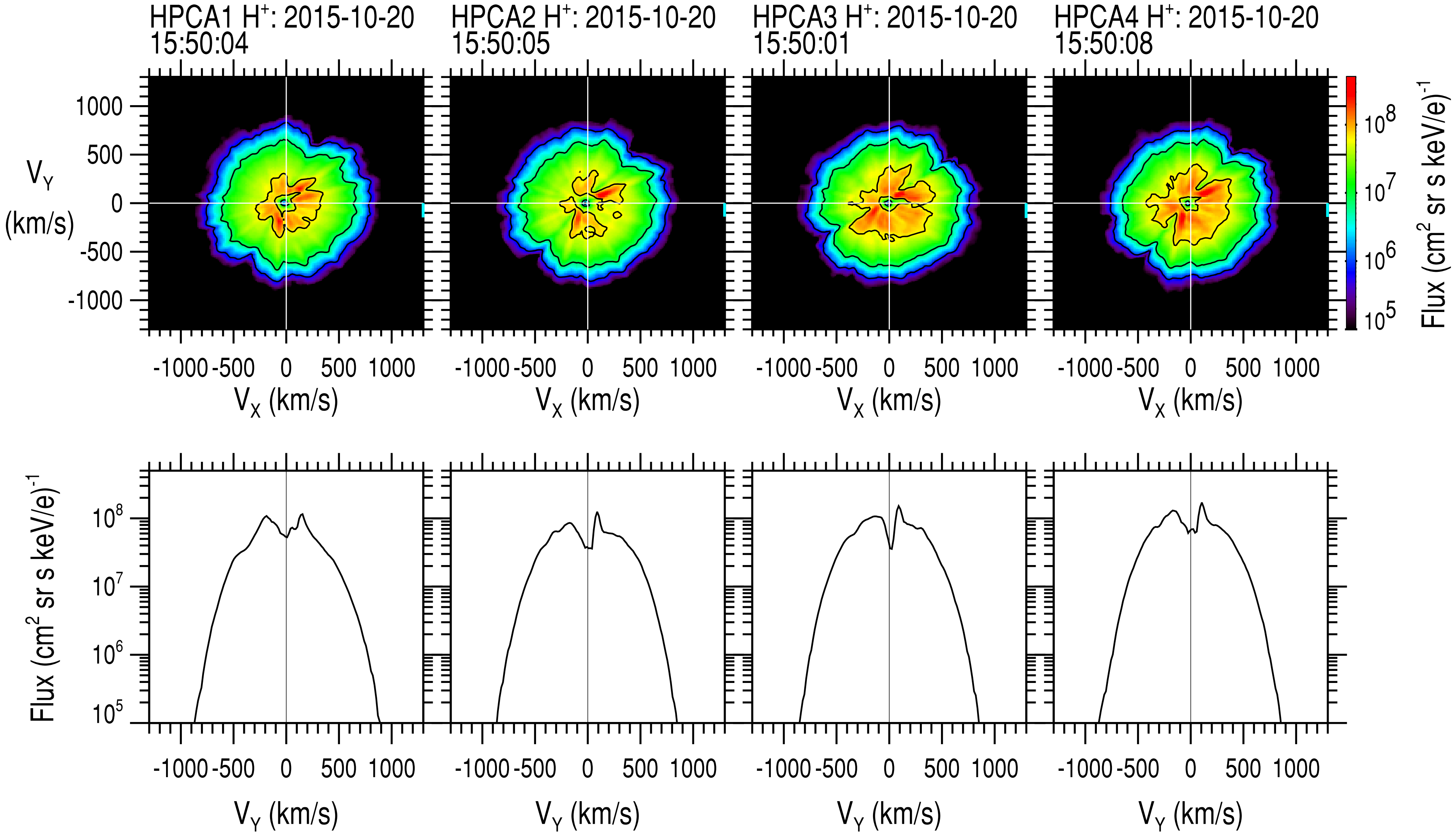
HPCA1 H+: 2015-10-20 15:50:04
HPCA2 H+: 2015-10-20 15:50:05
HPCA3 H+: 2015-10-20 15:50:01
HPCA4 H+: 2015-10-20 15:50:08
VY (km/s)
VX (km/s)
Flux (cm2 sr s keV/e)-1
VY (km/s)
Flux (cm2 sr s keV/e)-1

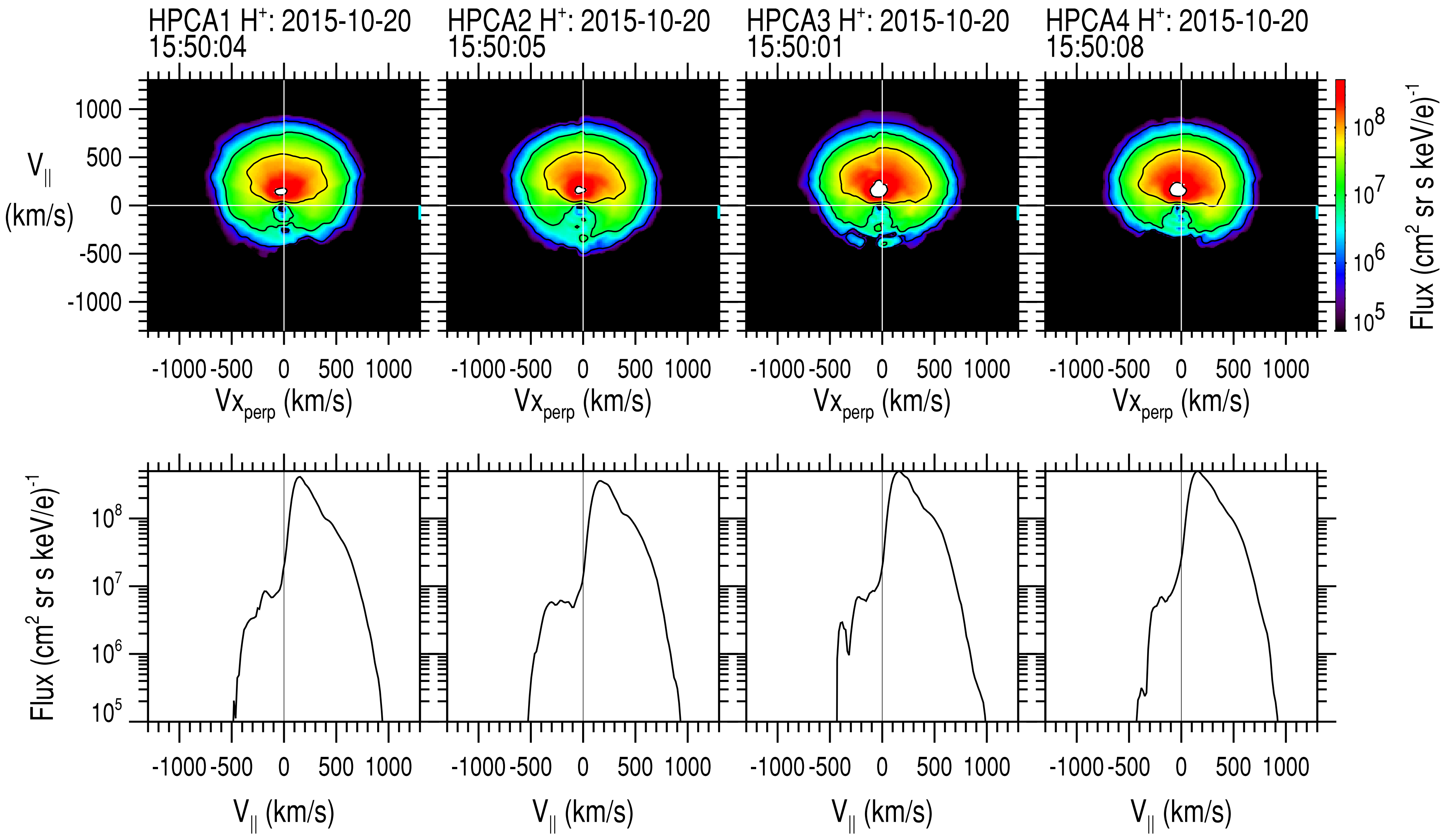
HPCA1 H+: 2015-10-20 15:50:04
HPCA2 H+: 2015-10-20 15:50:05
HPCA3 H+: 2015-10-20 15:50:01
HPCA4 H+: 2015-10-20 15:50:08
V|| (km/s)
Vxperp (km/s)
Flux (cm2 sr s keV/e)-1

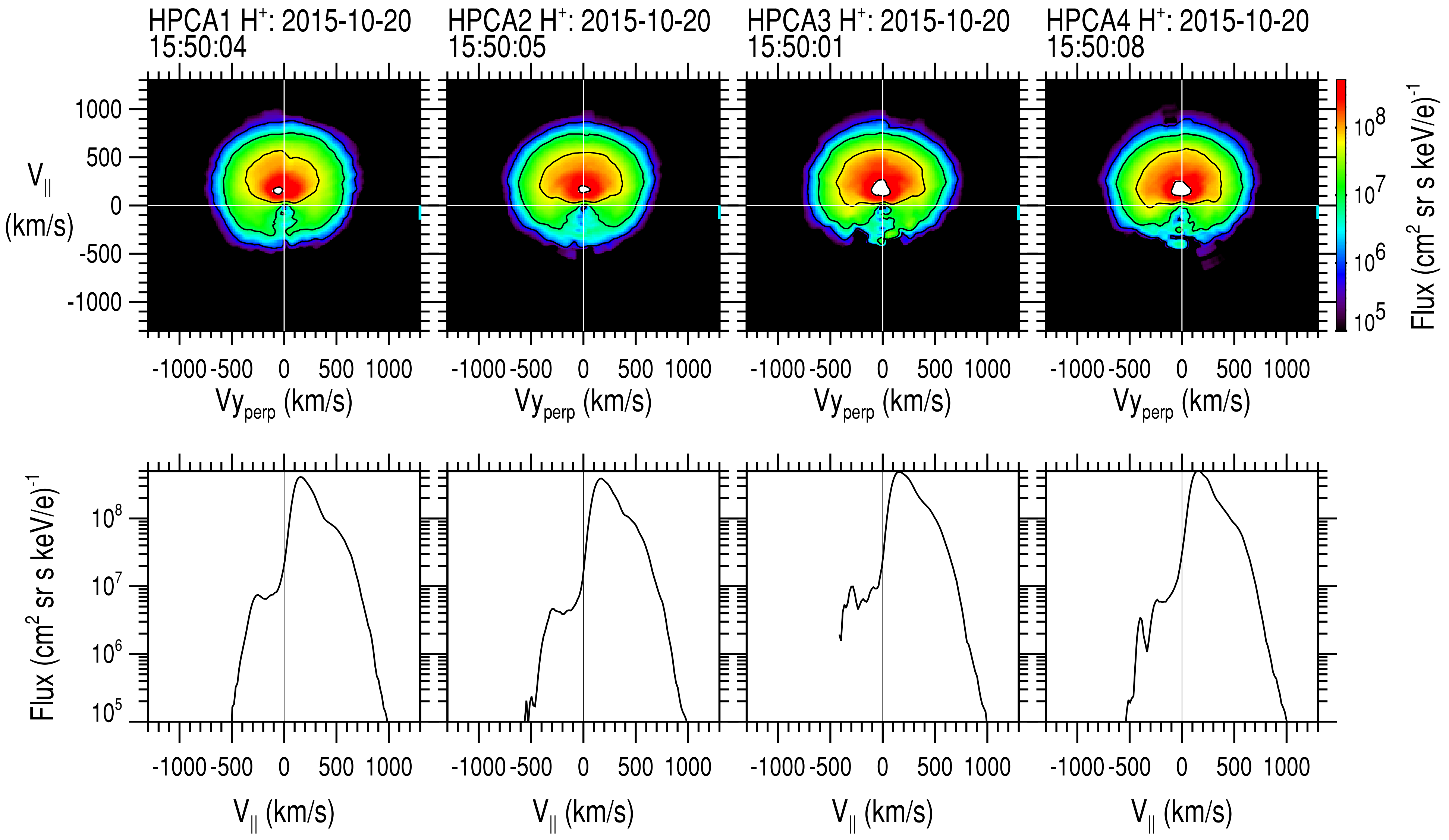

HPCA1 H+: 2015-10-20 15:50:04
HPCA2 H+: 2015-10-20 15:50:05
HPCA3 H+: 2015-10-20 15:50:01
HPCA4 H+: 2015-10-20 15:50:08
V|| (km/s)
Vyperp (km/s)
Flux (cm2 sr s keV/e)-1
V|| (km/s)

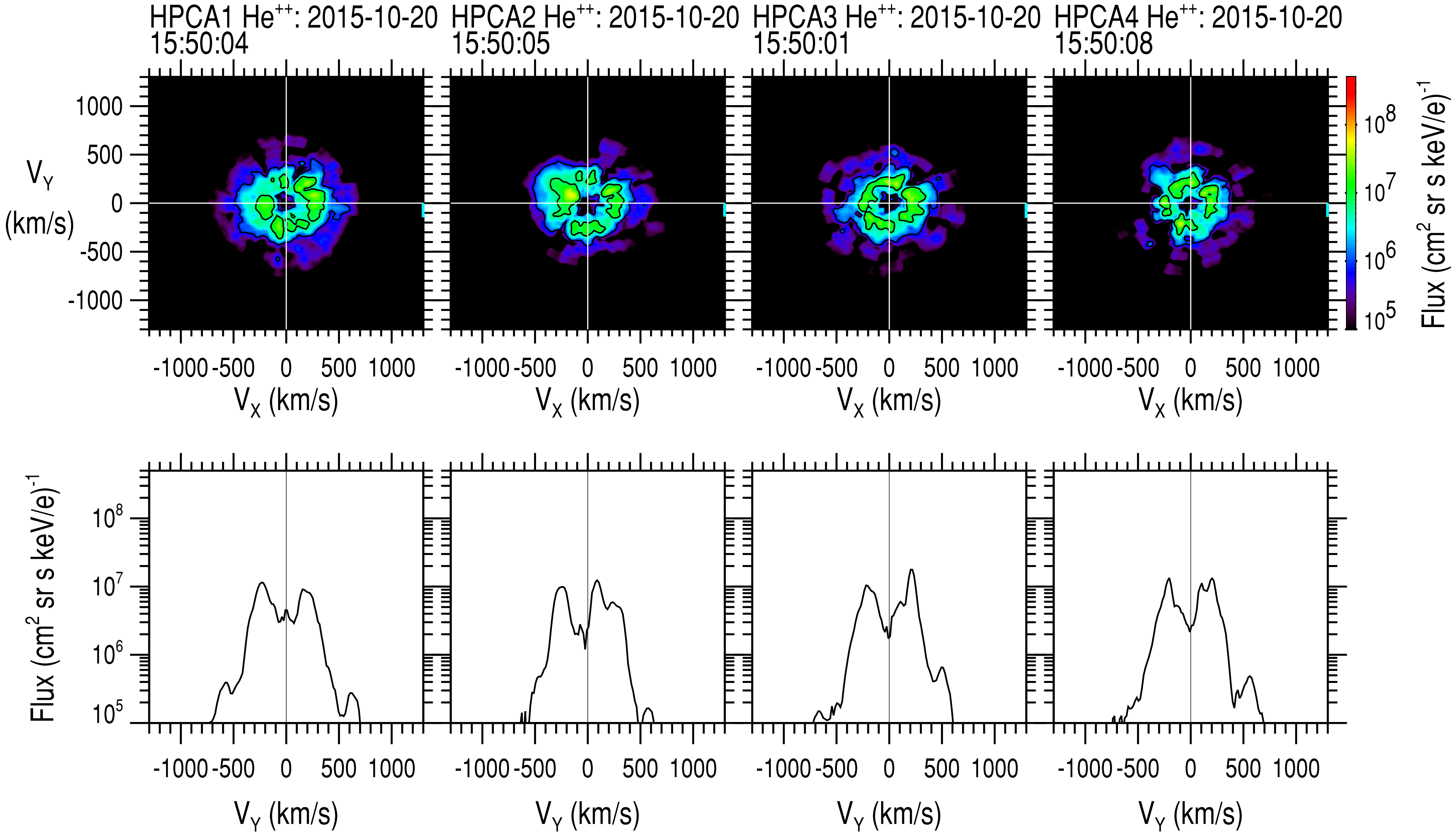

HPCA1 He++: 2015-10-20 15:50:04
HPCA2 He++: 2015-10-20 15:50:05
HPCA3 He++: 2015-10-20 15:50:01
HPCA4 He++: 2015-10-20 15:50:08
VY (km/s)
VX (km/s)
Flux (cm2 sr s keV/e)-1
VY (km/s)

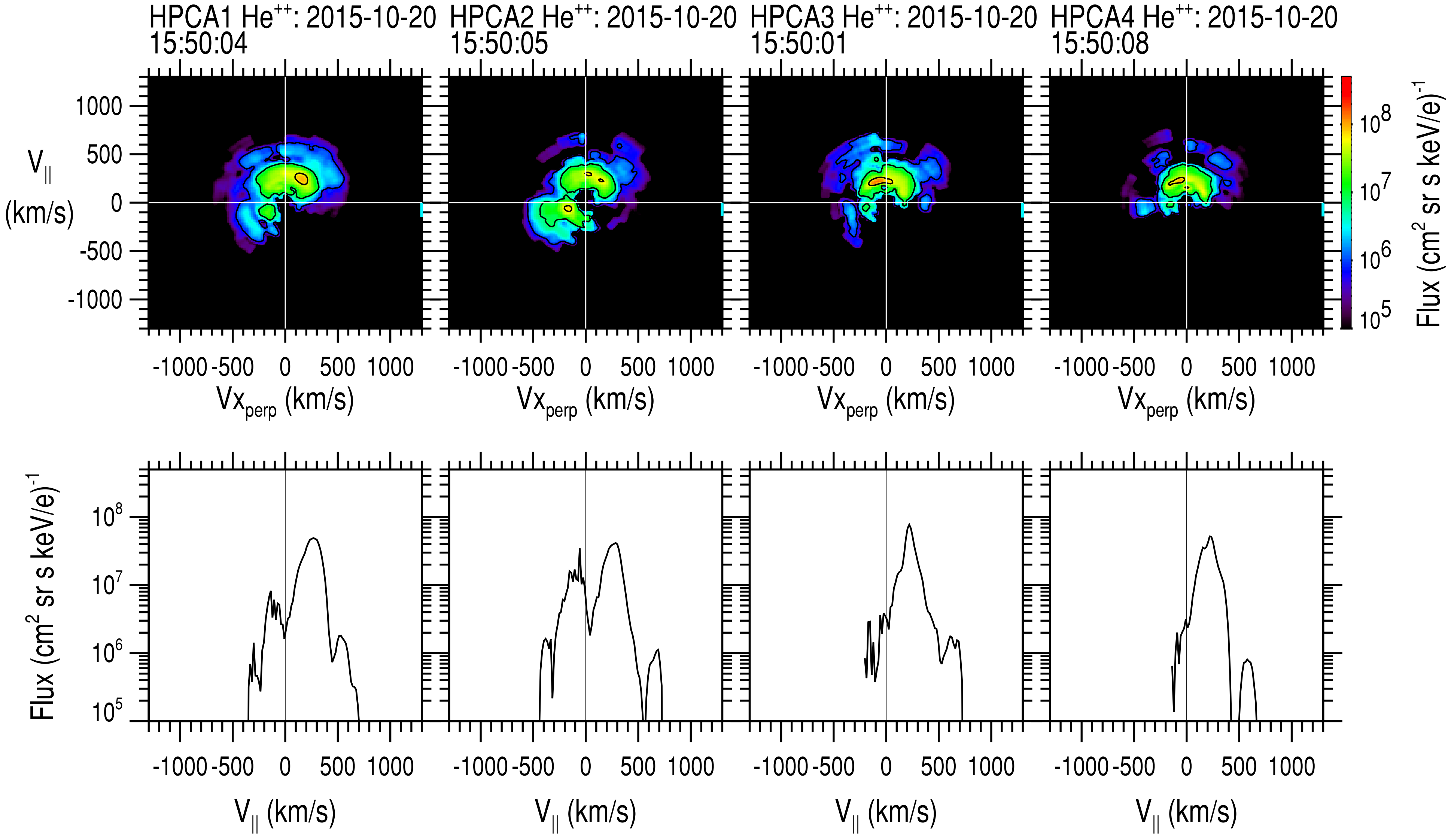
HPCA1 He++: 2015-10-20 15:50:04
HPCA2 He++: 2015-10-20 15:50:05
HPCA3 He++: 2015-10-20 15:50:01
HPCA4 He++: 2015-10-20 15:50:08
V|| (km/s)
Vxperp (km/s)
Flux (cm2 sr s keV/e)-1
V|| (km/s)

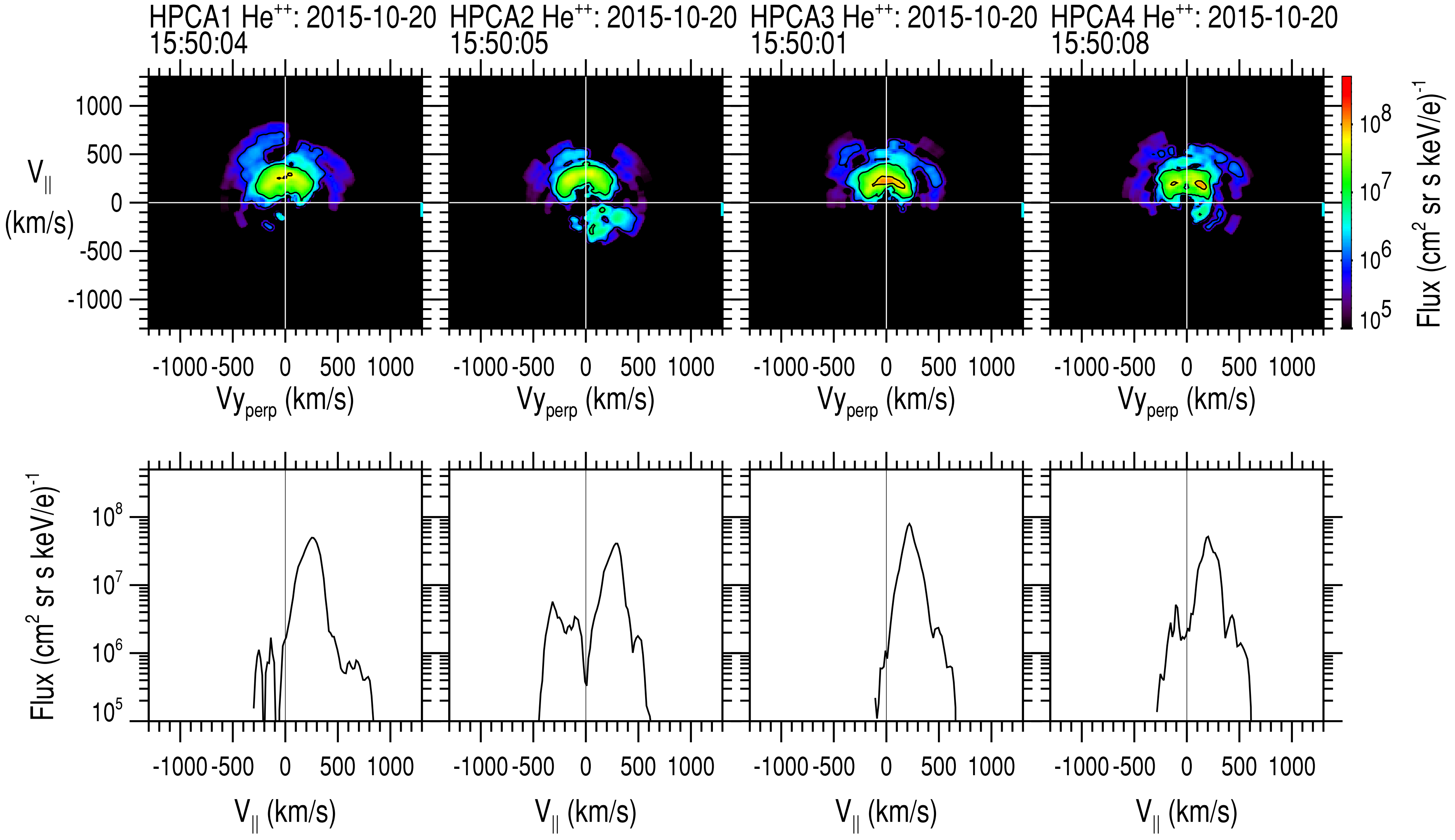

HPCA1 He++: 2015-10-20 15:50:04
HPCA2 He++: 2015-10-20 15:50:05
HPCA3 He++: 2015-10-20 15:50:01
HPCA4 He++: 2015-10-20 15:50:08
V|| (km/s)
Vyperp (km/s)
Flux (cm2 sr s keV/e)-1

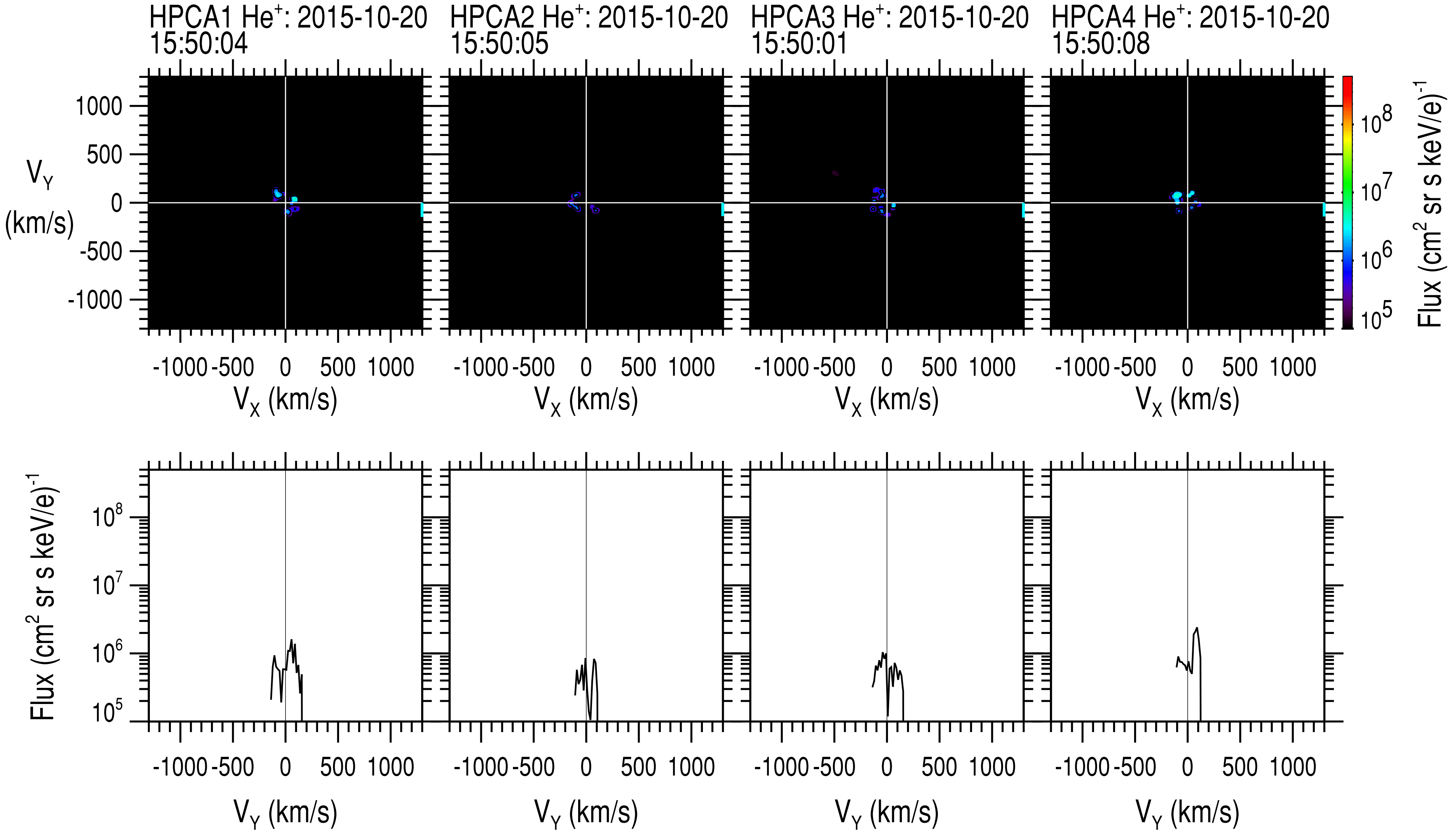

HPCA1 He+: 2015-10-20 15:50:04
HPCA2 He+: 2015-10-20 15:50:05
HPCA3 He+: 2015-10-20 15:50:01
HPCA4 He+: 2015-10-20 15:50:08
VY (km/s)
VX (km/s)
Flux (cm2 sr s keV/e)-1

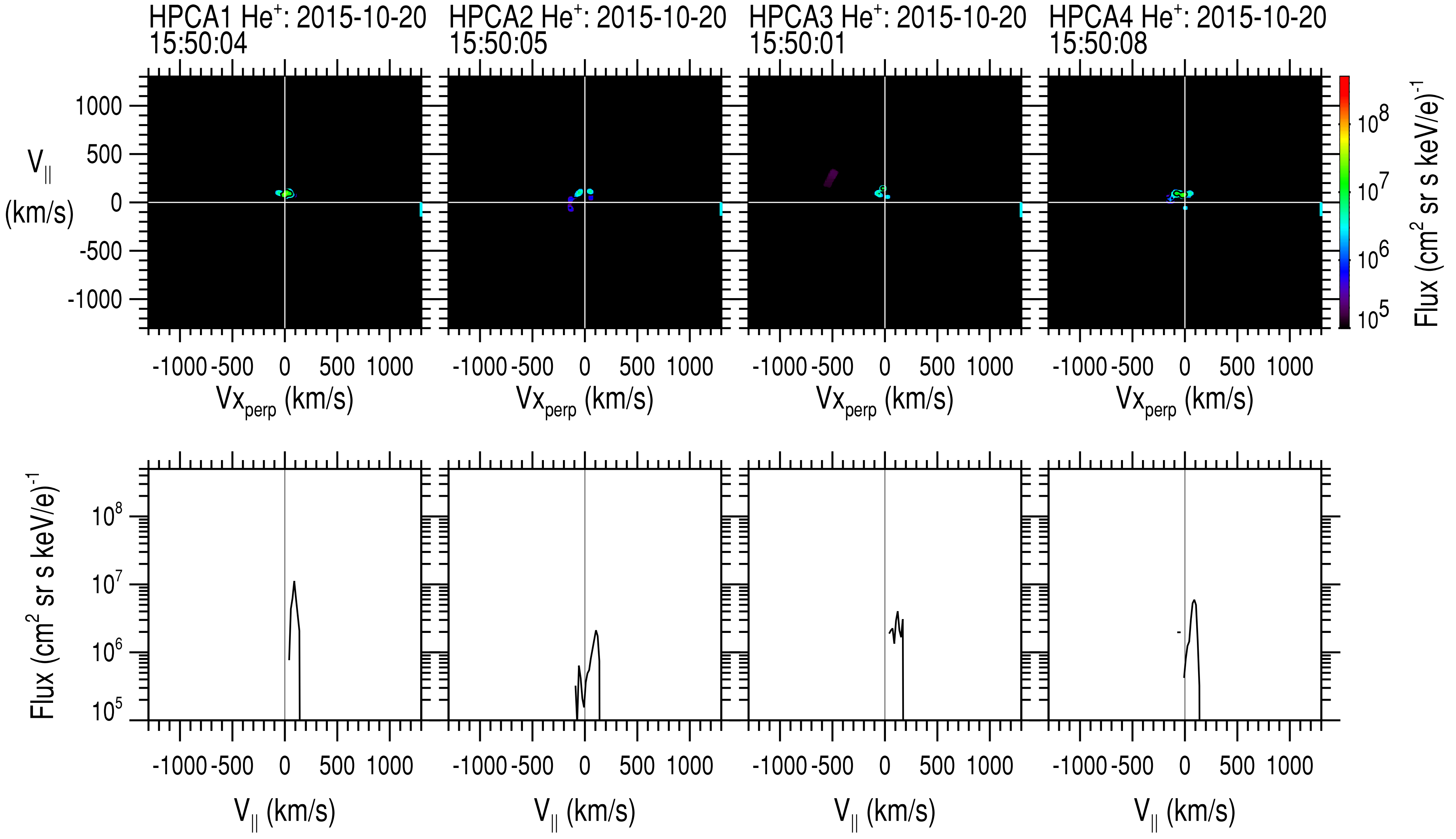
HPCA1 He+: 2015-10-20 15:50:04
HPCA2 He+: 2015-10-20 15:50:05
HPCA3 He+: 2015-10-20 15:50:01
HPCA4 He+: 2015-10-20 15:50:08
V|| (km/s)
Vxperp (km/s)
Flux (cm2 sr s keV/e)-1
-1000 -500 0 500 1000
10^5 10^6 10^7 10^8

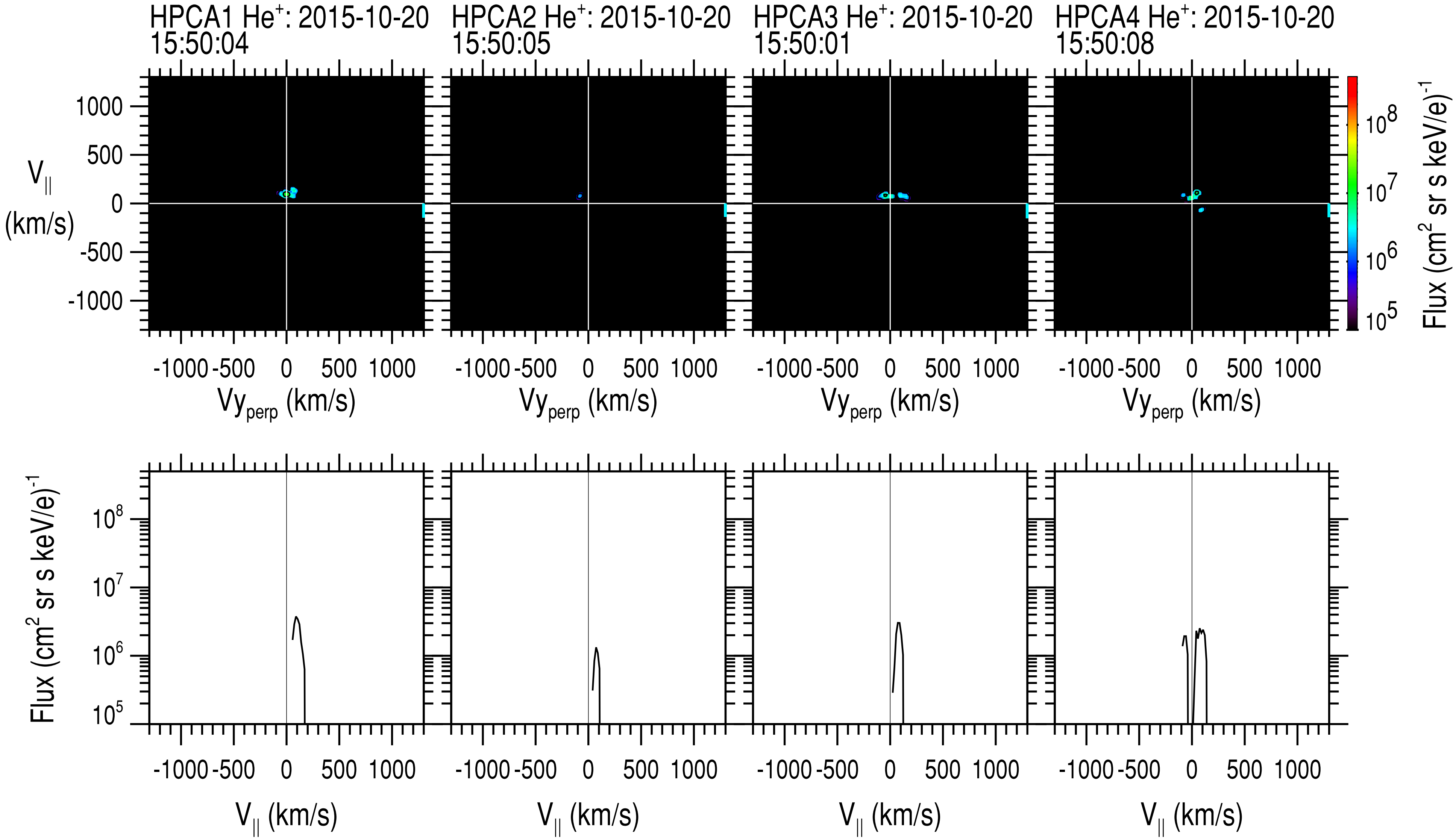
HPCA1 He+: 2015-10-20
15:50:04
HPCA2 He+: 2015-10-20
15:50:05
HPCA3 He+: 2015-10-20
15:50:01
HPCA4 He+: 2015-10-20
15:50:08
V|| (km/s)
Vyperp (km/s)
Flux (cm2 sr s keV/e)-1
-1000 -500 0 500 1000
10^5 10^6 10^7 10^8

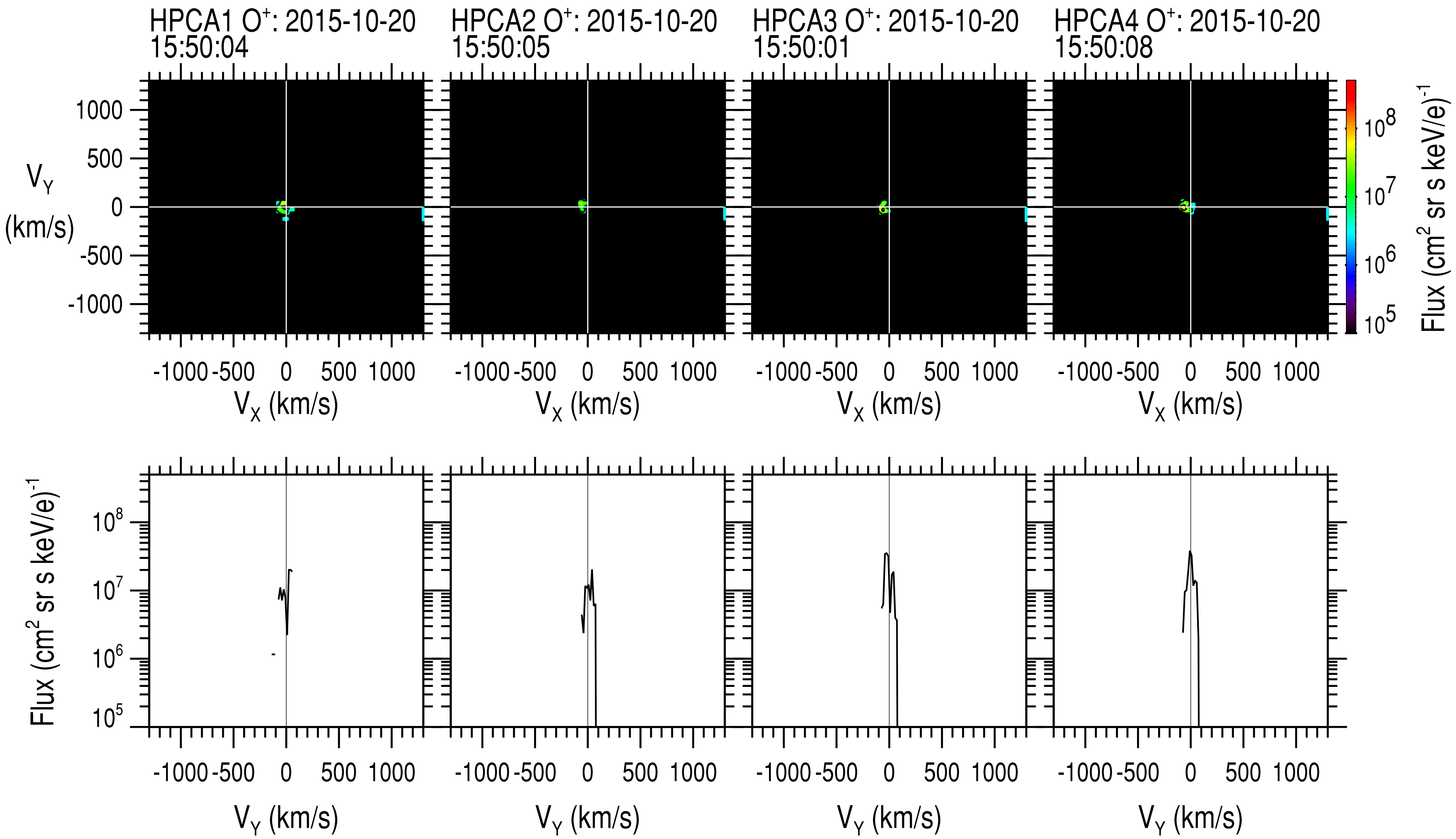

HPCA1 O+: 2015-10-20 15:50:04
HPCA2 O+: 2015-10-20 15:50:05
HPCA3 O+: 2015-10-20 15:50:01
HPCA4 O+: 2015-10-20 15:50:08
VY (km/s)
VX (km/s)
Flux (cm2 sr s keV/e)-1

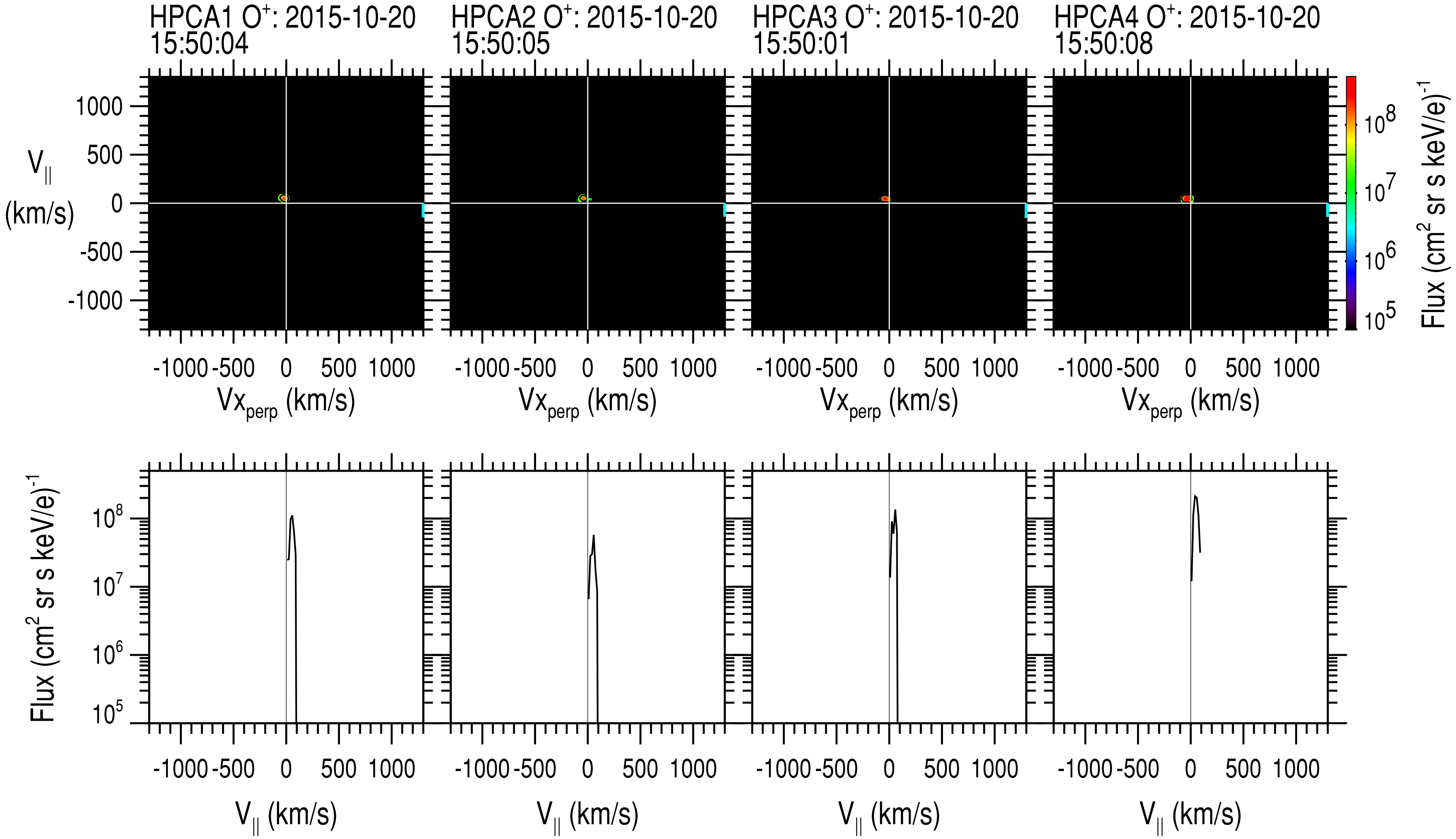
HPCA1 O+: 2015-10-20 15:50:04
HPCA2 O+: 2015-10-20 15:50:05
HPCA3 O+: 2015-10-20 15:50:01
HPCA4 O+: 2015-10-20 15:50:08
V|| (km/s)
Vxperp (km/s)
Flux (cm2 sr s keV/e)-1
1000
500
0
-500
-1000
10^5
10^6
10^7
10^8

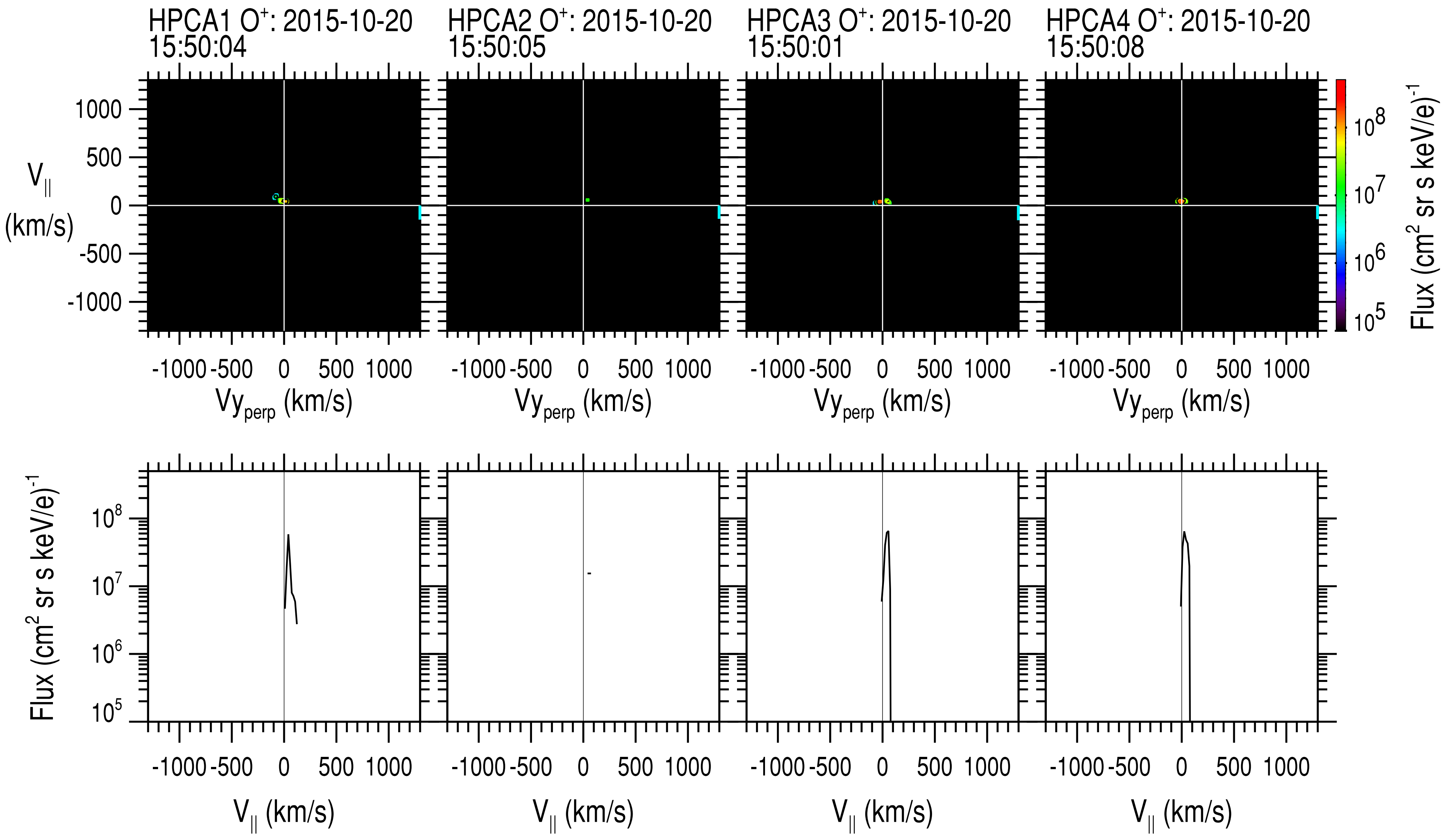

HPCA1 O+: 2015-10-20 15:50:04
HPCA2 O+: 2015-10-20 15:50:05
HPCA3 O+: 2015-10-20 15:50:01
HPCA4 O+: 2015-10-20 15:50:08
V|| (km/s)
Vyperp (km/s)
Flux (cm2 sr s keV/e)-1

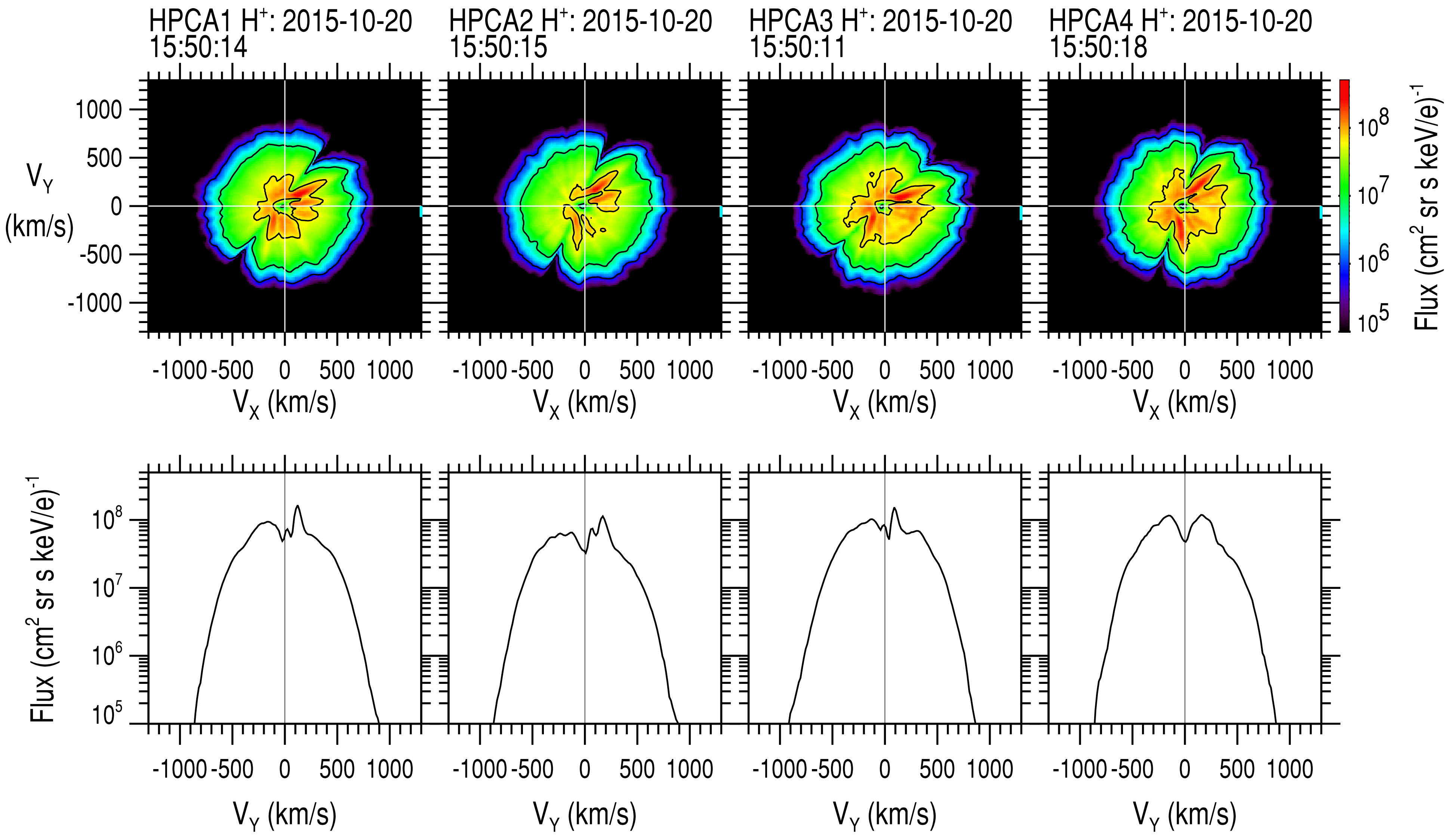

HPCA1 H+: 2015-10-20 15:50:14
HPCA2 H+: 2015-10-20 15:50:15
HPCA3 H+: 2015-10-20 15:50:11
HPCA4 H+: 2015-10-20 15:50:18
V_Y (km/s)
V_X (km/s)
Flux (cm2 sr s keV/e)-1

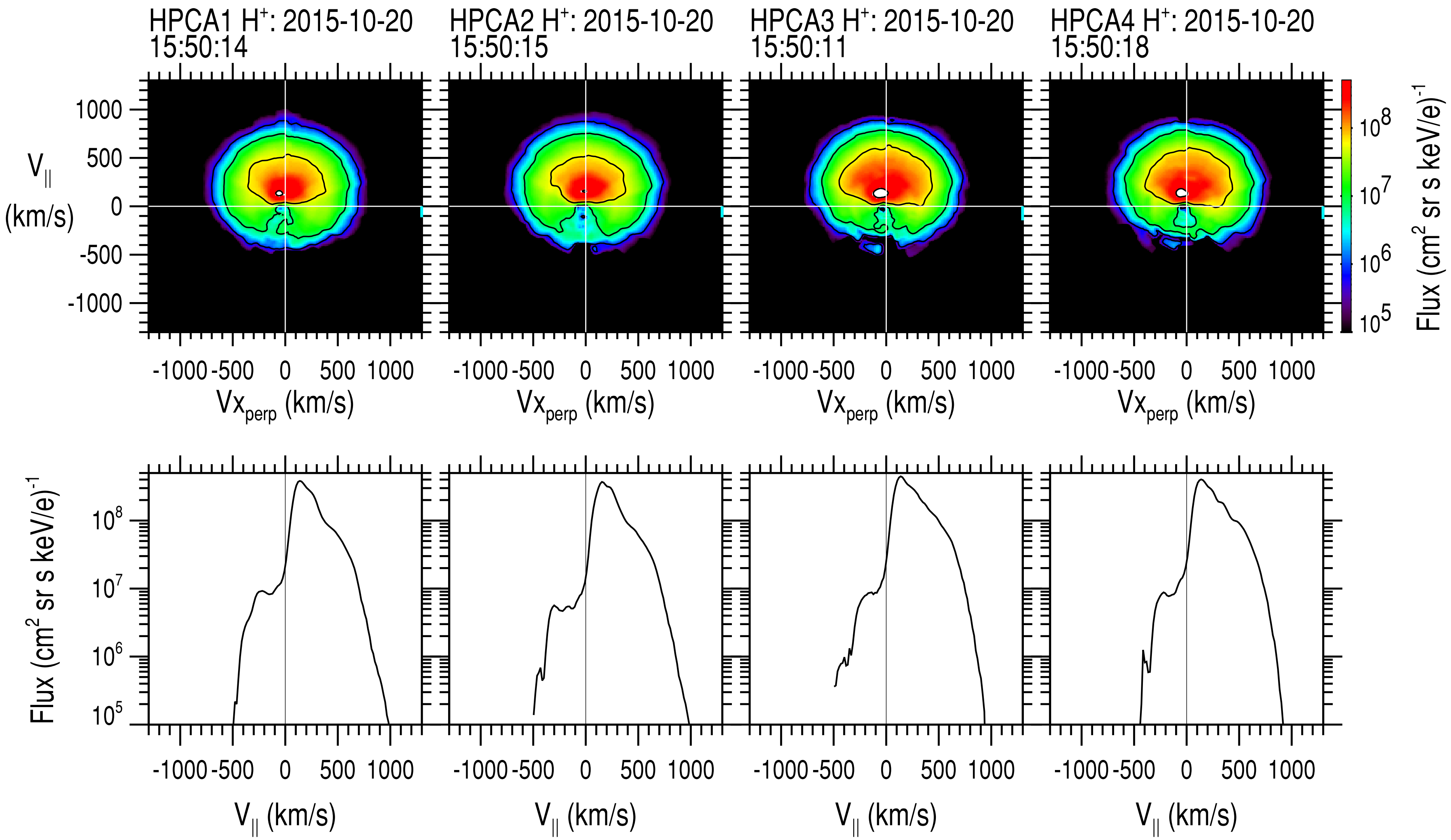

HPCA1 H+: 2015-10-20 15:50:14
HPCA2 H+: 2015-10-20 15:50:15
HPCA3 H+: 2015-10-20 15:50:11
HPCA4 H+: 2015-10-20 15:50:18
V|| (km/s)
Vxperp (km/s)
Flux (cm2 sr s keV/e)-1

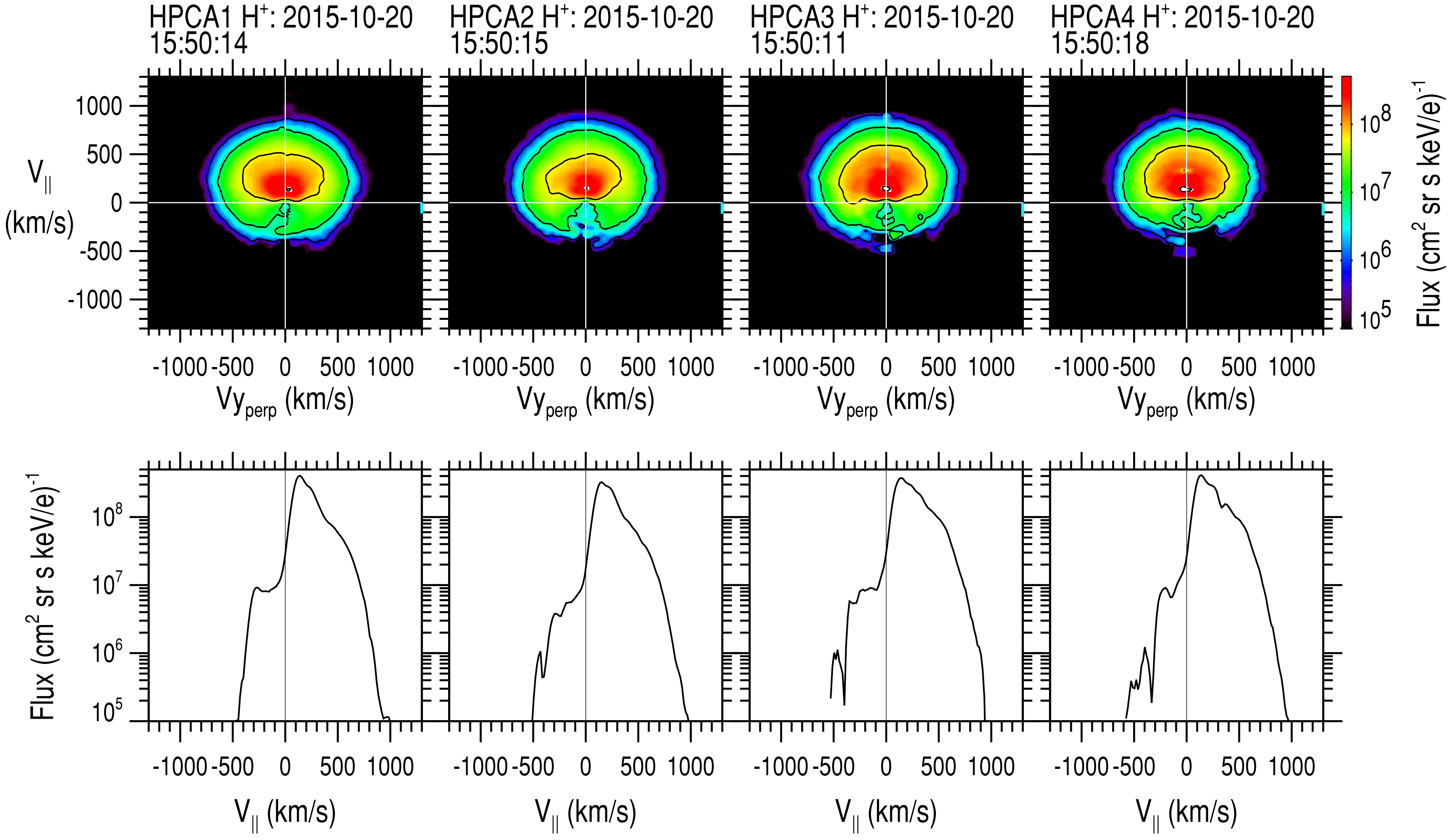

HPCA1 H+: 2015-10-20 15:50:14
HPCA2 H+: 2015-10-20 15:50:15
HPCA3 H+: 2015-10-20 15:50:11
HPCA4 H+: 2015-10-20 15:50:18
V|| (km/s)
Vyperp (km/s)
Flux (cm2 sr s keV/e)-1

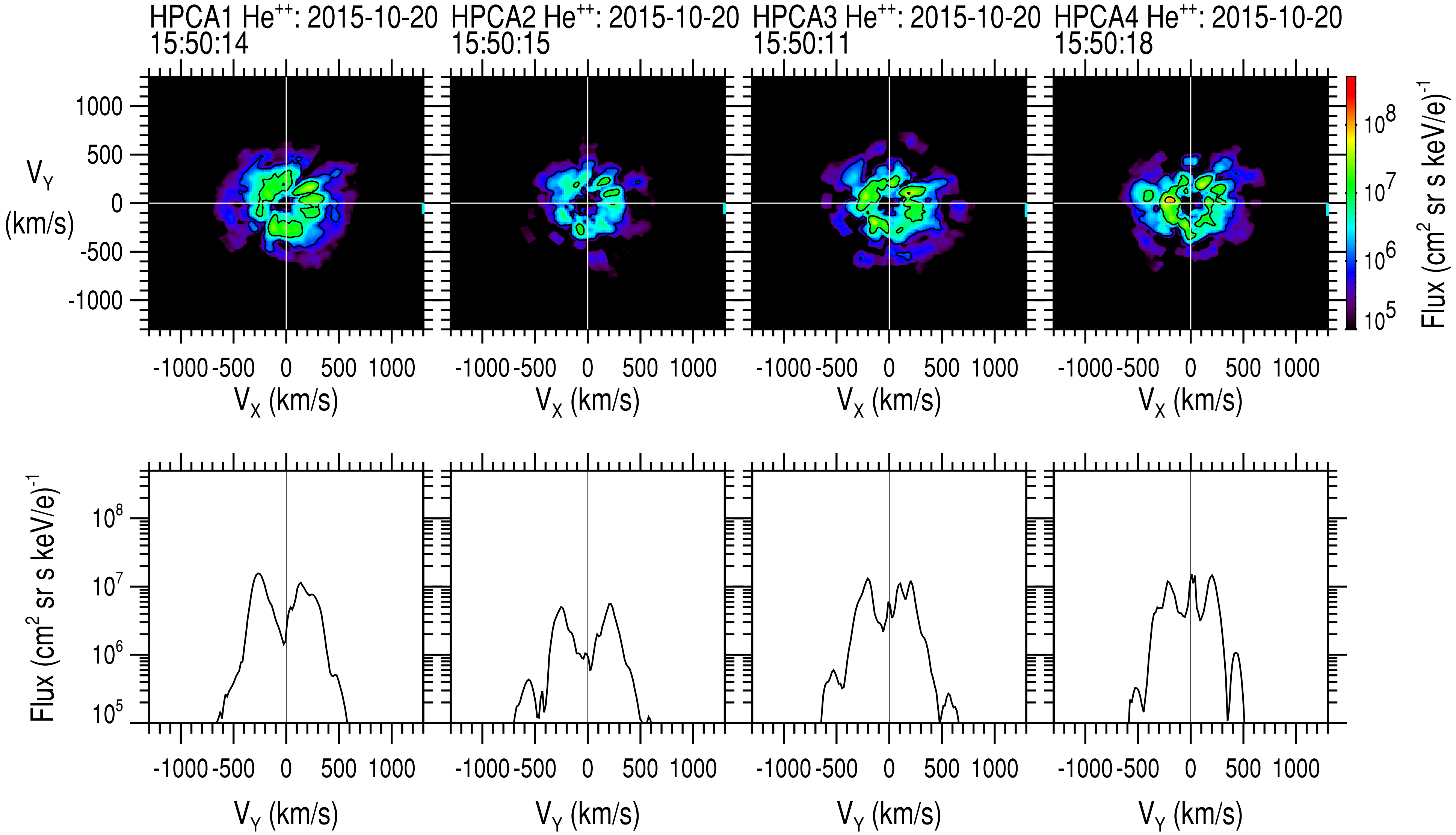
HPCA1 He++: 2015-10-20
15:50:14
HPCA2 He++: 2015-10-20
15:50:15
HPCA3 He++: 2015-10-20
15:50:11
HPCA4 He++: 2015-10-20
15:50:18
V_Y (km/s)
V_X (km/s)
V_Y (km/s)
-1000 -500 0 500 1000
Flux (cm^2 sr s keV/e)^-1
10^5
10^6
10^7
10^8

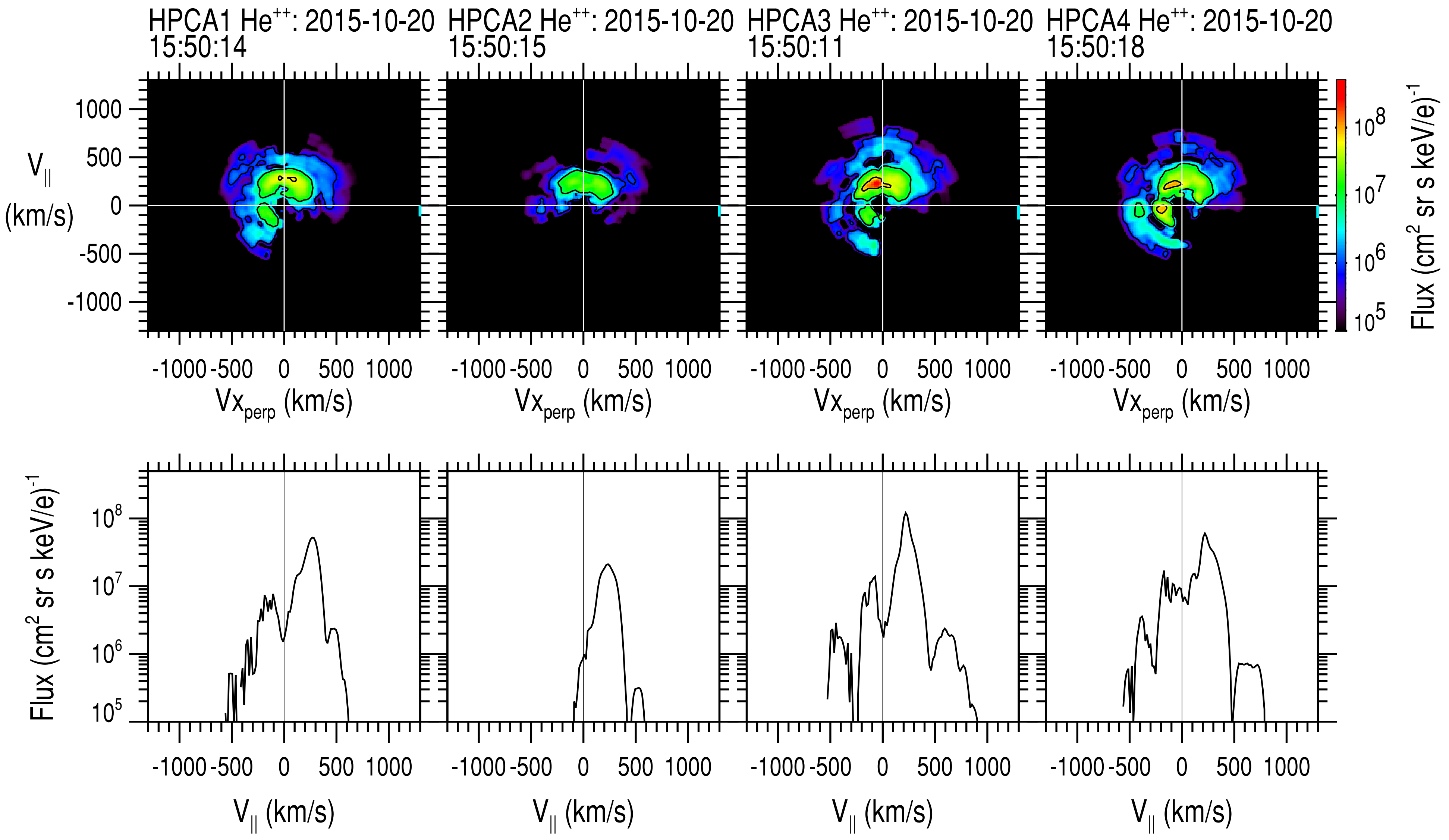

HPCA1 He++: 2015-10-20 15:50:14
HPCA2 He++: 2015-10-20 15:50:15
HPCA3 He++: 2015-10-20 15:50:11
HPCA4 He++: 2015-10-20 15:50:18
V|| (km/s)
Vxperp (km/s)
Flux (cm2 sr s keV/e)-1

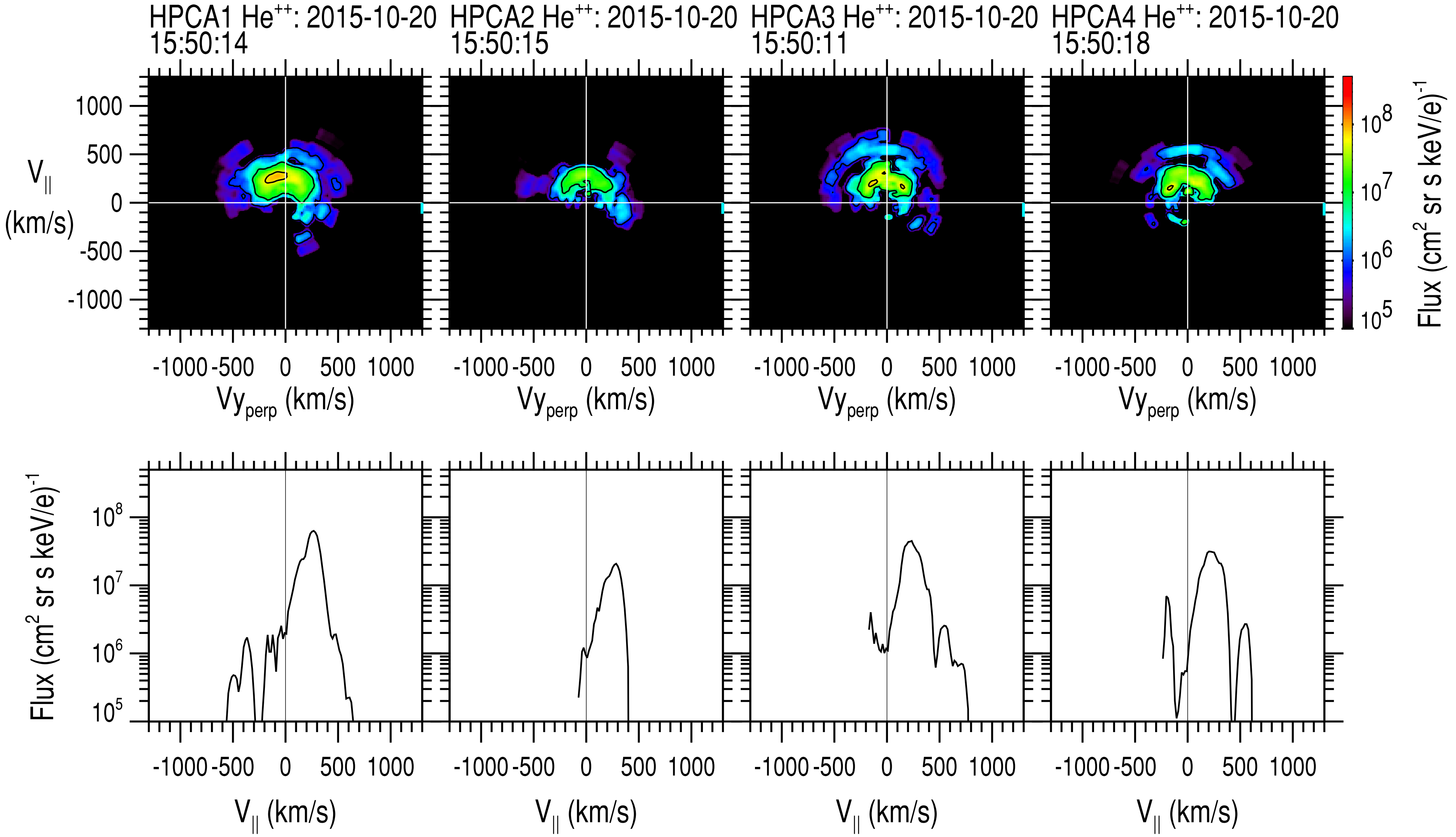
HPCA1 He++: 2015-10-20 15:50:14
HPCA2 He++: 2015-10-20 15:50:15
HPCA3 He++: 2015-10-20 15:50:11
HPCA4 He++: 2015-10-20 15:50:18
V|| (km/s)
1000
500
0
-500
-1000
-1000 -500 0 500 1000
Vyperp (km/s)
Flux (cm2 sr s keV/e)-1
10^5 10^6 10^7 10^8
V|| (km/s)

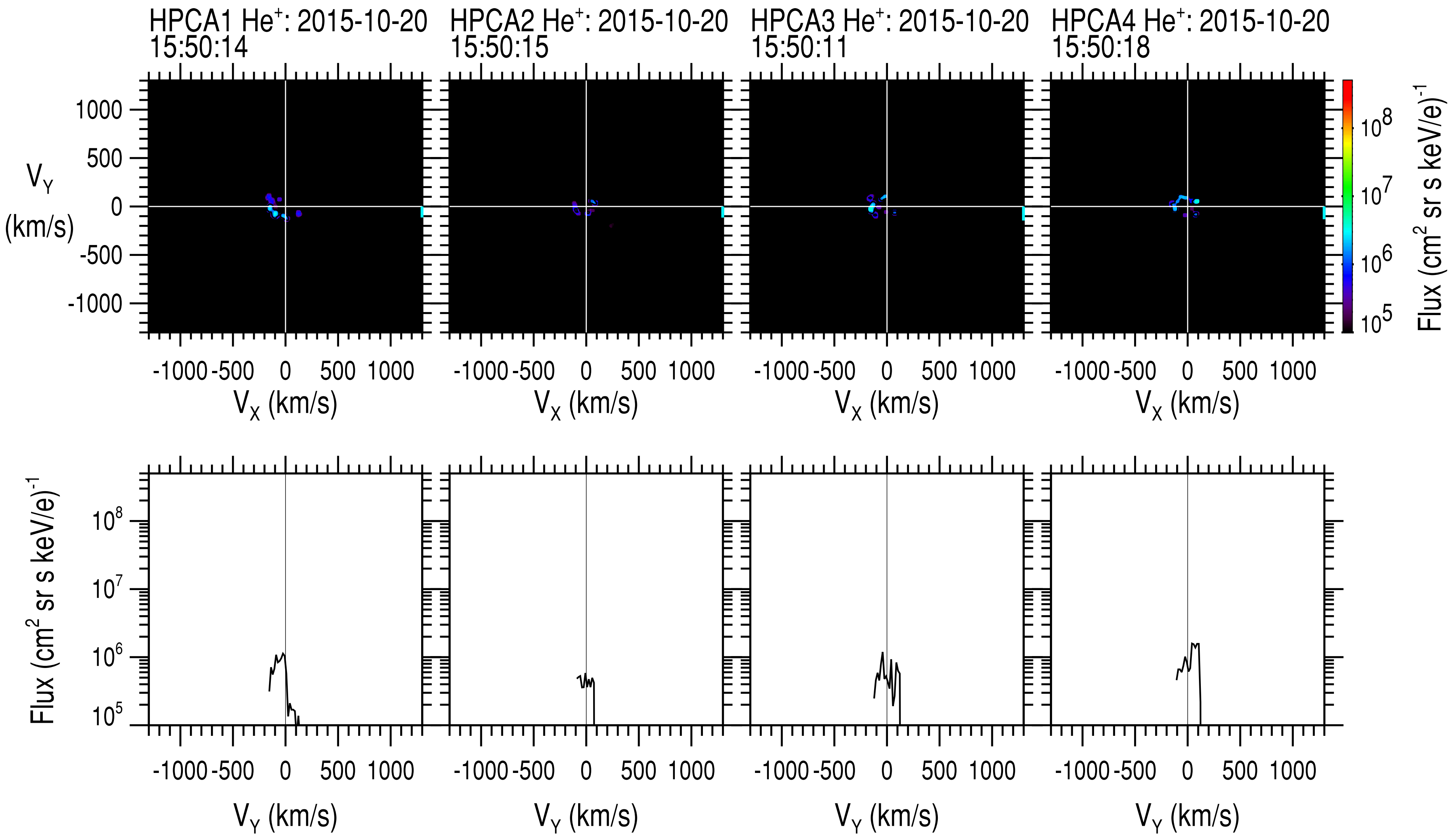

HPCA1 He+: 2015-10-20 15:50:14
HPCA2 He+: 2015-10-20 15:50:15
HPCA3 He+: 2015-10-20 15:50:11
HPCA4 He+: 2015-10-20 15:50:18
VY (km/s)
VX (km/s)
Flux (cm2 sr s keV/e)-1

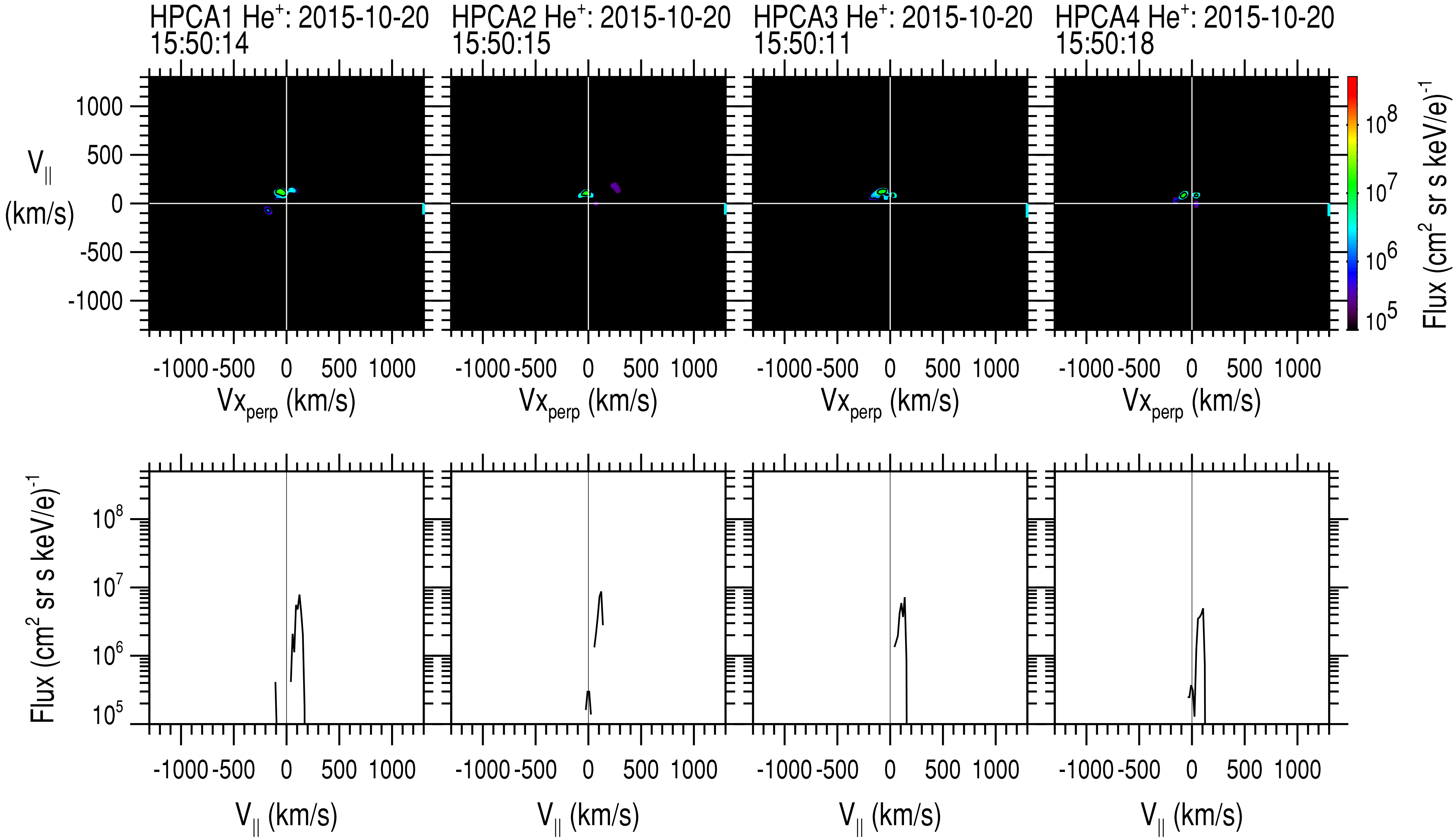

HPCA1 He+: 2015-10-20 15:50:14
HPCA2 He+: 2015-10-20 15:50:15
HPCA3 He+: 2015-10-20 15:50:11
HPCA4 He+: 2015-10-20 15:50:18
V|| (km/s)
Vxperp (km/s)
Flux (cm2 sr s keV/e)-1

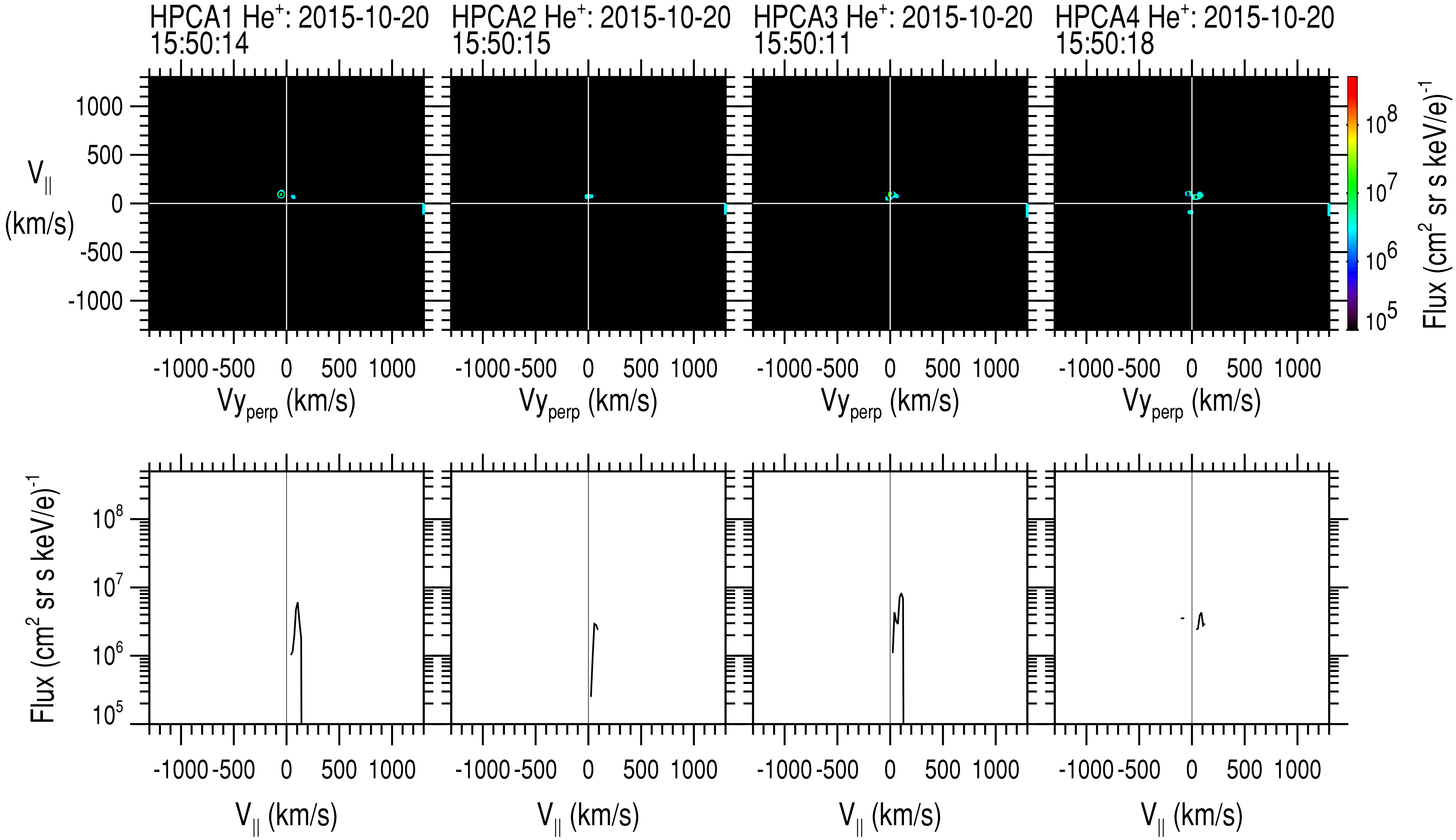
HPCA1 He+: 2015-10-20 15:50:14
HPCA2 He+: 2015-10-20 15:50:15
HPCA3 He+: 2015-10-20 15:50:11
HPCA4 He+: 2015-10-20 15:50:18
V|| (km/s)
Vyperp (km/s)
Flux (cm2 sr s keV/e)-1
-1000 -500 0 500 1000
10^5 10^6 10^7 10^8

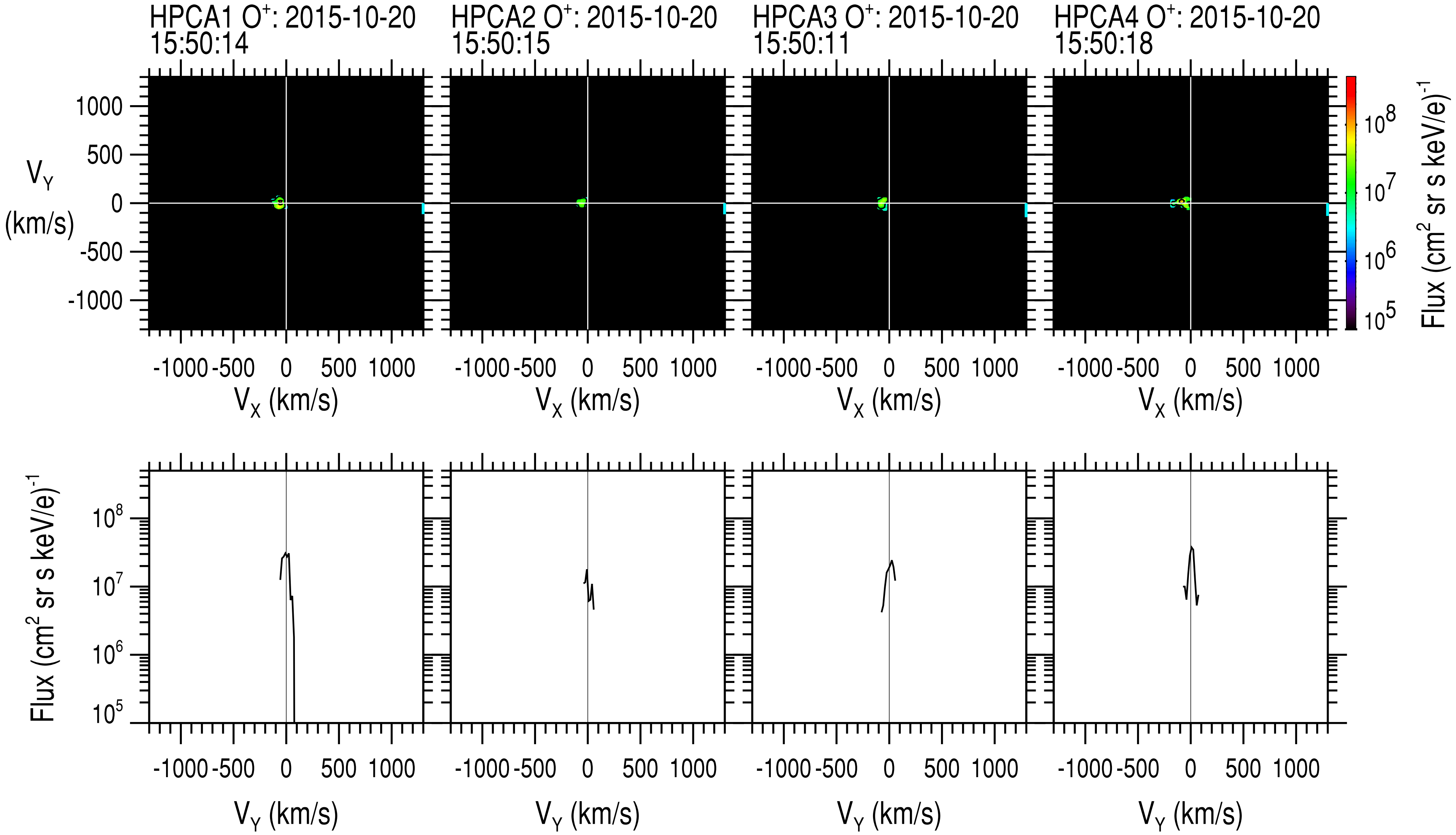
HPCA1 O+: 2015-10-20
15:50:14
HPCA2 O+: 2015-10-20
15:50:15
HPCA3 O+: 2015-10-20
15:50:11
HPCA4 O+: 2015-10-20
15:50:18
V_Y (km/s)
V_X (km/s)
V_Y (km/s)
Flux (cm^2 sr s keV/e)^-1

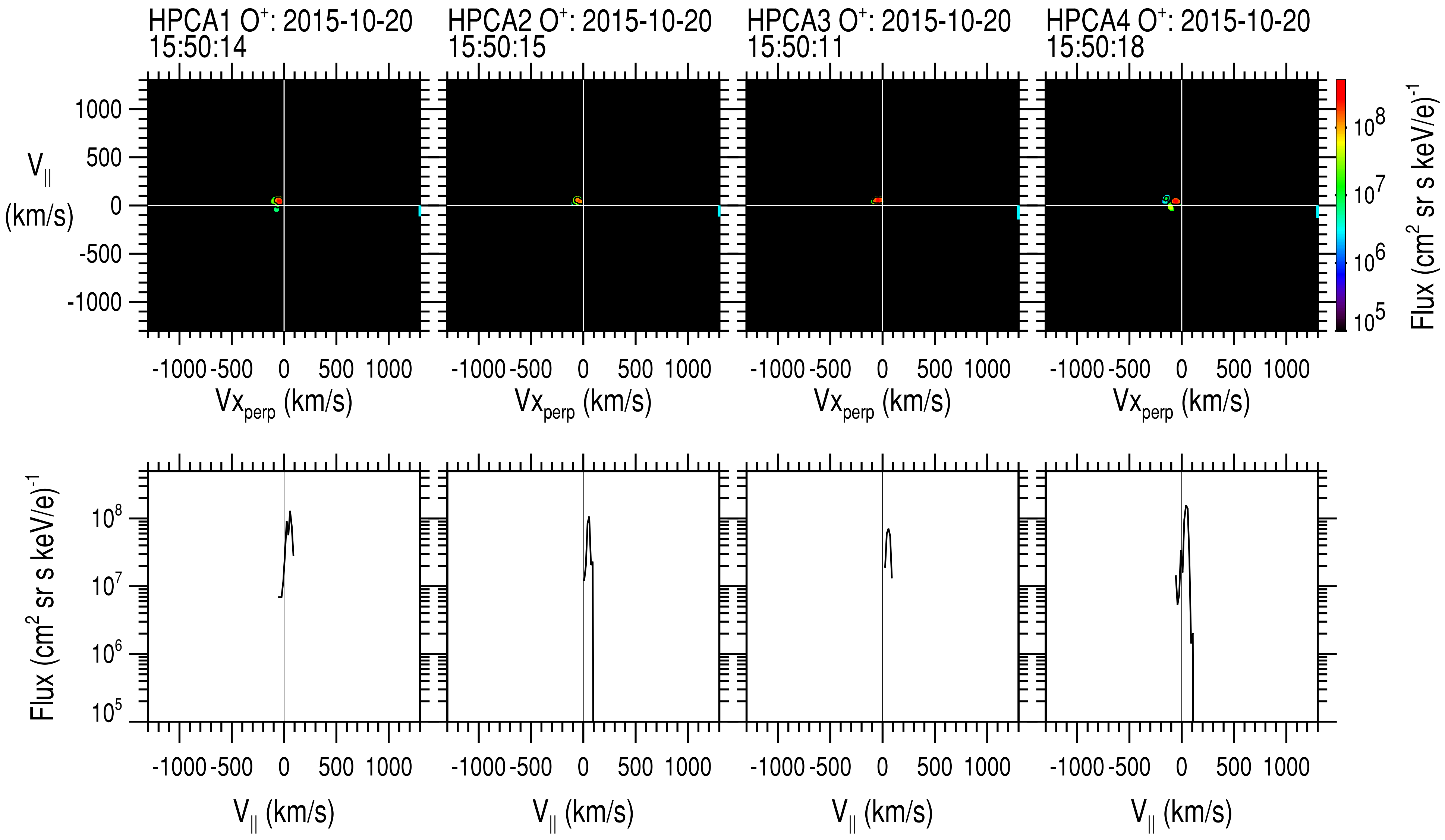

HPCA1 O+: 2015-10-20 15:50:14
HPCA2 O+: 2015-10-20 15:50:15
HPCA3 O+: 2015-10-20 15:50:11
HPCA4 O+: 2015-10-20 15:50:18
V|| (km/s)
Vxperp (km/s)
Flux (cm2 sr s keV/e)-1

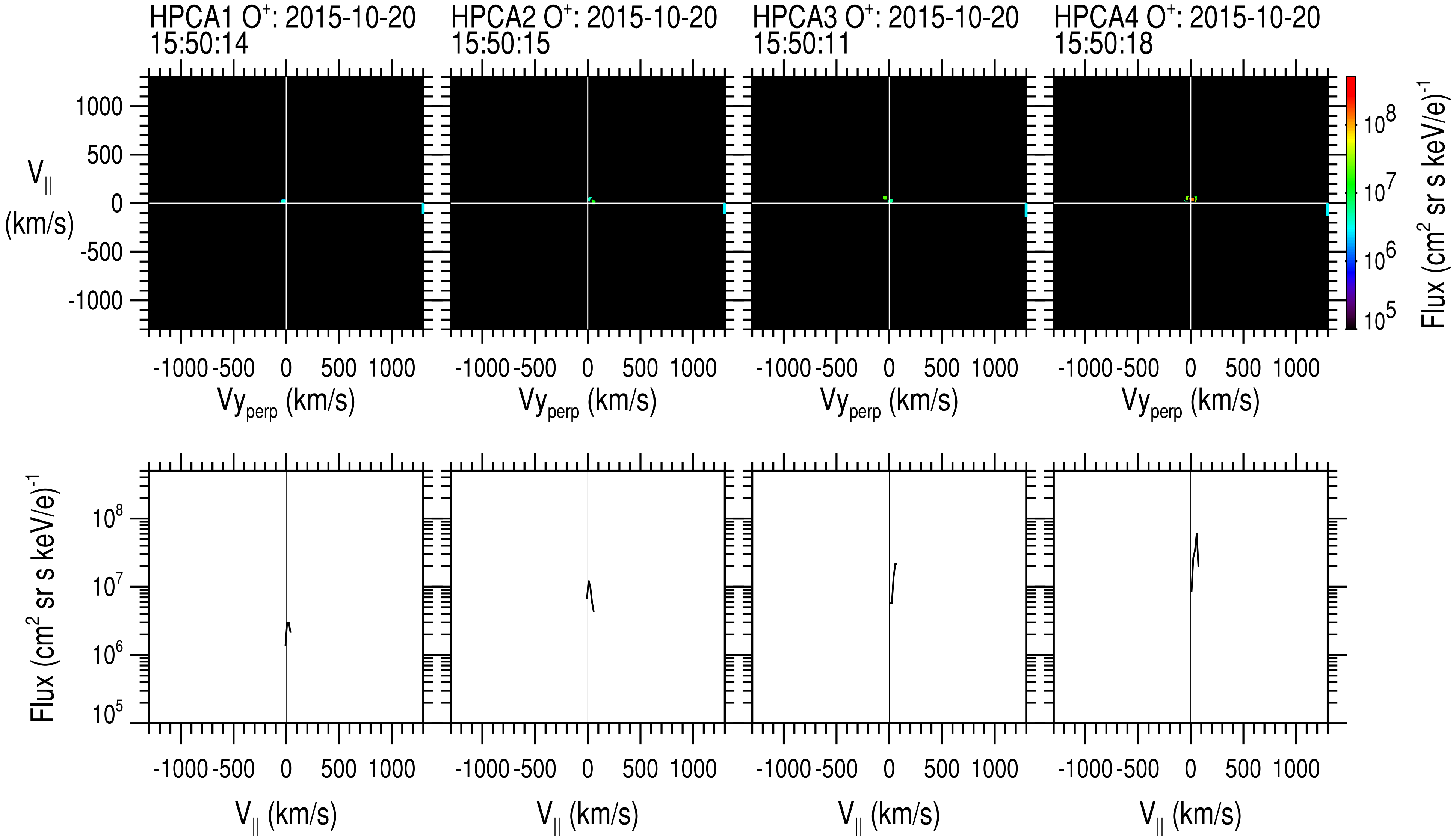
HPCA1 O+: 2015-10-20
15:50:14
HPCA2 O+: 2015-10-20
15:50:15
HPCA3 O+: 2015-10-20
15:50:11
HPCA4 O+: 2015-10-20
15:50:18
V|| (km/s)
Vyperp (km/s)
Flux (cm2 sr s keV/e)-1
V|| (km/s)
-1000 -500 0 500 1000
10^5 10^6 10^7 10^8

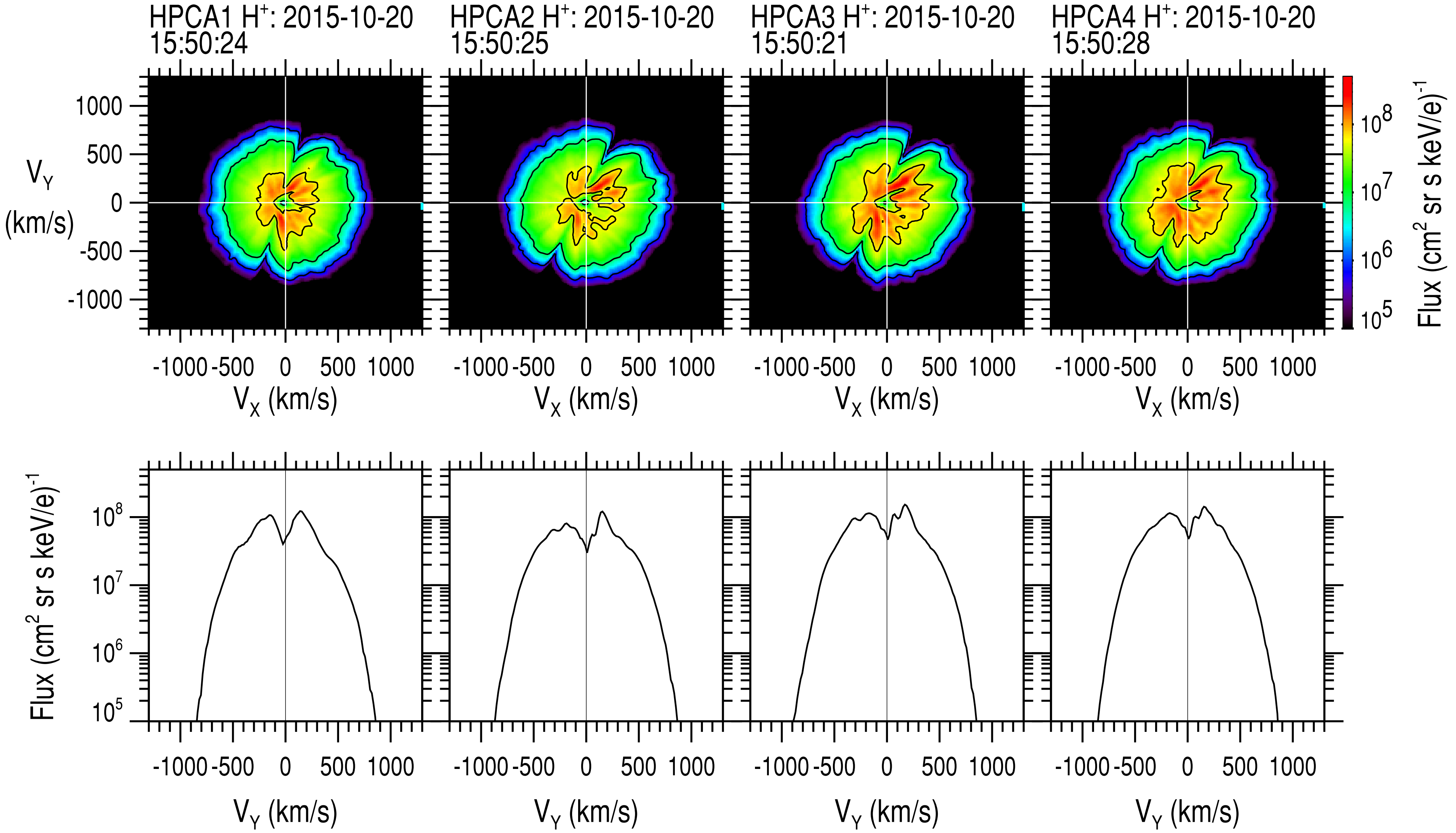
HPCA1 H+: 2015-10-20 15:50:24
HPCA2 H+: 2015-10-20 15:50:25
HPCA3 H+: 2015-10-20 15:50:21
HPCA4 H+: 2015-10-20 15:50:28
VY (km/s)
VX (km/s)
Flux (cm2 sr s keV/e)-1
VY (km/s)

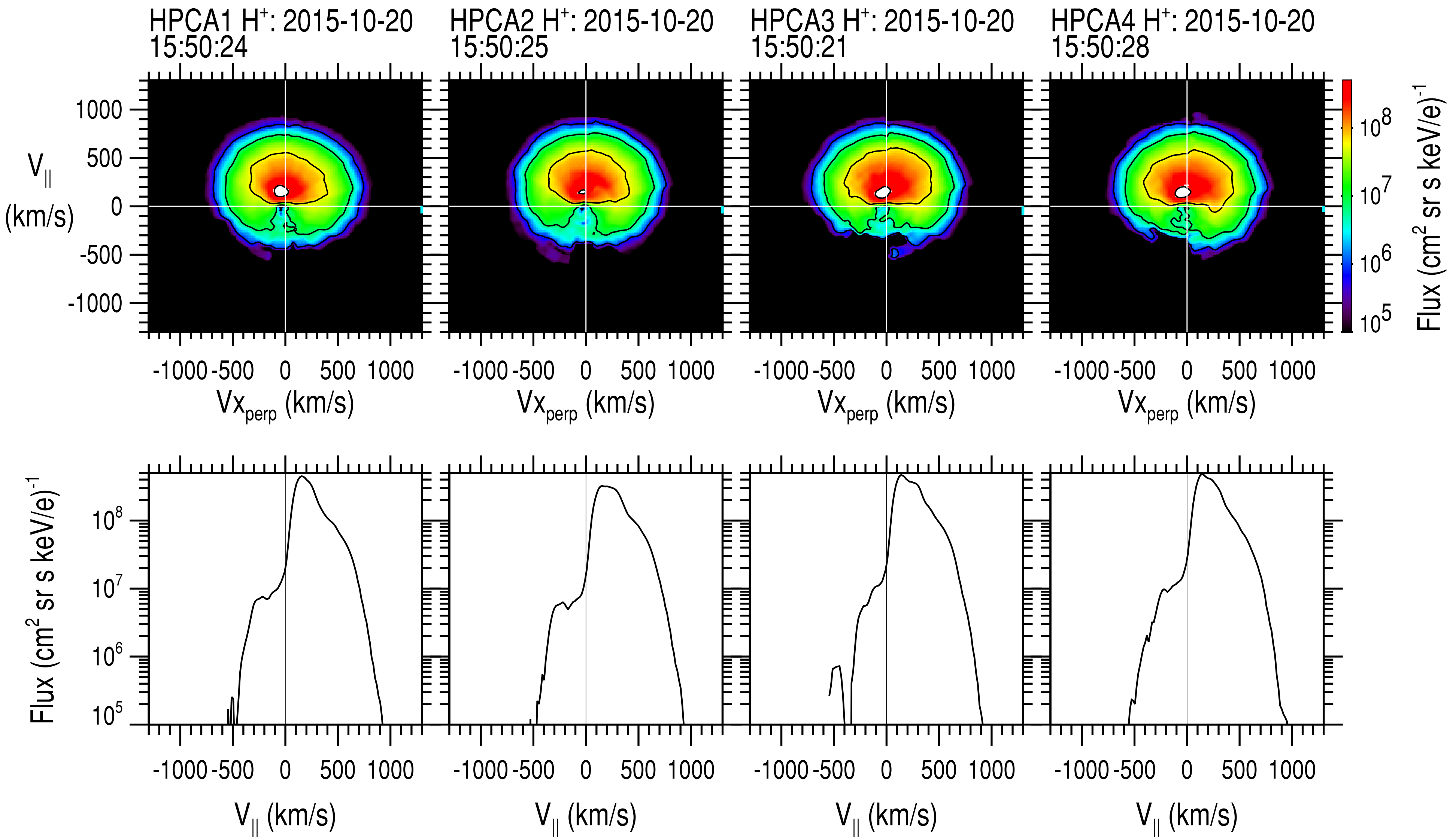
HPCA1 H+: 2015-10-20 15:50:24
HPCA2 H+: 2015-10-20 15:50:25
HPCA3 H+: 2015-10-20 15:50:21
HPCA4 H+: 2015-10-20 15:50:28
V|| (km/s)
Vxperp (km/s)
Flux (cm2 sr s keV/e)-1
1000
500
0
-500
-1000
10^5
10^6
10^7
10^8

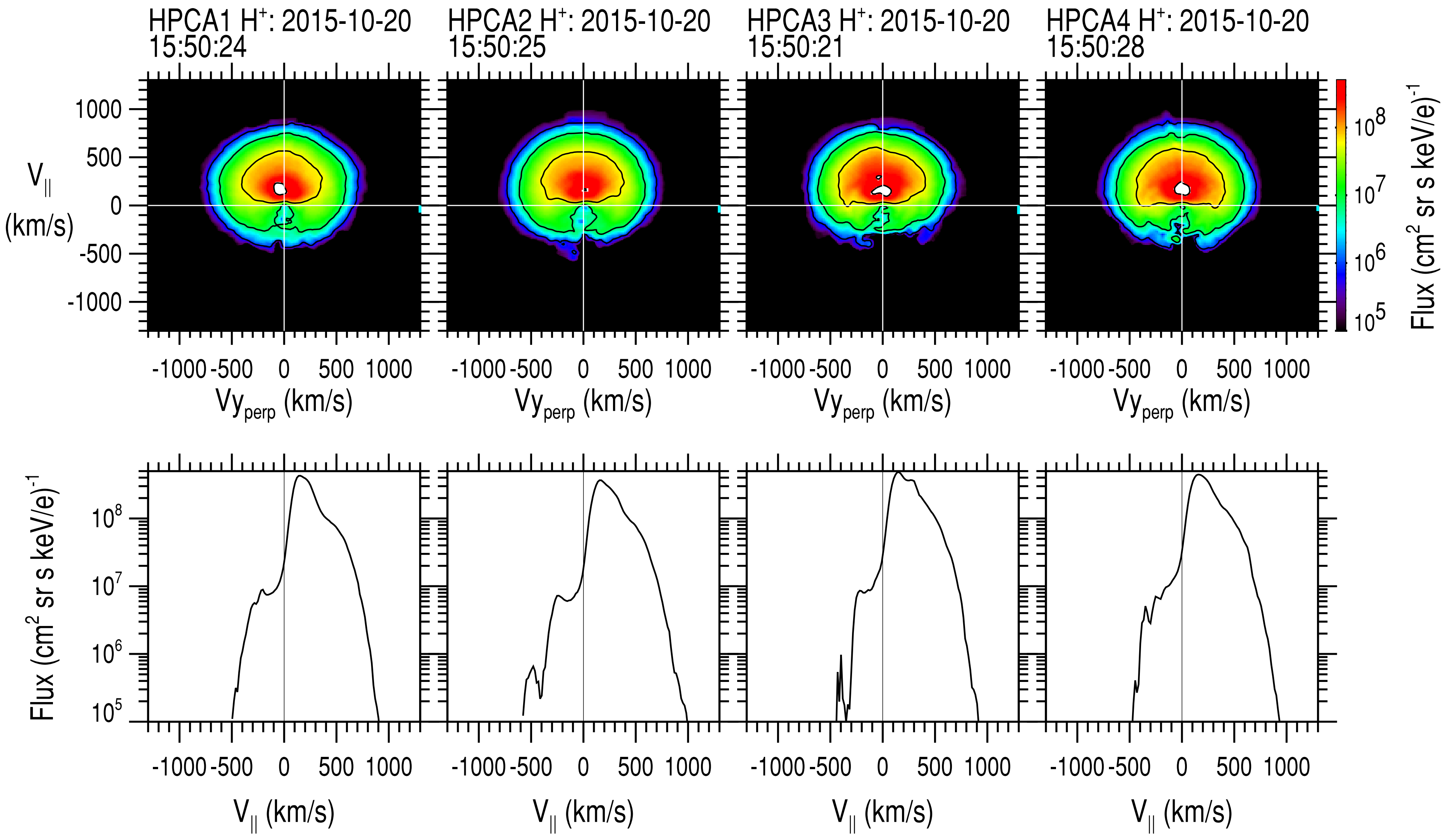
HPCA1 H+: 2015-10-20 15:50:24
HPCA2 H+: 2015-10-20 15:50:25
HPCA3 H+: 2015-10-20 15:50:21
HPCA4 H+: 2015-10-20 15:50:28
V|| (km/s)
Vyperp (km/s)
Flux (cm2 sr s keV/e)-1
V|| (km/s)

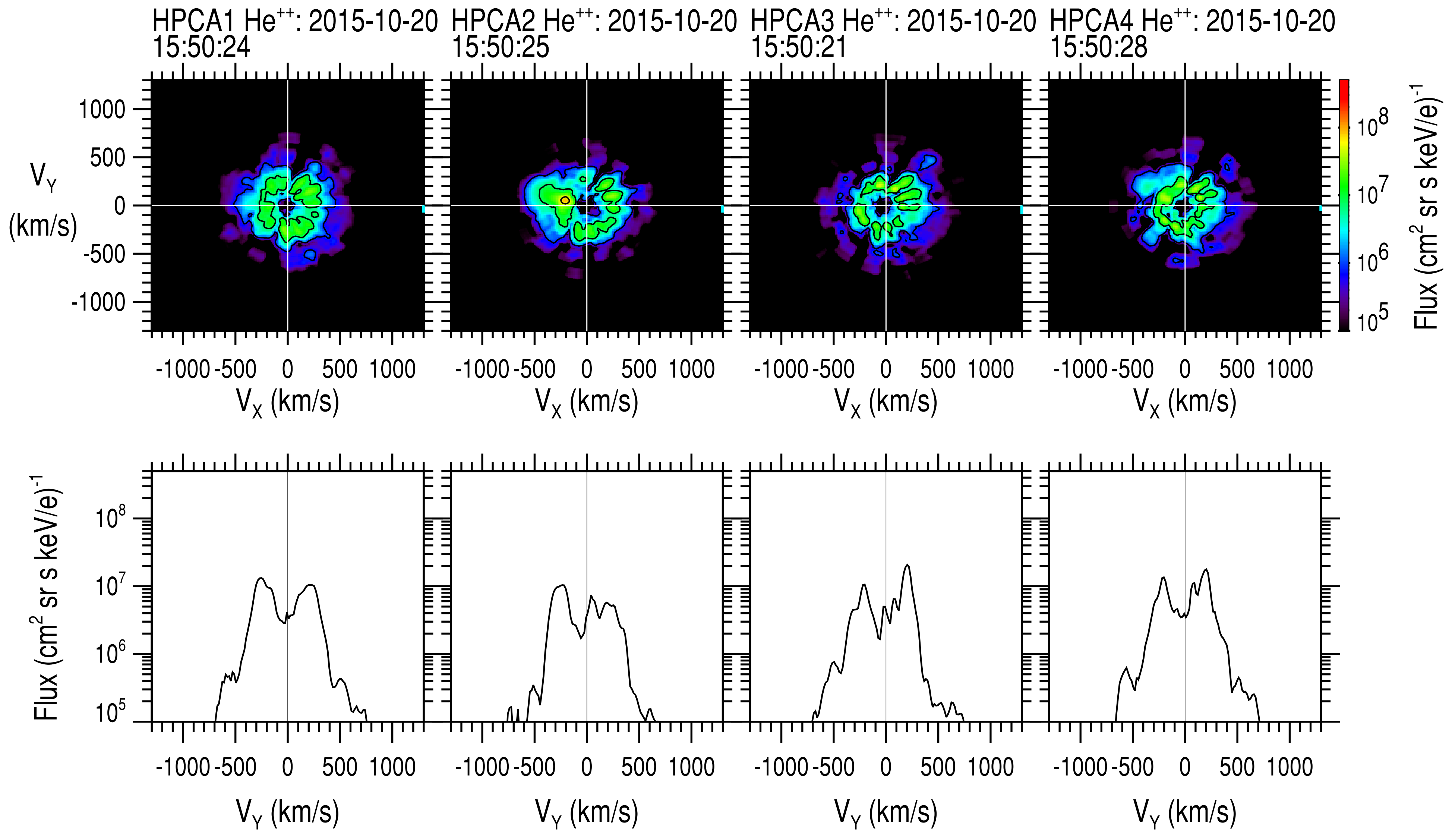

HPCA1 He++: 2015-10-20 15:50:24
HPCA2 He++: 2015-10-20 15:50:25
HPCA3 He++: 2015-10-20 15:50:21
HPCA4 He++: 2015-10-20 15:50:28
V_Y (km/s)
V_X (km/s)
Flux (cm^2 sr s keV/e)^-1
V_Y (km/s)

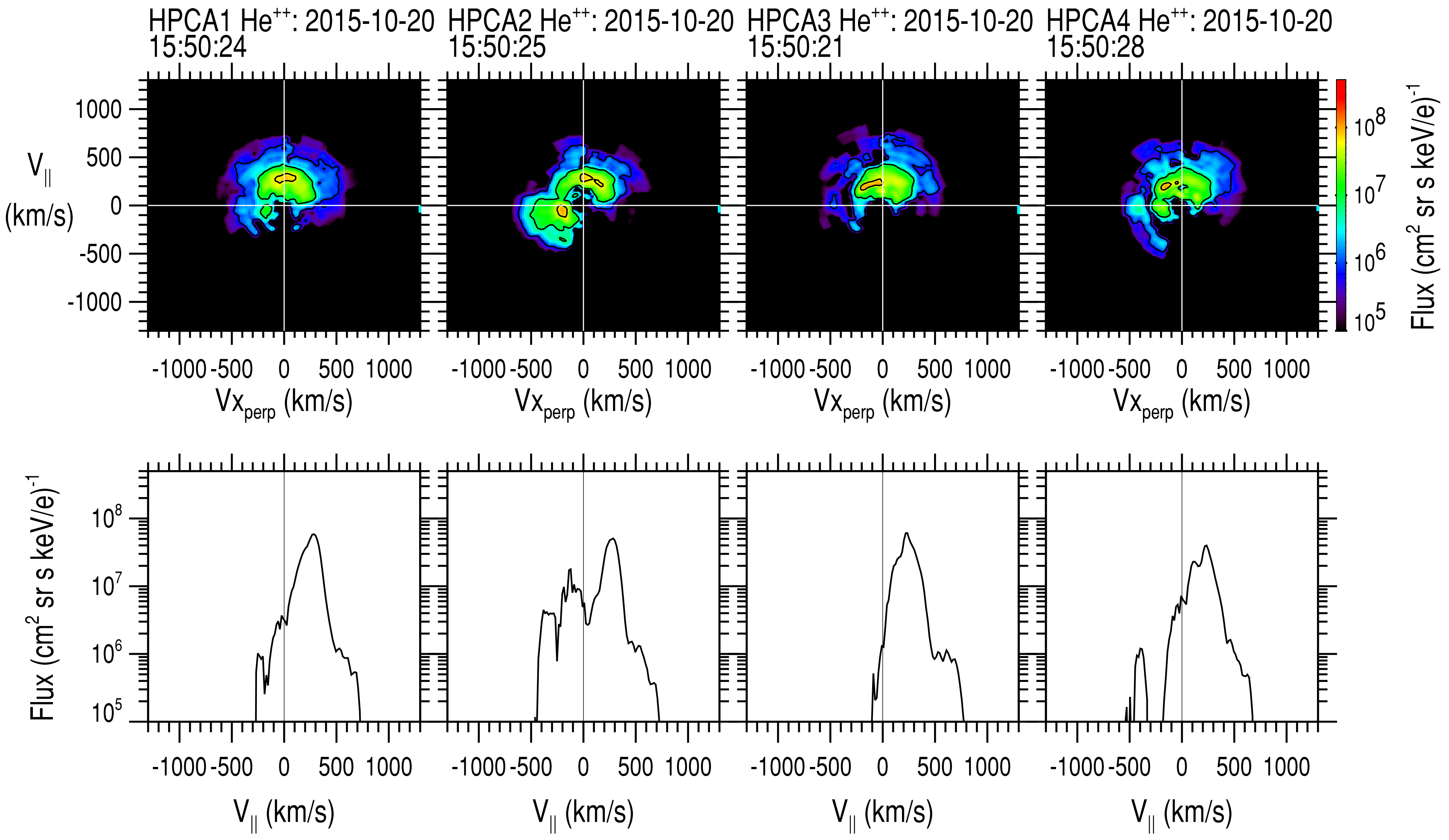
HPCA1 He++: 2015-10-20 15:50:24
HPCA2 He++: 2015-10-20 15:50:25
HPCA3 He++: 2015-10-20 15:50:21
HPCA4 He++: 2015-10-20 15:50:28
V|| (km/s)
Vxperp (km/s)
Flux (cm2 sr s keV/e)-1
V|| (km/s)

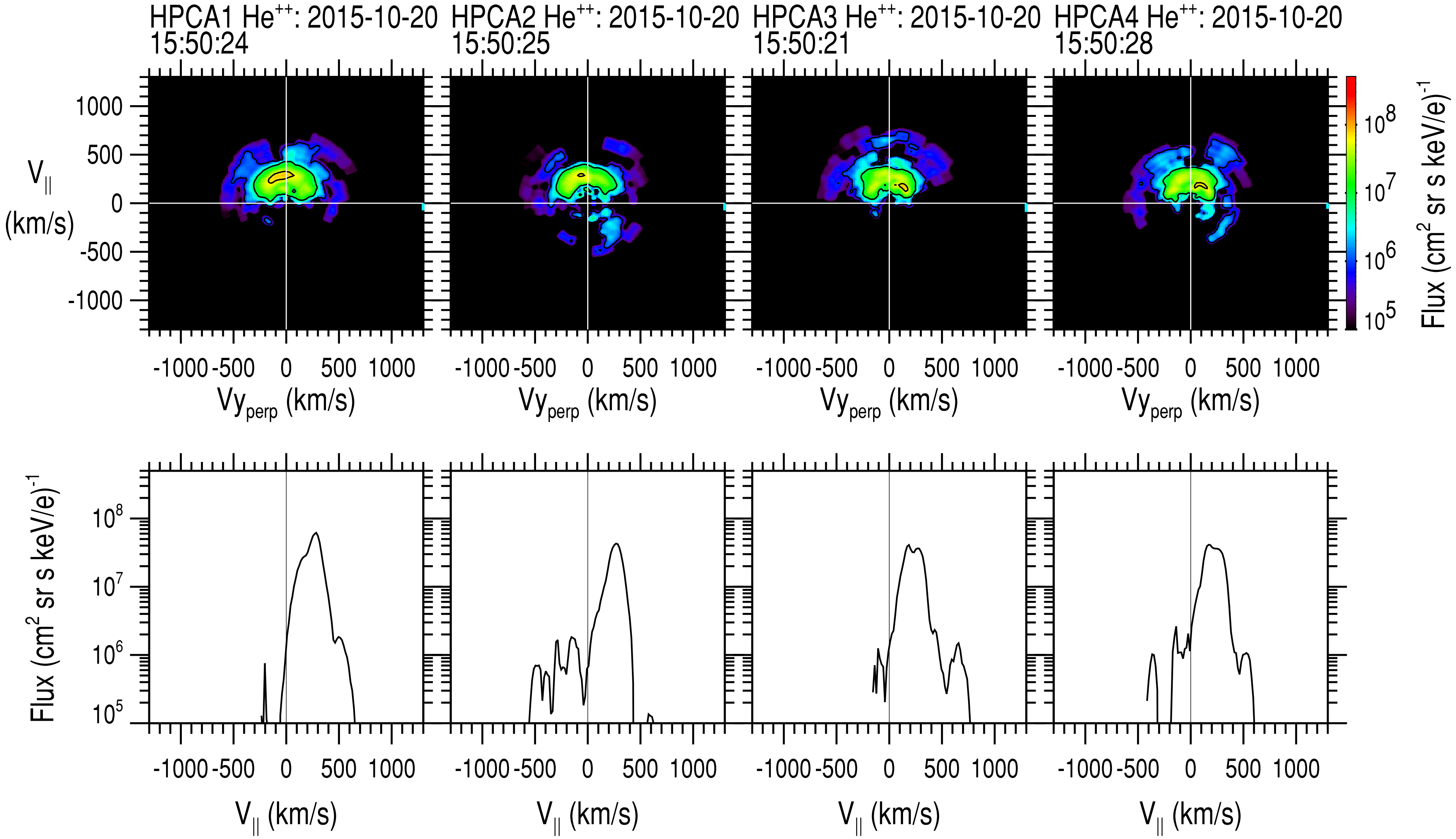

HPCA1 He++: 2015-10-20 15:50:24
HPCA2 He++: 2015-10-20 15:50:25
HPCA3 He++: 2015-10-20 15:50:21
HPCA4 He++: 2015-10-20 15:50:28
V|| (km/s)
Vyperp (km/s)
Flux (cm2 sr s keV/e)-1

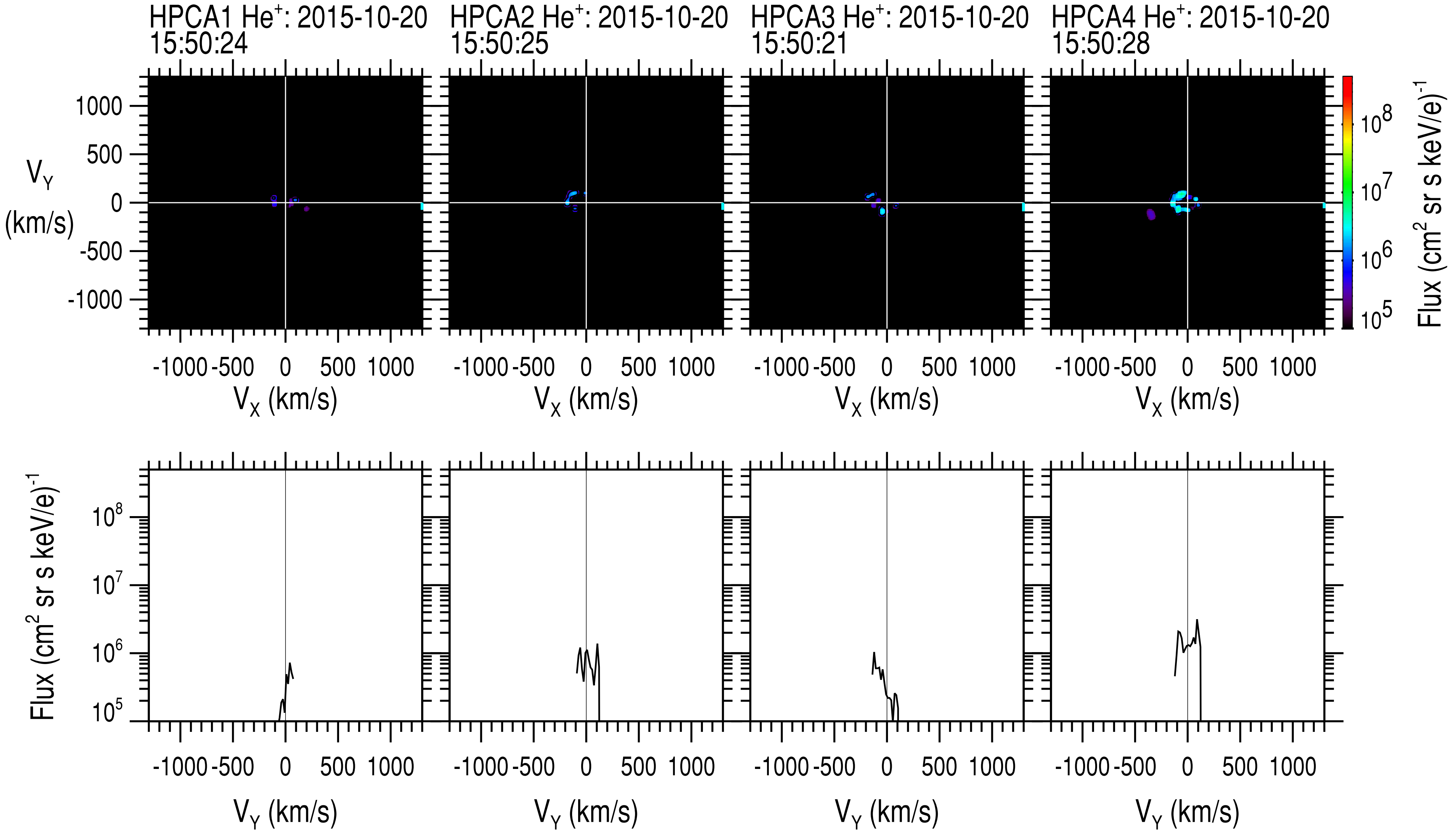

HPCA1 He+: 2015-10-20 15:50:24
HPCA2 He+: 2015-10-20 15:50:25
HPCA3 He+: 2015-10-20 15:50:21
HPCA4 He+: 2015-10-20 15:50:28
V_Y (km/s)
V_X (km/s)
Flux (cm2 sr s keV/e)-1

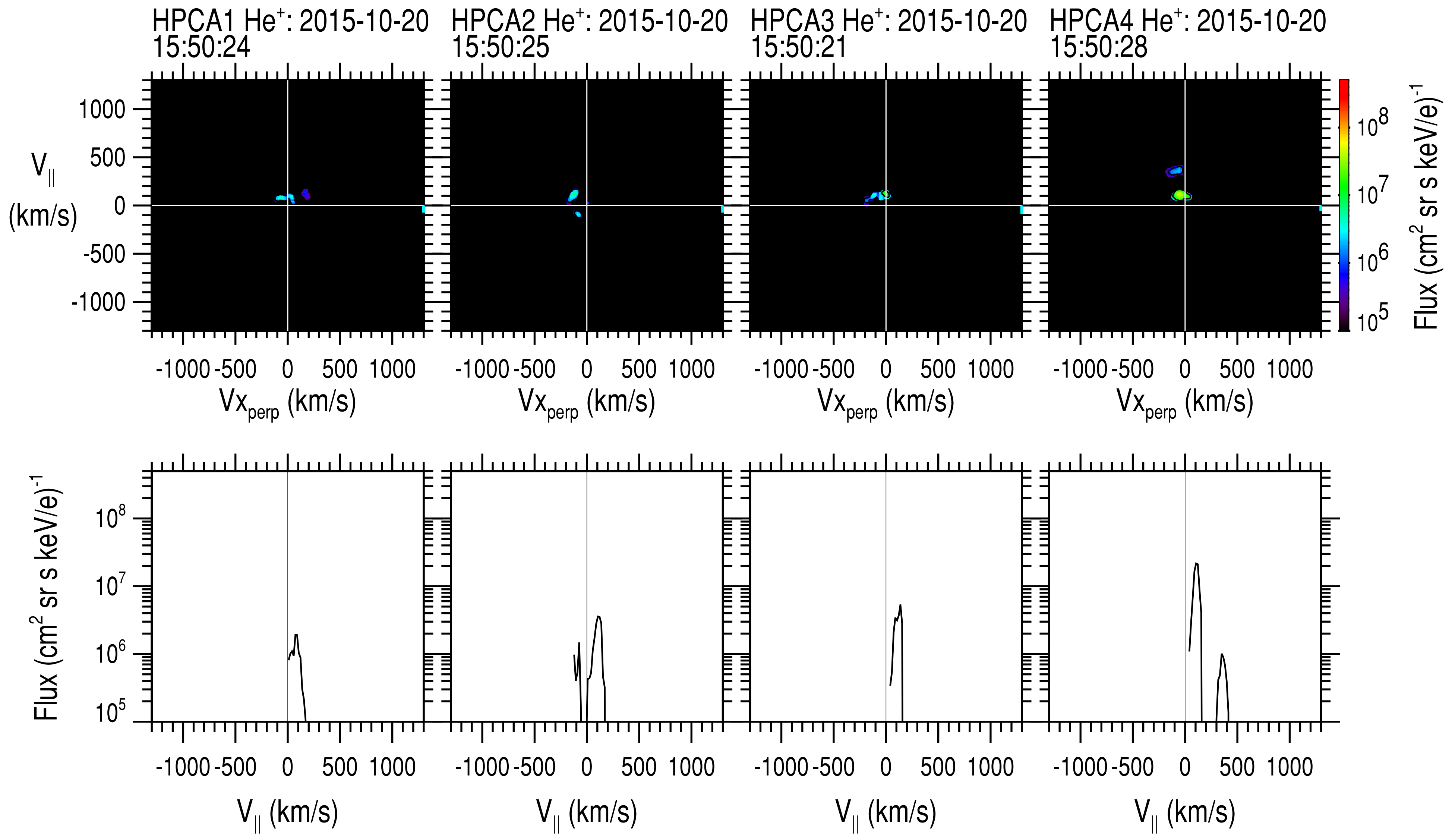
HPCA1 He+: 2015-10-20 15:50:24
HPCA2 He+: 2015-10-20 15:50:25
HPCA3 He+: 2015-10-20 15:50:21
HPCA4 He+: 2015-10-20 15:50:28
V|| (km/s)
Vxperp (km/s)
Flux (cm2 sr s keV/e)-1
V|| (km/s)

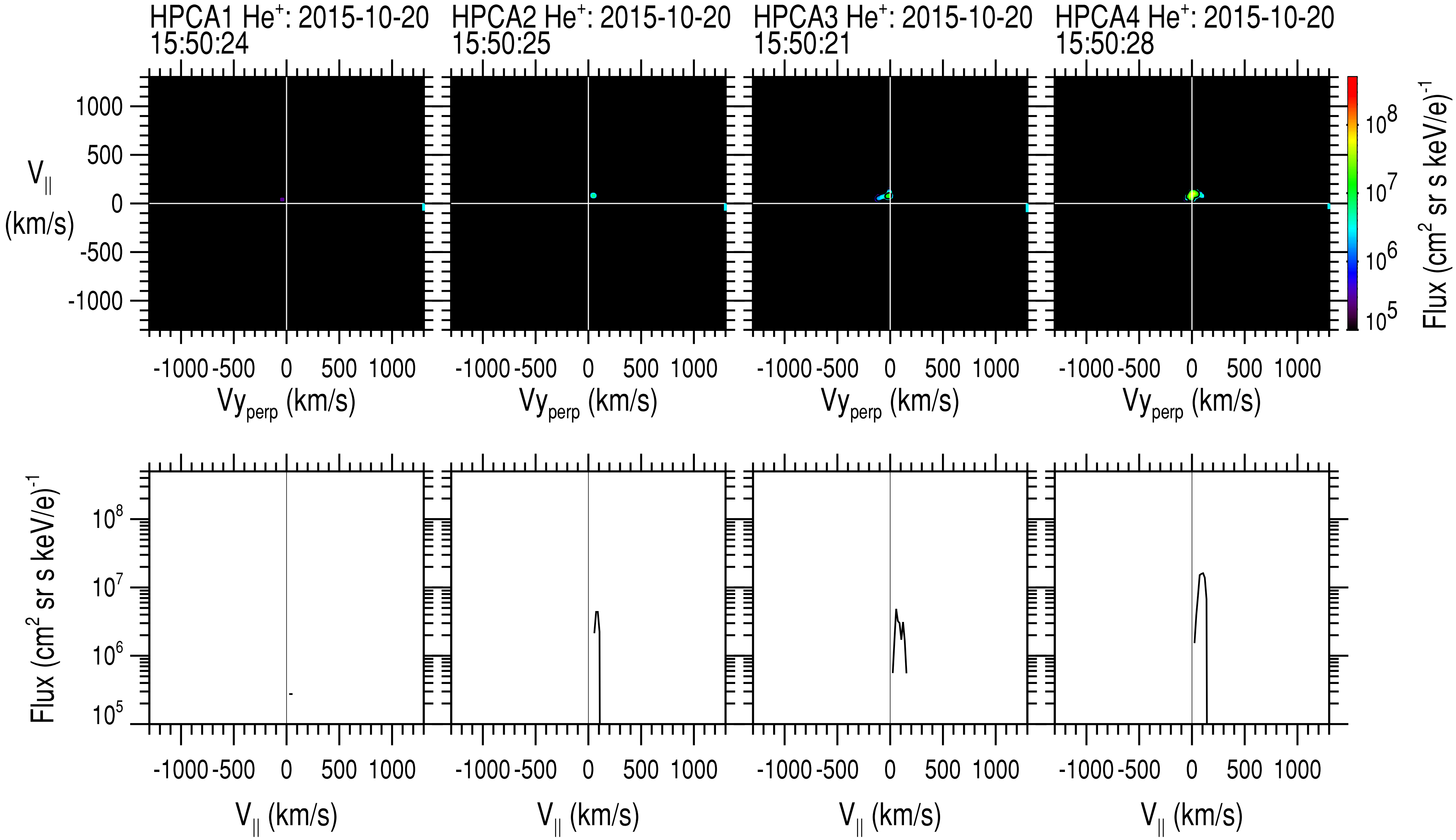
HPCA1 He+: 2015-10-20 15:50:24
HPCA2 He+: 2015-10-20 15:50:25
HPCA3 He+: 2015-10-20 15:50:21
HPCA4 He+: 2015-10-20 15:50:28
V|| (km/s)
Vyperp (km/s)
Flux (cm2 sr s keV/e)-1
1000
500
0
-500
-1000
10^5
10^6
10^7
10^8

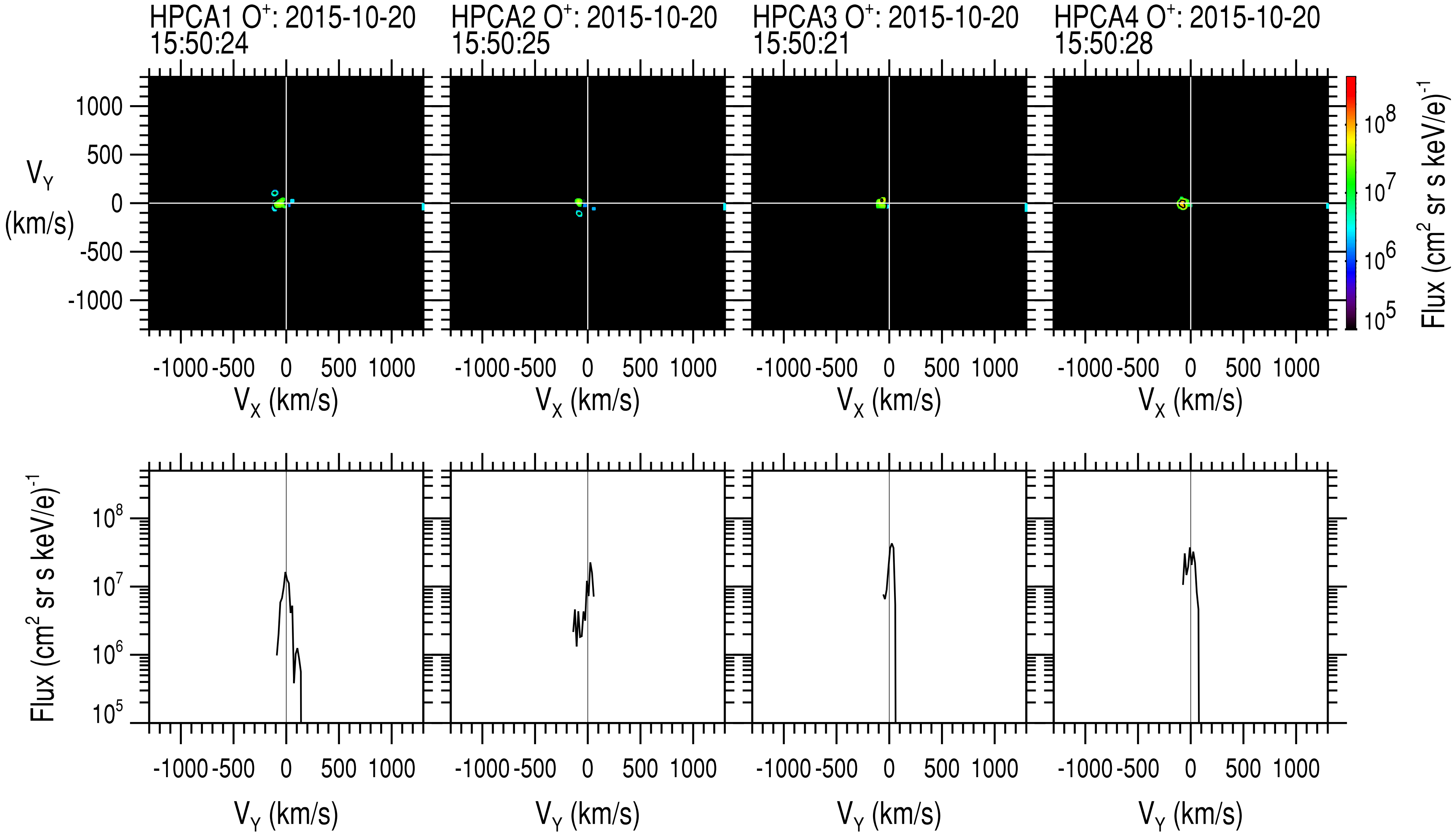
HPCA1 O+: 2015-10-20 15:50:24
HPCA2 O+: 2015-10-20 15:50:25
HPCA3 O+: 2015-10-20 15:50:21
HPCA4 O+: 2015-10-20 15:50:28
VY (km/s)
VX (km/s)
Flux (cm2 sr s keV/e)-1
VY (km/s)
Flux (cm2 sr s keV/e)-1

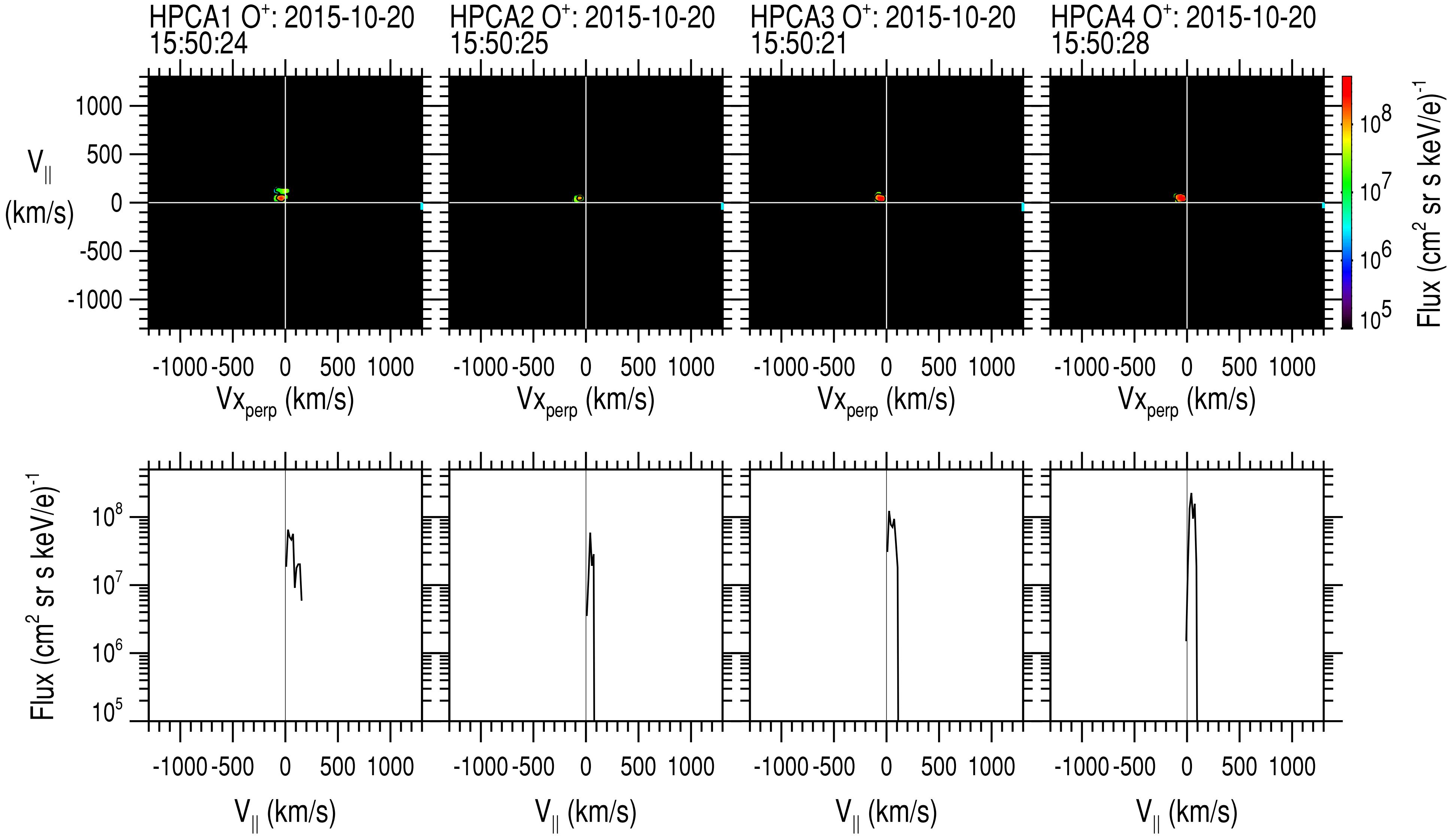

HPCA1 O+: 2015-10-20 15:50:24
HPCA2 O+: 2015-10-20 15:50:25
HPCA3 O+: 2015-10-20 15:50:21
HPCA4 O+: 2015-10-20 15:50:28
V|| (km/s)
Vxperp (km/s)
Flux (cm2 sr s keV/e)-1

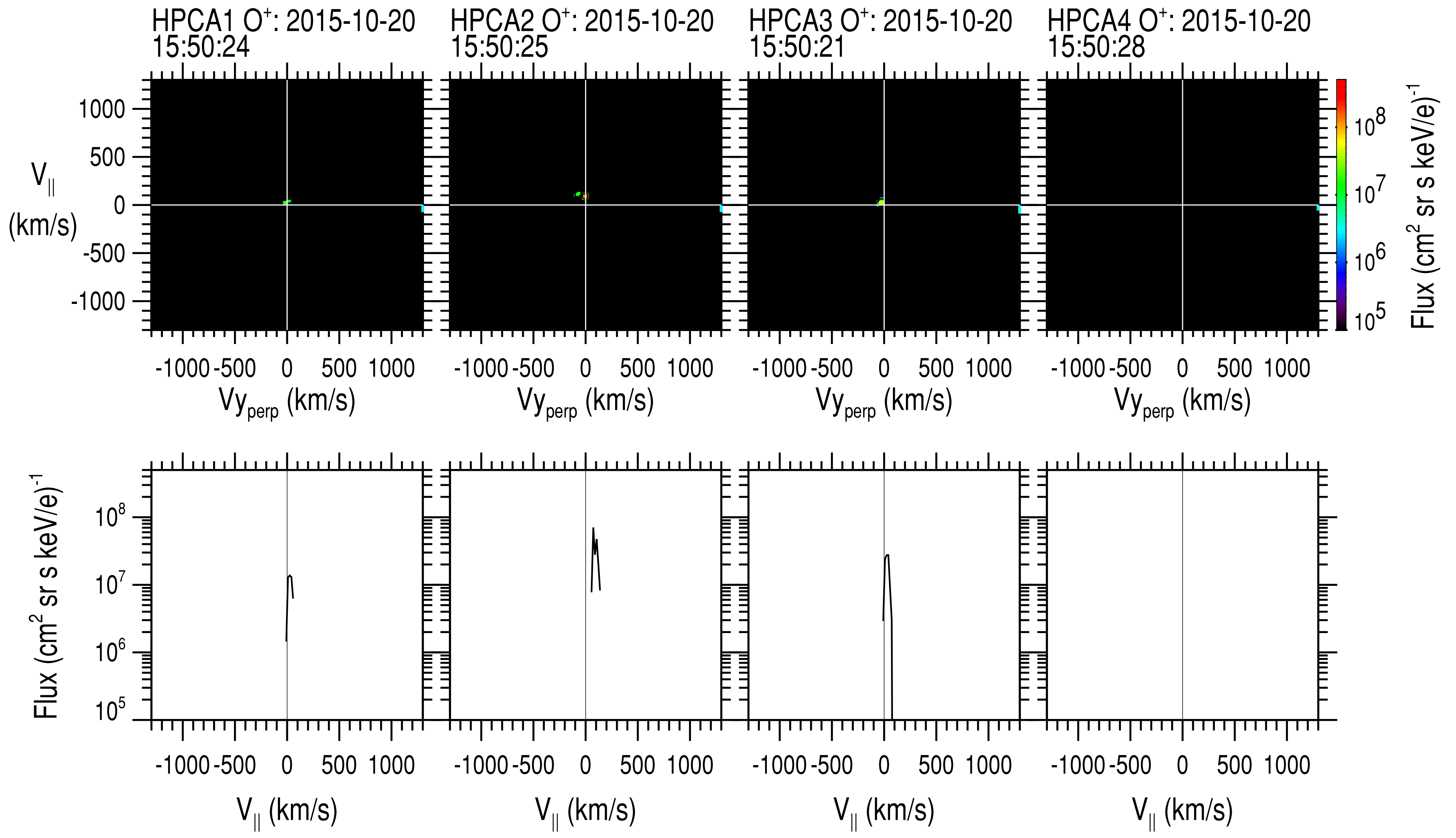
HPCA1 O+: 2015-10-20 15:50:24
HPCA2 O+: 2015-10-20 15:50:25
HPCA3 O+: 2015-10-20 15:50:21
HPCA4 O+: 2015-10-20 15:50:28
V|| (km/s)
Vyperp (km/s)
Flux (cm2 sr s keV/e)-1
V|| (km/s)

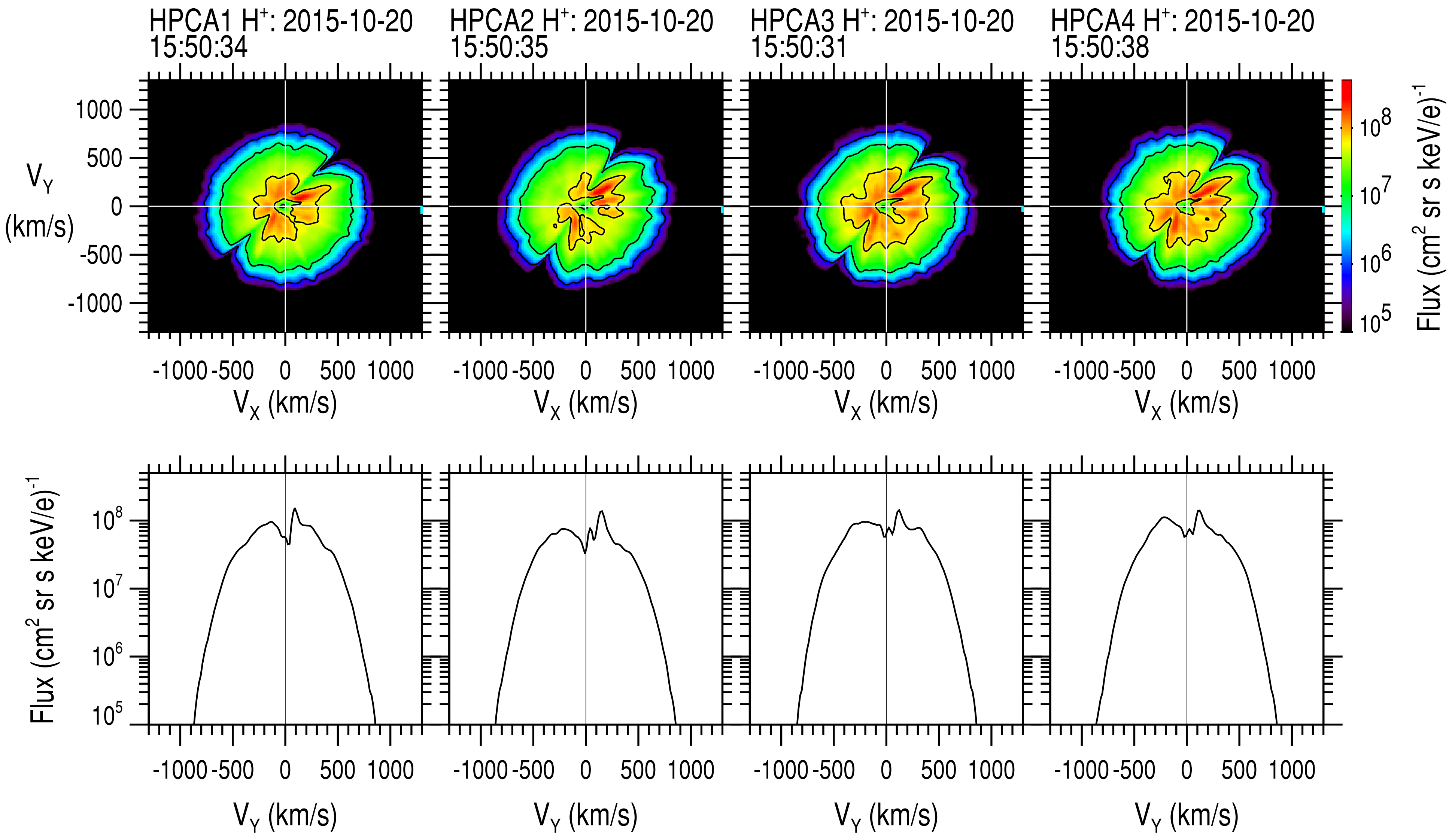
HPCA1 H+: 2015-10-20 15:50:34
HPCA2 H+: 2015-10-20 15:50:35
HPCA3 H+: 2015-10-20 15:50:31
HPCA4 H+: 2015-10-20 15:50:38
V_Y (km/s)
V_X (km/s)
Flux (cm^2 sr s keV/e)^-1
V_Y (km/s)

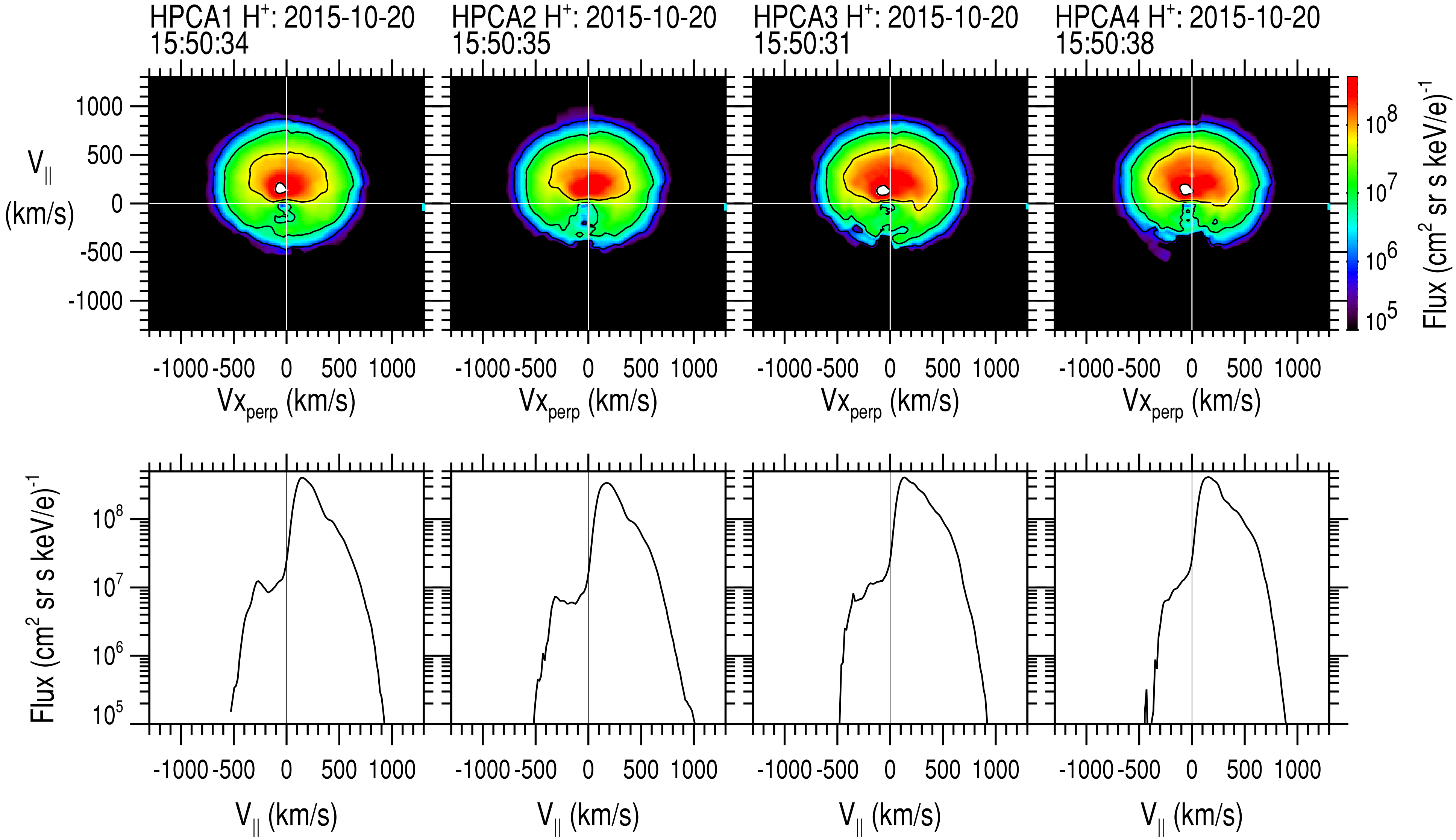

HPCA1 H+: 2015-10-20 15:50:34
HPCA2 H+: 2015-10-20 15:50:35
HPCA3 H+: 2015-10-20 15:50:31
HPCA4 H+: 2015-10-20 15:50:38
V|| (km/s)
Vxperp (km/s)
Flux (cm2 sr s keV/e)-1

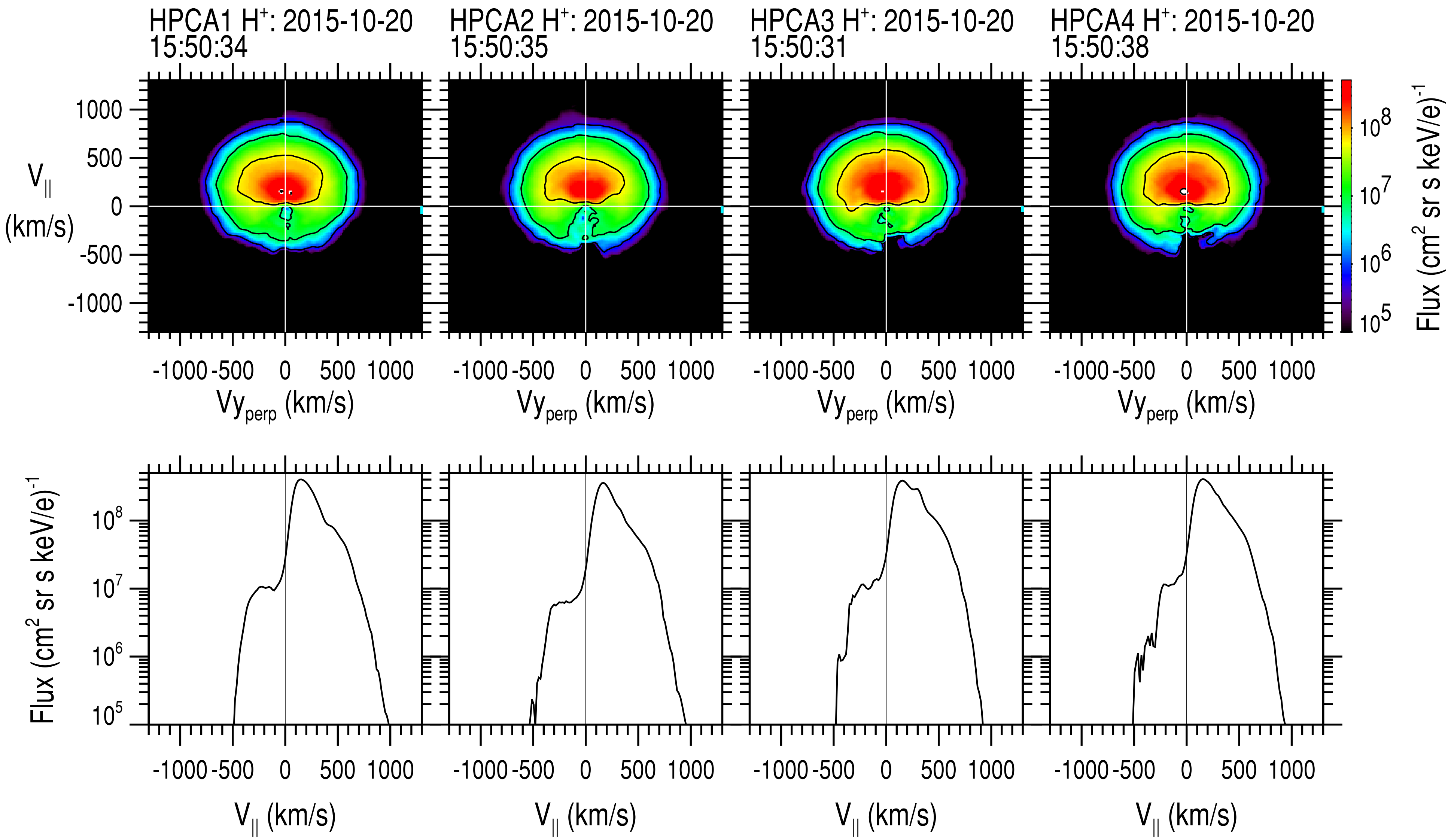

HPCA1 H+: 2015-10-20
15:50:34
HPCA2 H+: 2015-10-20
15:50:35
HPCA3 H+: 2015-10-20
15:50:31
HPCA4 H+: 2015-10-20
15:50:38
V|| (km/s)
Vyperp (km/s)
Flux (cm2 sr s keV/e)-1
V|| (km/s)

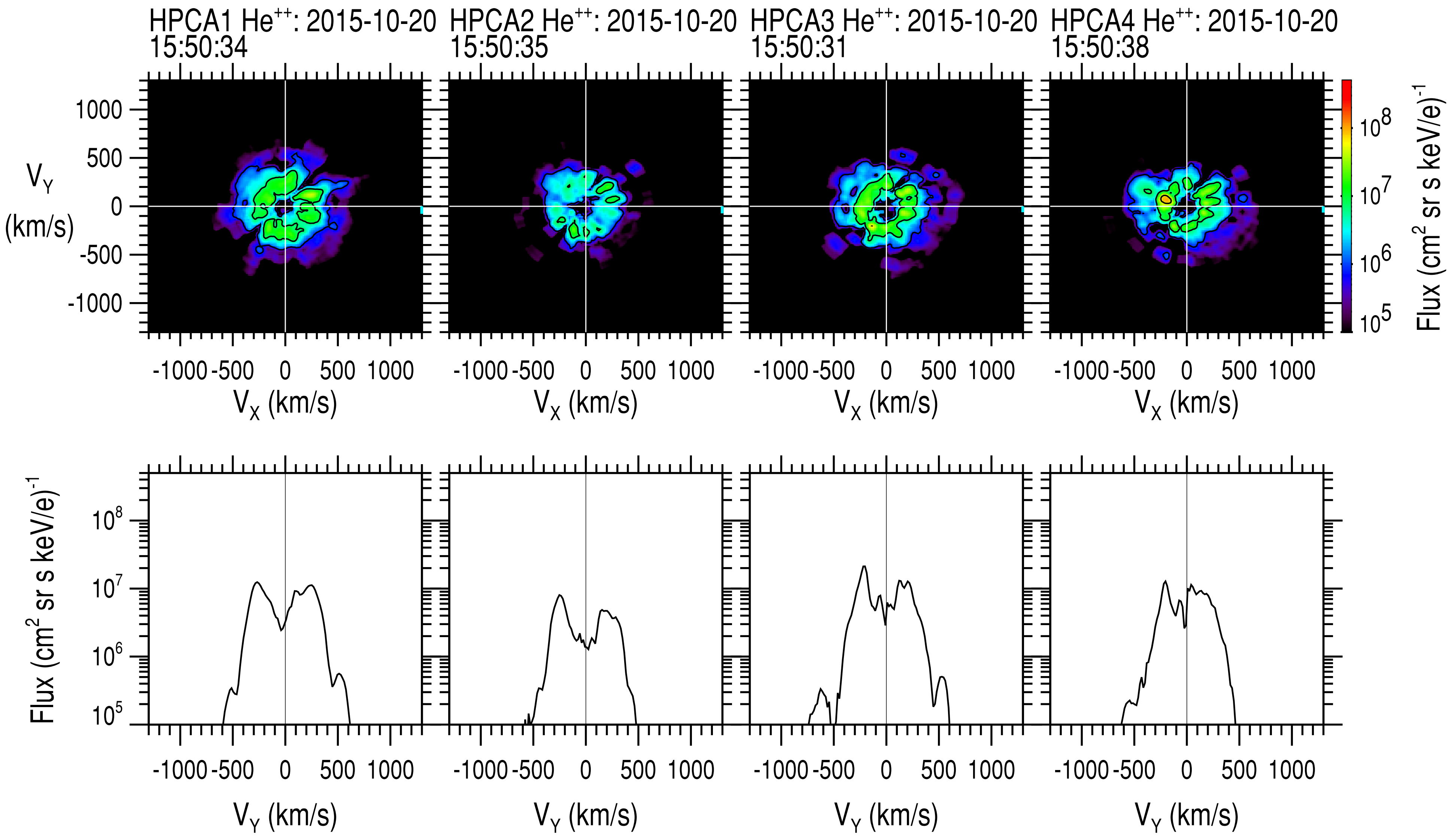
HPCA1 He++: 2015-10-20 15:50:34
HPCA2 He++: 2015-10-20 15:50:35
HPCA3 He++: 2015-10-20 15:50:31
HPCA4 He++: 2015-10-20 15:50:38
V_Y (km/s)
V_X (km/s)
Flux (cm^2 sr s keV/e)^-1
-1000 -500 0 500 1000
10^5 10^6 10^7 10^8

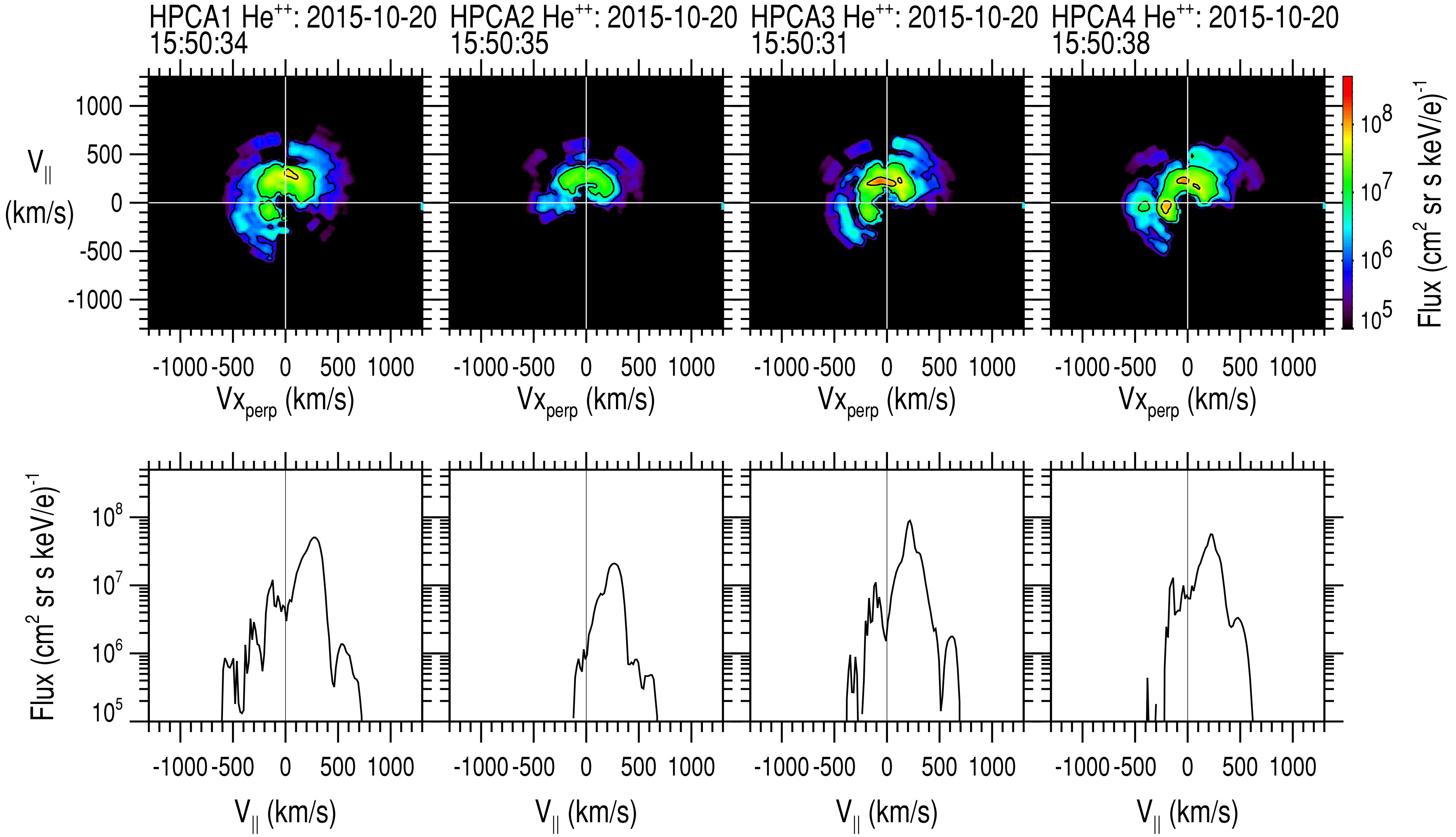

HPCA1 He++: 2015-10-20 15:50:34
HPCA2 He++: 2015-10-20 15:50:35
HPCA3 He++: 2015-10-20 15:50:31
HPCA4 He++: 2015-10-20 15:50:38
V|| (km/s)
Vxperp (km/s)
Flux (cm2 sr s keV/e)-1
1000
500
0
-500
-1000
10^5
10^6
10^7
10^8

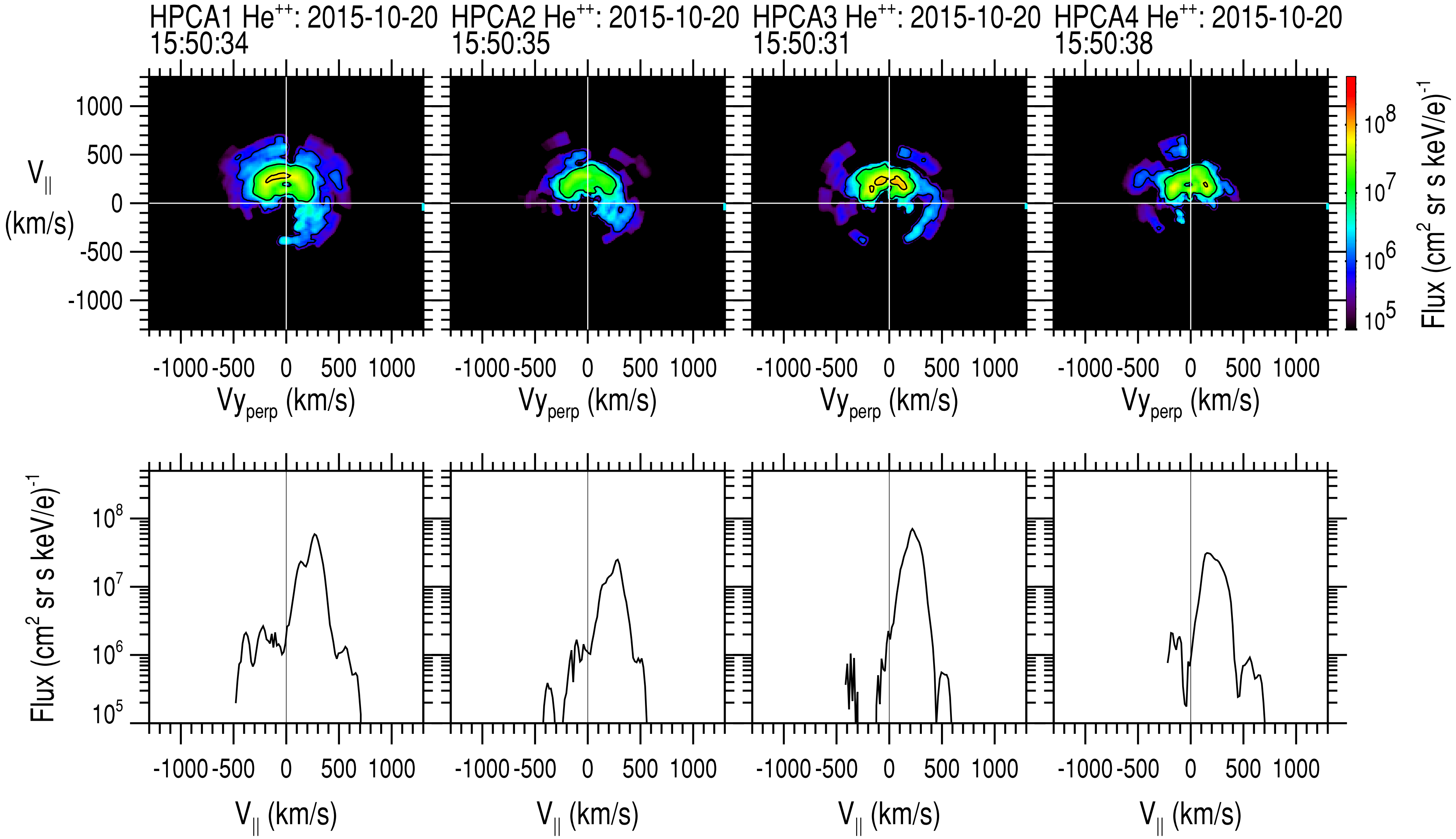
HPCA1 He++: 2015-10-20 15:50:34
HPCA2 He++: 2015-10-20 15:50:35
HPCA3 He++: 2015-10-20 15:50:31
HPCA4 He++: 2015-10-20 15:50:38
V|| (km/s)
Vyperp (km/s)
Flux (cm2 sr s keV/e)-1
1000
500
0
-500
-1000
10^5
10^6
10^7
10^8

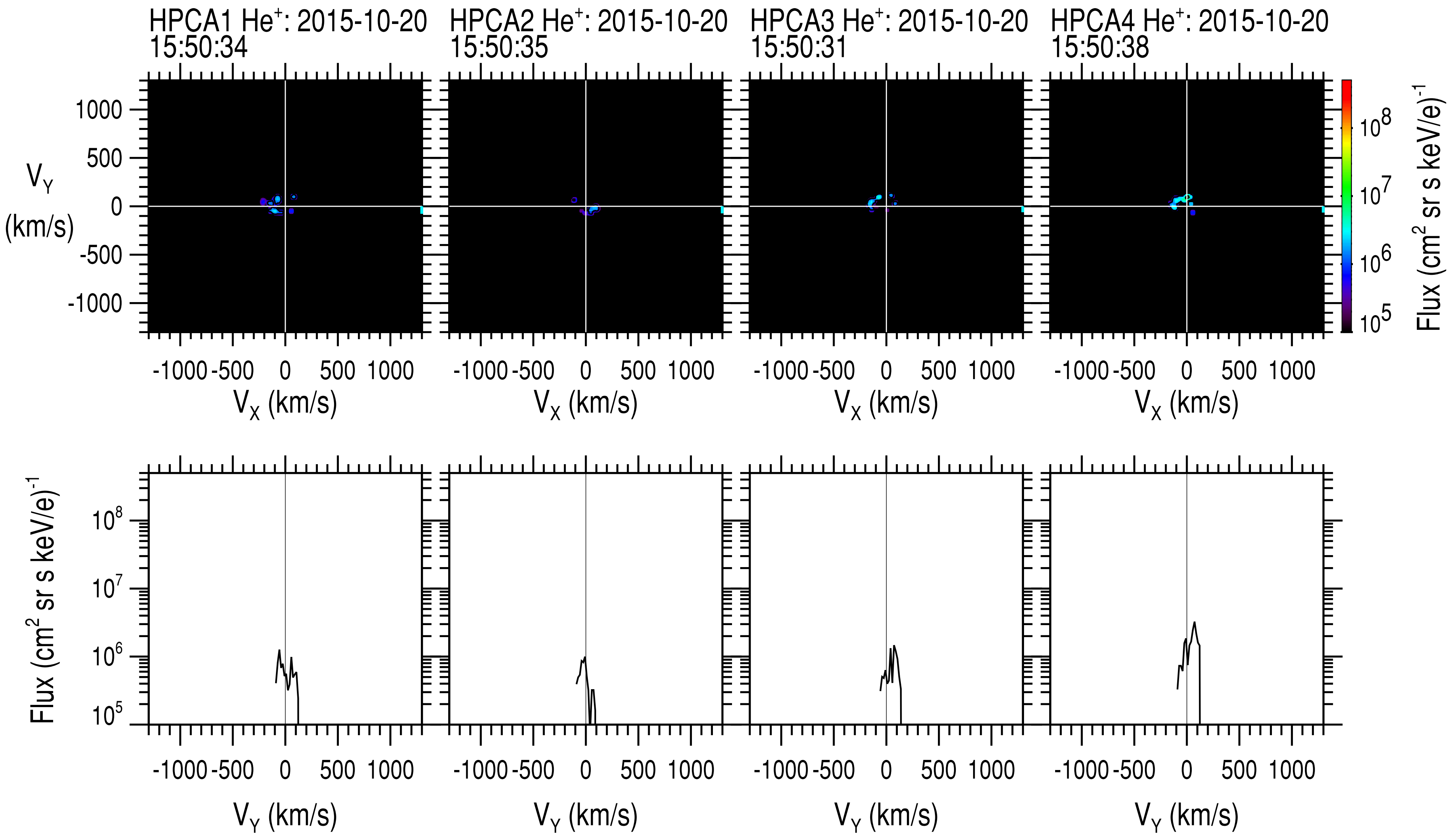

HPCA1 He+: 2015-10-20 15:50:34
HPCA2 He+: 2015-10-20 15:50:35
HPCA3 He+: 2015-10-20 15:50:31
HPCA4 He+: 2015-10-20 15:50:38
V_Y (km/s)
V_X (km/s)
Flux (cm2 sr s keV/e)-1
V_Y (km/s)

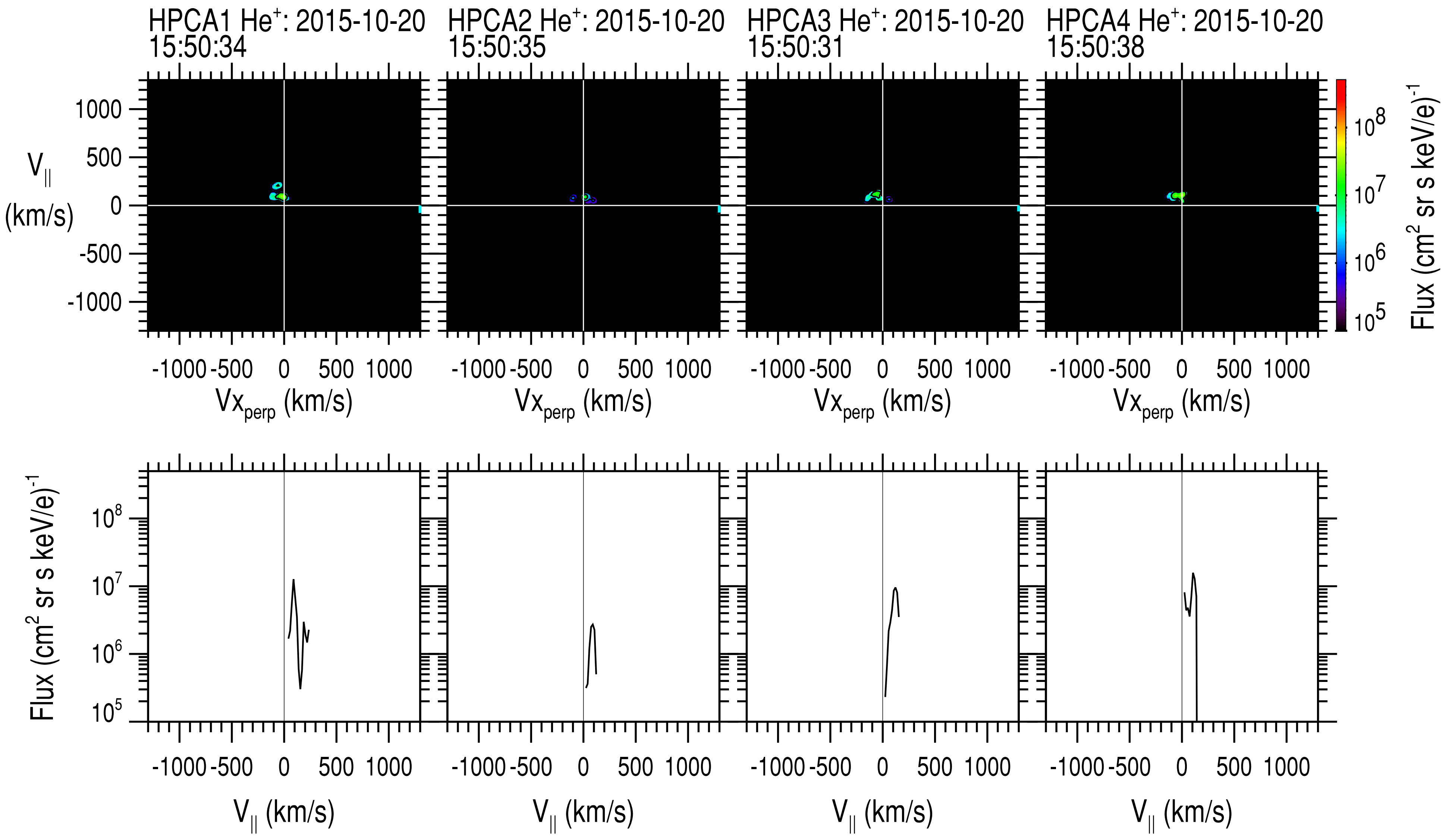

HPCA1 He⁺: 2015-10-20 15:50:34
HPCA2 He⁺: 2015-10-20 15:50:35
HPCA3 He⁺: 2015-10-20 15:50:31
HPCA4 He⁺: 2015-10-20 15:50:38
V|| (km/s)
Vx_perp (km/s)
Flux (cm^2 sr s keV/e)^-1

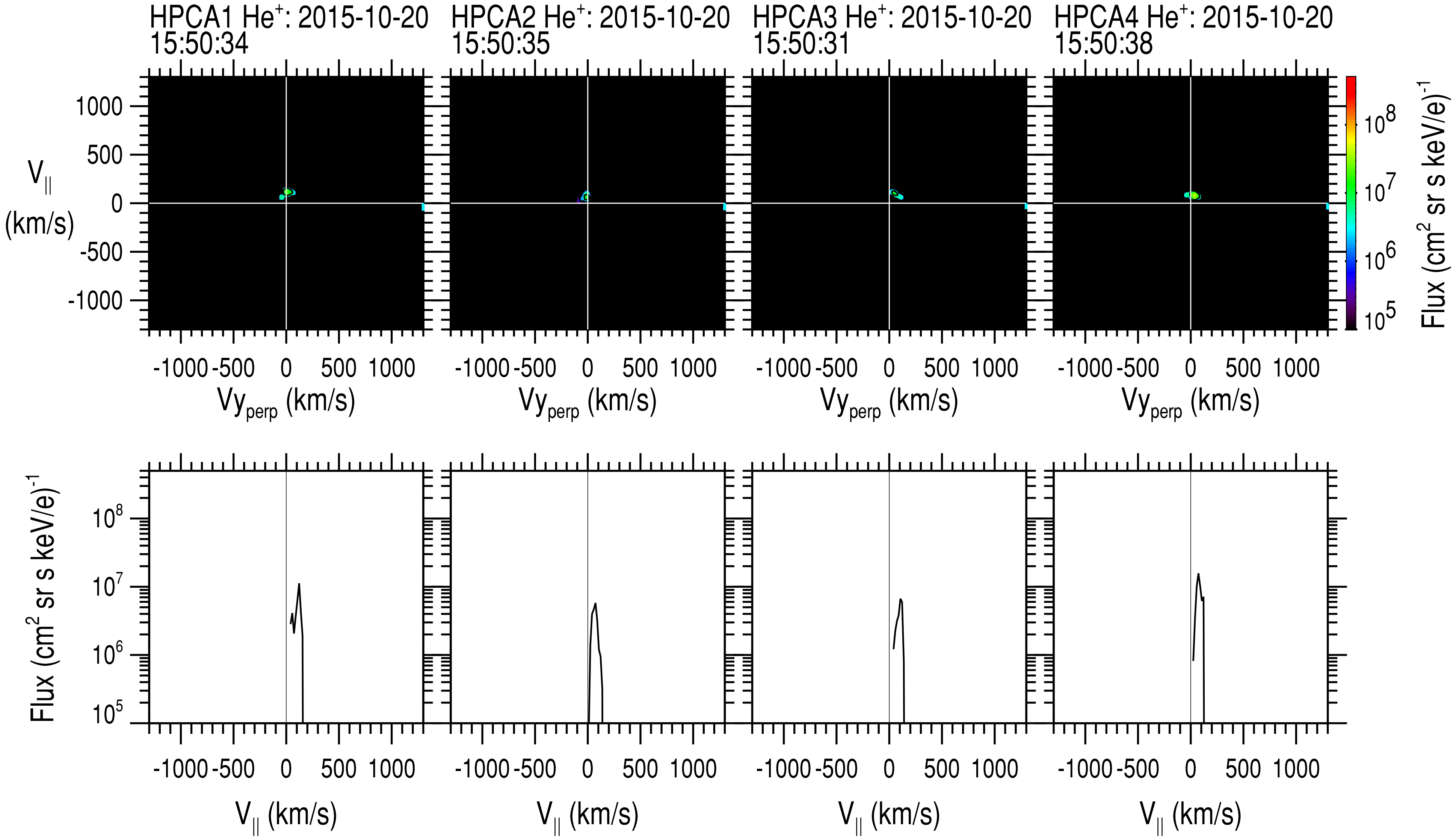

HPCA1 He+: 2015-10-20 15:50:34
HPCA2 He+: 2015-10-20 15:50:35
HPCA3 He+: 2015-10-20 15:50:31
HPCA4 He+: 2015-10-20 15:50:38
V|| (km/s)
Vy_perp (km/s)
Flux (cm2 sr s keV/e)-1
1000
500
0
-500
-1000
10^5
10^6
10^7
10^8

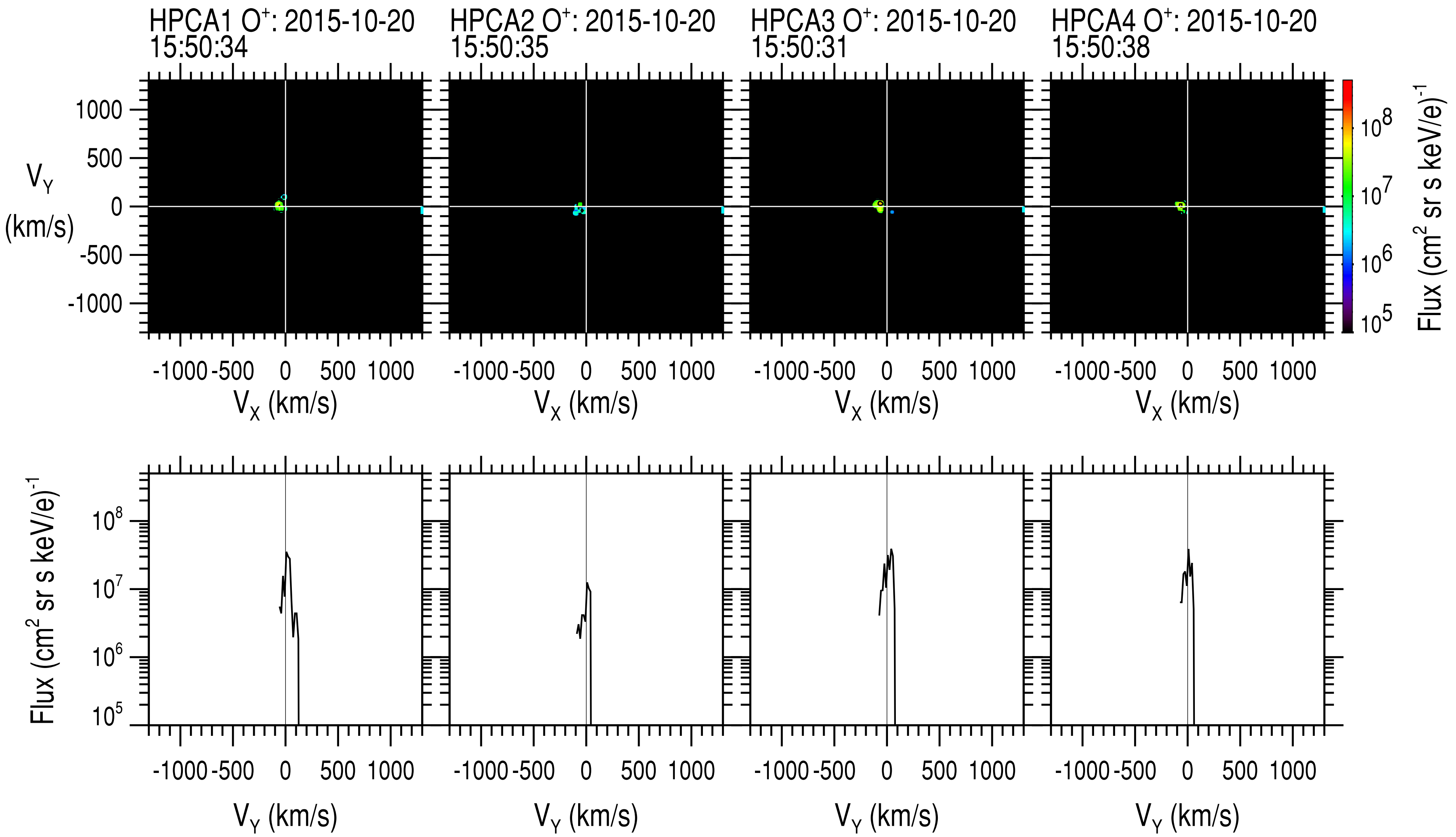
HPCA1 O+: 2015-10-20 15:50:34
HPCA2 O+: 2015-10-20 15:50:35
HPCA3 O+: 2015-10-20 15:50:31
HPCA4 O+: 2015-10-20 15:50:38
V_Y (km/s)
V_X (km/s)
Flux $(cm^2$ sr s keV/e$)^{-1}$
10^5
10^6
10^7
10^8
-1000
-500
0
500
1000

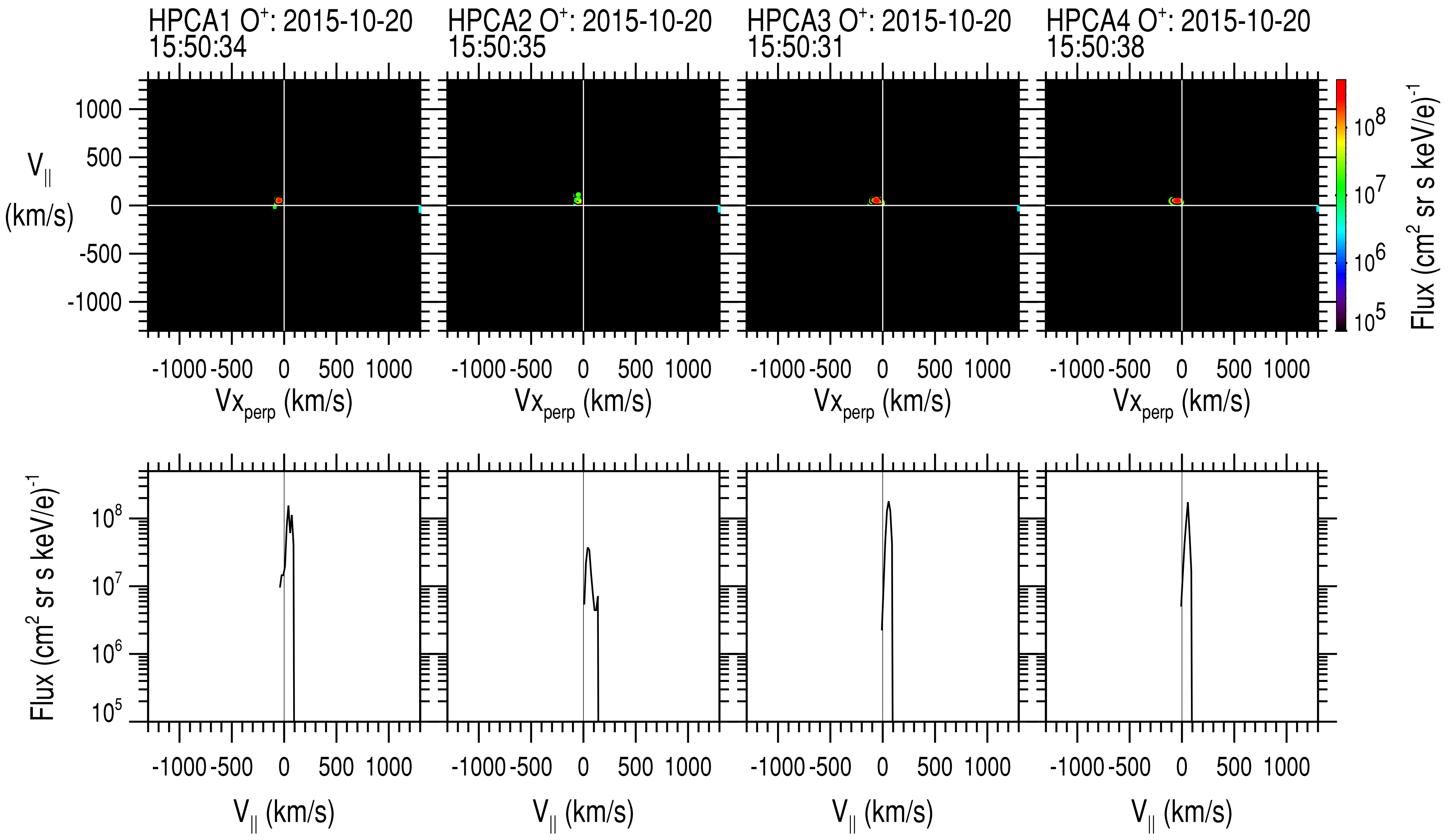

HPCA1 O+: 2015-10-20 15:50:34
HPCA2 O+: 2015-10-20 15:50:35
HPCA3 O+: 2015-10-20 15:50:31
HPCA4 O+: 2015-10-20 15:50:38
V|| (km/s)
Vxperp (km/s)
Flux (cm2 sr s keV/e)-1

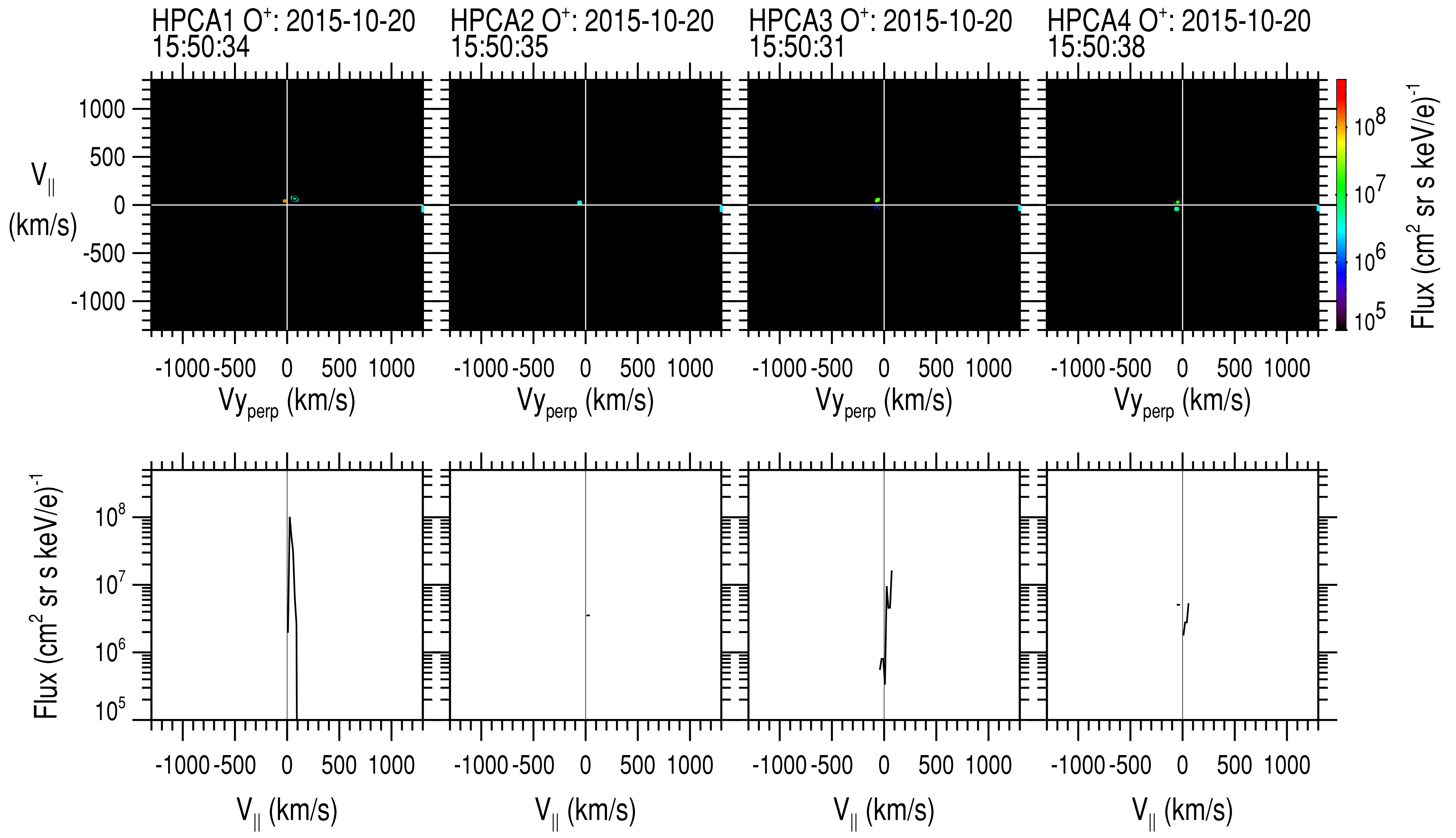

HPCA1 O+: 2015-10-20 15:50:34
HPCA2 O+: 2015-10-20 15:50:35
HPCA3 O+: 2015-10-20 15:50:31
HPCA4 O+: 2015-10-20 15:50:38
V|| (km/s)
Vyperp (km/s)
Flux (cm2 sr s keV/e)-1
